# An experimental approach in revisiting the magnetic orientation of cattle

**DOI:** 10.1371/journal.pone.0187848

**Published:** 2018-04-11

**Authors:** Debby Weijers, Lia Hemerik, Ignas M. A. Heitkönig

**Affiliations:** 1 Resource Ecology Group, Wageningen University, Wageningen, The Netherlands; 2 Biometris, Wageningen University and Research, Department of Mathematical and Statistical Methods, Wageningen, The Netherlands; Syddansk Universitet, DENMARK

## Abstract

In response to the increasing number of observational studies on an apparent south-north orientation in non-homing, non-migrating terrestrial mammals, we experimentally tested the alignment hypothesis using strong neodymium magnets on the resting orientation of individual cattle in Portugal. Contrary to the hypothesis, the 34 cows in the experiment showed no directional preference, neither with, nor without a strong neodymium magnet fixed to their collar. The concurrently performed 2,428 daytime observations—excluding the hottest part of the day—of 659 resting individual cattle did not show a south-north alignment when at rest either. The preferred compass orientation of these cows was on average 130 degrees from the magnetic north (i.e., south east). Cow compass orientation correlated significantly with sun direction, but not with wind direction. In as far as we can determine, this is the first experimental test on magnetic orientation in larger, non-homing, non-migrating mammals. These experimental and observational findings do not support previously published suggestions on the magnetic south-north alignment in these mammals.

## Introduction

The ability of animals to sense the Earth’s magnetic field has been demonstrated by field observations for several animal taxa, including insects, e.g. ([1], [2], [3]), fishes, e.g. ([4], [5], [6], [7], [8]), amphibians and reptiles, e.g.([9], [10], [11], [12], [13]), birds, e.g. ([14], [15], [16], [17], [18], [19], [20]), small mammals e.g. ([21], [22], [23], [24], [25], [26], [27], [28]) and larger mammals like dogs [29], foxes [30] and wild pigs [31], but not horses [32]. While the majority of these studies are based on behavioural and tracking observations in the field, strong experimental evidence for sensing the Earth’s magnetic field is provided for relatively small animals in studies for homing, e.g. [19] and migrating species, e.g. ([1], [9], [10], [14], [28], [30]). We have been unable to find an experimental study for large mammals, which, at the time of observation, are neither homing nor migrating.

In a study using high resolution Google Earth images and snow prints [33], it was suggested that large mammals, i.e., cattle and deer, also respond to the Earth’s magnetic field. They would preferentially align their bodies in a south-north direction when at rest [33]. In 2009, a follow-up study [34] demonstrated that extremely low-frequency electromagnetic fields, which are generated by high-voltage power lines, disrupt the north-south alignment of ruminants: body orientation of cattle and deer was random on pastures under or in the close proximity to power lines. Further away from the power lines a south-north body alignment could again be detected [34]. The result from the latter study suggests that large herbivores also possess the ability to sense the Earth’s magnetic field. Moreover, it would make a magnetic-orientation-hypothesis, and magnetism-aided homing and navigation processes in large mammals more plausible ([31], [33], [35]). However, when the earlier study [32] was replicated [36], no alignment of cattle along south-north geomagnetic field lines was found. Although a re-analysis of the critical data [36] in a subsequent publication [37] suggested that the south-north alignment was evident, the latter conclusion remains strongly contested [38].

To date, it seems that these large mammal studies have not yet shown an experiment involving a treatment and a control group. Furthermore, the positioning of the head or tail of the animal has been used only in some studies that support the hypothesis of a north-south alignment. Our study aims to experimentally test the magnetic alignment hypothesis on cattle with close, direct observations on the body orientation with the position of the head known. We performed our experiment on 34 individual cattle, with or without a magnet, to test the hypothesis that cattle align their bodies in a south-north direction along the geomagnetic lines when at rest, as suggested by the literature. We concurrently conducted observations (*n* = 2428) on the orientation of a large number of cattle (*n* = 659) at rest to re-evaluate the alignment hypothesis from earlier observational studies.

To determine possible other environmental influences on body orientation, we correlated wind direction and sun direction with the compass orientation of large mammals. These influences were found to be non-significant in earlier magnetic alignment papers [33] or were not tested [31].

In the experimental part of our study, we aimed to disrupt the possible geo-magnetic orientation by fixing a strong neodymium magnet to the collar of individual cattle, and also measured their orientation when at rest. We applied circular statistics [39] on bootstrapped subsets of our data sets to test (1) deviations from random and (2) deviations from north-south.

## Results

### Experimental test on cow compass orientation

**Fig 1:**
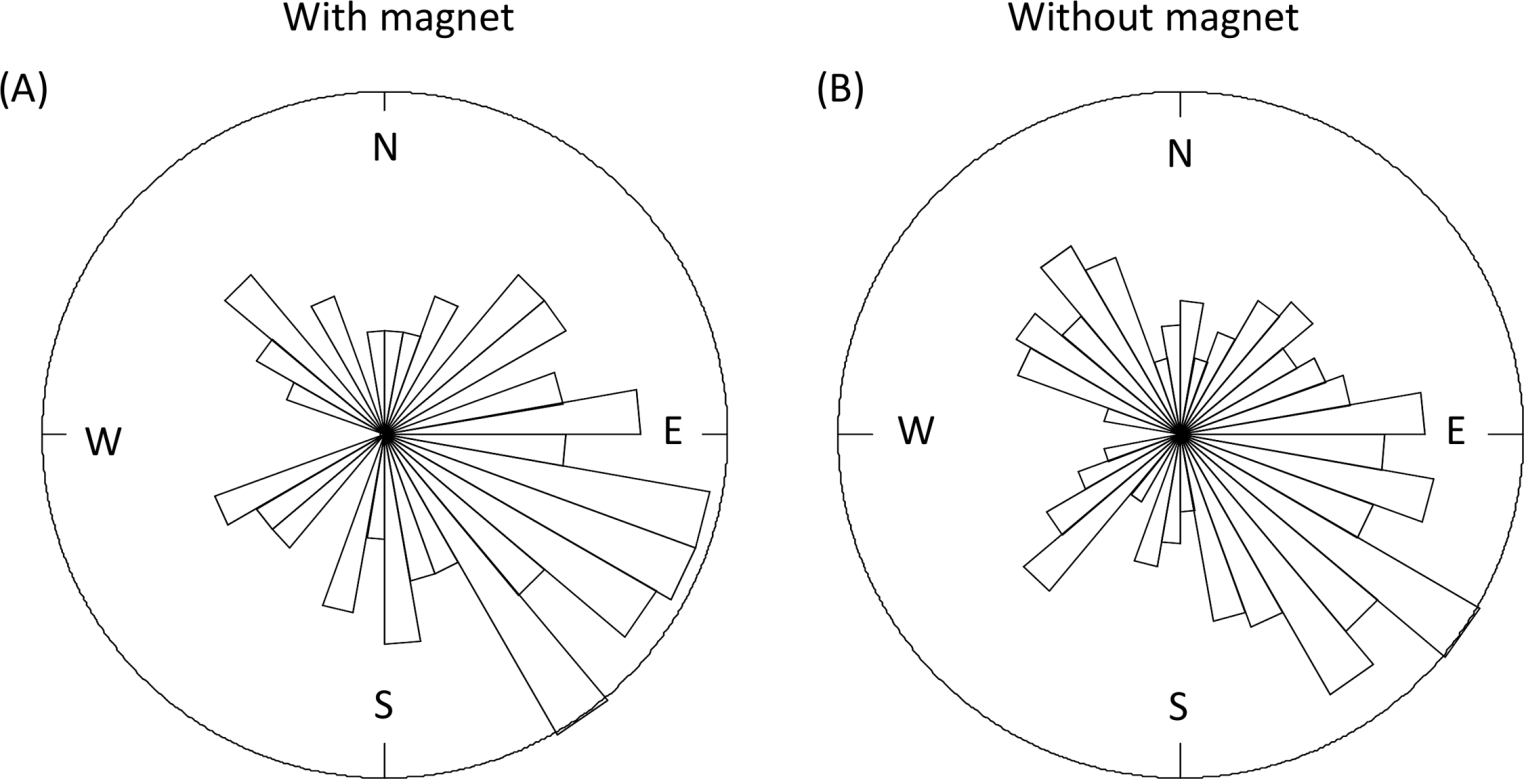
Rose diagram (full data set): Orientation of cows wearing and not wearing a magnet. Rose diagrams: (*A*) the full data set of cow orientations with a magnet in the experiment (*n =* 97 measurements from 34 individual cows), (*B*) the full data set of cow orientations without a neodymium magnet in the experiment (*n =* 177 measurements from 34 cows).

**Fig 2:**
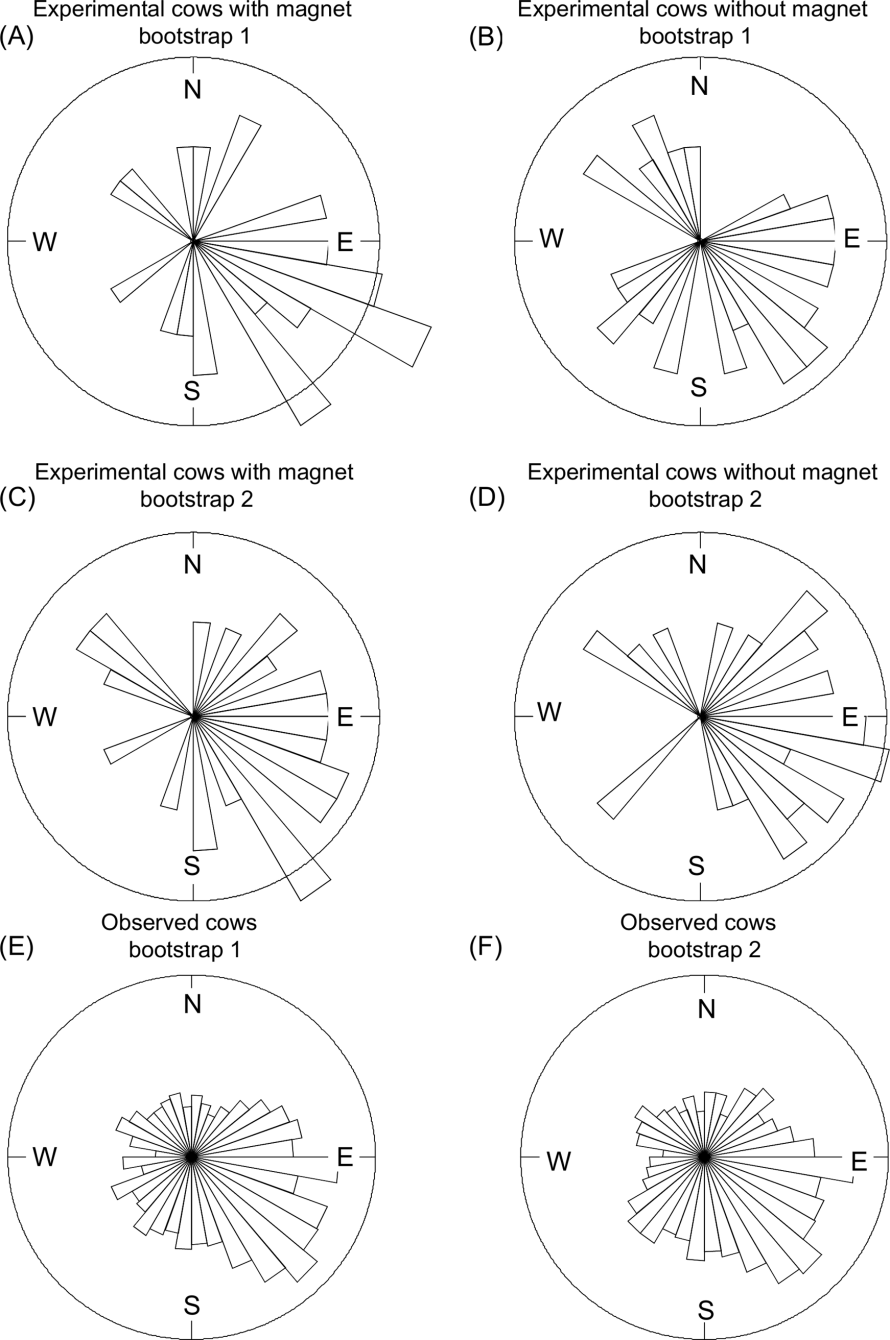
Rose diagram (bootstrap data set): Orientation of cows wearing and not wearing a magnet. Rose diagrams, each representing one realisation of a bootstrap data set of (*A*) and (*B*) 659 cows observed in the field, (*C*) and (*E*) 34 cows with a magnet in the experiment, and (*D*) and (*F*) 34 cows without a magnet in the experiment.

In the experiment, cows without a magnet showed a direction deviating from random in only 8 out of 500 bootstrap datasets (1.6%). Similarly, when a magnet was attached, their direction differed from random in only 7 out of 500 bootstrap datasets (1.4%). The few significant deviations from random were sometimes positive and sometimes negative, with the same occurrence rate. There was no obvious correlation between compass orientation of cows with or without a magnet. Overall, the mean direction of the 34 cows was not significantly different from a random distribution, regardless of whether the cows were wearing a magnet (*n =* 97 measurements) or not (*n =* 177 measurements; Fig 1). Hence, we could not find evidence to support the hypothesis that the preferred direction is towards north in the observed orientations in the groups with and without magnet (Table 1). Bootstrapped subsets of the range of compass directions are shown in Fig 2A–2D.

**Table 1:** Results of the bootstrap analysis on cattle with or without a magnet attached. The mean cow direction is given in degrees (+/- standard deviation). The “Rayleigh north” is the mean result of the Rayleigh test (+/- standard deviation), testing for a deviation from north. “Rayleigh random” is the mean result of the Rayleigh test (+/- standard deviation), testing for a deviation from random distribution.

Test	Without magnet	With magnet
Mean cow direction	33.56 (+/- 92.80)	24.92 (+/- 127.80)
Rayleigh north	0.01 (+/- 0.10)	-0.07 (+/- 0.09)
P-value north	0.48 (+/- 0.26)	0.67 (+/- 0.23)
Rayleigh random	0.14 (+/- 0.07)	0.13 (+/- 0.07)
P-value random	0.56 (+/- 0.27)	0.57 (+/- 0.27)

Testing the correlation between cow compass orientation of cows without an attached magnet, and wind direction or sun direction, respectively, results in four non-significant correlations out of 1000 bootstrap sets (Table 2). The correlations between cow direction and sun direction or wind direction show only significant correlations in very few (0.6 to 3.6%) of the total number of bootstrap datasets.

When we performed a Rayleigh test on the difference between the paired data of the same cow with or without a magnet the mean difference in all bootstrap data sets was -14.9° (S.D. 76.1°; and the range was between -178.4° and 176.6°; test statistic = 0.15, S.D. 0.08, mean P-value = 0.51). These differences thus did not differ from zero.

**Table 2:** Results of the bootstrap analysis of correlation between cow compass direction and wind direction or sun direction while wearing or not wearing a magnet. The mean correlations are shown +/- standard deviation and represent the correlation between cow compass orientation and wind direction or sun direction, respectively. The mean test statistic and the mean P-value are also given (+/- standard deviation).

Test	Without magnet	With magnet
**Correlation wind**	0.00 (+/- 0.18)	0.03 (+/- 0.15)
**Test stat correlation wind**	0.00 (+/- 0.92)	0.16 (+/- 0.86)
**P-value (correlation wind)**	0.53 (+/- 0.29)	0.53 (+/- 0.26)
**Correlation sun**	0.00 (+/- 0.18)	0.02 (+/- 0.16)
**Test stat correlation sun**	0.00 (+/- 0.88)	0.11 (+/- 0.78)
**P-value (correlation sun)**	0.54 (+/- 0.28)	0.57 (+/- 0.27)

### Orientation of cattle in the field

**Fig 3:**
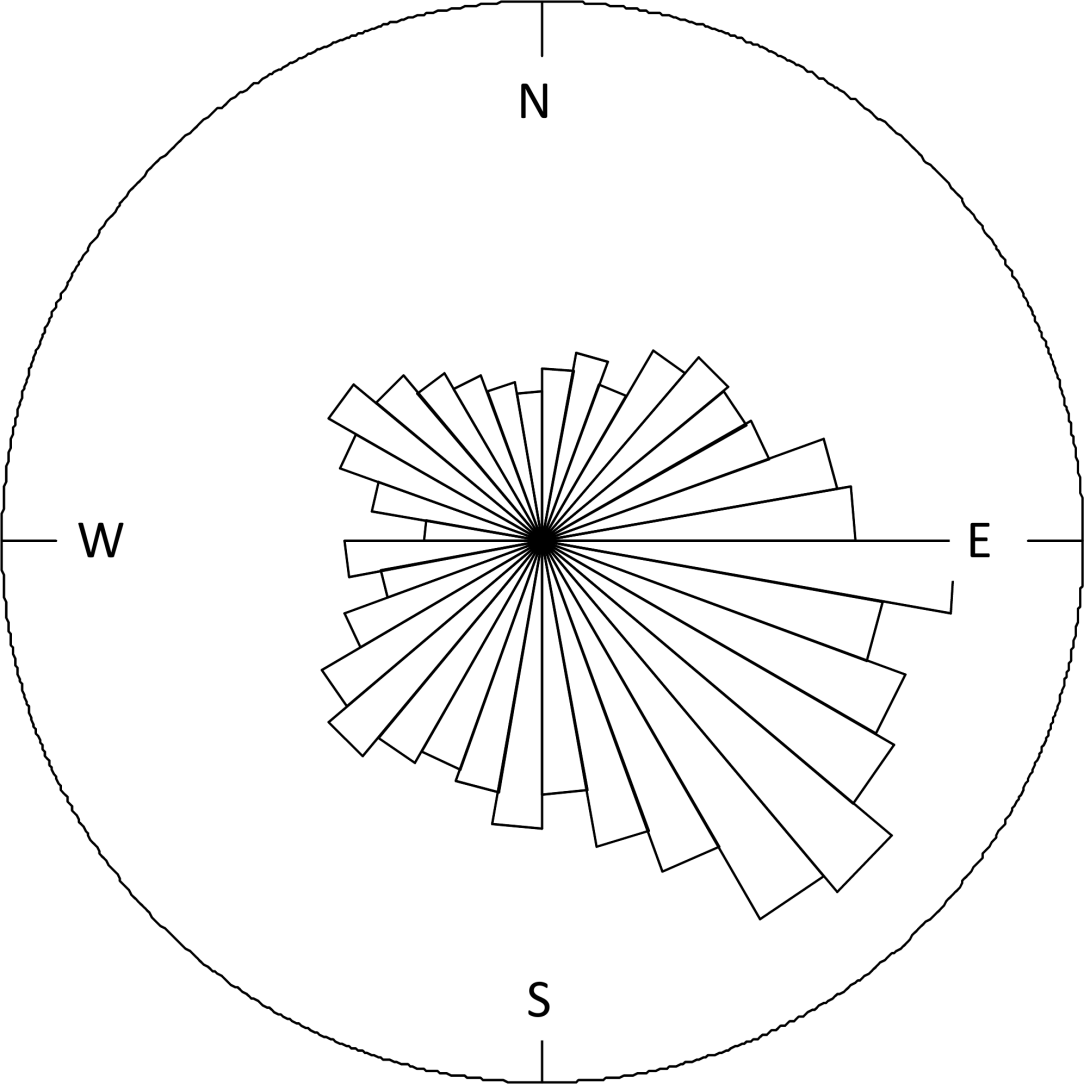
Rose diagram: the full data set of cow orientations in the field. *n =* 2428 observations on 659 individual cows from 6 different farms in southwest Portugal.

Concurrent observations of cow compass orientations in the field (n = 2428 observations on 659 individual cows) showed, in bootstrapped data sets, a direction towards the south-east, at approximately 130.2 degrees (+/- 2.3 degrees) from the magnetic north (all data are shown in Fig 3). The Rayleigh test on bootstrapped data showed that this orientation followed a von Mises distribution with a preferred direction and was not randomly distributed around the circle (P << 0.001; Table 3). When the observed orientations were tested against the reference point “north”, we found a significant deviation from north (Rayleigh test, P >> 0.05, Table 3). The field observations resulted in a range of compass directions, of which bootstrapped subsets are shown in Fig 2E and 2F.

**Table 3:** Results from the bootstrap for orientation of cattle in the field. The mean cow direction is the mean direction of the cow in degrees clock-wise from the magnetic north. The “Rayleigh north” is the result of the Rayleigh test, testing the deviation from north. The “P-value north” is the P-value corresponding with this Rayleigh test. “Rayleigh random” is the result of the Rayleigh test, testing the deviation from a random distribution. The “P-value random” is the corresponding P-value. We also report the minimum, maximum and the standard deviation, as we have 1000 bootstrap data sets (S1 File).

	Mean cow direction	Rayleigh north	P-value north	Rayleigh random	P-value random
**Mean**	130.21	-0.26	1	0.40	<< 0.001
**S.D.**	2.44	0.02	0	0.02	<< 0.001
**Minimum**	123.37	-0.30	1	0.35	<< 0.001
**Maximum**	137.86	-0.21	1	0.46	<< 0.001

Testing the correlation between cow compass orientation and wind direction or sun direction in 1000 bootstrapped data sets, resulted in correlations of 0.09 (+/- 0.03) and 0.19 (+/- 0.03), respectively. The correlation between cow compass orientation and sun direction was significant (P < 0.001;Table 4), but not with wind direction (P > 0.05).

**Table 4:** Results of the analysis of correlation between cow compass direction and wind direction or sun direction, on bootstrapped data. “Correlation wind” and “correlation sun” represent the correlation between cow direction and wind direction or sun direction, respectively. “Test stat wind” and “Test stat sun” represent the respective test statistics. The “P-value” represents the significance of the correlation between cow compass orientation and wind direction or sun direction, respectively.

	Correlation wind	Test stat wind	P-value (wind)	Correlation sun	Test stat sun	P-value (sun)
**Mean**	0.09	2.26	0.07	0.19	4.47	< 0.001
**SD**	0.03	0.72		0.03	0.69	
**Minimum**	-0.01	-0.23		0.10	2.25	
**Maximum**	0.18	4.35		0.28	6.53	

## Discussion

The subset of 34 cattle in our experimental set-up showed neither a significant deviation from a random orientation when at rest, and a significant deviation from a south-north alignment, regardless of whether the cows were equipped with a strong magnet or not. This experiment confirms the conclusions in the observational study ([36], [38]) on the lack of a south-north alignment in cattle. Similarly, in an observational horse study, the expected N-S alignment was absent [32]. Furthermore, the statistical analyses of the magnetic orientation data in swine and warthogs, in our opinion, showed a 20° deviation from south-north (even though the authors reported a significant north-south preference in their abstract, for which a test was lacking) [31]. In contrast to the studies on the orientation of large mammals ([32], [33], [37]), our observational results showed that the cattle’s tail-to-head orientation at rest aligned preferentially to a north-west to south-east compass direction, significantly deviating from random and also from north. Although the mean directions in our experimental and observational data set differ to some extent, as they do in the experimental subsets with and without a magnet, there never was an indication of north-south orientation. In our observational study, the orientation of the large set of cattle at rest correlated significantly with the sun’s direction, but not with wind direction.

Our study tried to improve the methods used in earlier studies on magnetic orientation in non-homing, non-migrating large mammals. First, we took care to only consider individual resting cattle, which are likely not directionally attracted to food or water sources. Hence, we managed to avoid that the local resource availability and the animals’ quest for food or water were overruling possible magnetic signals. Previous studies mostly used indirect observations, i.e., data from Google Earth, and therefore could not distinguish resting from moving animals in a herd, nor head-tail from tail-head orientation ([33], [34]). Secondly, a topic that we wished to take into consideration was the density of the cattle. High density cattle herds (over 12 individuals per 1000 m^2^) have been observed not to have a preferential direction [40]. Because a cow is a herd animal, fellow cattle can influence each other strongly when density is high. Indeed, we found that cattle clustered together to ruminate around midday. Therefore, it was our intention to not carry out any measurements on the high density animals during this time span, *cf*. [40]. However, our data do not show a contrast in variation of orientation between low and high cattle densities (S1 Fig). Furthermore, in our statistical analyses we also statistically tested whether a deviation from north-south was significant.

One of the topics that we wished to take into consideration, which a few earlier studies have not considered or not tested for, is the possible influence of environmental factors (wind, sun). Begall et al. [33] stated that “it is unlikely that effective direction of each of the factors (wind, sun and temperature) was a common key factor of the alignment in all places and times”. We found no correlation between wind direction and cattle axial orientation, although we did not test for the possible influence of wind strength, due to lack of data. Although the detailed dates of the satellite images are not provided by Google Earth, “most views were apparently made on cloudless sunny days, judging from short shades, mainly around midday” [33]. We found a significant correlation between the animal’s orientation and the direction of the sun, minimizing sun radiation on the animal’s body, similar to what was observed in horses [32]. Indeed, if most views were made on cloudless sunny days, then the animals were possibly reducing their body exposure to the sun. We thus believe that the cattle’s reported south-north alignment observed in earlier studies could perhaps, at least in part, be attributed to thermoregulation, possibly overriding a magnetic alignment. Similar sun-reducing behaviour has been observed in springbok [41] and in black wildebeest [42], and was demonstrated to be linked to thermoregulation in those studies.

We conclude that the individual animals in our experimental and observational study clearly show a lack of south-north orientation, in contrast to the studies using Google Earth pictures or snow prints. Instead, the resting body alignment of these animals tended to reduce the exposure to the sun, suggesting that this environmental factor influences their resting orientation in the field. As long as observations are done during daytime, this factor is present and can disguise a possible magnetic alignment.

## Material and methods

### Study area

Our study was conducted in the Beja district, Alentejo, southwest Portugal (strength of earth magnetic field: 43.184 nT, inclination 51.24 degrees, declination −2.95 degrees). This area has several characteristics that make it suitable for this research: first, the landscape is mainly flat so the influence of slopes is excluded. Secondly, the proximity of the Santa Clara reservoir makes year-round irrigation possible, which provides the opportunity of keeping cattle outside all year-round. Thirdly, due to the proximity of the Atlantic Ocean, the climate is fairly mild which contributes to the right circumstances for the cows to be outside 24 hours a day, 365 days a year. We confirmed that there were no deviations from the earth’s magnetic field in each of the six farmyards used, by checking the orientation of the compass on line transects in each of them.

### Observations on cattle

Concurrently with the experiment and in the experiment in the next paragraph, observations were made at a 3-10m distance from a resting, lying-down cow without disturbing it. If a cow decided to stand up (which only happened occasionally), its data was discarded. Resting cow compass orientation has been measured using a Bresser compass (Art. No. 49–7000, analogous) as was done in [31]. Observations were made between 8:45 h and 17:00 h over a period of 3 months, excluding the hottest part of the day when the animals tended to cluster together when resting. This compass was also used for recording wind direction and sun direction. When a large herbivore lays down to rest, its body is a little bent. To get an unambiguous, accurate measurement every single time, an imaginary straight line has been drawn from the withers and torso vertebrae backwards when standing behind the cow. This method resulted in axial data (angles from 0 to 360 degrees) in the direction of the front of the animal. Measurements were carried out at a substantial distance from power lines (> 150 m, see [37]), settlements or communication poles (> 25 m) to prevent interference from extremely low magnetic fields. Sun direction was measured as the compass angle between the position of the sun and north. Wind direction was denoted as the direction from which the wind blew.

### Experimental test on cow compass orientation

**Fig 4:**
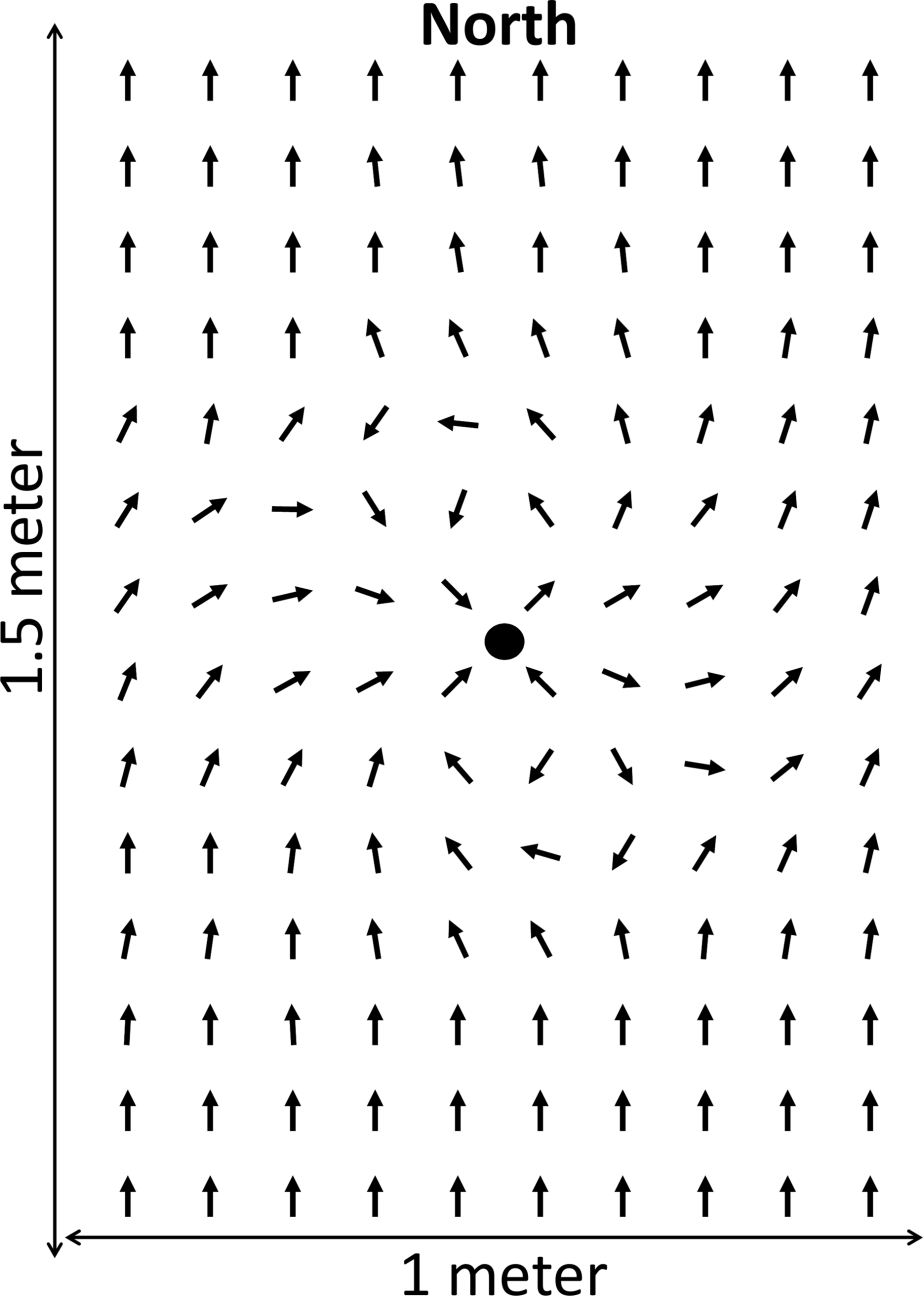
Deviation of the compass needle from the magnetic north. Deviation of the compass needle from the magnetic north (top of Fig) when exposed to one of the neodymium magnets used in the experiment (black dot). The dotted circle represents an area with a radius of 45 cm in which a clear deviation from the compass needle is visible.

In a field trial, we compared the resting, i.e., lying down or standing, orientation of cattle either wearing a magnet or not, at different times. To this end, we attached strong neodymium magnets (type 35, 22 mm in diameter and 10 mm in height, weight 24 g, stalling load of 11 kg) to the neck collar of cattle. In total 34 individuals were involved in the magnet experiment which resulted in 97 measurements when wearing a magnet and 177 when not wearing a magnet. The influence of the magnets on the earth’s magnetic field has been established by measuring compass orientations on a 10 by 10 cm grid surrounding such a magnet. This shows a strong localized influence within 45 cm of the magnet (Fig 4), a distance which includes most of the cow’s head. We found no data on the location of sensory cells in the bodies of large mammals, so we presumed that—if present—the head would be the most likely location: in birds such sensors were suggested to be located in the beak [43], and in mole rats in the cornea [44]. Two mechanisms could explain magnetic alignment behaviour in animals, namely a magnetite-based sensor and/or the radical pair mechanism ([45], [46]). However, the magnetite hypothesis of magneto-sensation is recently contested by some ([47], [48]), and the workings of the radical pair mechanism are unclear. The attached magnets did not influence the compass orientation when determining the cow compass orientation from a distance of ~1.5 m.

### Compass orientation of cattle in the field

We determined compass orientation of 659 individual resting cows, which led to 2428 observations together with covariates of wind direction and sun direction. The individuals were housed at 6 different private farms. All farm owners explicitly gave permission to conduct the study on their site. We studied dairy cows, 596 of them being of the Frisian Holstein breed, and 63 being a crossbreed of Fleckvieh and Montbeliard. On most farms the density was between 1.7 and 10.4 cows/1000 m^2^ with two exceptions of 27.1 and 65 cows/1000 m^2^. No density effect was detected here (S1 Fig).

### Data analysis

#### Experimental test on orientation

Of the 34 cows in the experiment some were measured once with or without a magnet, while others were measured more frequently. Here, we resampled the data 500 times and assessed the mean angle (mean cow compass orientation), the difference from a random Von Mises distribution (chapter 15 in [39]), the difference from north, the correlation between cow compass orientation and wind direction, and the correlation between cow compass orientation and sun direction. All these analyses were done for the same cows when wearing a magnet and when not wearing a magnet.

To test whether there is a distinction from a random distribution when resting, and assessing a preferred direction if present, a Rayleigh’s test has been performed, assuming a random Von Mises distribution. To test whether there is a deviation from north, the Rayleigh’s test has been performed again now comparing the cow compass orientation to reference point “north”. The paired test was based on data obtained by subtracting the resting orientations of the same cow after from before wearing a magnet, and performed with a Rayleigh’s test.

For the correlation between the orientation of the cows and the wind direction or the sun direction we used the correlation coefficient for angular variables that is programmed in the program circ.cor in the library CircStats in R 2.12.2 [49]. The formula for this sample correlation coefficient for *n* data points ($\alpha_{i},\;\beta_{i}$) with *i* = 1,…, *n* is *r*_*c*,*n*_ [50].

$$r_{c,n}=\frac{\sum_{i=1}^{n}\sin\left(\alpha_{i}-\bar{\alpha }\right)\cos\left(\beta_{i}-\bar{\beta }\right)}{\sqrt{\sum_{i=1}^{n}\sin^{2}\left(\alpha_{i}-\bar{\alpha }\right)\cos^{2}\left(\beta_{i}-\bar{\beta }\right)}}$$ (1)

In the formula $\overset{-}{\alpha }$ and $\overset{-}{\beta }$ represent the sample mean directions. The significance of this correlation coefficient was computed as the significance of the normal Pearson’s correlation coefficient.

#### Analysis of cow compass body orientations

Because we used the same animals repeatedly for our observations, we risk pseudo-replication. One could argue that pseudo-replication is present for animals within the same pasture, and more easily leads to significant deviations from the null hypothesis (elevated Type I error). In case no significant difference is found with all (pseudo-replicated) data, this also holds for aggregated data.

We used a Monte Carlo bootstrap method to assess whether the different measurements on the same cow influenced our statistical outcomes. Therefore, we resampled the data from the different cows 1000 times. In each dataset each of the 596 different cows is present once; the 350 cows that are only present with one measurement are thus always in the sample data set. With these sample data sets we assessed the mean angle (mean orientation), the difference from a random Von Mises distribution (which can be regarded as the circular analogue of the uniform distribution on the line, chapter 15 in [39]), the difference from north, the correlation between cow compass orientation and wind direction, and the correlation between cow compass orientation and sun direction.

## Supporting information

S1 File S1 File.pdf Rose diagrams for 1000 bootstrap data sets. (PDF)

S1 Fig S1 Fig.pdf Boxplot of orientation of cows per density on the farm. (PDF)
