# Supplementary material for: An experimental approach in revisiting the magnetic orientation of cattle
Source: PLoS One. 2018 Apr 11;13(4):e0187848. doi: 10.1371/journal.pone.0187848 (PMC5894954; doi:10.1371/journal.pone.0187848)
Supplement: S1 Fig — (PDF) [file pone.0187848.s002.pdf]

Boxplot of orientation of cows per density on the farm (Weijers et al. 2017)

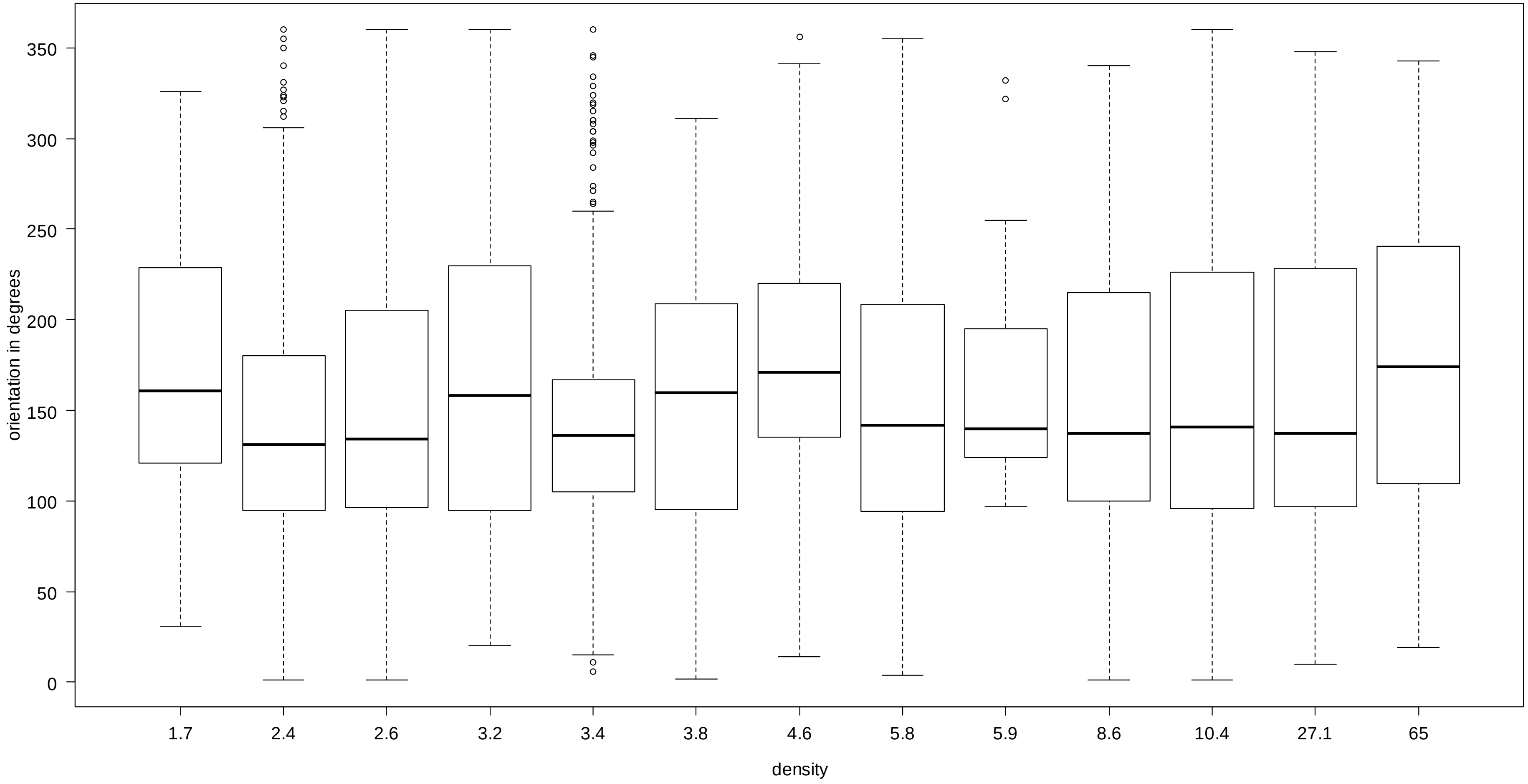

S1 Fig: The orientation of individual cows is plotted for the different densities of the cows on the farms; no relationship with density could be detected.
