# Supplementary material for: An experimental approach in revisiting the magnetic orientation of cattle
Source: PLoS One. 2018 Apr 11;13(4):e0187848. doi: 10.1371/journal.pone.0187848 (PMC5894954; doi:10.1371/journal.pone.0187848)

bootstrap 1

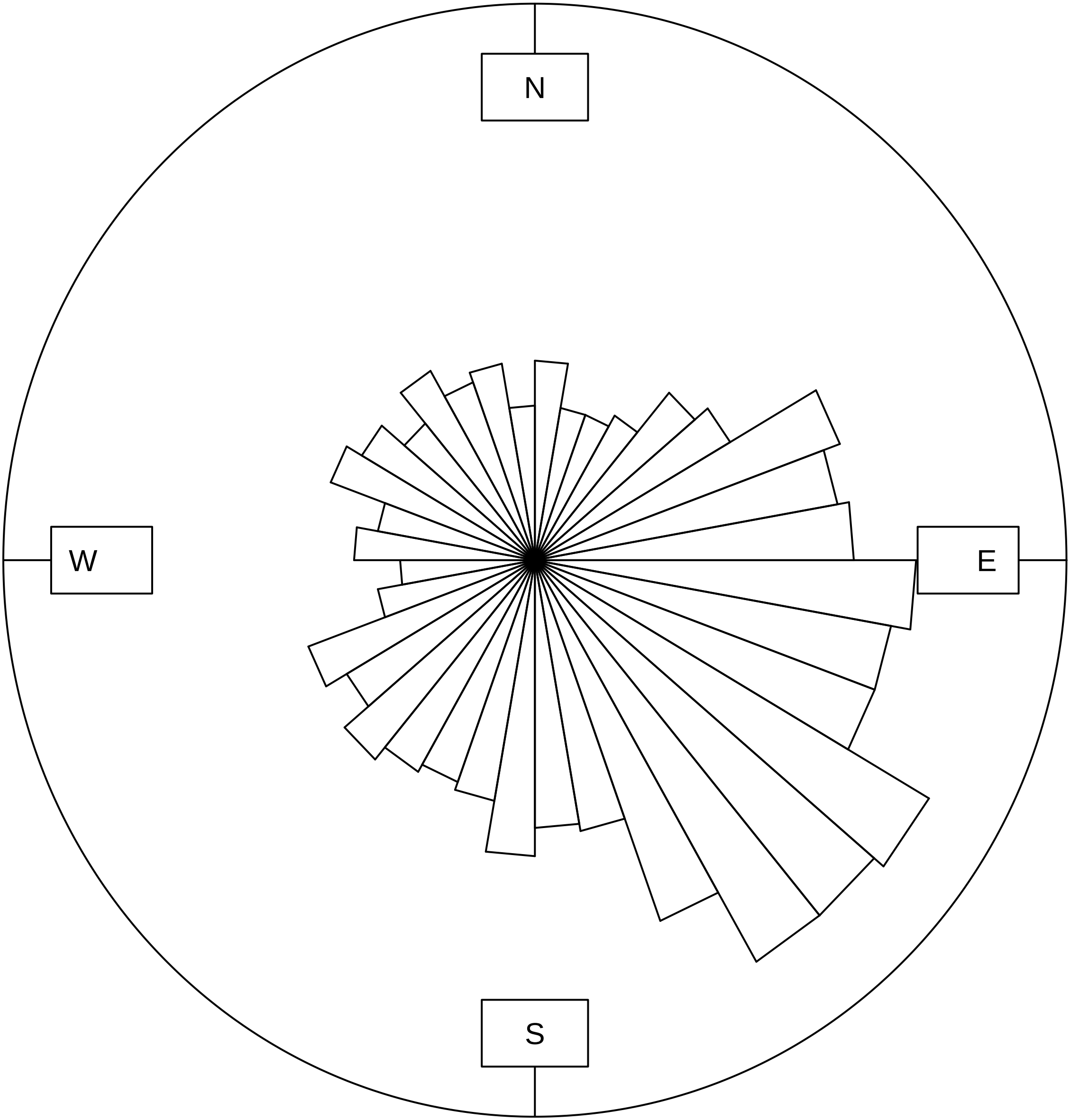

bootstrap 2

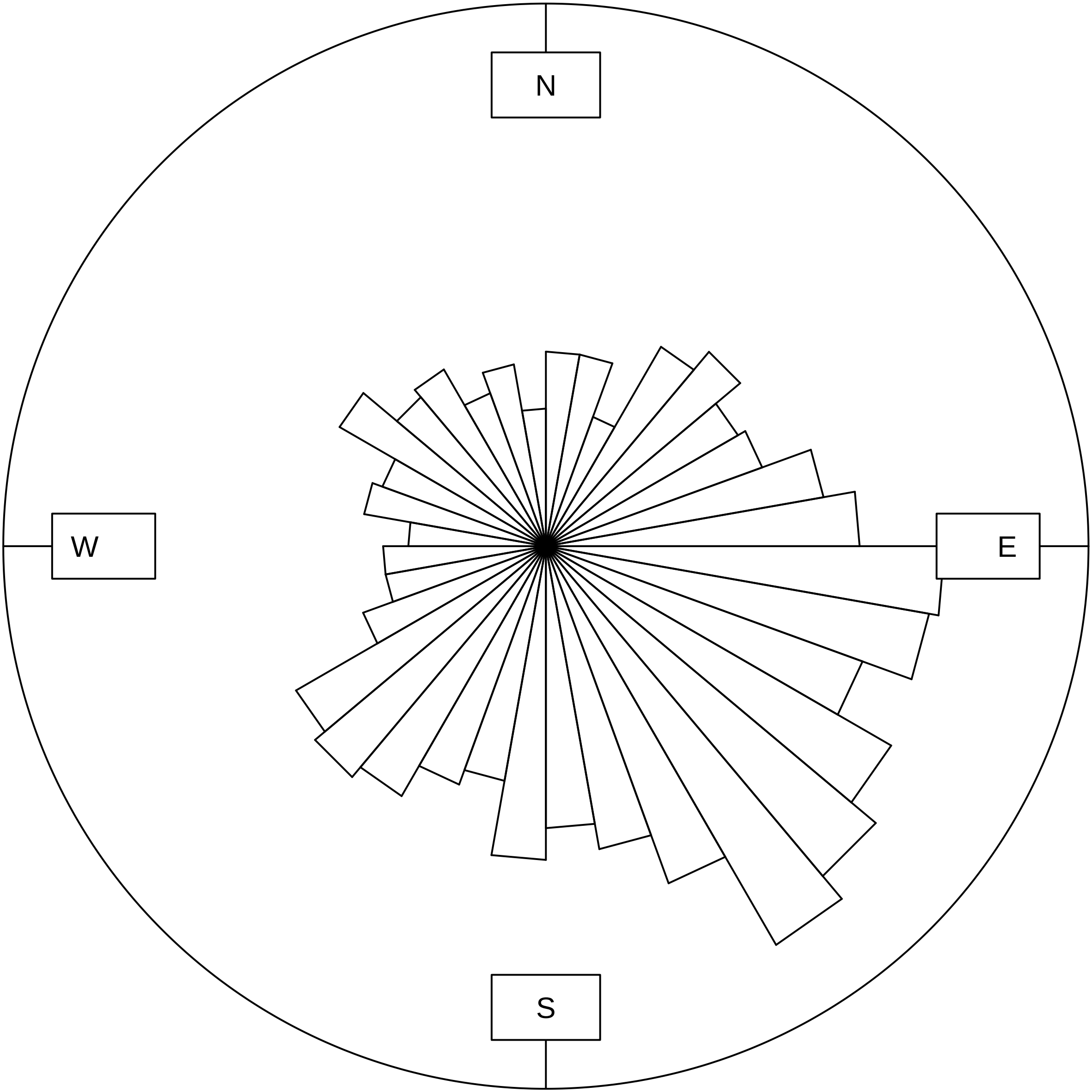

bootstrap 3

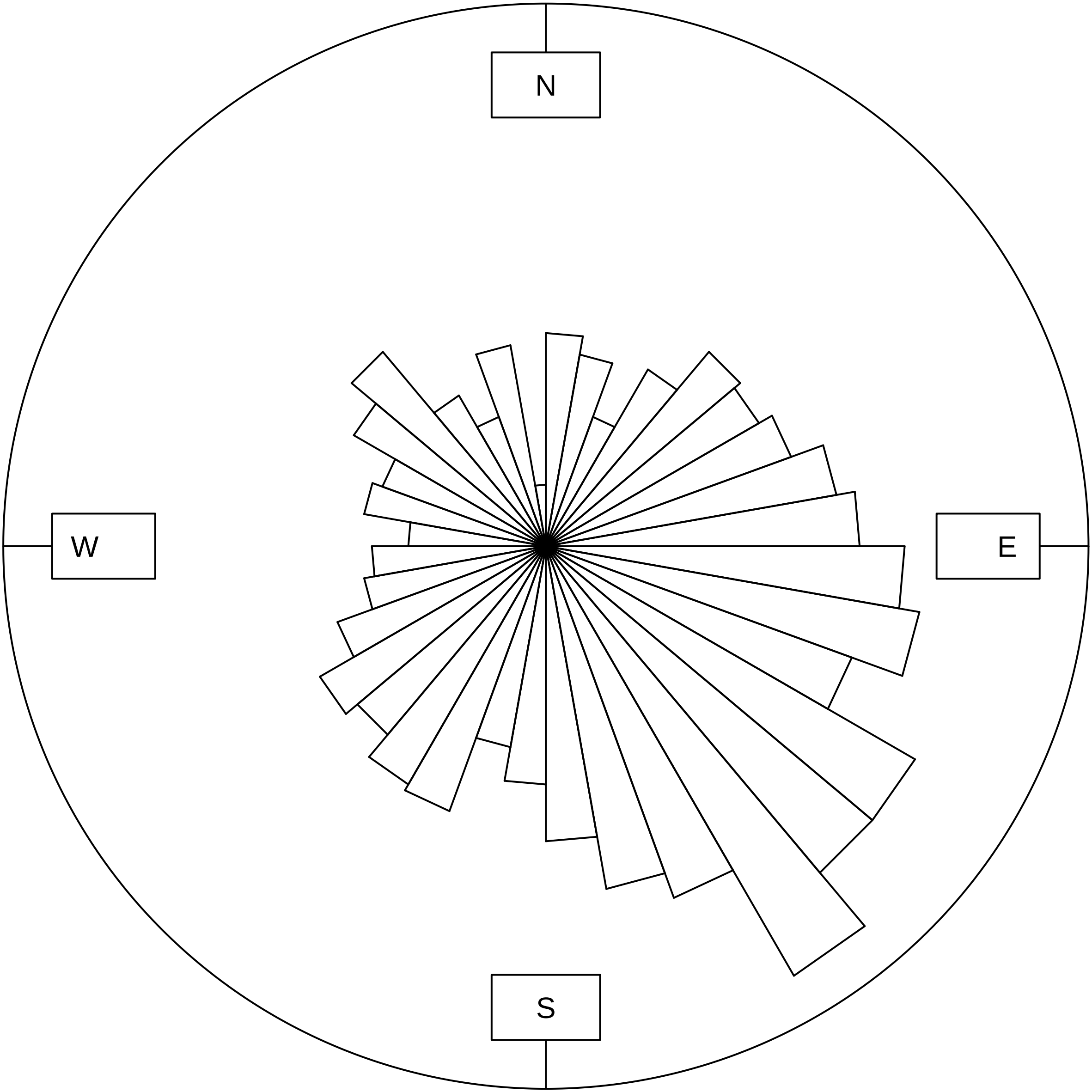

bootstrap 4

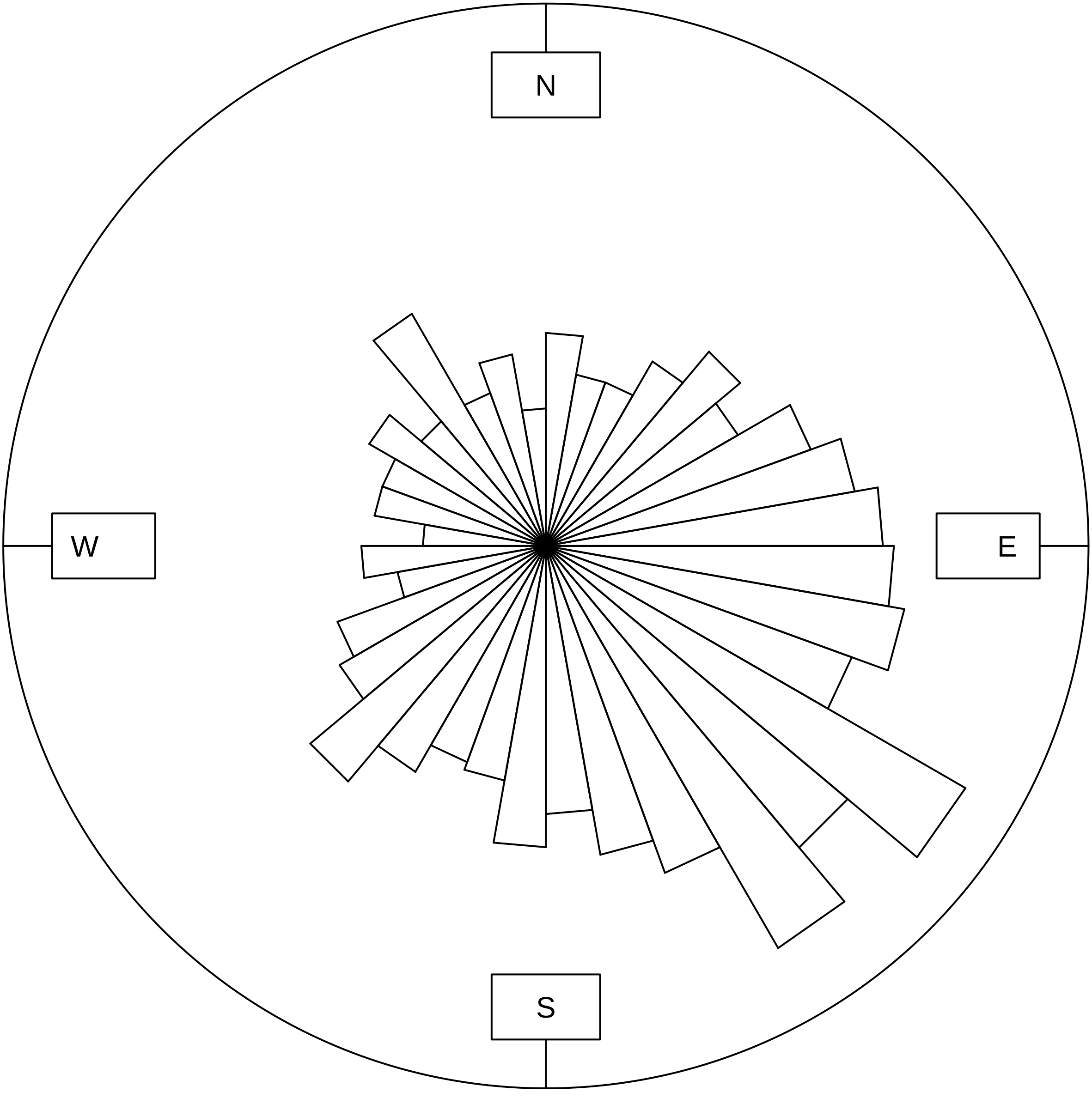

bootstrap 5

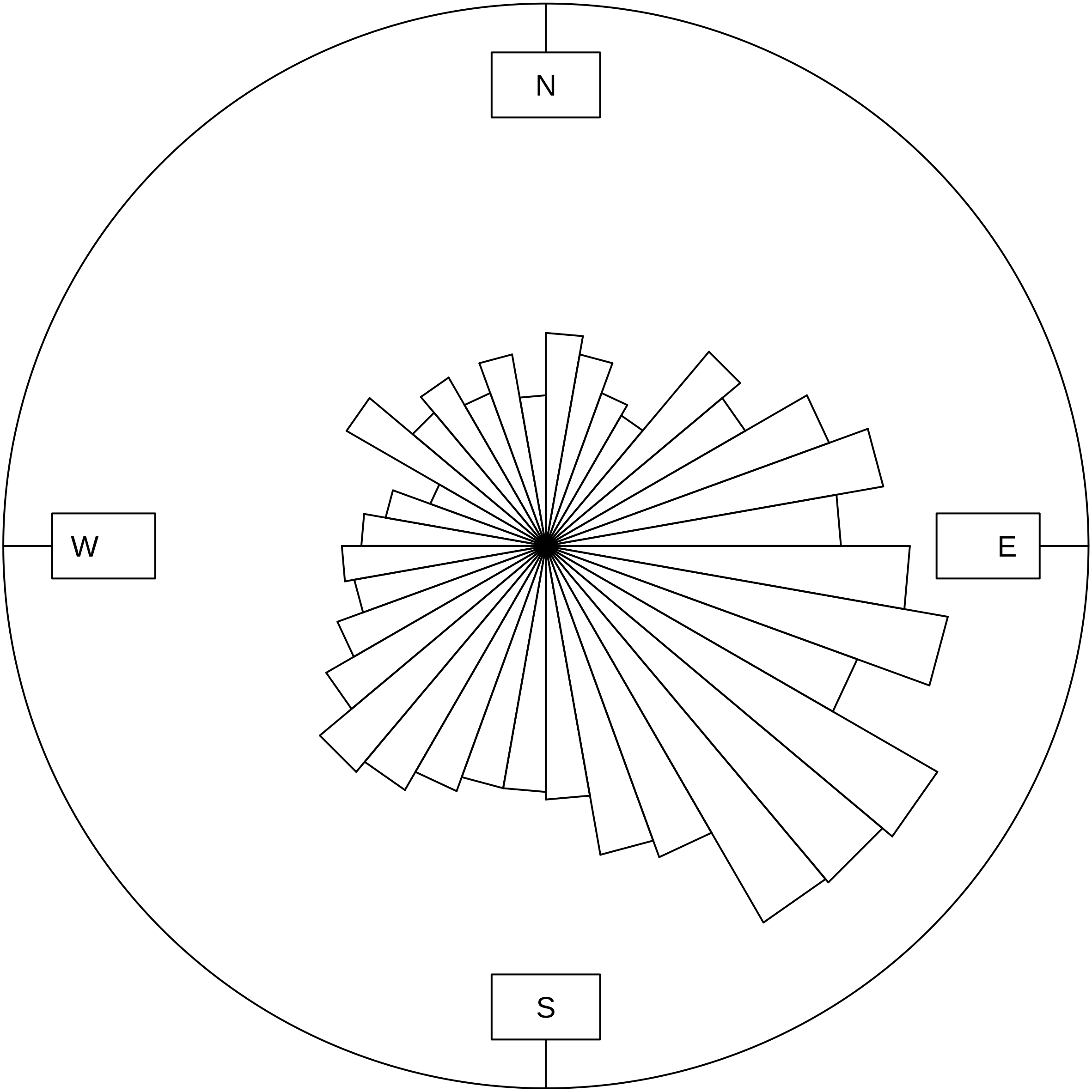

bootstrap 6

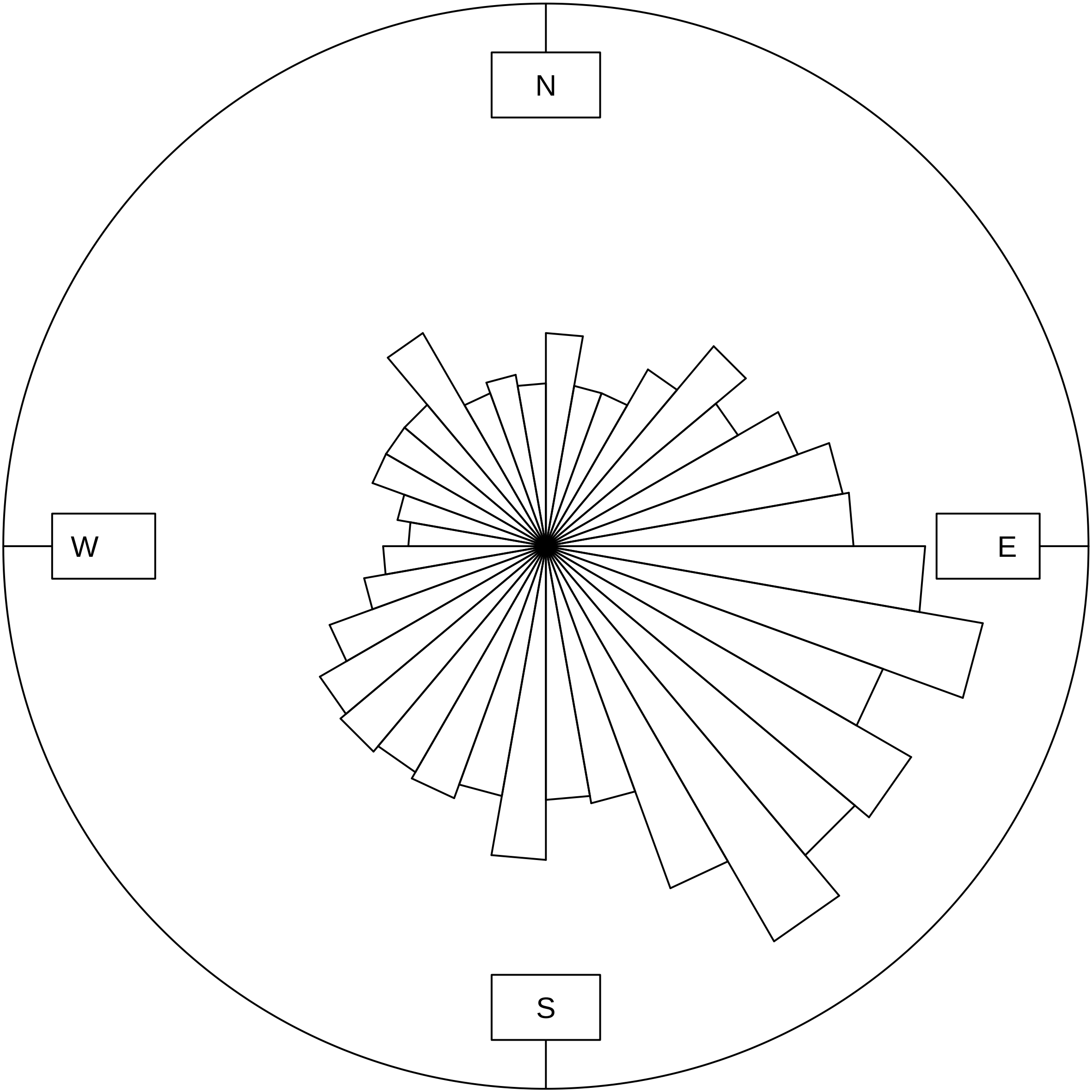

bootstrap 7

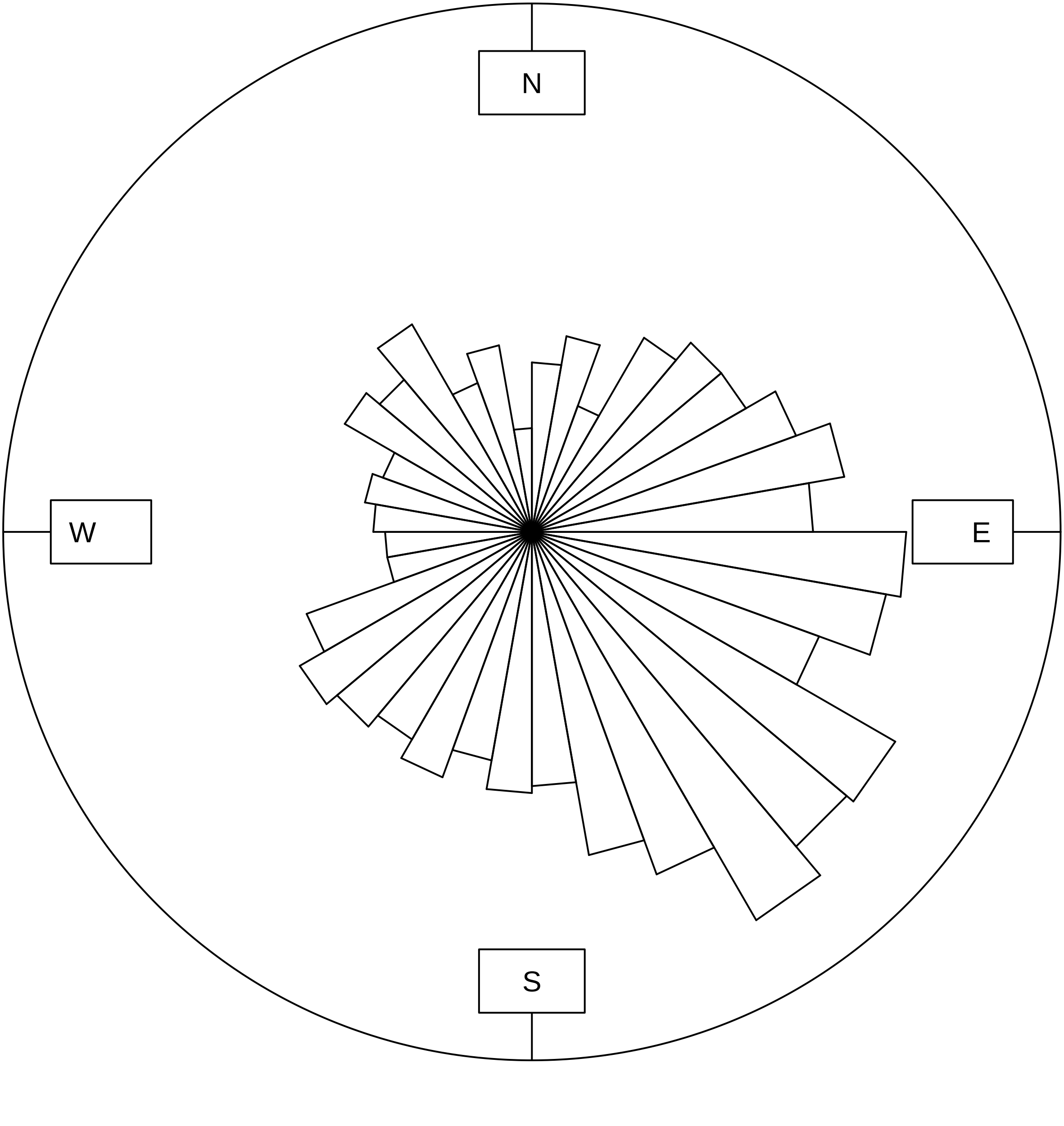

bootstrap 8

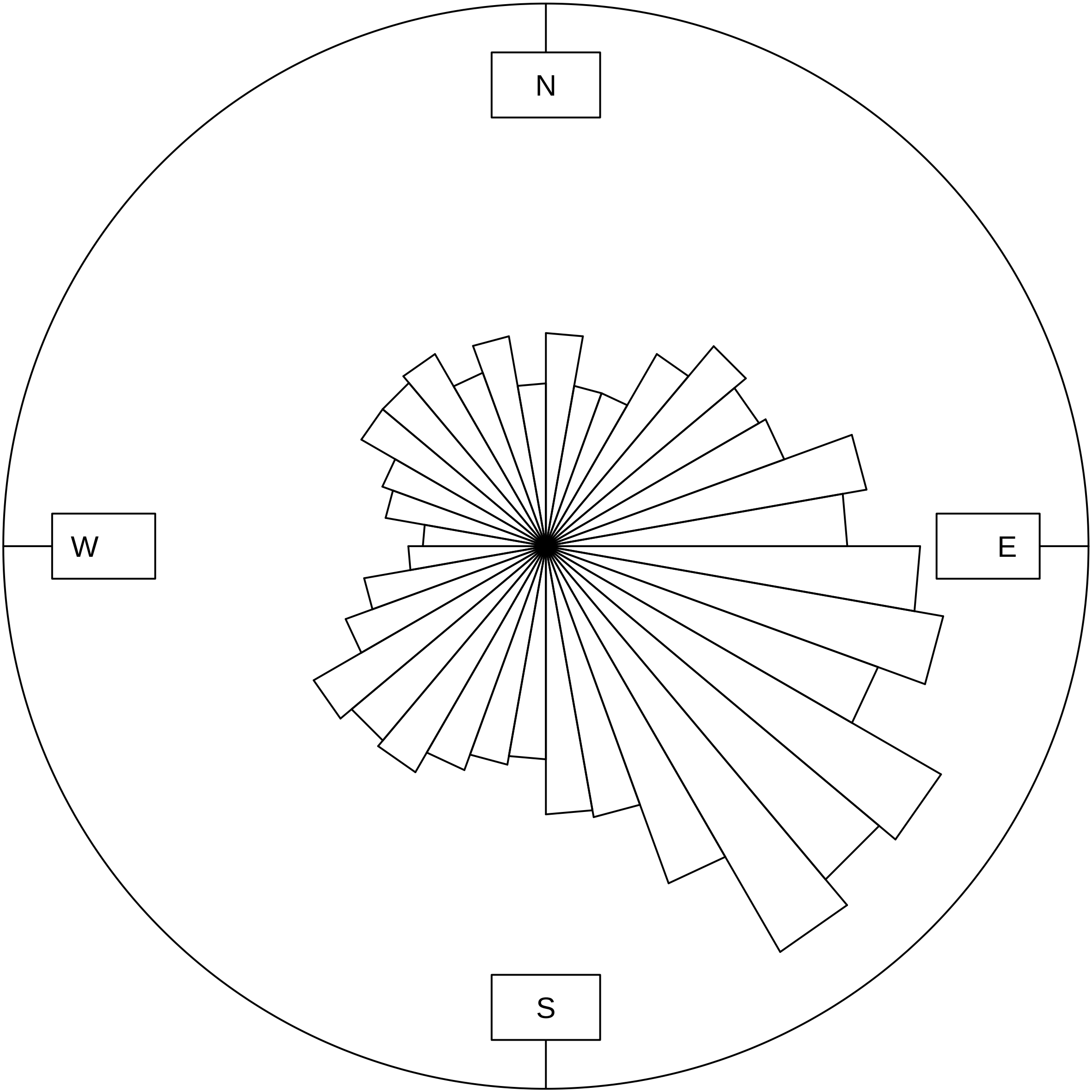

bootstrap 9

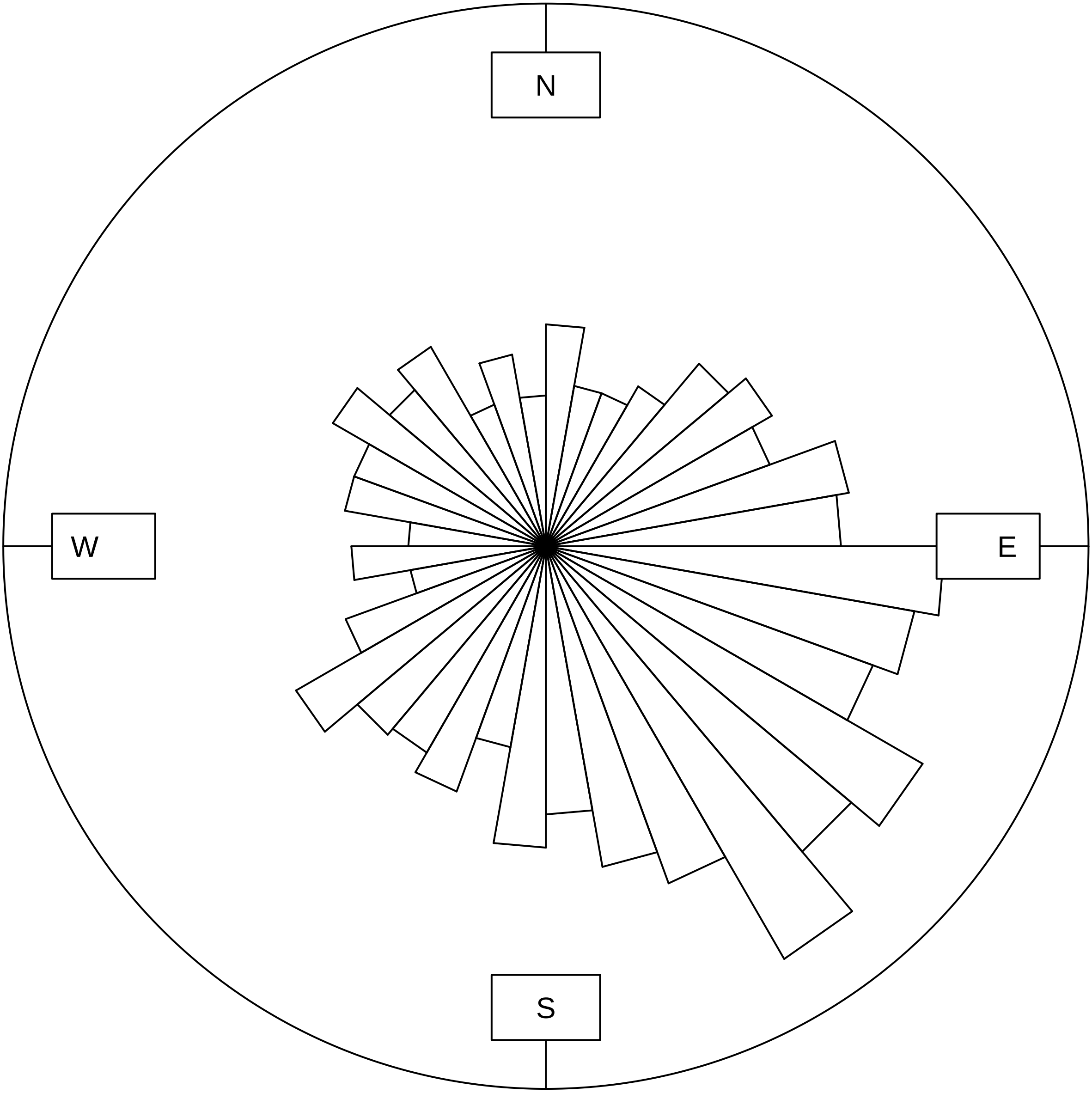

bootstrap 10

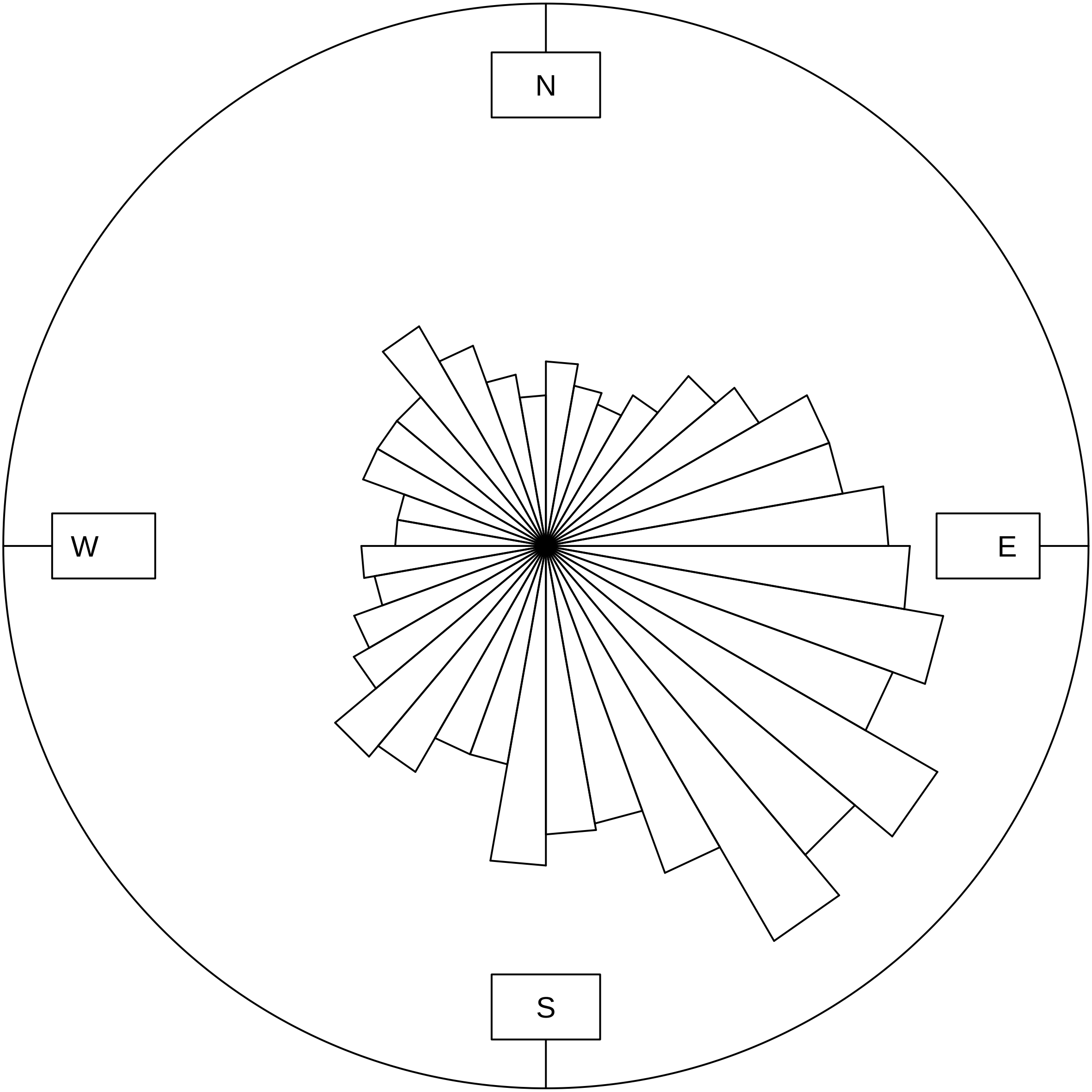

bootstrap 11

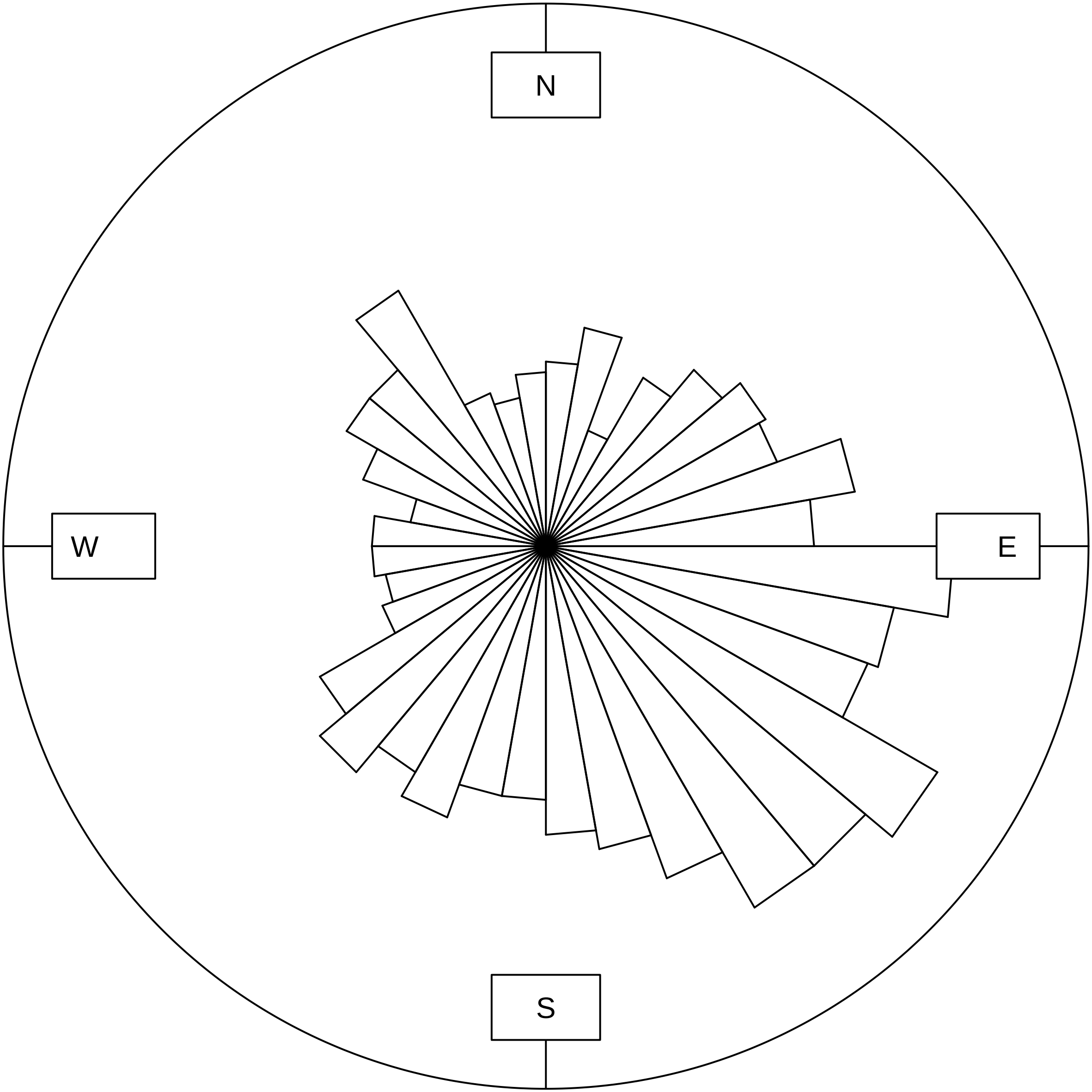

bootstrap 12

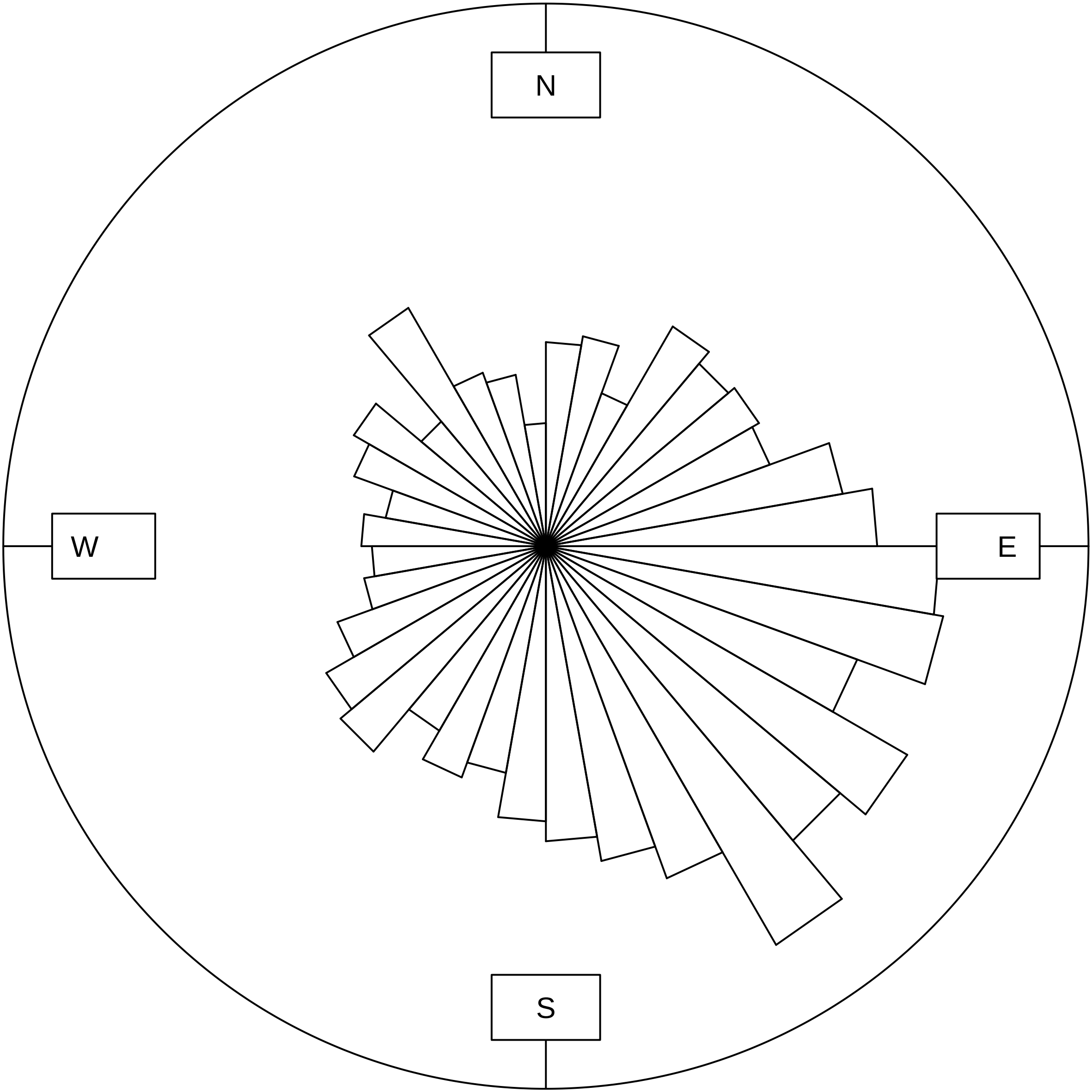

bootstrap 13

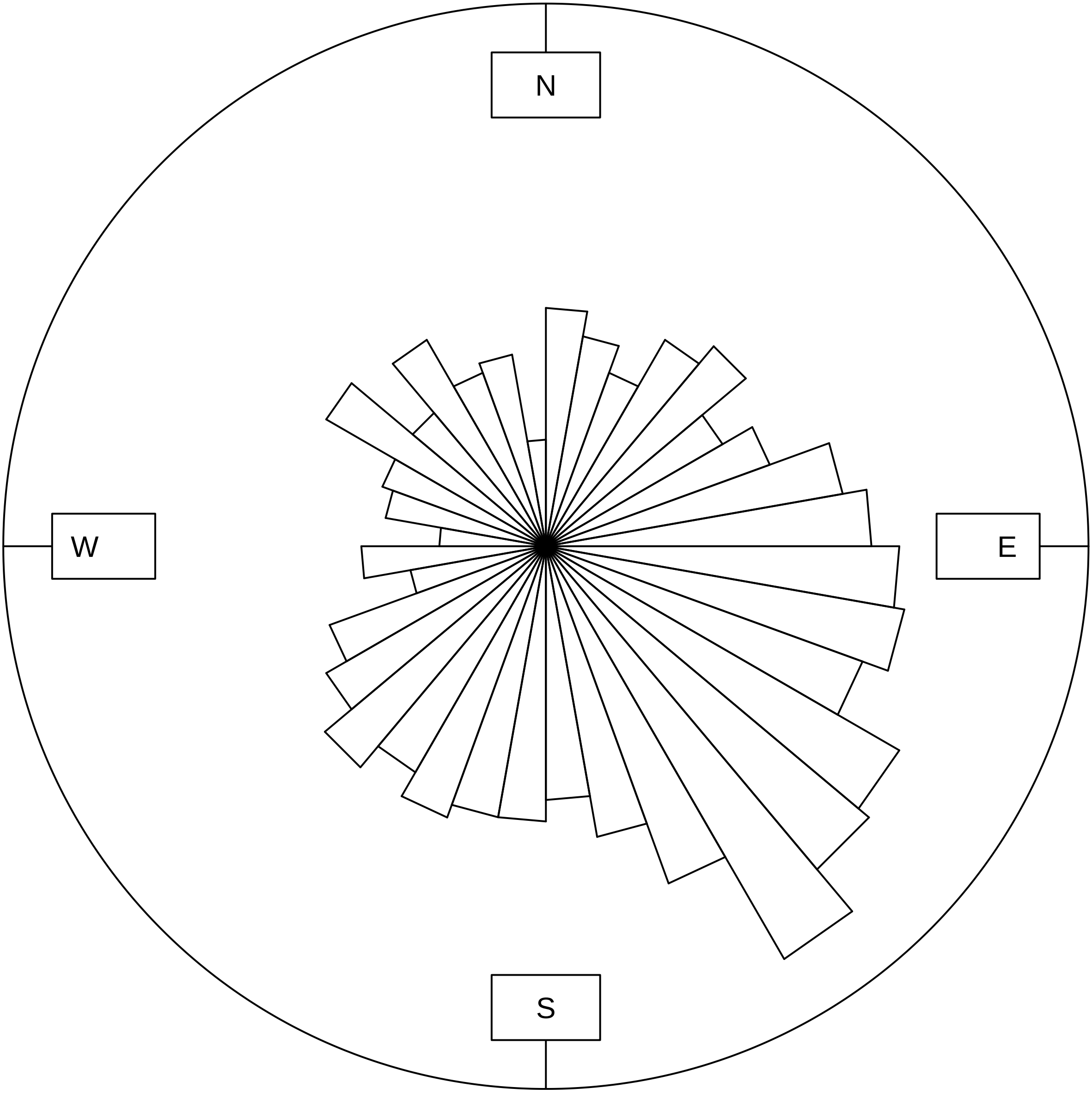

bootstrap 14

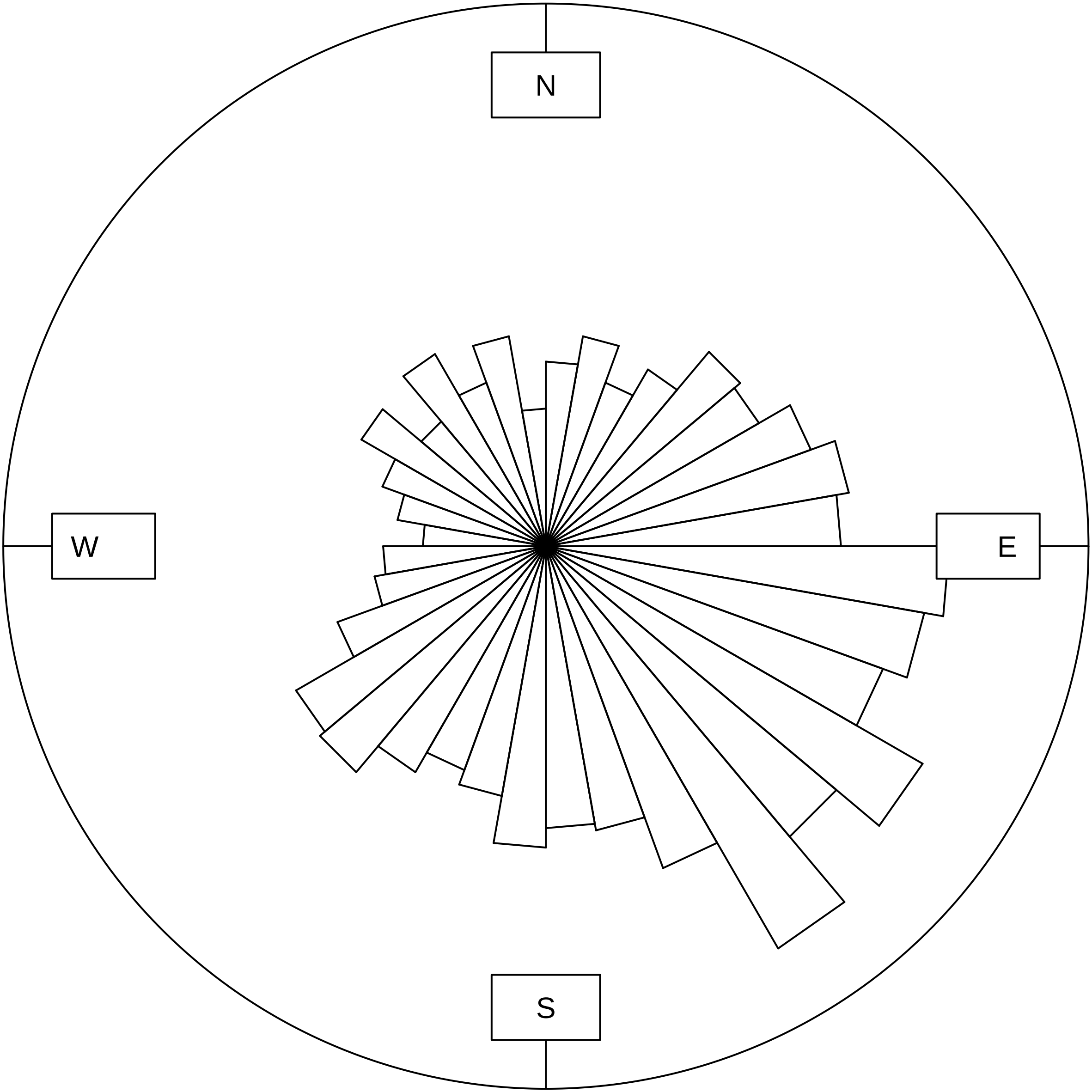

bootstrap 15

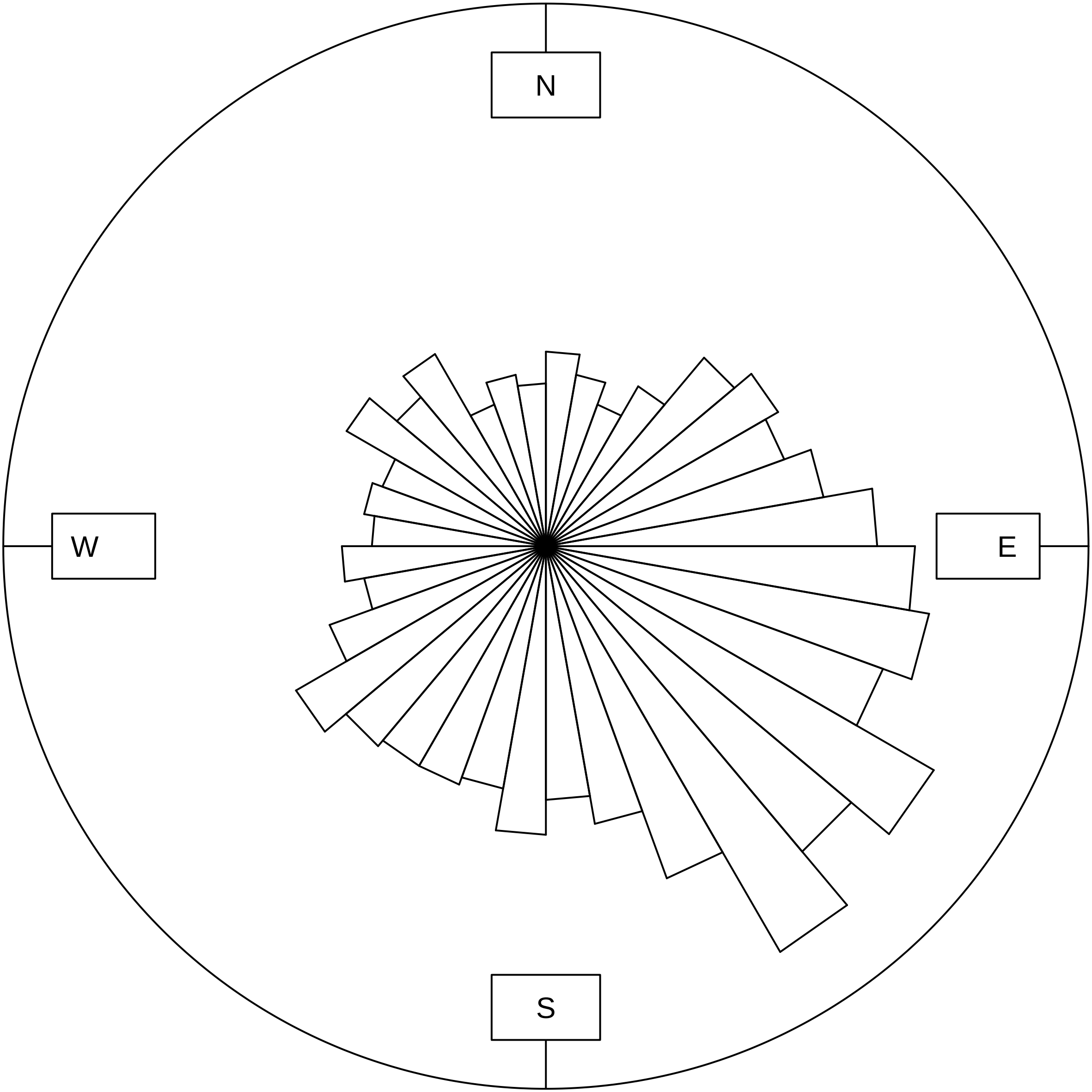

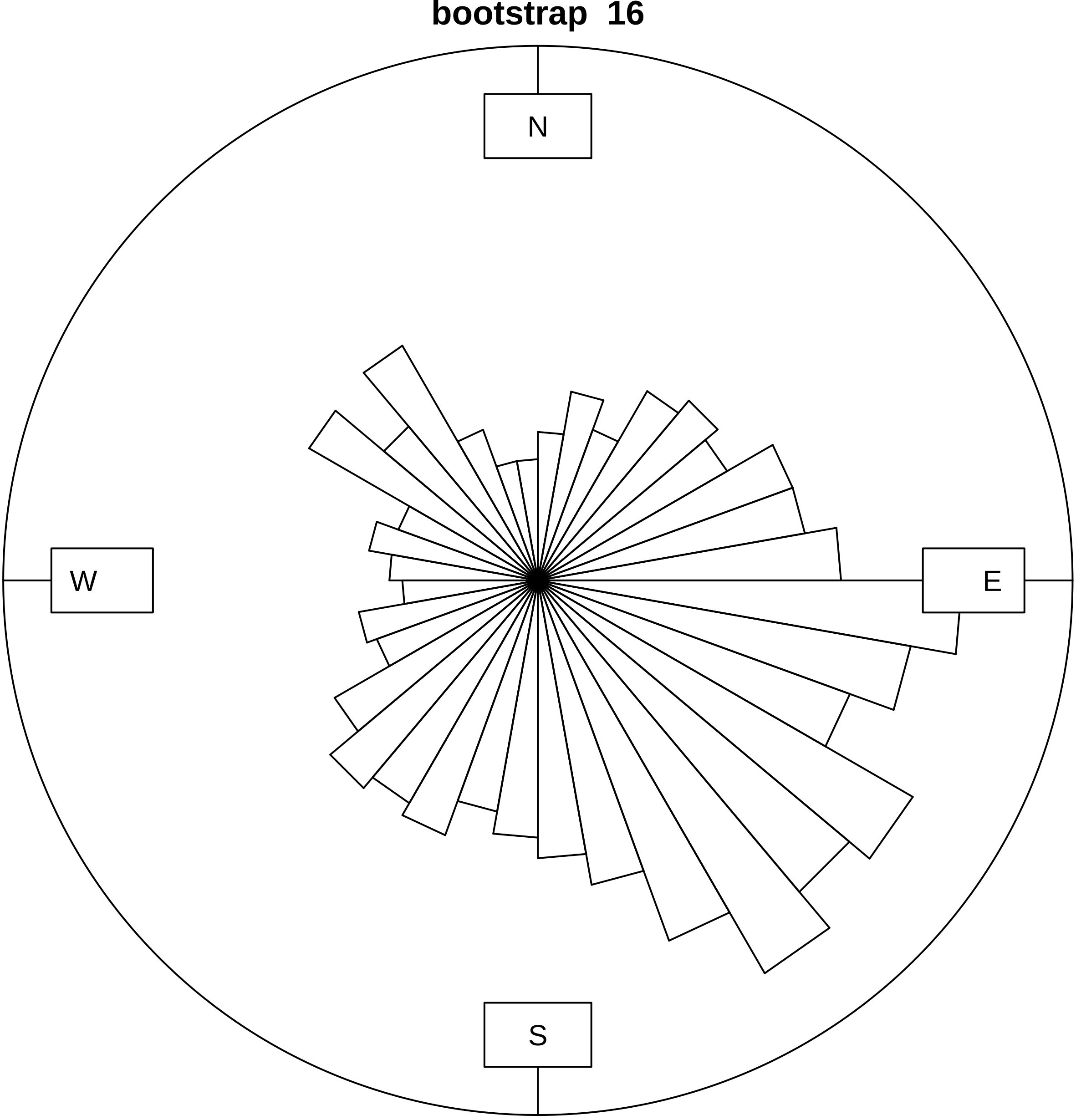
bootstrap 16
N
W
E
S

bootstrap 17

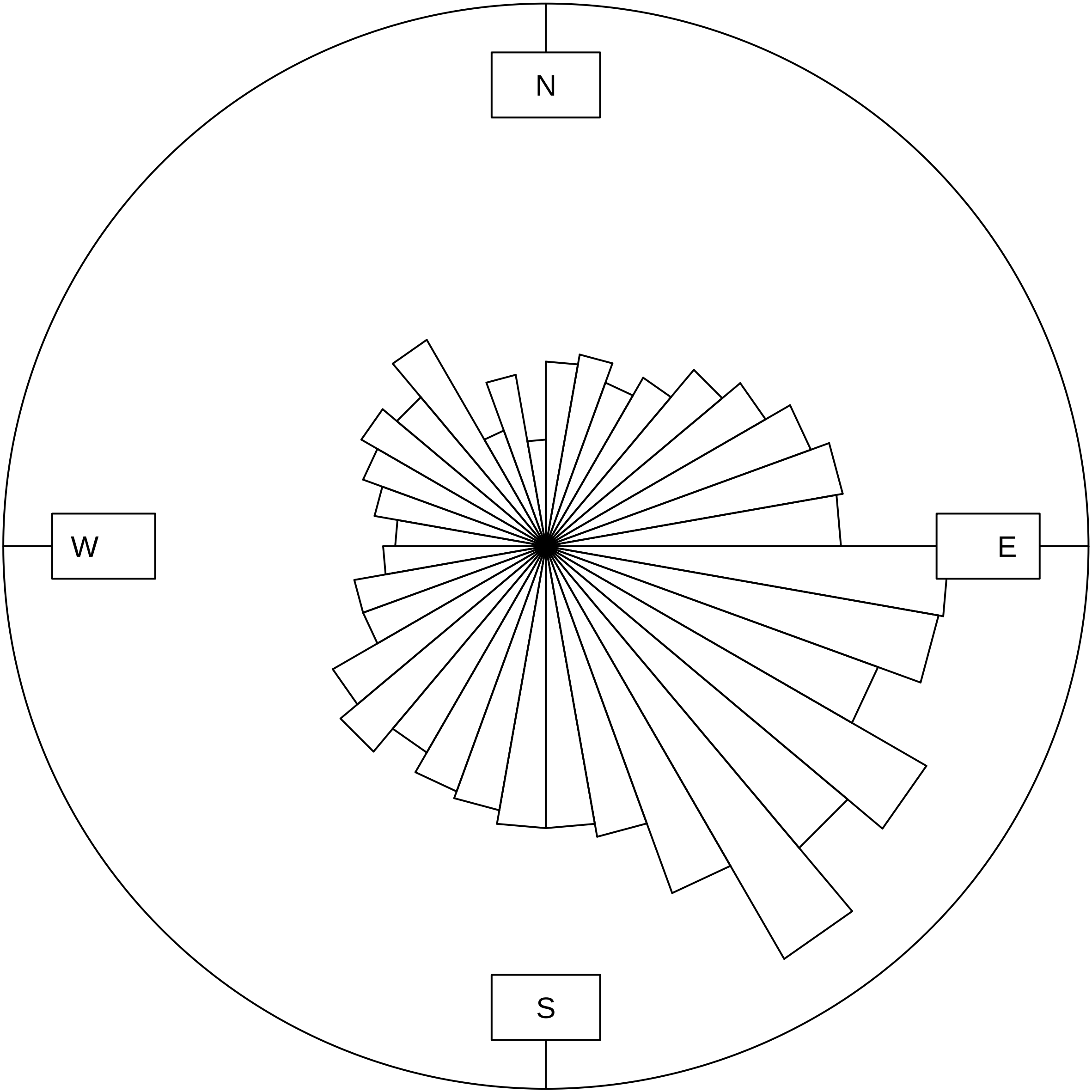

bootstrap 18

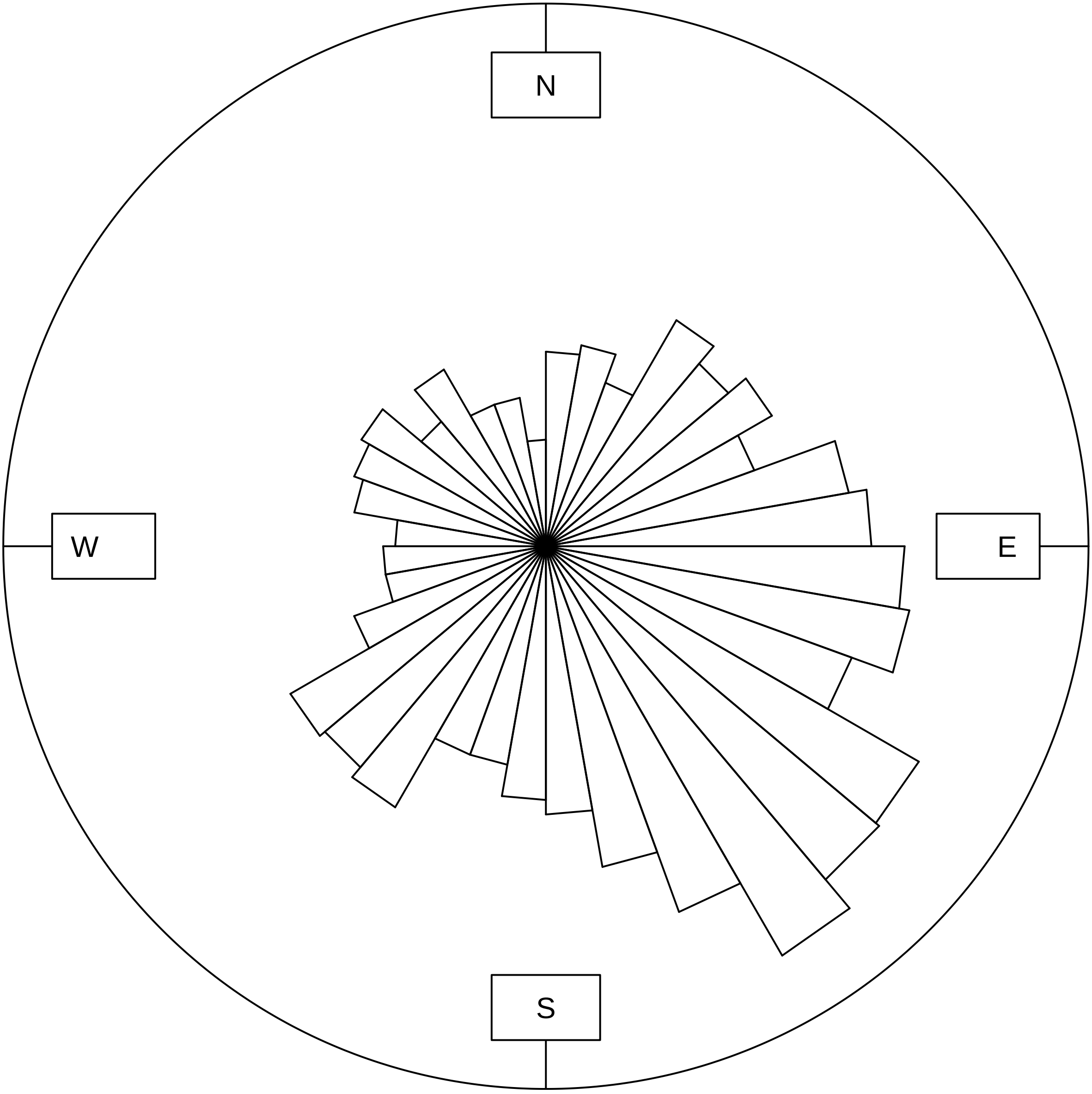

bootstrap 19

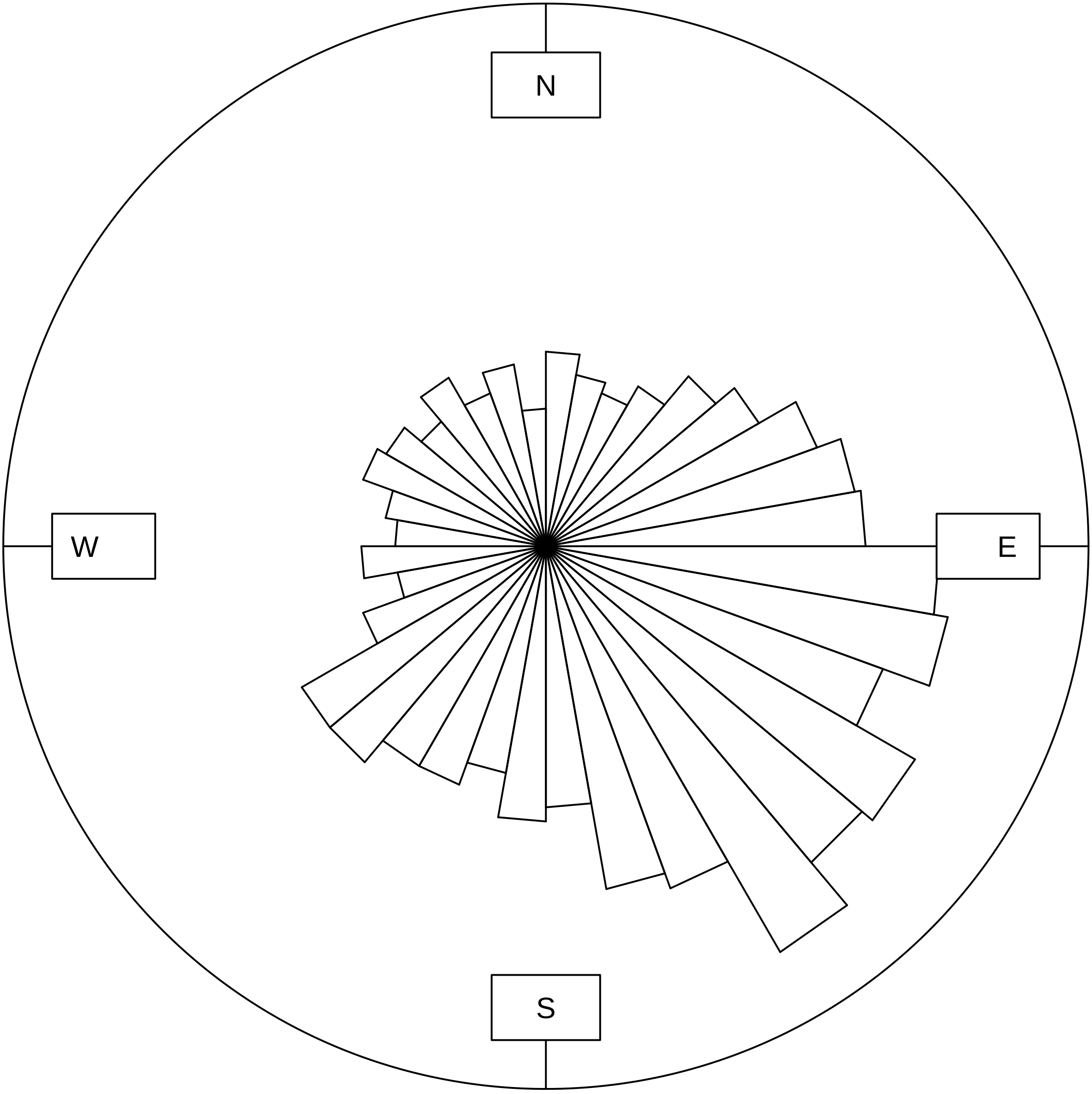

bootstrap 20

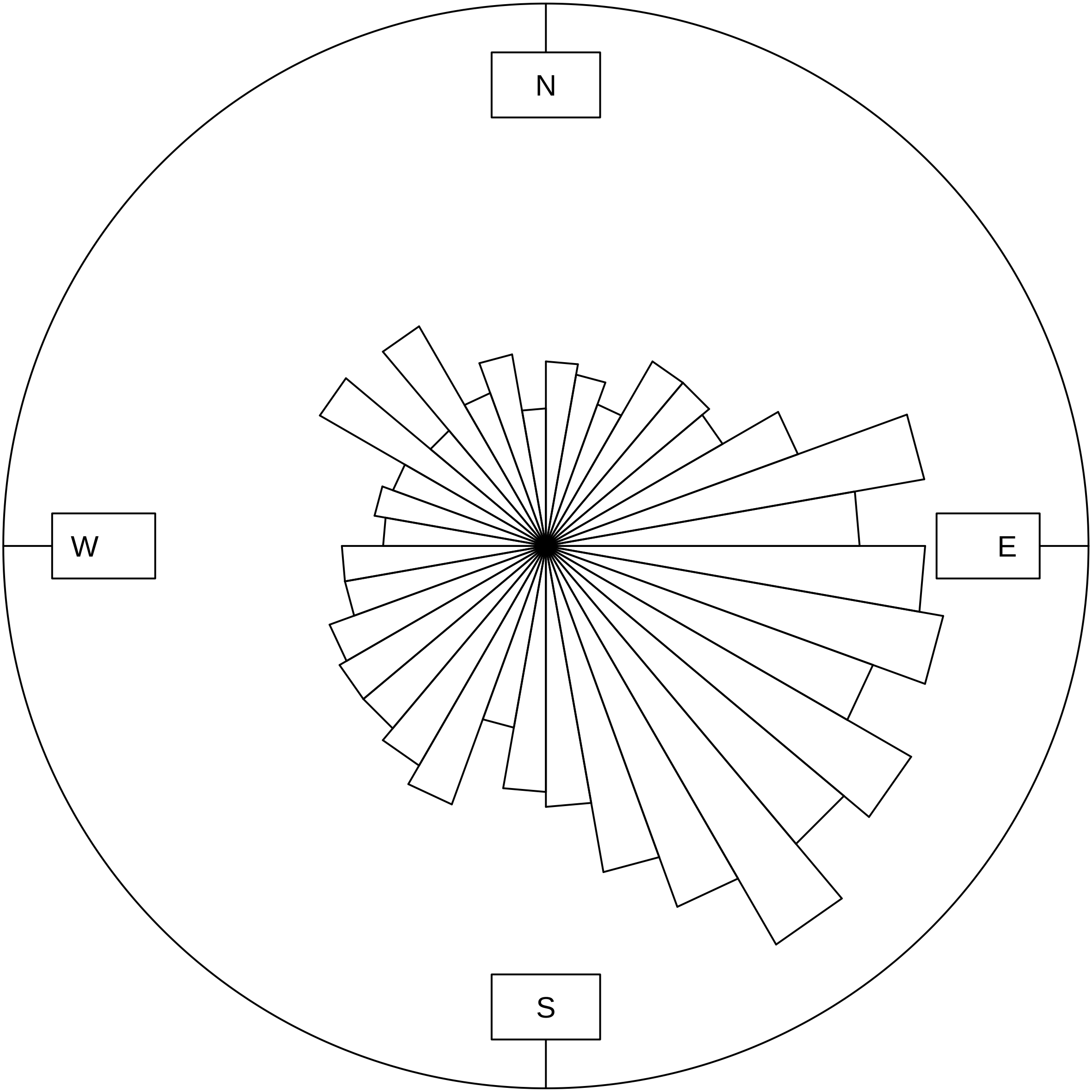

bootstrap 21

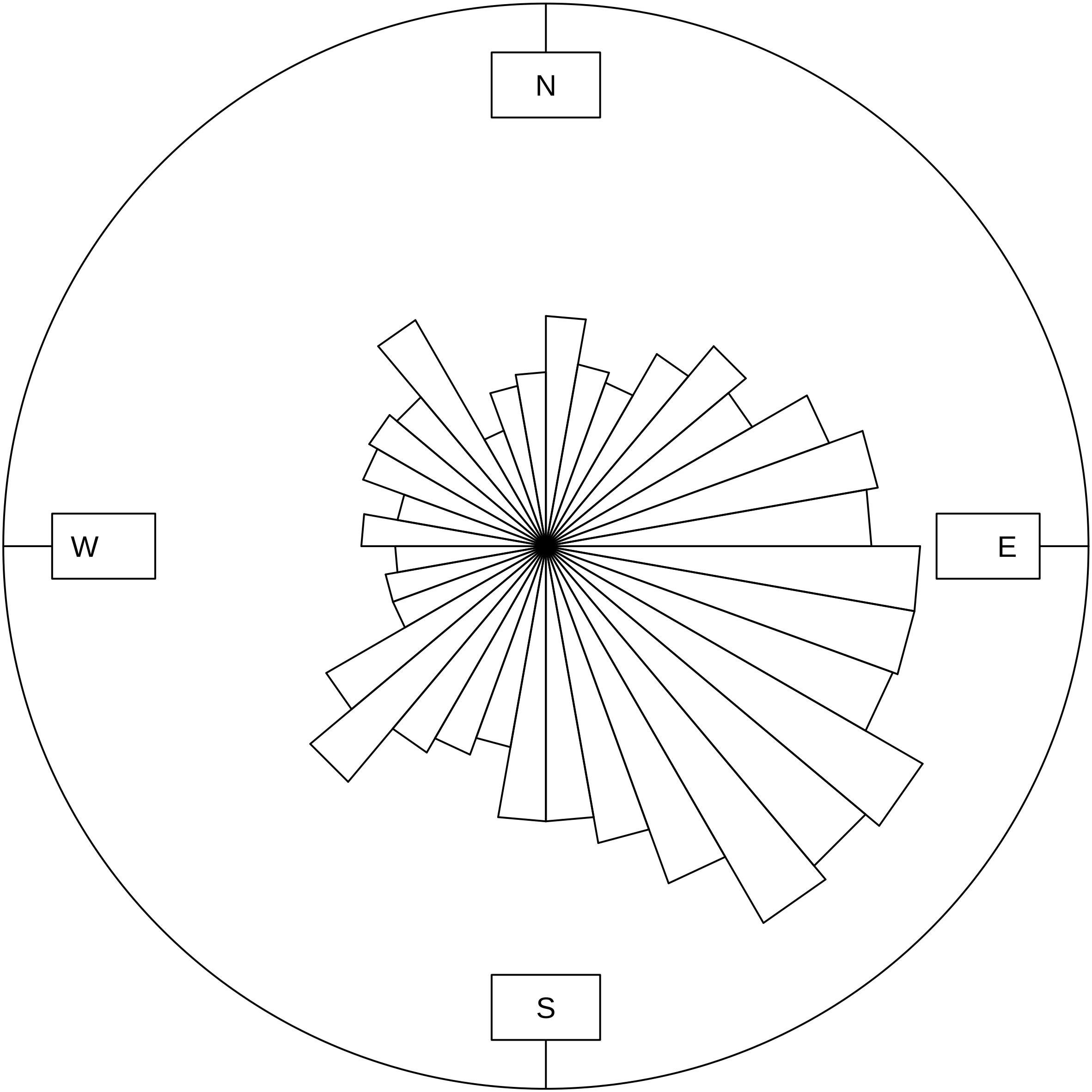

bootstrap 22

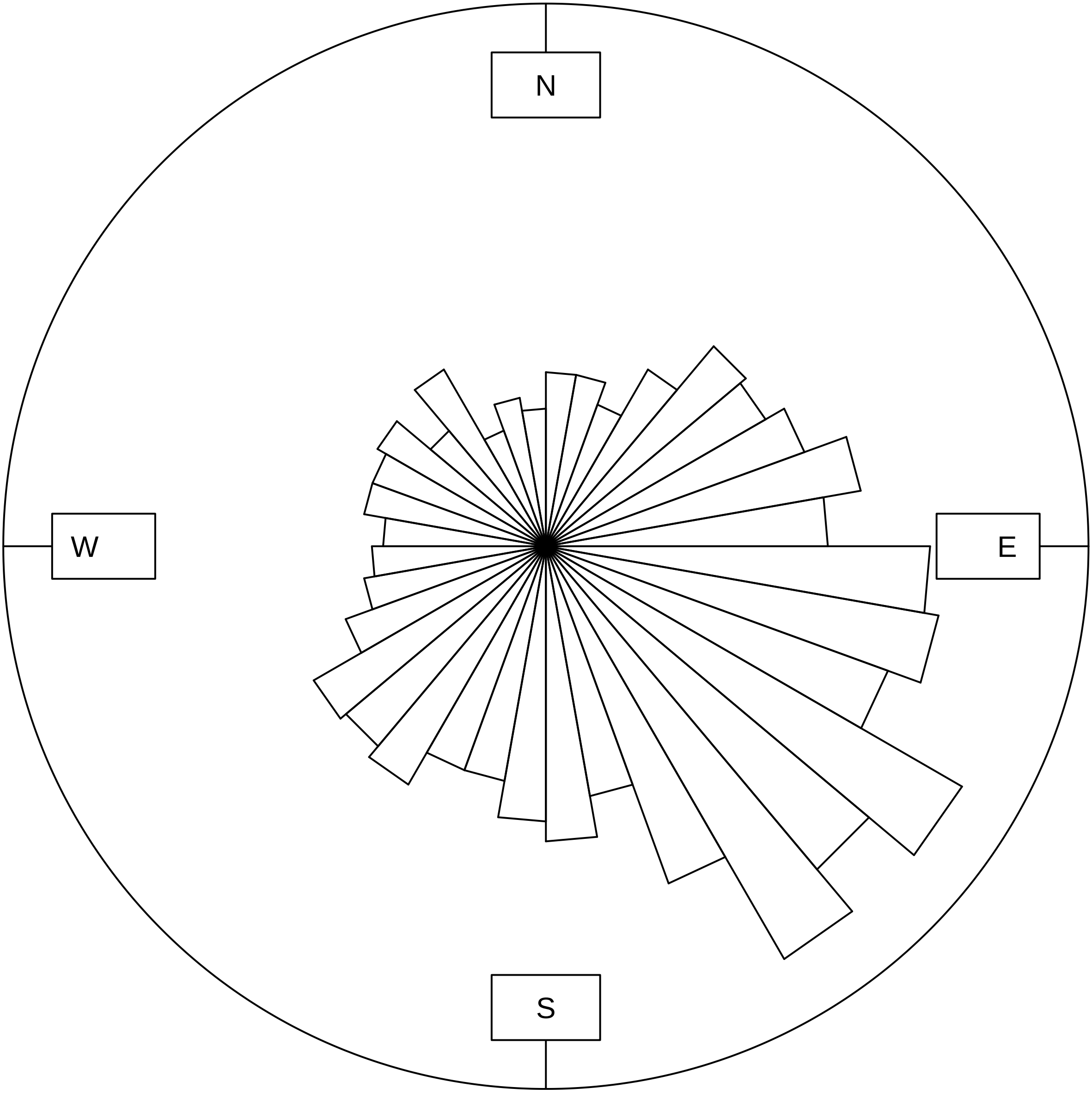

bootstrap 23

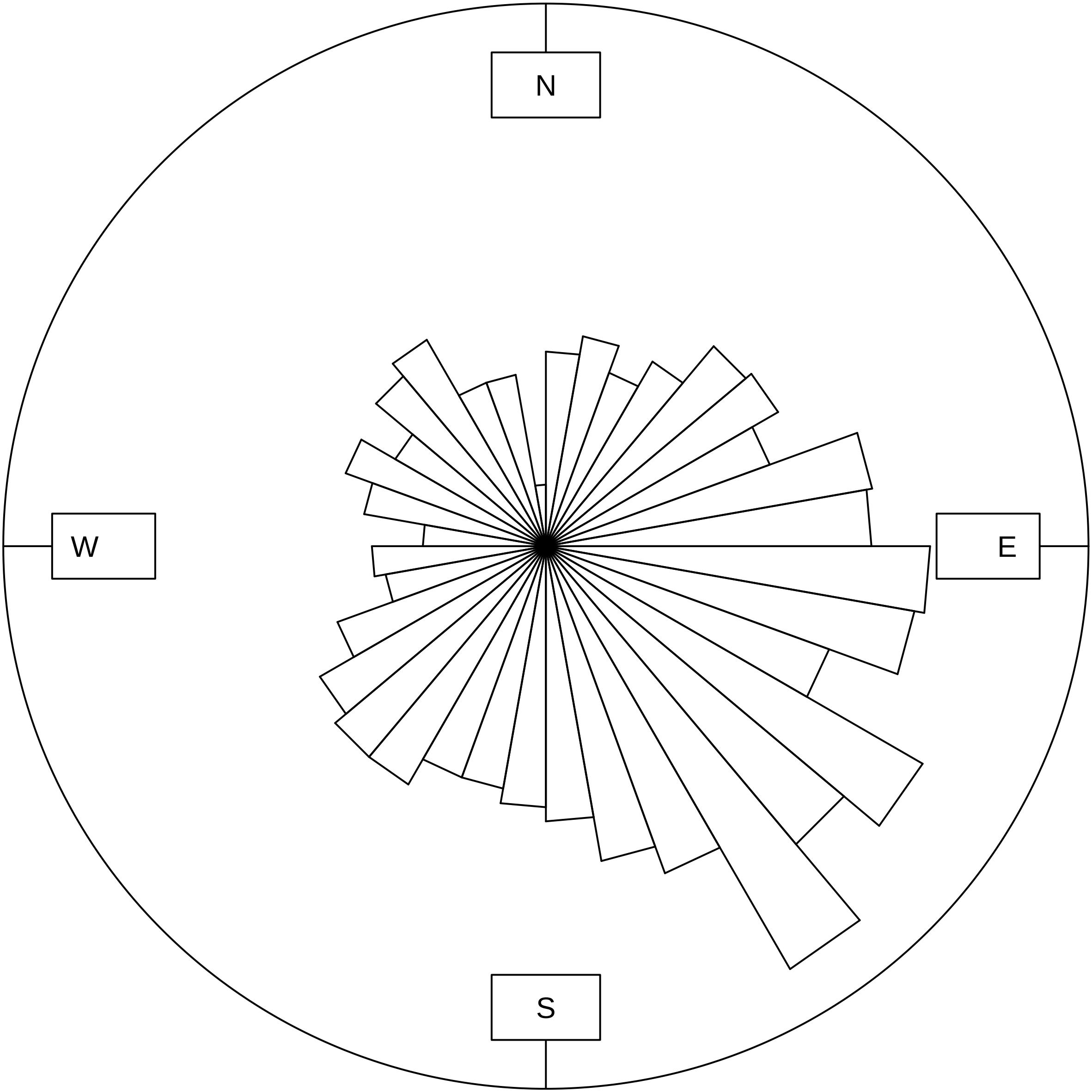

bootstrap 24

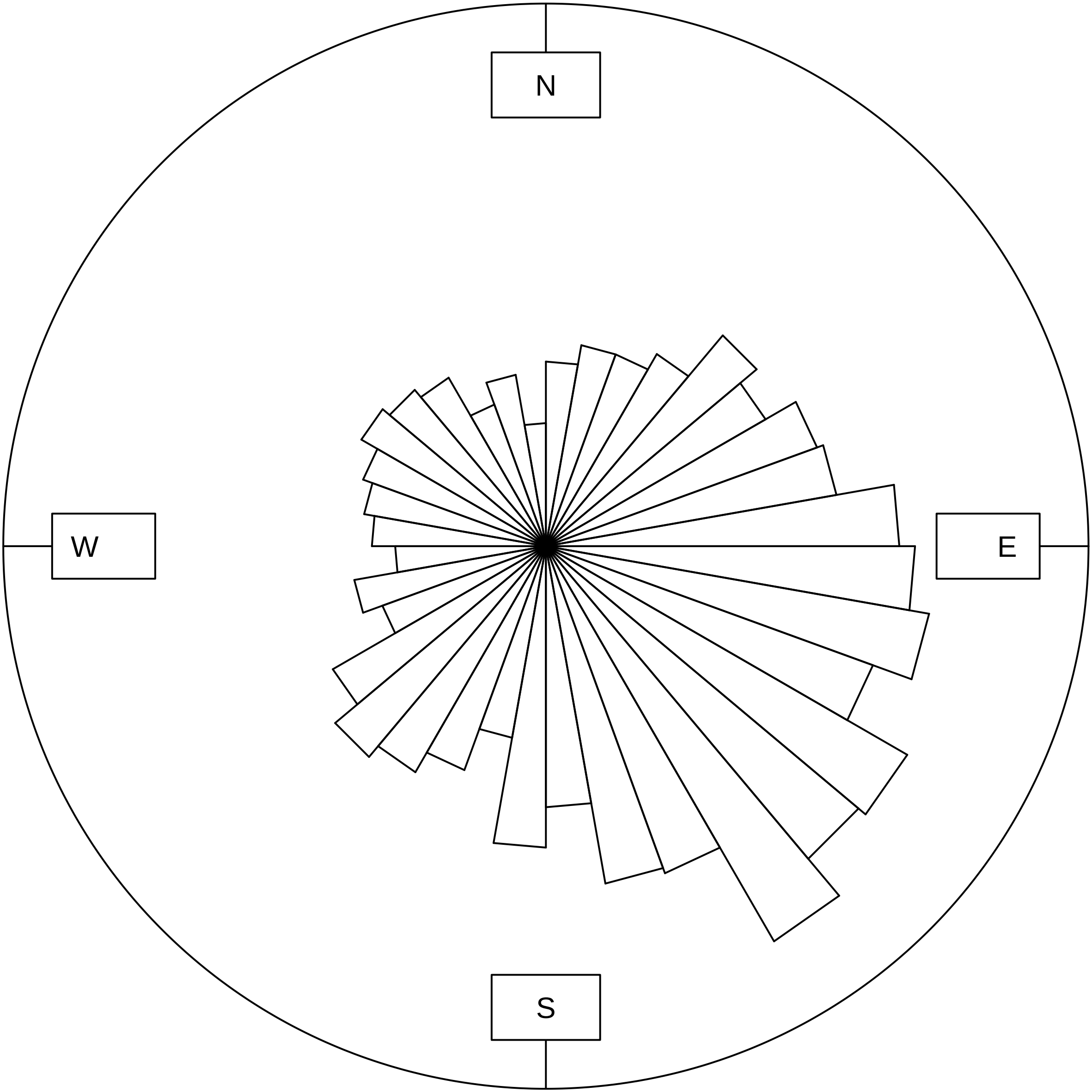

bootstrap 25

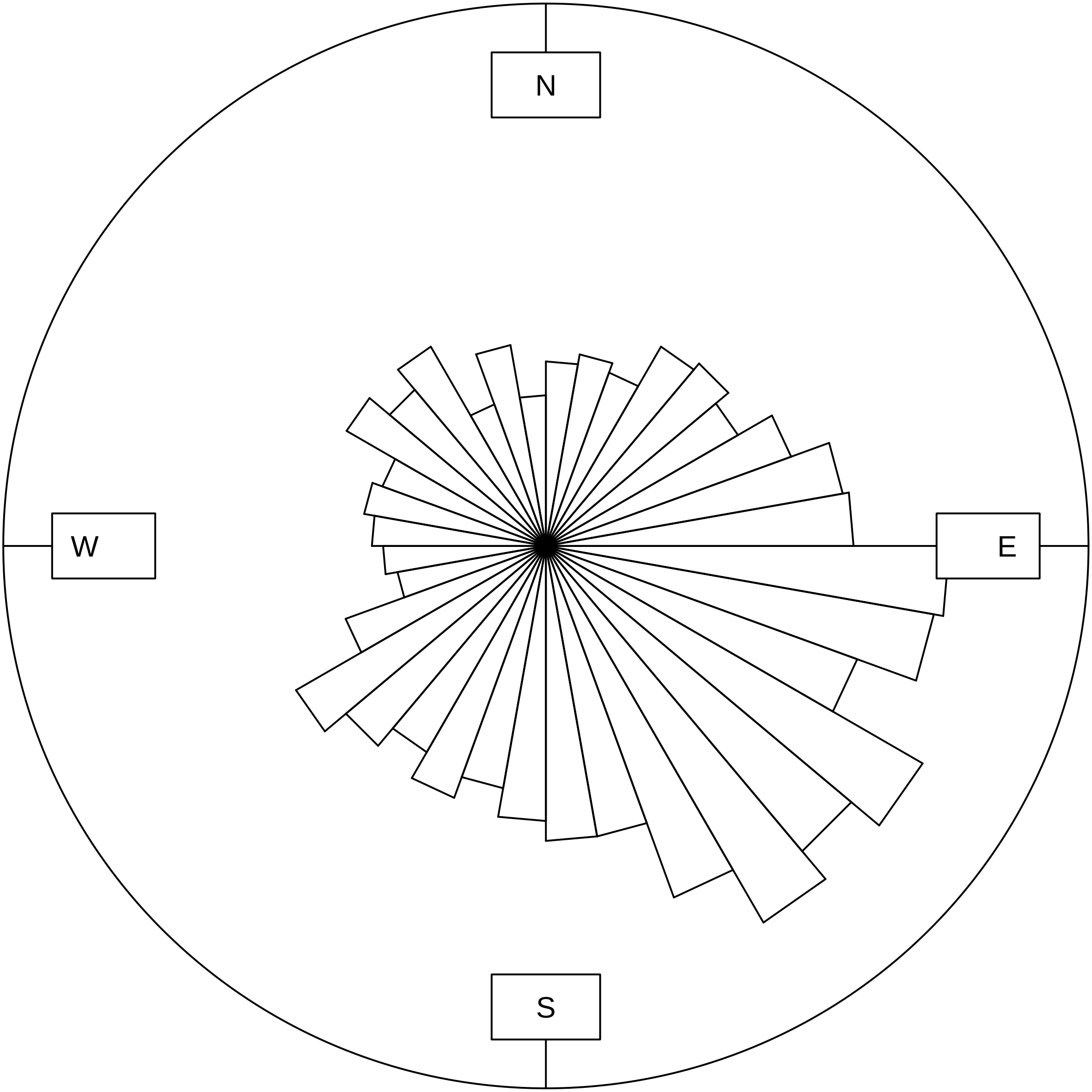

bootstrap 26

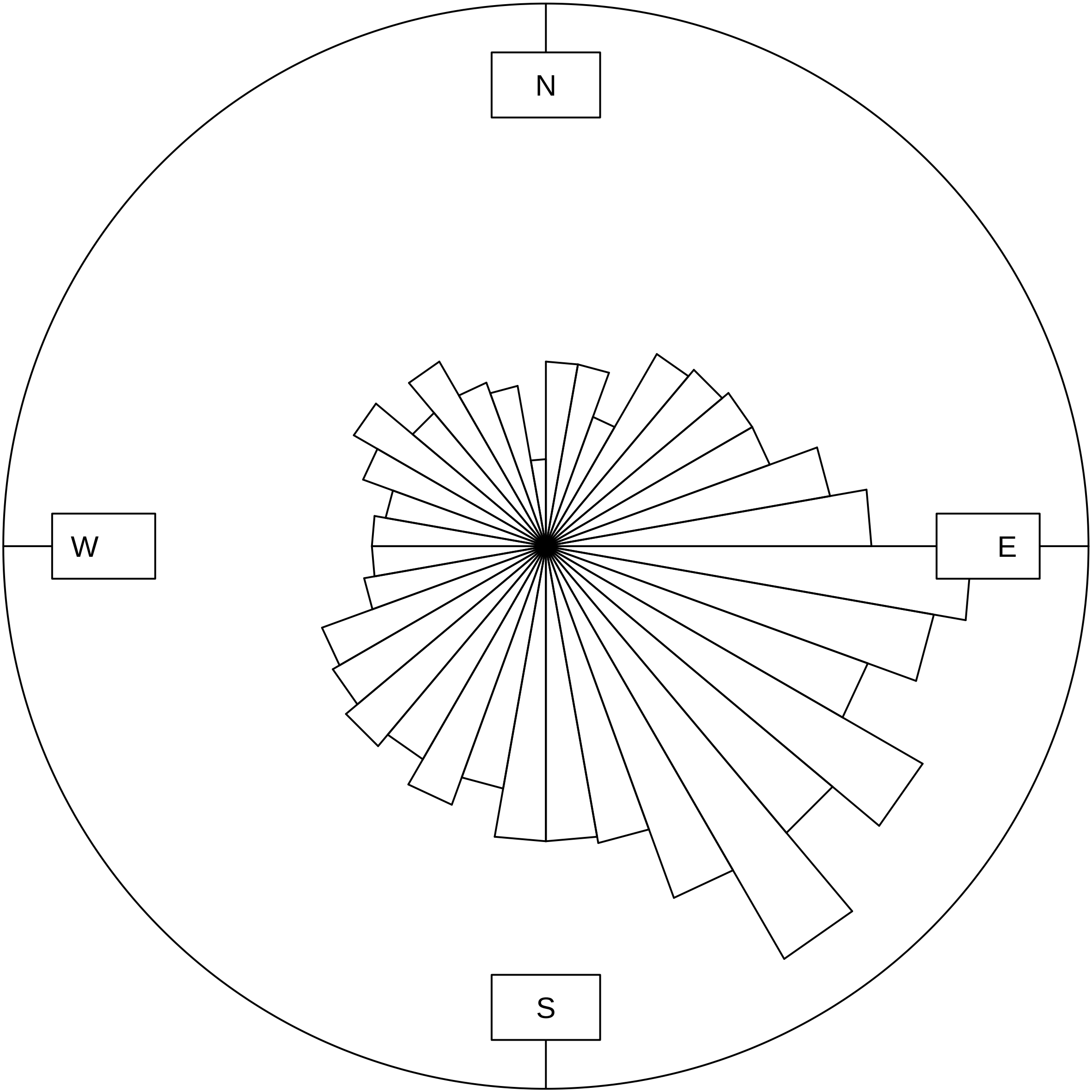

bootstrap 27

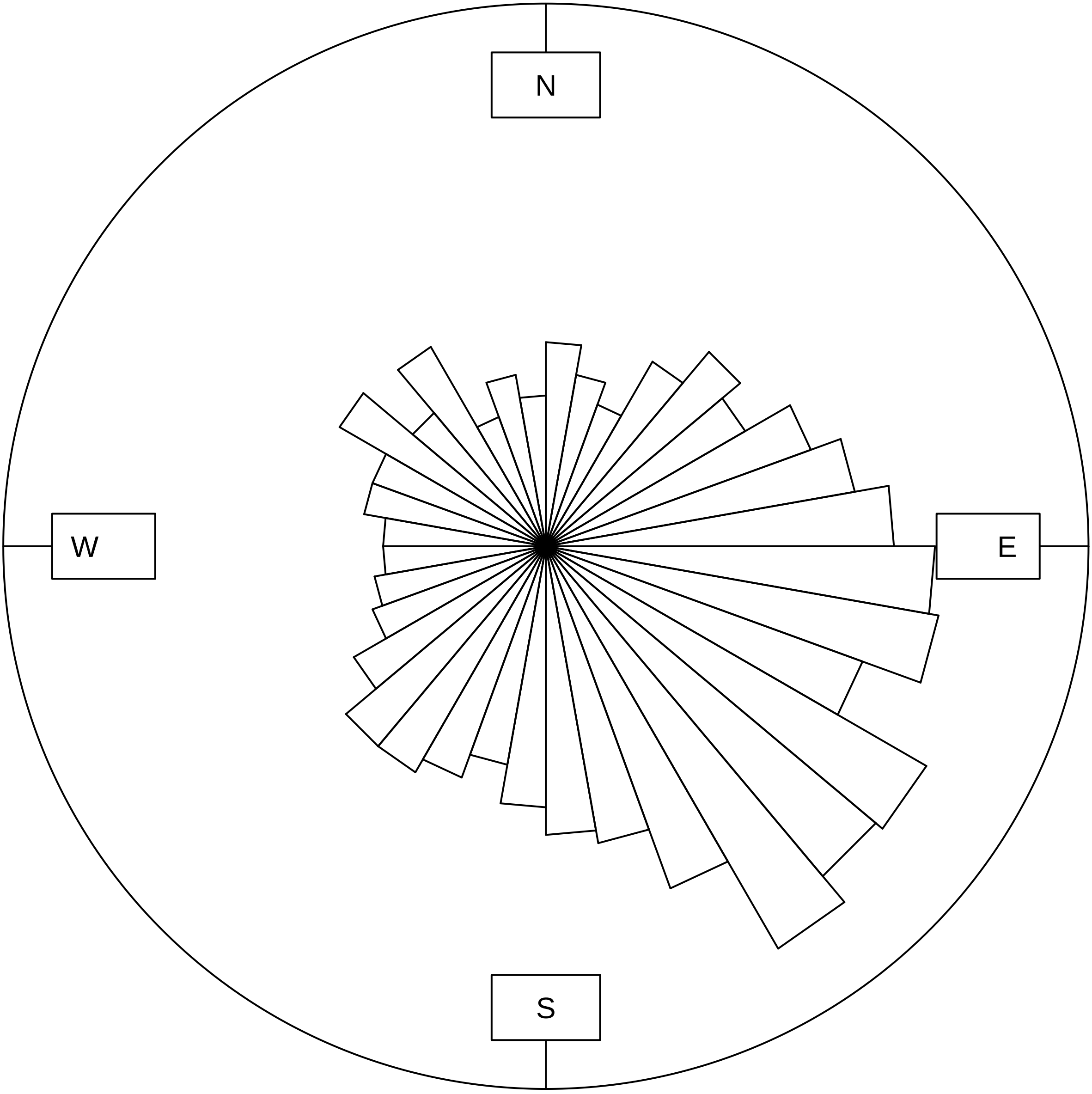

bootstrap 28

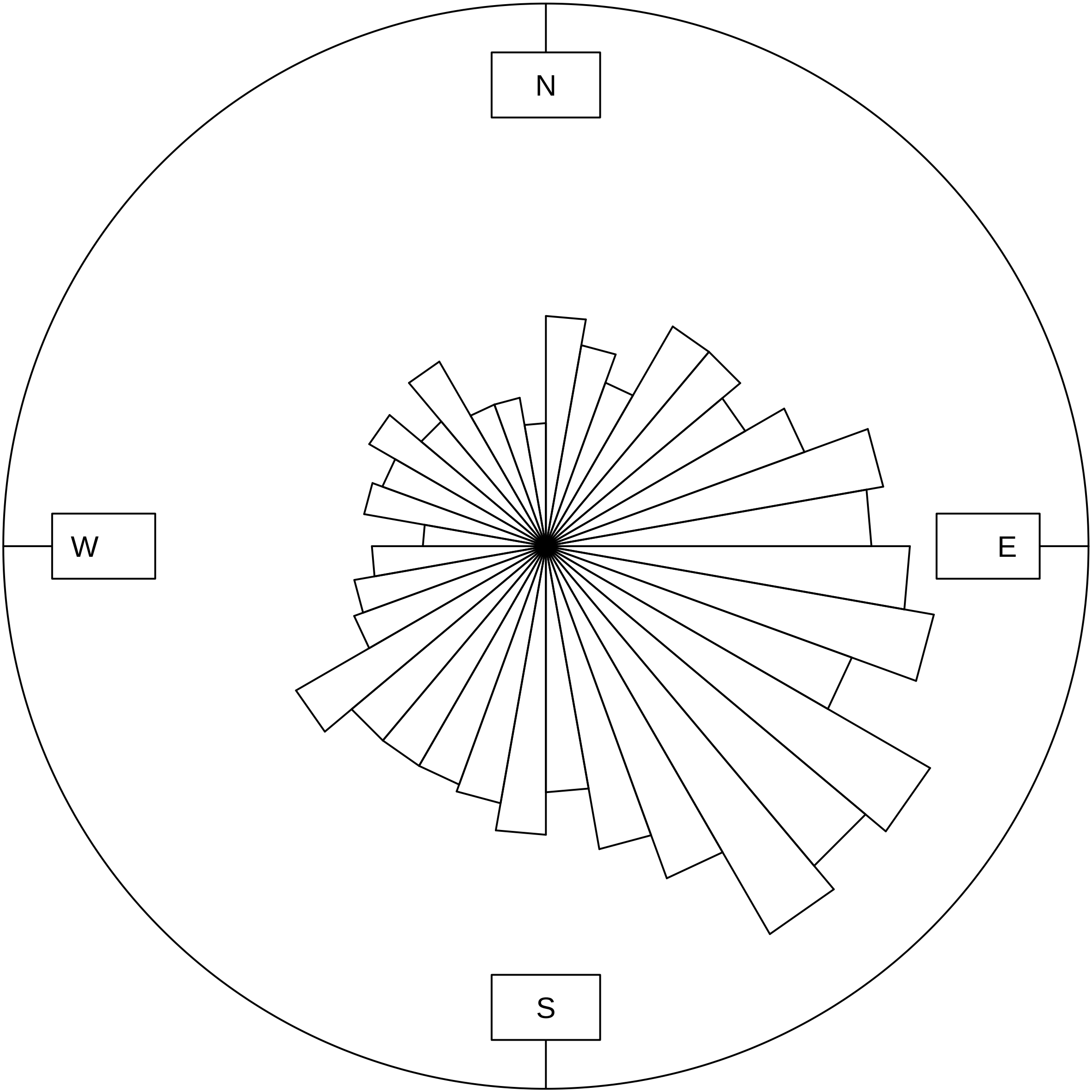

bootstrap 29

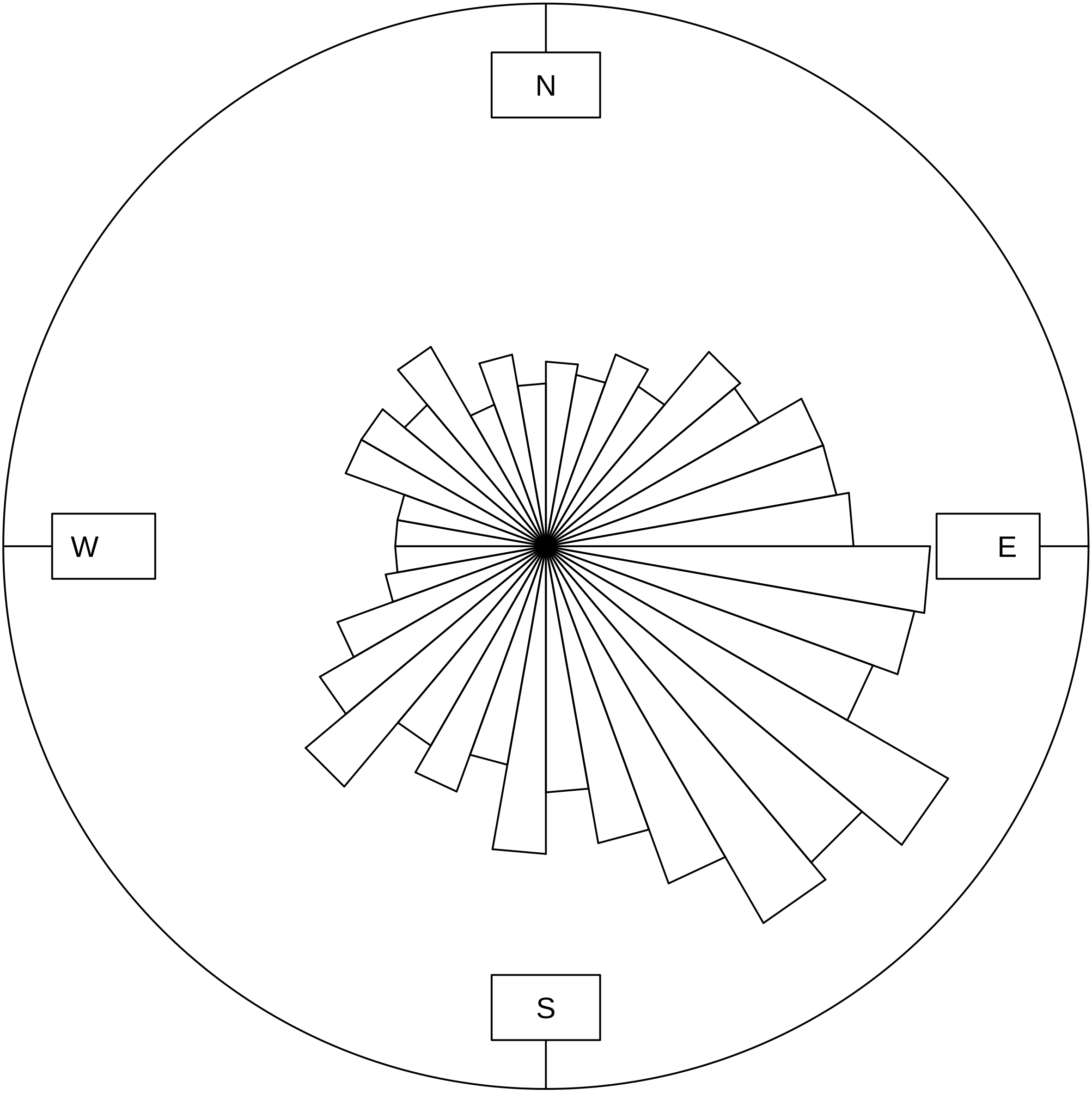

bootstrap 30

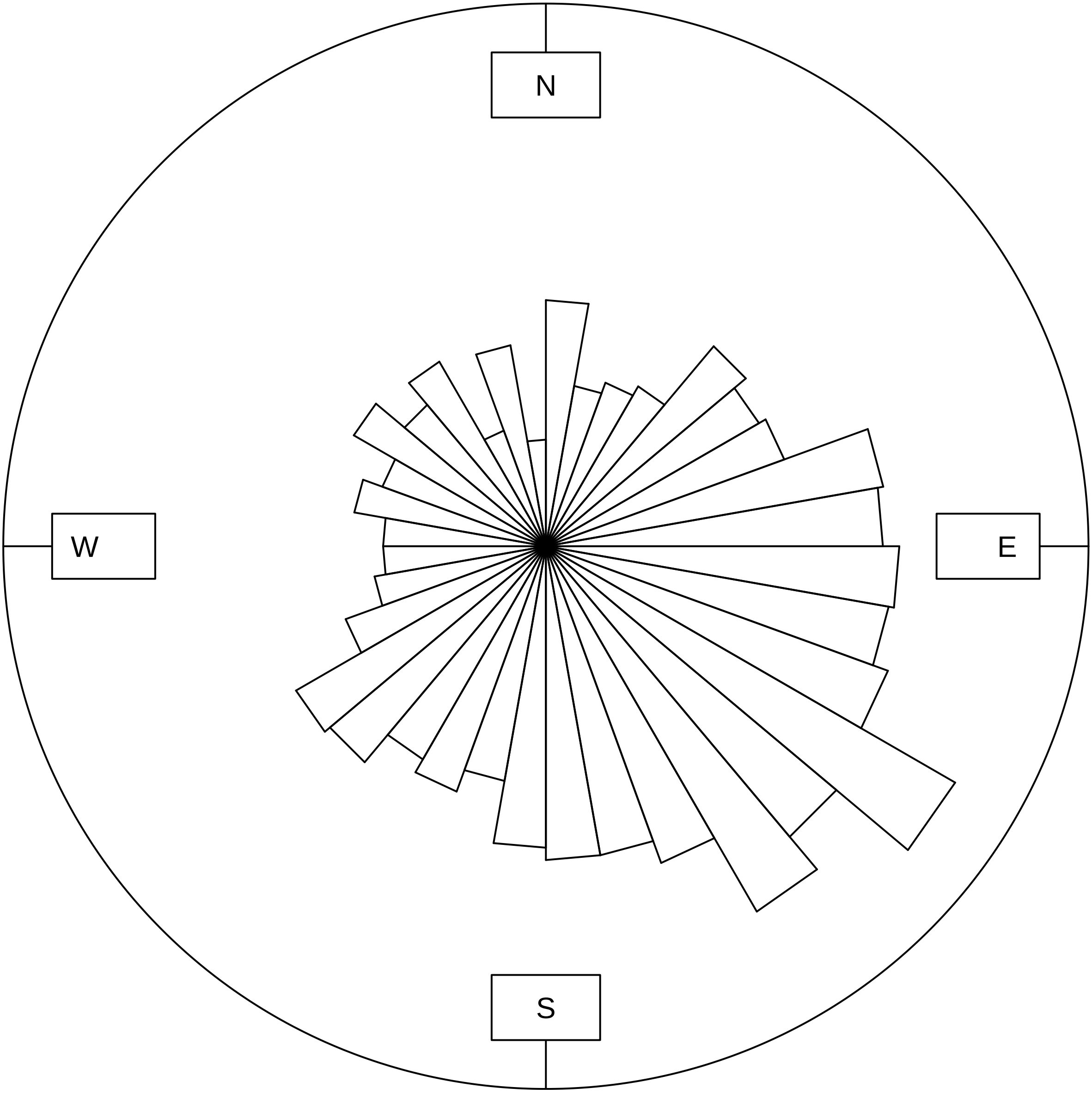

bootstrap 31

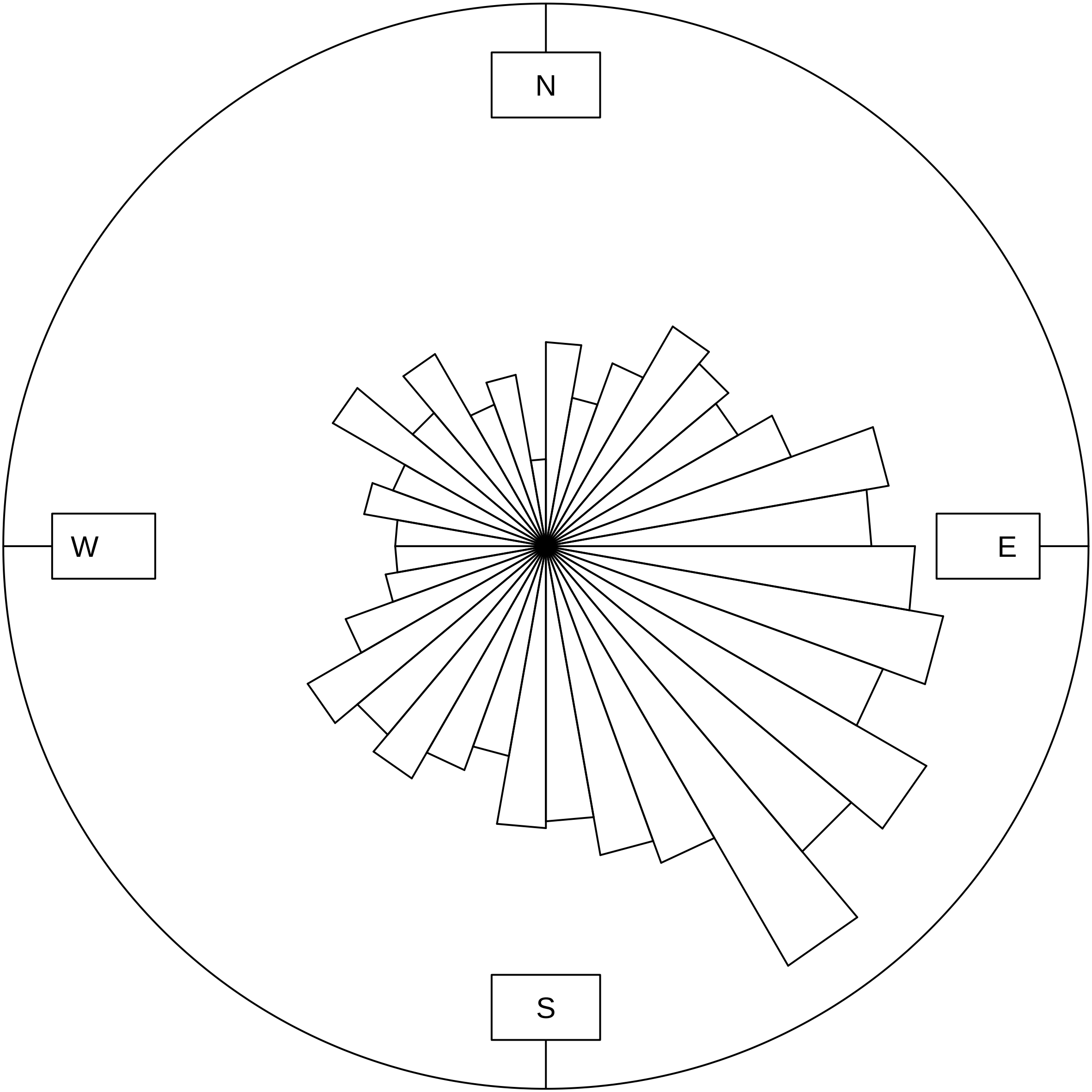

bootstrap 32

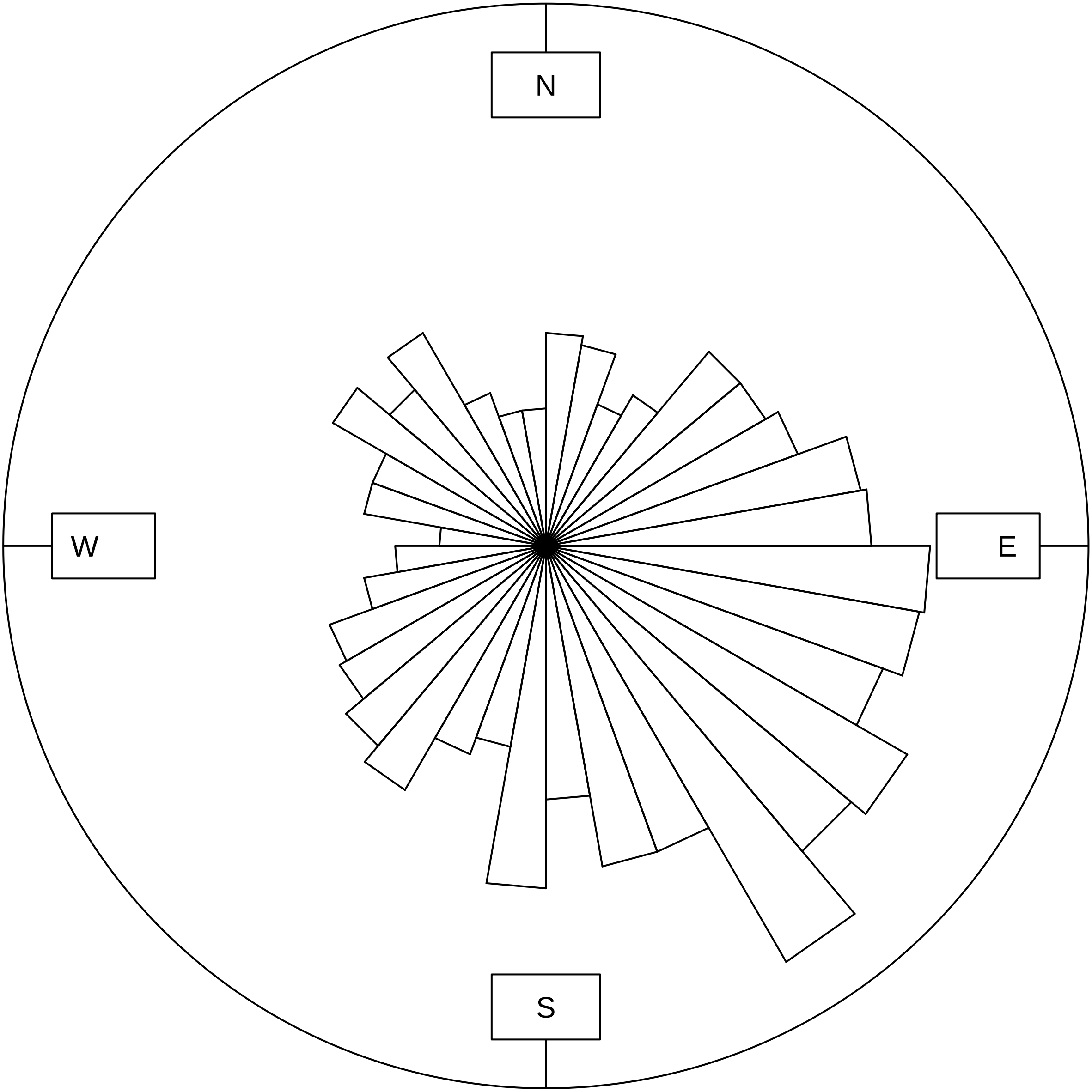

bootstrap 33

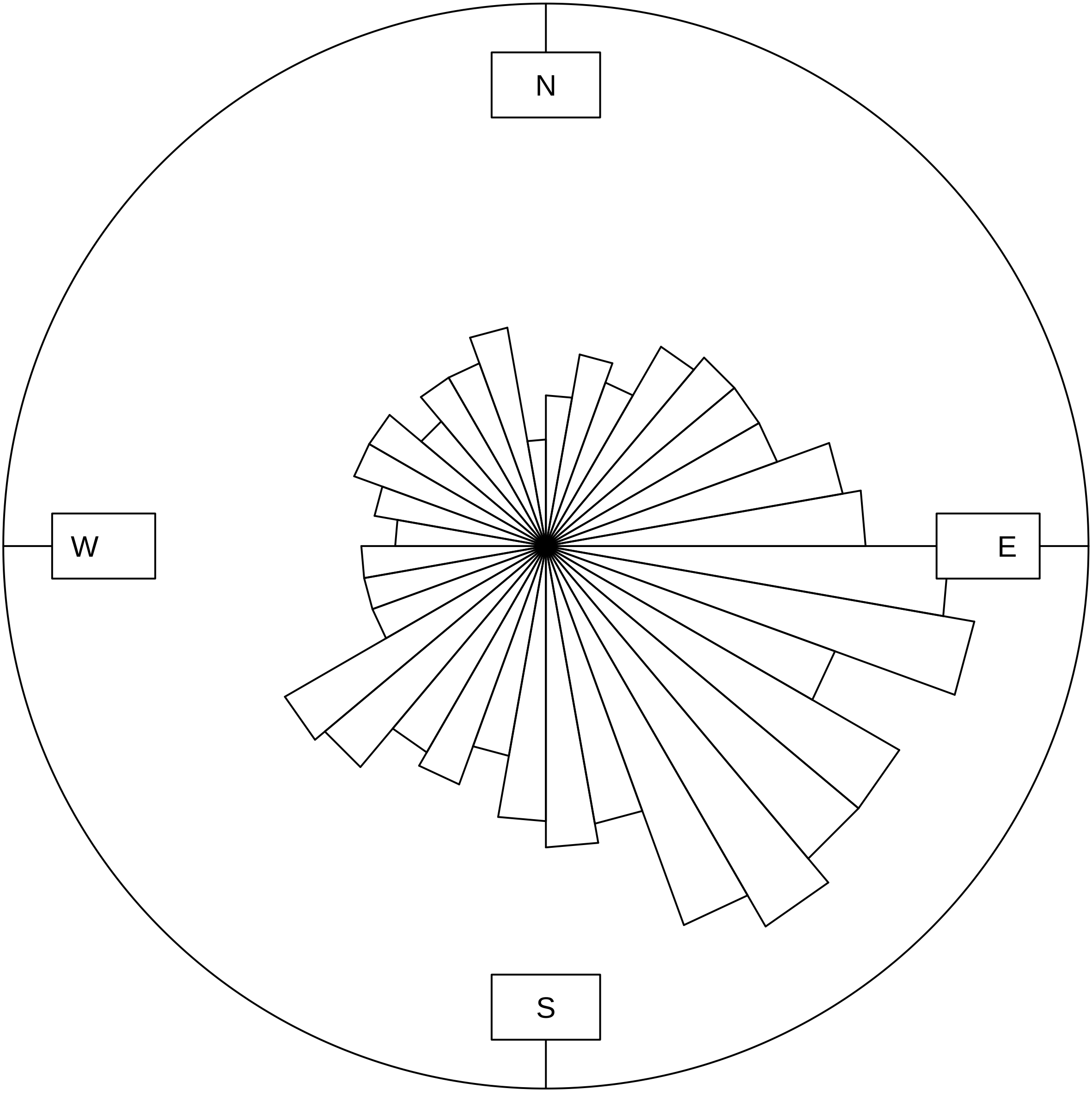

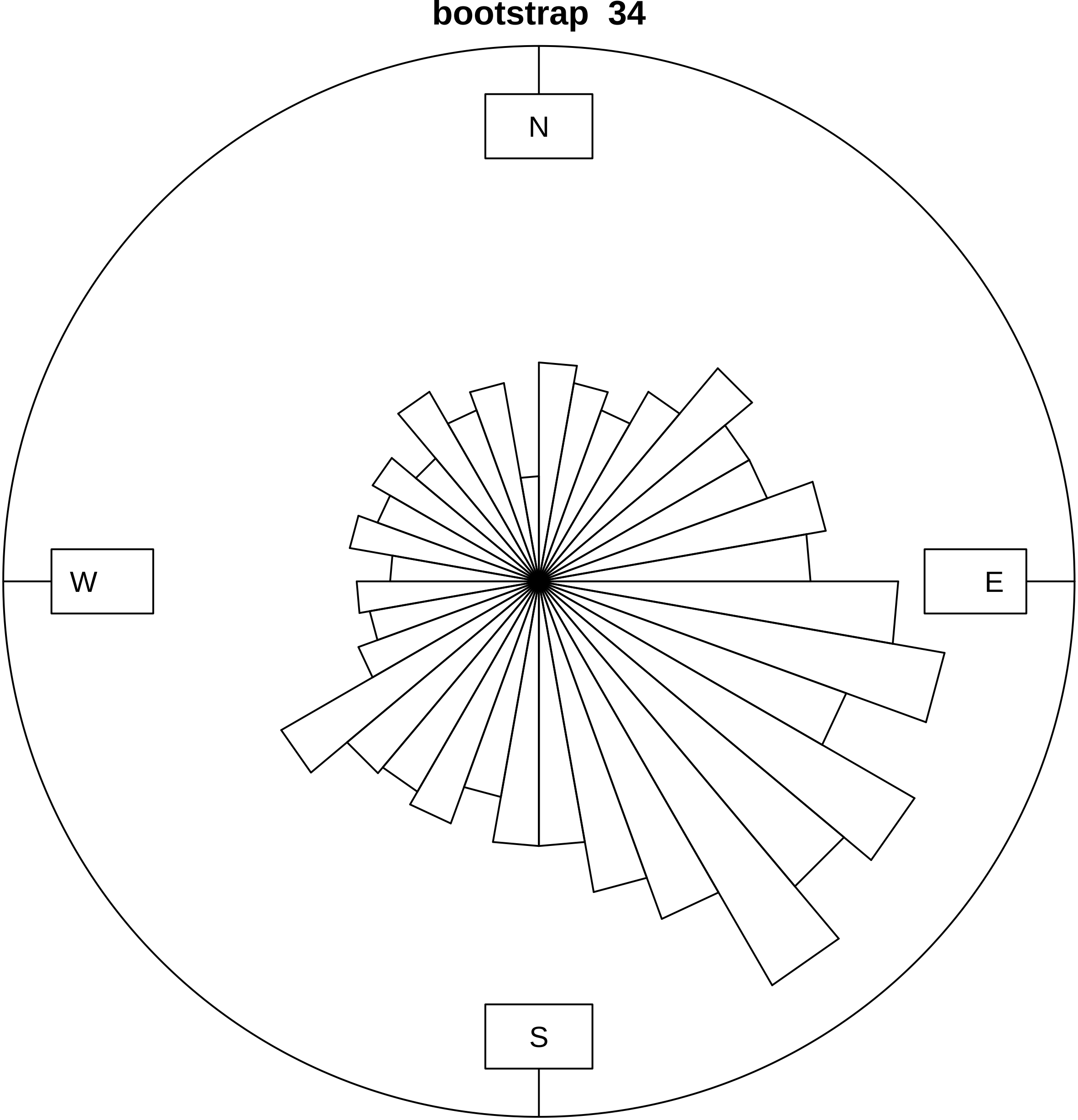
bootstrap 34
N
W
E
S

bootstrap 35

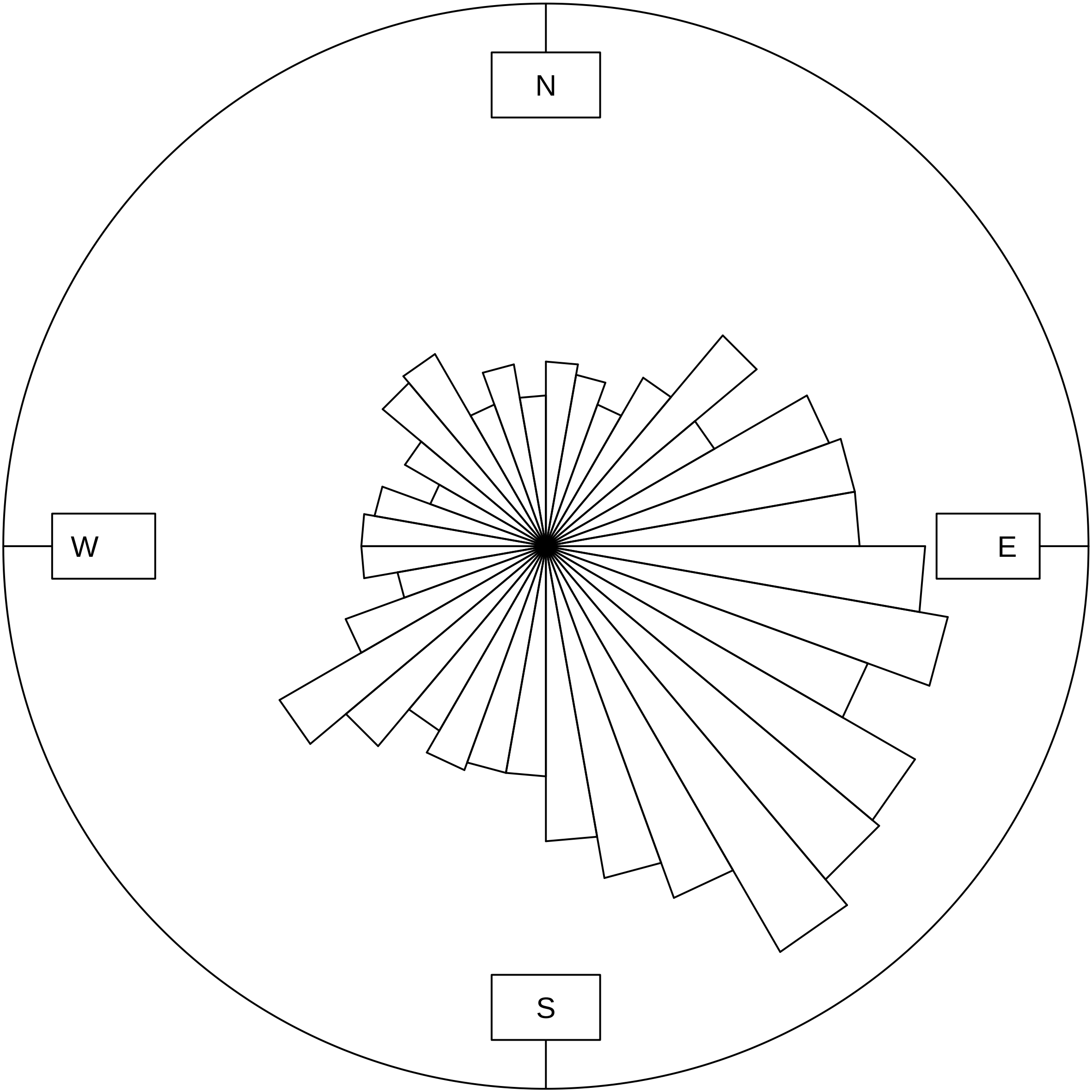

bootstrap 36

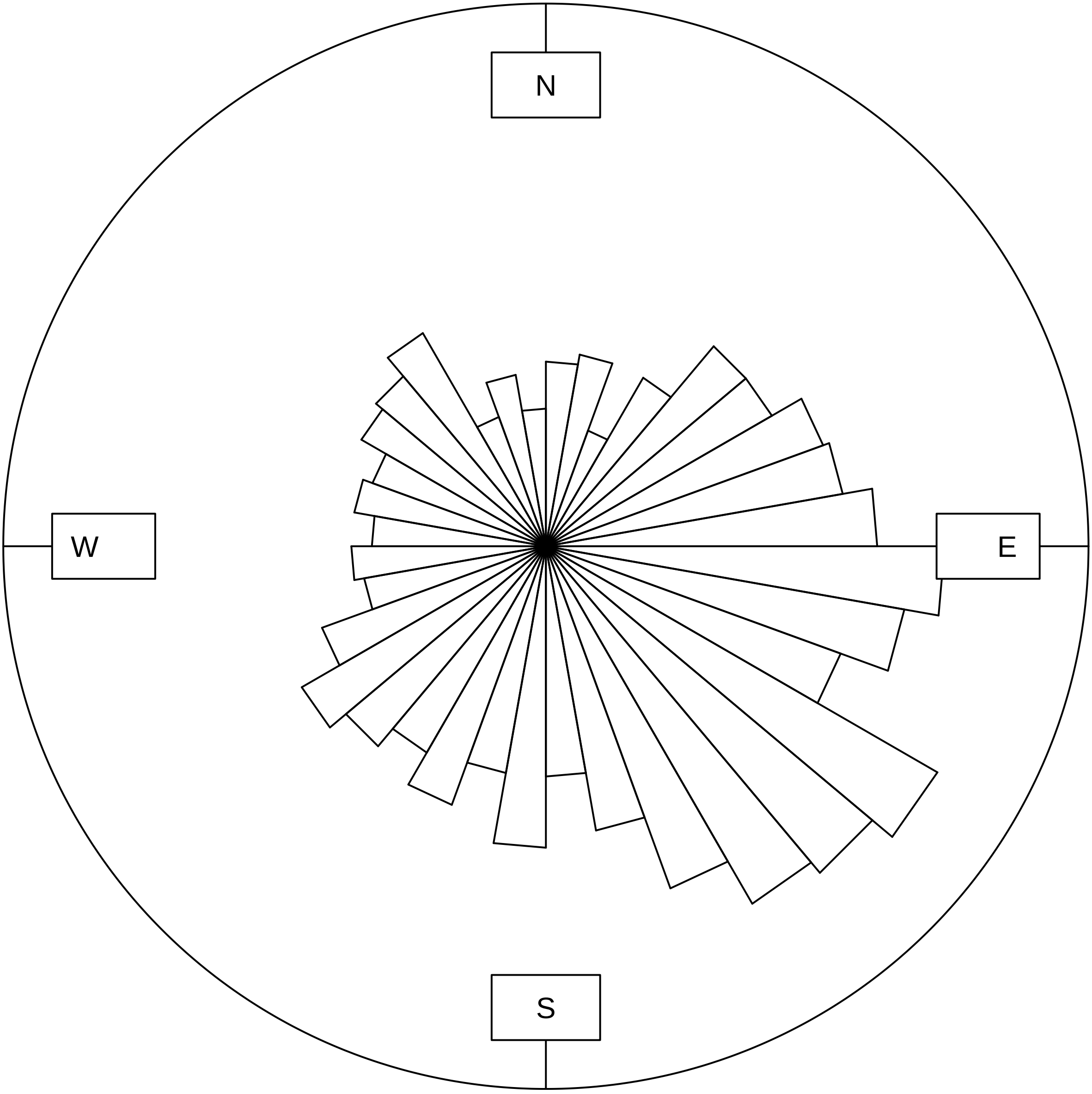

bootstrap 37

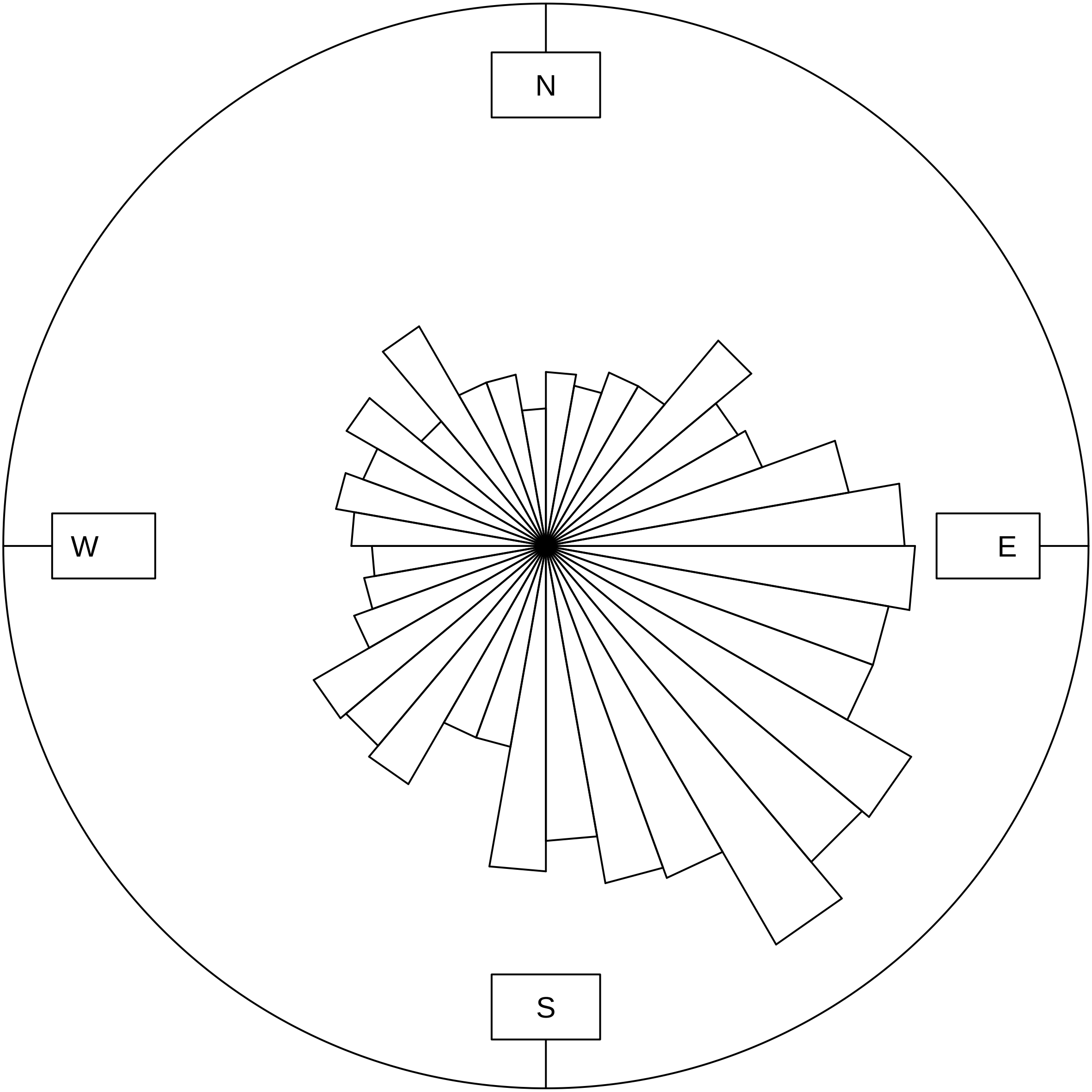

bootstrap 38

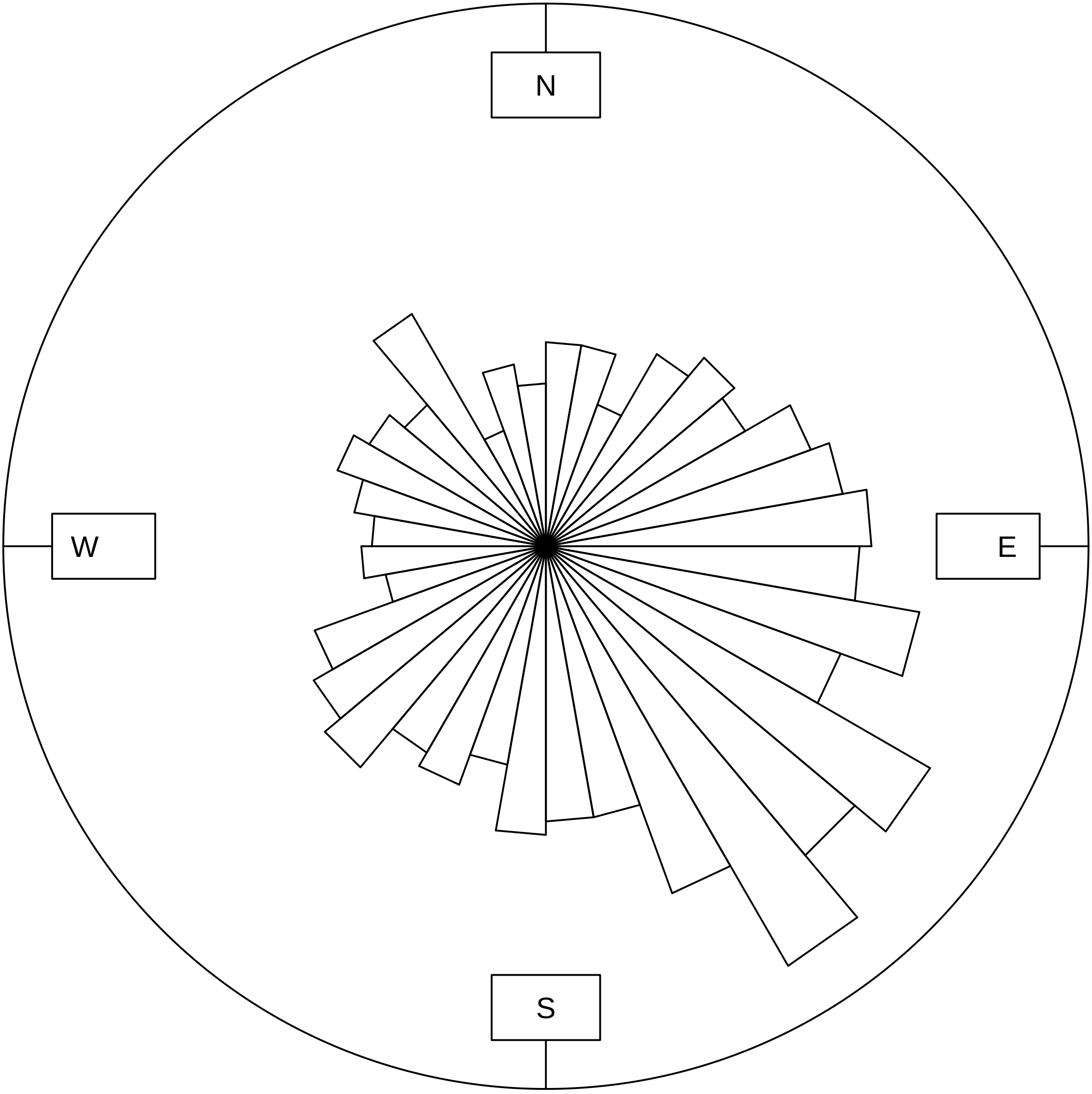

bootstrap 39

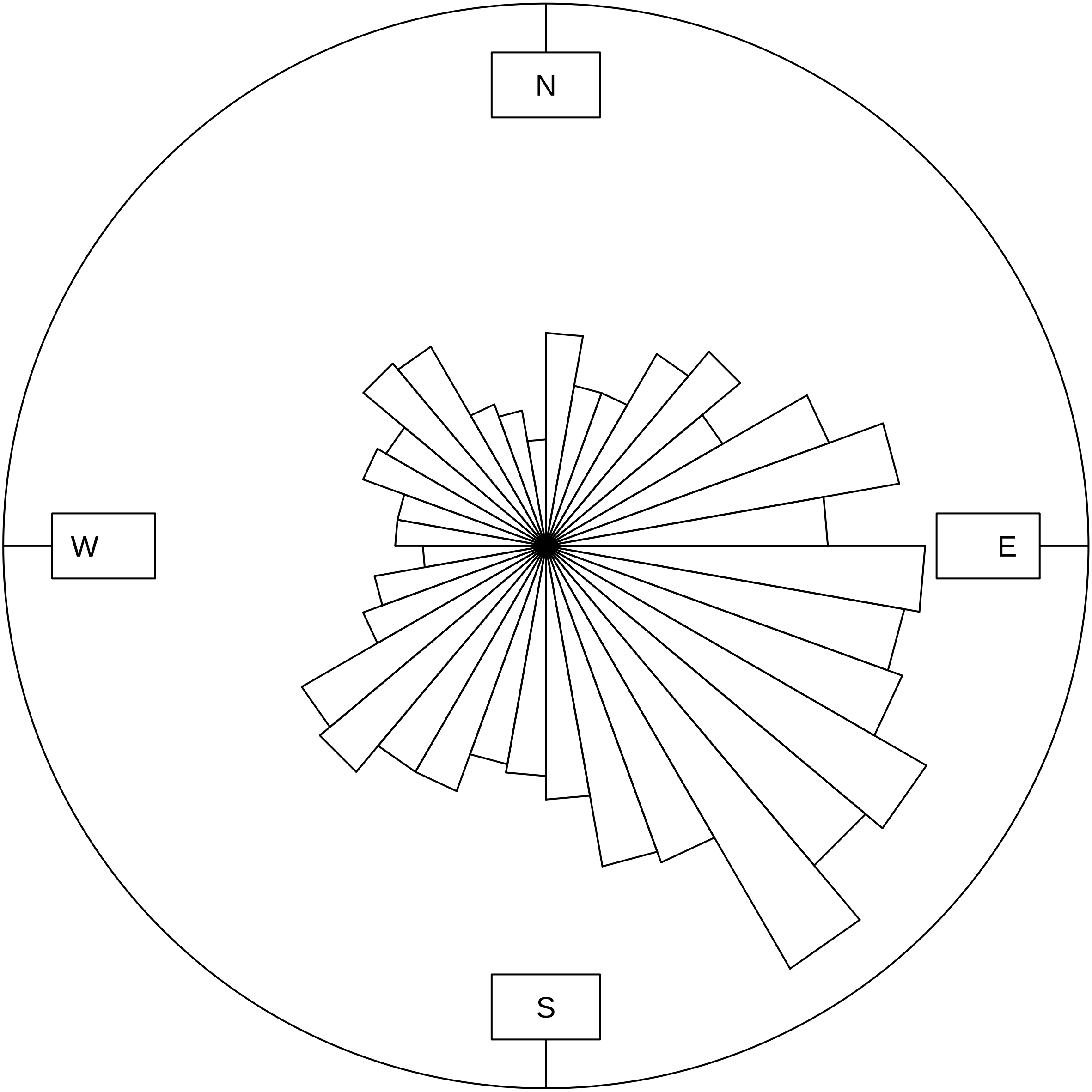

bootstrap 40

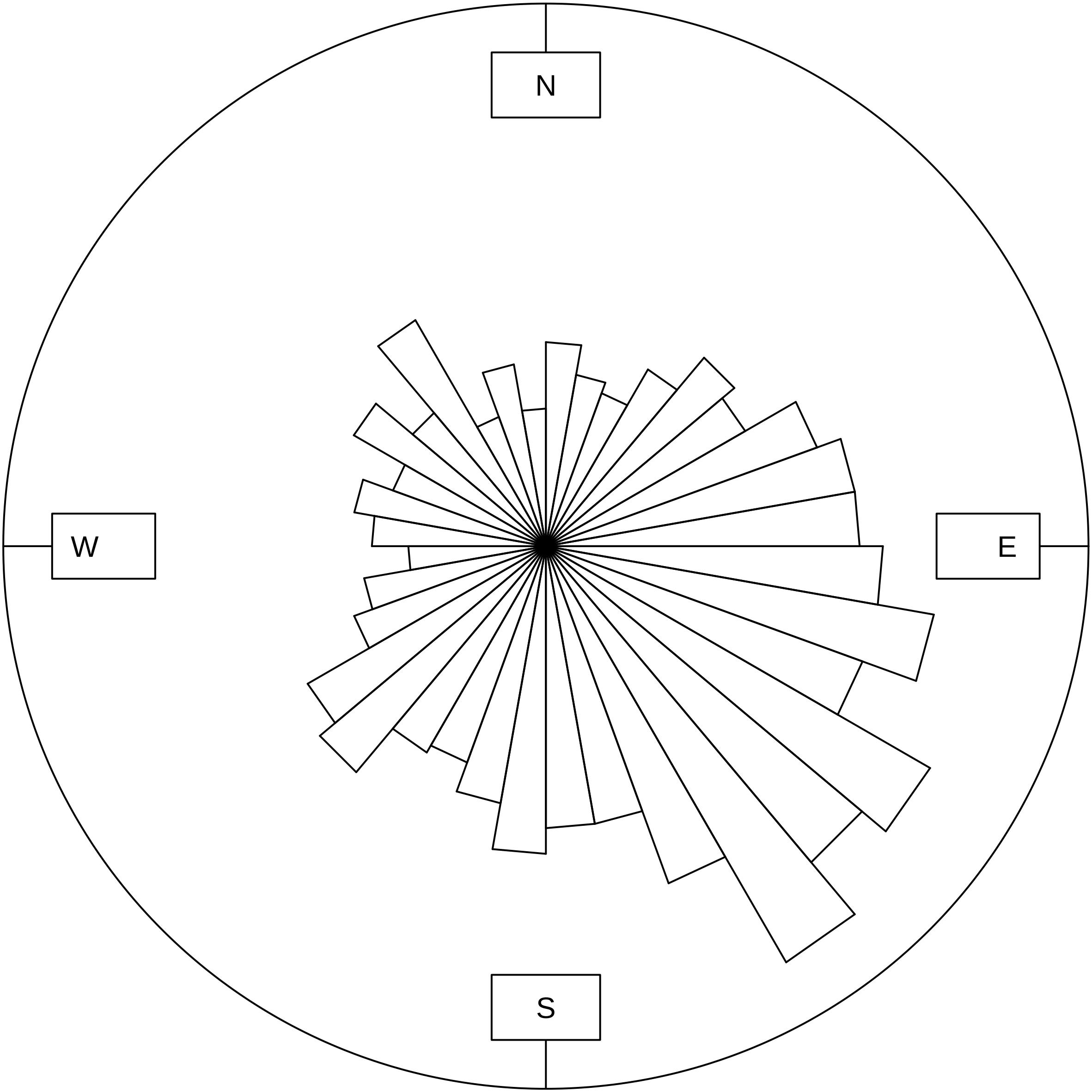

bootstrap 41

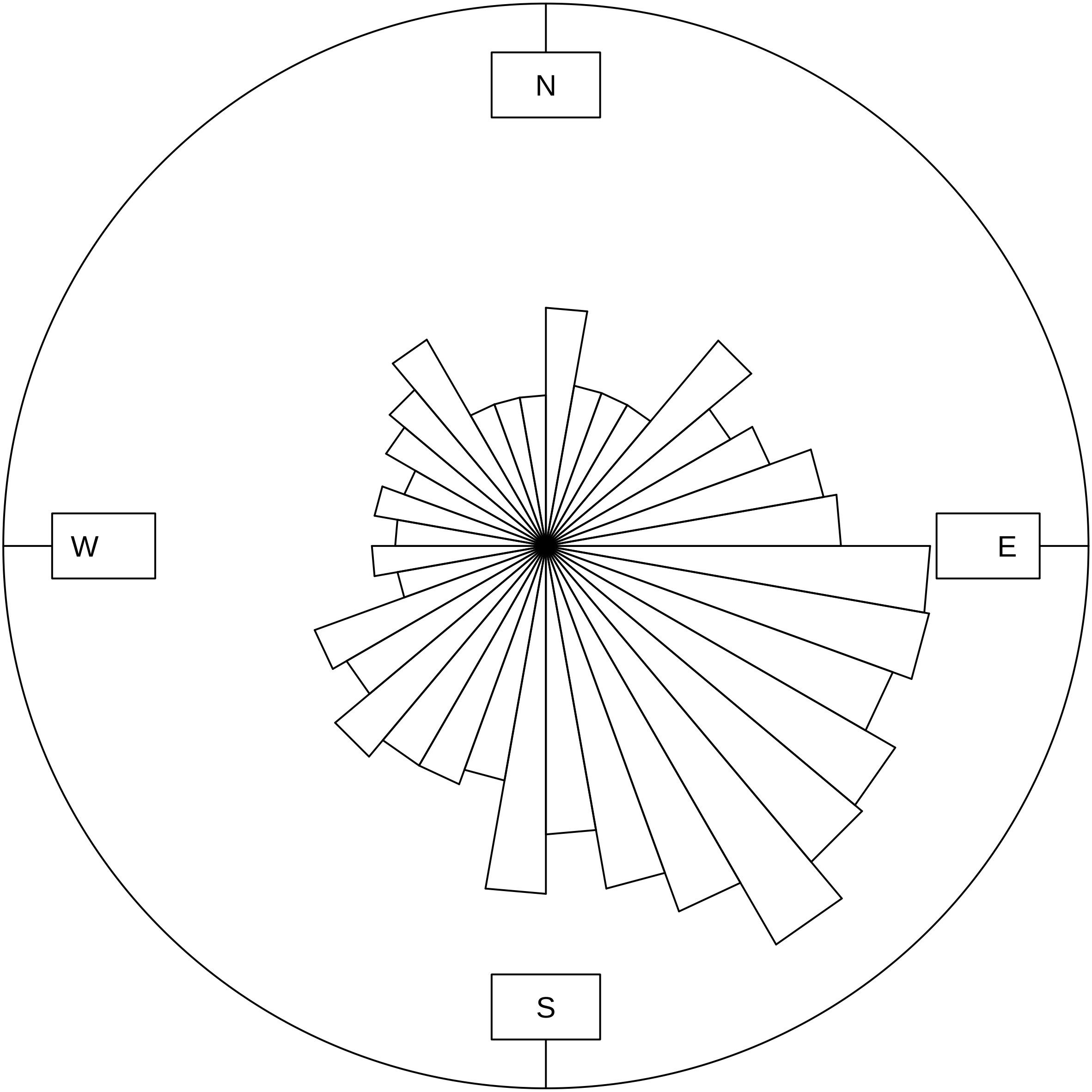

bootstrap 42

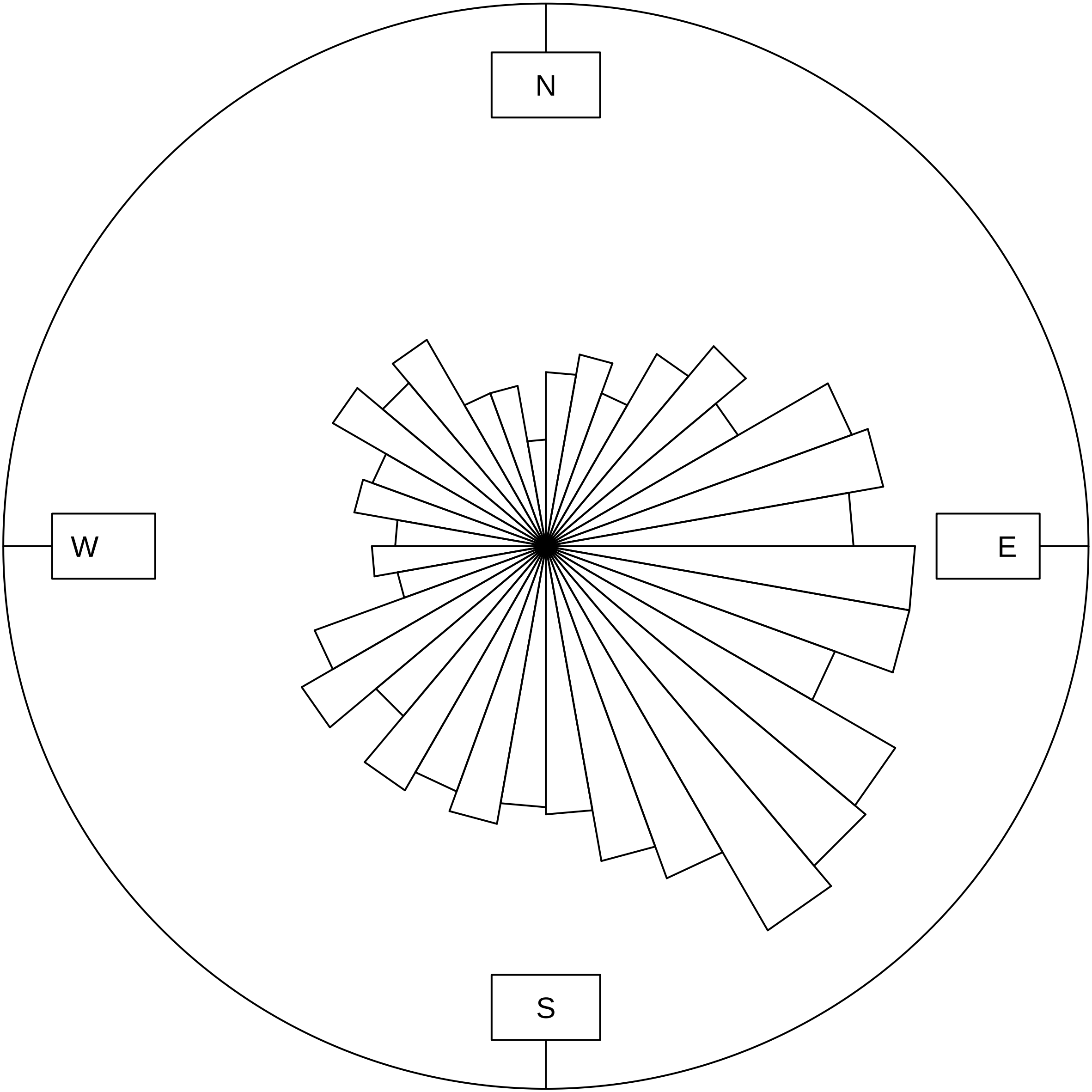

bootstrap 43

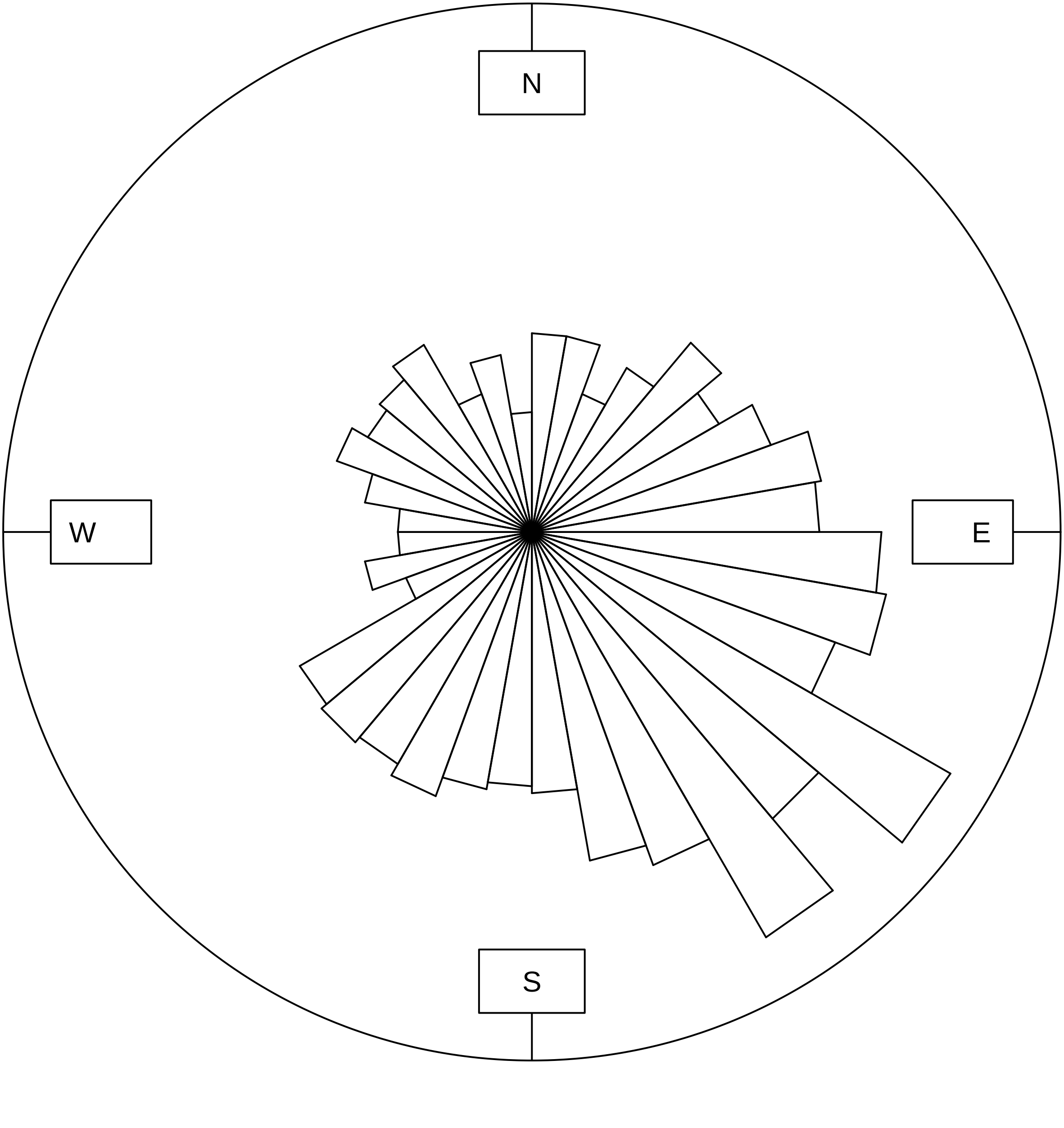

bootstrap 44

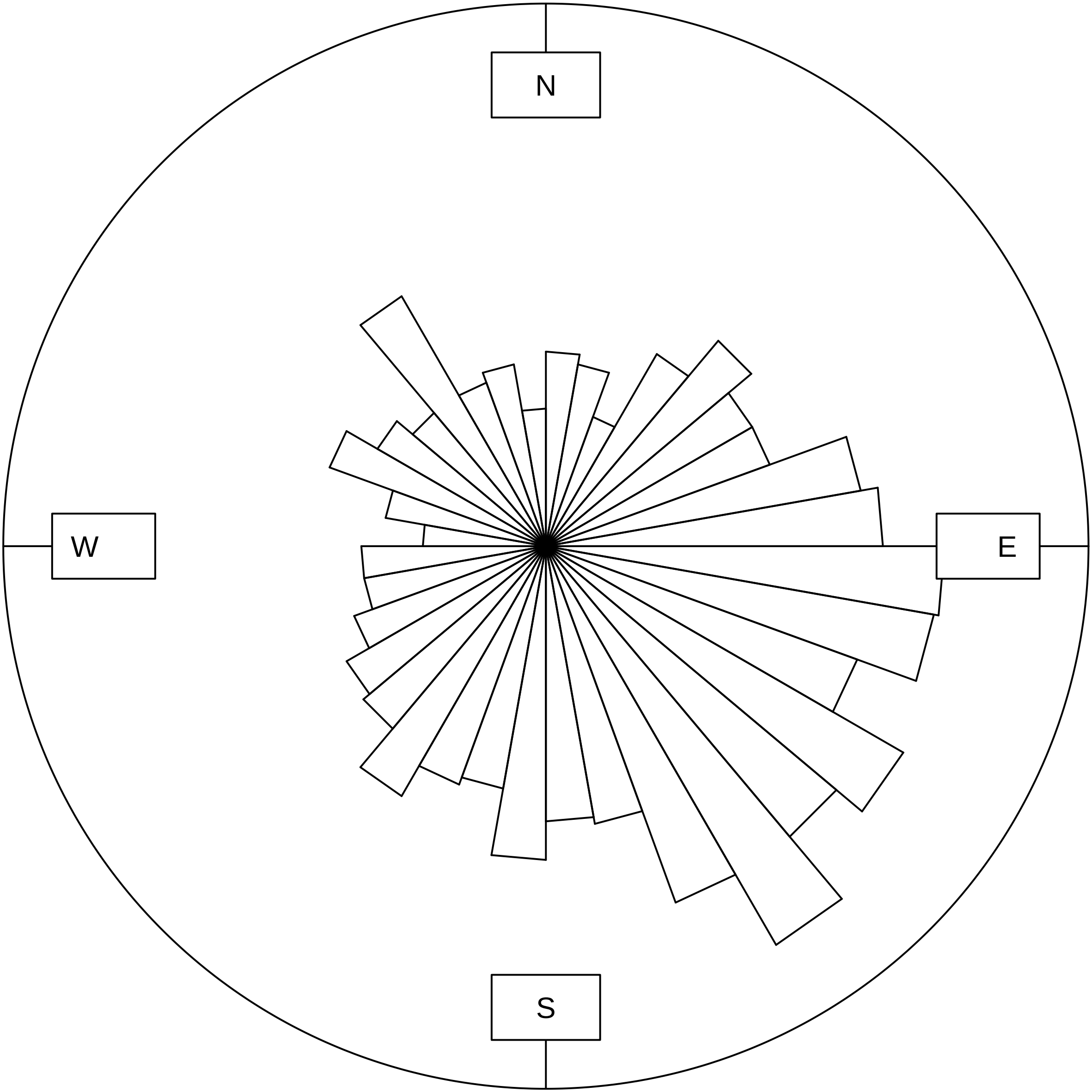

bootstrap 45

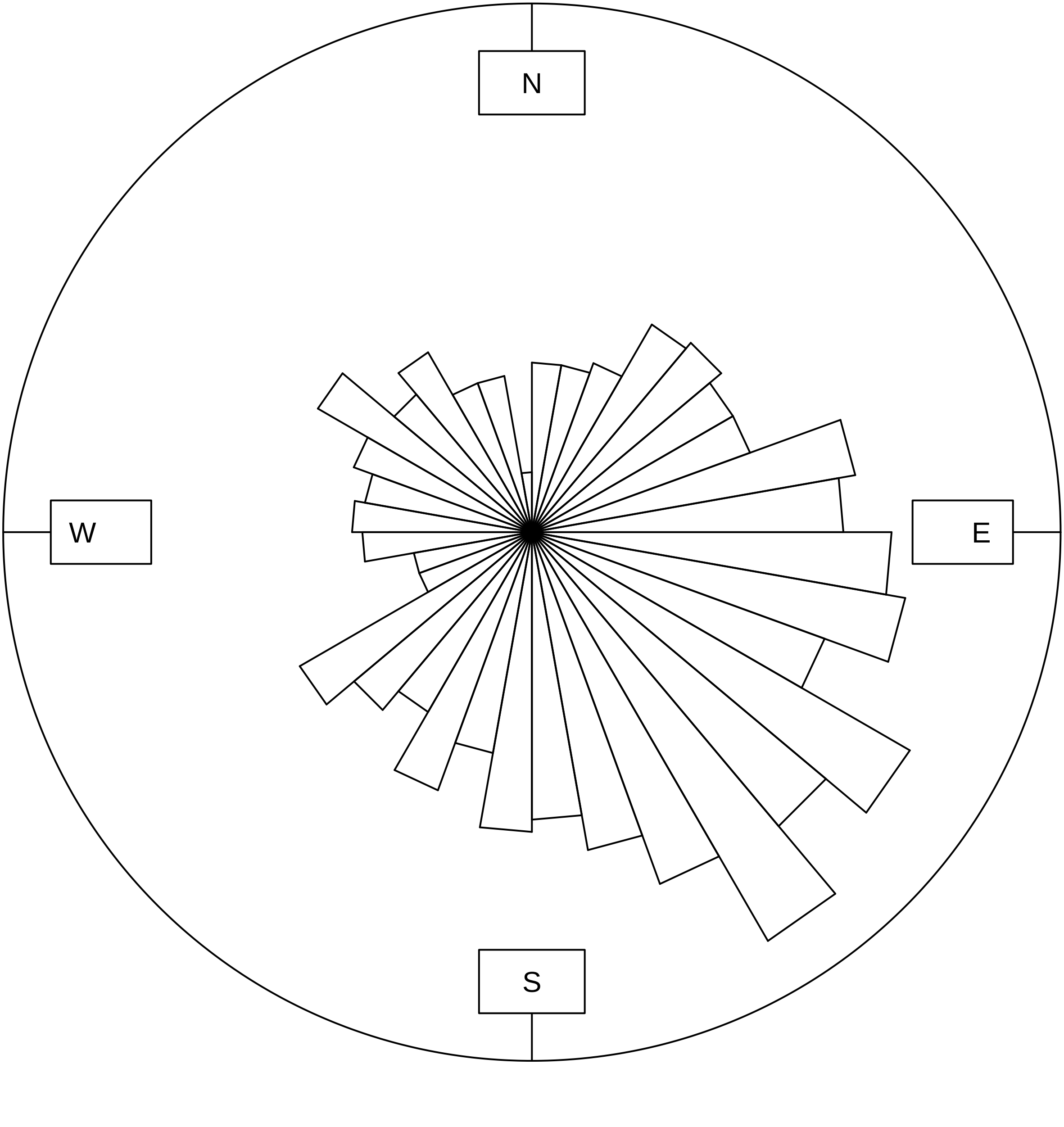

bootstrap 46

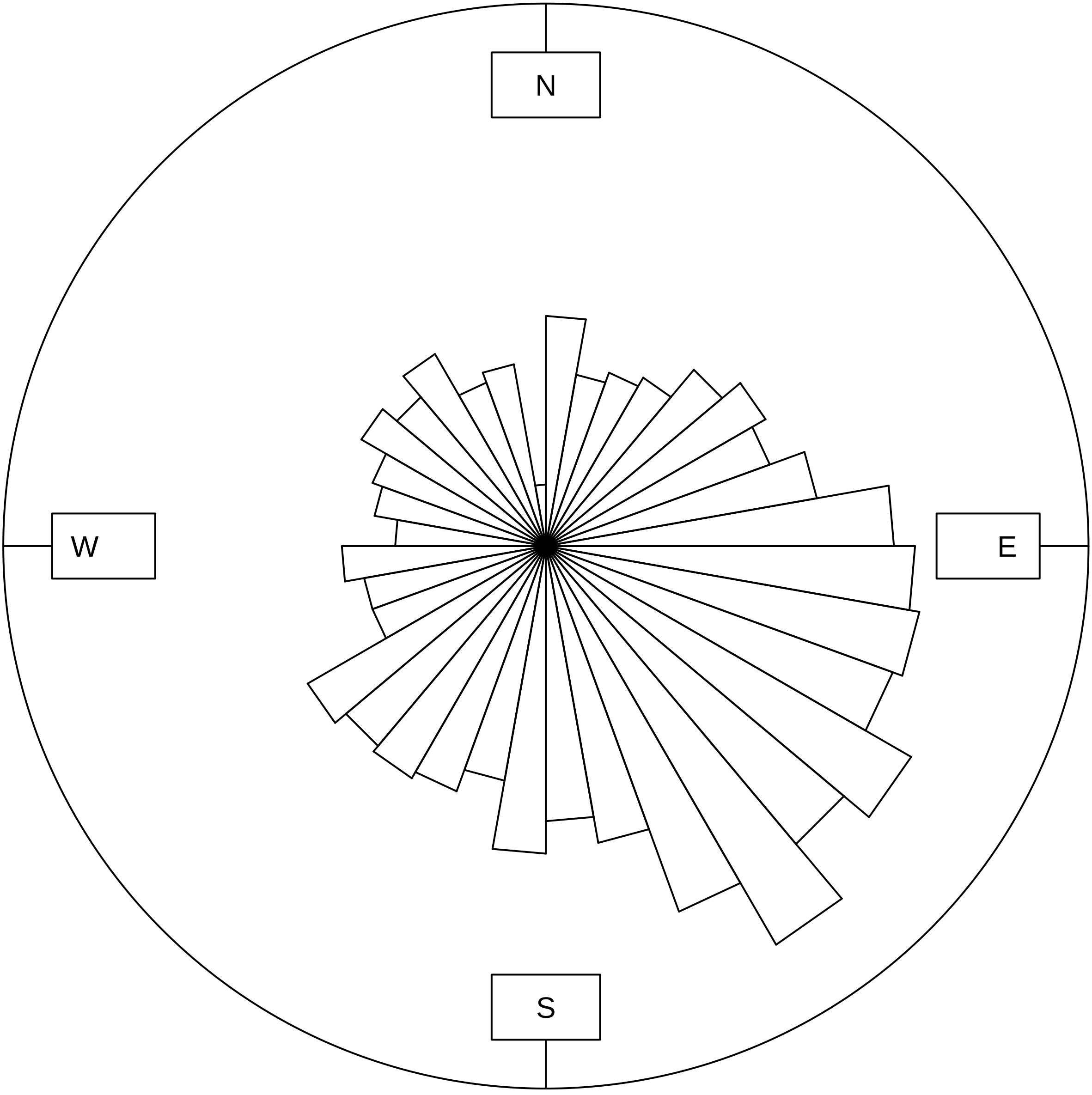

bootstrap 47

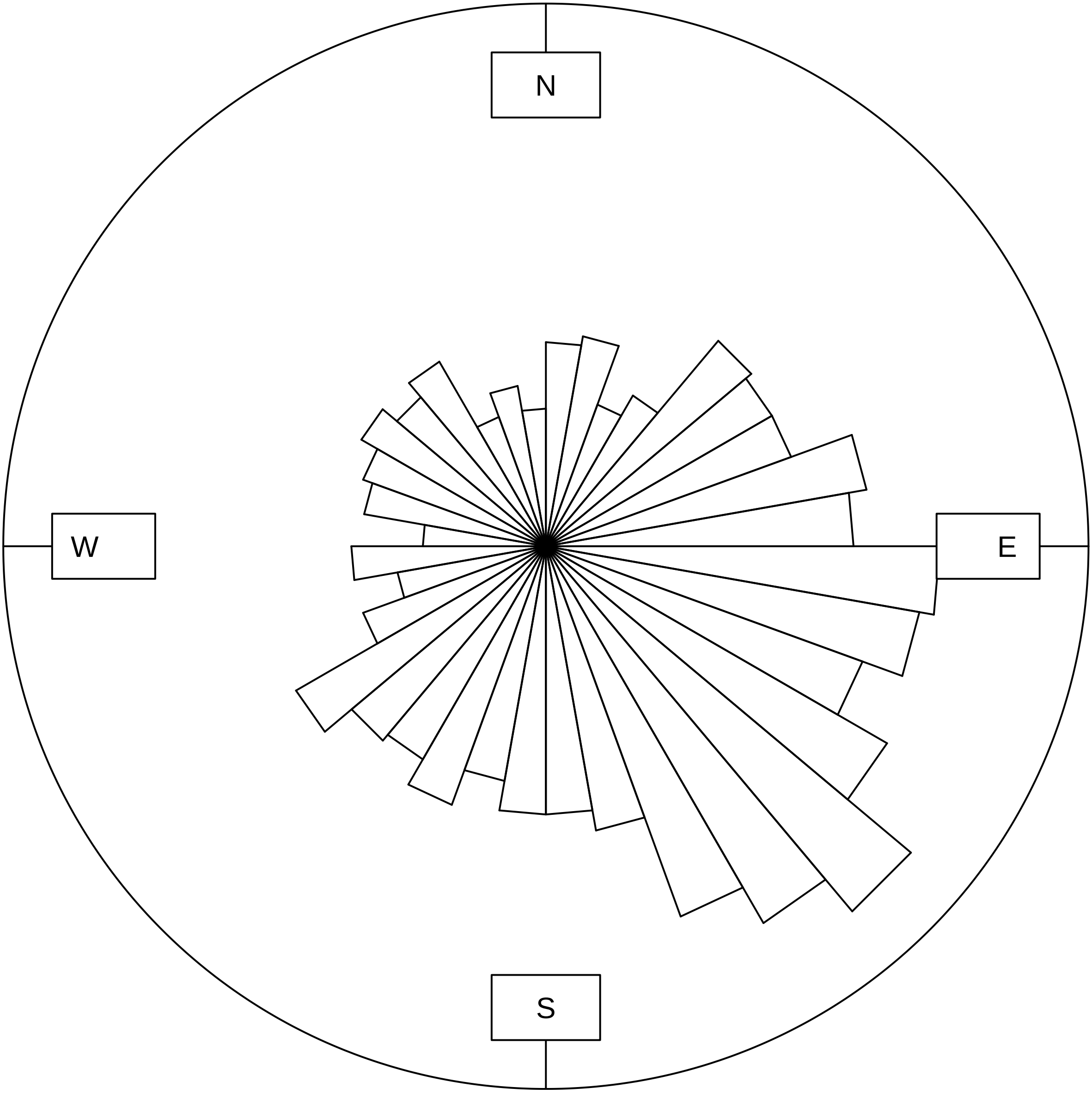

bootstrap 48

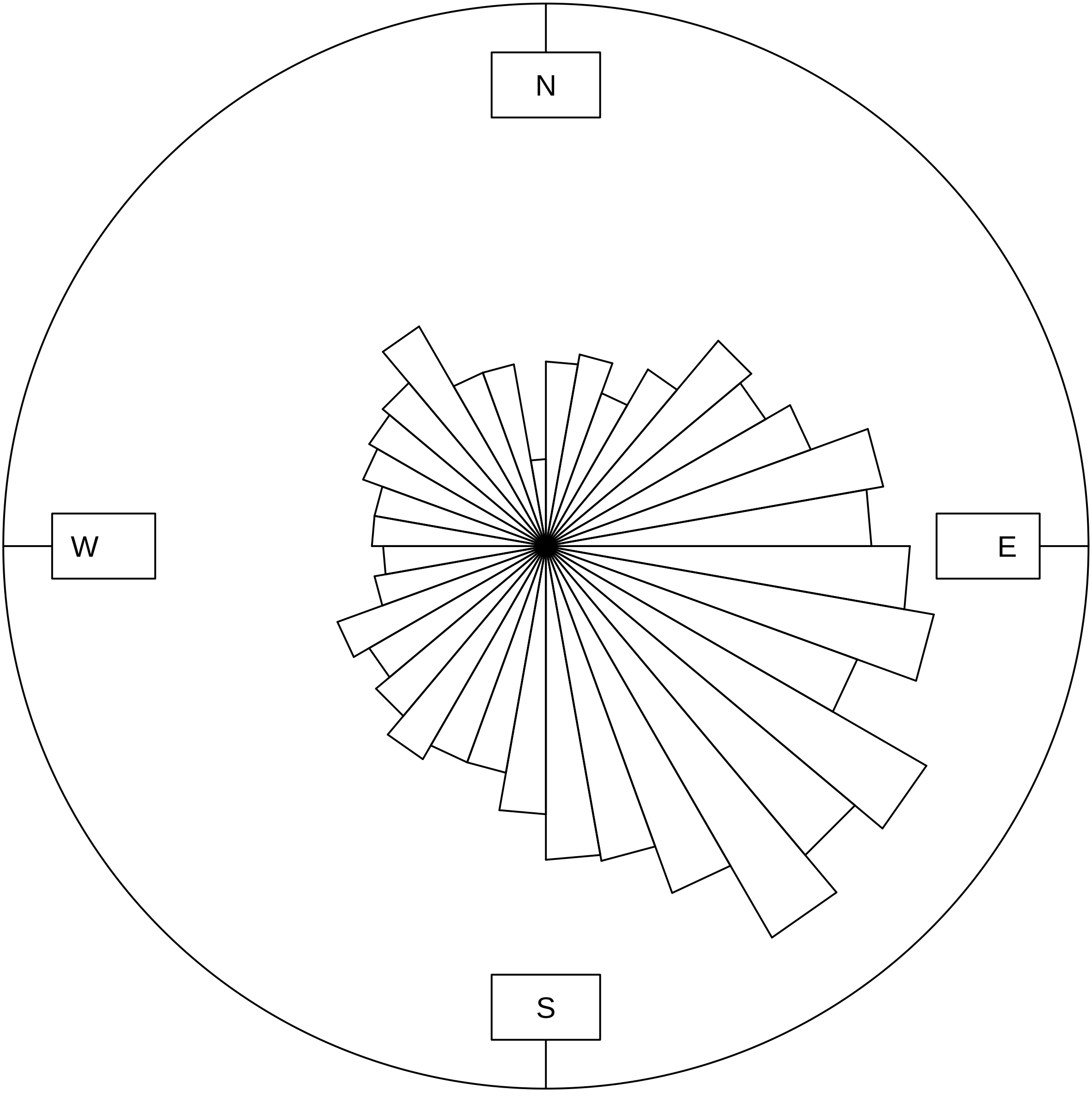

bootstrap 49

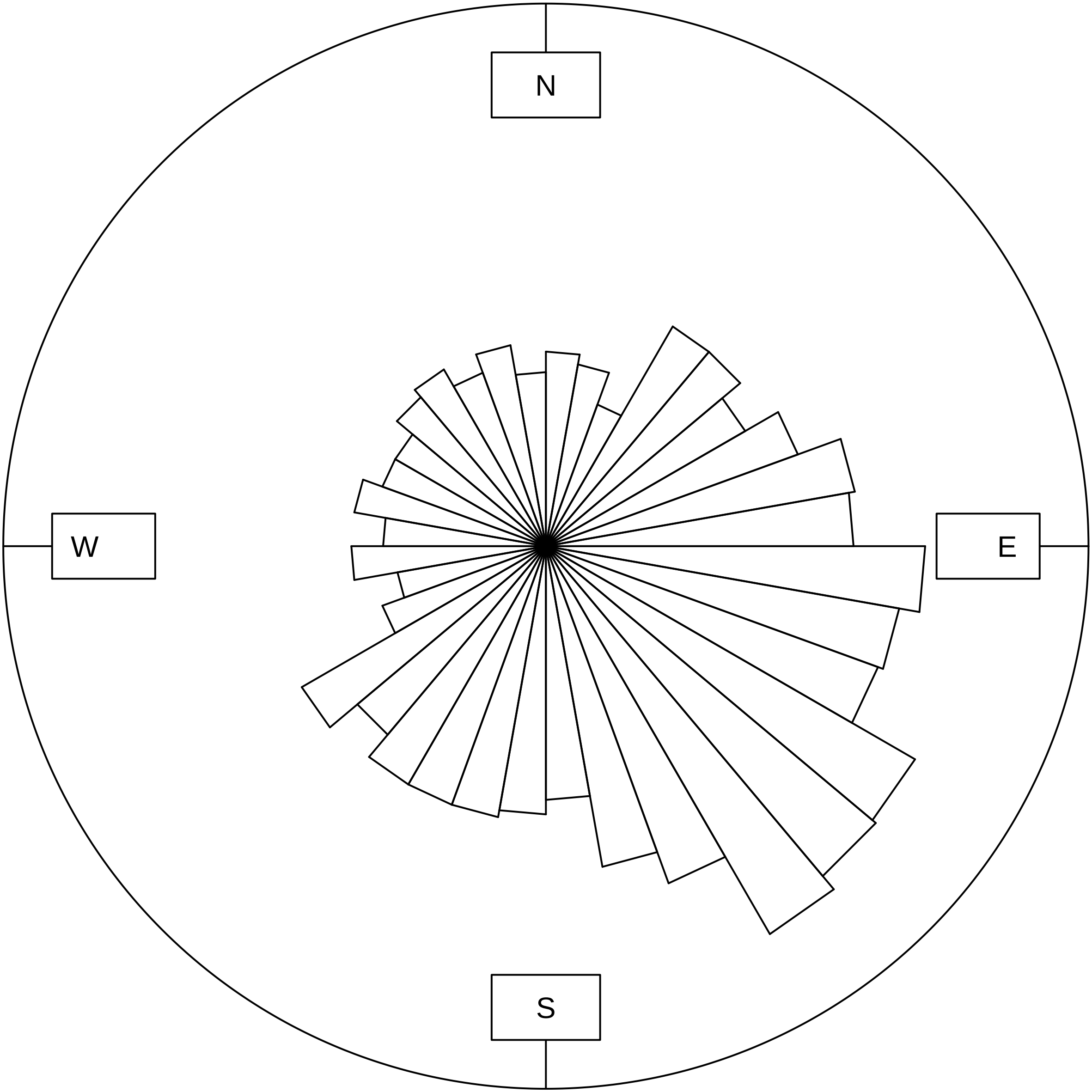

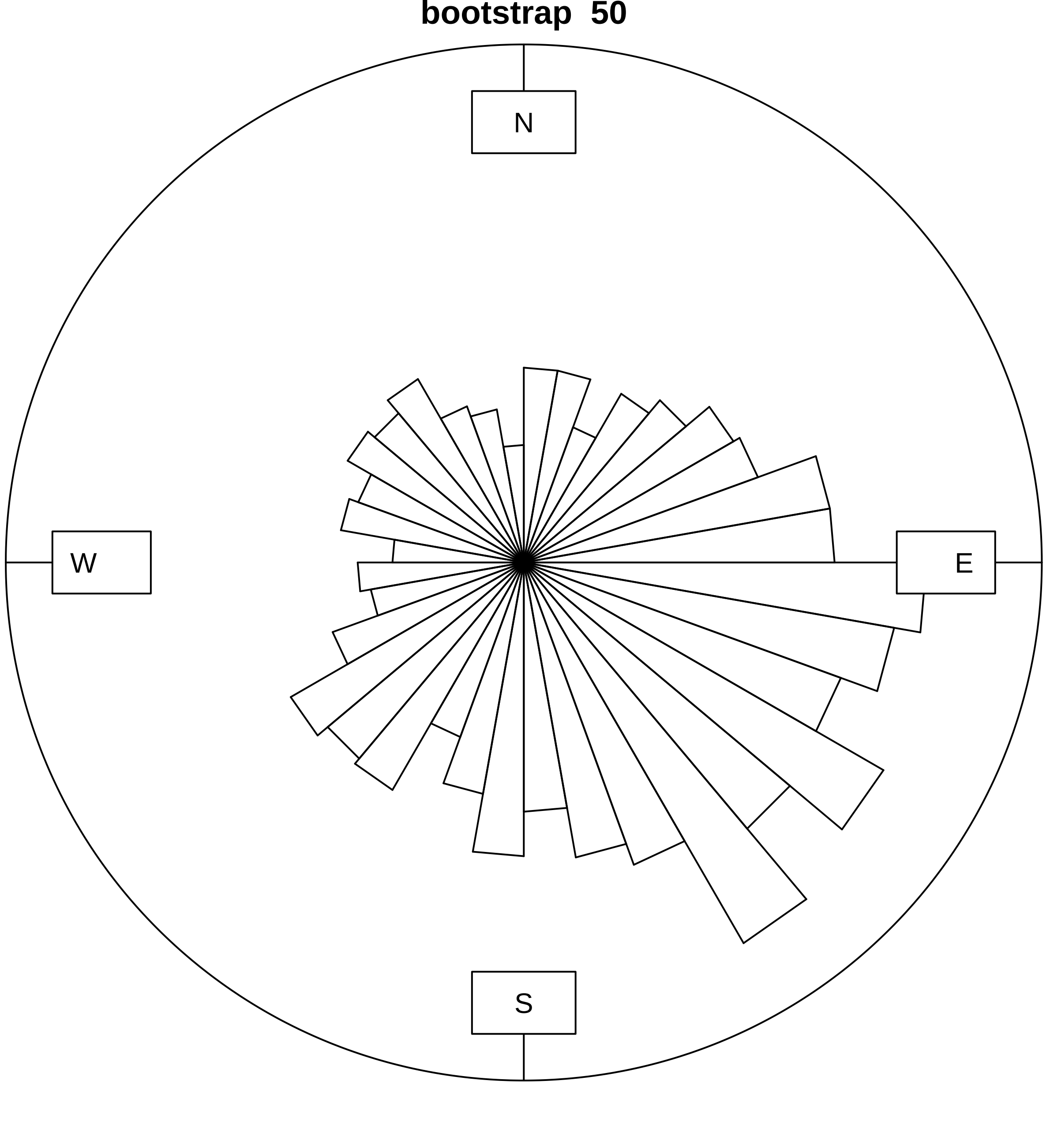
bootstrap 50
N
W
E
S

bootstrap 51

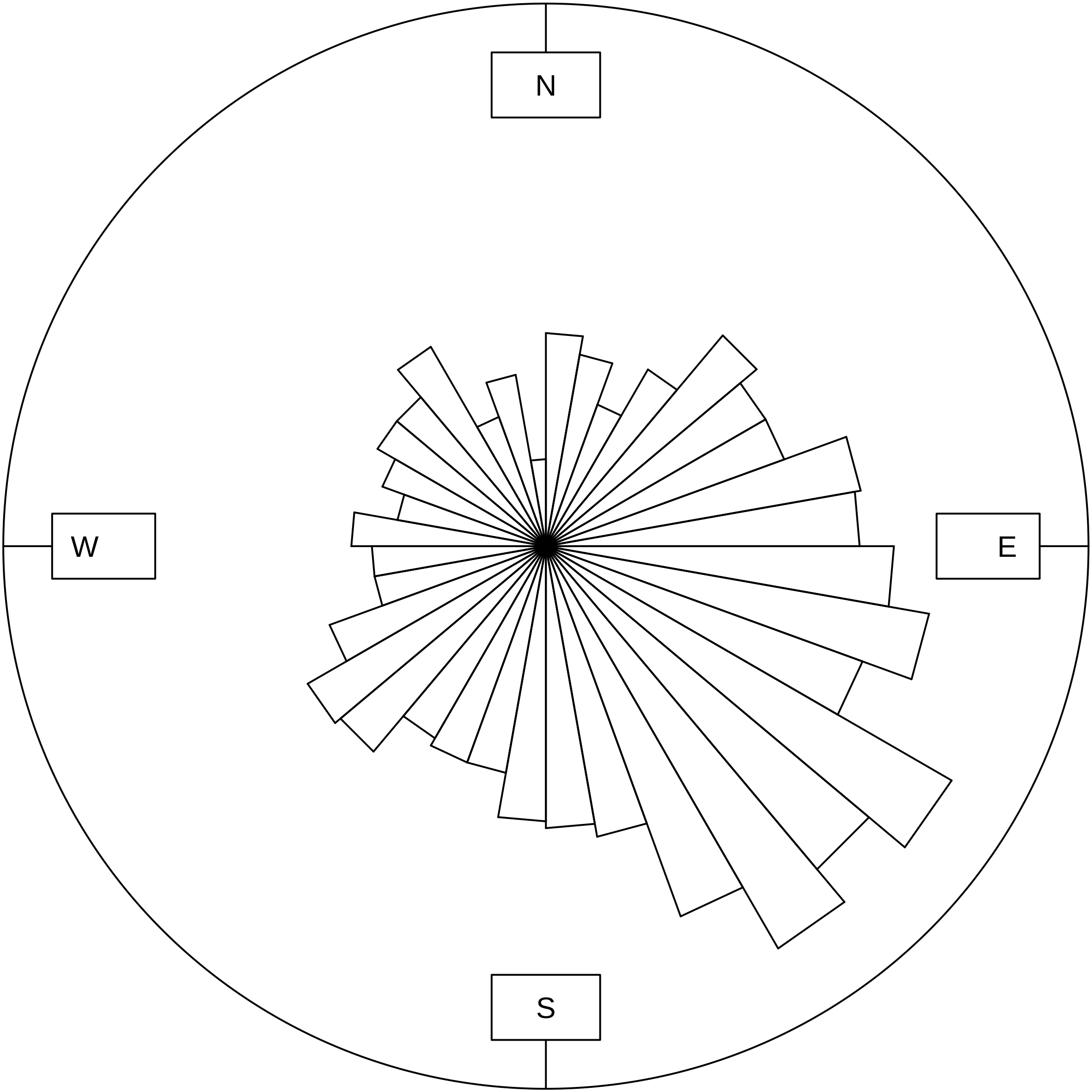

bootstrap 52

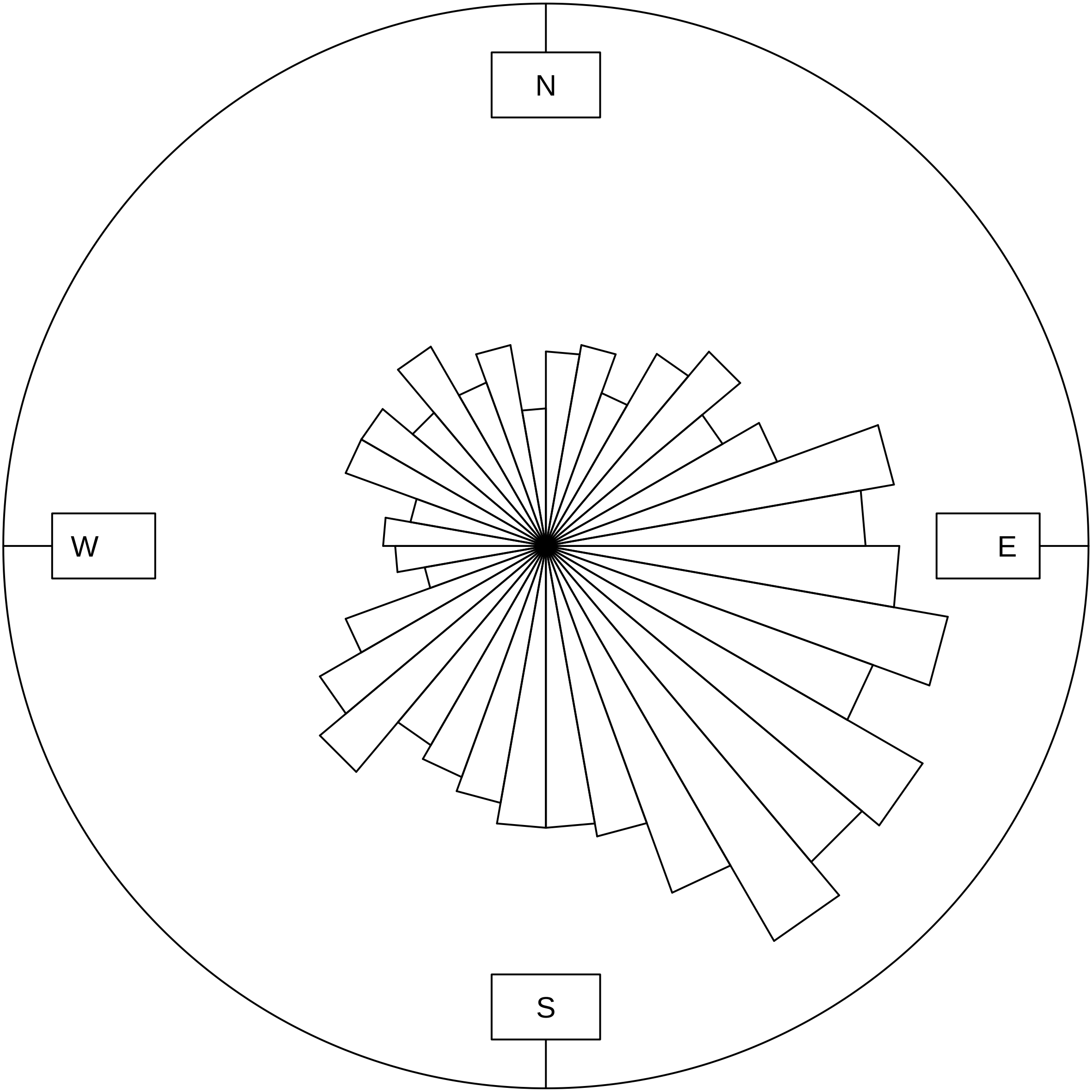

bootstrap 53

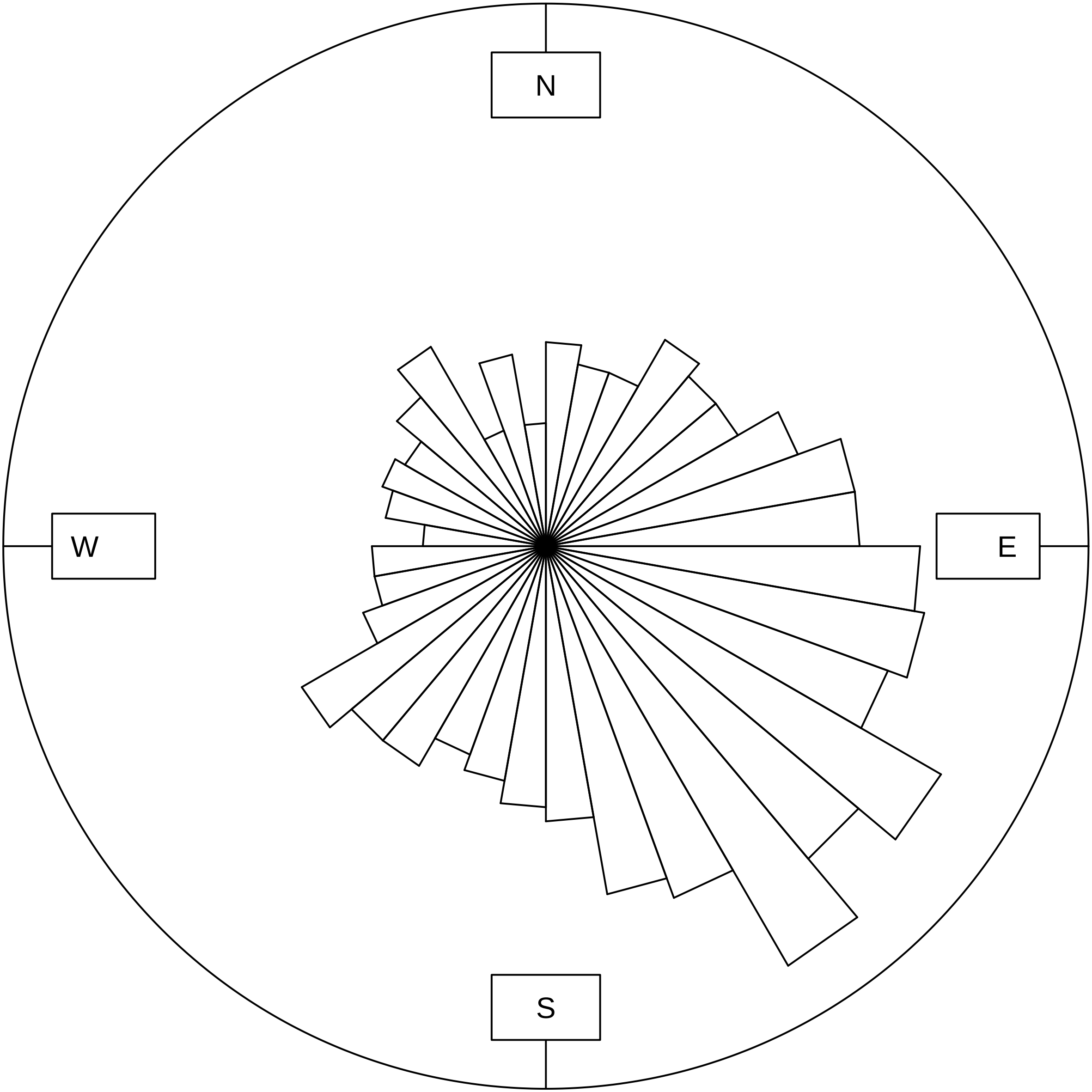

bootstrap 54

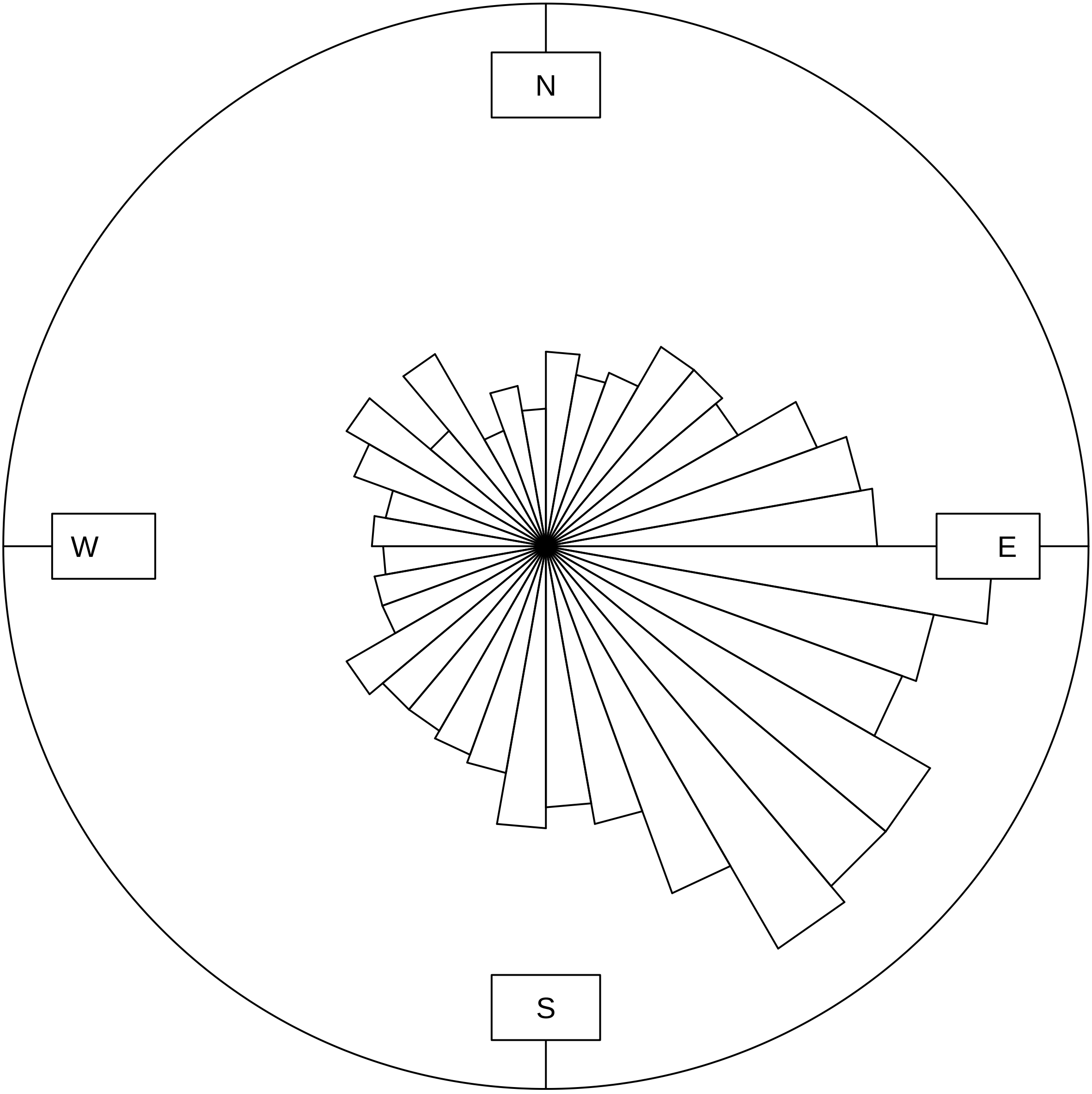

bootstrap 55

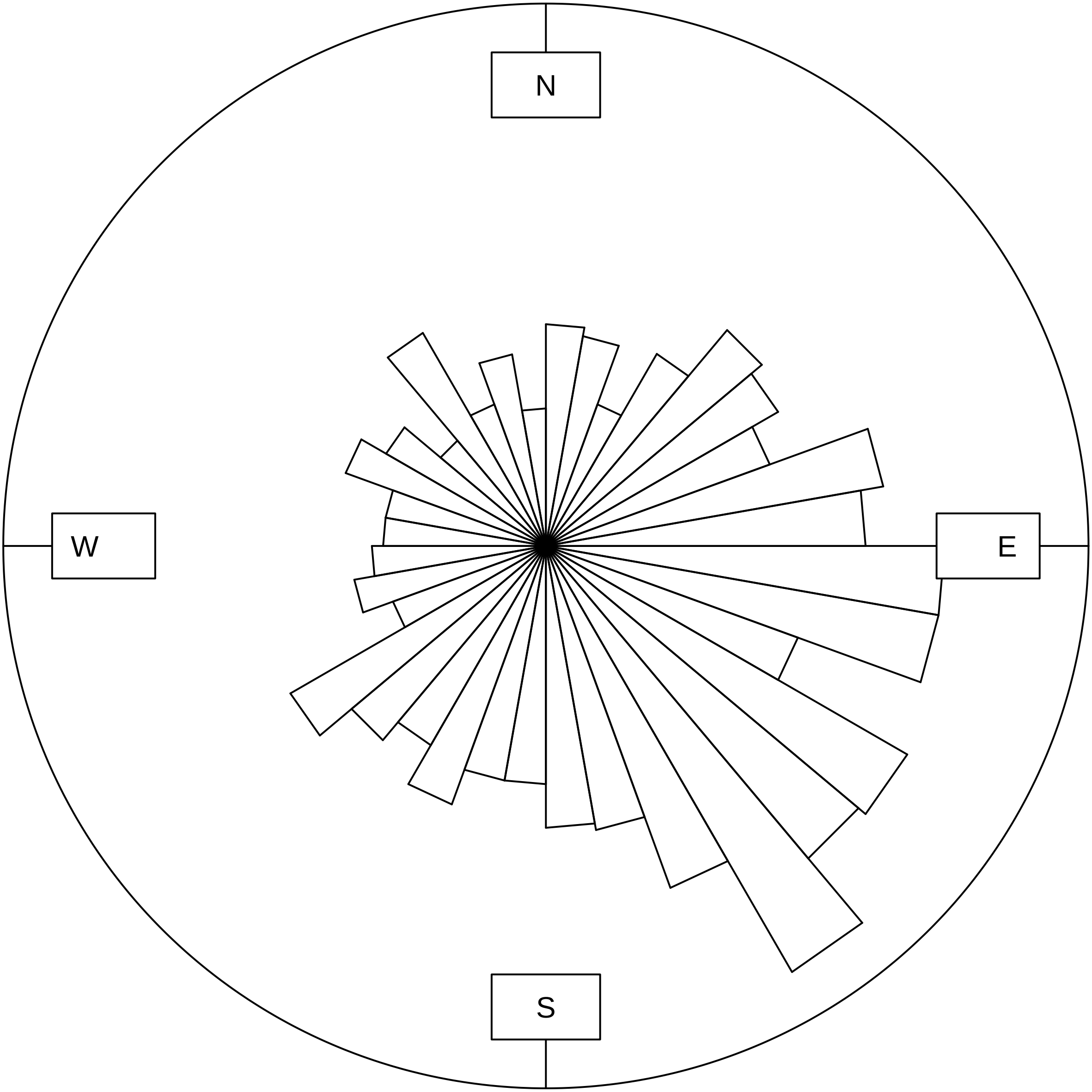

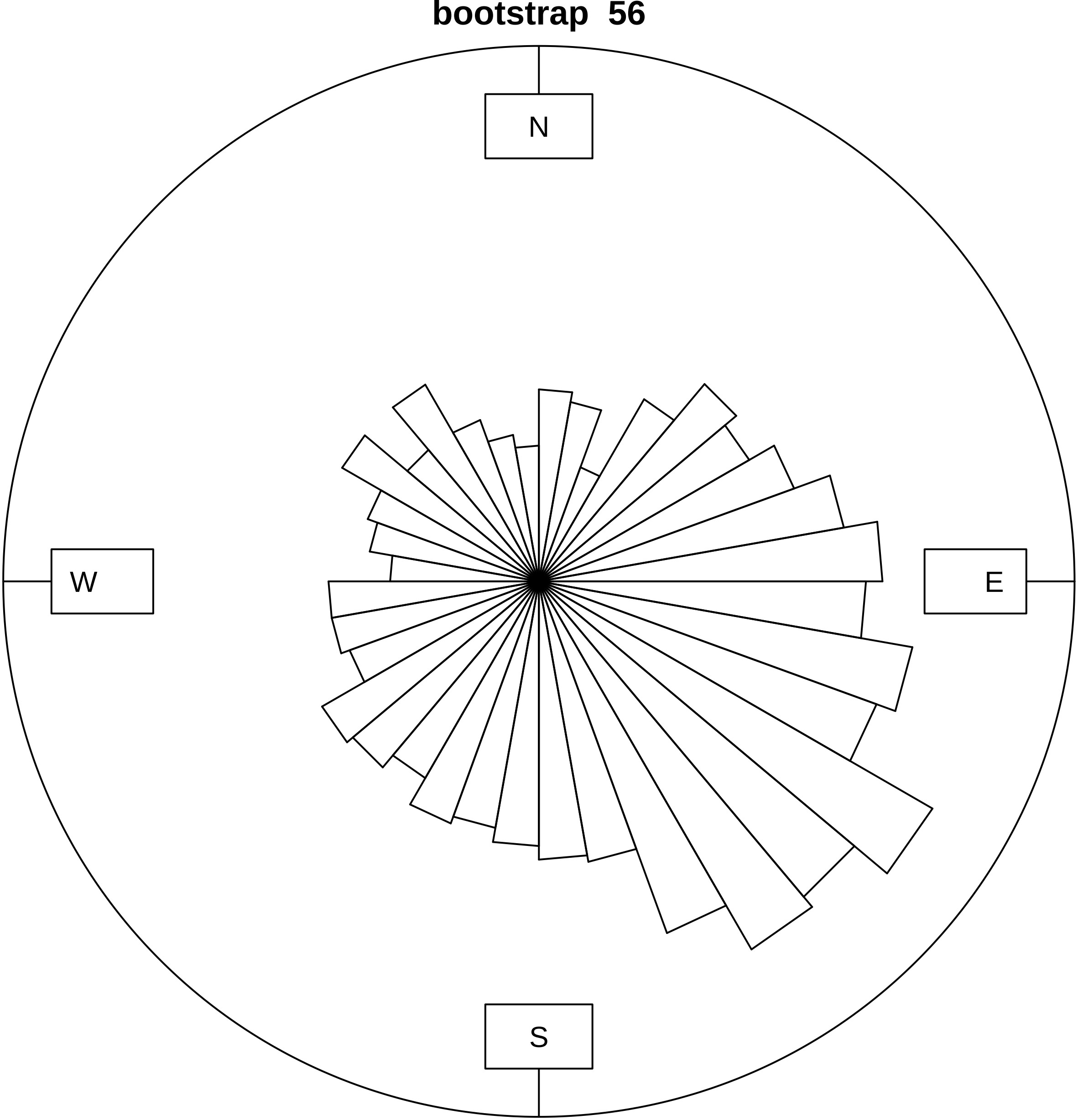
bootstrap 56
N
W
E
S

bootstrap 57

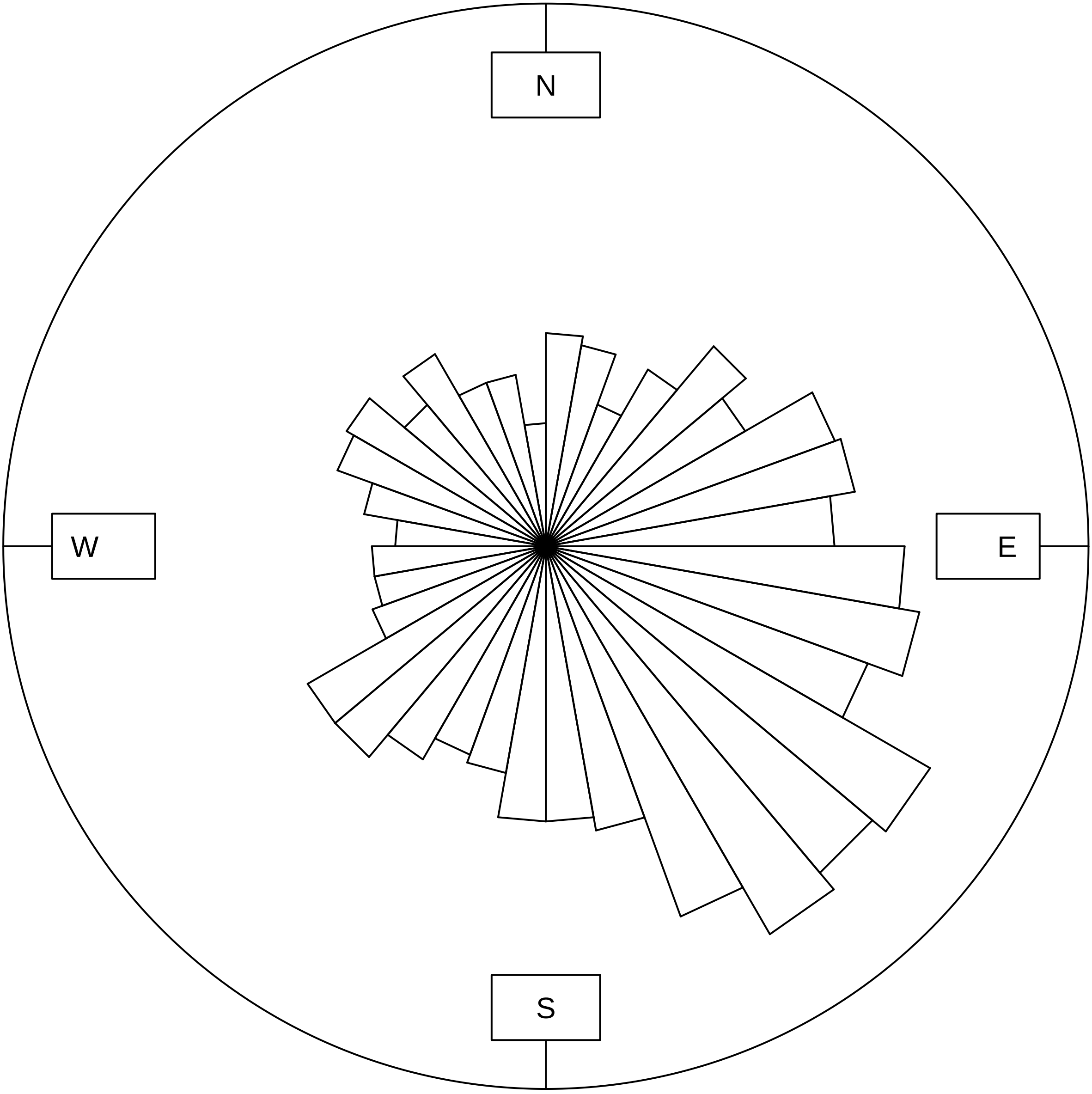

bootstrap 58

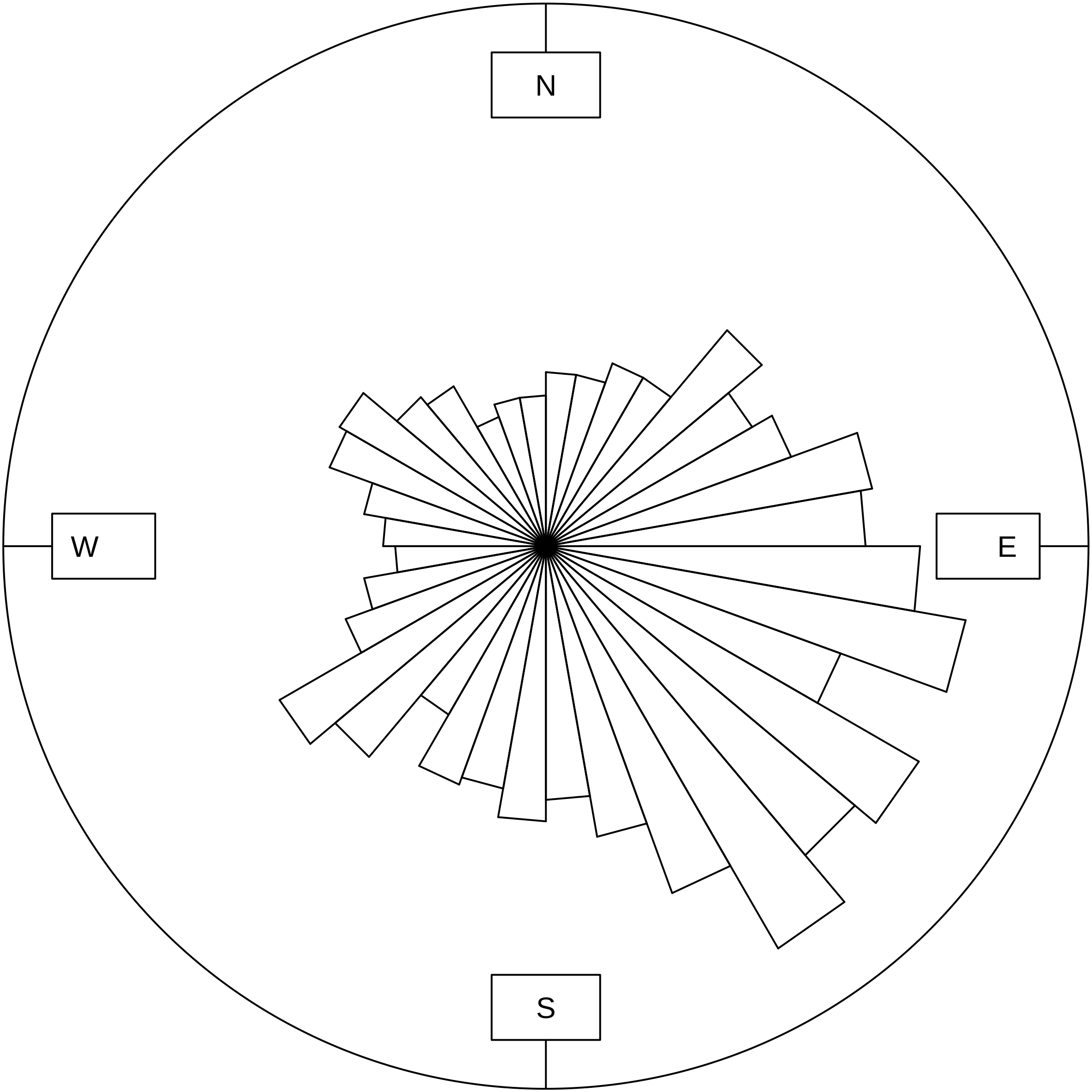

bootstrap 59

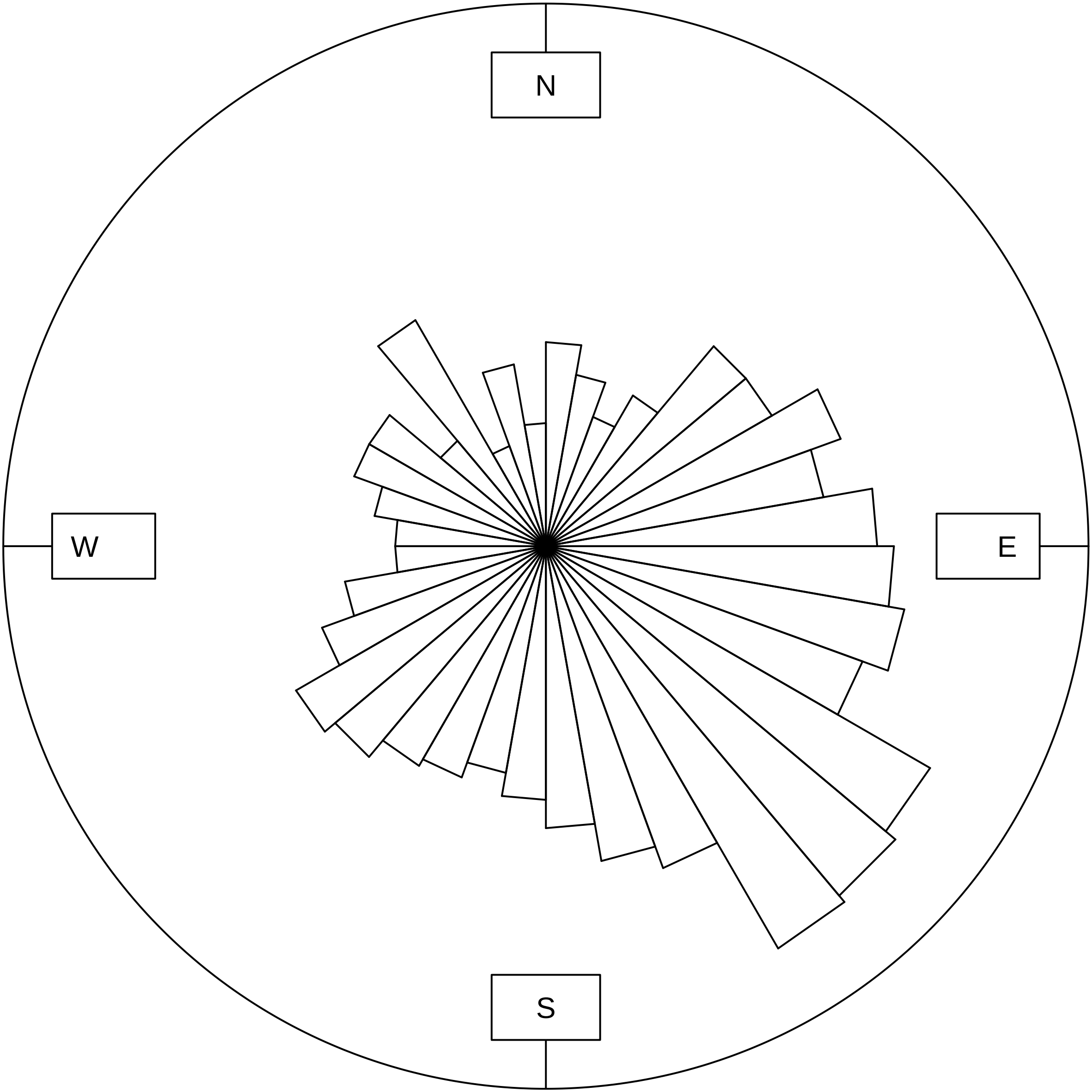

bootstrap 60

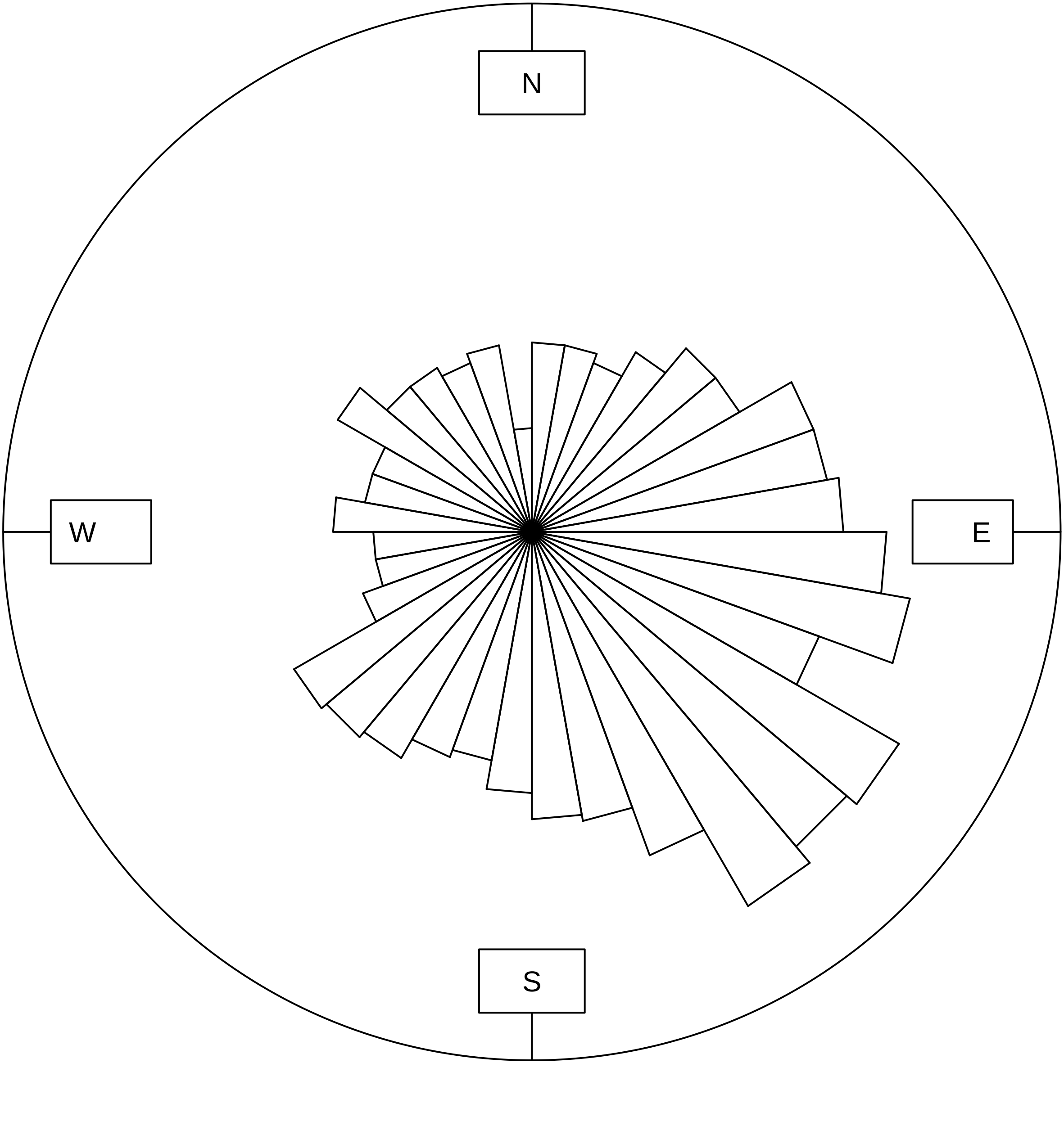

bootstrap 61

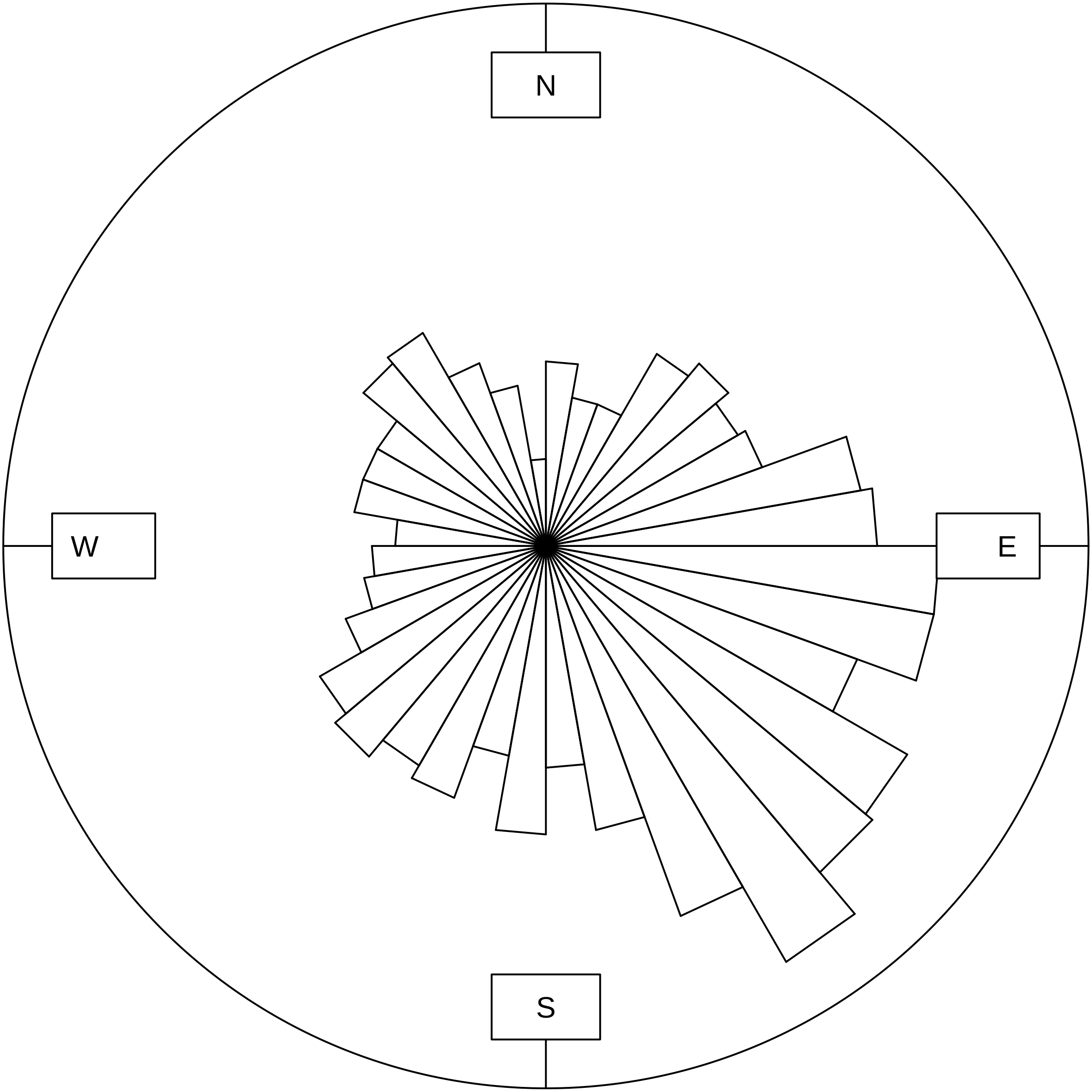

bootstrap 62

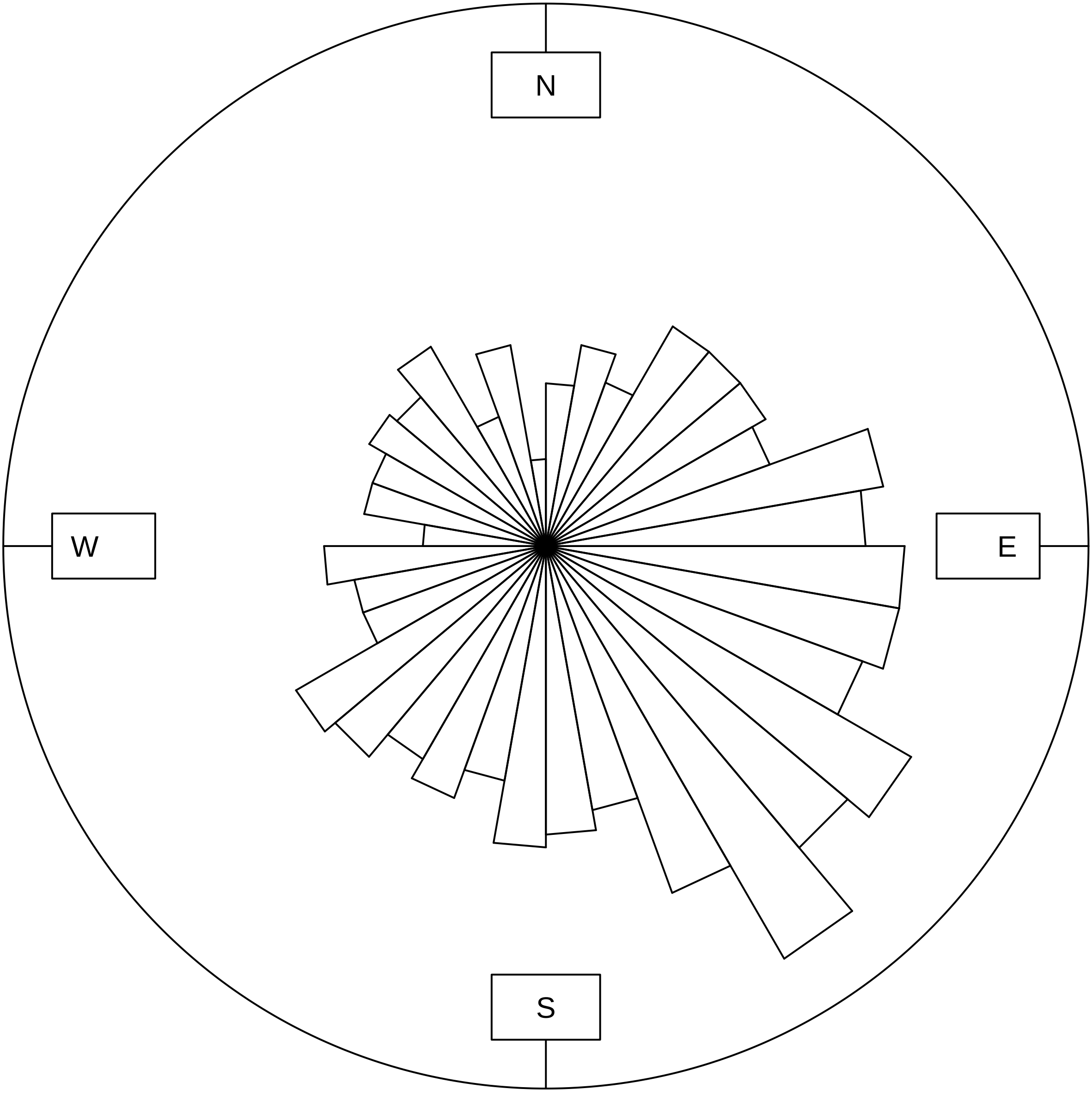

bootstrap 63

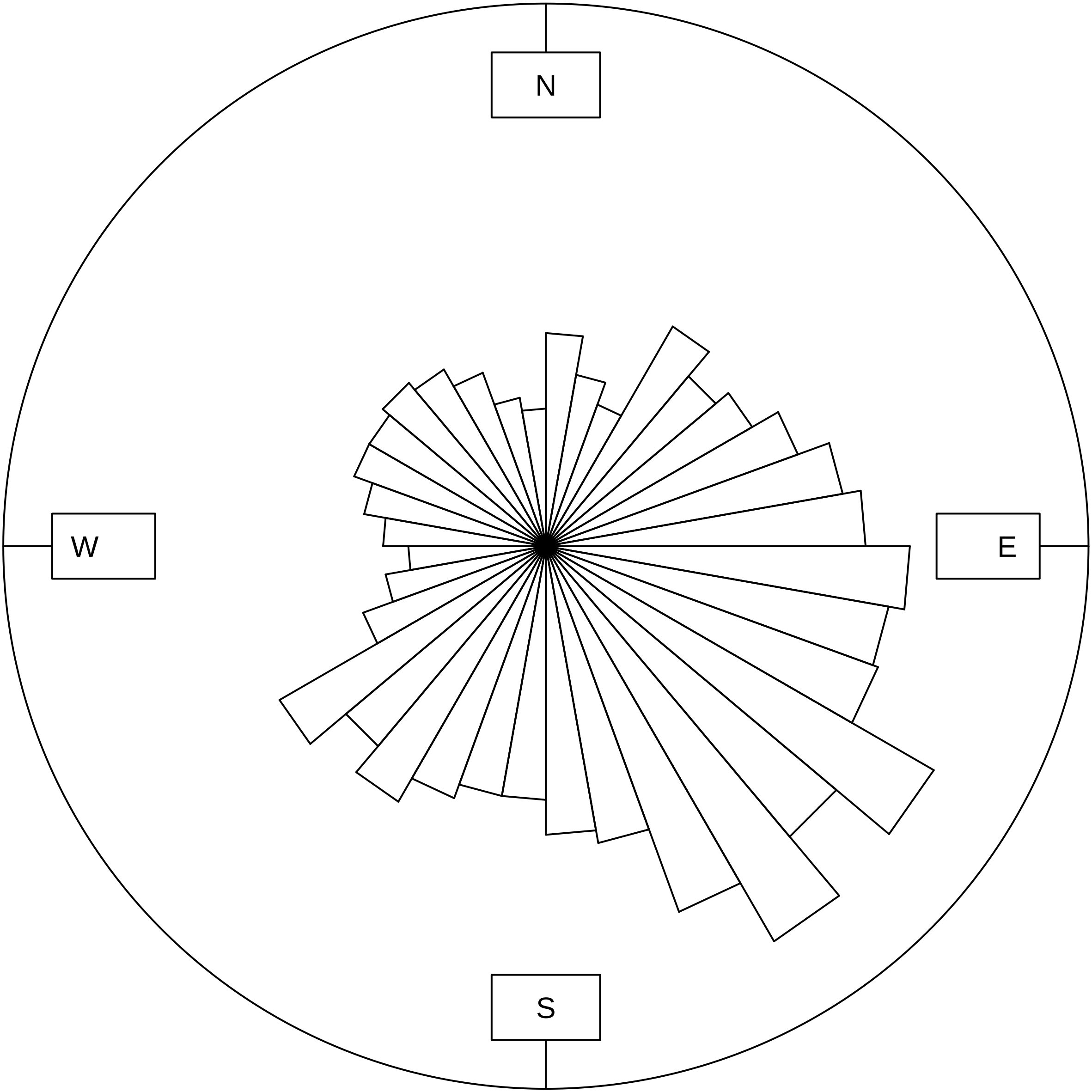

bootstrap 64

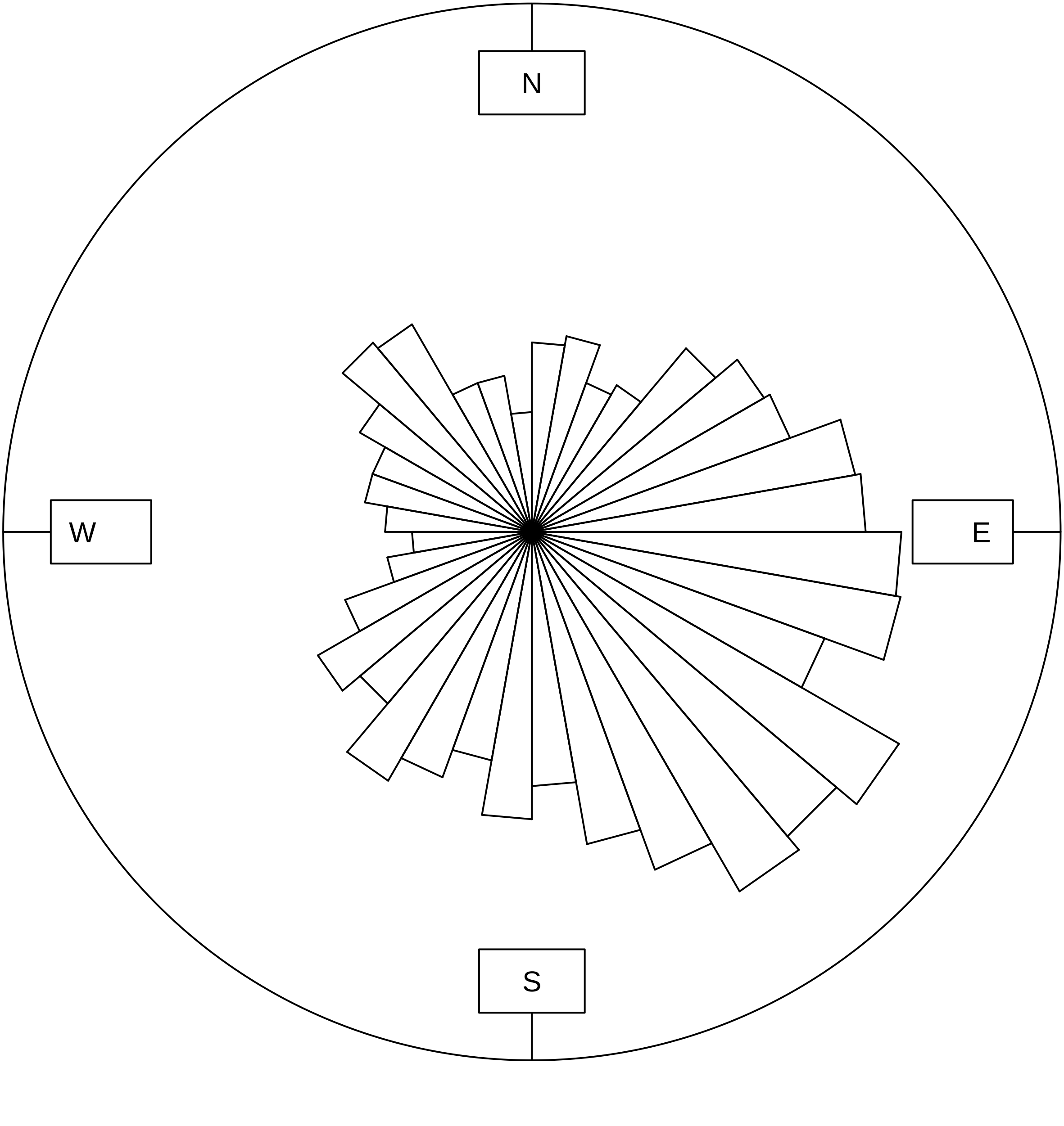

bootstrap 65

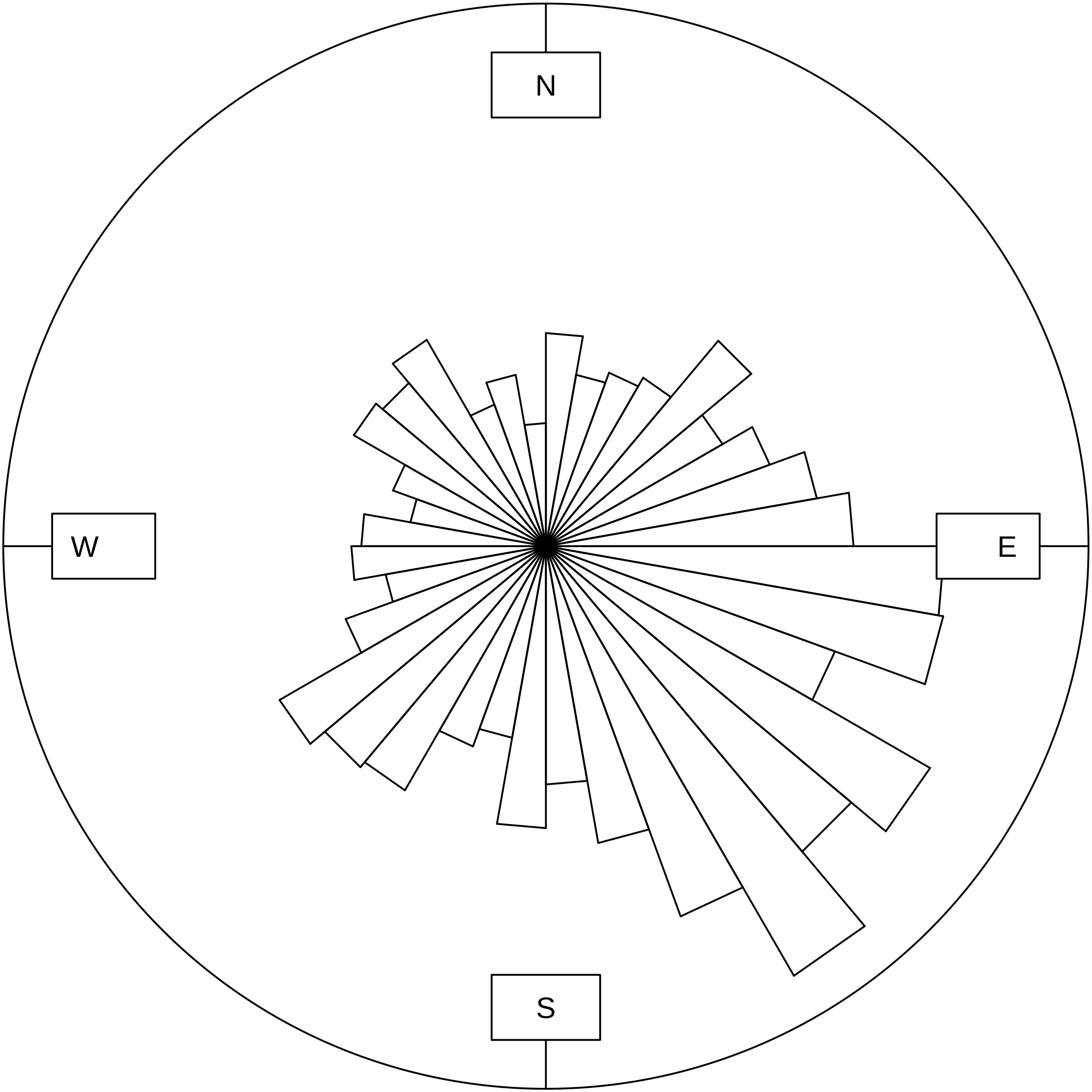

bootstrap 66

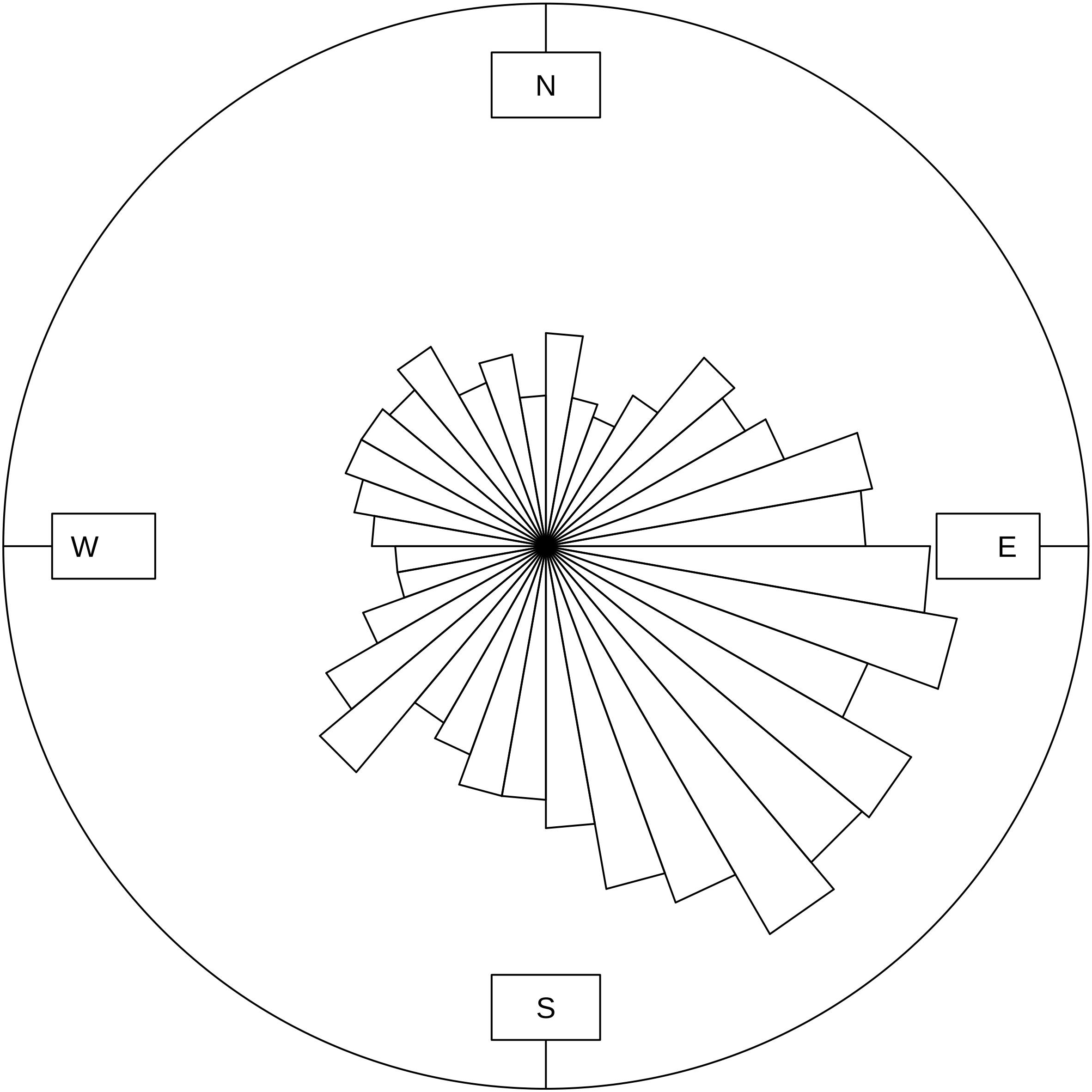

bootstrap 67

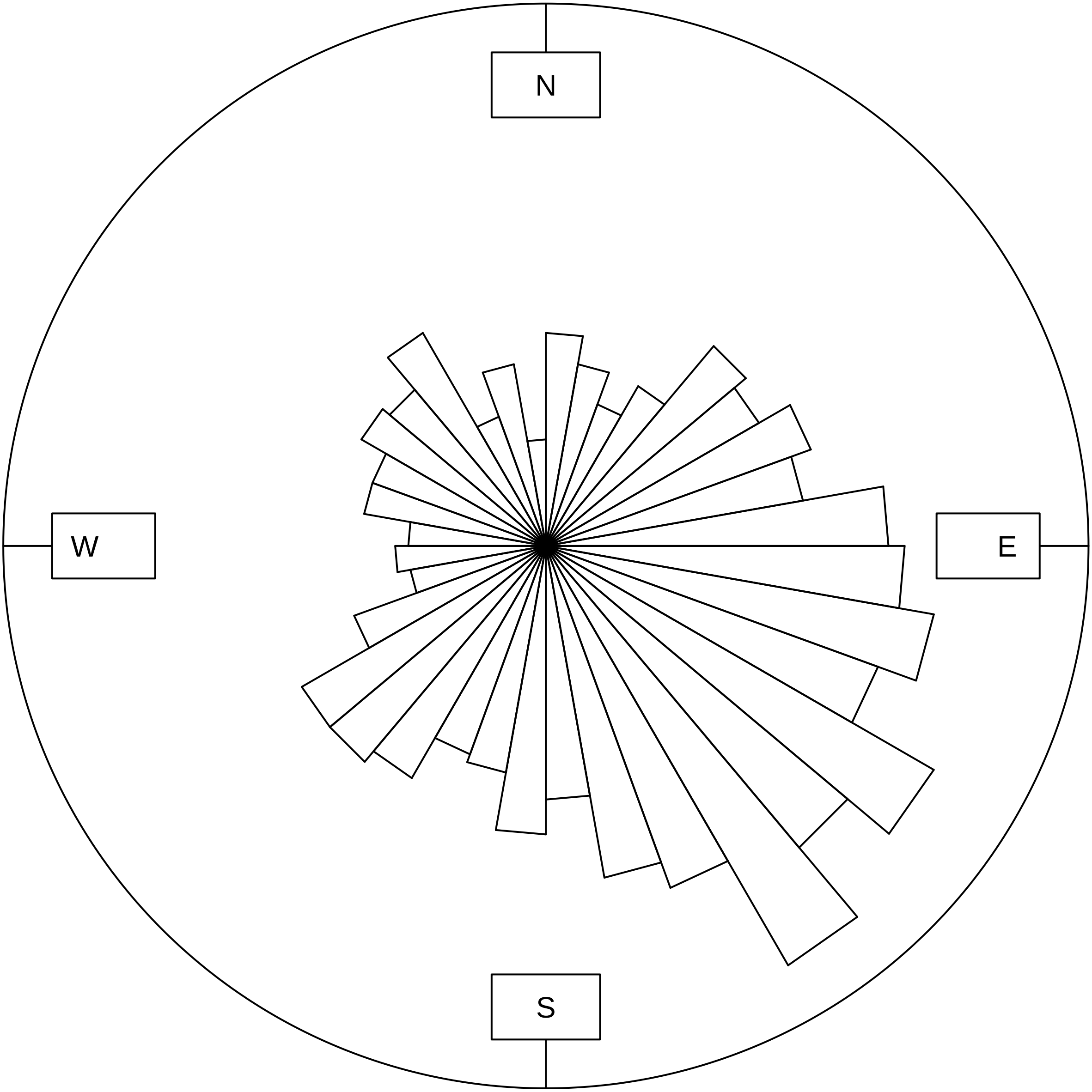

bootstrap 68

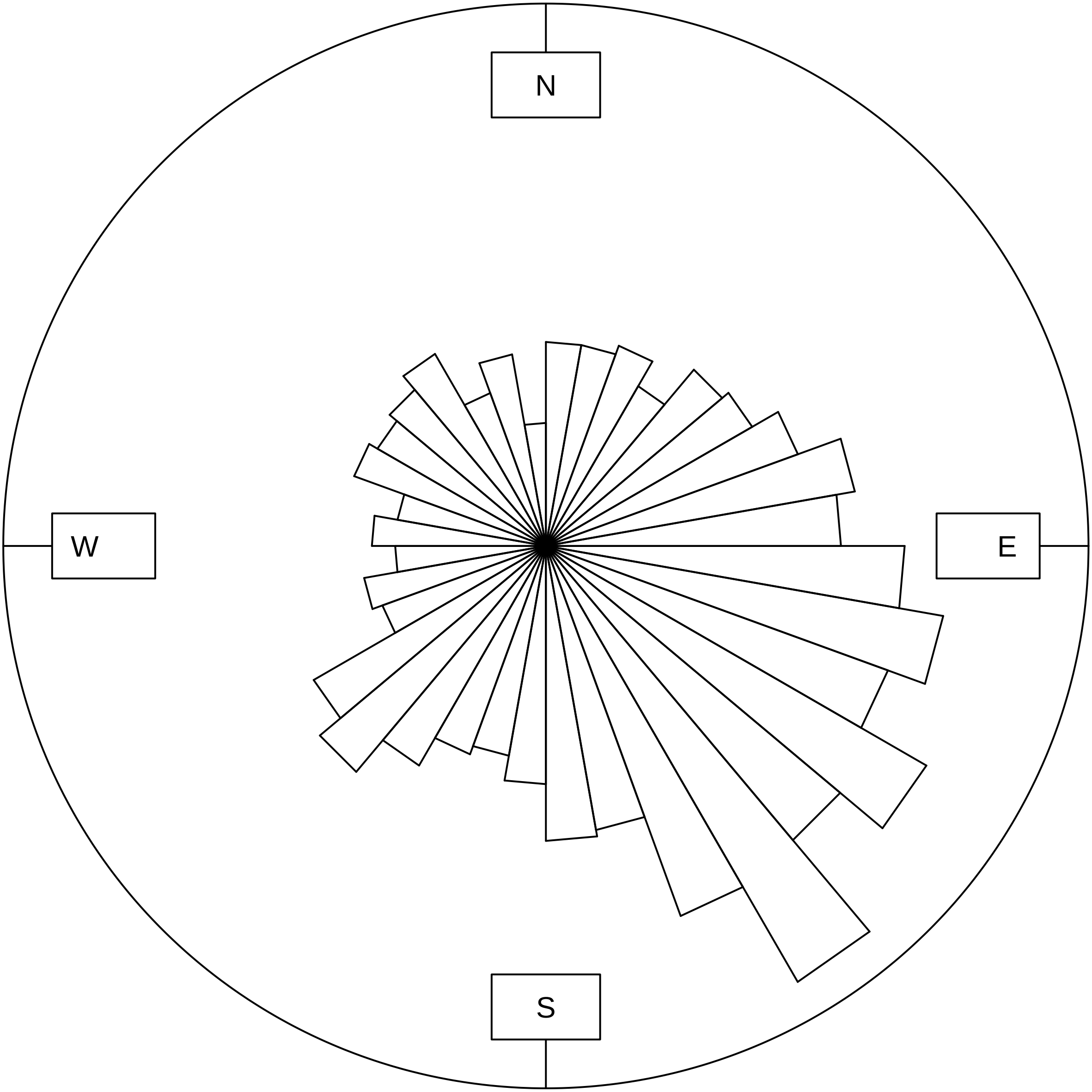

bootstrap 69

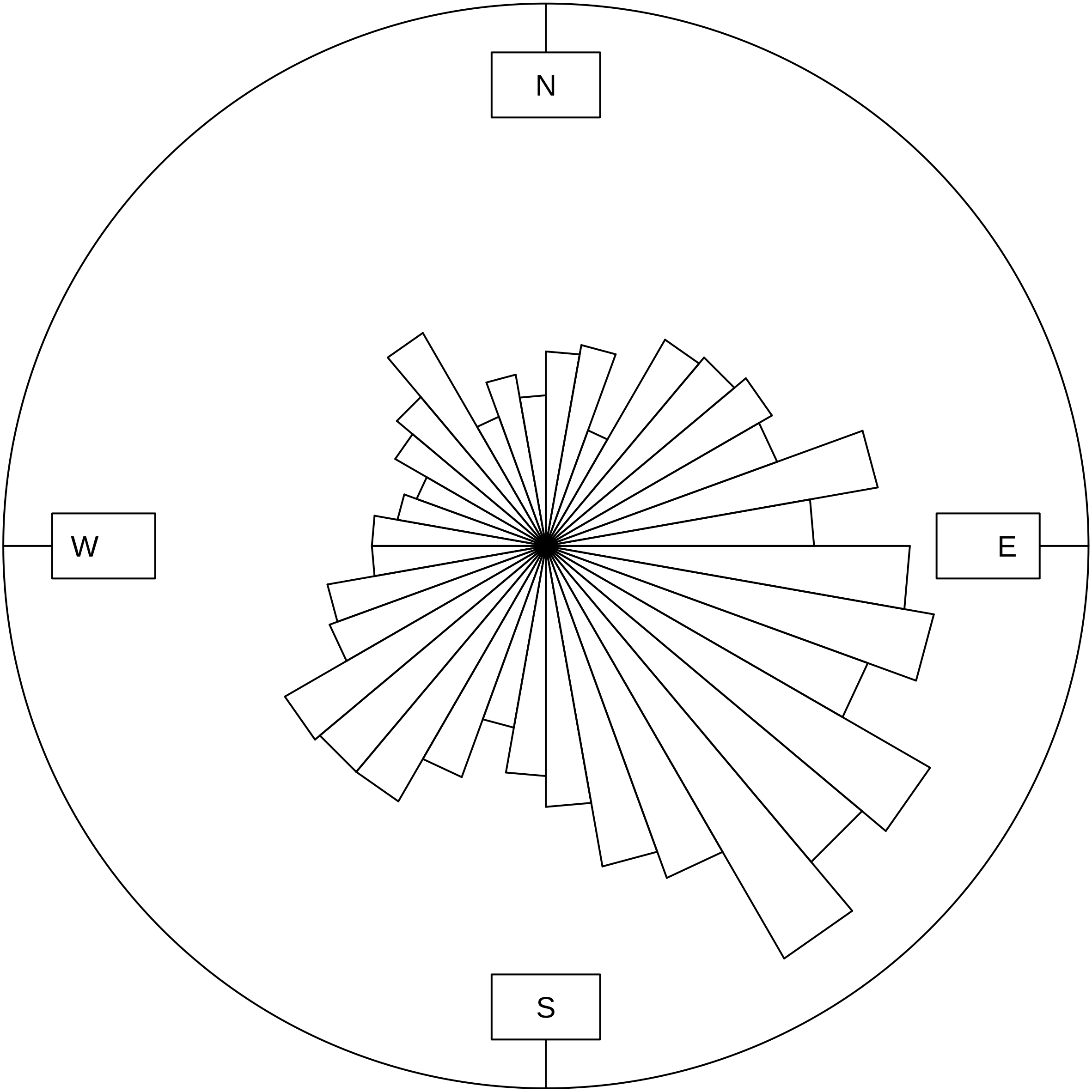

bootstrap 70

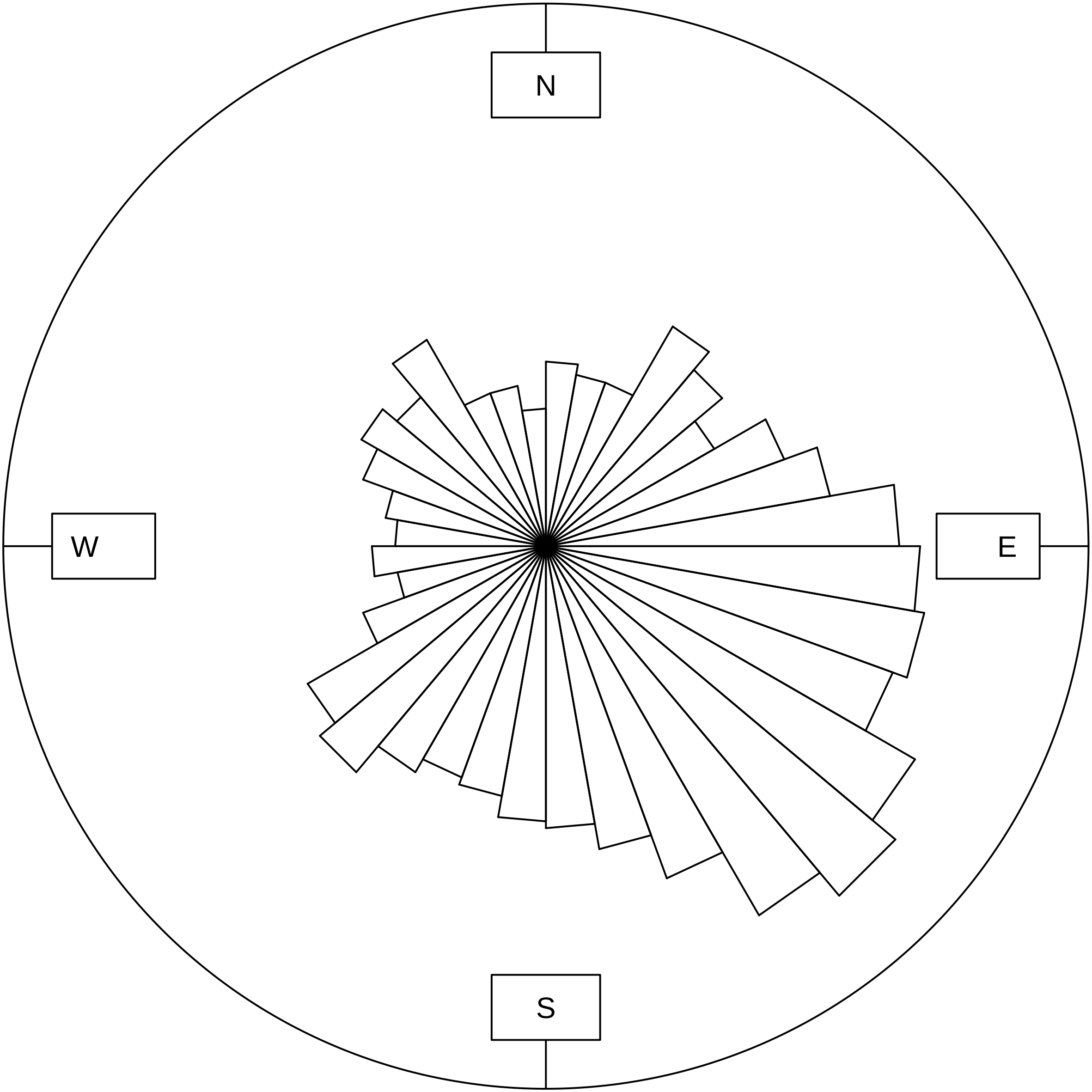

bootstrap 71

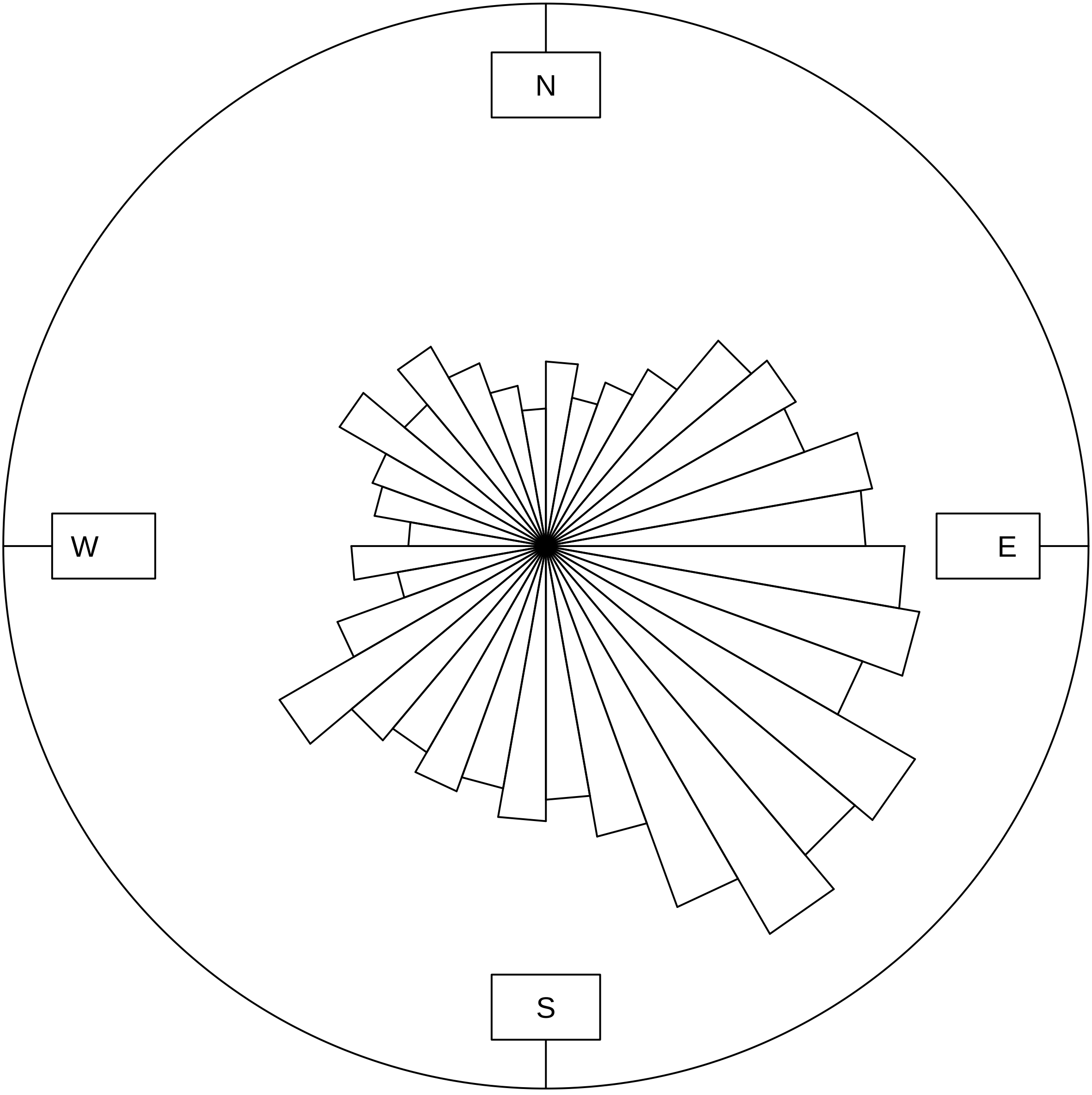

bootstrap 72

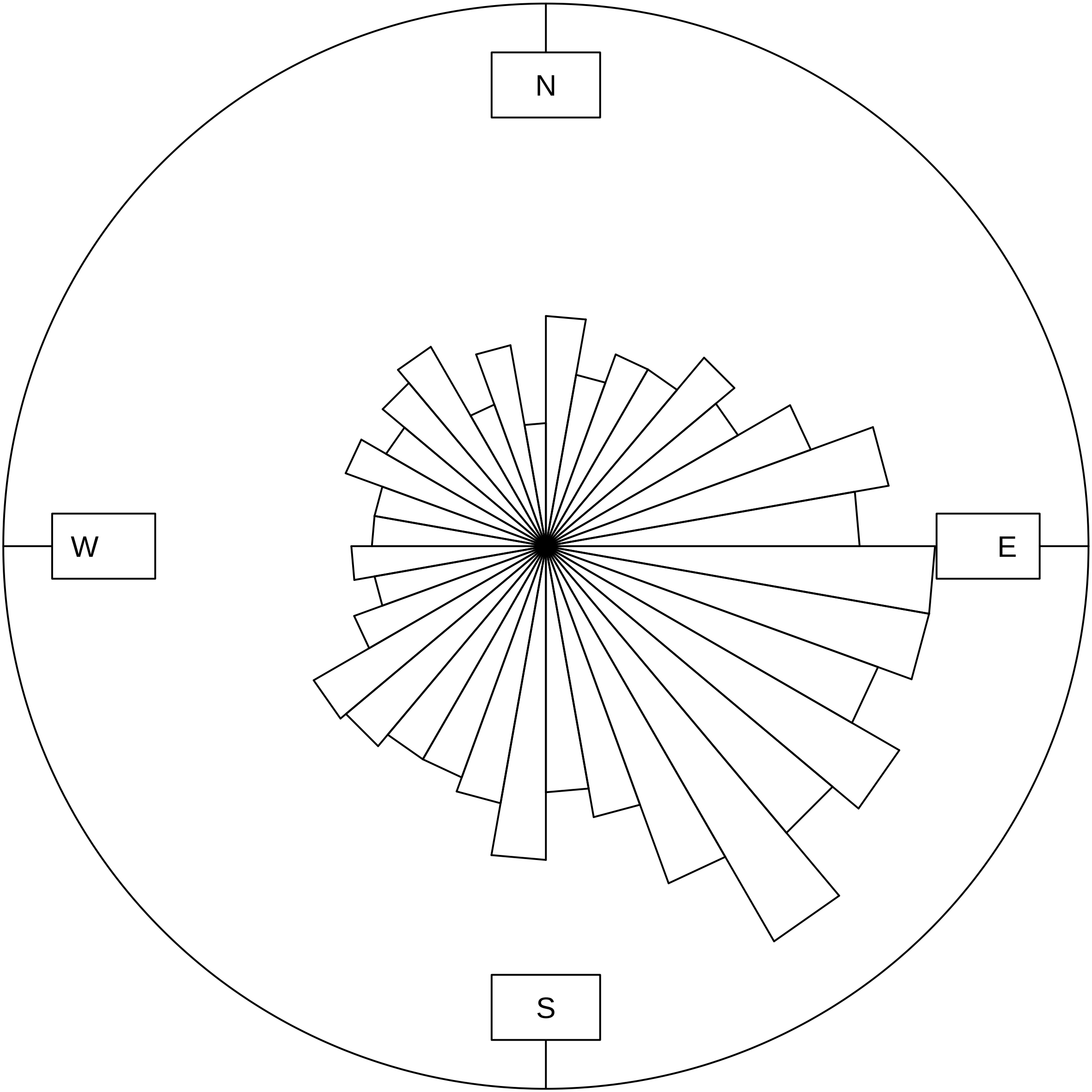

bootstrap 73

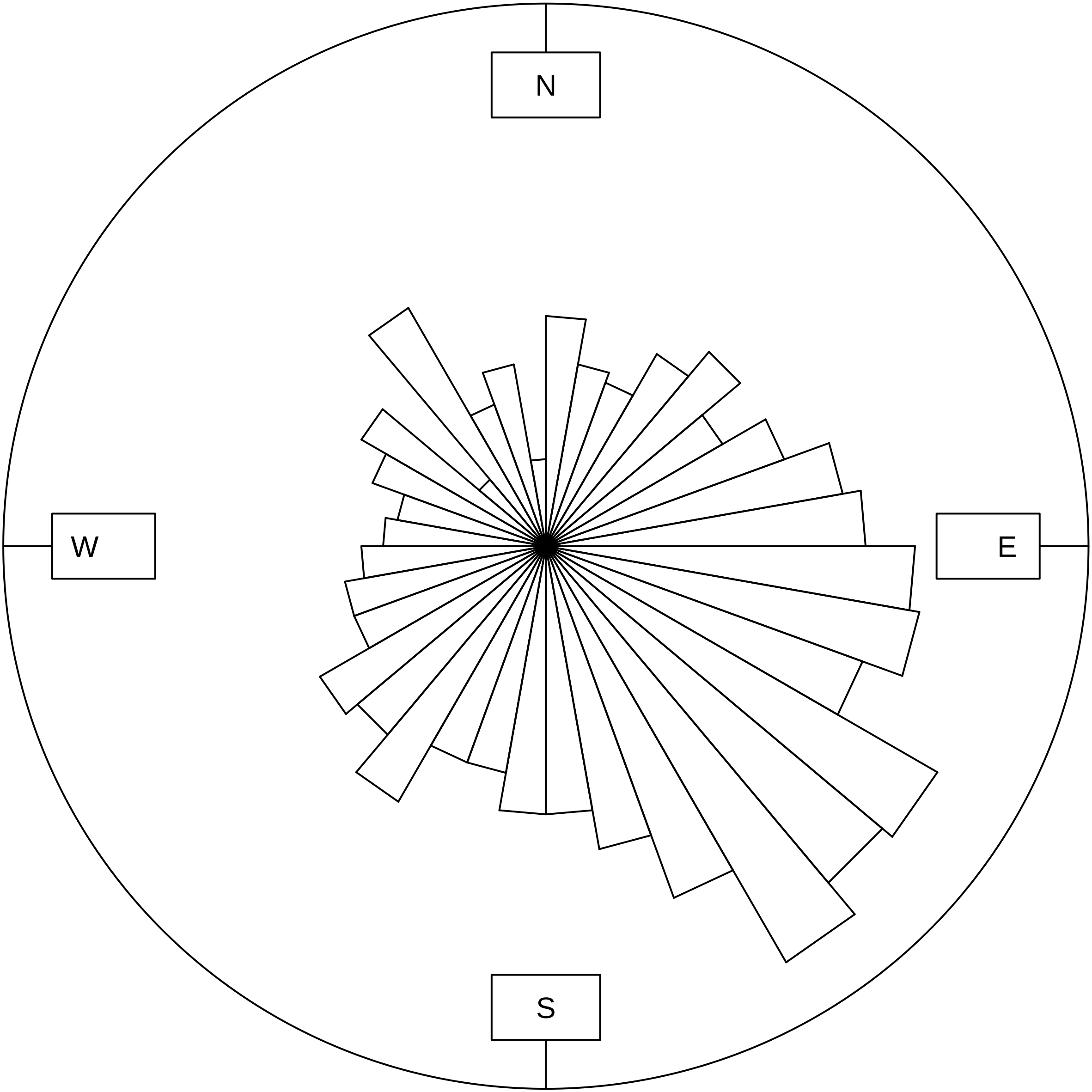

bootstrap 74

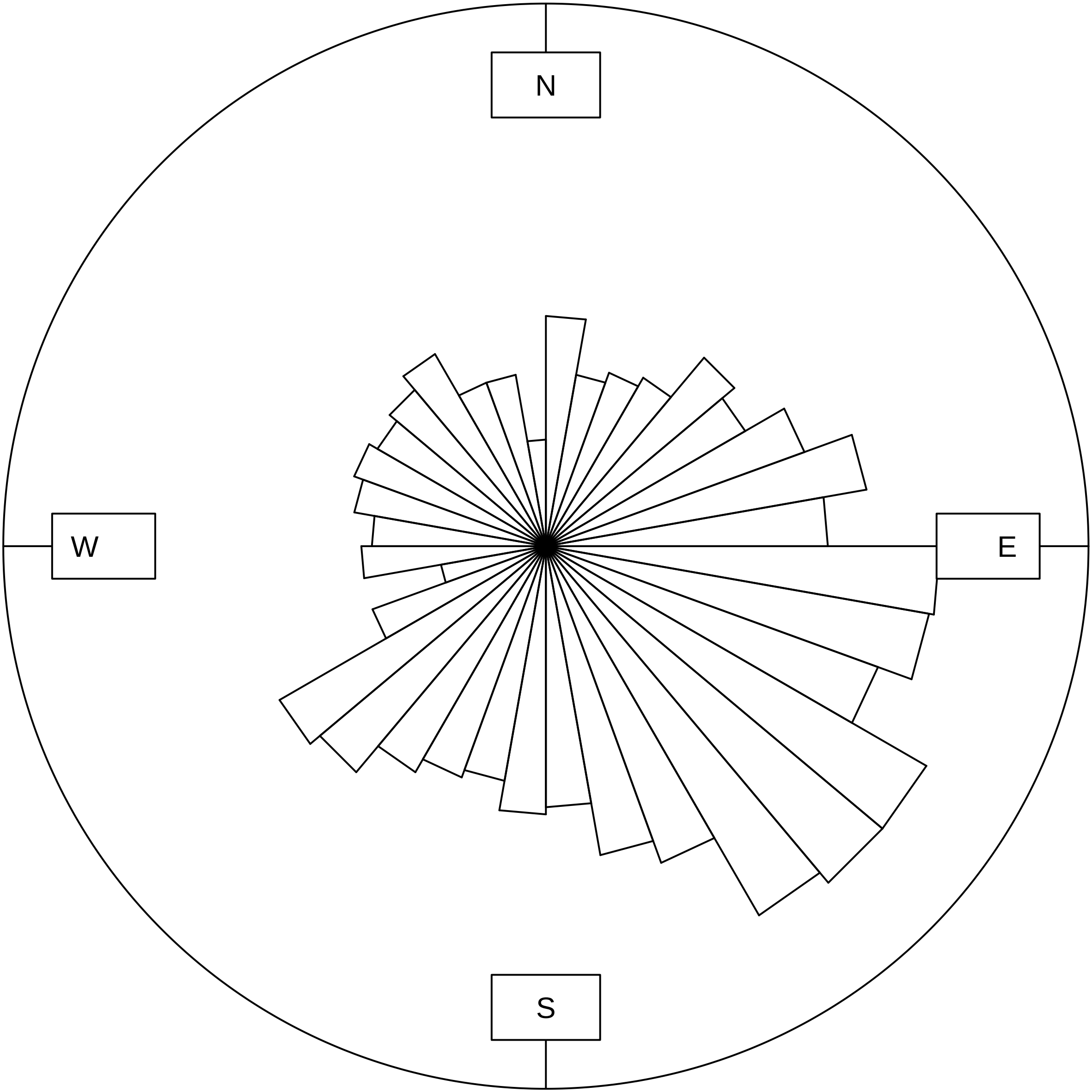

bootstrap 75

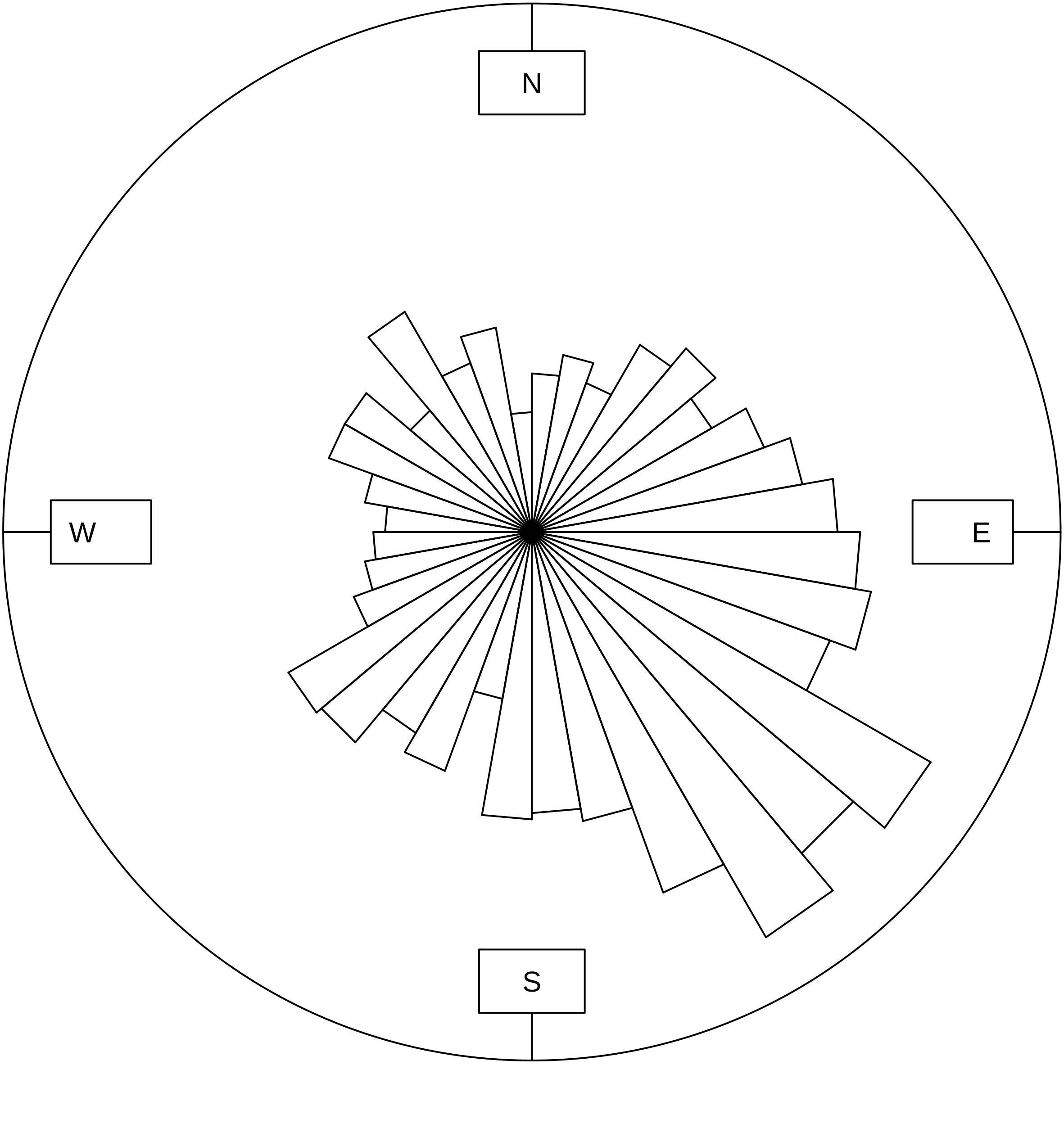

bootstrap 76

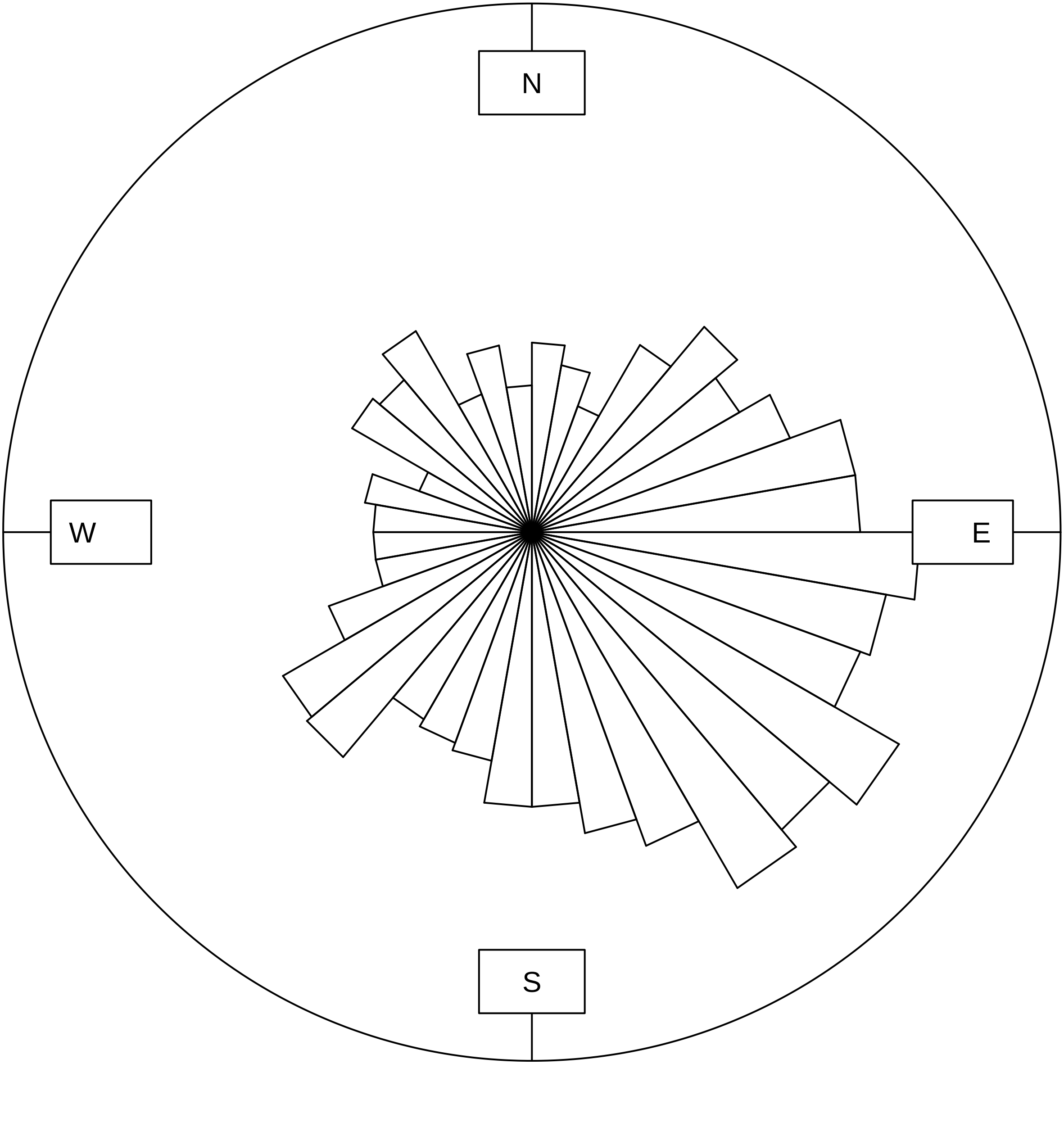

bootstrap 77

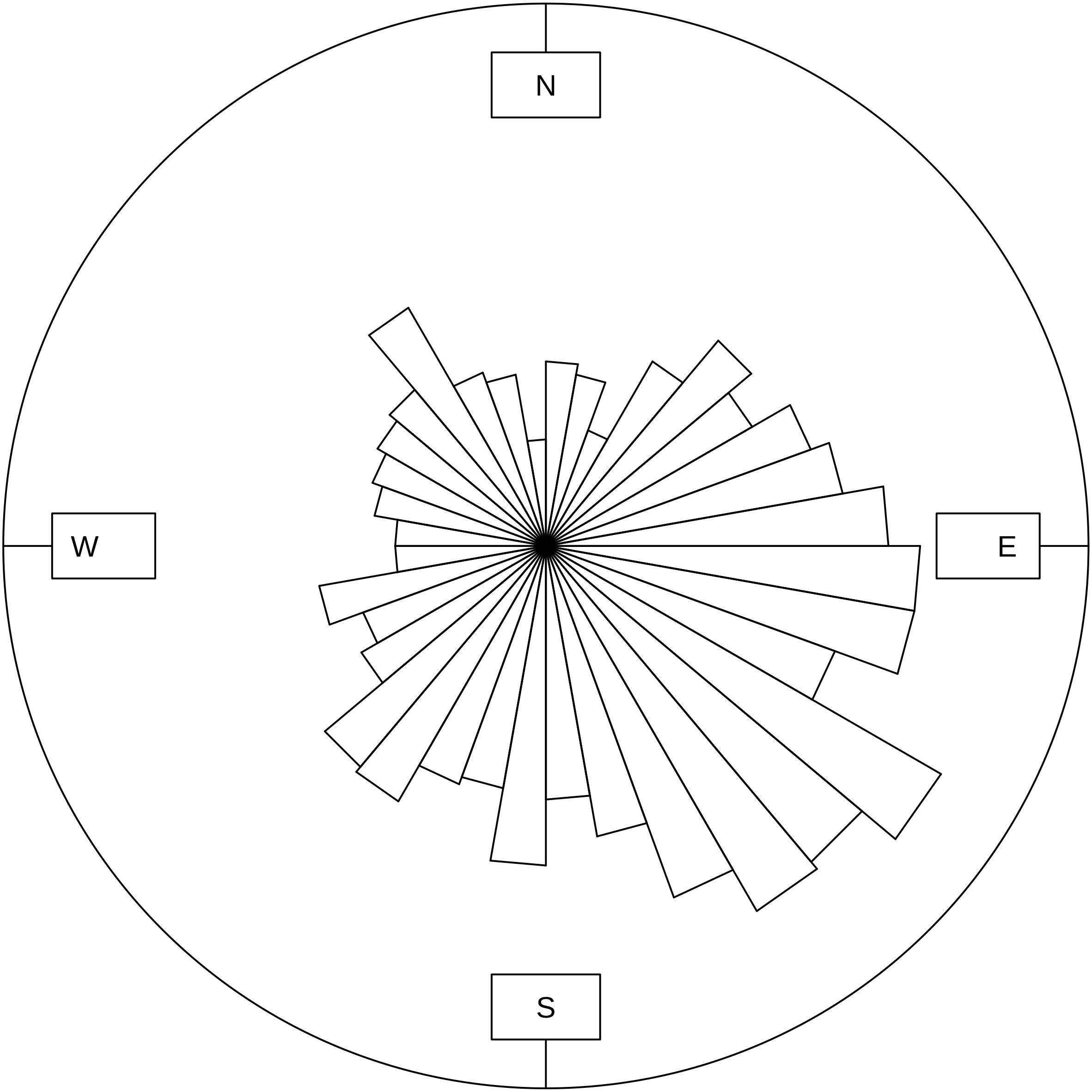

bootstrap 78

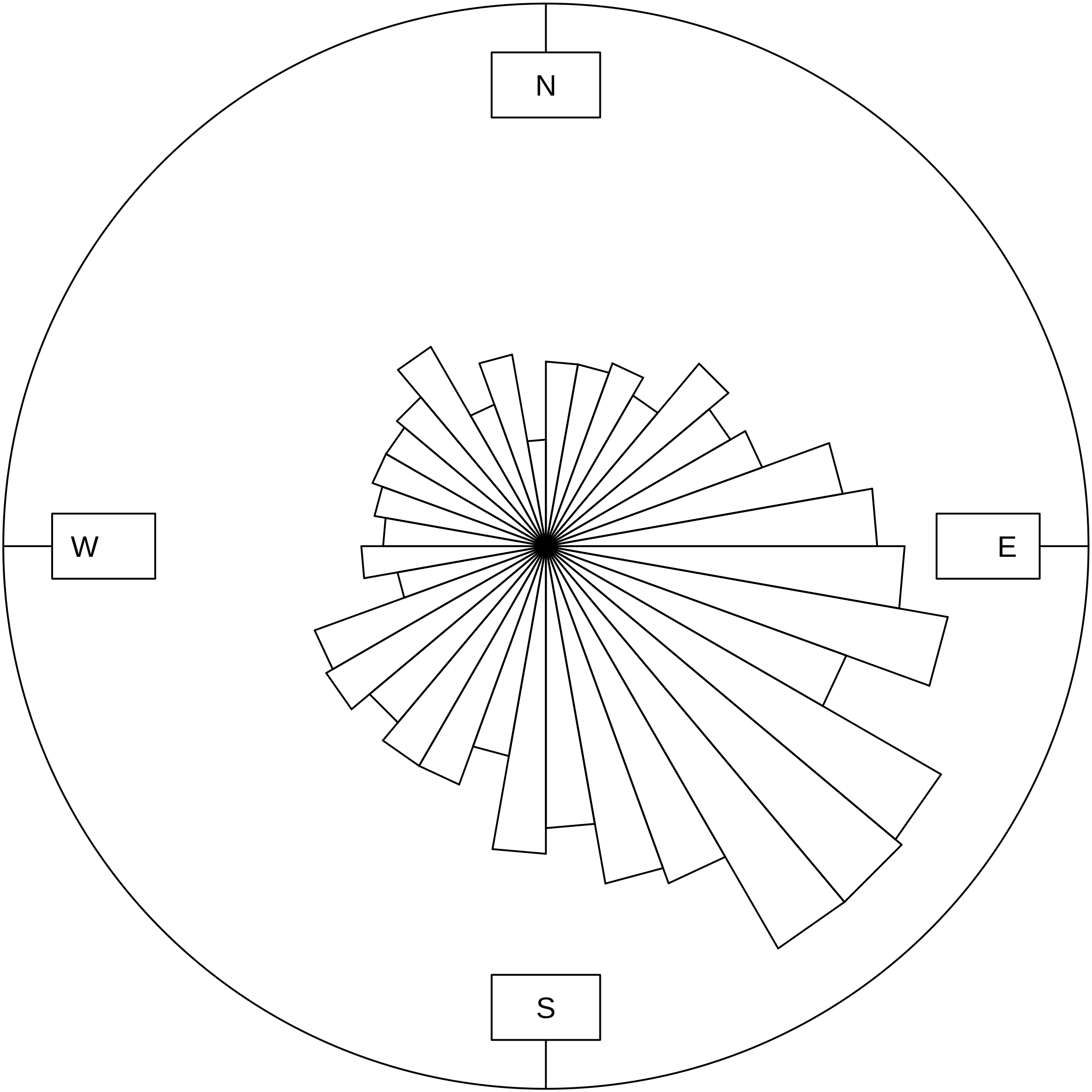

bootstrap 79

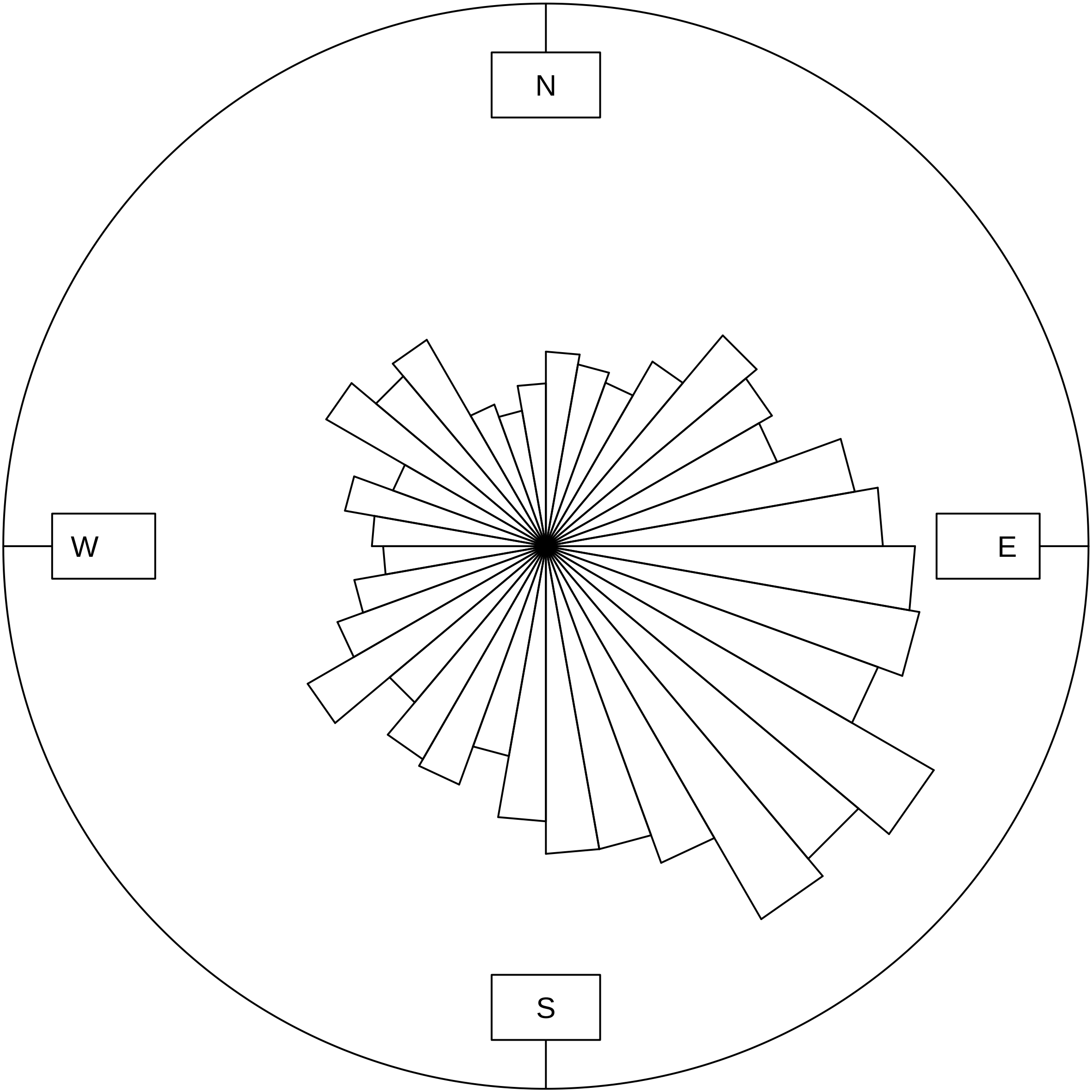

bootstrap 80

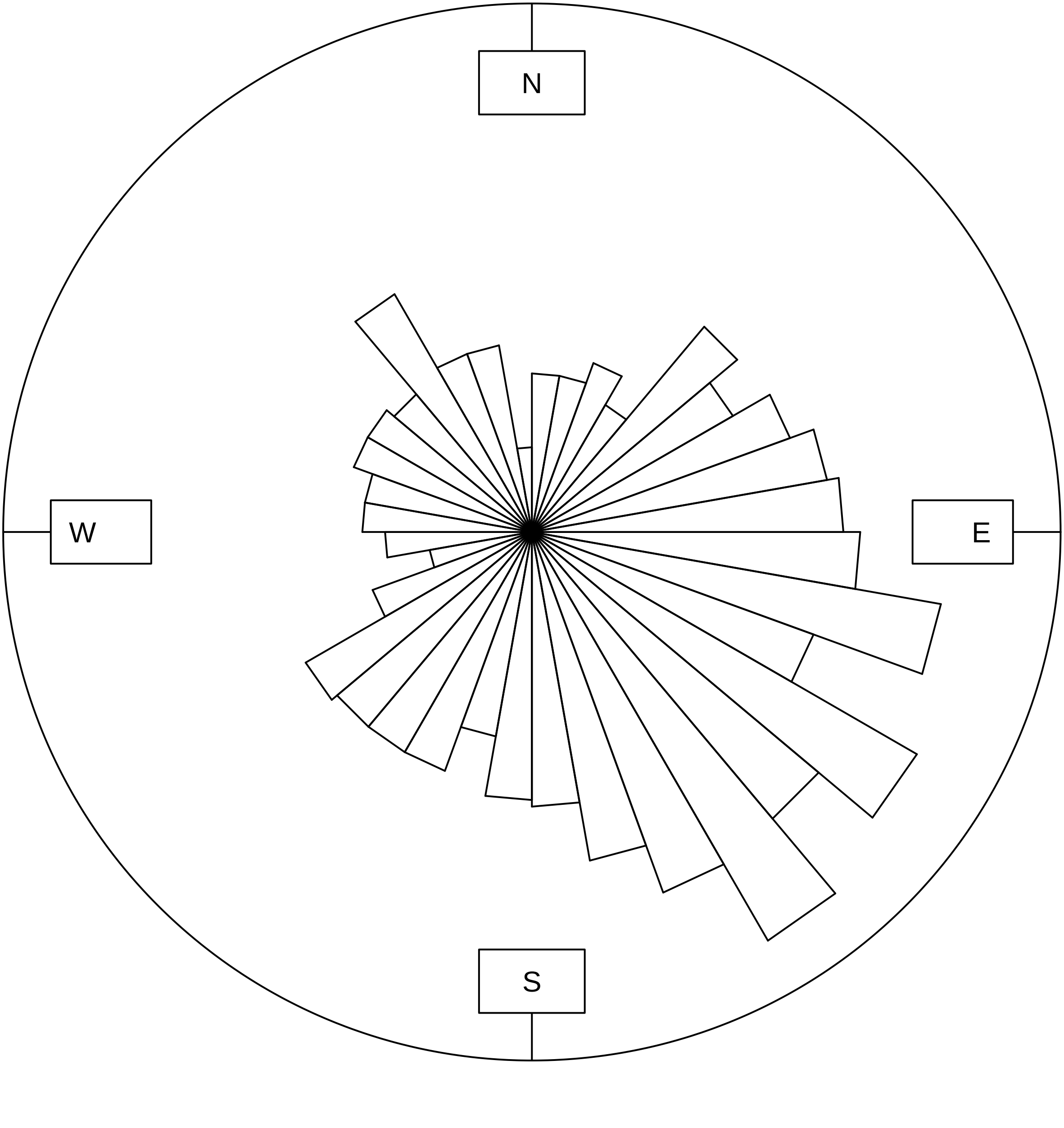

bootstrap 81

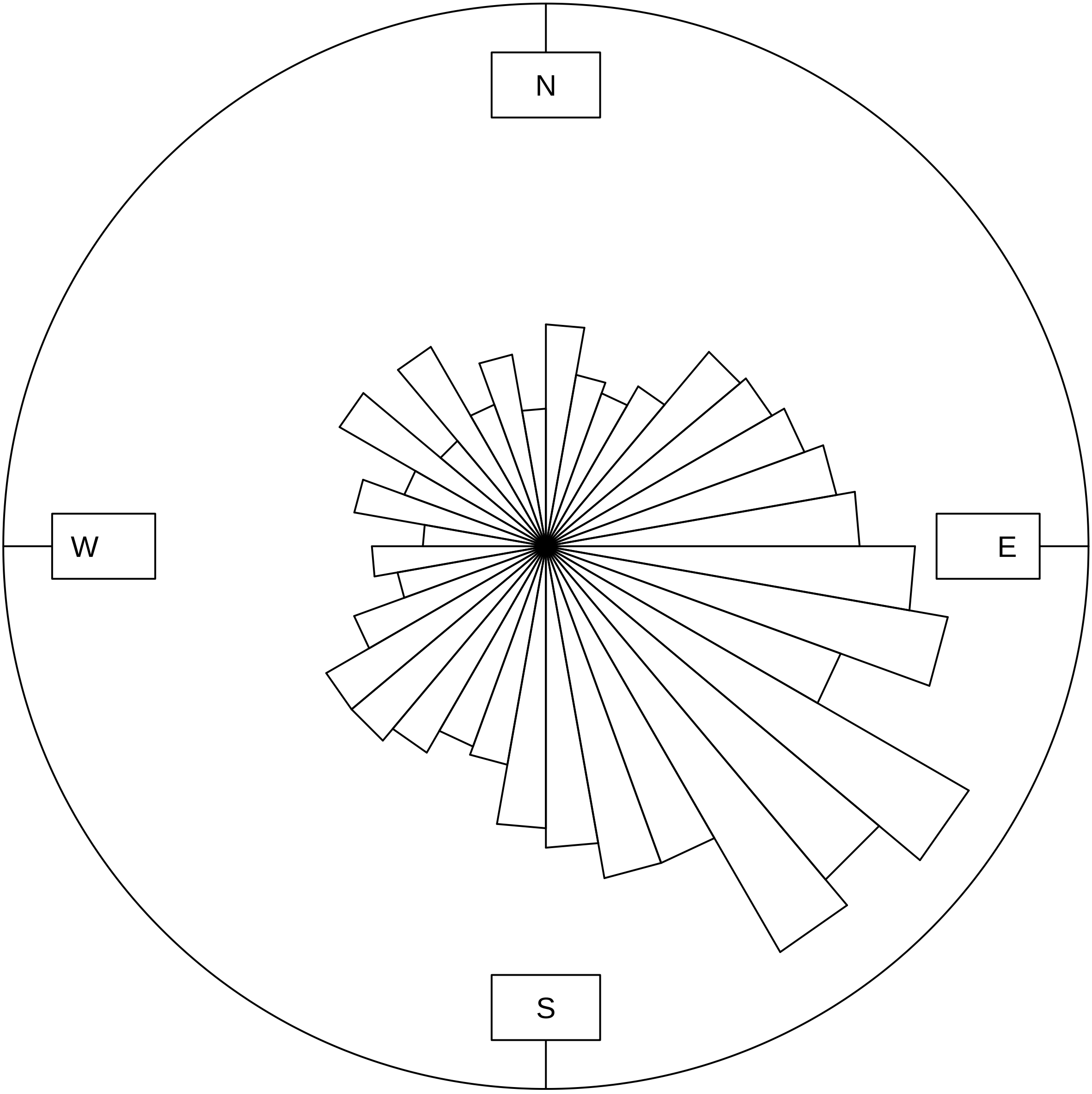

bootstrap 82

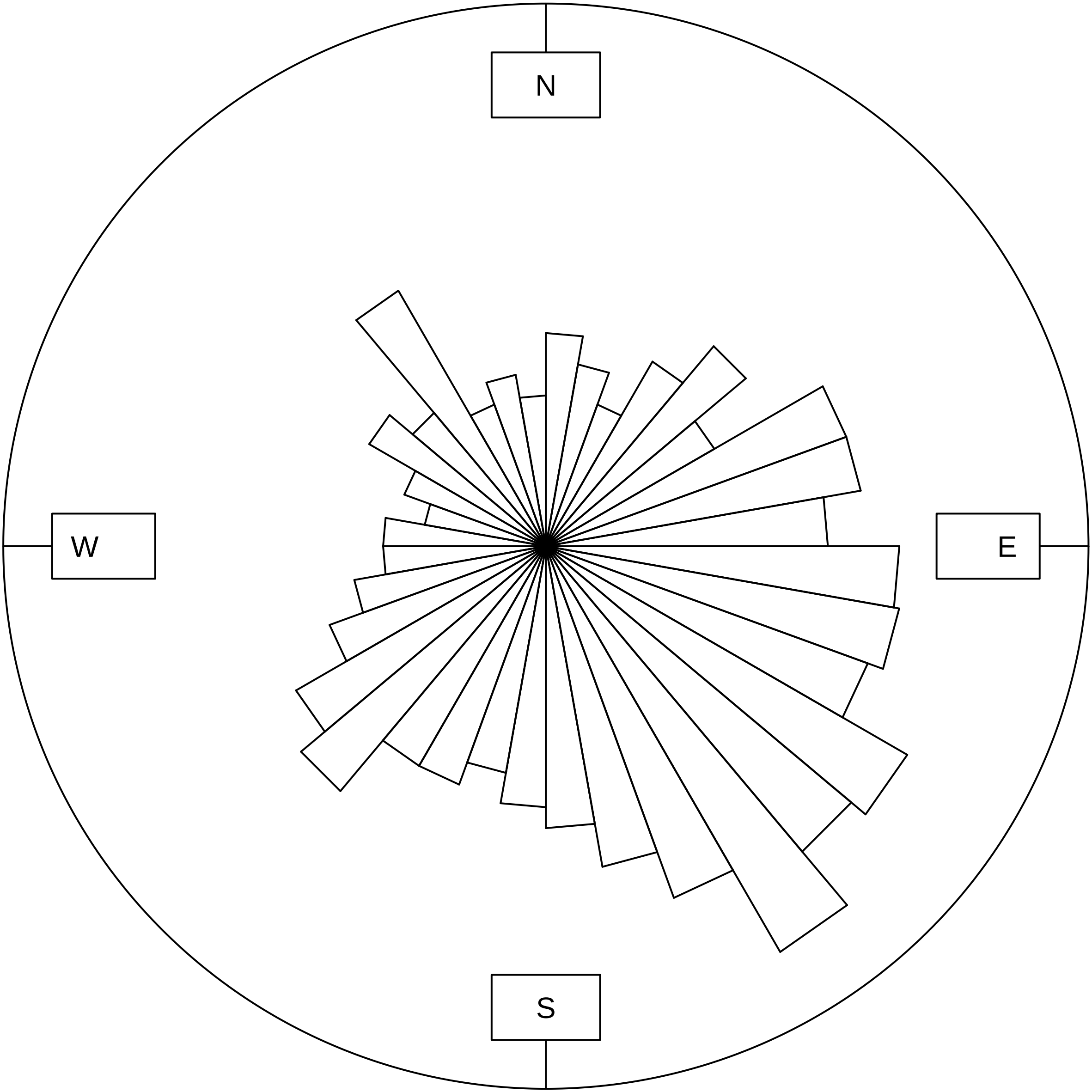

bootstrap 83

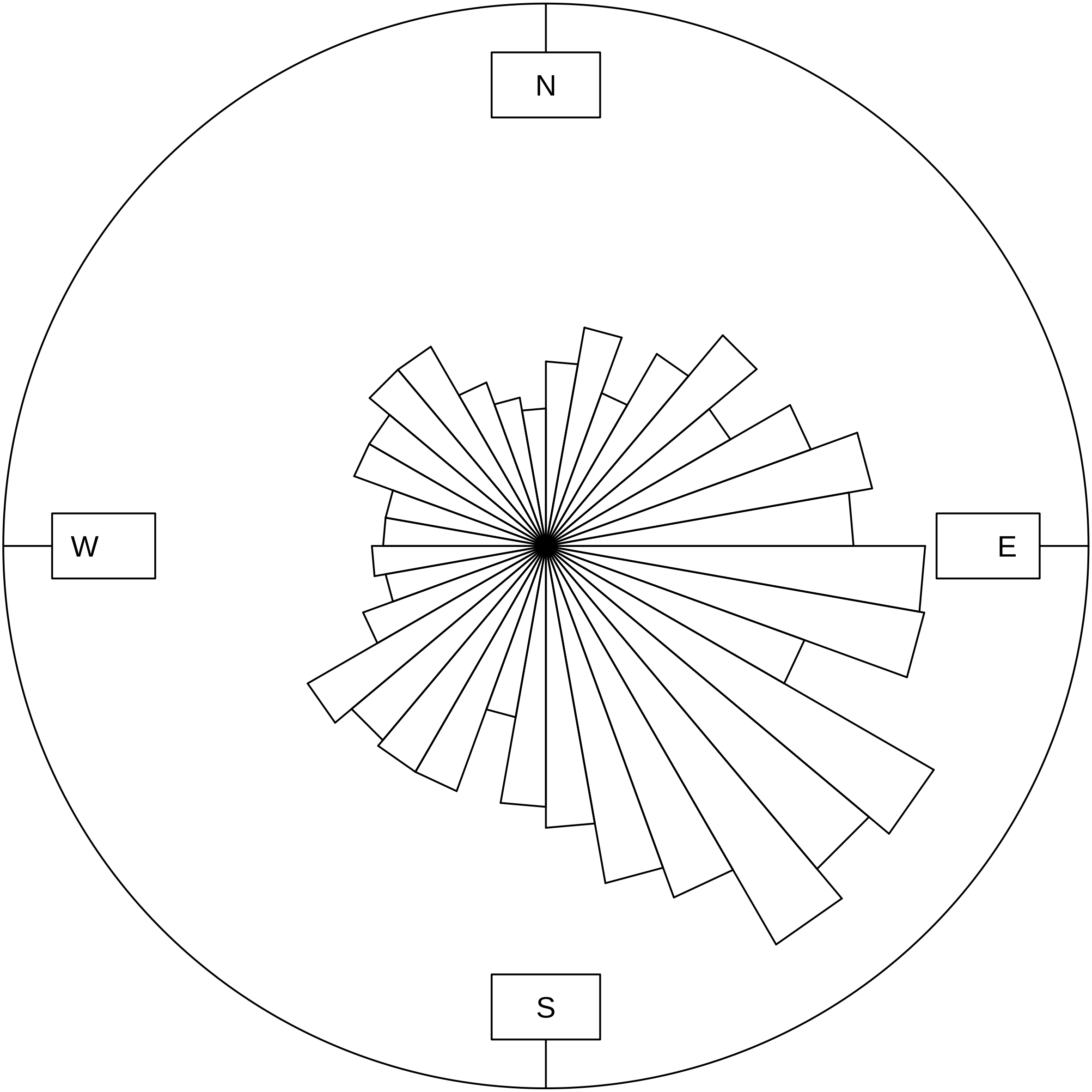

bootstrap 84

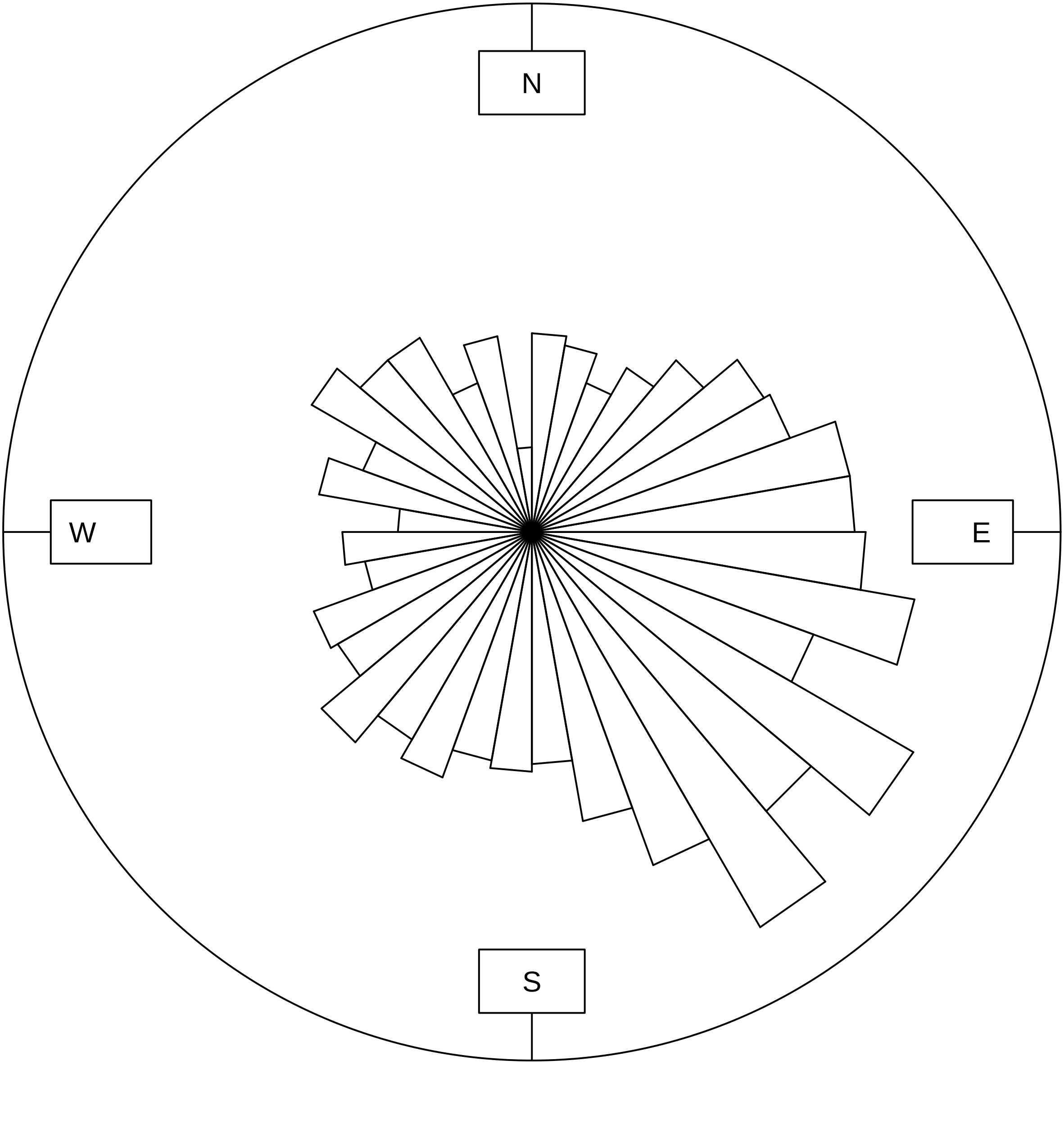

bootstrap 85

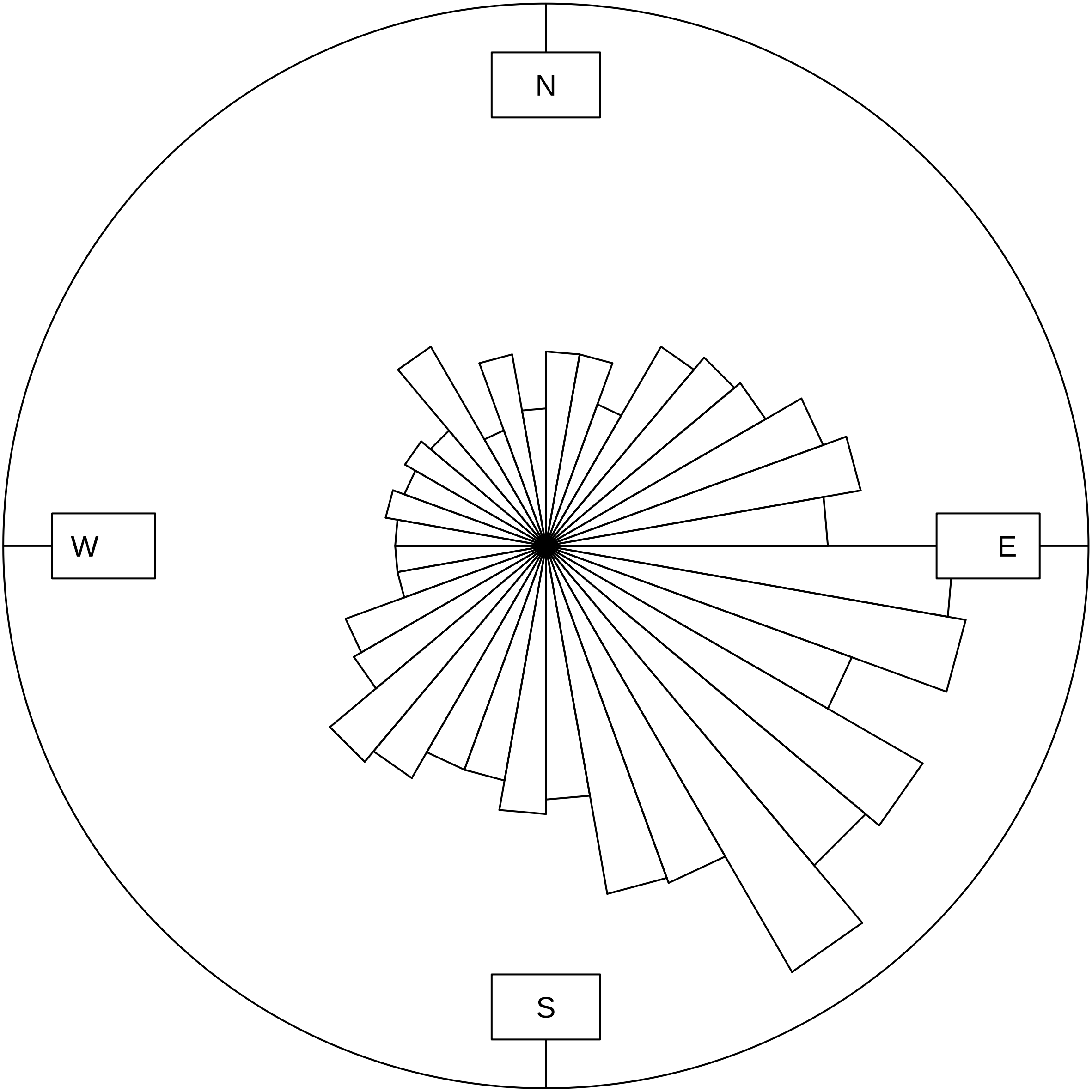

bootstrap 86

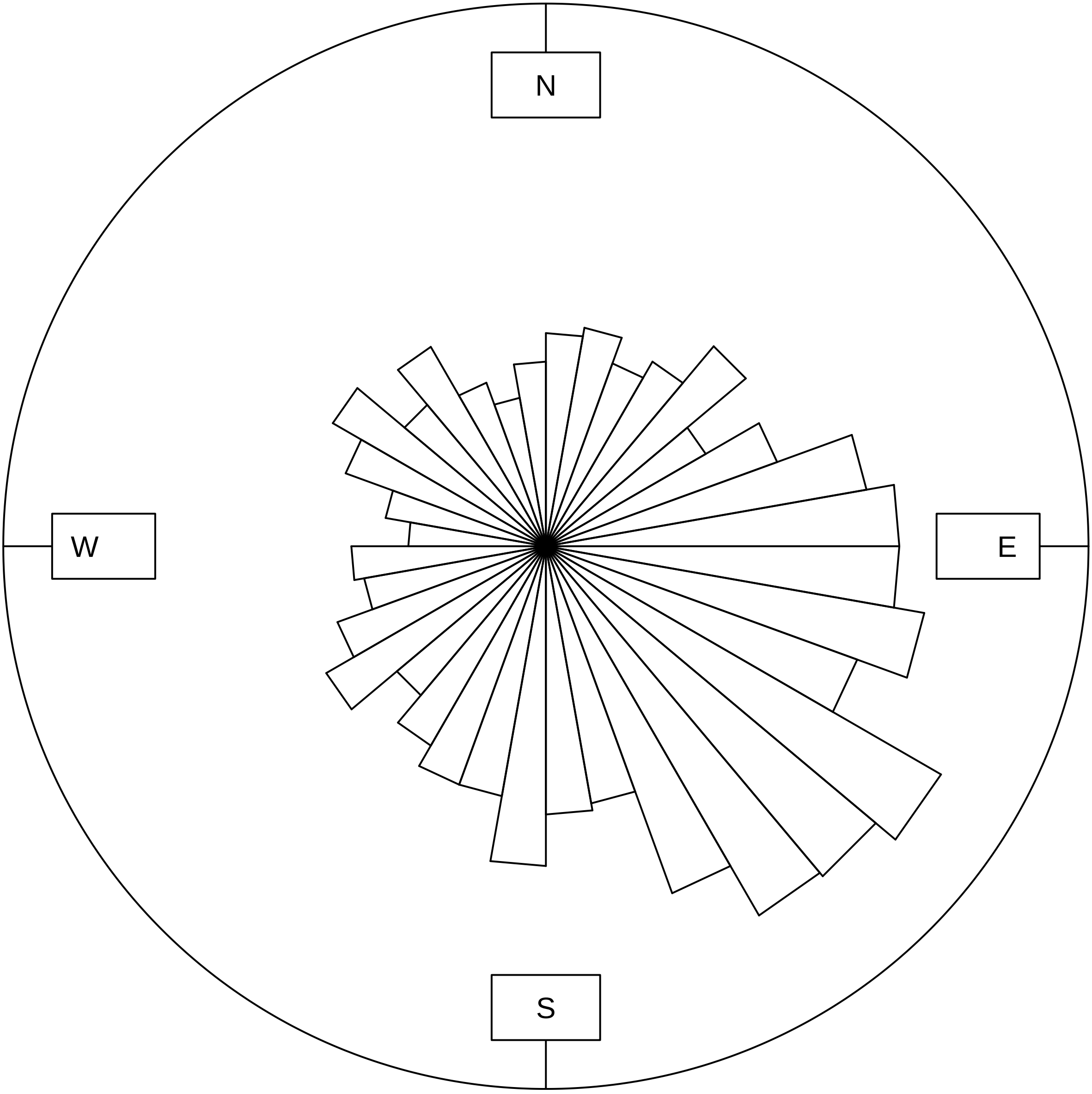

bootstrap 87

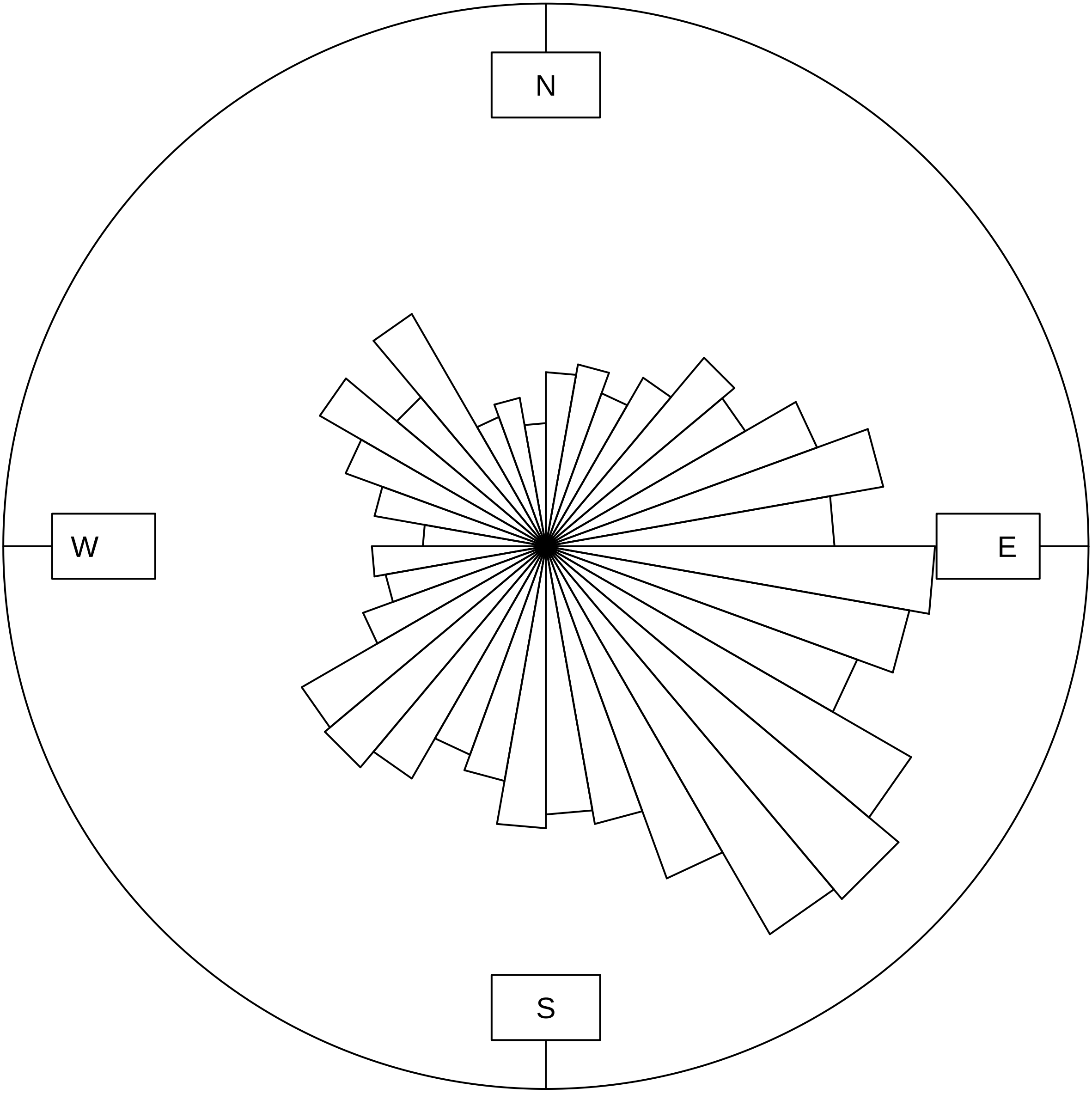

bootstrap 88

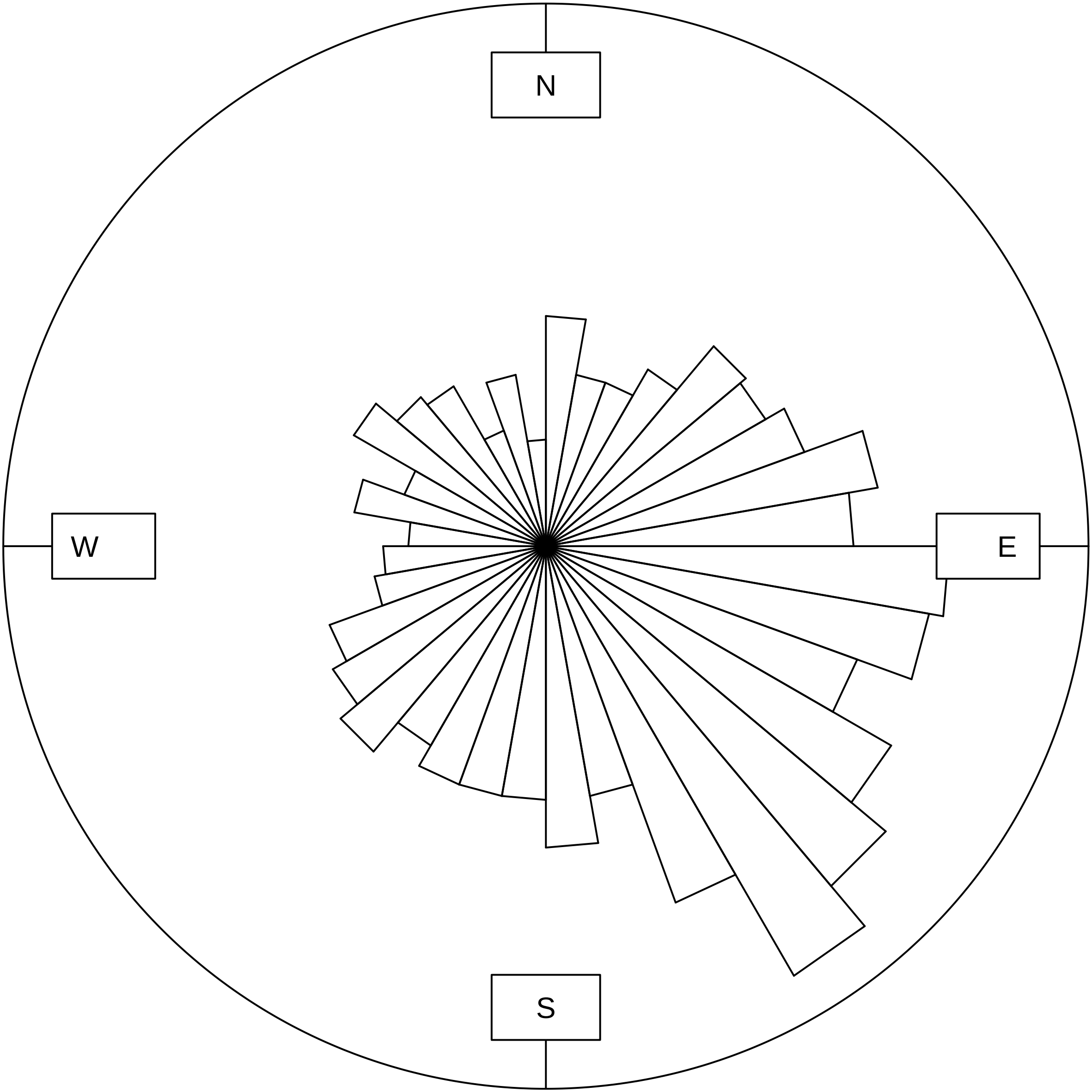

bootstrap 89

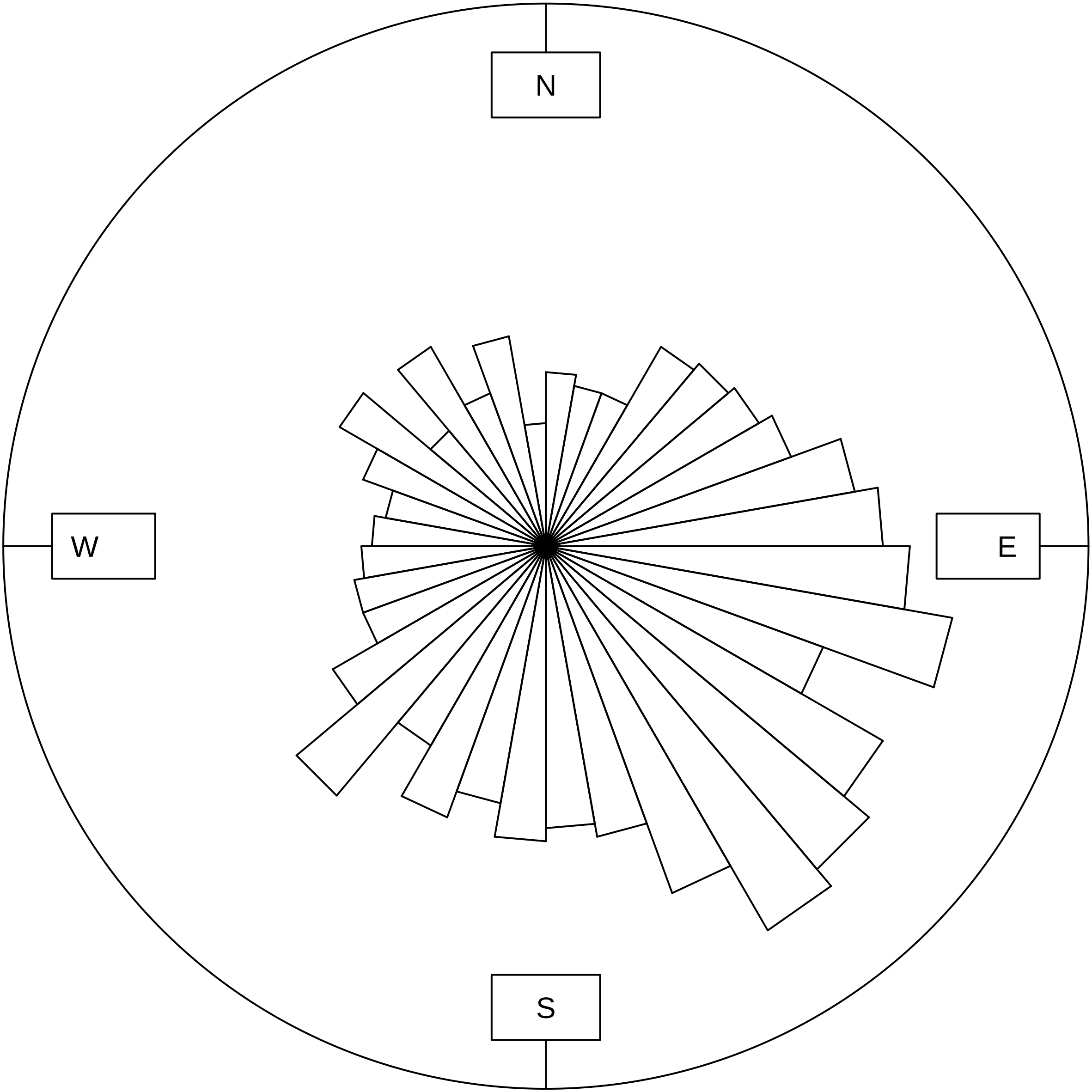

bootstrap 90

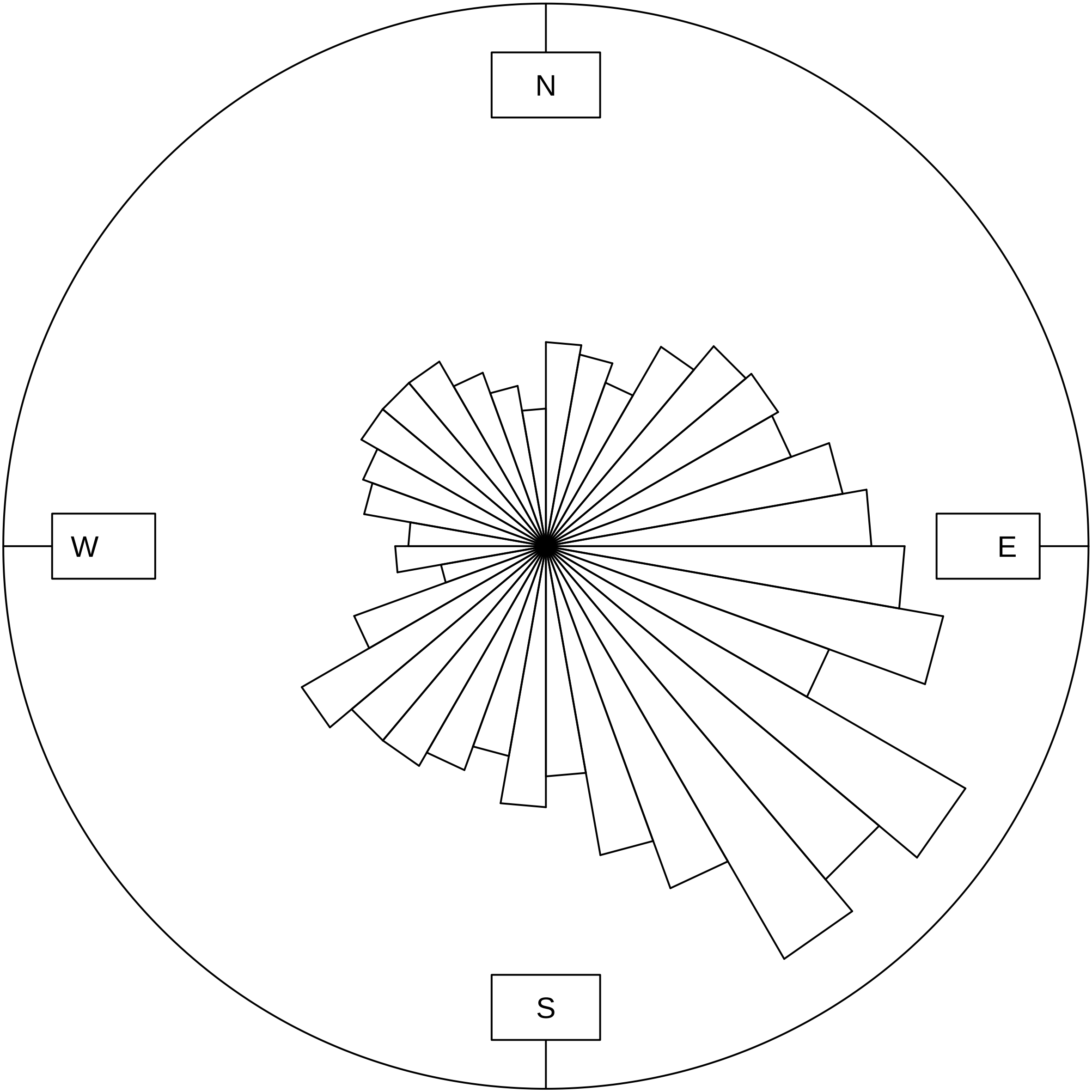

bootstrap 91

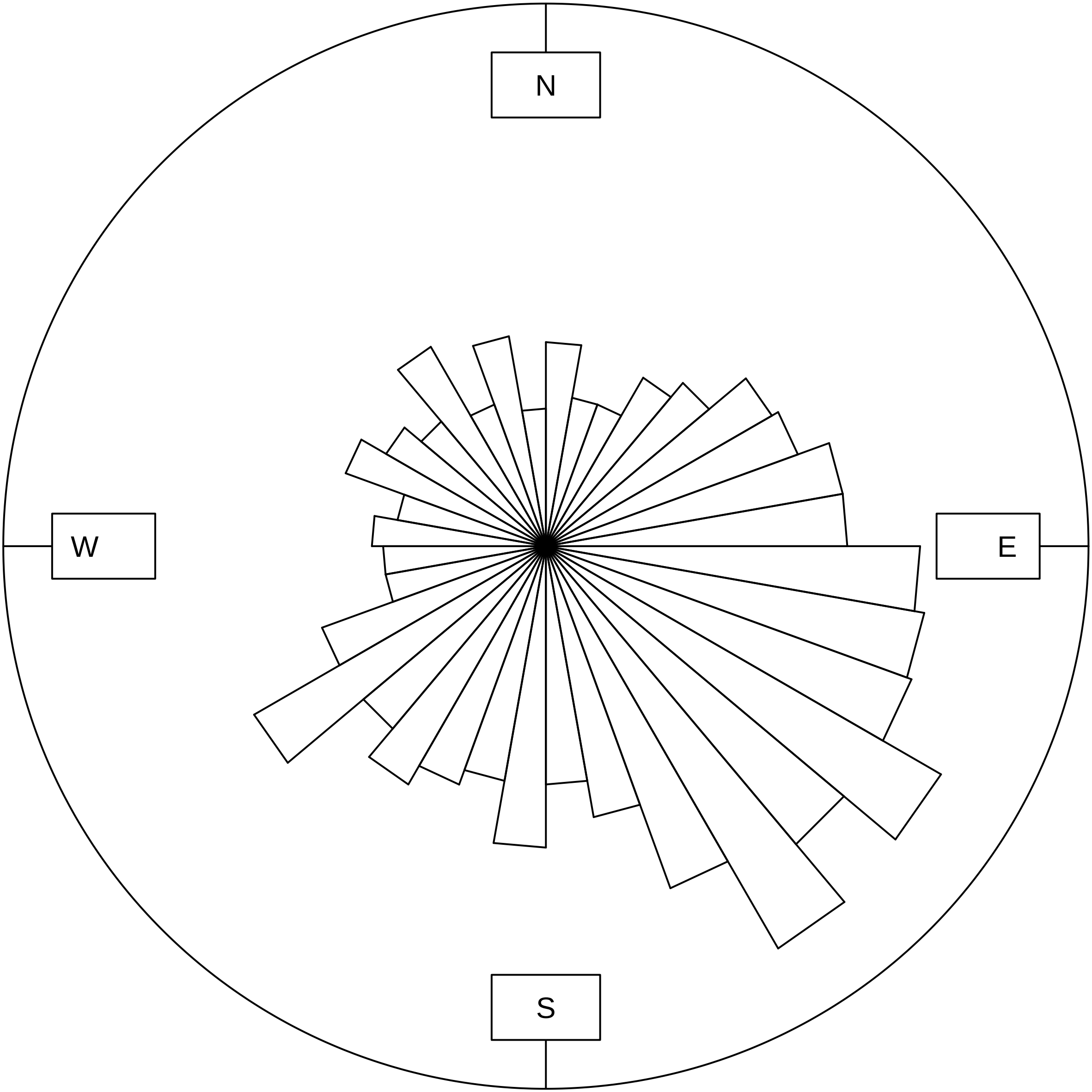

bootstrap 92

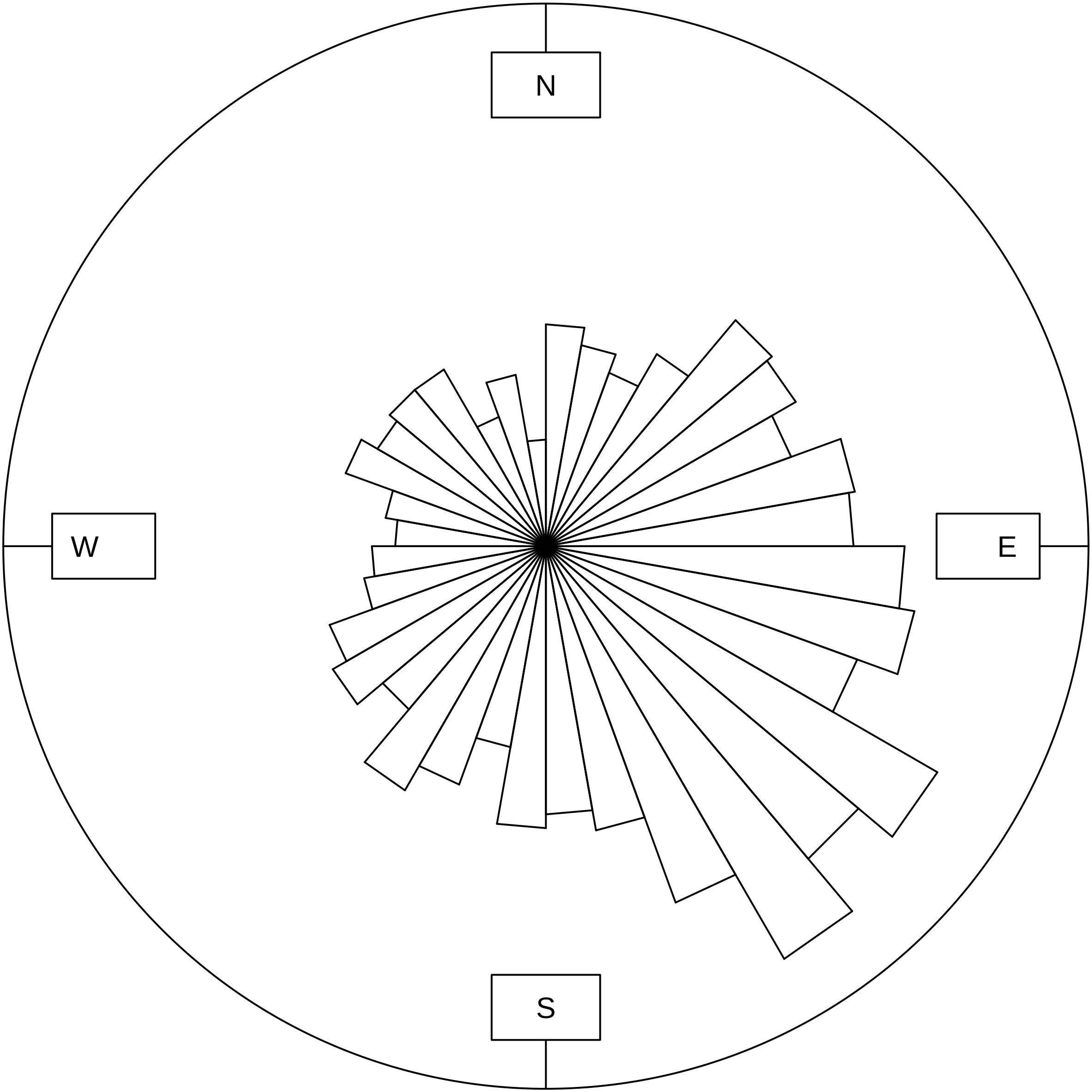

bootstrap 93

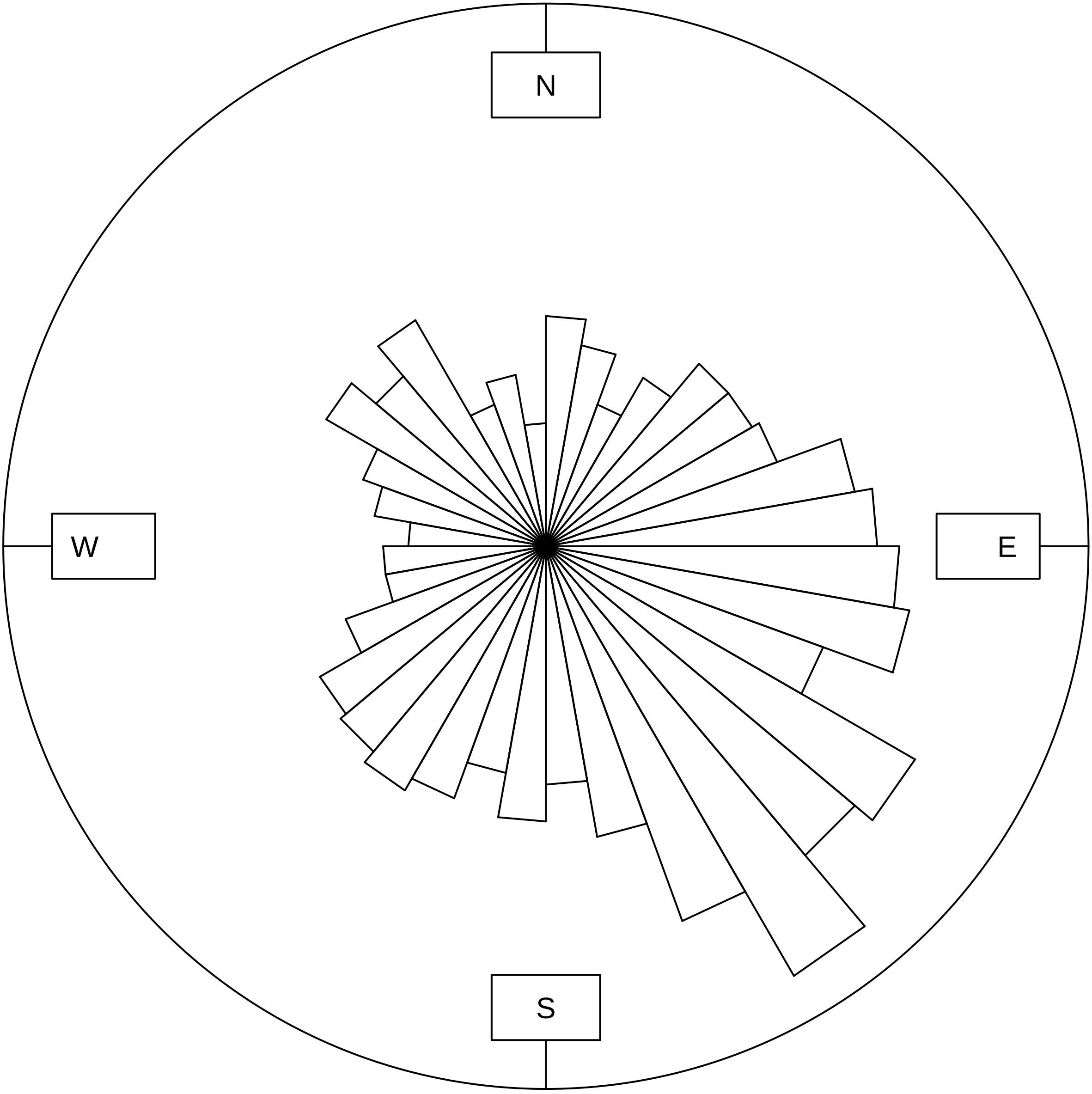

bootstrap 94

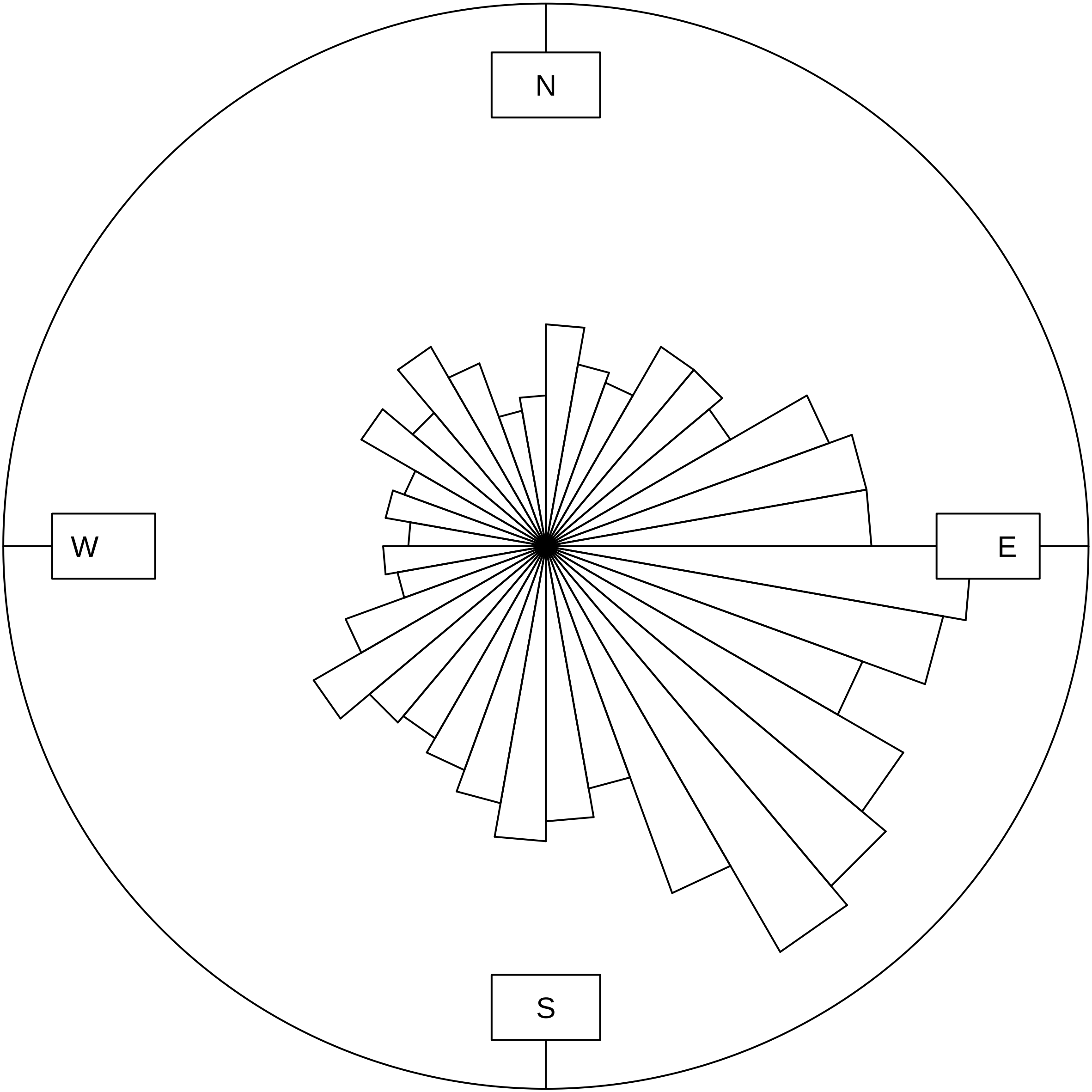

bootstrap 95

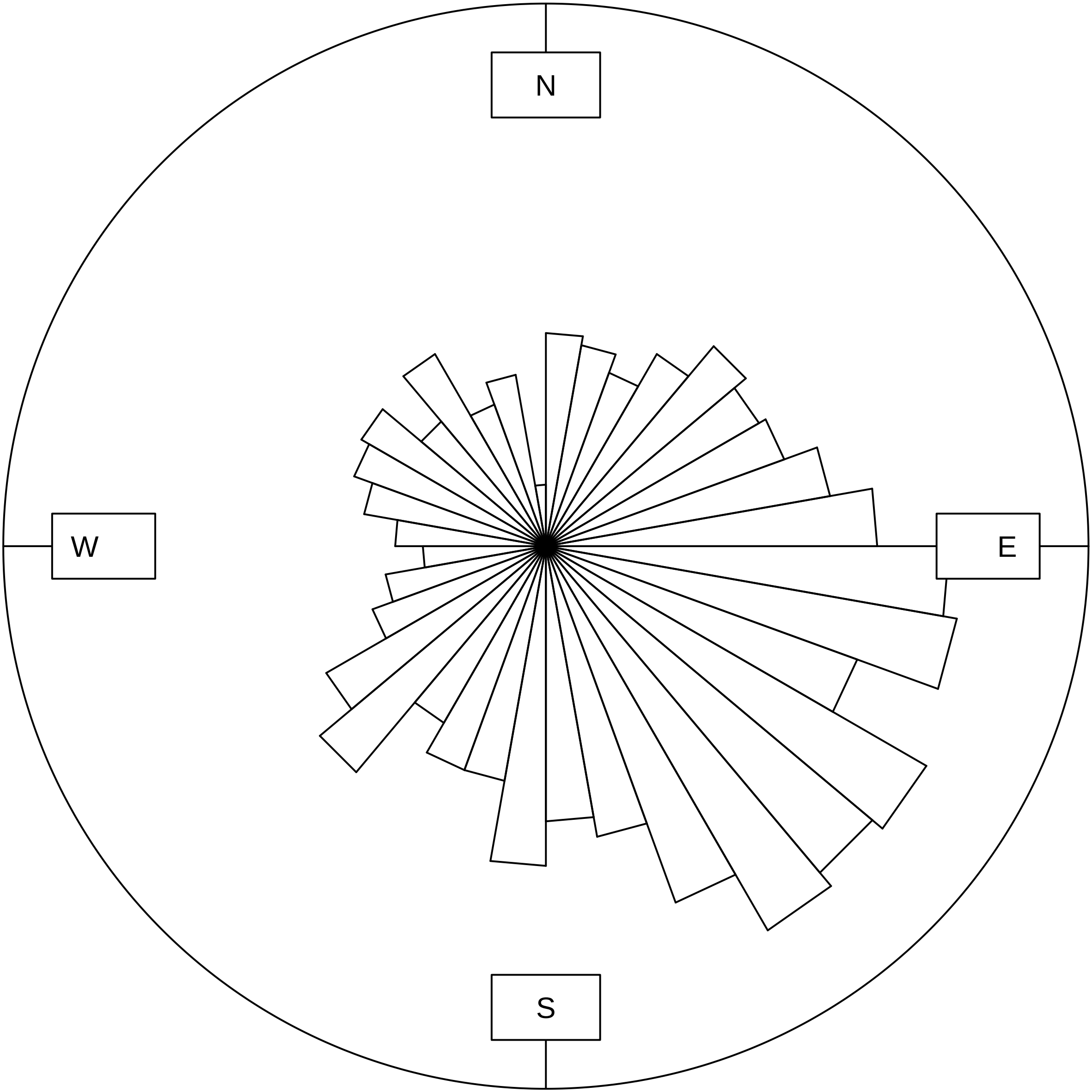

bootstrap 96

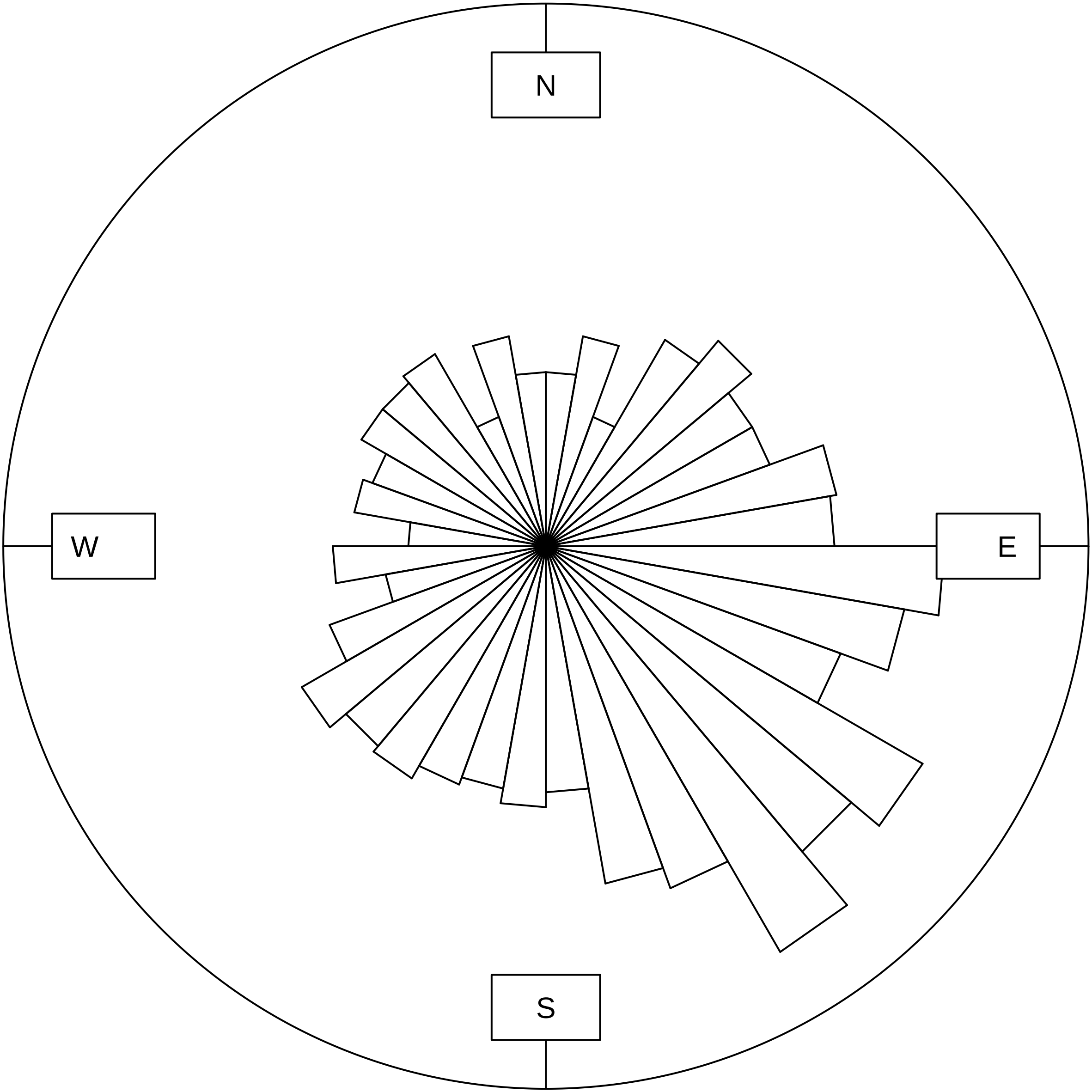

bootstrap 97

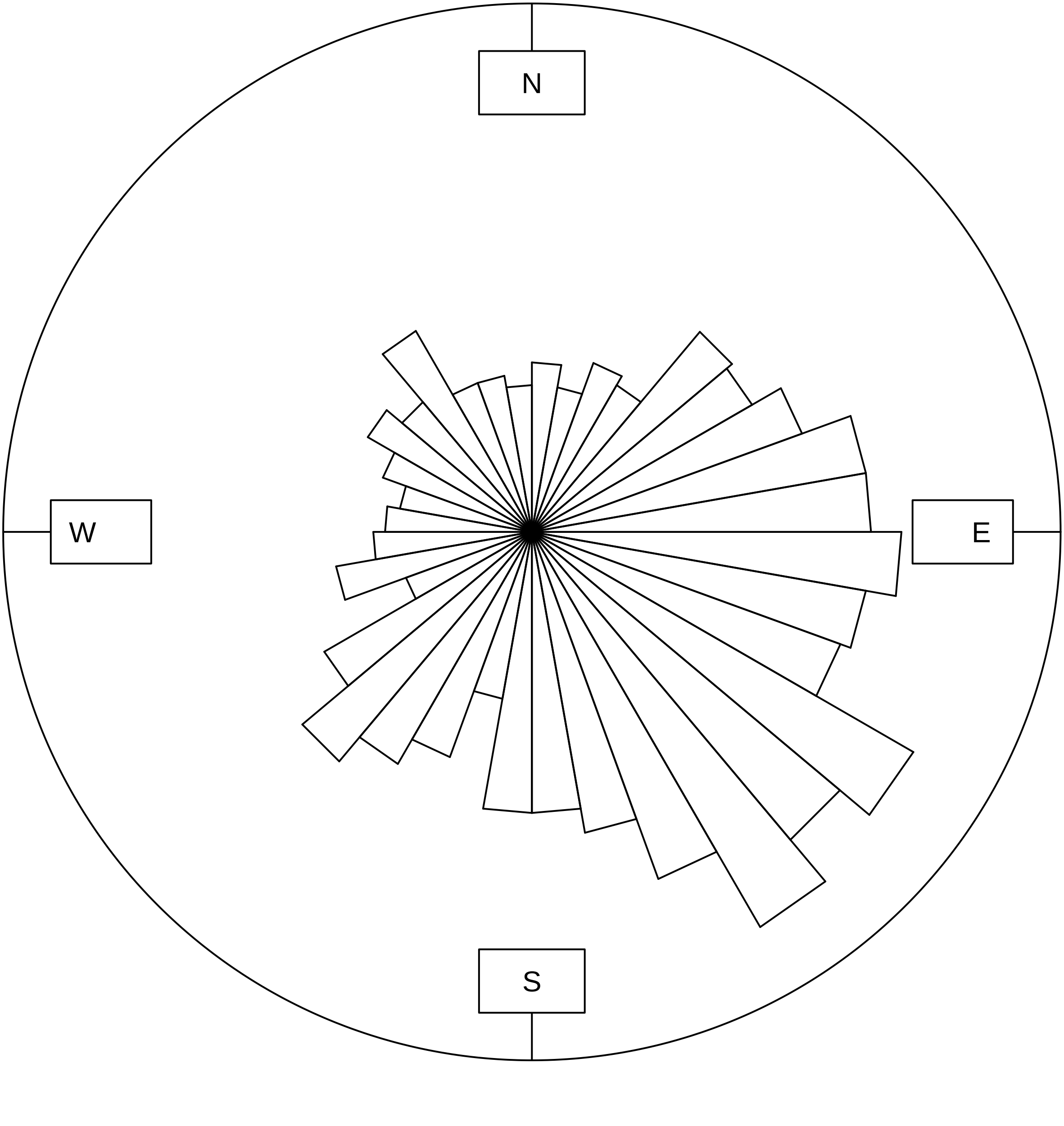

bootstrap 98

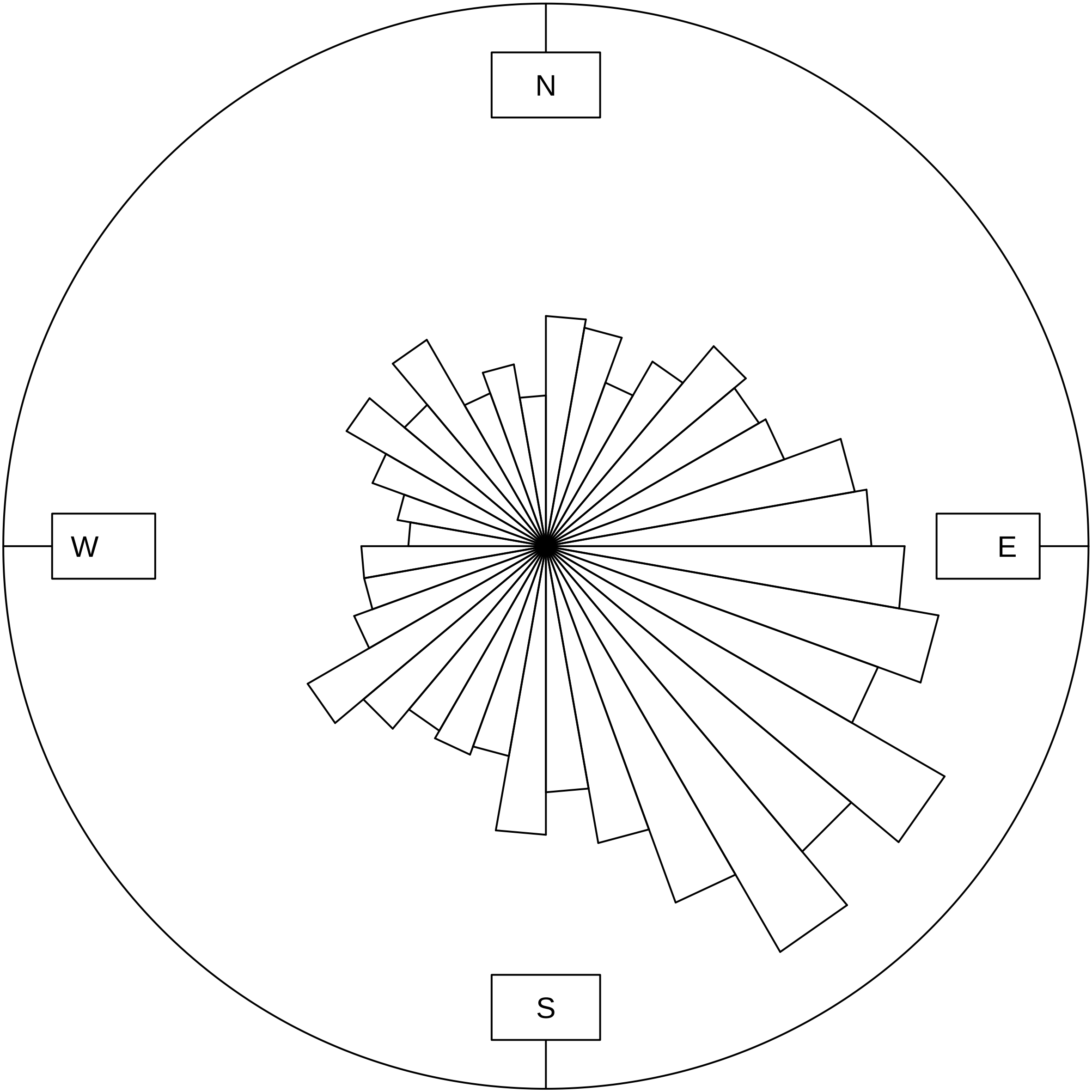

bootstrap 99

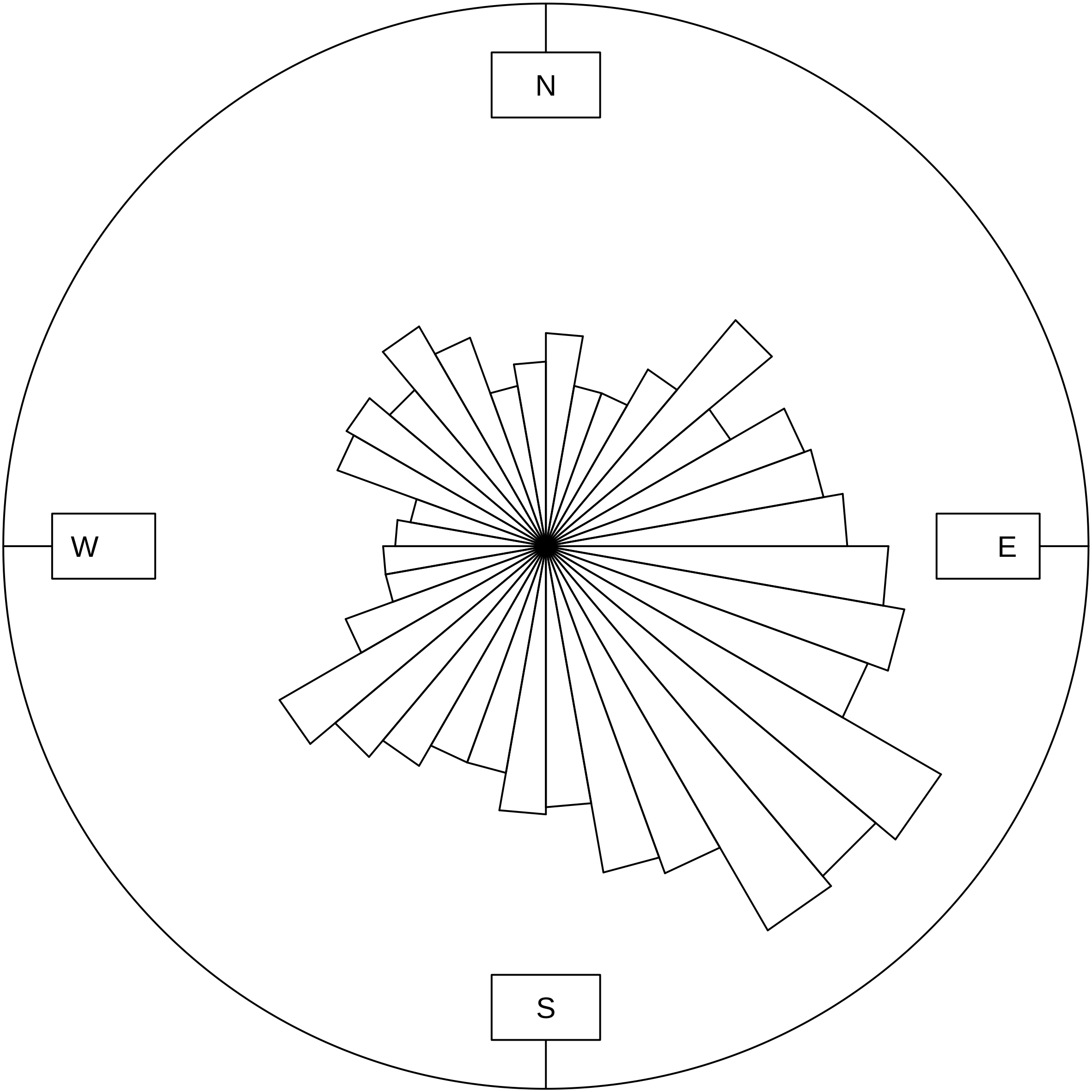

bootstrap 100

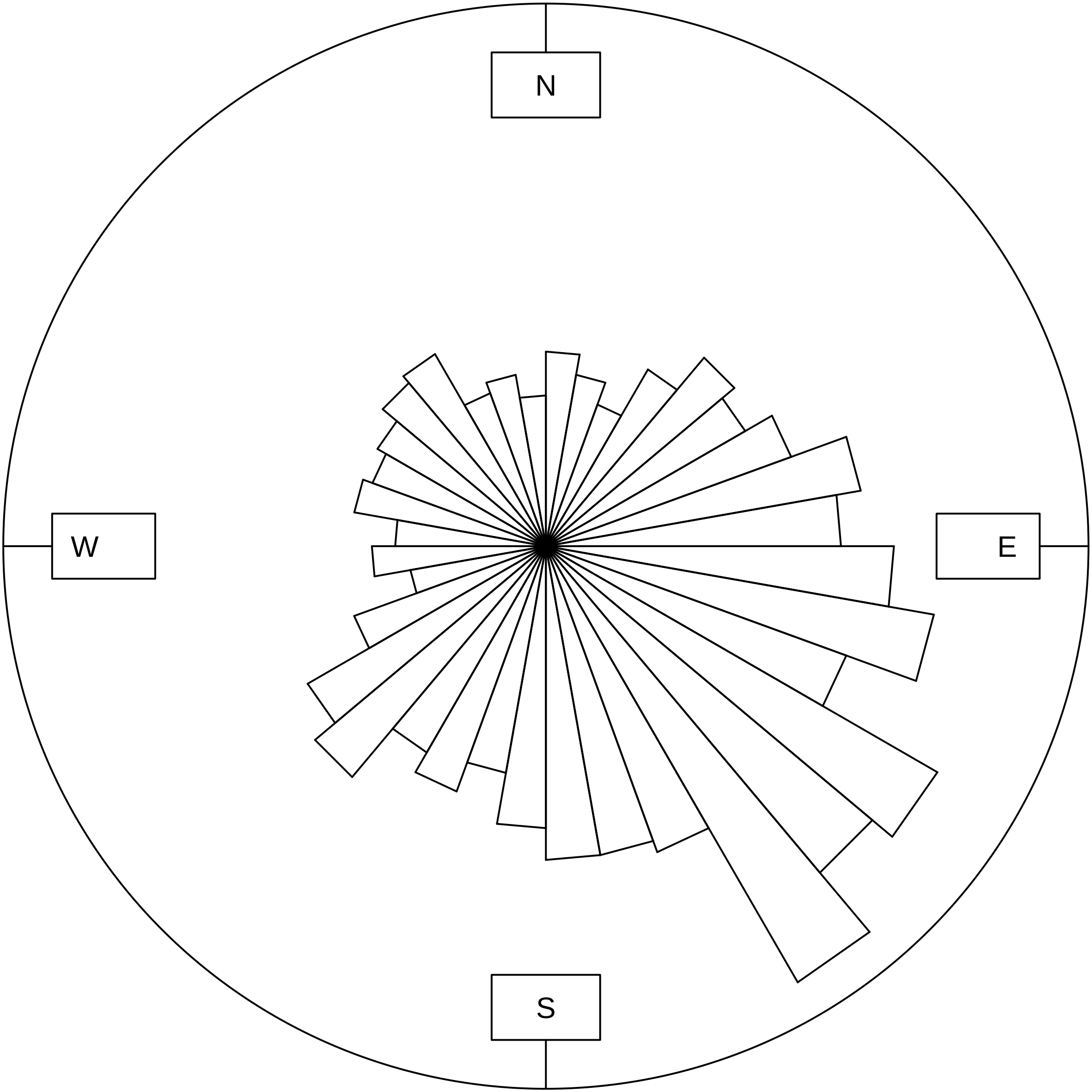

bootstrap 101

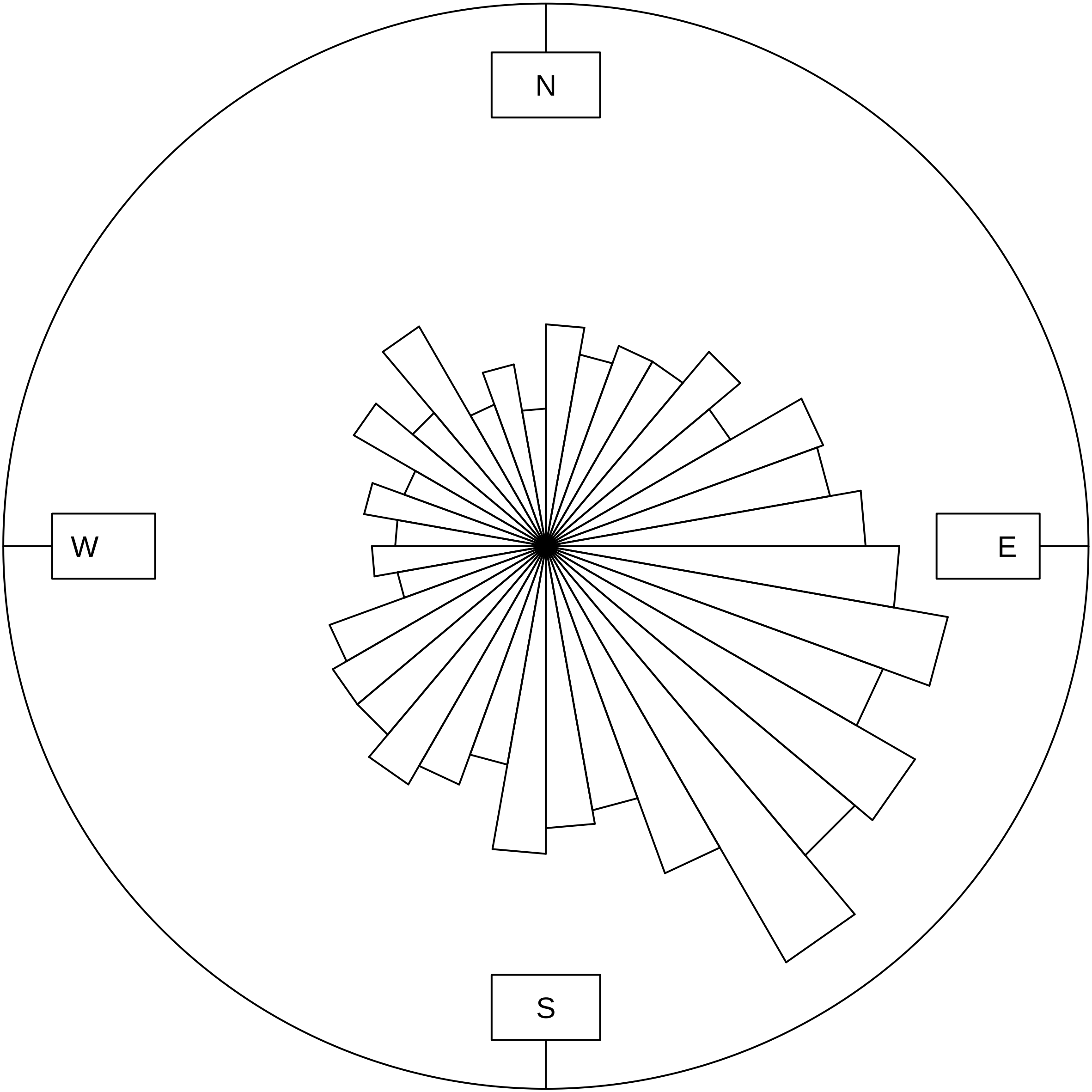

bootstrap 102

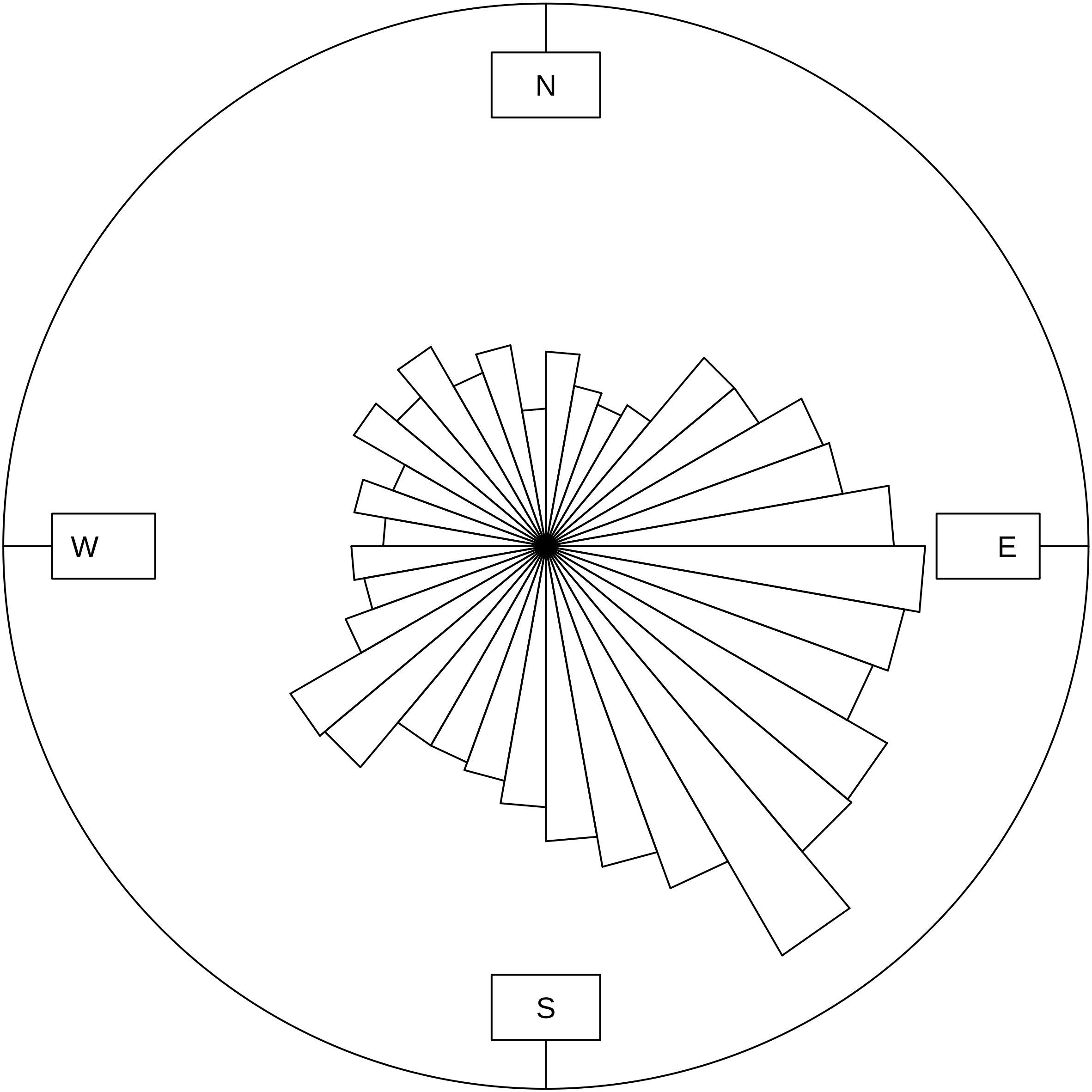

bootstrap 103

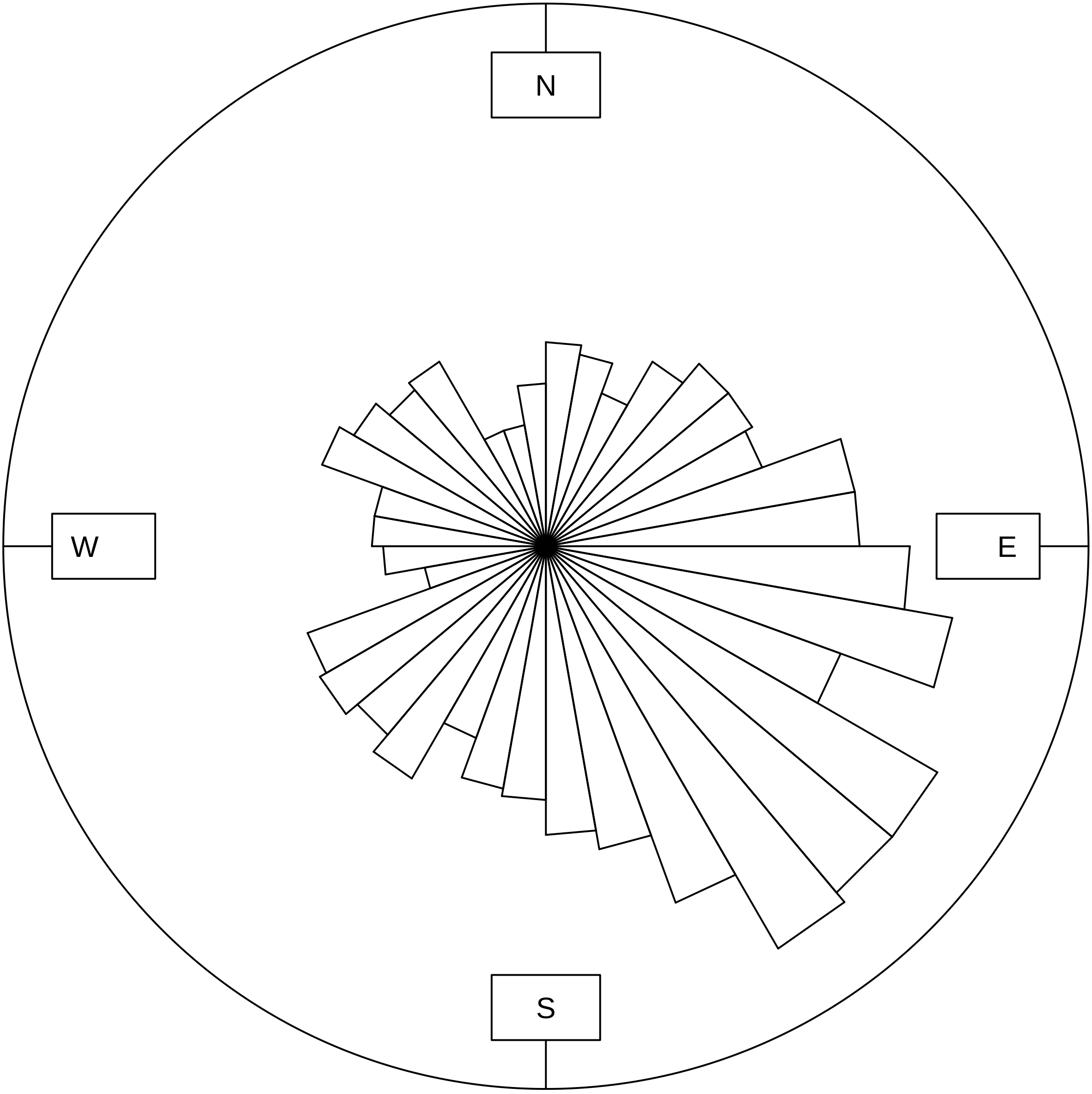

bootstrap 104

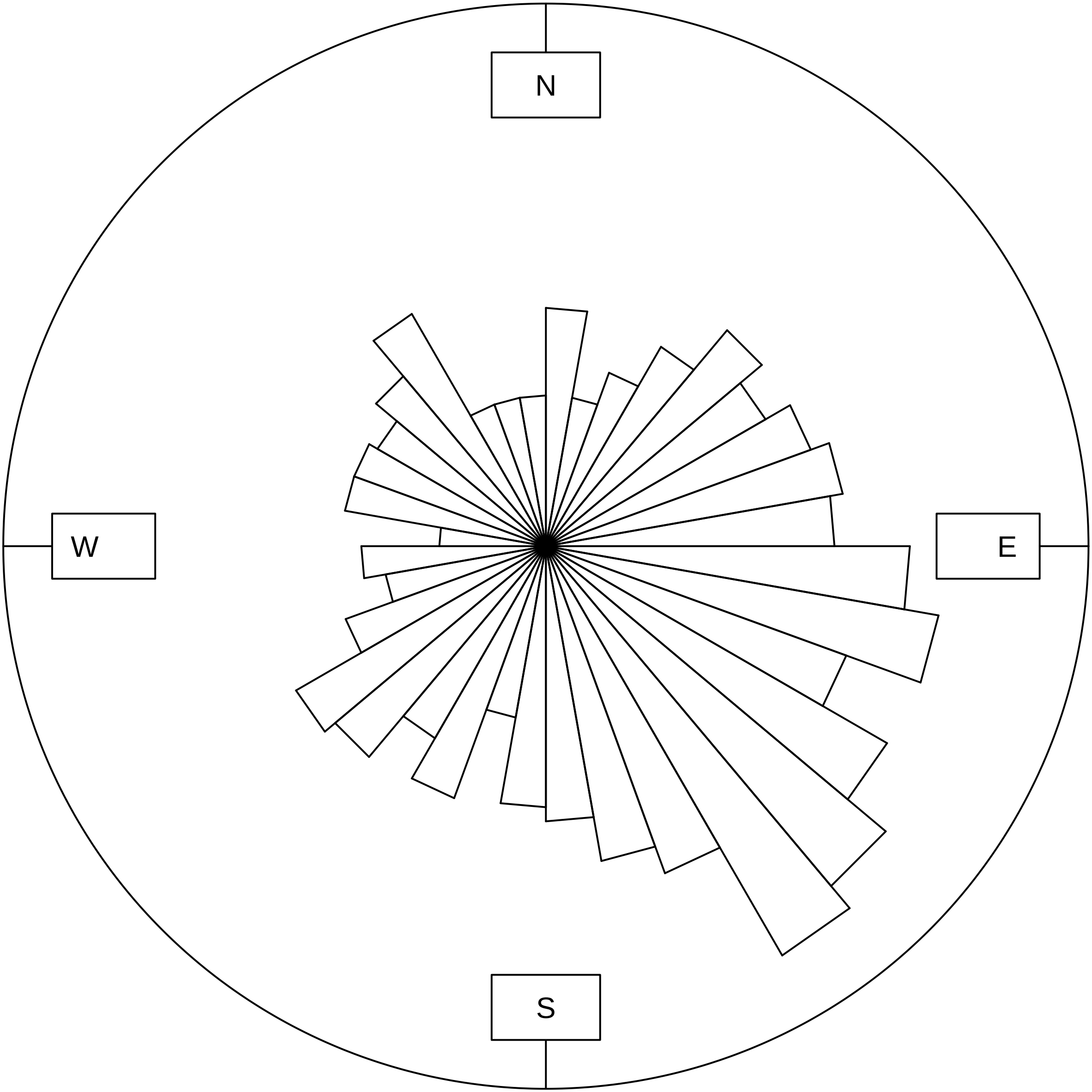

bootstrap 105

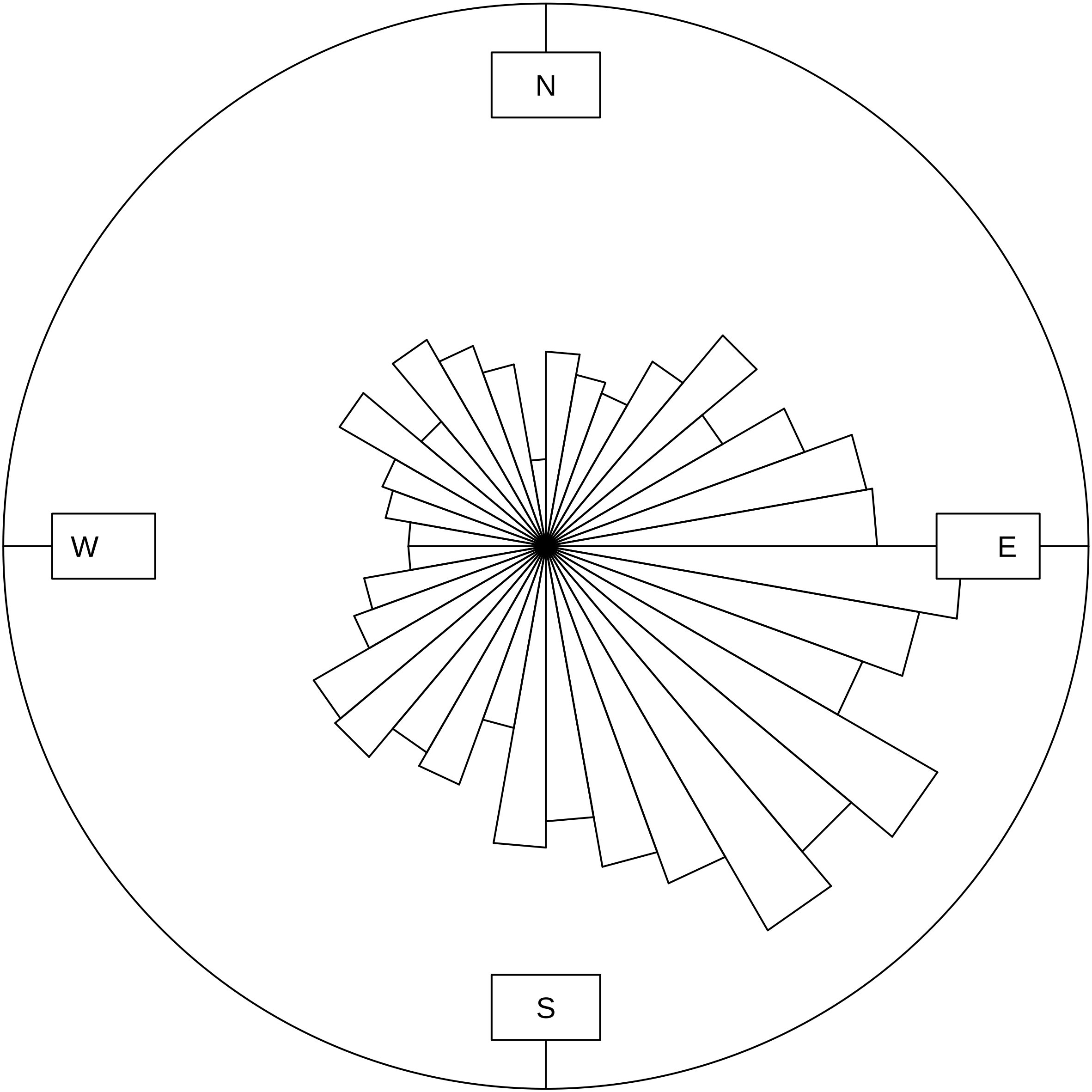

bootstrap 106

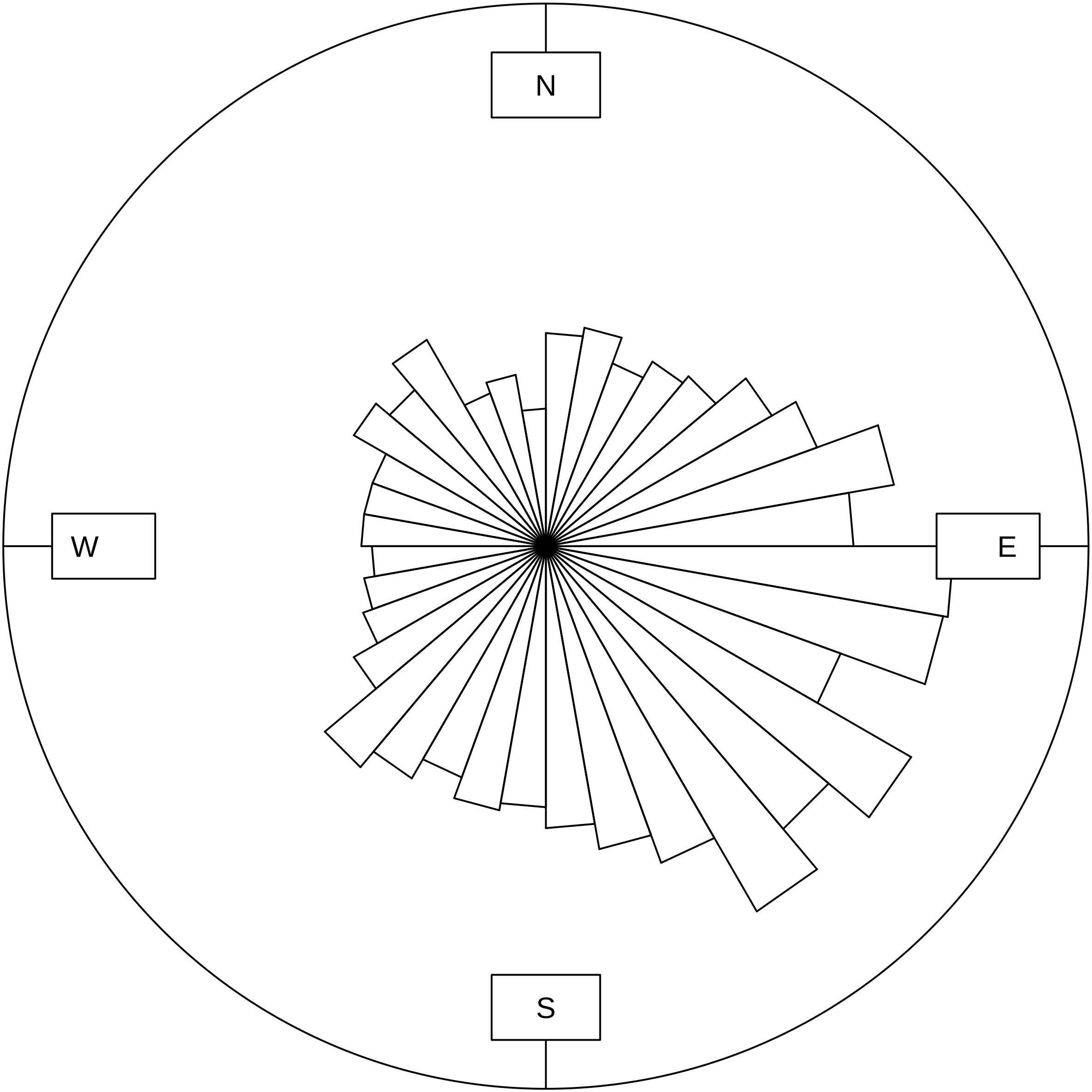

bootstrap 107

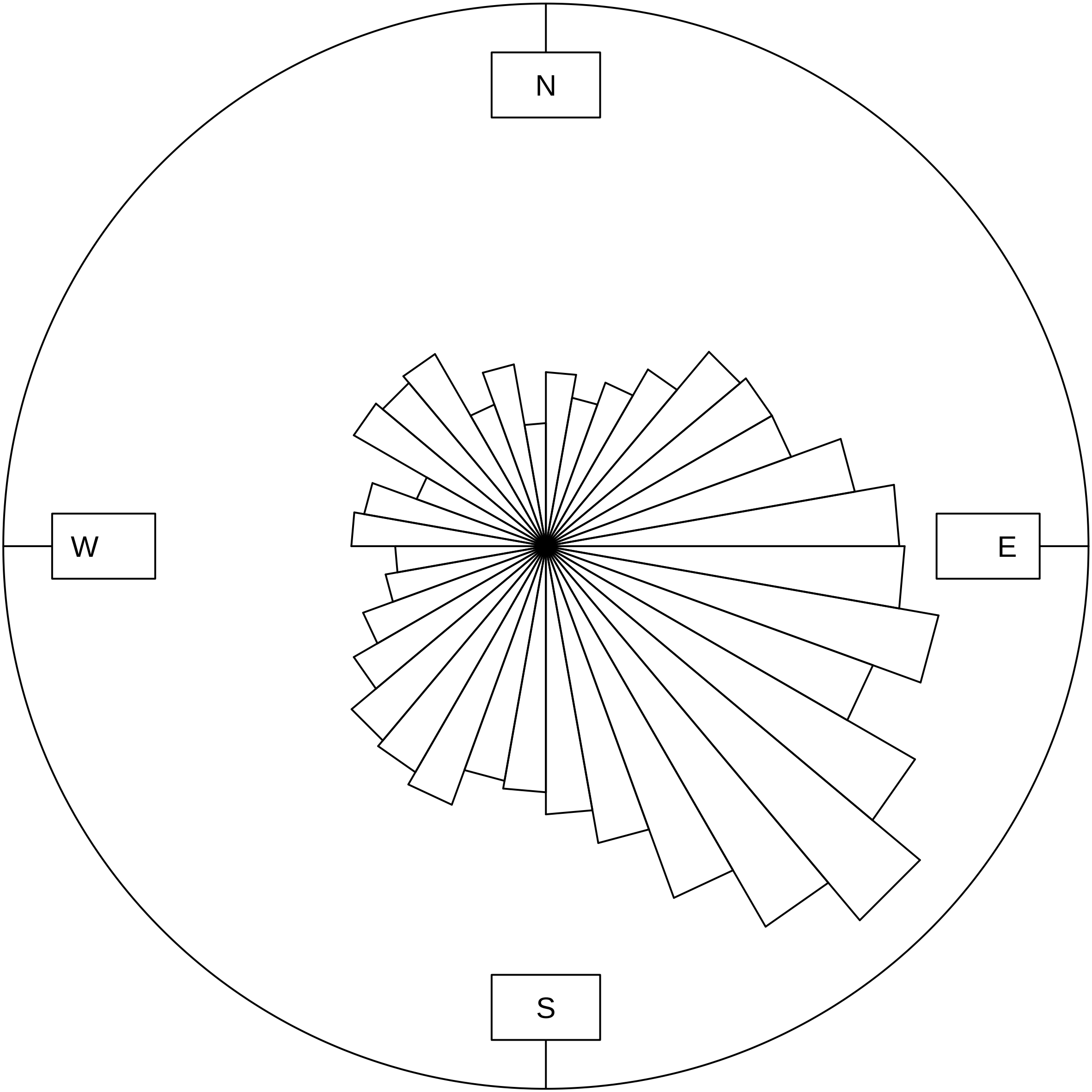

bootstrap 108

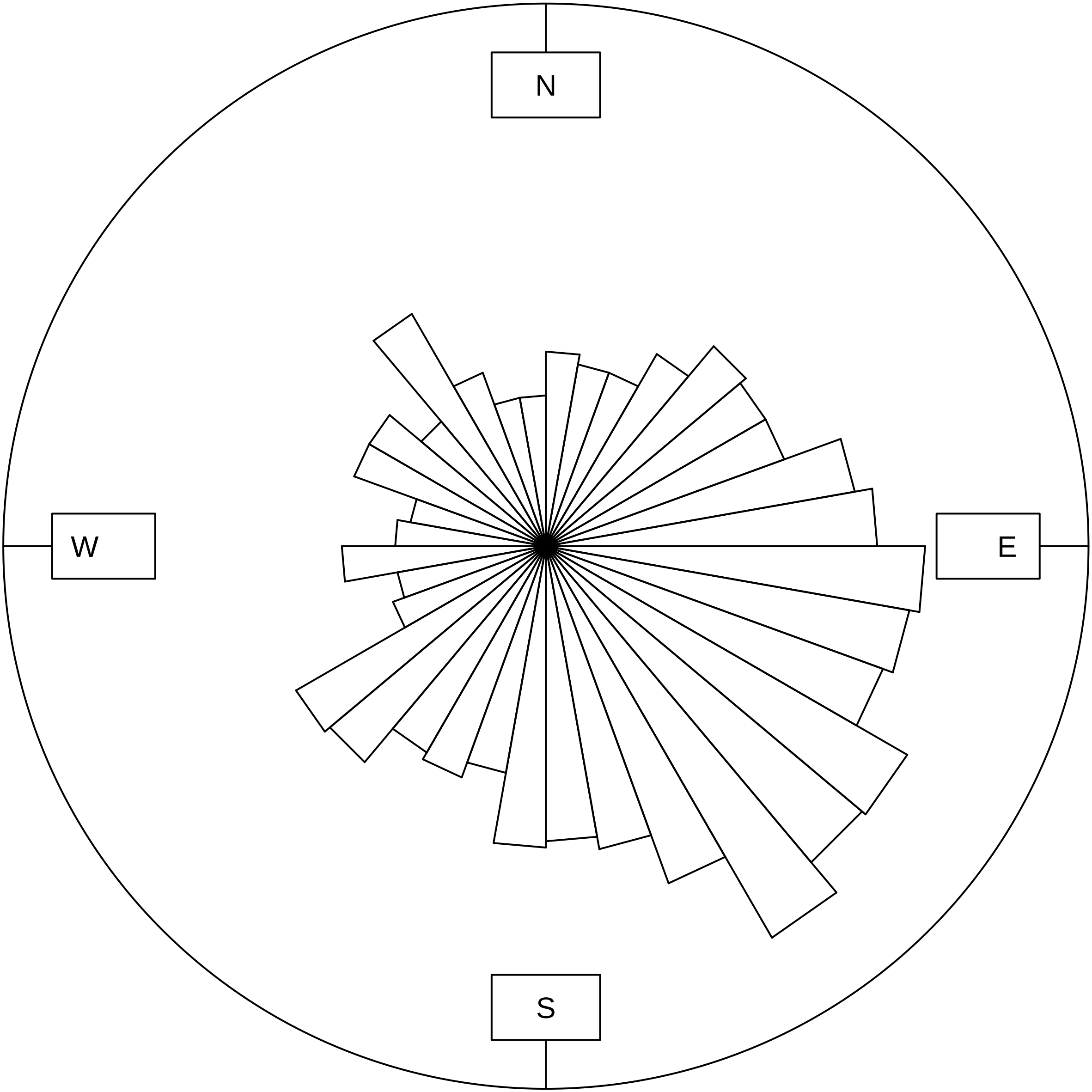

bootstrap 109

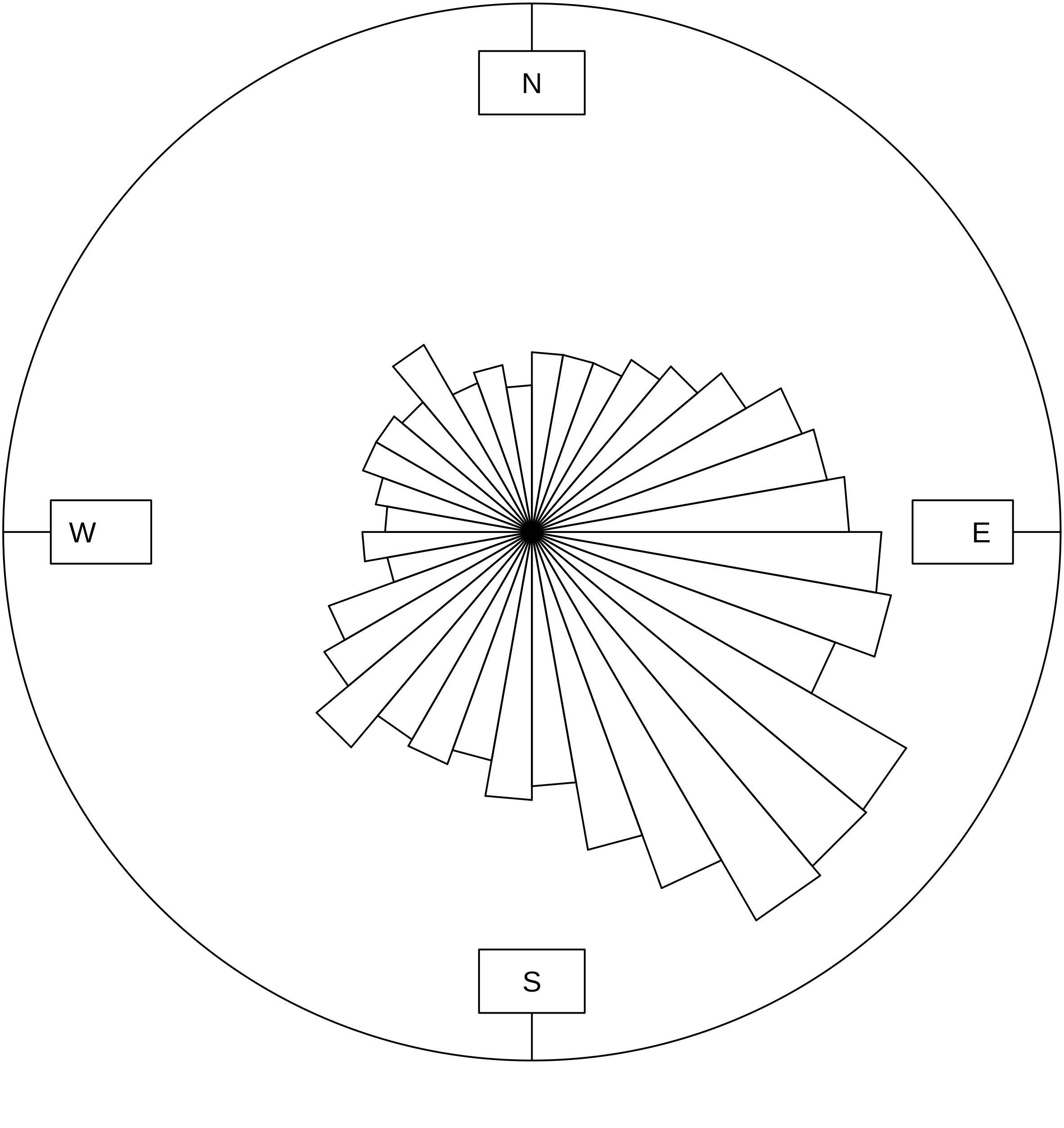

bootstrap 110

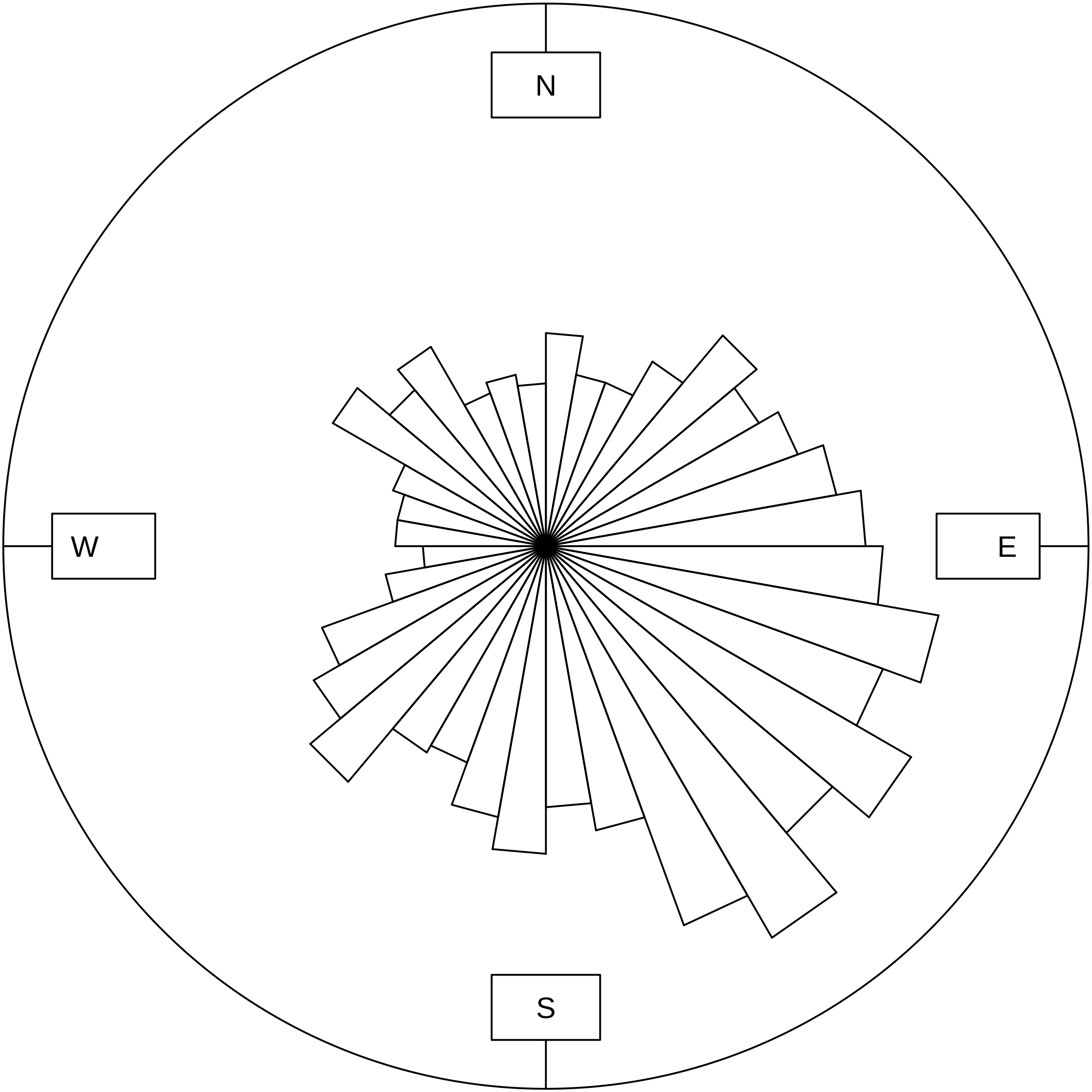

bootstrap 111

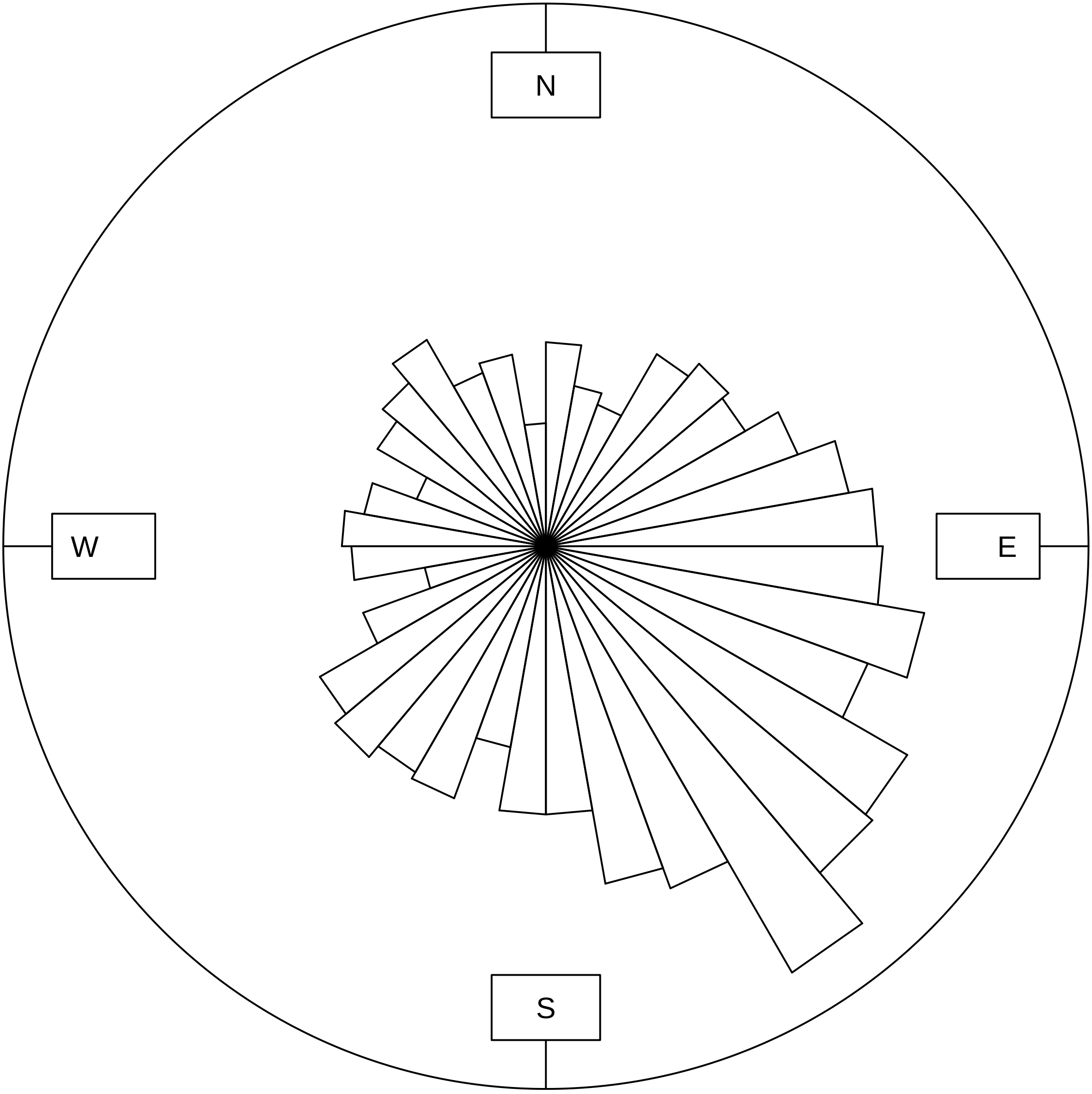

bootstrap 112

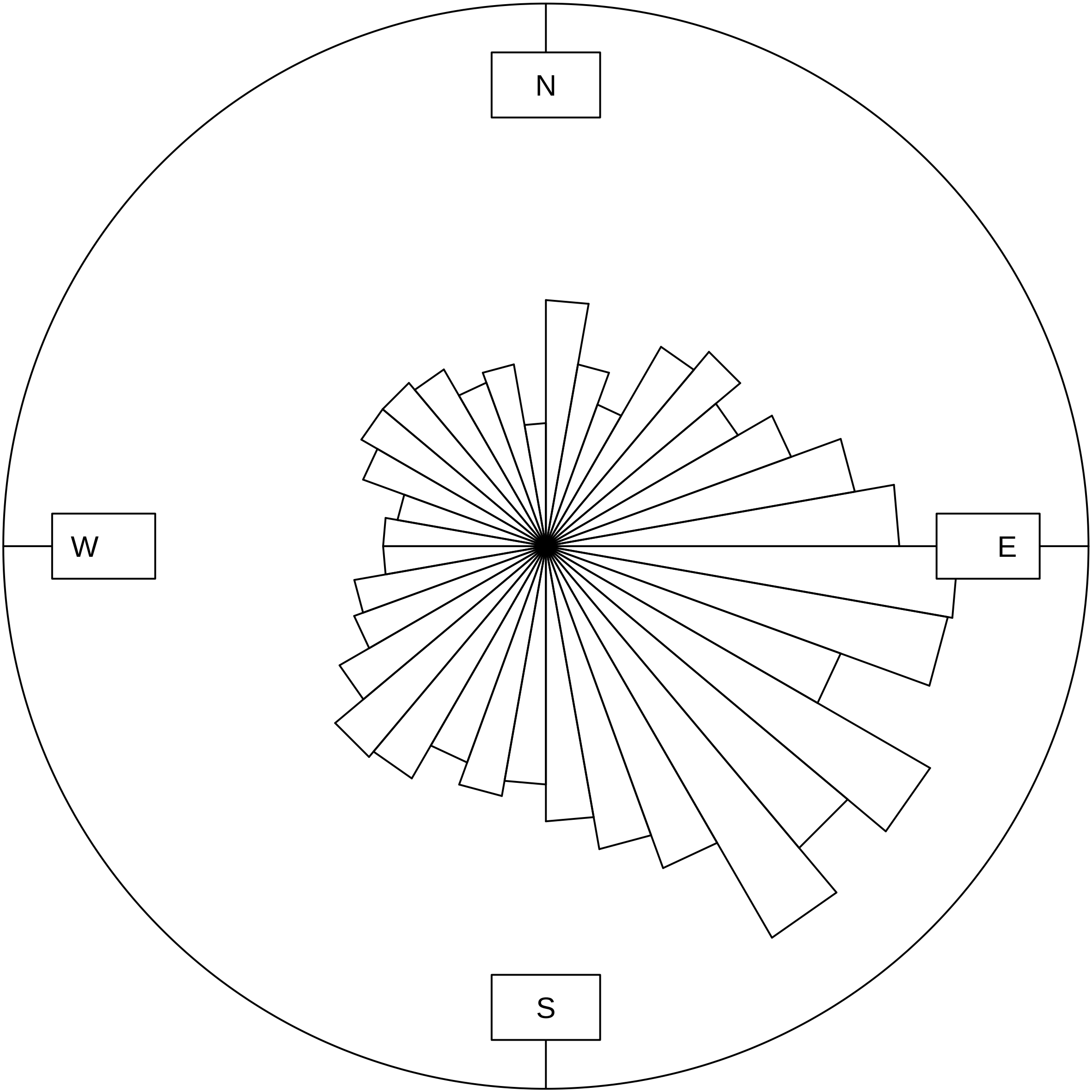

bootstrap 113

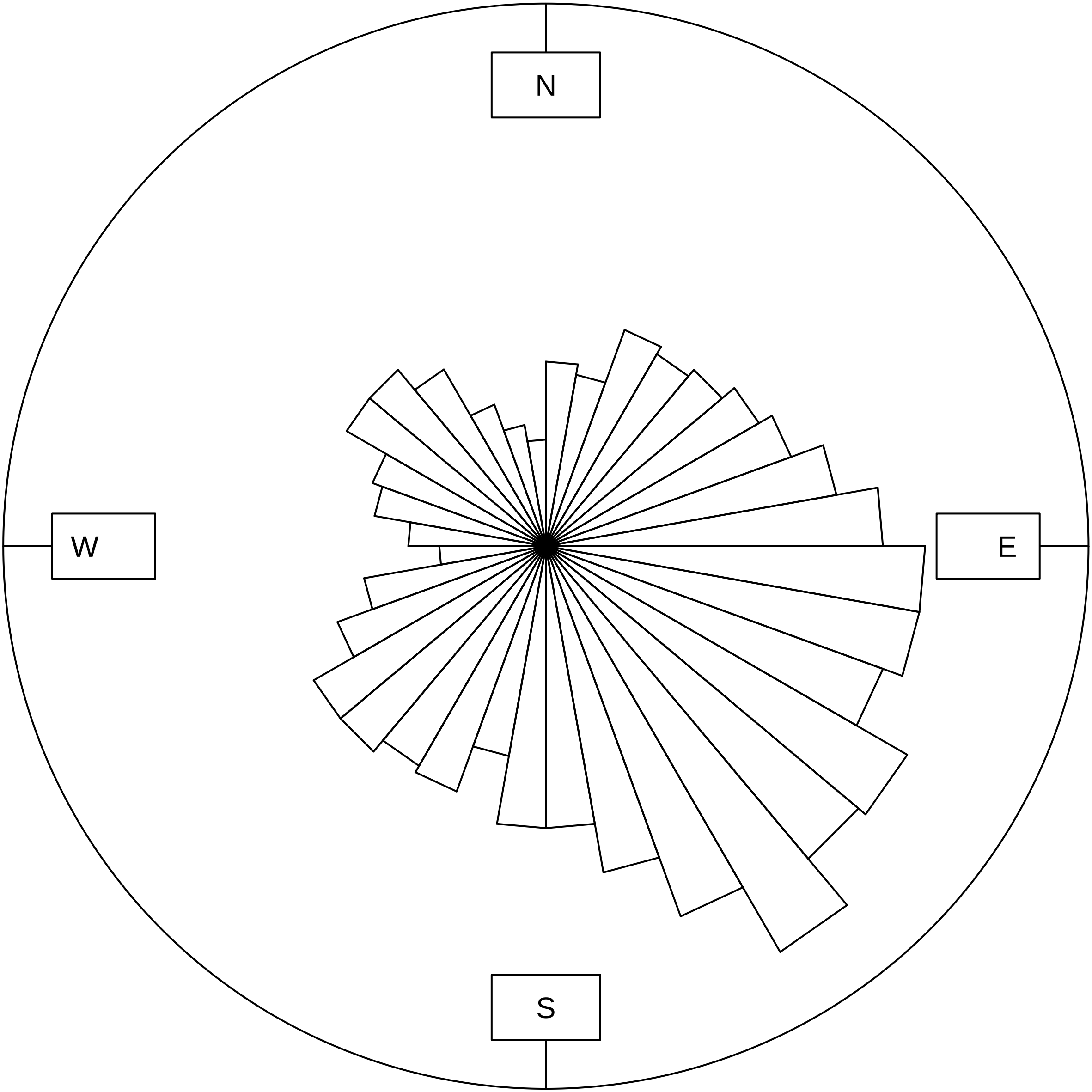

bootstrap 114

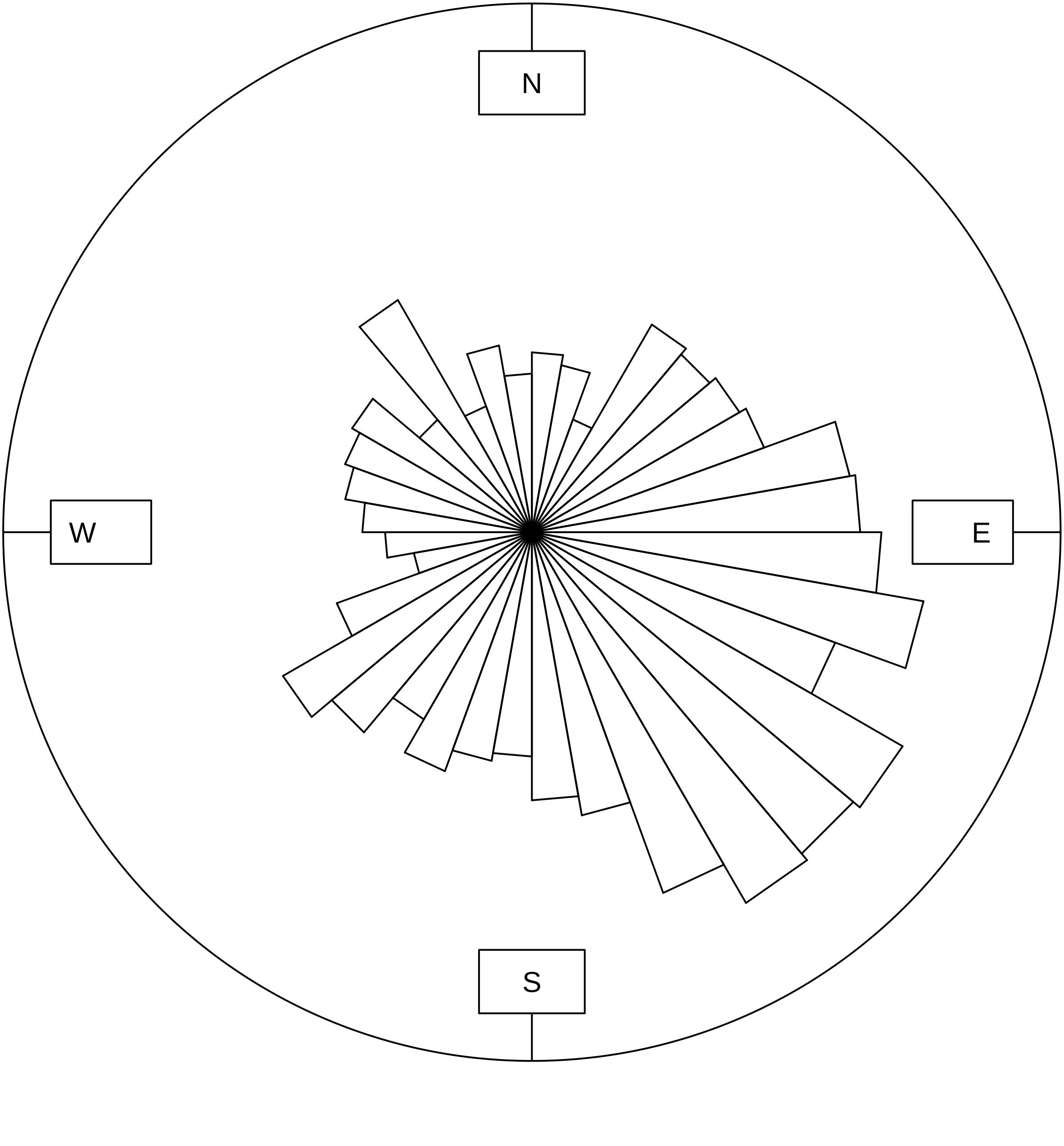

bootstrap 115

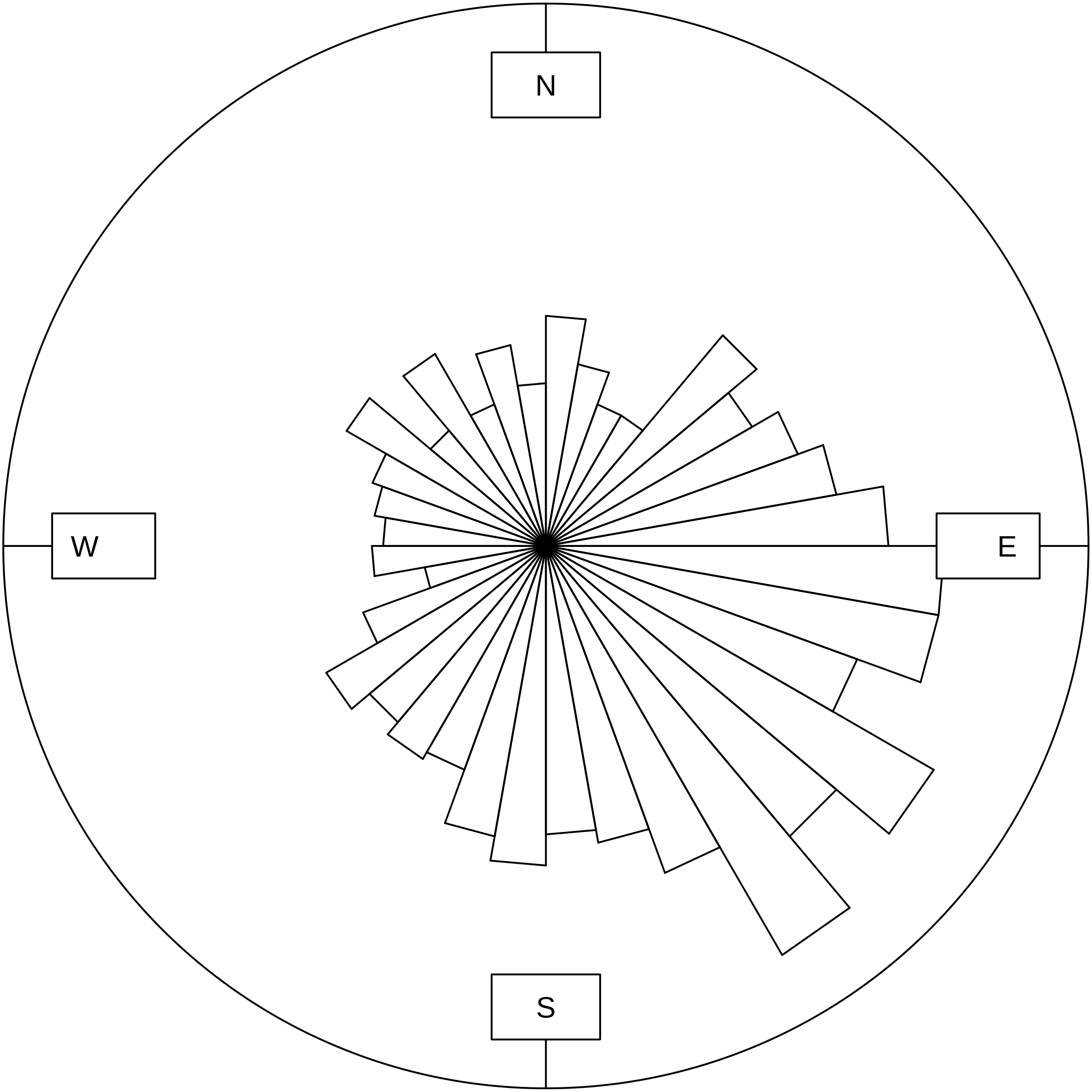

bootstrap 116

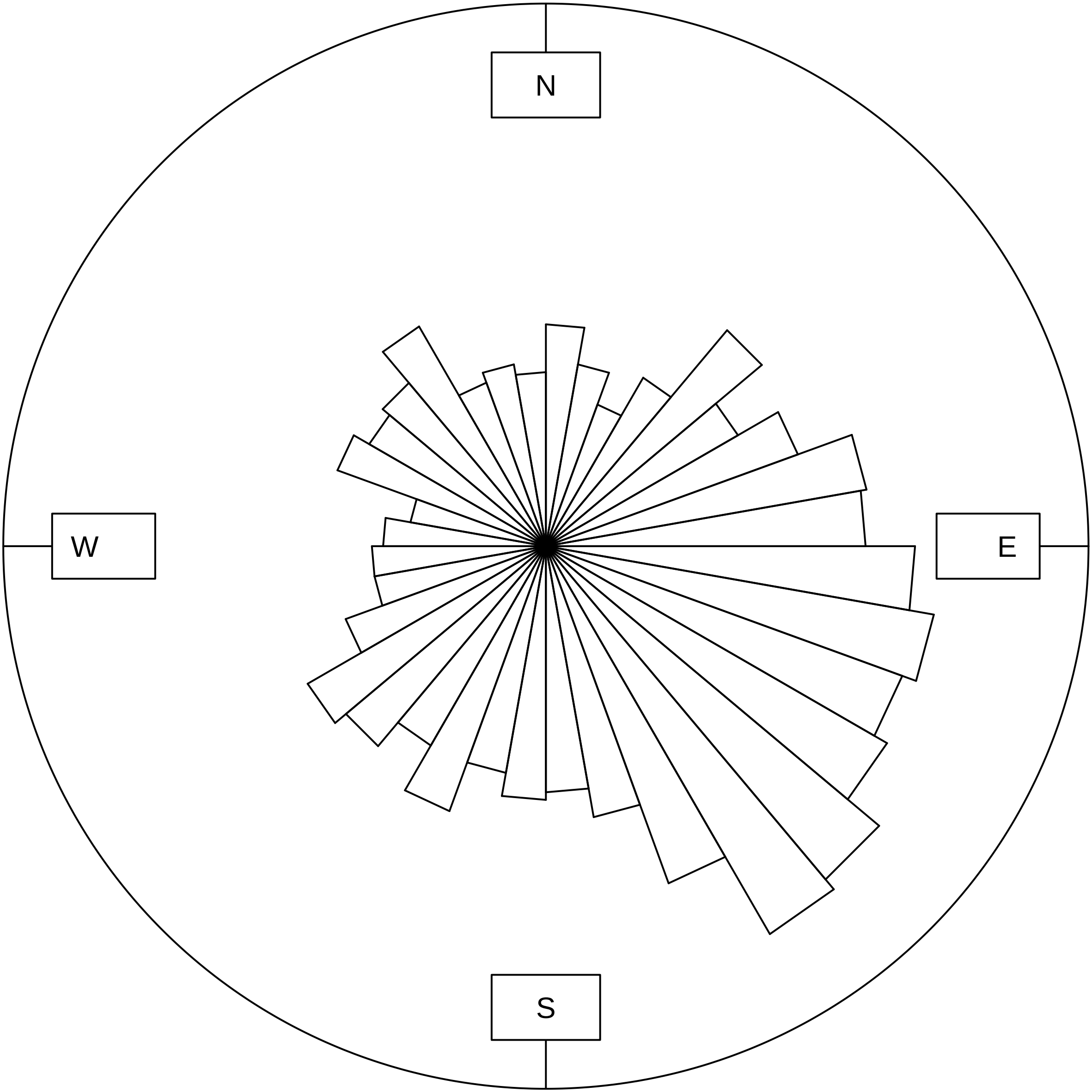

bootstrap 117

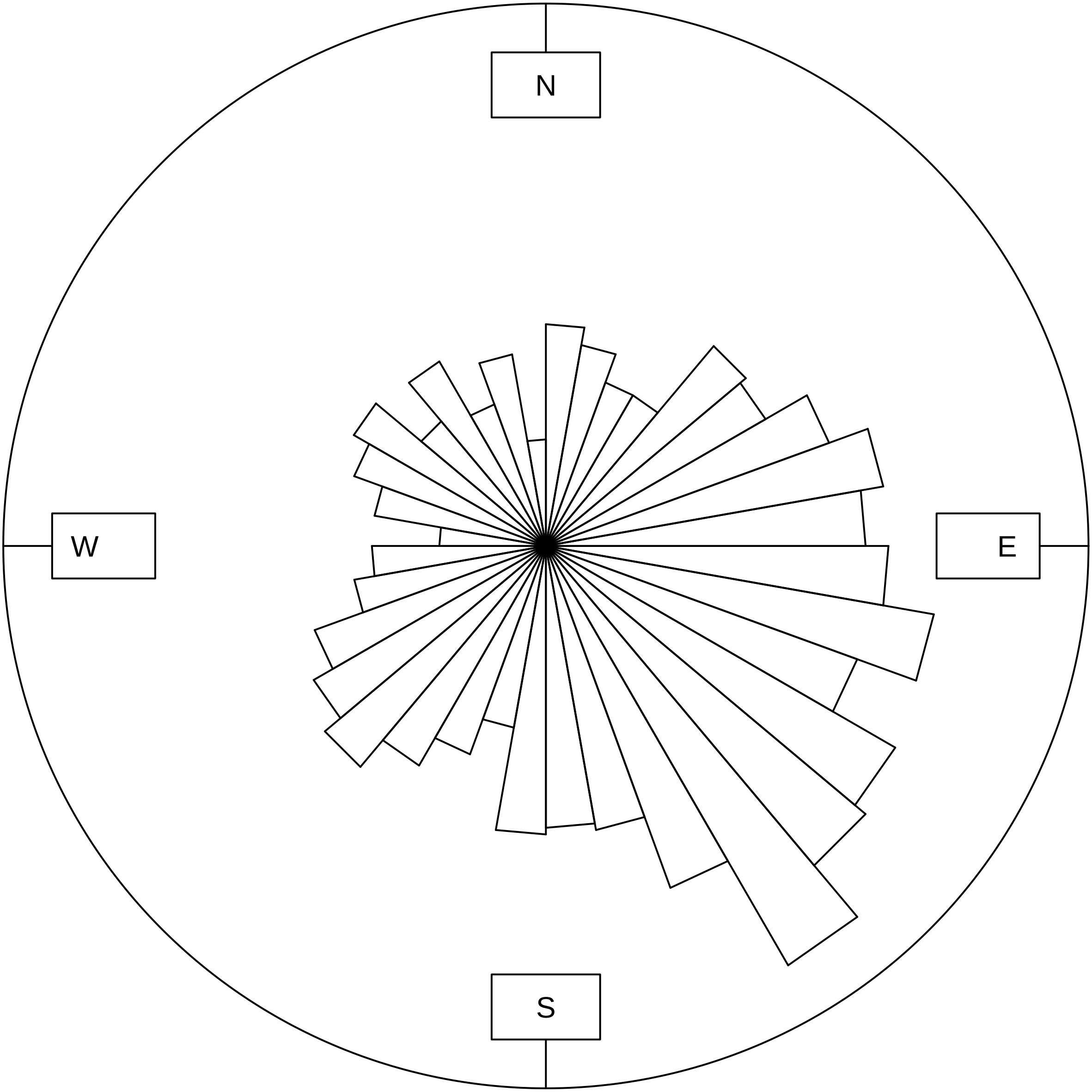

bootstrap 118

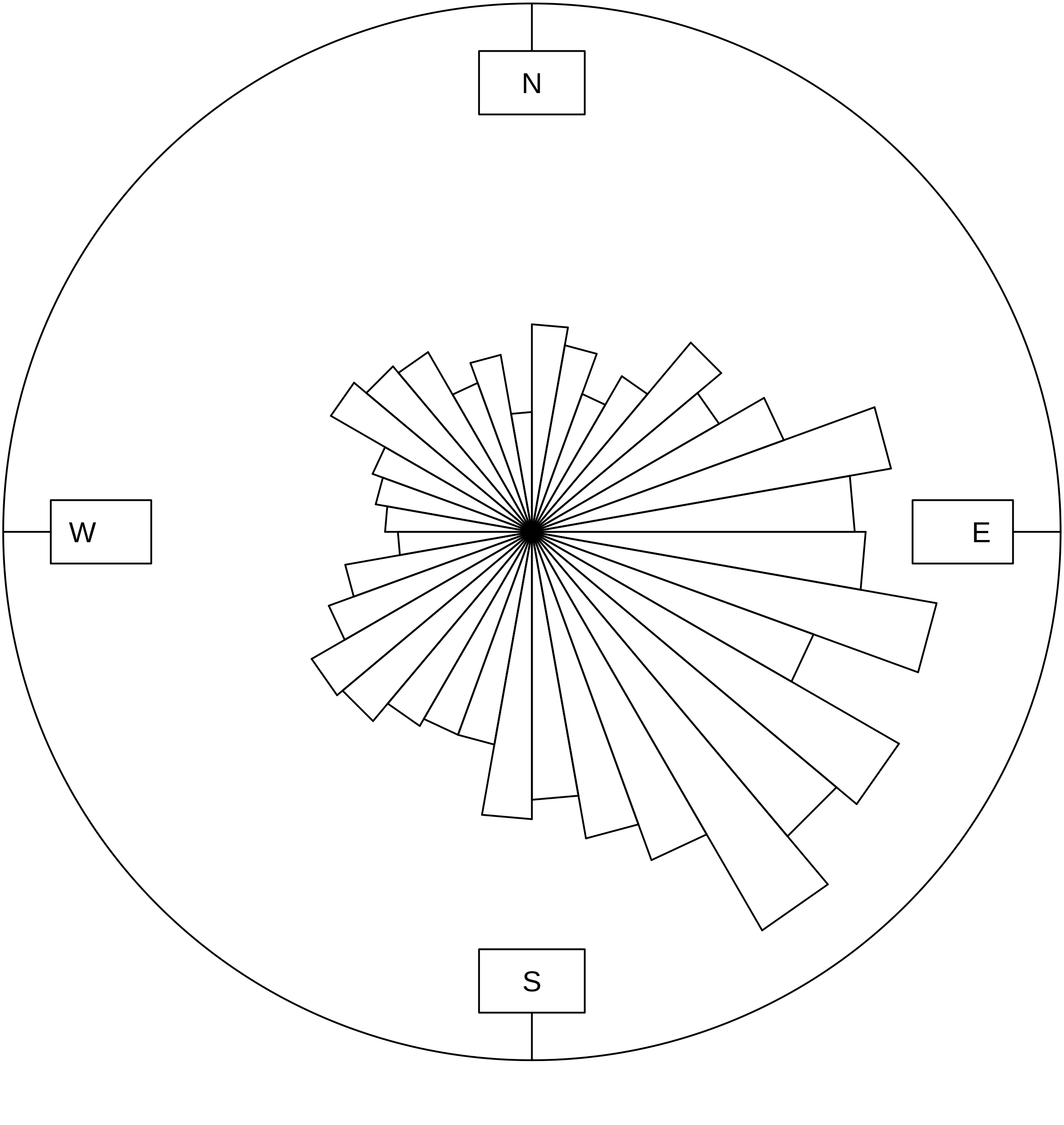

bootstrap 119

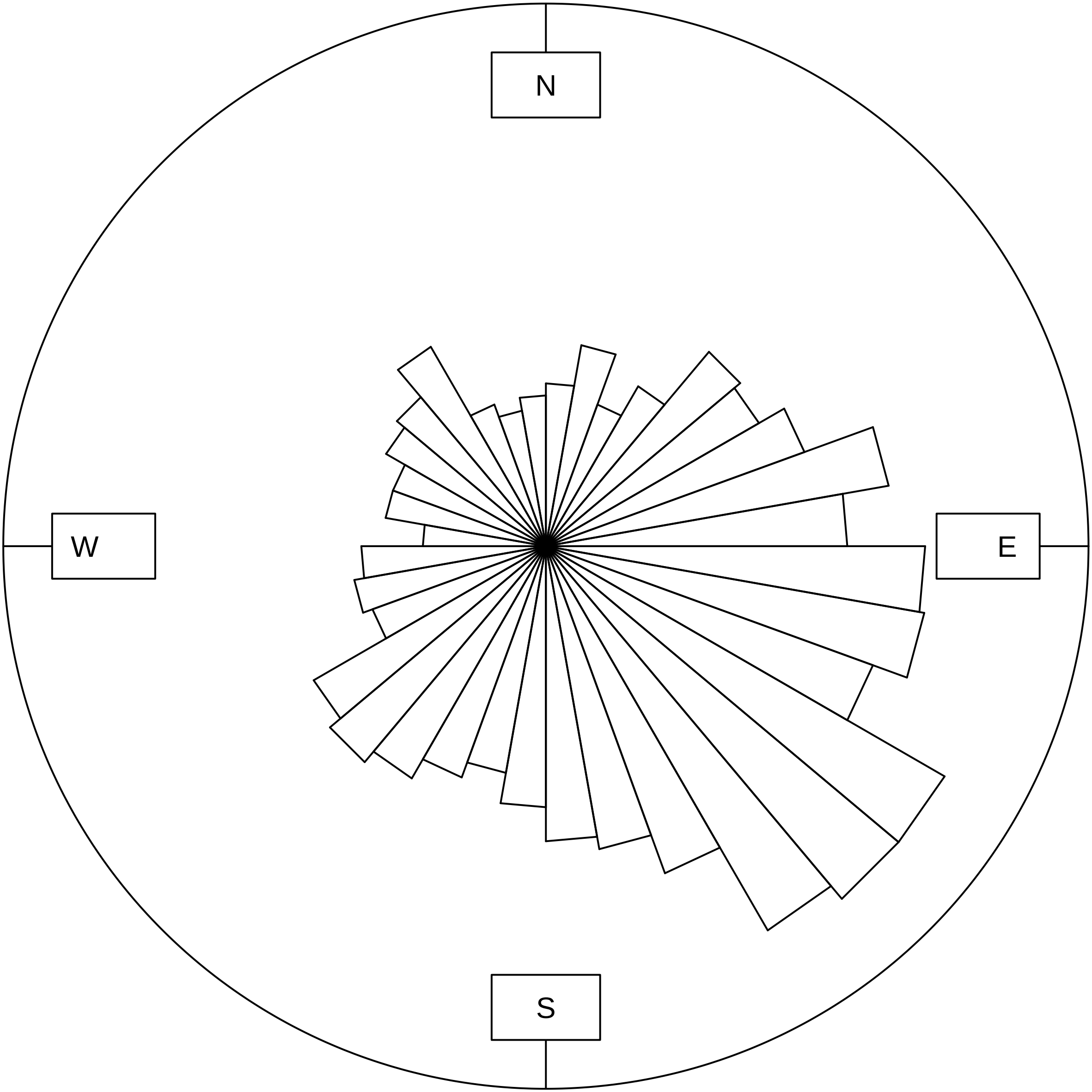

bootstrap 120

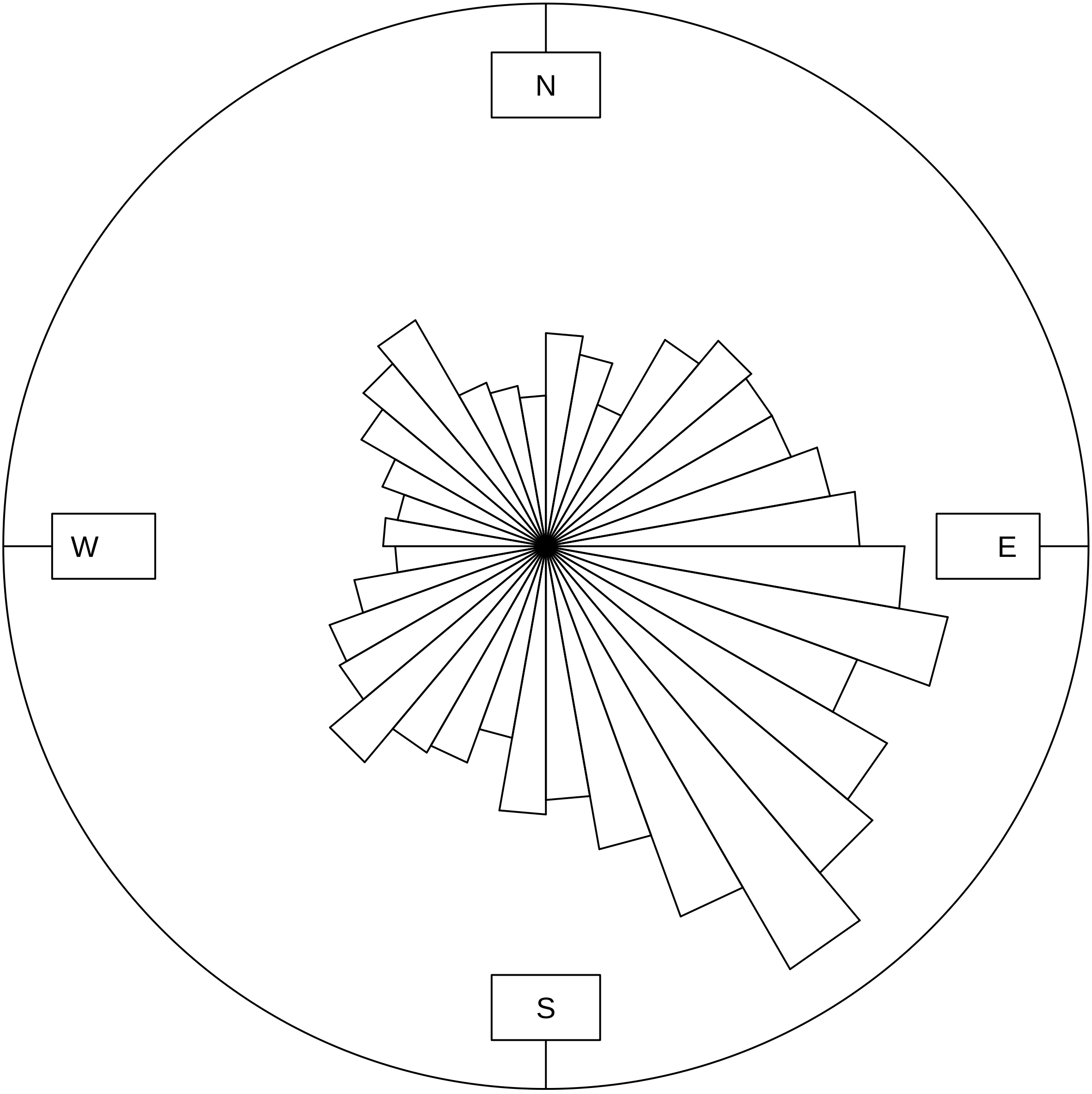

bootstrap 121

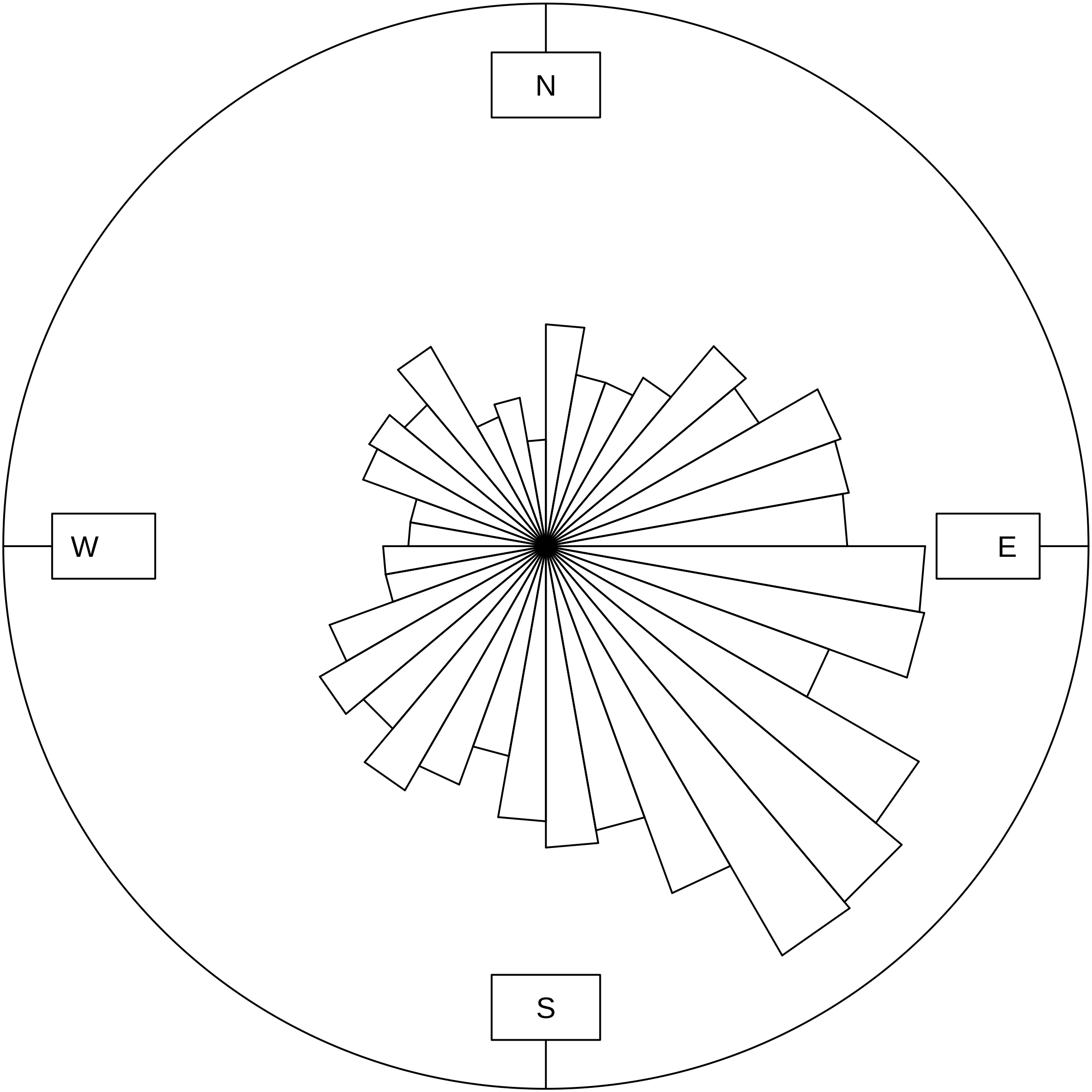

bootstrap 122

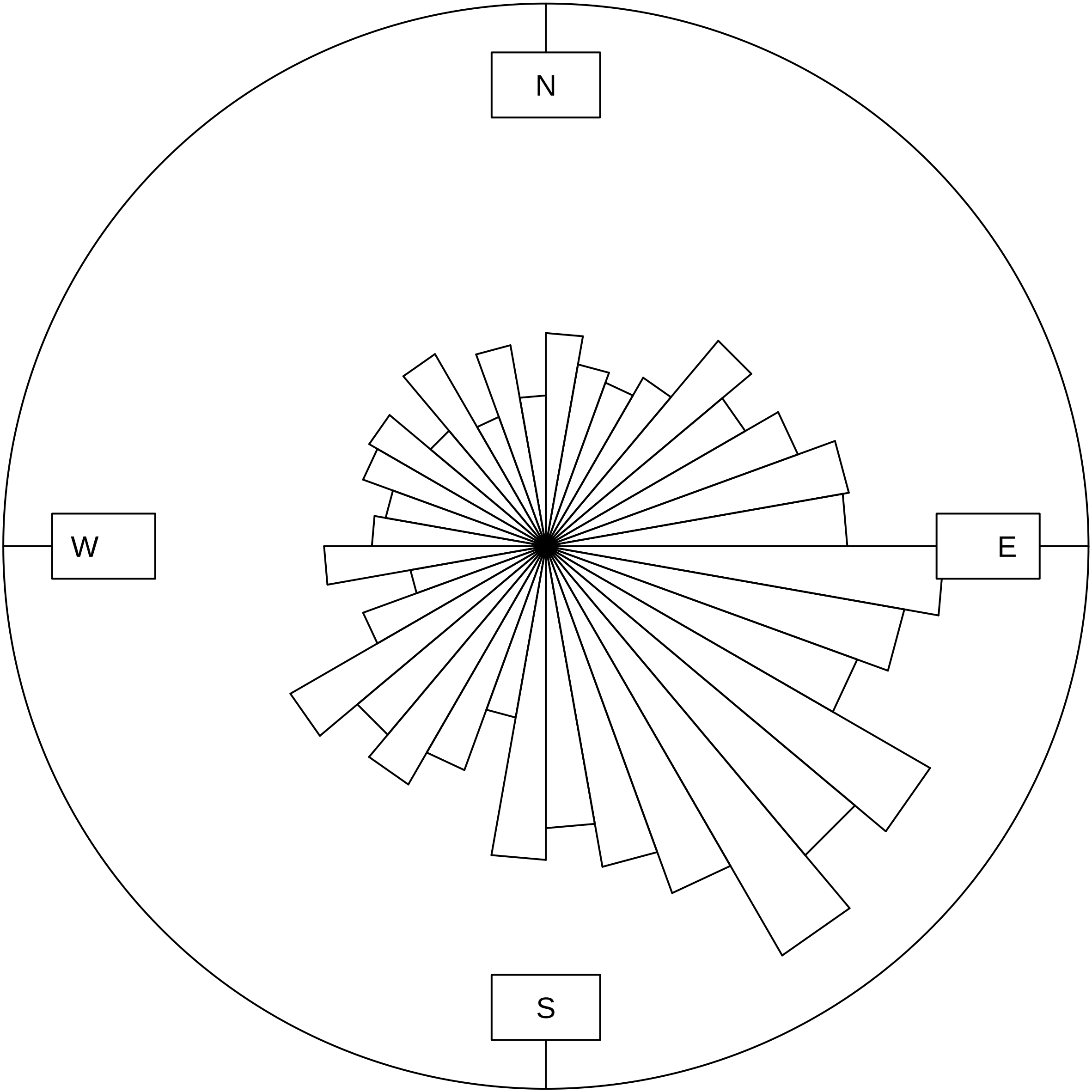

bootstrap 123

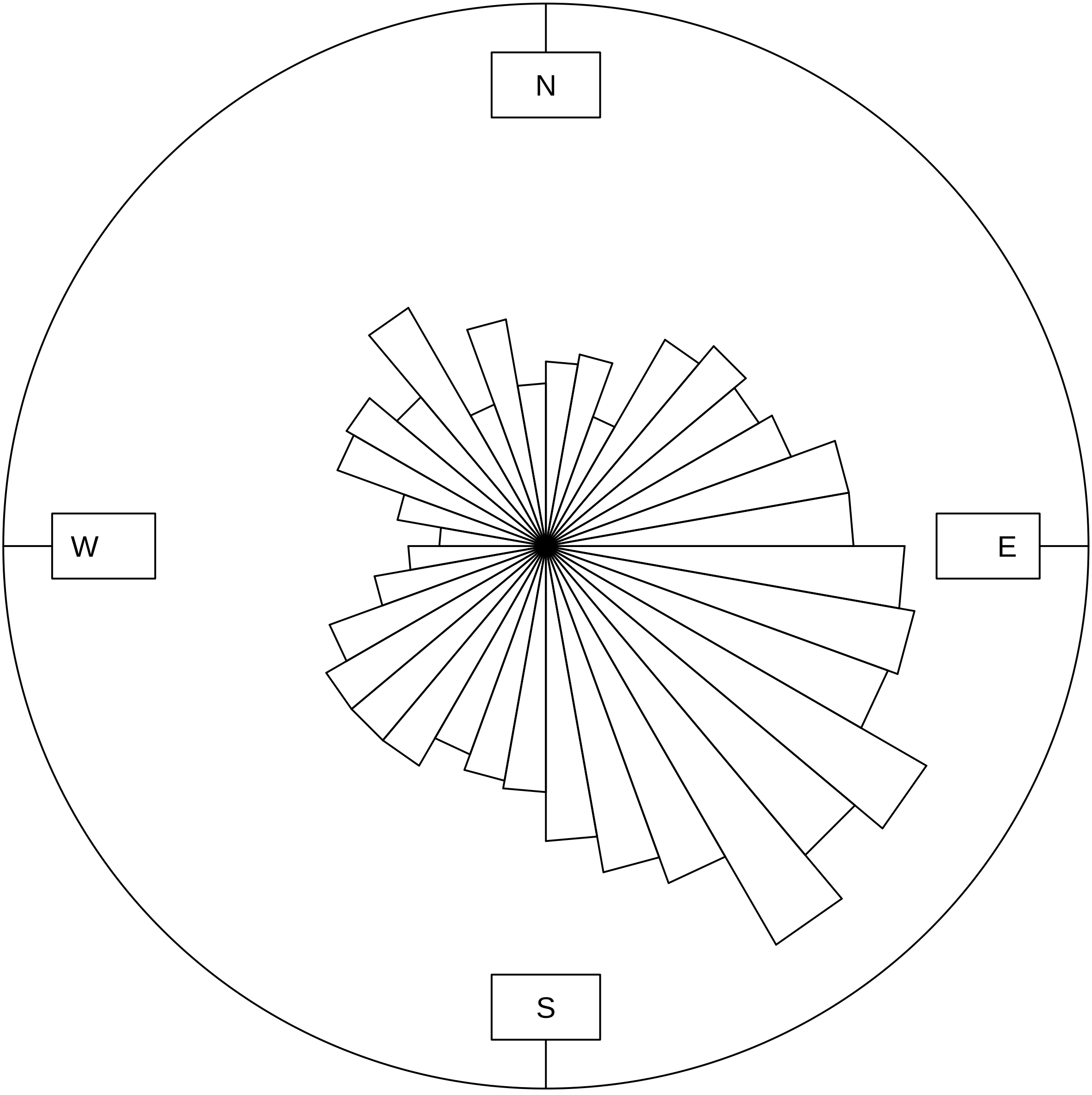

bootstrap 124

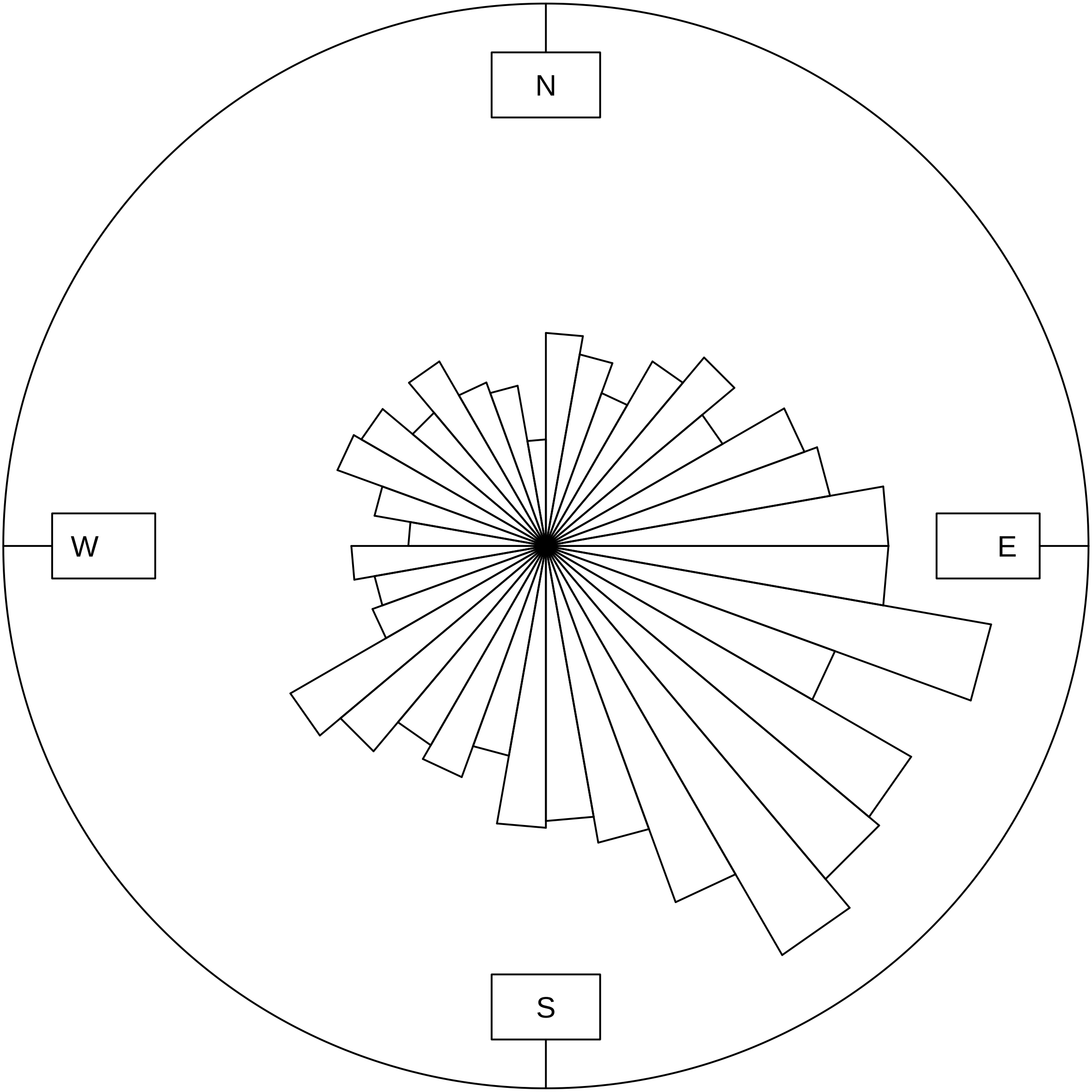

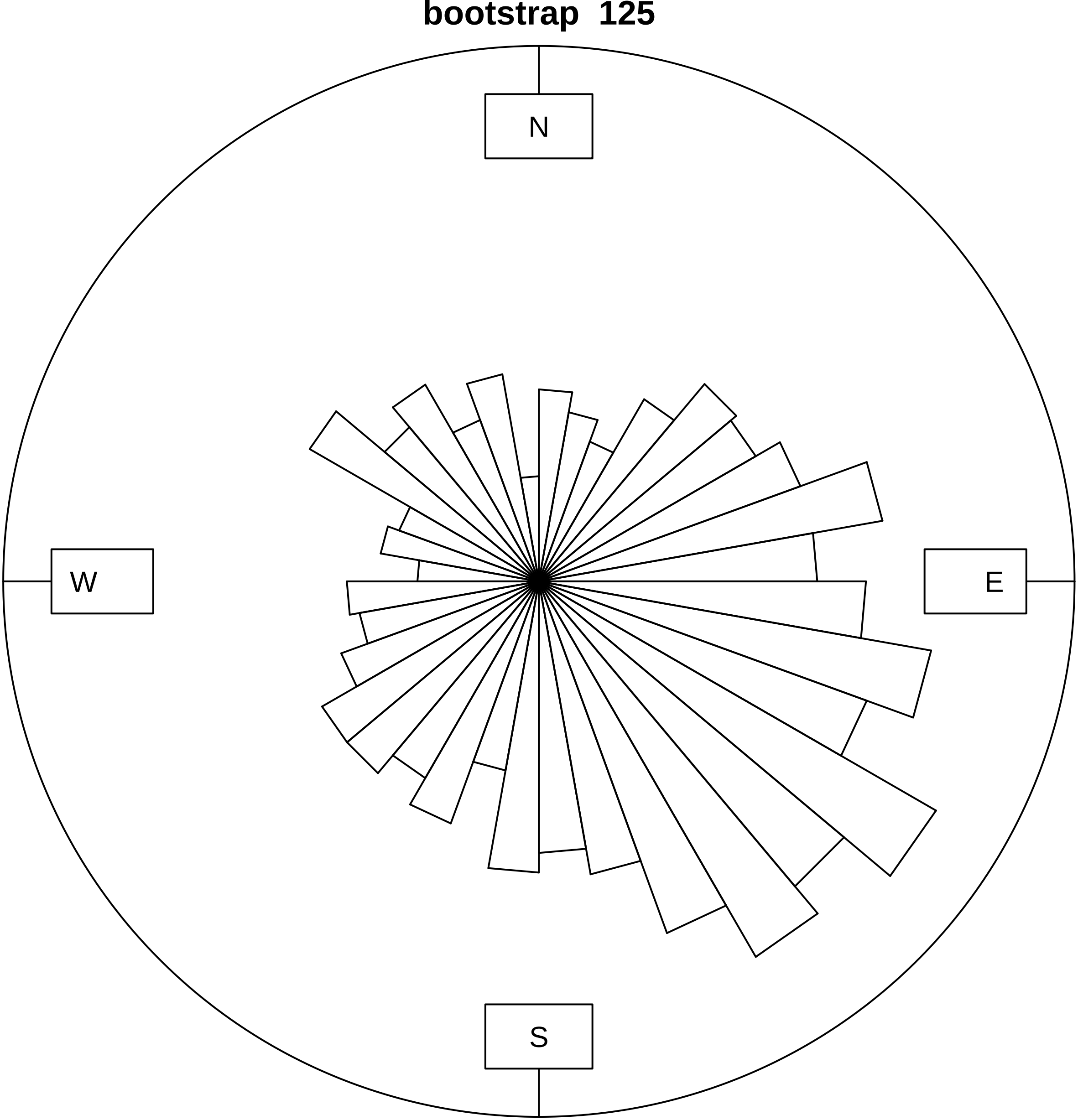
bootstrap 125
N
W
E
S

bootstrap 126

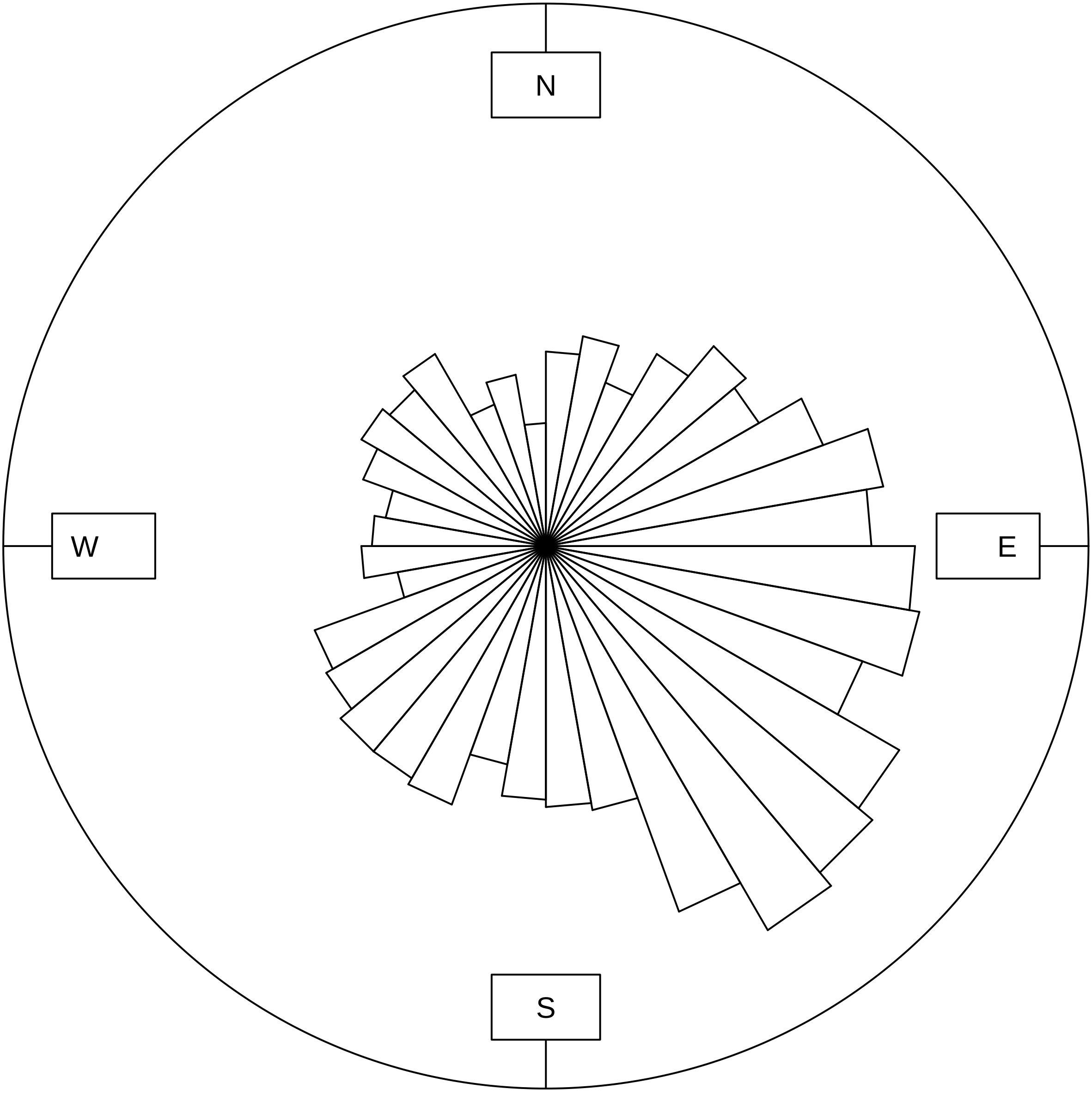

bootstrap 127

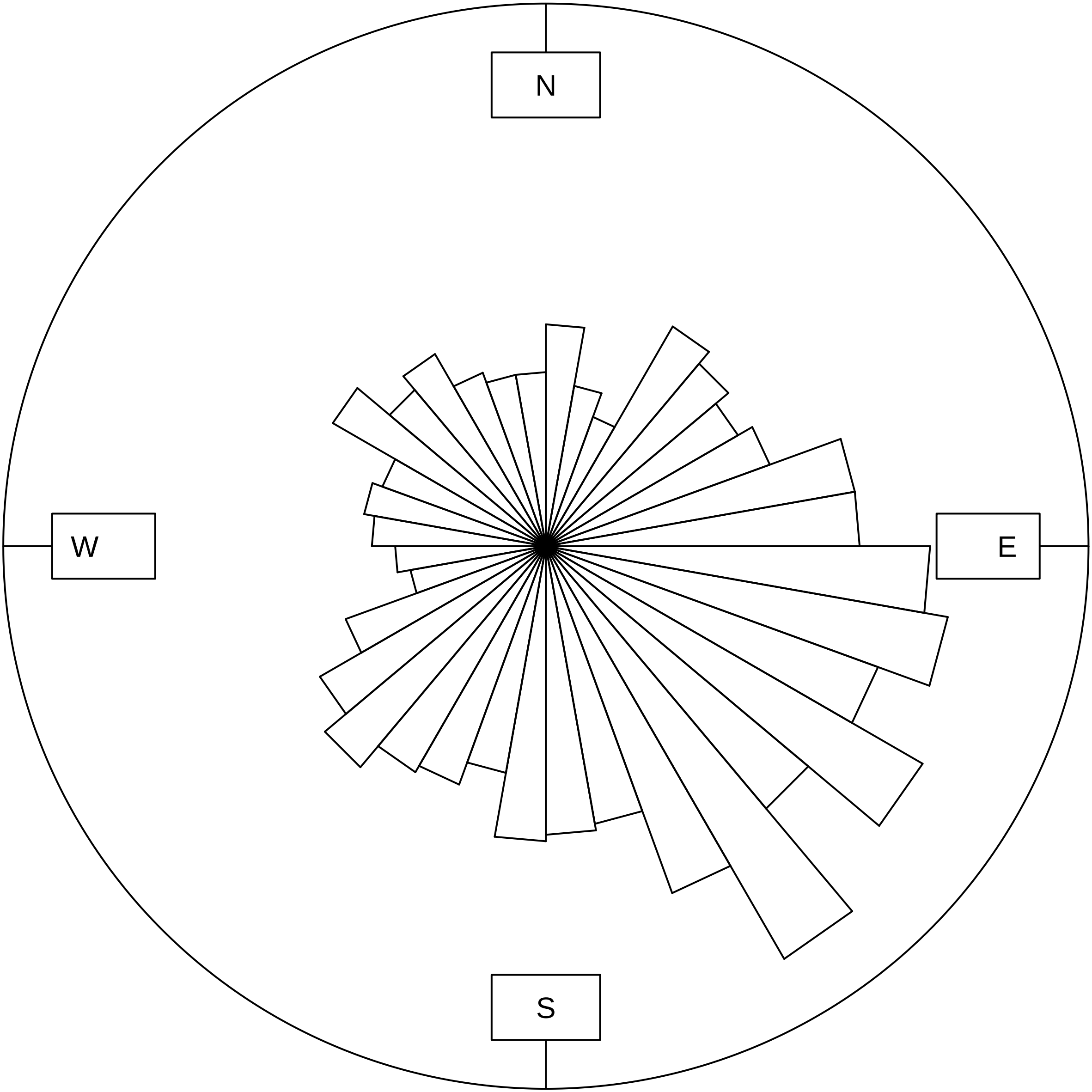

bootstrap 128

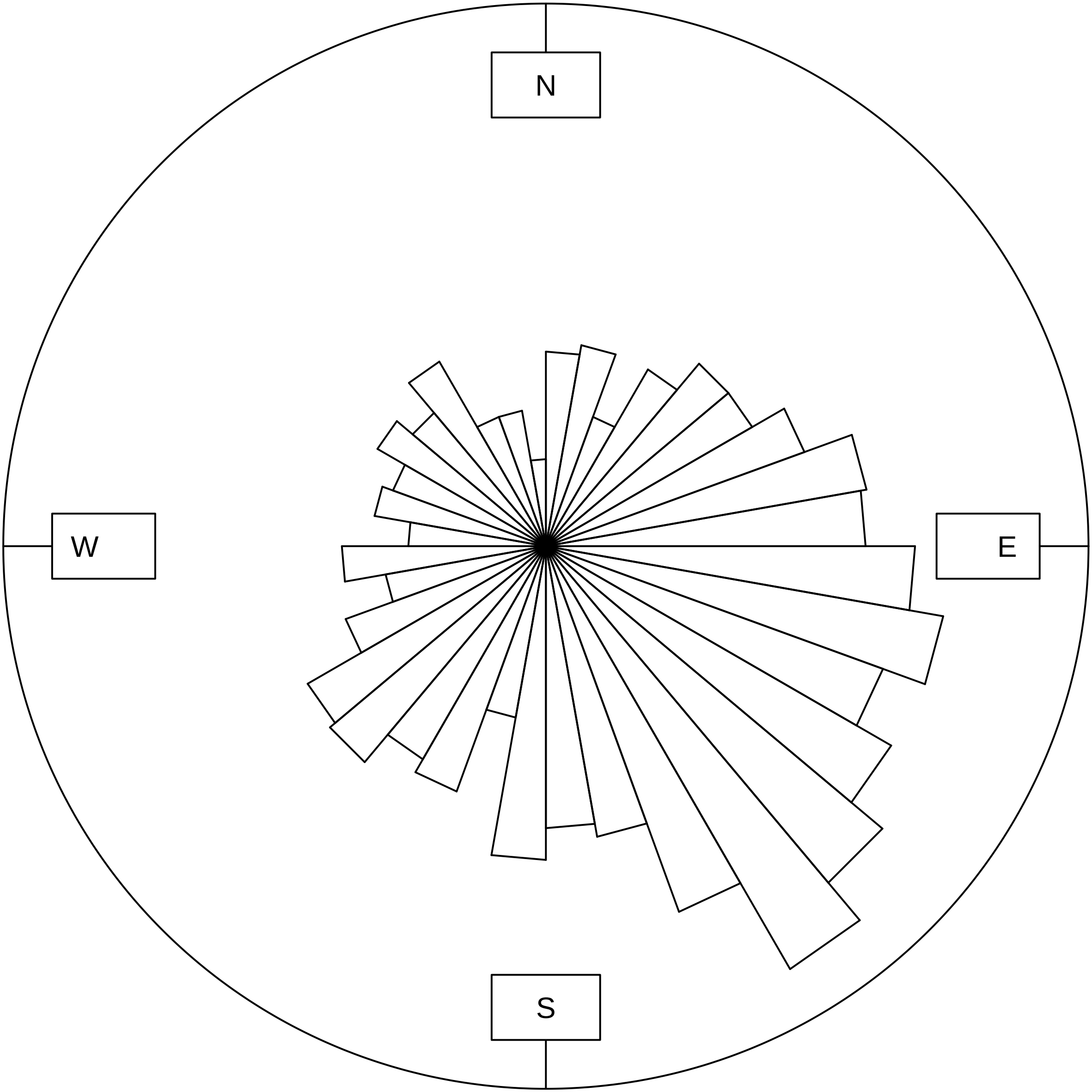

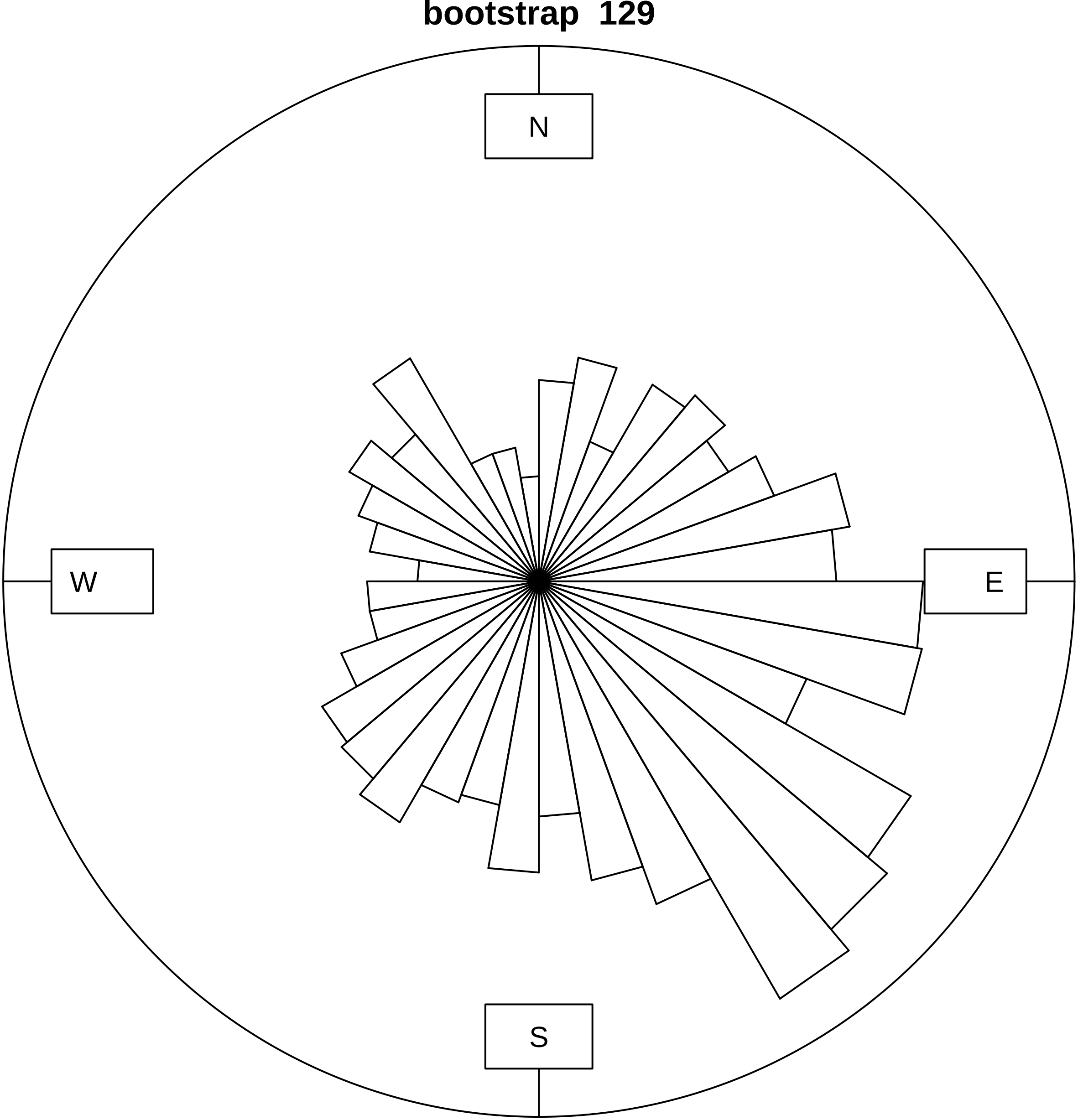
bootstrap 129
N
W
E
S

bootstrap 130

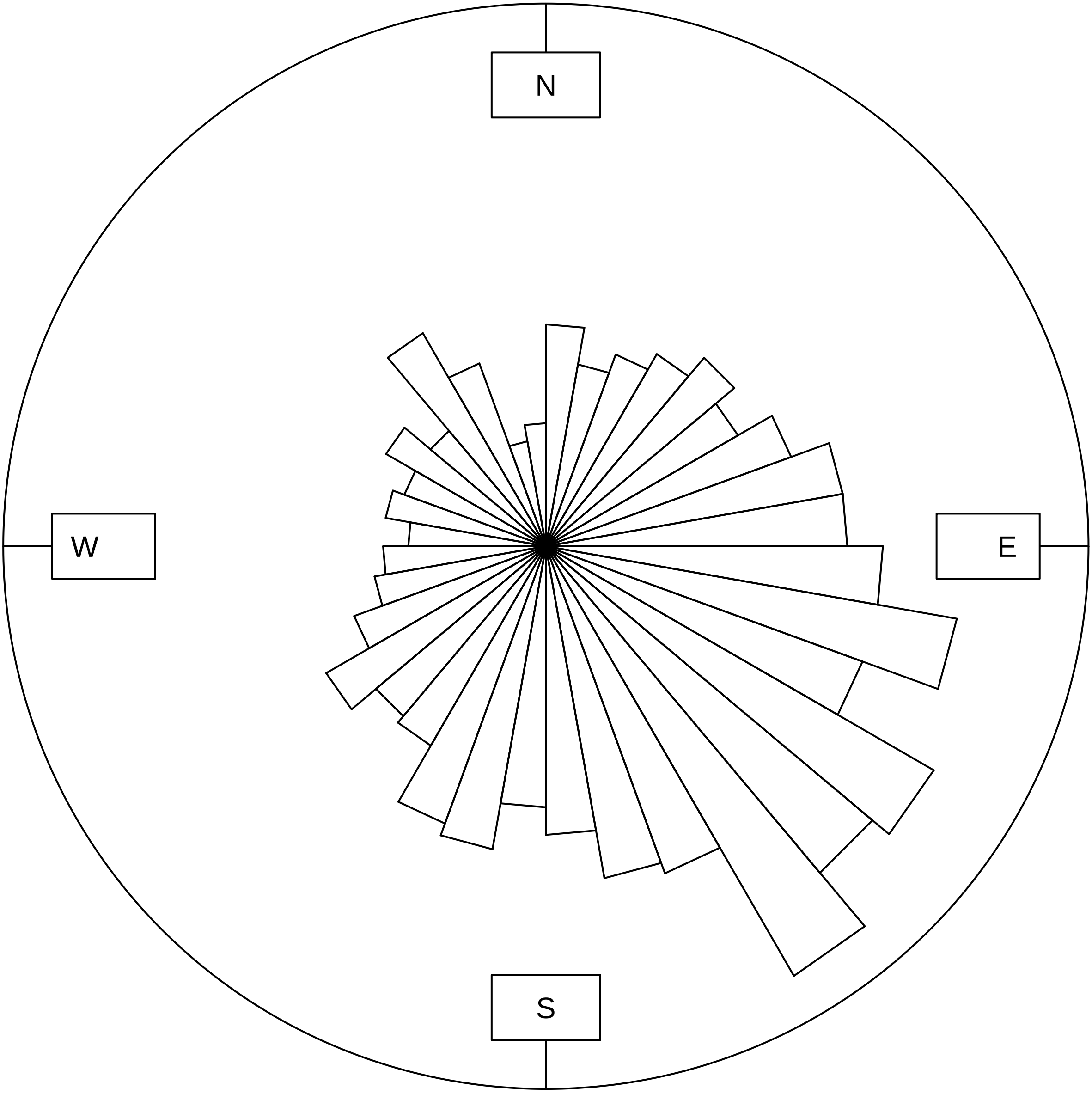

bootstrap 131

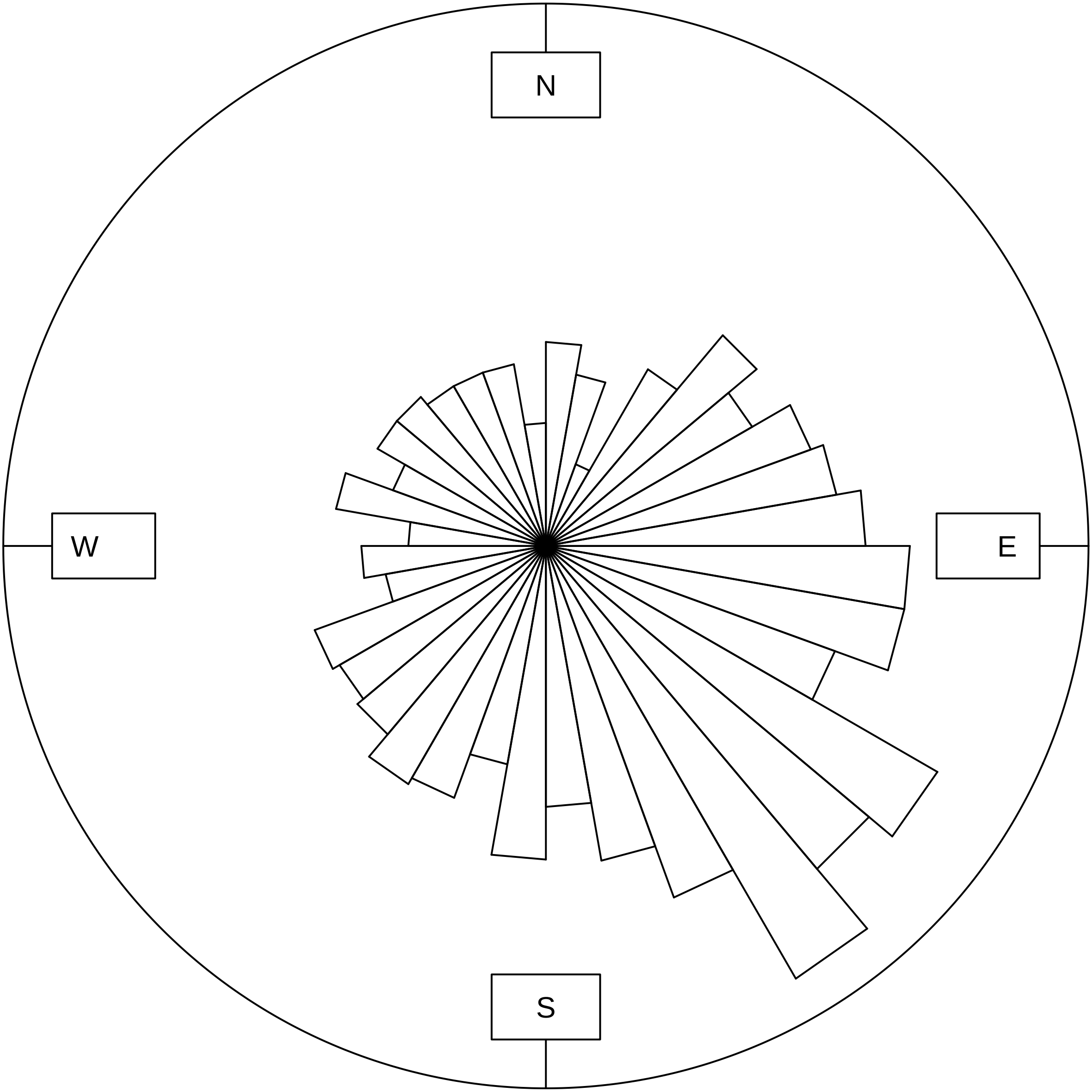

bootstrap 132

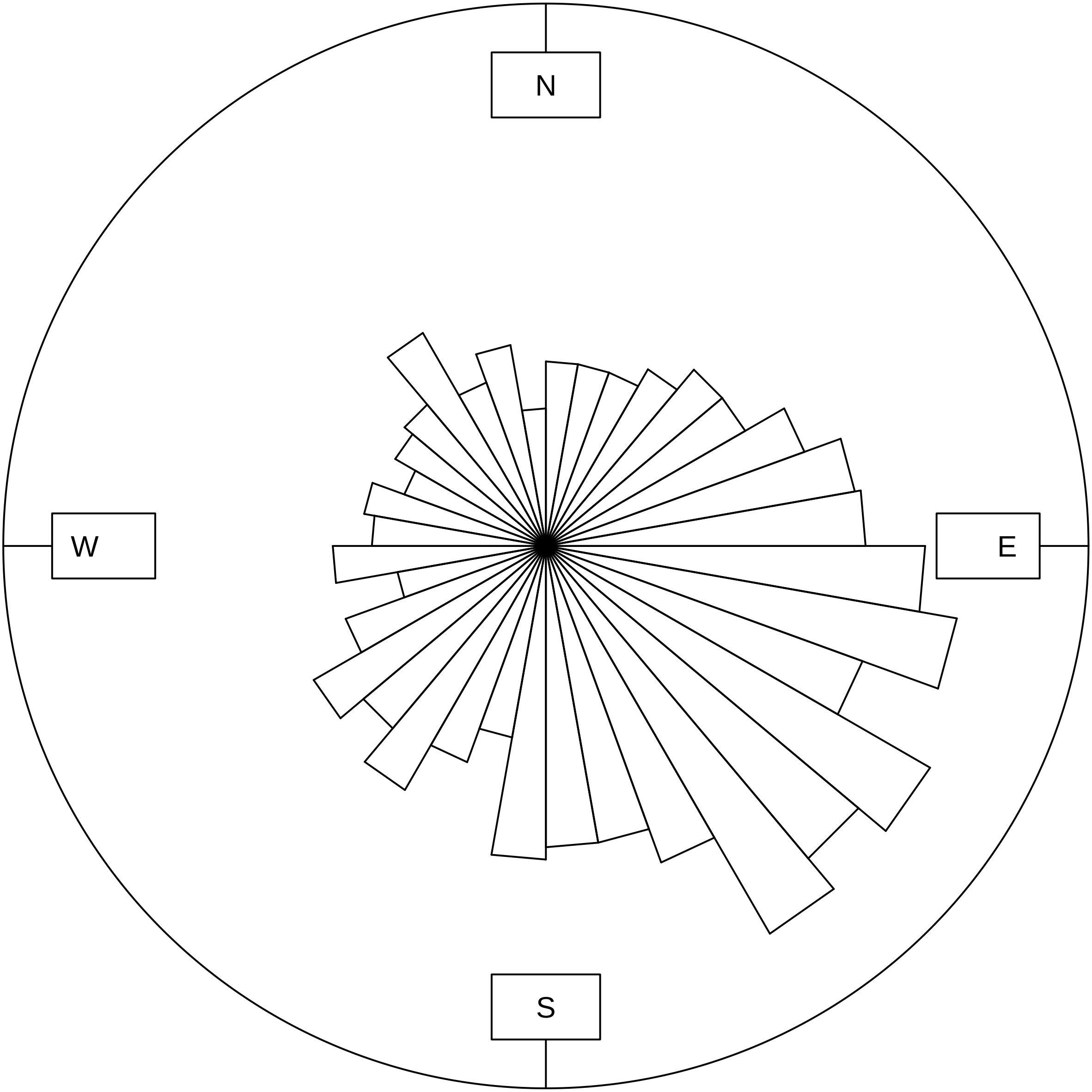

bootstrap 133

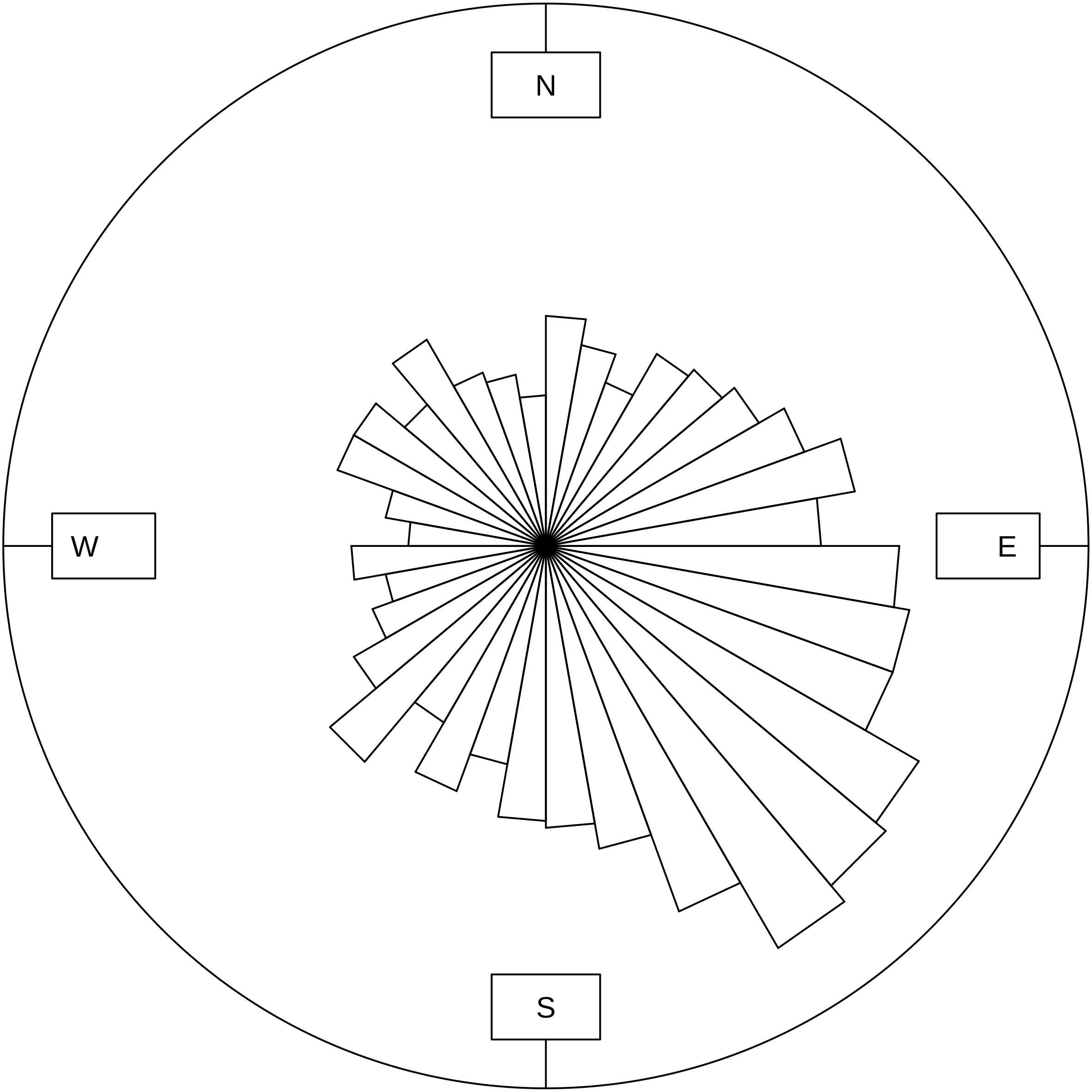

bootstrap 134

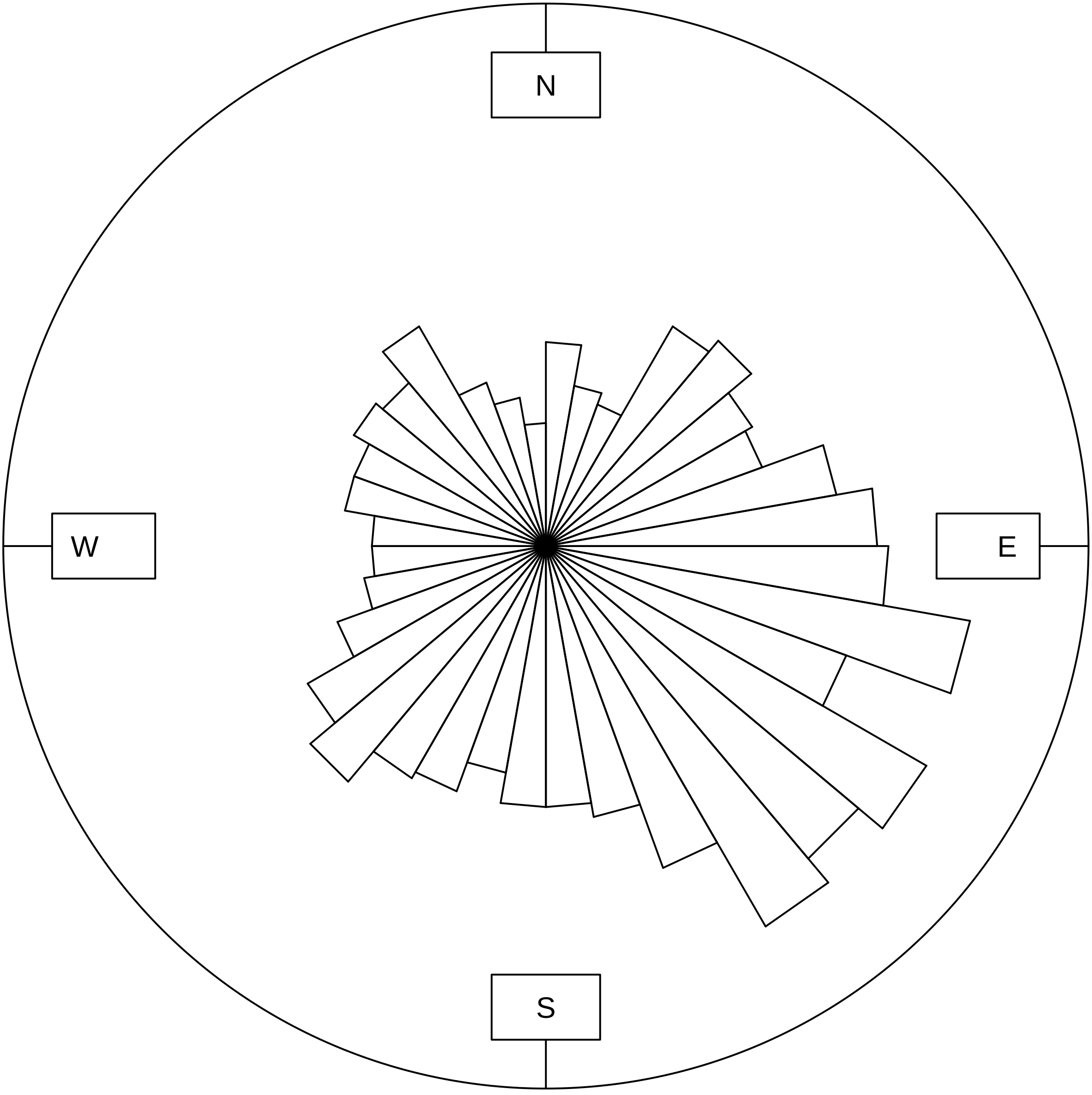

bootstrap 135

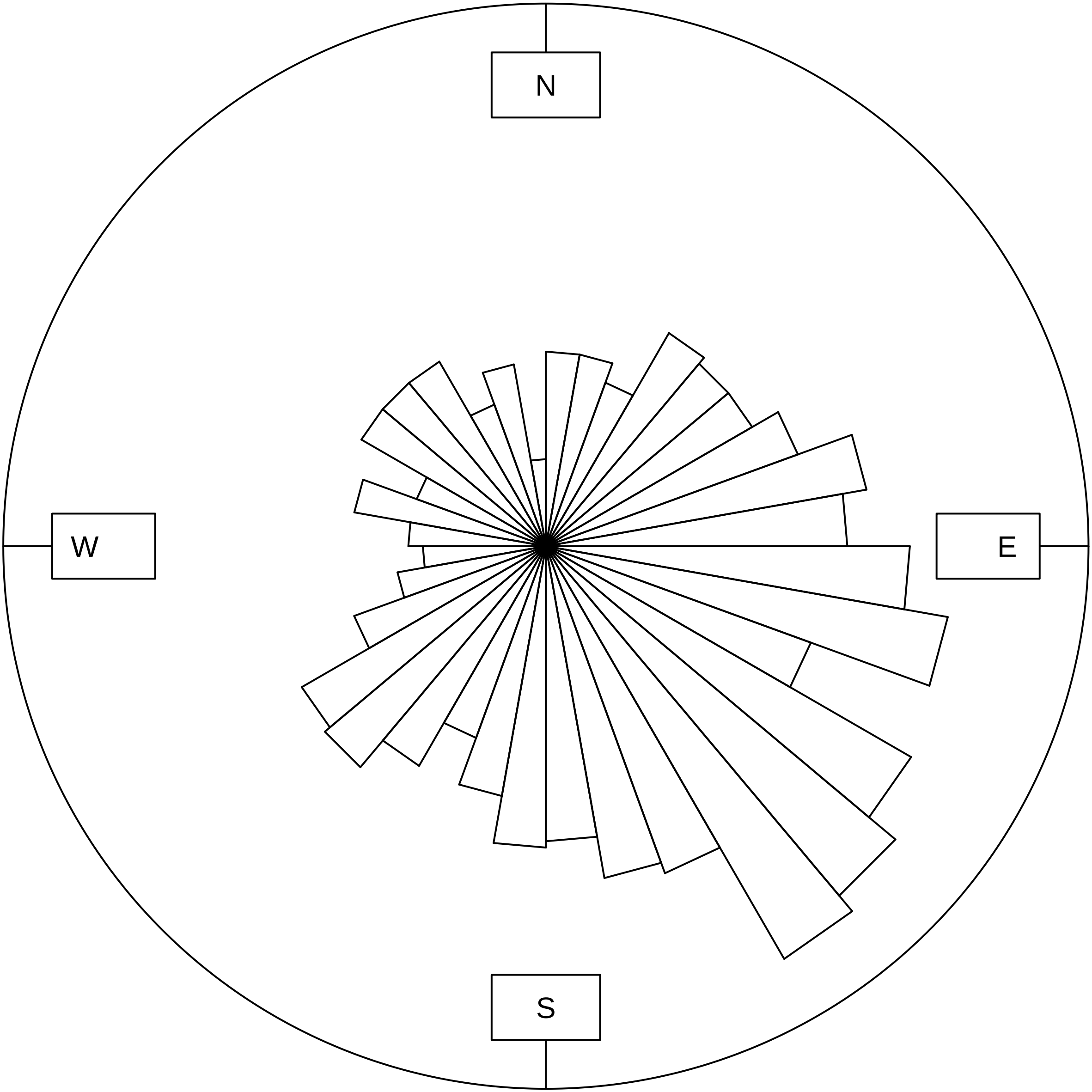

bootstrap 136

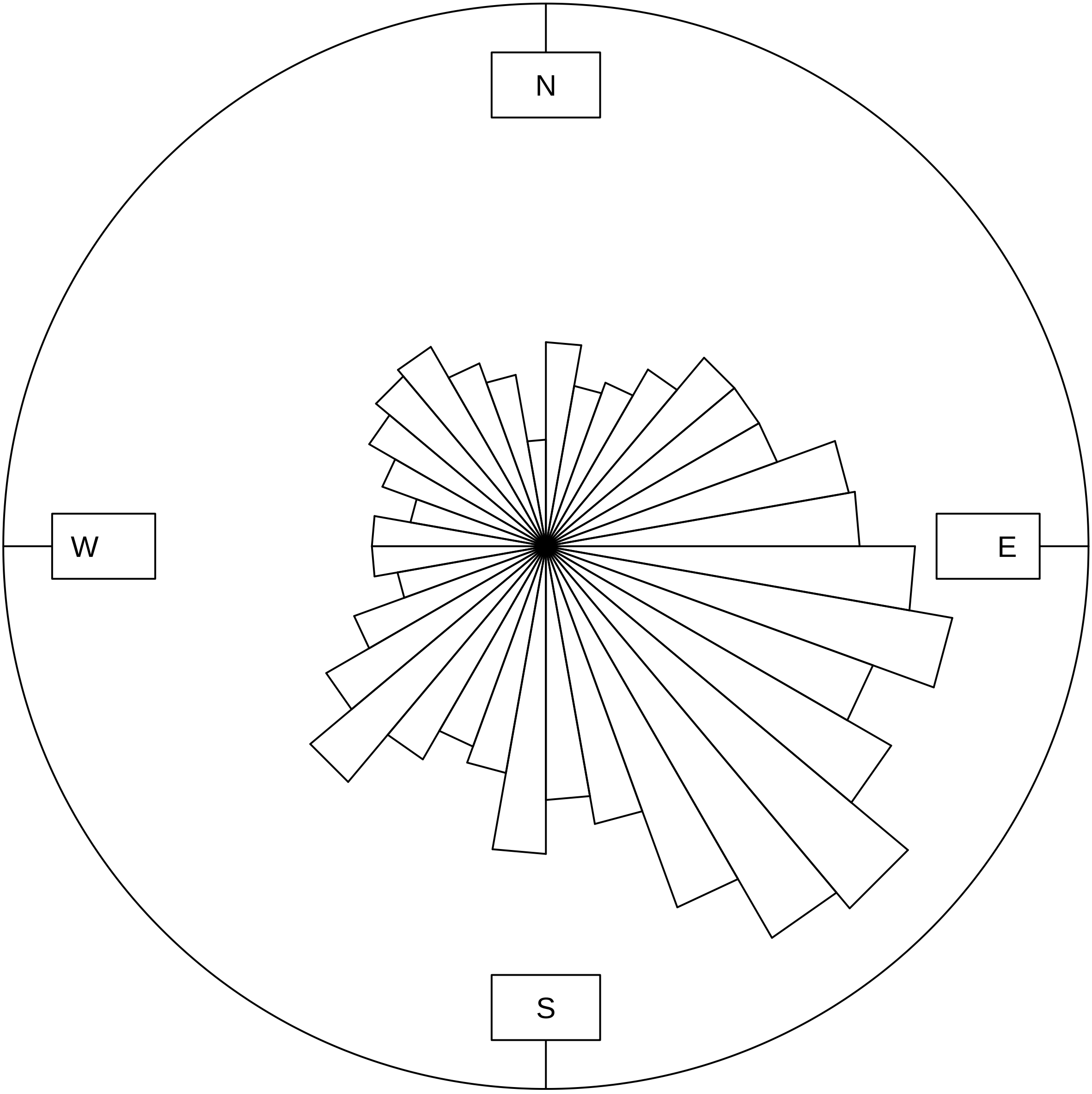

bootstrap 137

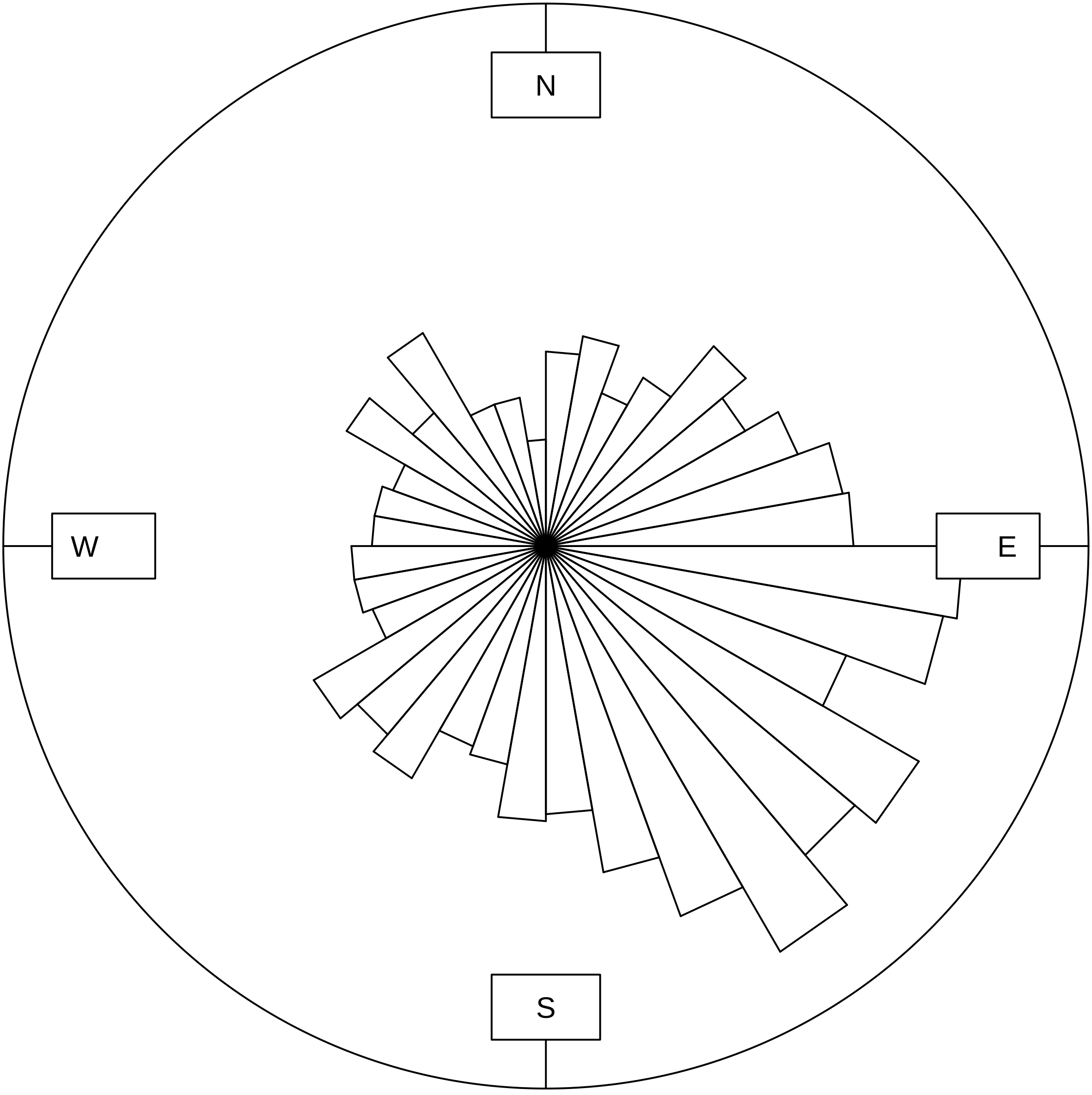

bootstrap 138

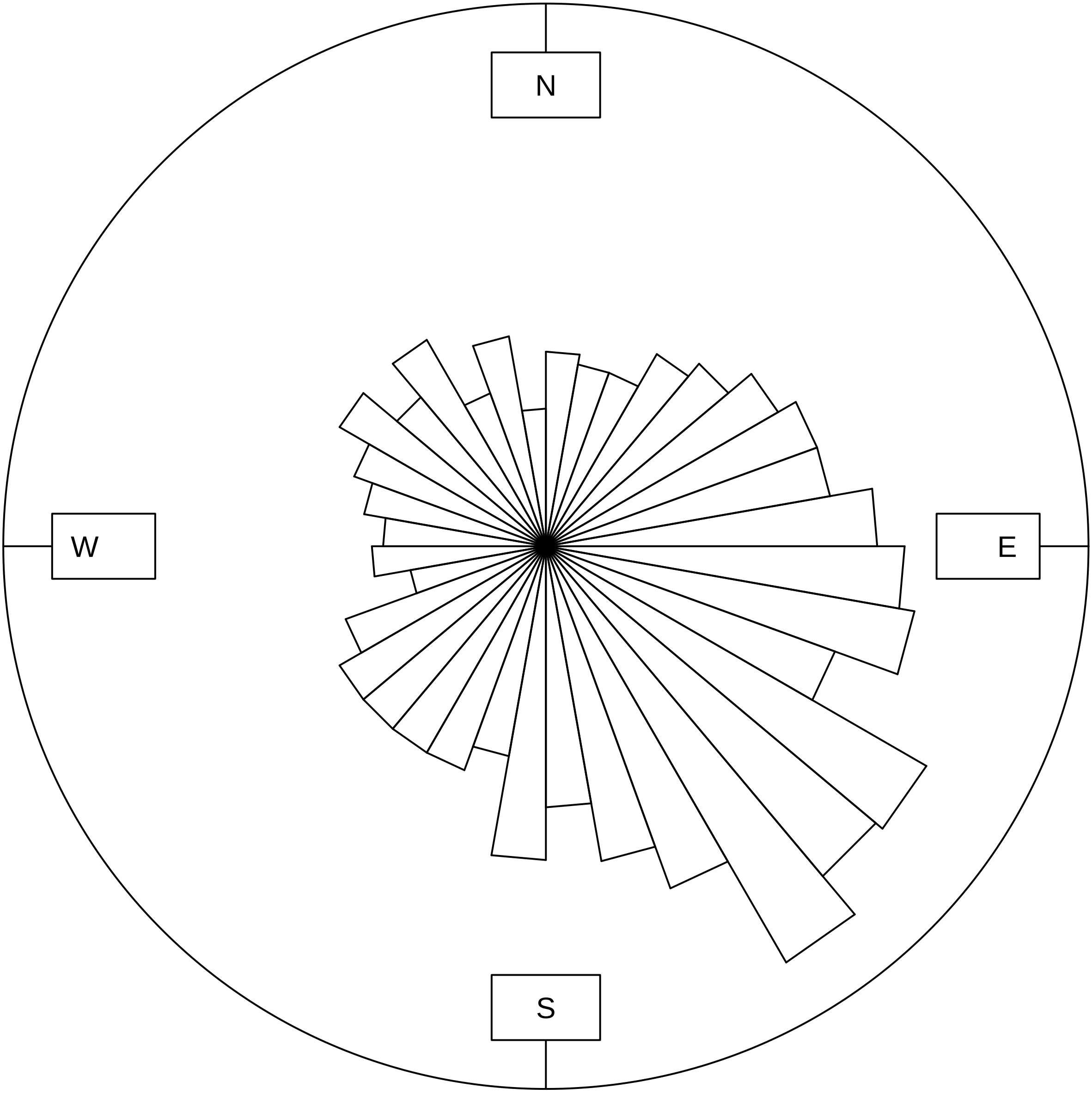

bootstrap 139

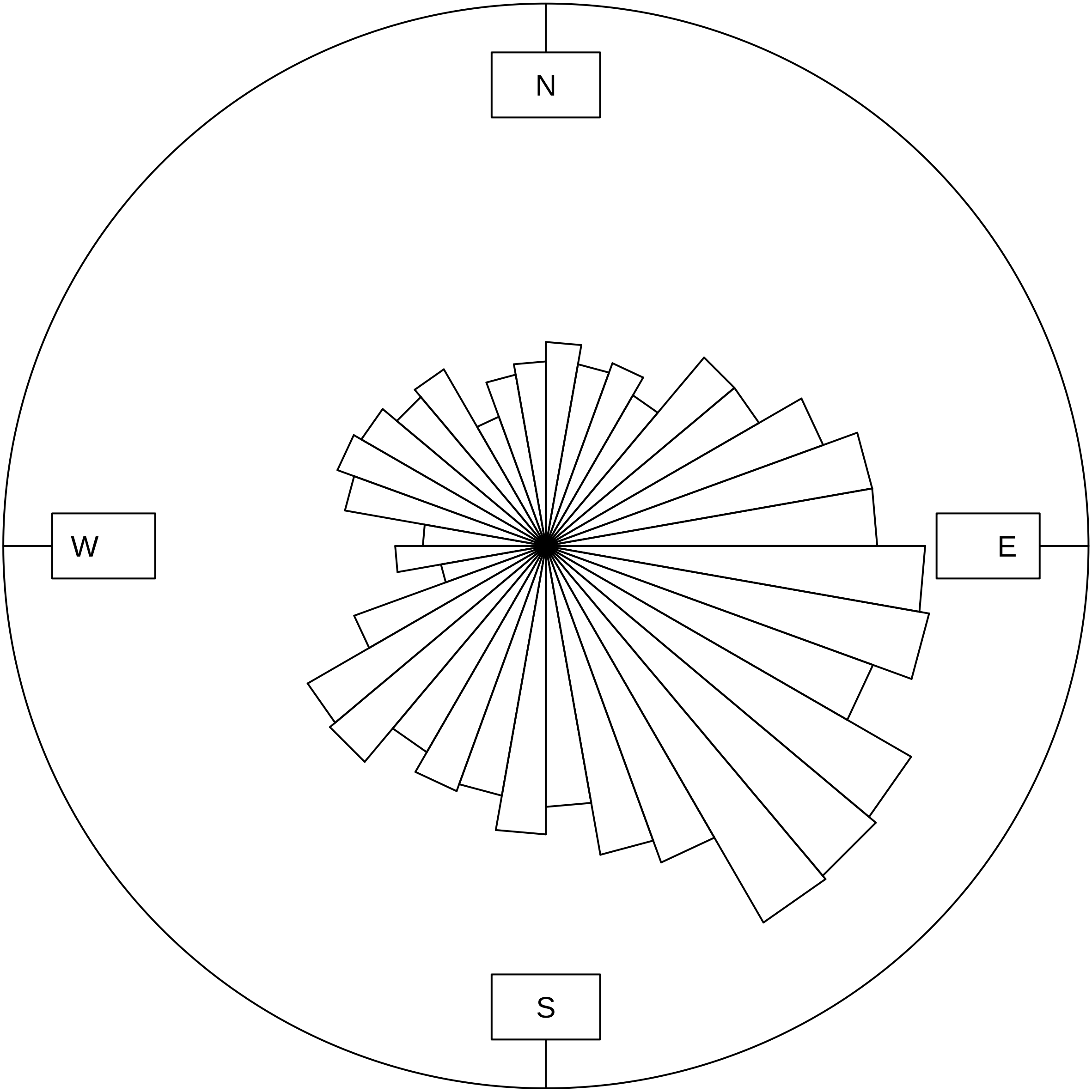

bootstrap 140

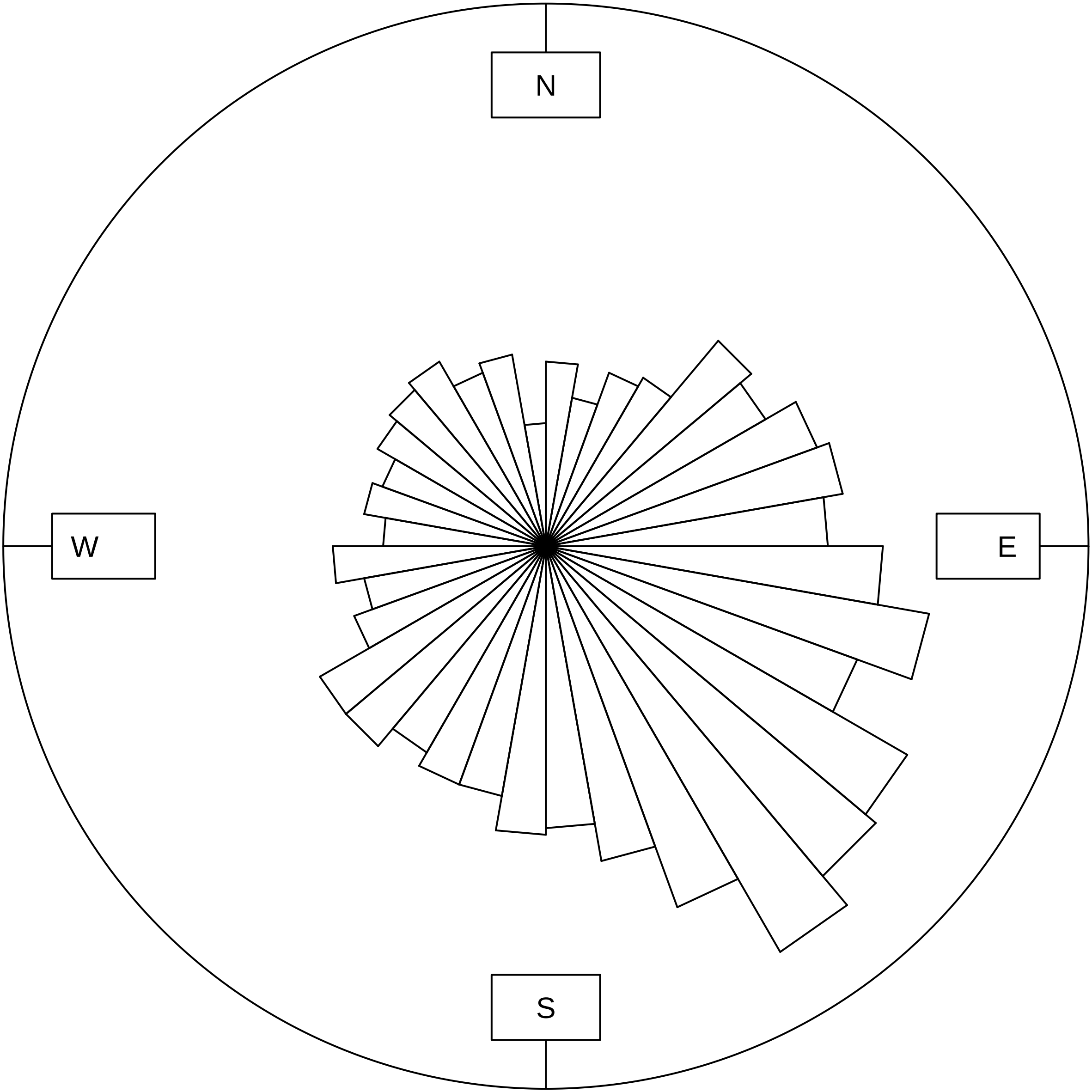

bootstrap 141

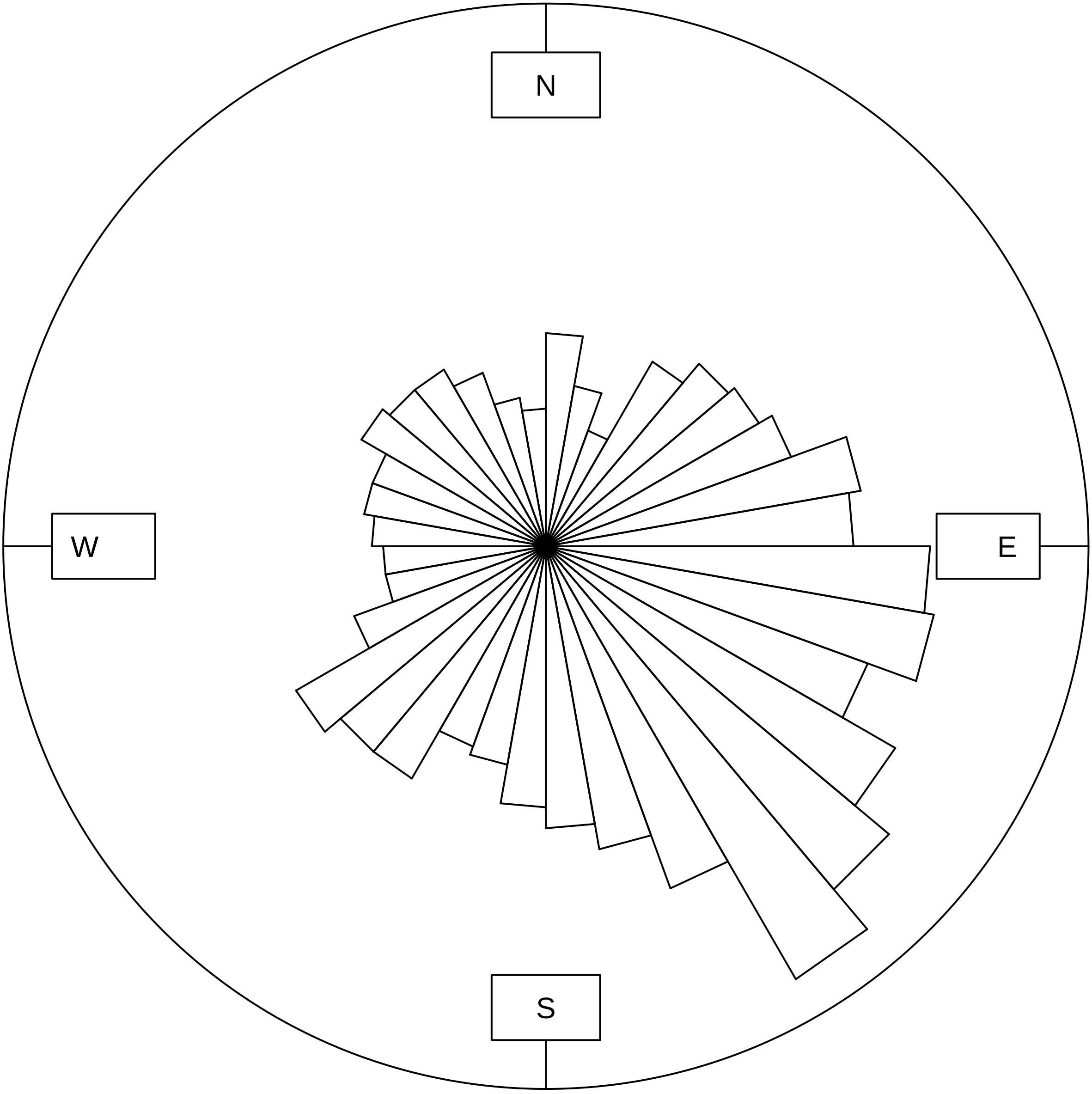

bootstrap 142

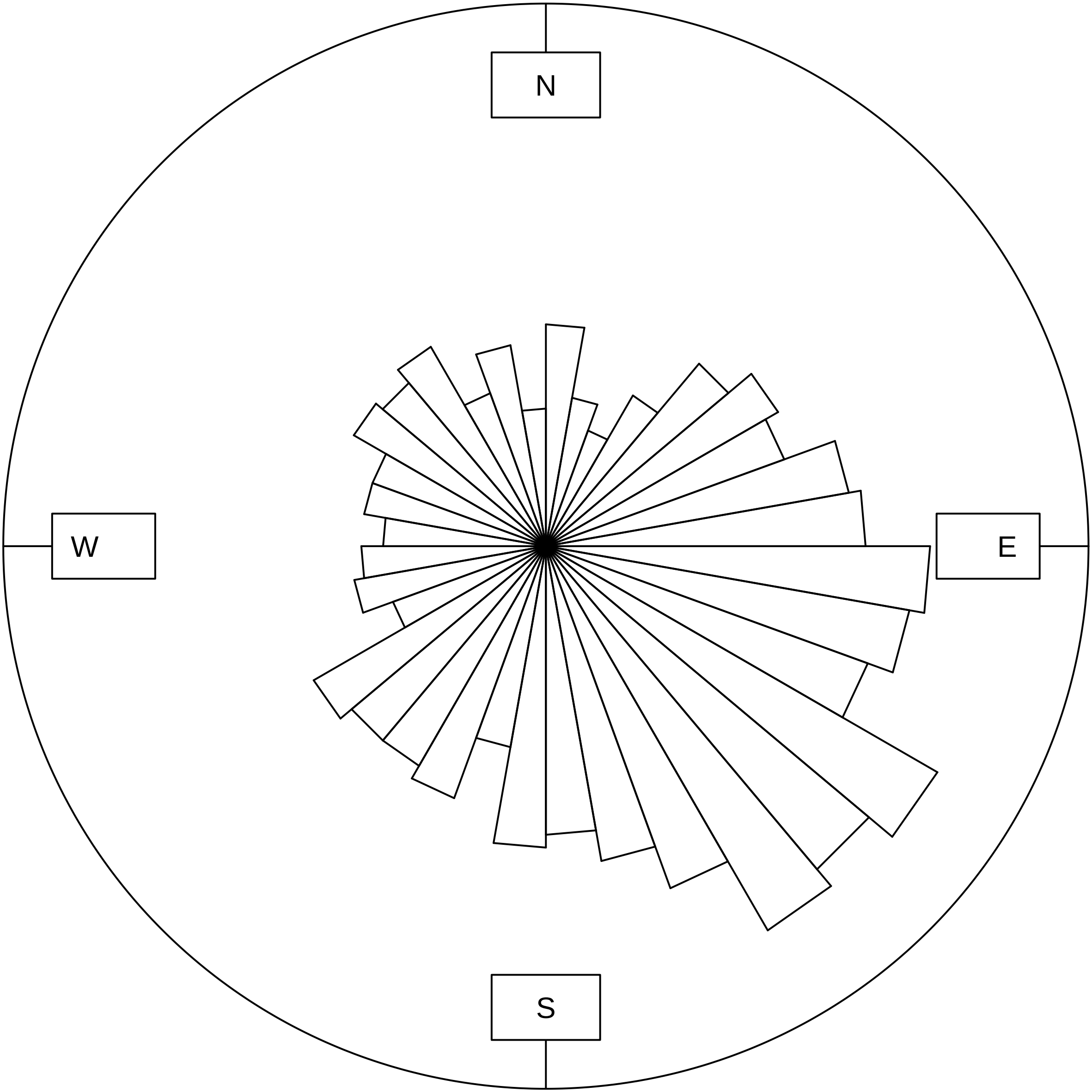

bootstrap 143

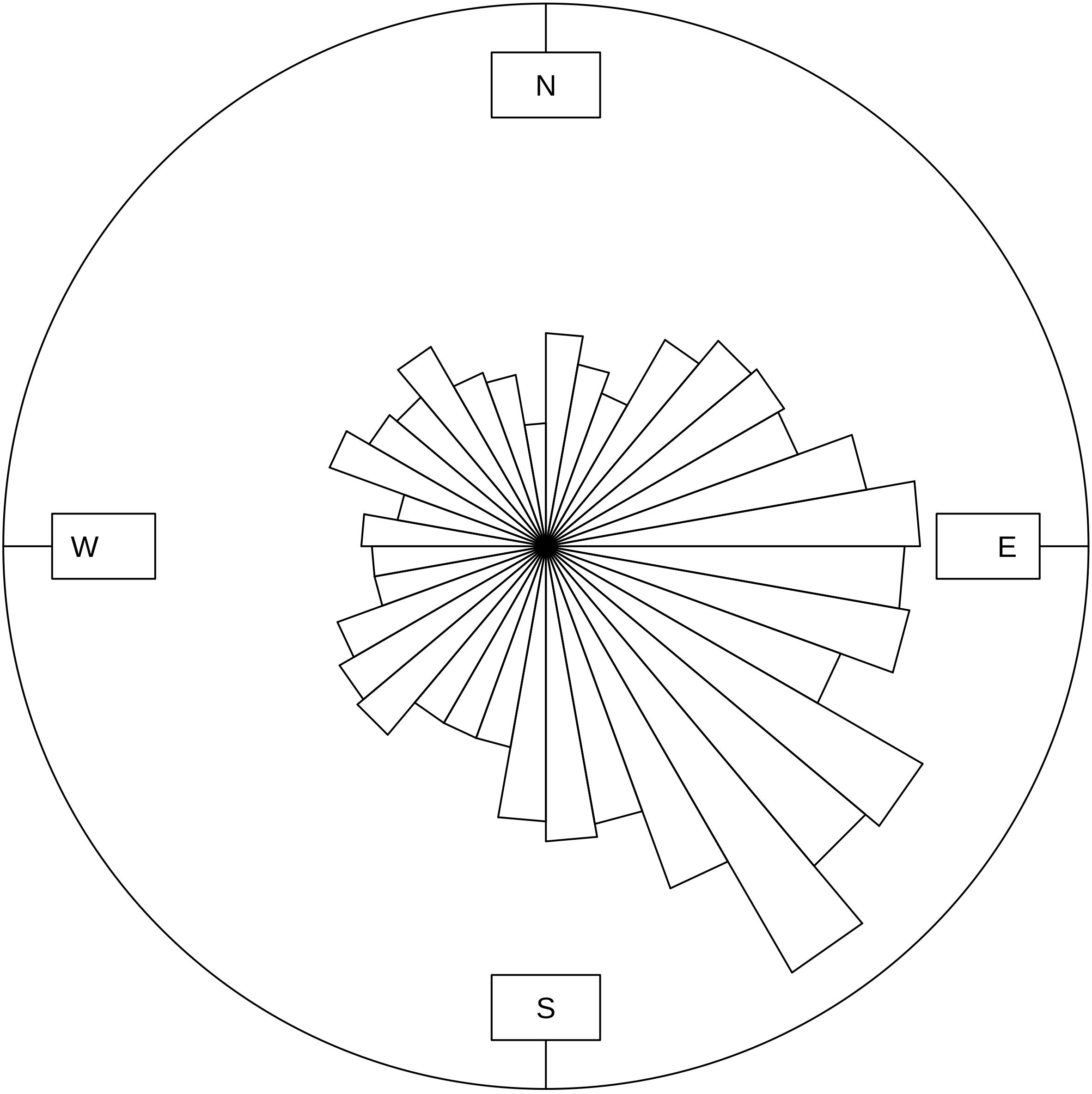

bootstrap 144

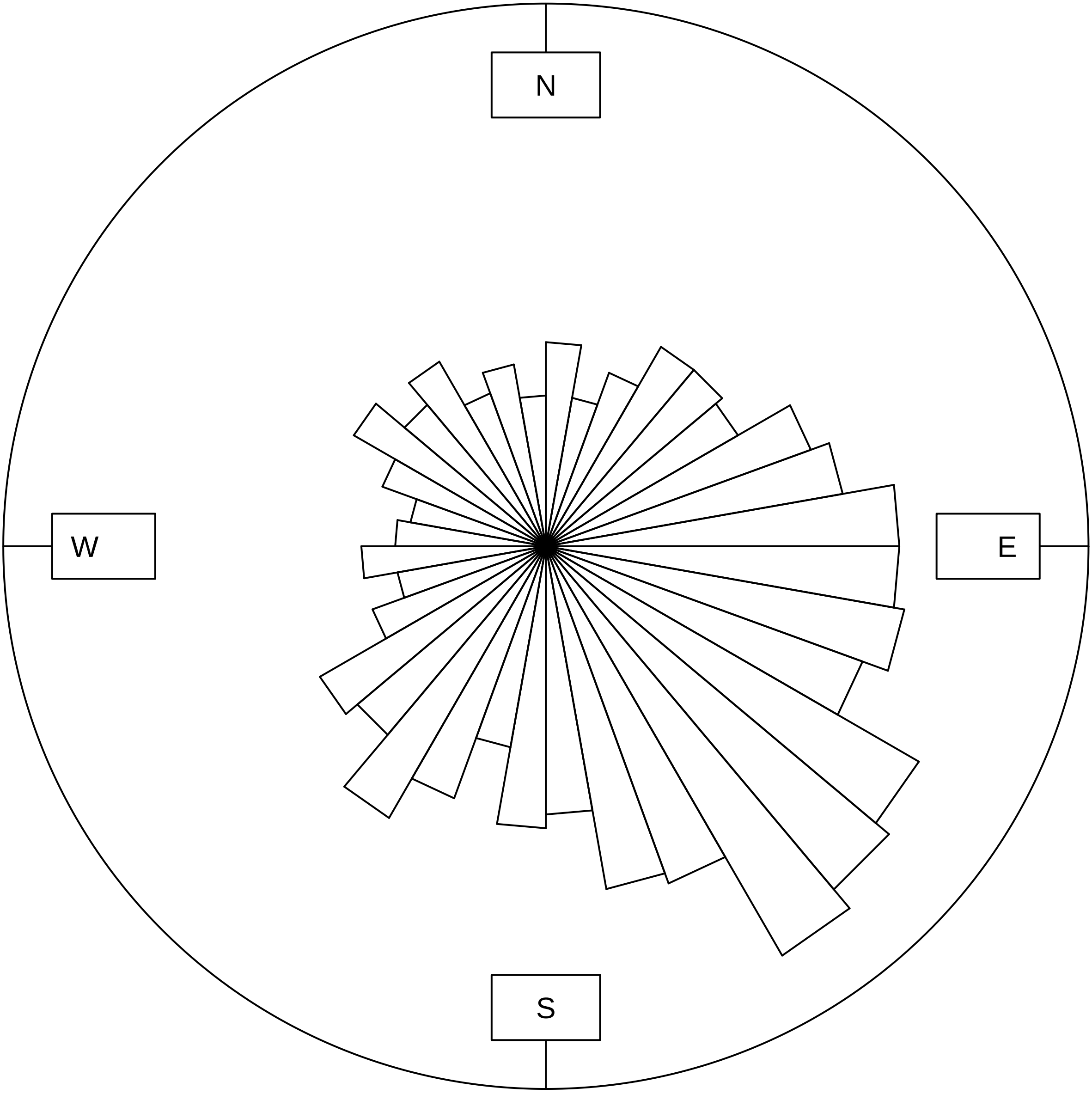

bootstrap 145

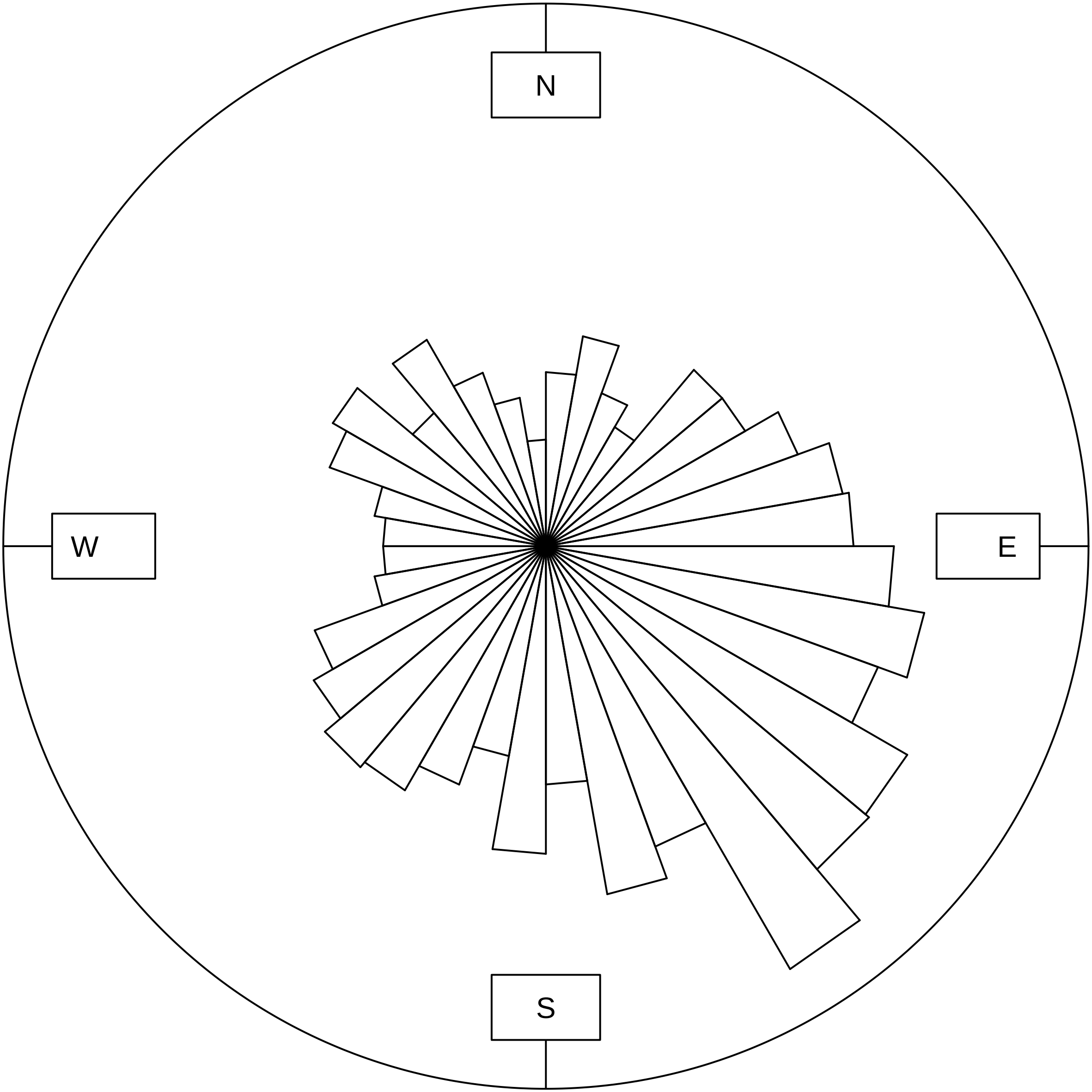

bootstrap 146

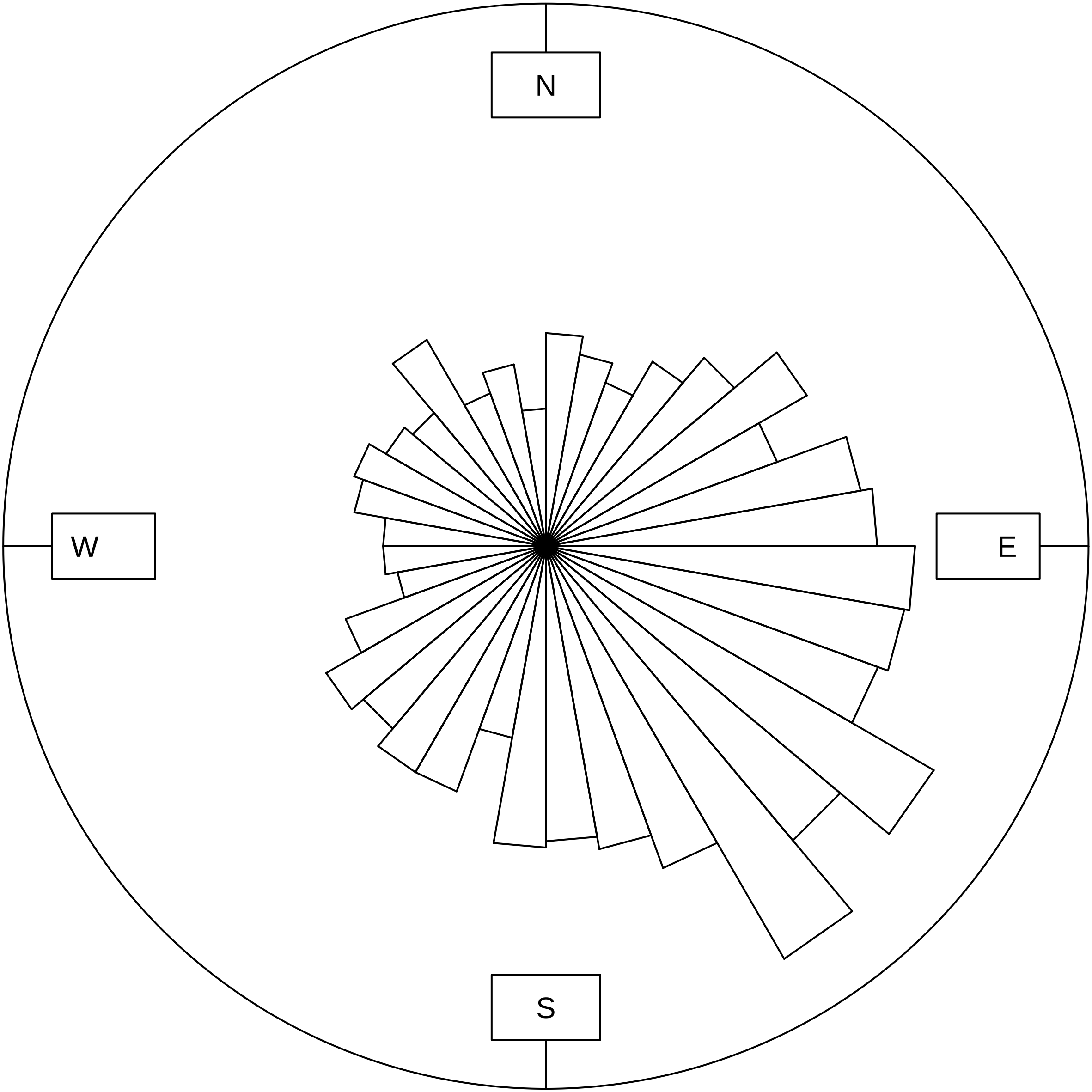

bootstrap 147

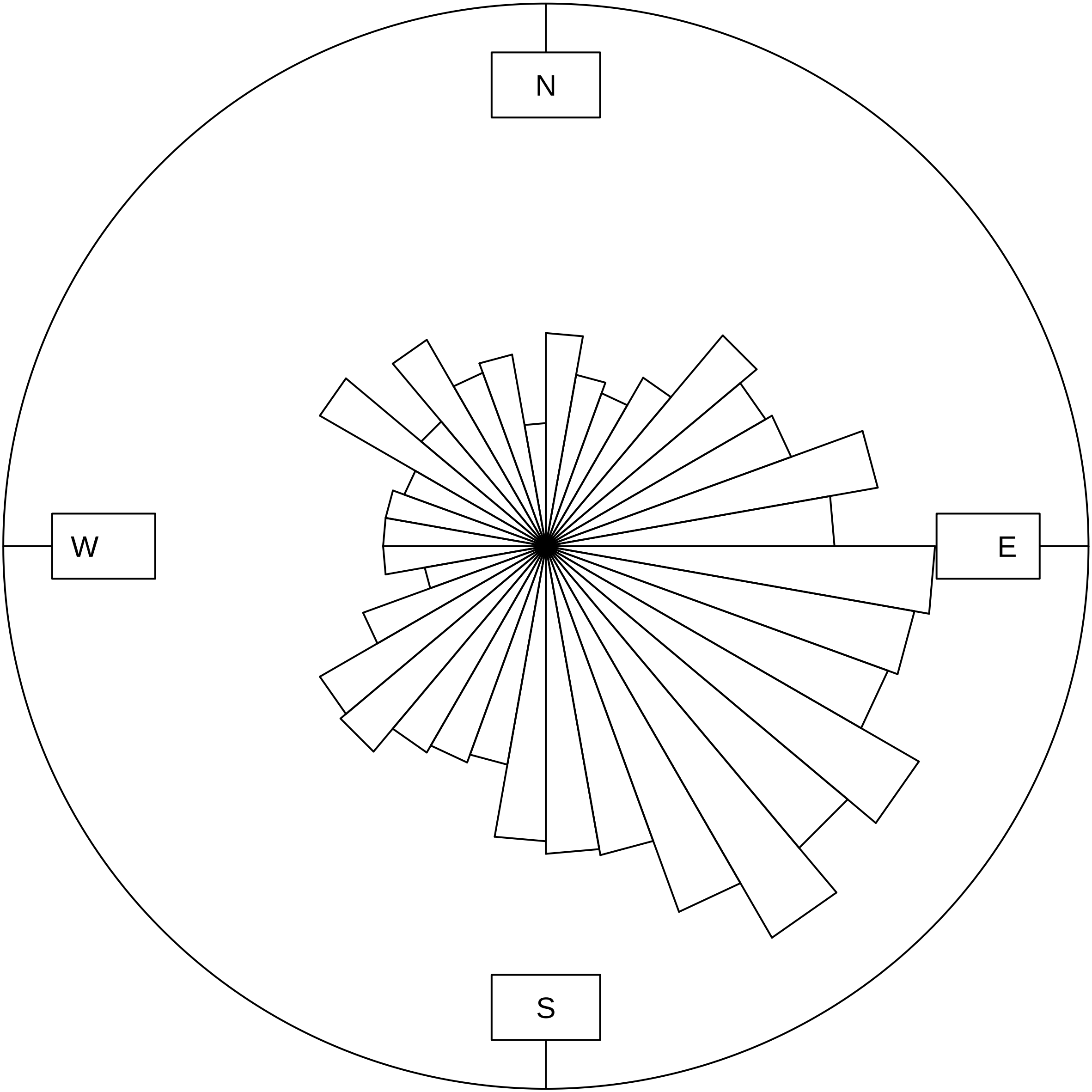

bootstrap 148

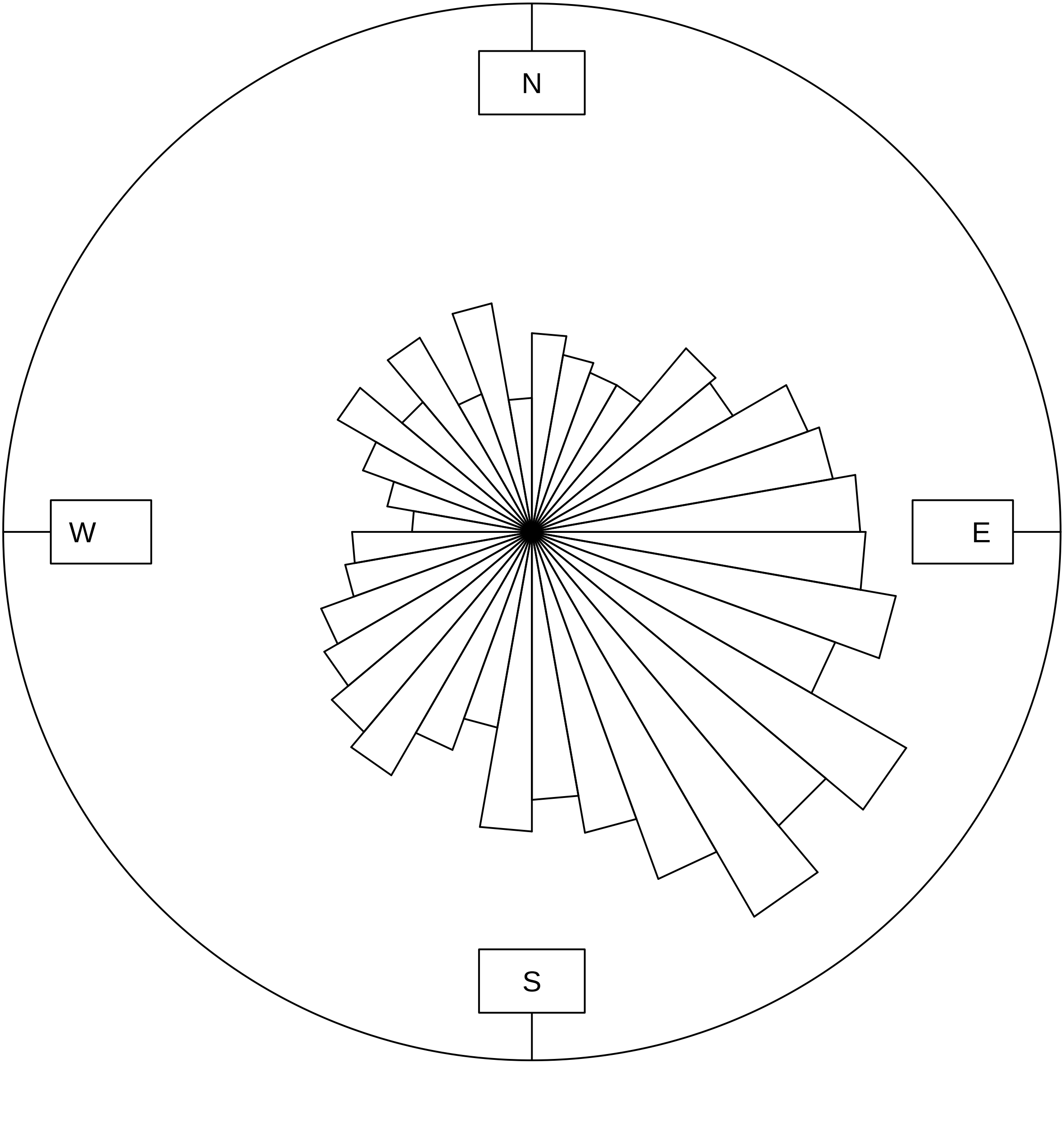

bootstrap 149

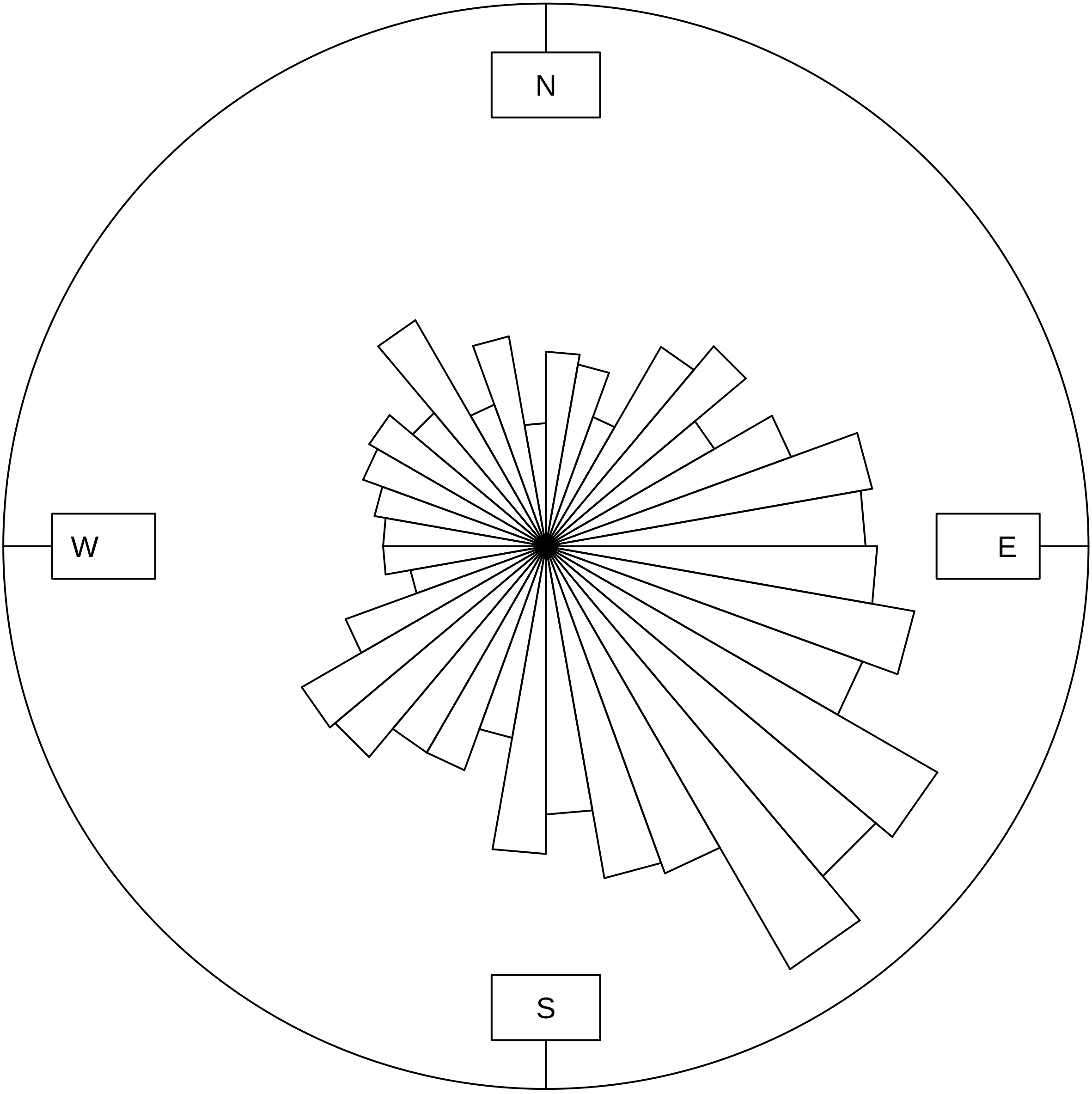

bootstrap 150

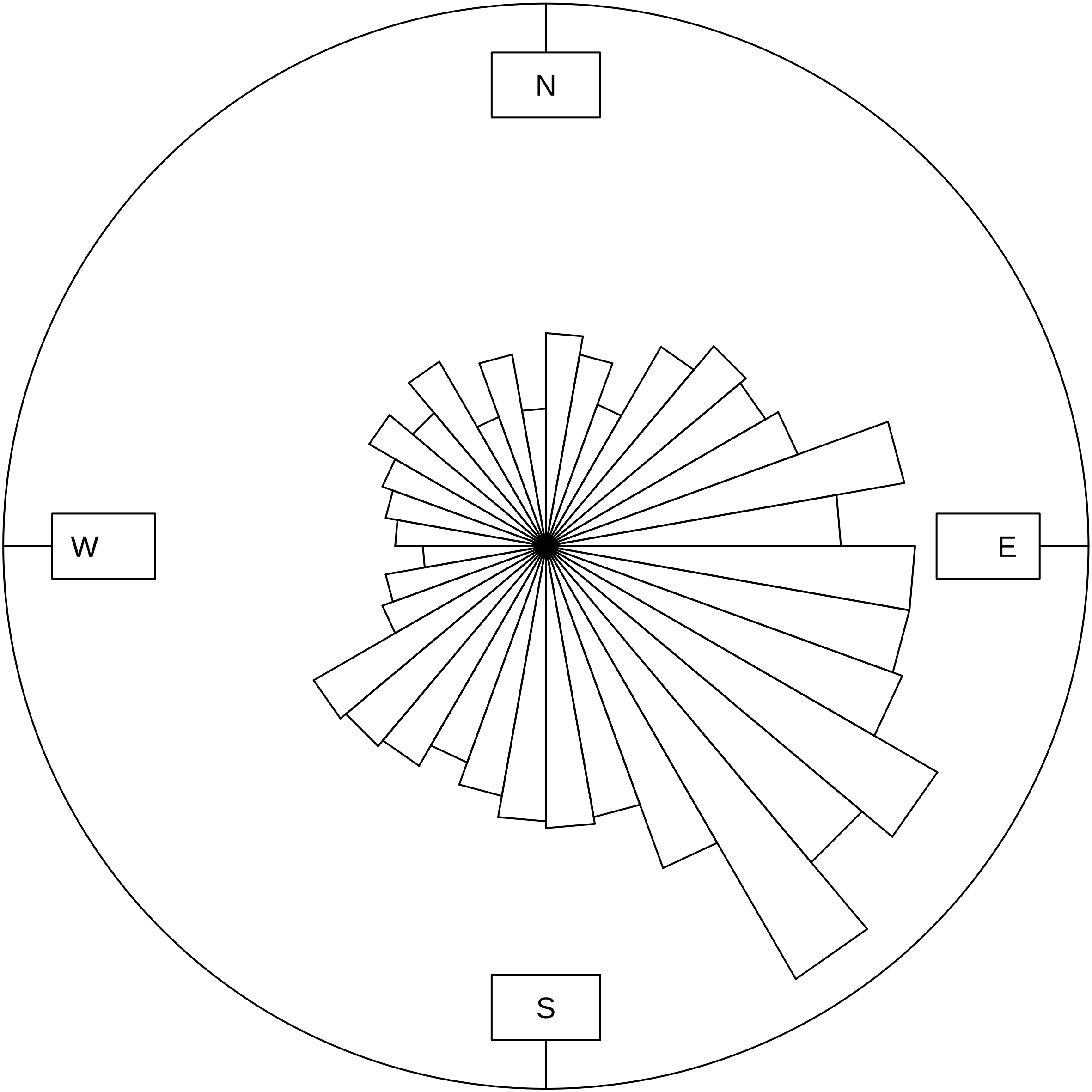

bootstrap 151

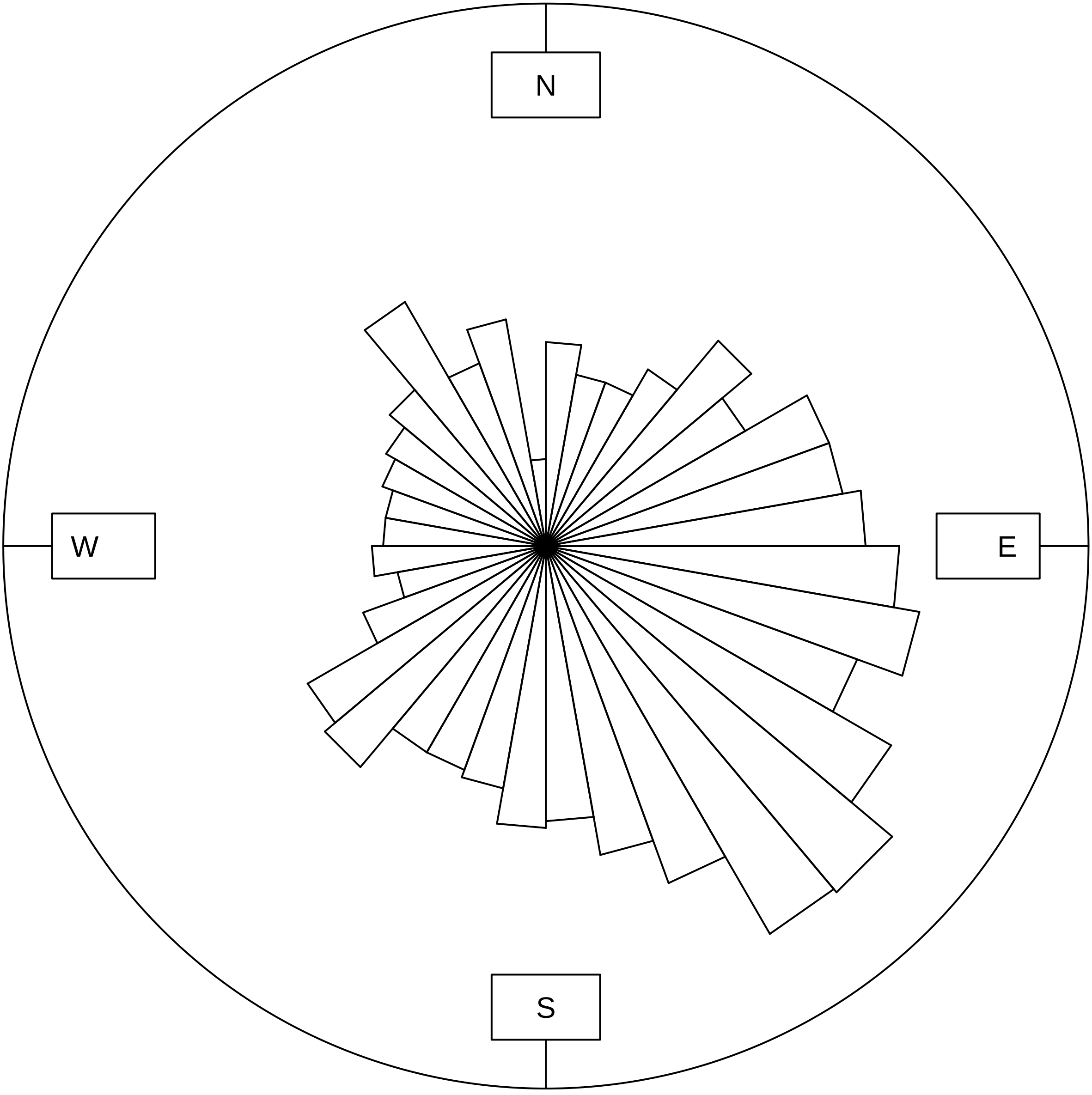

bootstrap 152

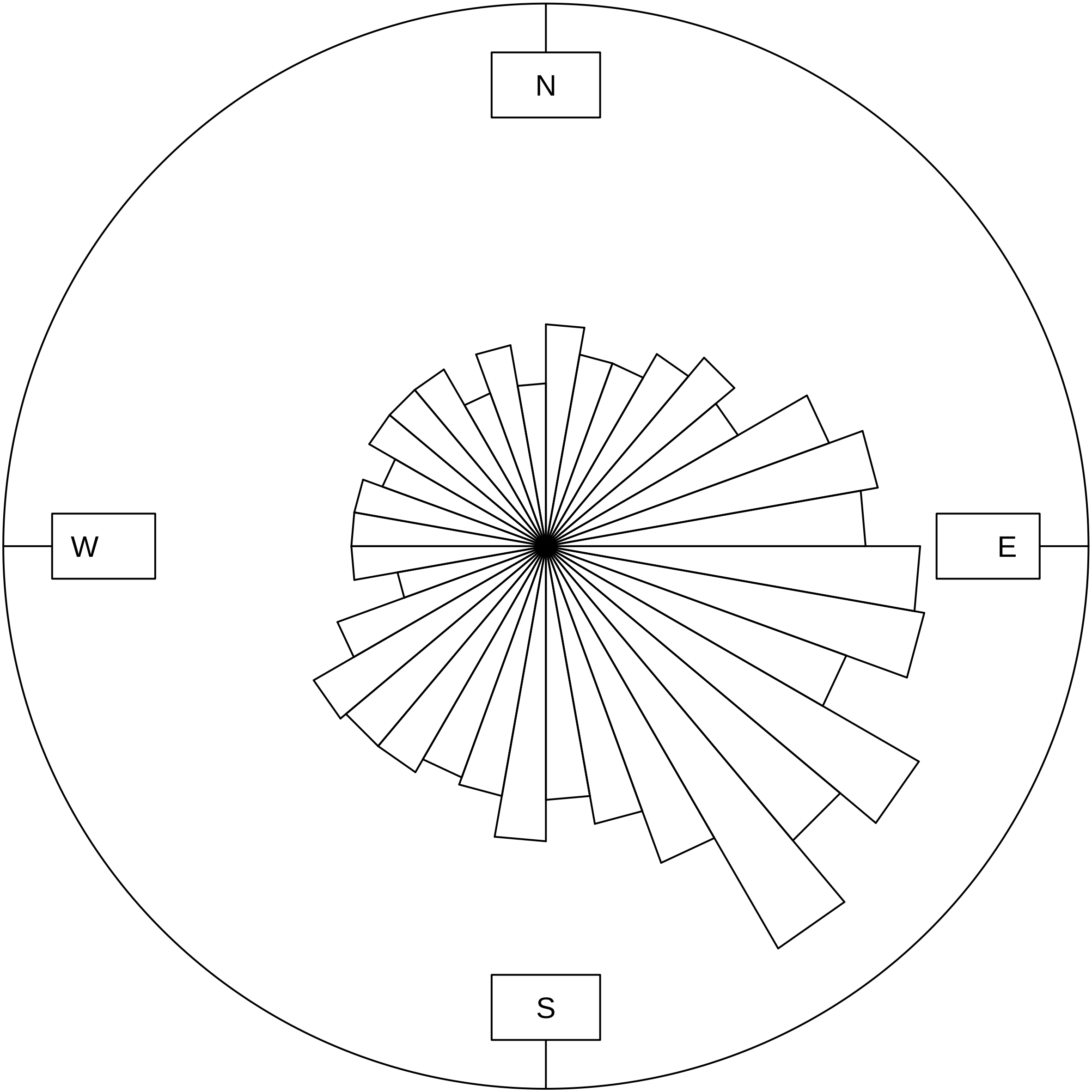

bootstrap 153

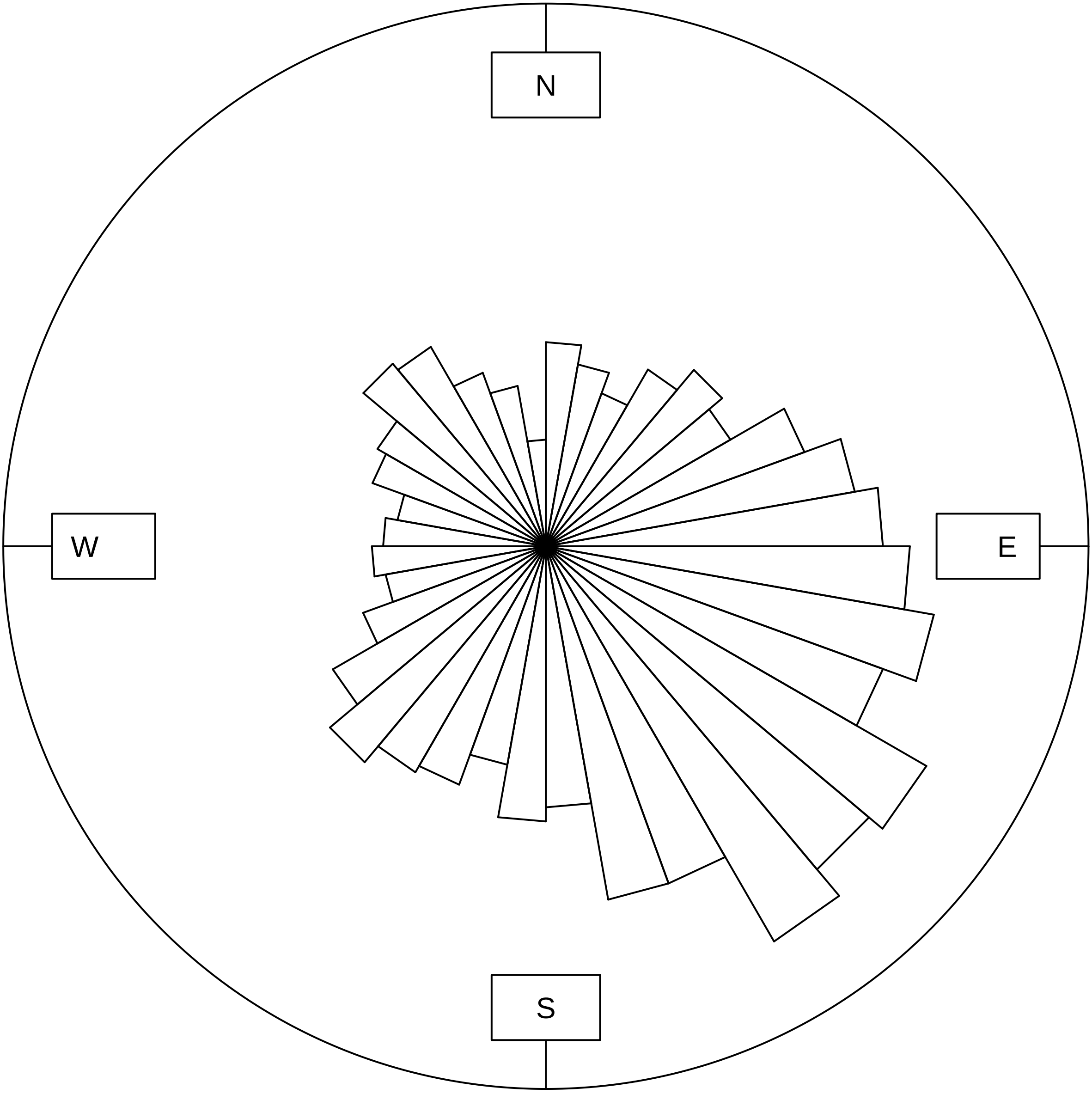

bootstrap 154

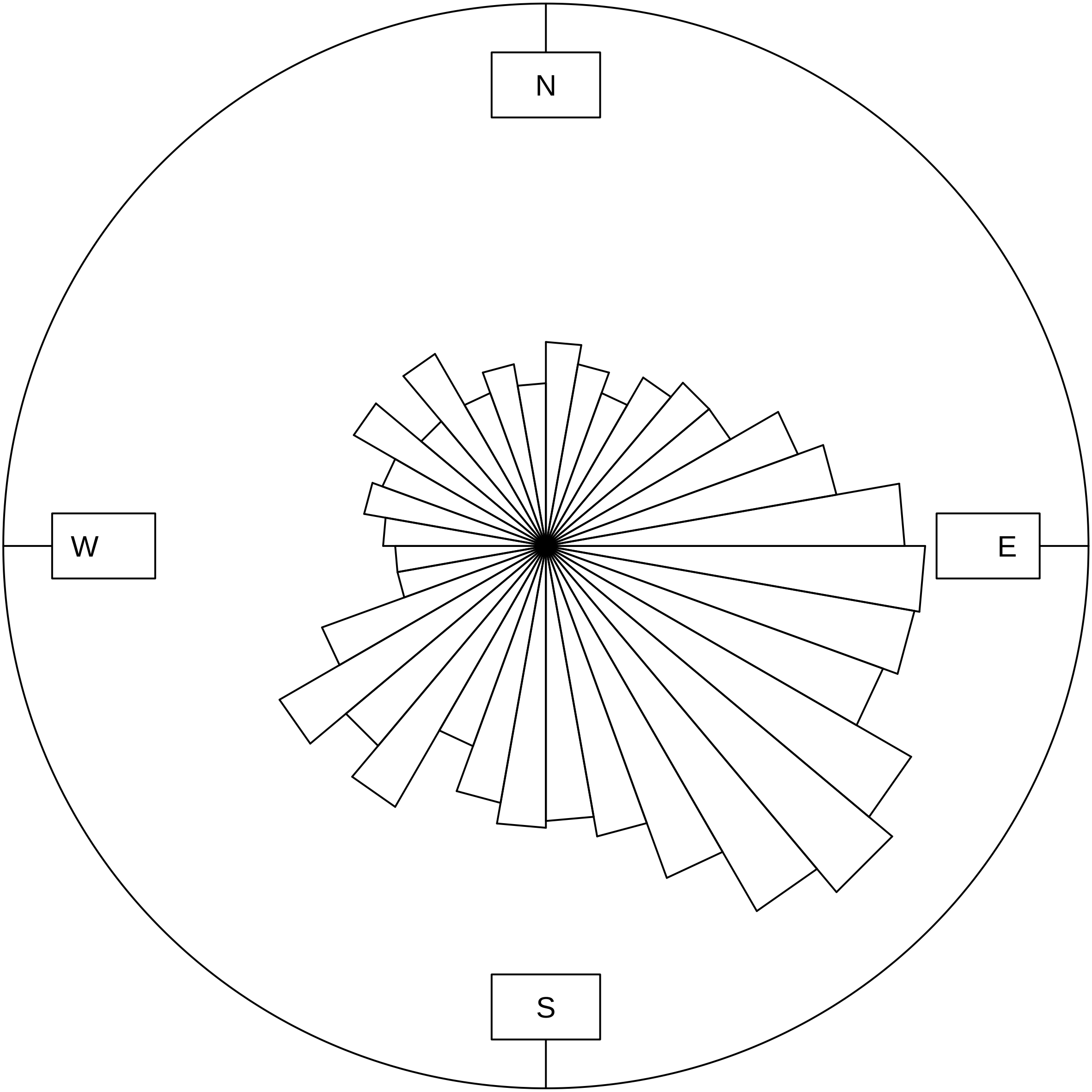

bootstrap 155

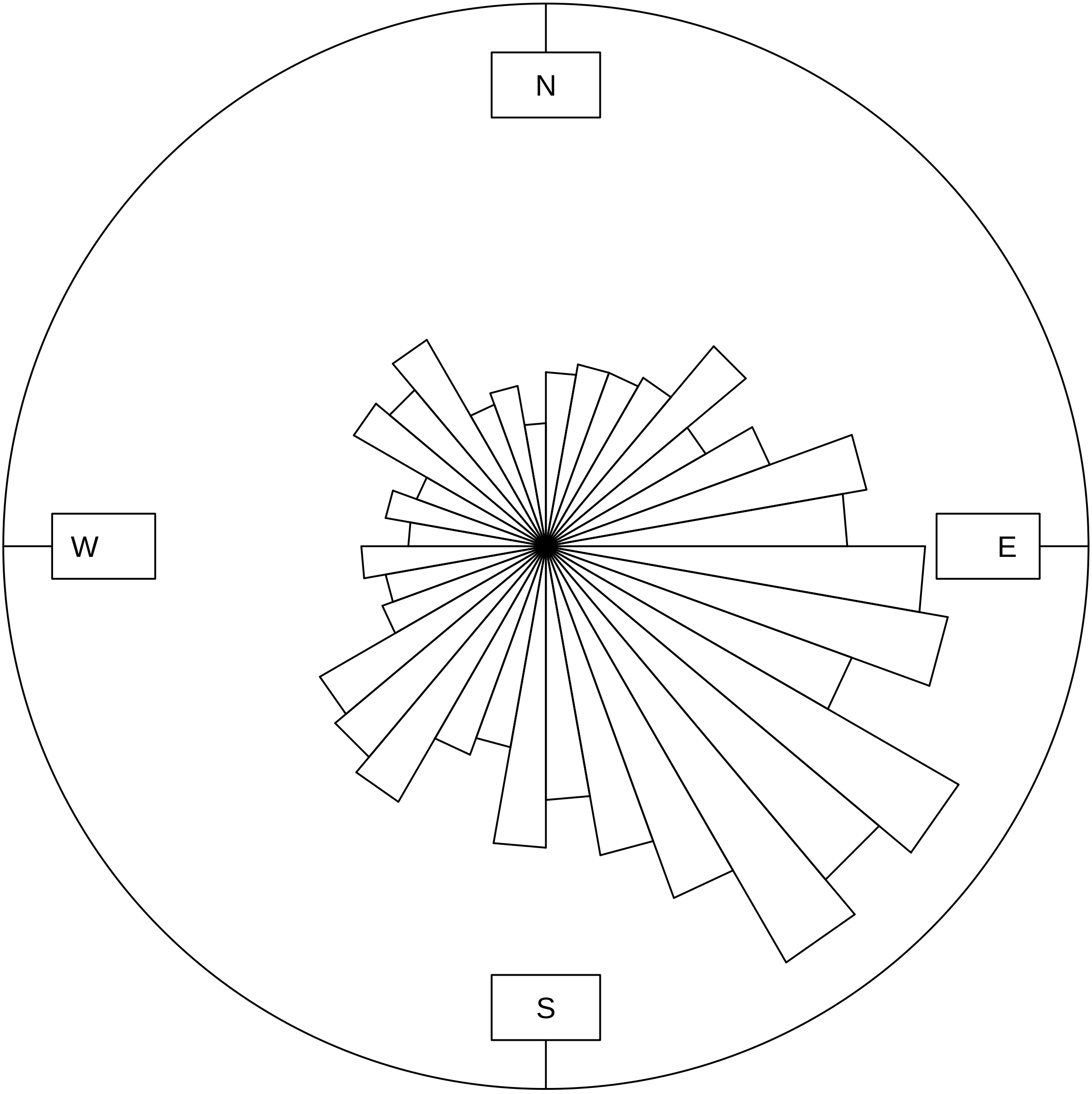

bootstrap 156

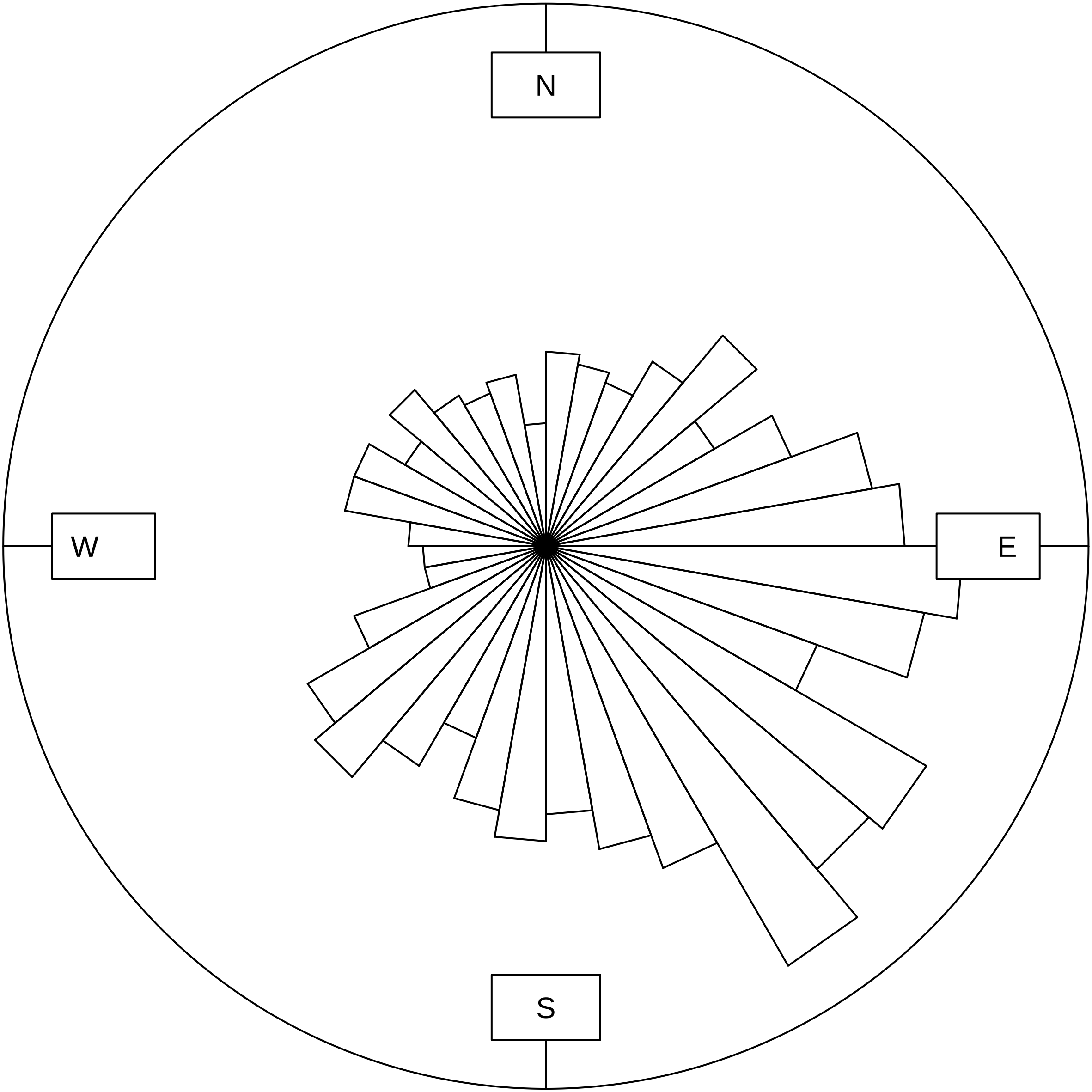

bootstrap 157

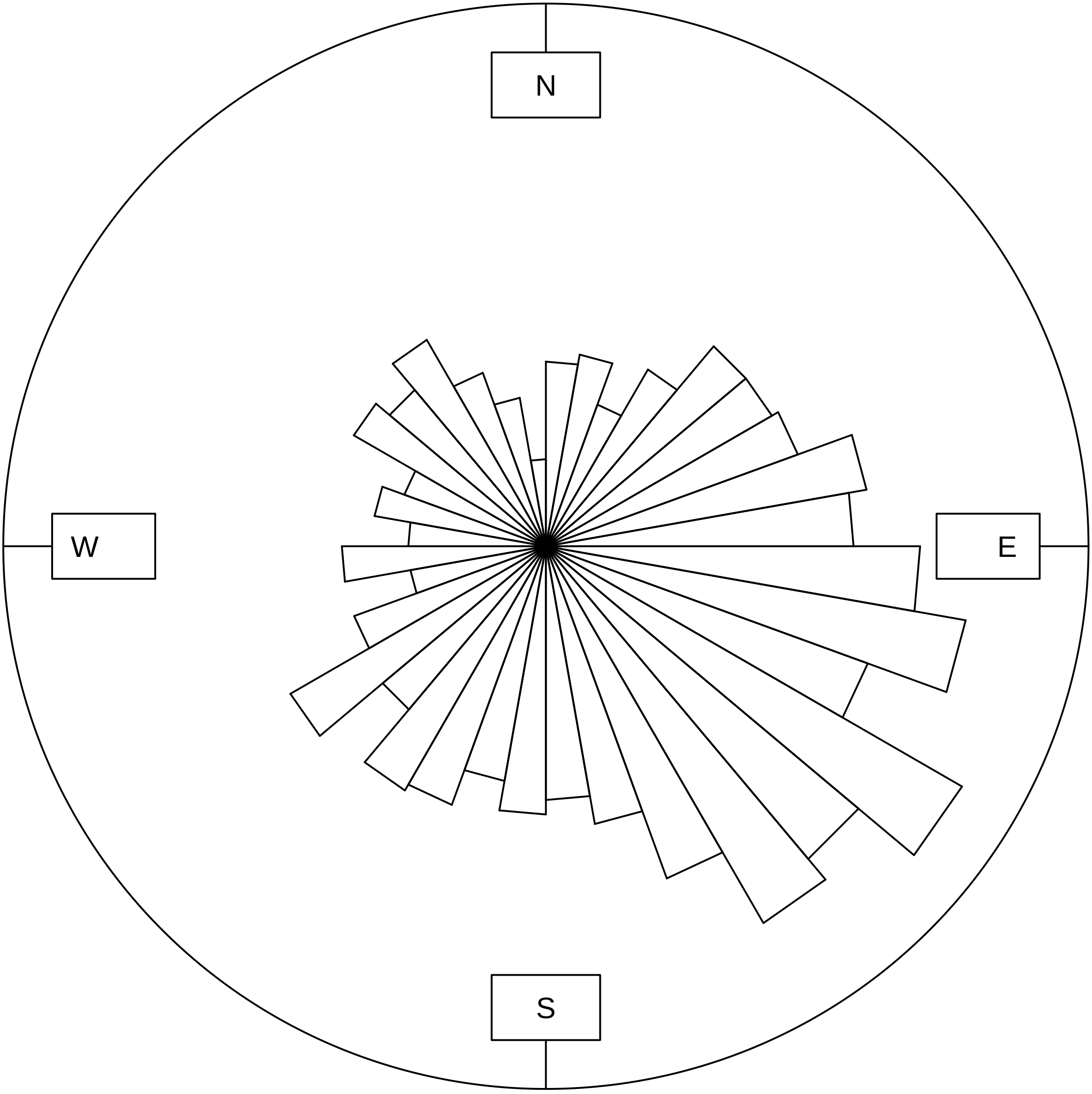

bootstrap 158

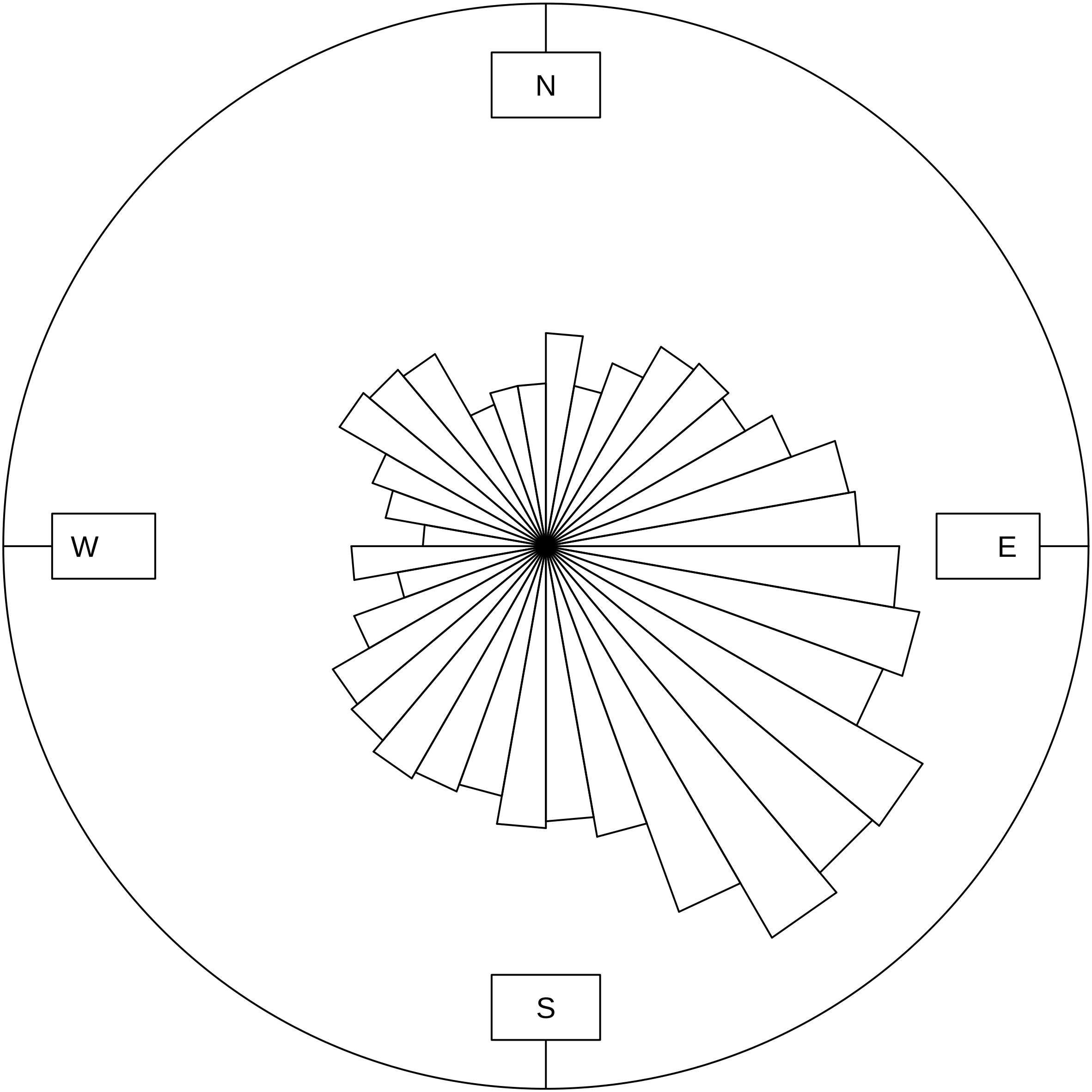

bootstrap 159

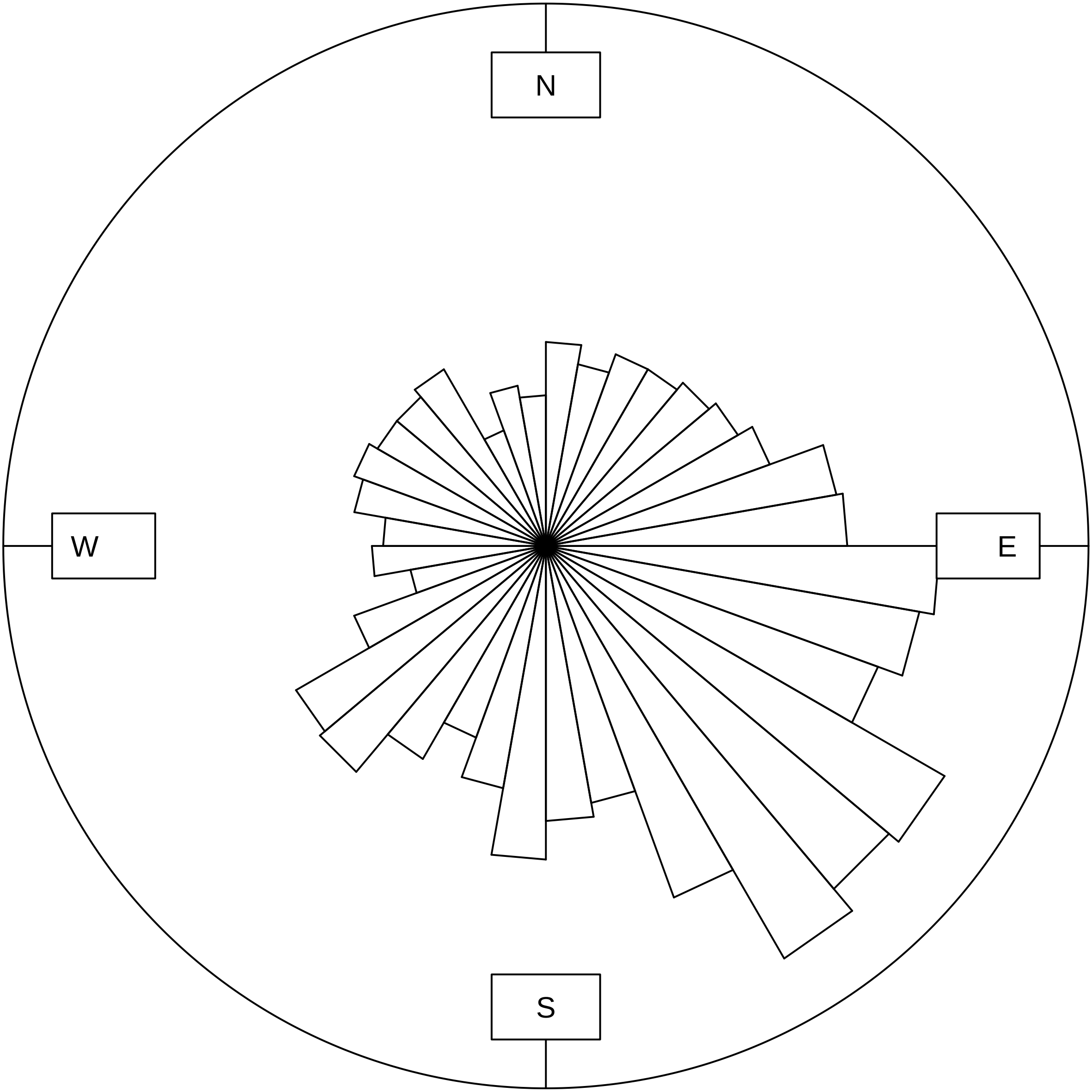

bootstrap 160

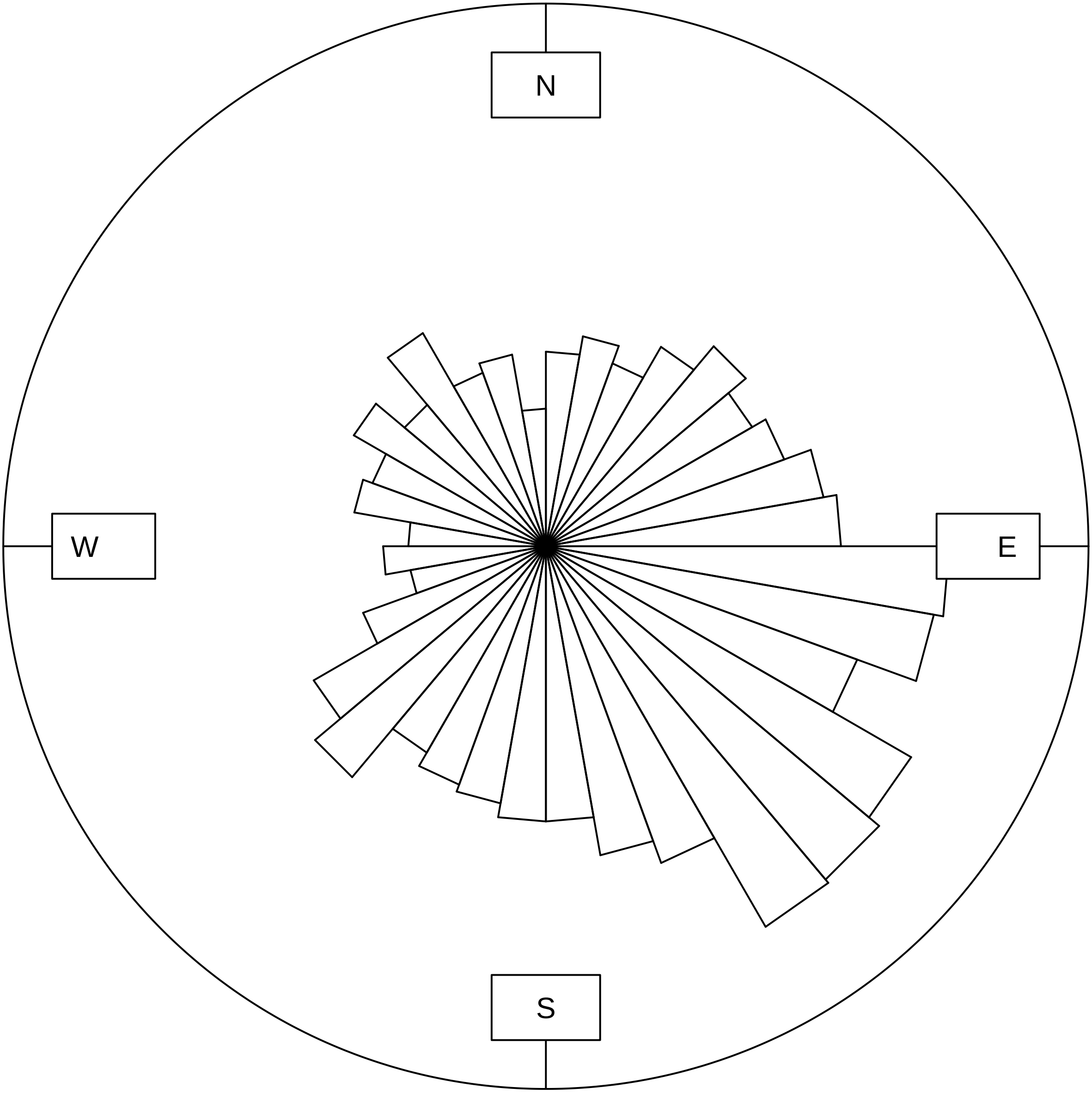

bootstrap 161

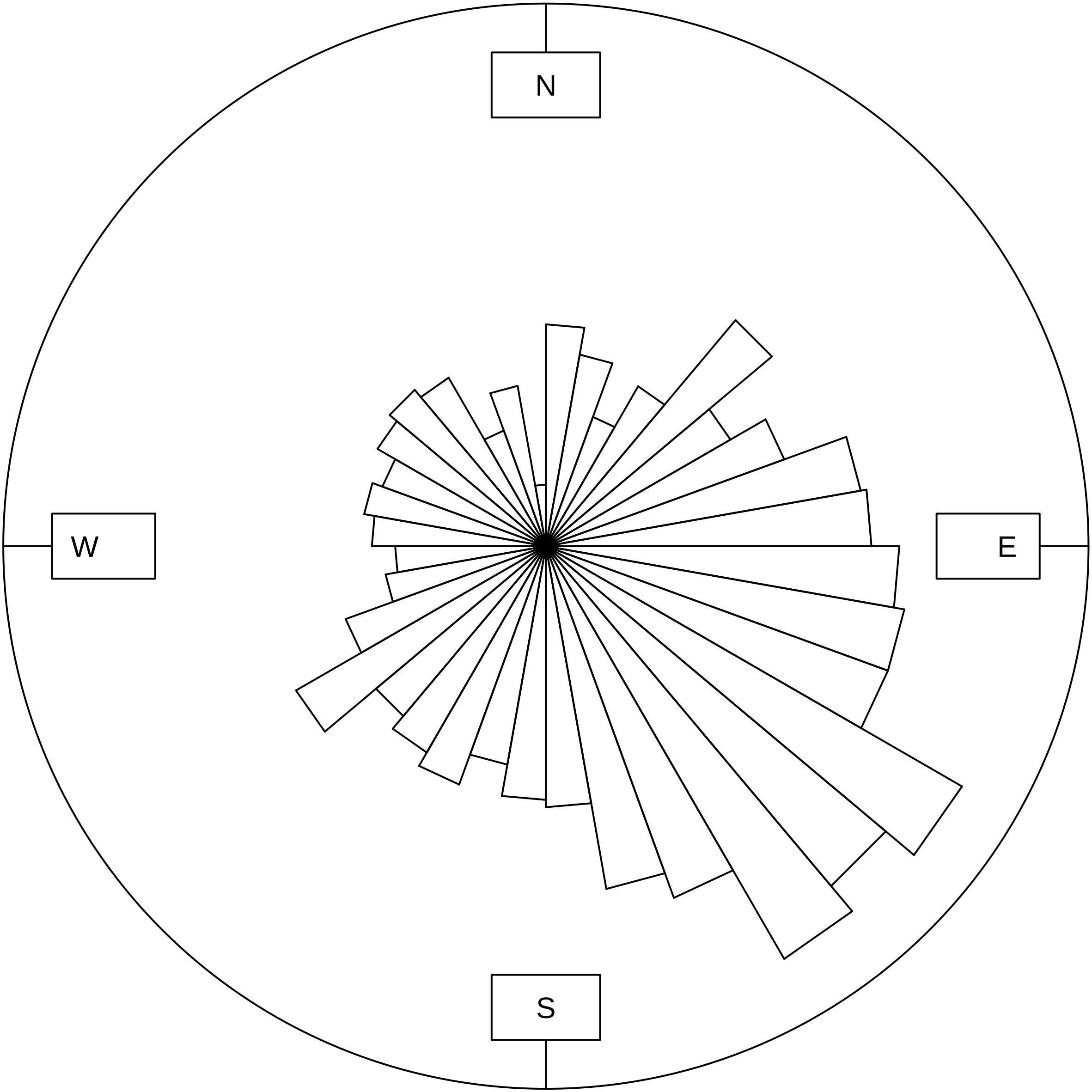

bootstrap 162

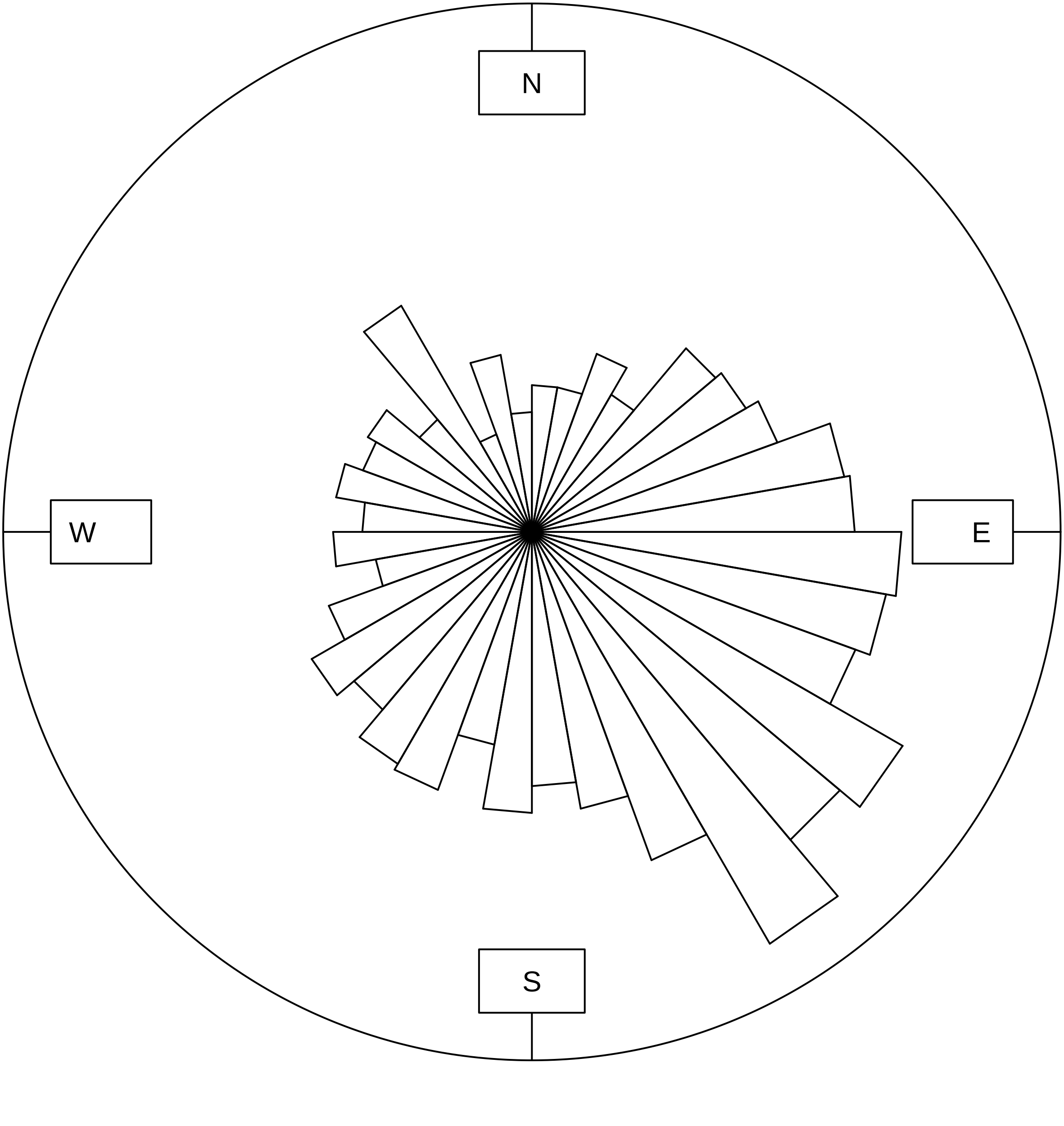

bootstrap 163

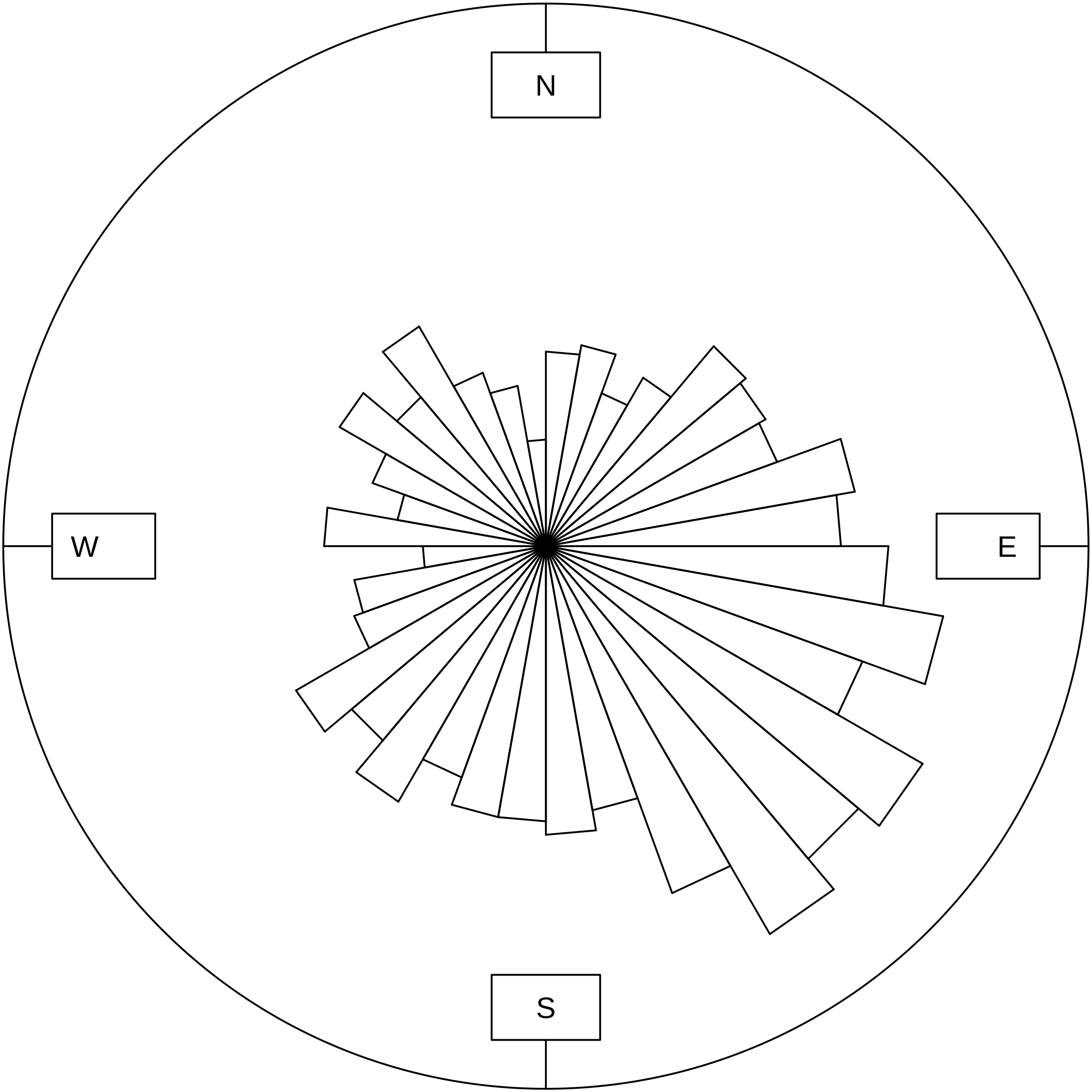

bootstrap 164

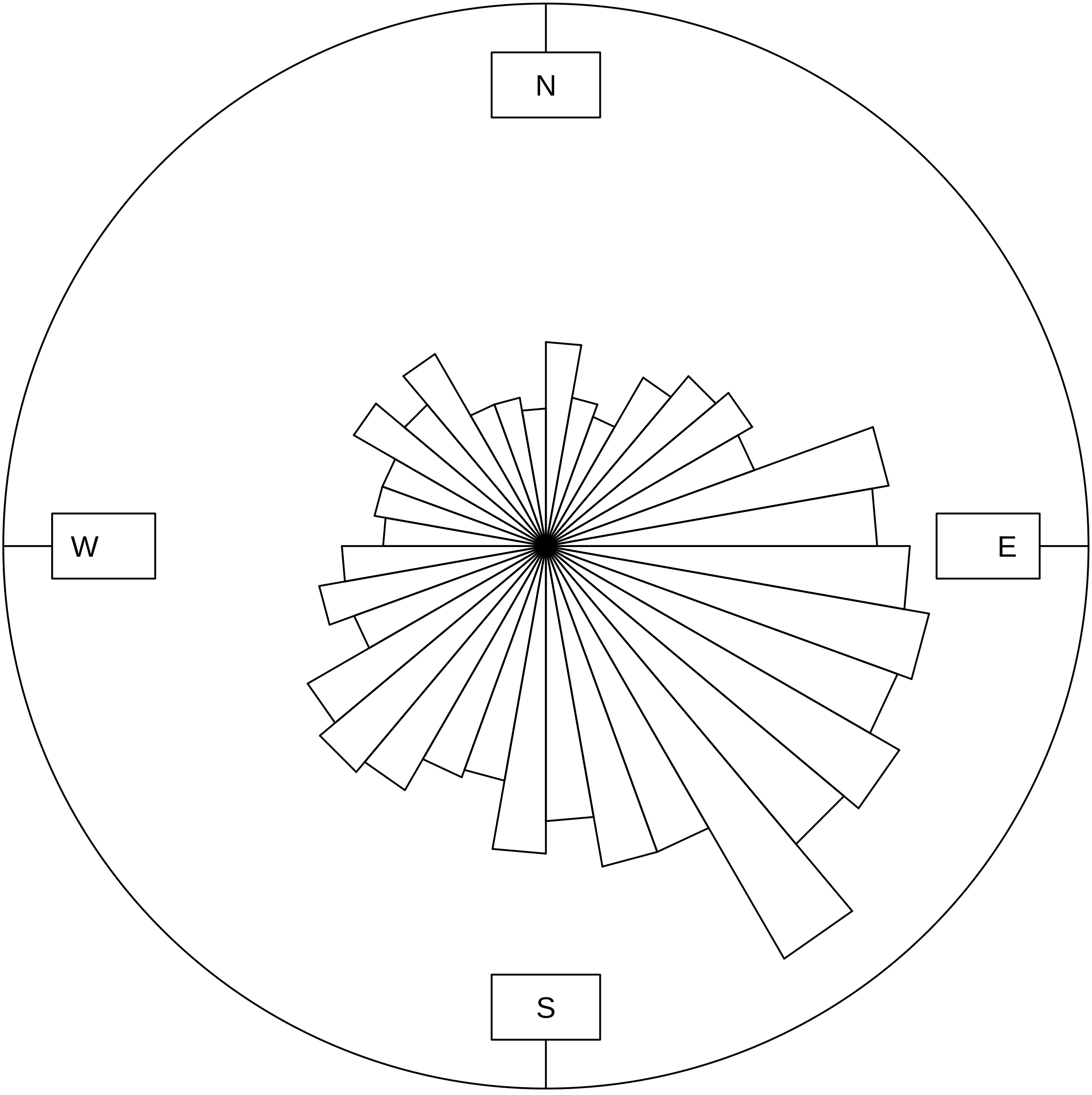

bootstrap 165

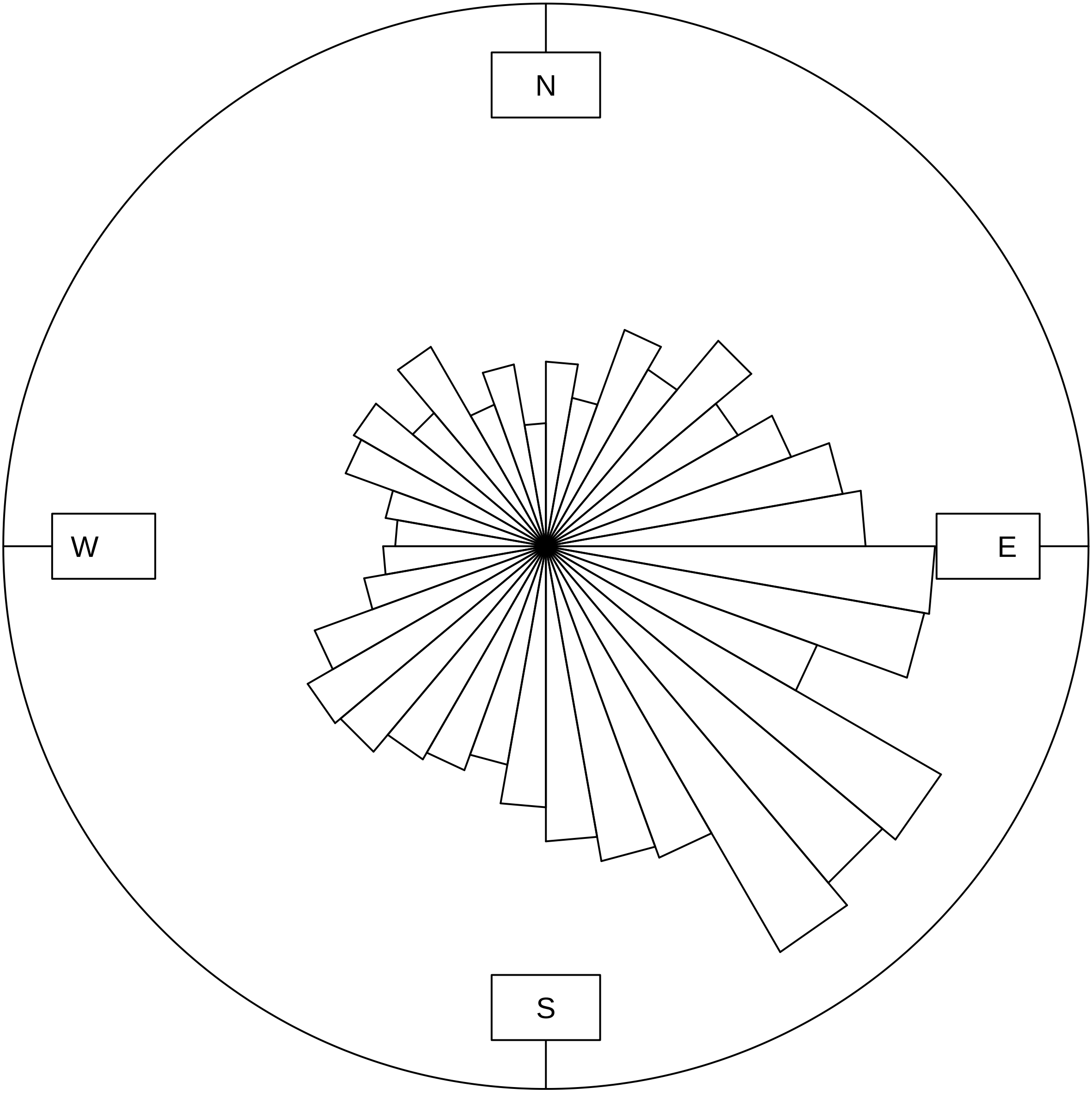

bootstrap 166

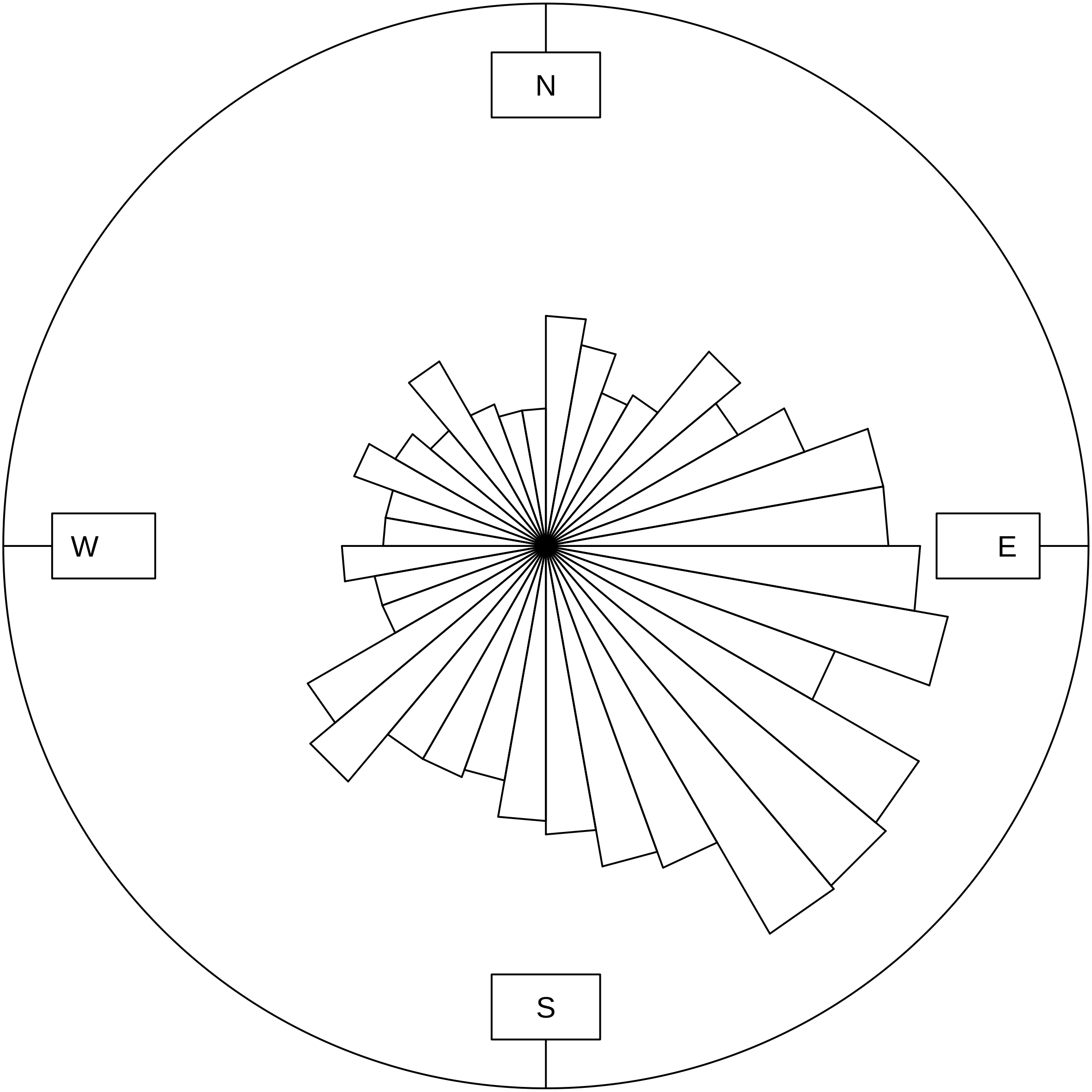

bootstrap 167

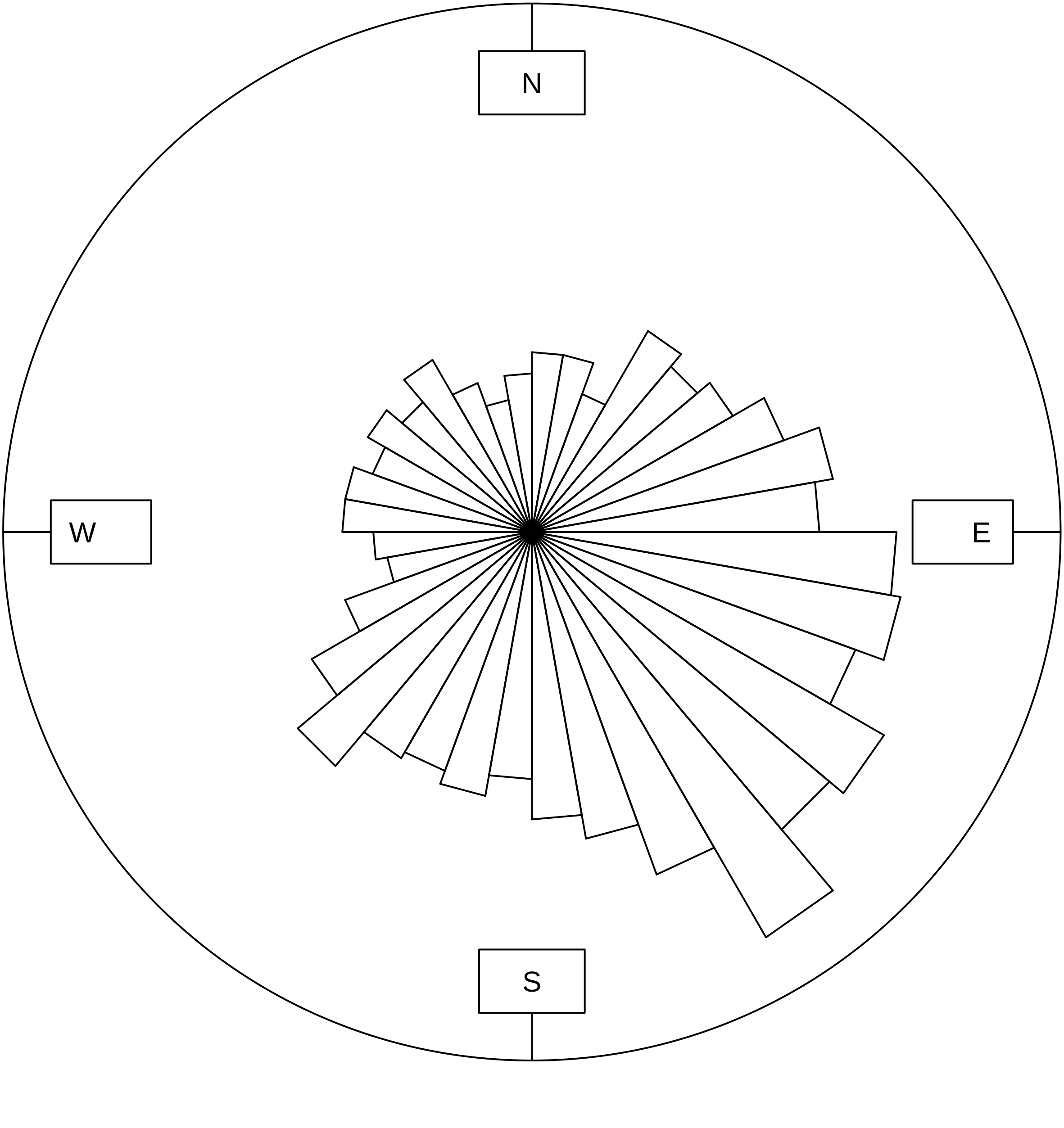

bootstrap 168

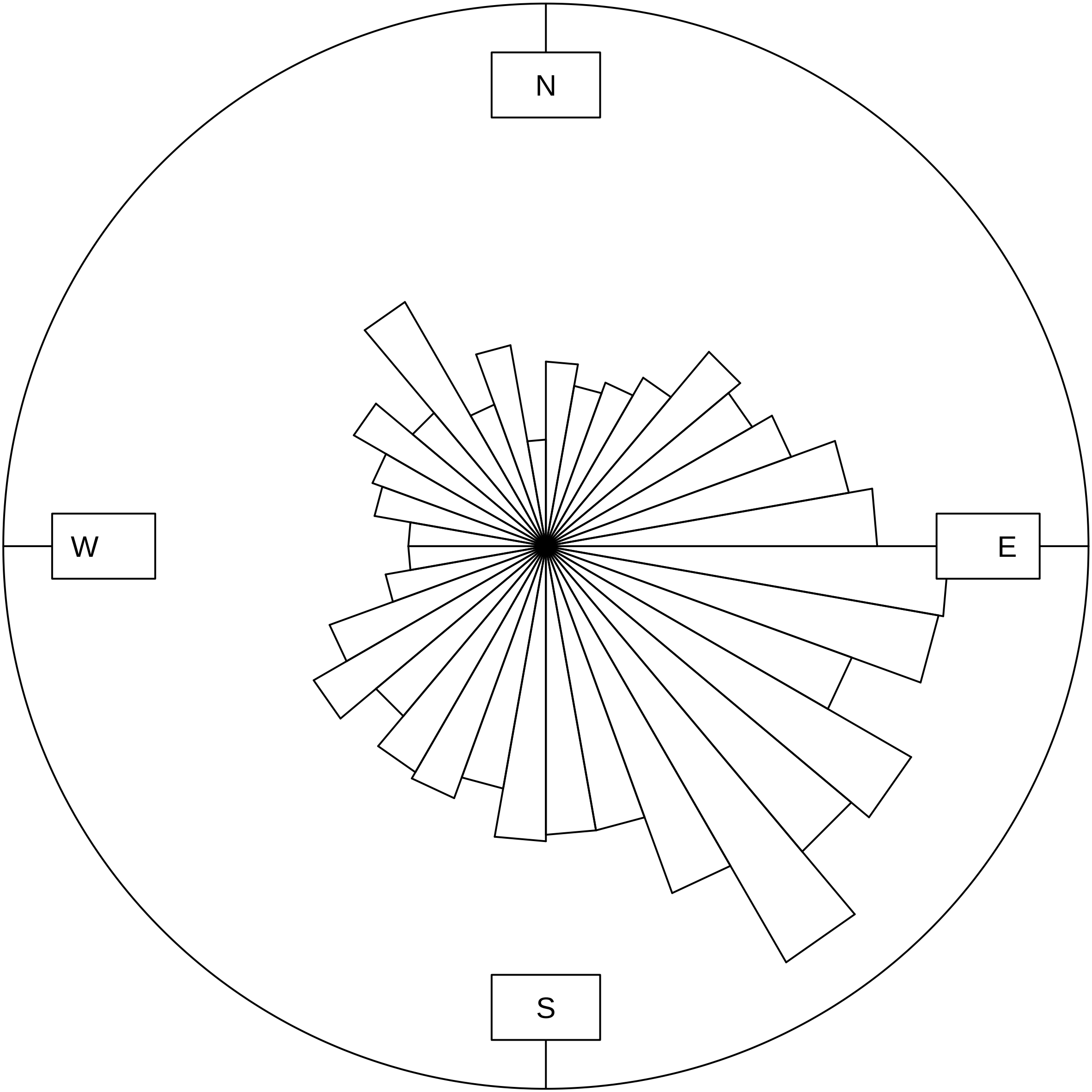

bootstrap 169

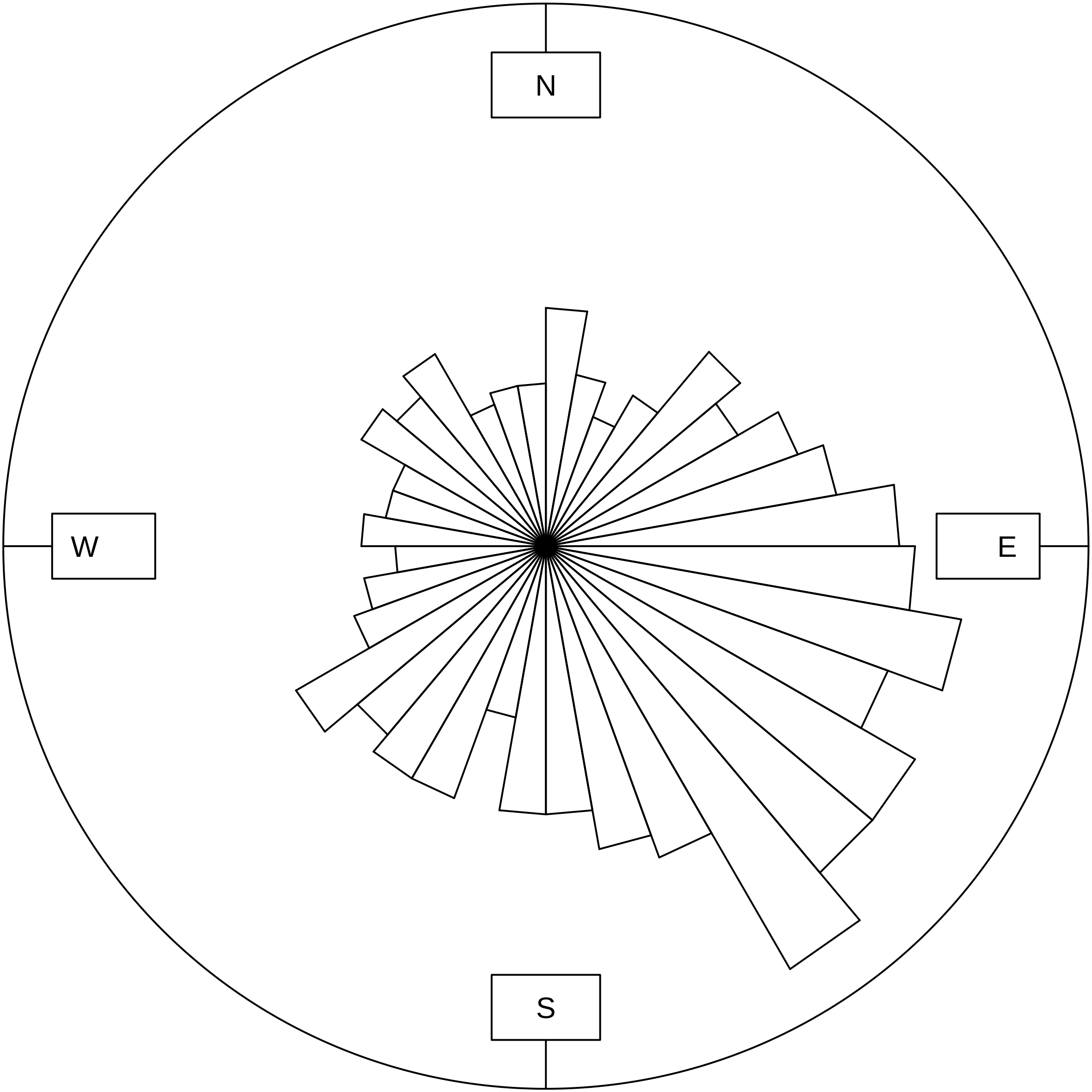

bootstrap 170

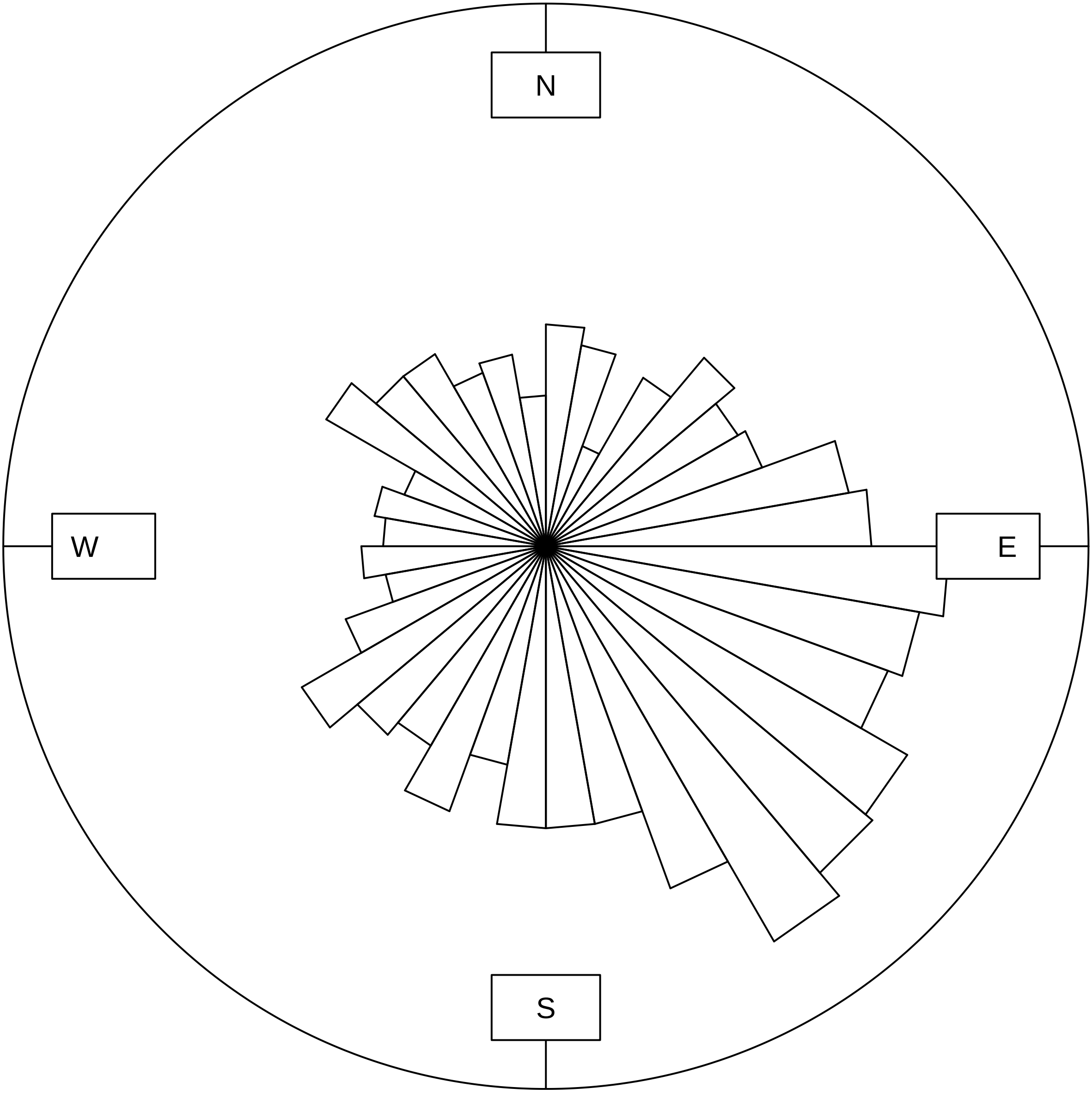

bootstrap 171

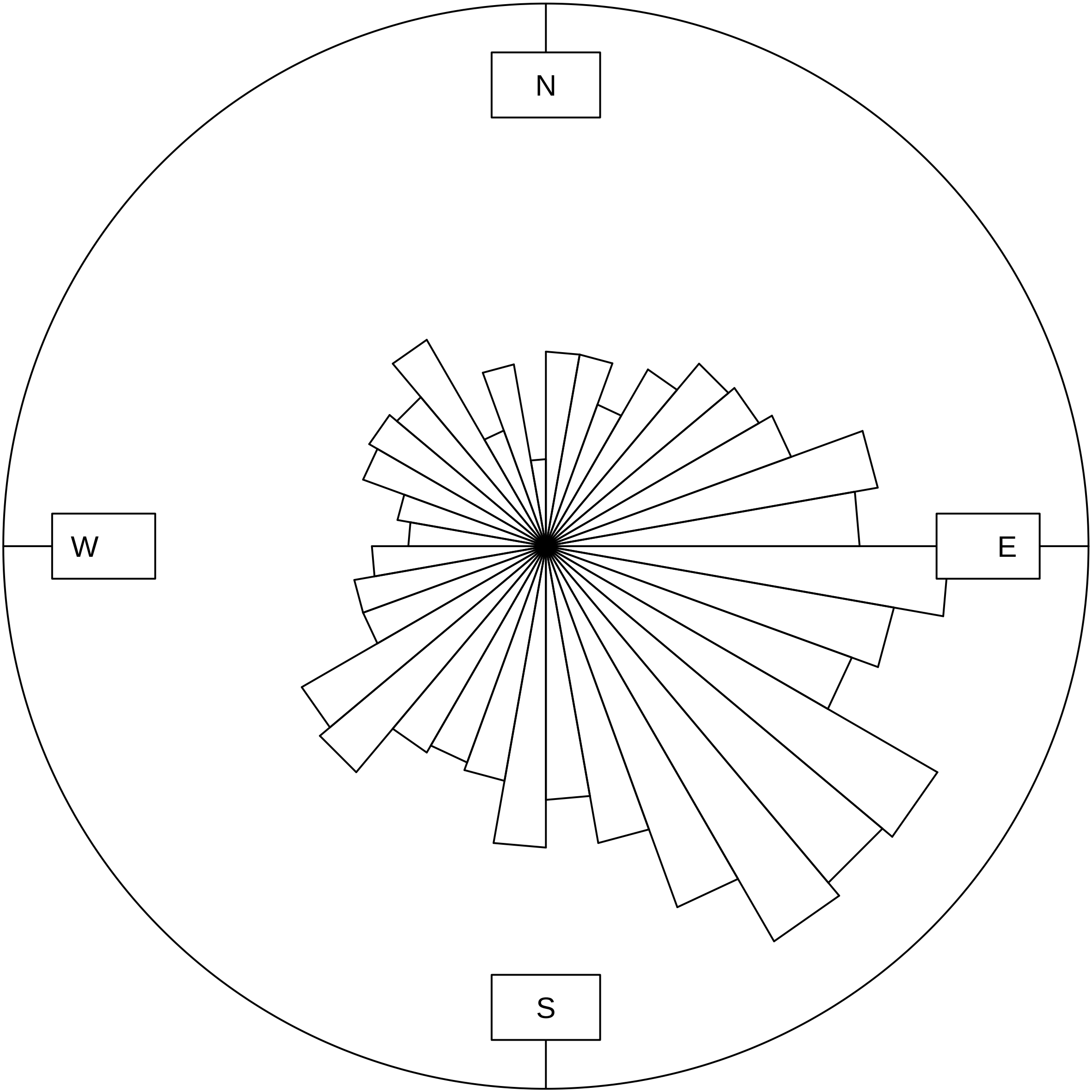

bootstrap 172

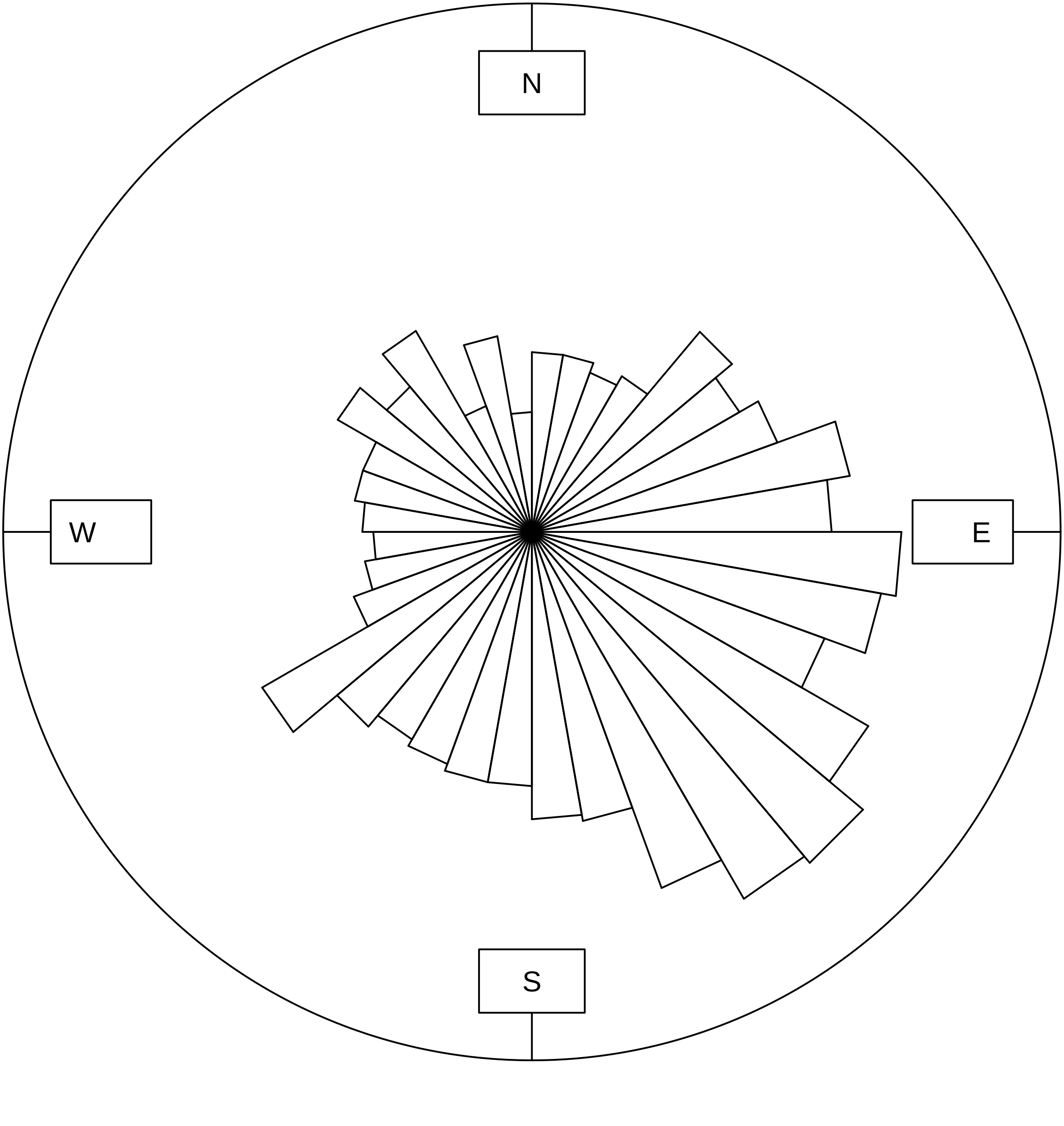

bootstrap 173

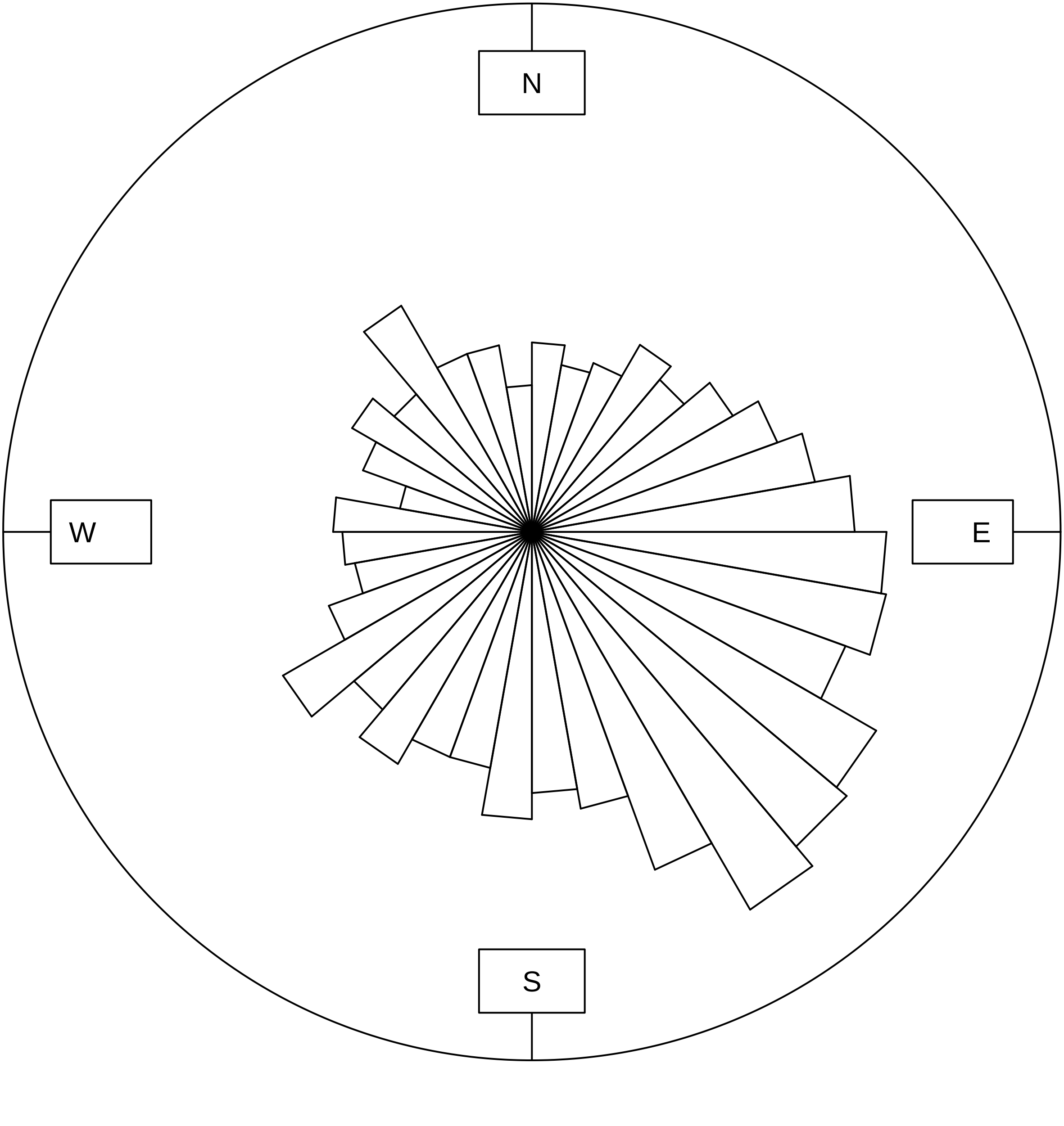

bootstrap 174

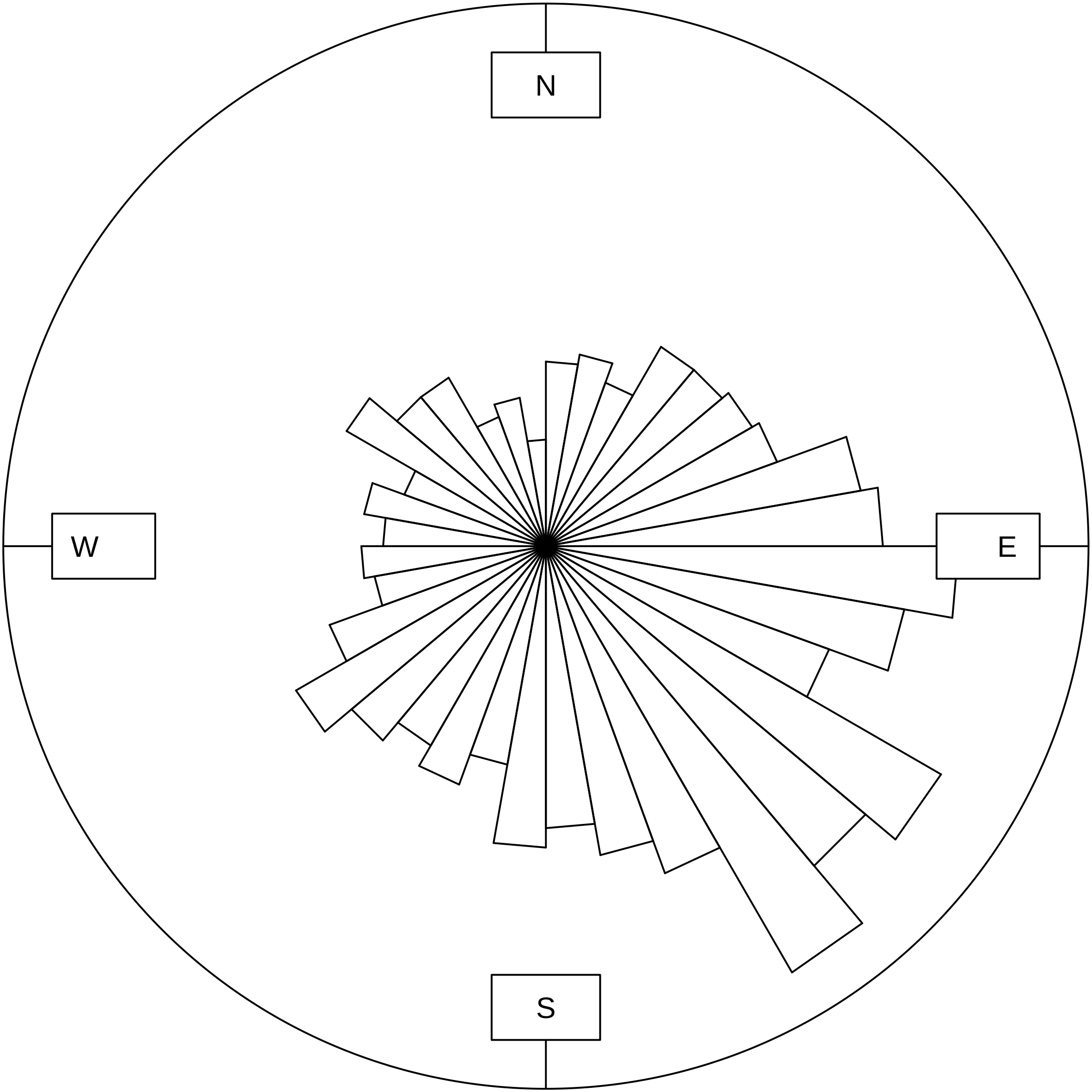

bootstrap 175

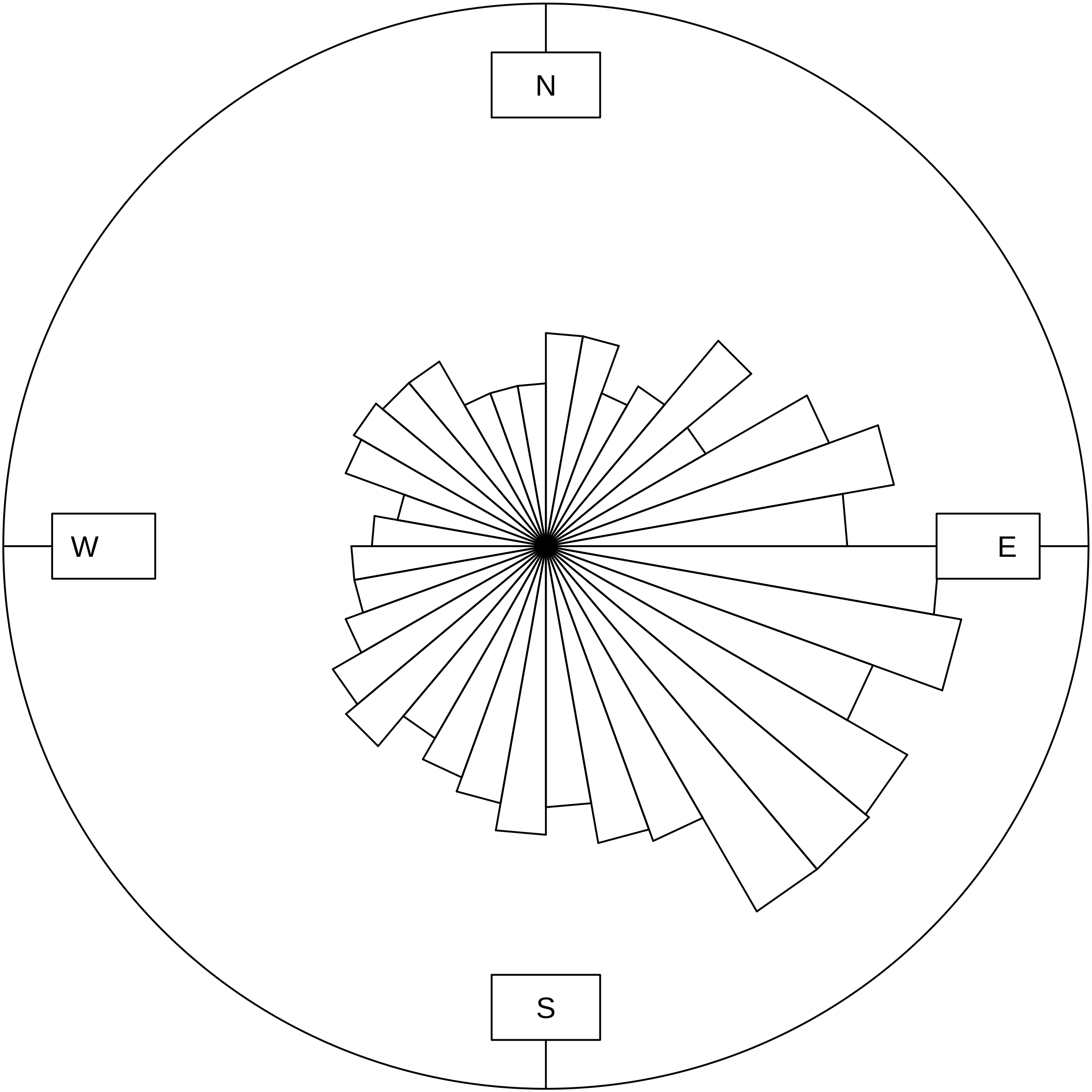

bootstrap 176

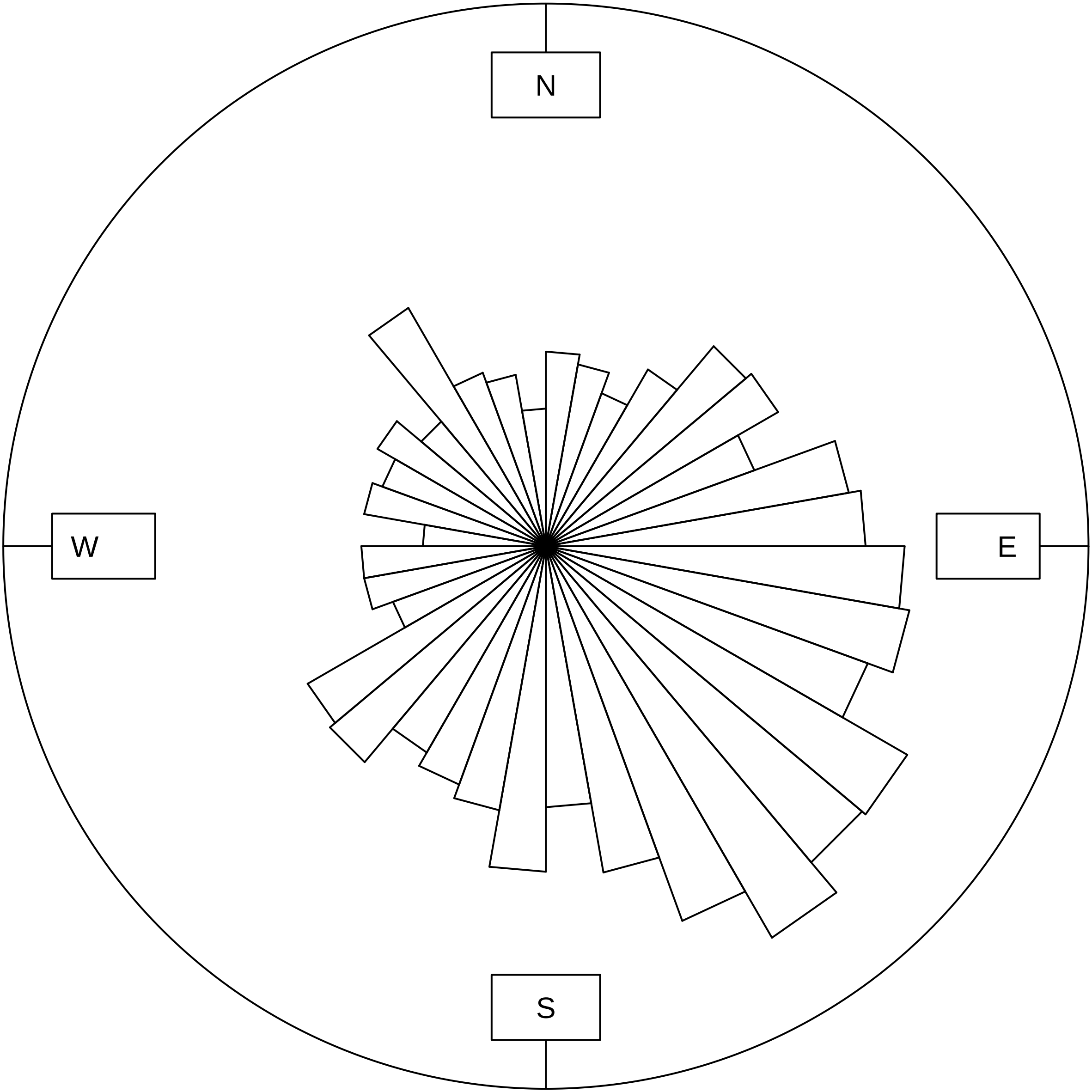

bootstrap 177

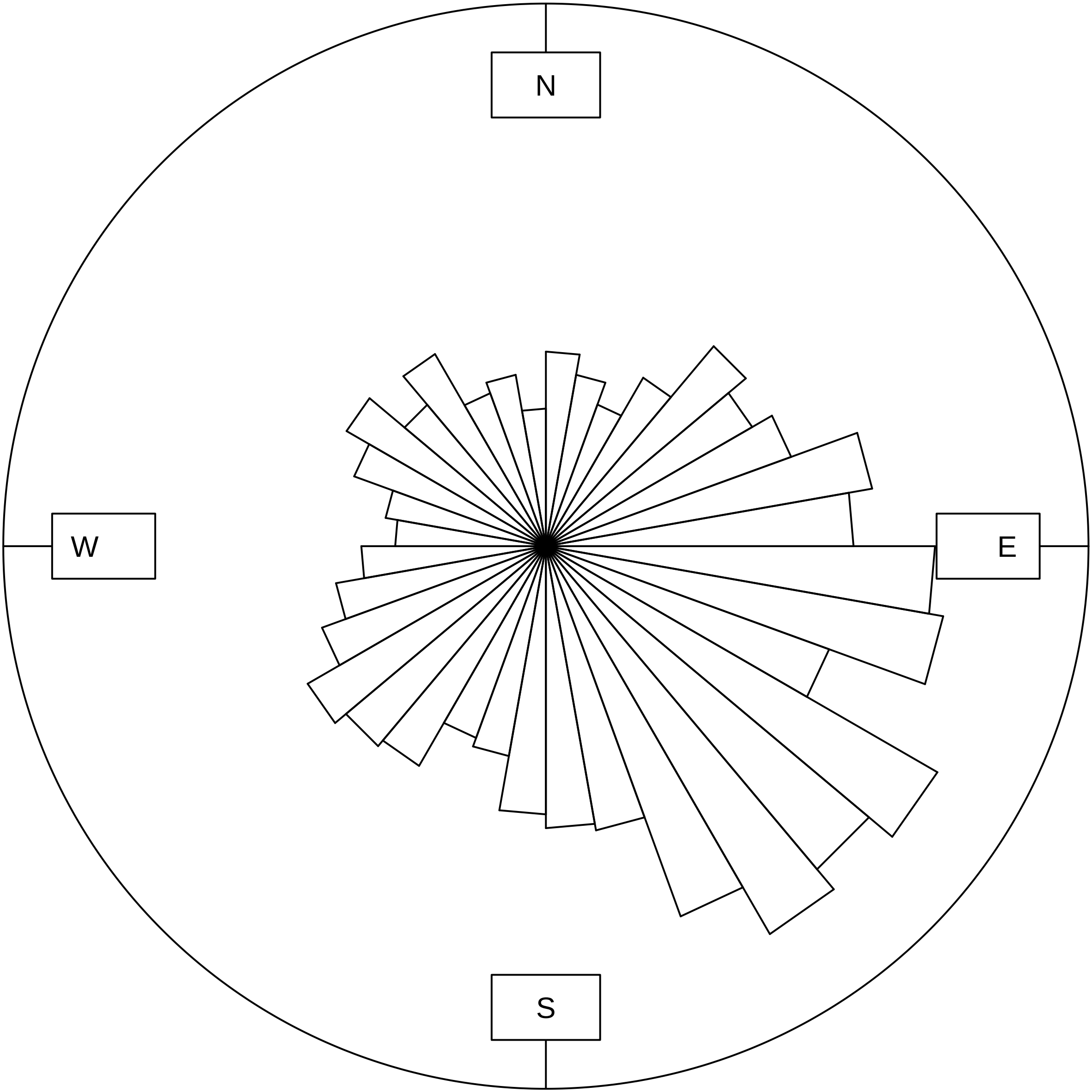

bootstrap 178

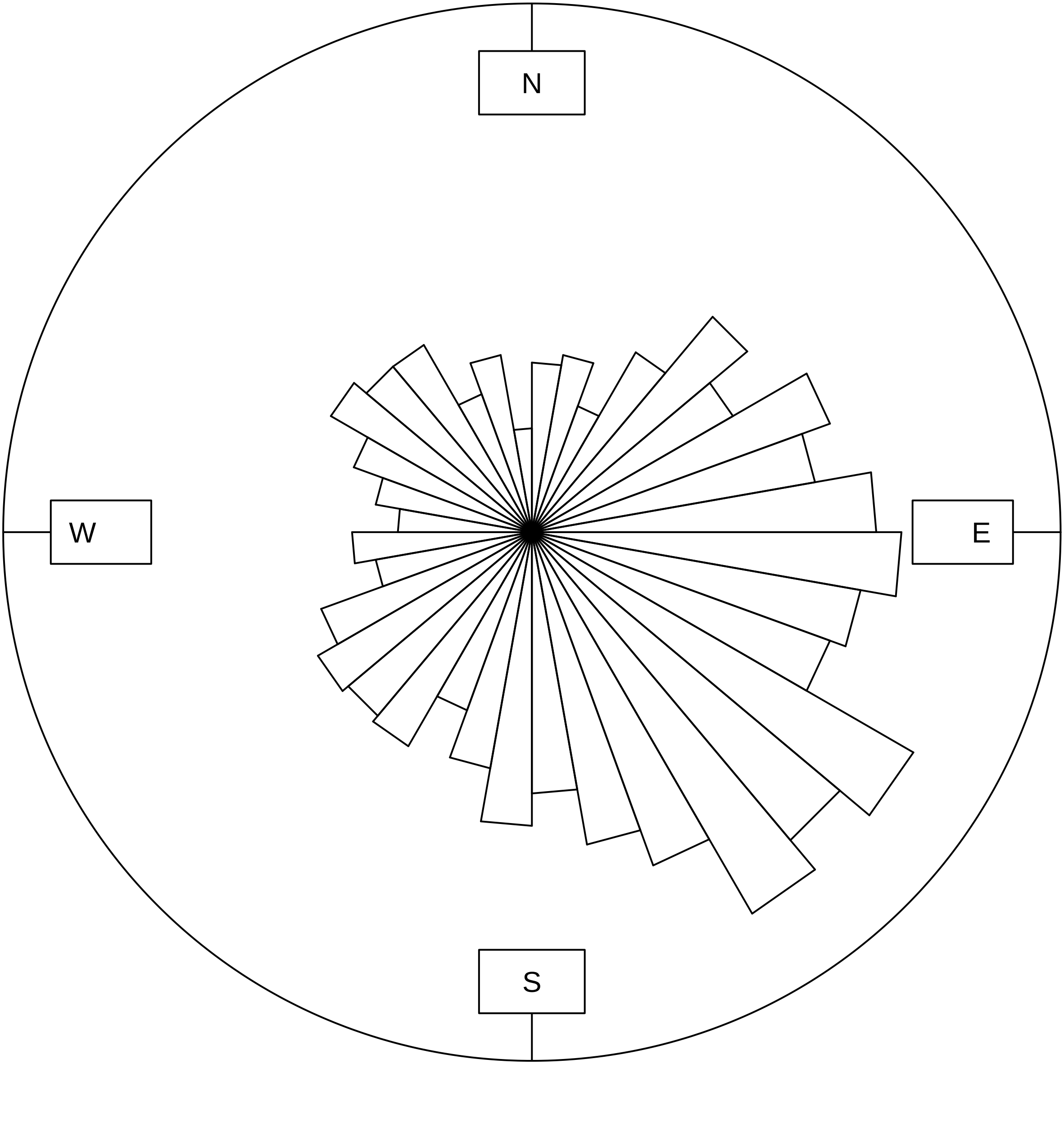

bootstrap 179

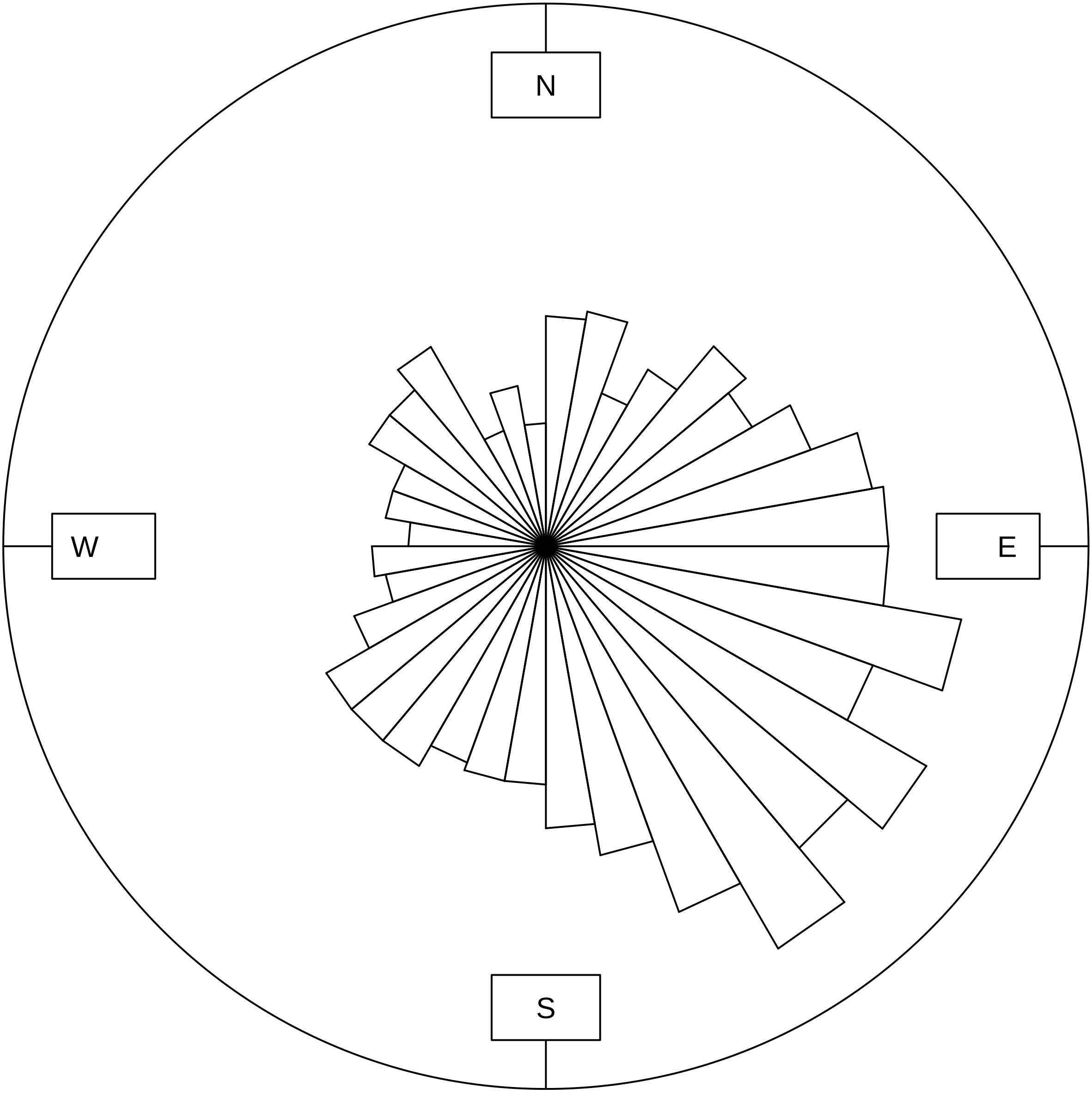

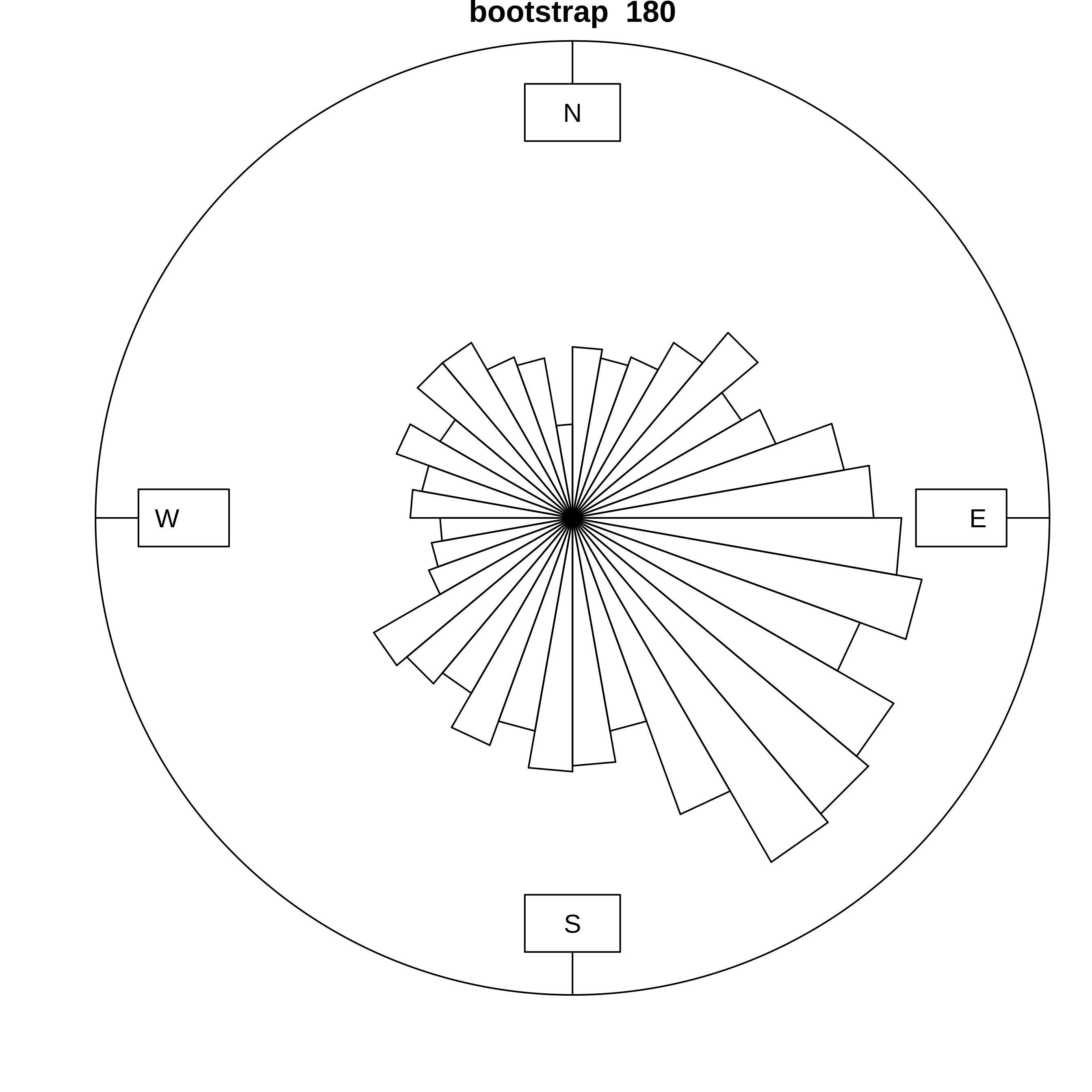
bootstrap 180
N
W
E
S

bootstrap 181

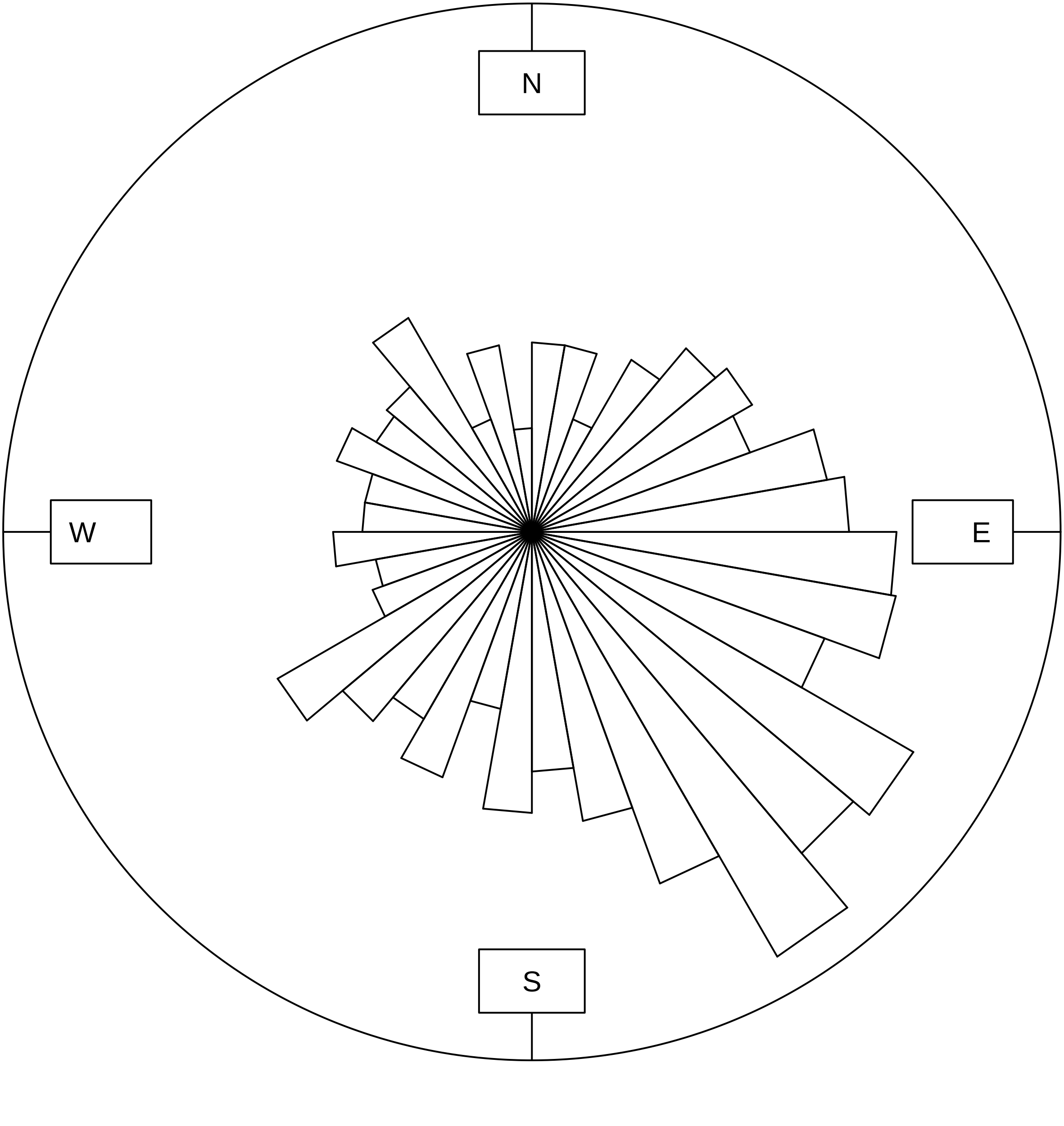

bootstrap 182

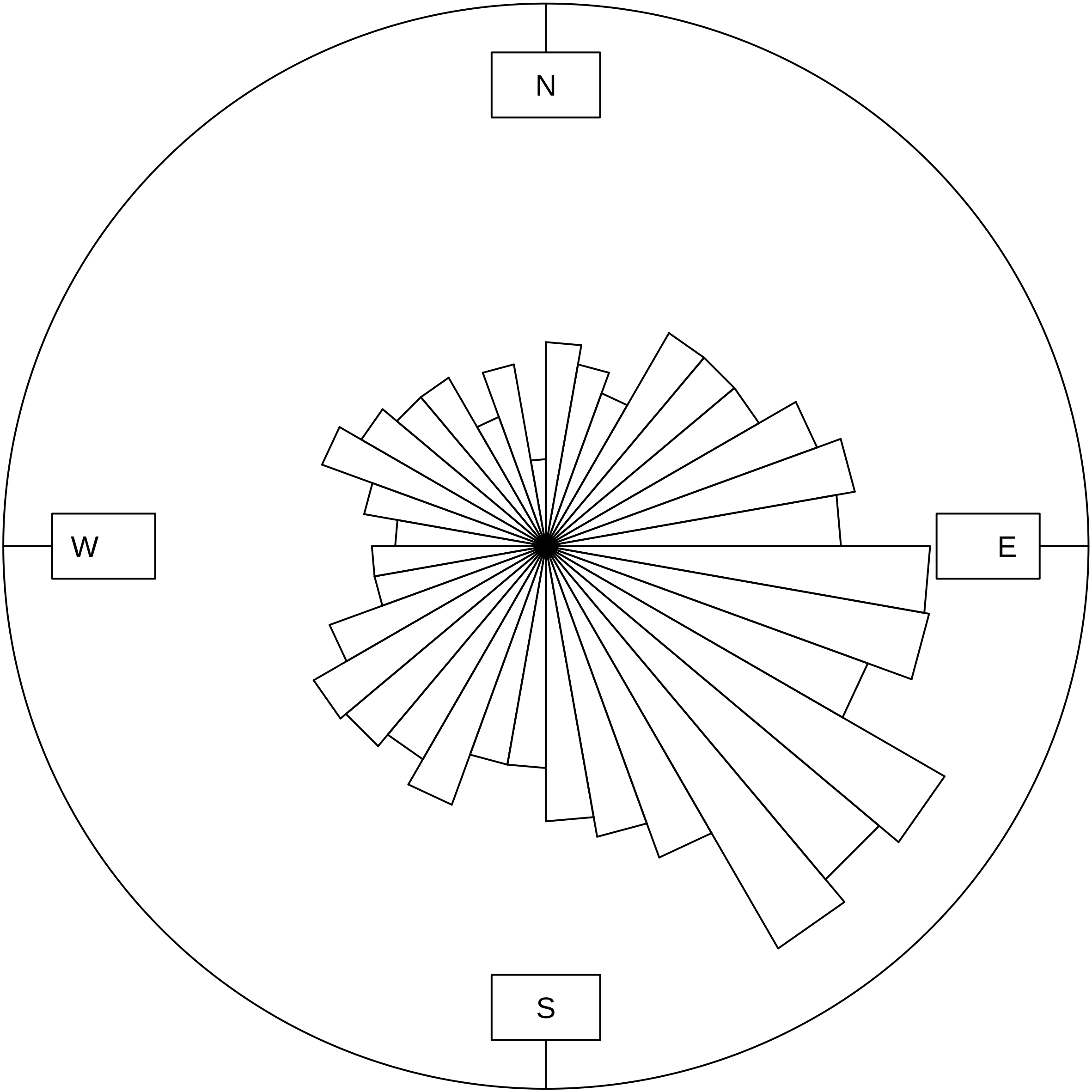

bootstrap 183

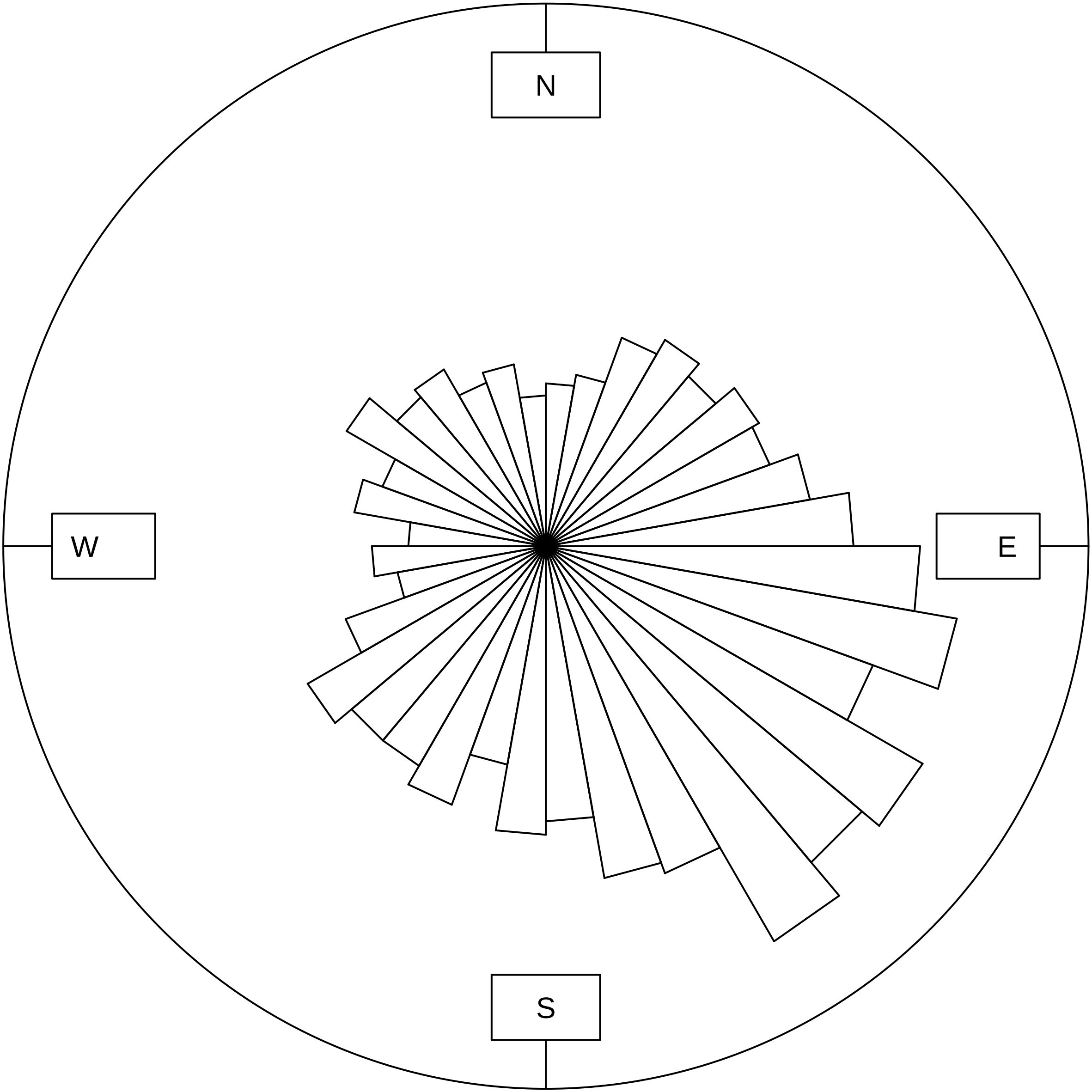

bootstrap 184

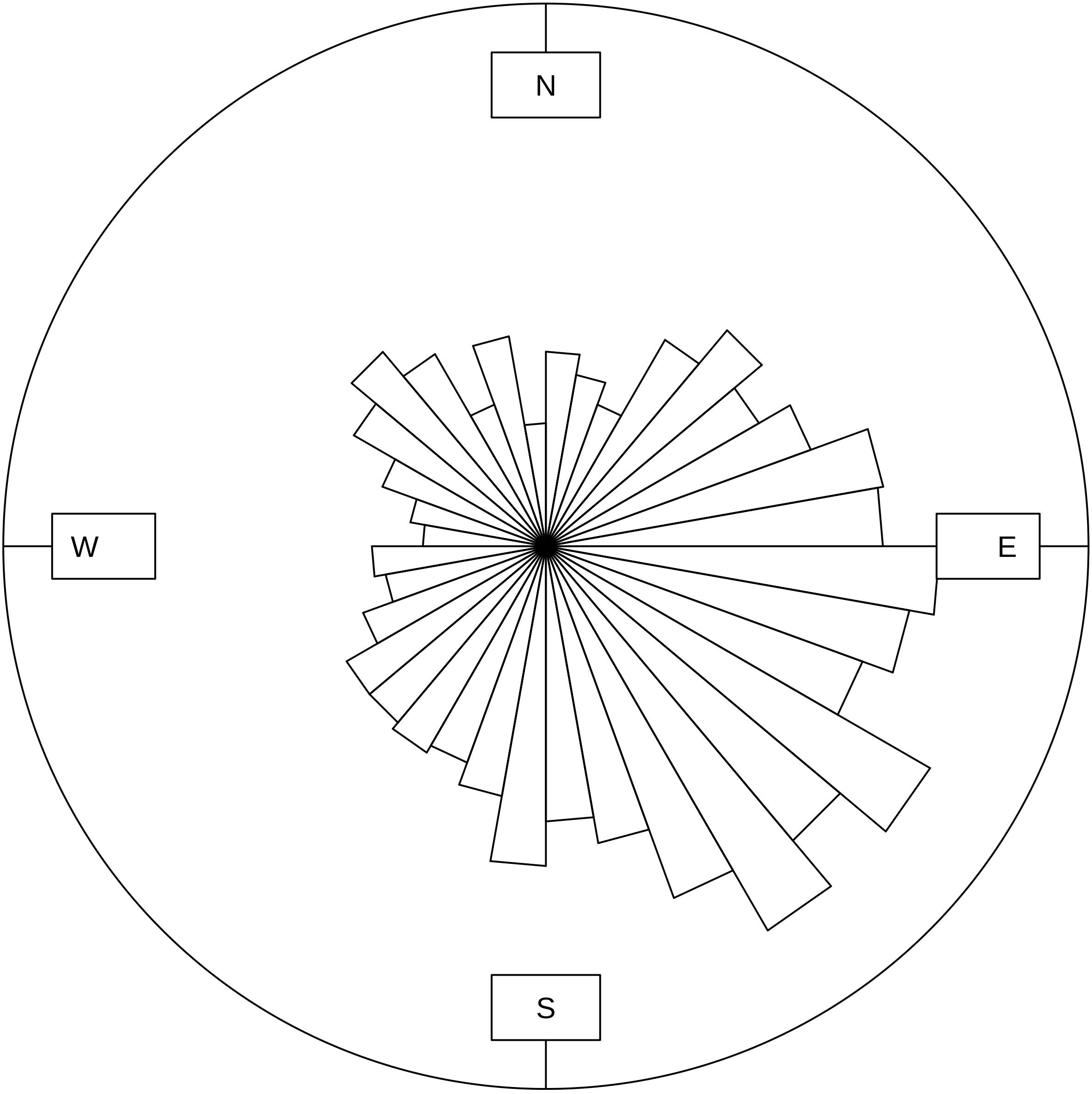

bootstrap 185

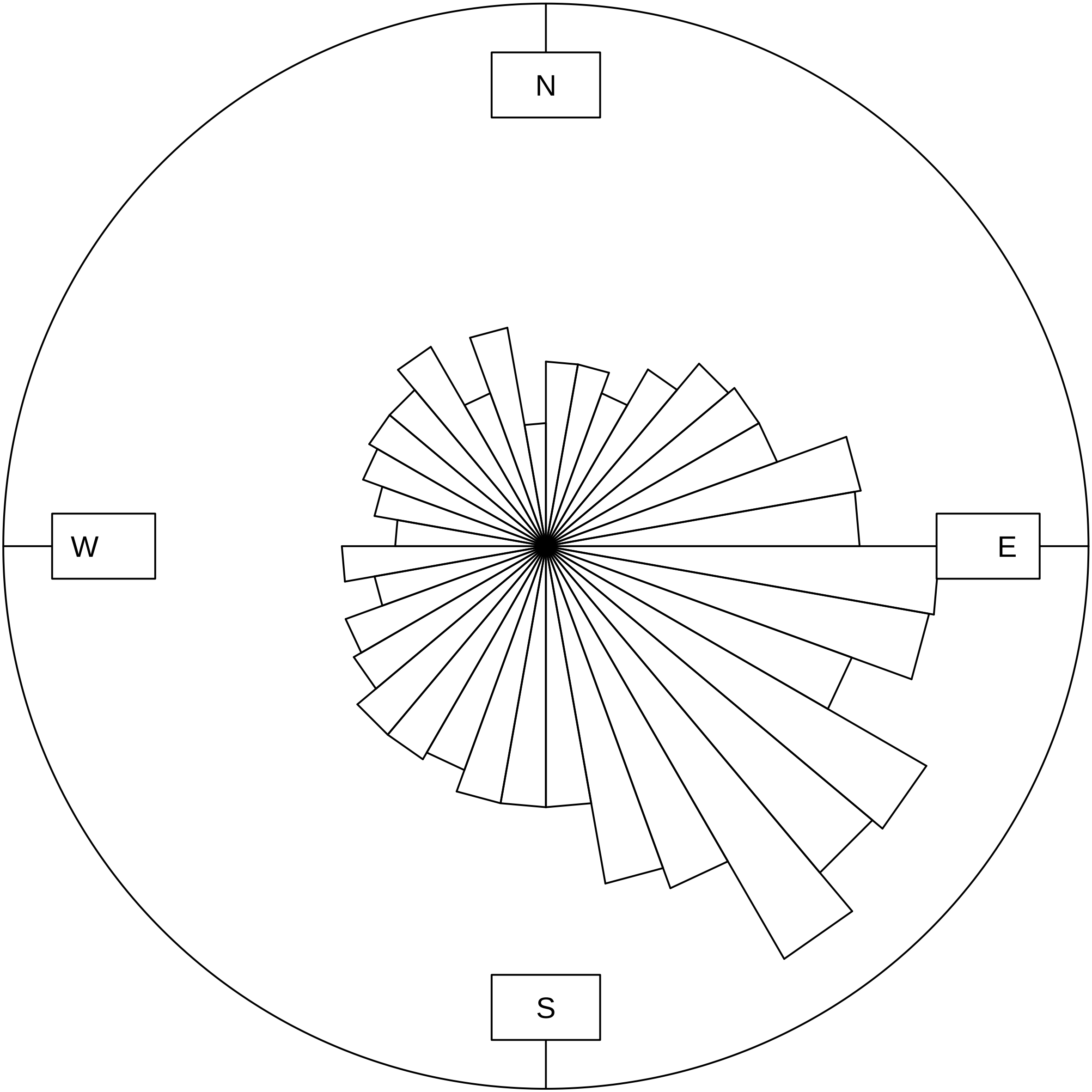

bootstrap 186

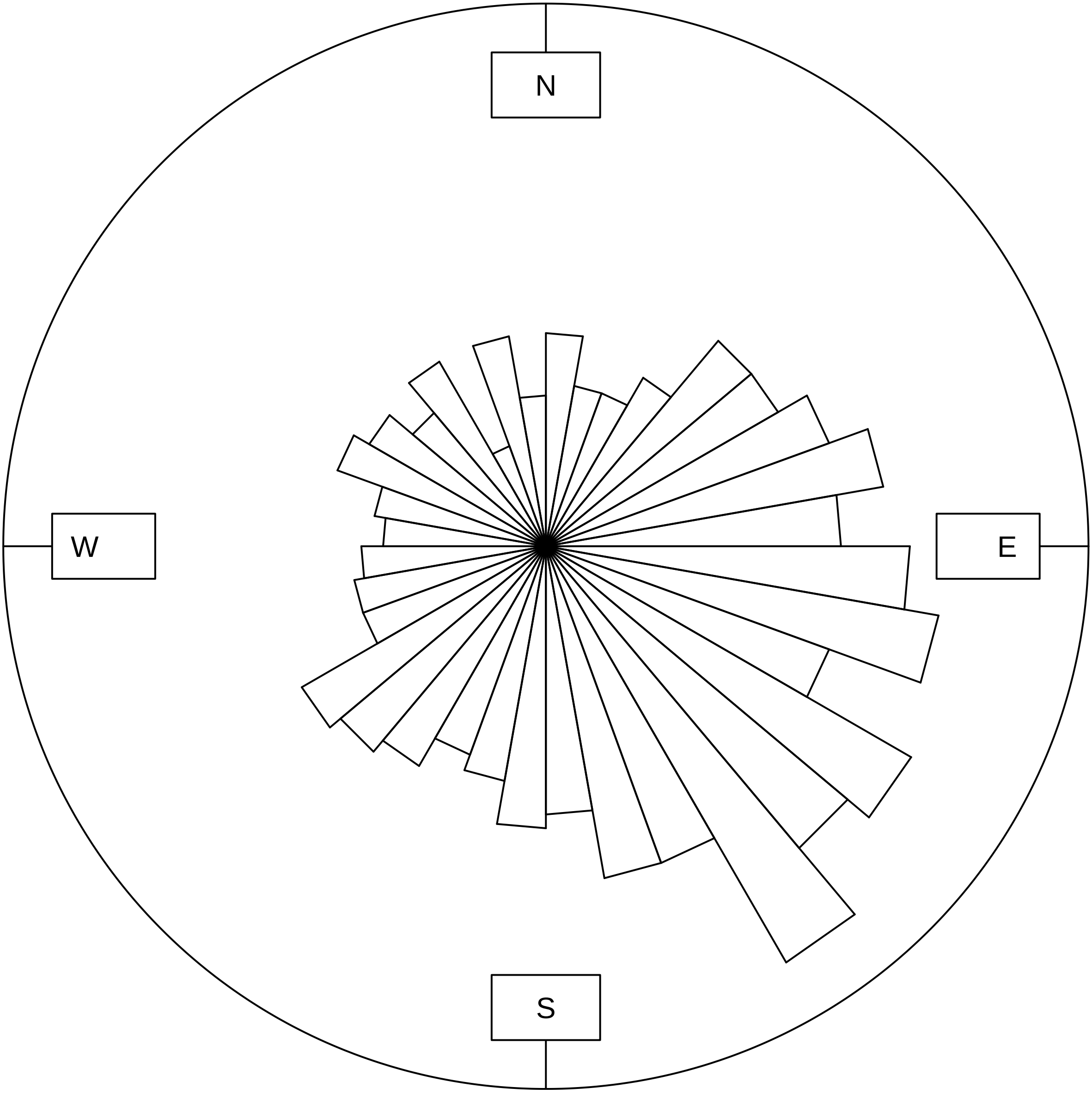

bootstrap 187

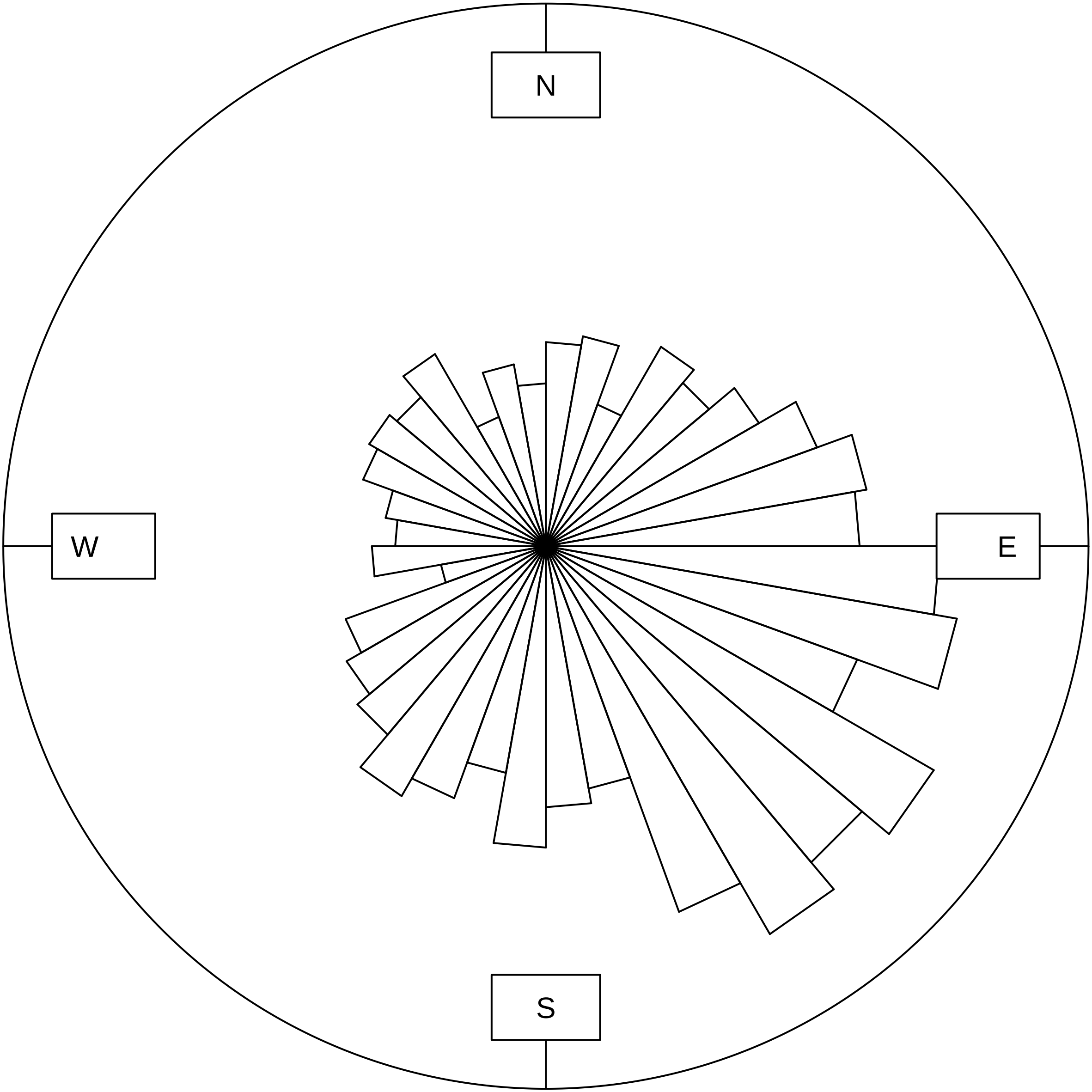

bootstrap 188

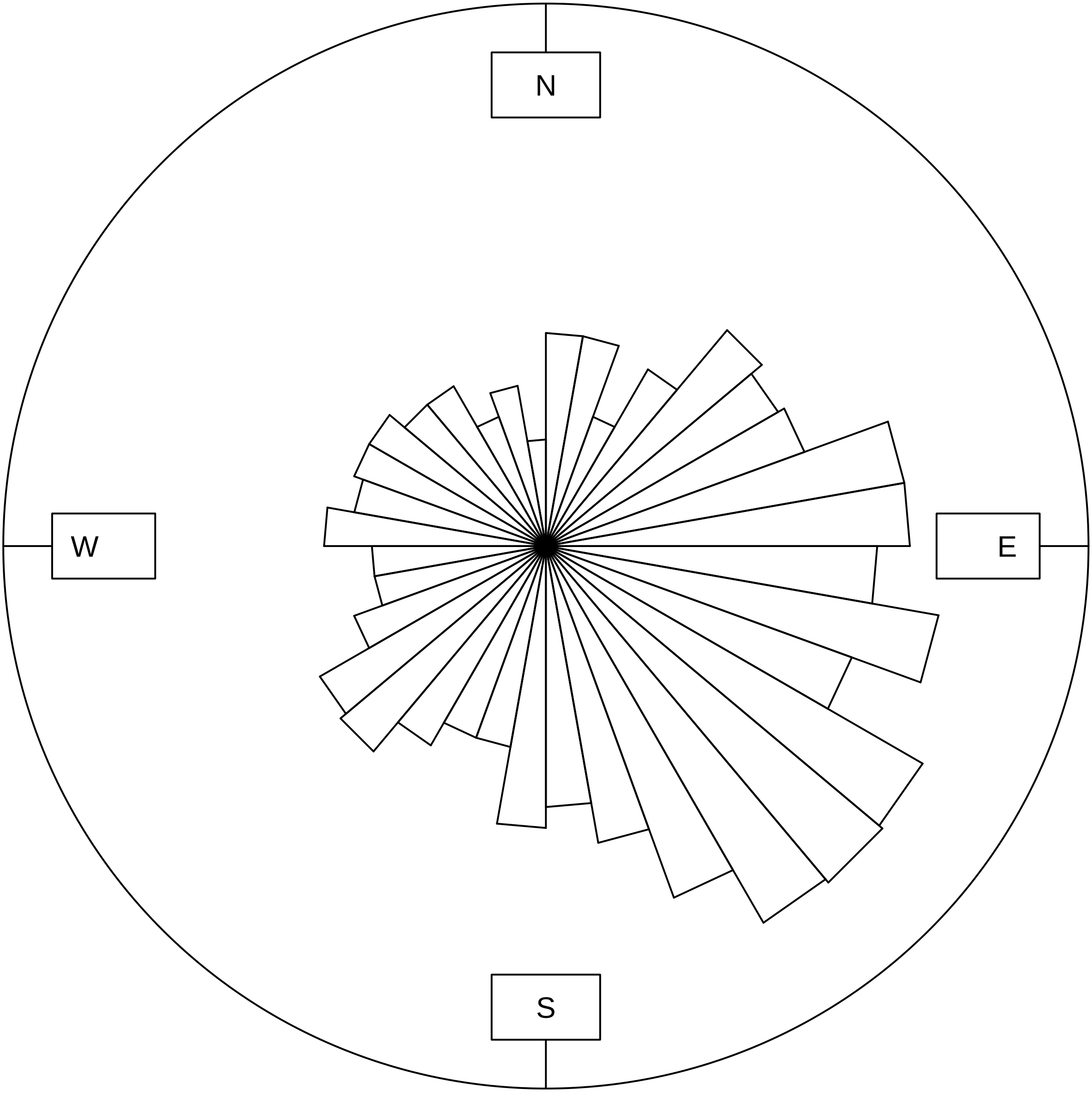

bootstrap 189

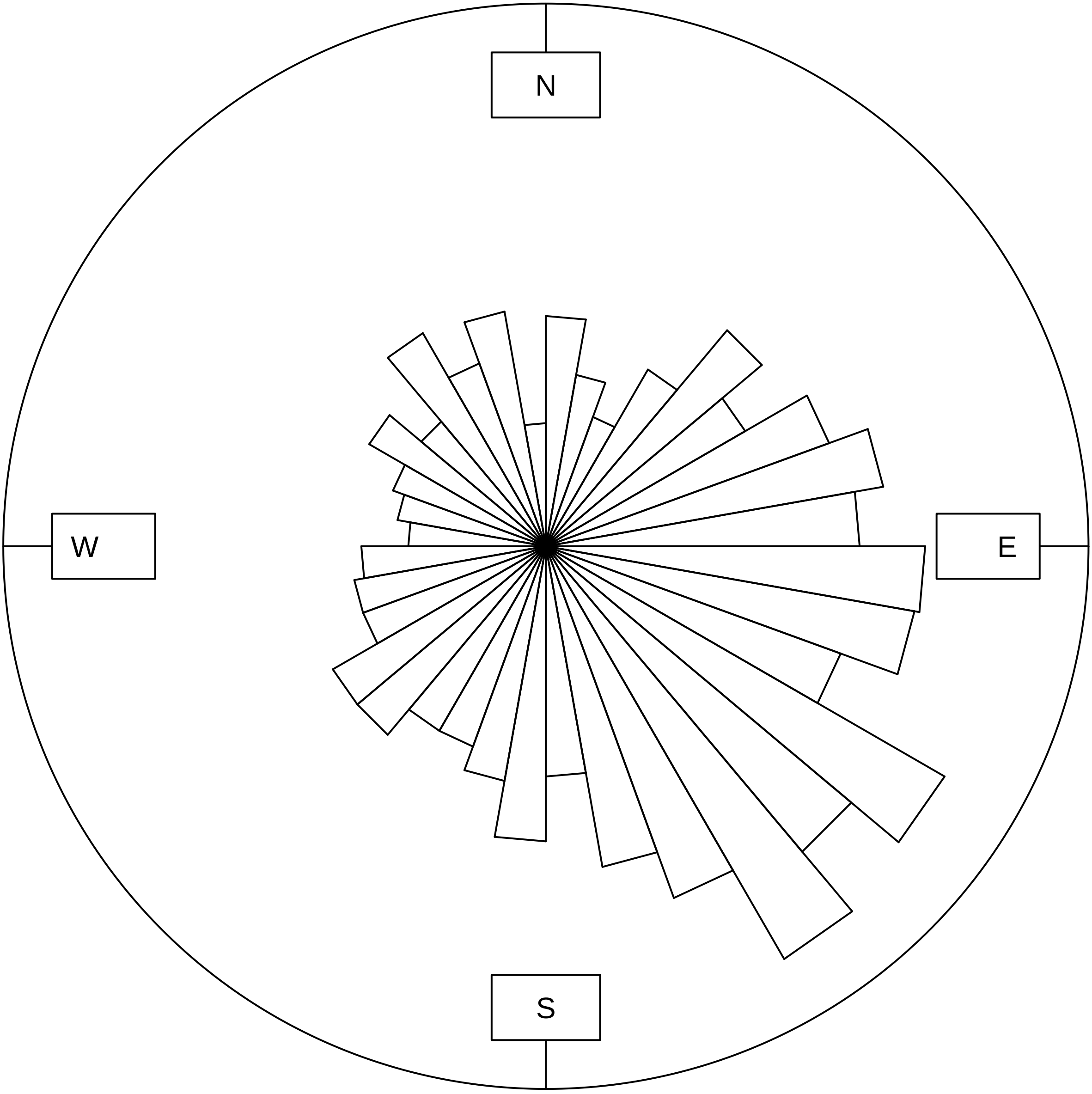

bootstrap 190

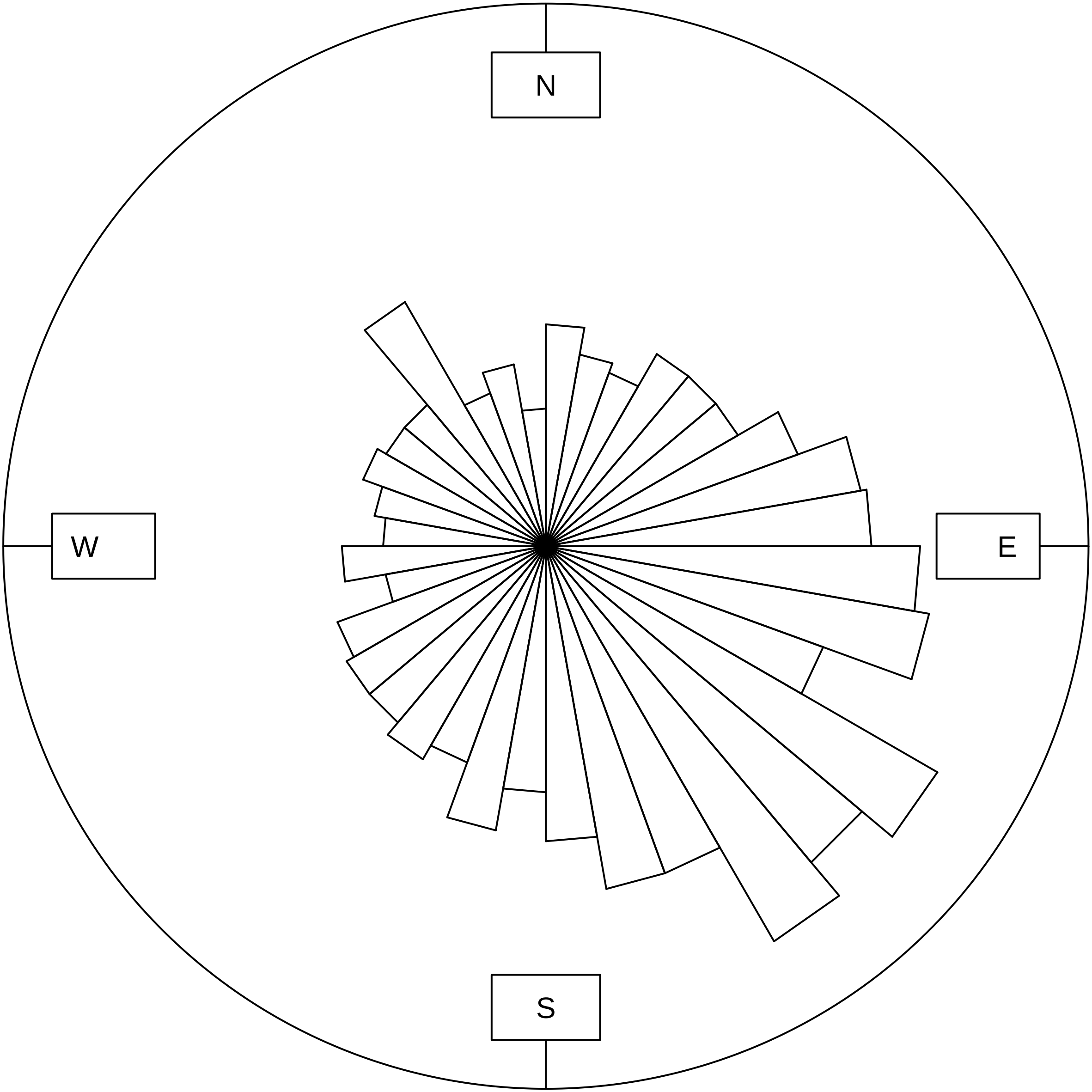

bootstrap 191

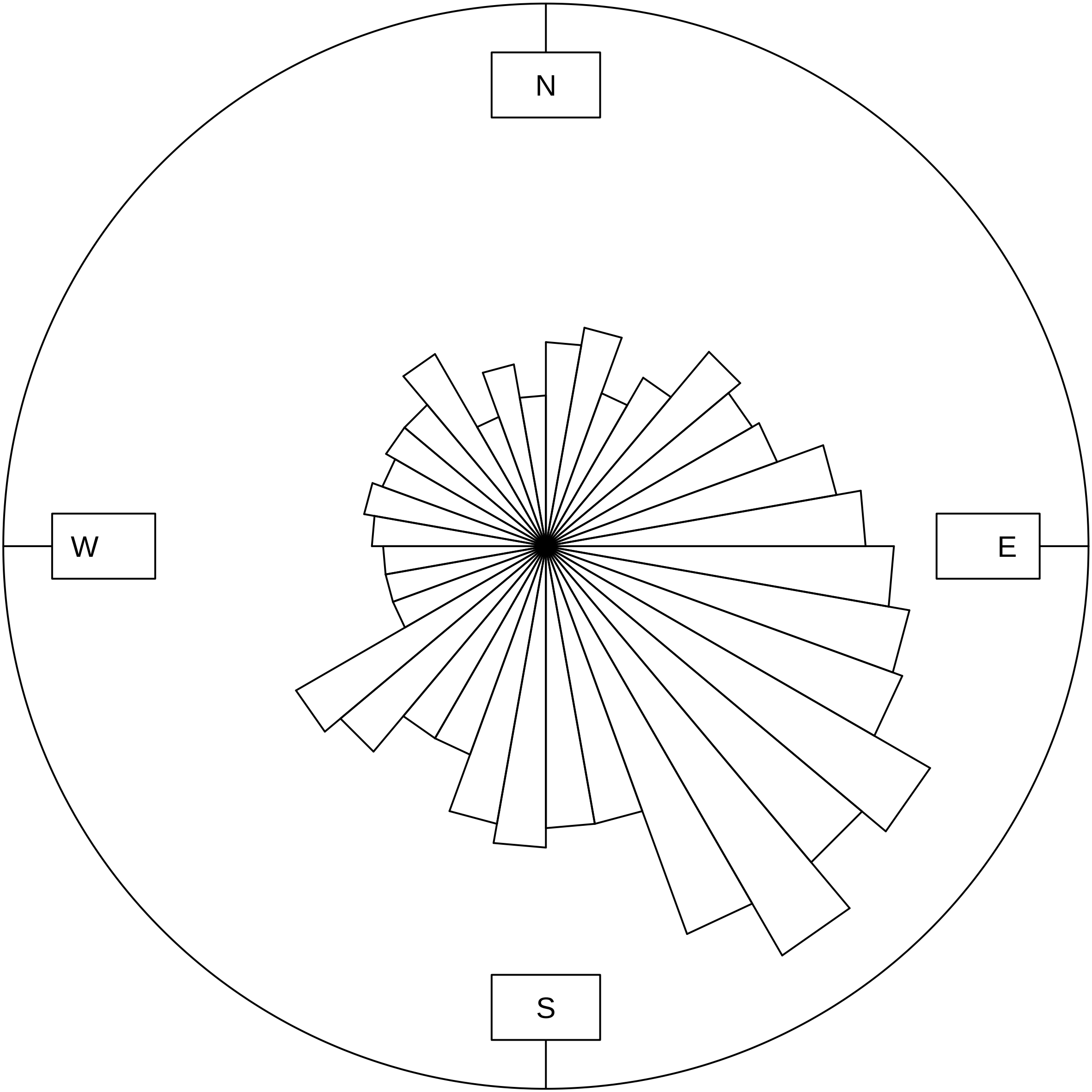

bootstrap 192

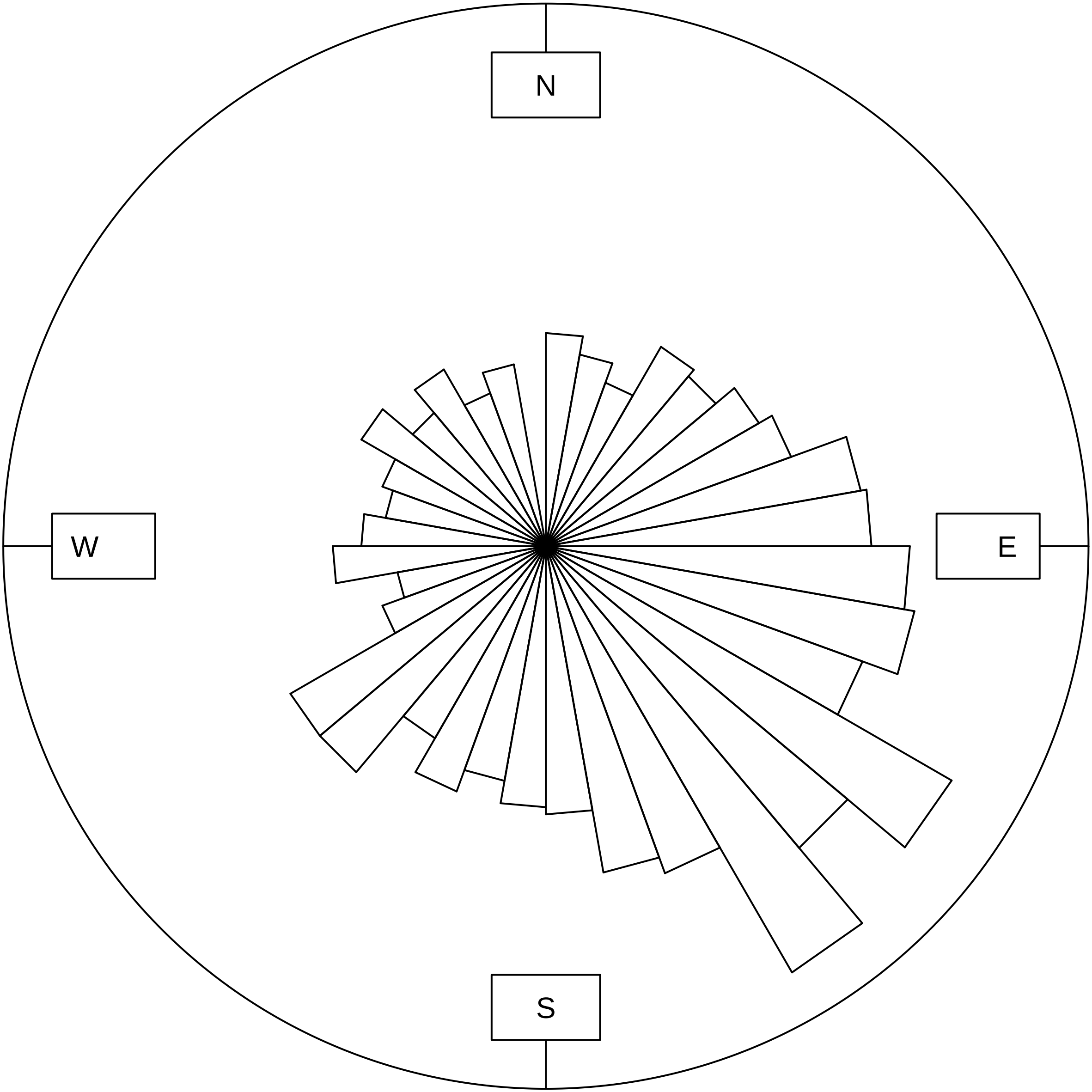

bootstrap 193

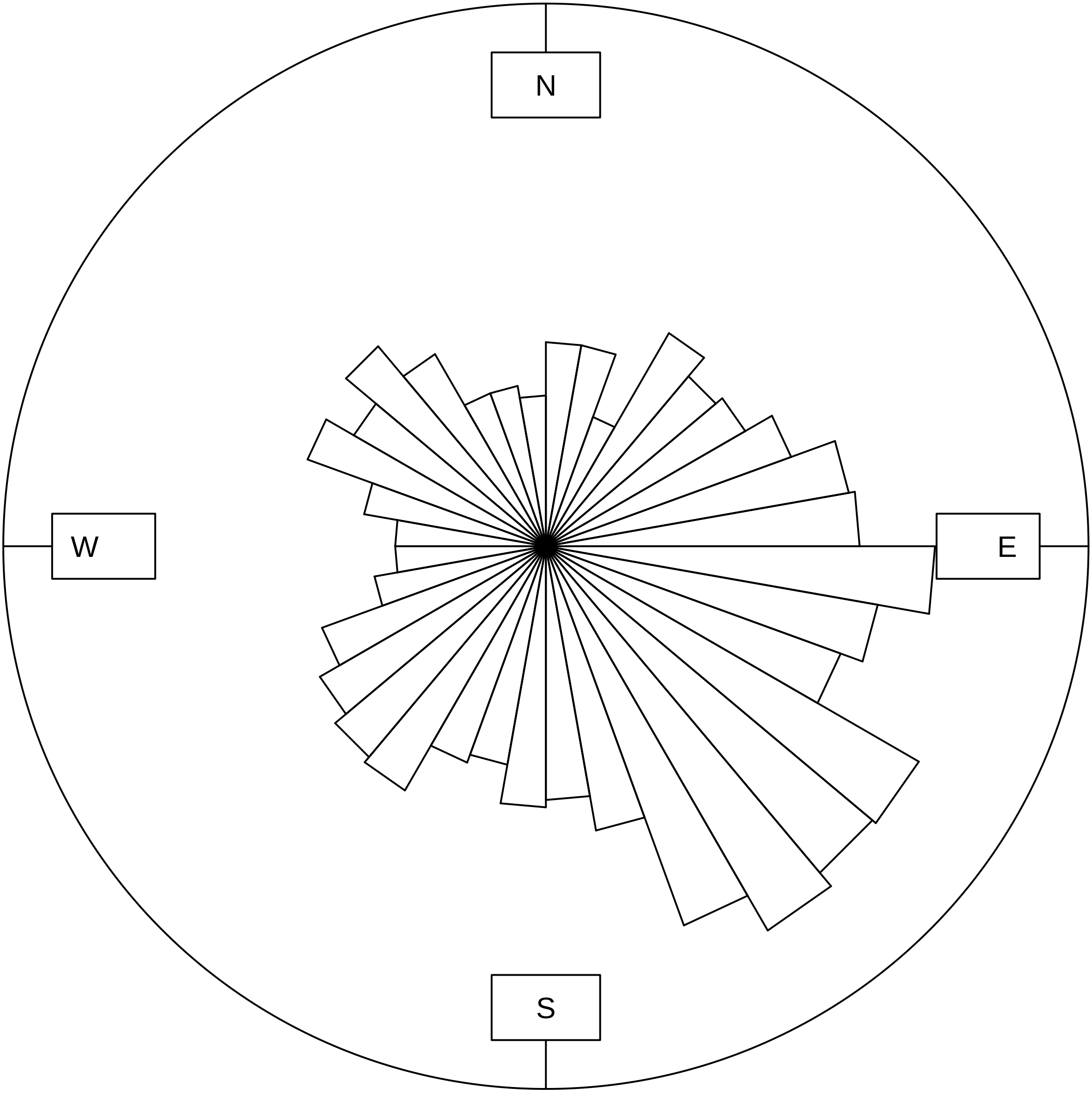

bootstrap 194

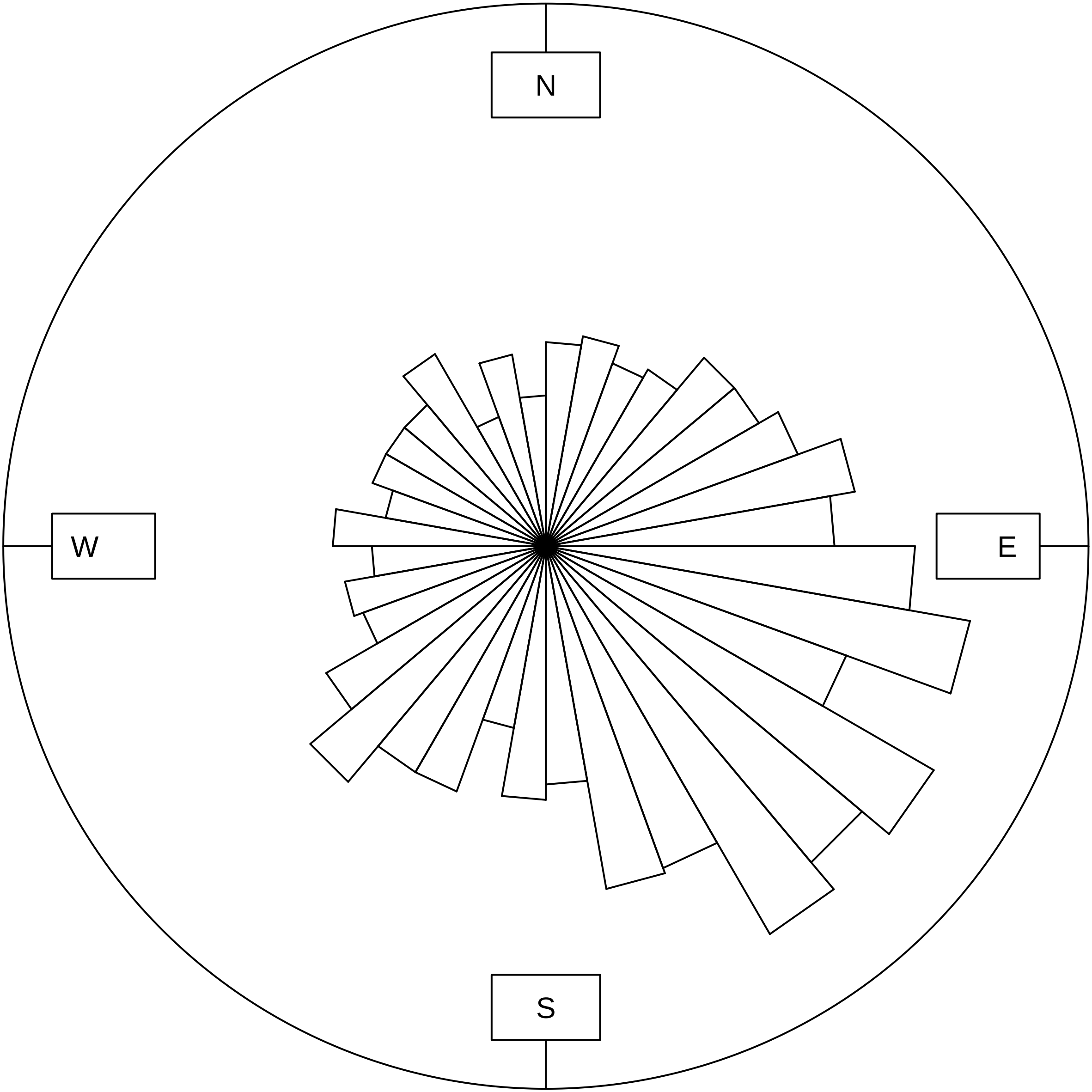

bootstrap 195

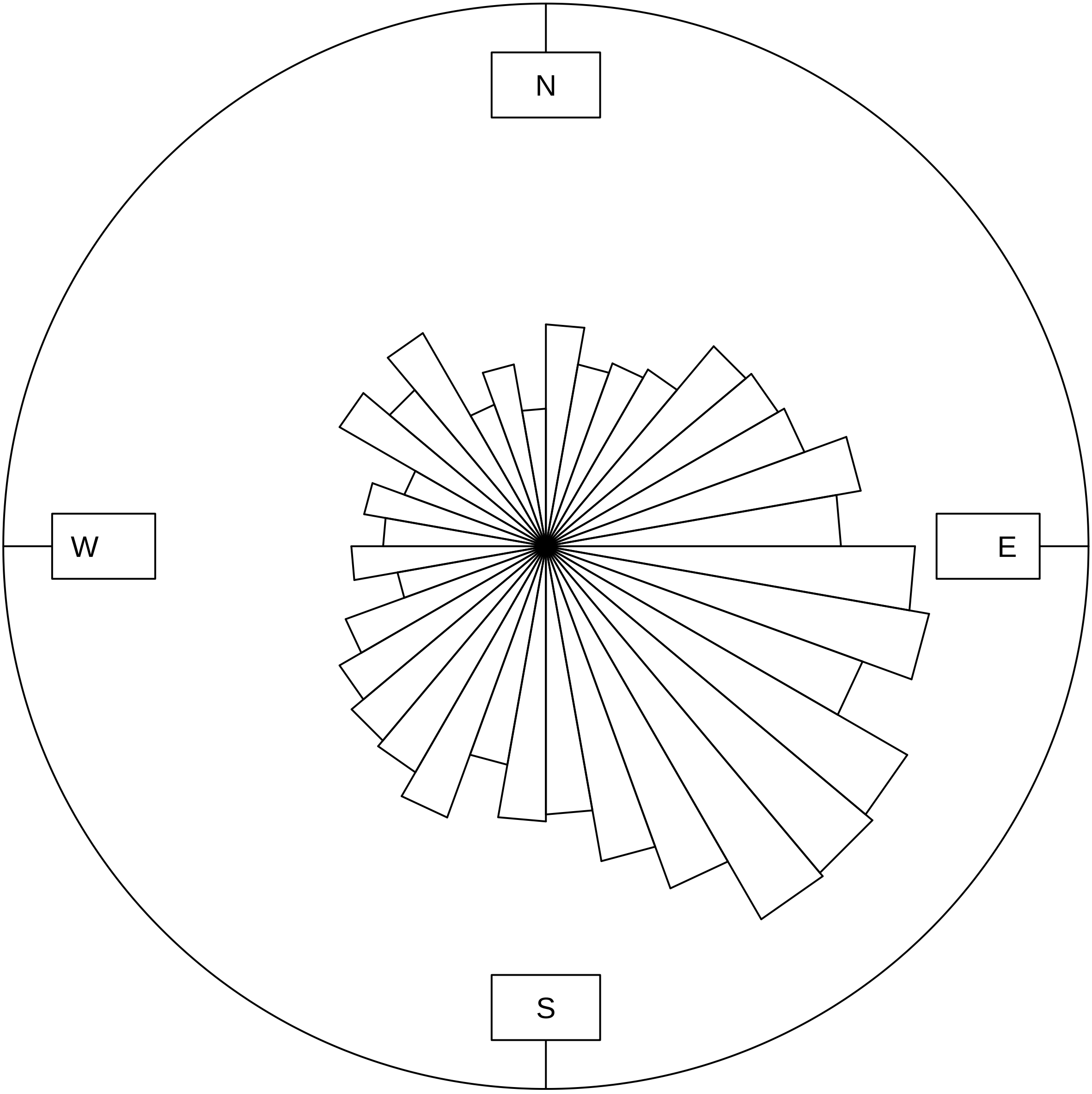

bootstrap 196

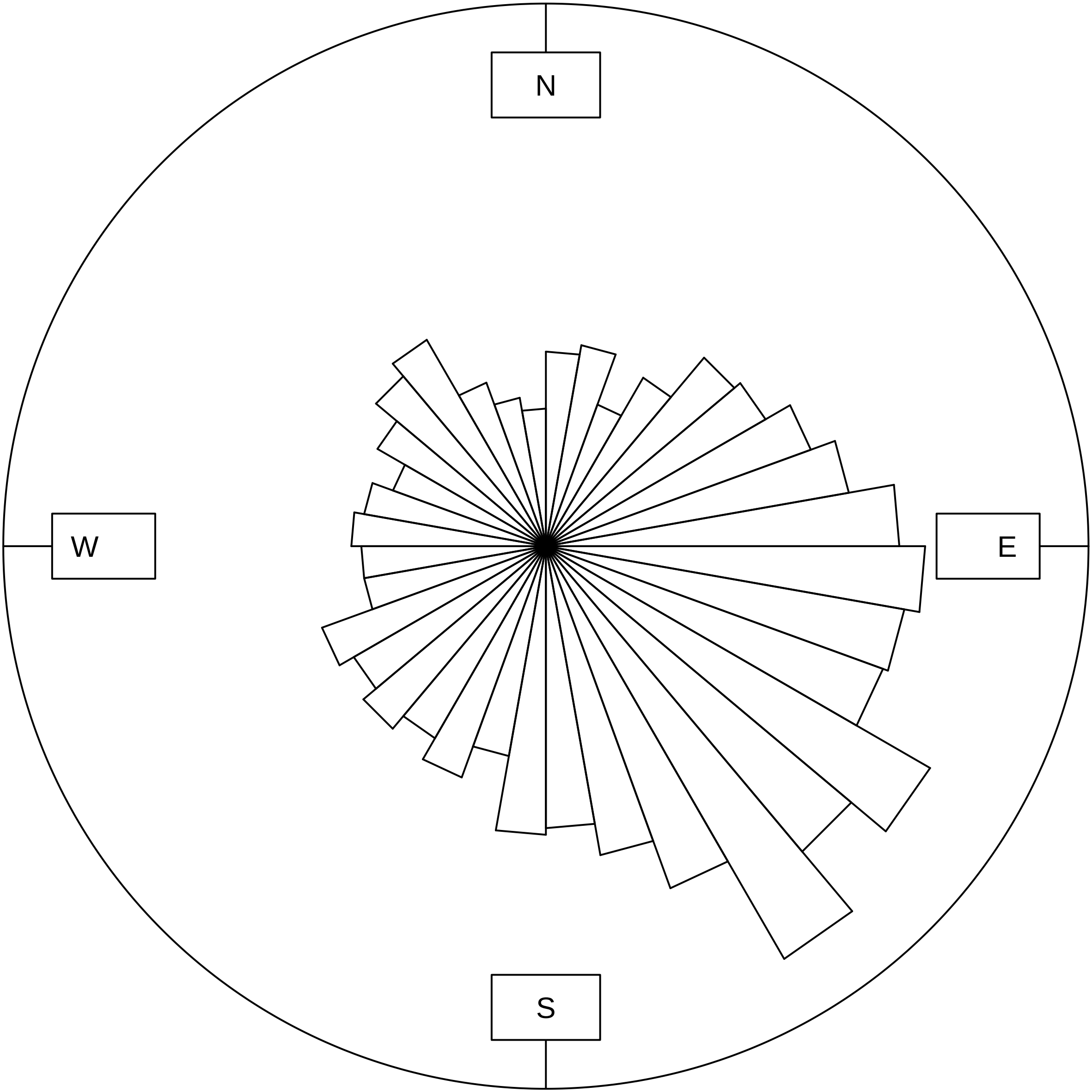

bootstrap 197

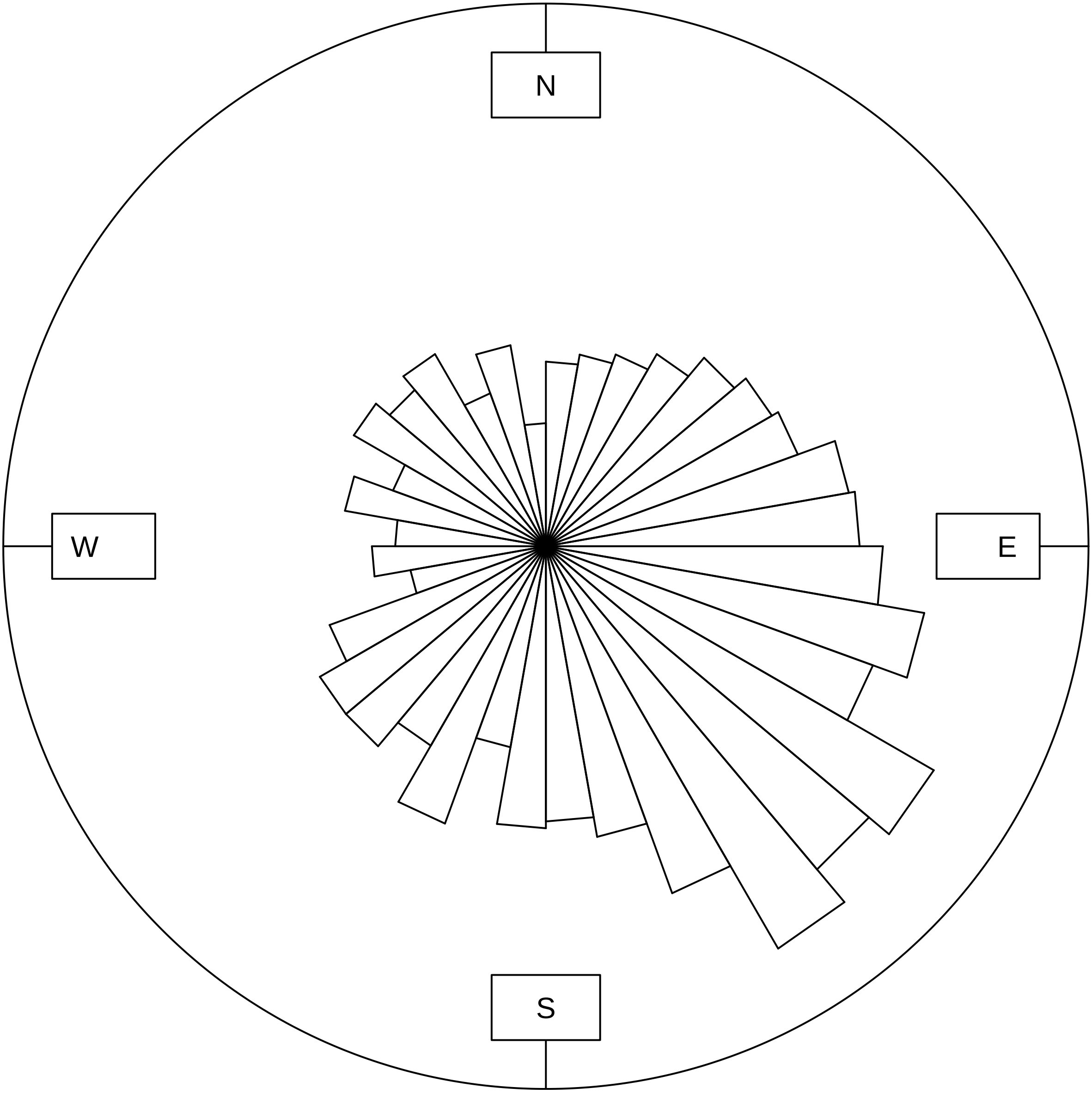

bootstrap 198

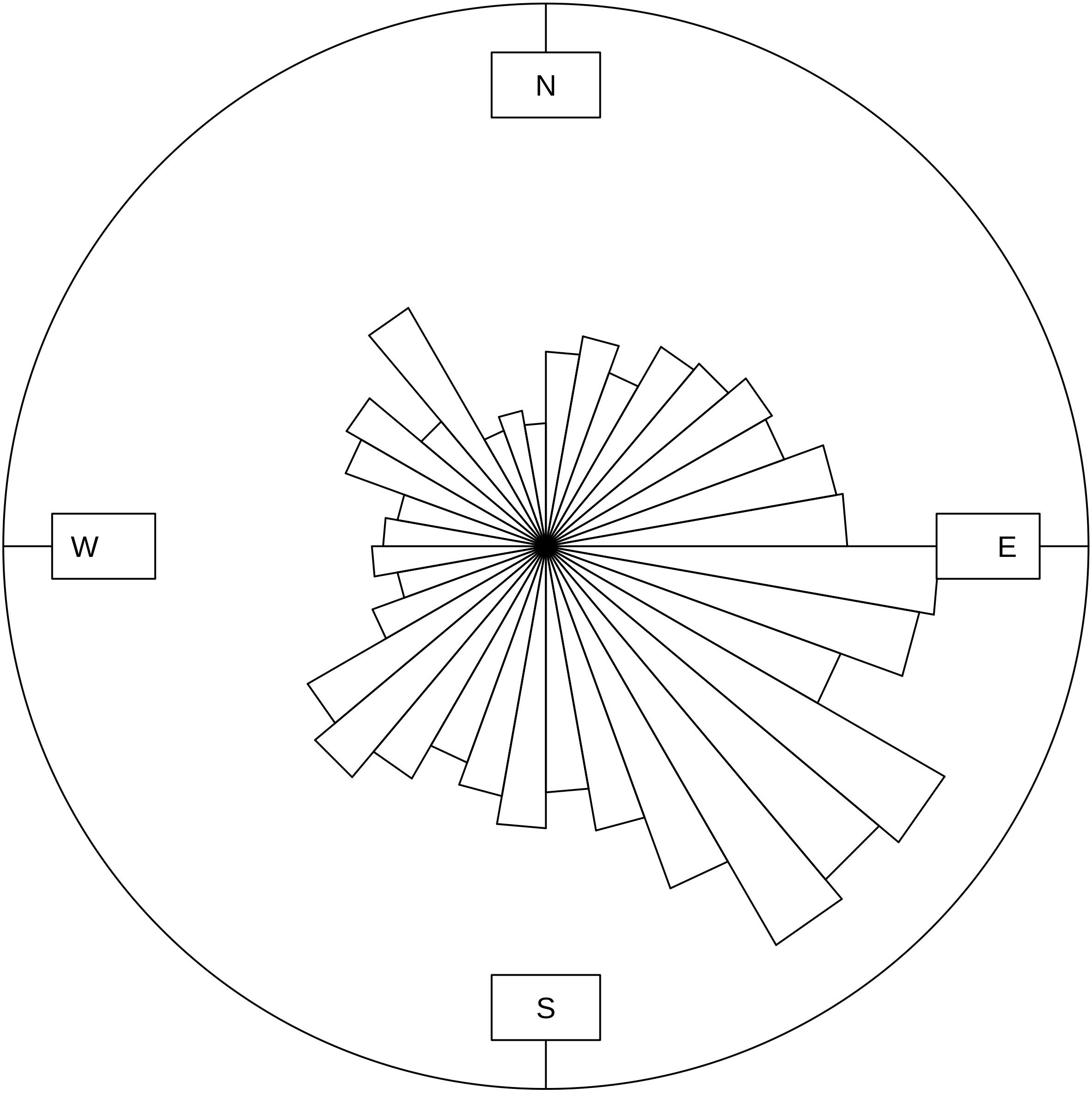

bootstrap 199

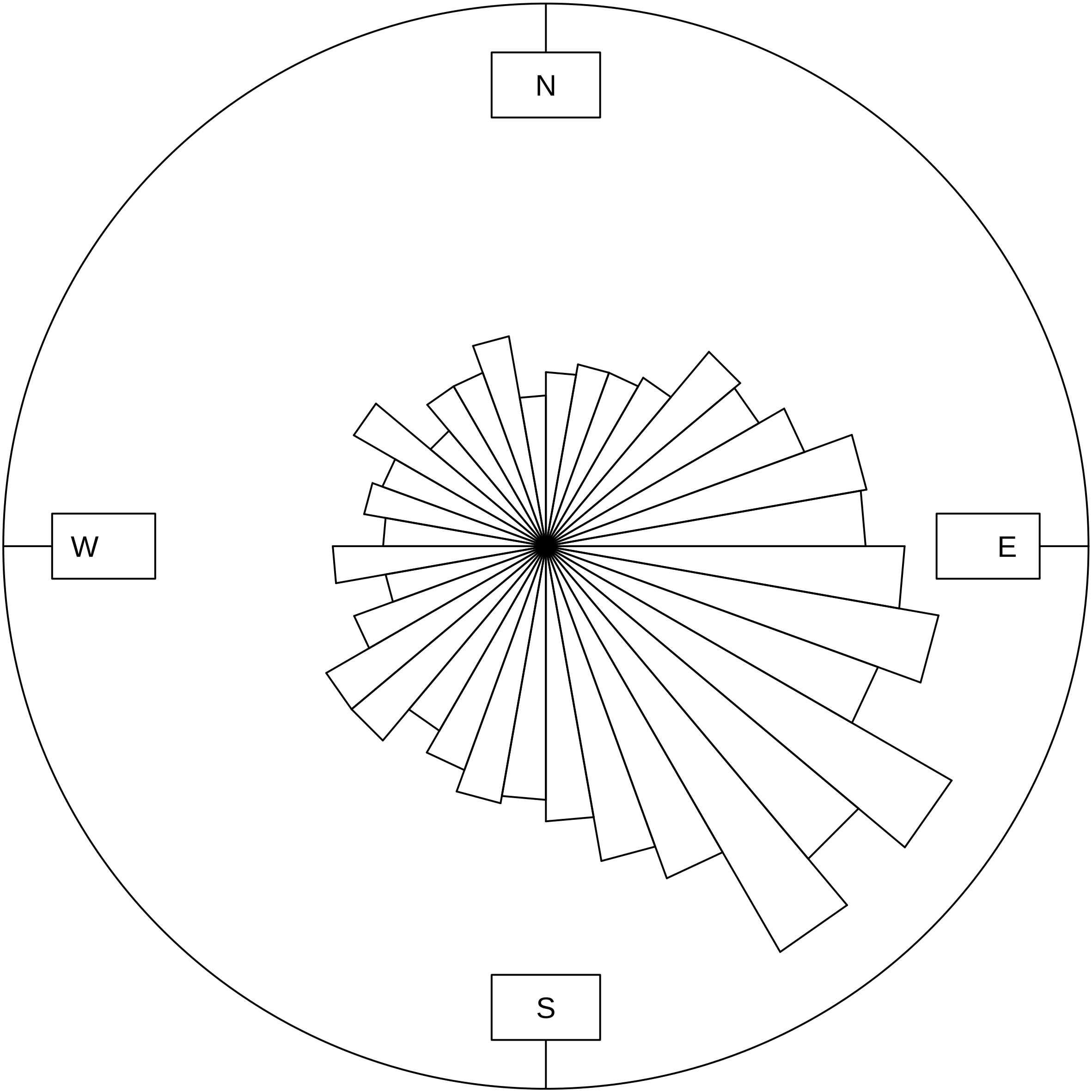

bootstrap 200

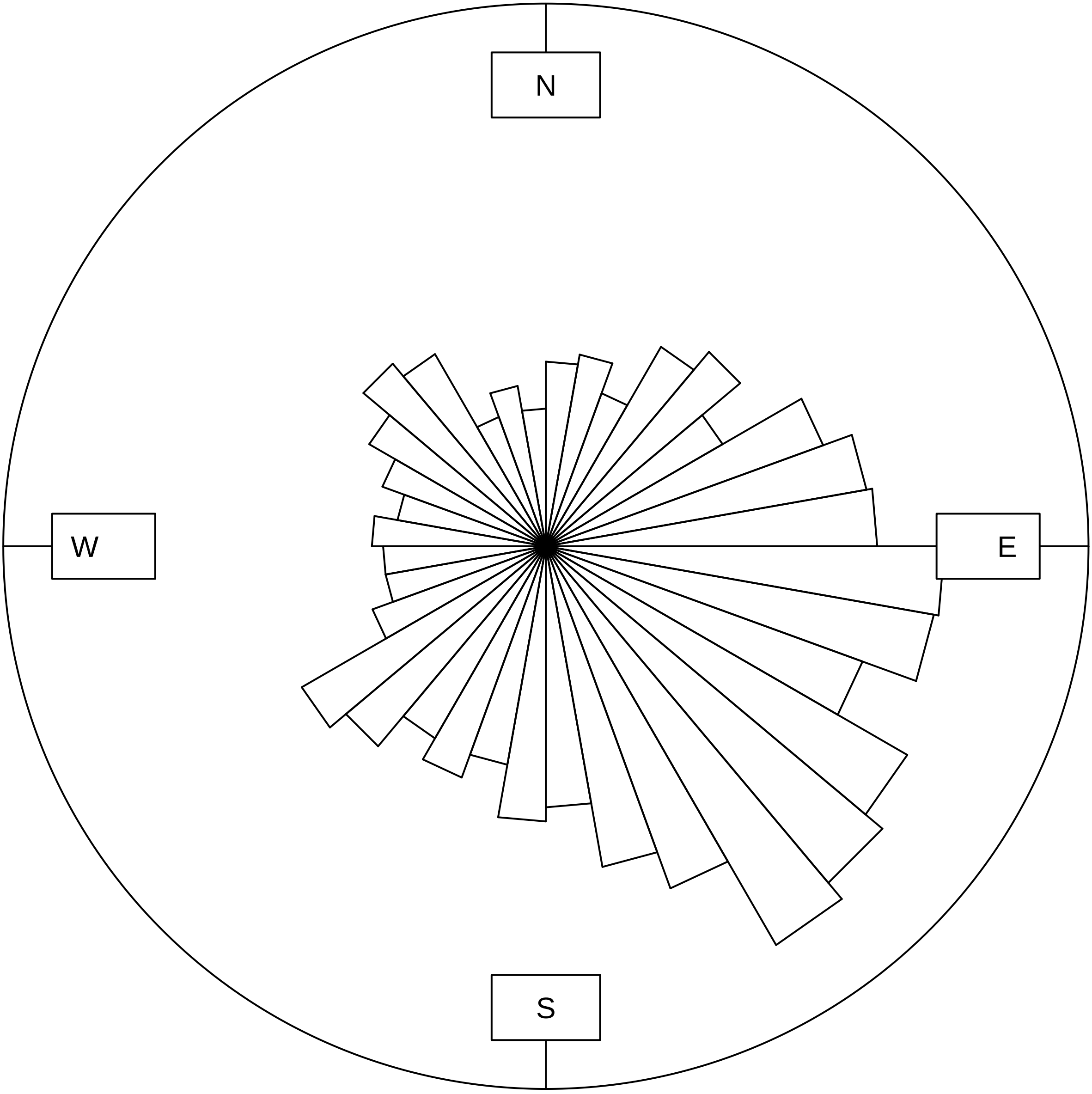

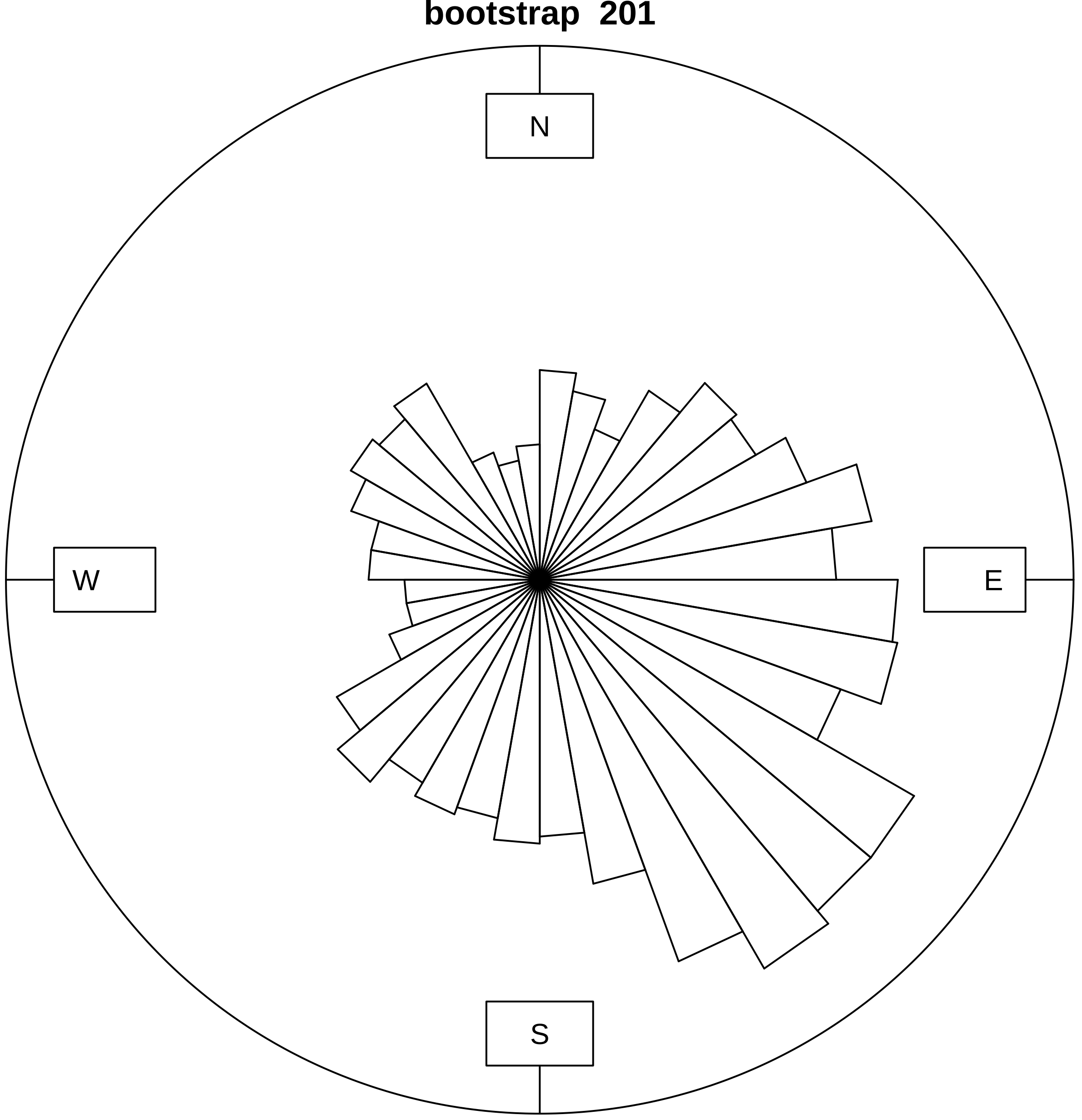
bootstrap 201
N
W
E
S

bootstrap 202

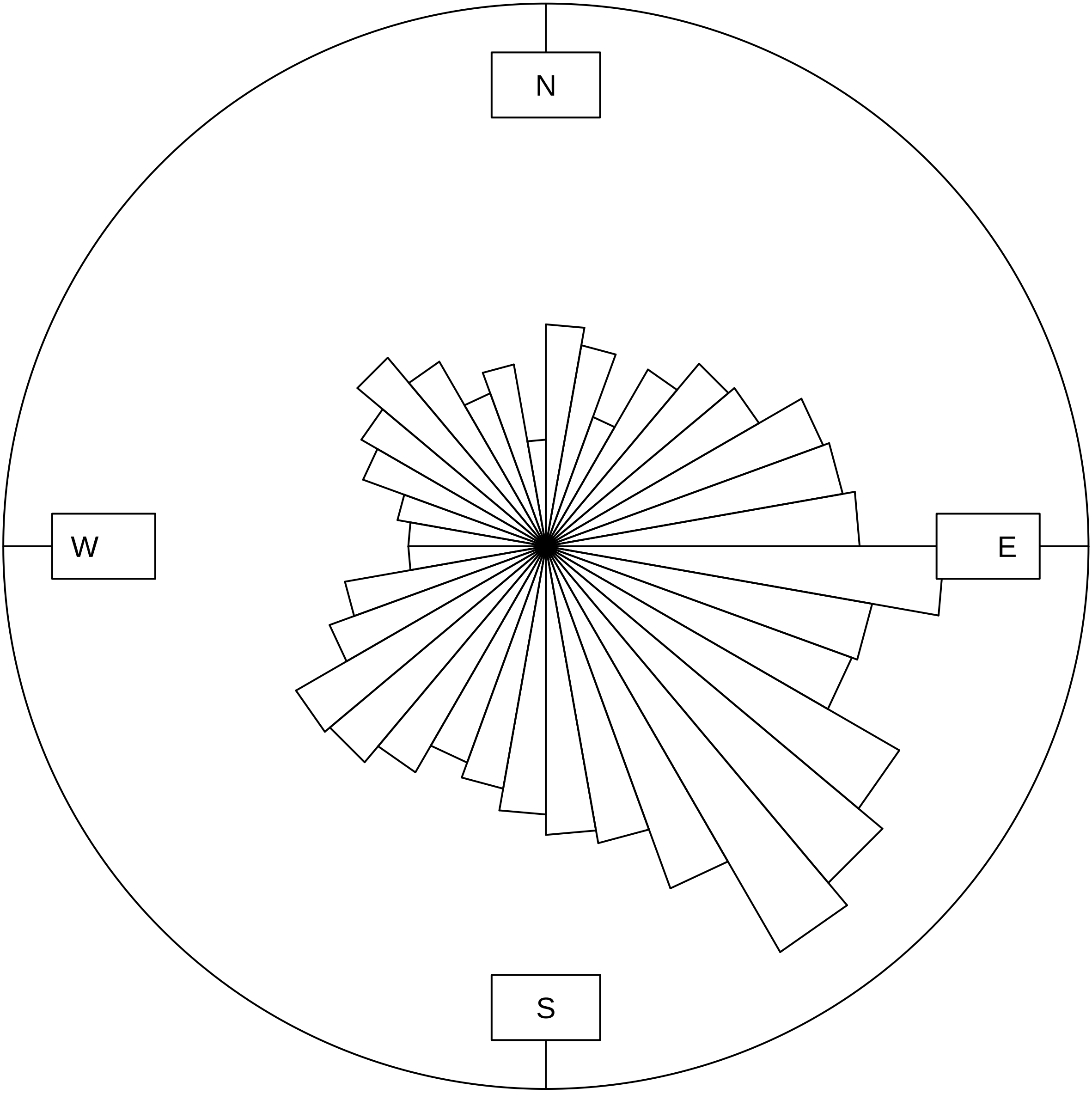

bootstrap 203

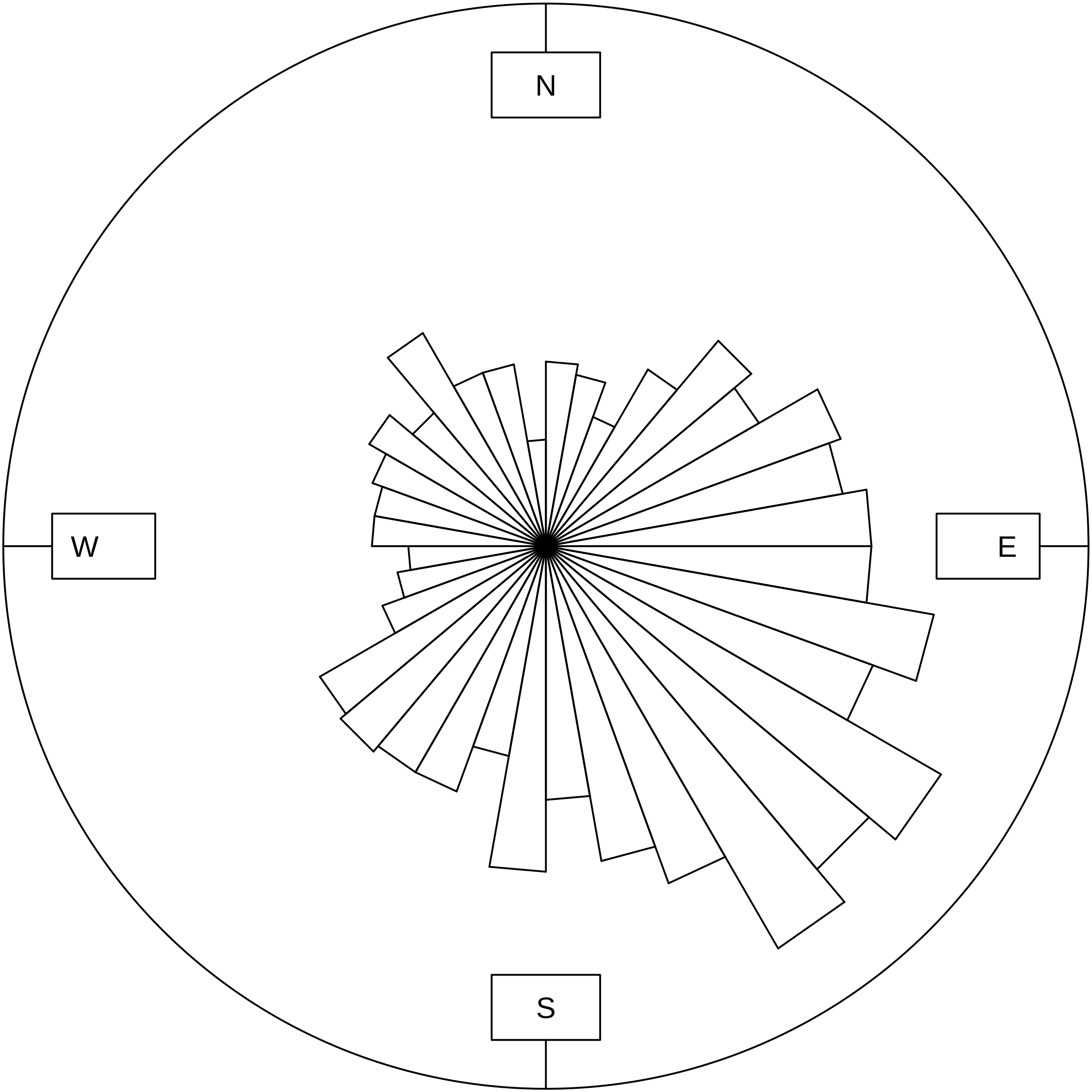

bootstrap 204

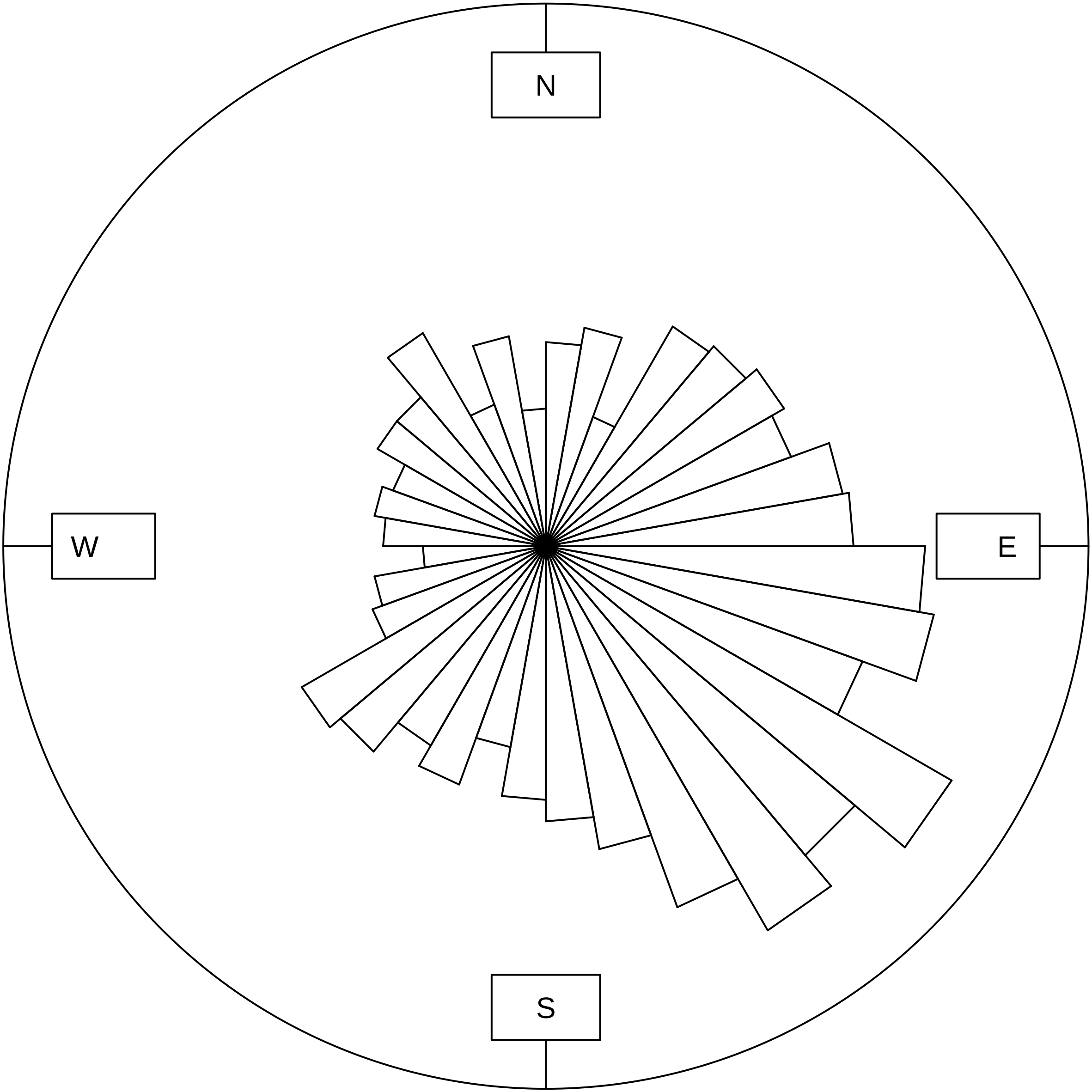

bootstrap 205

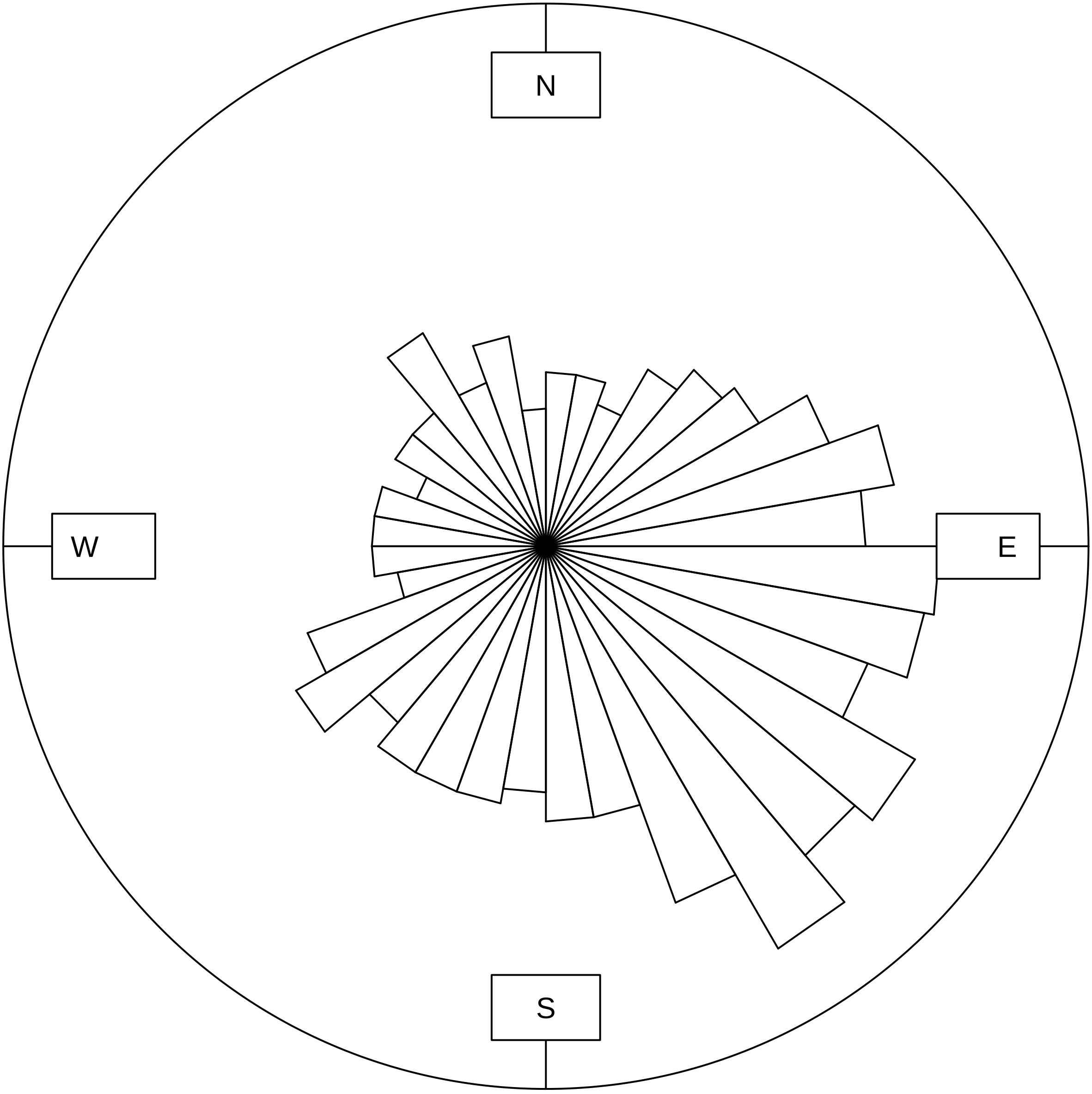

bootstrap 206

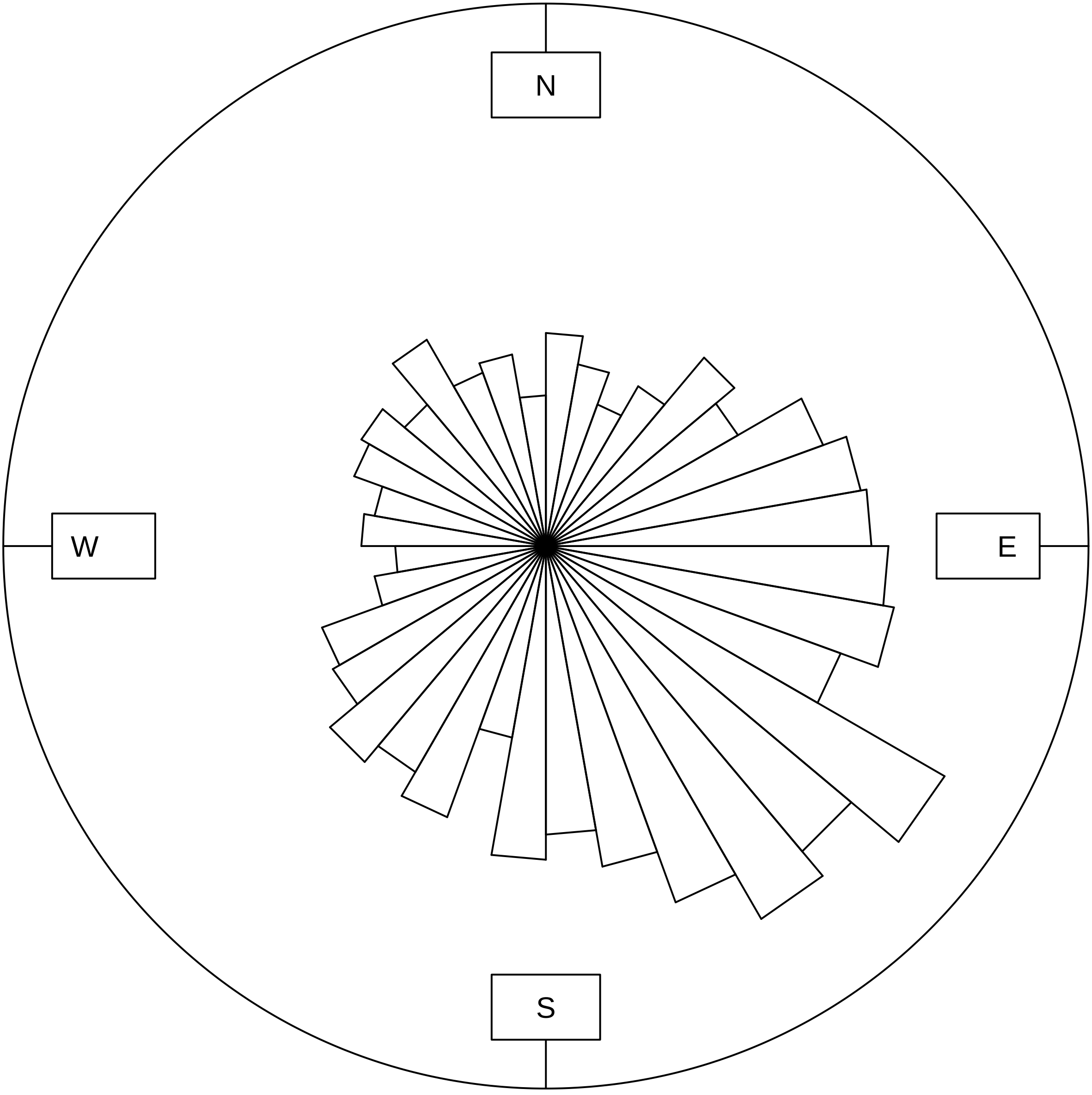

bootstrap 207

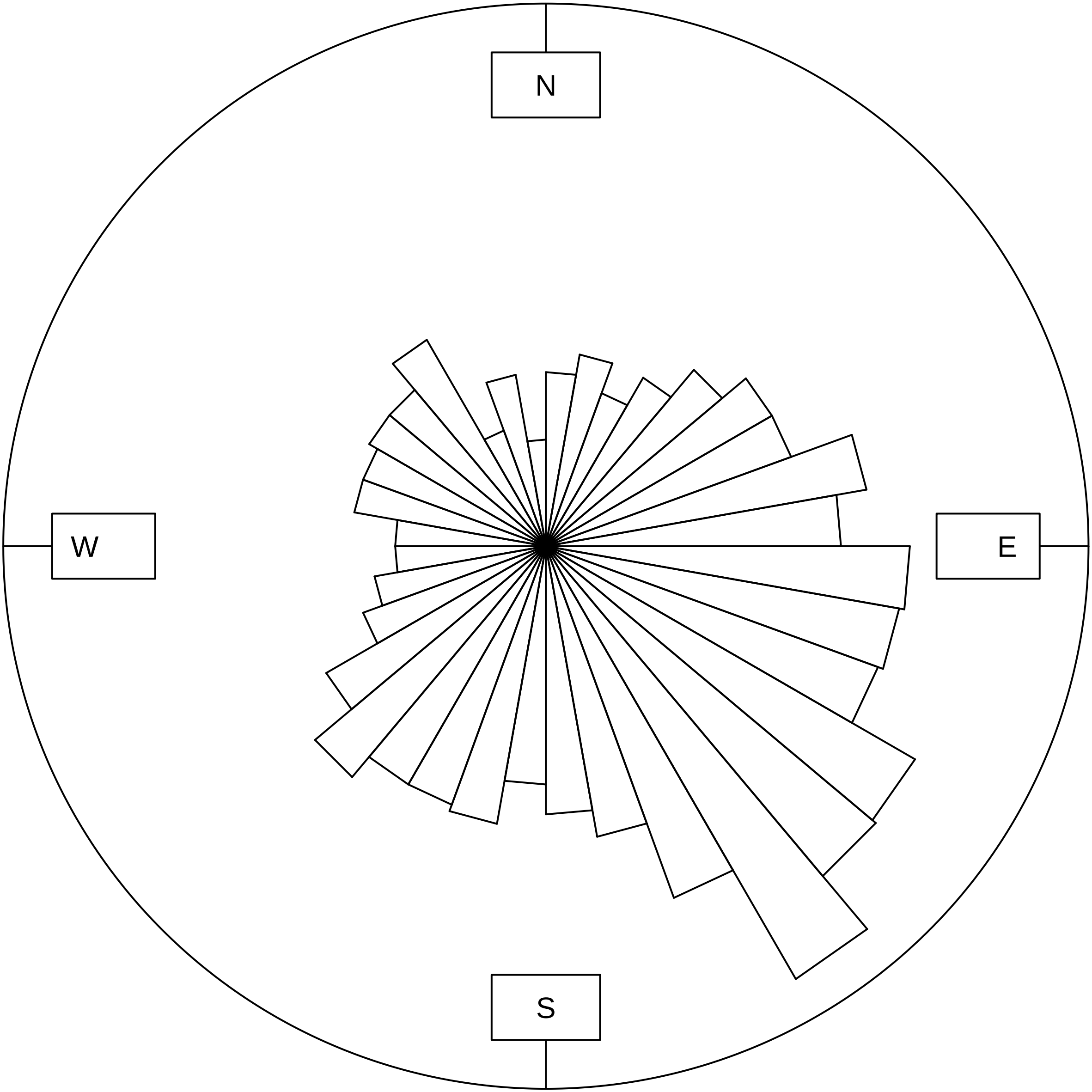

bootstrap 208

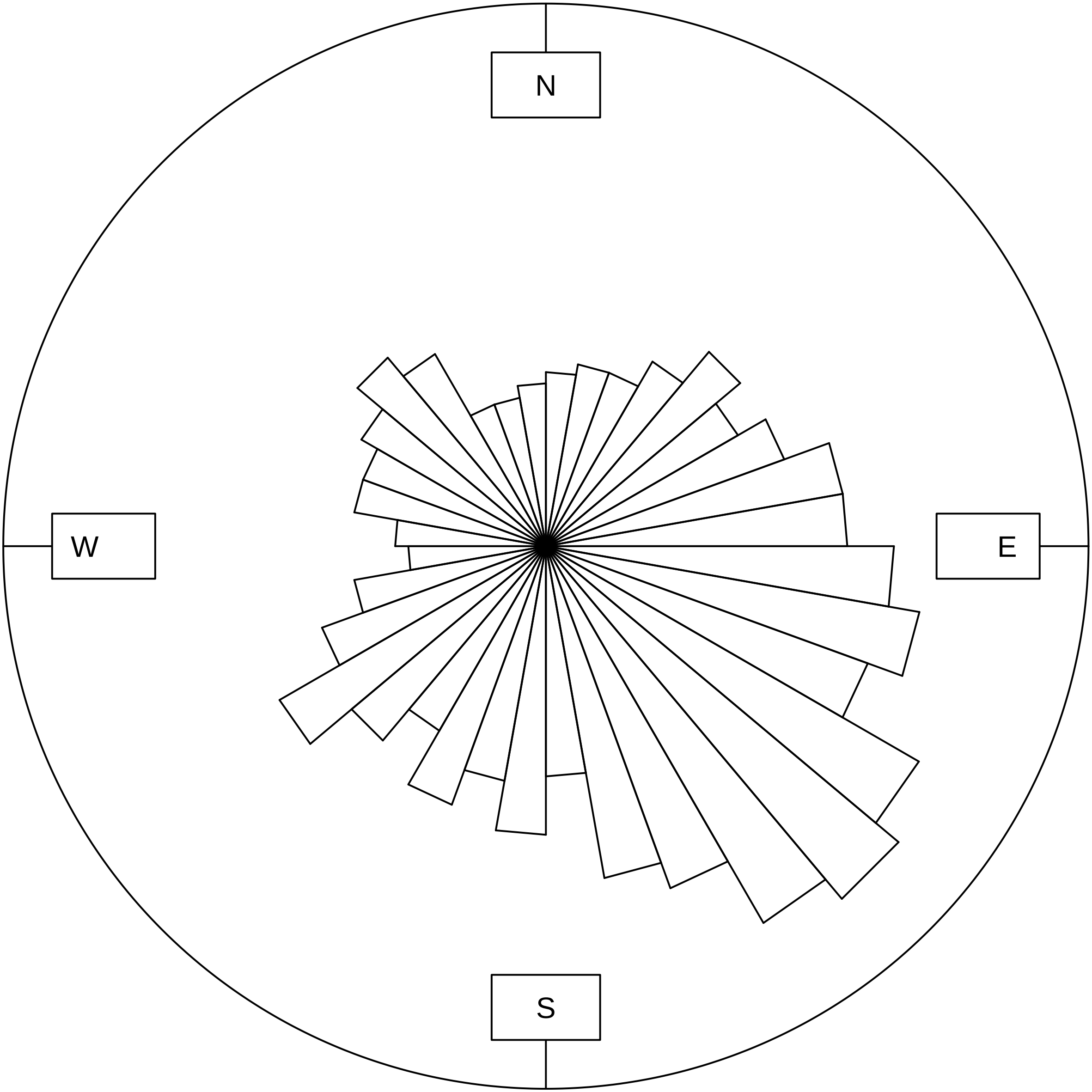

bootstrap 209

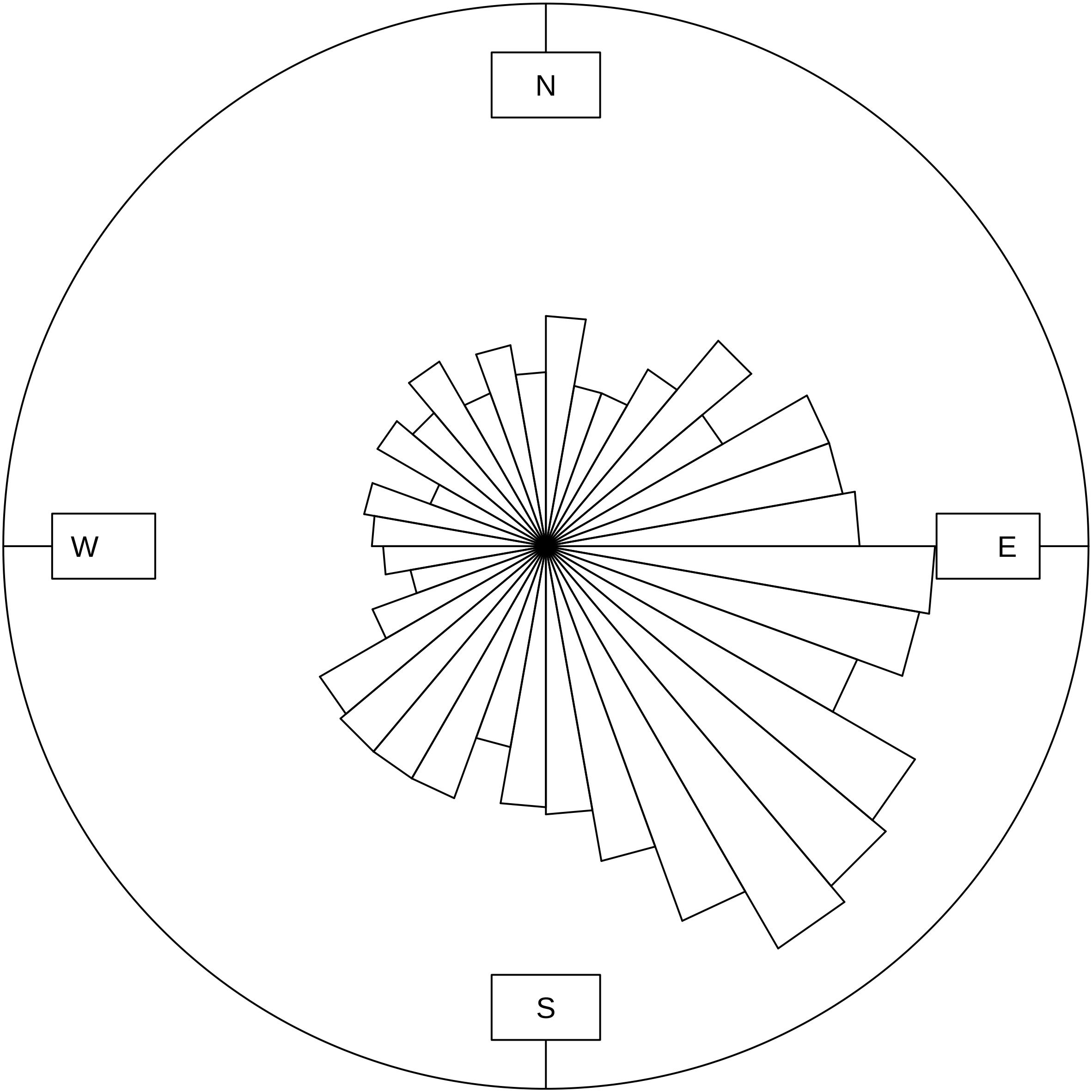

bootstrap 210

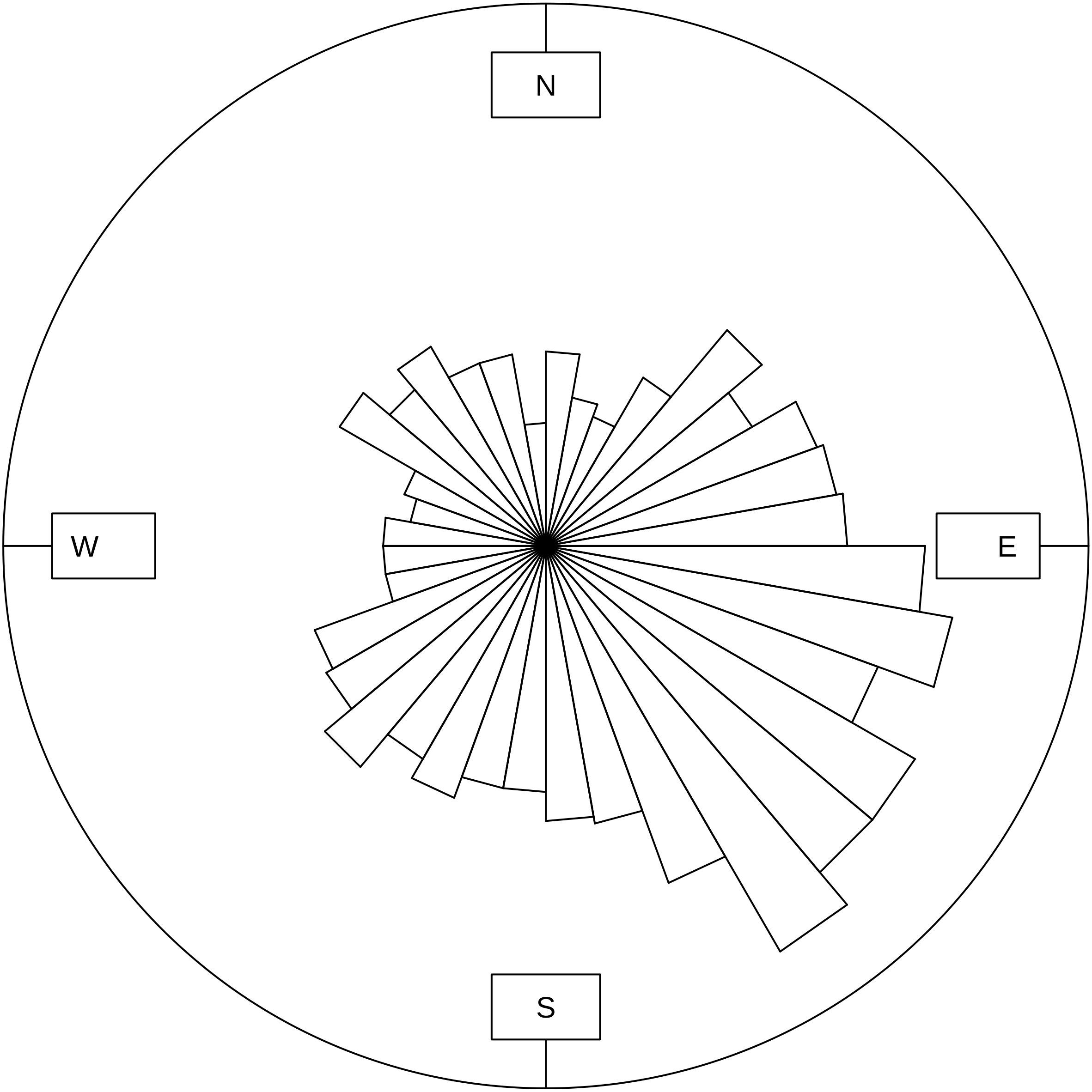

bootstrap 211

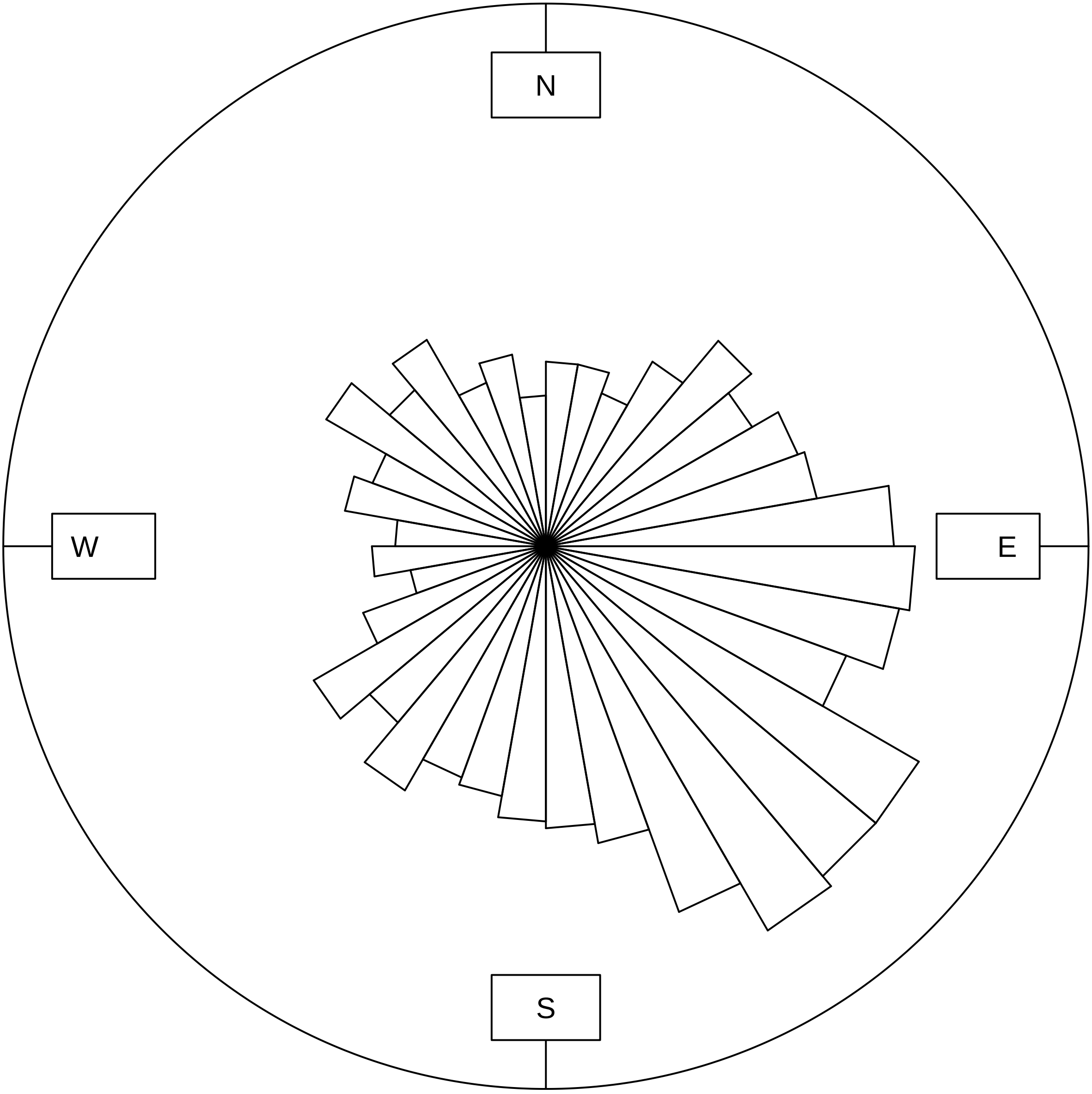

bootstrap 212

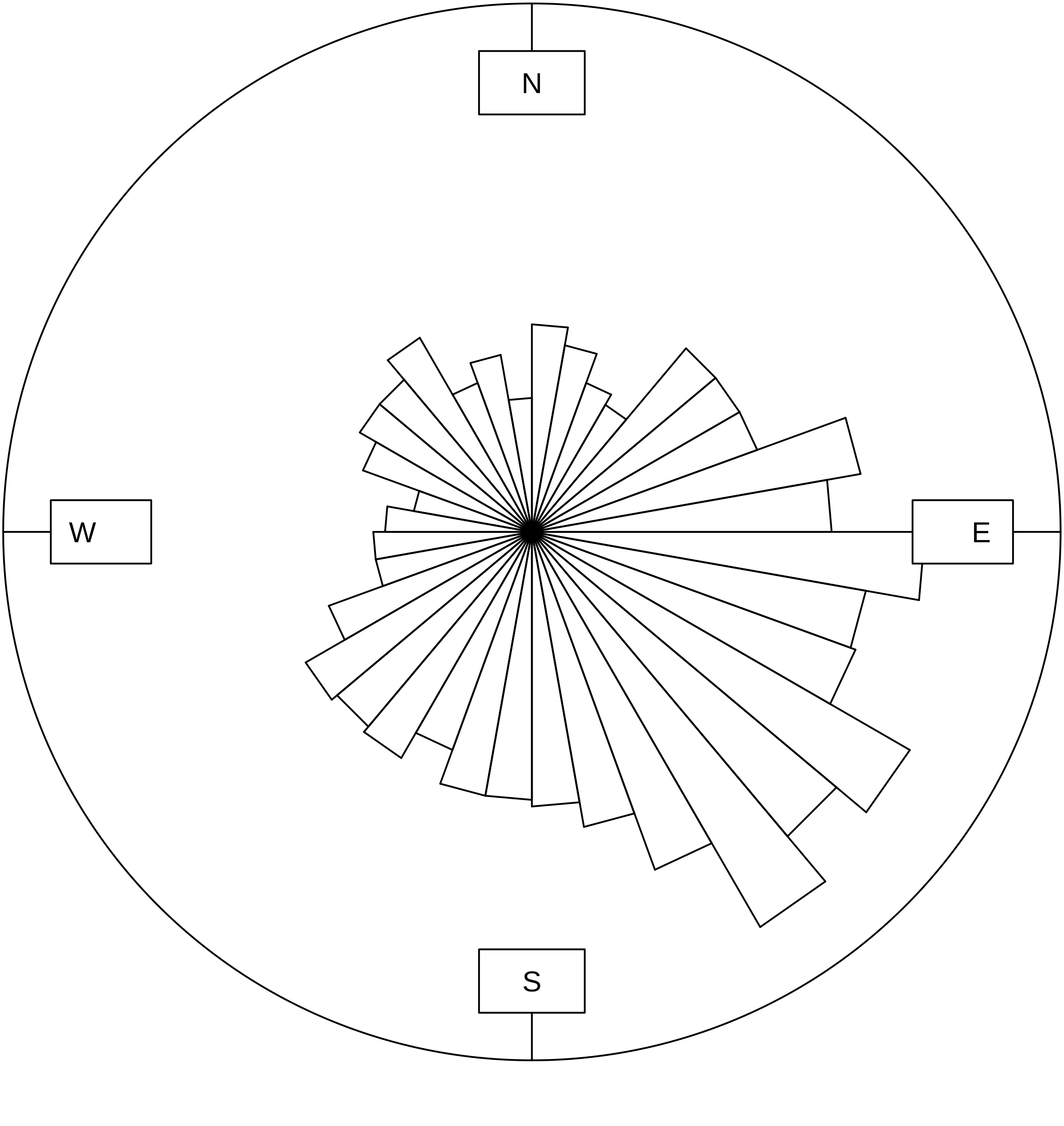

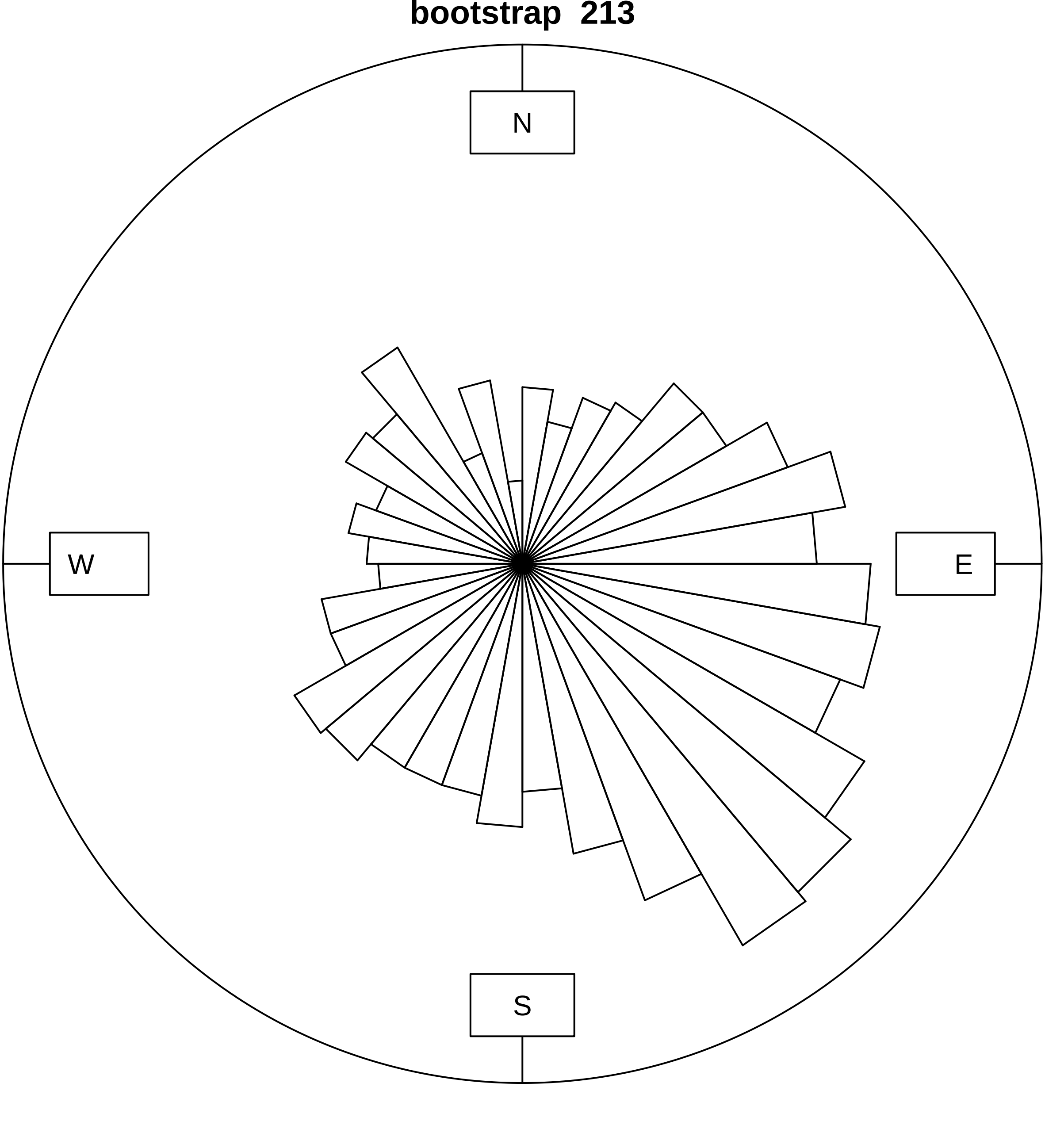

bootstrap 213
N
W
E
S

bootstrap 214

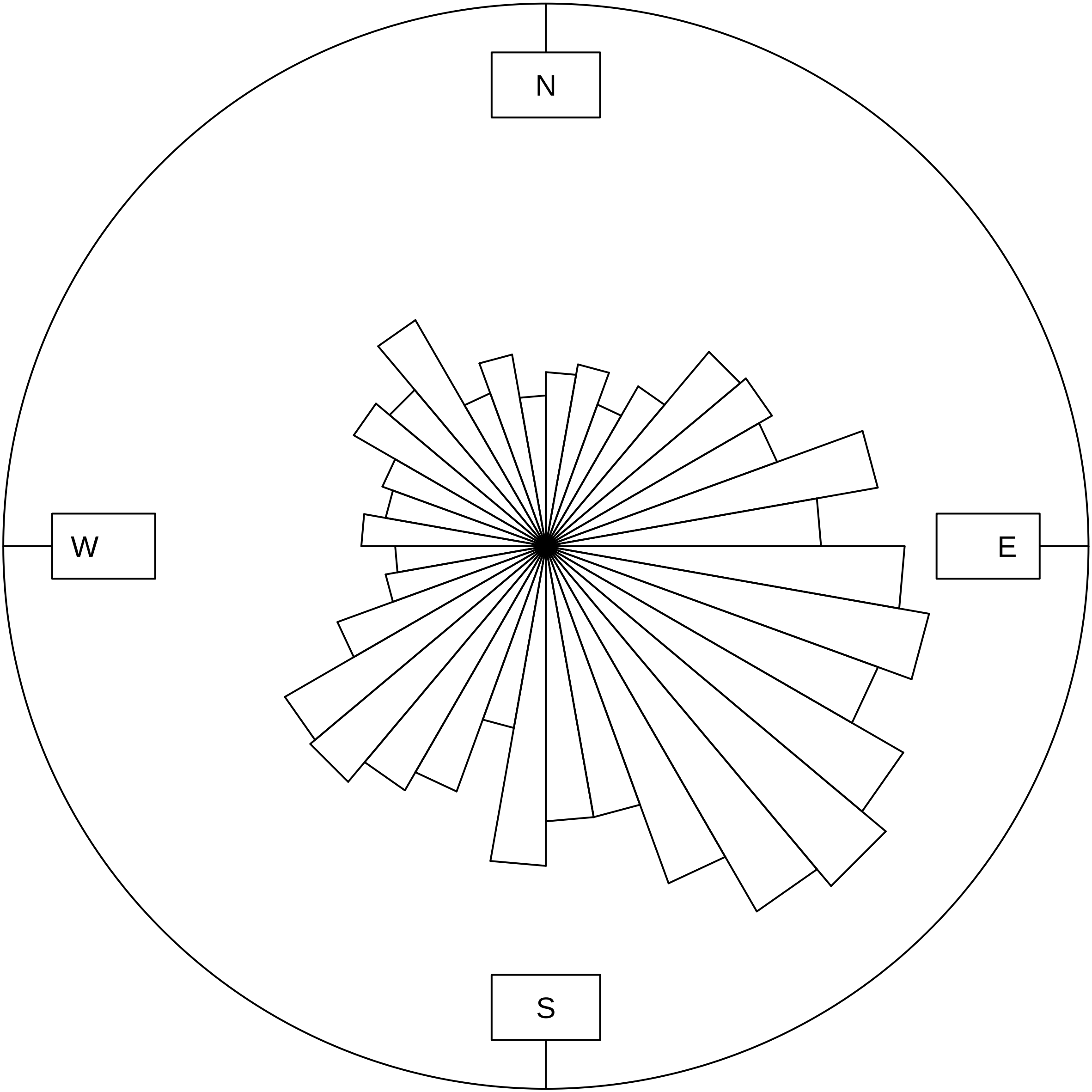

bootstrap 215

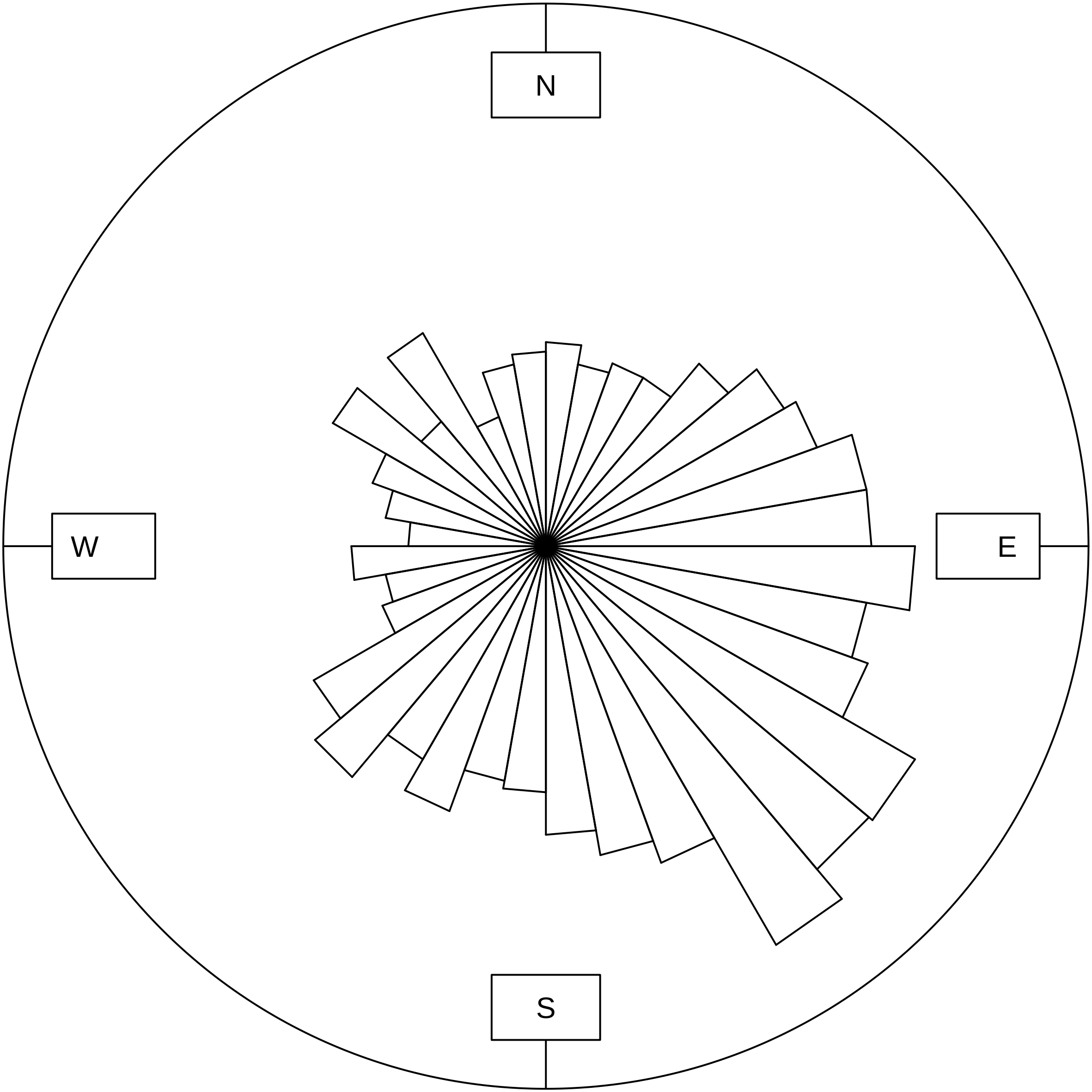

bootstrap 216

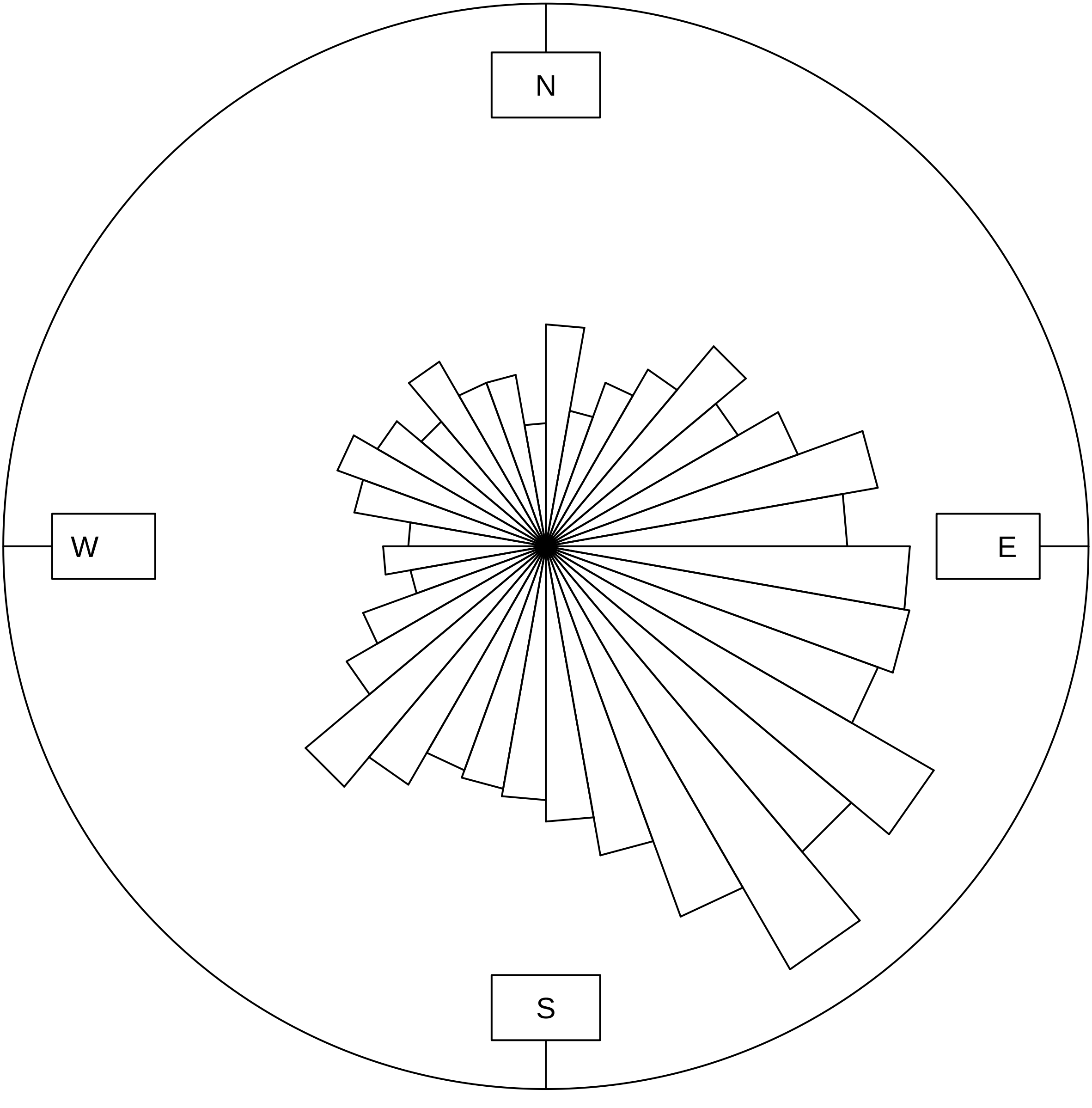

bootstrap 217

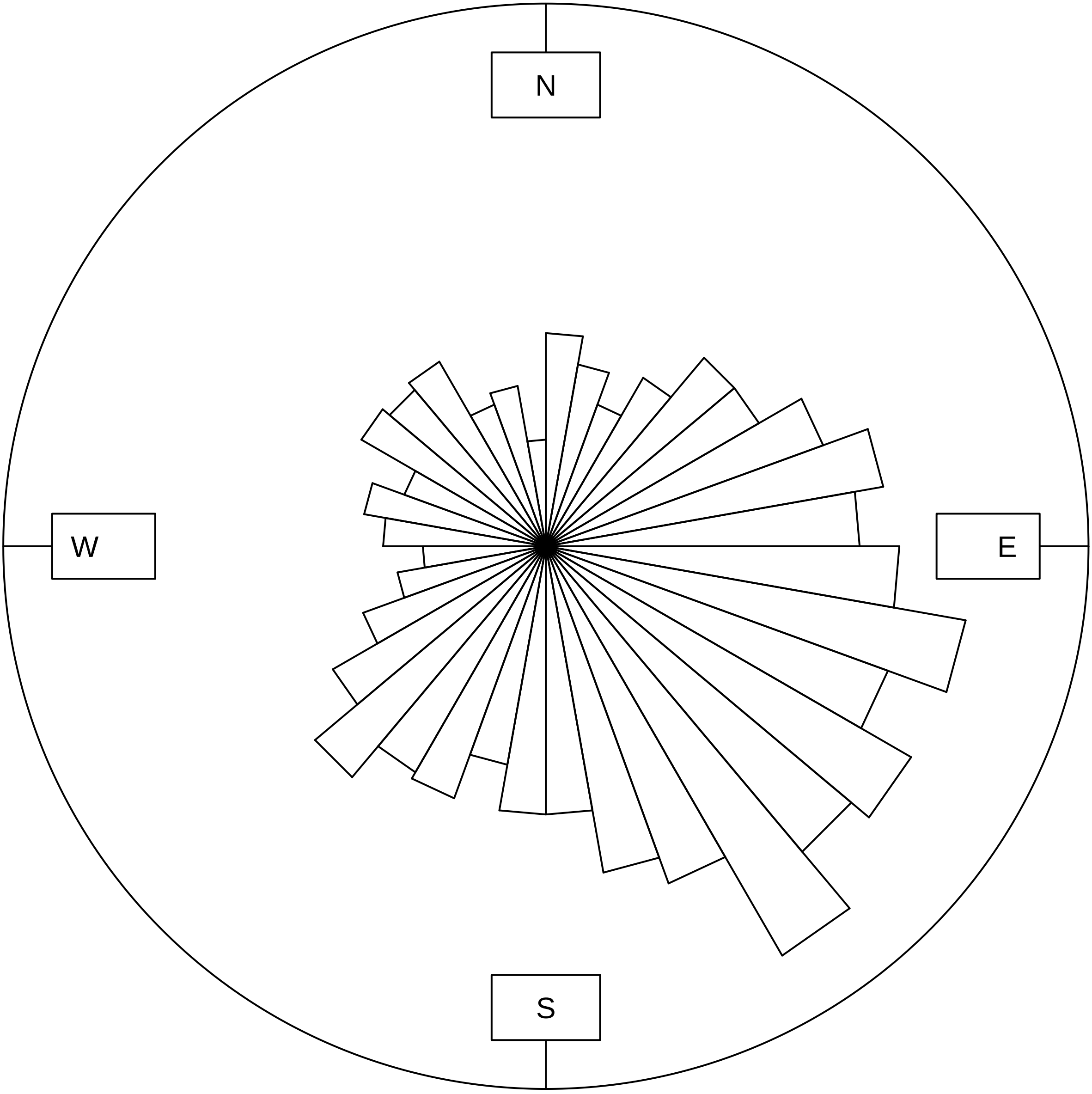

bootstrap 218

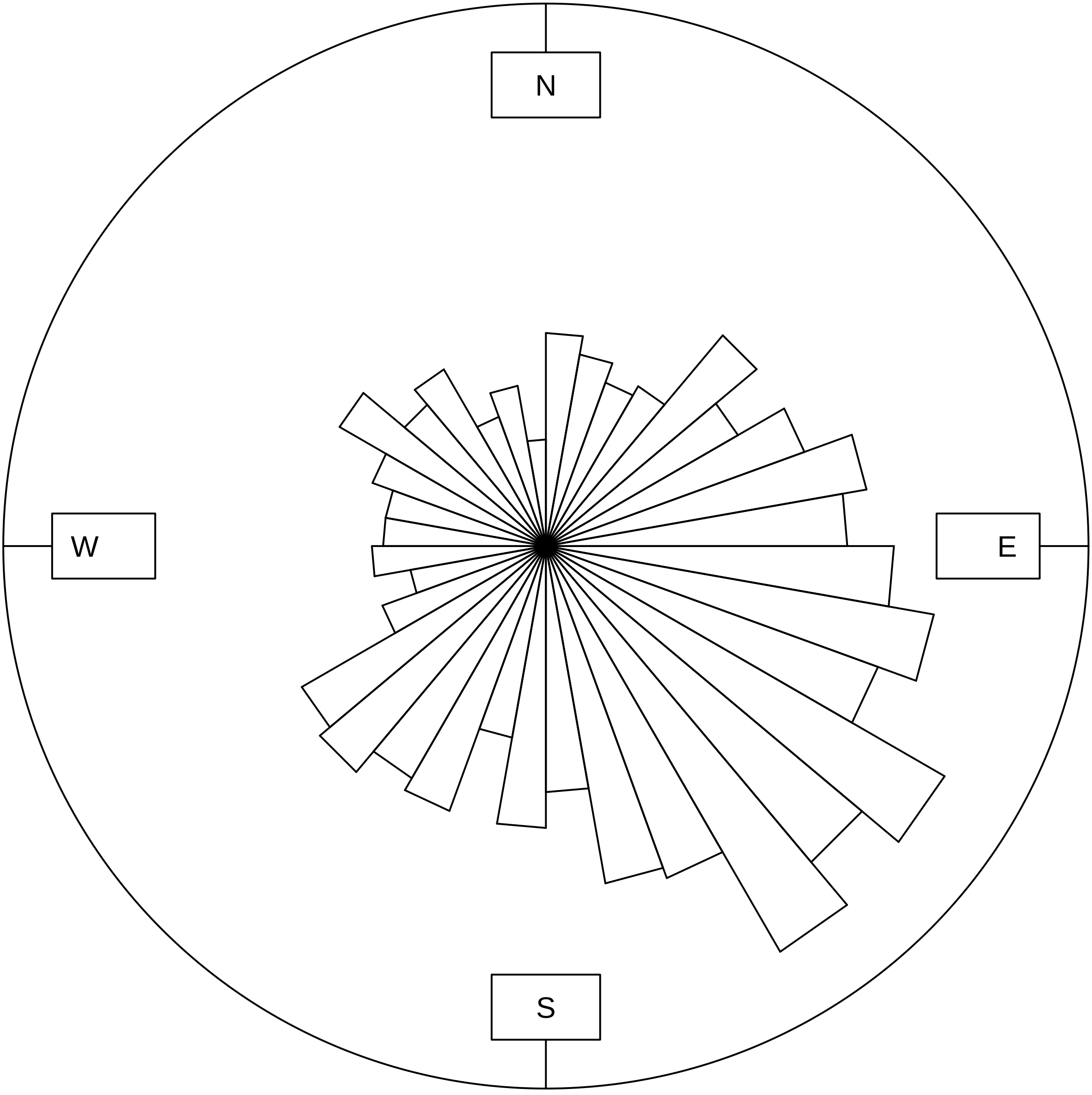

bootstrap 219

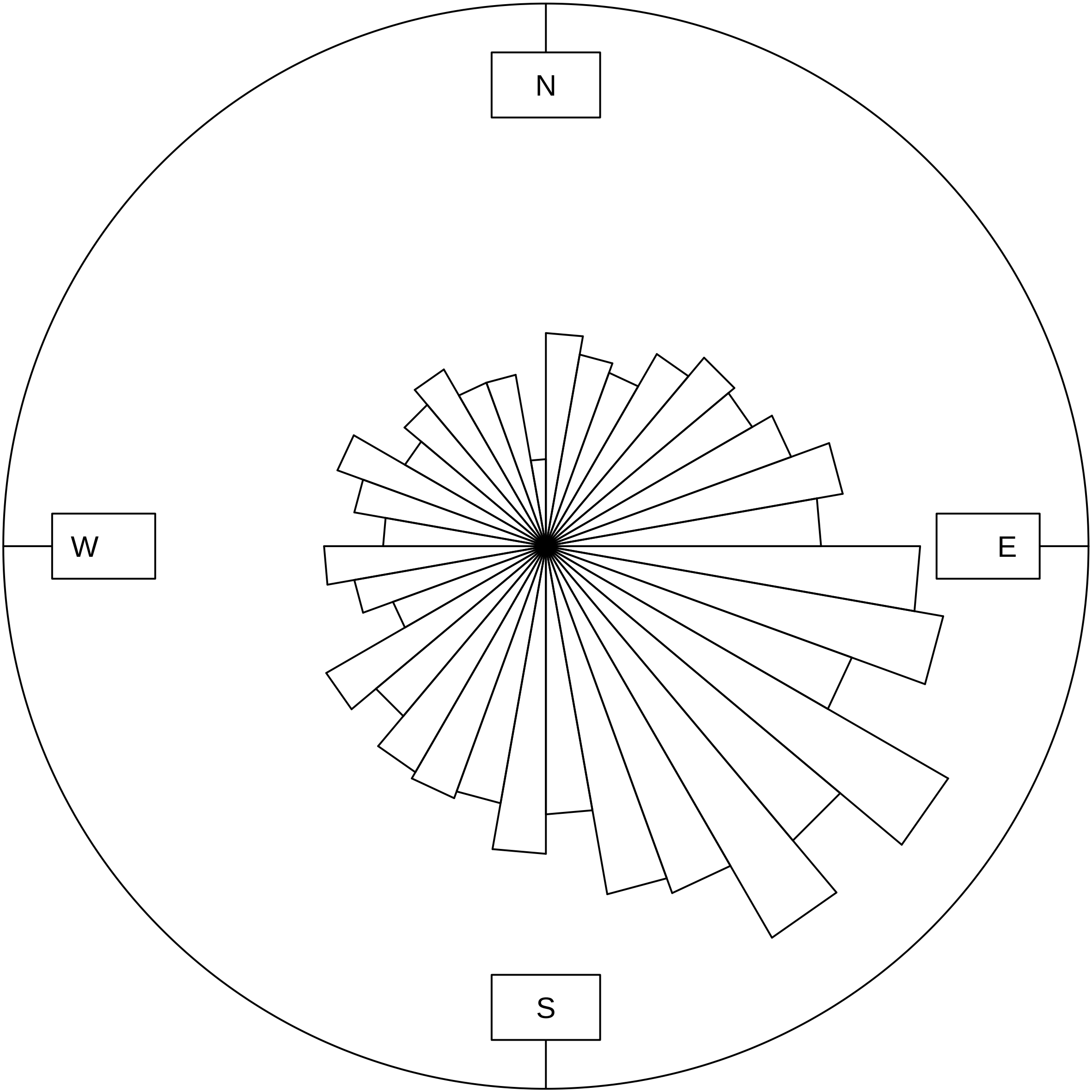

bootstrap 220

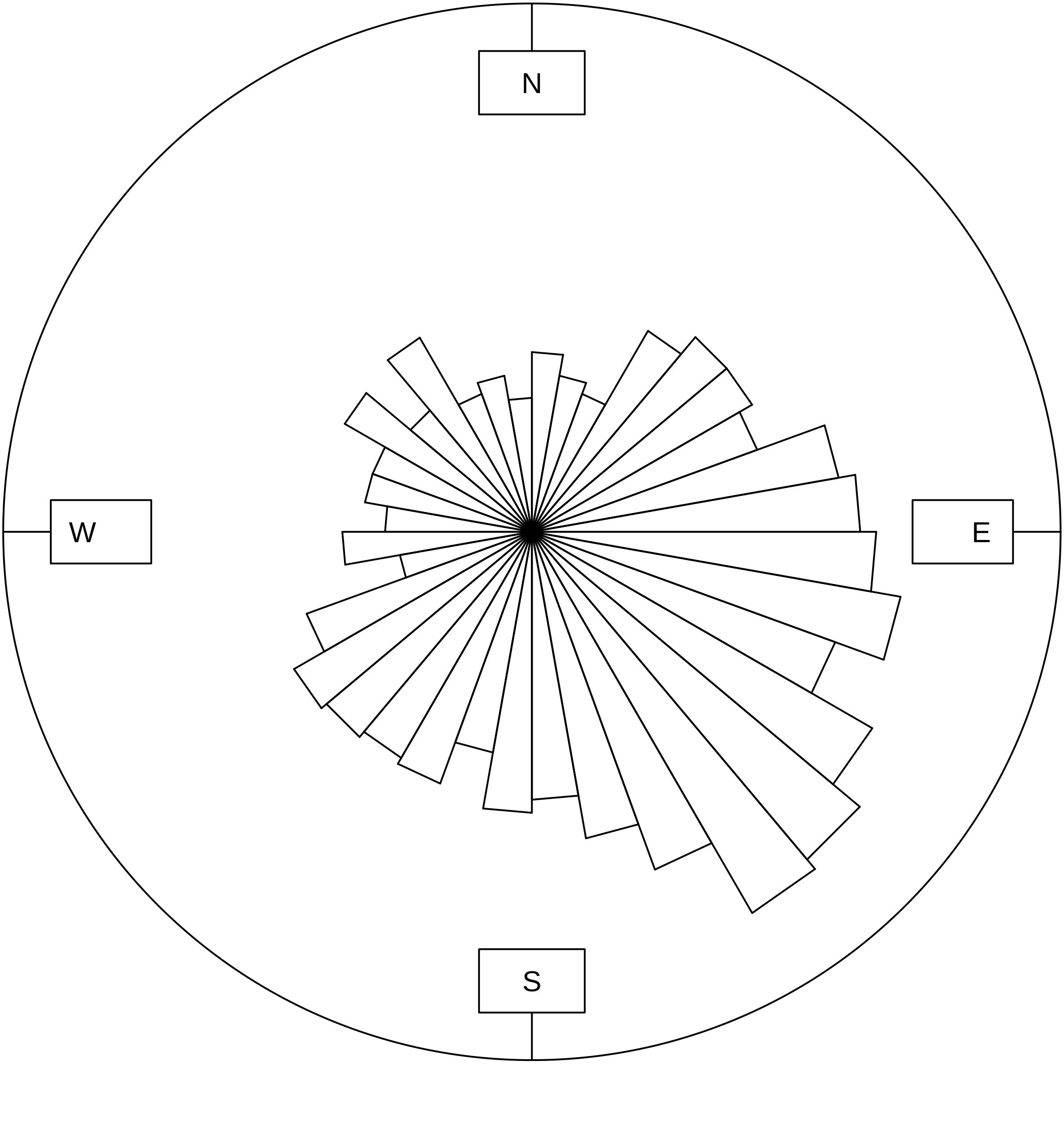

bootstrap 221

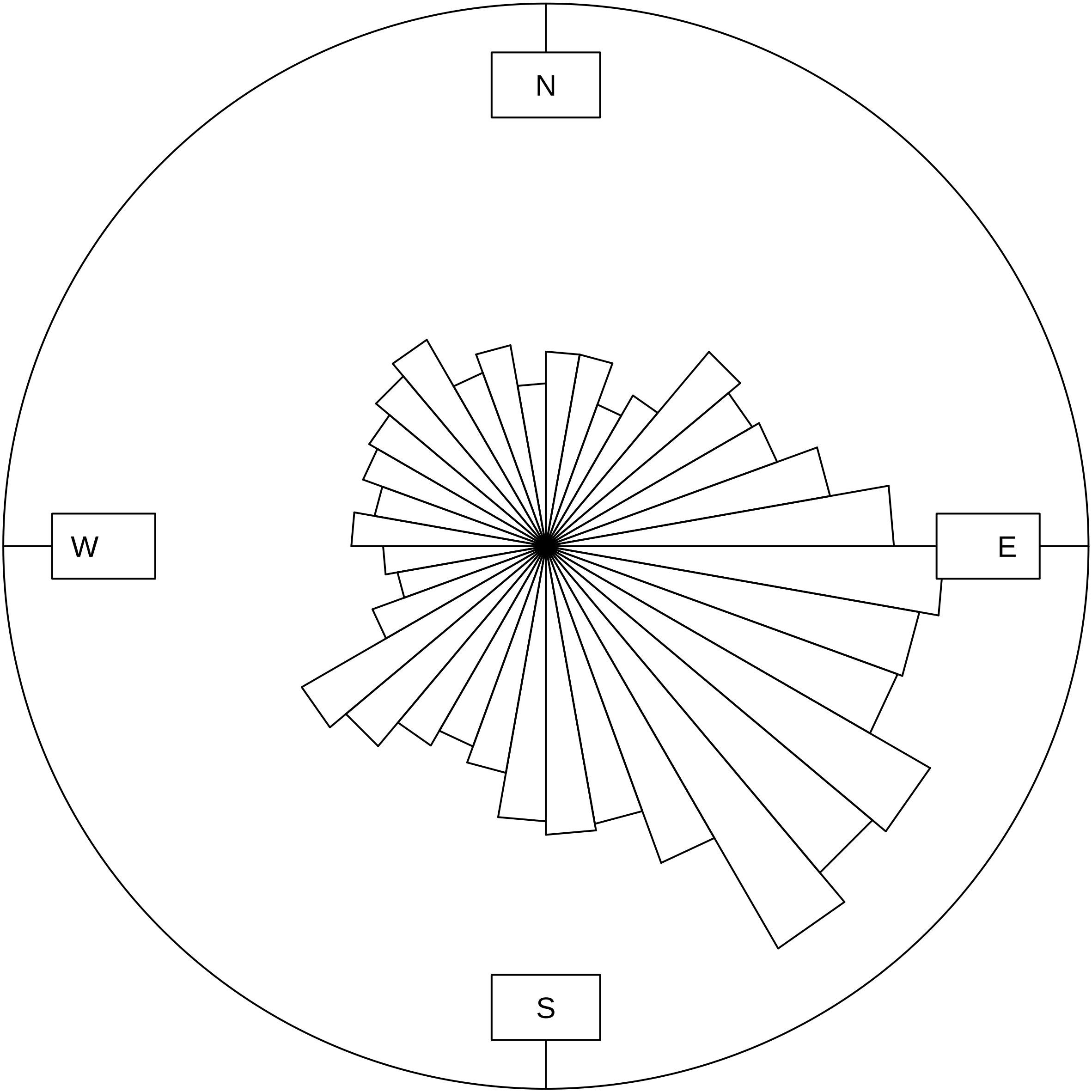

bootstrap 222

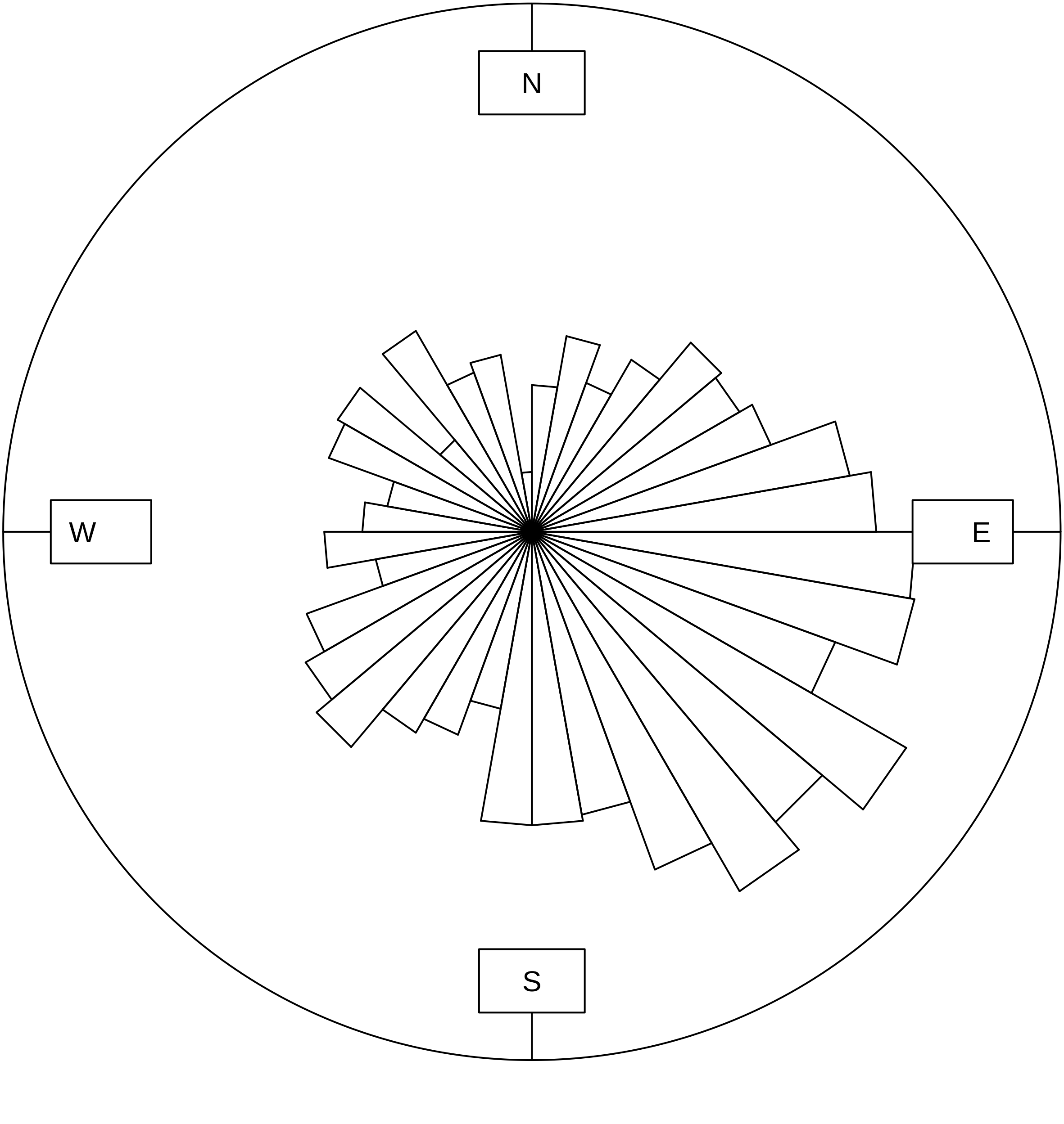

bootstrap 223

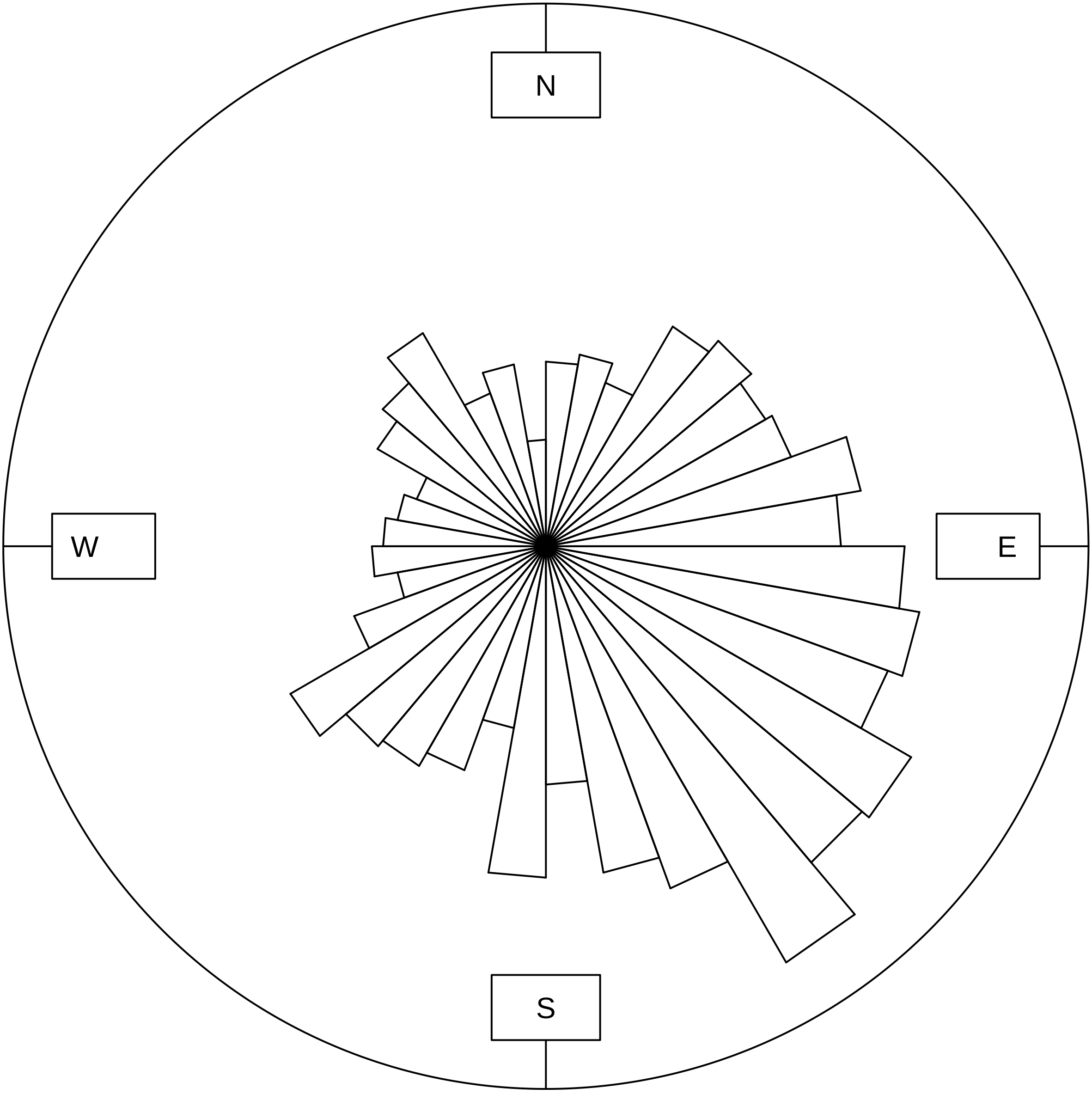

bootstrap 224

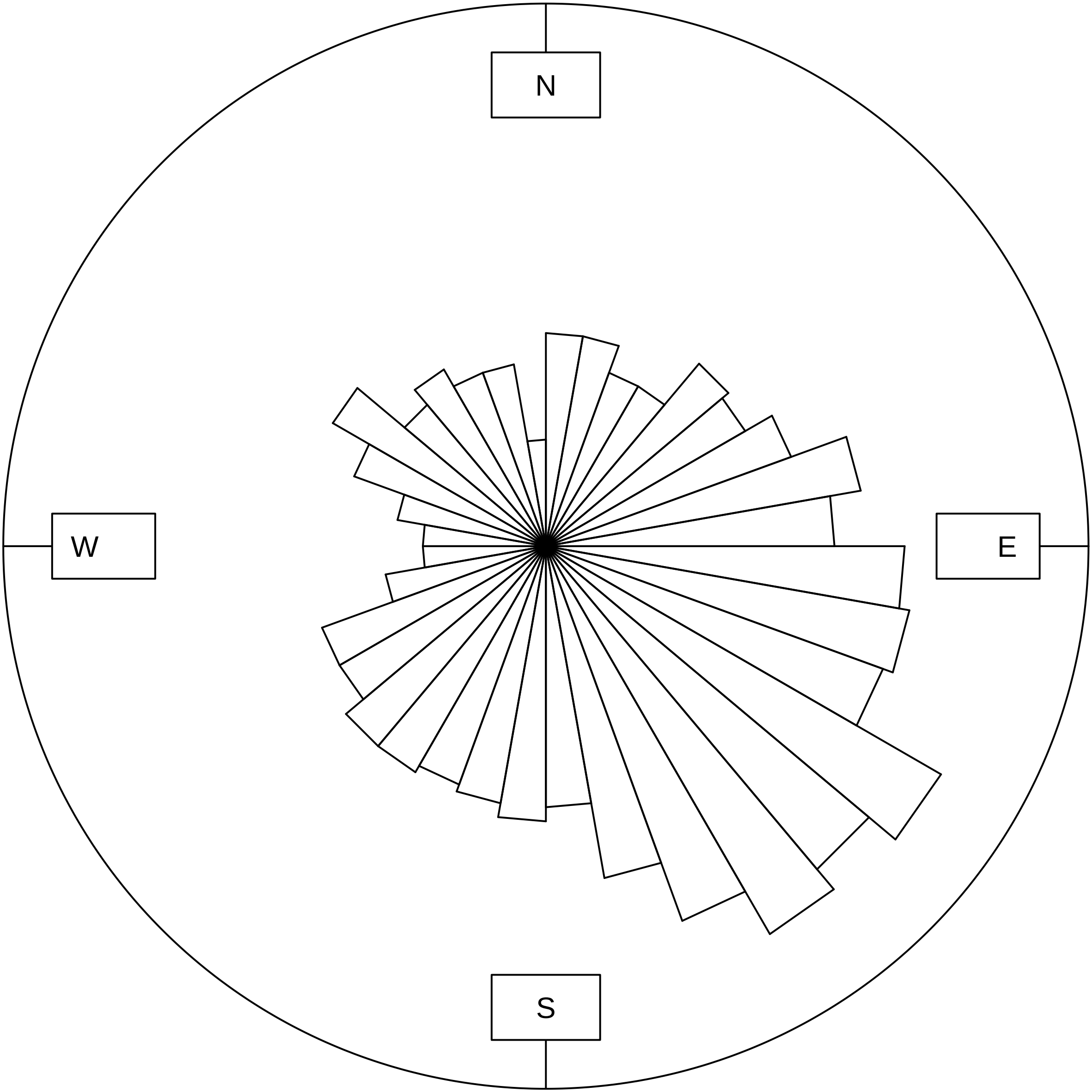

bootstrap 225

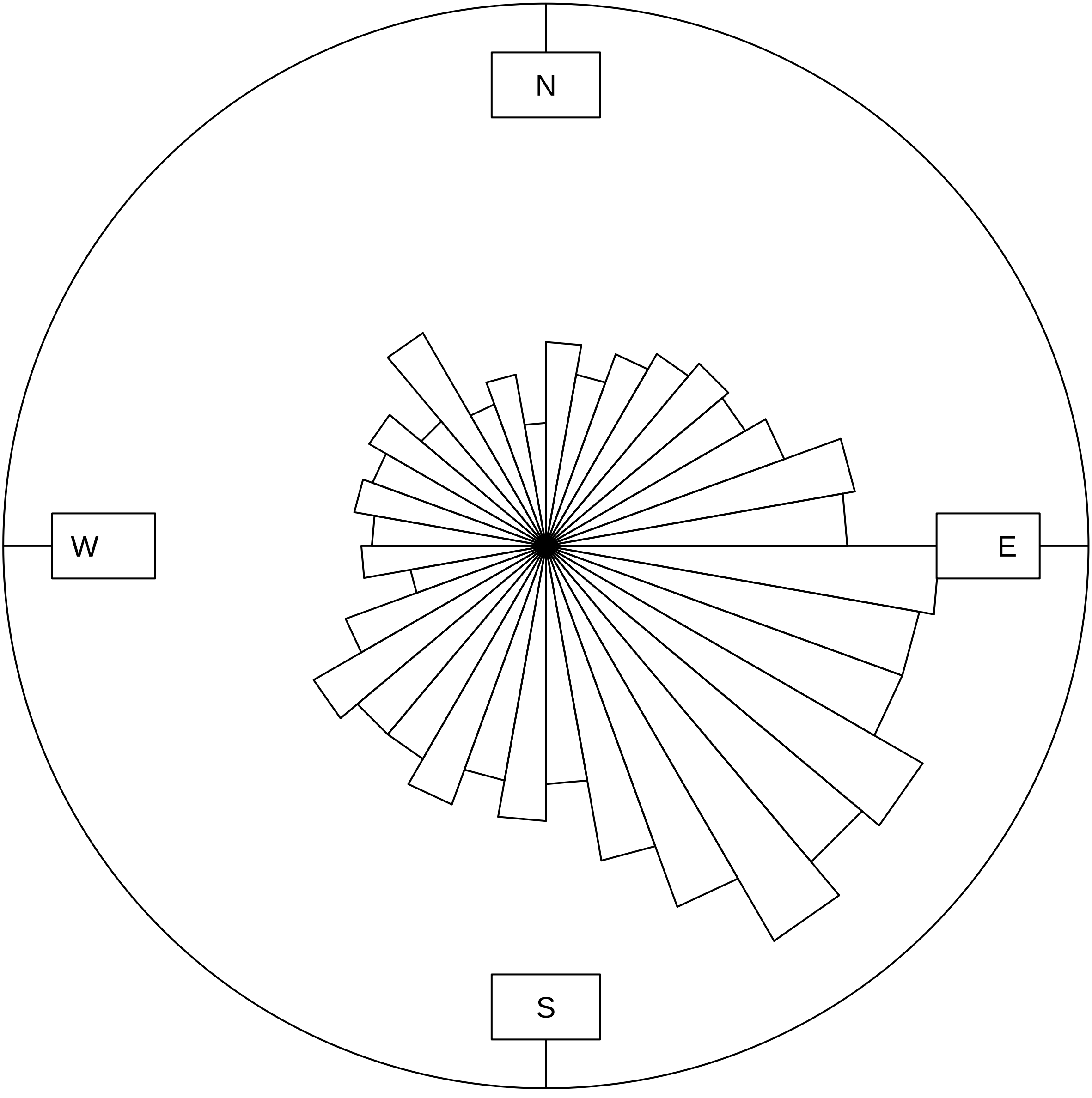

bootstrap 226

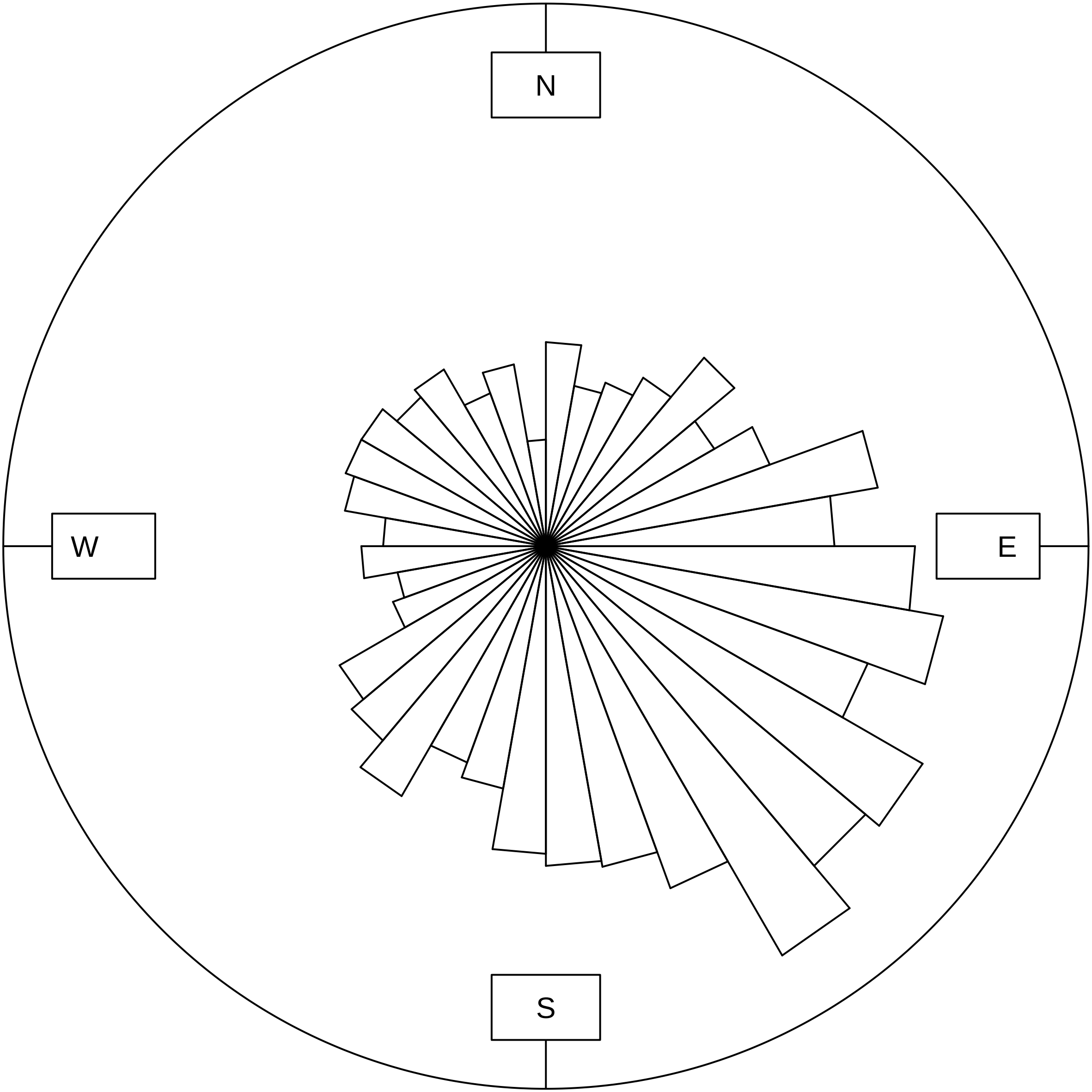

bootstrap 227

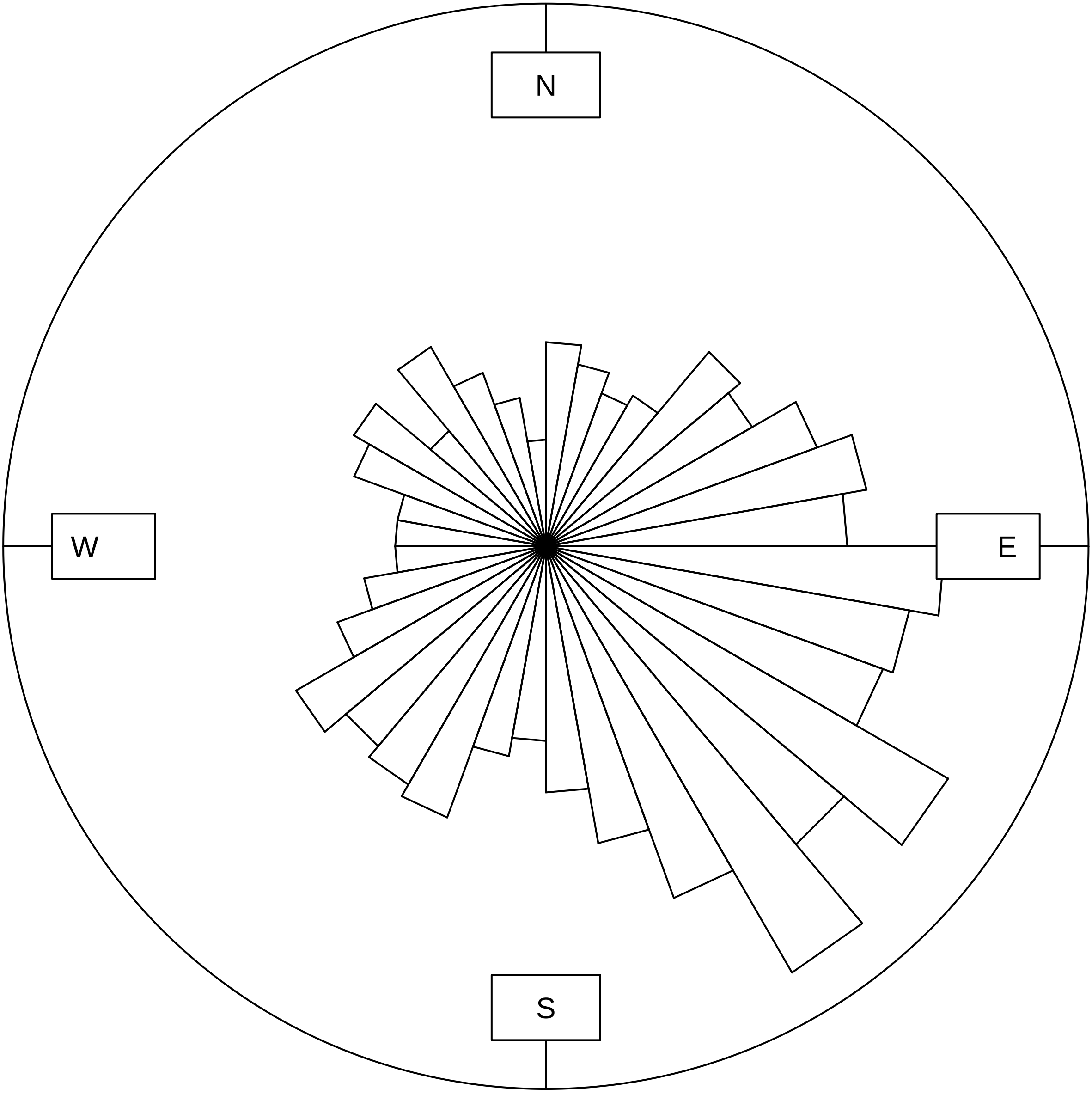

bootstrap 228

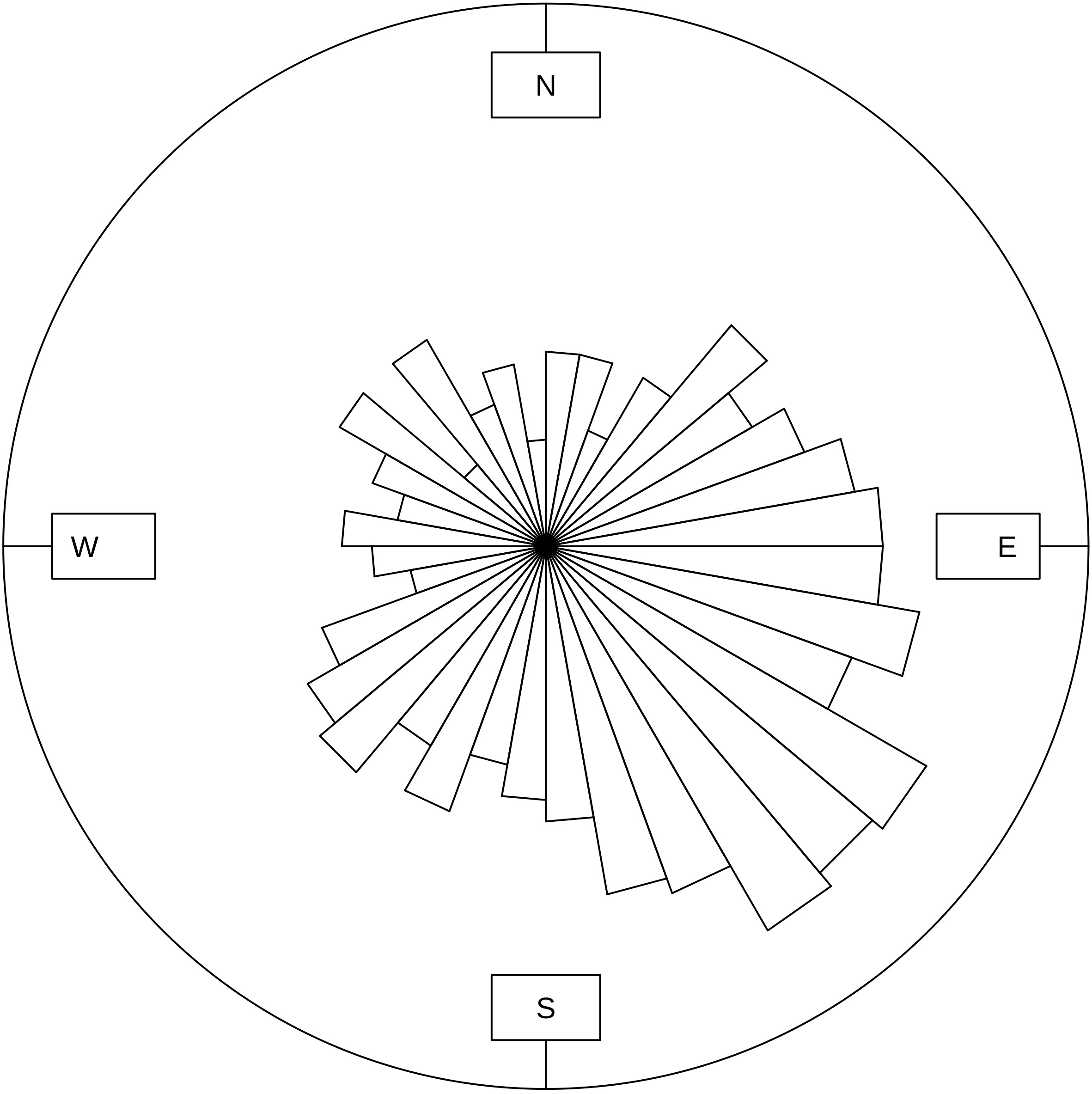

bootstrap 229

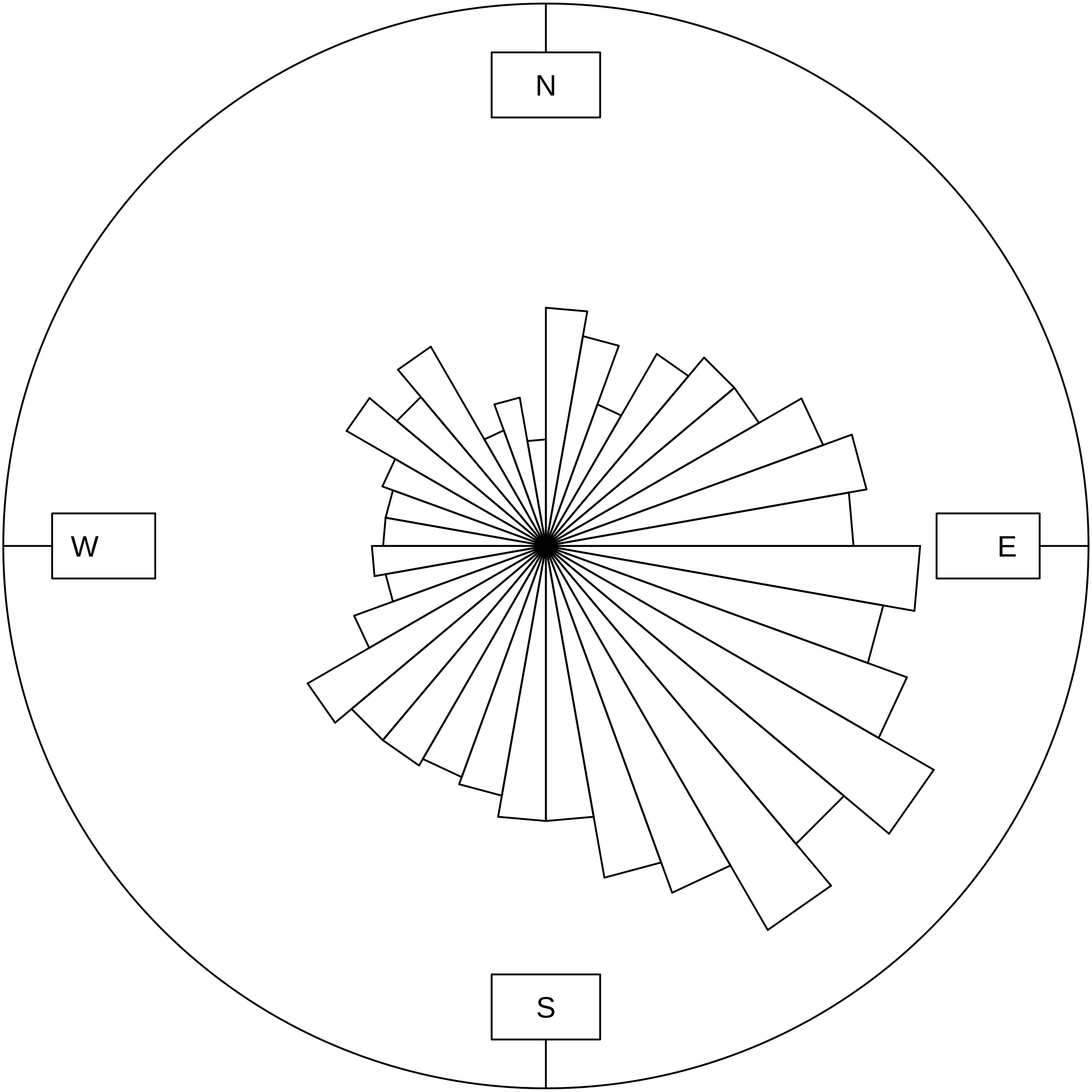

bootstrap 230

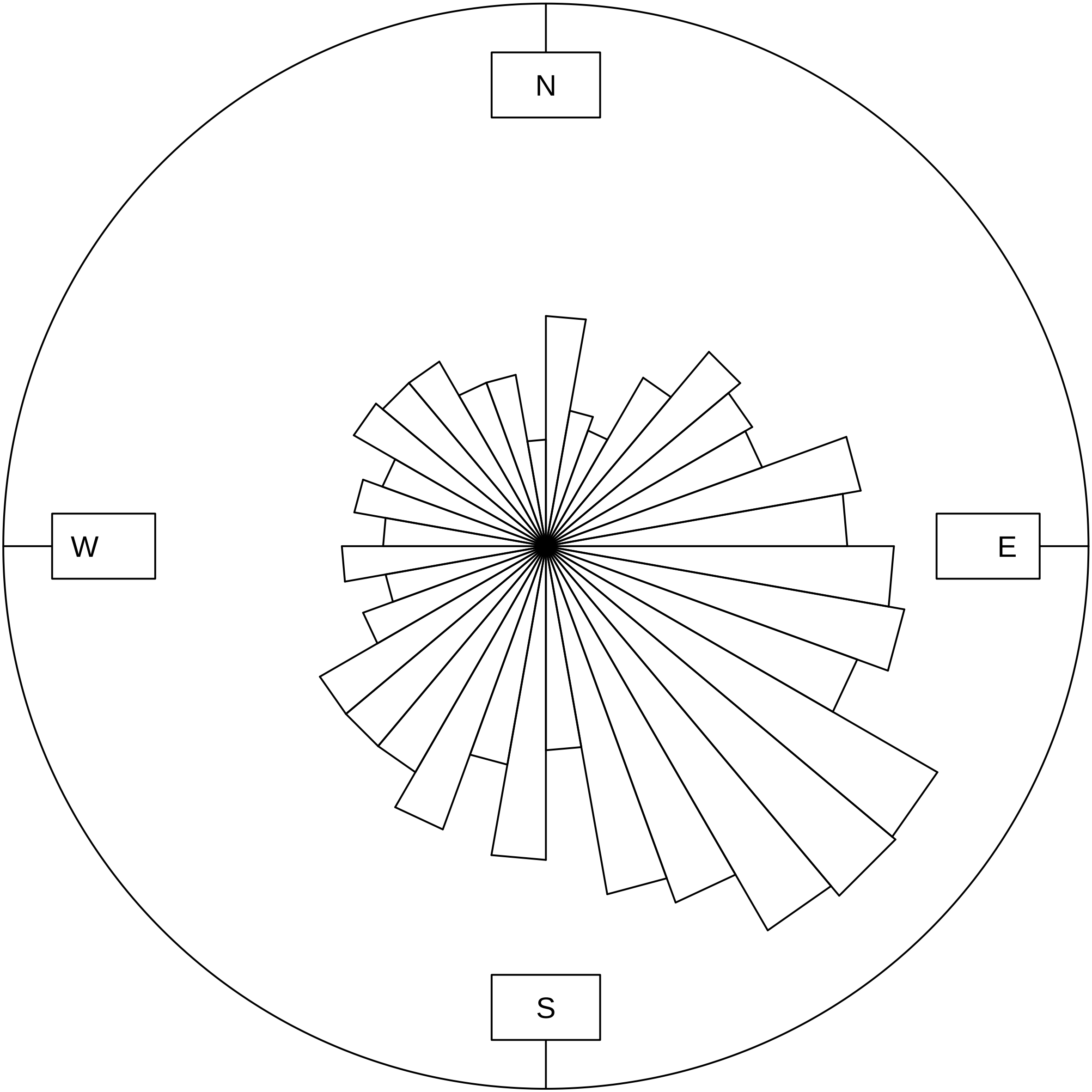

bootstrap 231

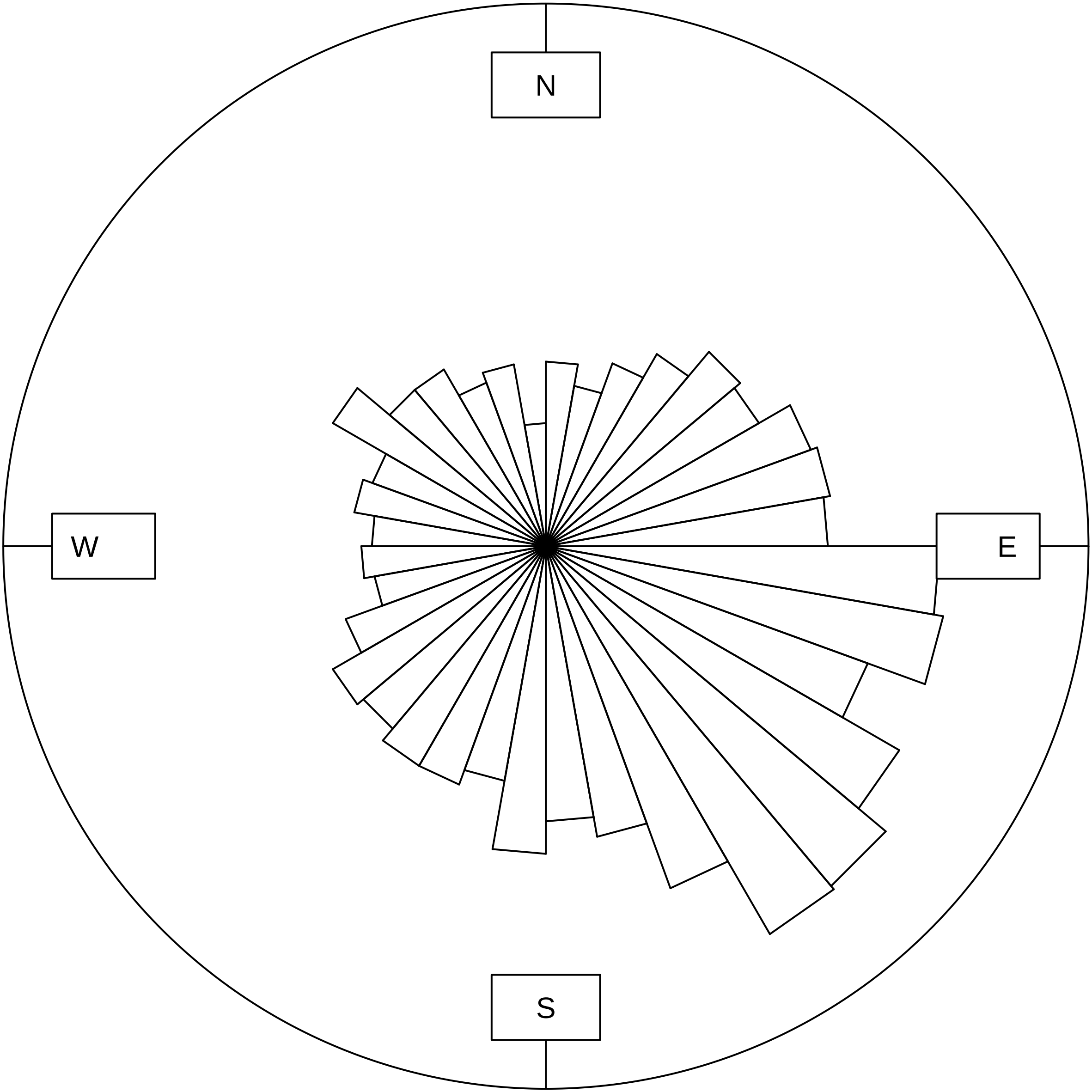

bootstrap 232

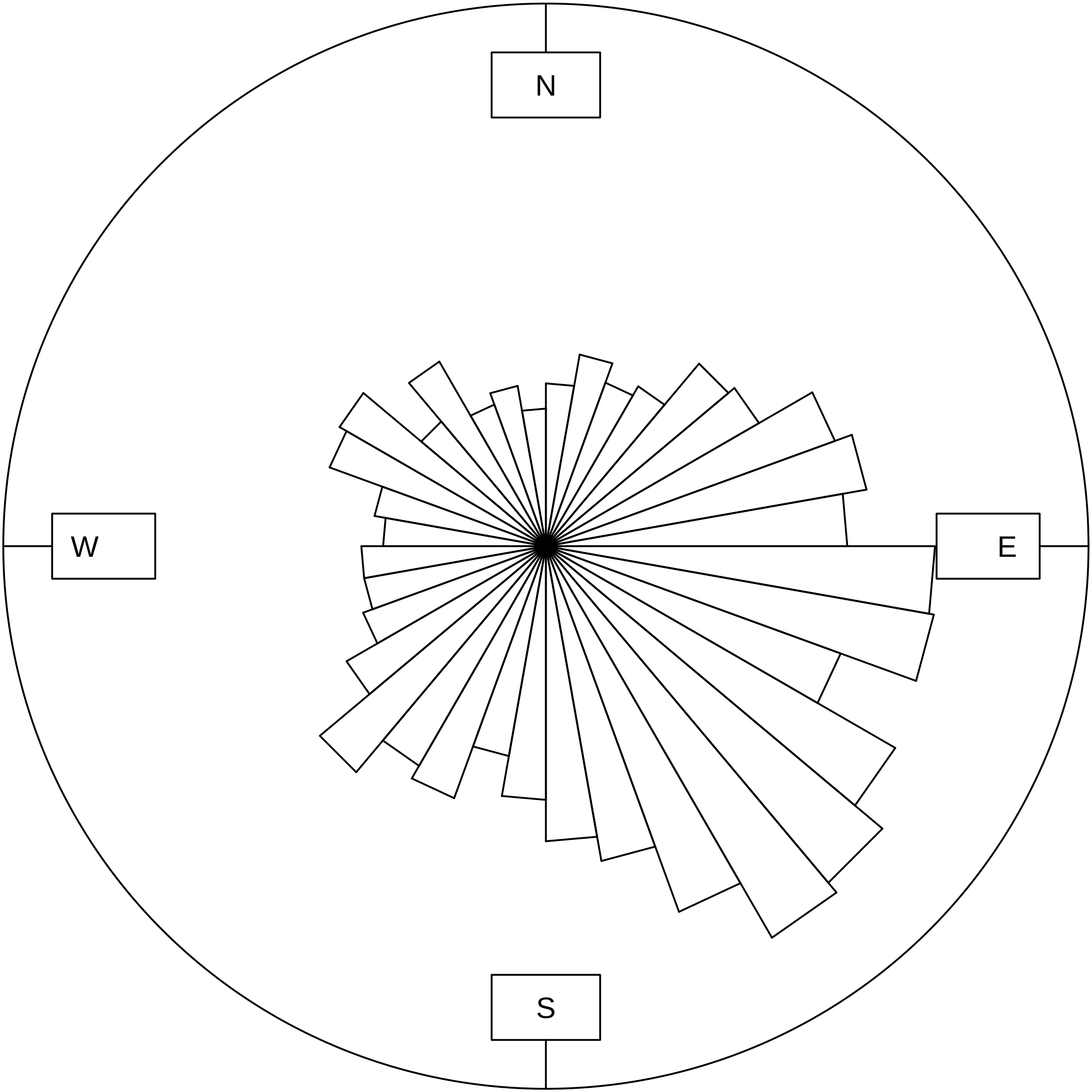

bootstrap 233

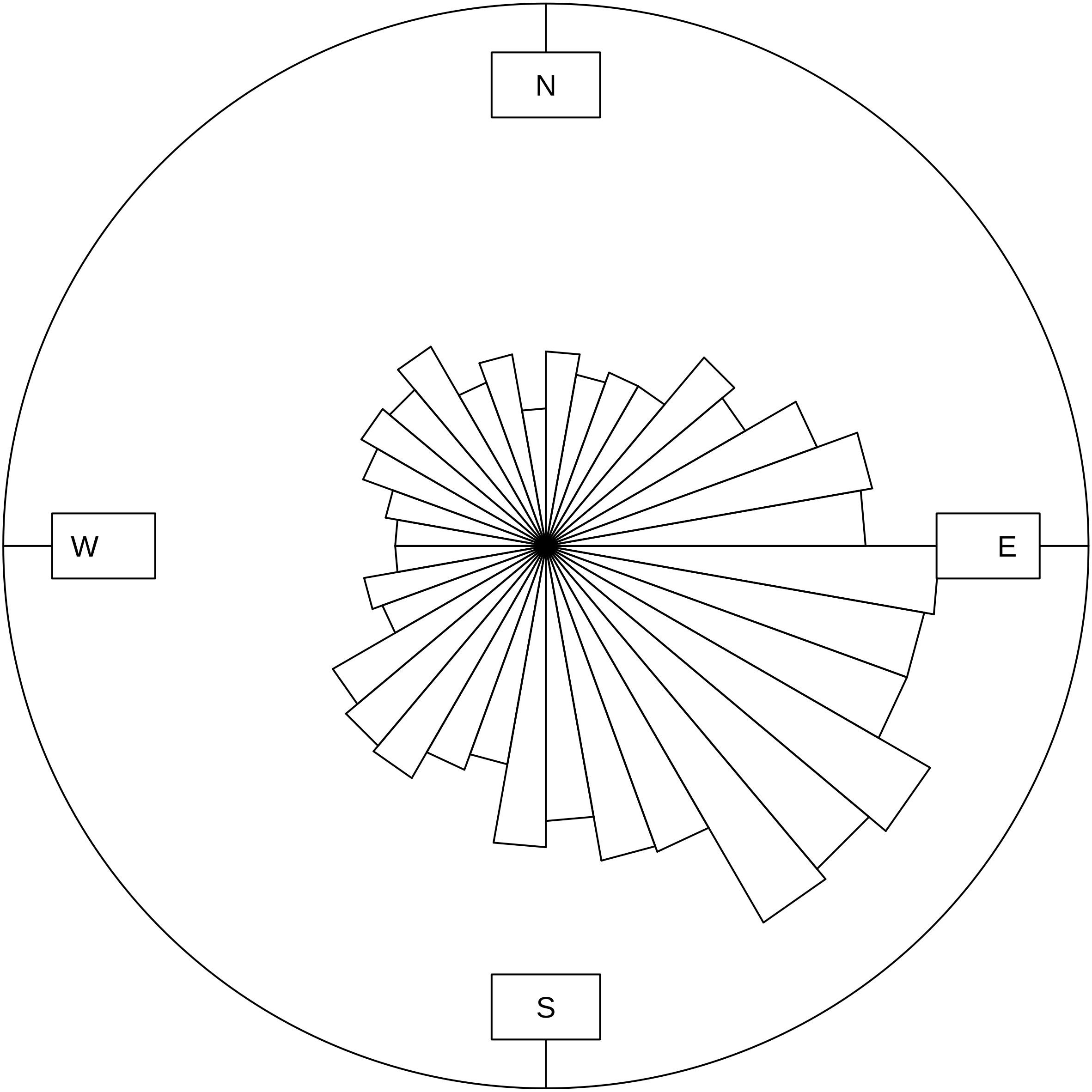

bootstrap 234

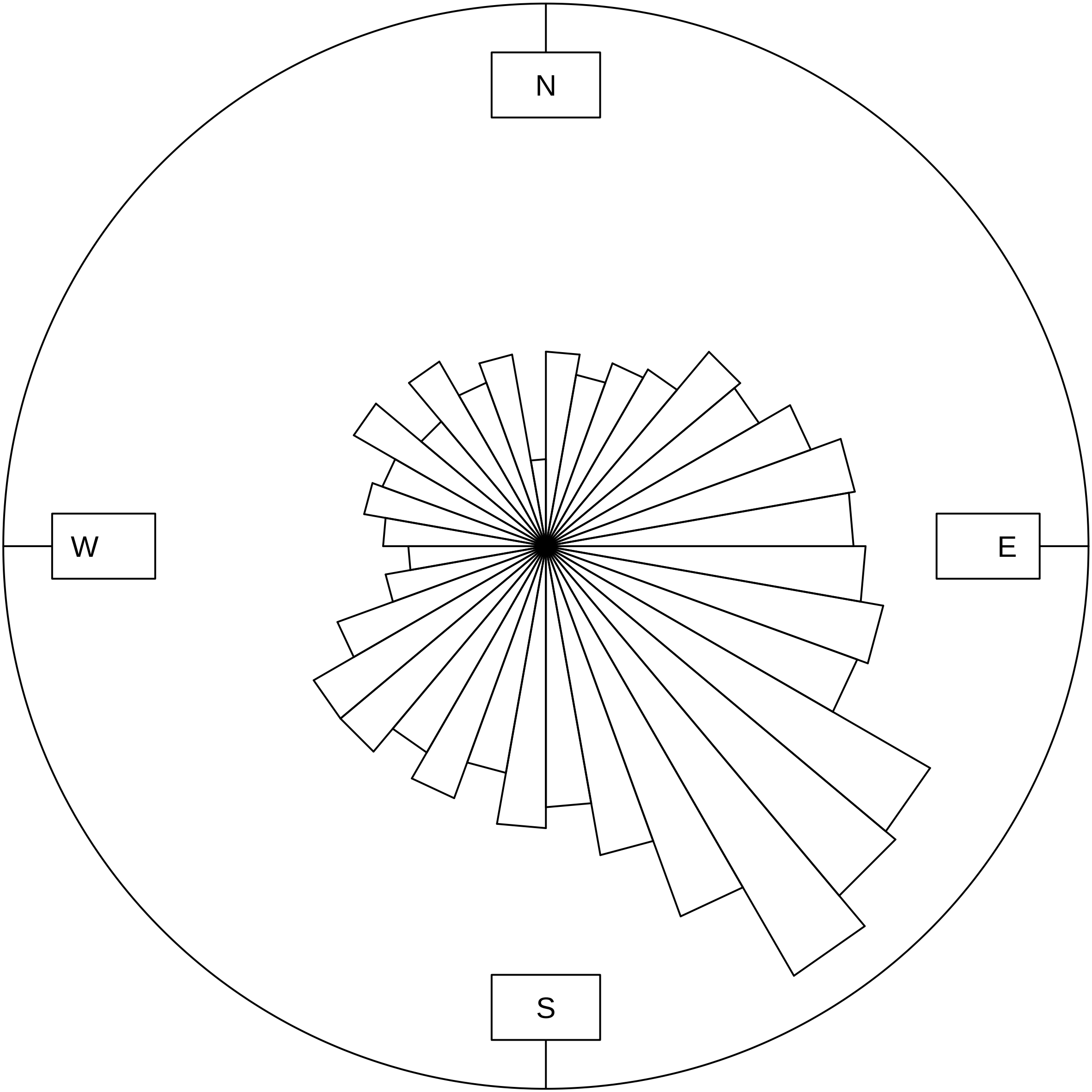

bootstrap 235

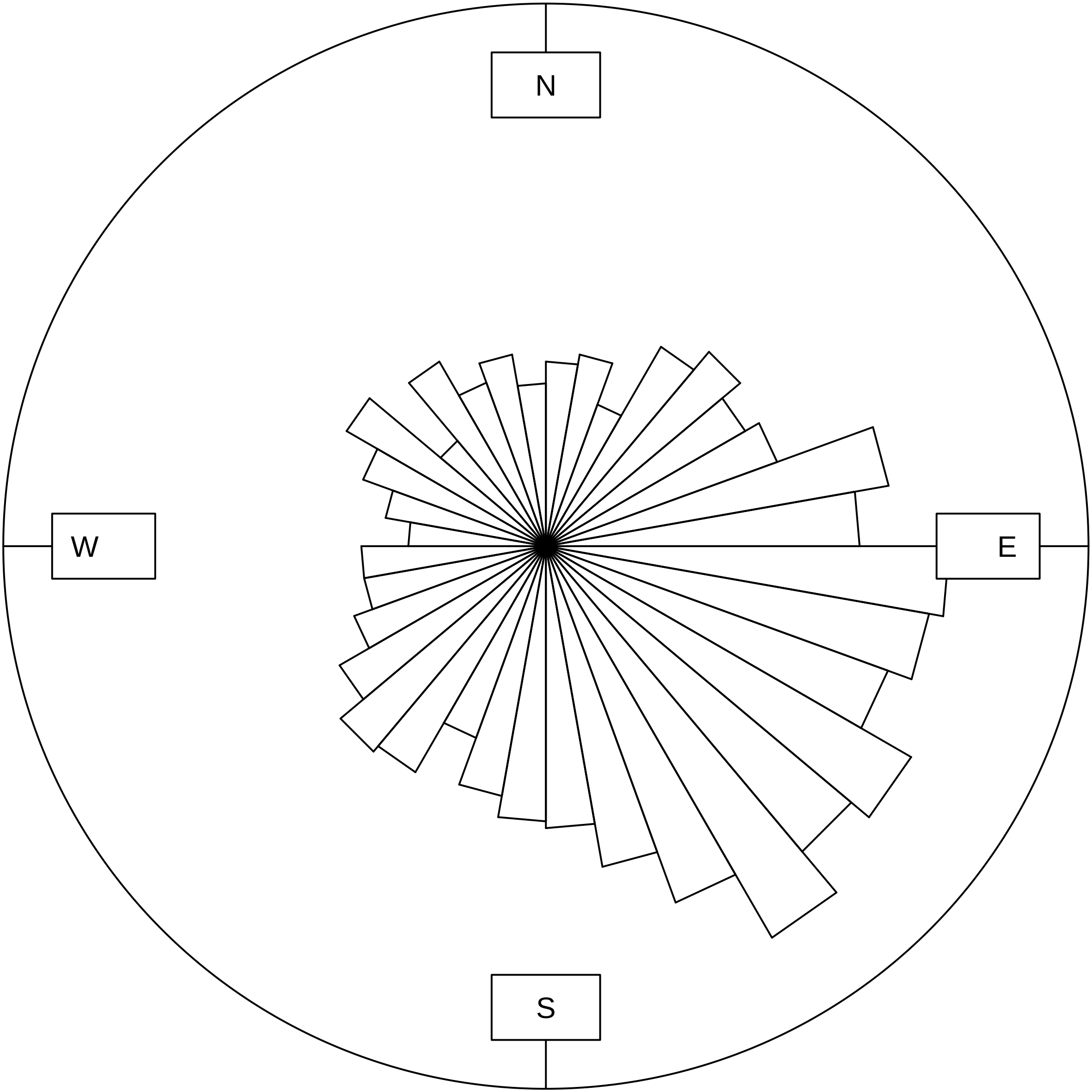

bootstrap 236

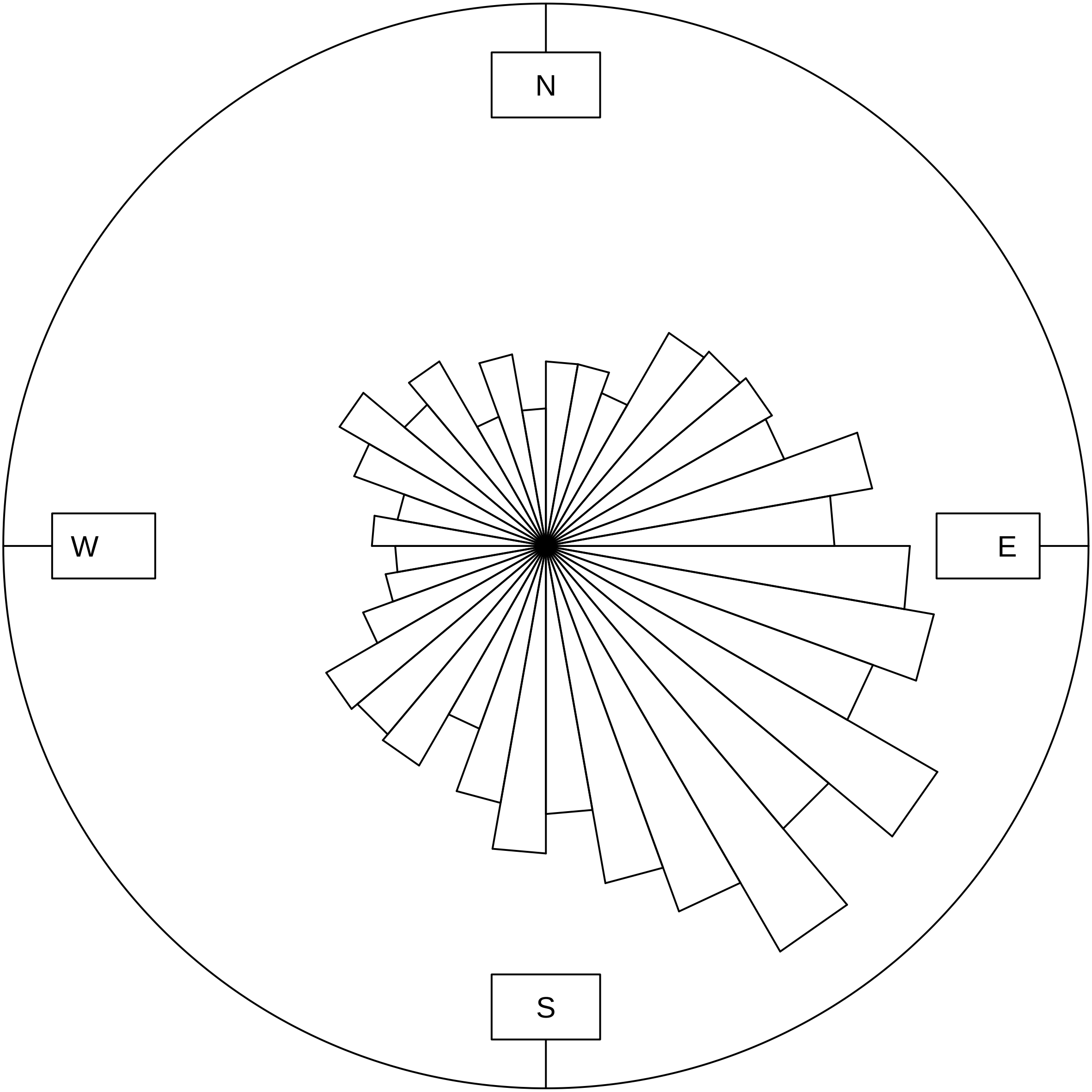

bootstrap 237

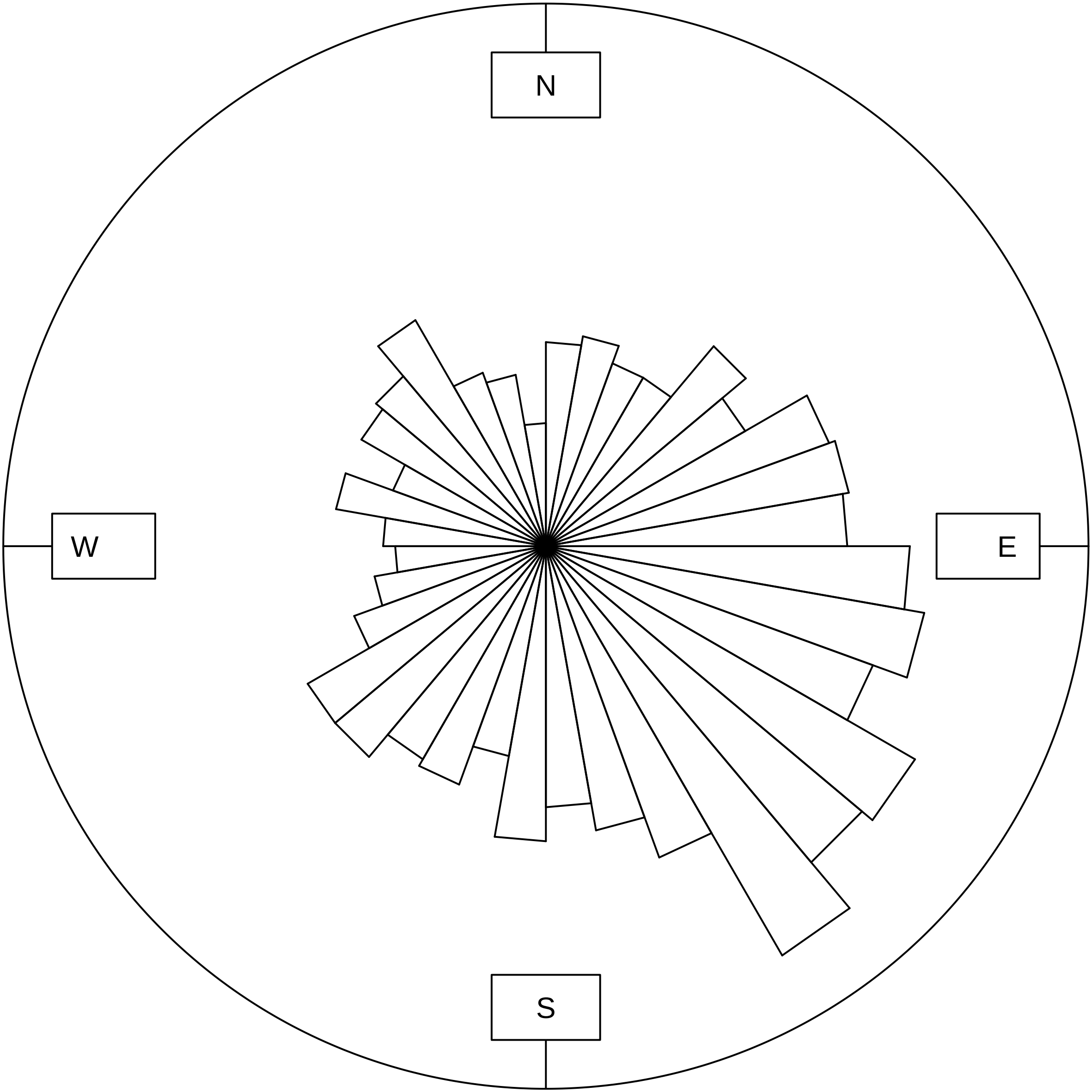

bootstrap 238

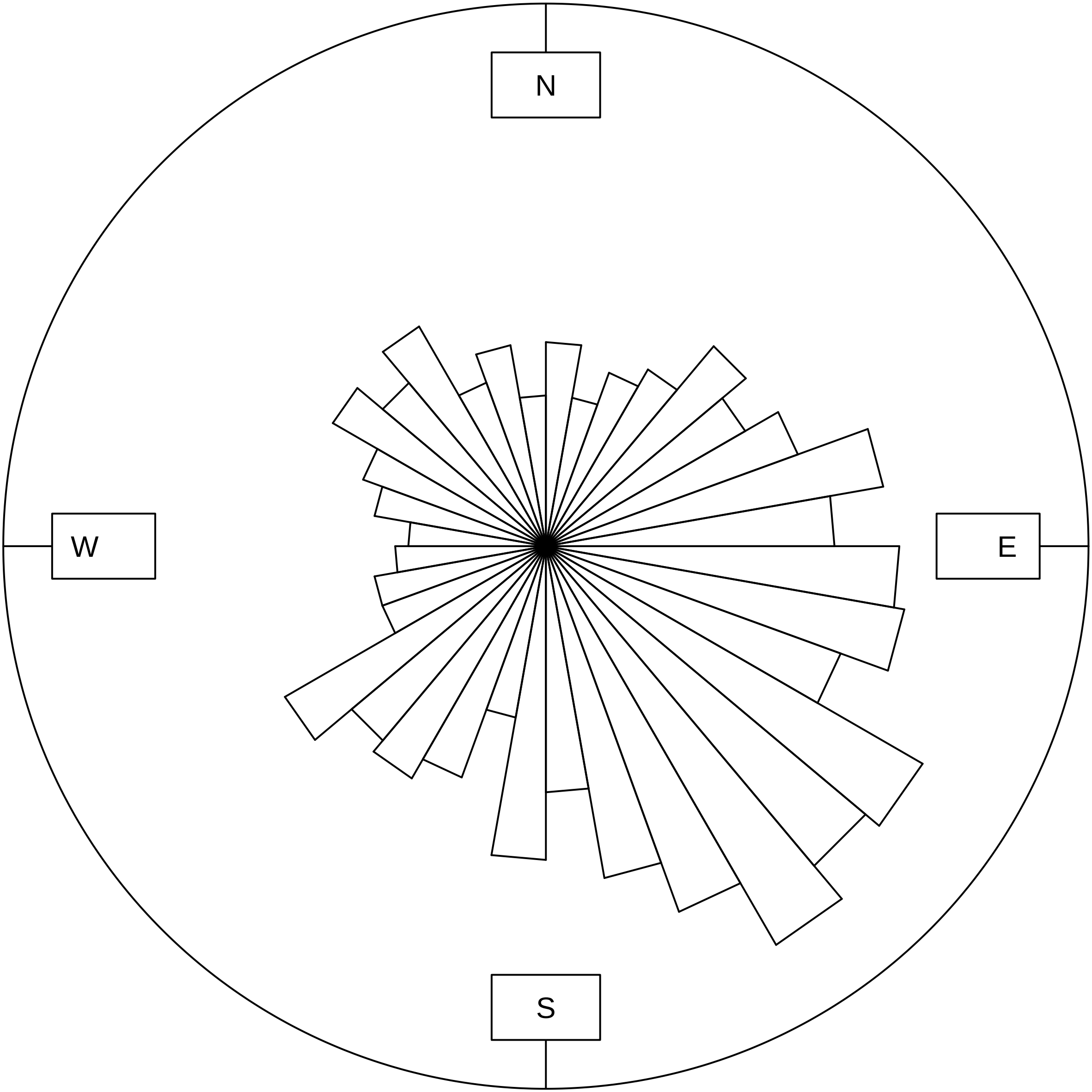

bootstrap 239

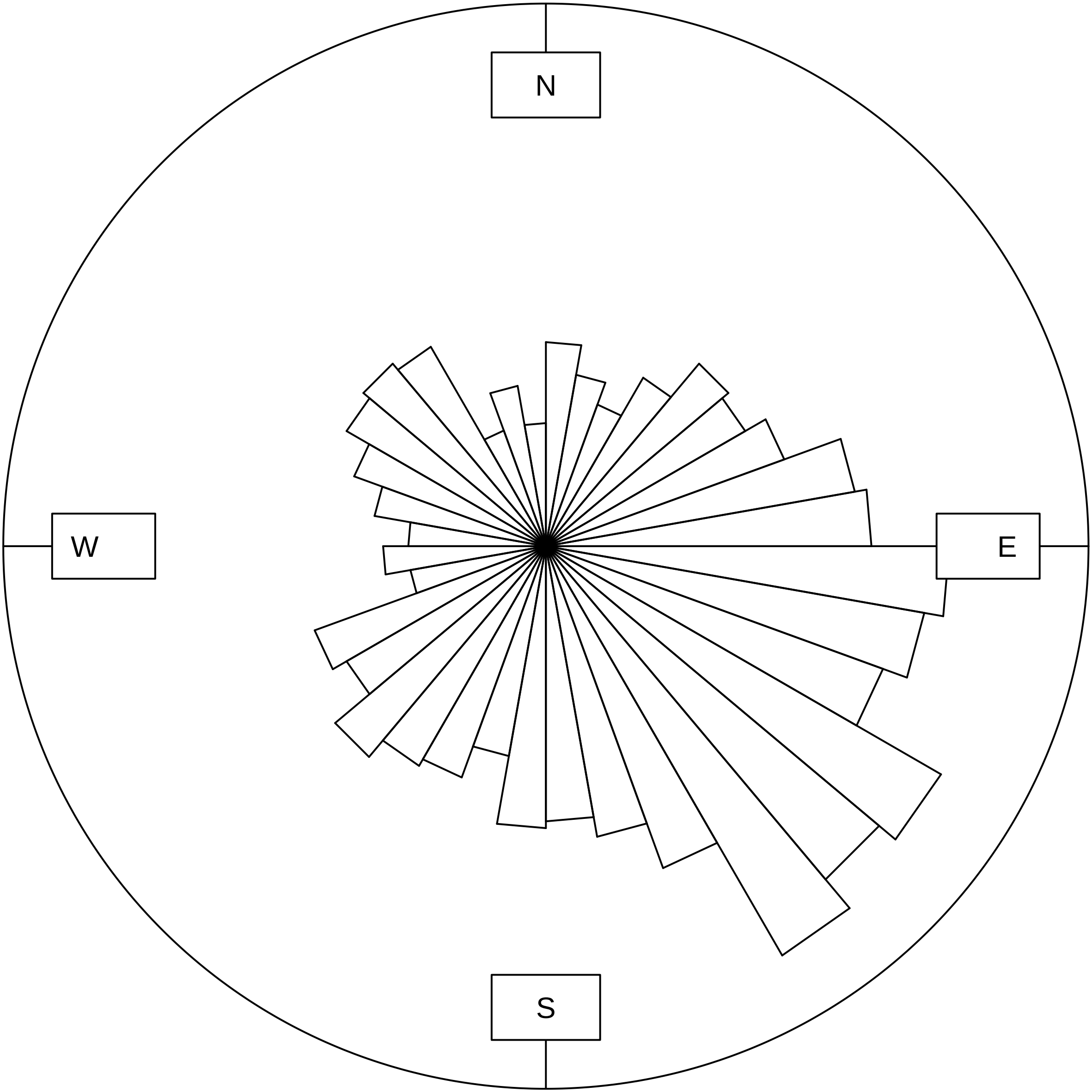

bootstrap 240

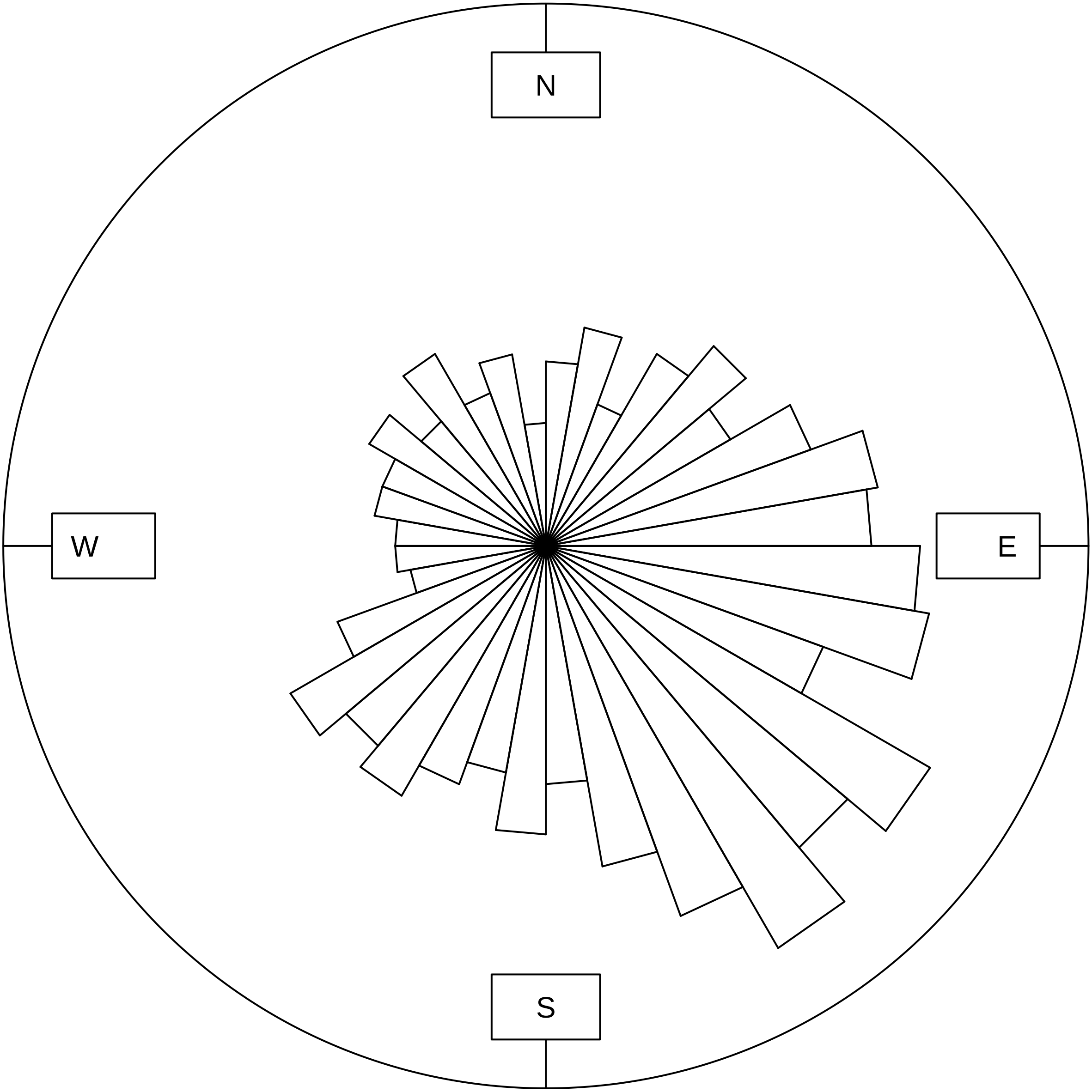

bootstrap 241

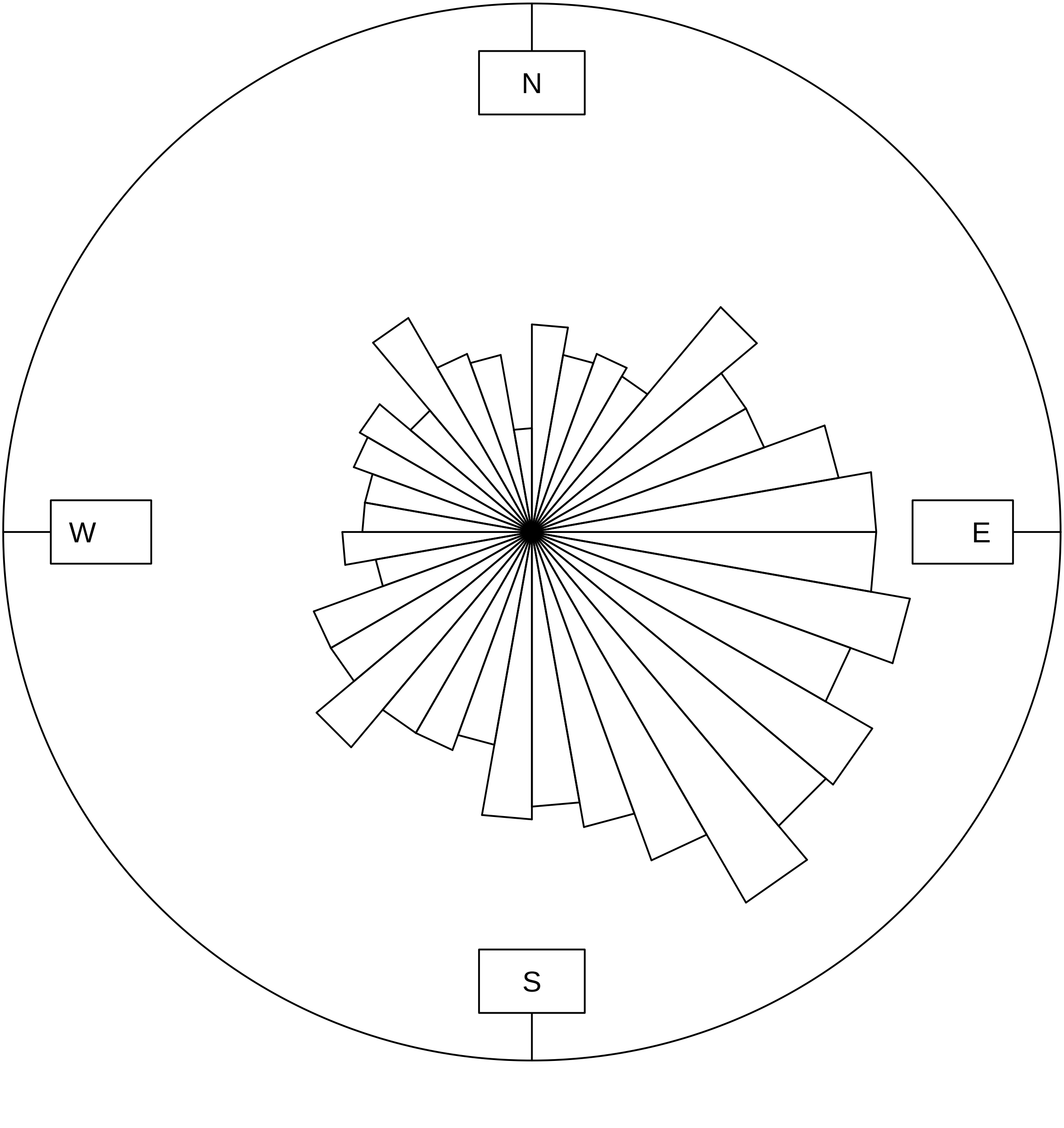

bootstrap 242

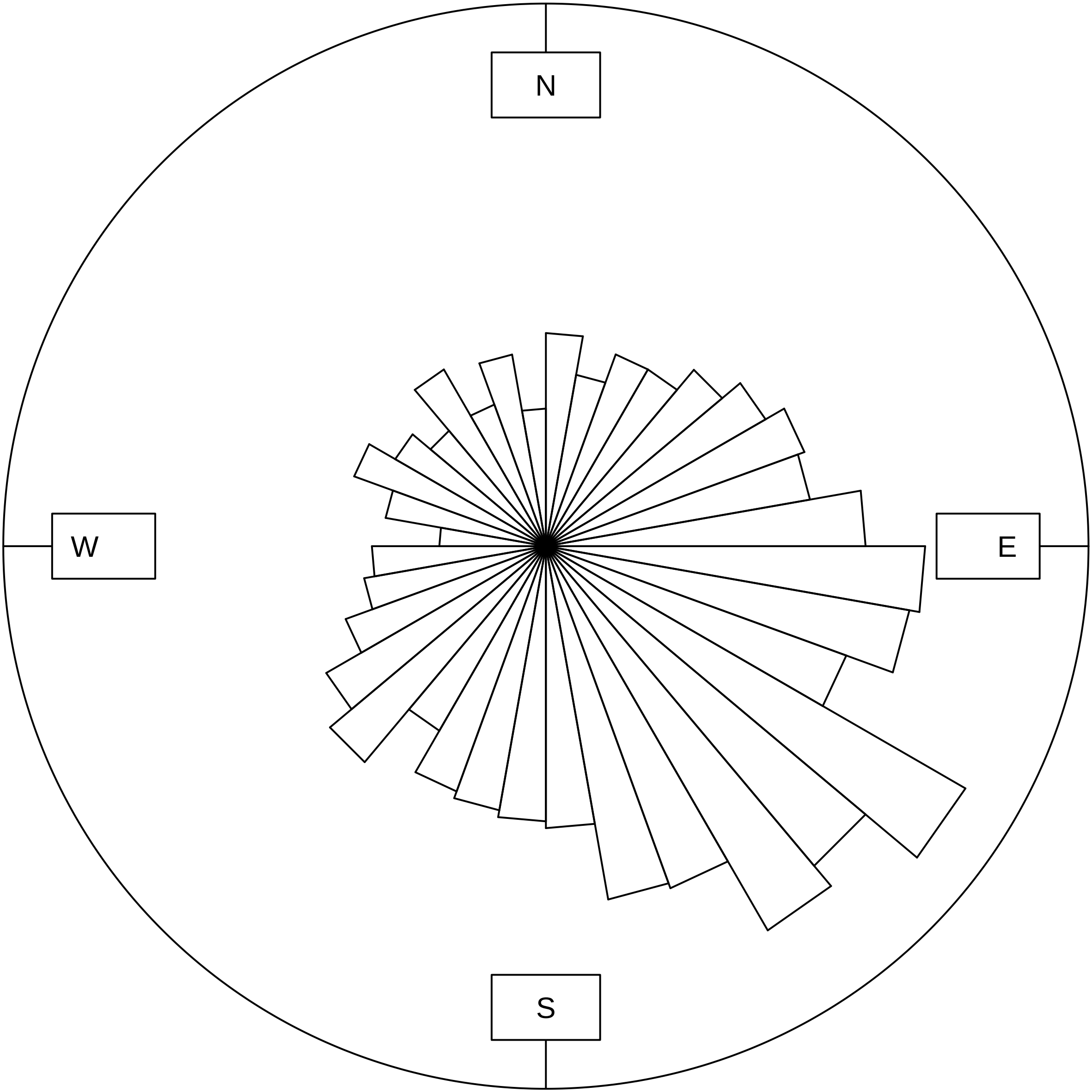

bootstrap 243

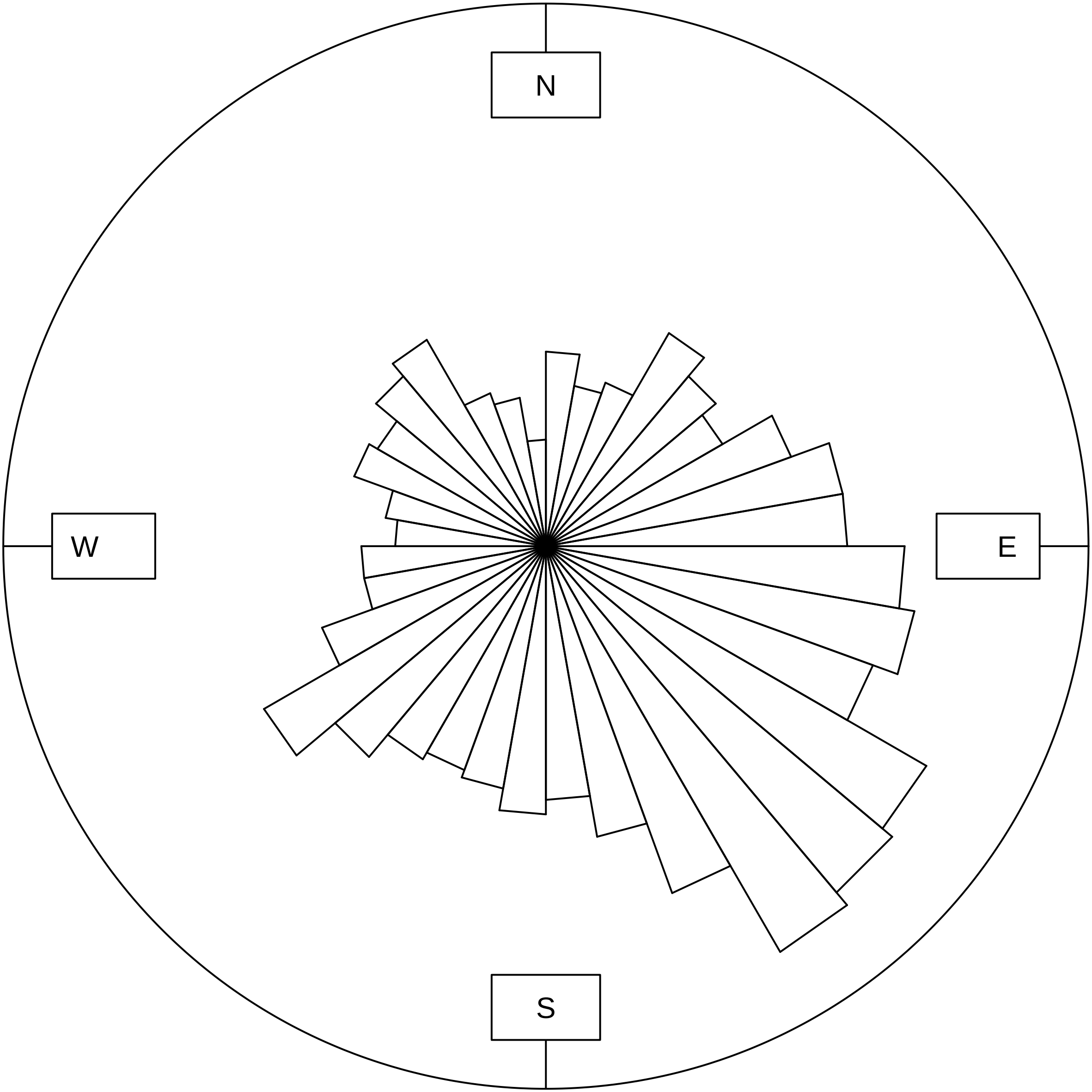

bootstrap 244

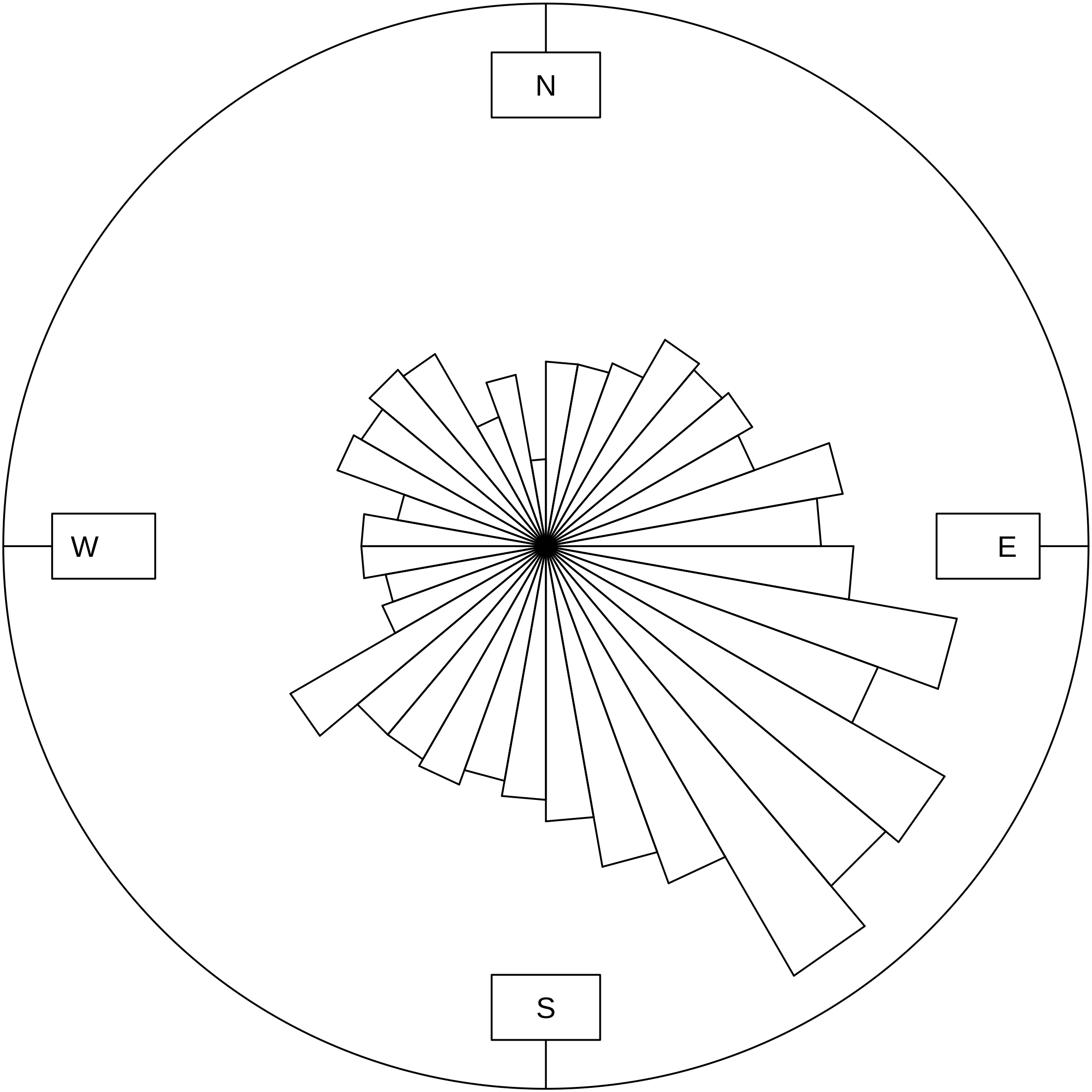

bootstrap 245

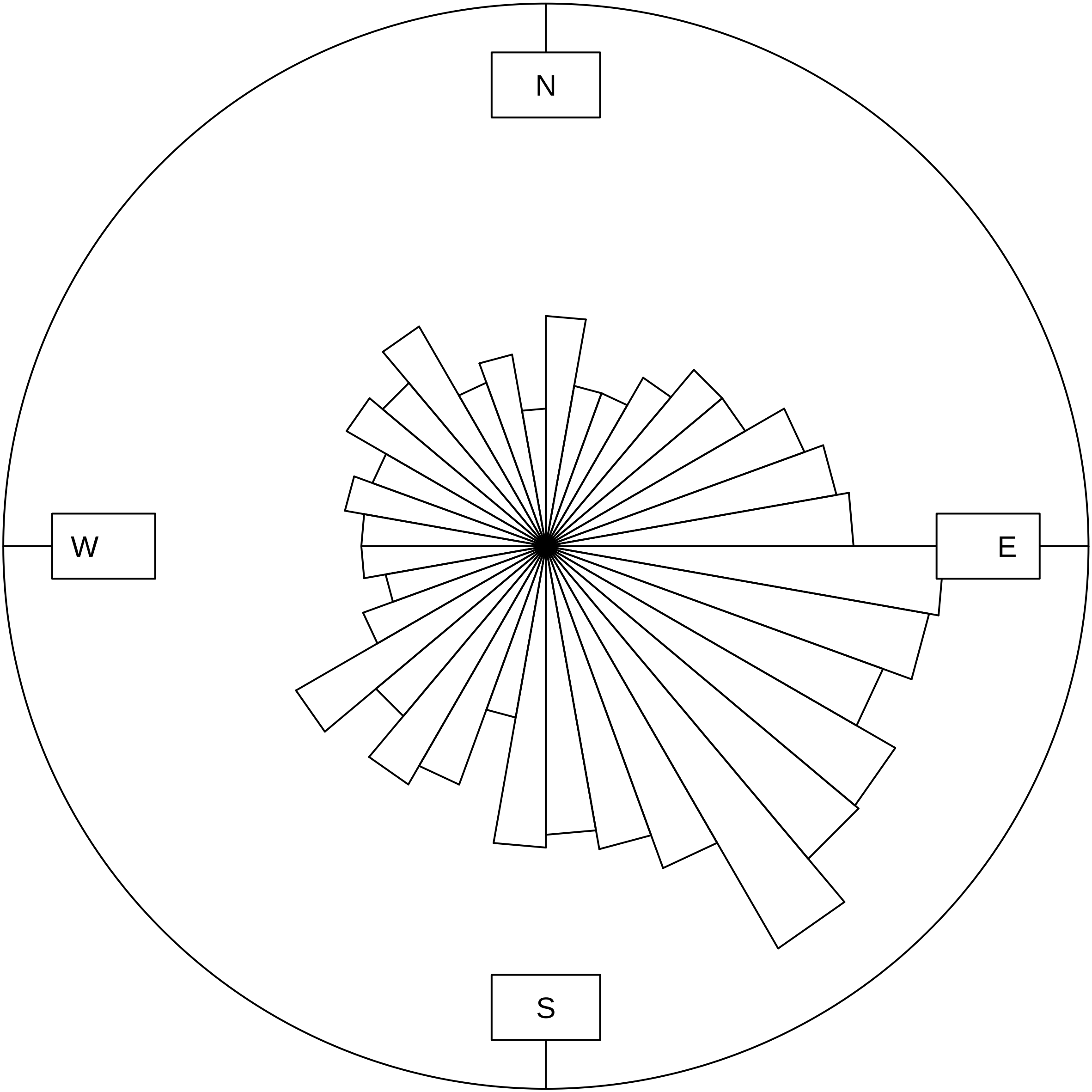

bootstrap 246

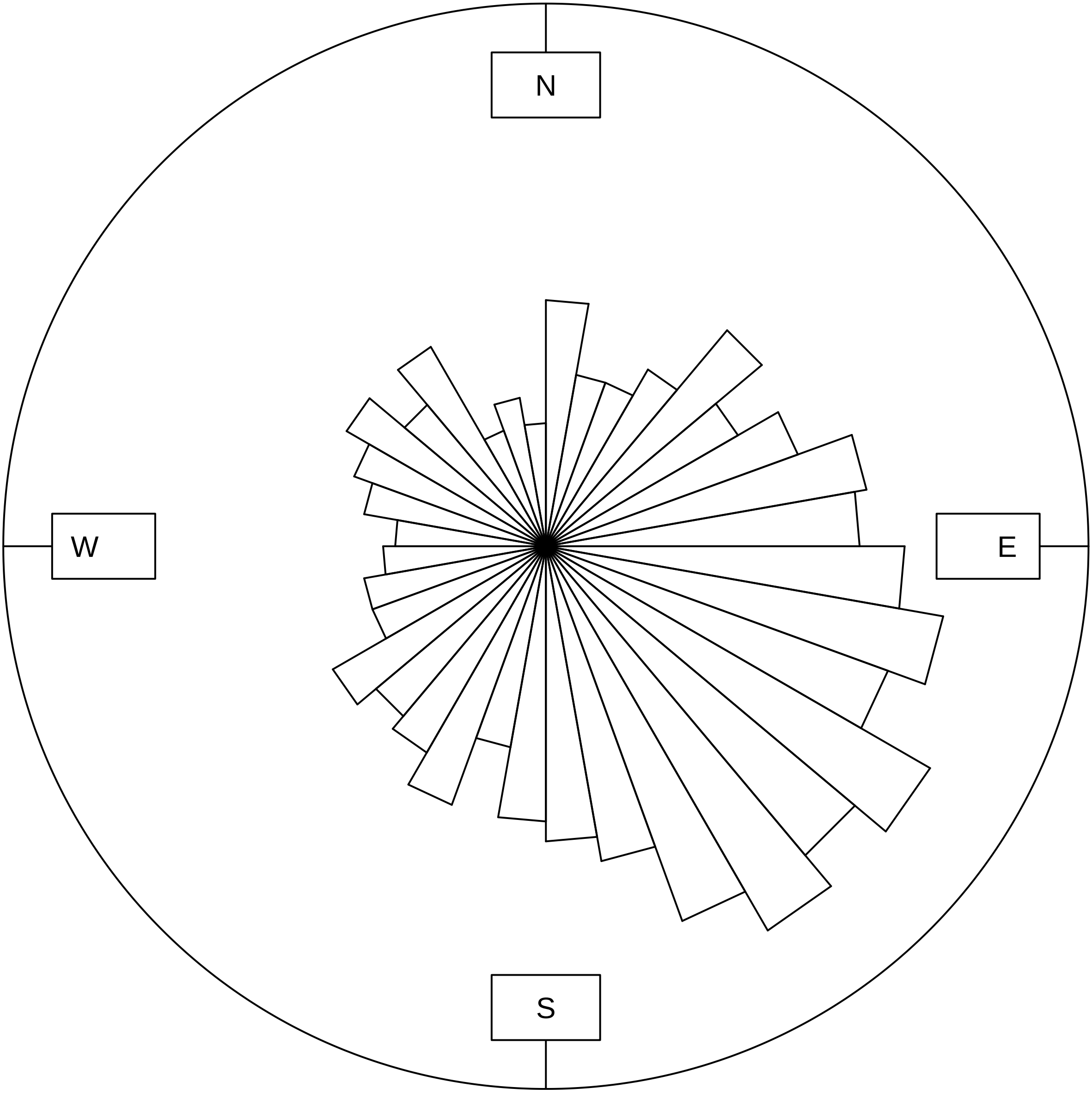

bootstrap 247

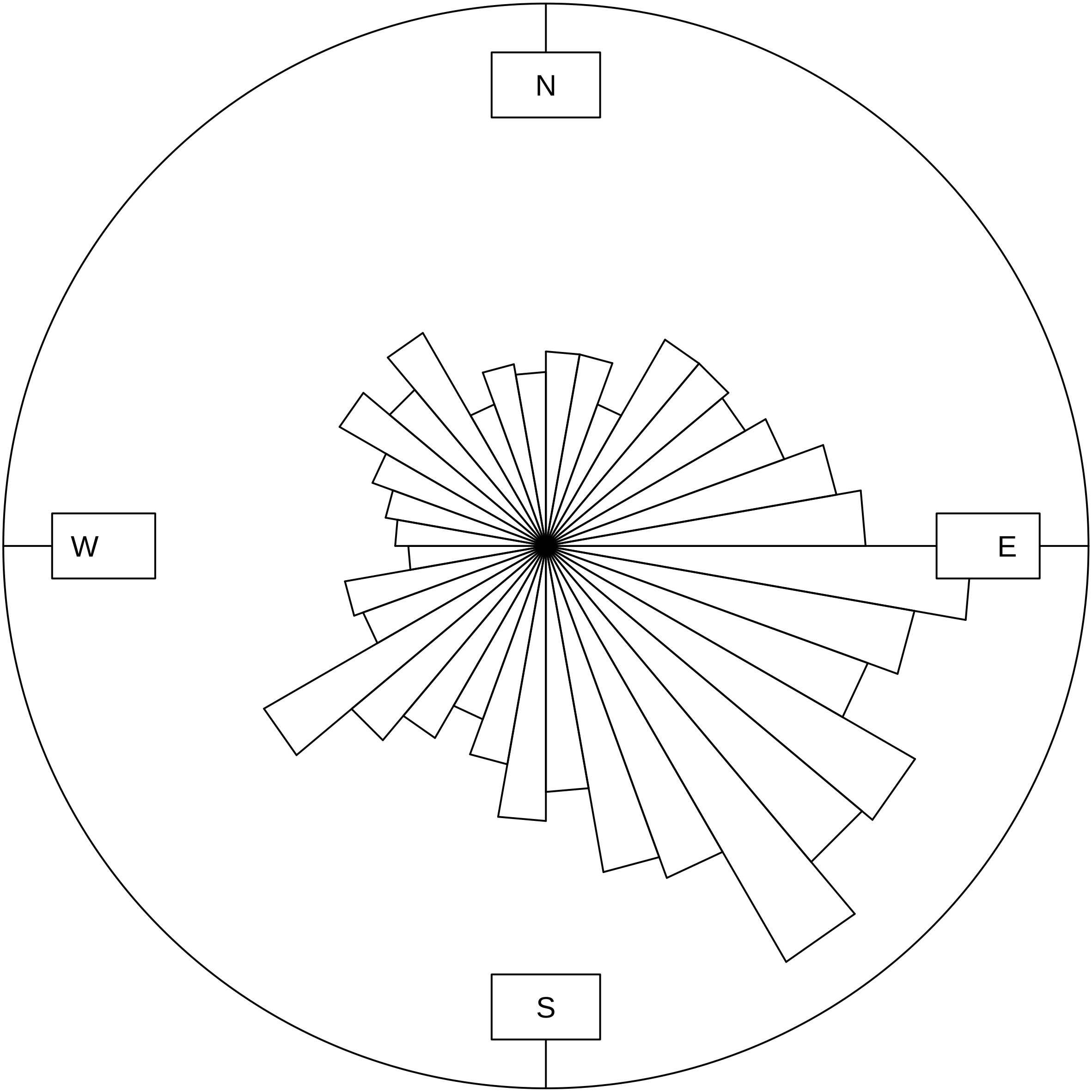

bootstrap 248

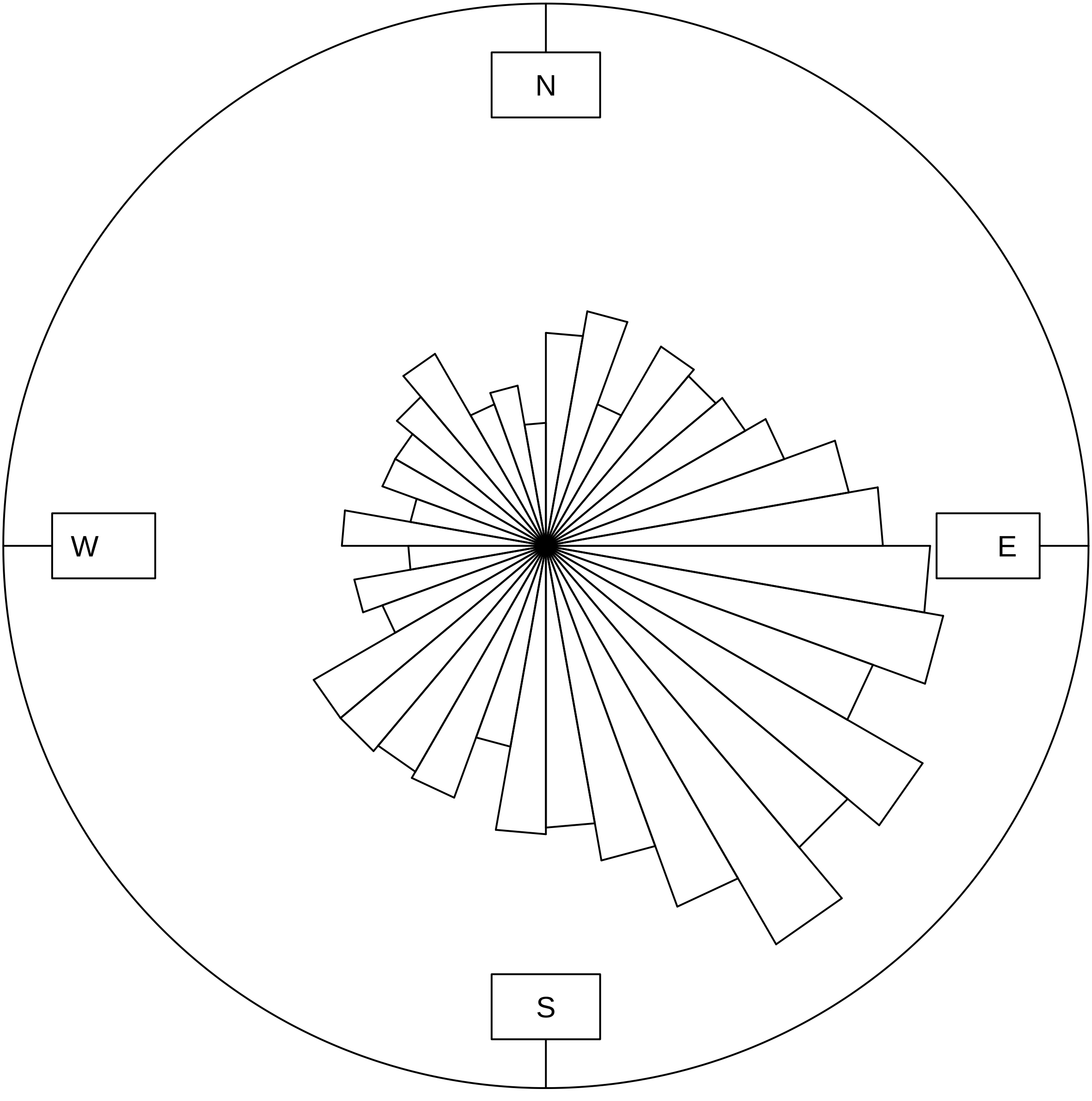

bootstrap 249

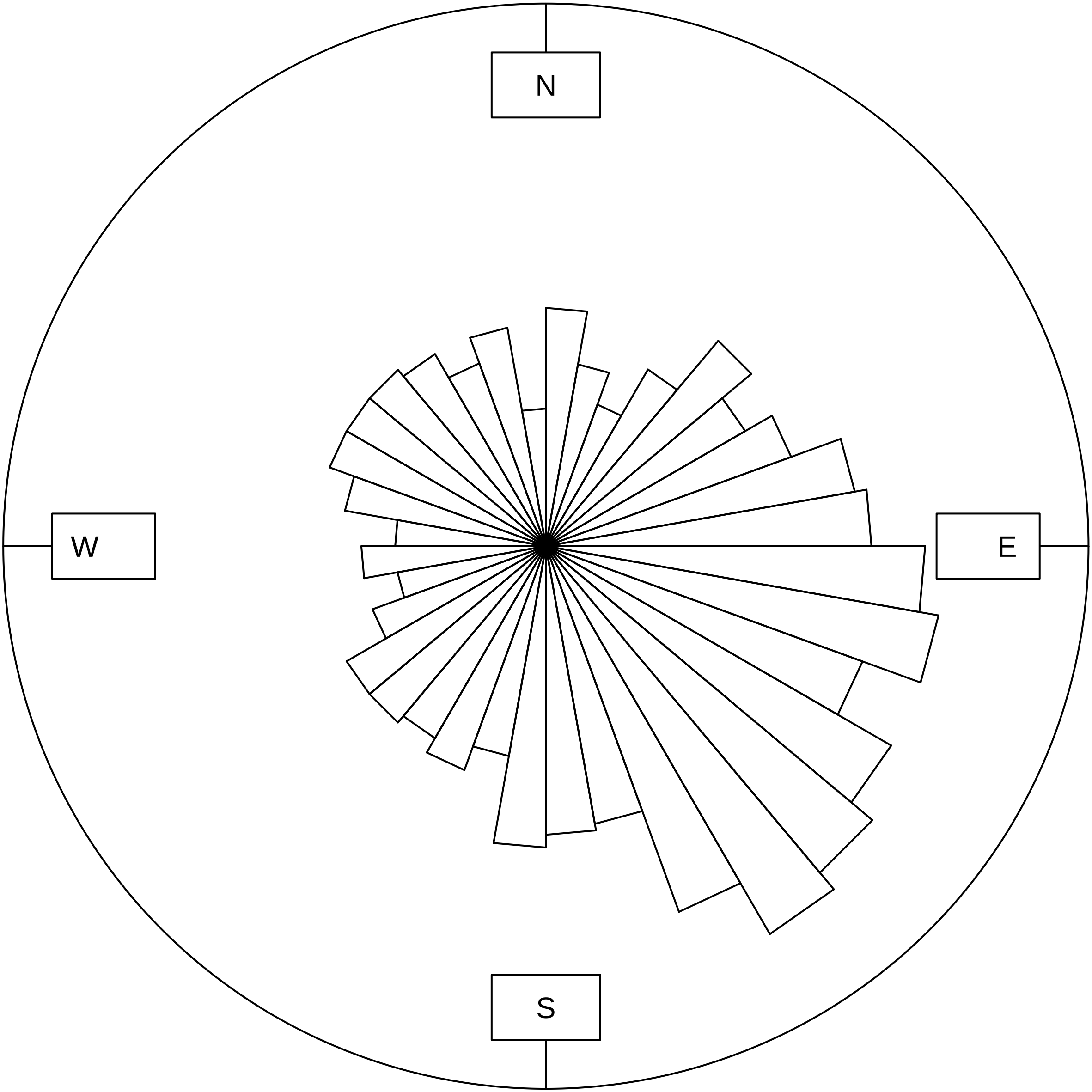

bootstrap 250

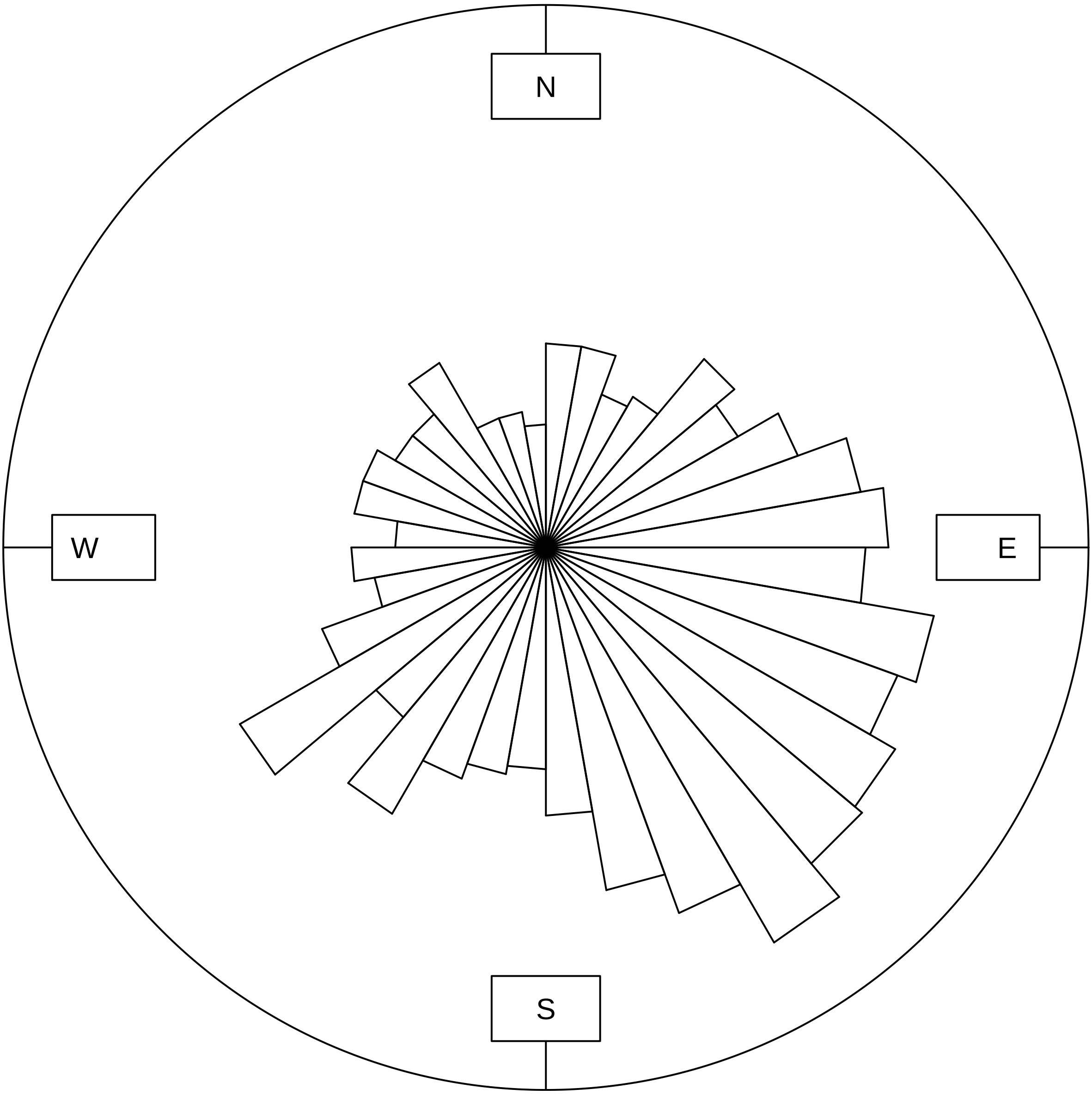

bootstrap 251

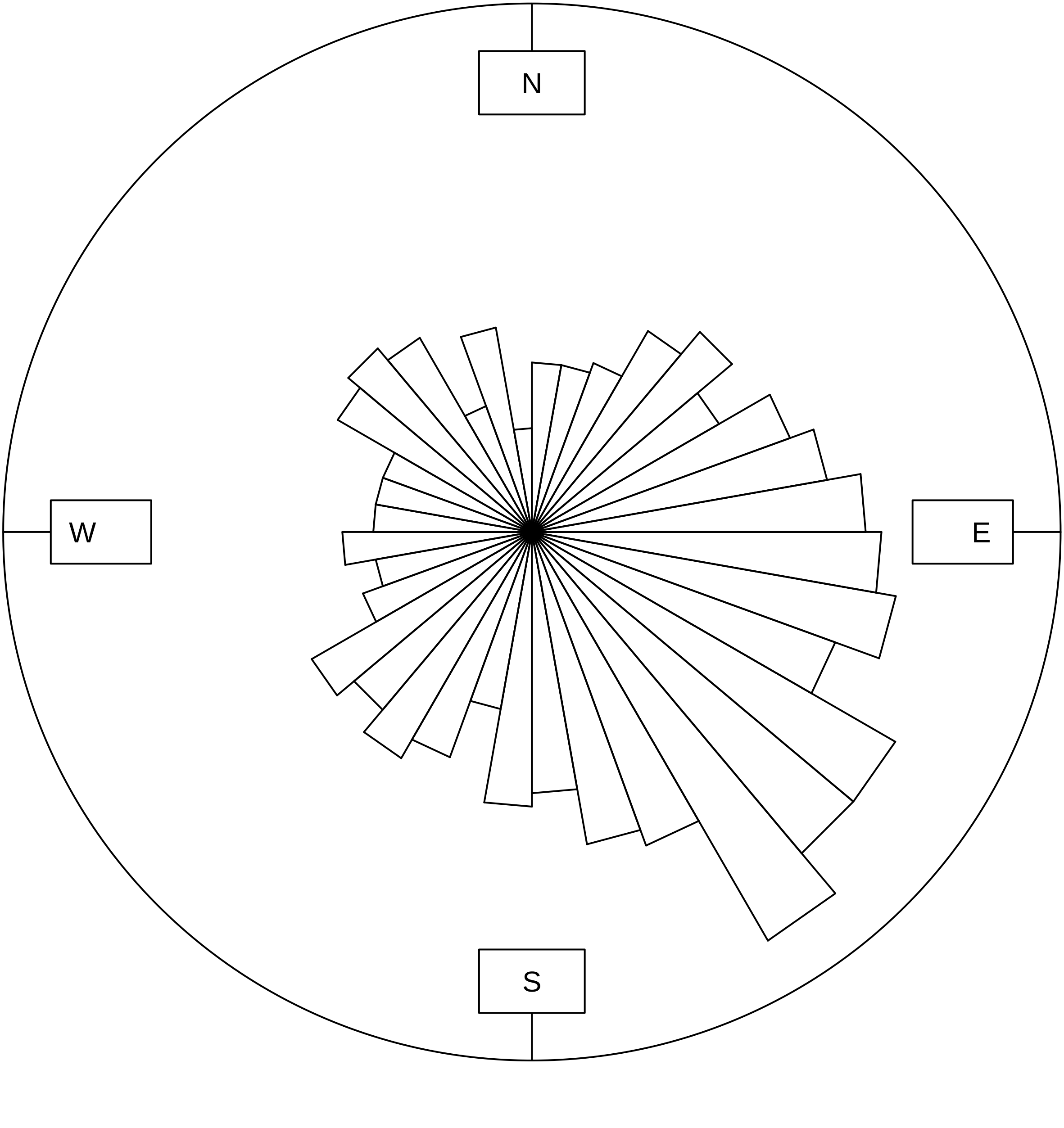

bootstrap 252

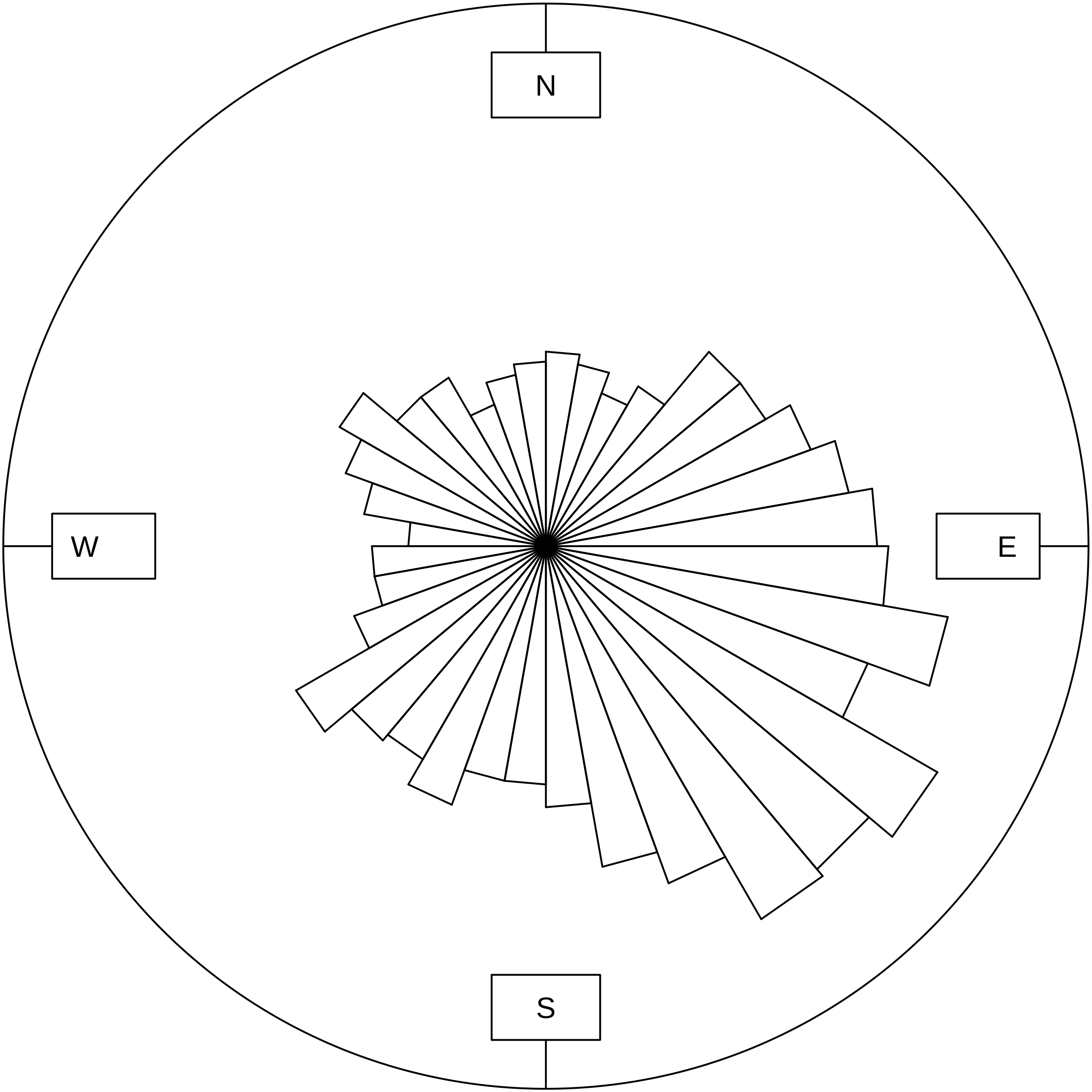

bootstrap 253

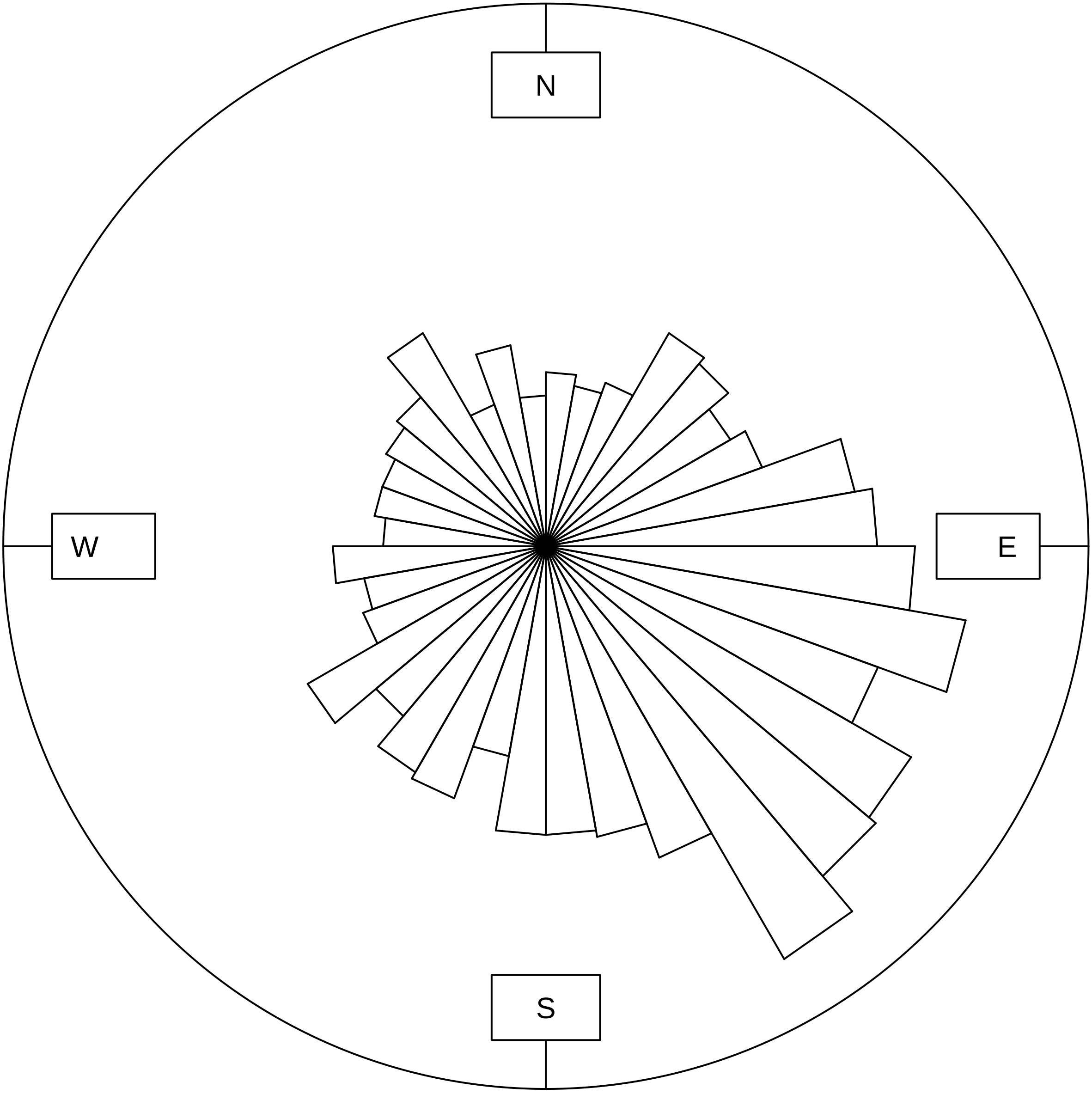

bootstrap 254

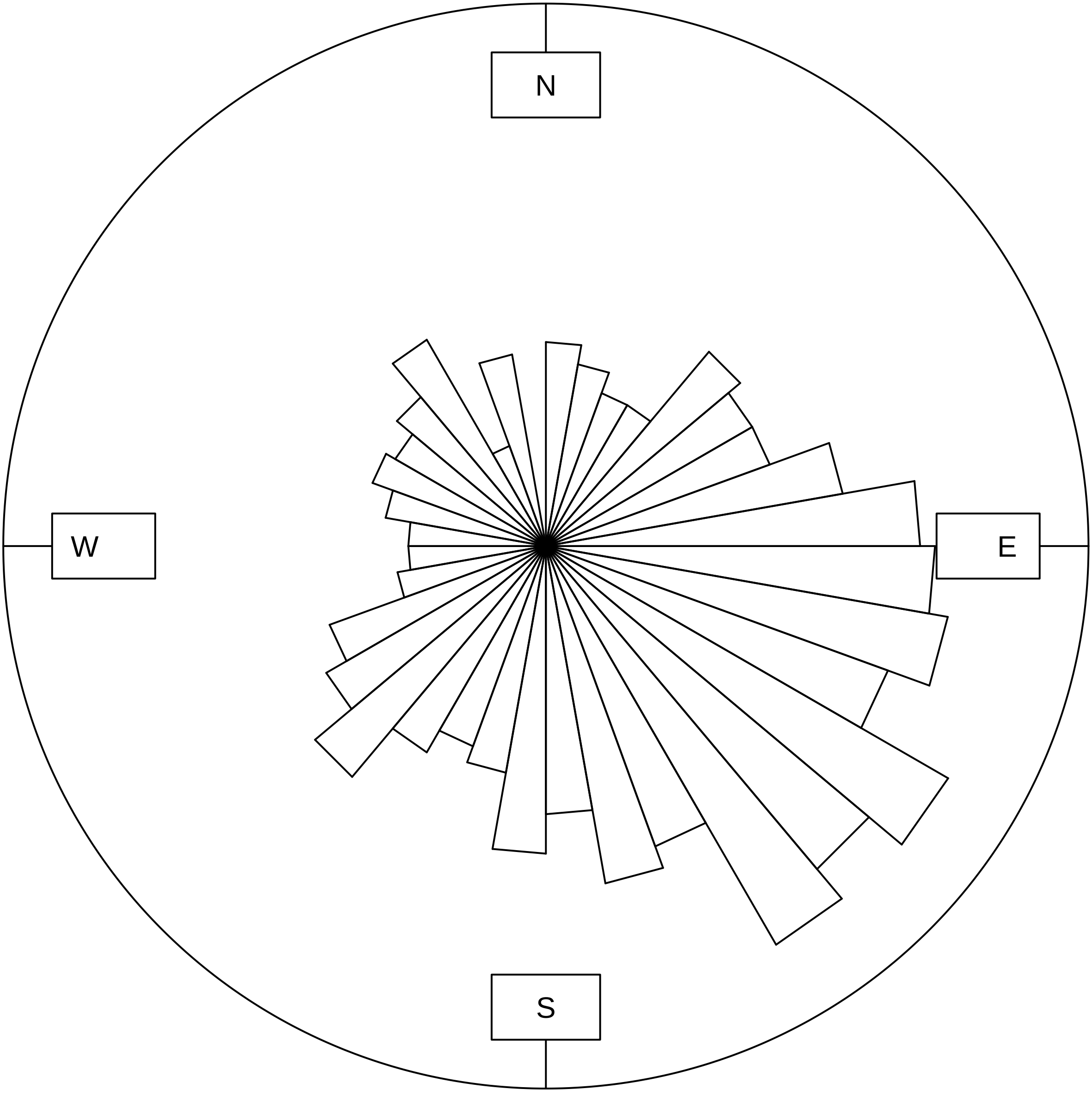

bootstrap 255

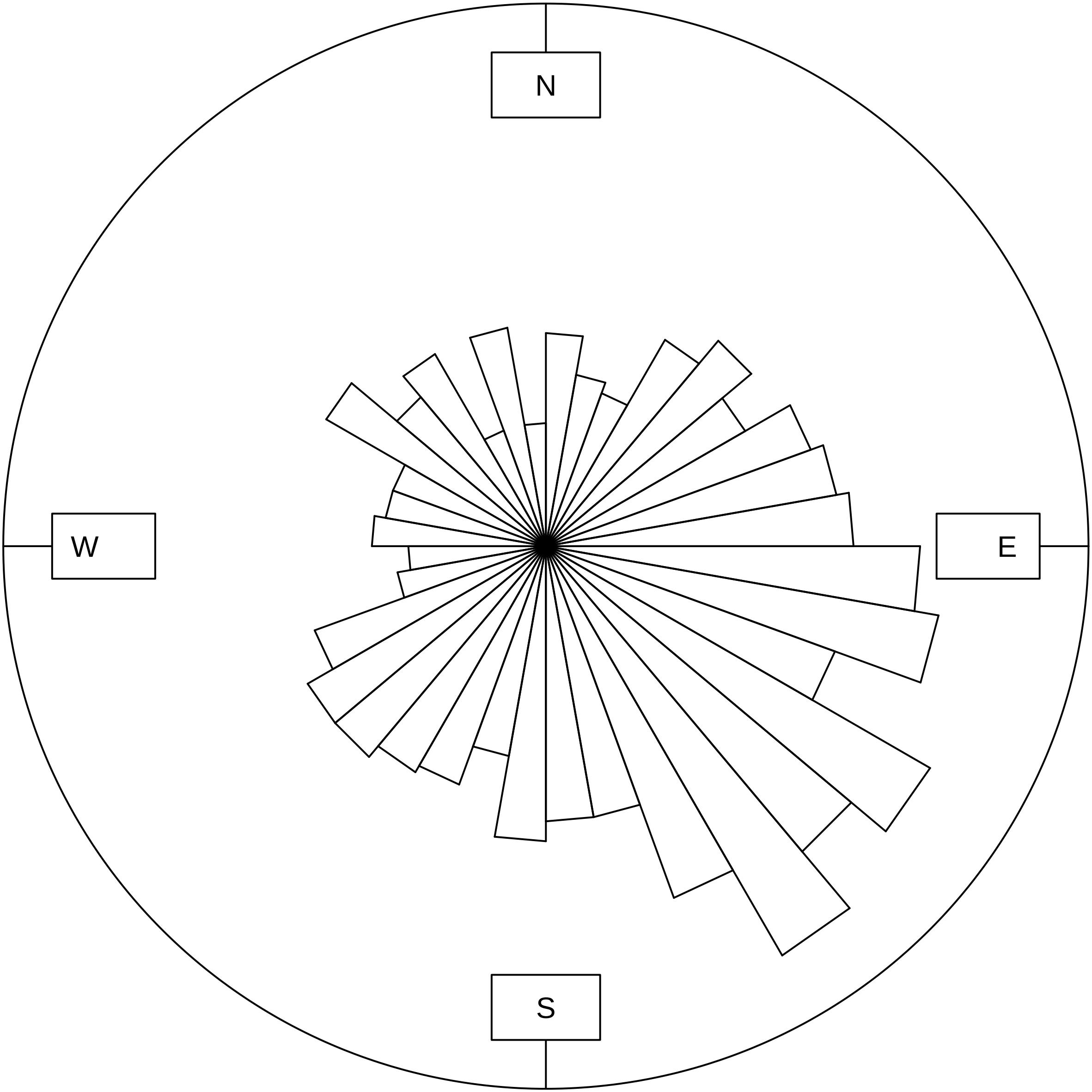

bootstrap 256

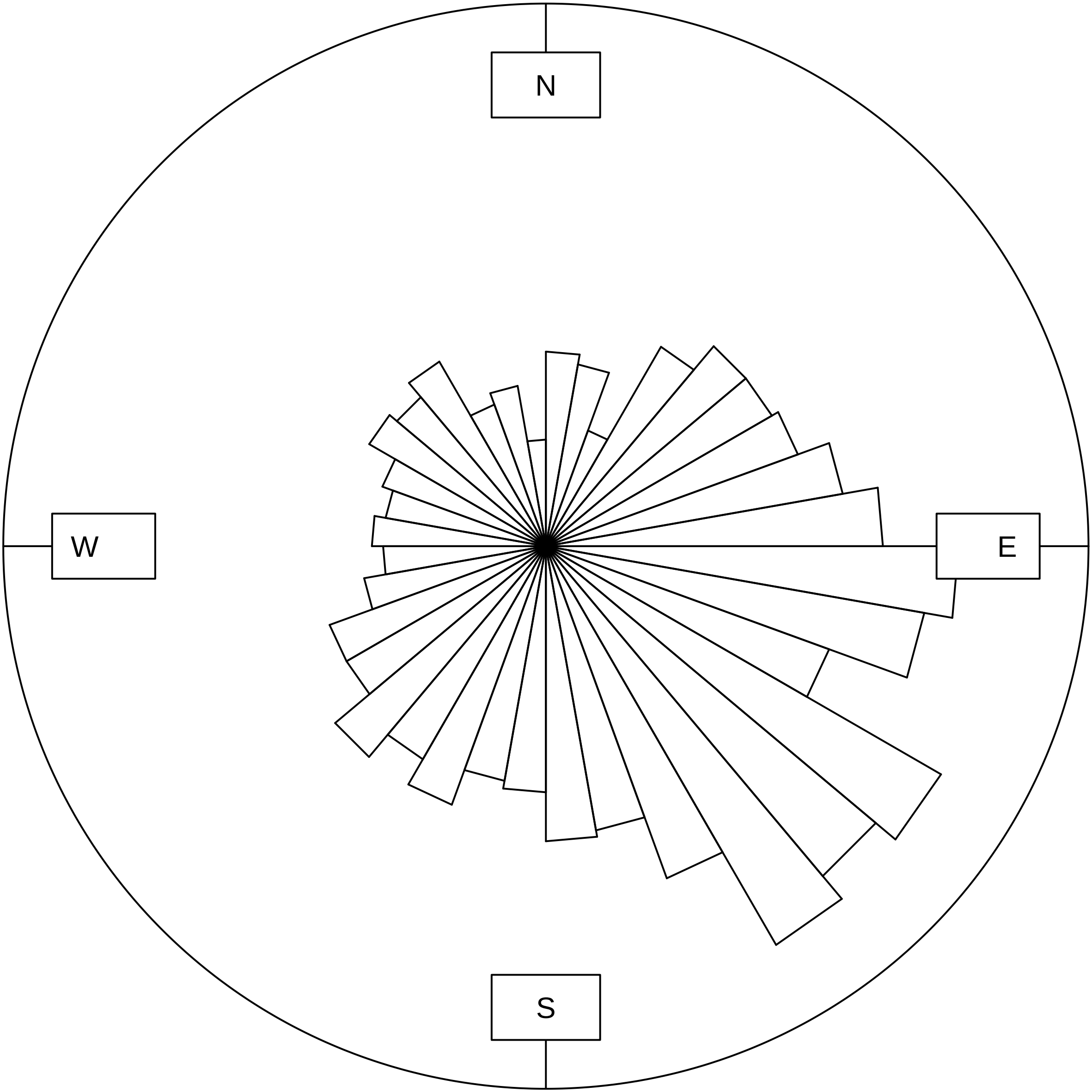

bootstrap 257

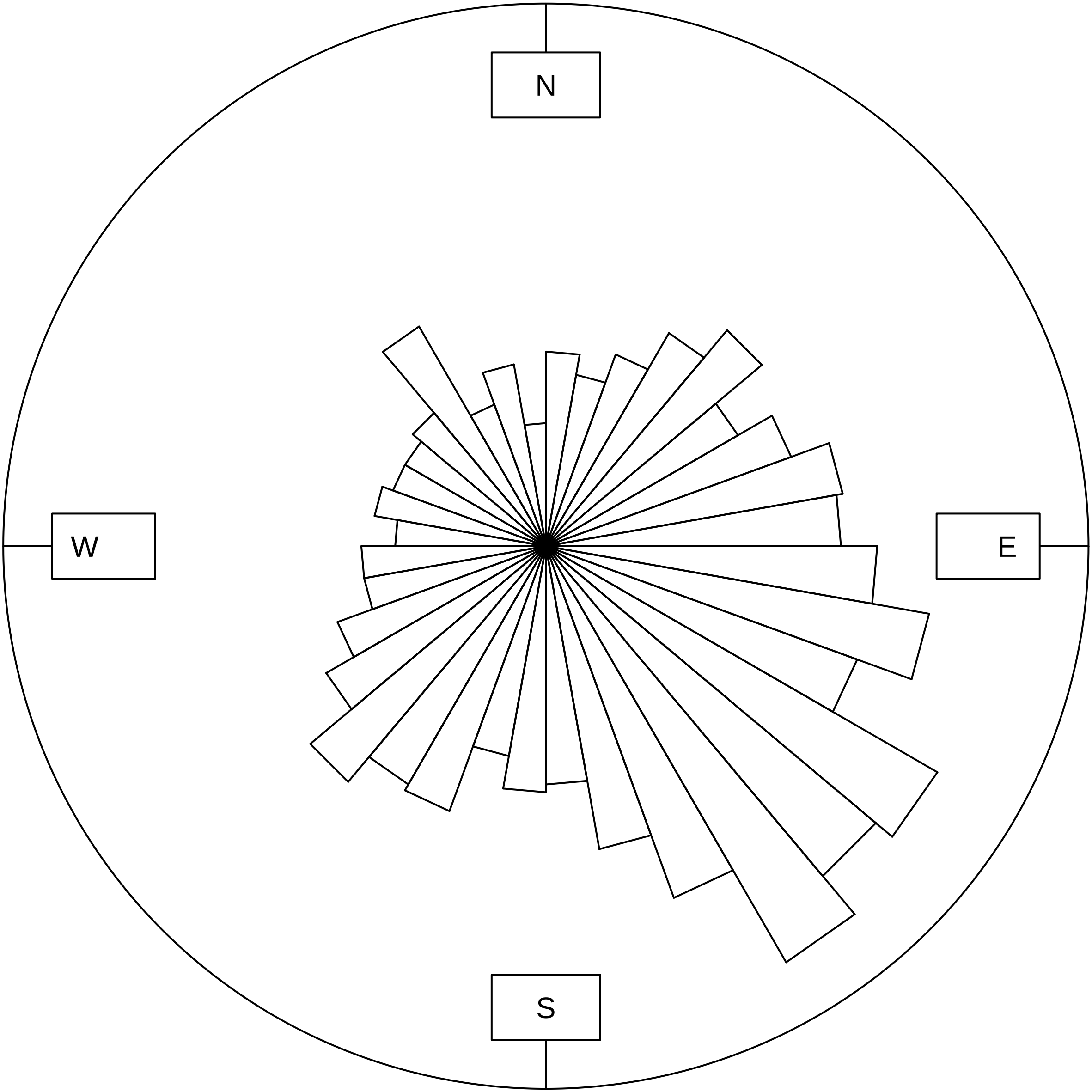

bootstrap 258

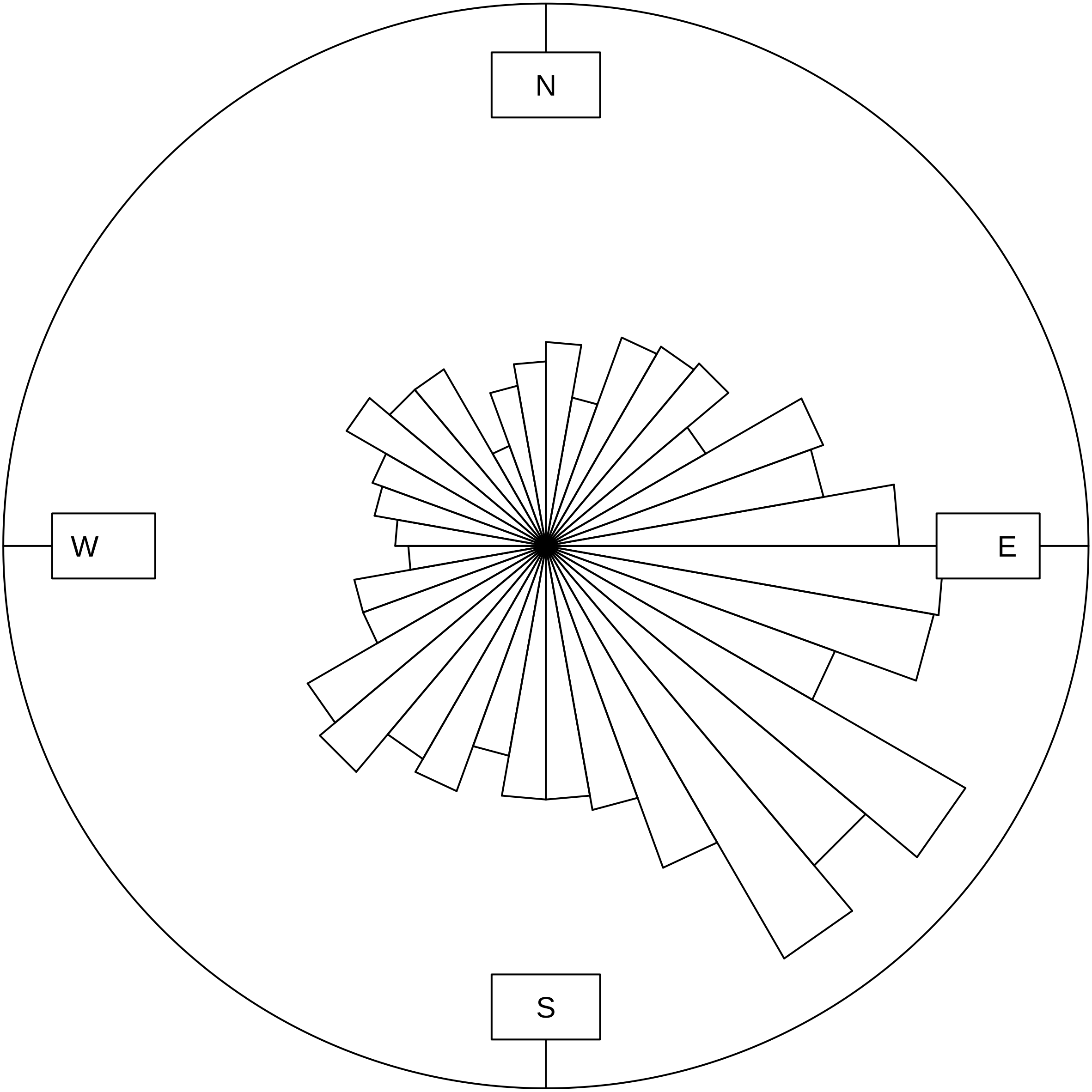

bootstrap 259

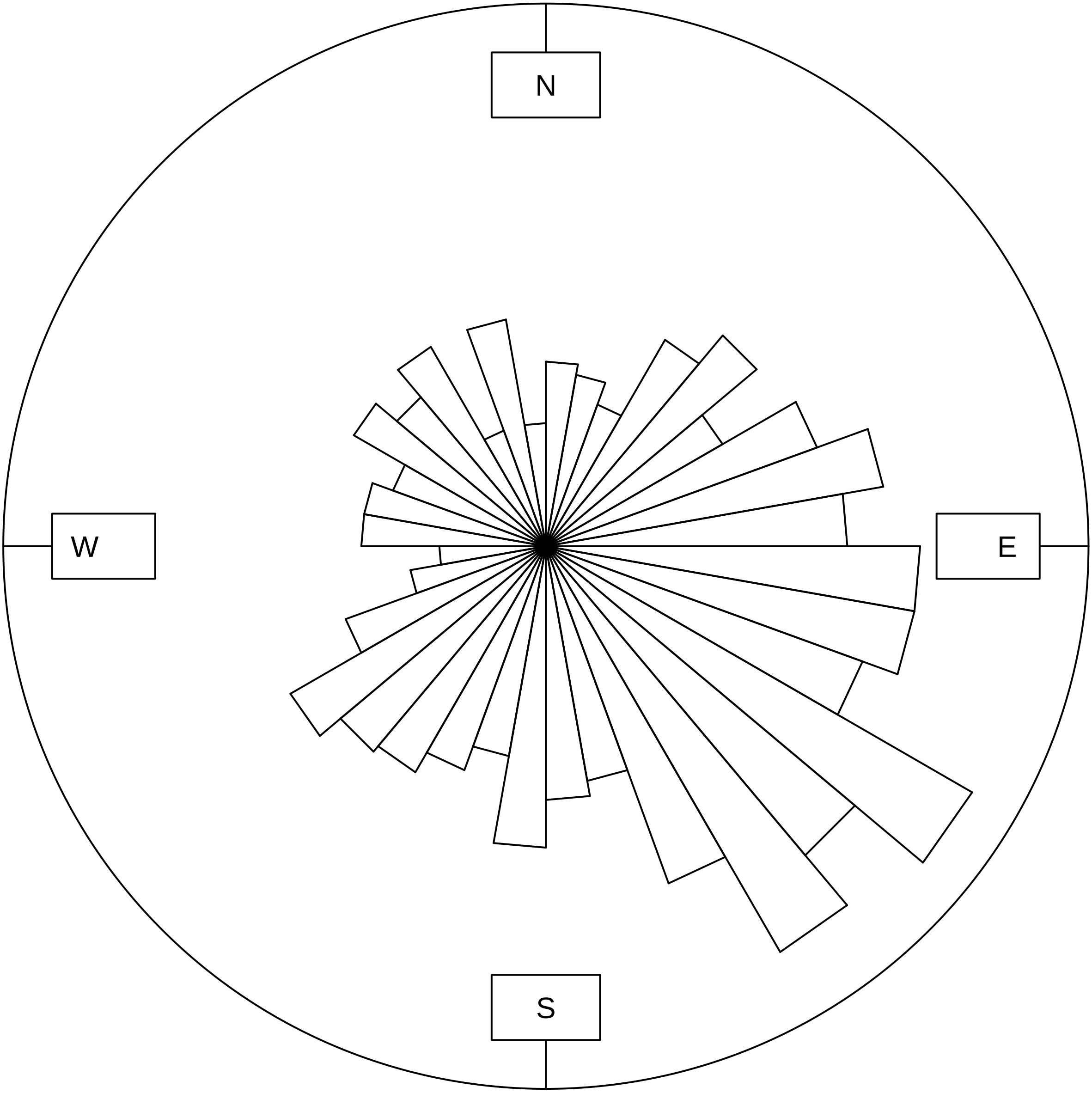

bootstrap 260

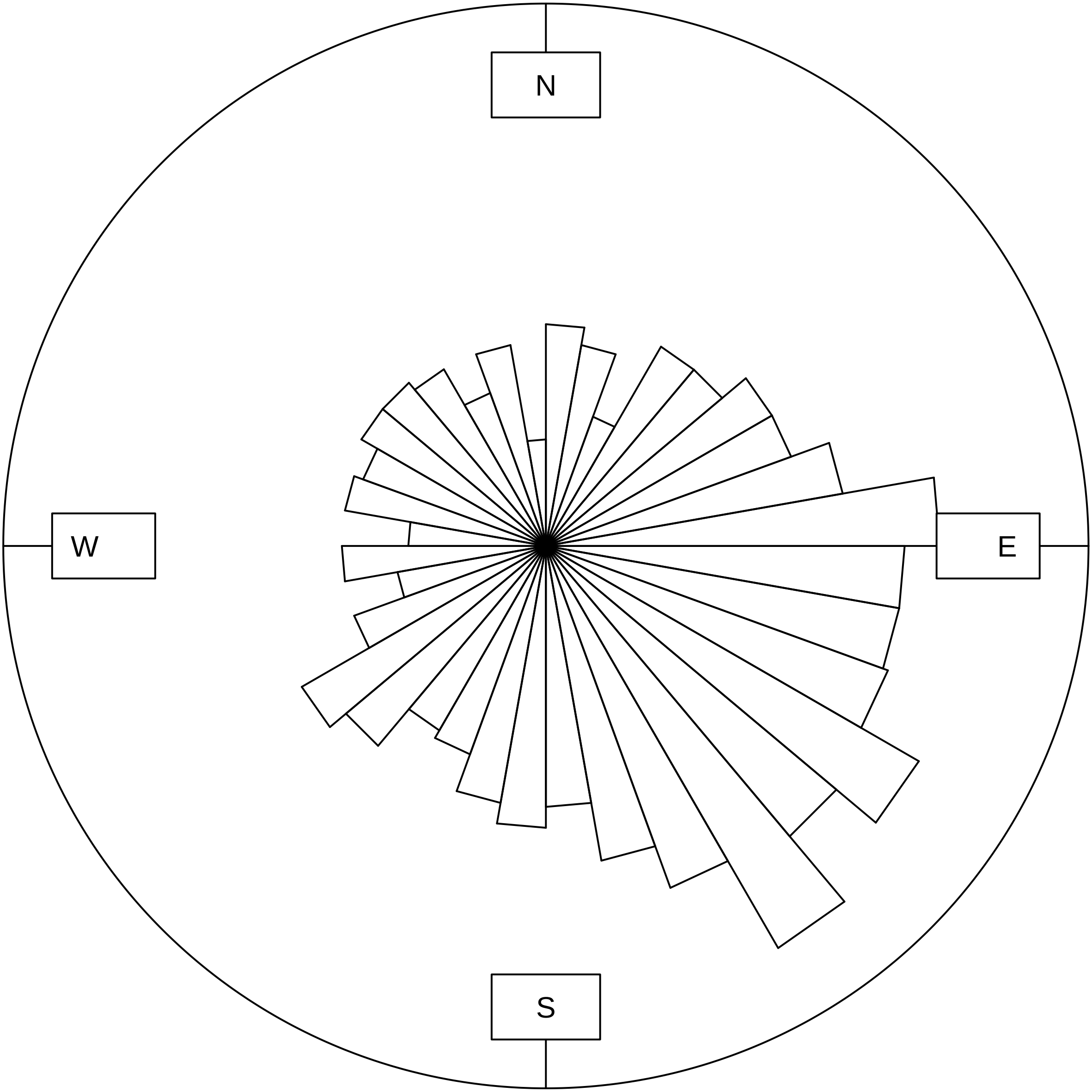

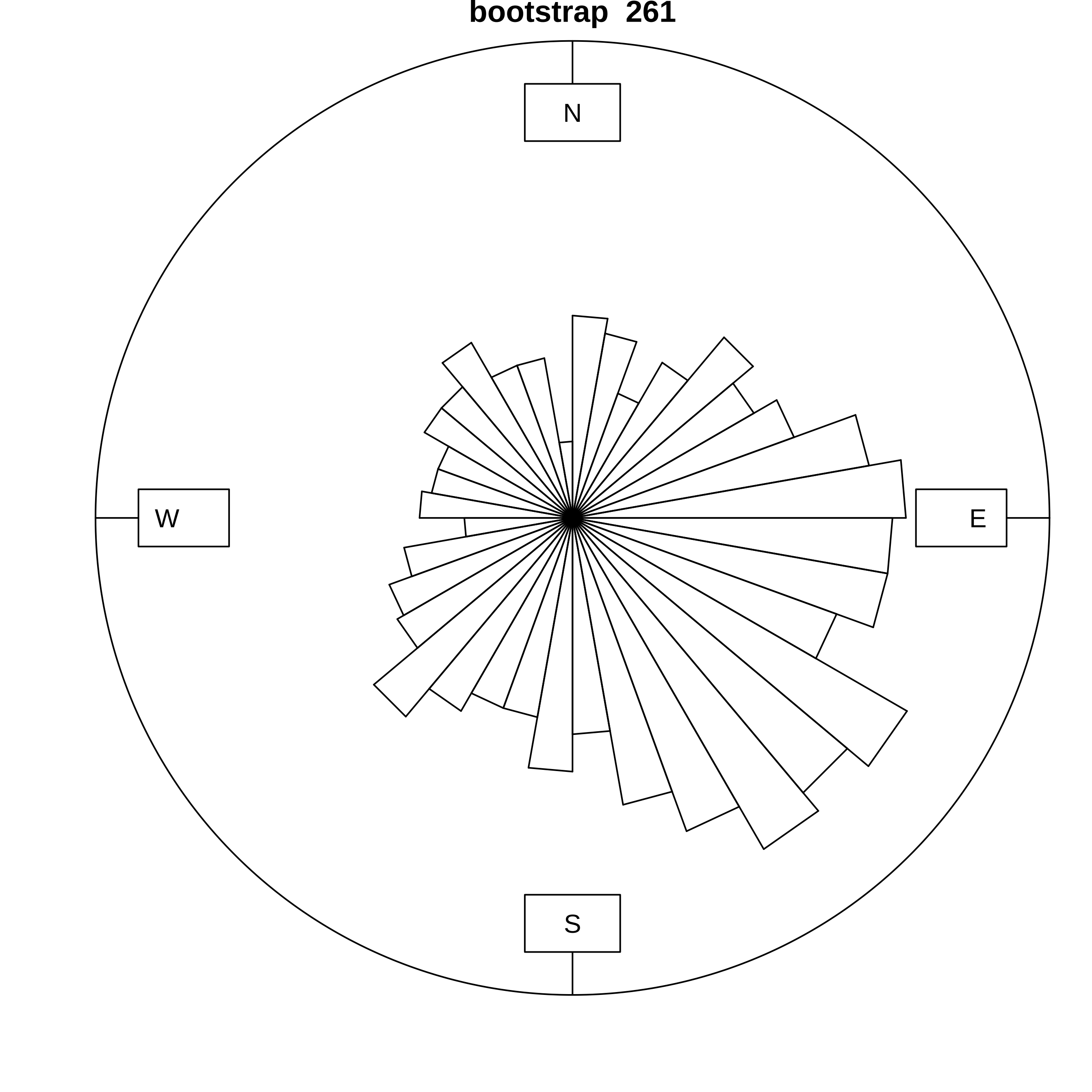

bootstrap 262

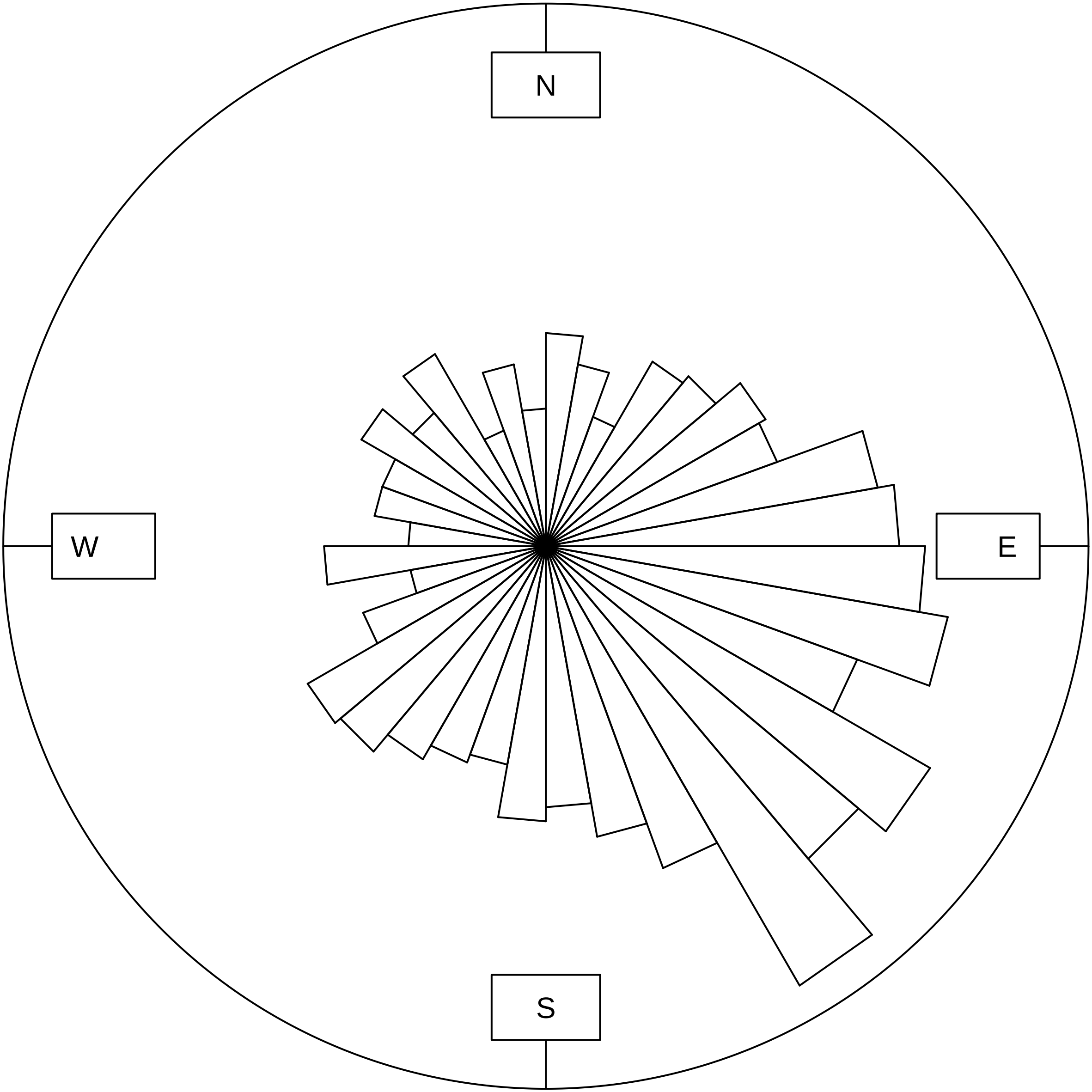

bootstrap 263

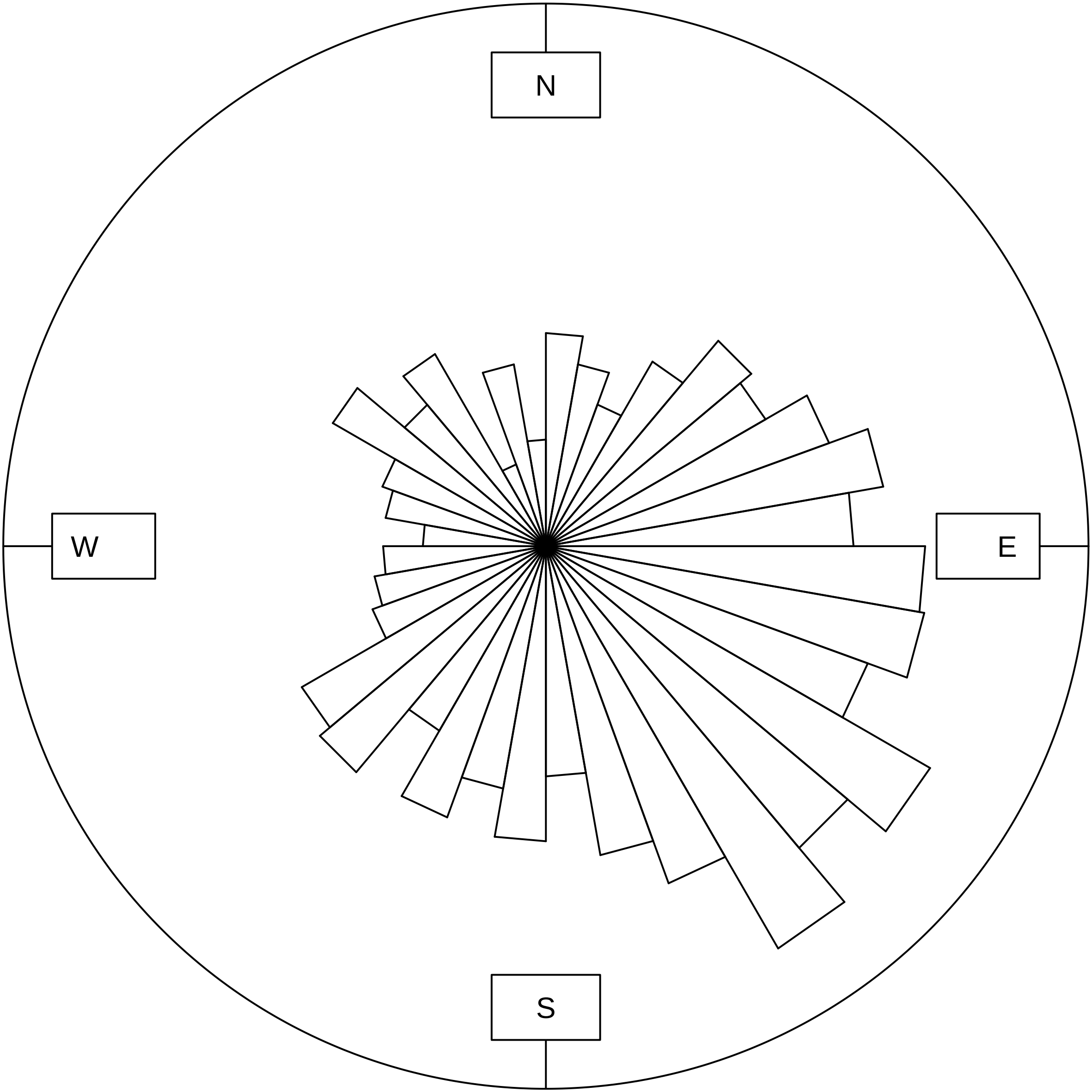

bootstrap 264

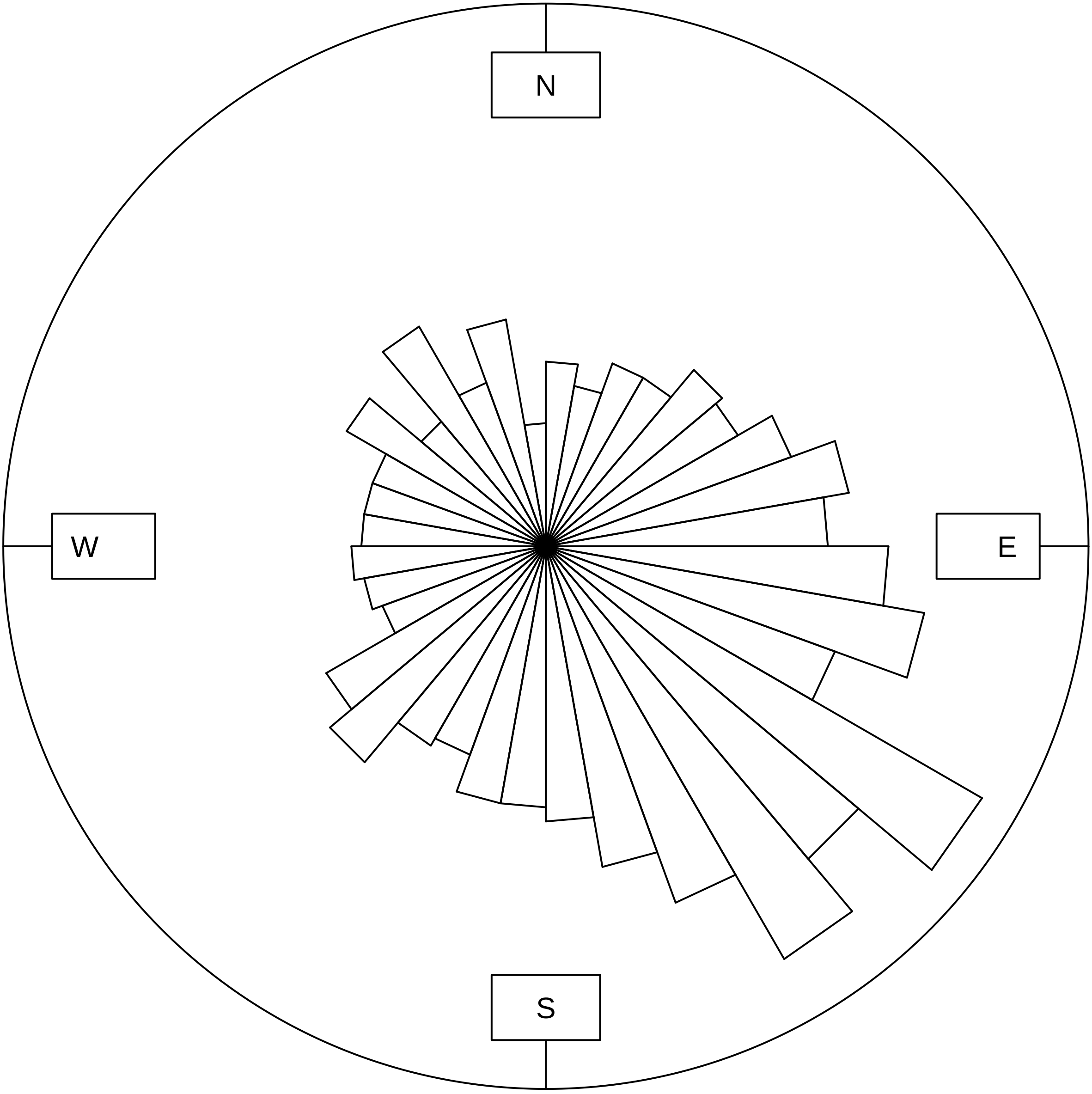

bootstrap 265

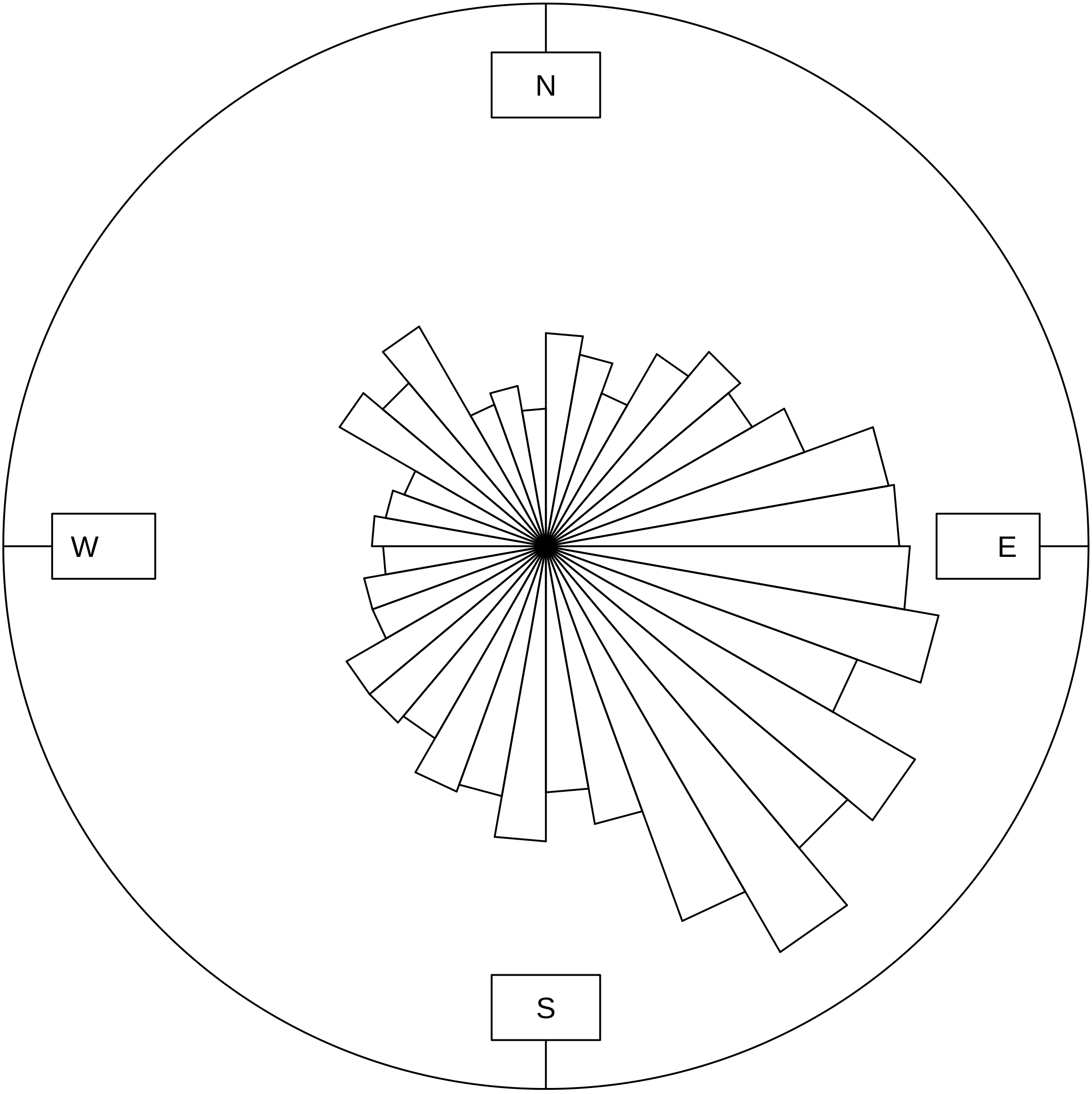

bootstrap 266

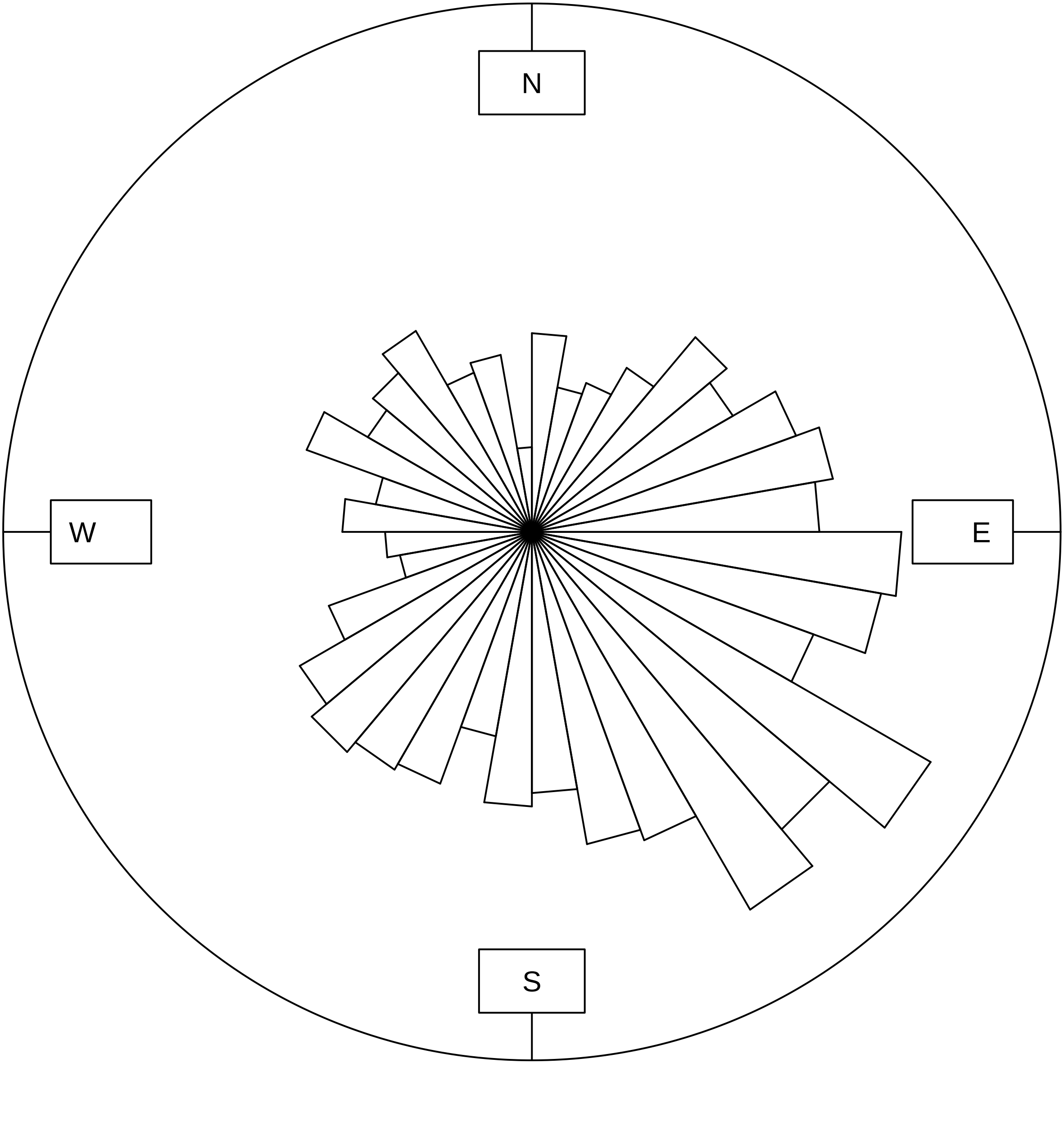

bootstrap 267

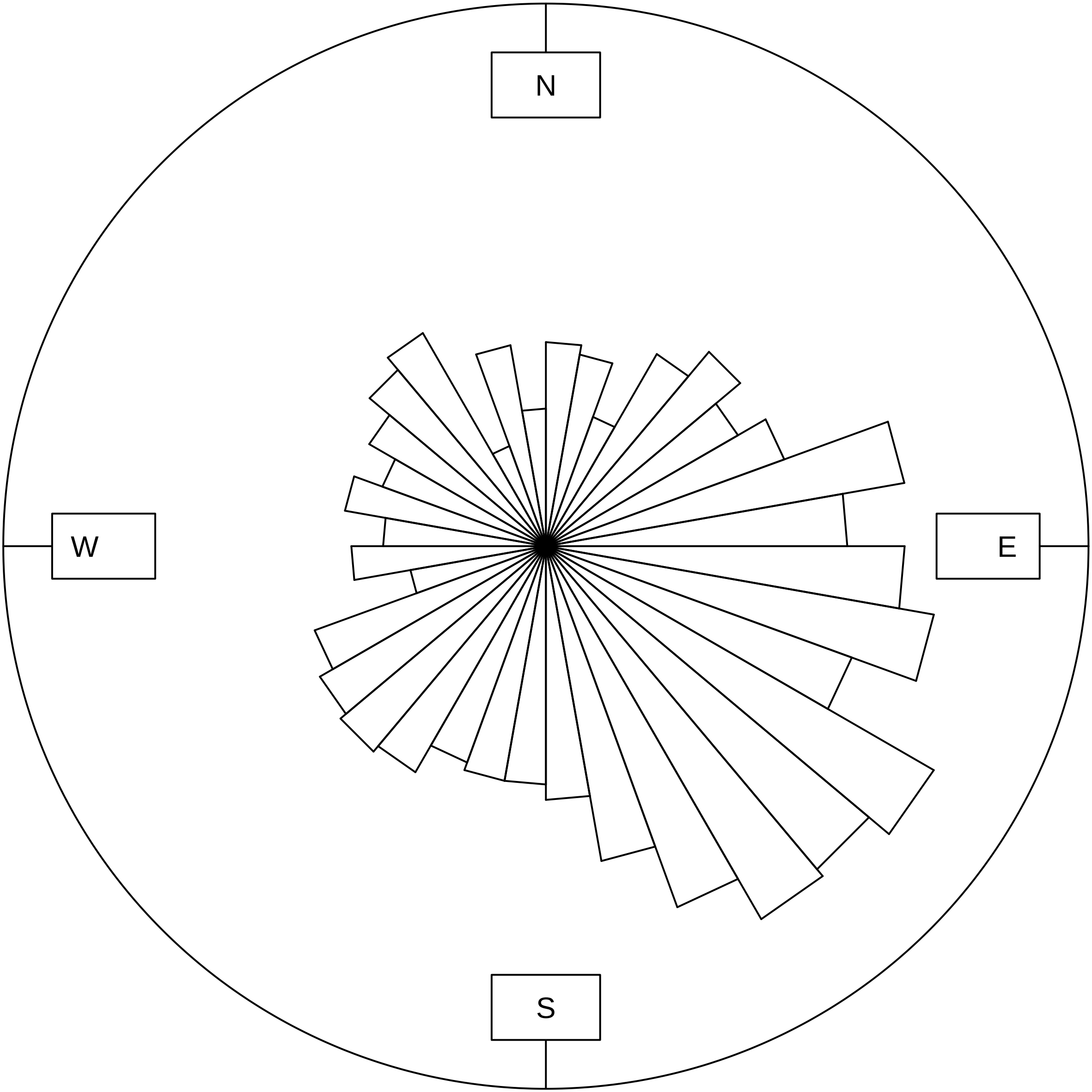

bootstrap 268

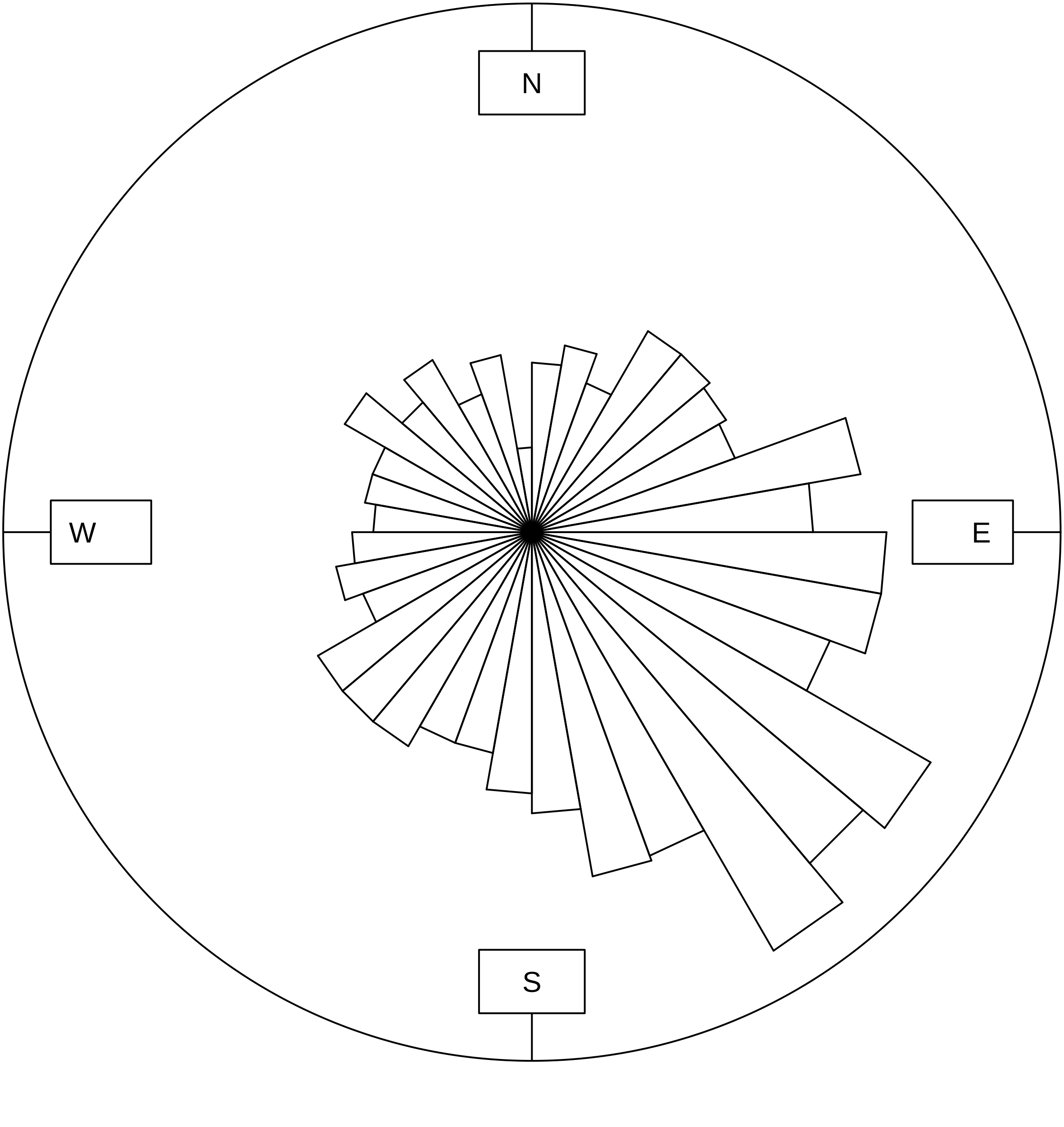

bootstrap 269

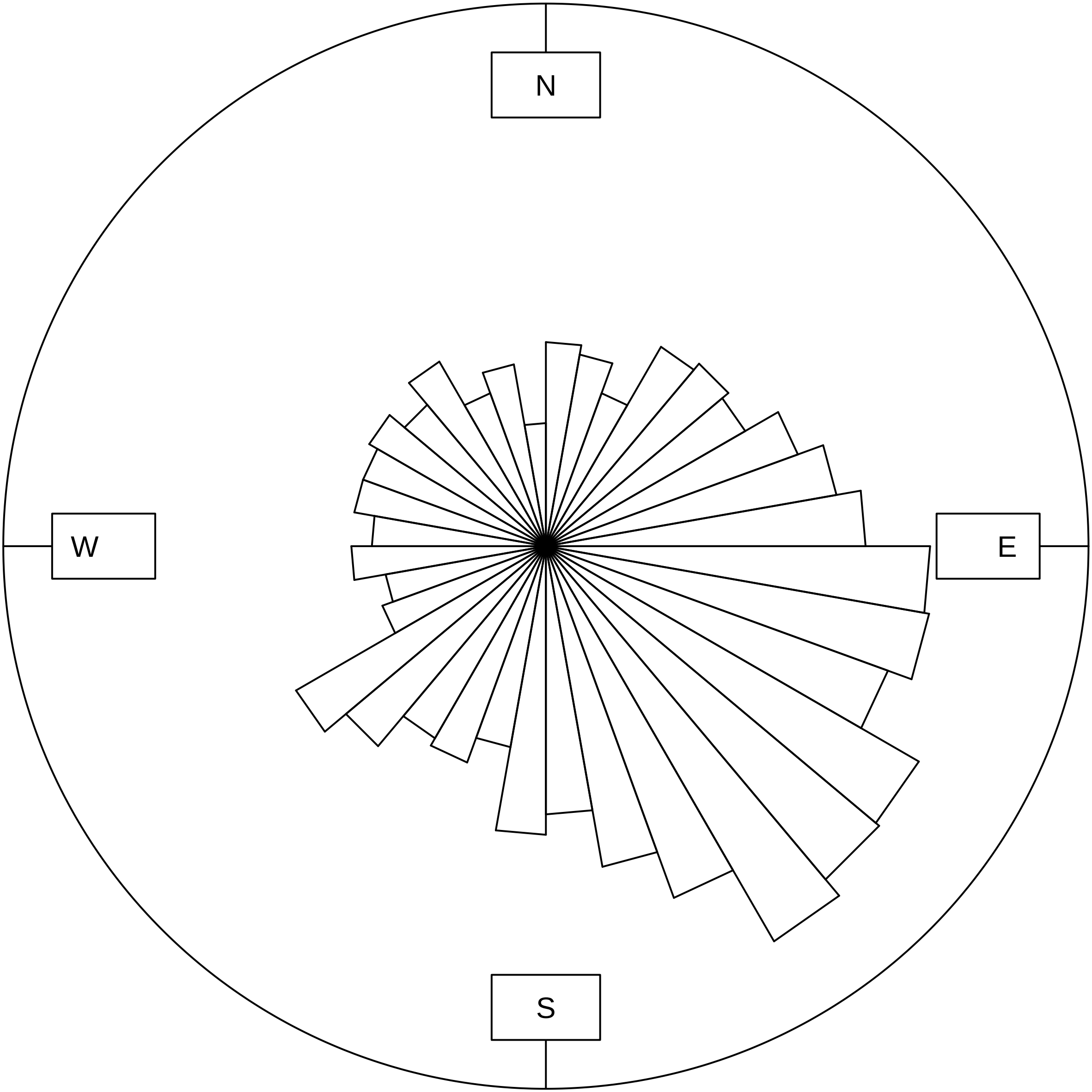

bootstrap 270

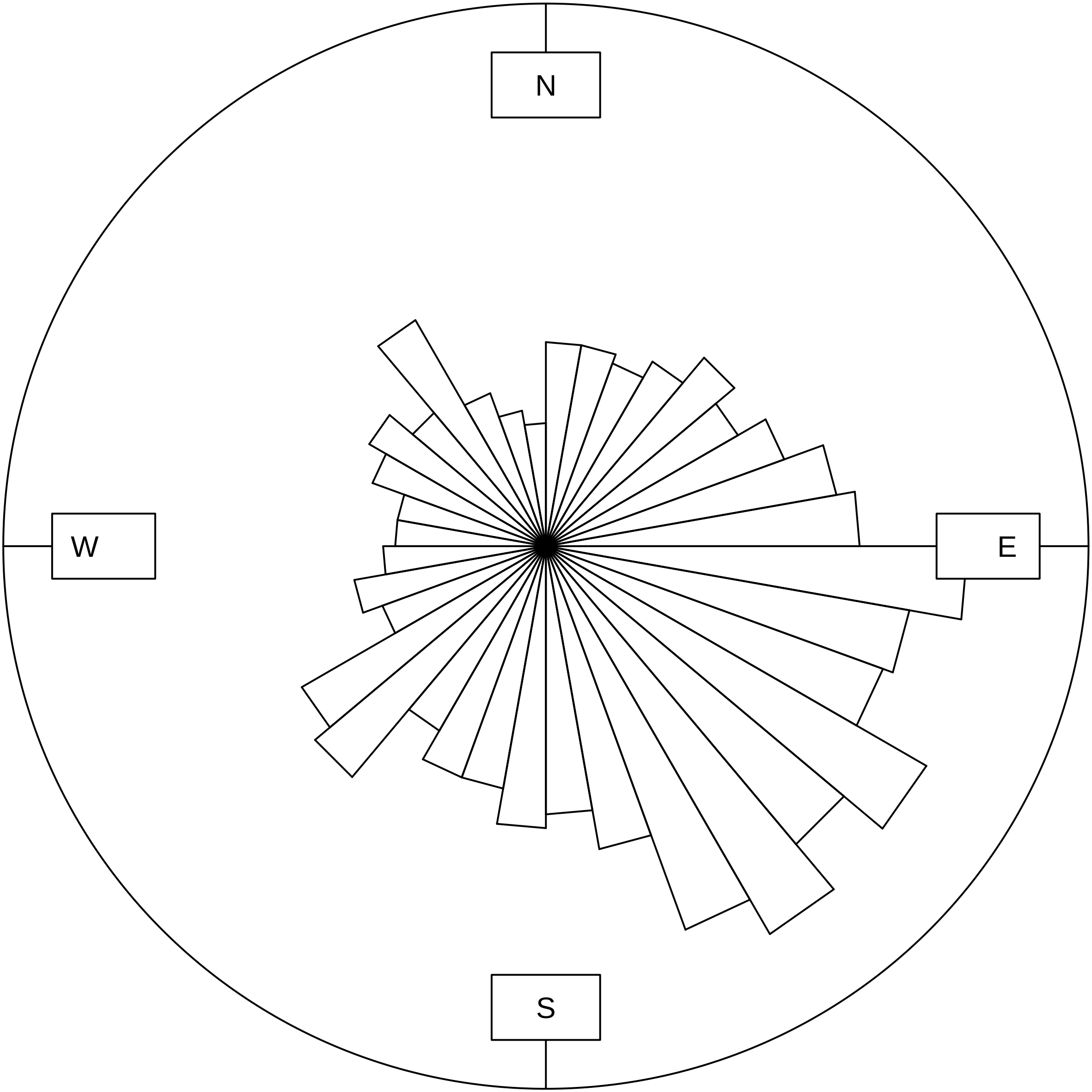

bootstrap 271

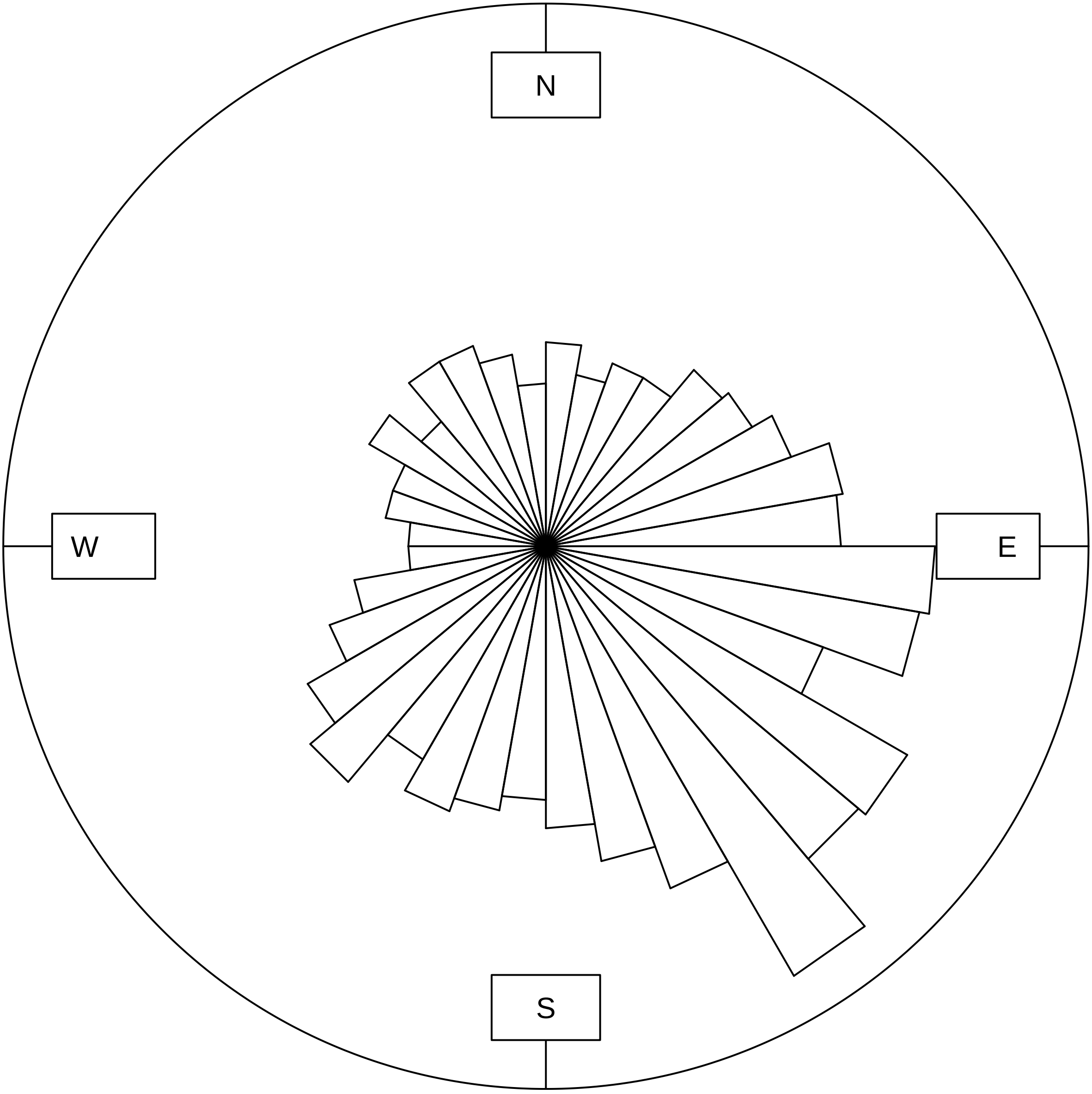

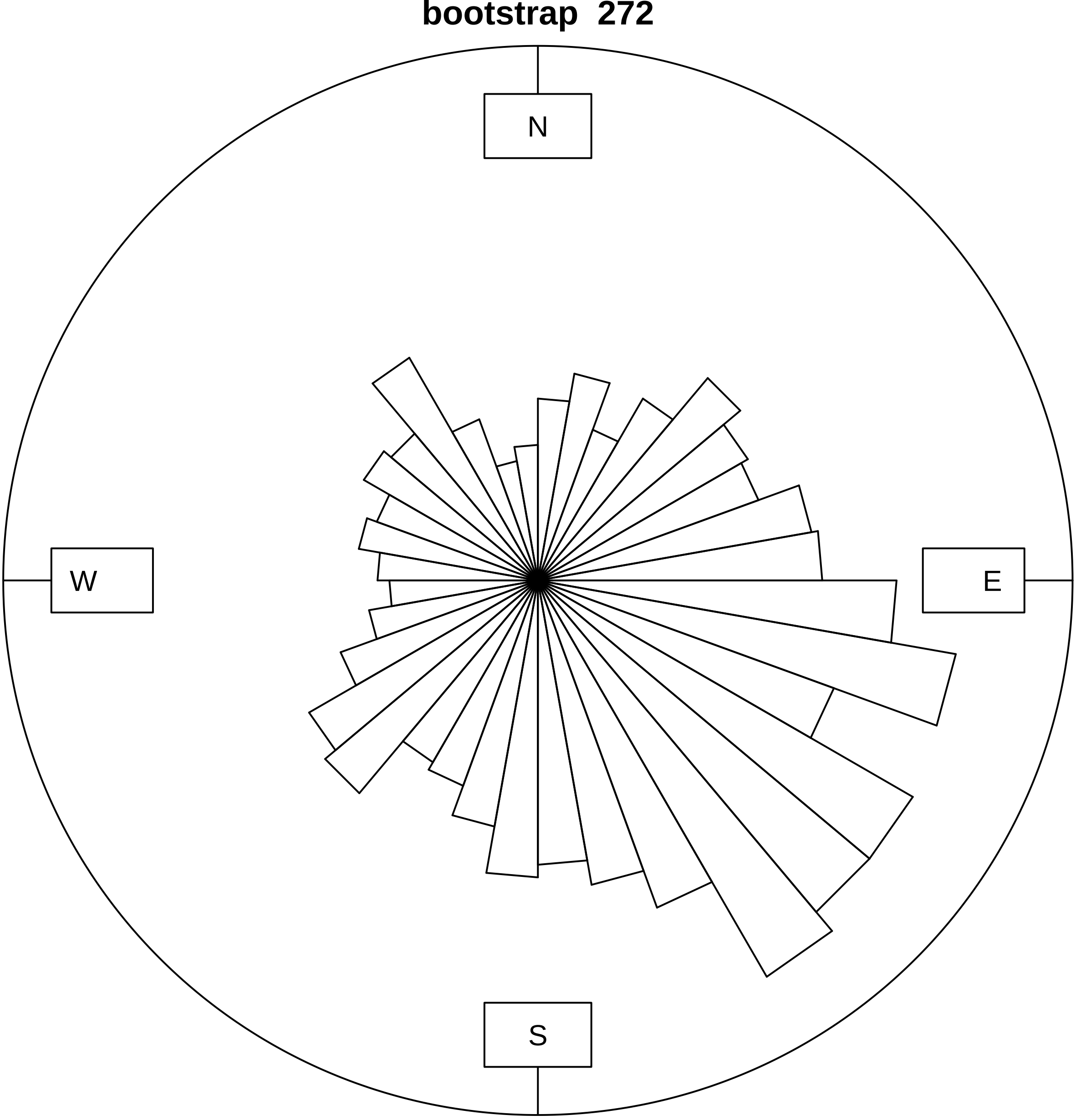
bootstrap 272
N
W
E
S

bootstrap 273

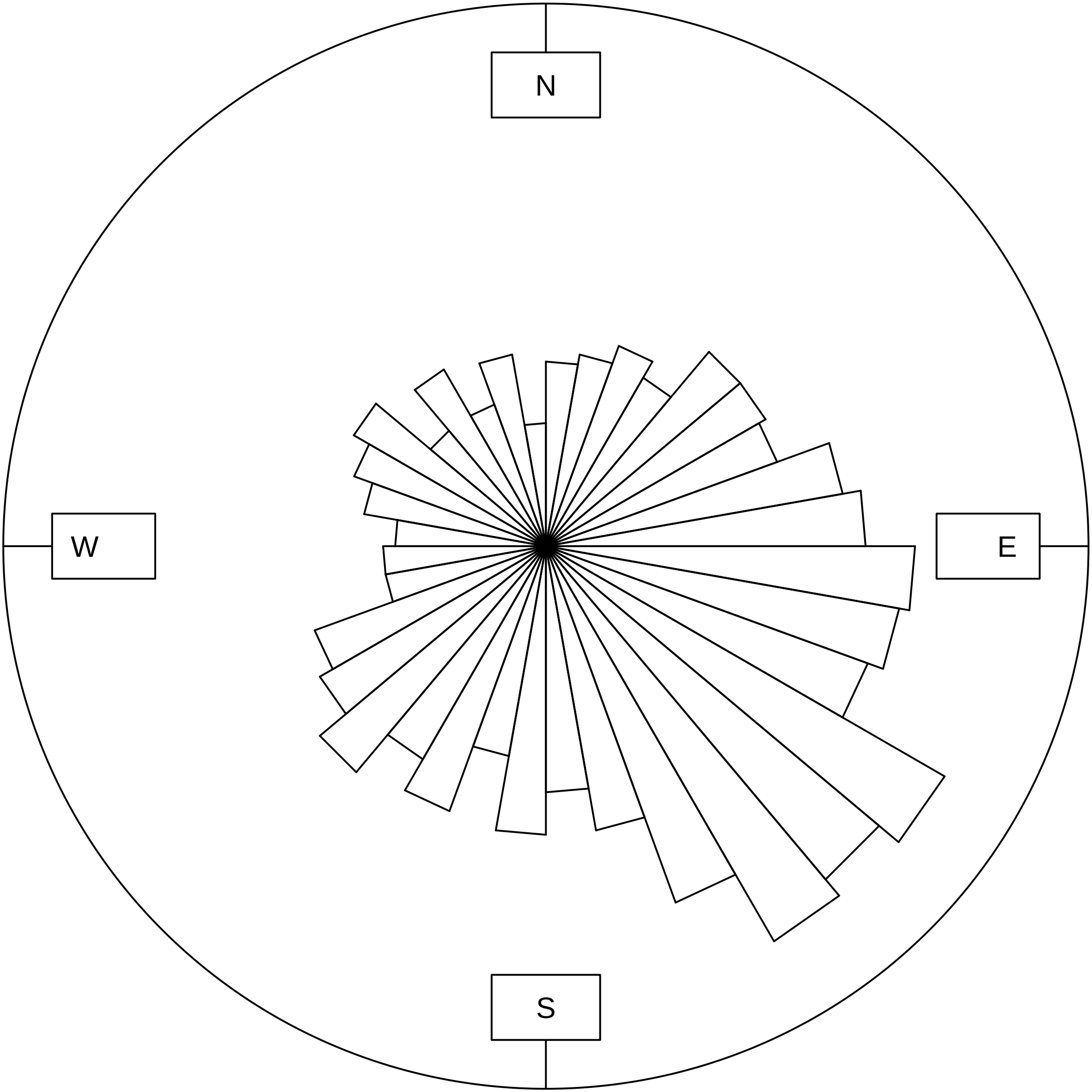

bootstrap 274

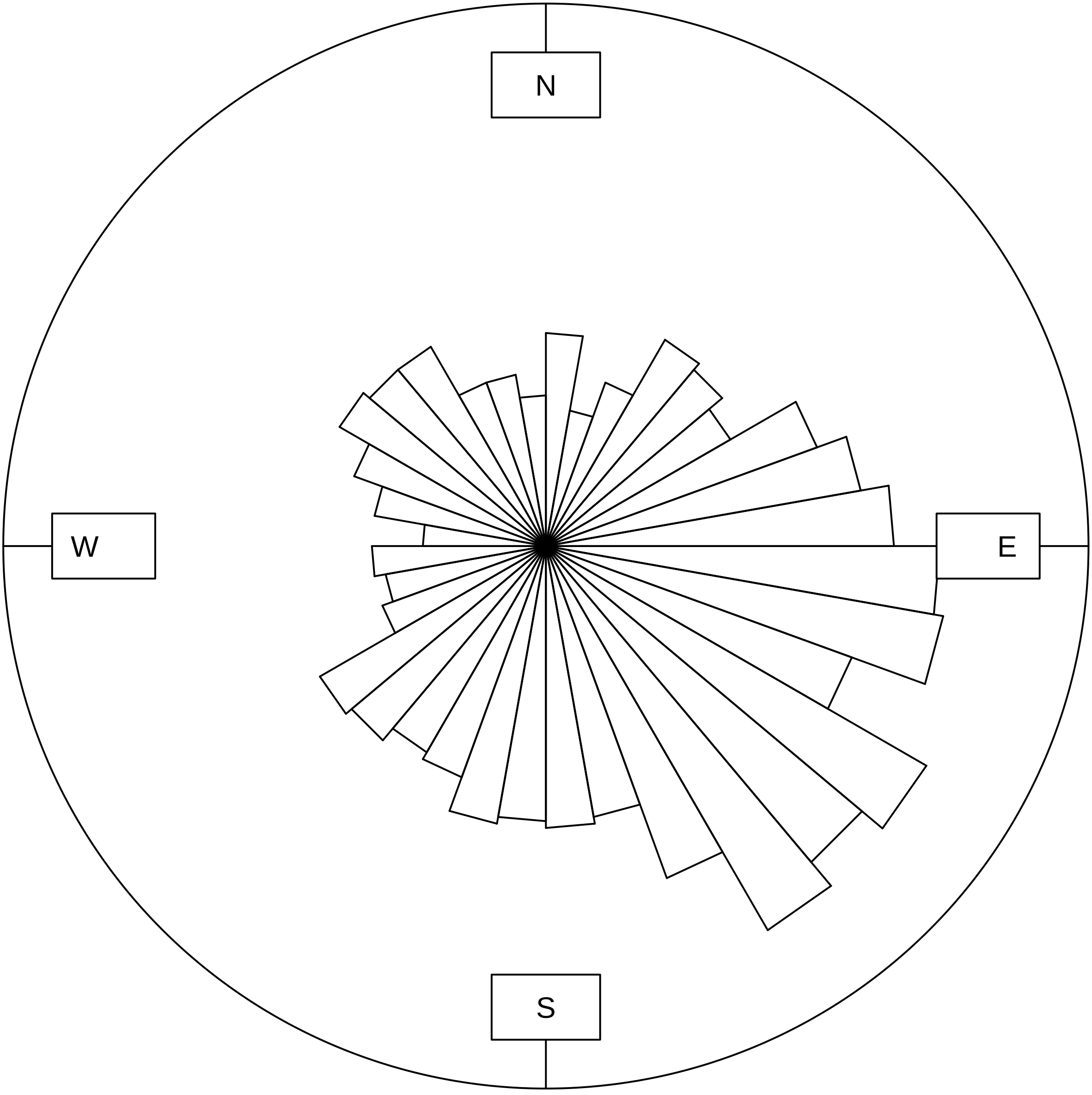

bootstrap 275

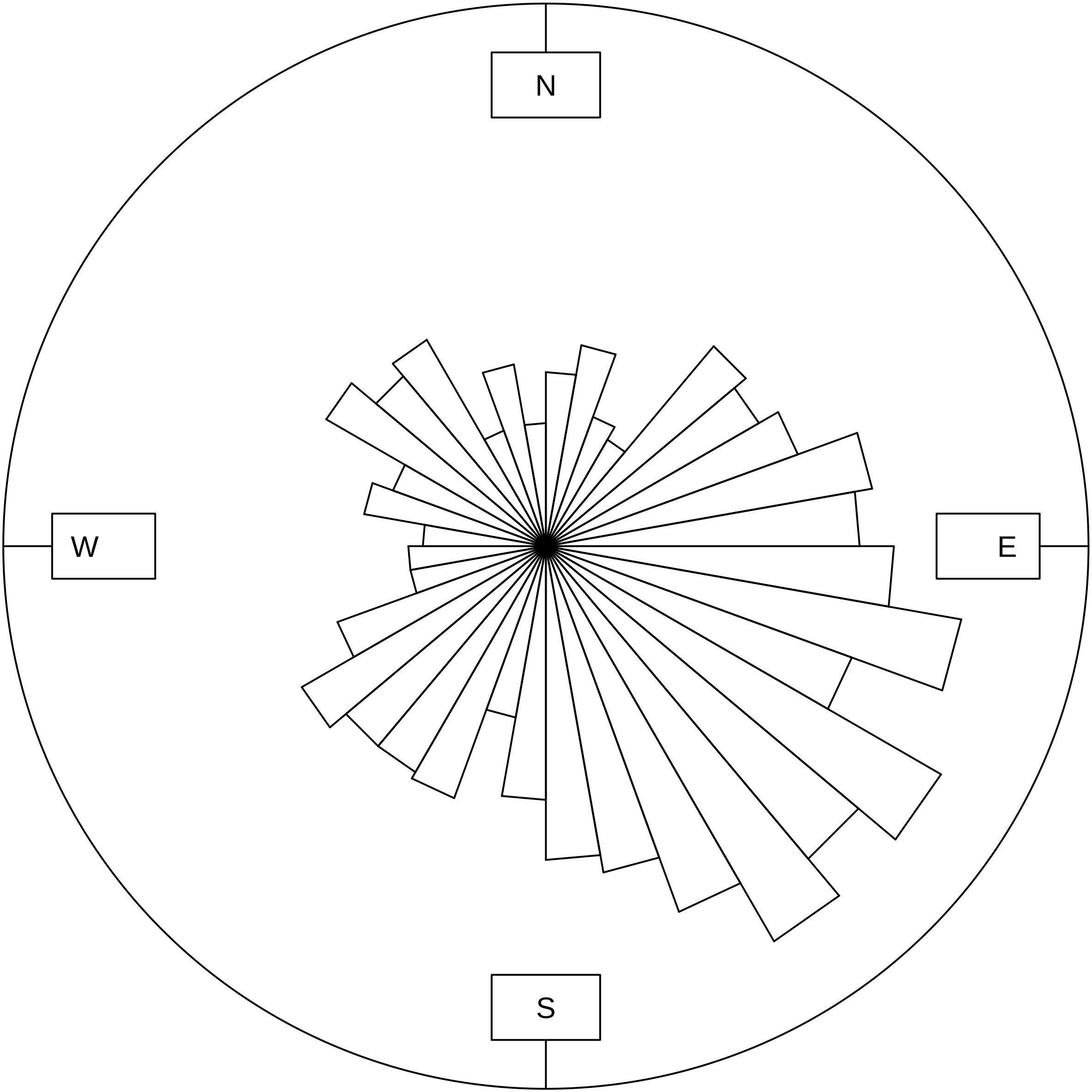

bootstrap 276

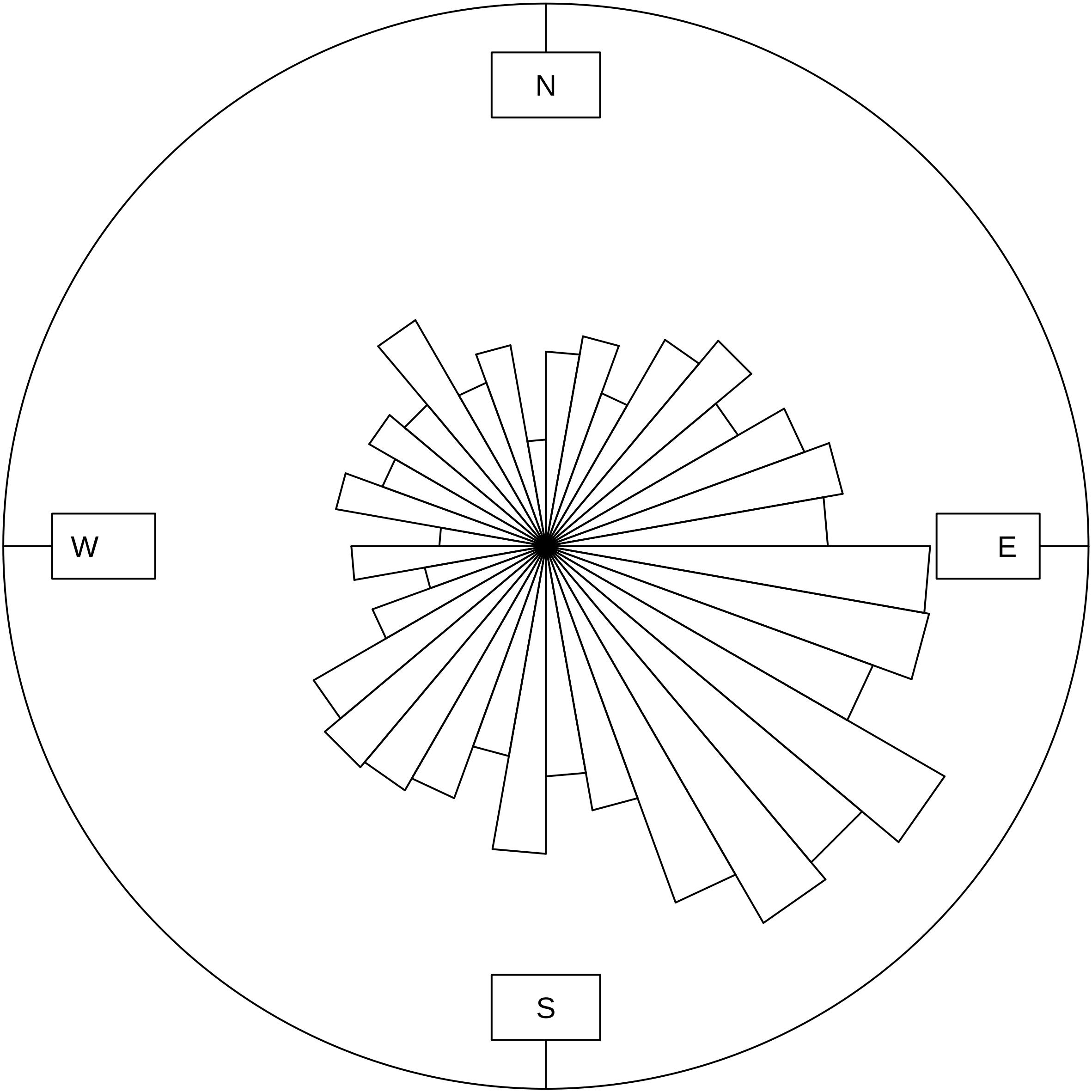

bootstrap 277

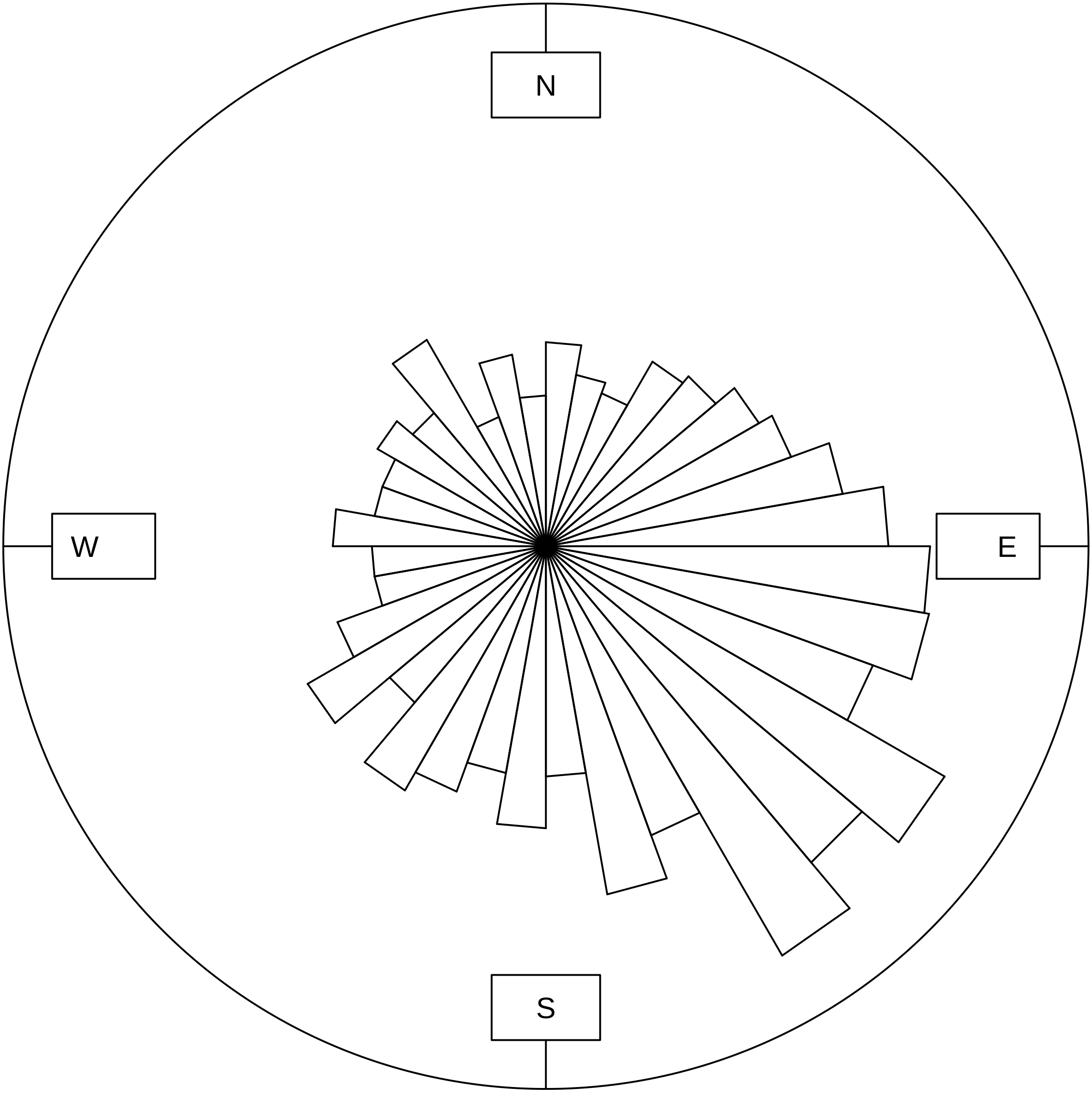

bootstrap 278

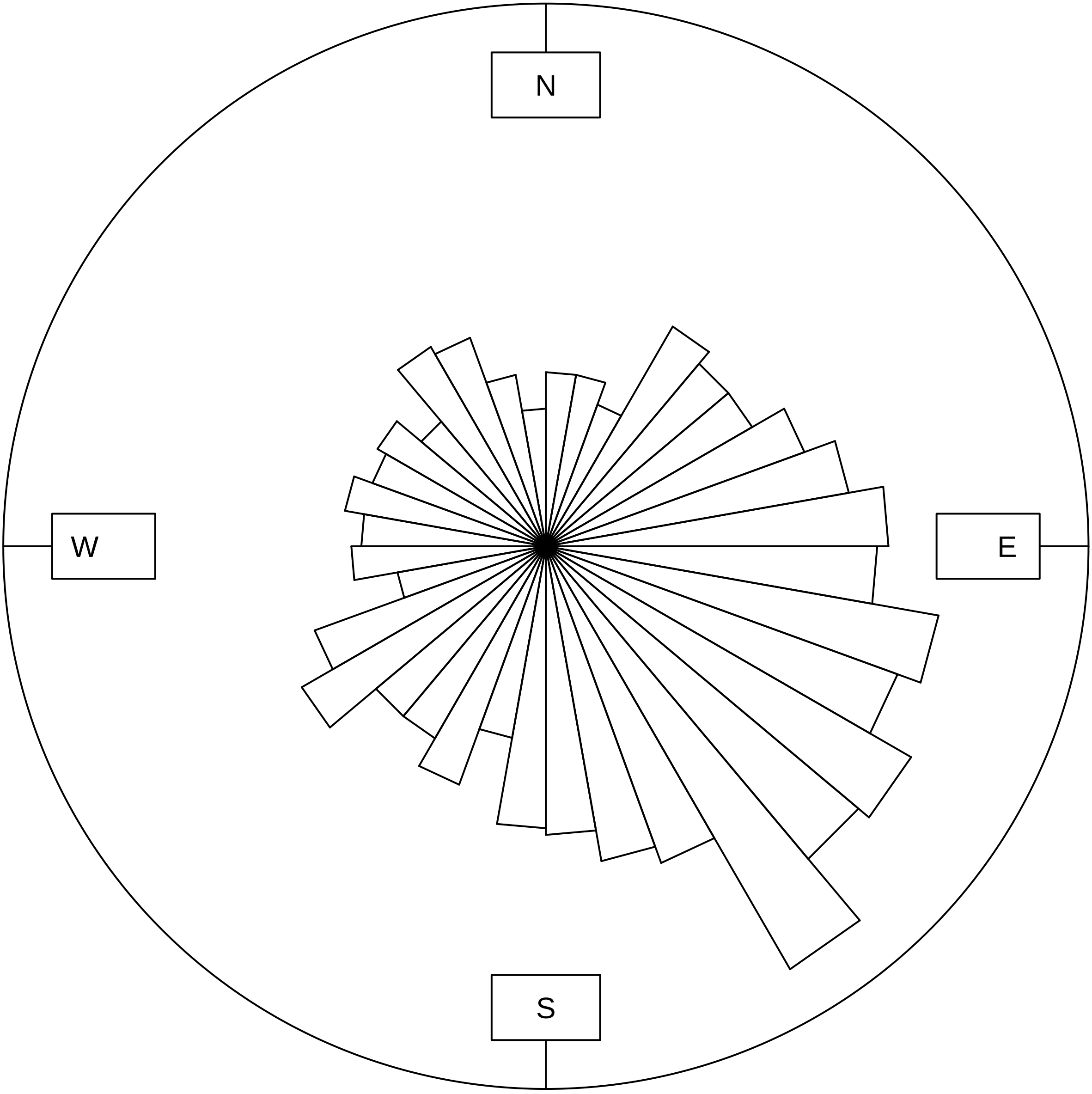

bootstrap 279

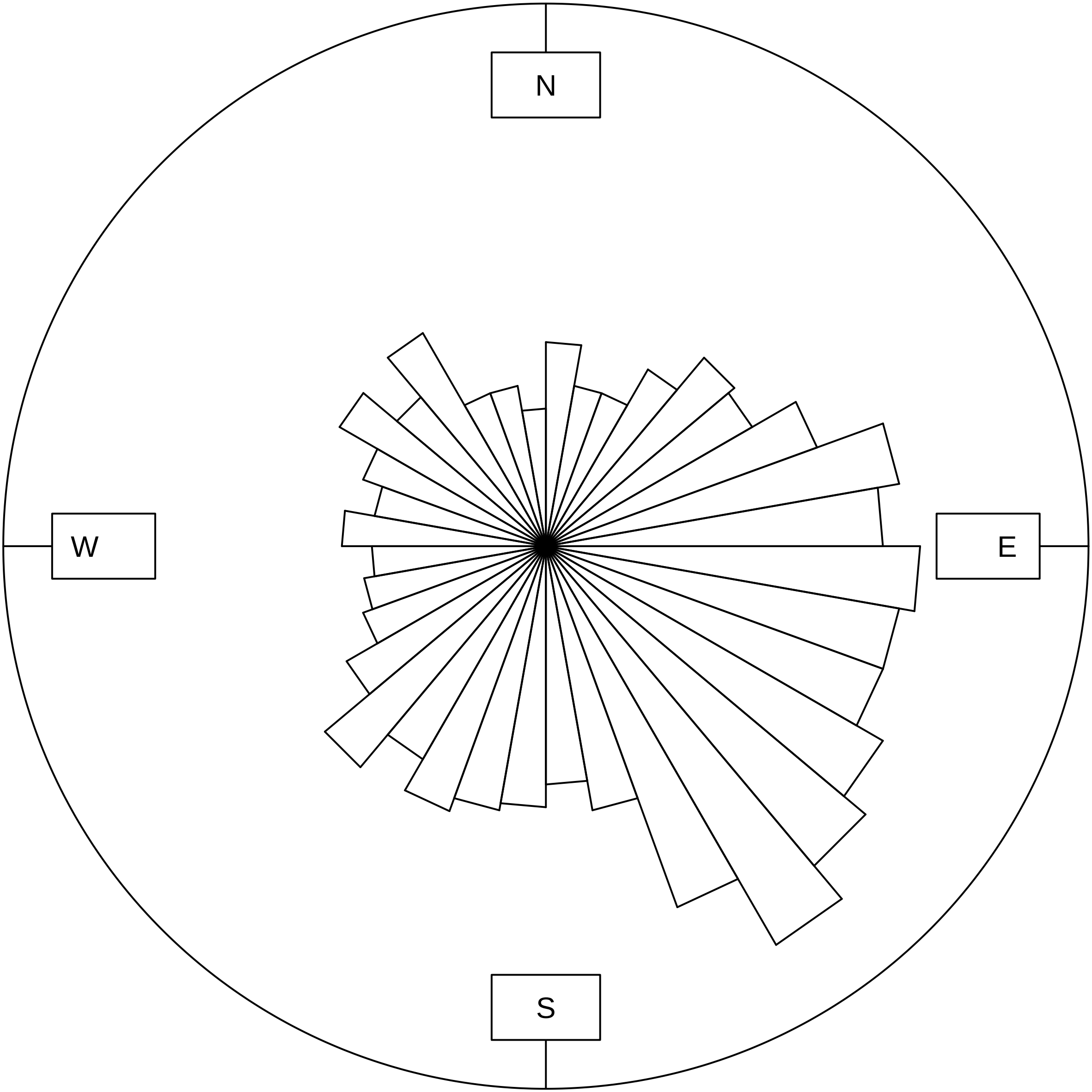

bootstrap 280

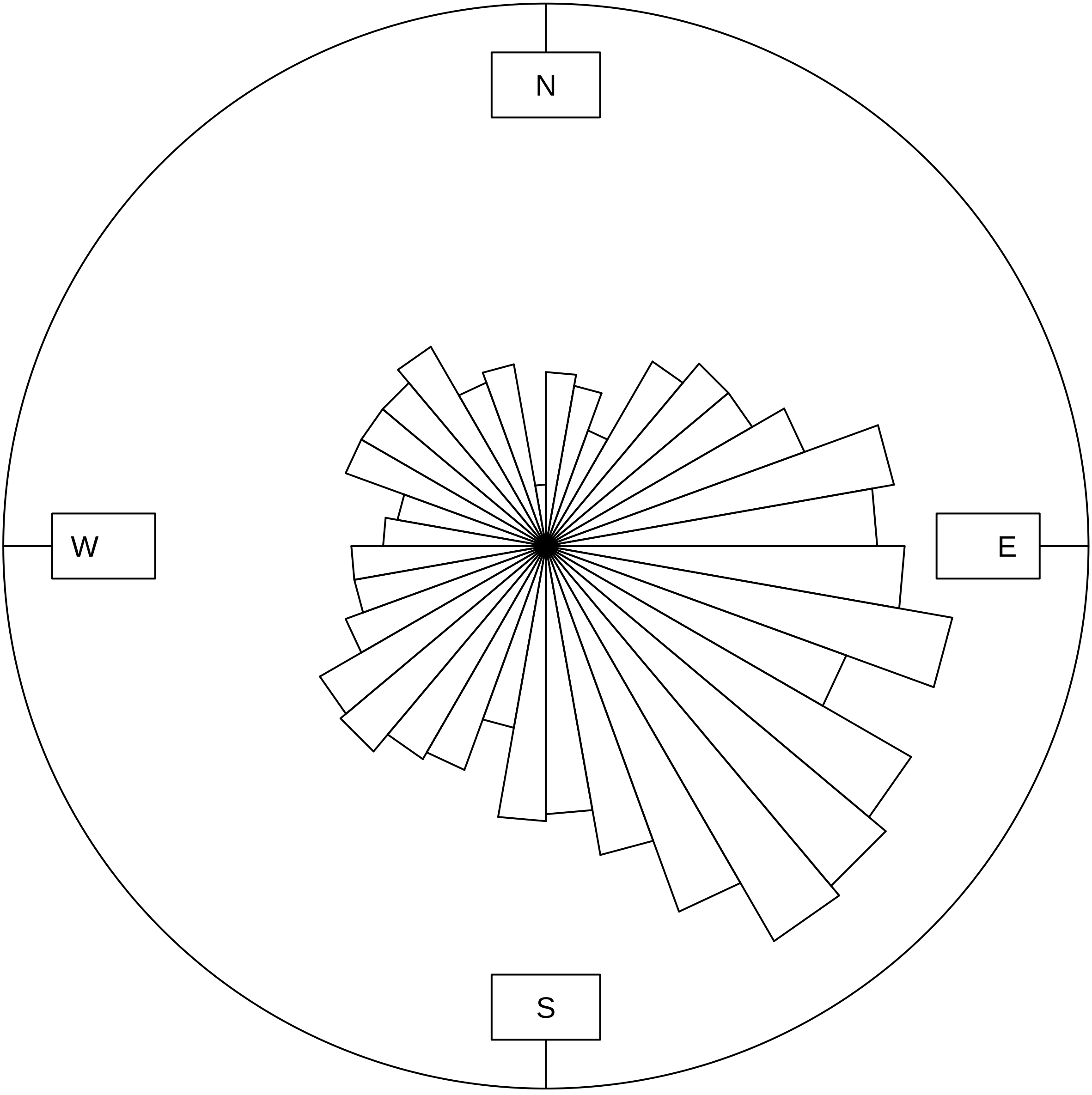

bootstrap 281

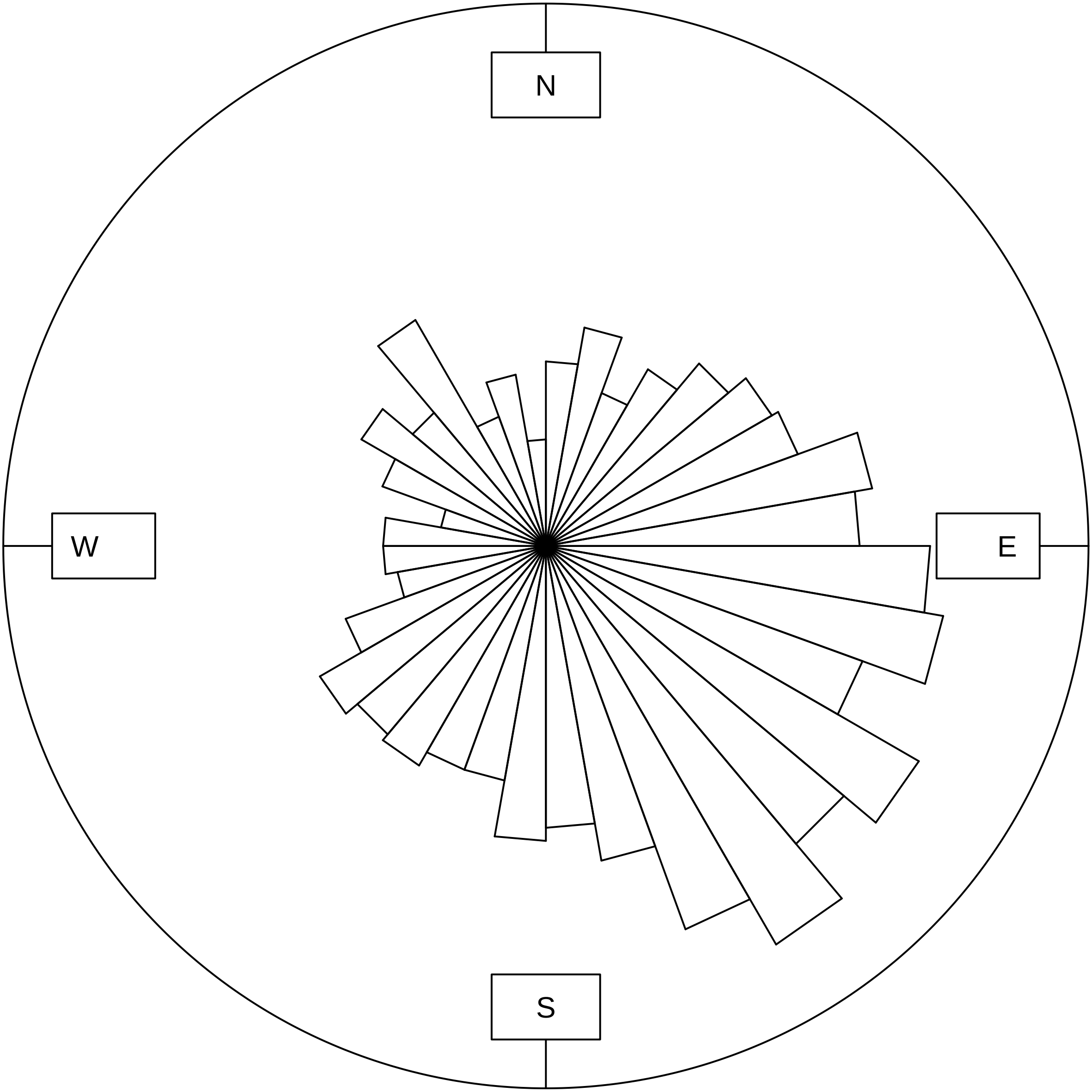

bootstrap 282

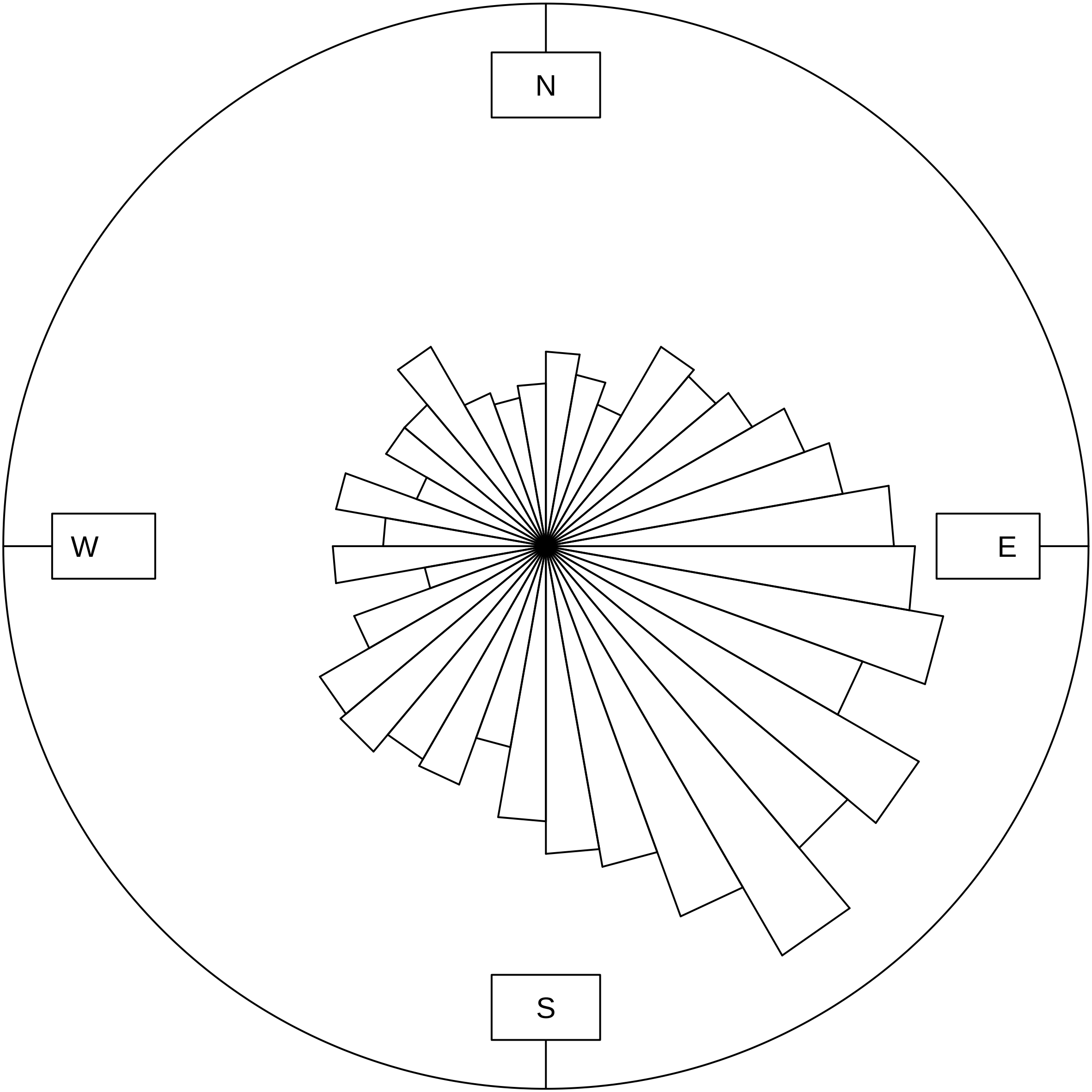

bootstrap 283

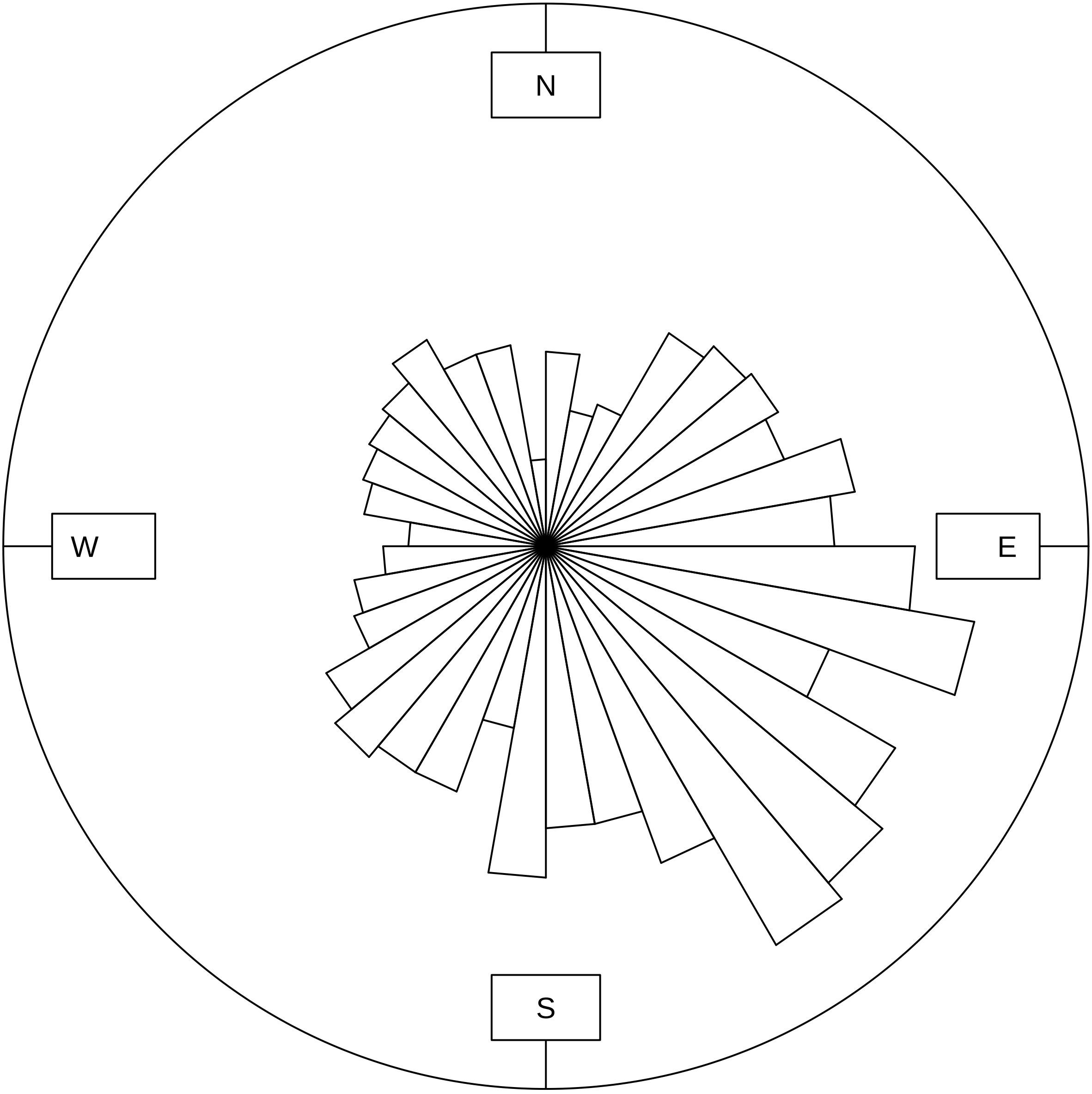

bootstrap 284

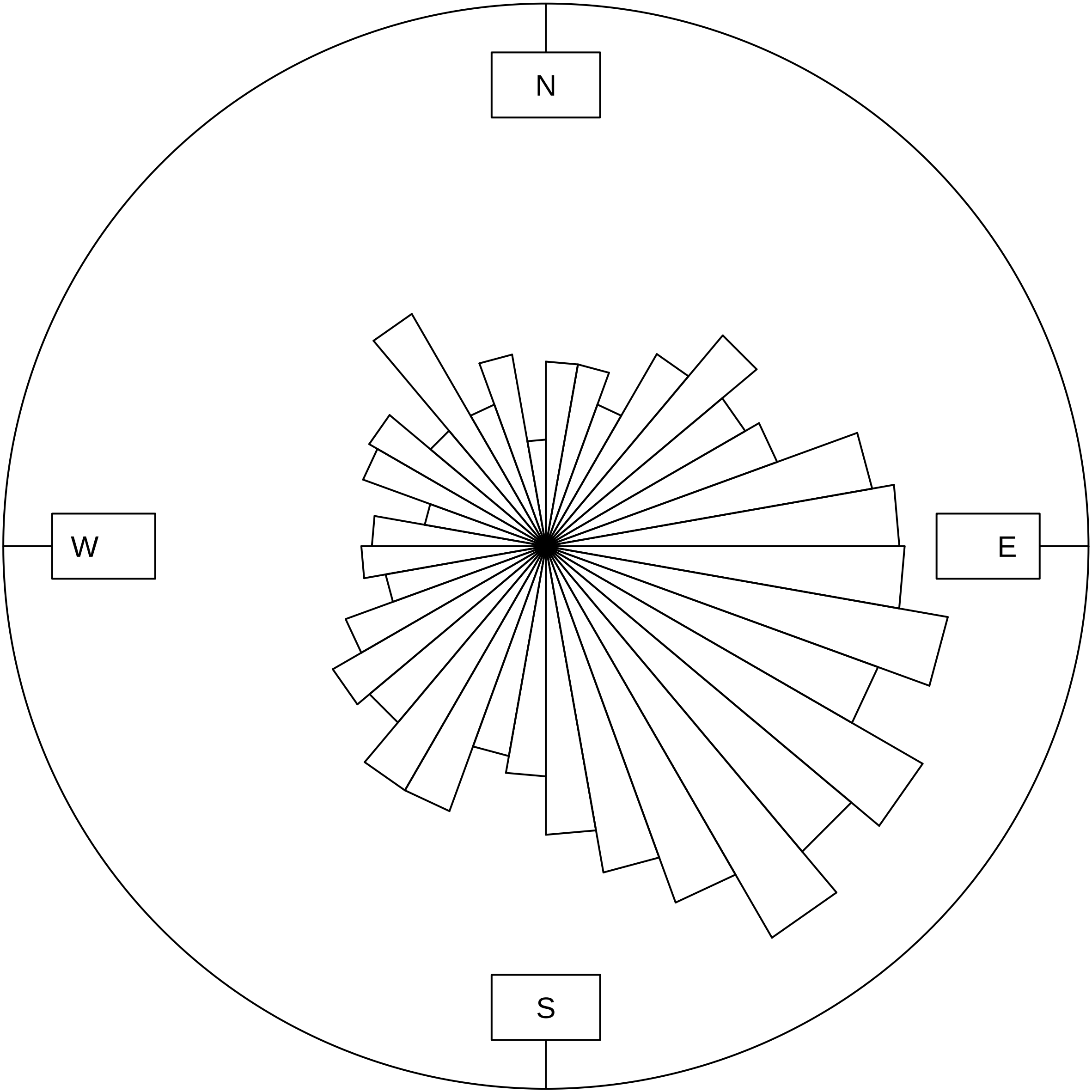

bootstrap 285

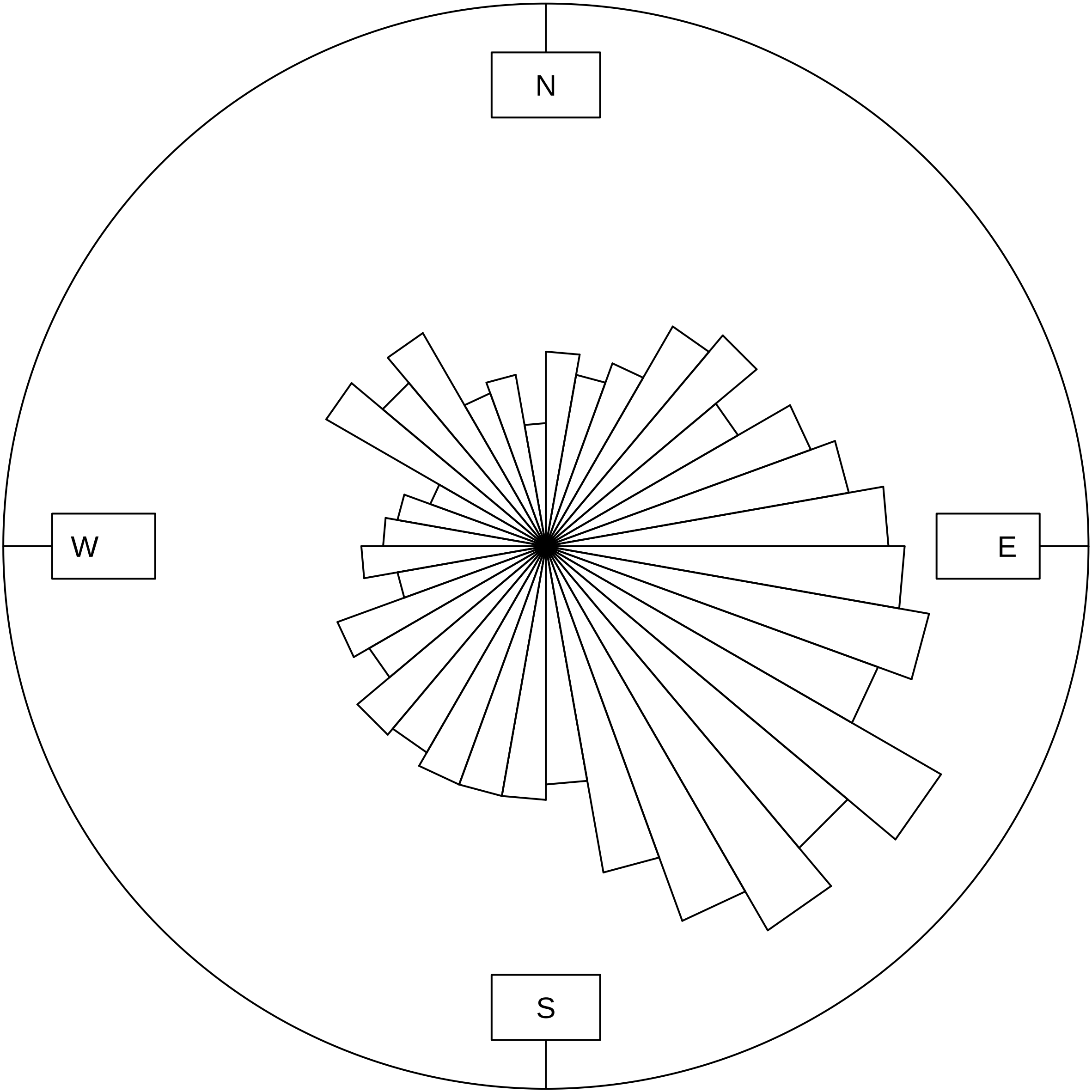

bootstrap 286

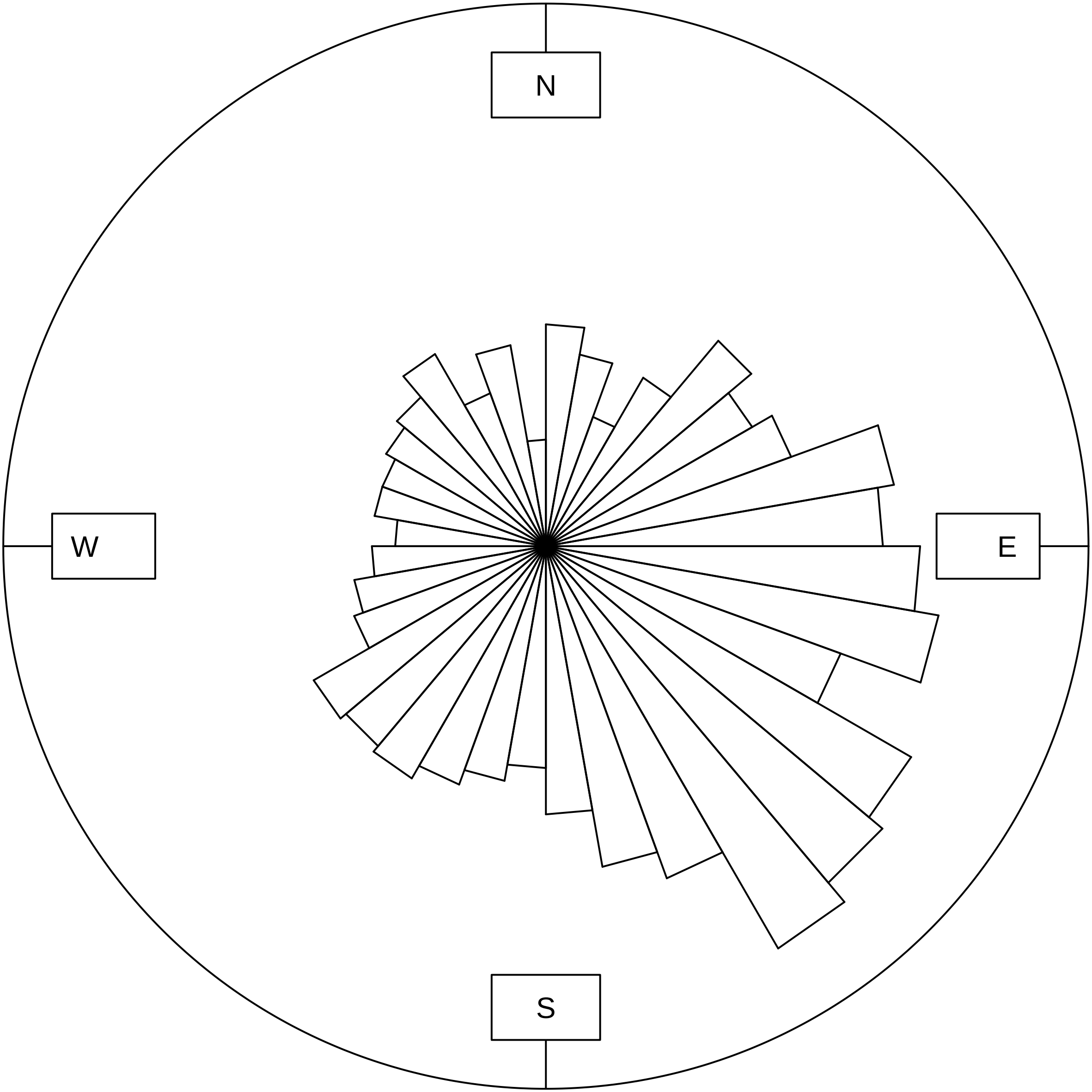

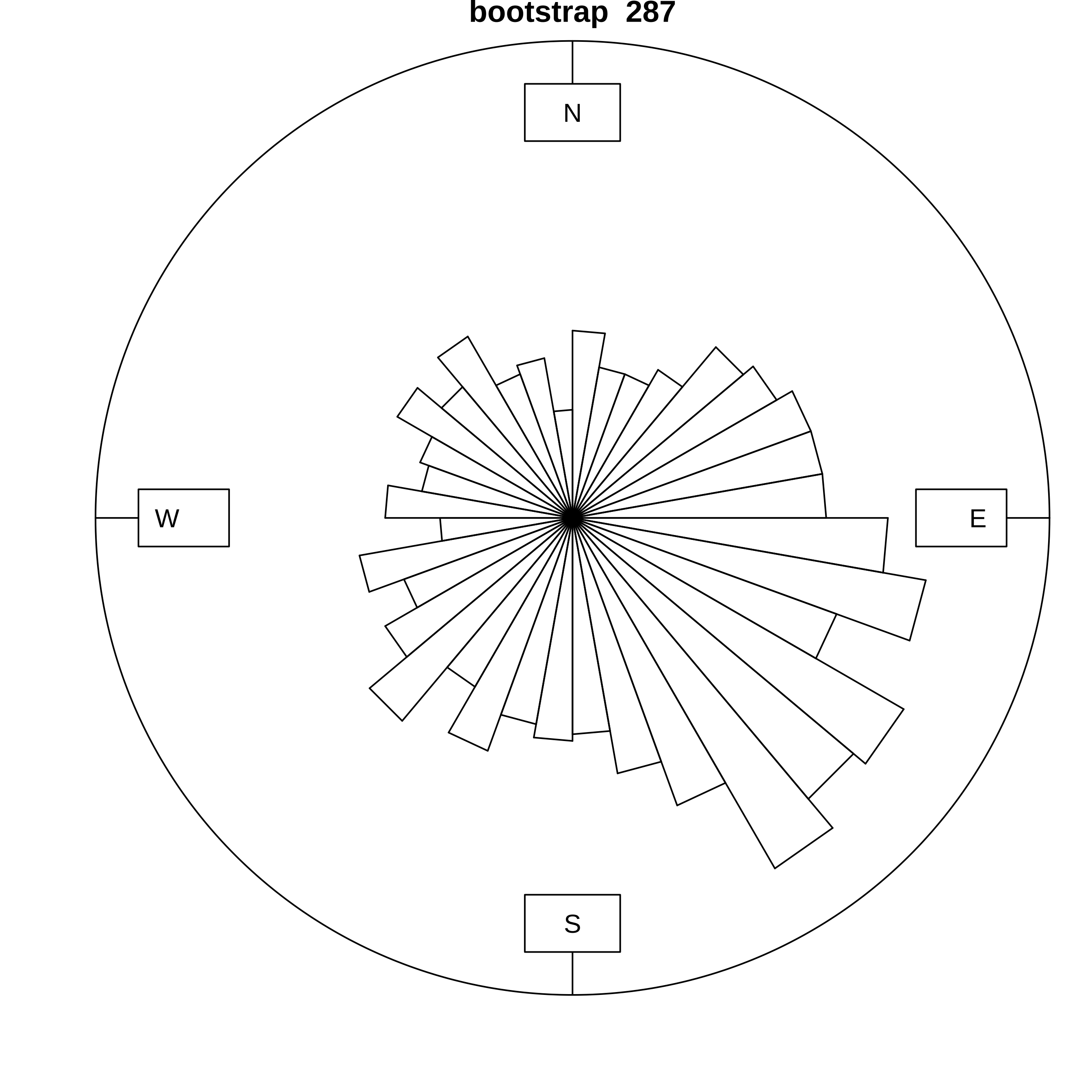
bootstrap 287
N
W
E
S

bootstrap 288

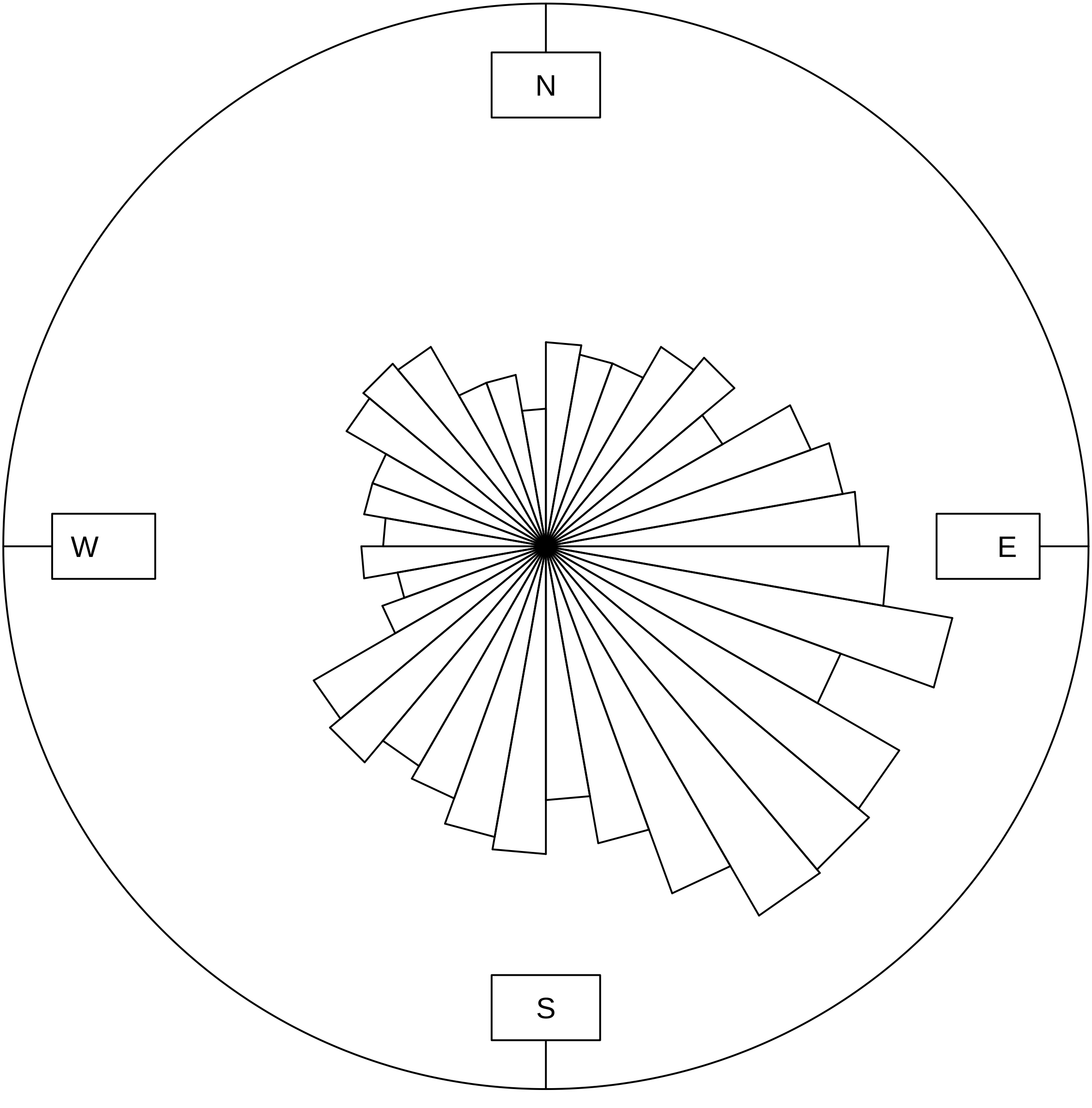

bootstrap 289

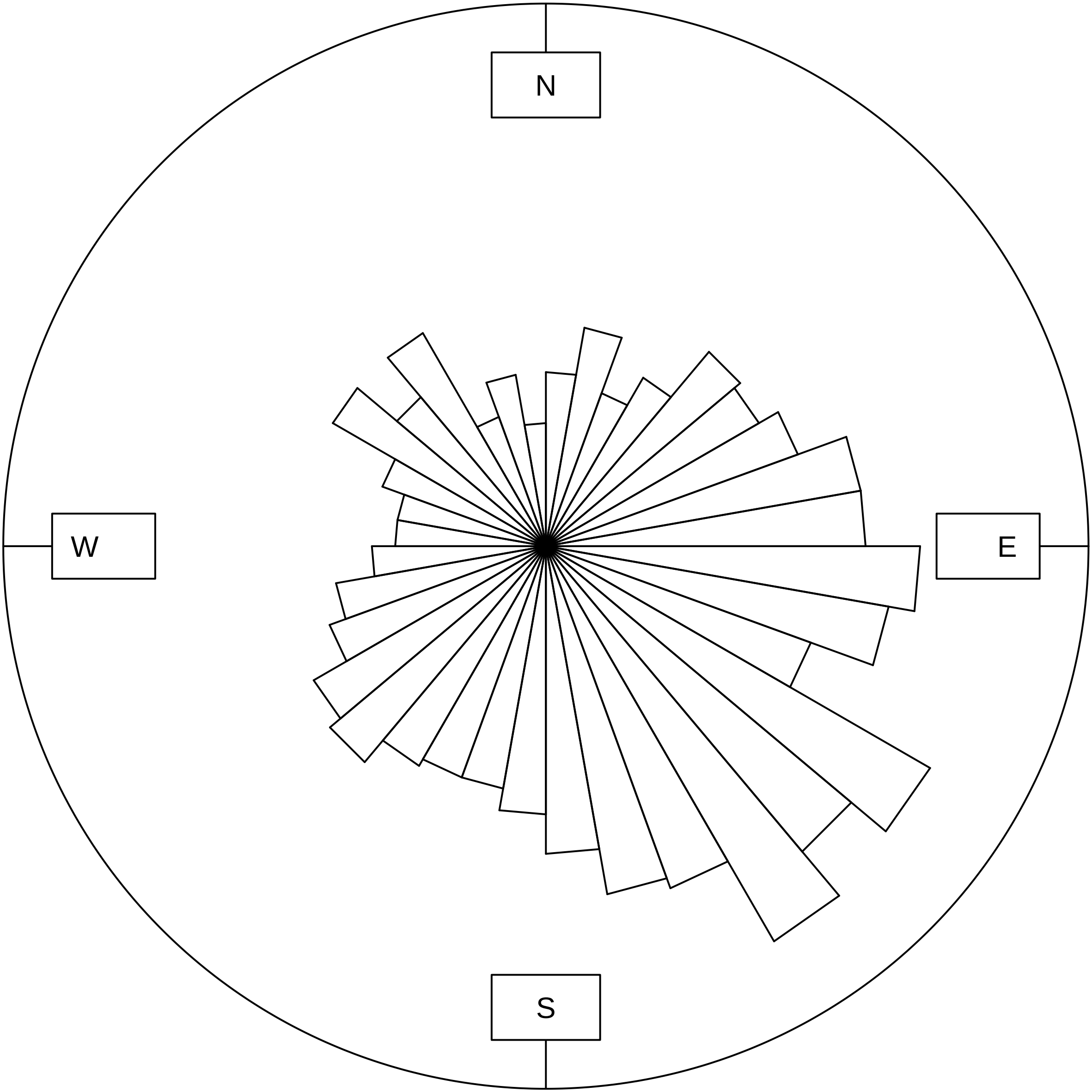

bootstrap 290

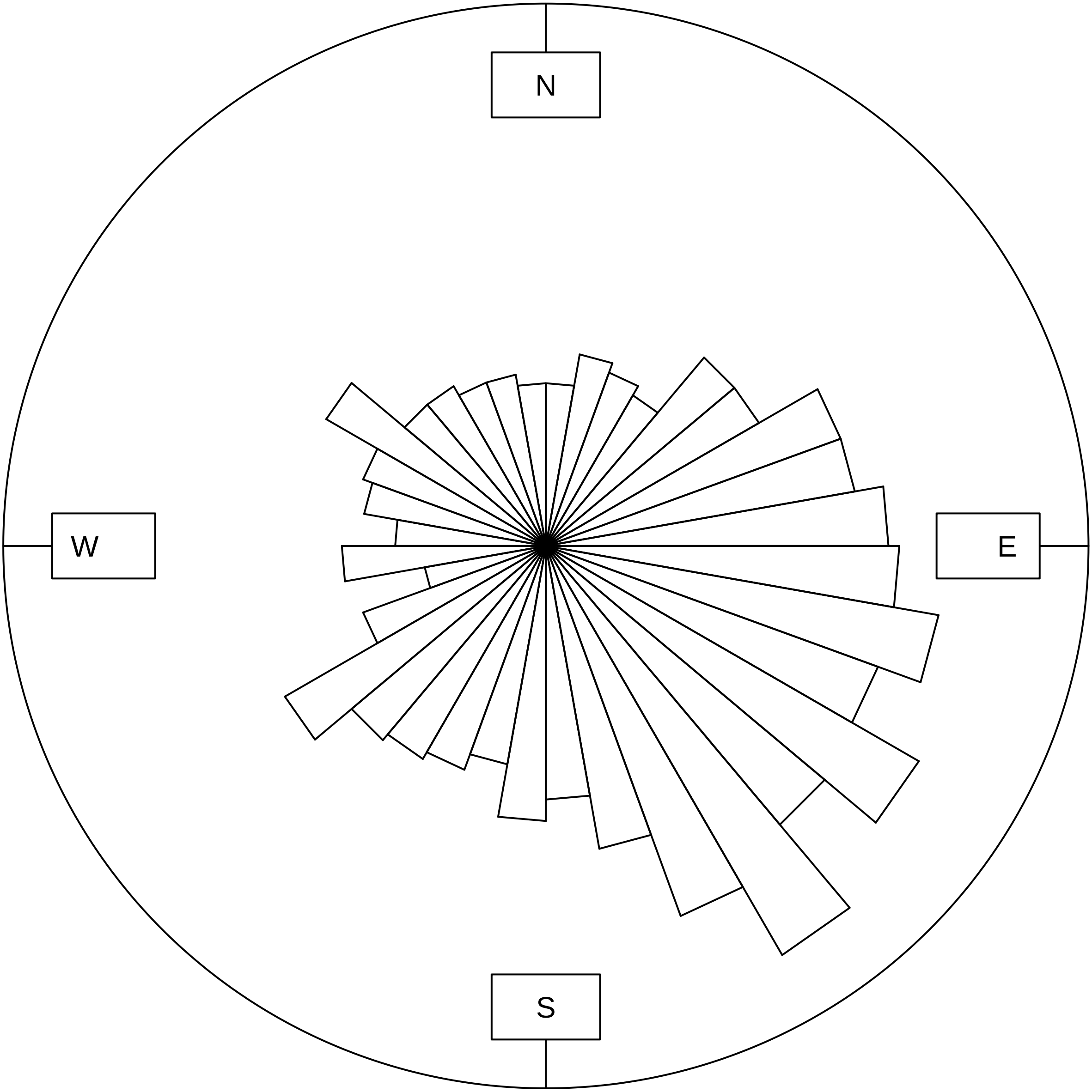

bootstrap 291

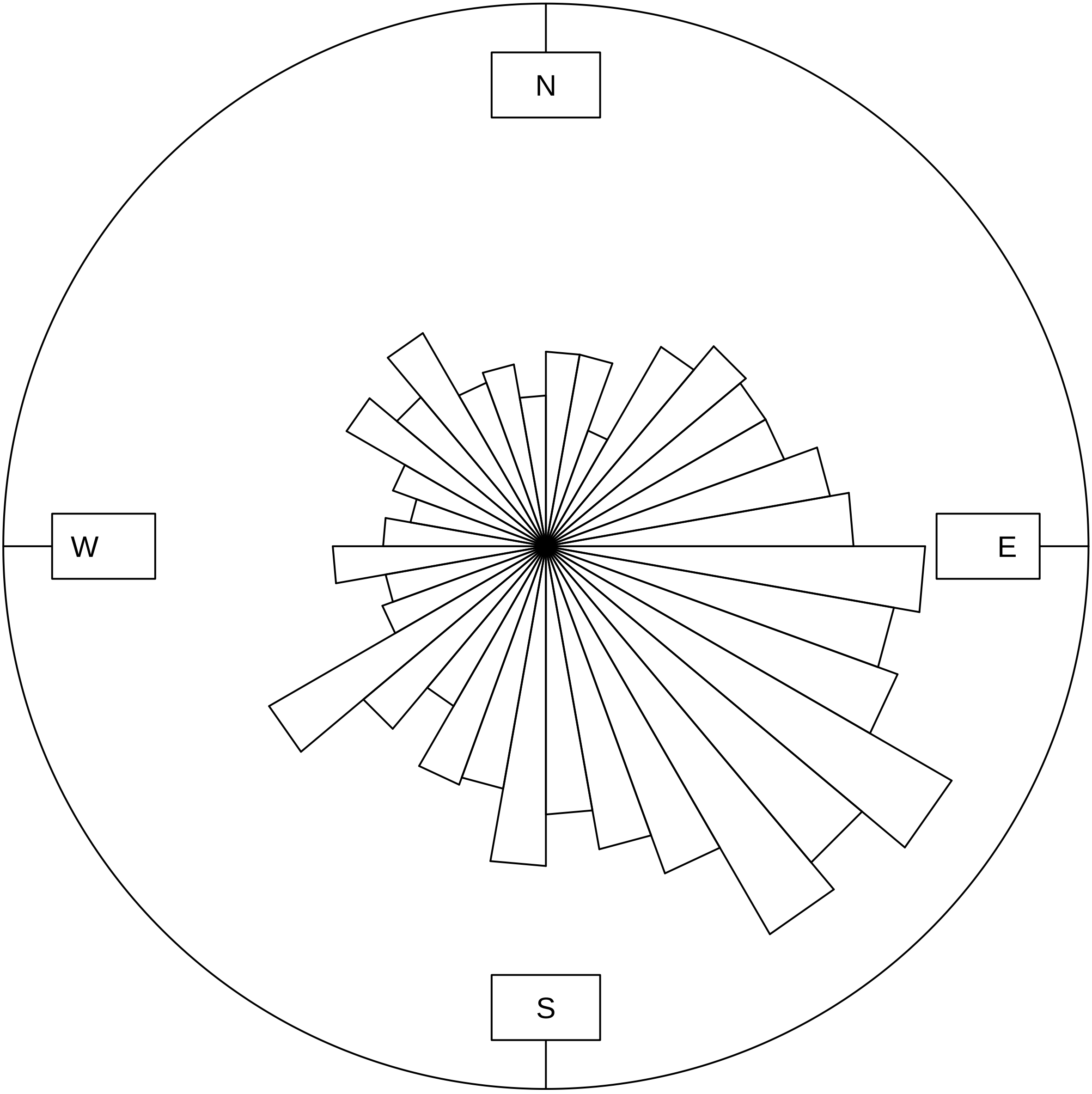

bootstrap 292

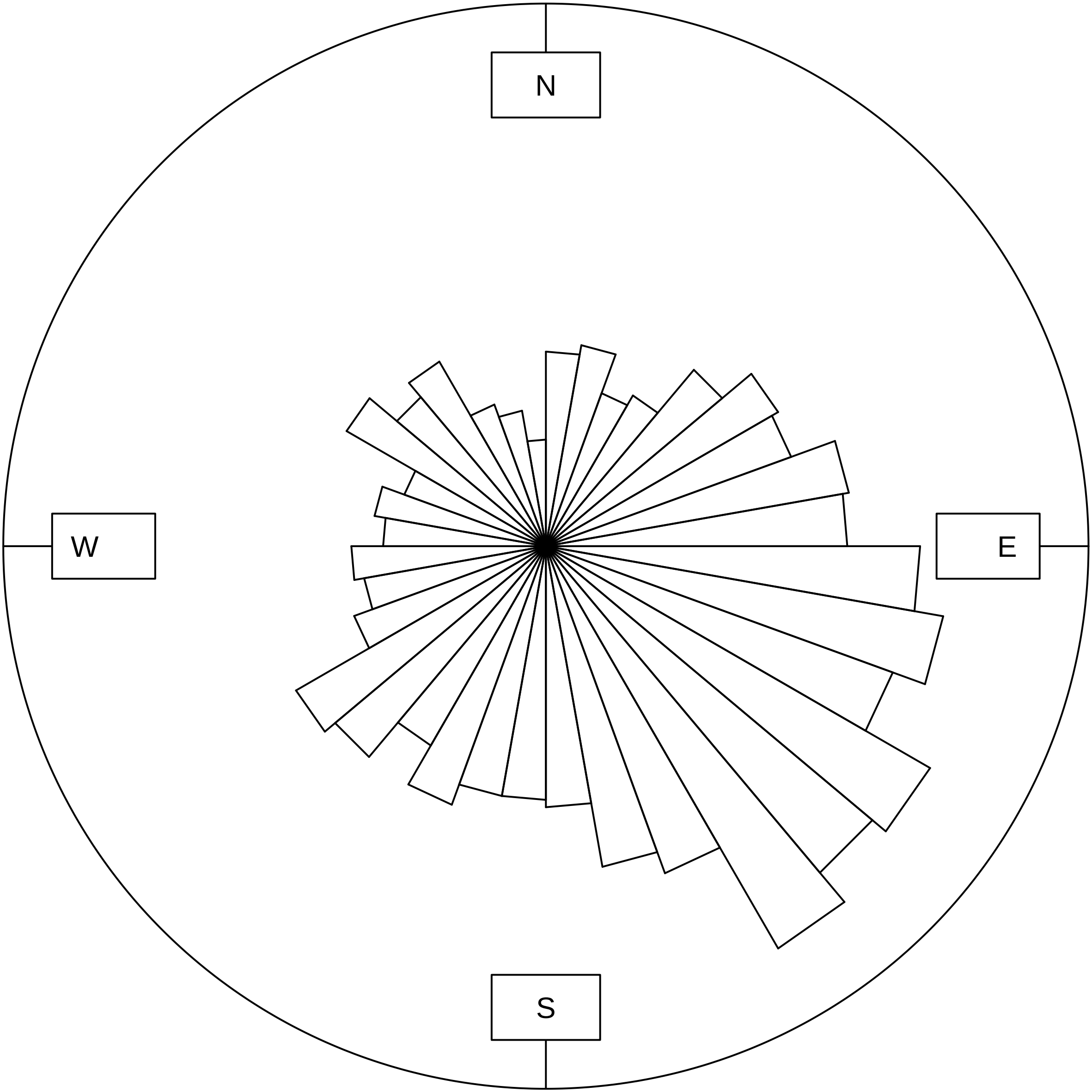

bootstrap 293

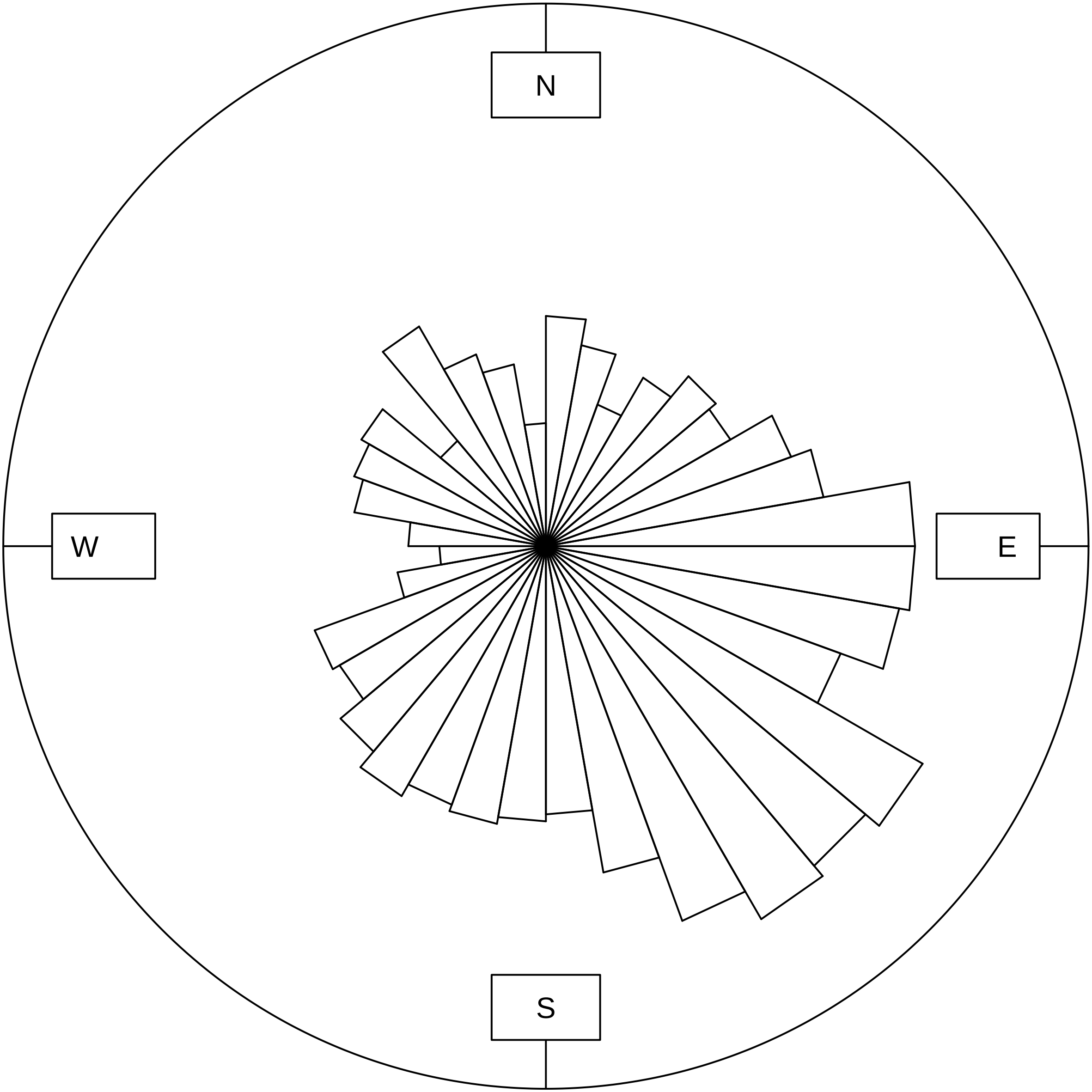

bootstrap 294

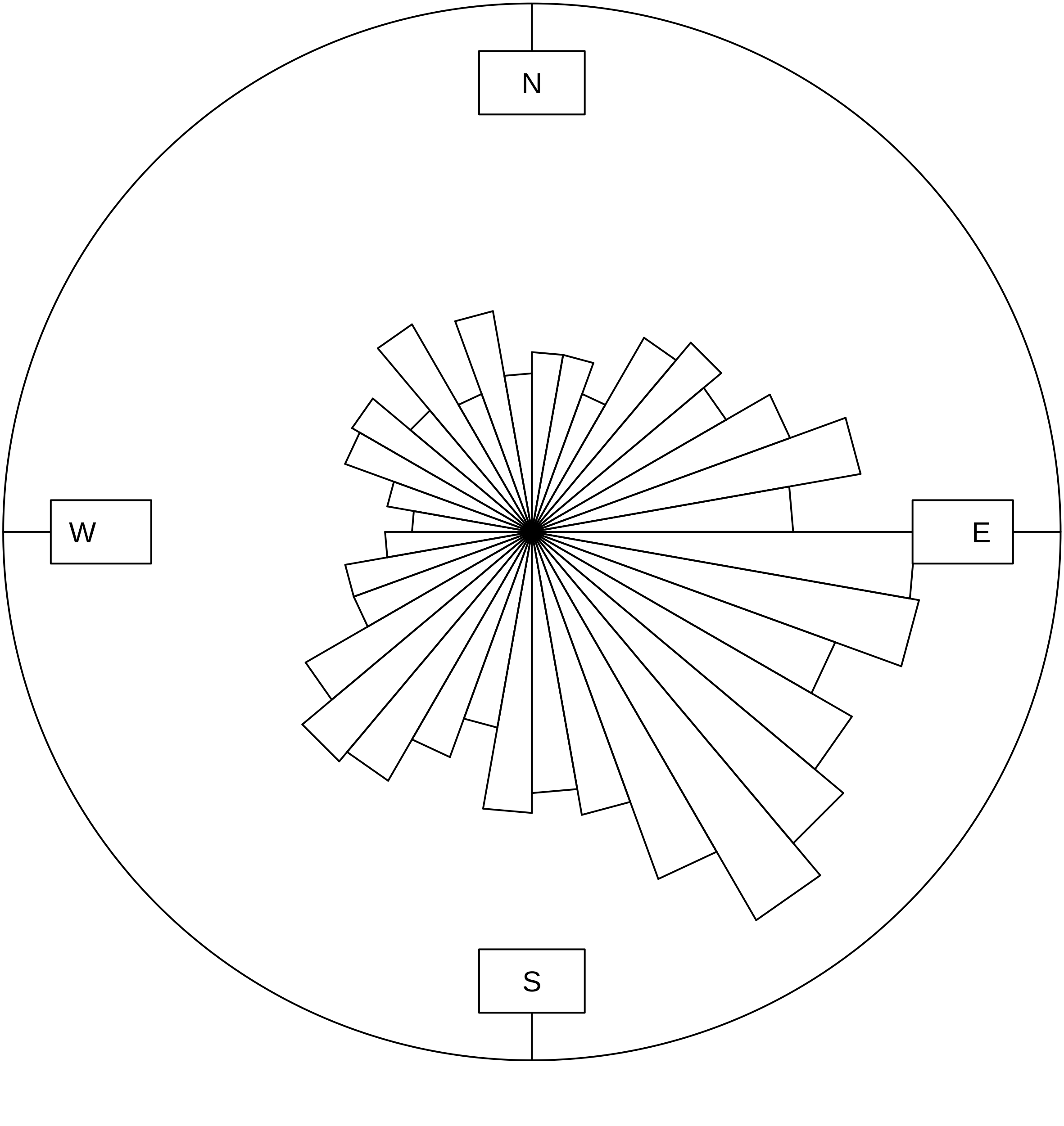

bootstrap 295

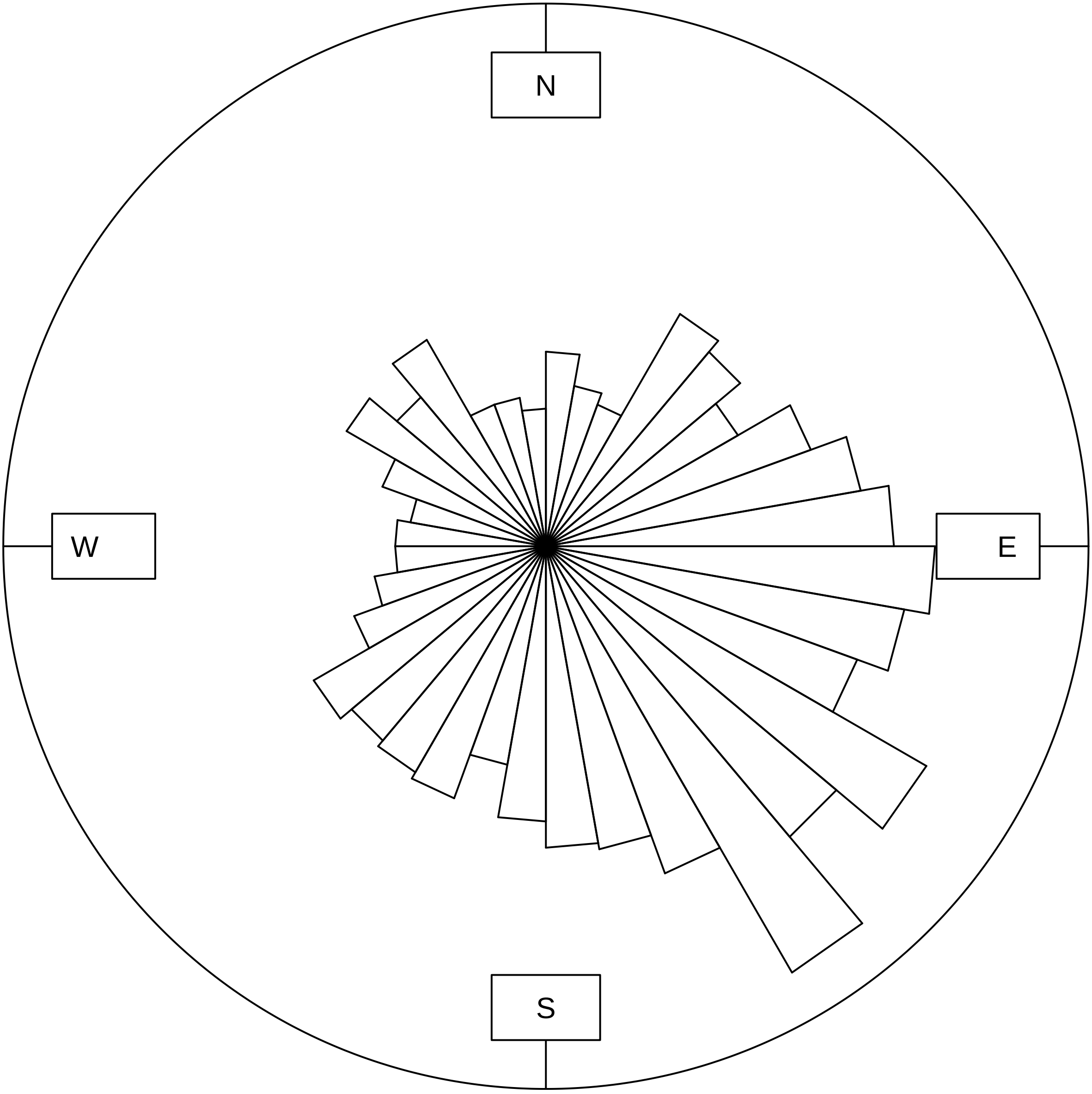

bootstrap 296

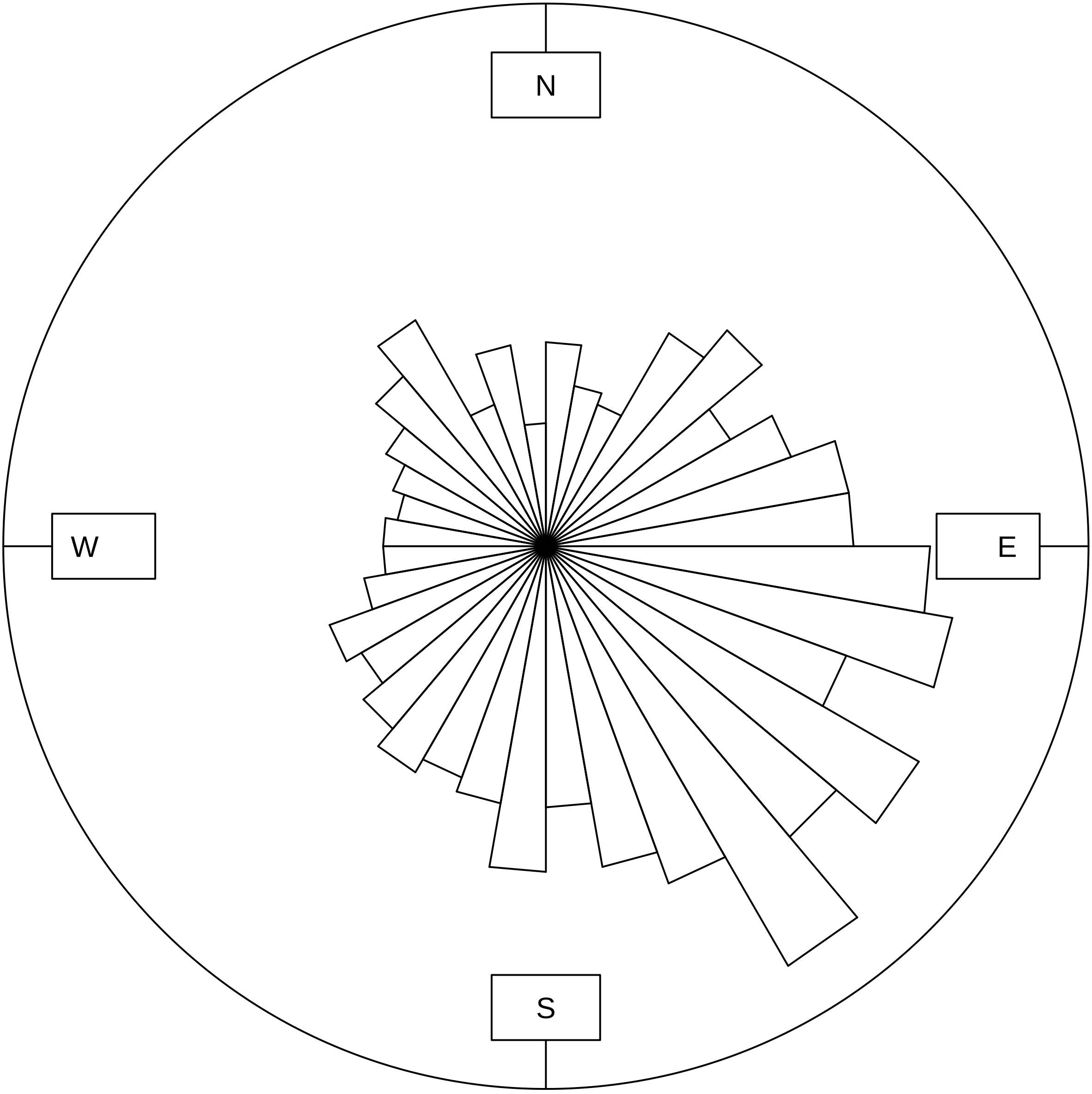

bootstrap 297

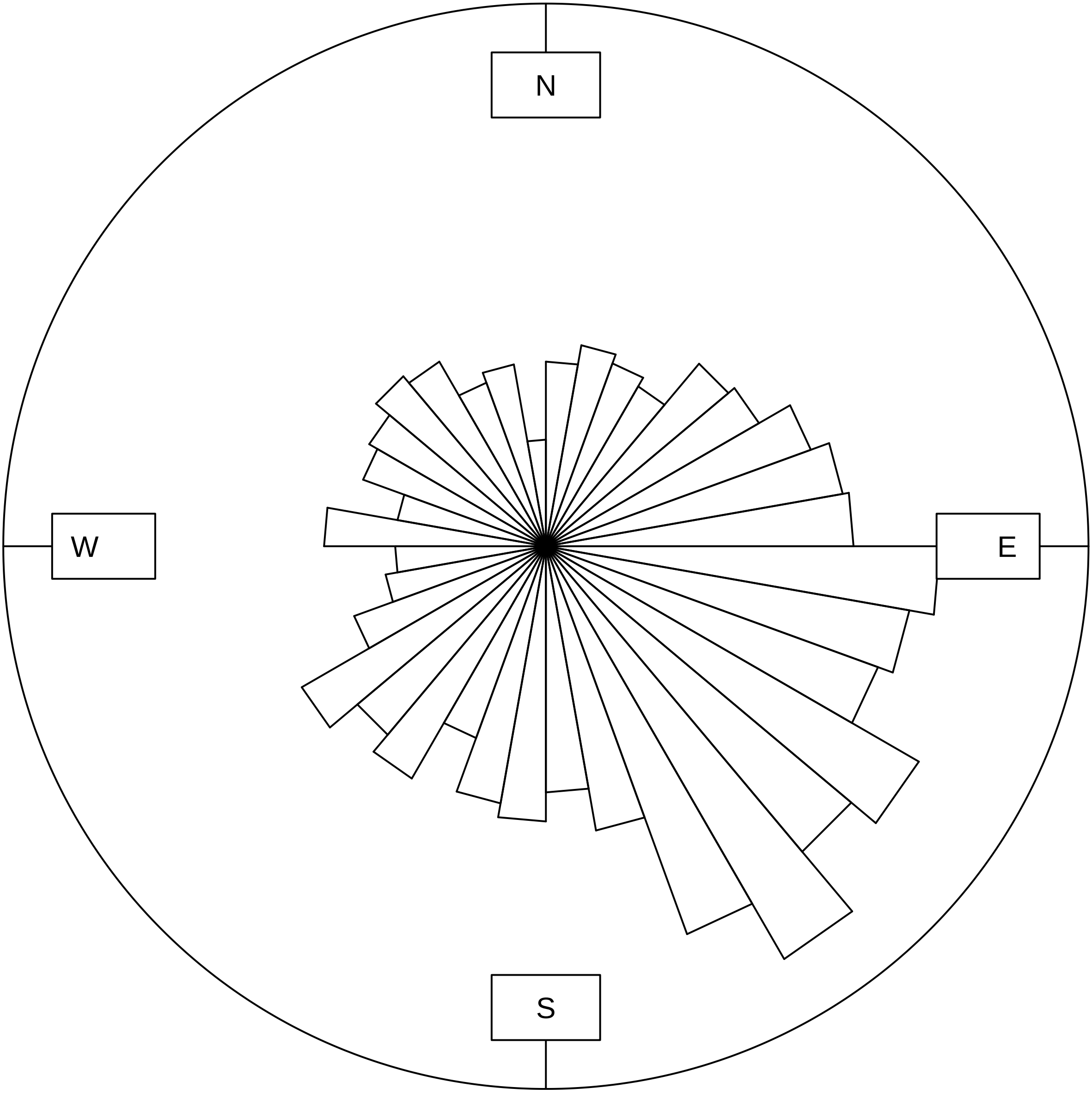

bootstrap 298

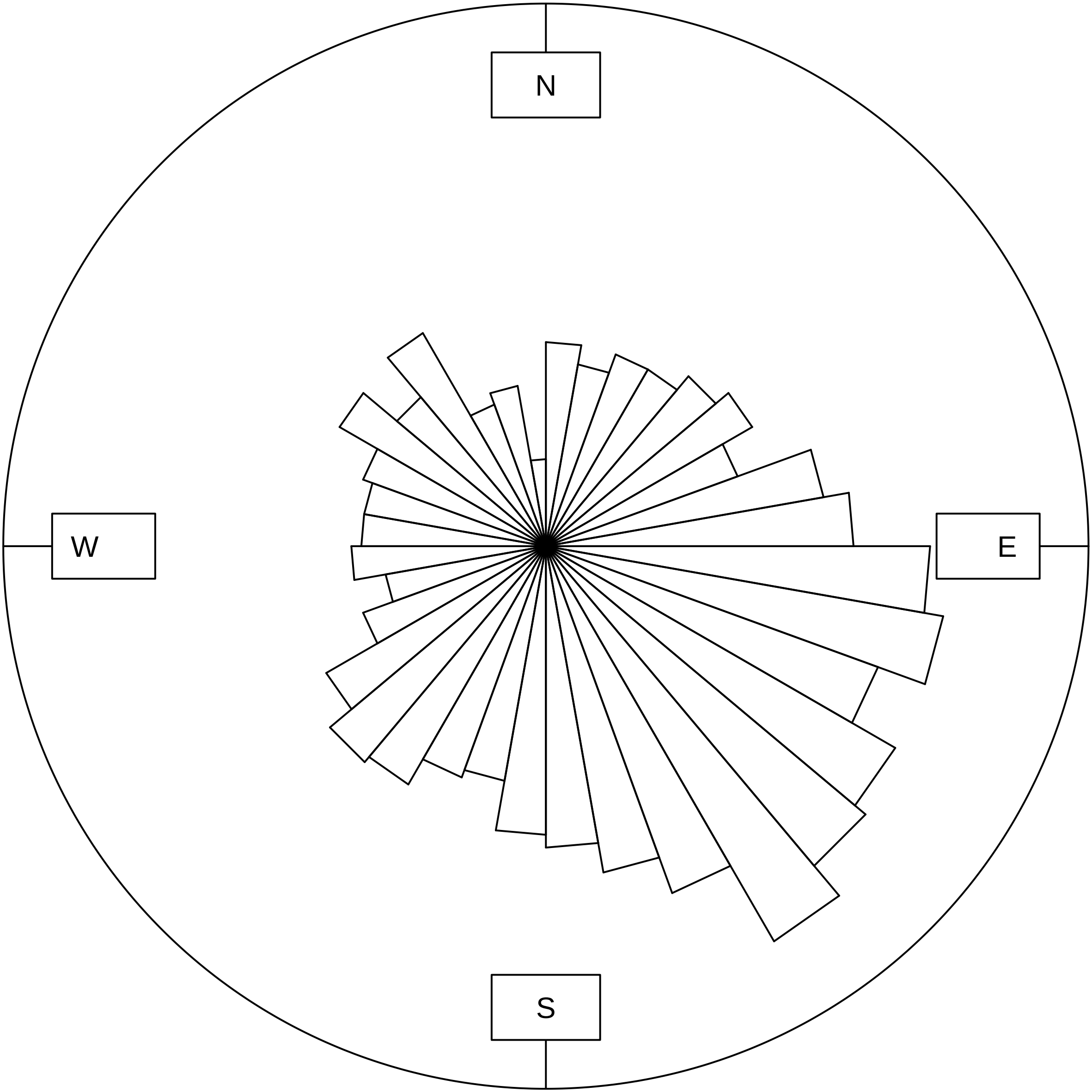

bootstrap 299

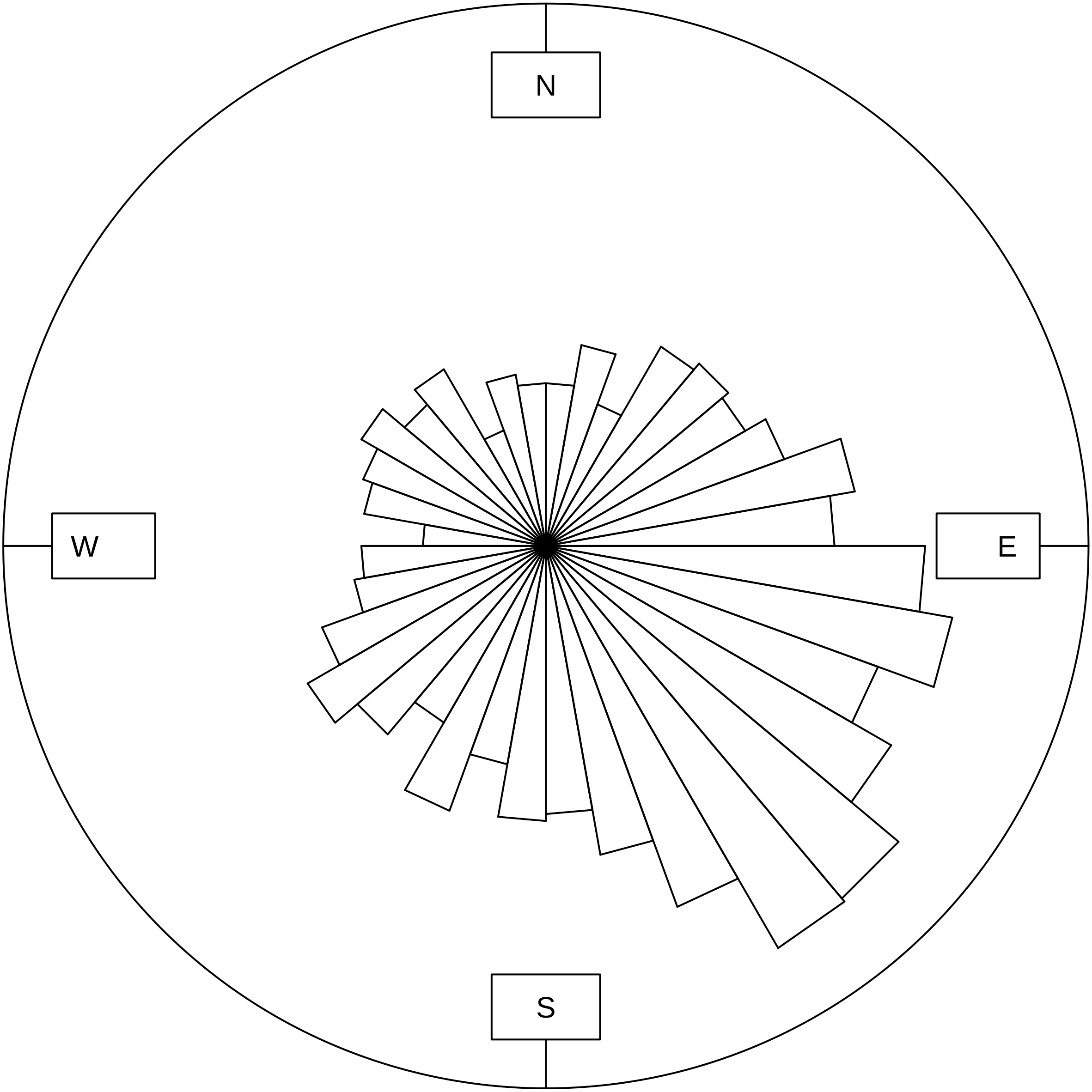

bootstrap 300

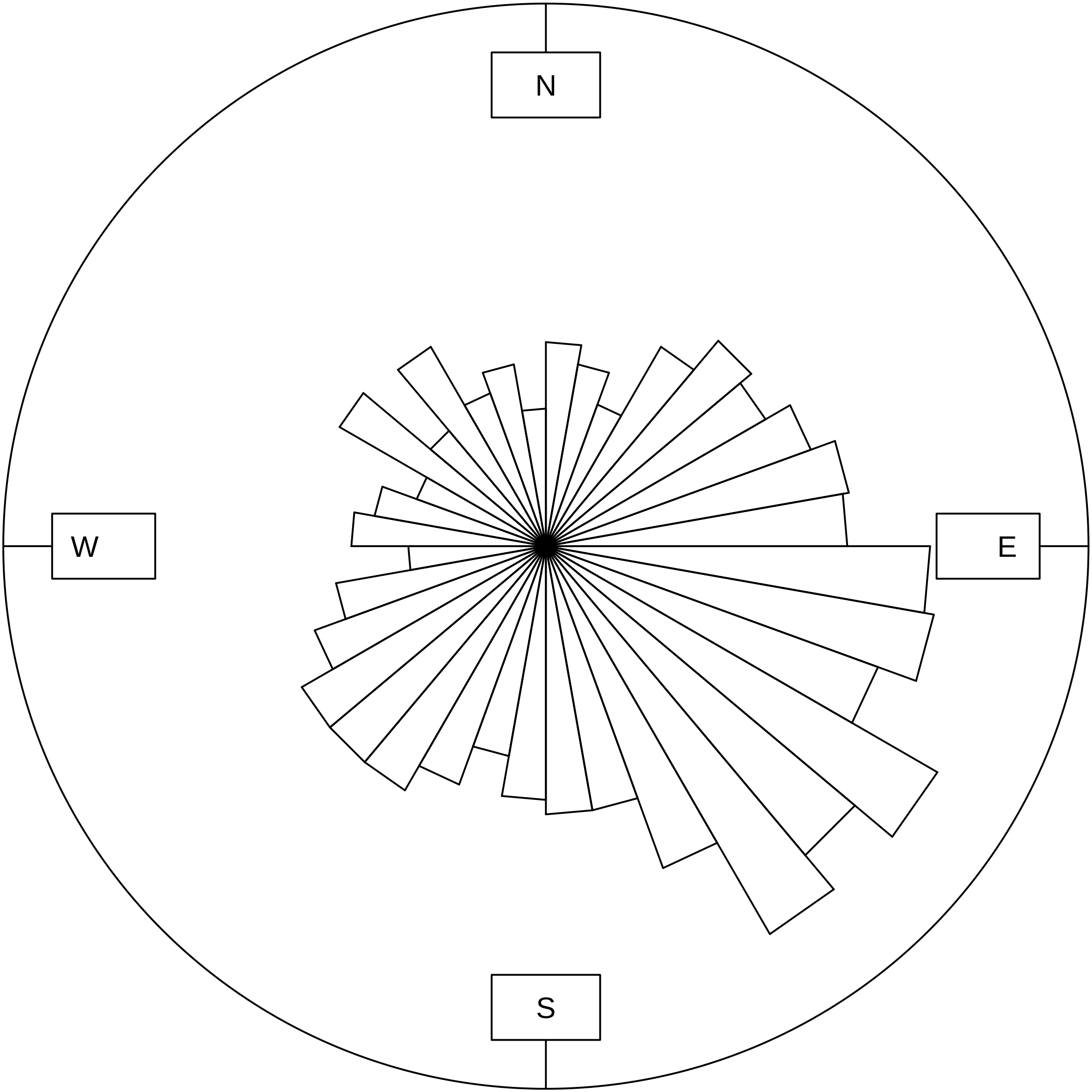

bootstrap 301

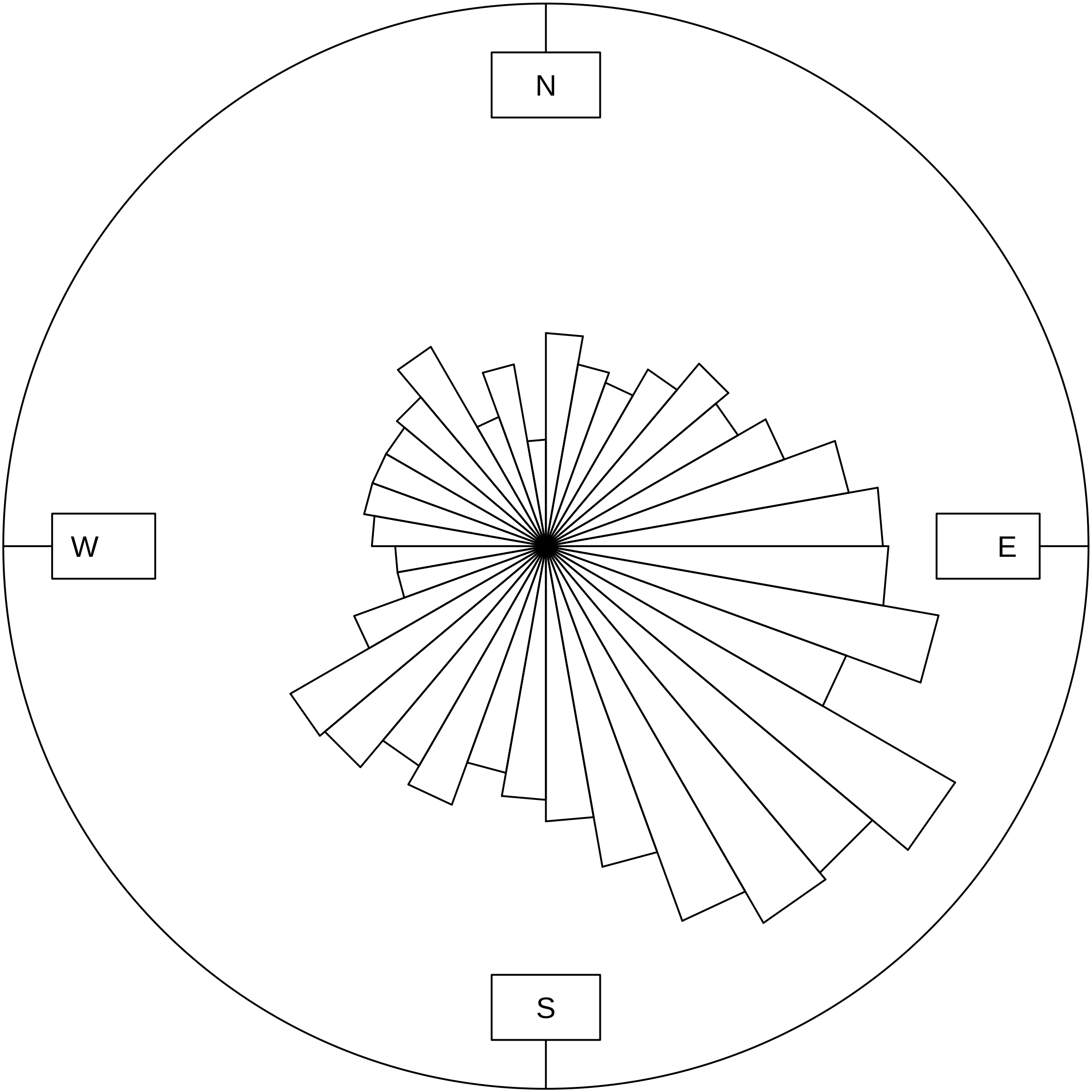

bootstrap 302

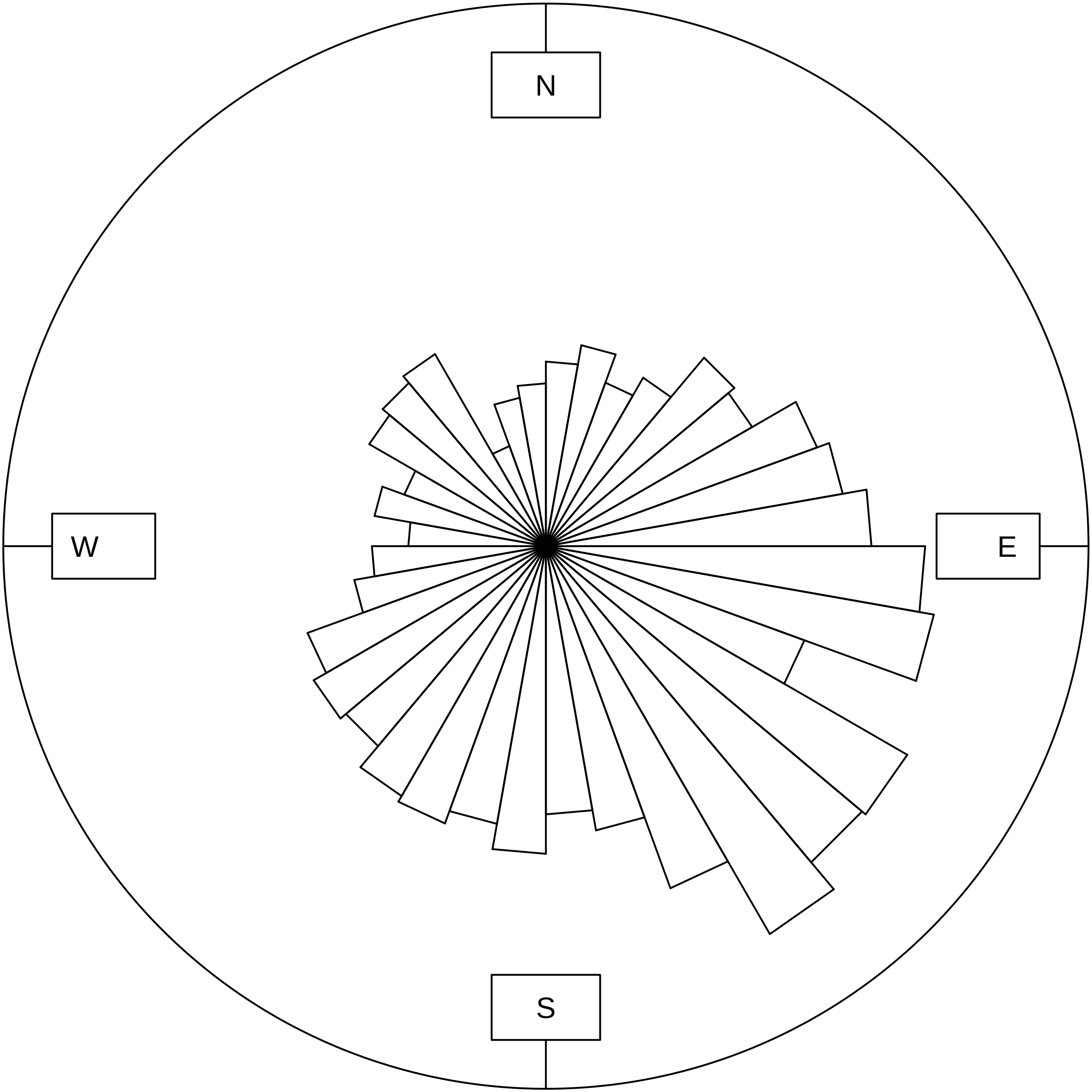

bootstrap 303

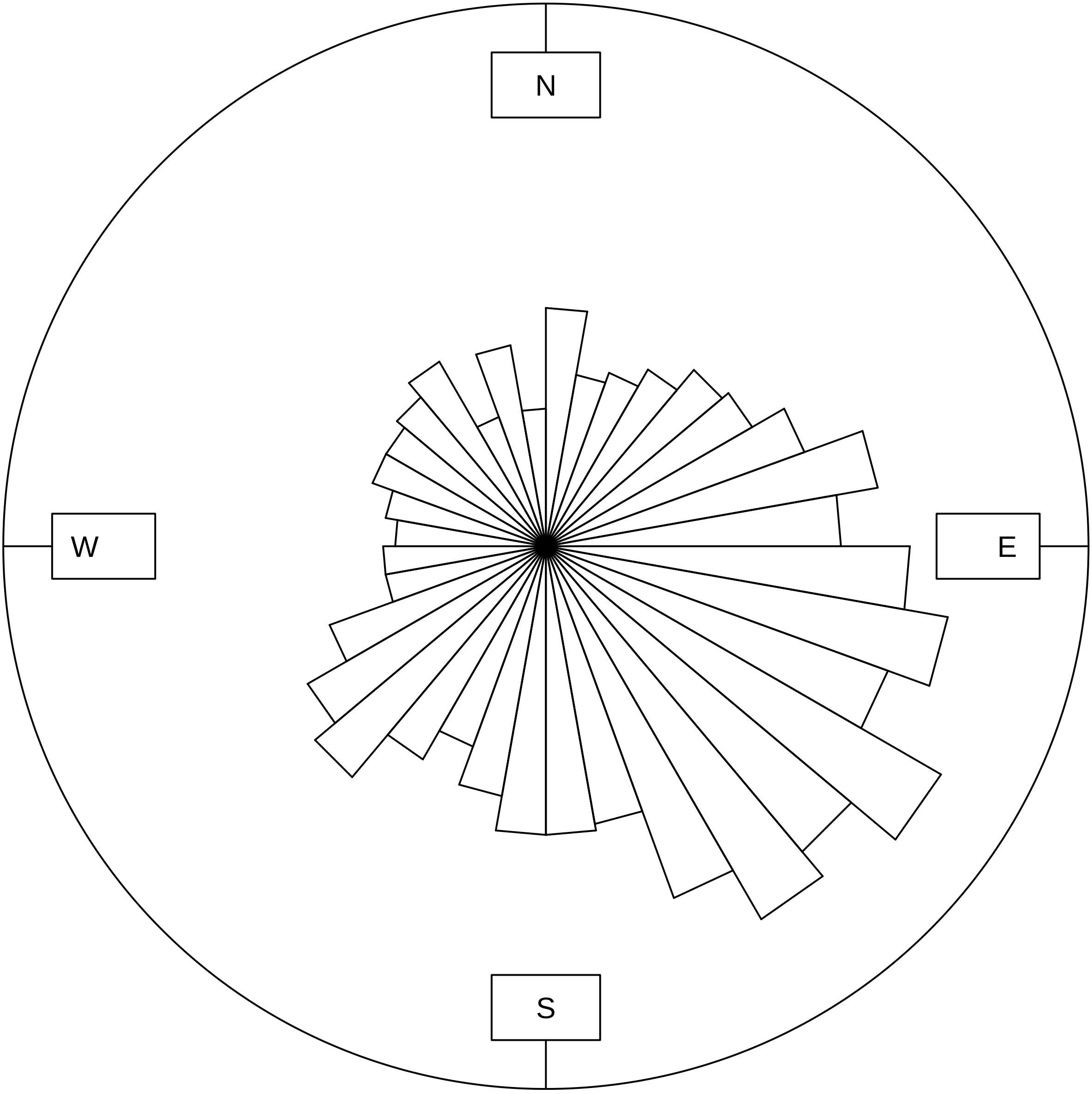

bootstrap 304

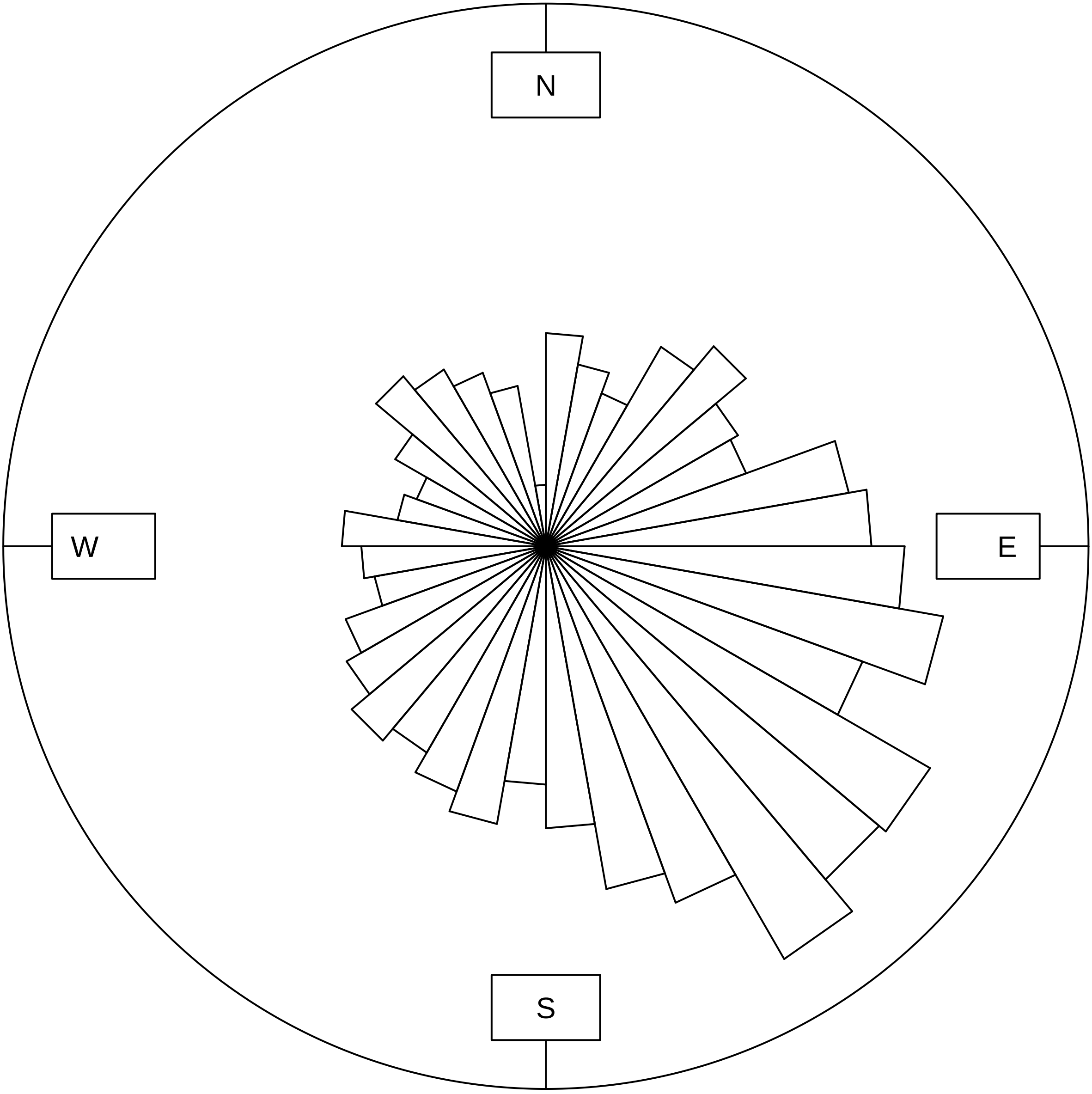

bootstrap 305

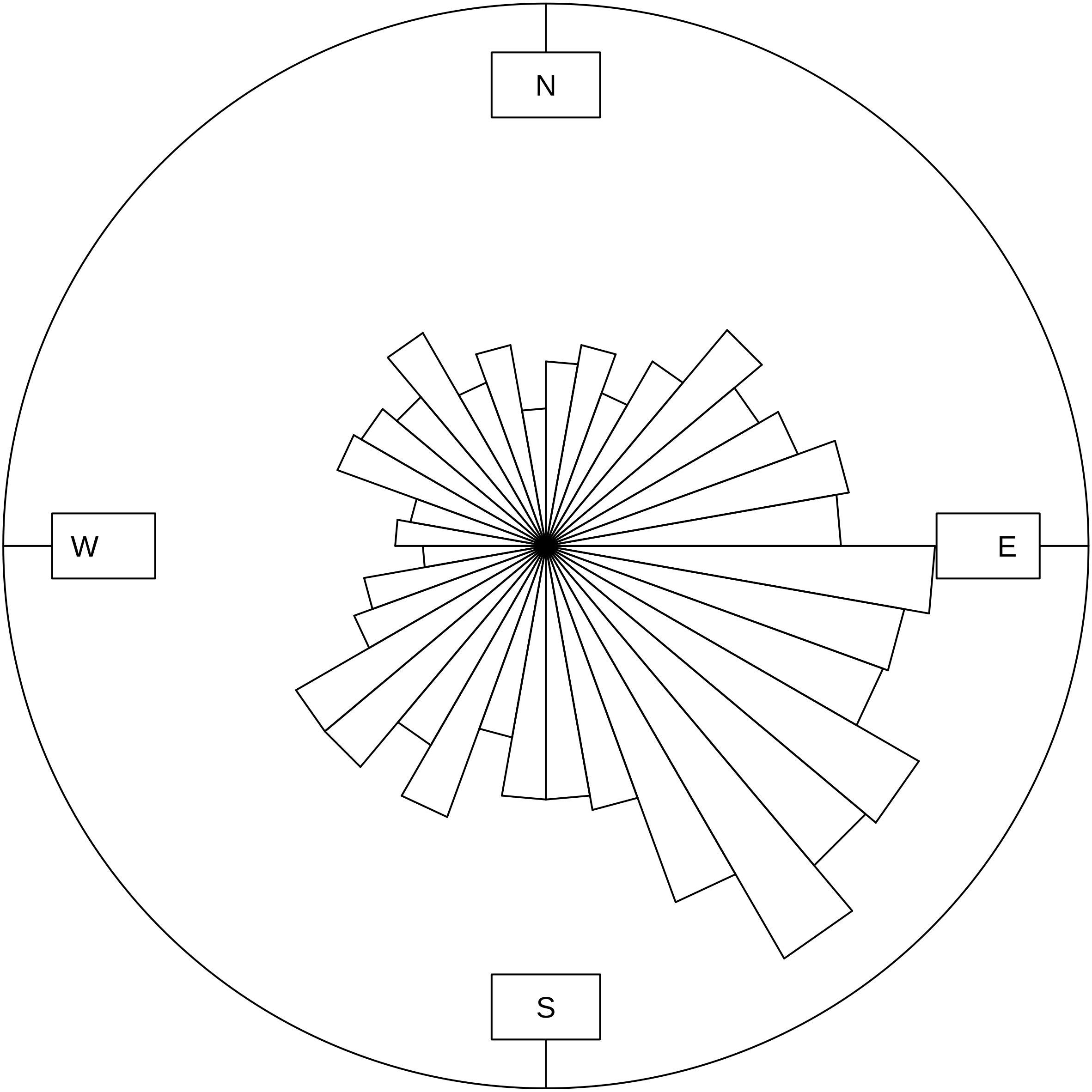

bootstrap 306

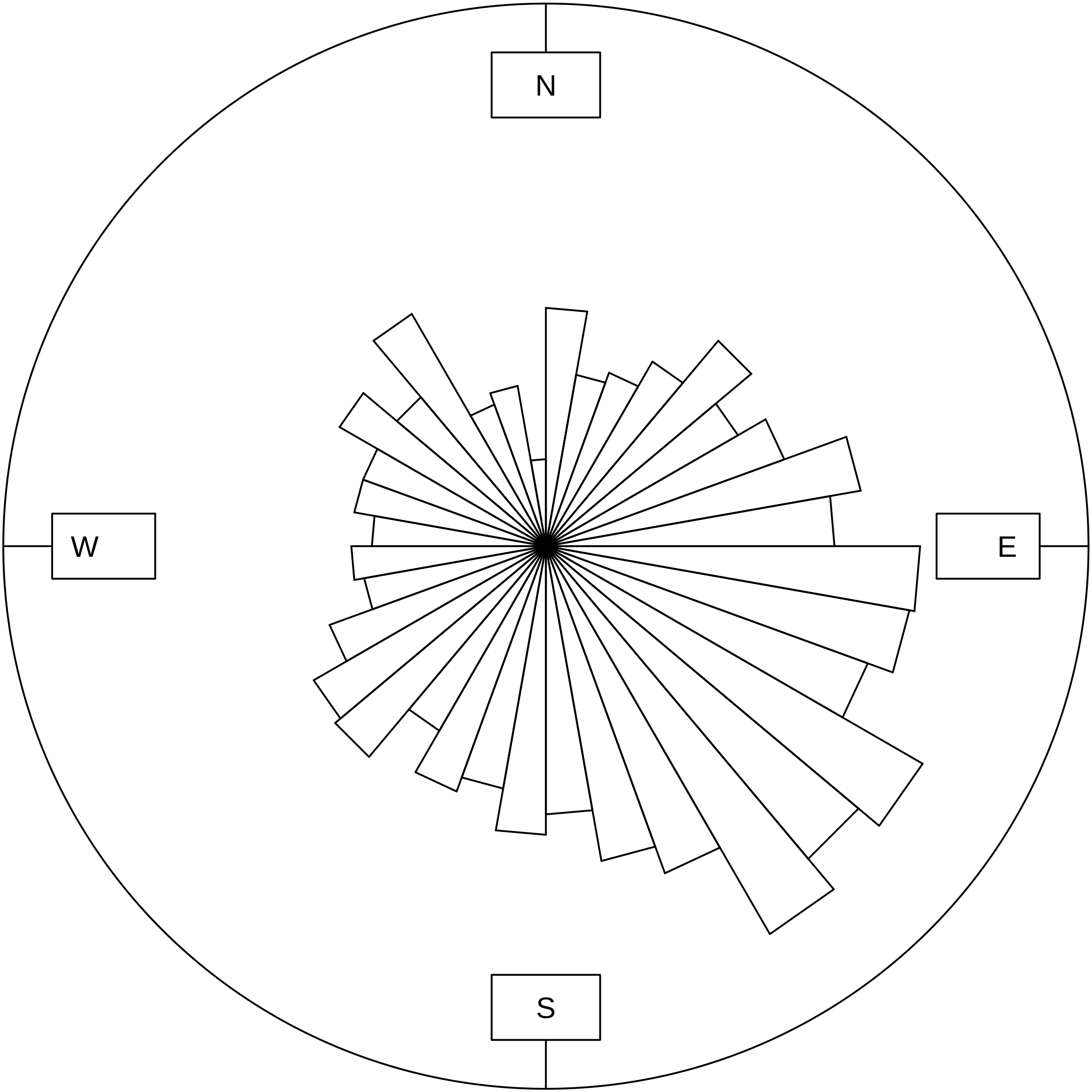

bootstrap 307

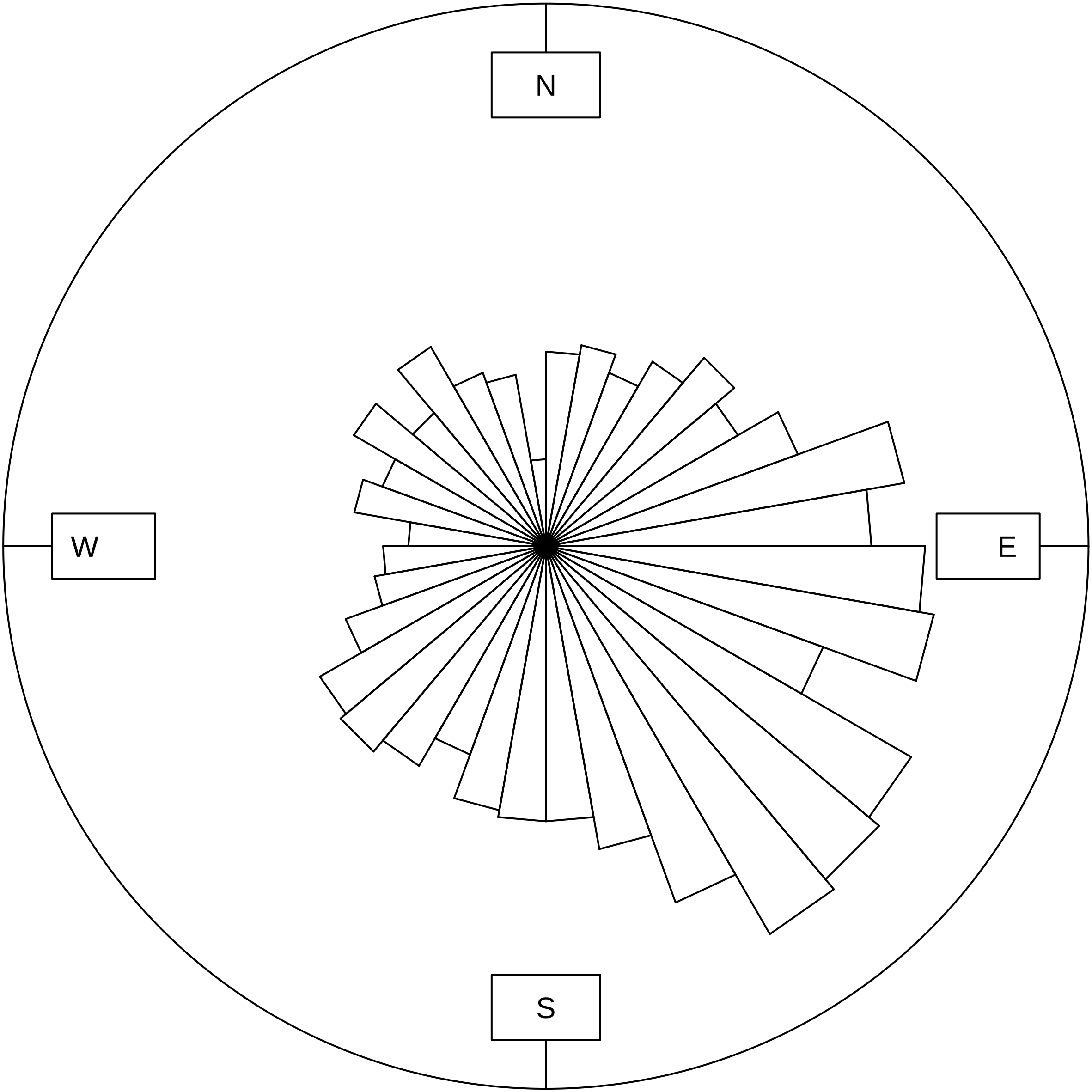

bootstrap 308

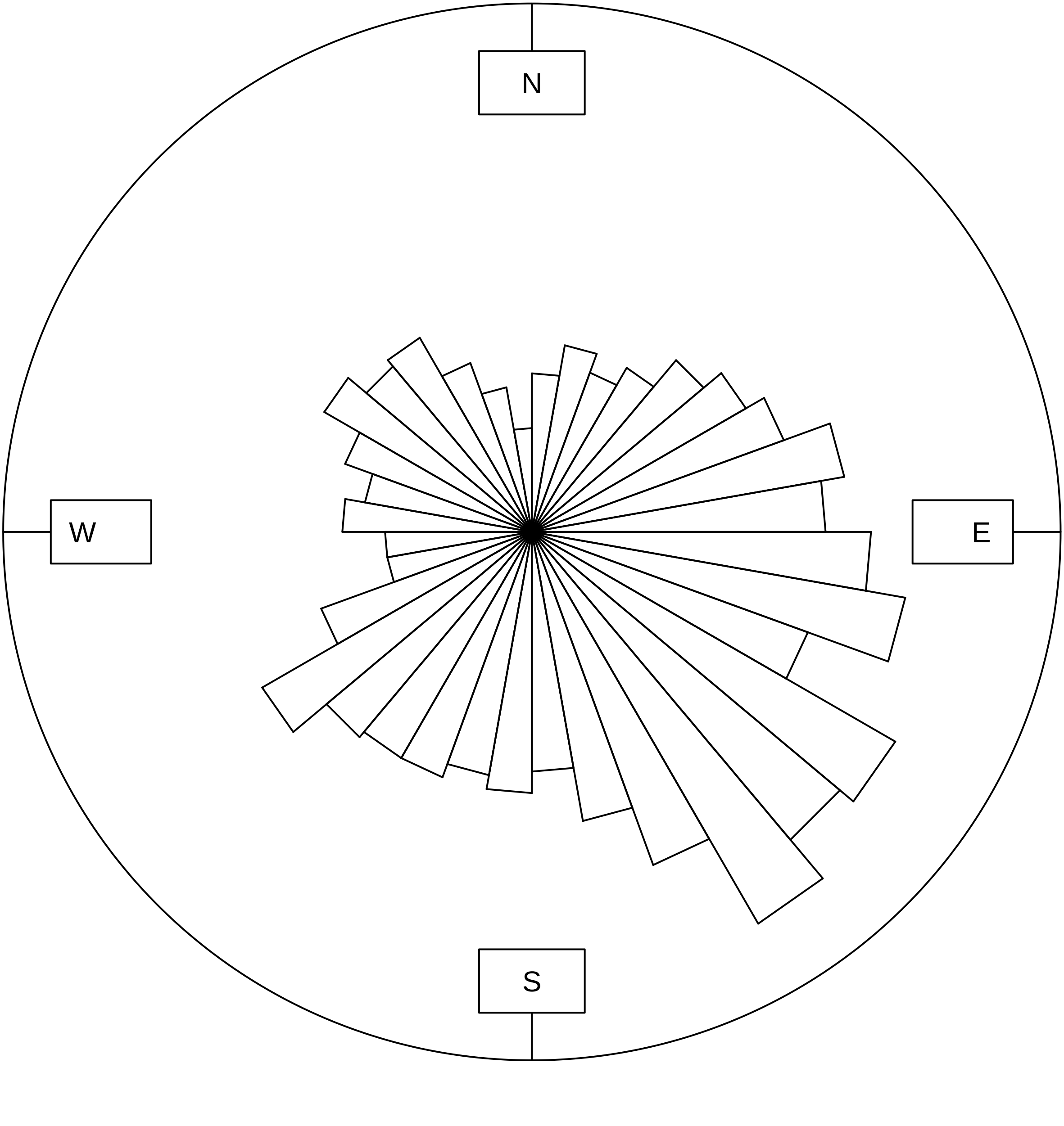

bootstrap 309

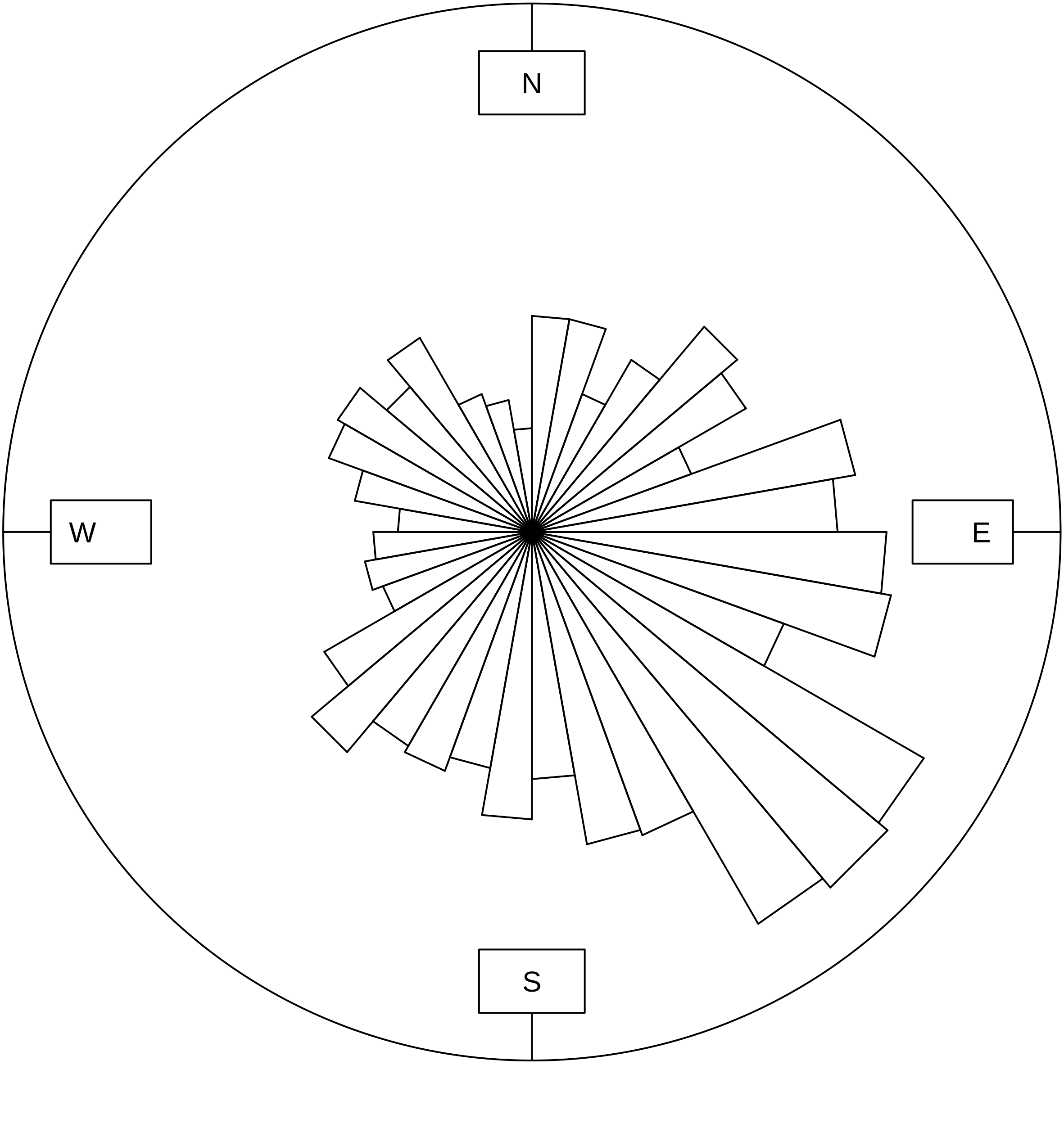

bootstrap 310

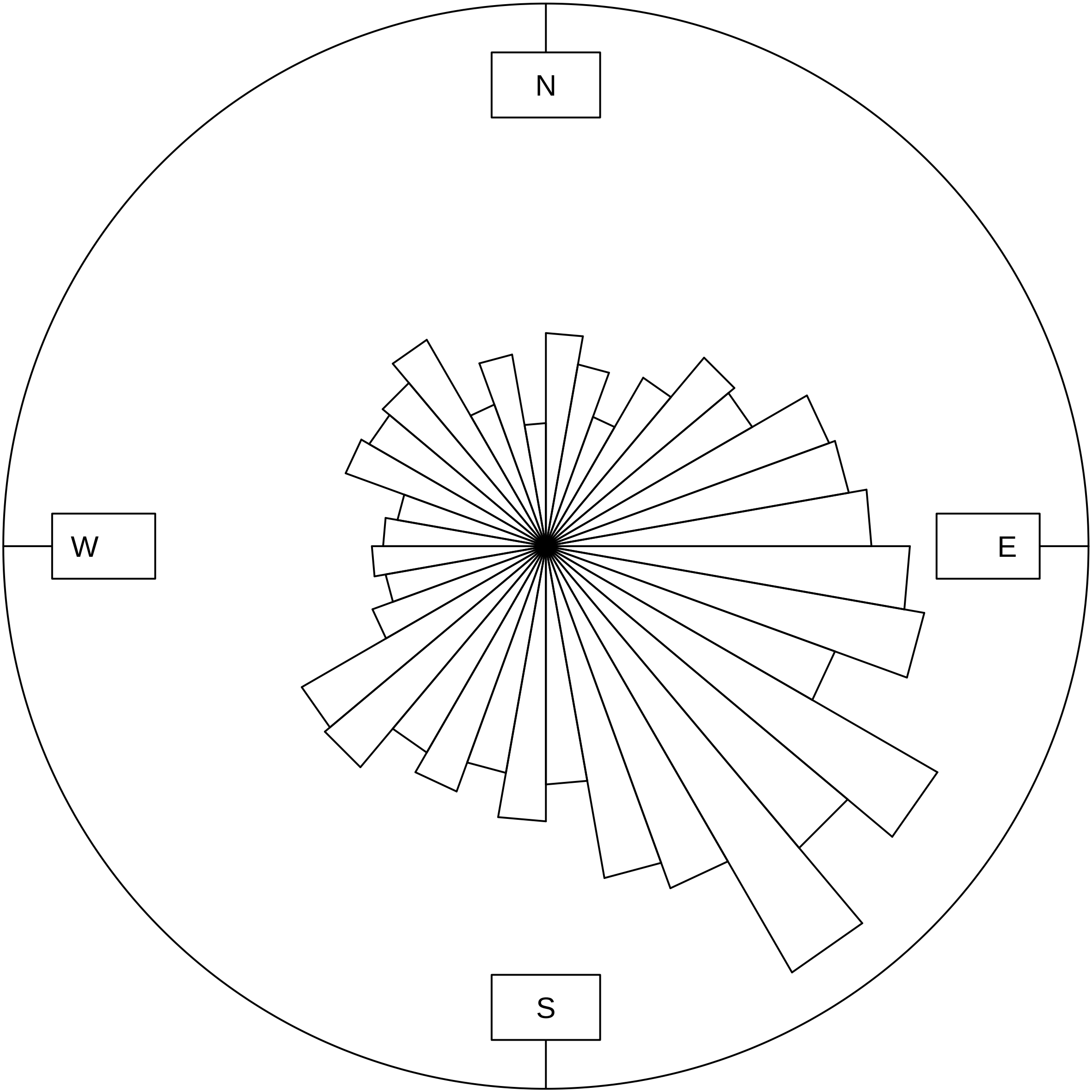

bootstrap 311

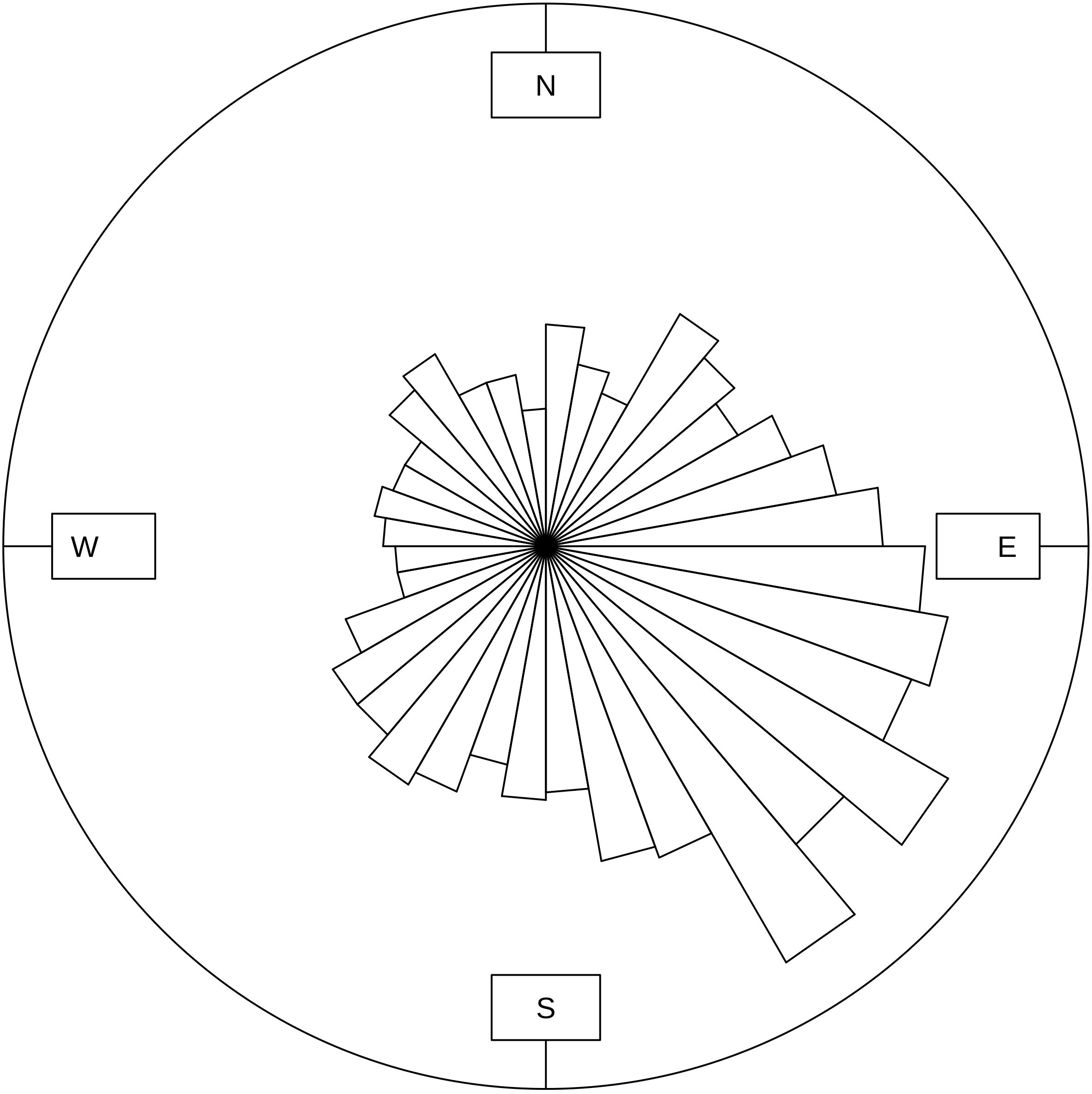

bootstrap 312

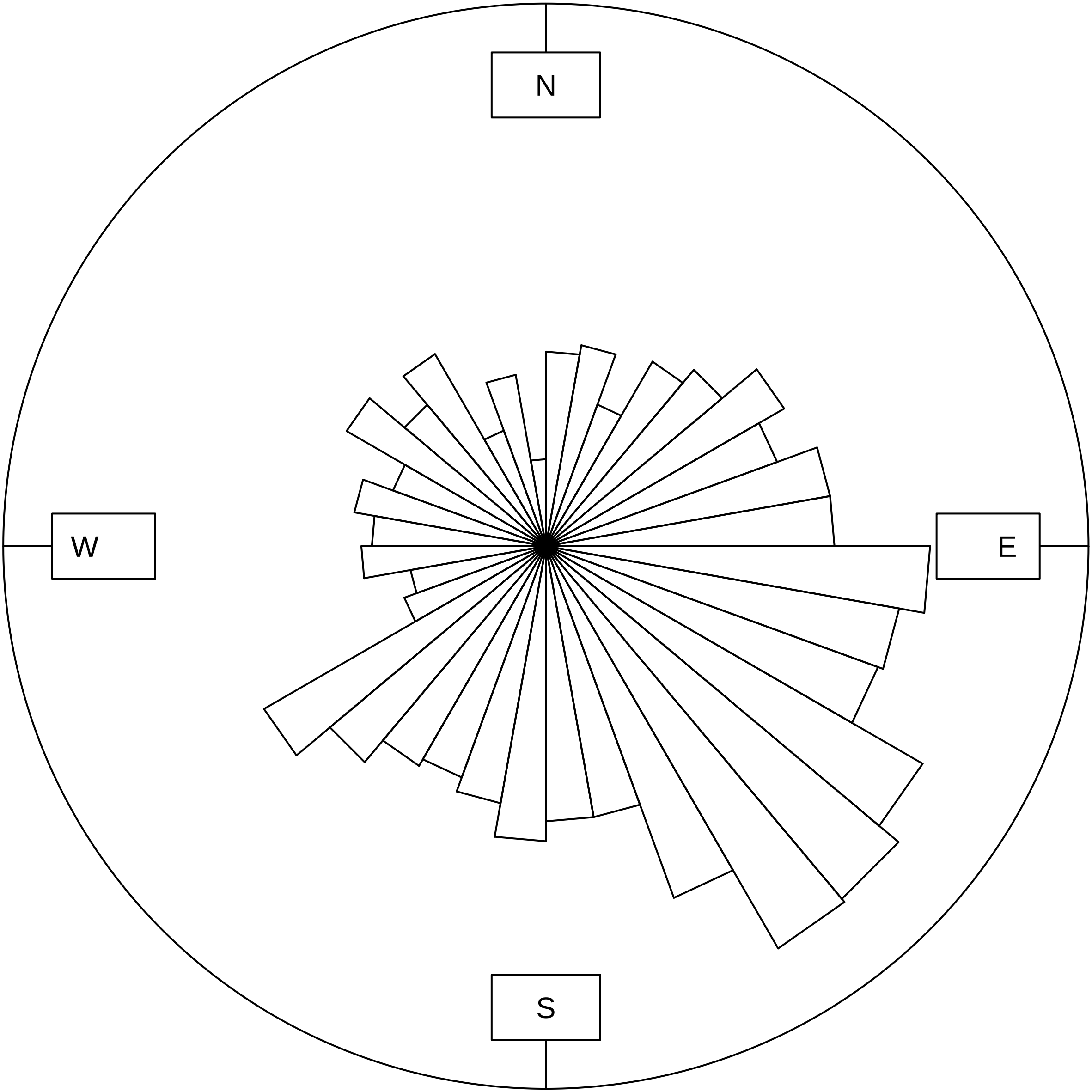

bootstrap 313

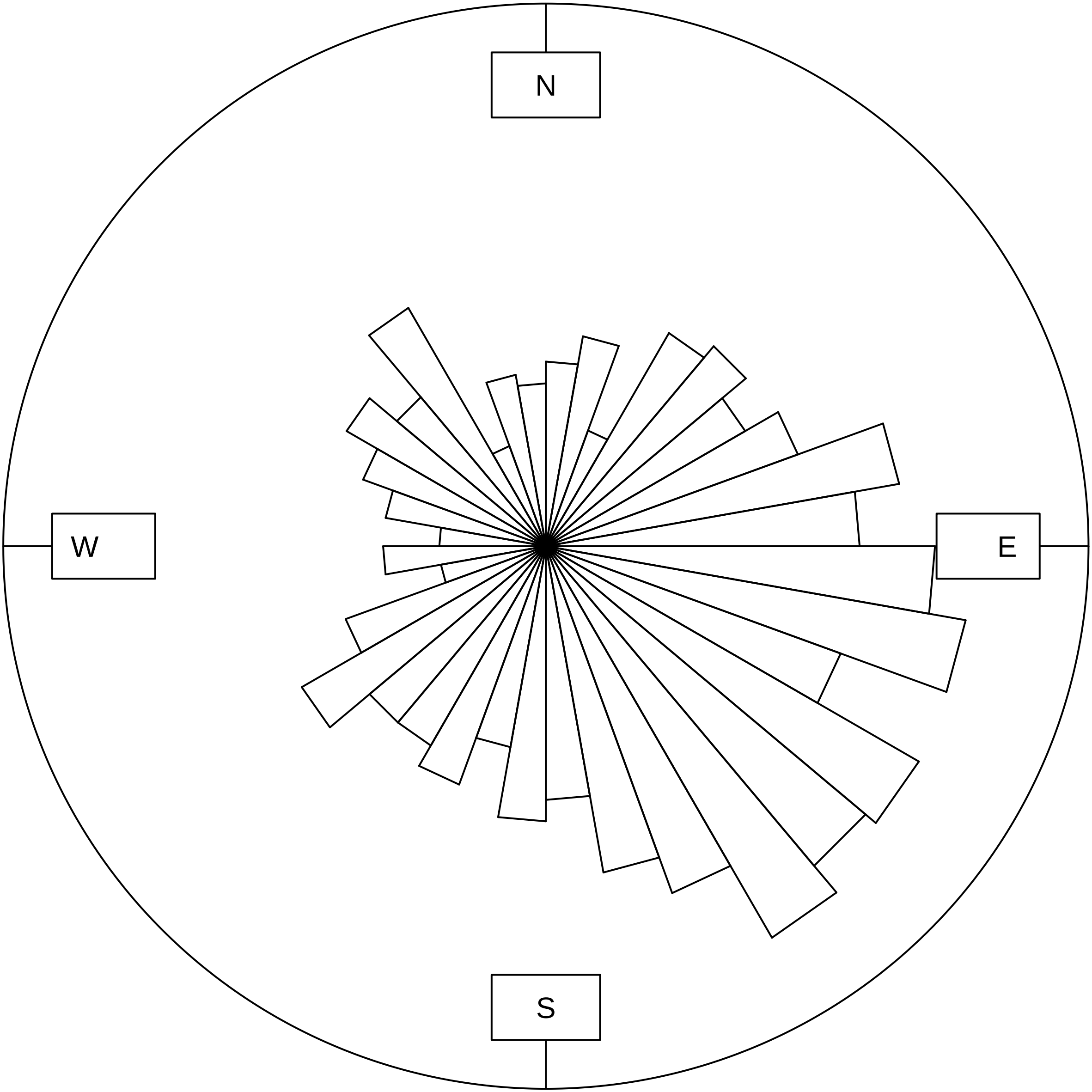

bootstrap 314

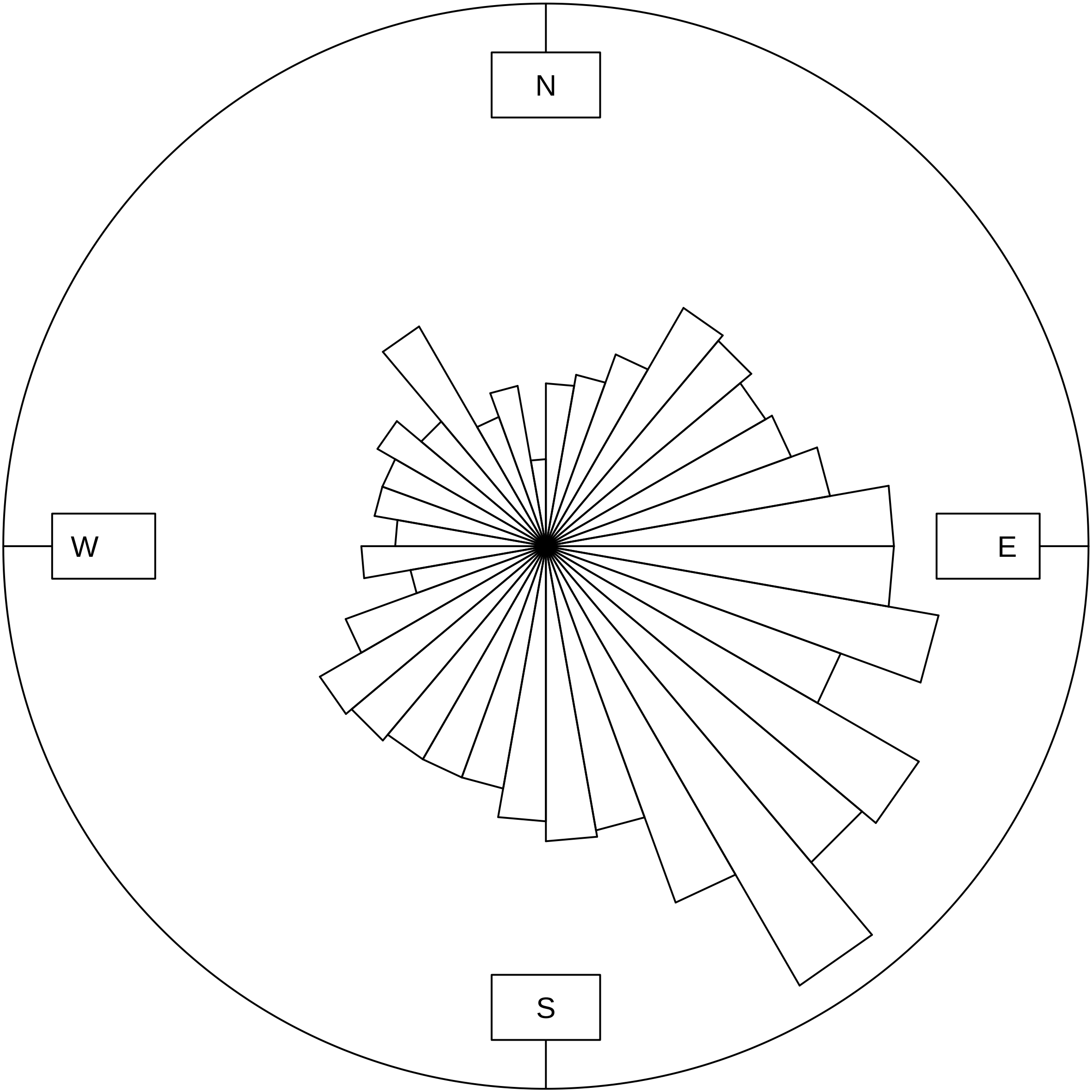

bootstrap 315

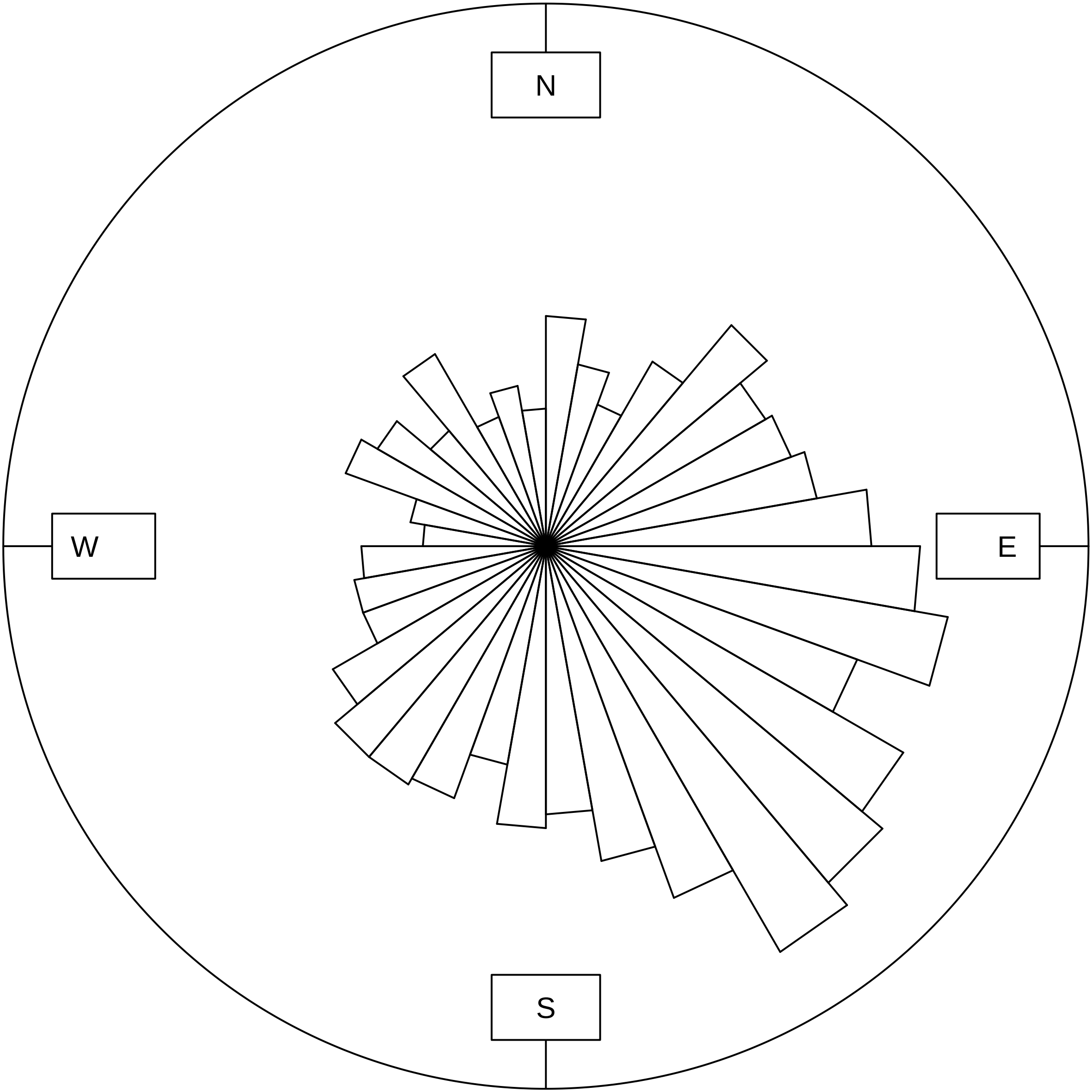

bootstrap 316

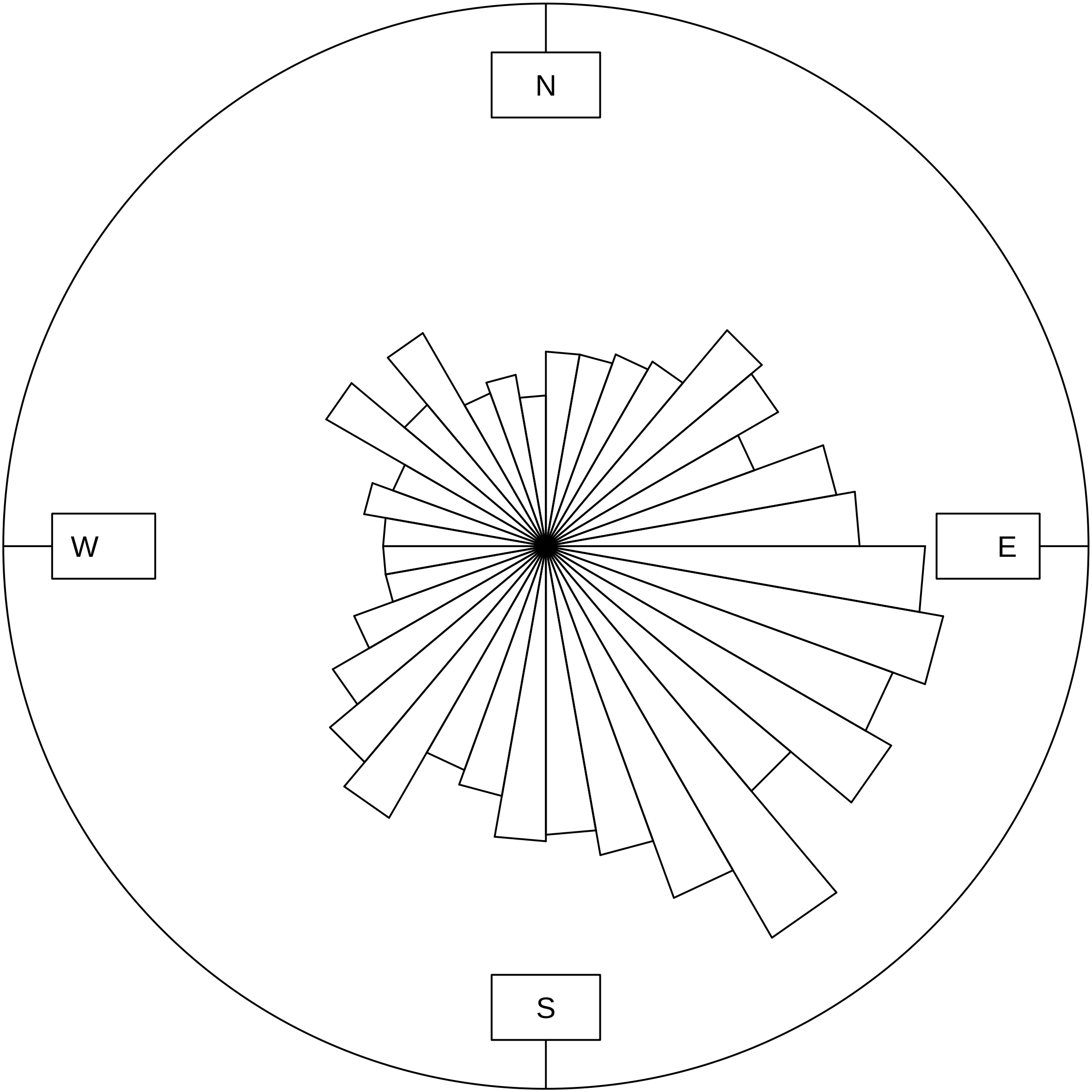

bootstrap 317

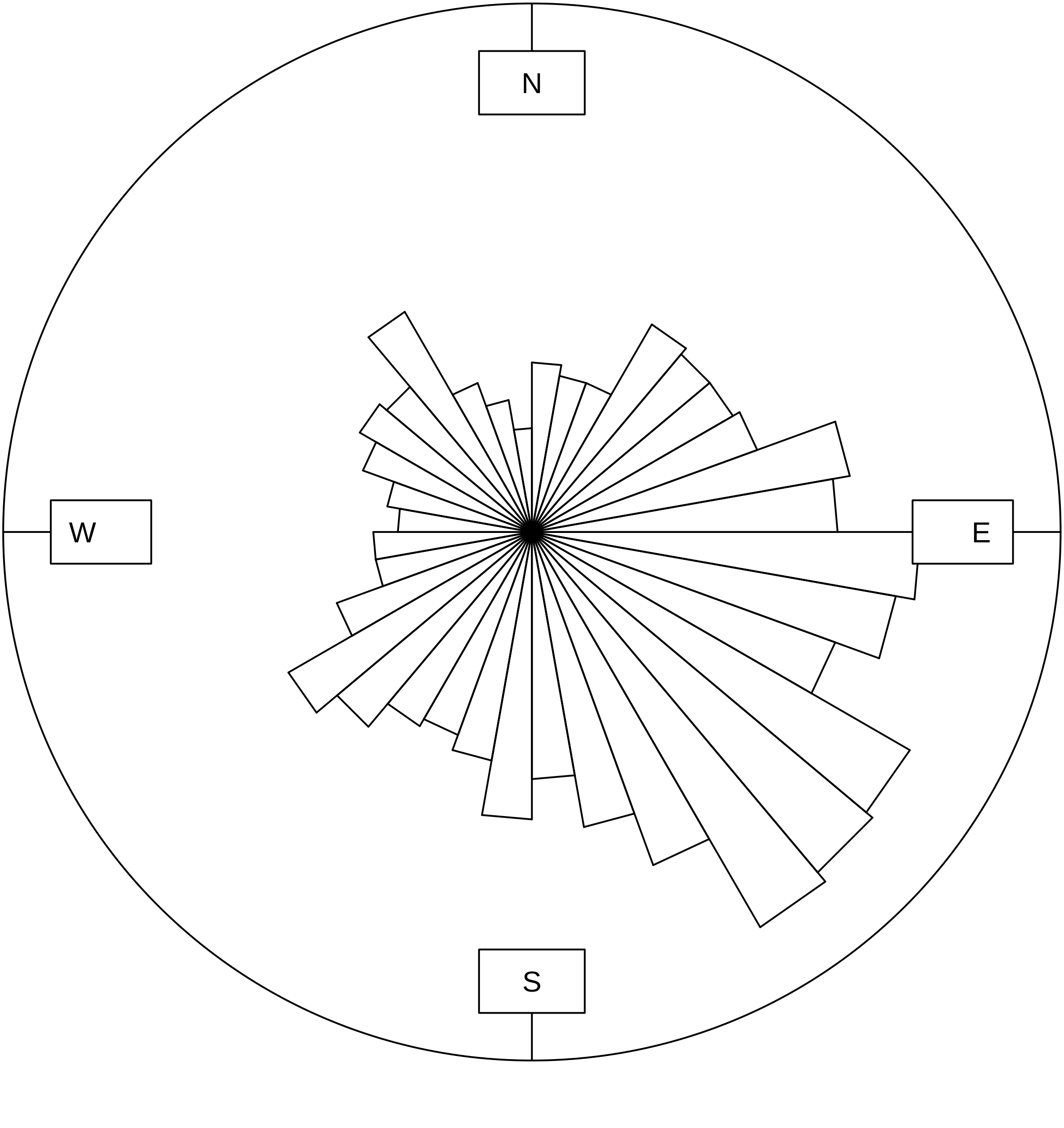

bootstrap 318

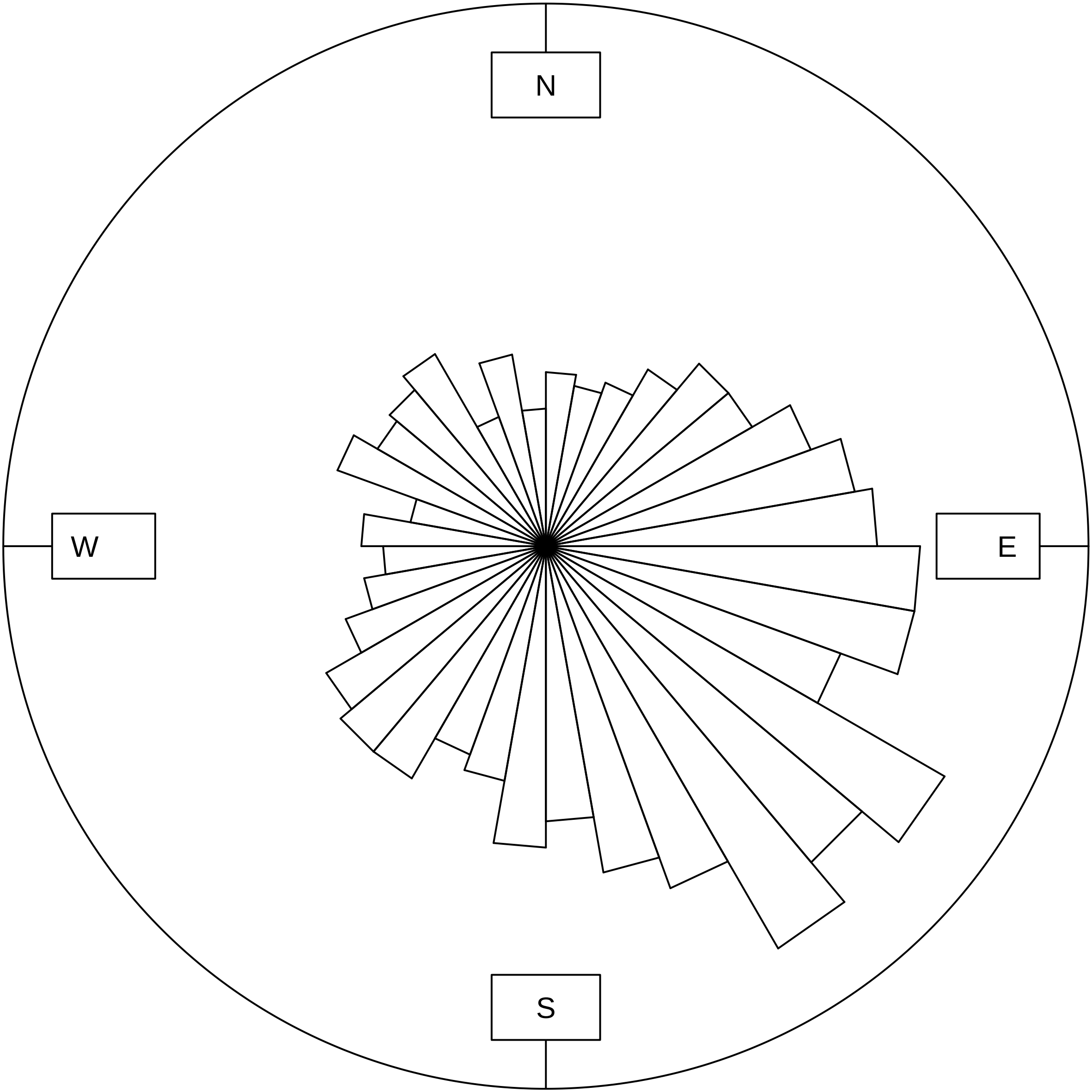

bootstrap 319

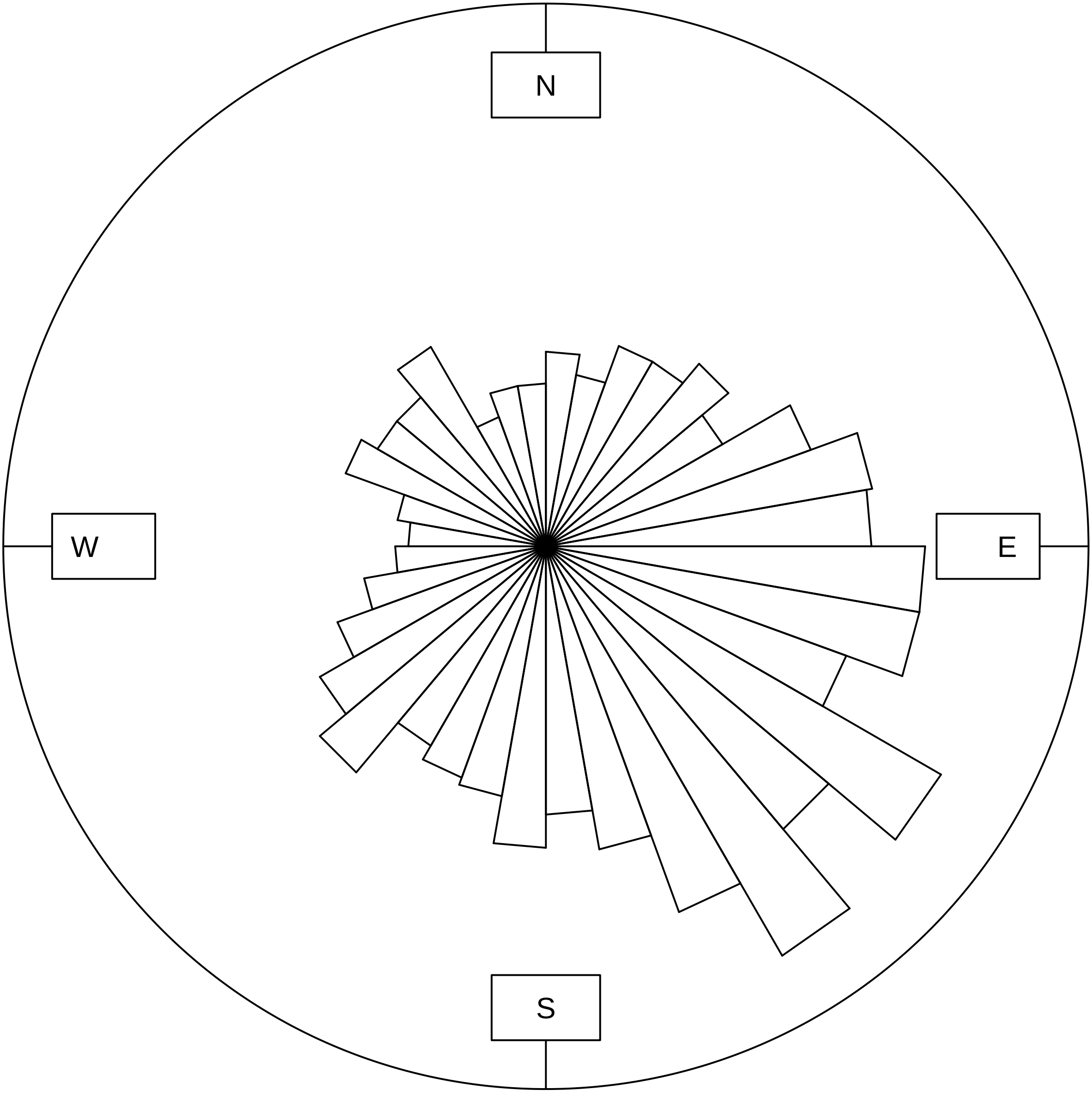

bootstrap 320

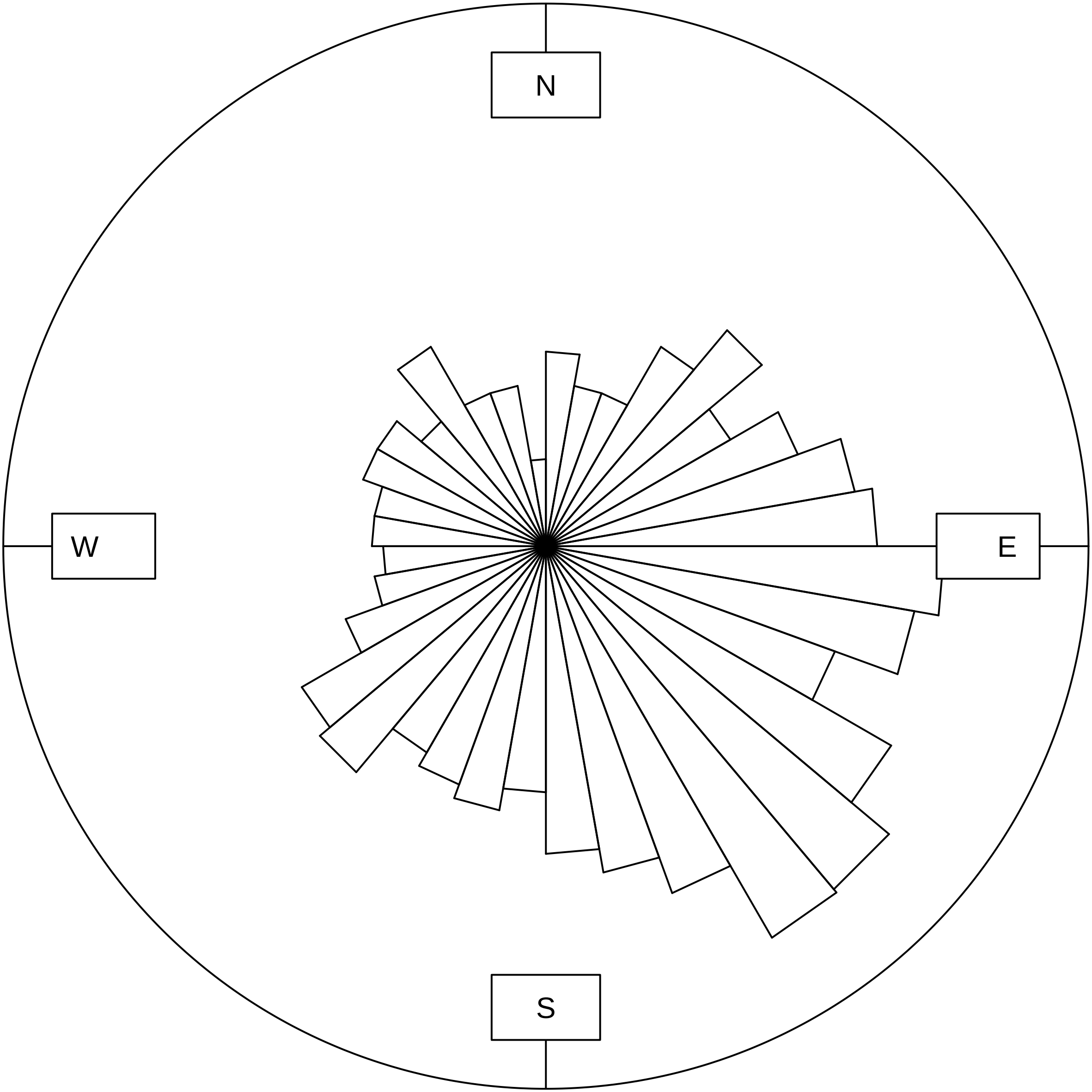

bootstrap 321

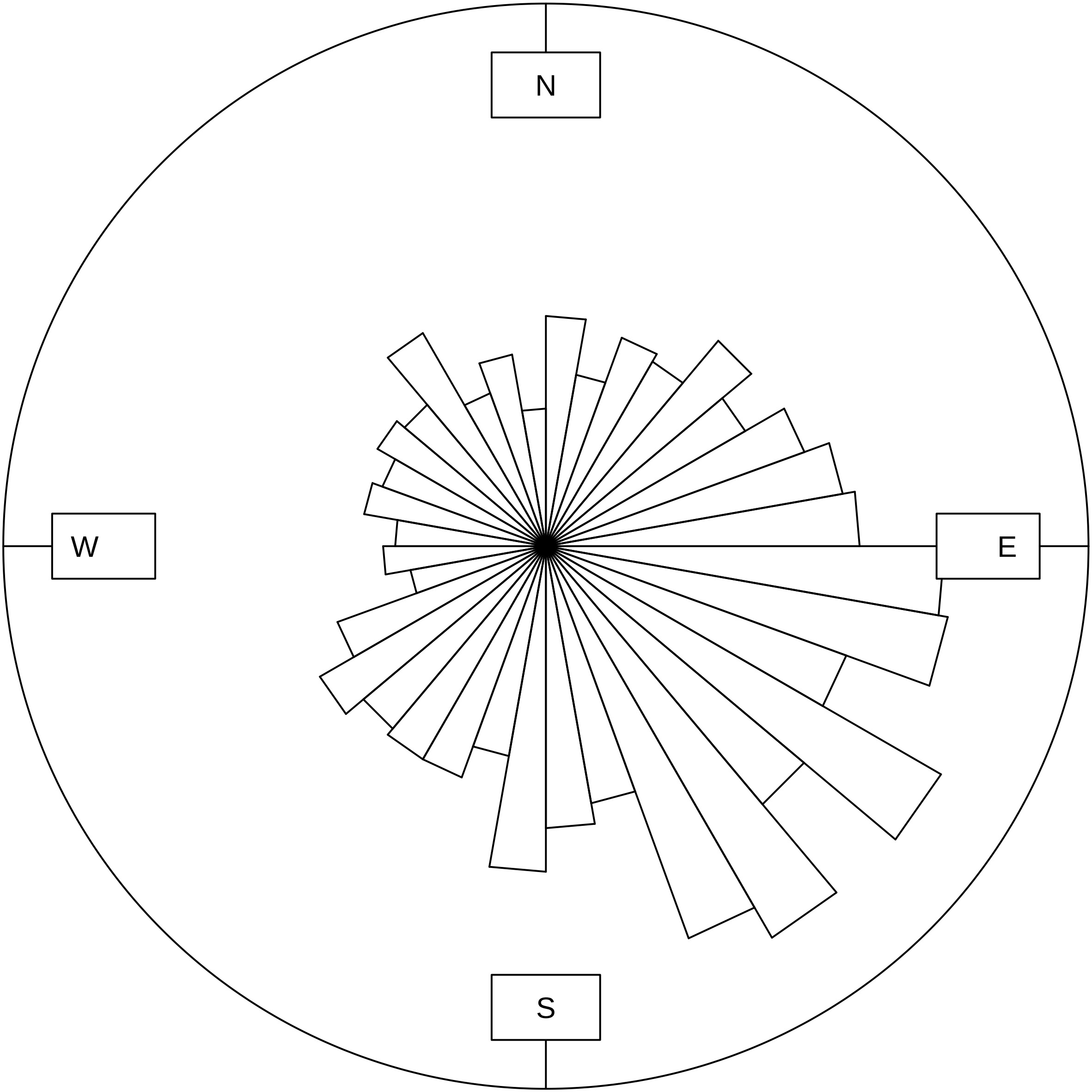

bootstrap 322

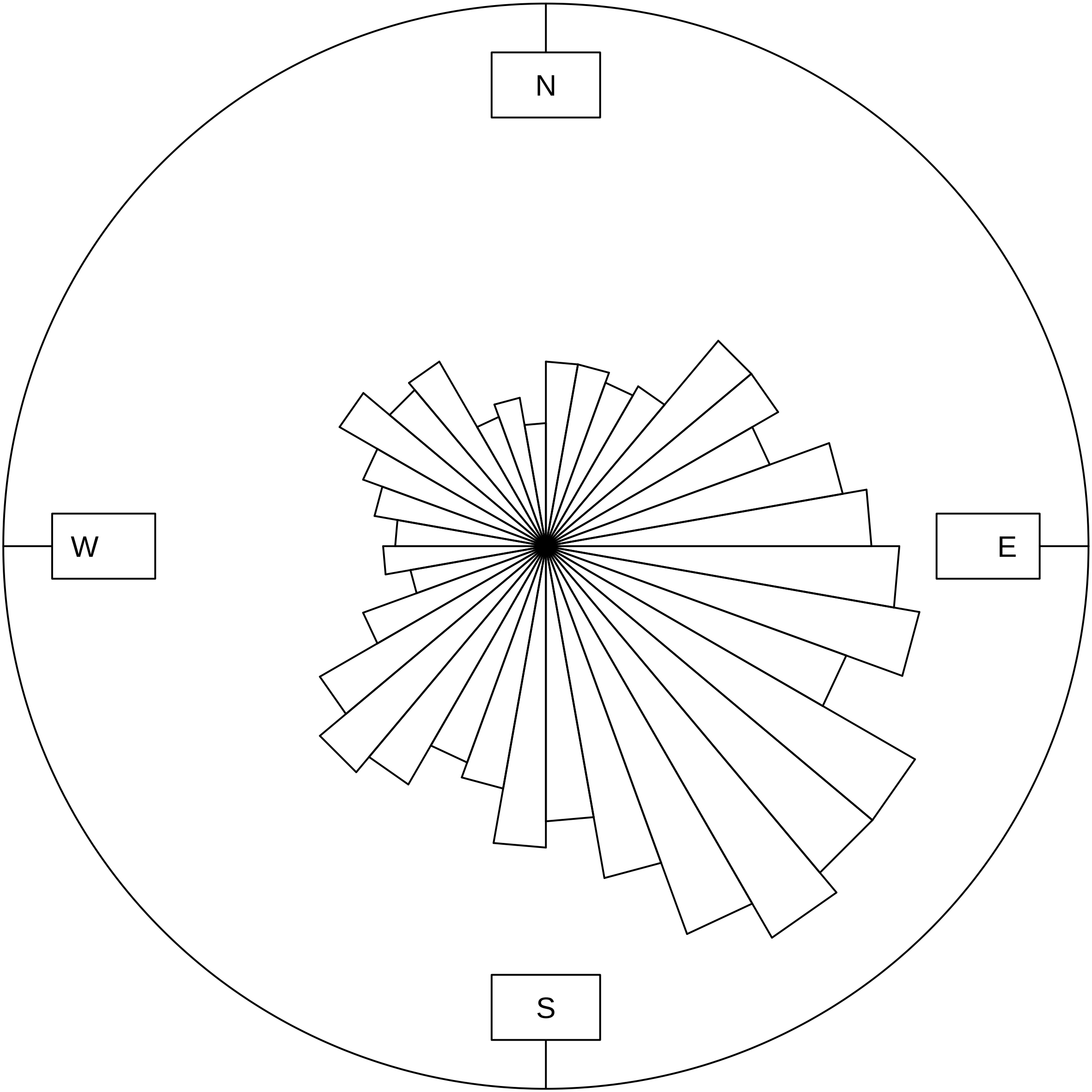

bootstrap 323

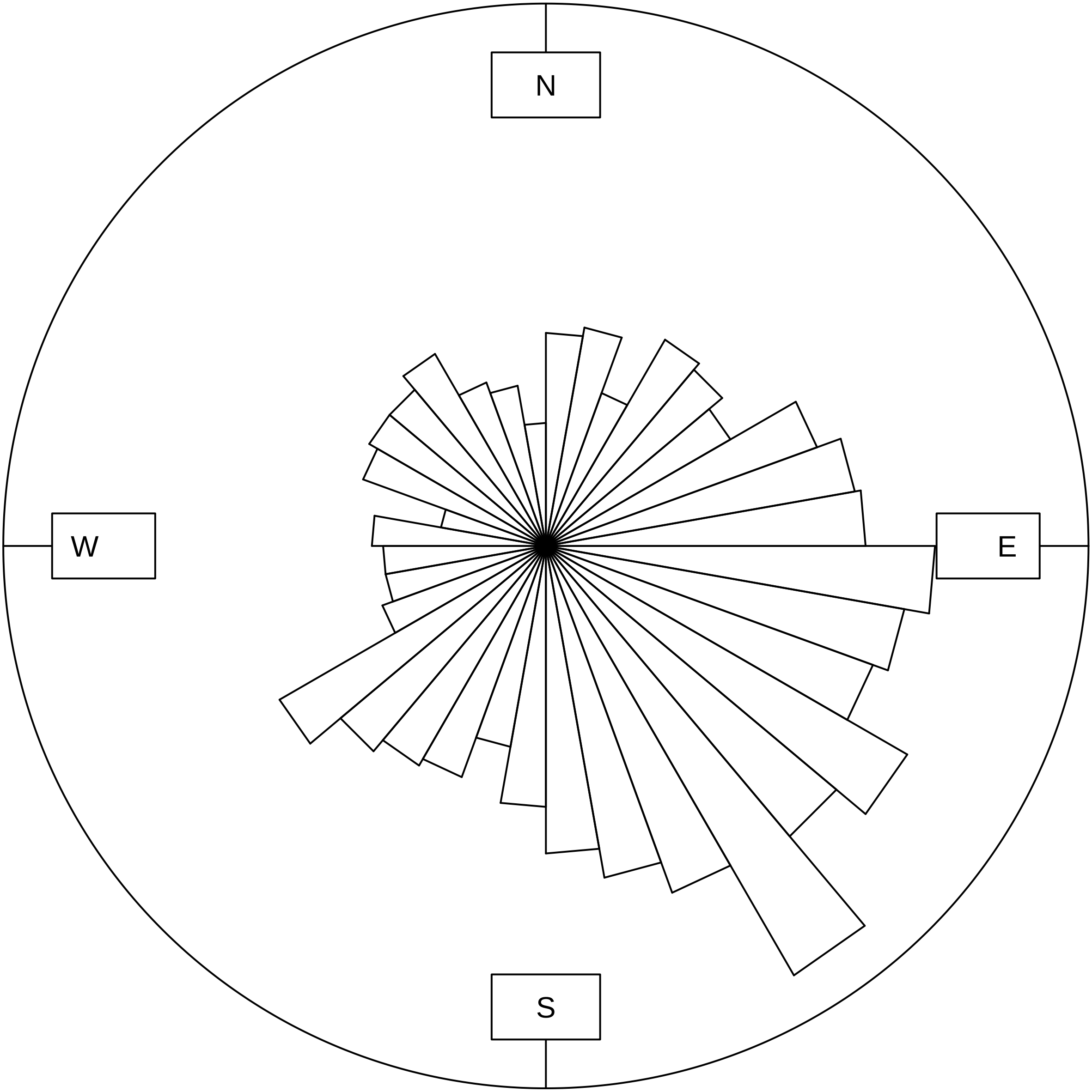

bootstrap 324

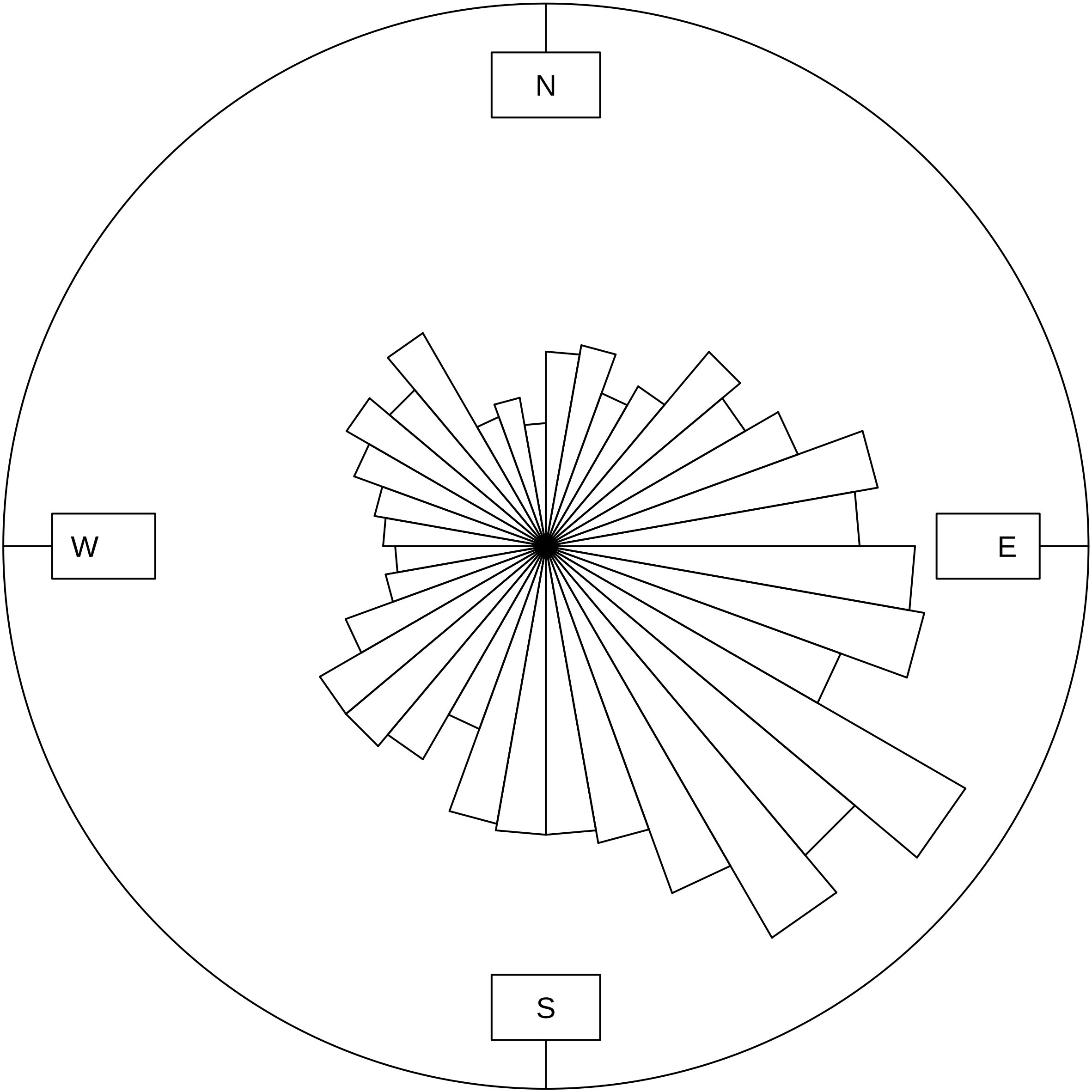

bootstrap 325

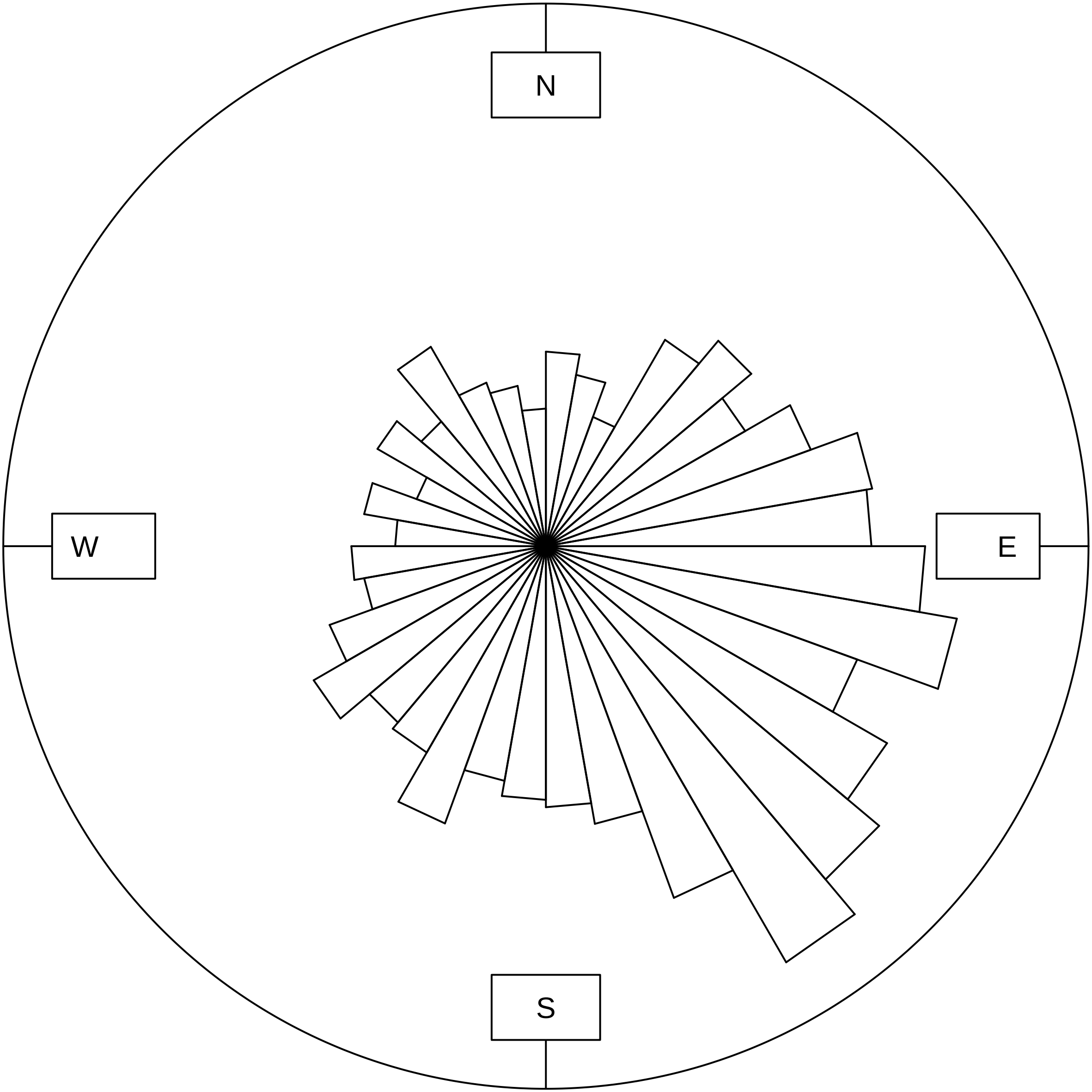

bootstrap 326

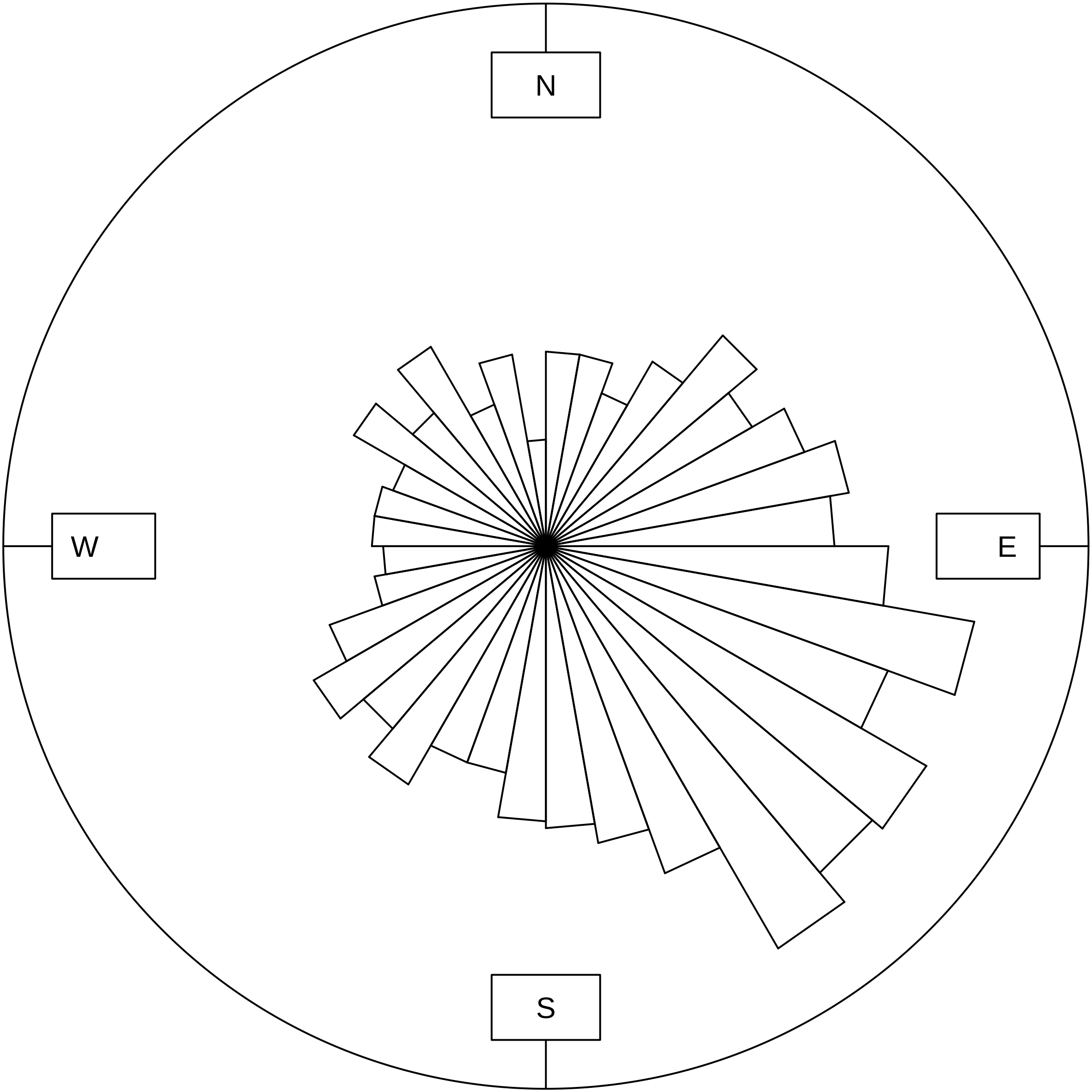

bootstrap 327

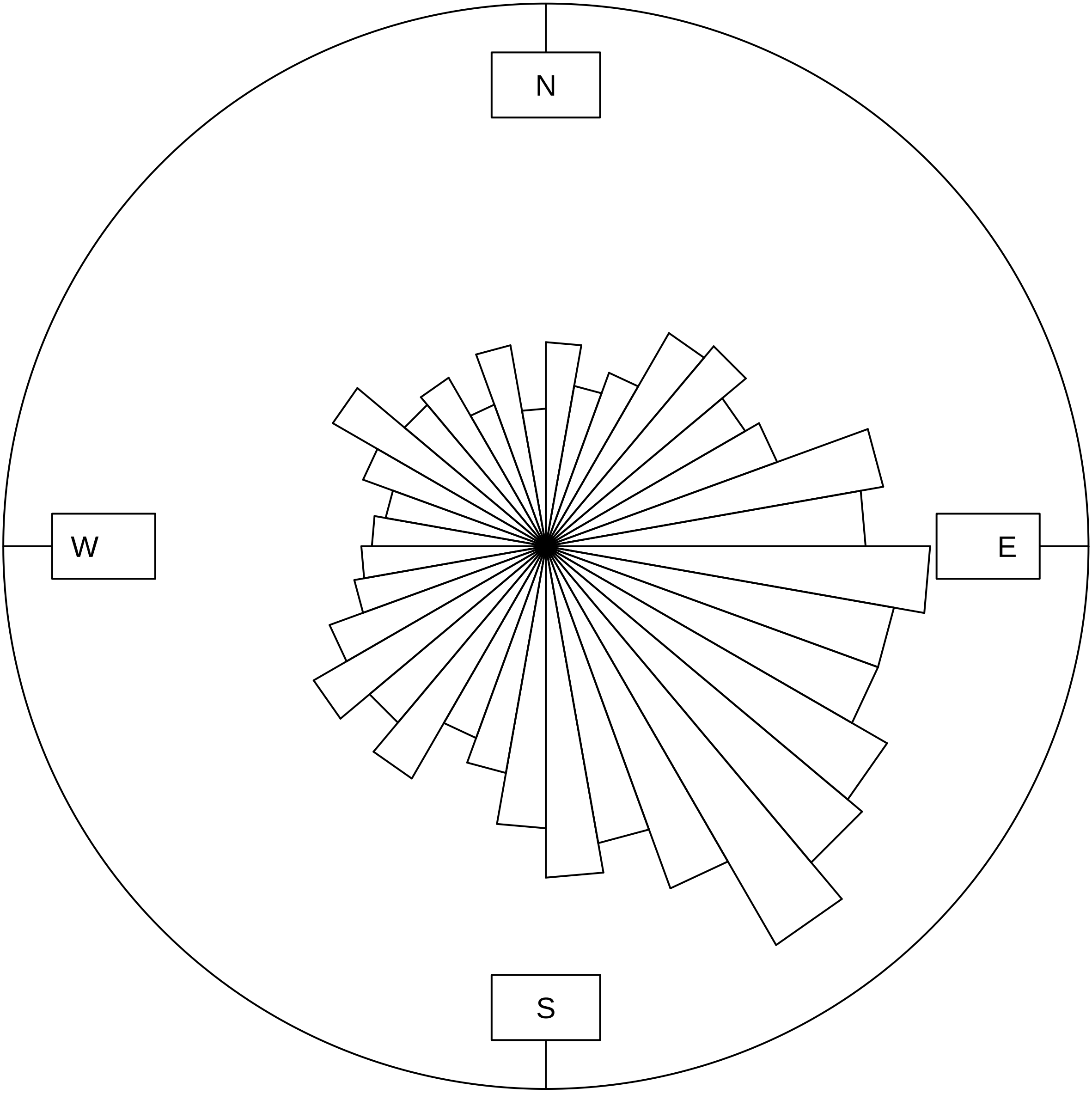

bootstrap 328

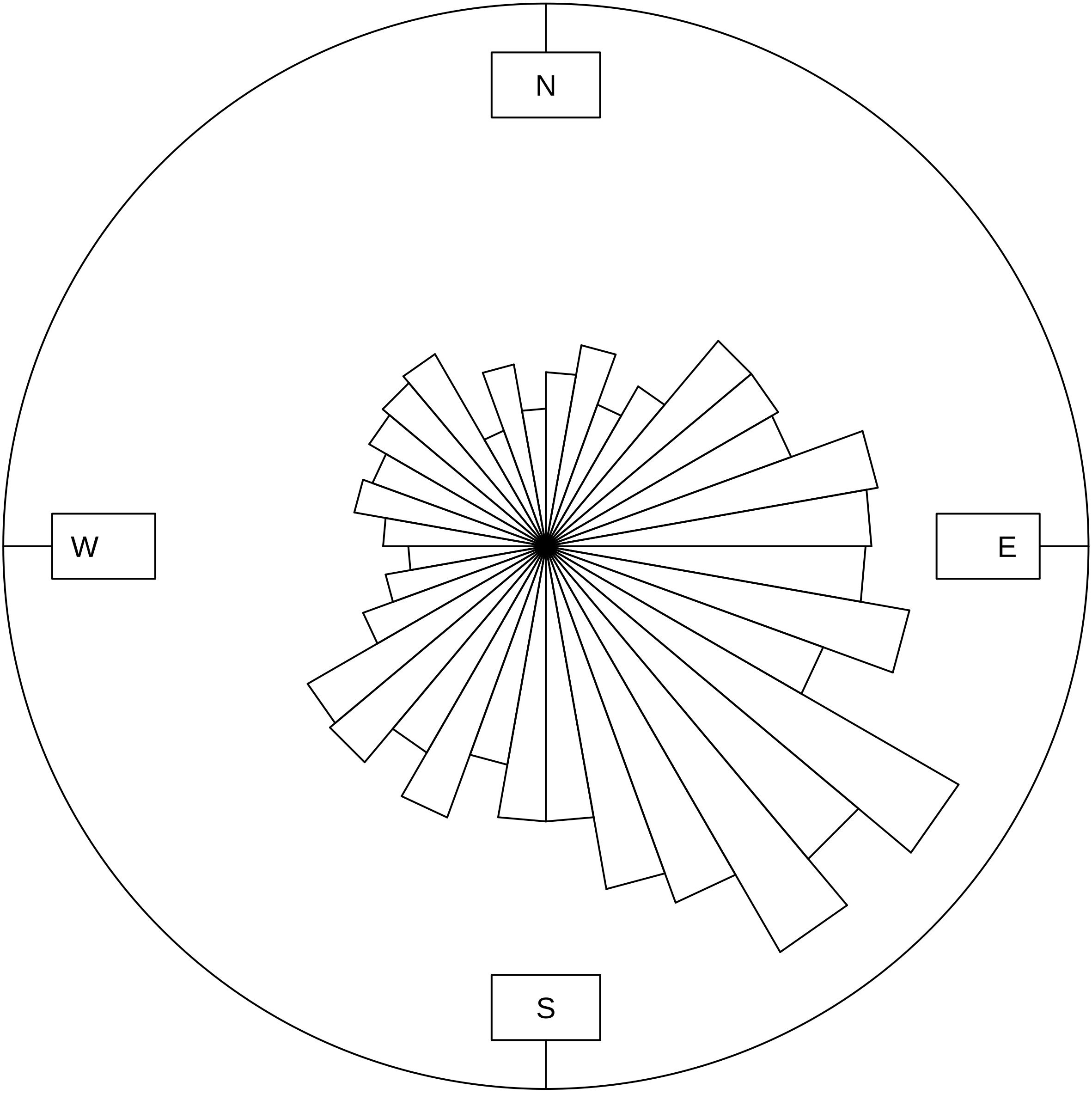

bootstrap 329

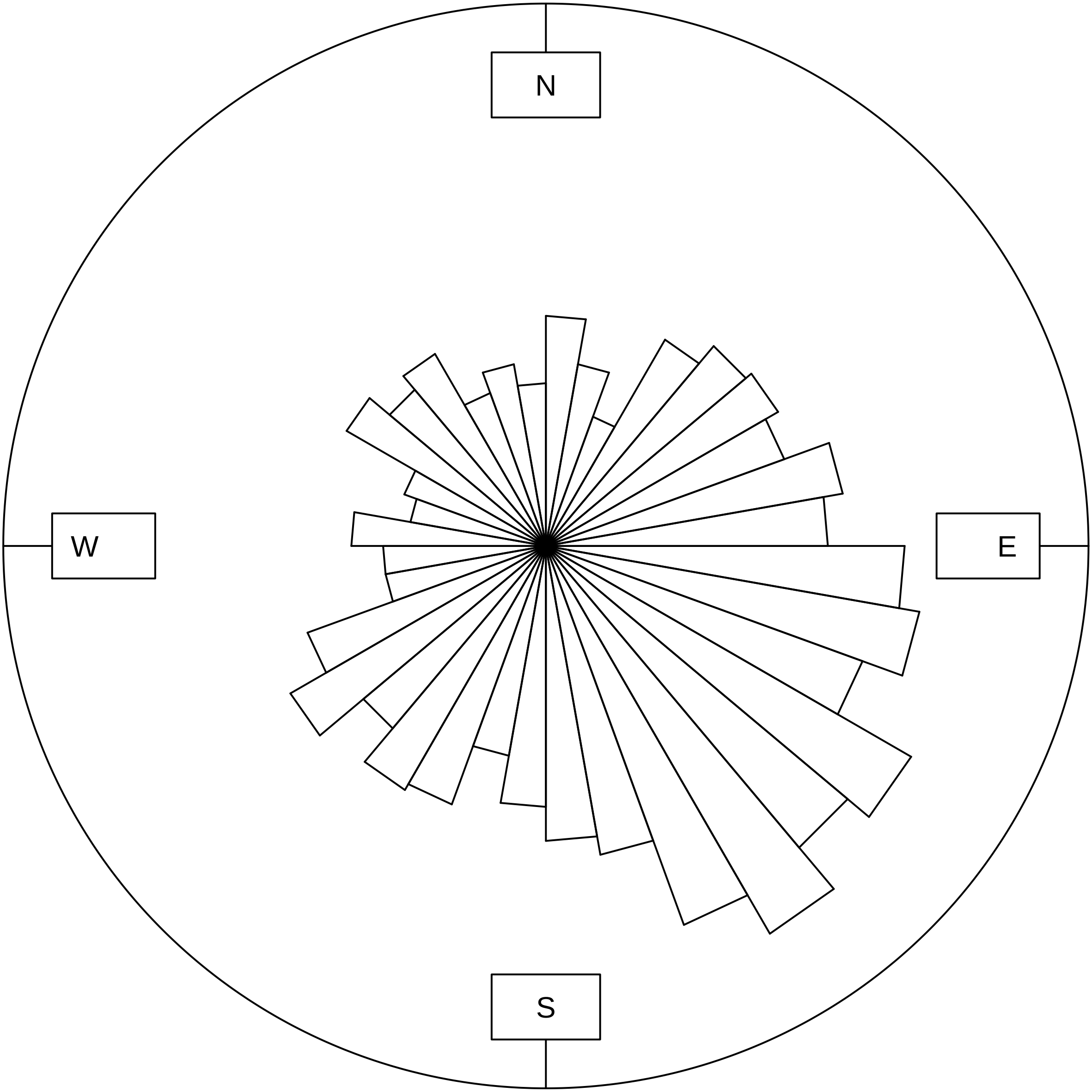

bootstrap 330

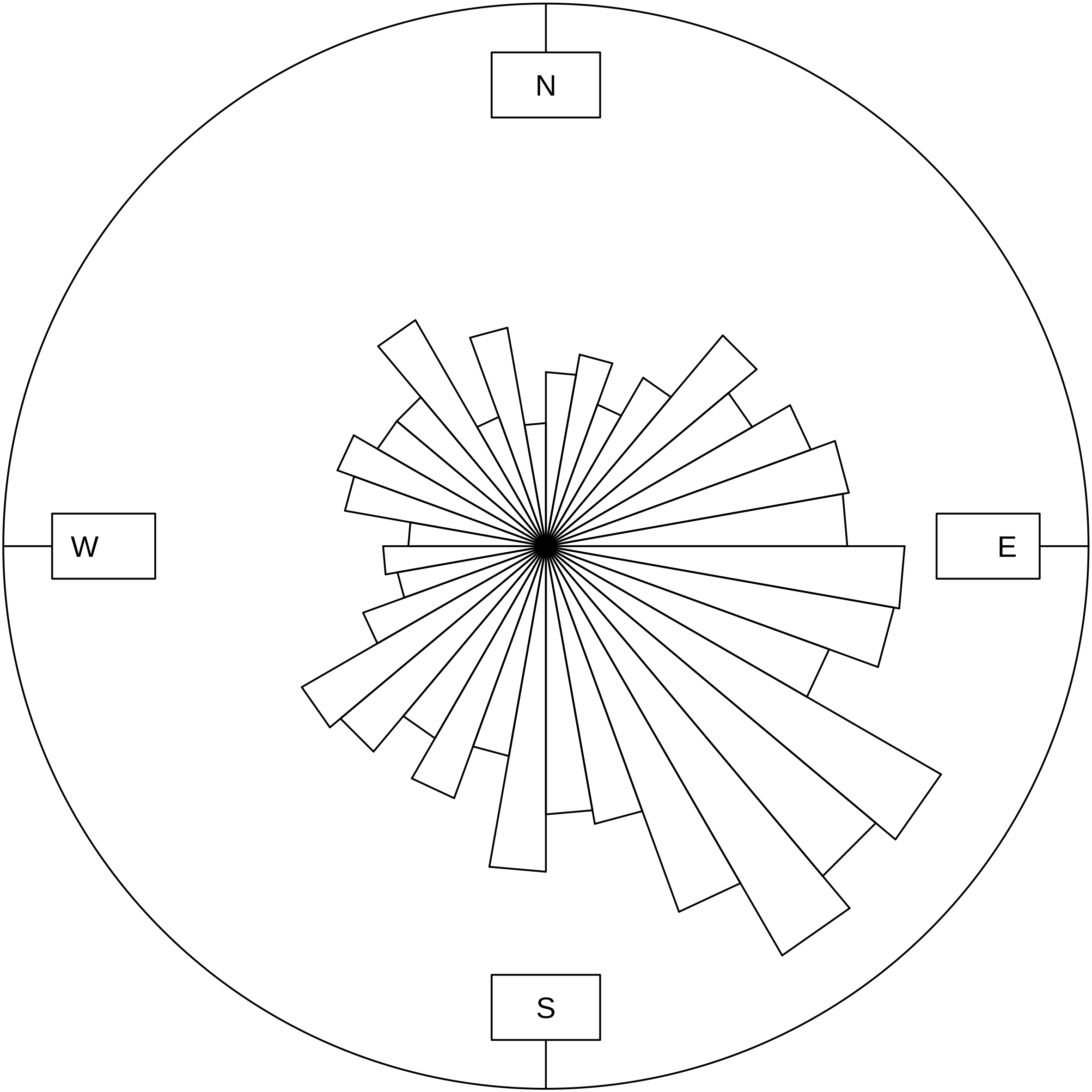

bootstrap 331

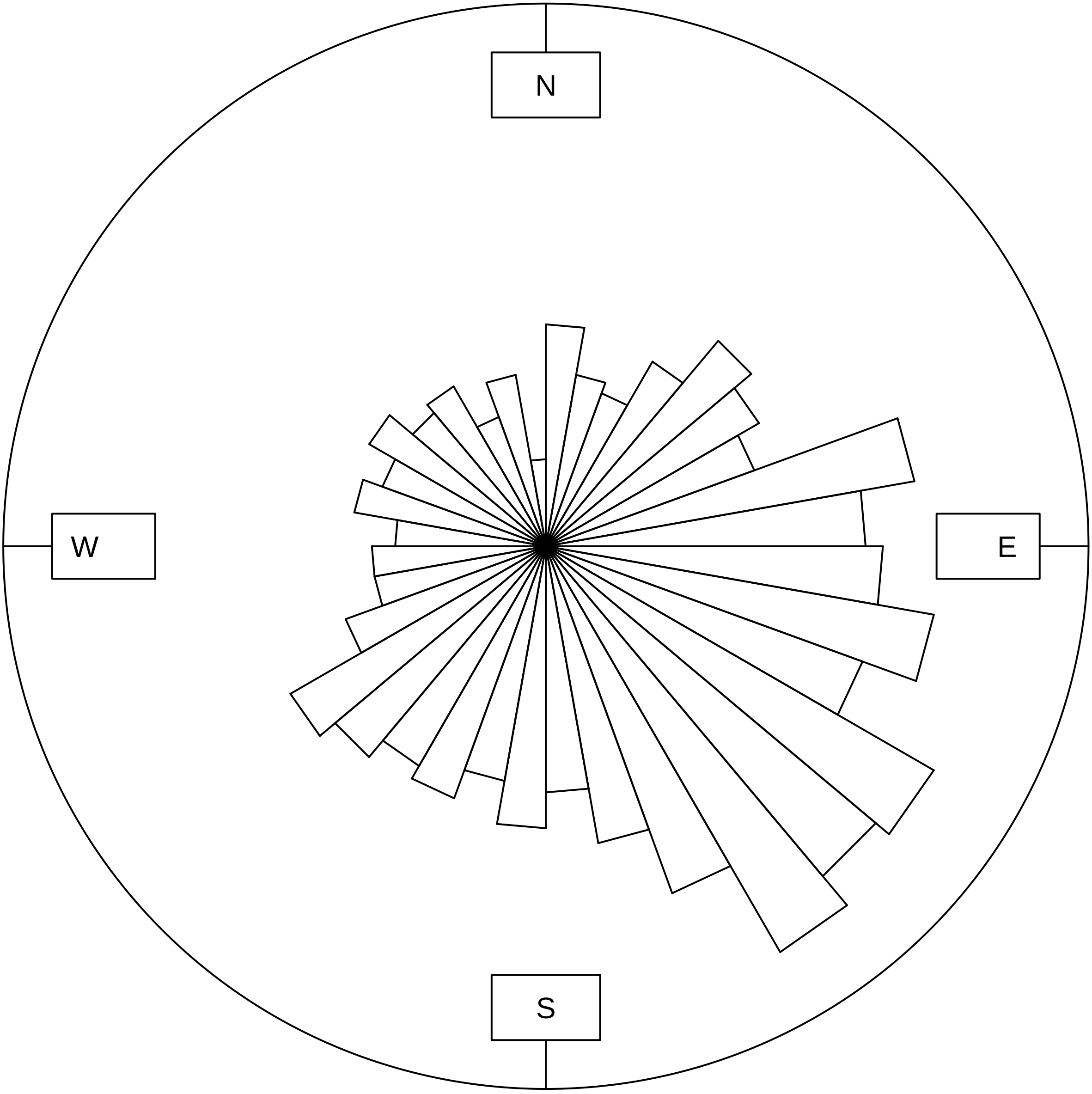

bootstrap 332

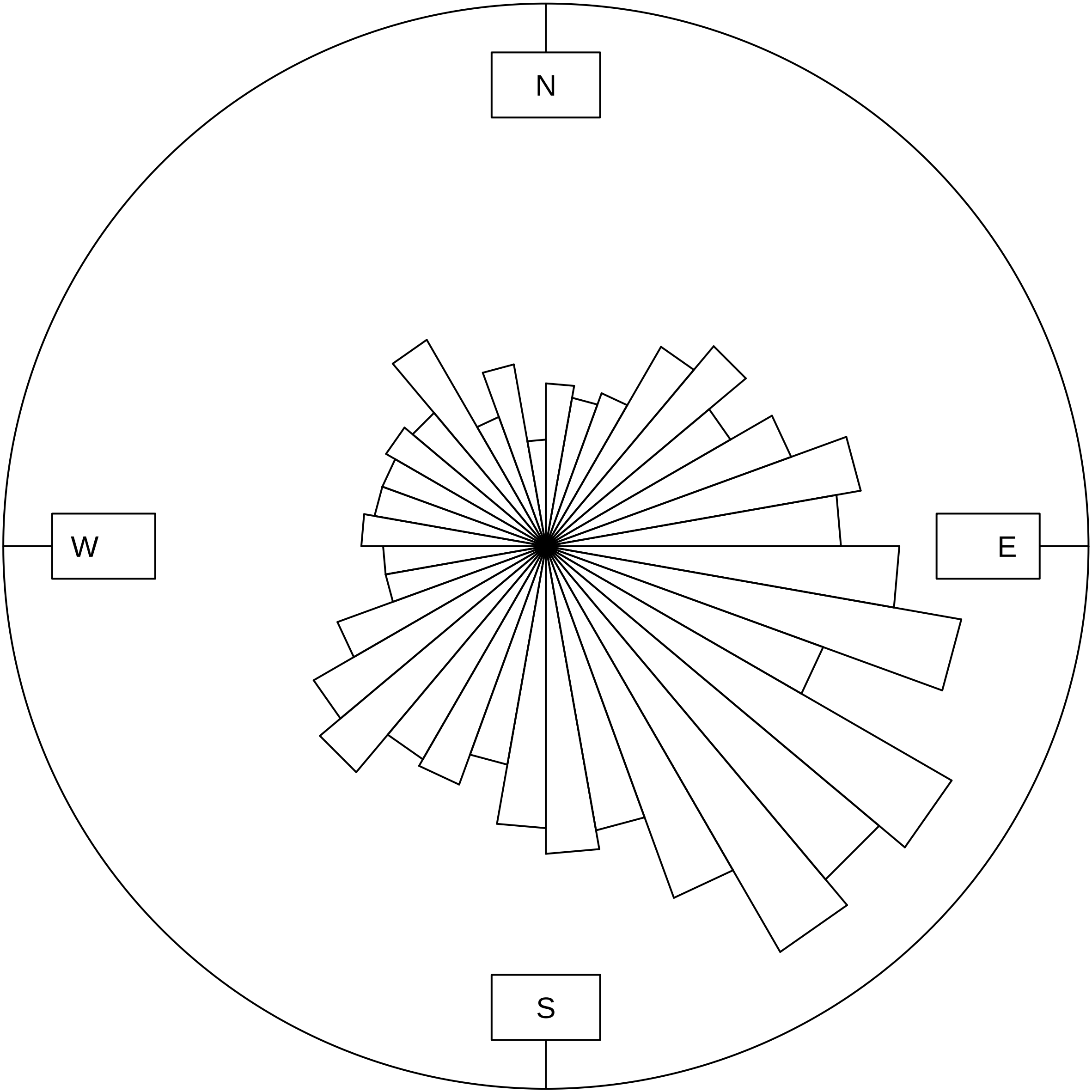

bootstrap 333

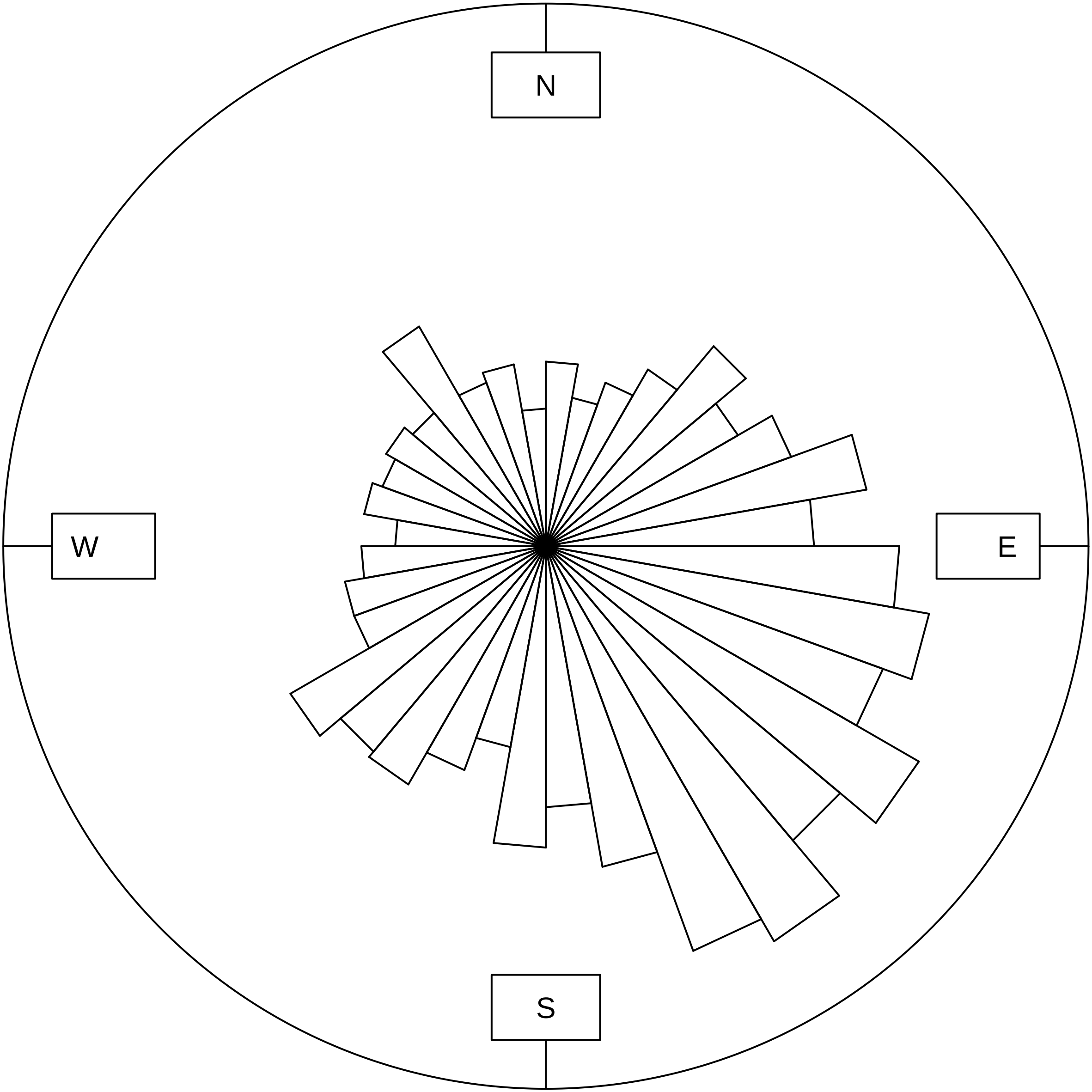

bootstrap 334

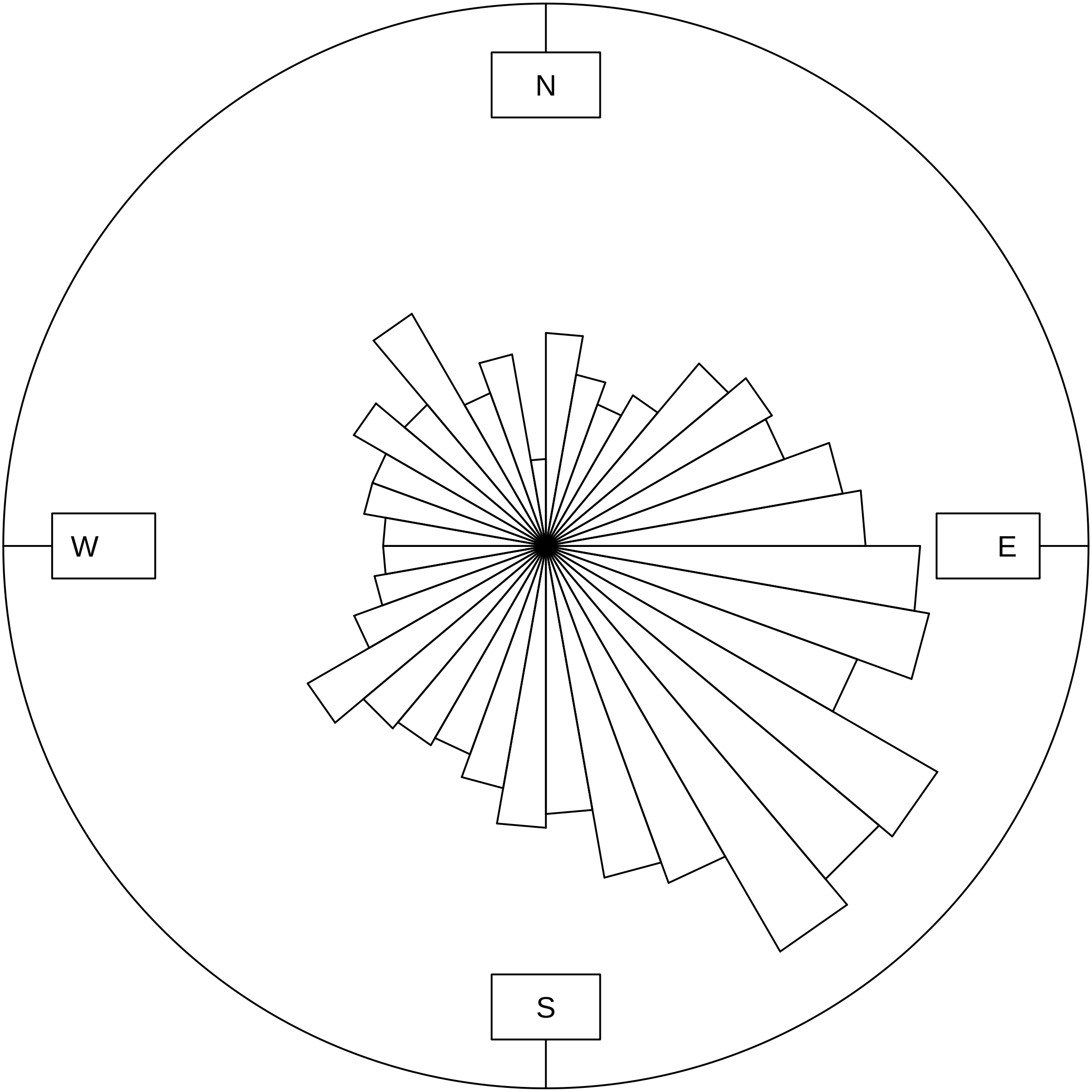

bootstrap 335

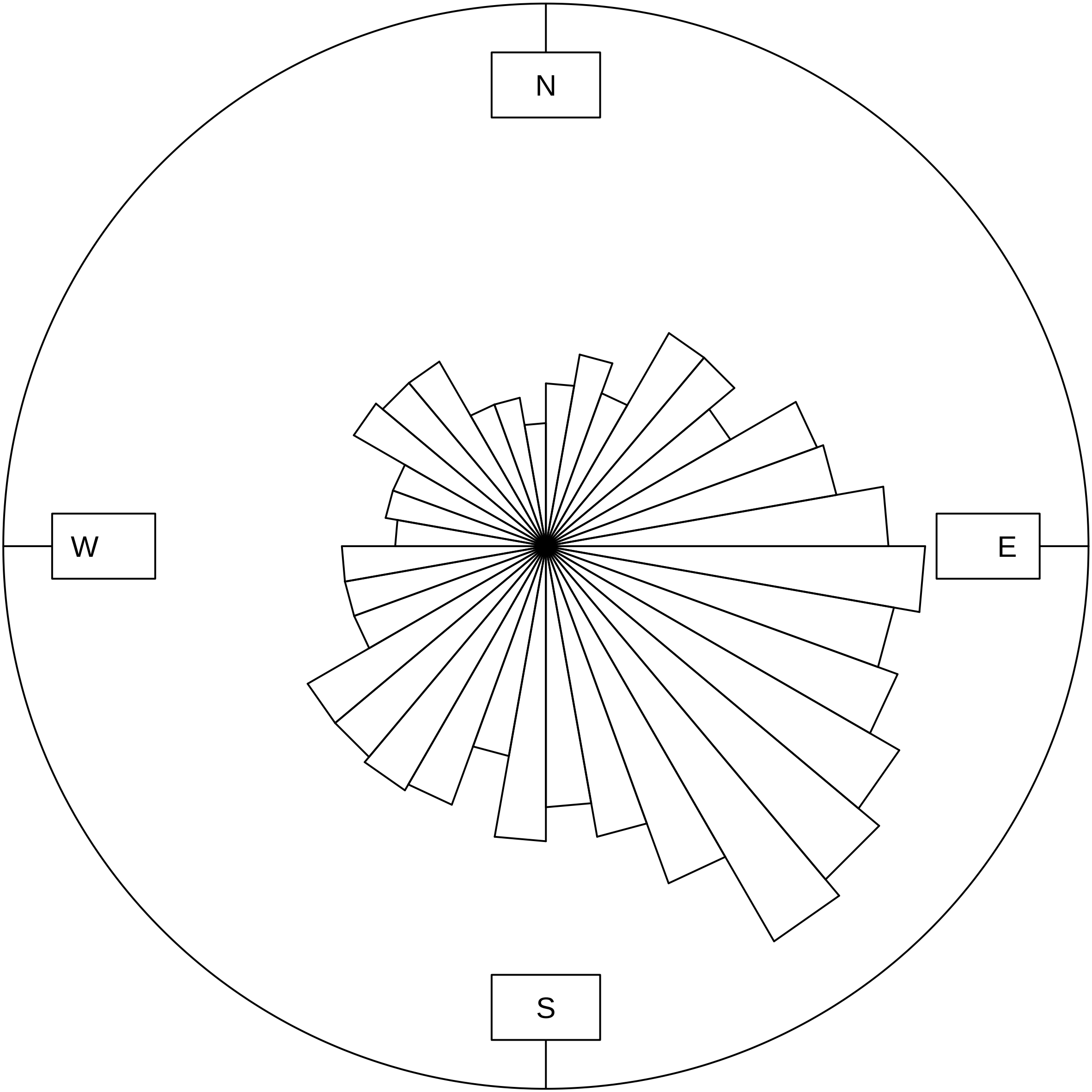

bootstrap 336

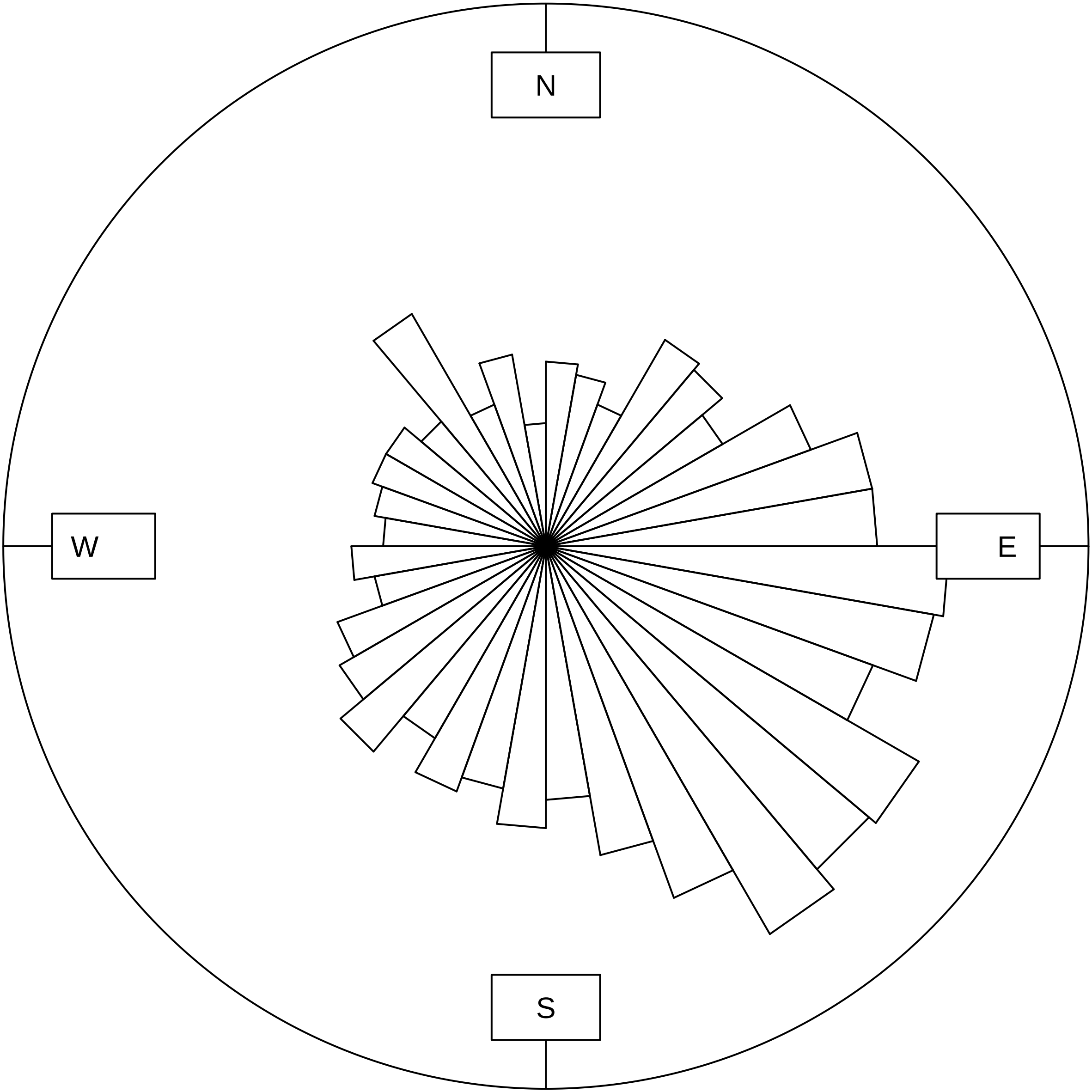

bootstrap 337

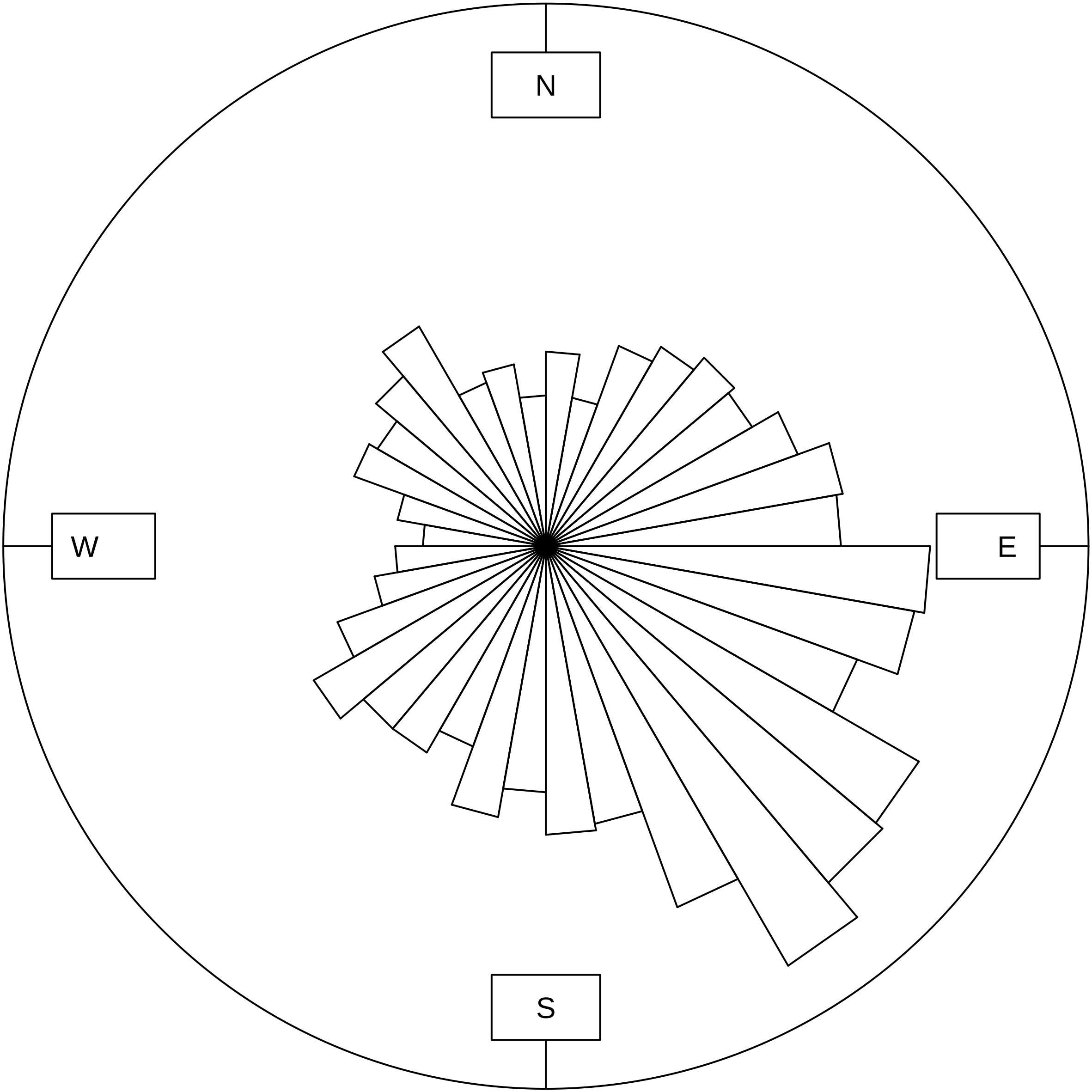

bootstrap 338

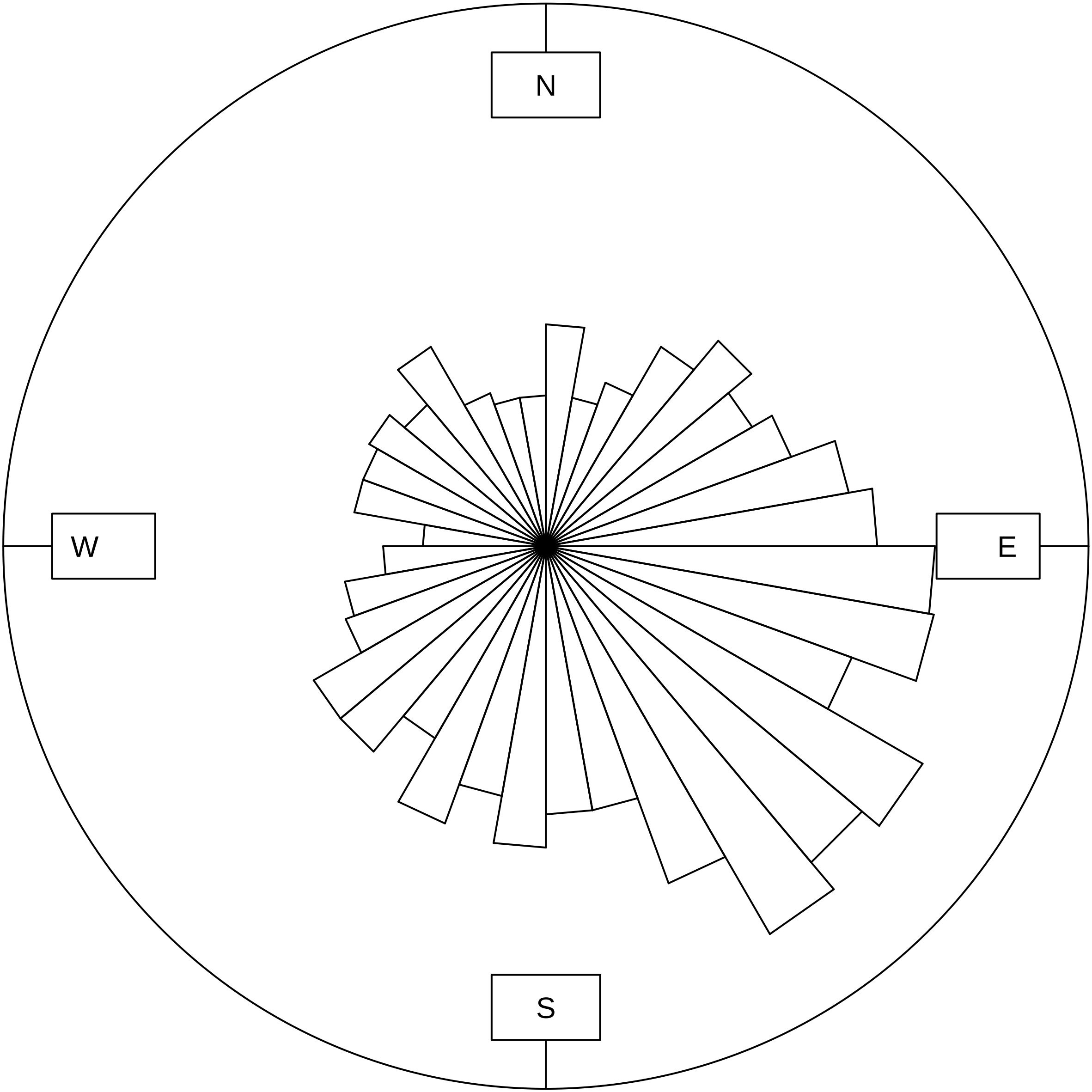

bootstrap 339

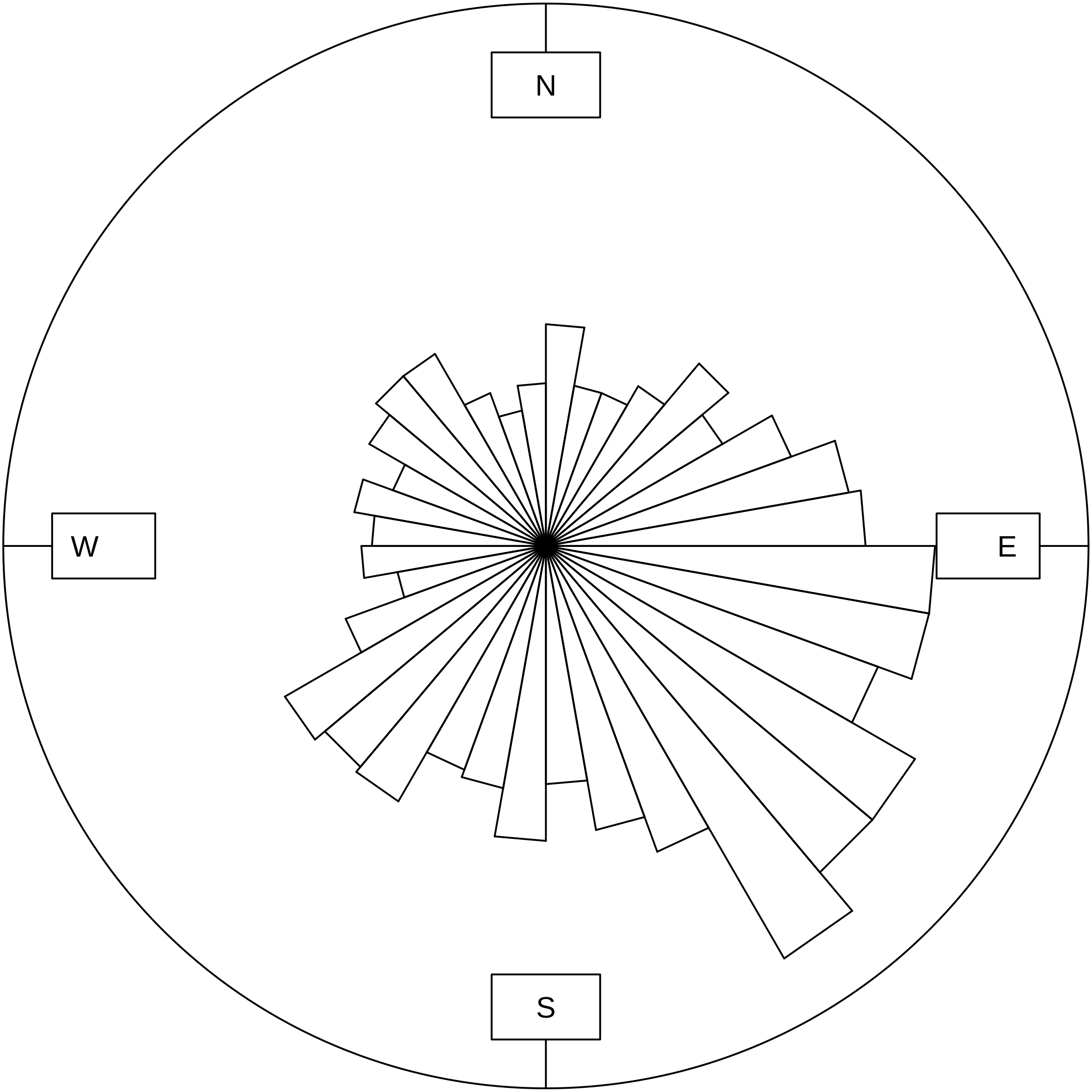

bootstrap 340

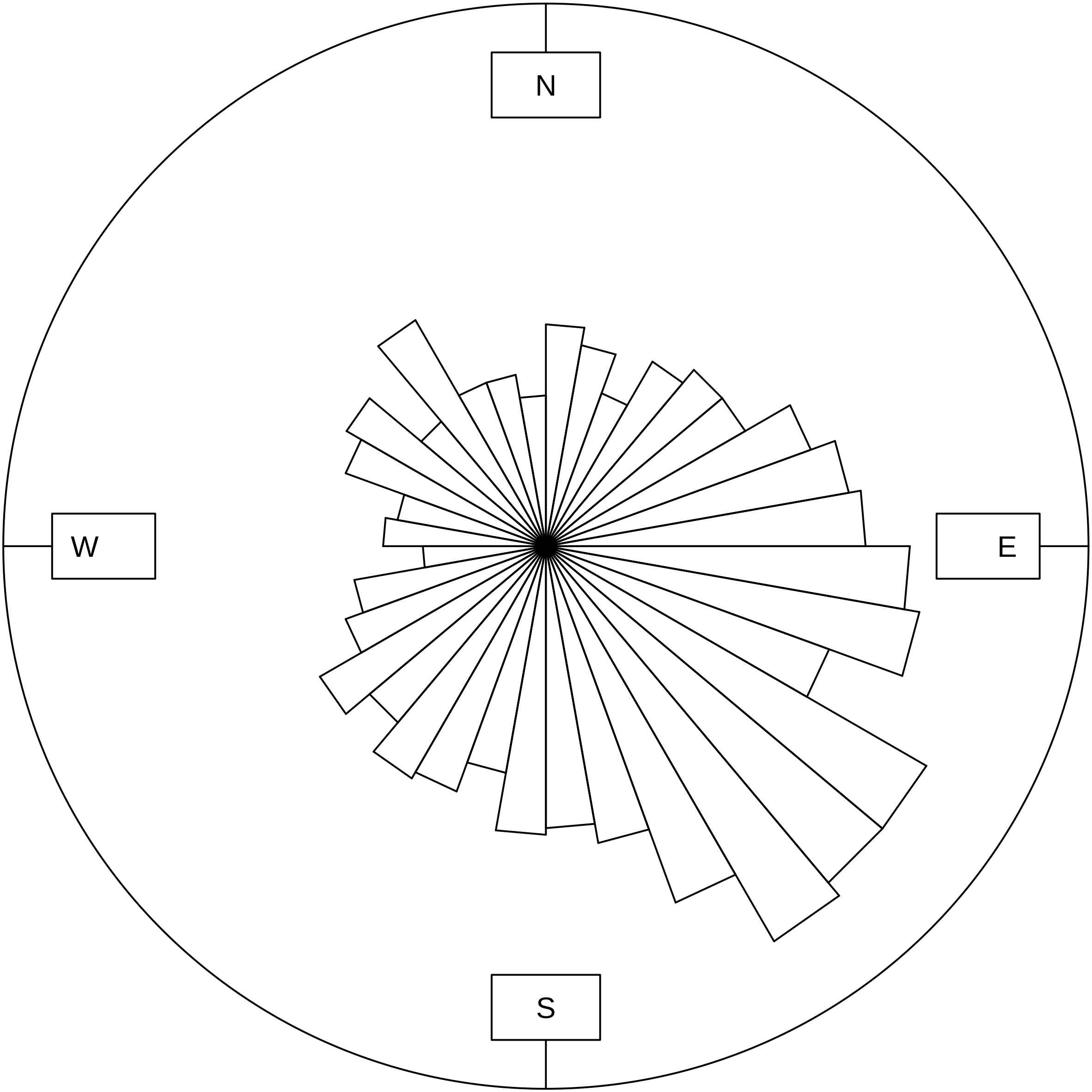

bootstrap 341

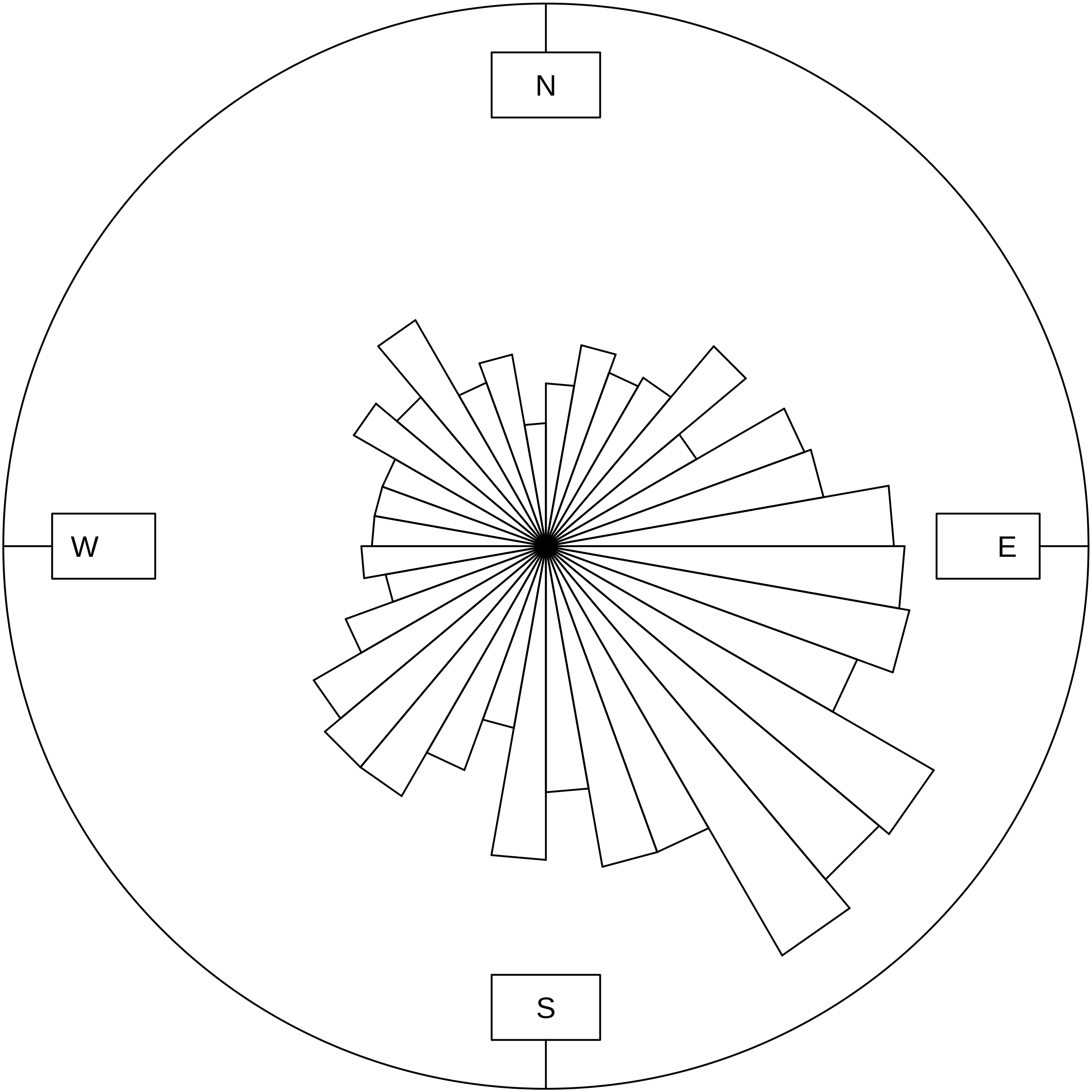

bootstrap 342

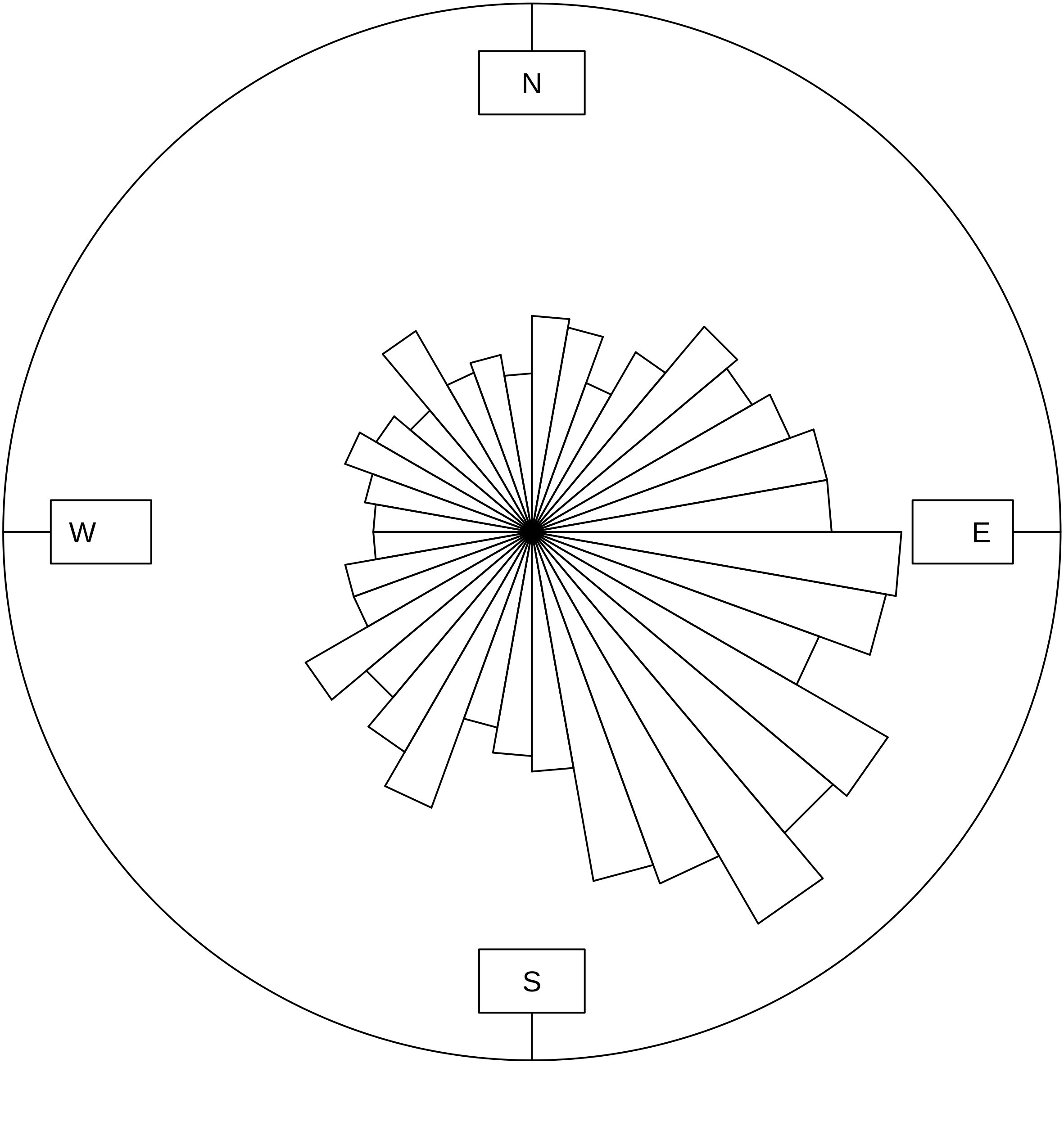

bootstrap 343

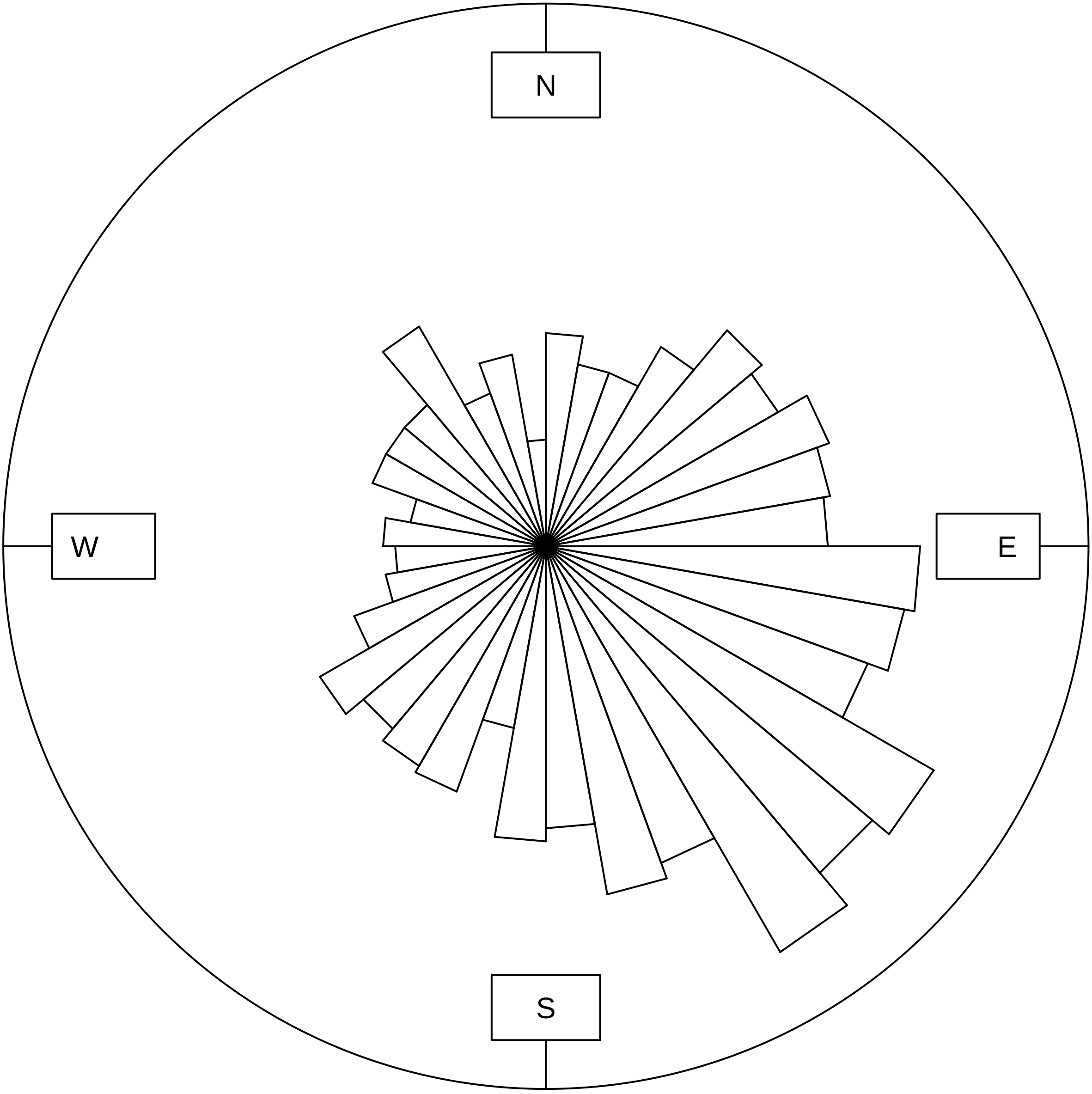

bootstrap 344

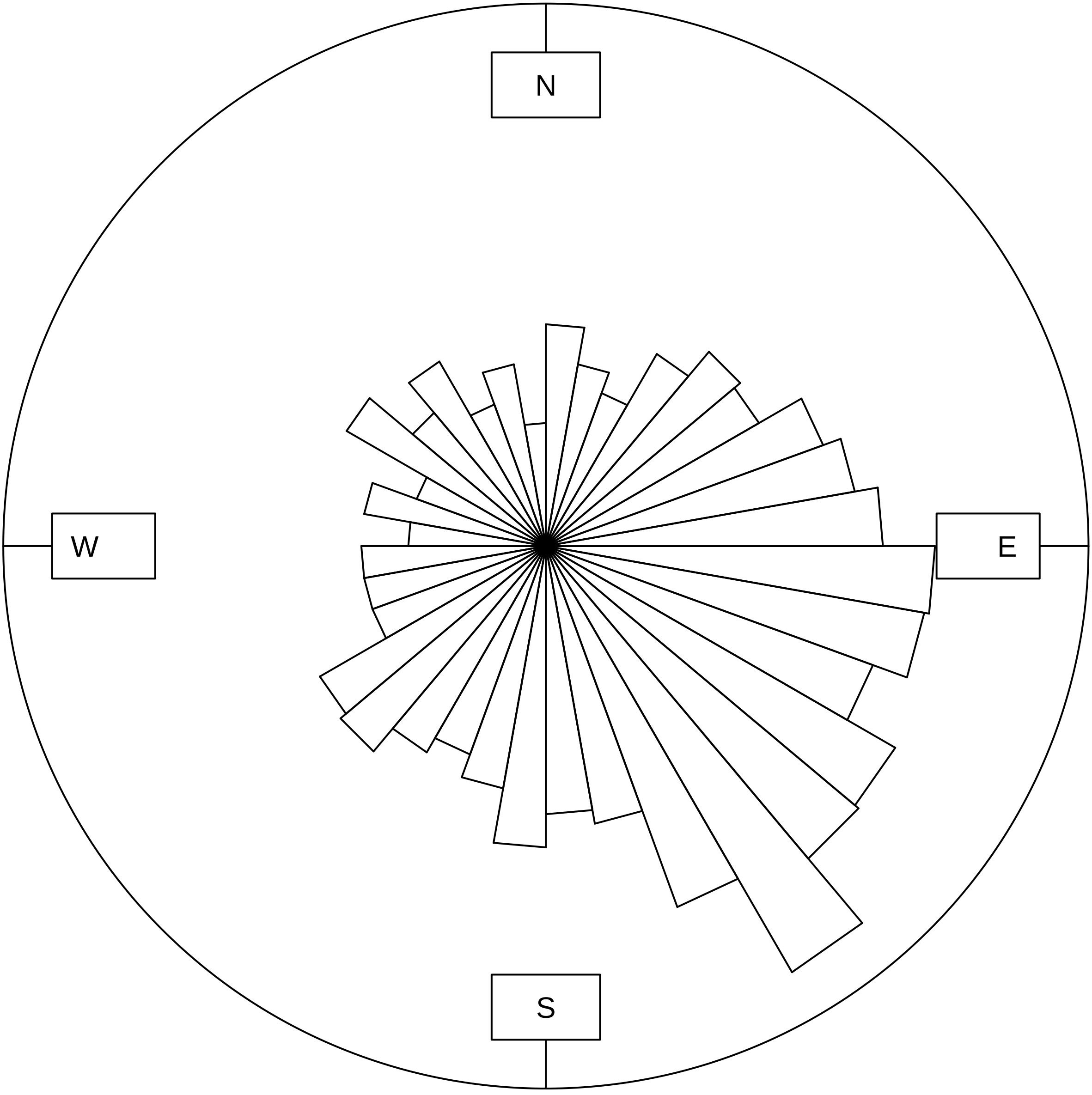

bootstrap 345

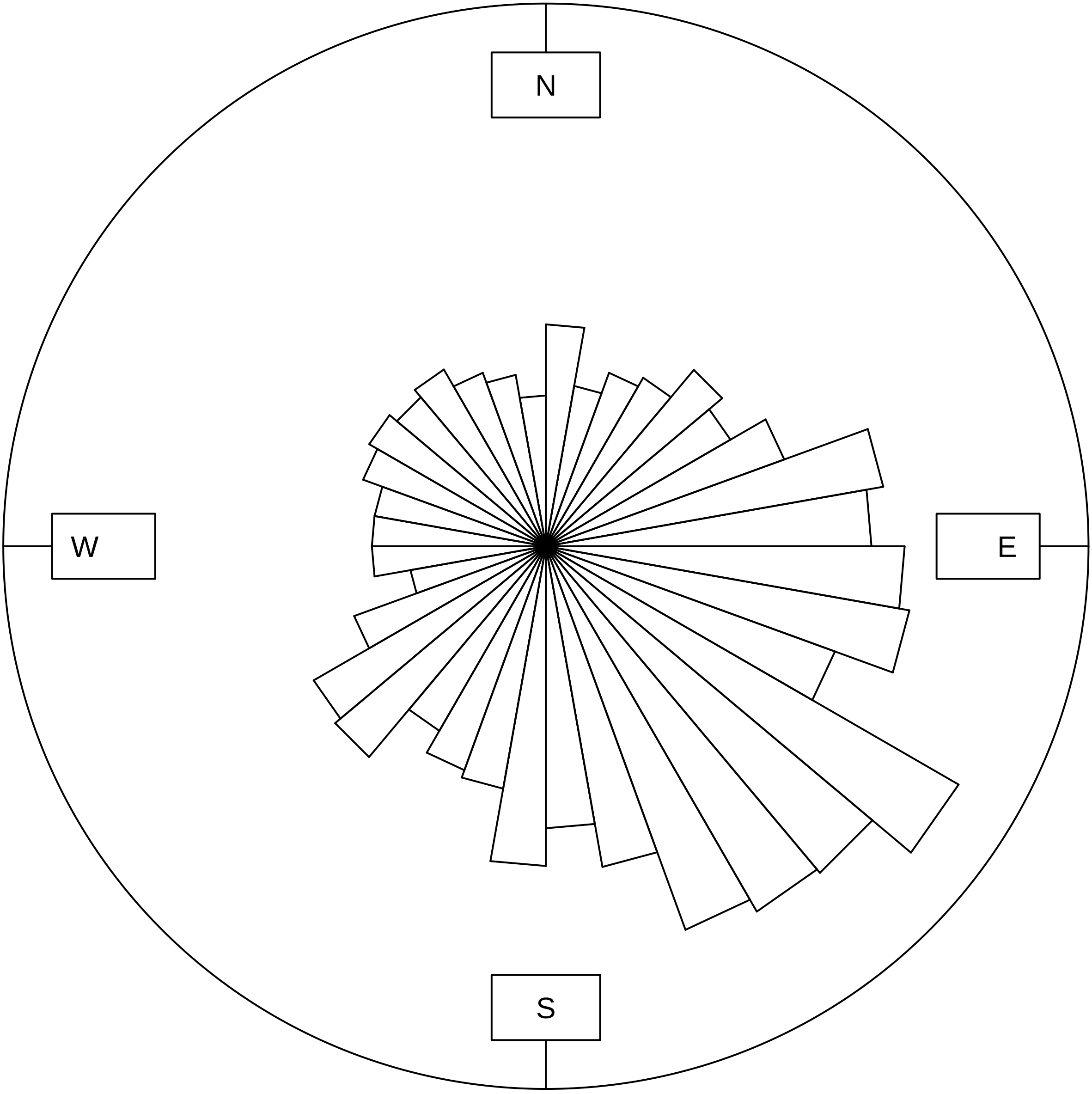

bootstrap 346

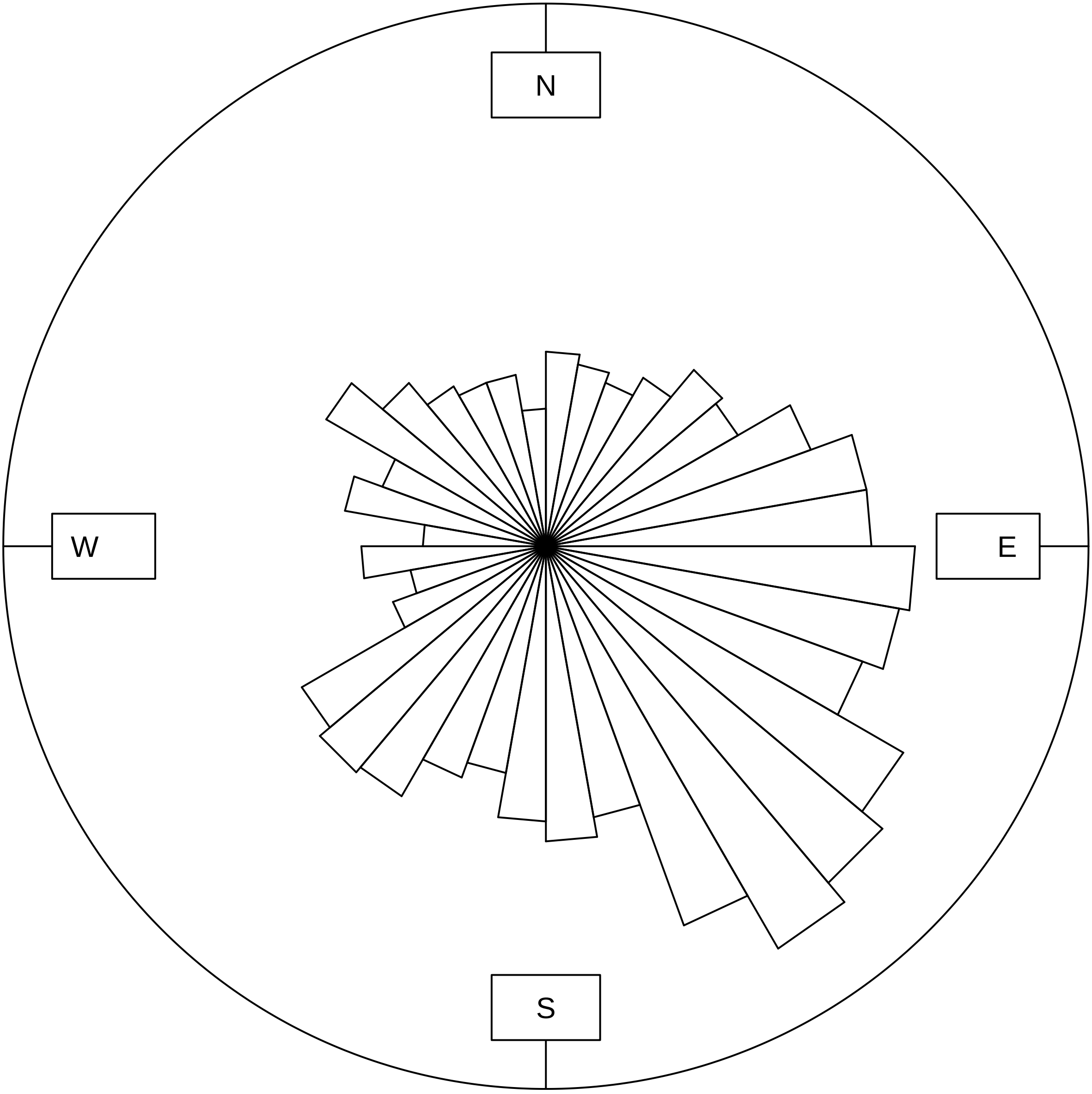

bootstrap 347

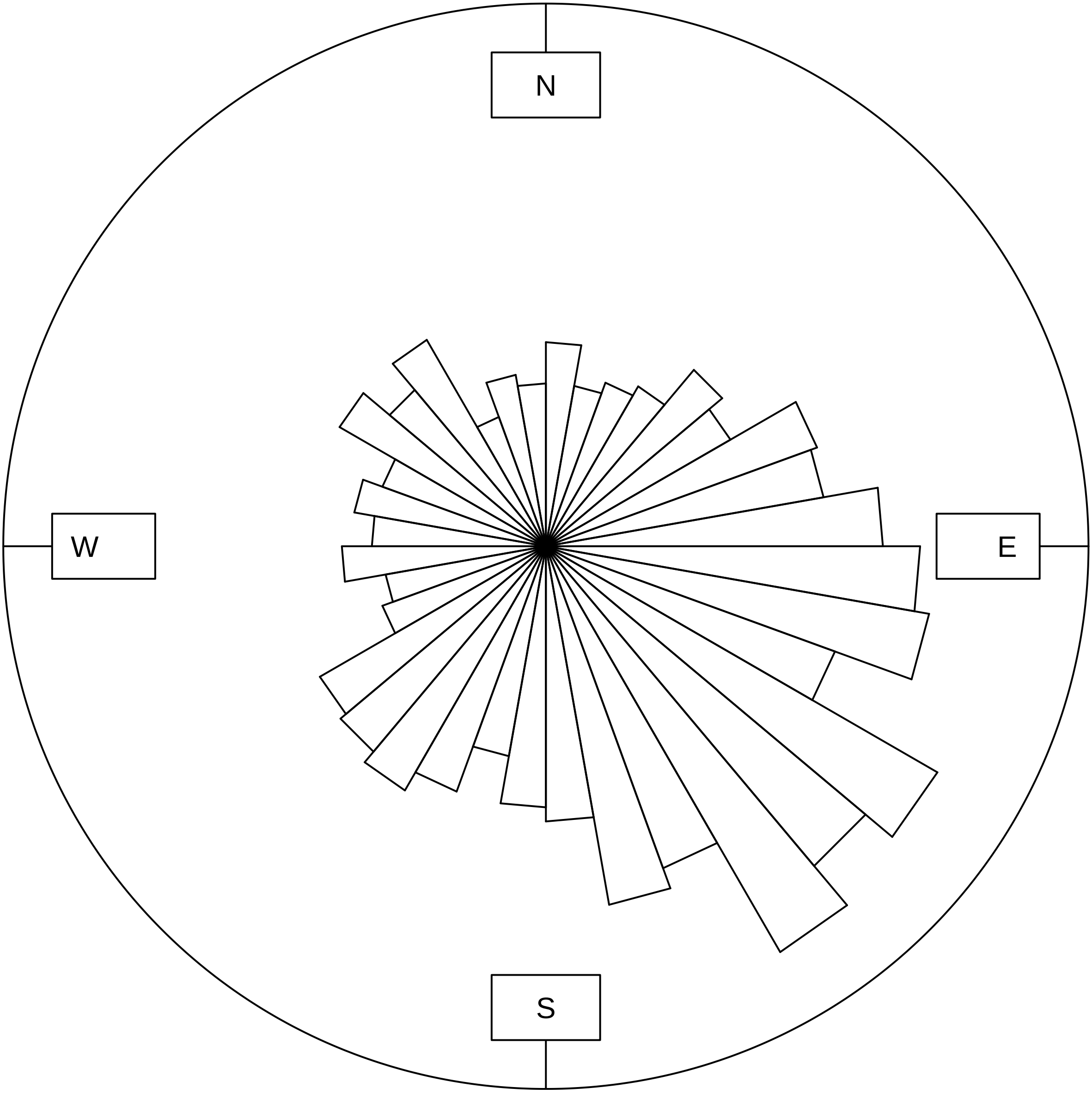

bootstrap 348

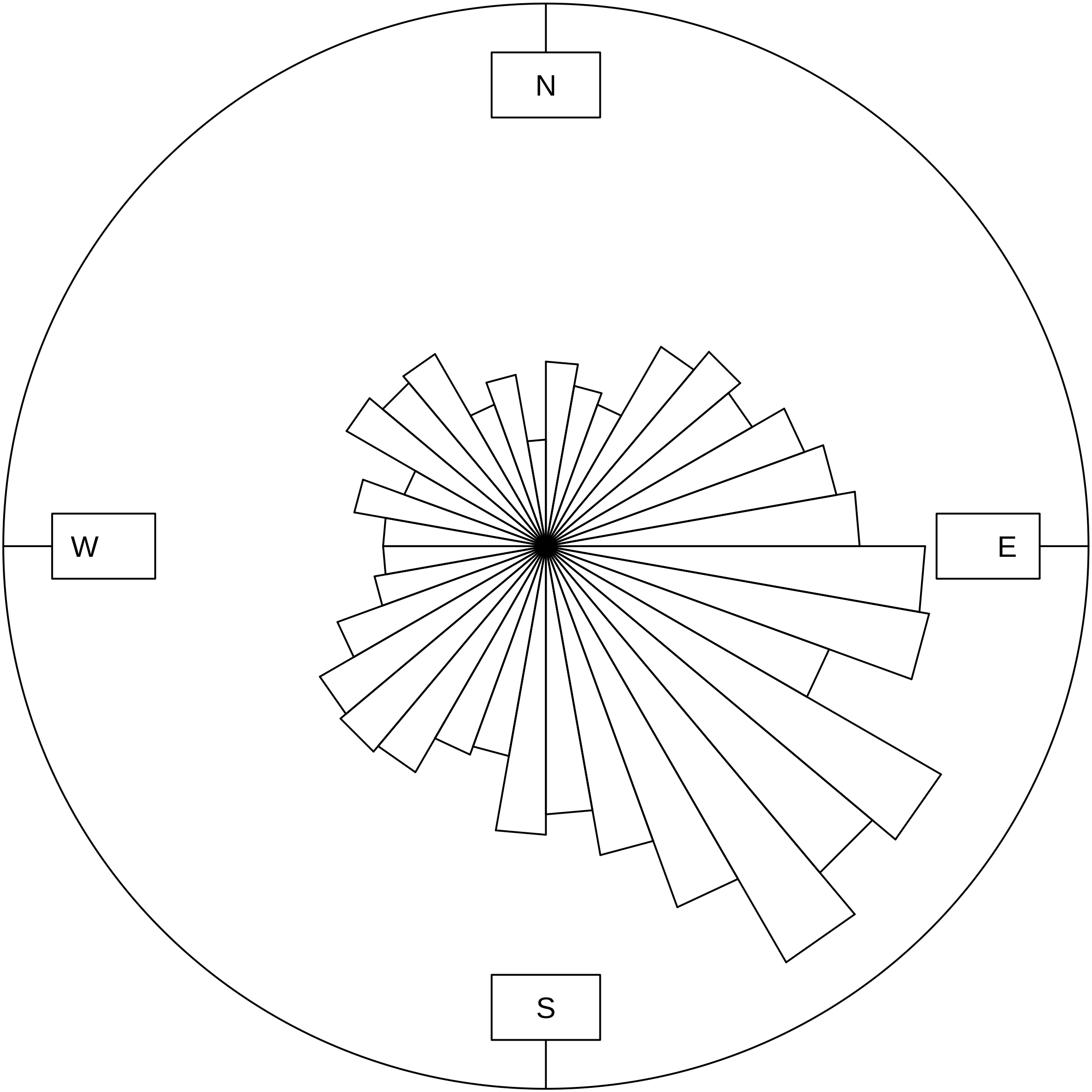

bootstrap 349

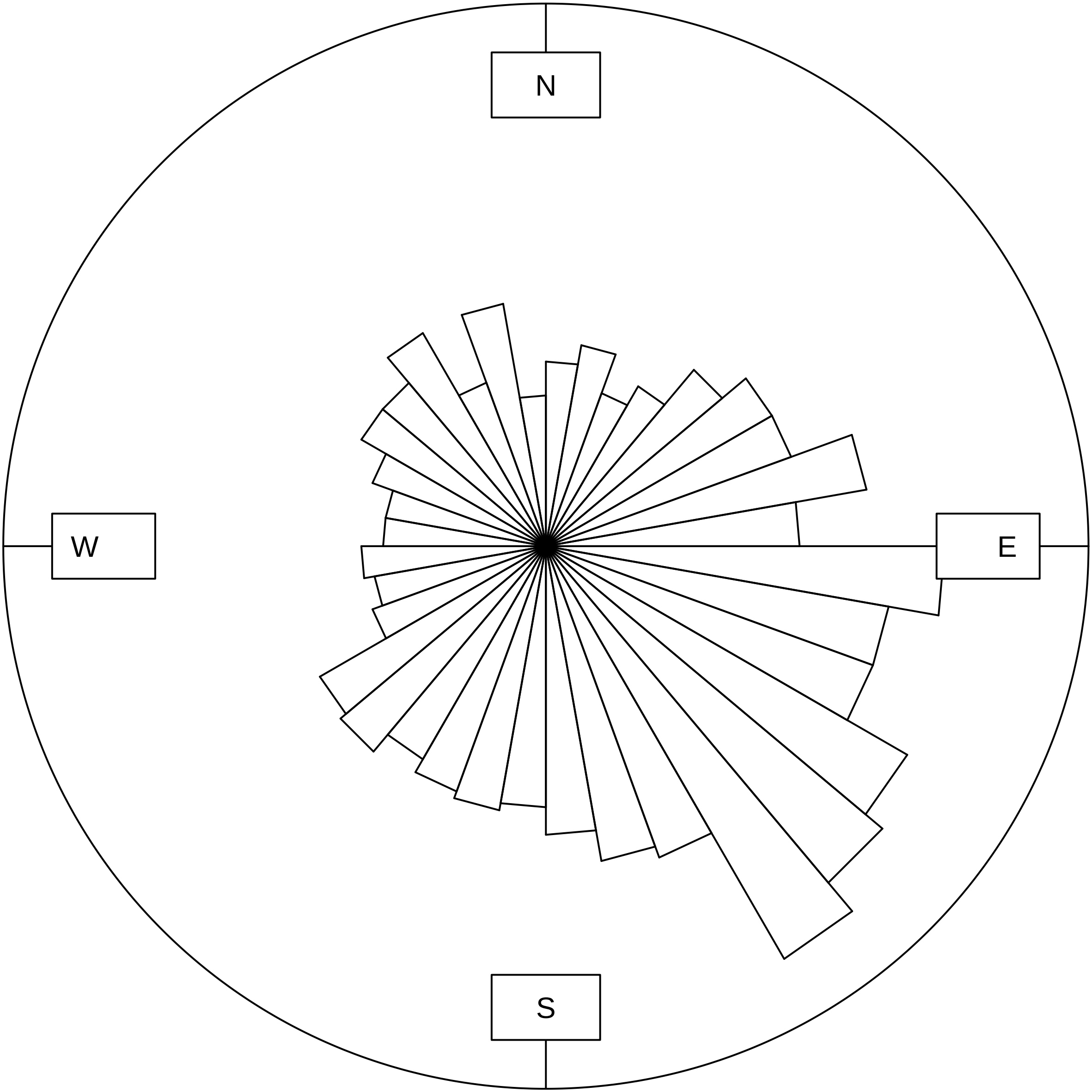

bootstrap 350

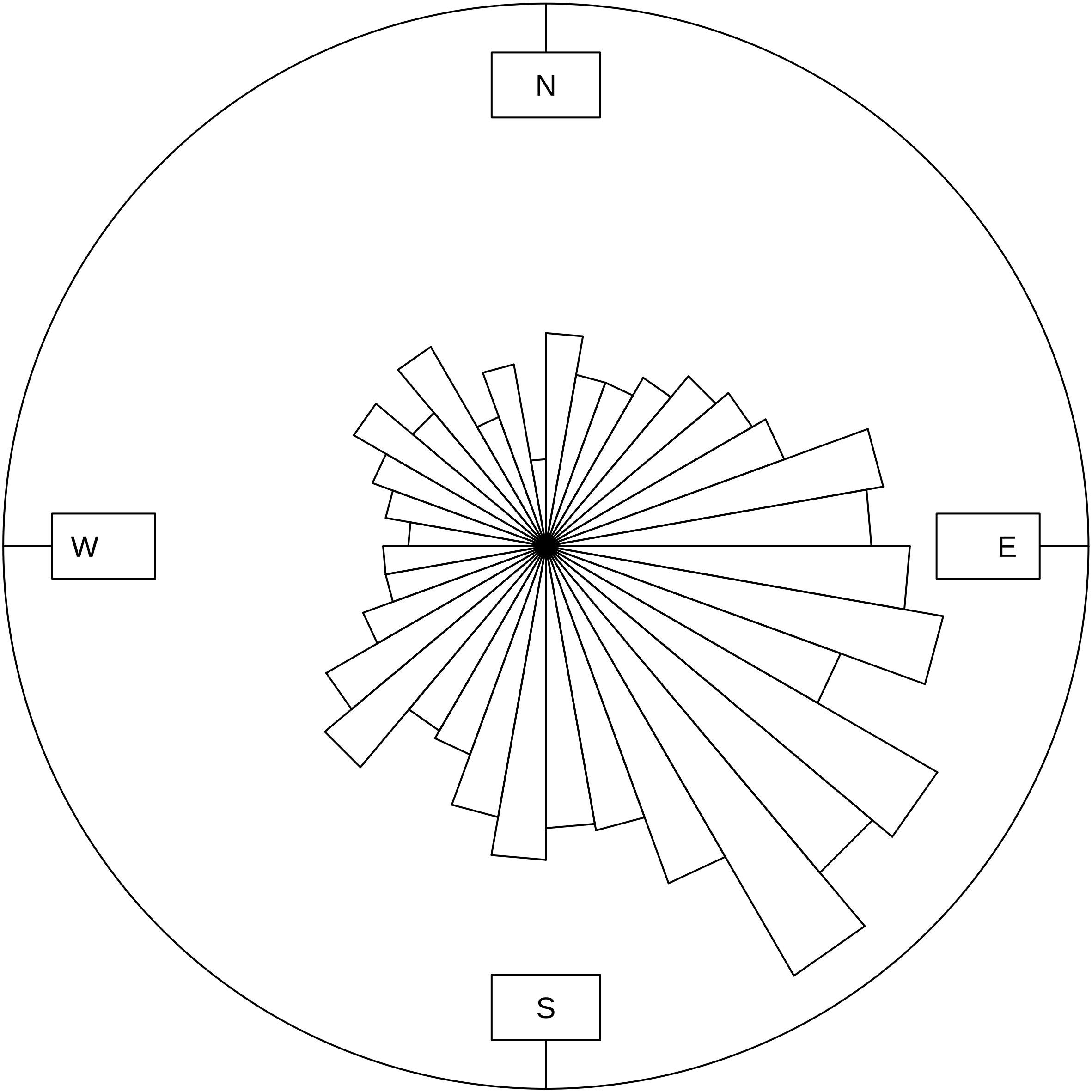

bootstrap 351

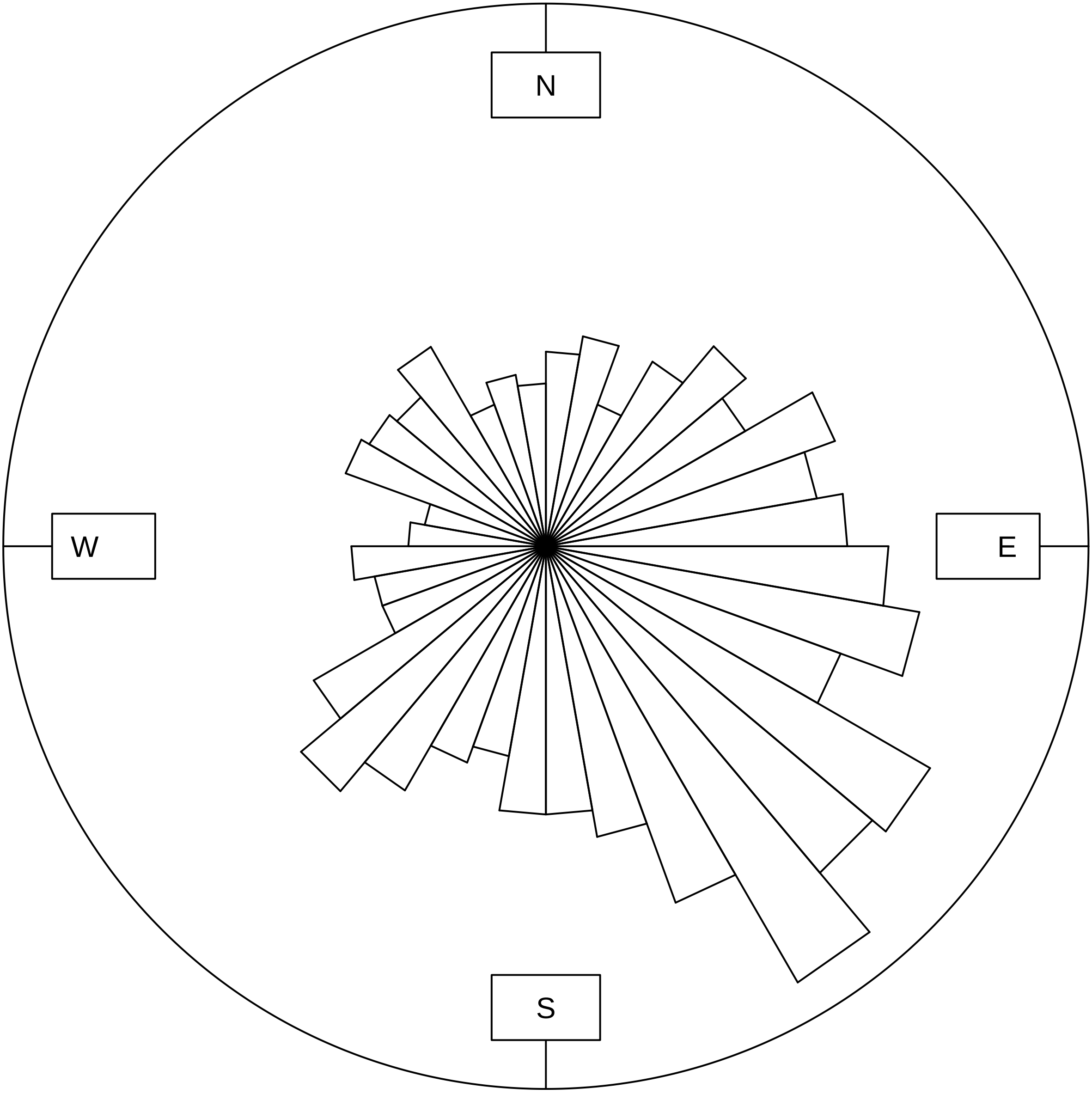

bootstrap 352

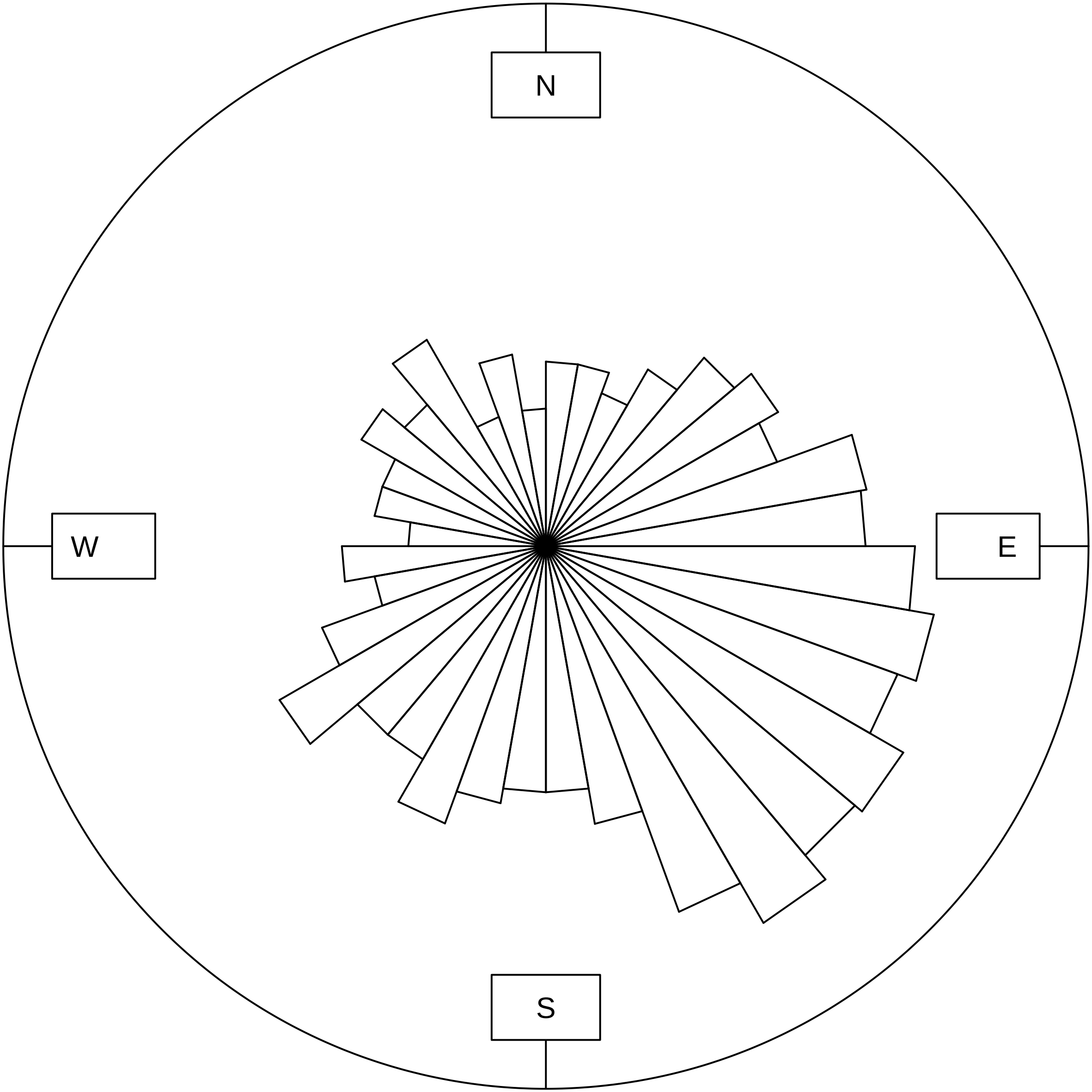

bootstrap 353

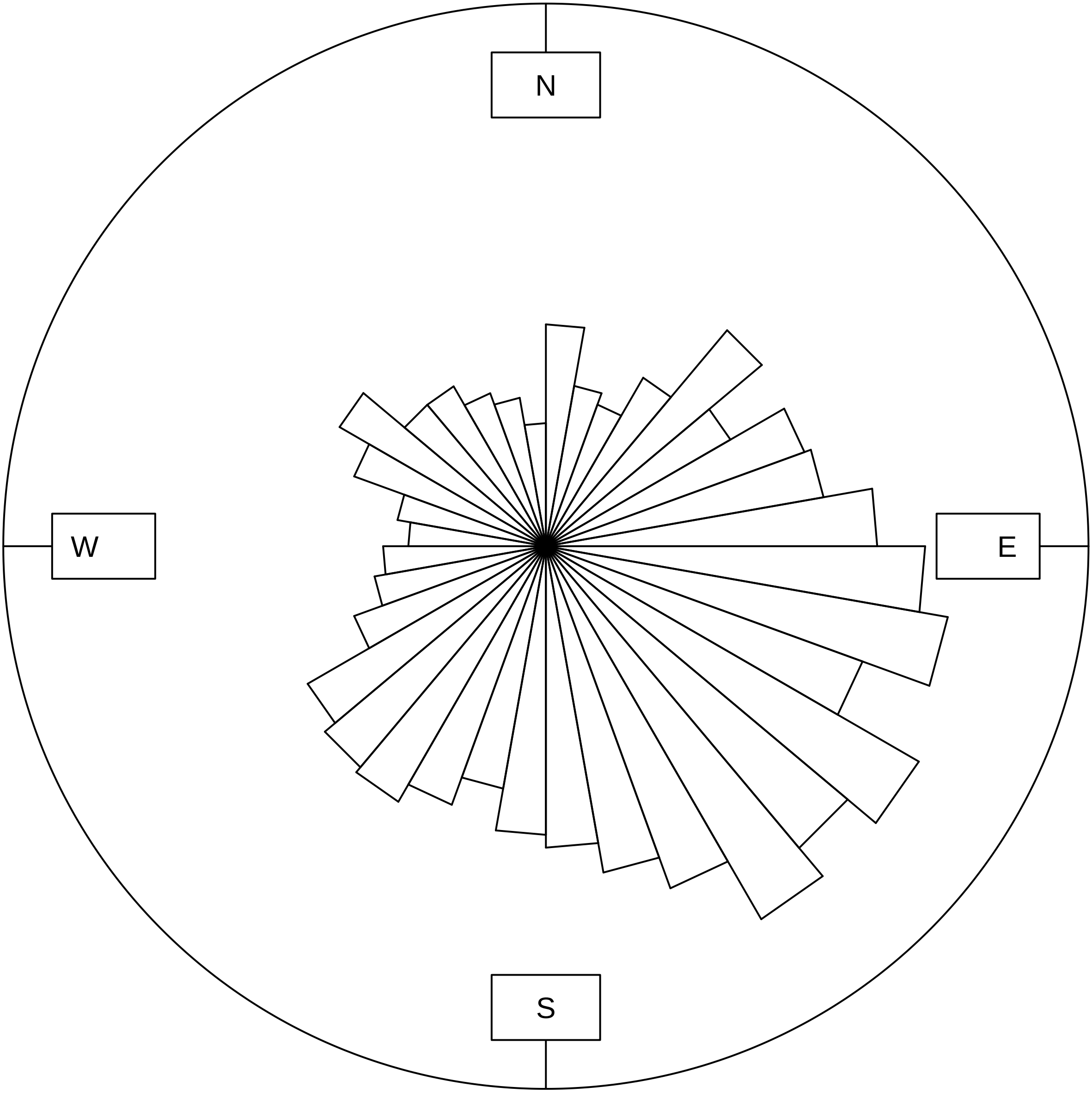

bootstrap 354

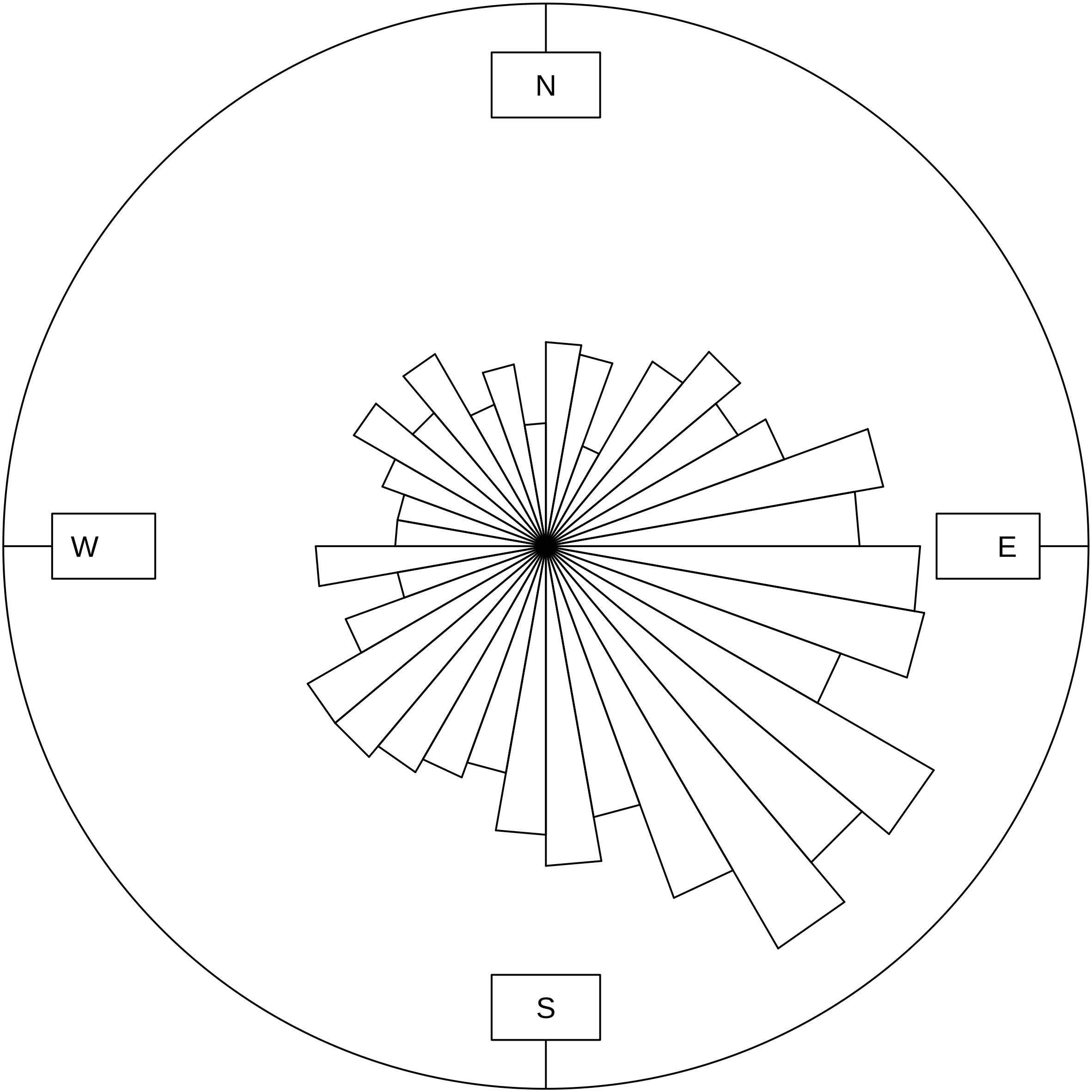

bootstrap 355

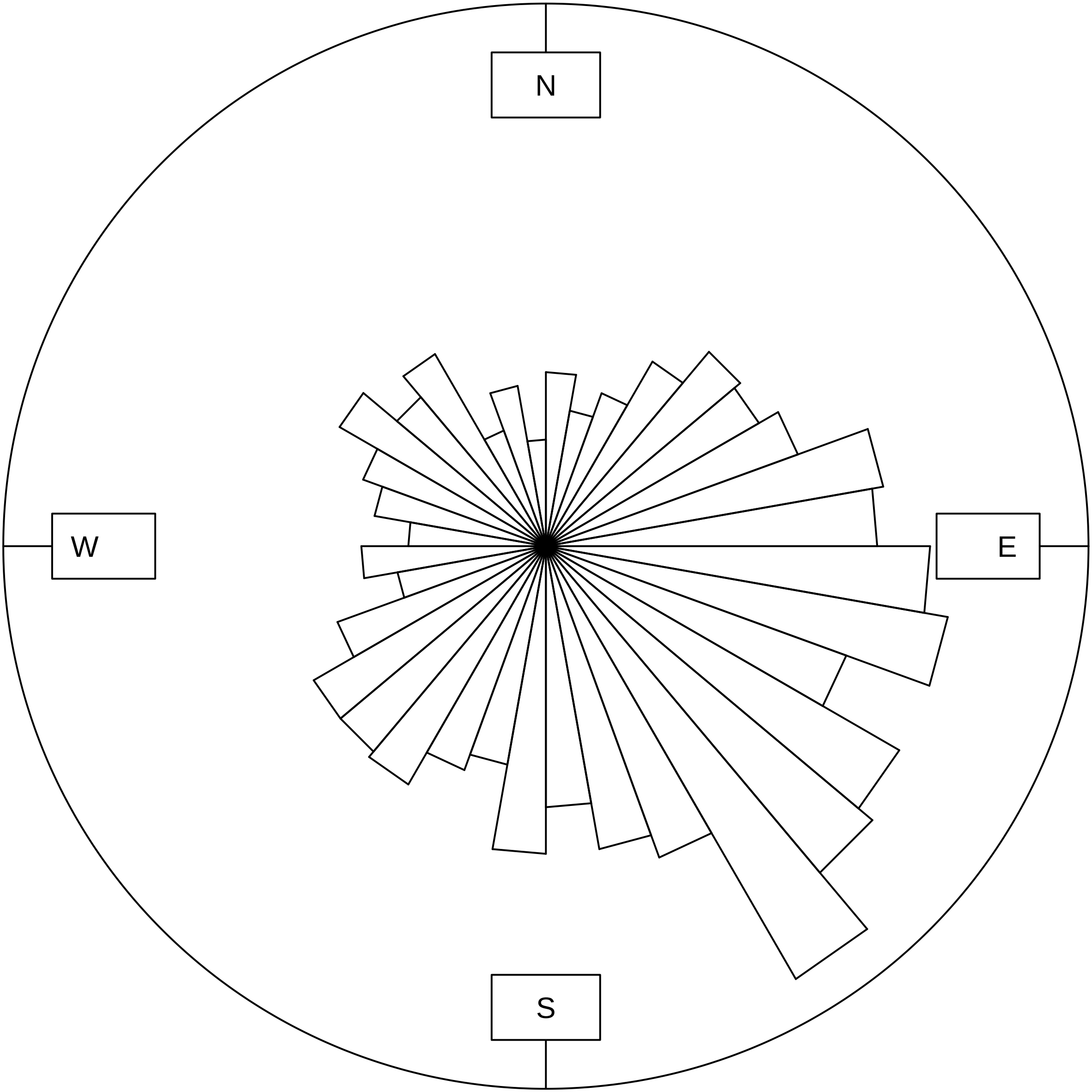

bootstrap 356

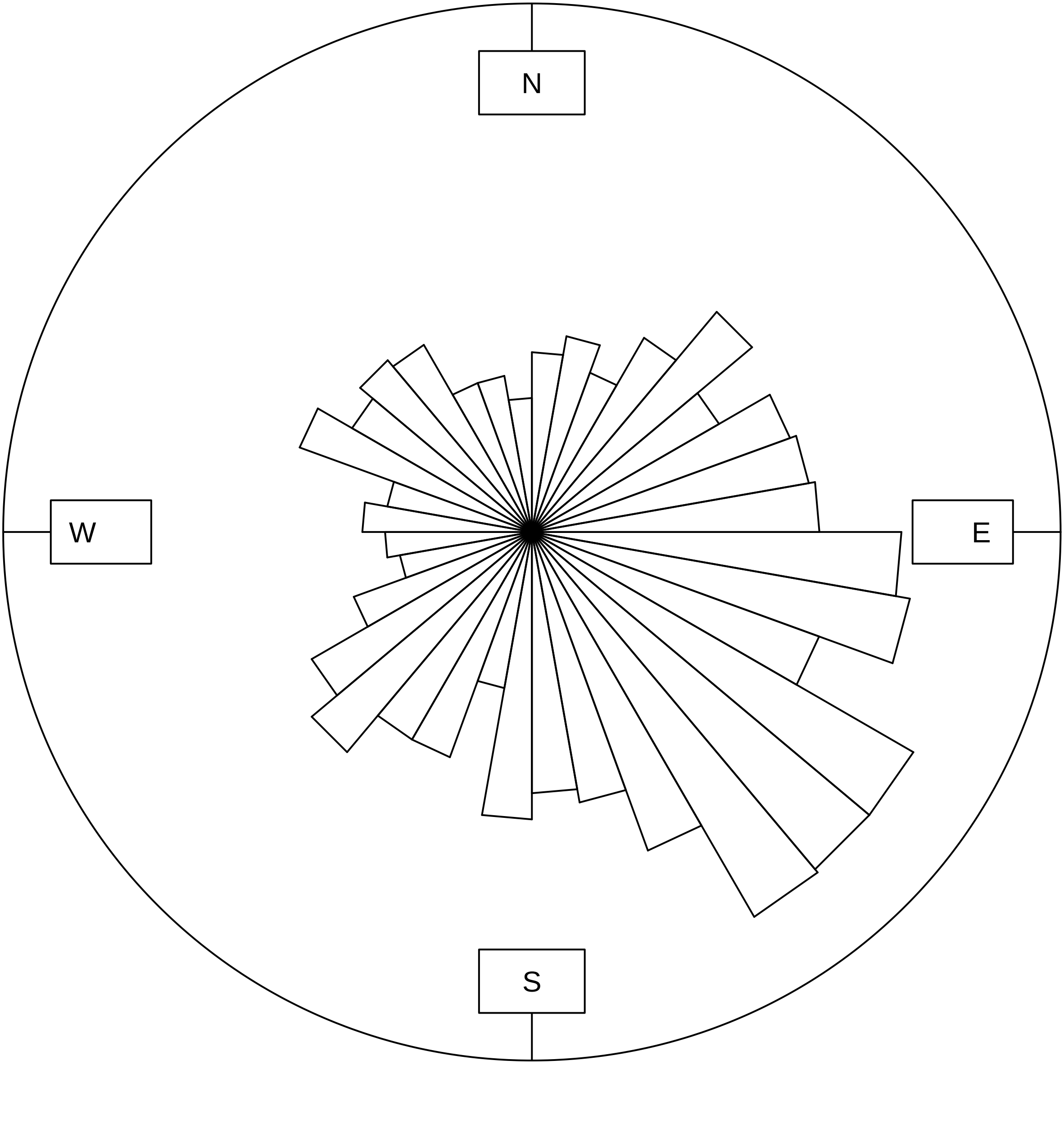

bootstrap 357

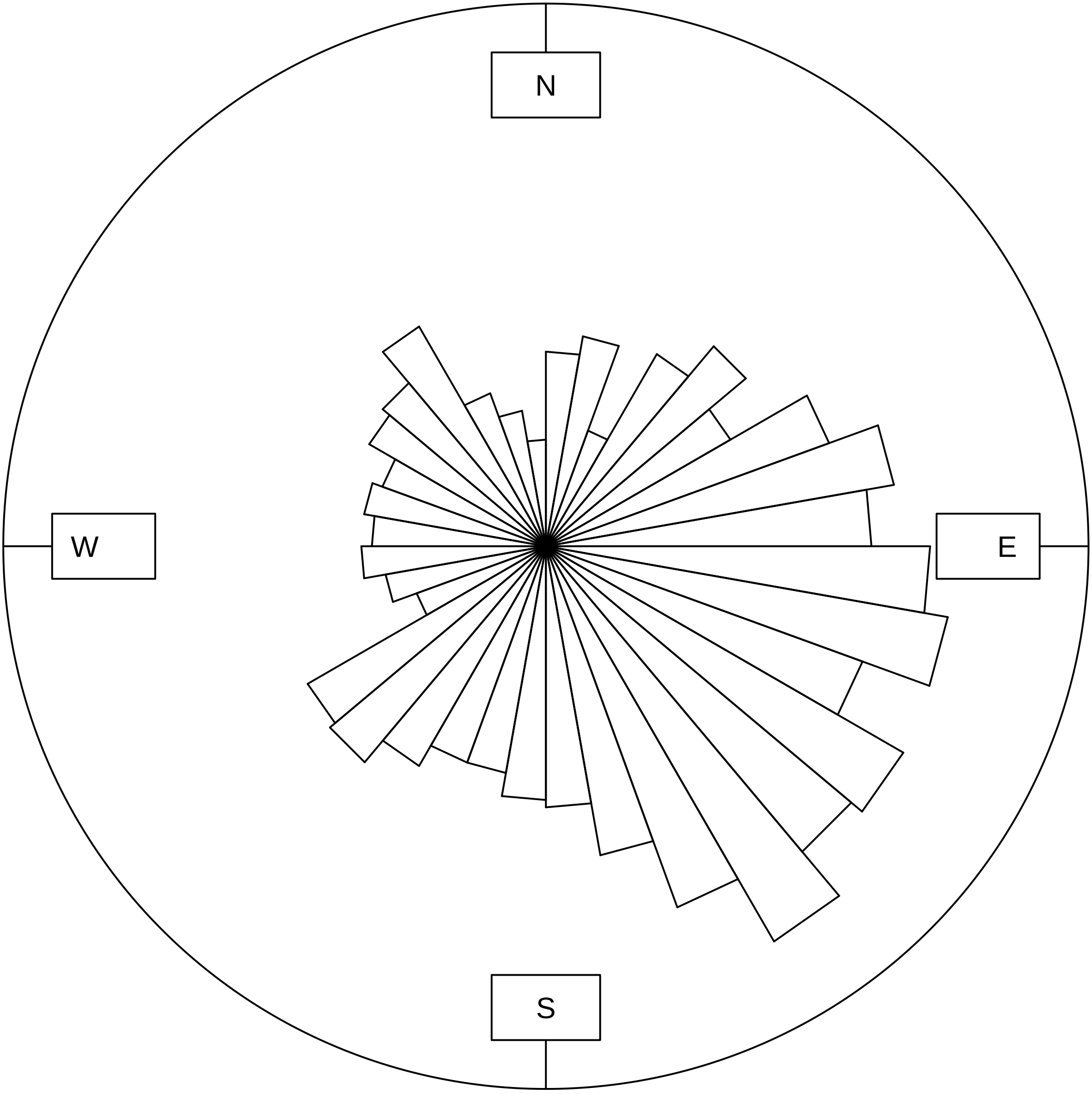

bootstrap 358

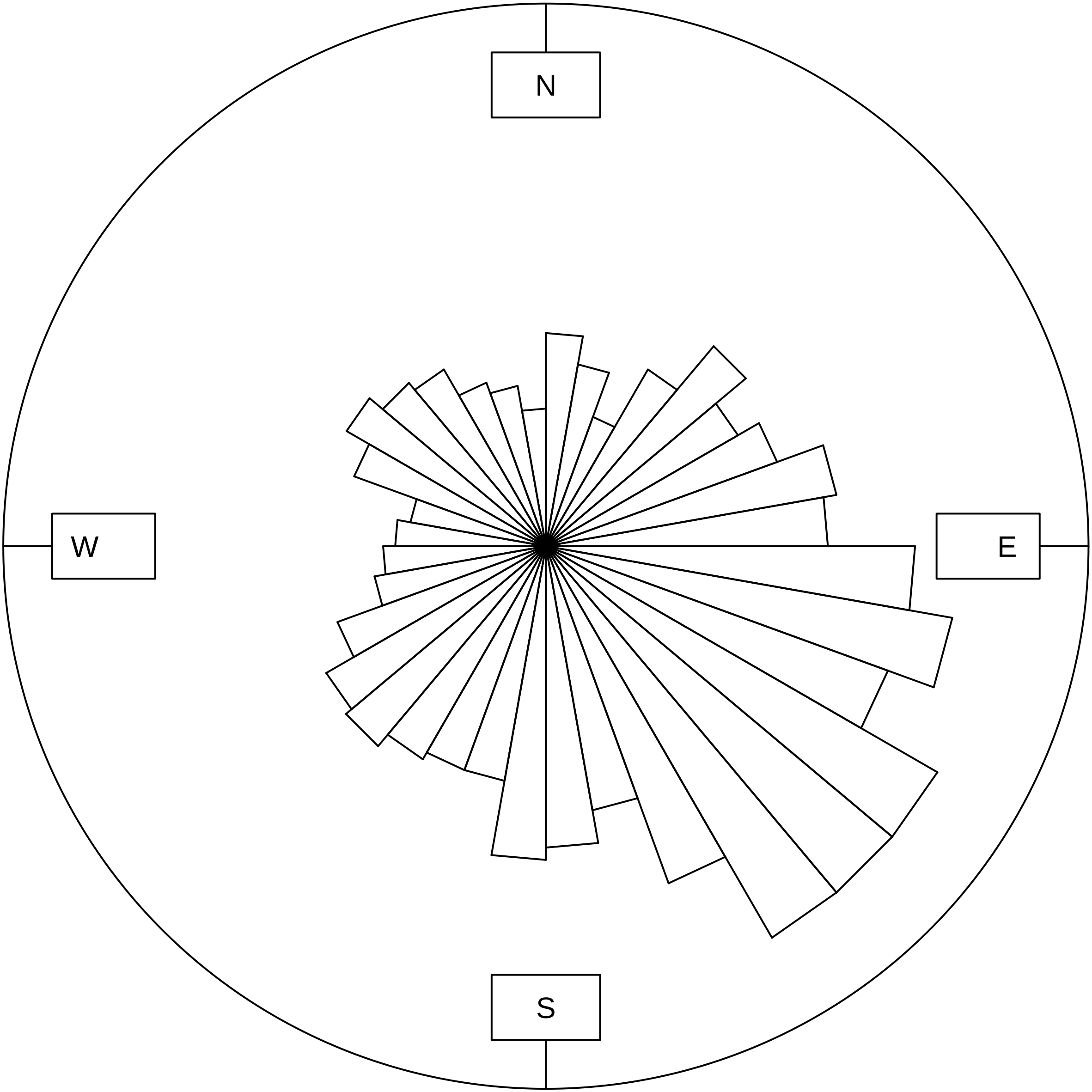

bootstrap 359

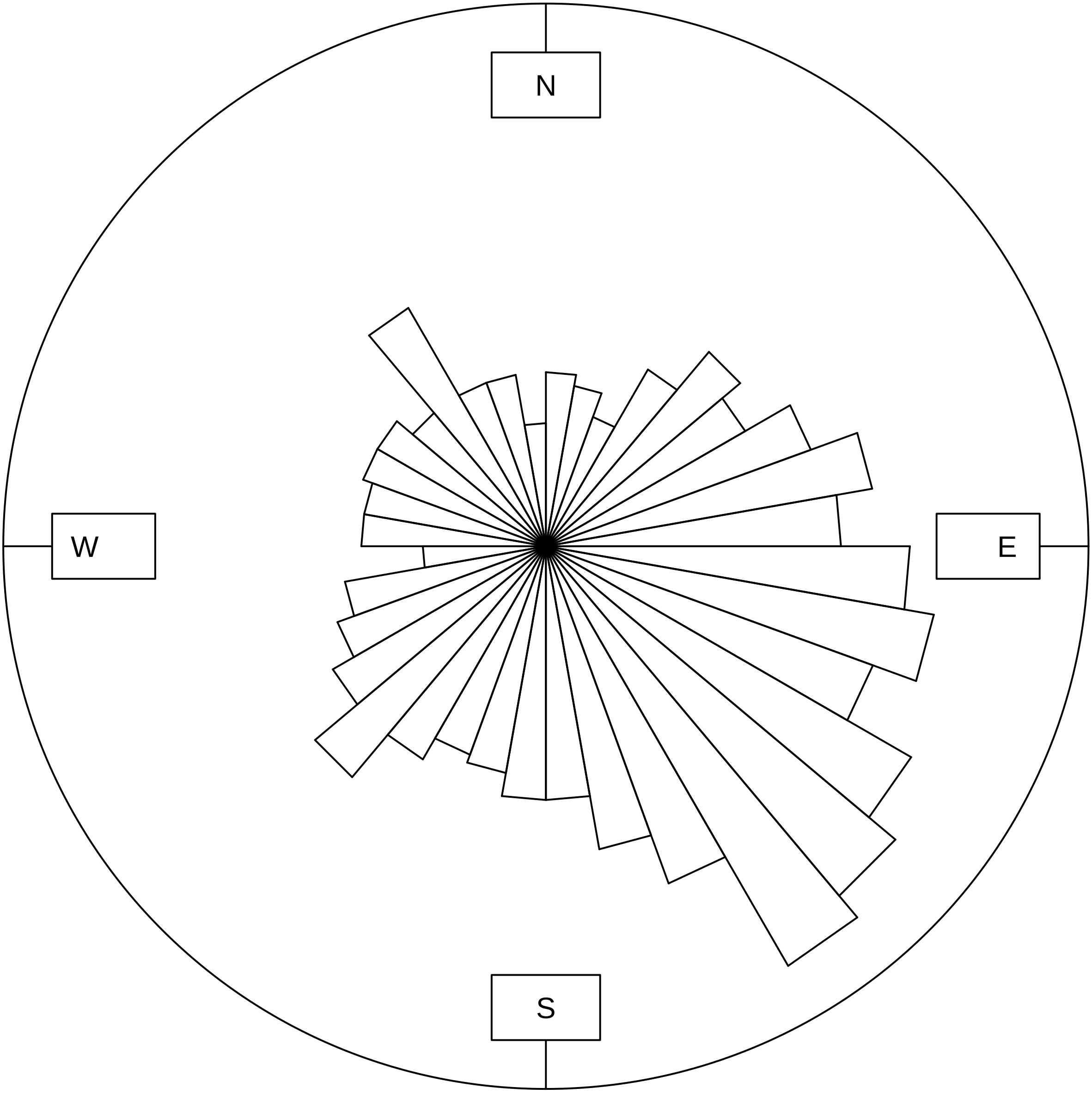

bootstrap 360

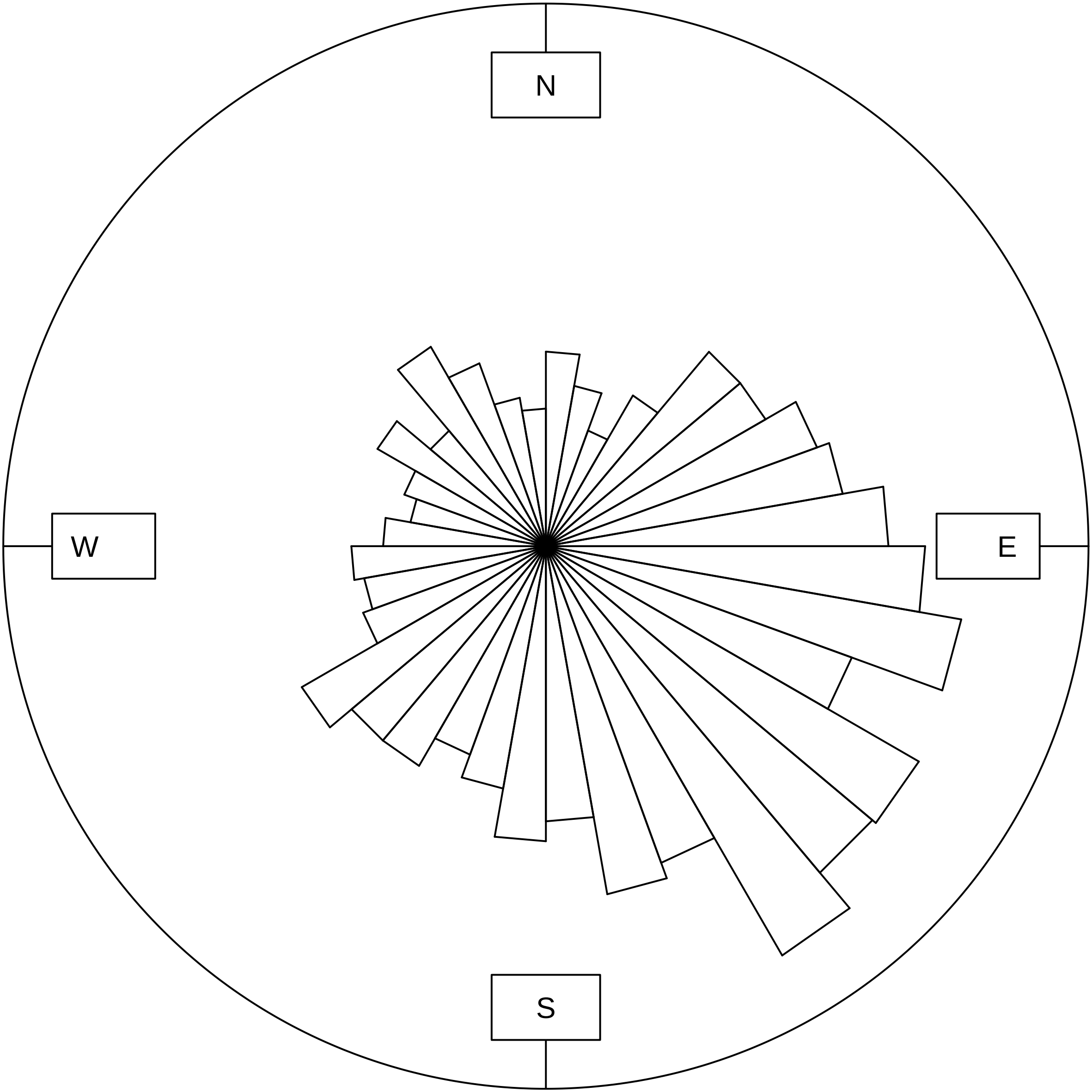

bootstrap 361

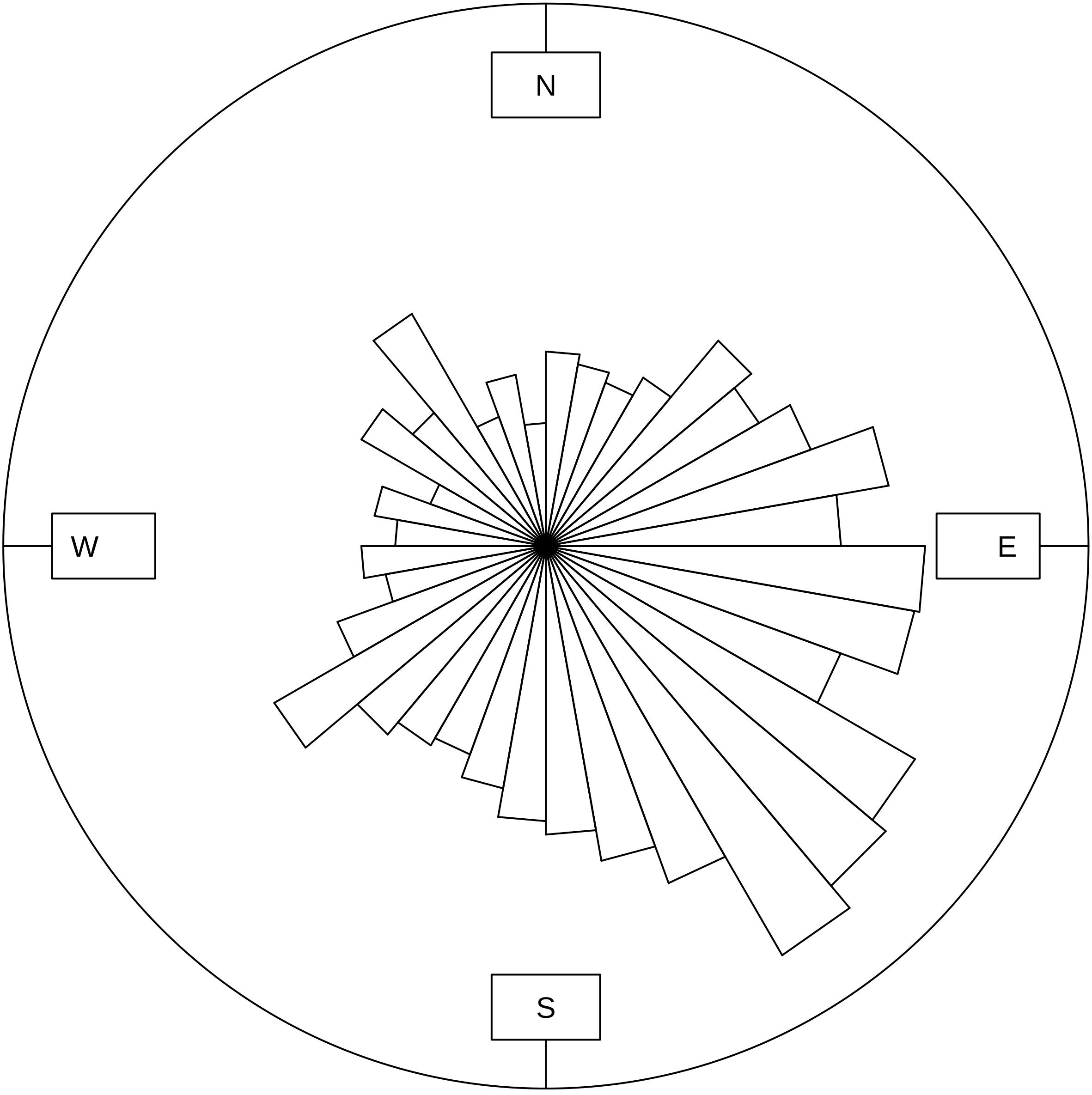

bootstrap 362

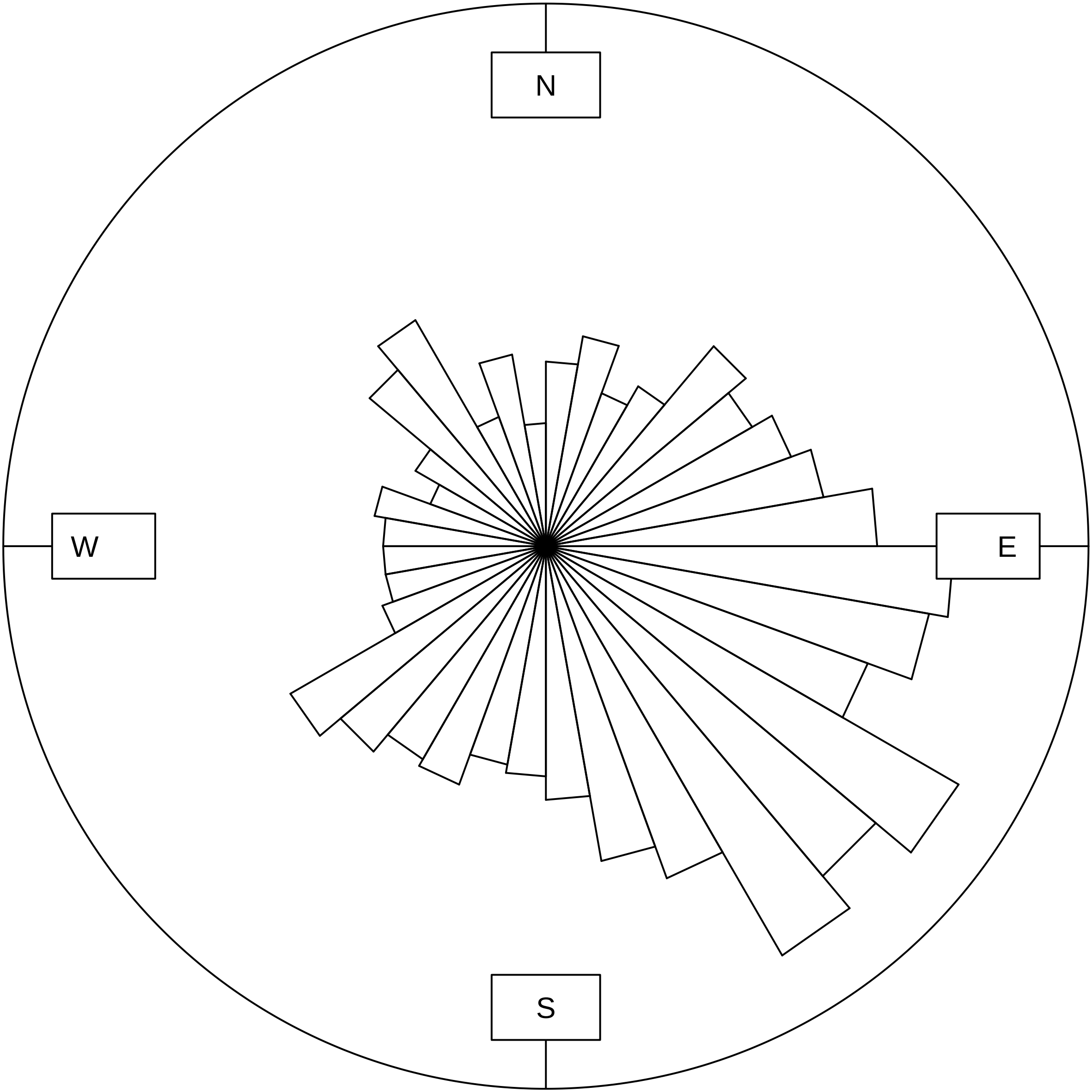

bootstrap 363

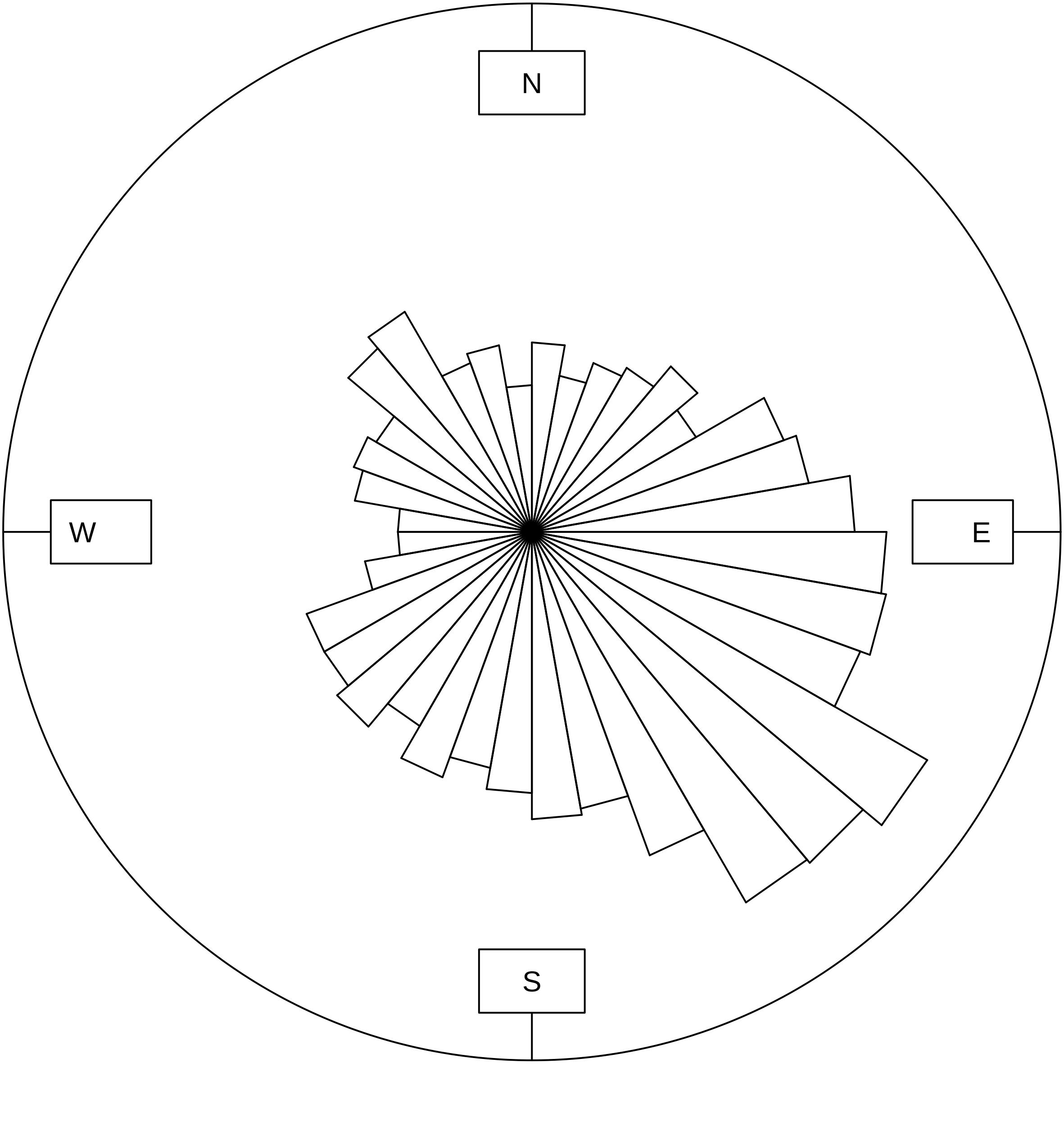

bootstrap 364

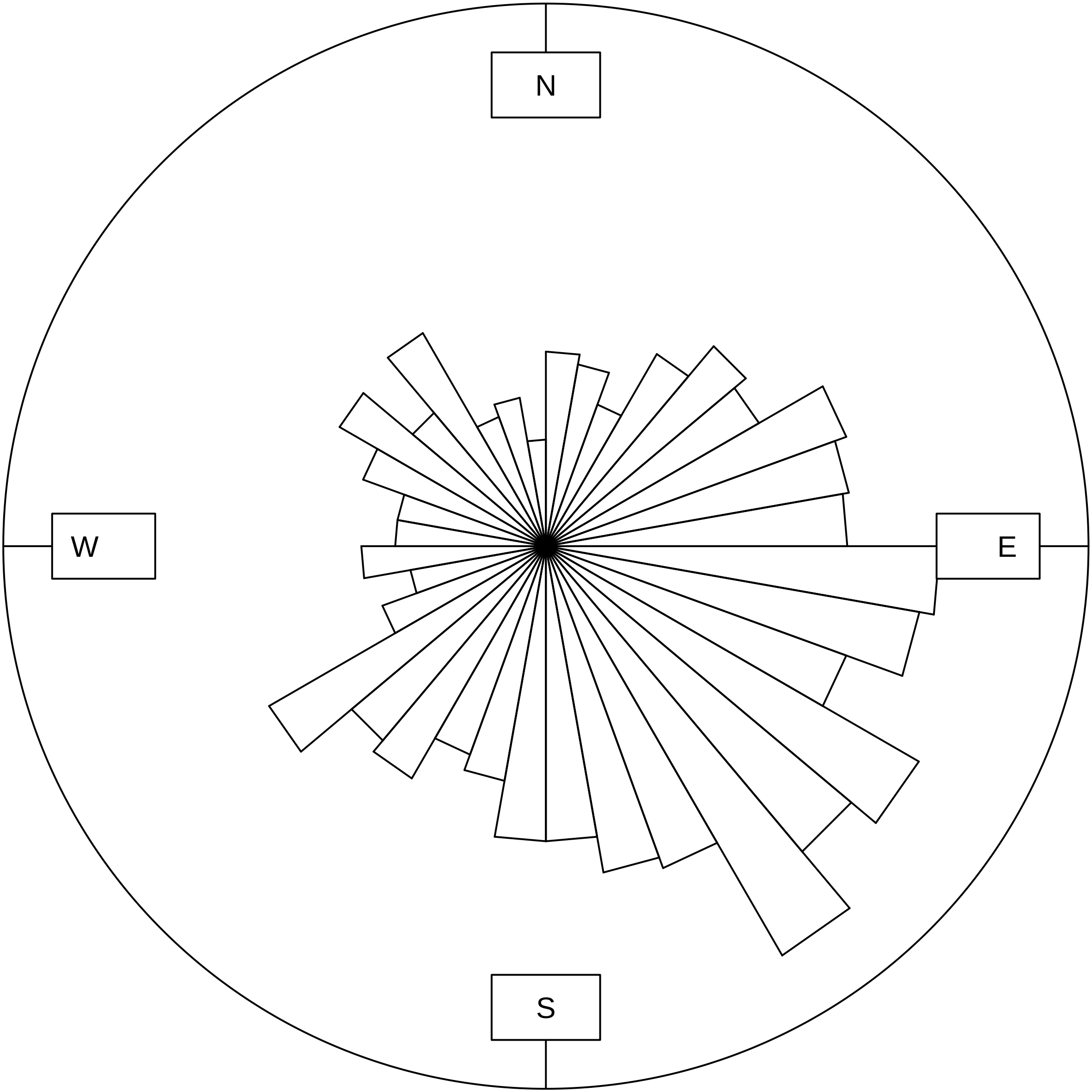

bootstrap 365

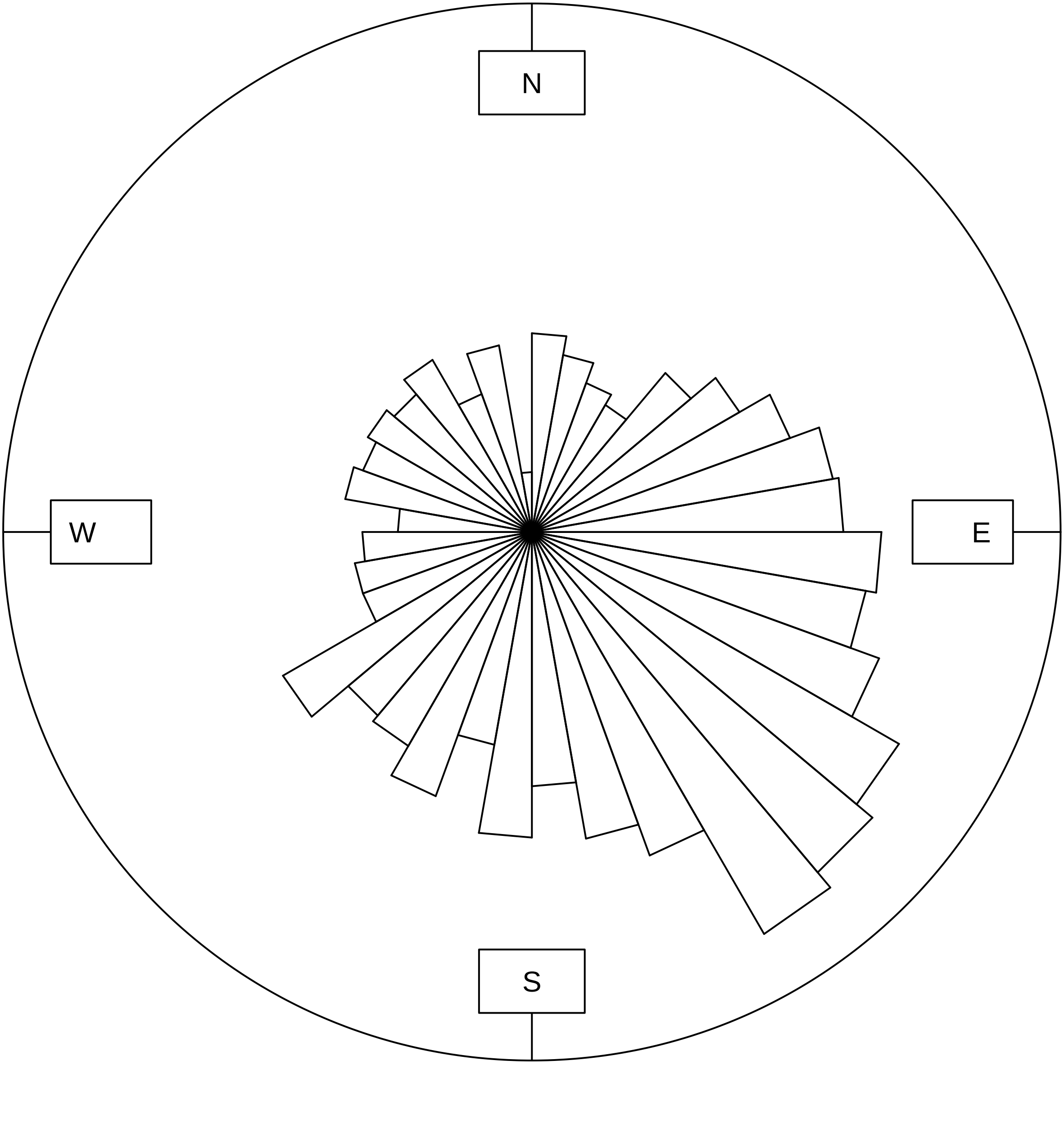

bootstrap 366

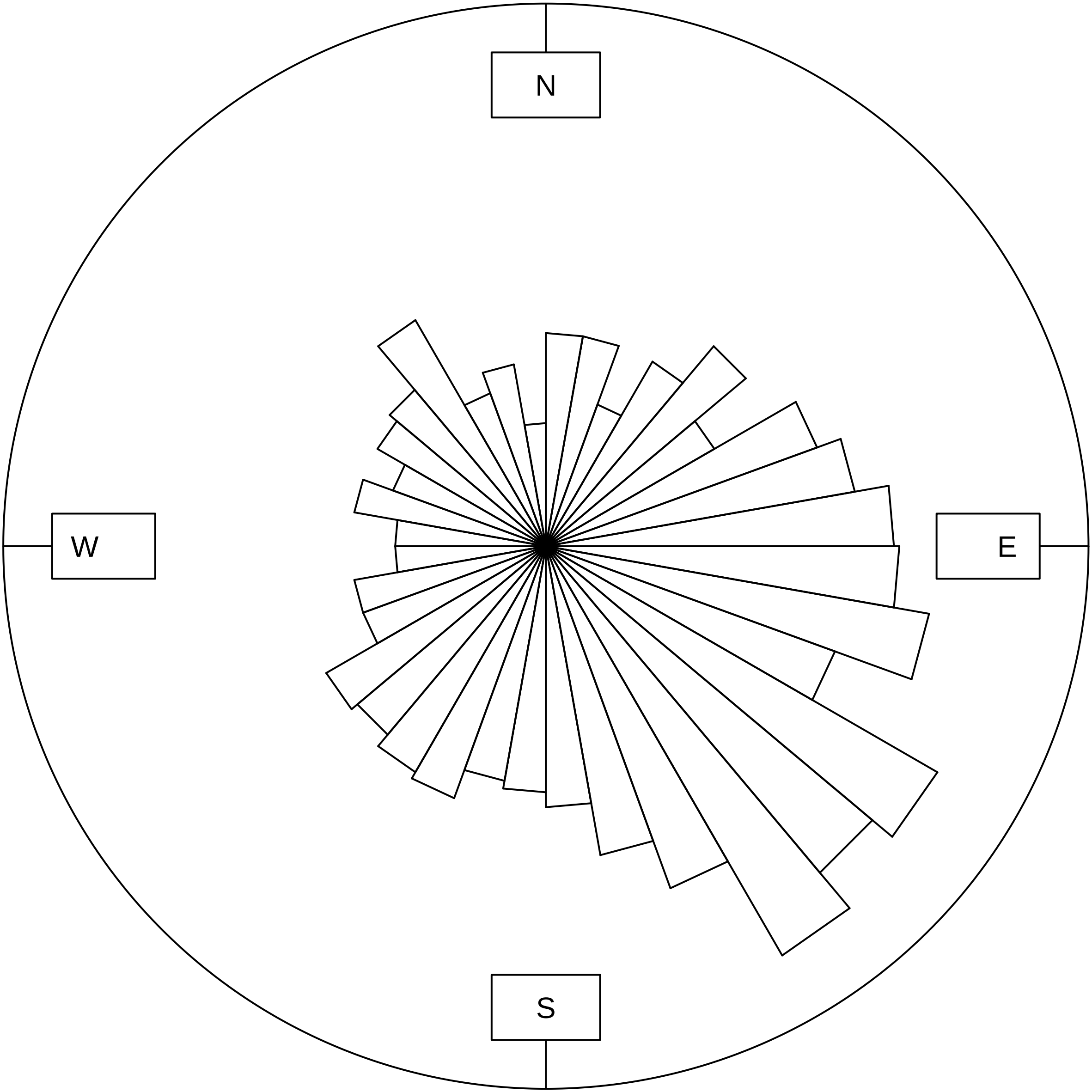

bootstrap 367

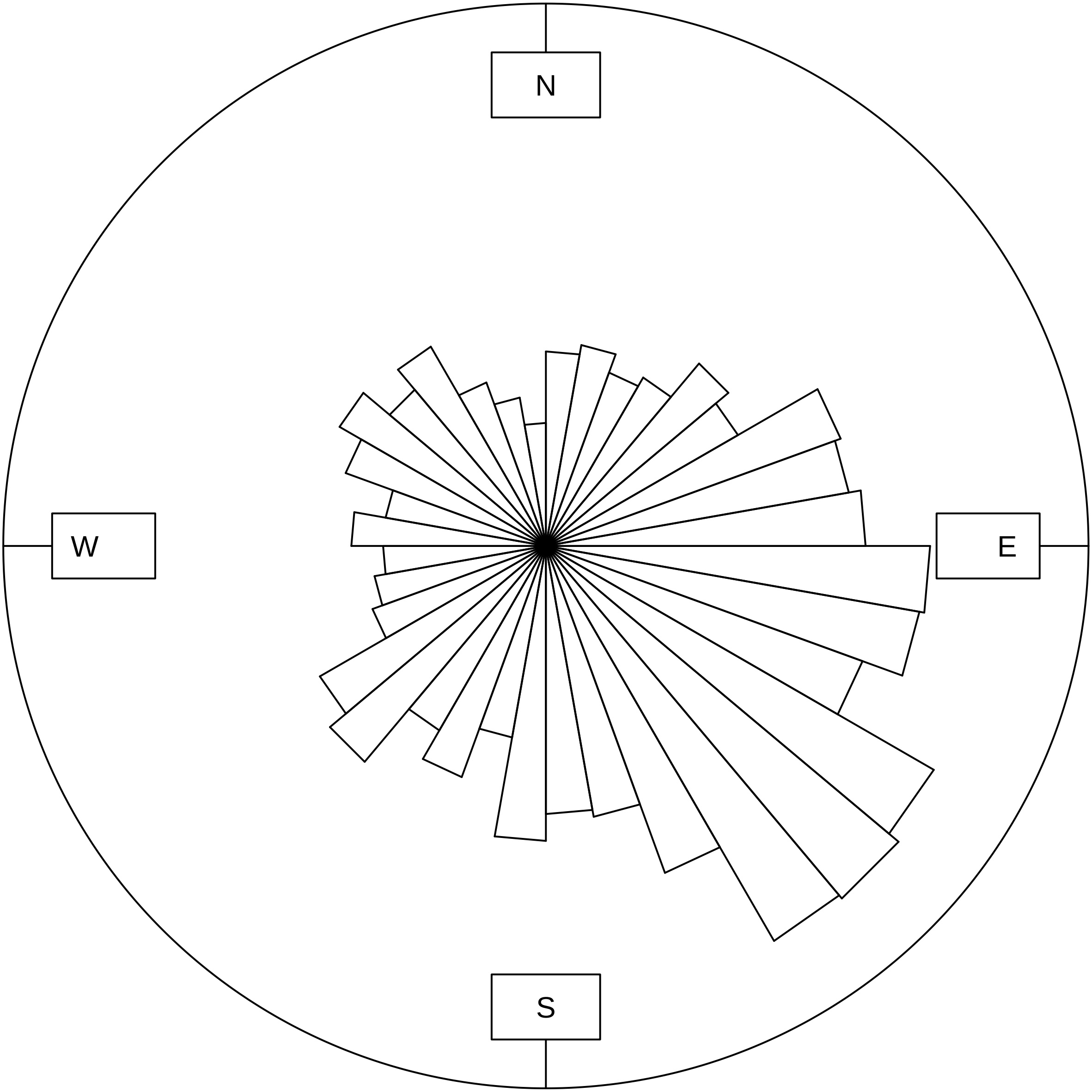

bootstrap 368

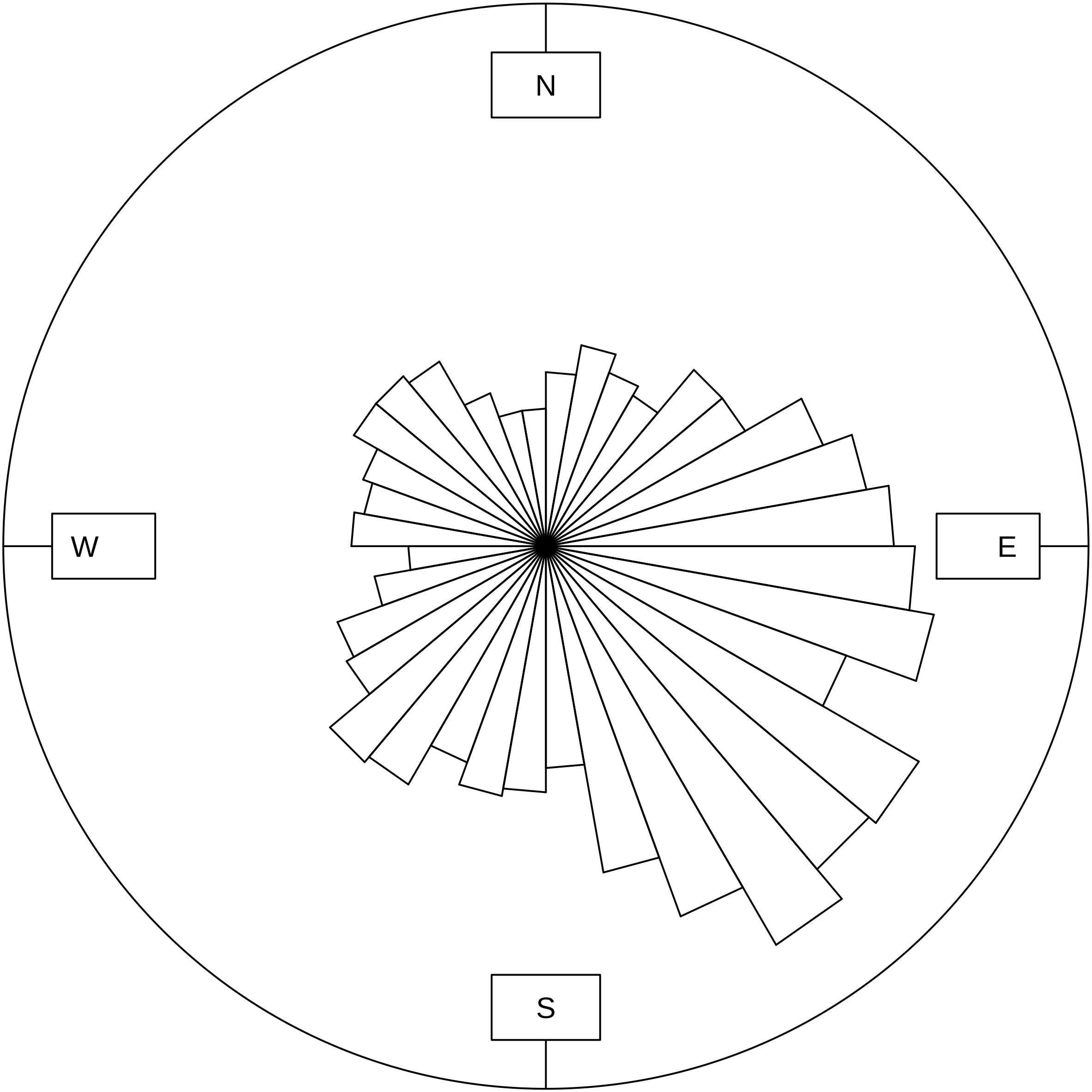

bootstrap 369

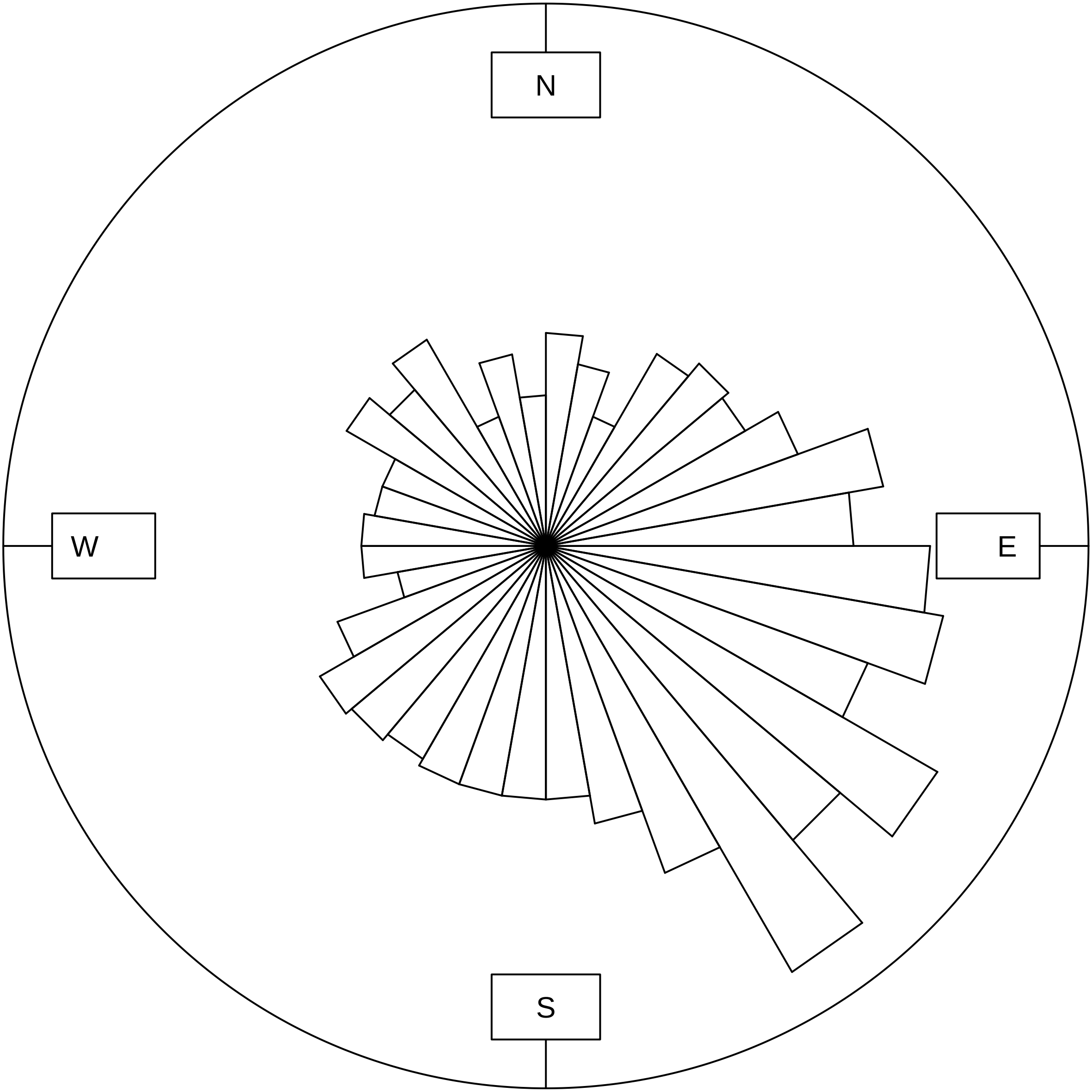

bootstrap 370

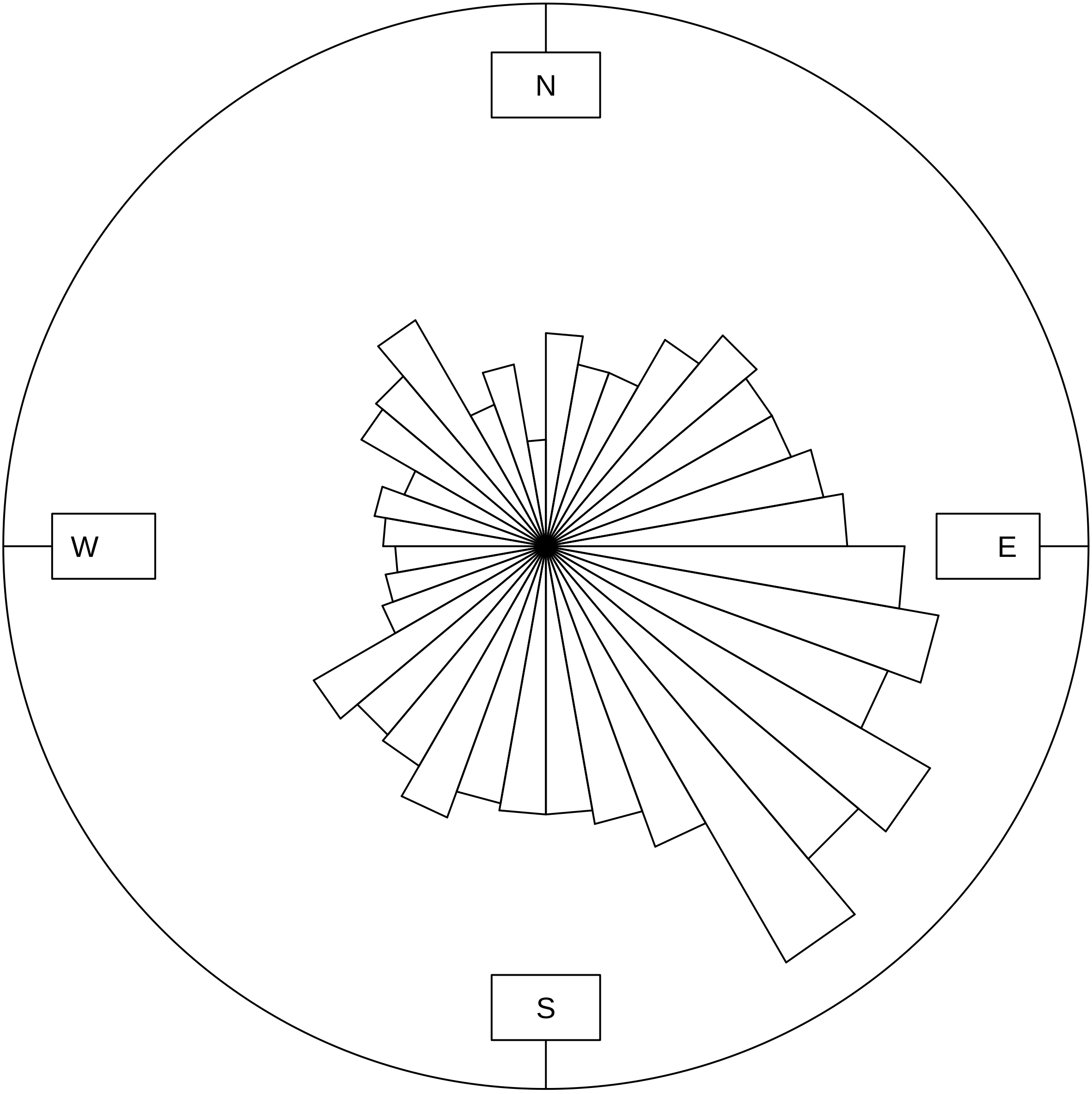

bootstrap 371

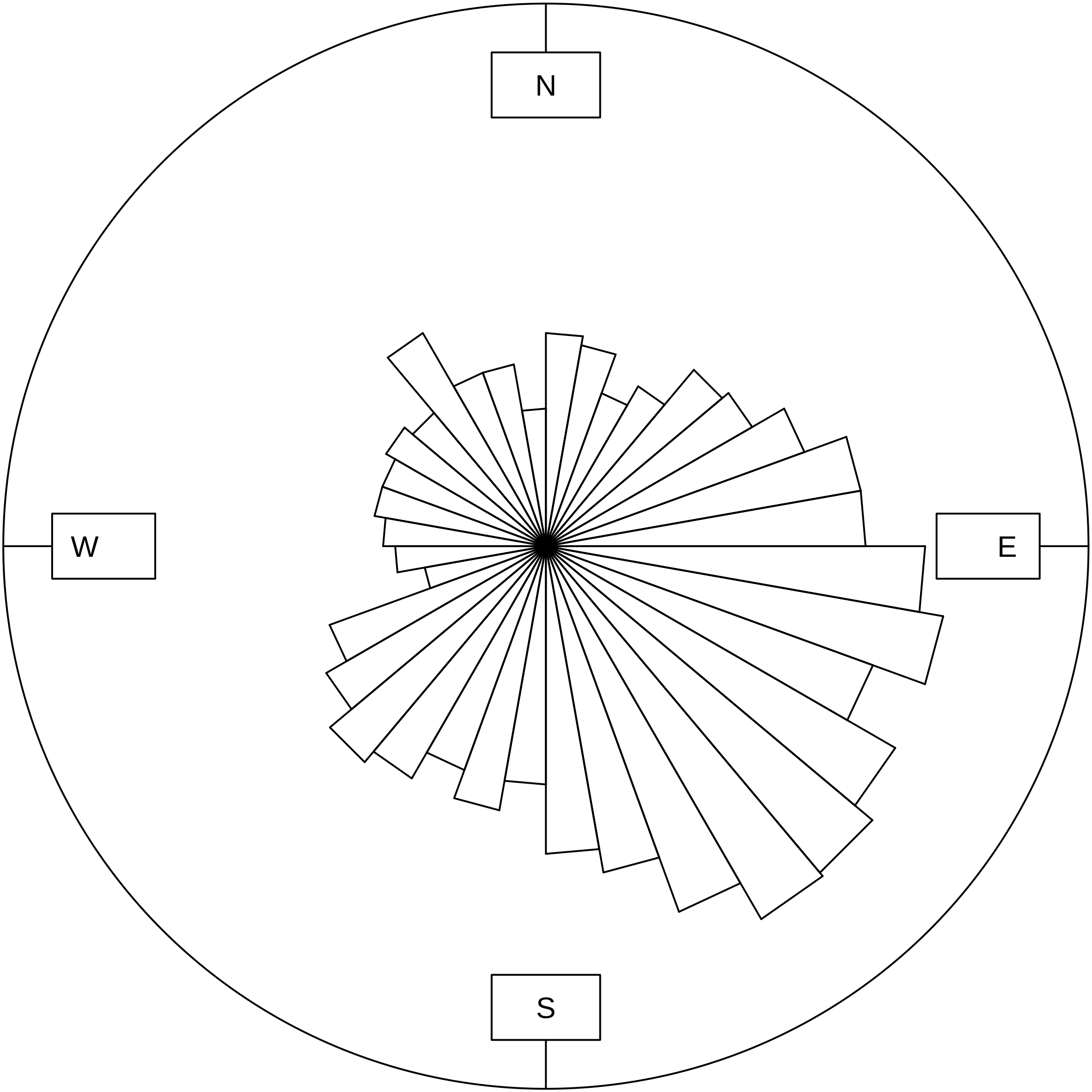

bootstrap 372

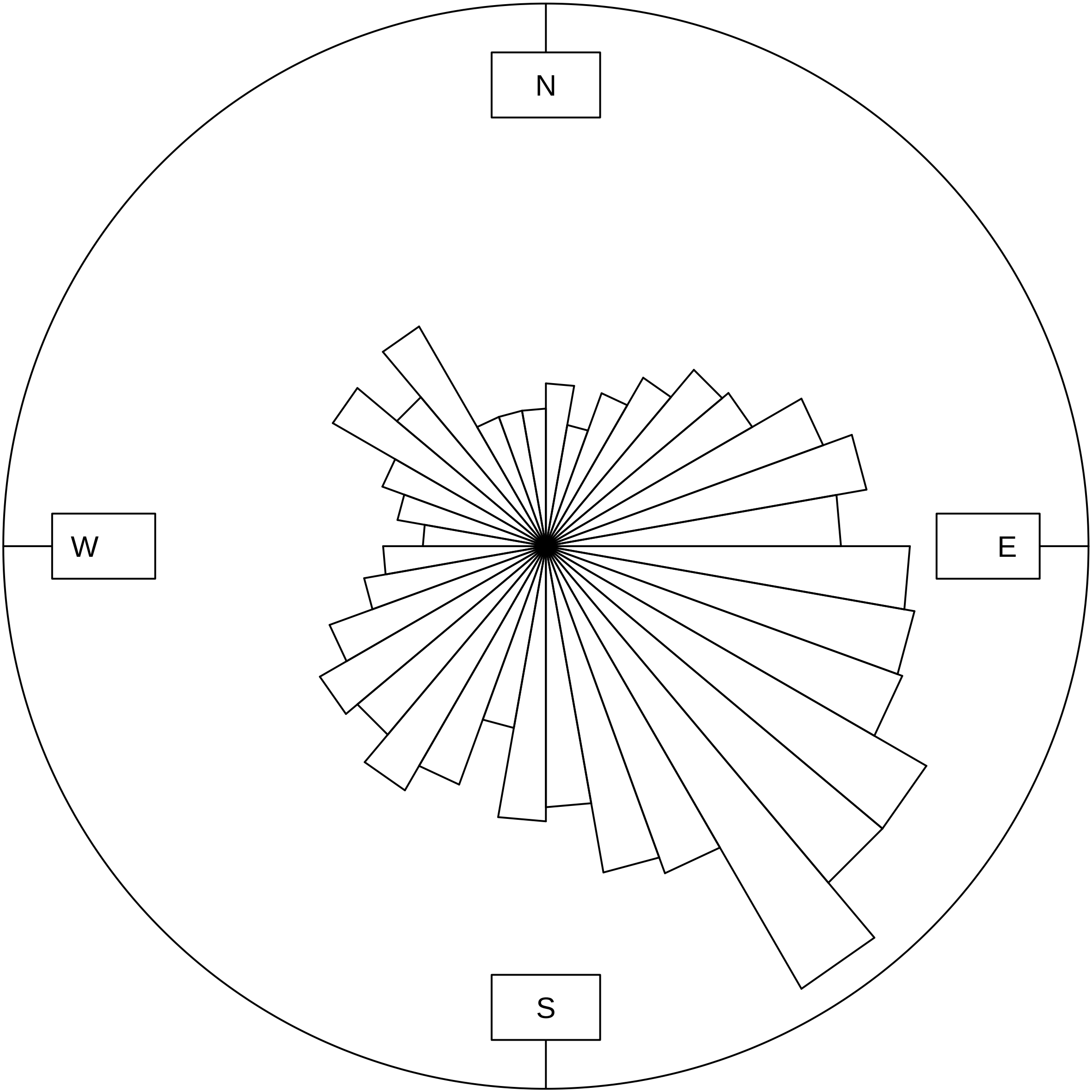

bootstrap 373

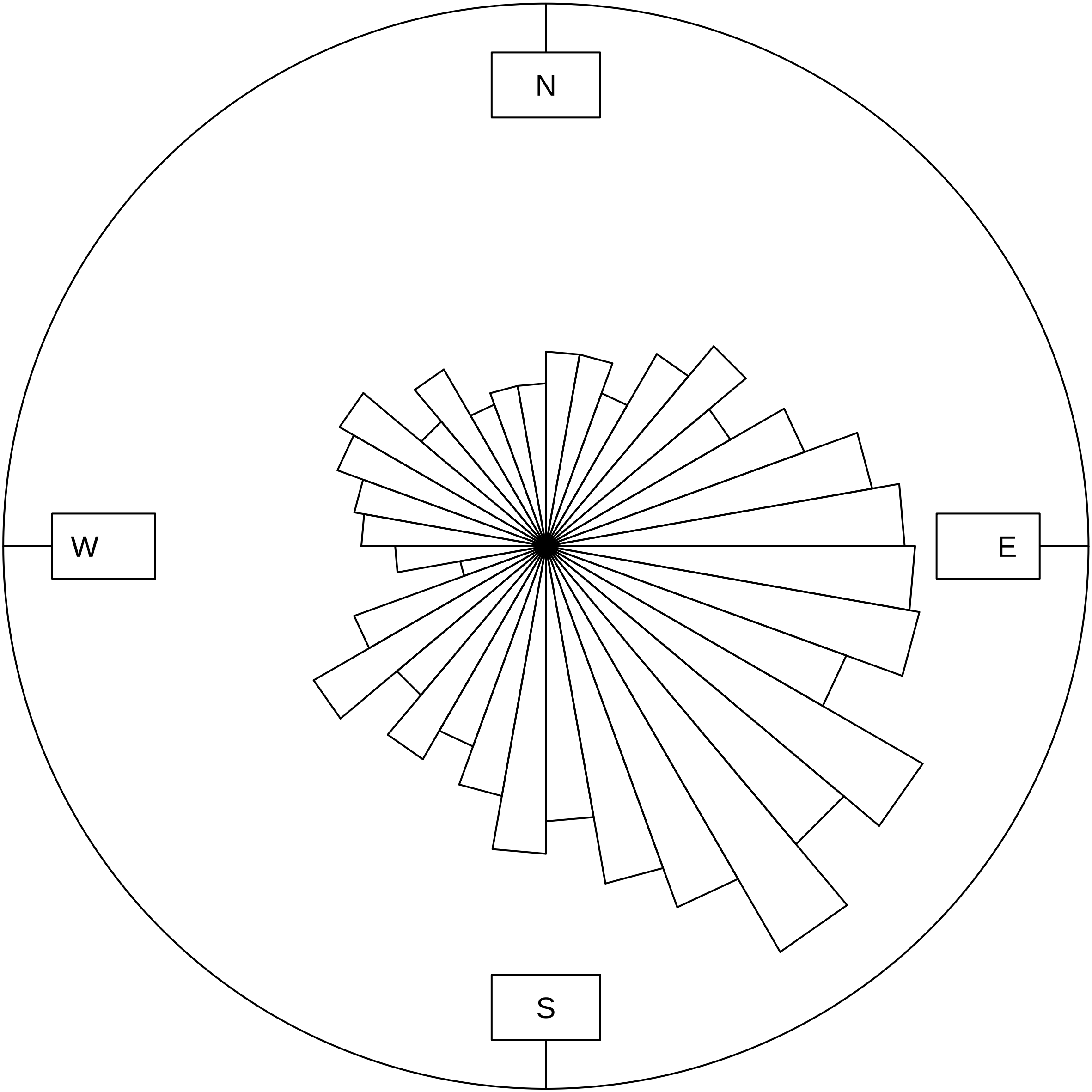

bootstrap 374

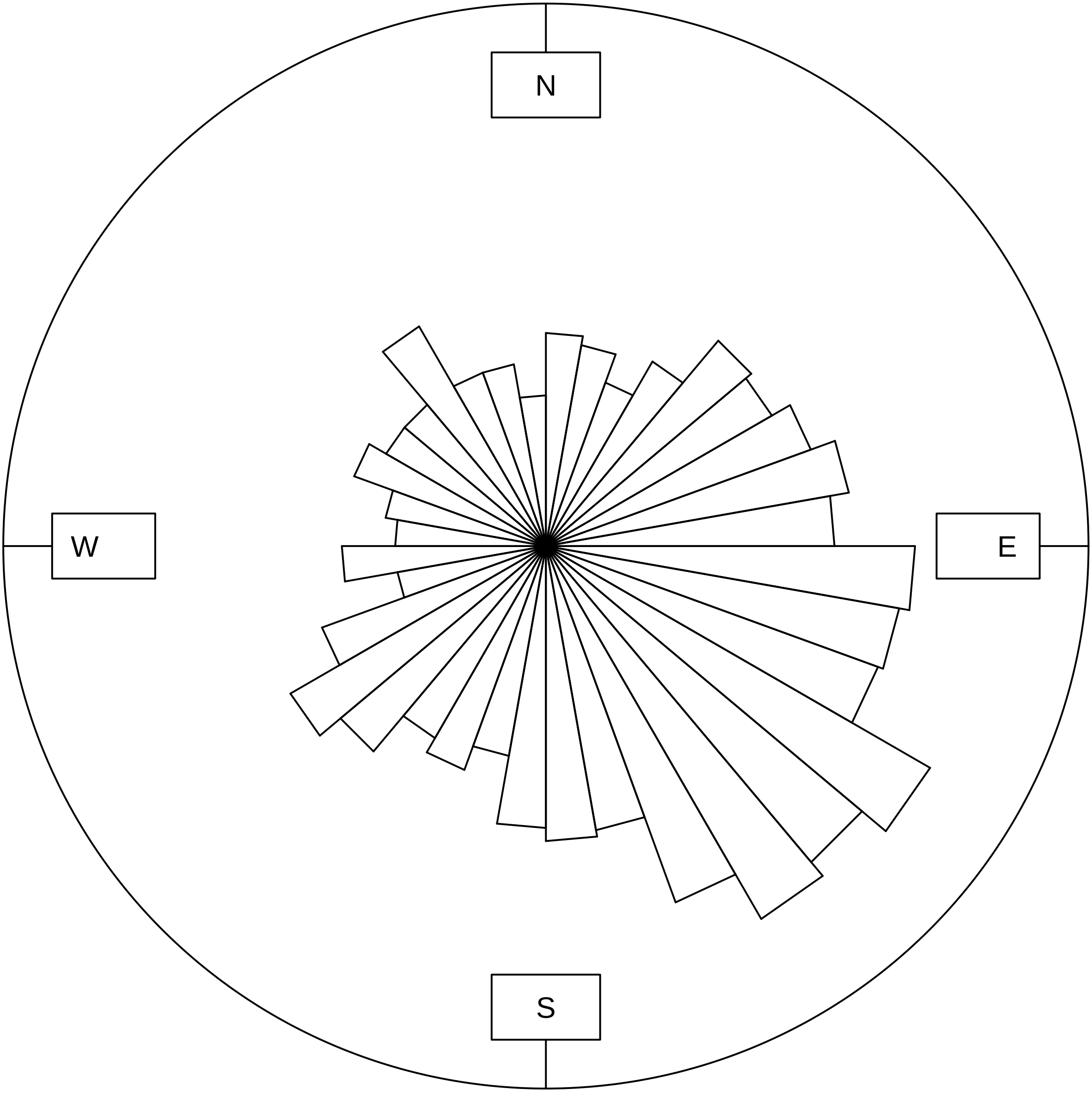

bootstrap 375

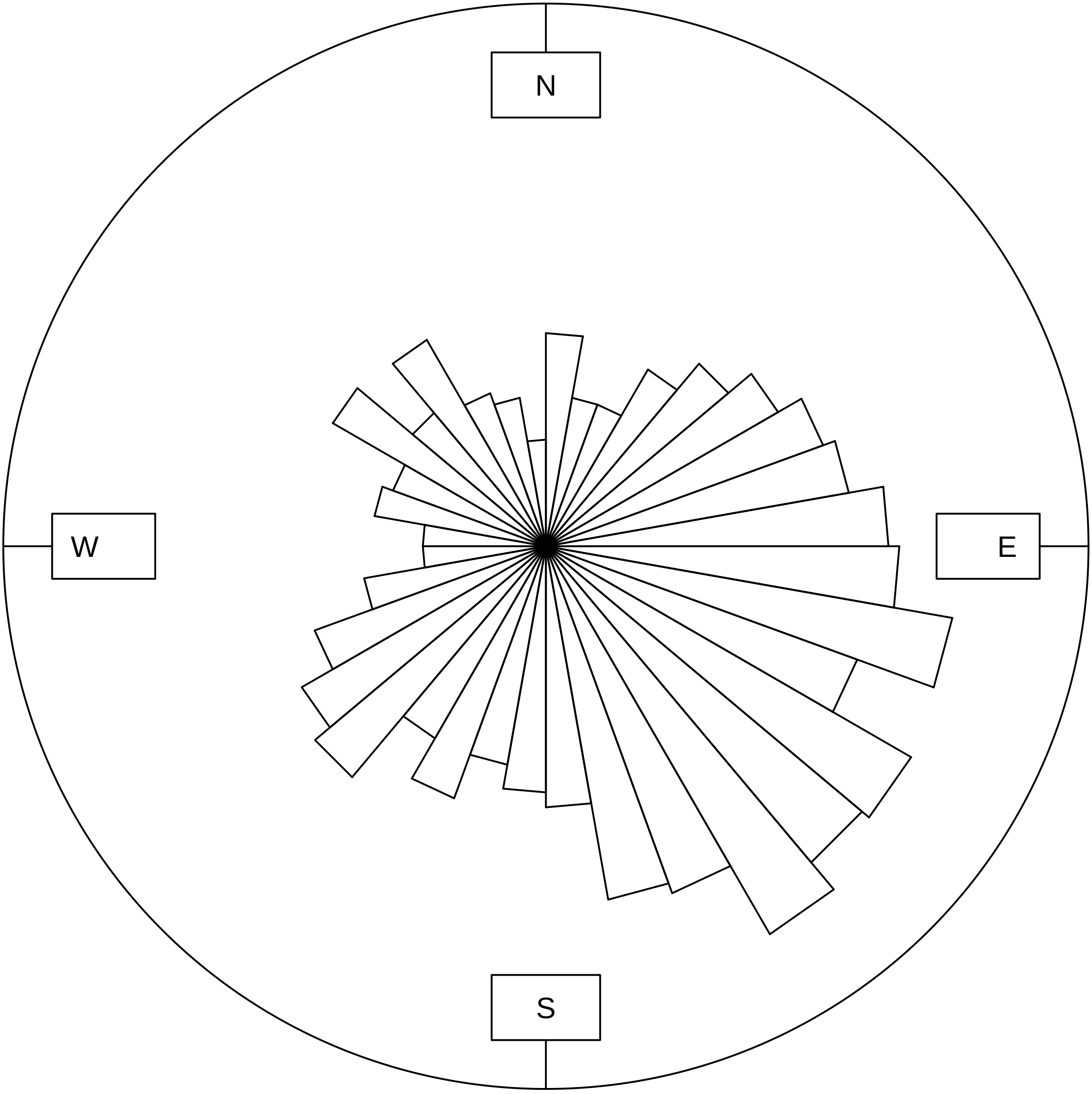

bootstrap 376

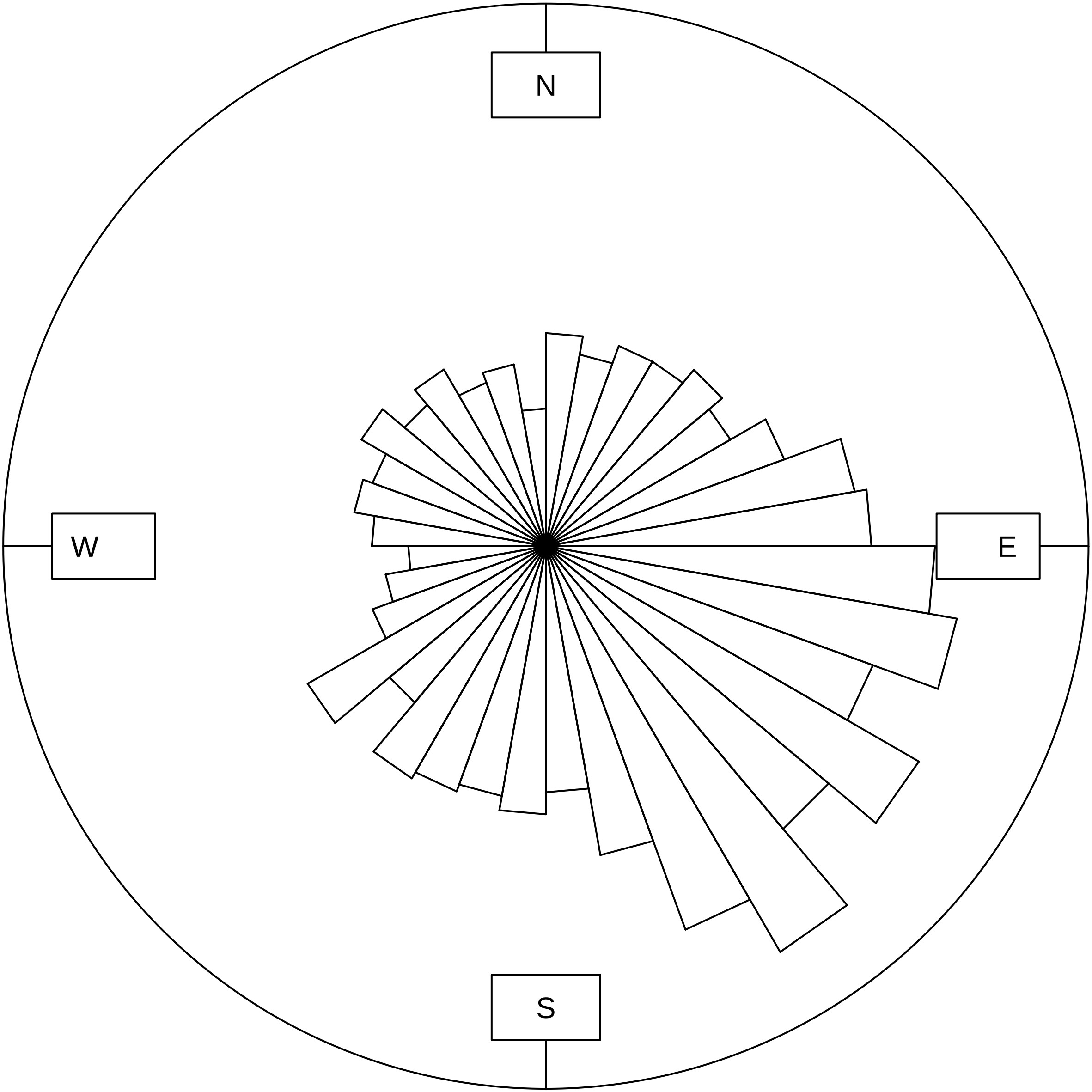

bootstrap 377

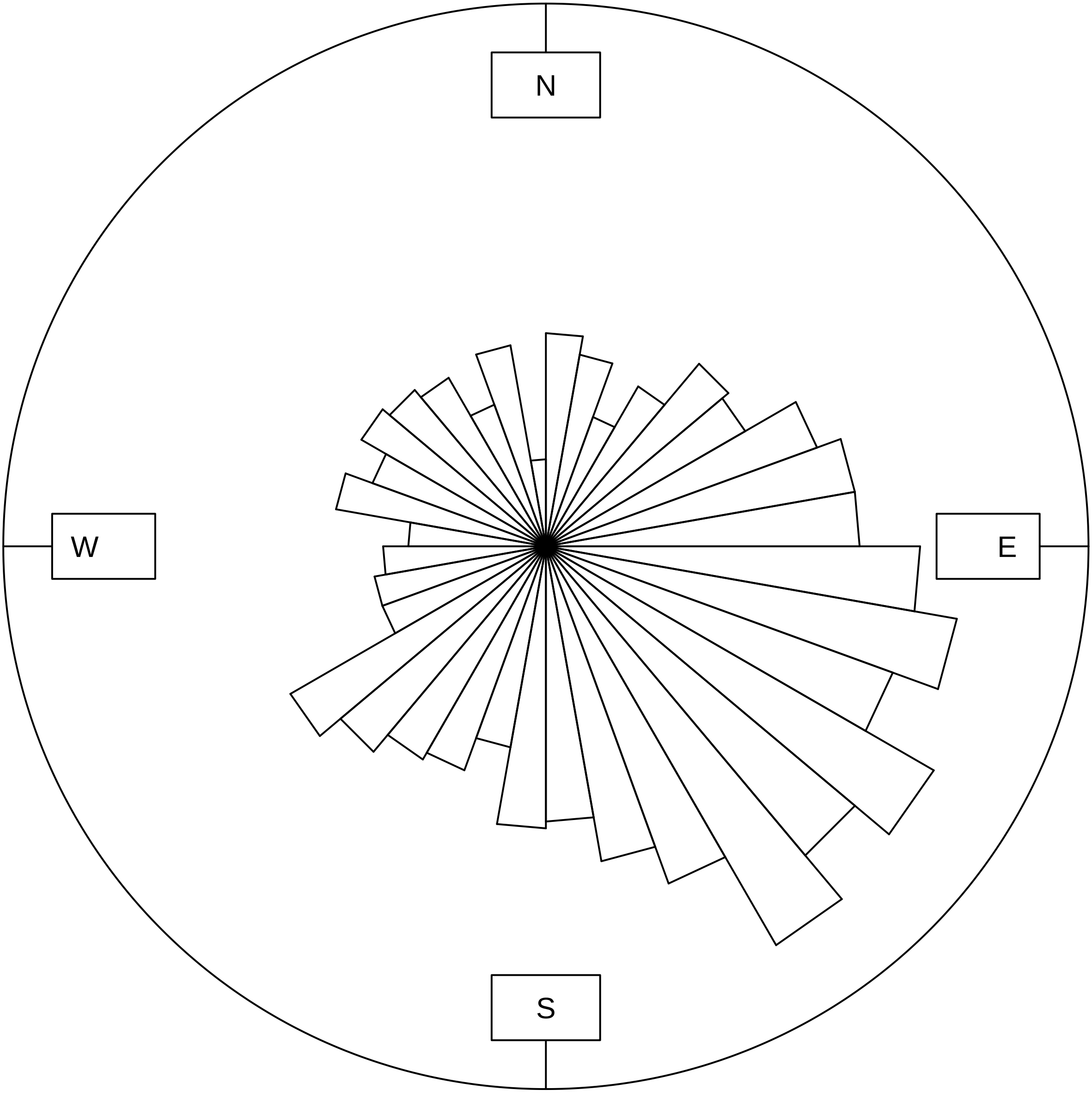

bootstrap 378

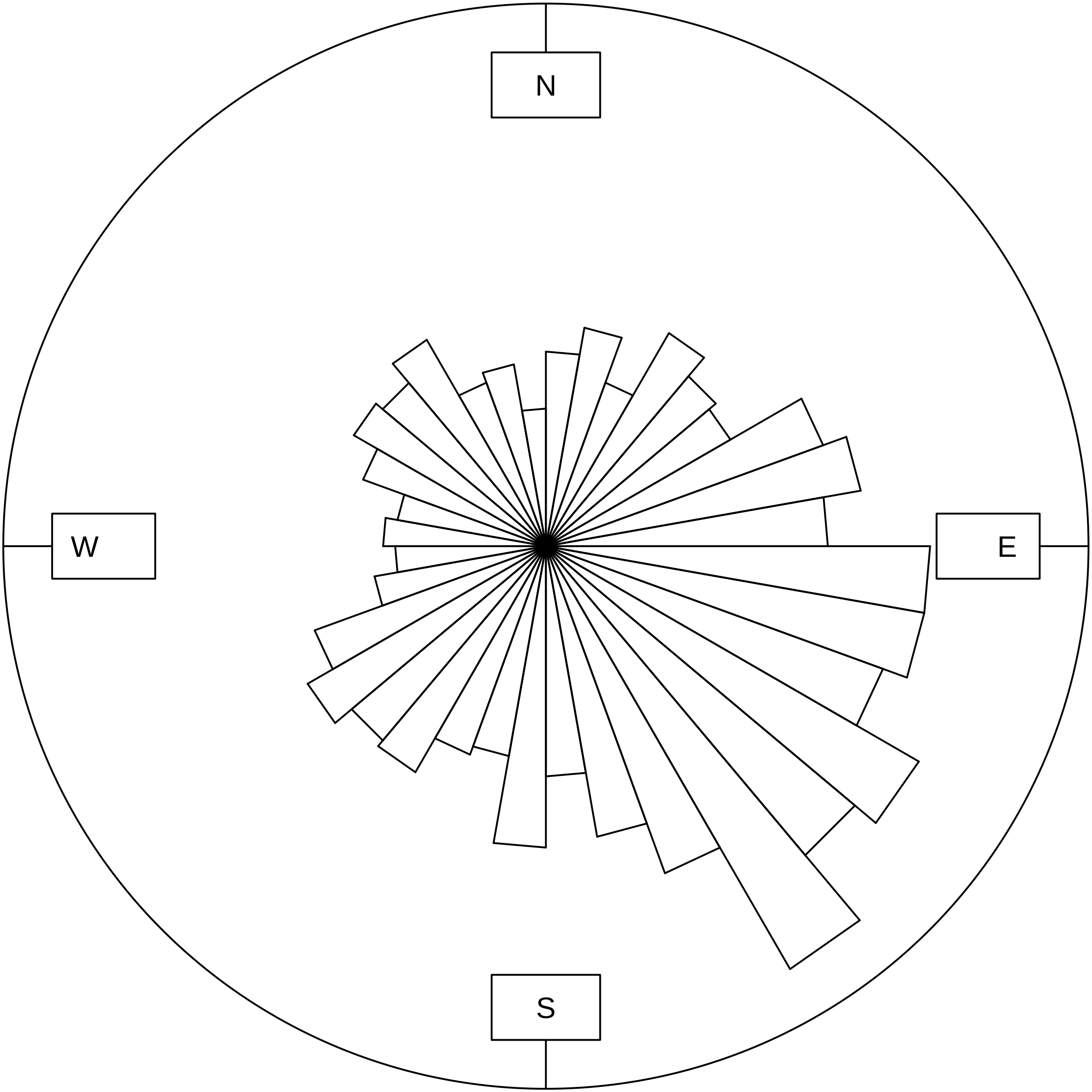

bootstrap 379

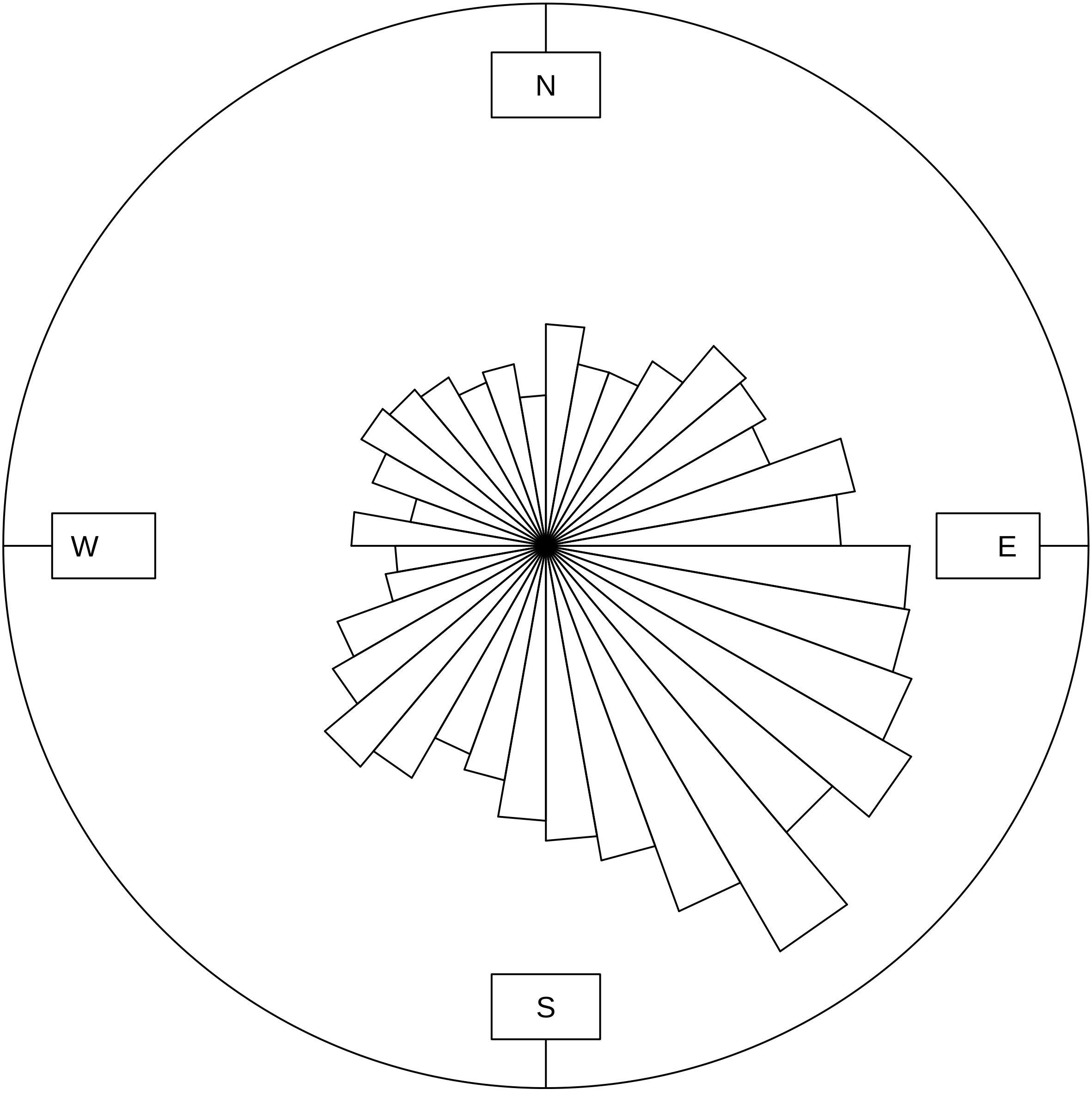

bootstrap 380

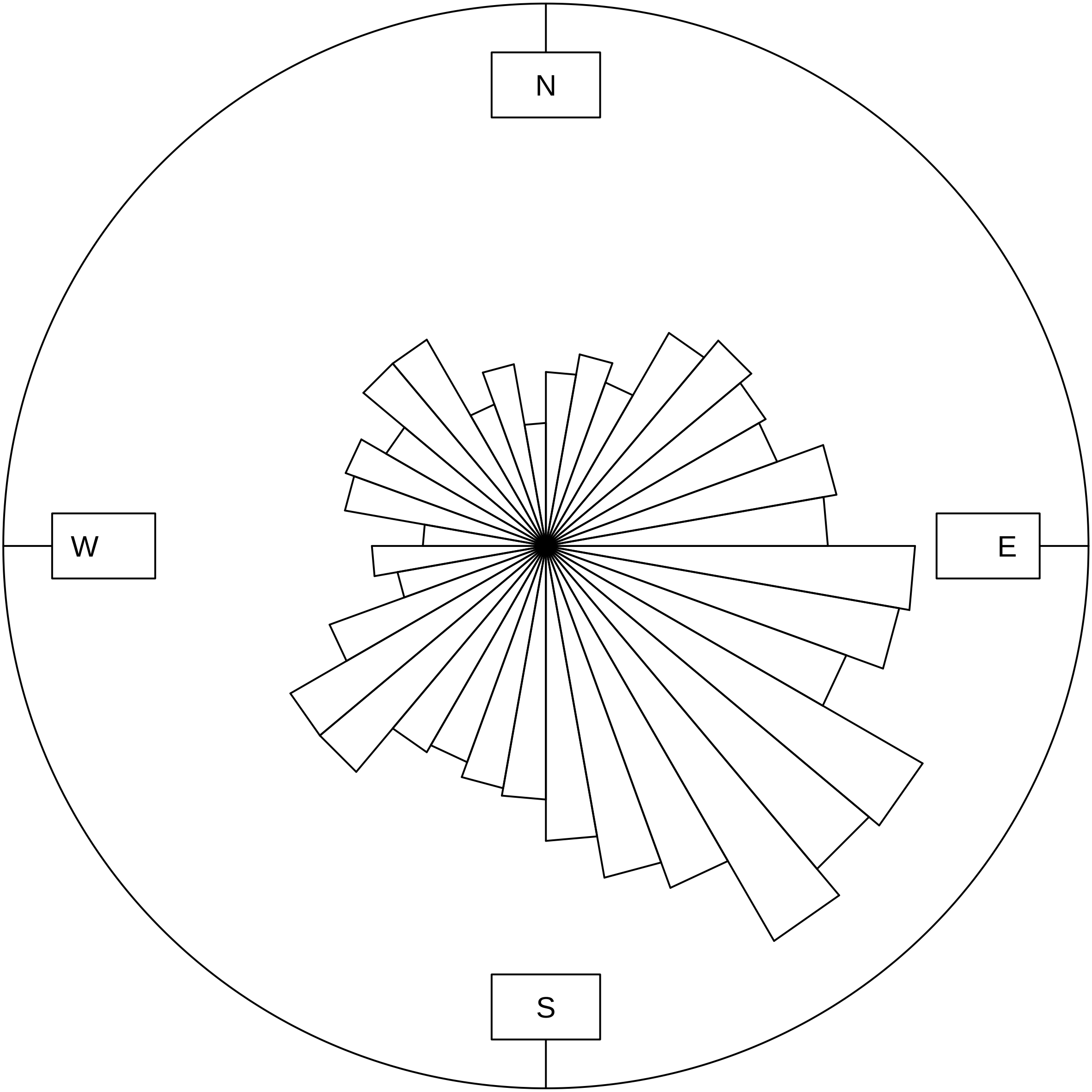

bootstrap 381

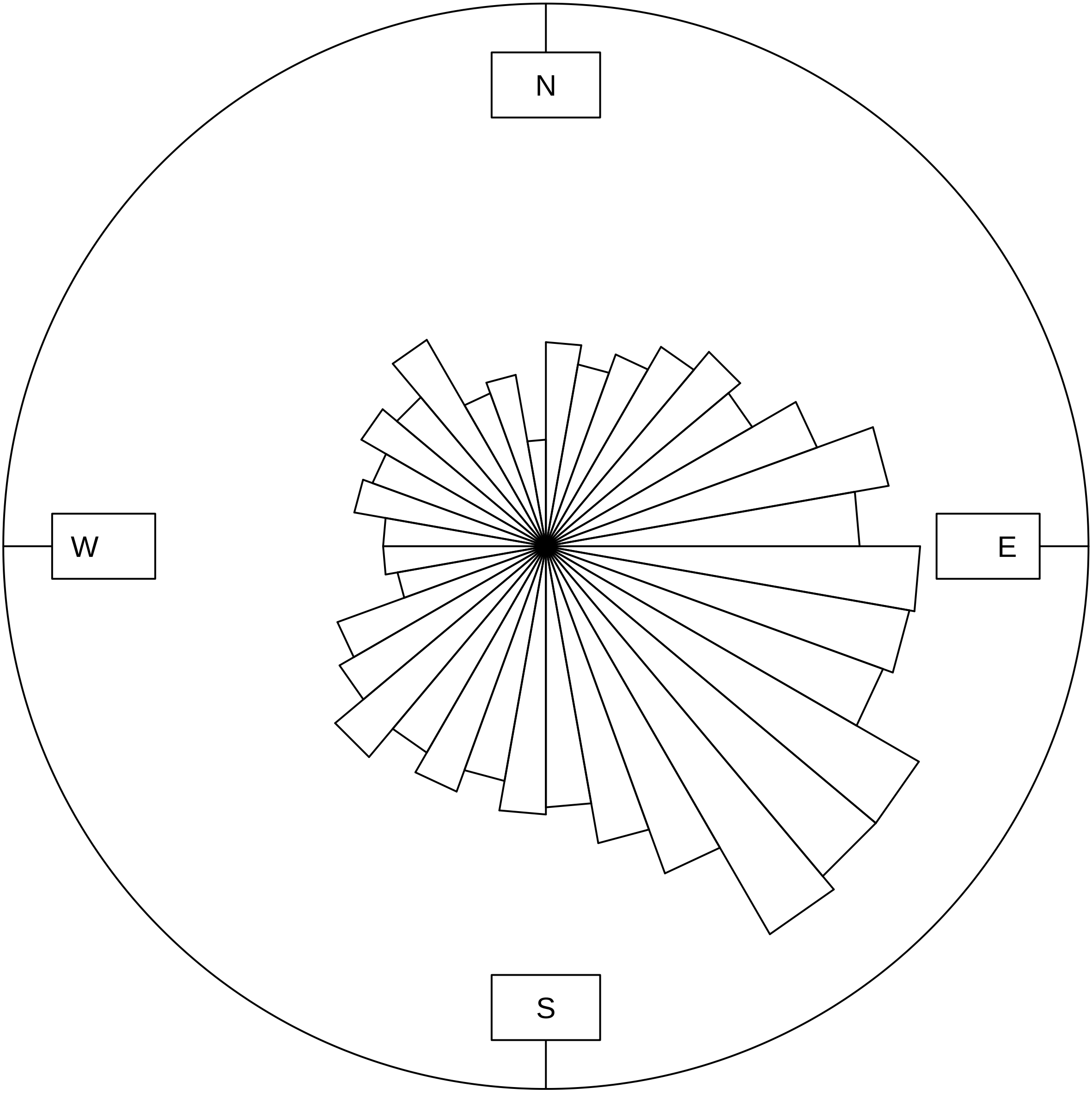

bootstrap 382

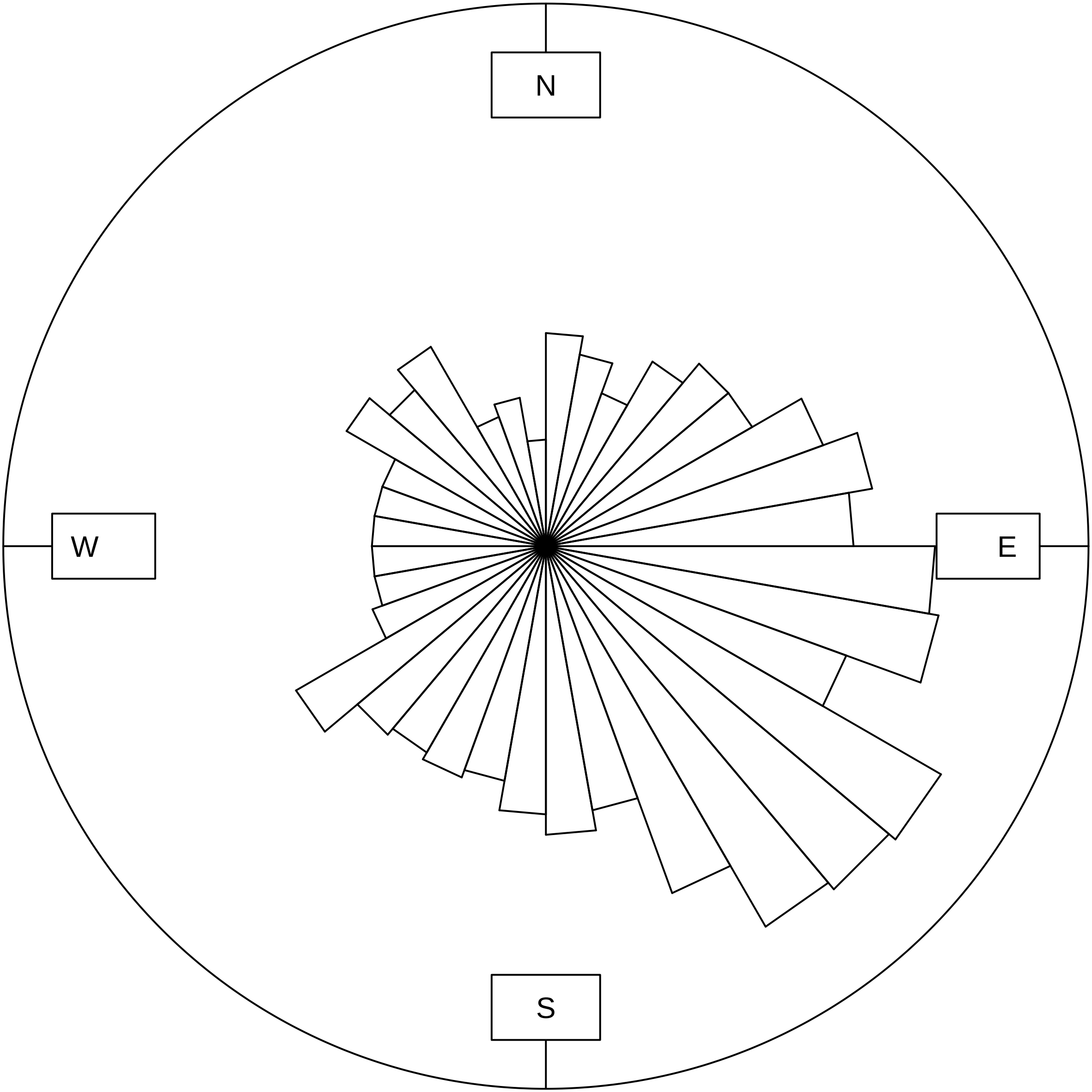

bootstrap 383

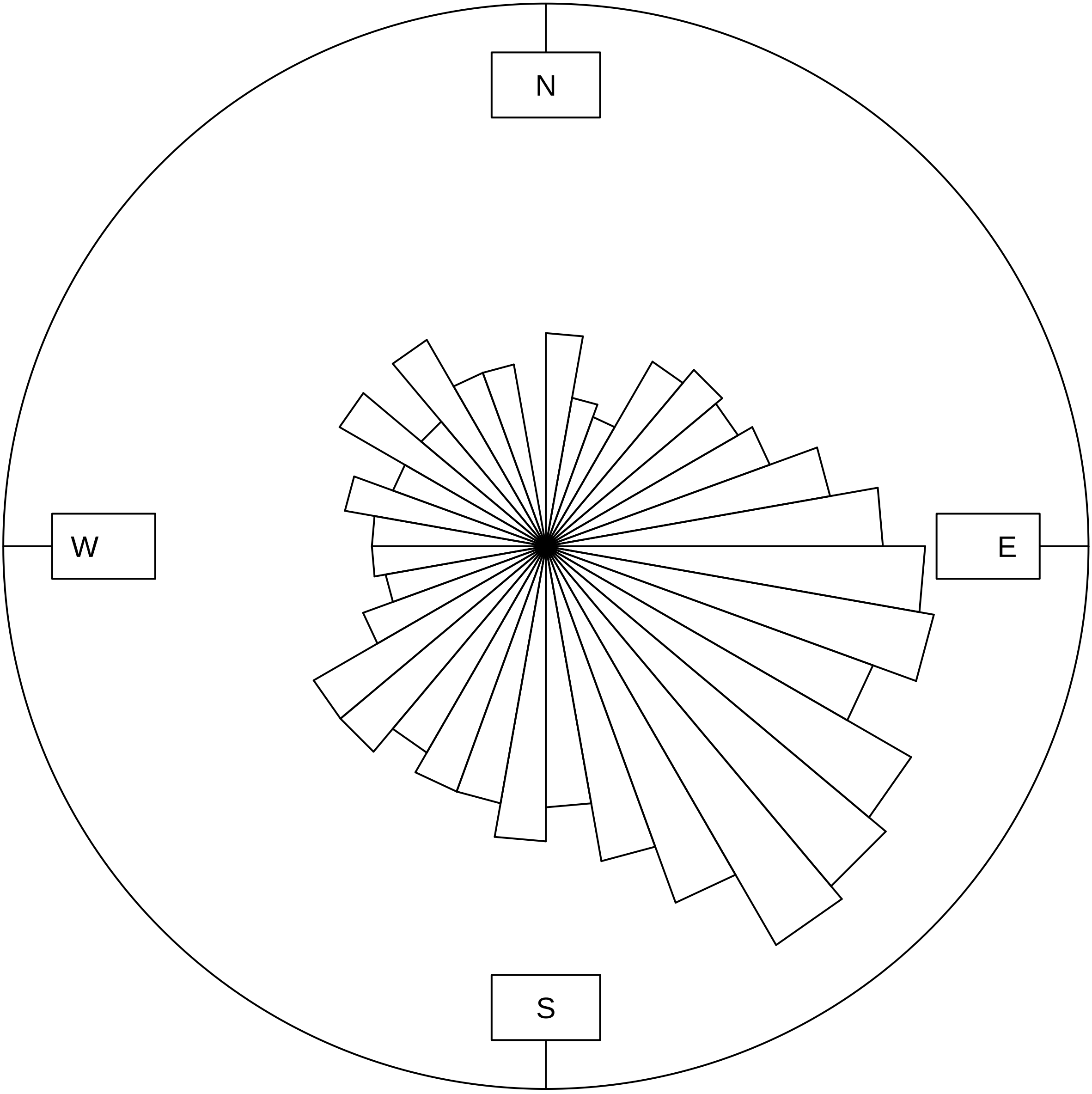

bootstrap 384

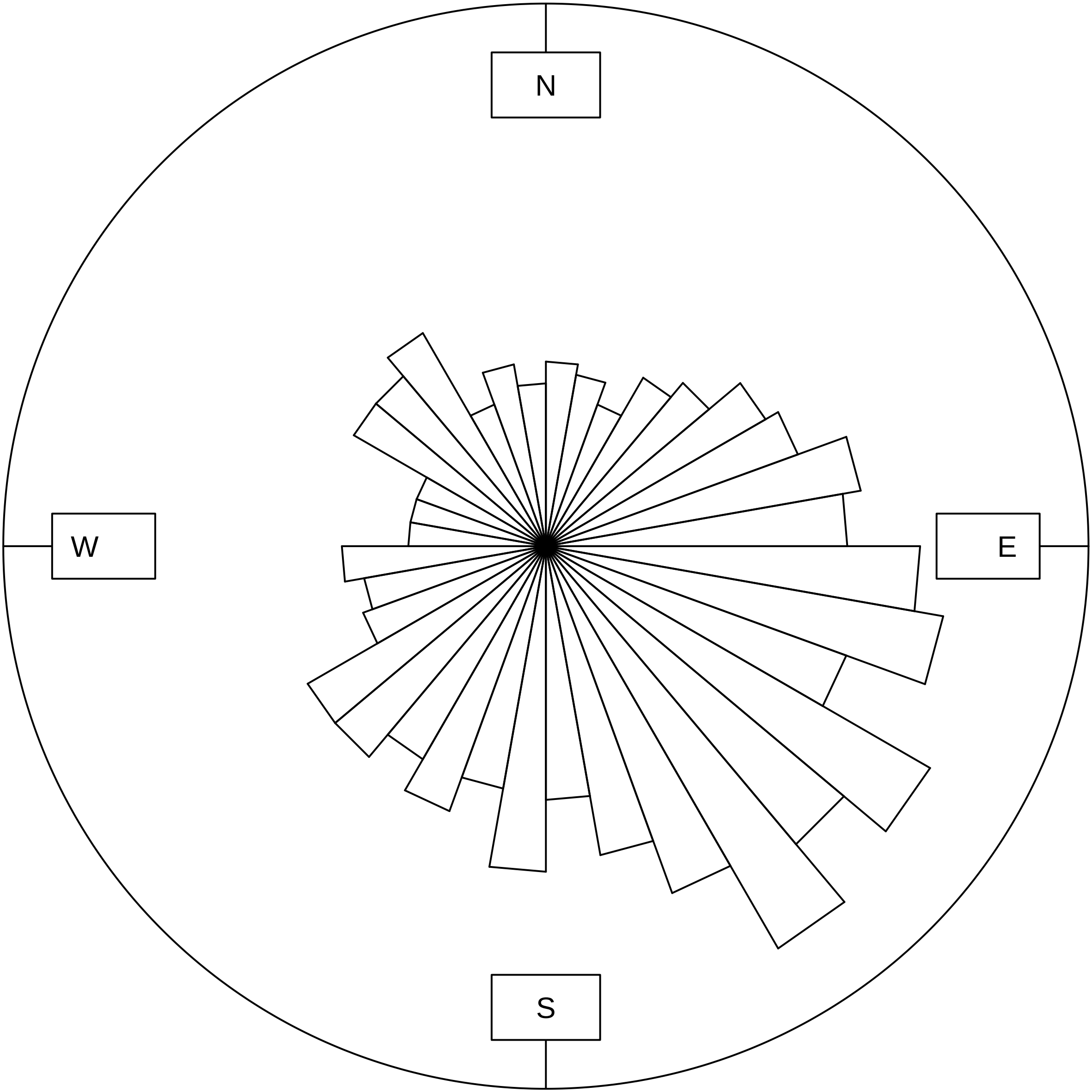

bootstrap 385

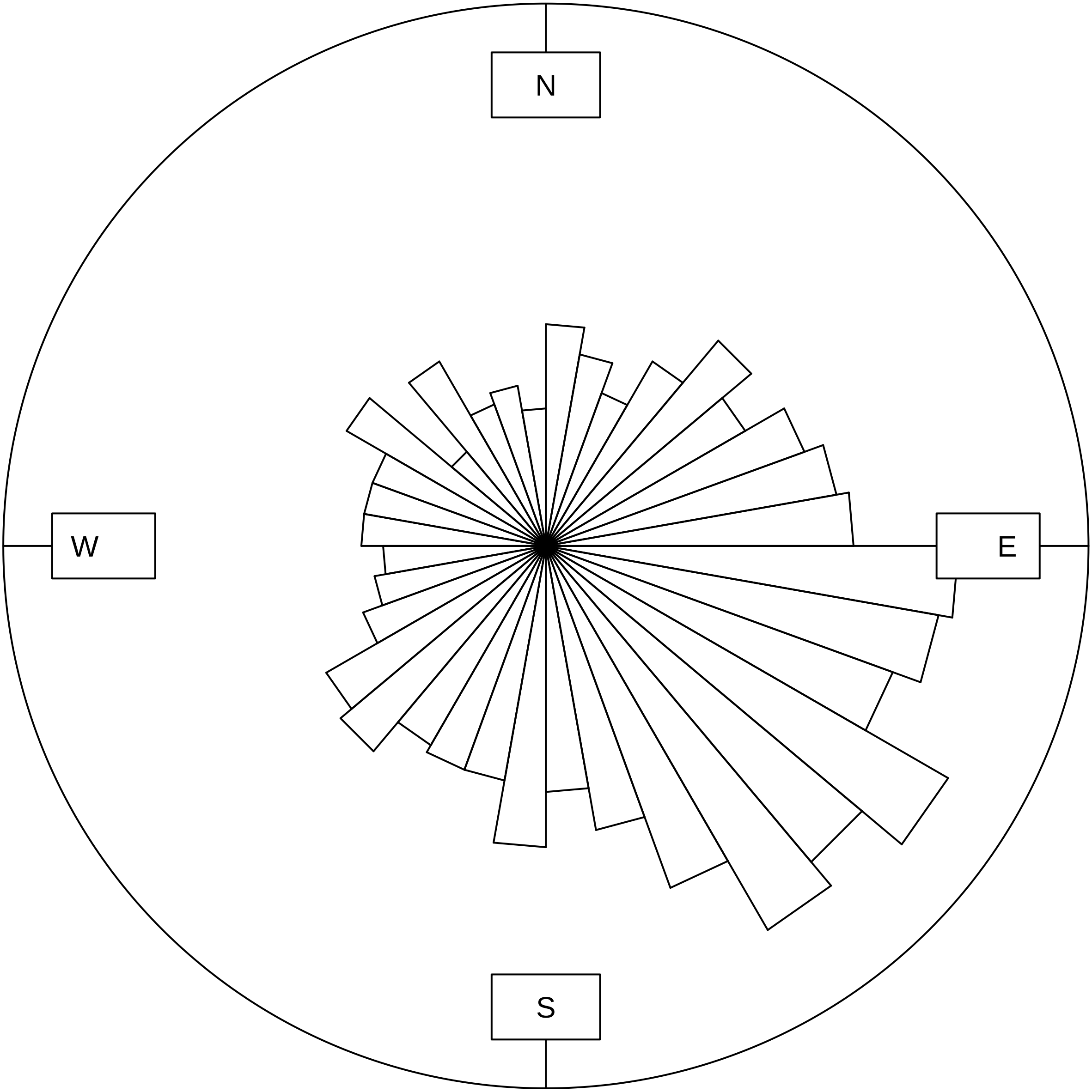

bootstrap 386

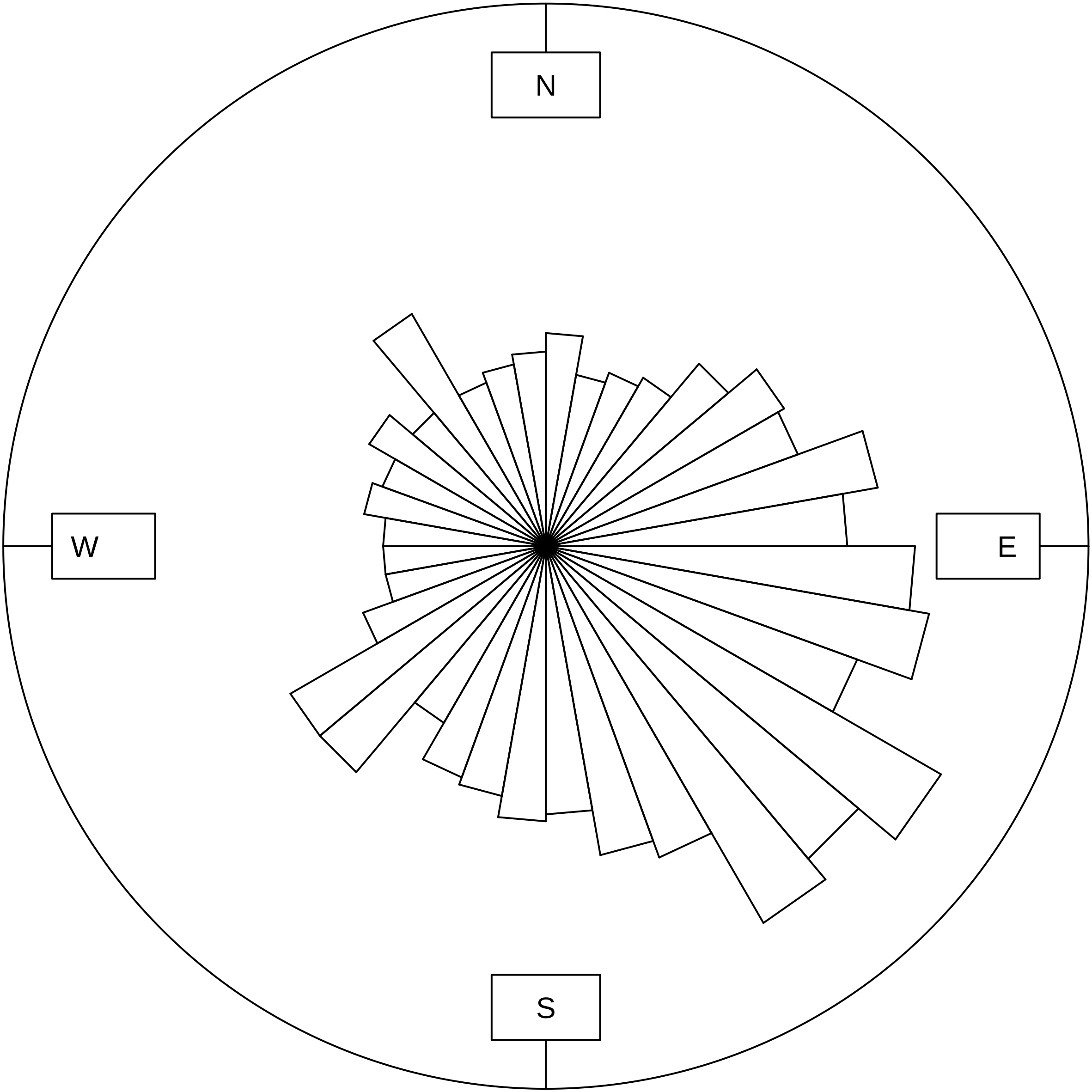

bootstrap 387

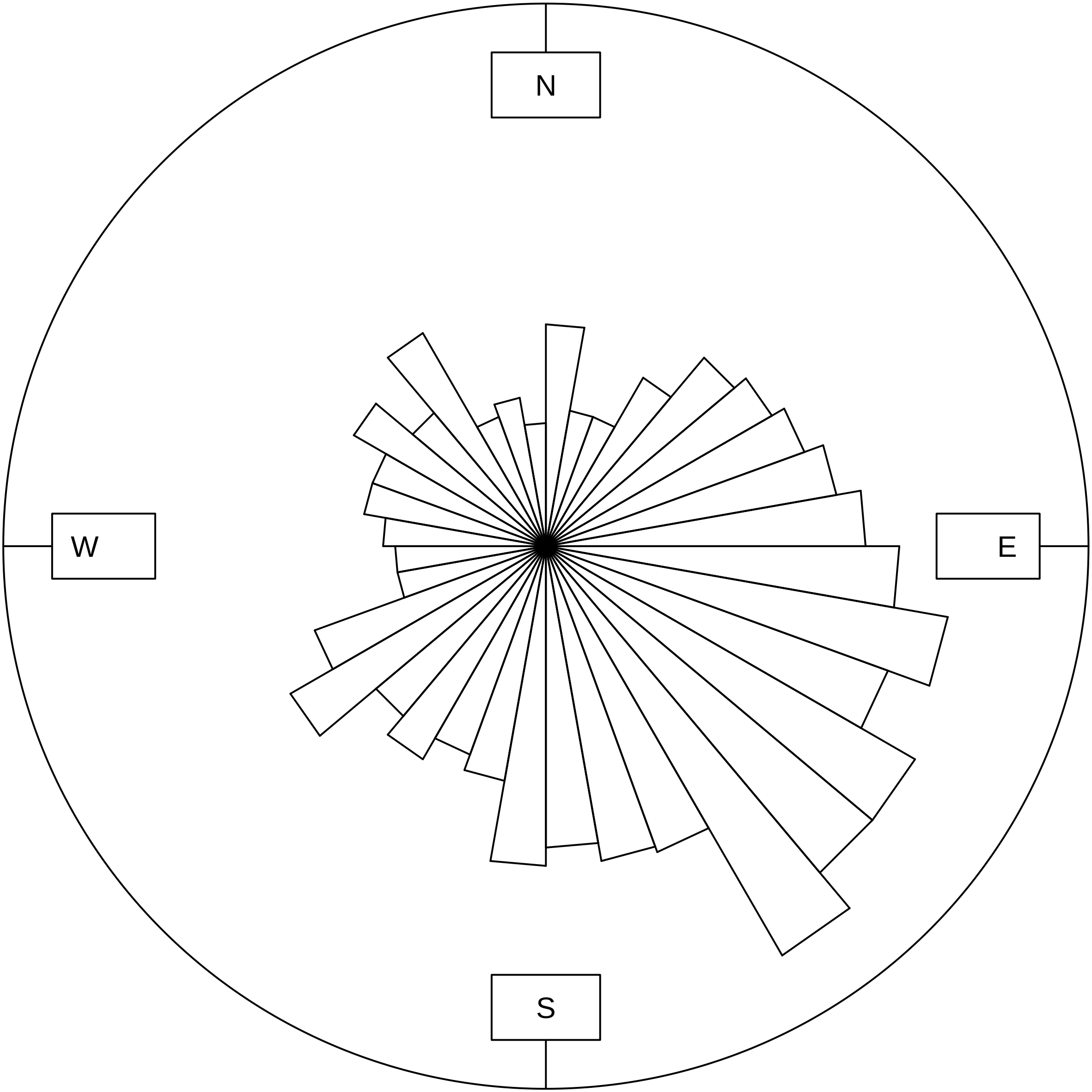

bootstrap 388

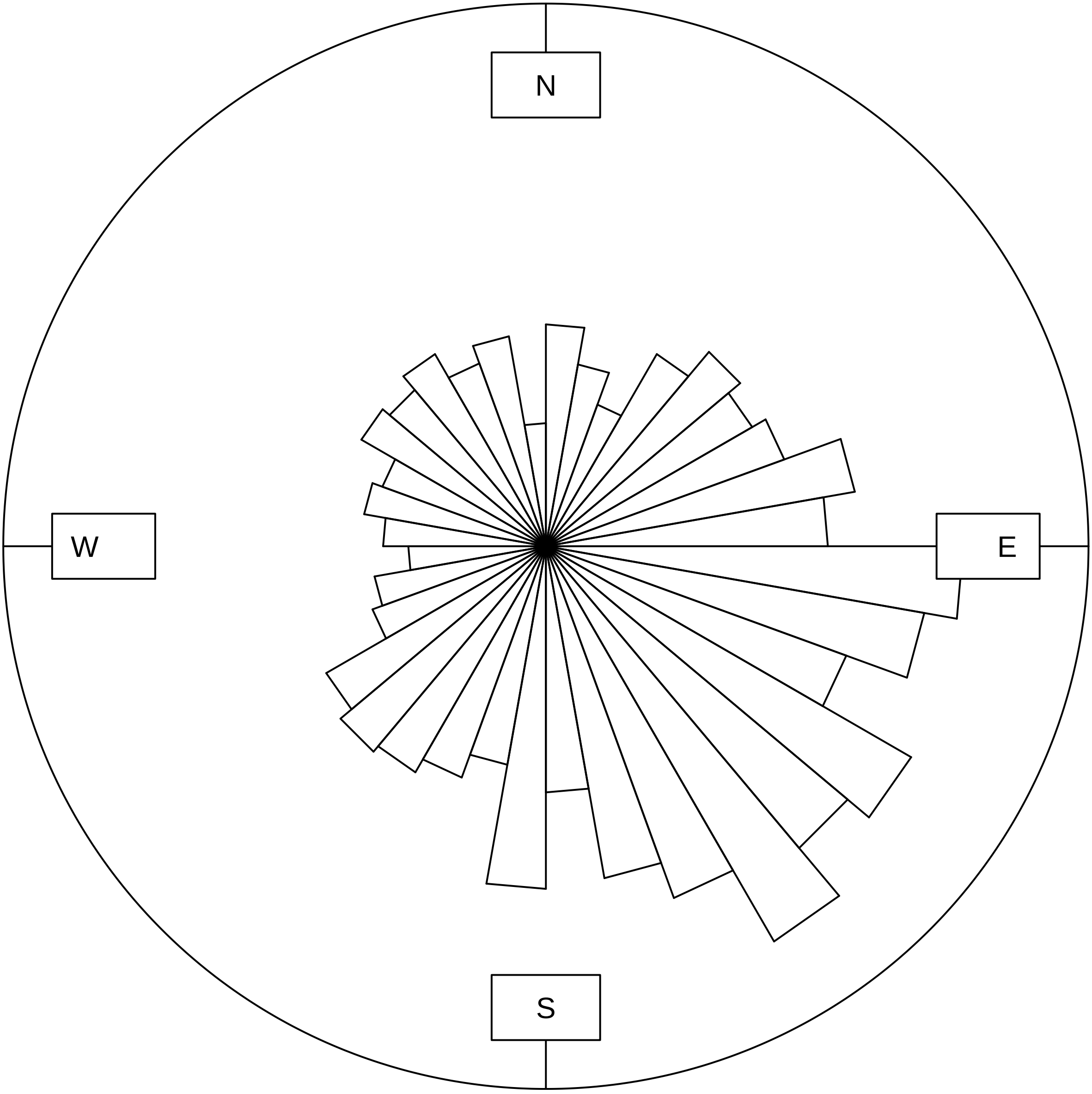

bootstrap 389

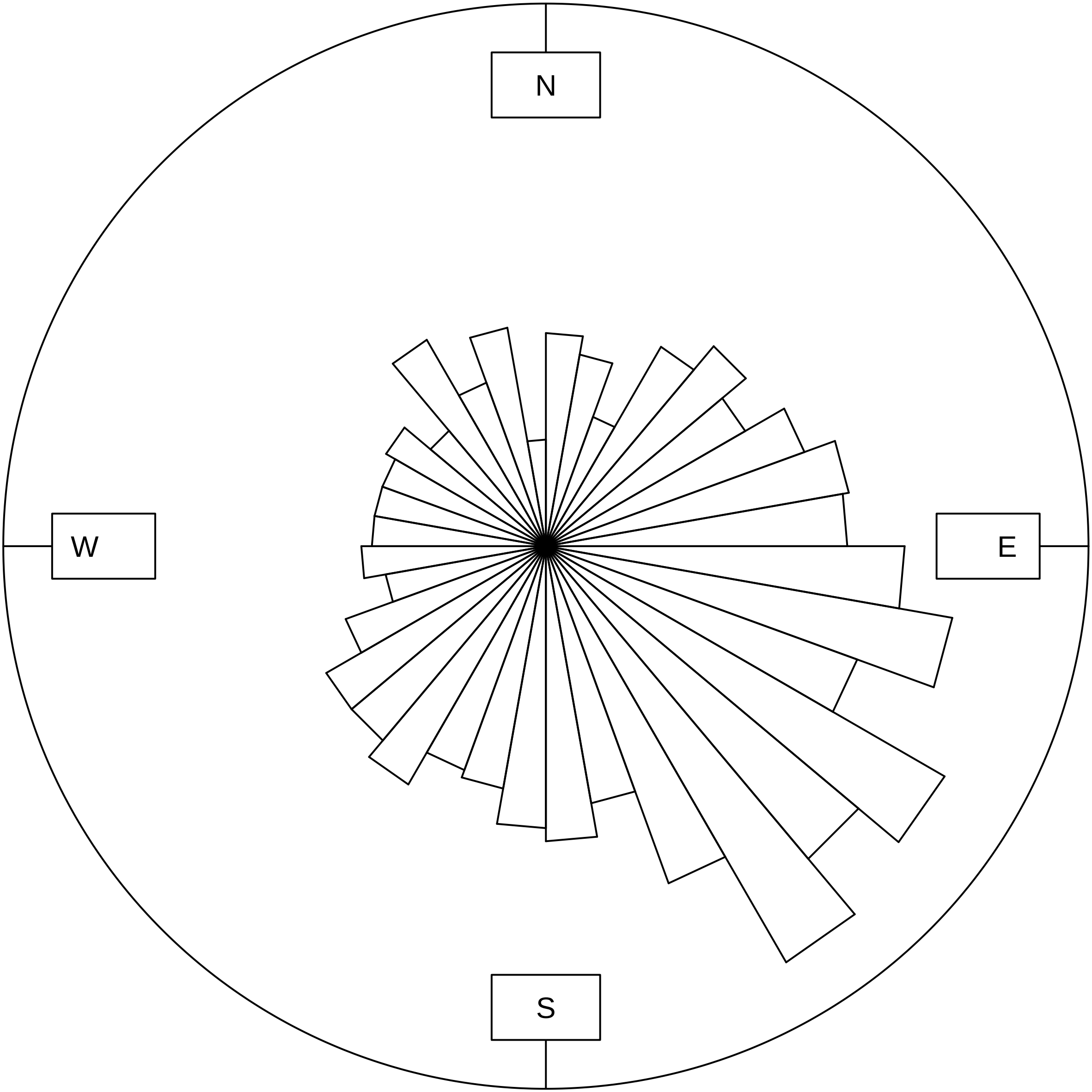

bootstrap 390

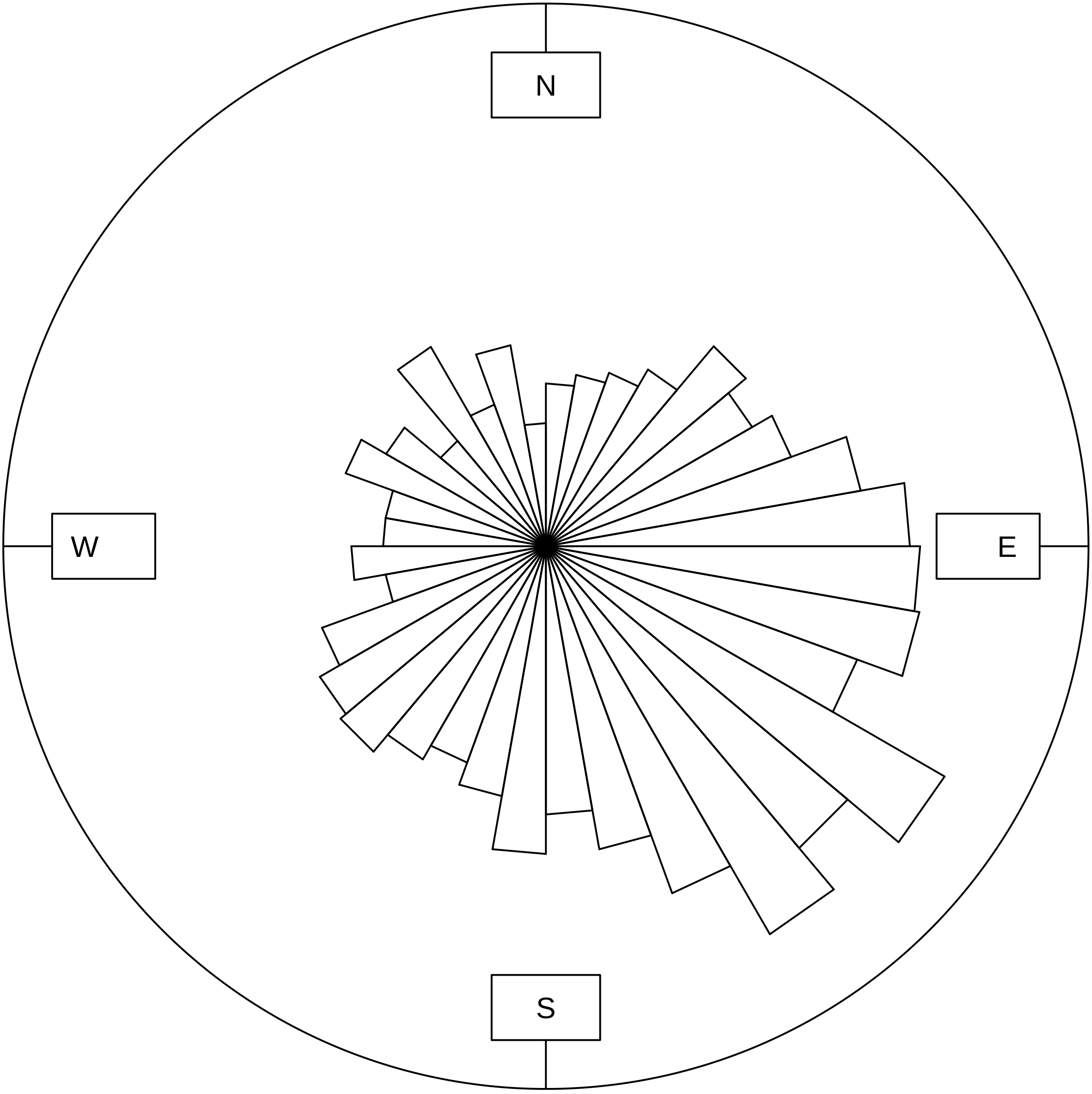

bootstrap 391

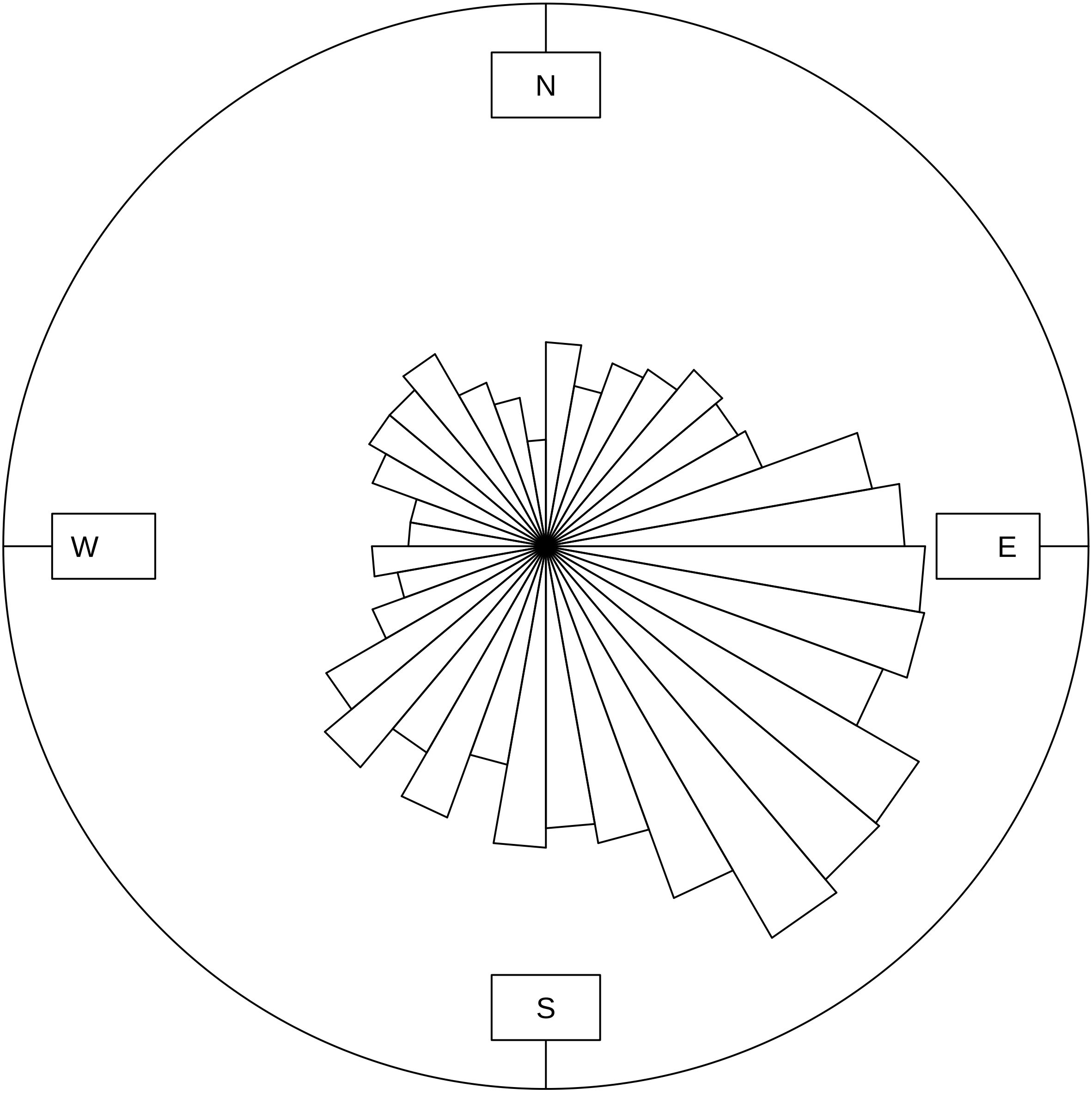

bootstrap 392

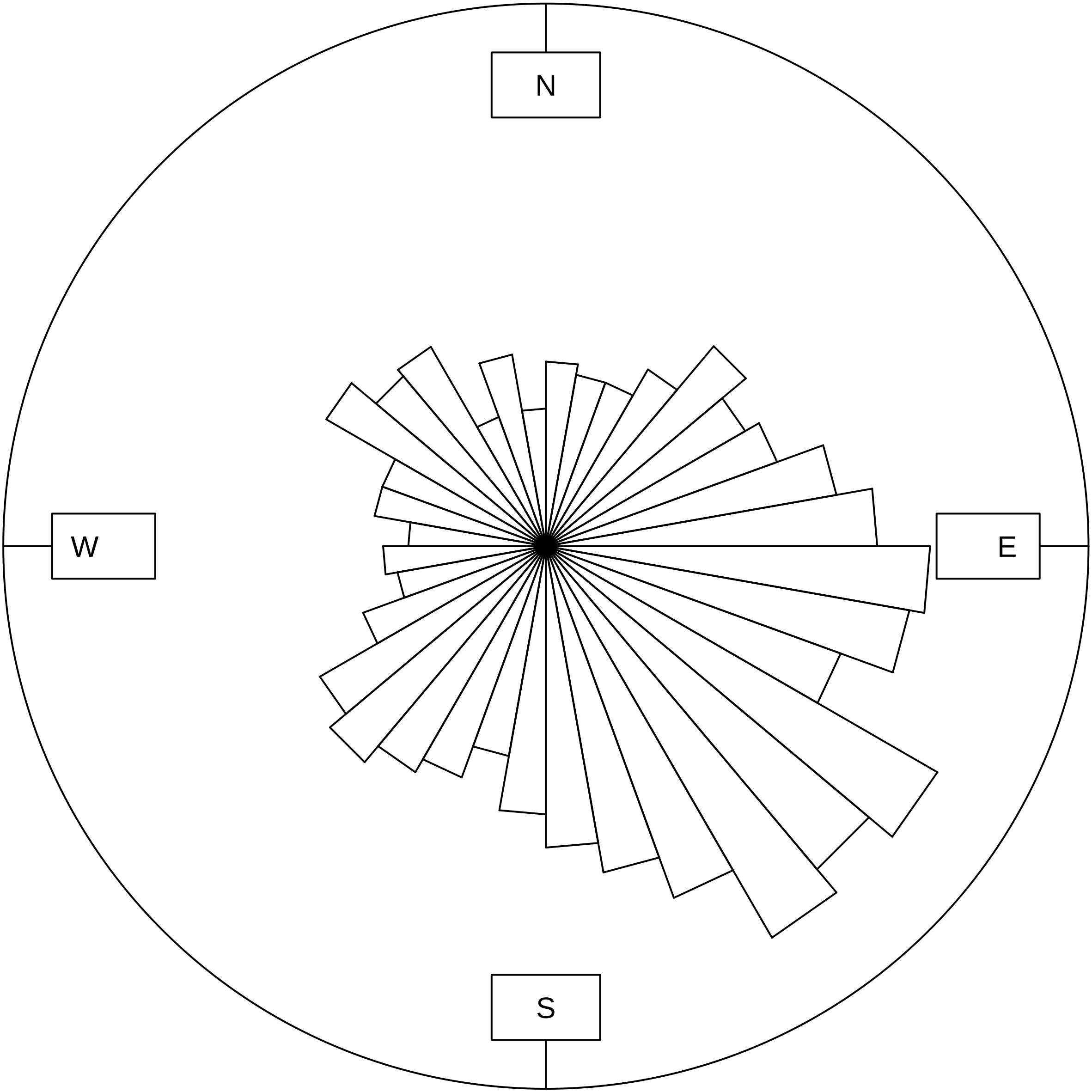

bootstrap 393

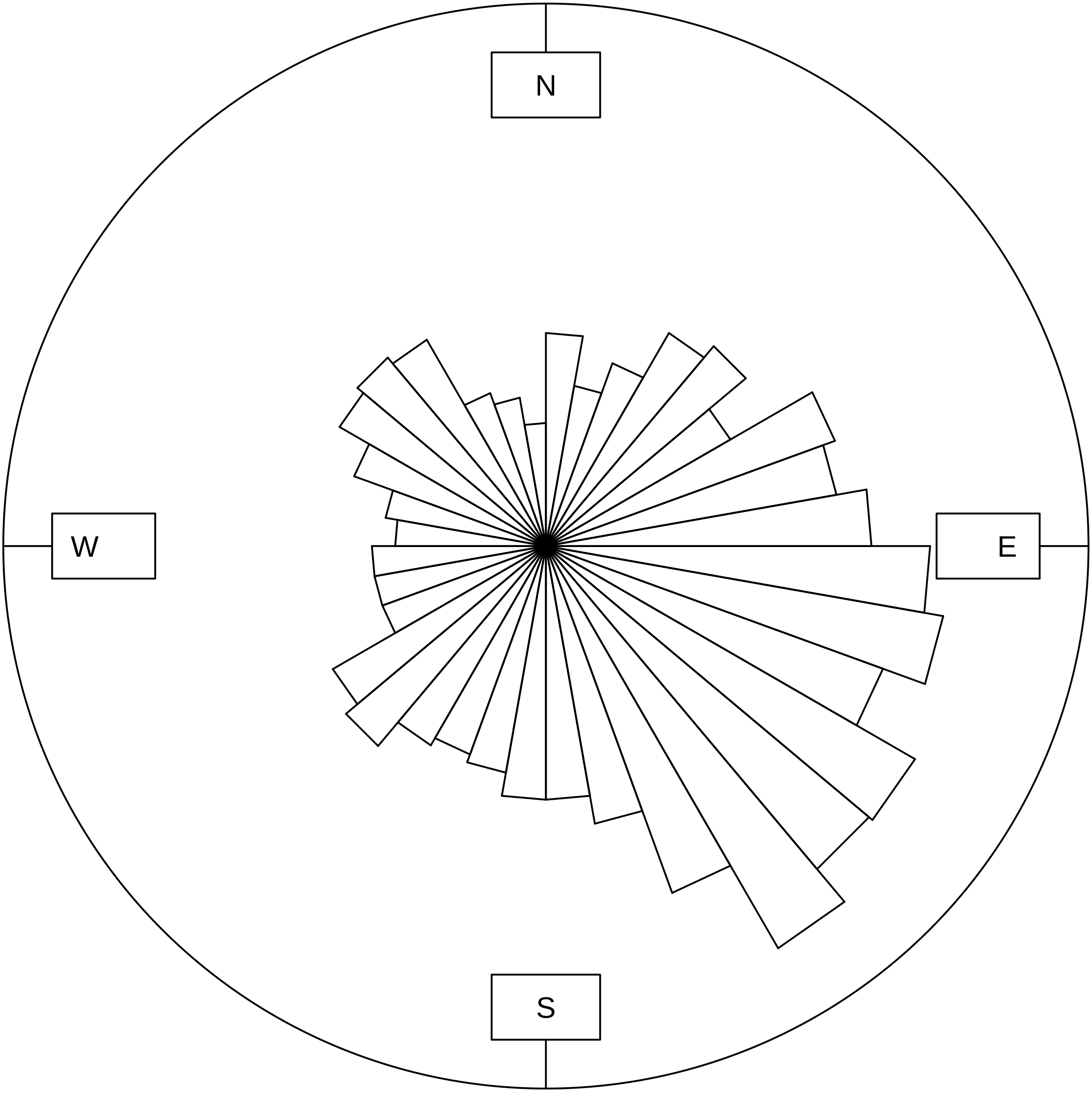

bootstrap 394

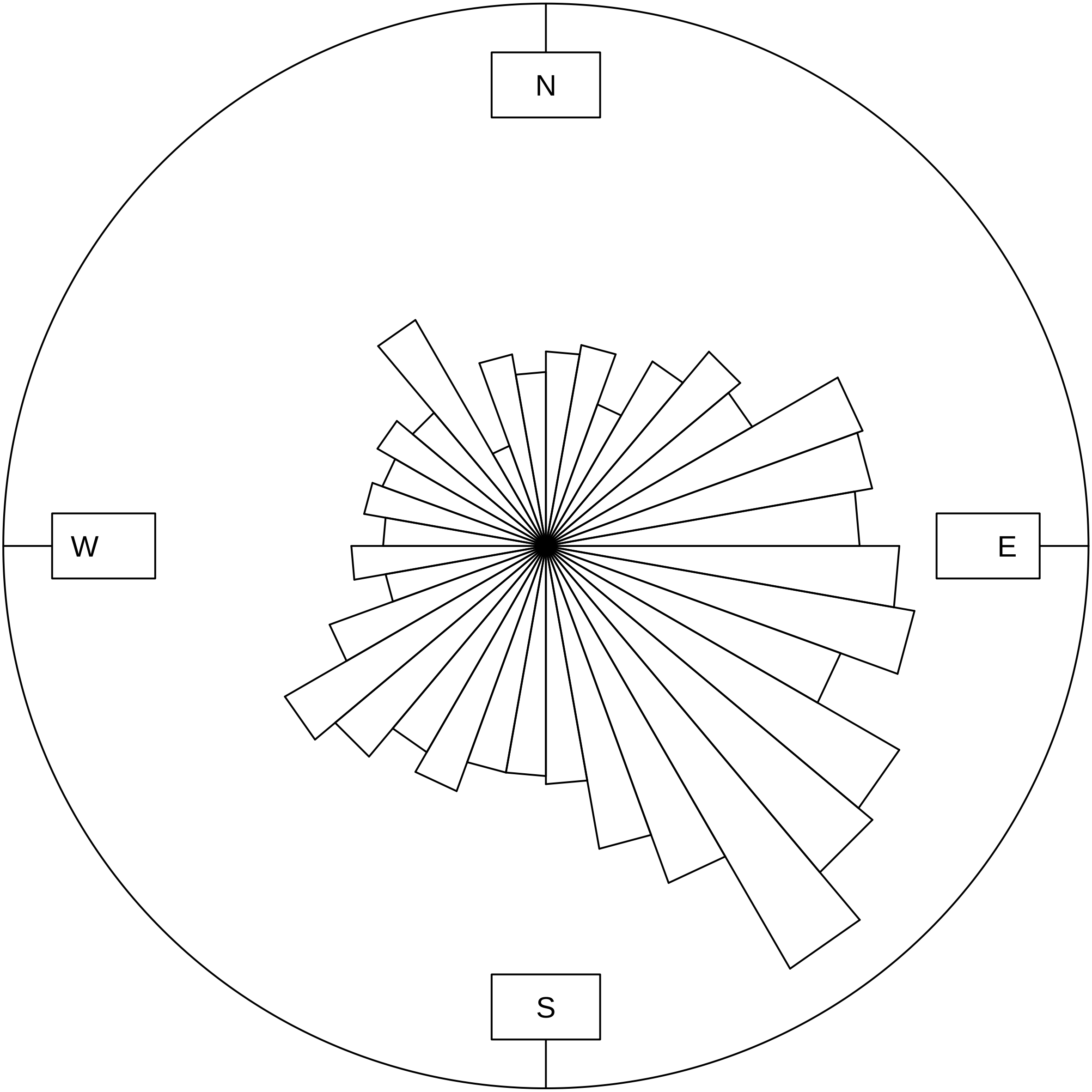

bootstrap 395

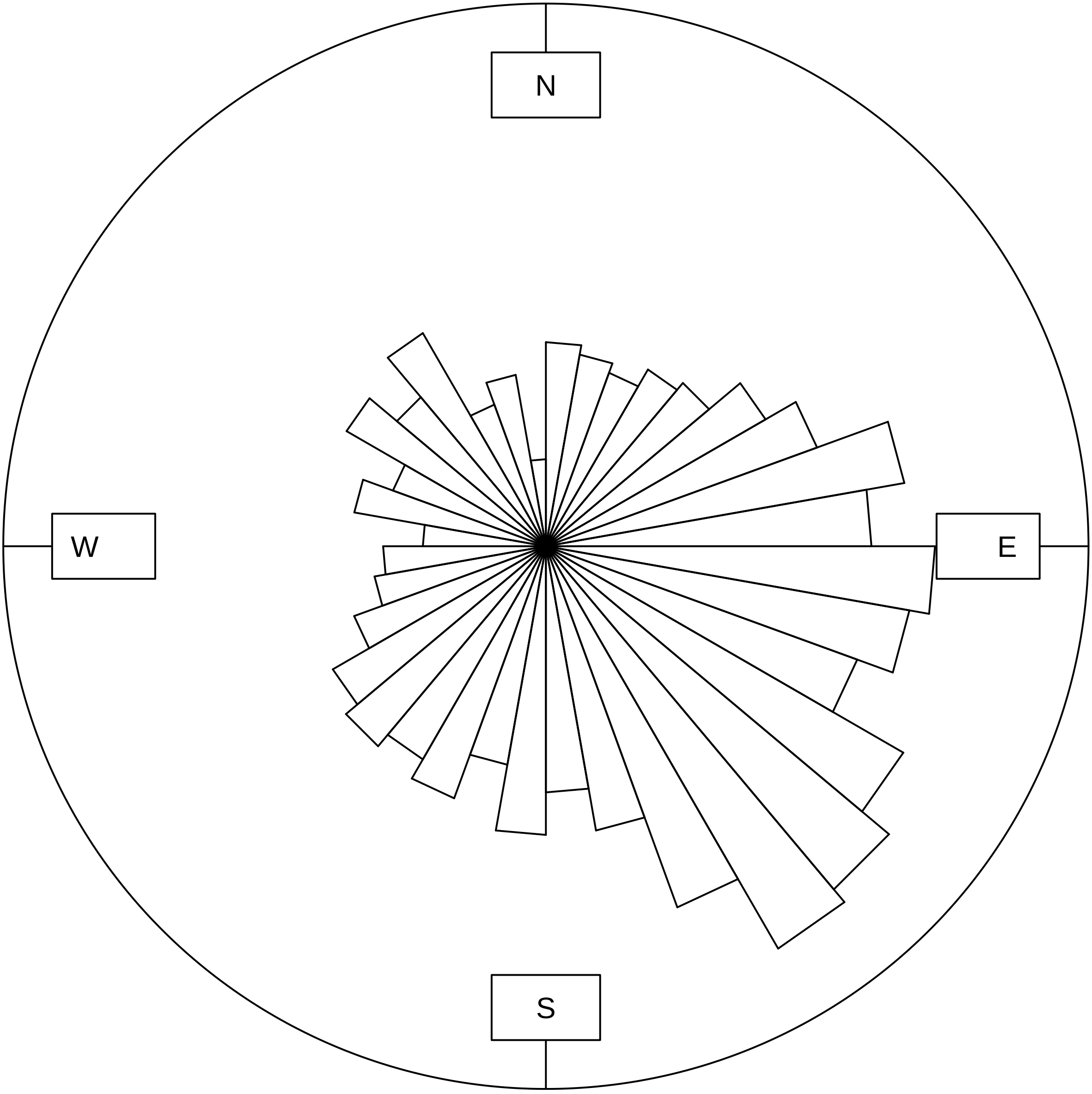

bootstrap 396

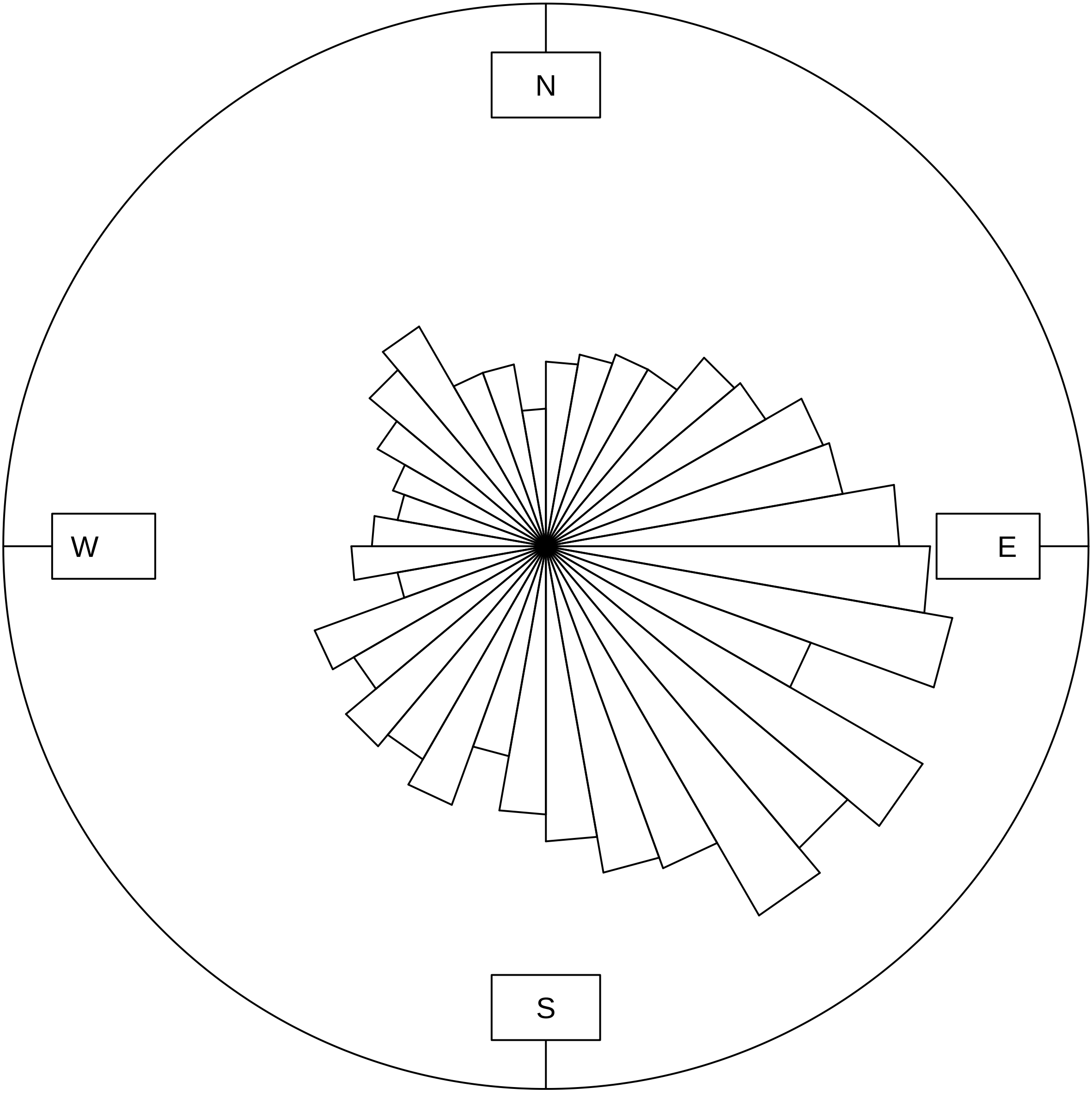

bootstrap 397

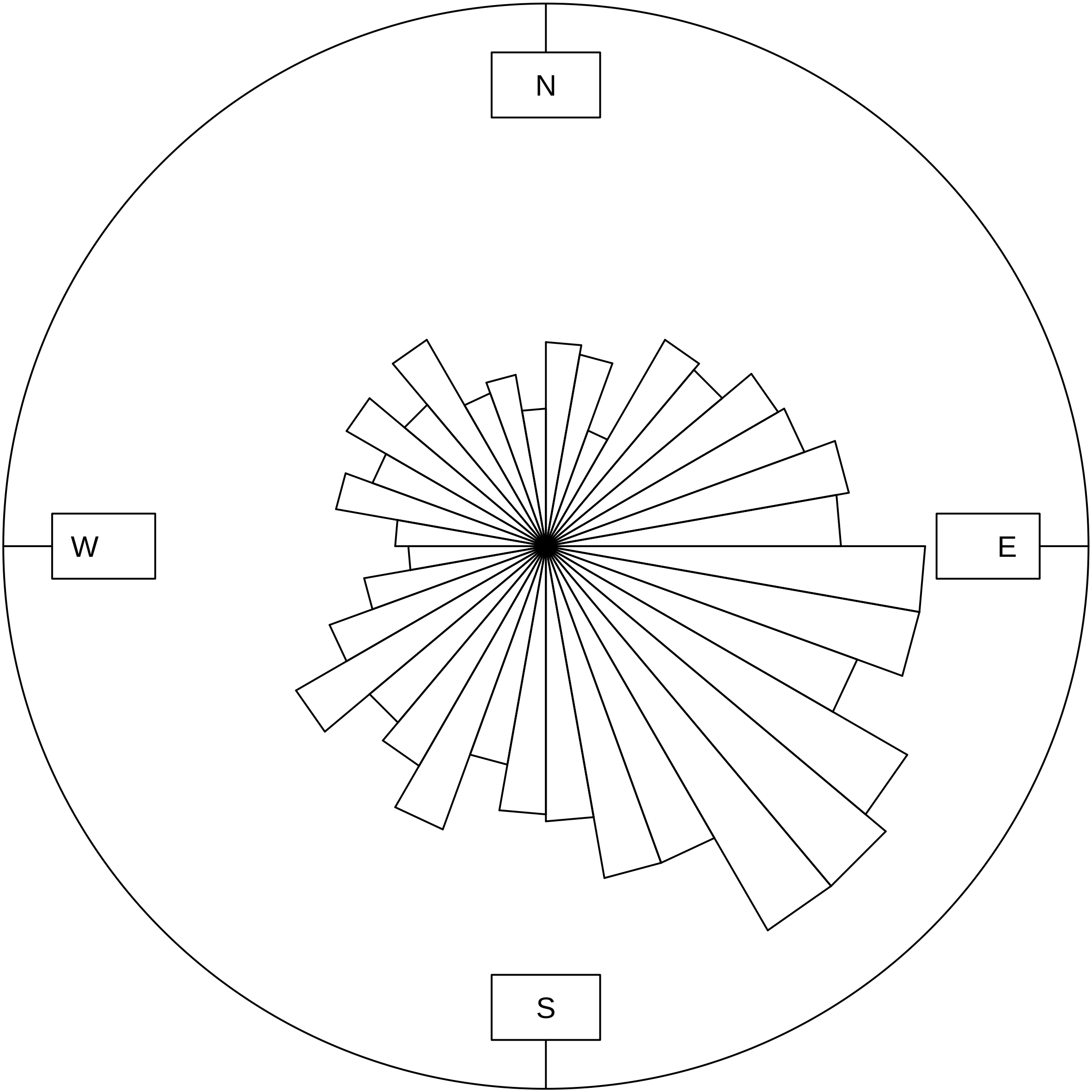

bootstrap 398

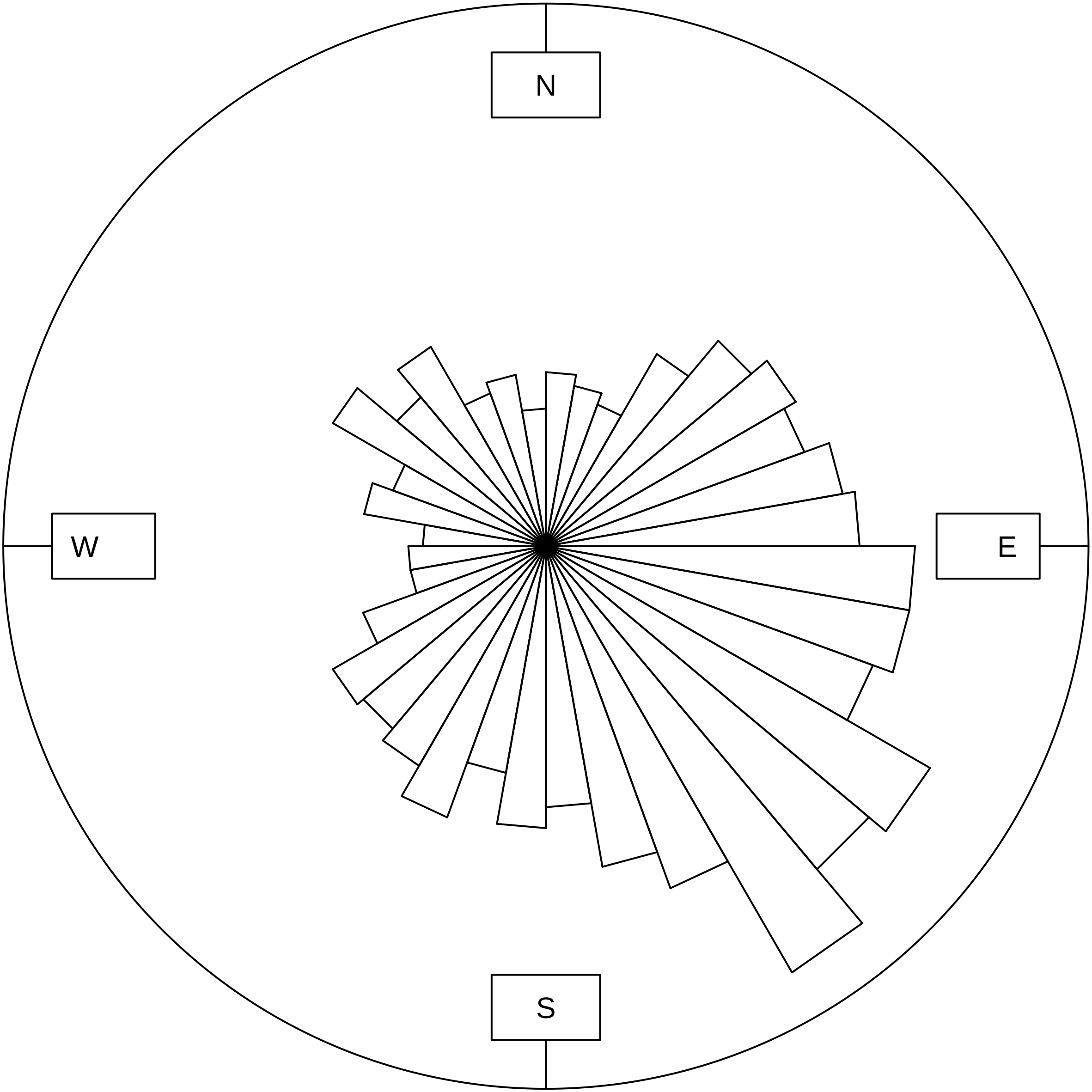

bootstrap 399

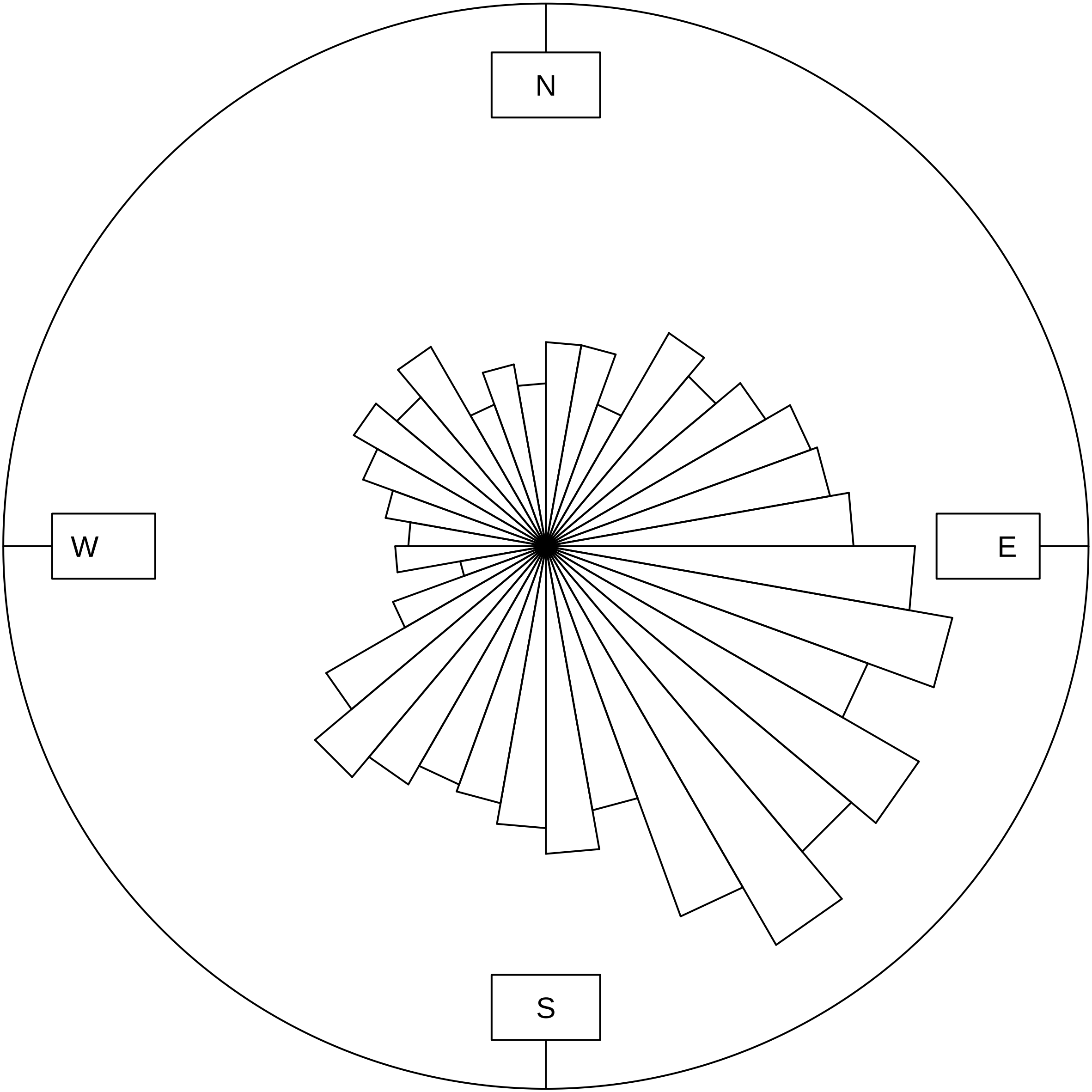

bootstrap 400

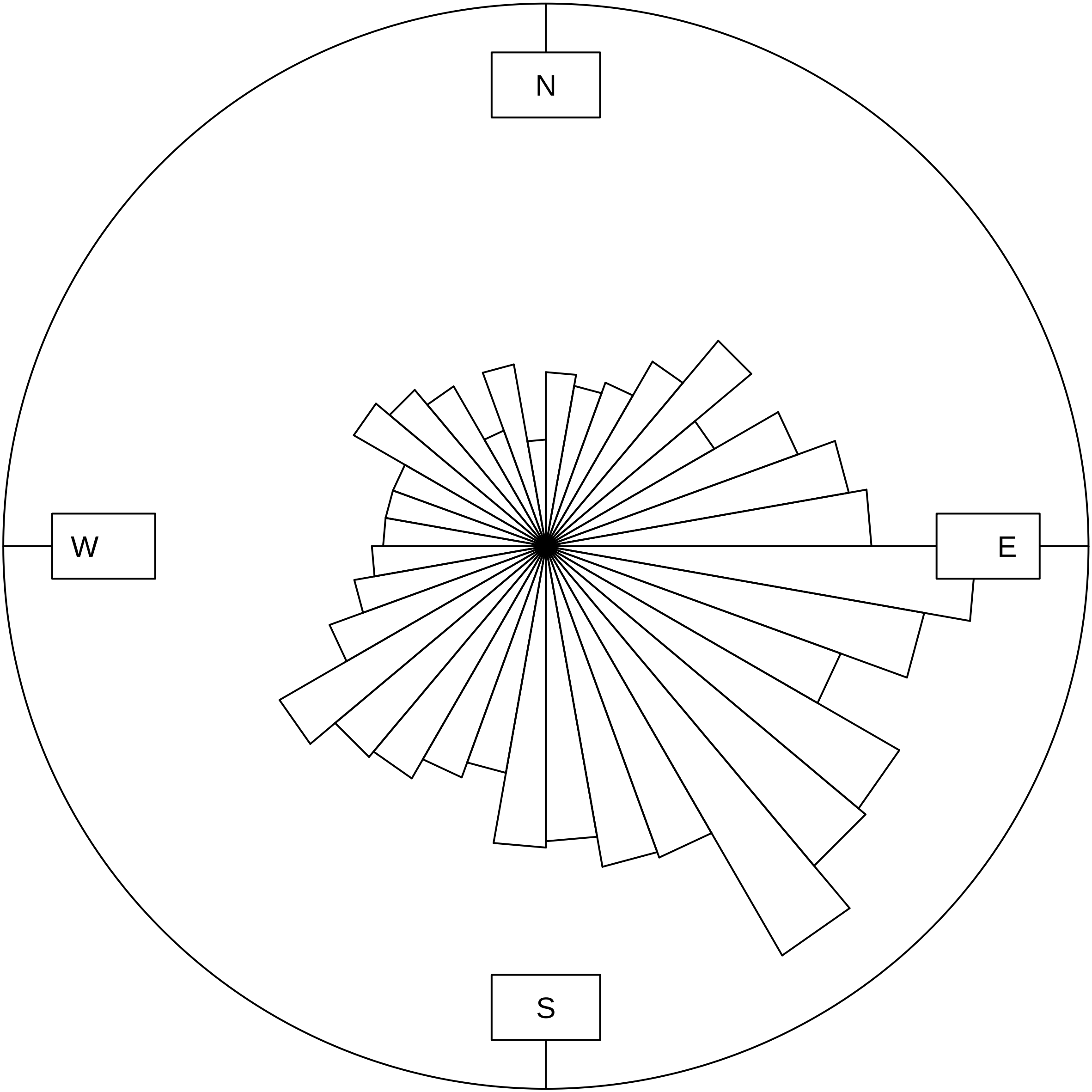

bootstrap 401

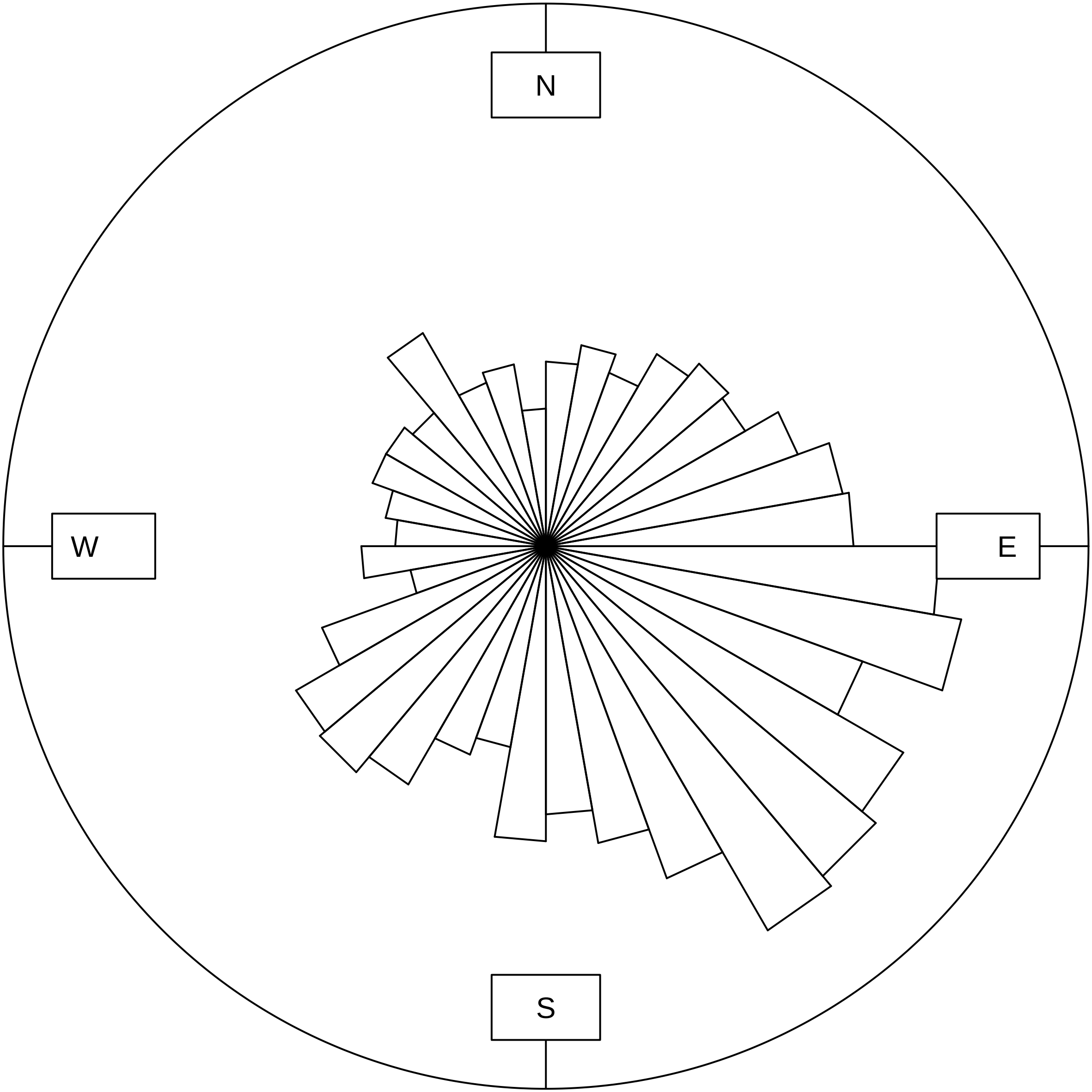

bootstrap 402

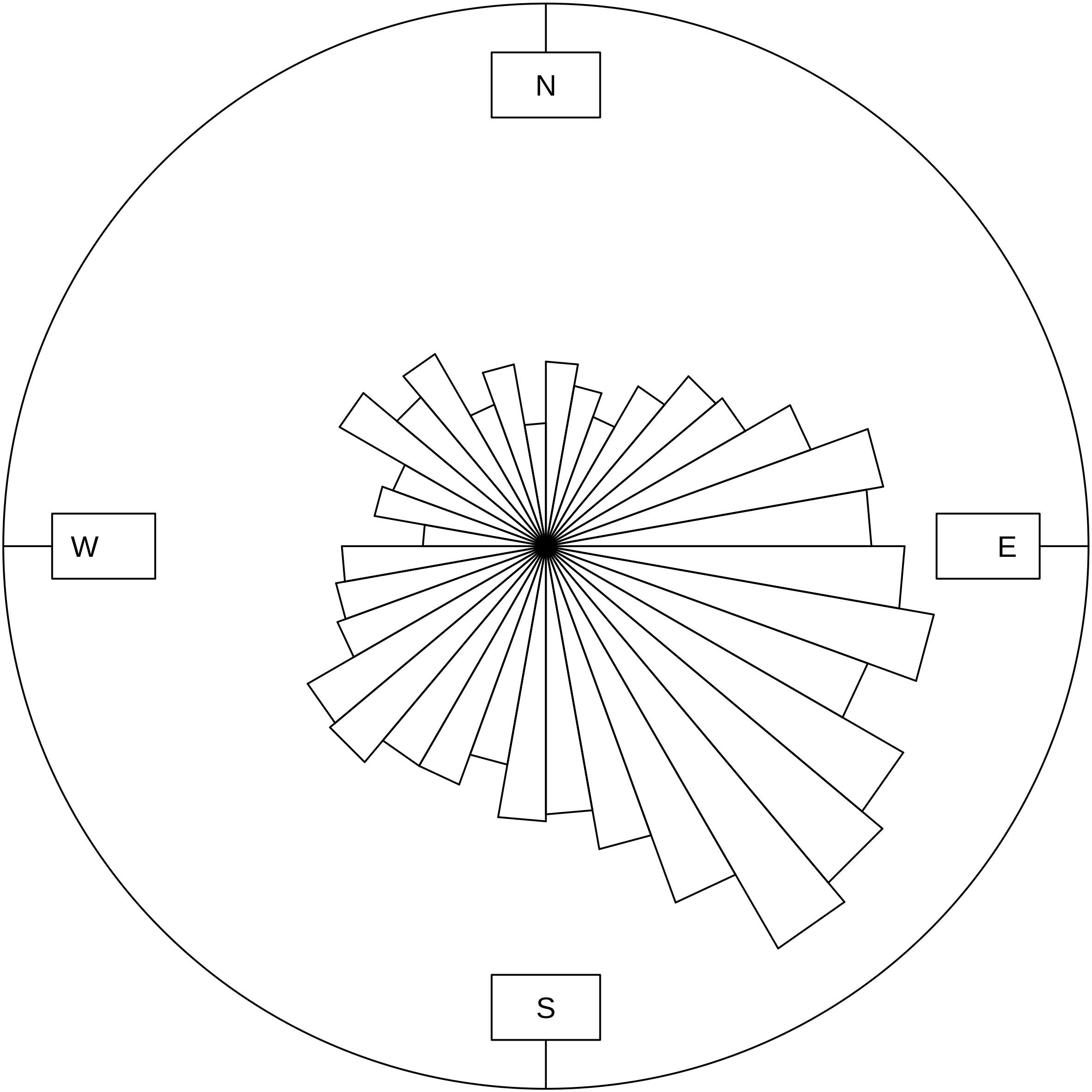

bootstrap 403

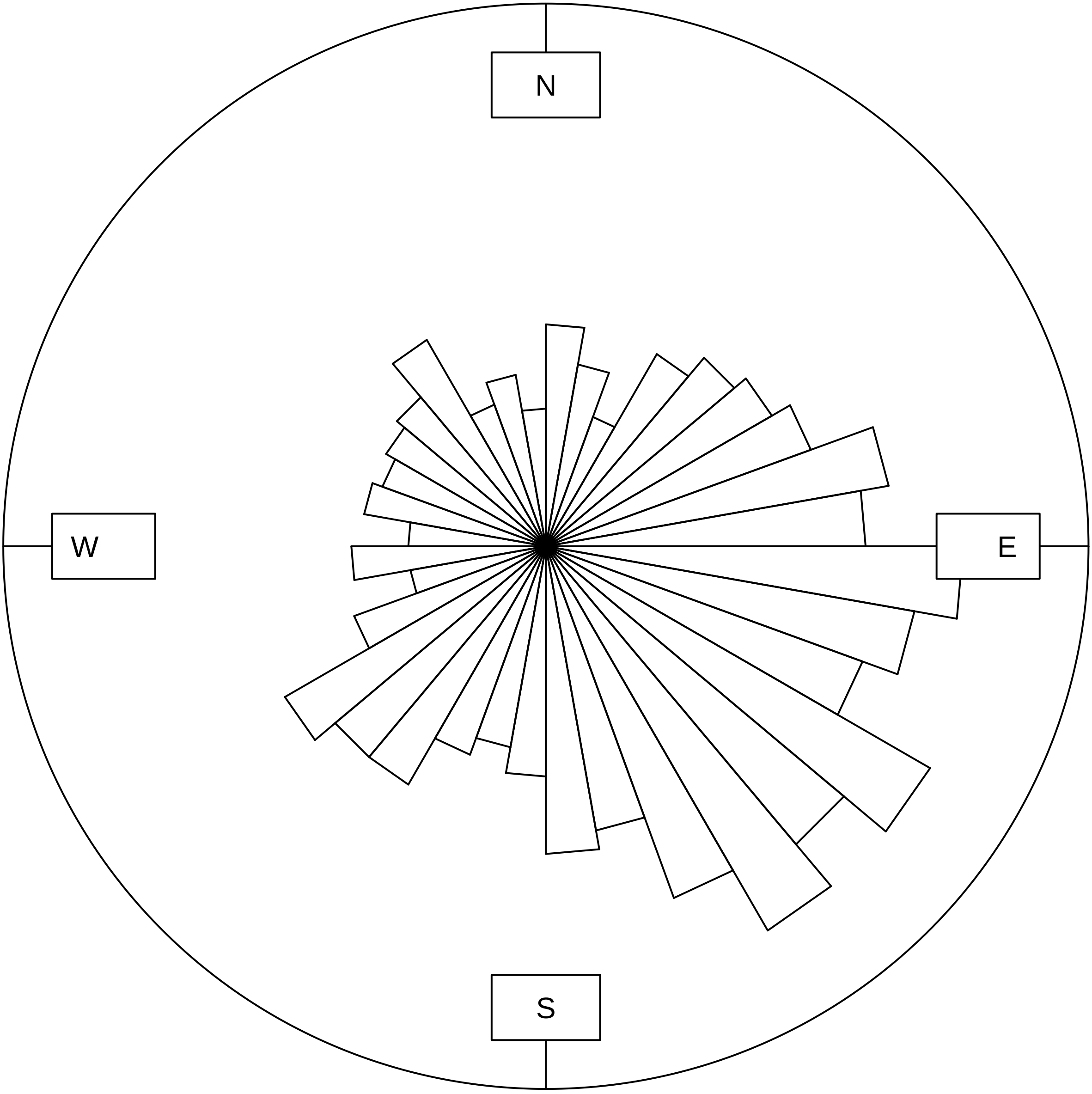

bootstrap 404

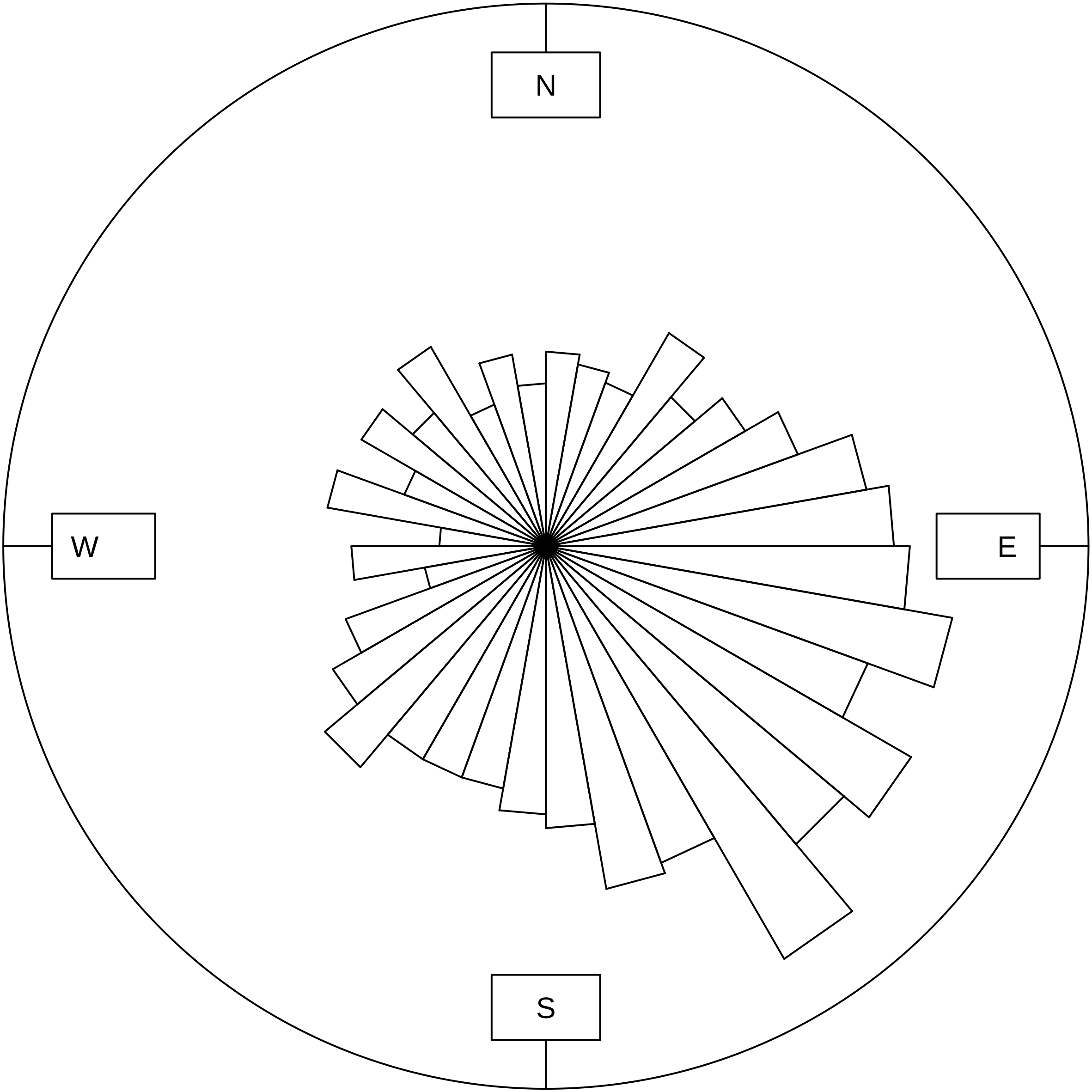

bootstrap 405

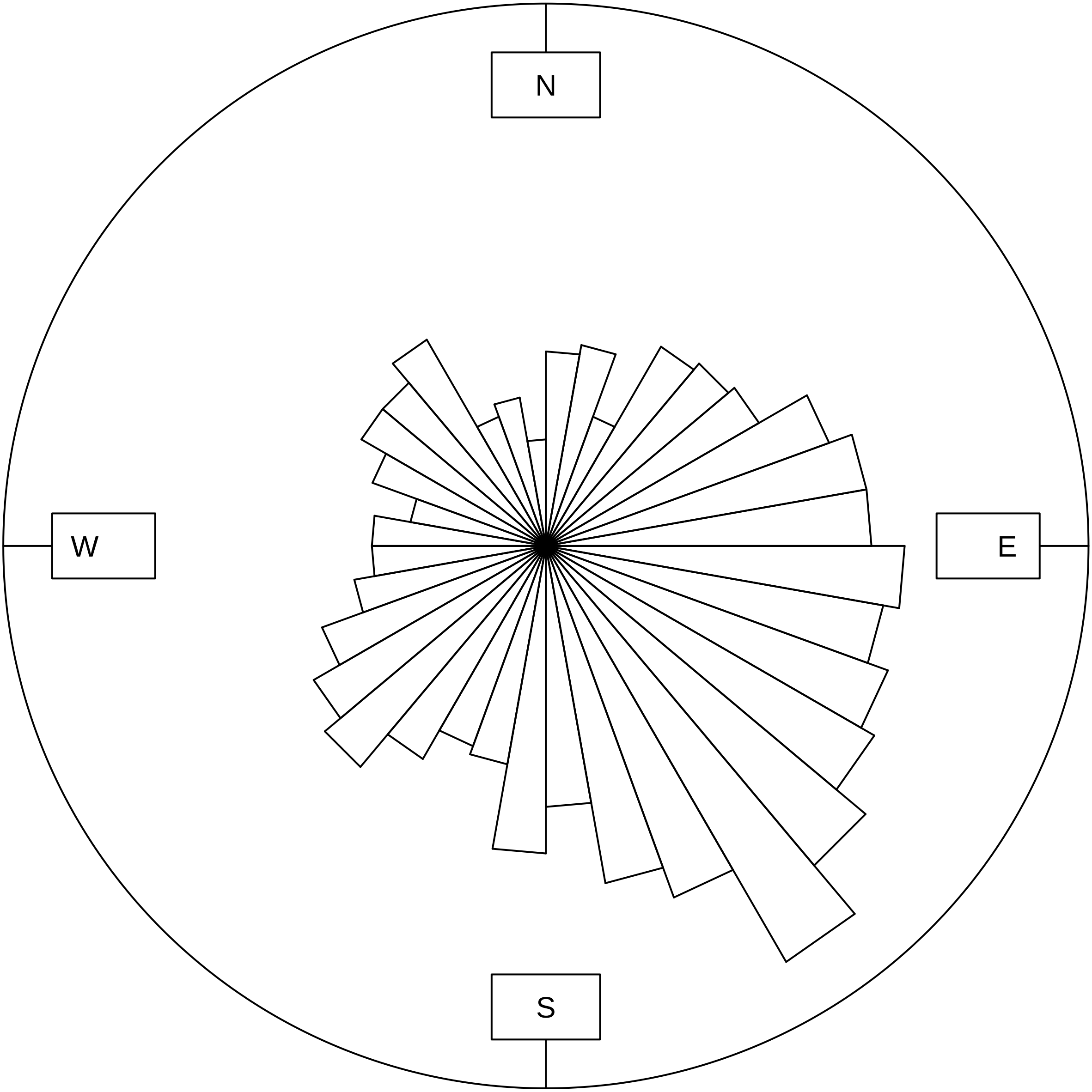

bootstrap 406

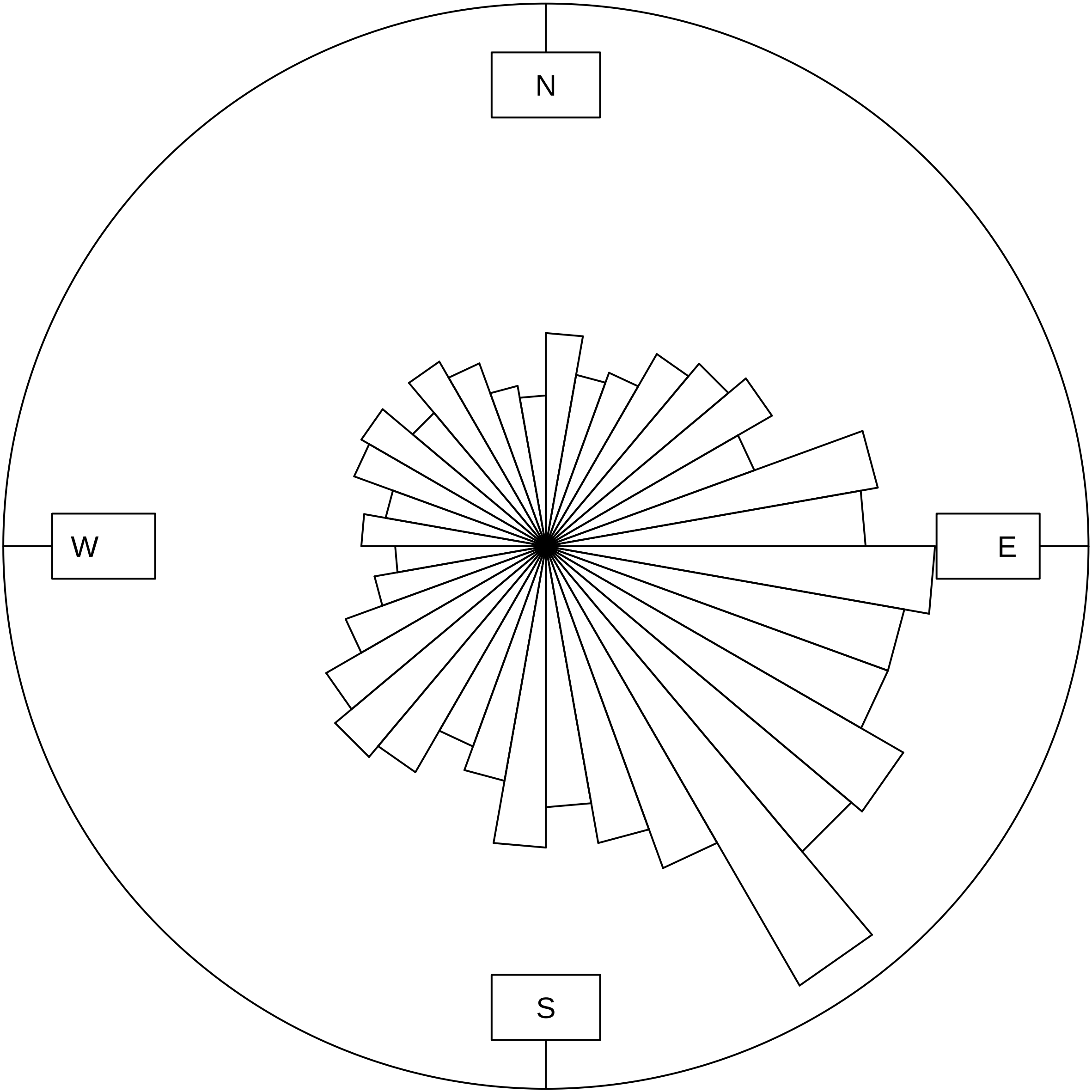

bootstrap 407

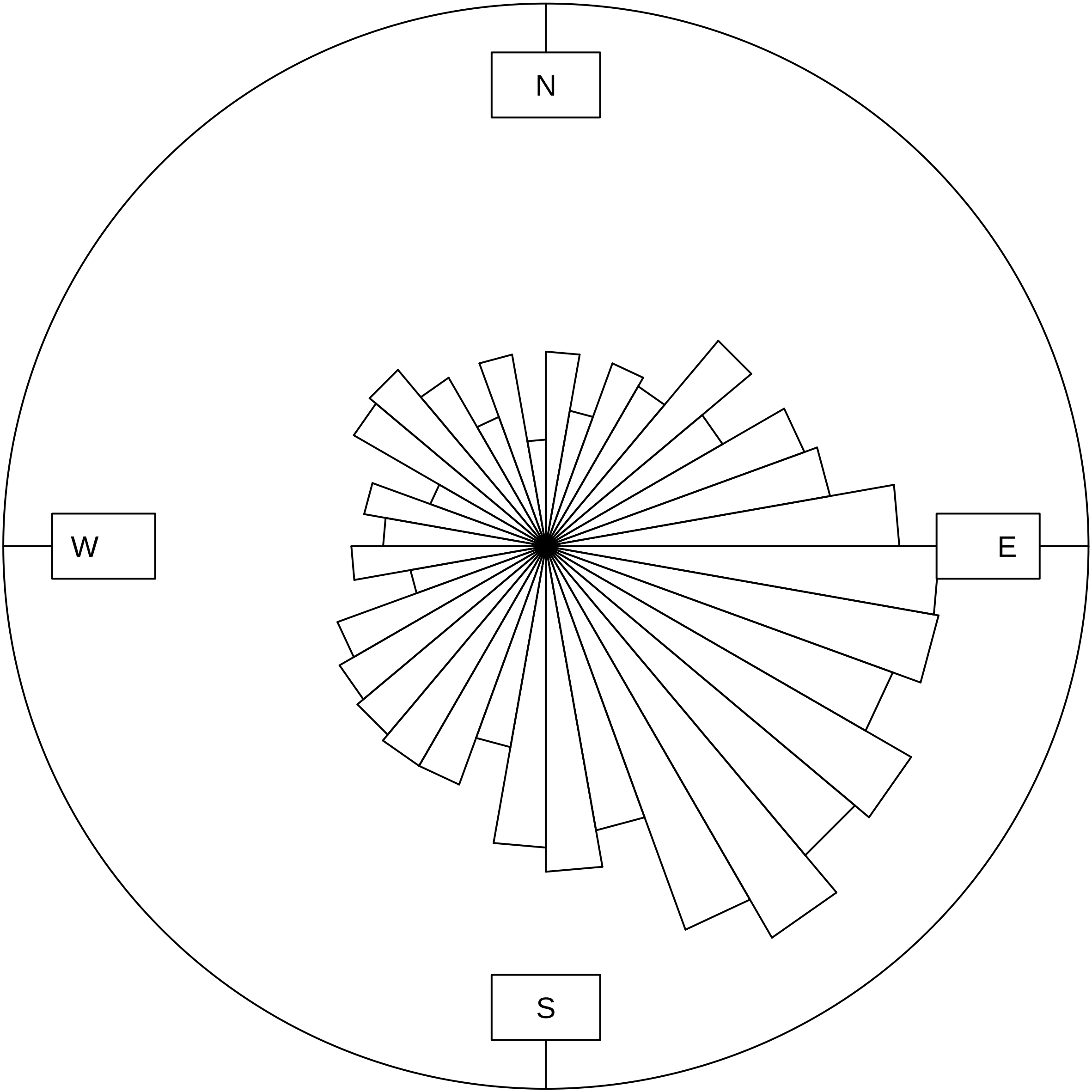

bootstrap 408

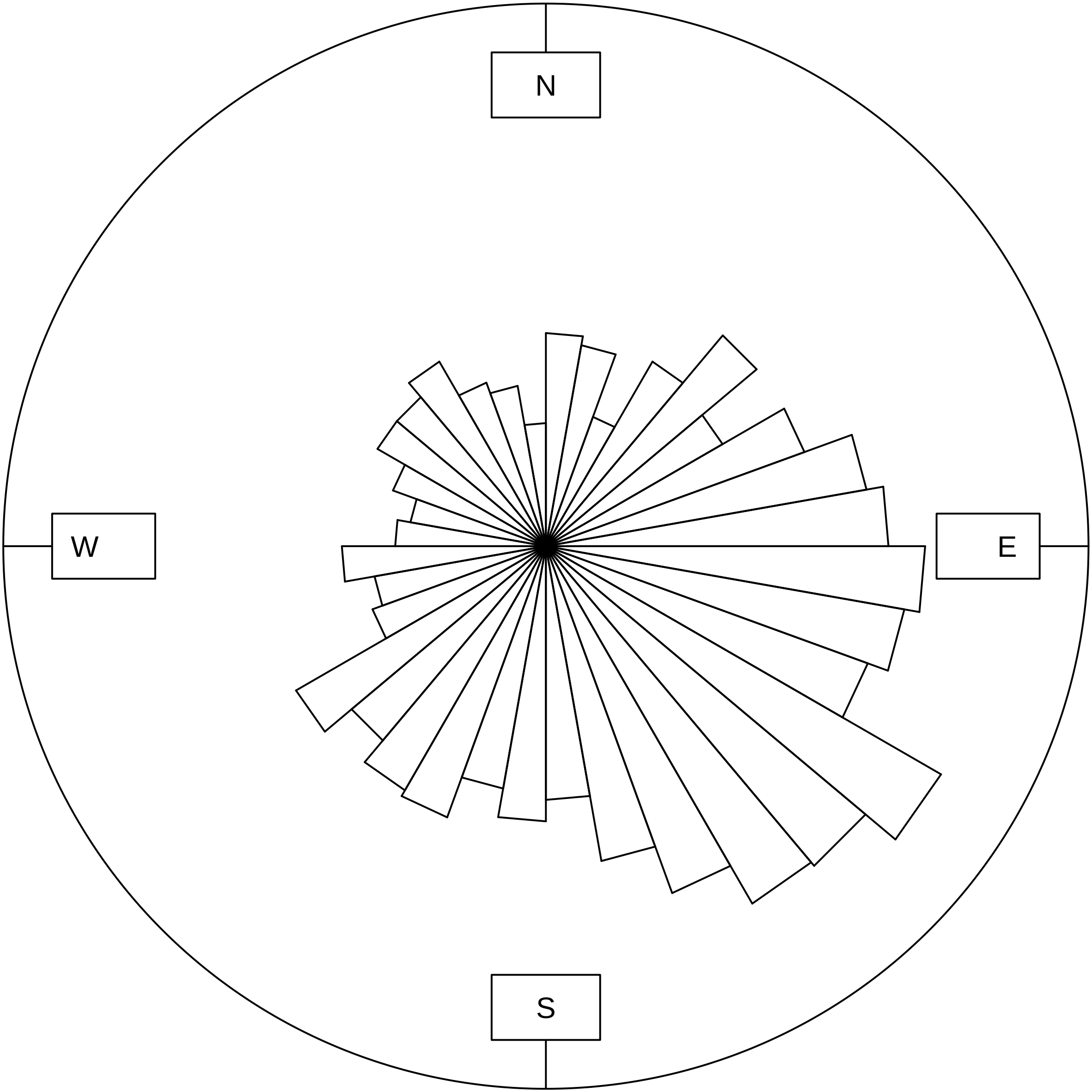

bootstrap 409

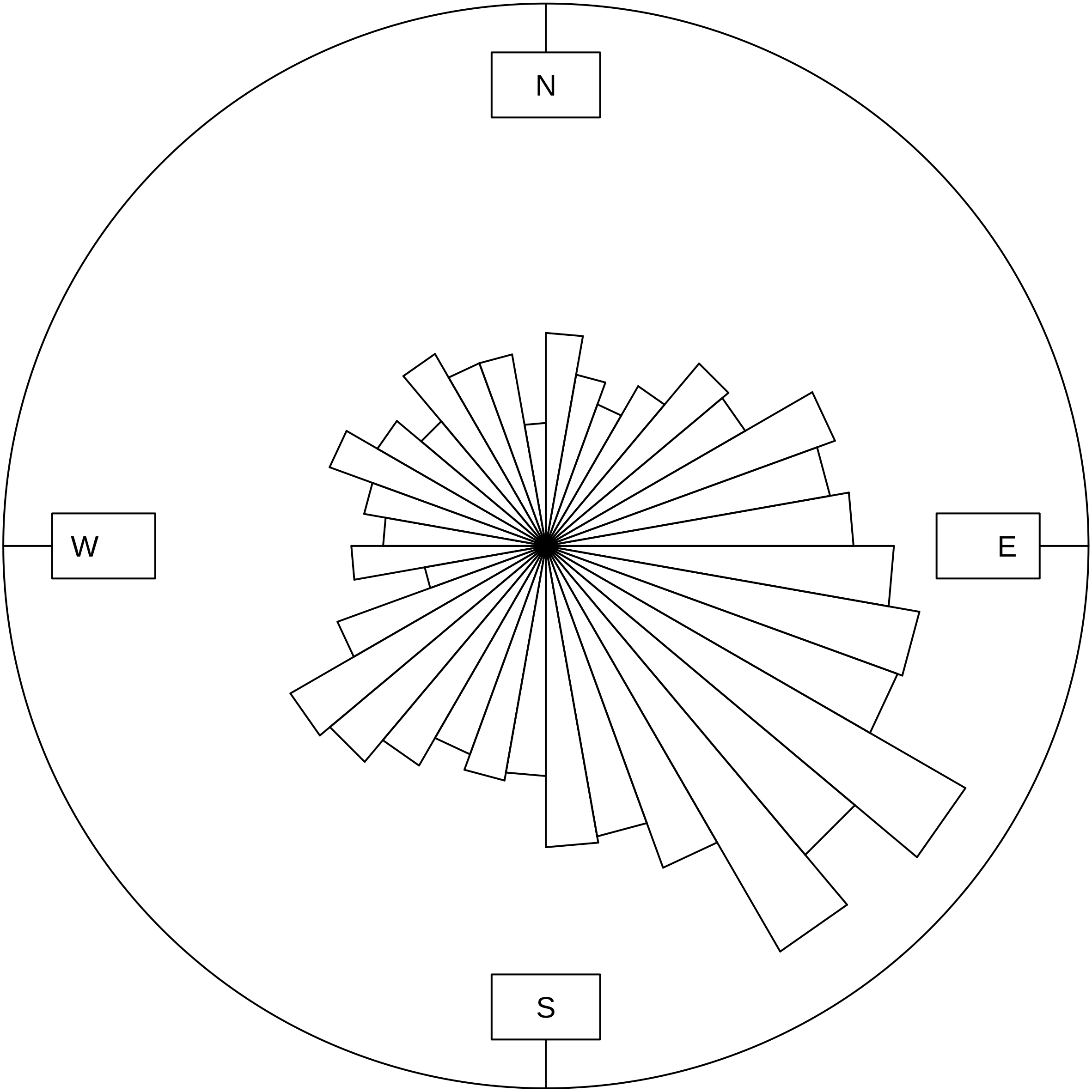

bootstrap 410

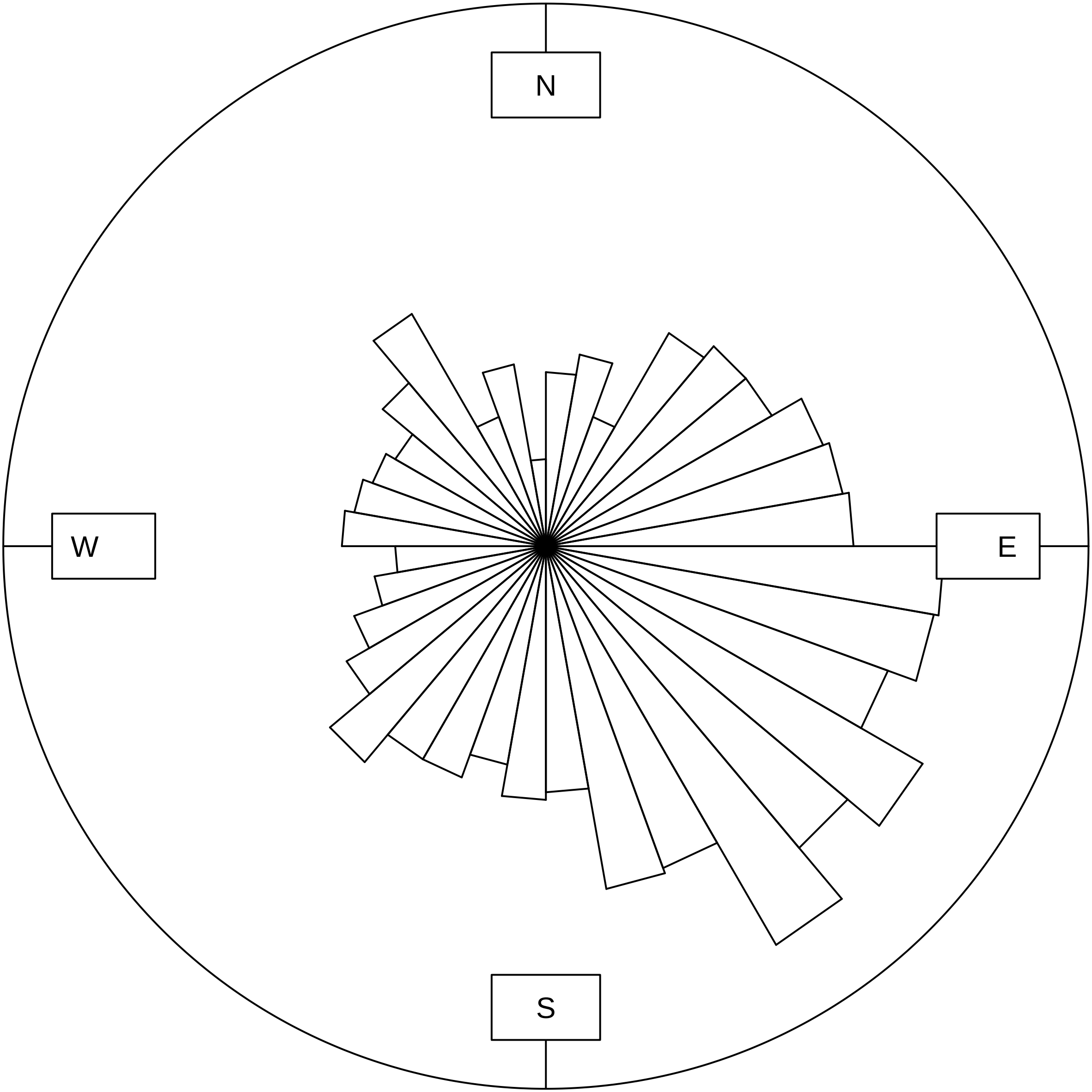

bootstrap 411

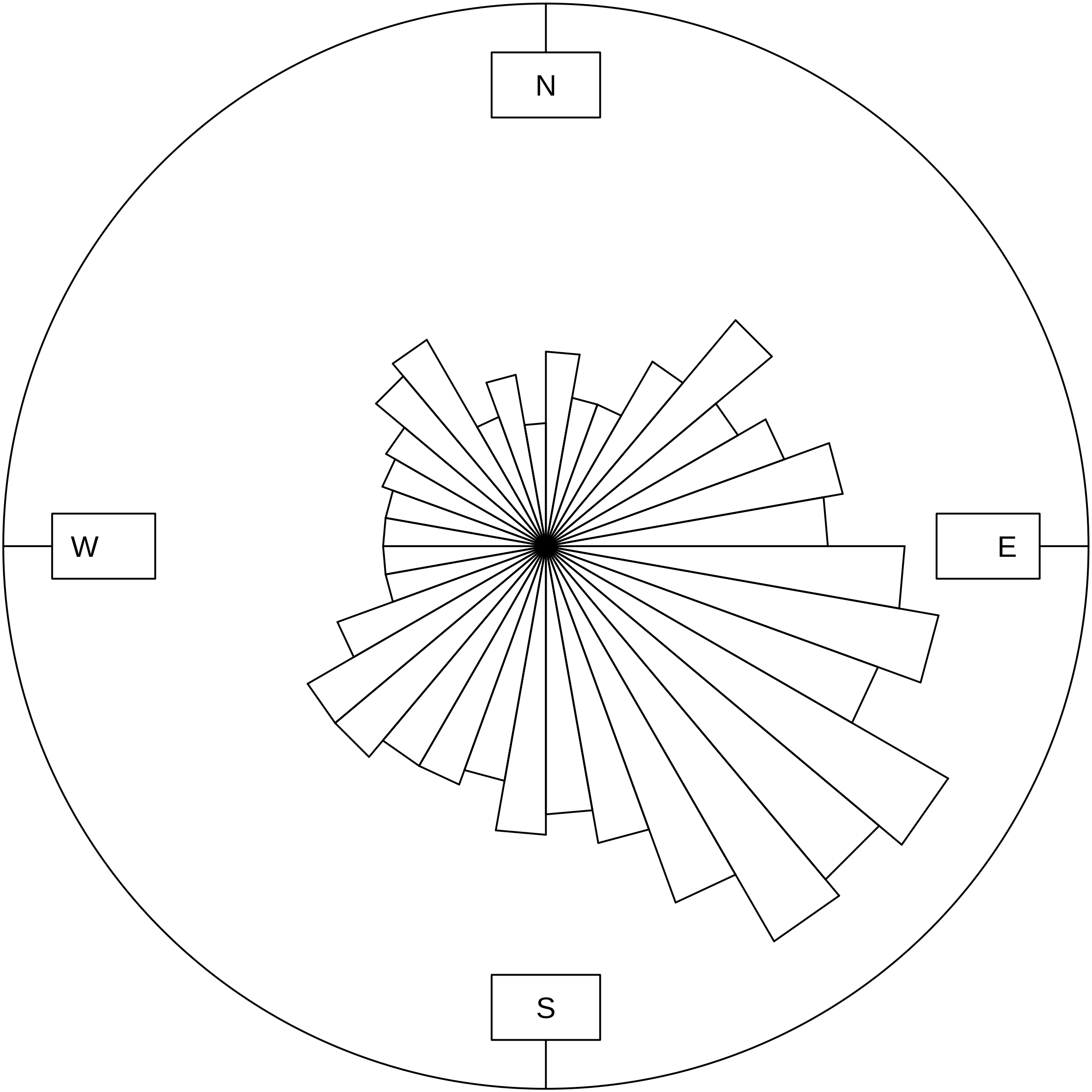

bootstrap 412

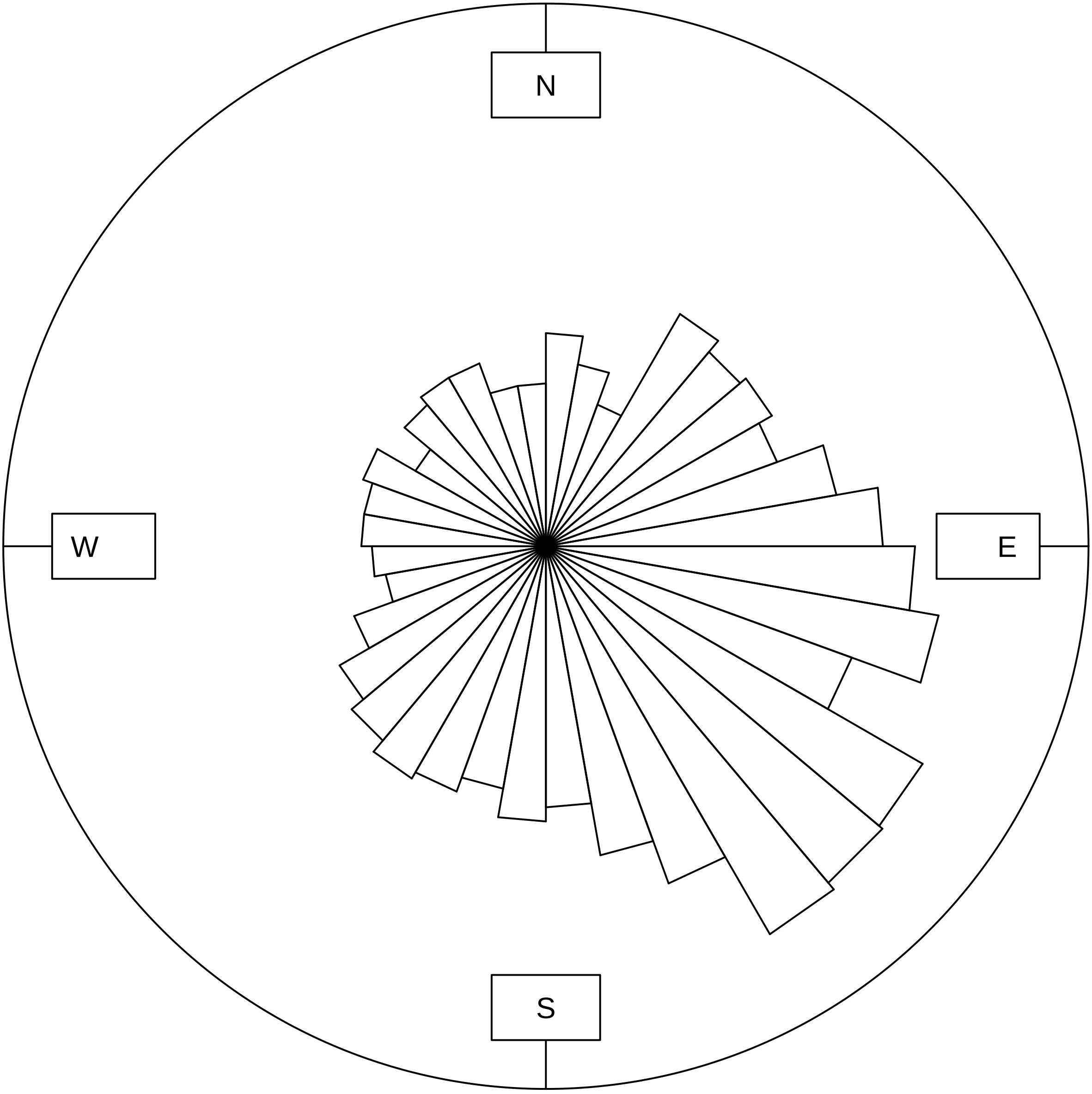

bootstrap 413

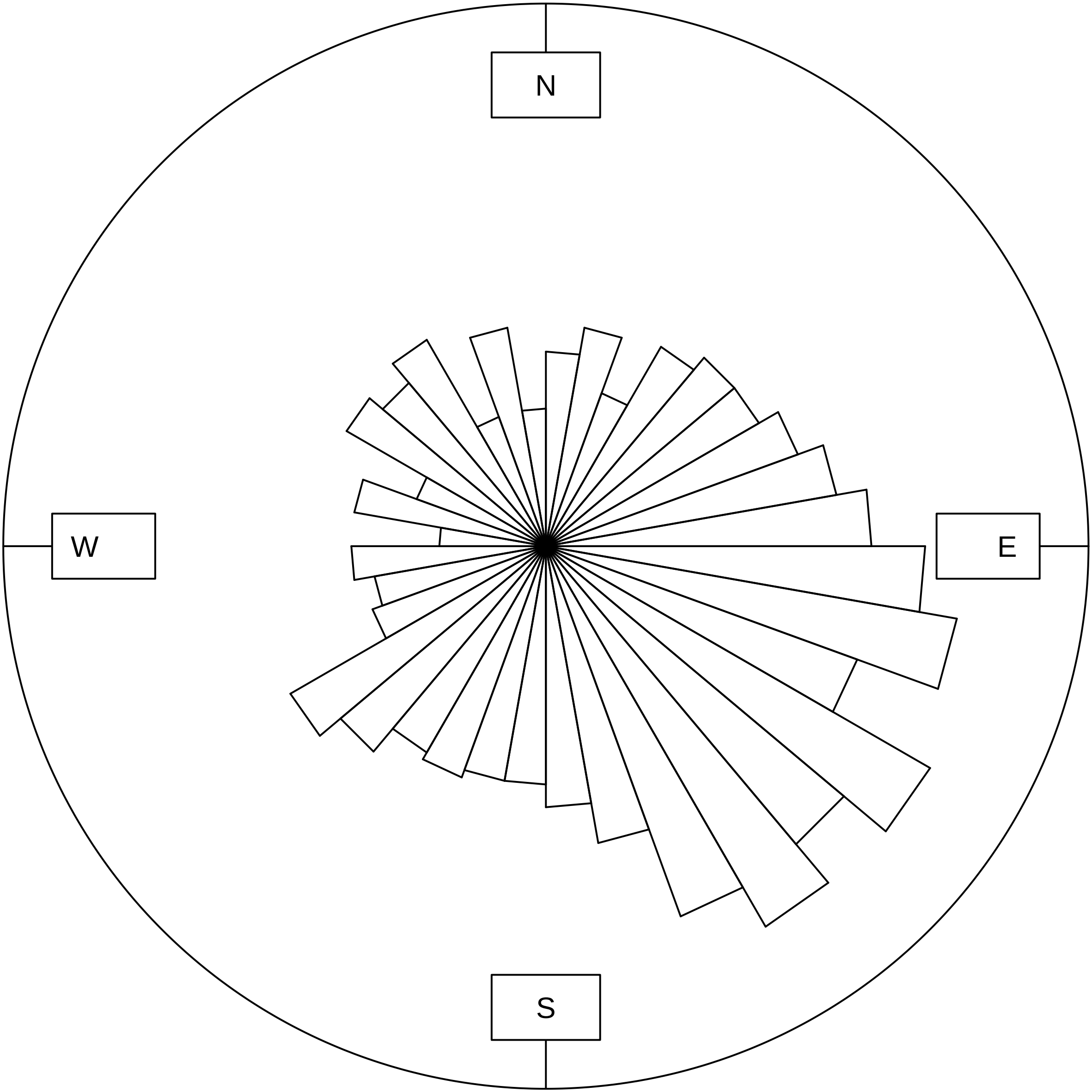

bootstrap 414

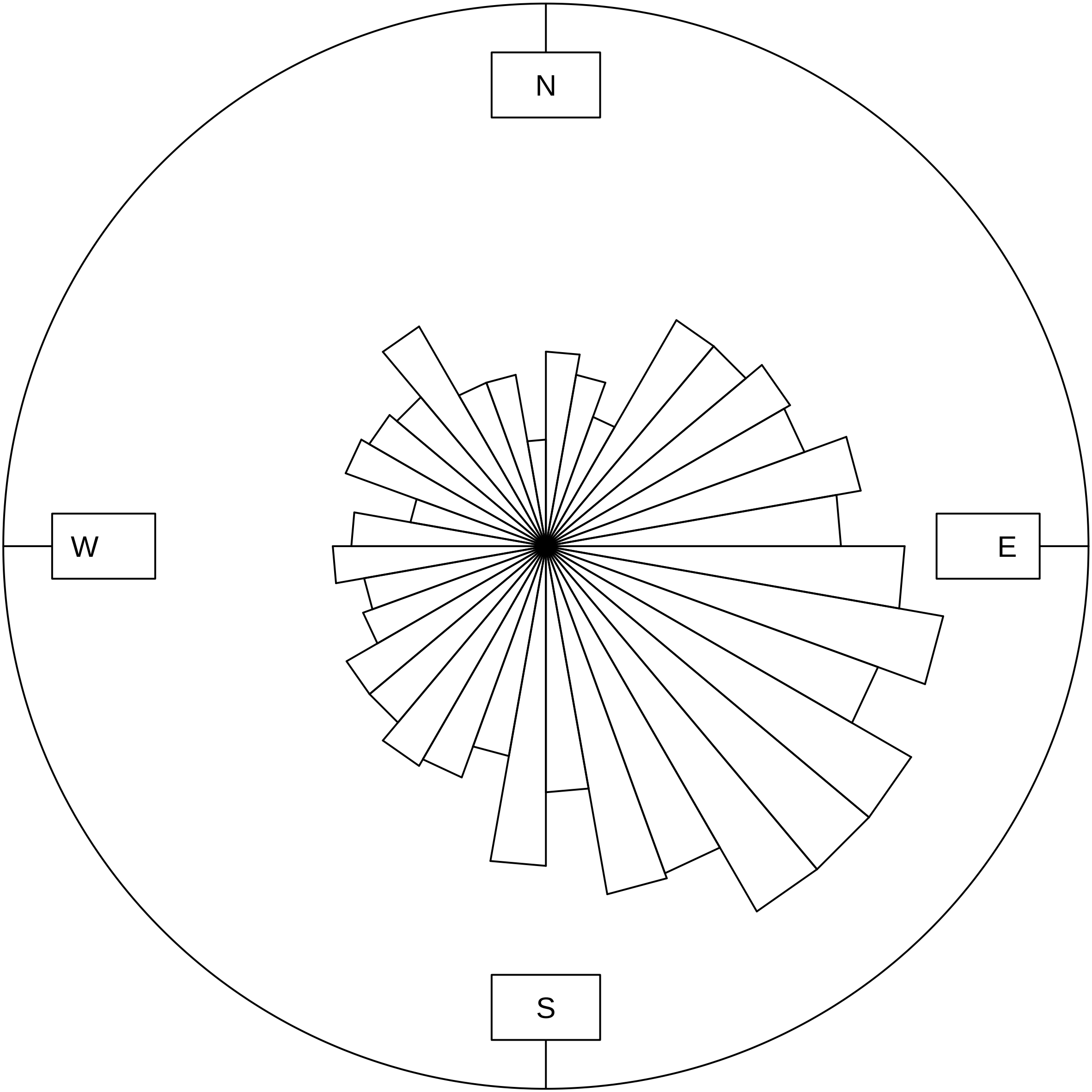

bootstrap 415

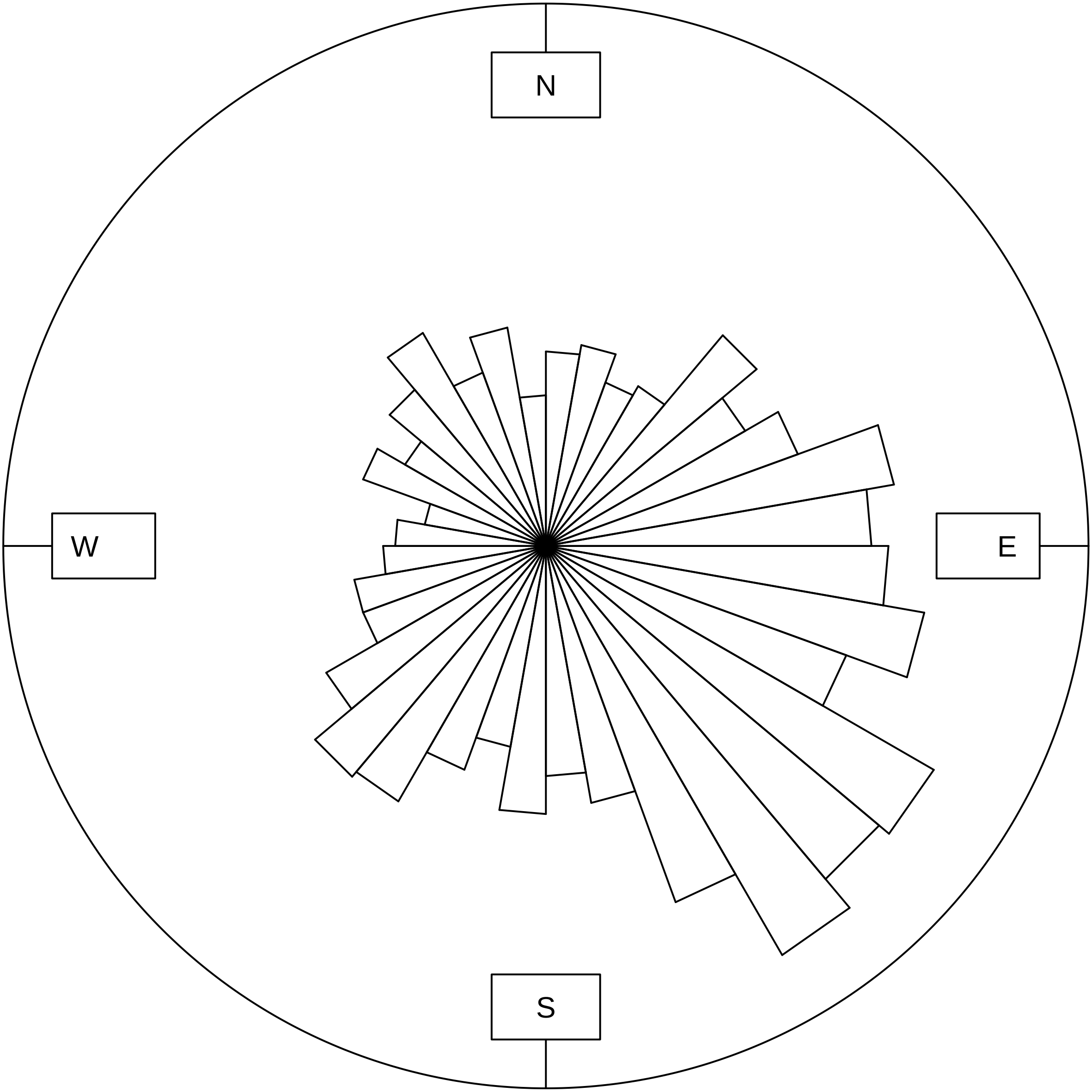

bootstrap 416

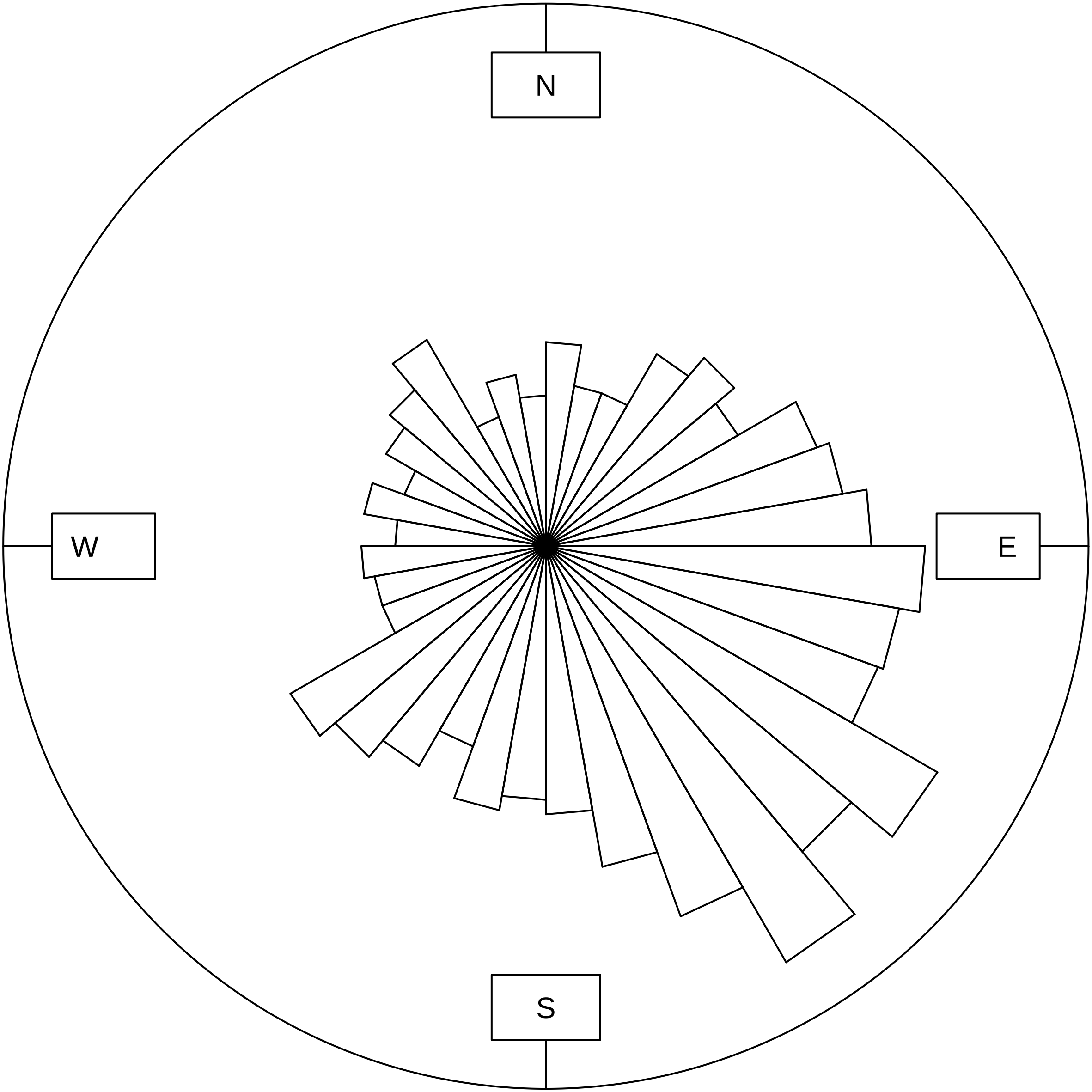

bootstrap 417

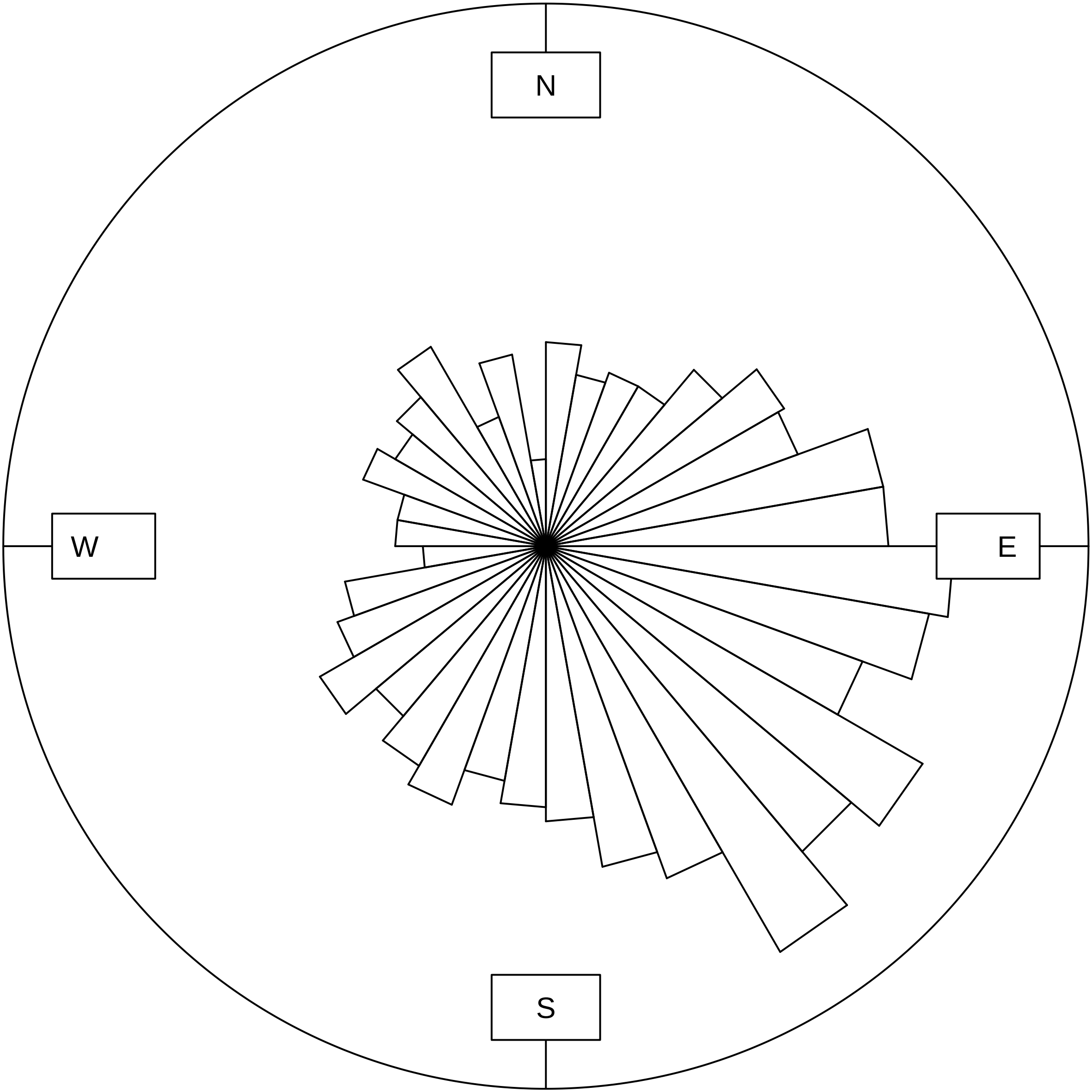

bootstrap 418

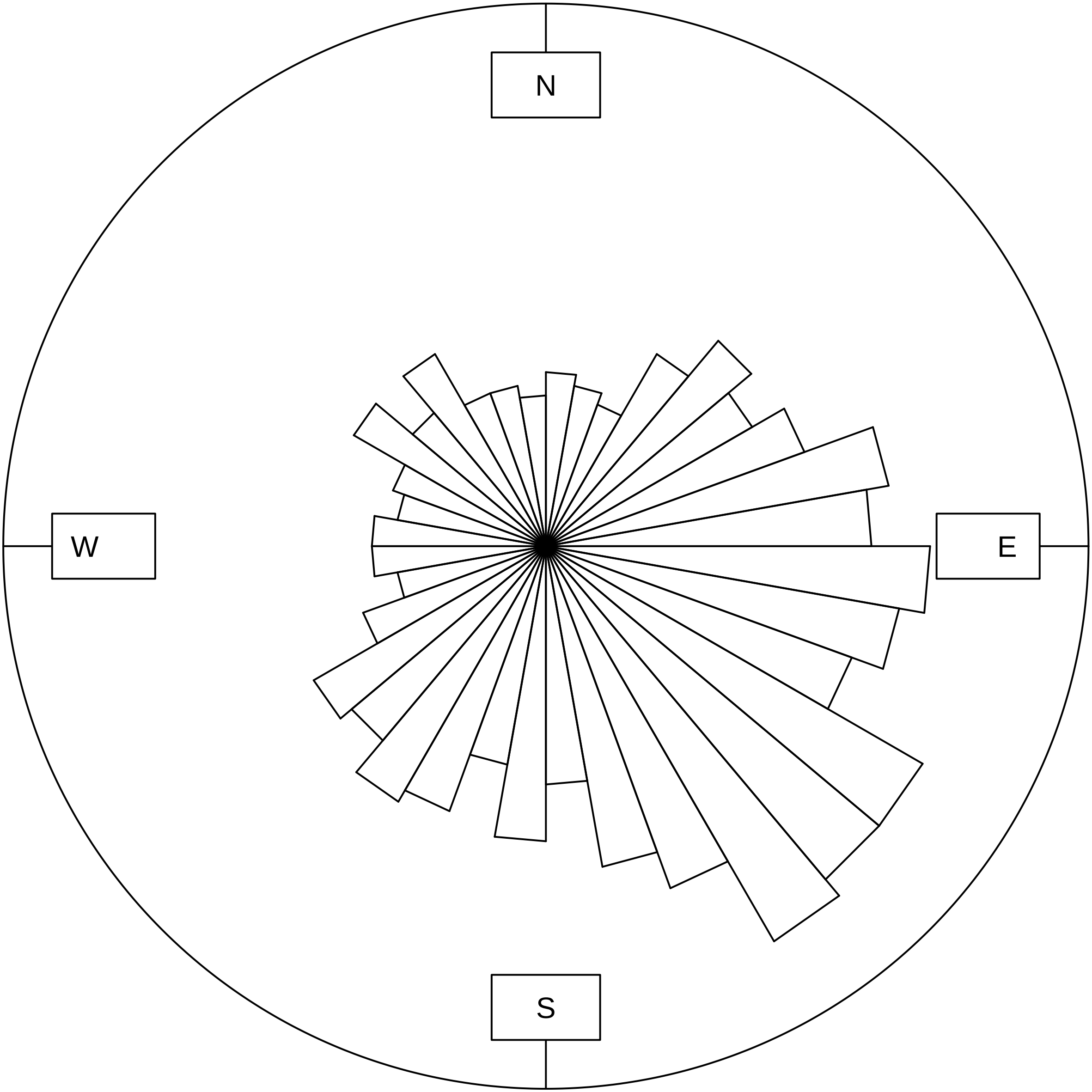

bootstrap 419

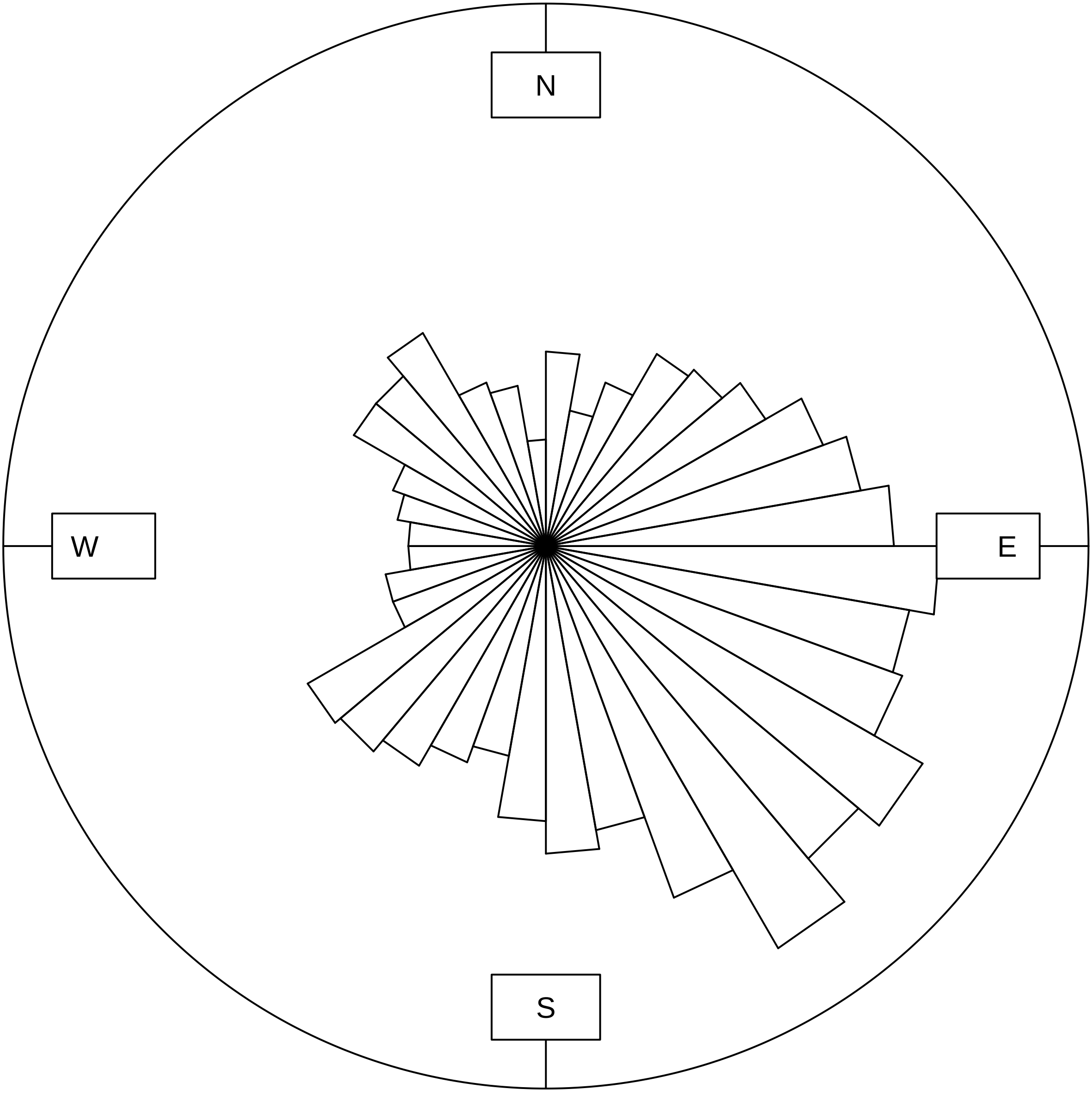

bootstrap 420

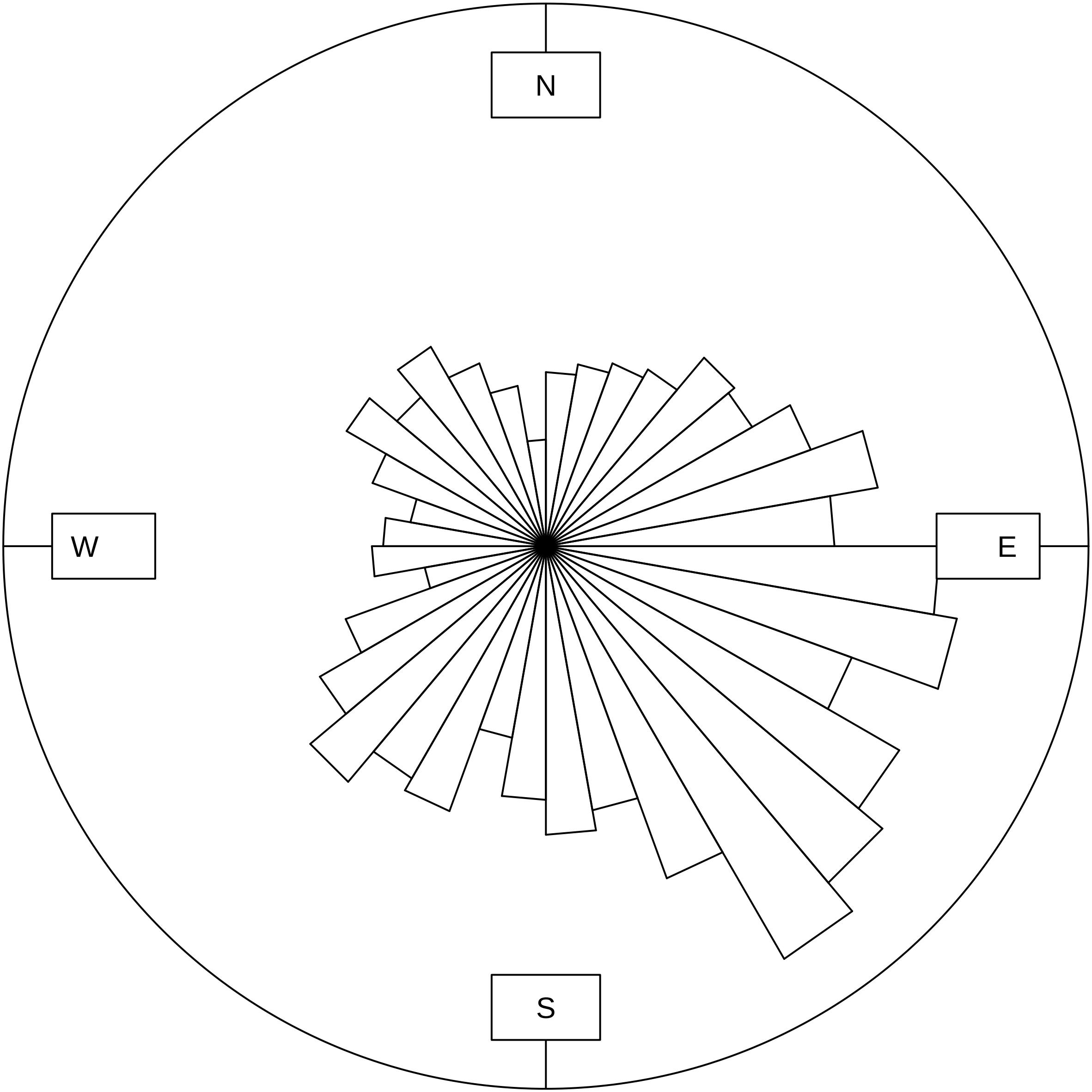

bootstrap 421

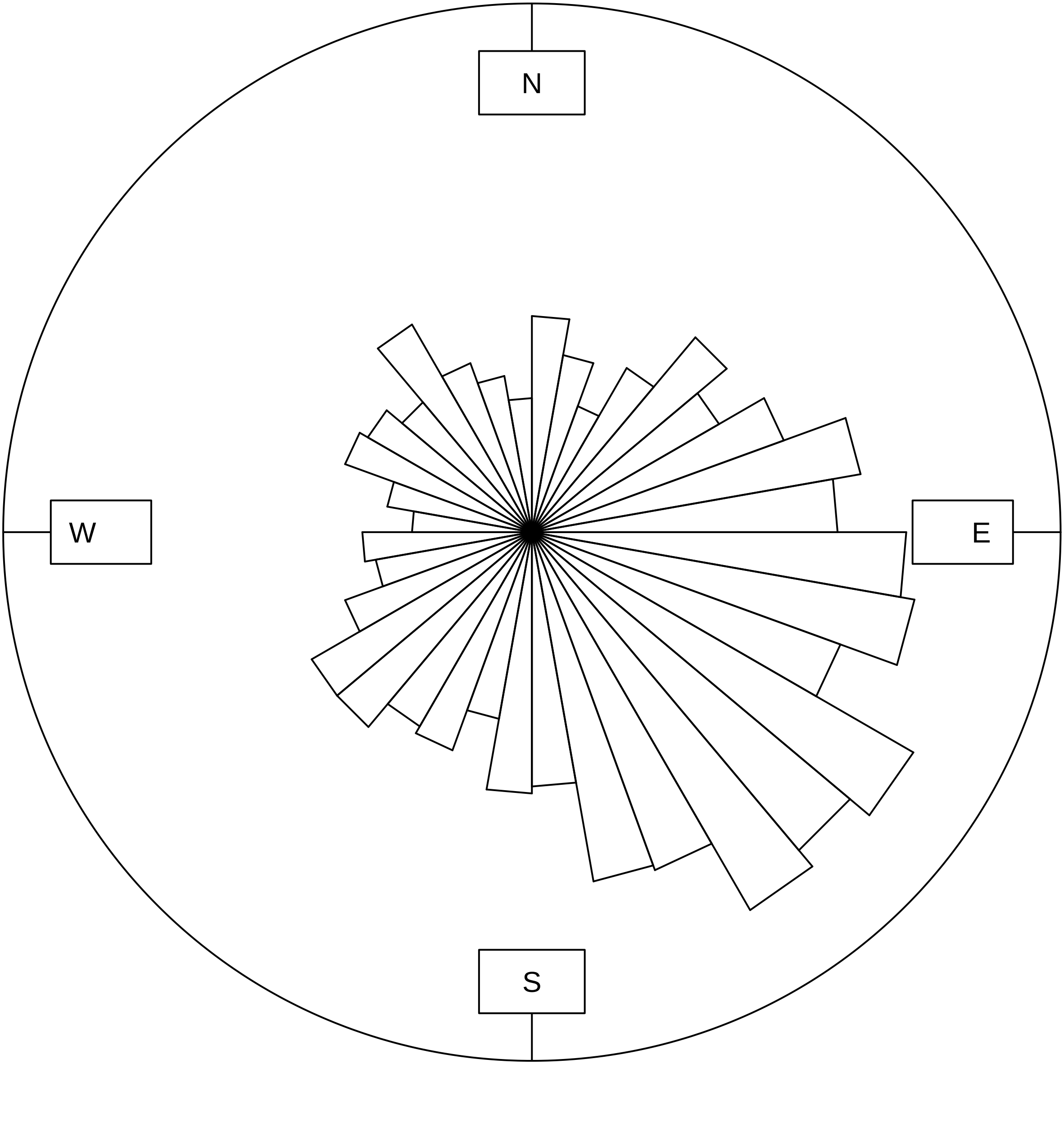

bootstrap 422

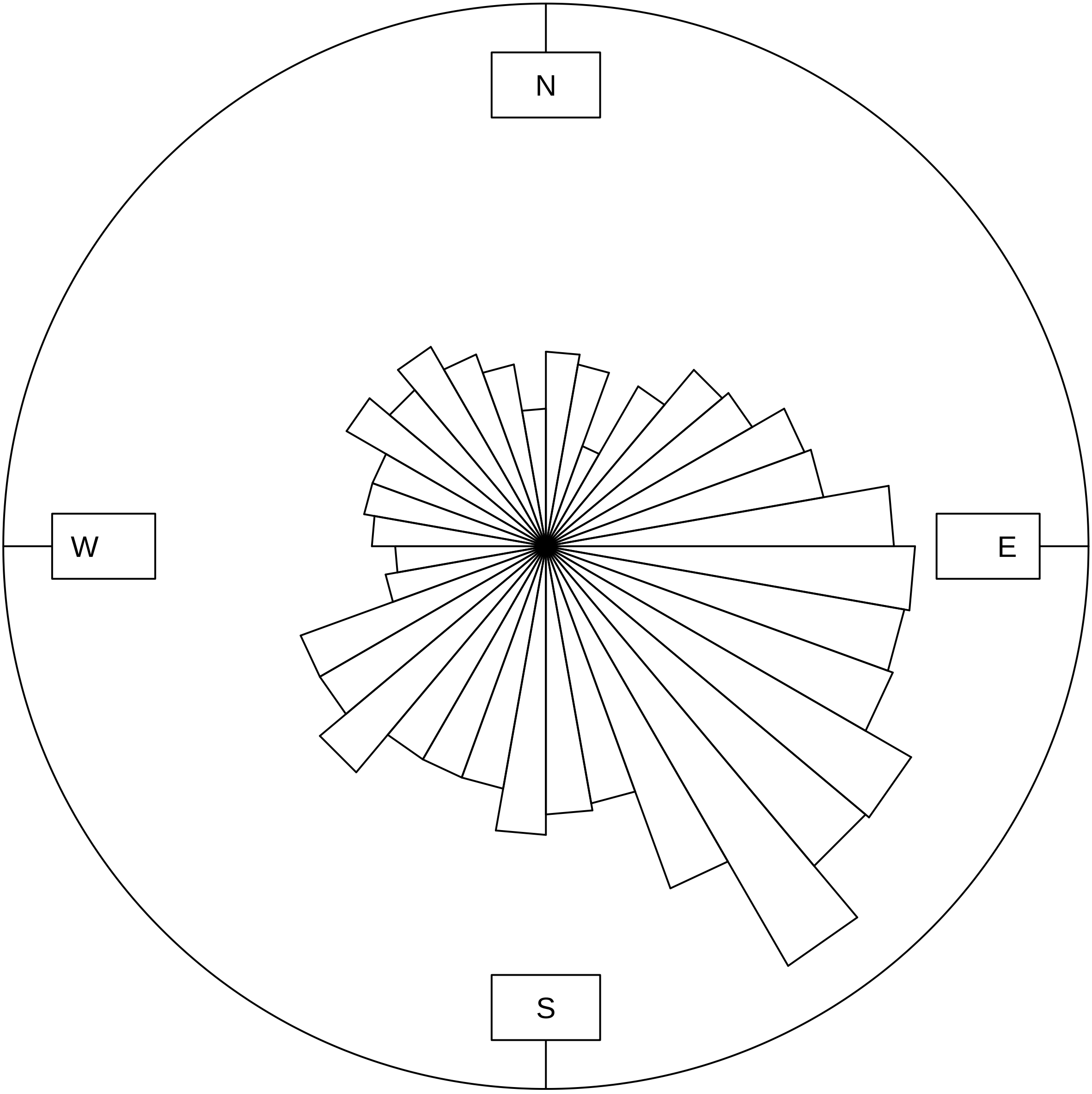

bootstrap 423

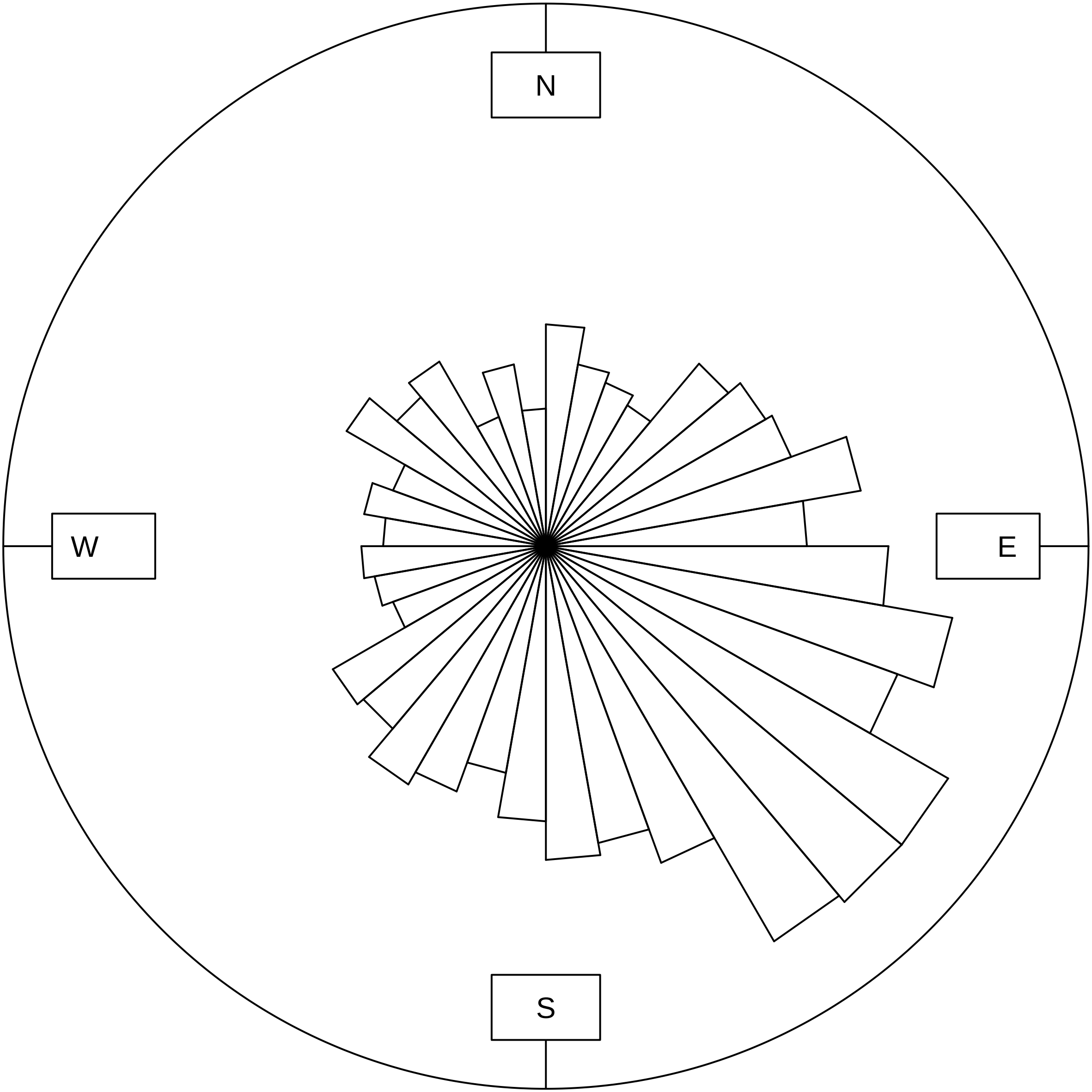

bootstrap 424

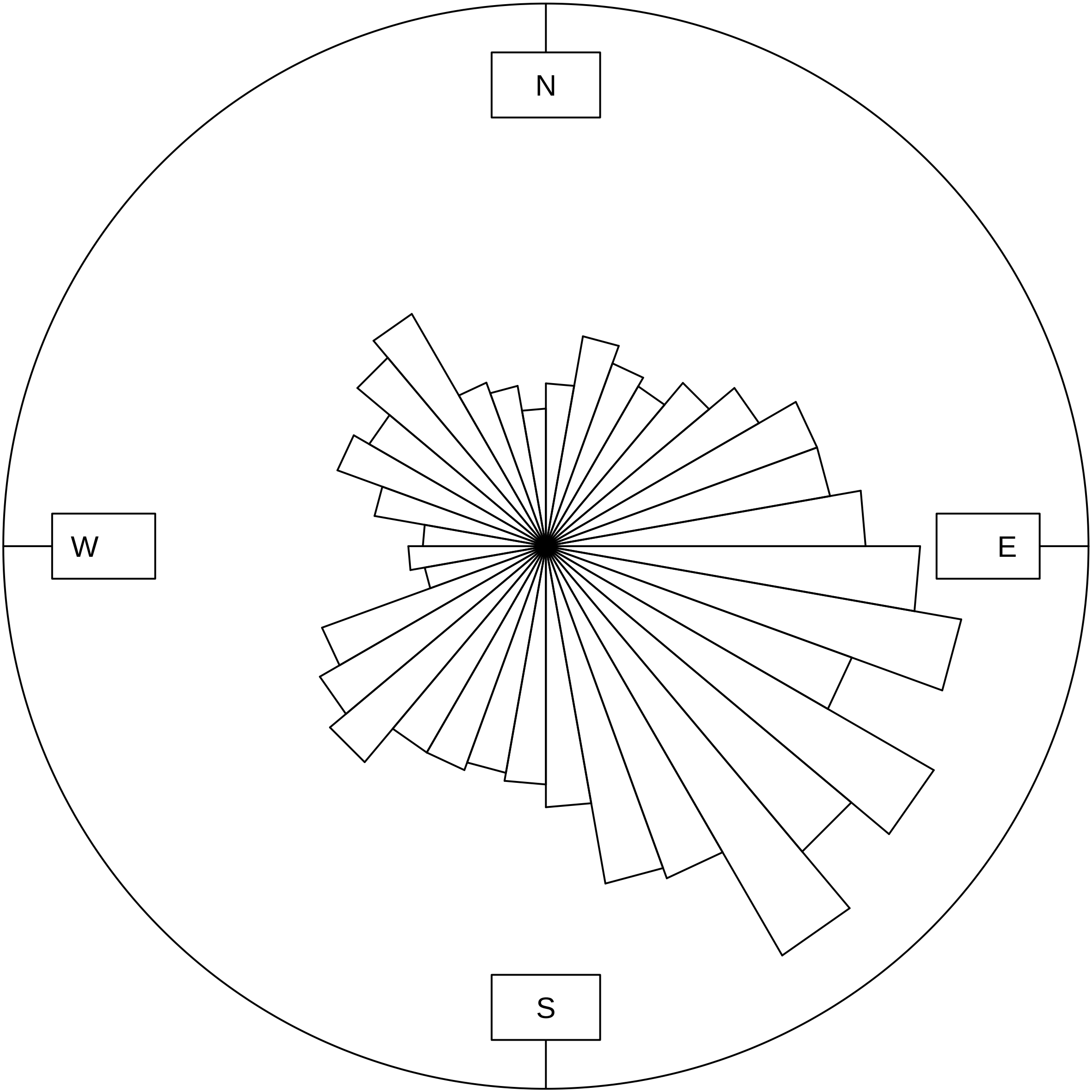

bootstrap 425

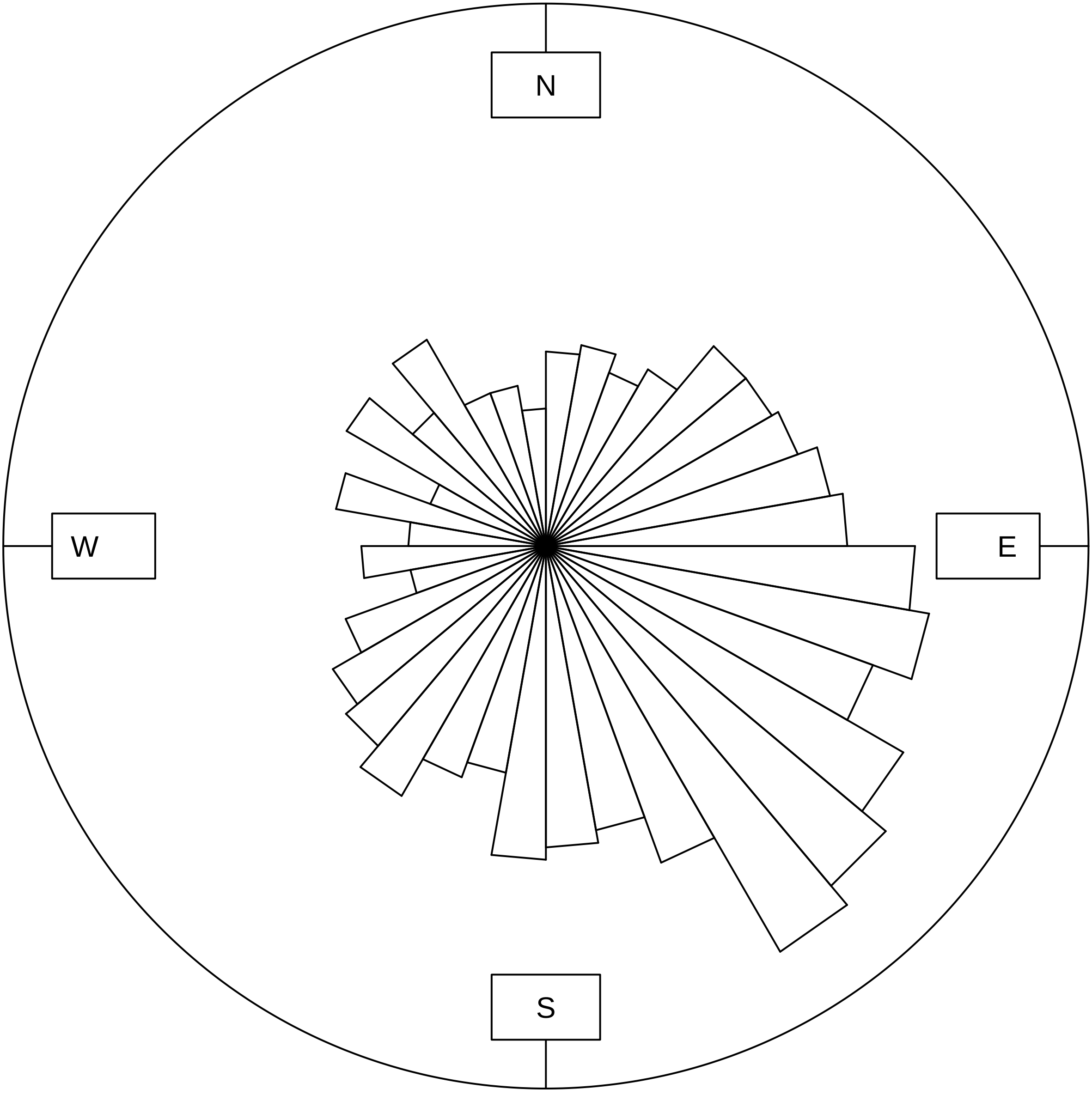

bootstrap 426

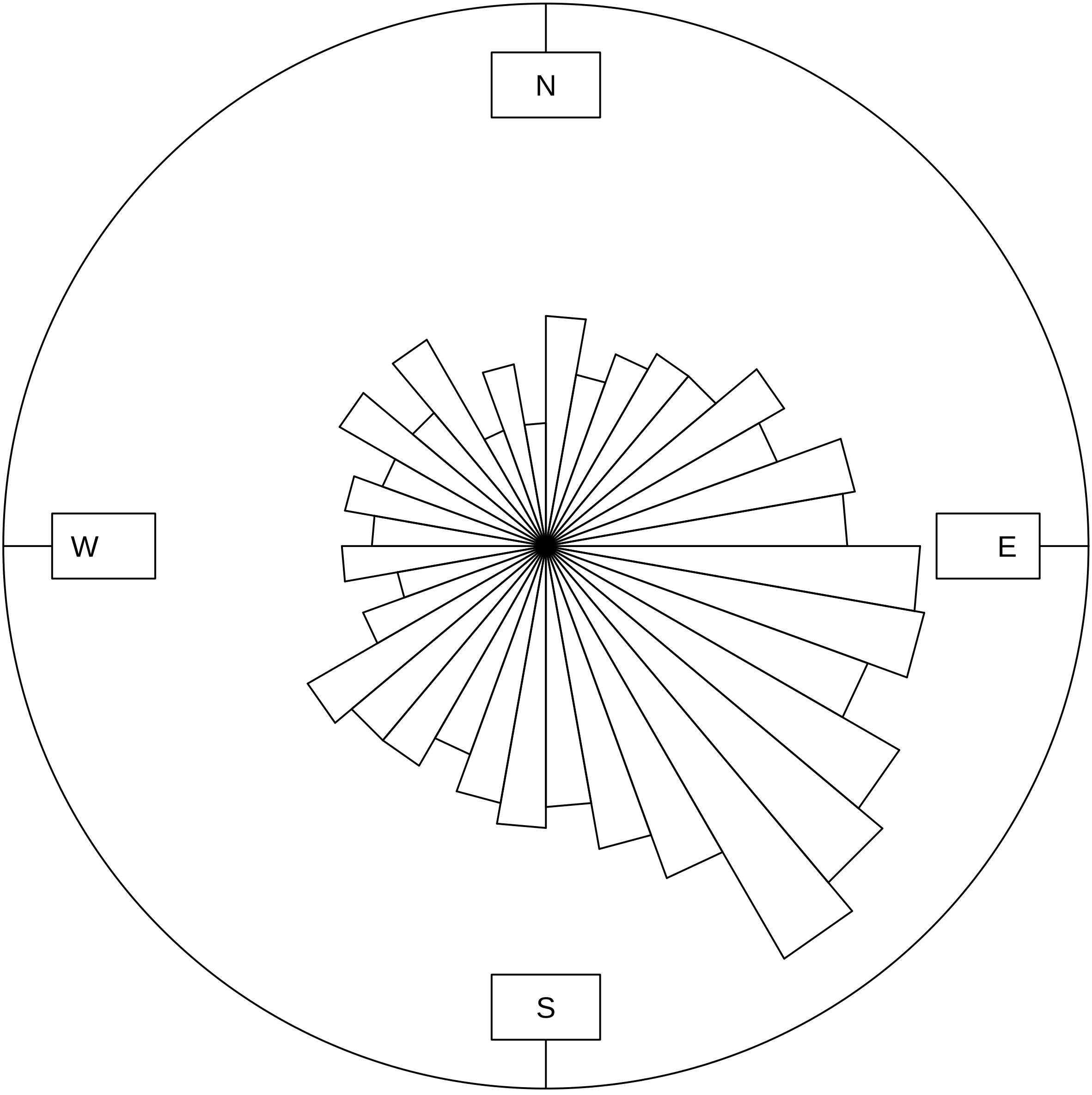

bootstrap 427

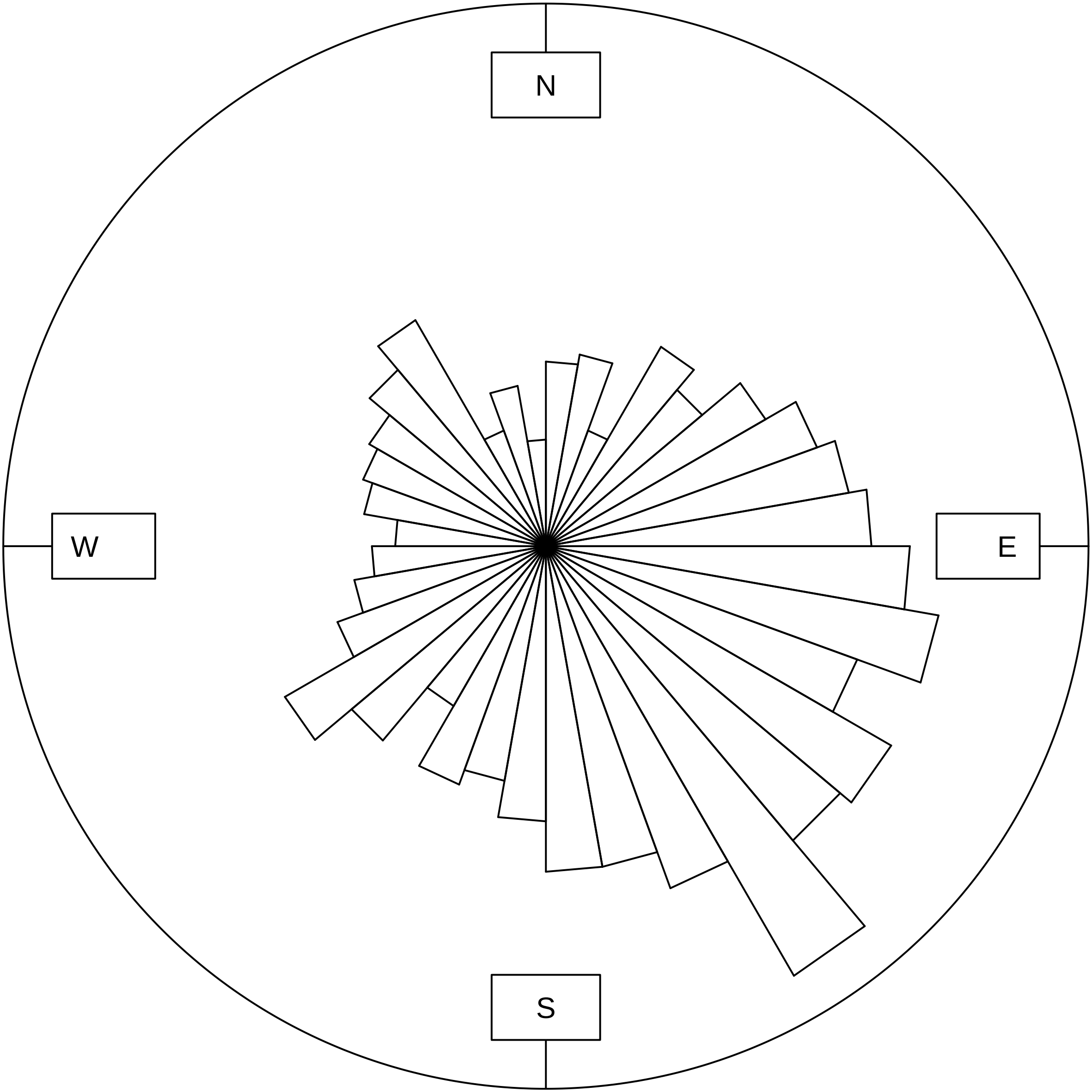

bootstrap 428

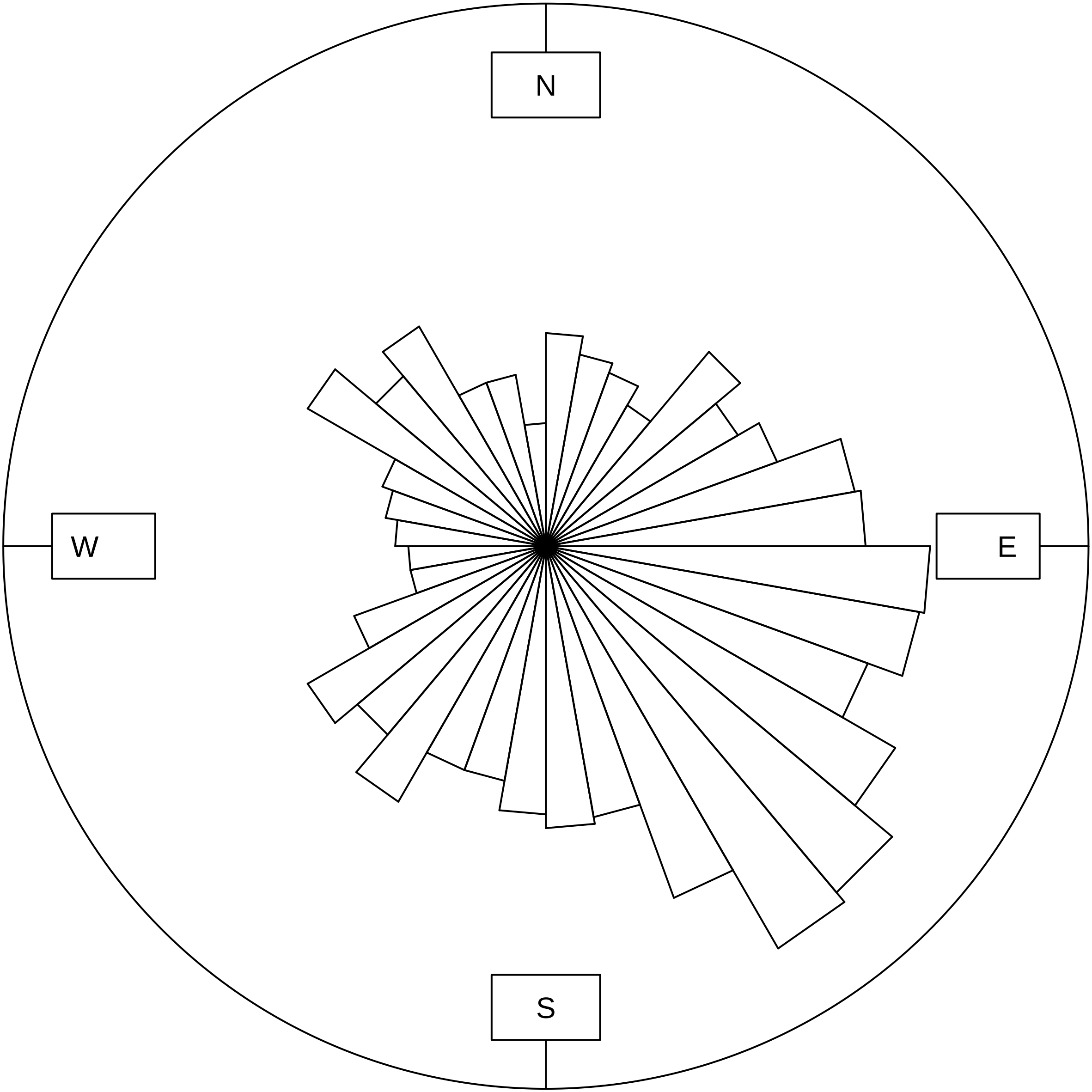

bootstrap 429

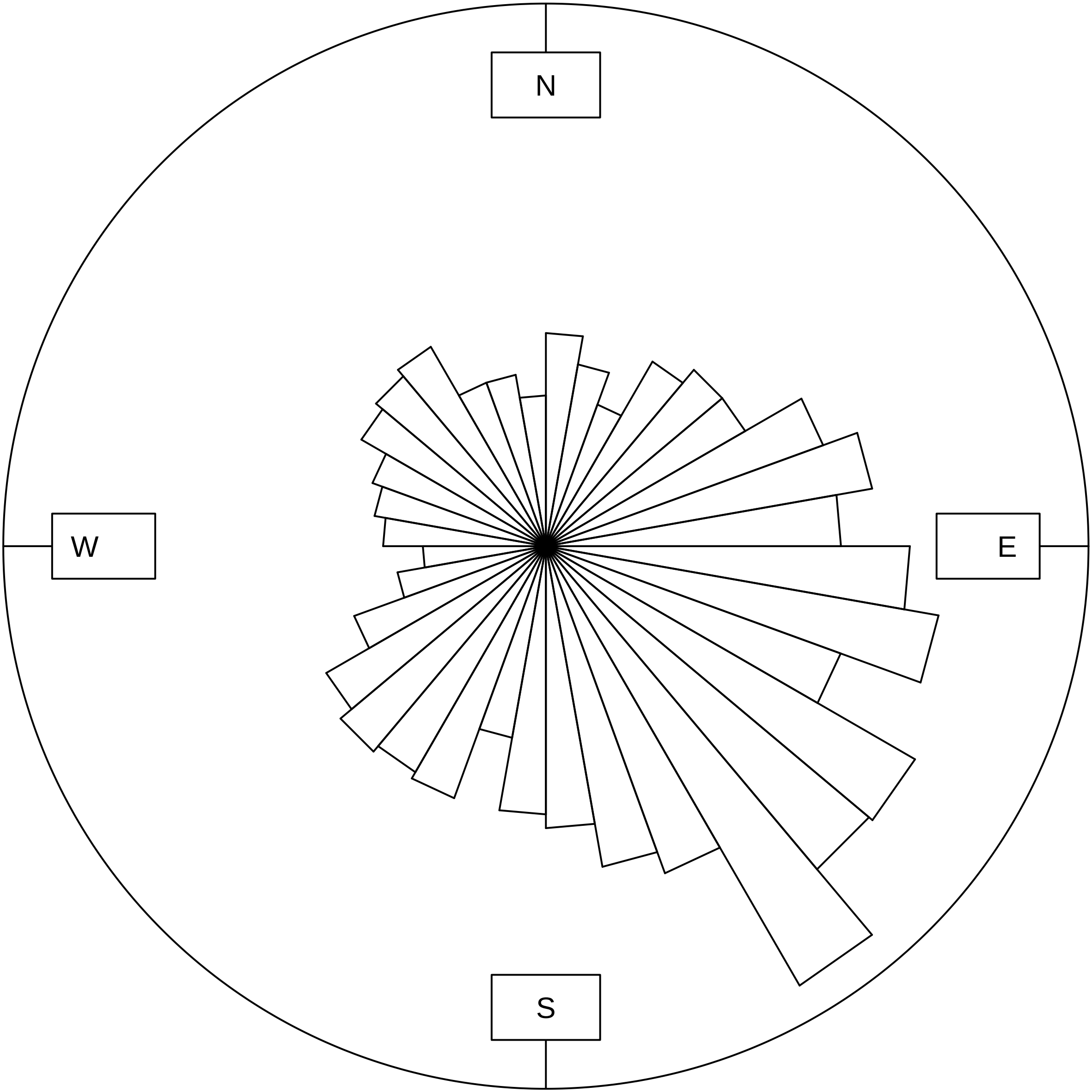

bootstrap 430

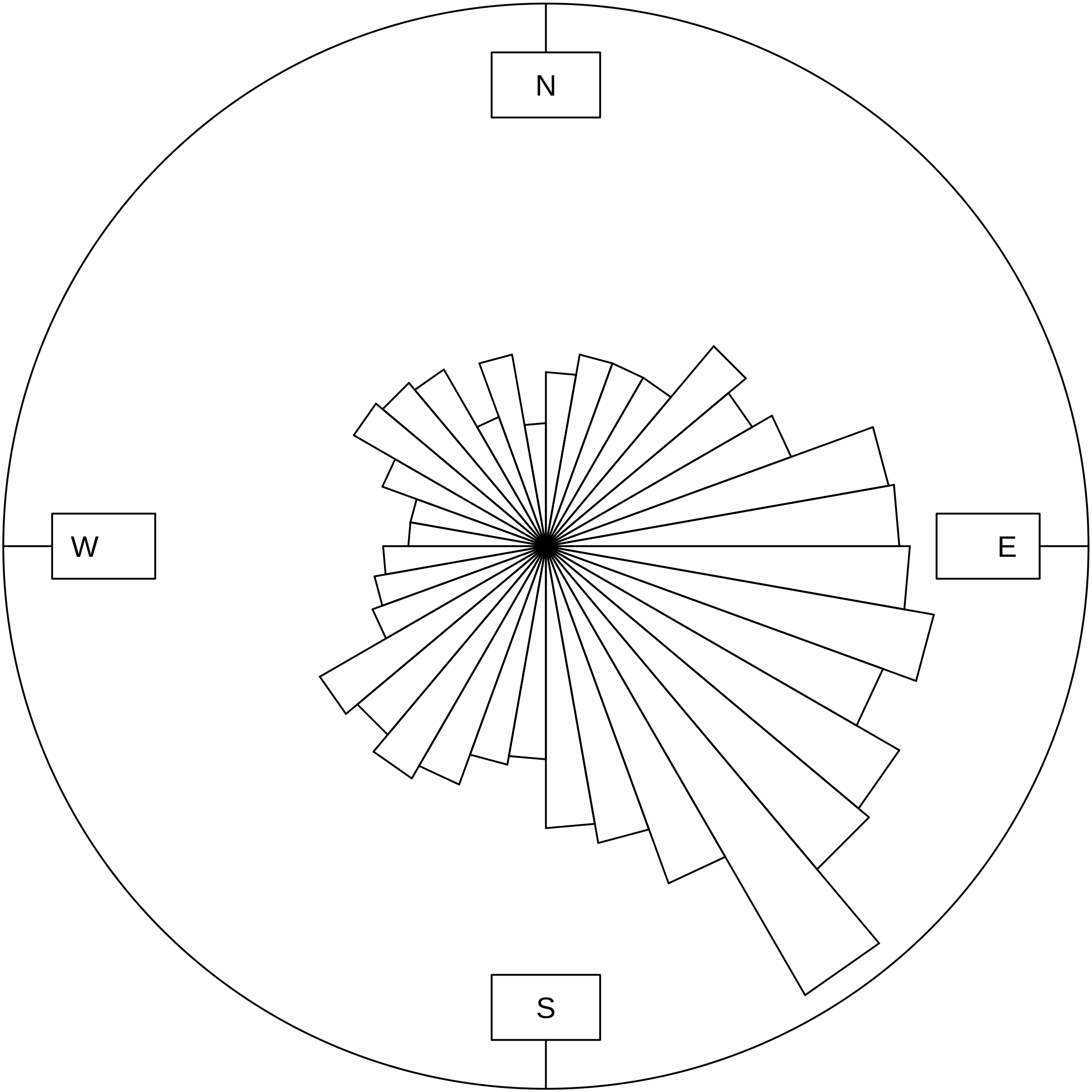

bootstrap 431

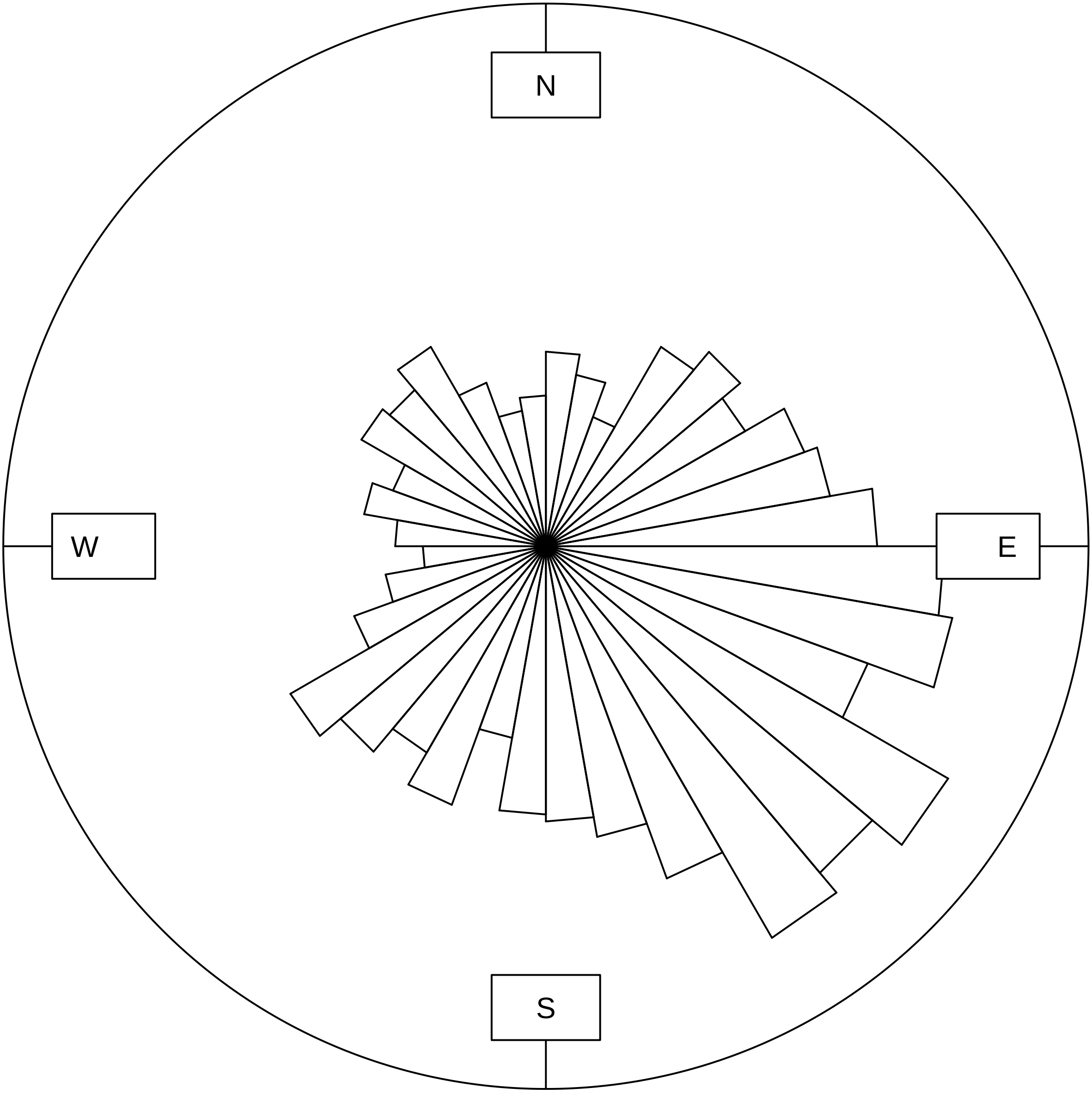

bootstrap 432

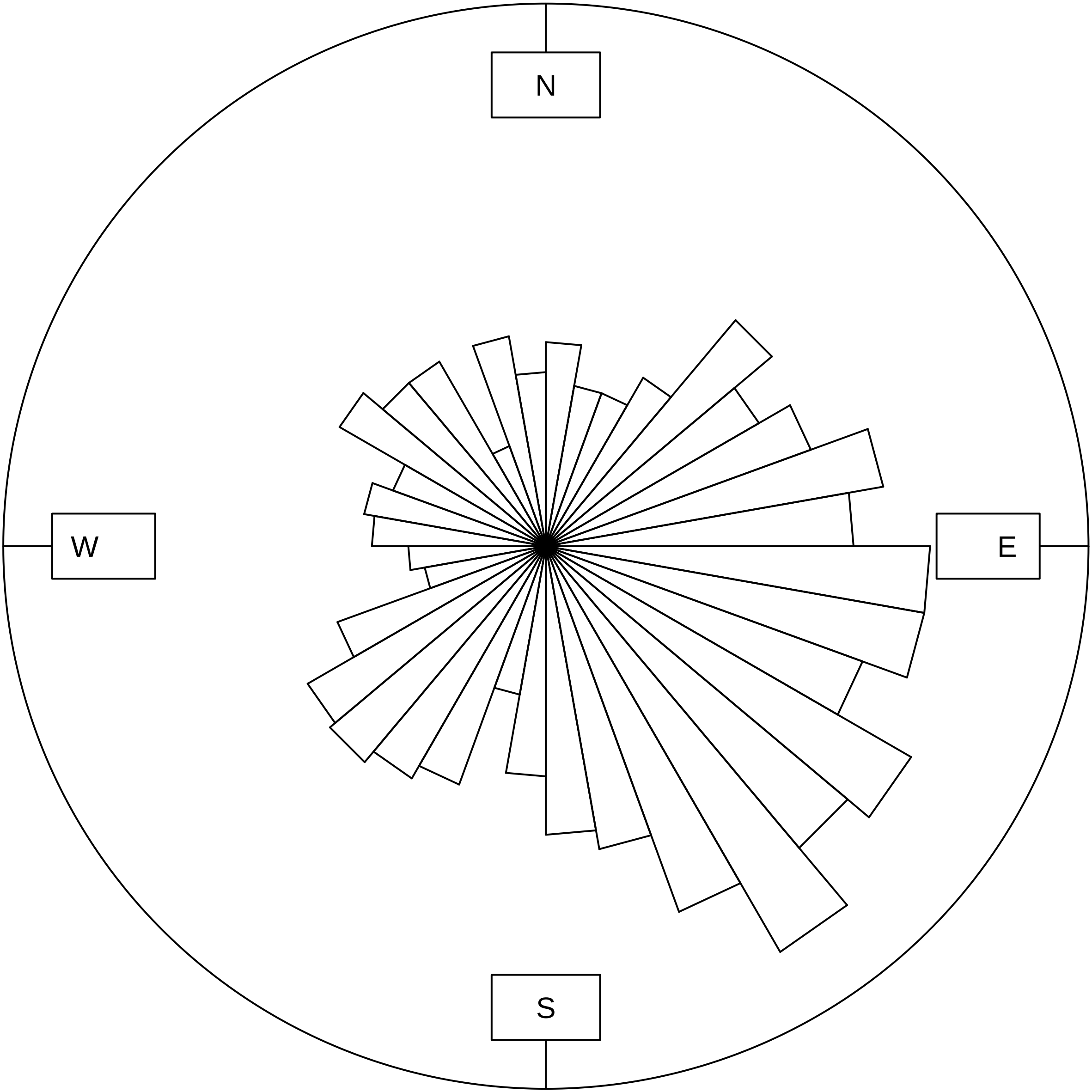

bootstrap 433

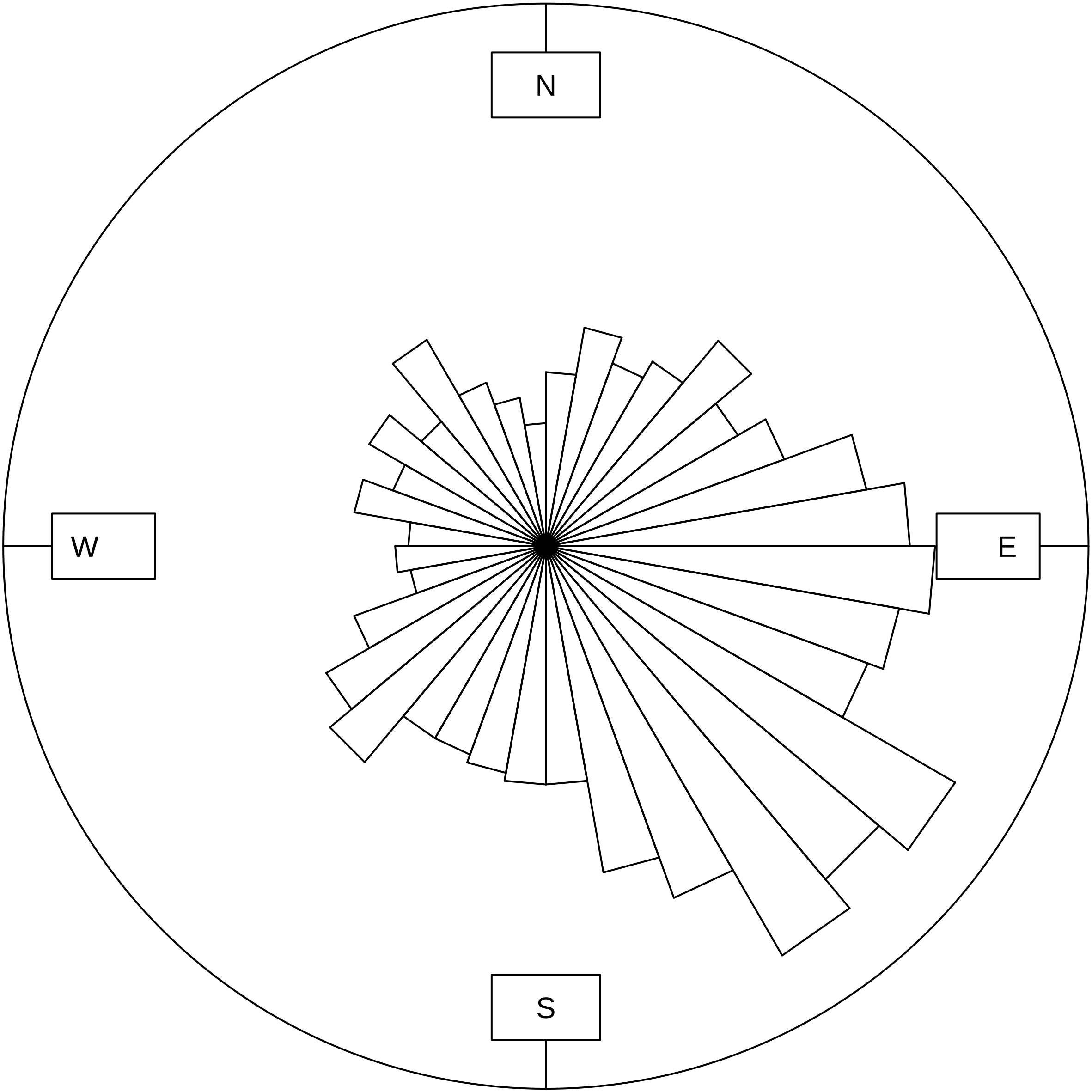

bootstrap 434

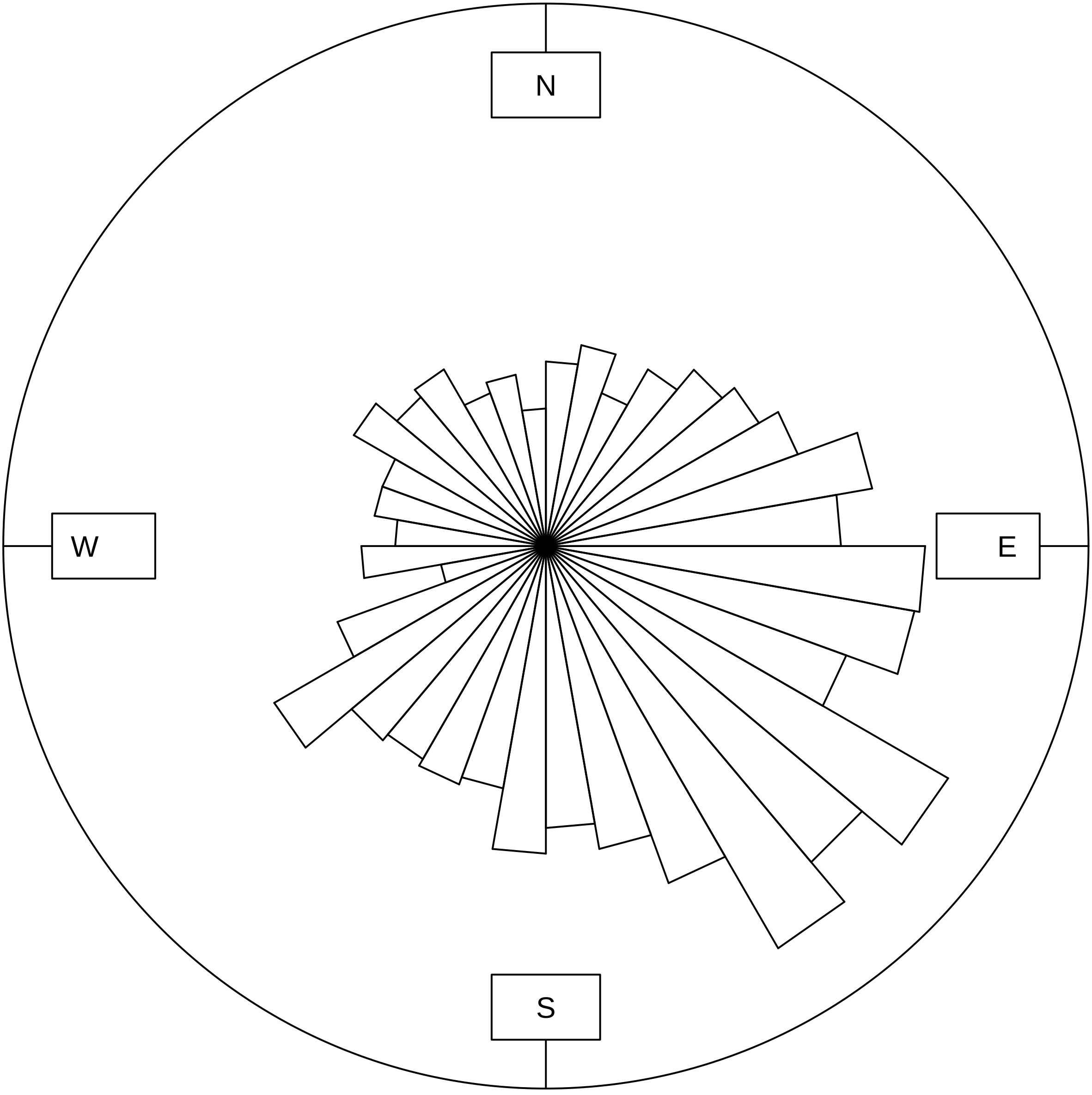

bootstrap 435

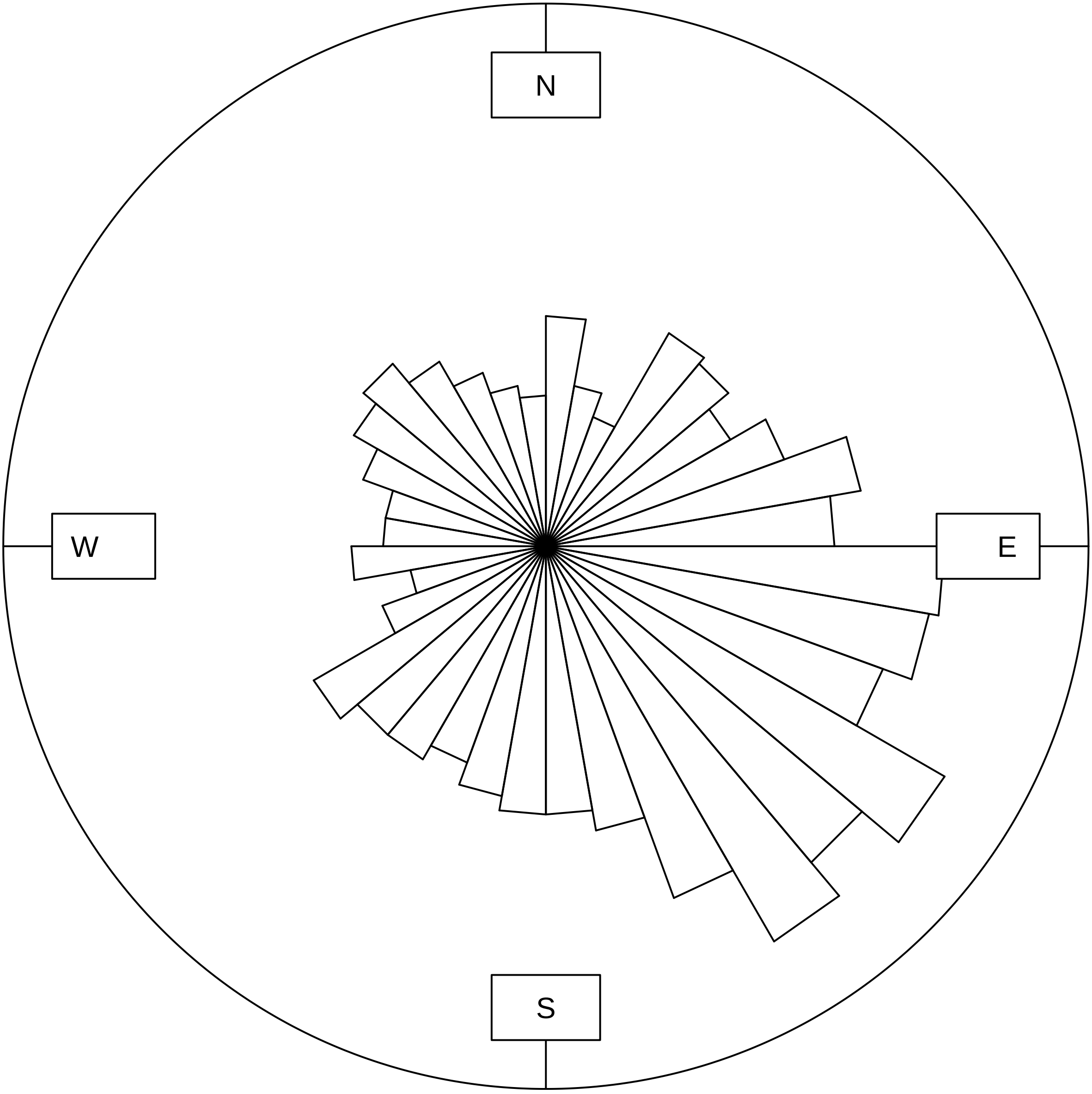

bootstrap 436

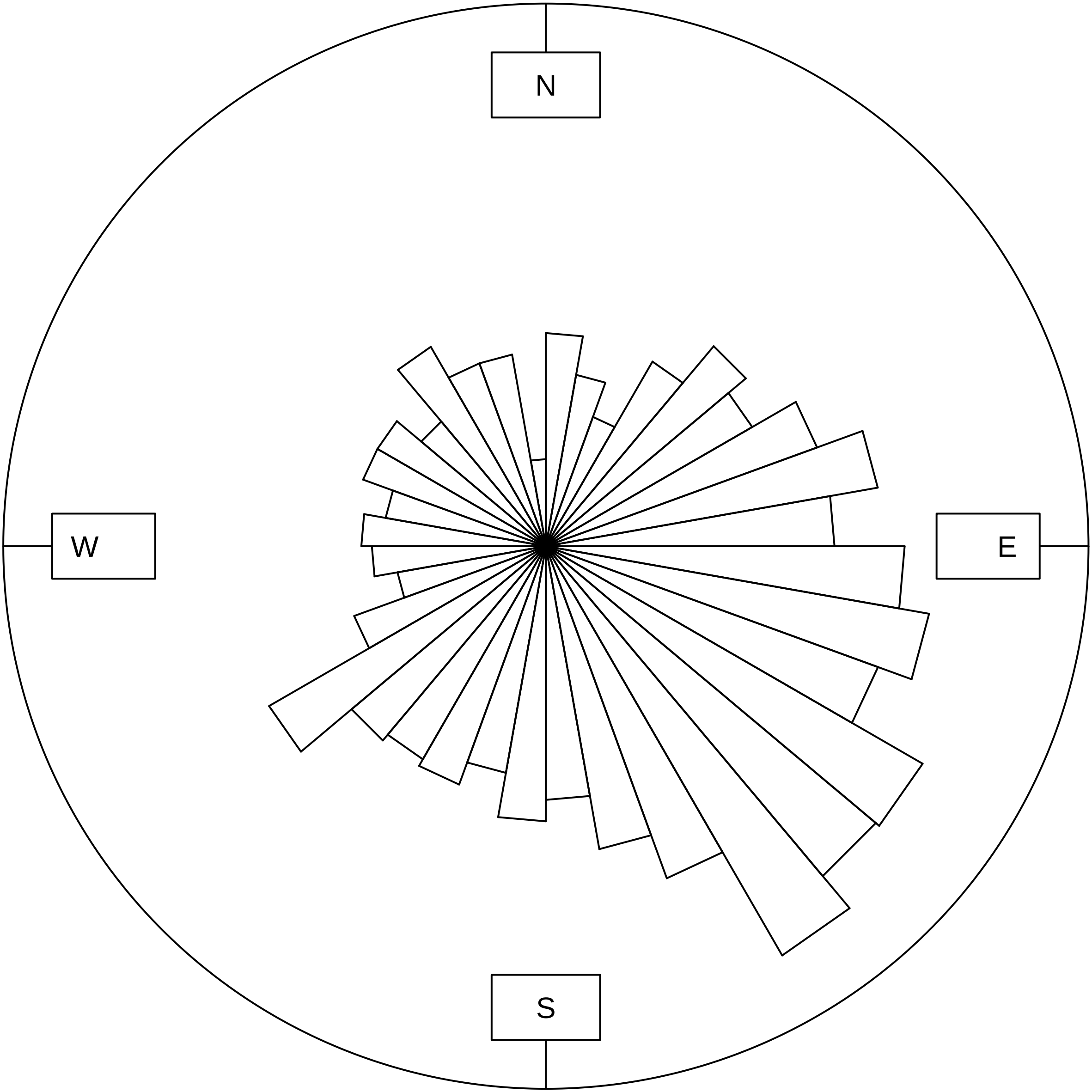

bootstrap 437

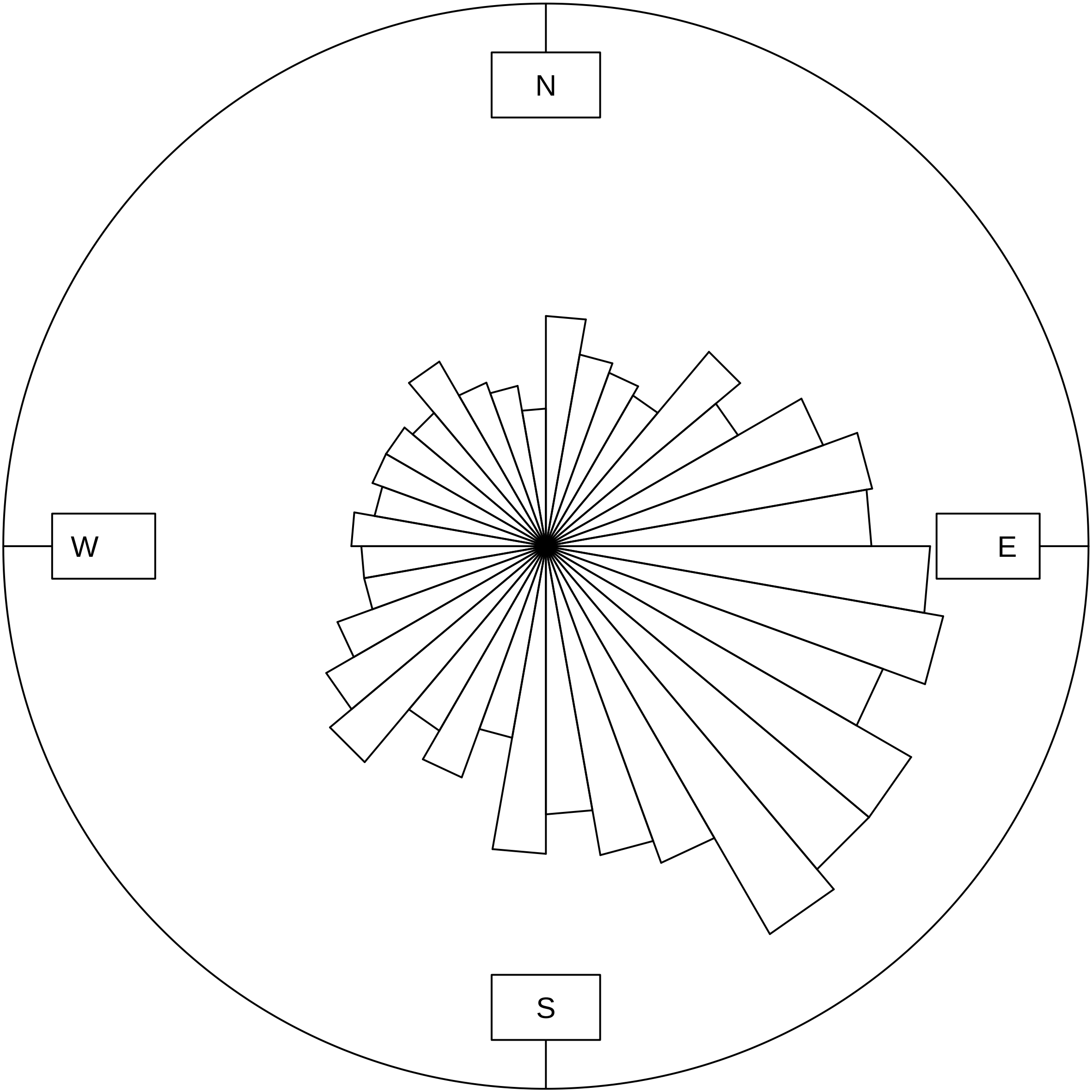

bootstrap 438

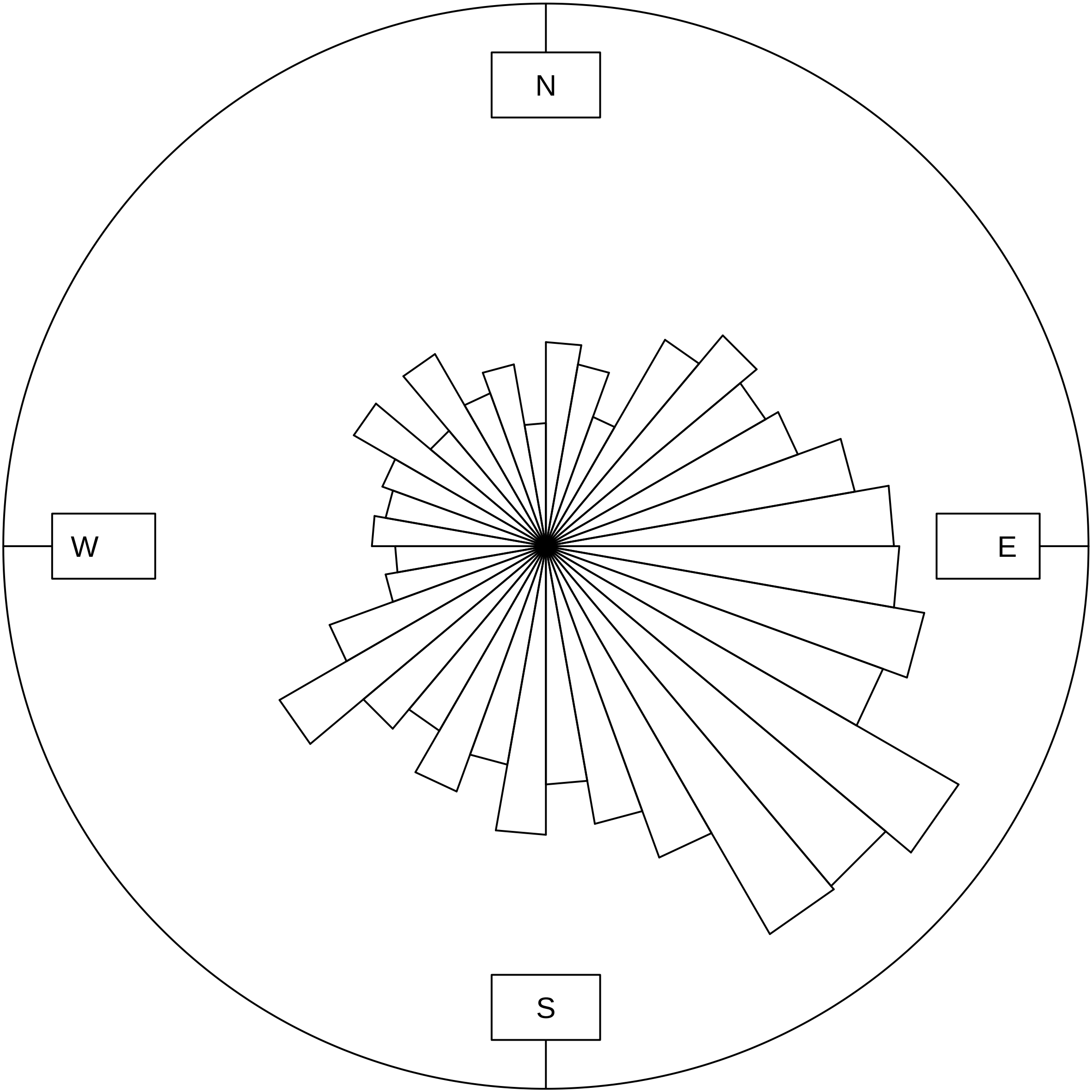

bootstrap 439

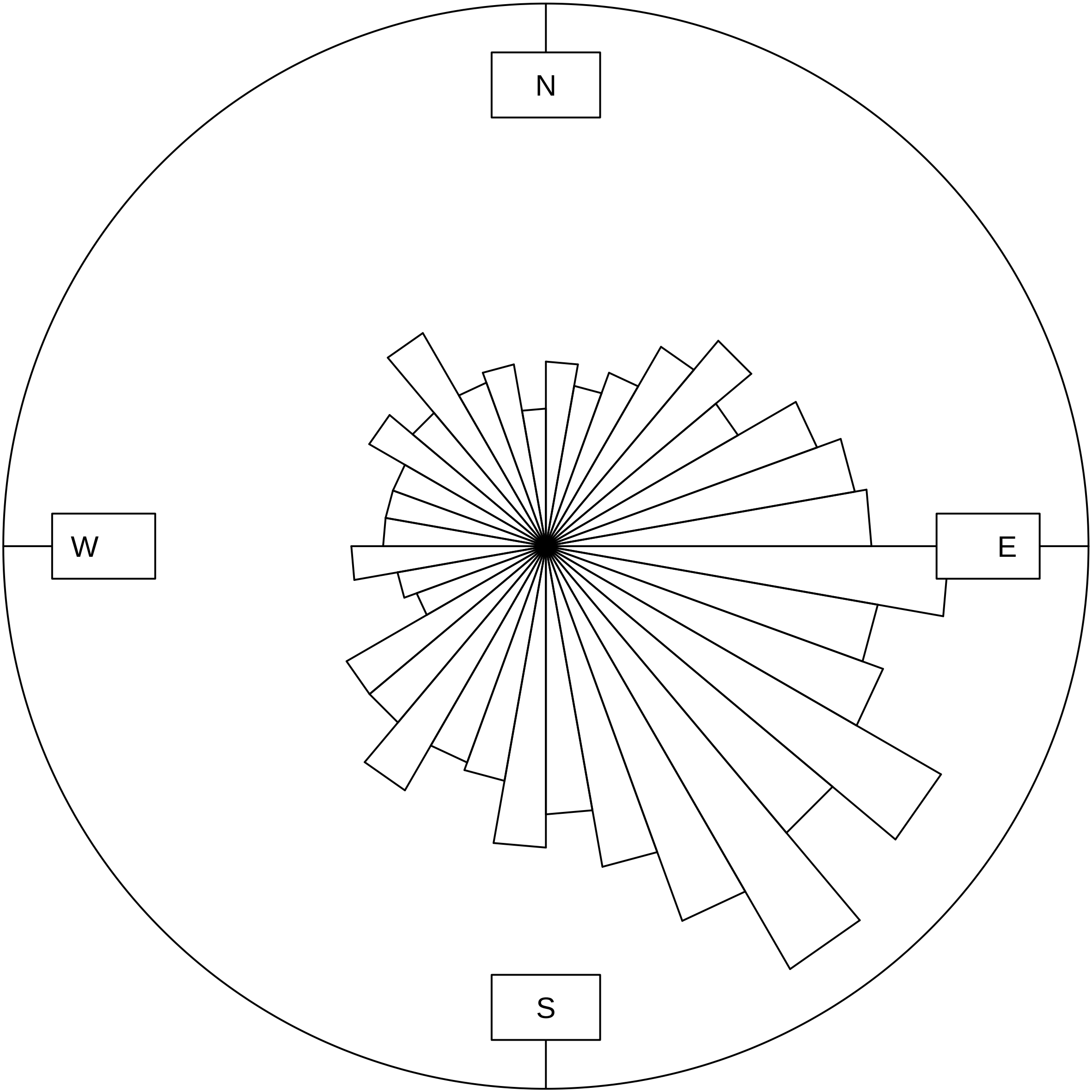

bootstrap 440

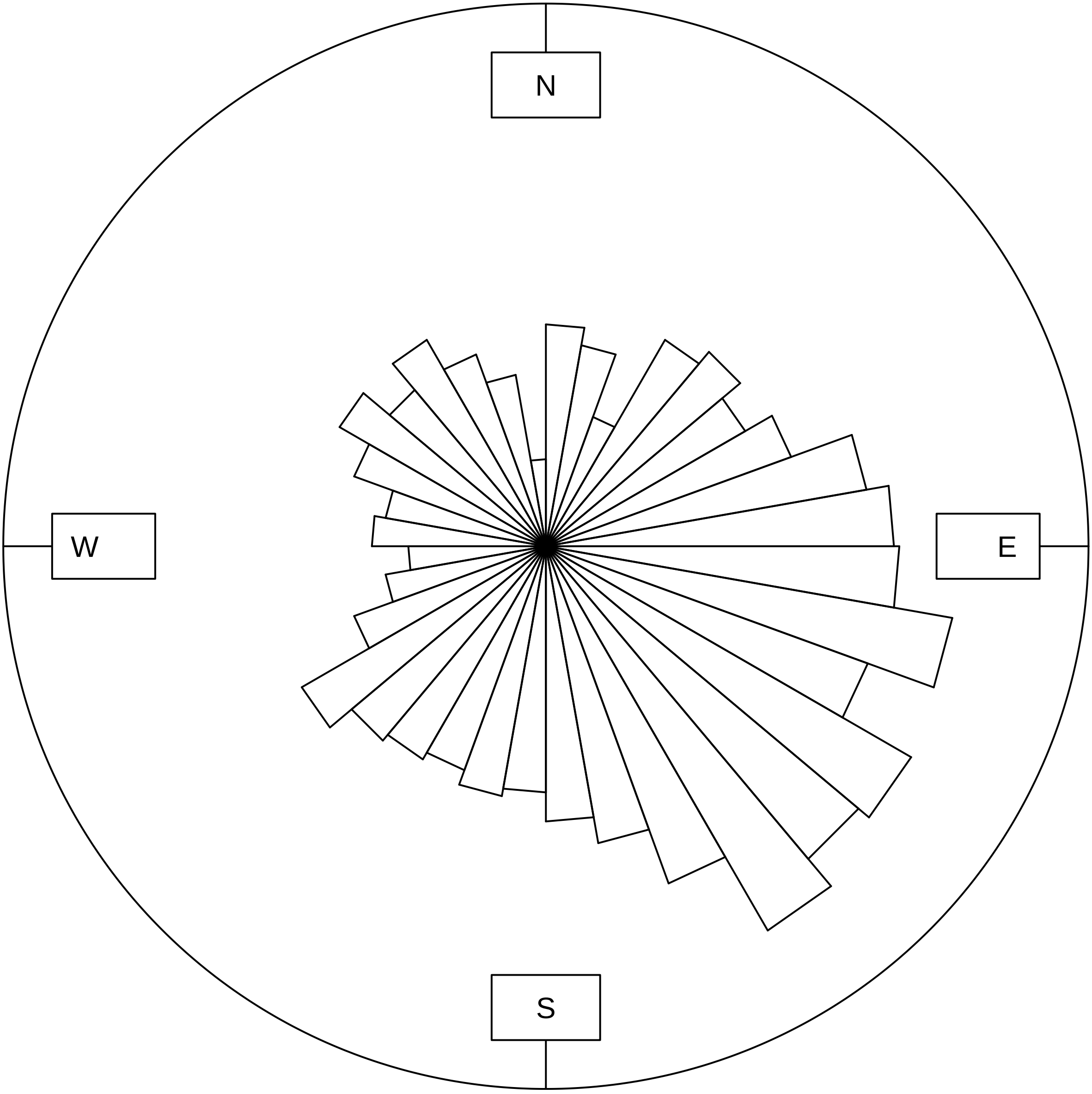

bootstrap 441

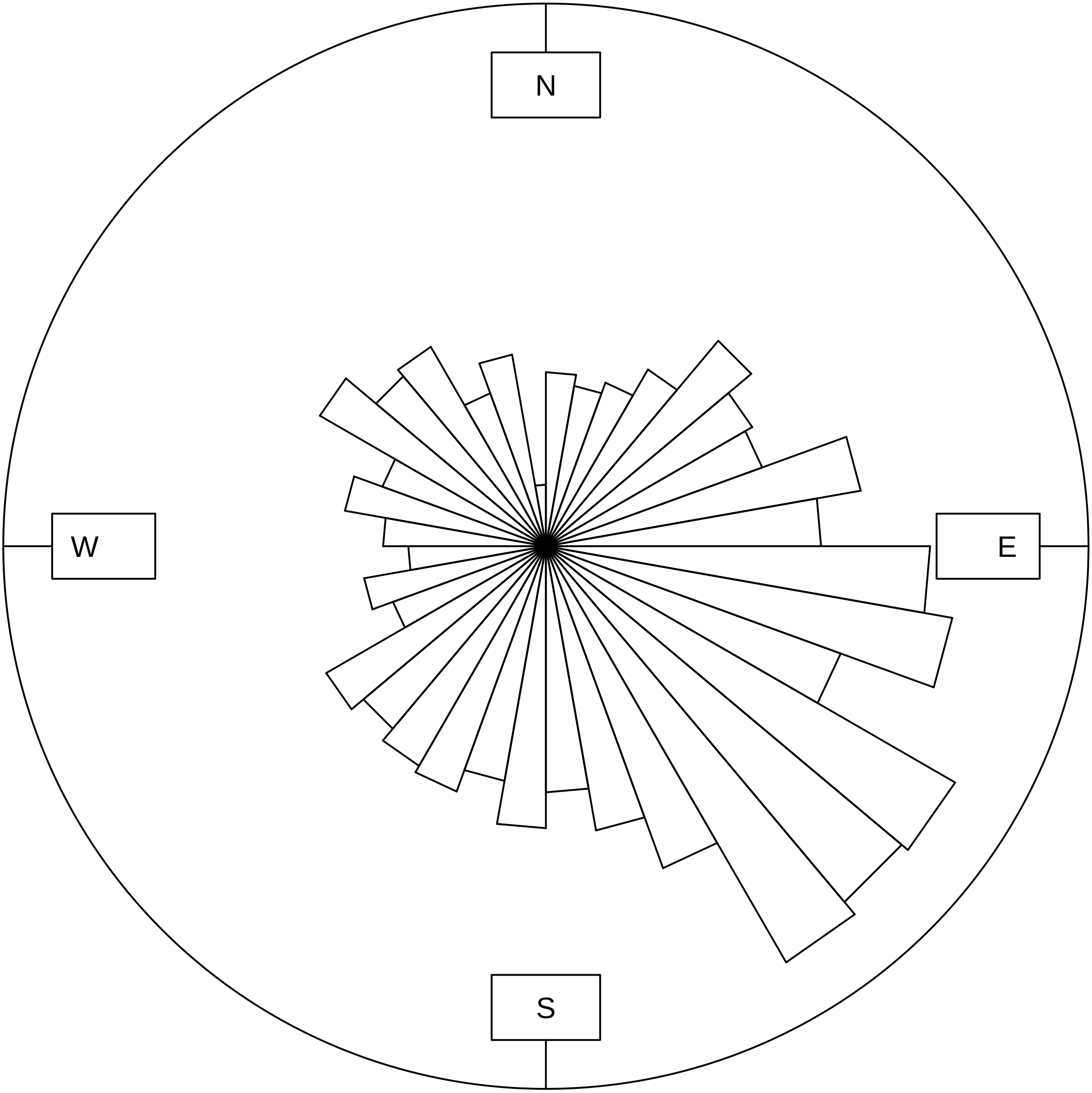

bootstrap 442

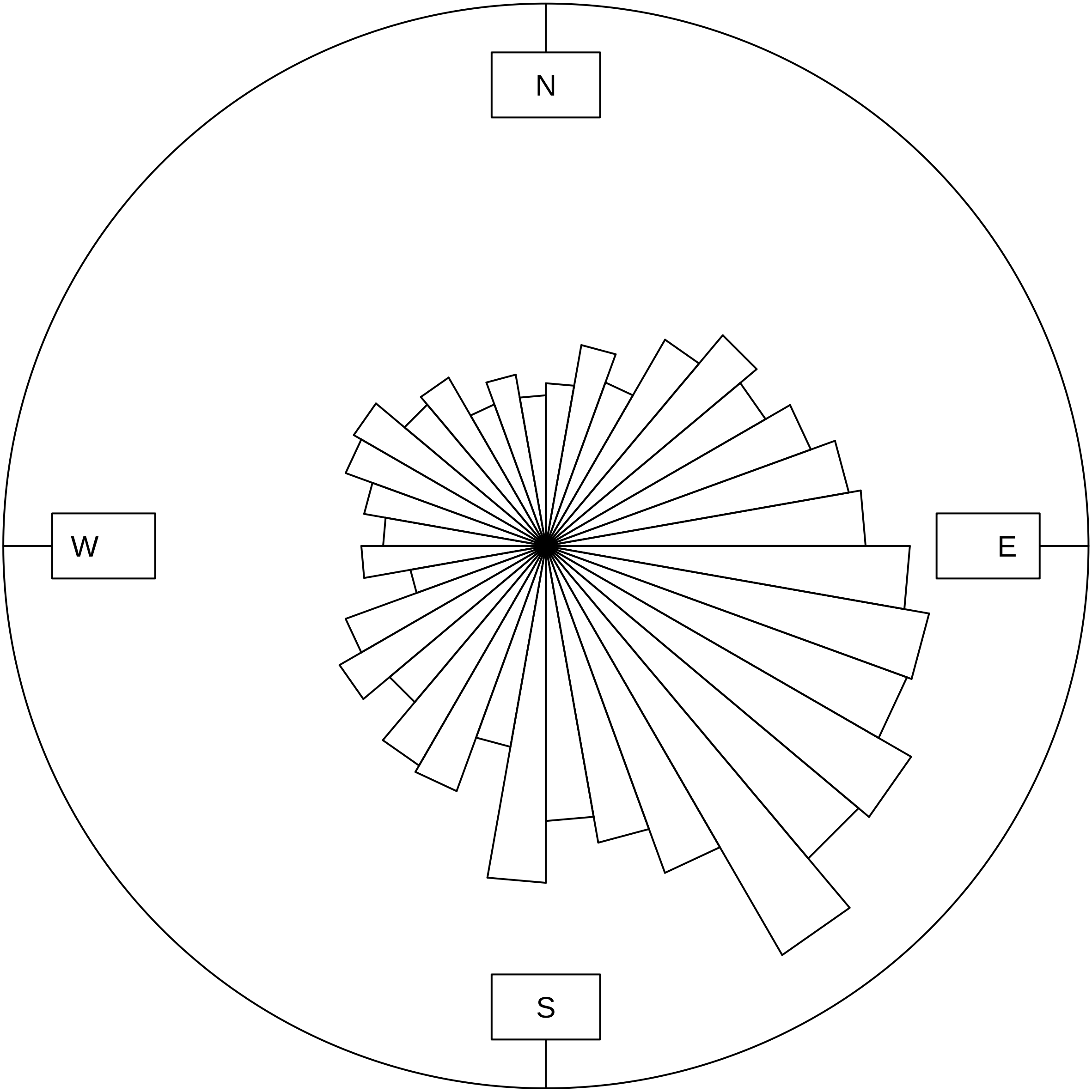

bootstrap 443

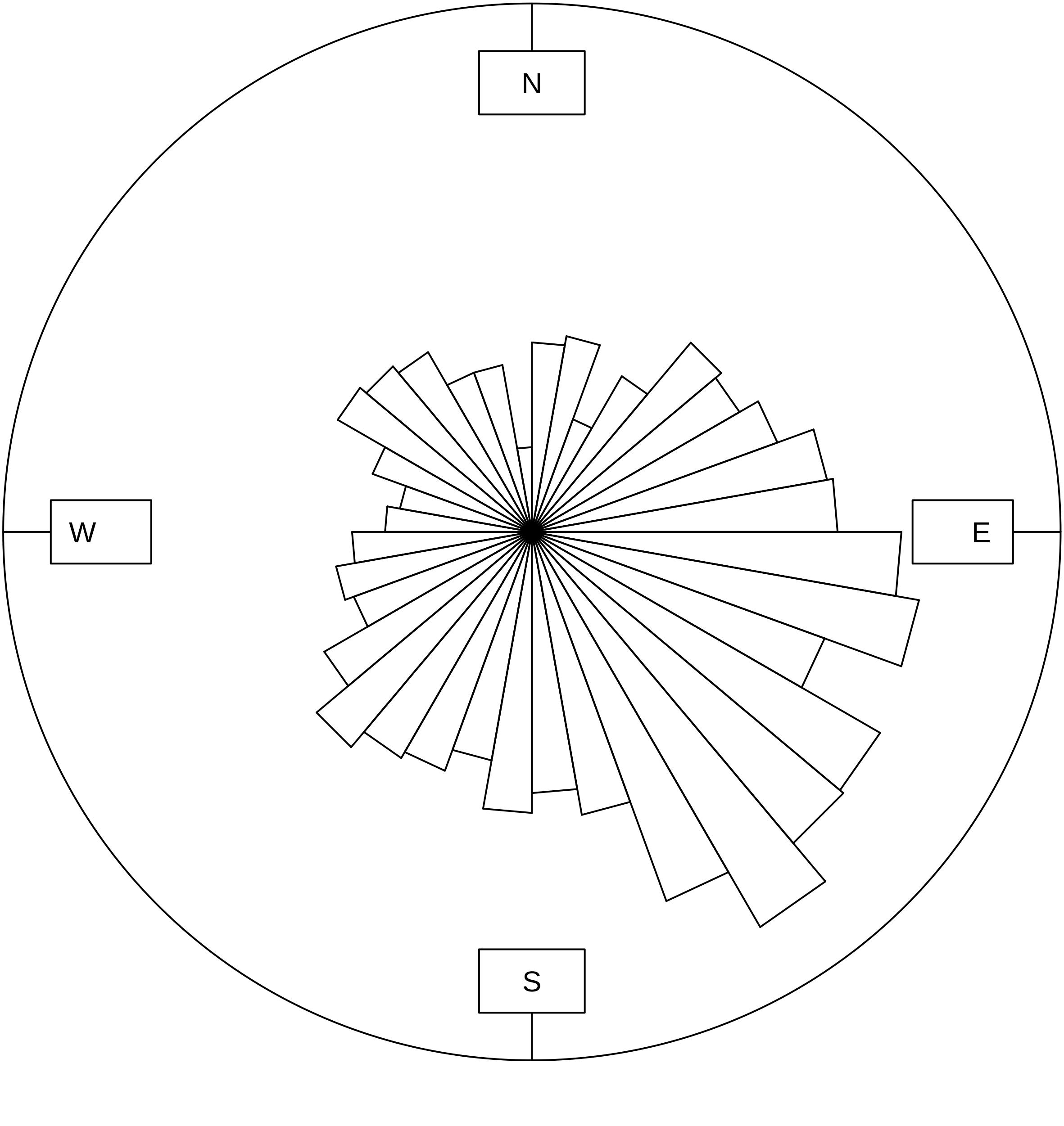

bootstrap 444

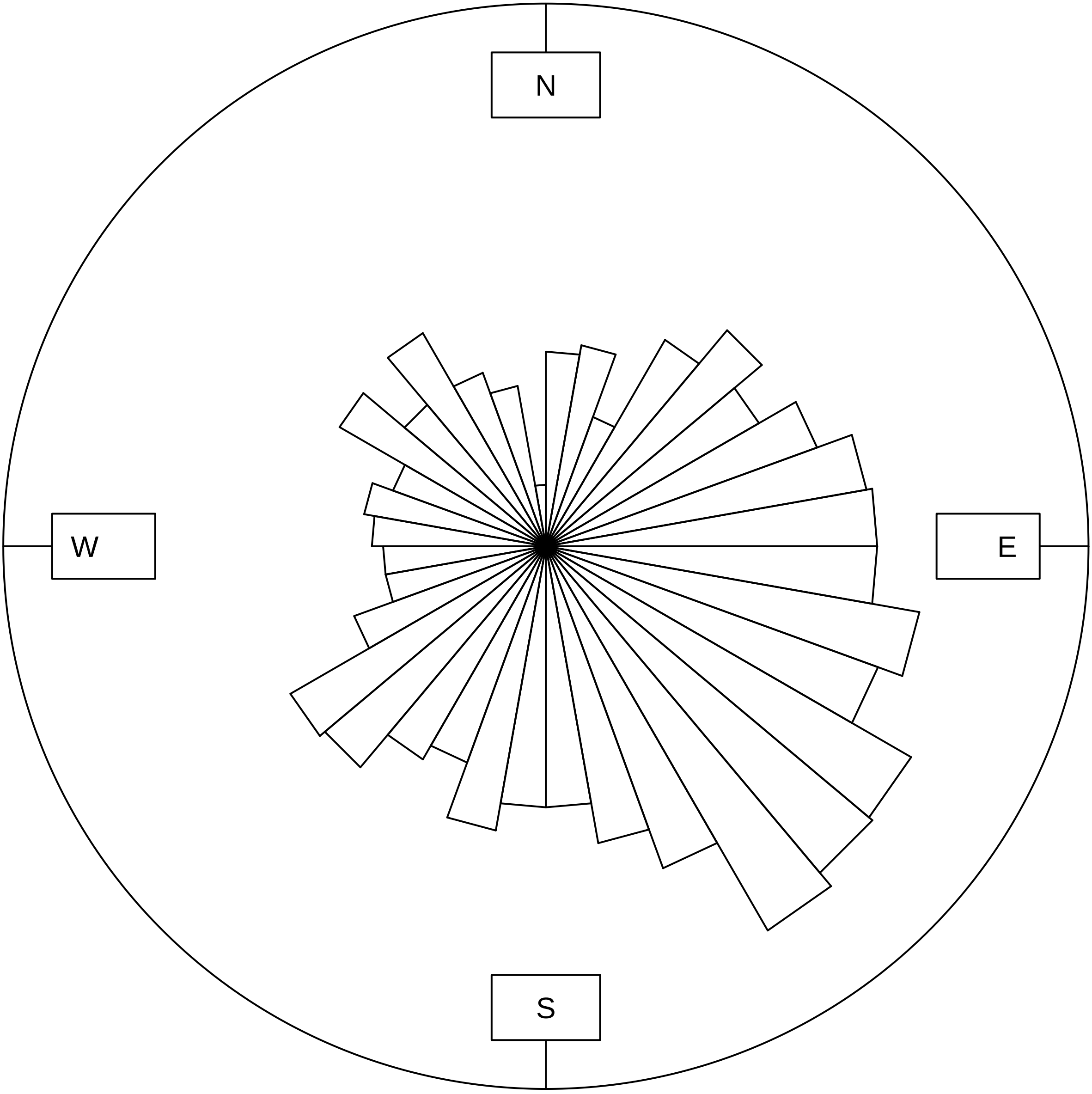

bootstrap 445

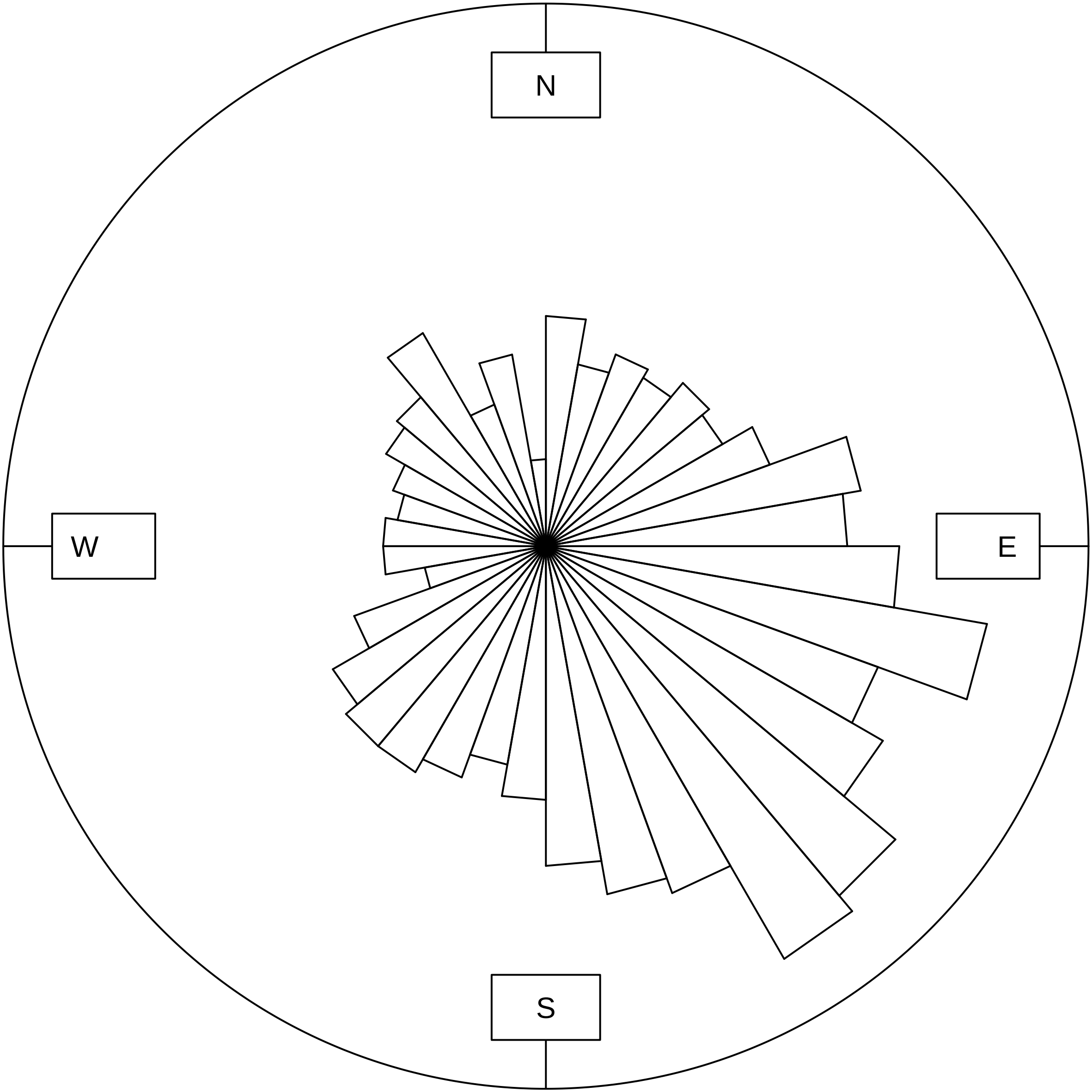

bootstrap 446

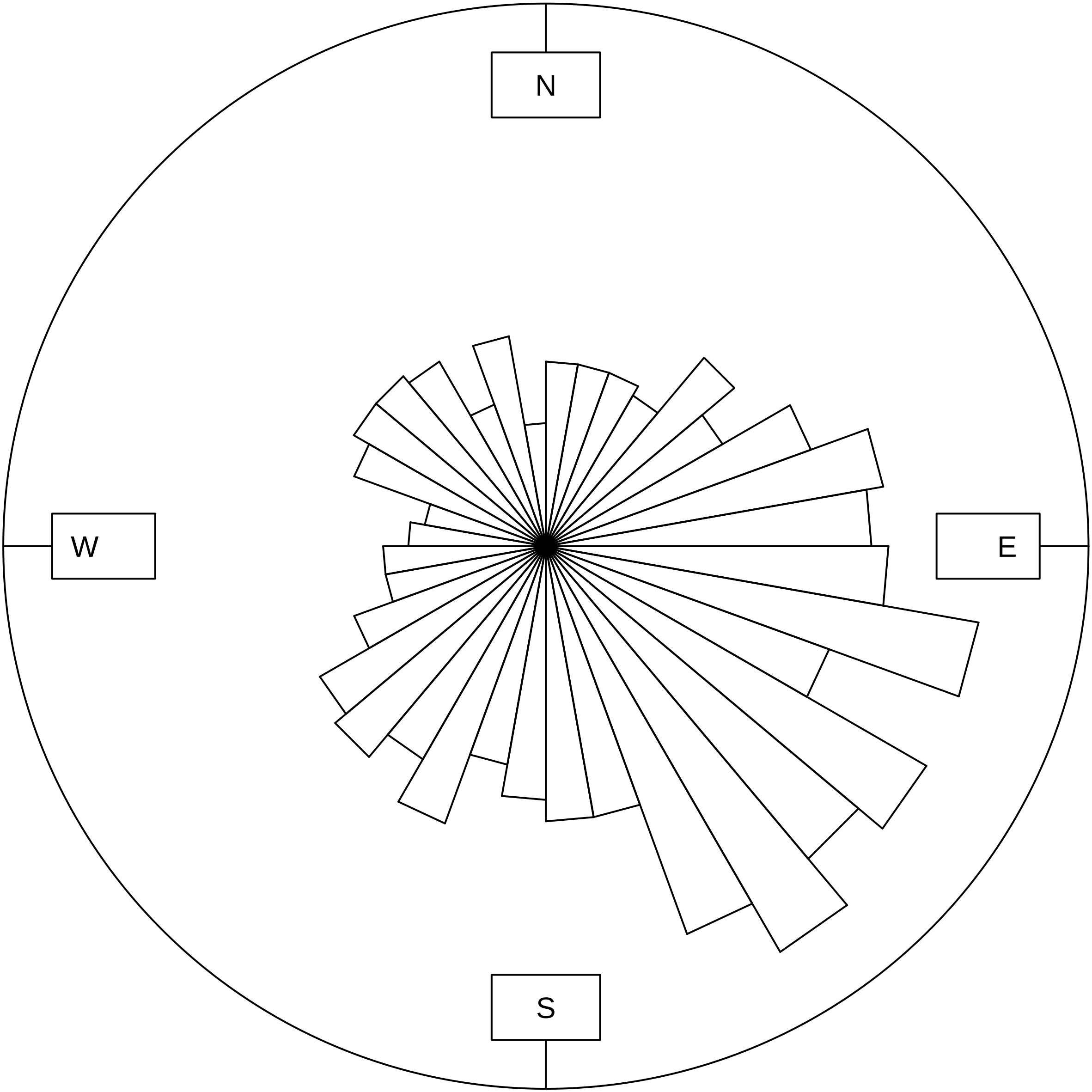

bootstrap 447

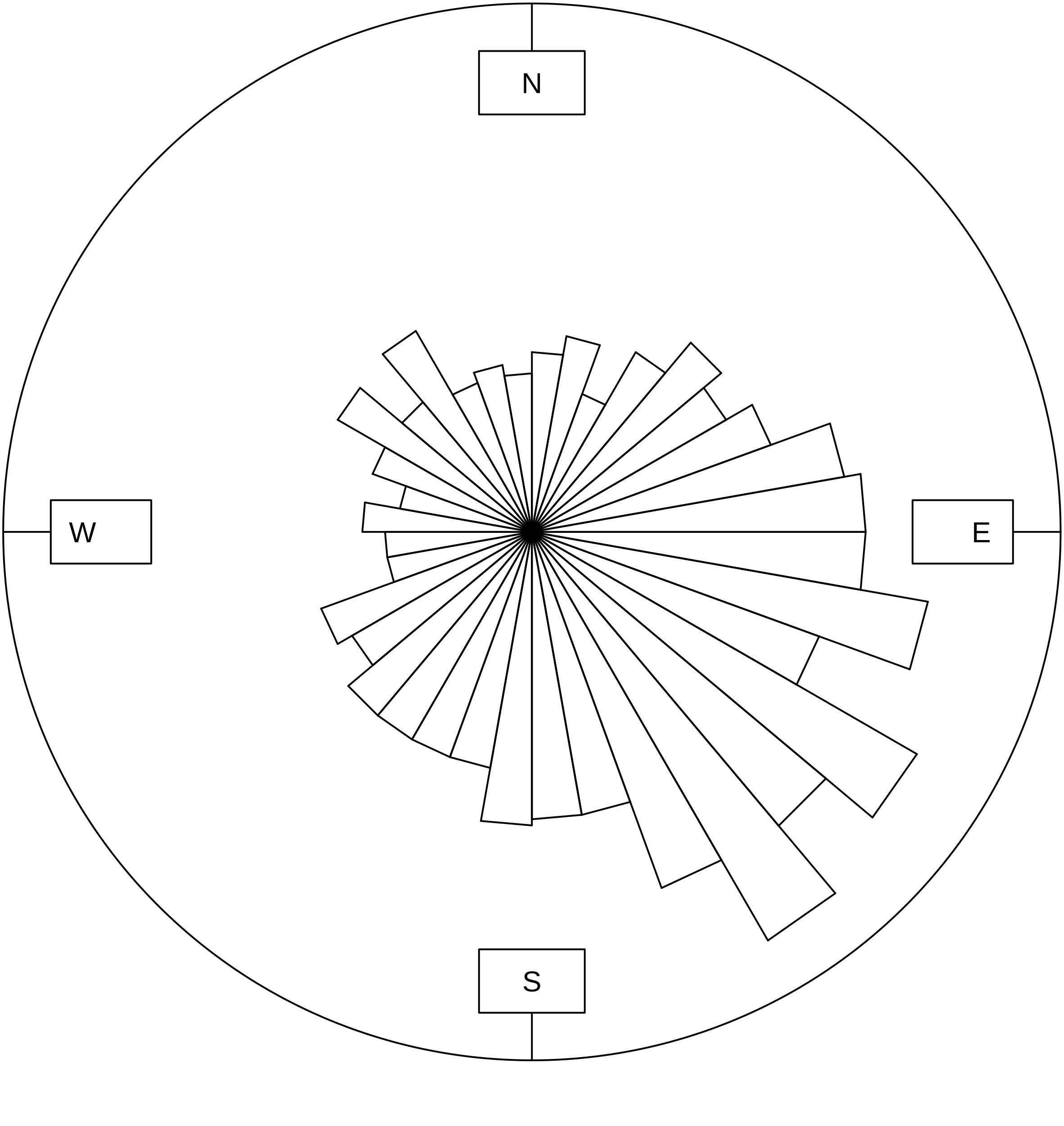

bootstrap 448

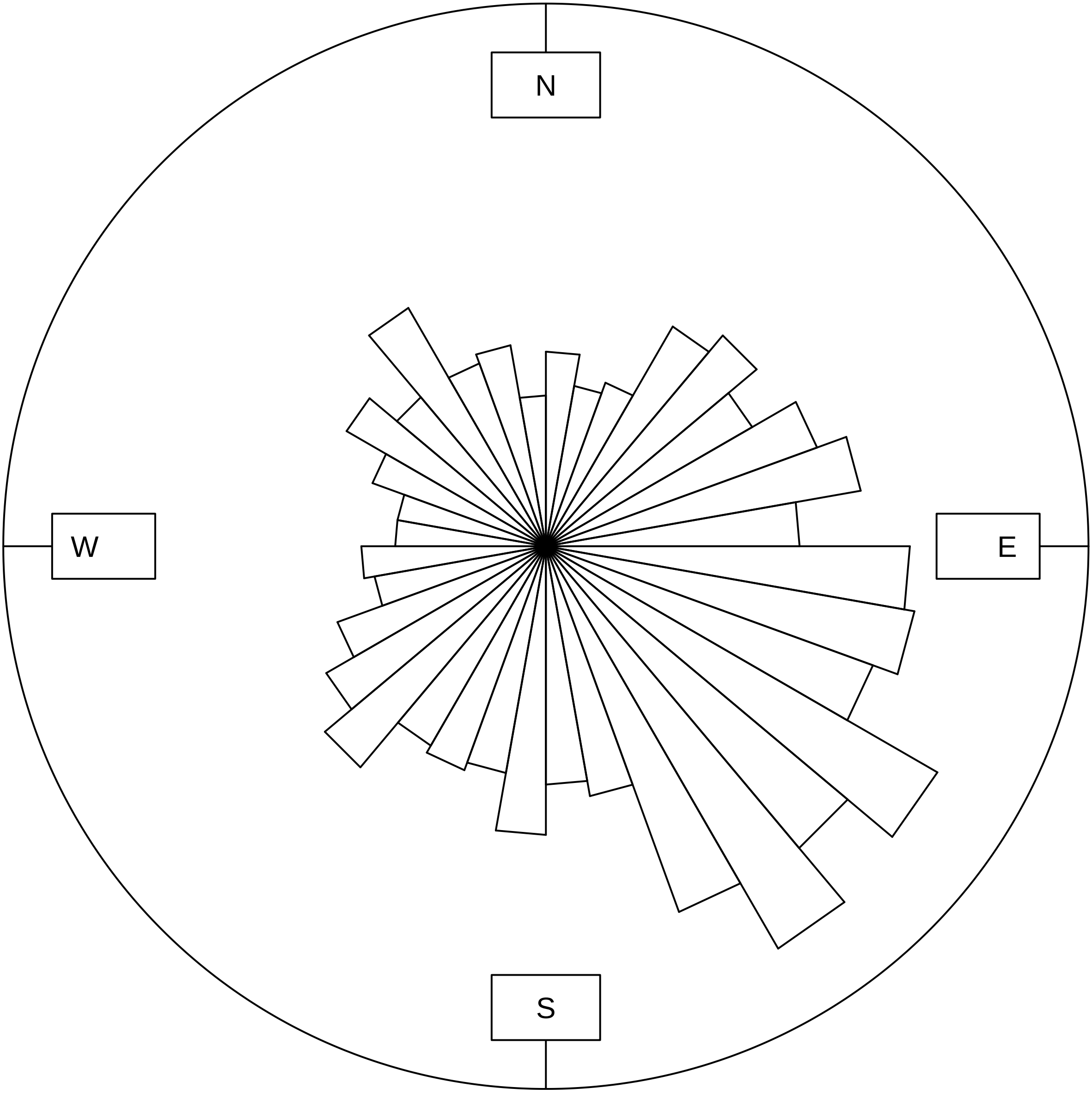

bootstrap 449

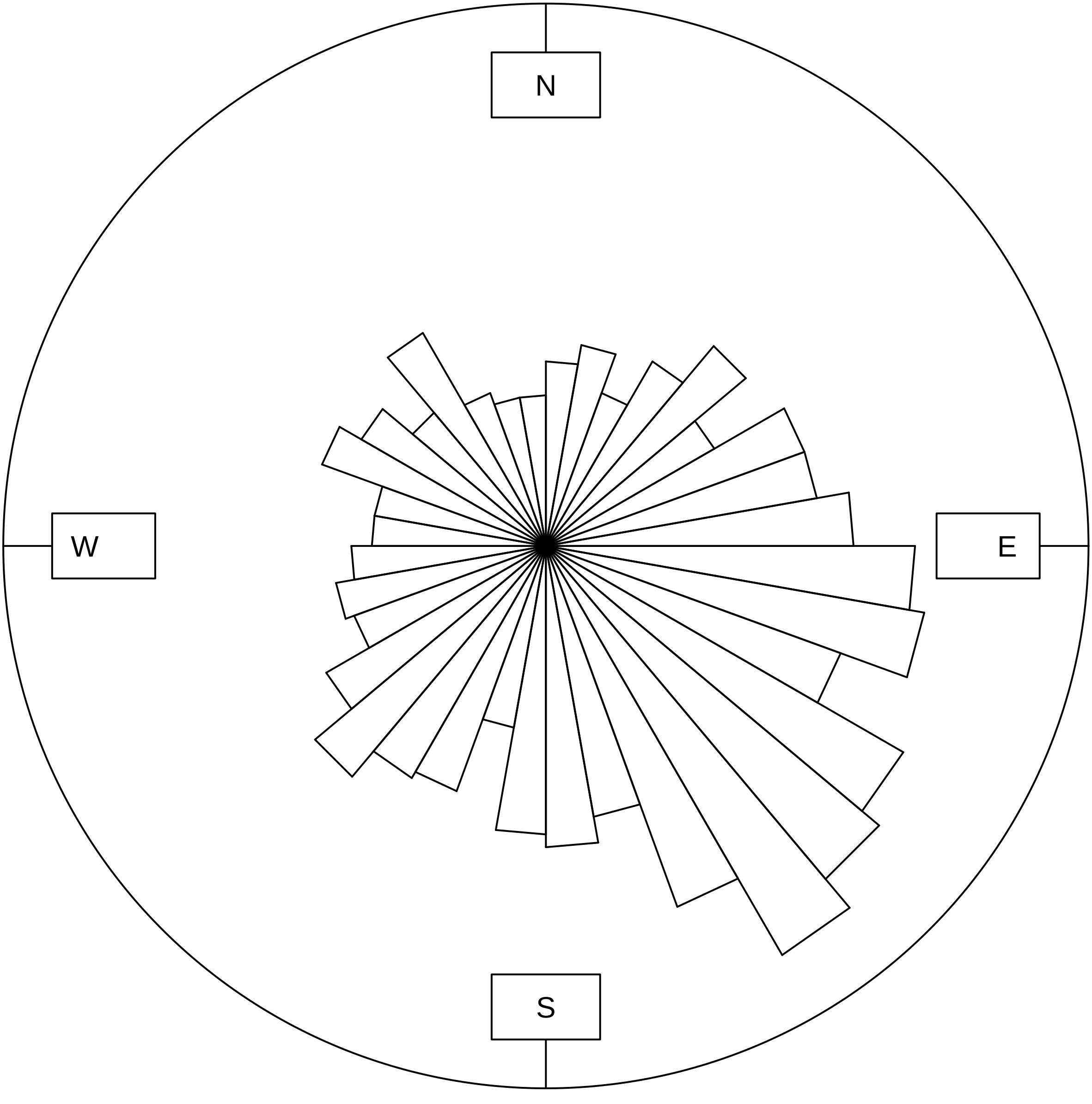

bootstrap 450

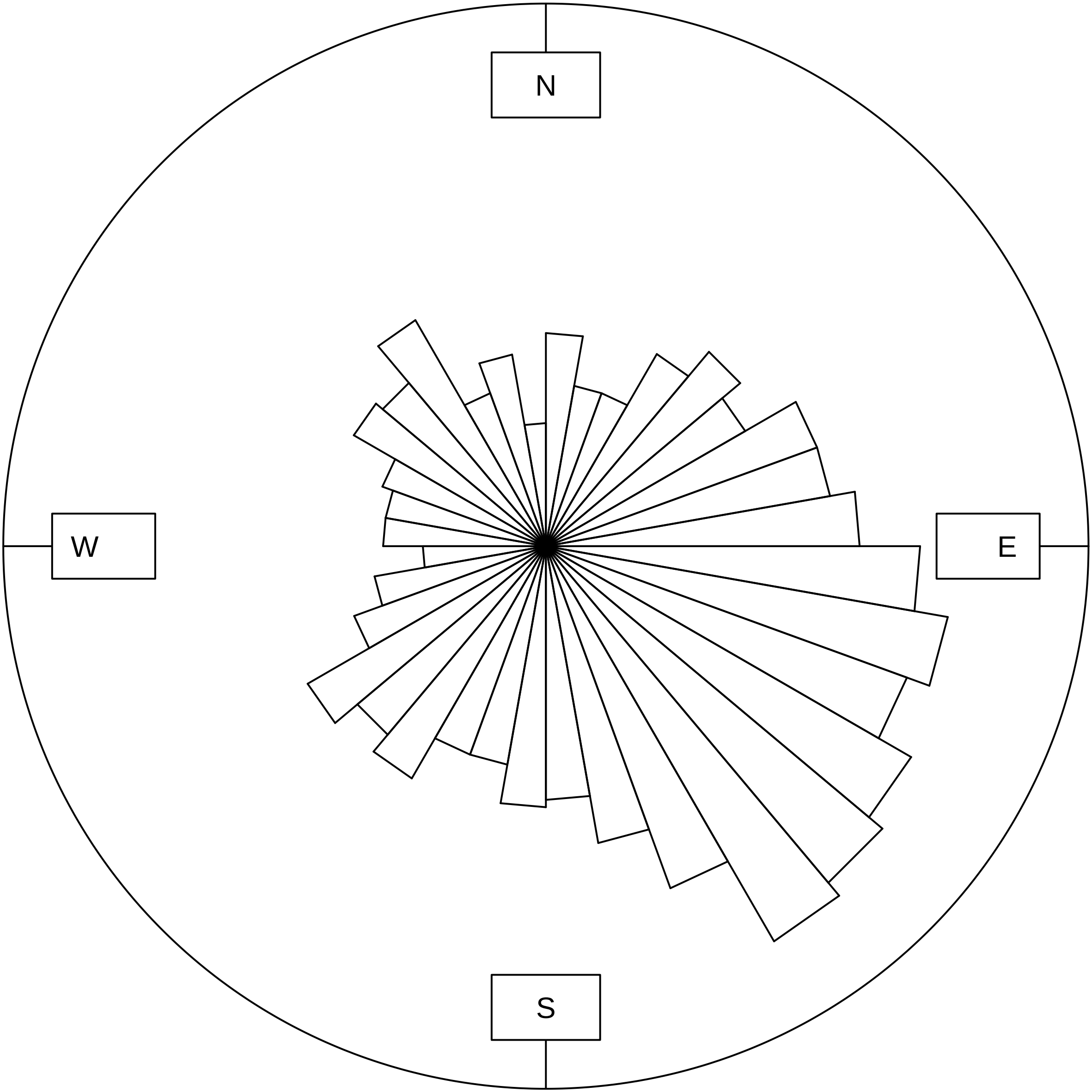

bootstrap 451

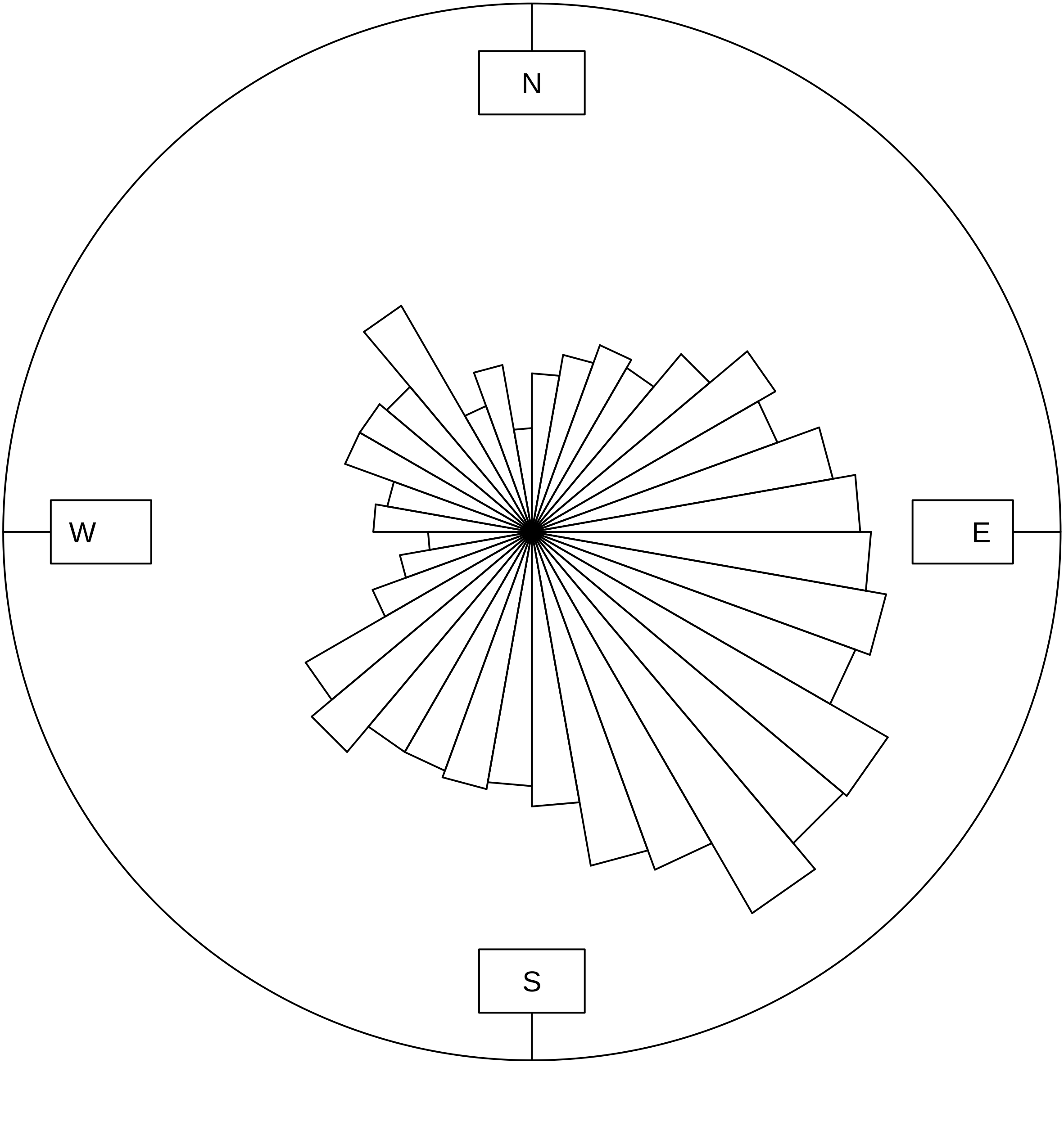

bootstrap 452

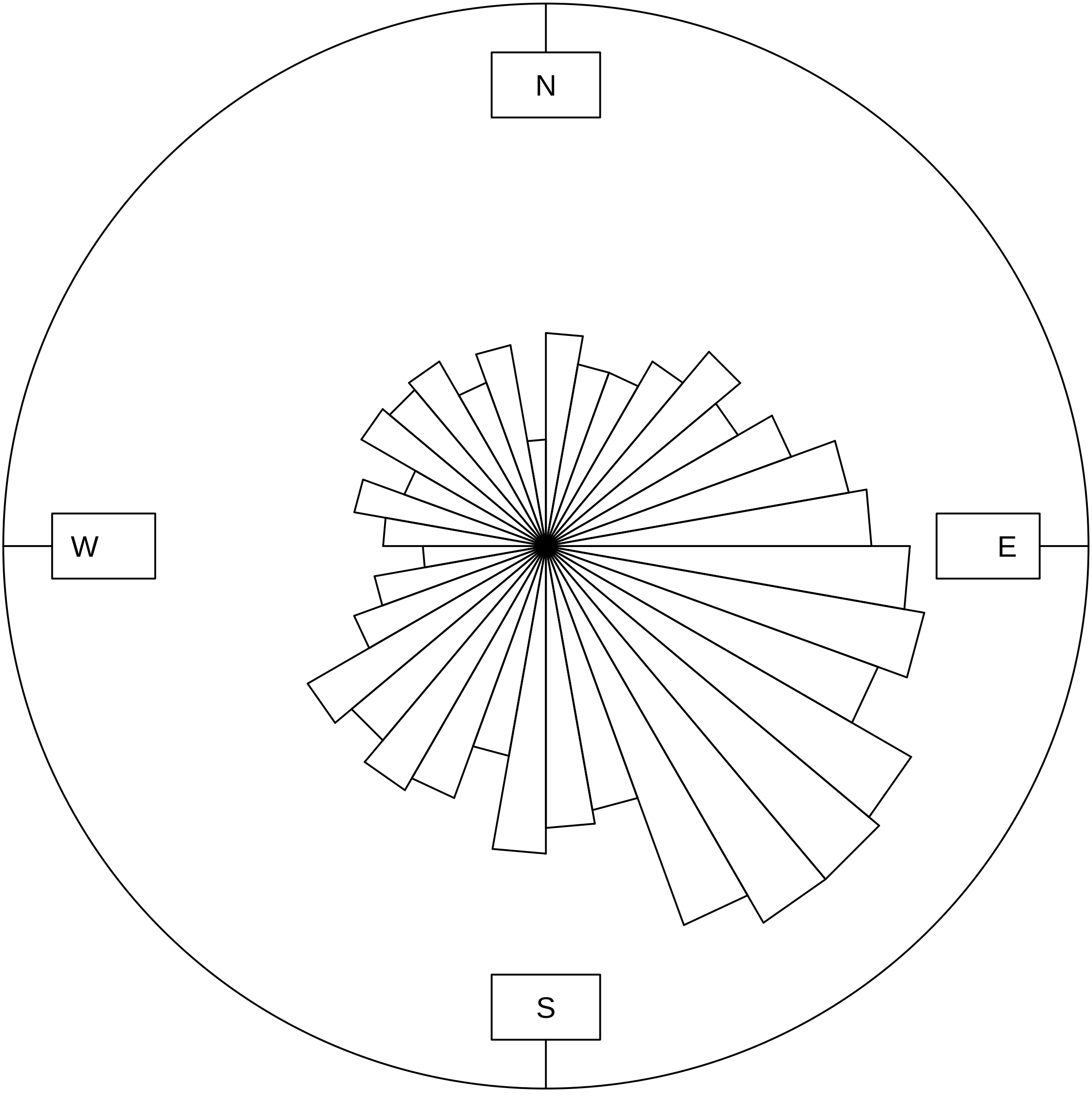

bootstrap 453

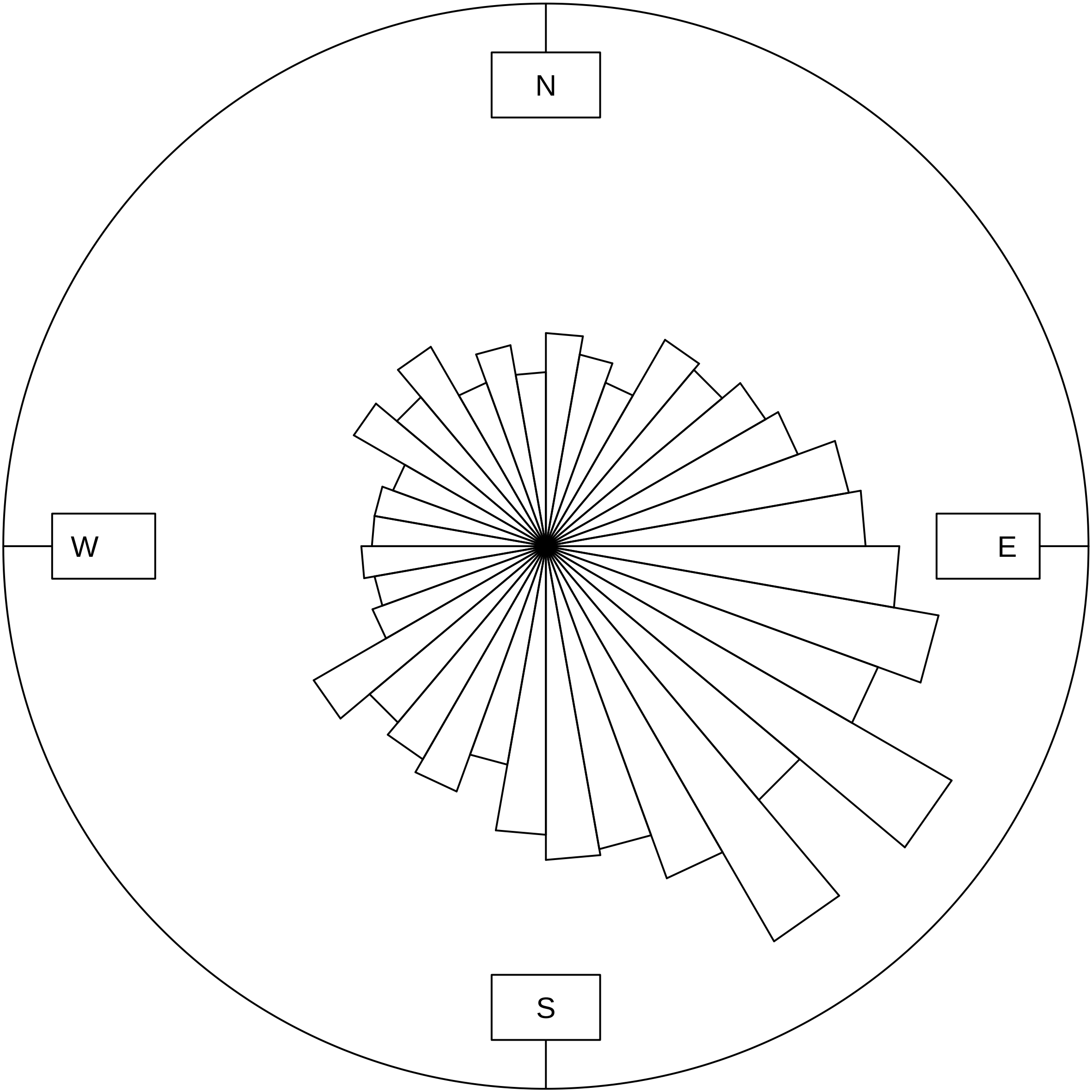

bootstrap 454

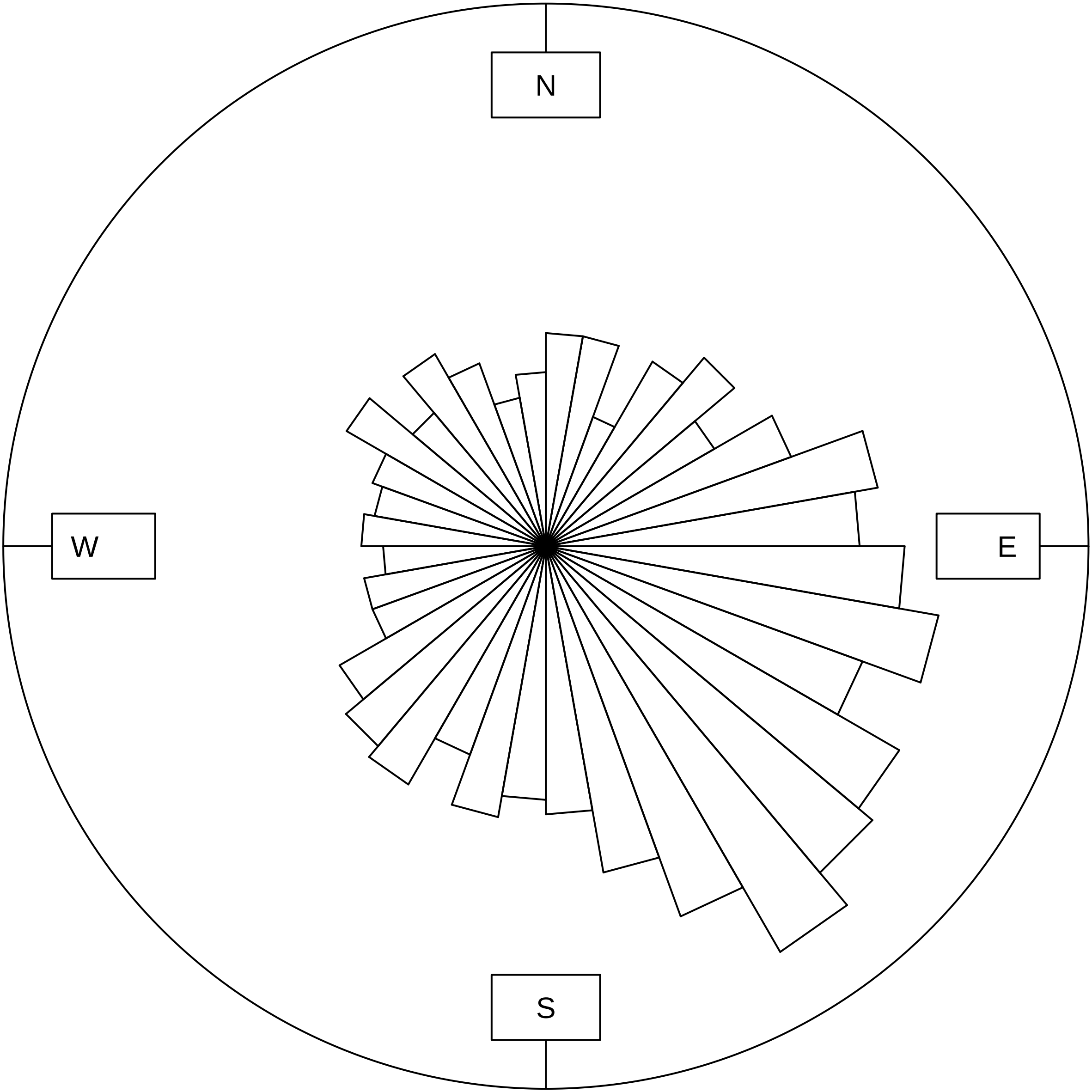

bootstrap 455

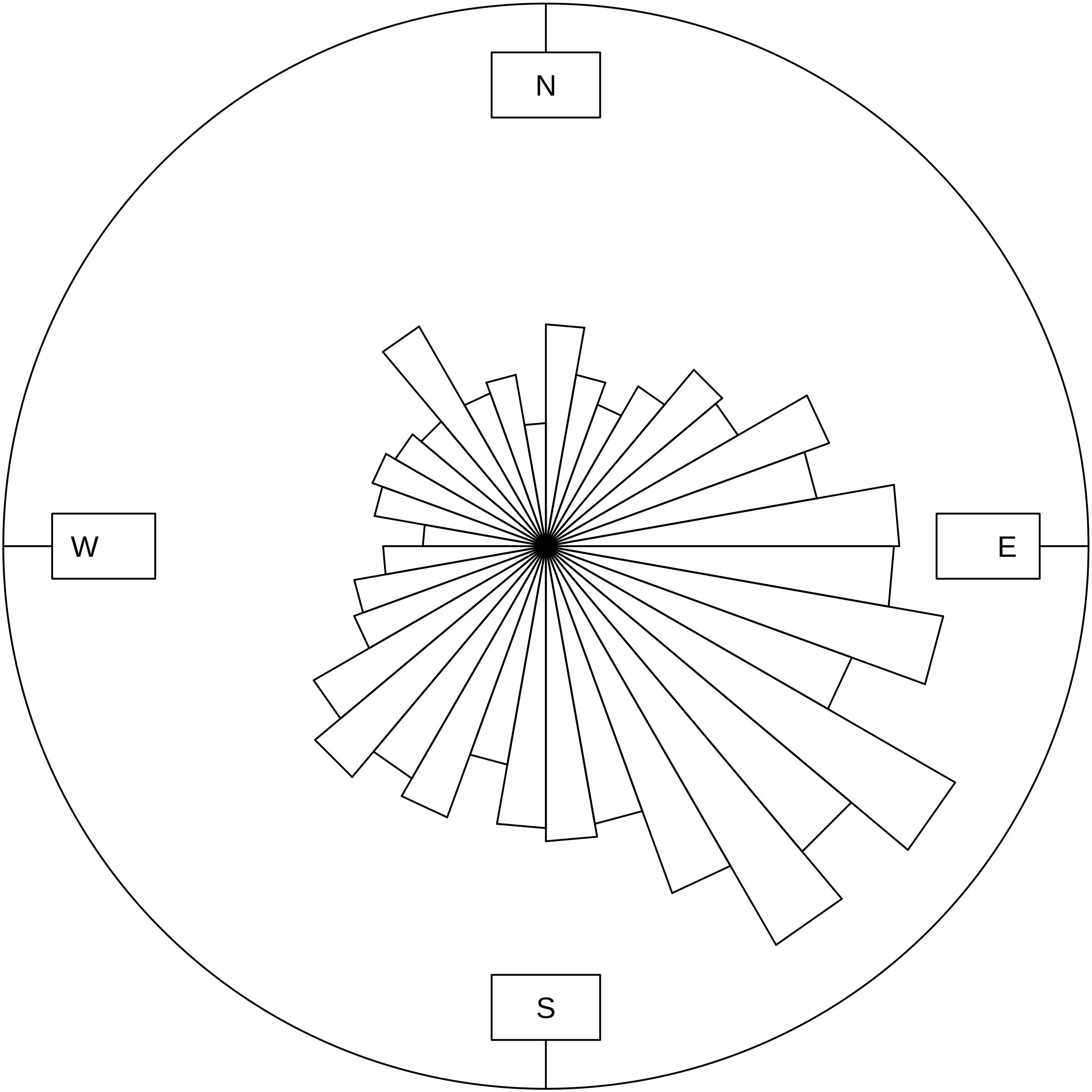

bootstrap 456

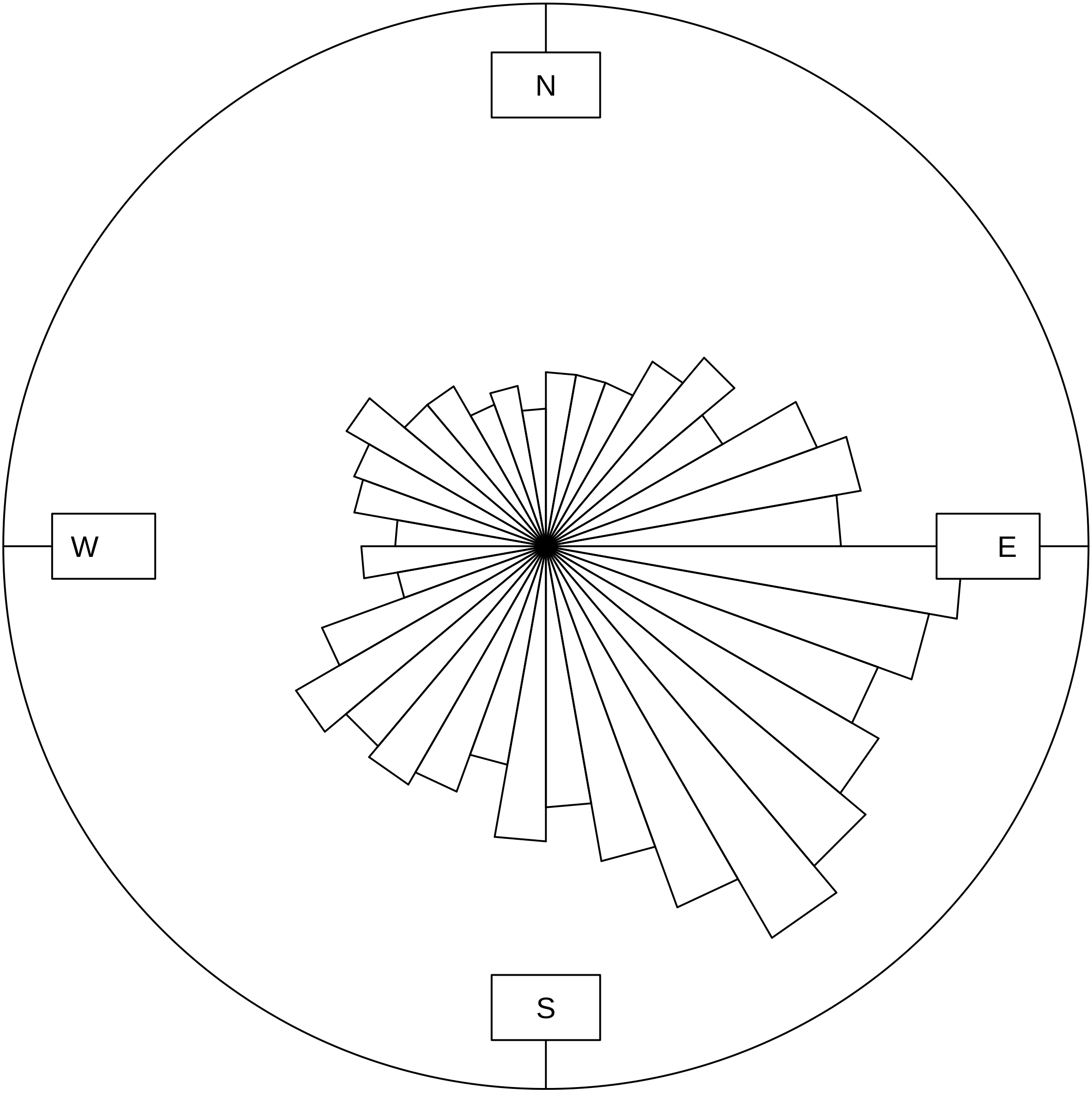

bootstrap 457

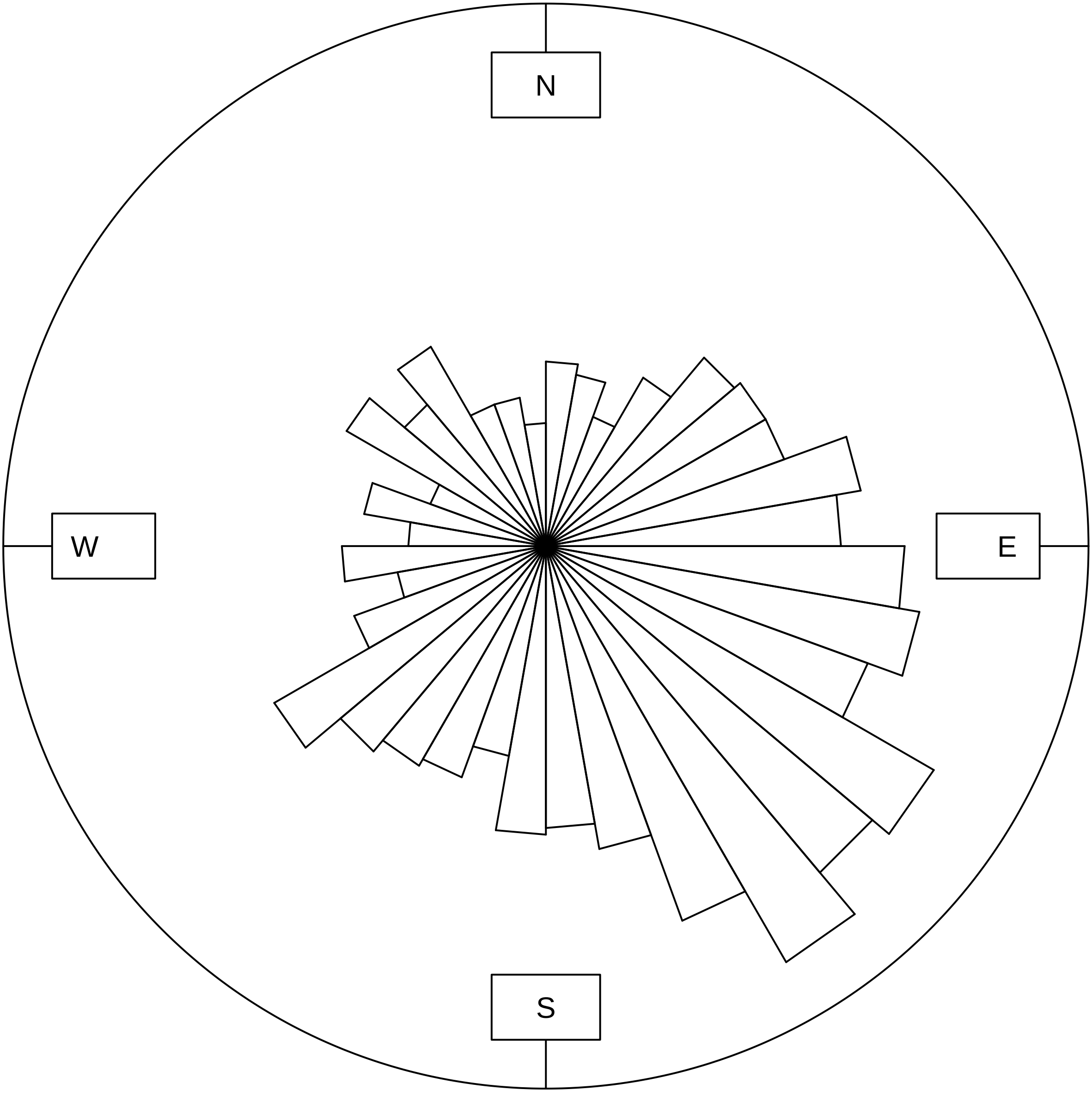

bootstrap 458

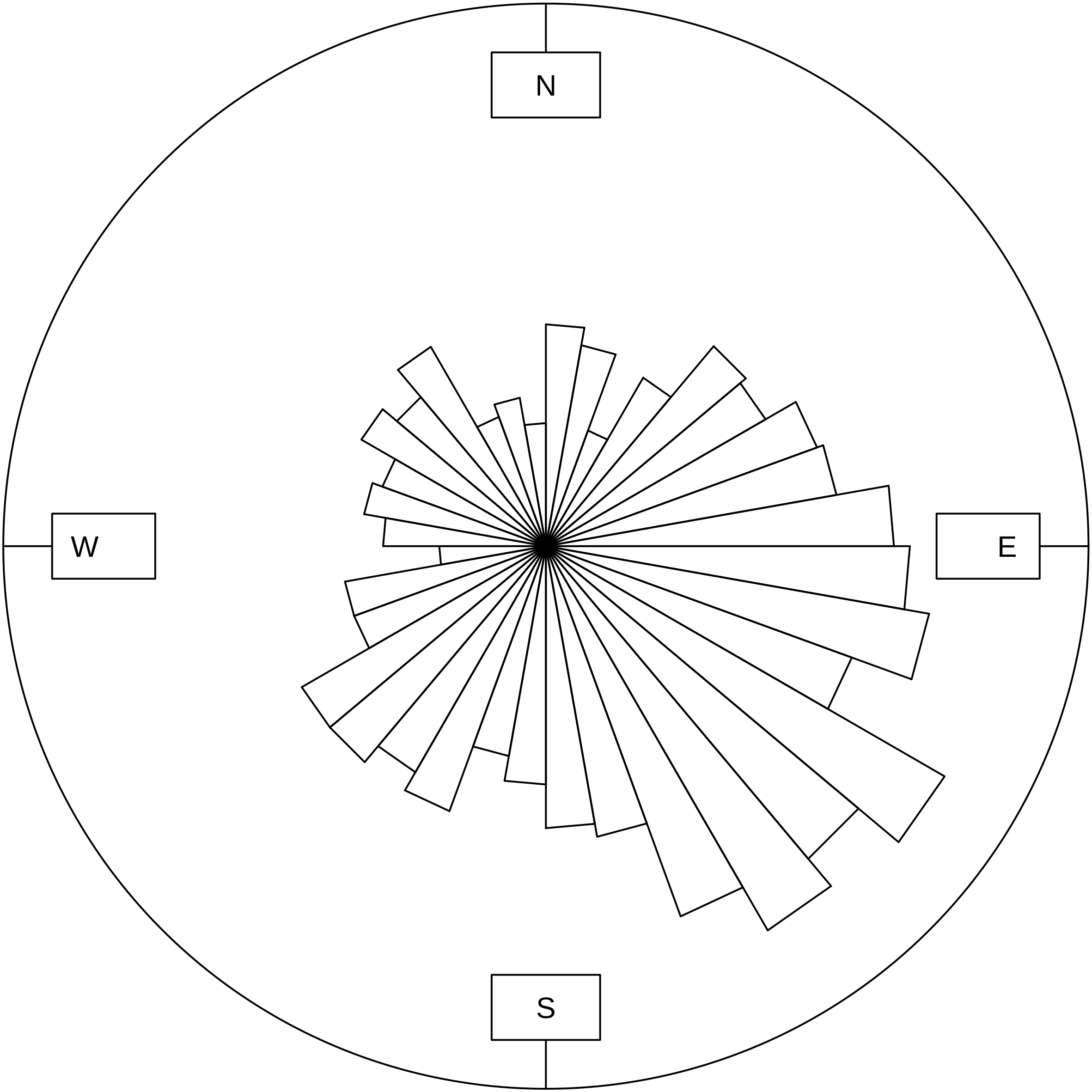

bootstrap 459

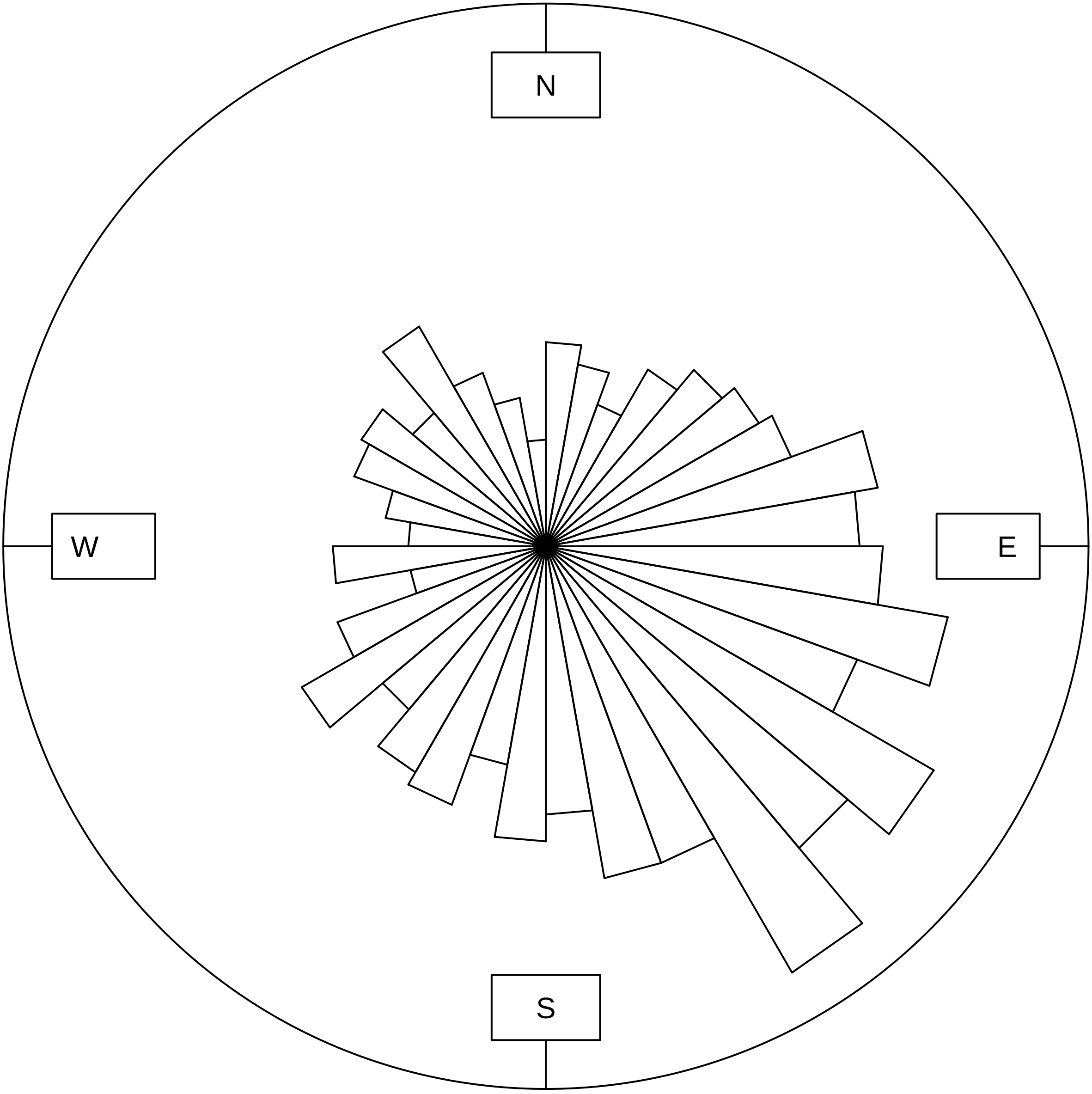

bootstrap 460

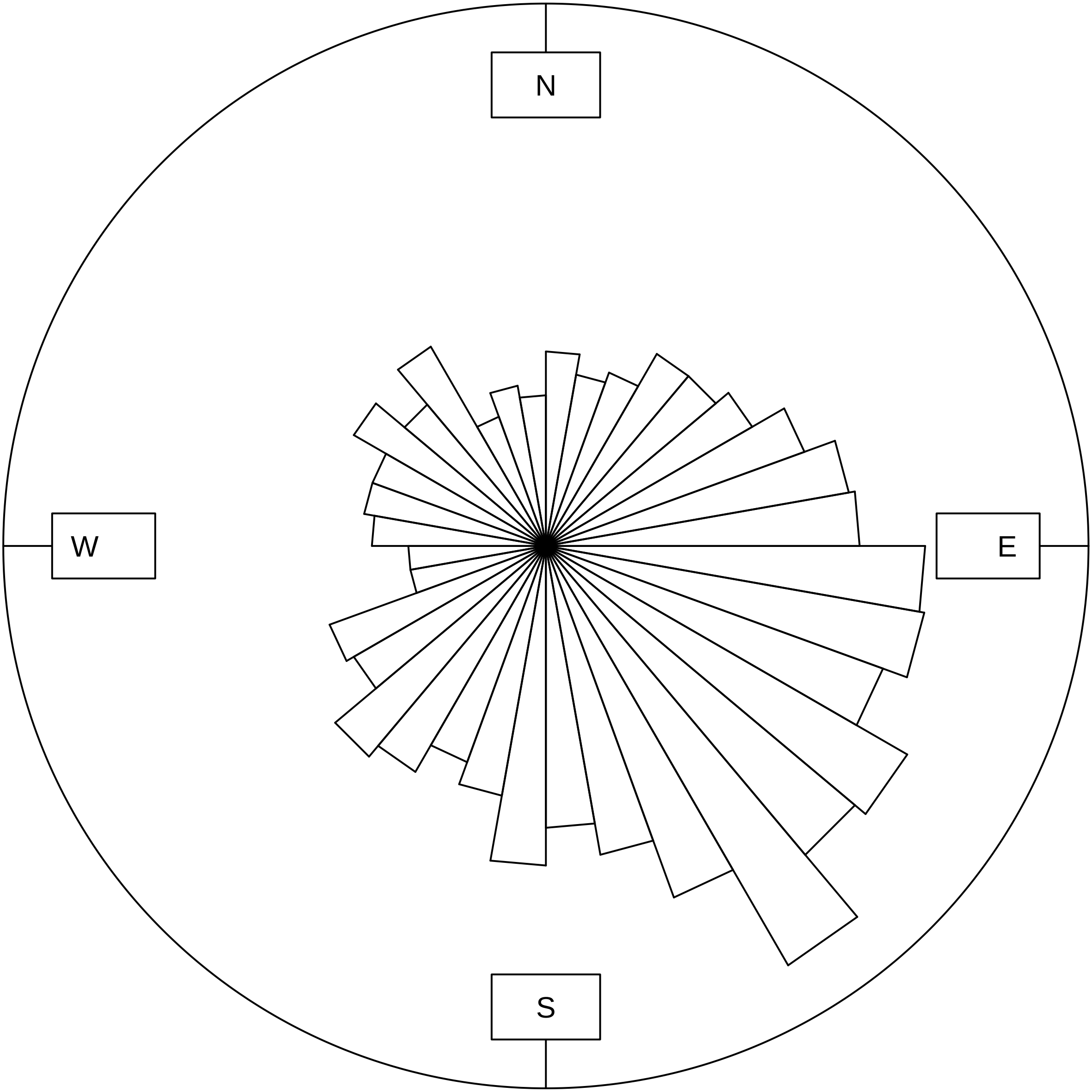

bootstrap 461

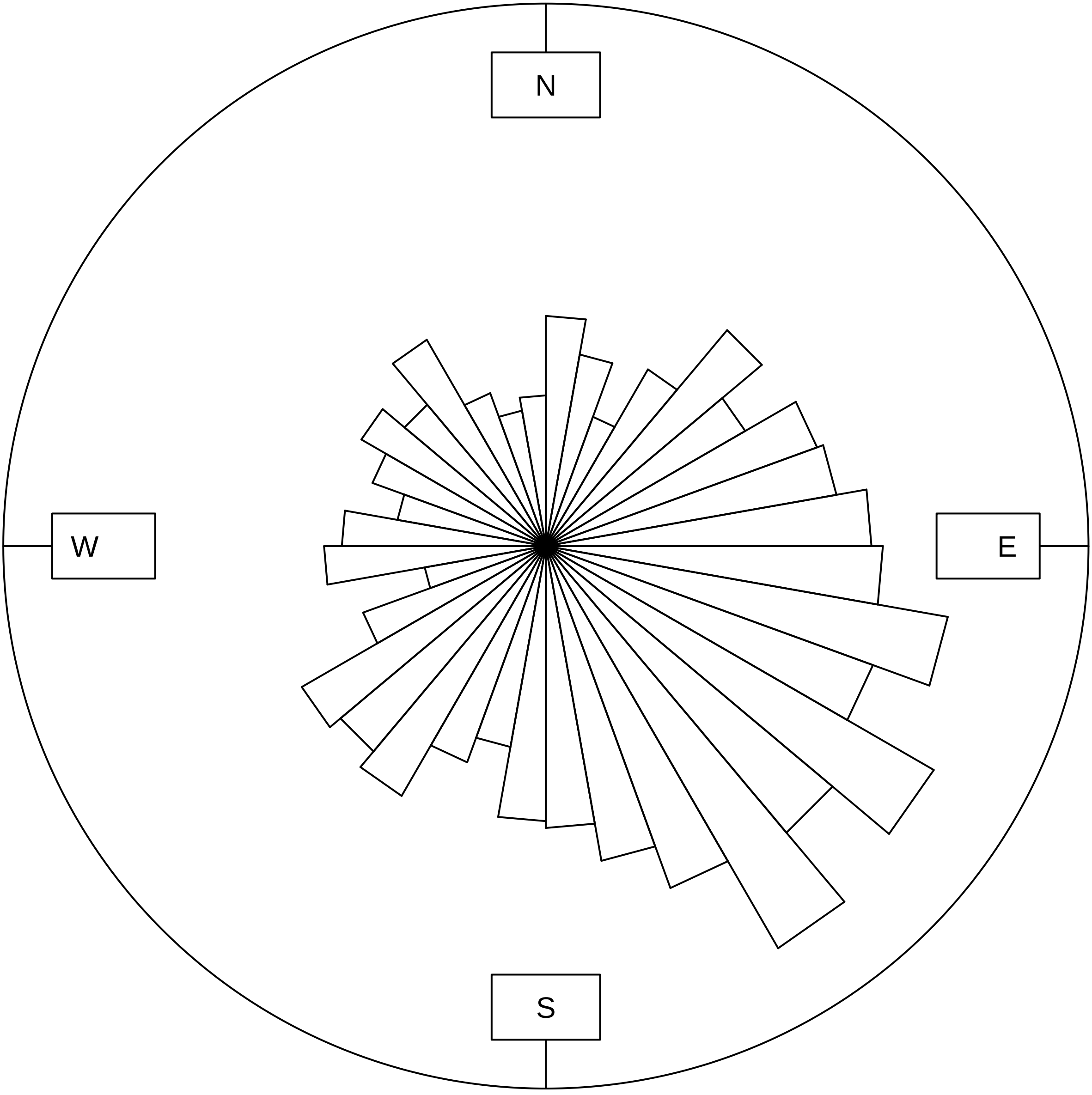

bootstrap 462

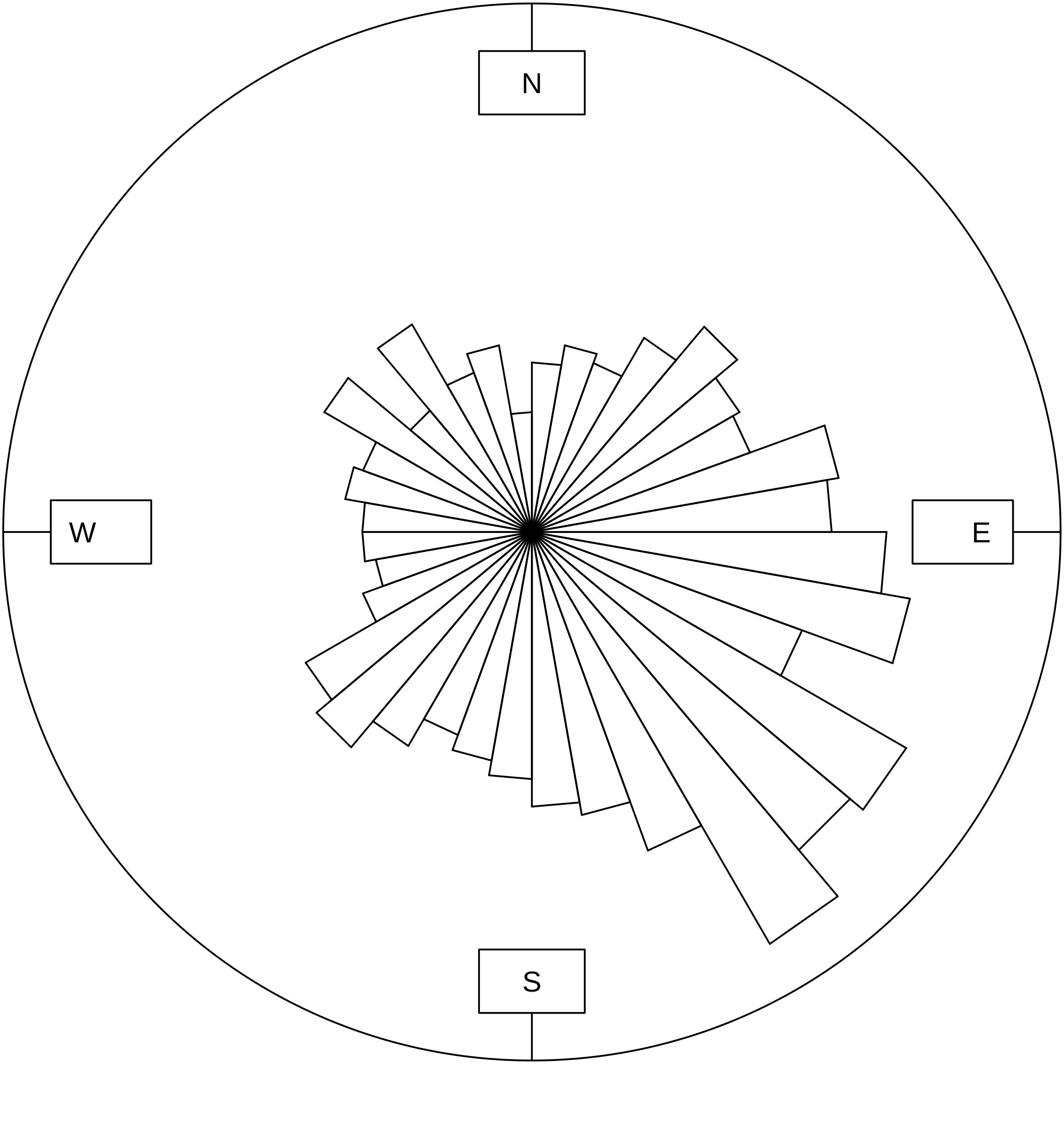

bootstrap 463

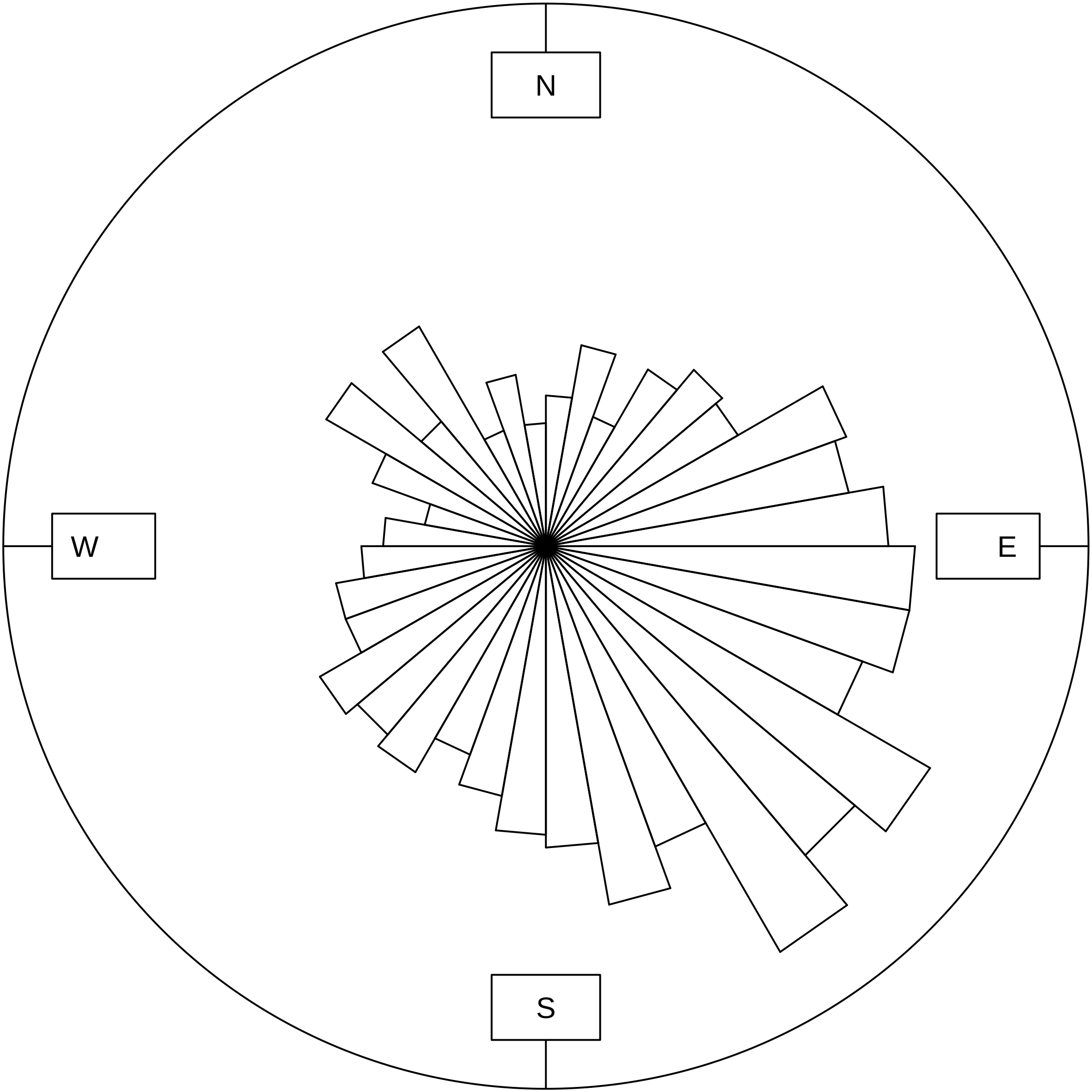

bootstrap 464

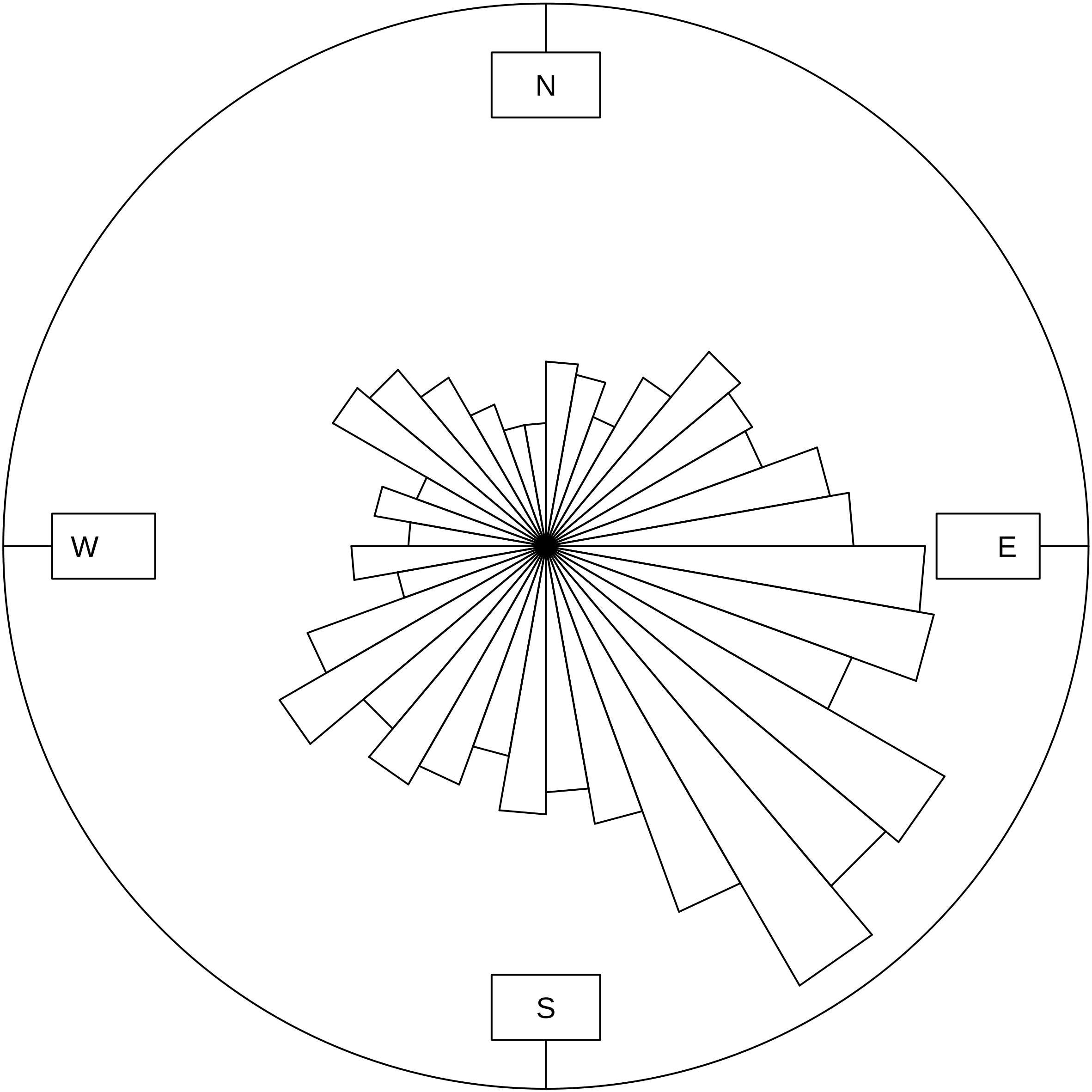

bootstrap 465

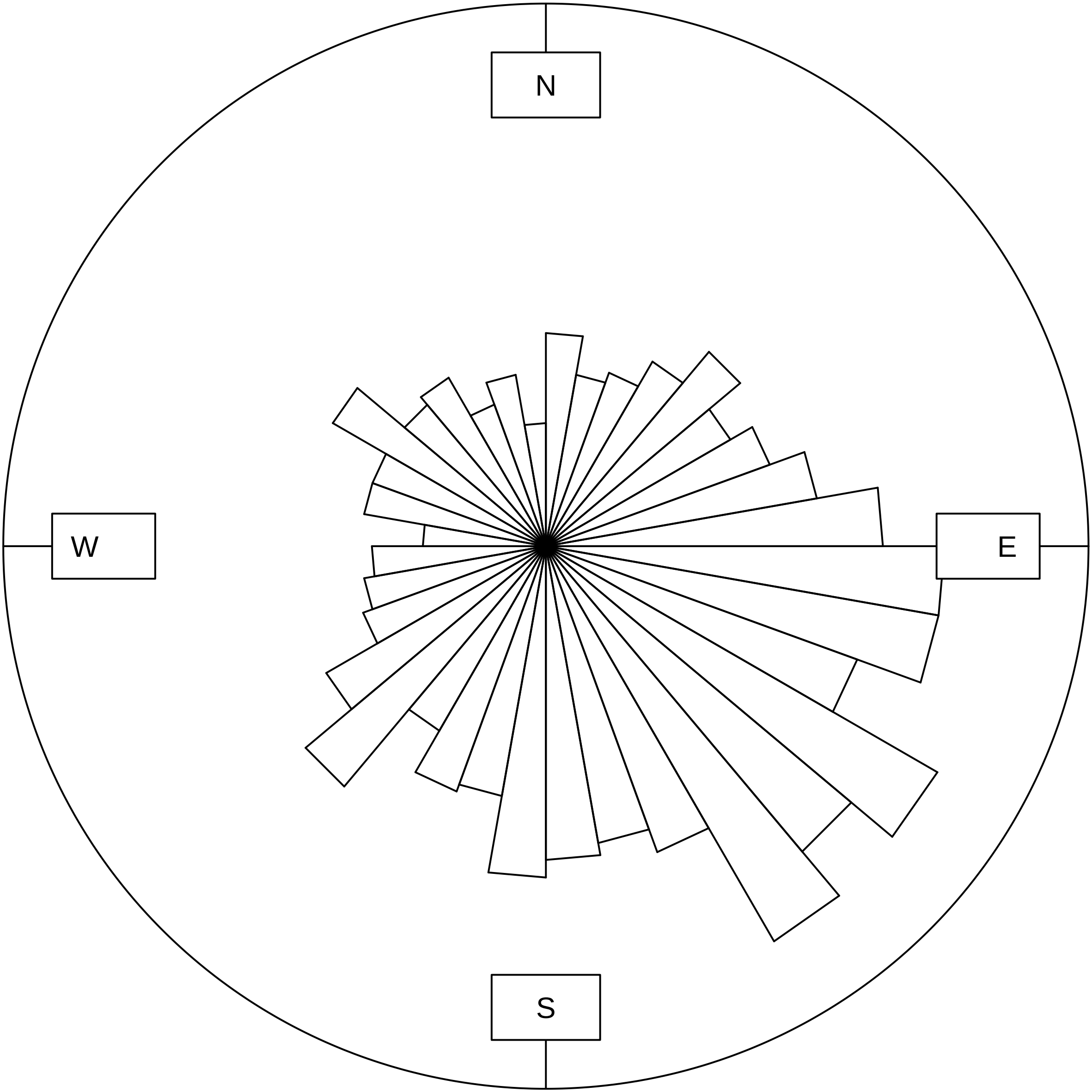

bootstrap 466

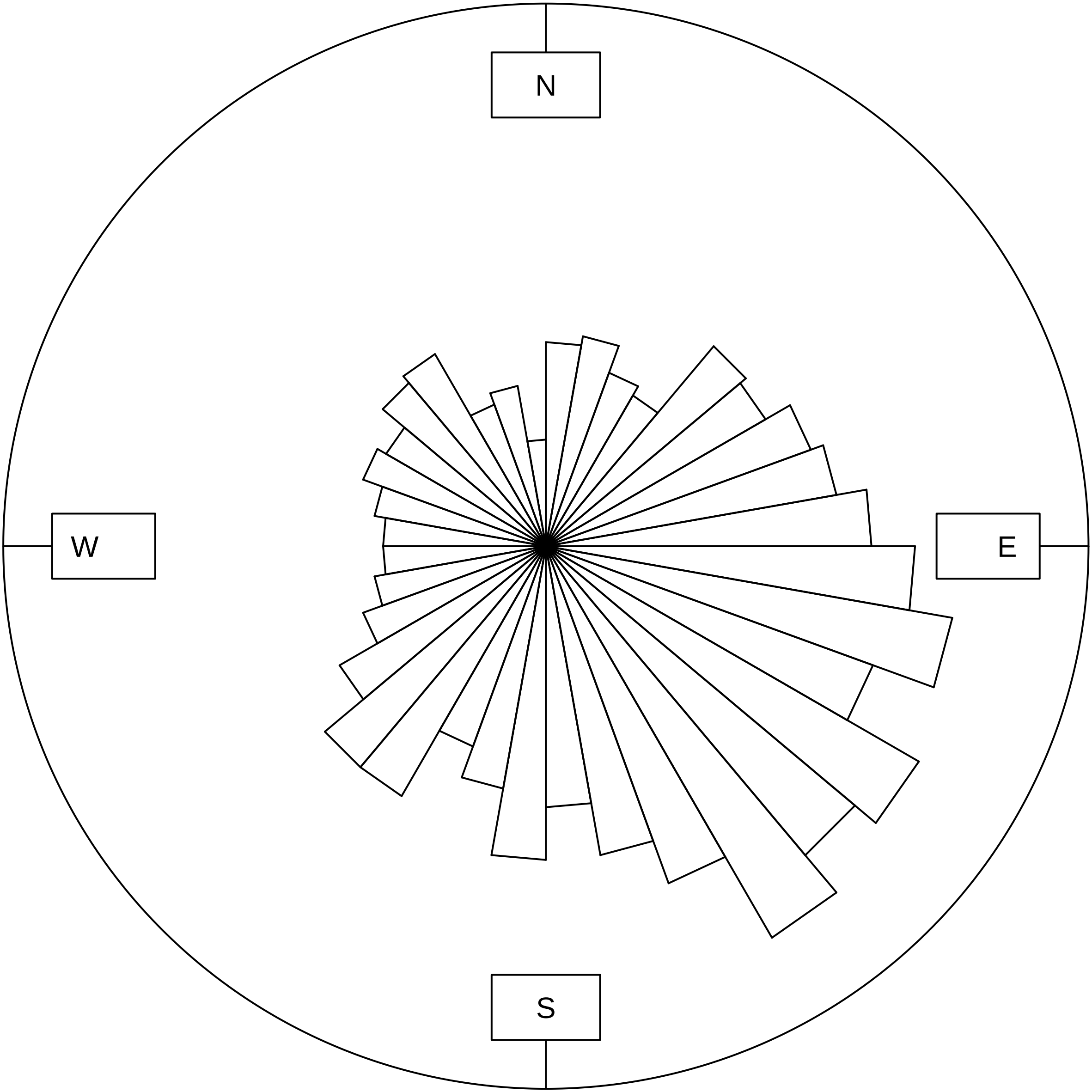

bootstrap 467

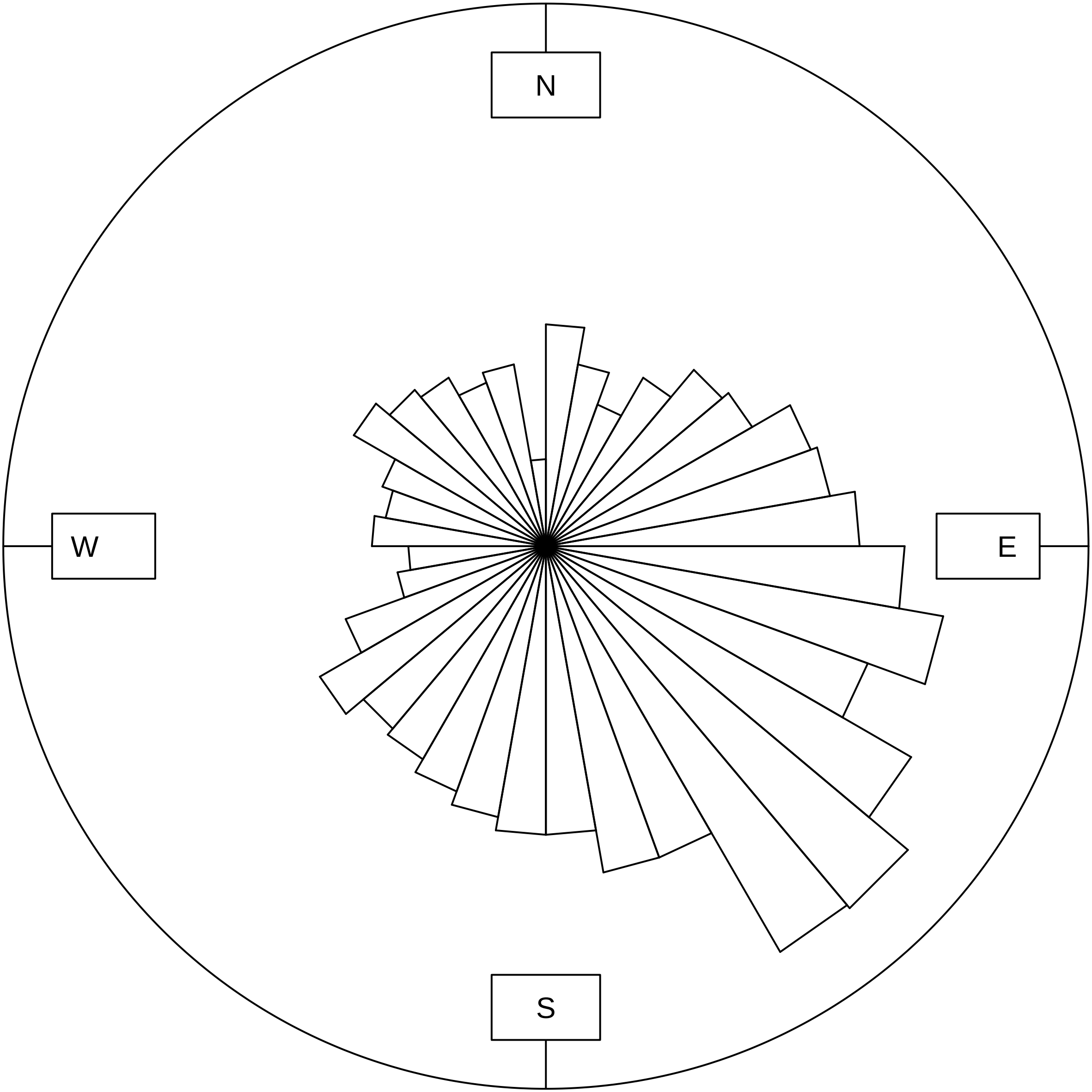

bootstrap 468

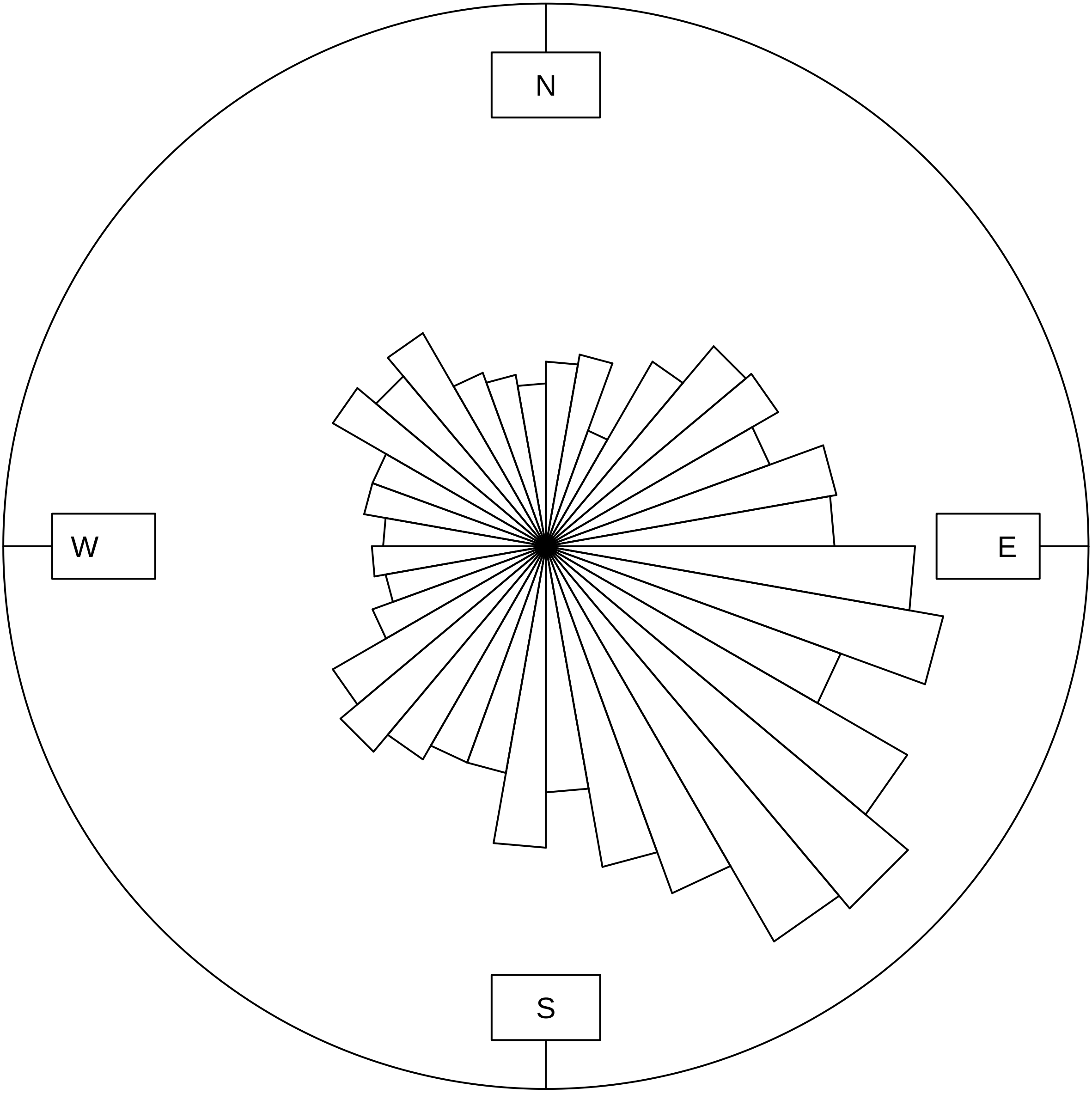

bootstrap 469

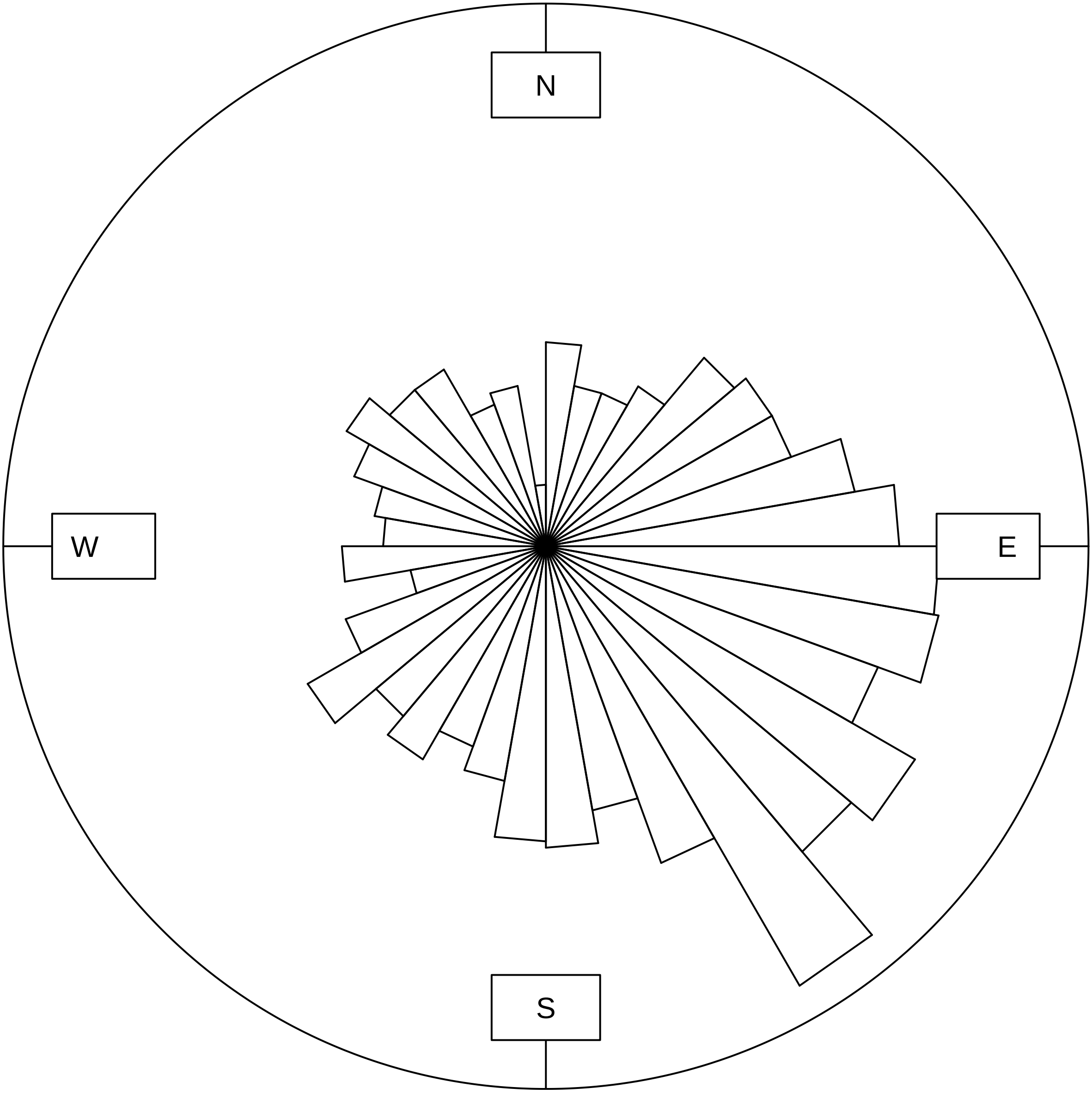

bootstrap 470

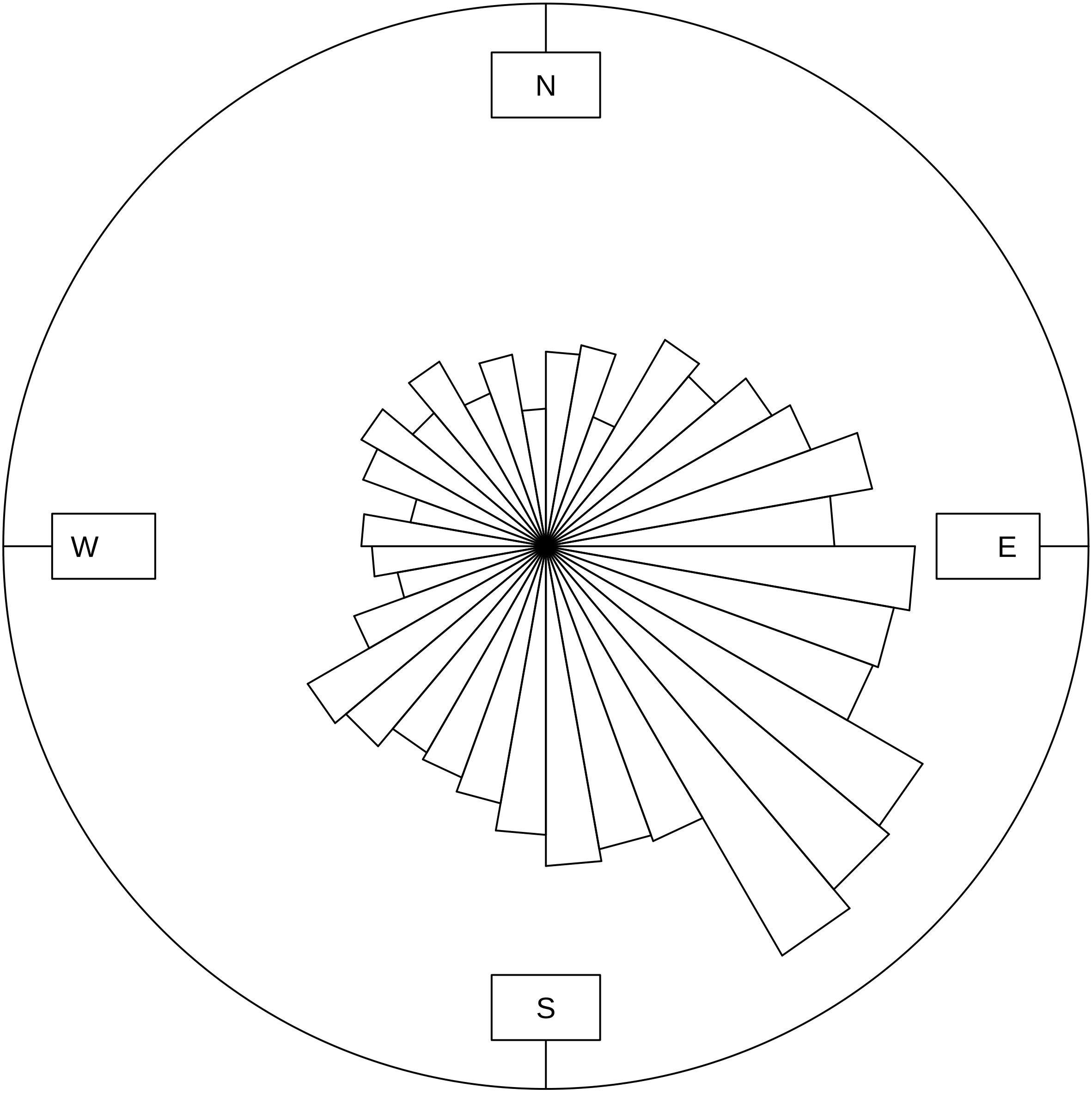

bootstrap 471

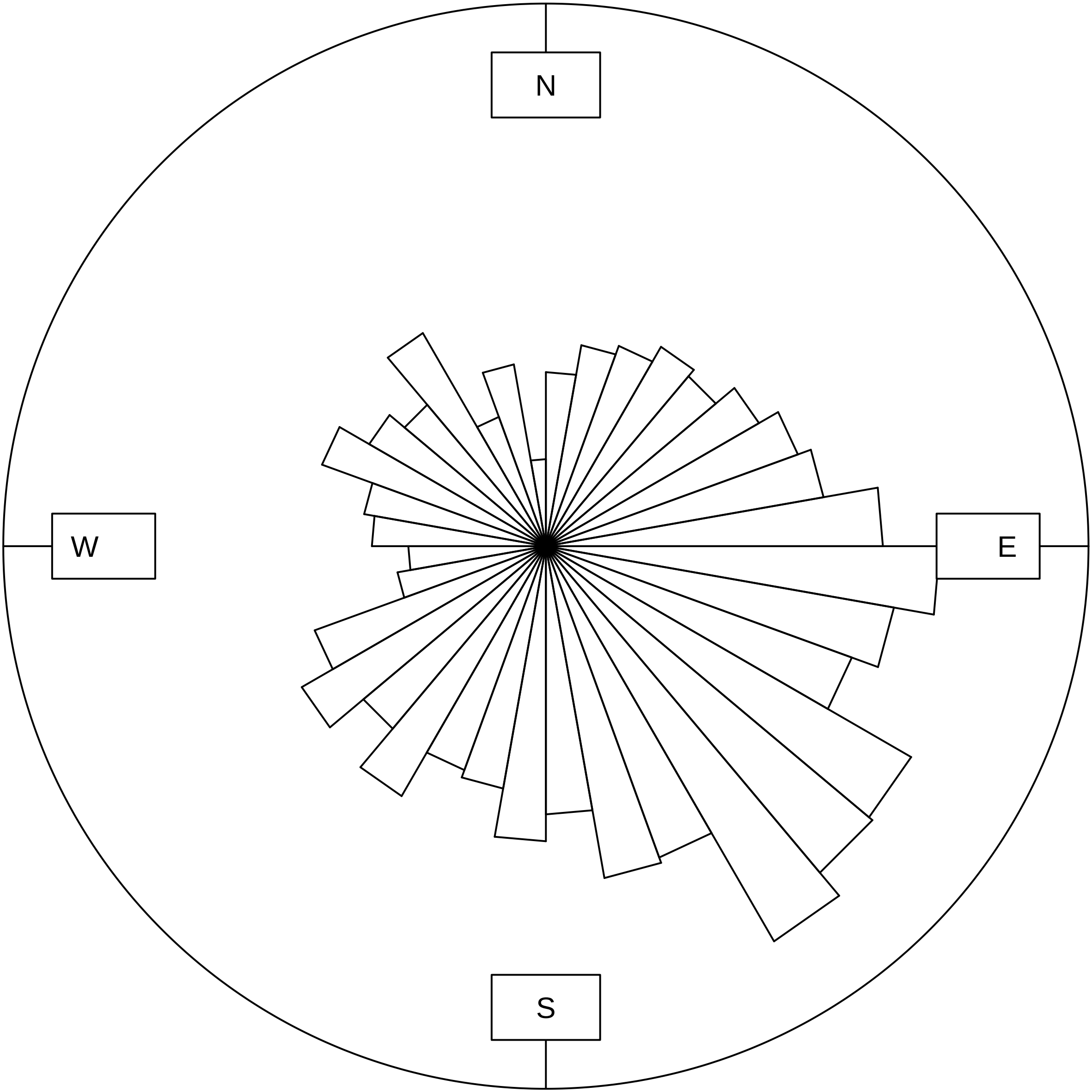

bootstrap 472

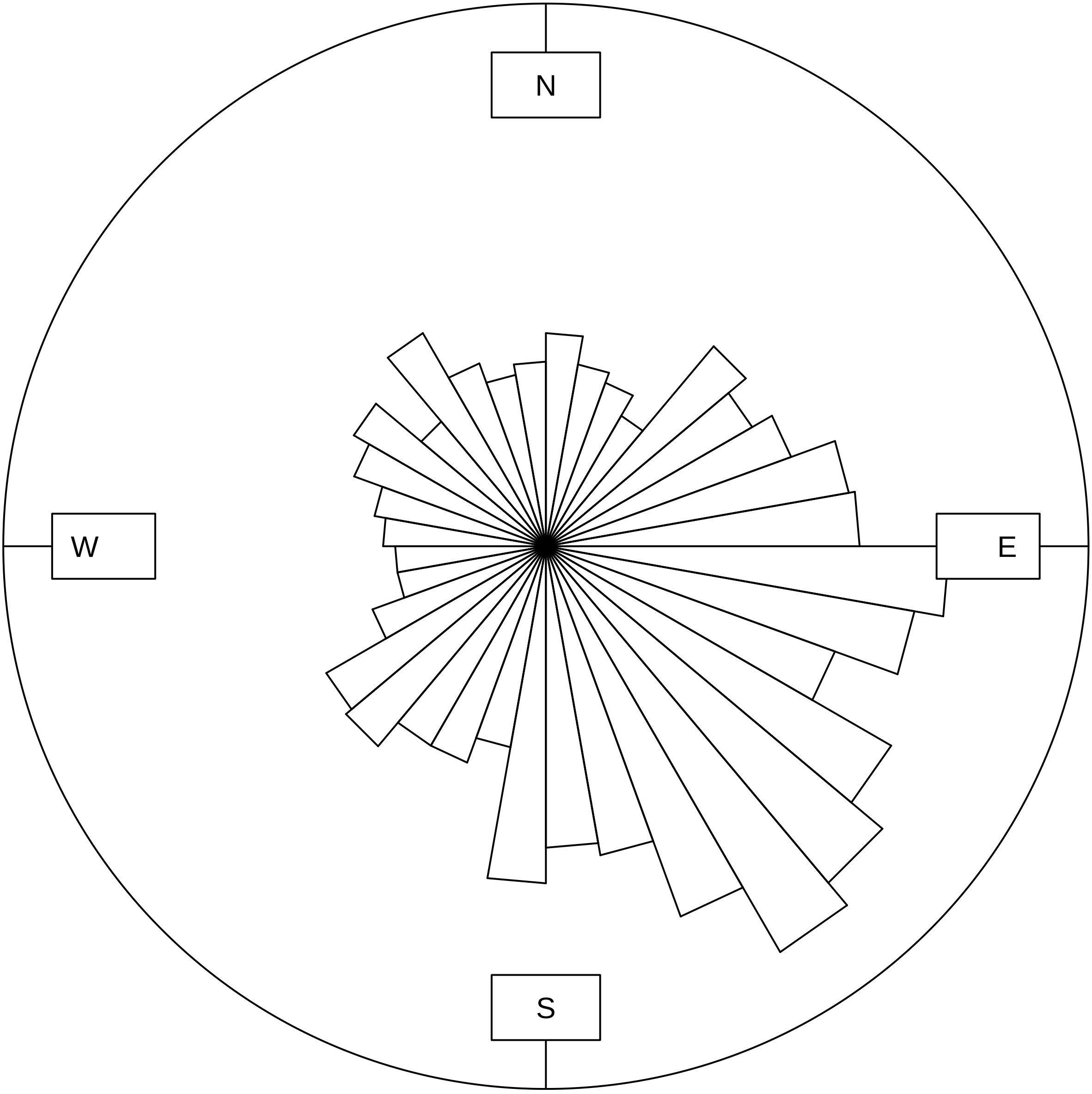

bootstrap 473

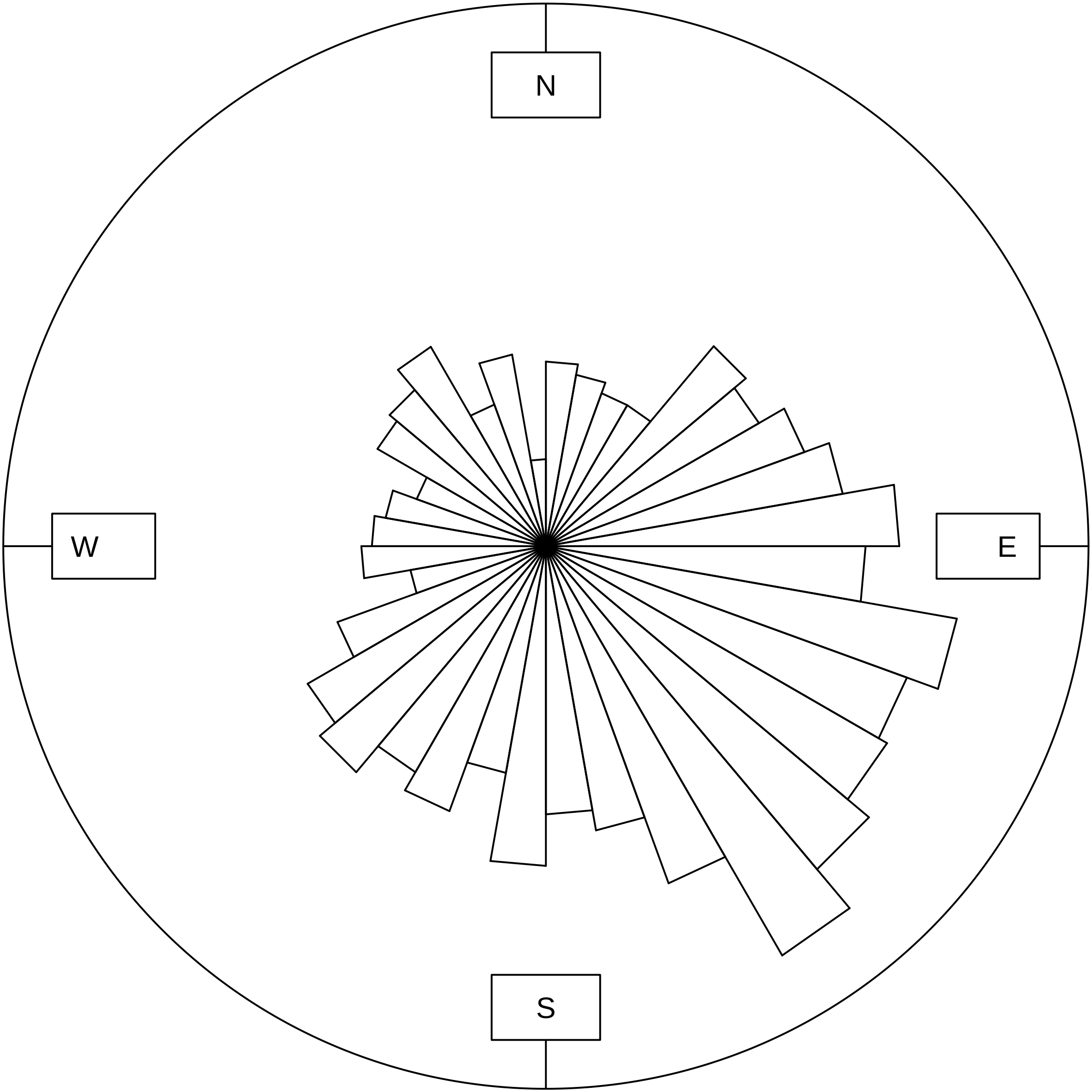

bootstrap 474

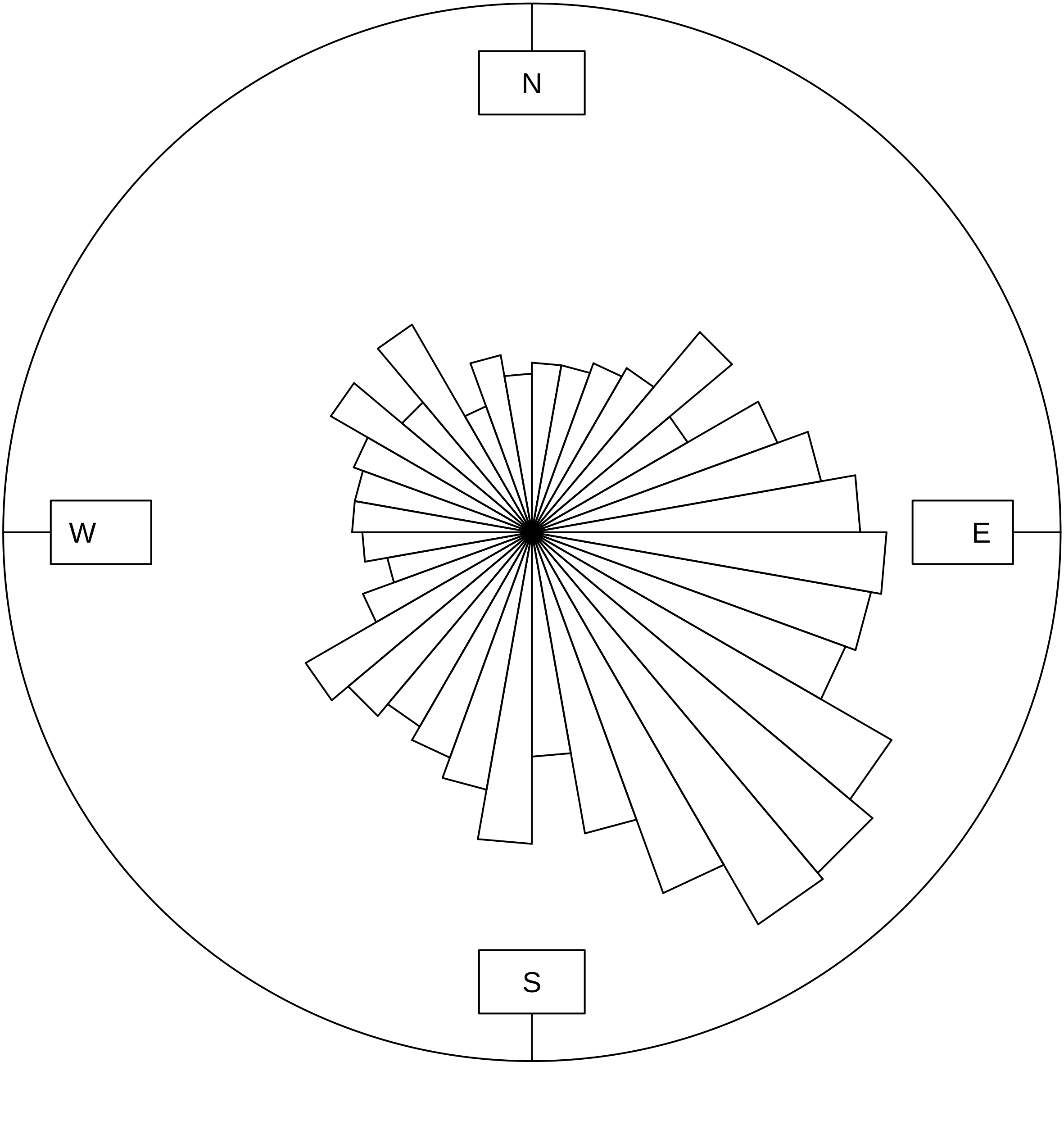

bootstrap 475

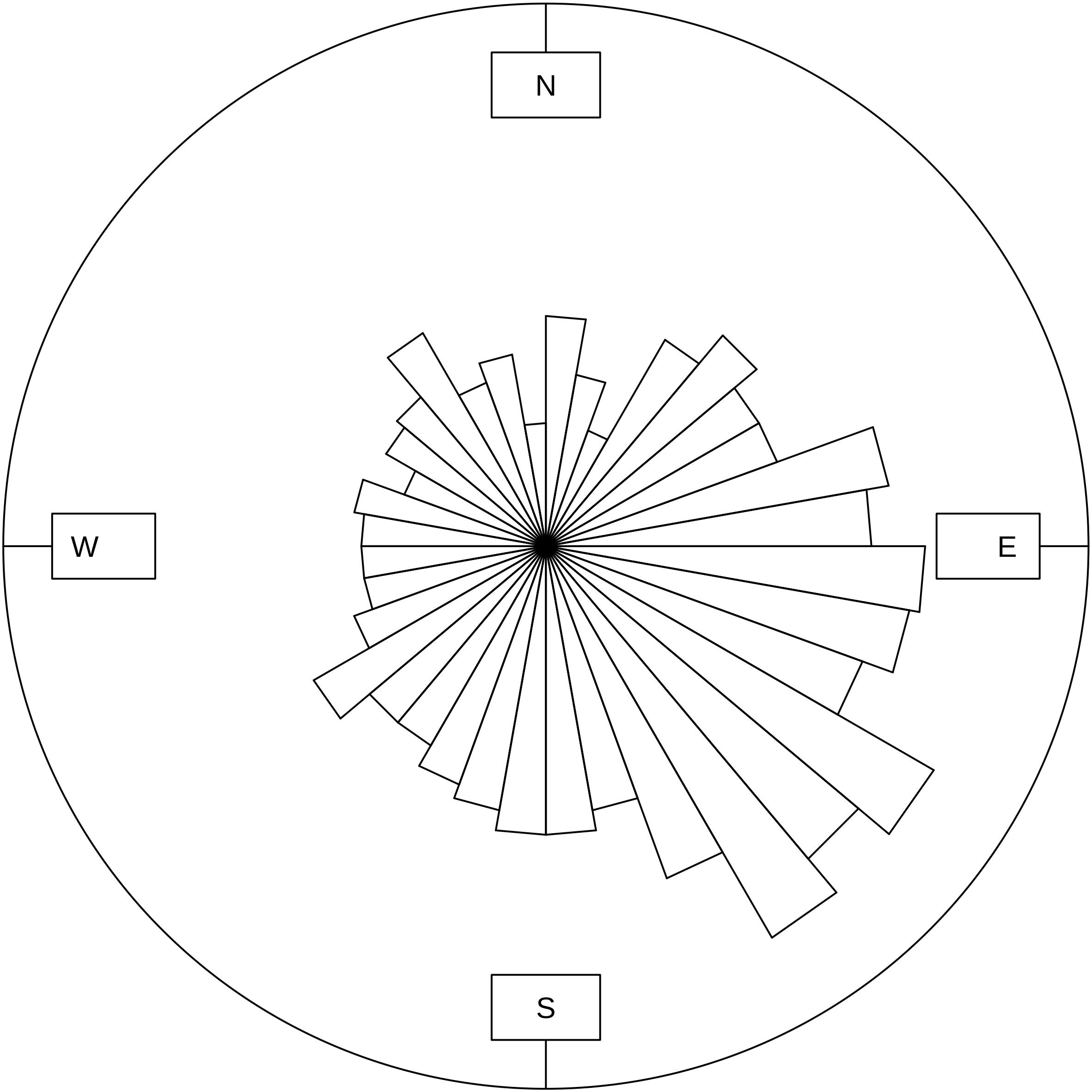

bootstrap 476

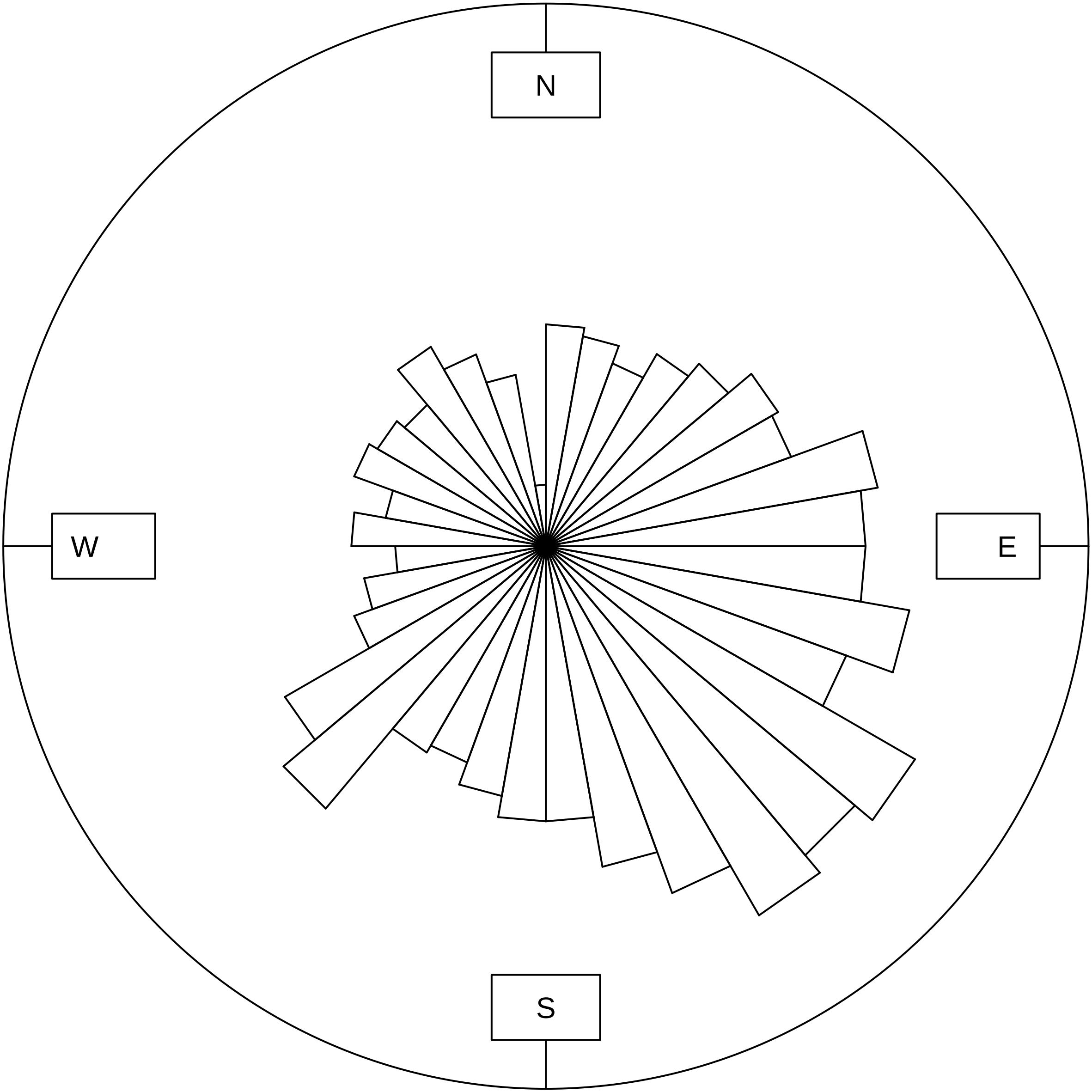

bootstrap 477

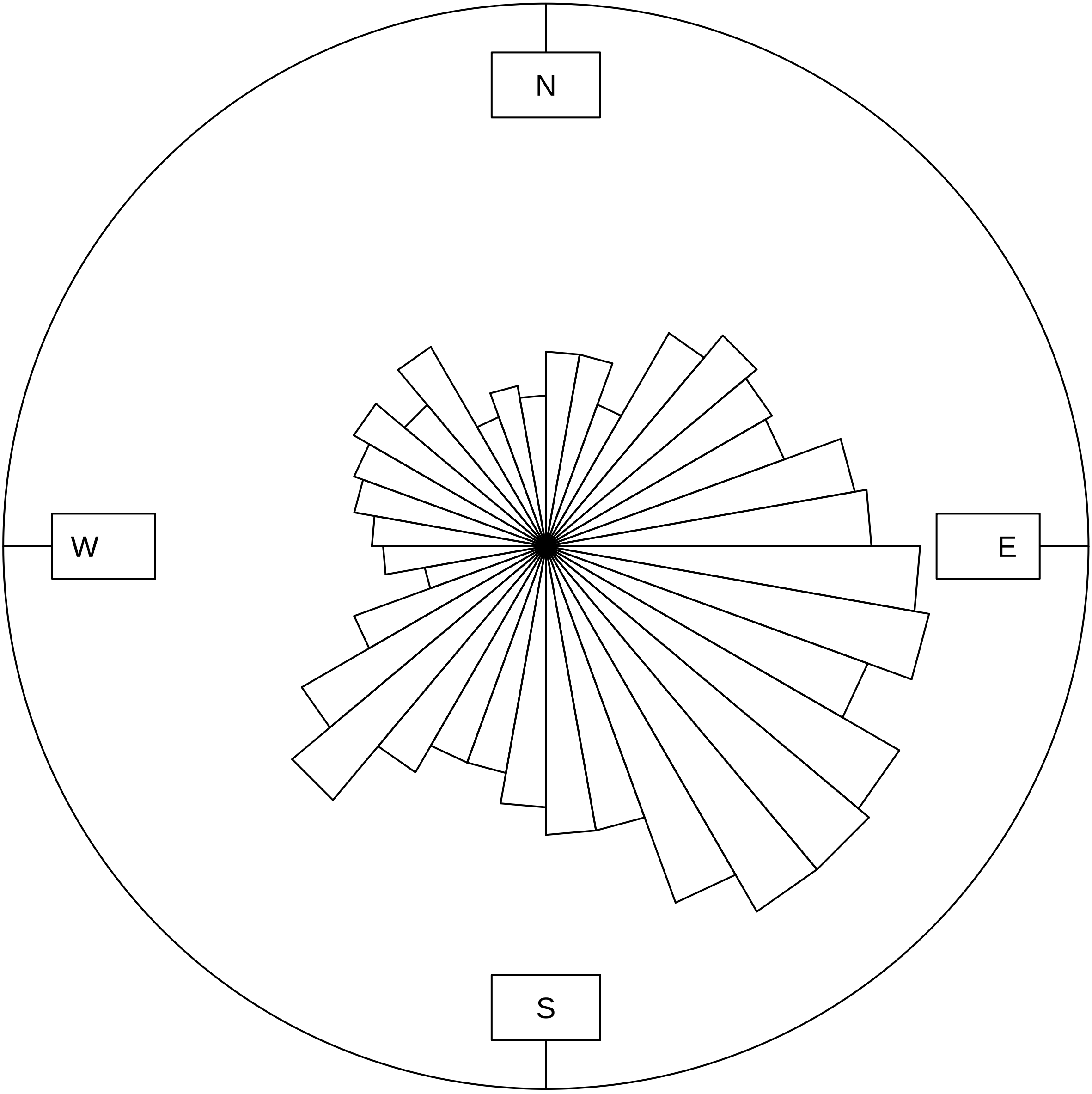

bootstrap 478

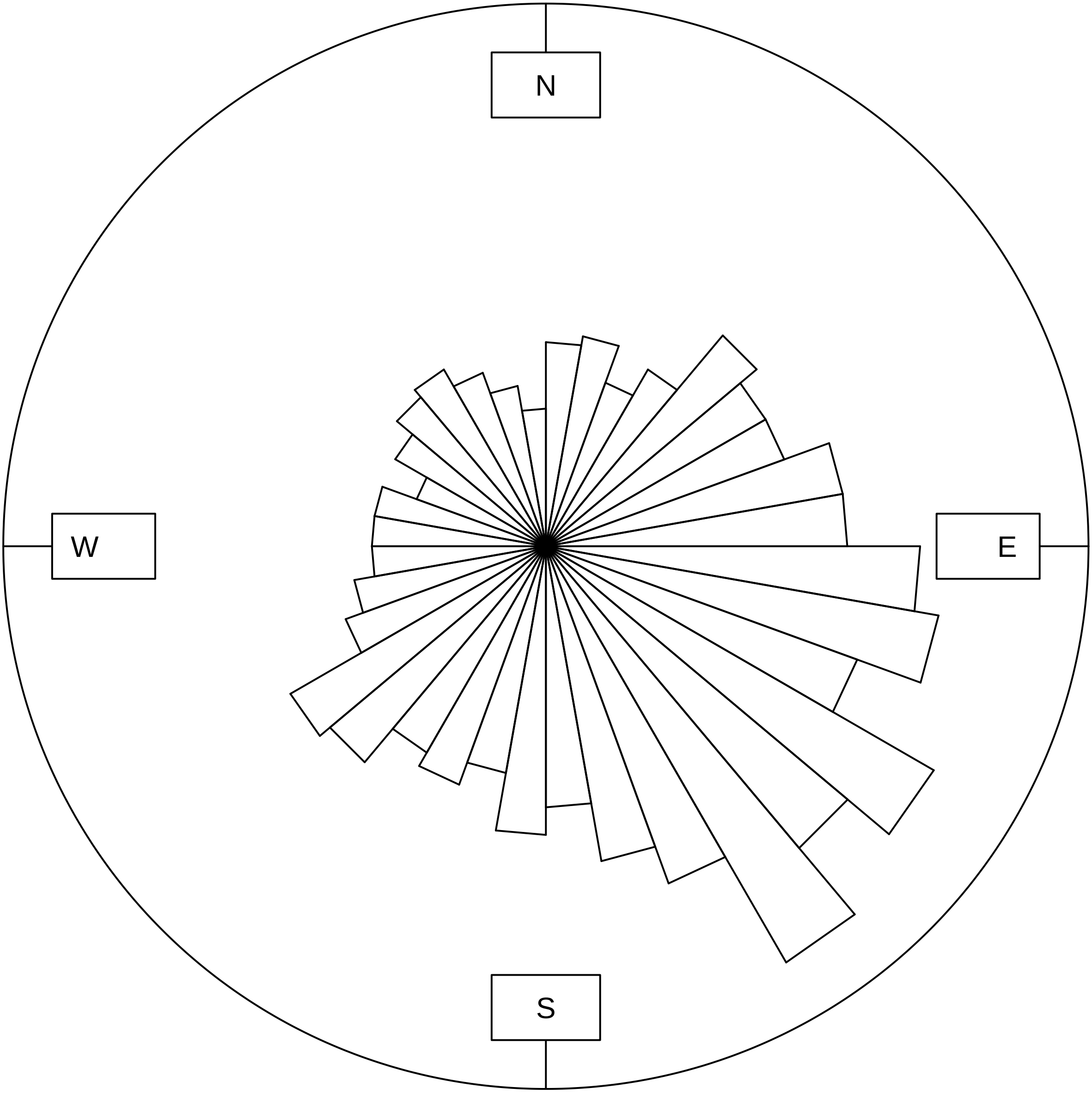

bootstrap 479

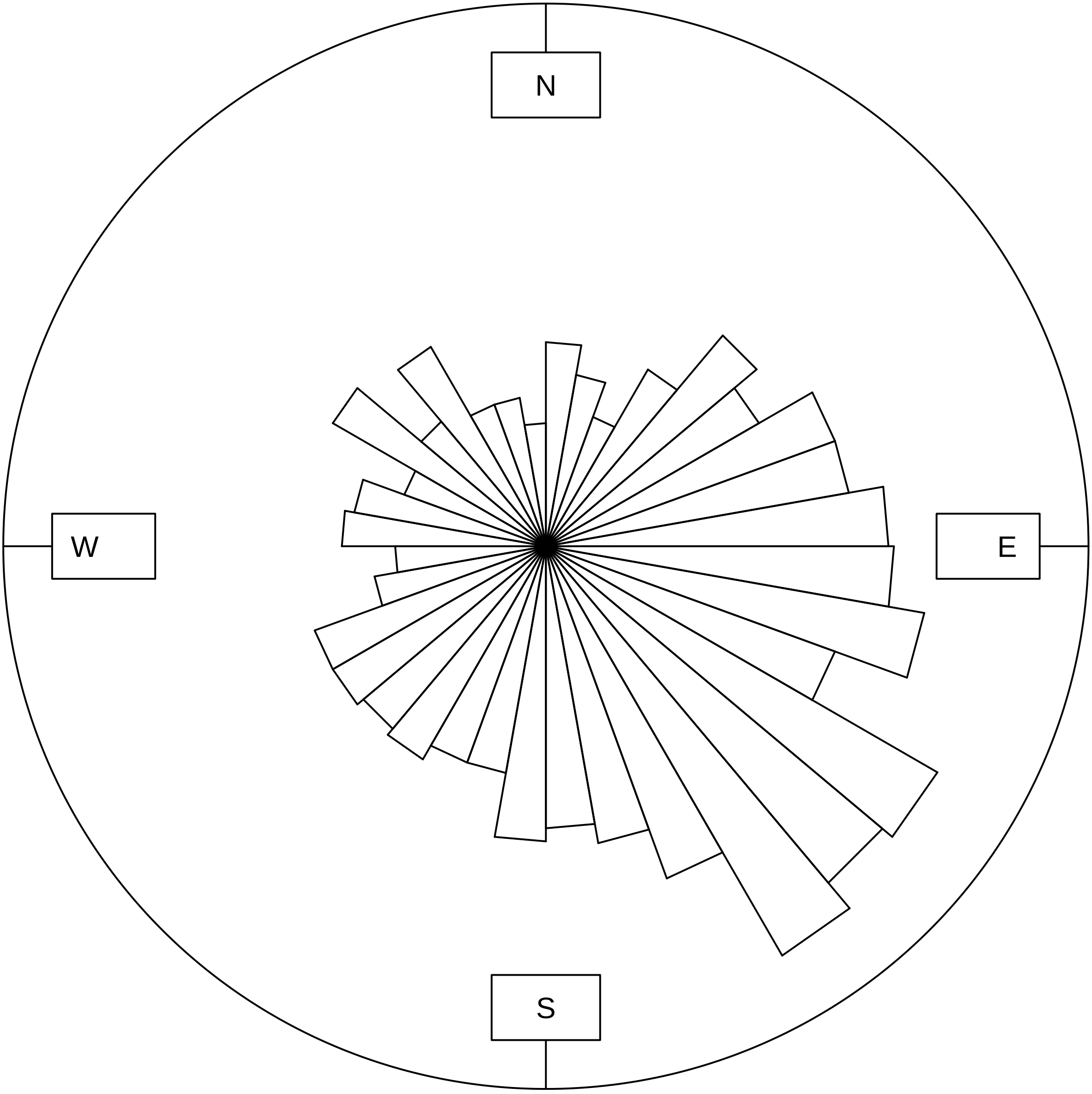

bootstrap 480

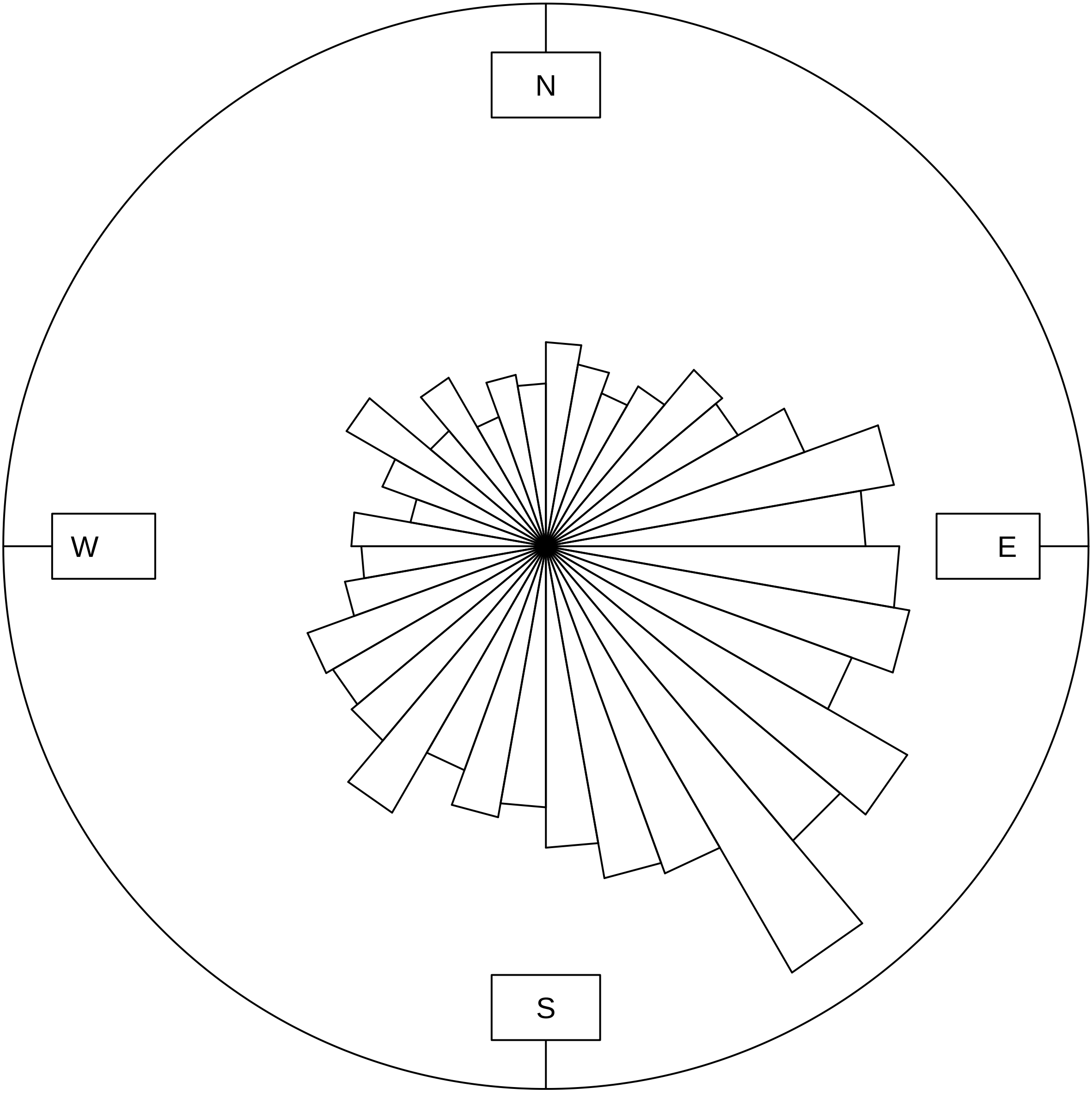

bootstrap 481

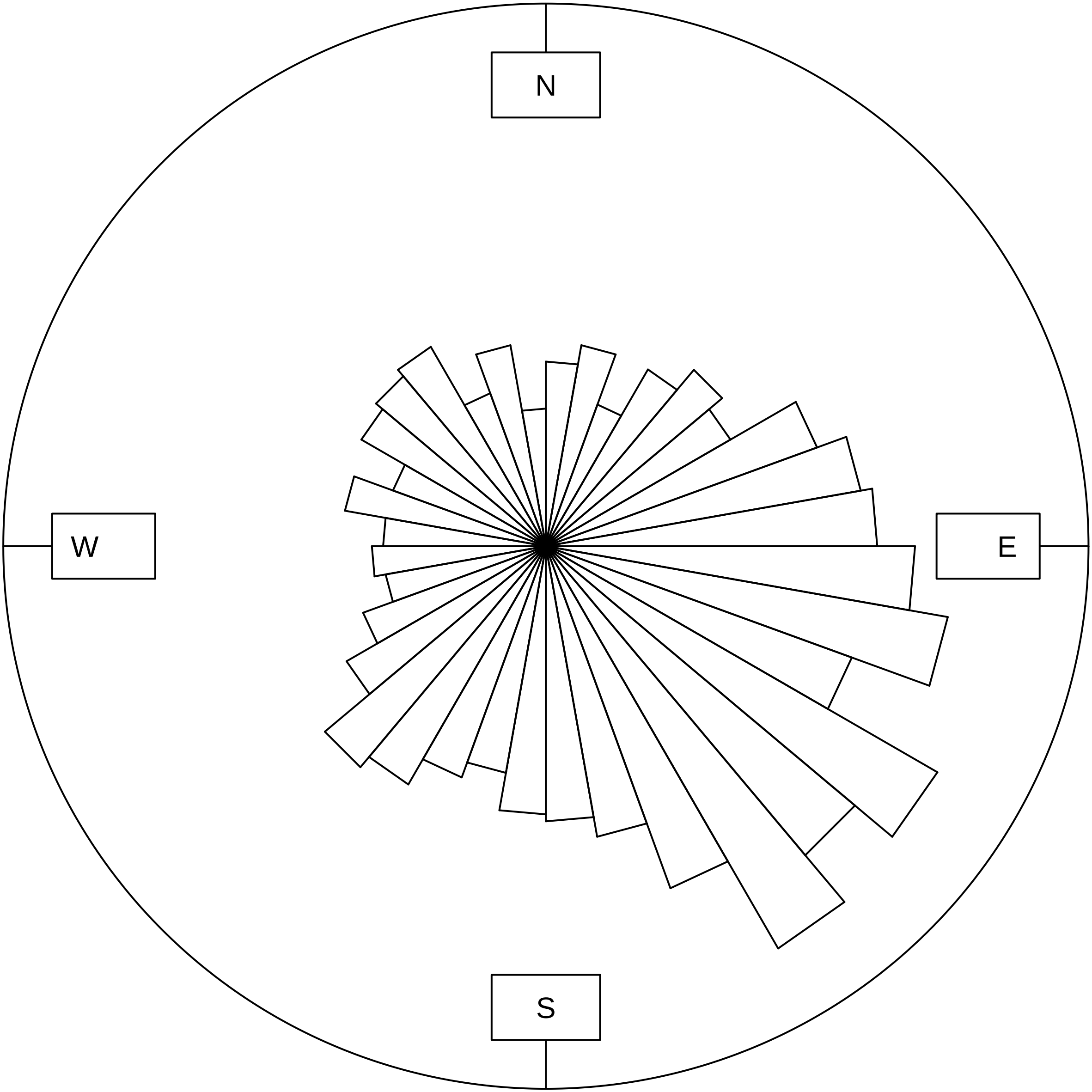

bootstrap 482

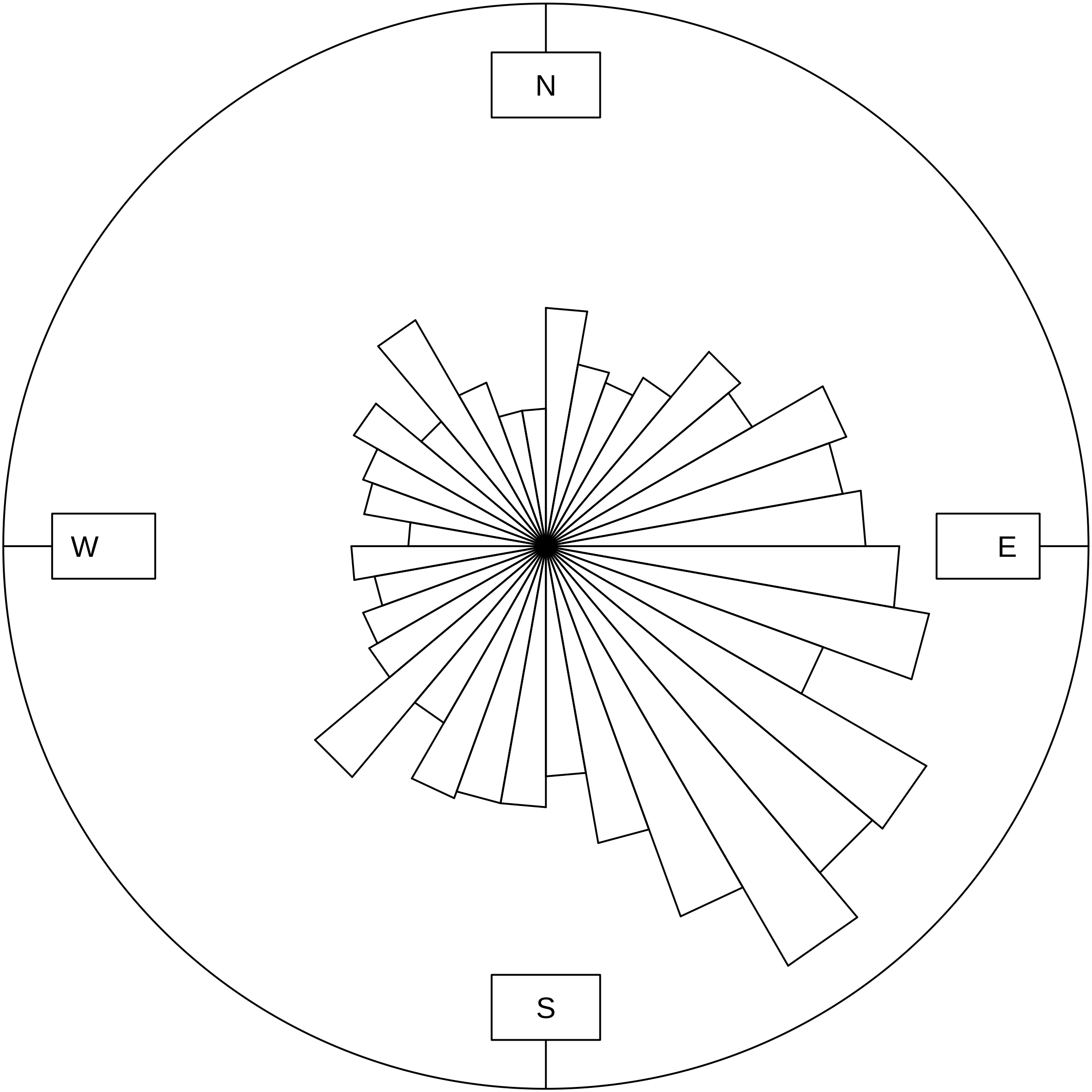

bootstrap 483

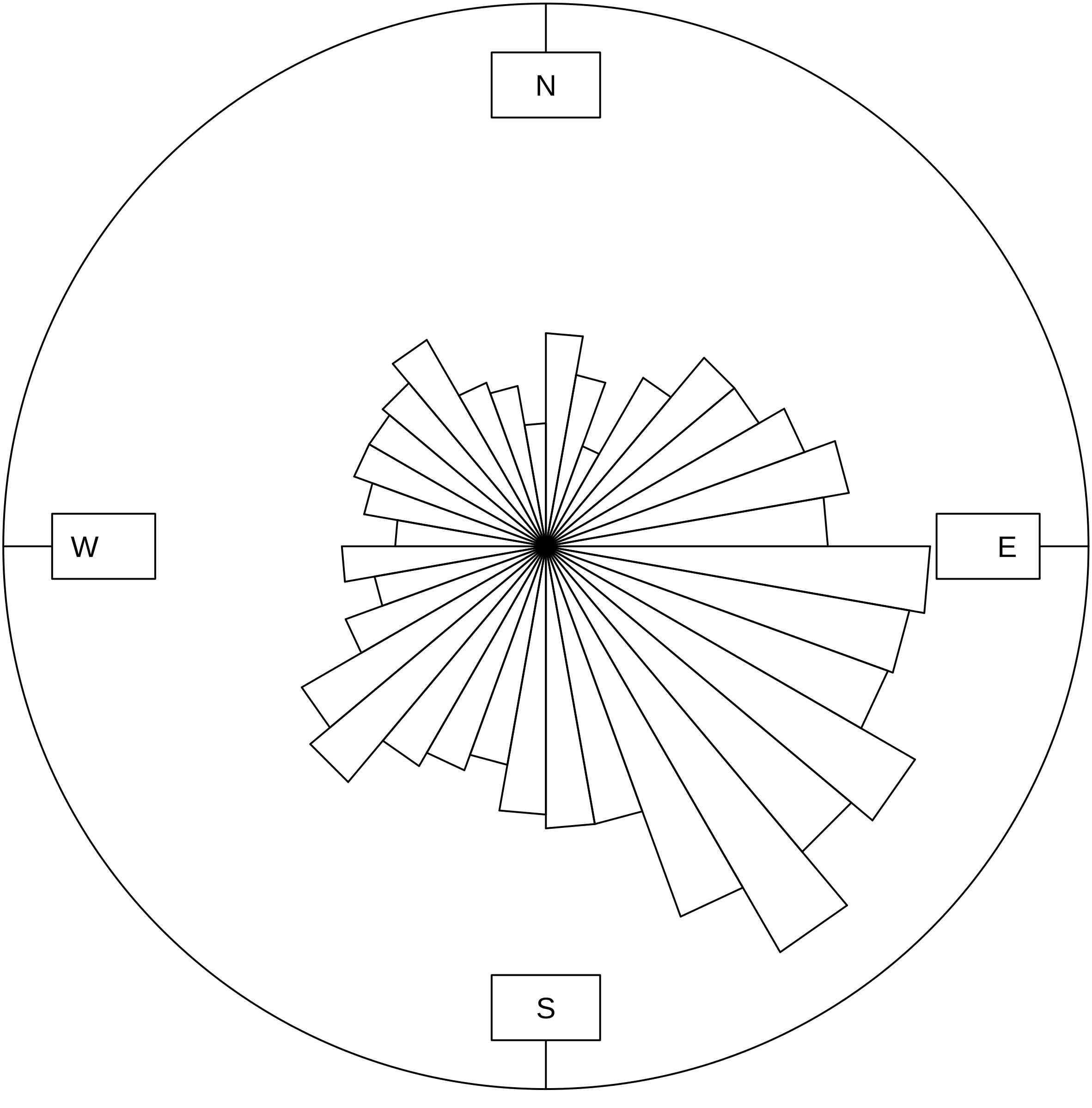

bootstrap 484

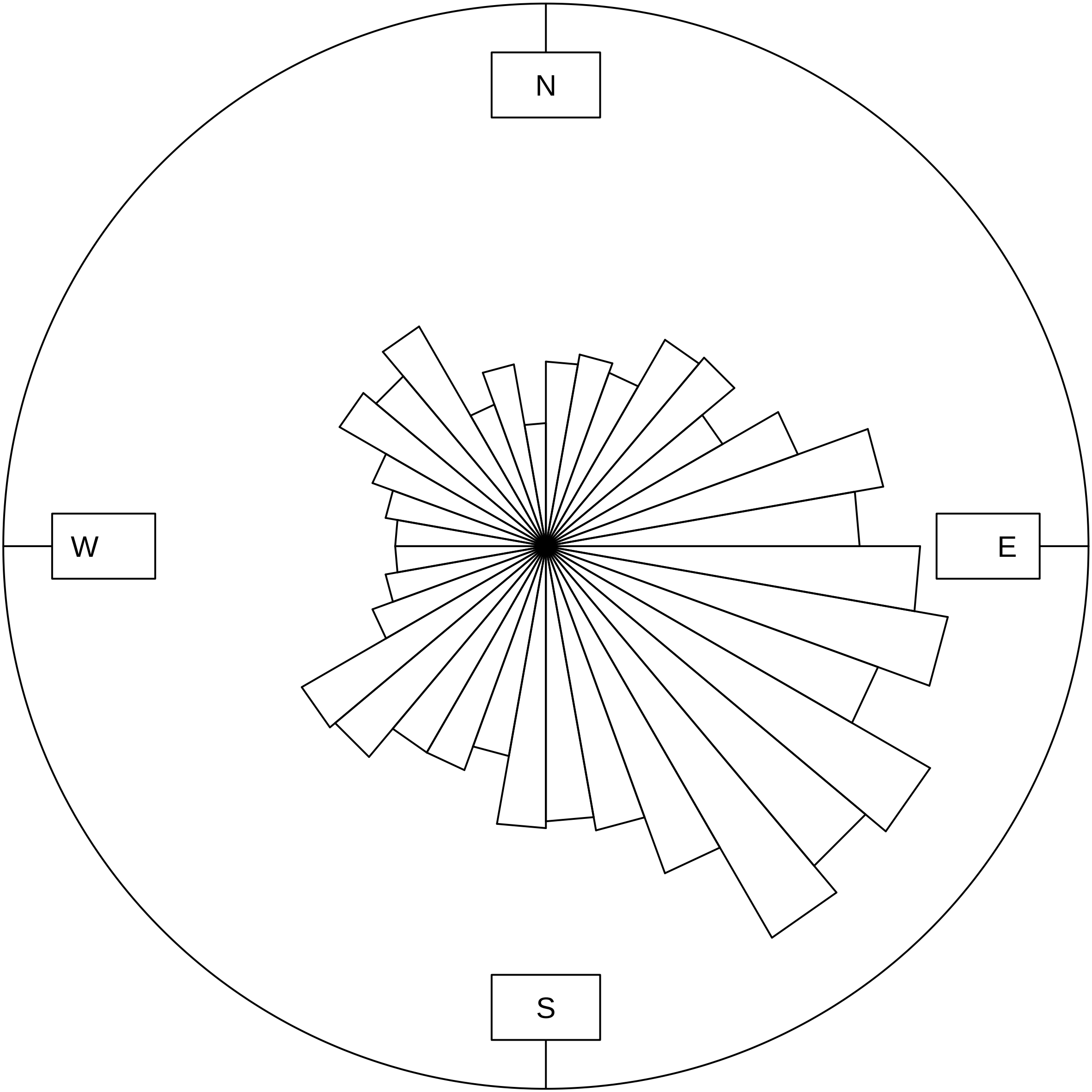

bootstrap 485

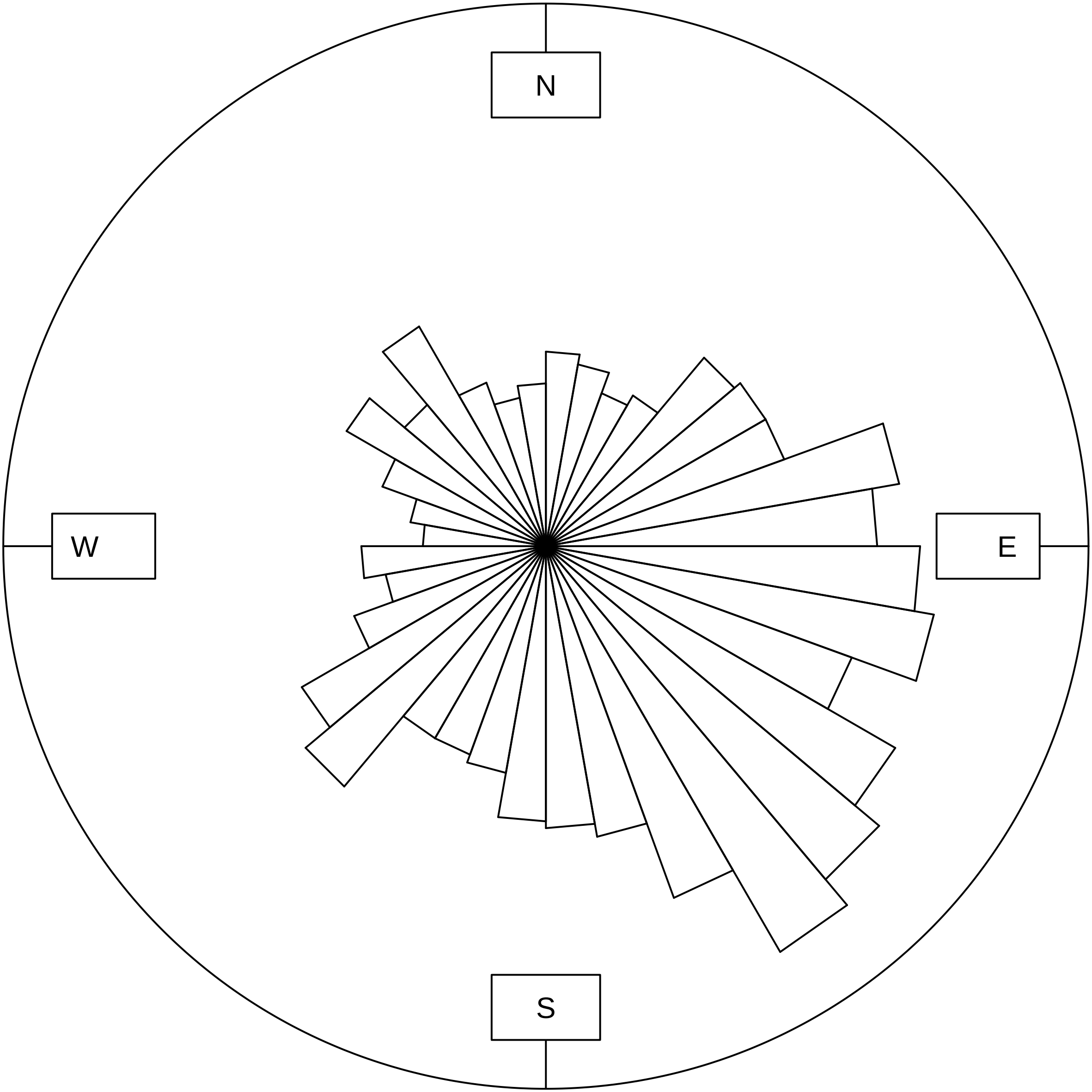

bootstrap 486

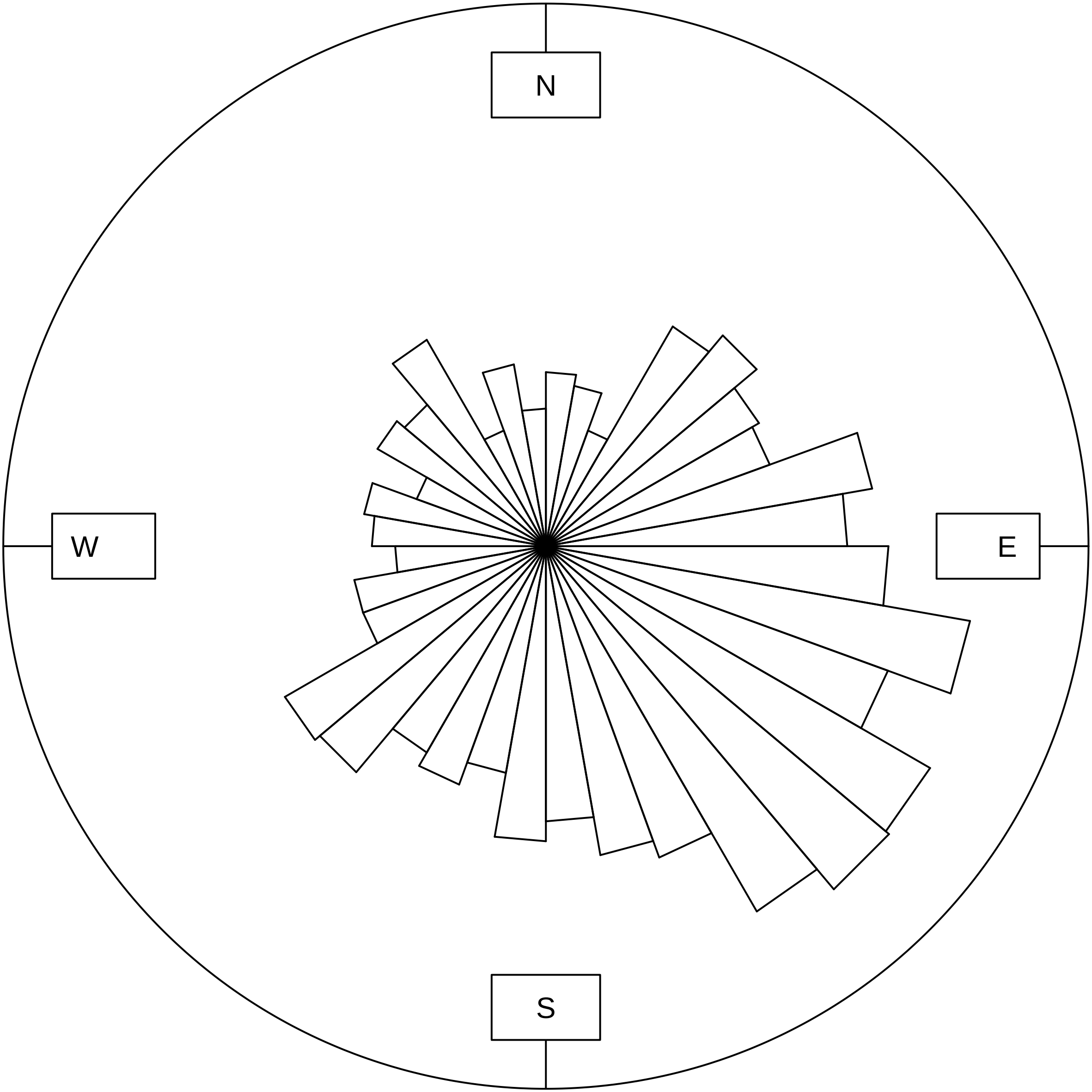

bootstrap 487

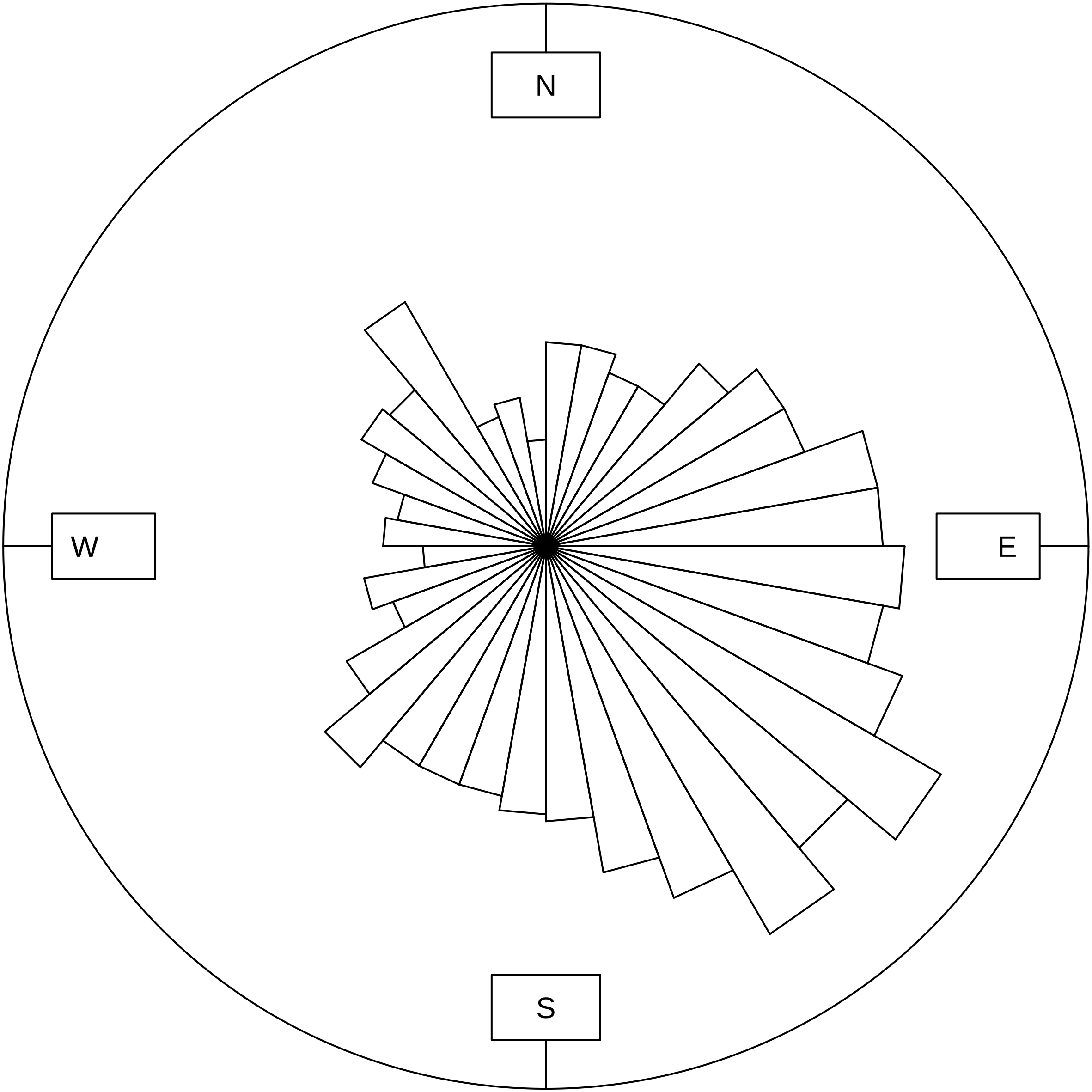

bootstrap 488

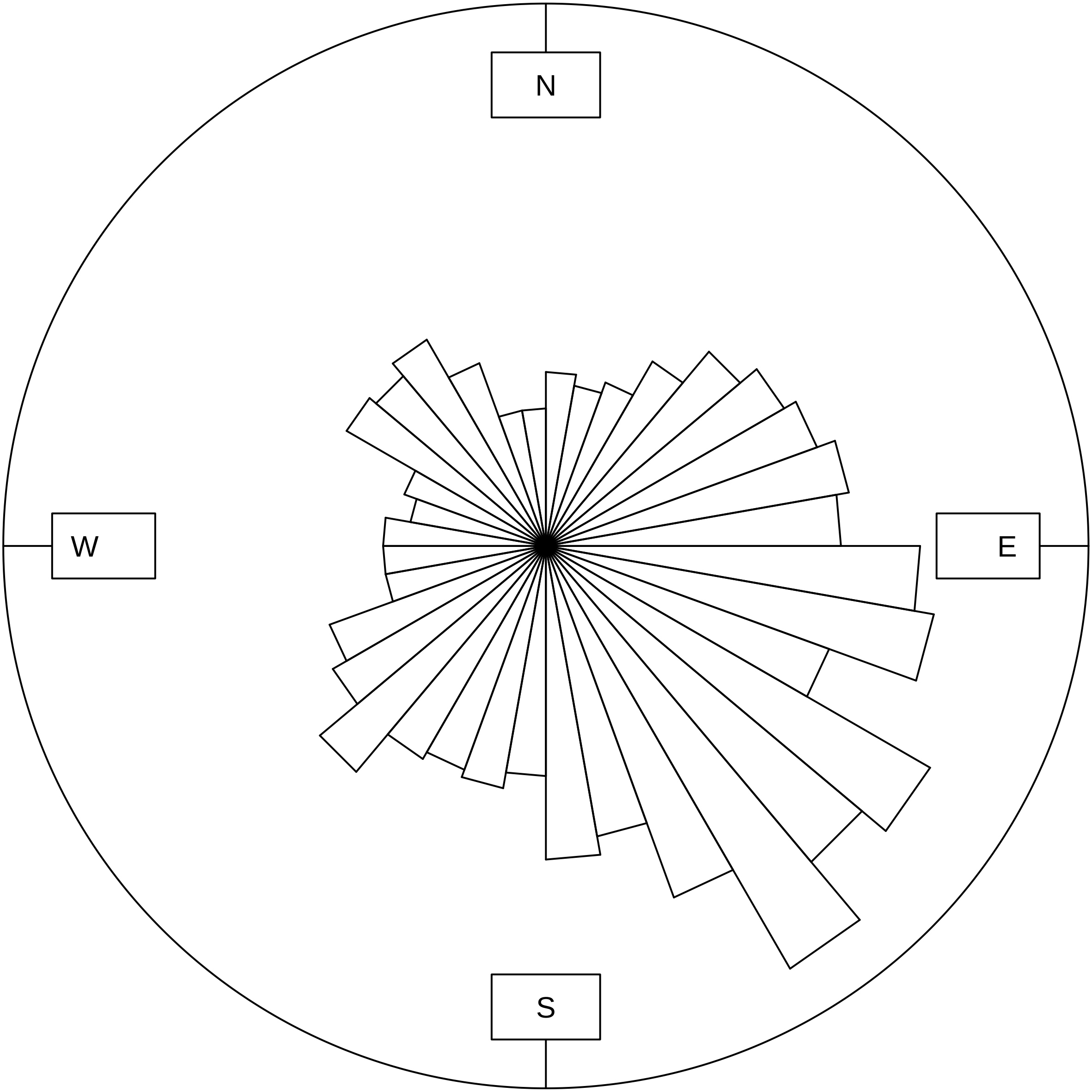

bootstrap 489

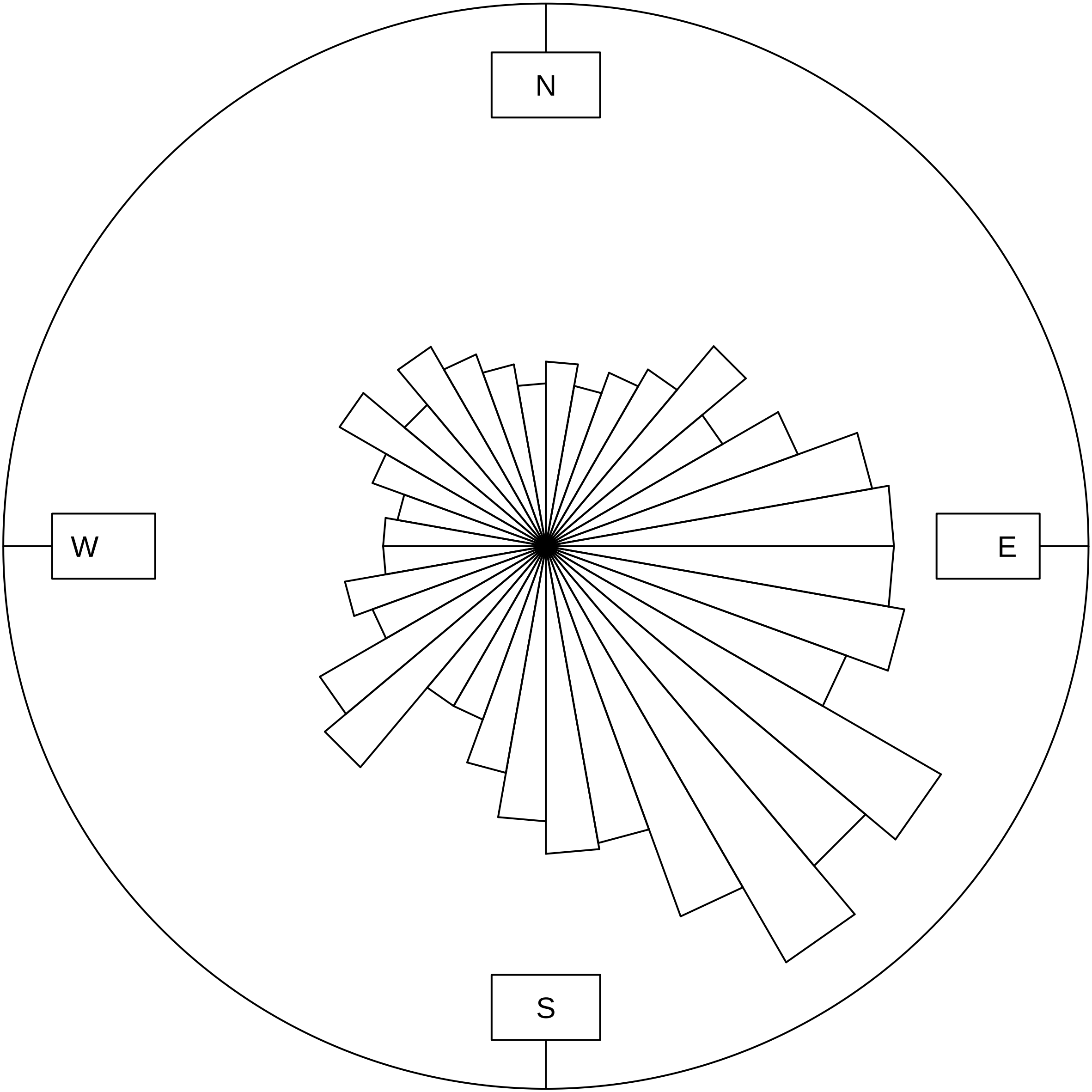

bootstrap 490

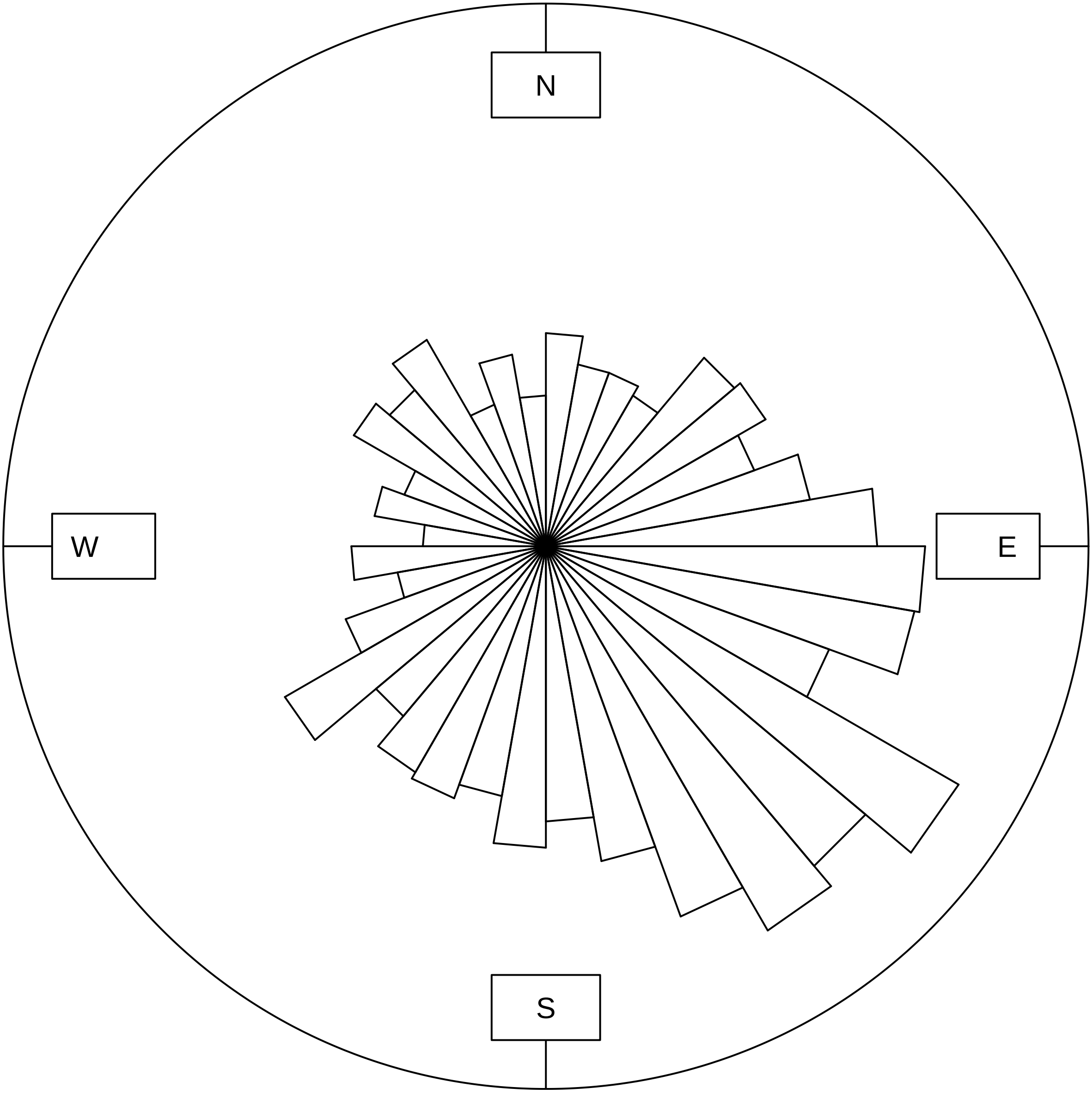

bootstrap 491

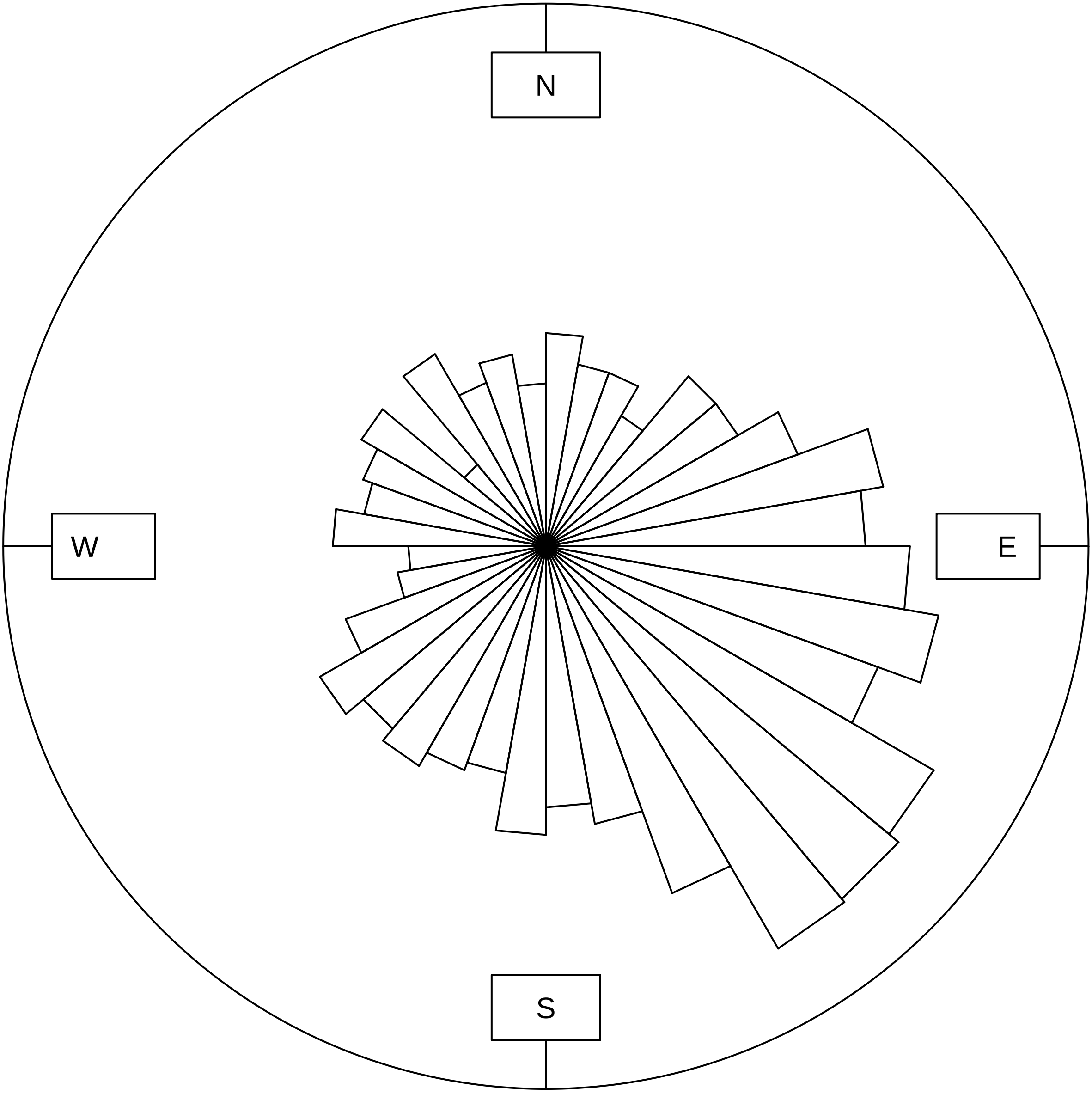

bootstrap 492

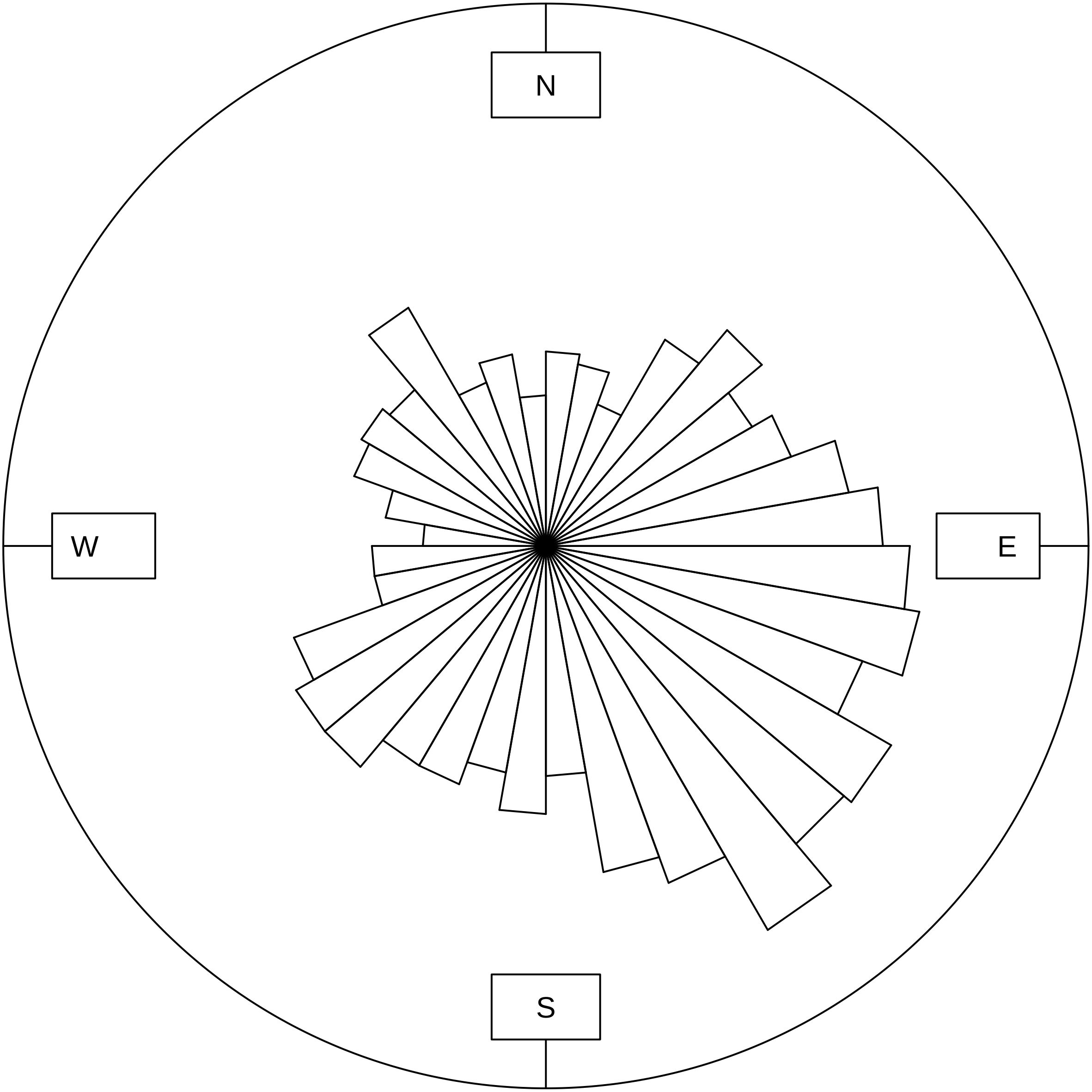

bootstrap 493

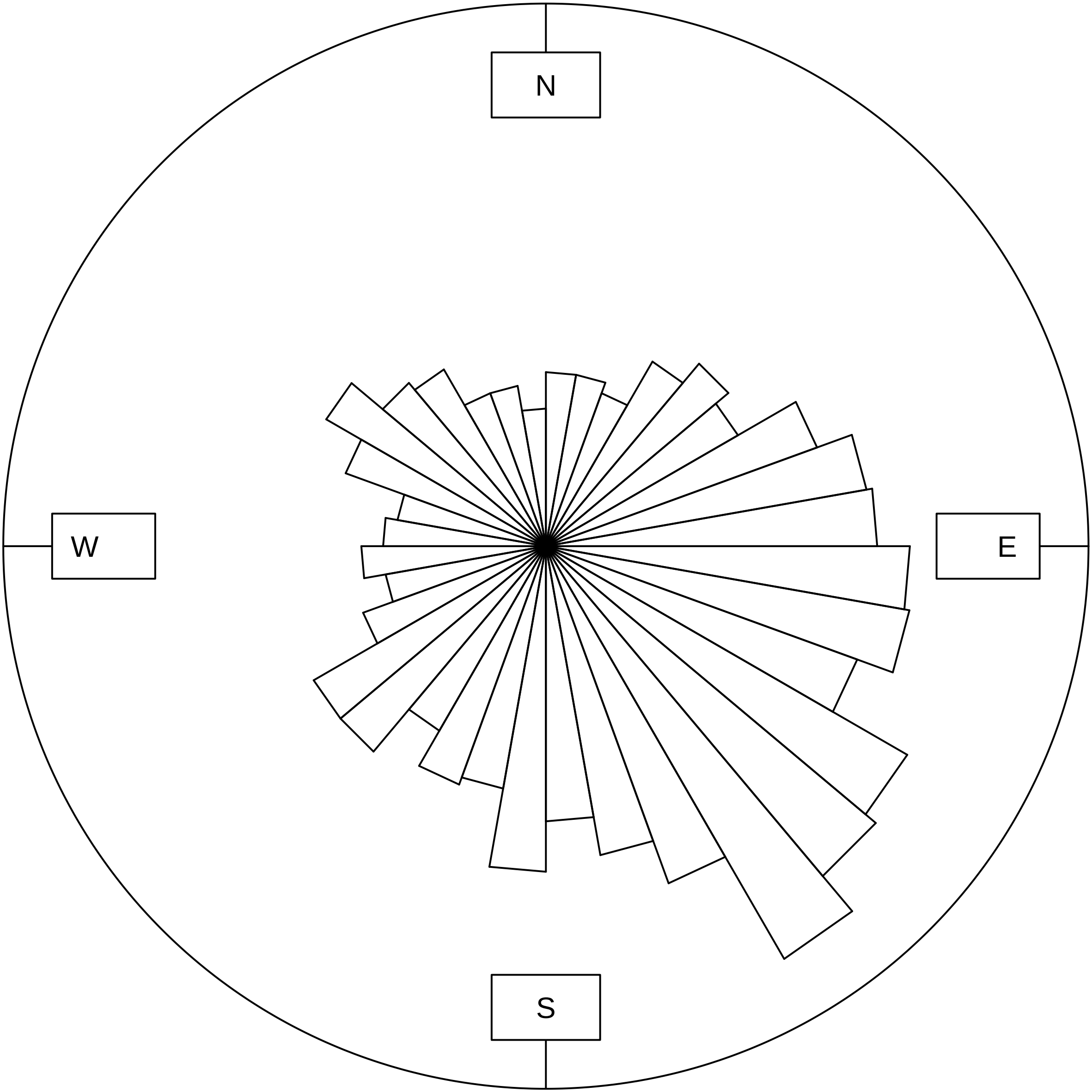

bootstrap 494

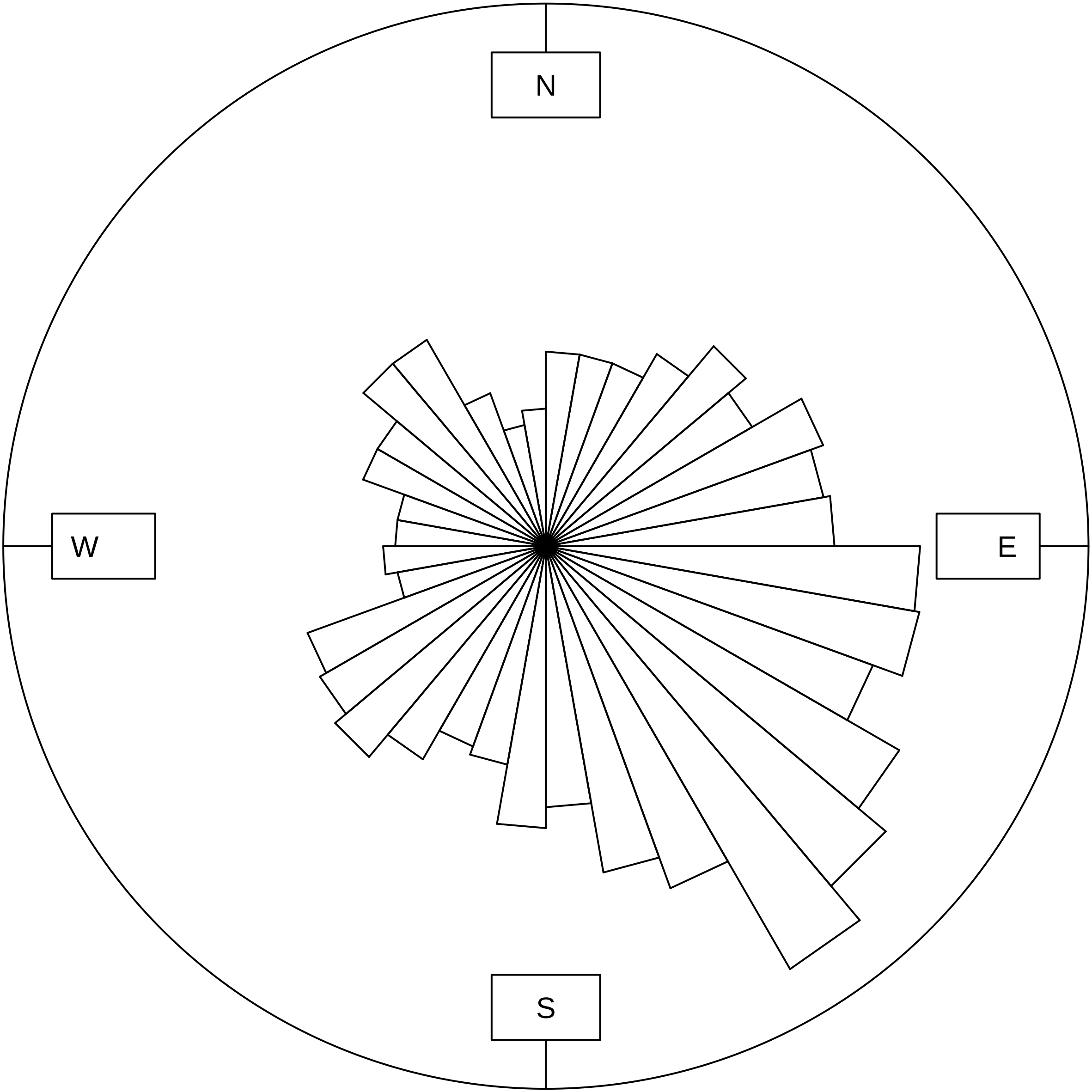

bootstrap 495

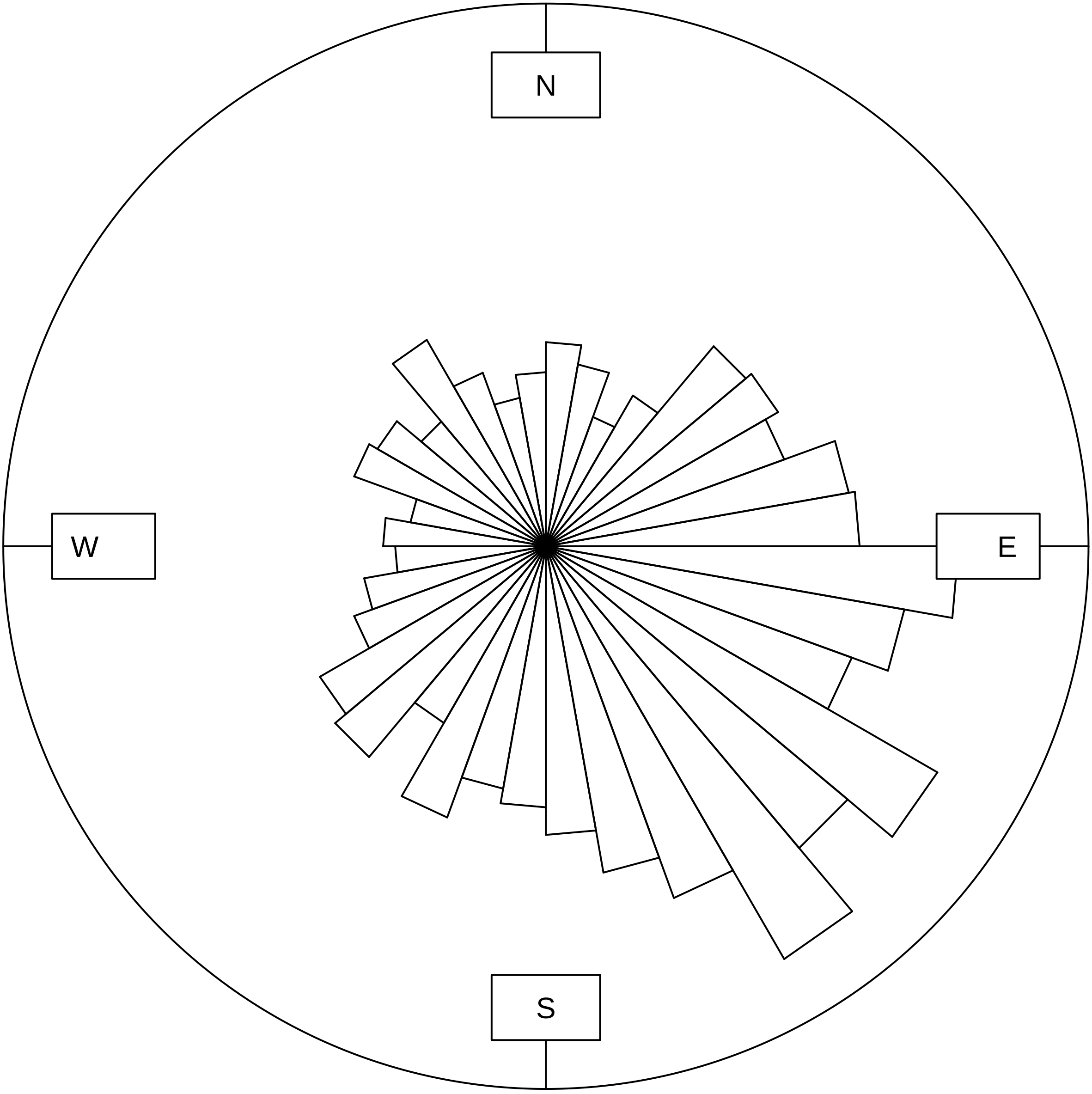

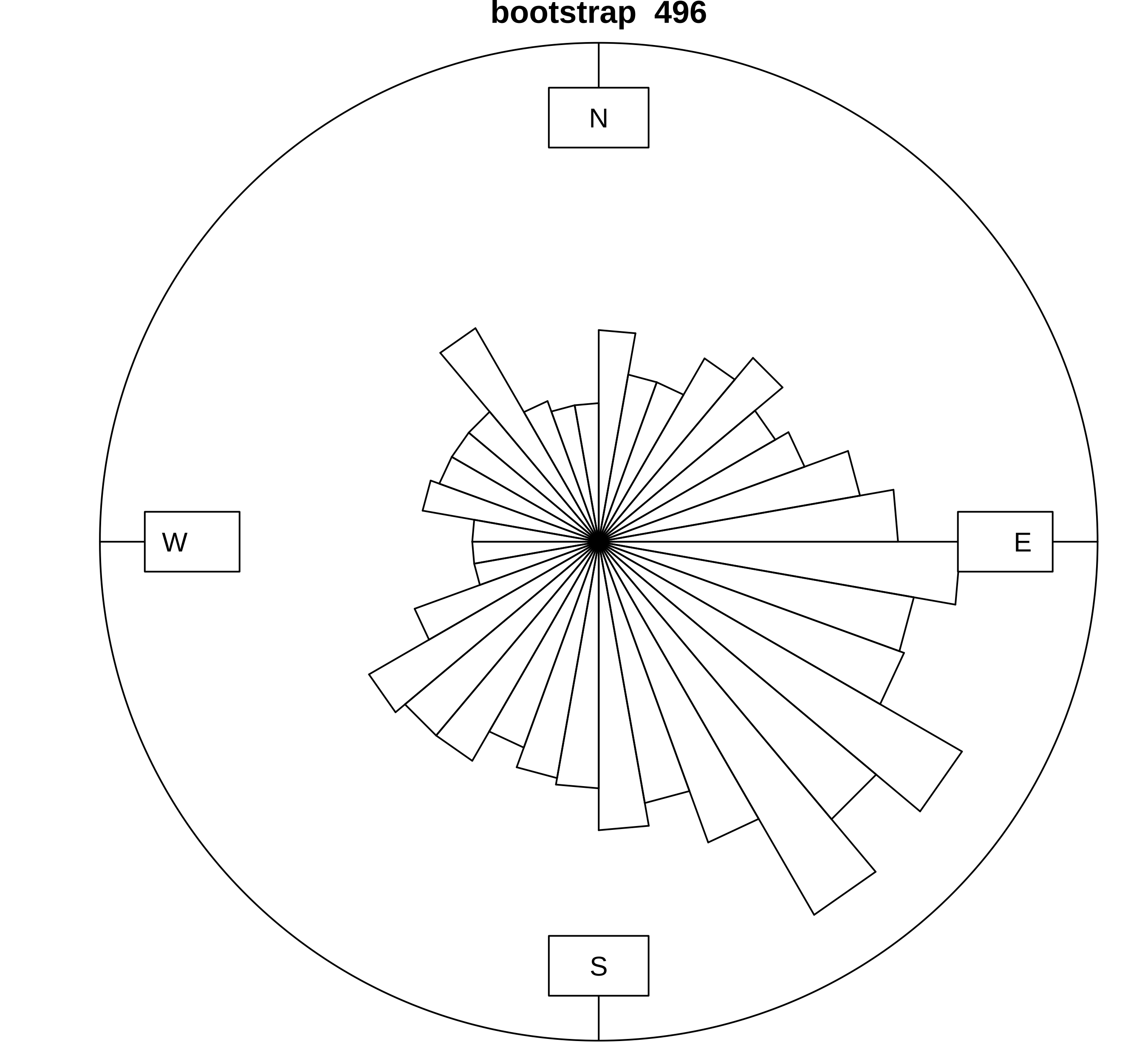
bootstrap 496
N
W
E
S

bootstrap 497

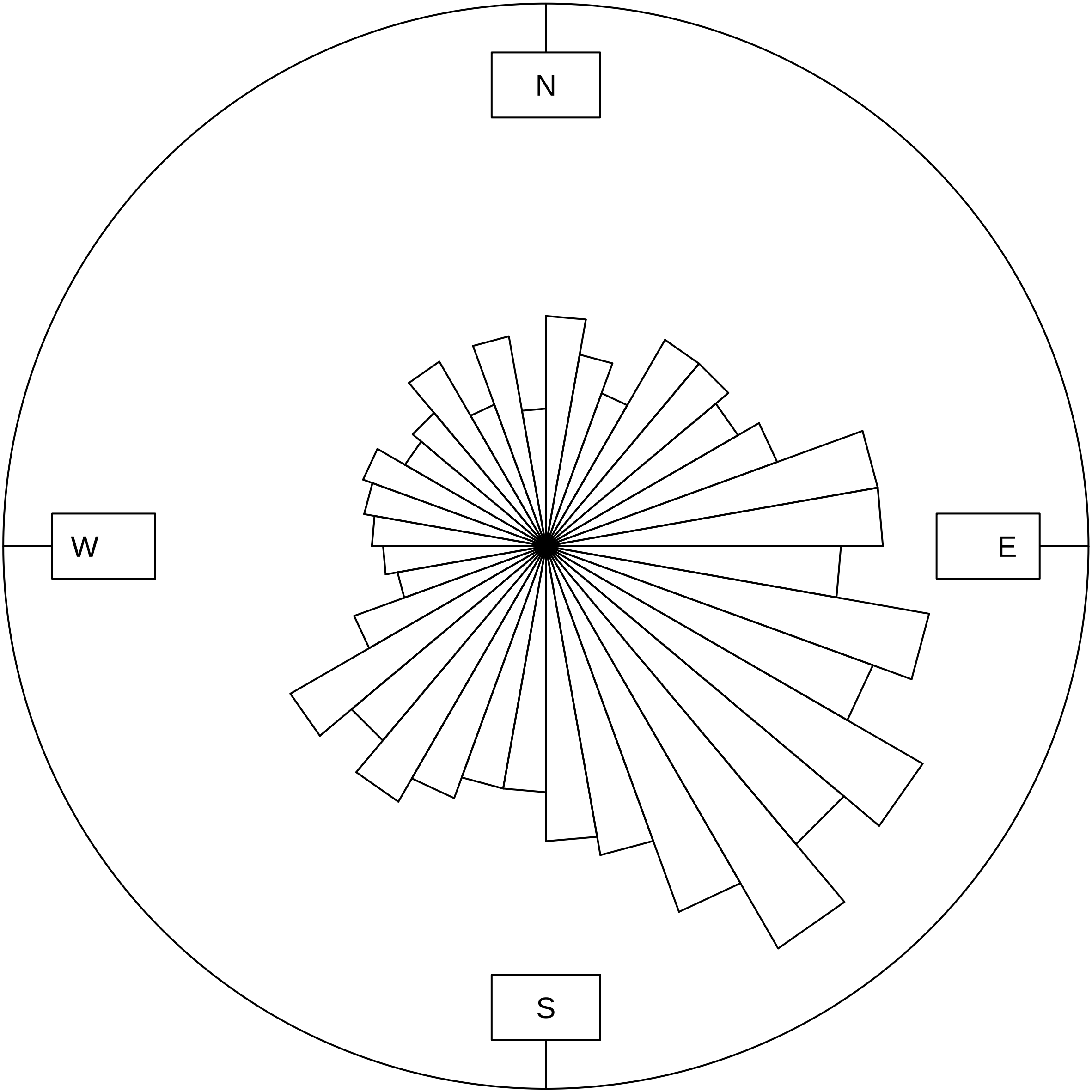

bootstrap 498

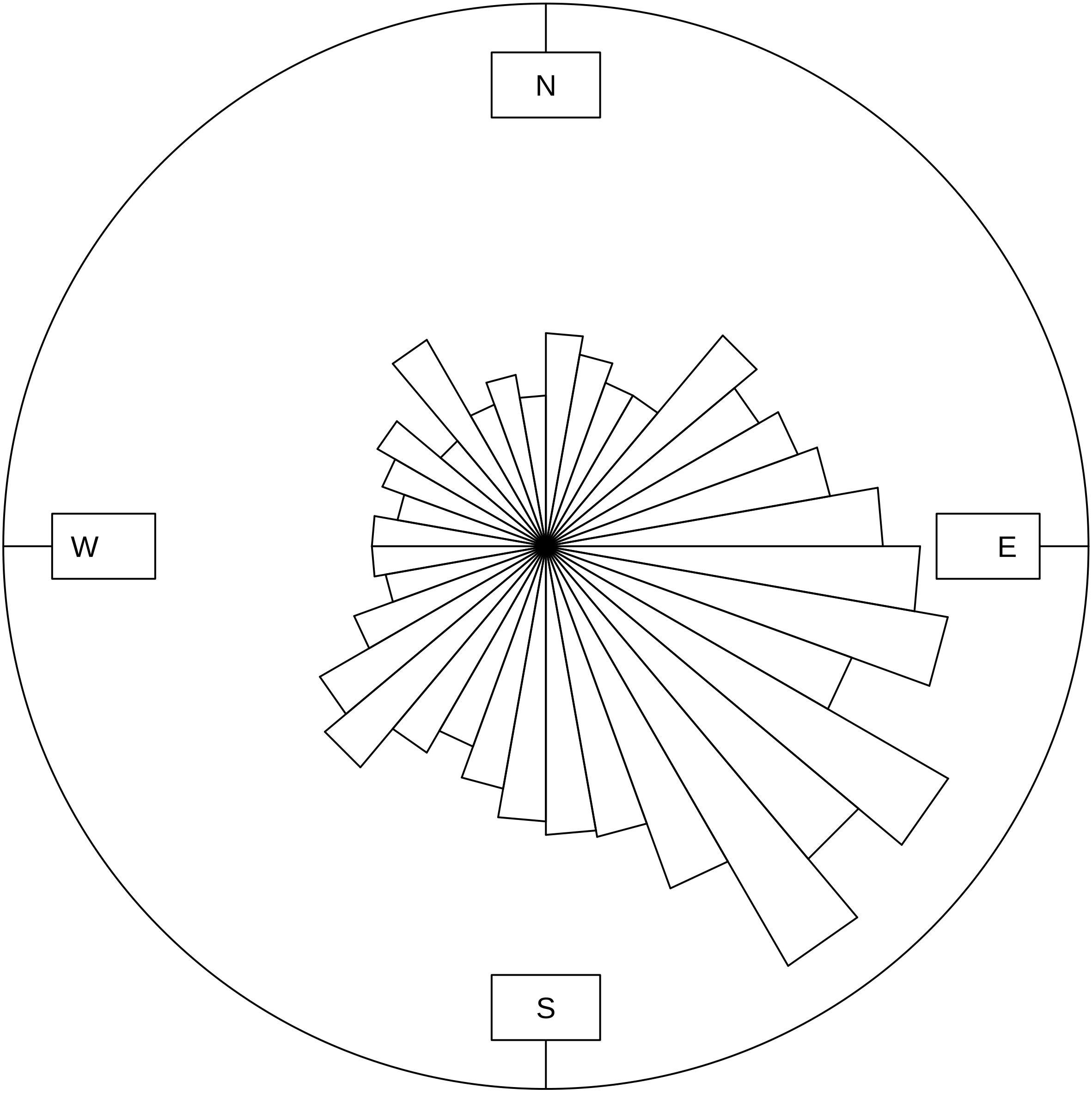

bootstrap 499

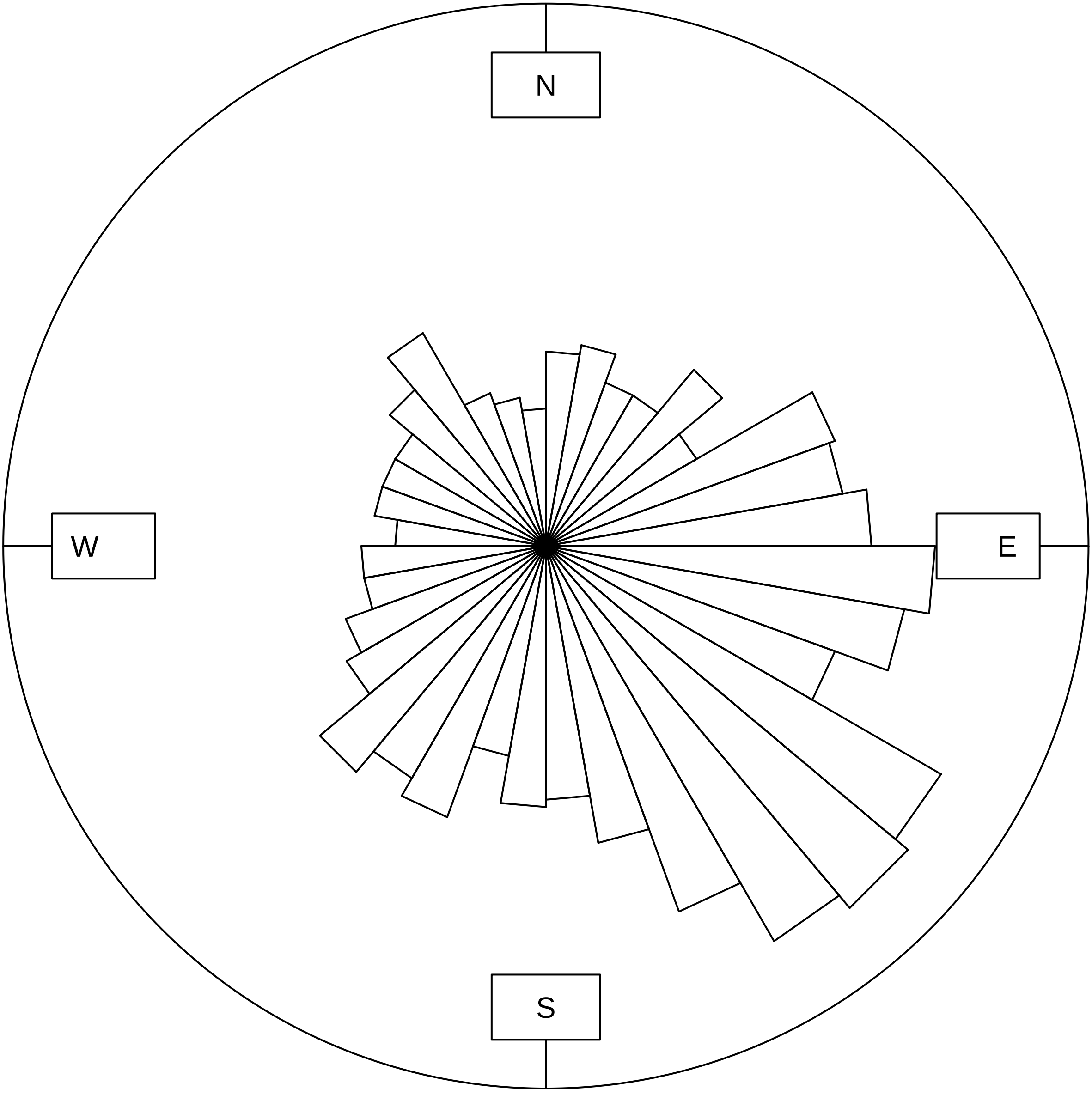

bootstrap 500

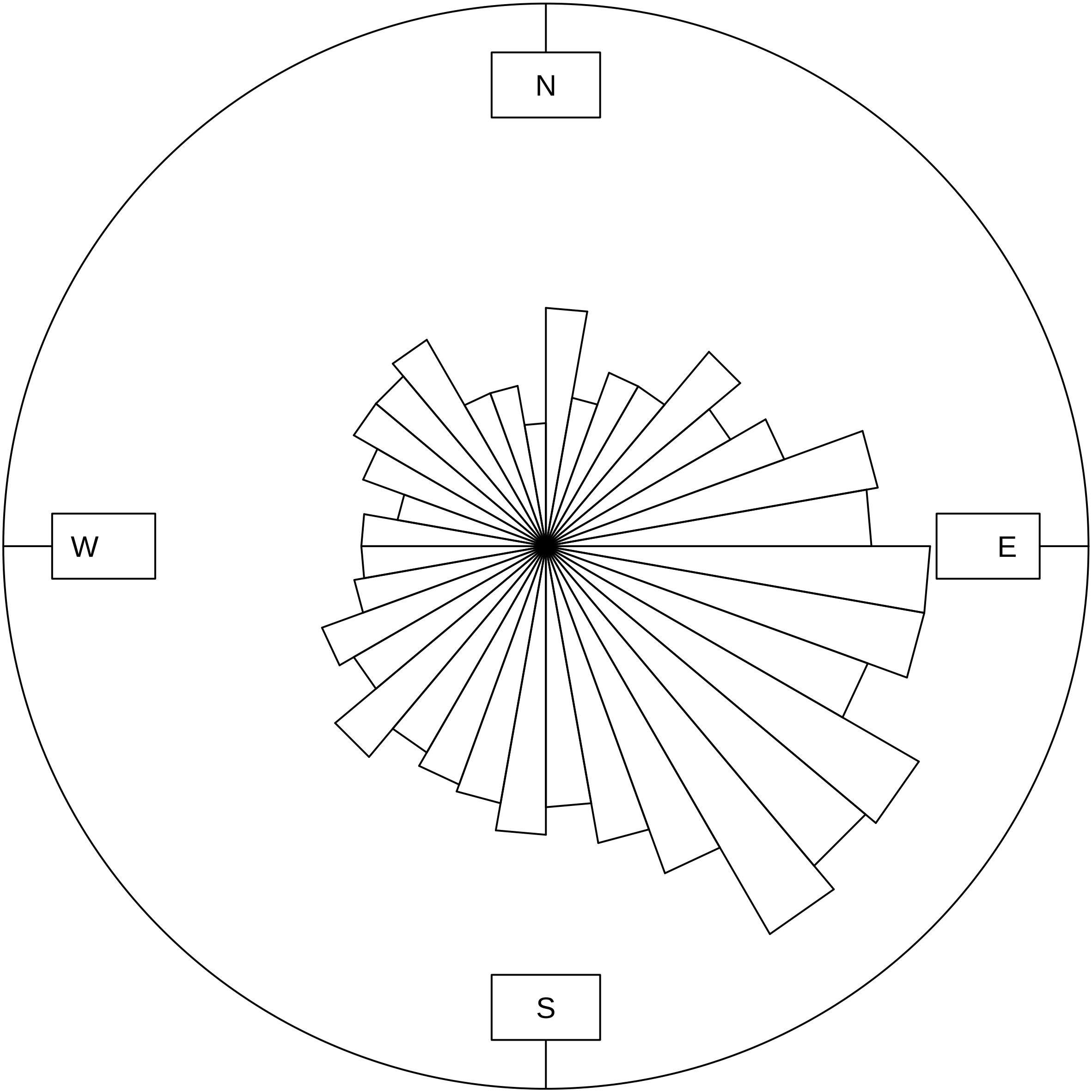

bootstrap 501

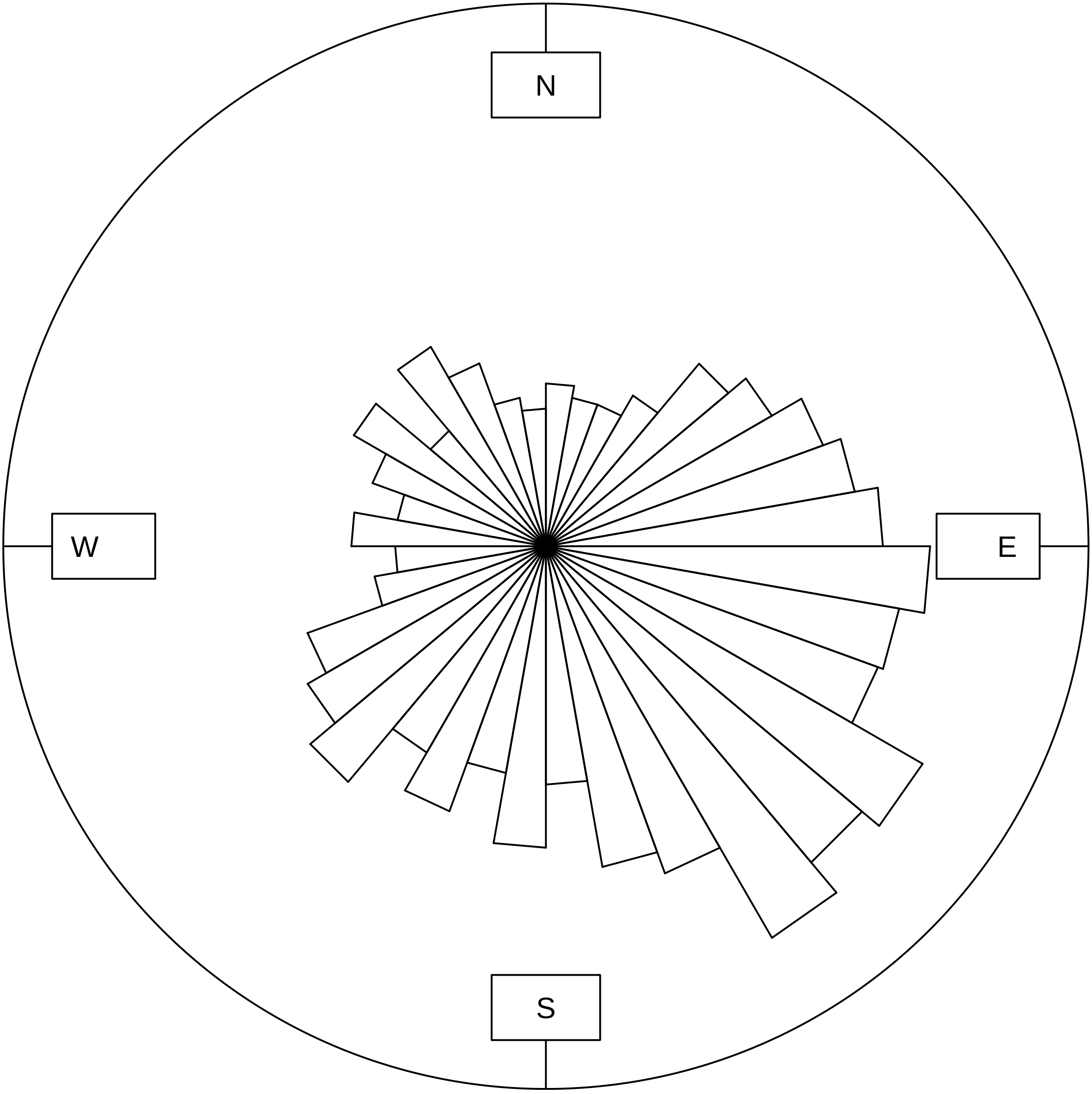

bootstrap 502

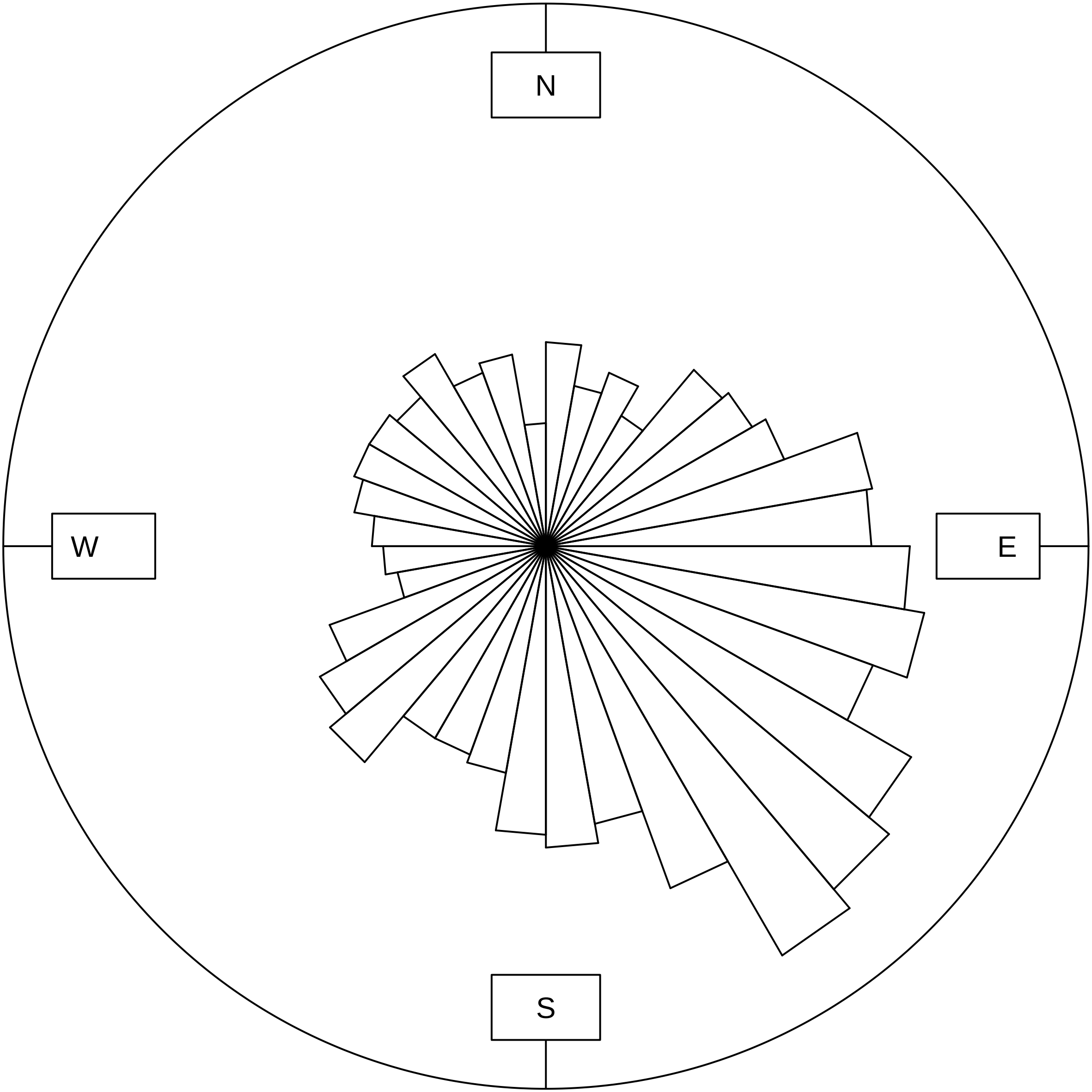

bootstrap 503

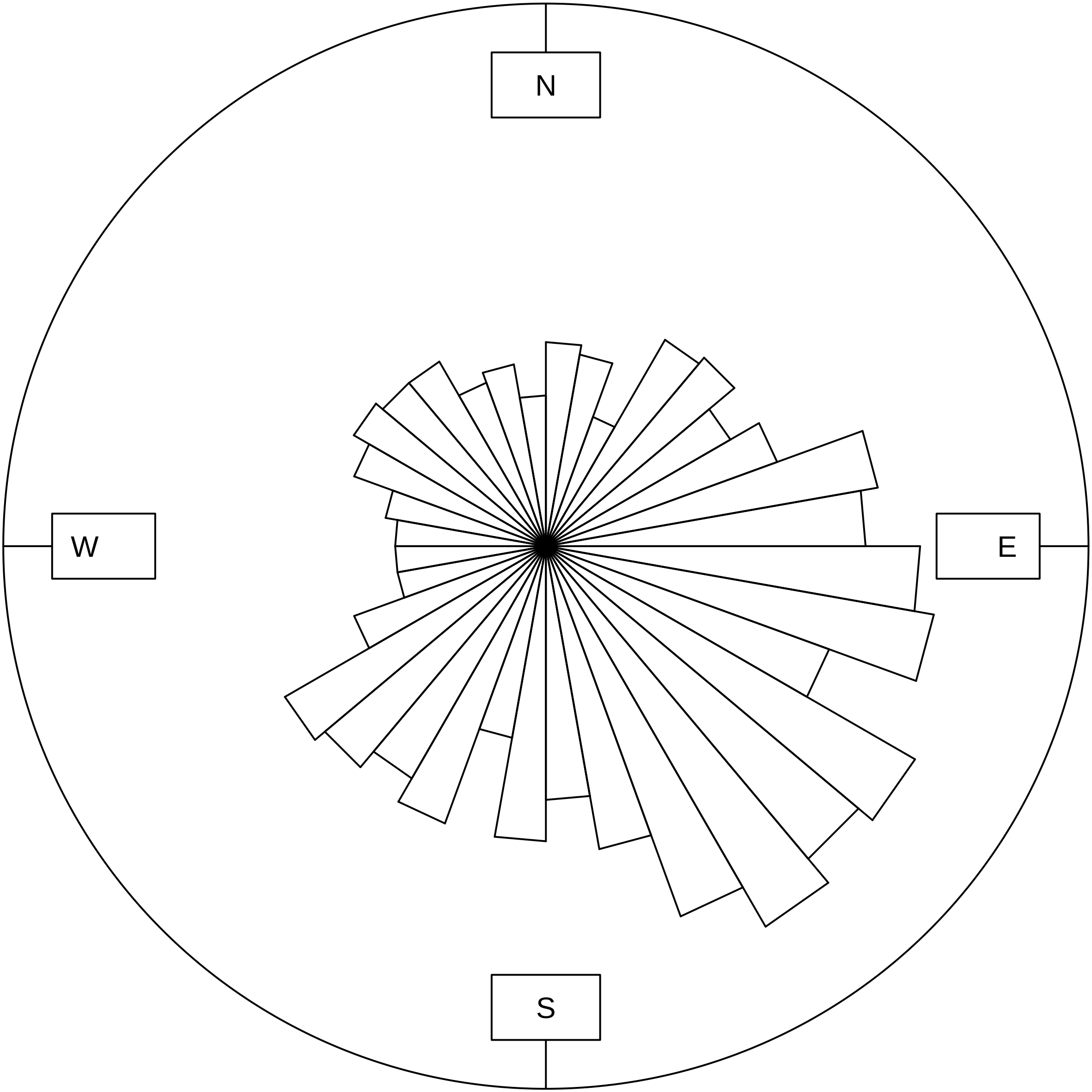

bootstrap 504

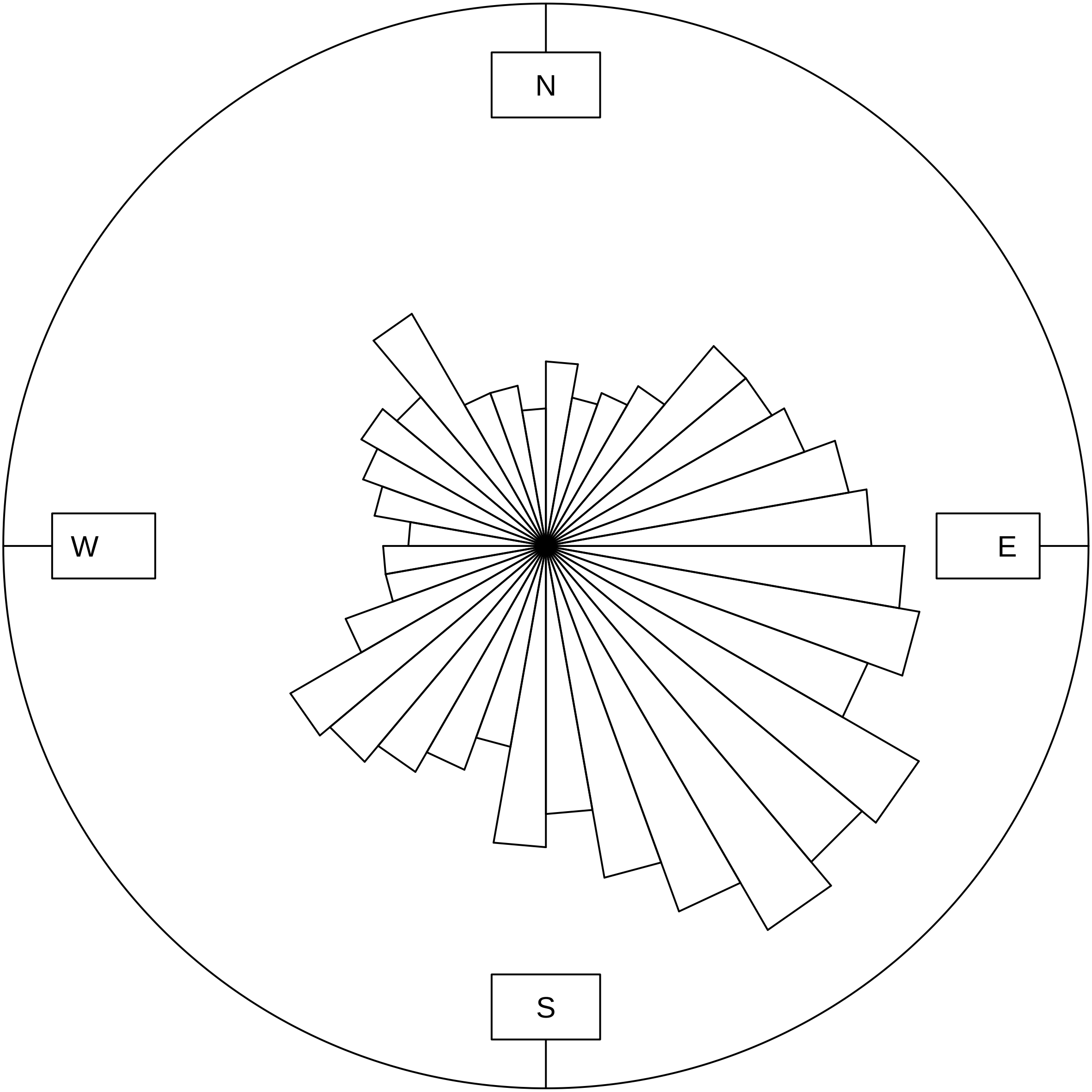

bootstrap 505

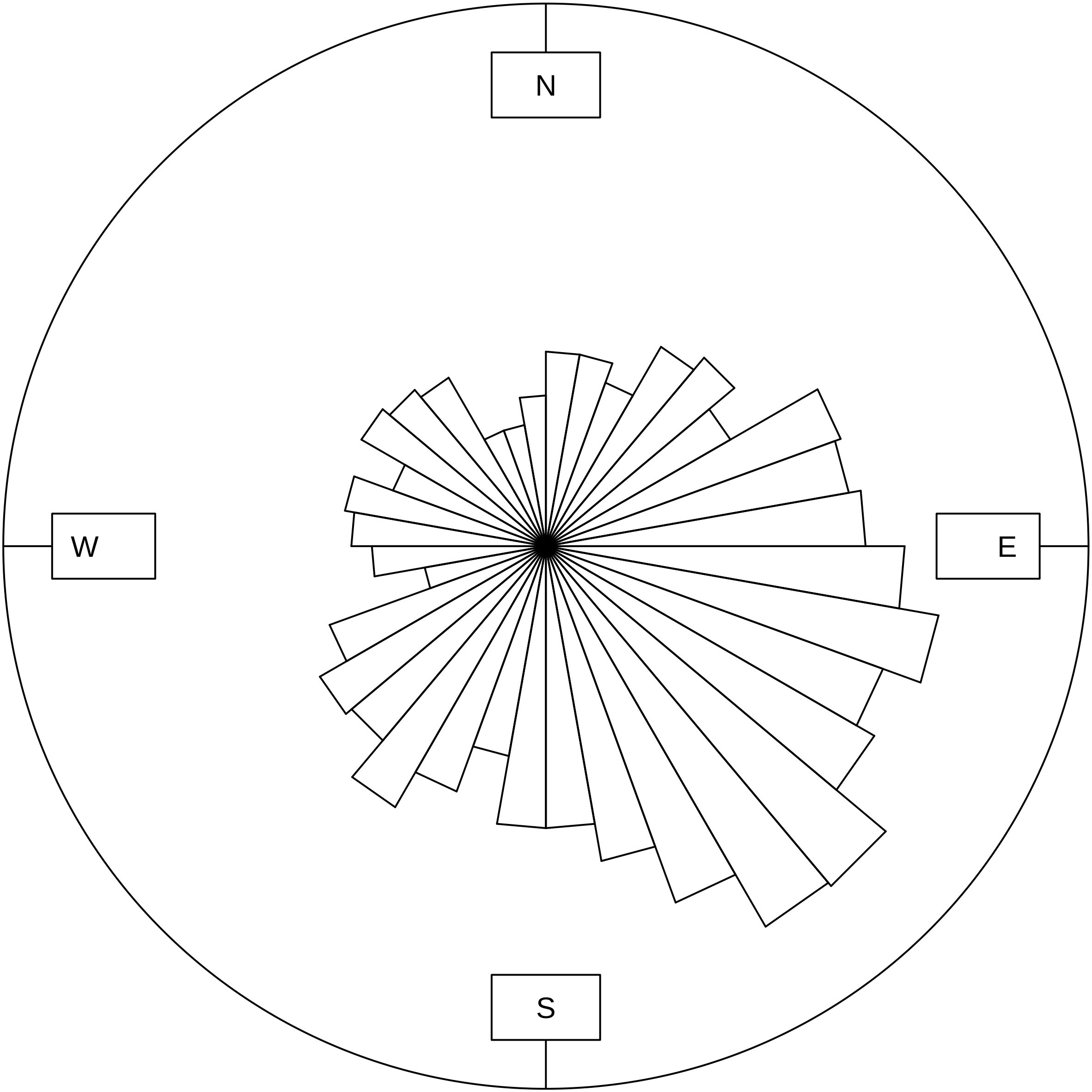

bootstrap 506

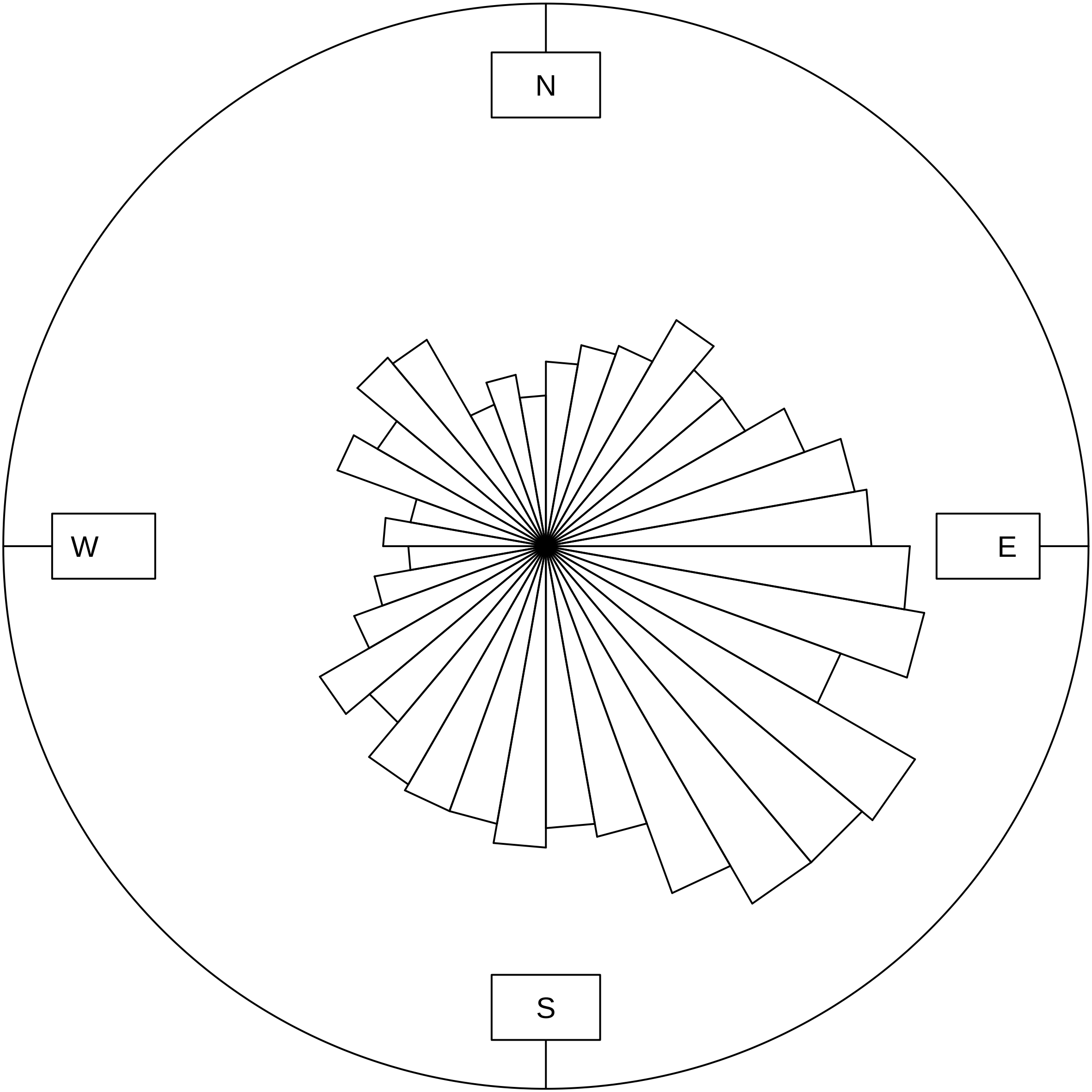

bootstrap 507

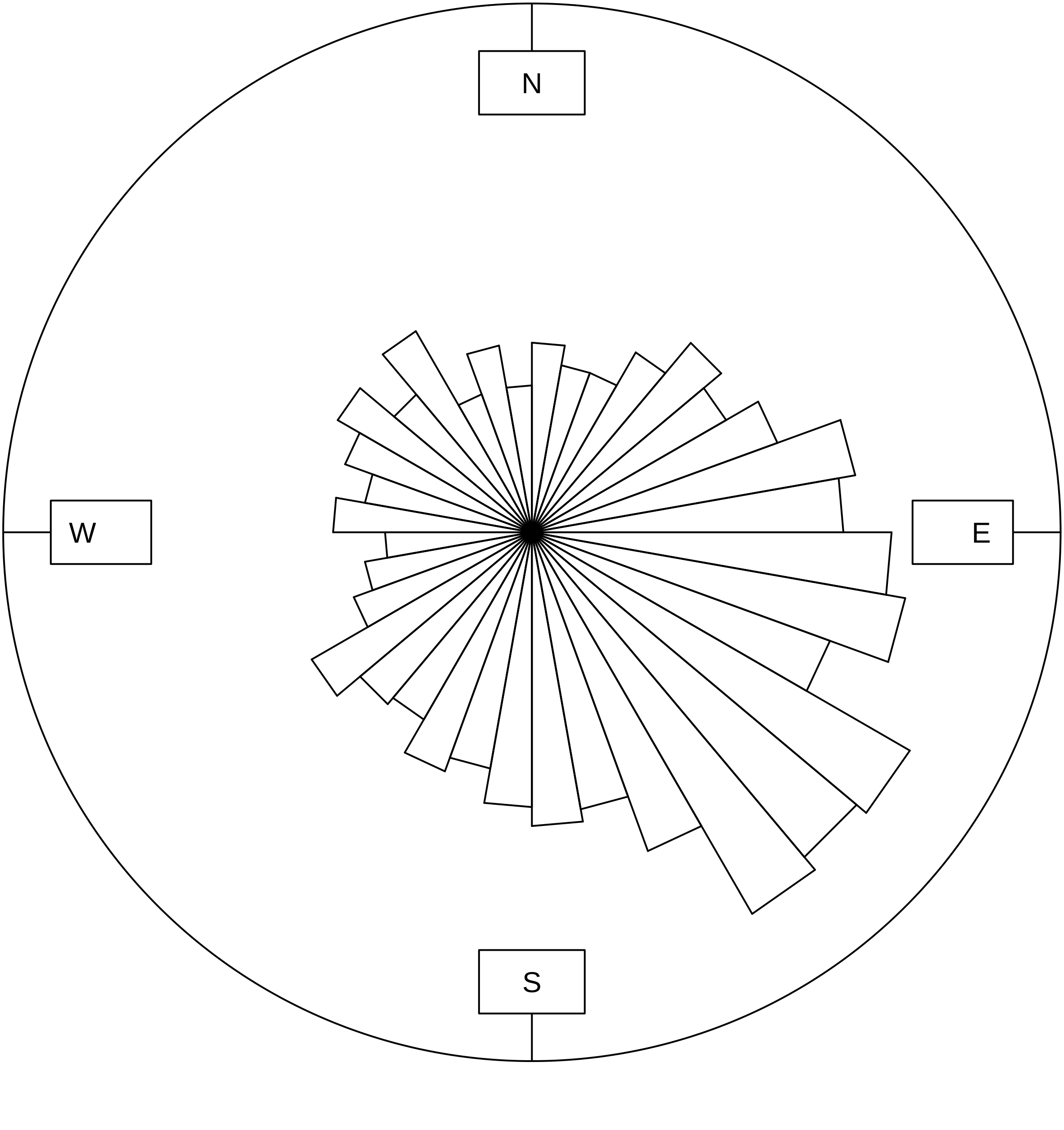

bootstrap 508

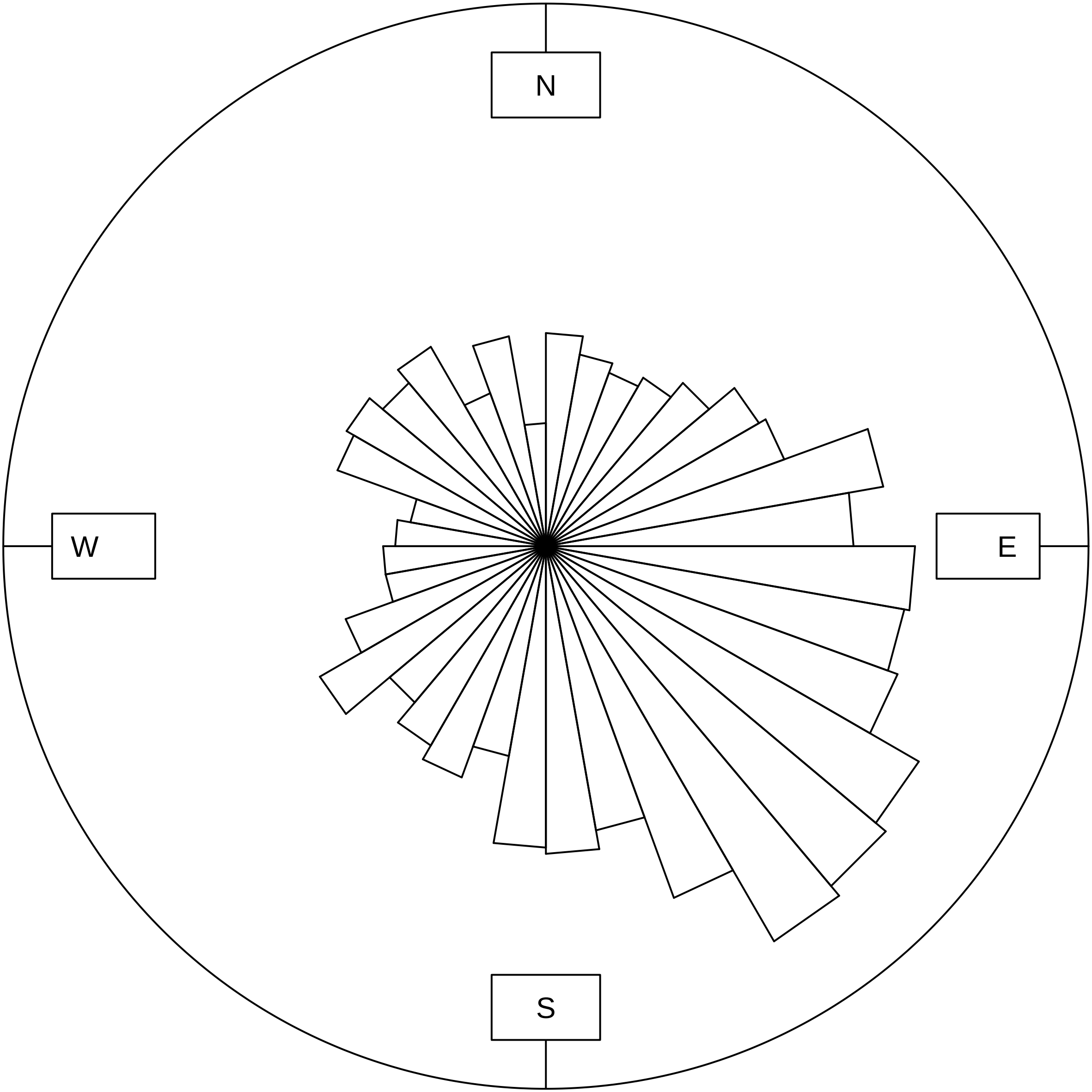

bootstrap 509

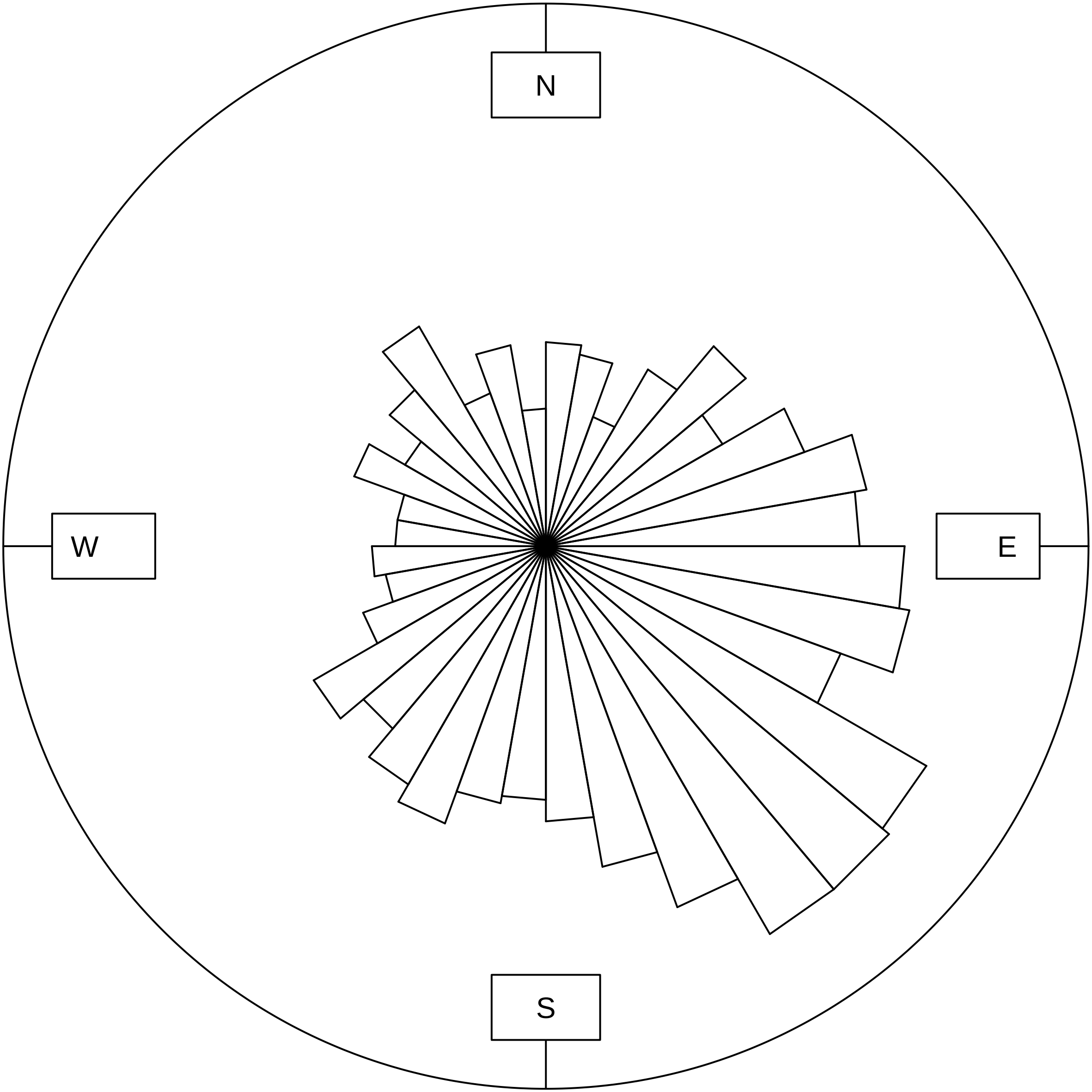

bootstrap 510

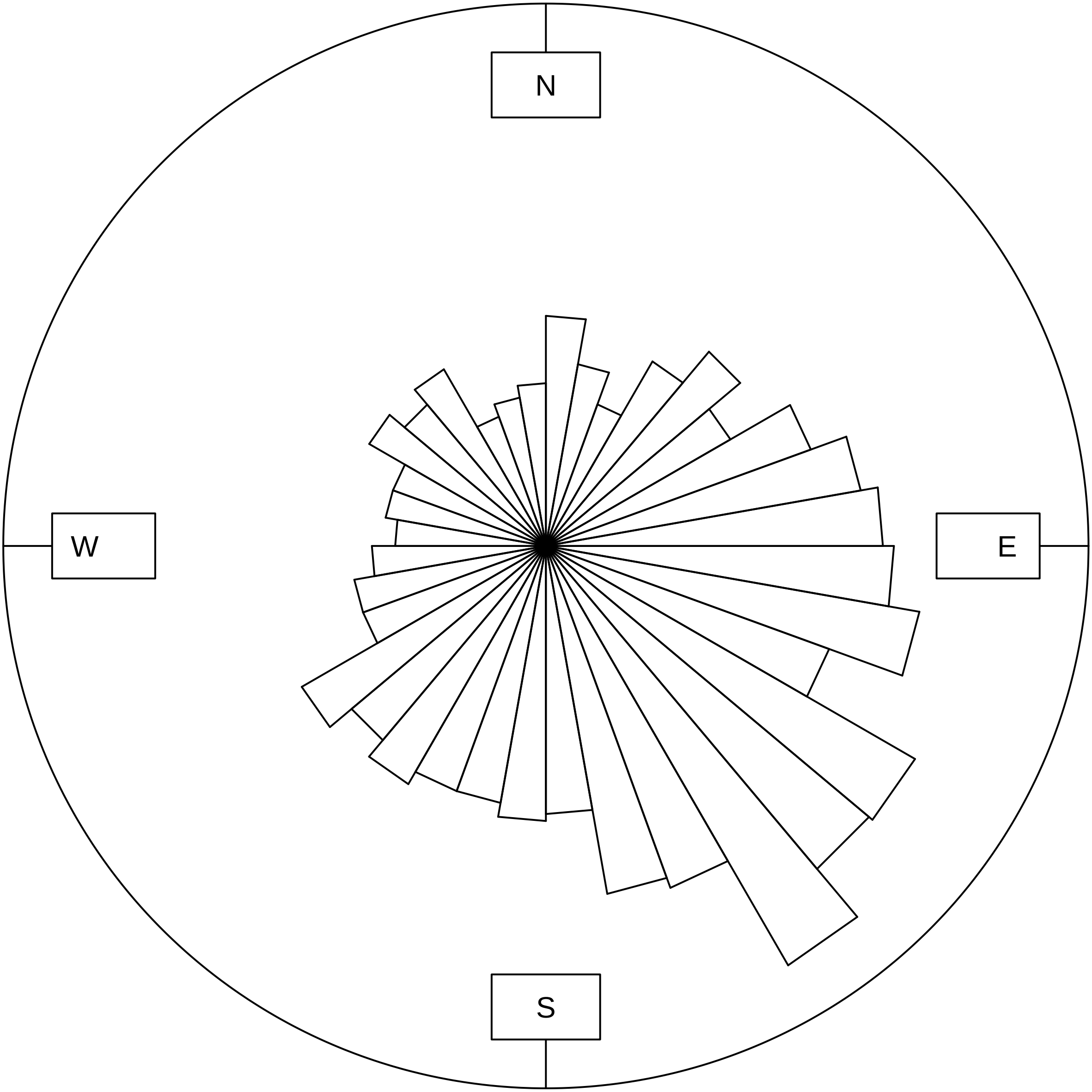

bootstrap 511

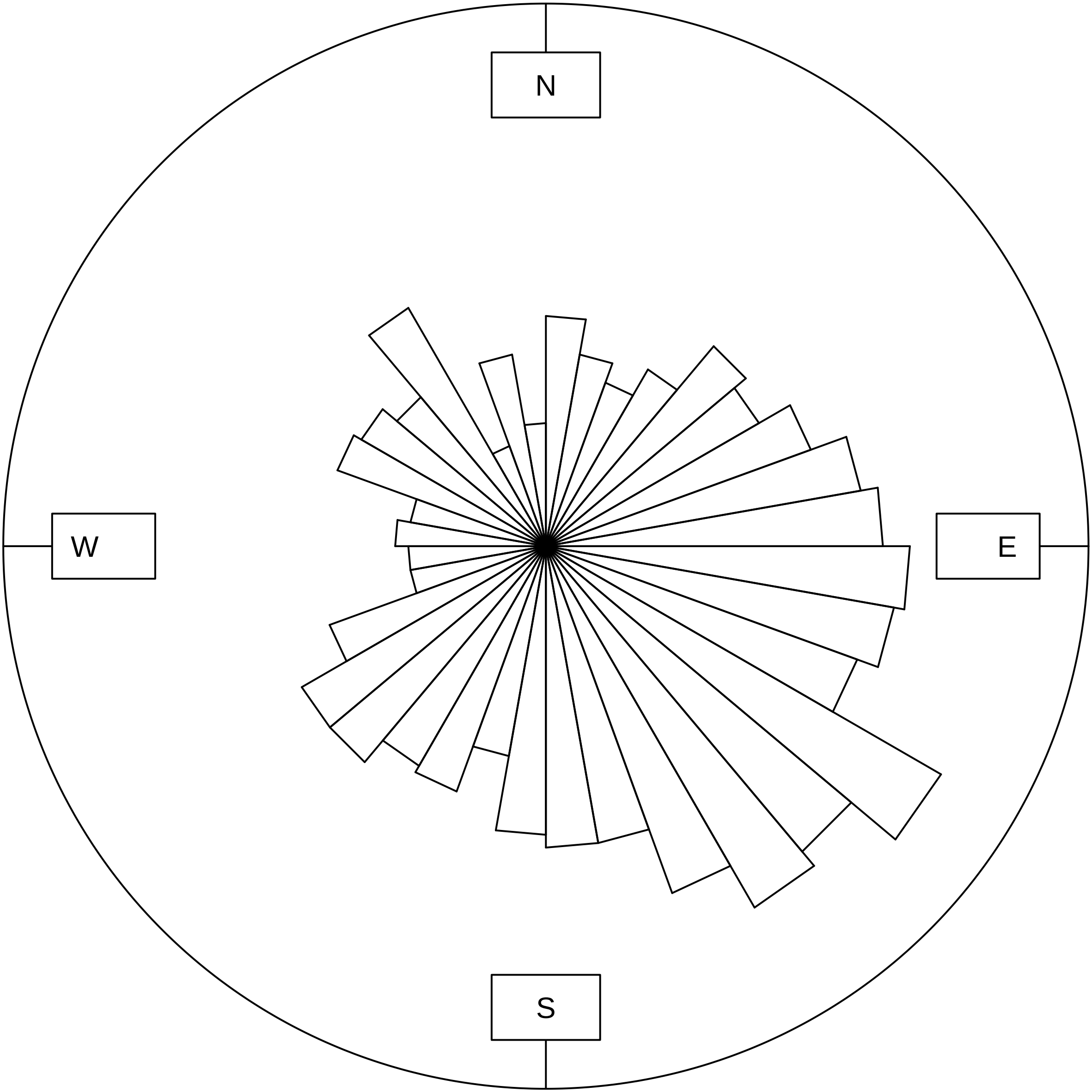

bootstrap 512

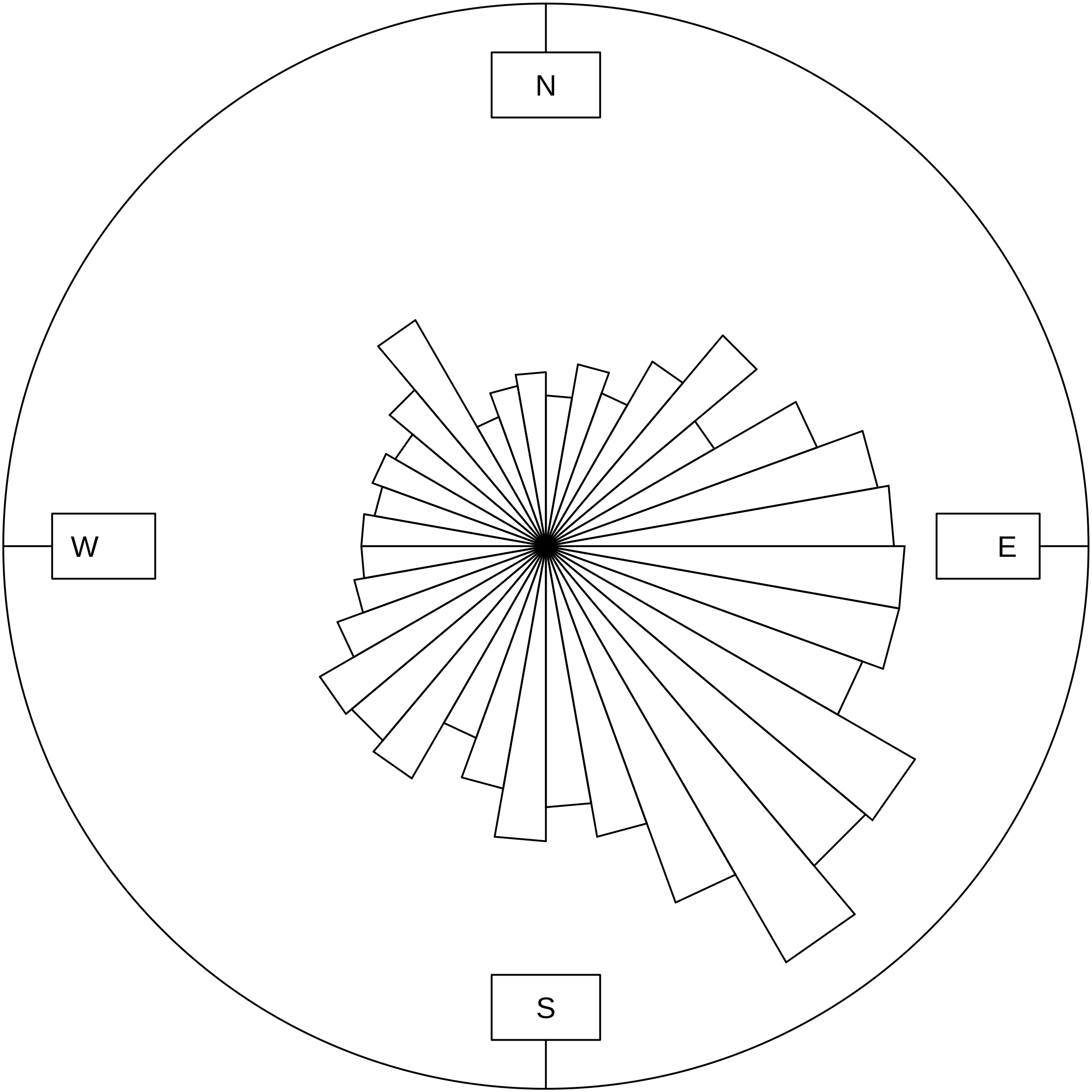

bootstrap 513

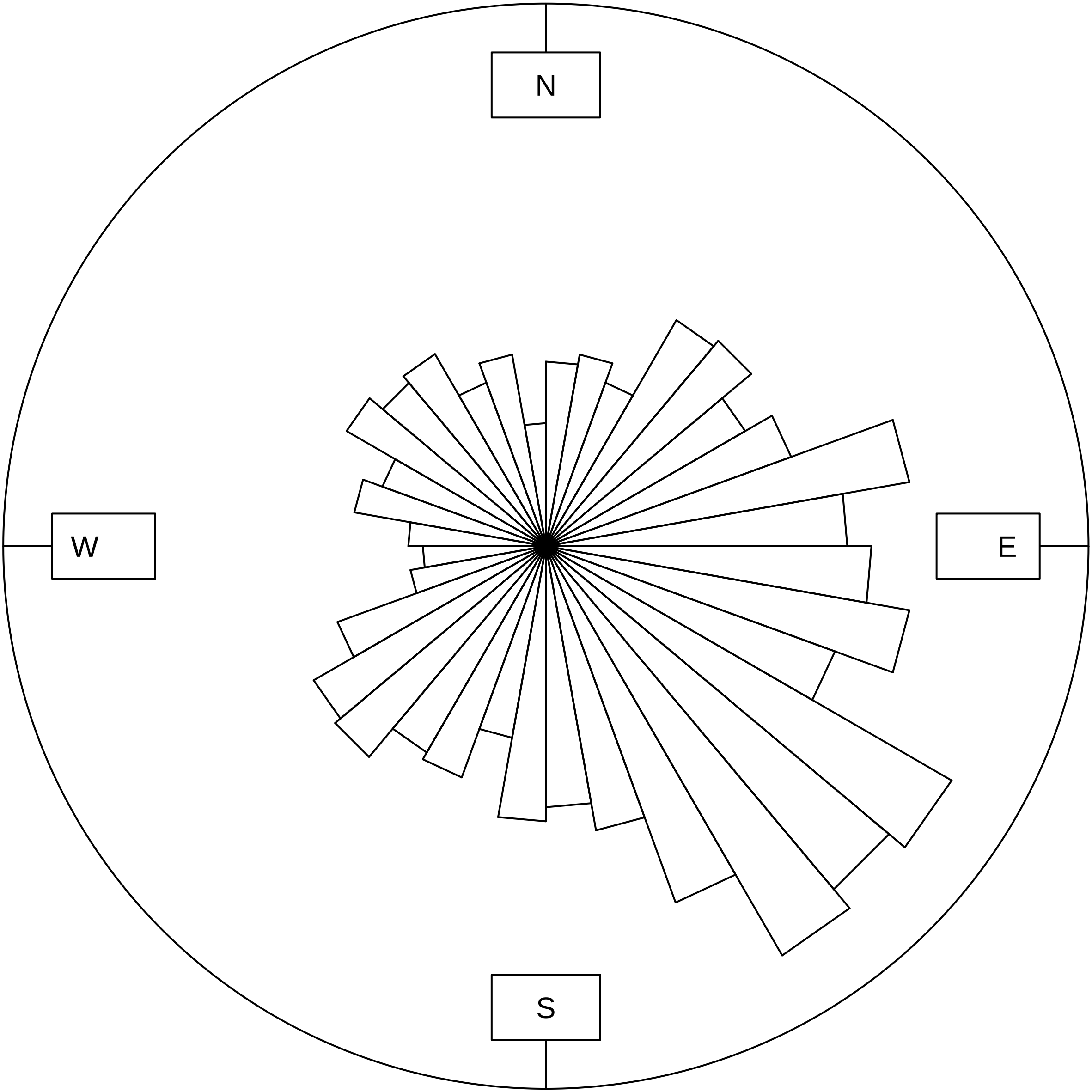

bootstrap 514

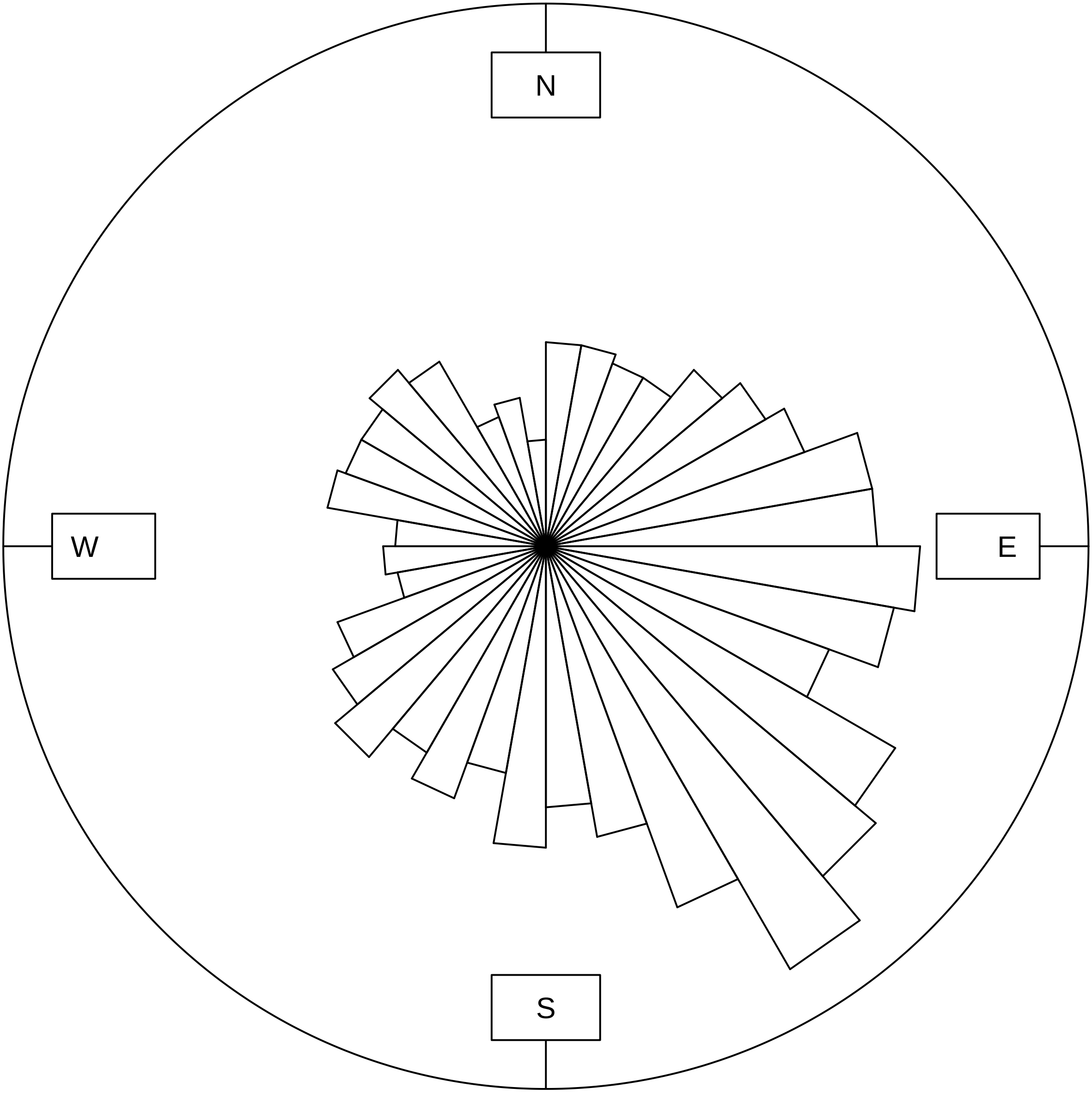

bootstrap 515

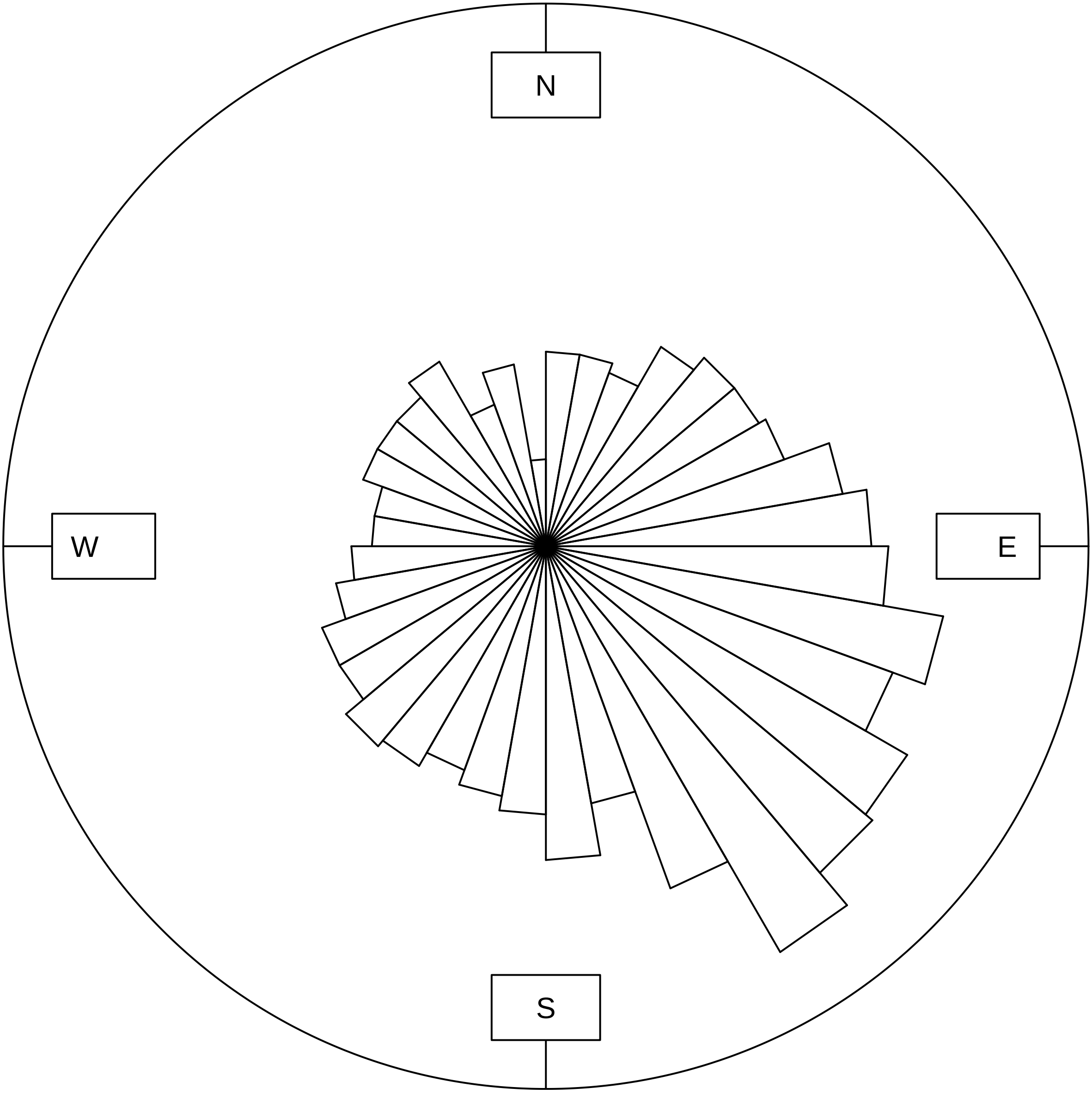

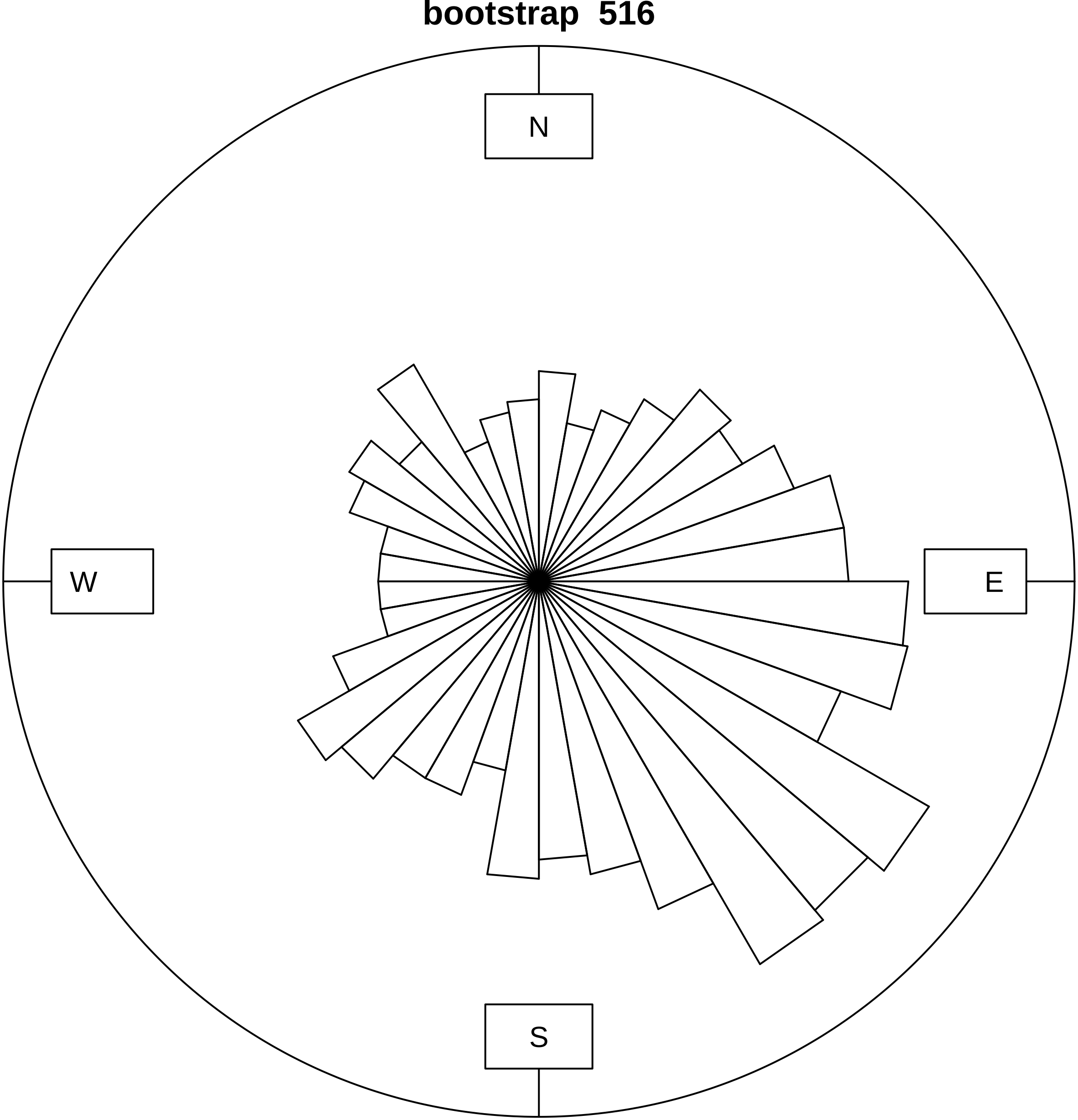
bootstrap 516
N
W
E
S

bootstrap 517

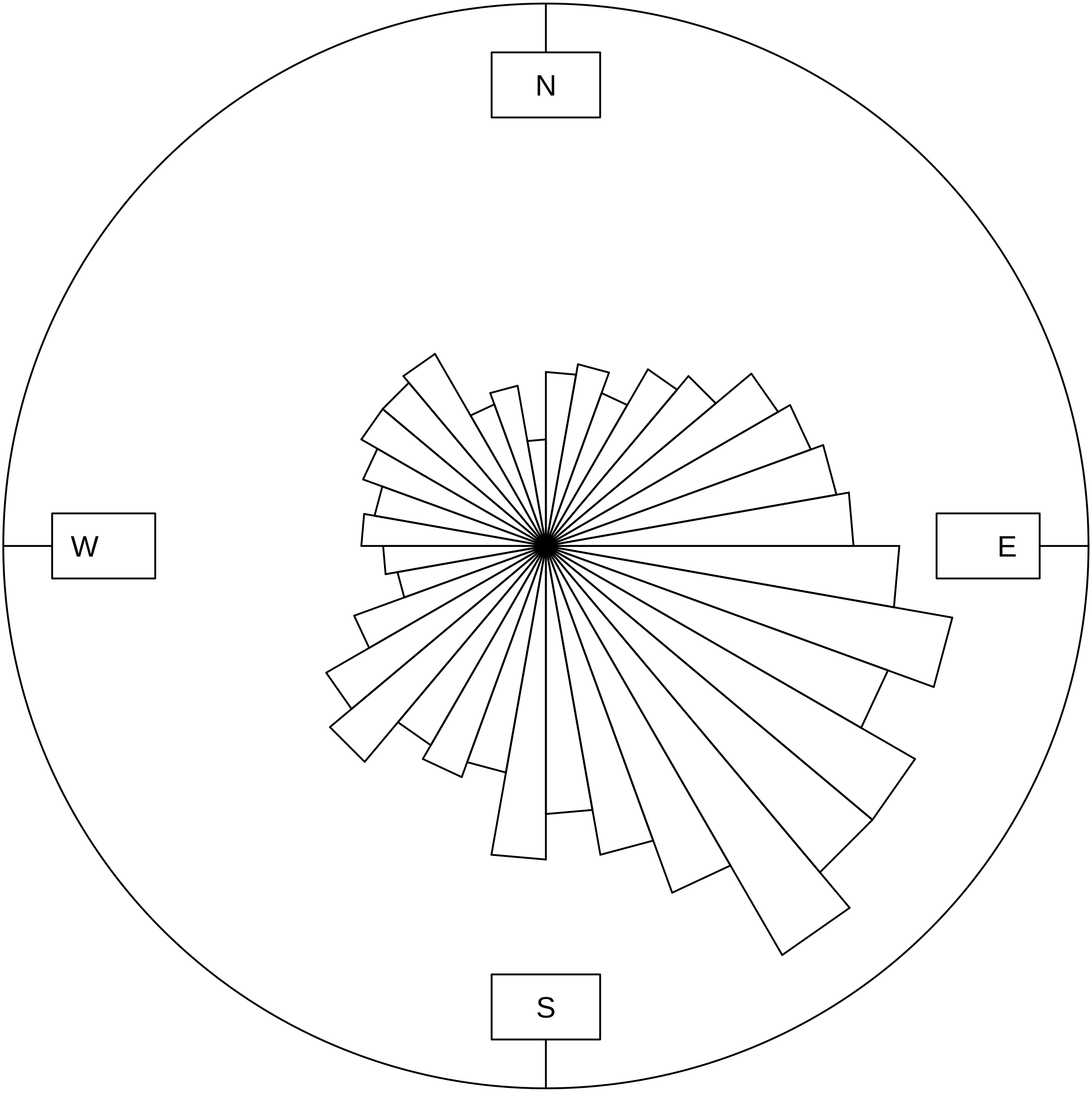

bootstrap 518

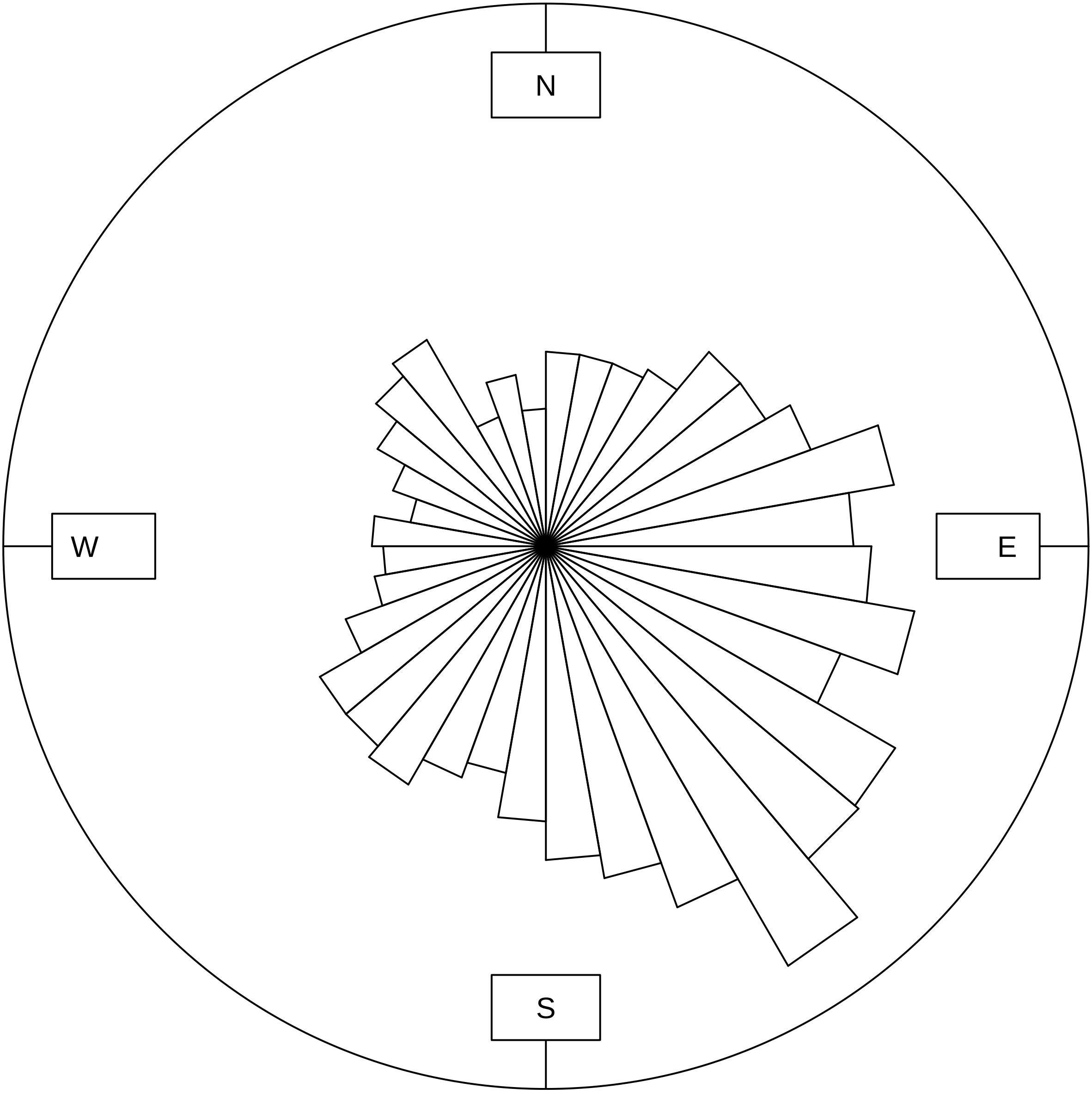

bootstrap 519

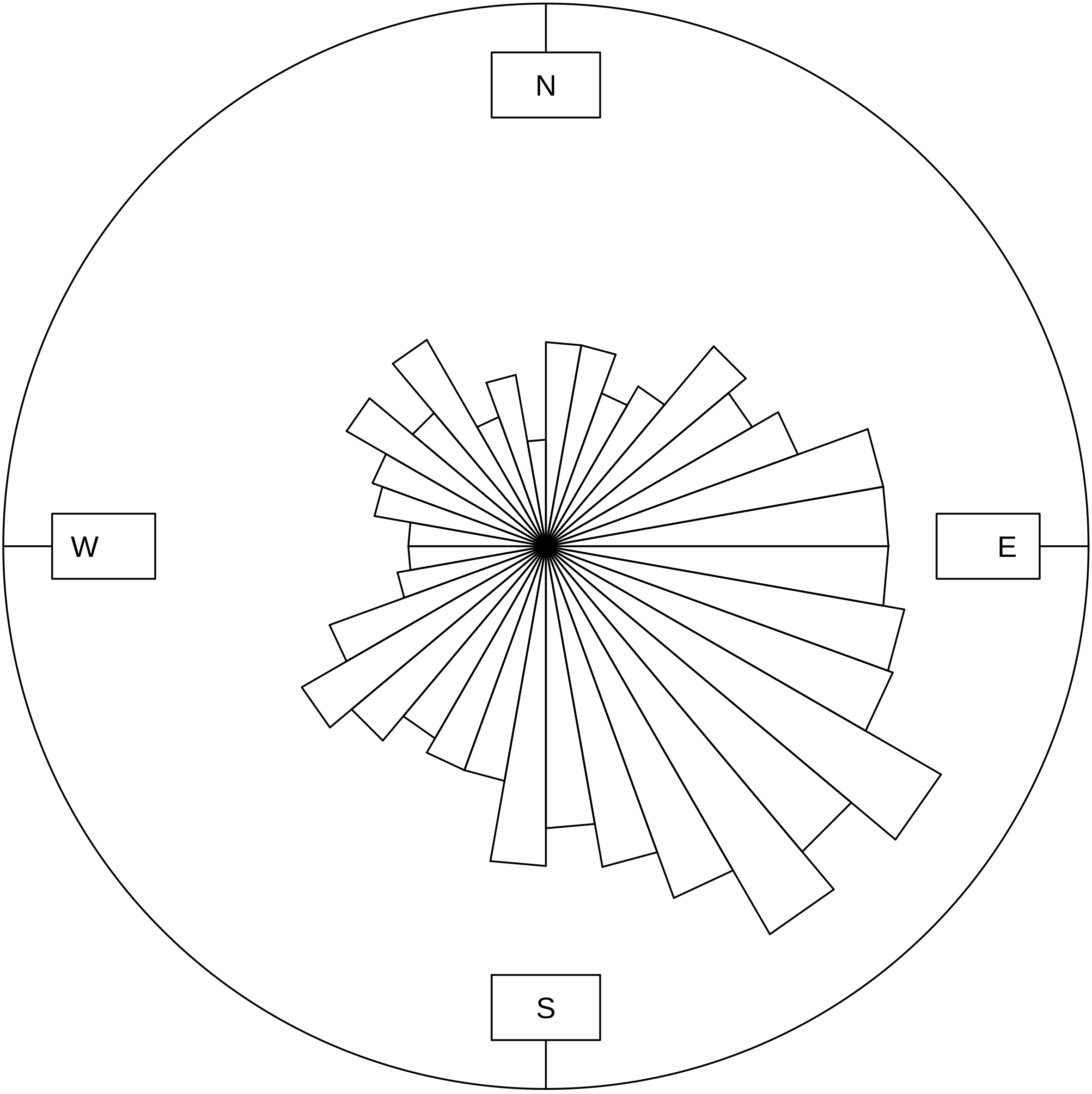

bootstrap 520

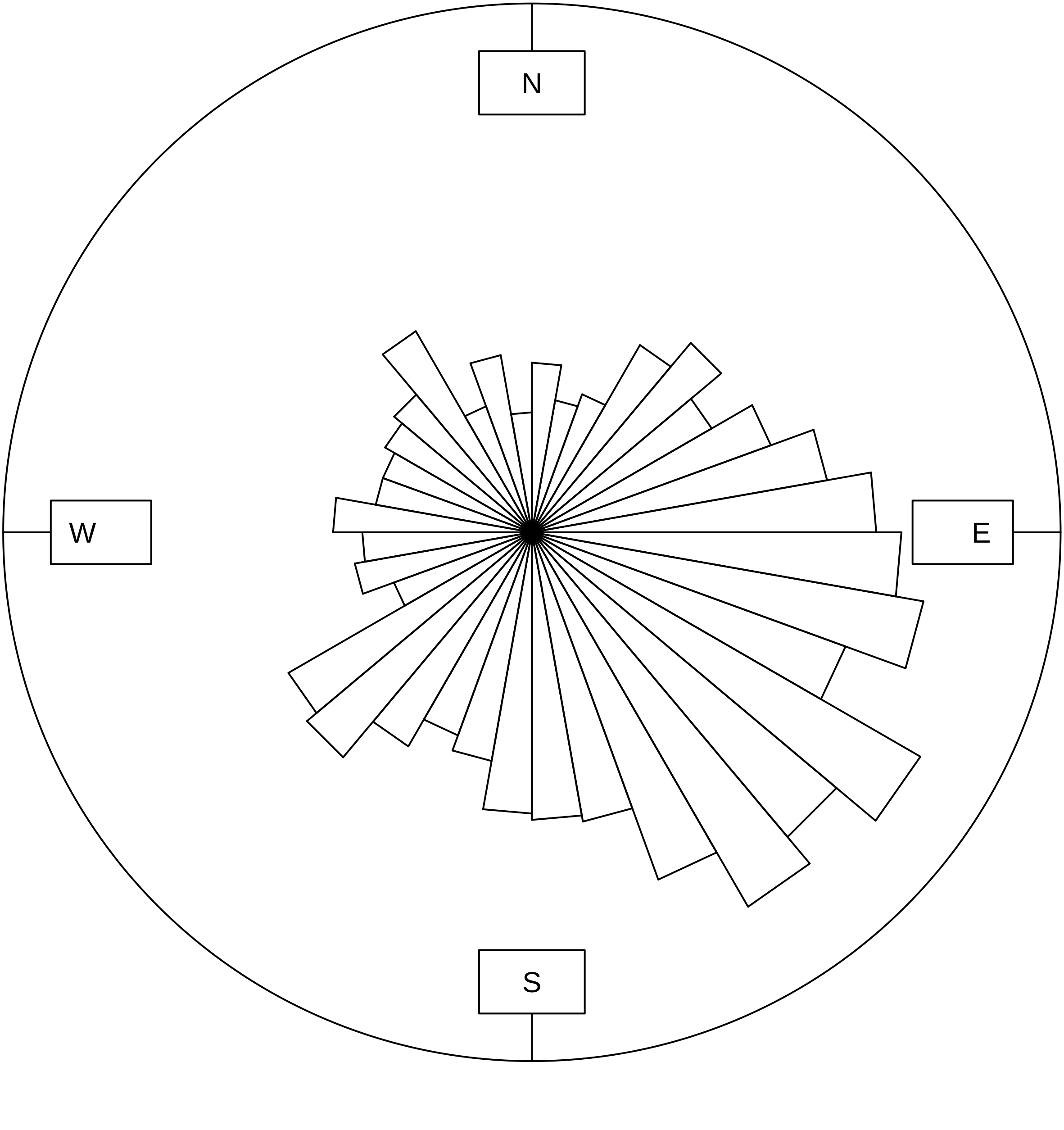

bootstrap 521

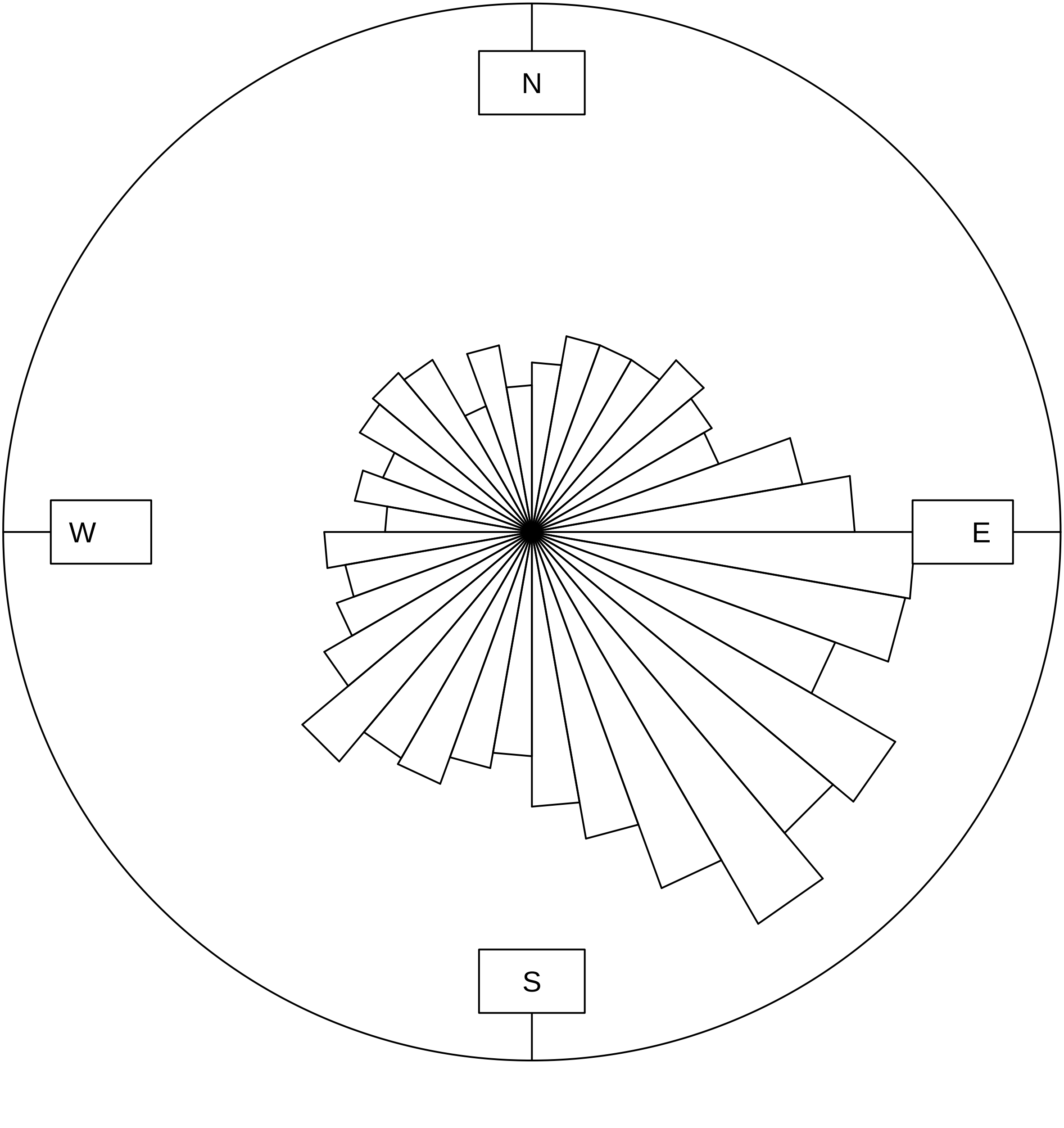

bootstrap 522

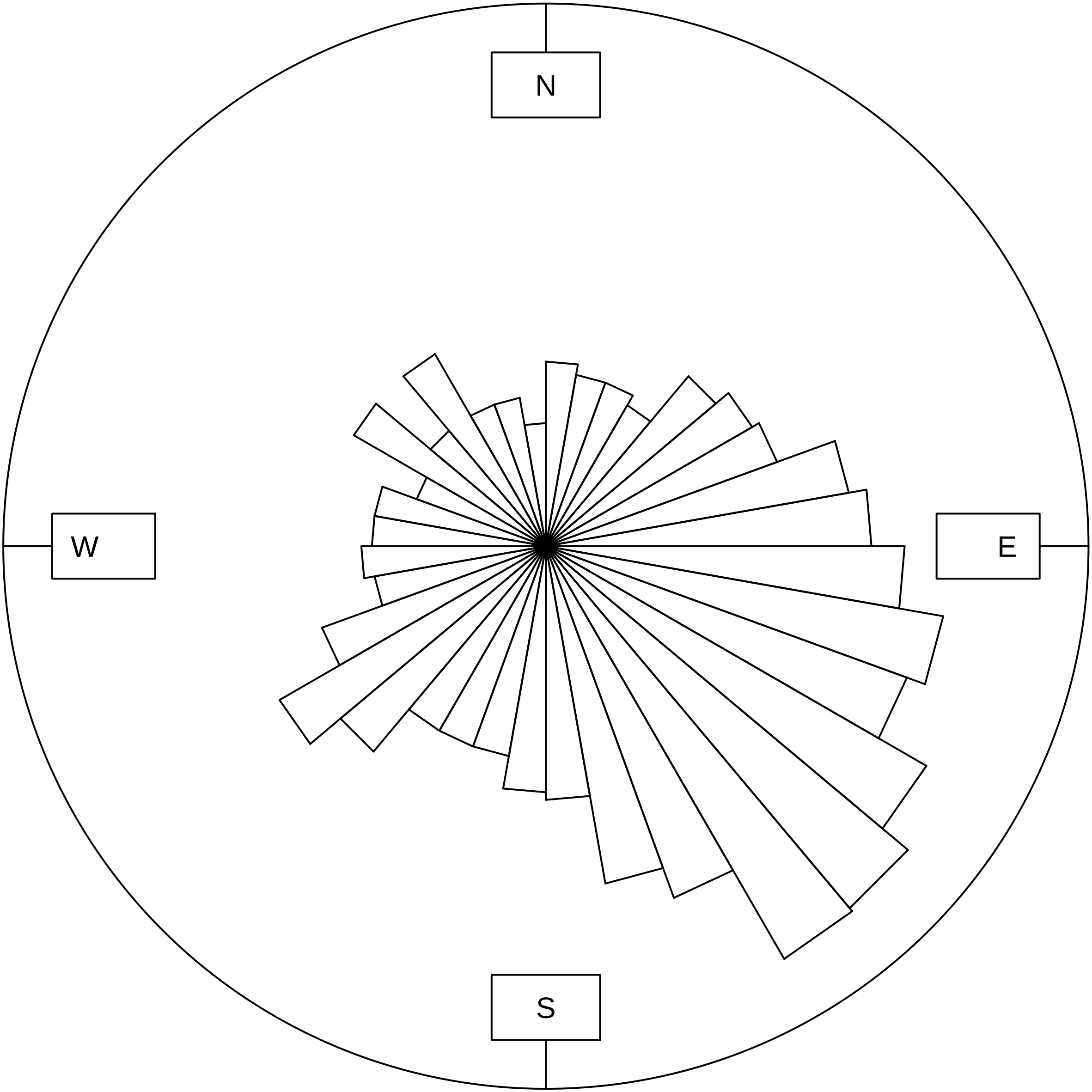

bootstrap 523

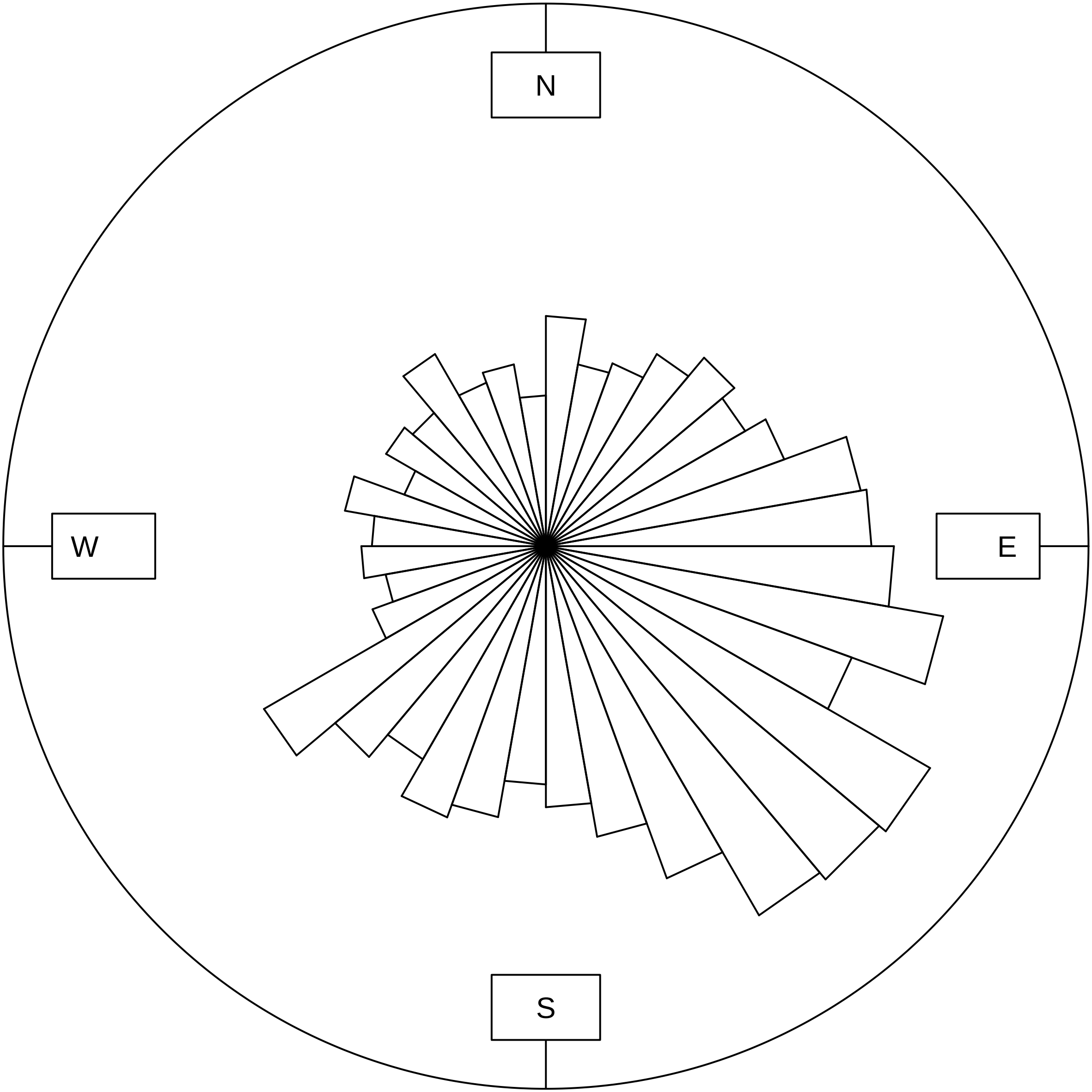

bootstrap 524

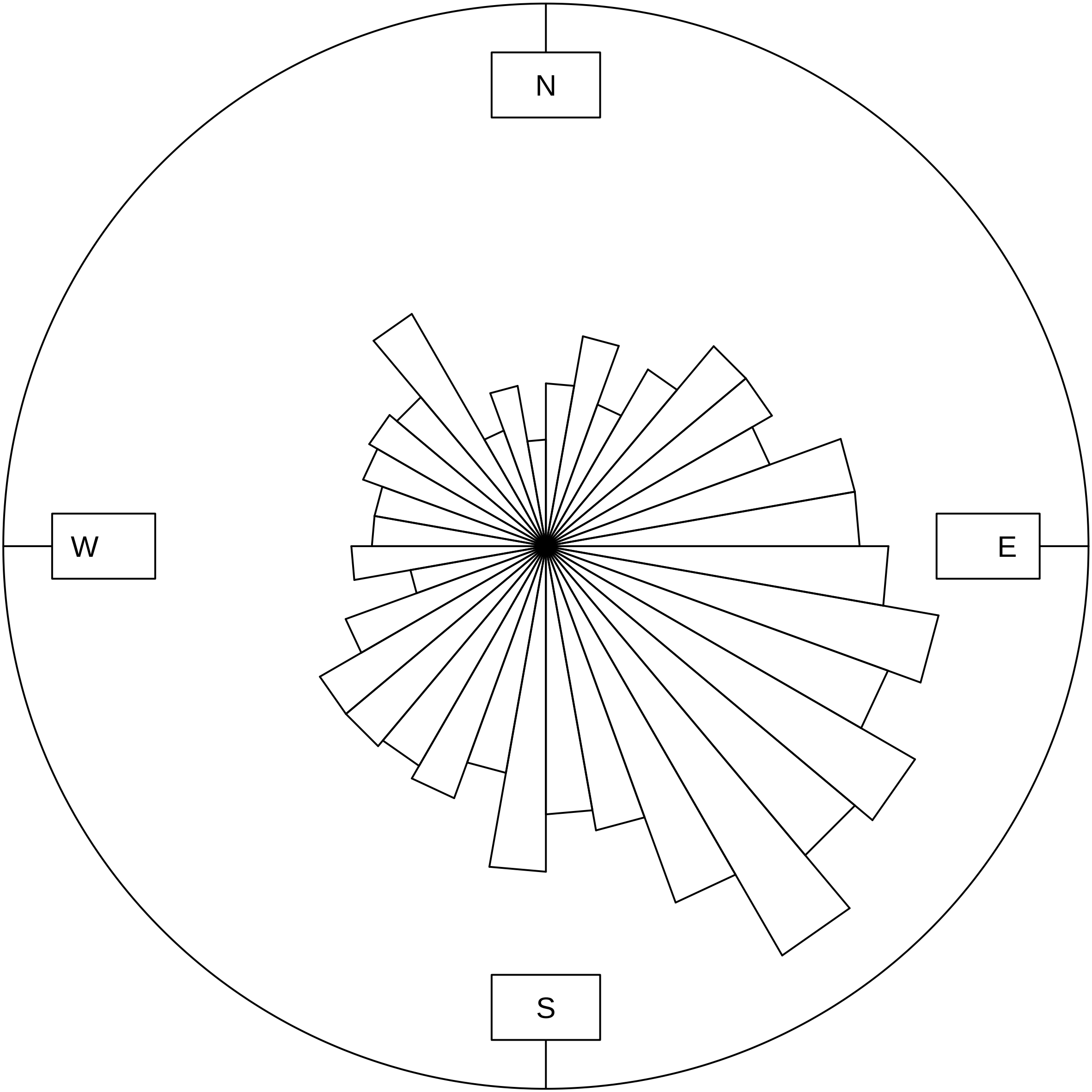

bootstrap 525

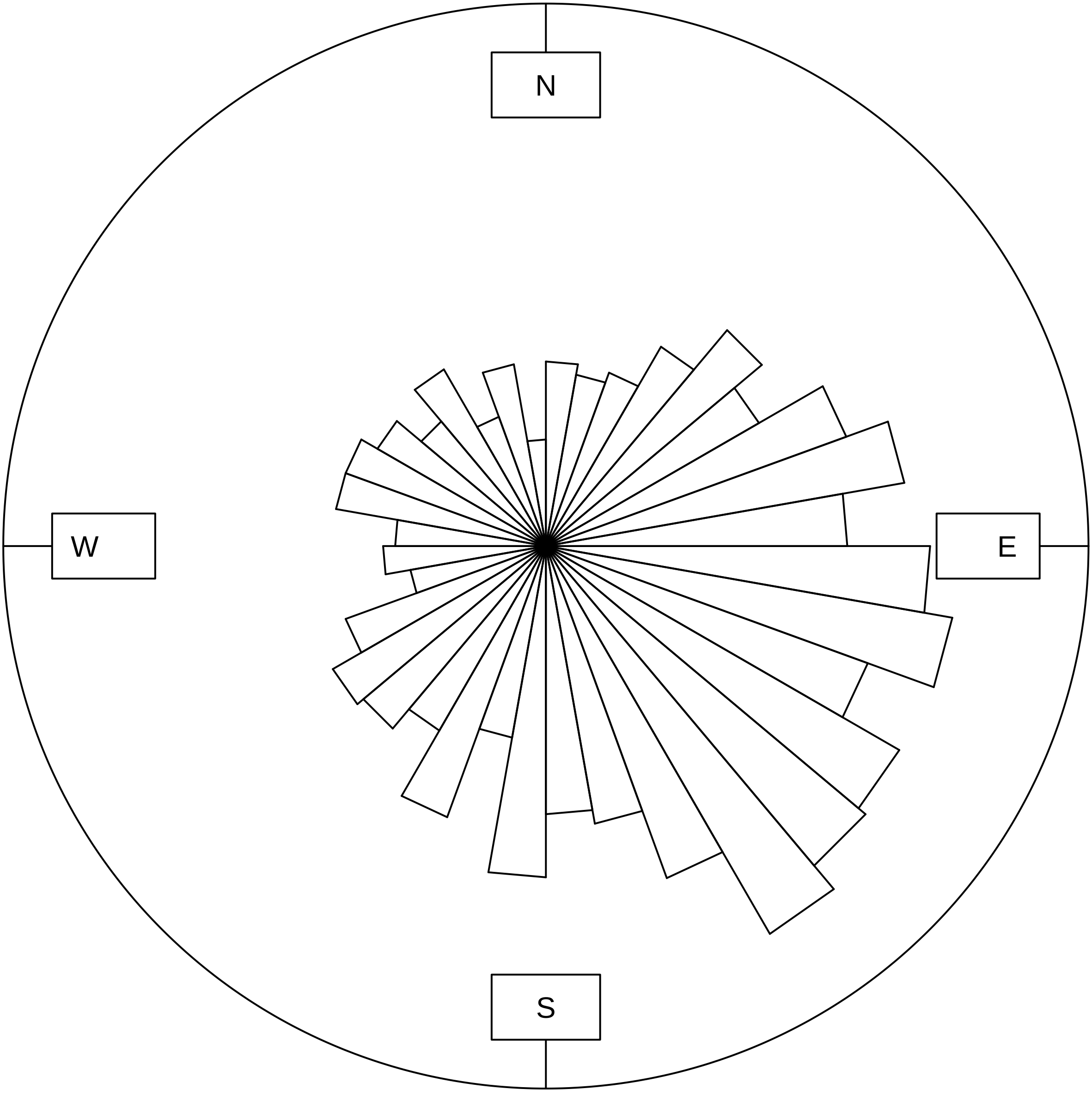

bootstrap 526

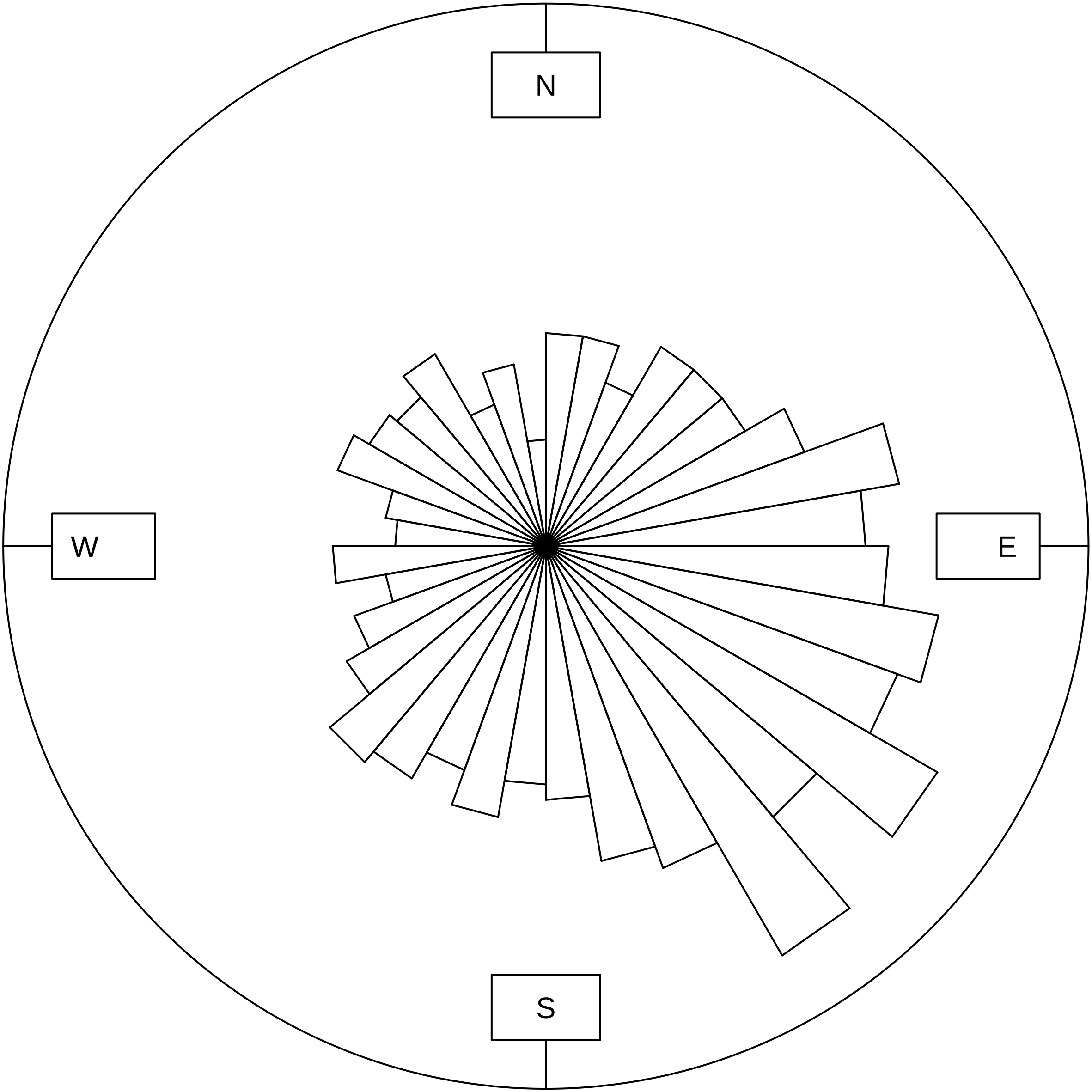

bootstrap 527

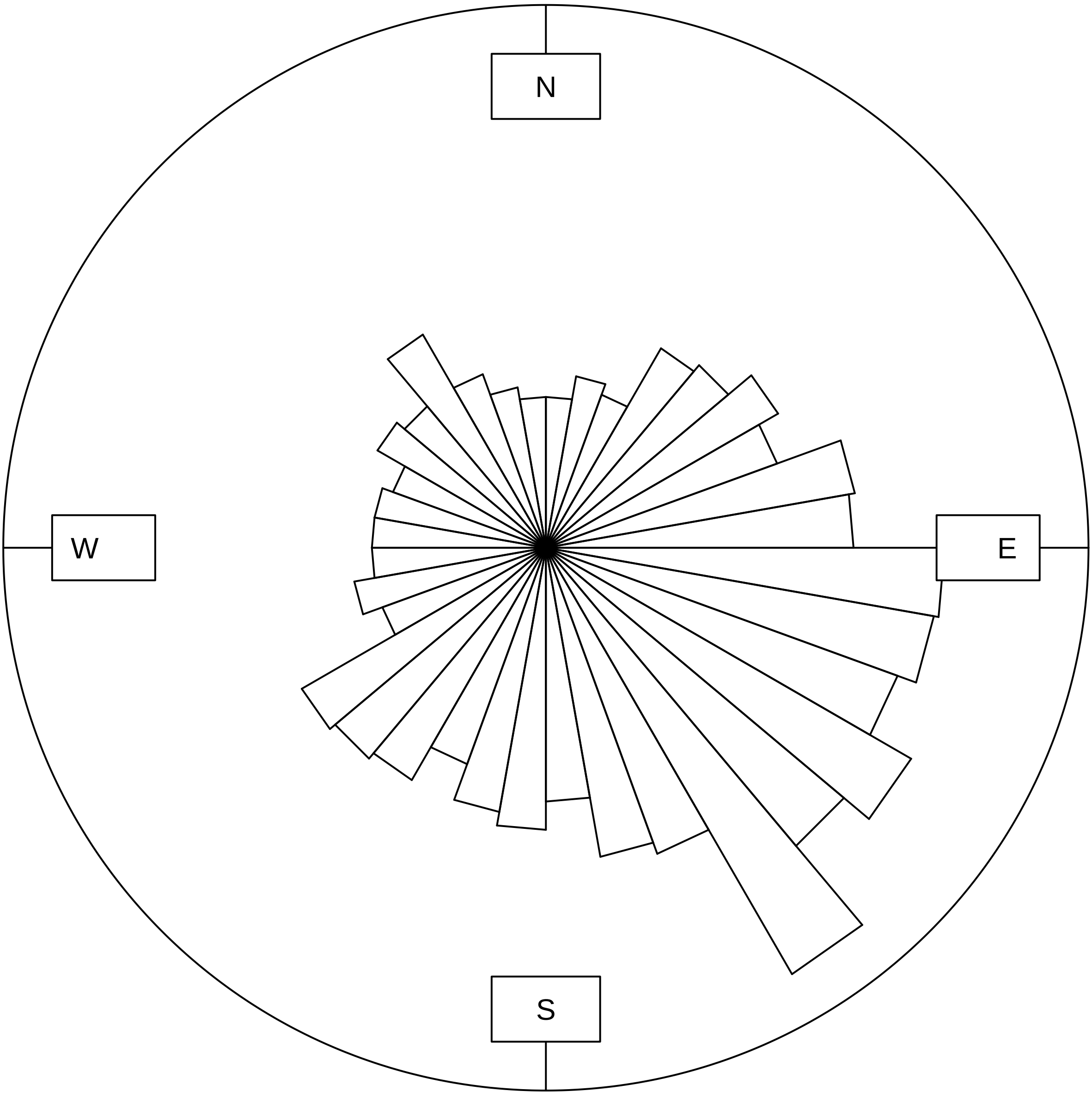

bootstrap 528

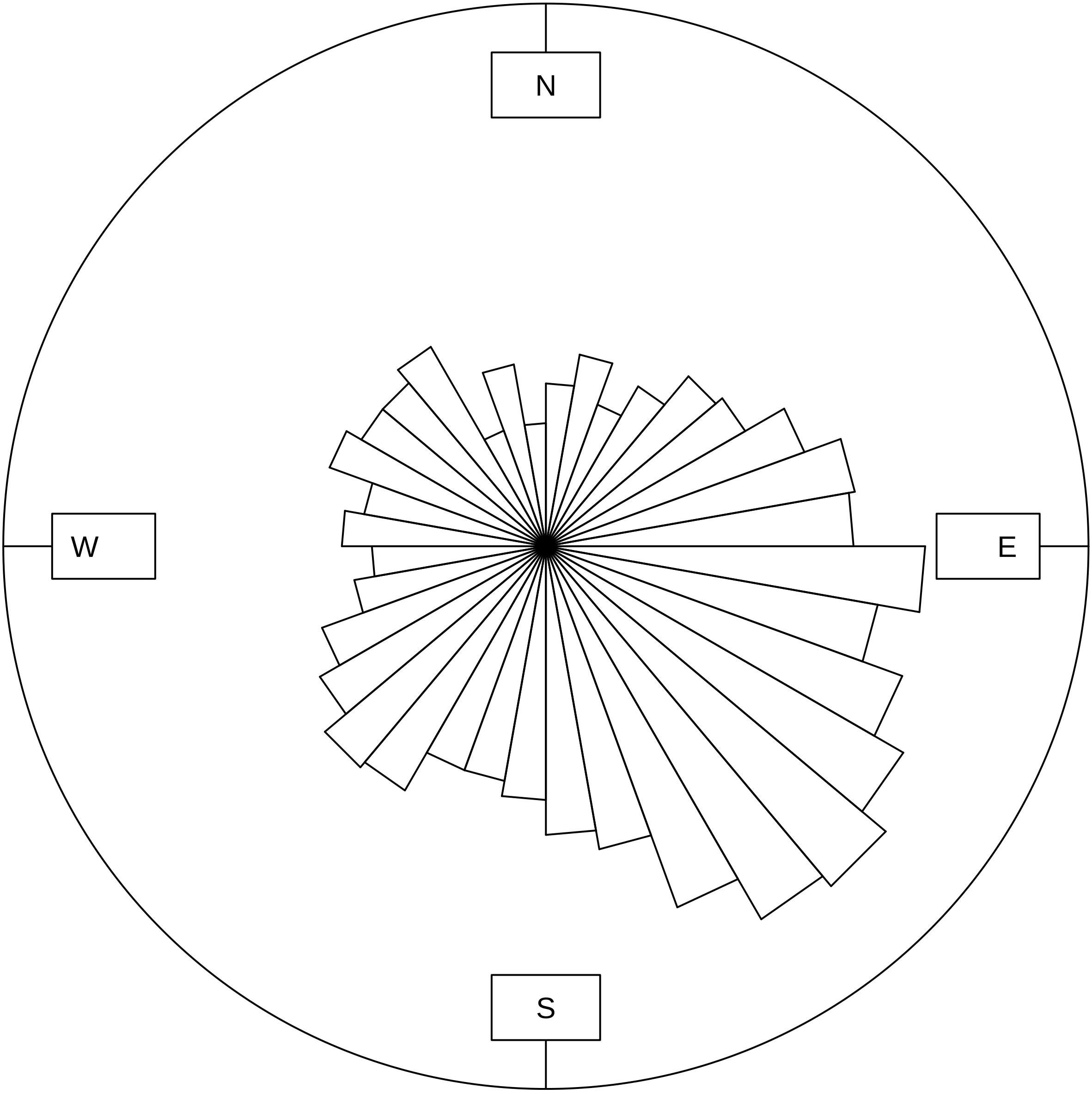

bootstrap 529

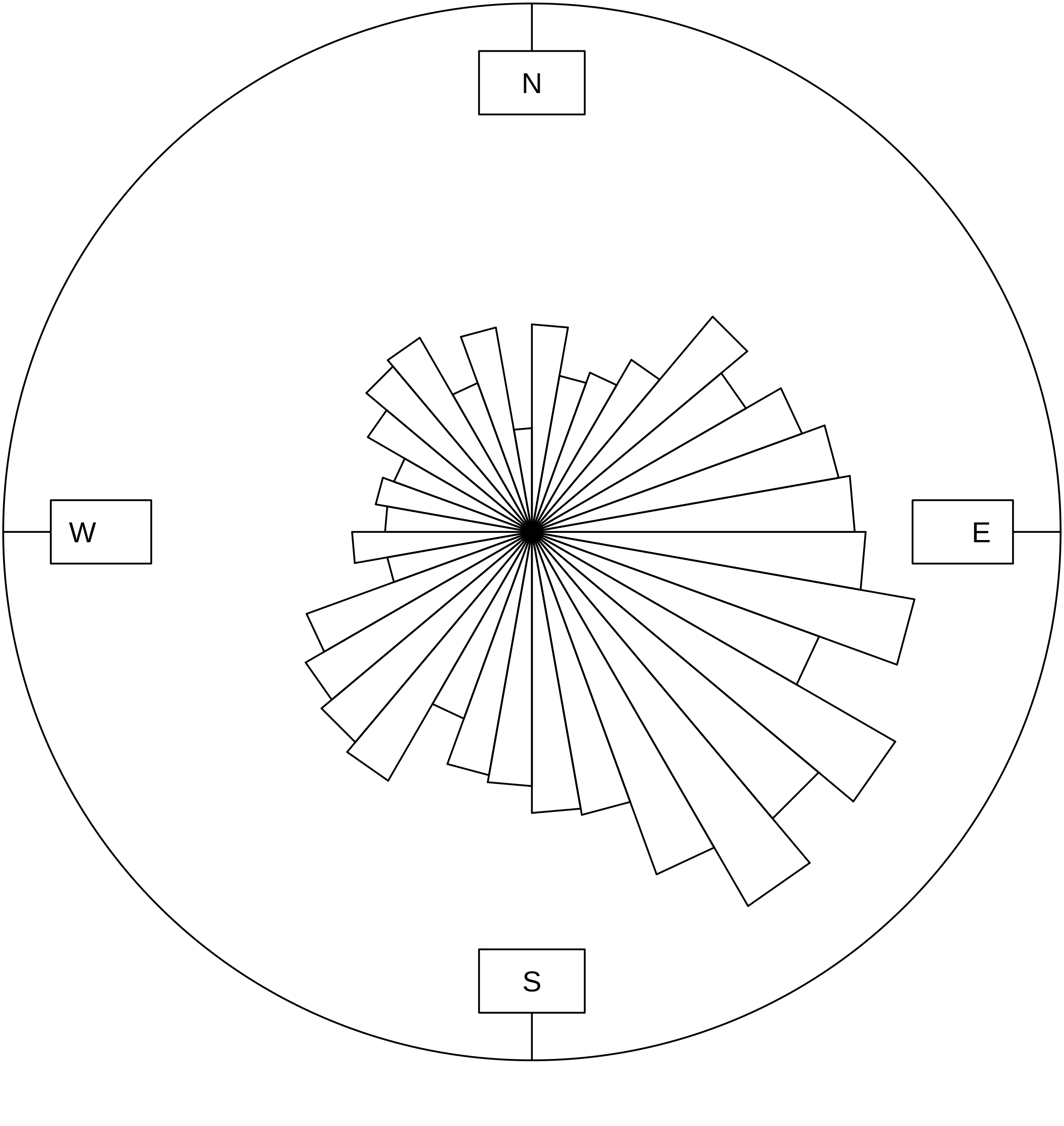

bootstrap 530

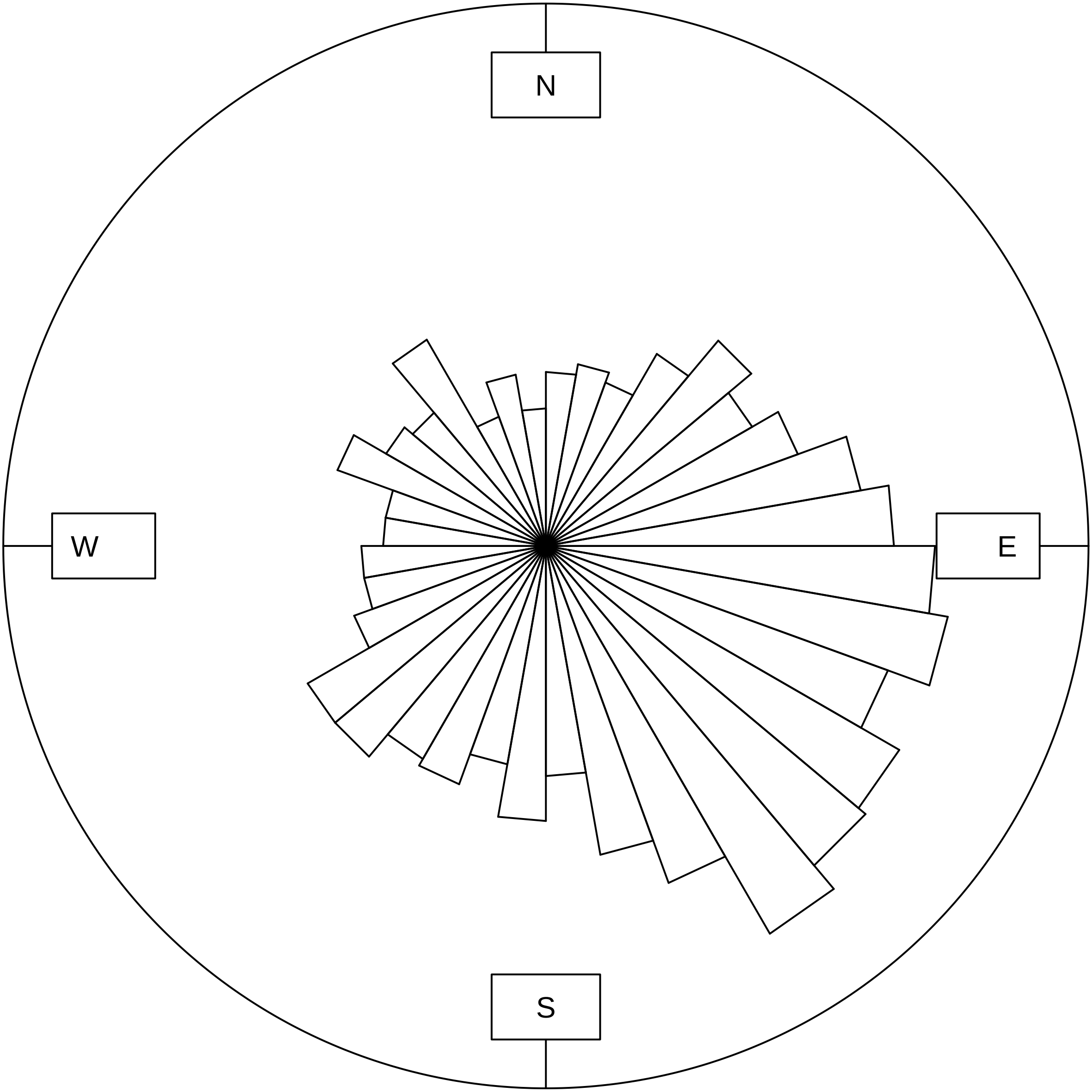

bootstrap 531

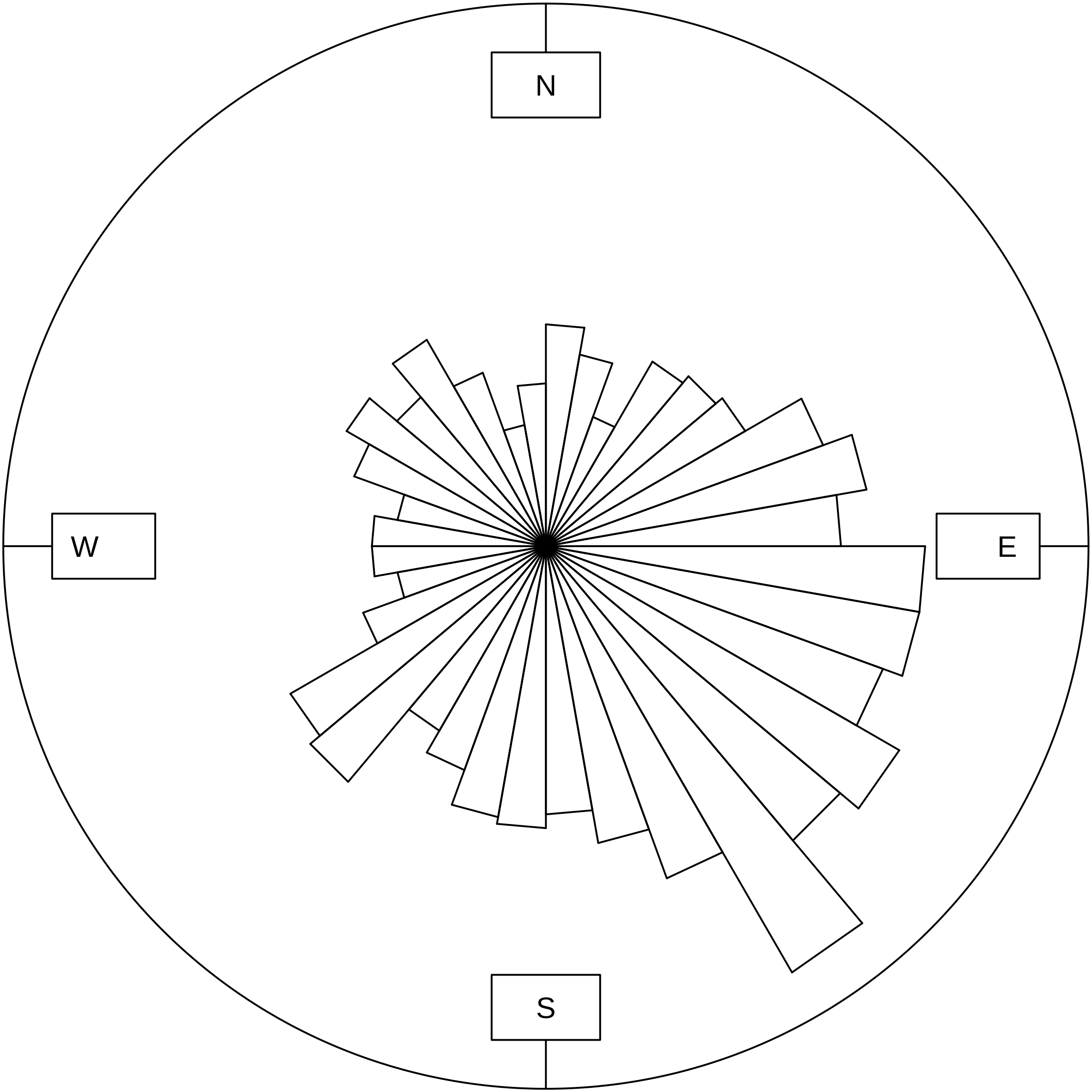

bootstrap 532

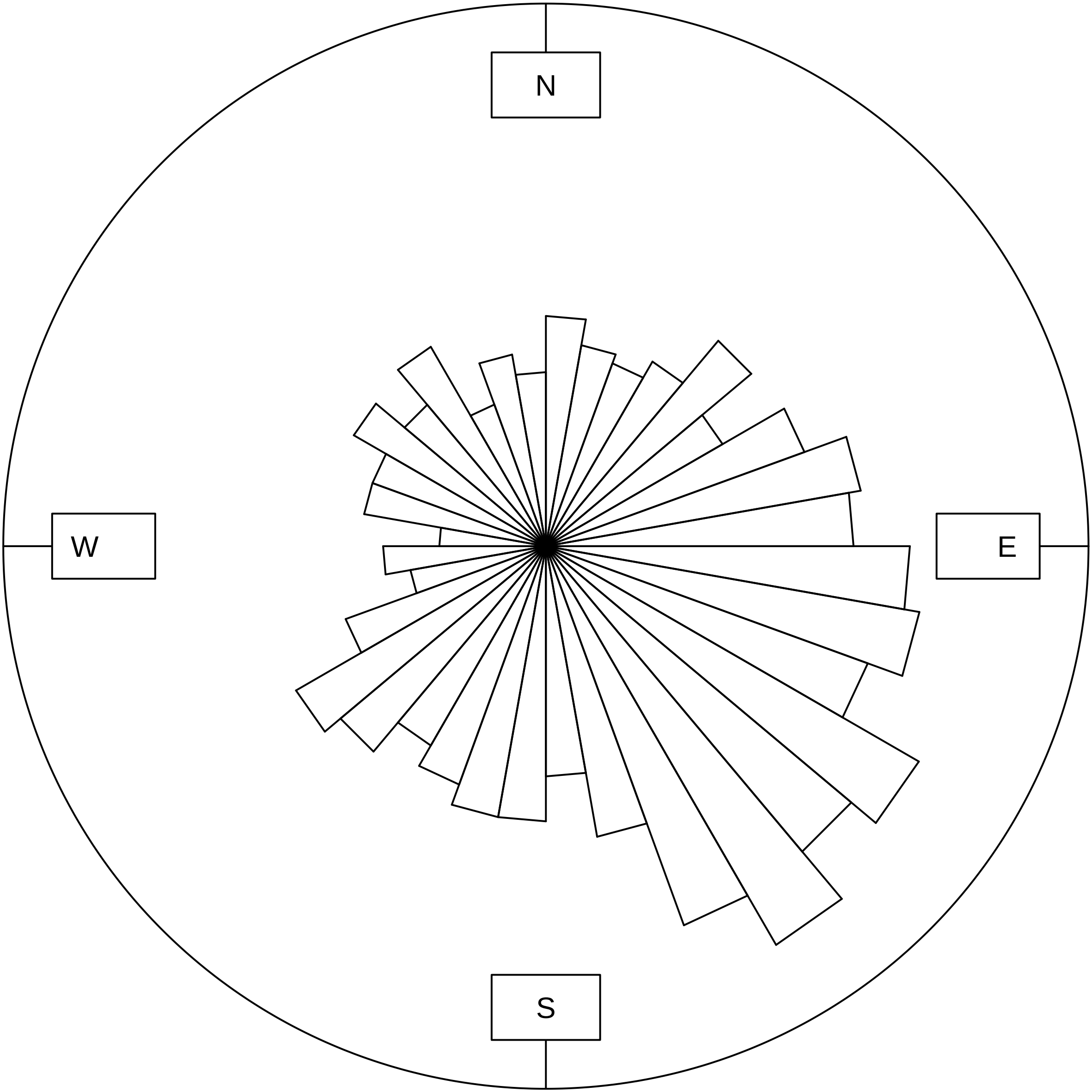

bootstrap 533

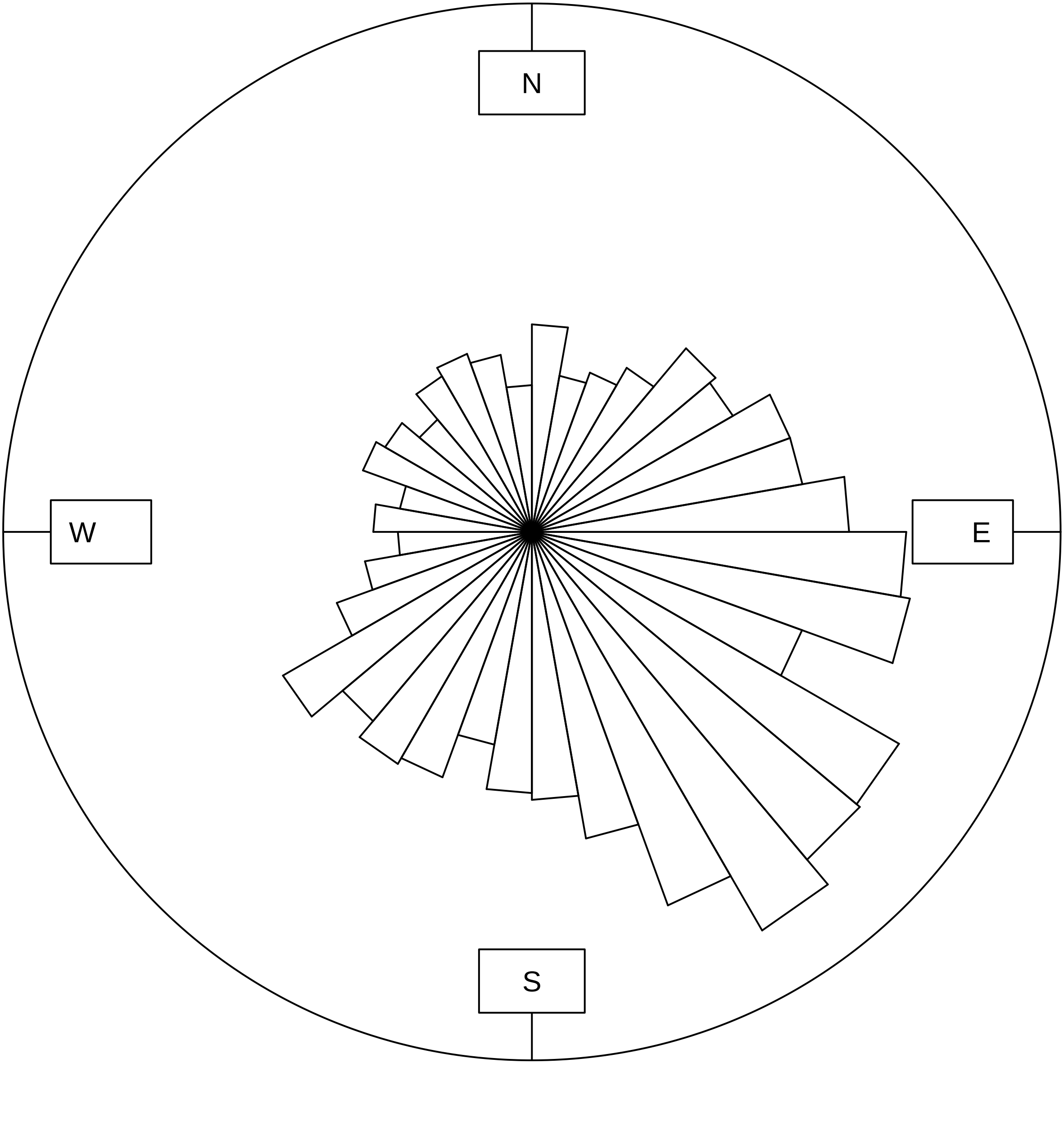

bootstrap 534

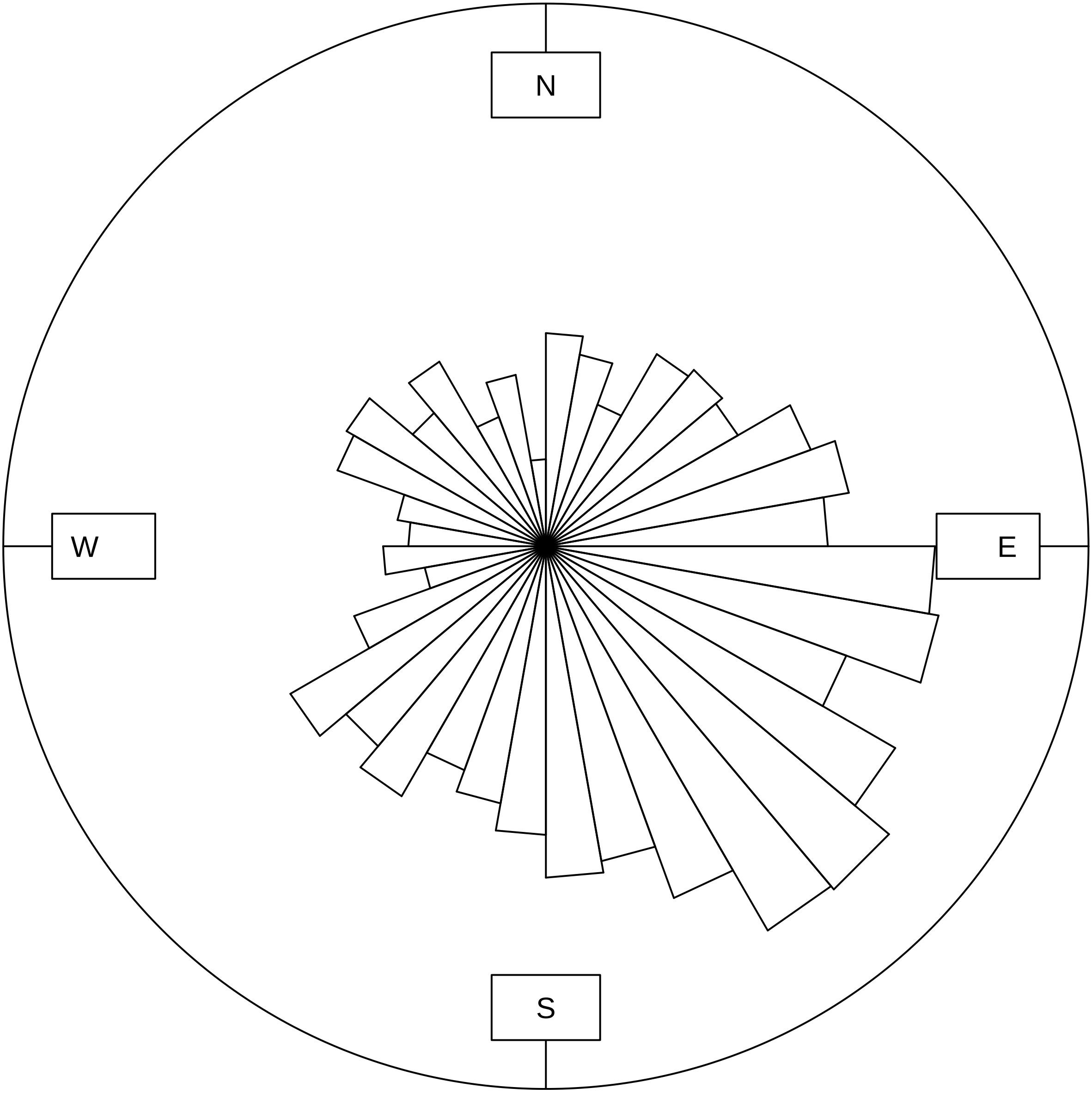

bootstrap 535

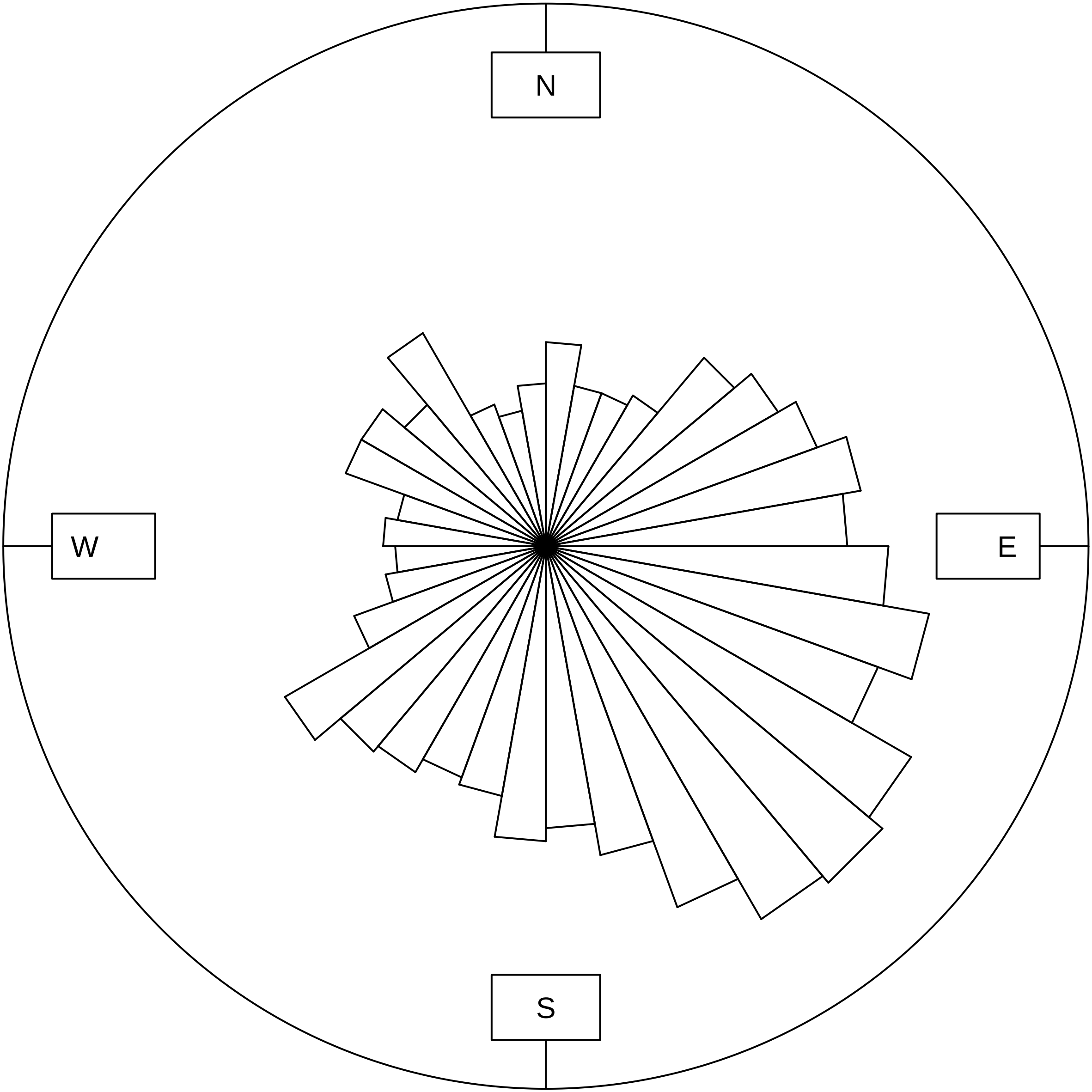

bootstrap 536

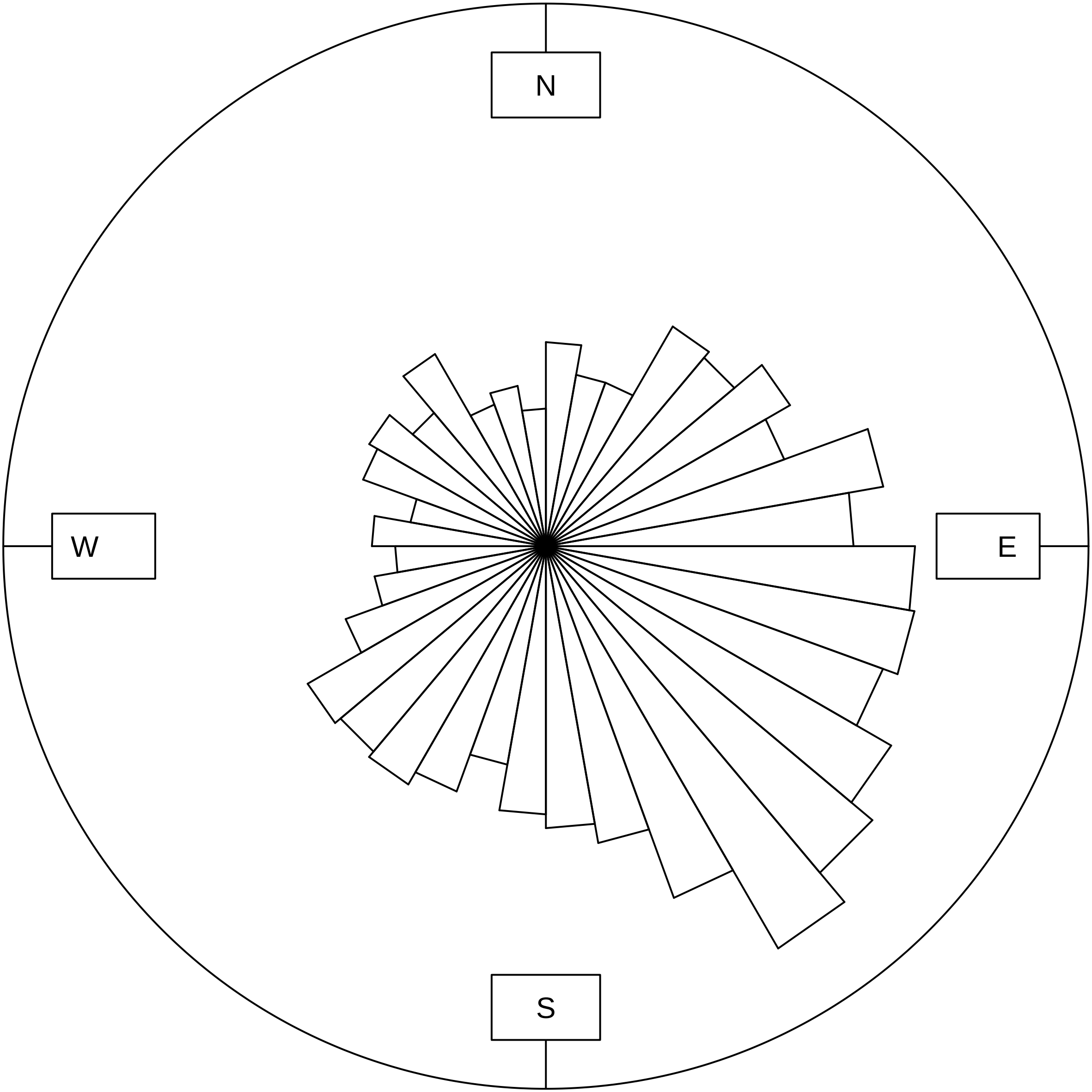

bootstrap 537

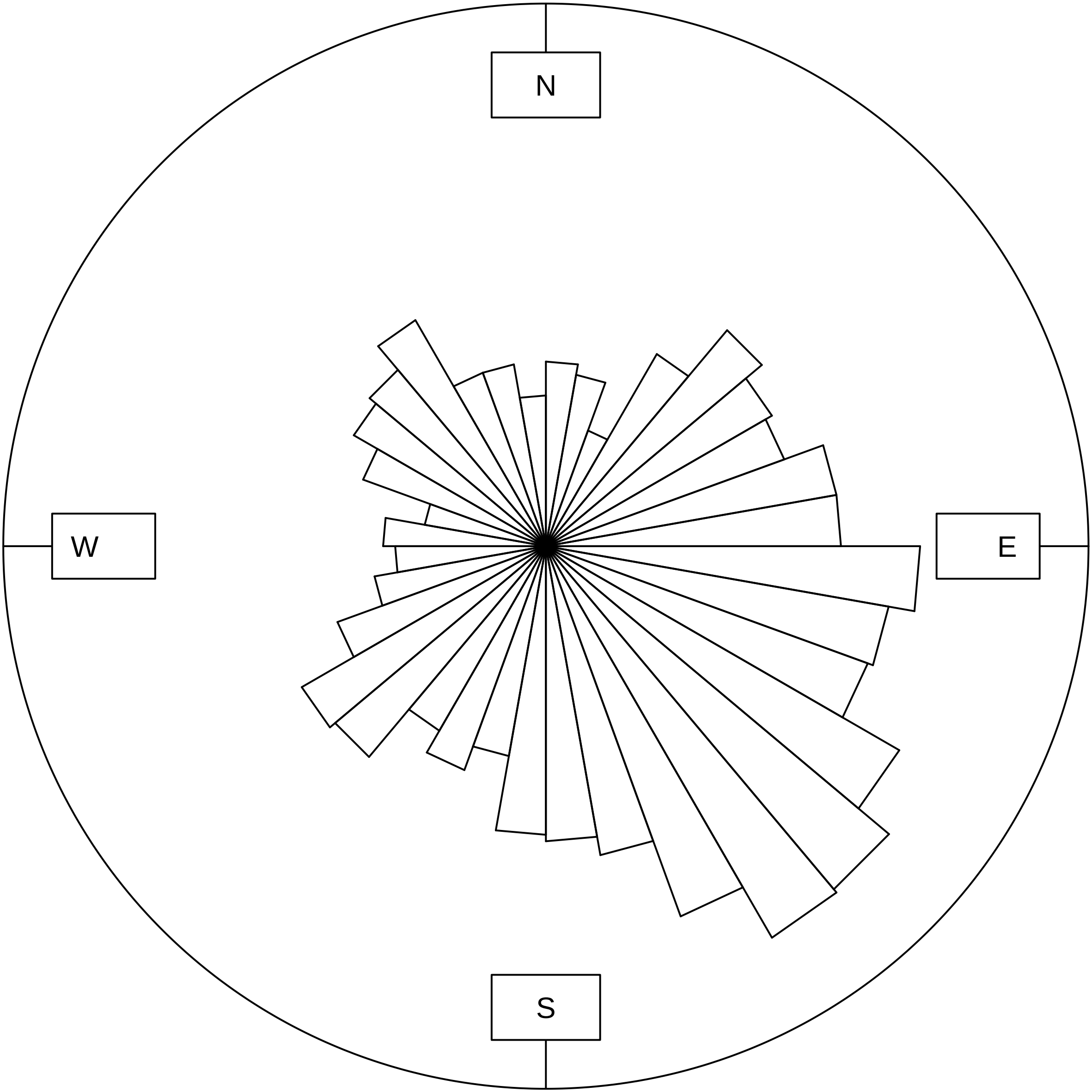

bootstrap 538

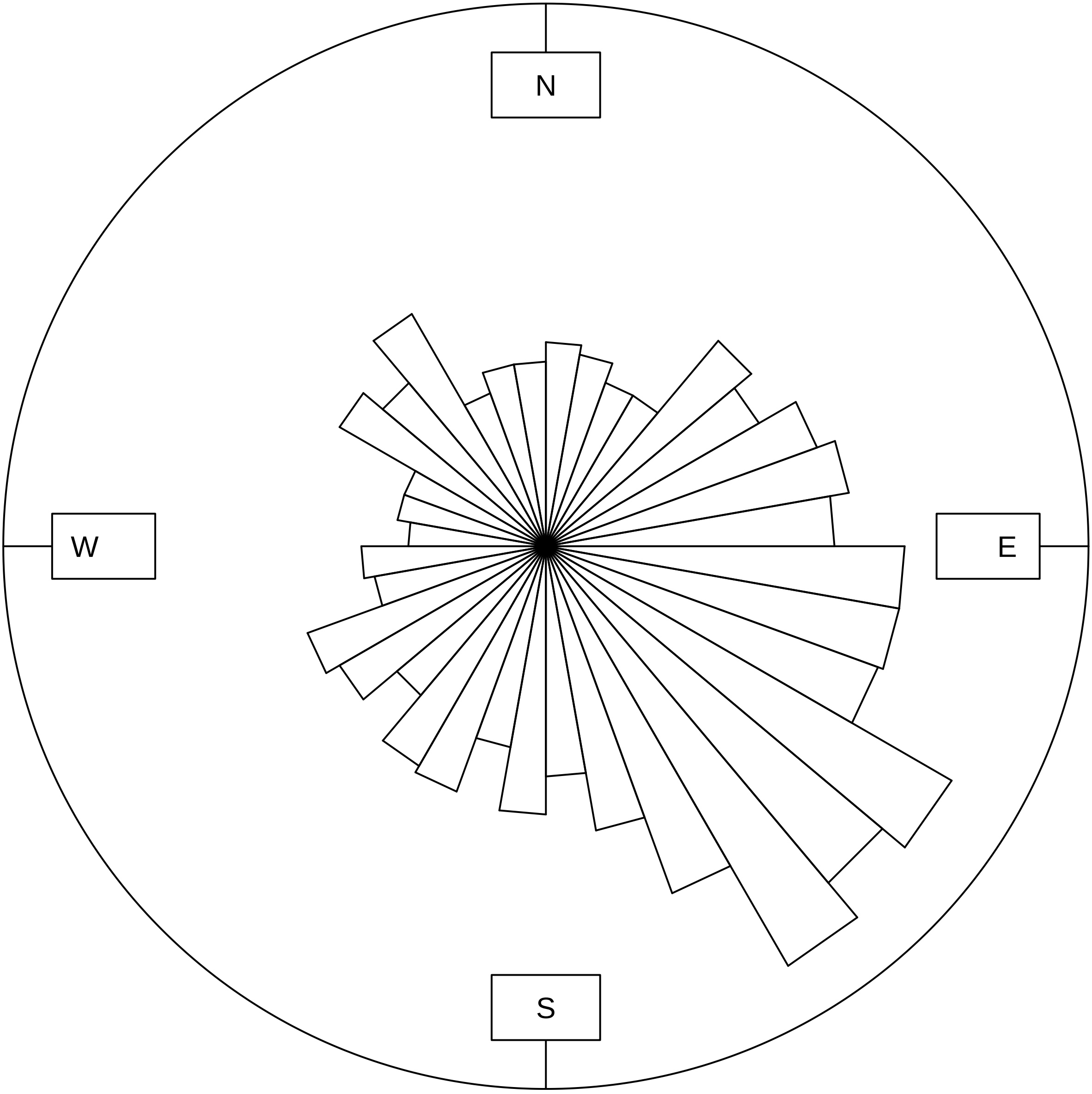

bootstrap 539

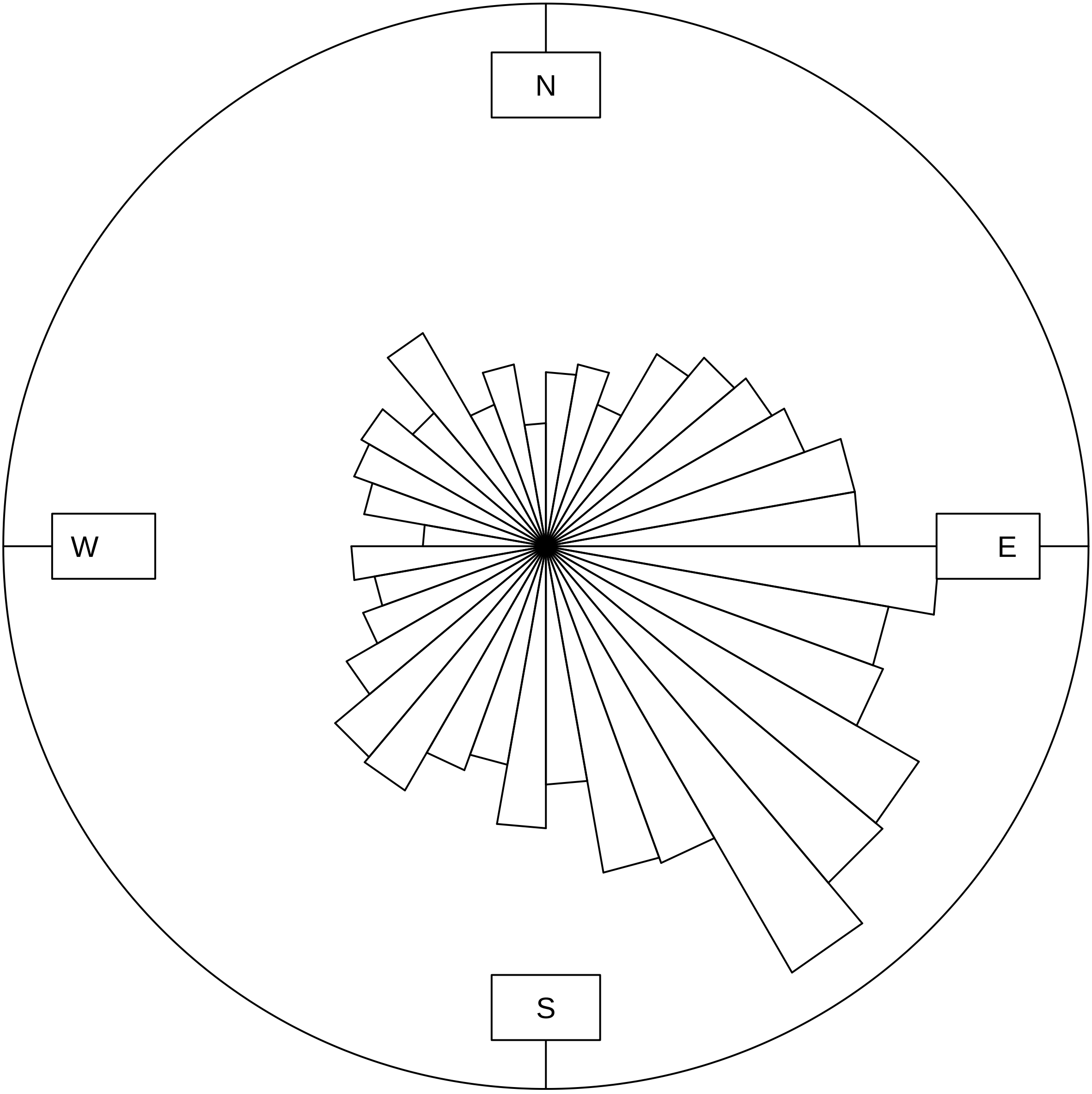

bootstrap 540

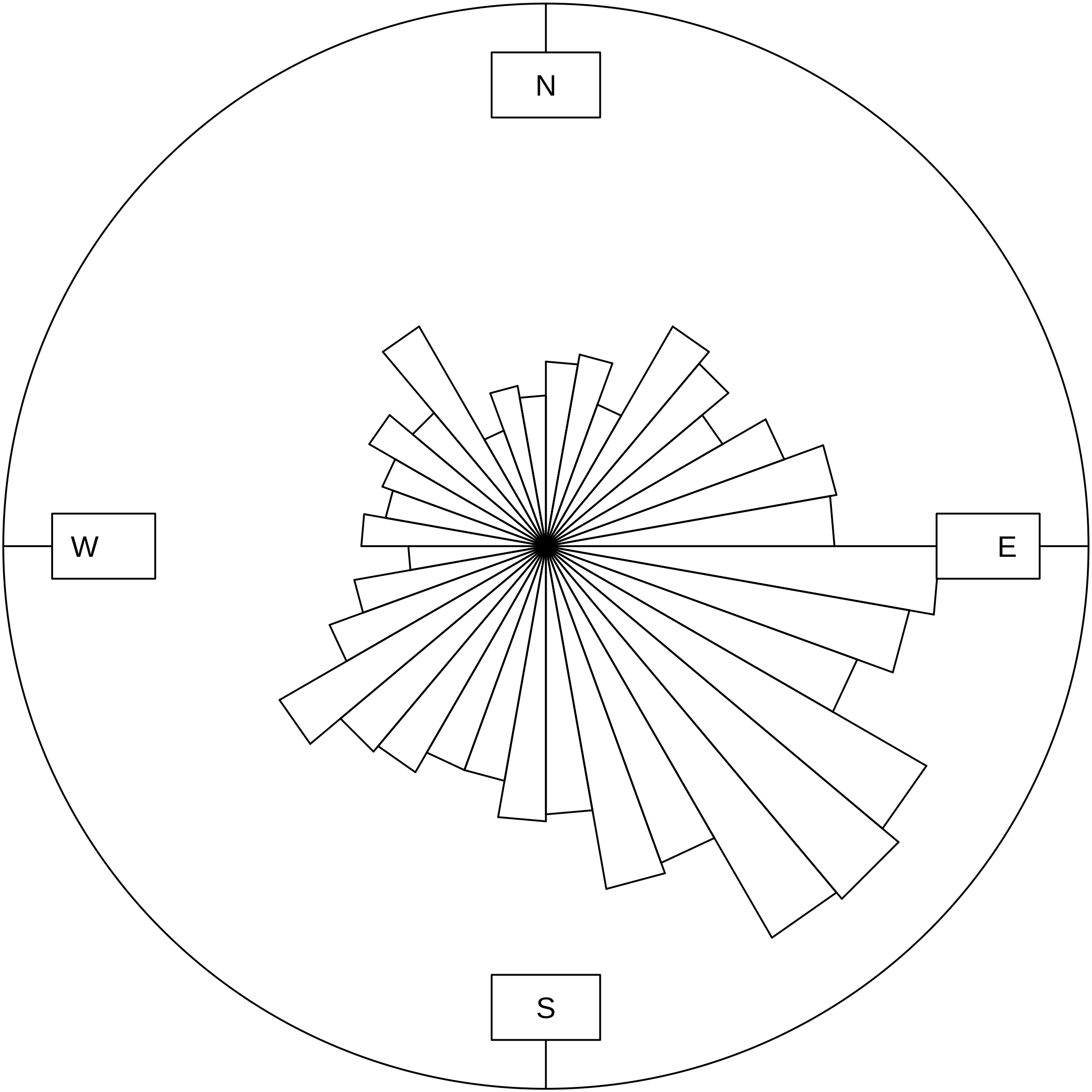

bootstrap 541

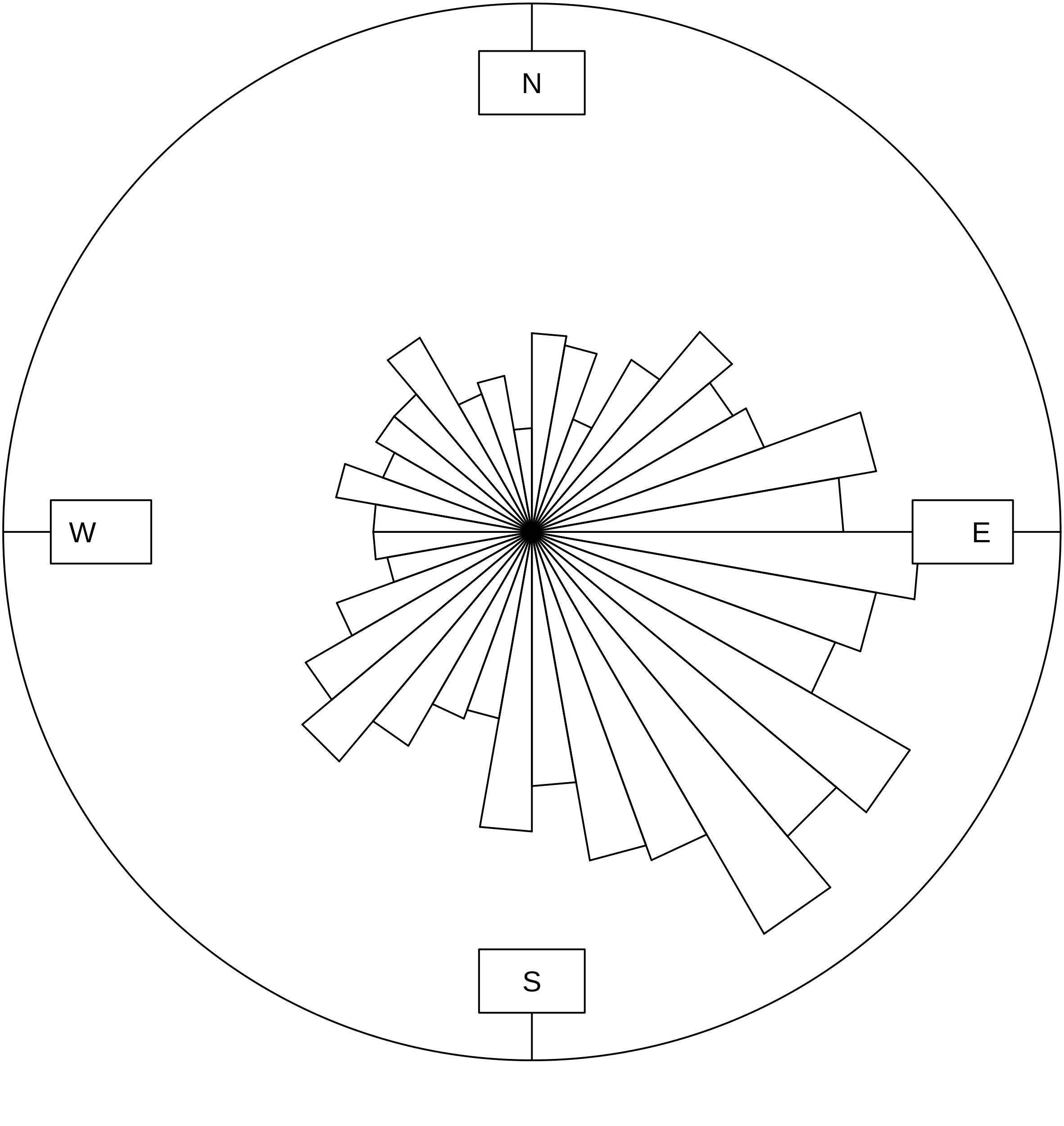

bootstrap 542

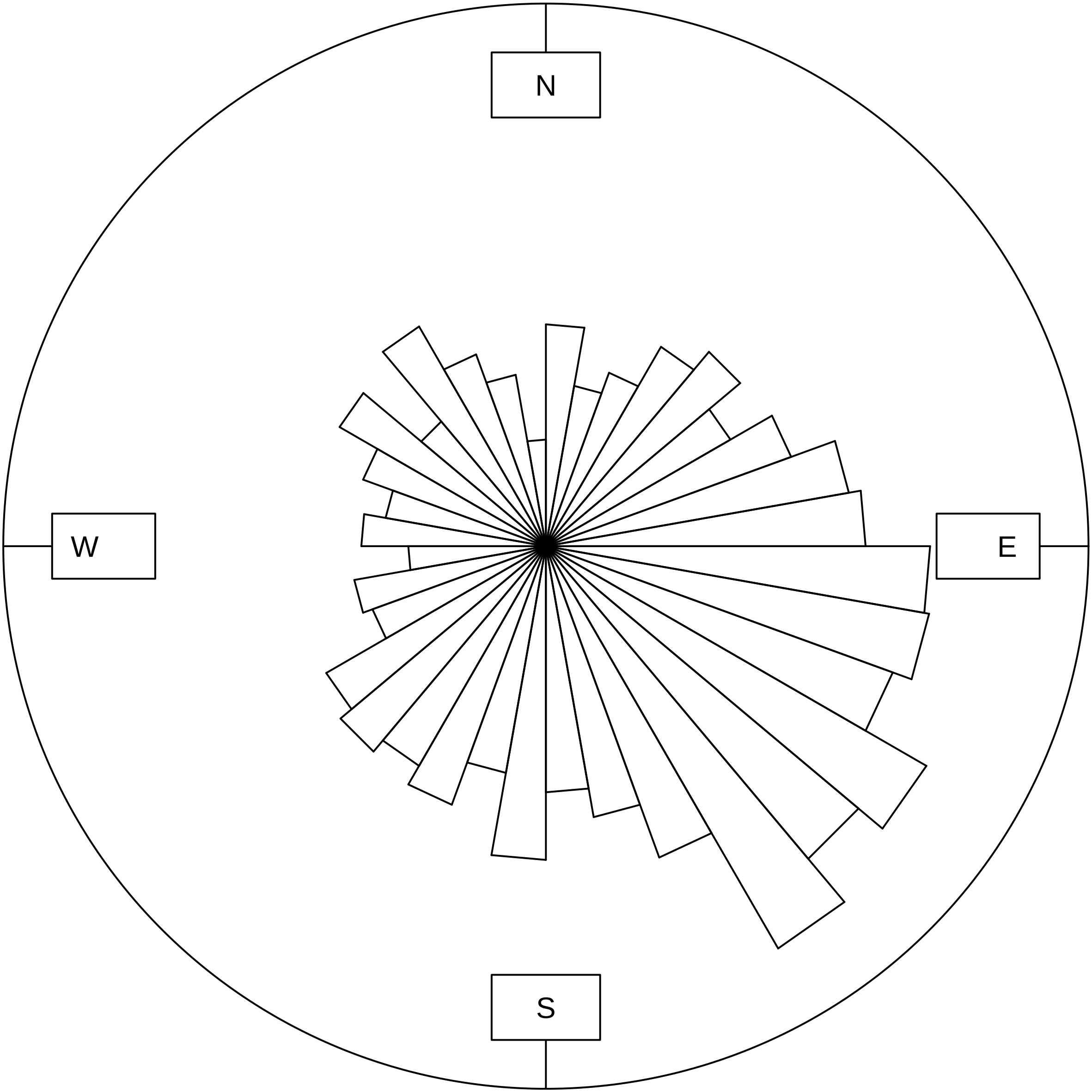

bootstrap 543

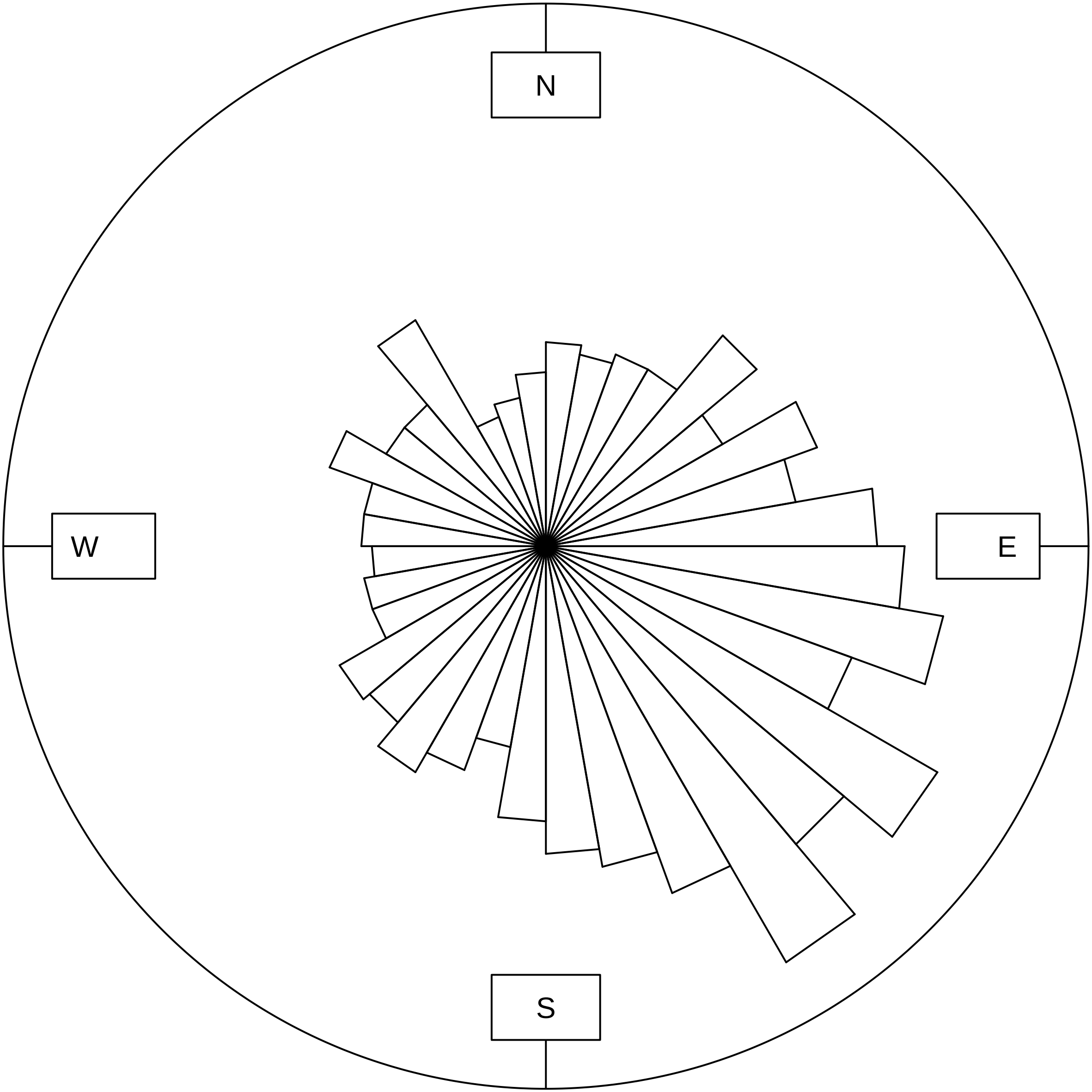

bootstrap 544

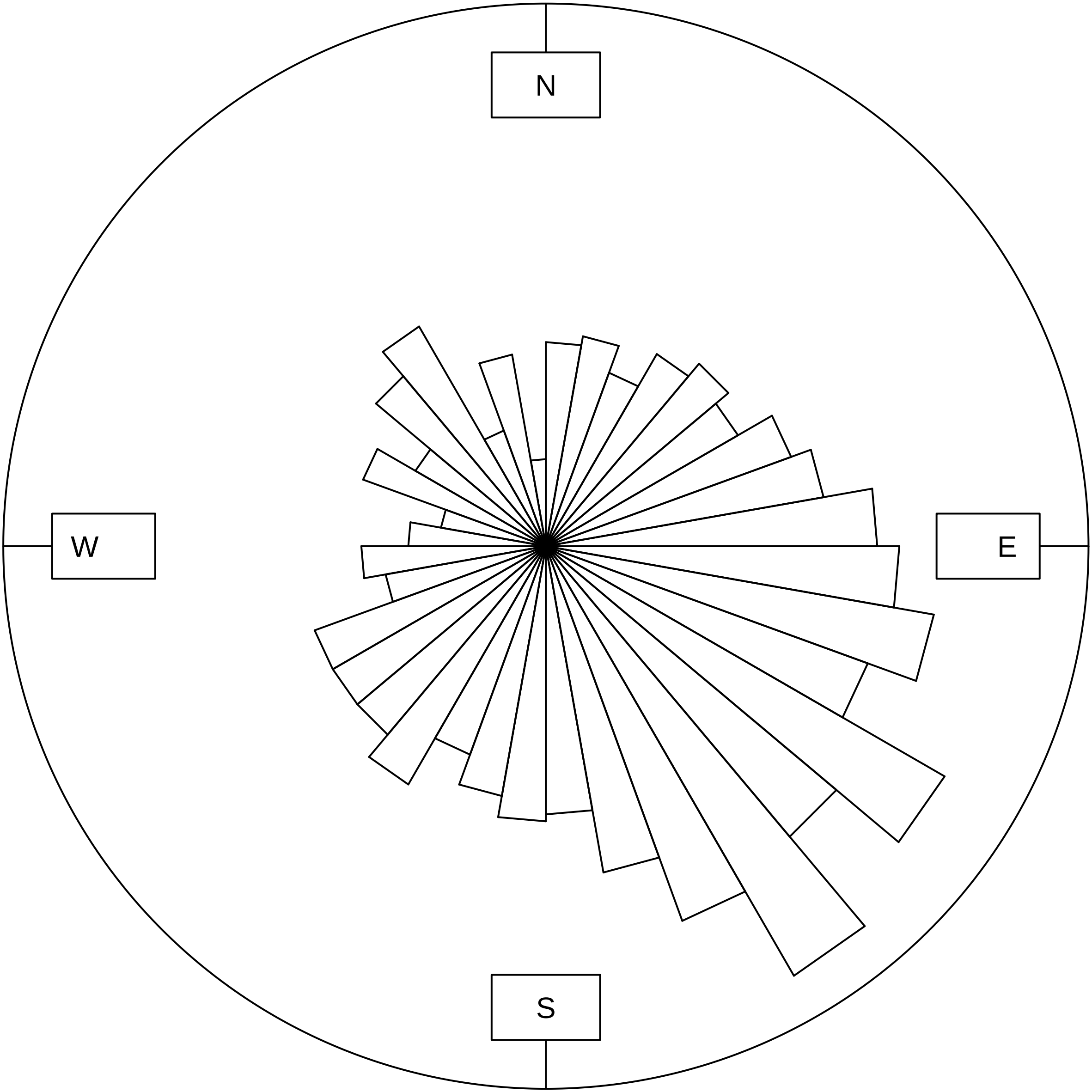

bootstrap 545

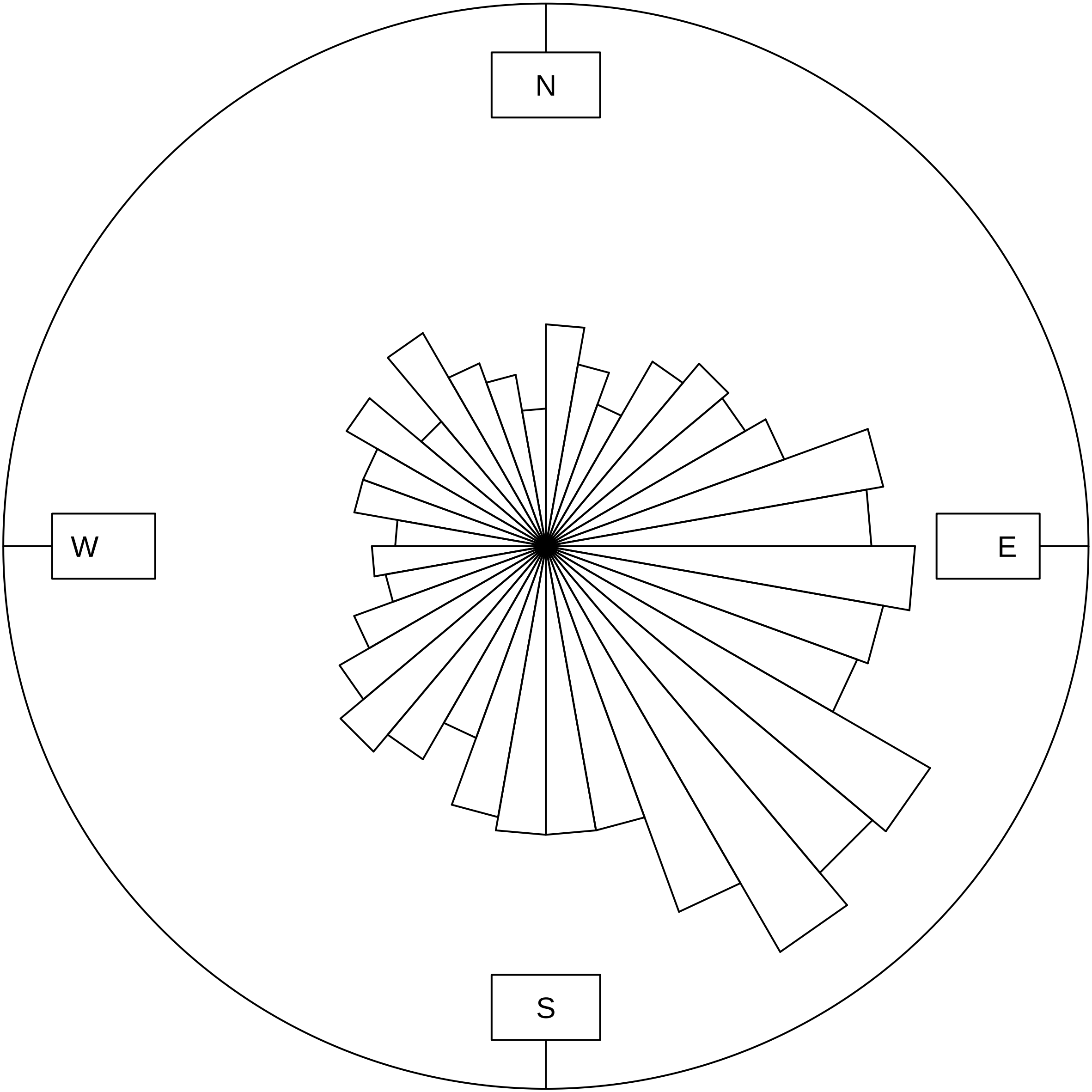

bootstrap 546

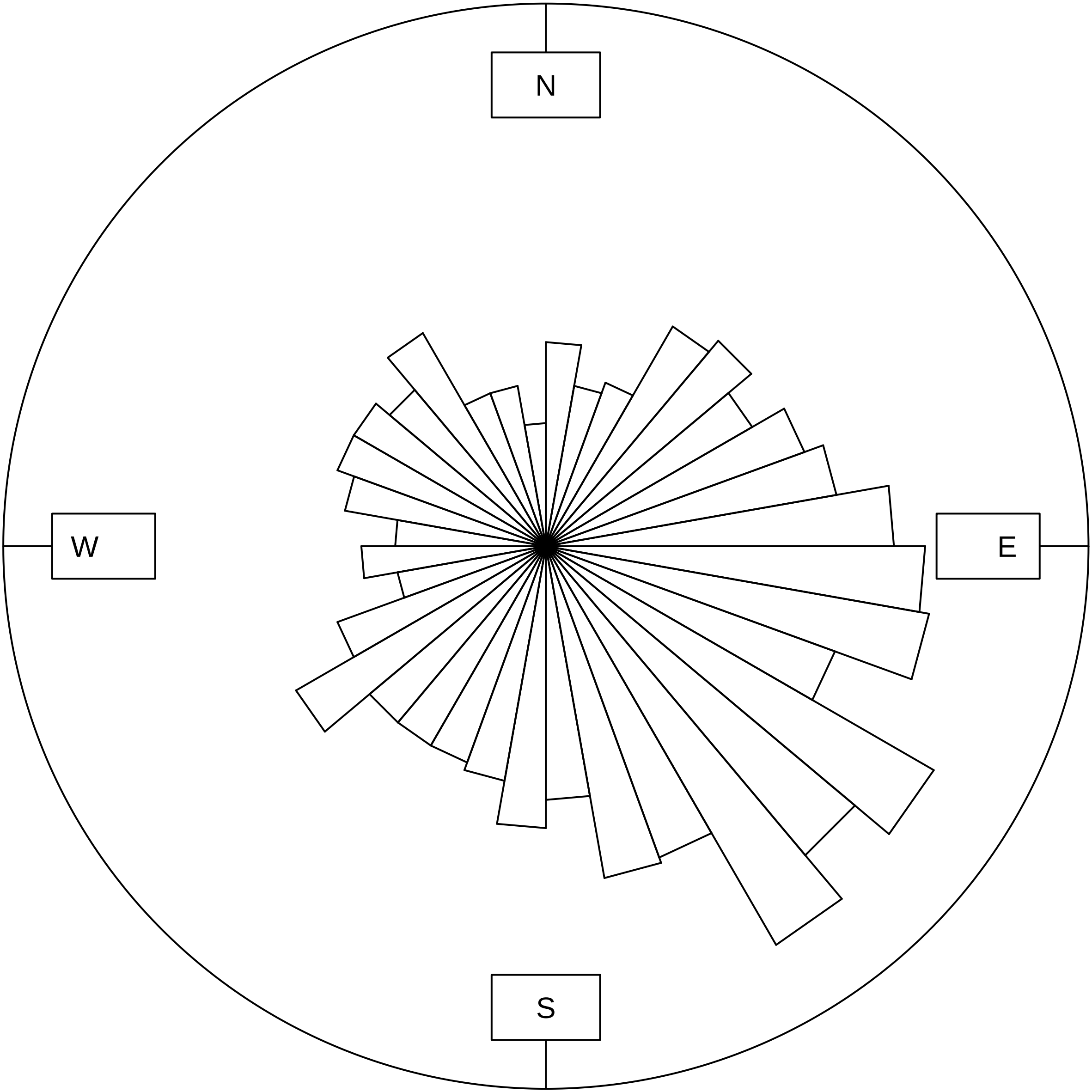

bootstrap 547

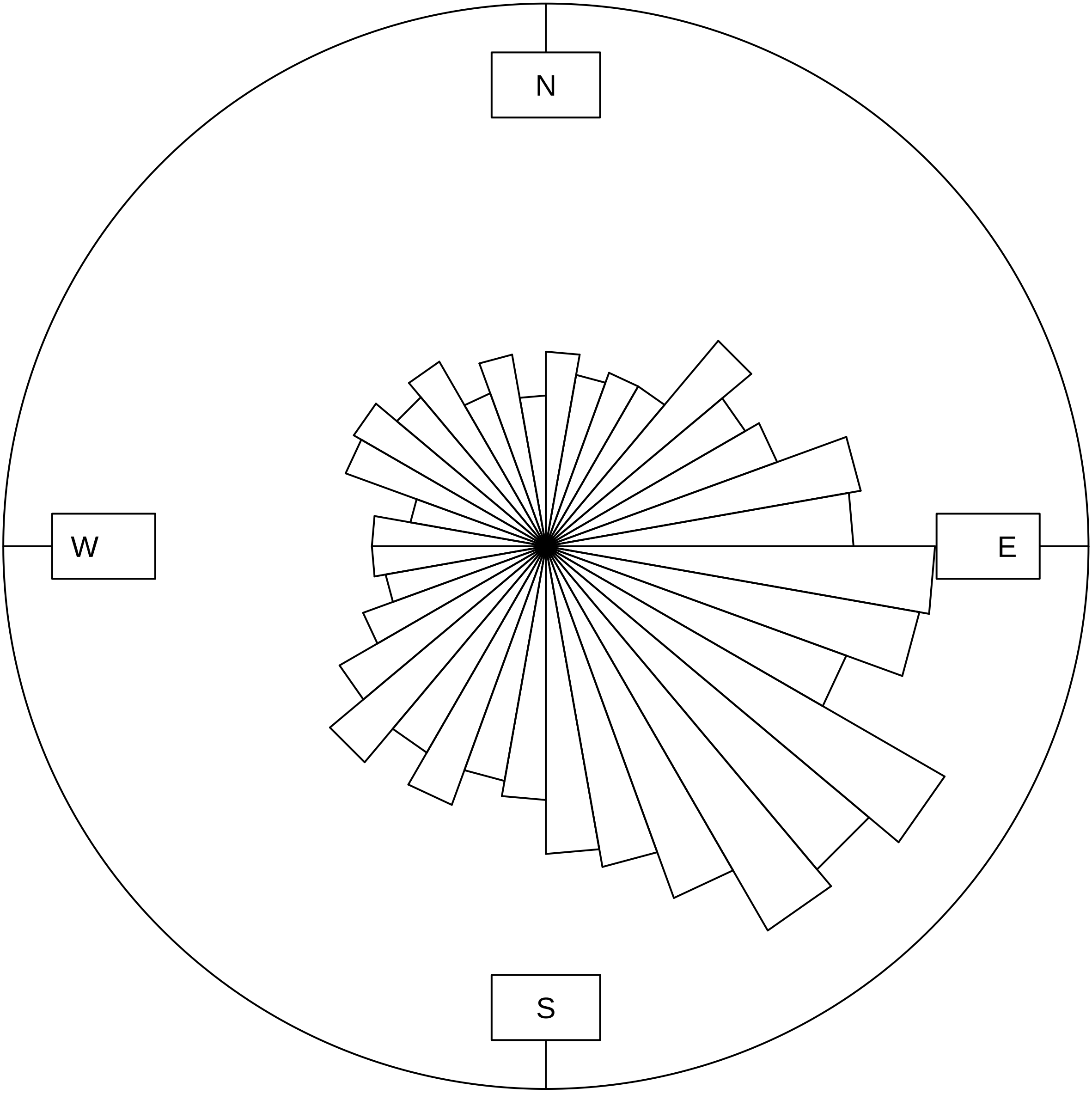

bootstrap 548

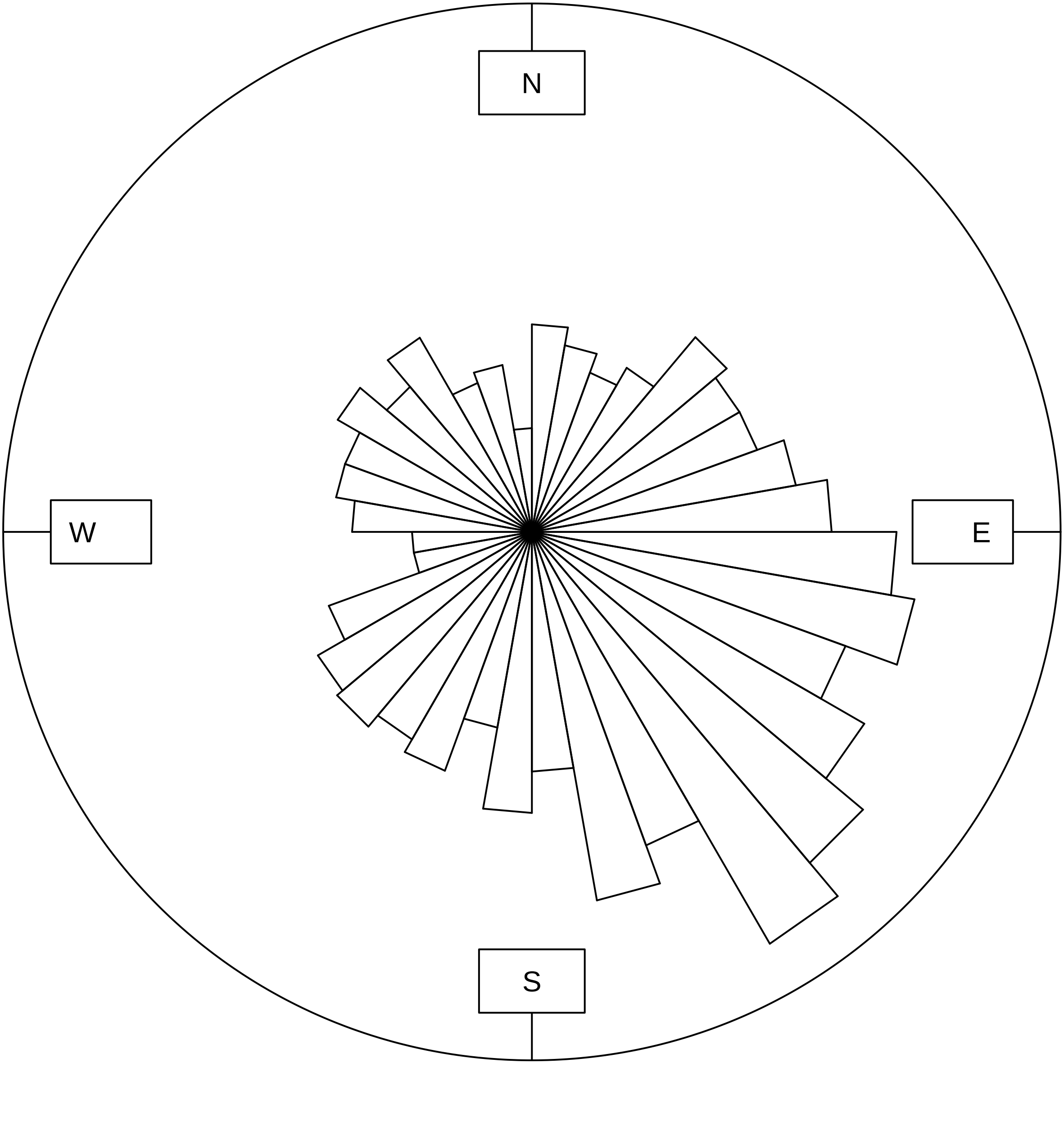

bootstrap 549

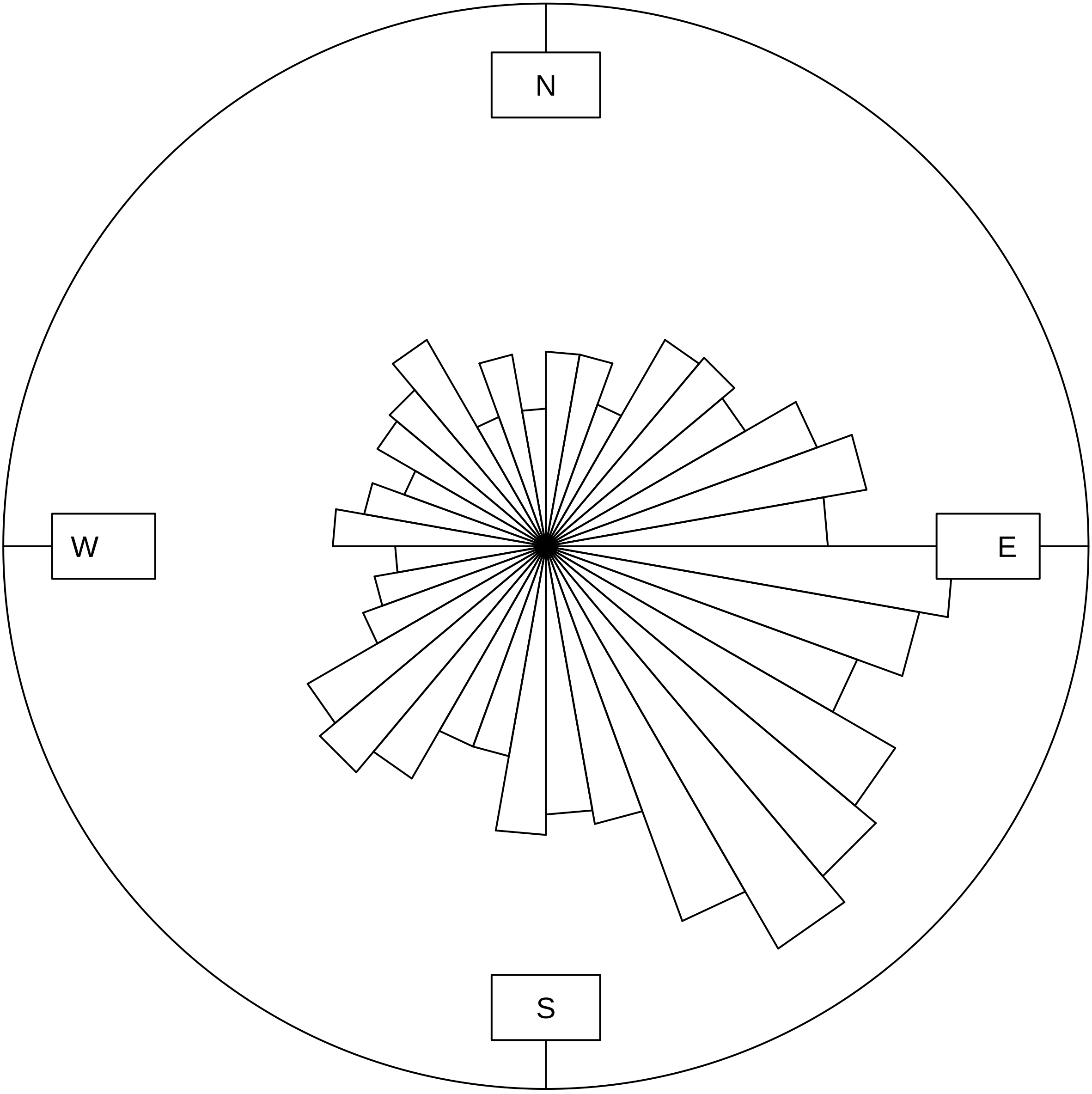

bootstrap 550

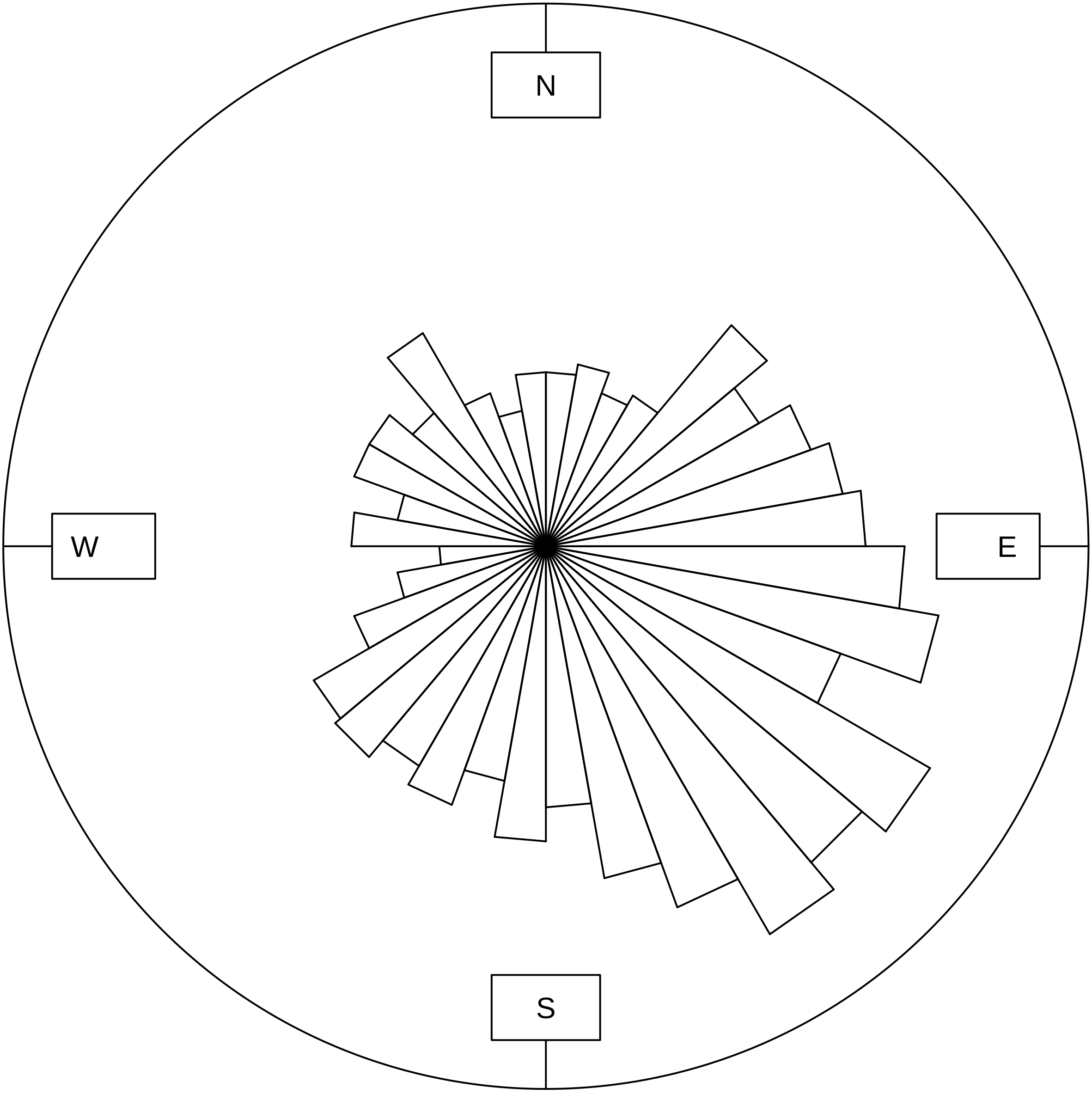

bootstrap 551

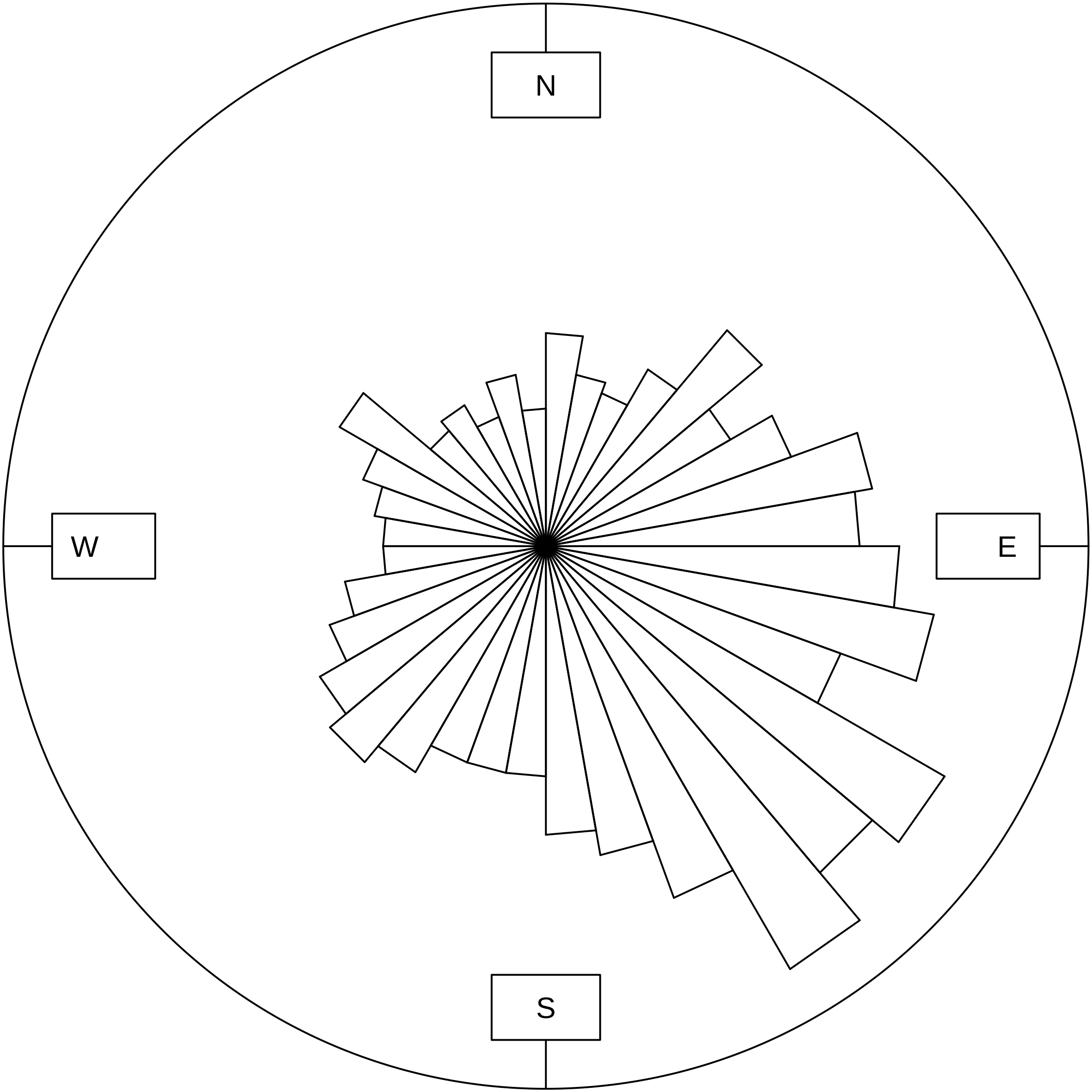

bootstrap 552

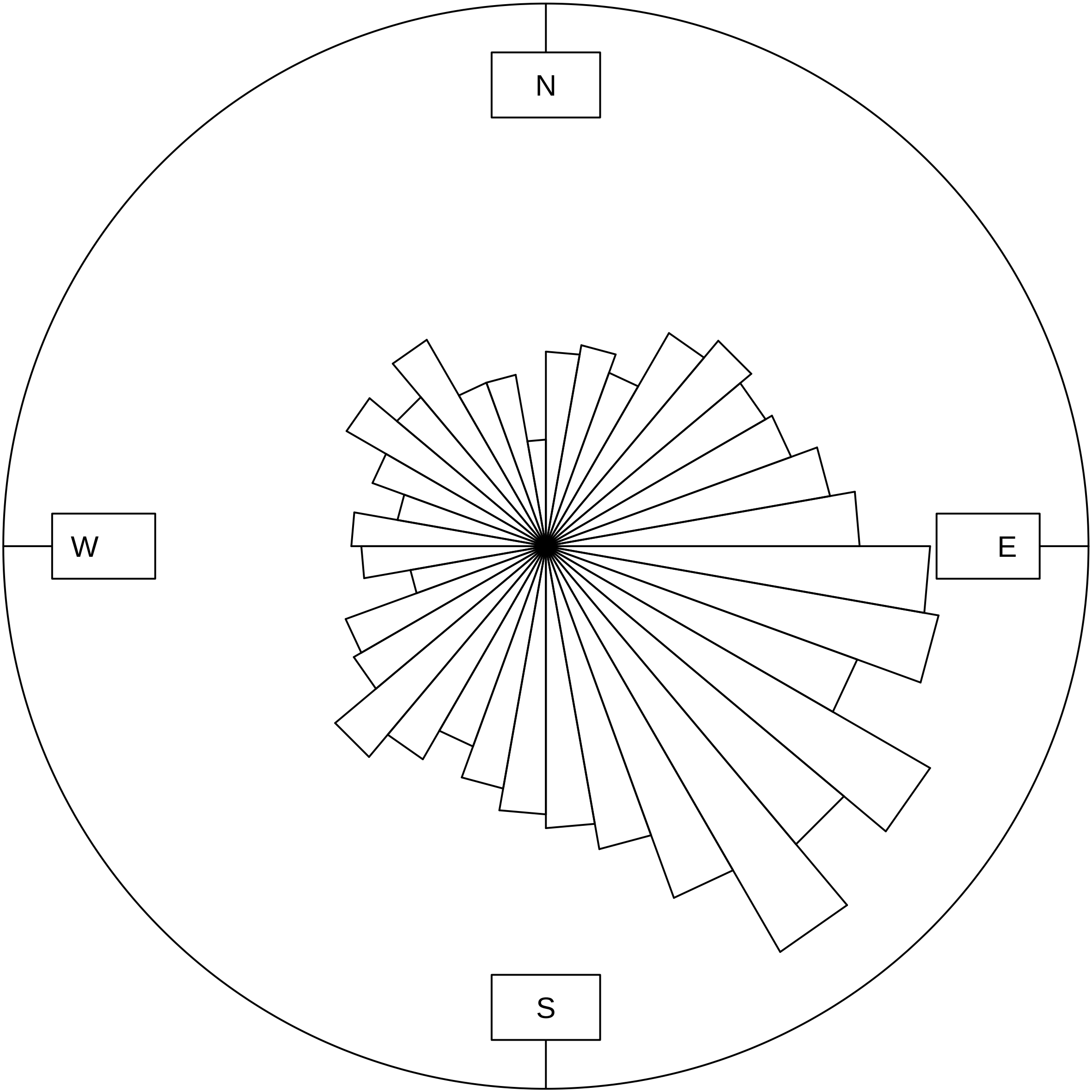

bootstrap 553

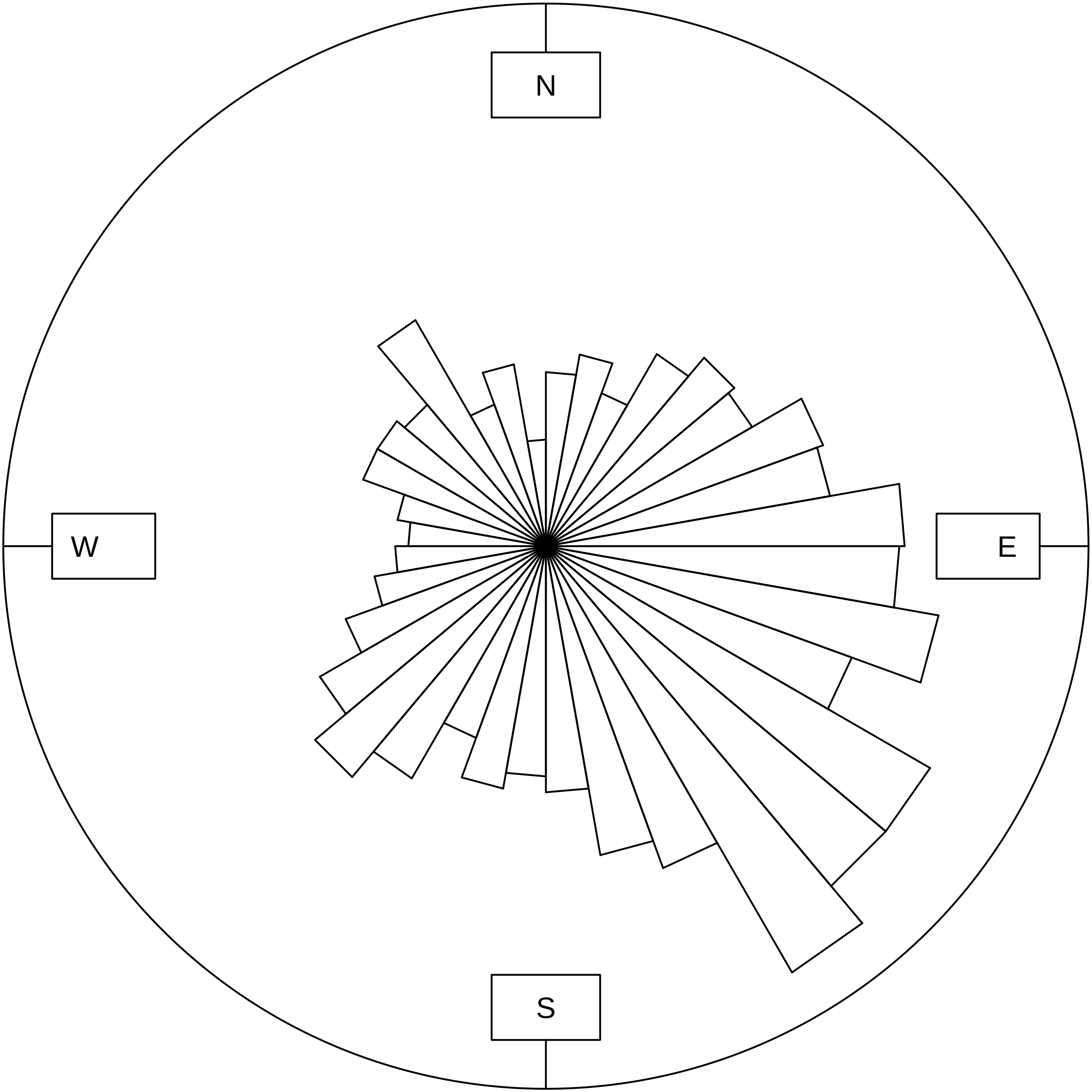

bootstrap 554

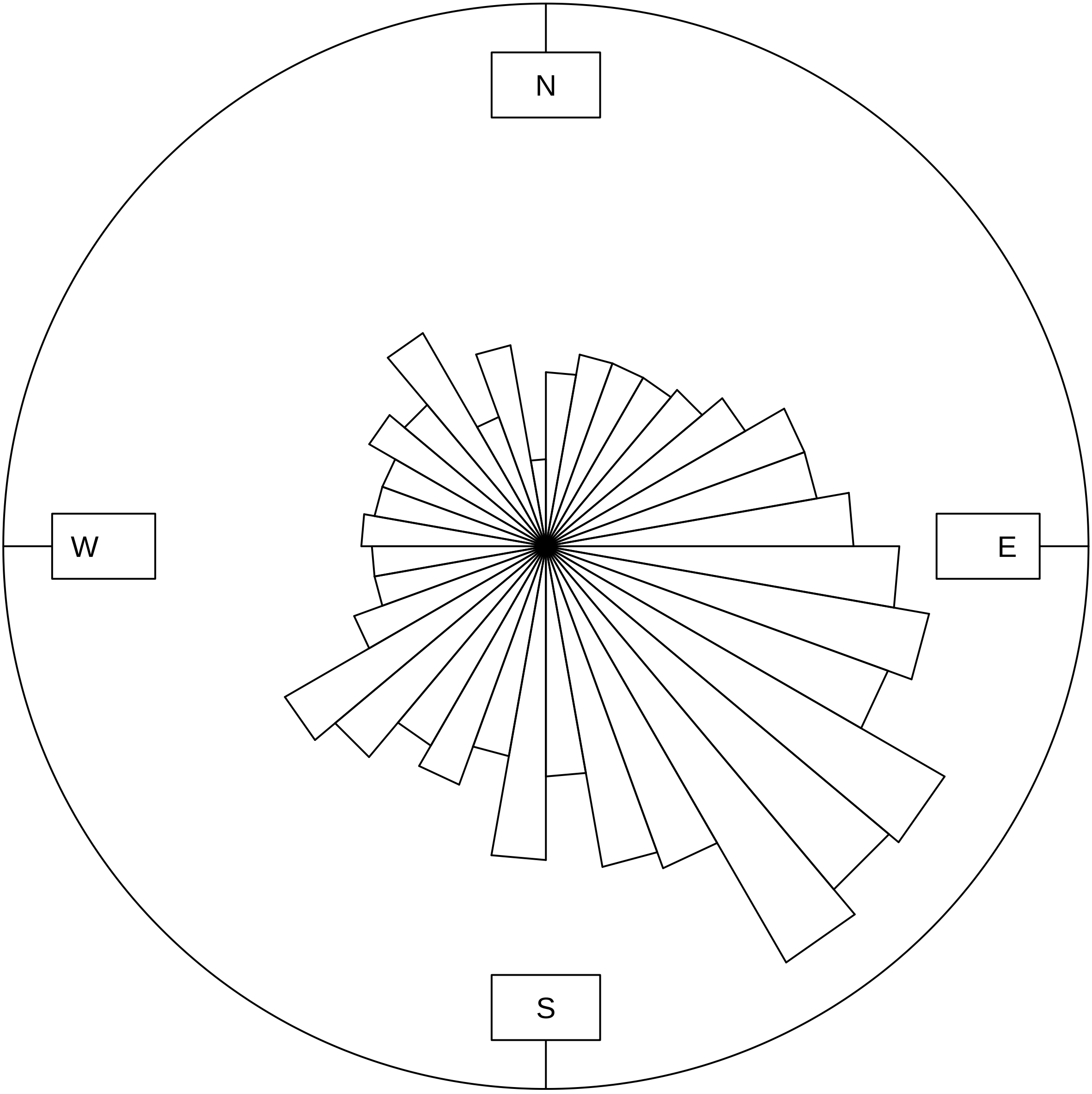

bootstrap 555

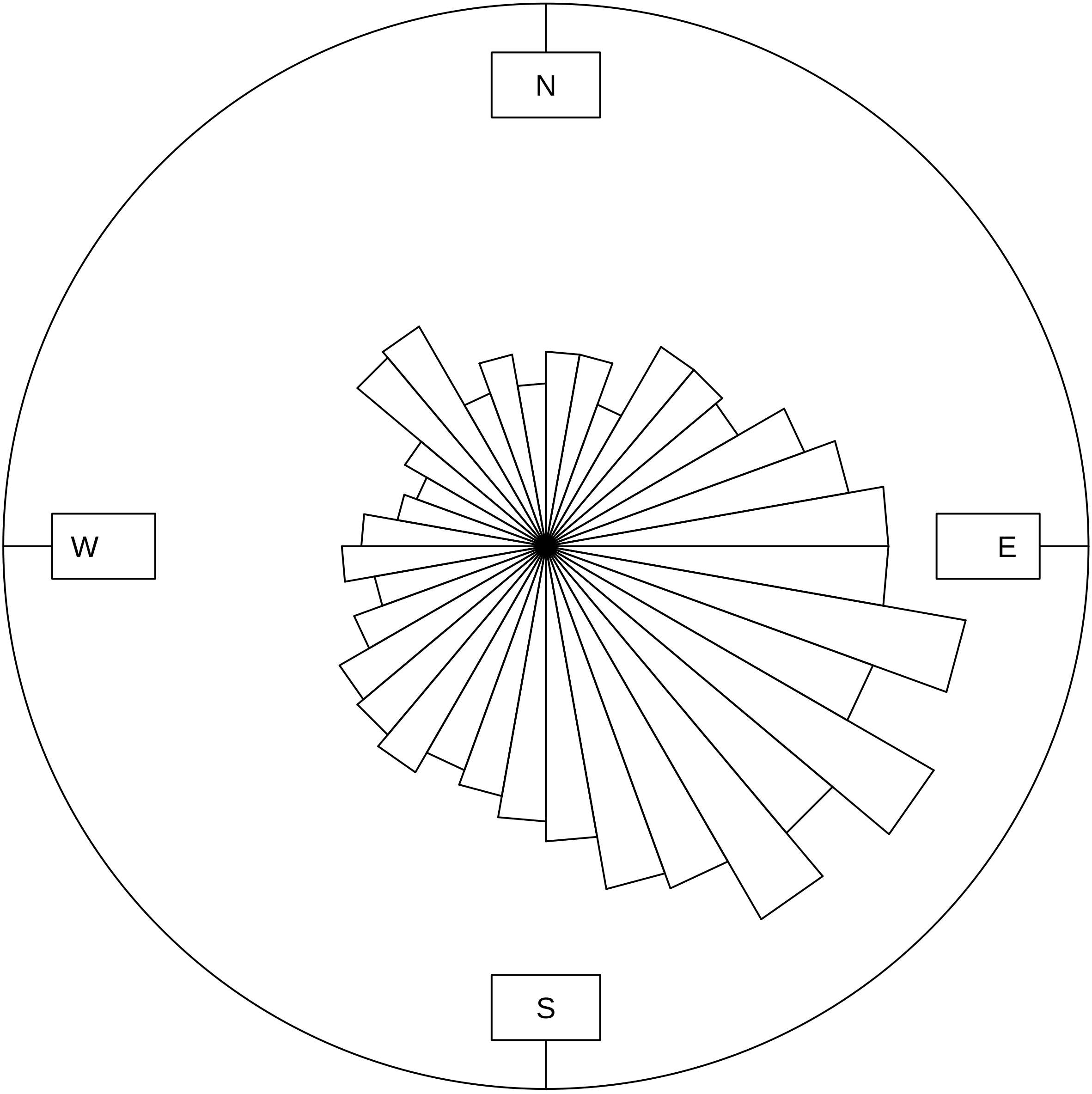

bootstrap 556

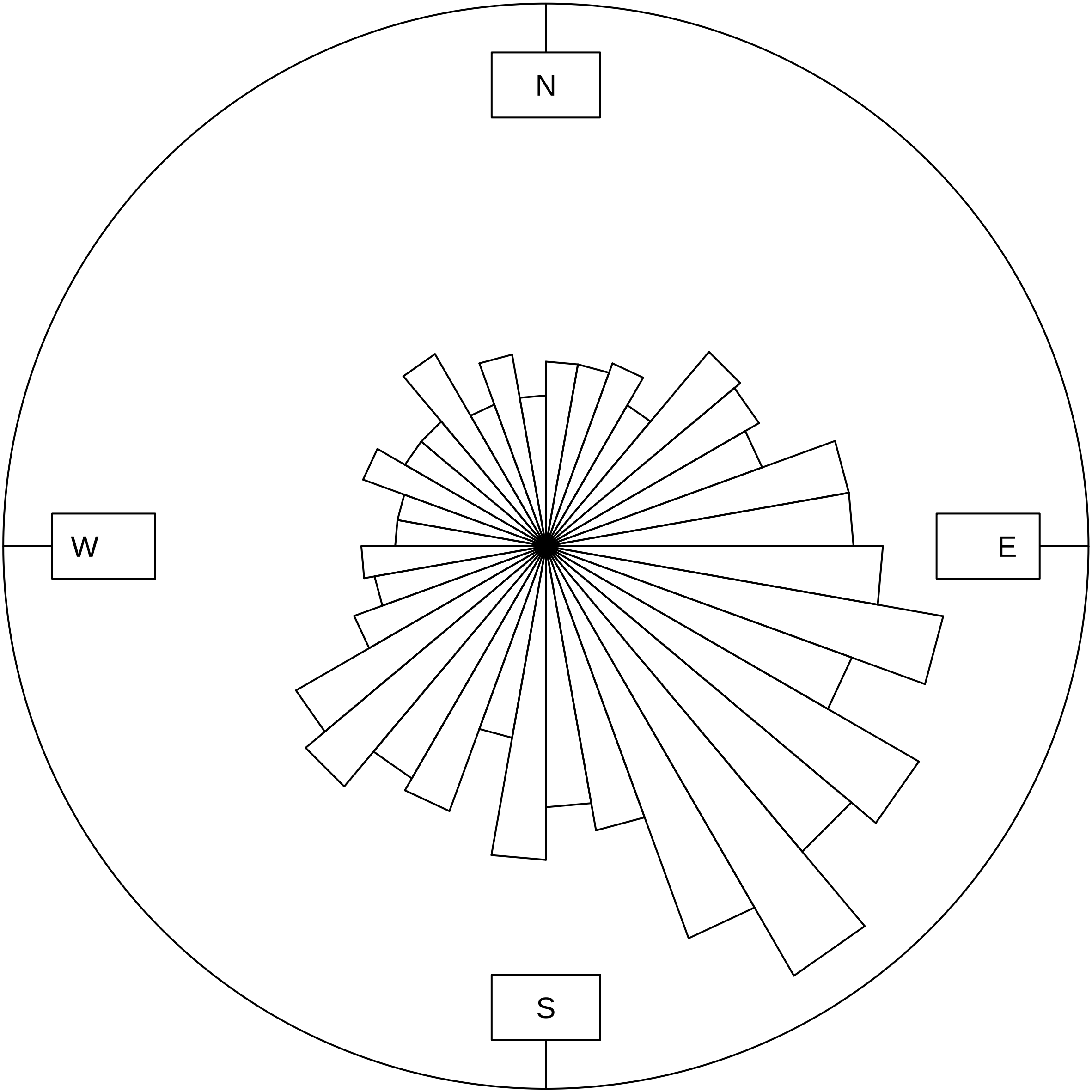

bootstrap 557

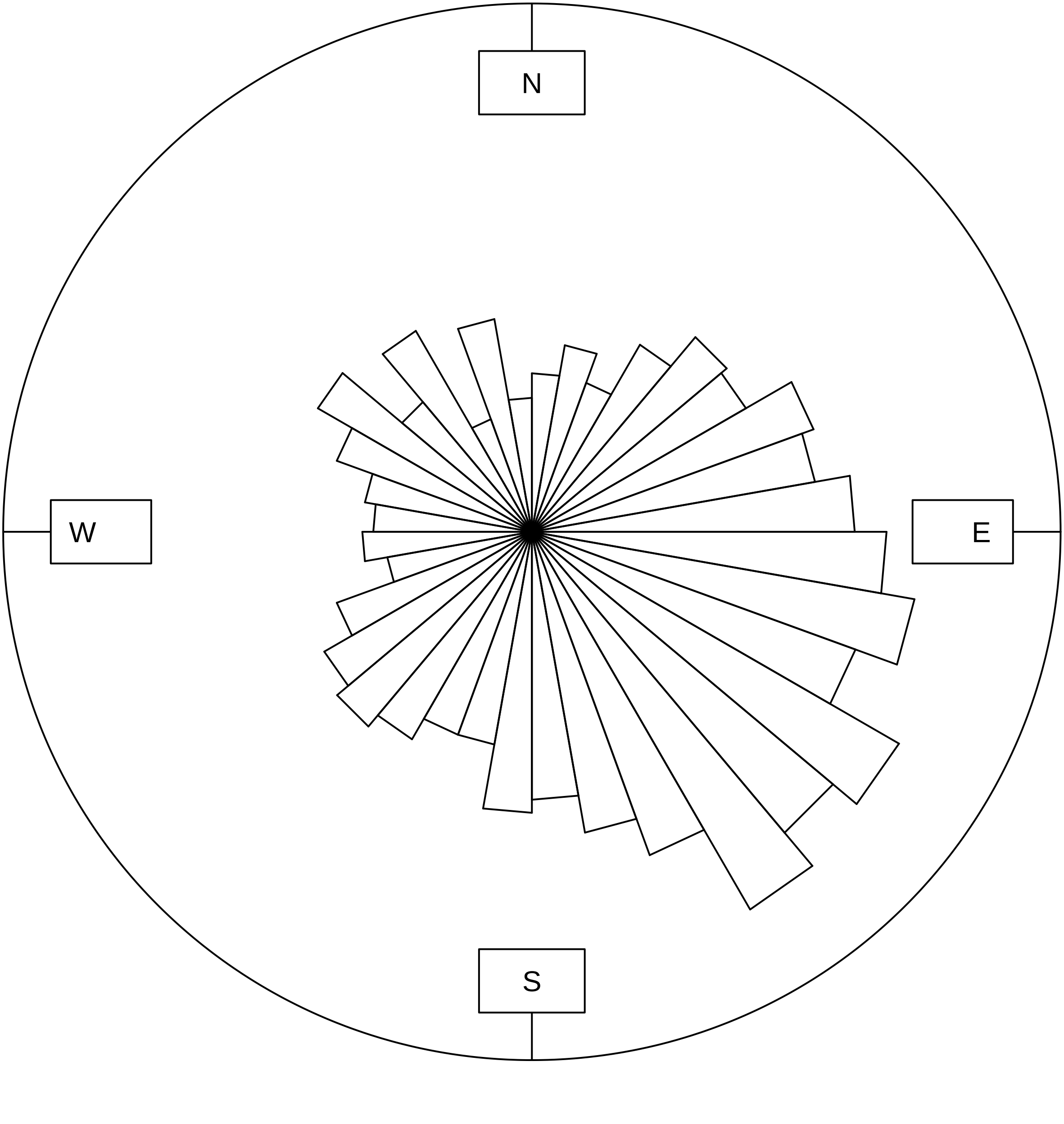

bootstrap 558

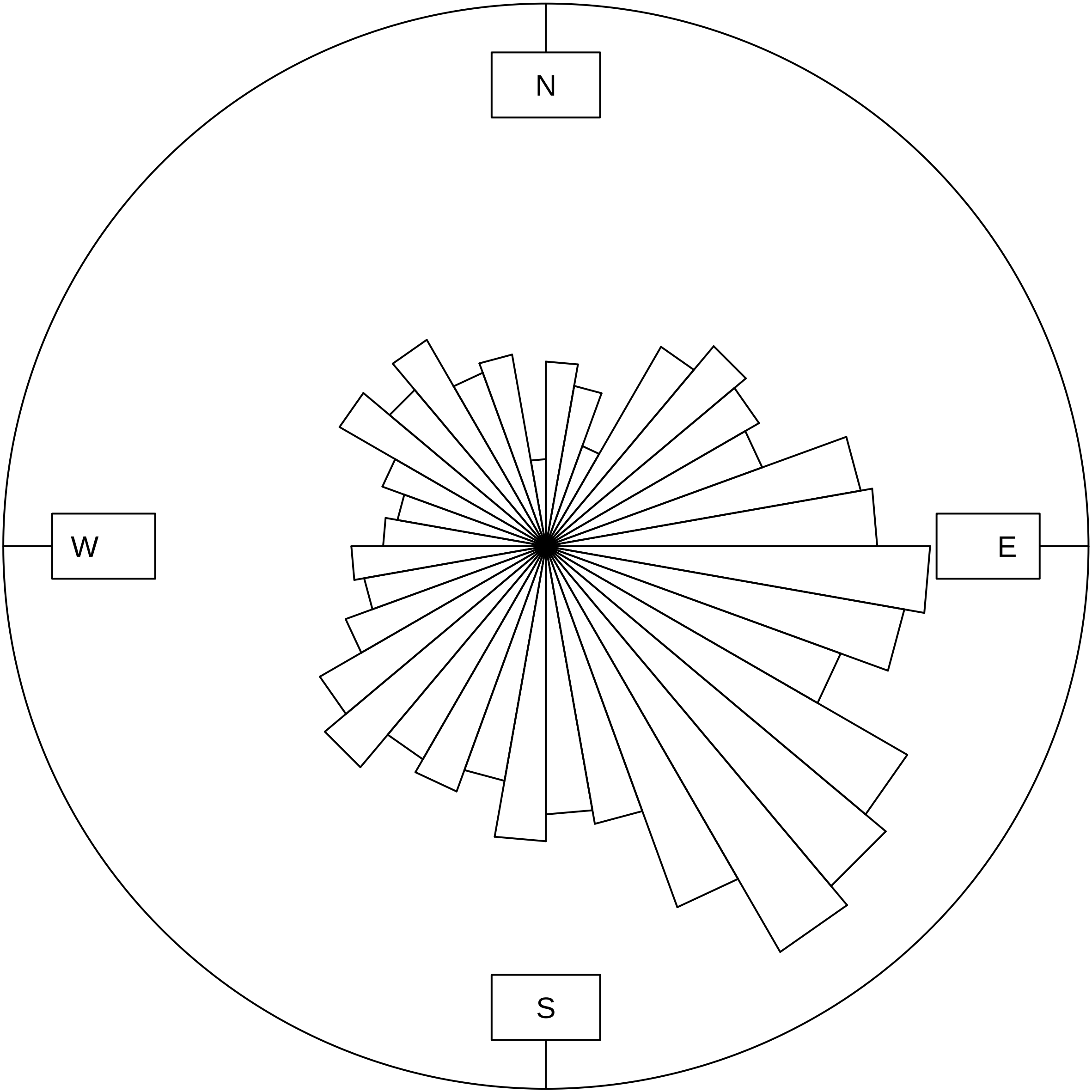

bootstrap 559

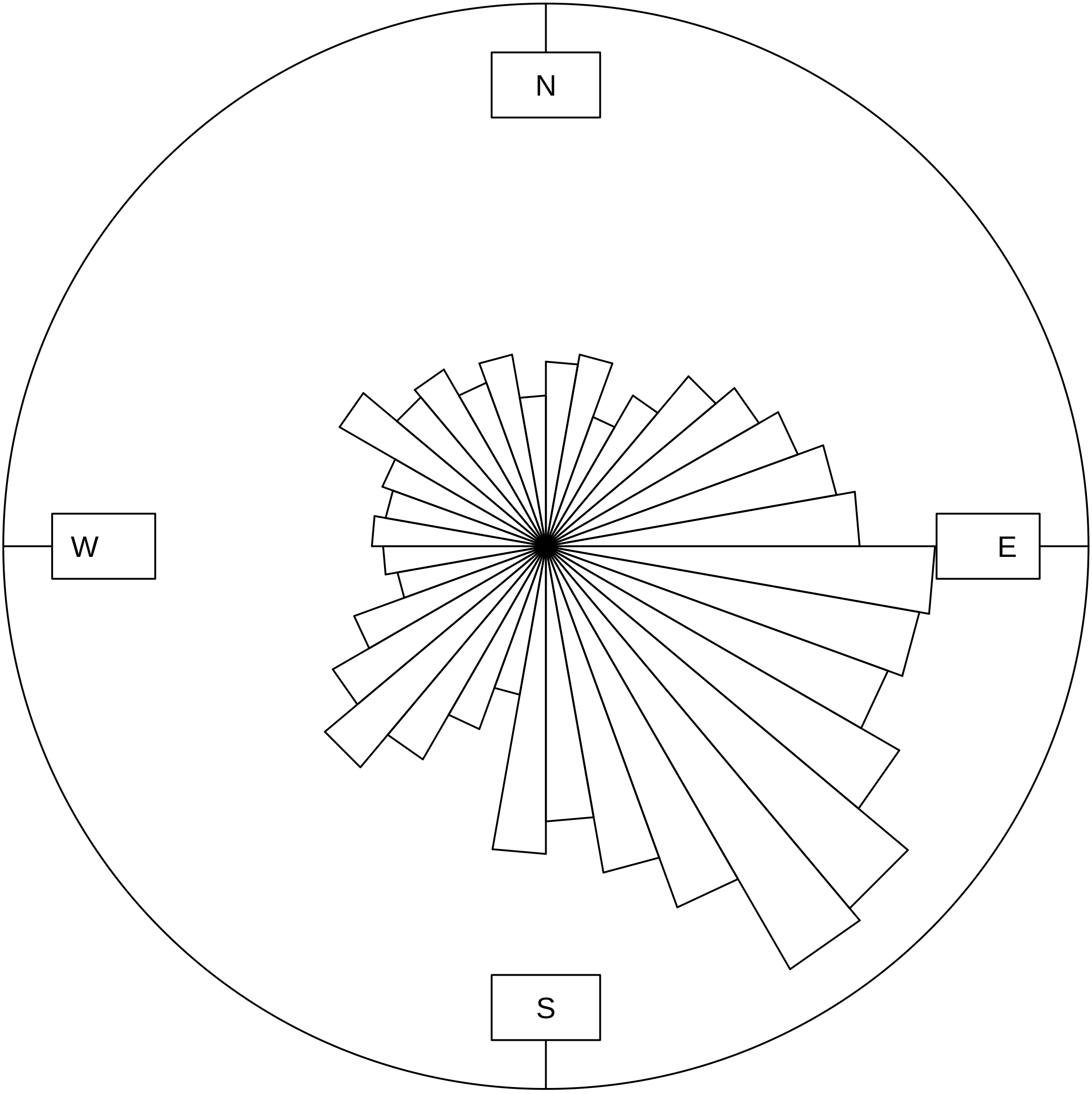

bootstrap 560

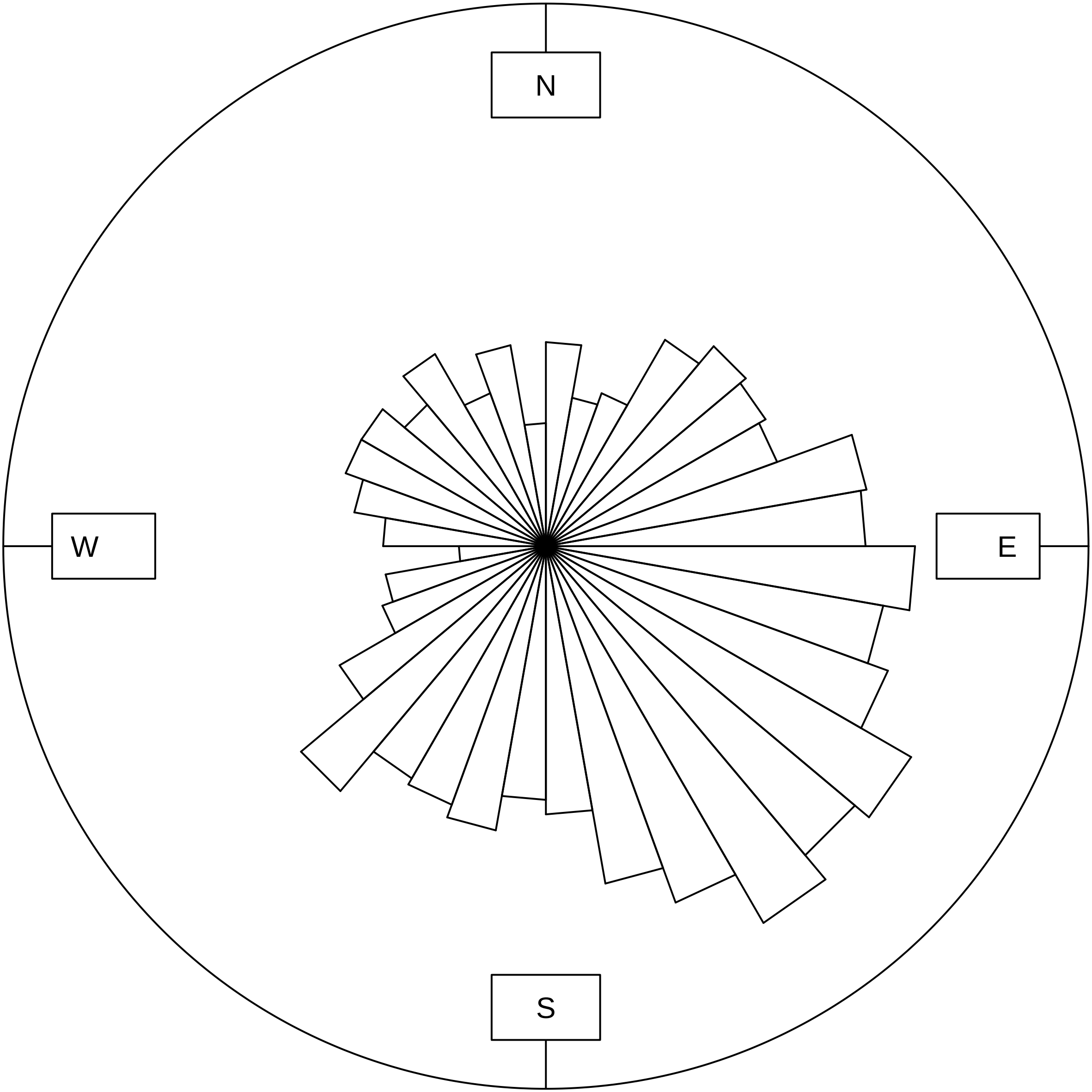

bootstrap 561

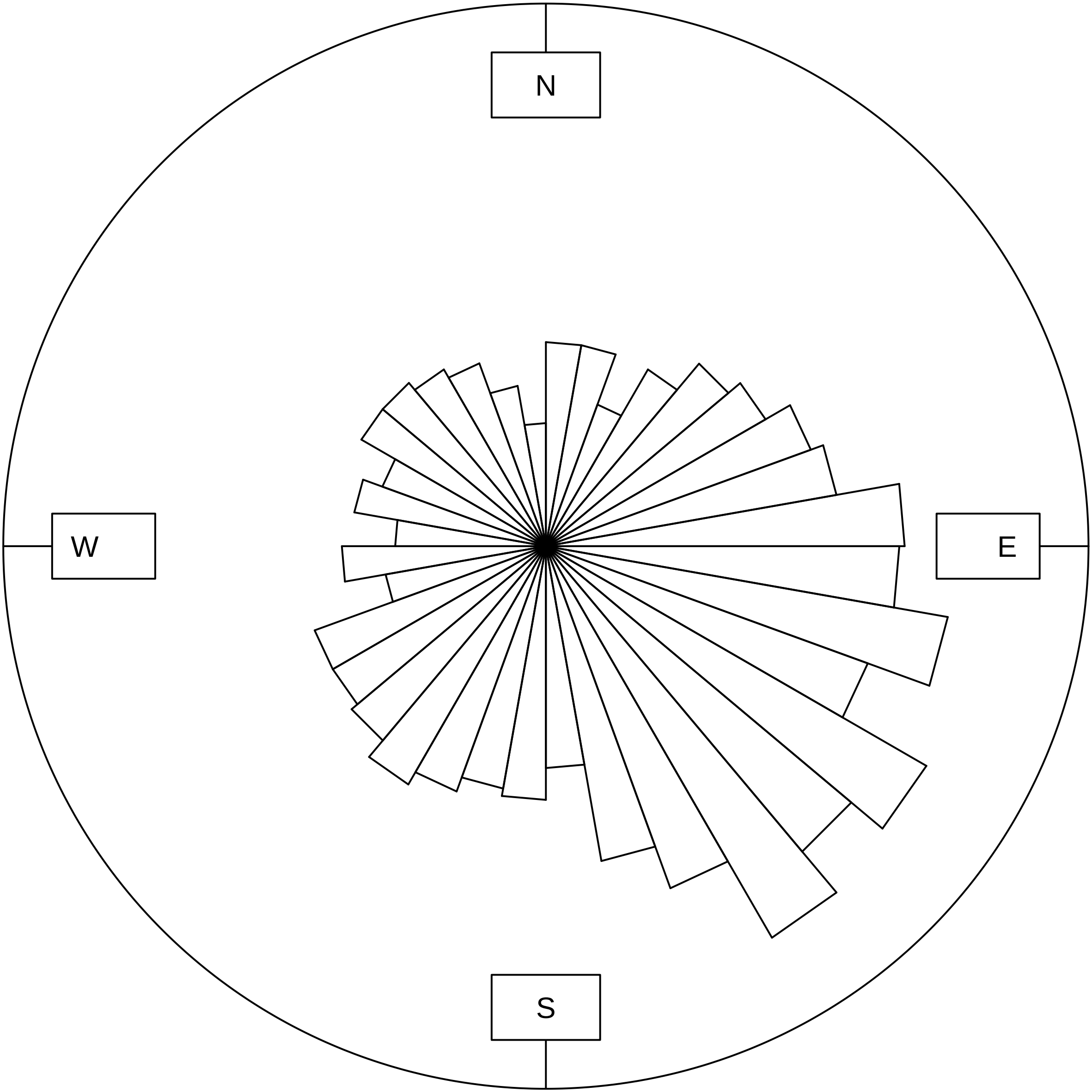

bootstrap 562

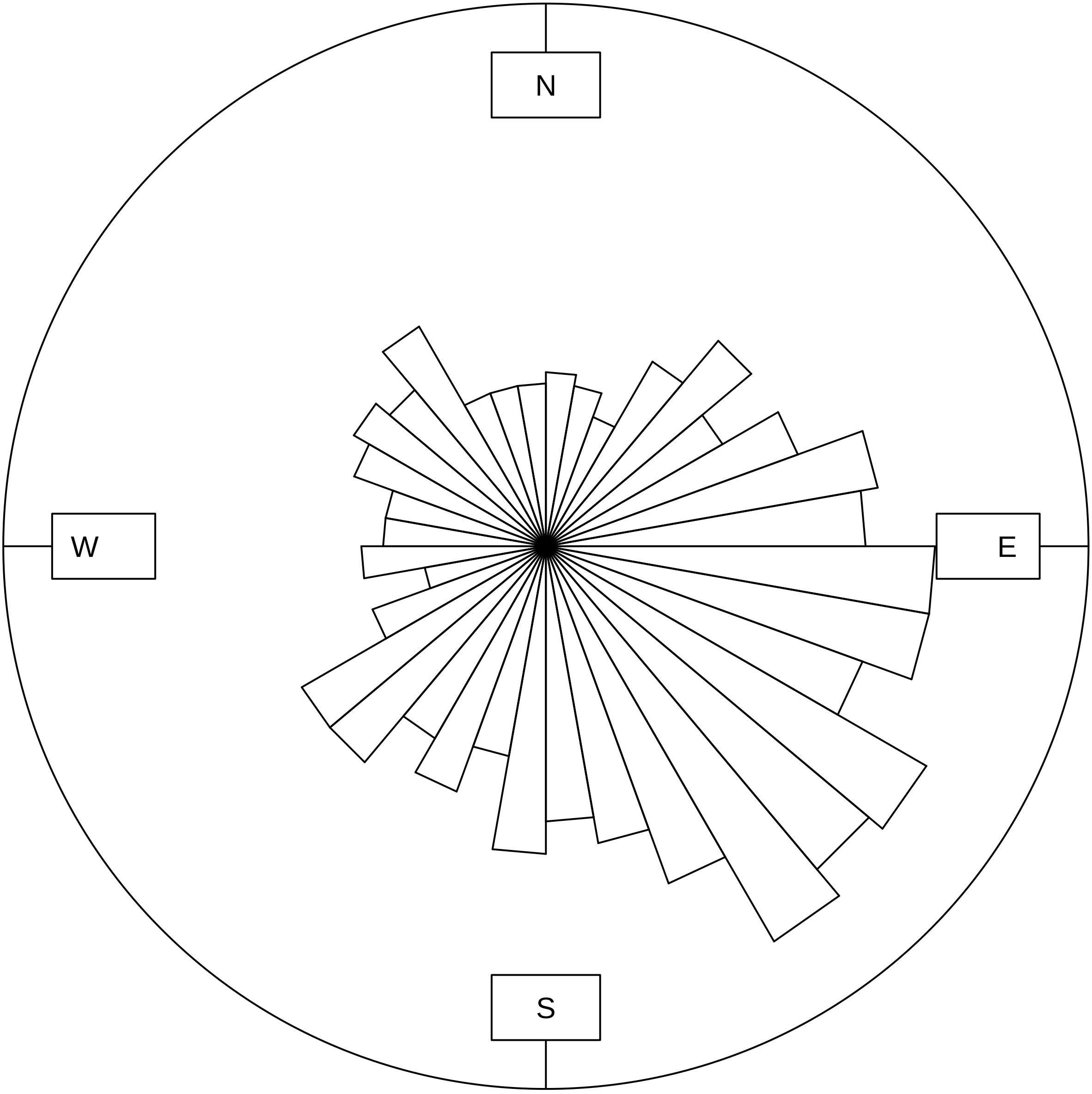

bootstrap 563

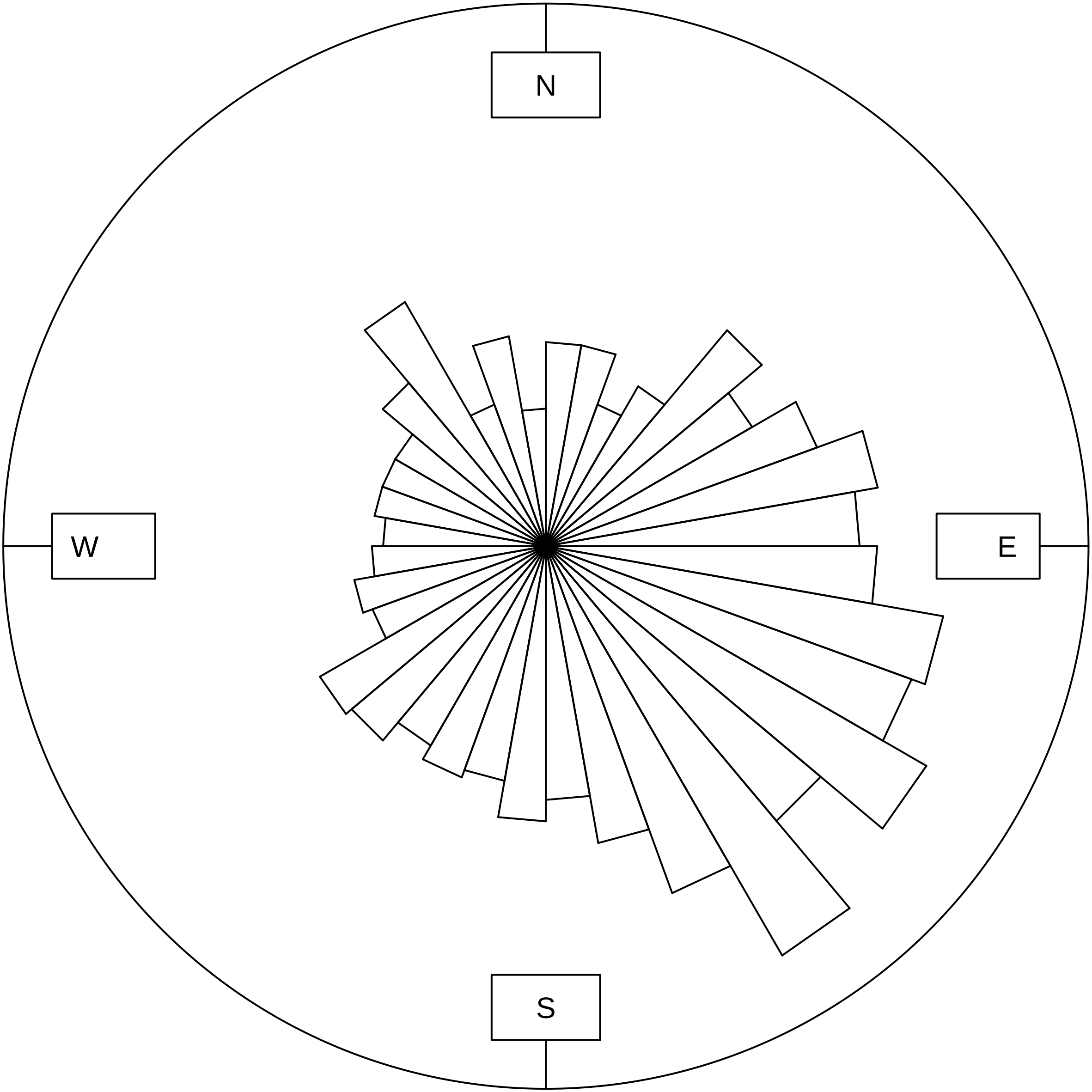

bootstrap 564

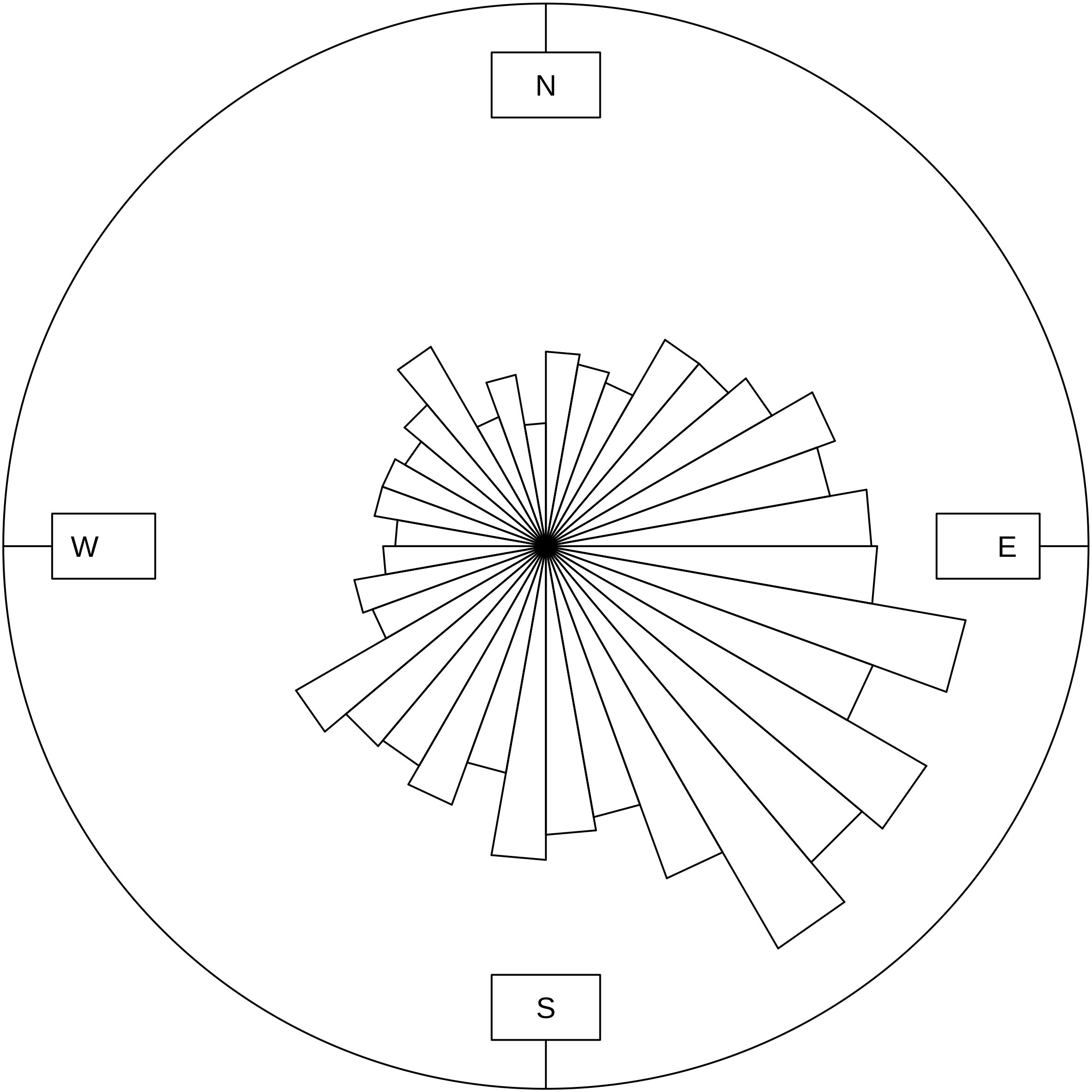

bootstrap 565

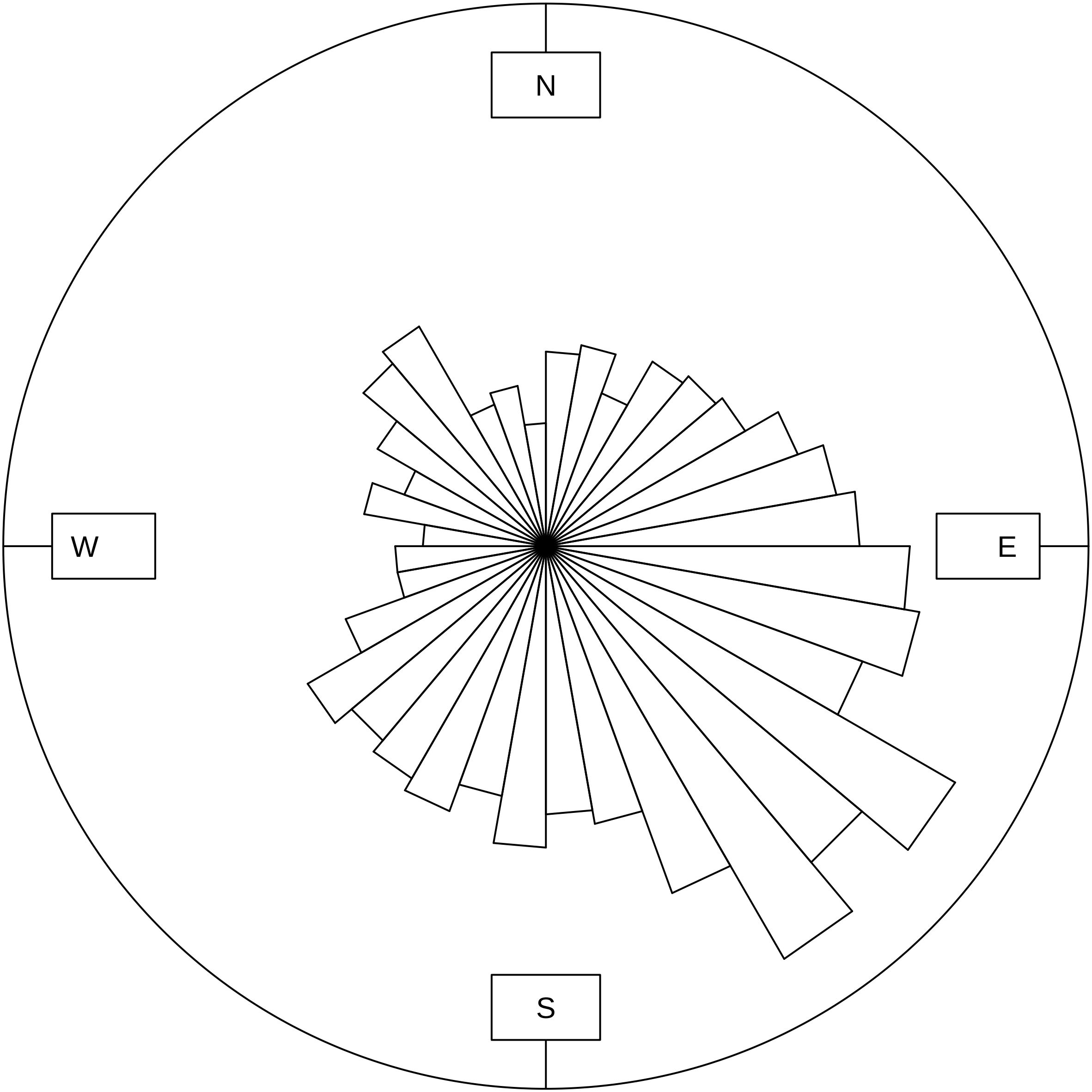

bootstrap 566

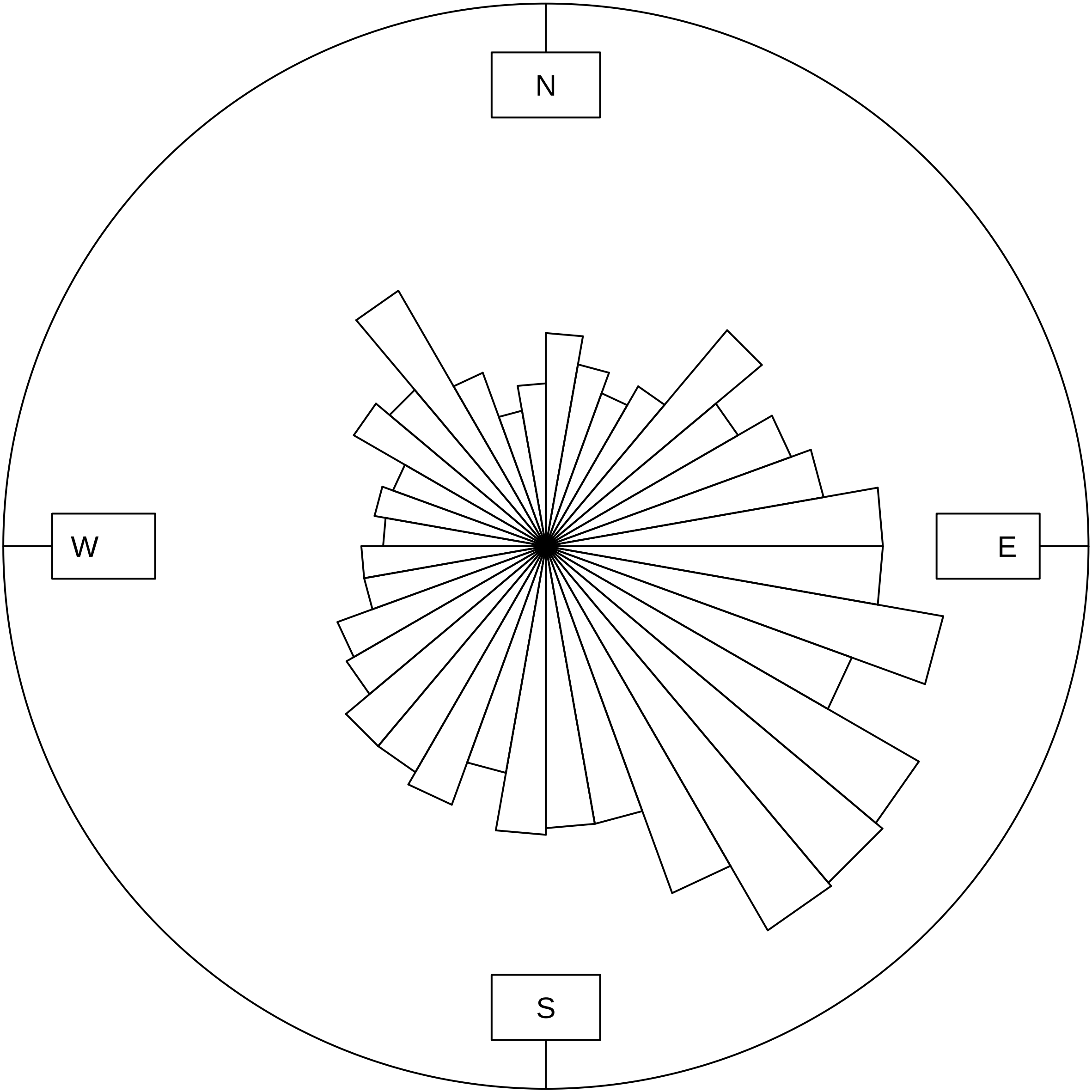

bootstrap 567

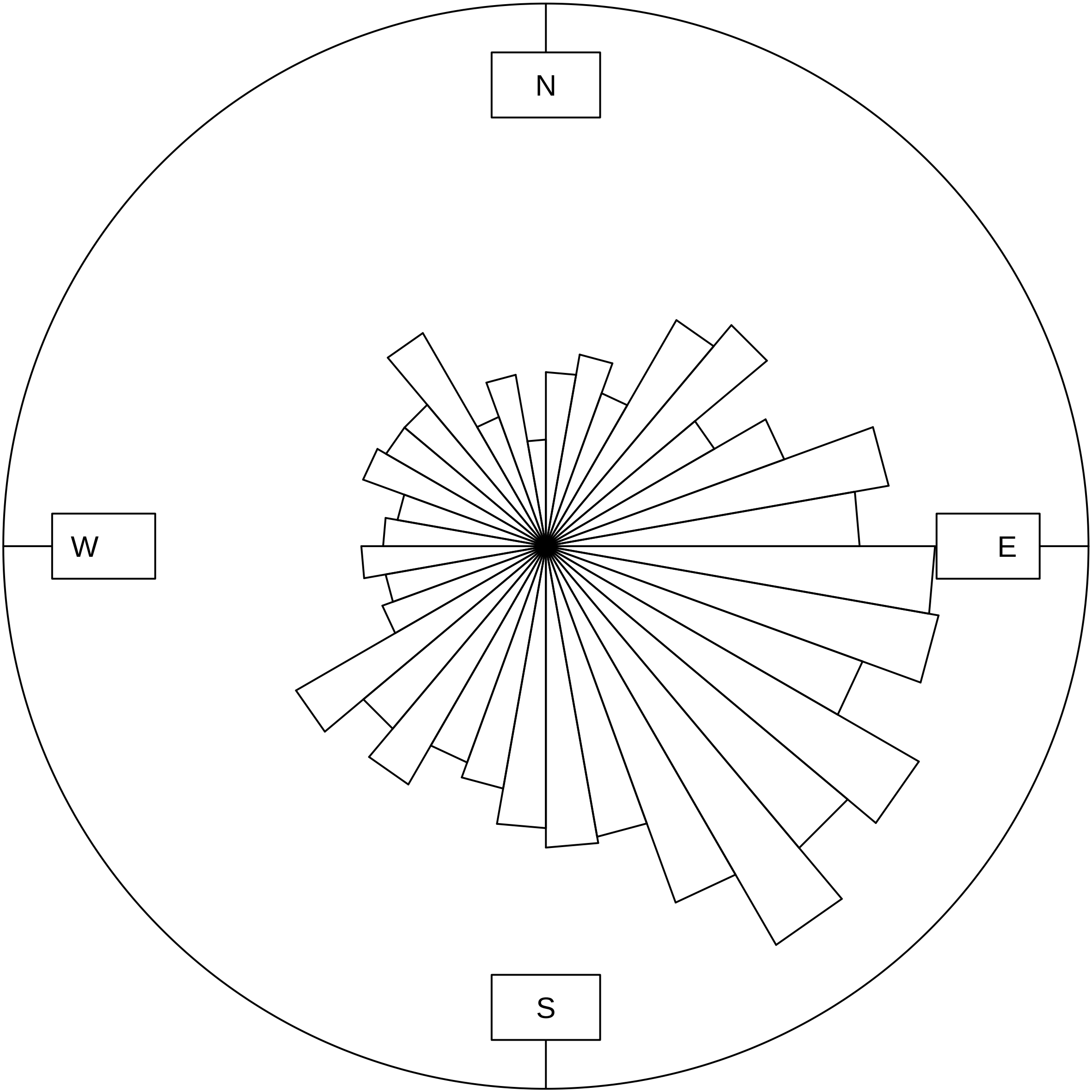

bootstrap 568

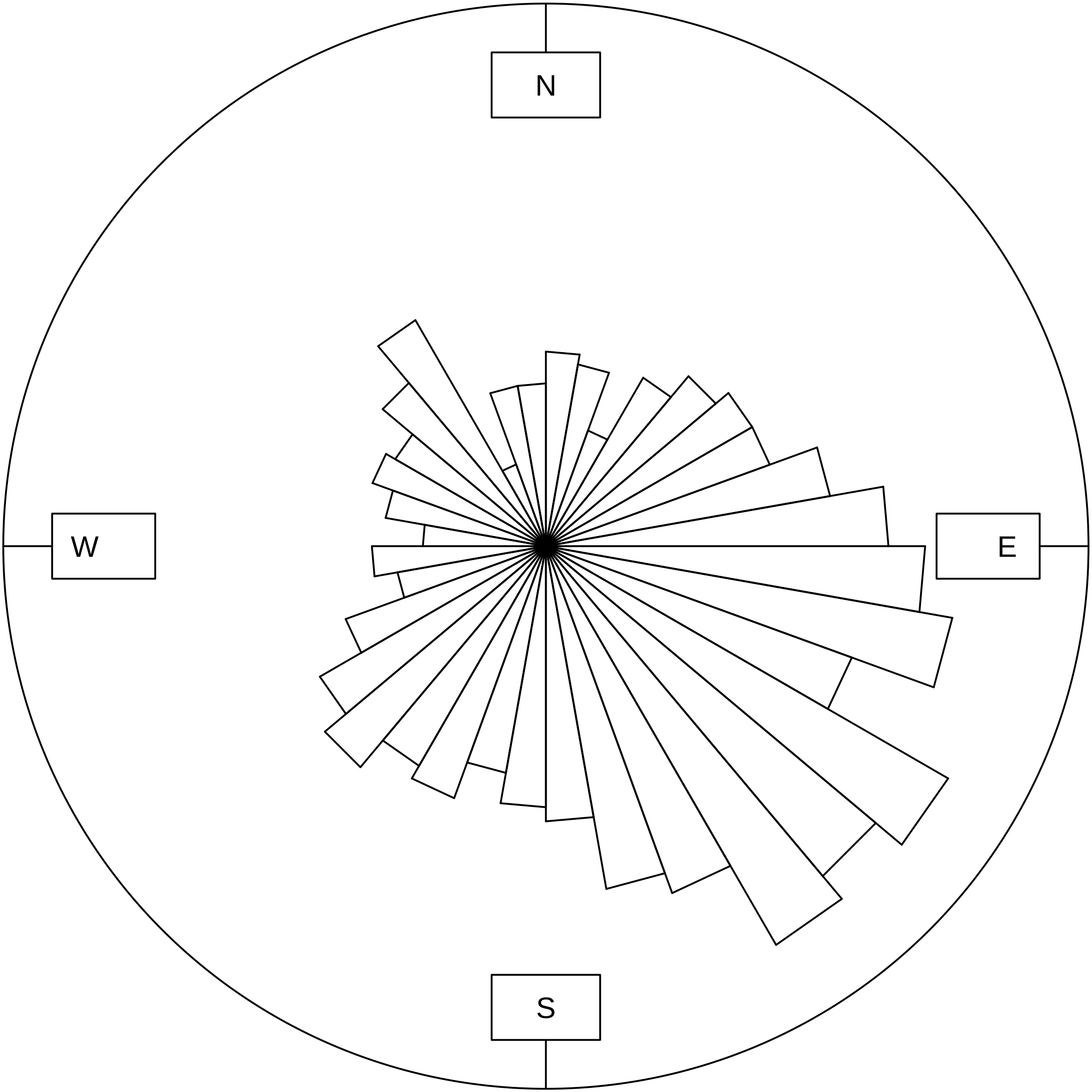

bootstrap 569

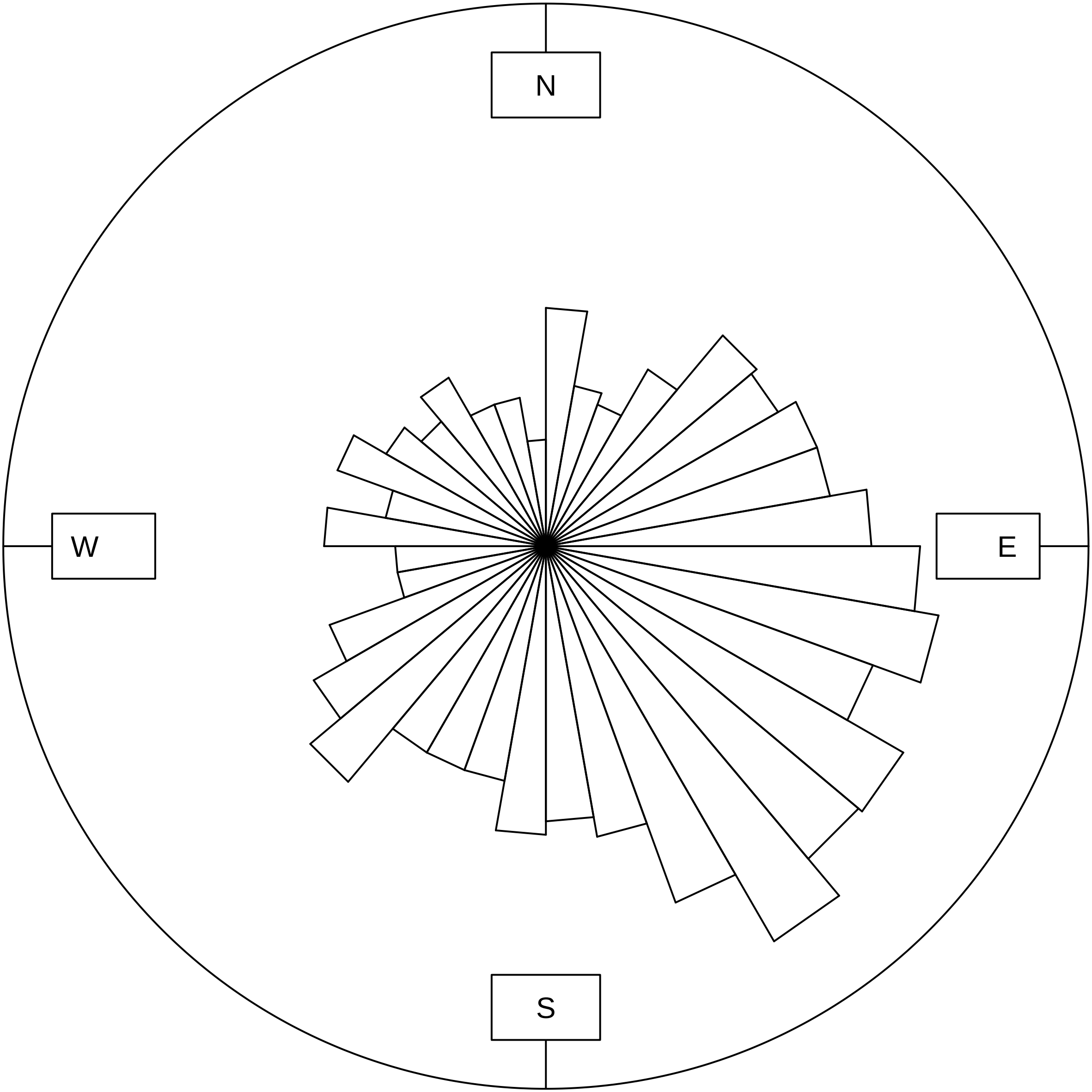

bootstrap 570

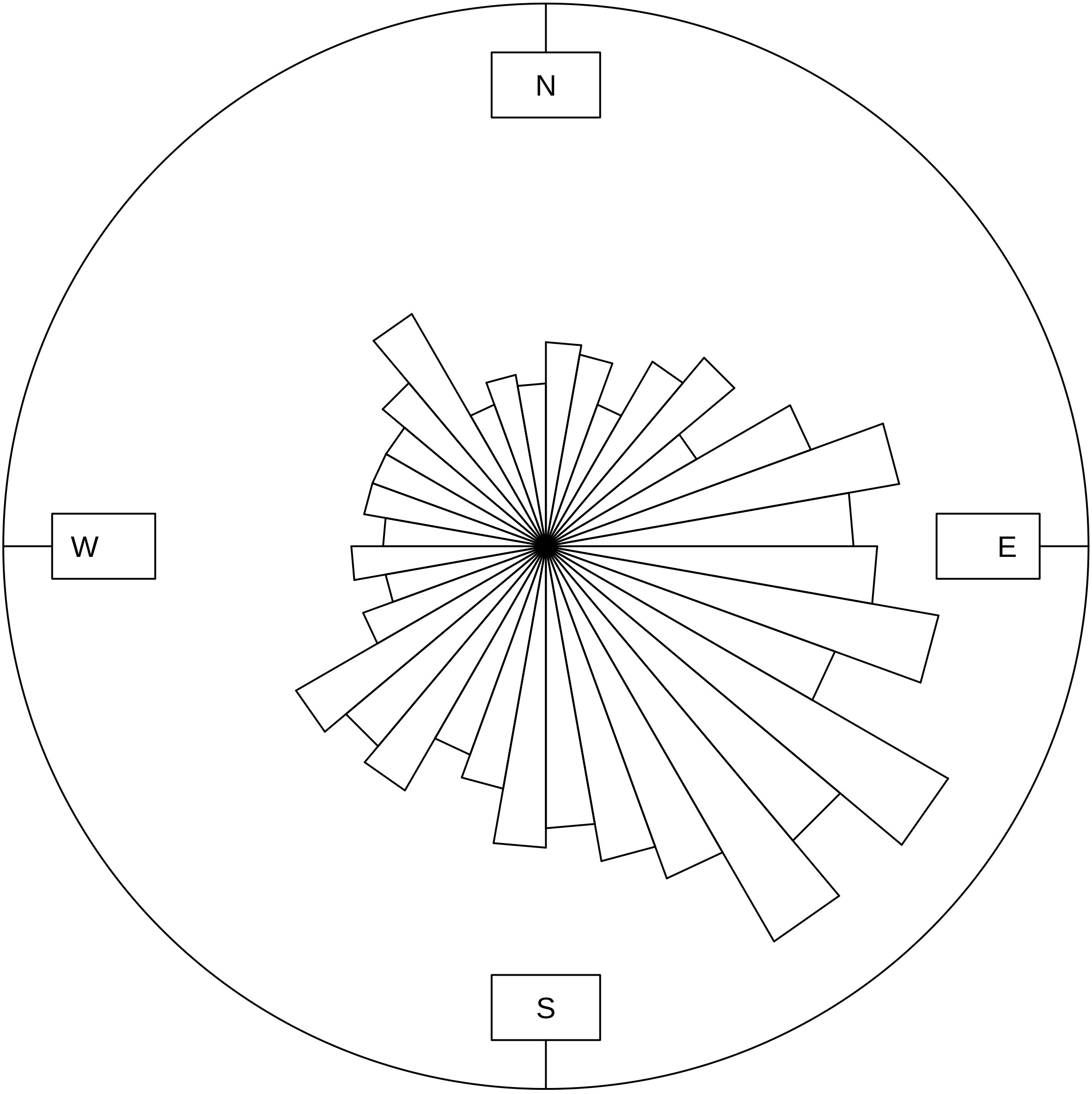

bootstrap 571

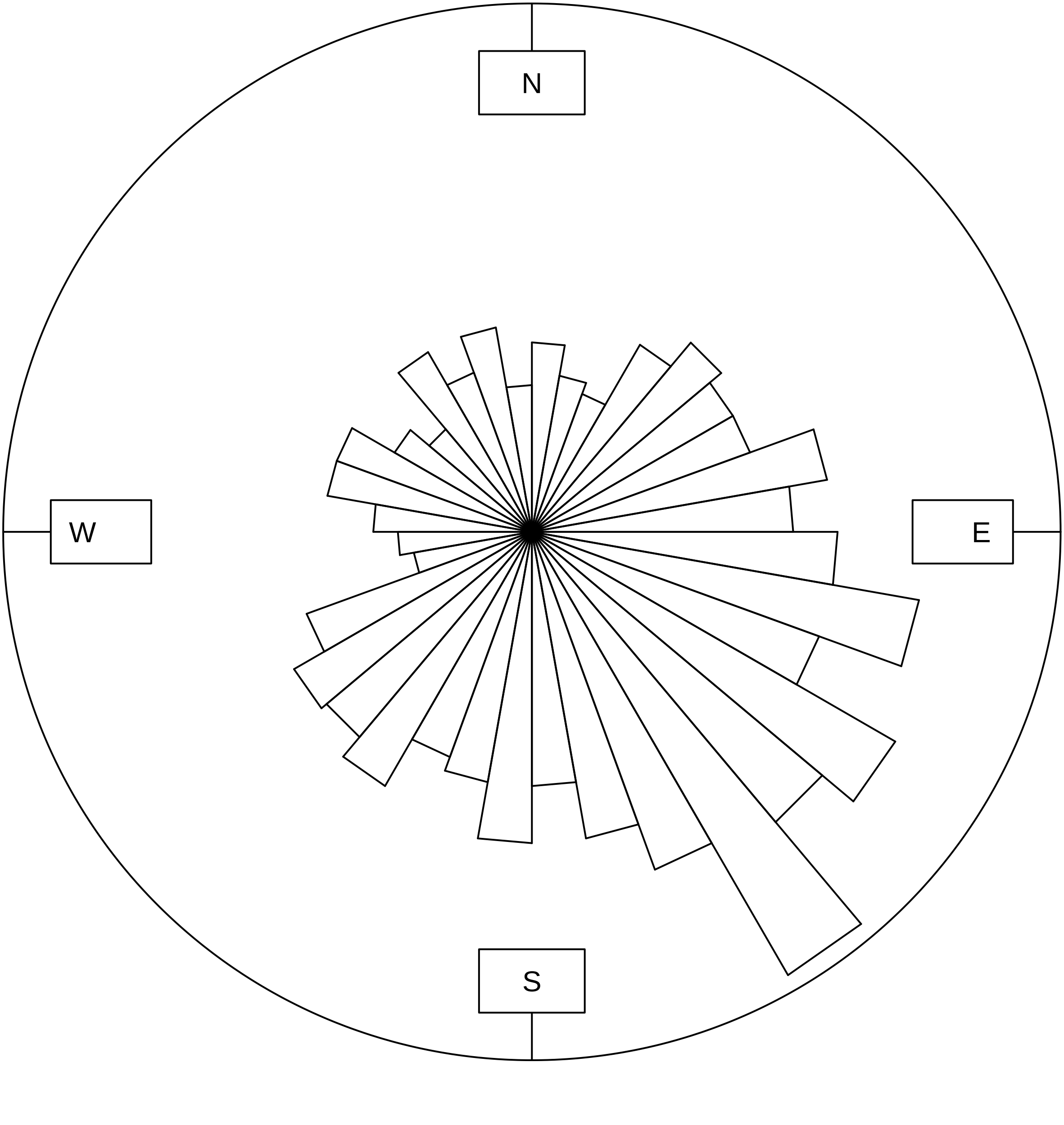

bootstrap 572

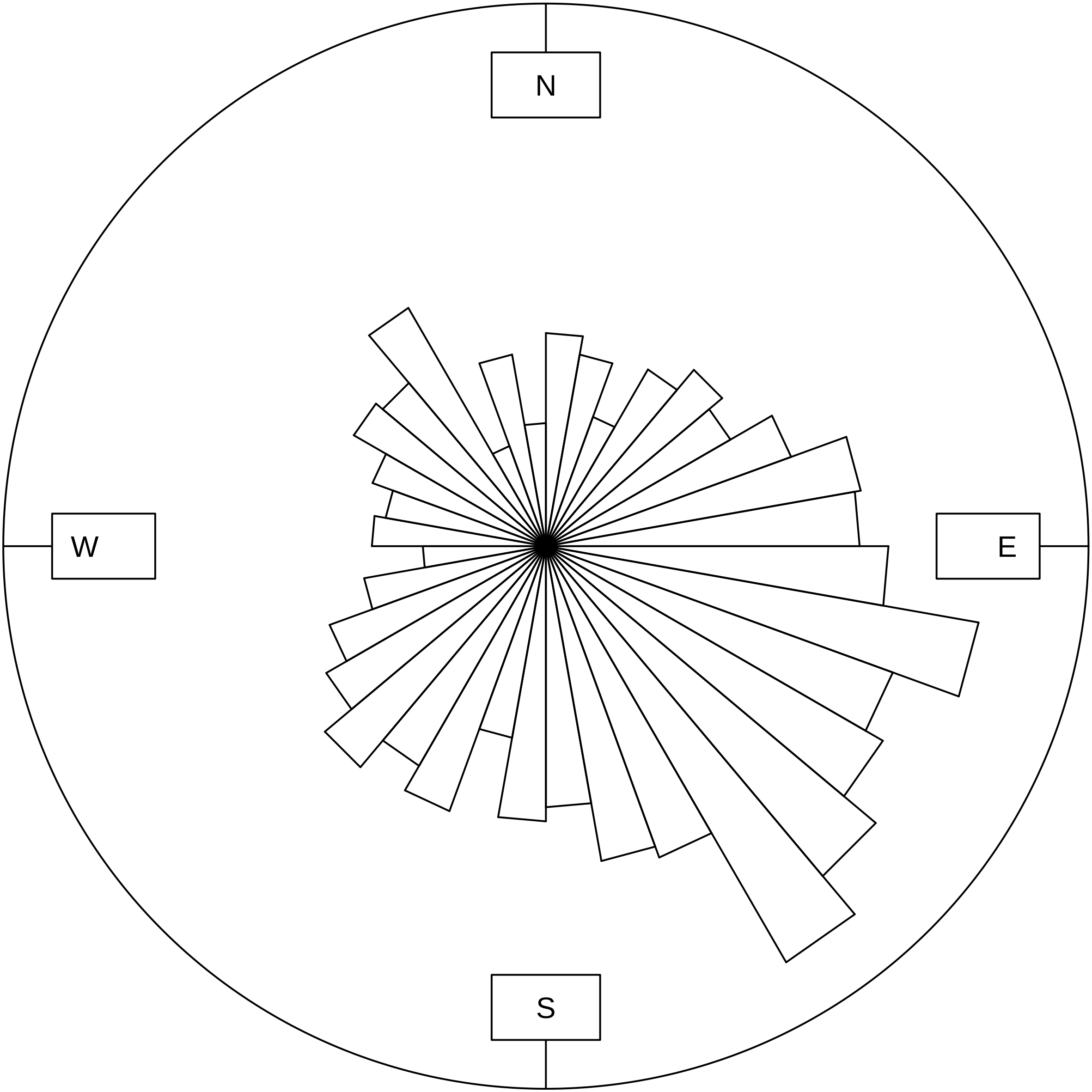

bootstrap 573

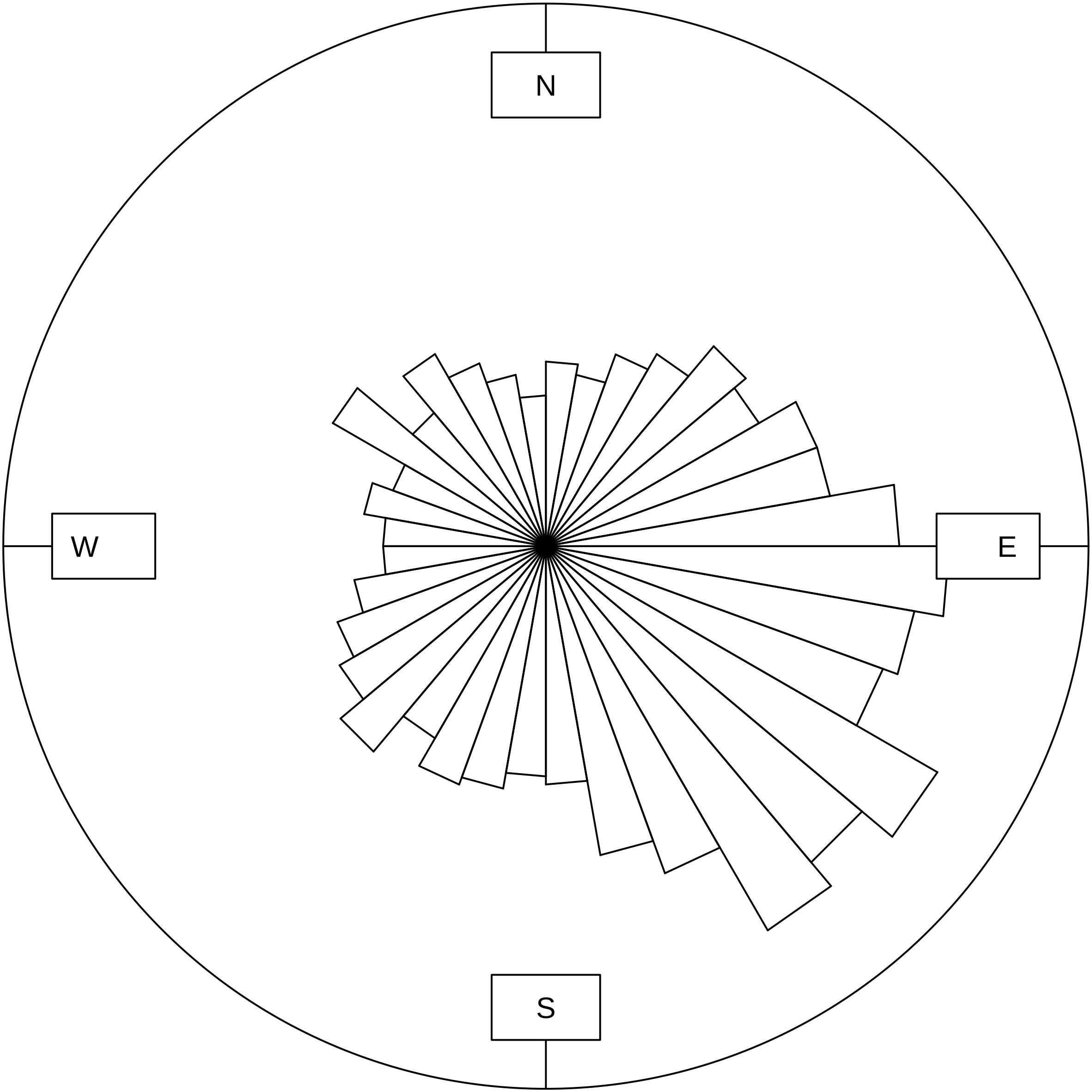

bootstrap 574

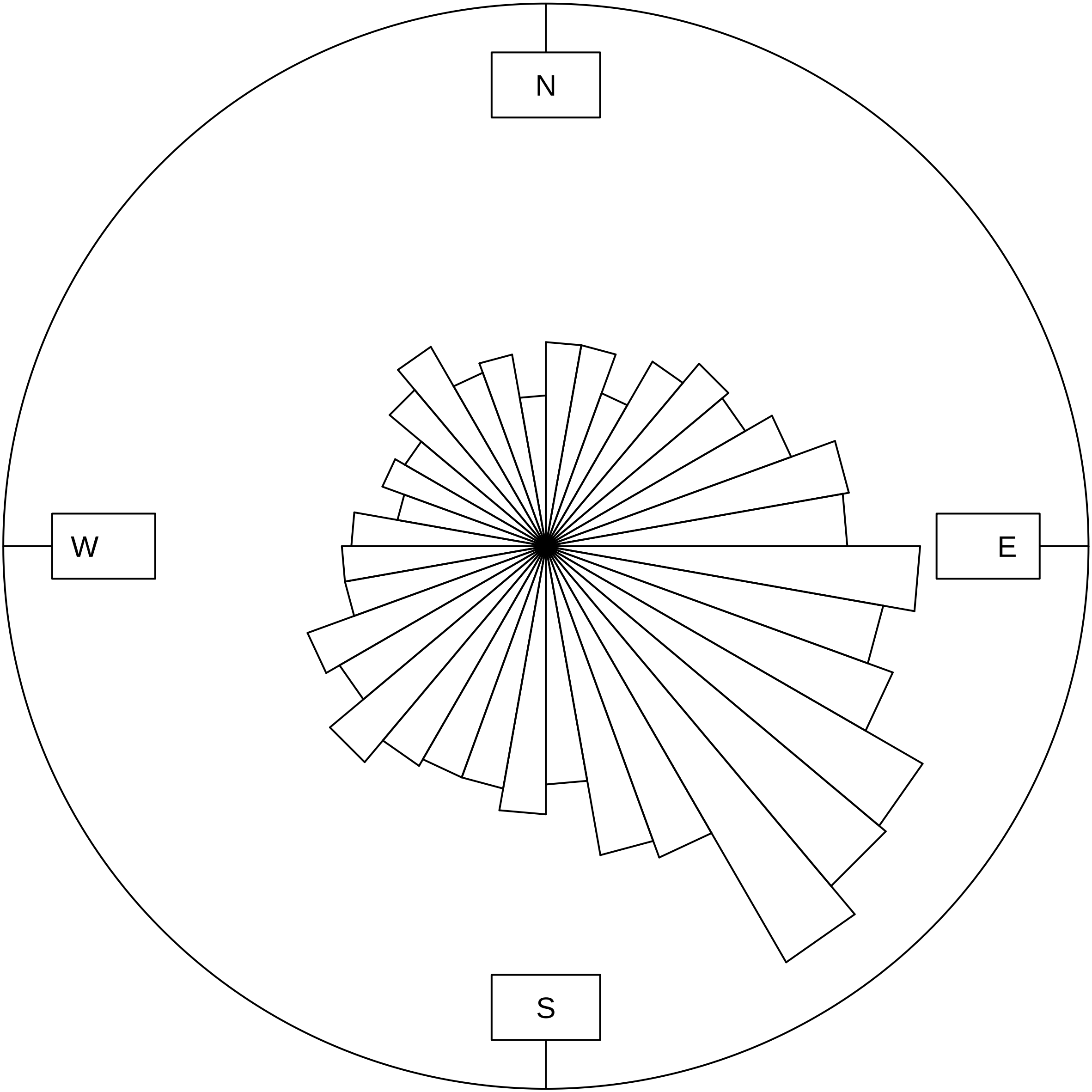

bootstrap 575

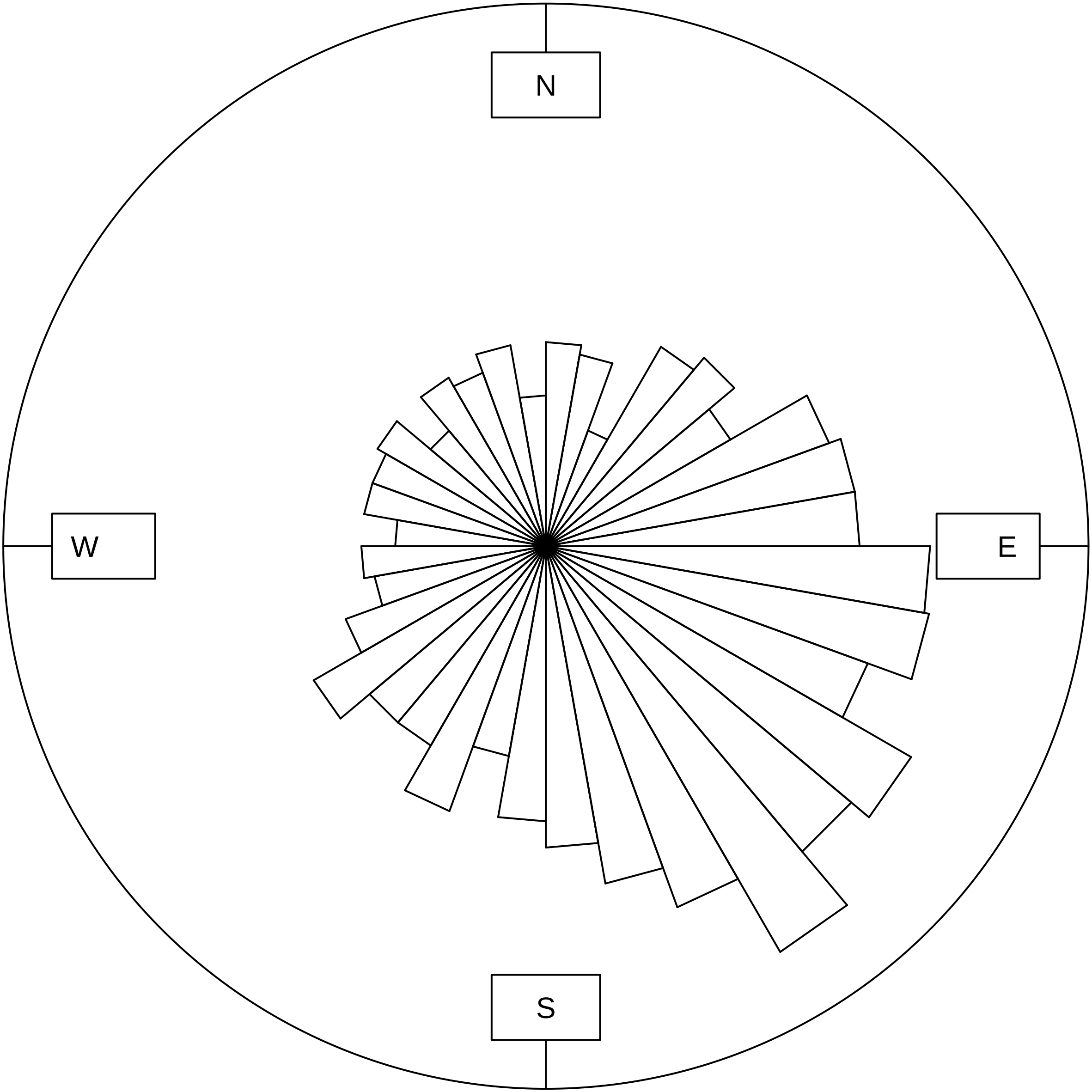

bootstrap 576

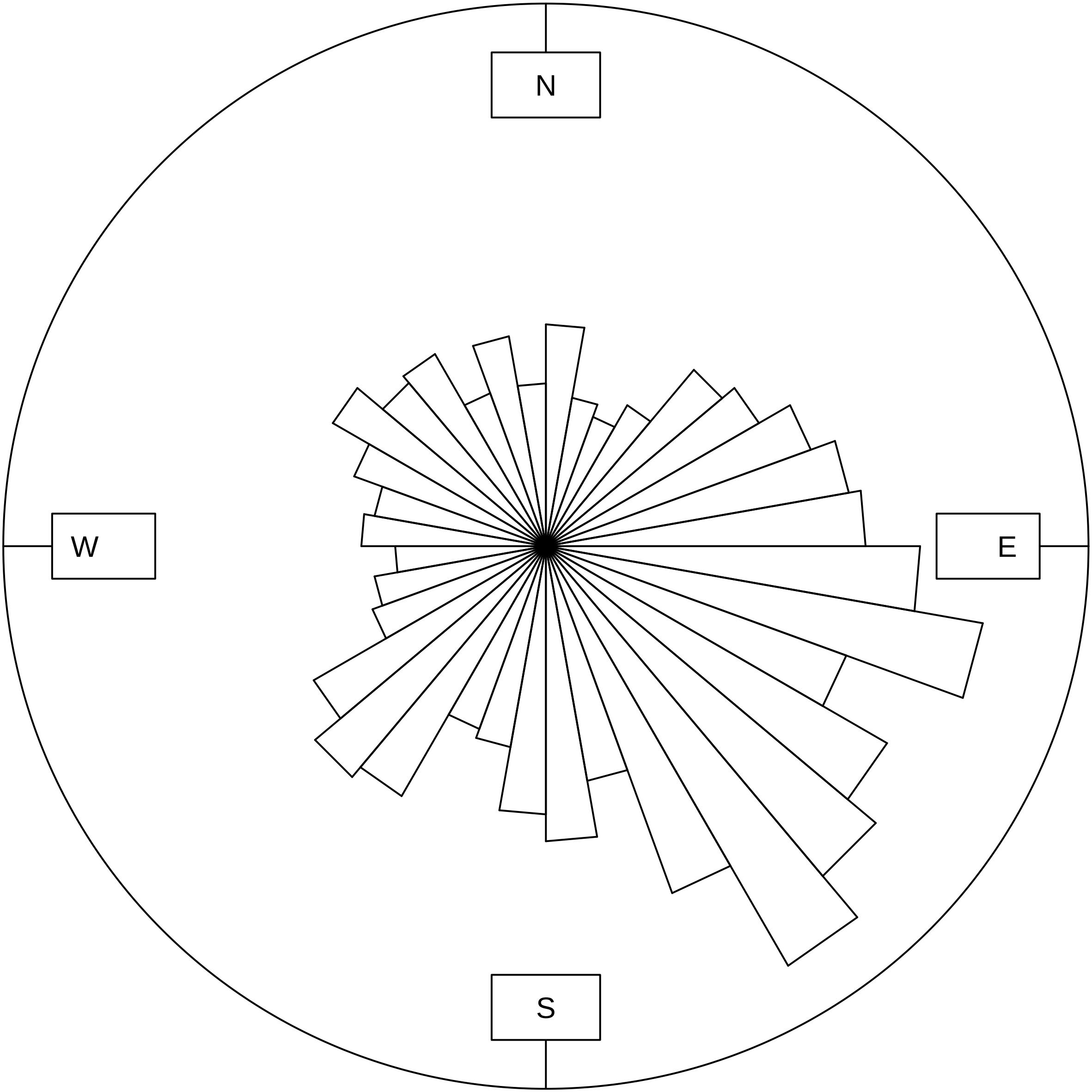

bootstrap 577

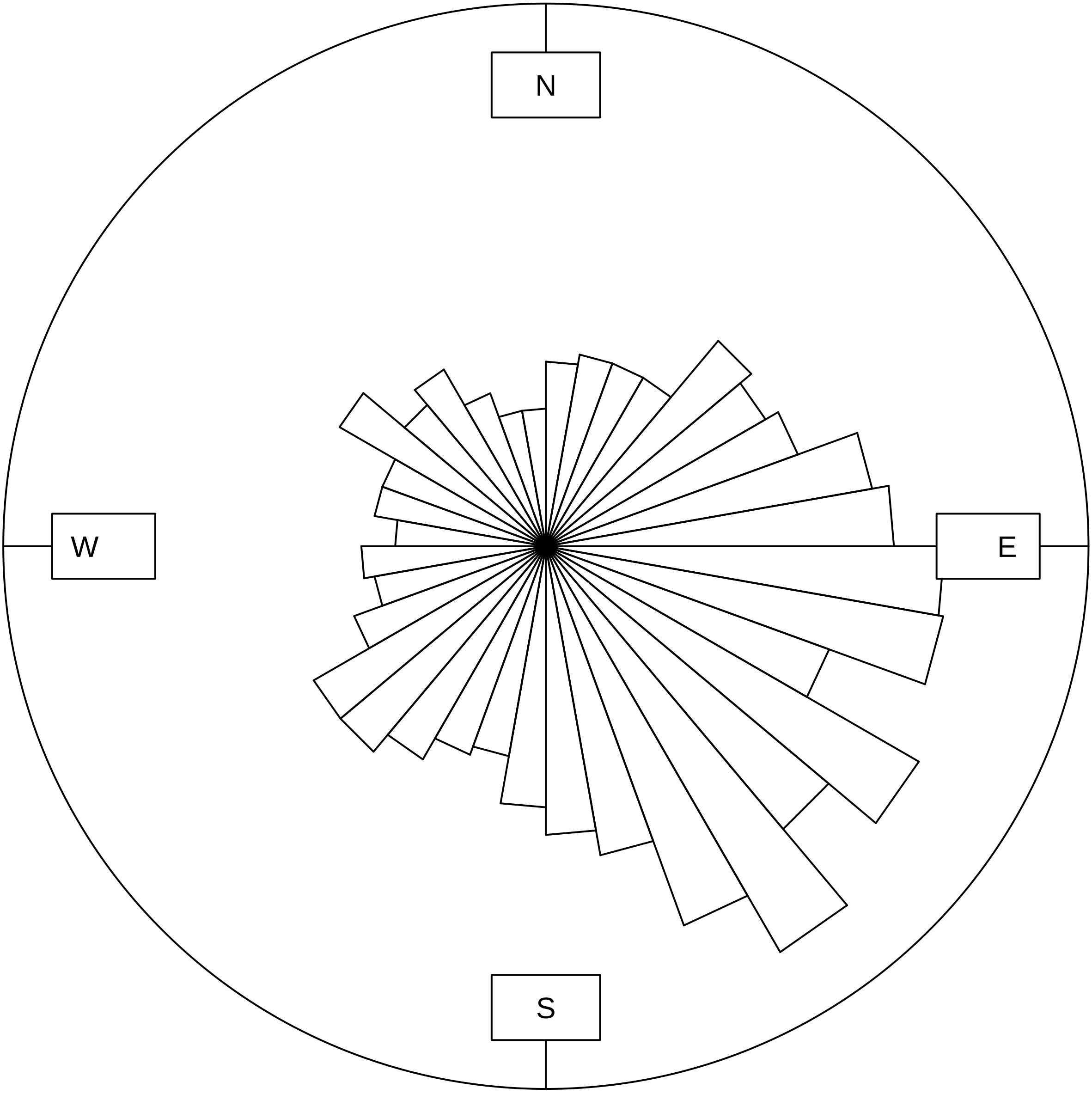

bootstrap 578

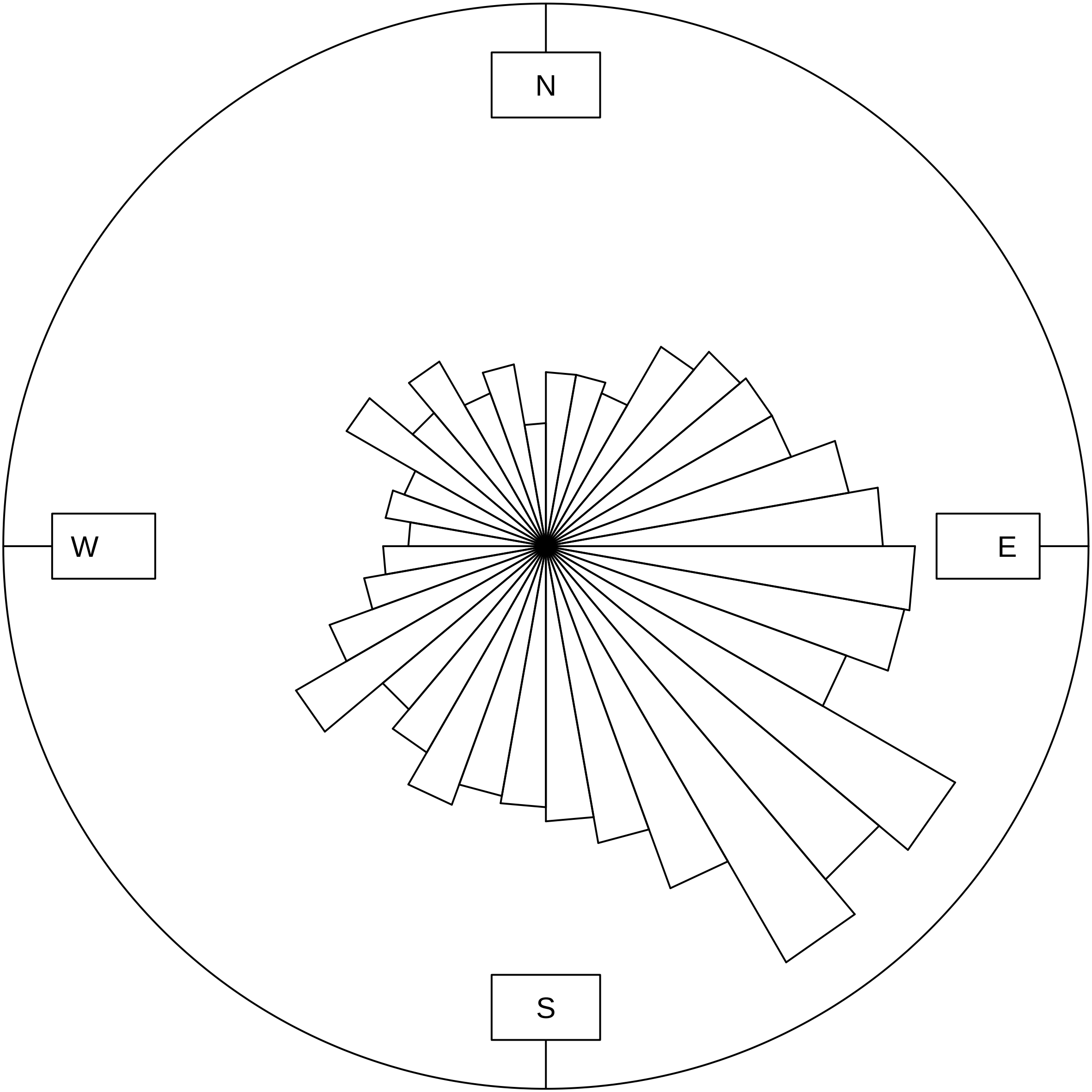

bootstrap 579

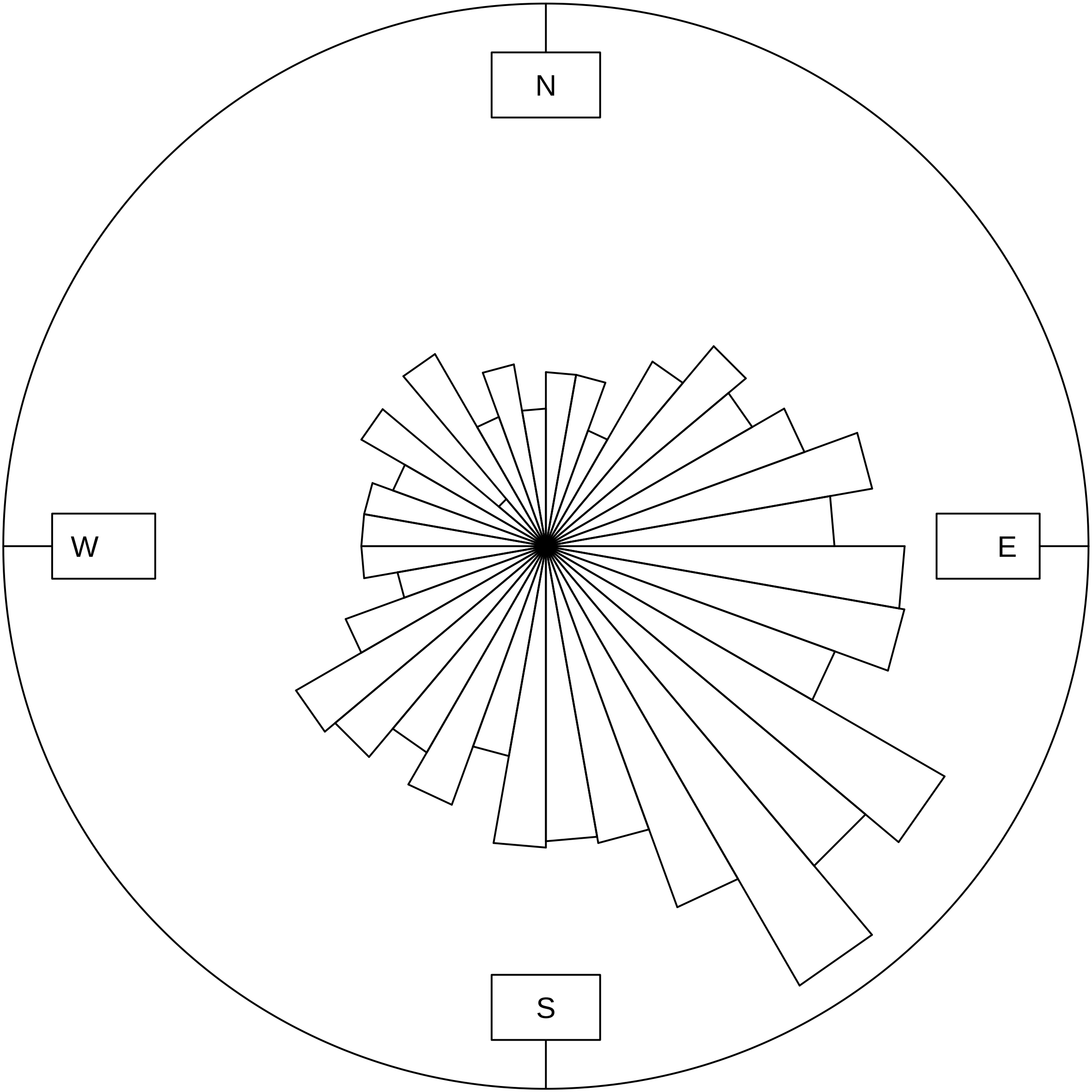

bootstrap 580

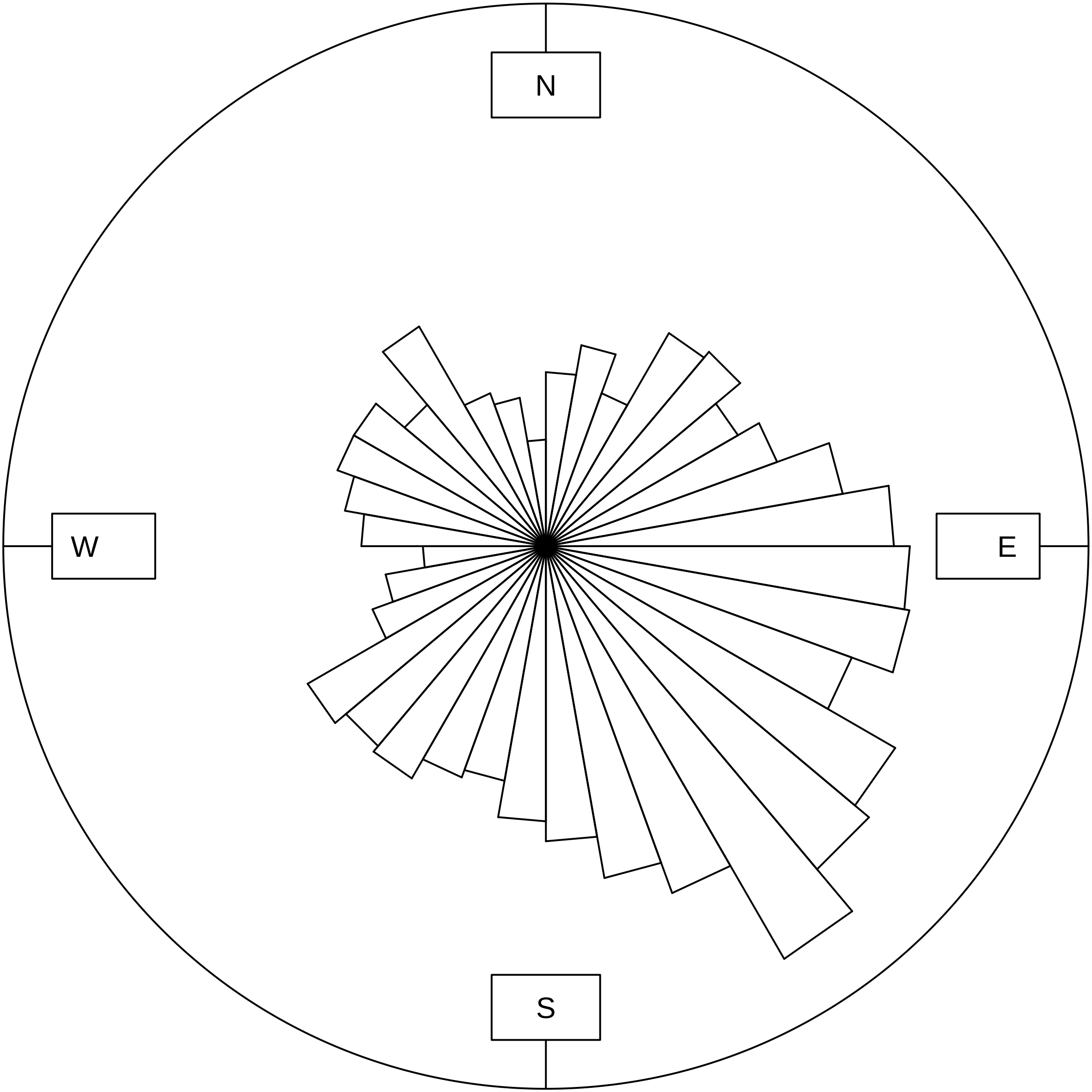

bootstrap 581

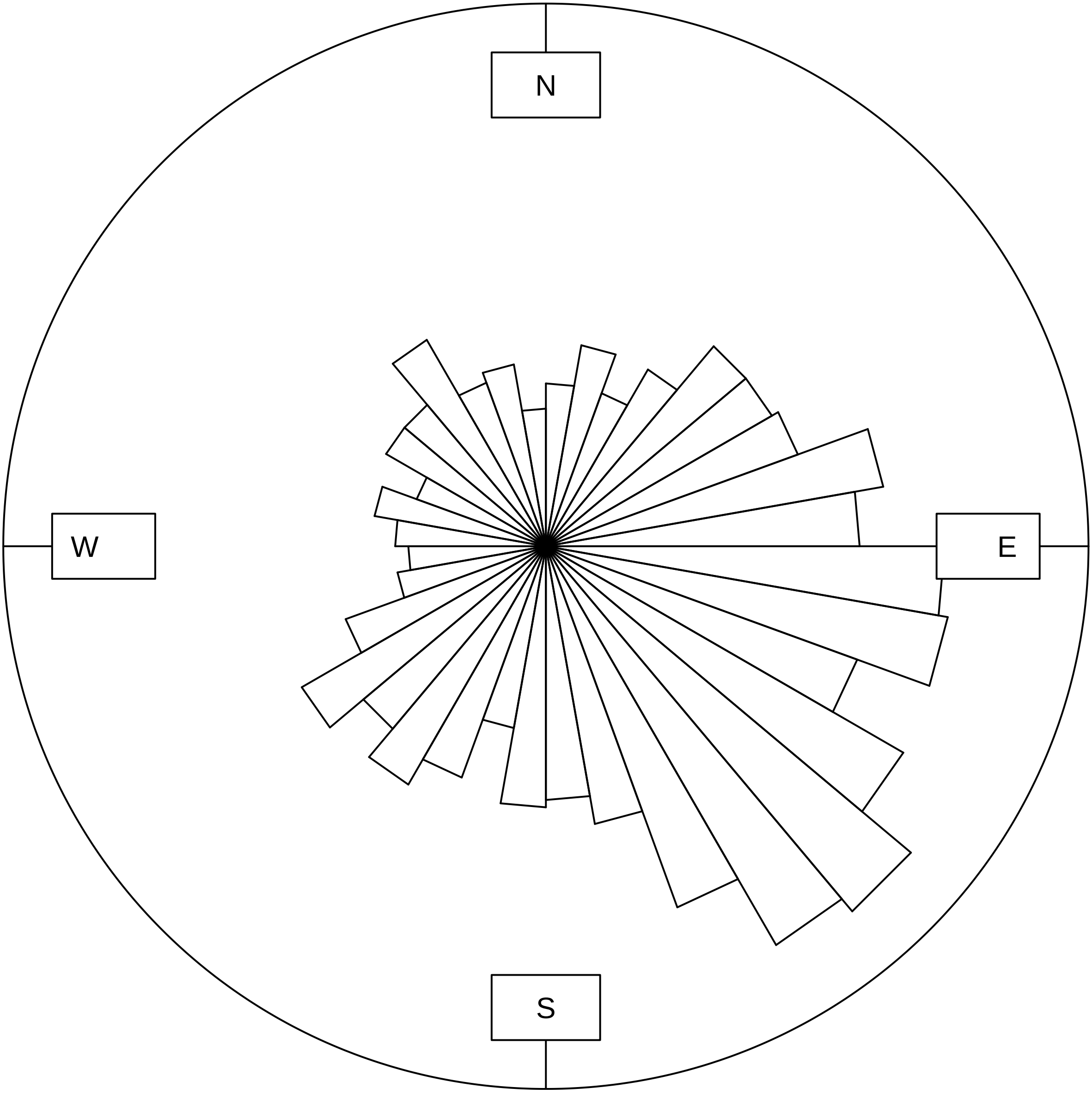

bootstrap 582

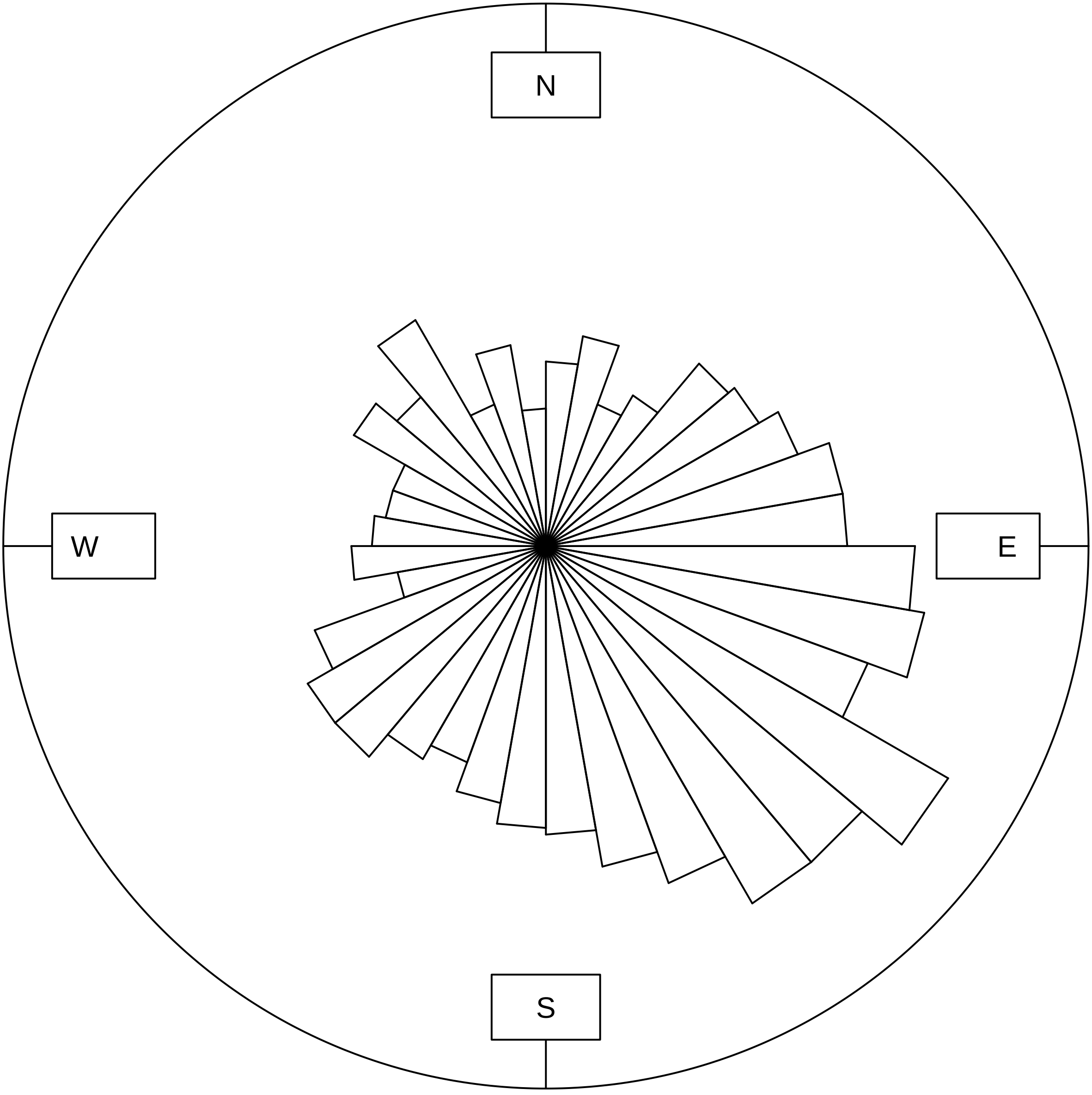

bootstrap 583

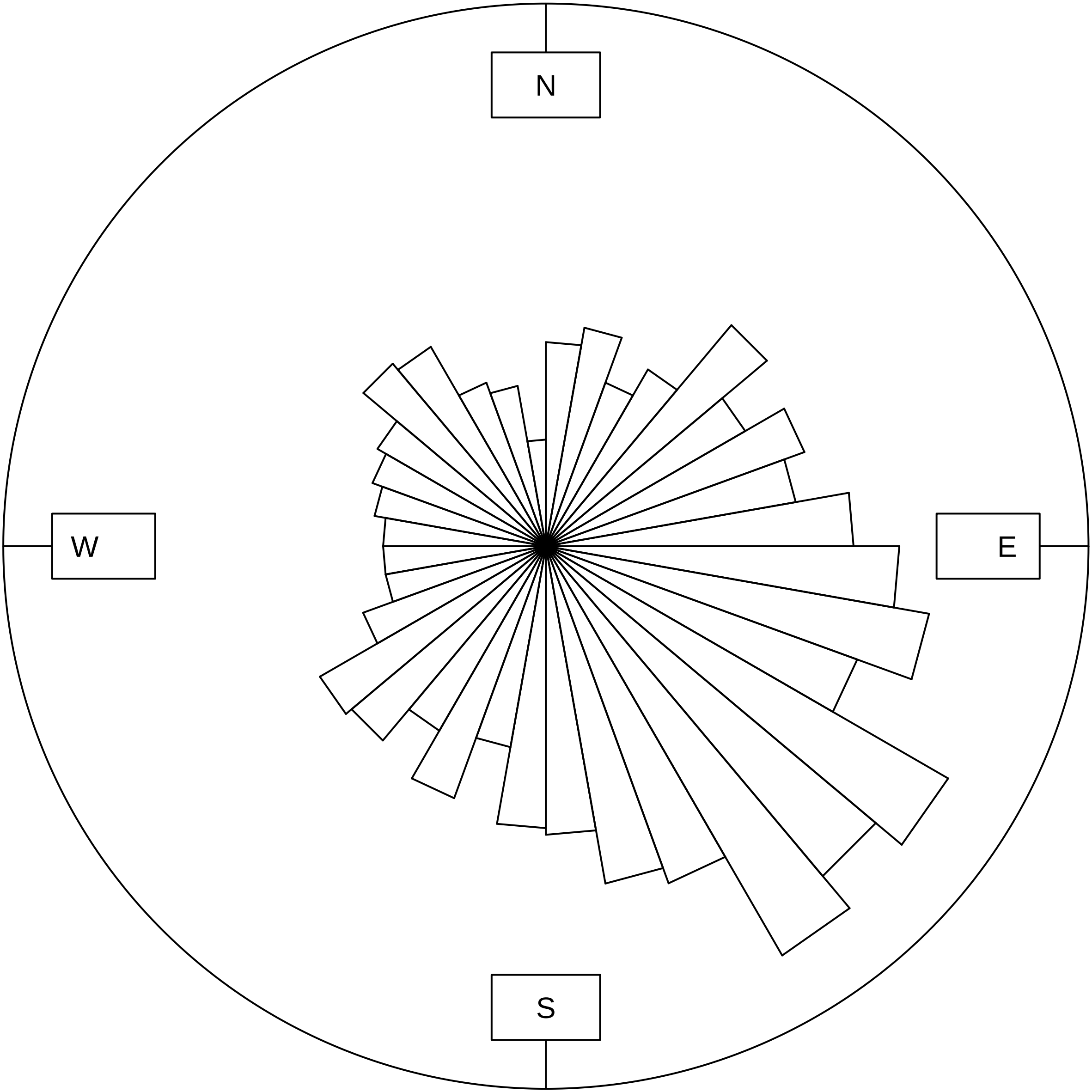

bootstrap 584

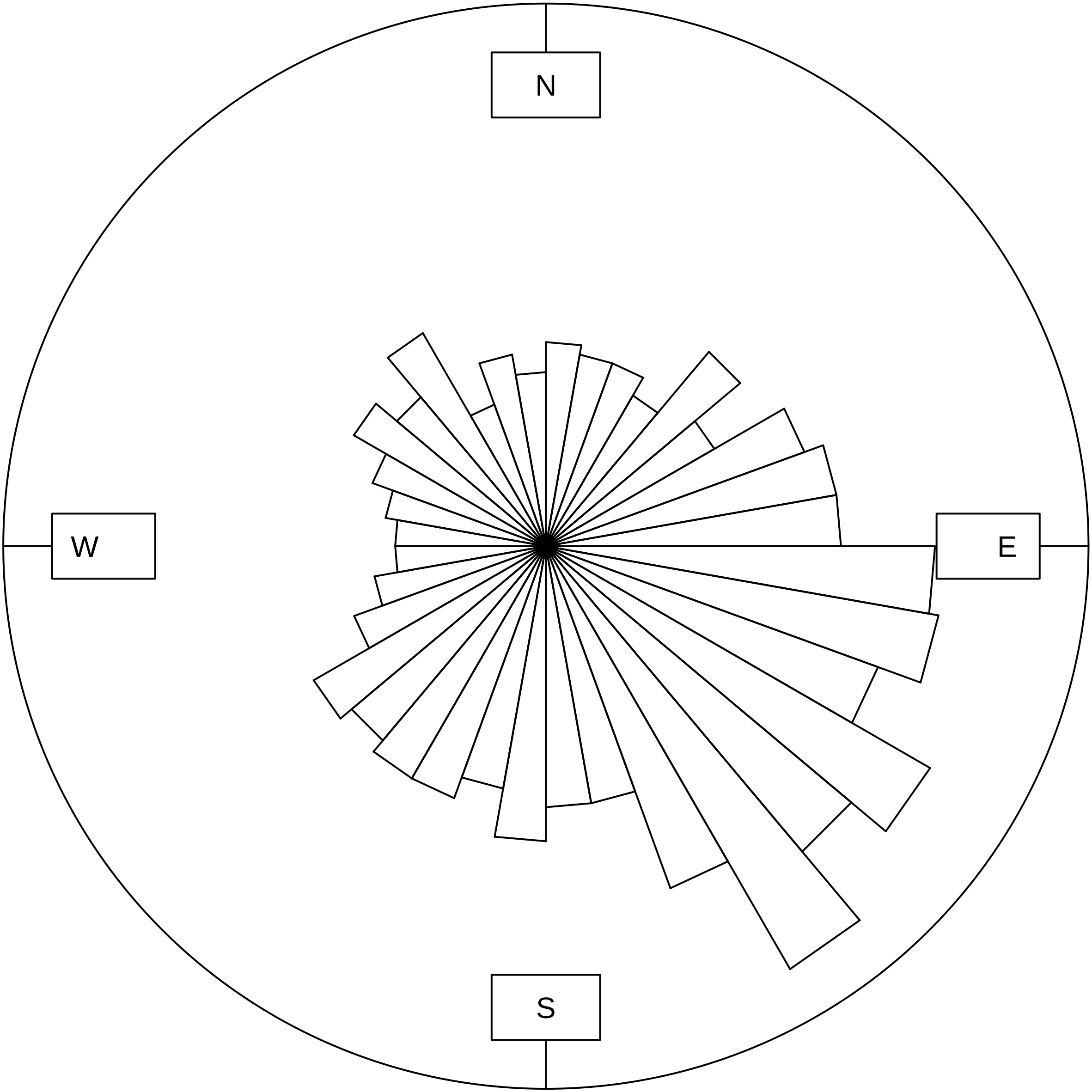

bootstrap 585

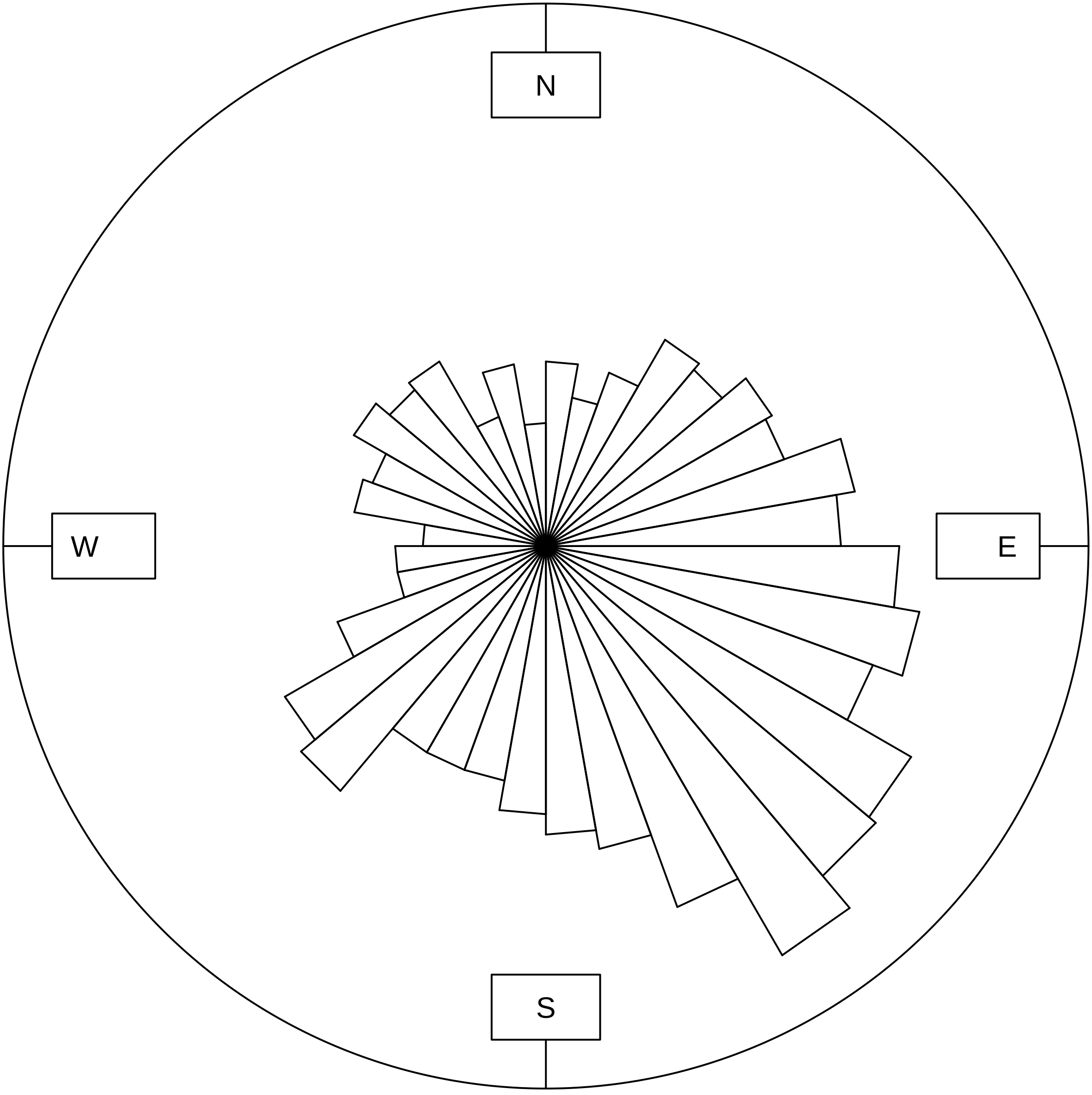

bootstrap 586

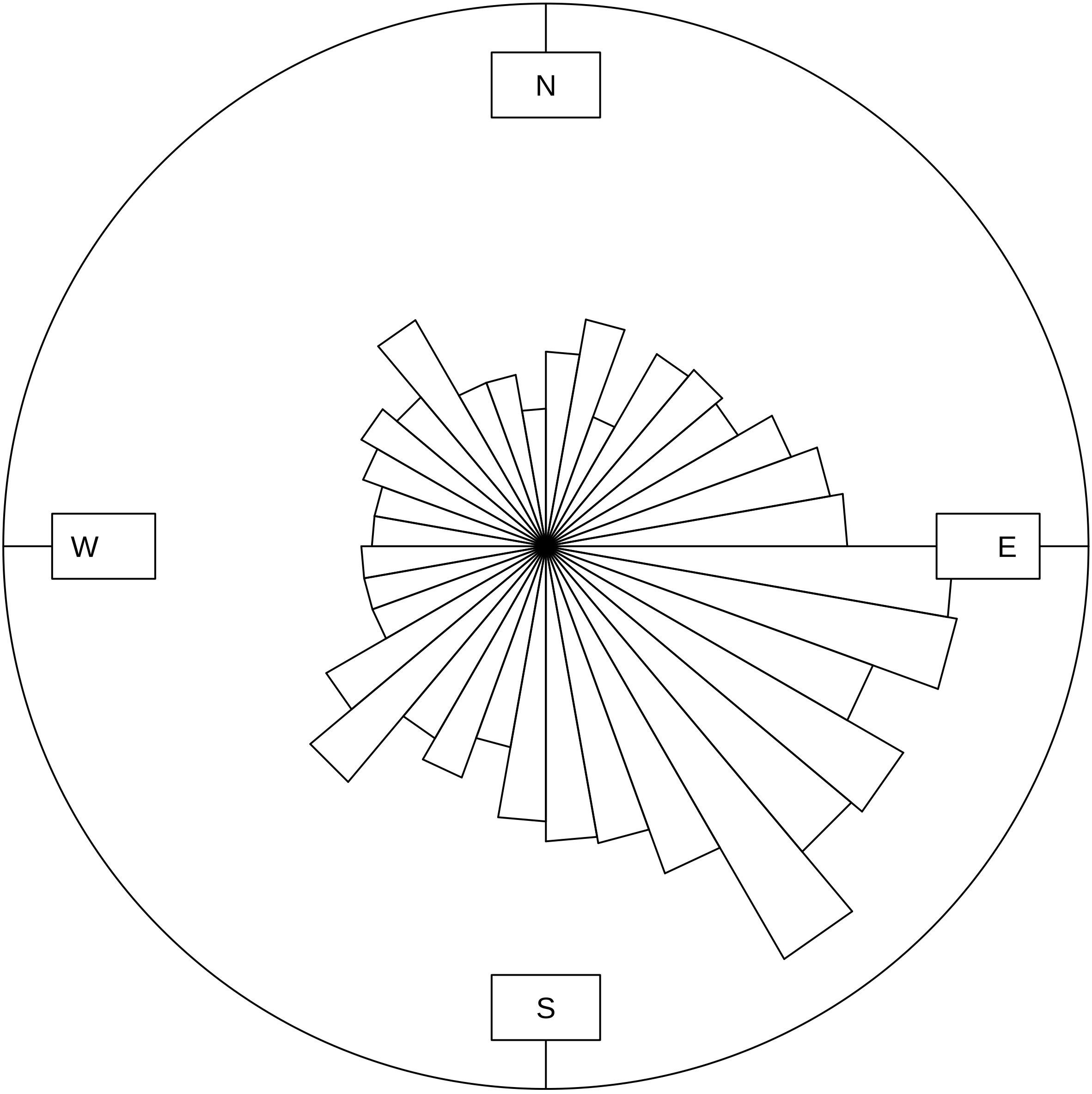

bootstrap 587

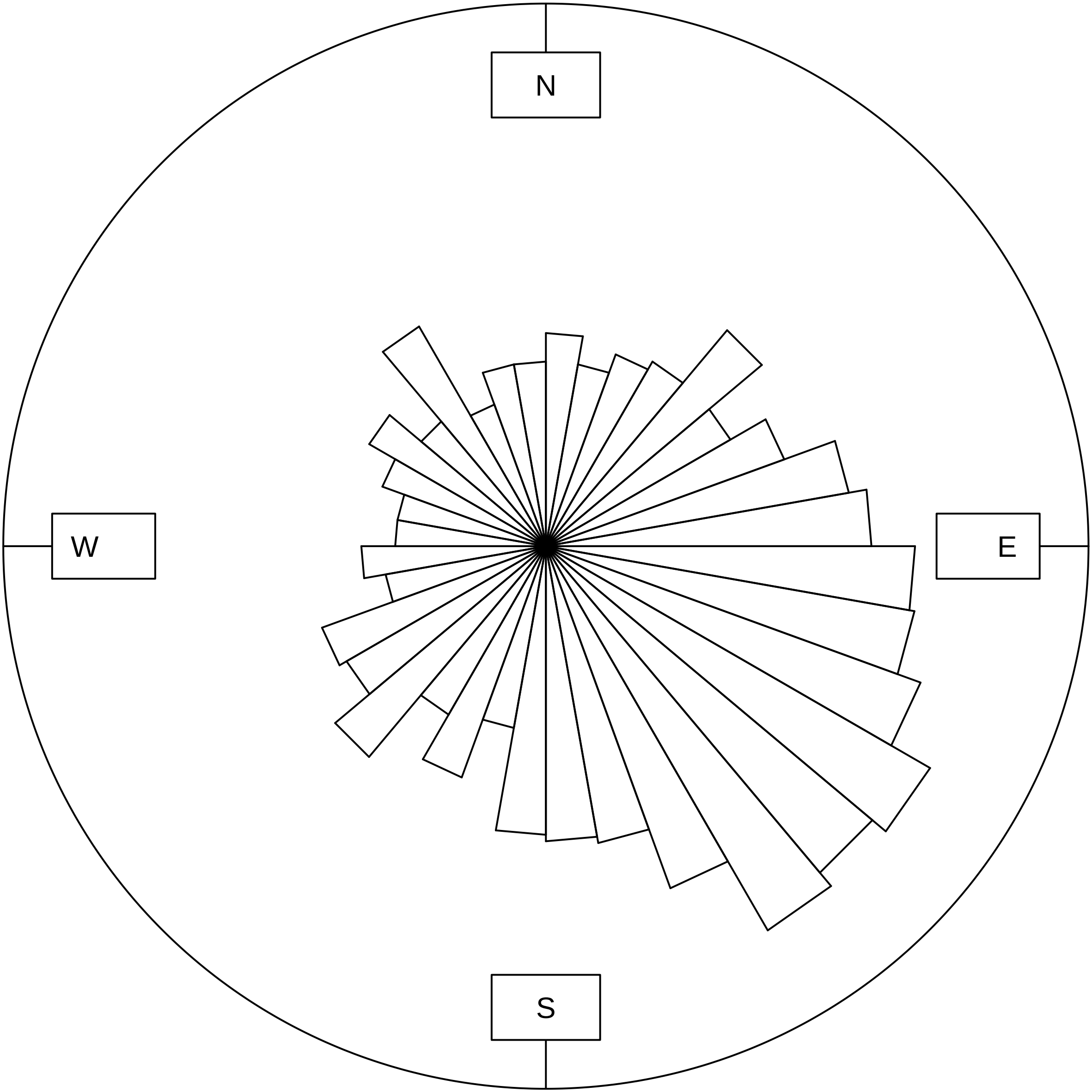

bootstrap 588

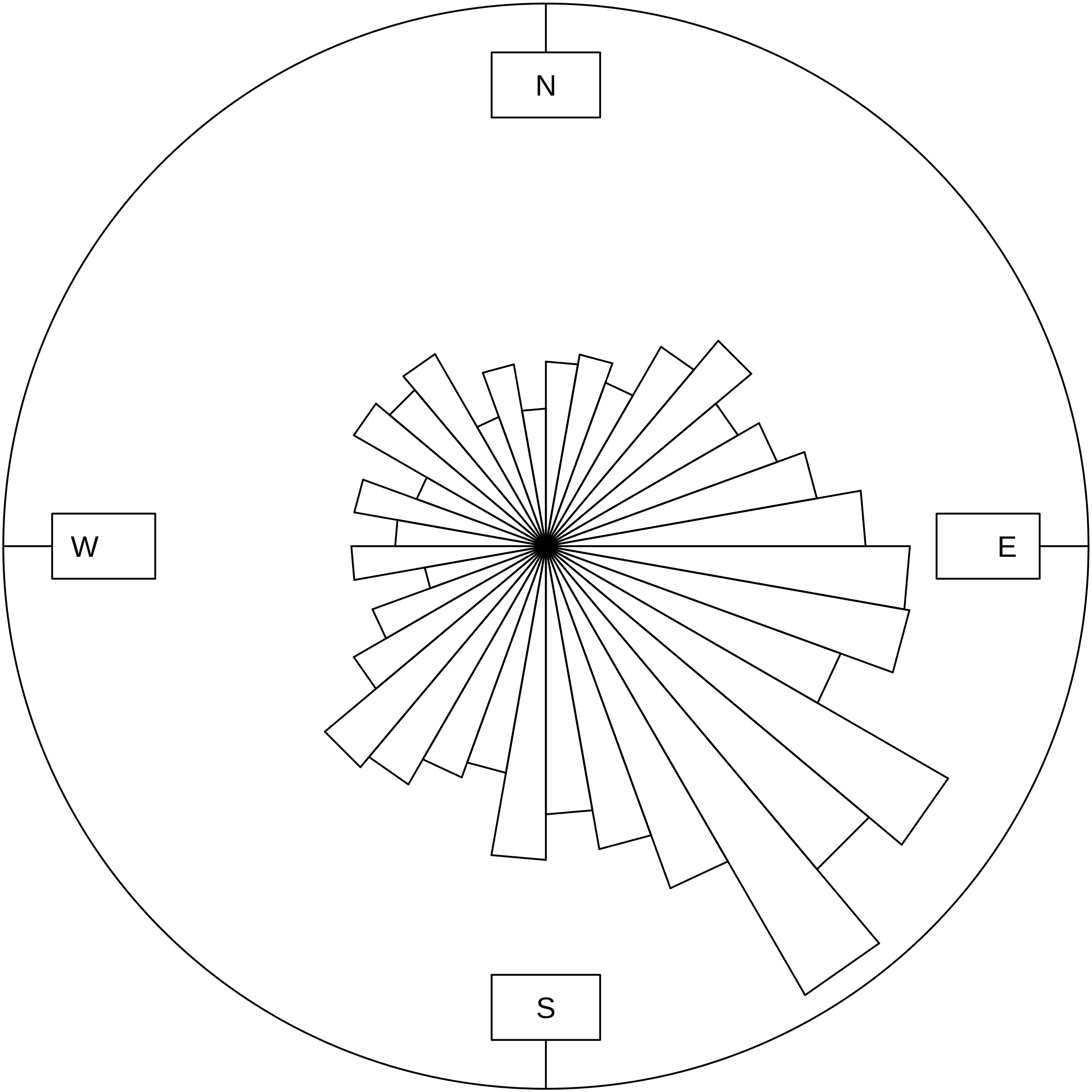

bootstrap 589

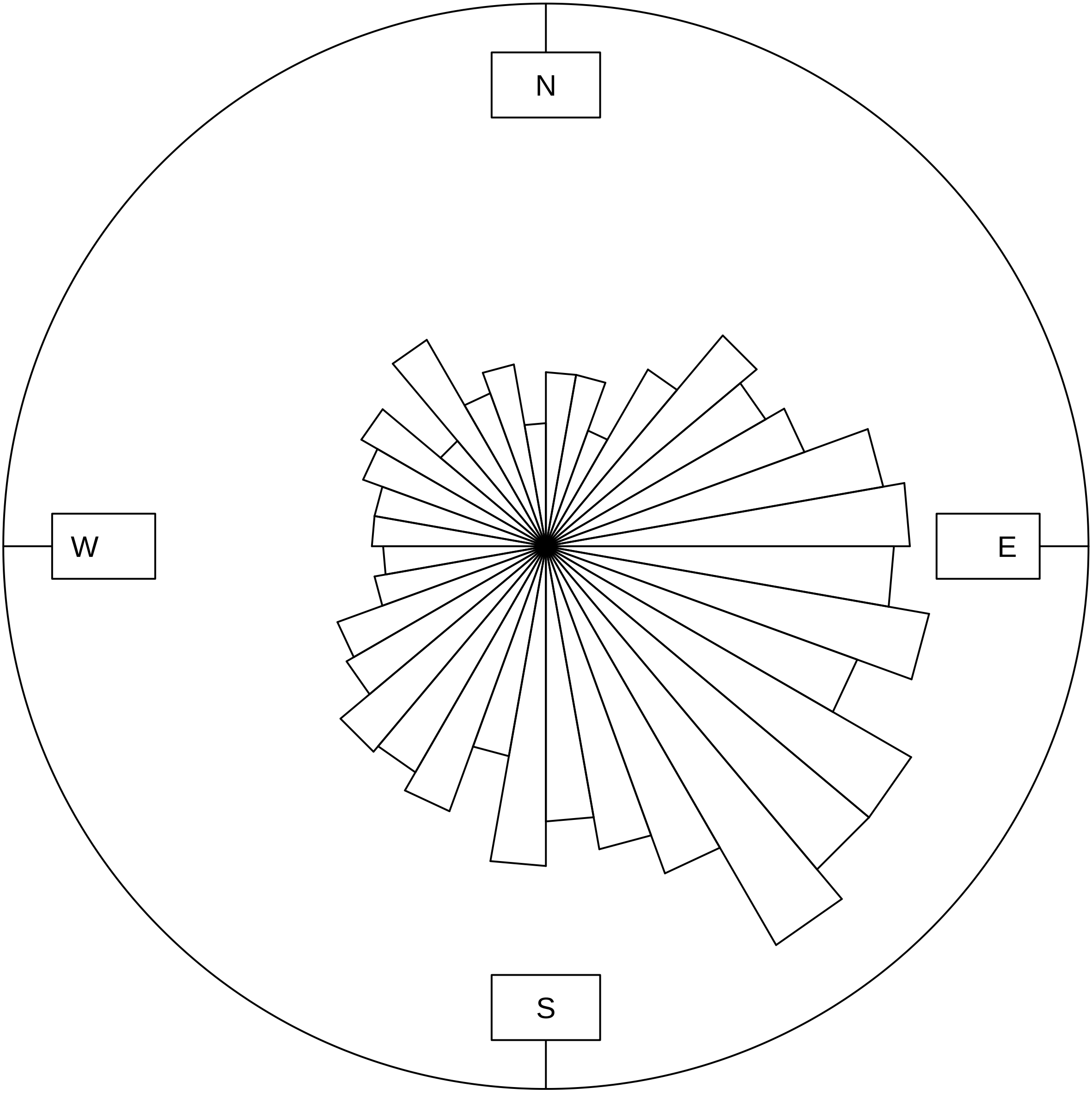

bootstrap 590

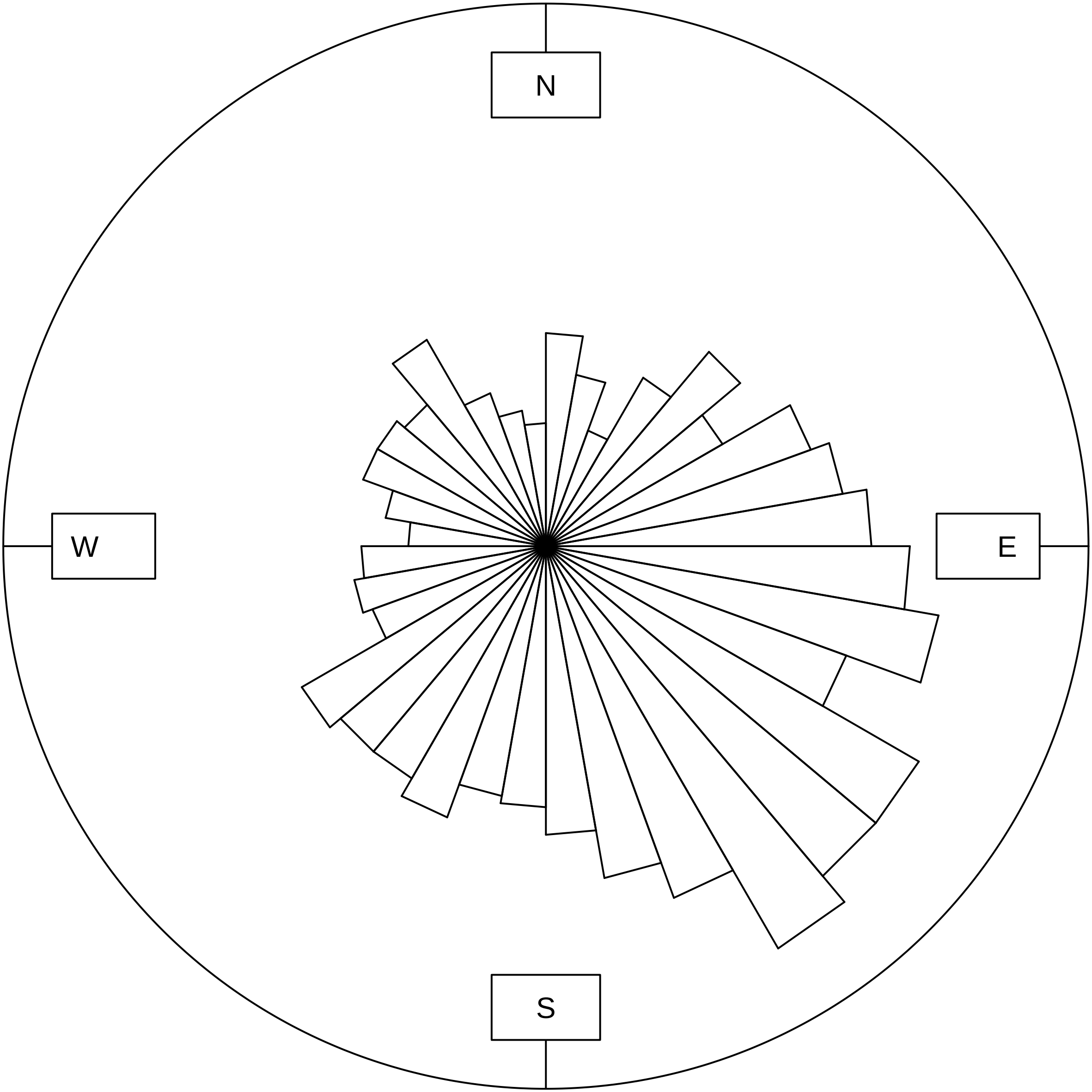

bootstrap 591

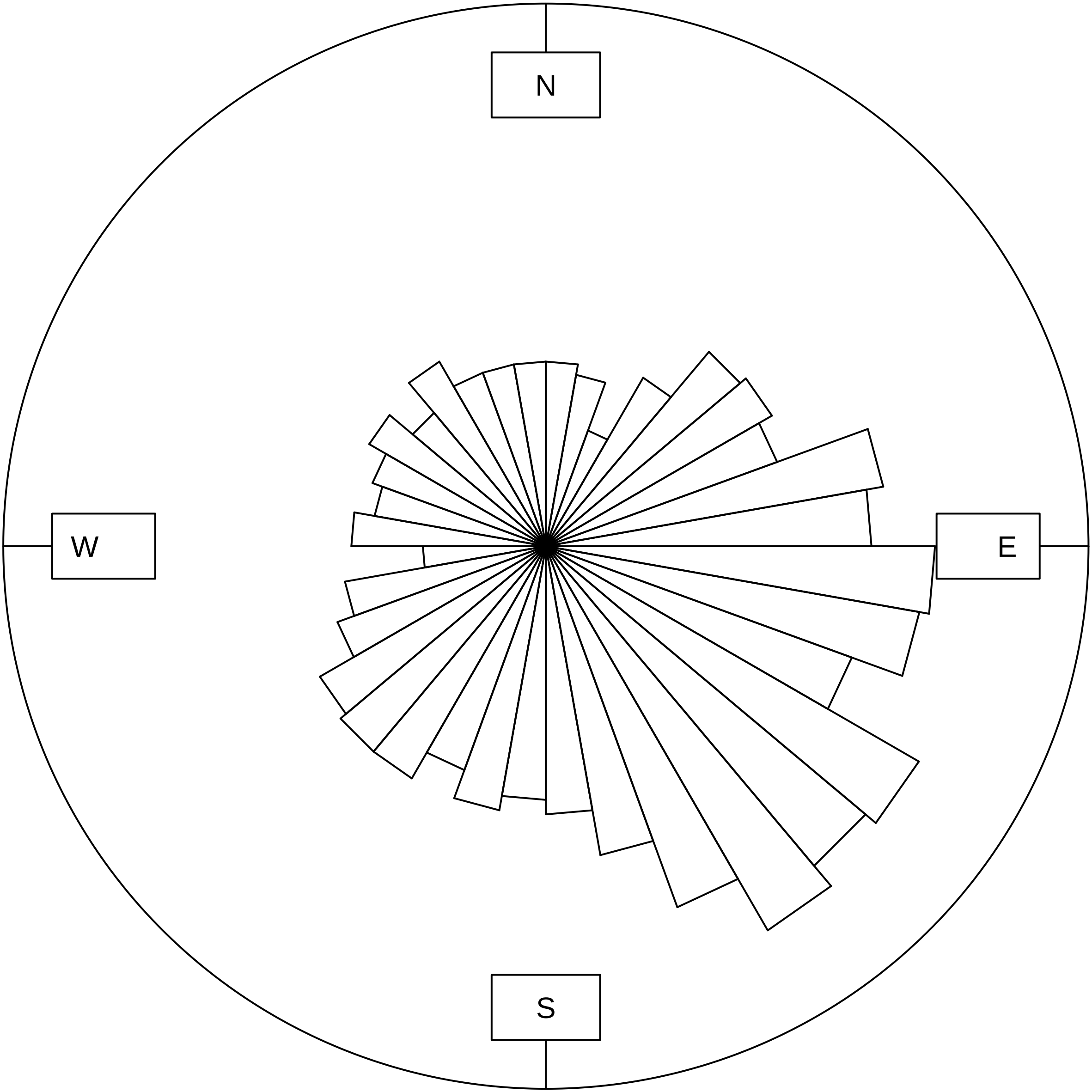

bootstrap 592

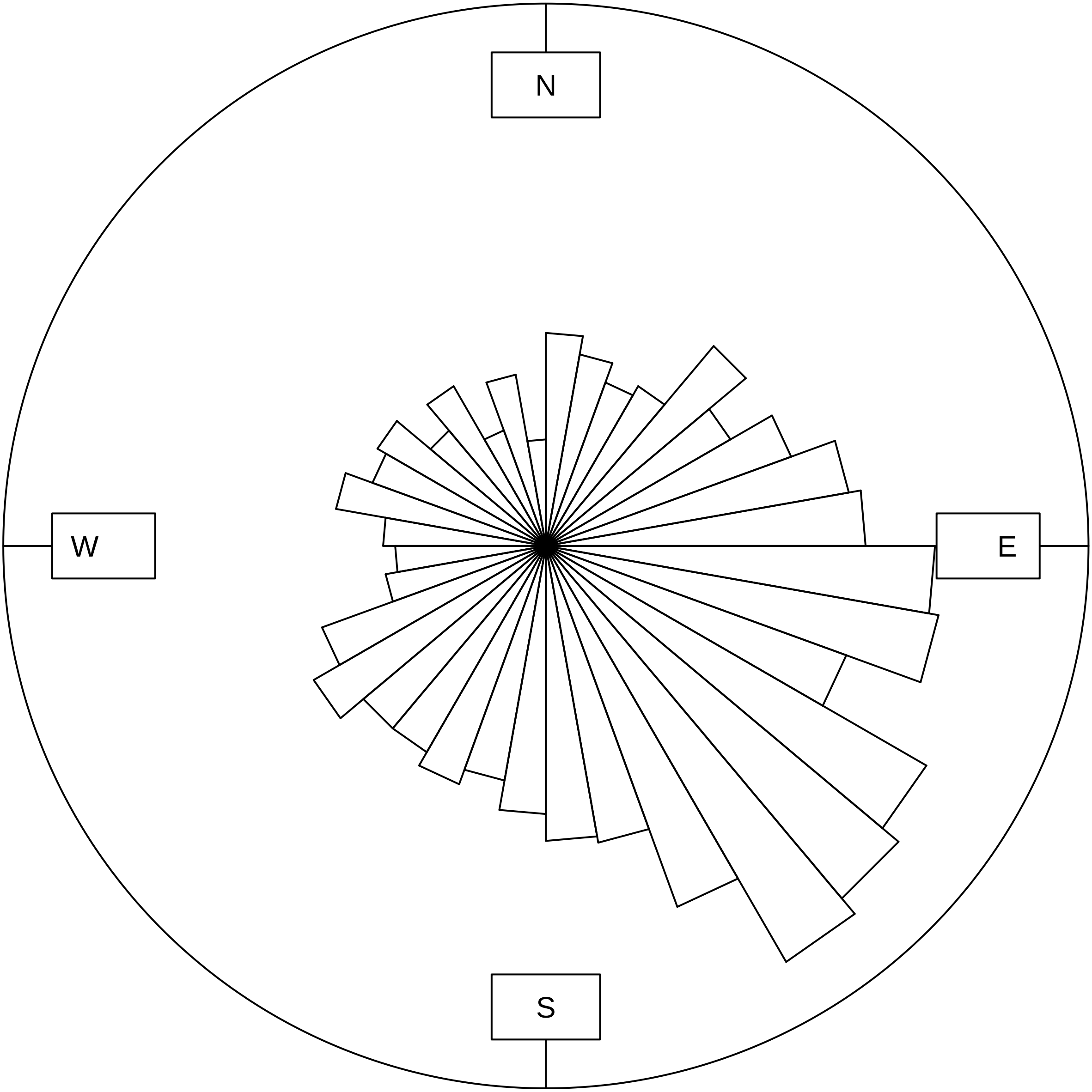

bootstrap 593

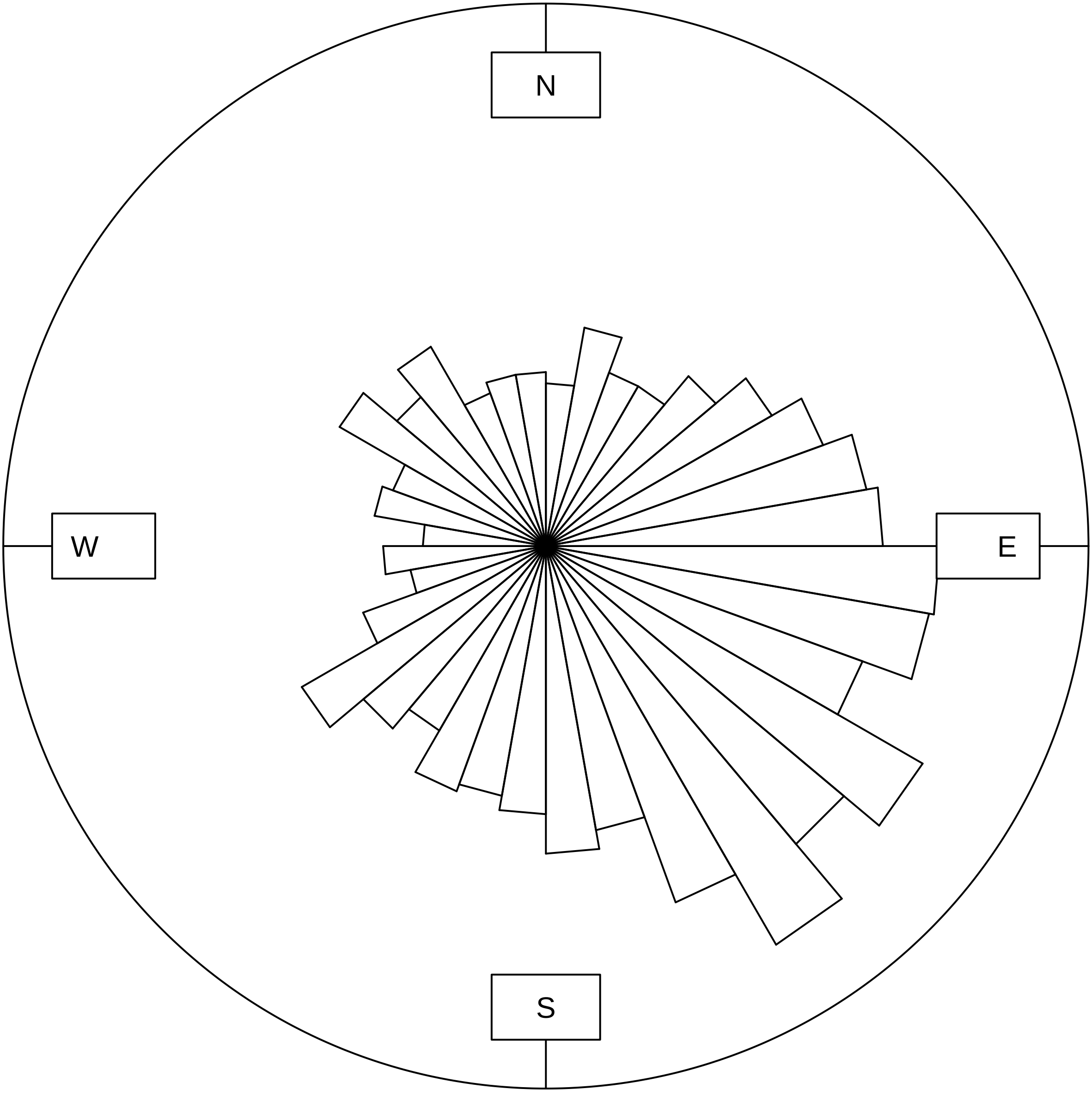

bootstrap 594

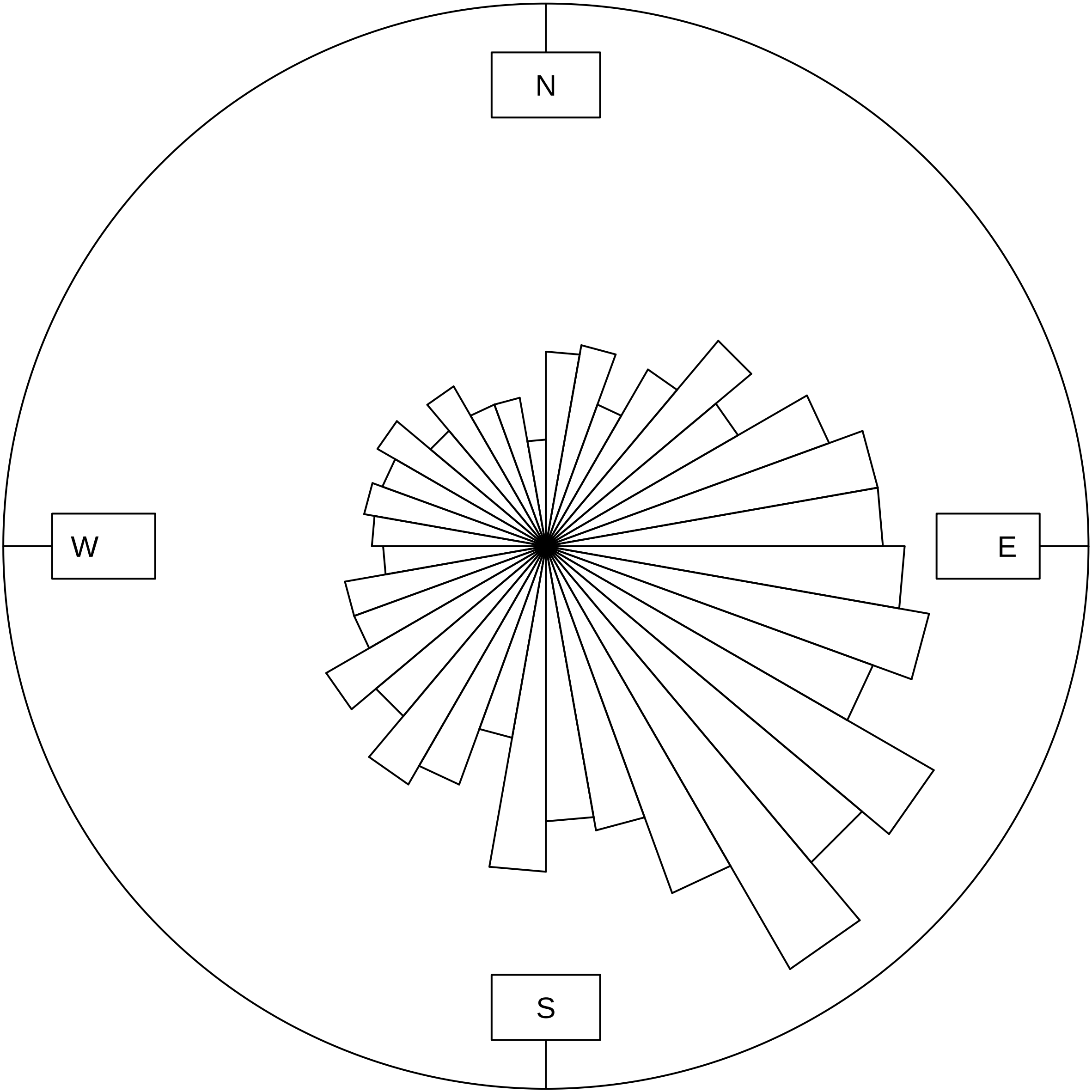

bootstrap 595

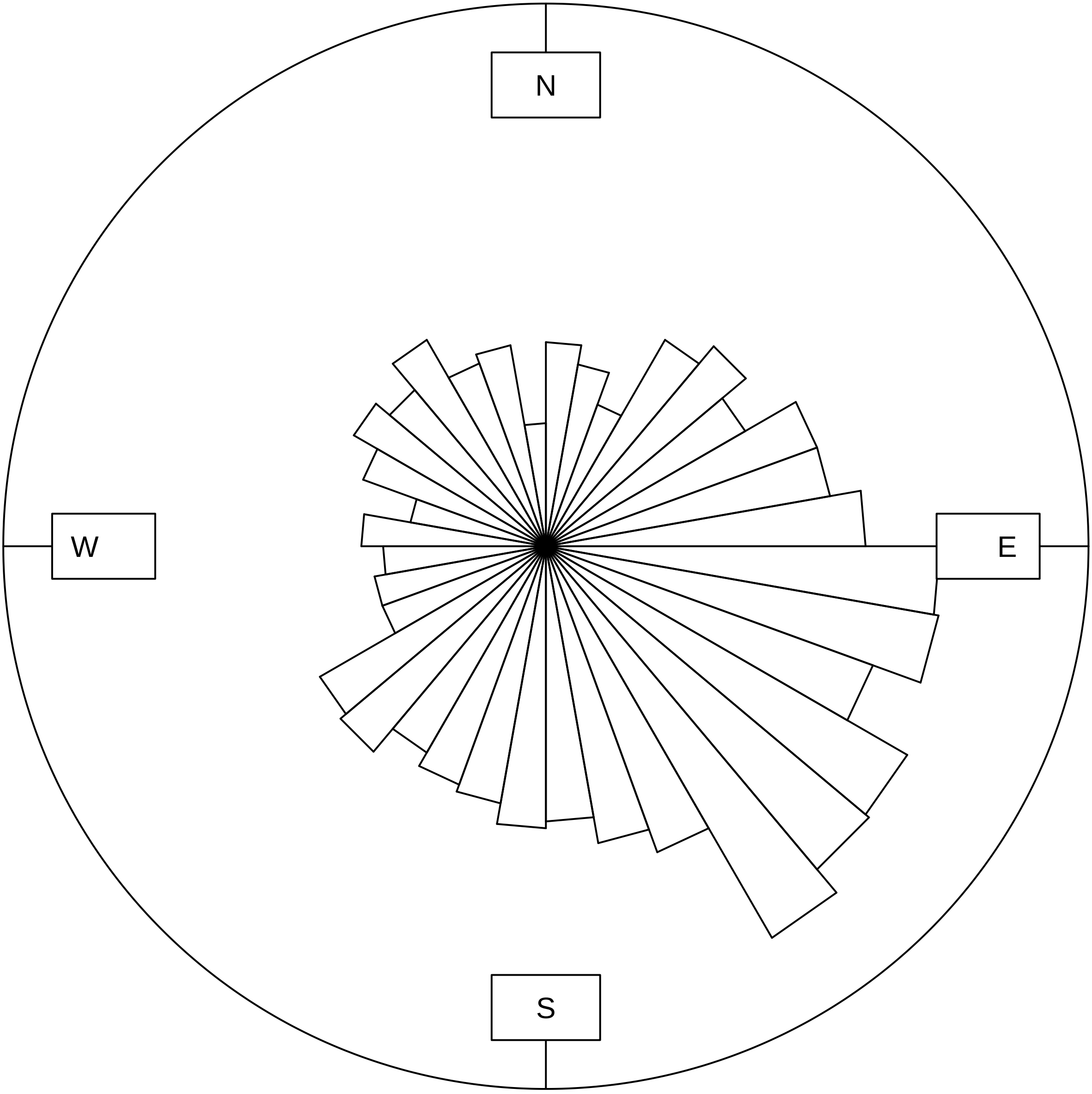

bootstrap 596

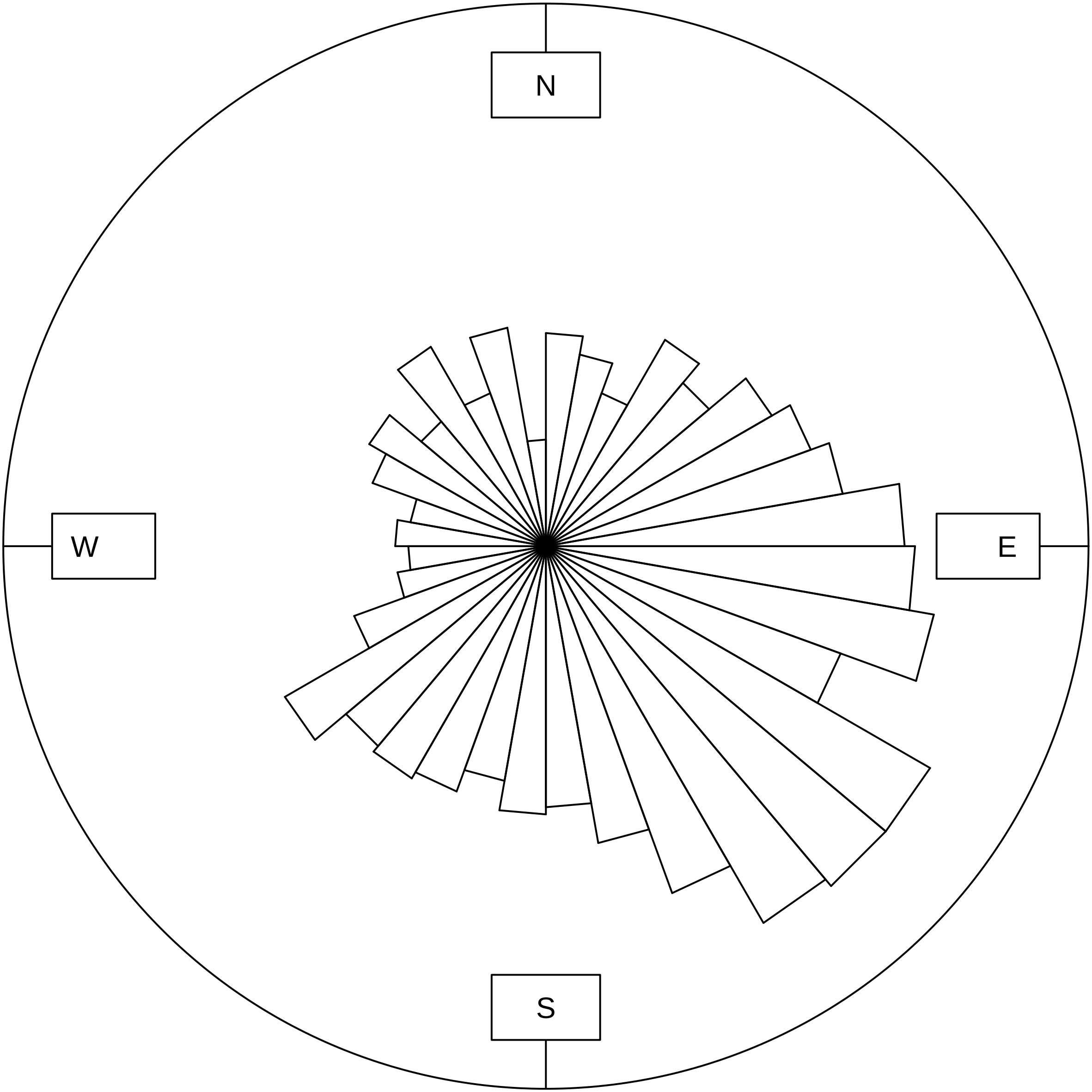

bootstrap 597

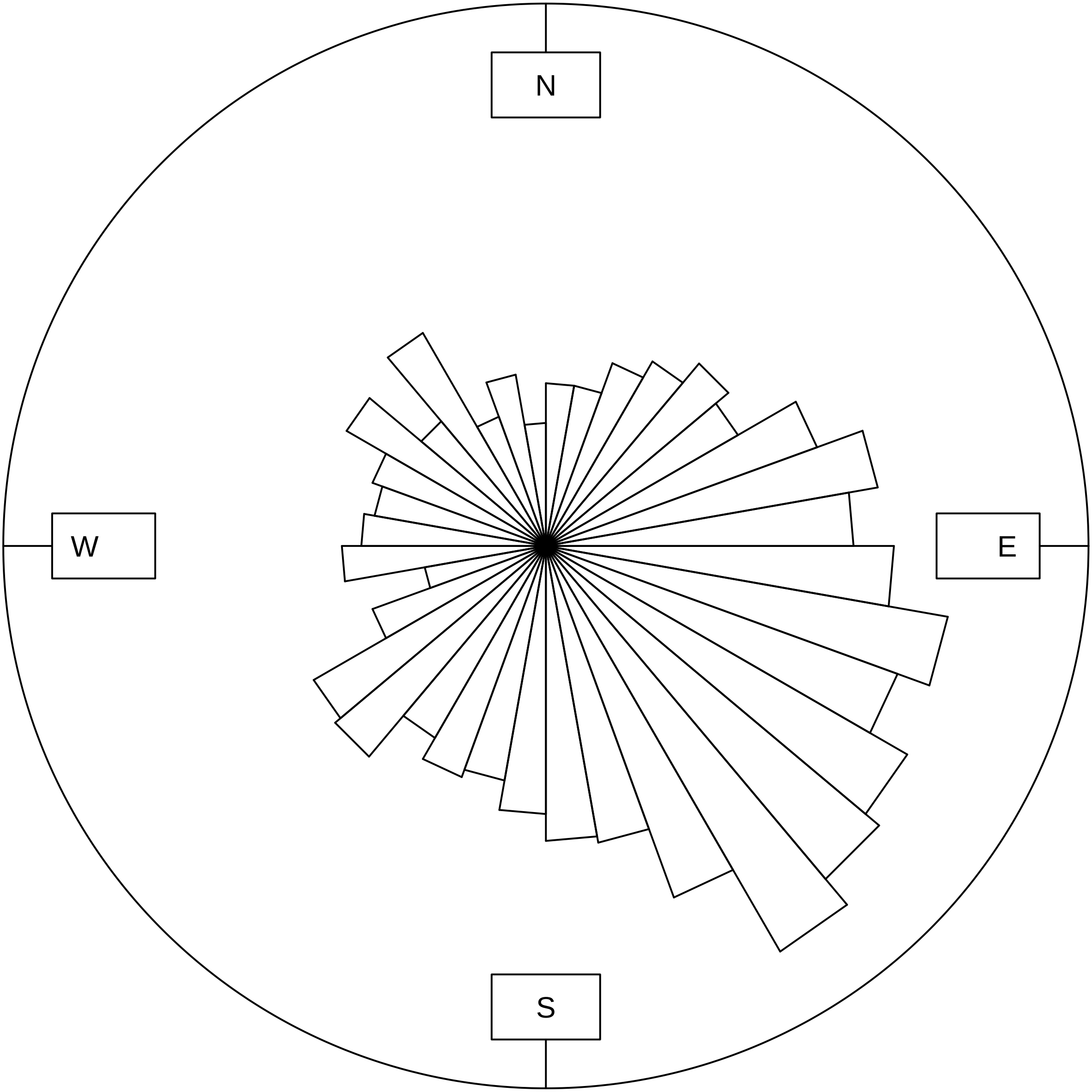

bootstrap 598

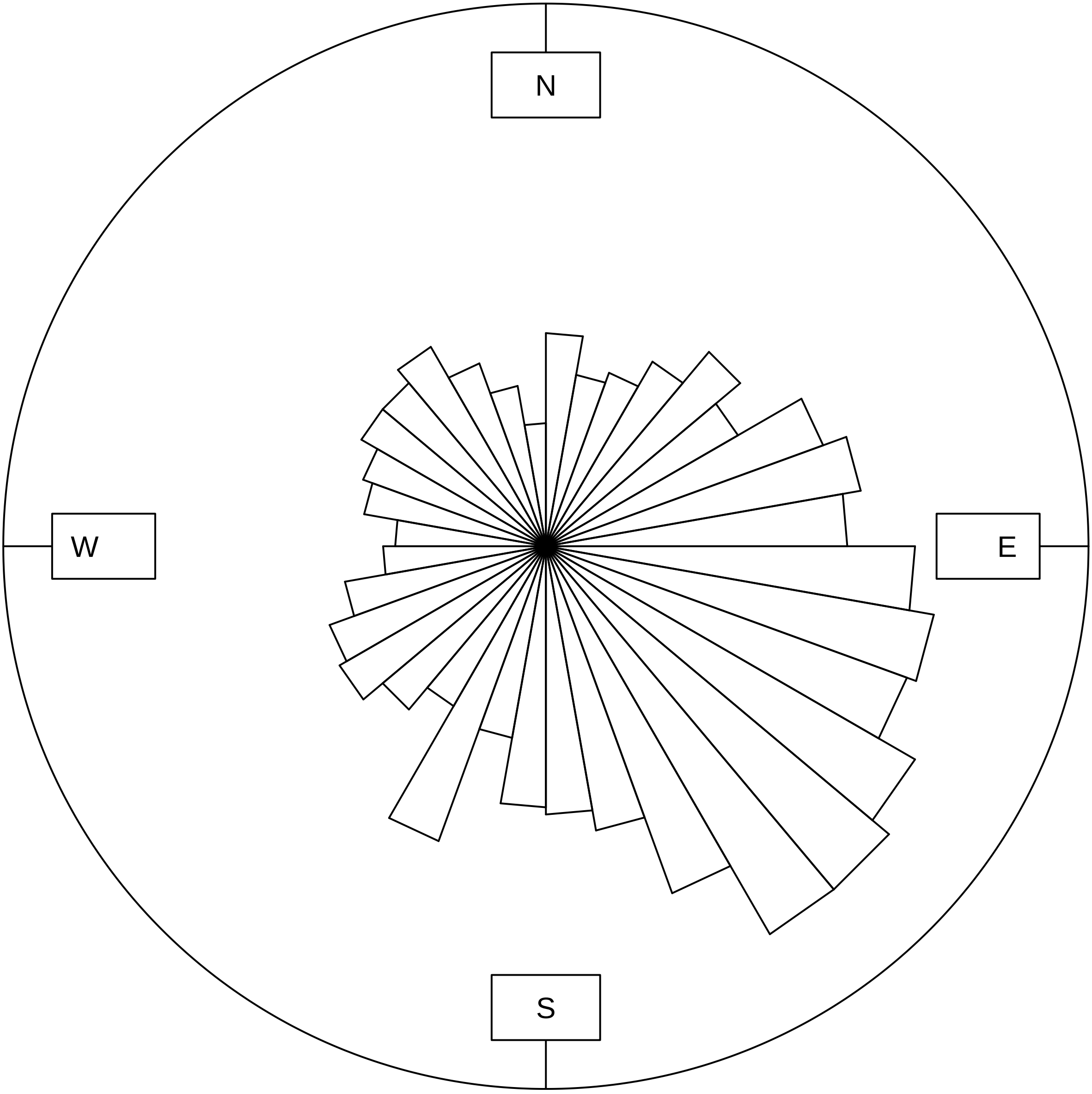

bootstrap 599

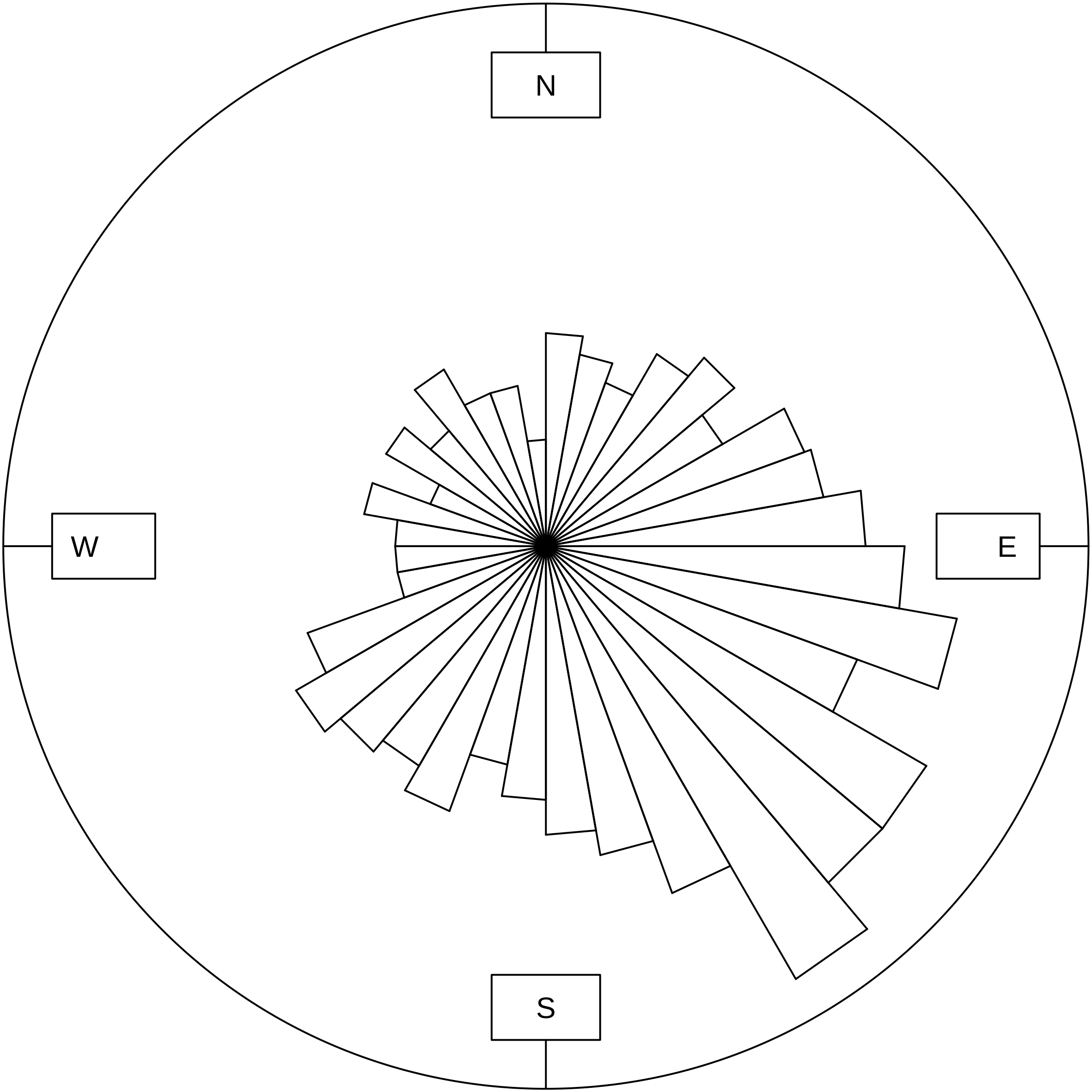

bootstrap 600

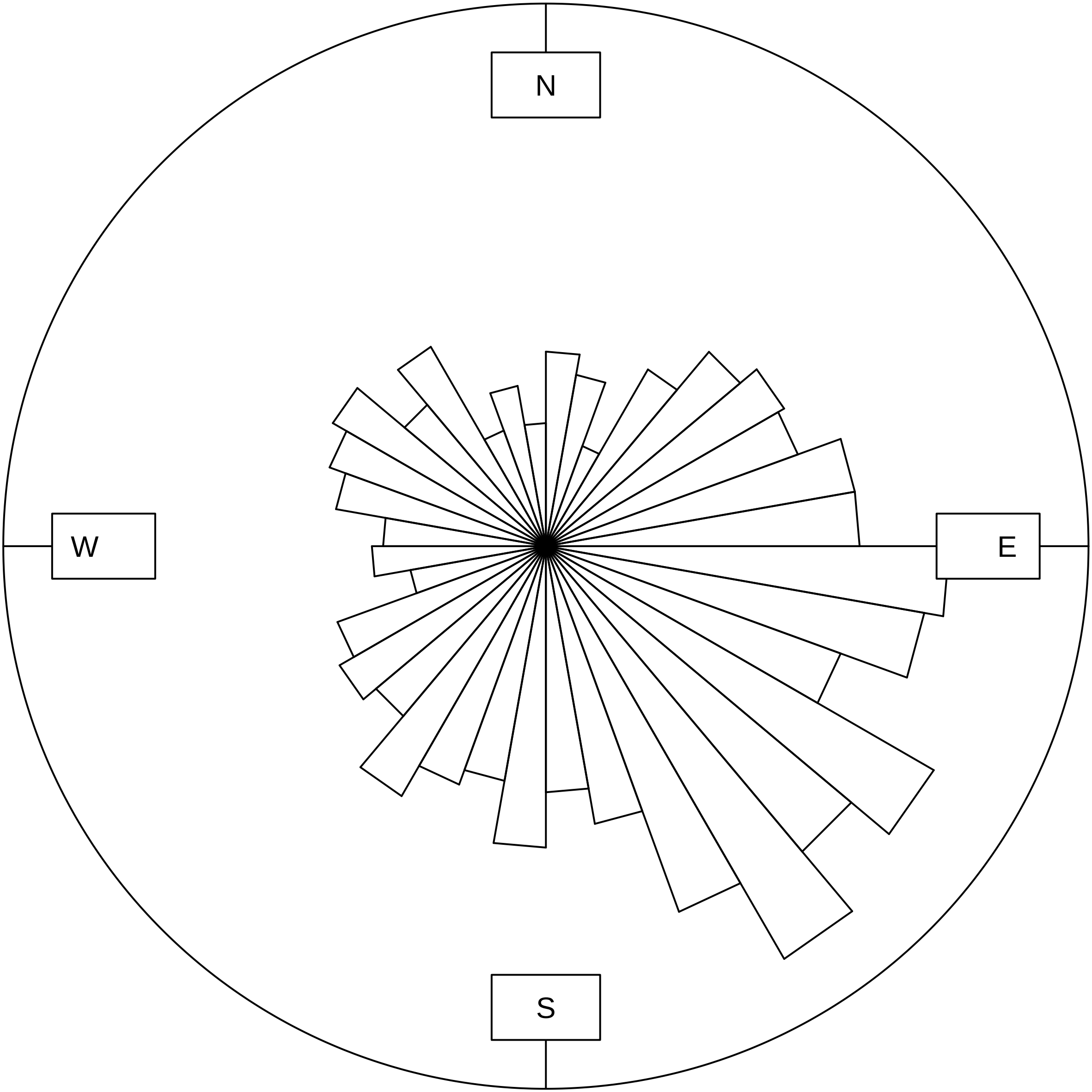

bootstrap 601

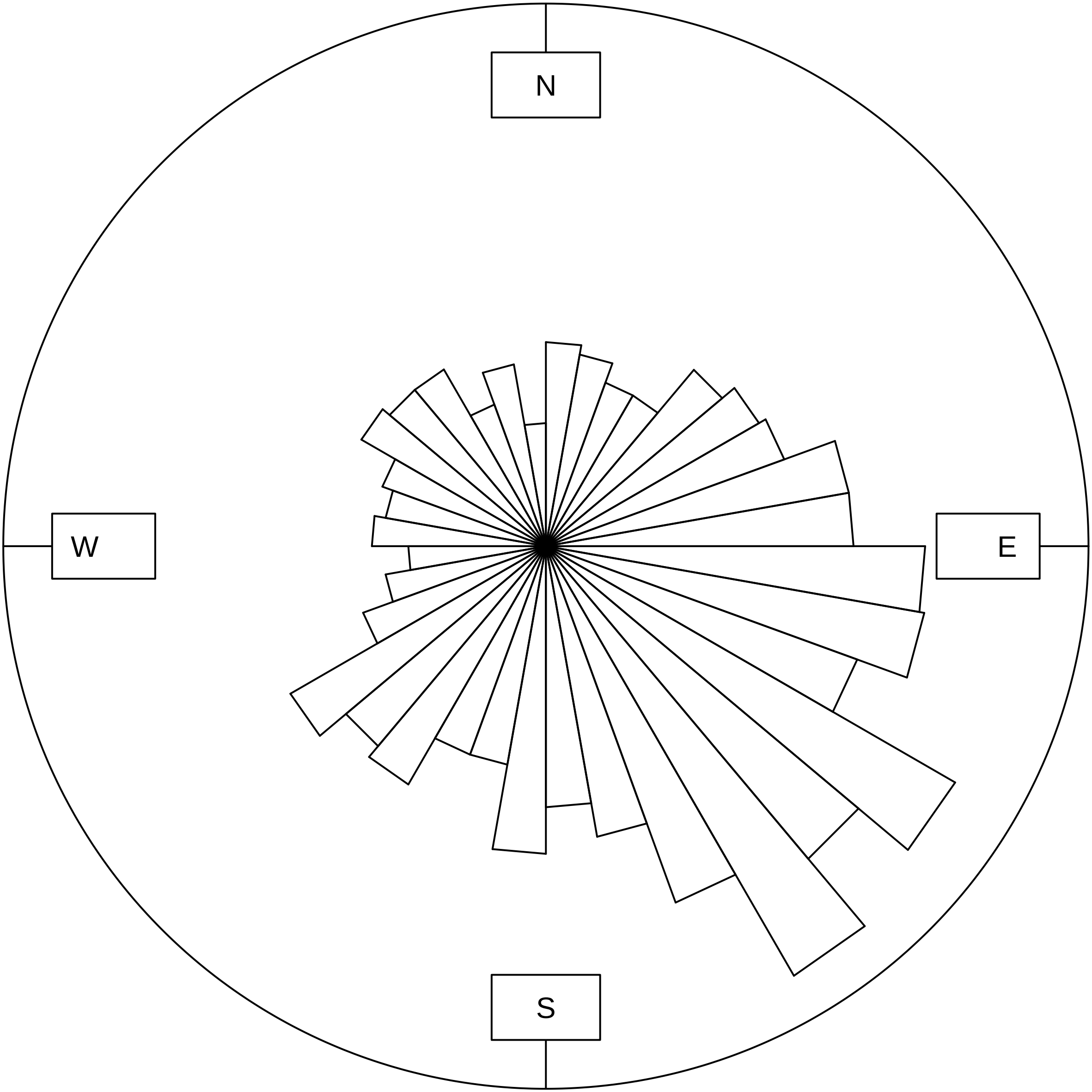

bootstrap 602

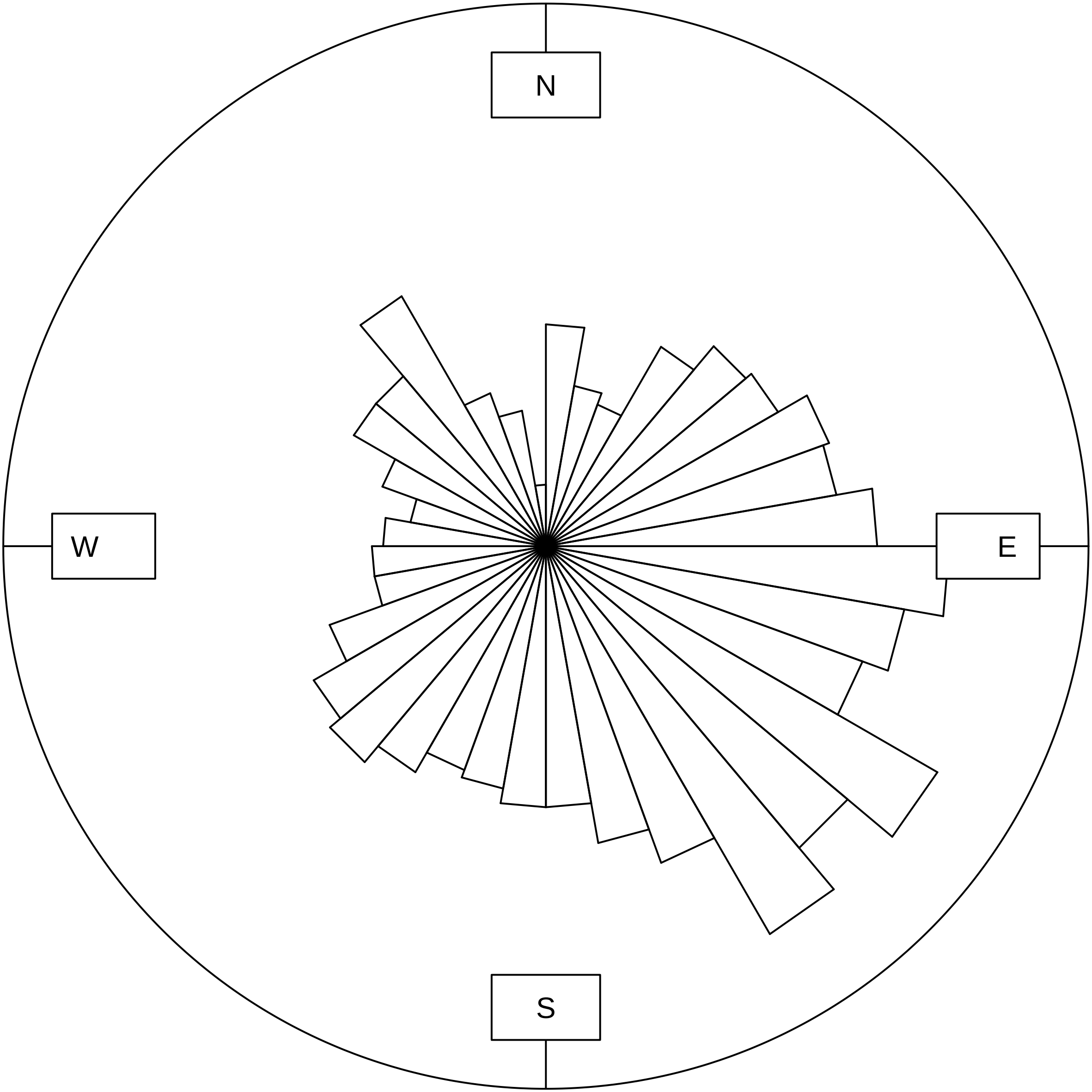

bootstrap 603

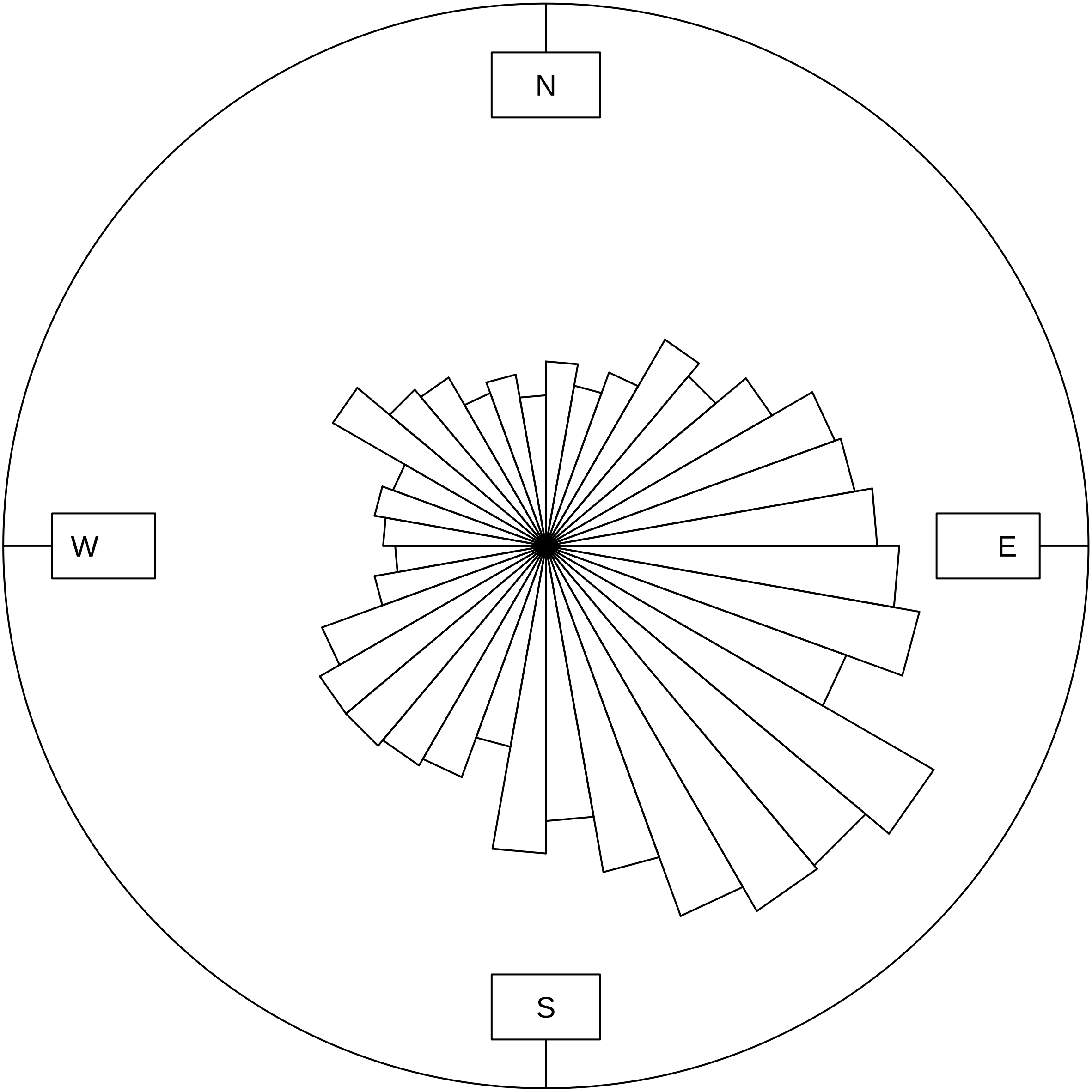

bootstrap 604

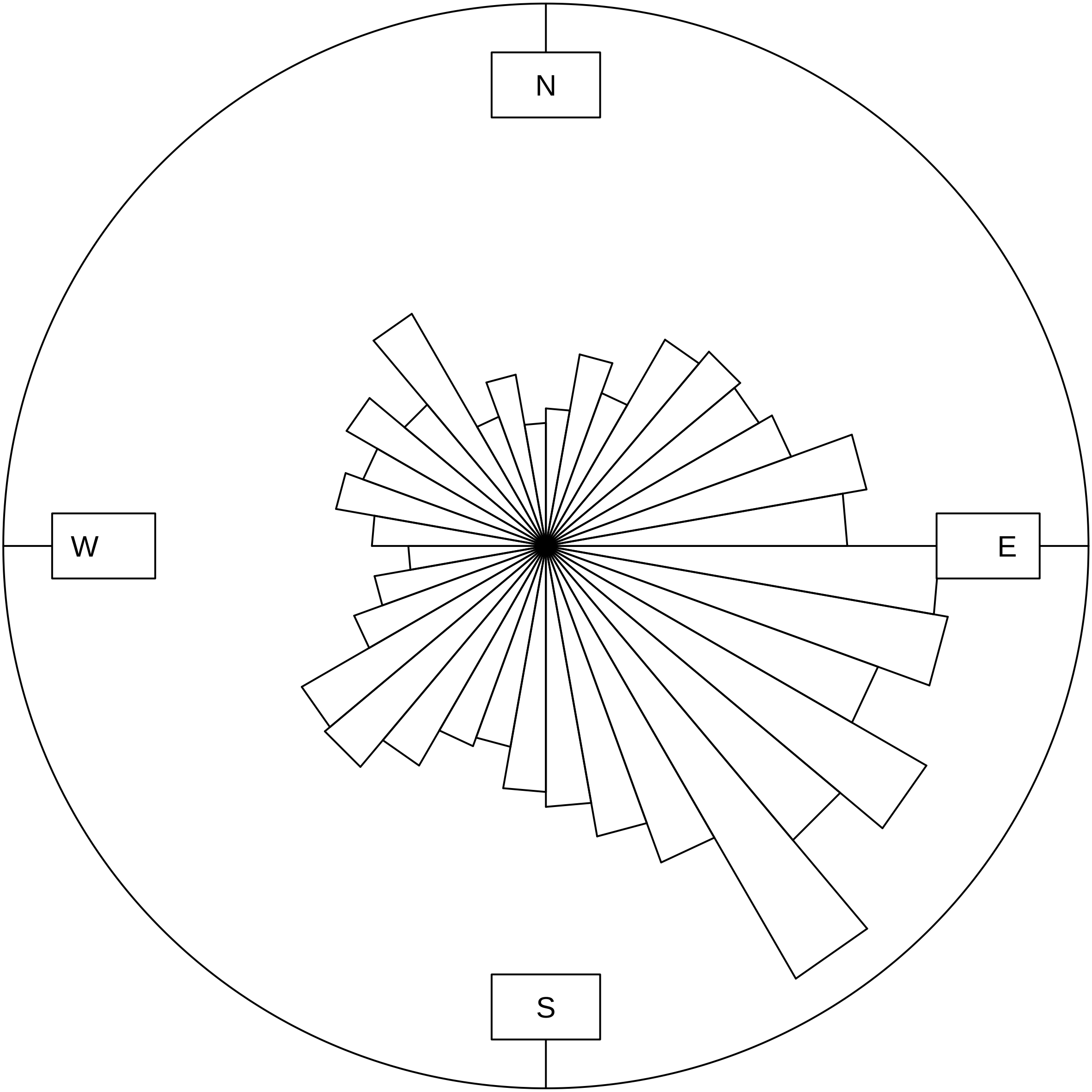

bootstrap 605

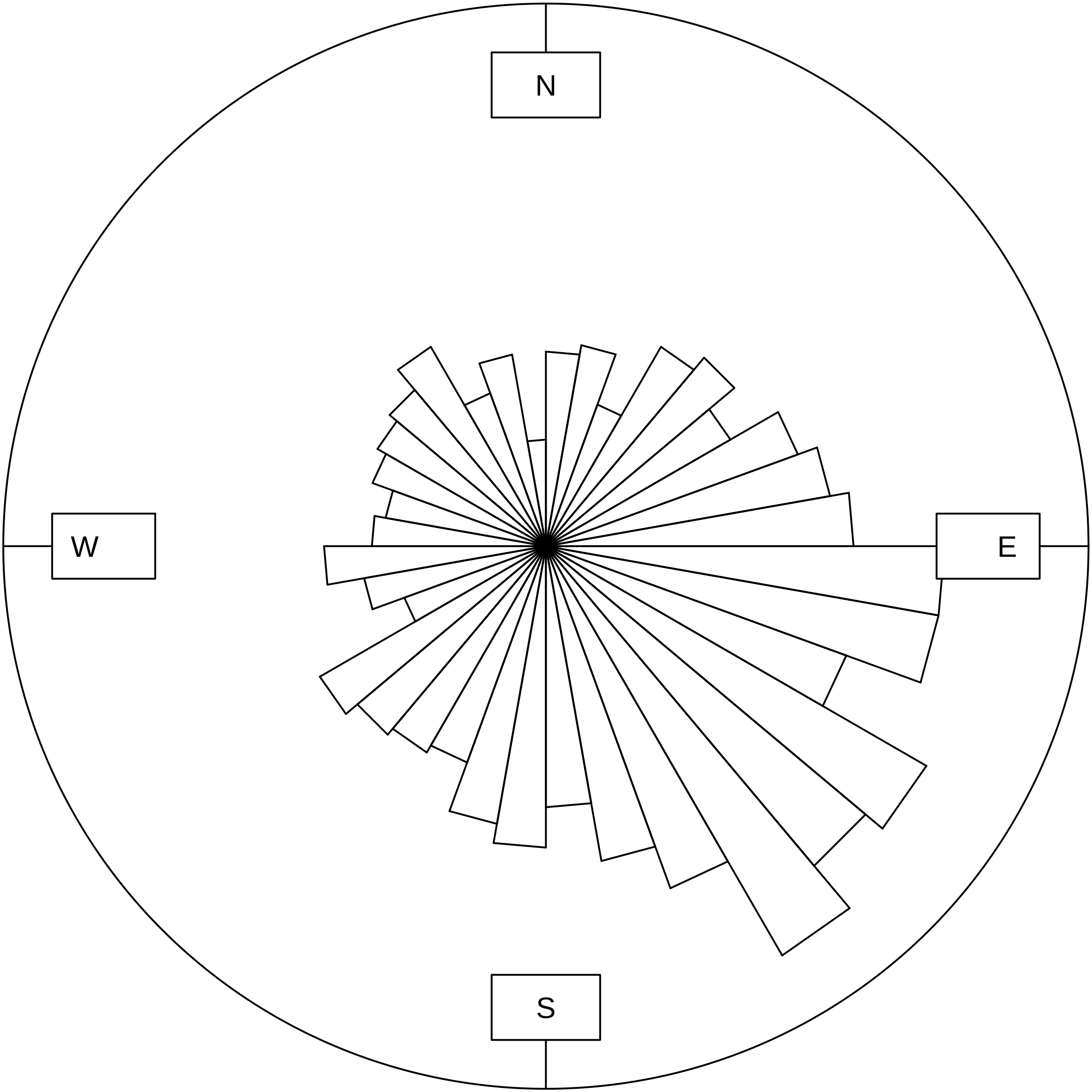

bootstrap 606

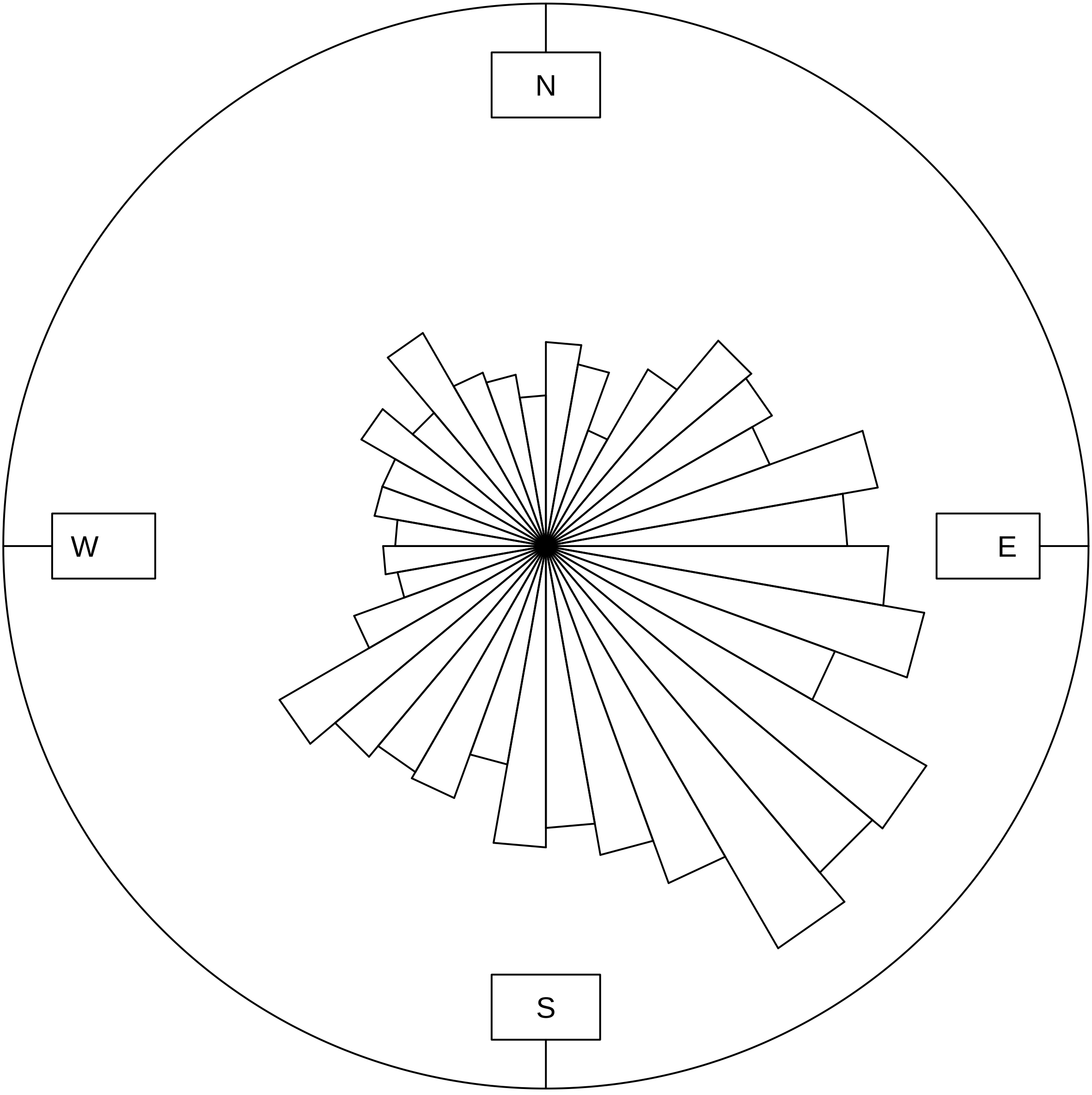

bootstrap 607

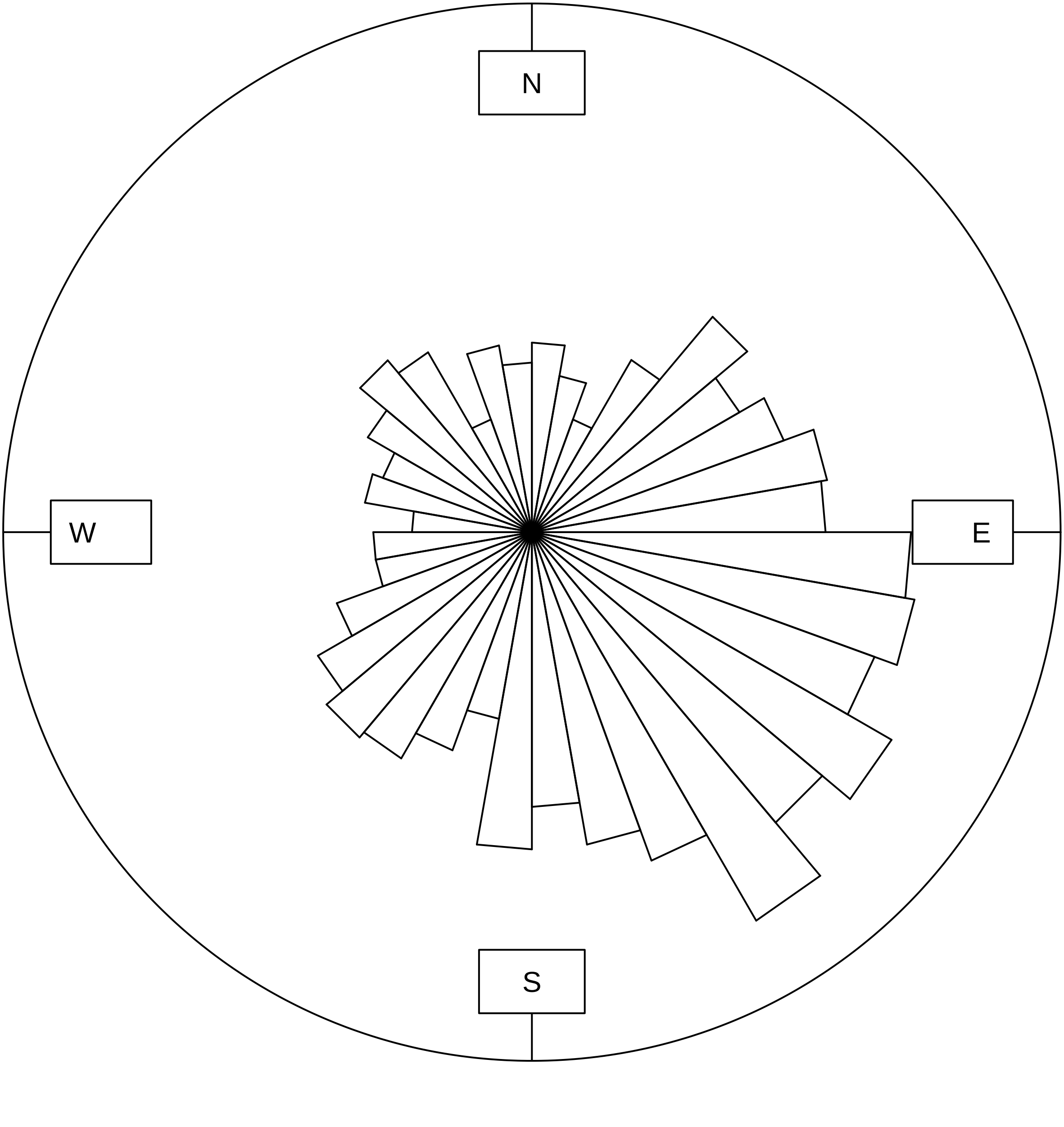

bootstrap 608

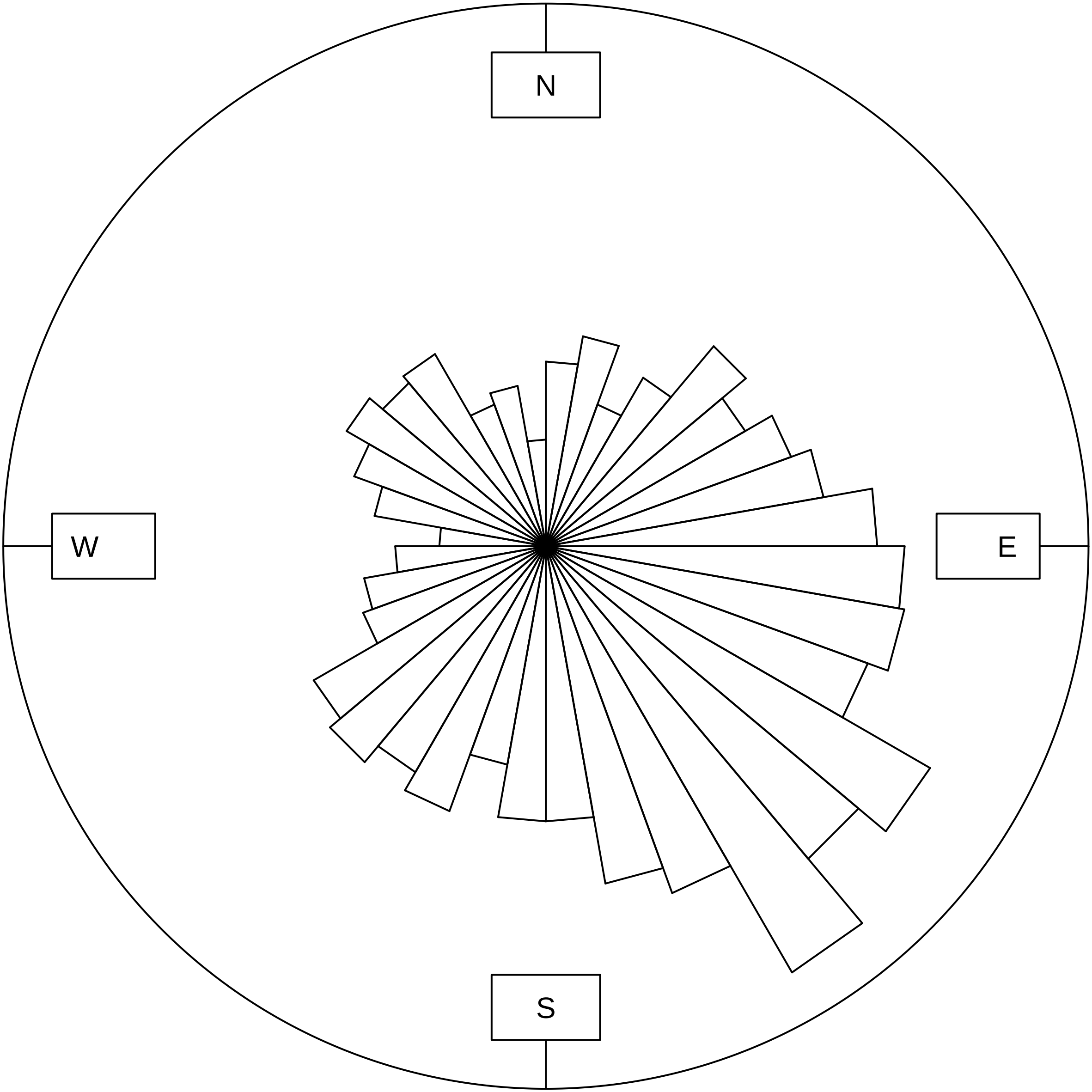

bootstrap 609

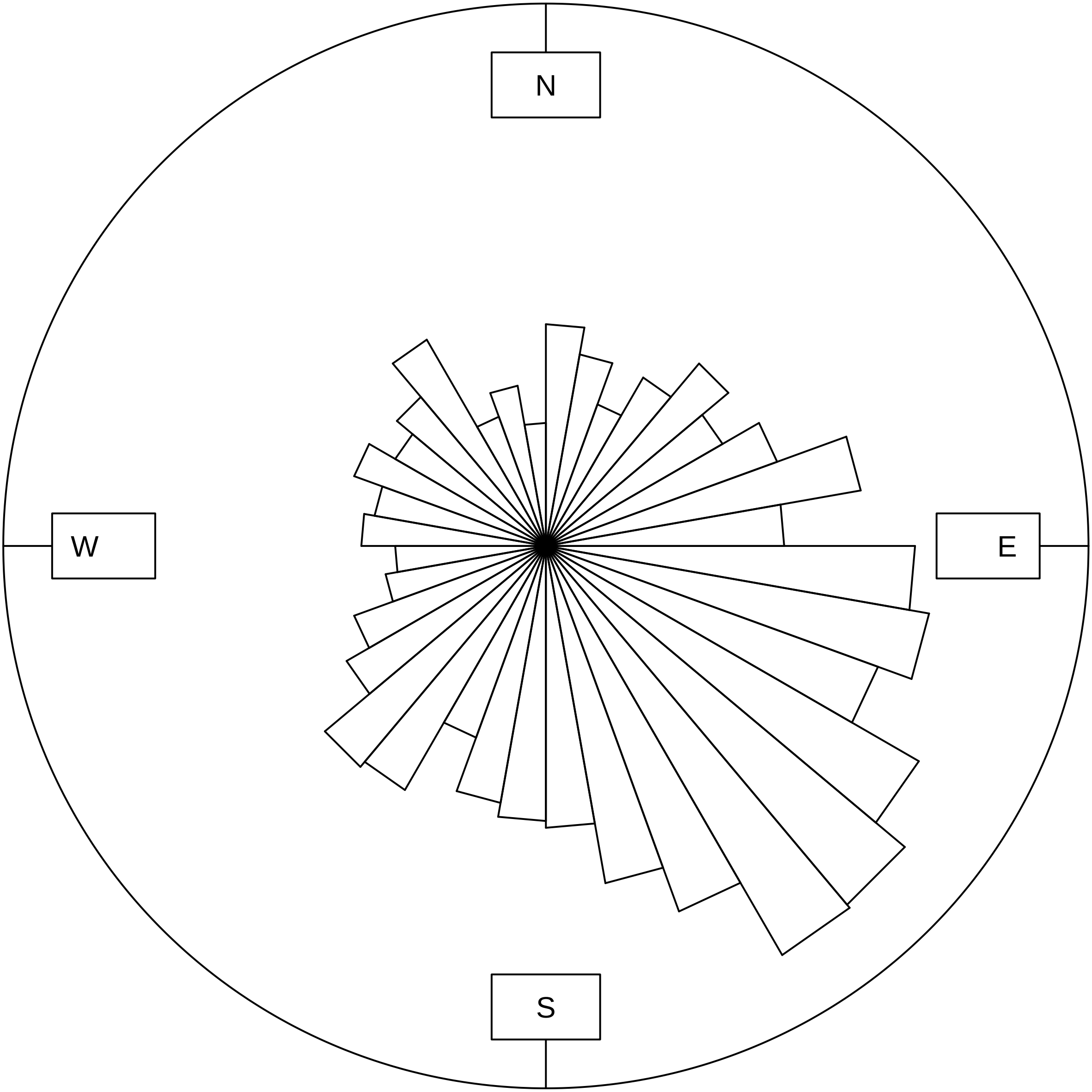

bootstrap 610

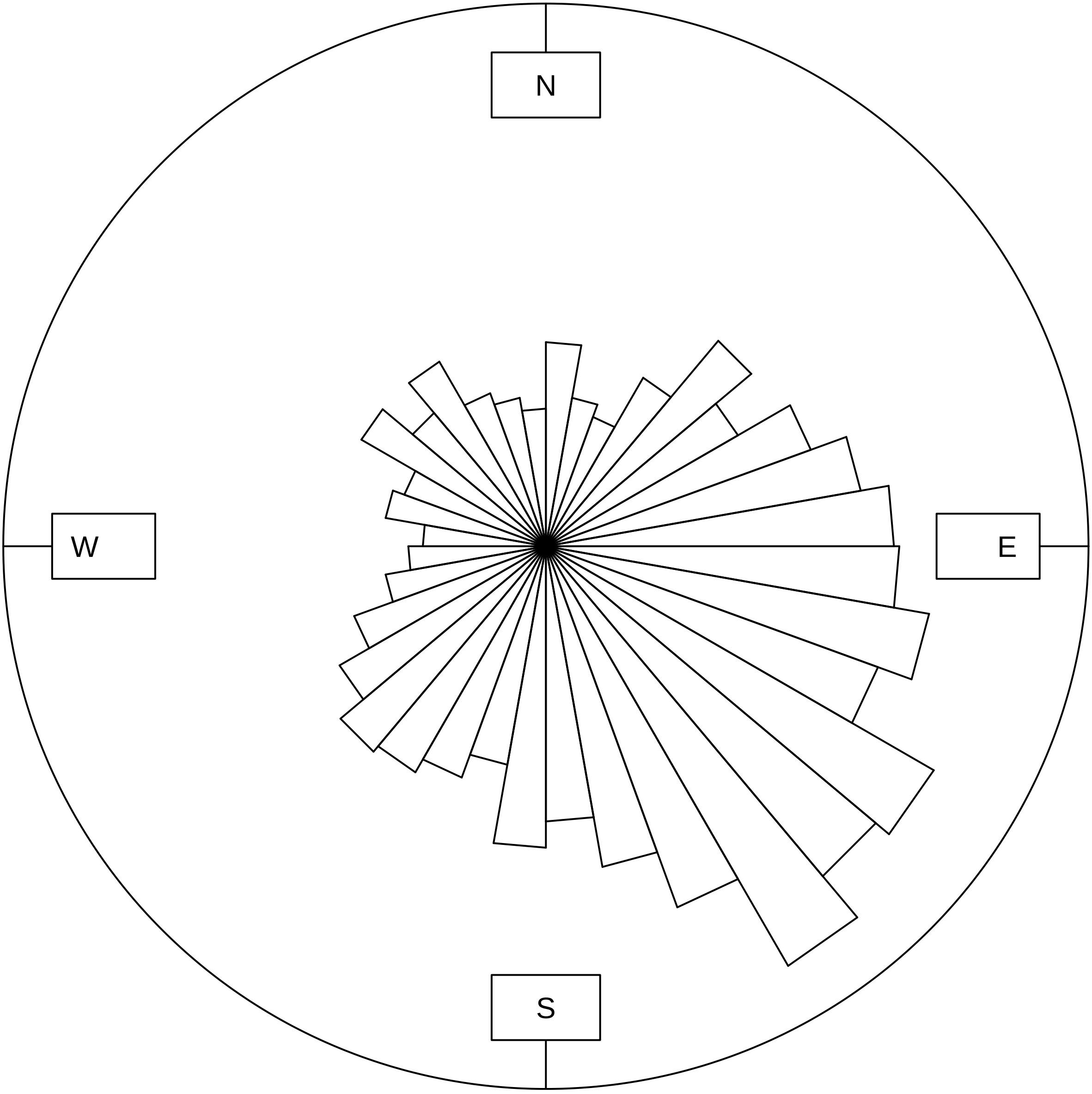

bootstrap 611

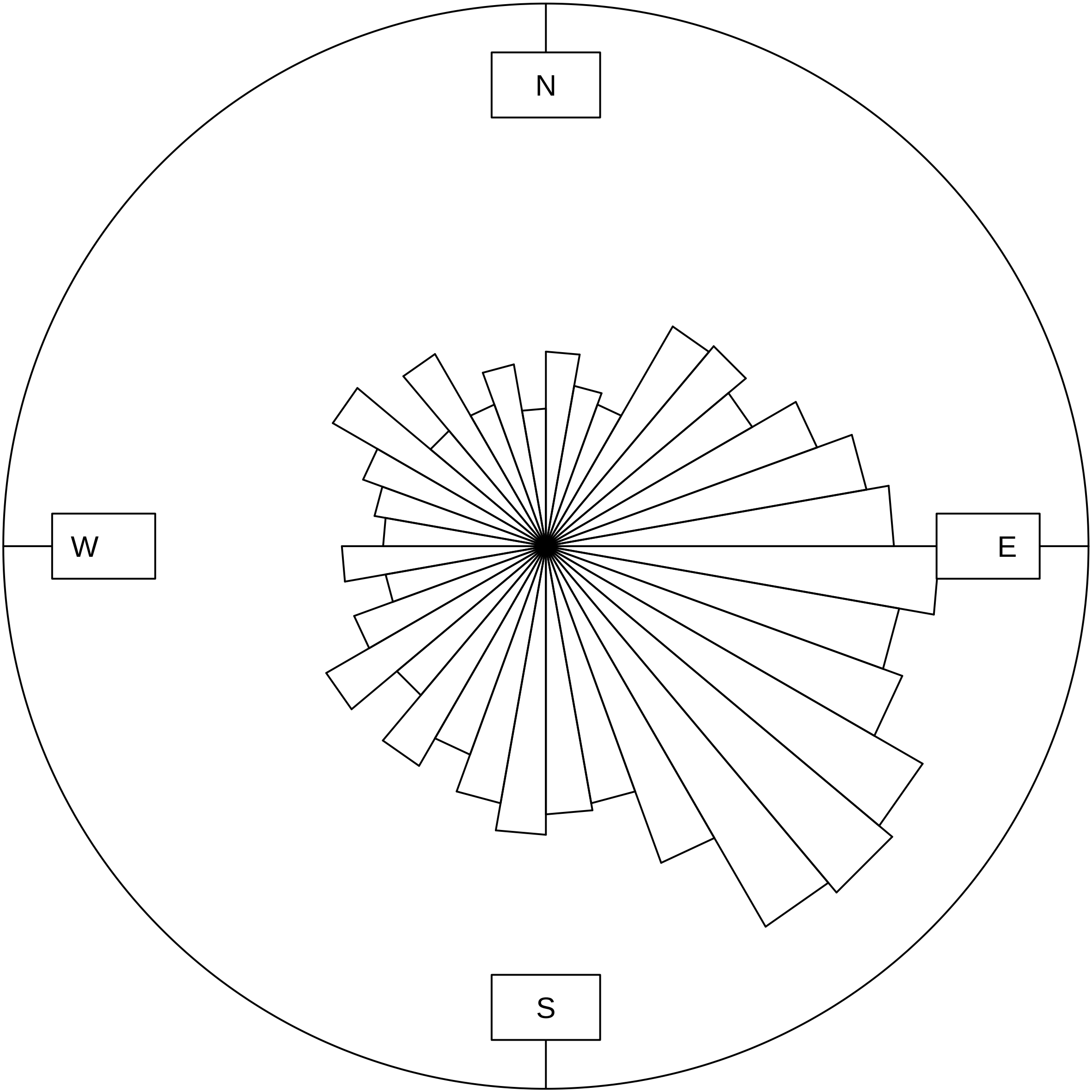

bootstrap 612

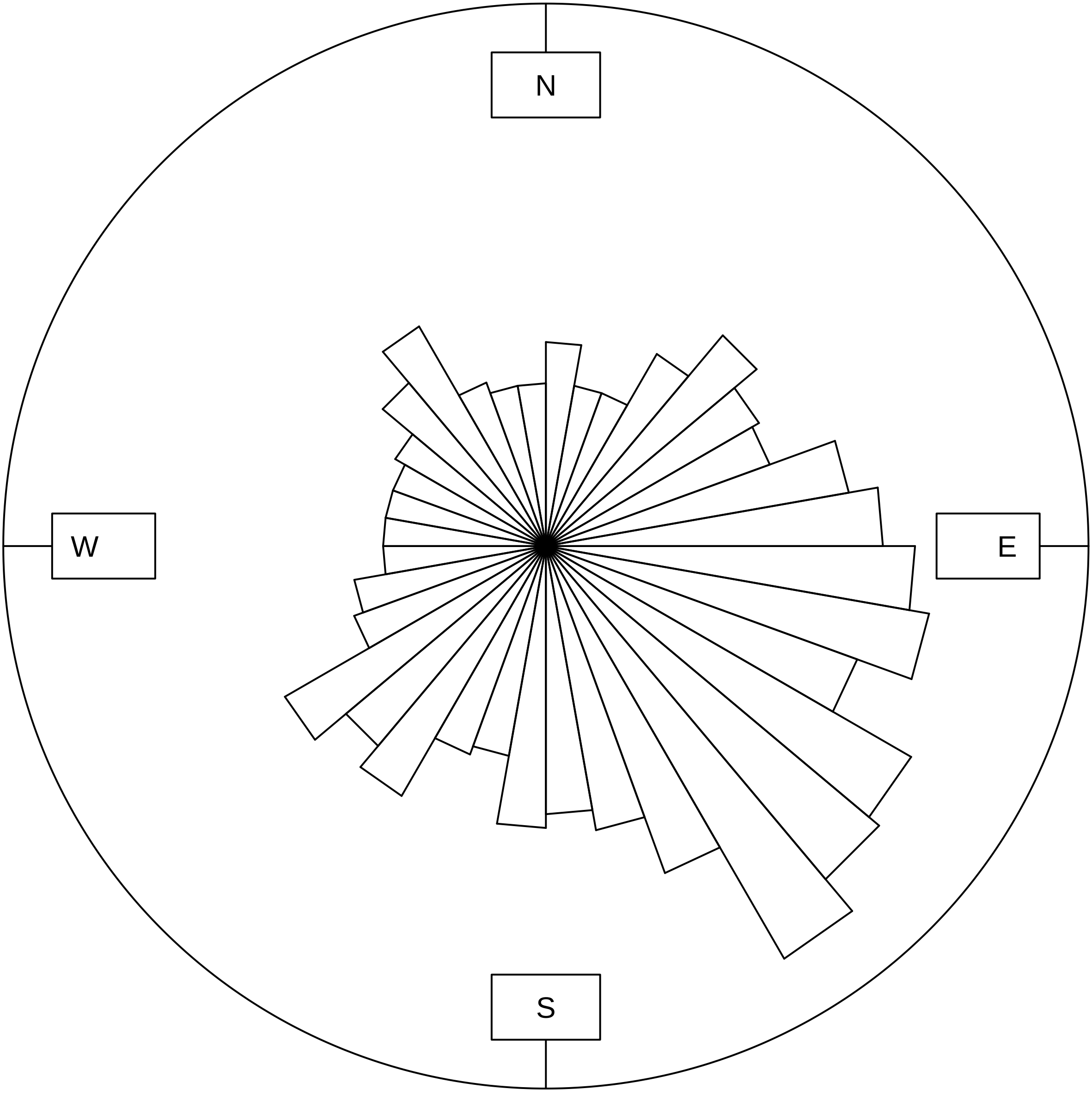

bootstrap 613

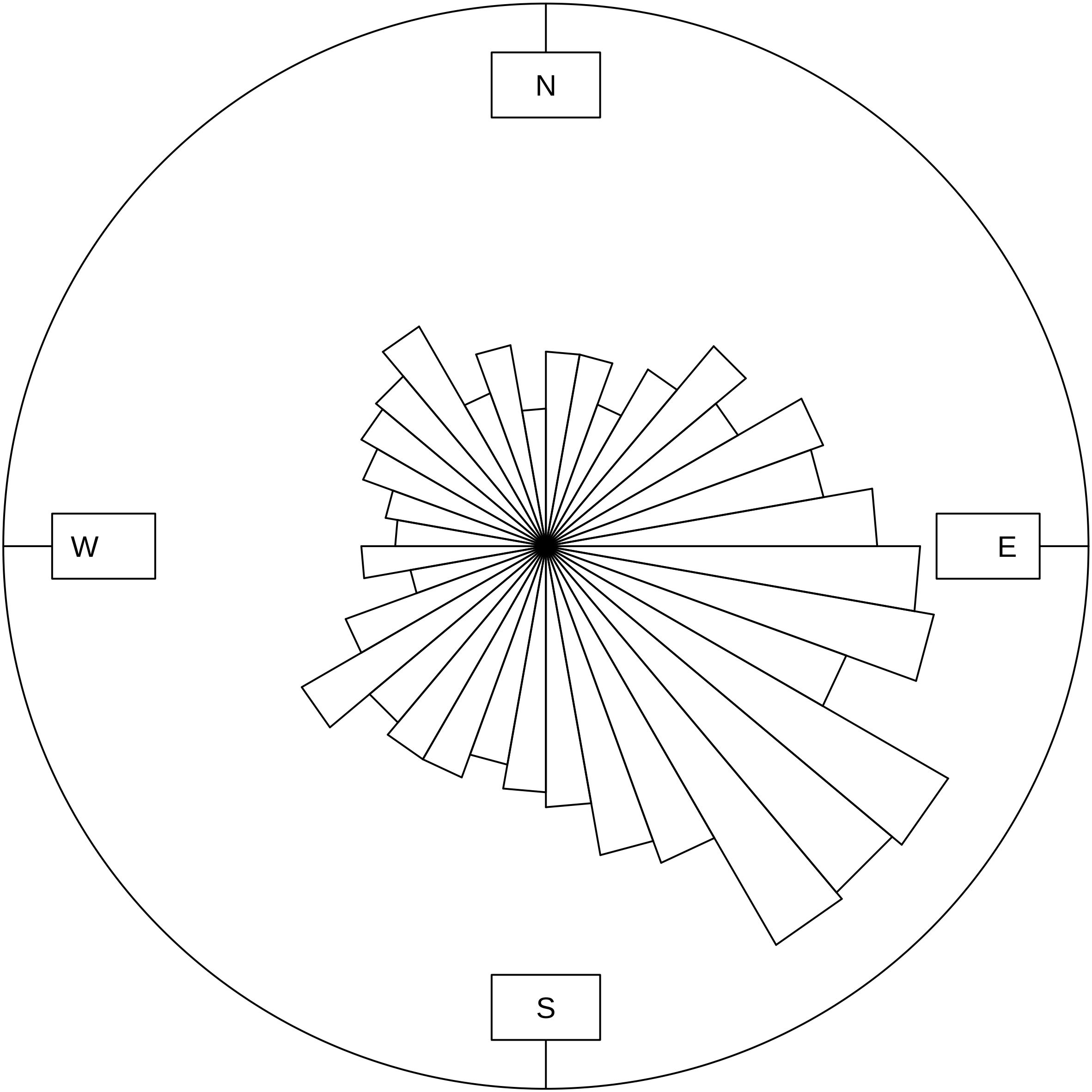

bootstrap 614

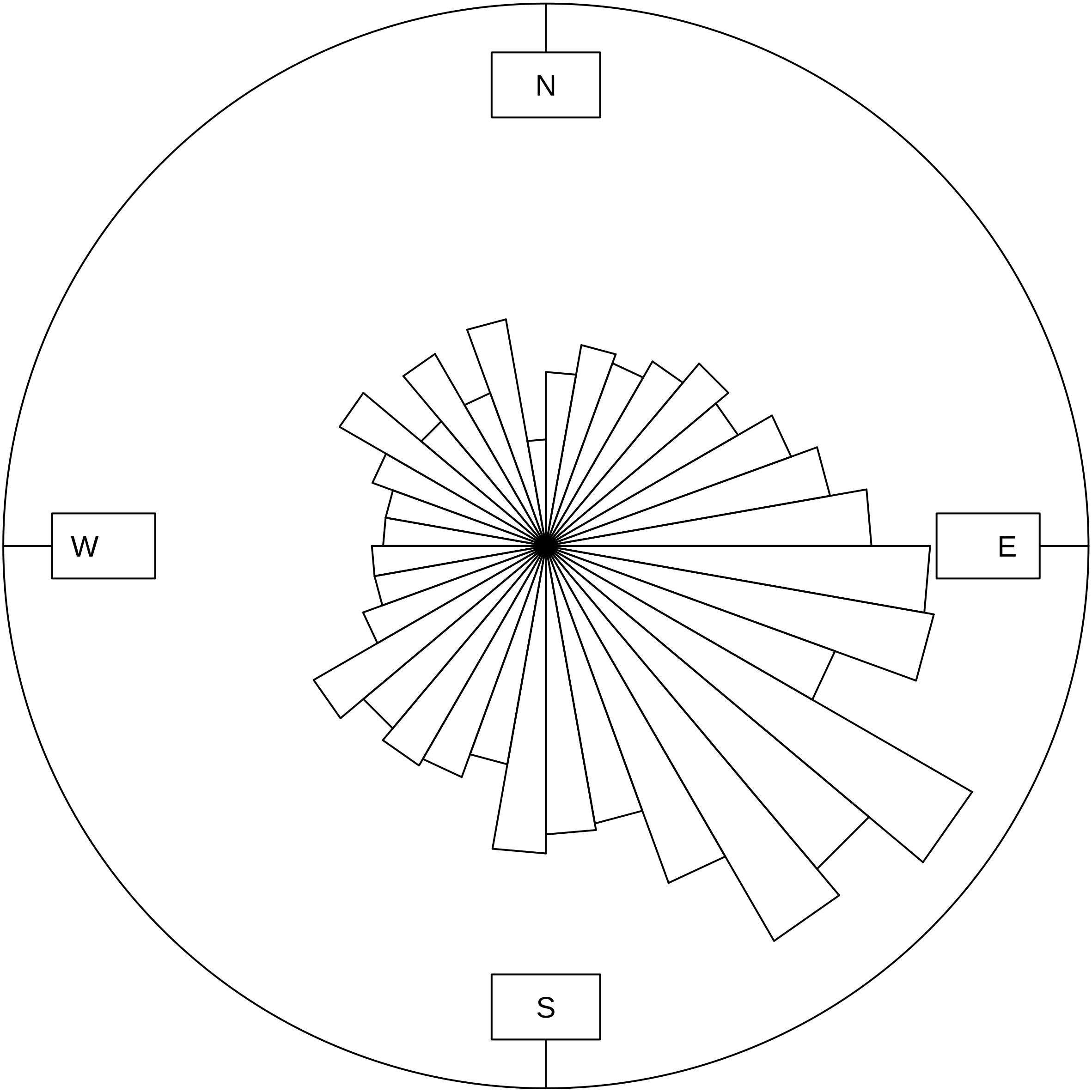

bootstrap 615

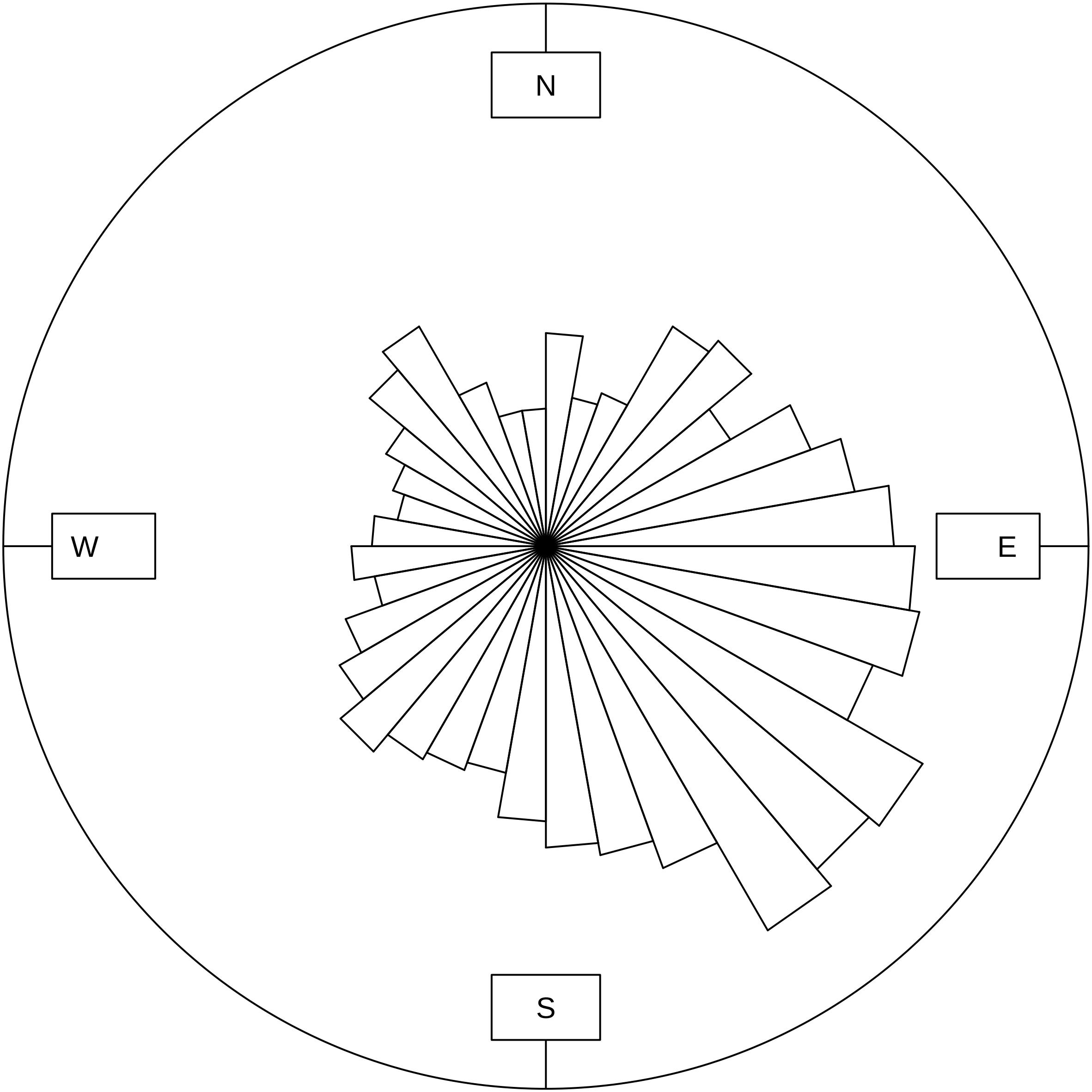

bootstrap 616

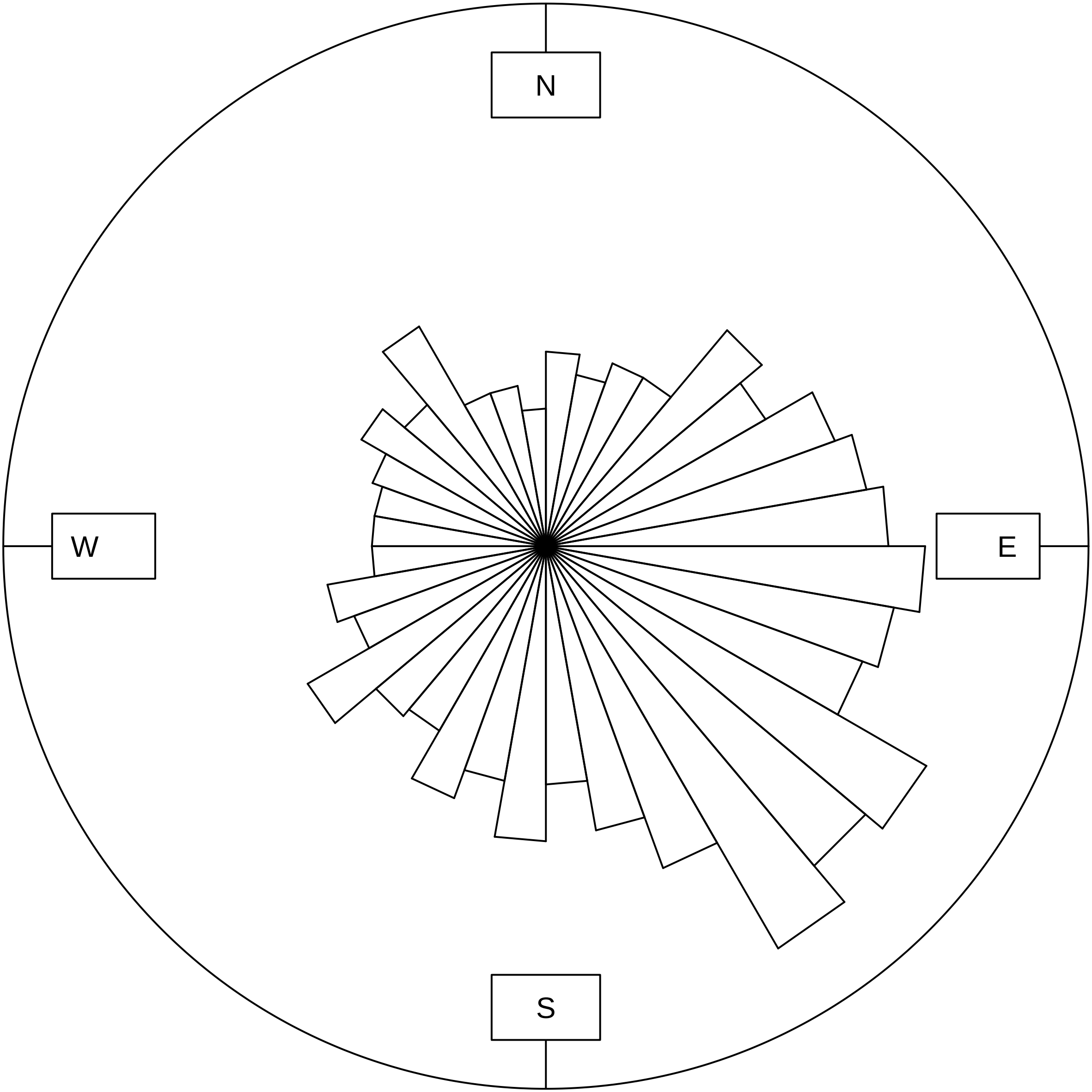

bootstrap 617

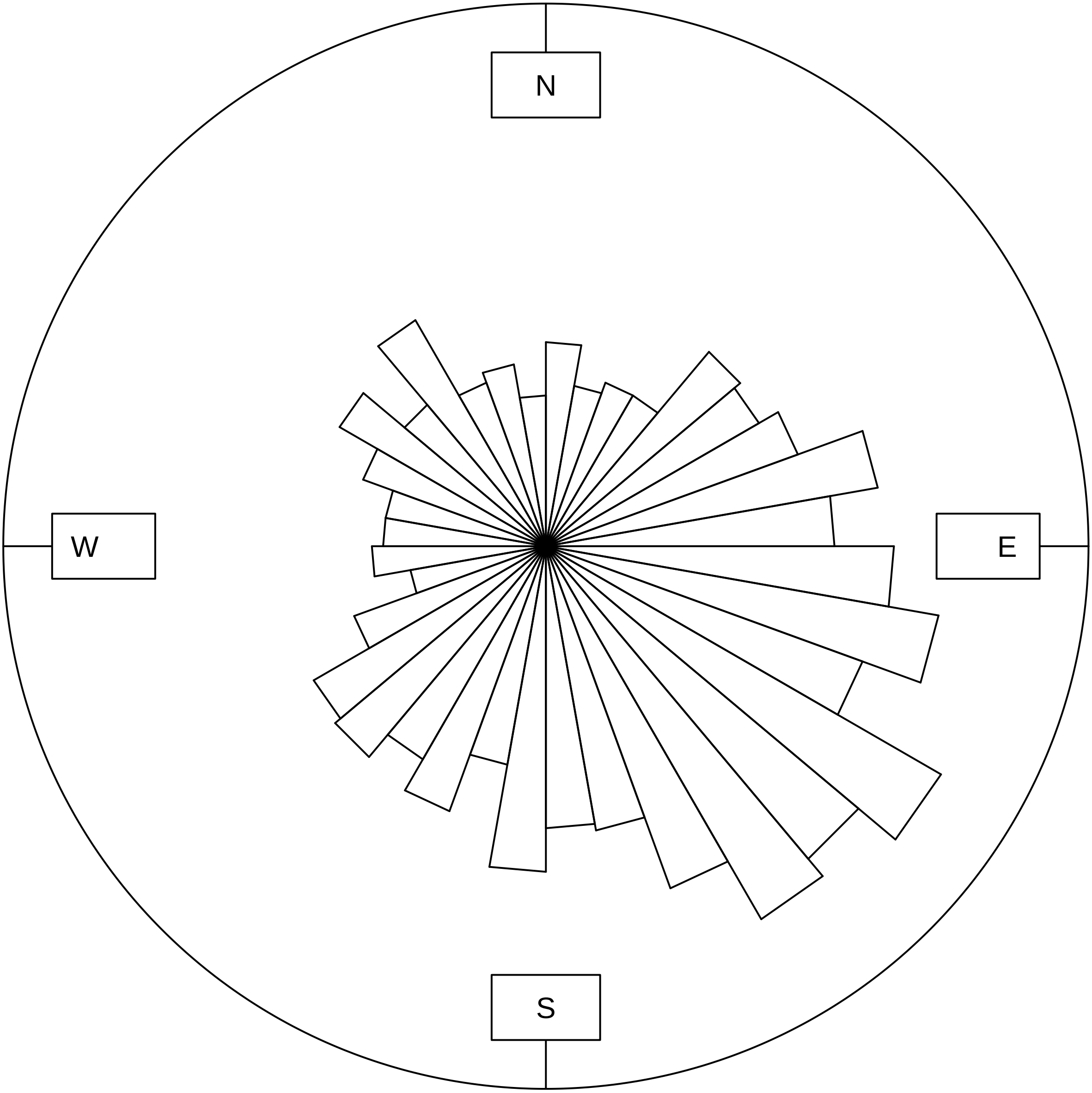

bootstrap 618

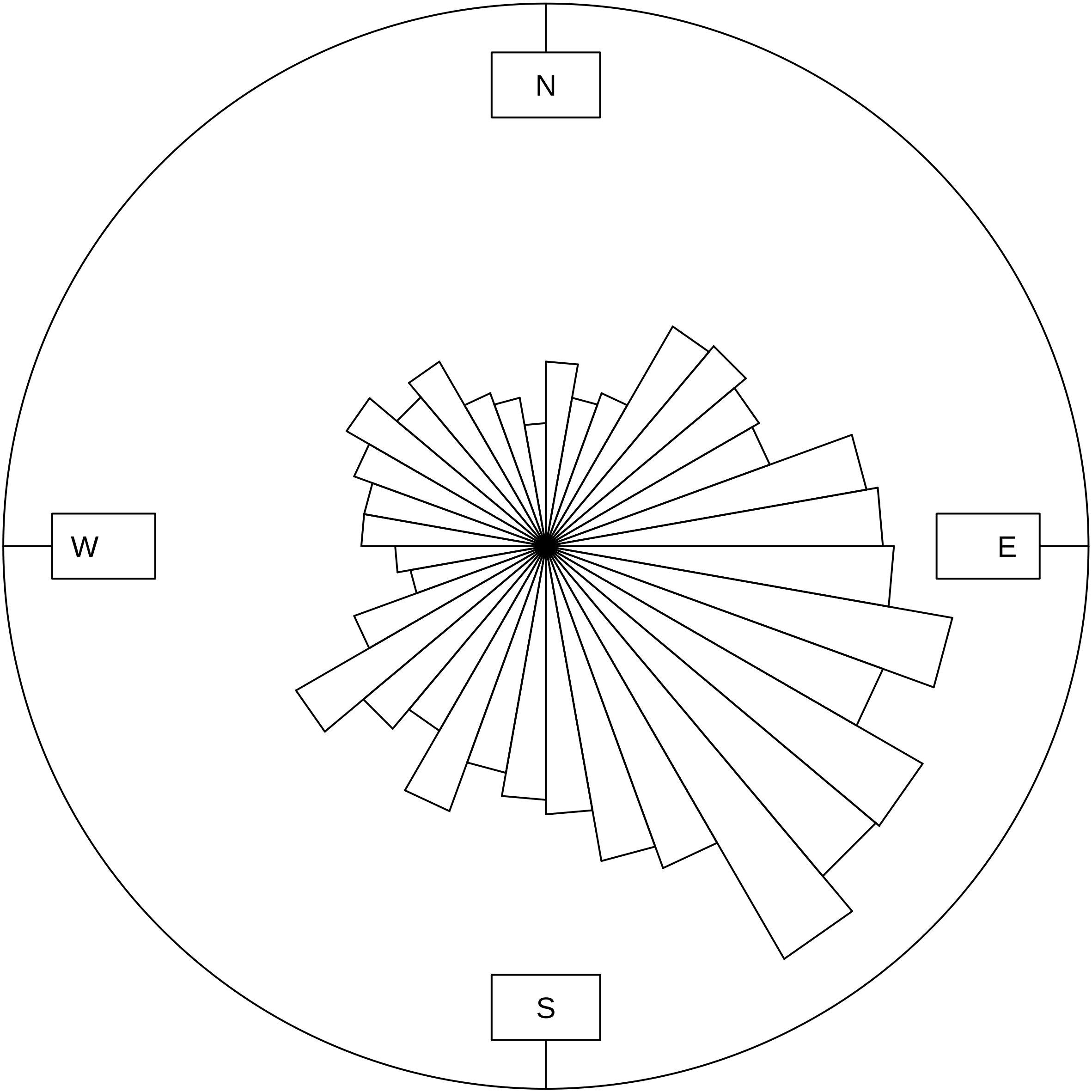

bootstrap 619

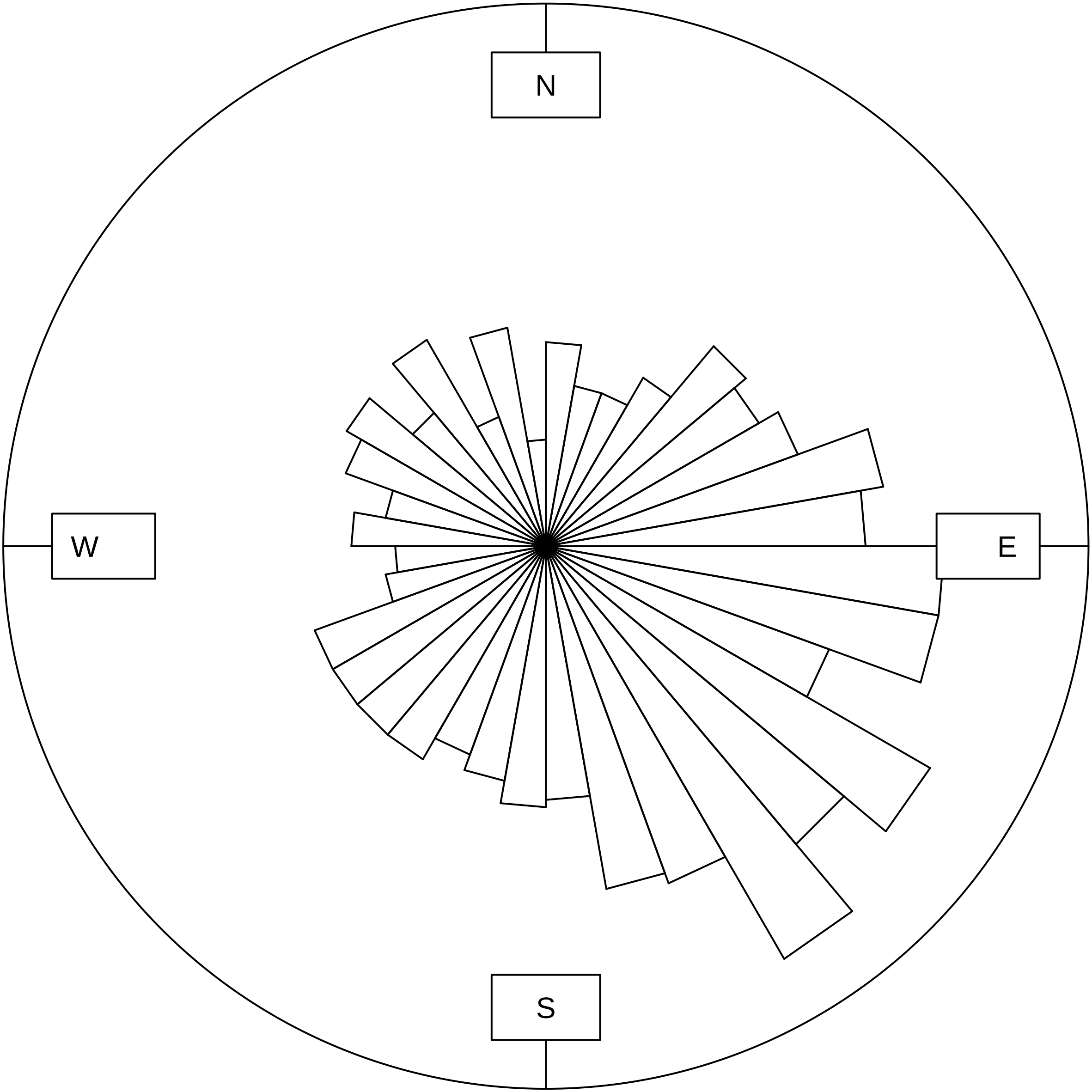

bootstrap 620

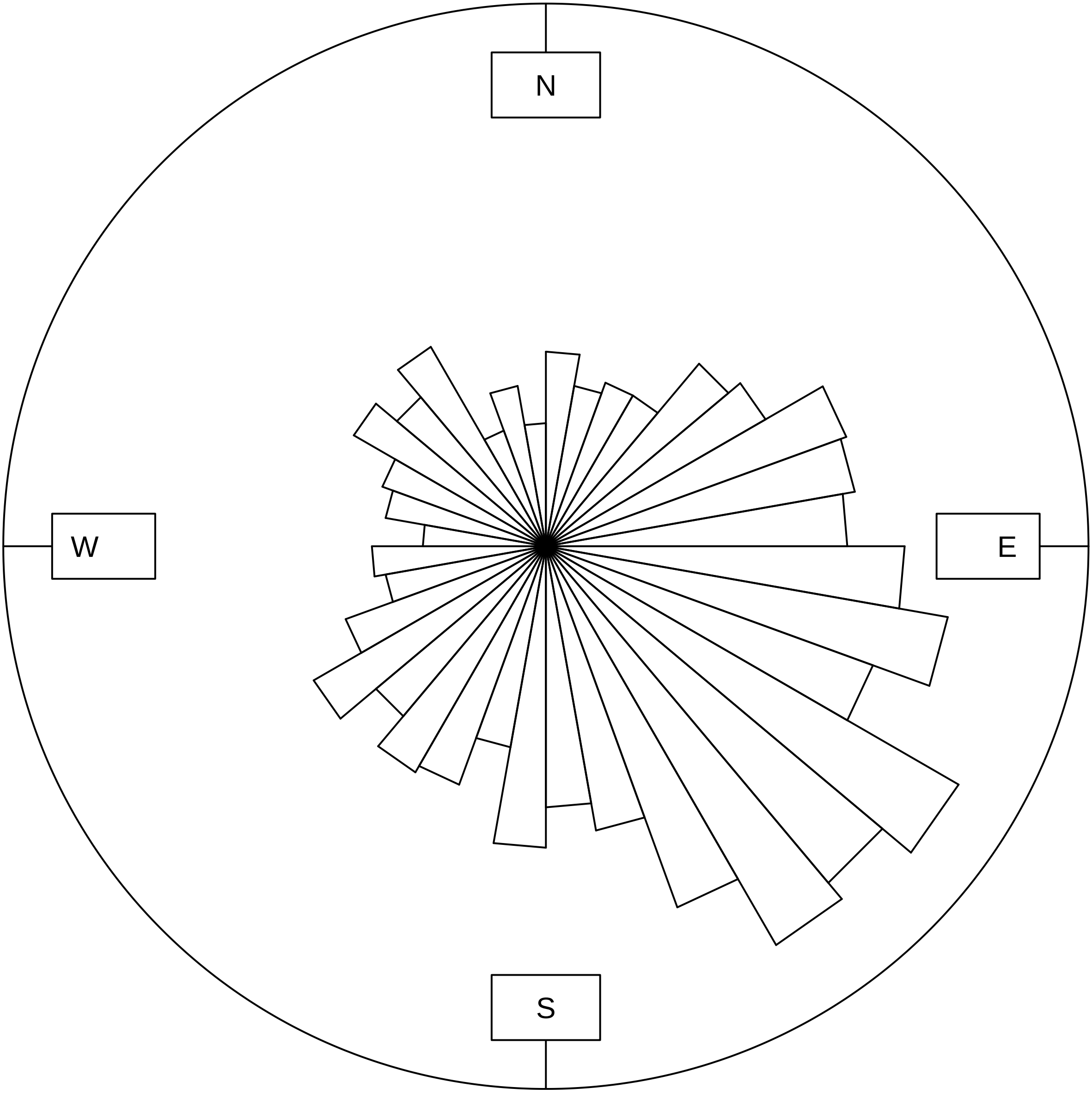

bootstrap 621

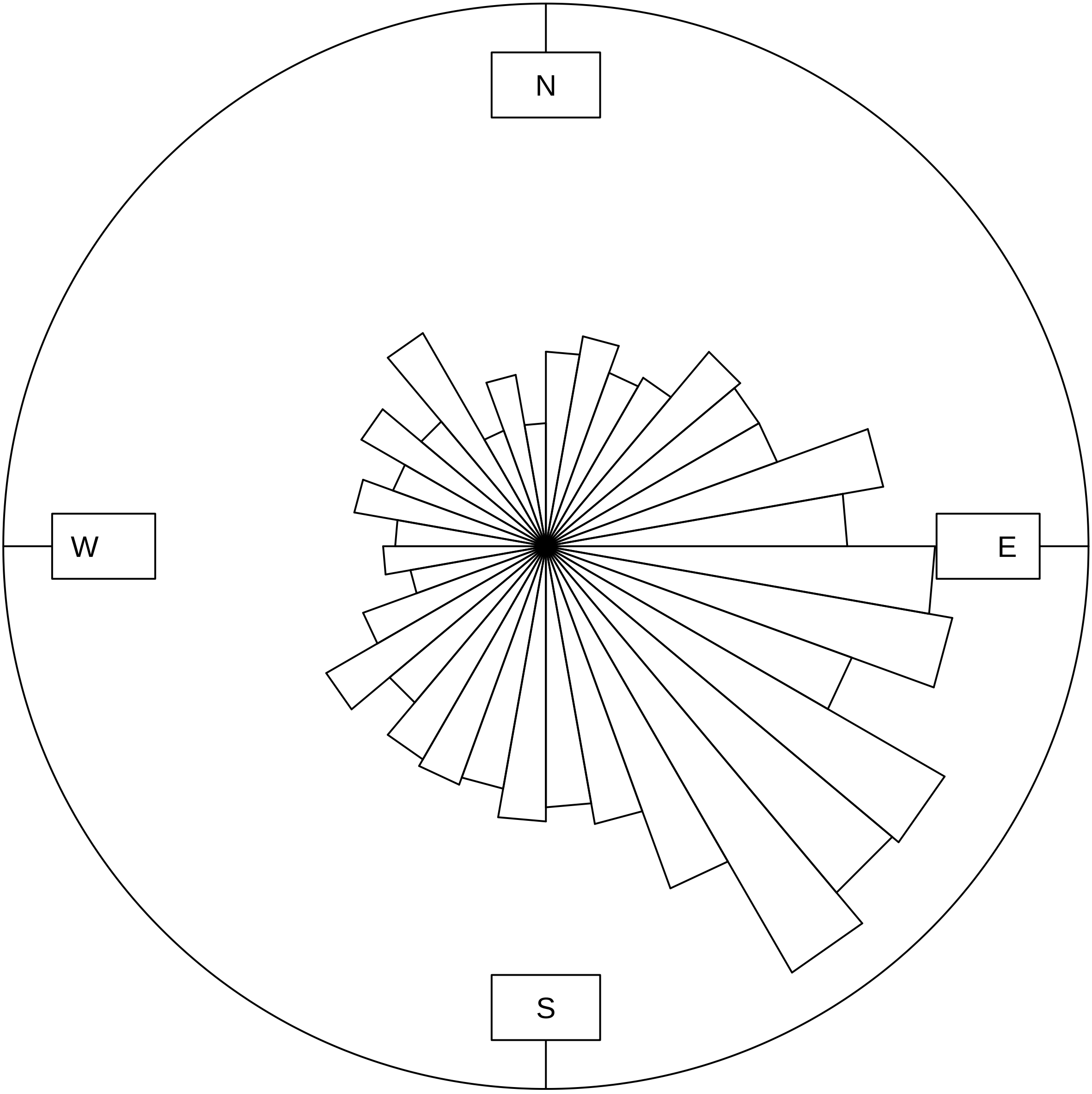

bootstrap 622

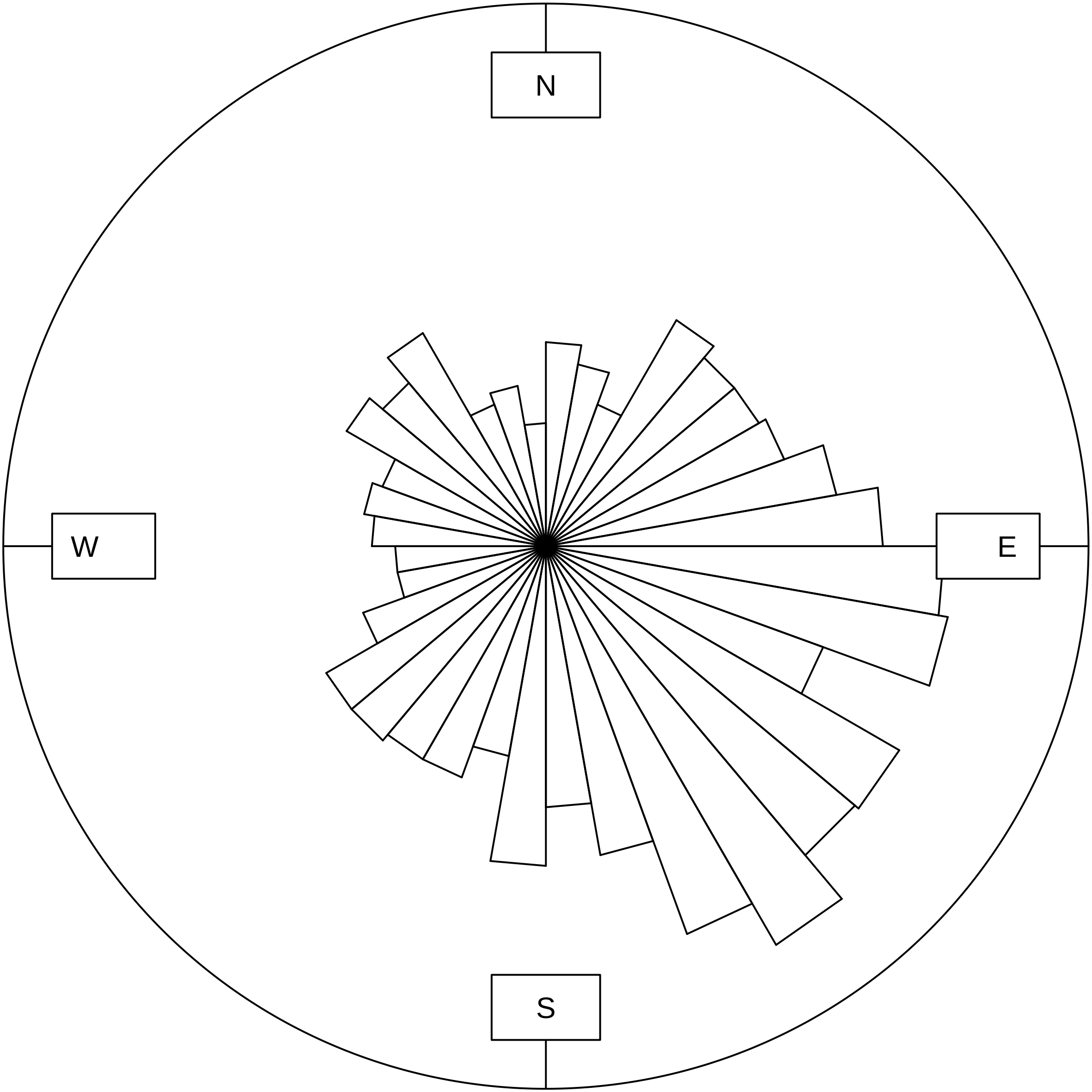

bootstrap 623

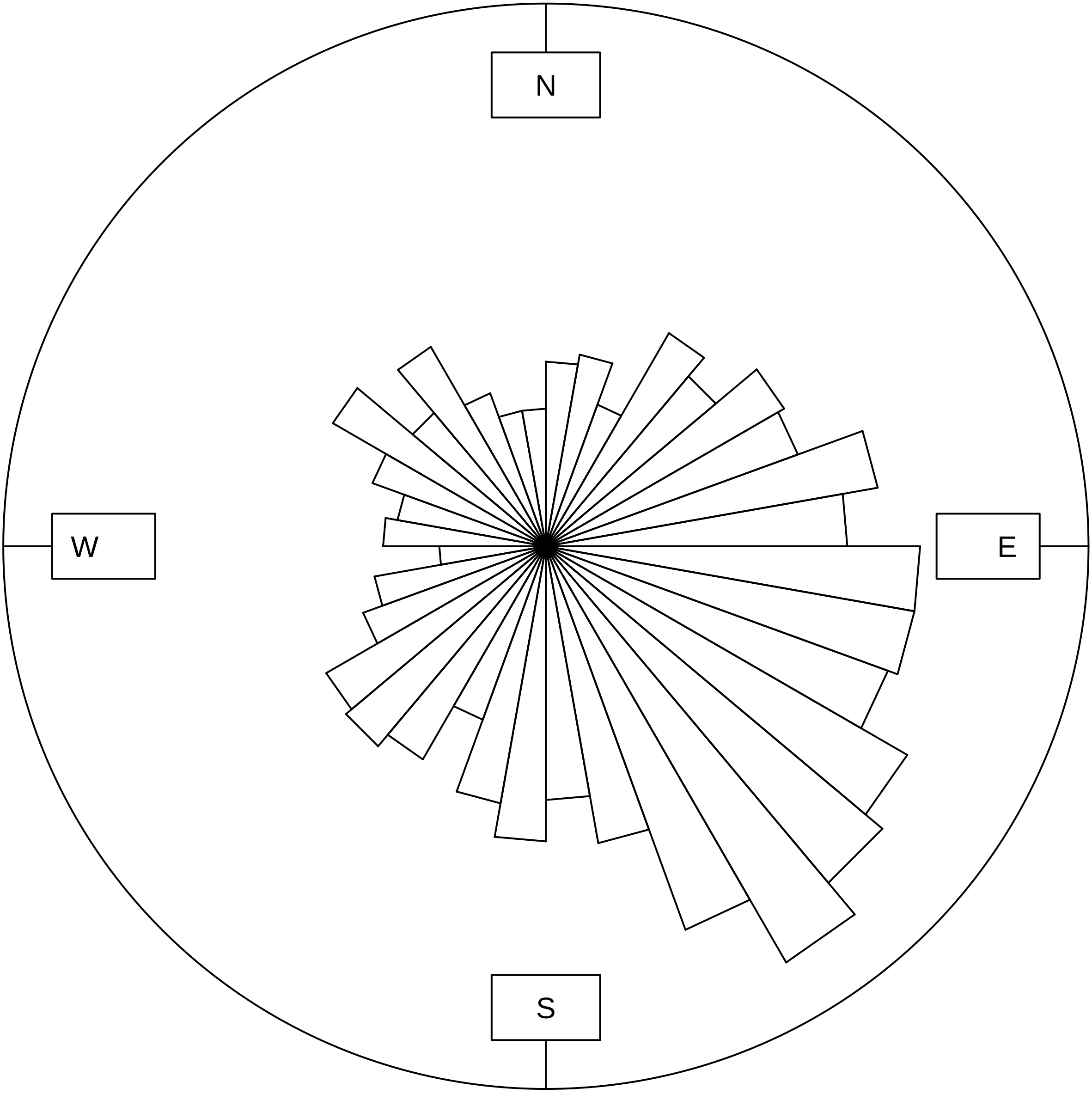

bootstrap 624

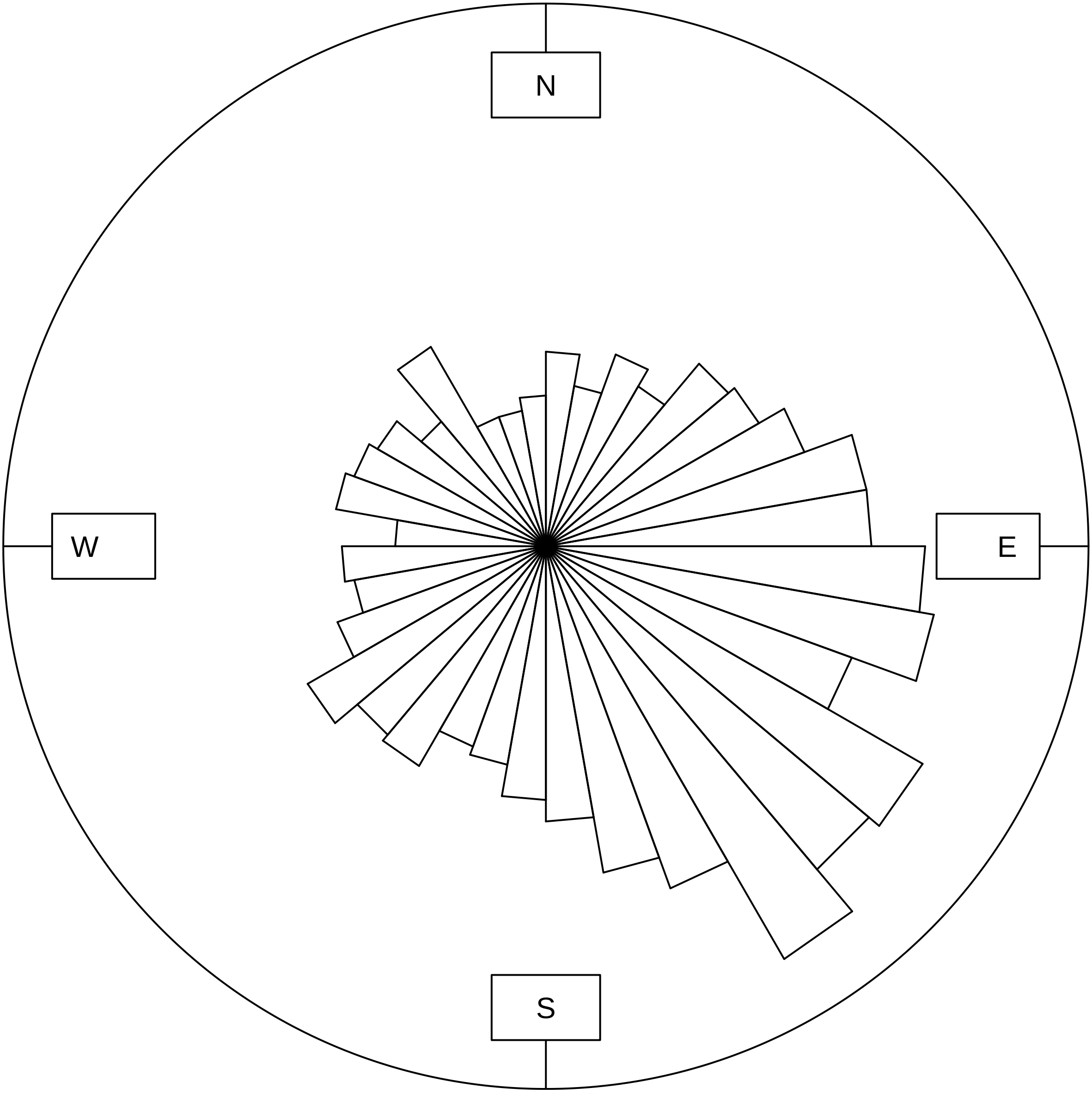

bootstrap 625

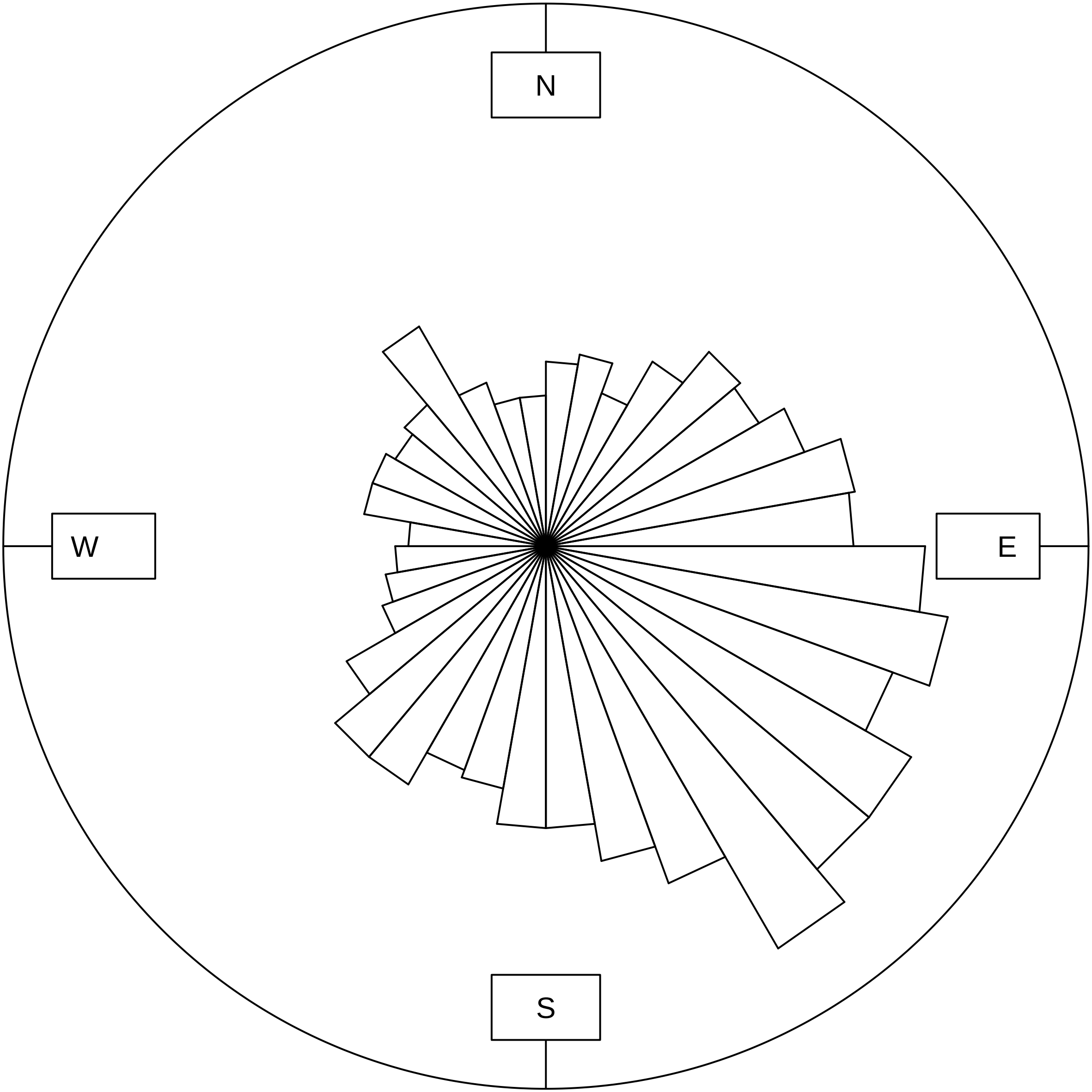

bootstrap 626

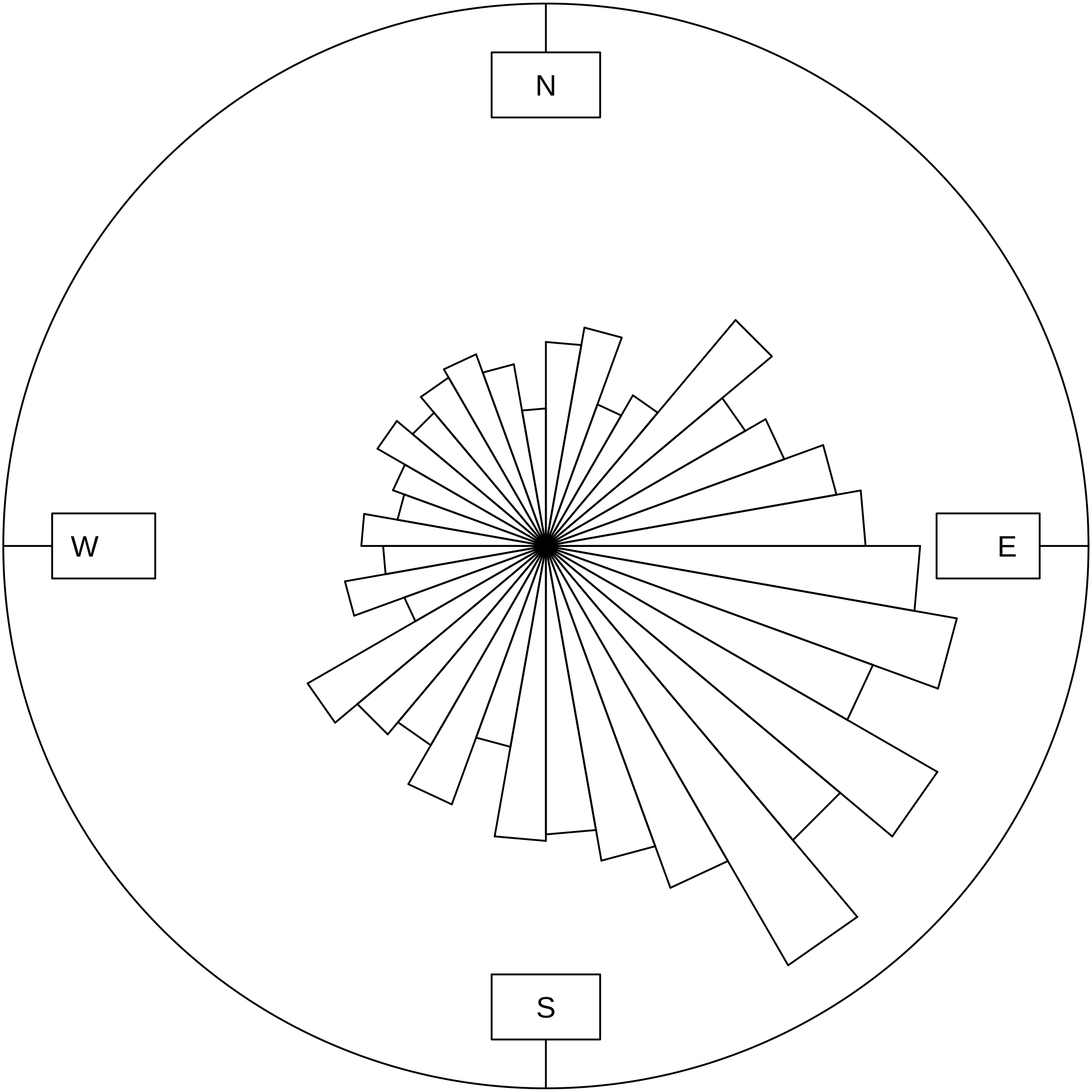

bootstrap 627

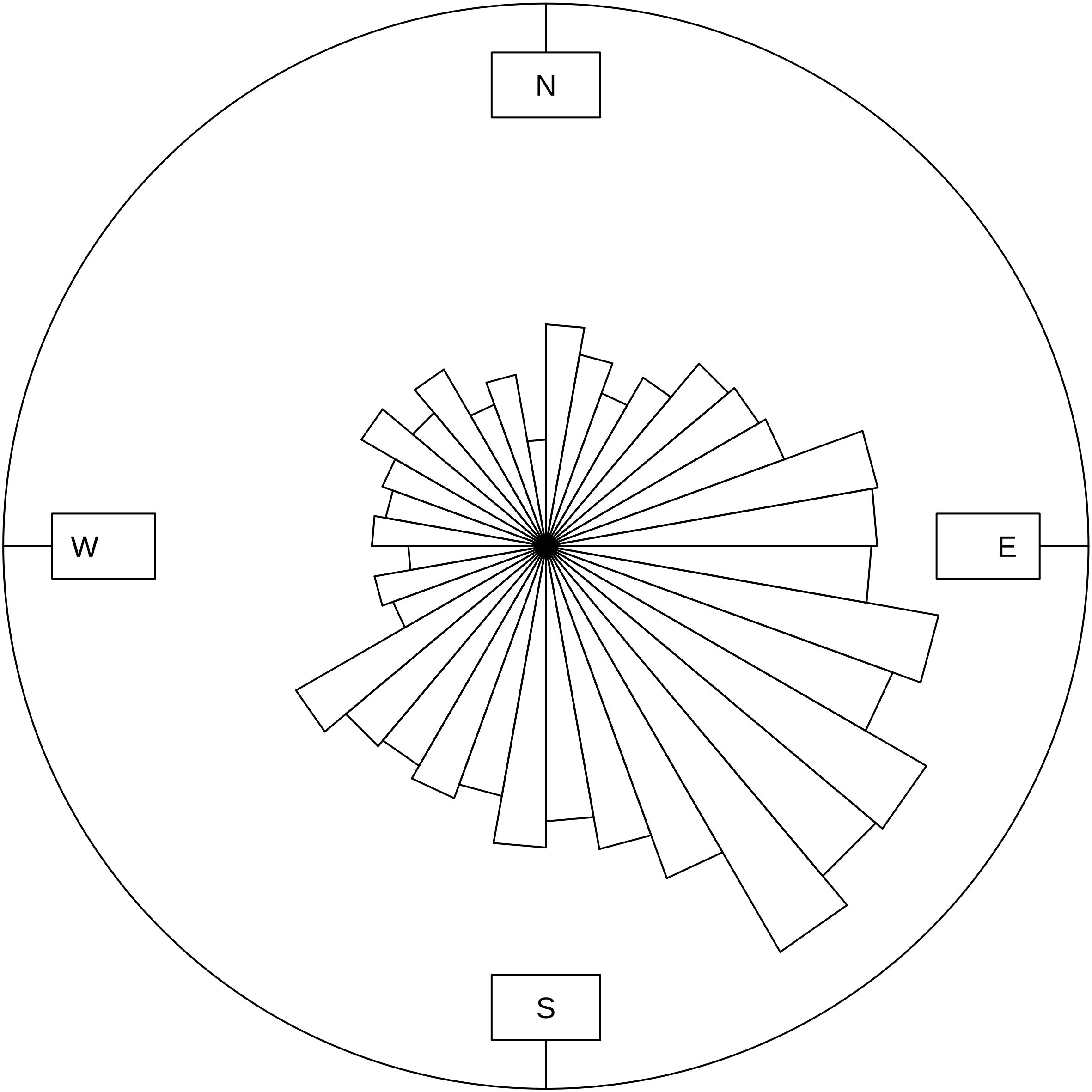

bootstrap 628

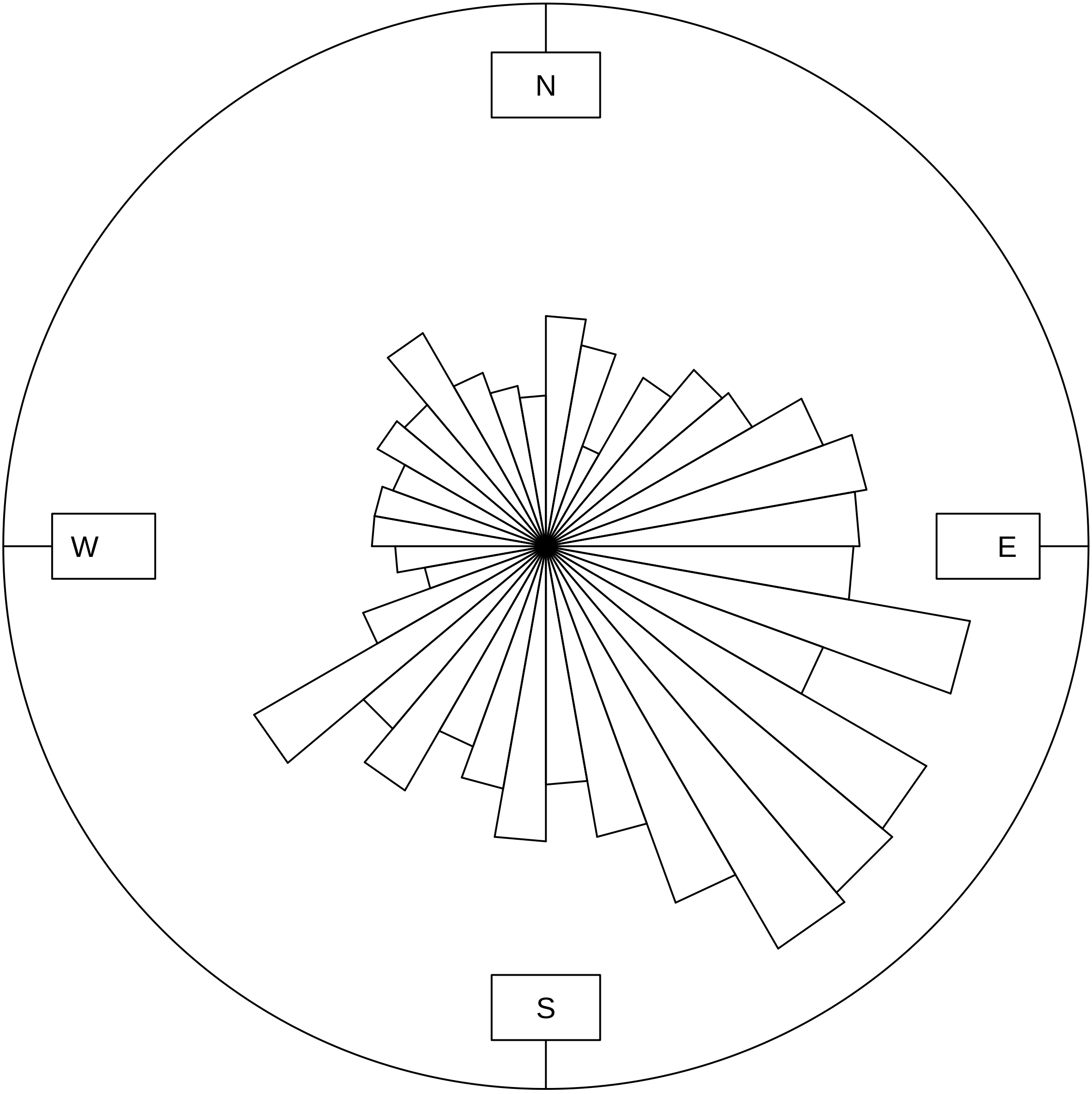

bootstrap 629

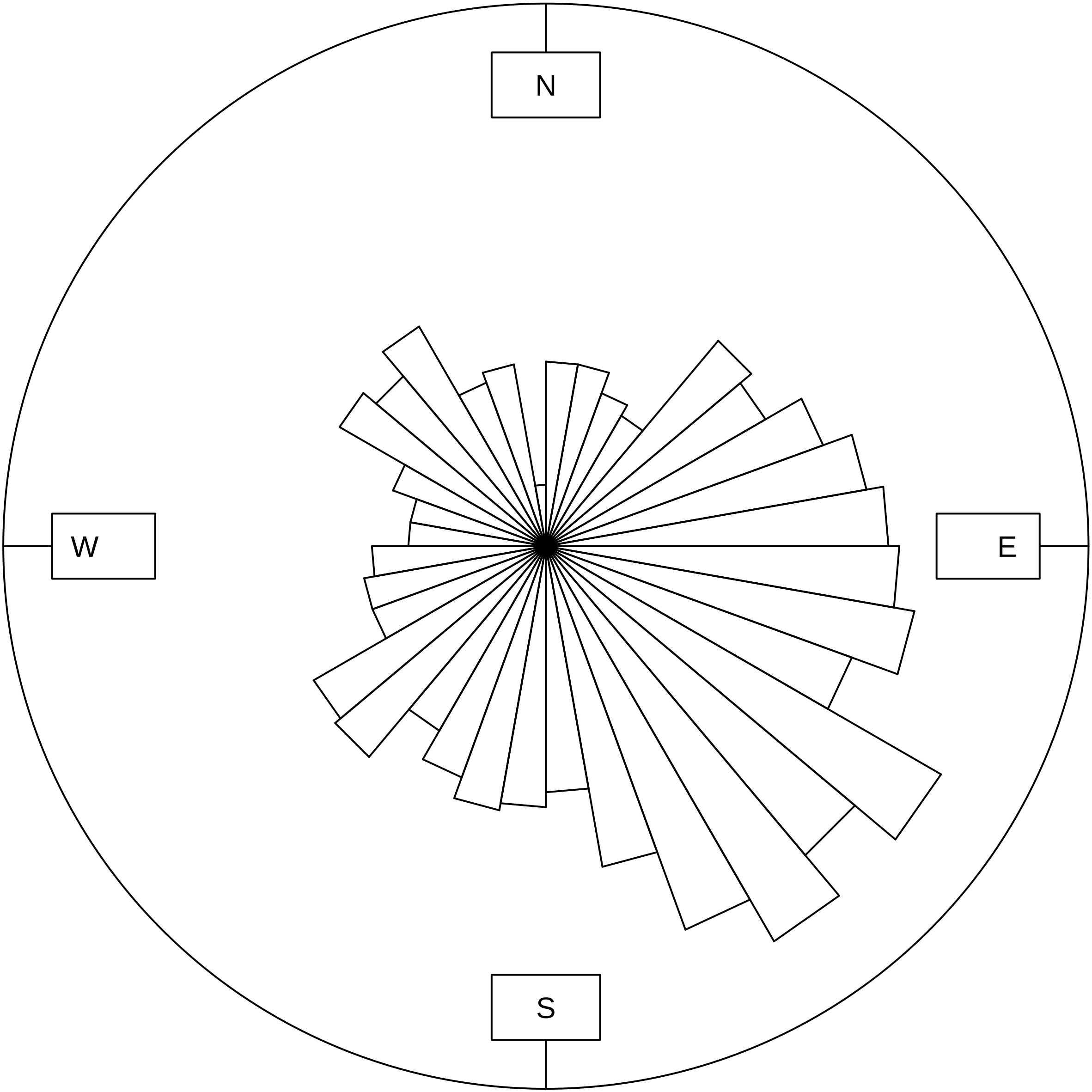

bootstrap 630

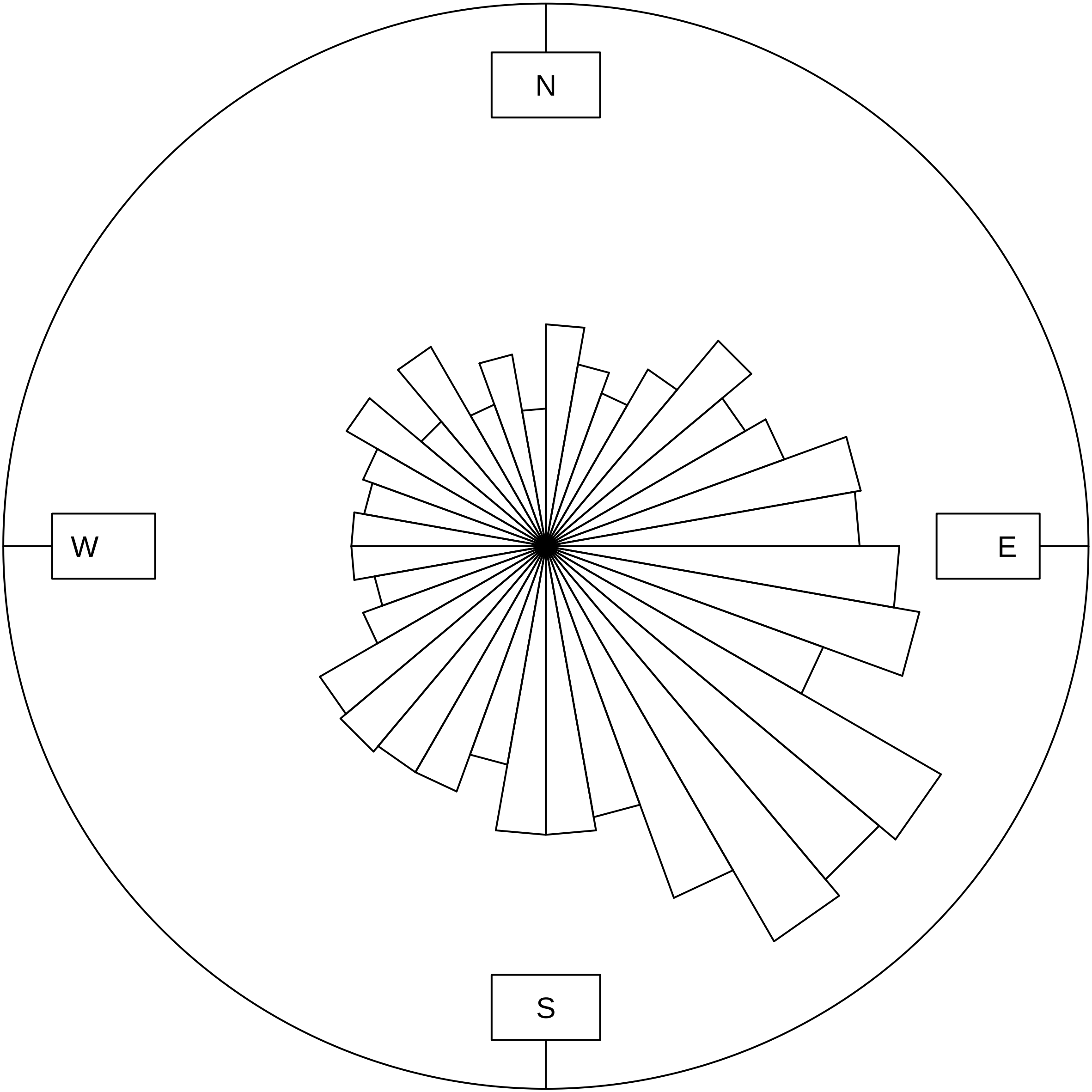

bootstrap 631

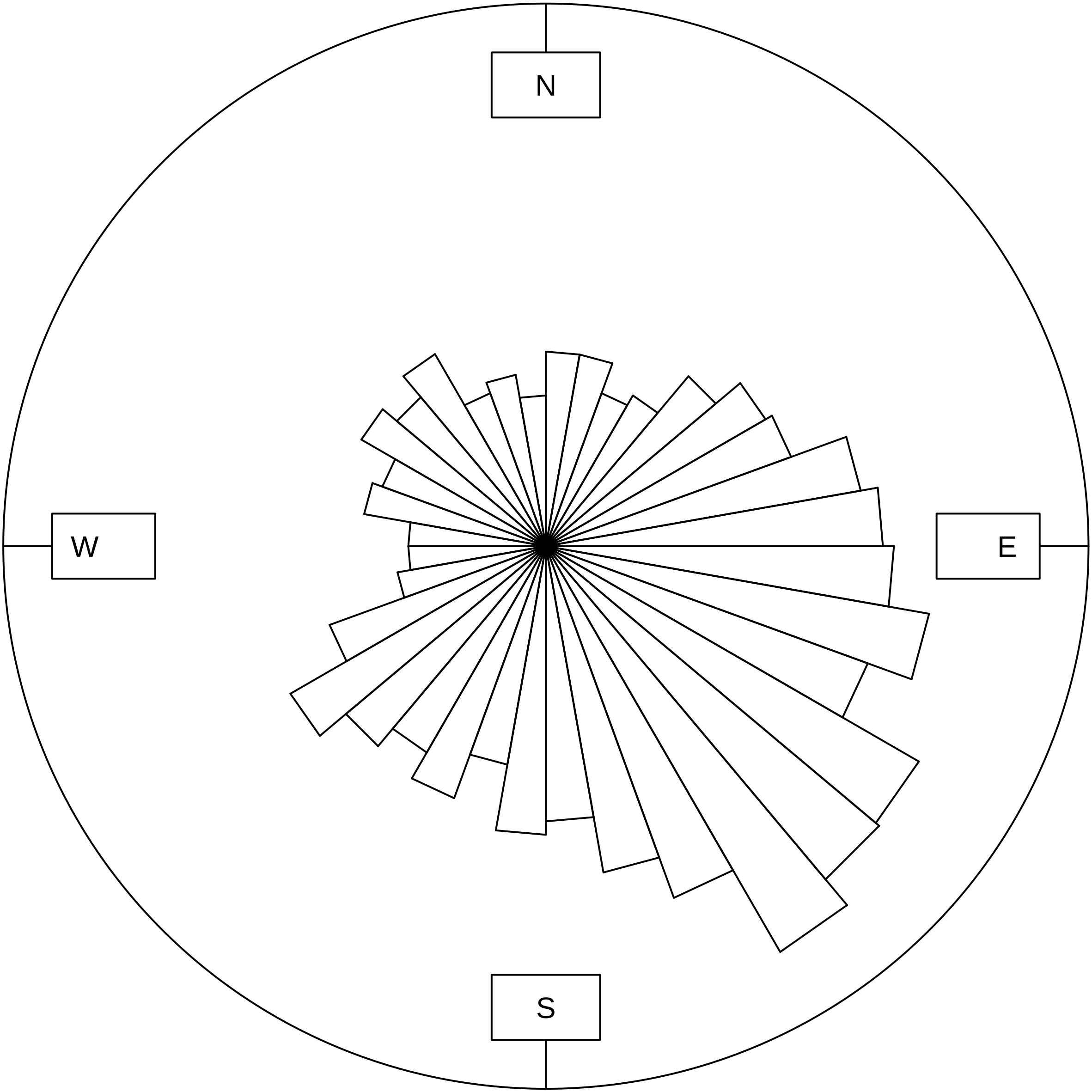

bootstrap 632

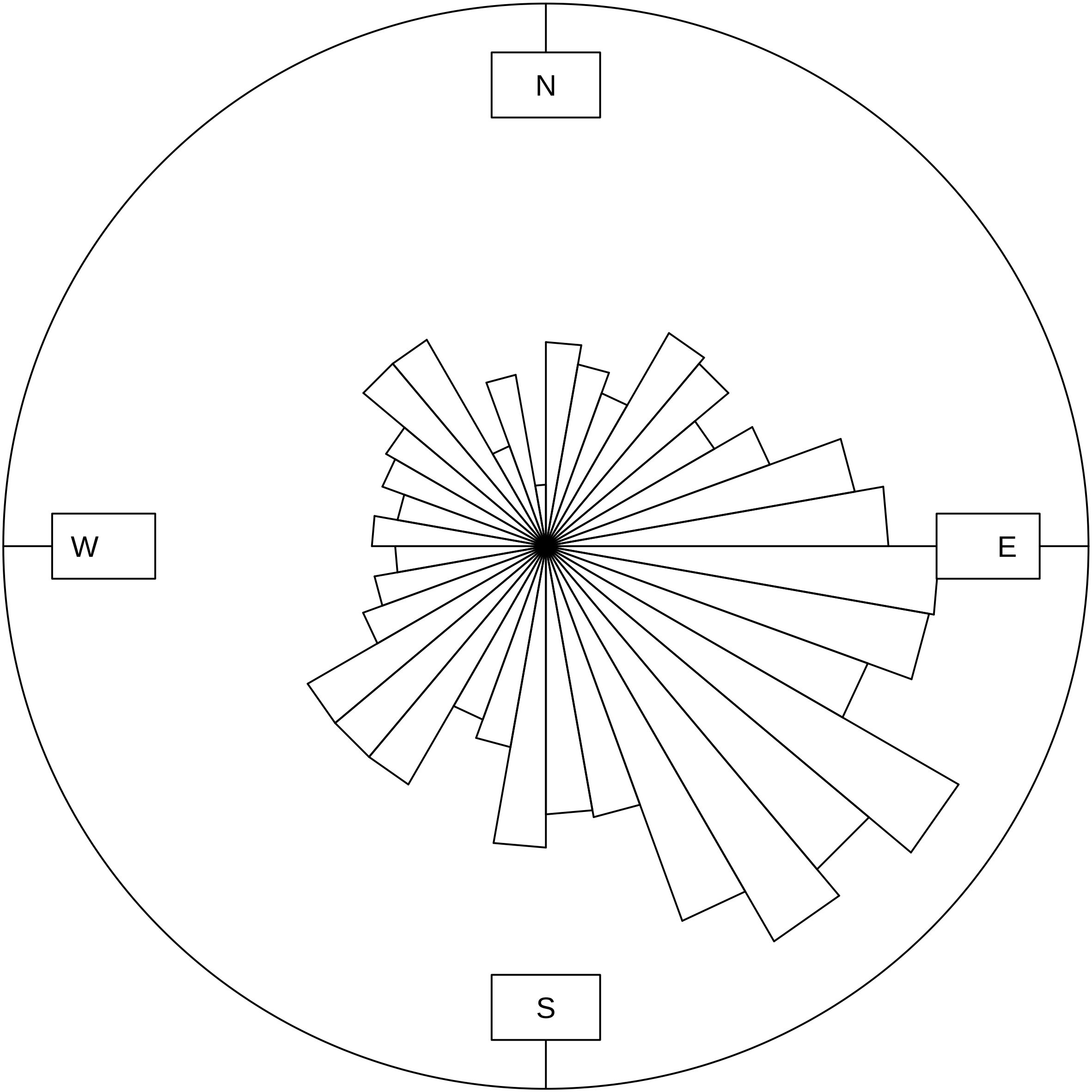

bootstrap 633

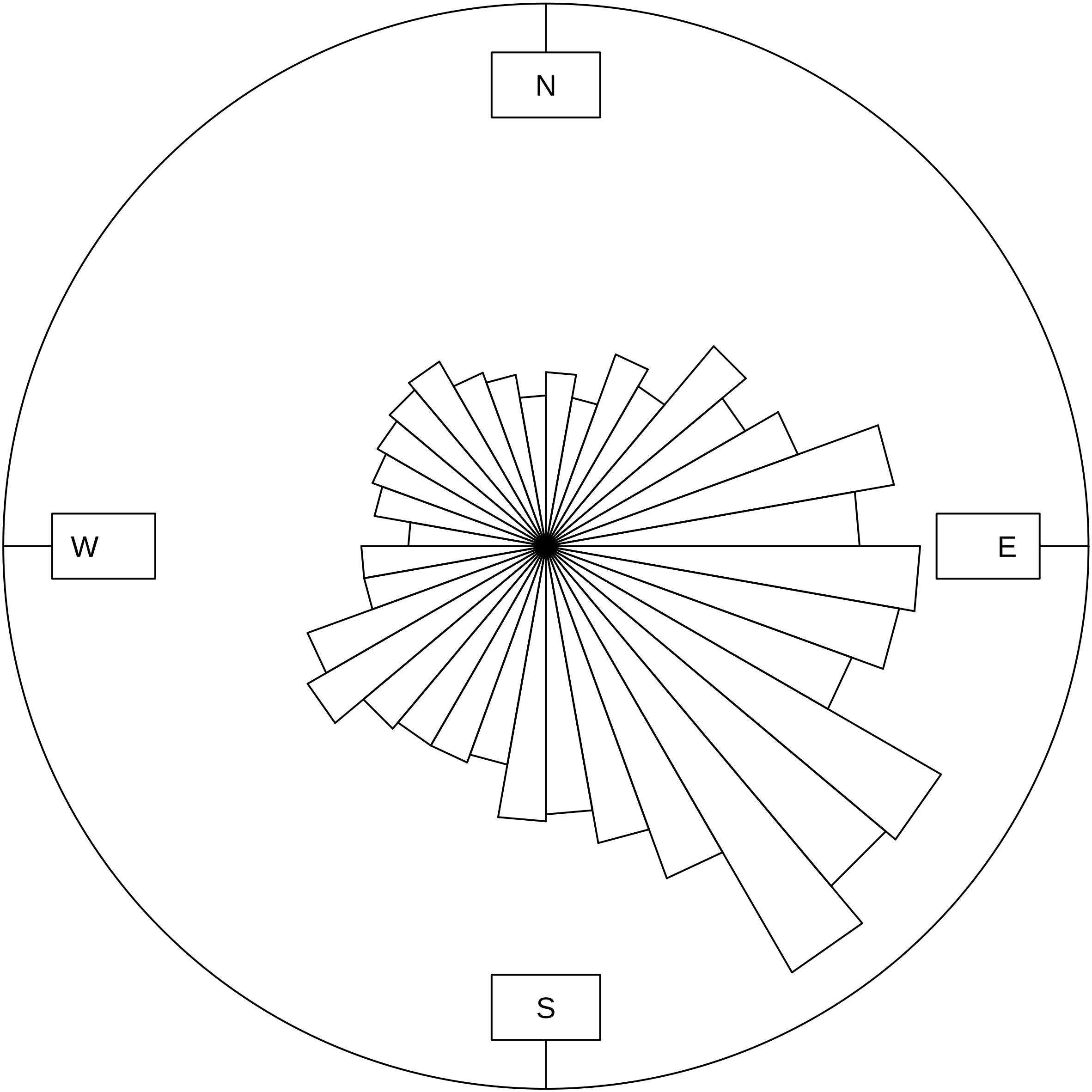

bootstrap 634

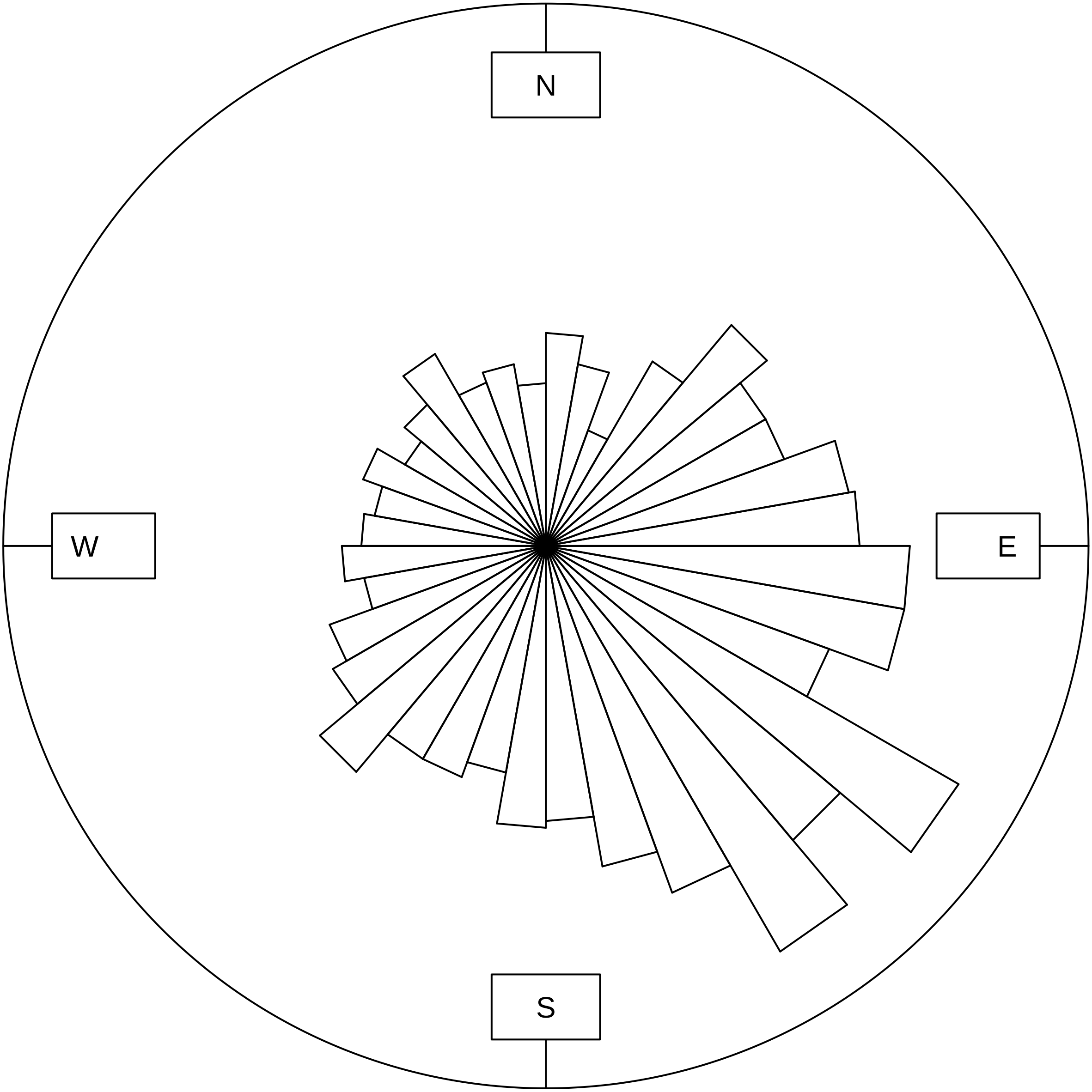

bootstrap 635

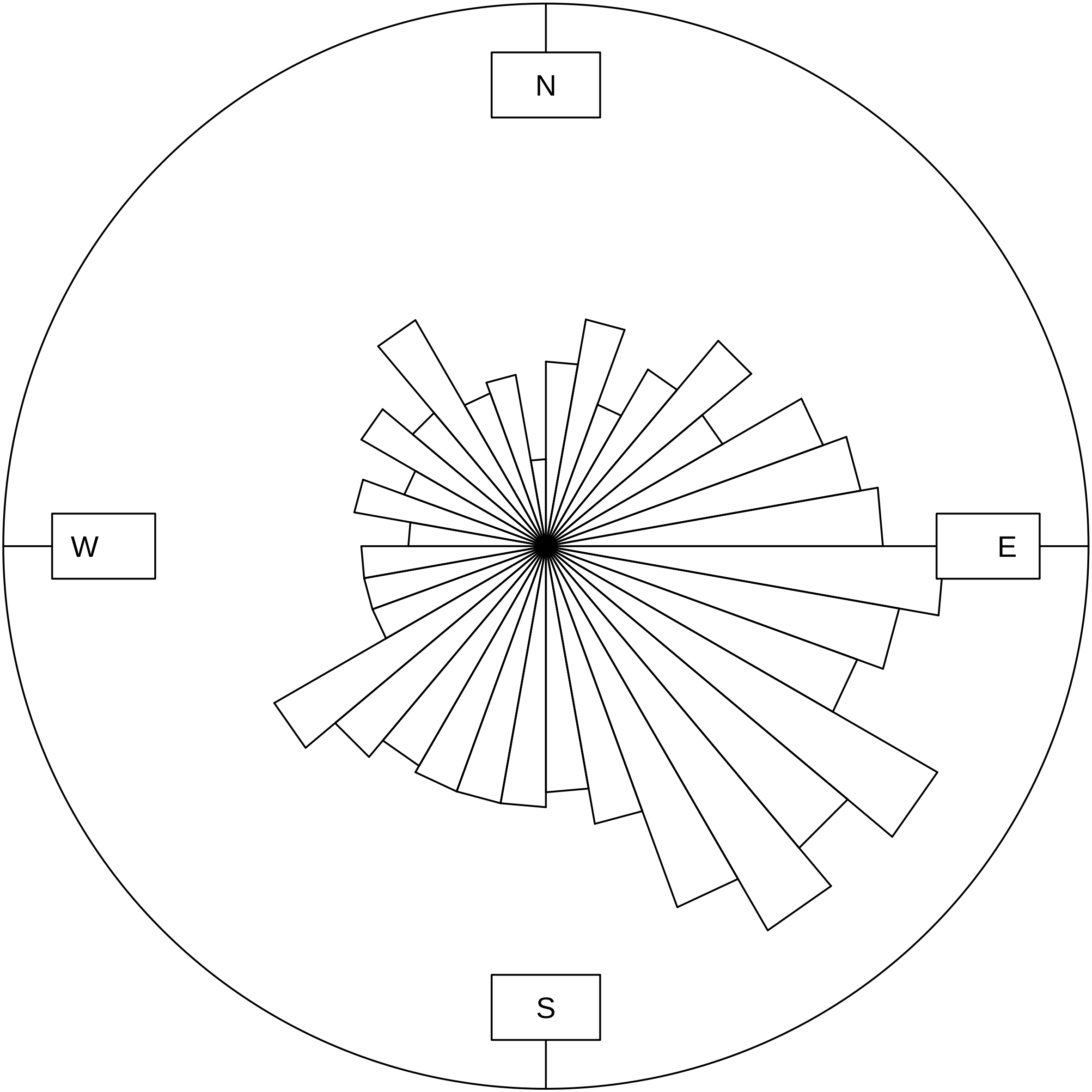

bootstrap 636

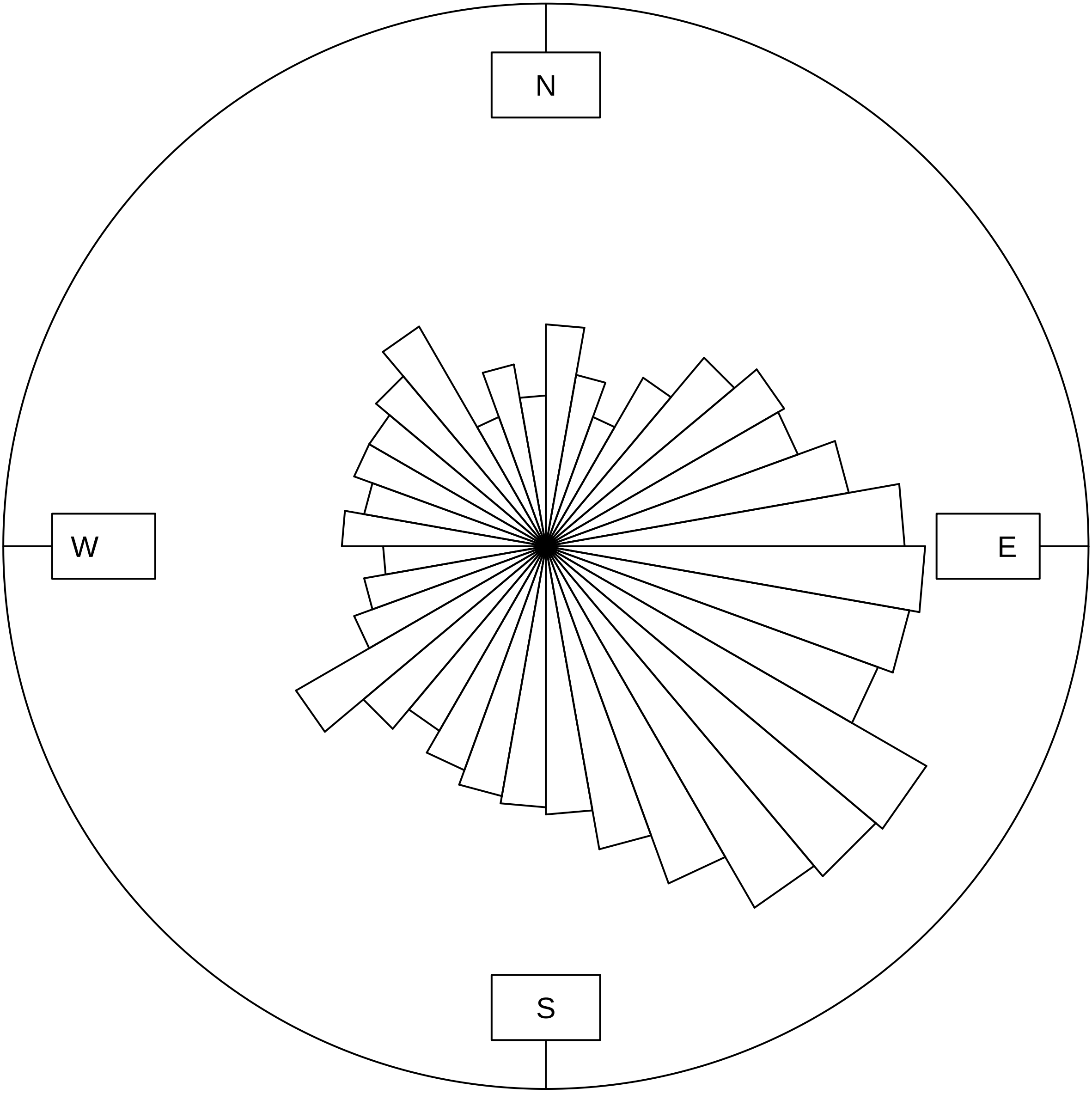

bootstrap 637

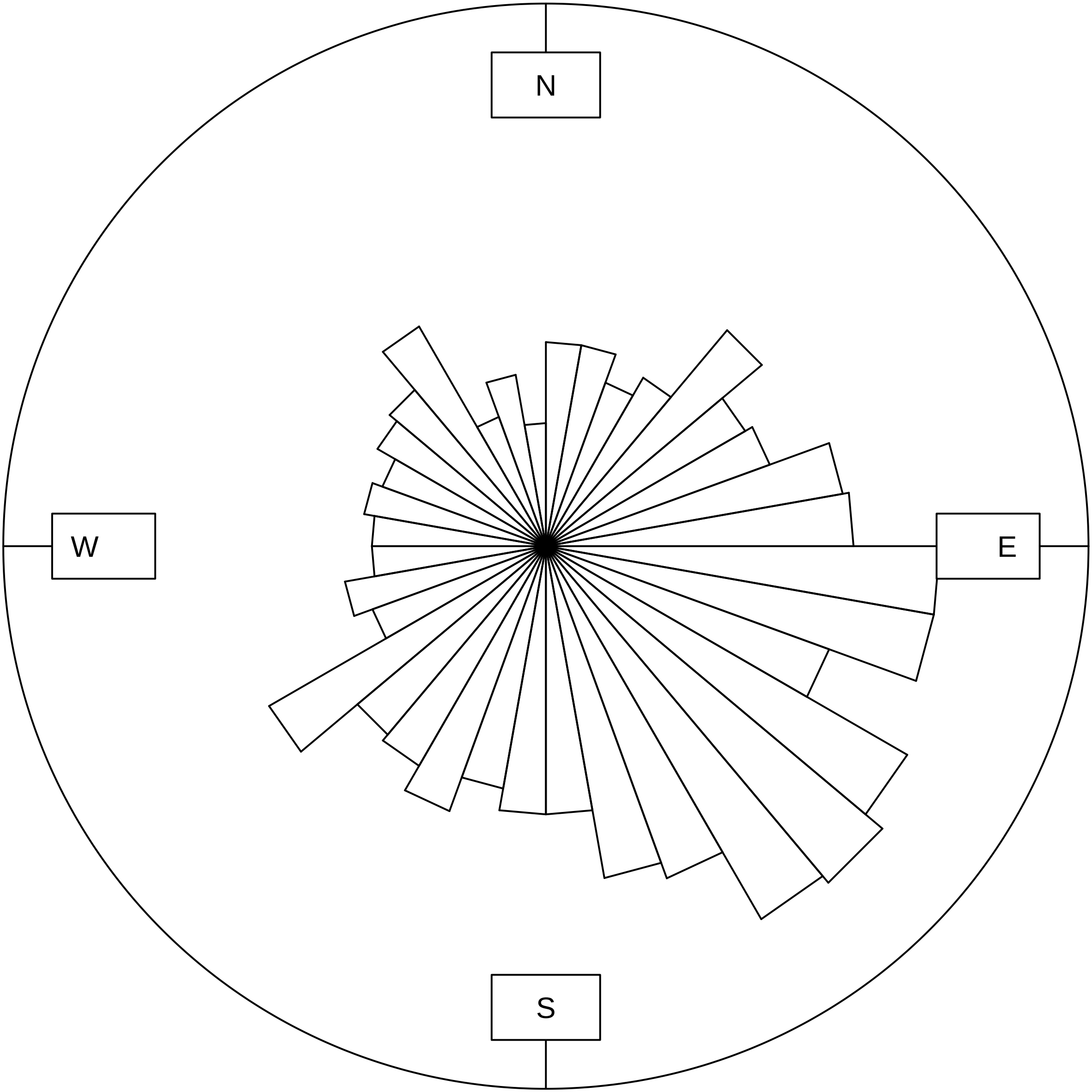

bootstrap 638

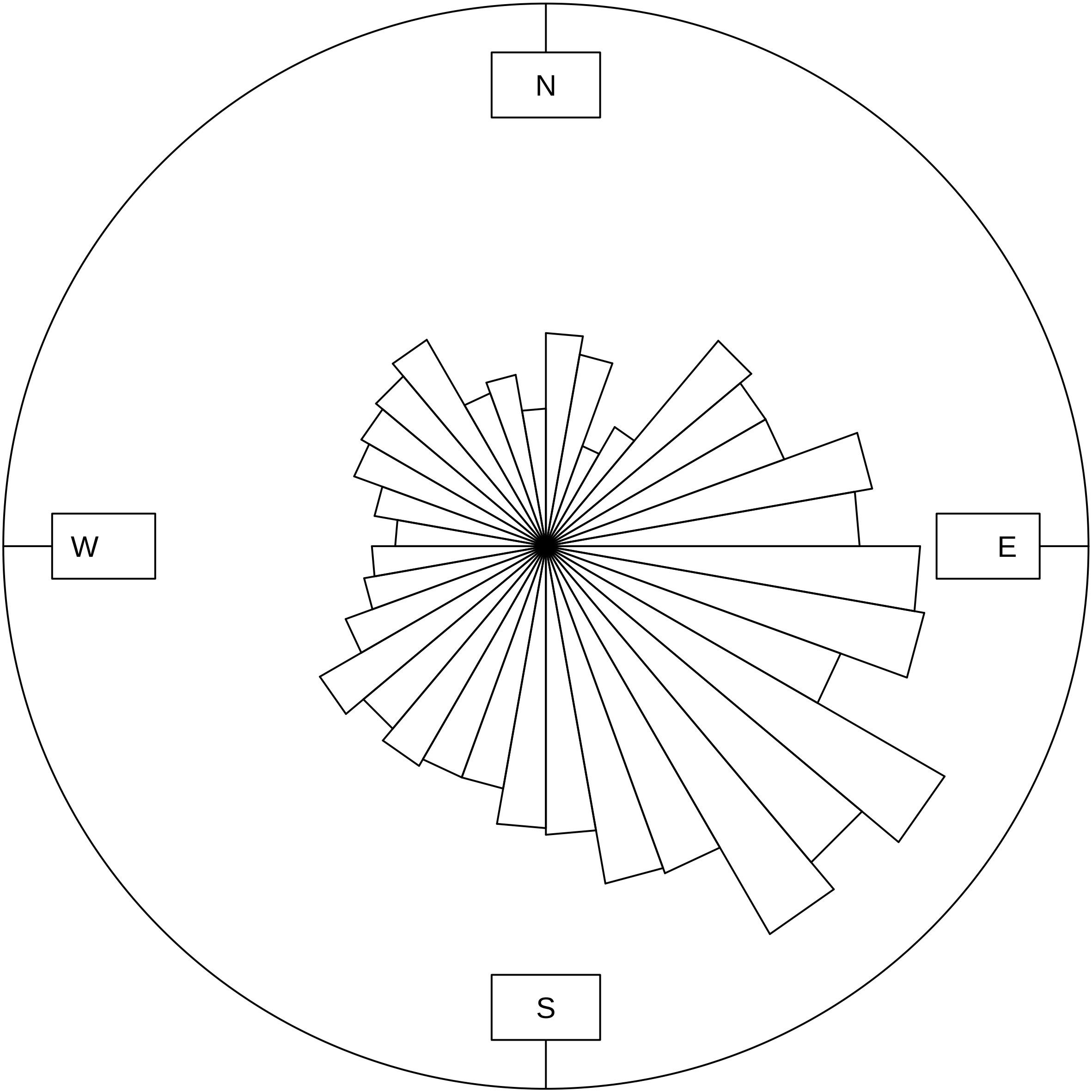

bootstrap 639

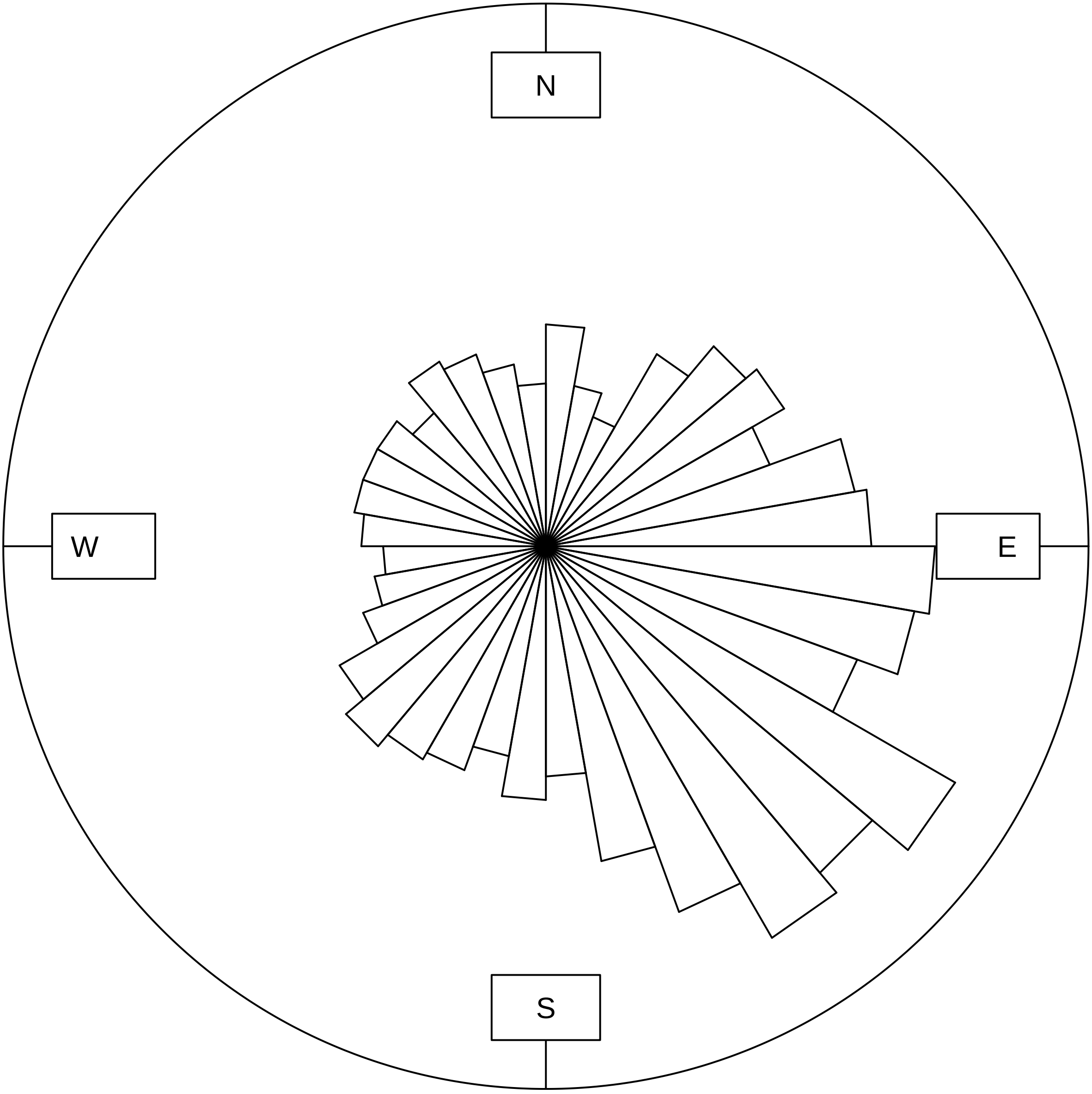

bootstrap 640

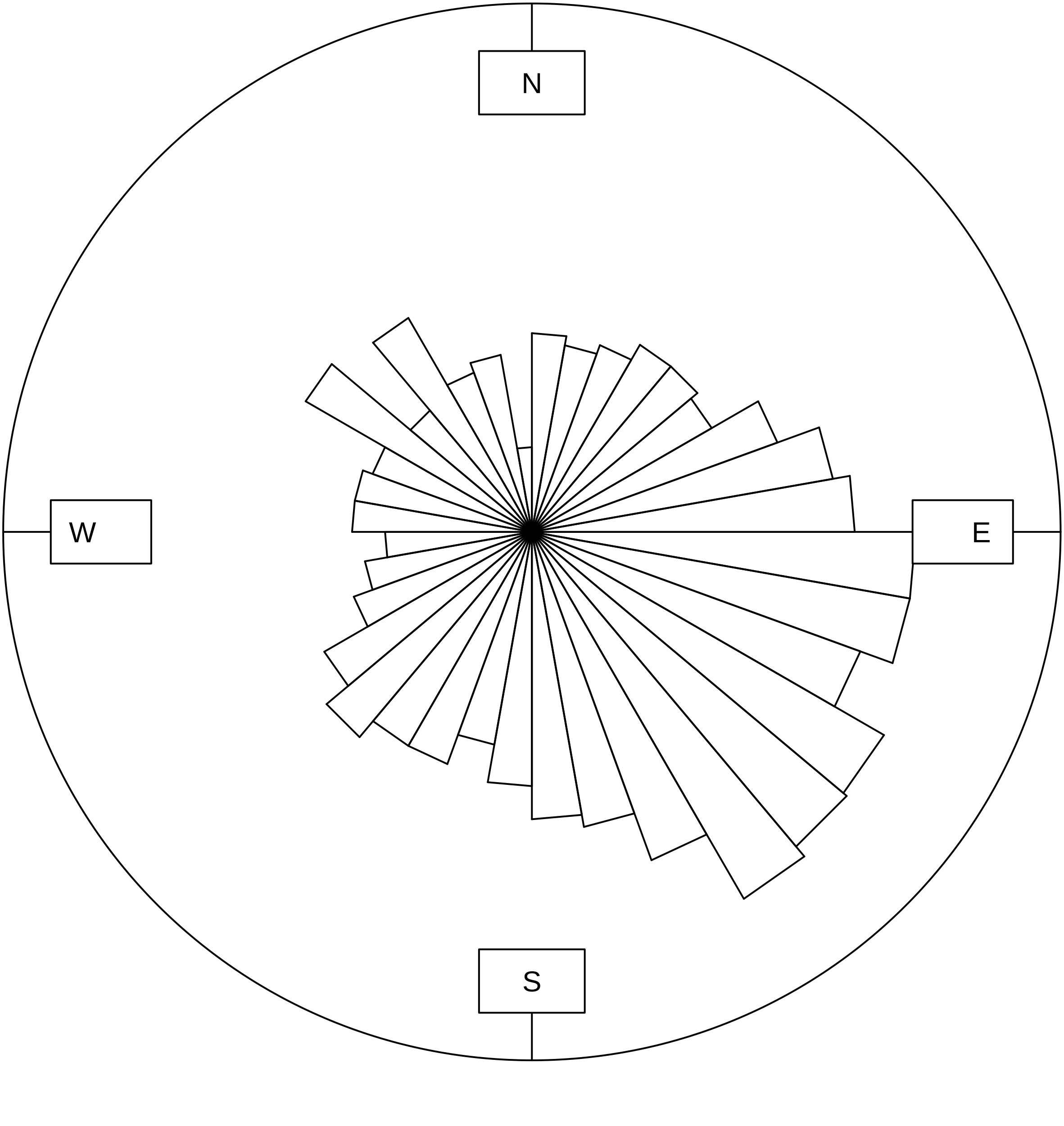

bootstrap 641

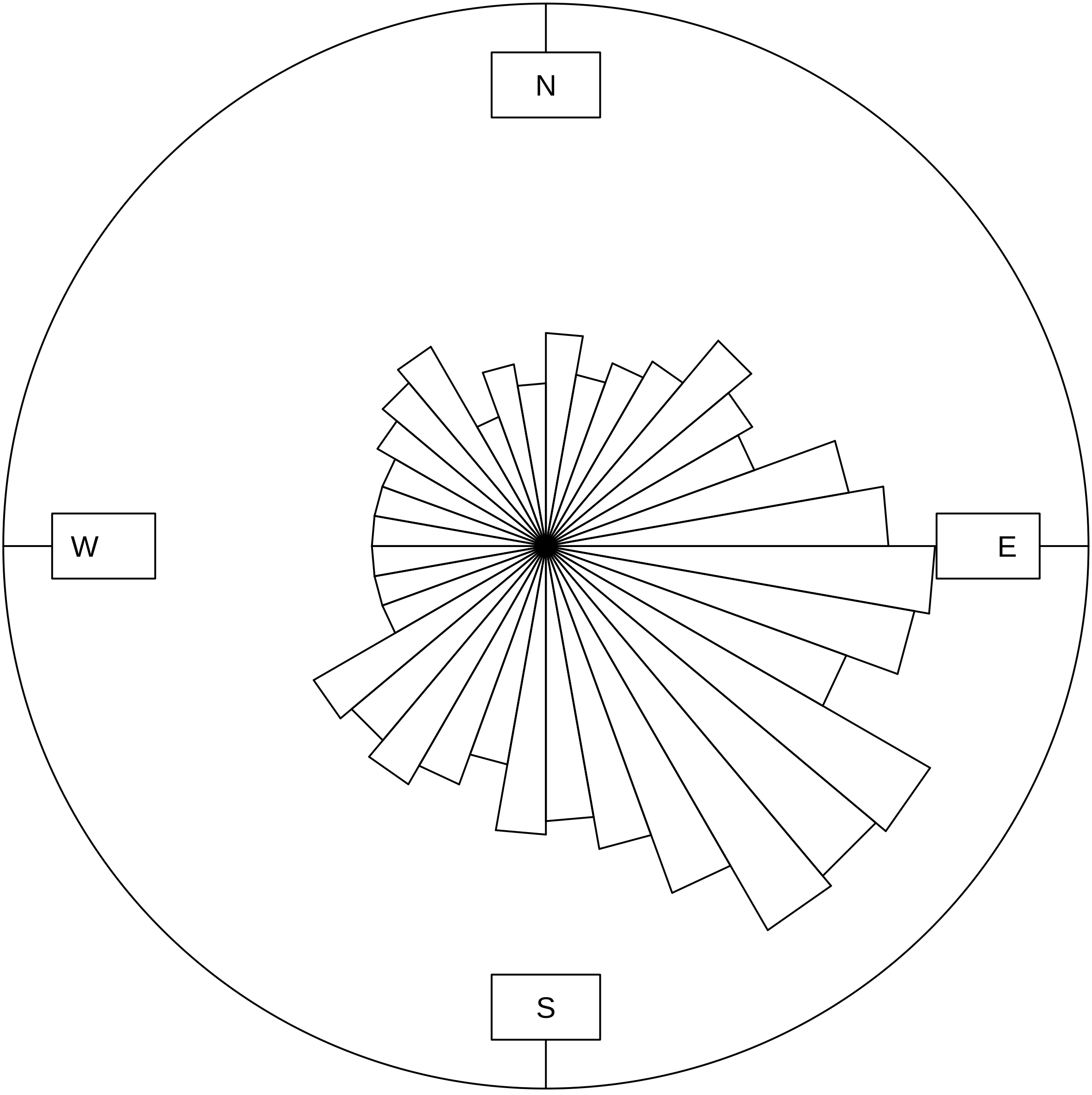

bootstrap 642

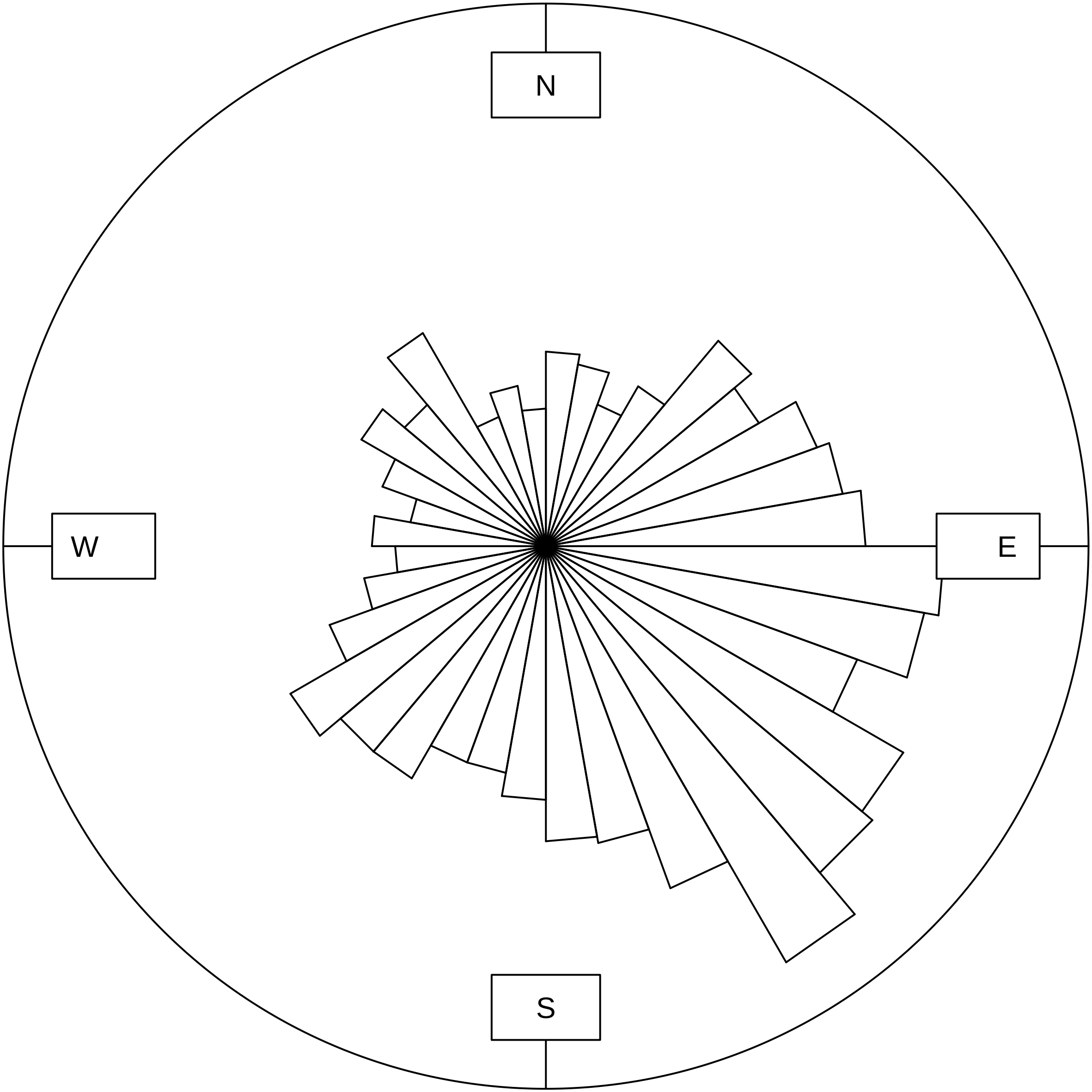

bootstrap 643

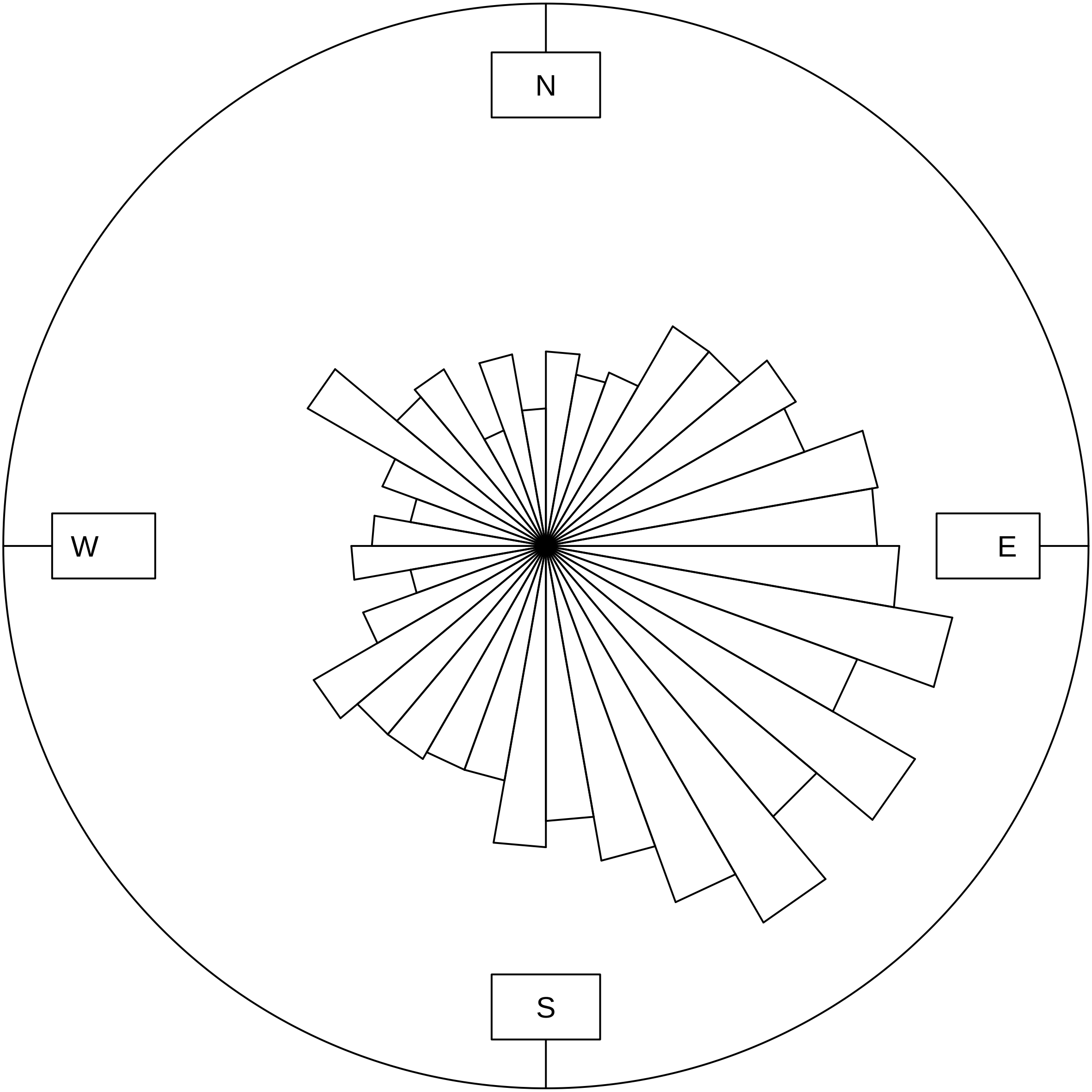

bootstrap 644

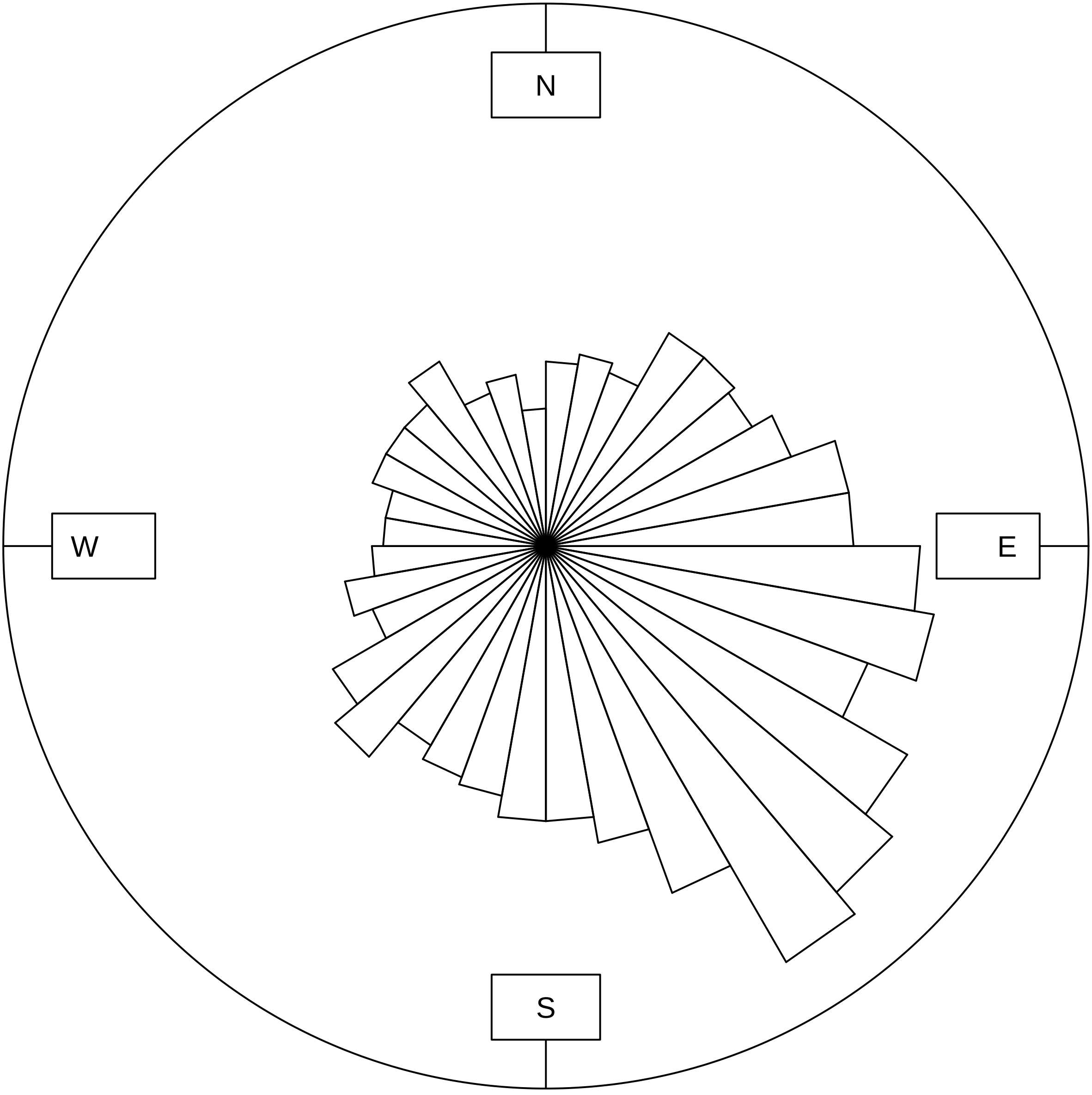

bootstrap 645

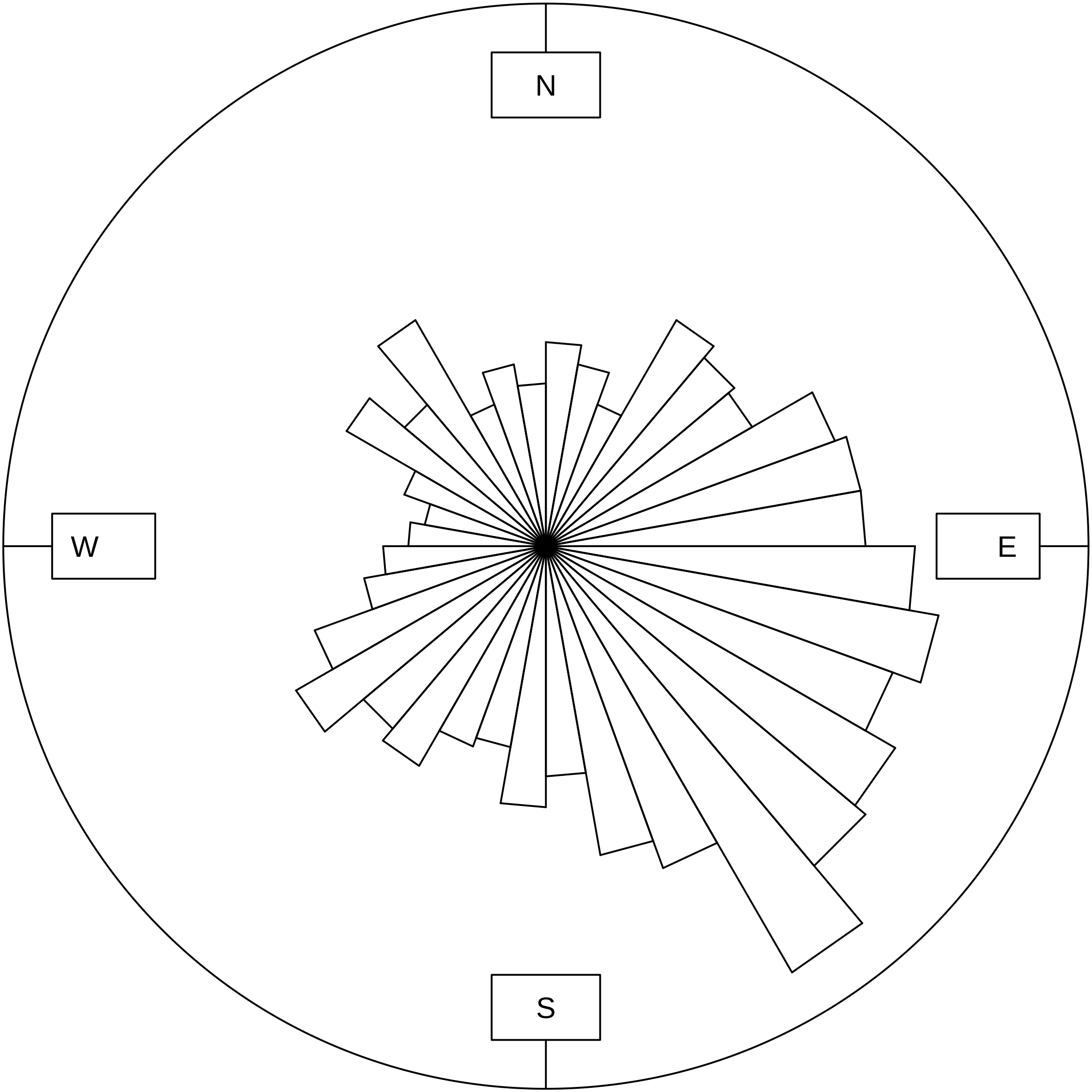

bootstrap 646

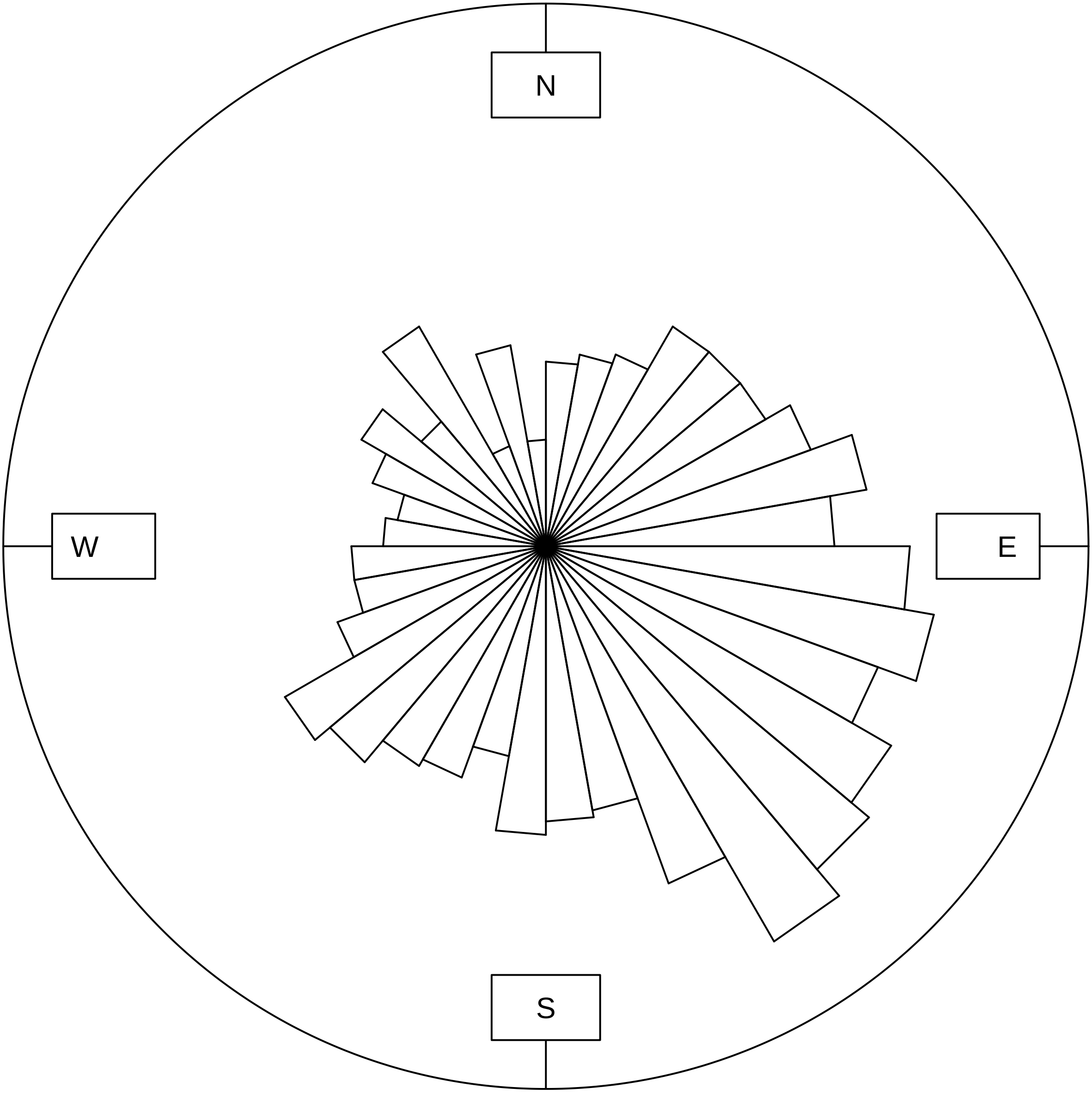

bootstrap 647

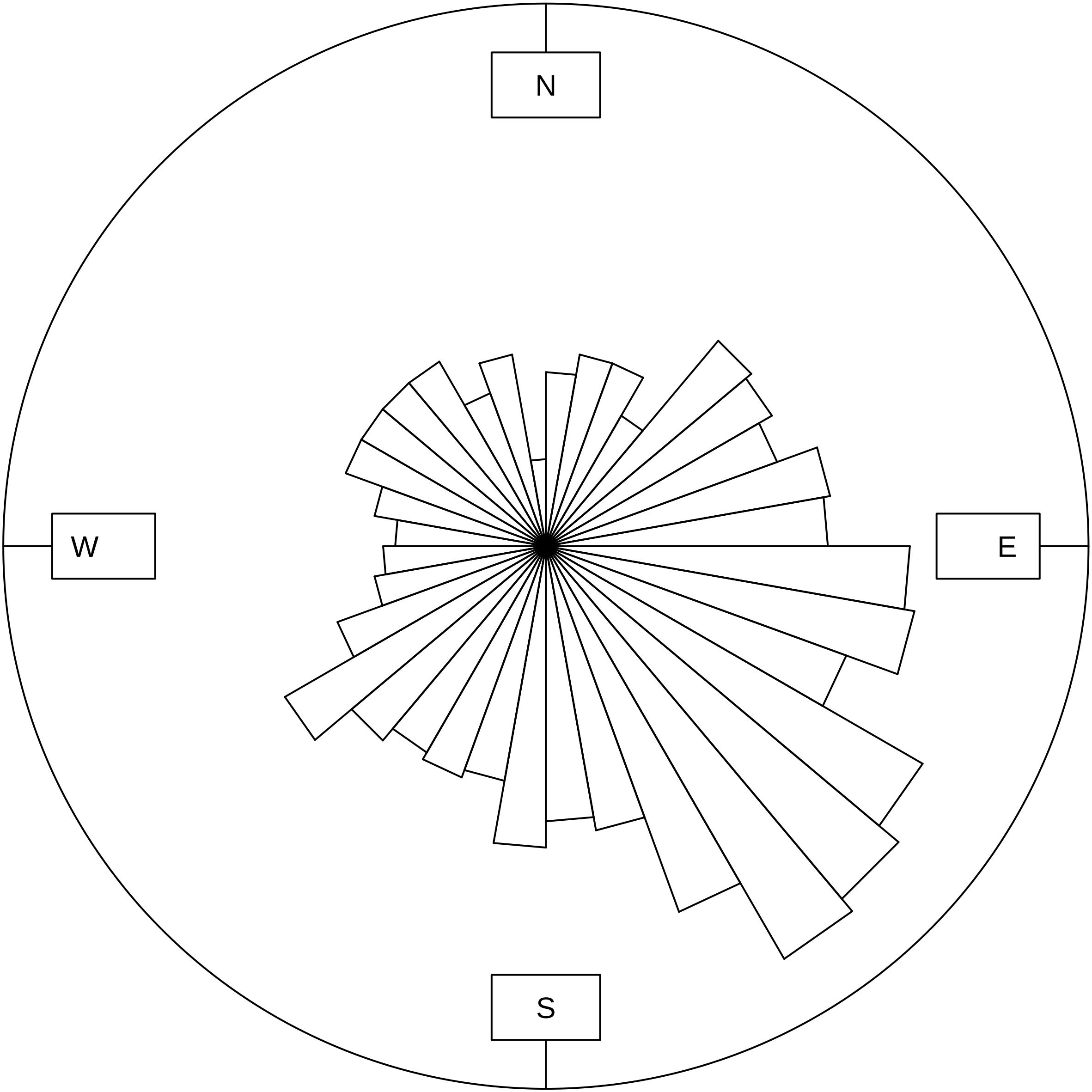

bootstrap 648

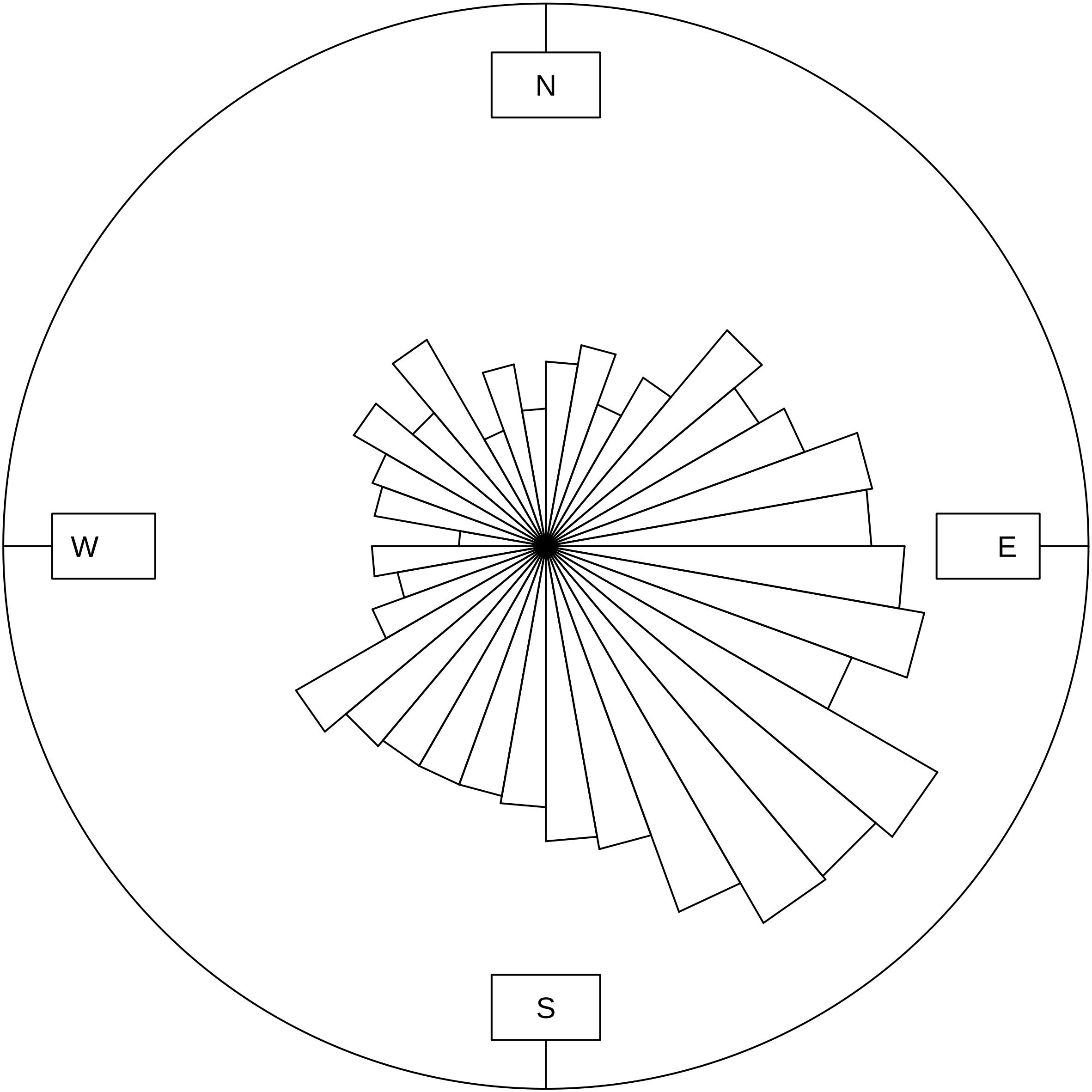

bootstrap 649

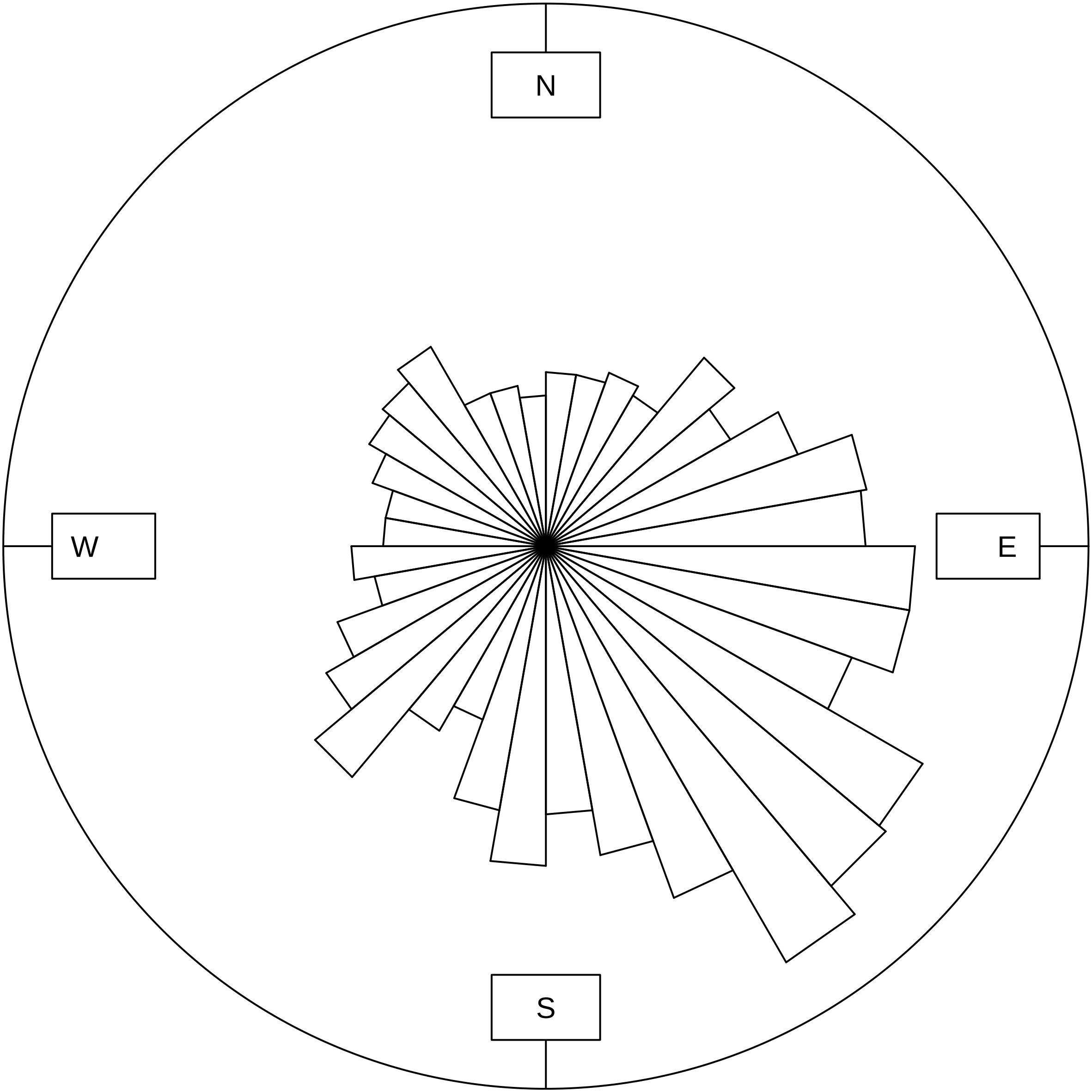

bootstrap 650

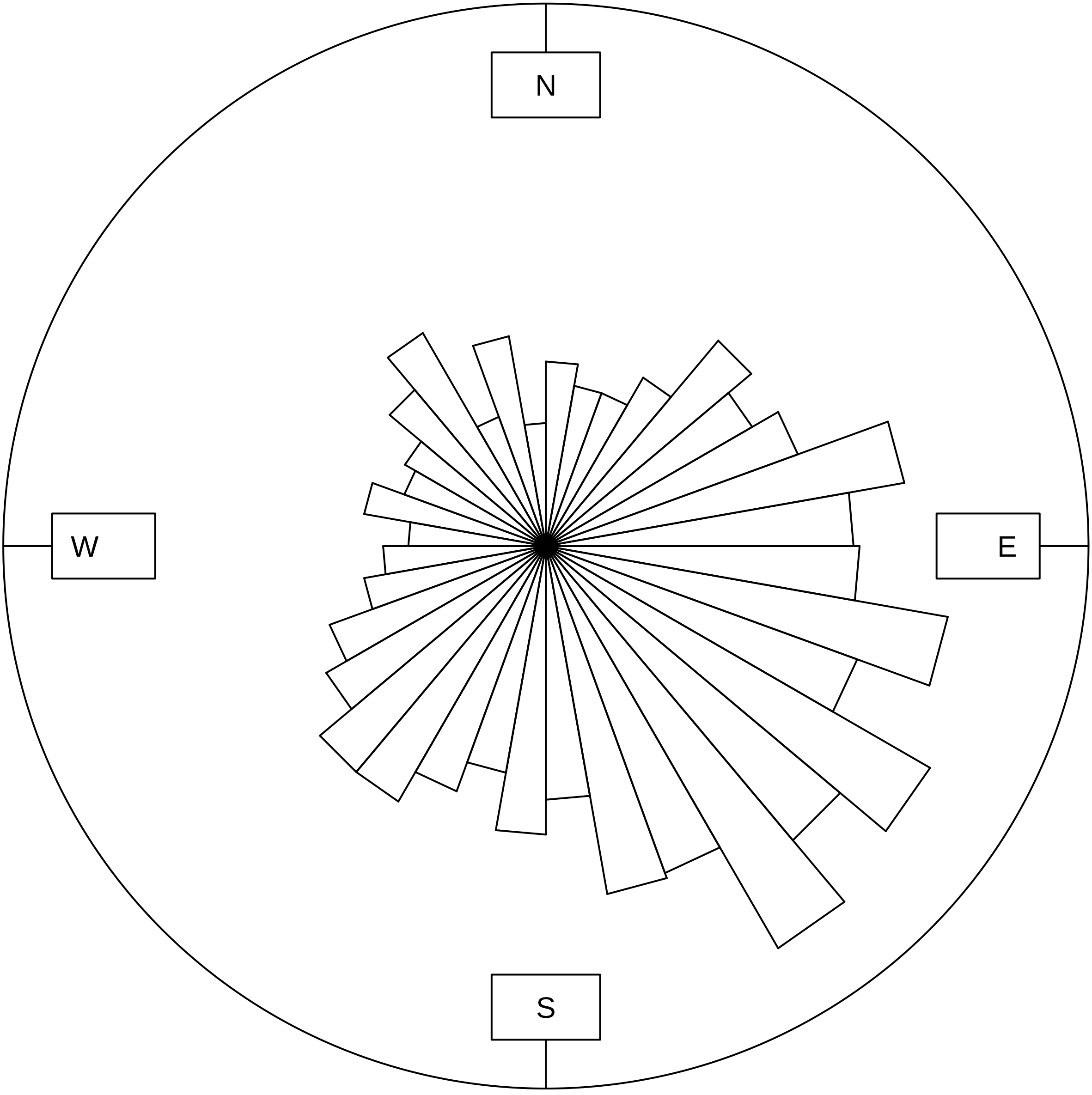

bootstrap 651

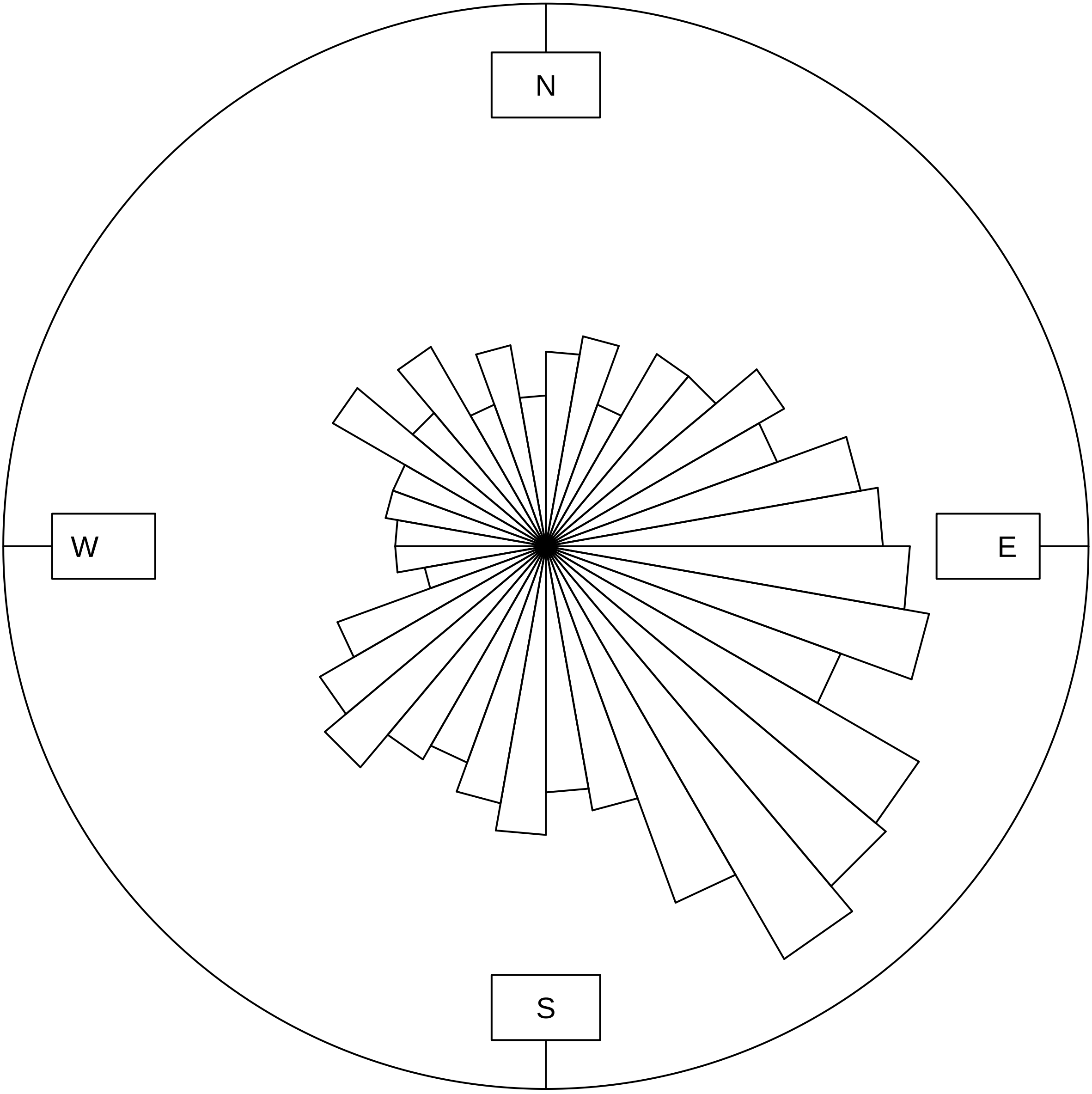

bootstrap 652

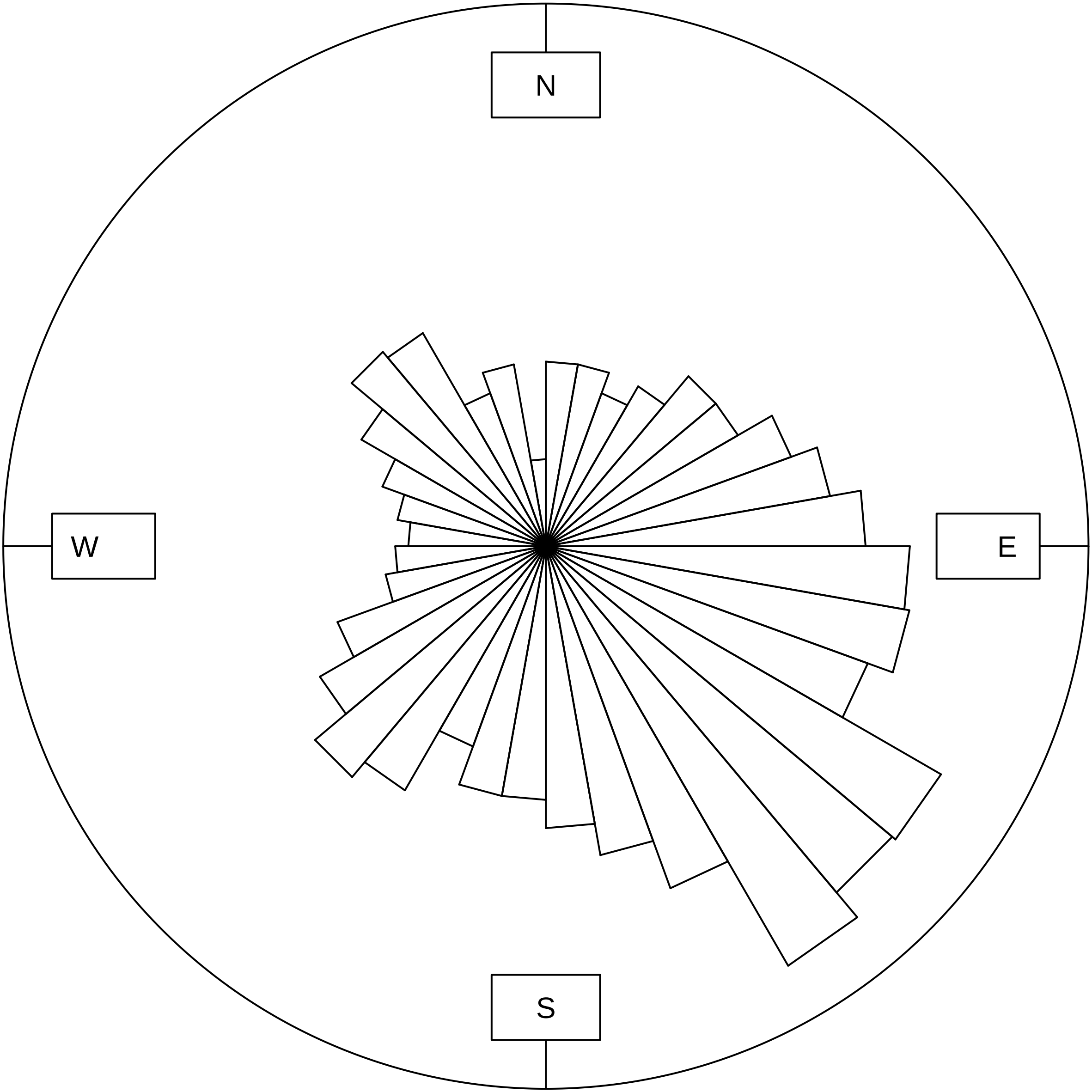

bootstrap 653

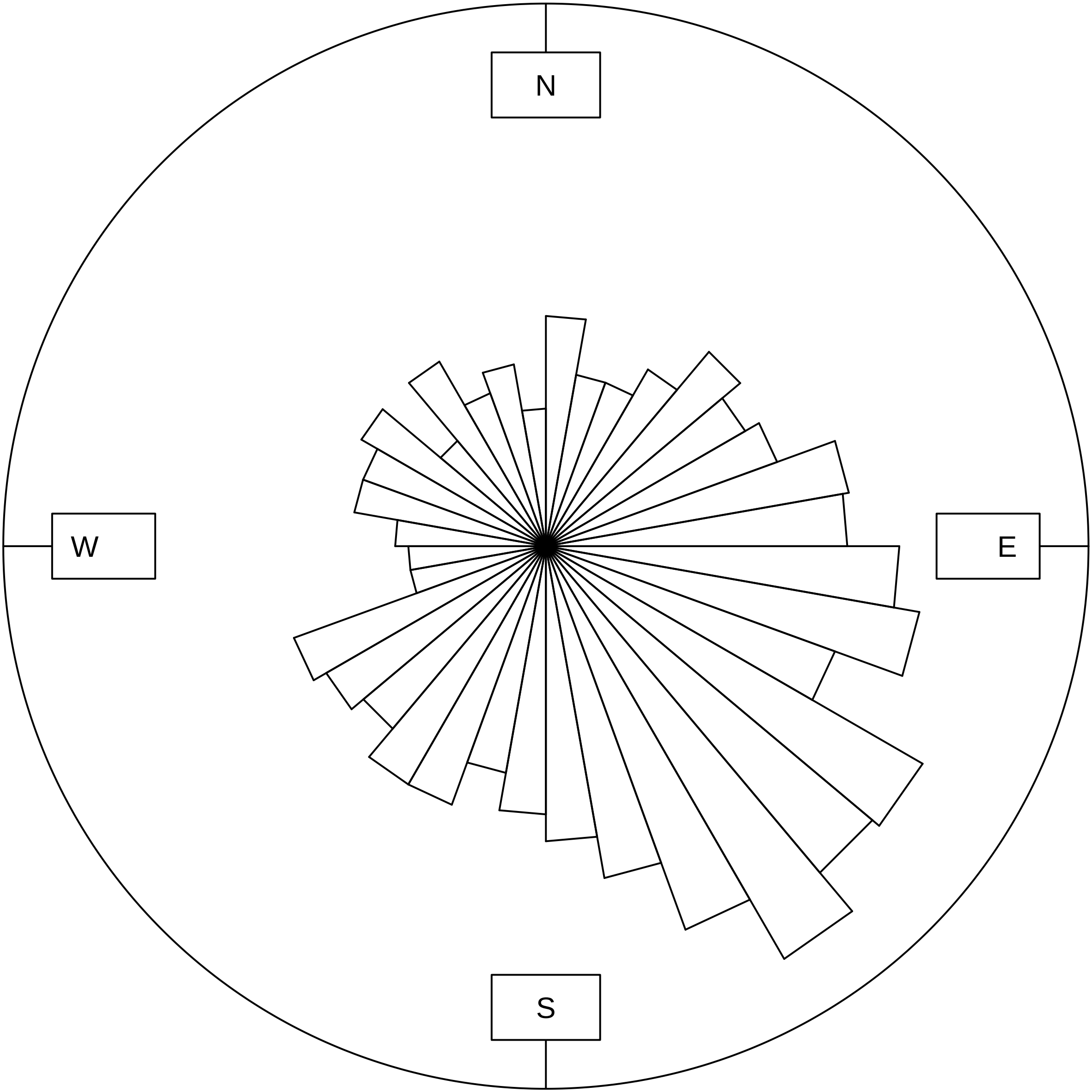

bootstrap 654

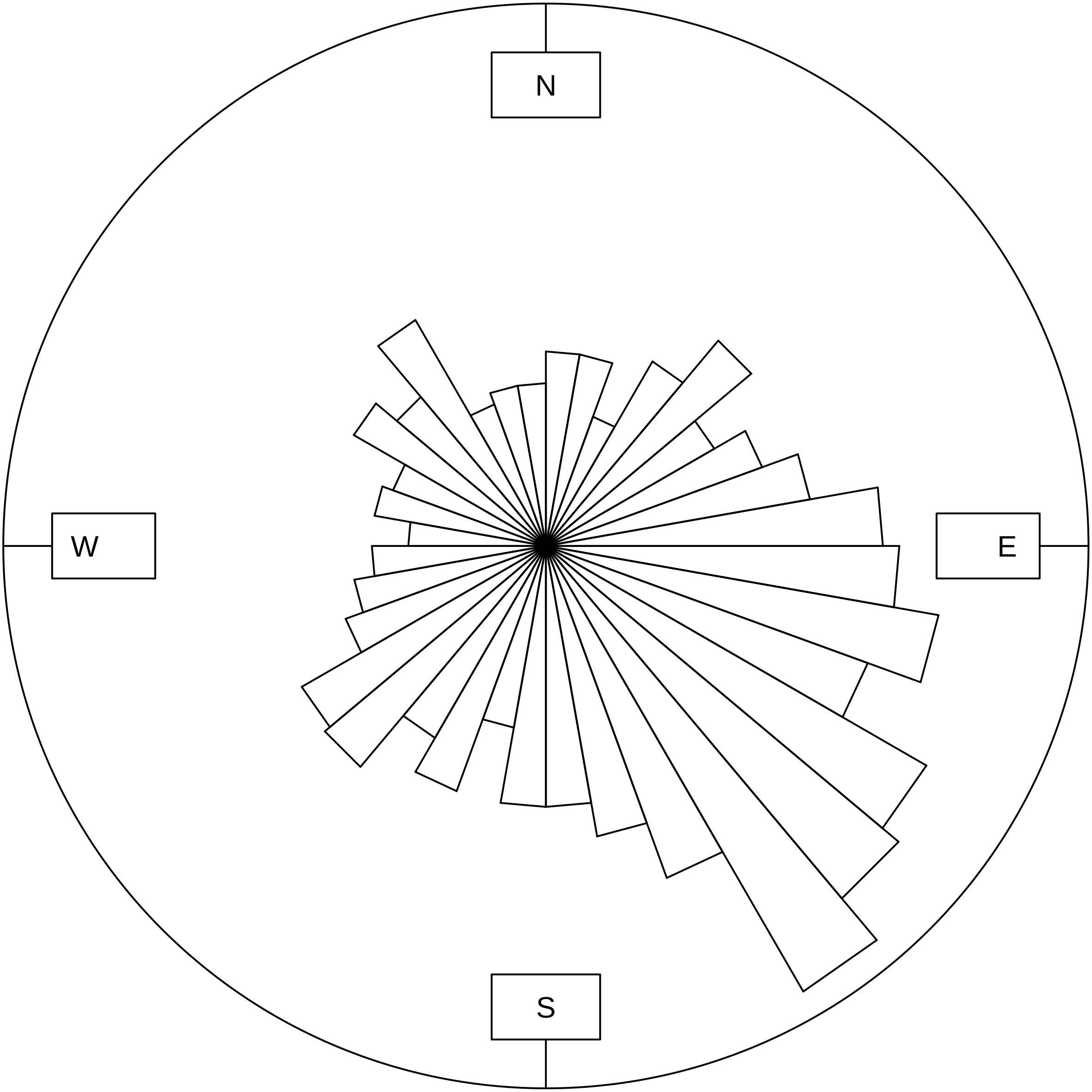

bootstrap 655

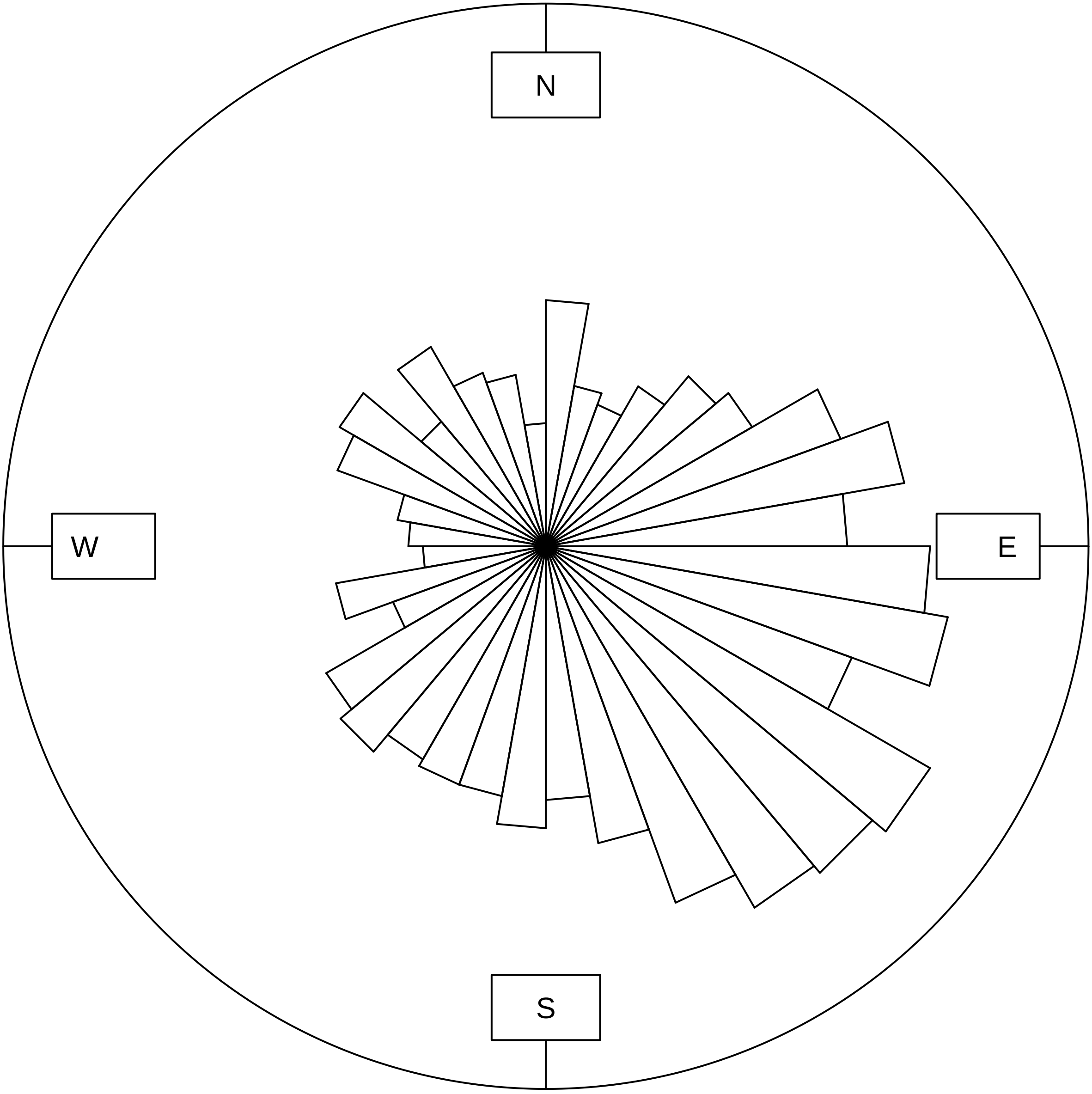

bootstrap 656

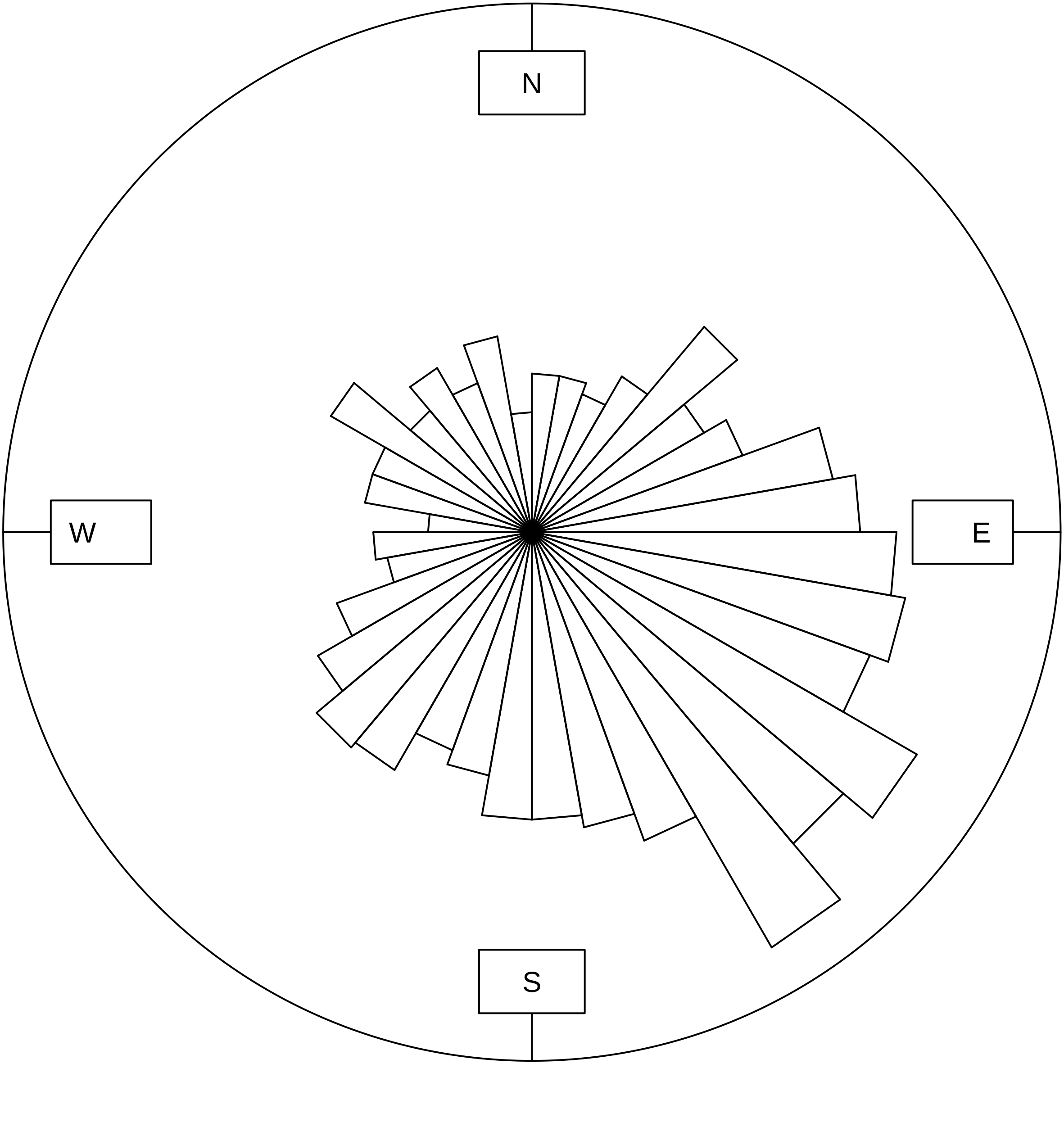

bootstrap 657

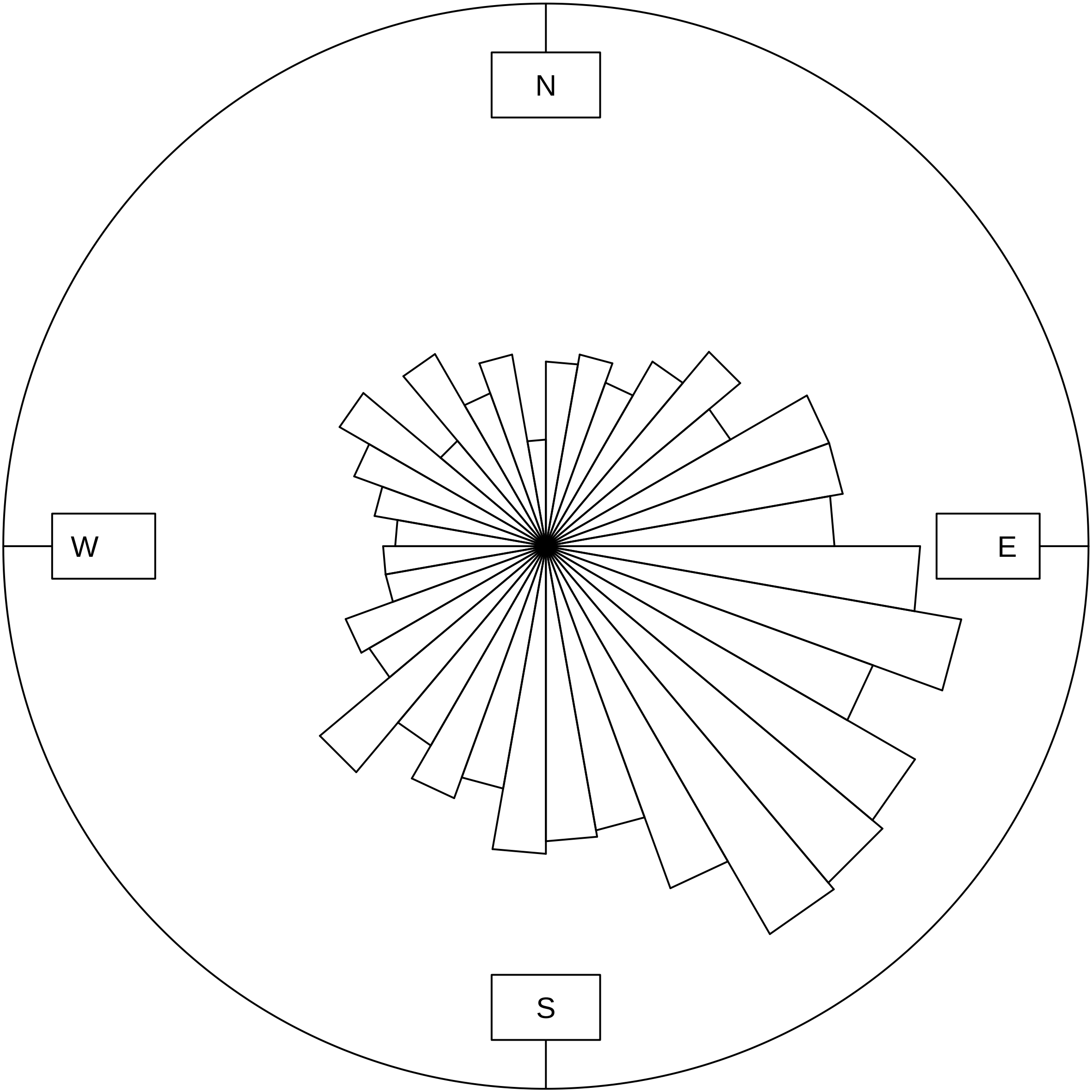

bootstrap 658

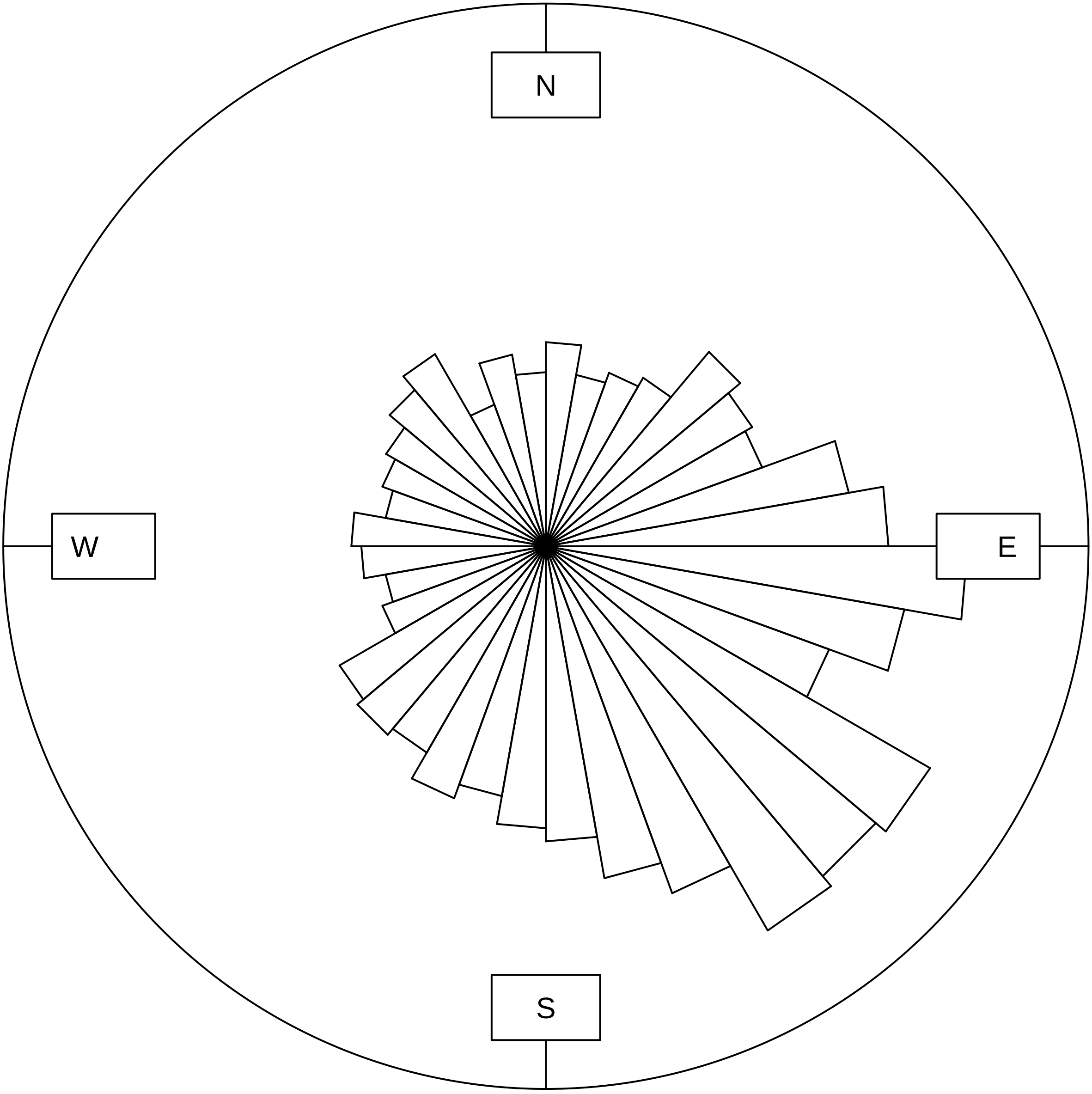

bootstrap 659

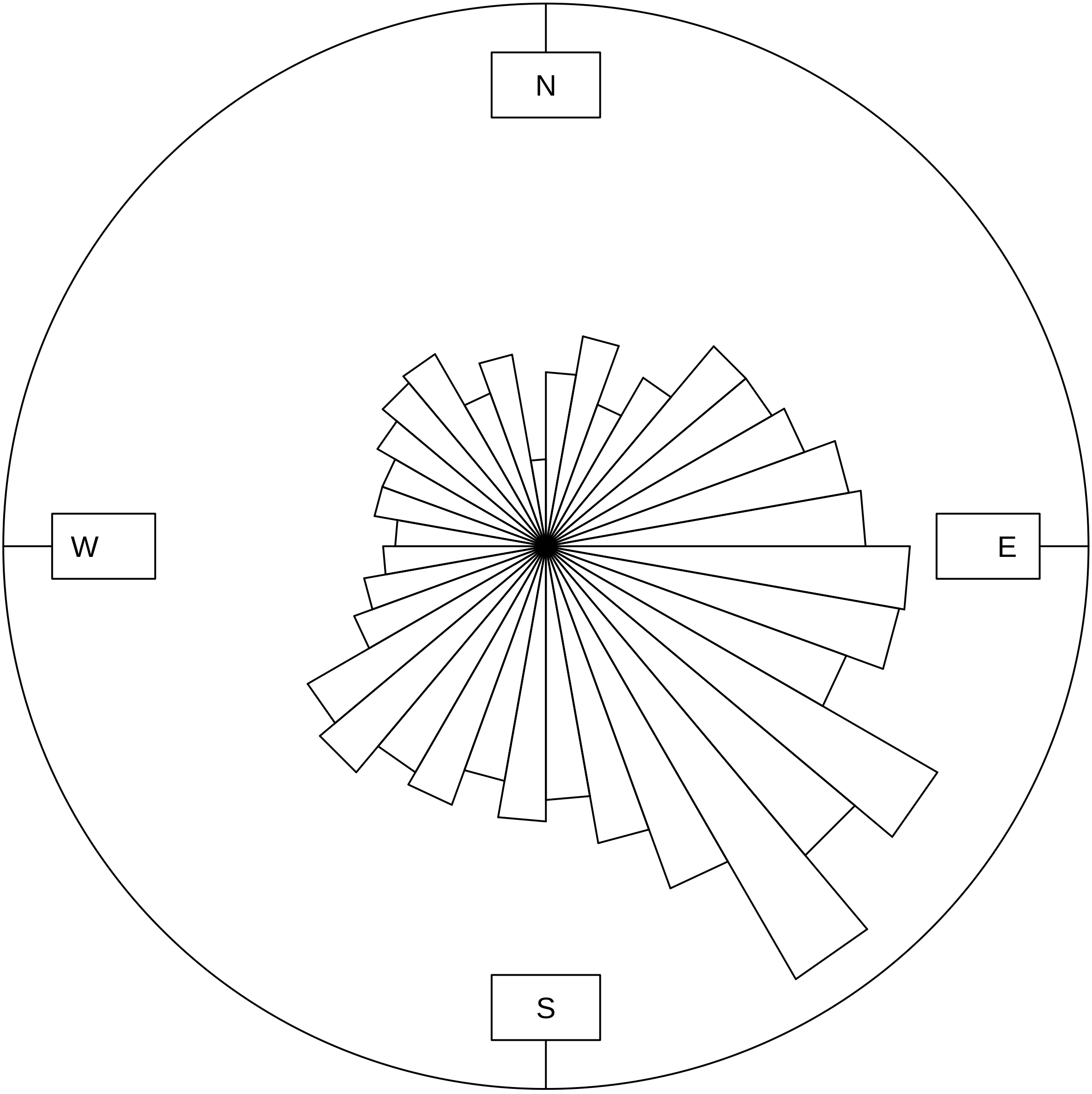

bootstrap 660

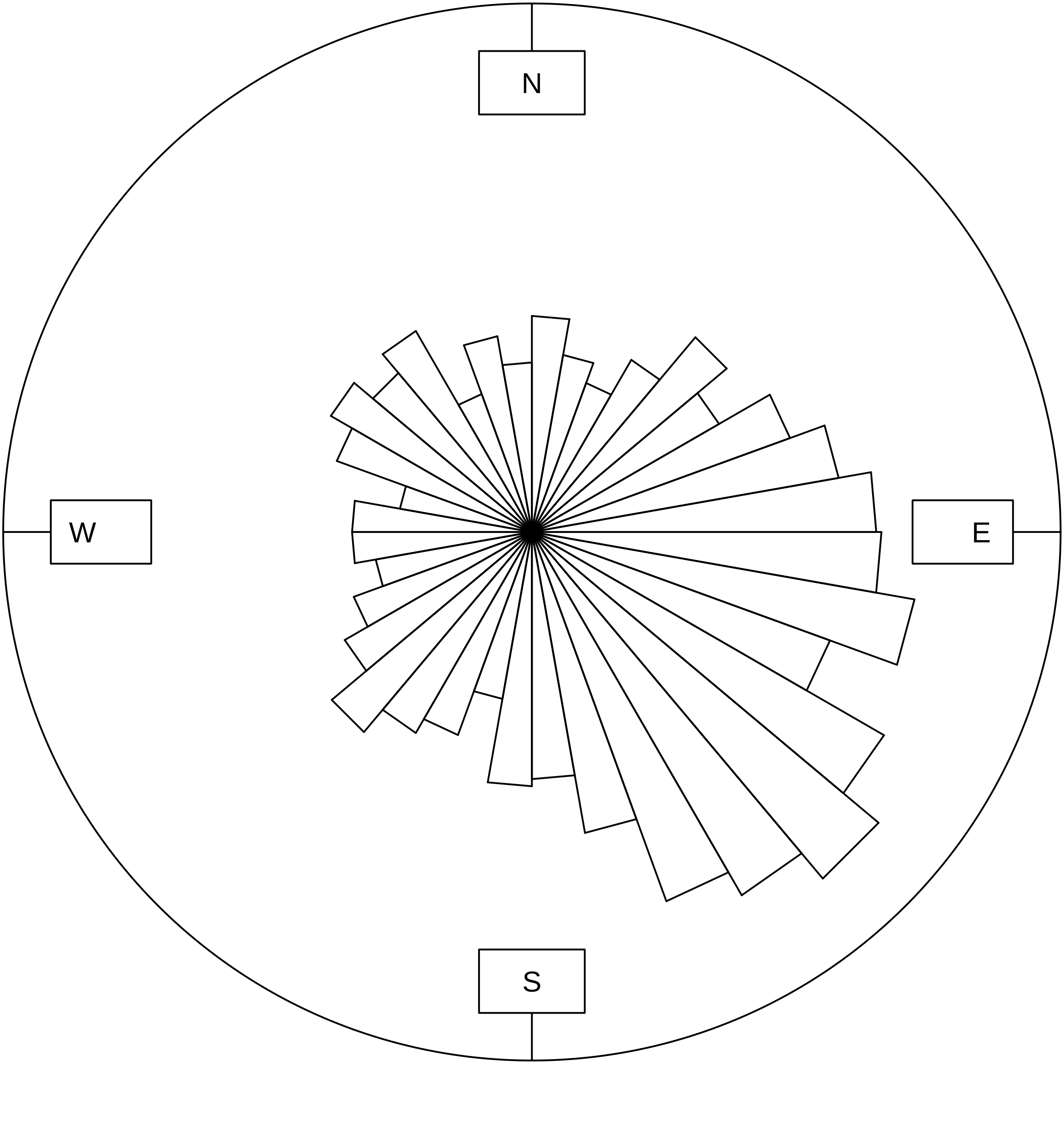

bootstrap 661

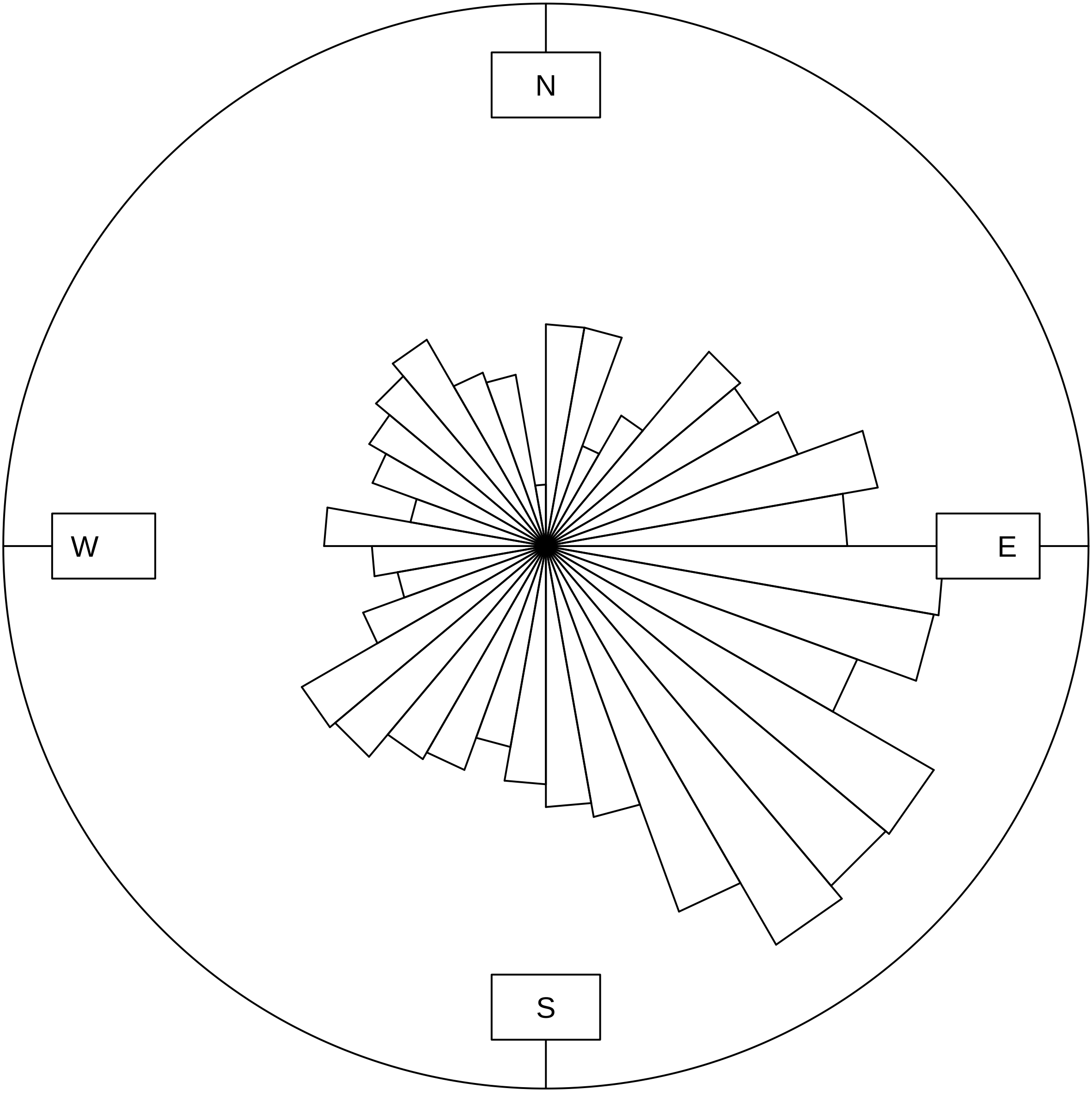

bootstrap 662

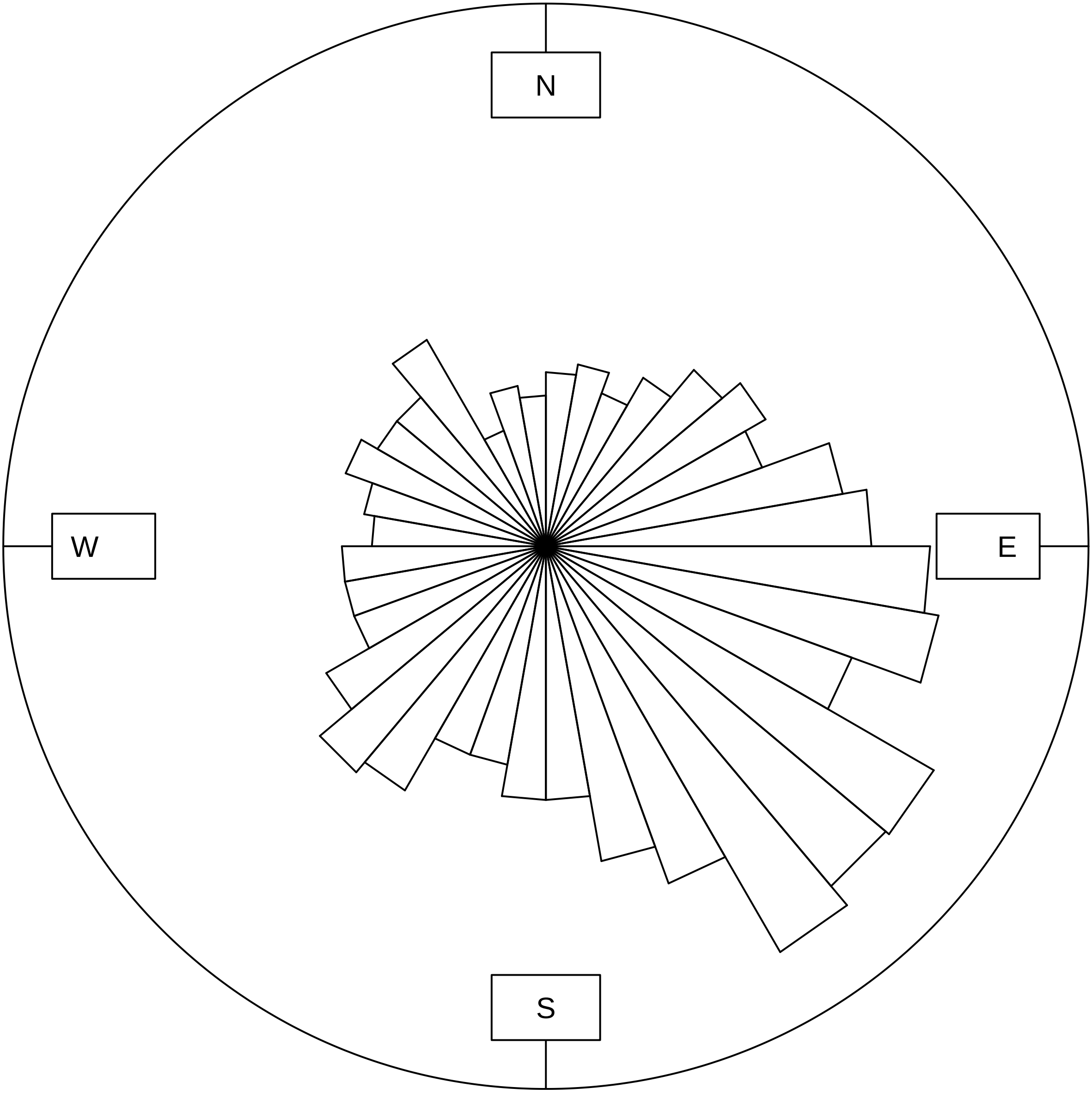

bootstrap 663

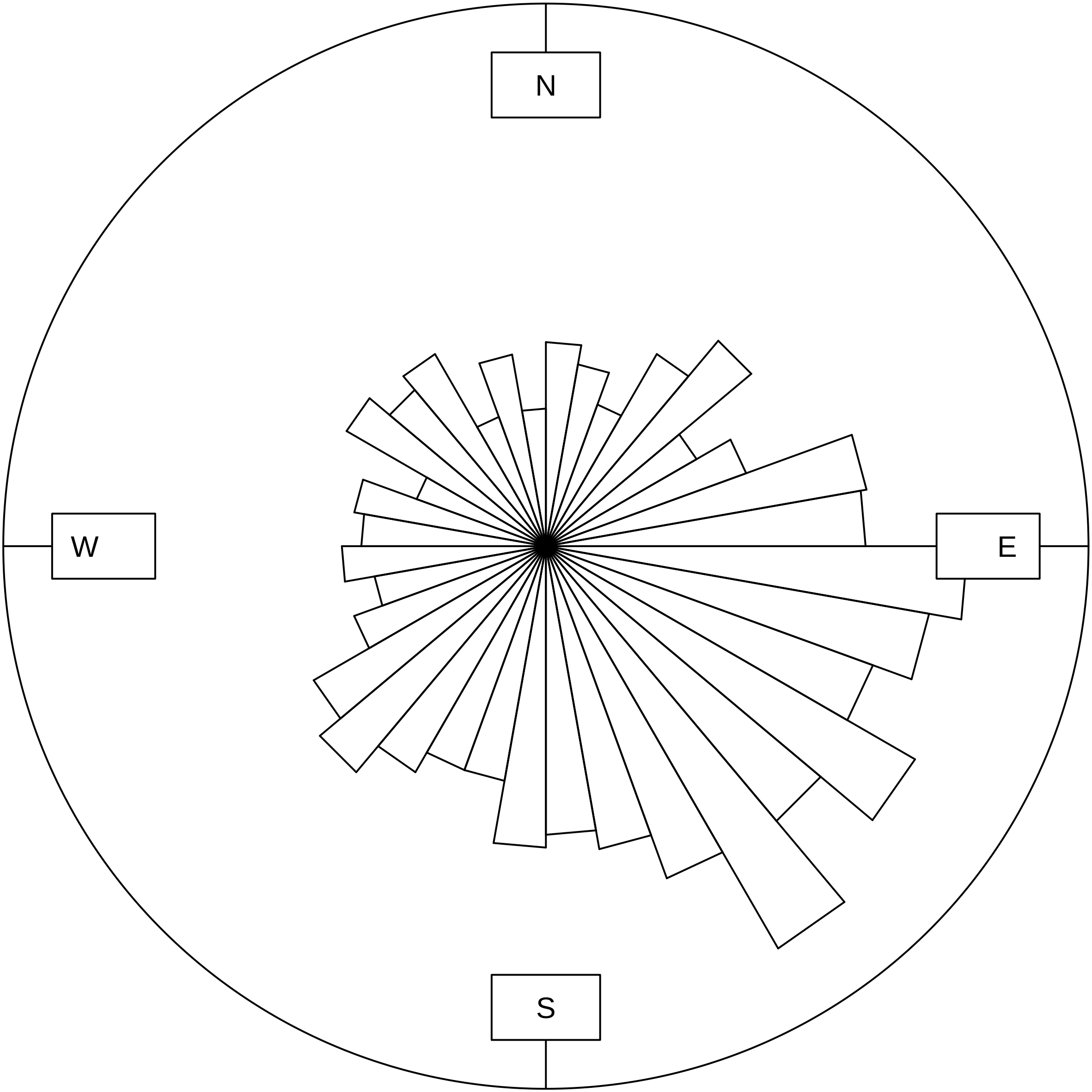

bootstrap 664

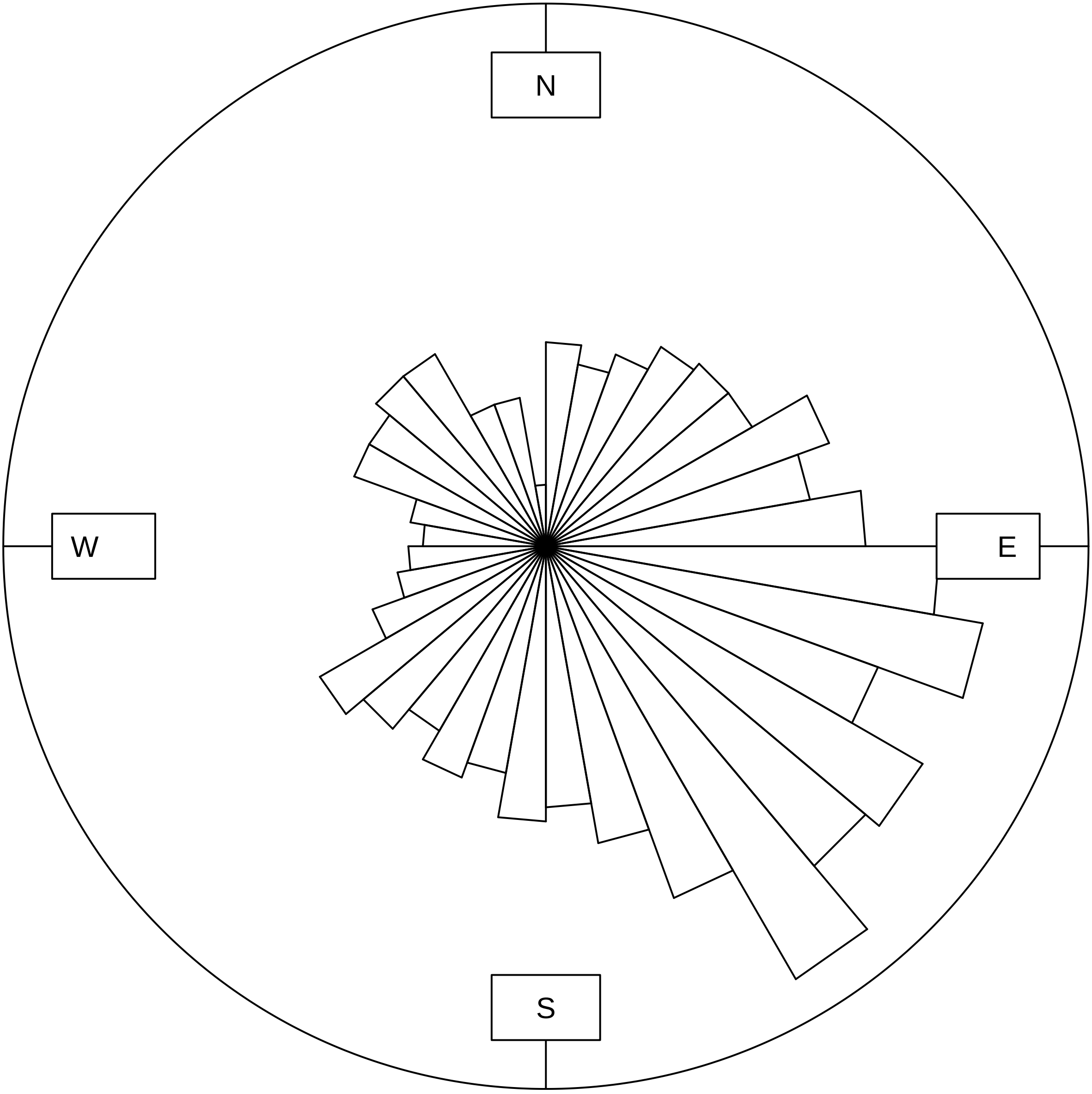

bootstrap 665

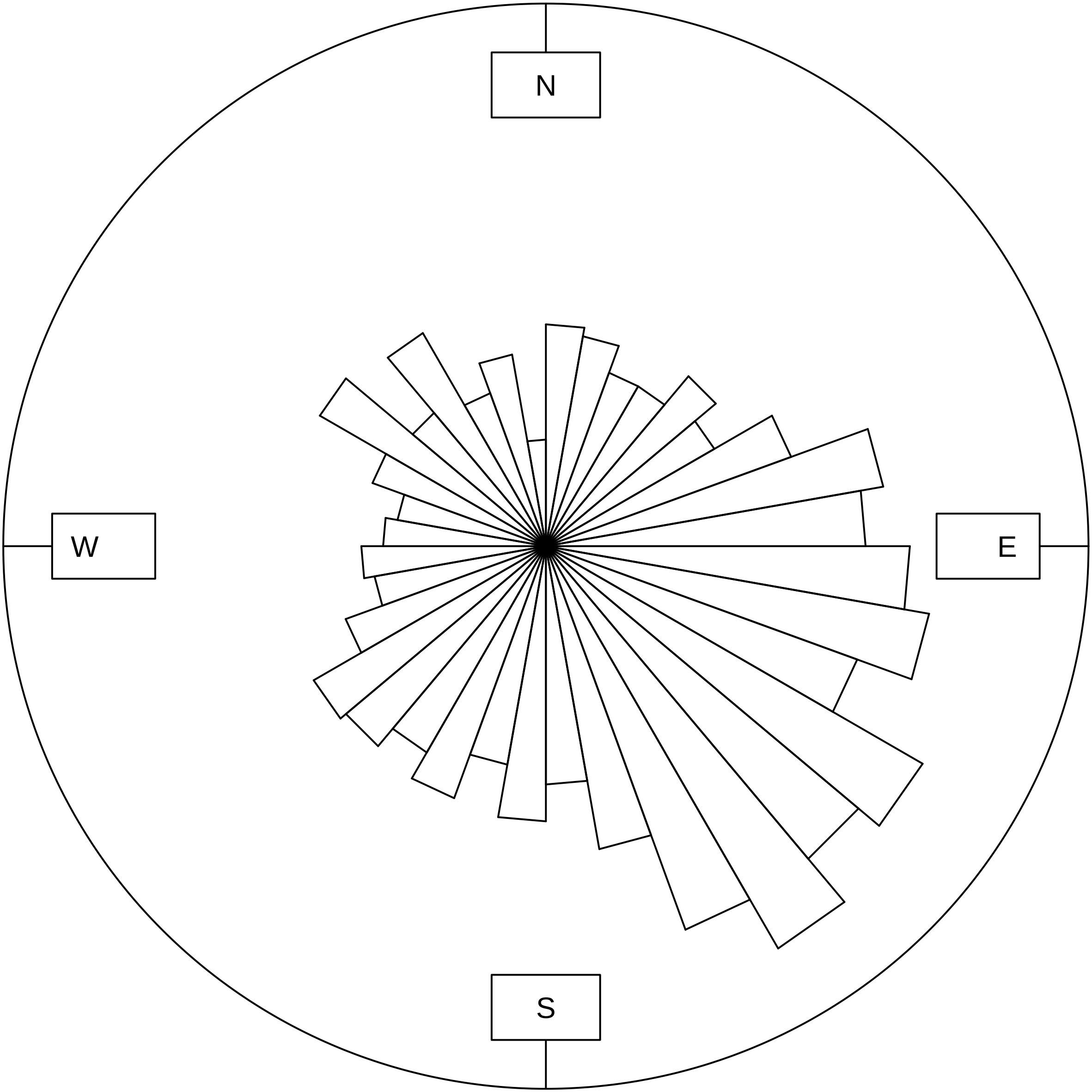

bootstrap 666

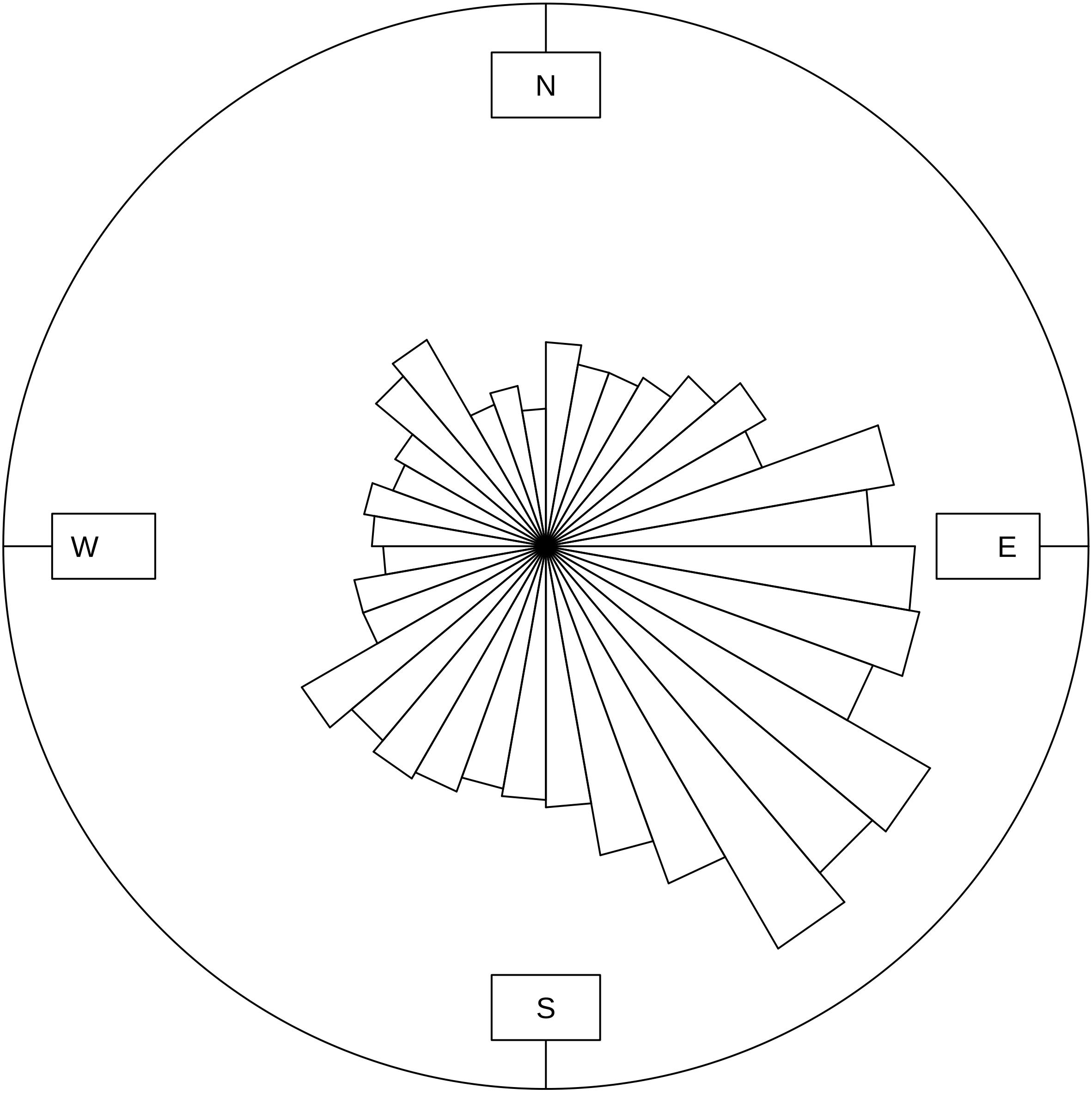

bootstrap 667

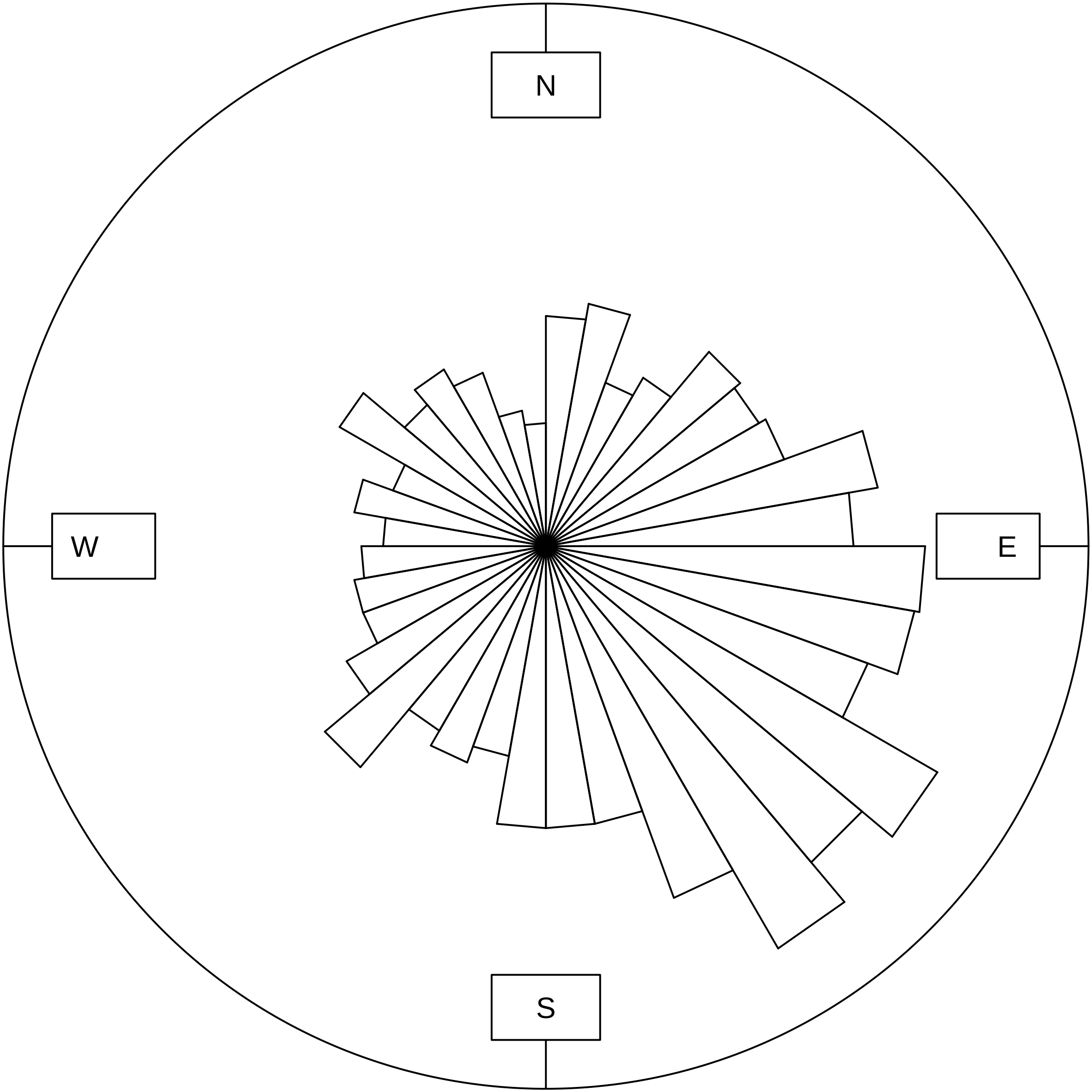

bootstrap 668

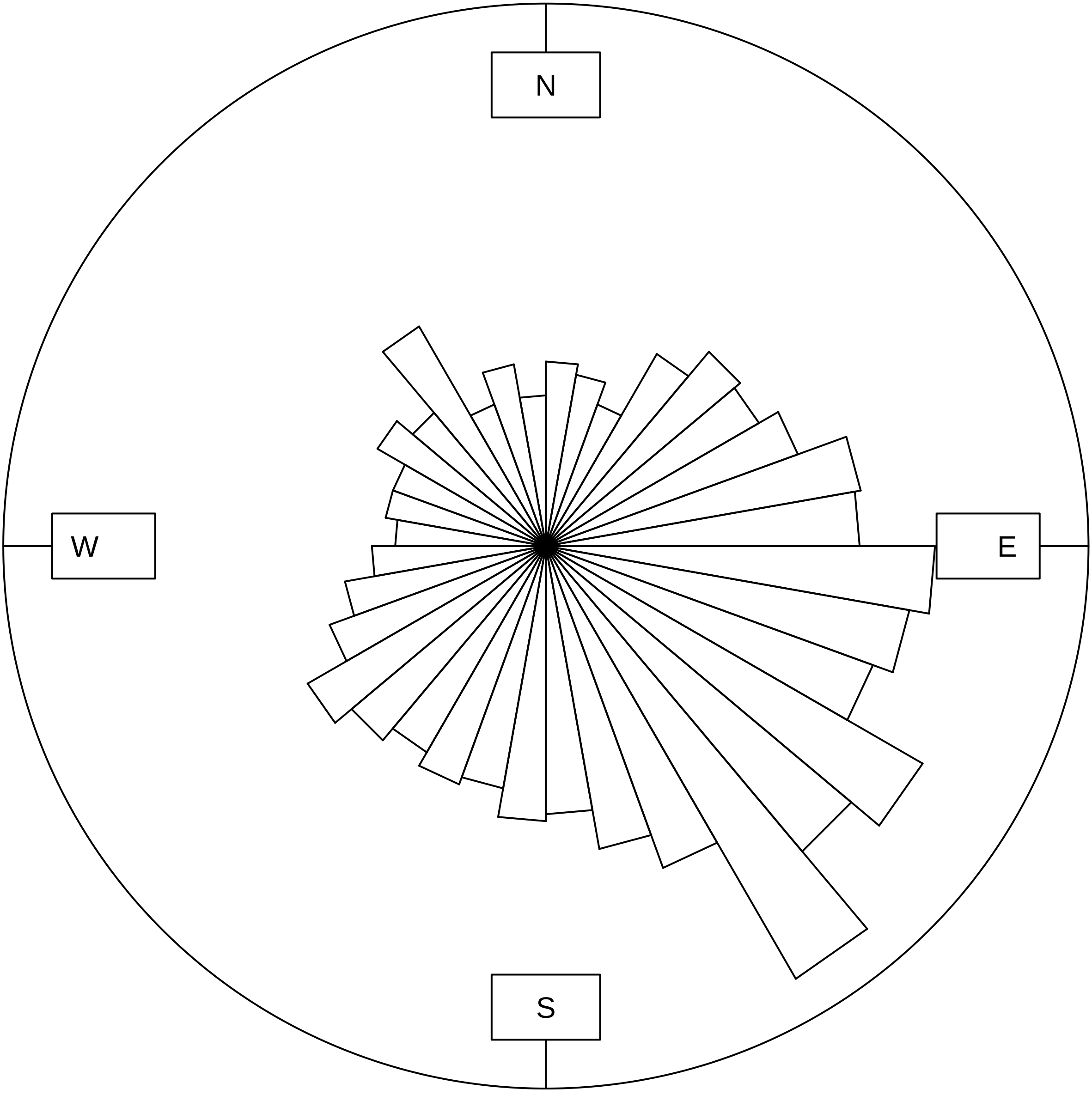

bootstrap 669

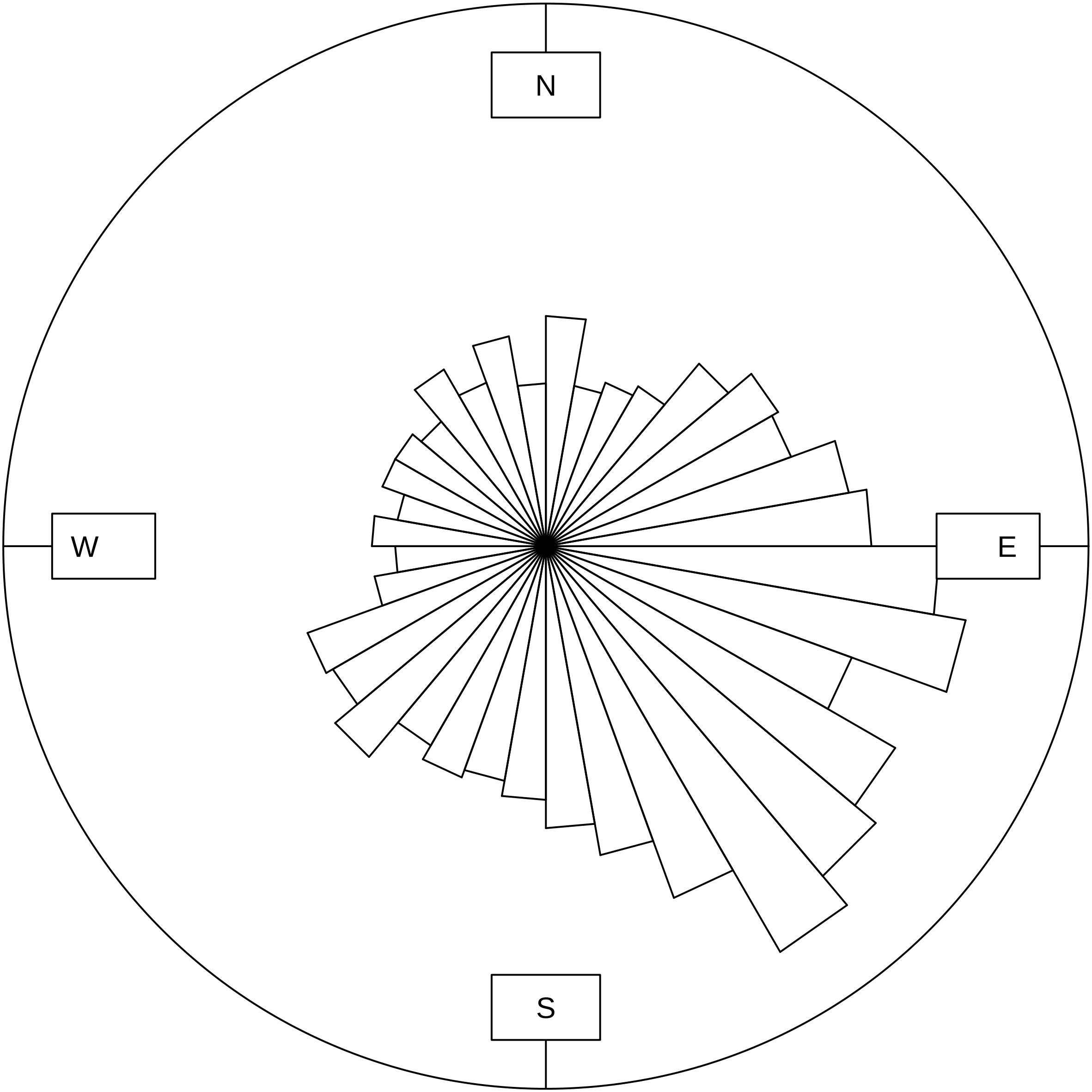

bootstrap 670

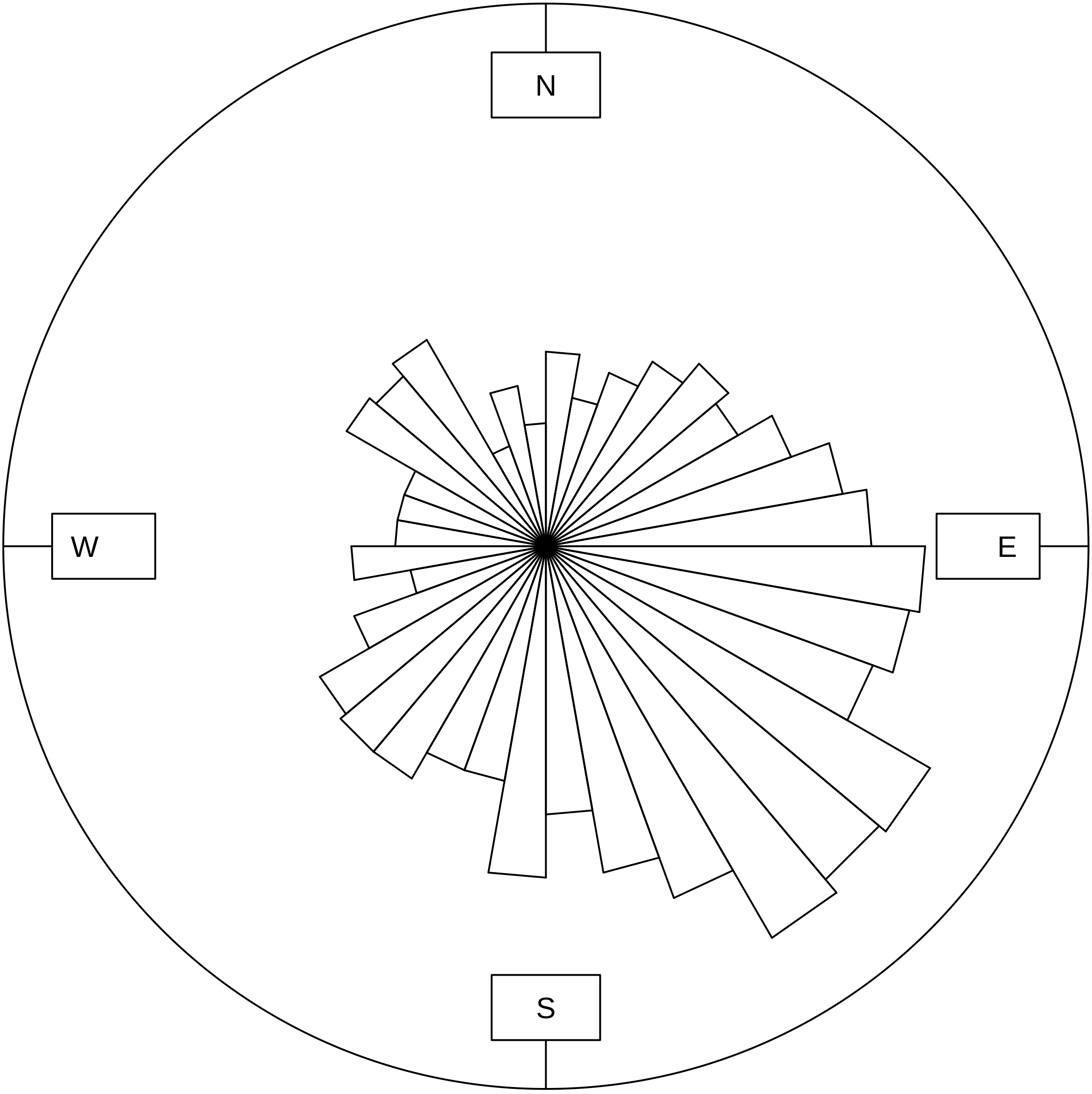

bootstrap 671

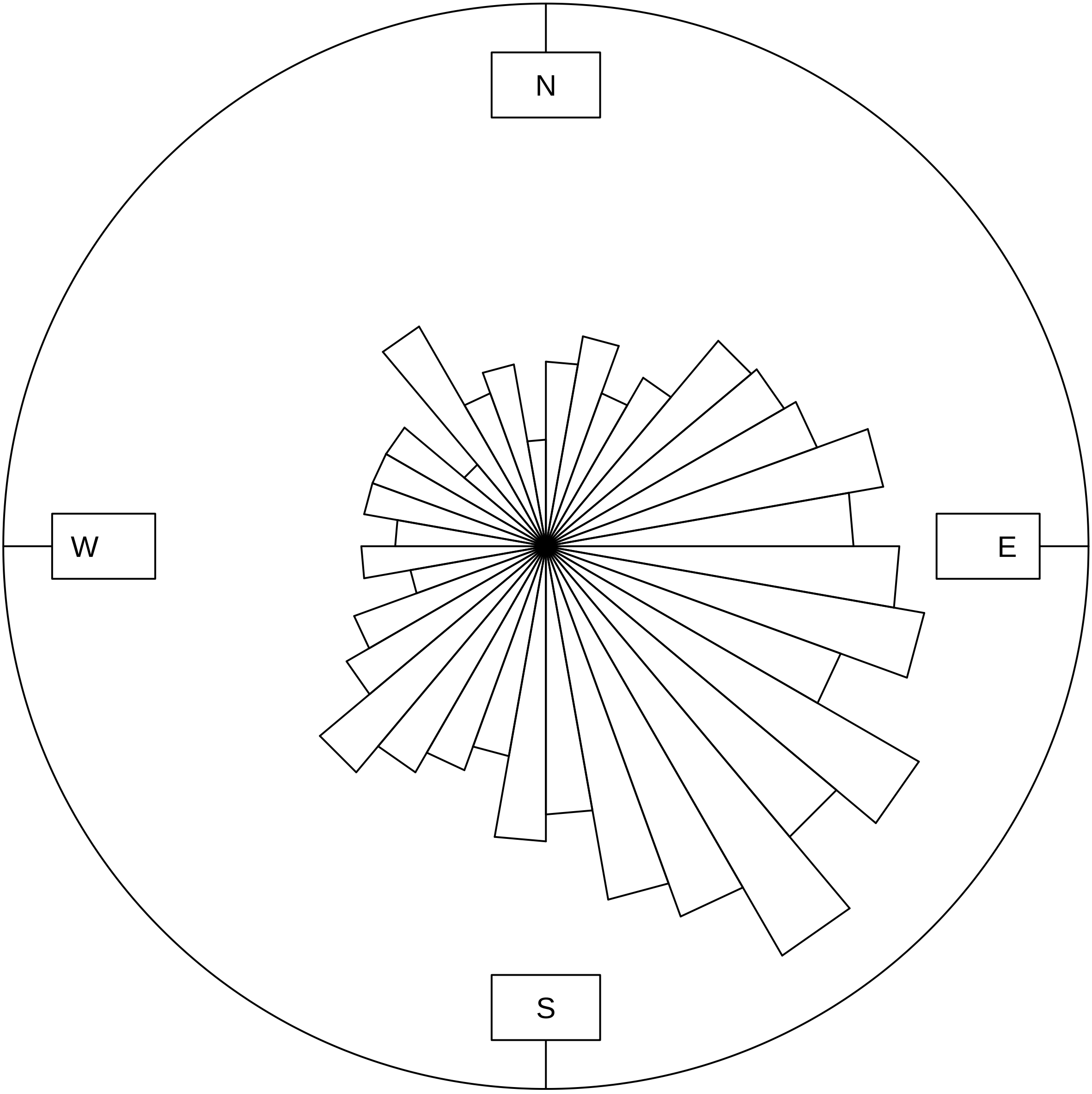

bootstrap 672

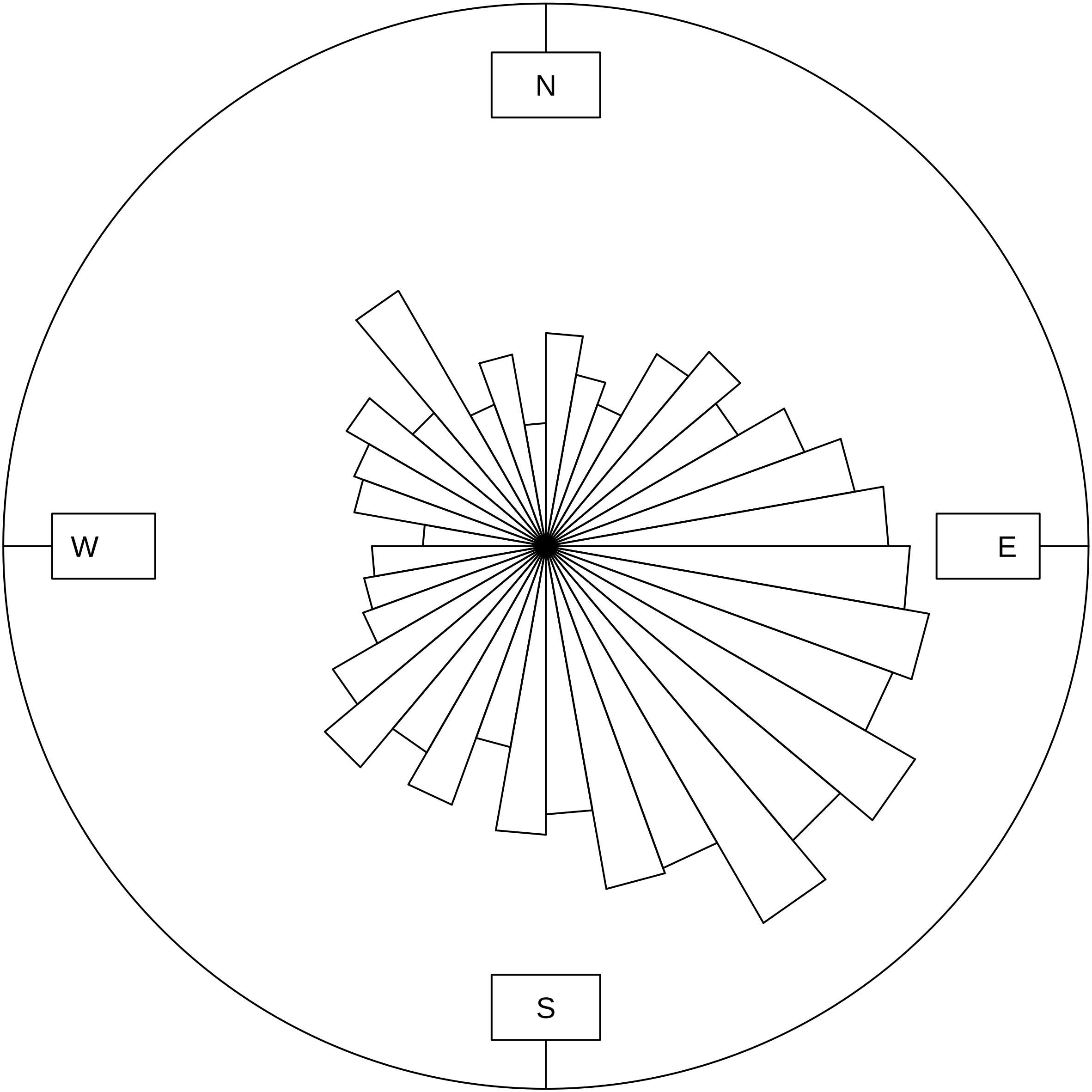

bootstrap 673

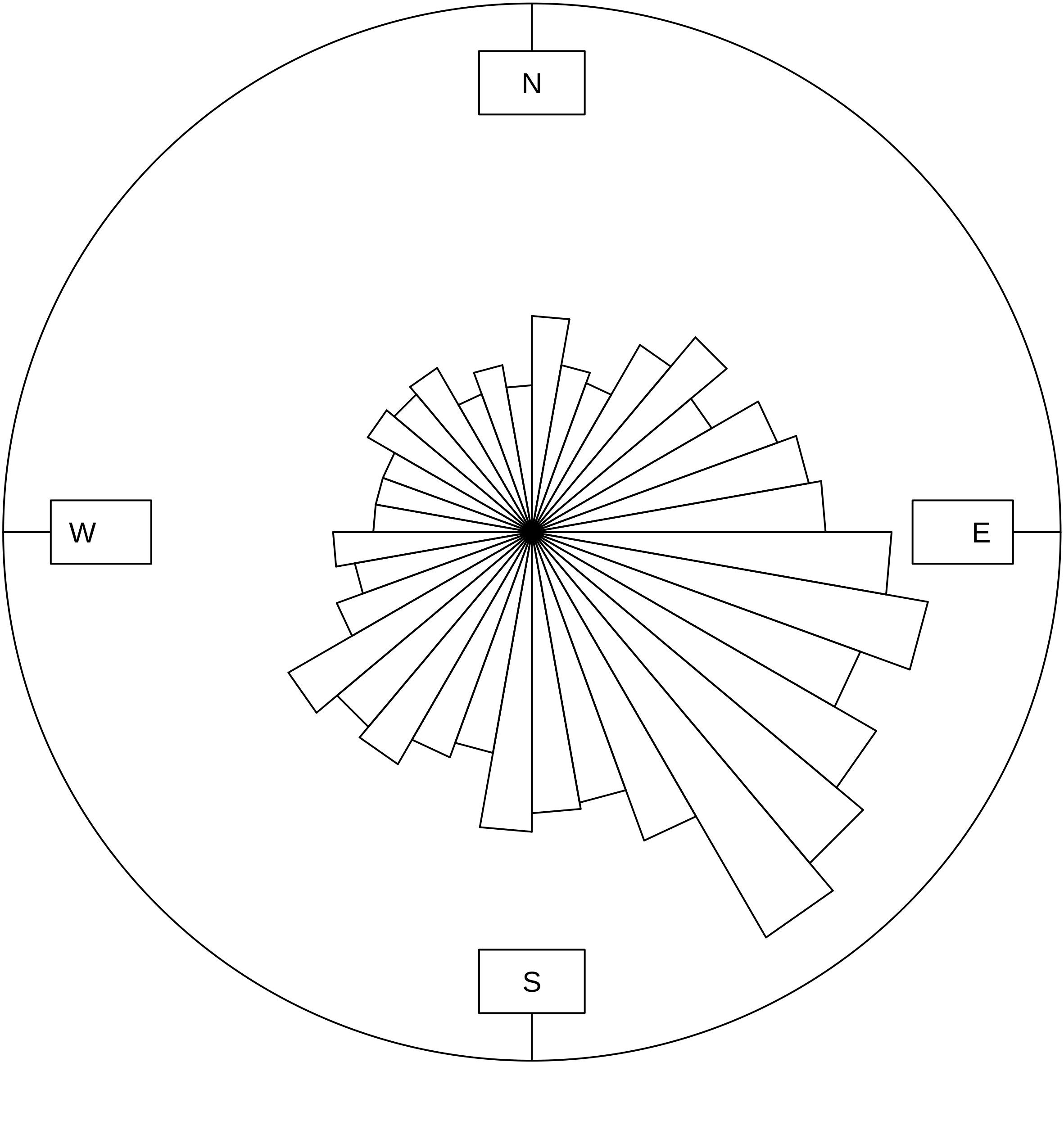

bootstrap 674

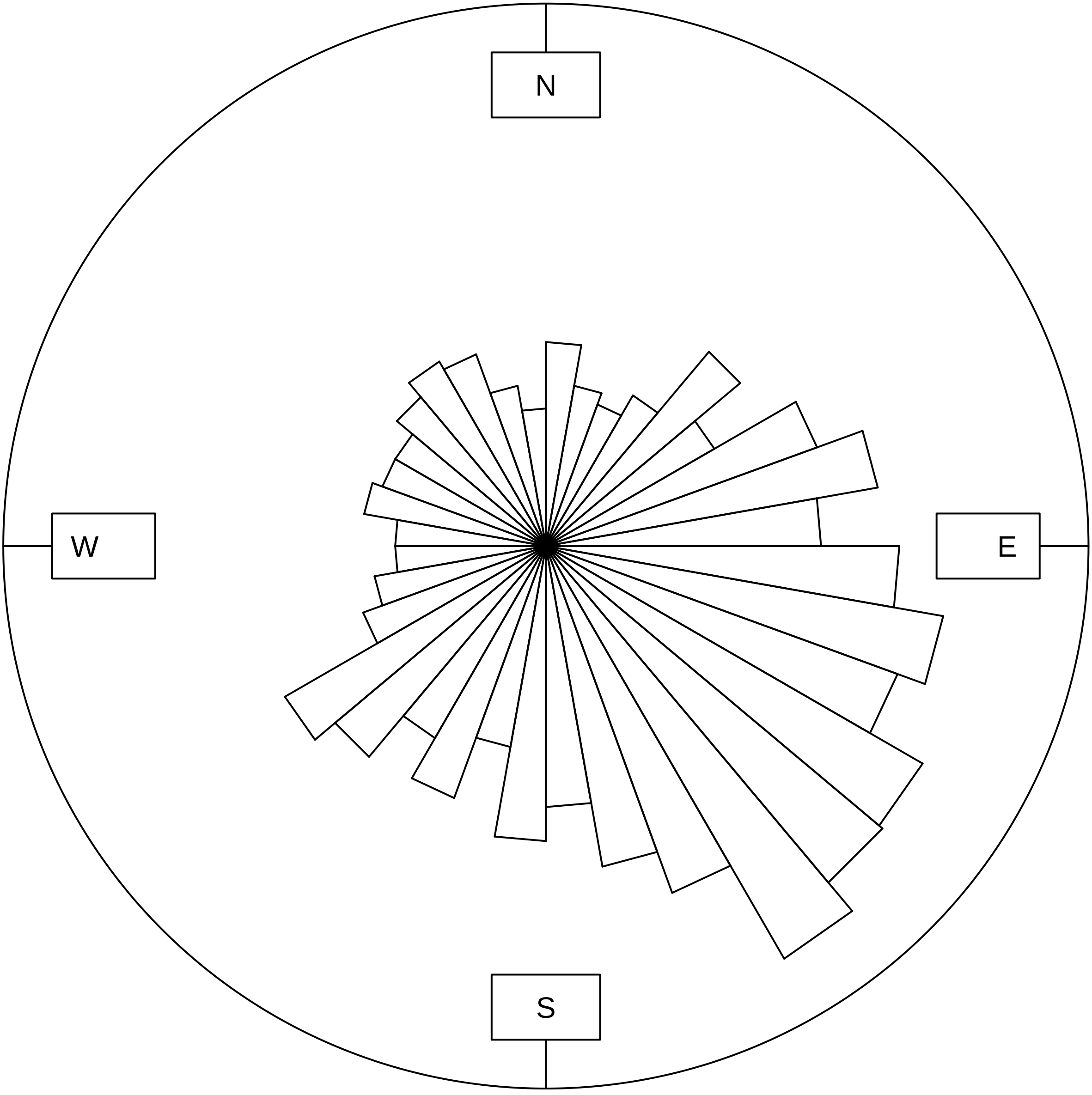

bootstrap 675

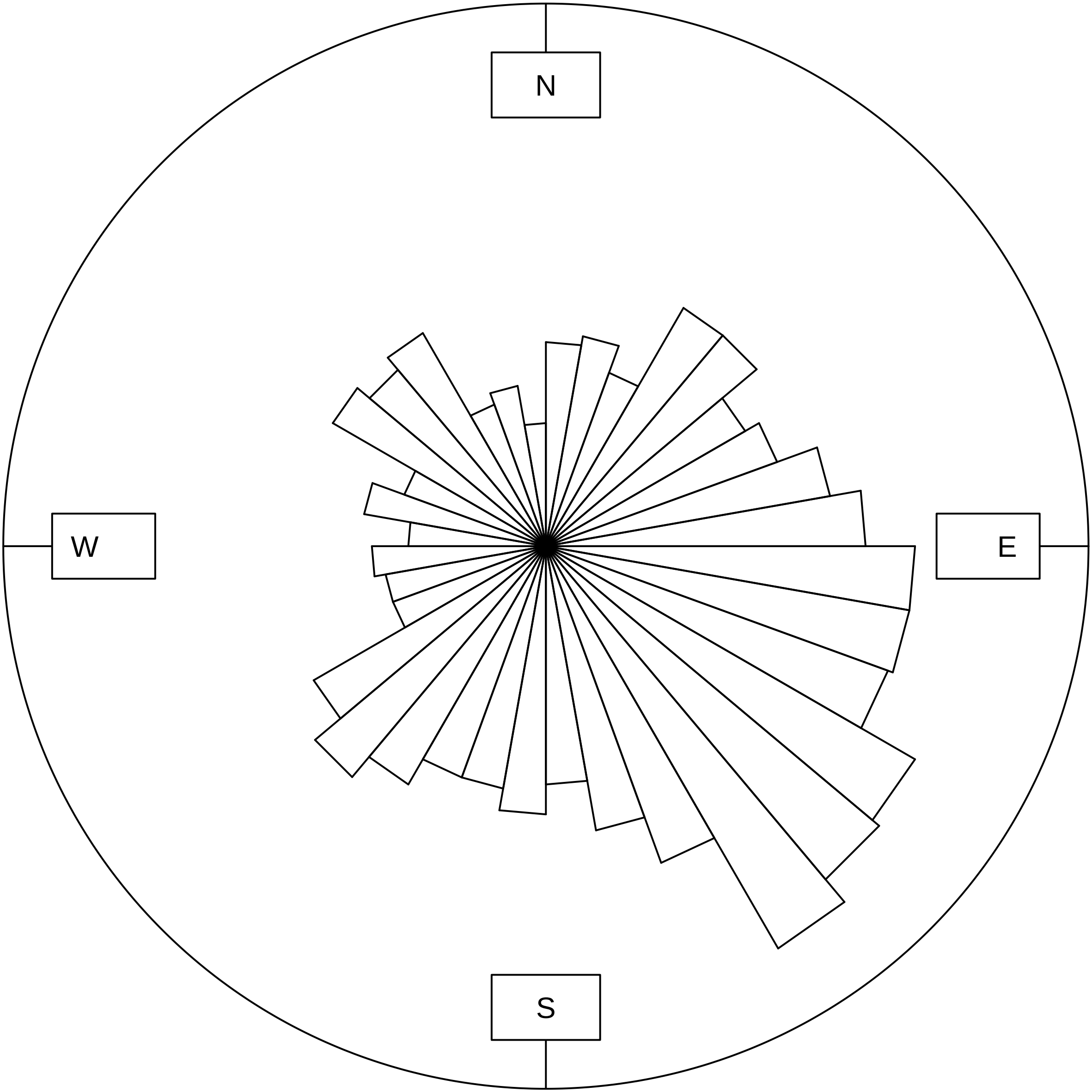

bootstrap 676

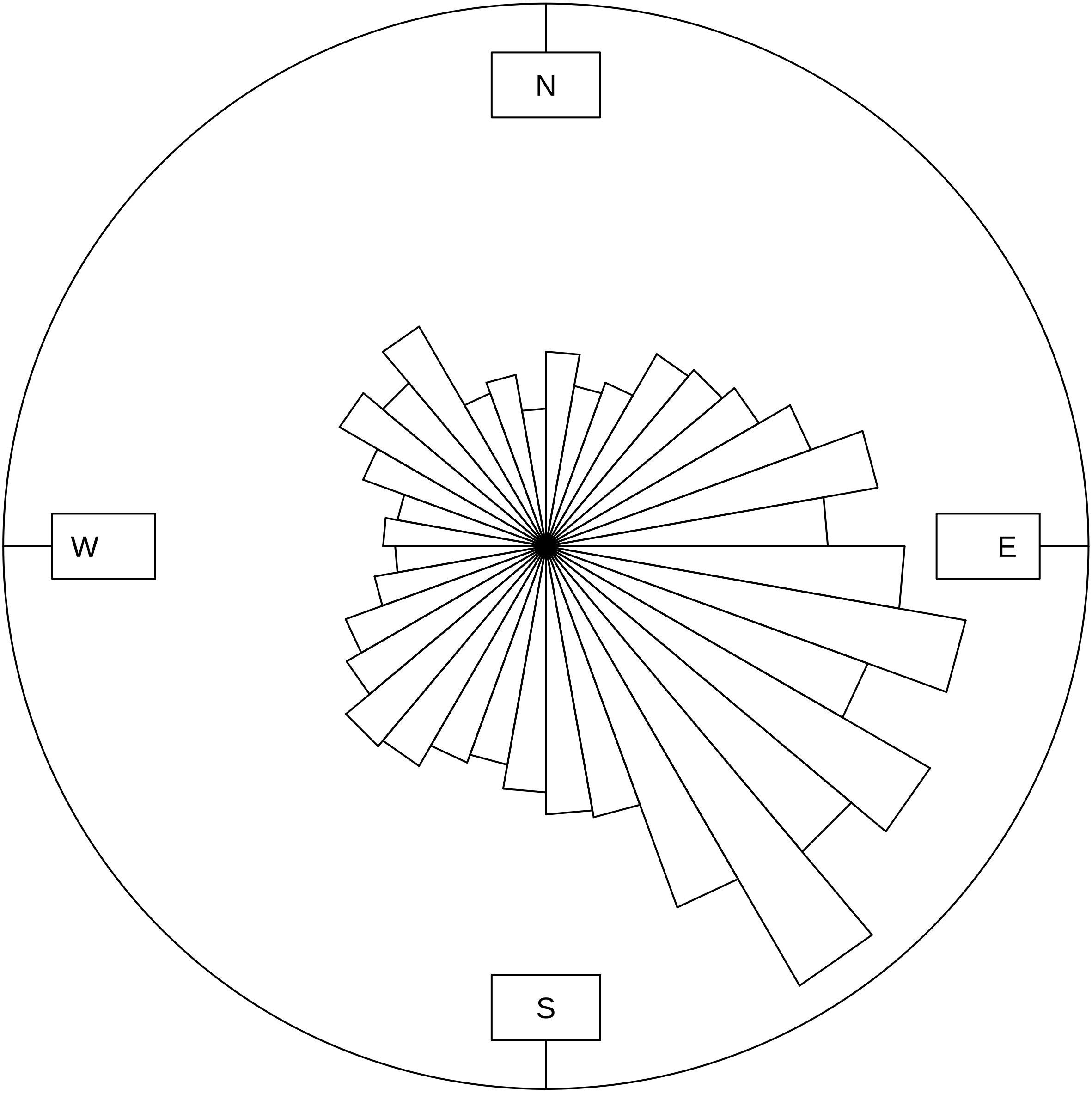

bootstrap 677

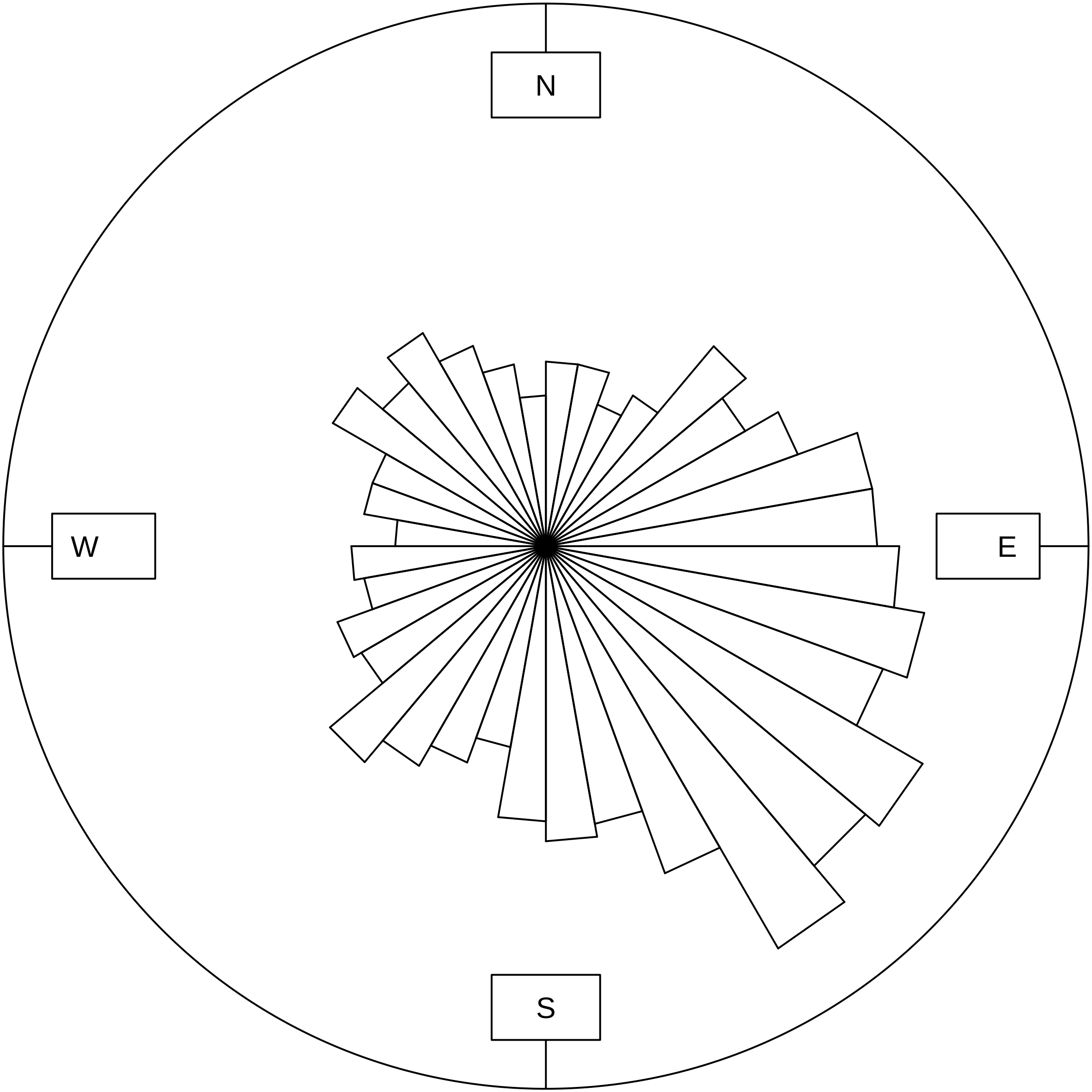

bootstrap 678

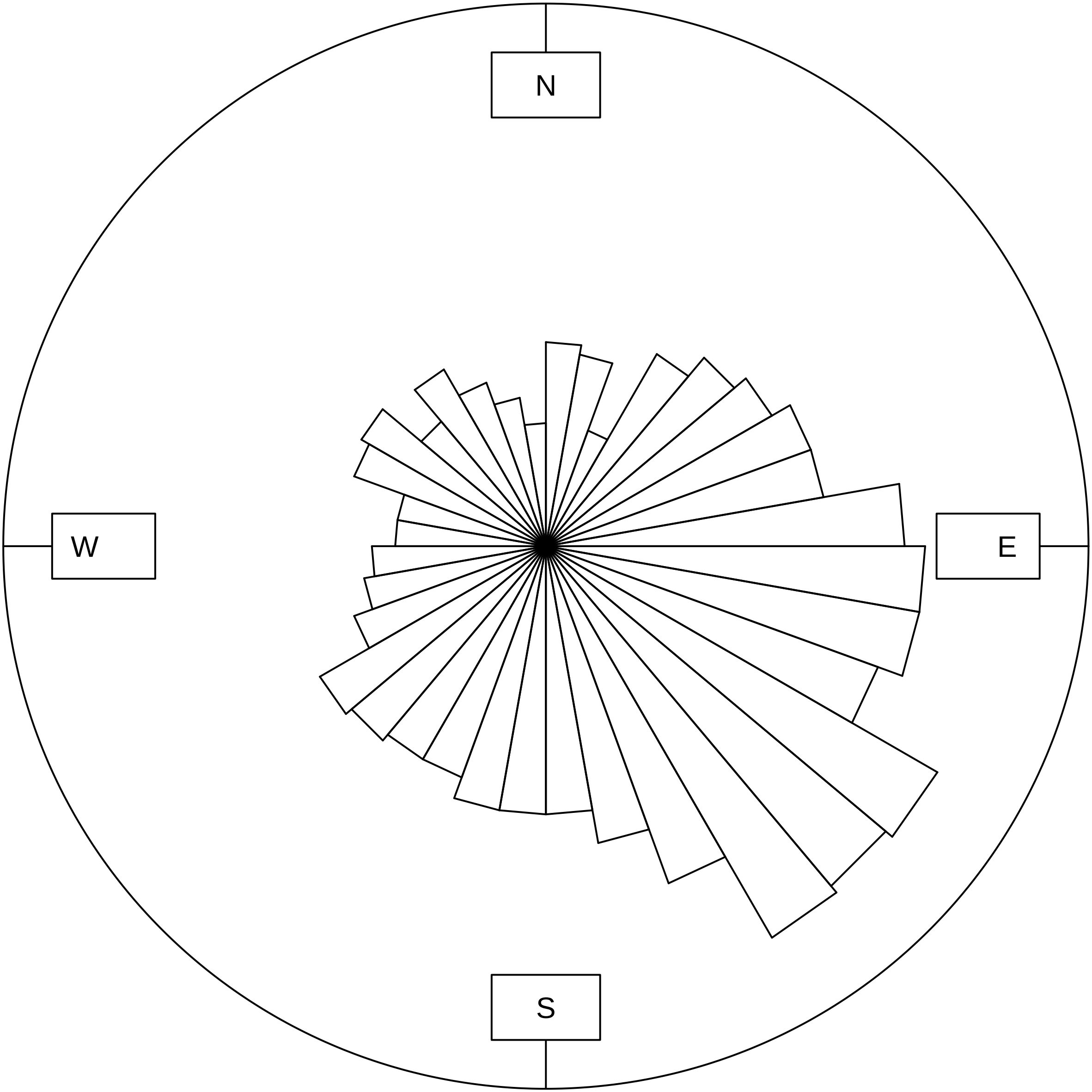

bootstrap 679

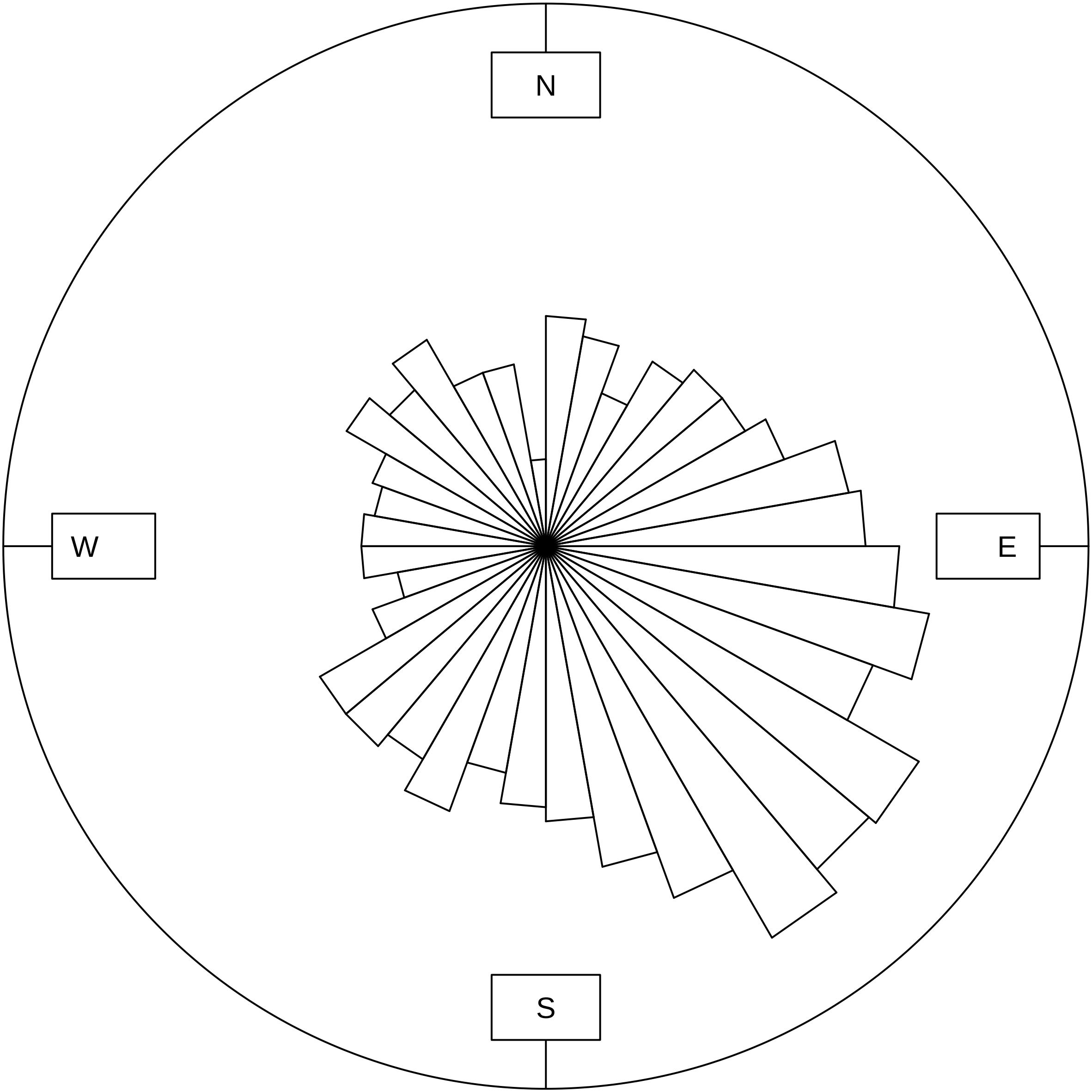

bootstrap 680

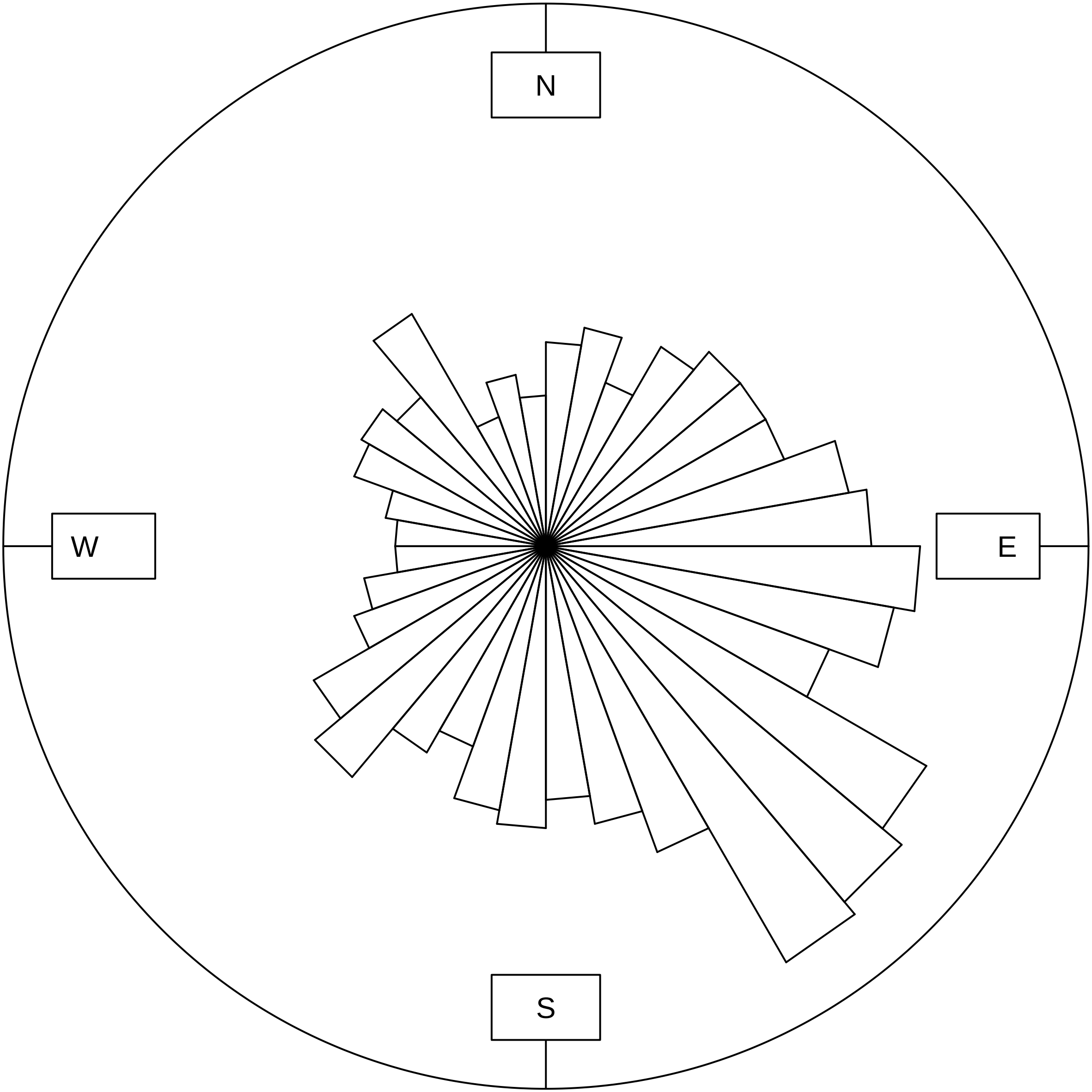

bootstrap 681

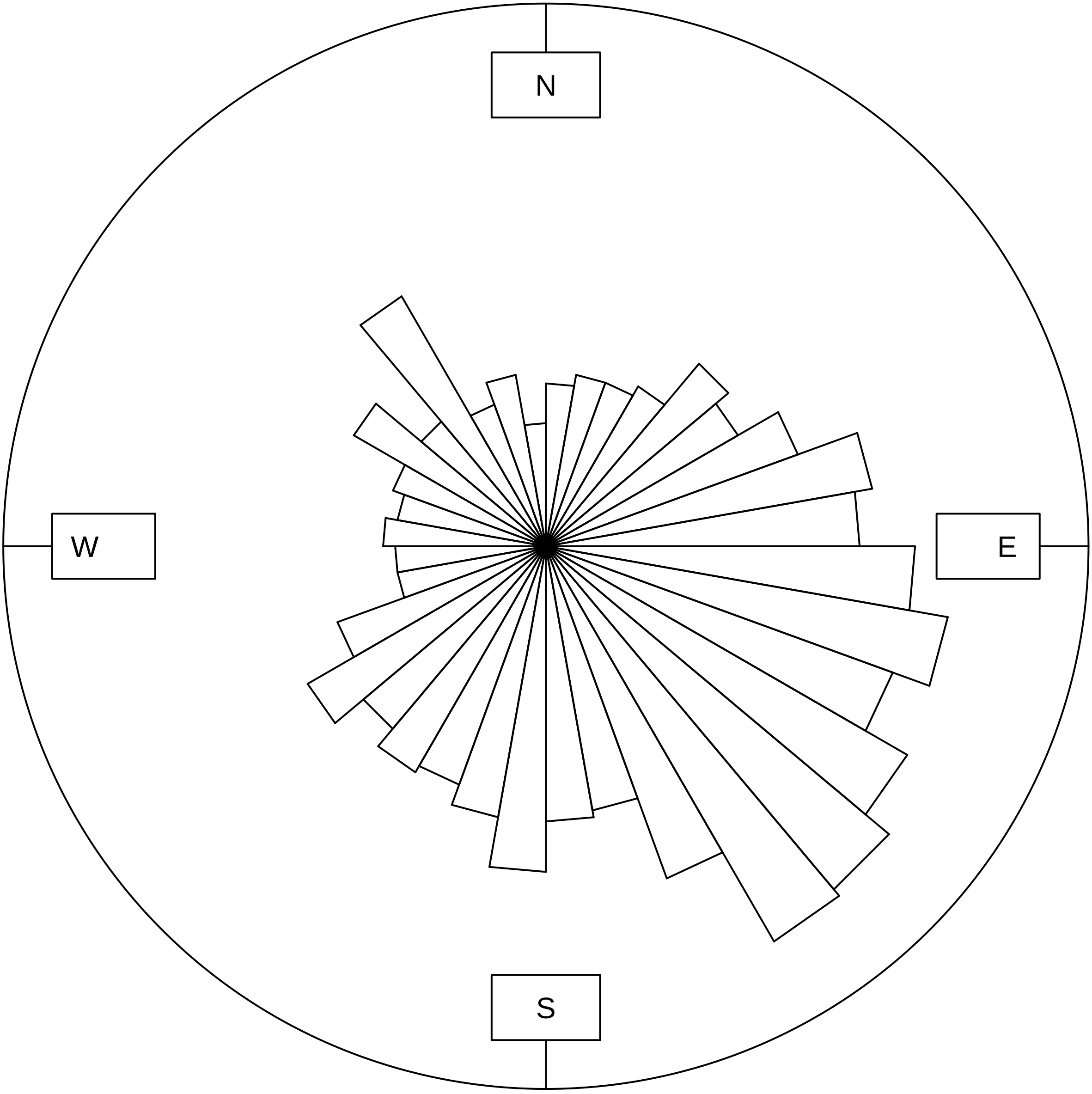

bootstrap 682

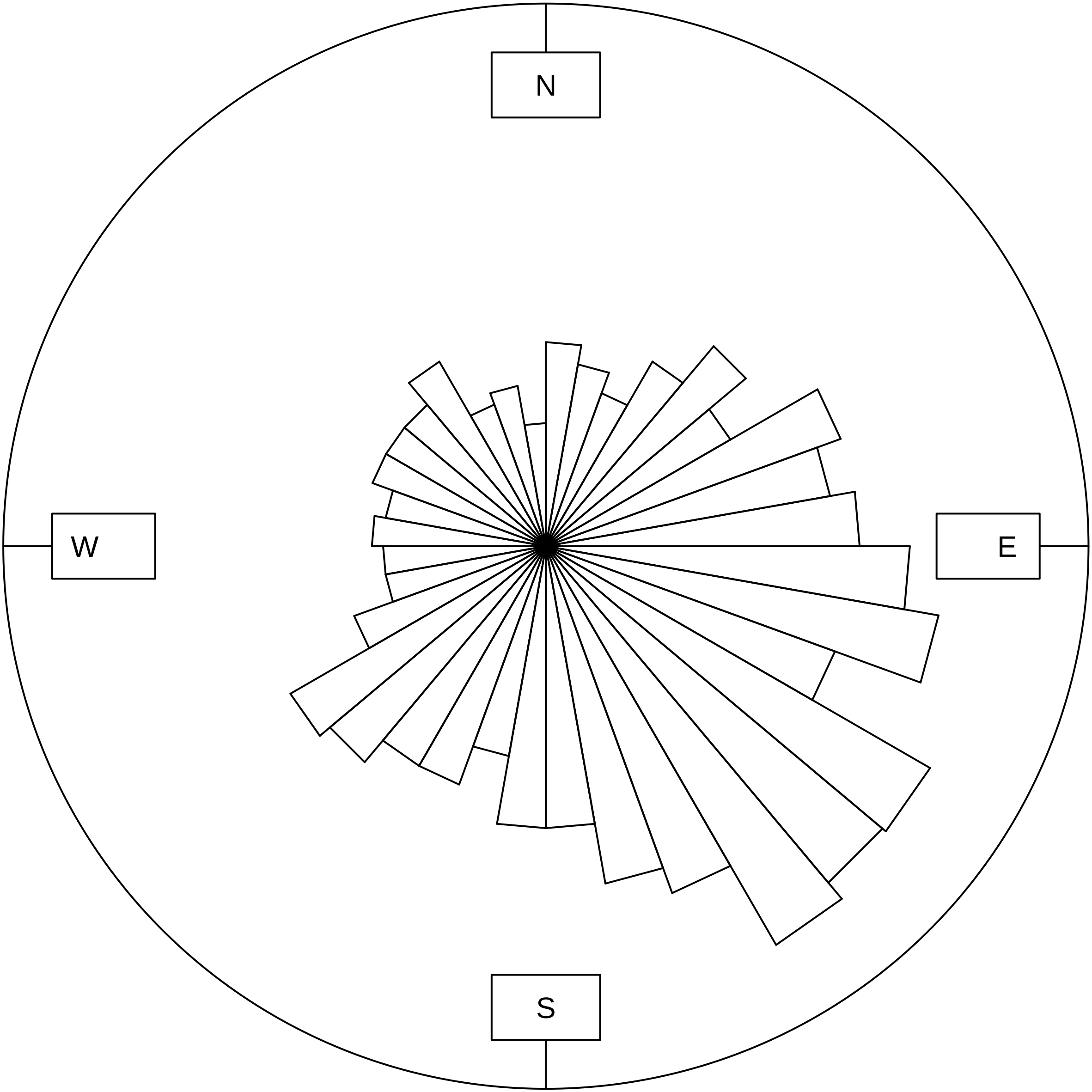

bootstrap 683

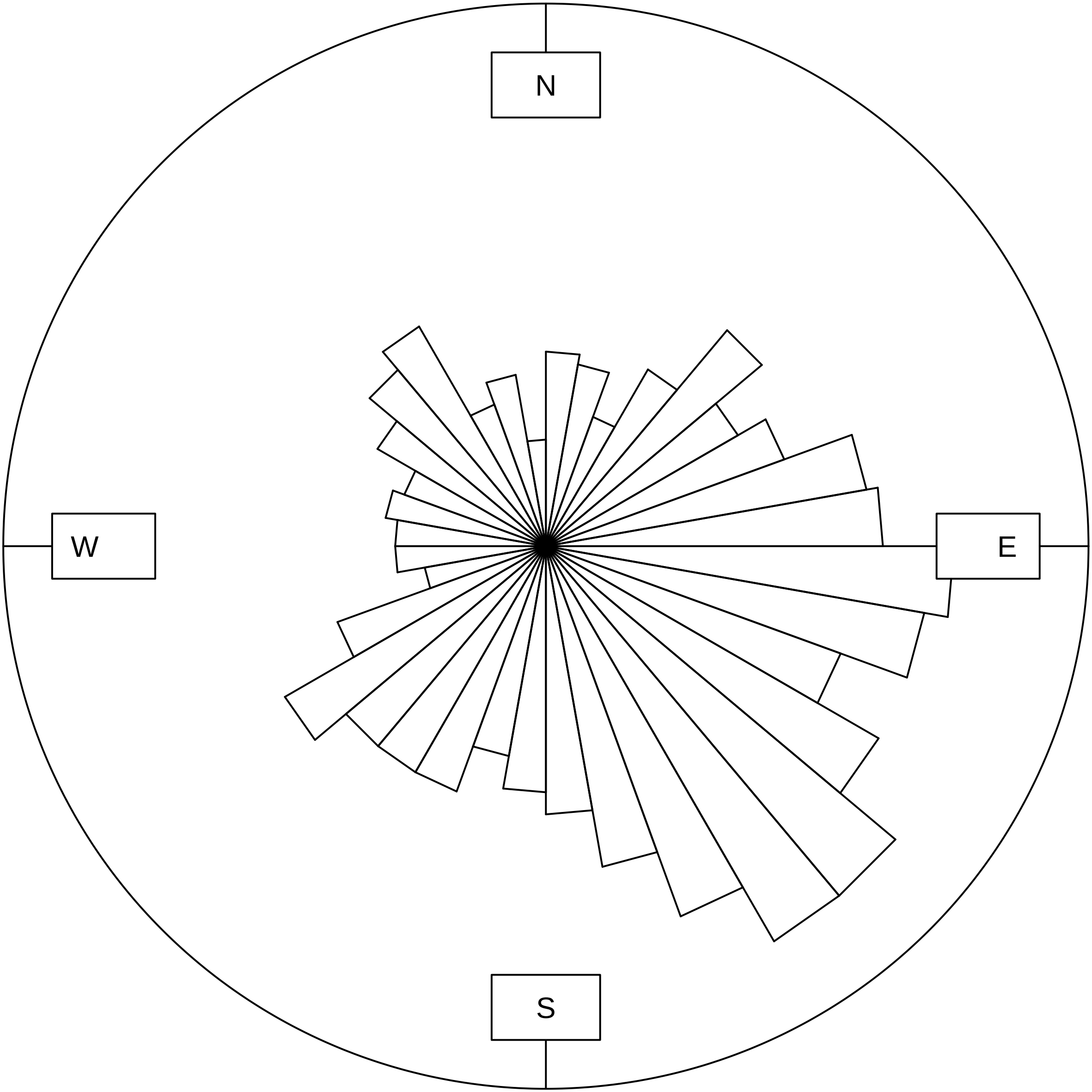

bootstrap 684

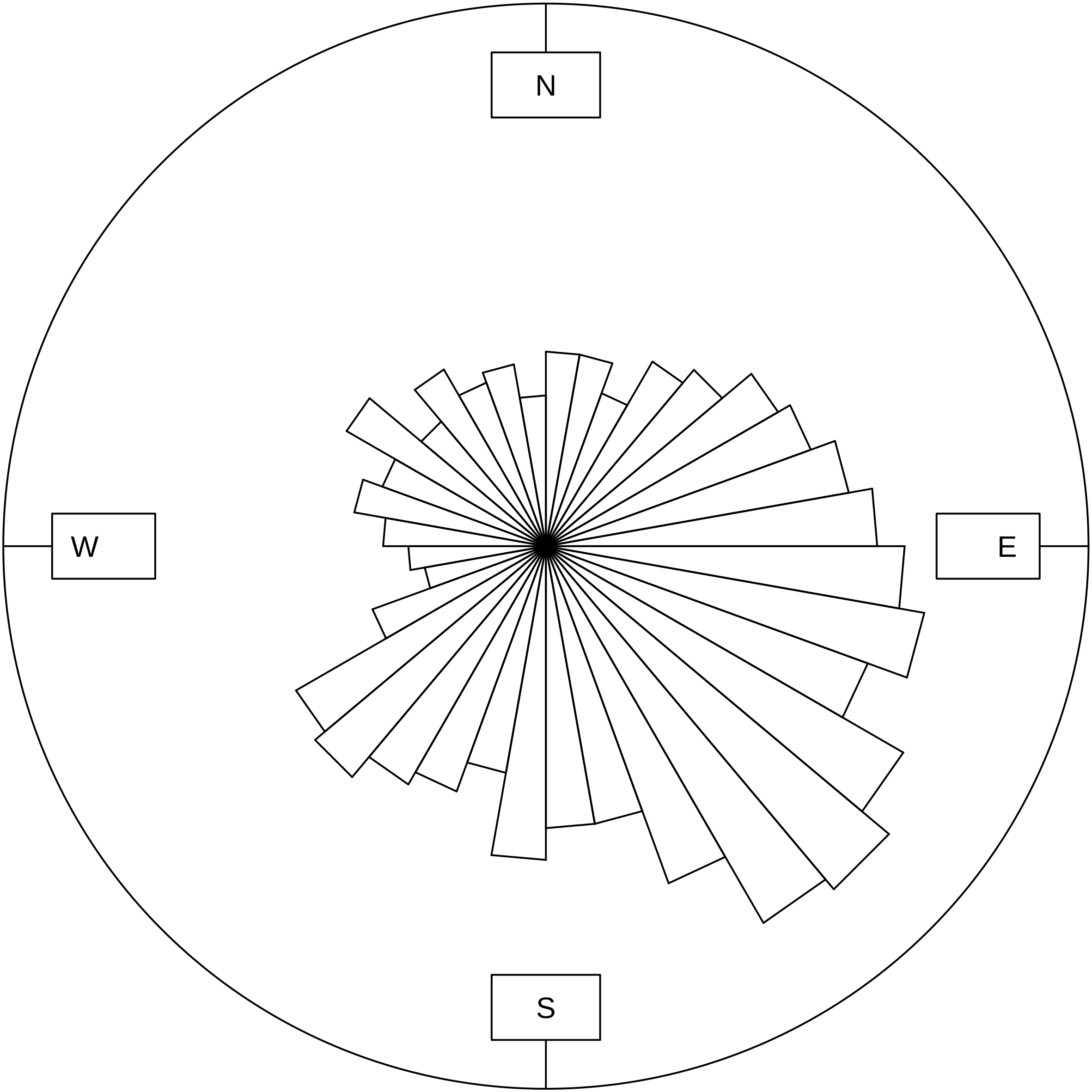

bootstrap 685

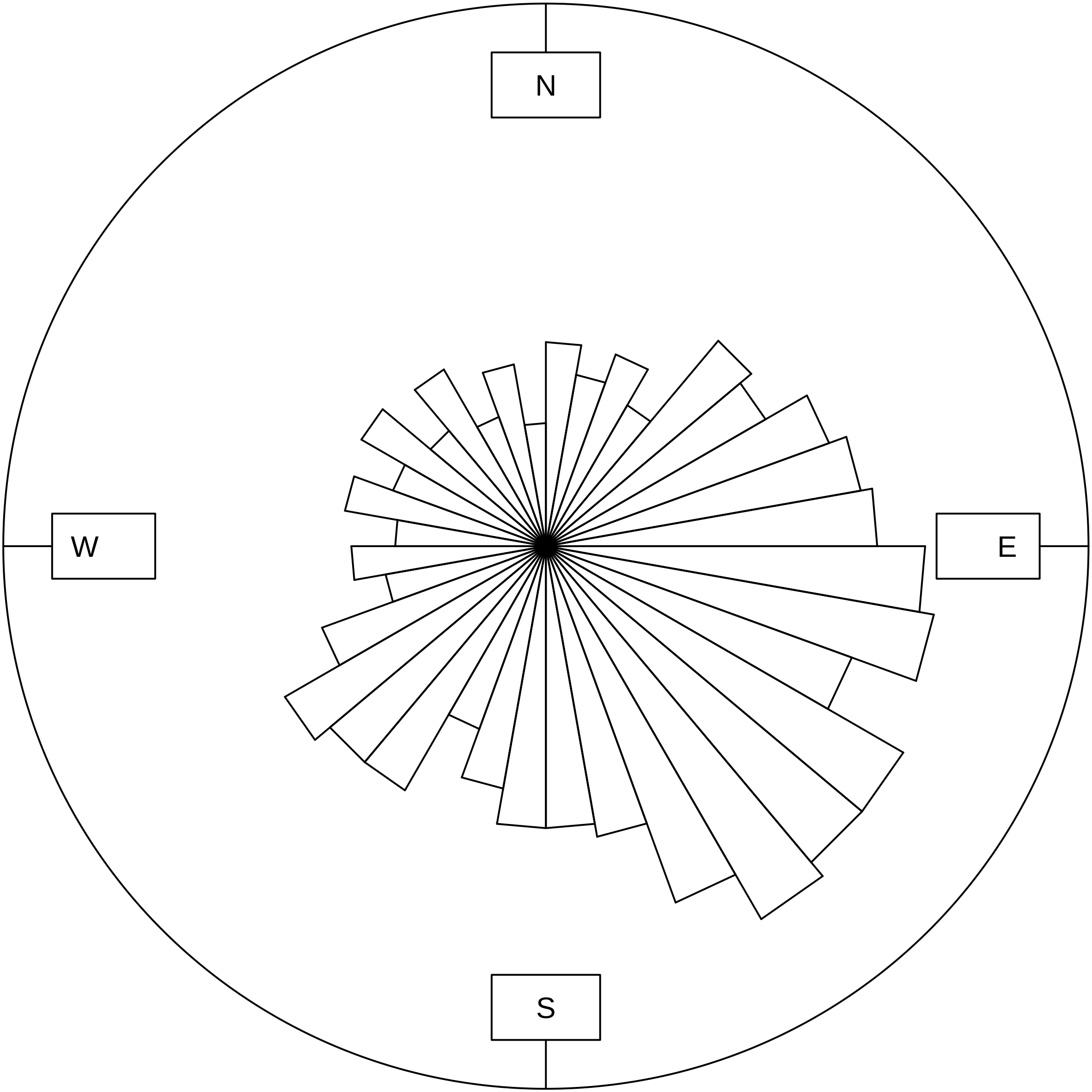

bootstrap 686

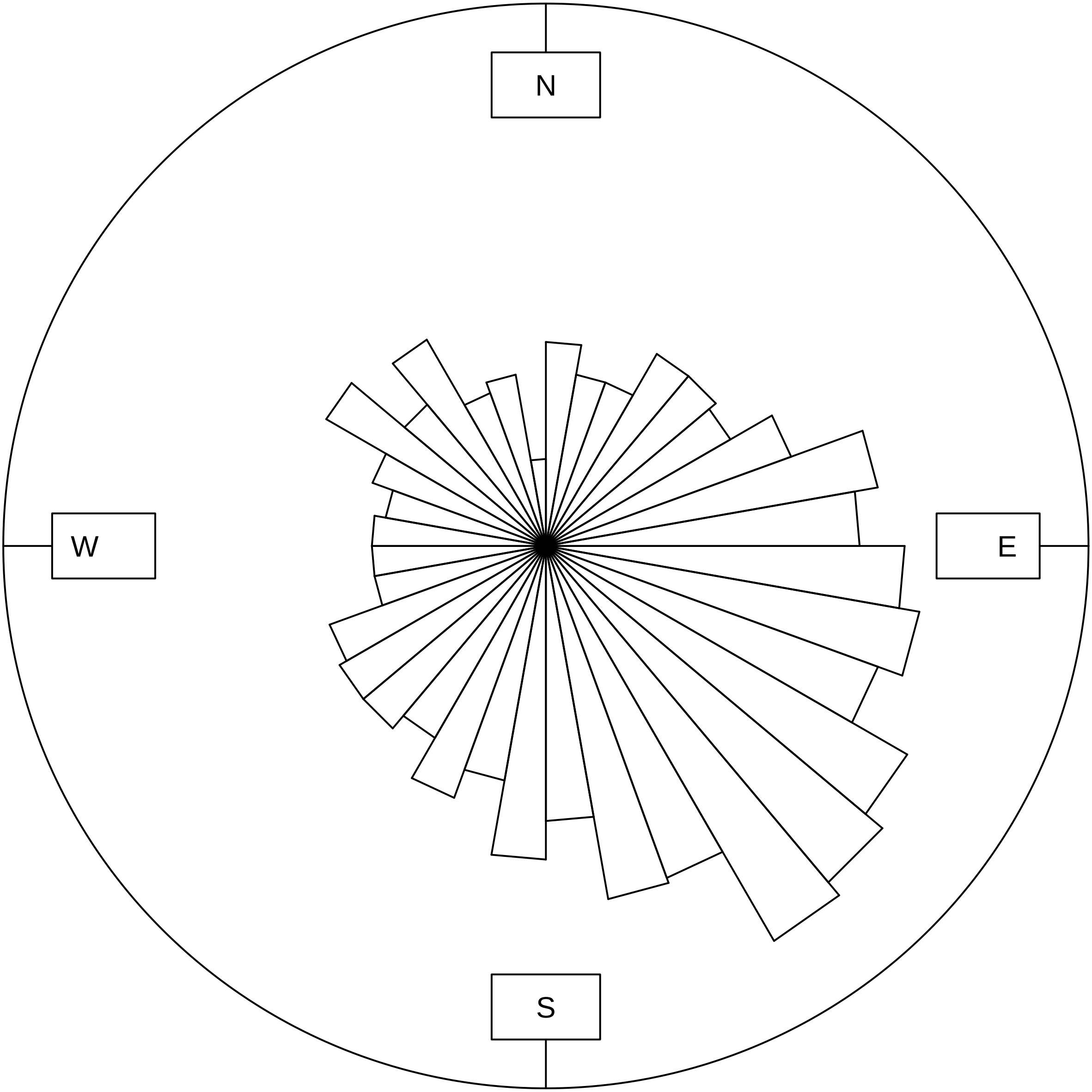

bootstrap 687

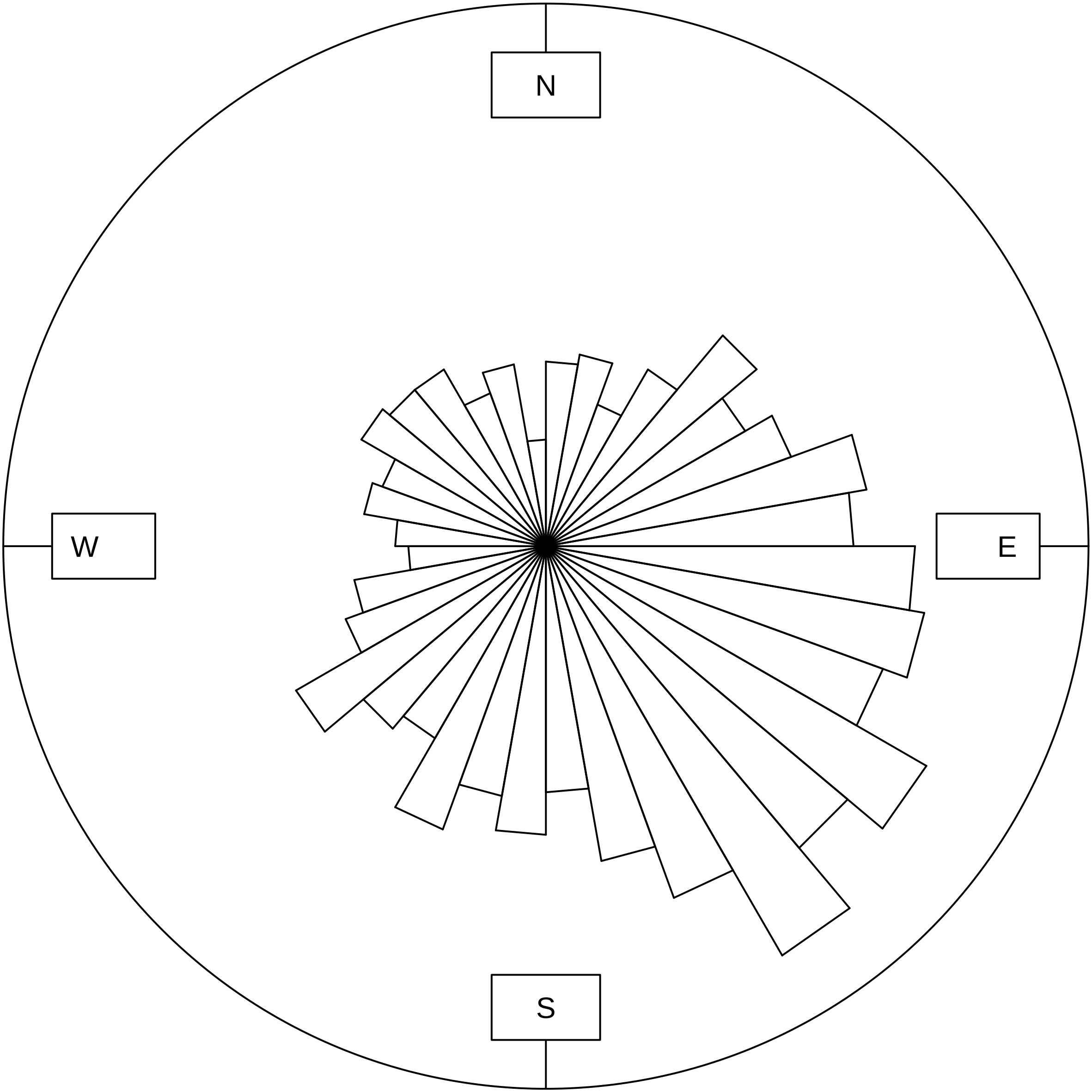

bootstrap 688

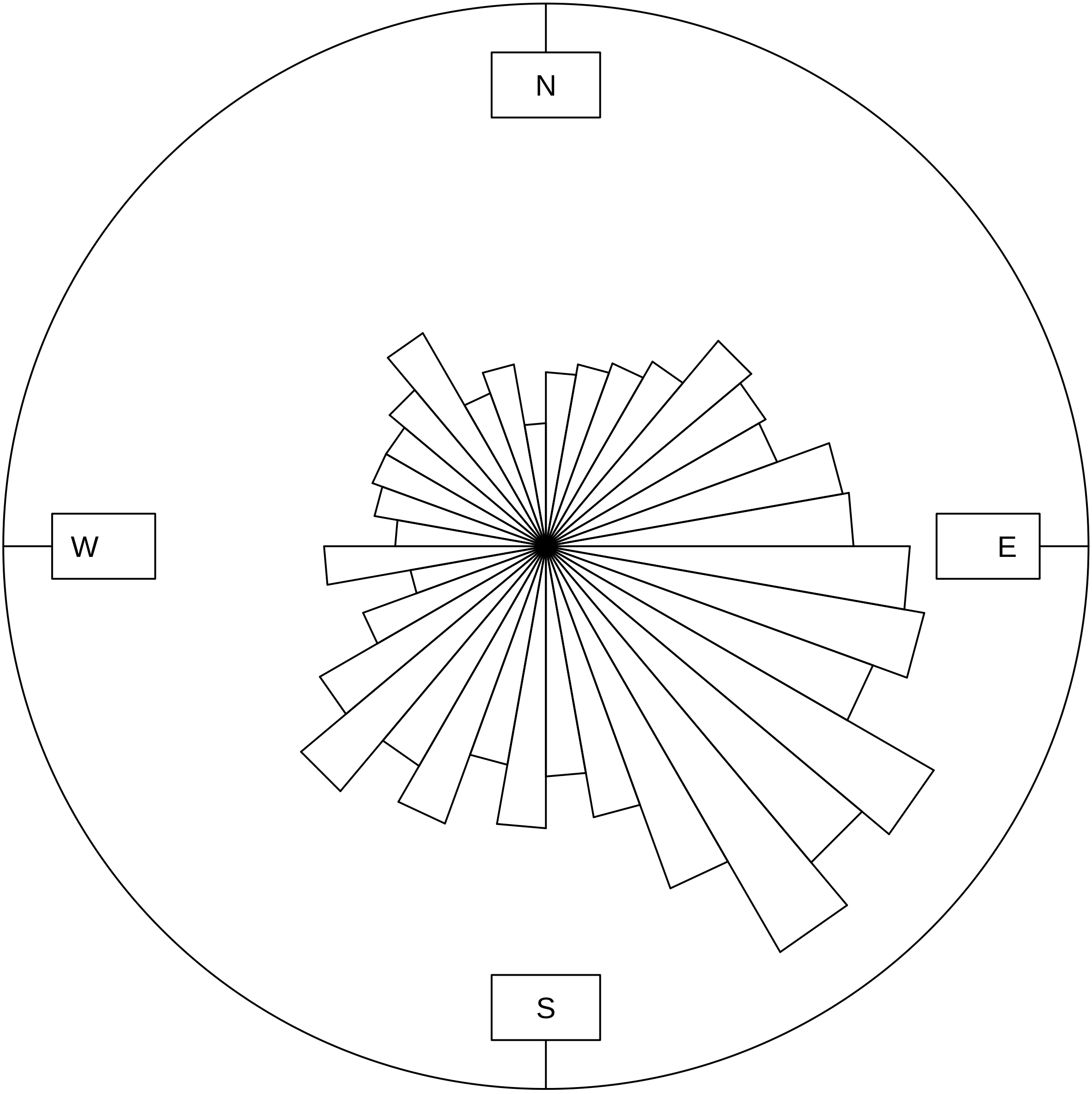

bootstrap 689

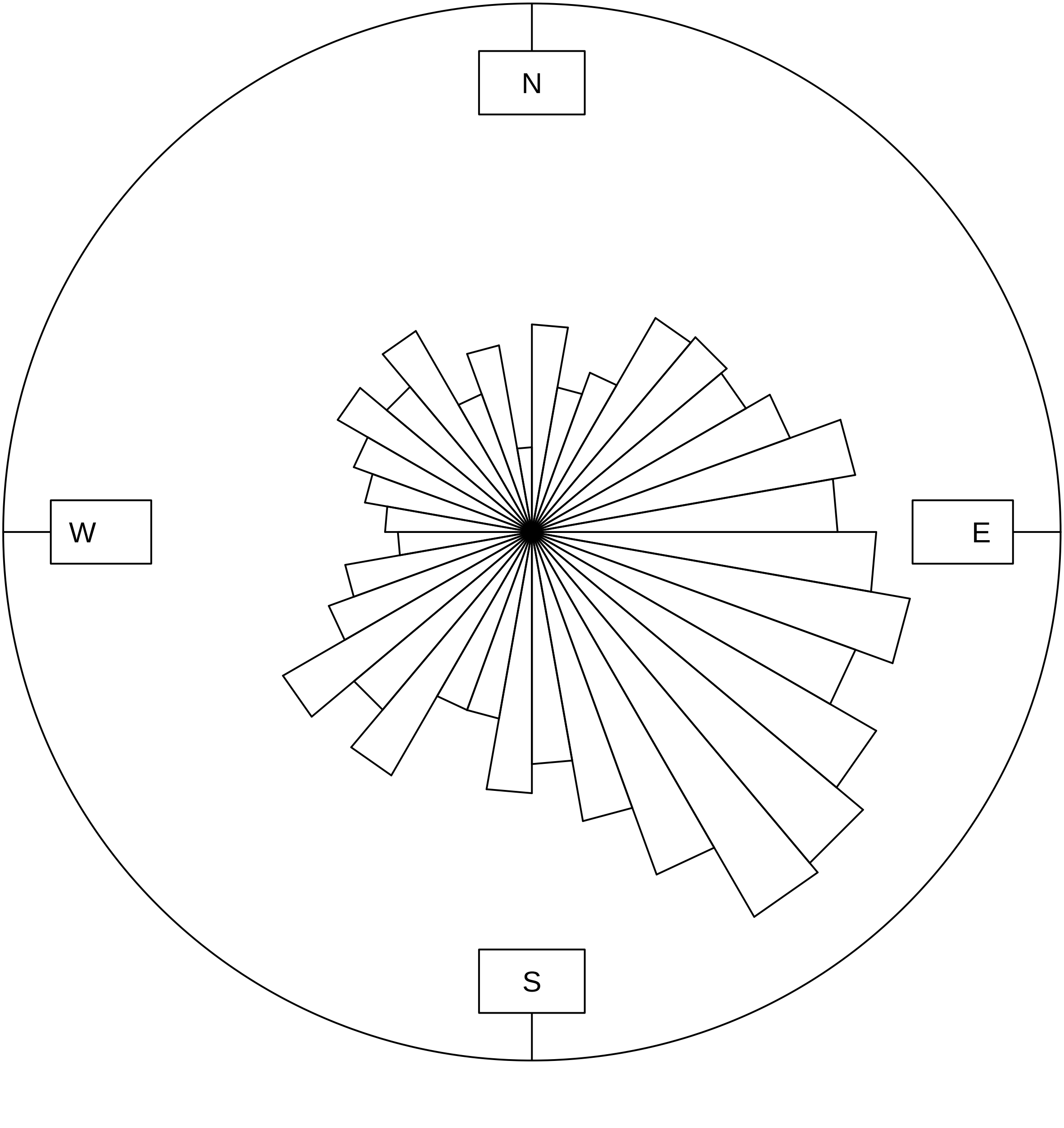

bootstrap 690

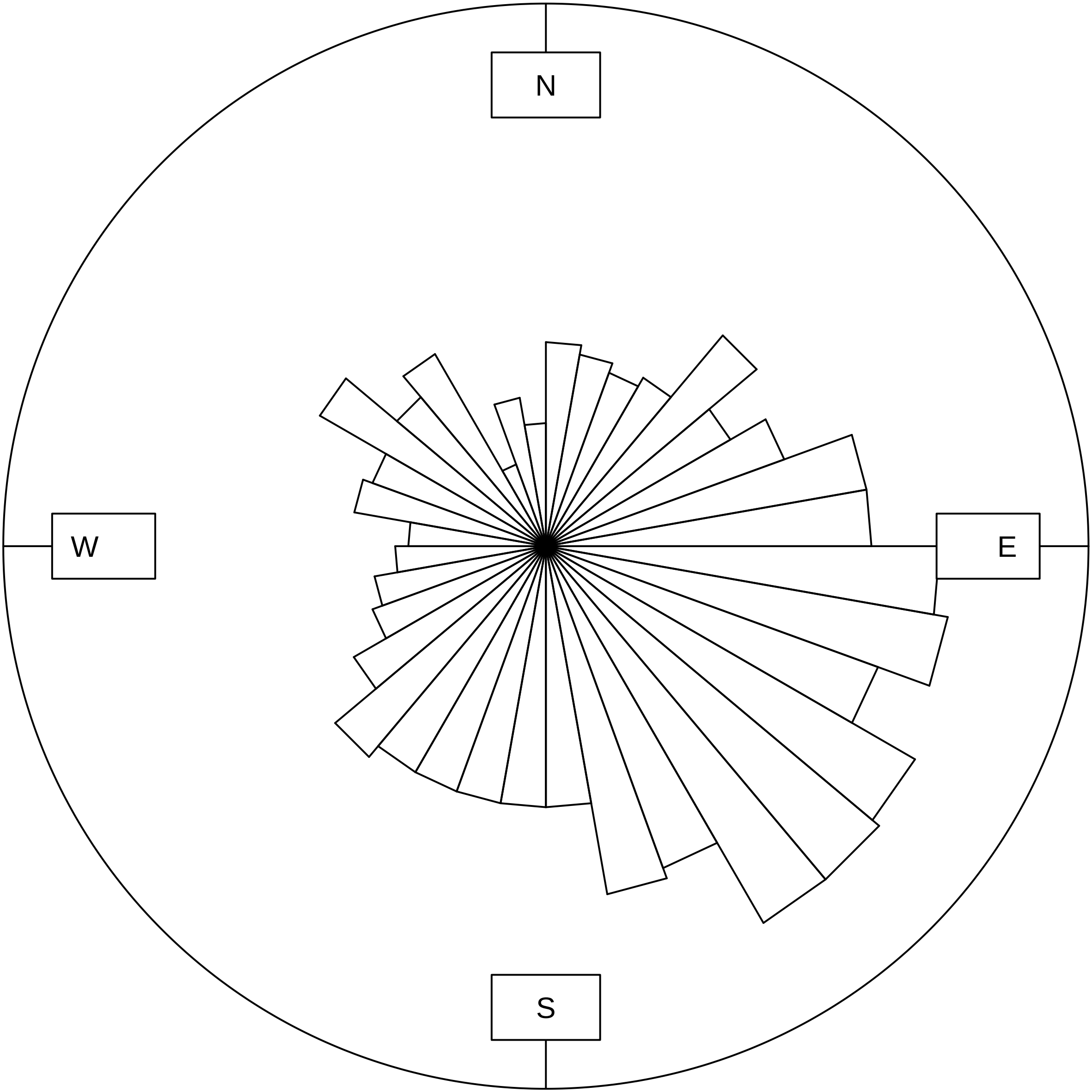

bootstrap 691

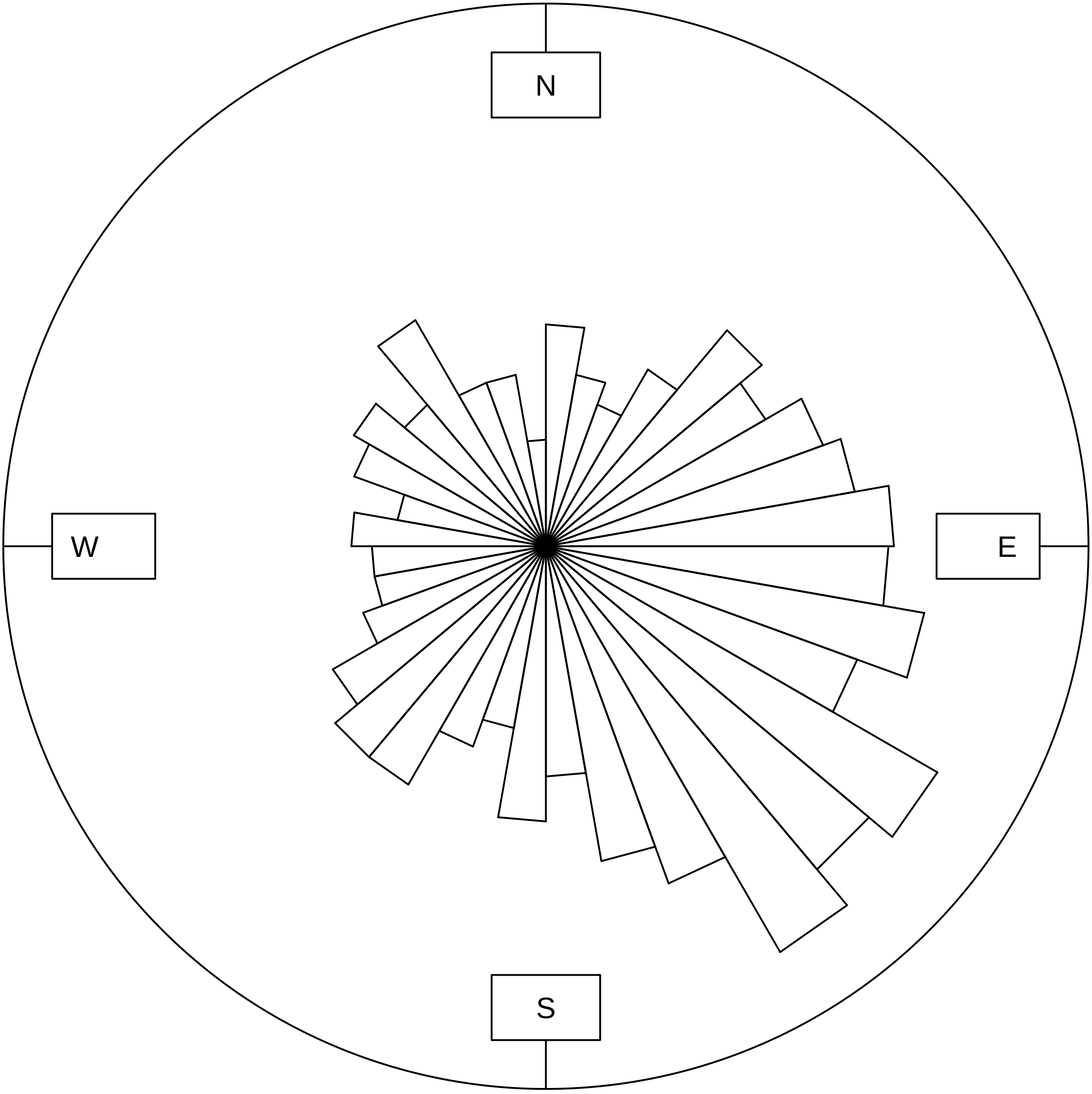

bootstrap 692

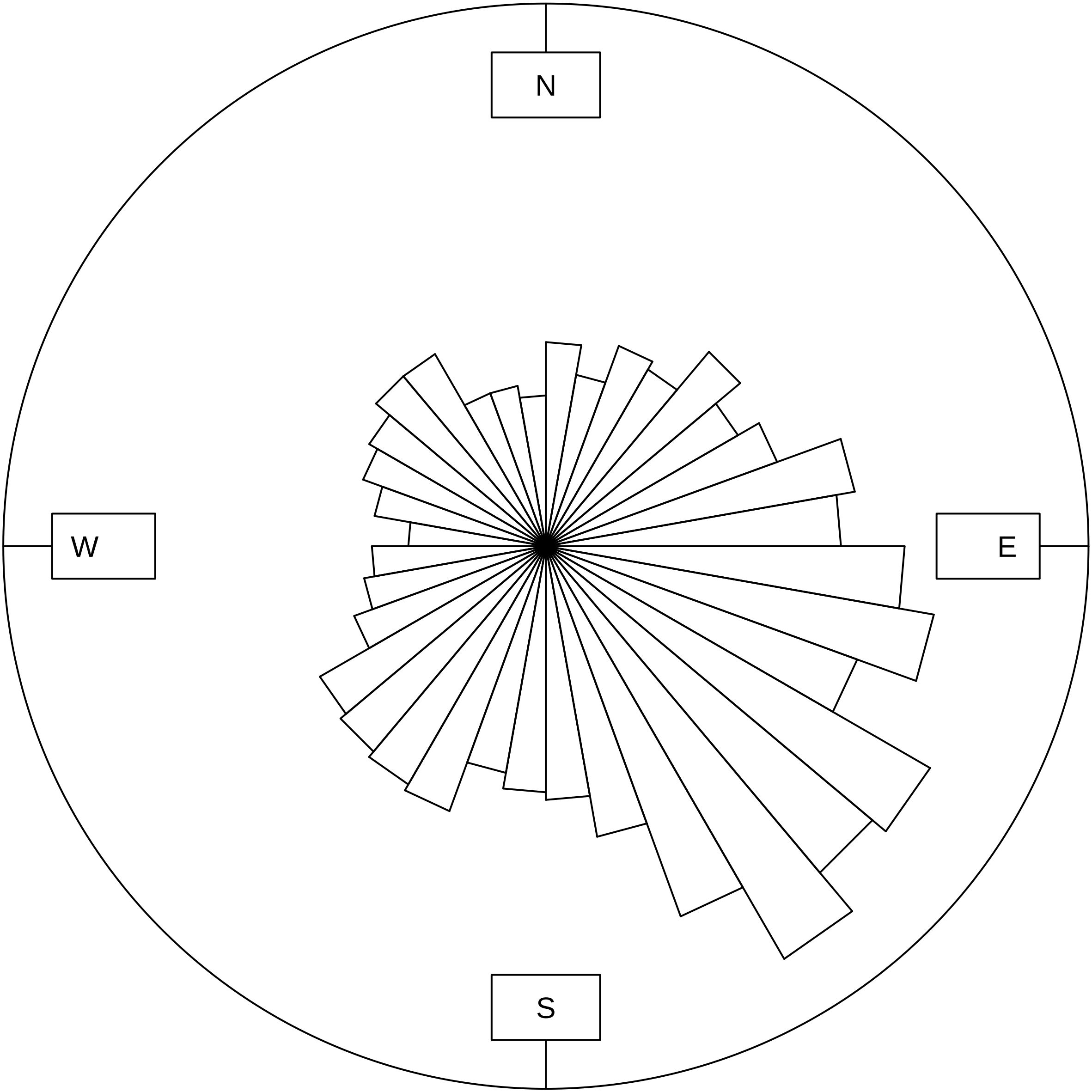

bootstrap 693

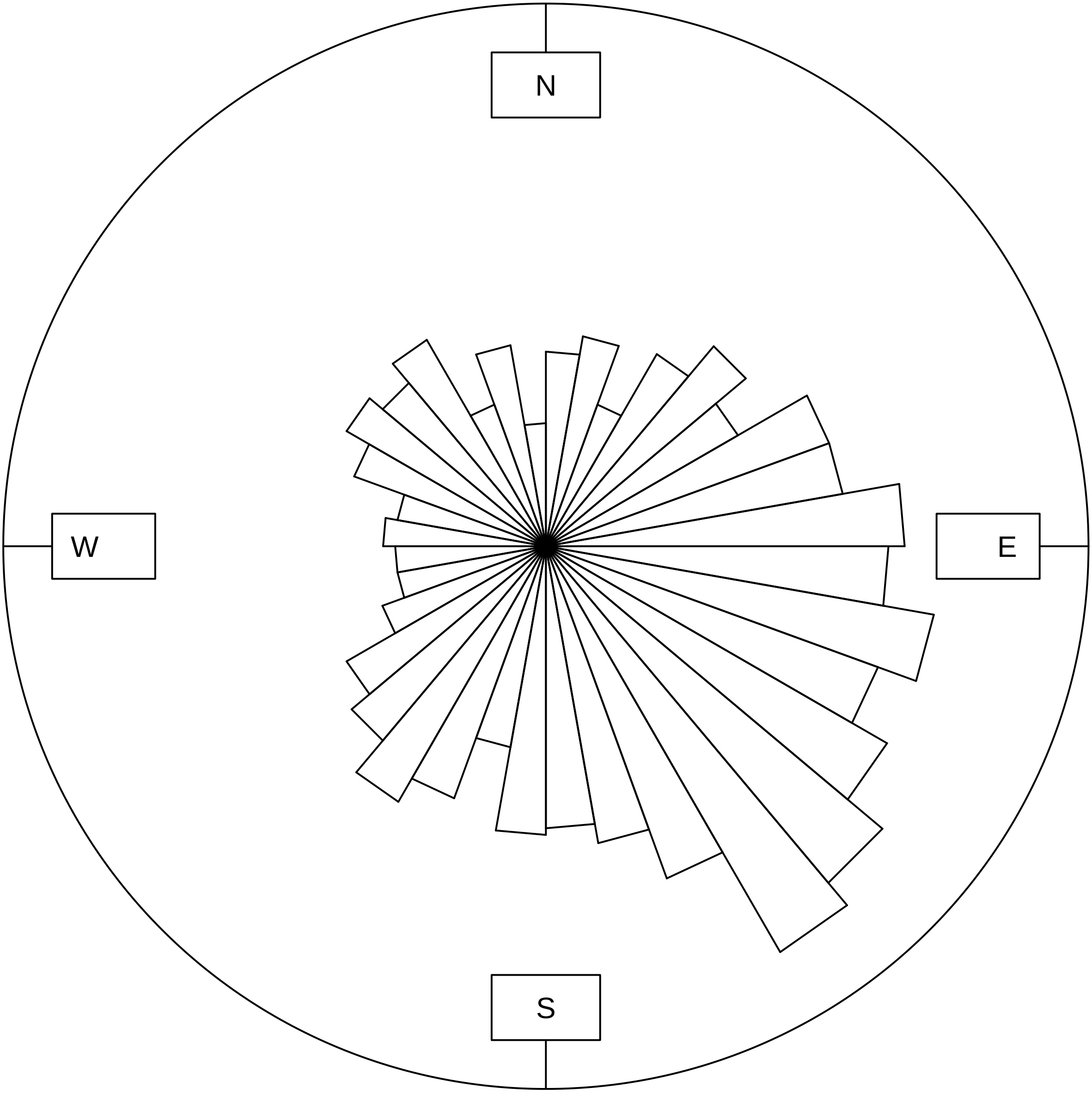

bootstrap 694

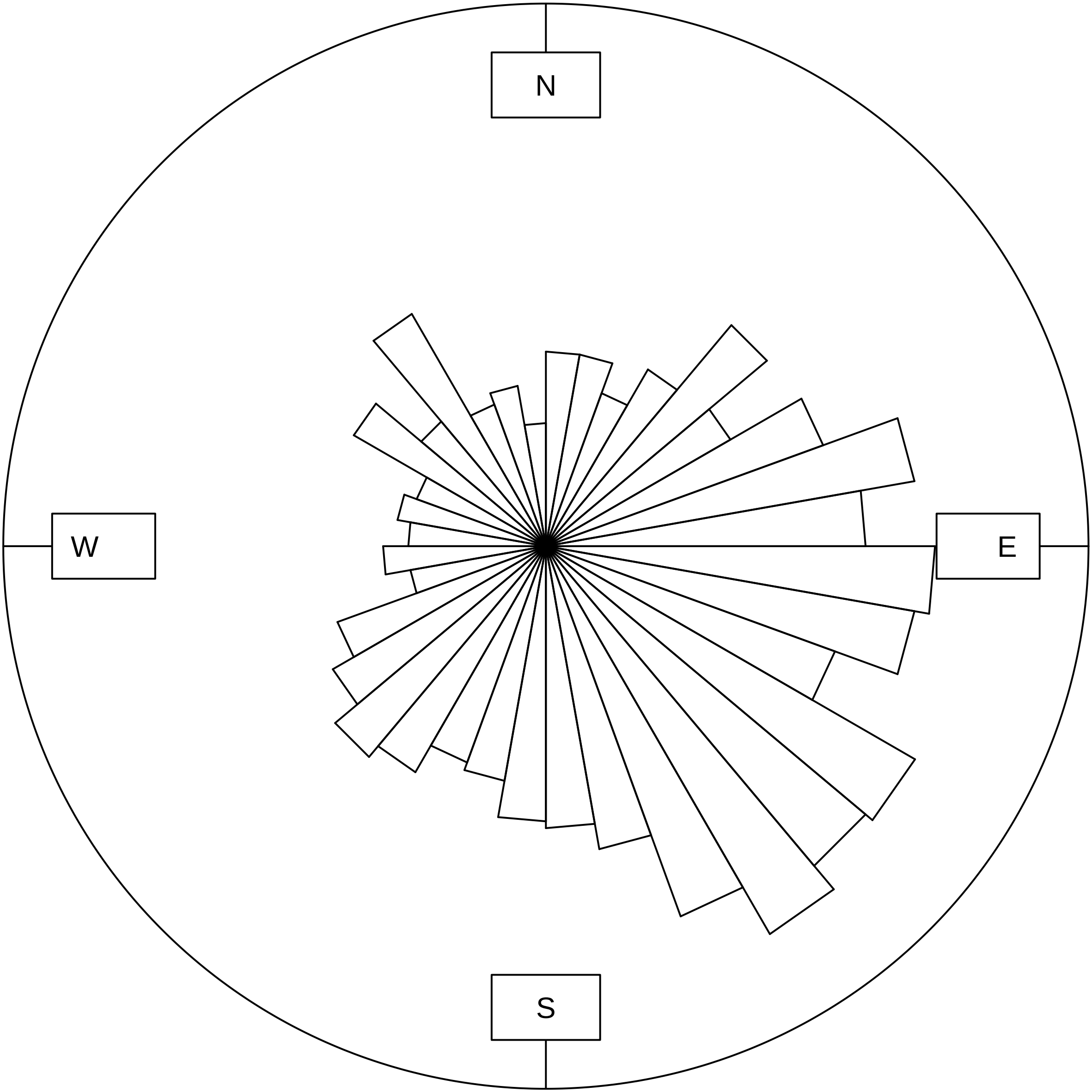

bootstrap 695

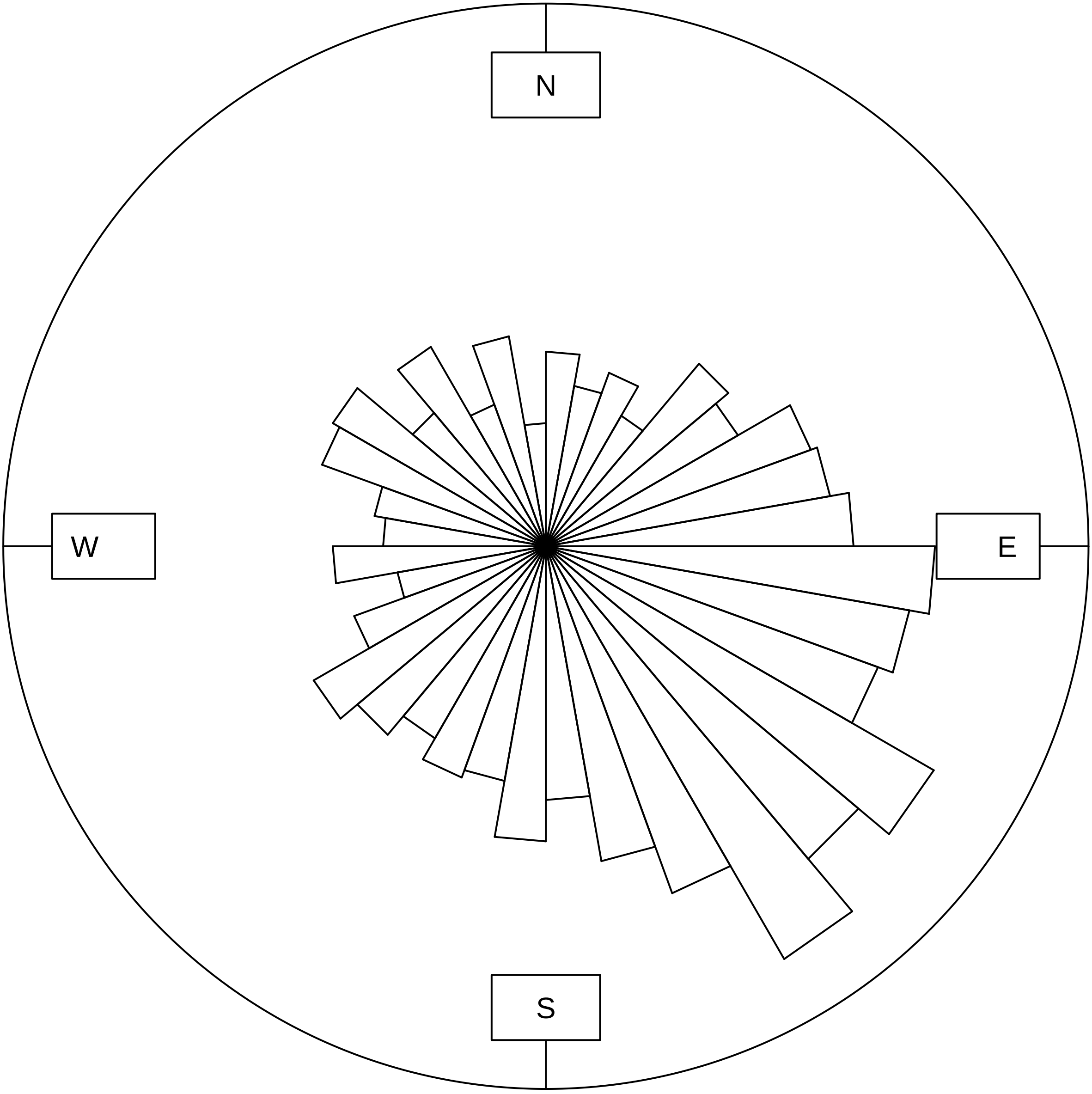

bootstrap 696

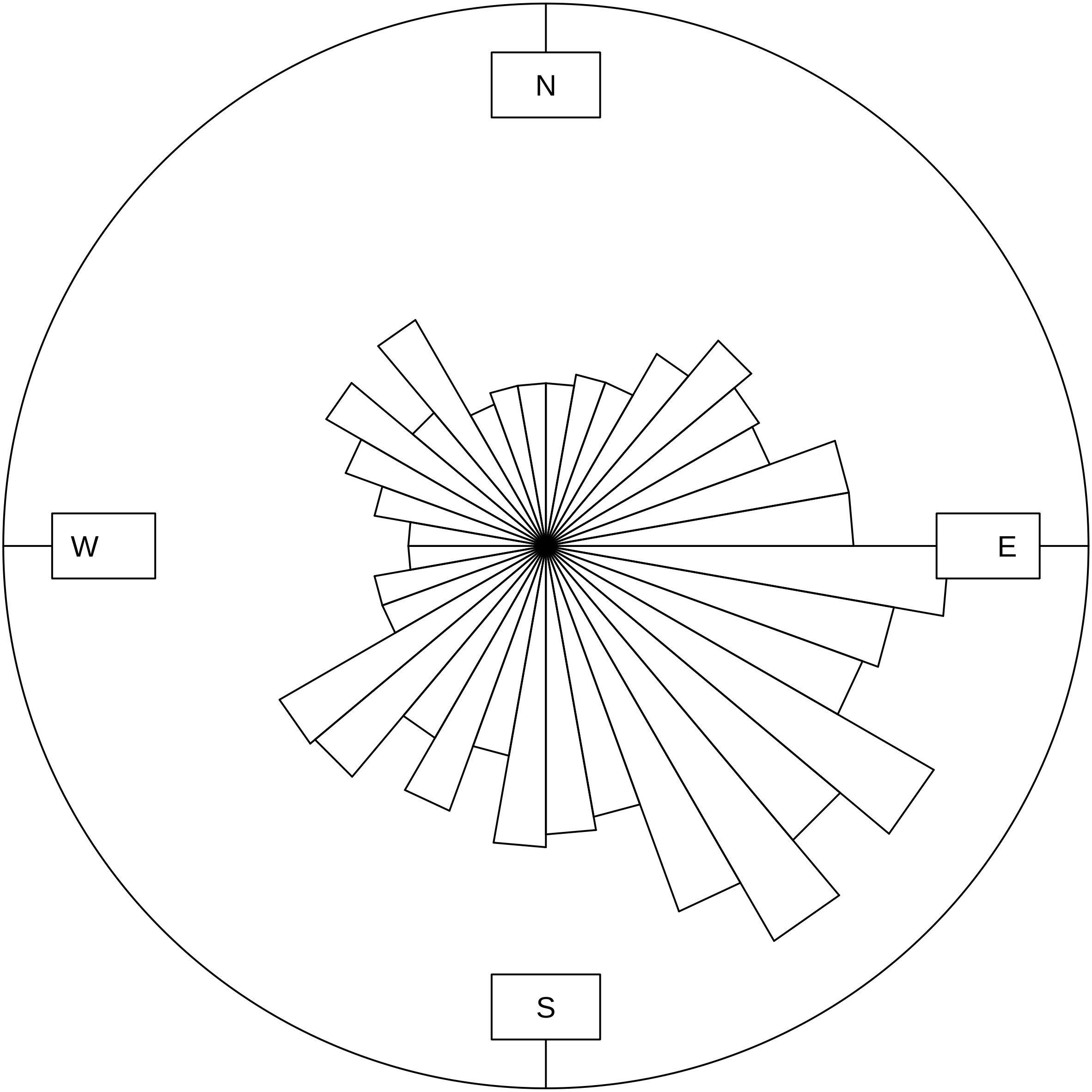

bootstrap 697

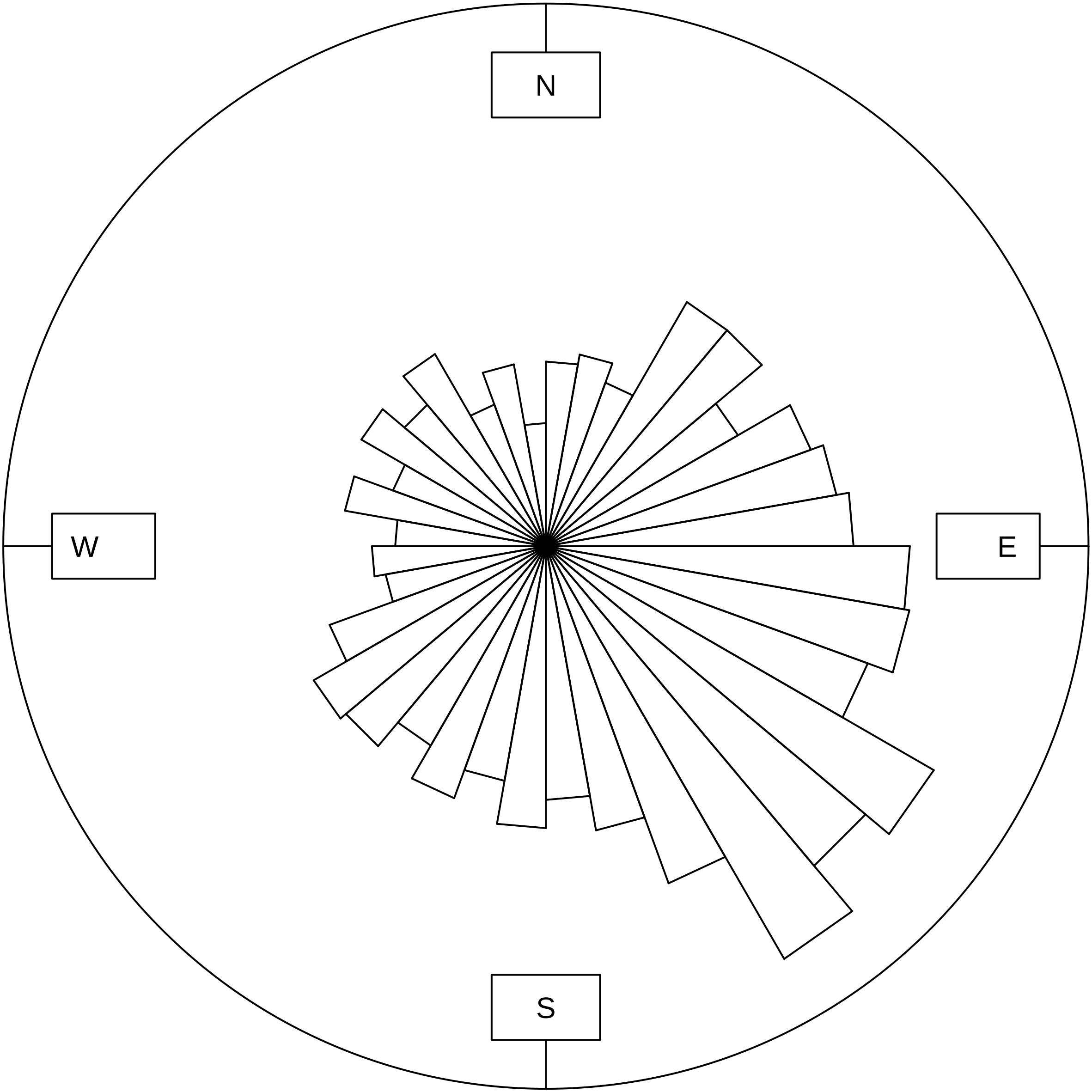

bootstrap 698

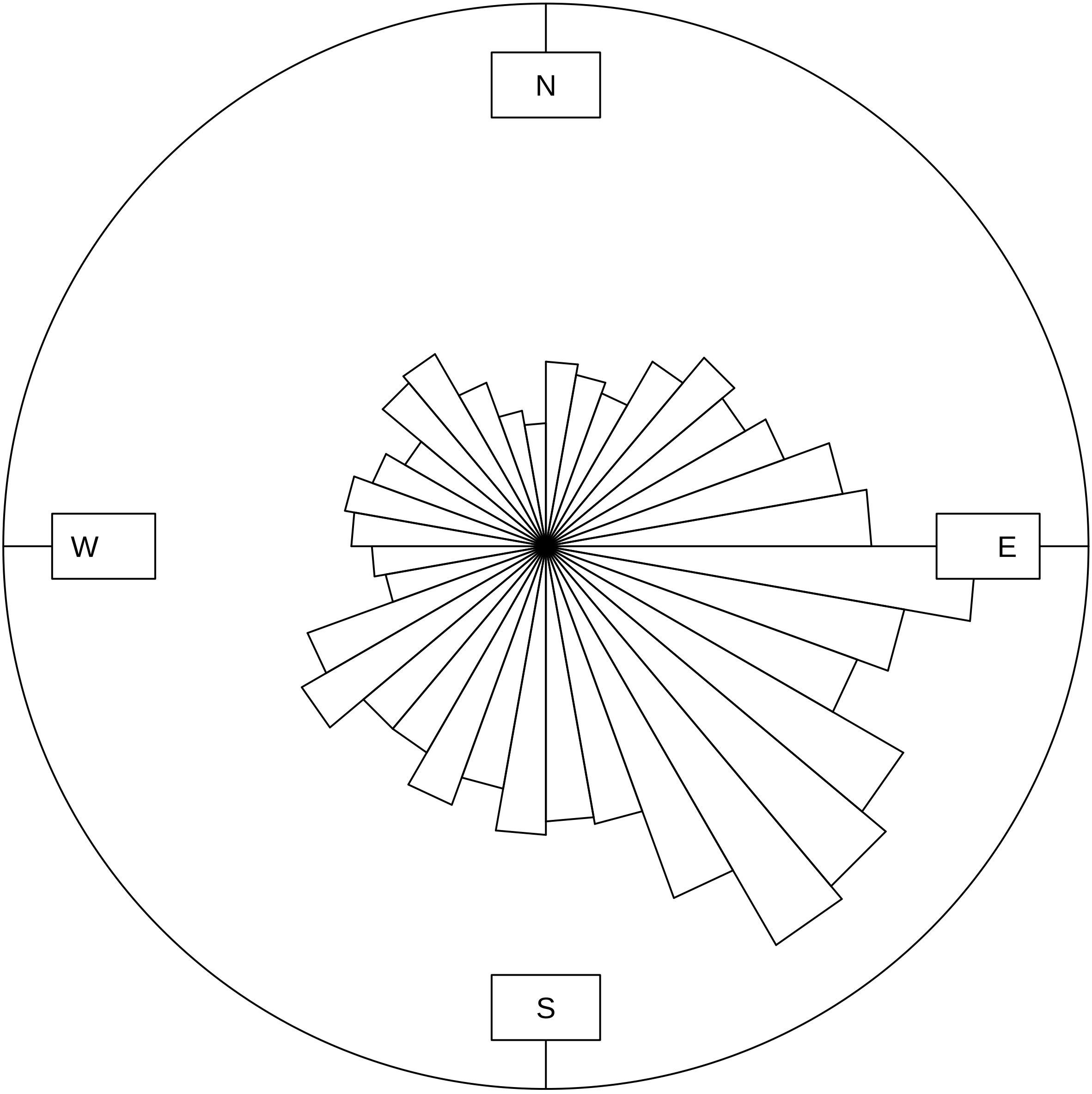

bootstrap 699

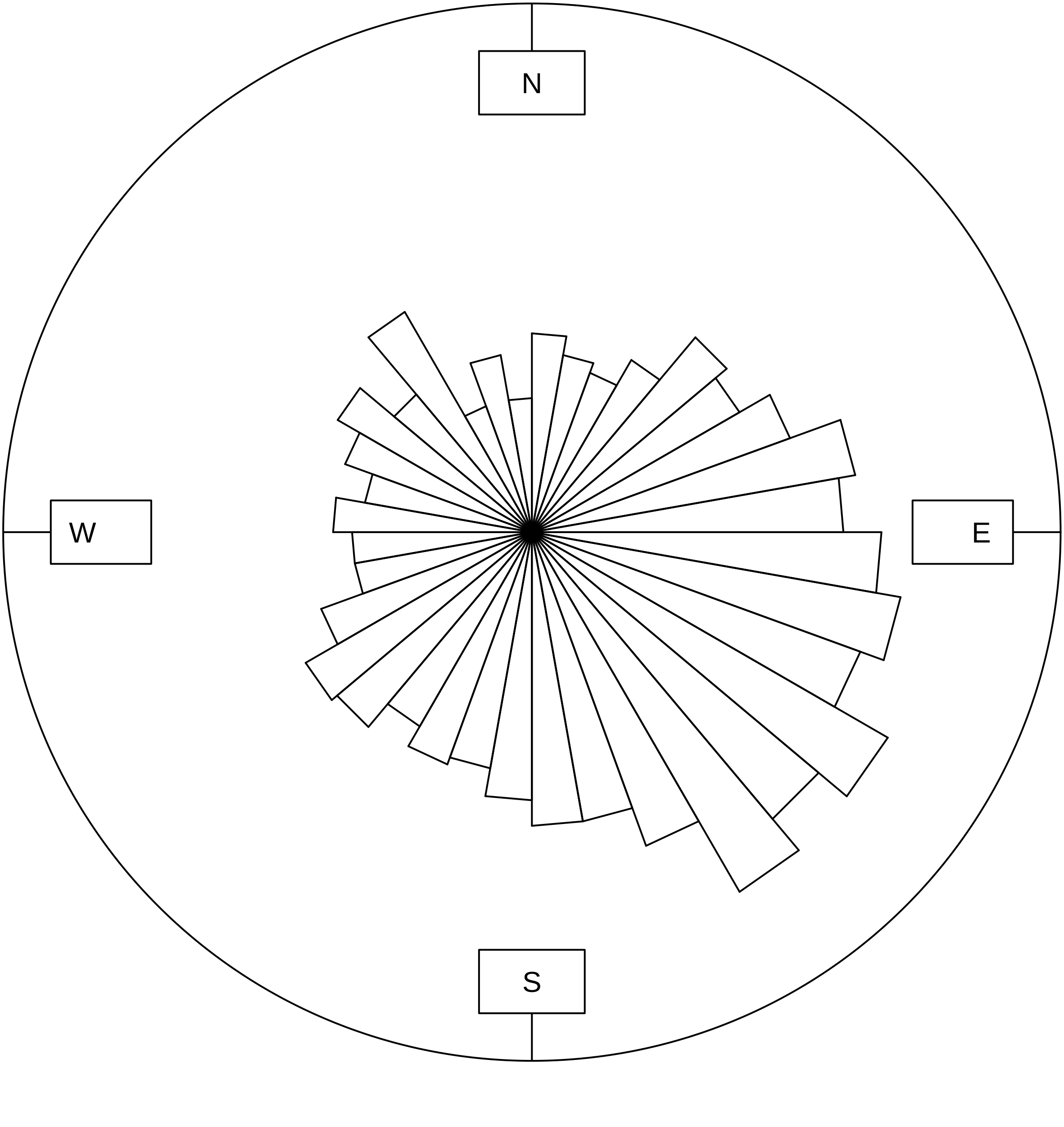

bootstrap 700

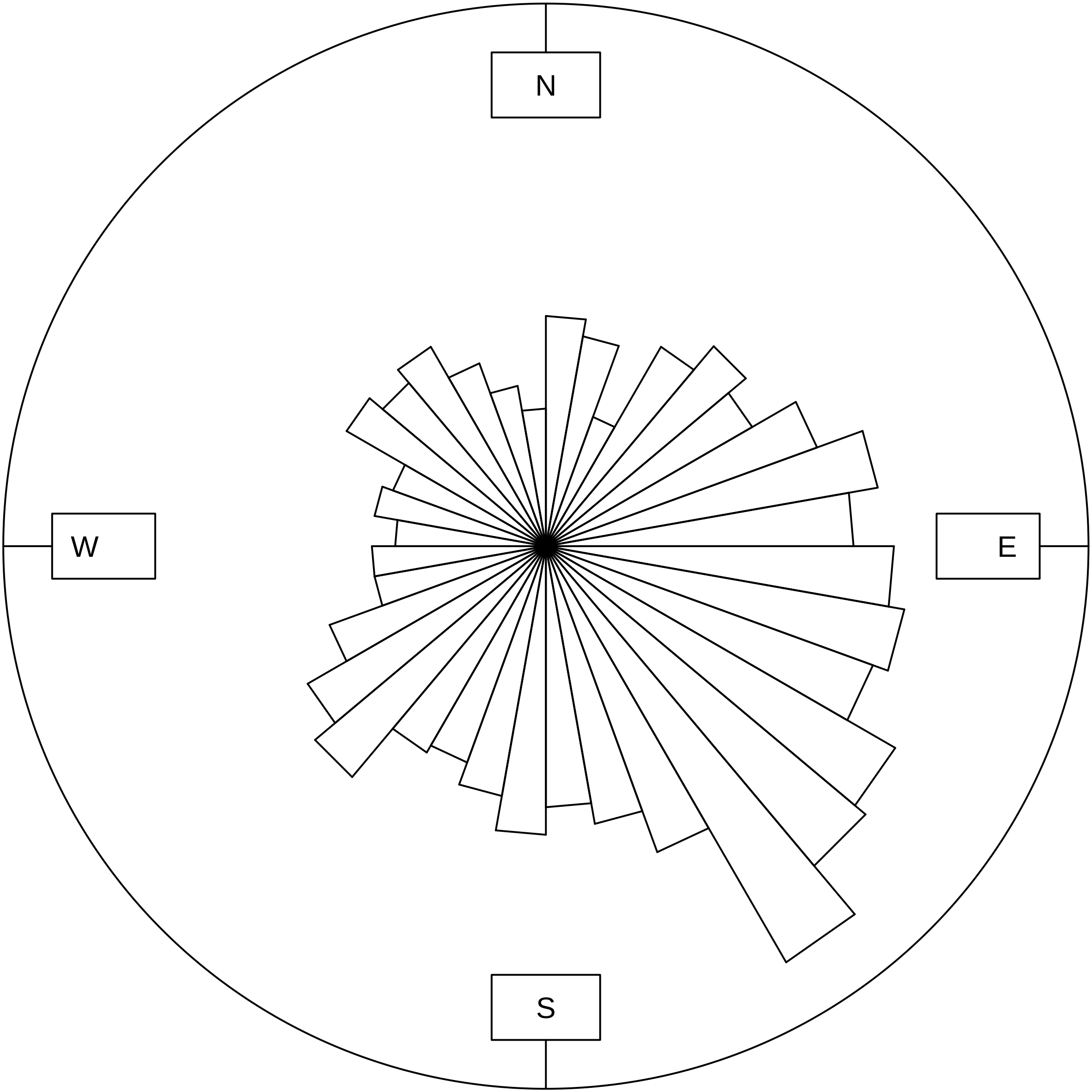

bootstrap 701

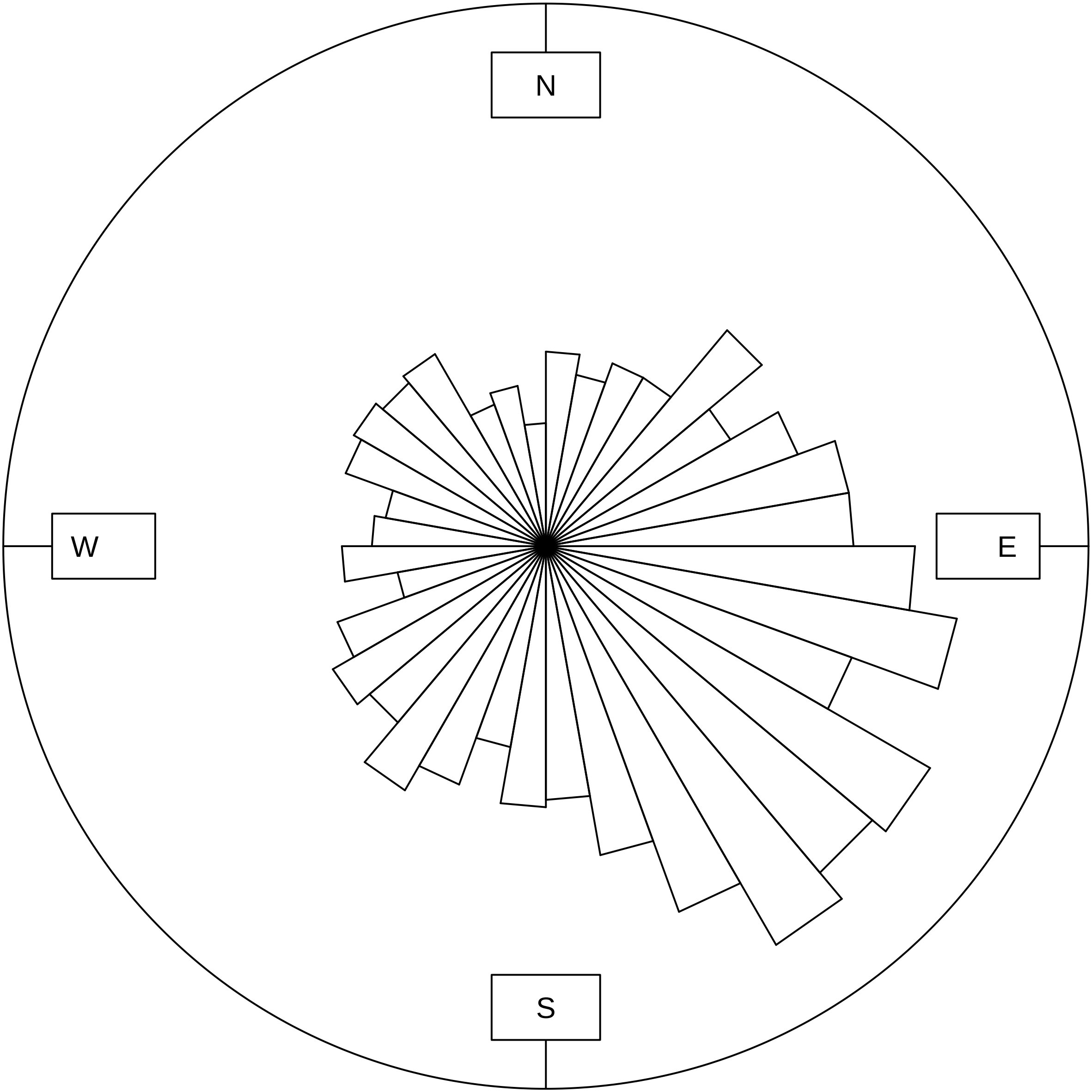

bootstrap 702

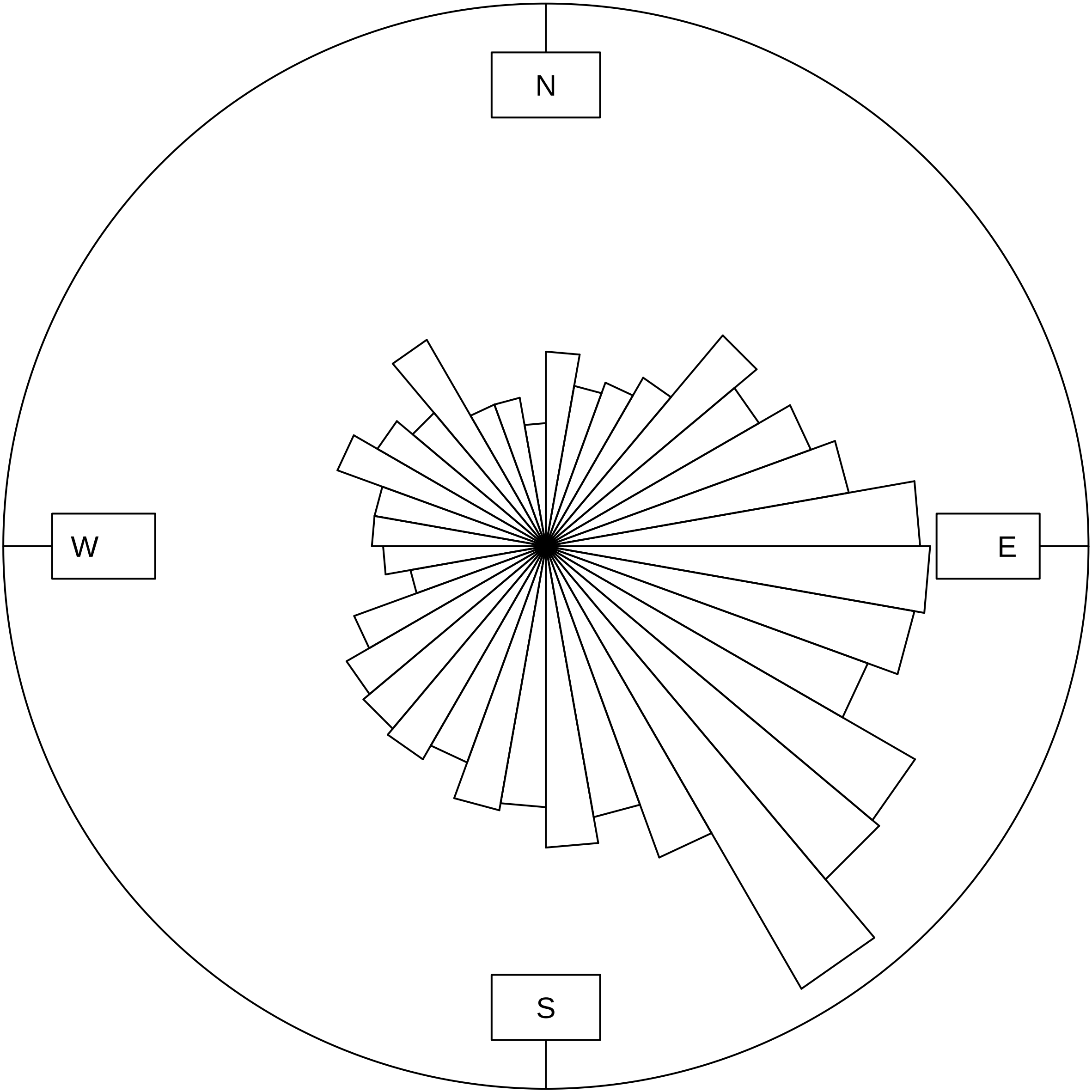

bootstrap 703

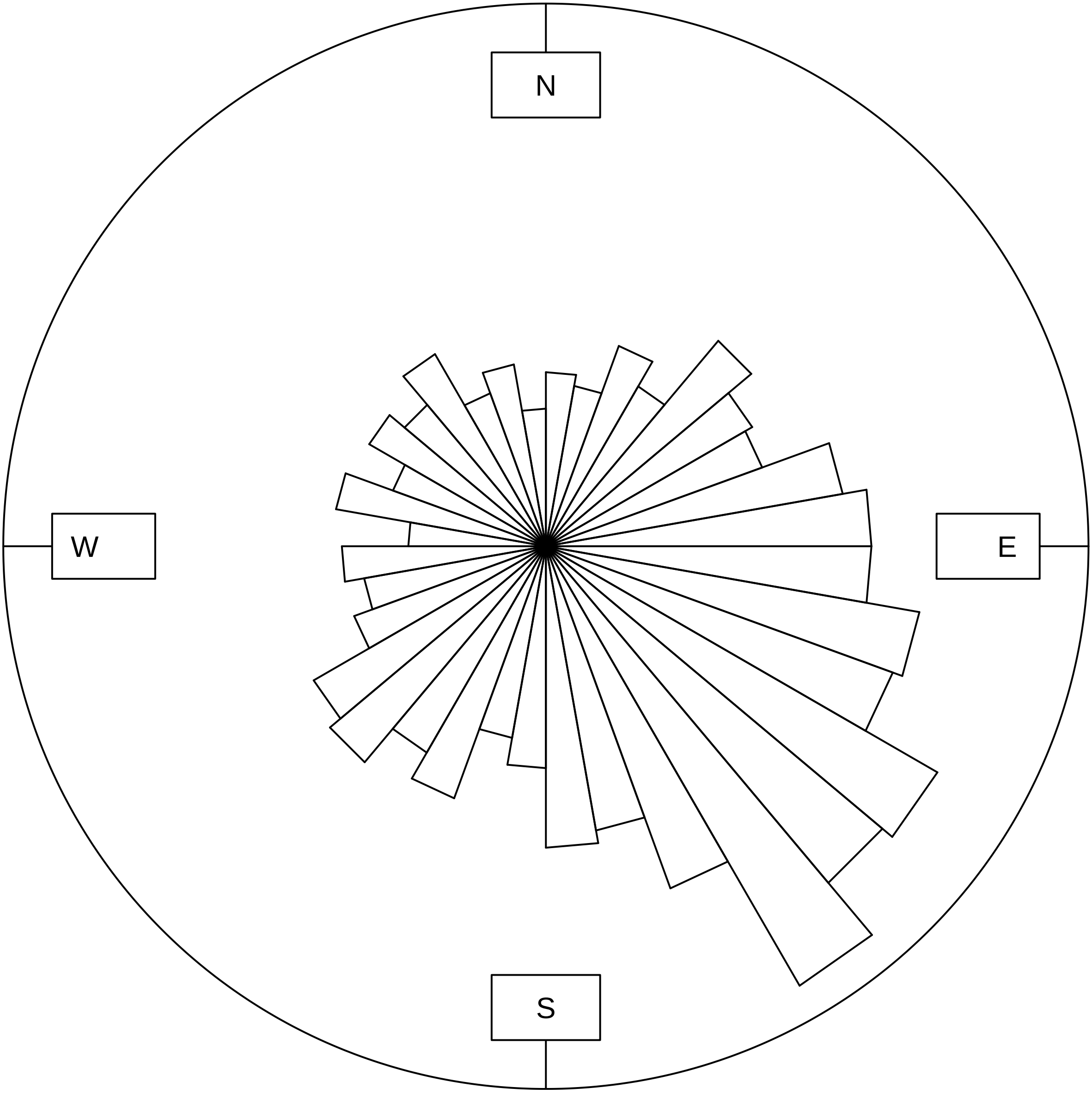

bootstrap 704

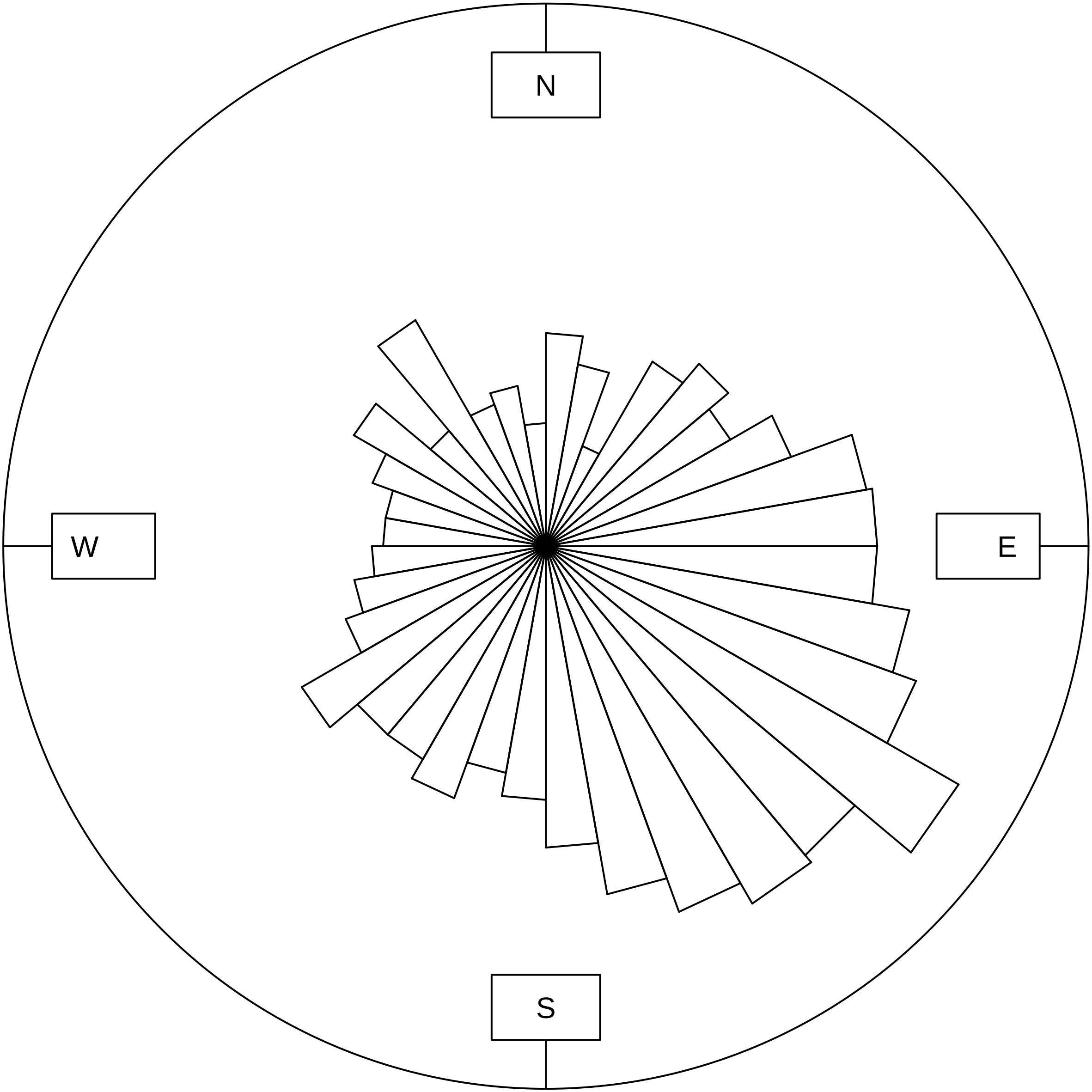

bootstrap 705

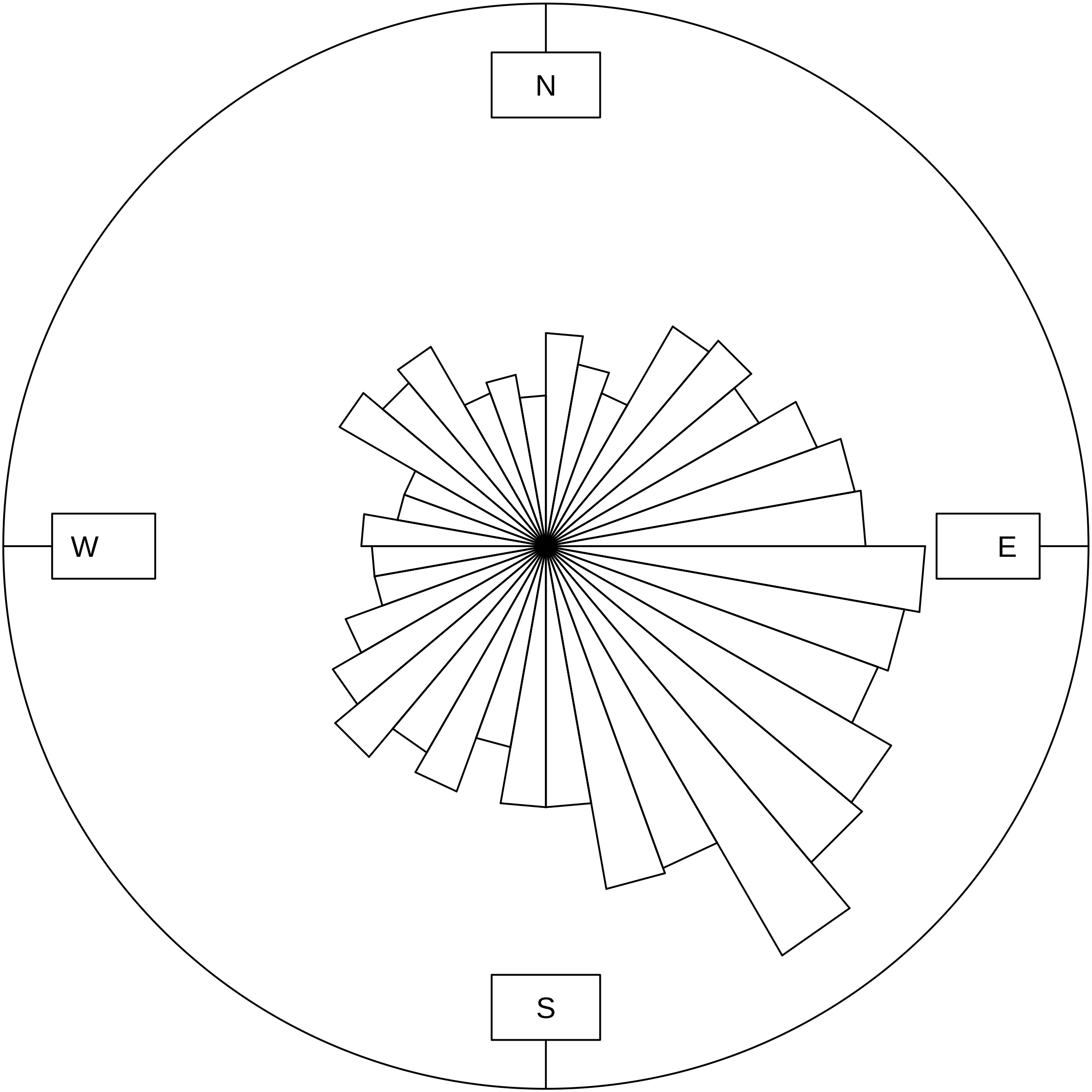

bootstrap 706

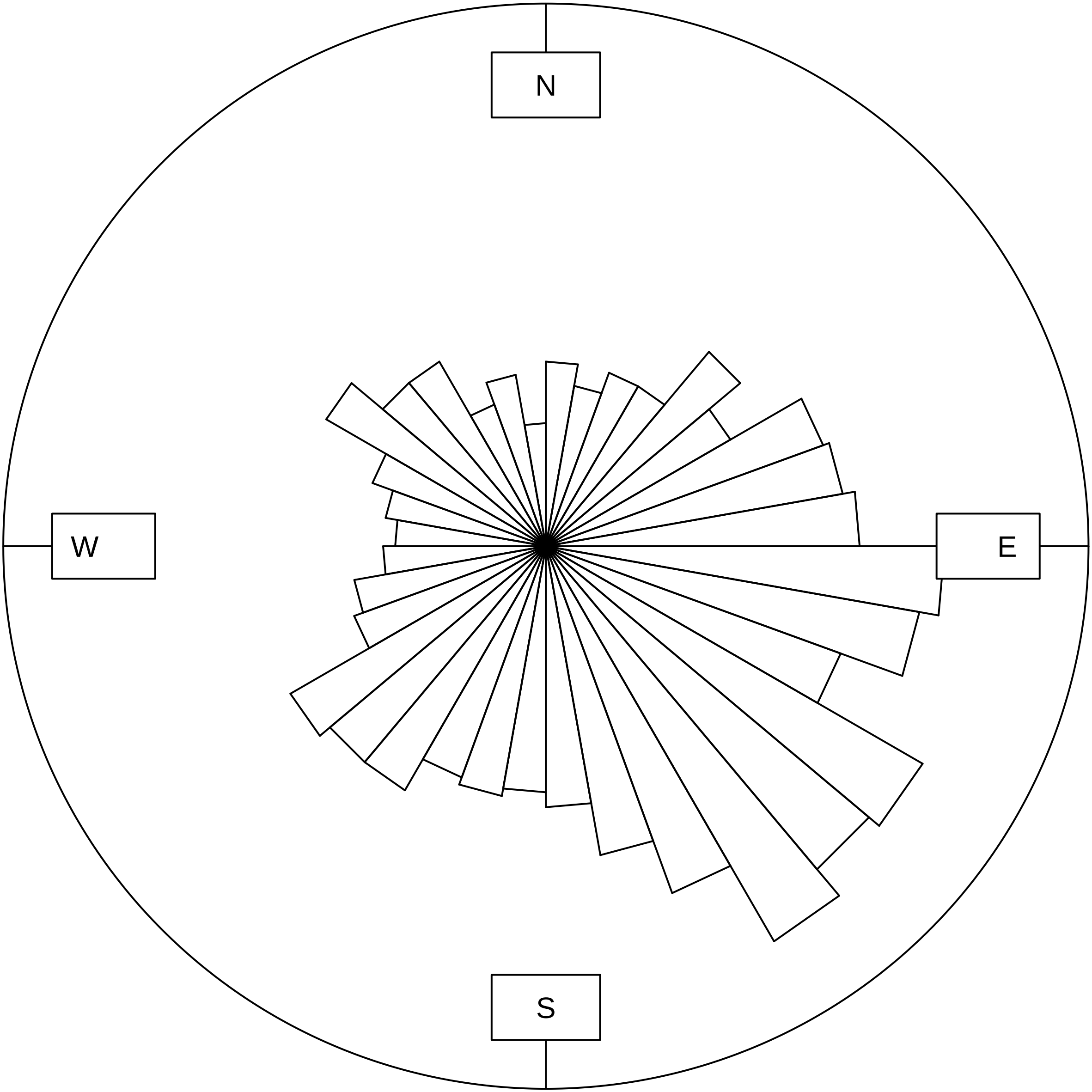

bootstrap 707

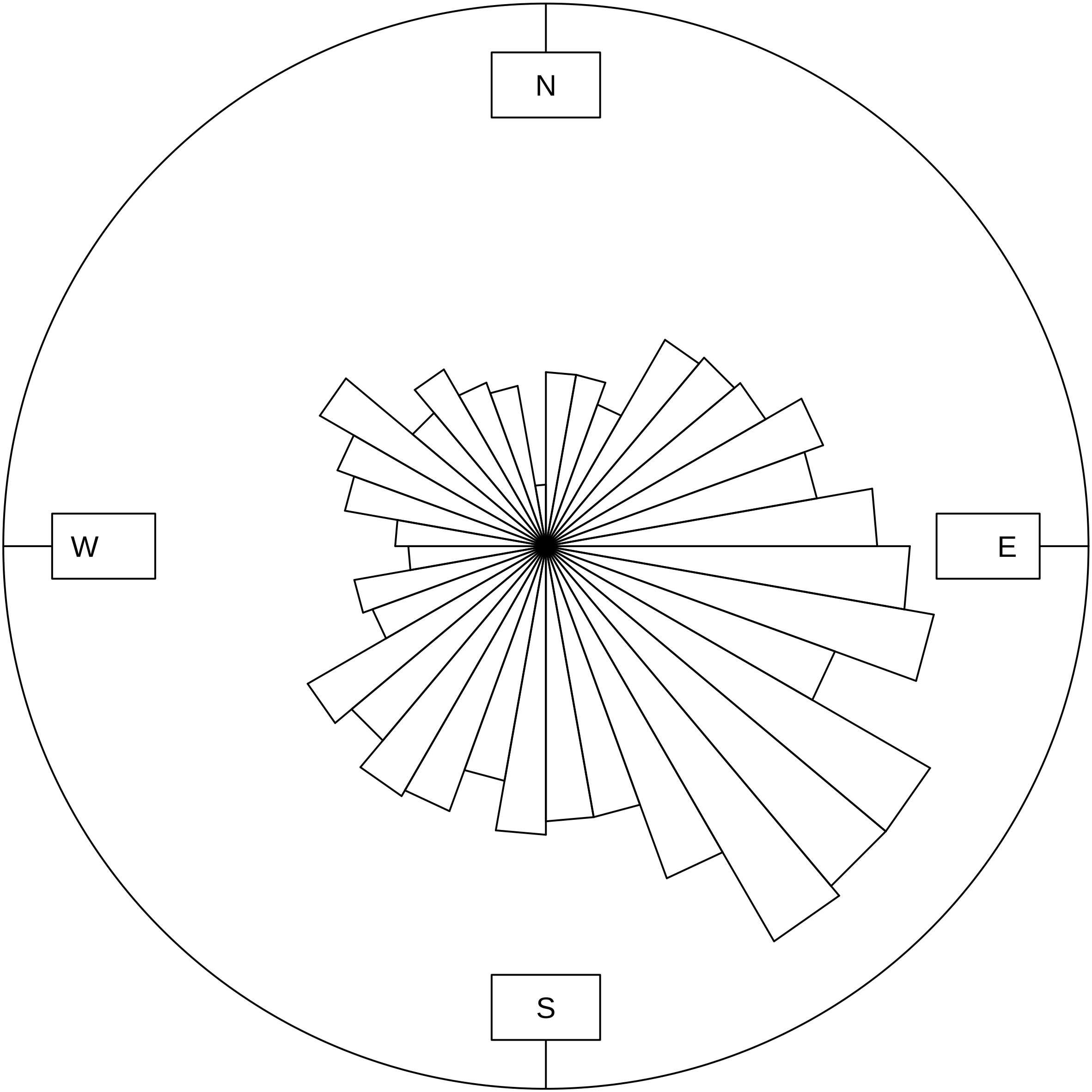

bootstrap 708

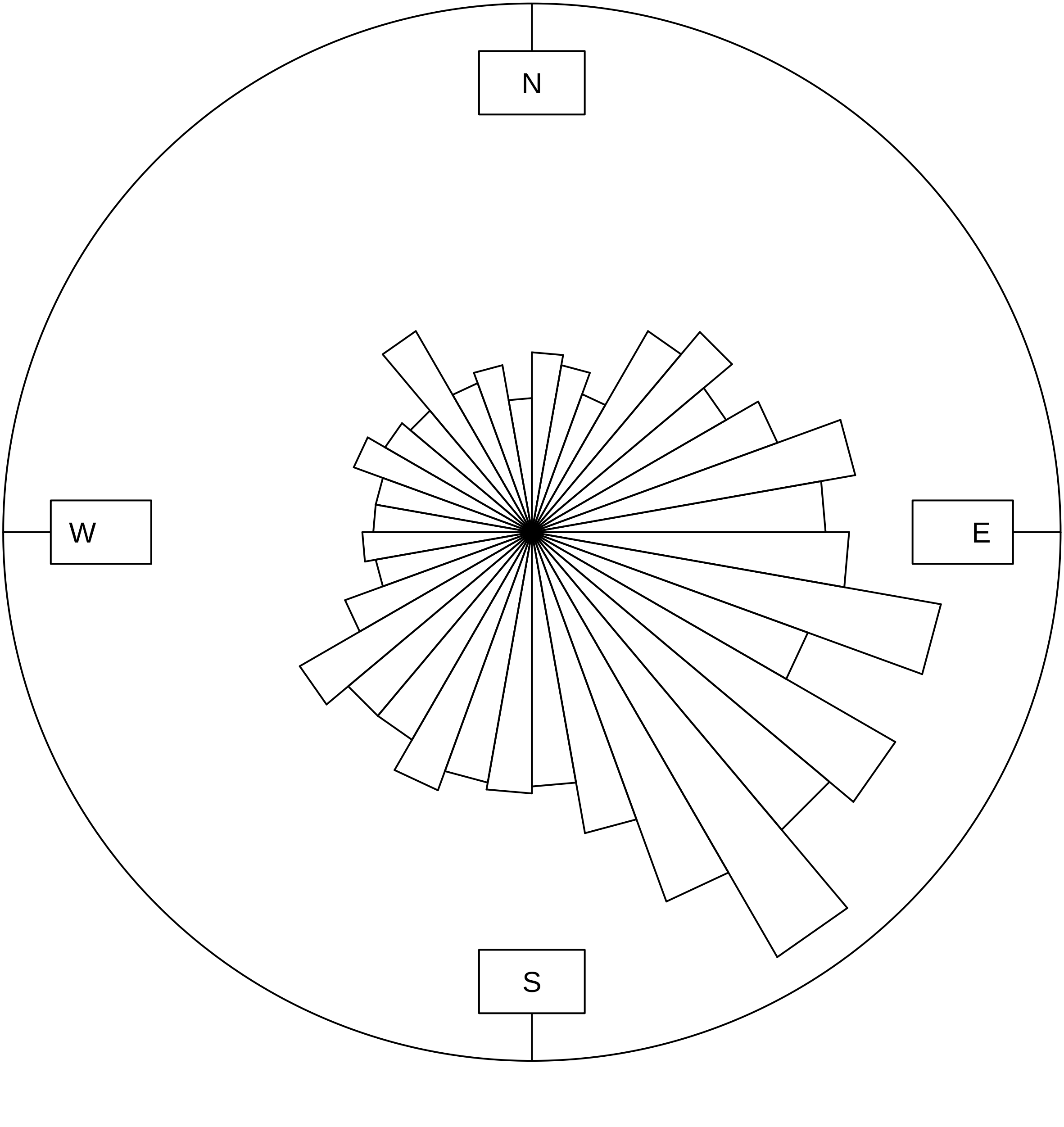

bootstrap 709

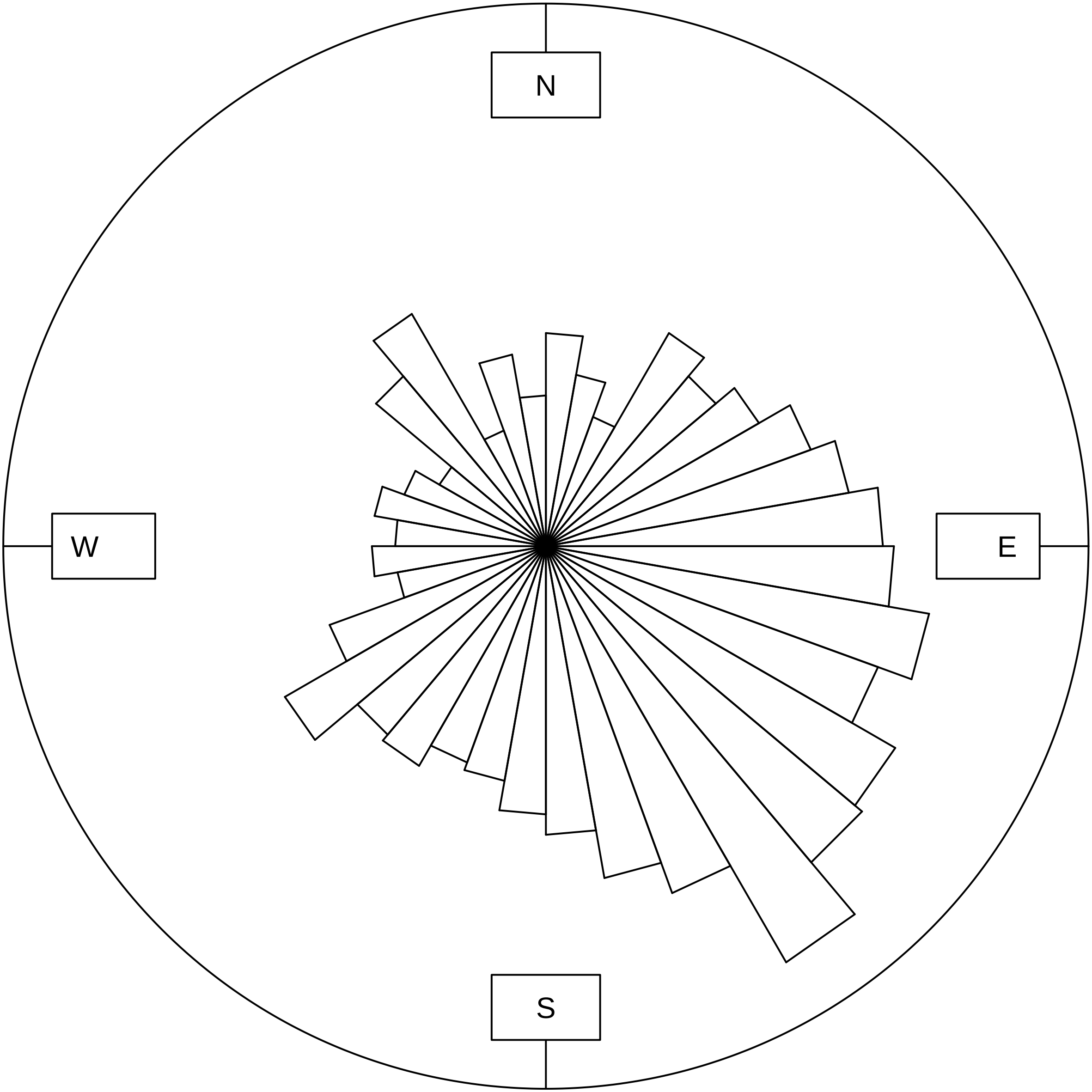

bootstrap 710

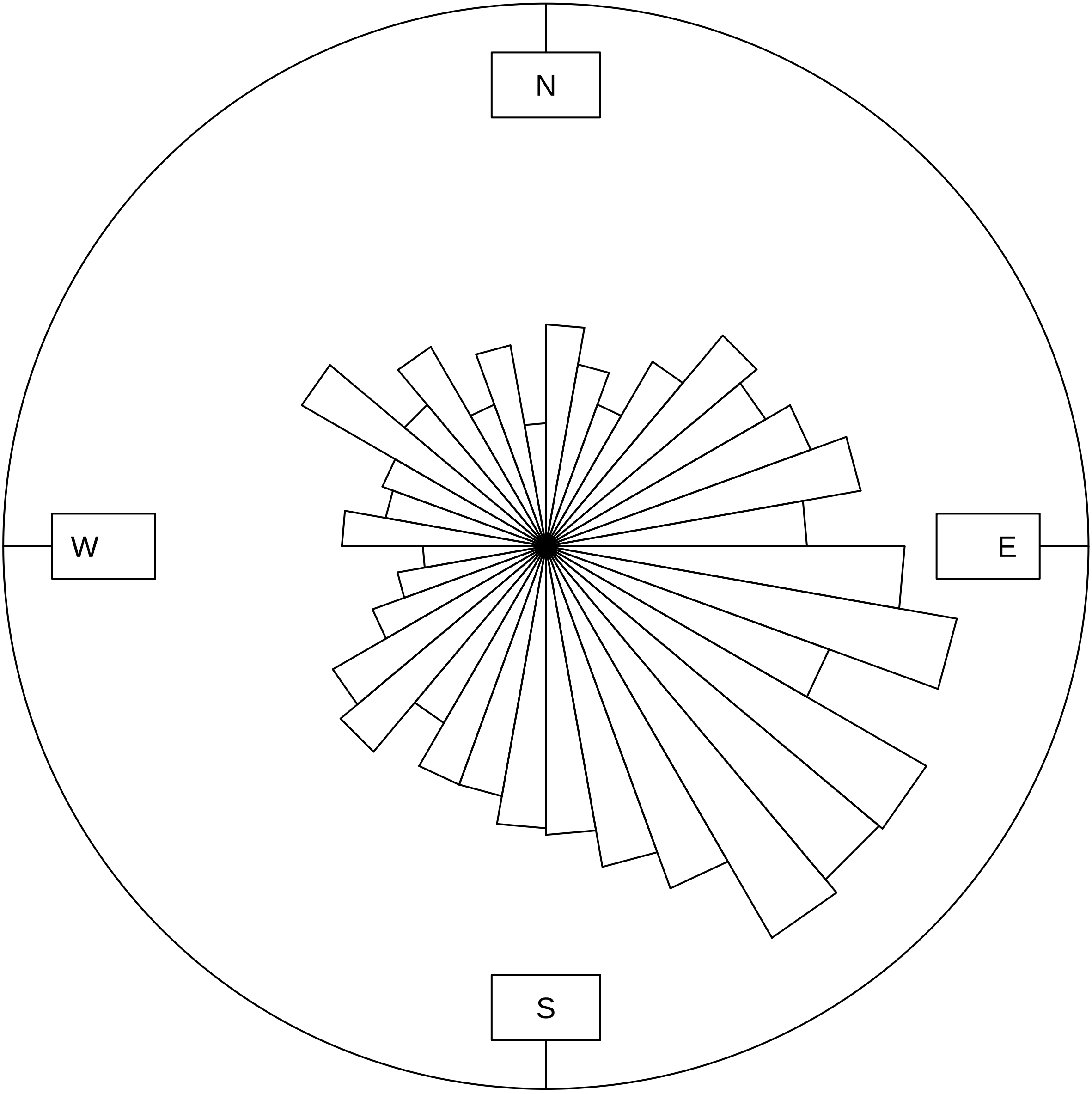

bootstrap 711

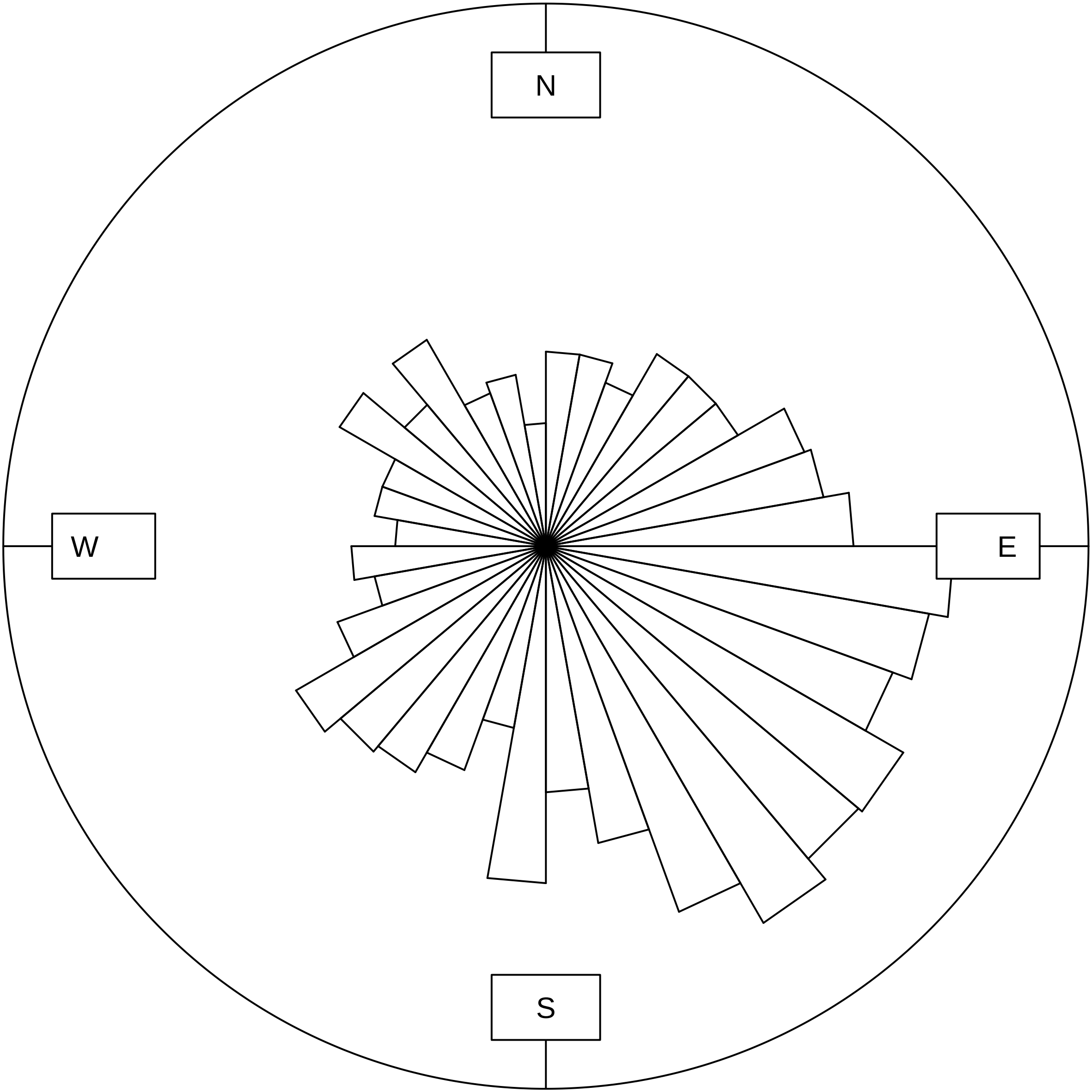

bootstrap 712

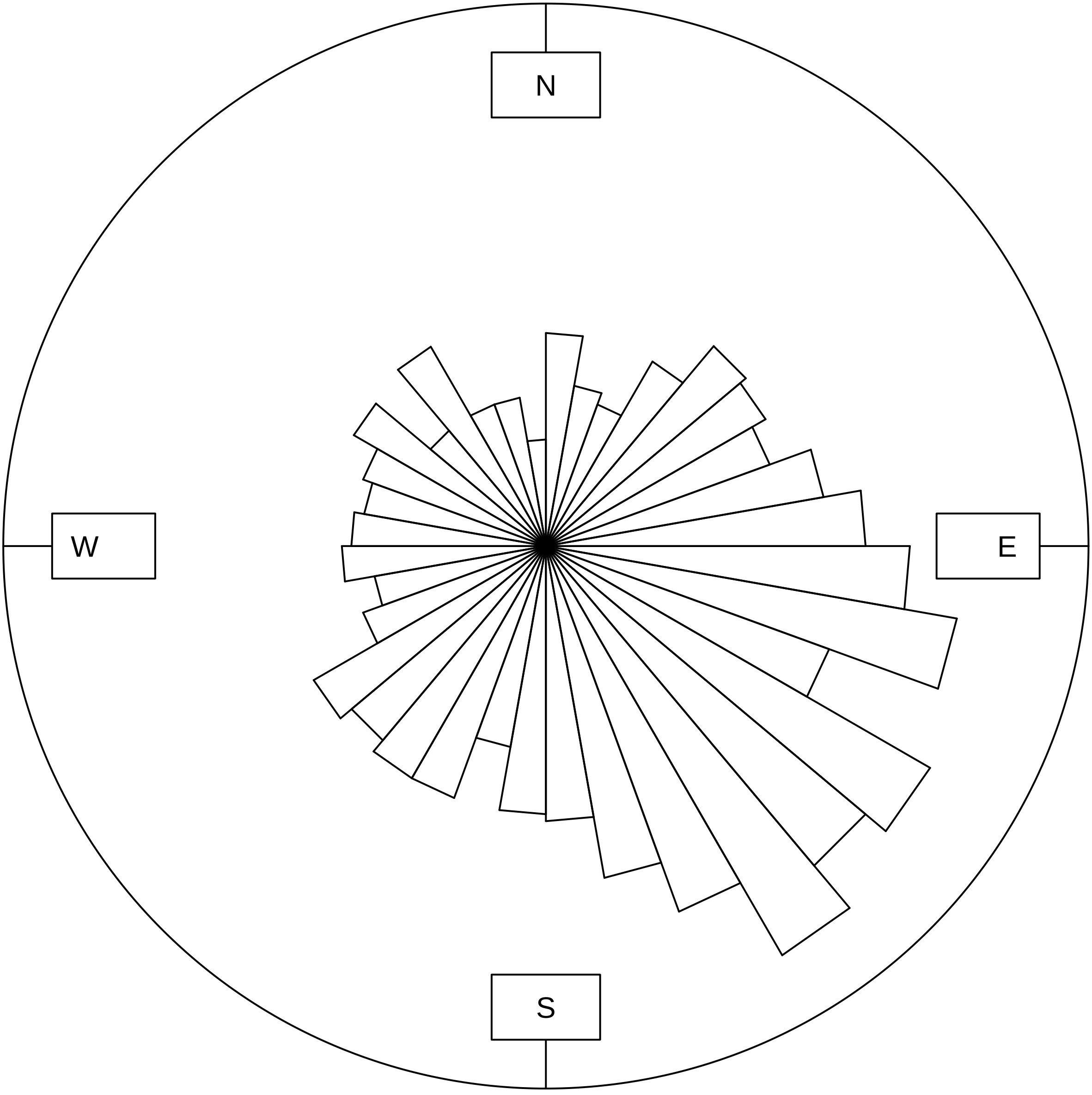

bootstrap 713

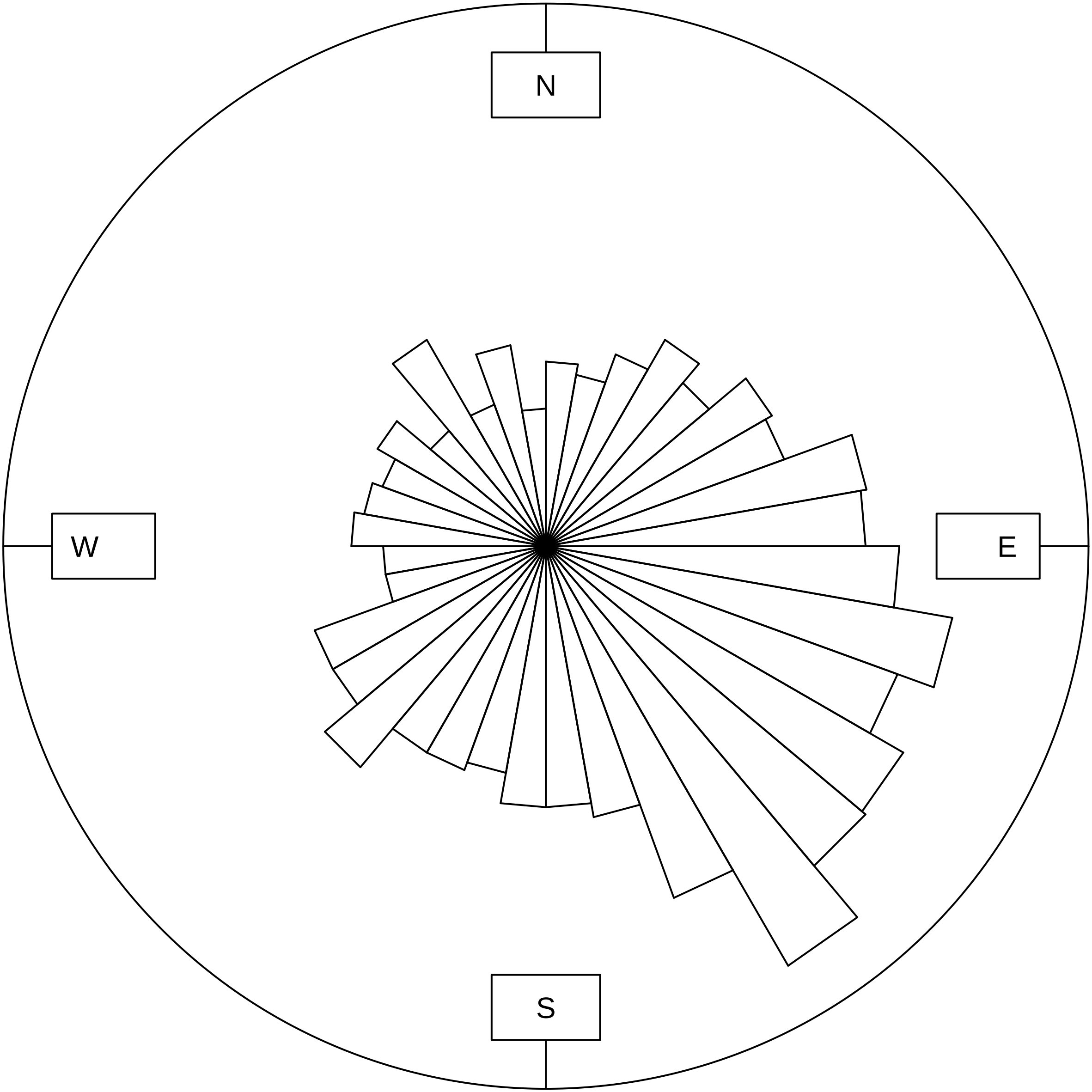

bootstrap 714

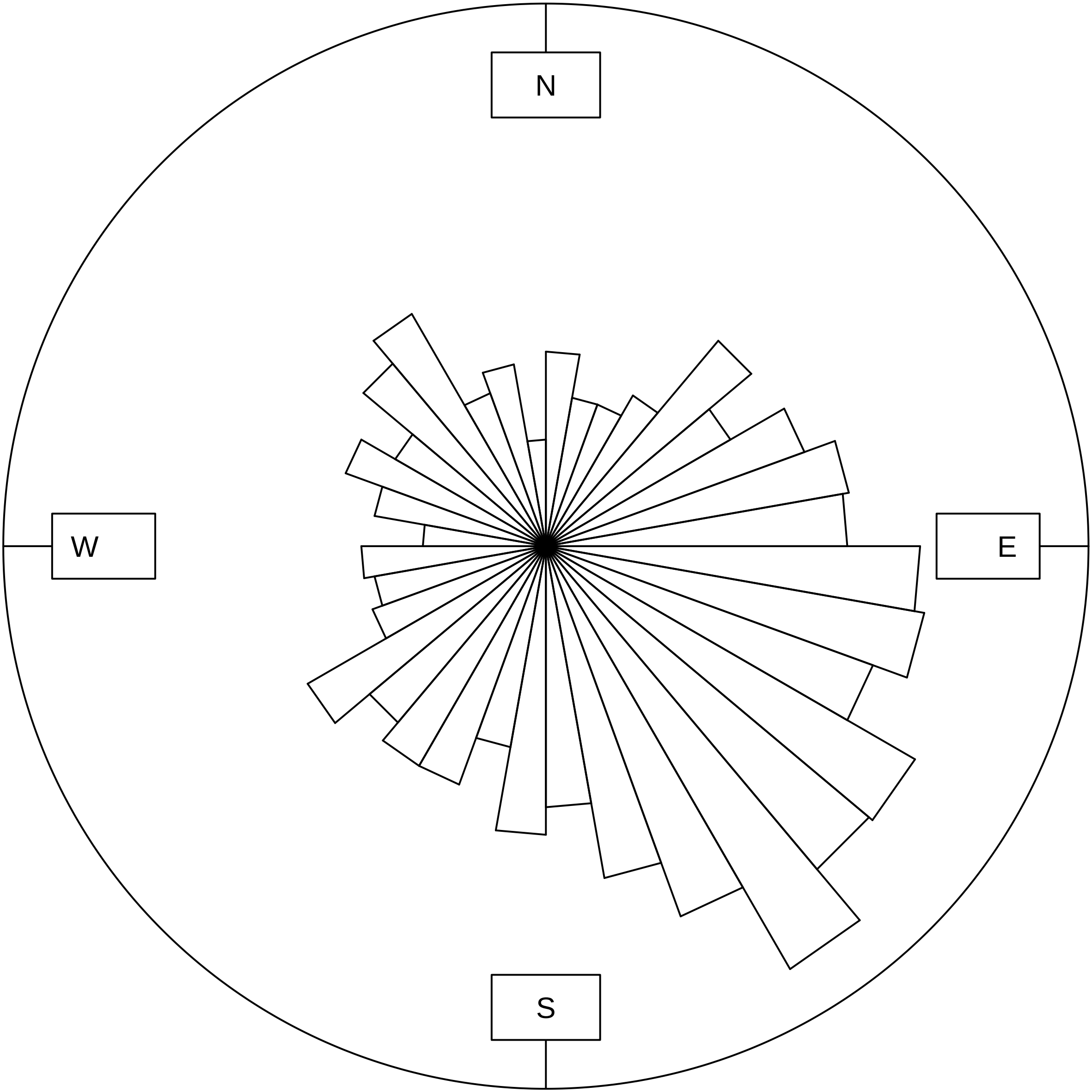

bootstrap 715

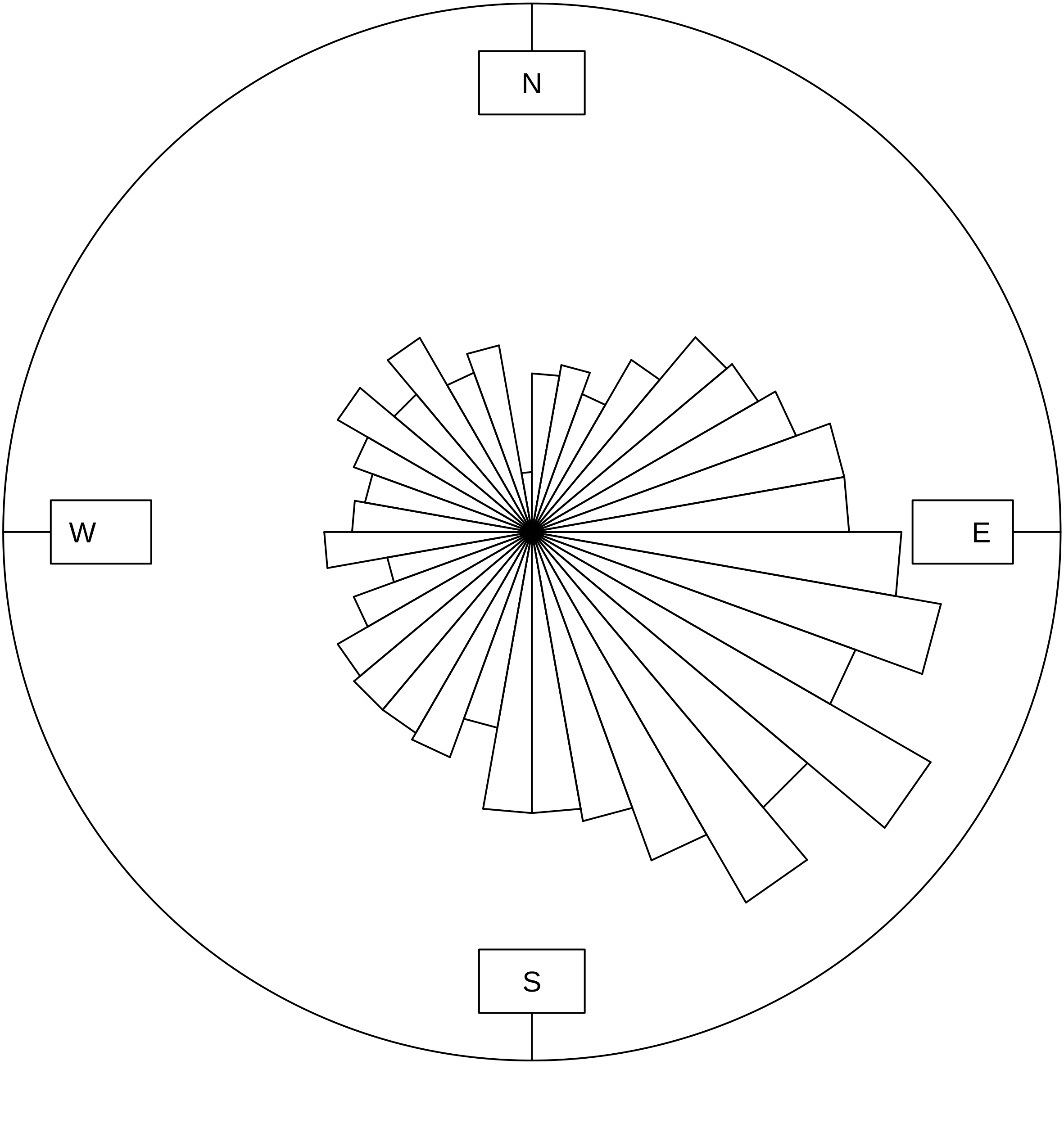

bootstrap 716

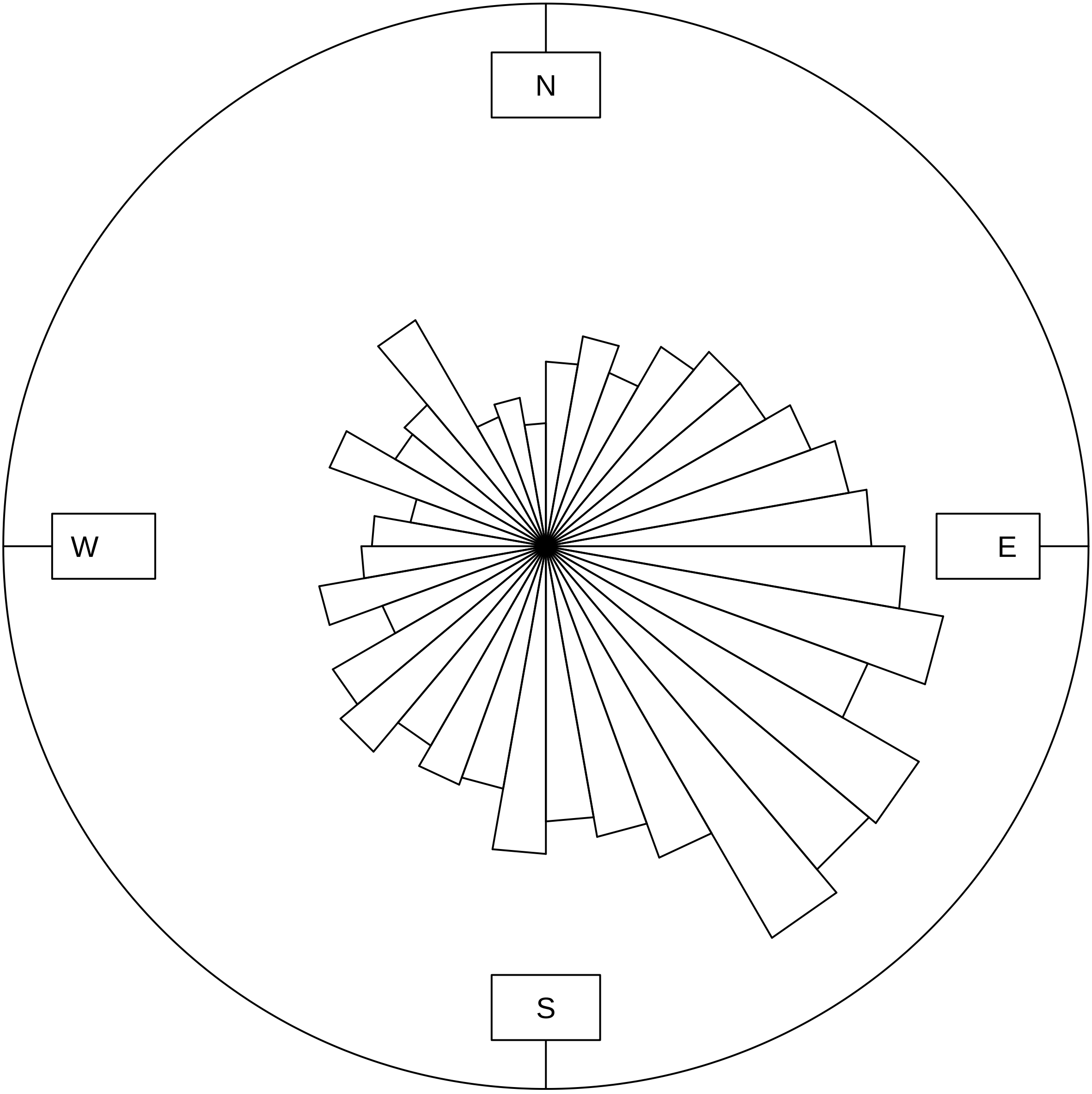

bootstrap 717

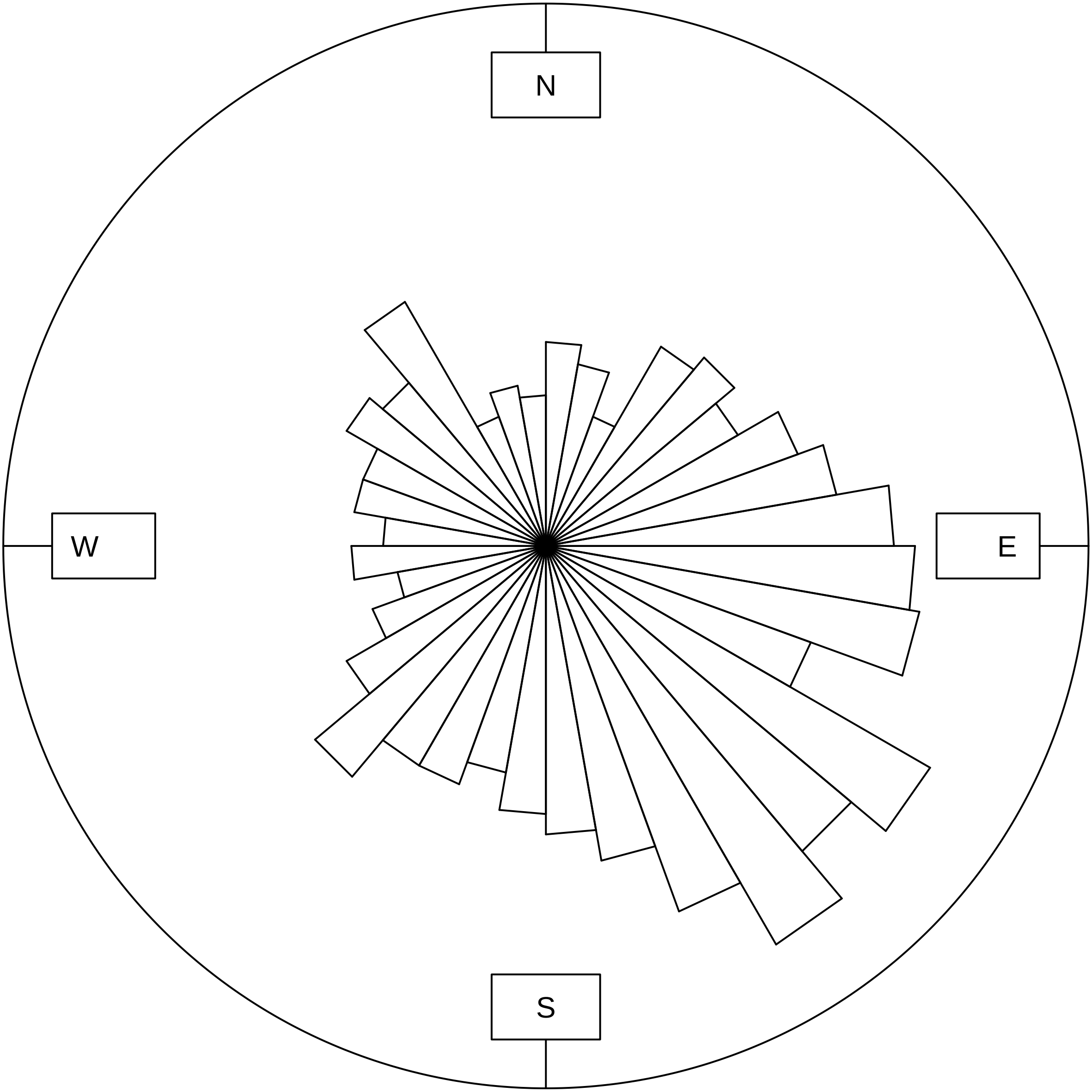

bootstrap 718

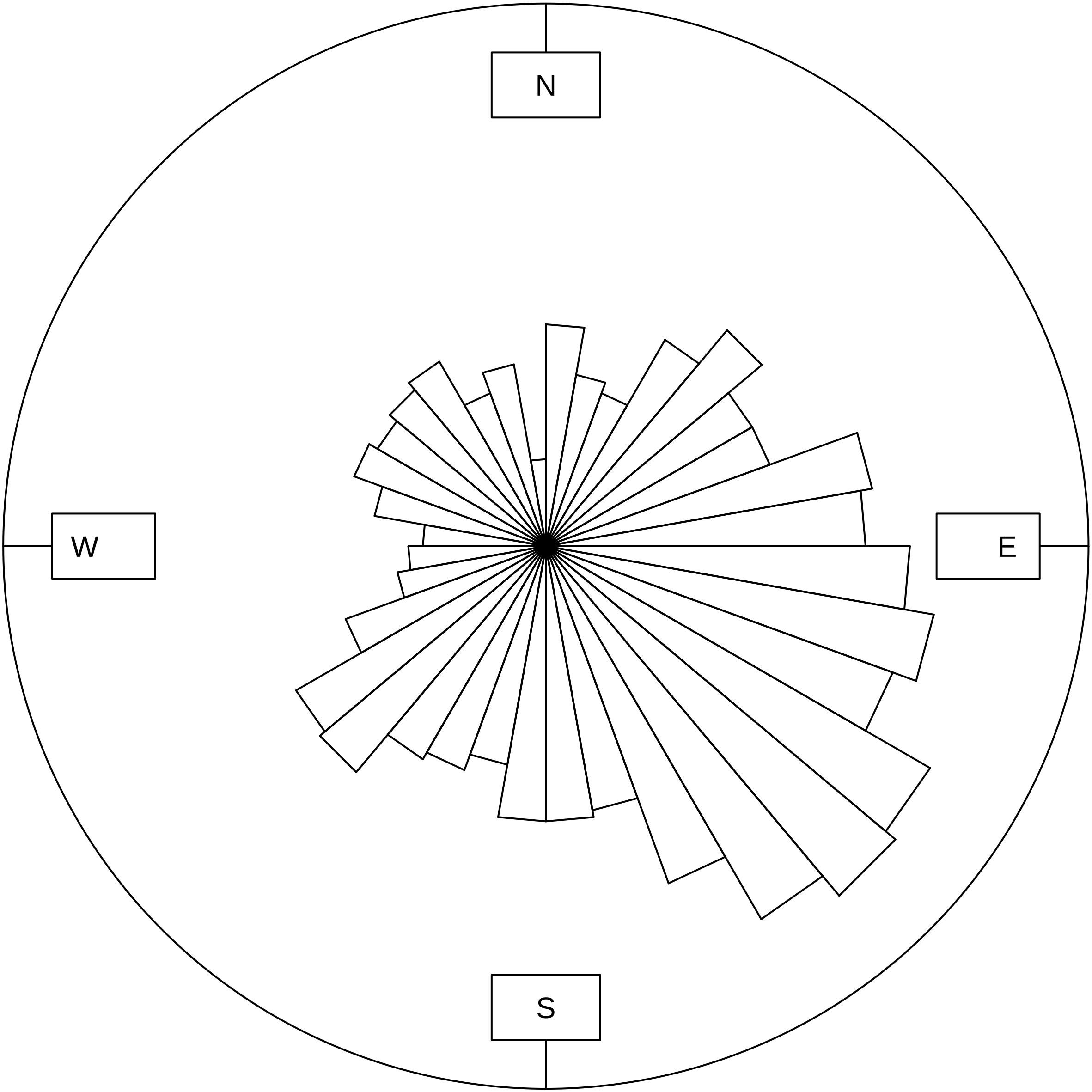

bootstrap 719

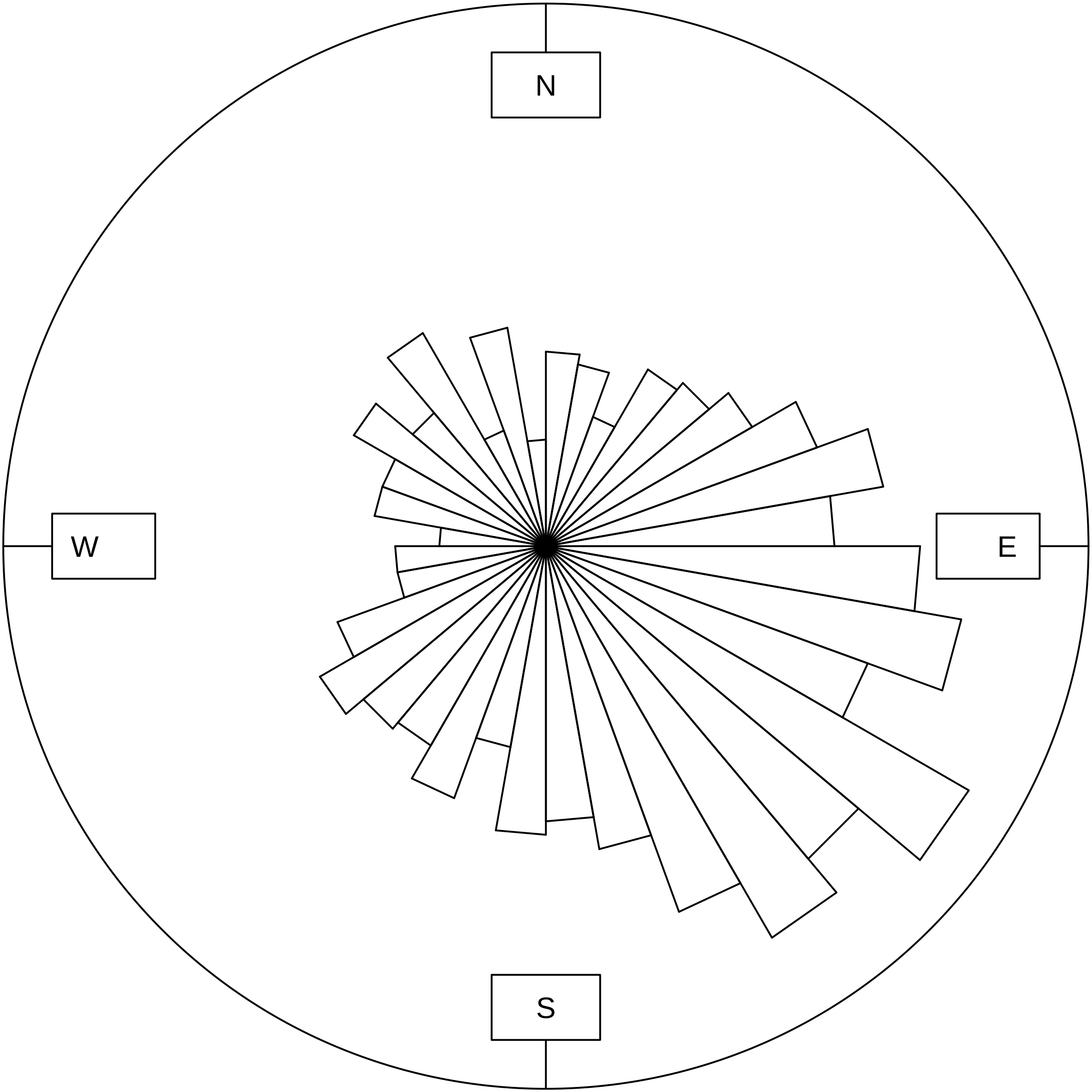

bootstrap 720

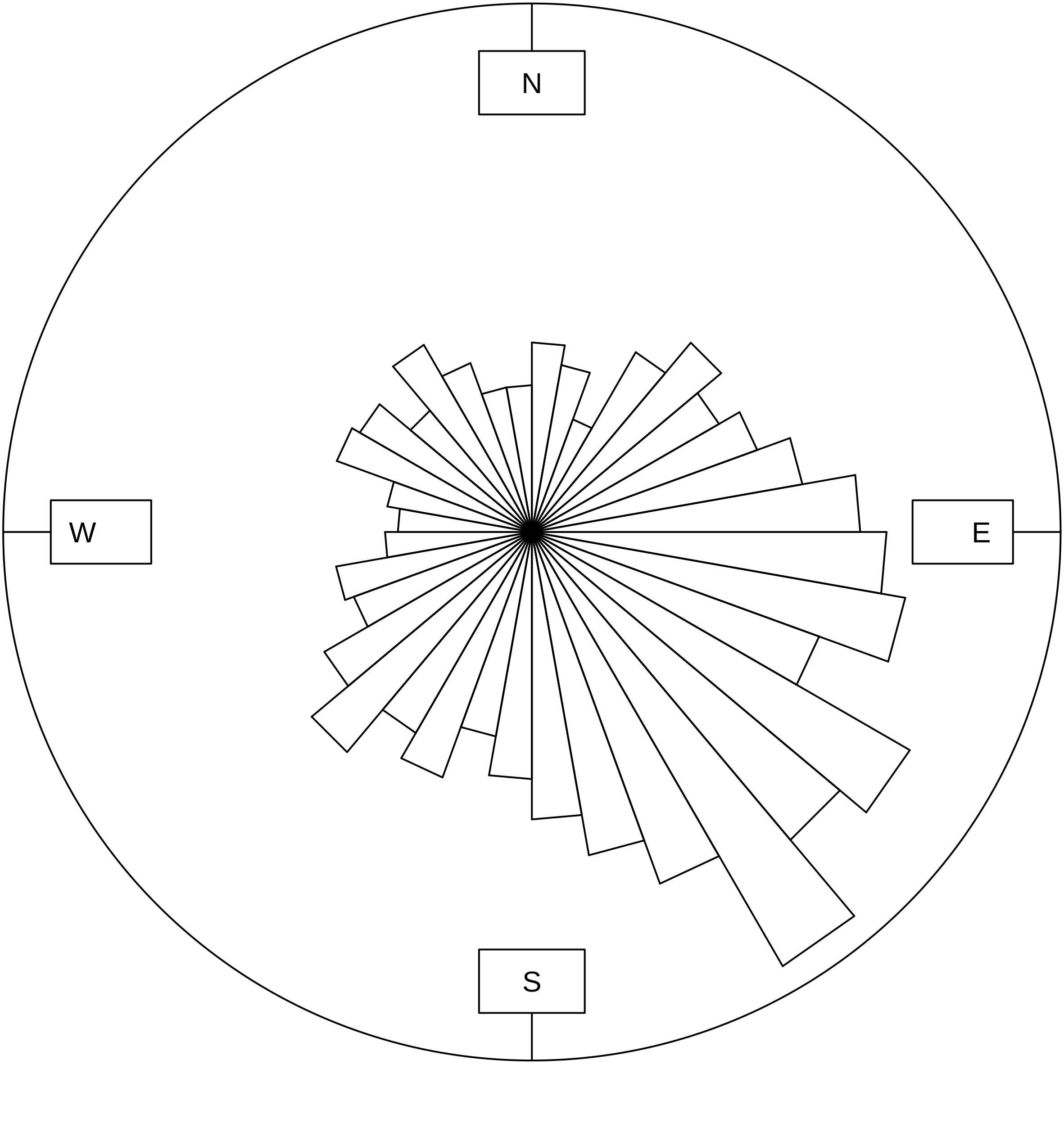

bootstrap 721

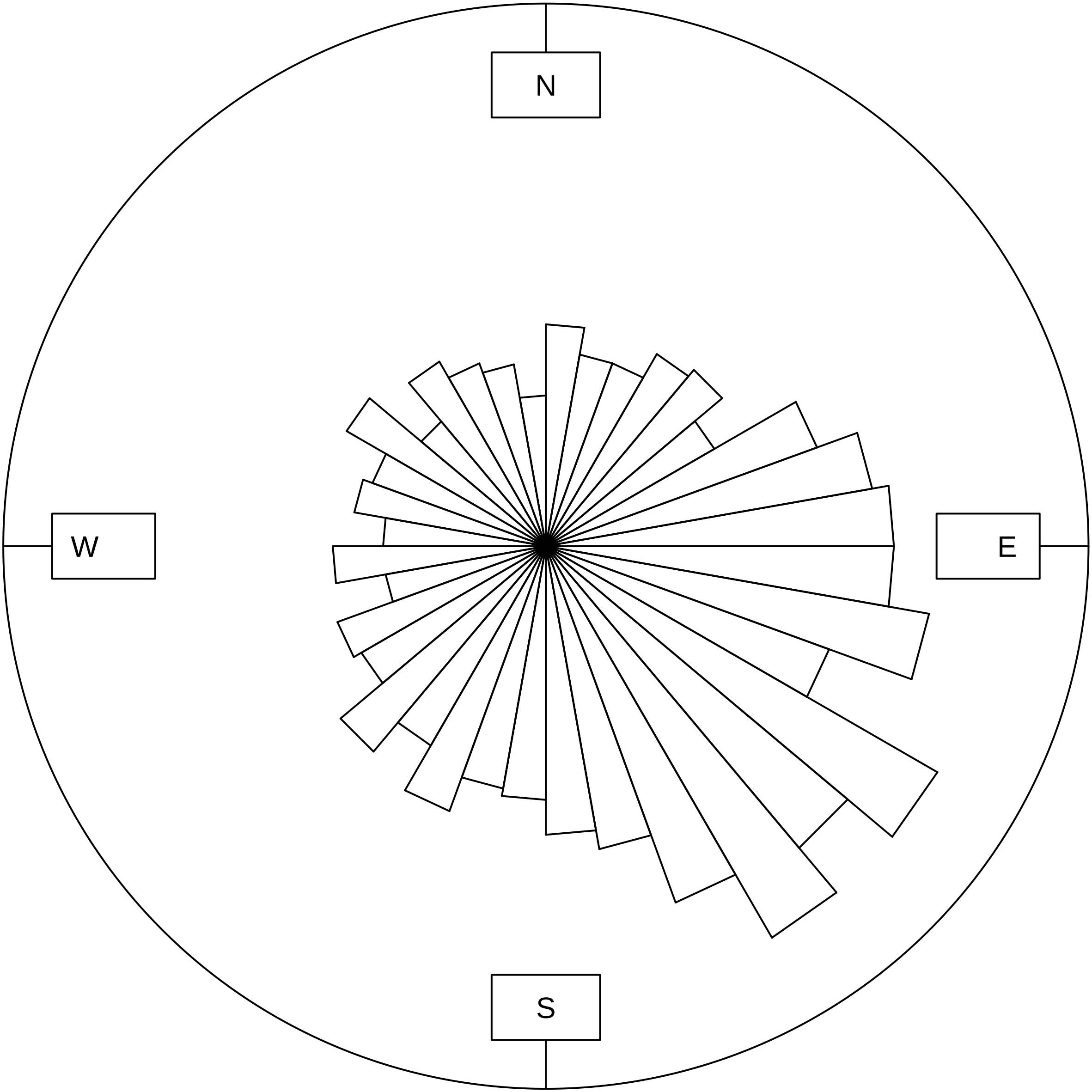

bootstrap 722

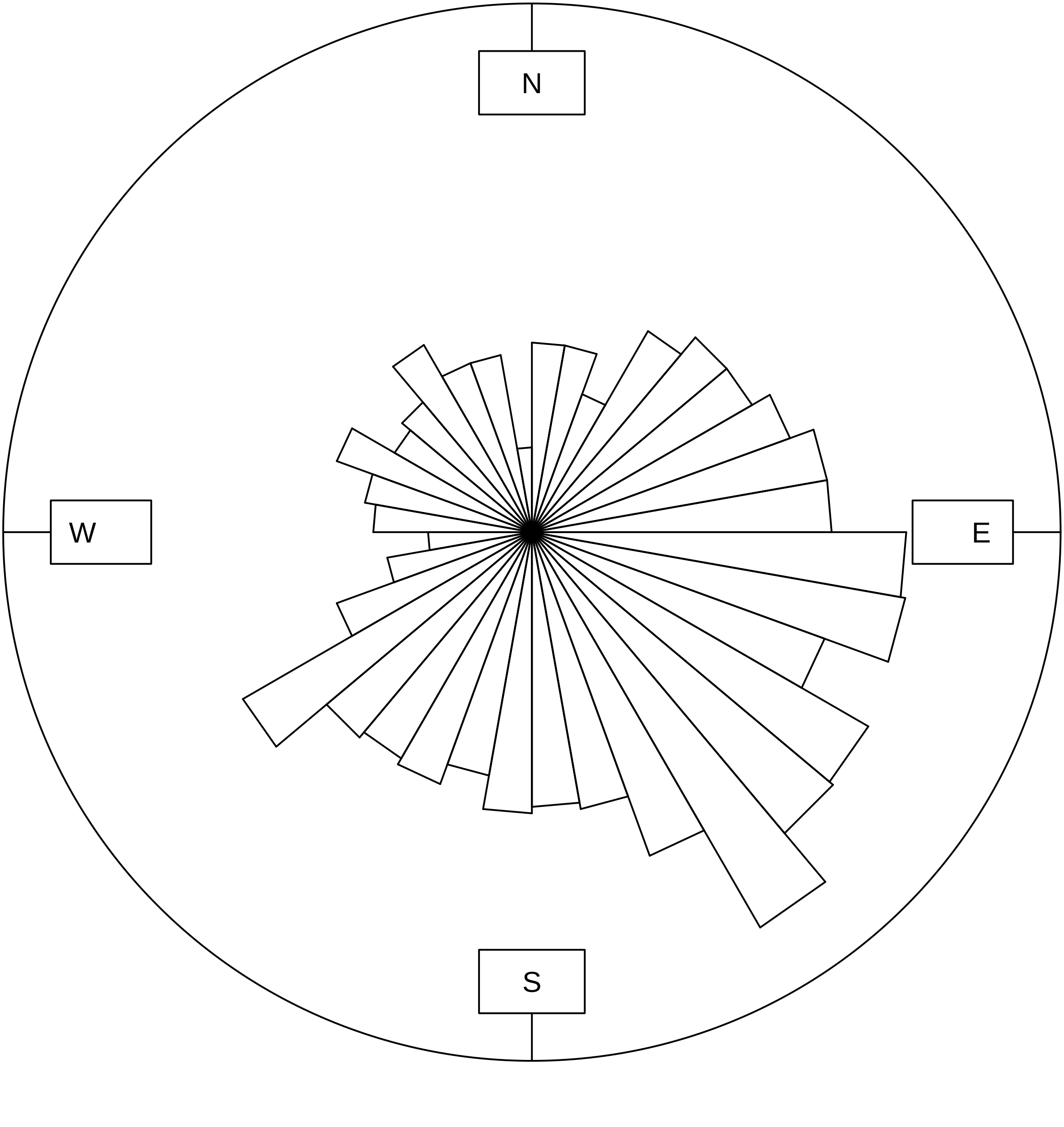

bootstrap 723

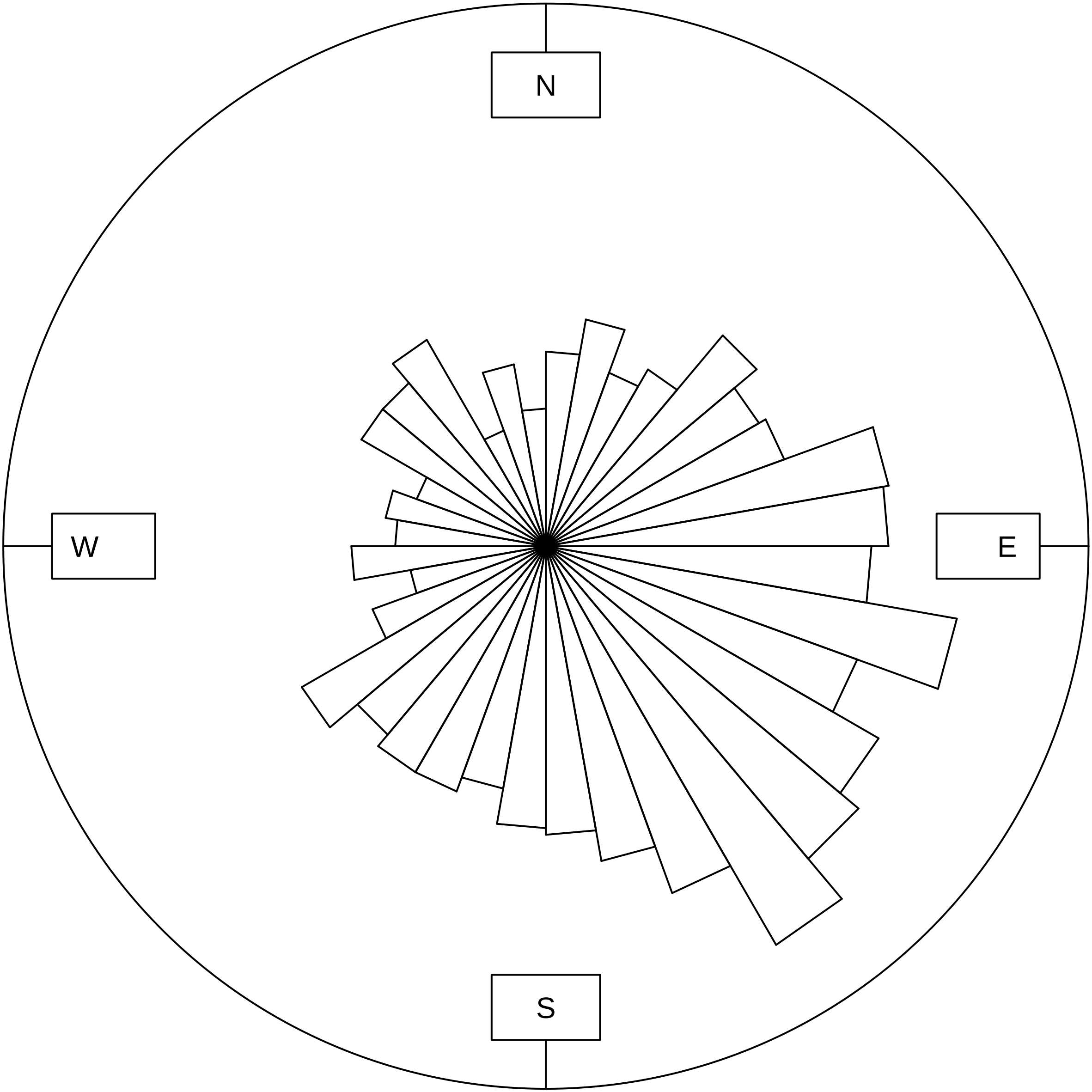

bootstrap 724

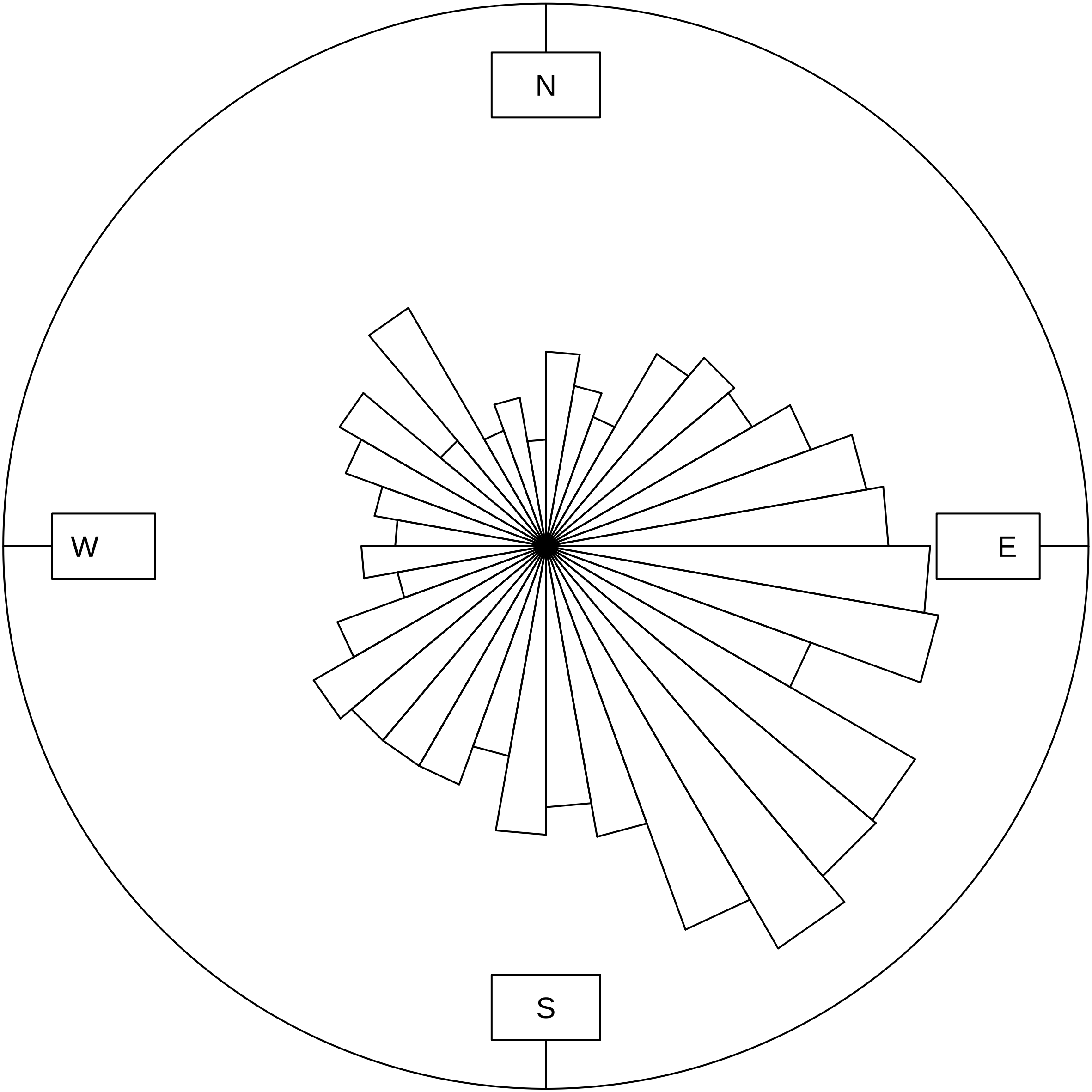

bootstrap 725

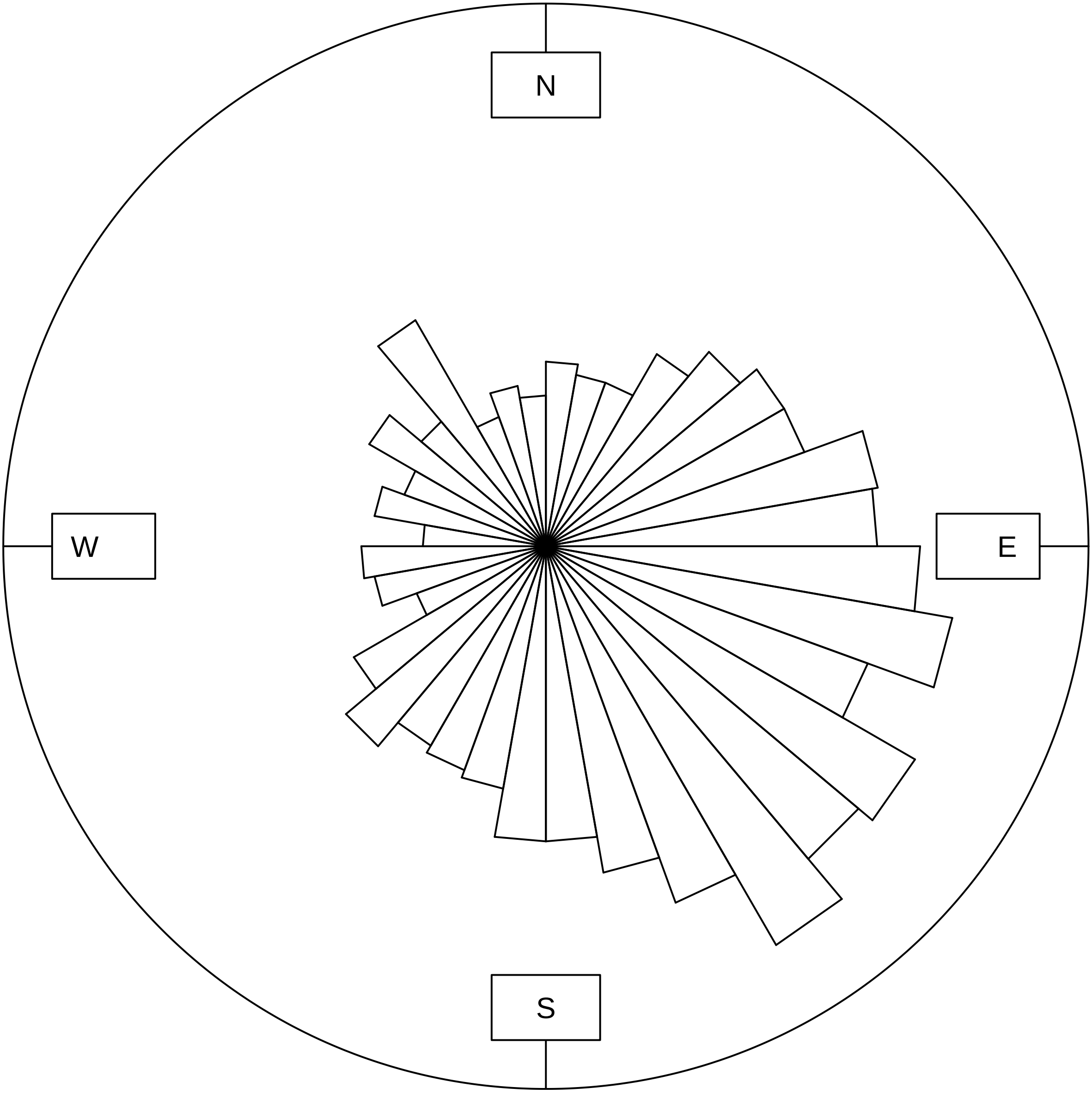

bootstrap 726

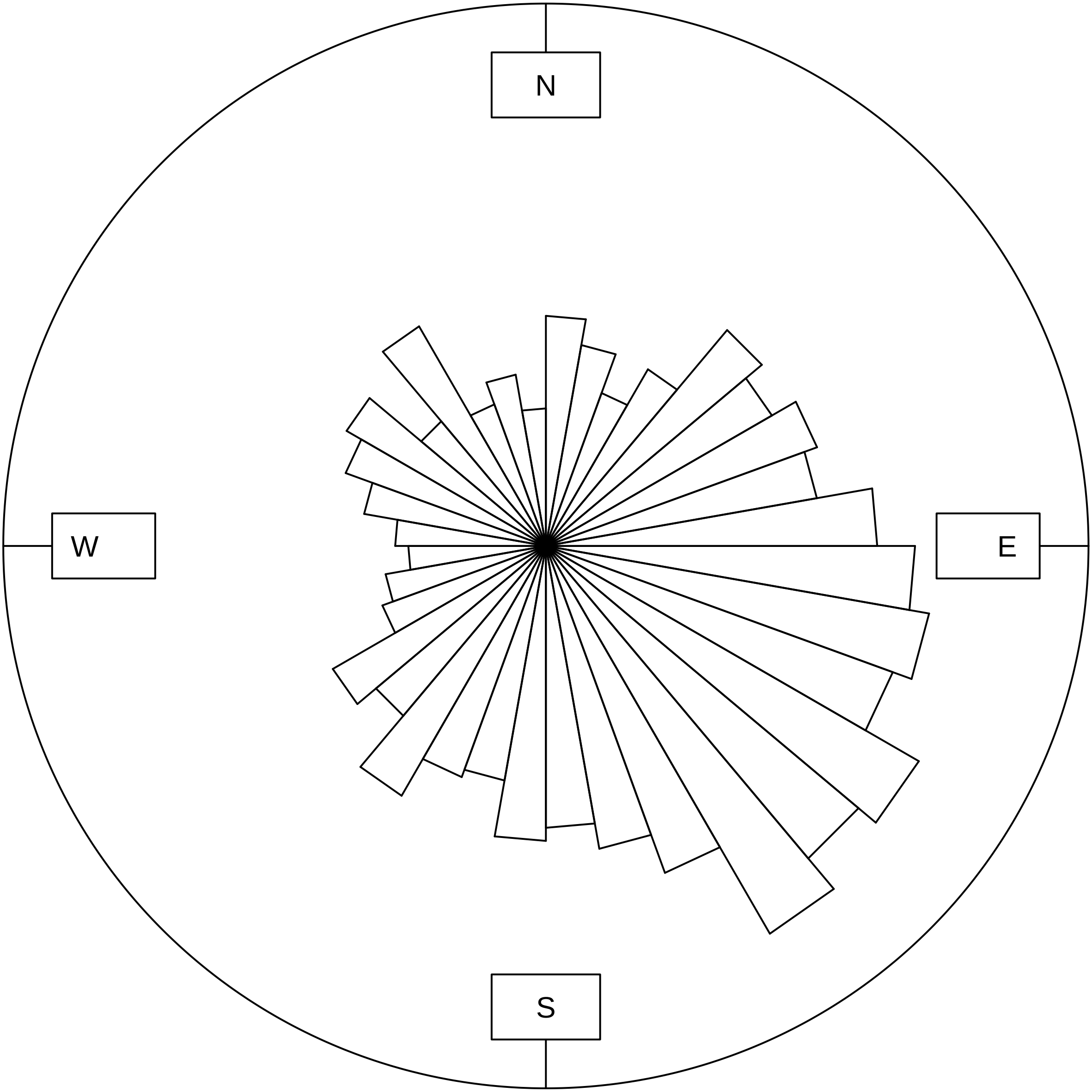

bootstrap 727

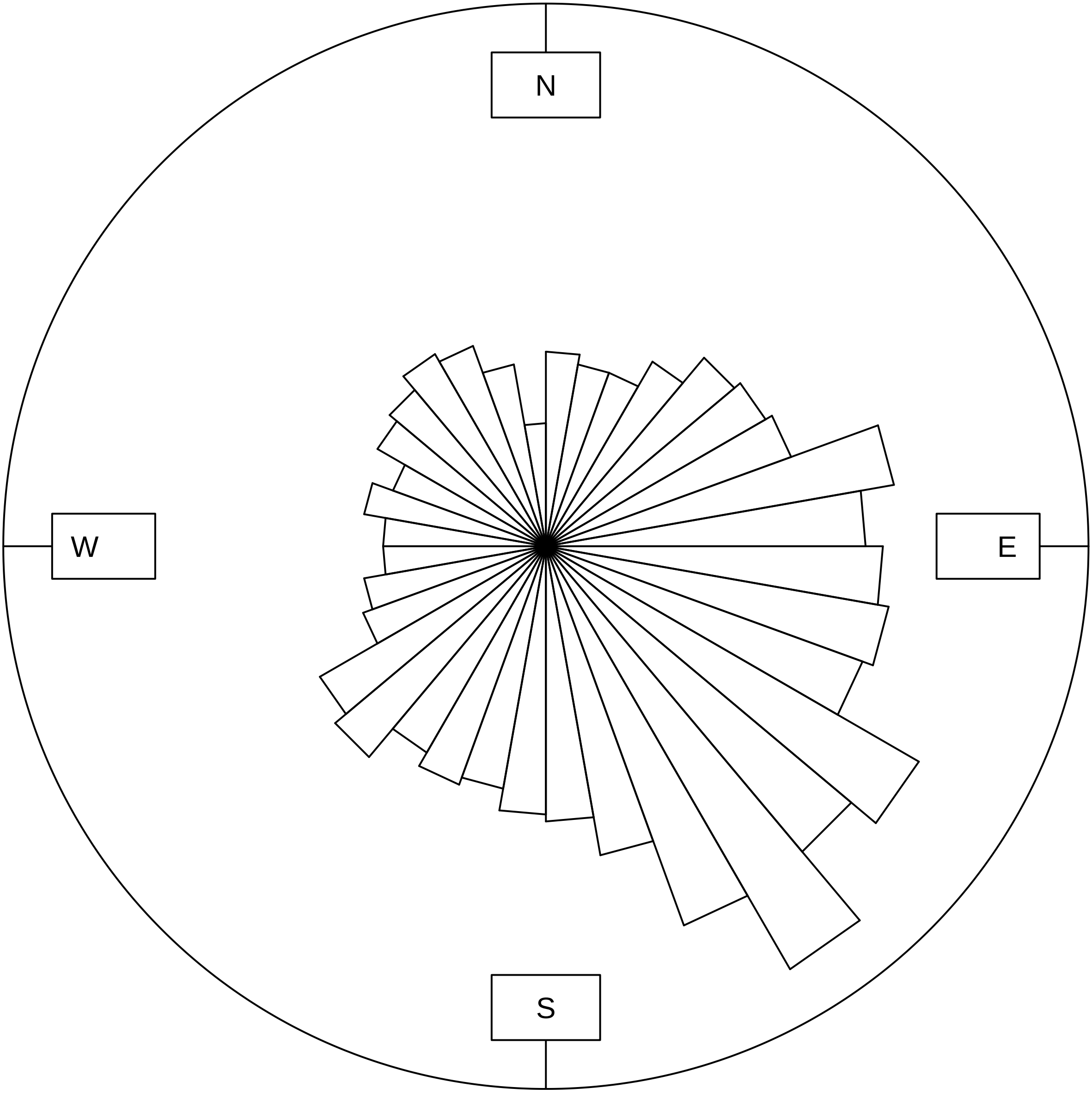

bootstrap 728

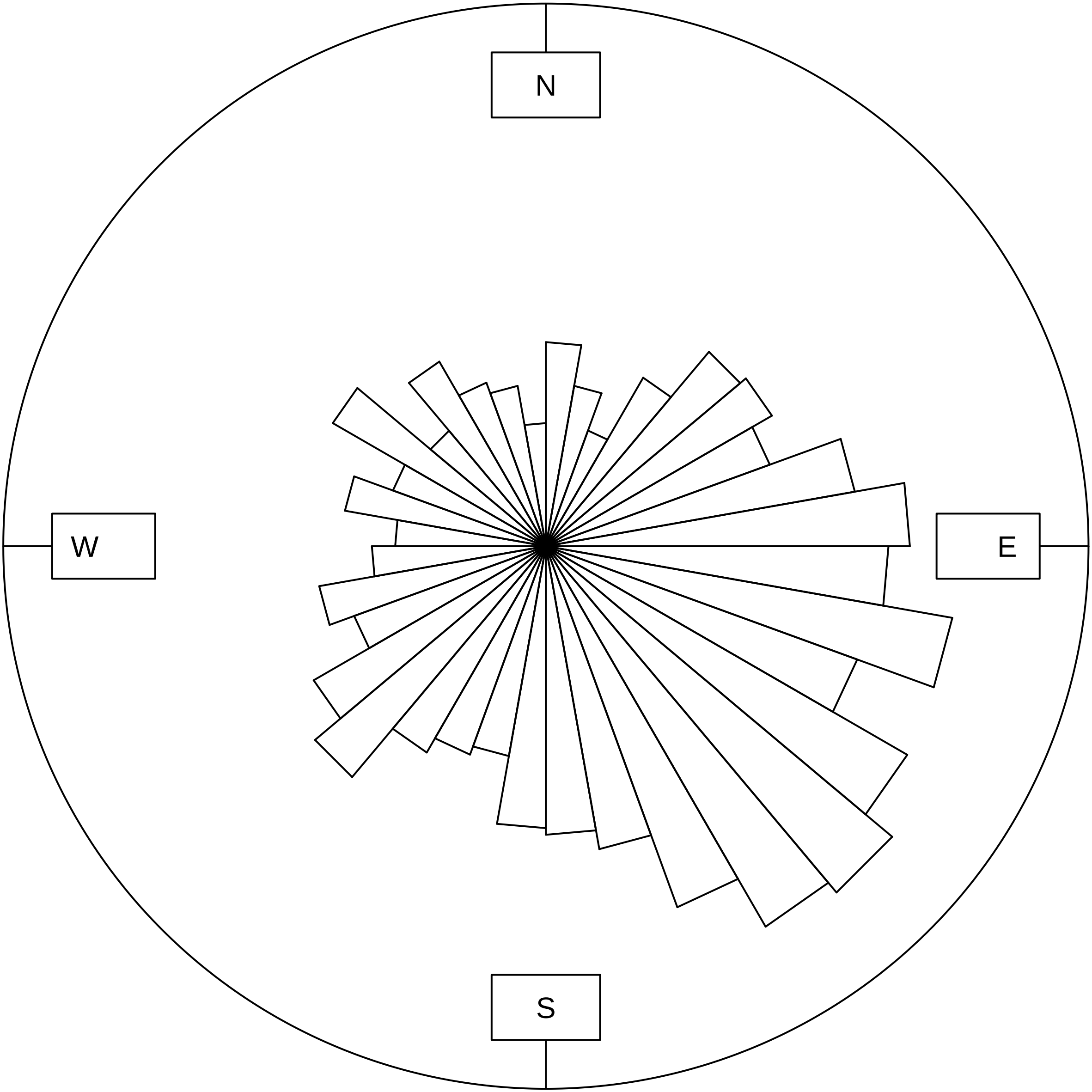

bootstrap 729

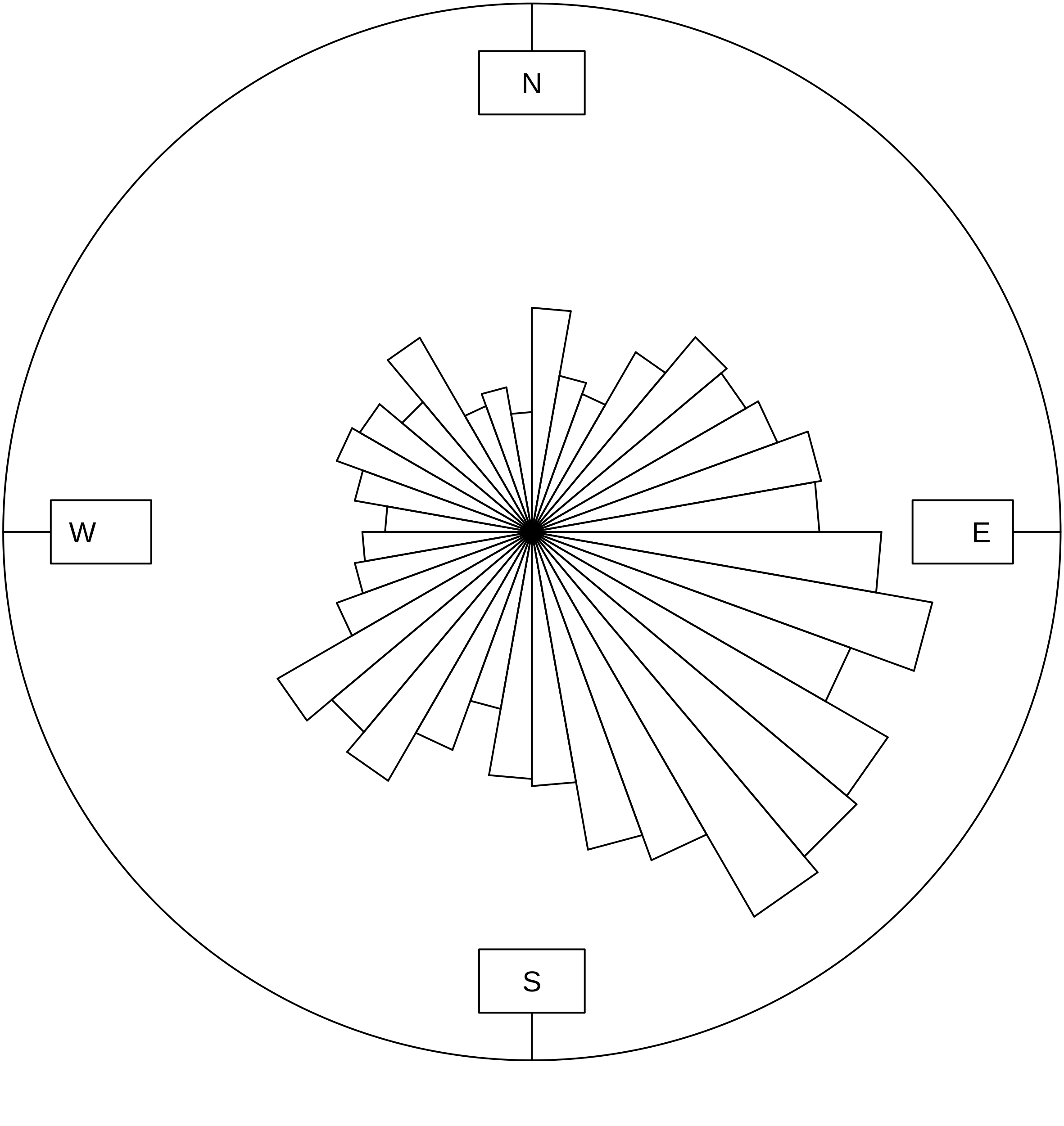

bootstrap 730

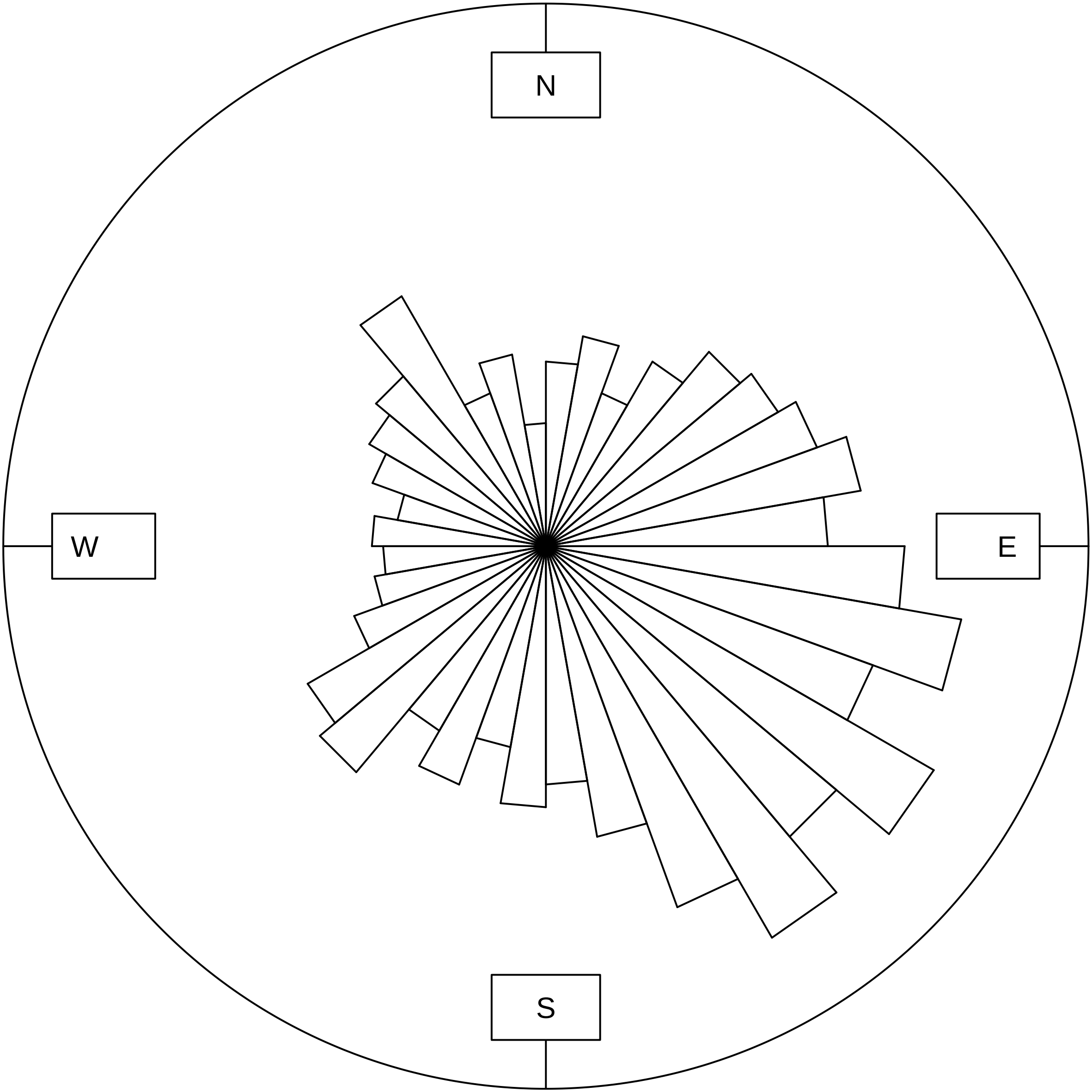

bootstrap 731

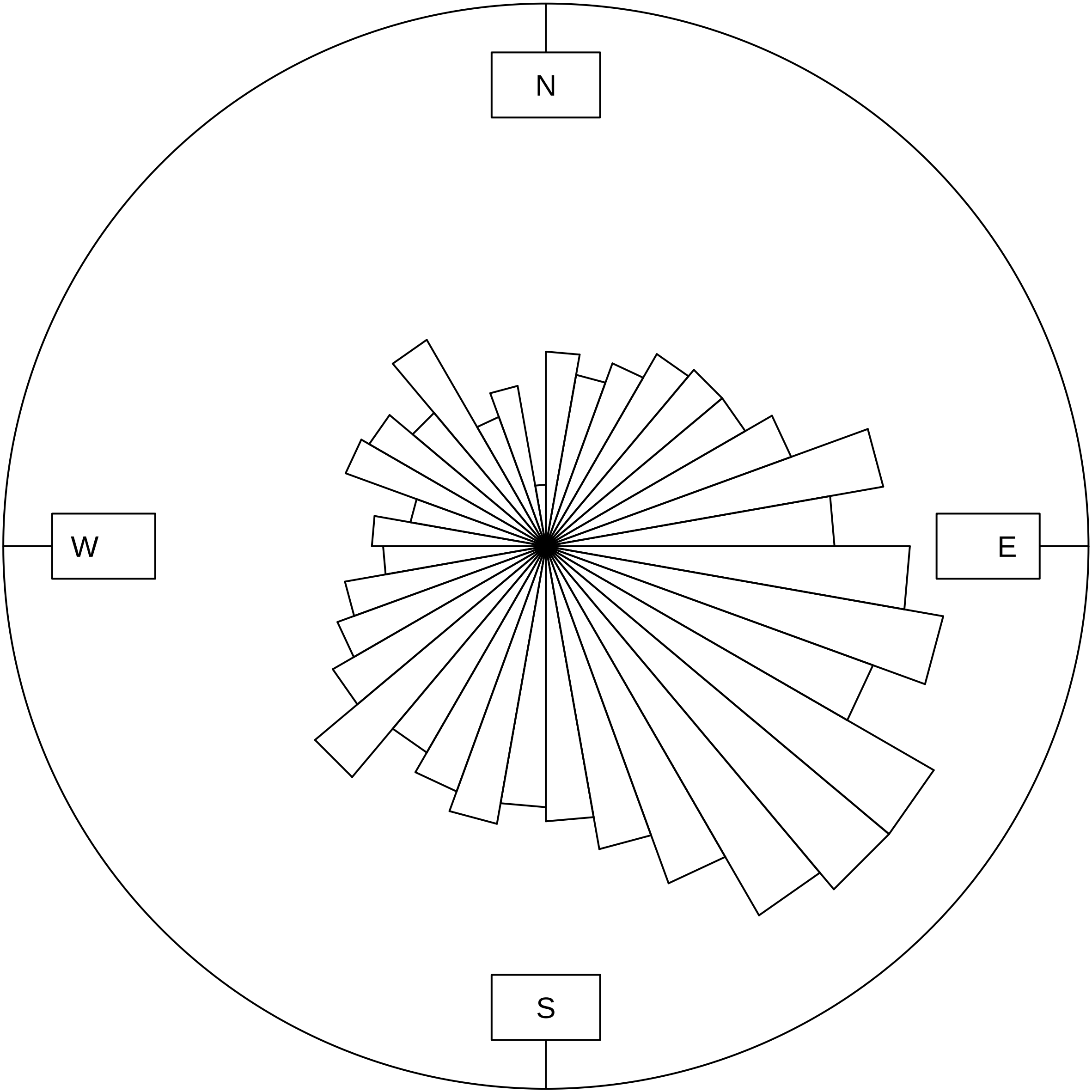

bootstrap 732

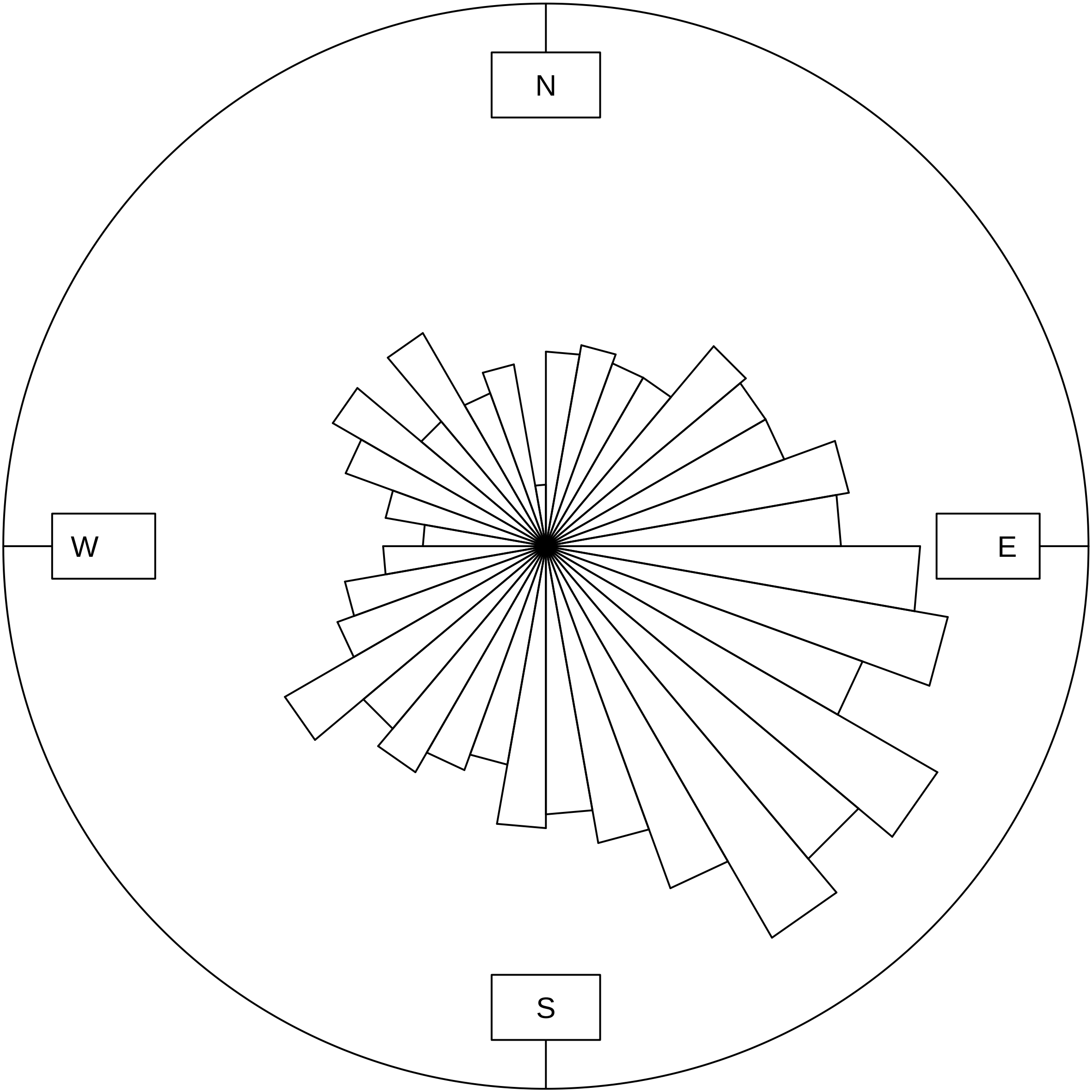

bootstrap 733

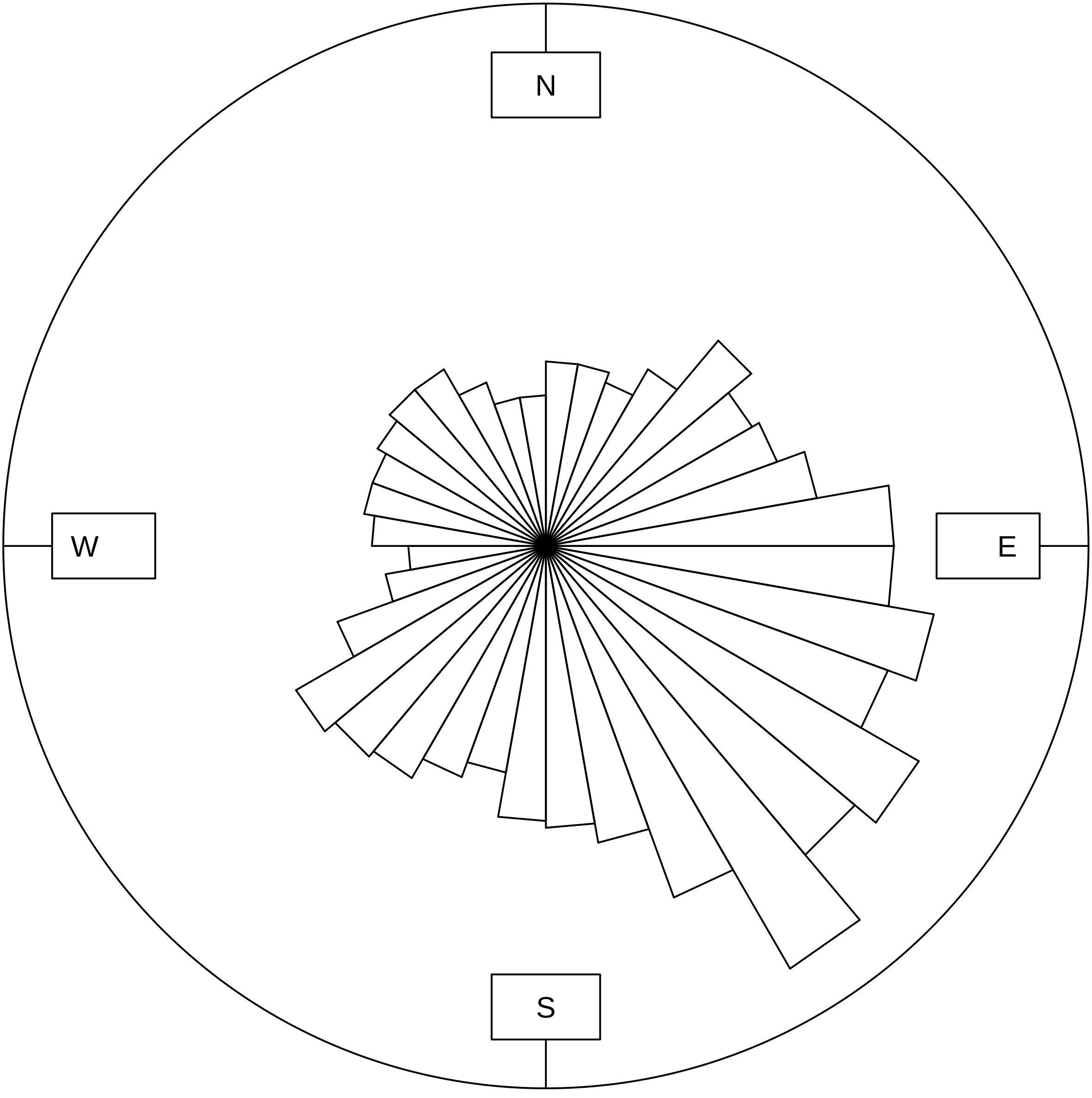

bootstrap 734

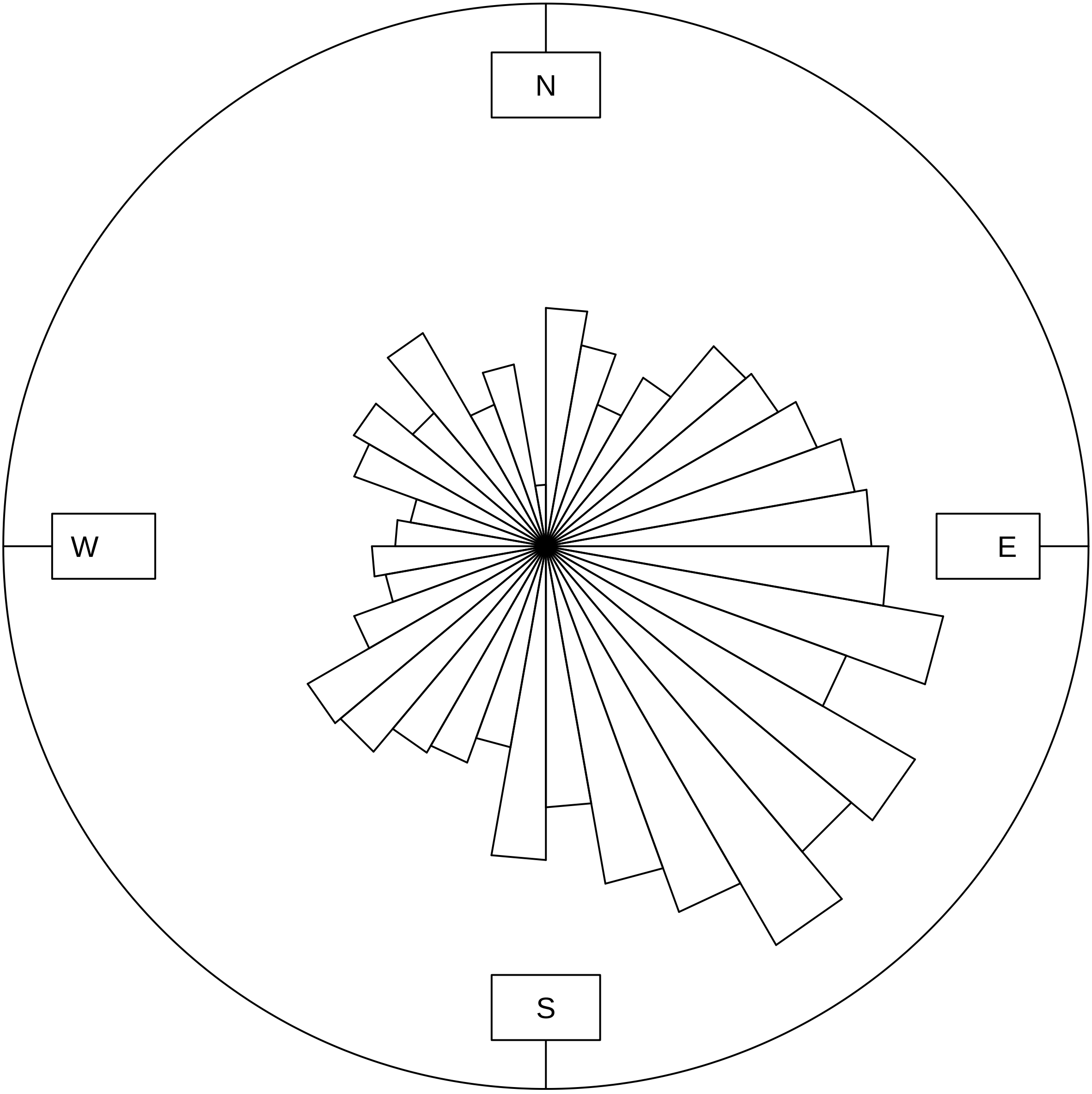

bootstrap 735

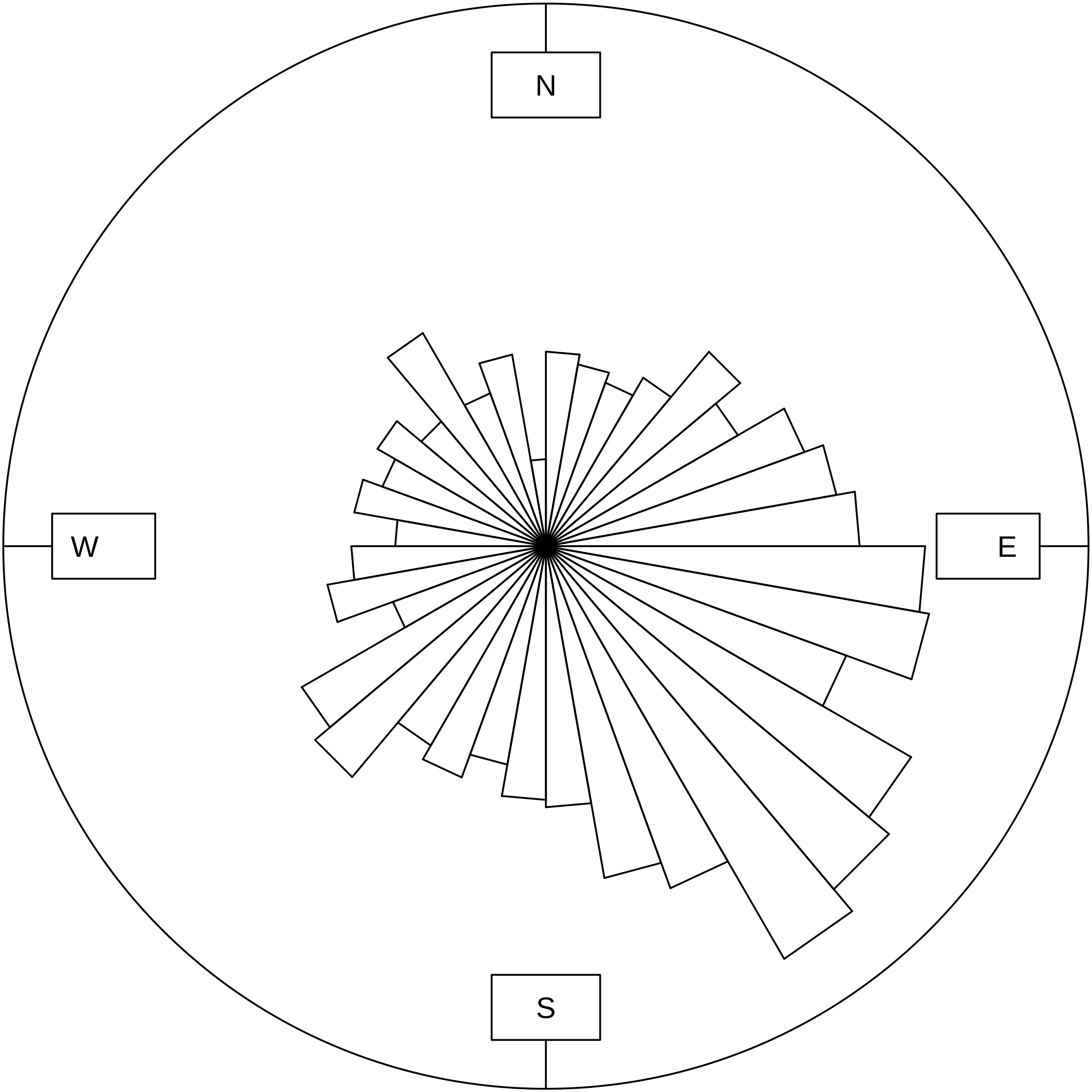

bootstrap 736

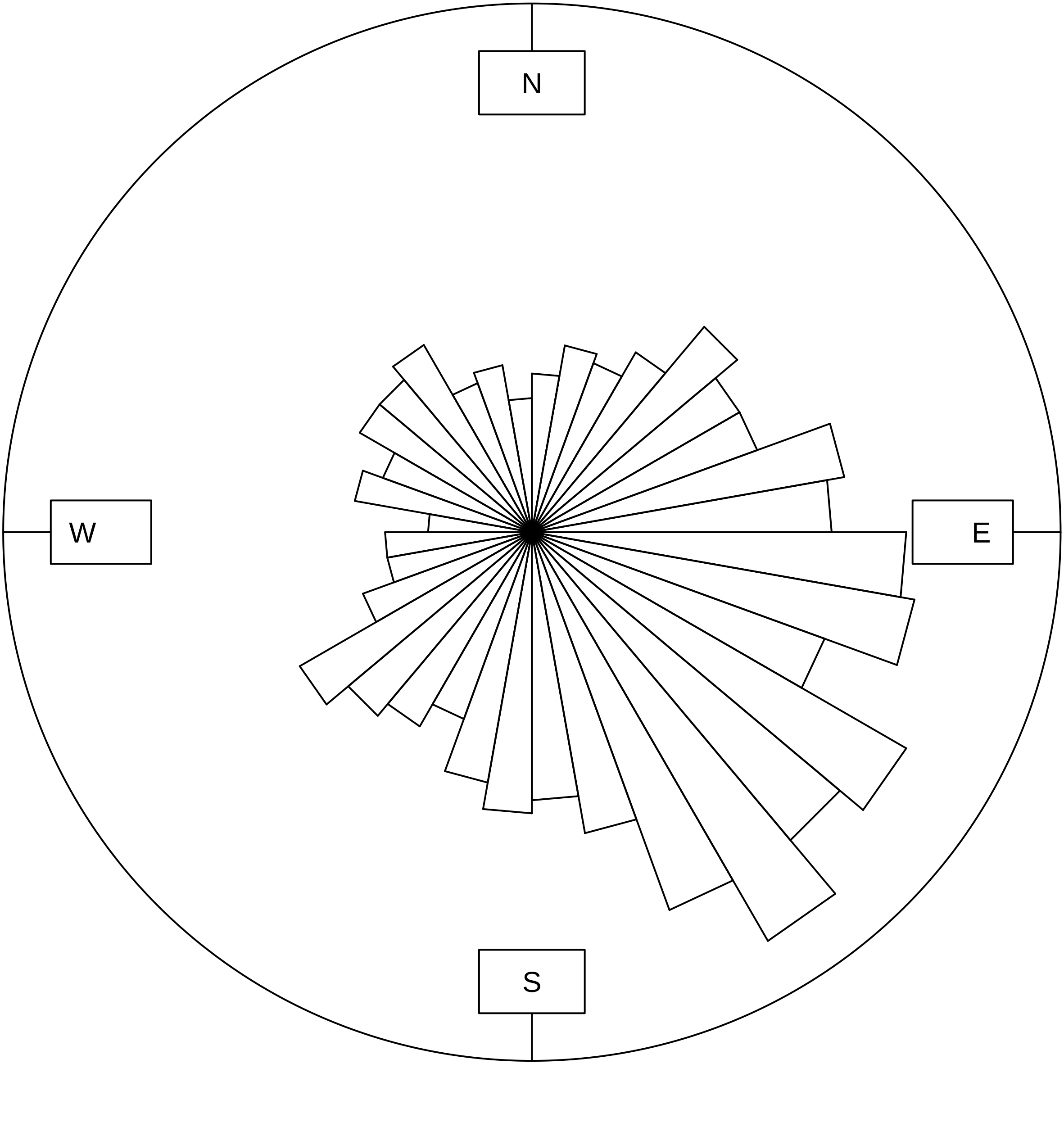

bootstrap 737

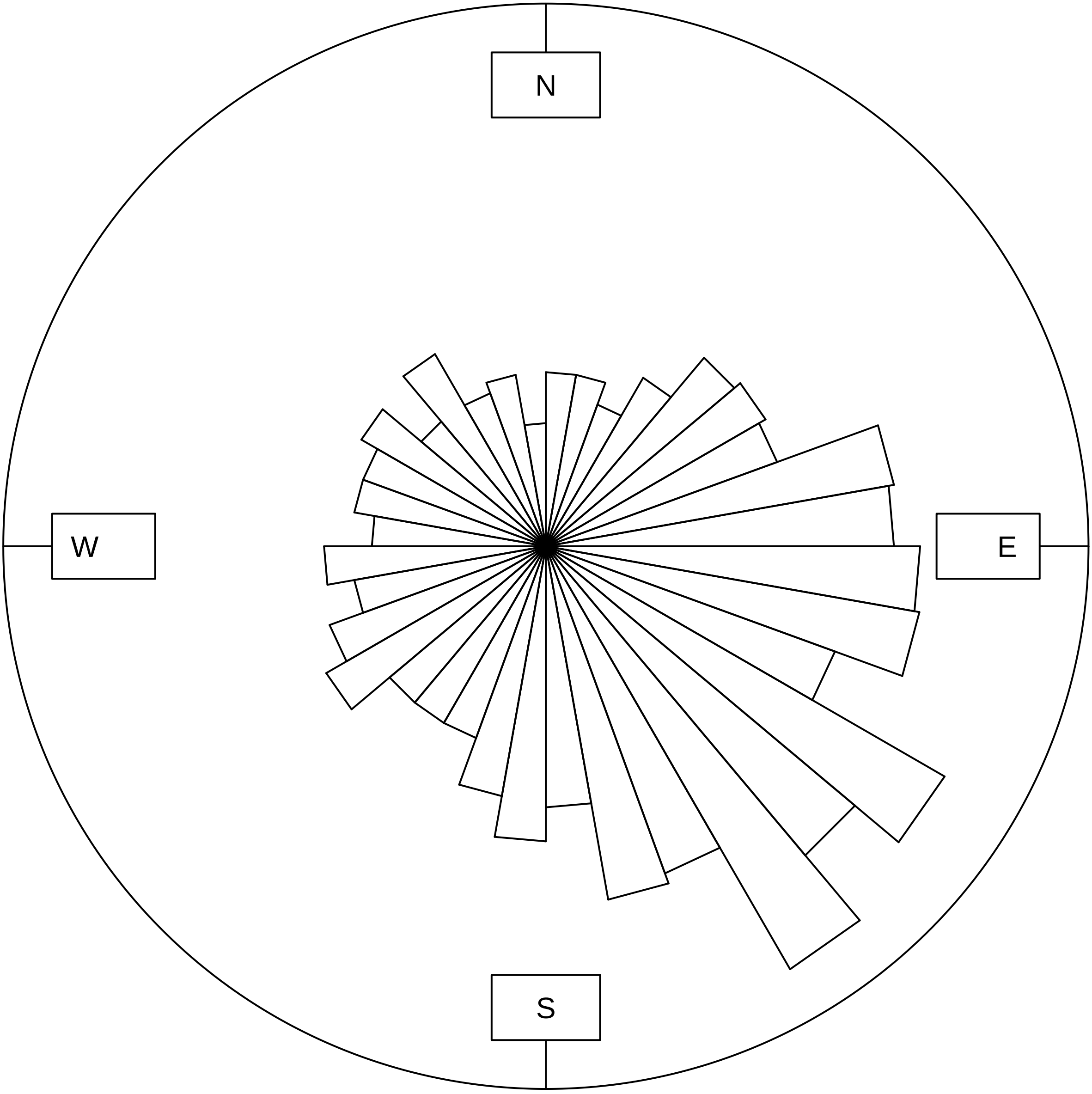

bootstrap 738

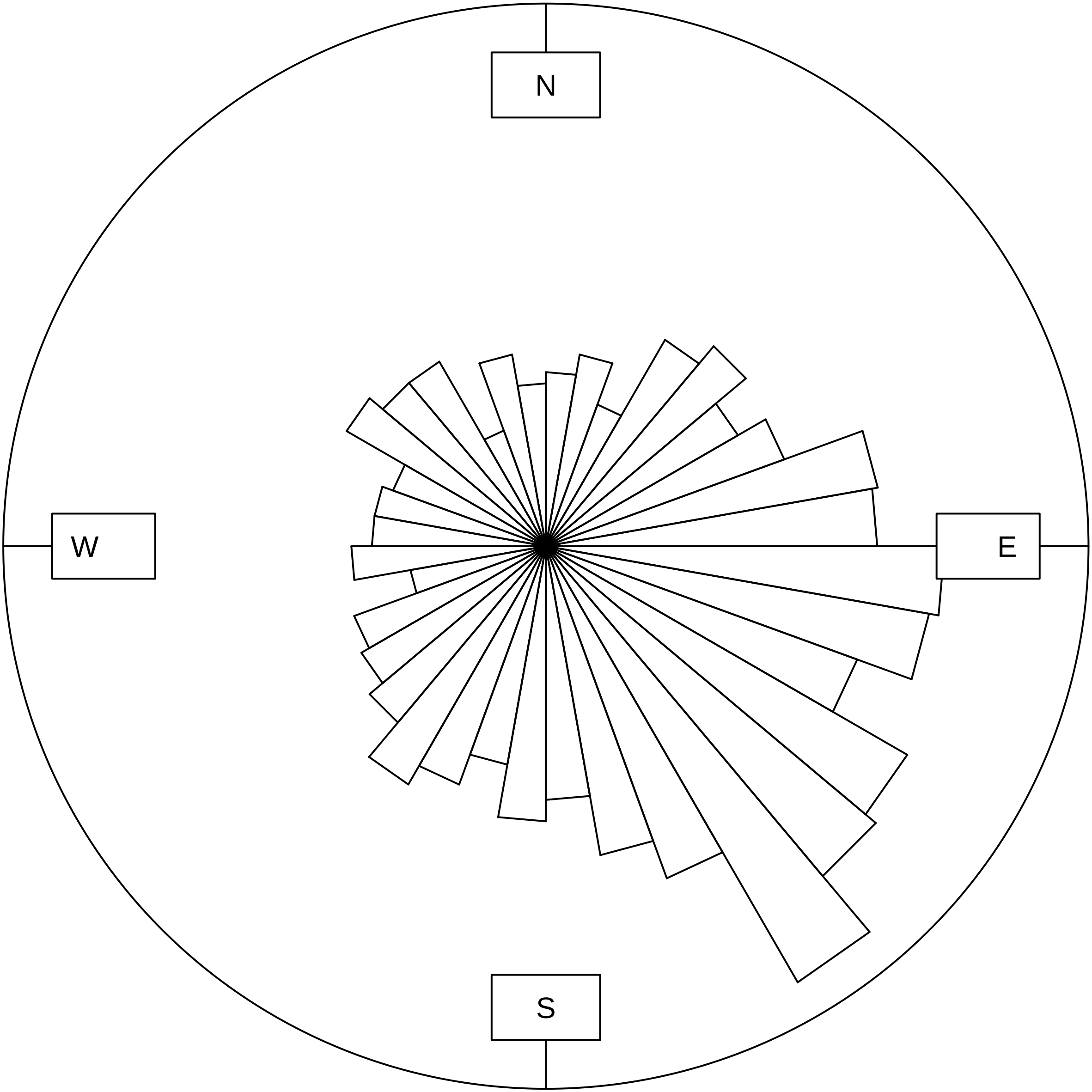

bootstrap 739

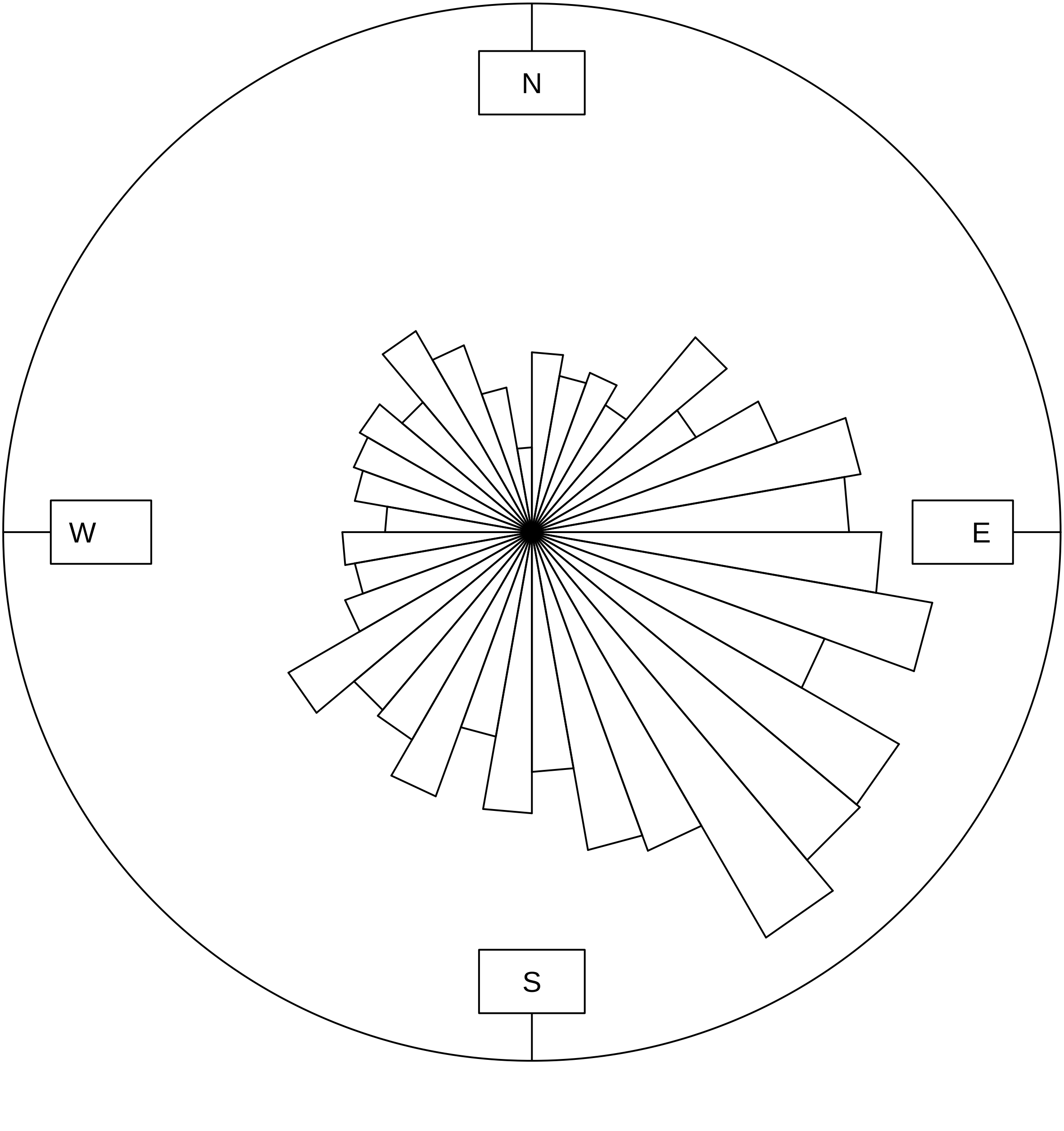

bootstrap 740

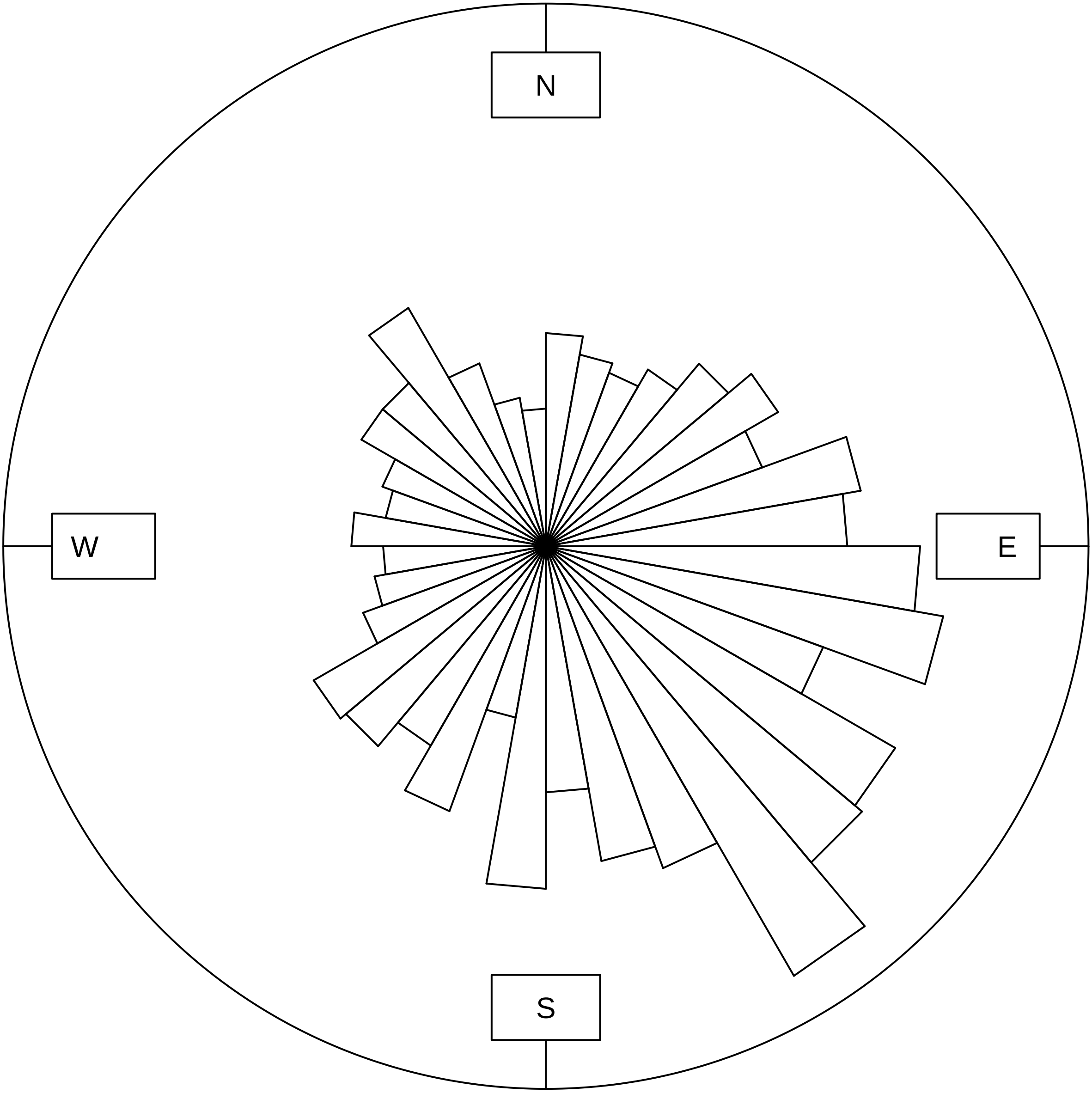

bootstrap 741

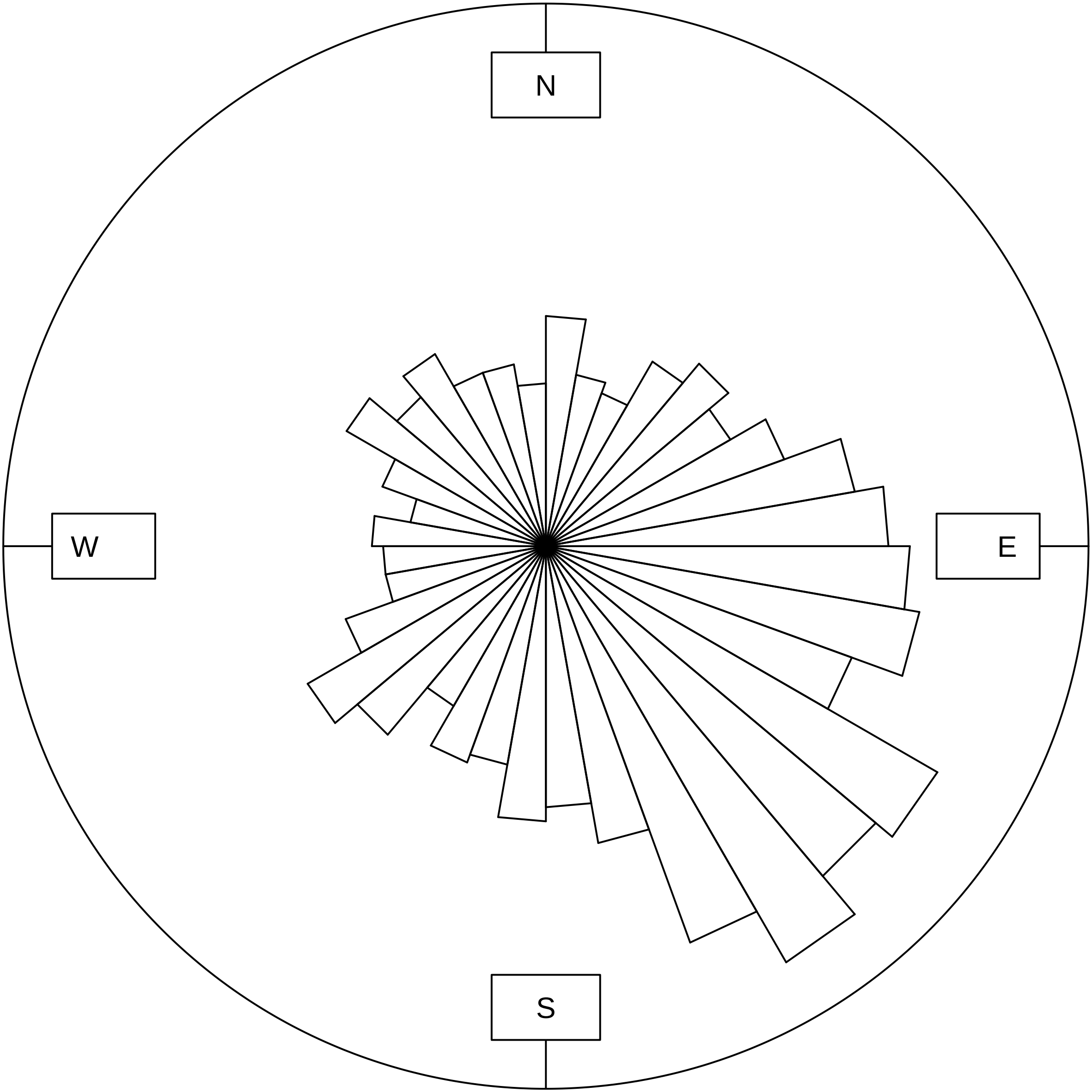

bootstrap 742

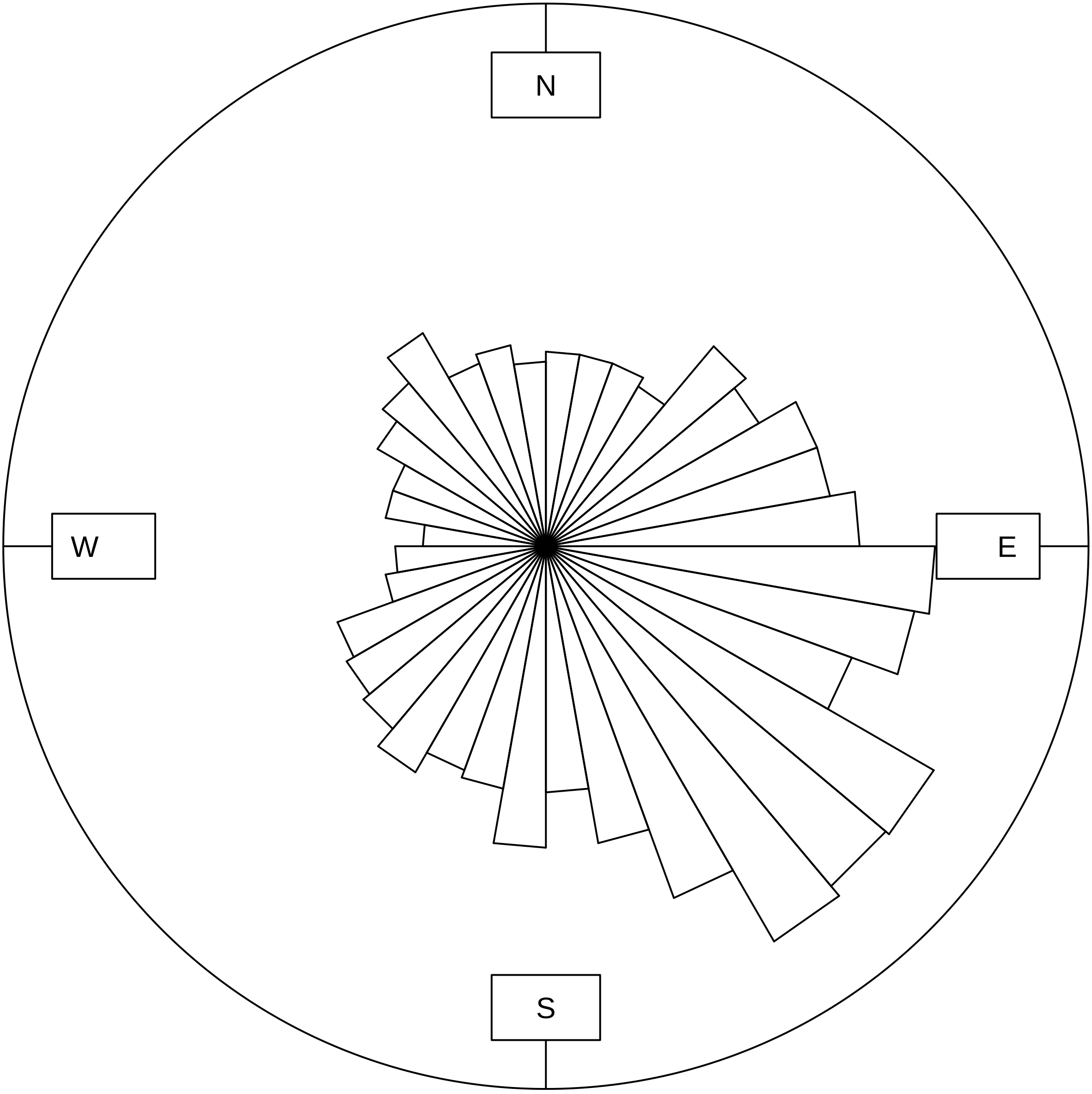

bootstrap 743

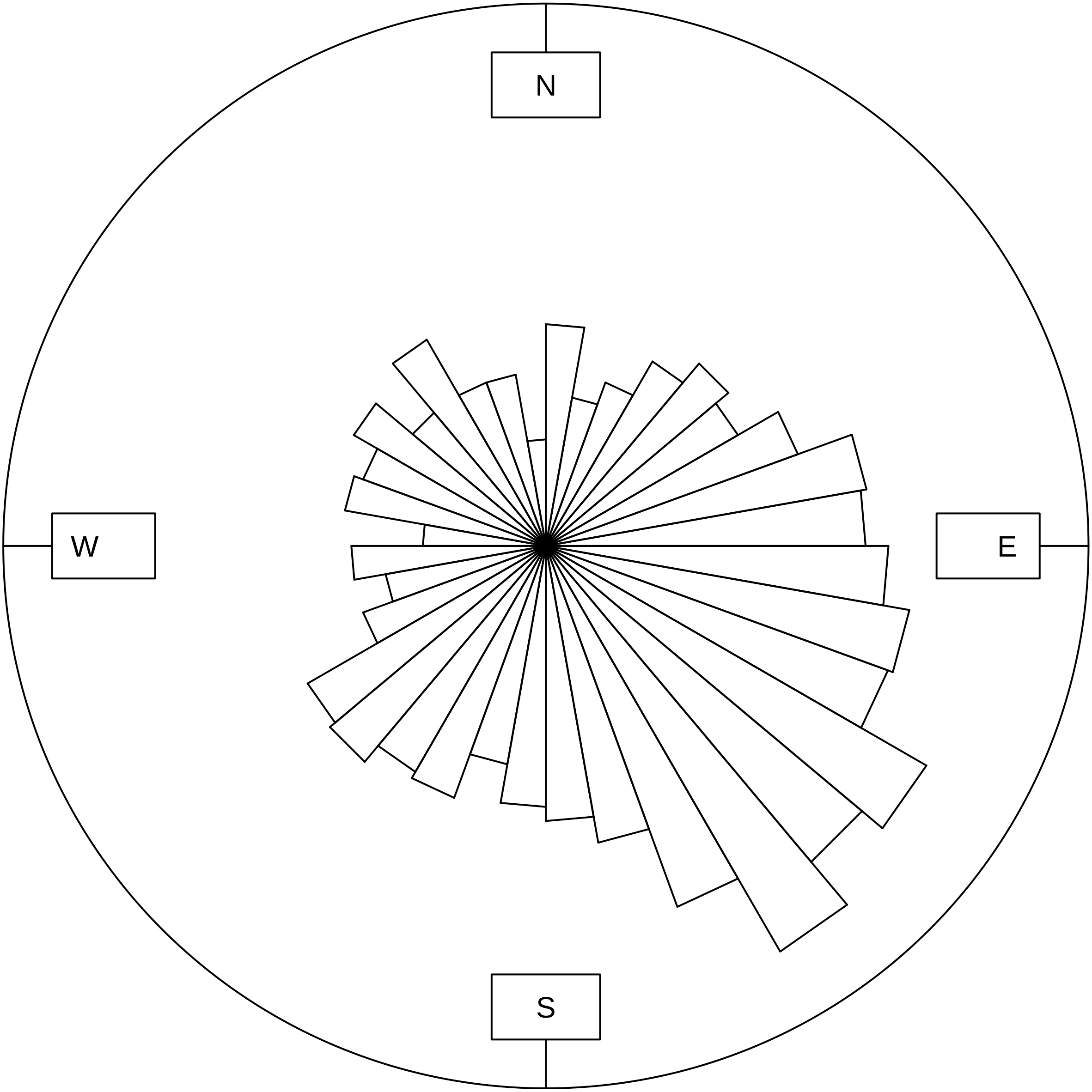

bootstrap 744

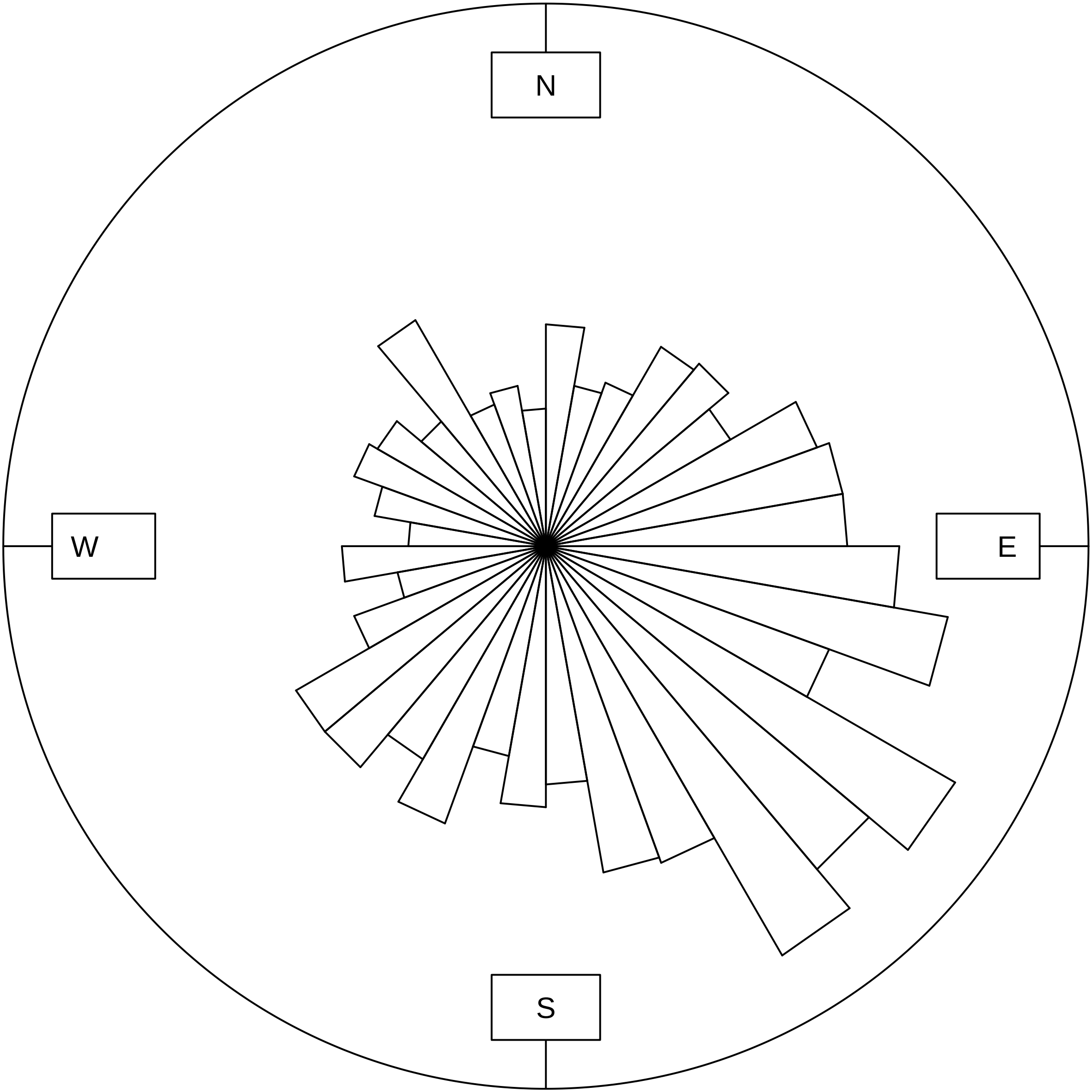

bootstrap 745

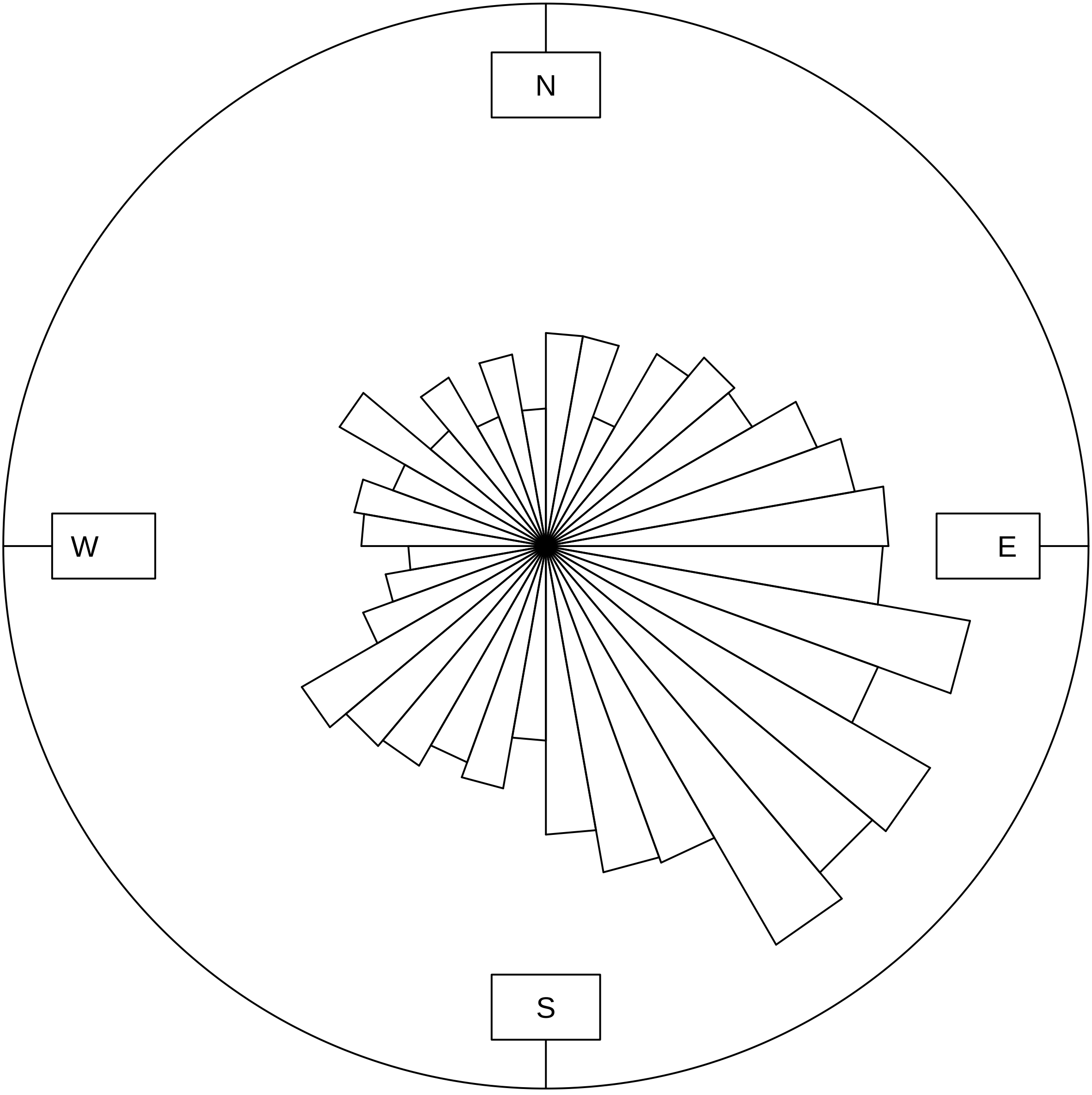

bootstrap 746

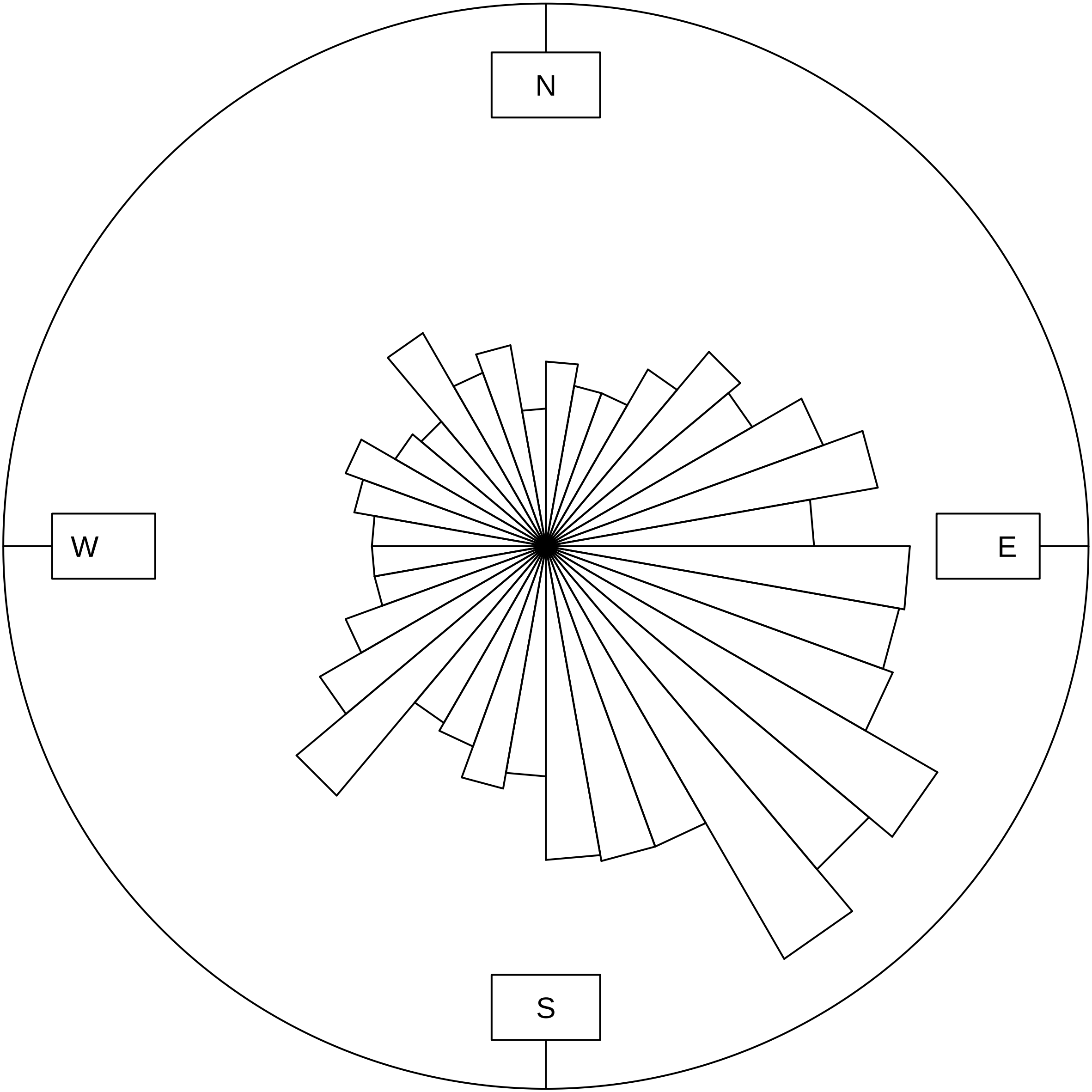

bootstrap 747

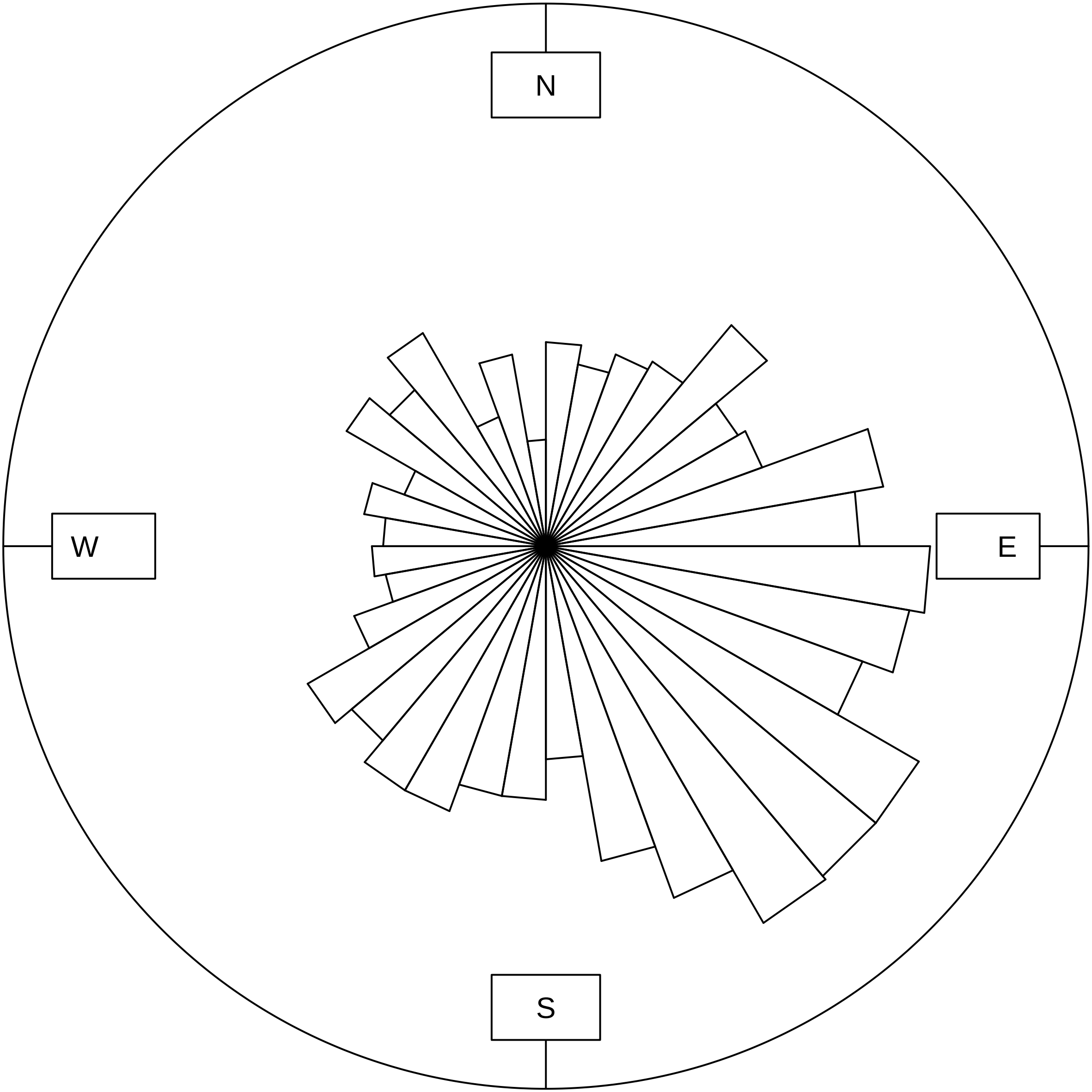

bootstrap 748

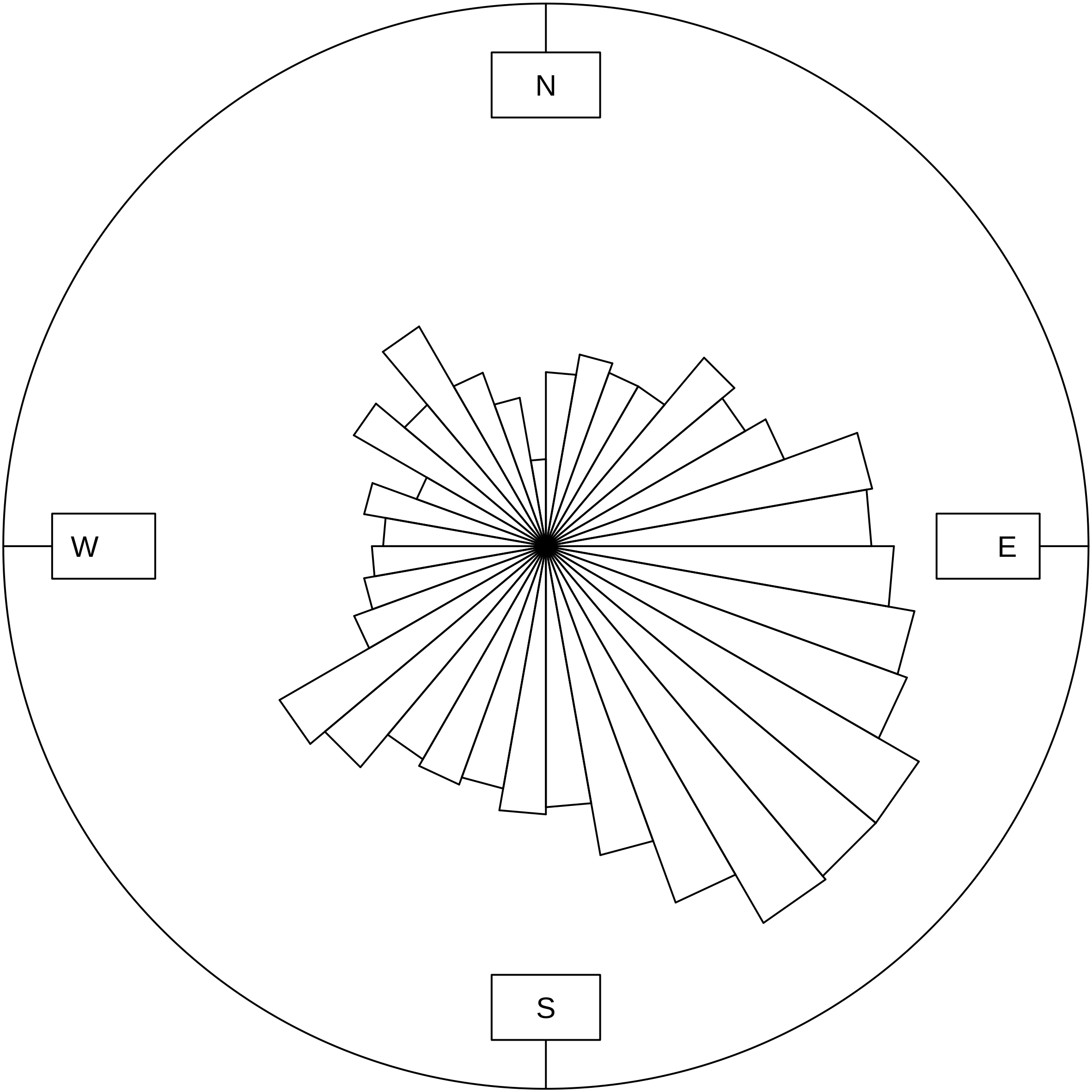

bootstrap 749

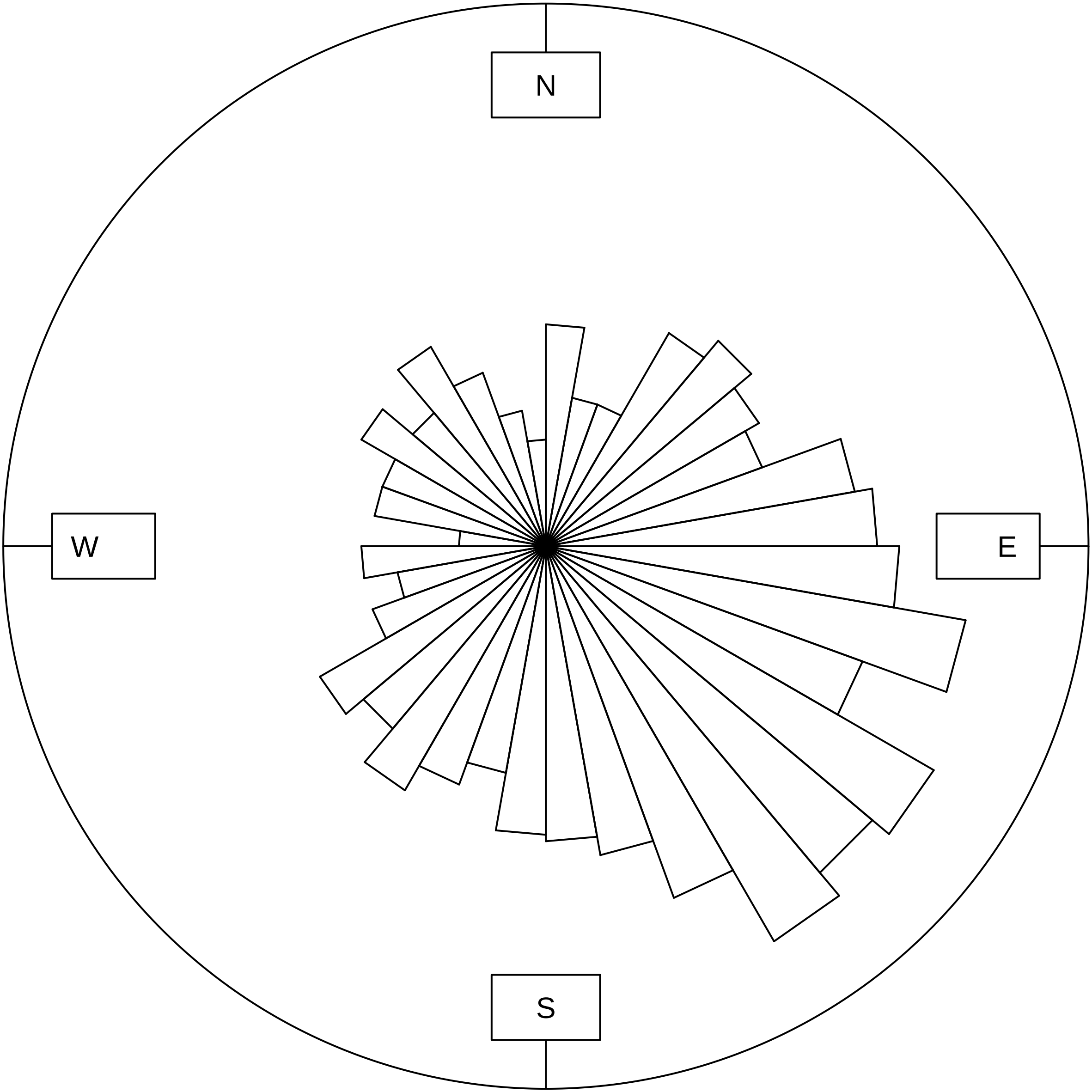

bootstrap 750

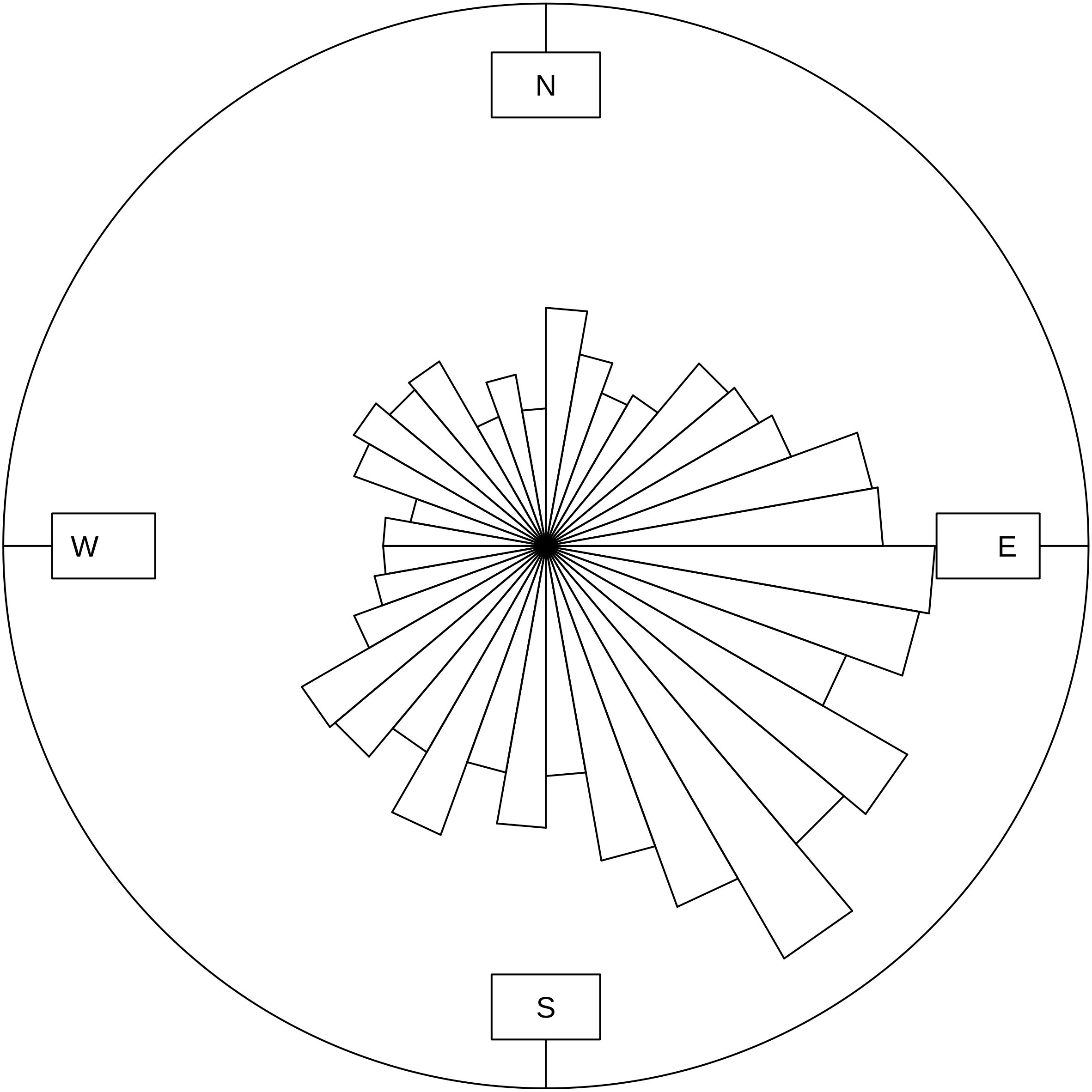

bootstrap 751

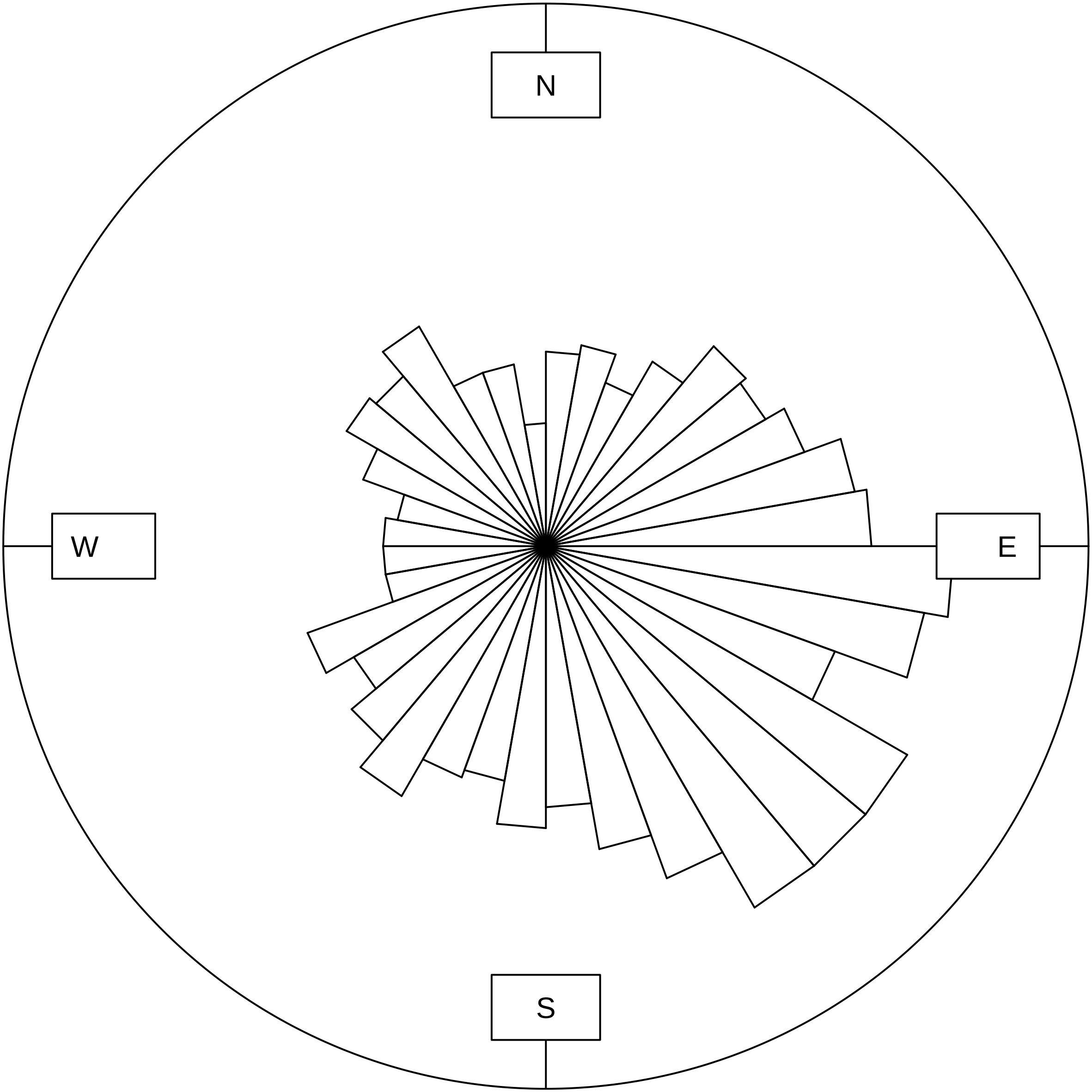

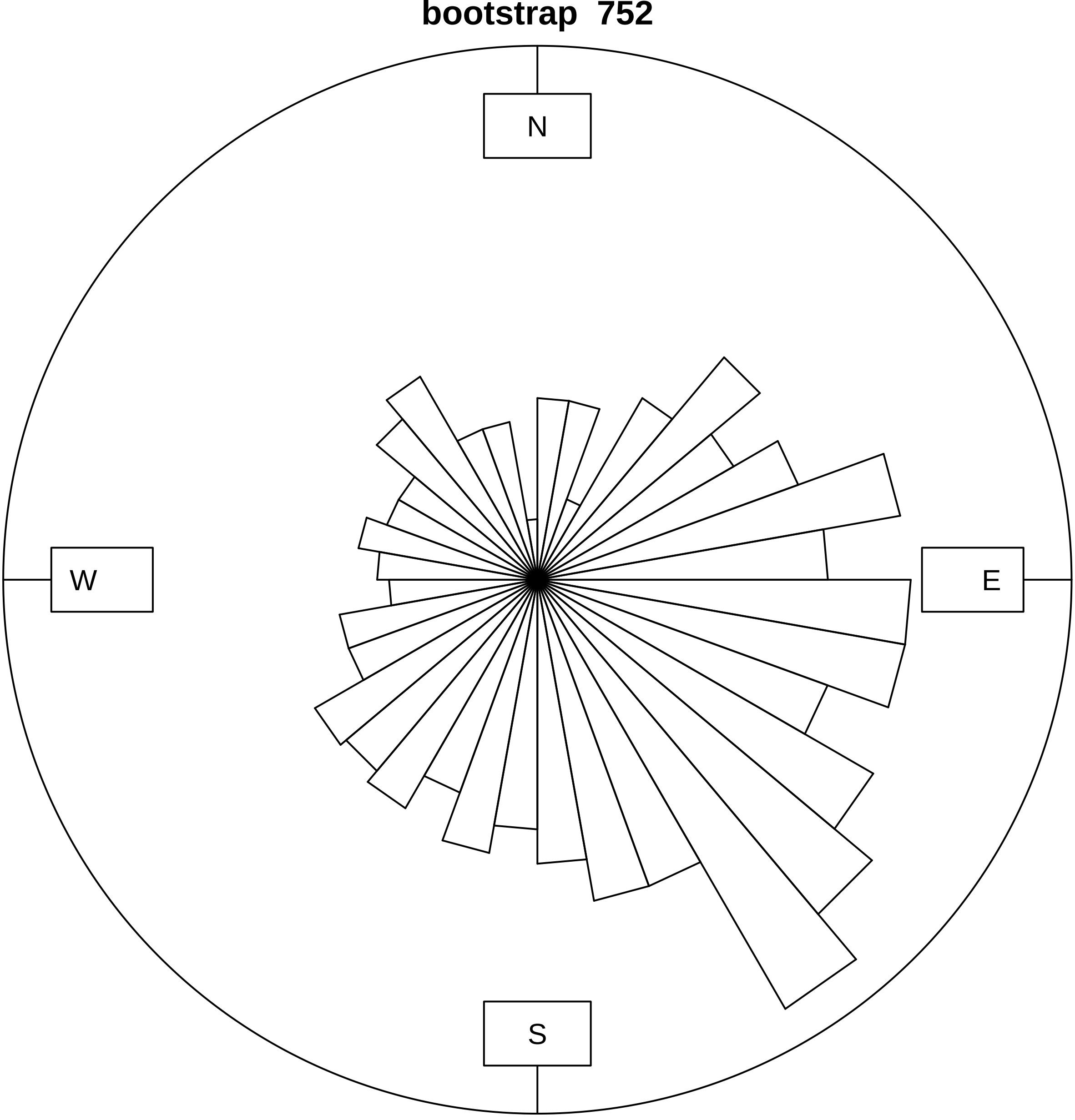
bootstrap 752
N
W
E
S

bootstrap 753

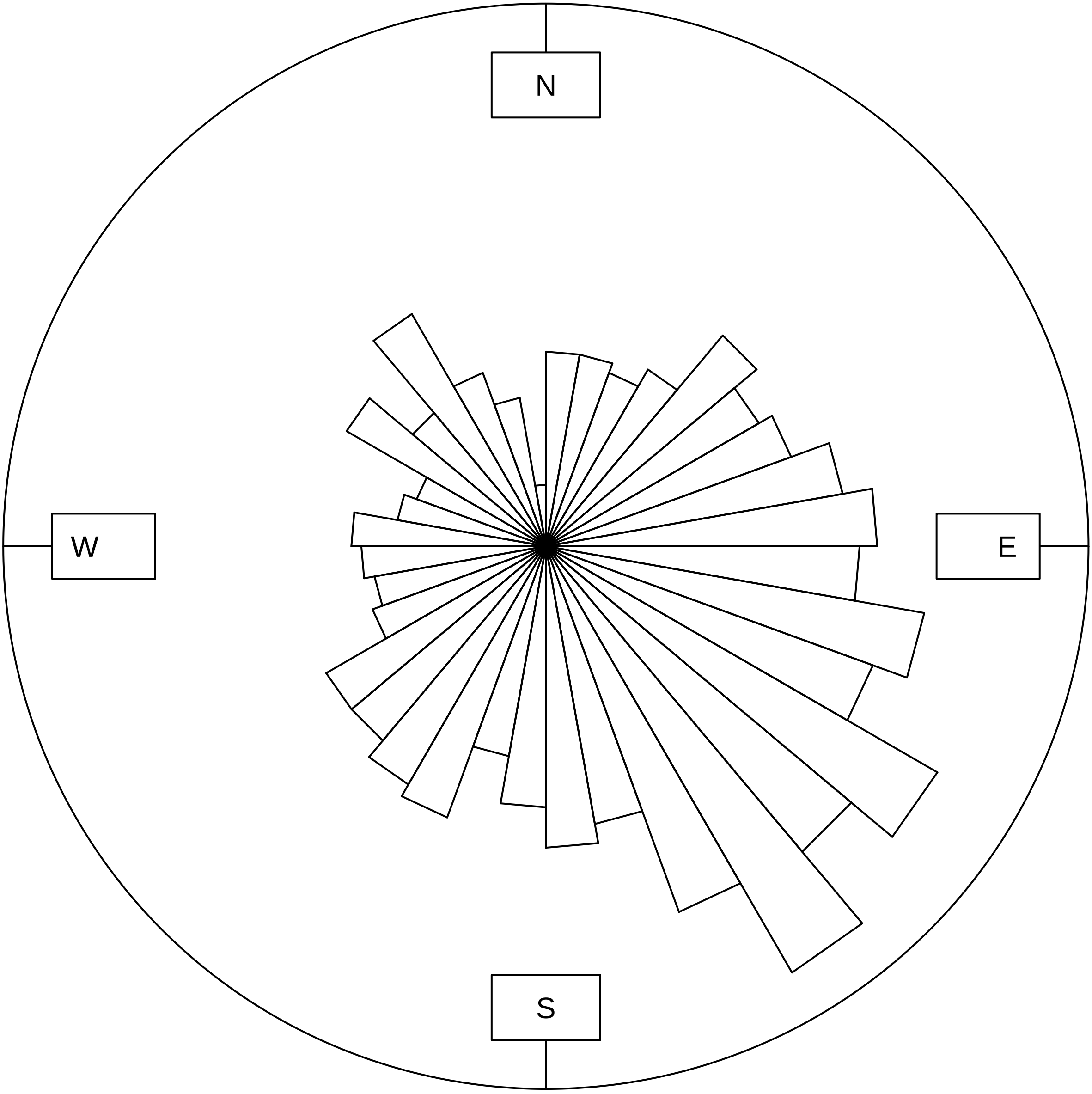

bootstrap 754

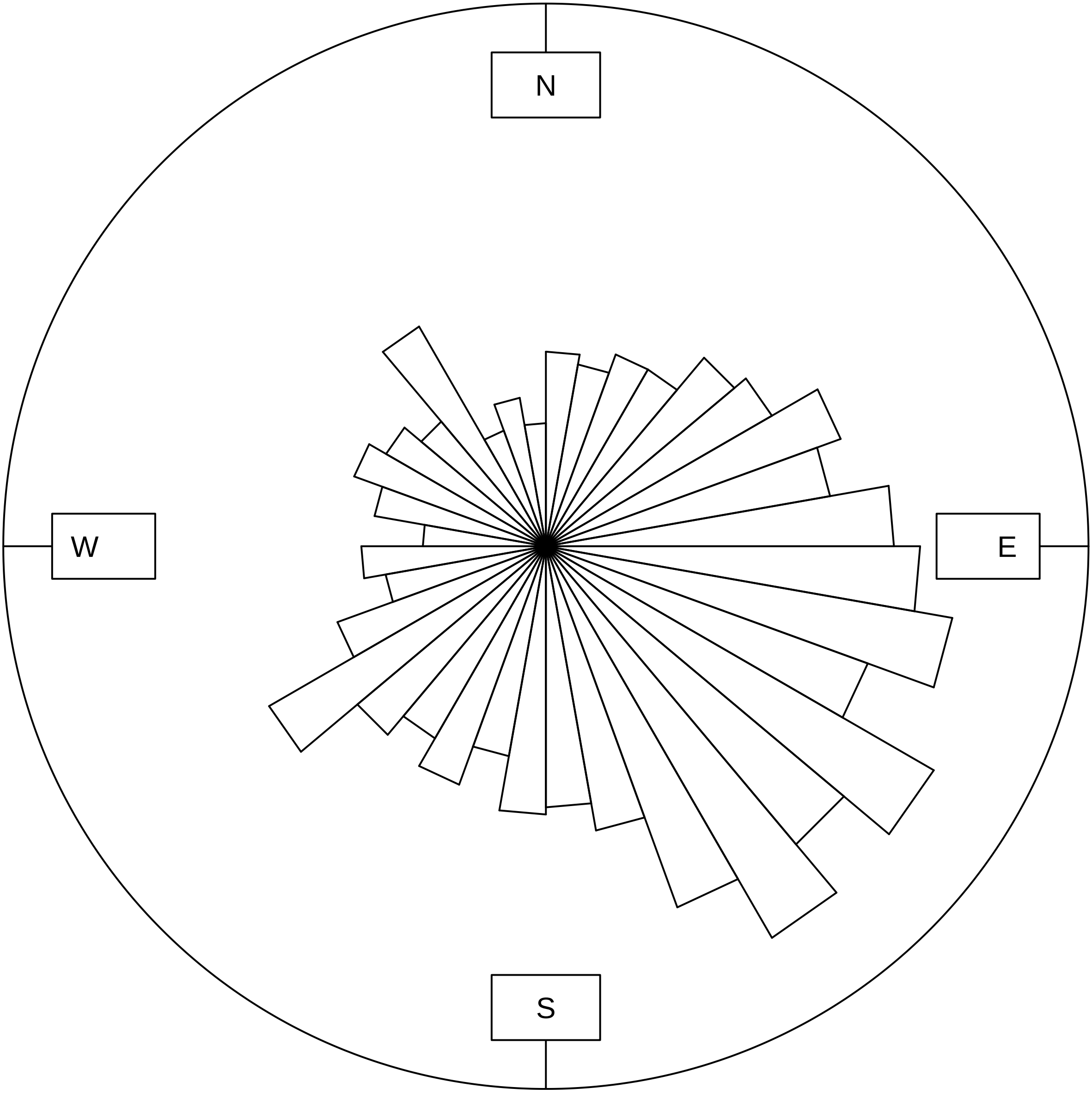

bootstrap 755

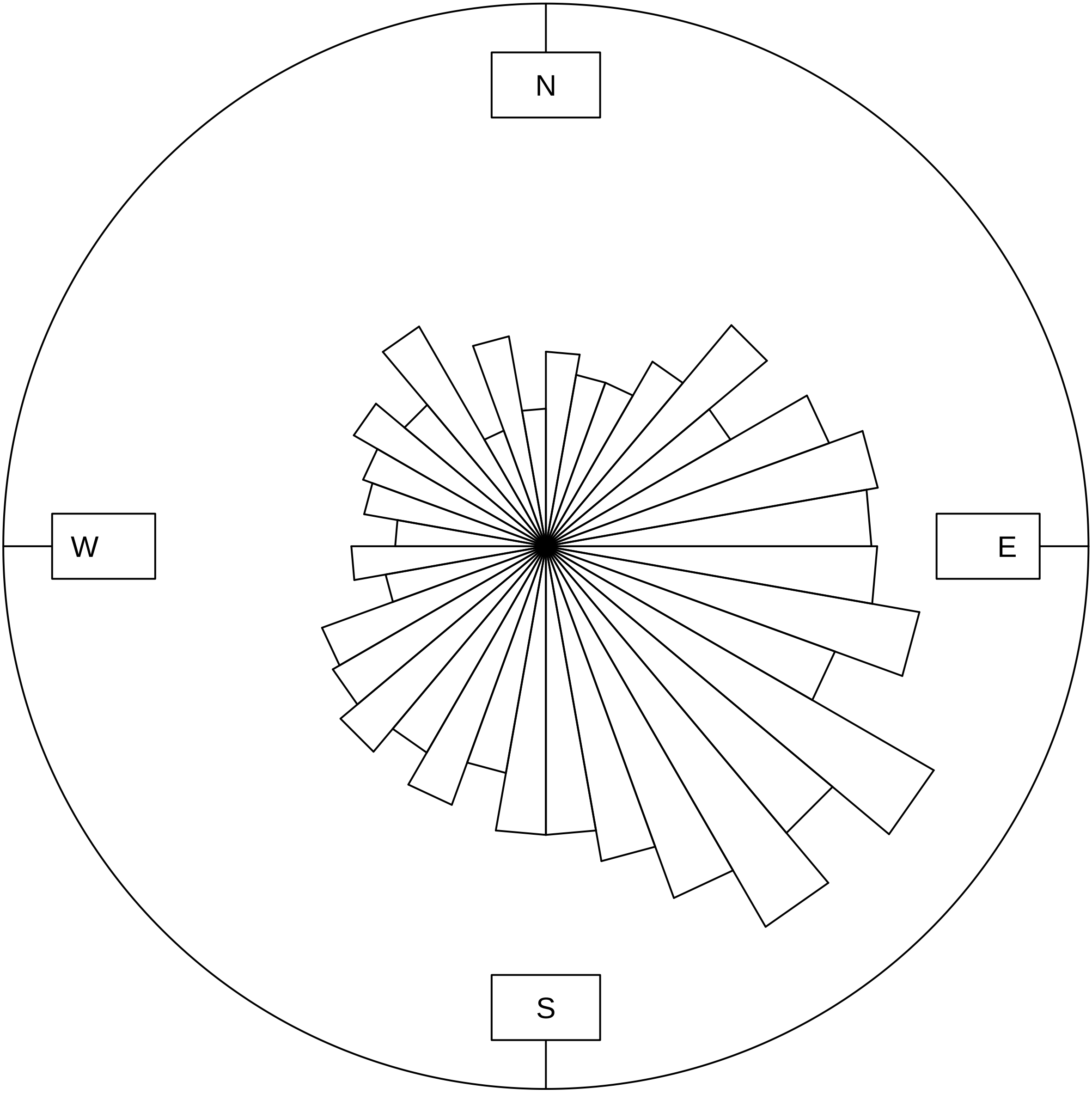

bootstrap 756

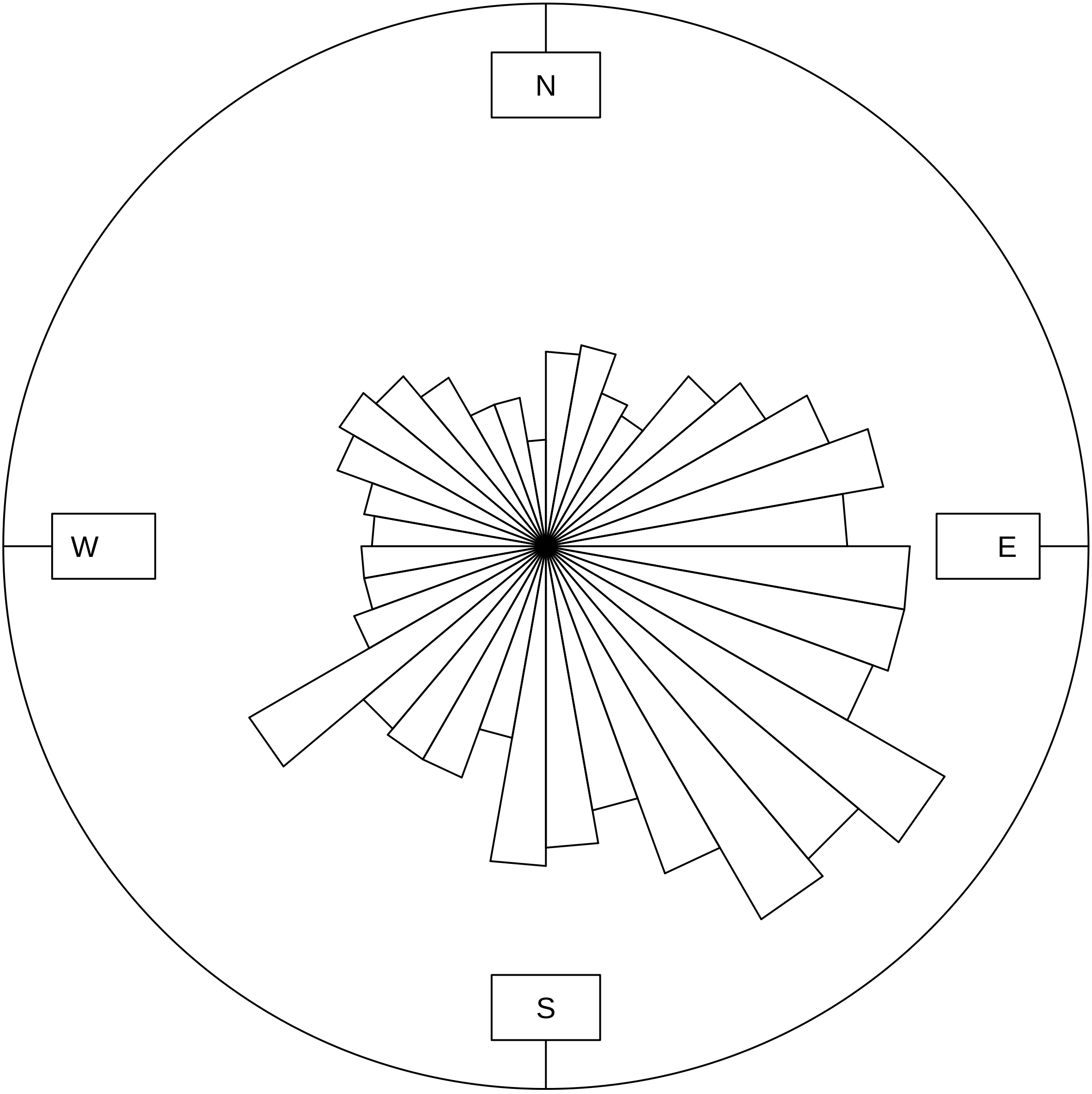

bootstrap 757

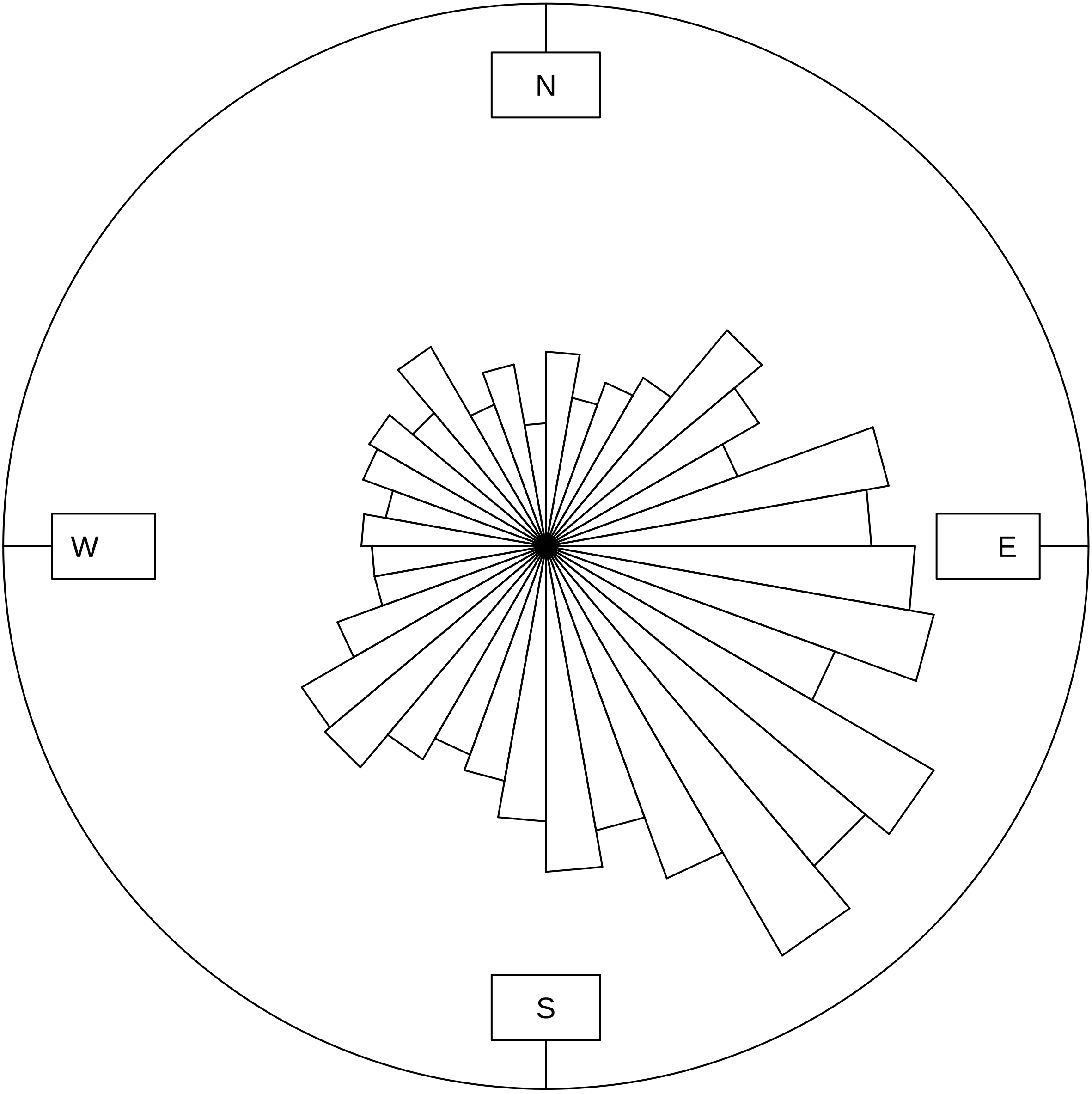

bootstrap 758

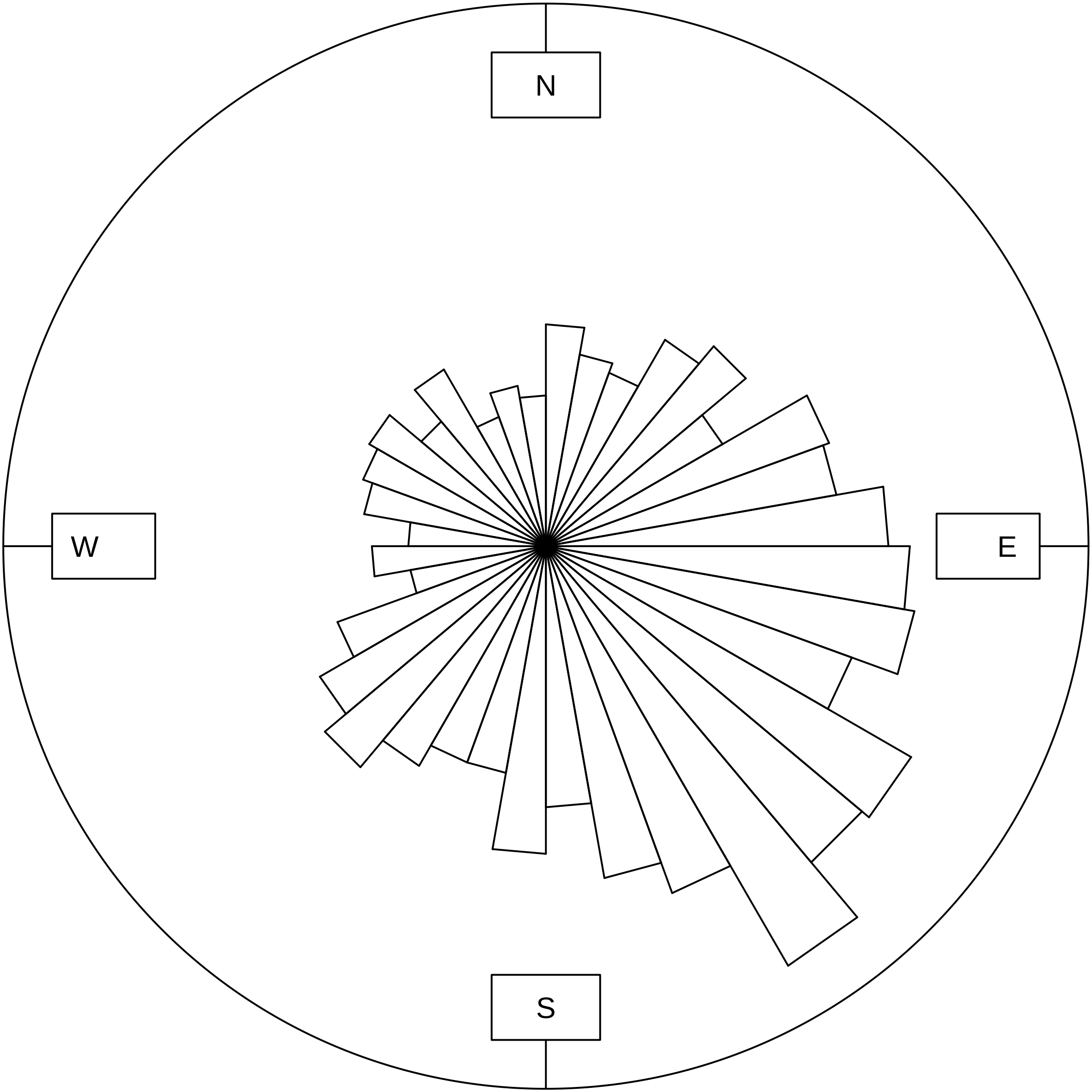

bootstrap 759

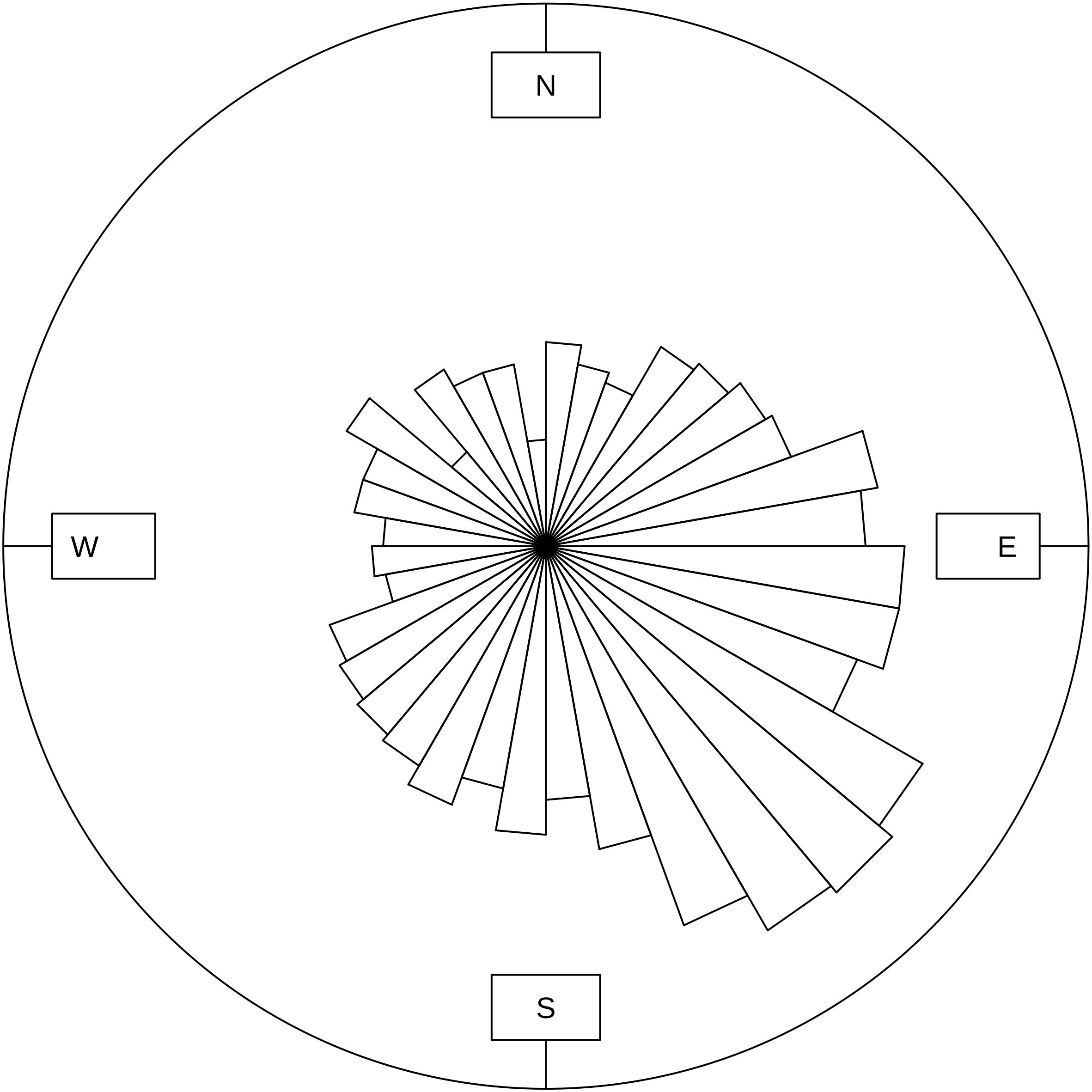

bootstrap 760

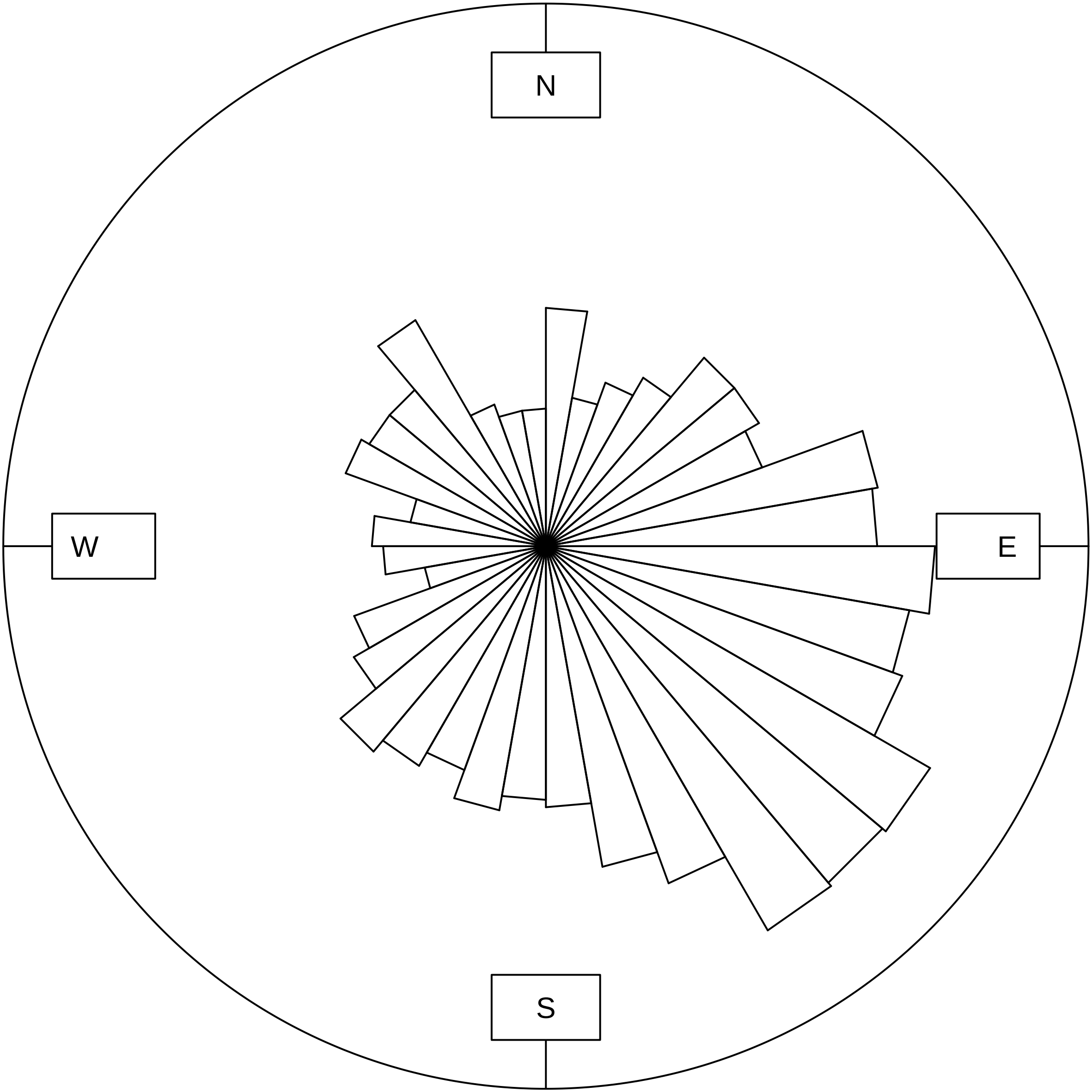

bootstrap 761

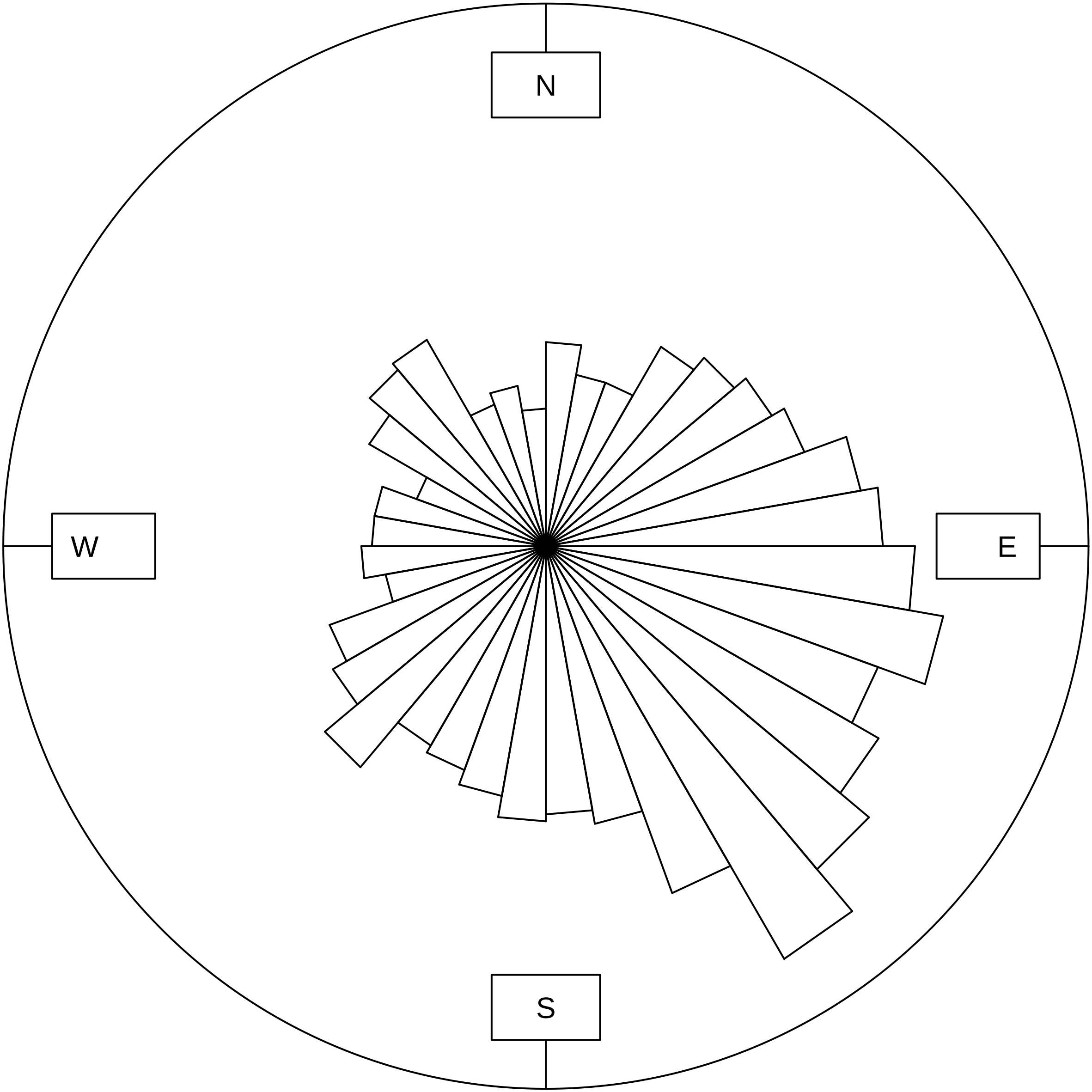

bootstrap 762

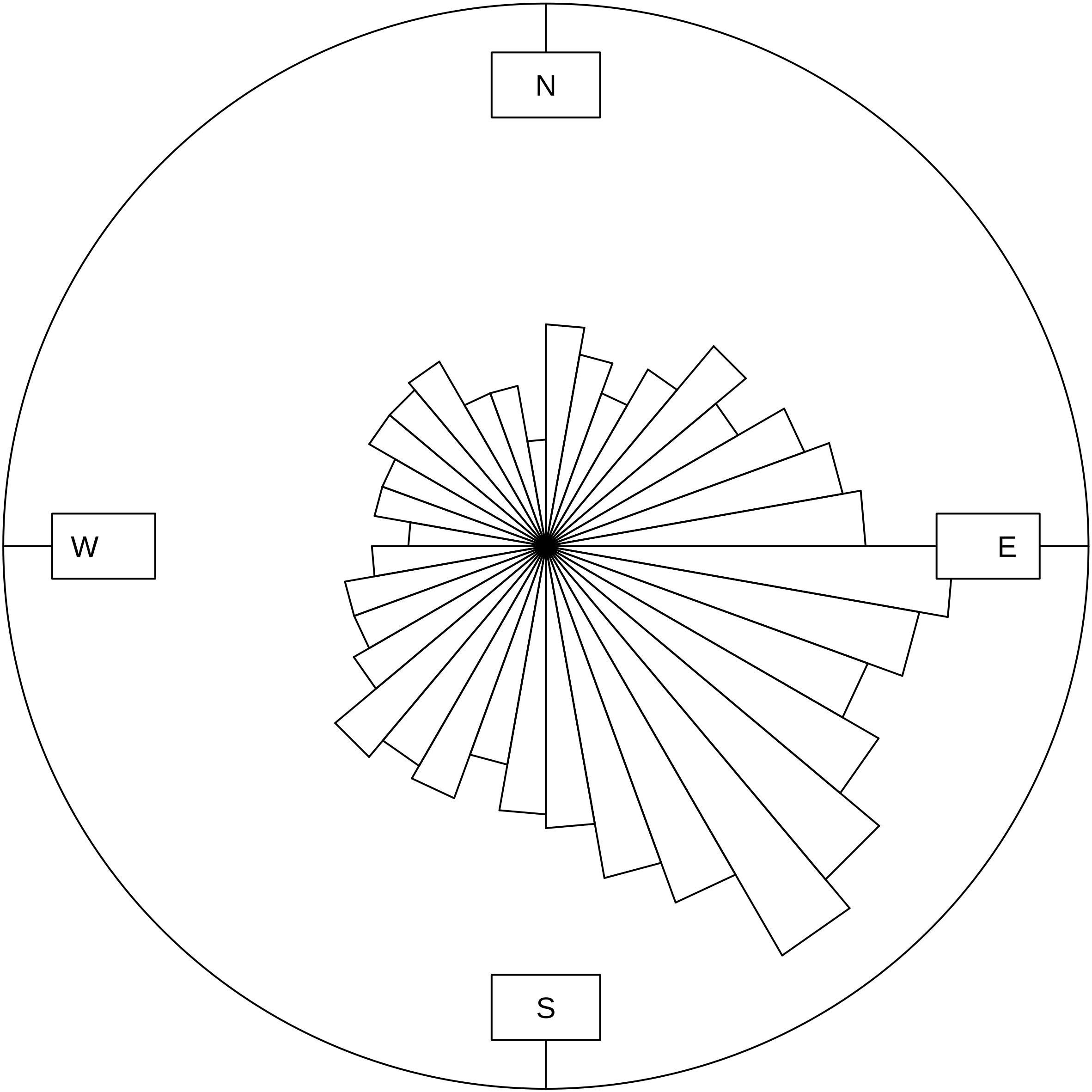

bootstrap 763

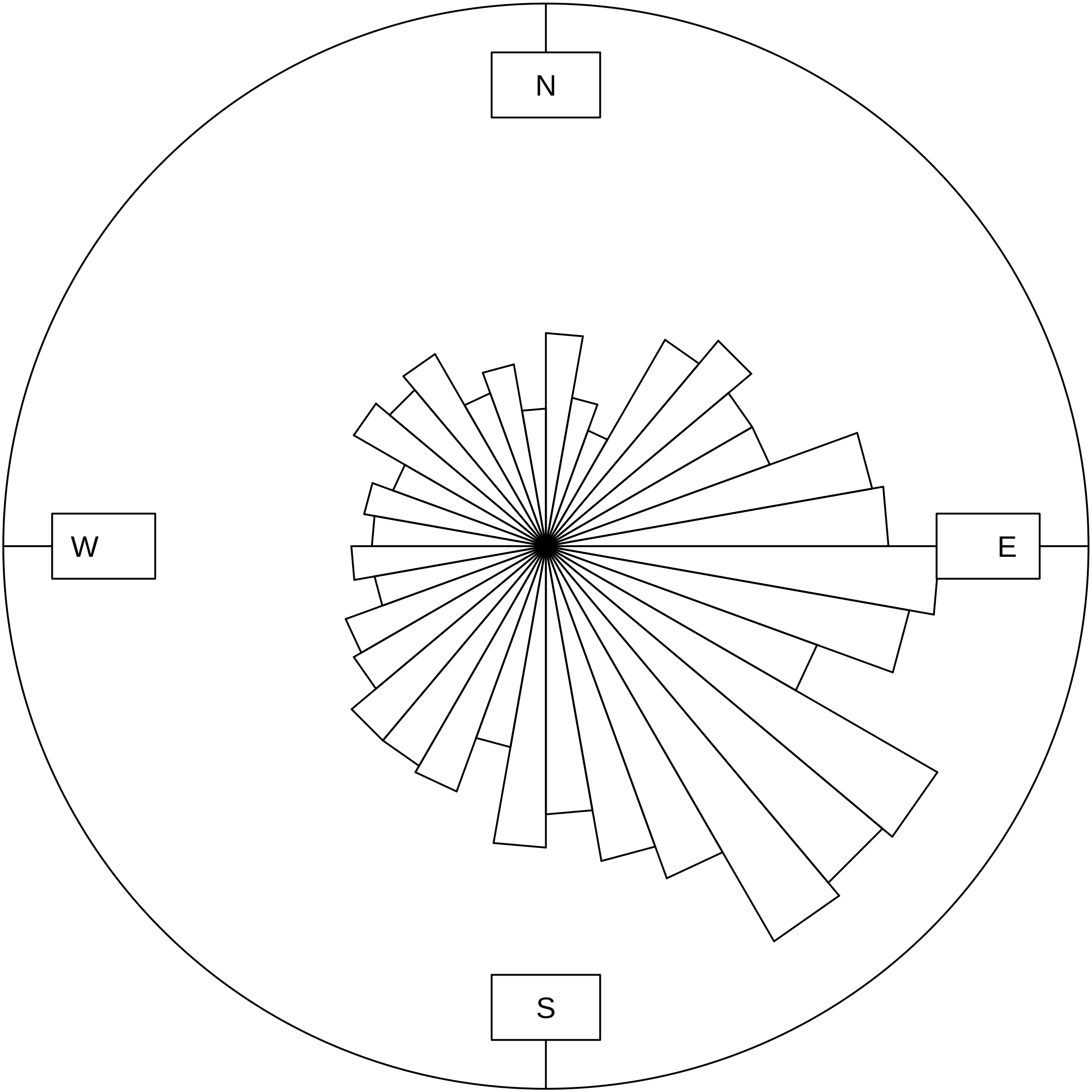

bootstrap 764

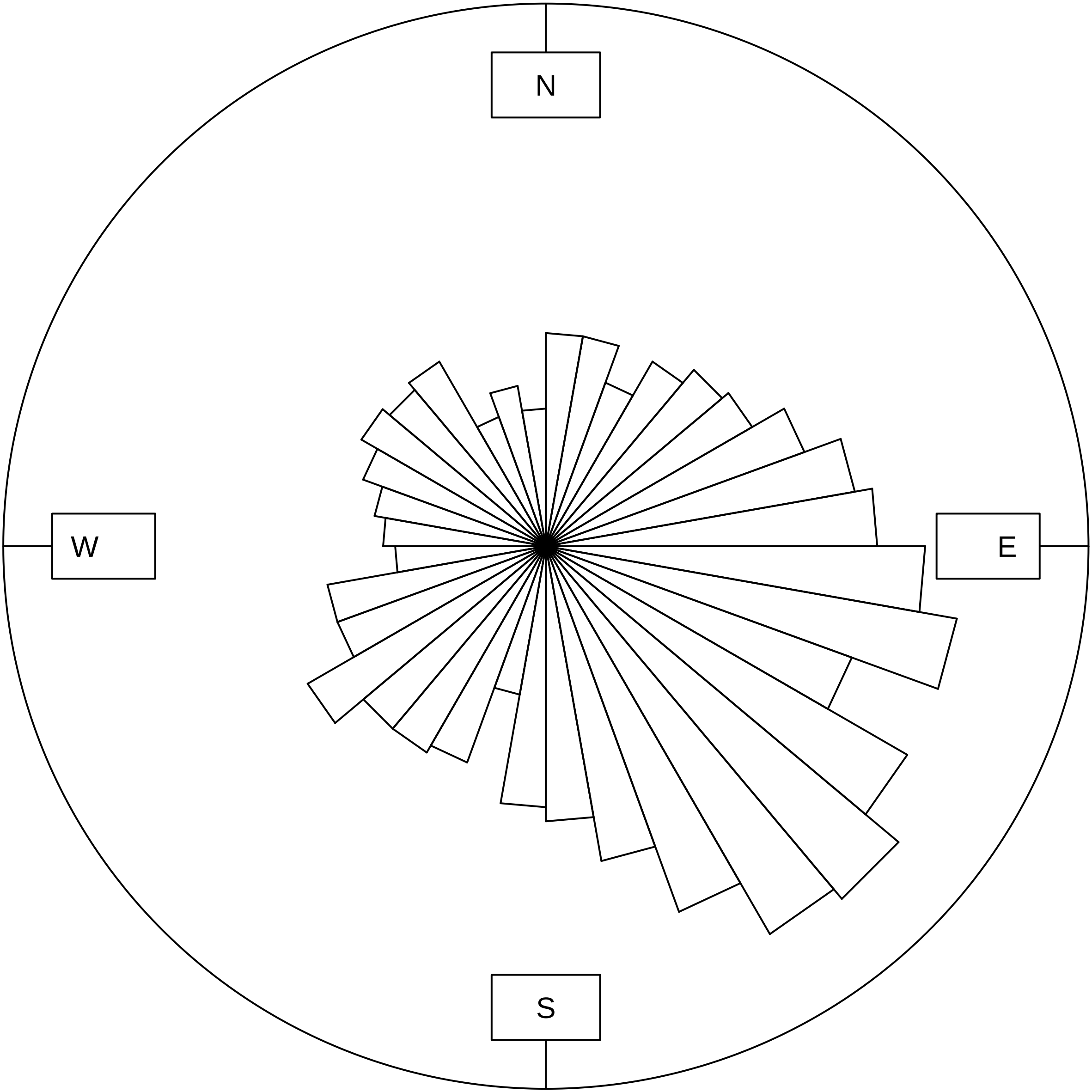

bootstrap 765

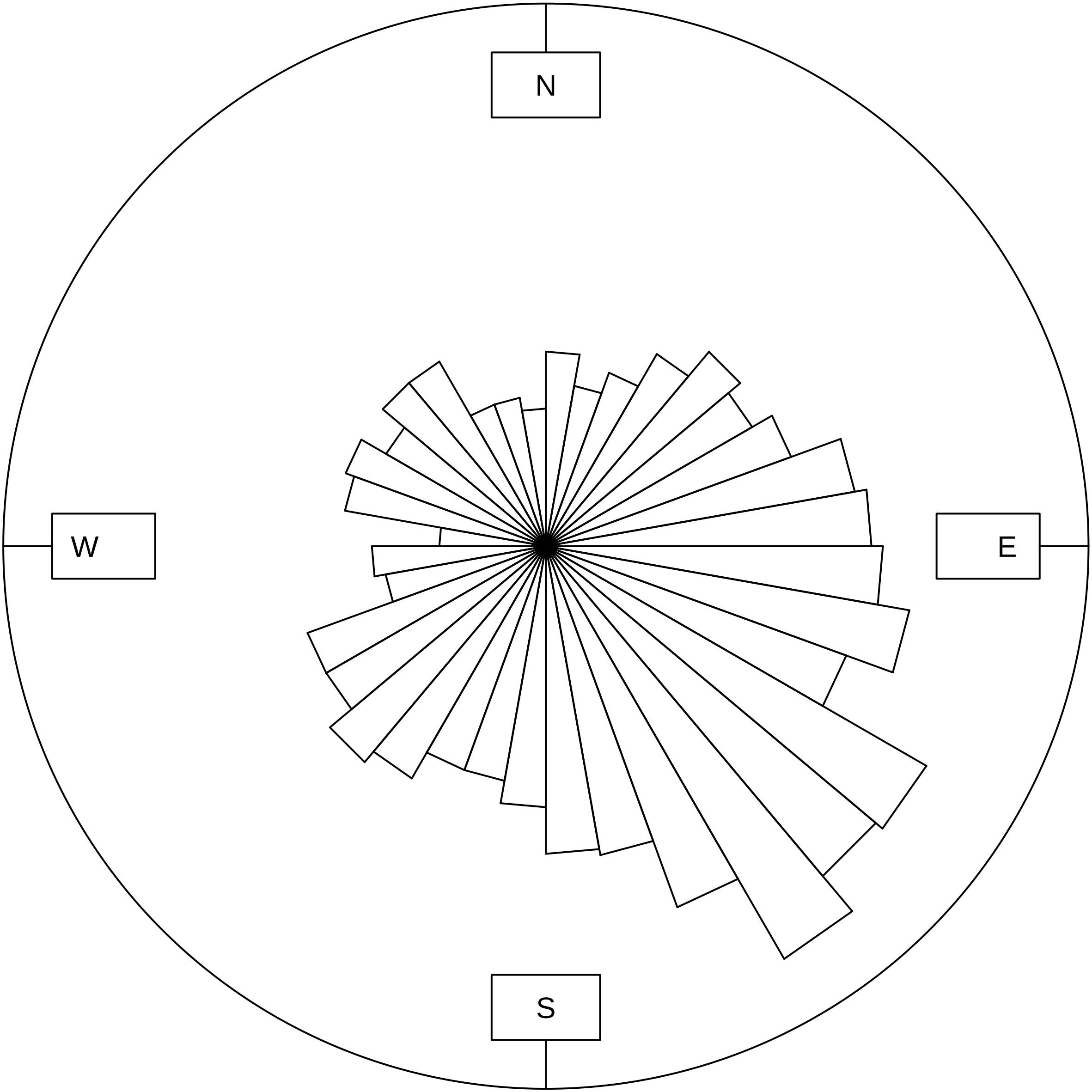

bootstrap 766

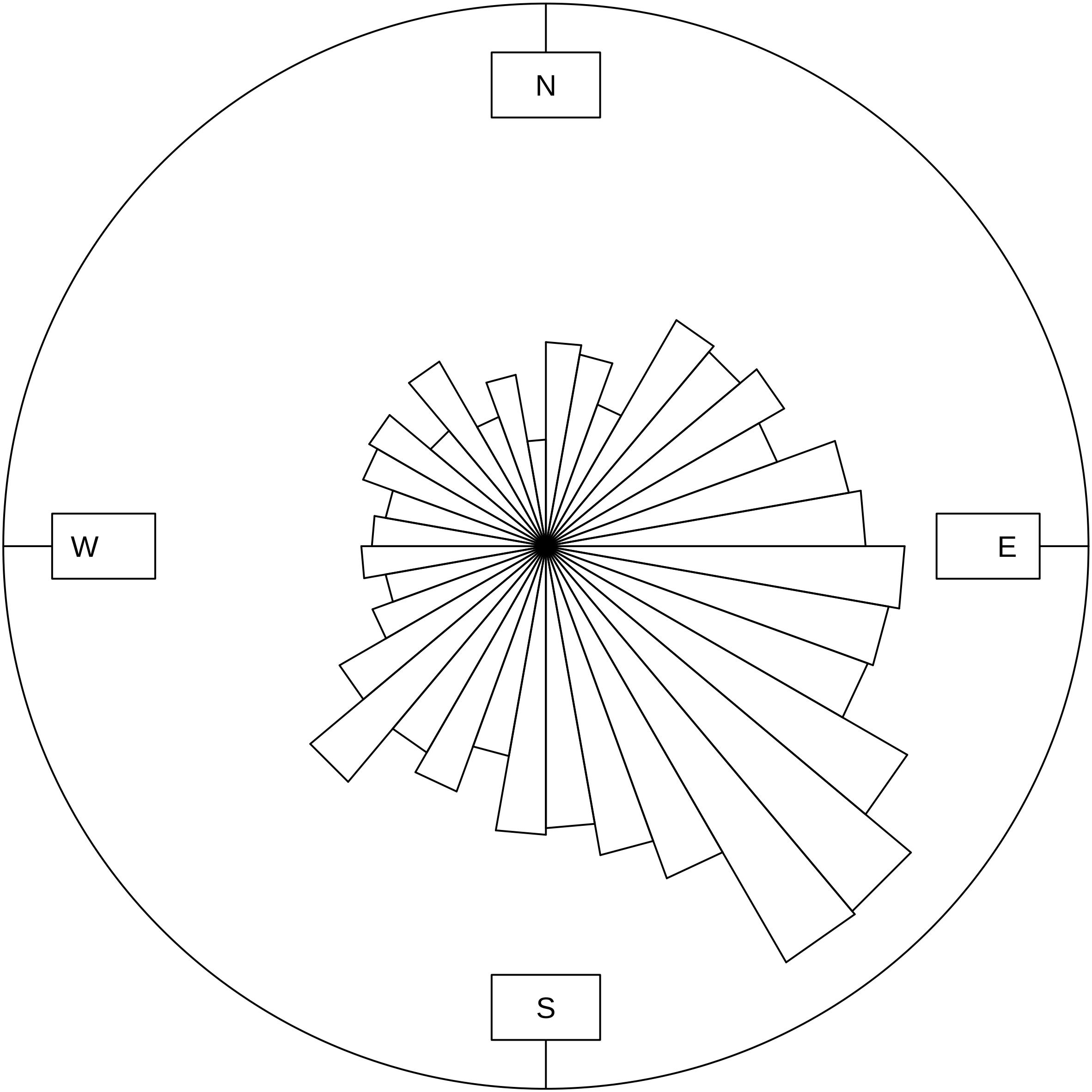

bootstrap 767

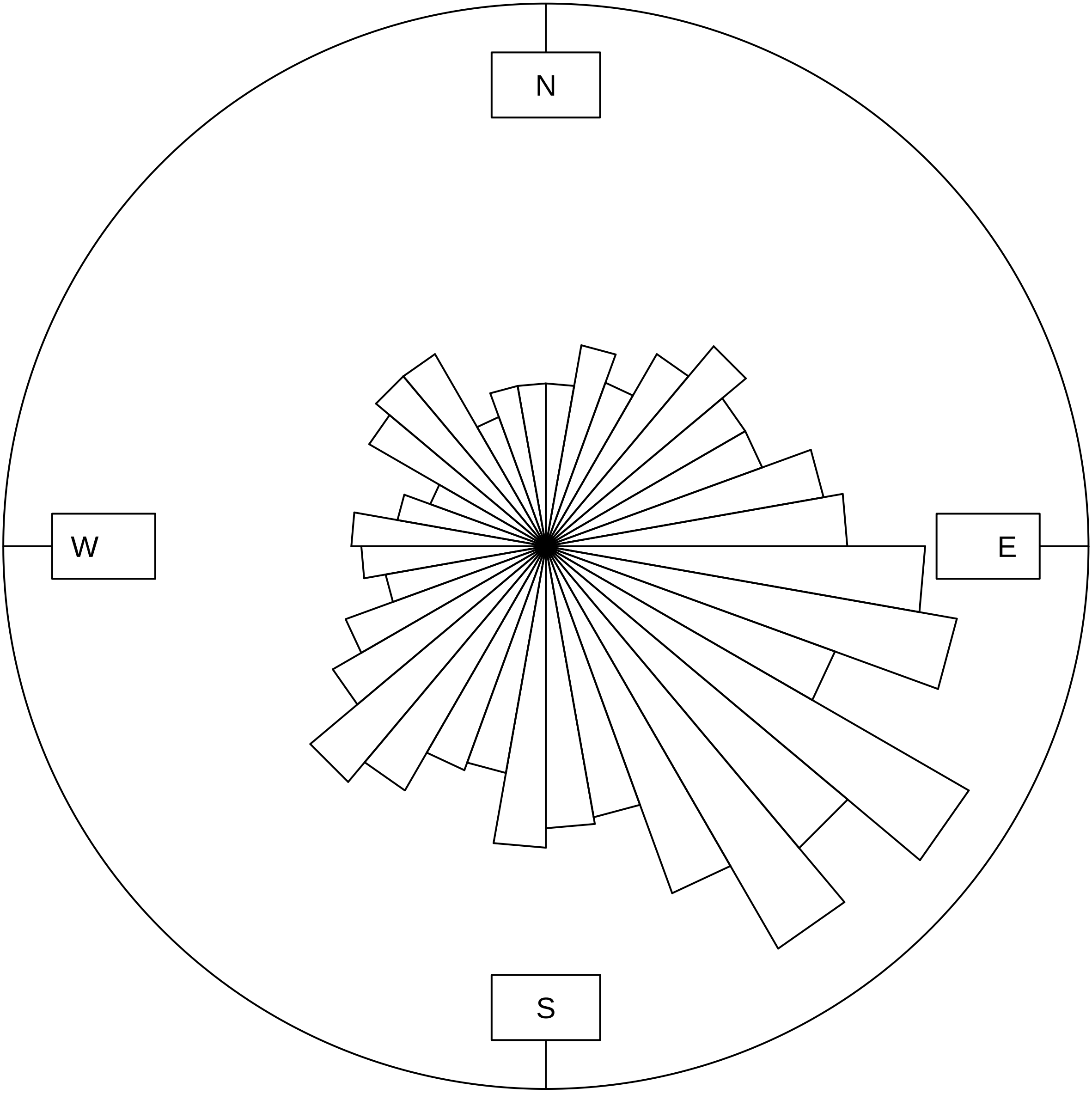

bootstrap 768

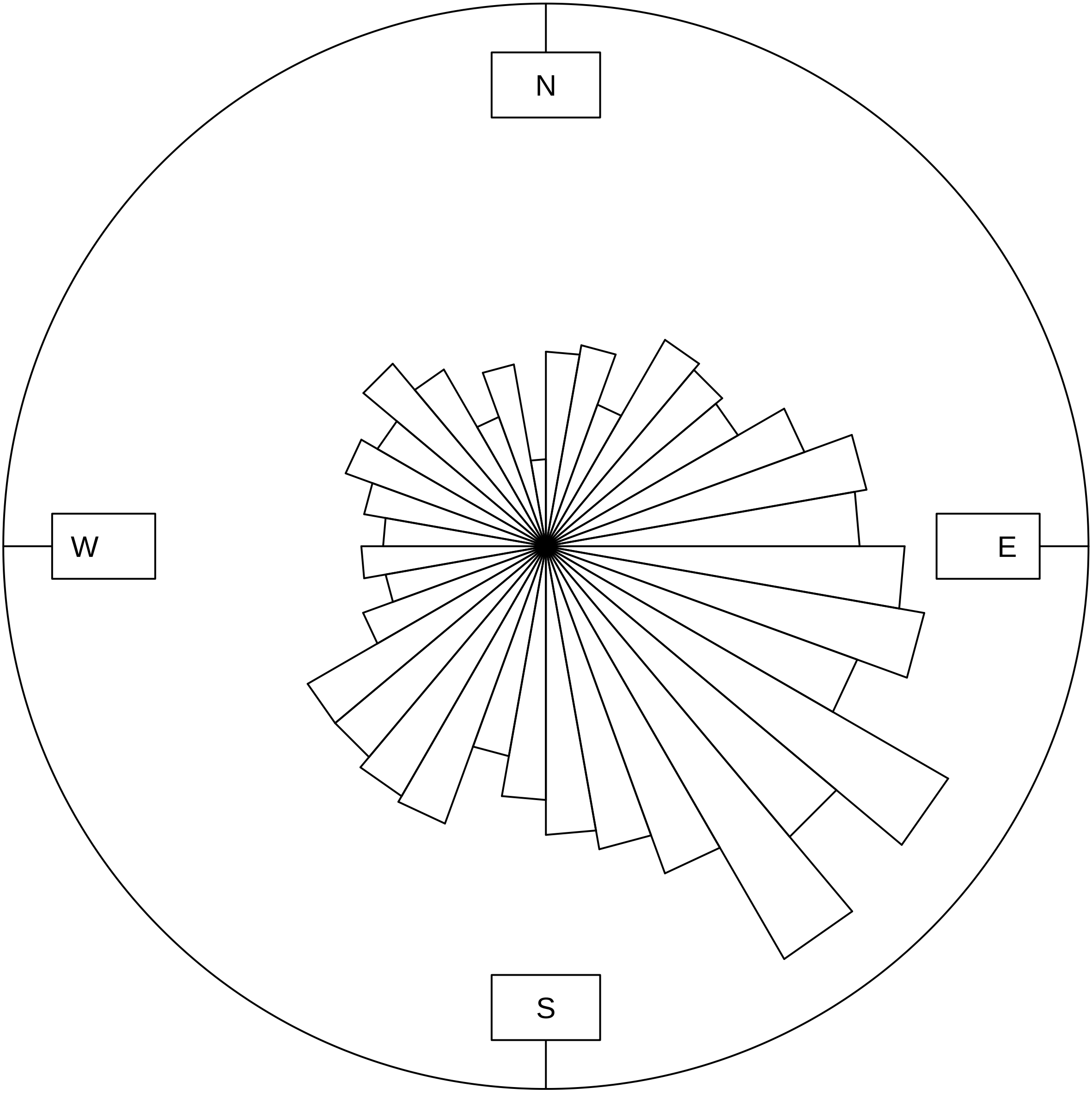

bootstrap 769

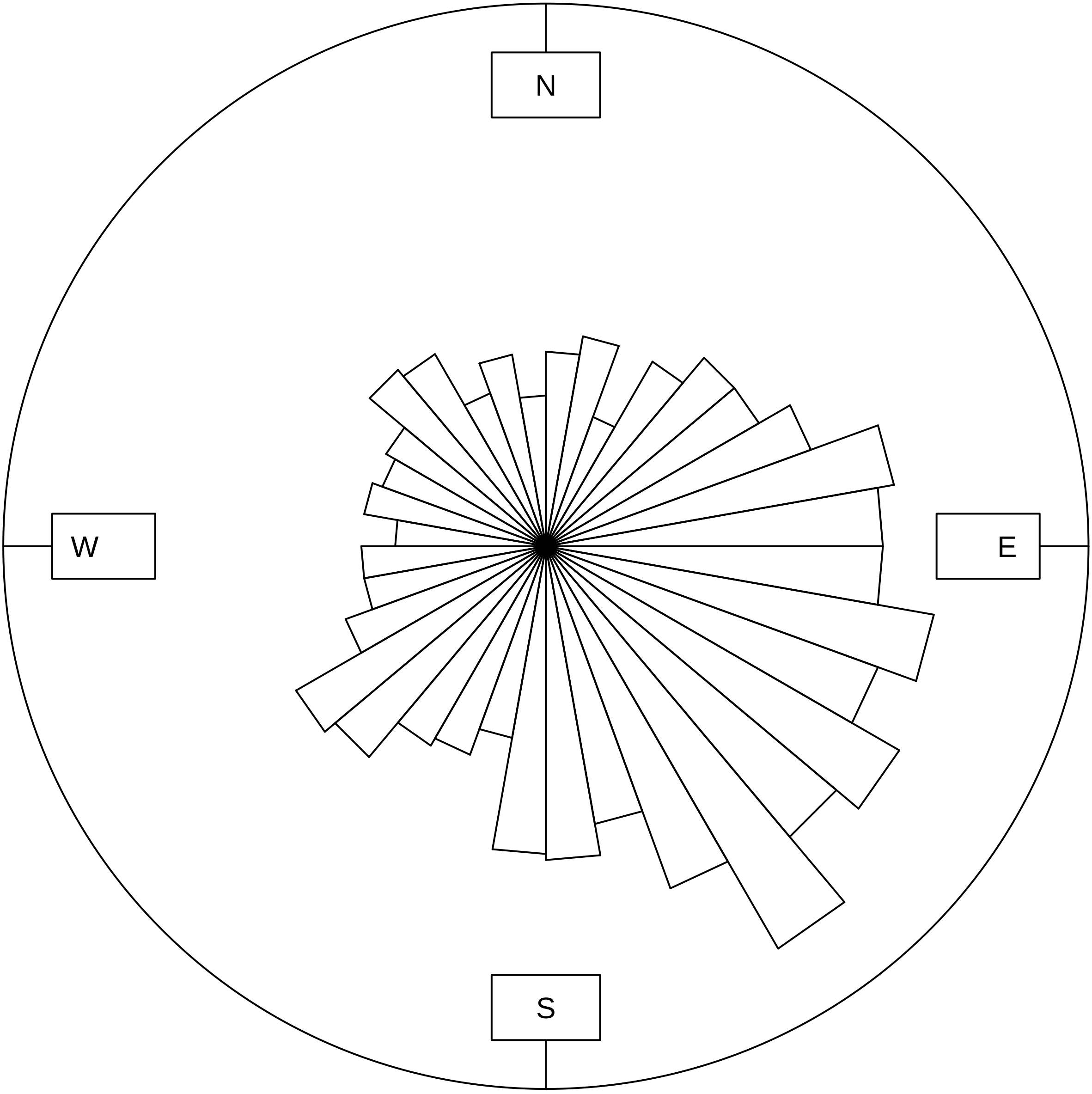

bootstrap 770

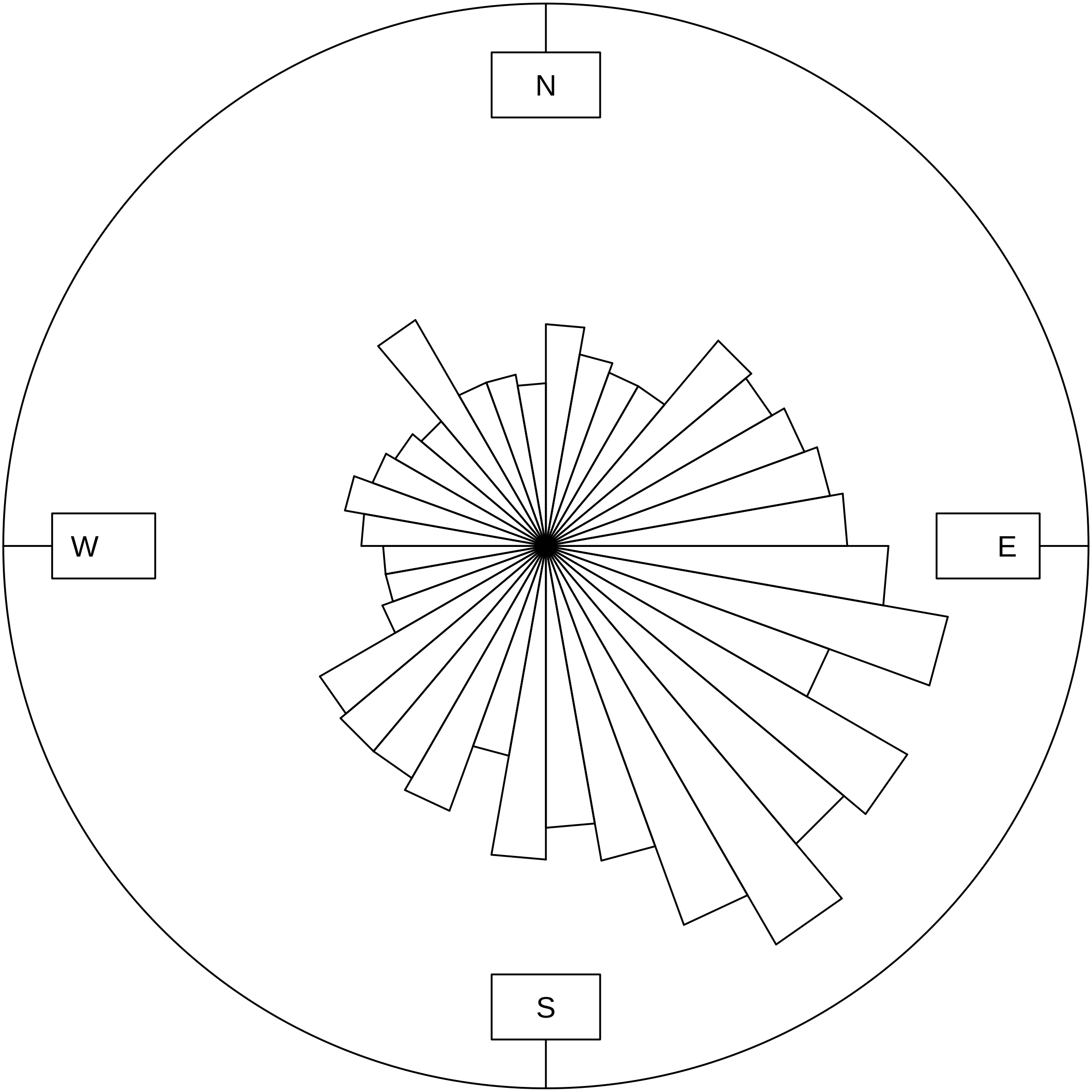

bootstrap 771

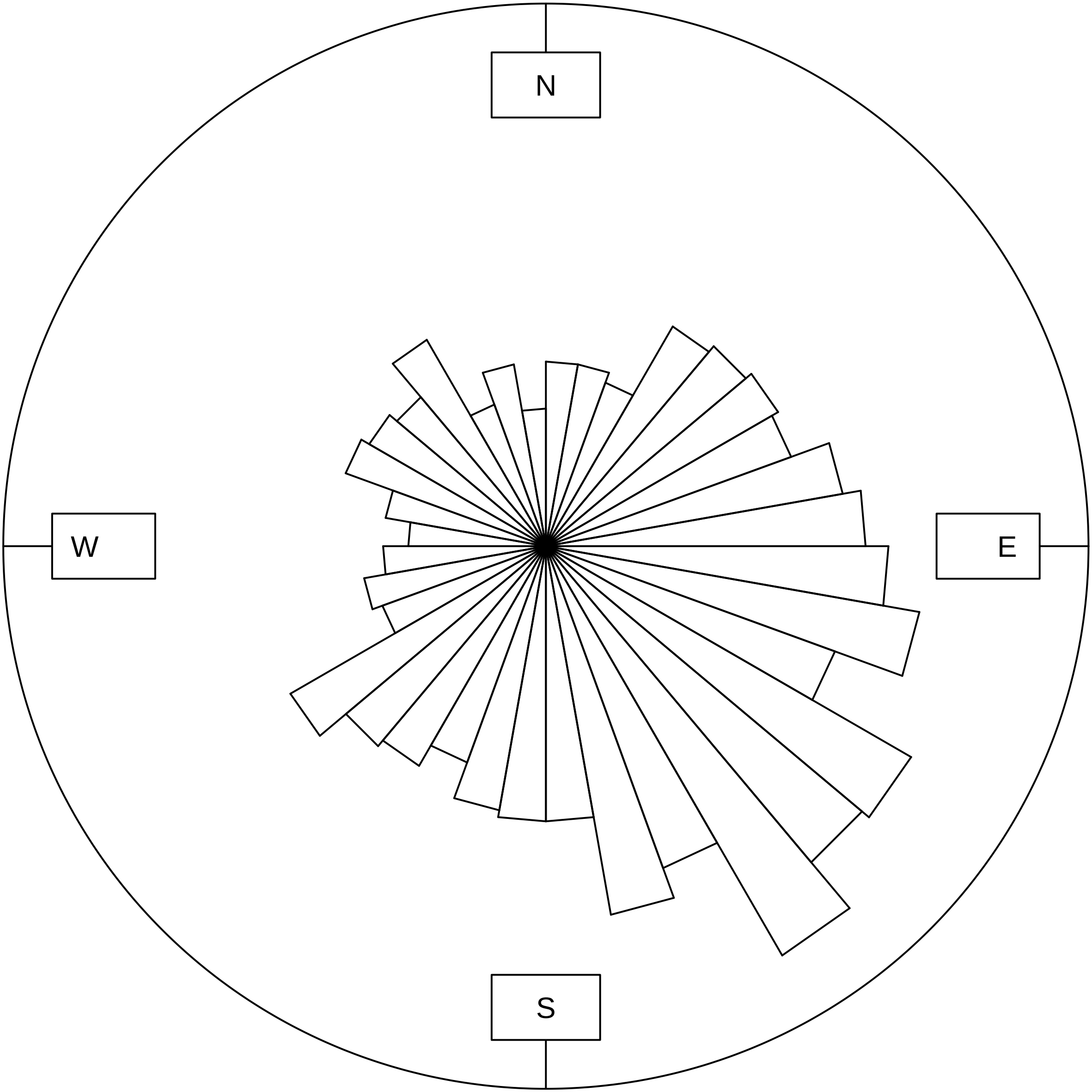

bootstrap 772

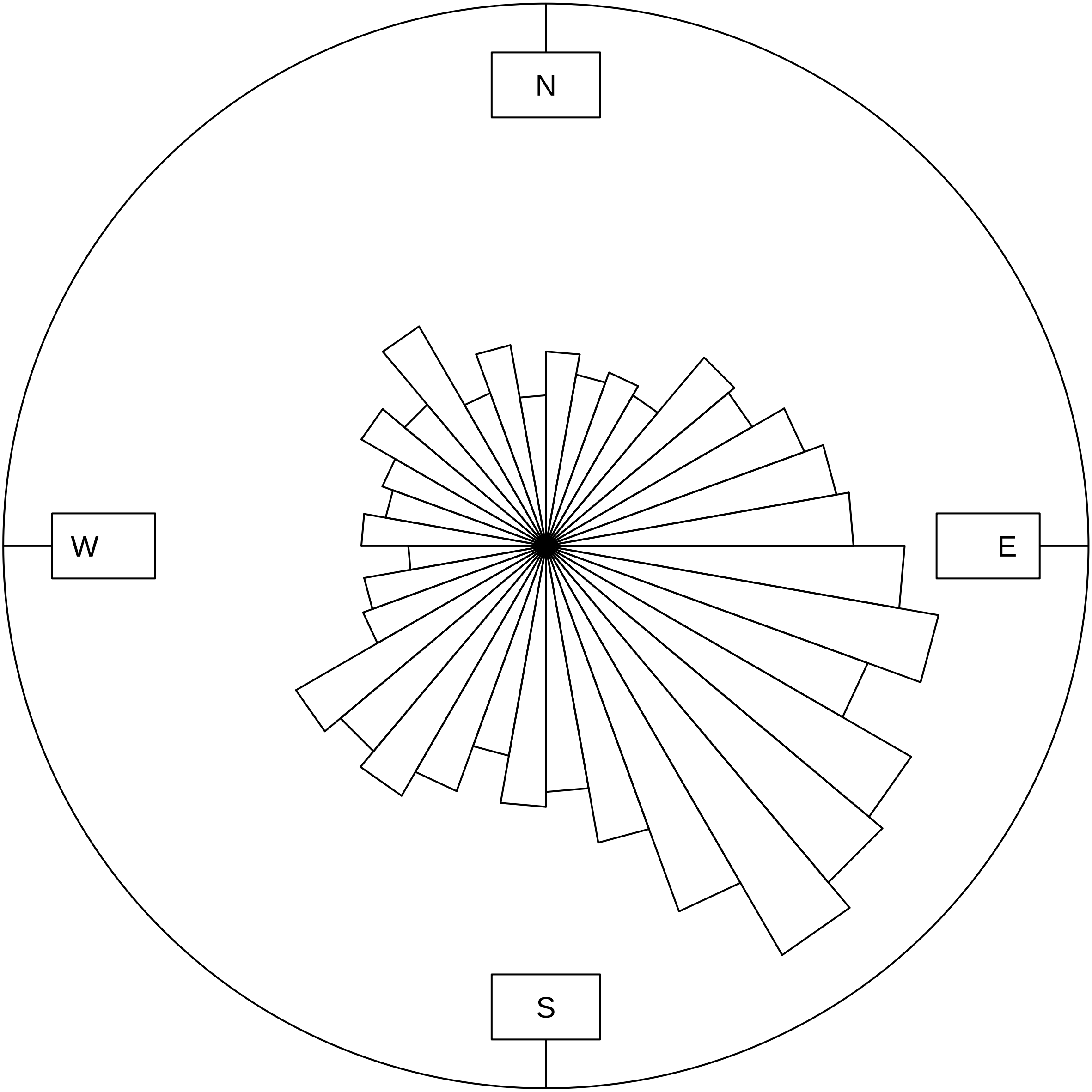

bootstrap 773

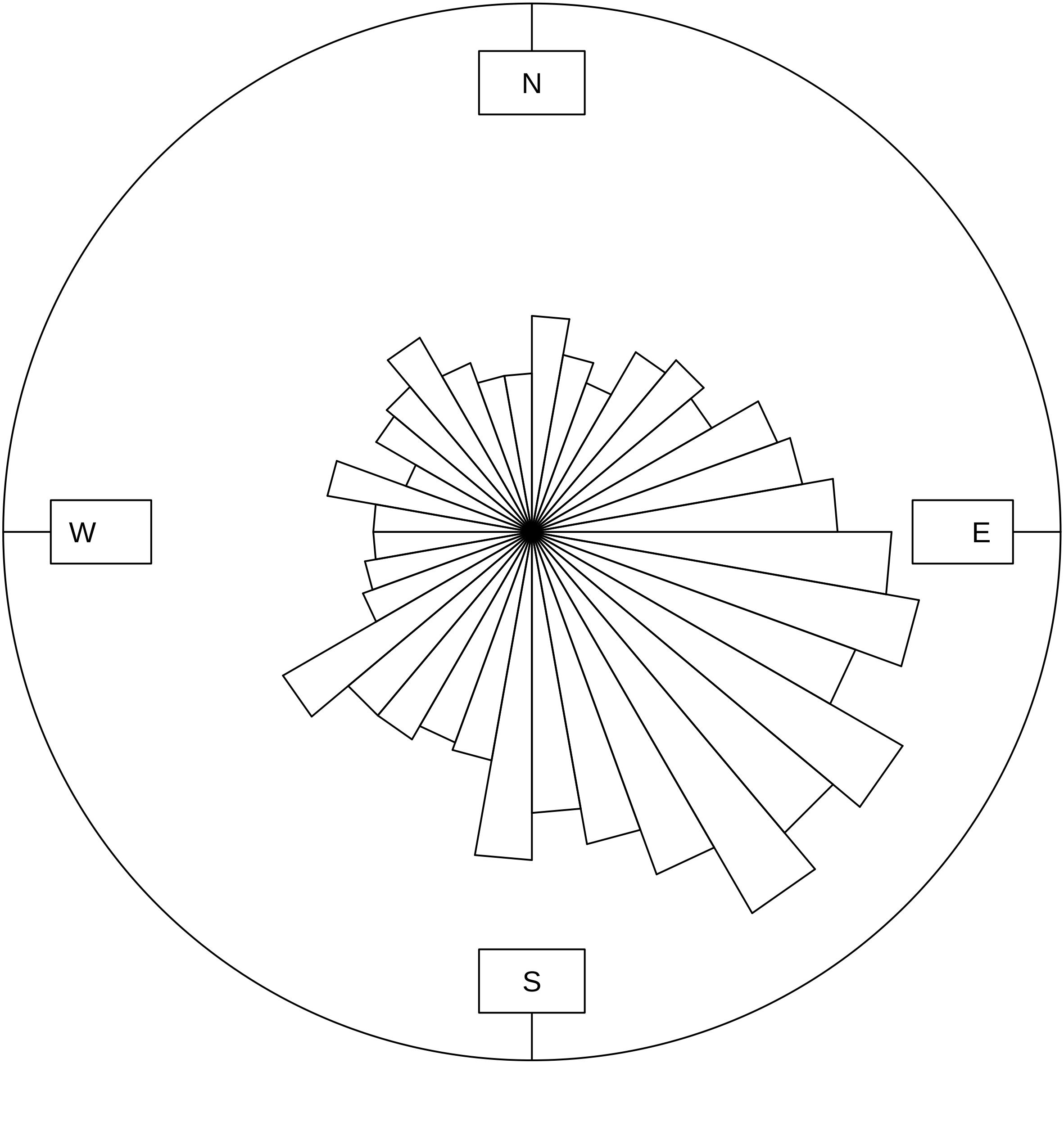

bootstrap 774

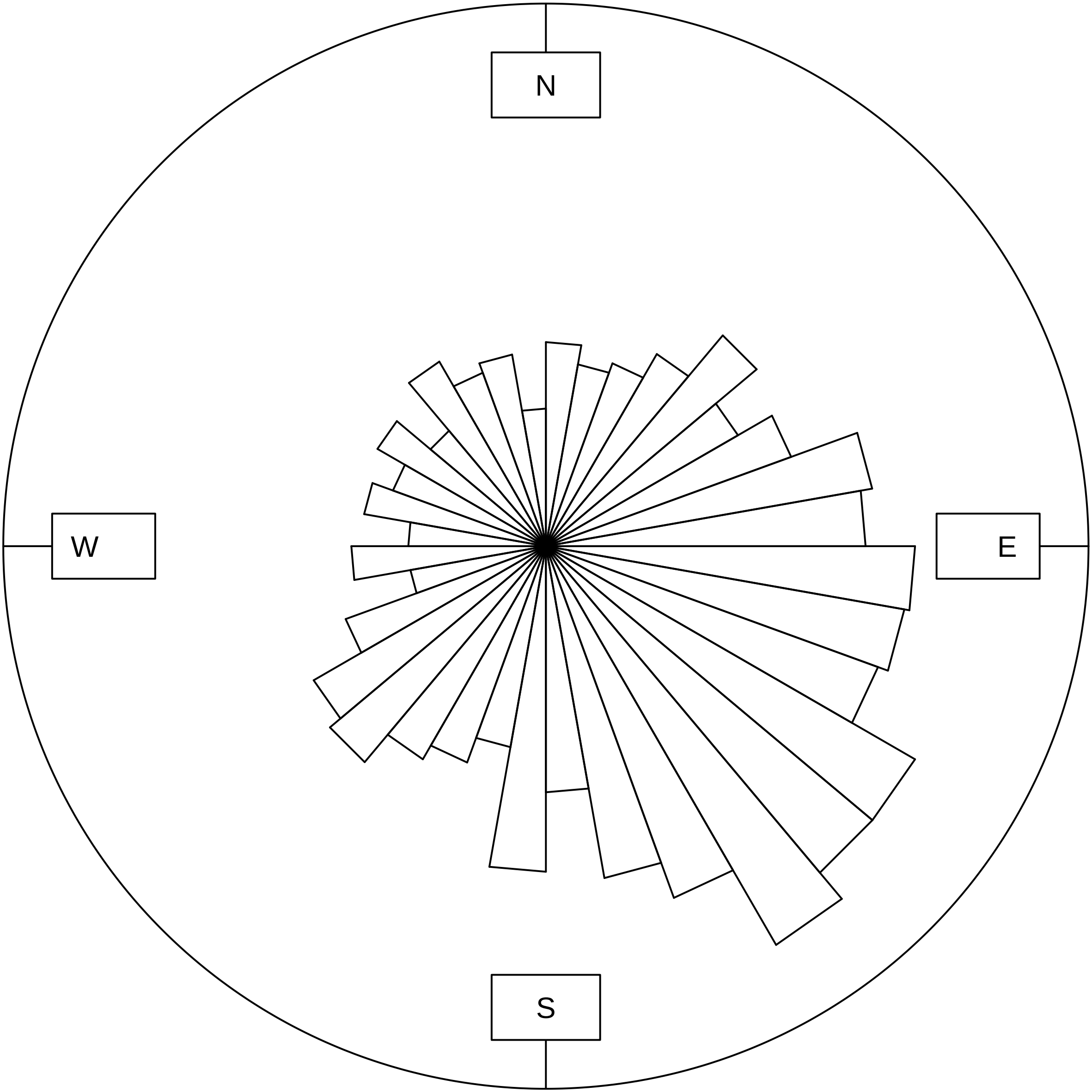

bootstrap 775

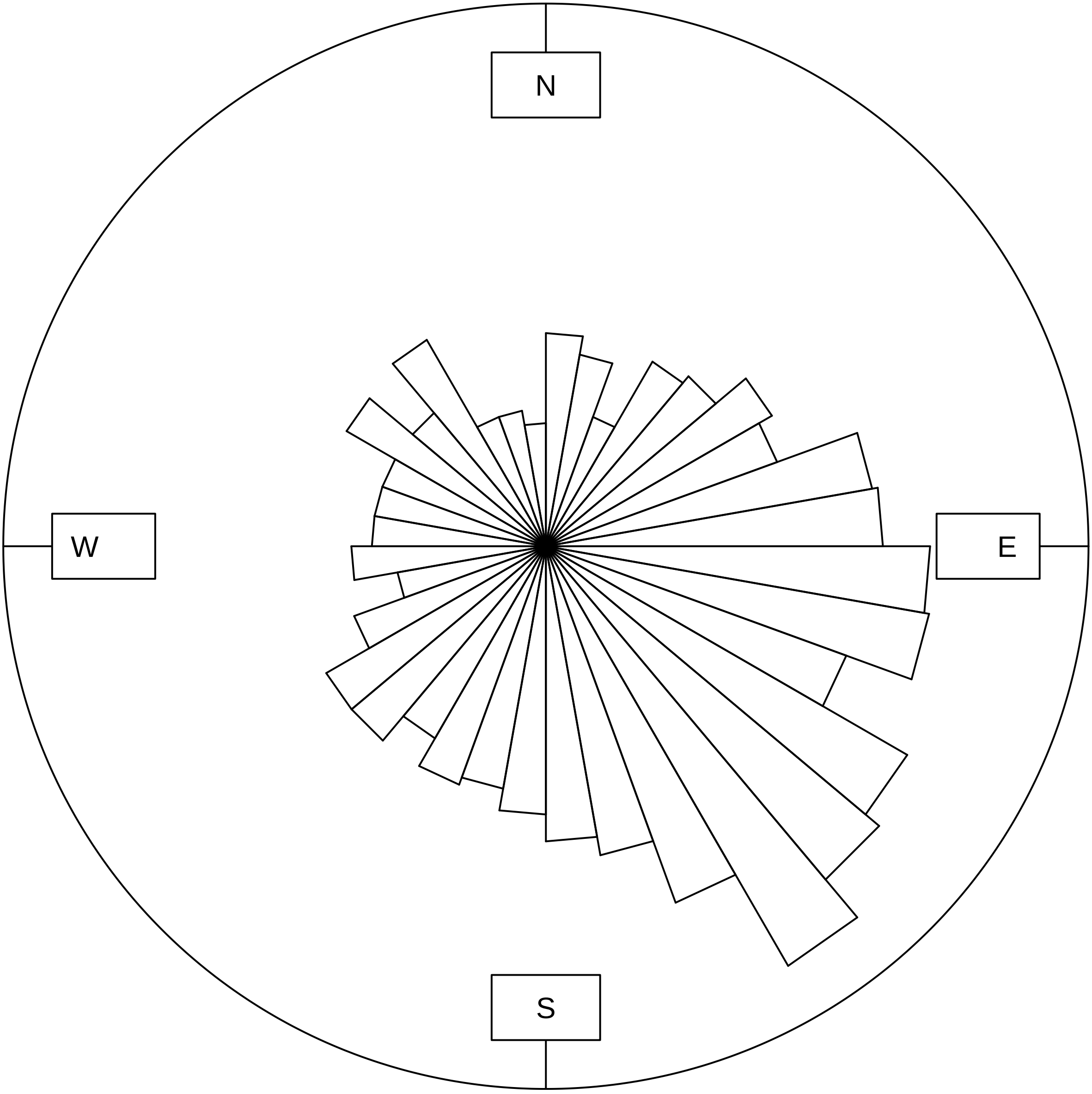

bootstrap 776

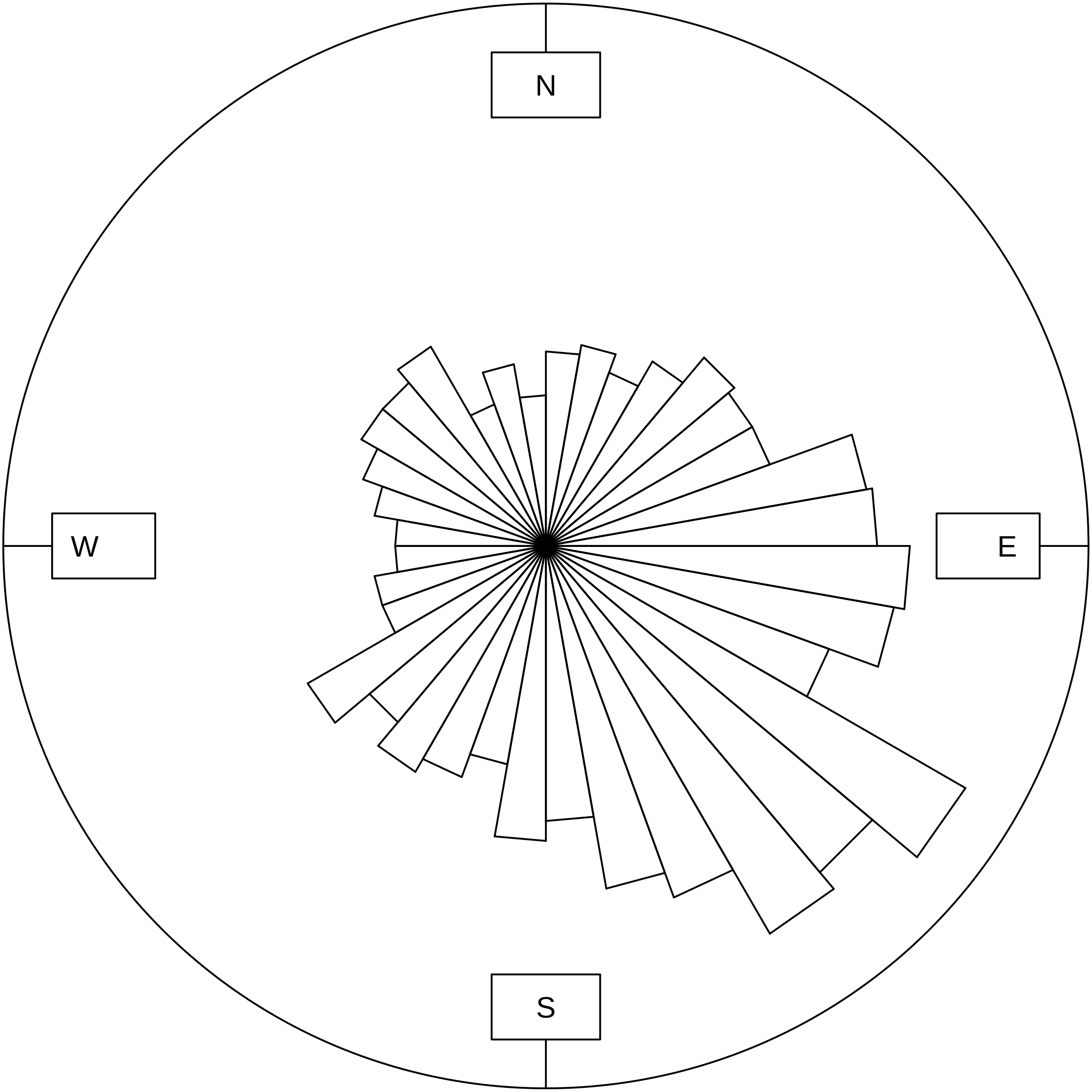

bootstrap 777

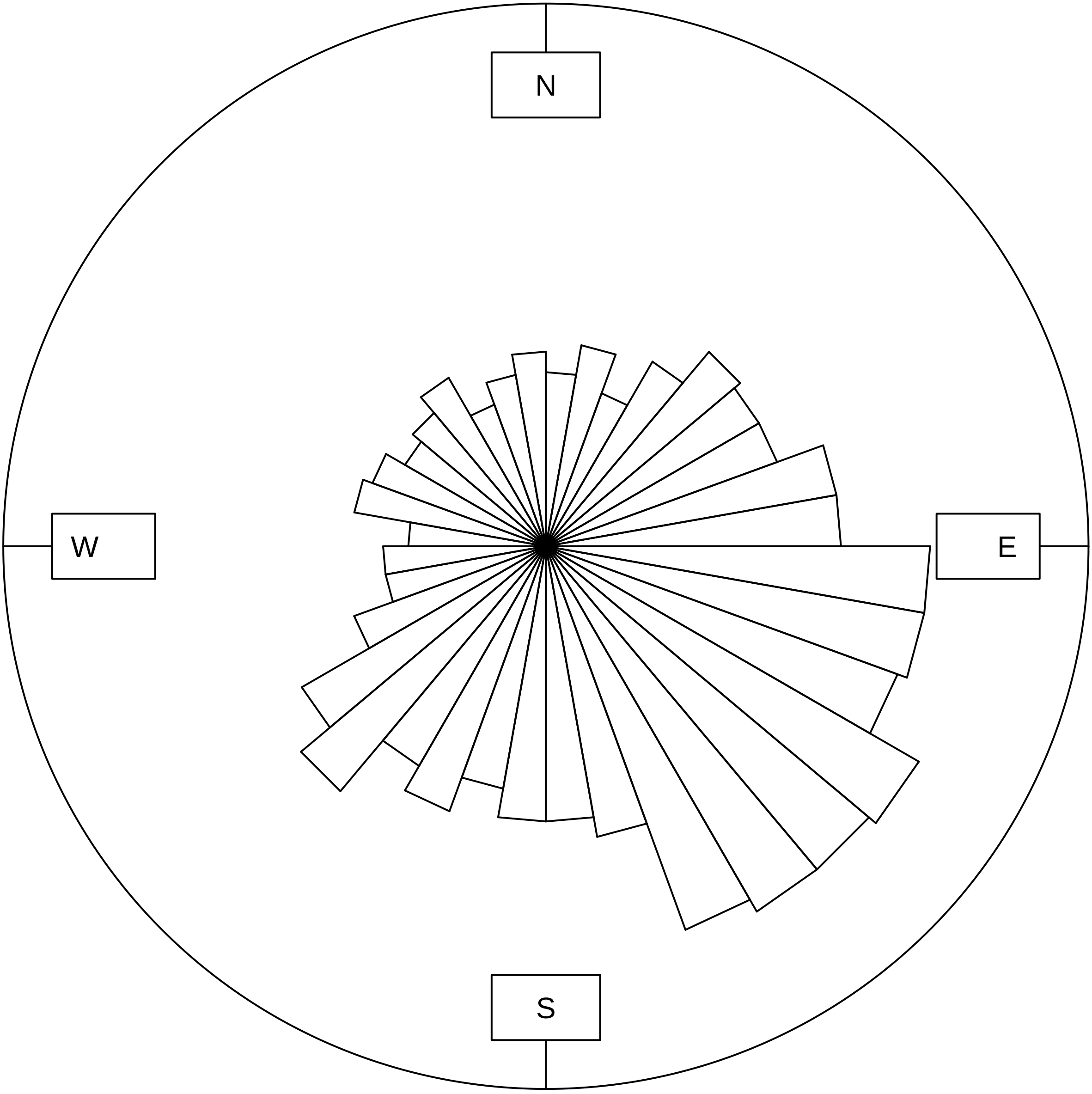

bootstrap 778

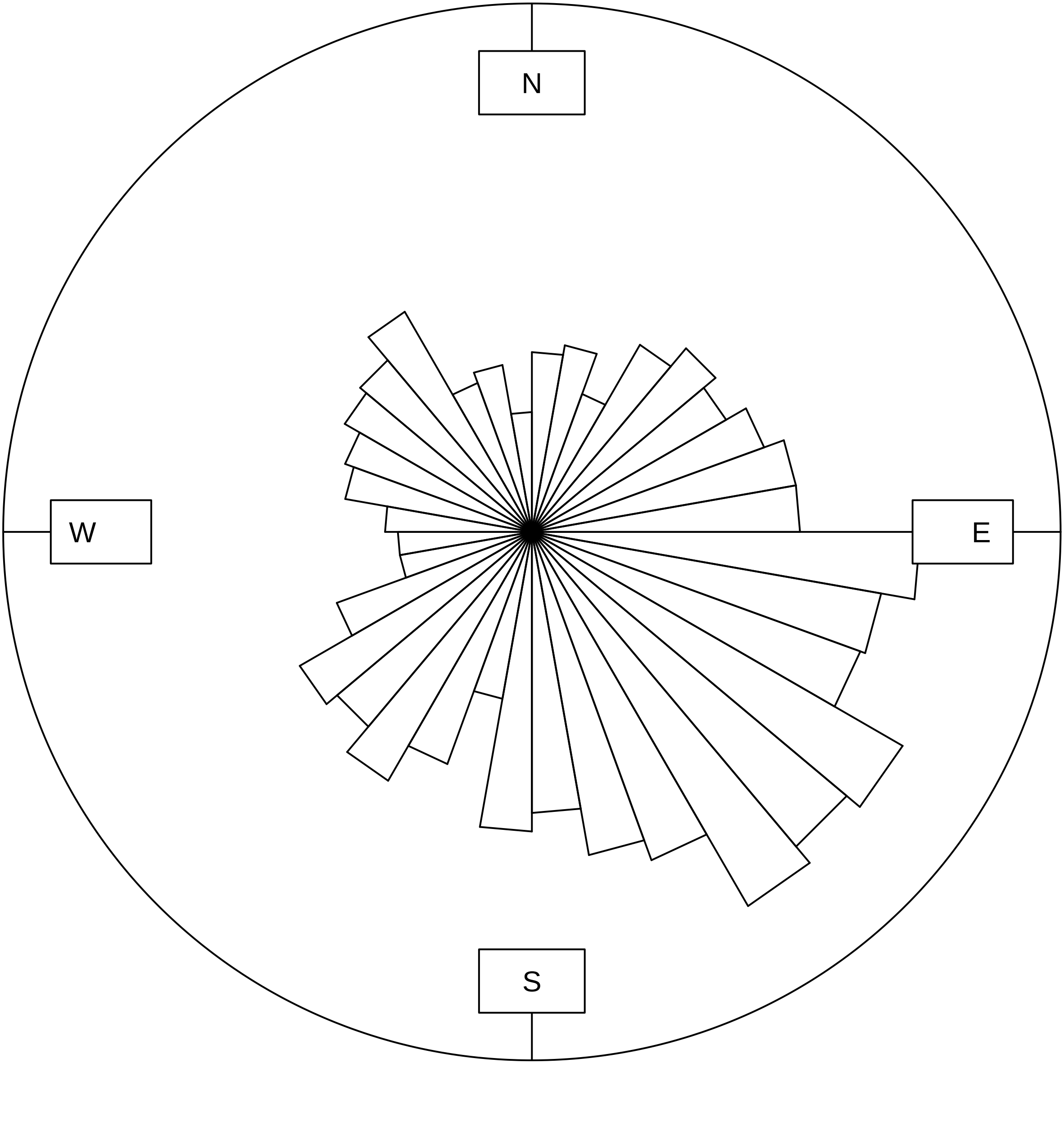

bootstrap 779

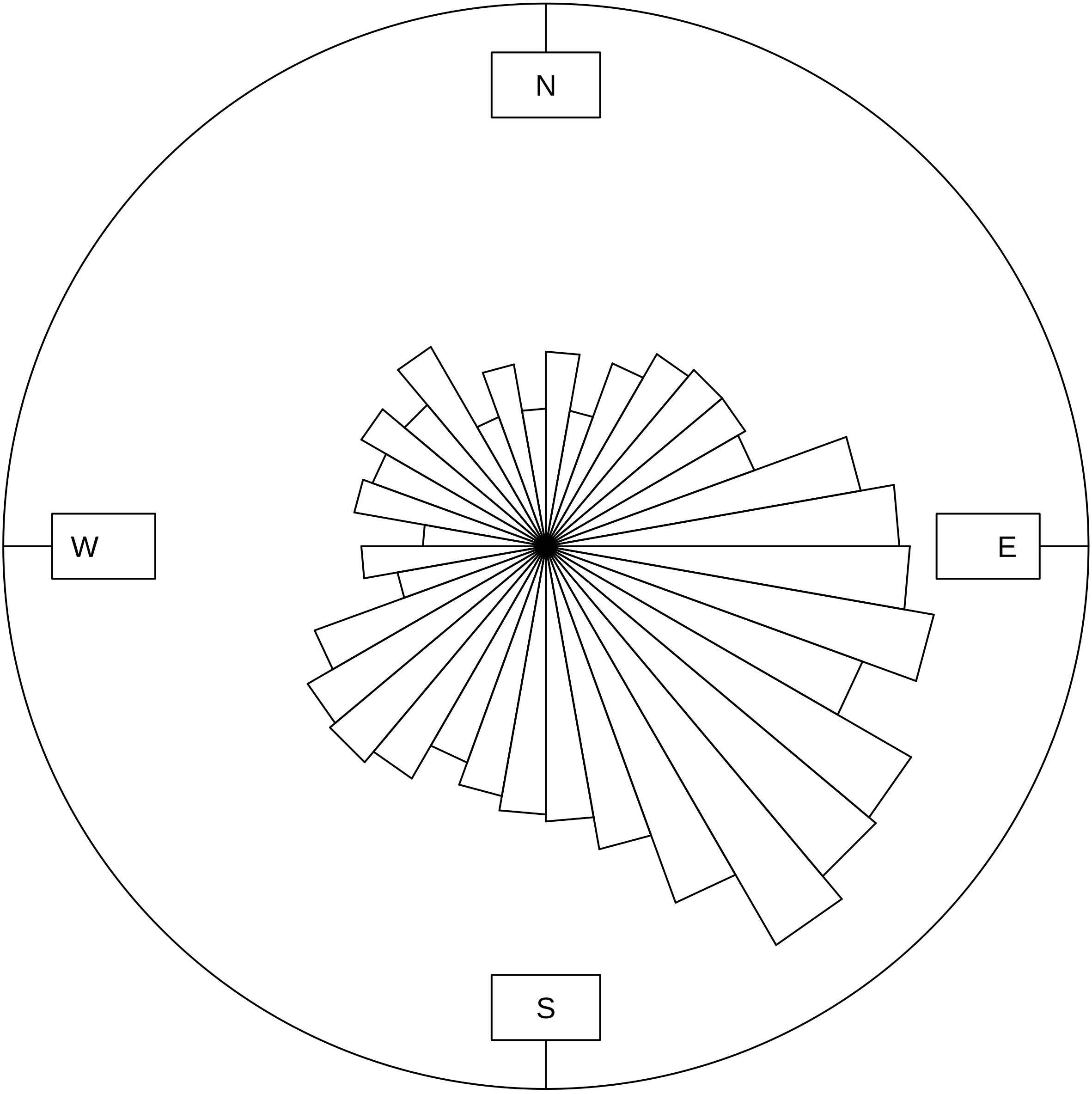

bootstrap 780

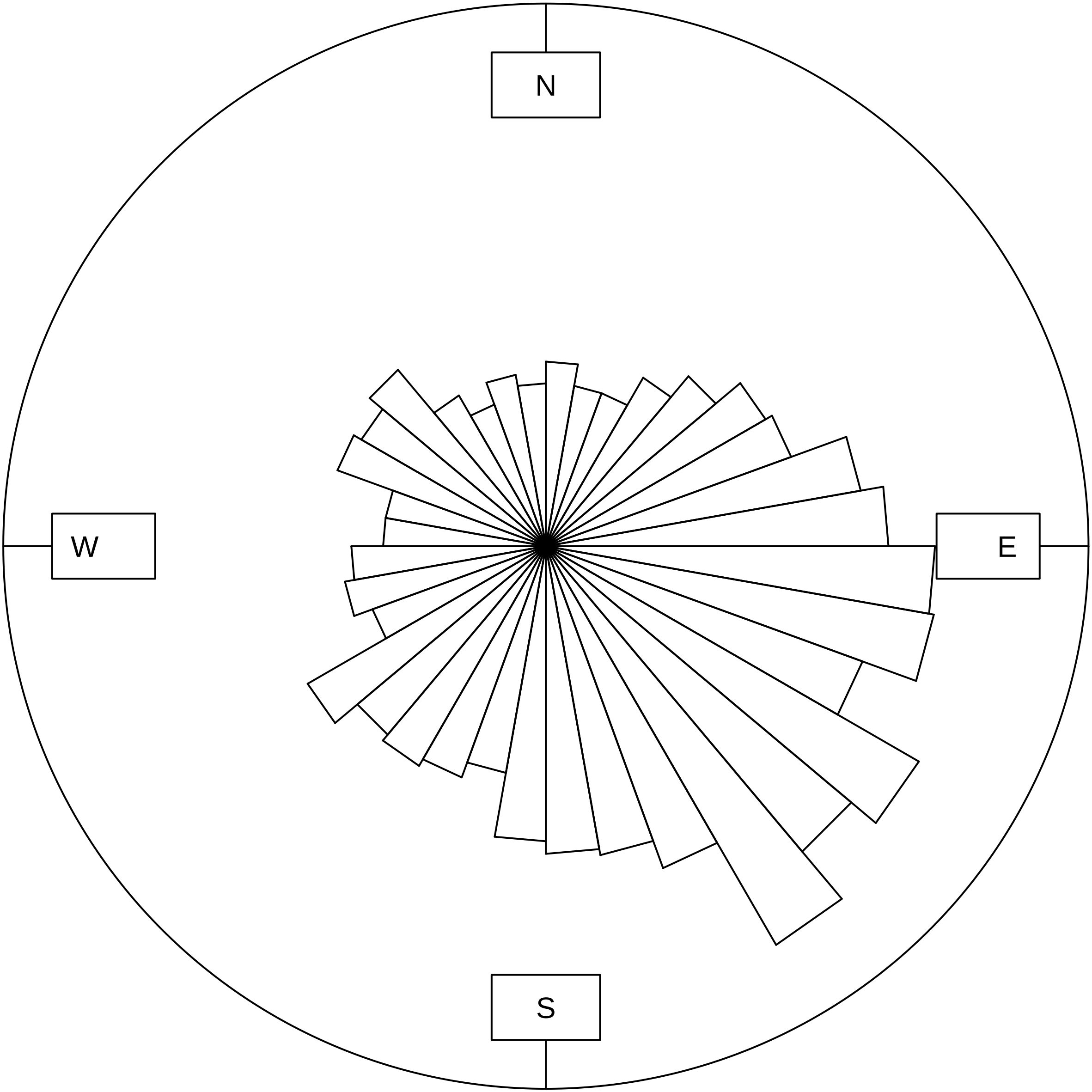

bootstrap 781

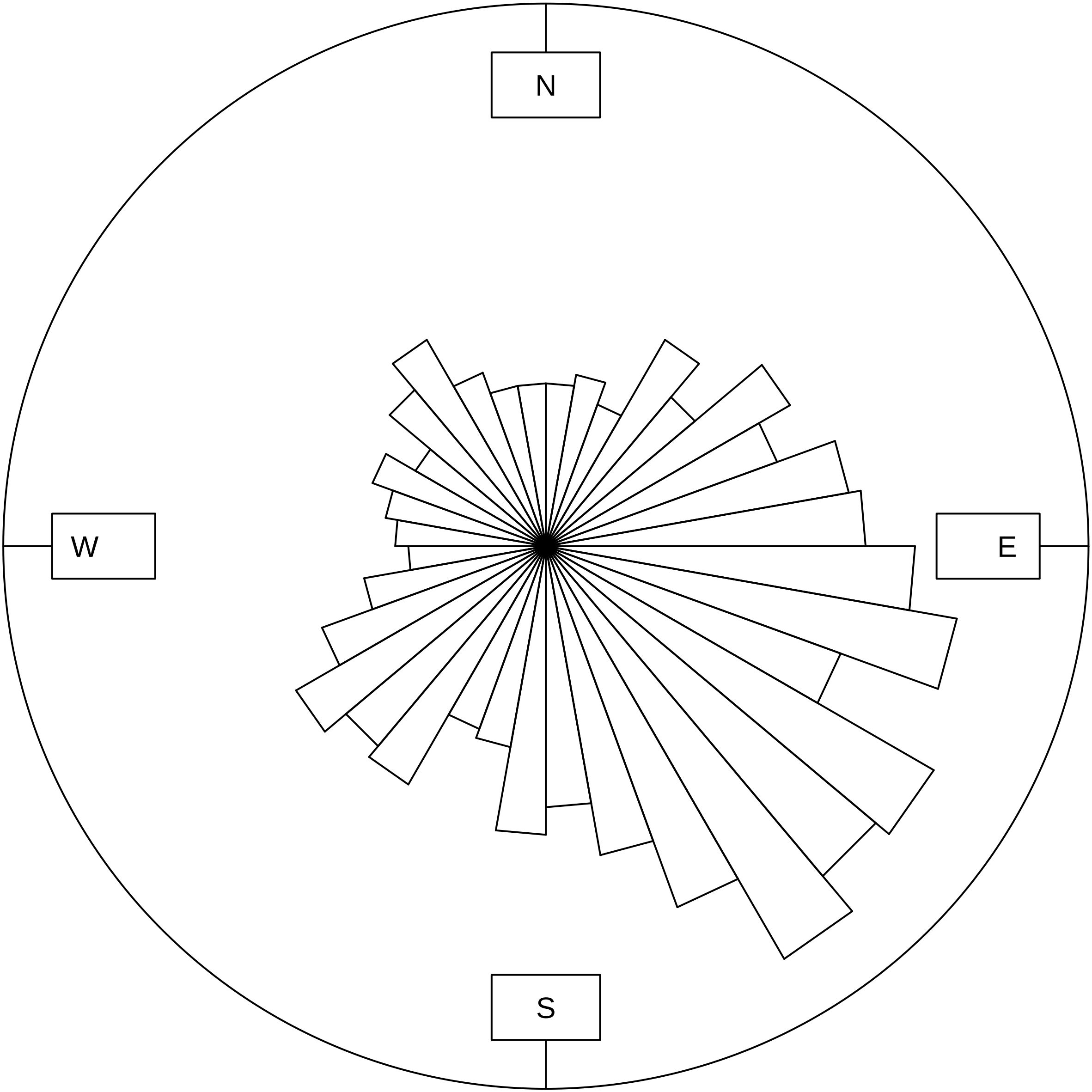

bootstrap 782

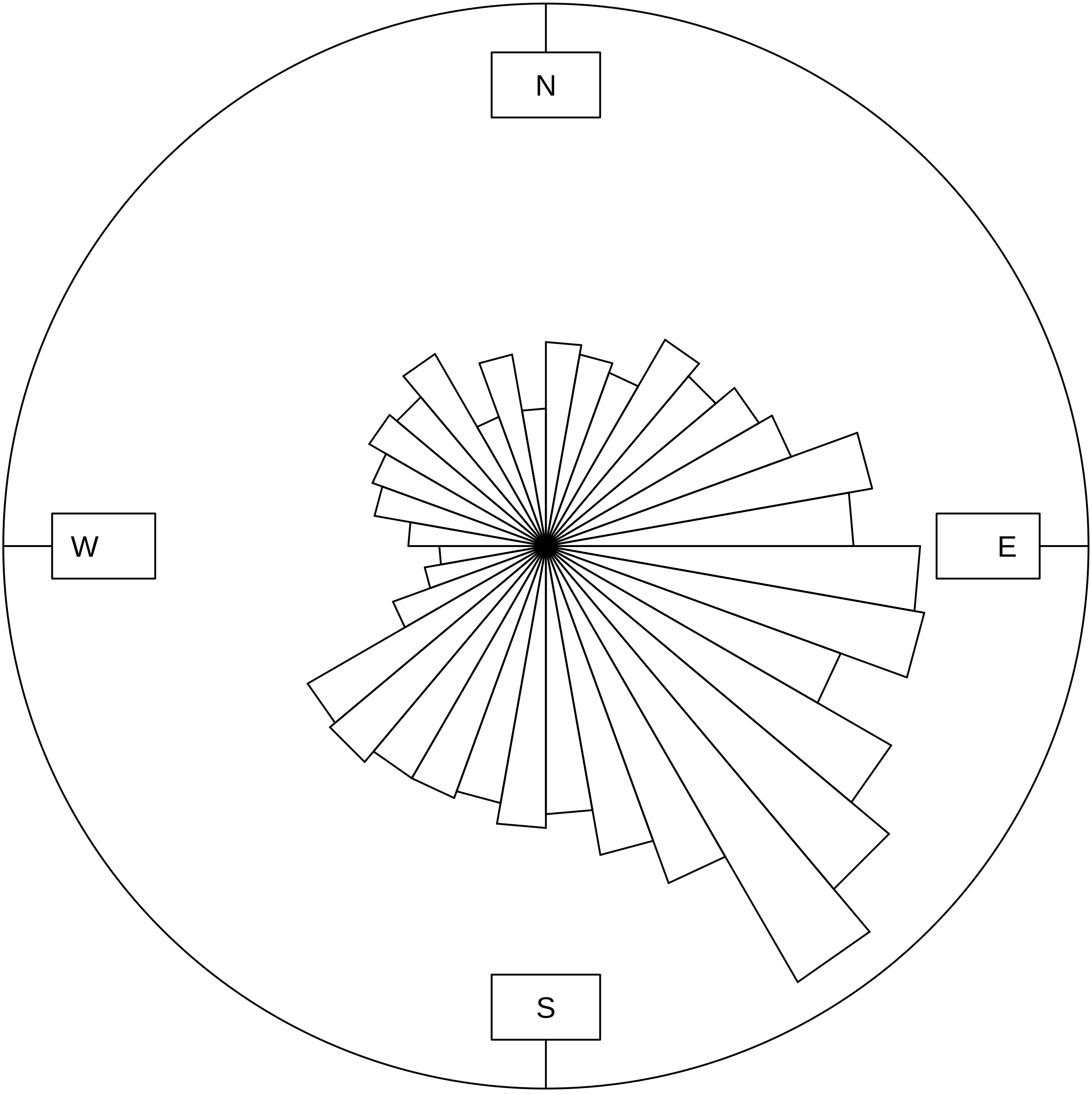

bootstrap 783

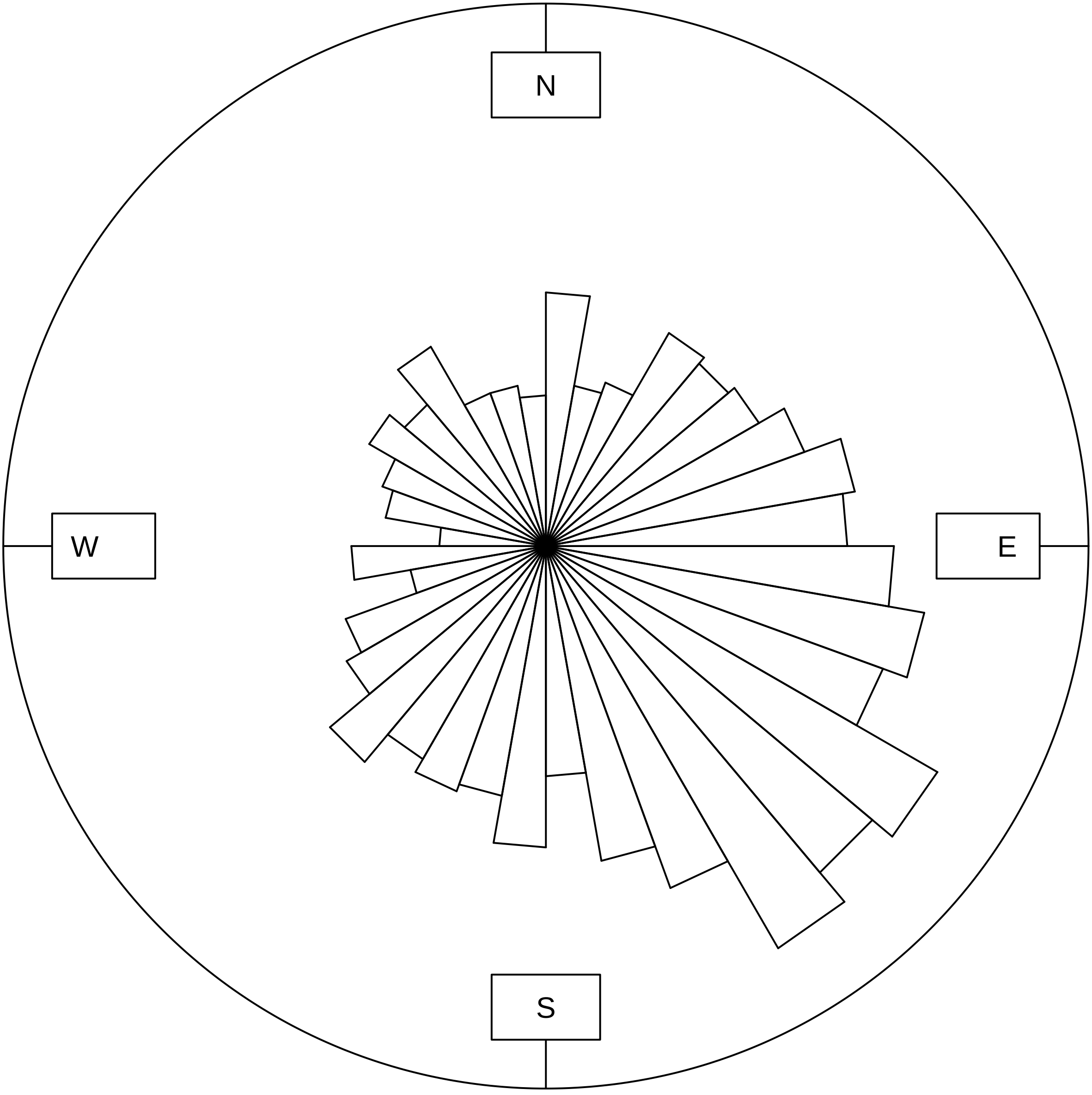

bootstrap 784

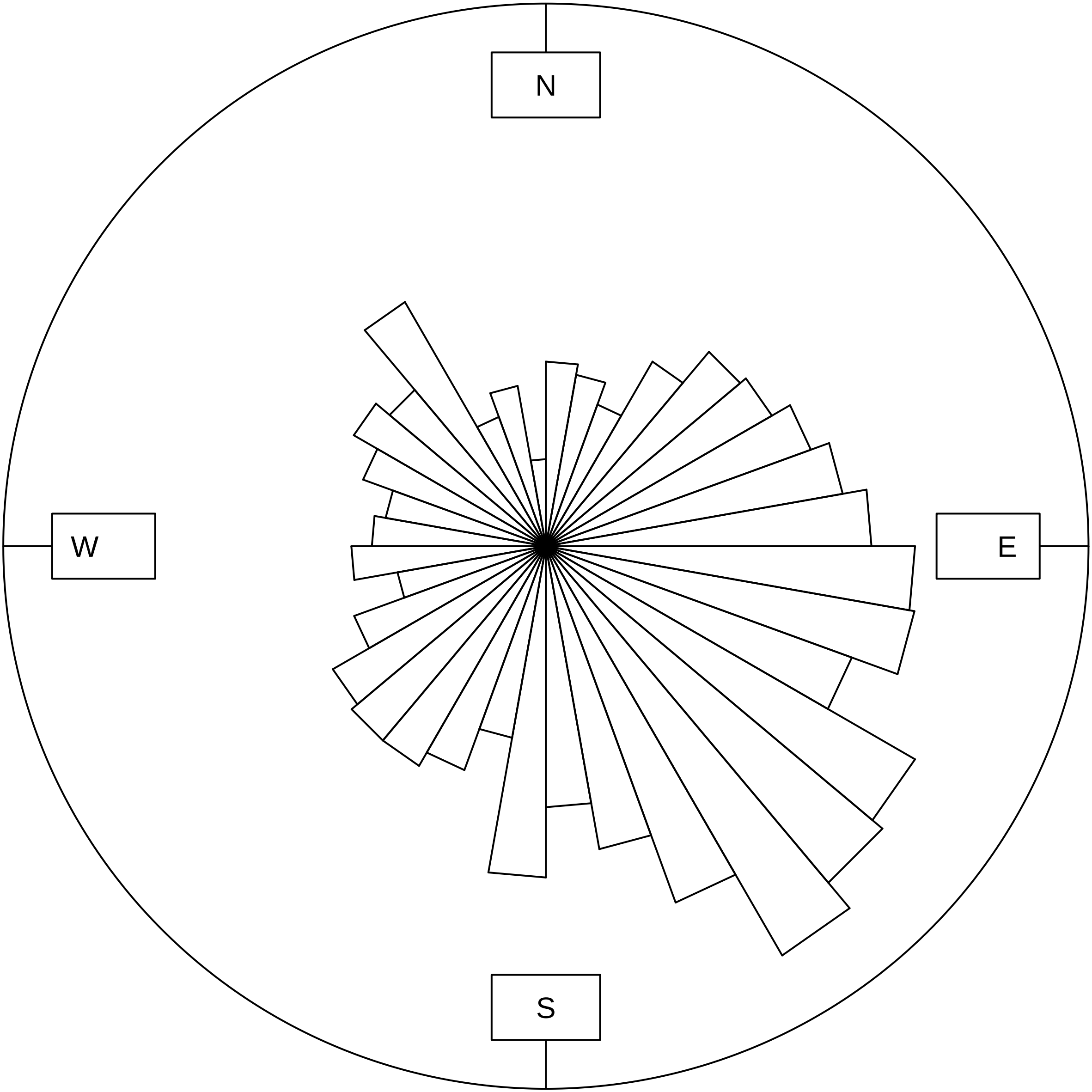

bootstrap 785

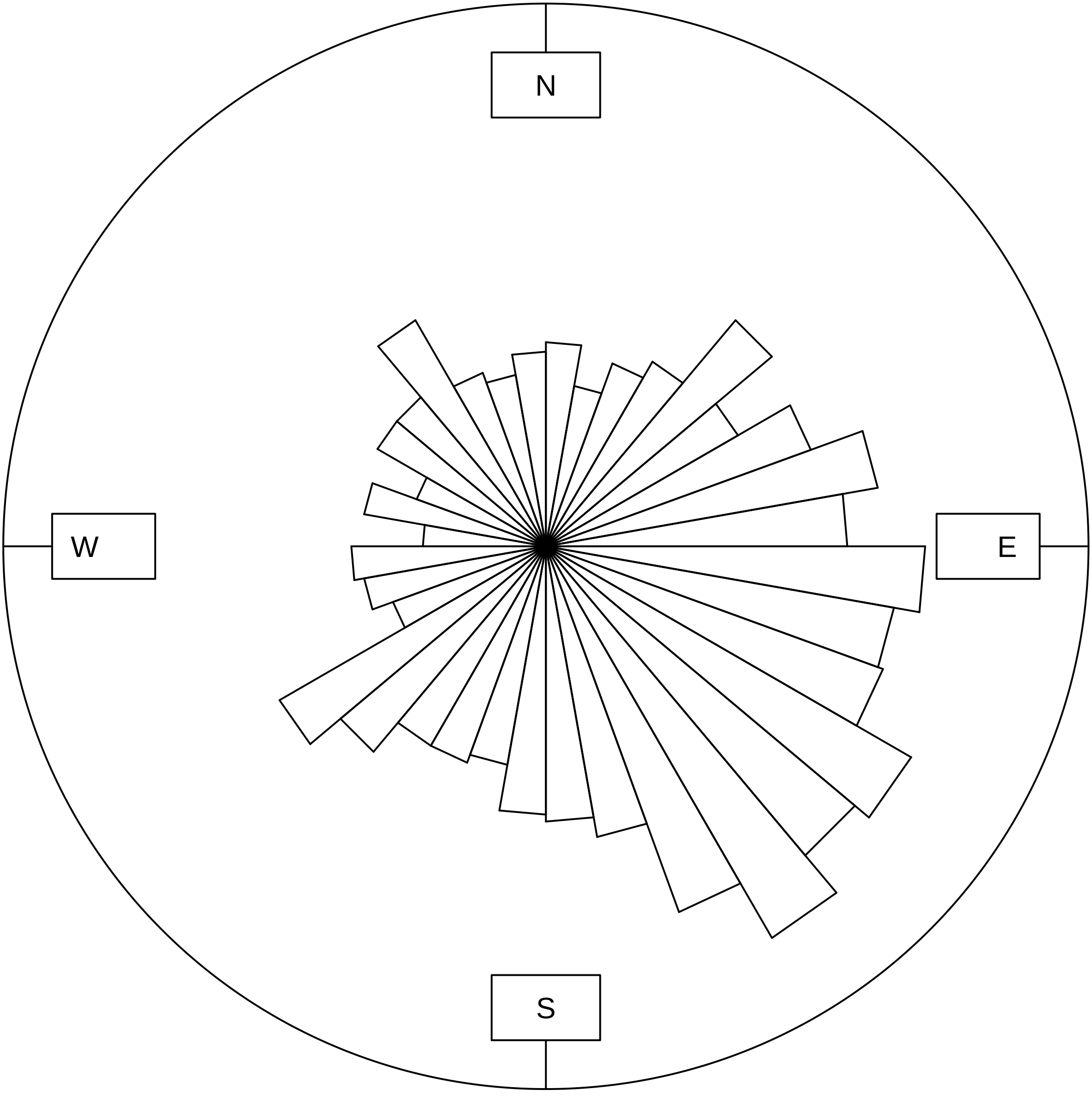

bootstrap 786

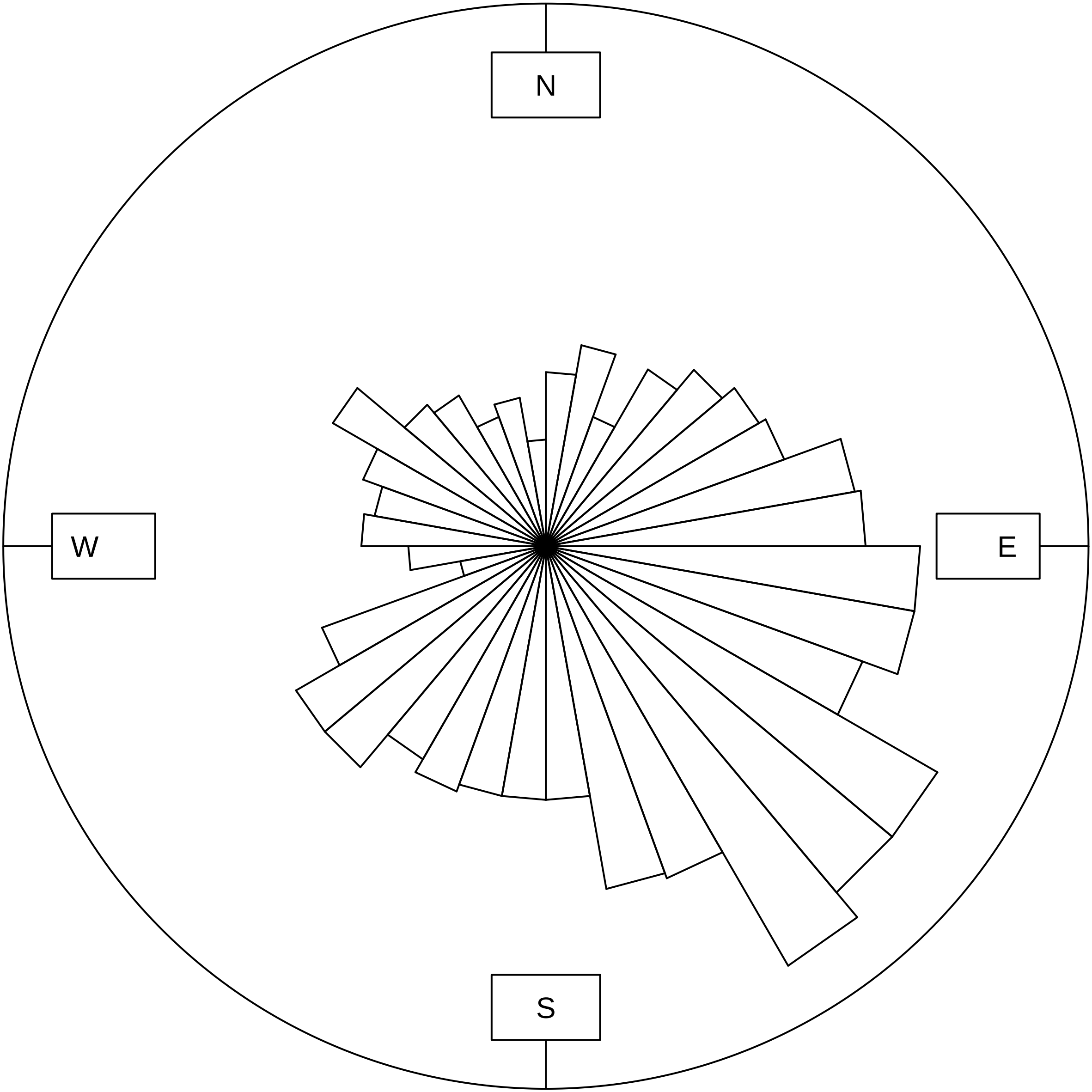

bootstrap 787

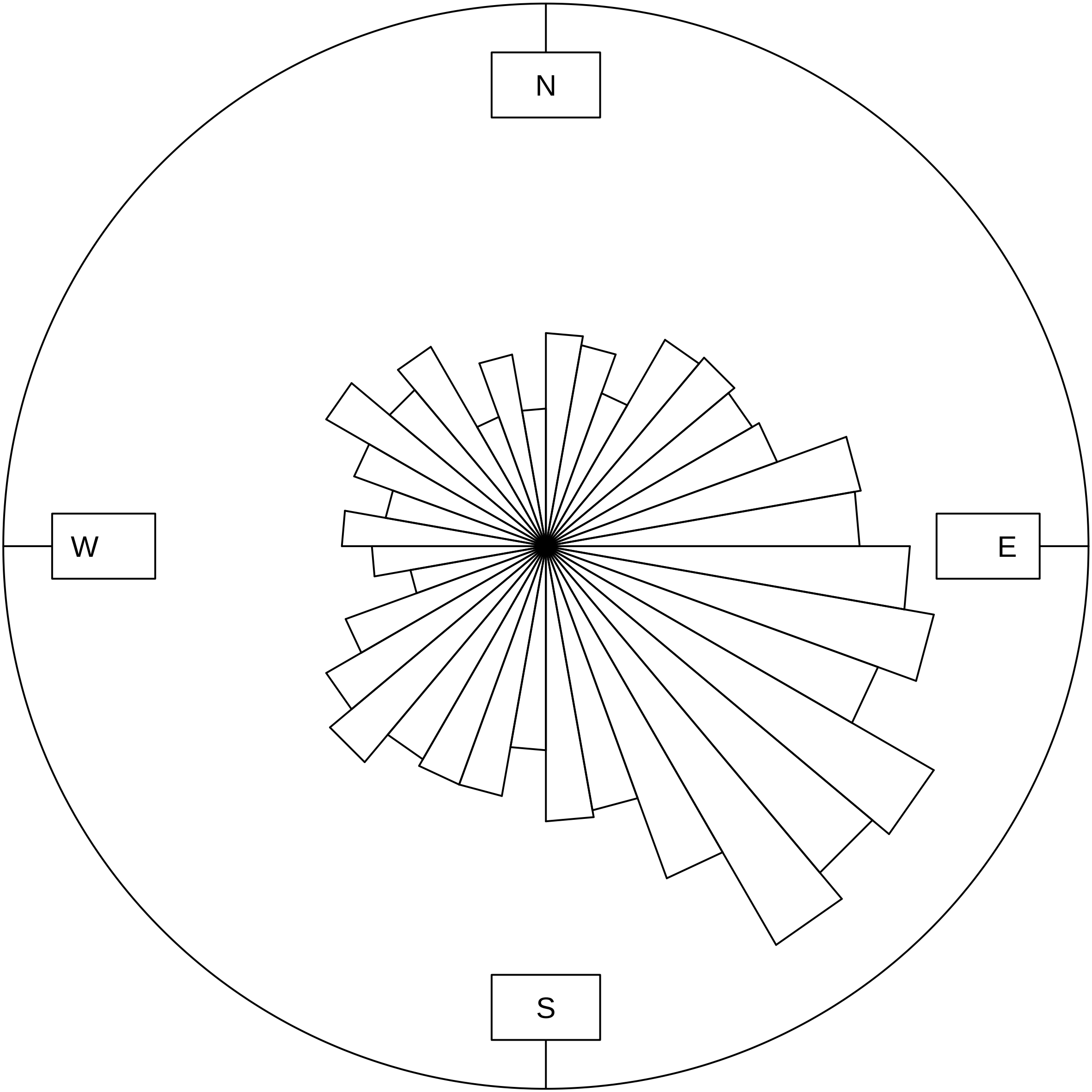

bootstrap 788

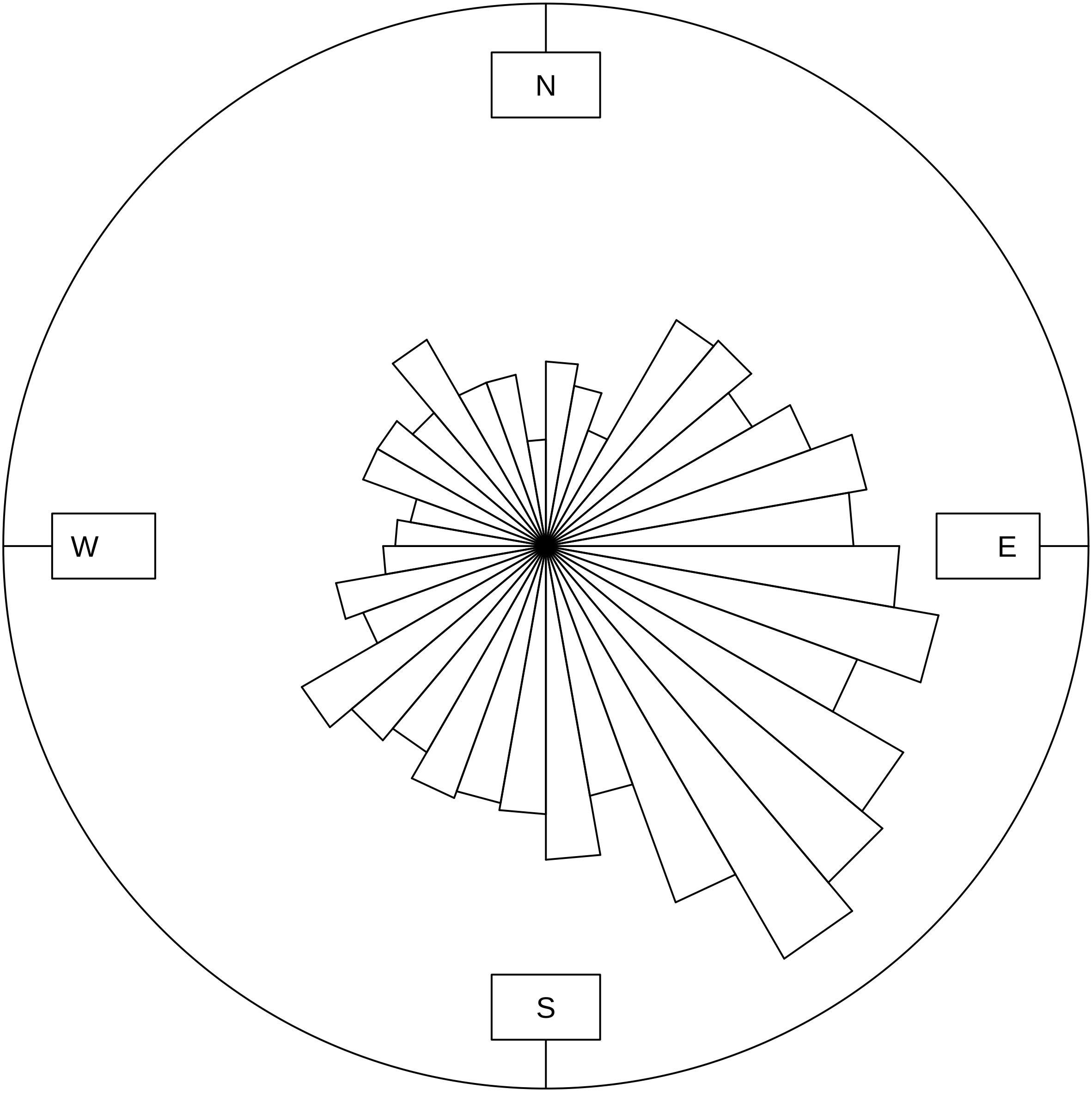

bootstrap 789

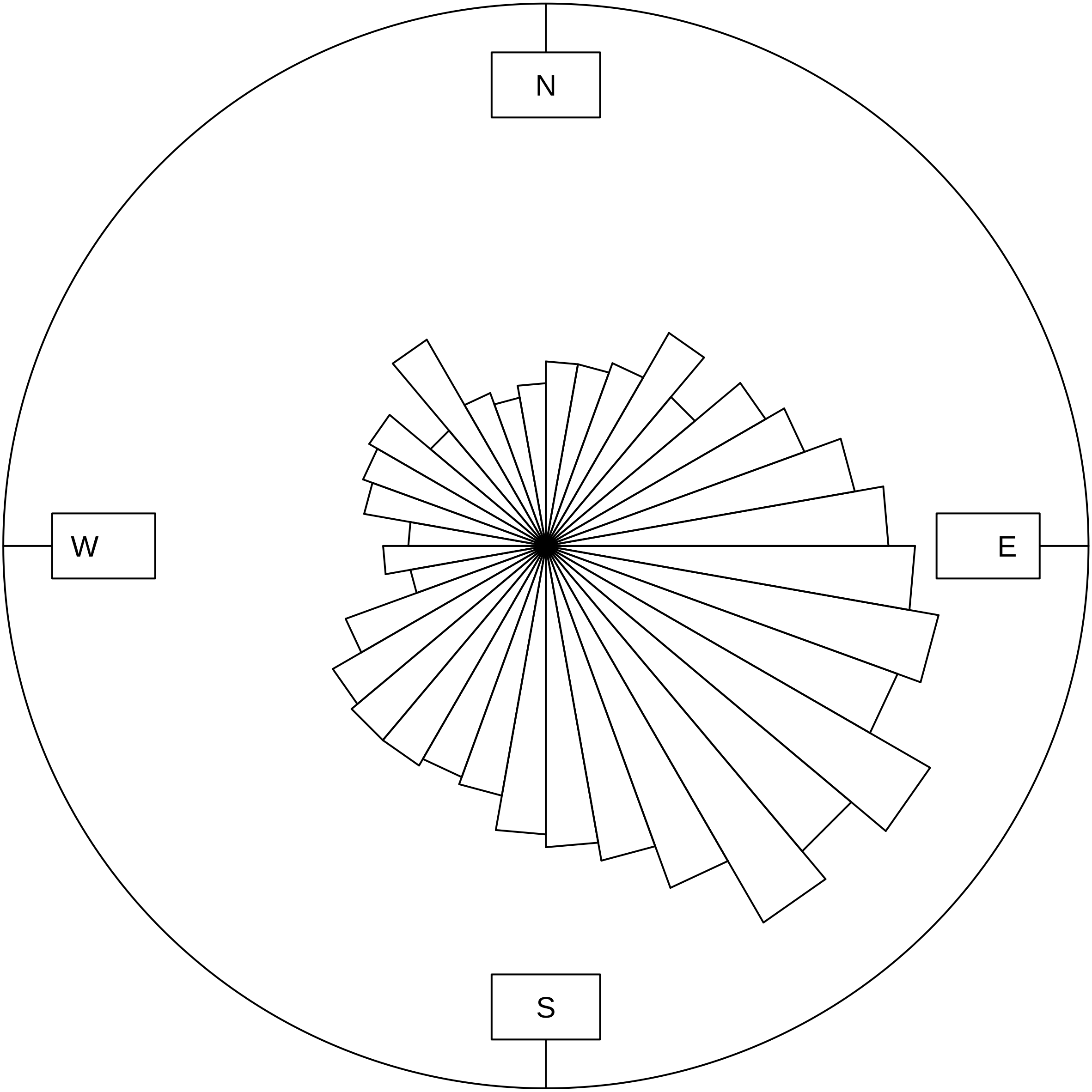

bootstrap 790

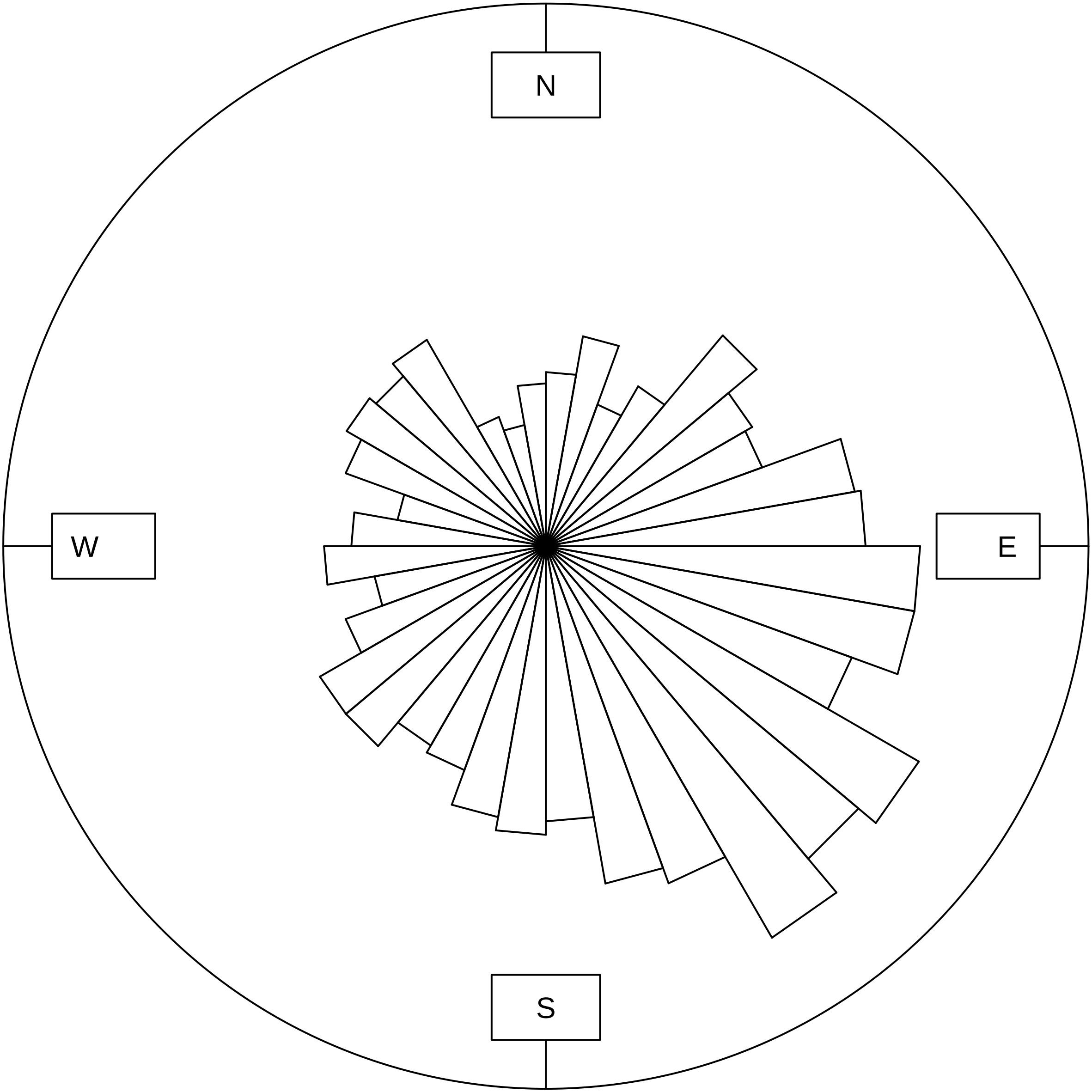

bootstrap 791

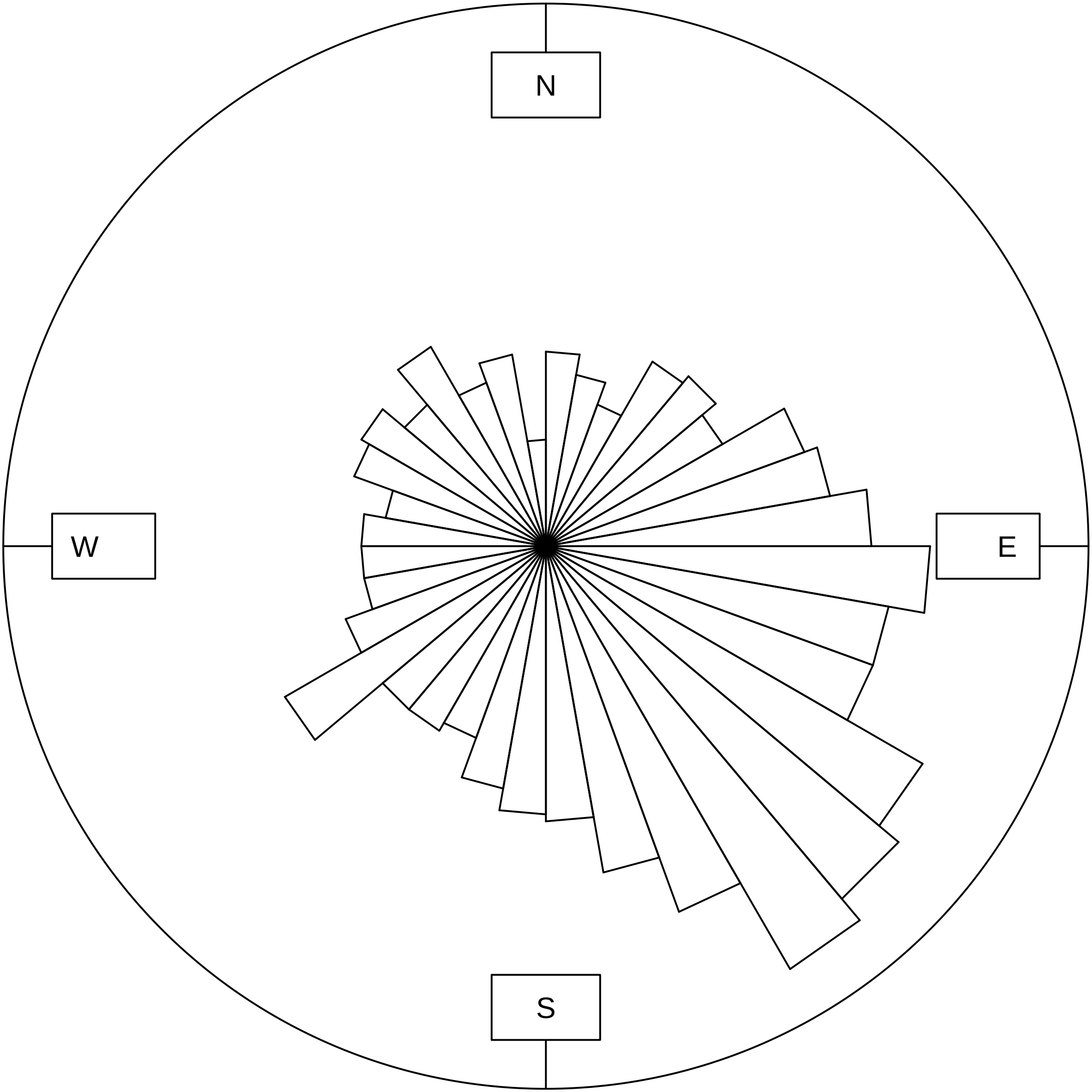

bootstrap 792

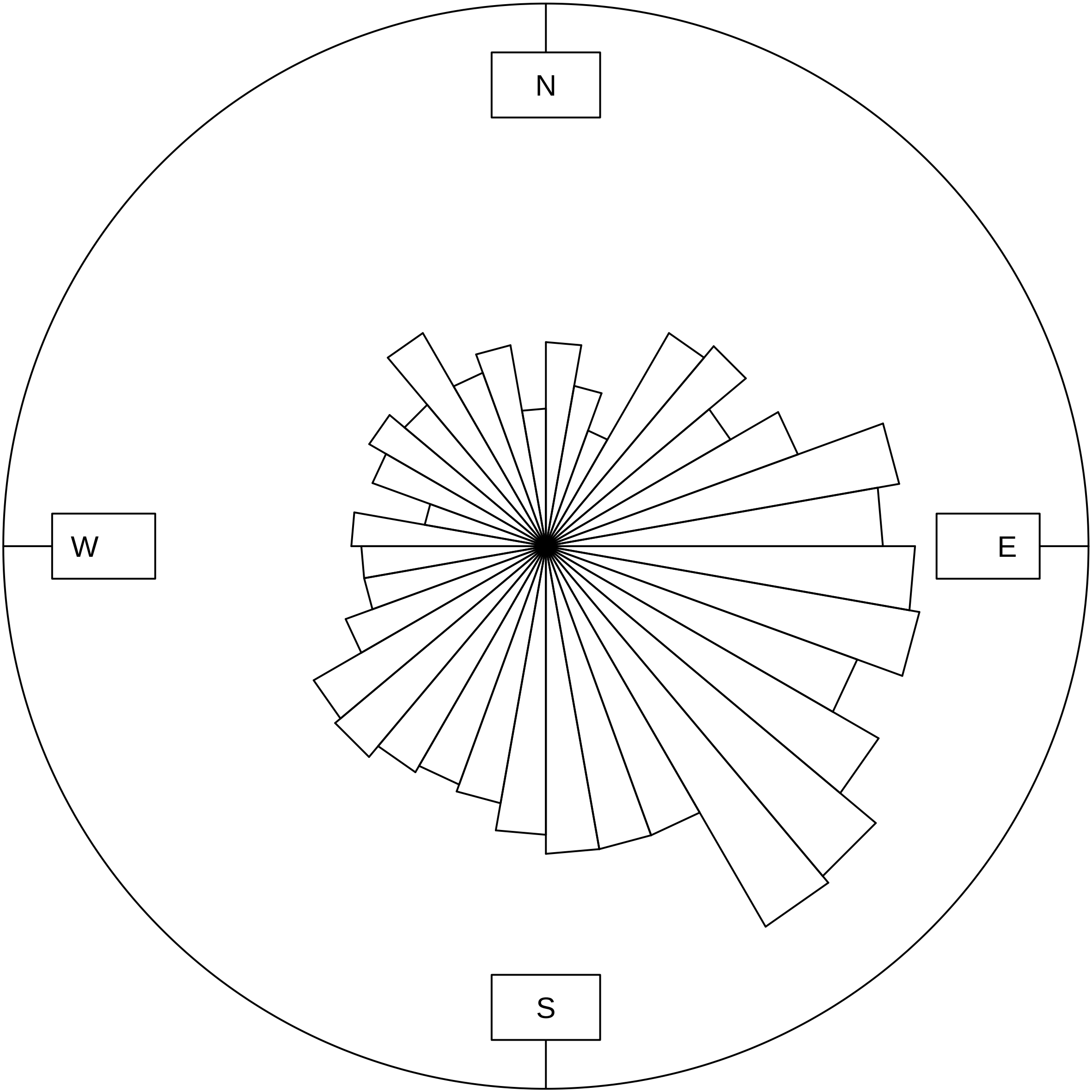

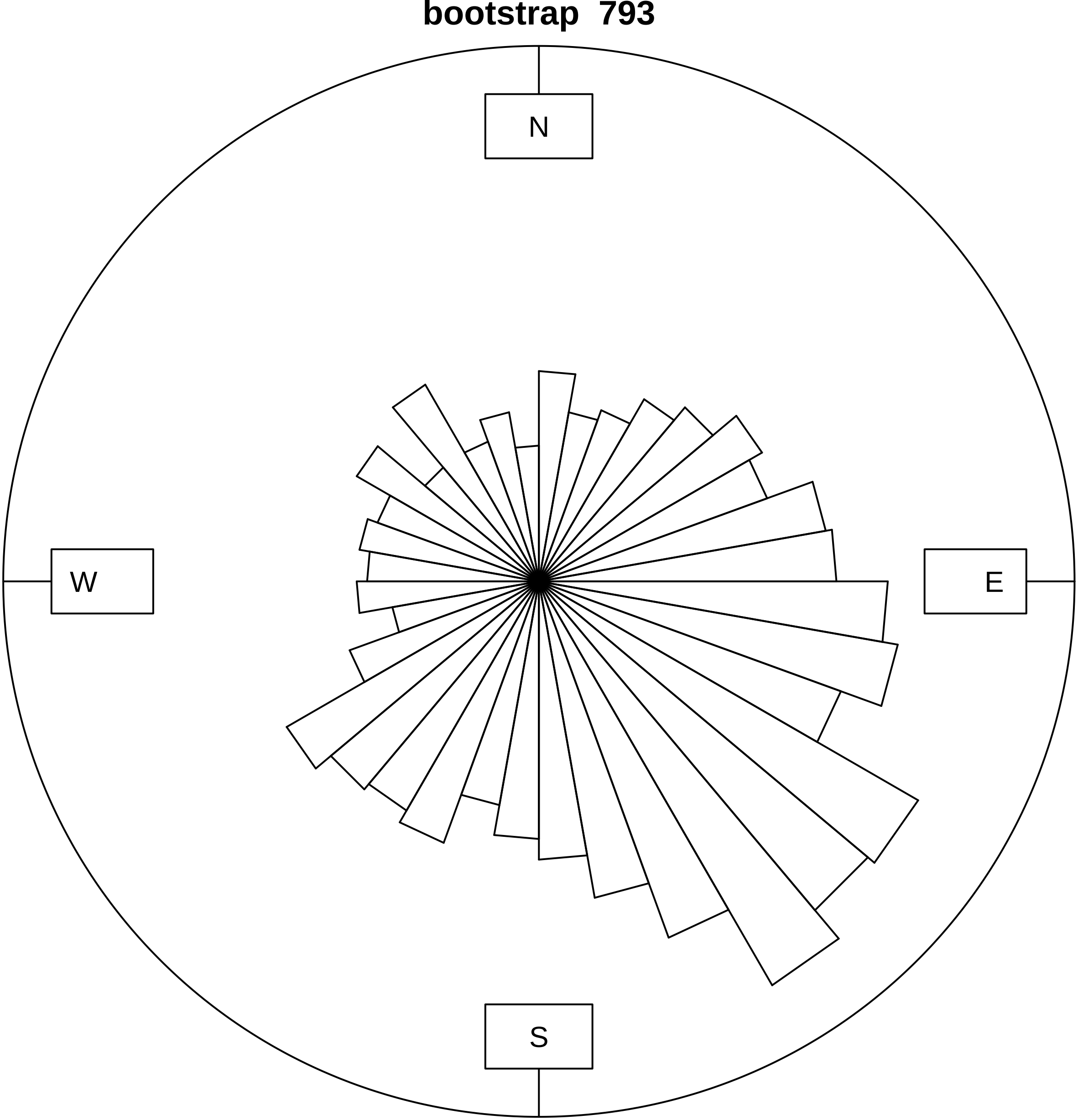
bootstrap 793
N
W
E
S

bootstrap 794

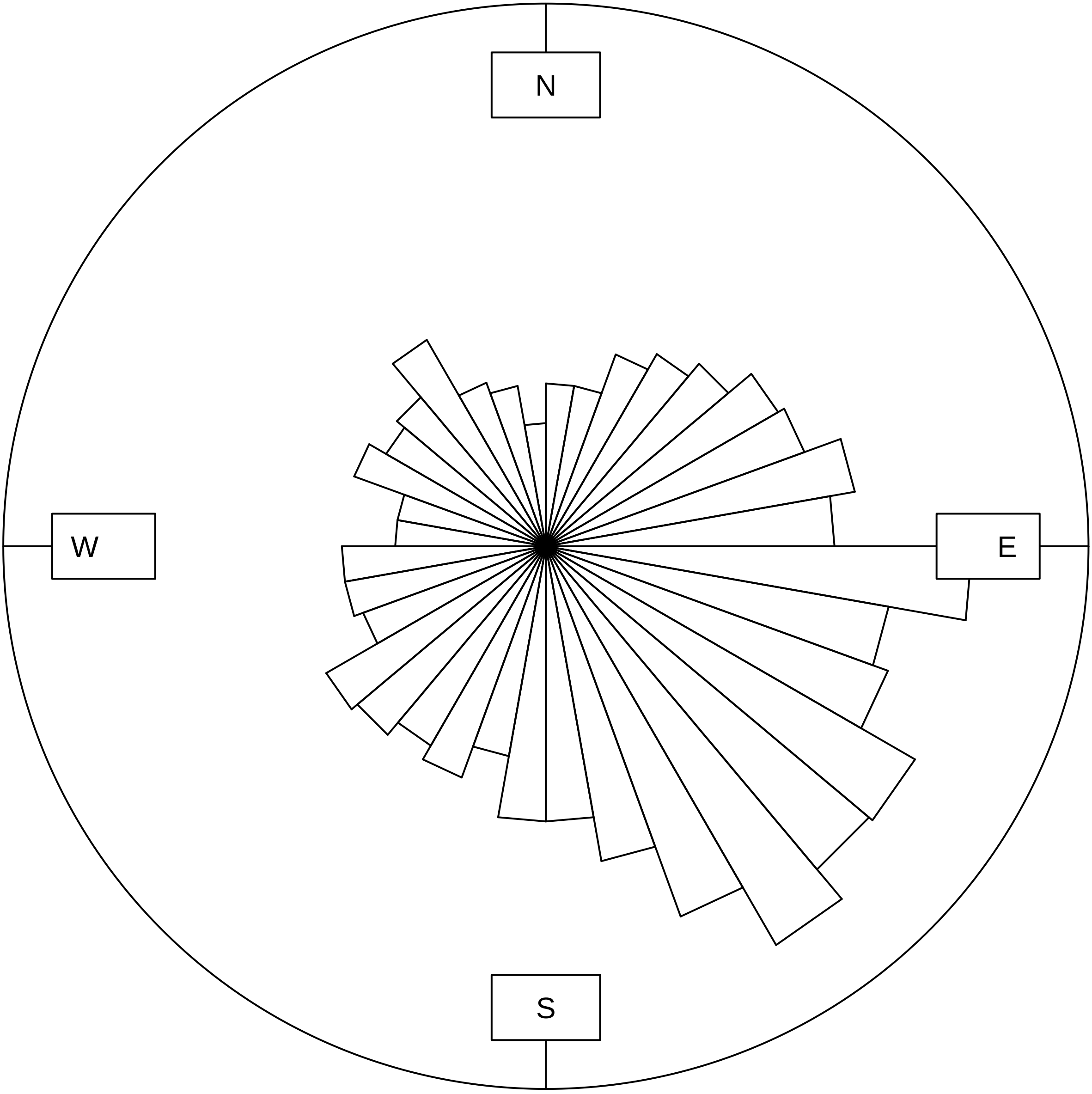

bootstrap 795

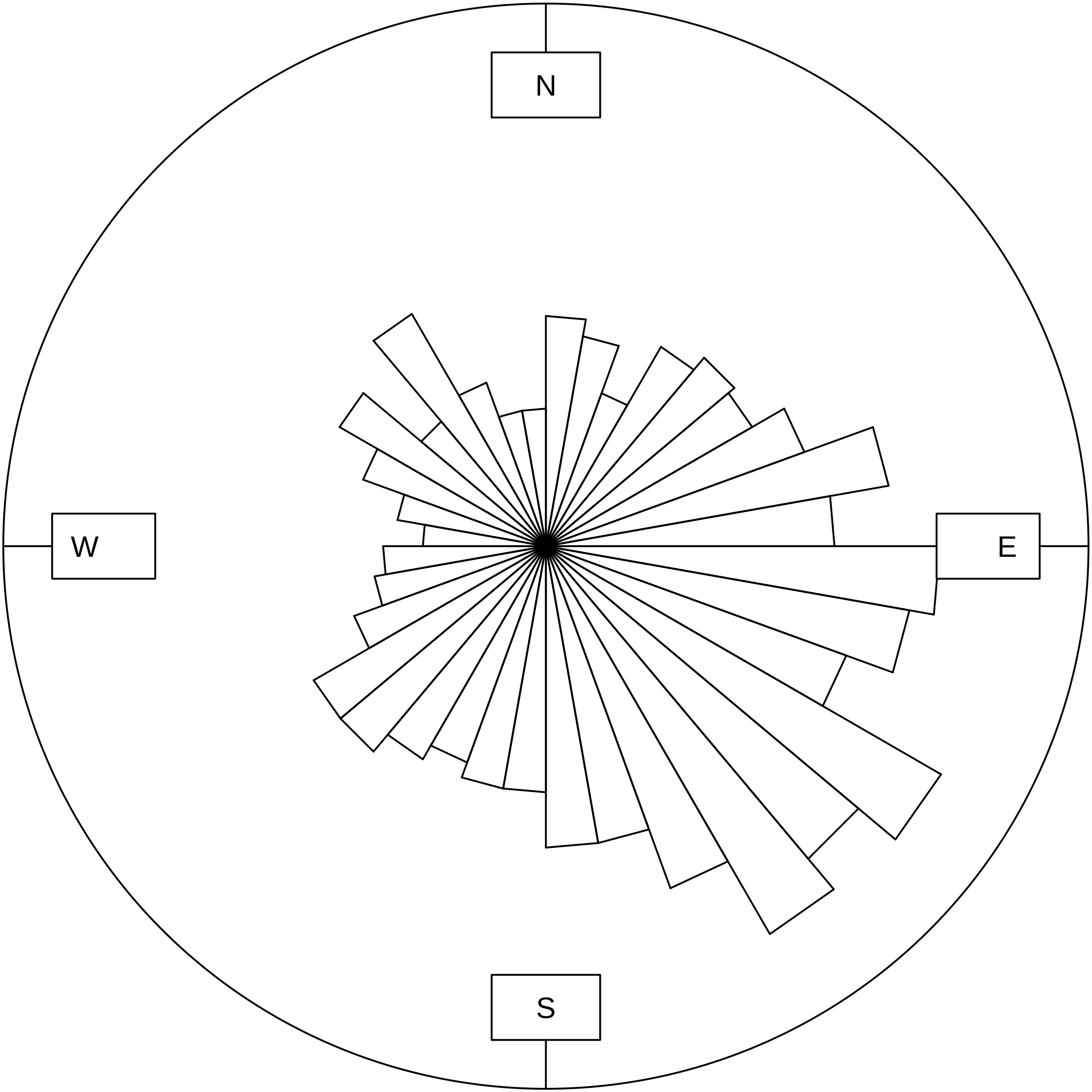

bootstrap 796

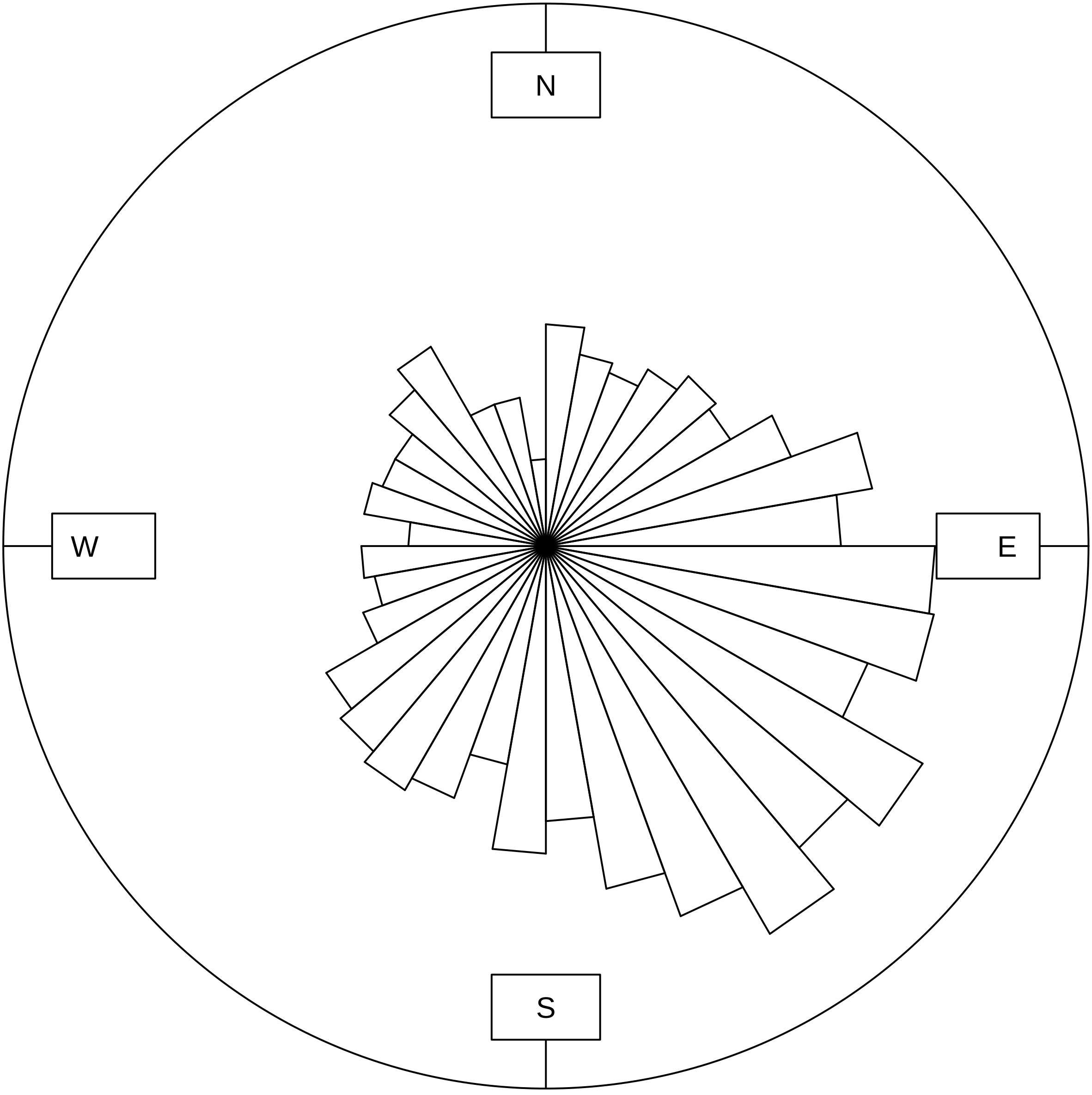

bootstrap 797

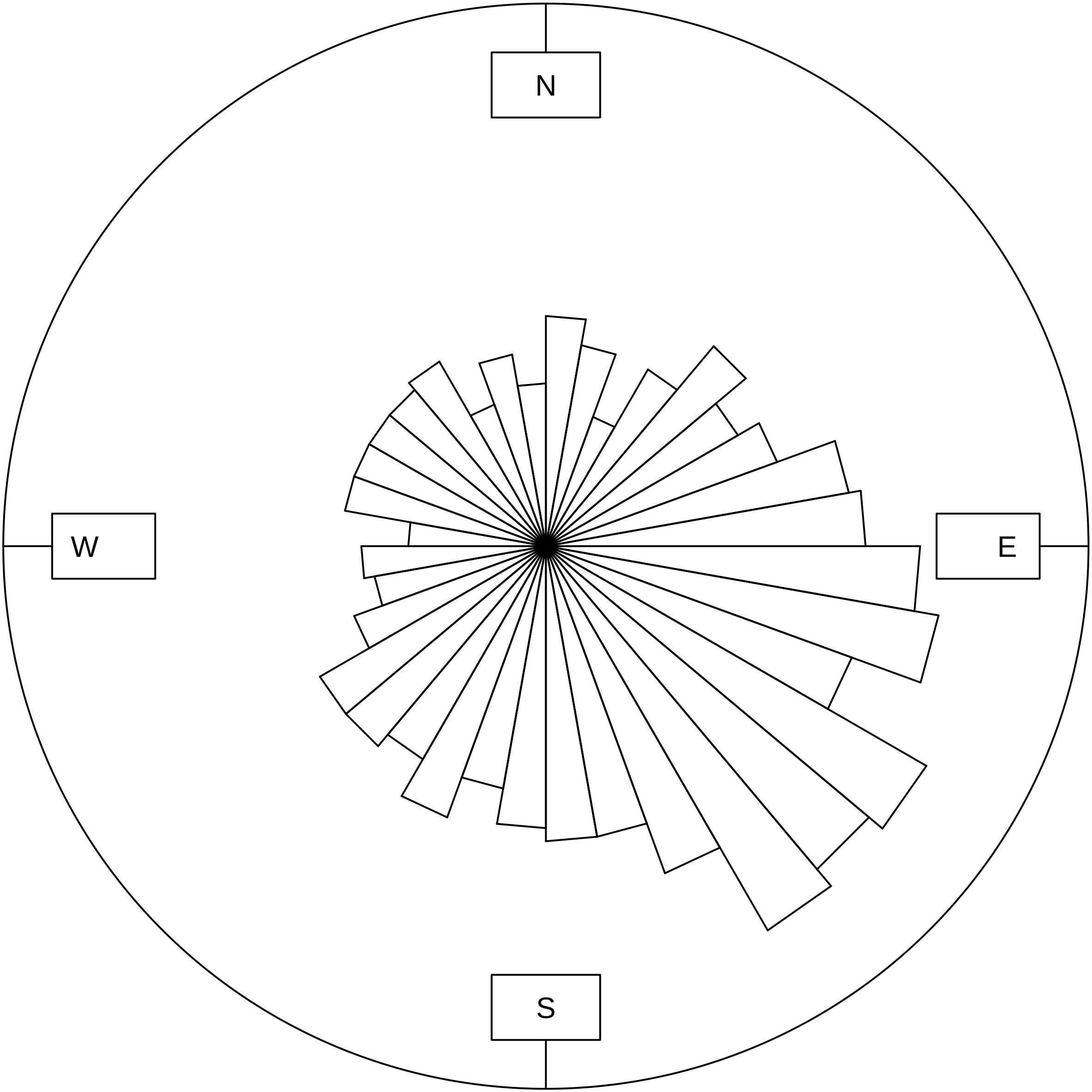

bootstrap 798

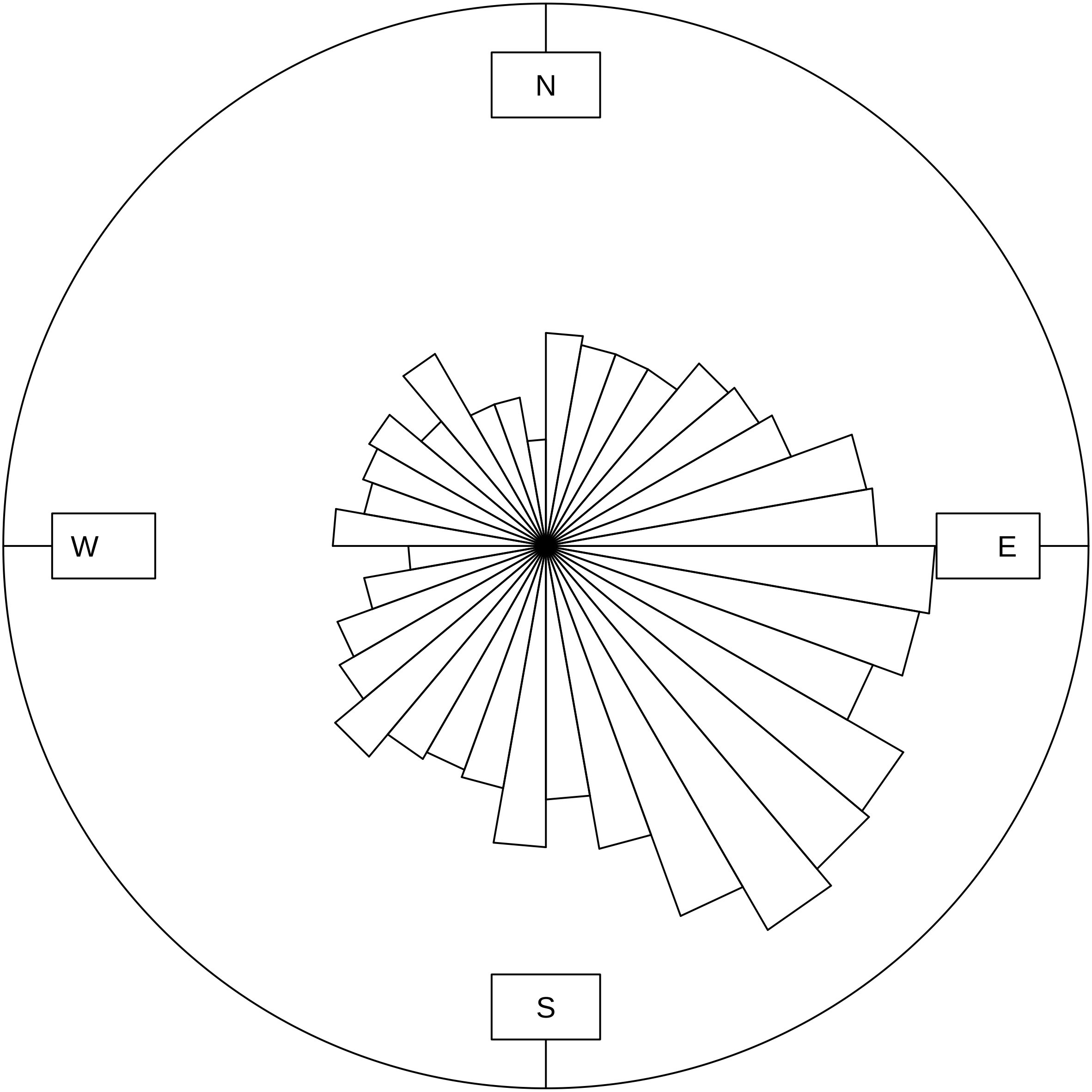

bootstrap 799

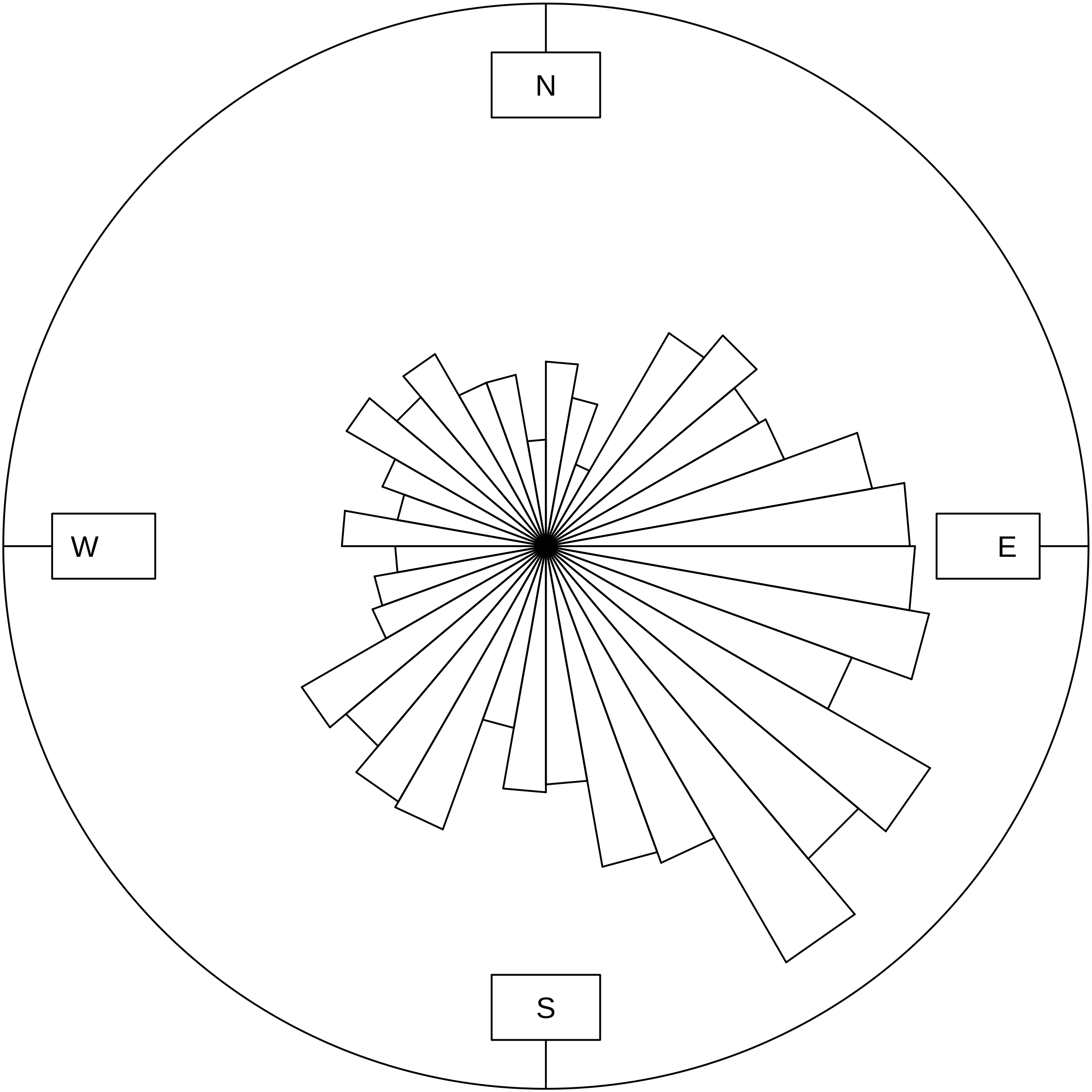

bootstrap 800

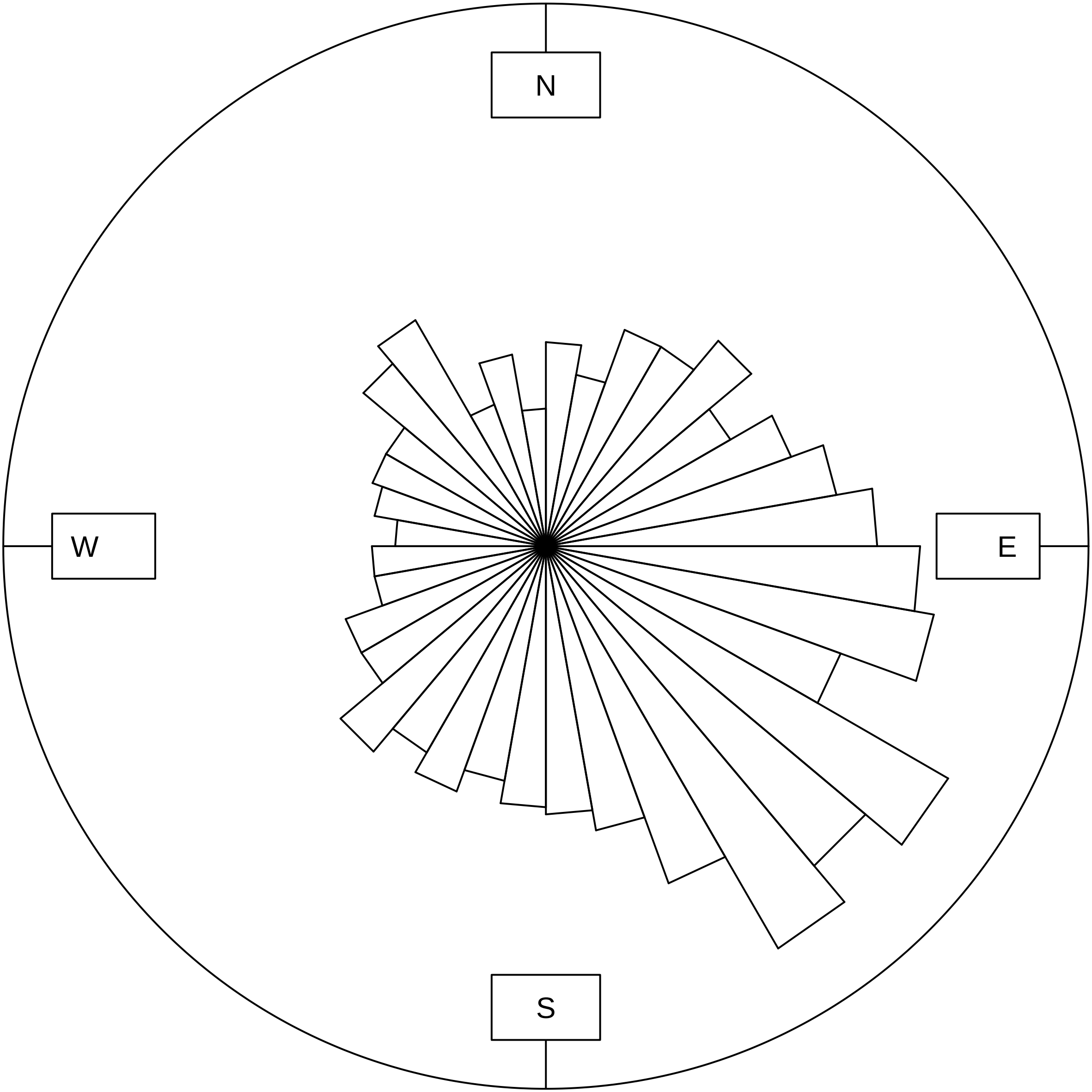

bootstrap 801

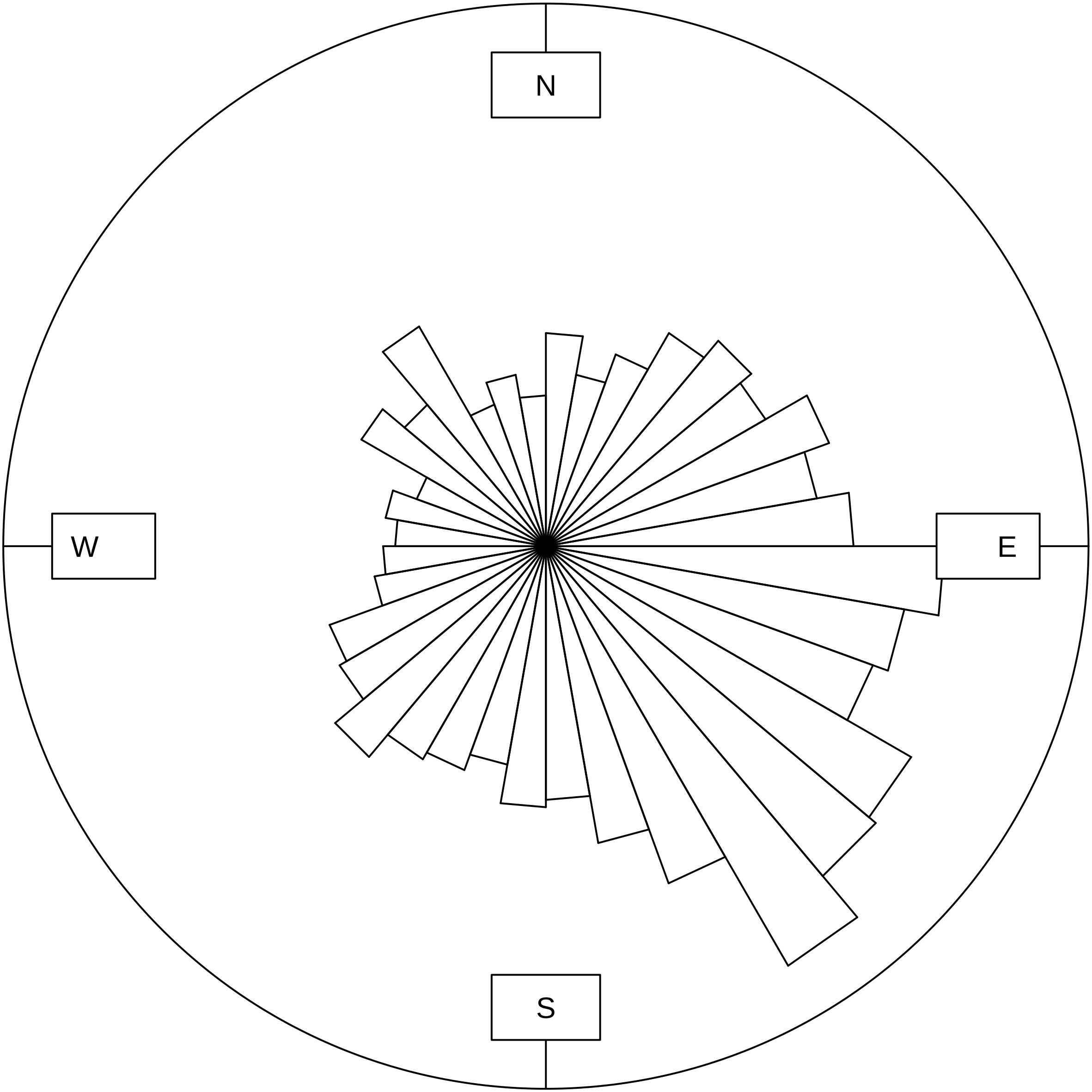

bootstrap 802

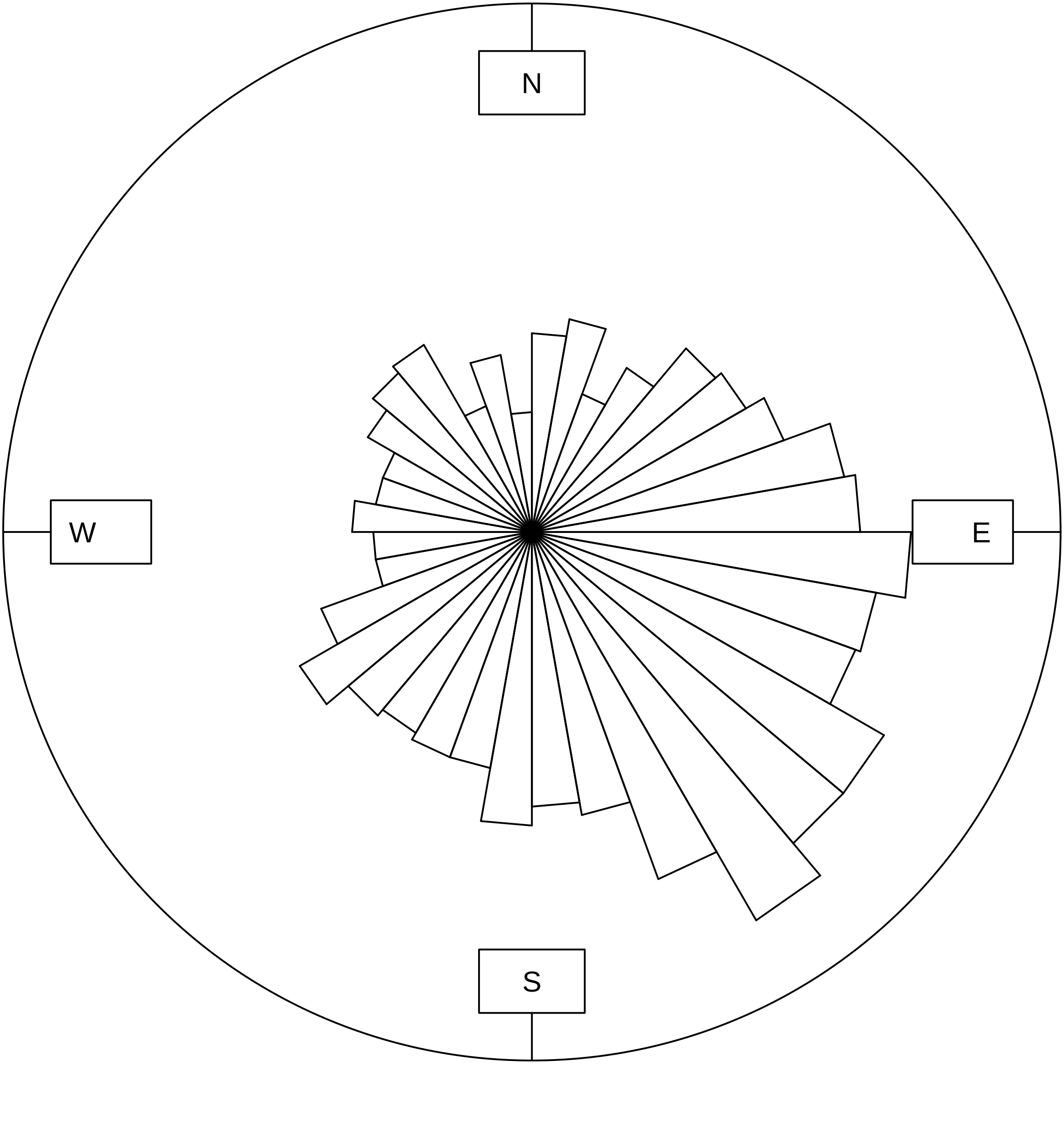

bootstrap 803

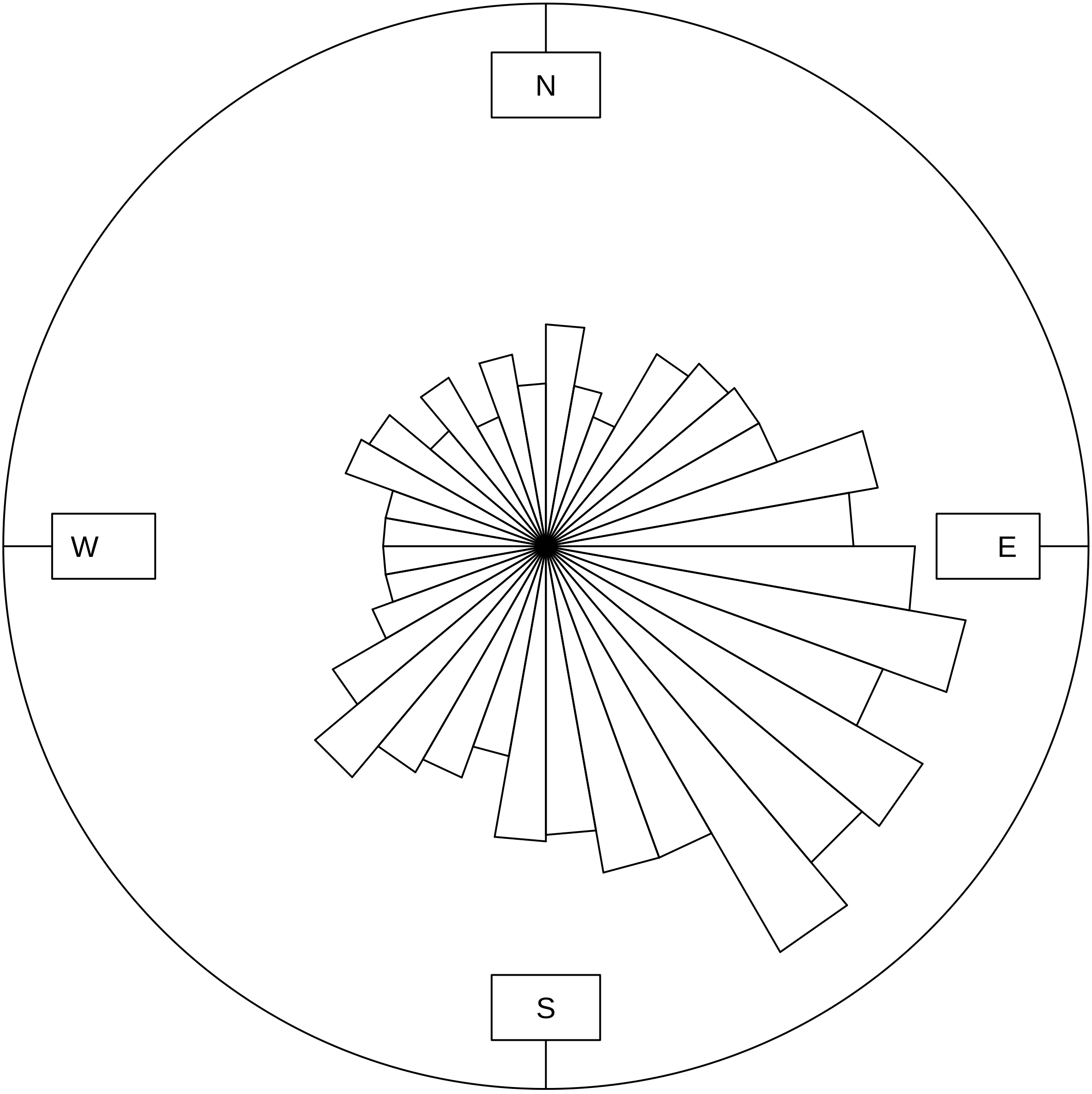

bootstrap 804

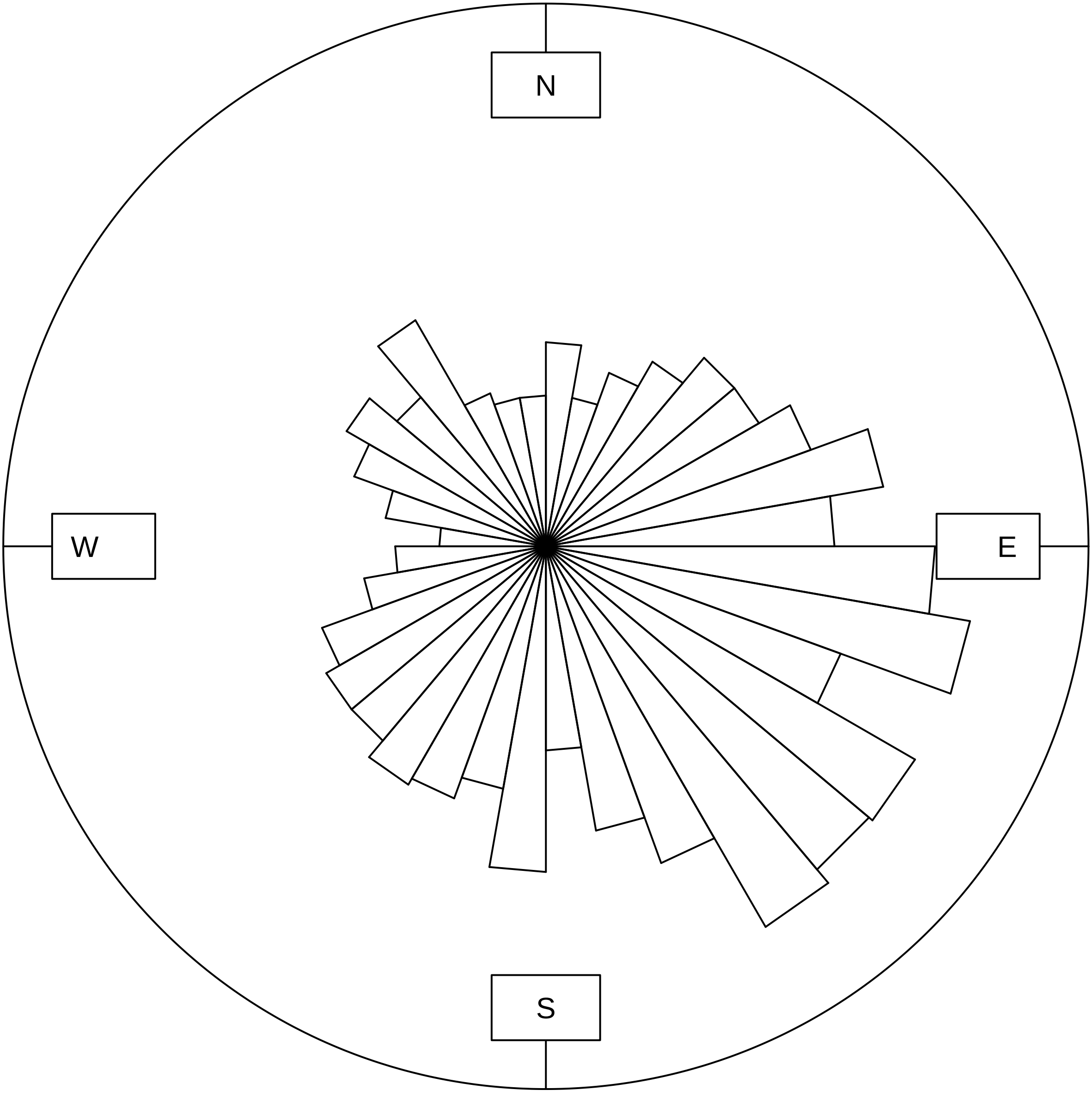

bootstrap 805

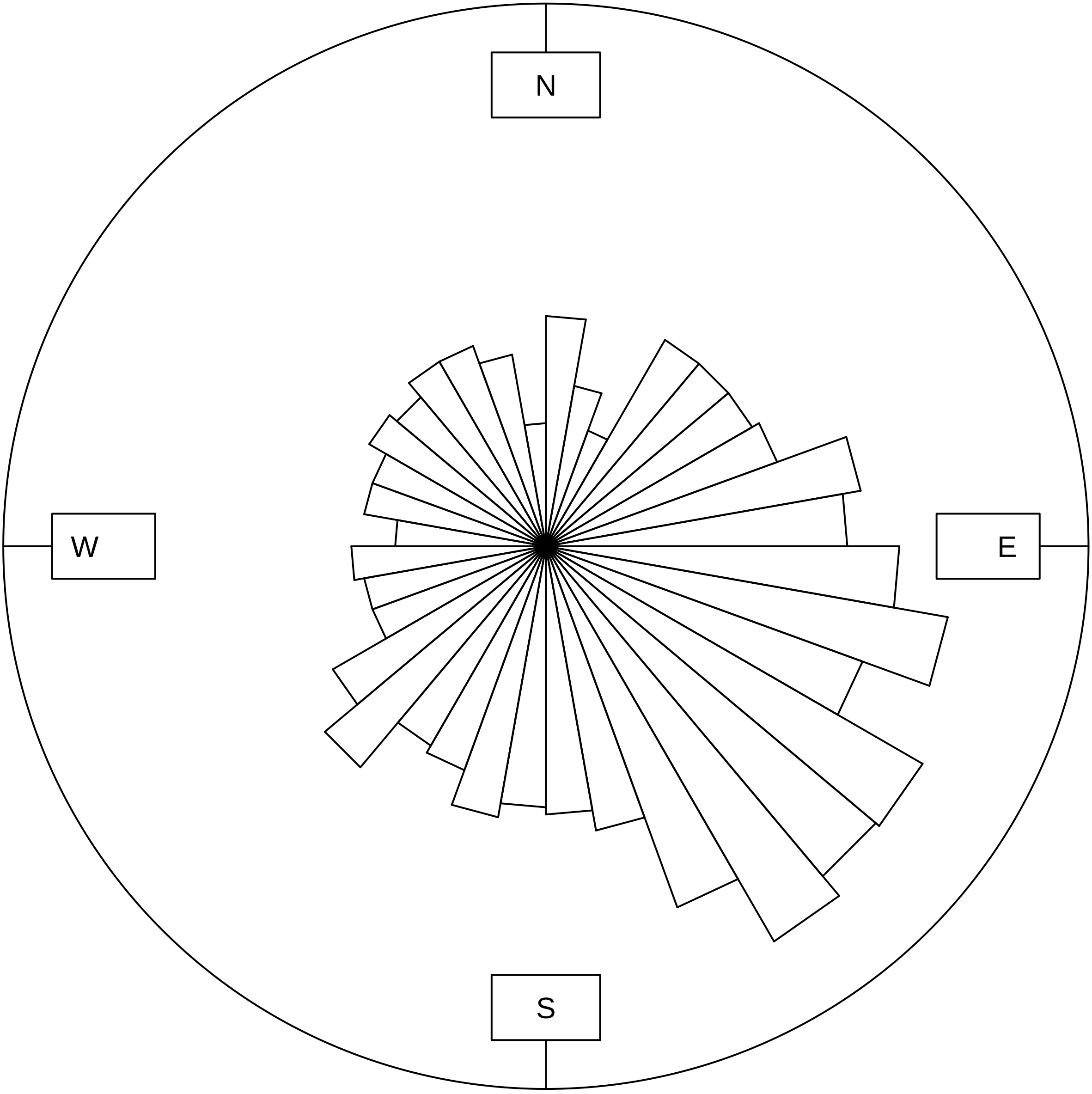

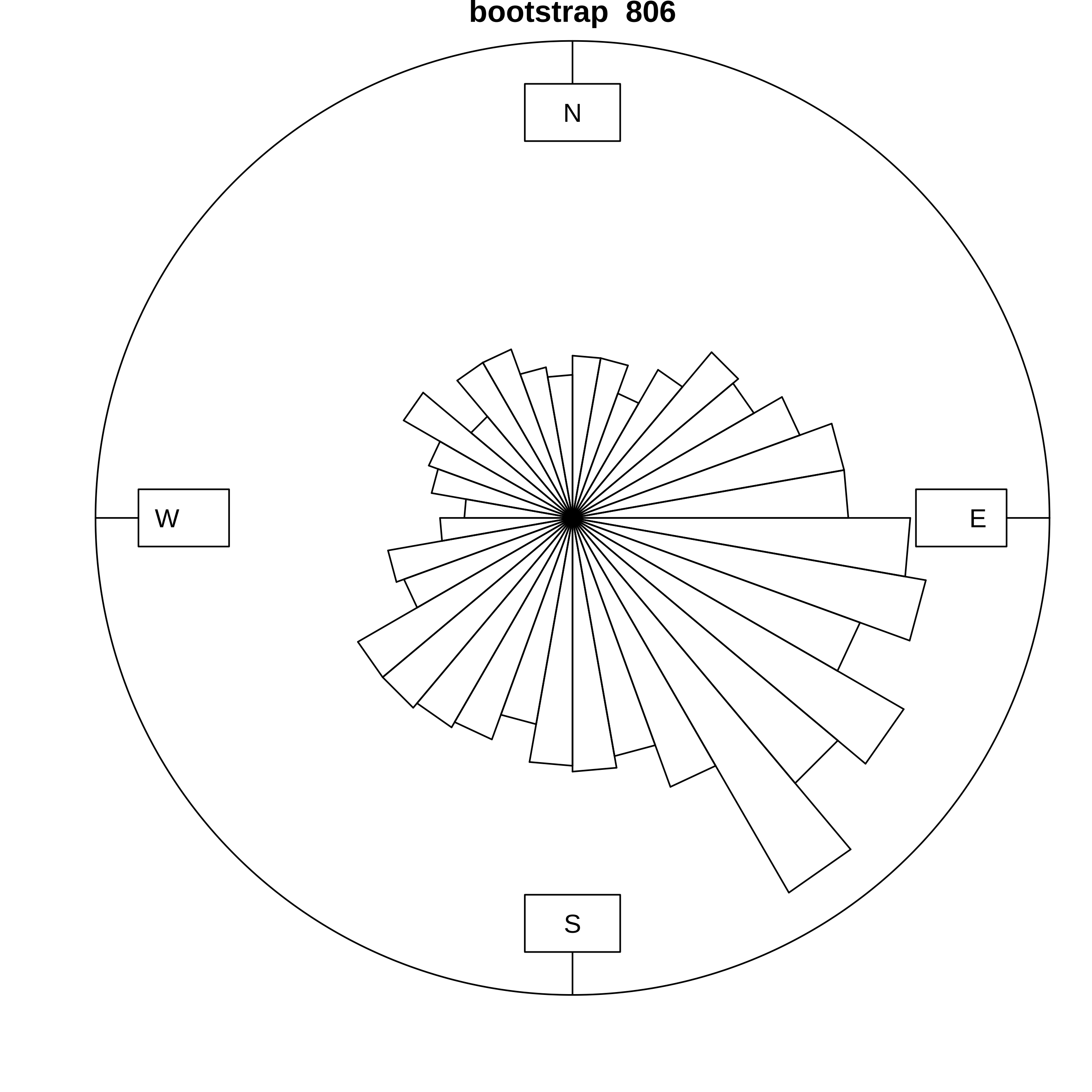
bootstrap 806
N
W
E
S

bootstrap 807

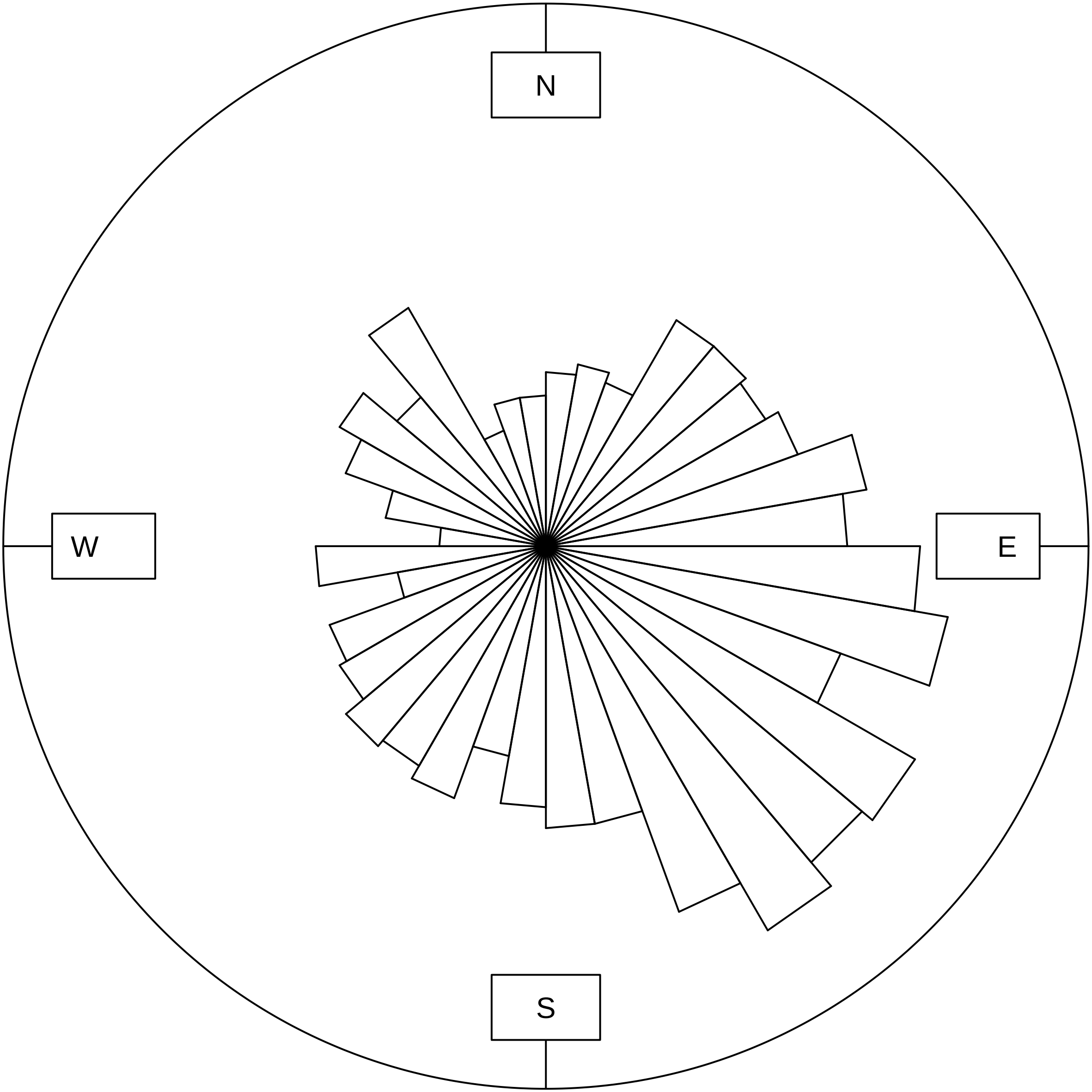

bootstrap 808

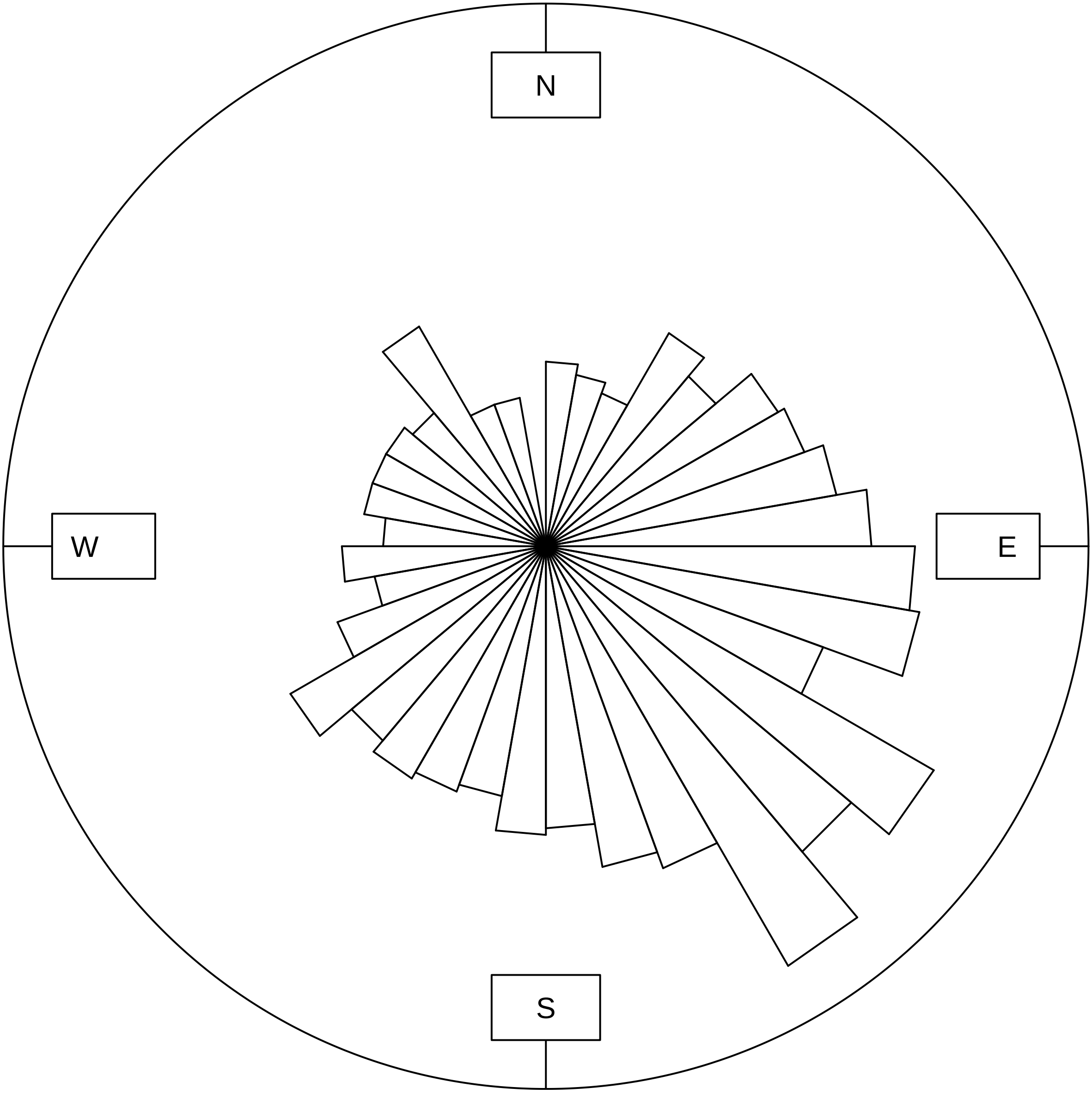

bootstrap 809

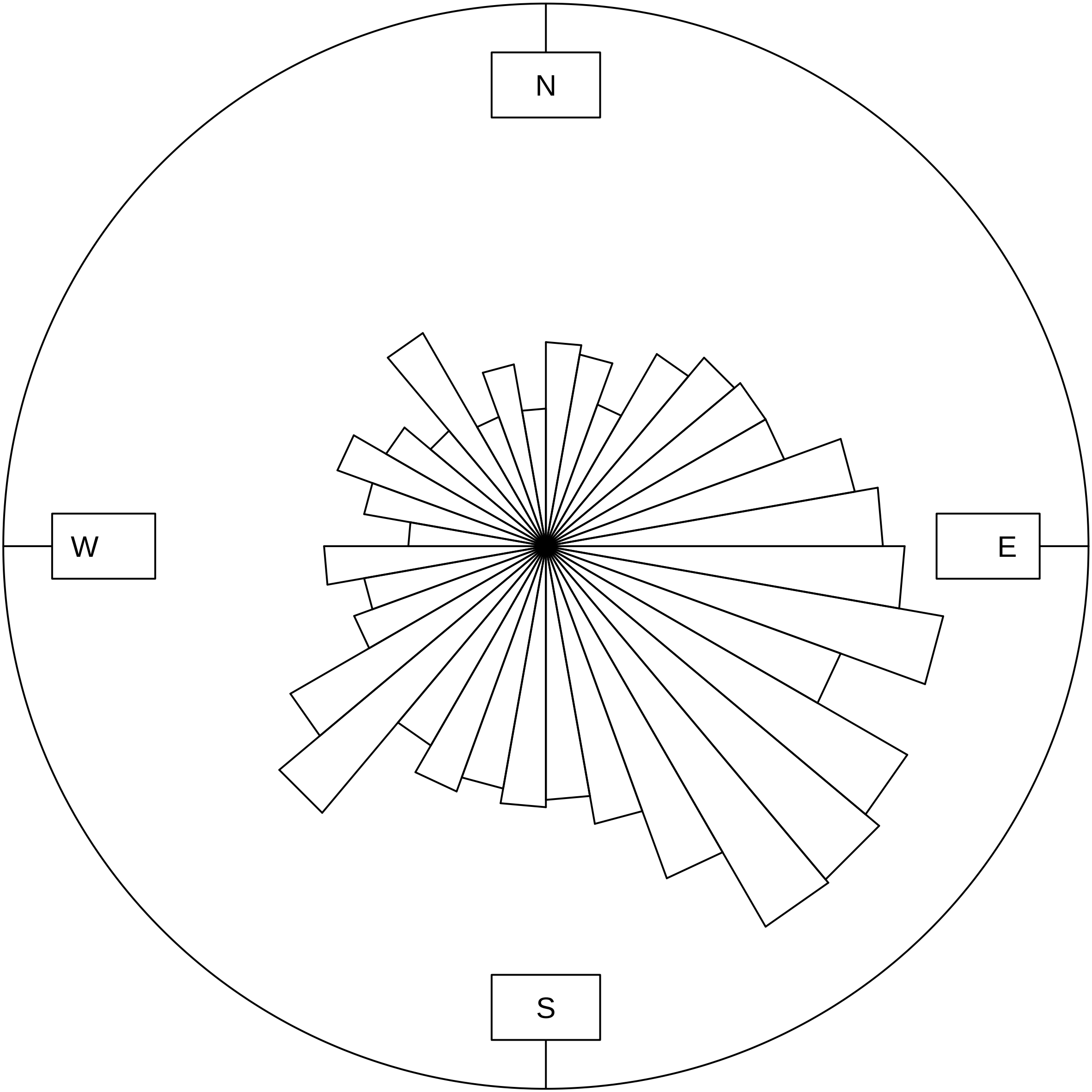

bootstrap 810

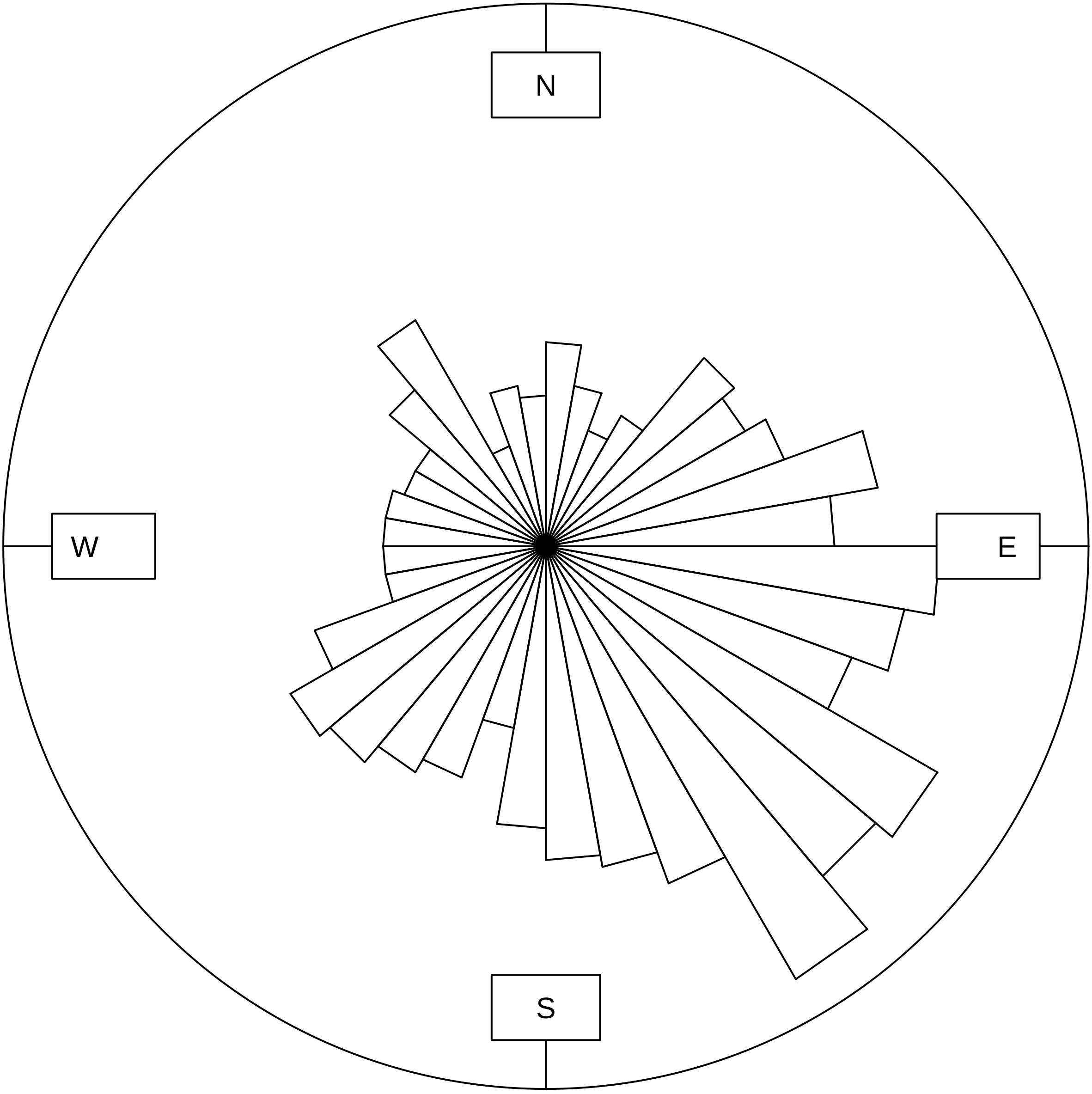

bootstrap 811

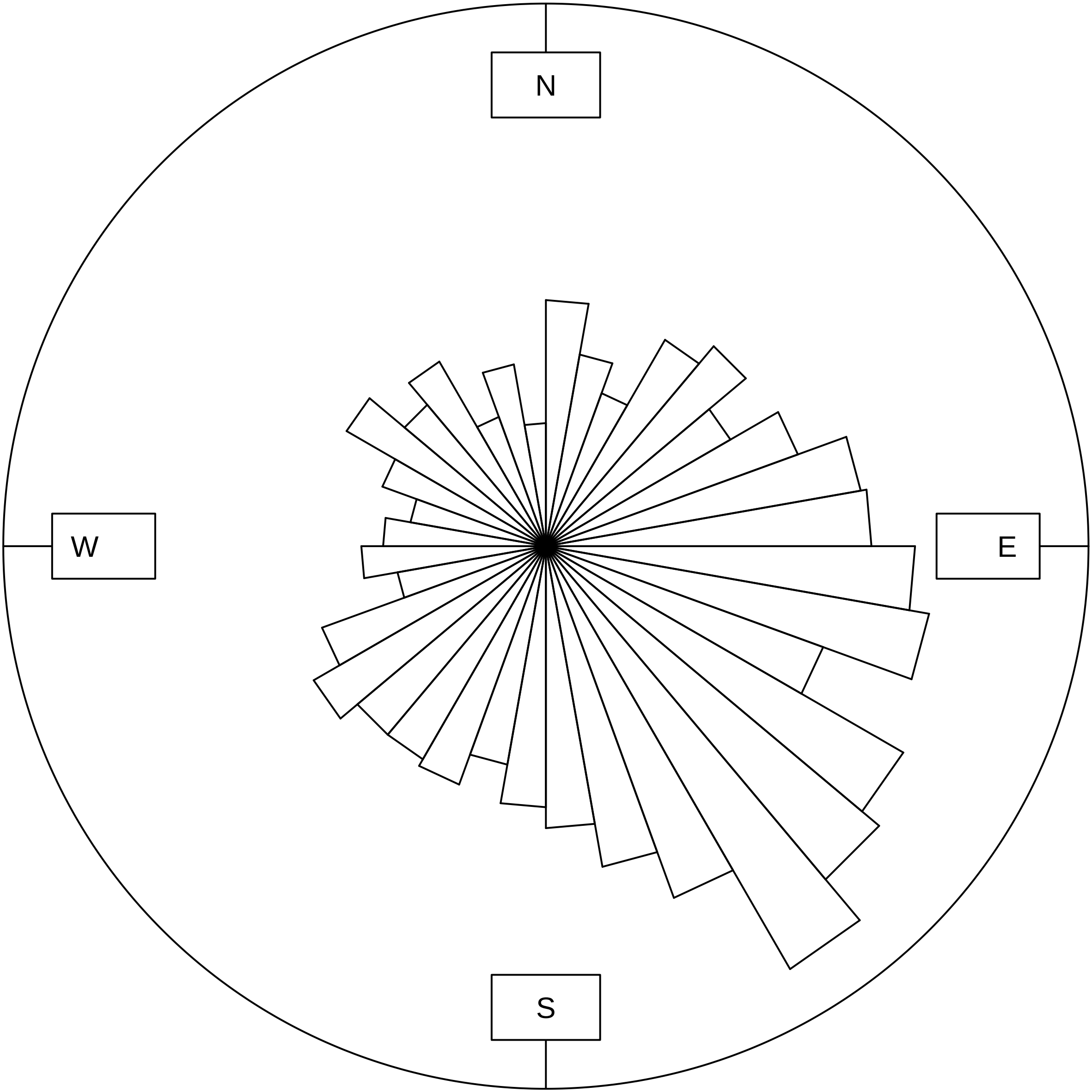

bootstrap 812

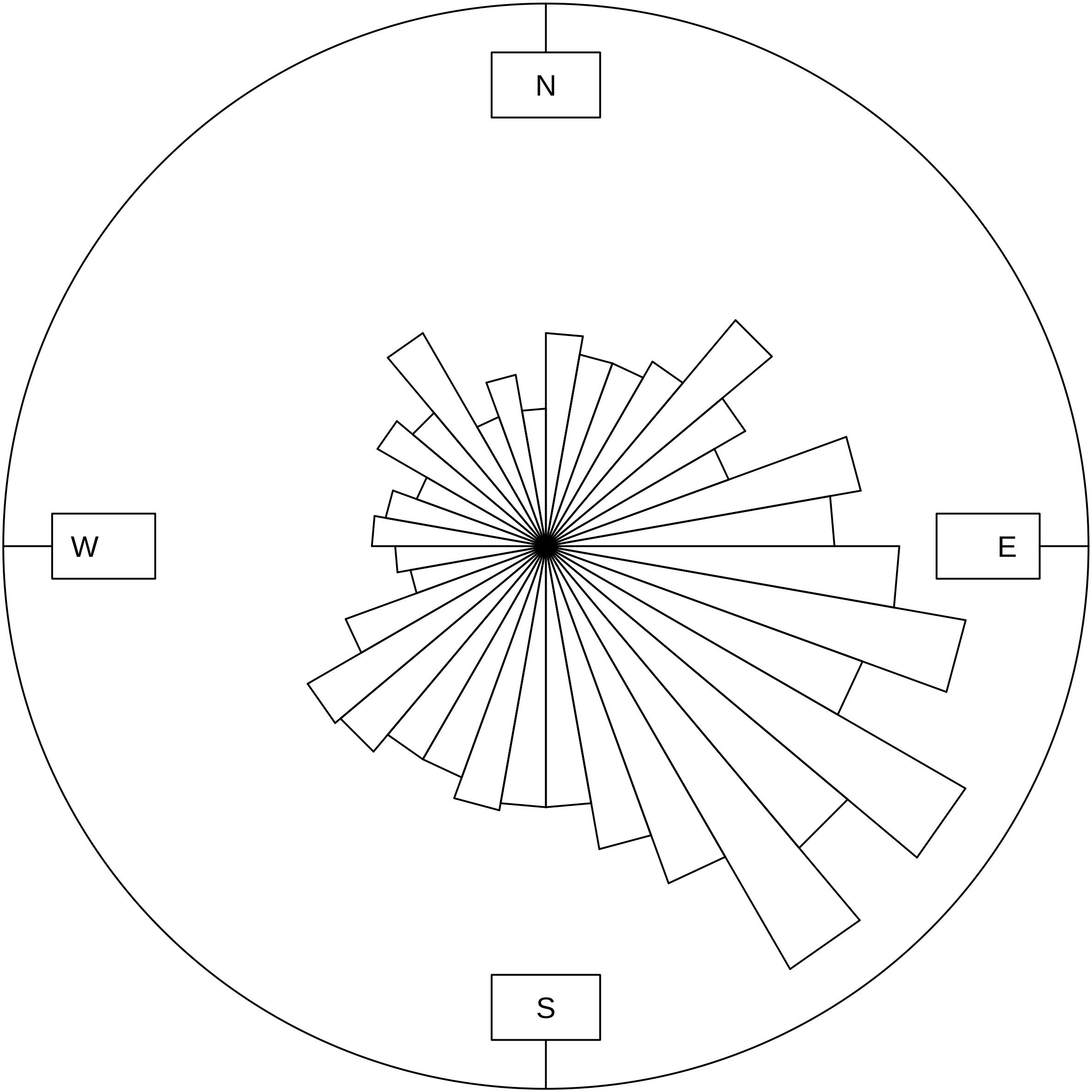

bootstrap 813

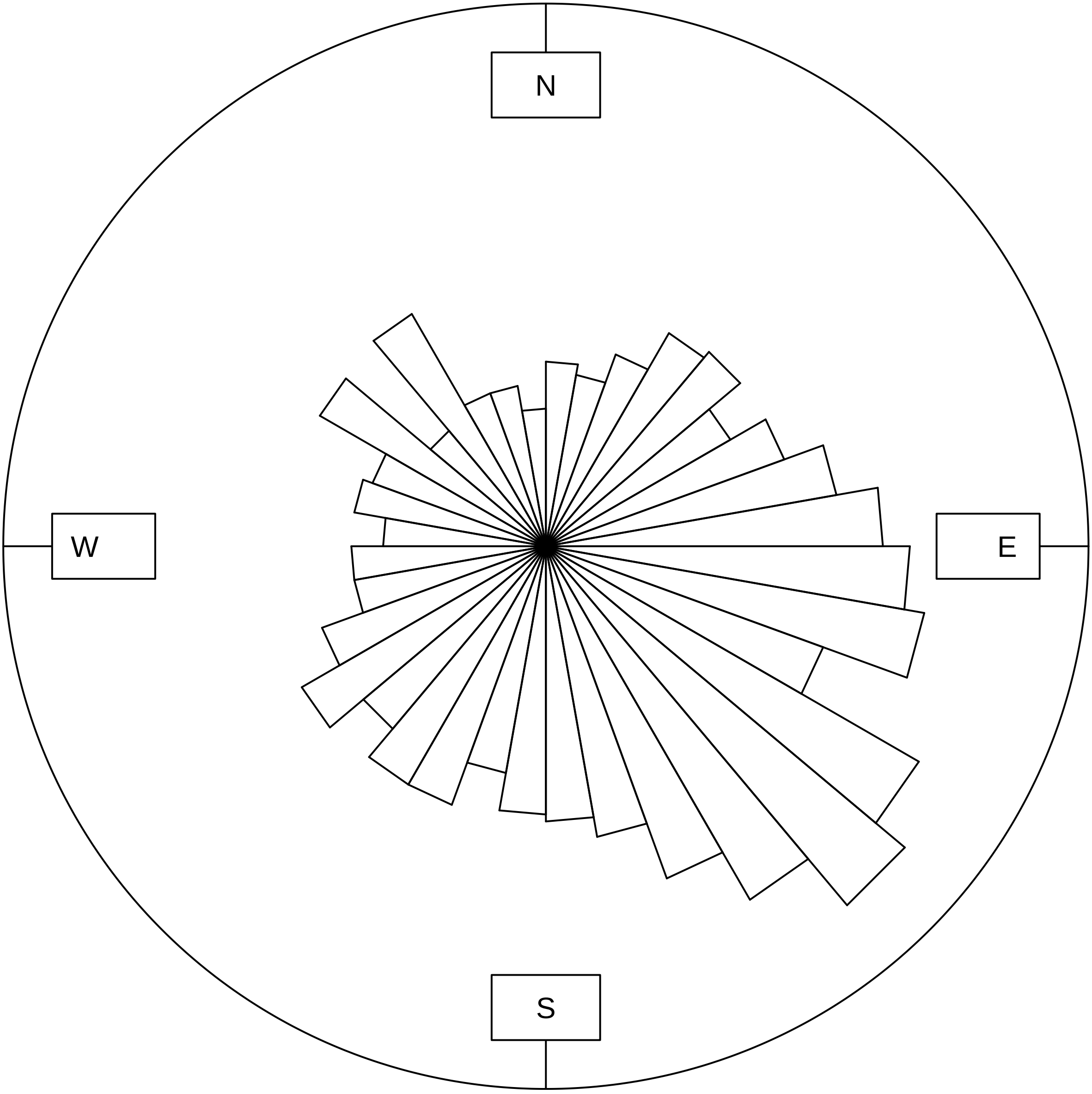

bootstrap 814

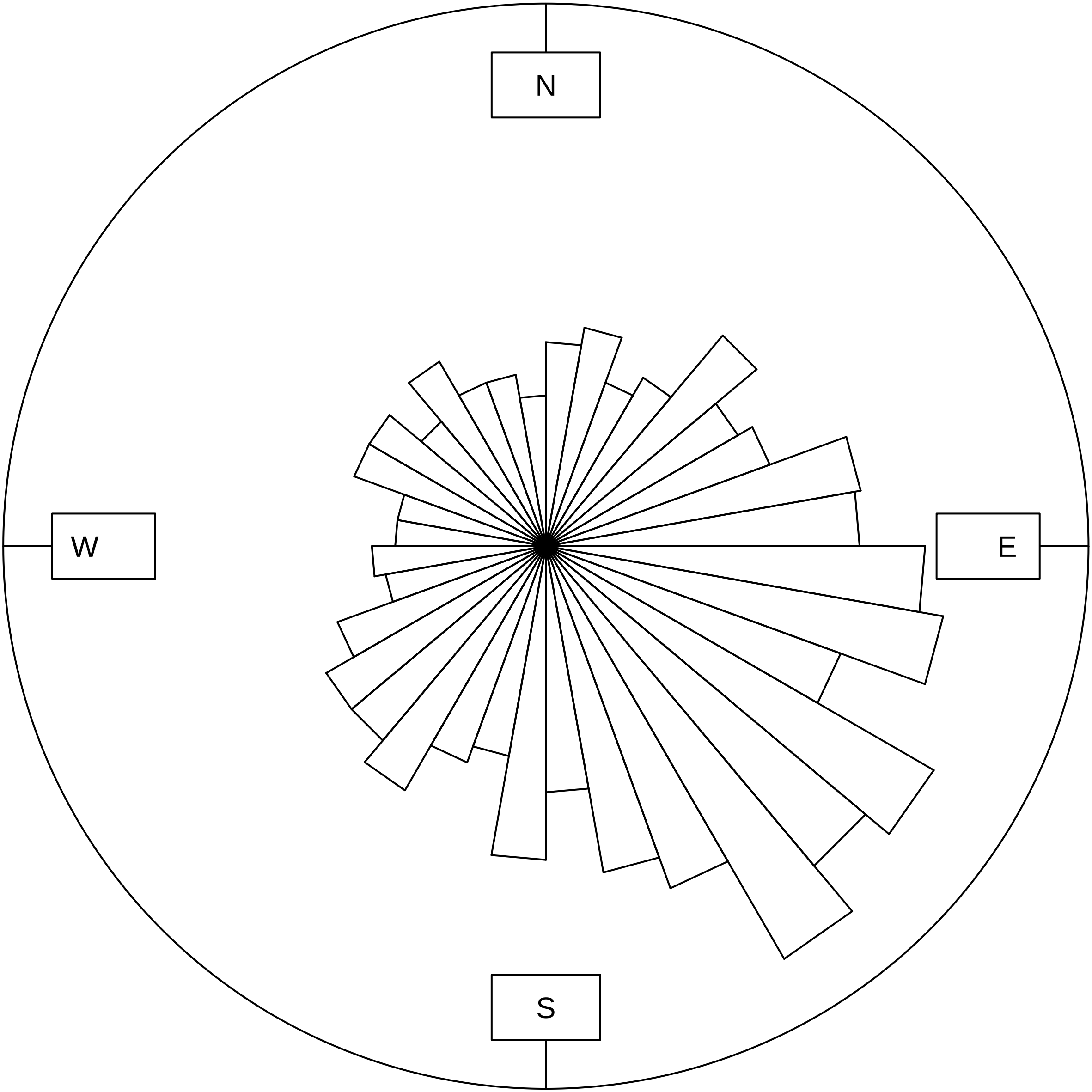

bootstrap 815

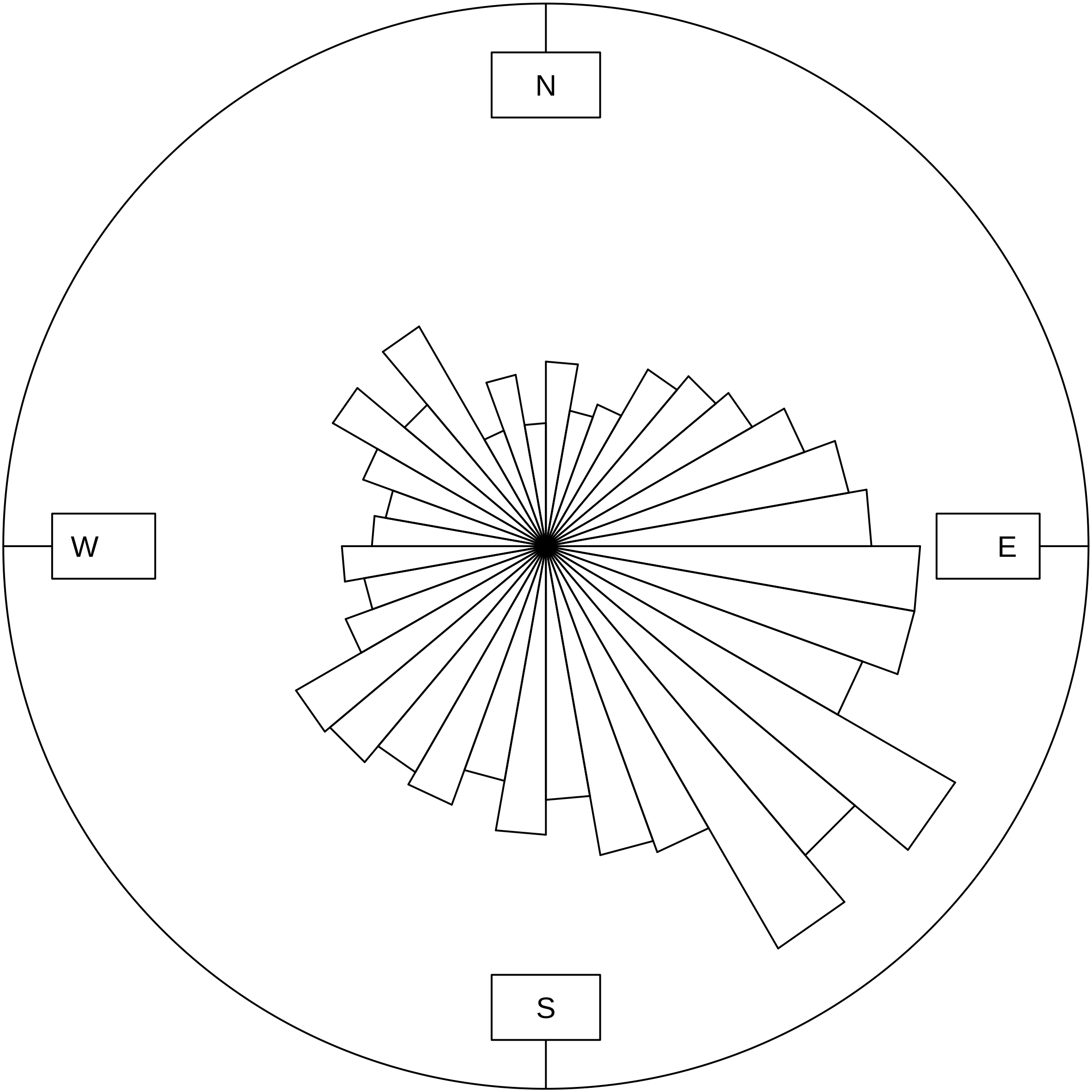

bootstrap 816

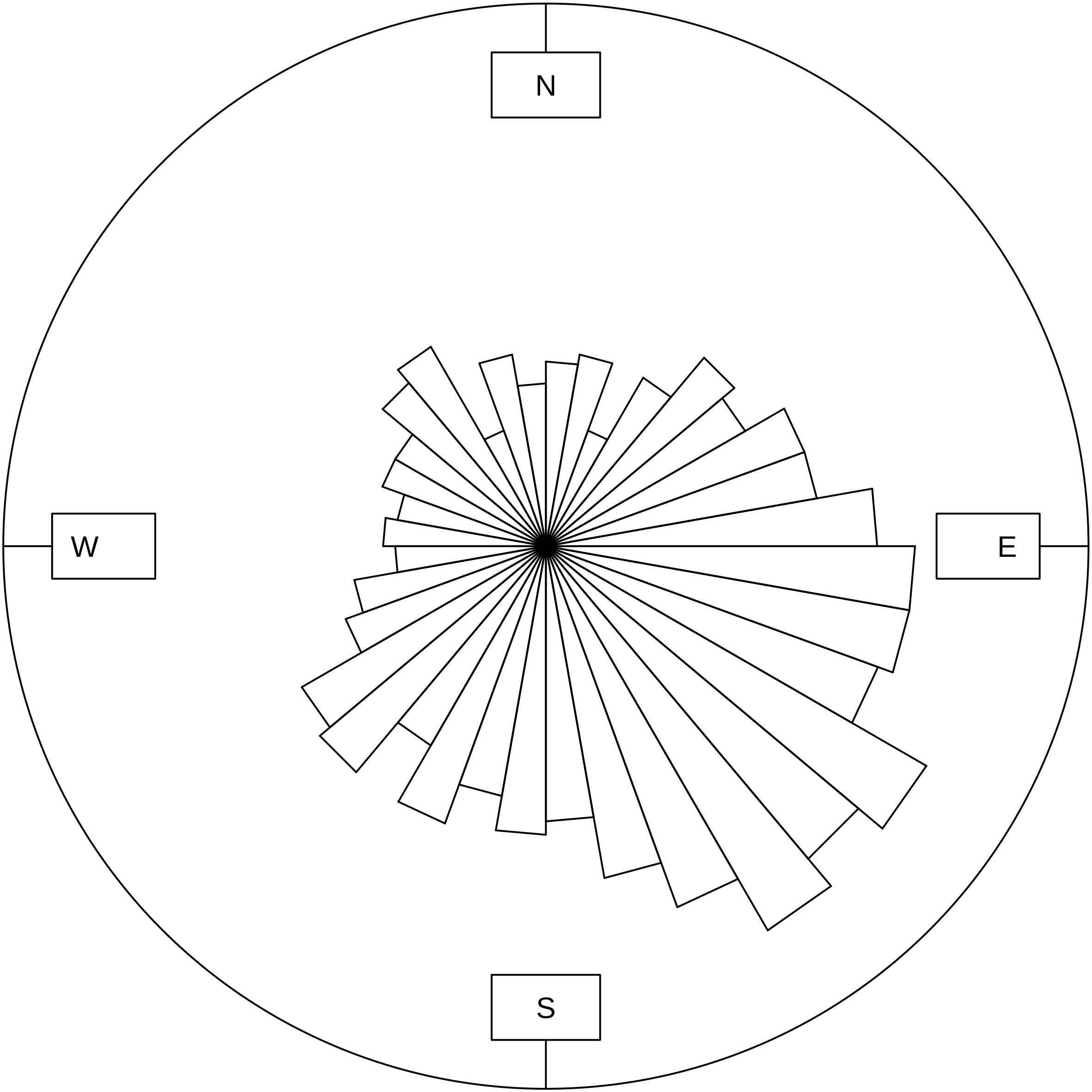

bootstrap 817

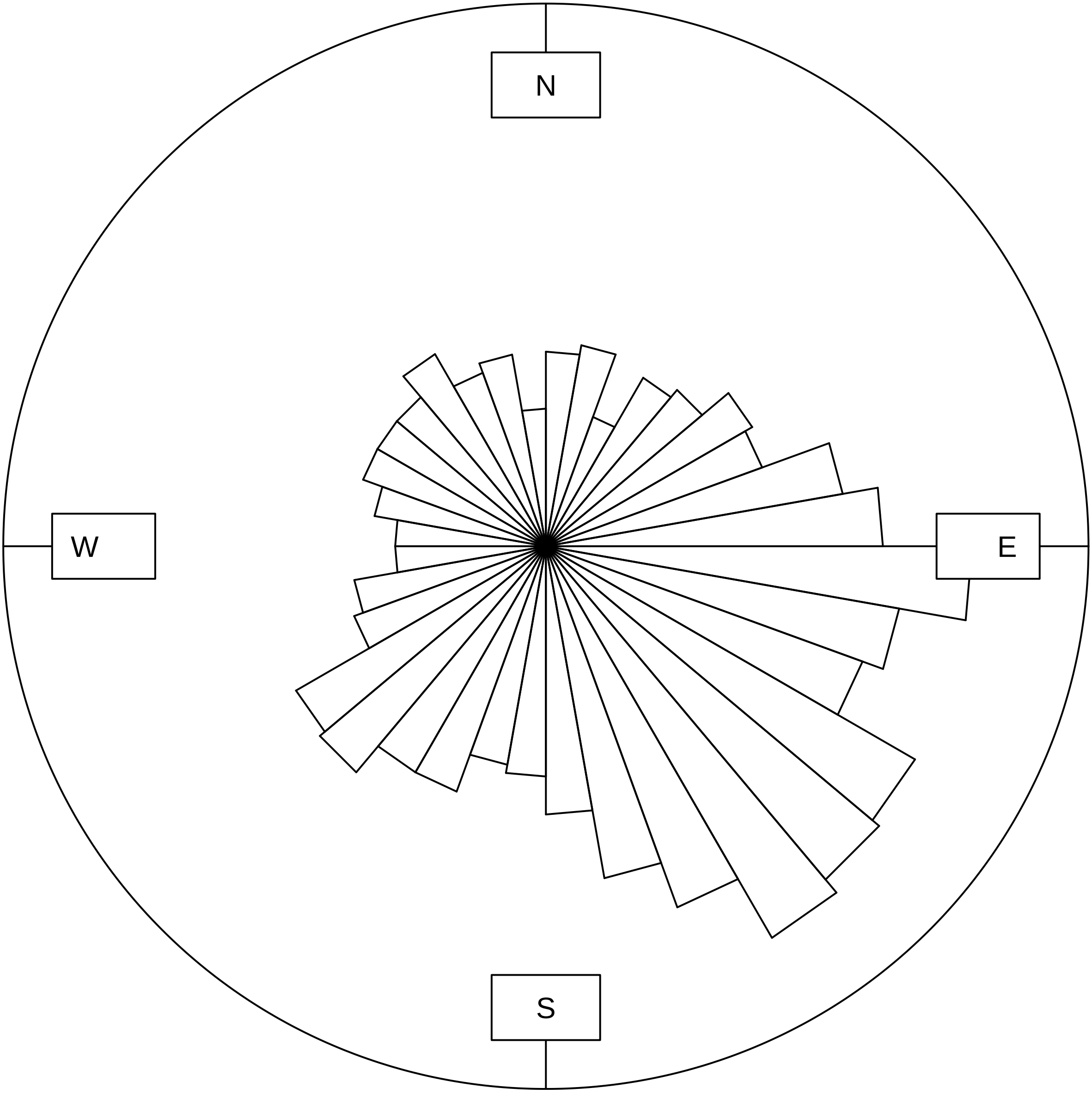

bootstrap 818

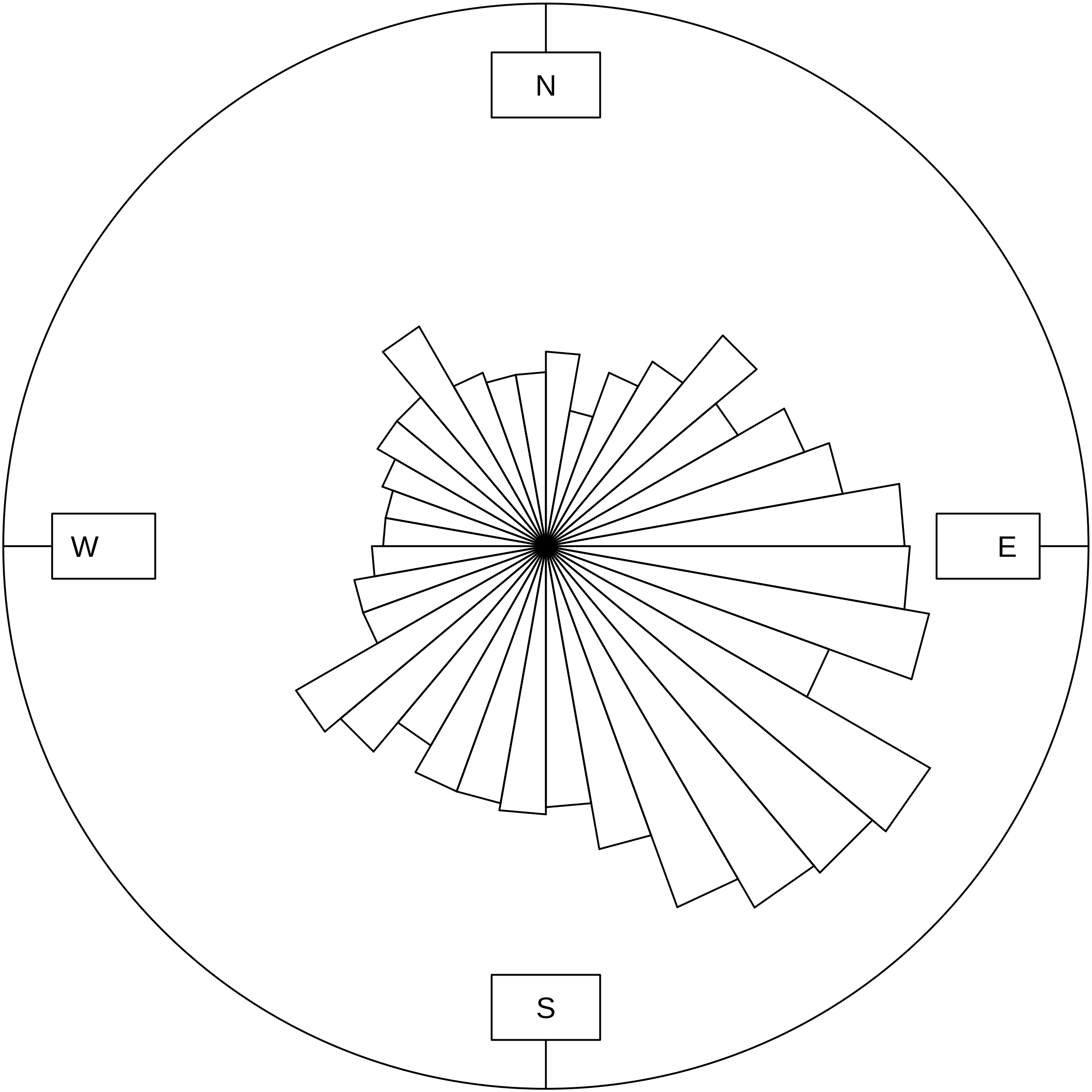

bootstrap 819

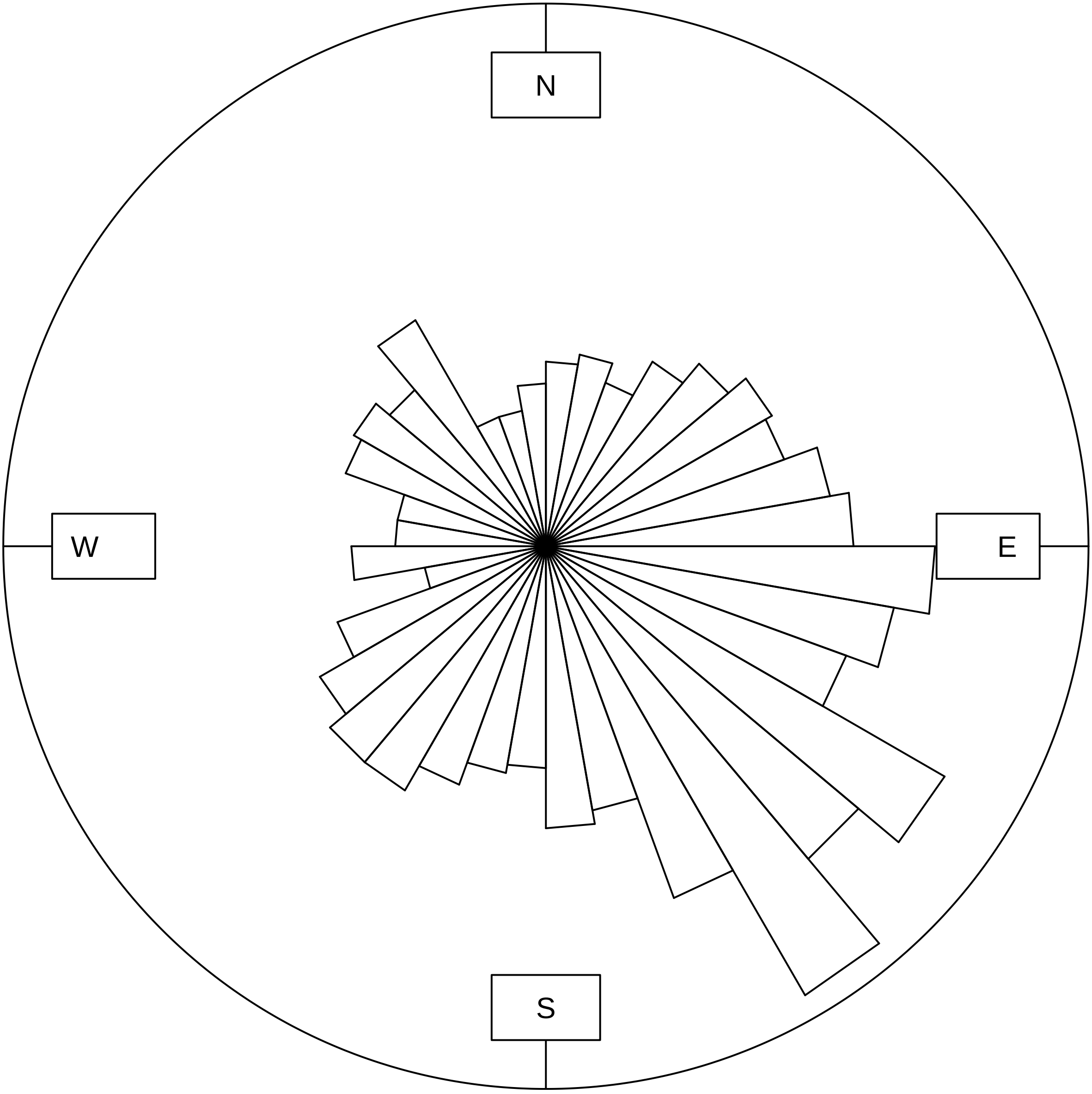

bootstrap 820

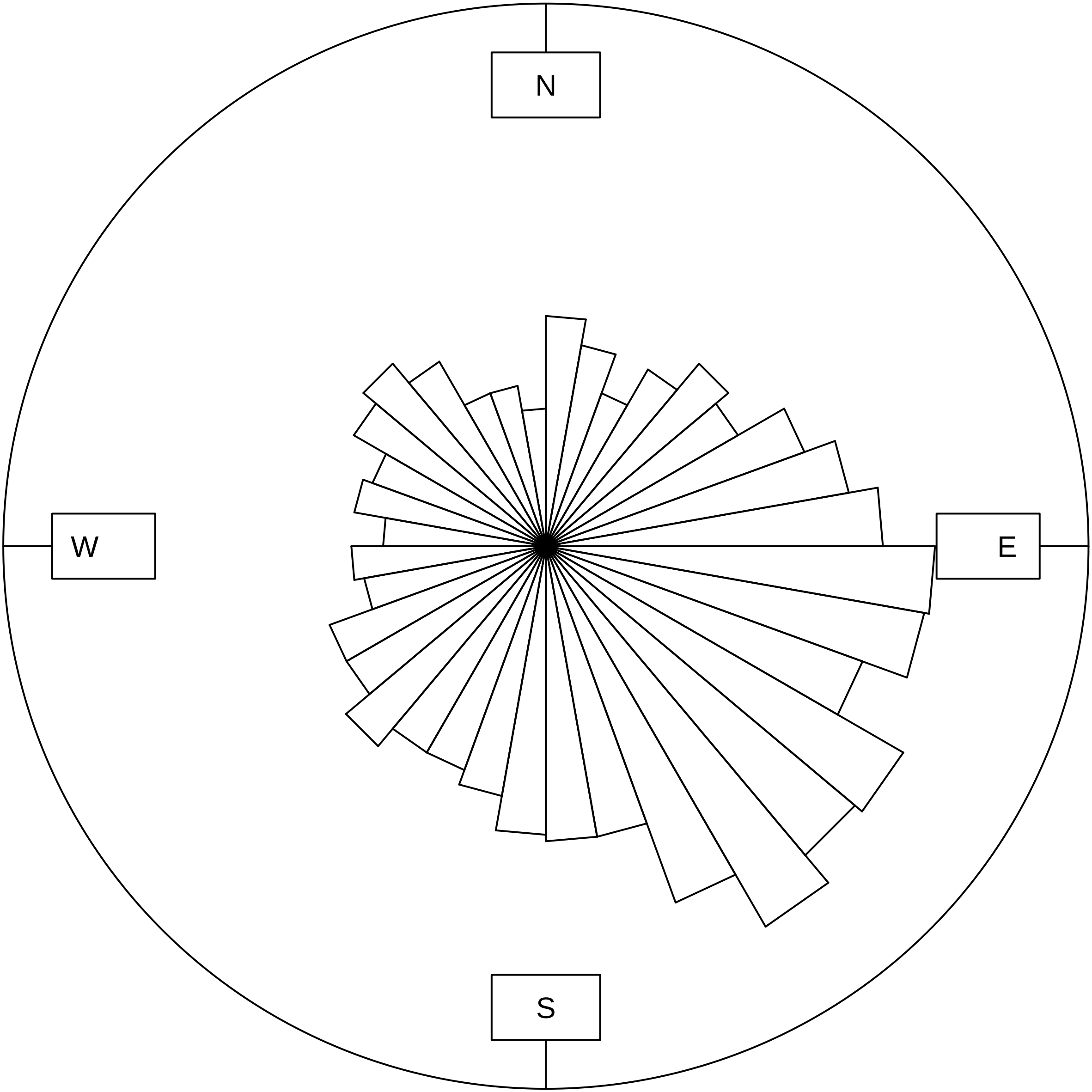

bootstrap 821

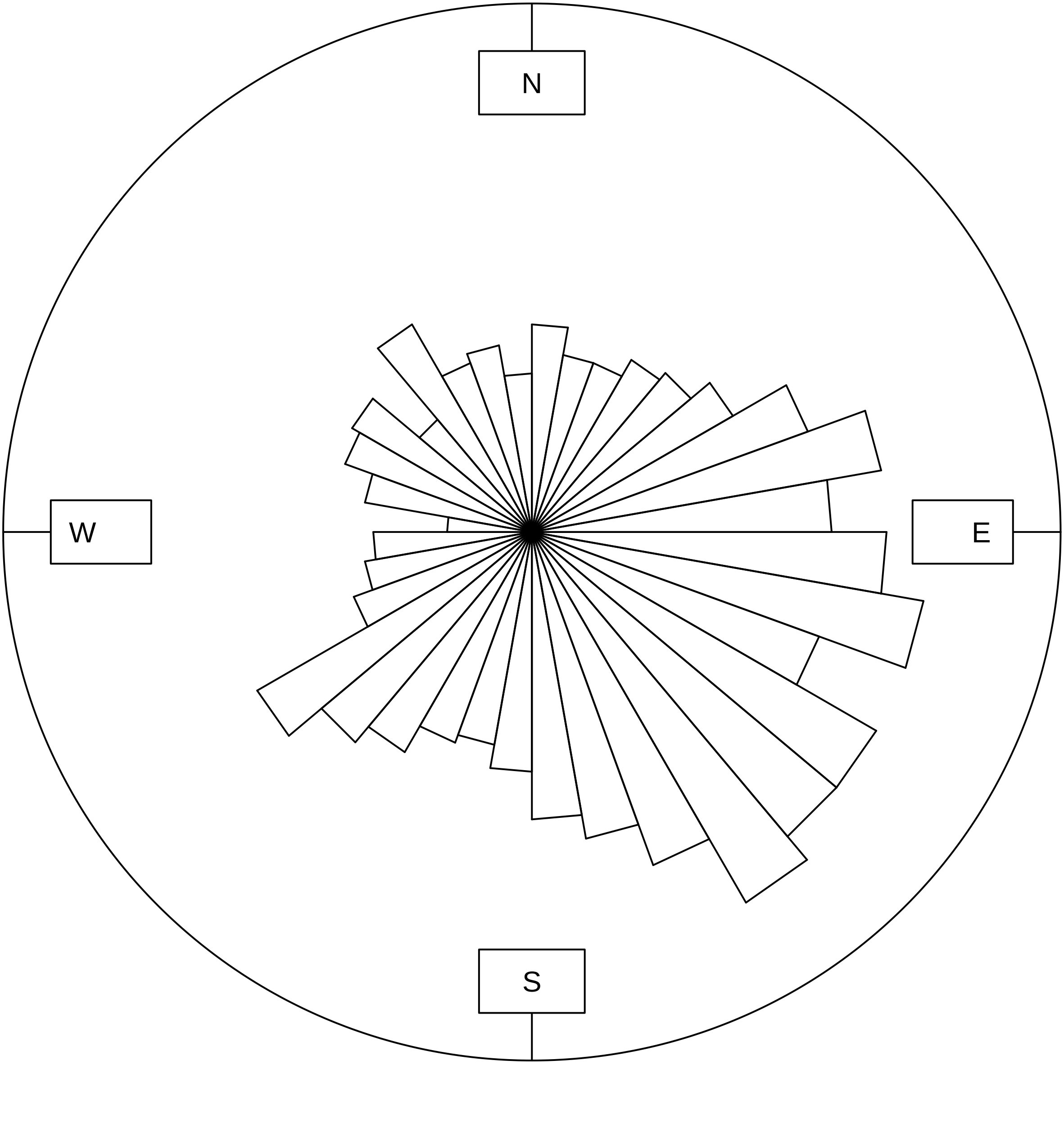

bootstrap 822

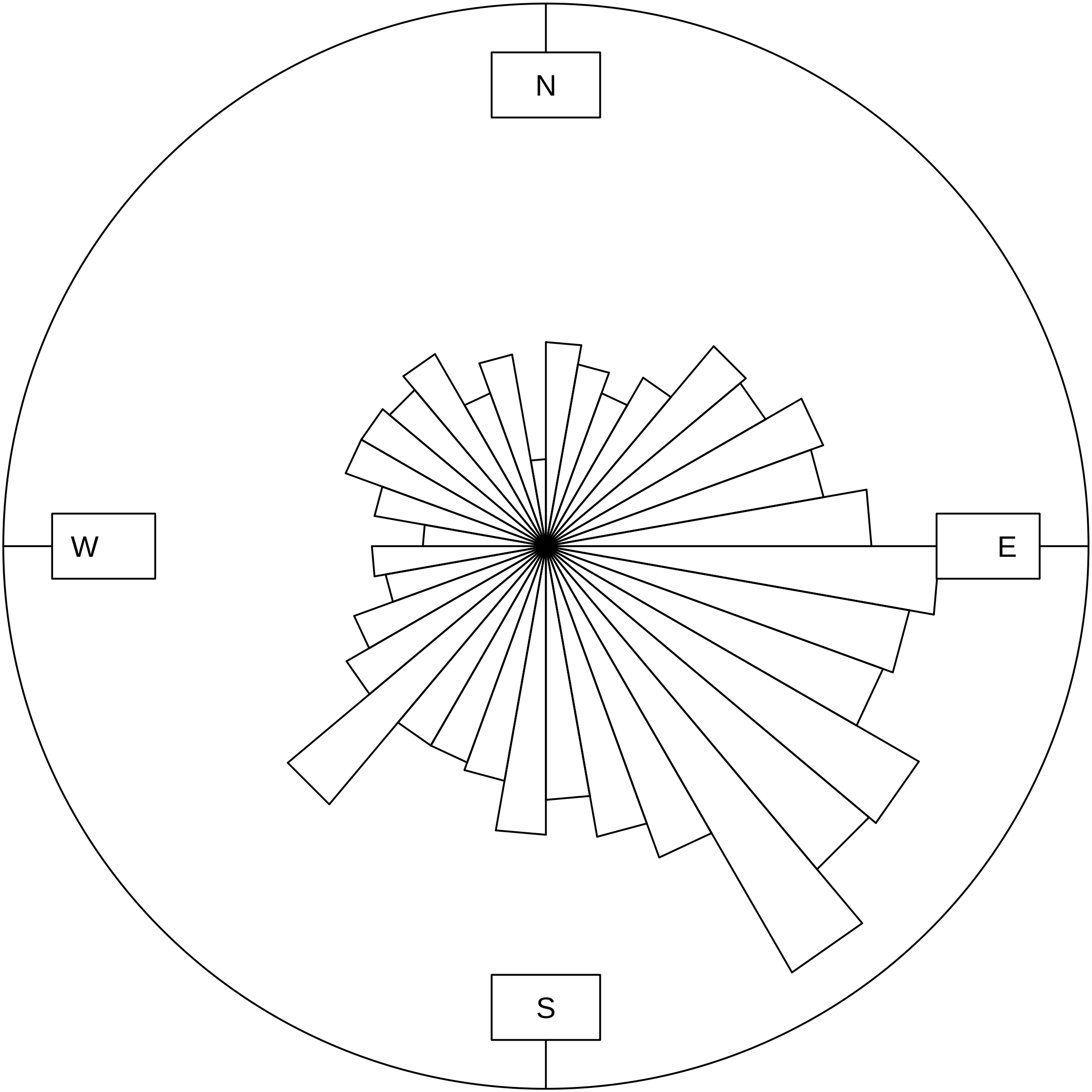

bootstrap 823

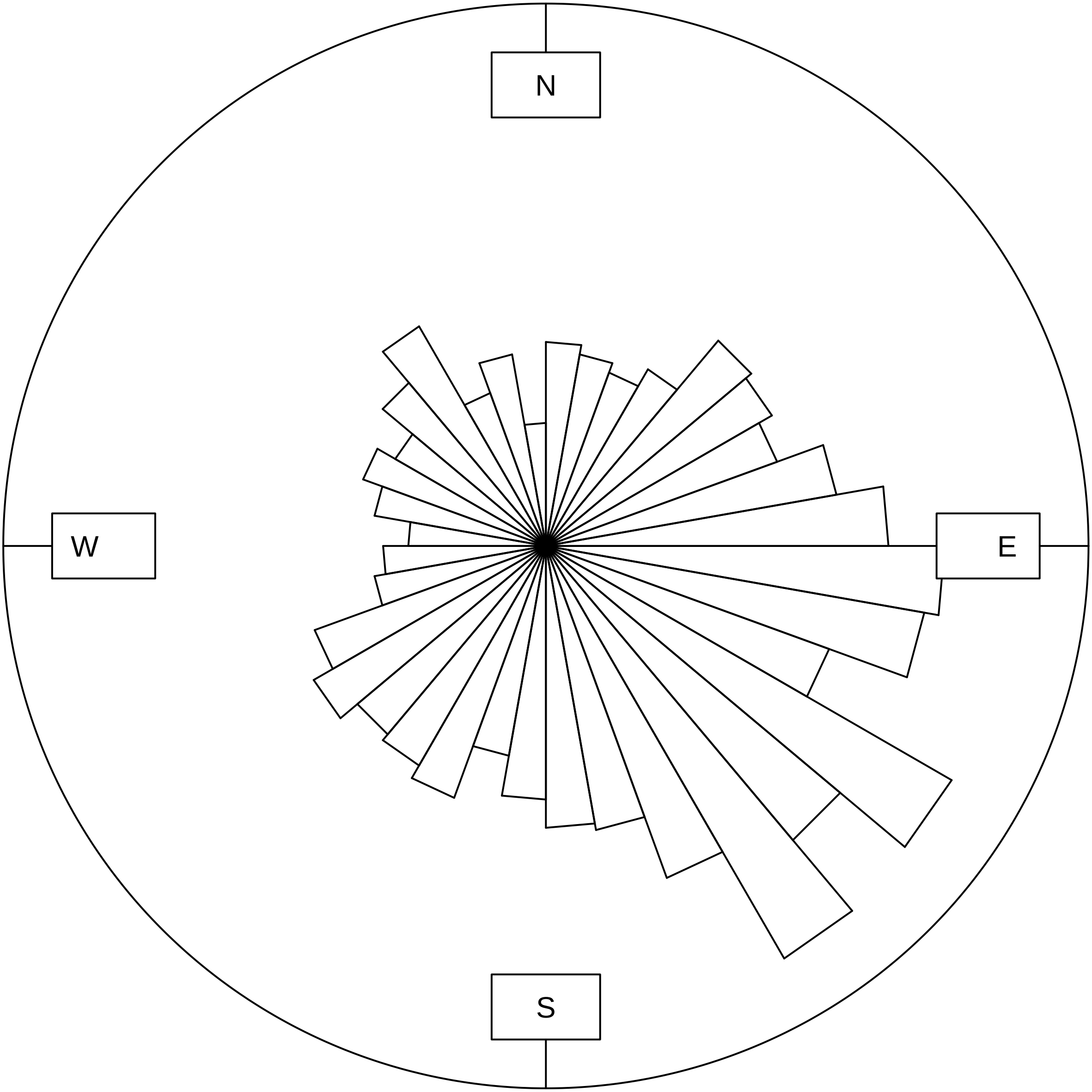

bootstrap 824

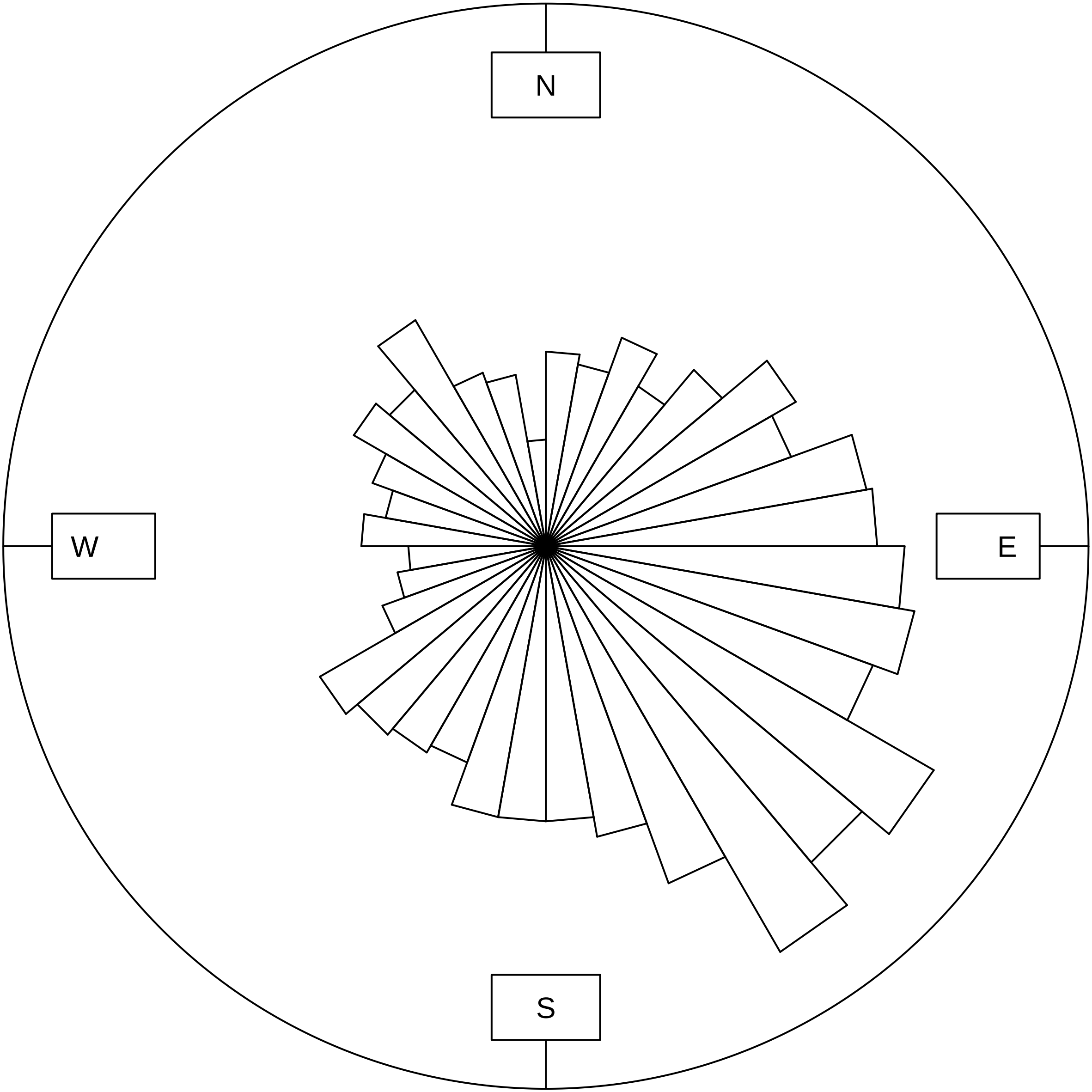

bootstrap 825

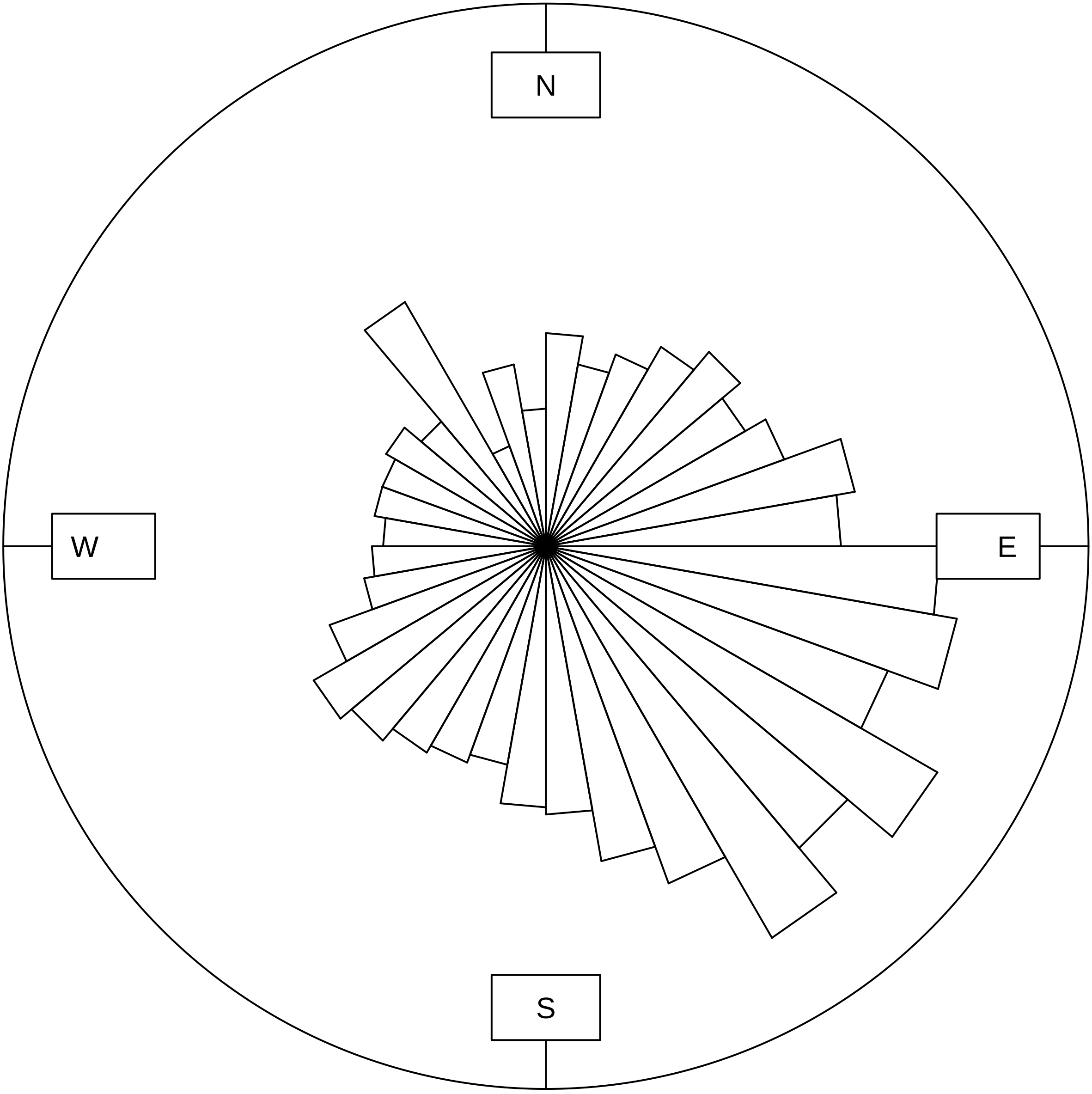

bootstrap 826

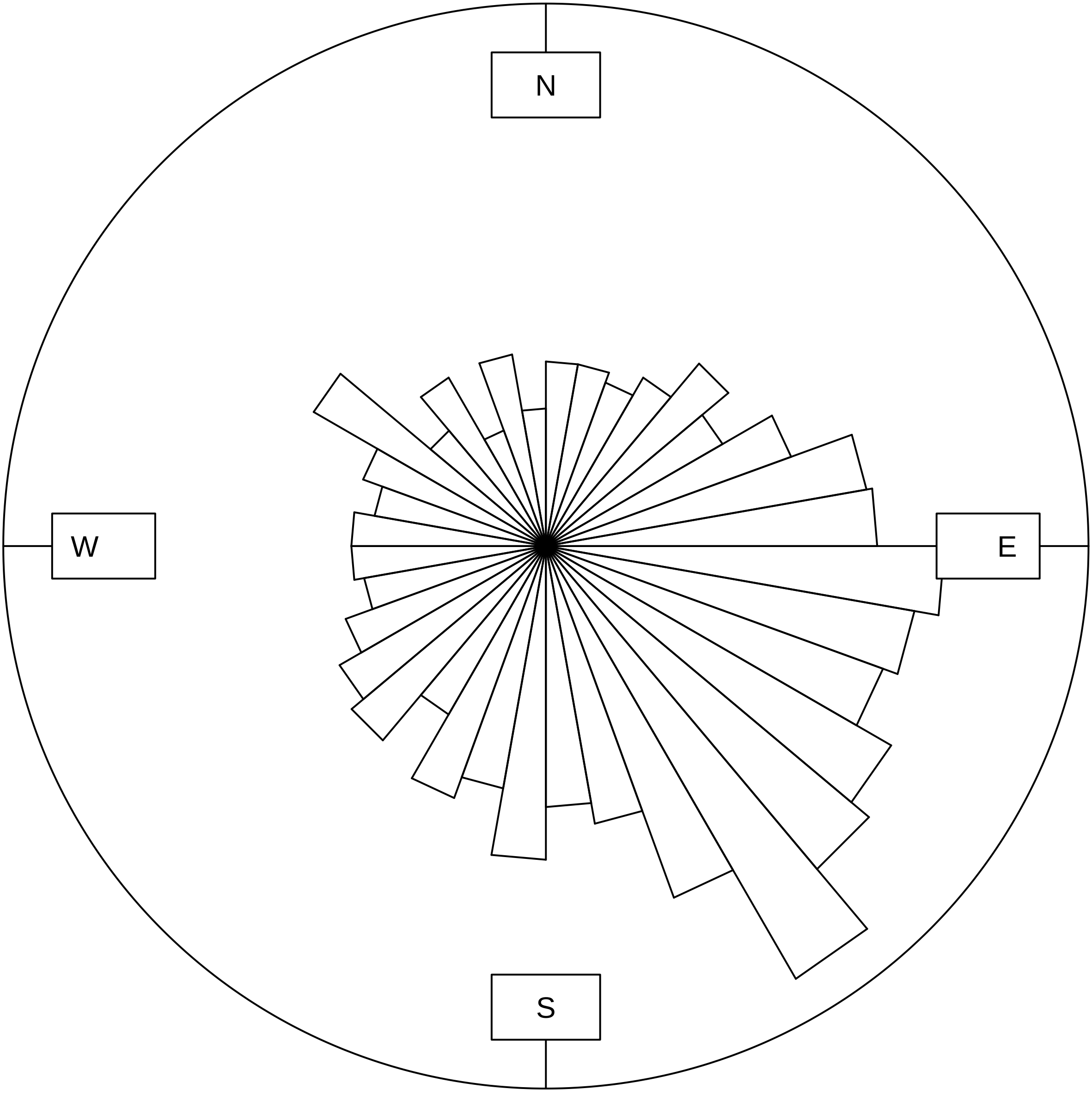

bootstrap 827

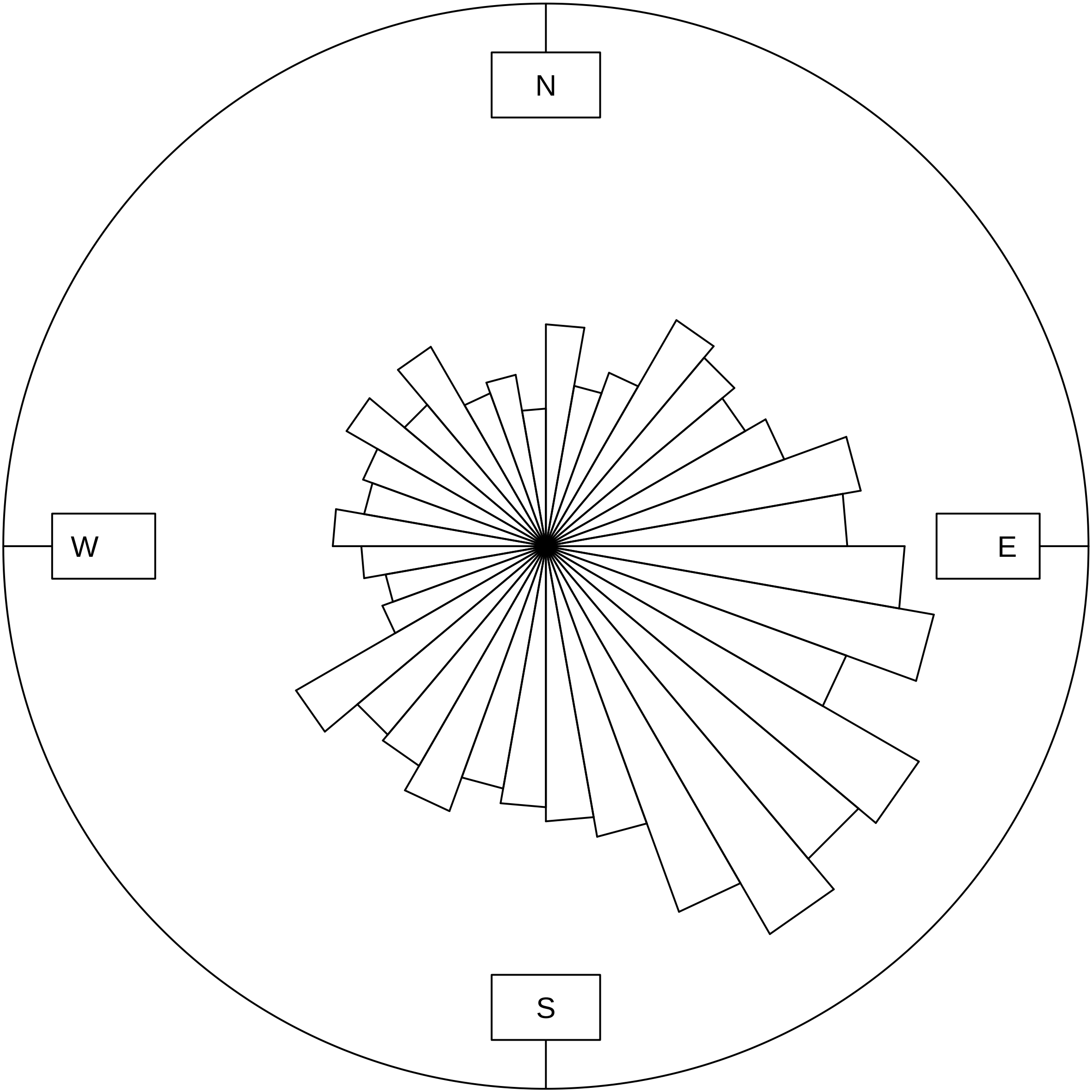

bootstrap 828

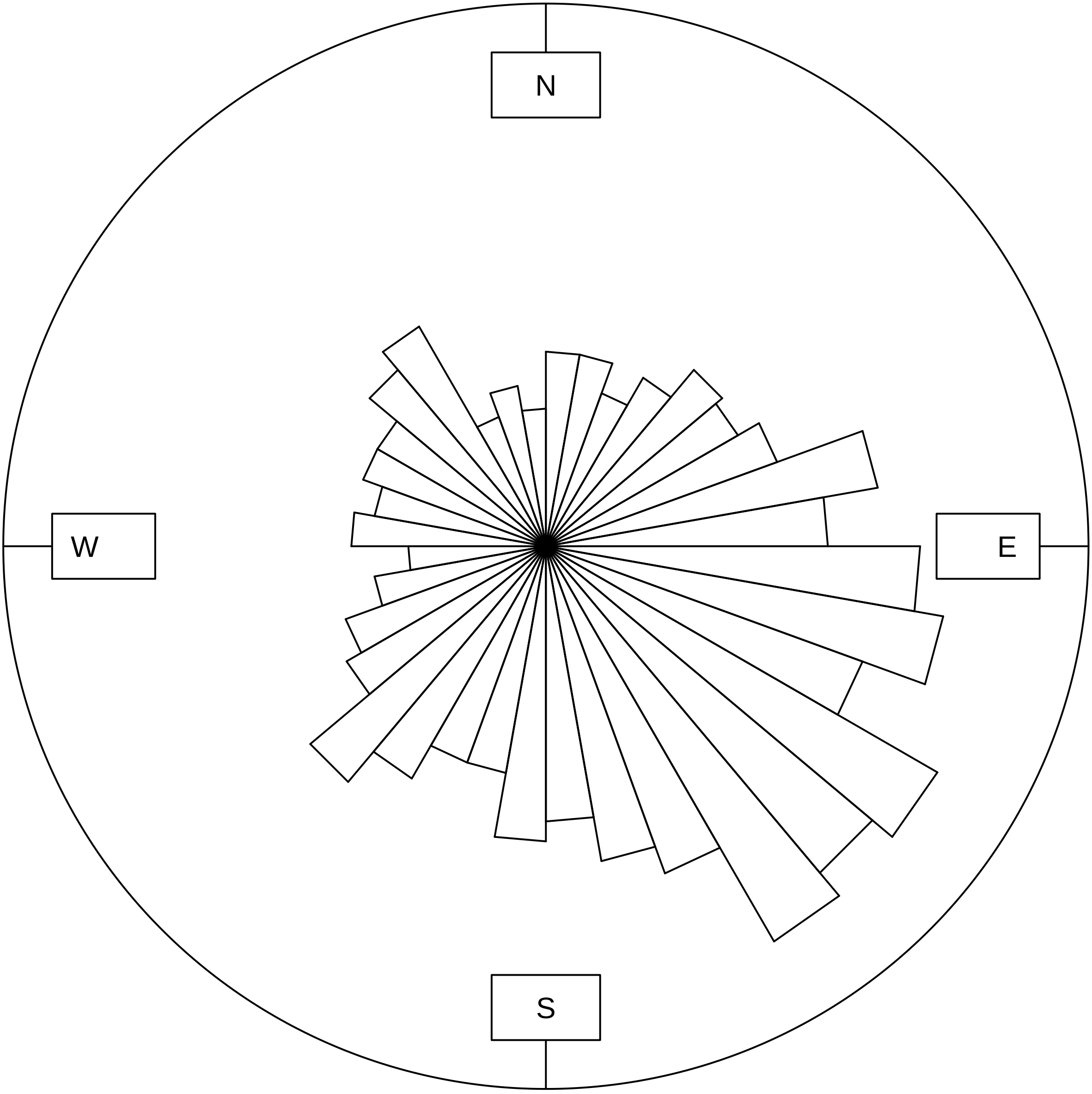

bootstrap 829

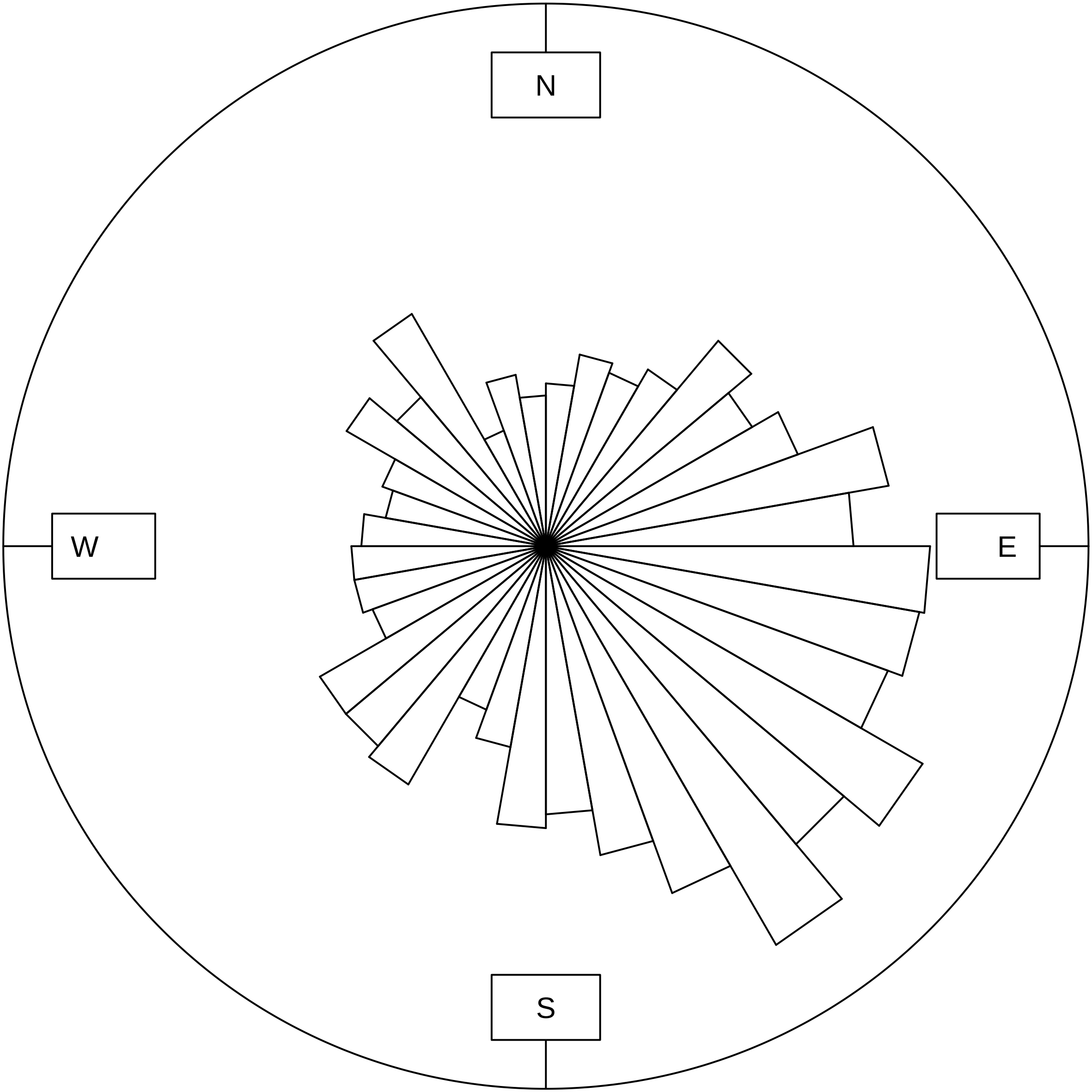

bootstrap 830

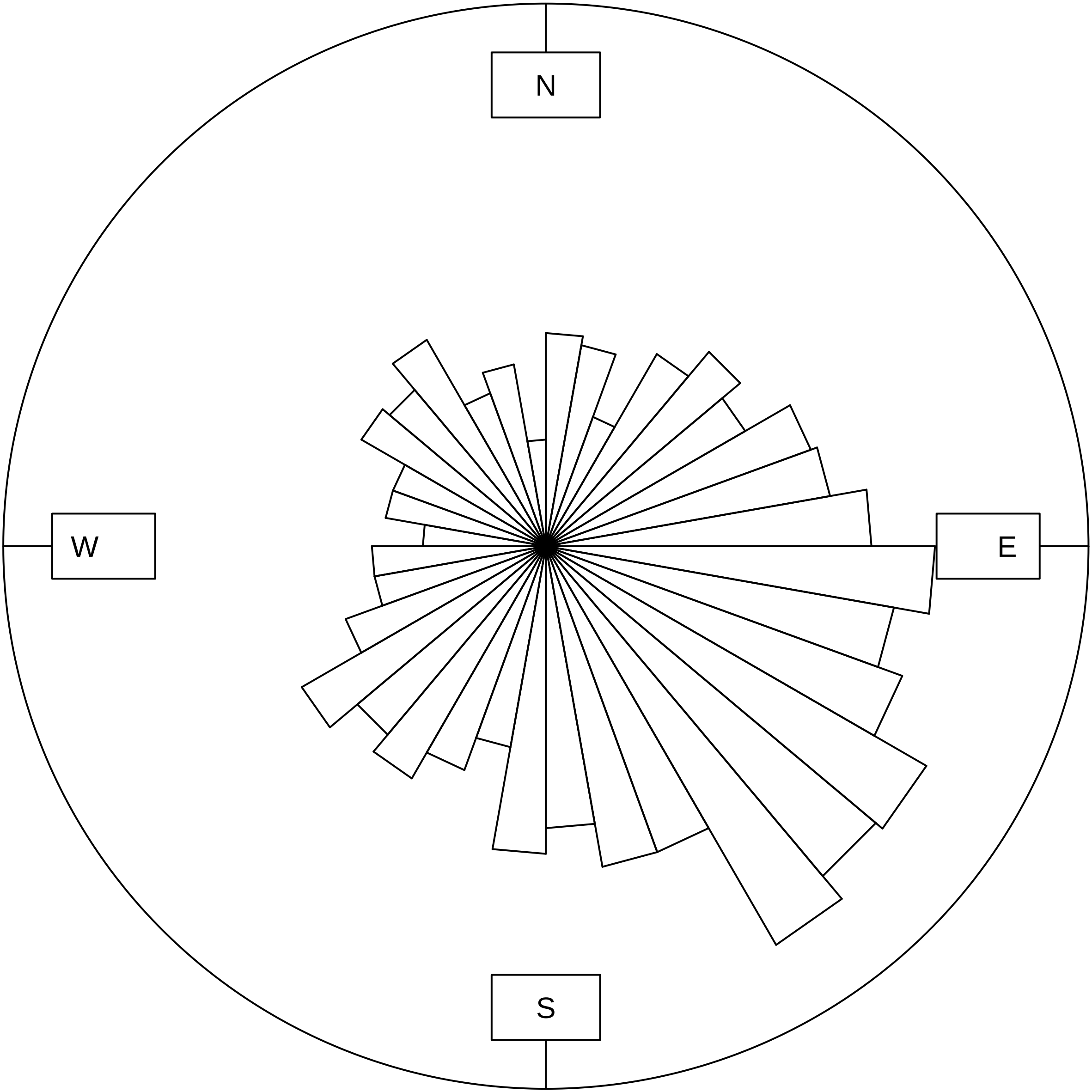

bootstrap 831

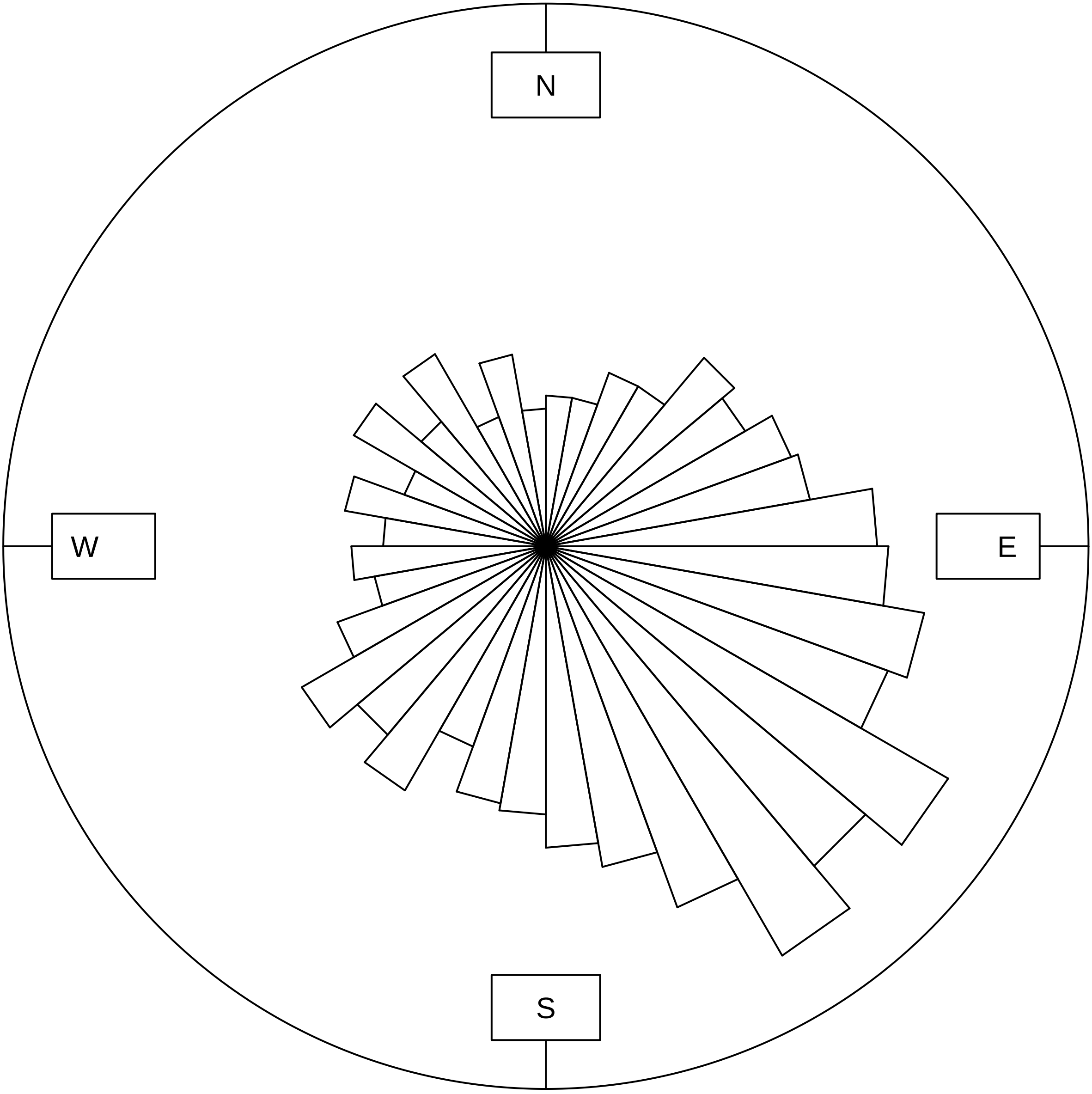

bootstrap 832

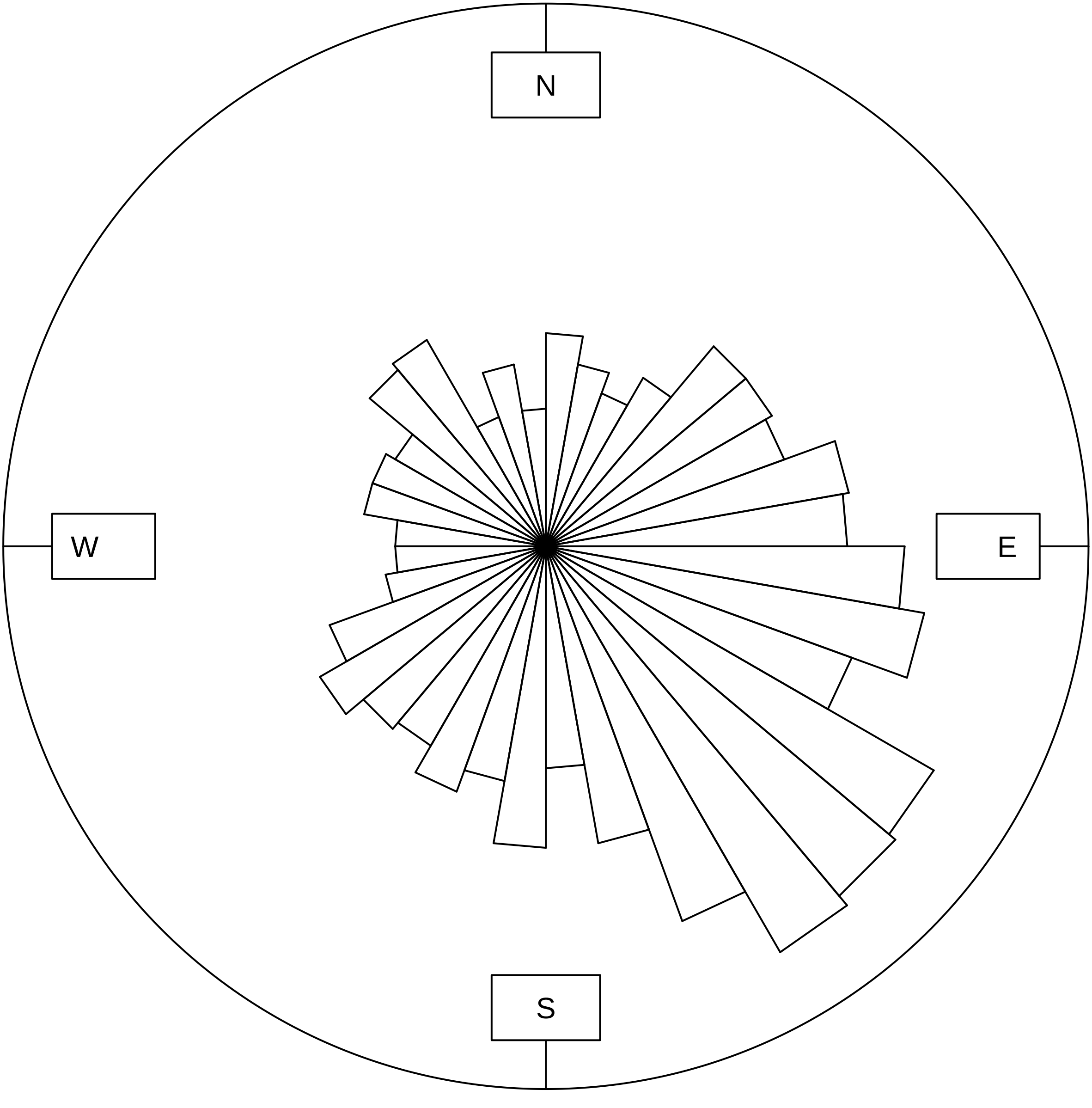

bootstrap 833

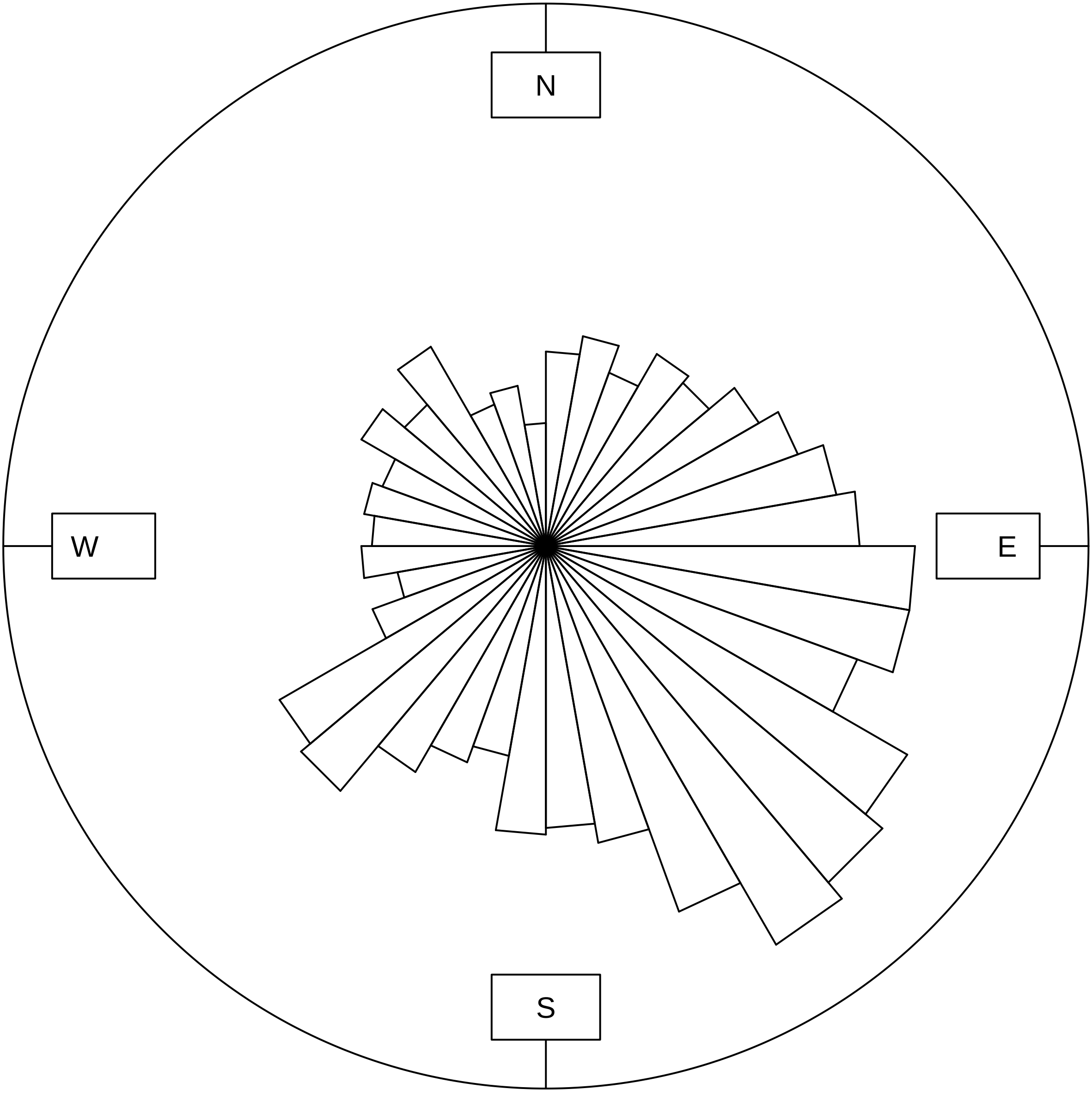

bootstrap 834

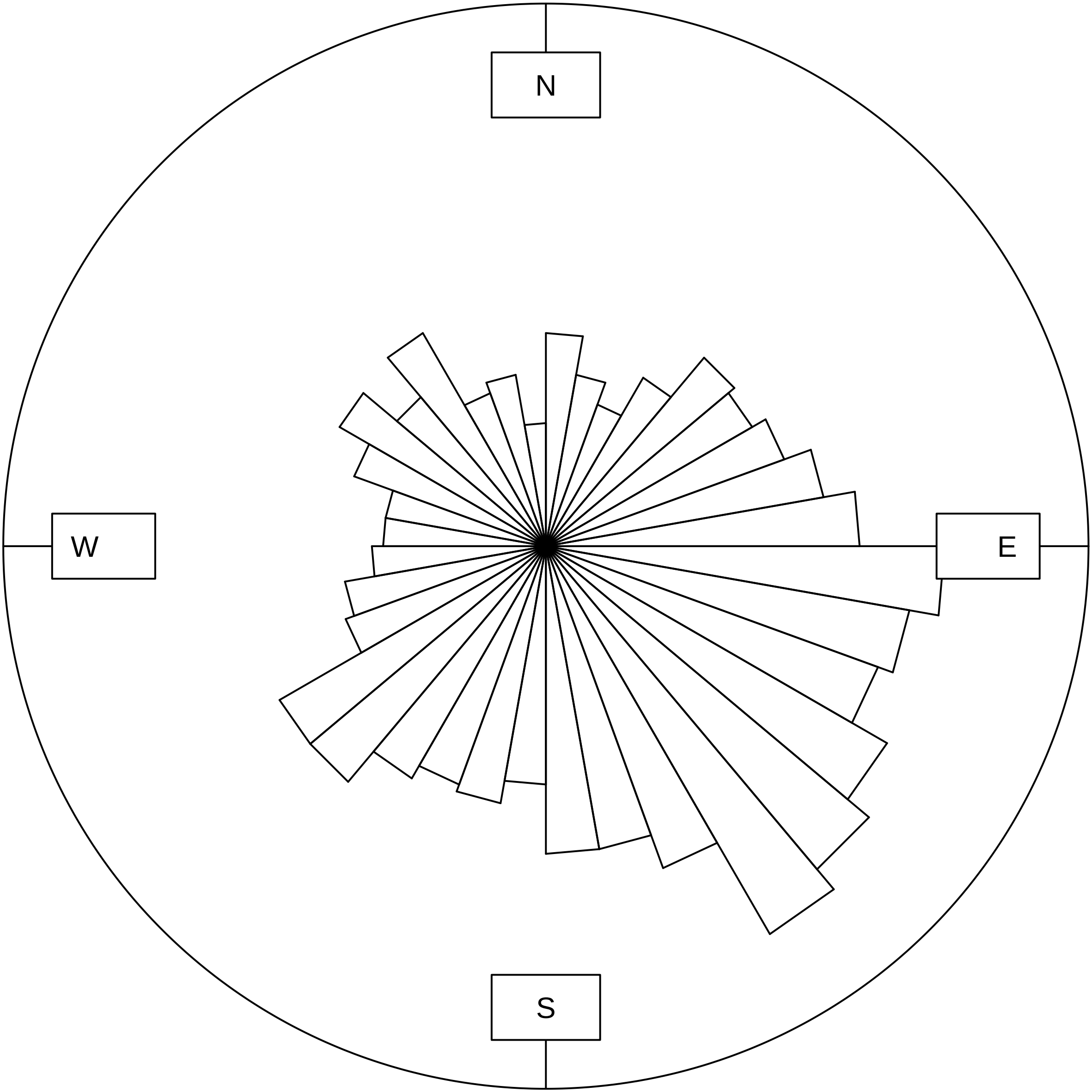

bootstrap 835

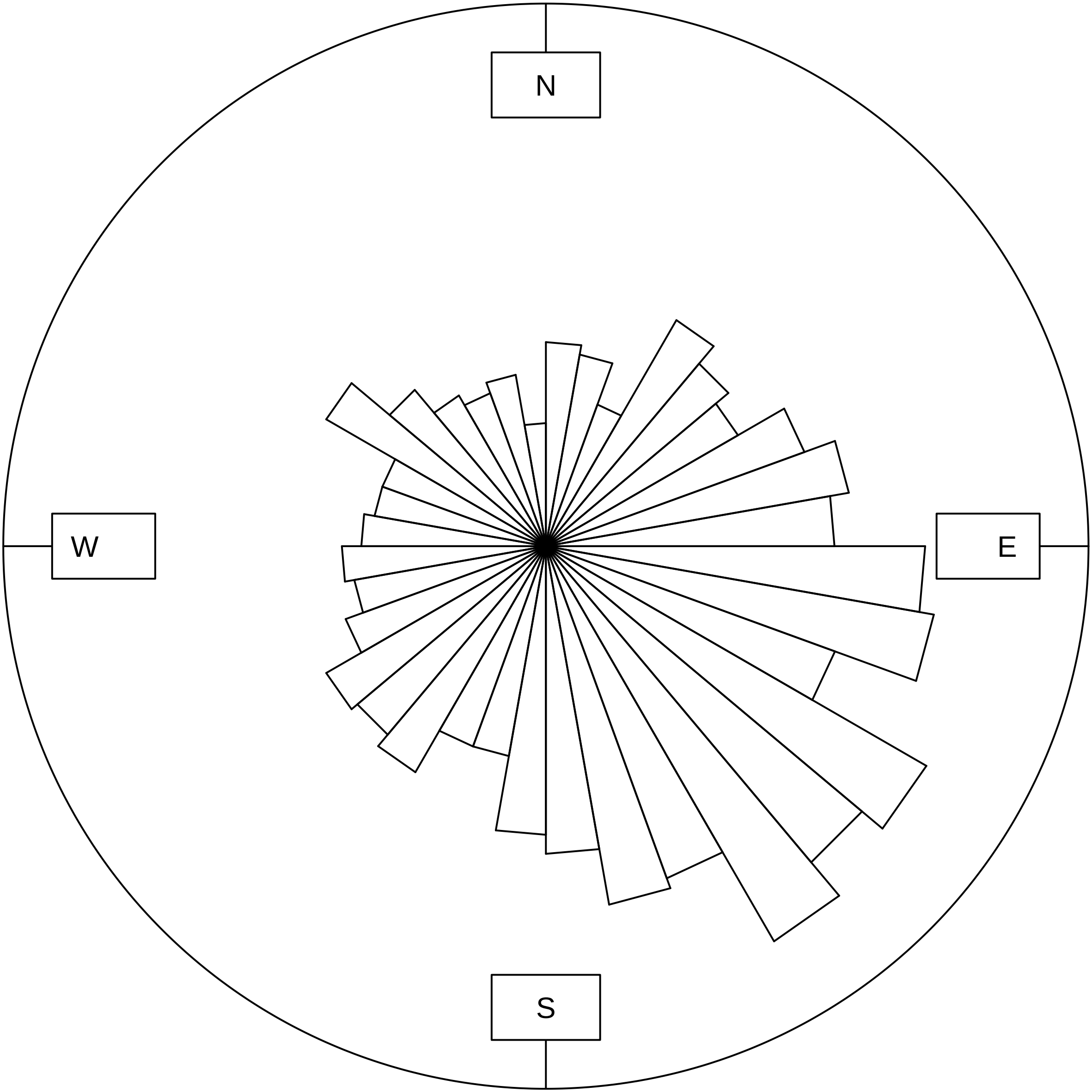

bootstrap 836

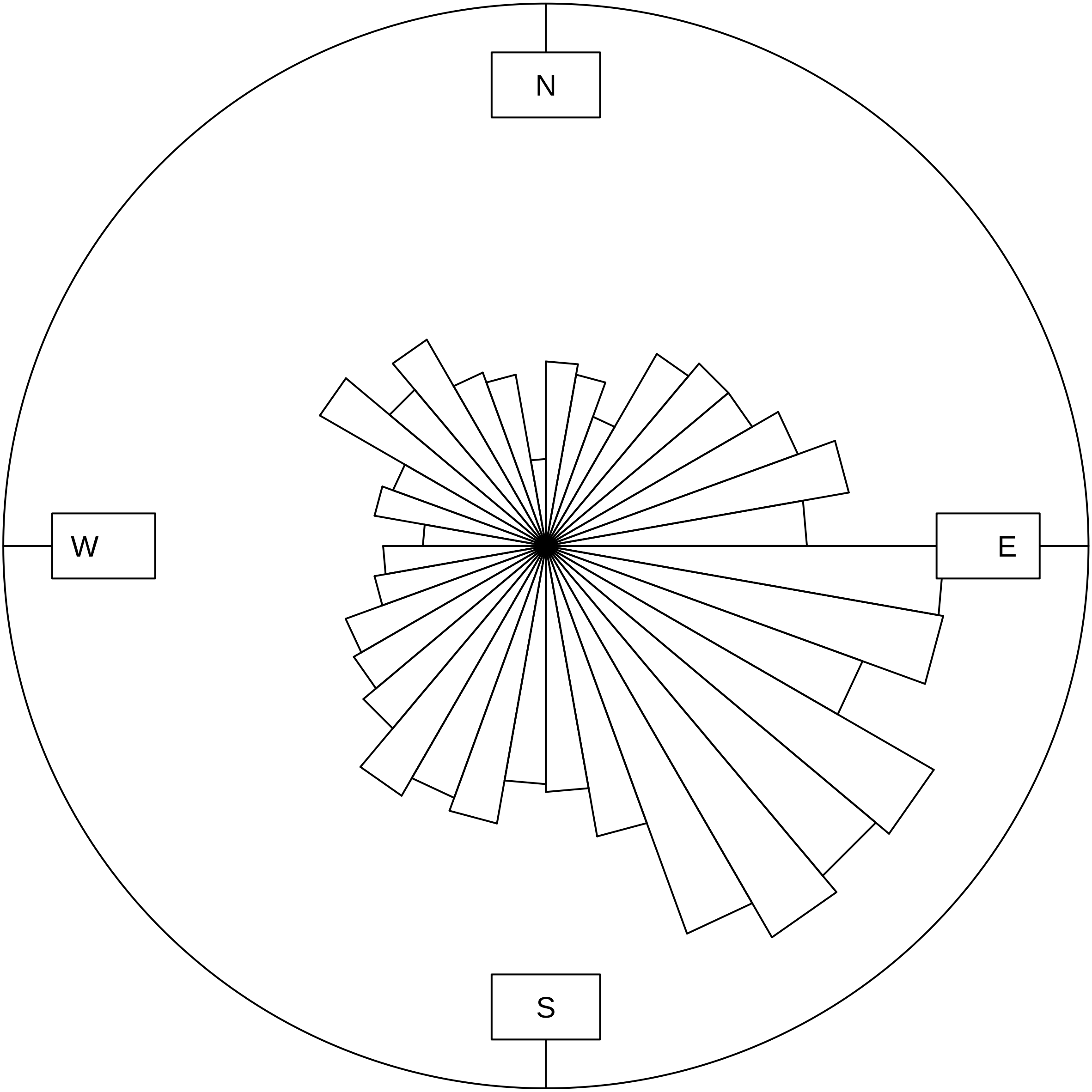

bootstrap 837

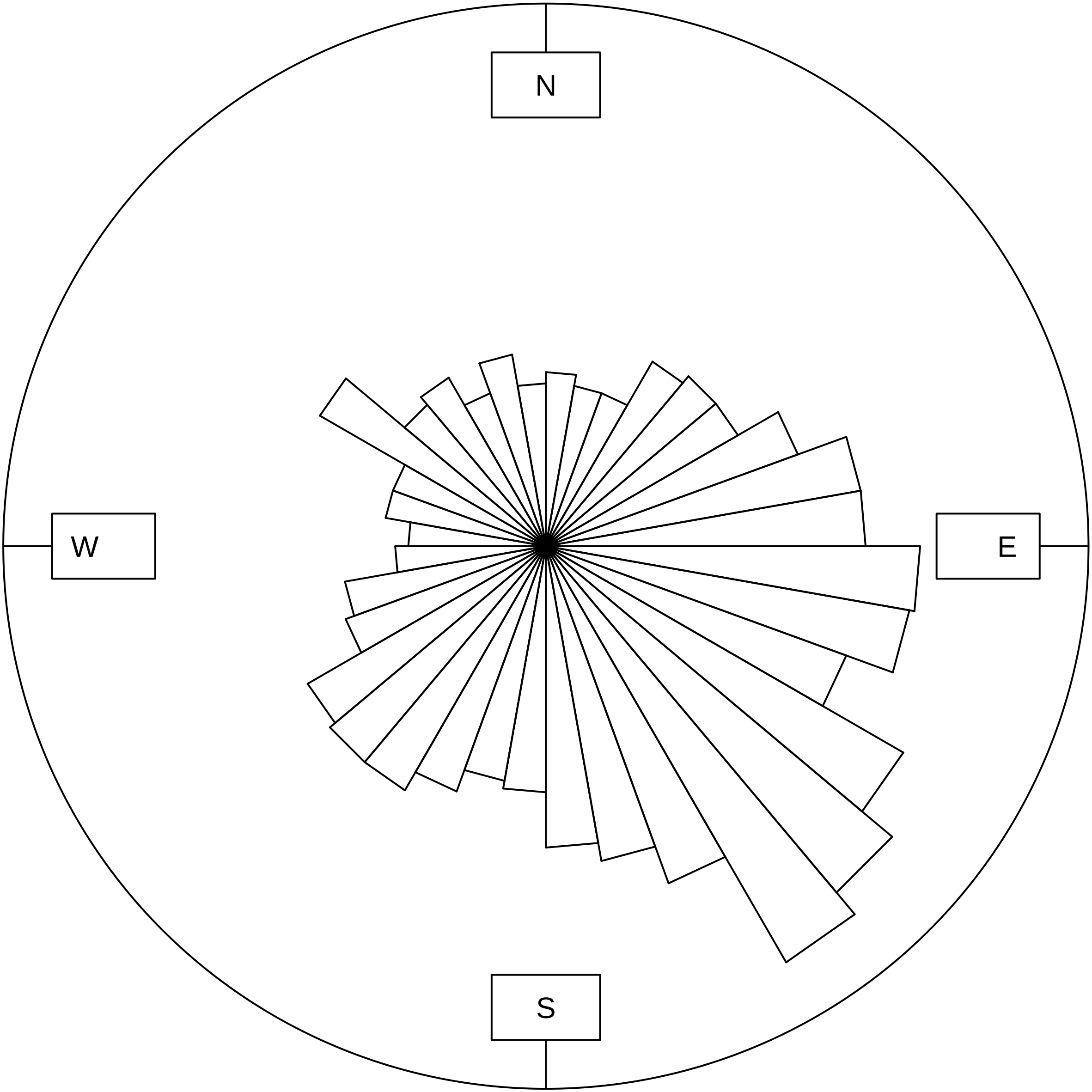

bootstrap 838

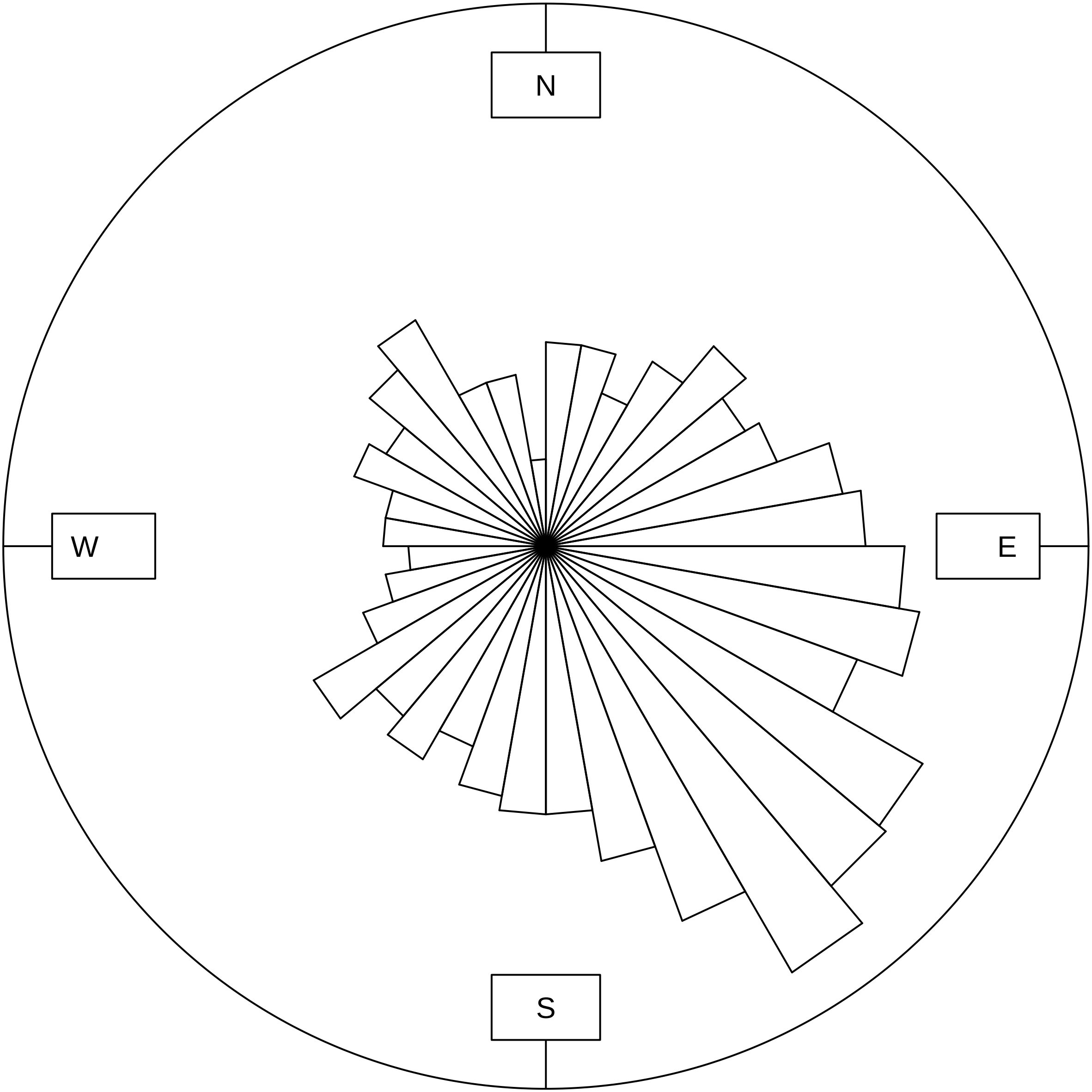

bootstrap 839

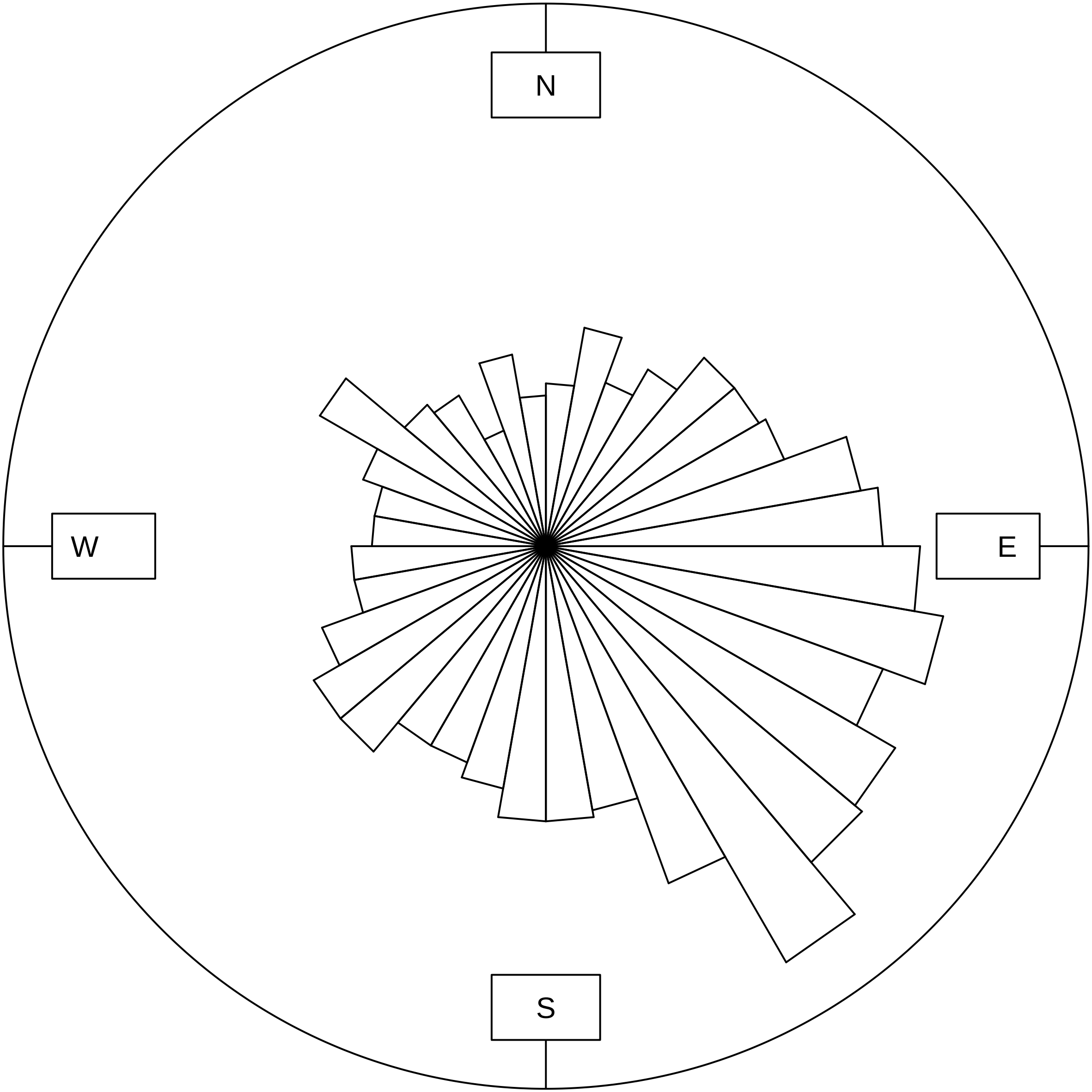

bootstrap 840

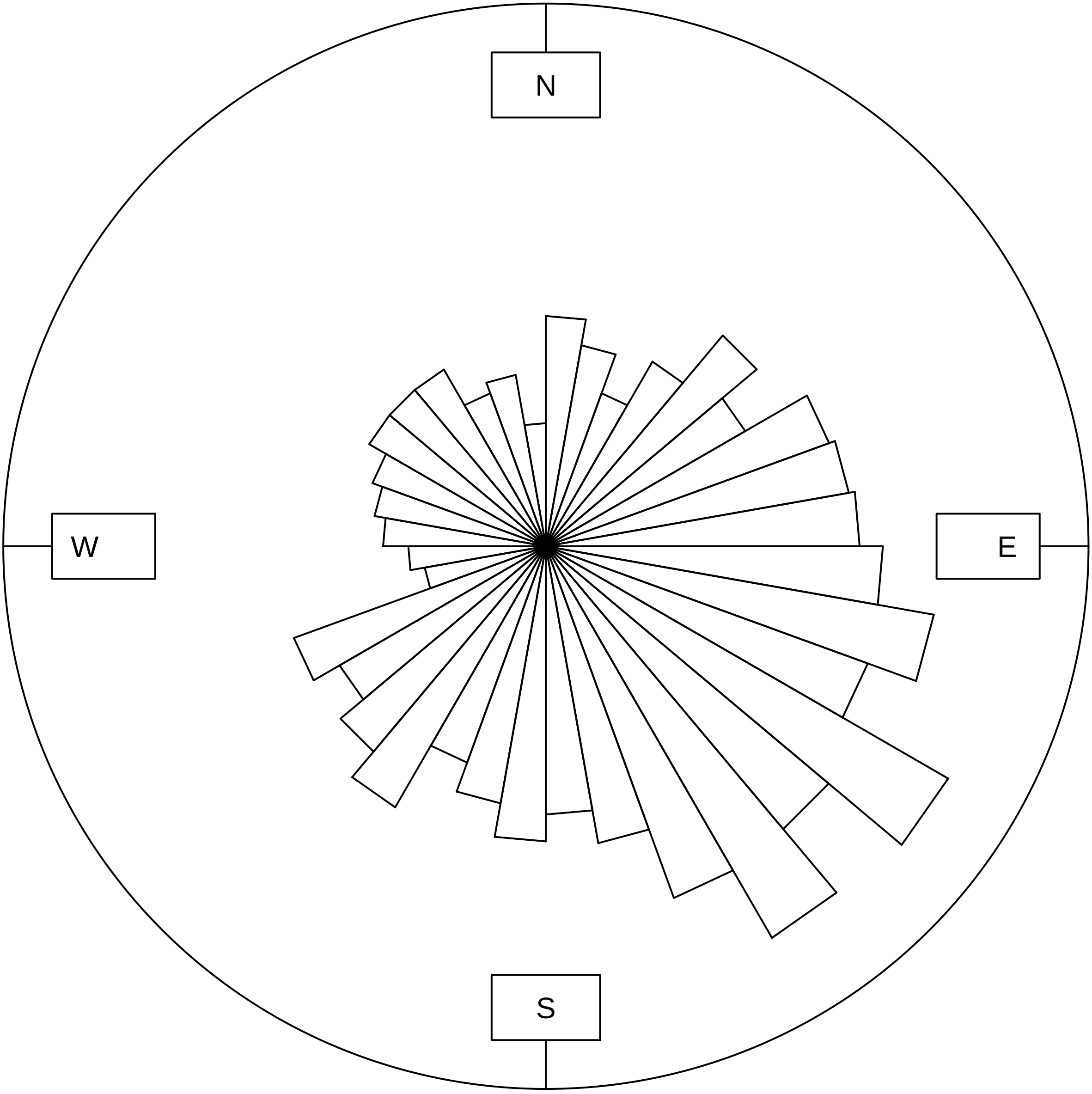

bootstrap 841

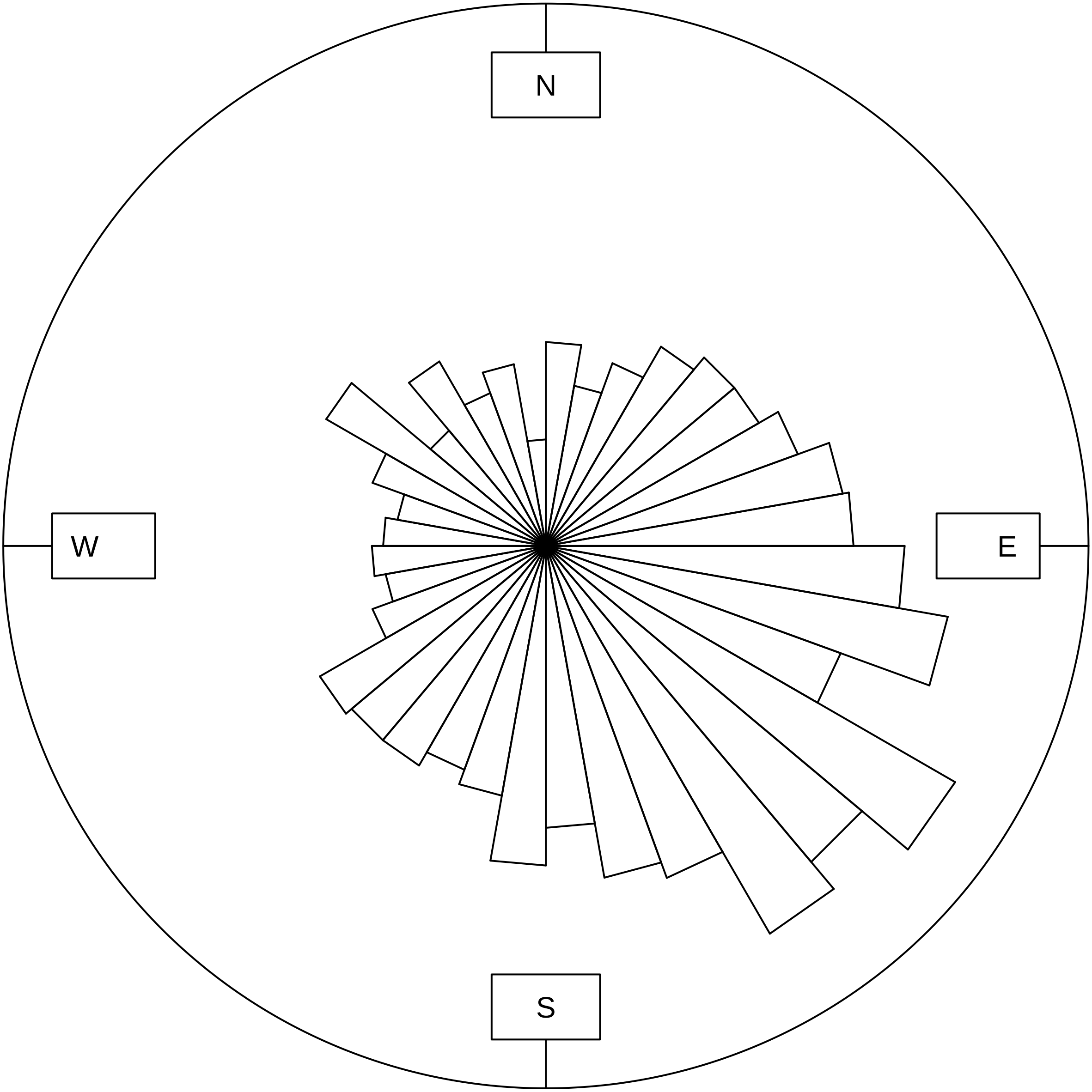

bootstrap 842

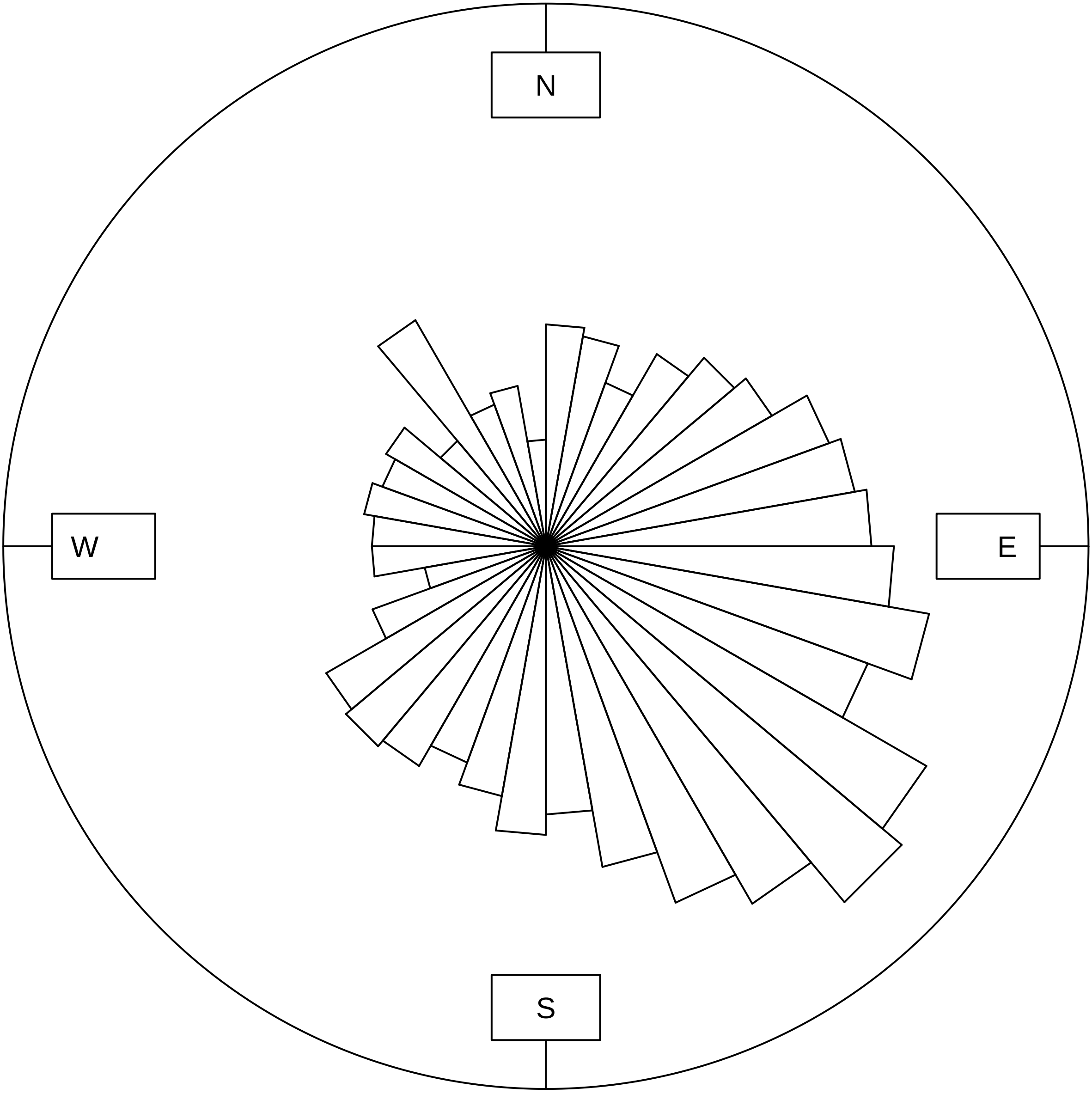

bootstrap 843

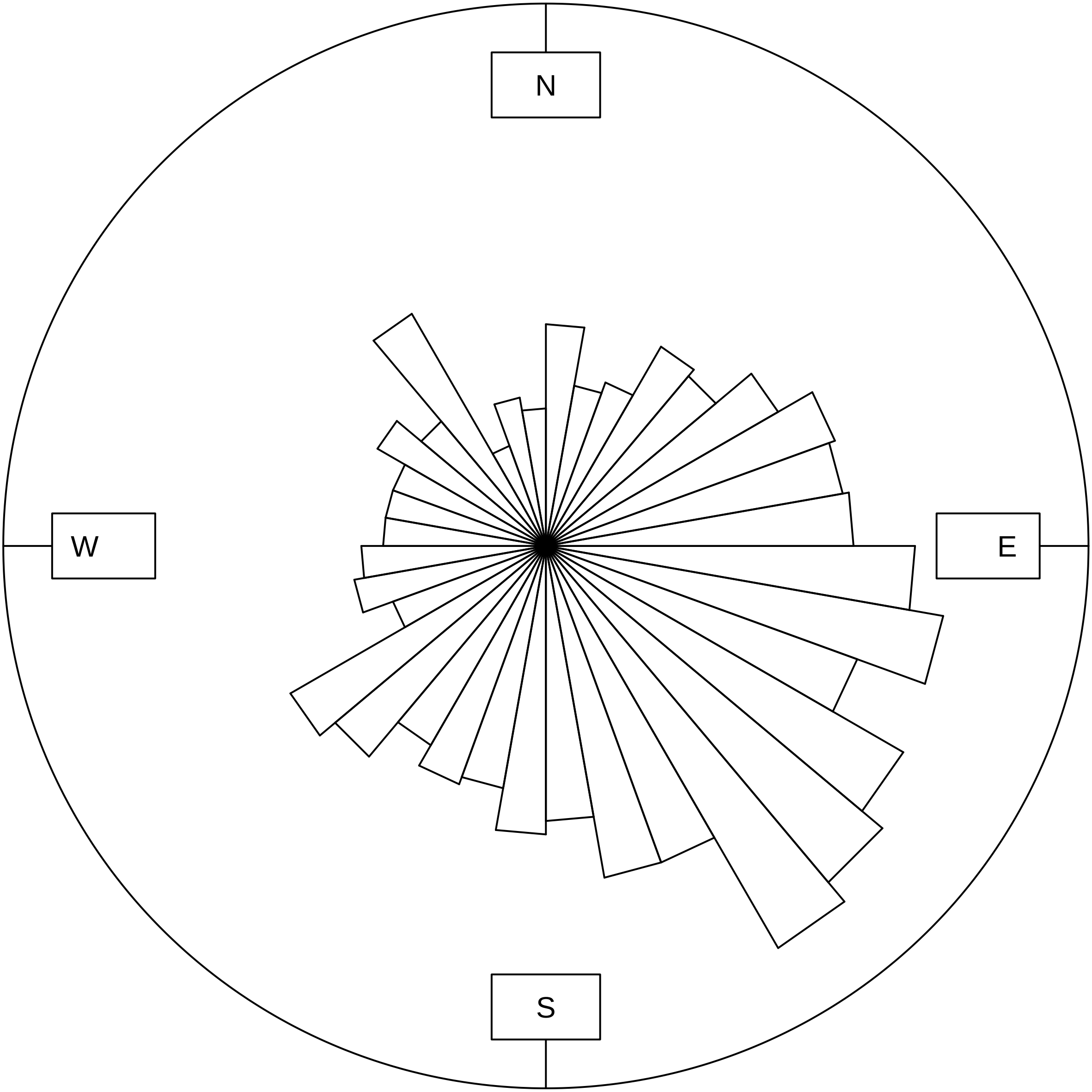

bootstrap 844

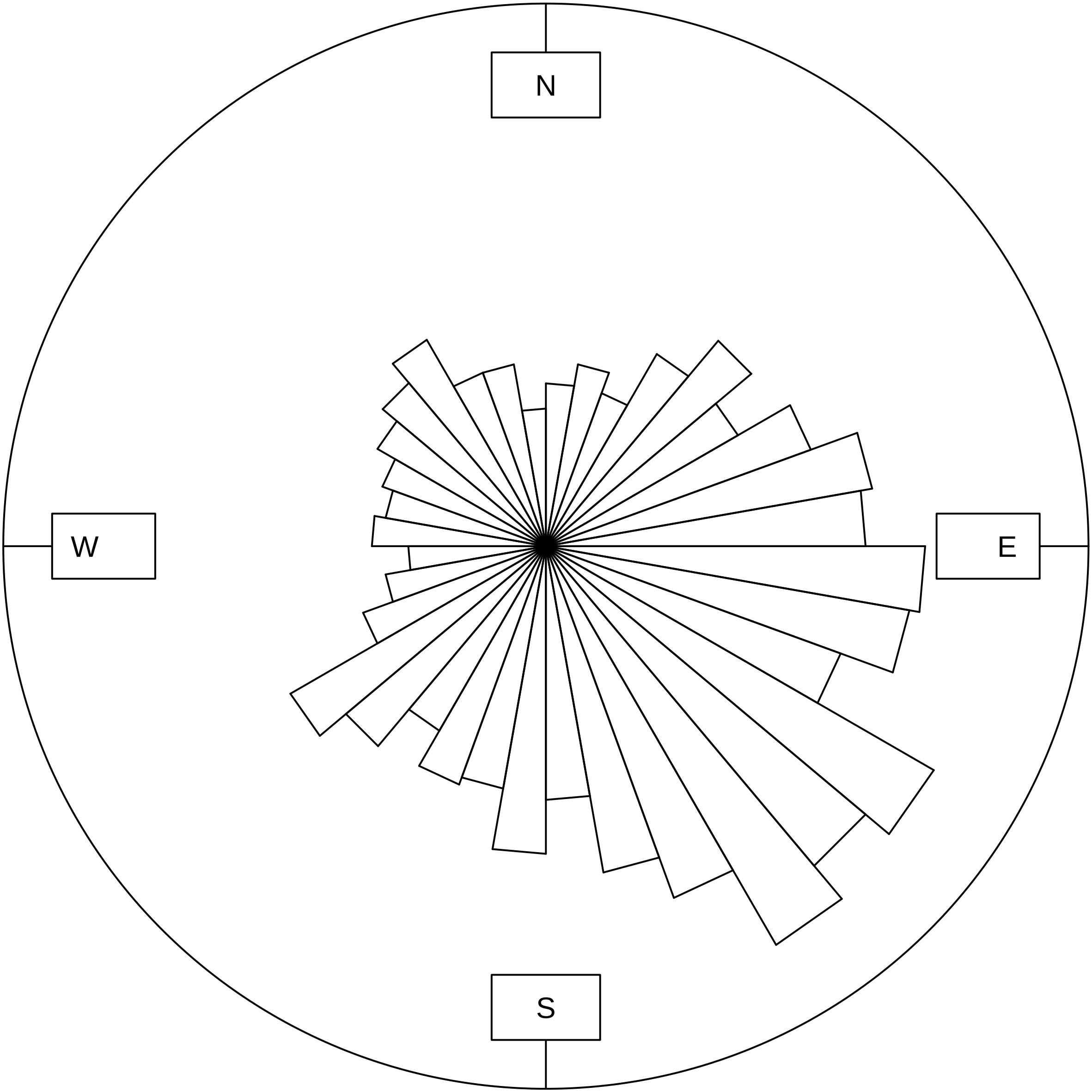

bootstrap 845

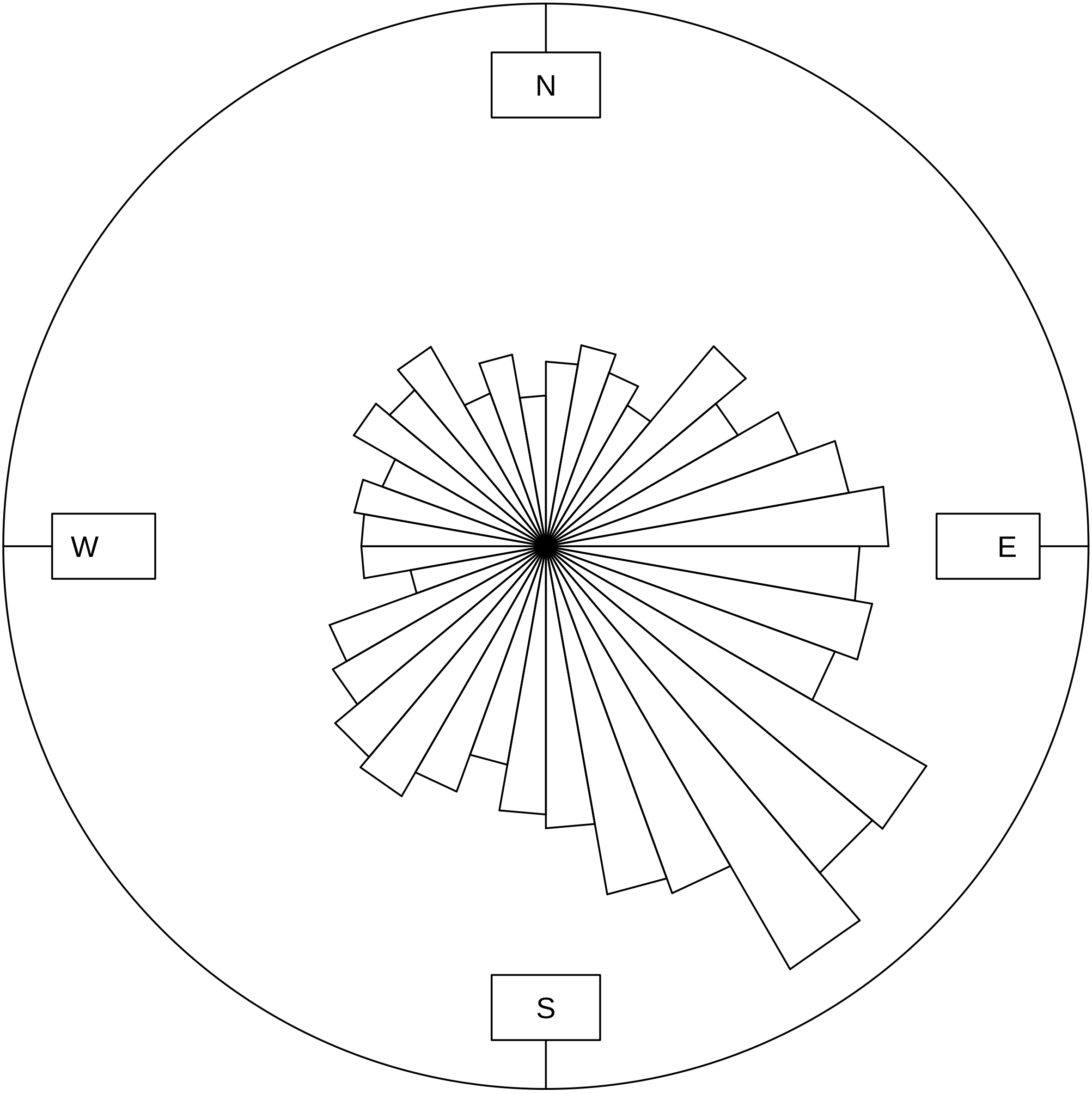

bootstrap 846

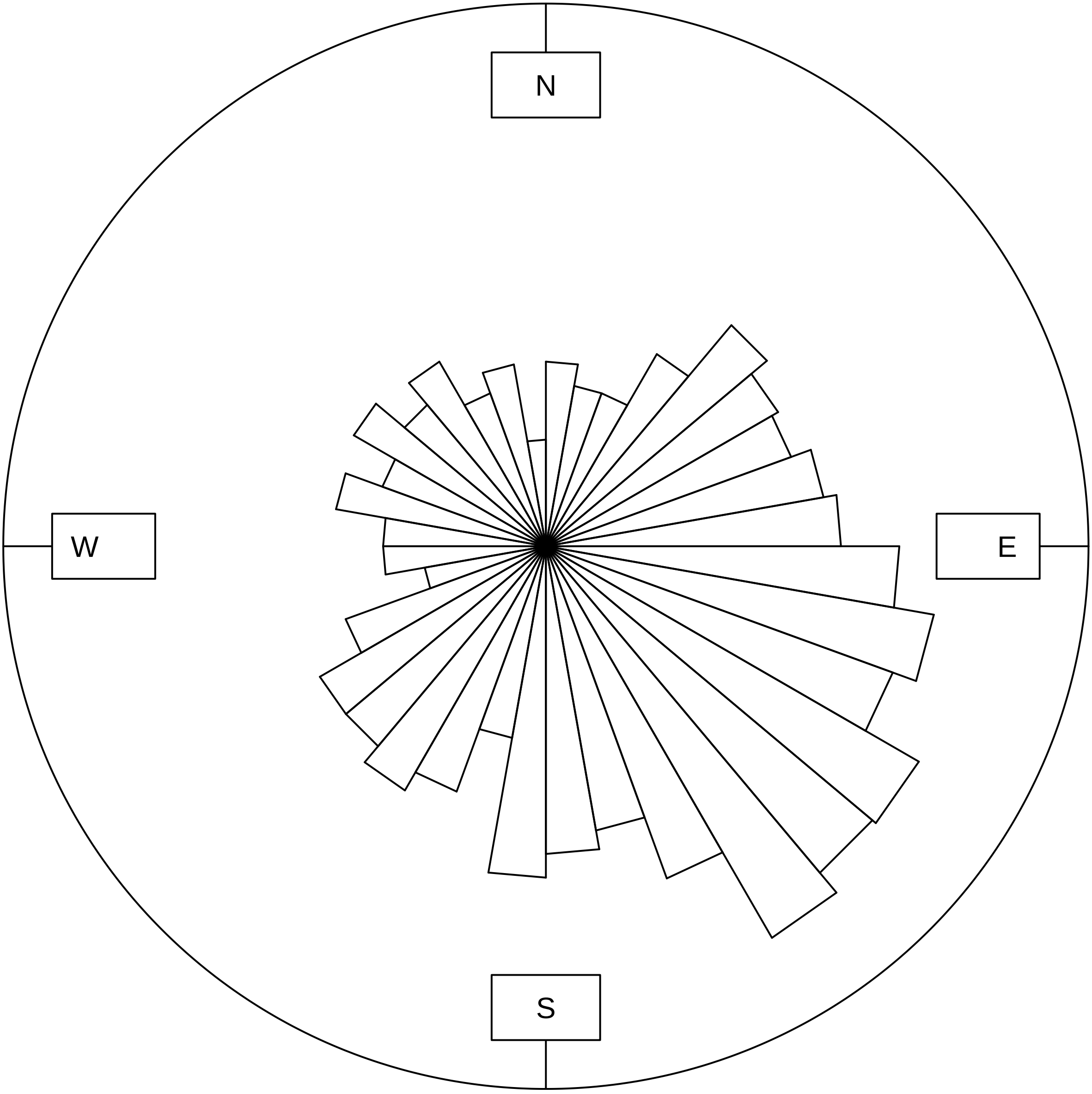

bootstrap 847

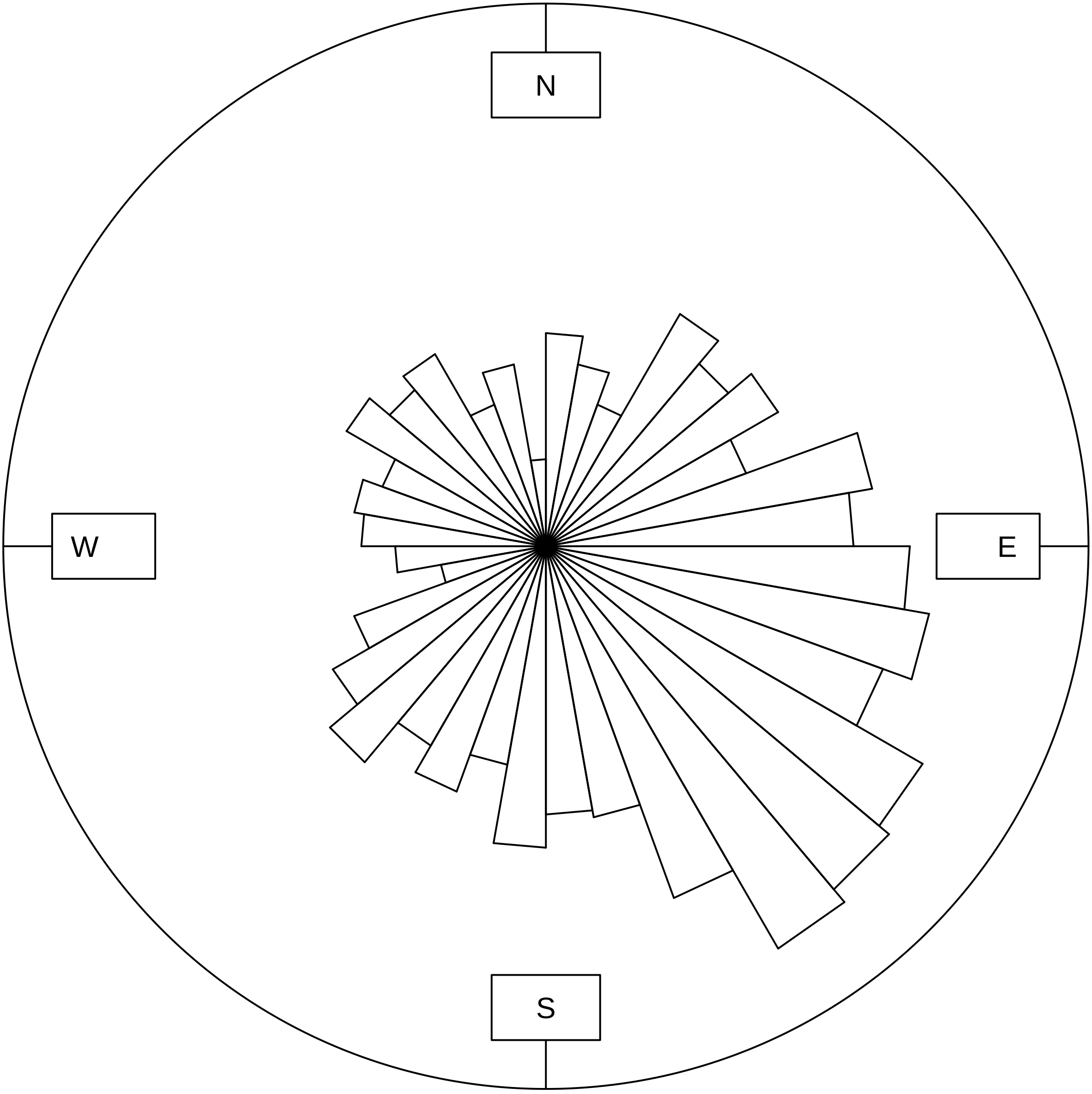

bootstrap 848

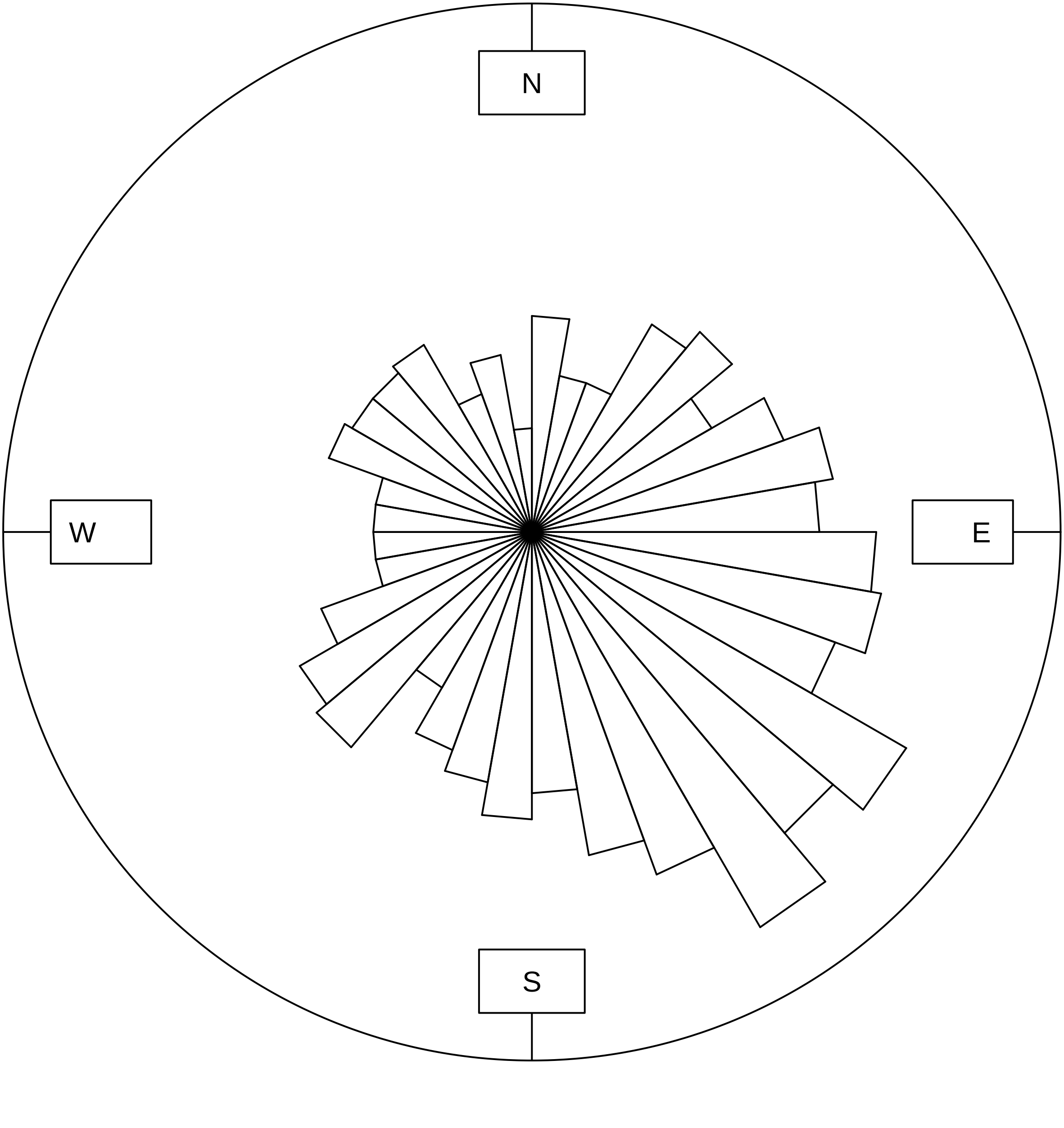

bootstrap 849

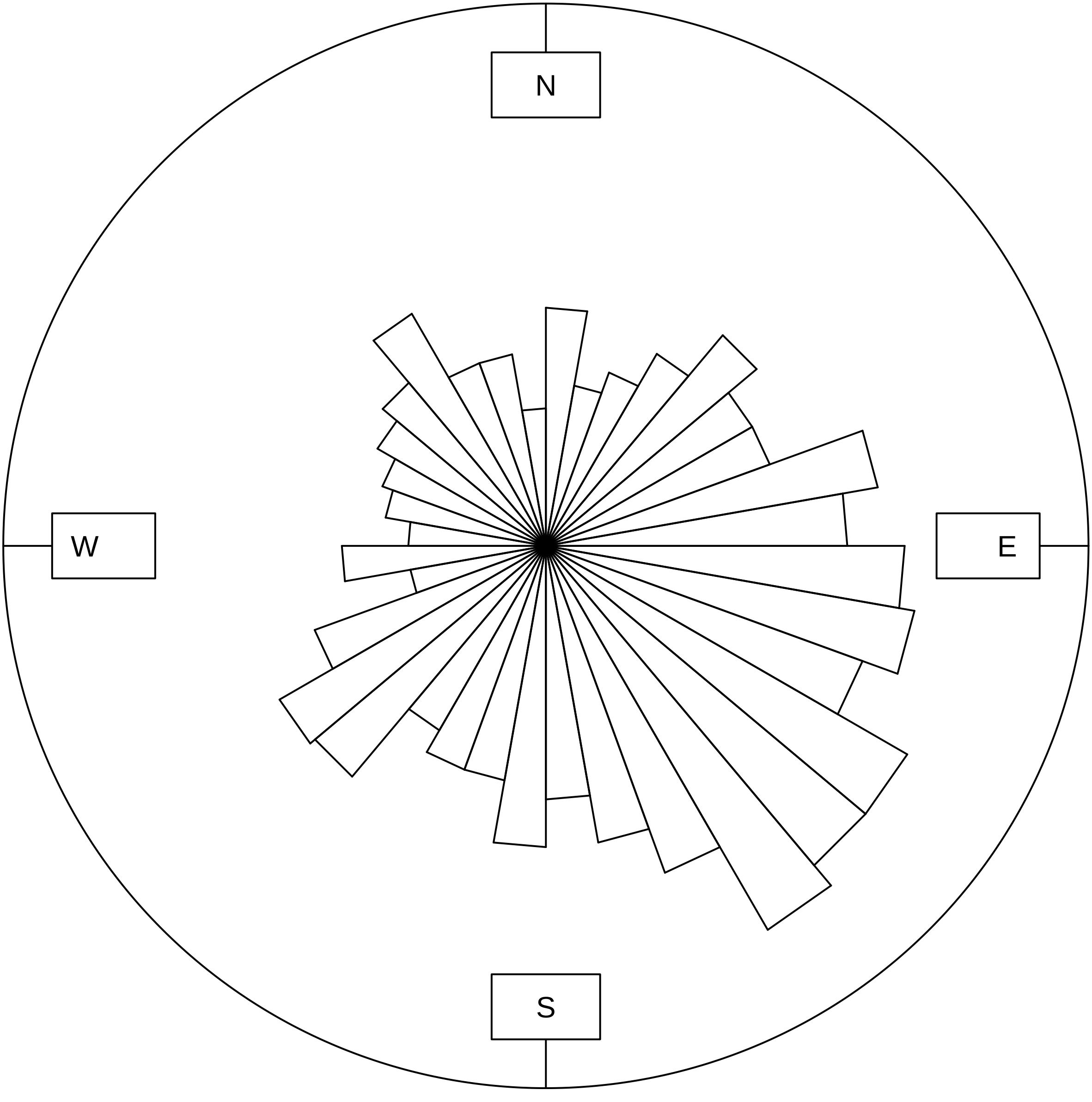

bootstrap 850

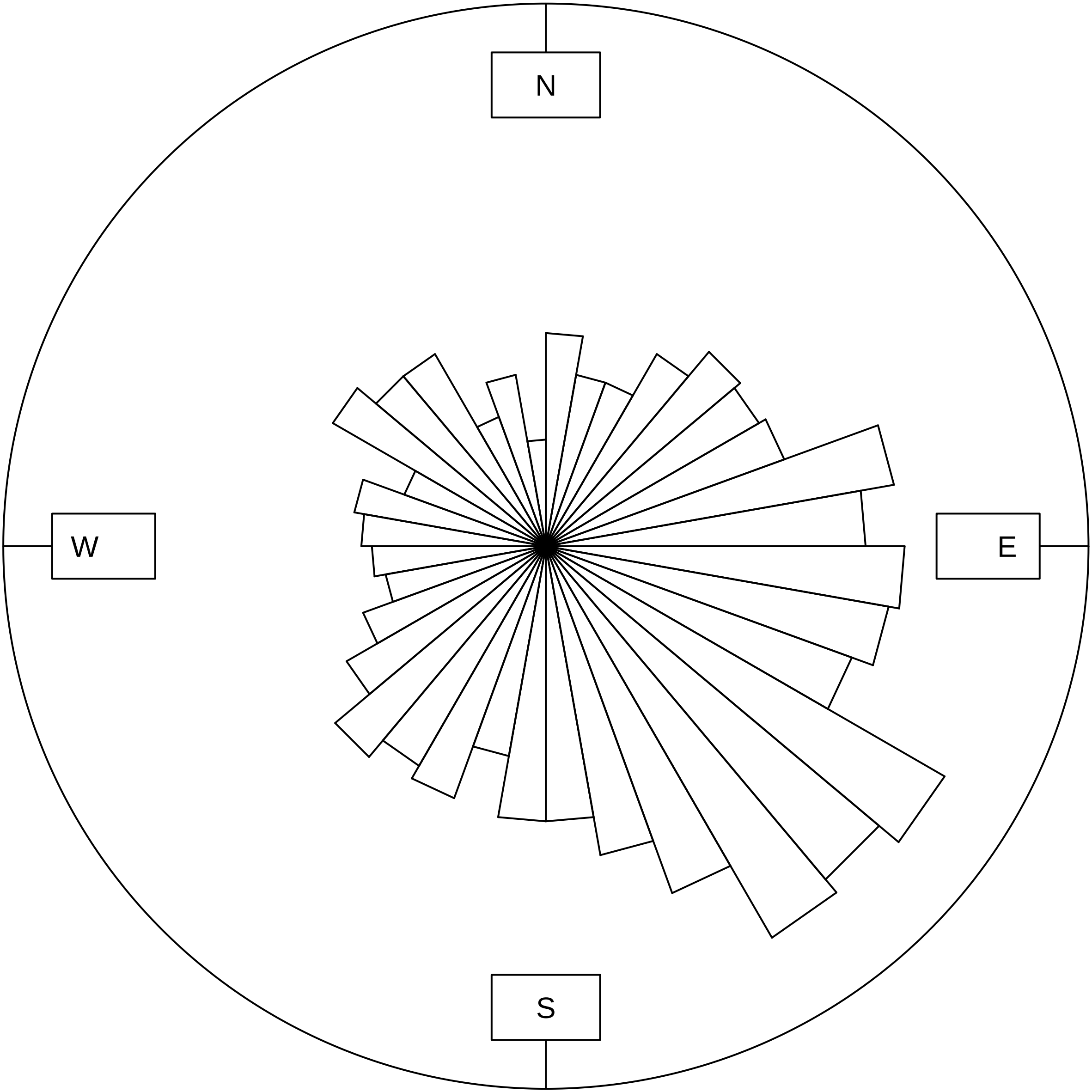

bootstrap 851

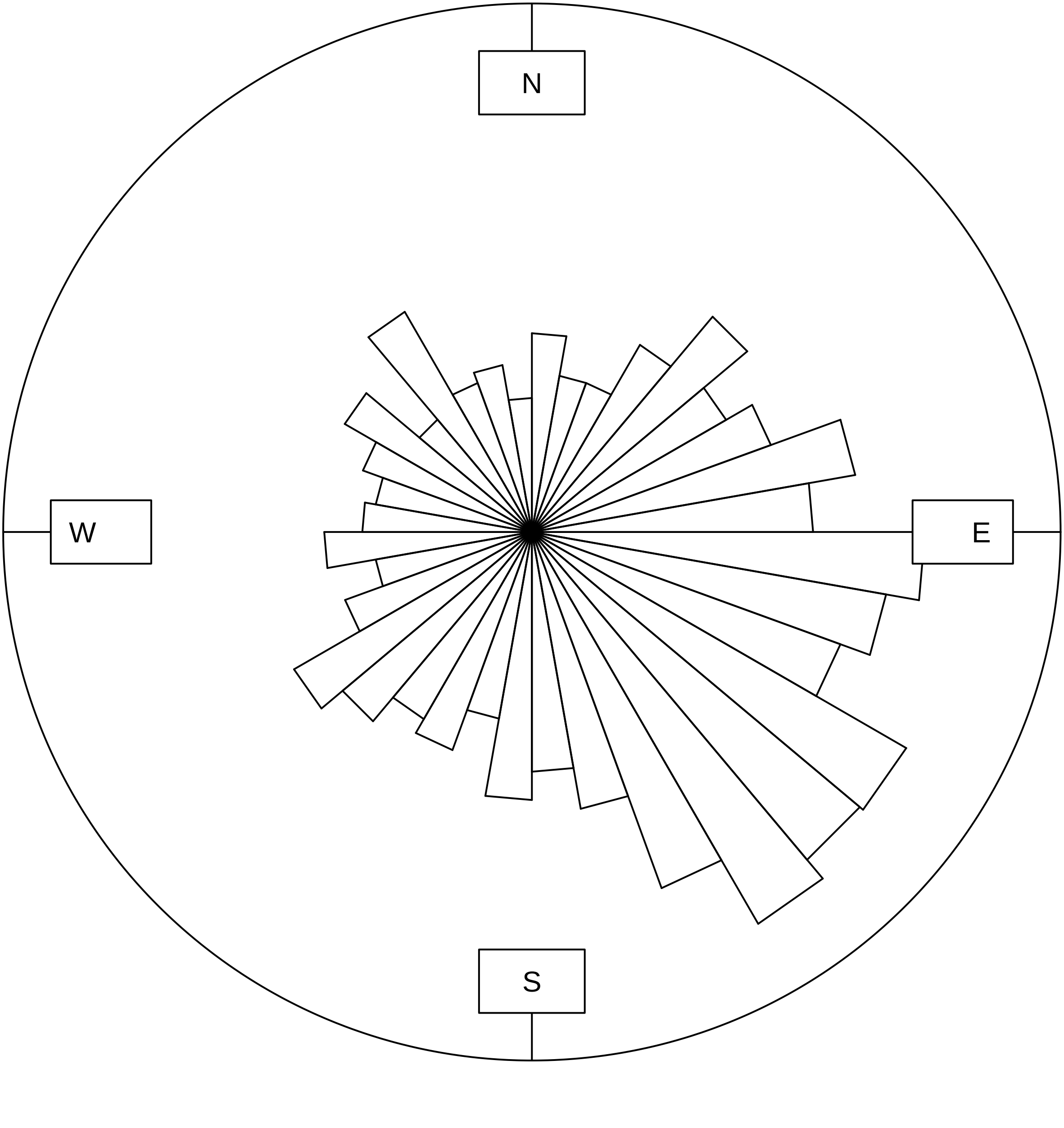

bootstrap 852

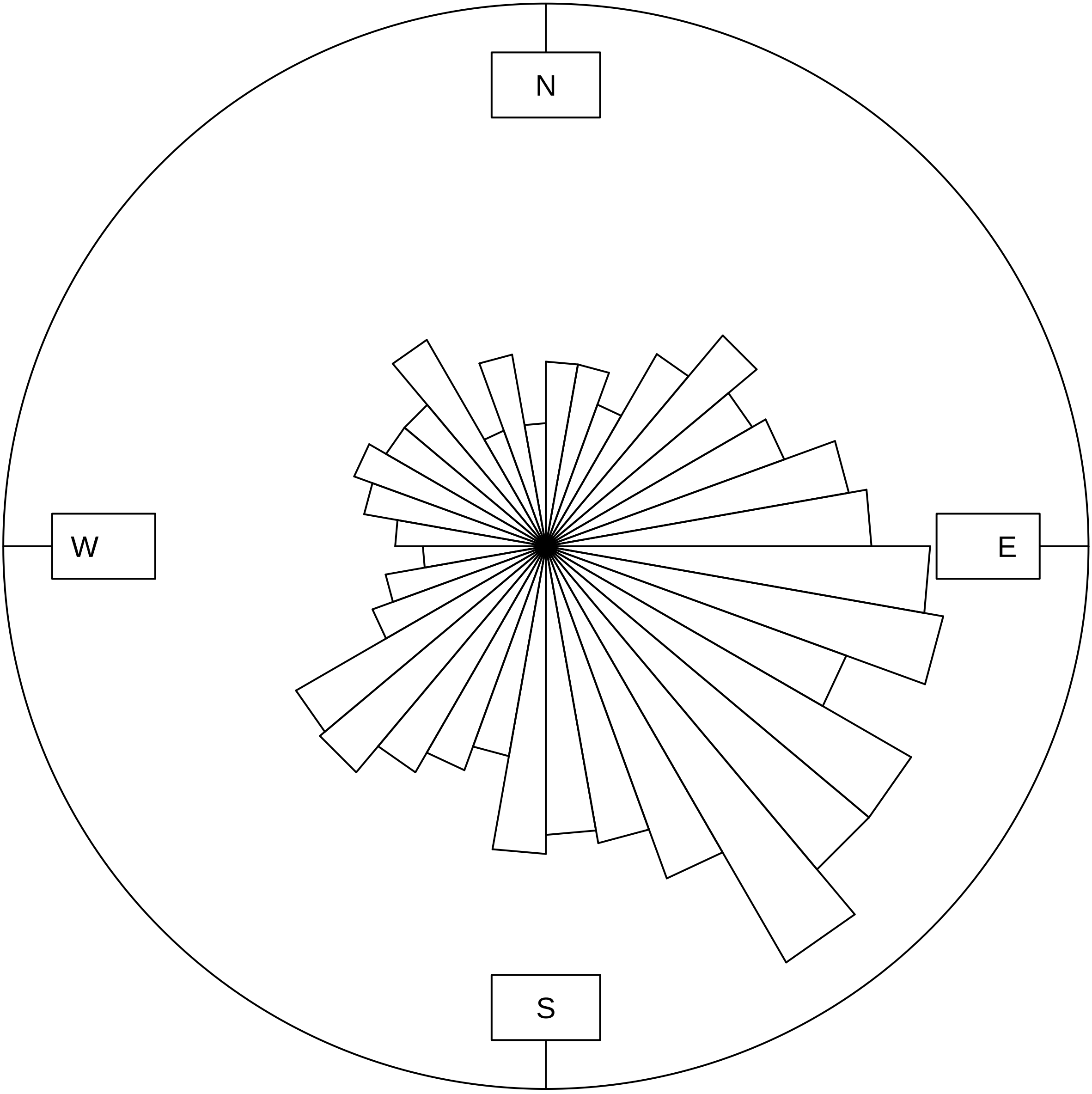

bootstrap 853

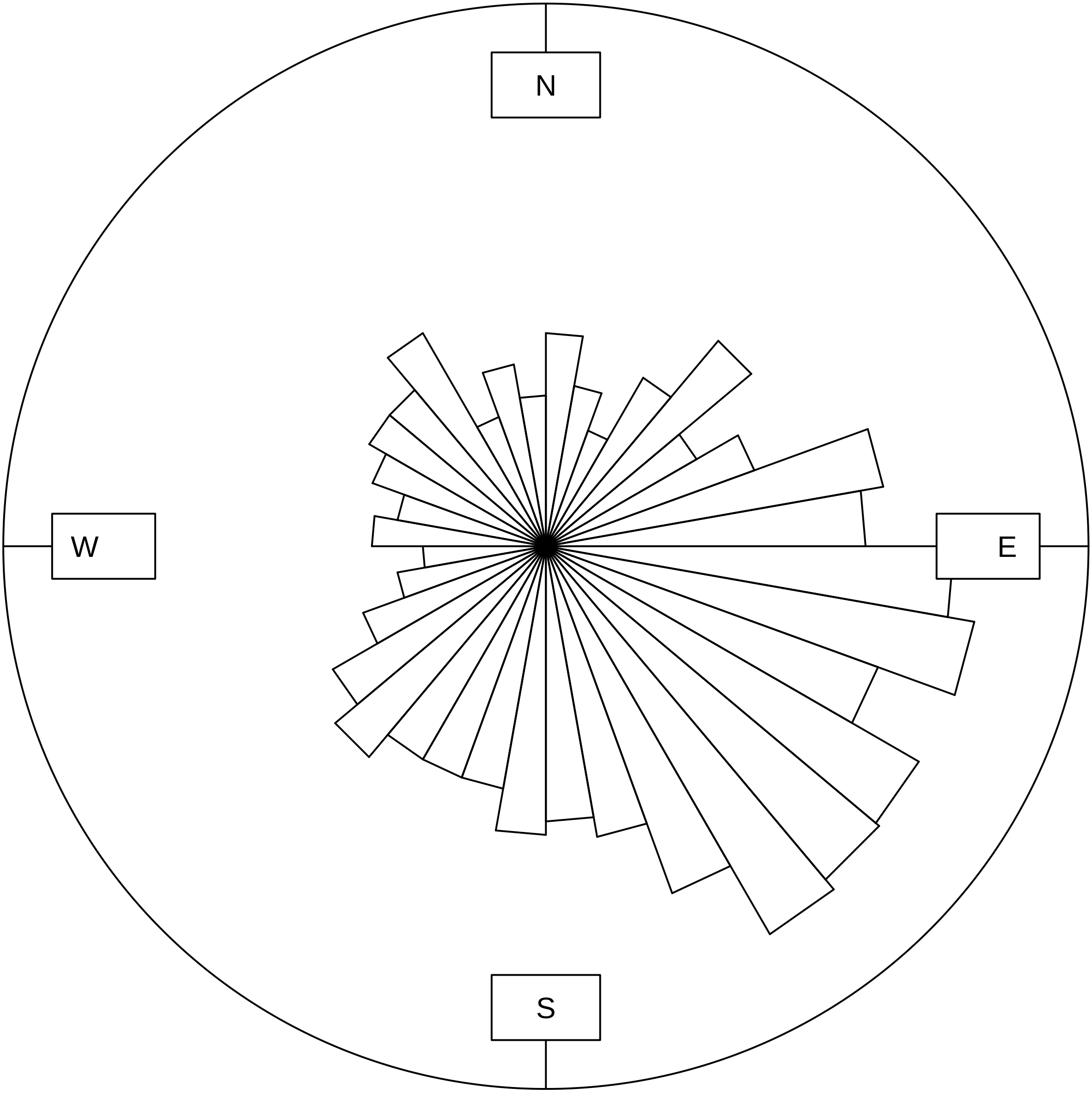

bootstrap 854

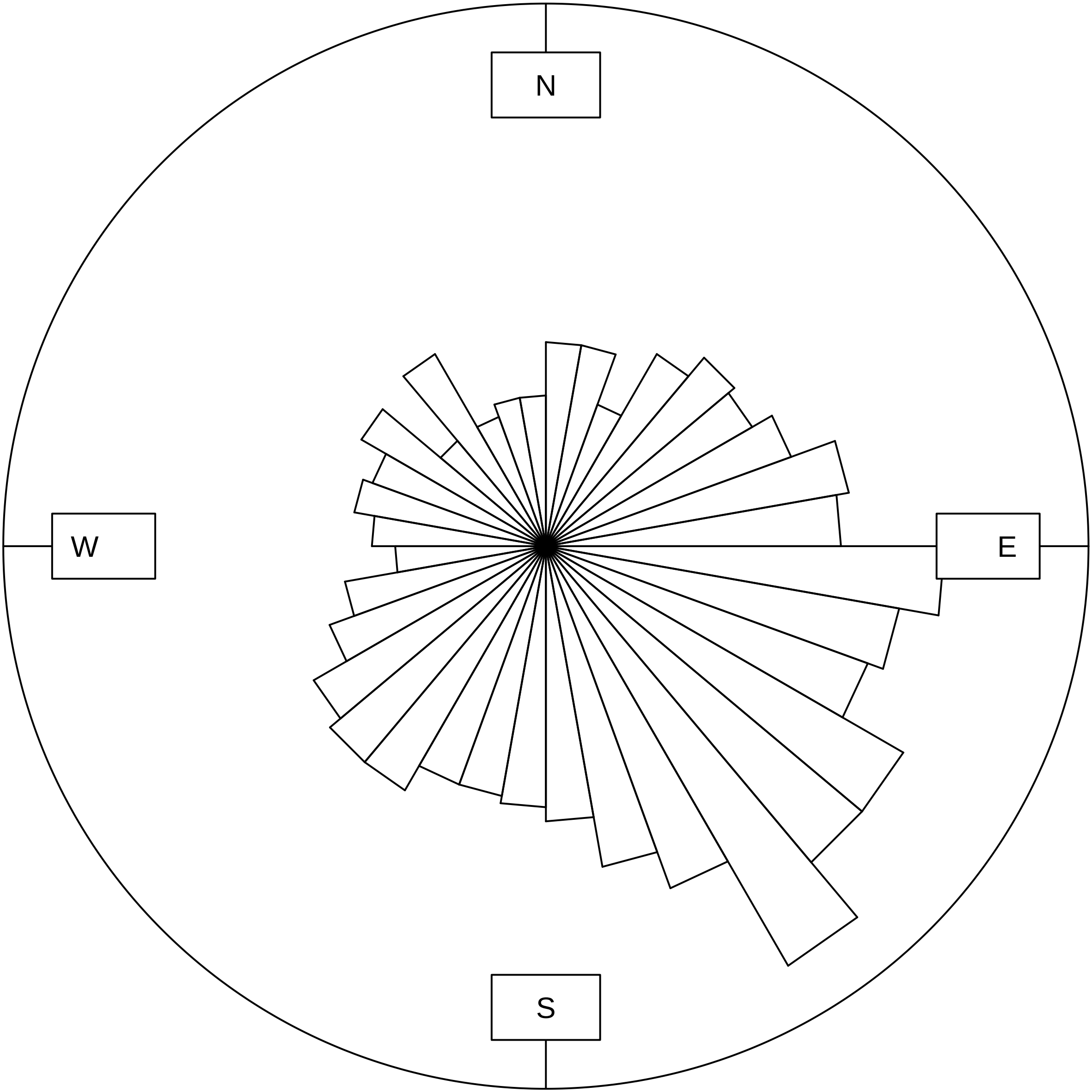

bootstrap 855

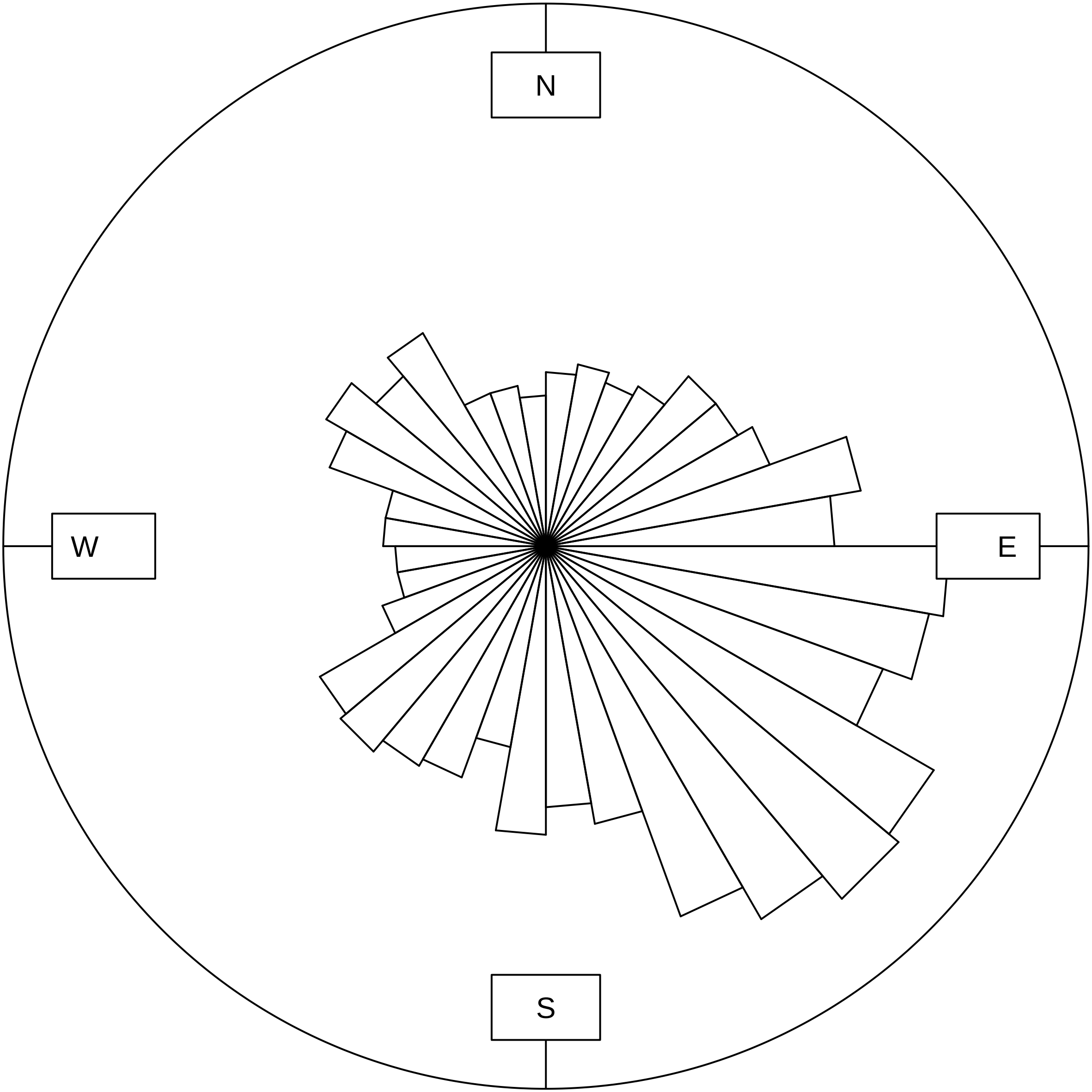

bootstrap 856

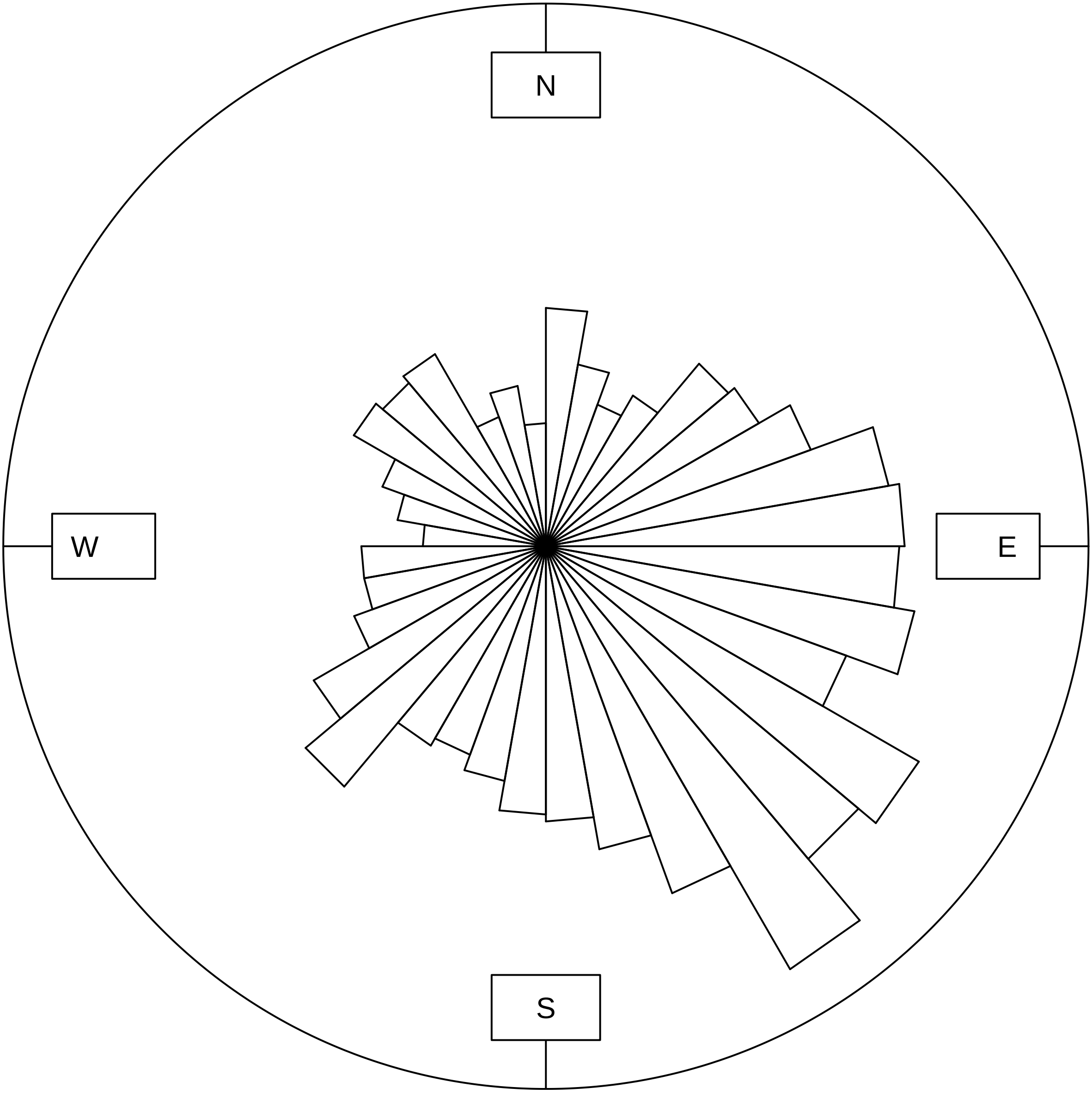

bootstrap 857

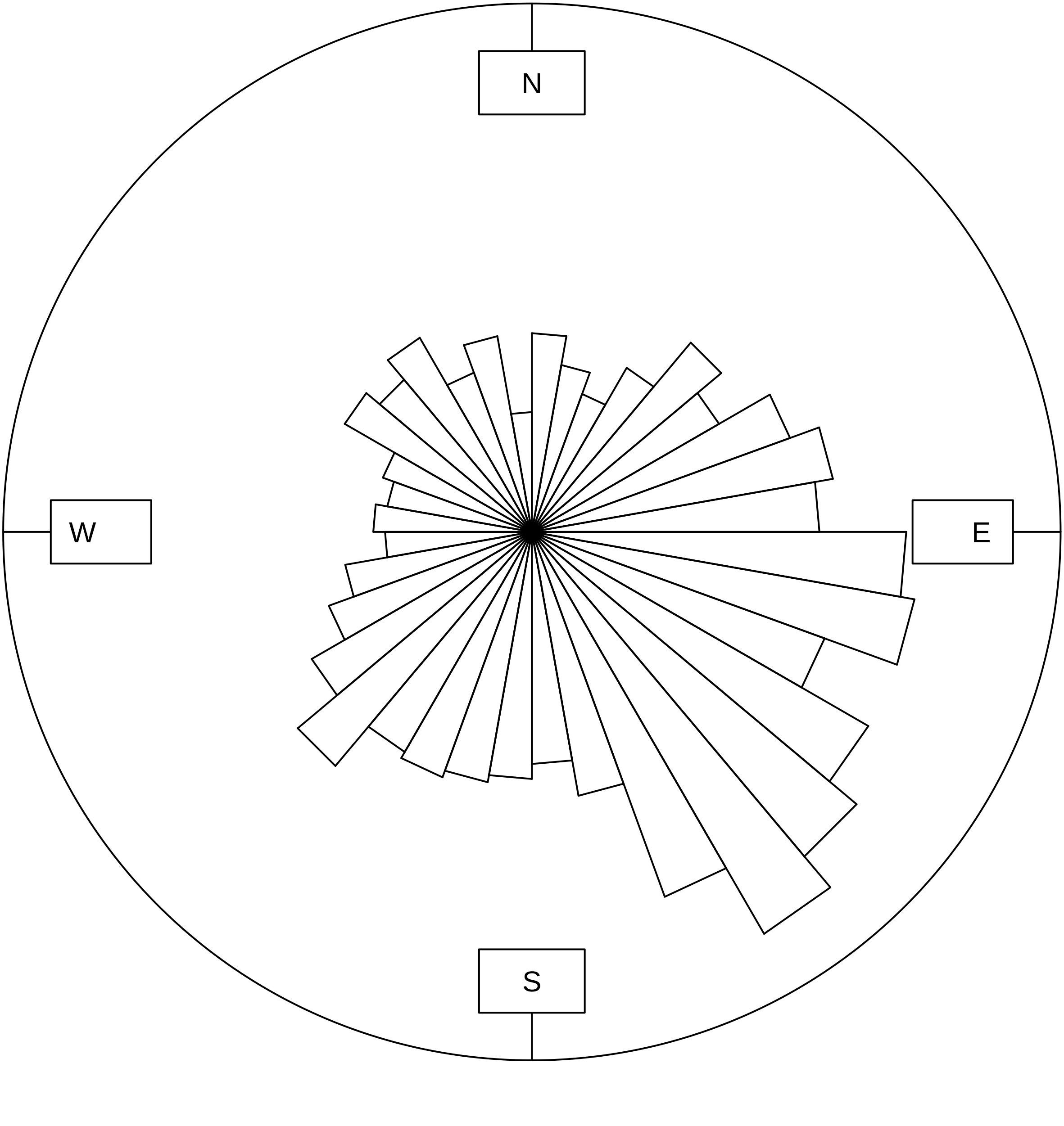

bootstrap 858

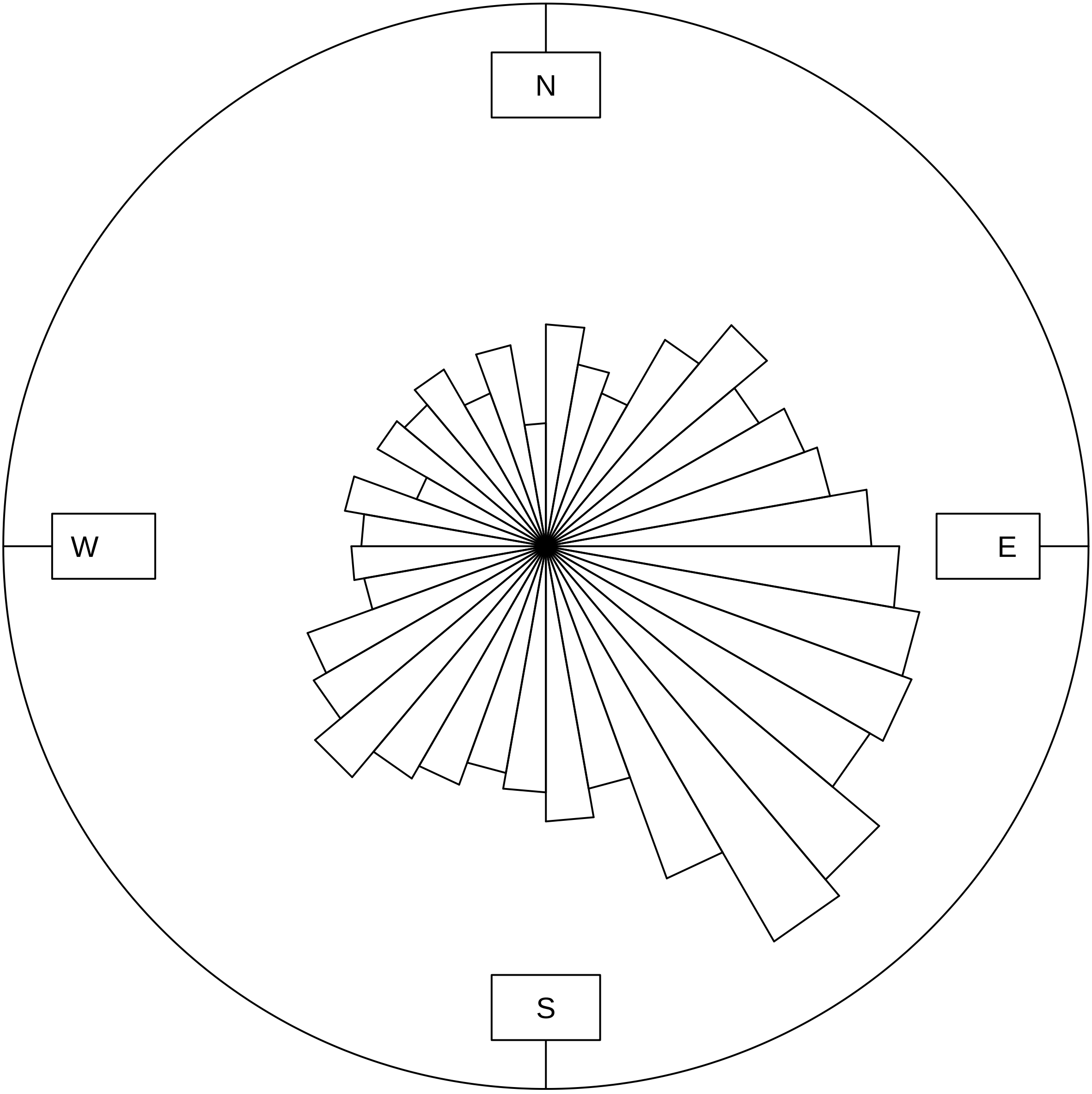

bootstrap 859

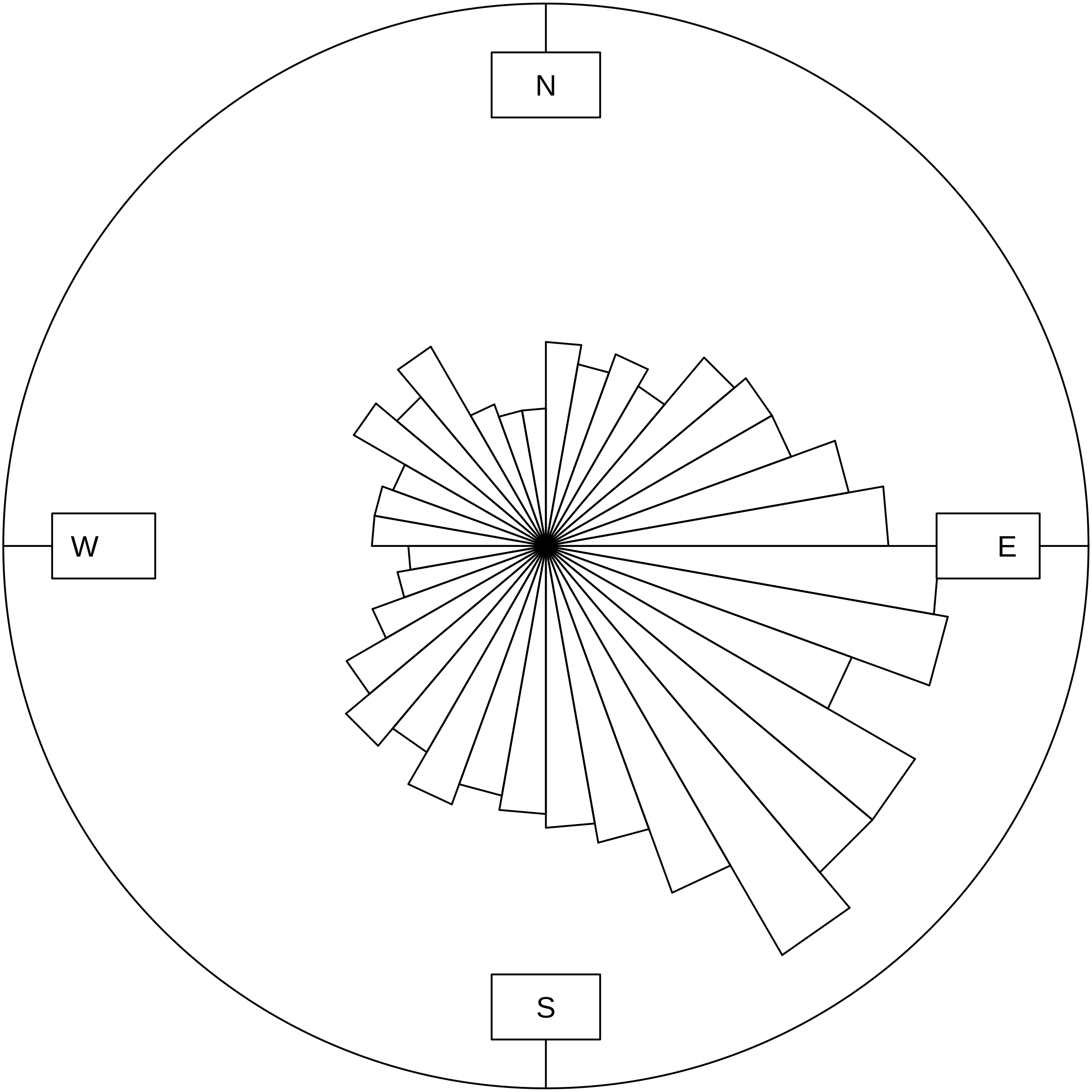

bootstrap 860

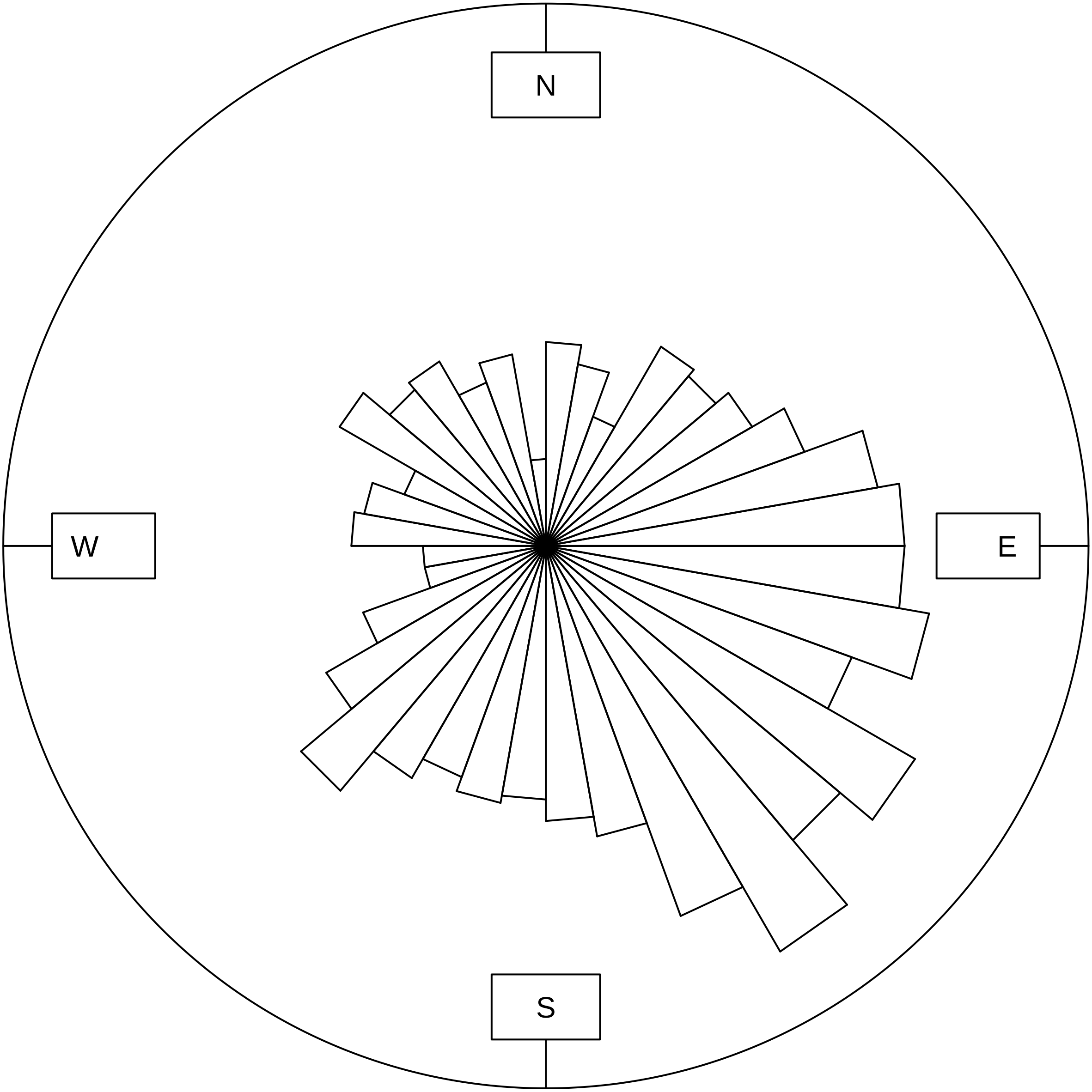

bootstrap 861

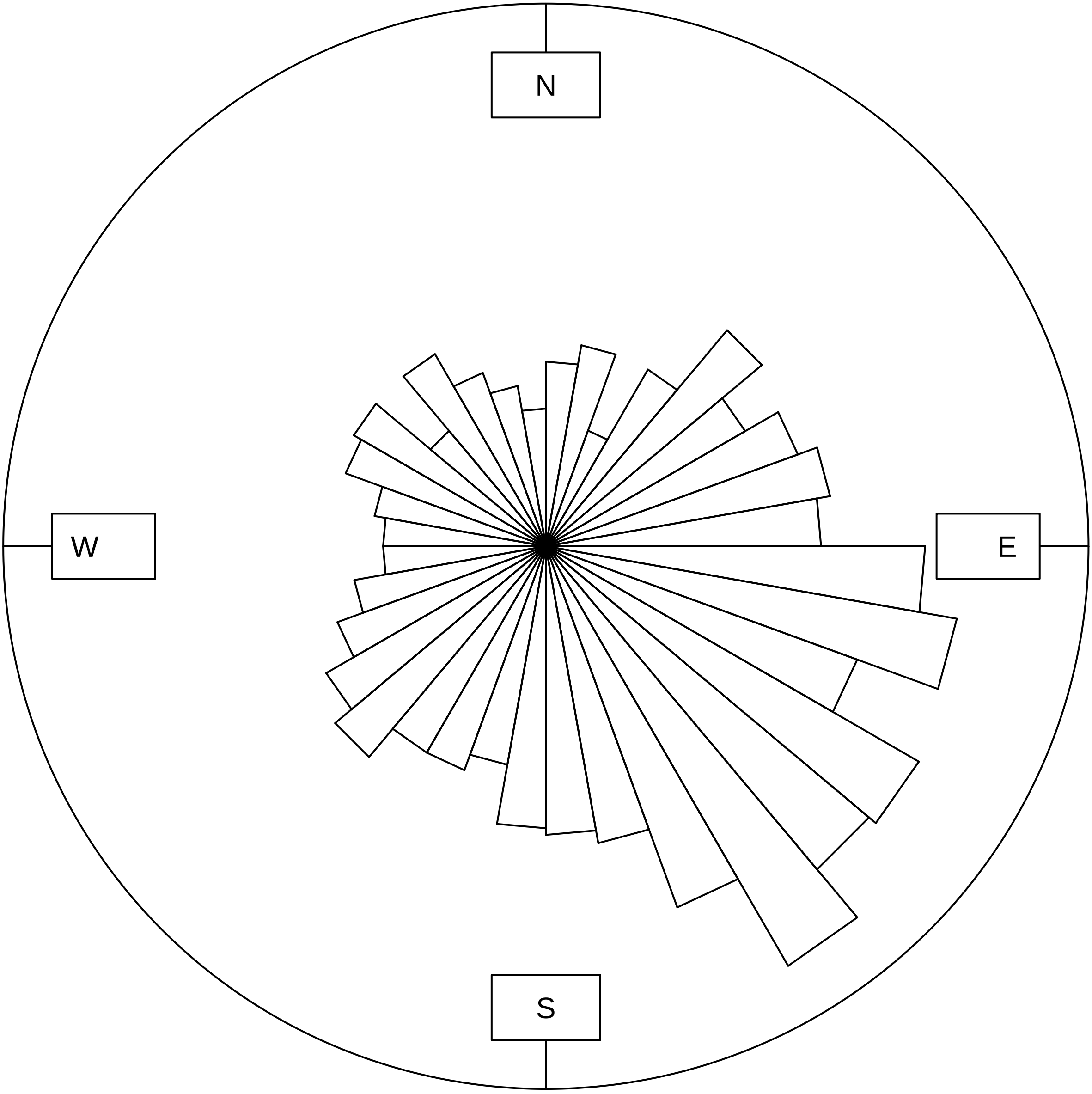

bootstrap 862

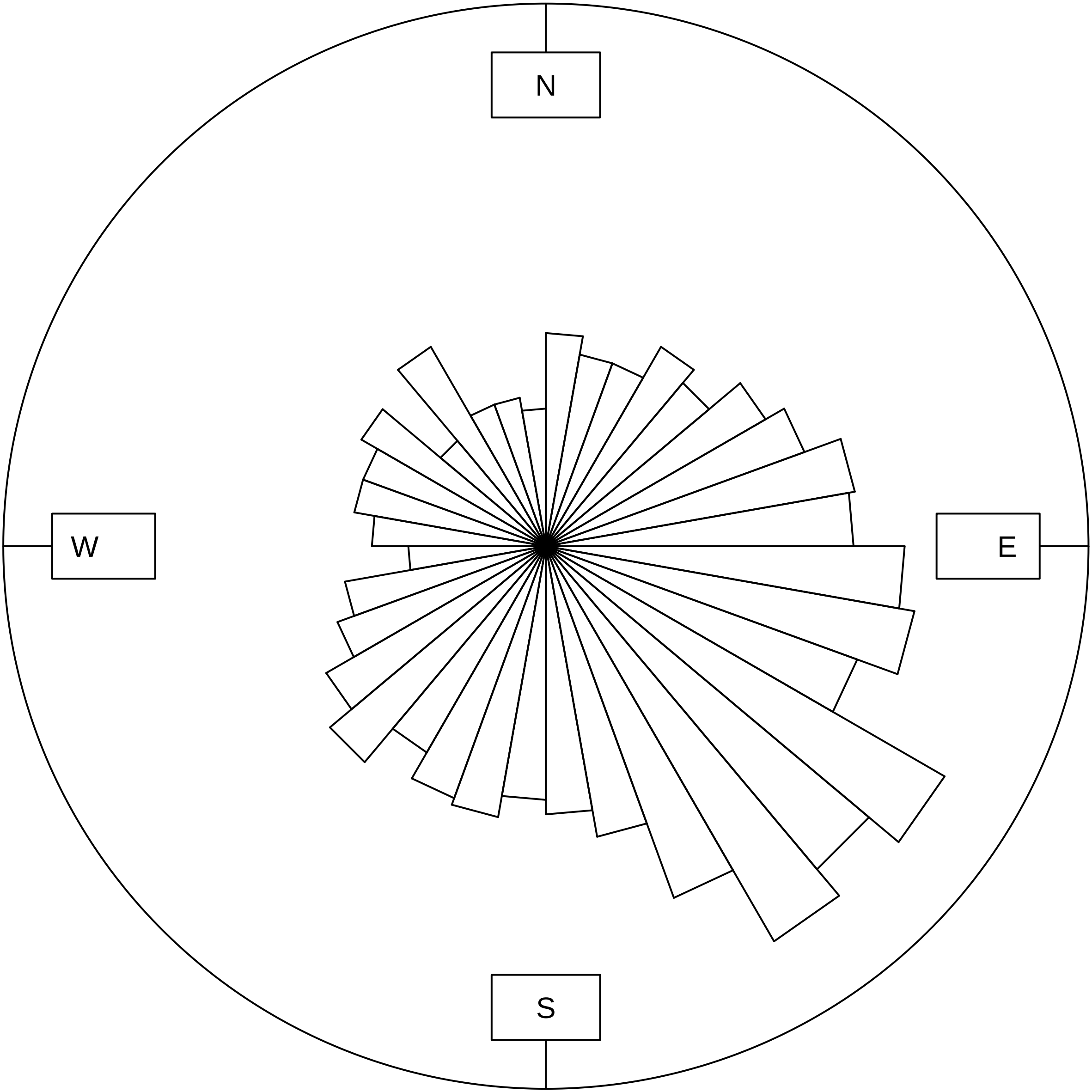

bootstrap 863

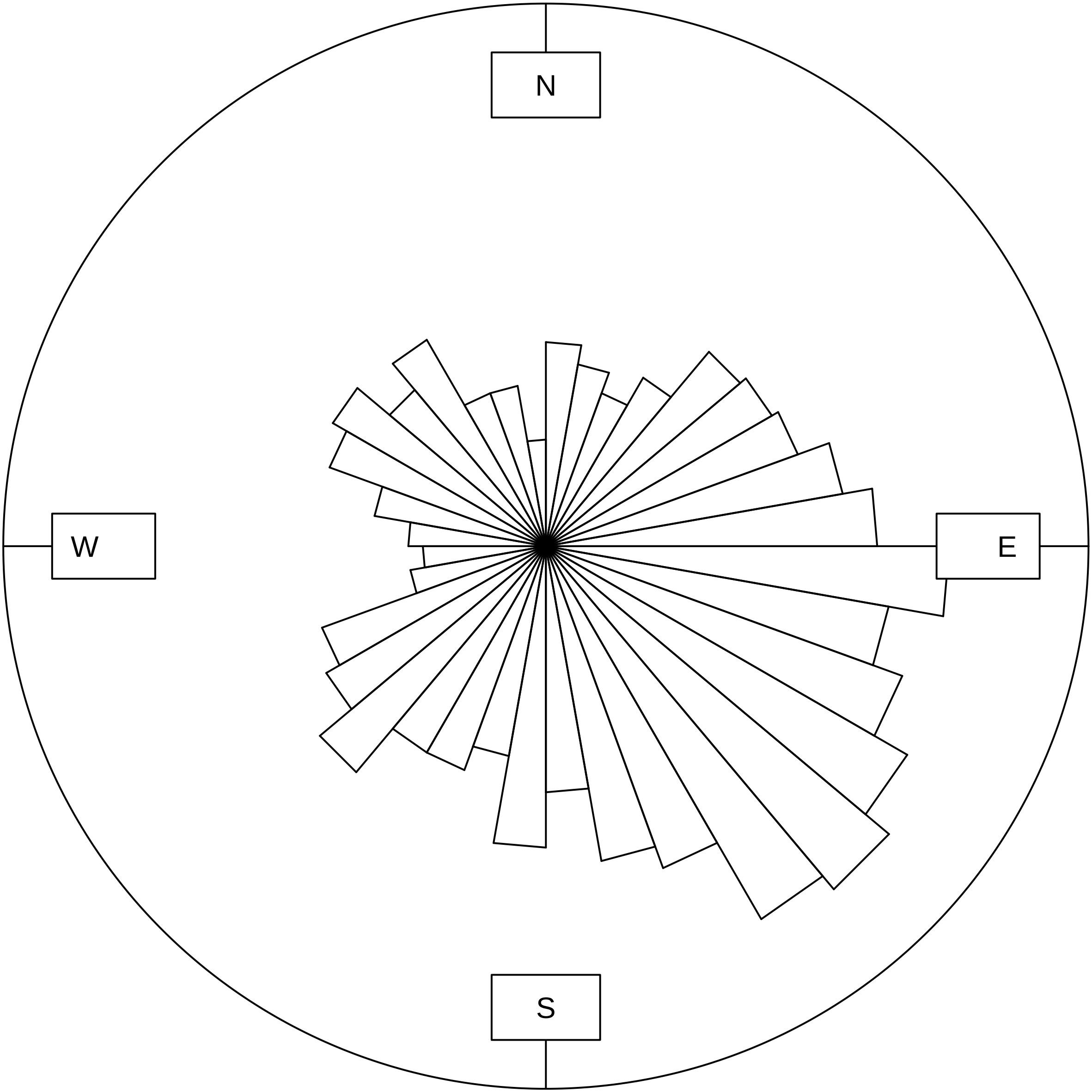

bootstrap 864

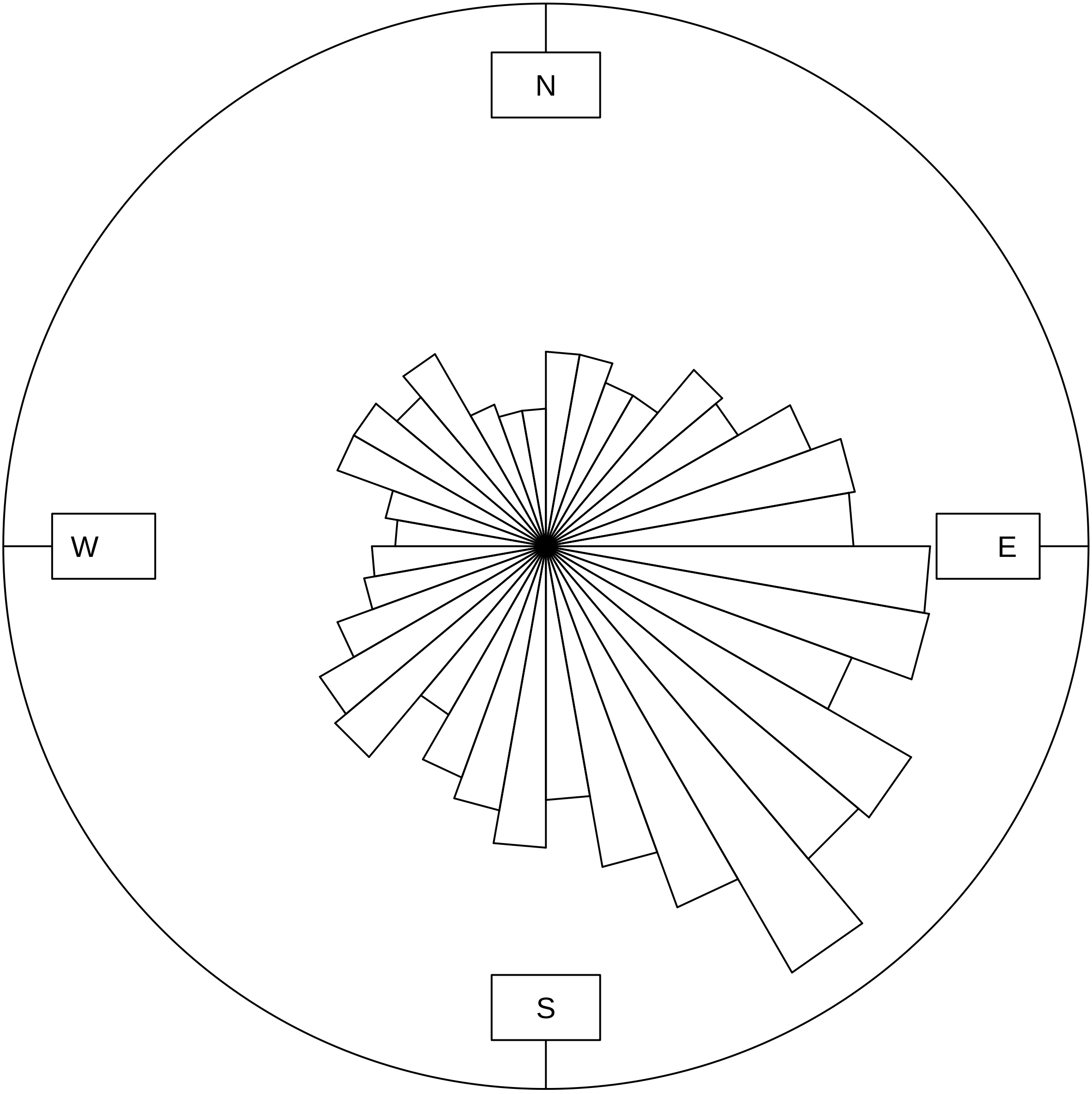

bootstrap 865

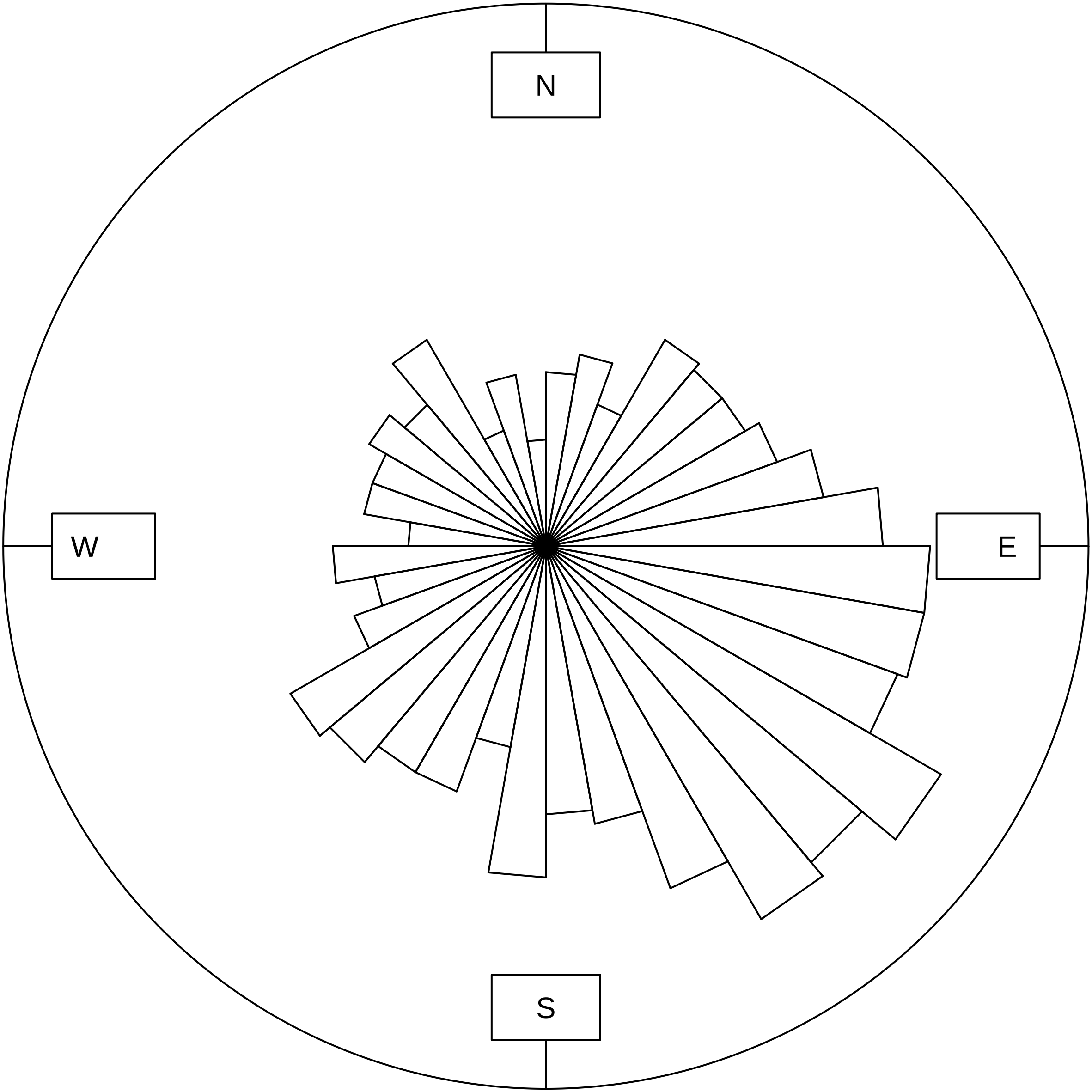

bootstrap 866

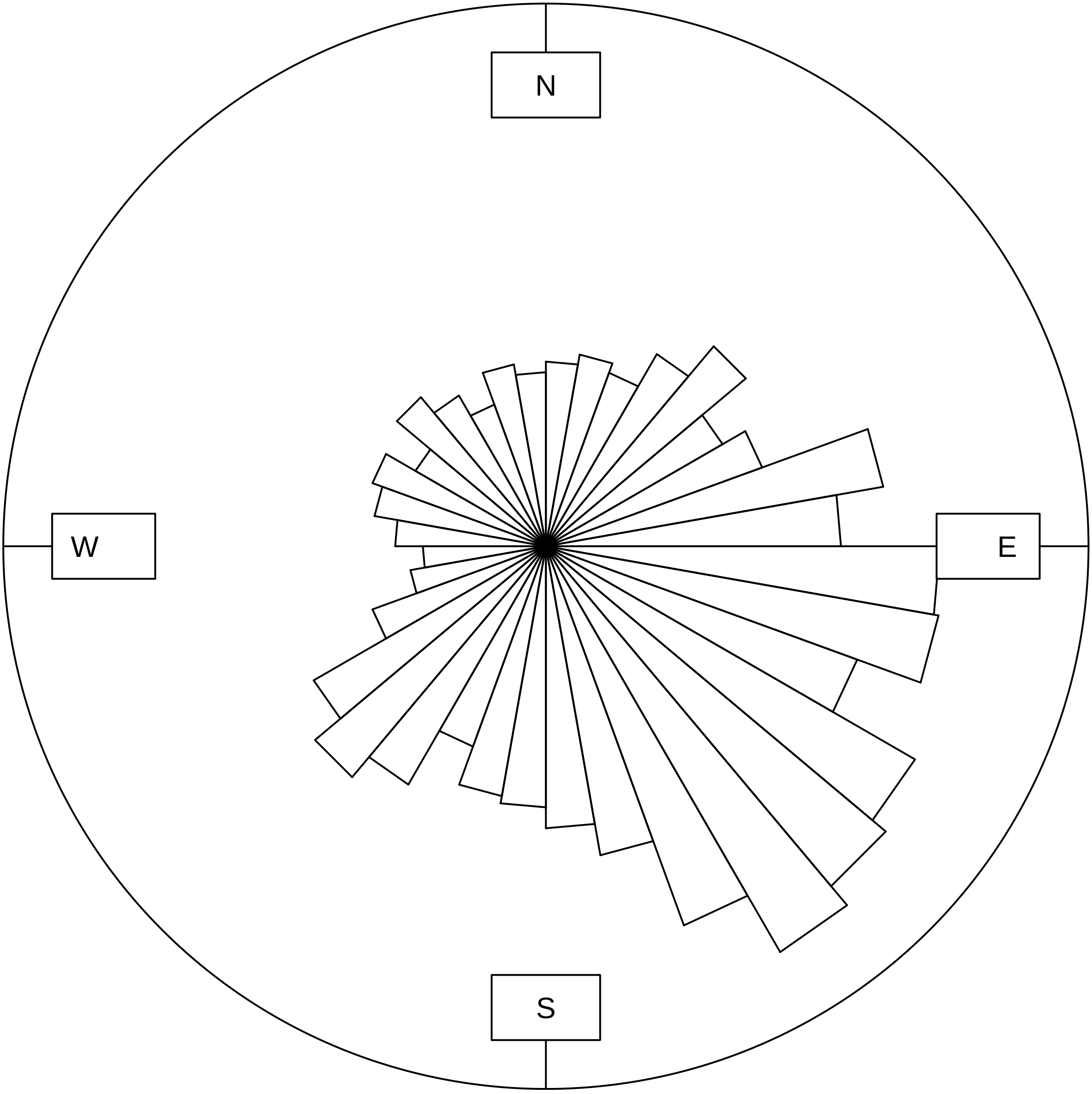

bootstrap 867

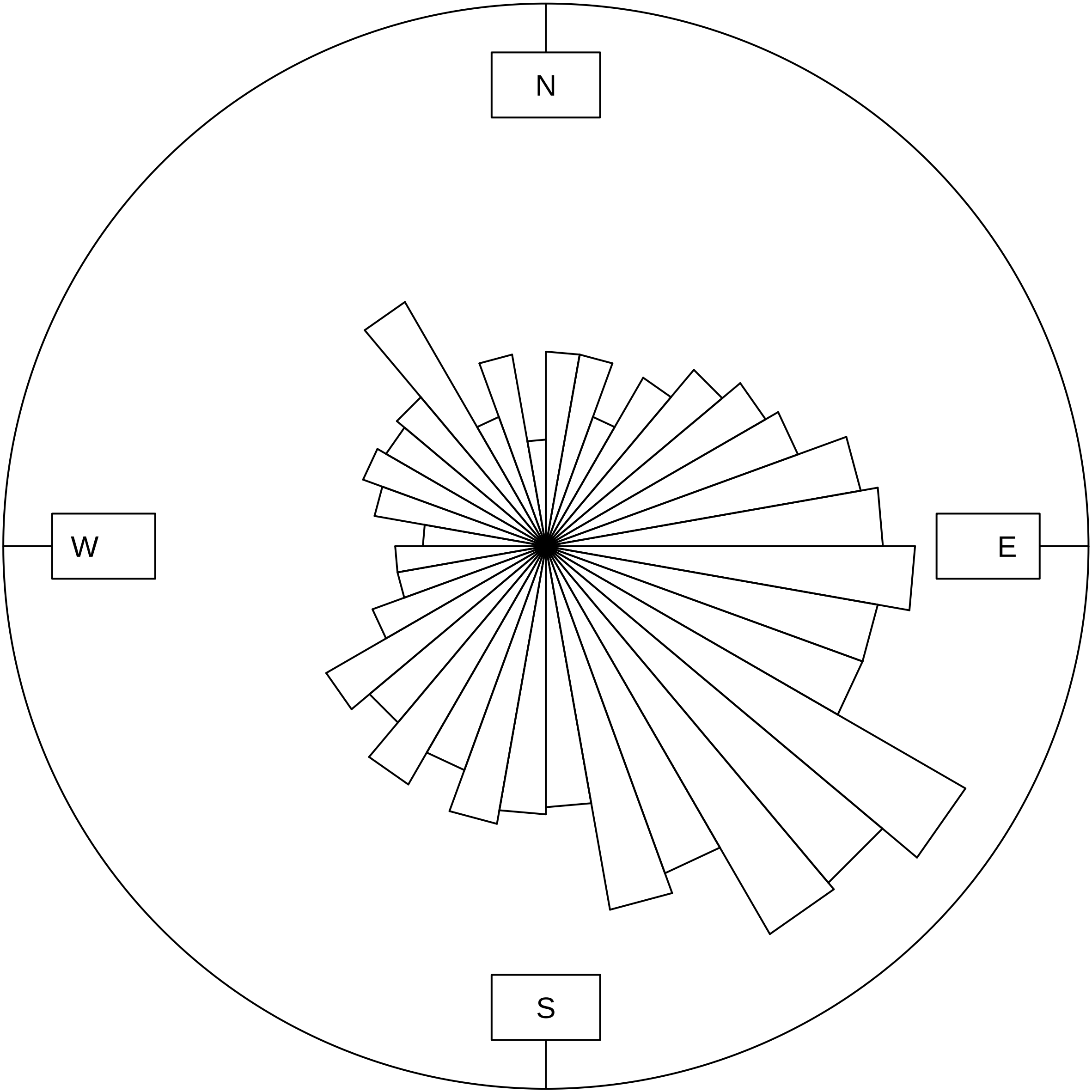

bootstrap 868

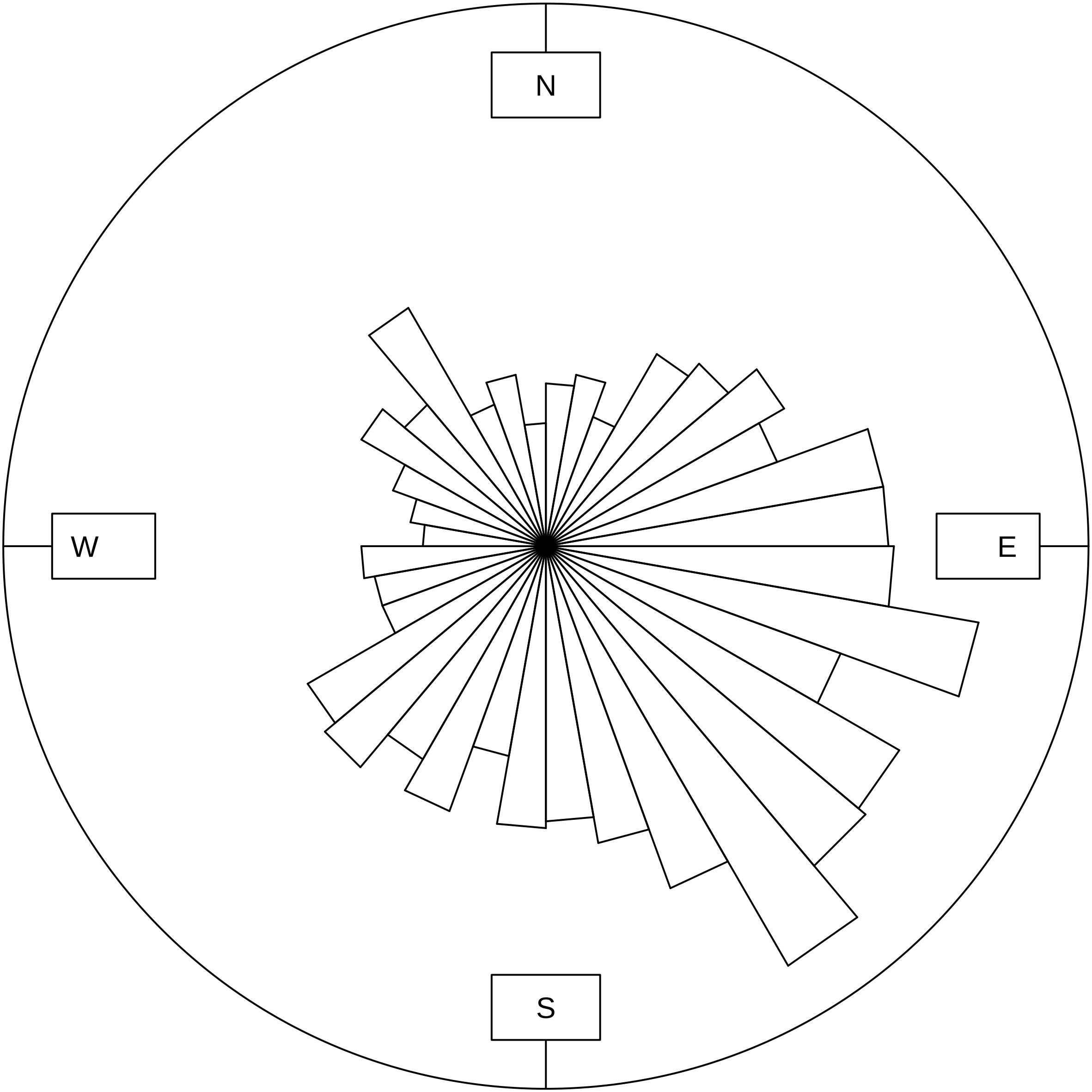

bootstrap 869

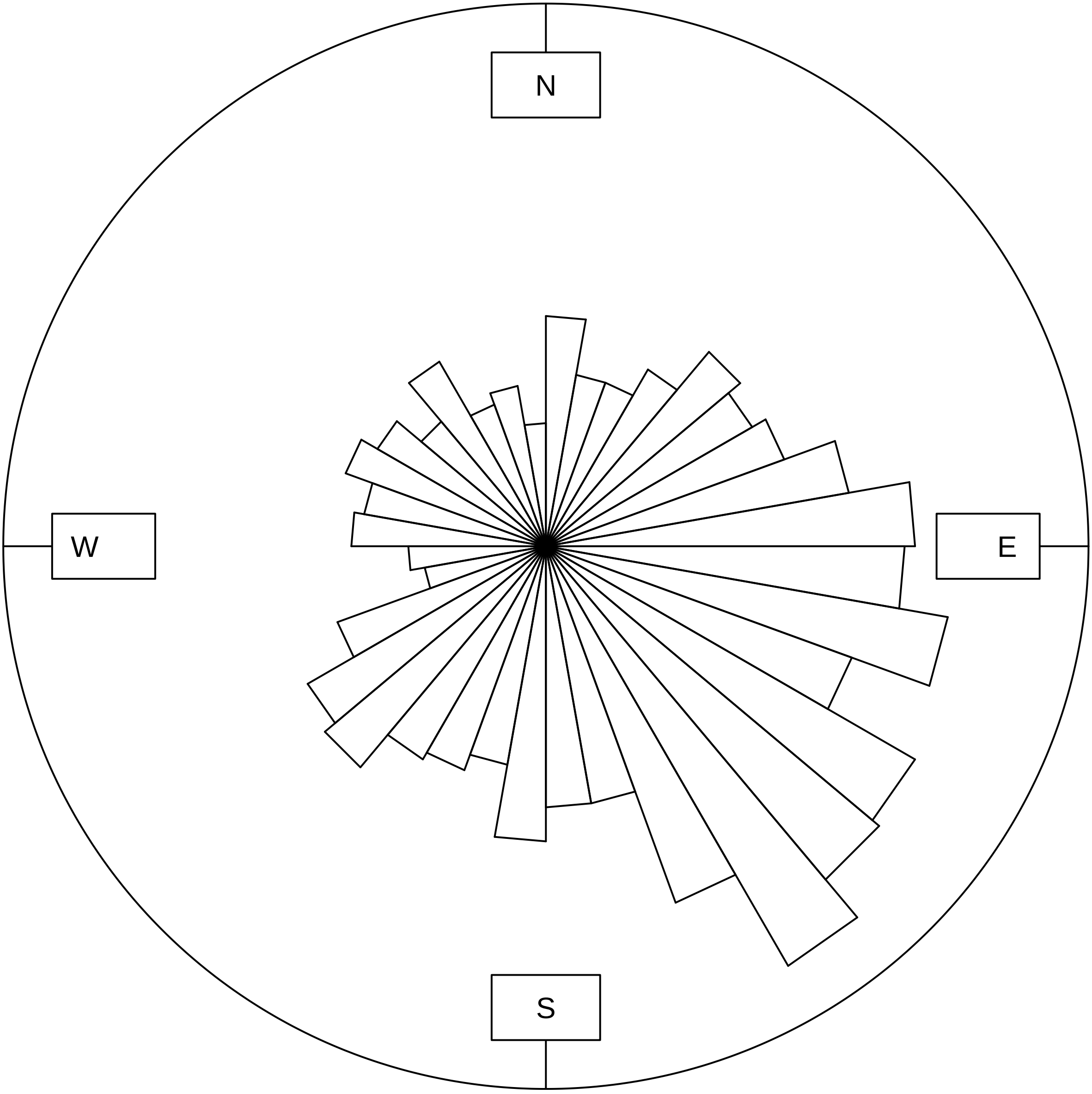

bootstrap 870

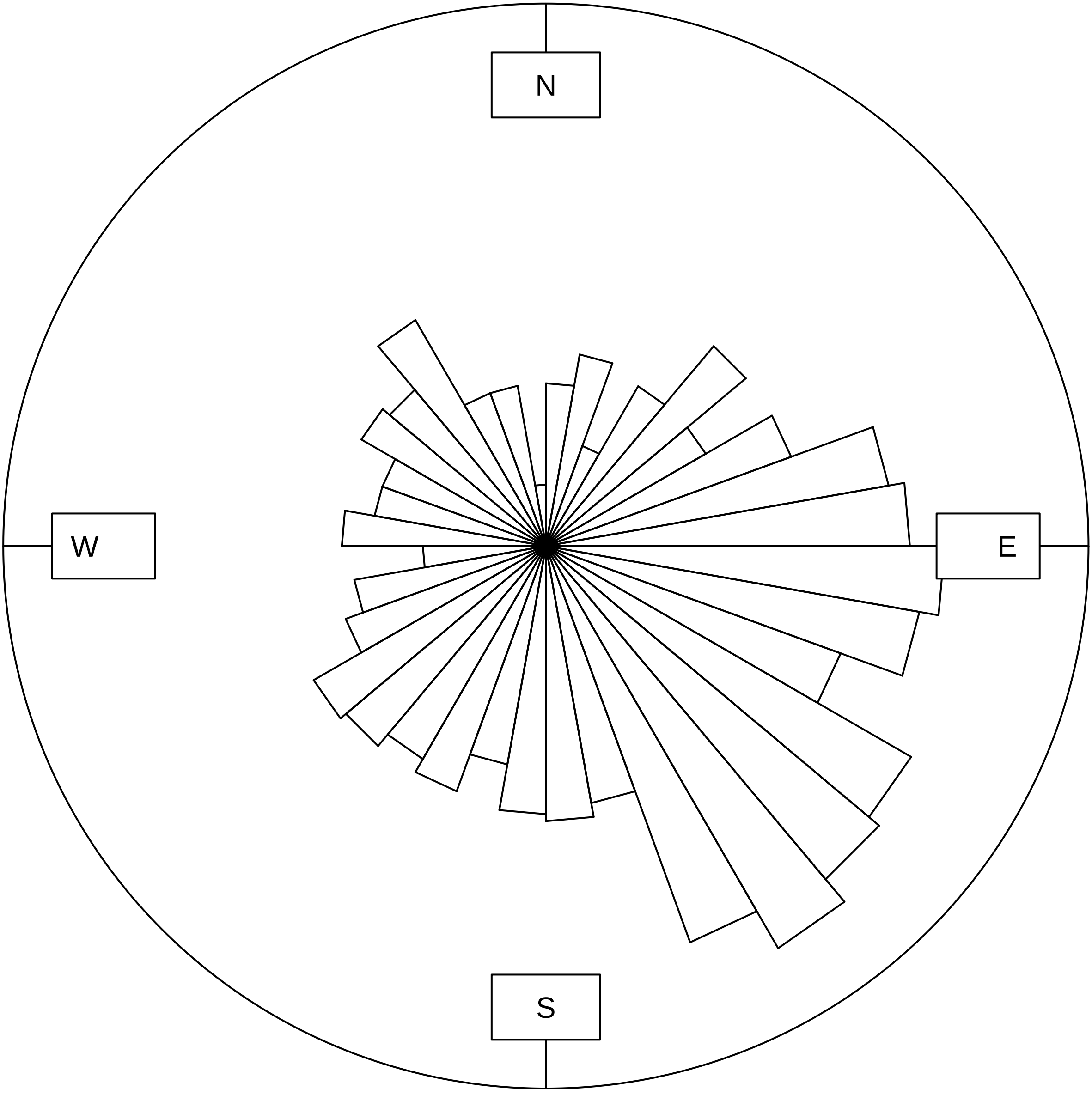

bootstrap 871

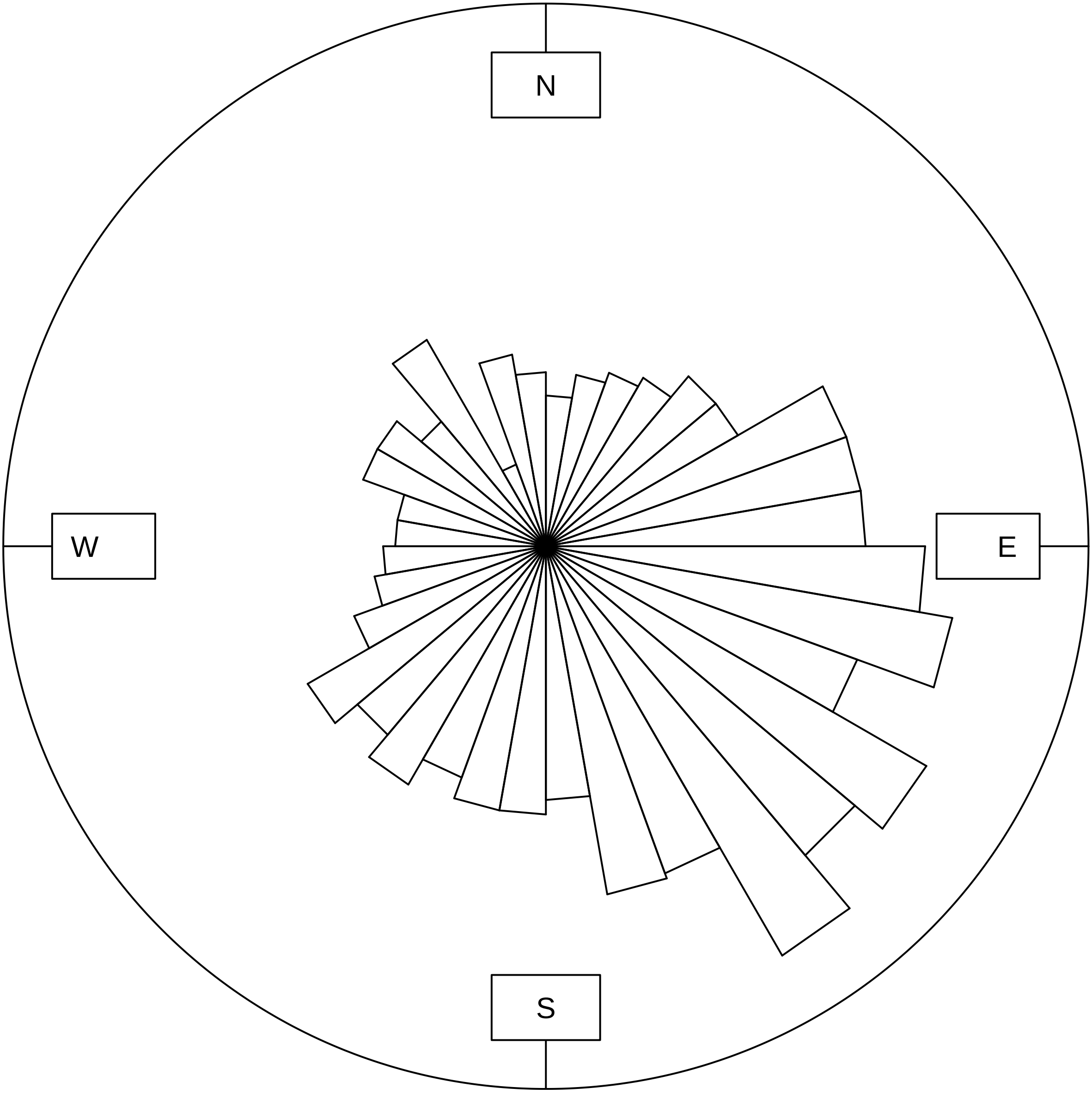

bootstrap 872

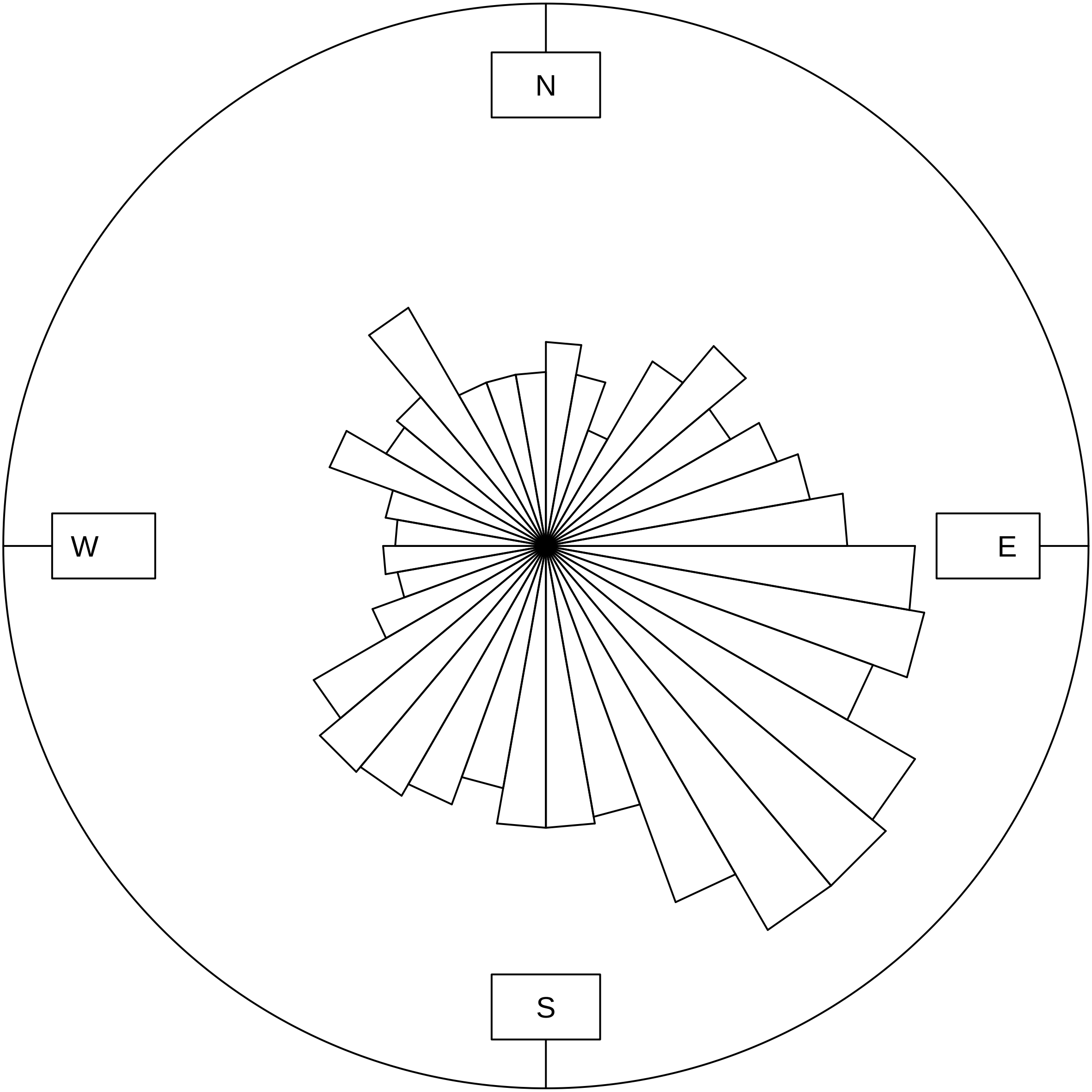

bootstrap 873

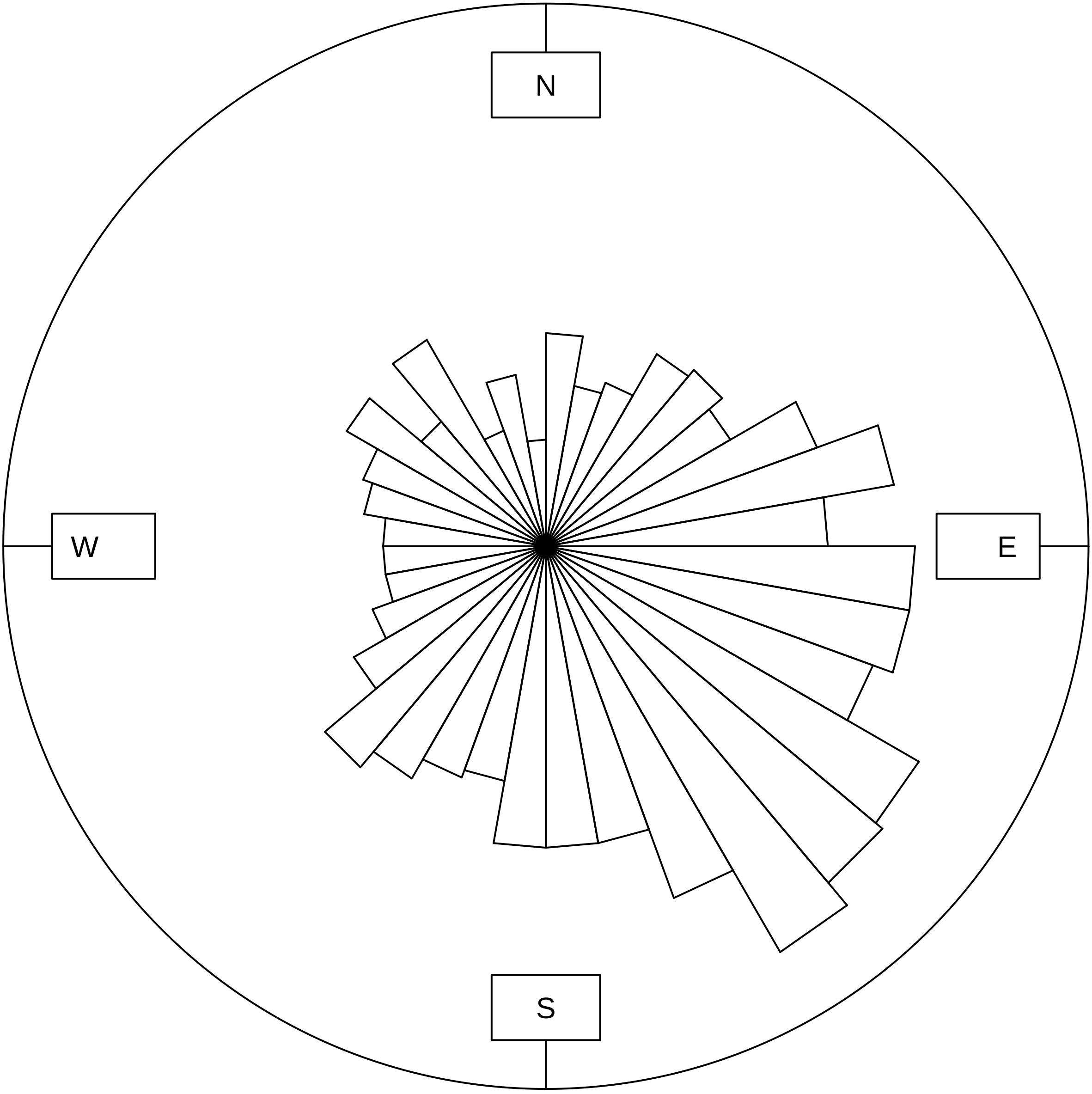

bootstrap 874

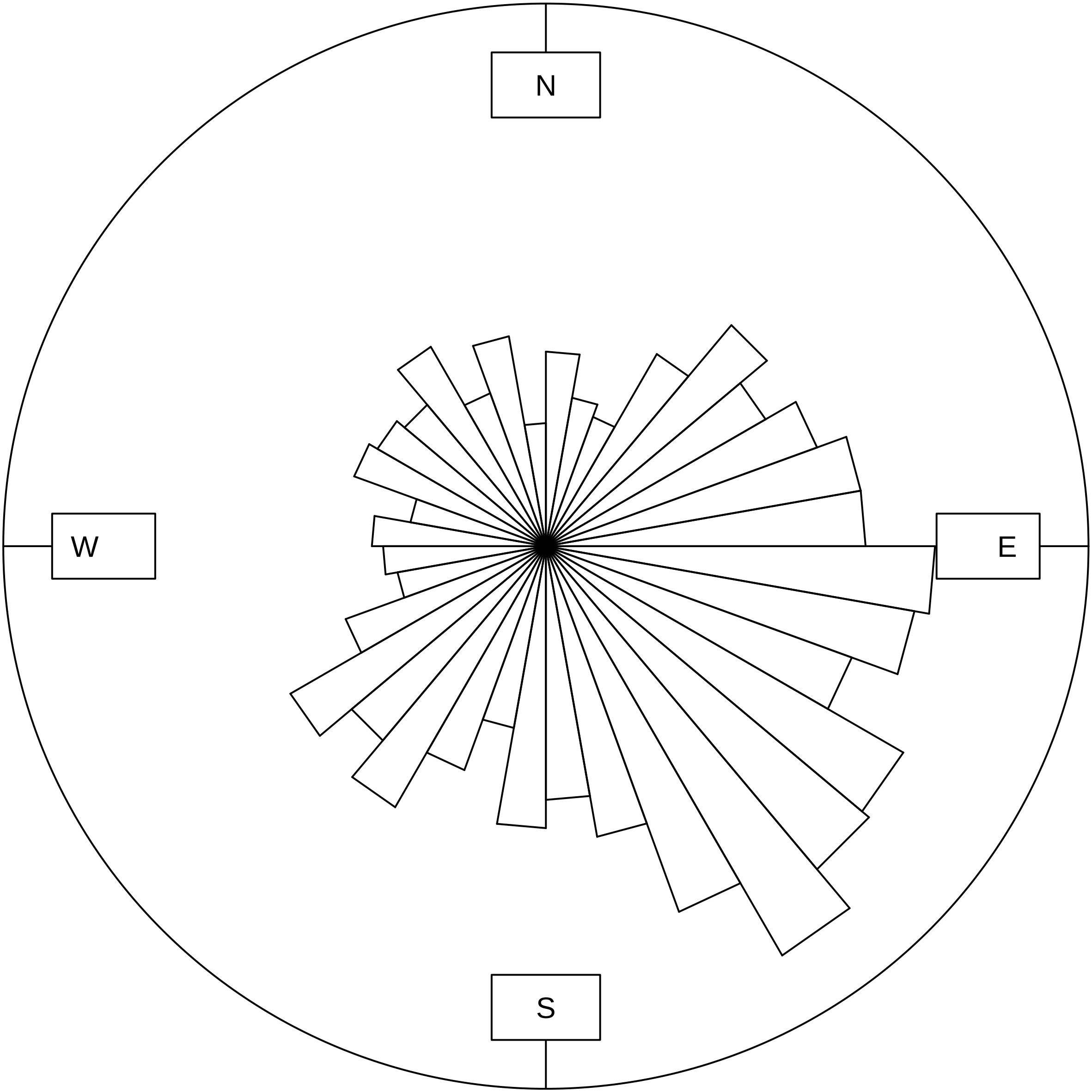

bootstrap 875

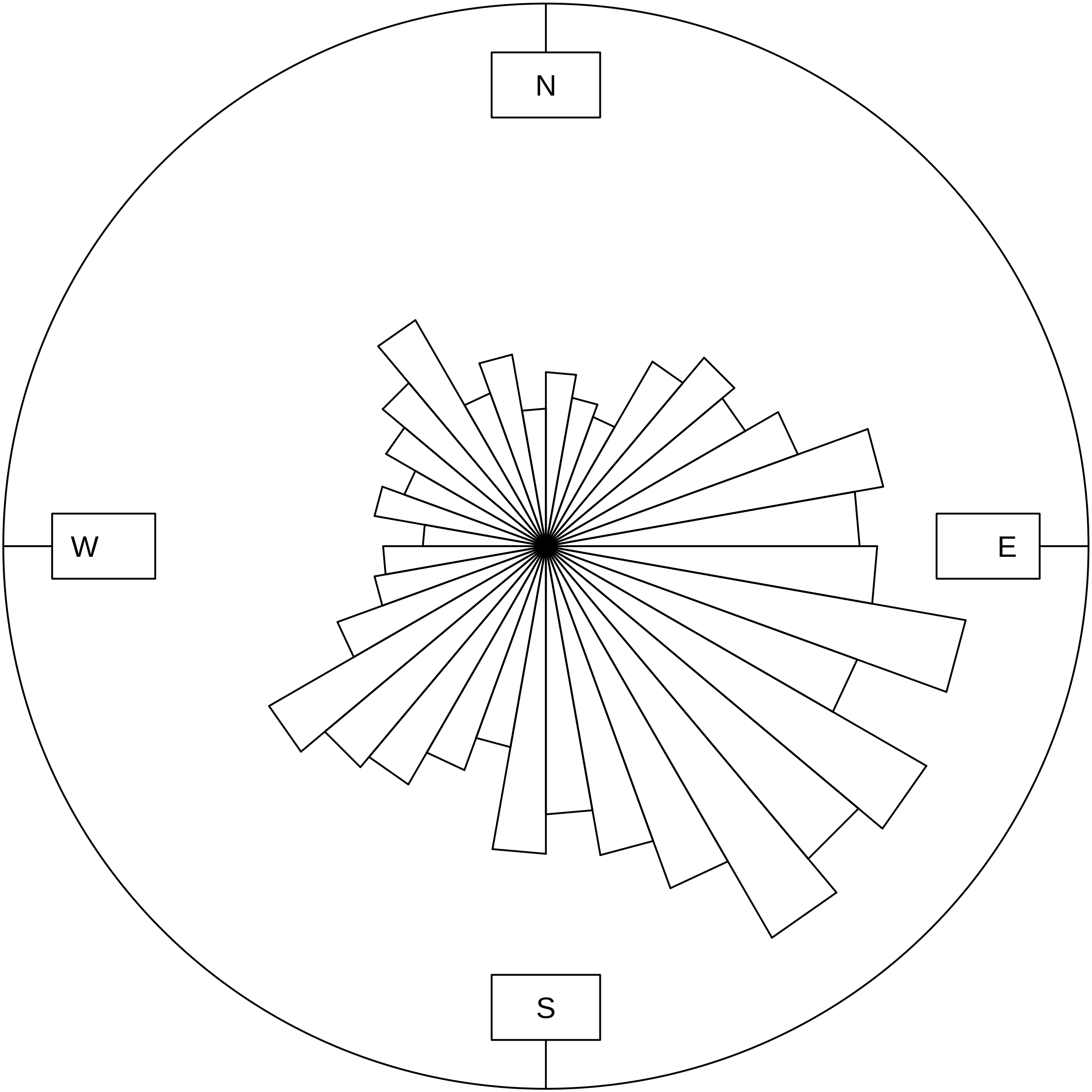

bootstrap 876

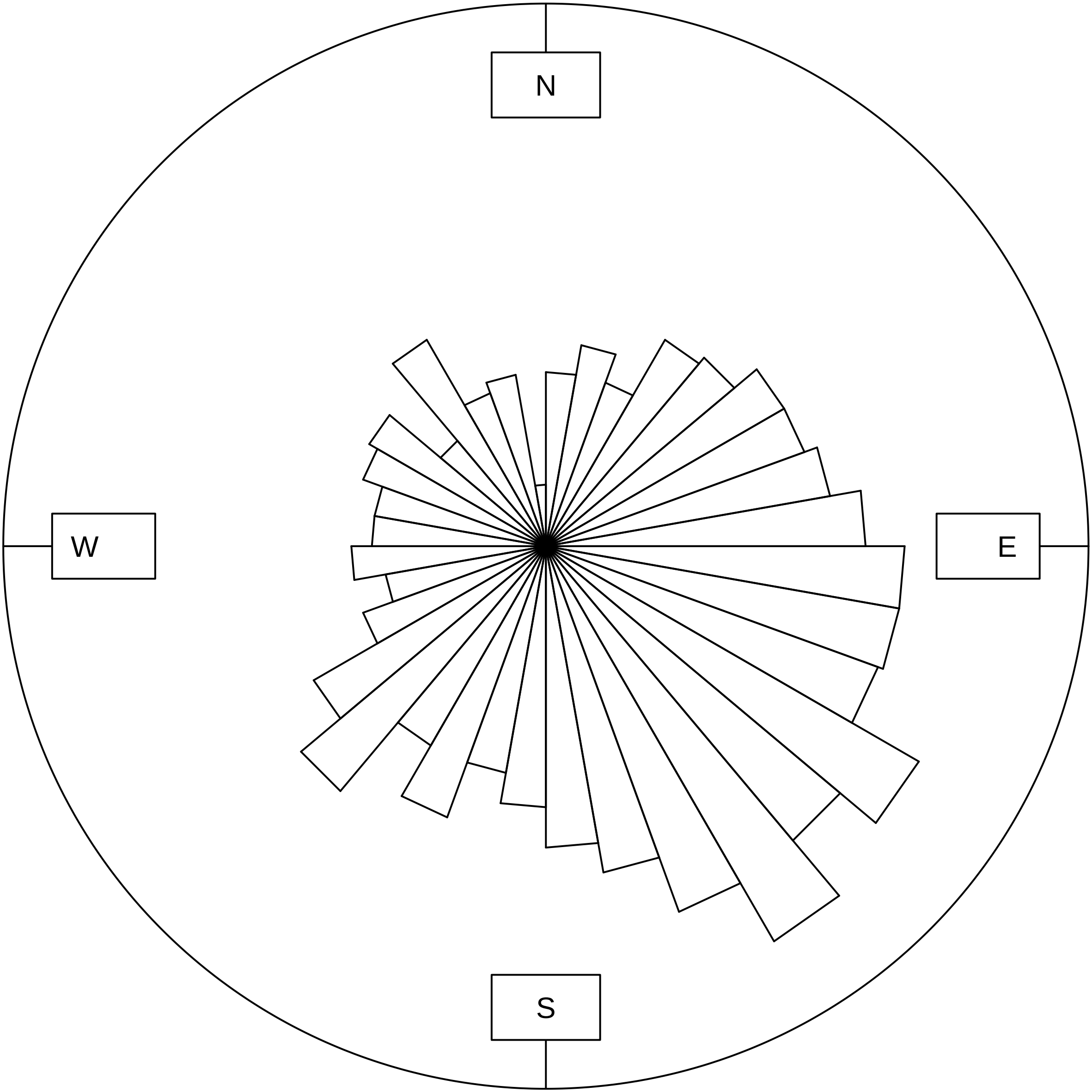

bootstrap 877

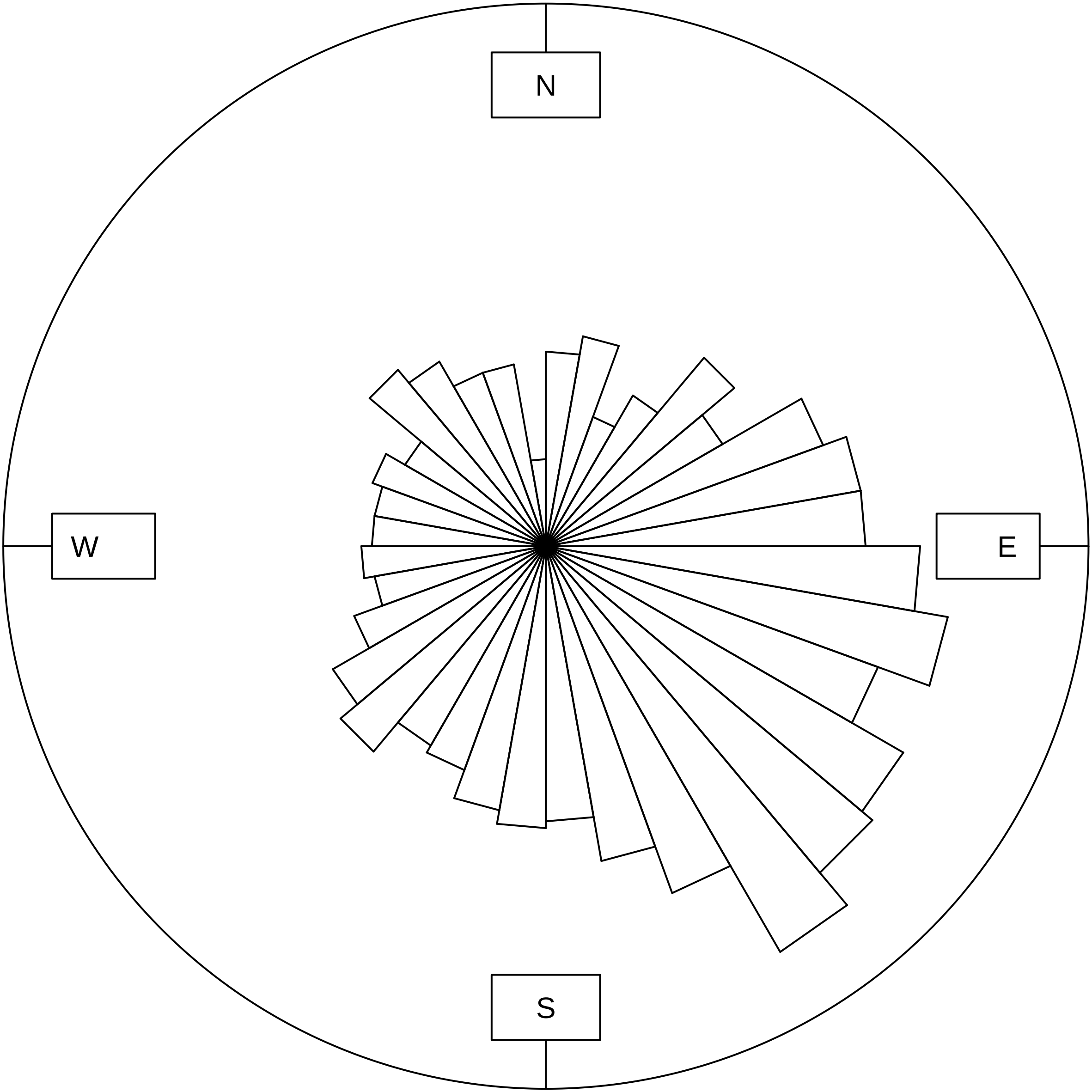

bootstrap 878

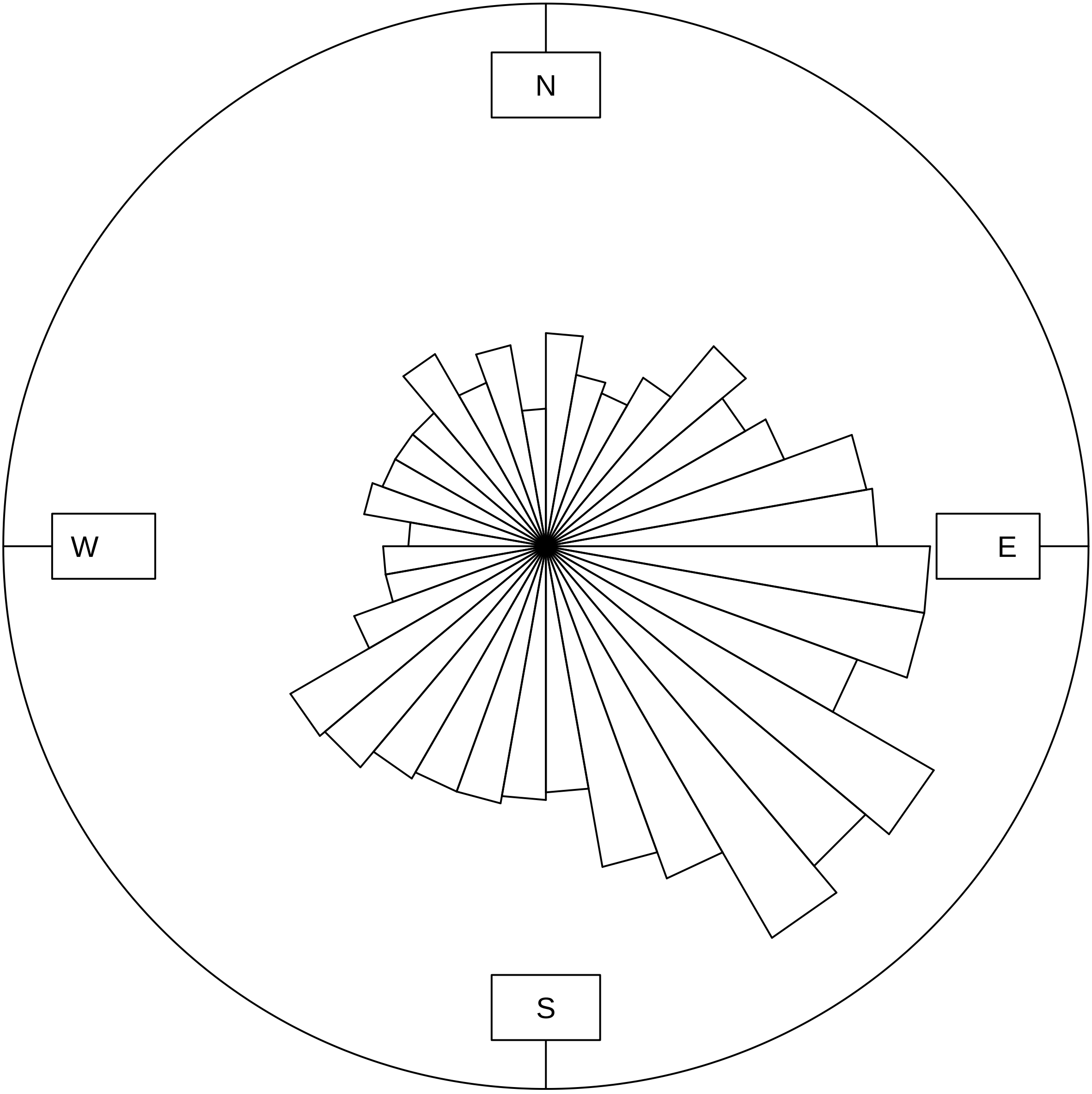

bootstrap 879

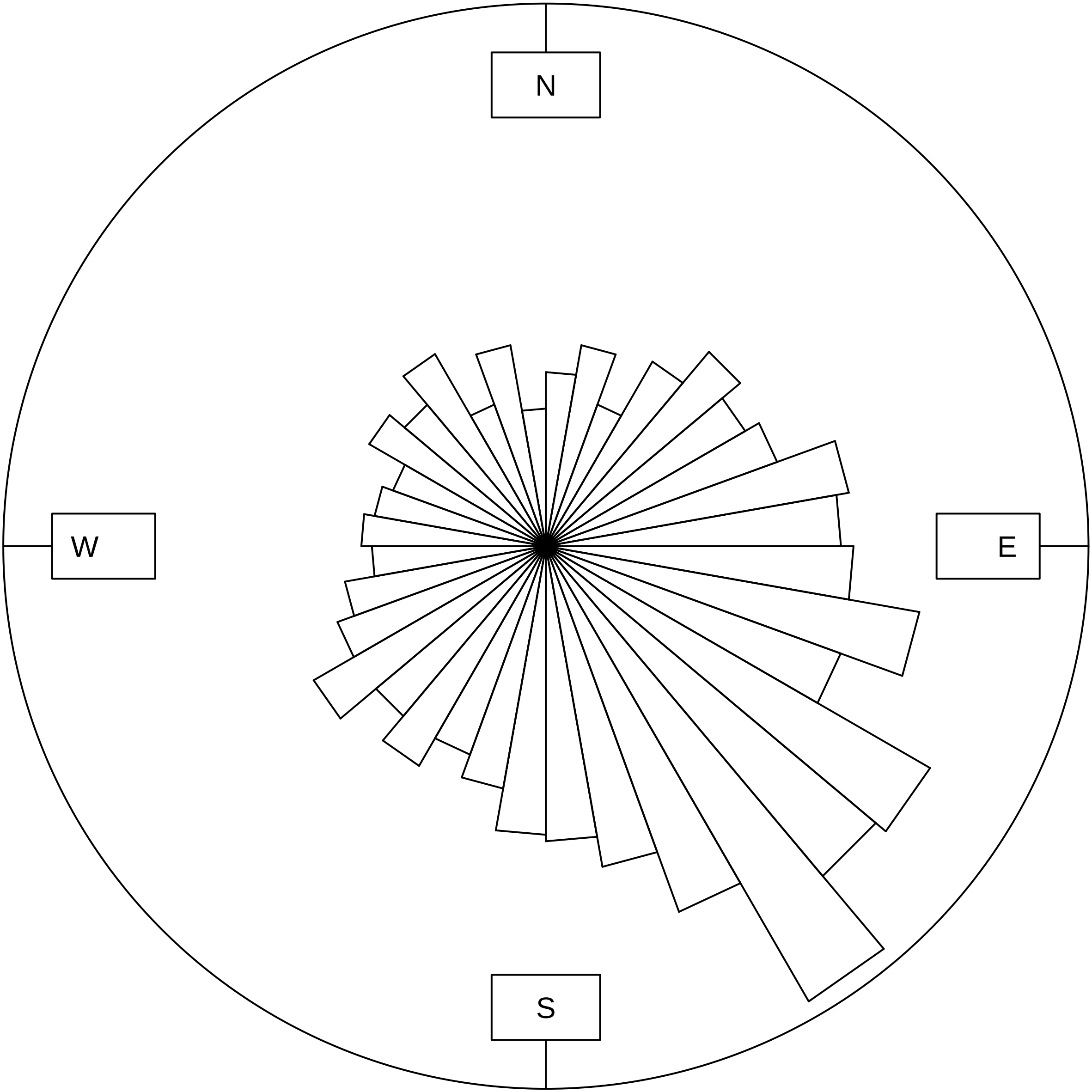

bootstrap 880

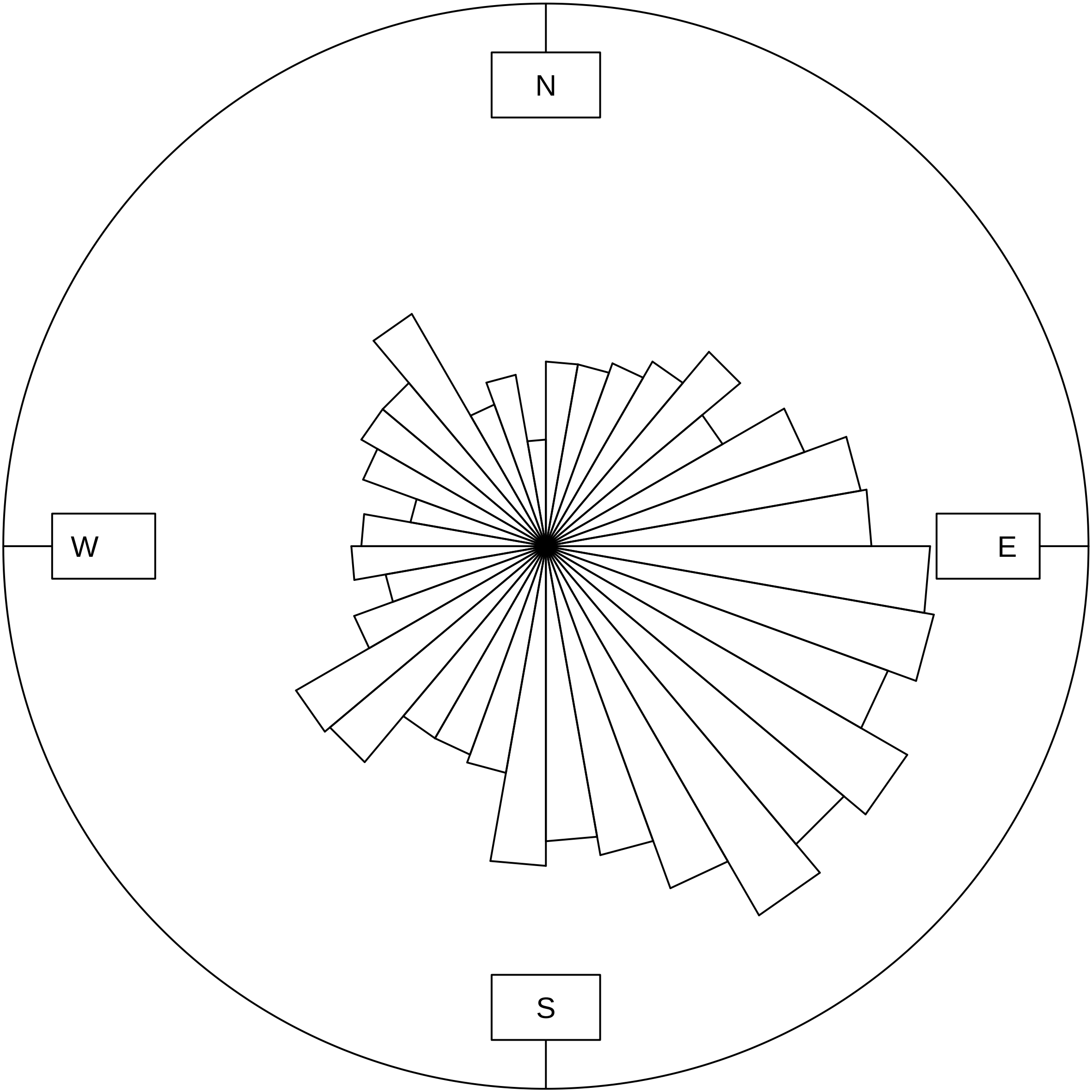

bootstrap 881

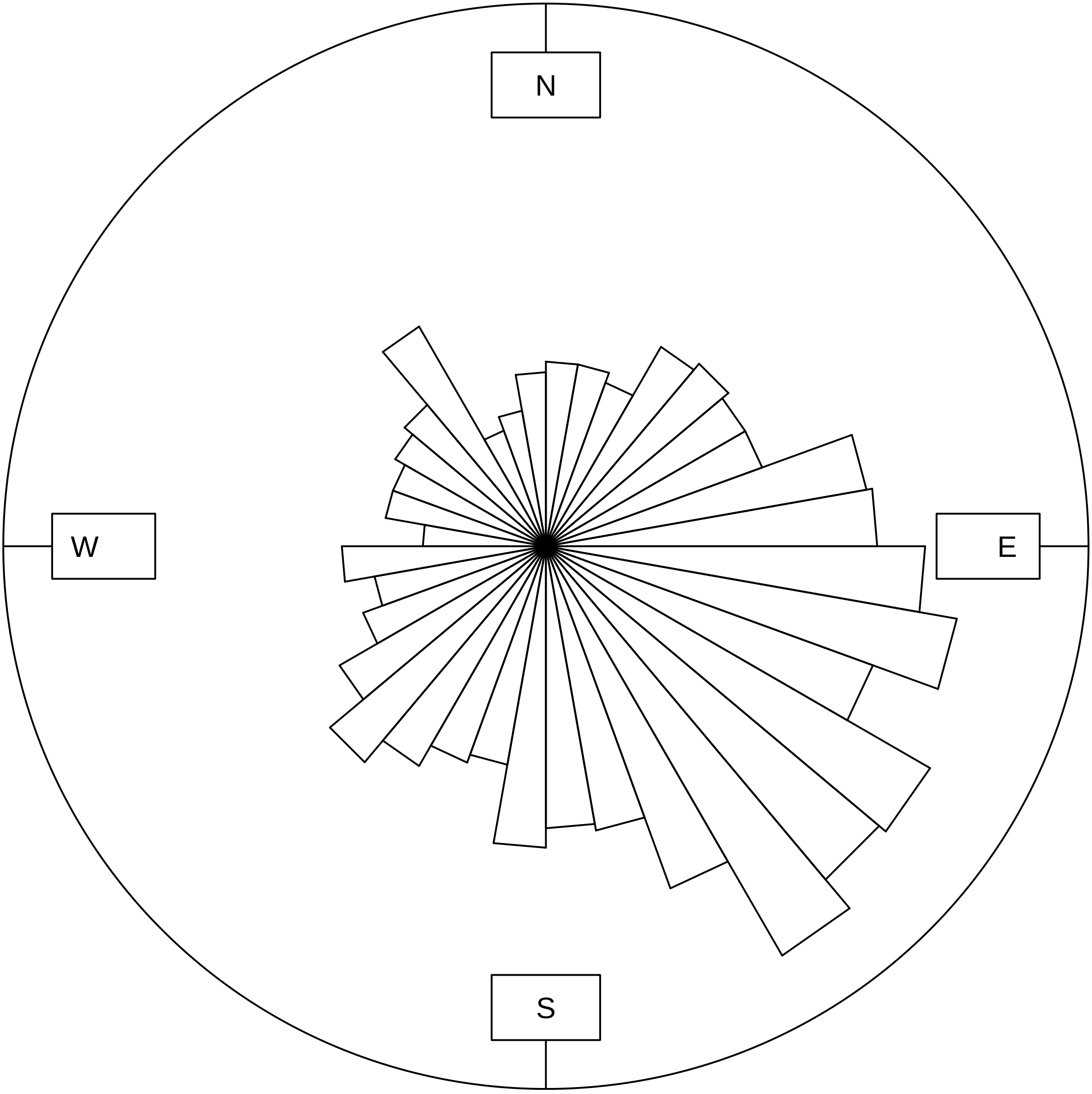

bootstrap 882

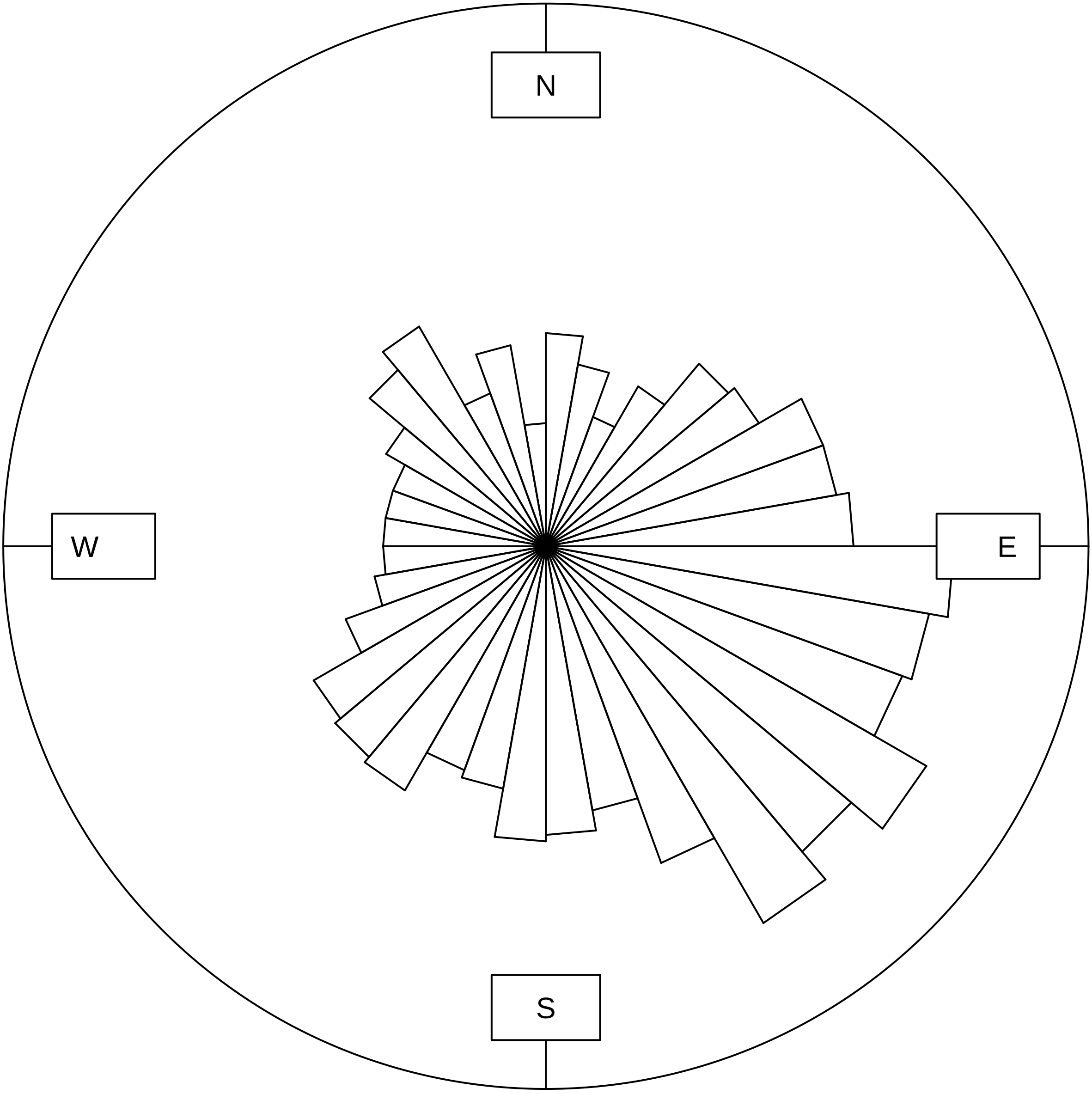

bootstrap 883

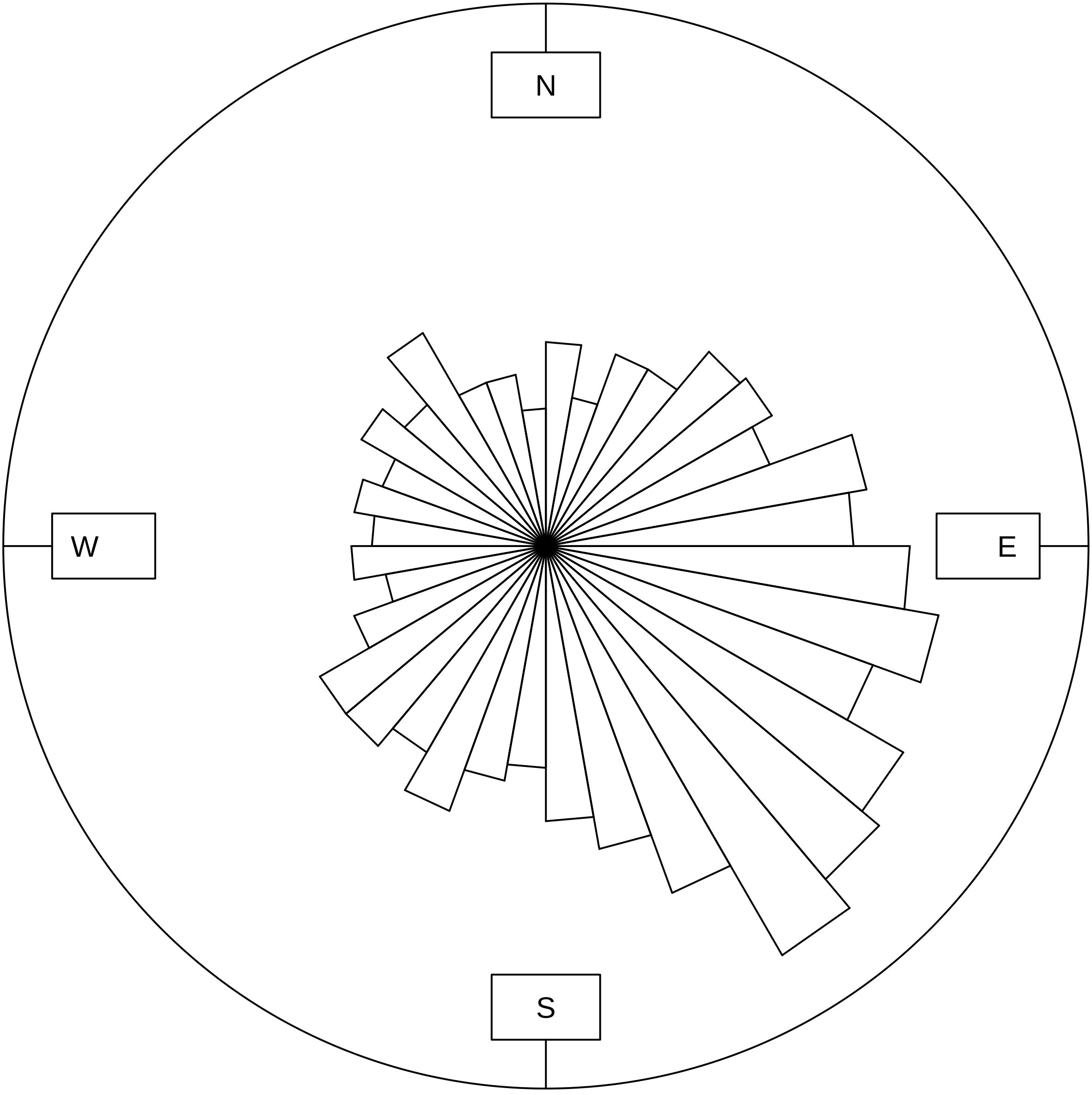

bootstrap 884

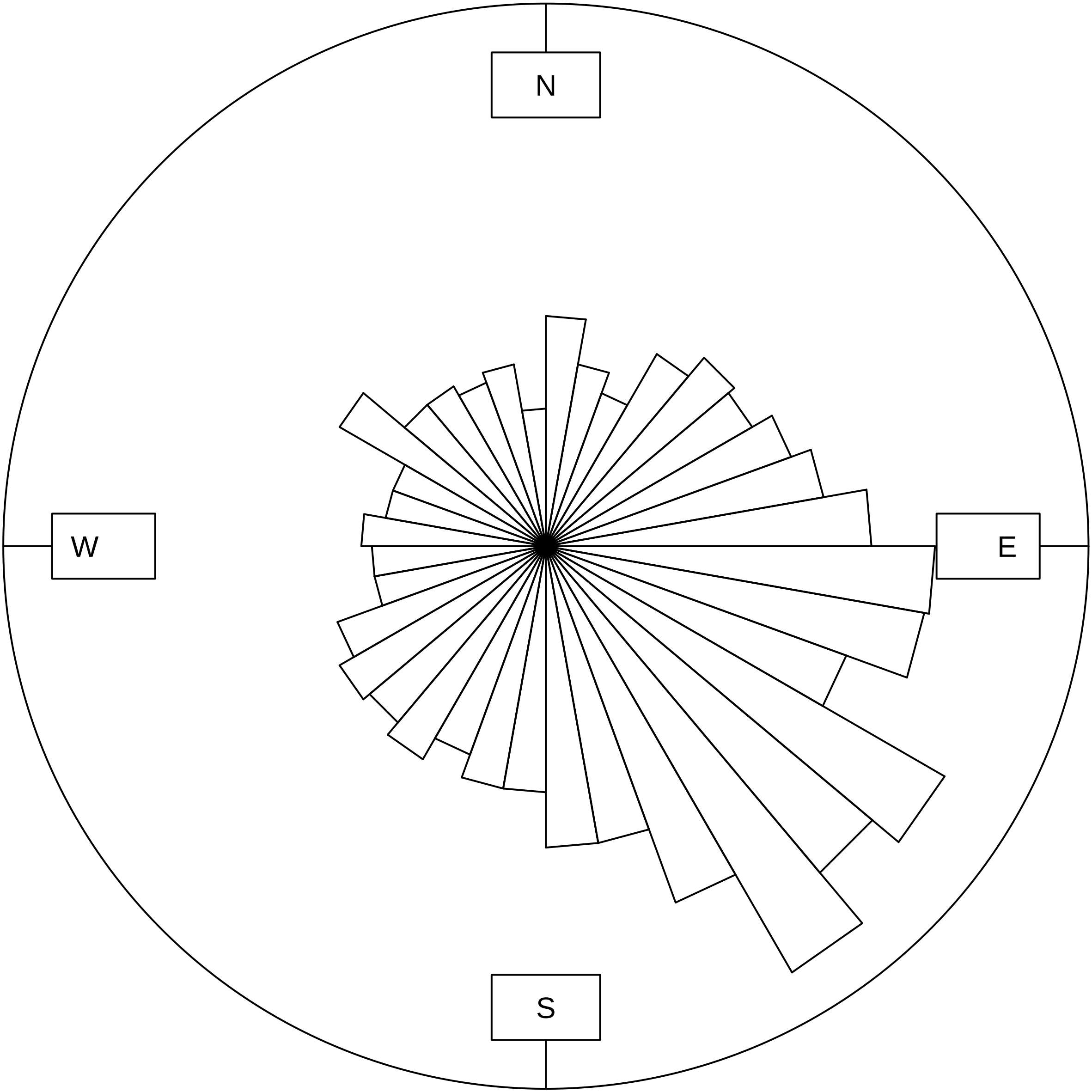

bootstrap 885

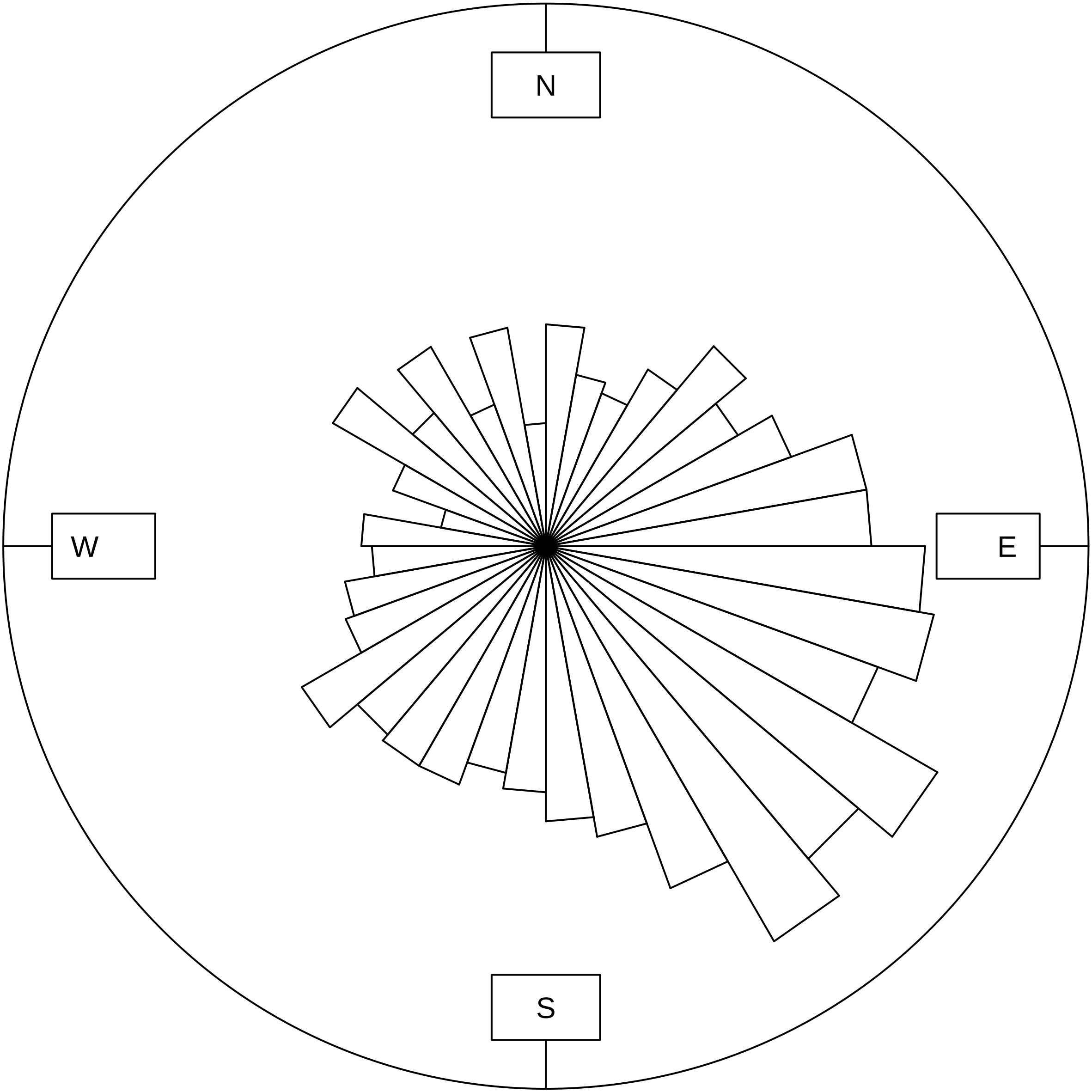

bootstrap 886

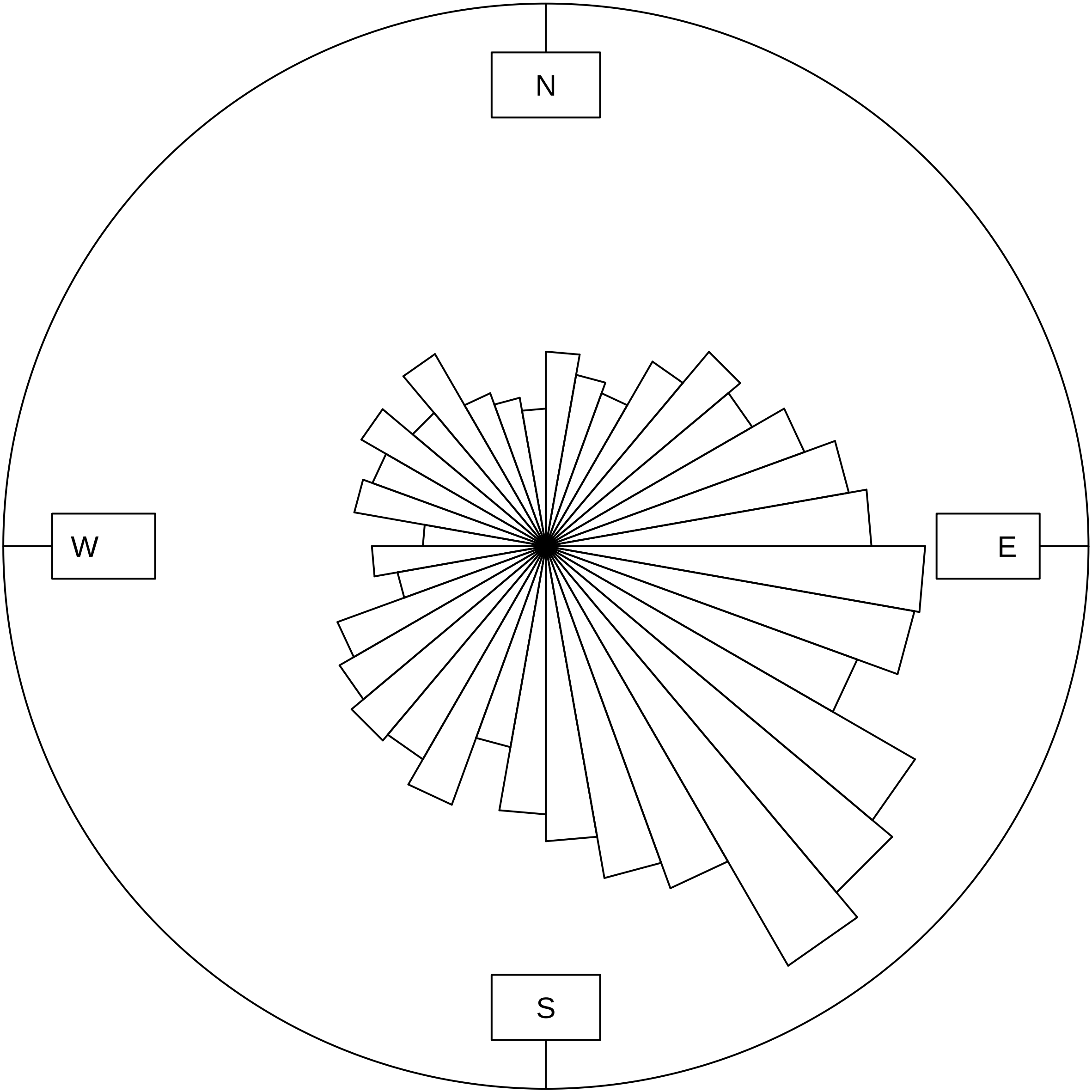

bootstrap 887

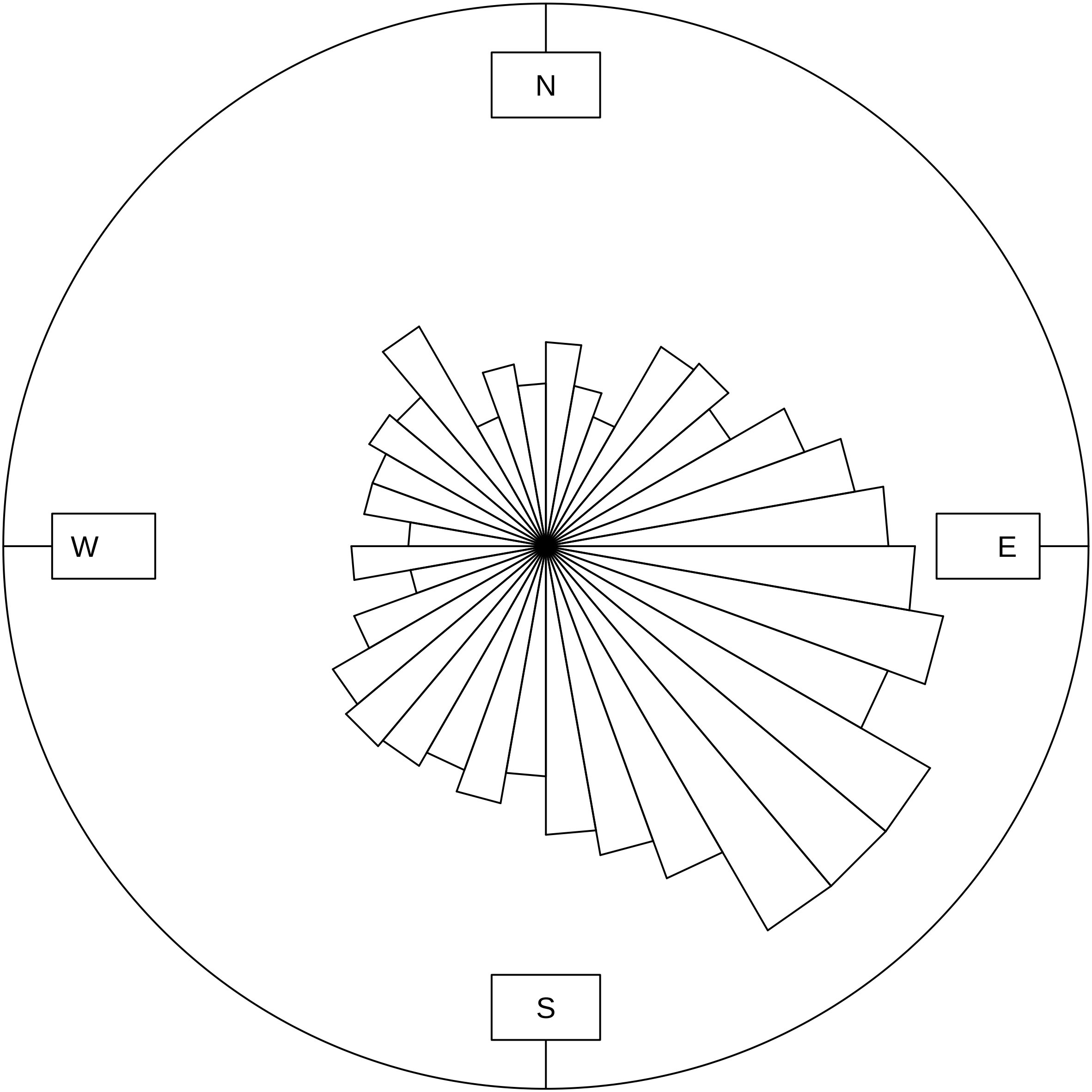

bootstrap 888

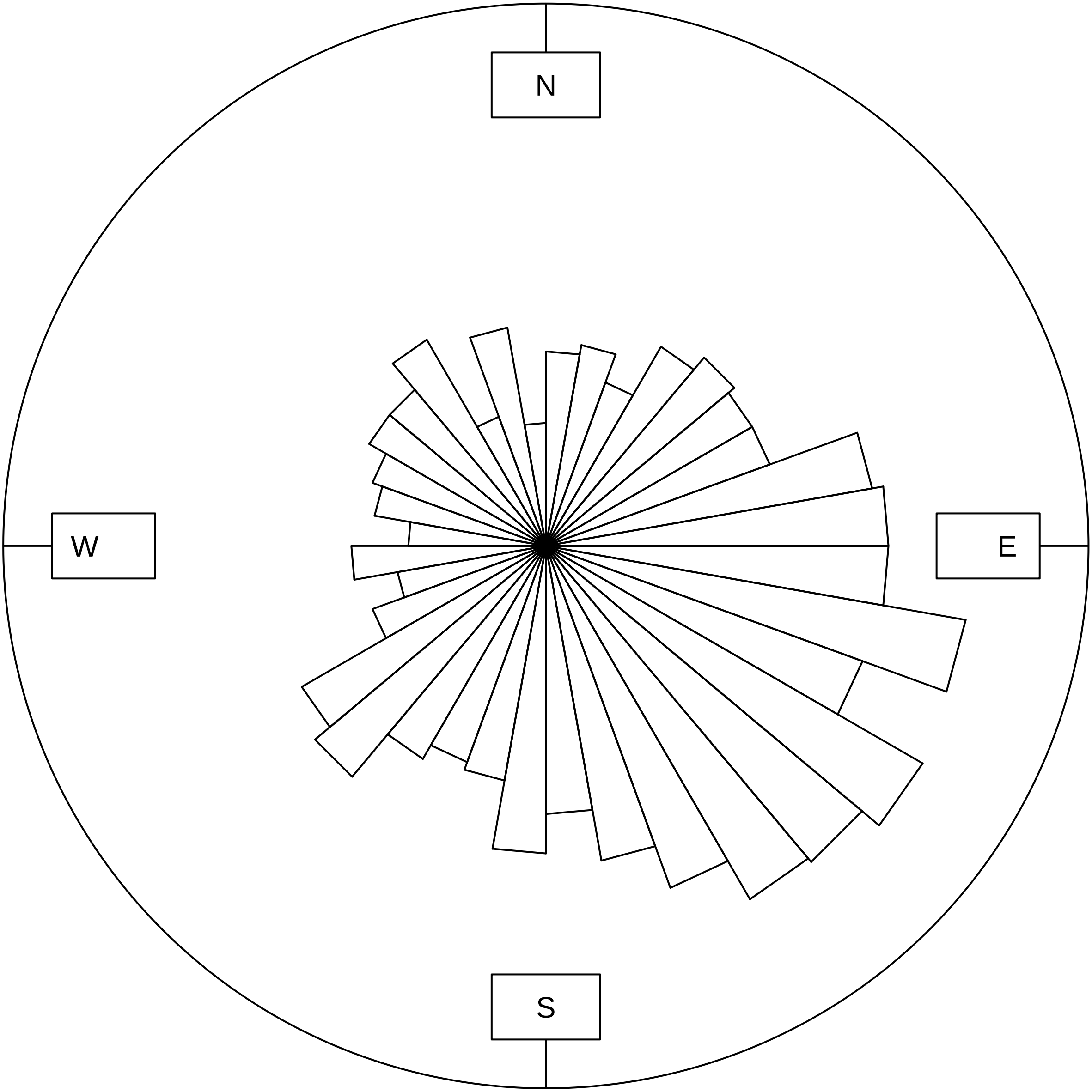

bootstrap 889

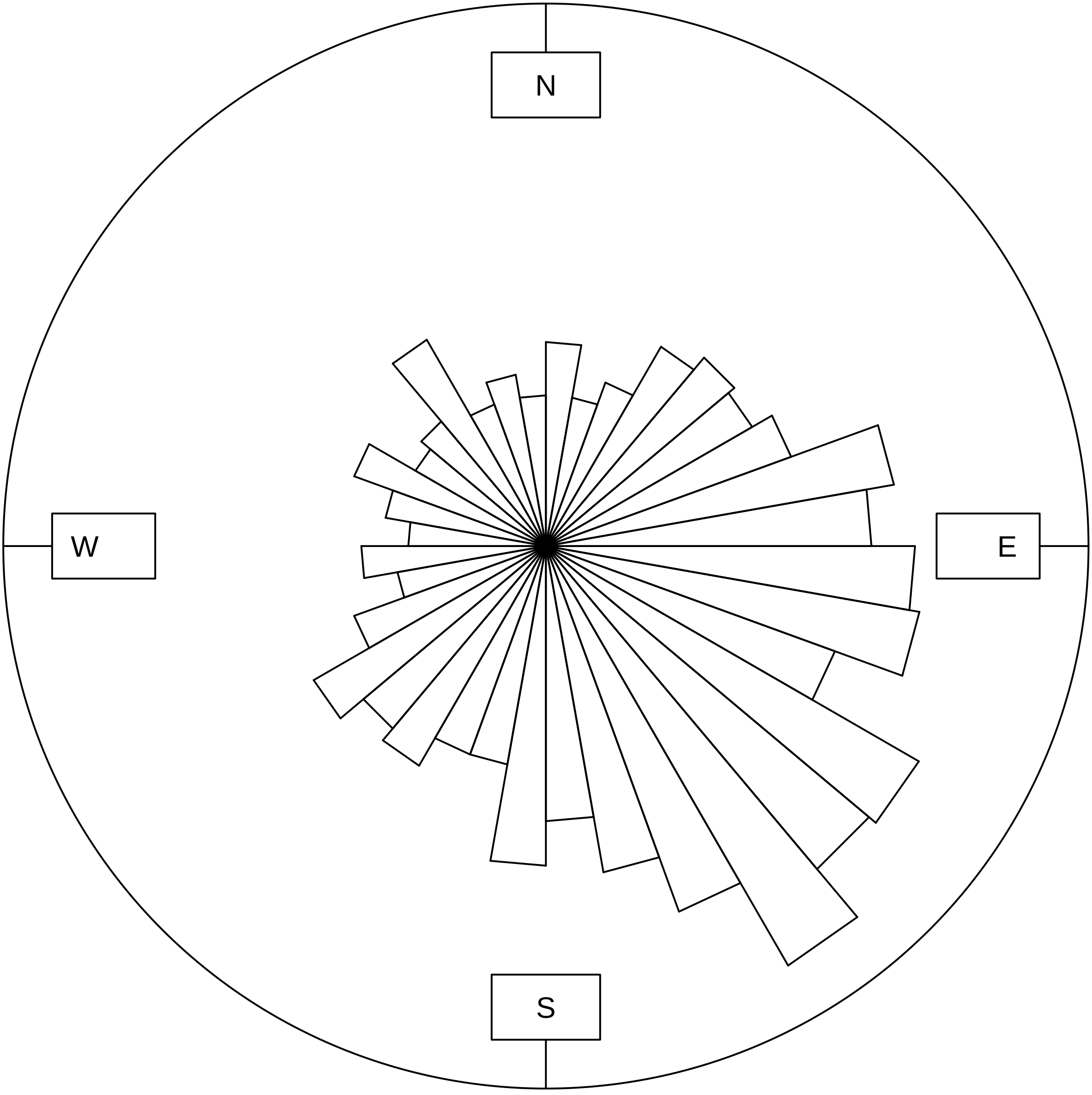

bootstrap 890

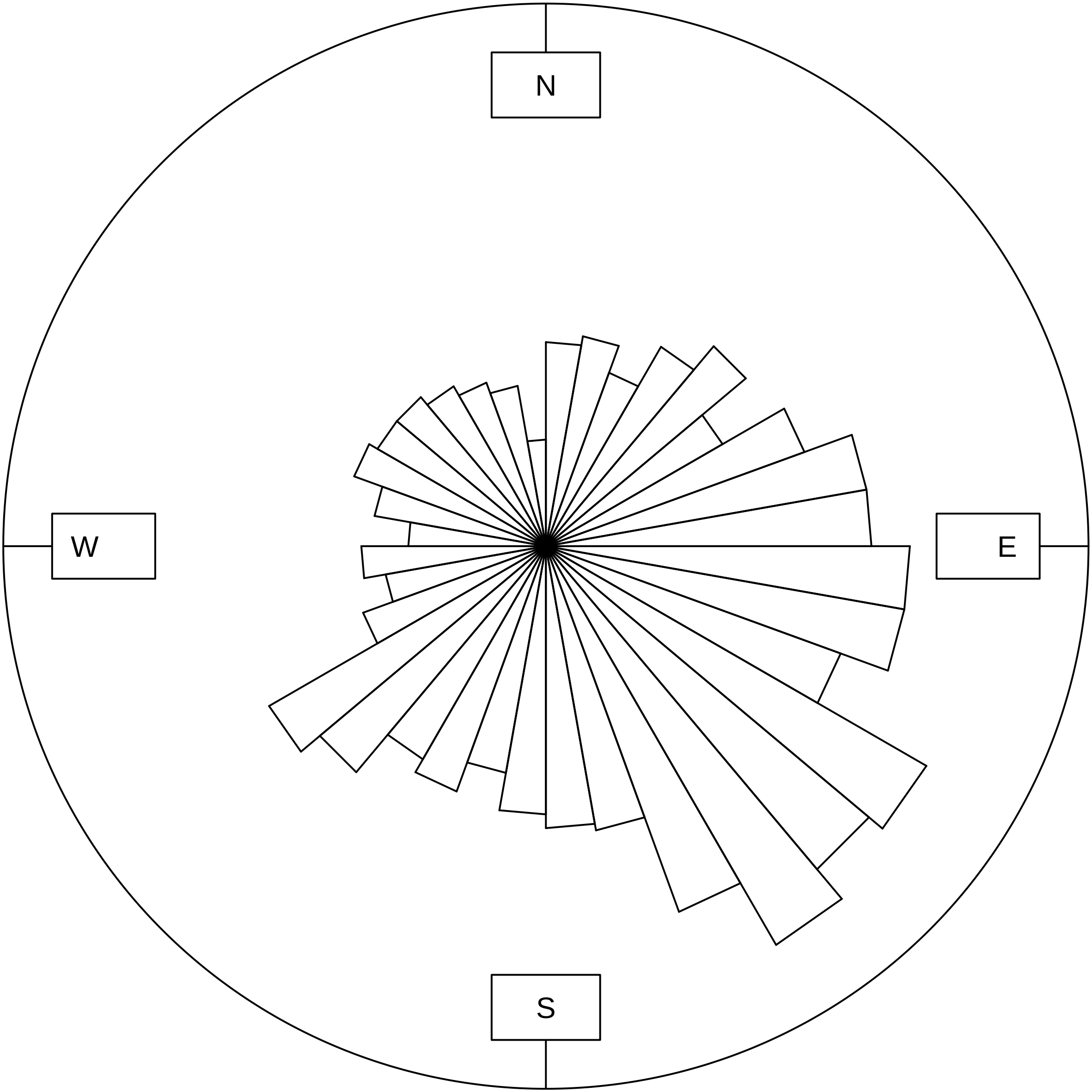

bootstrap 891

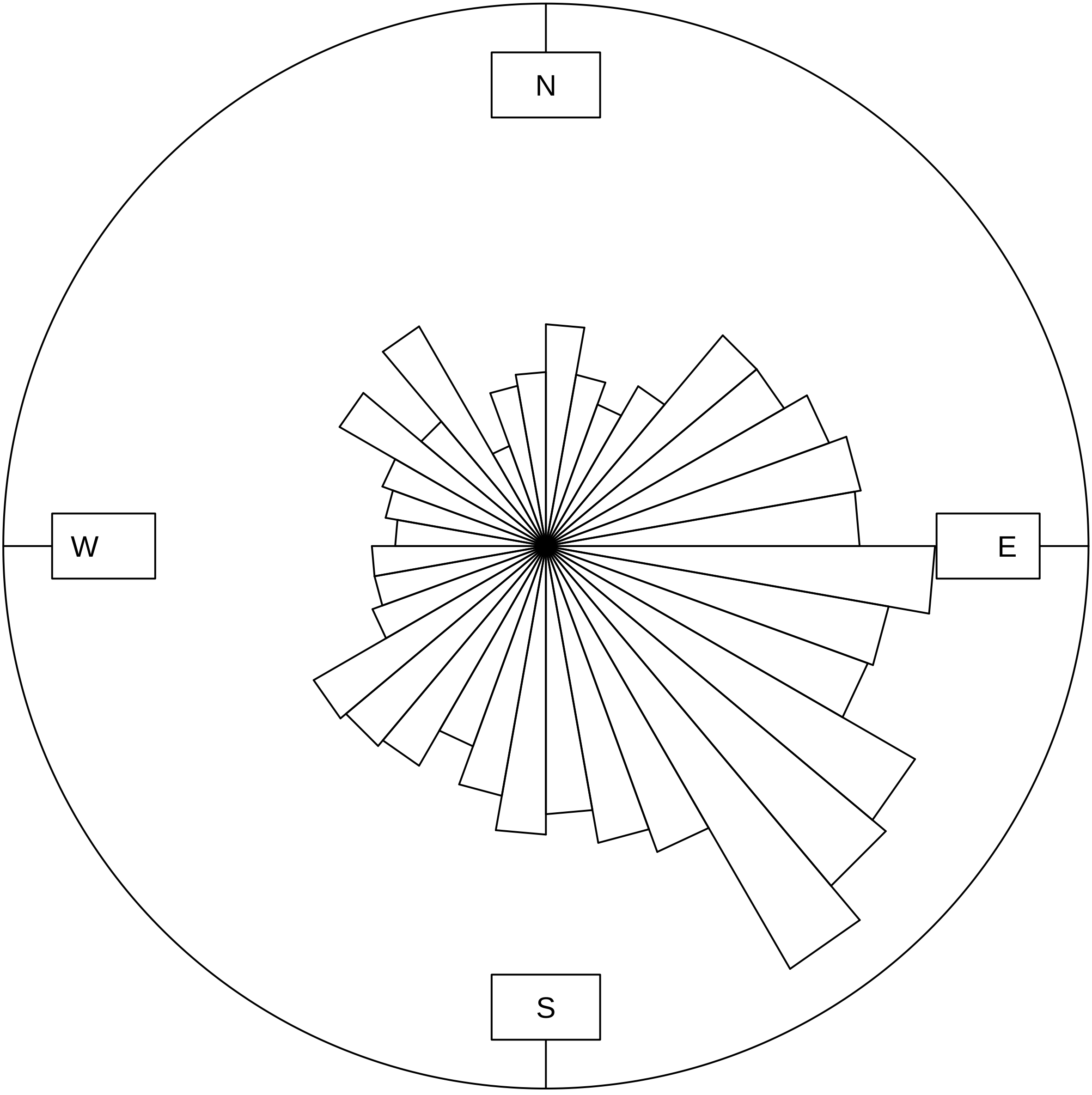

bootstrap 892

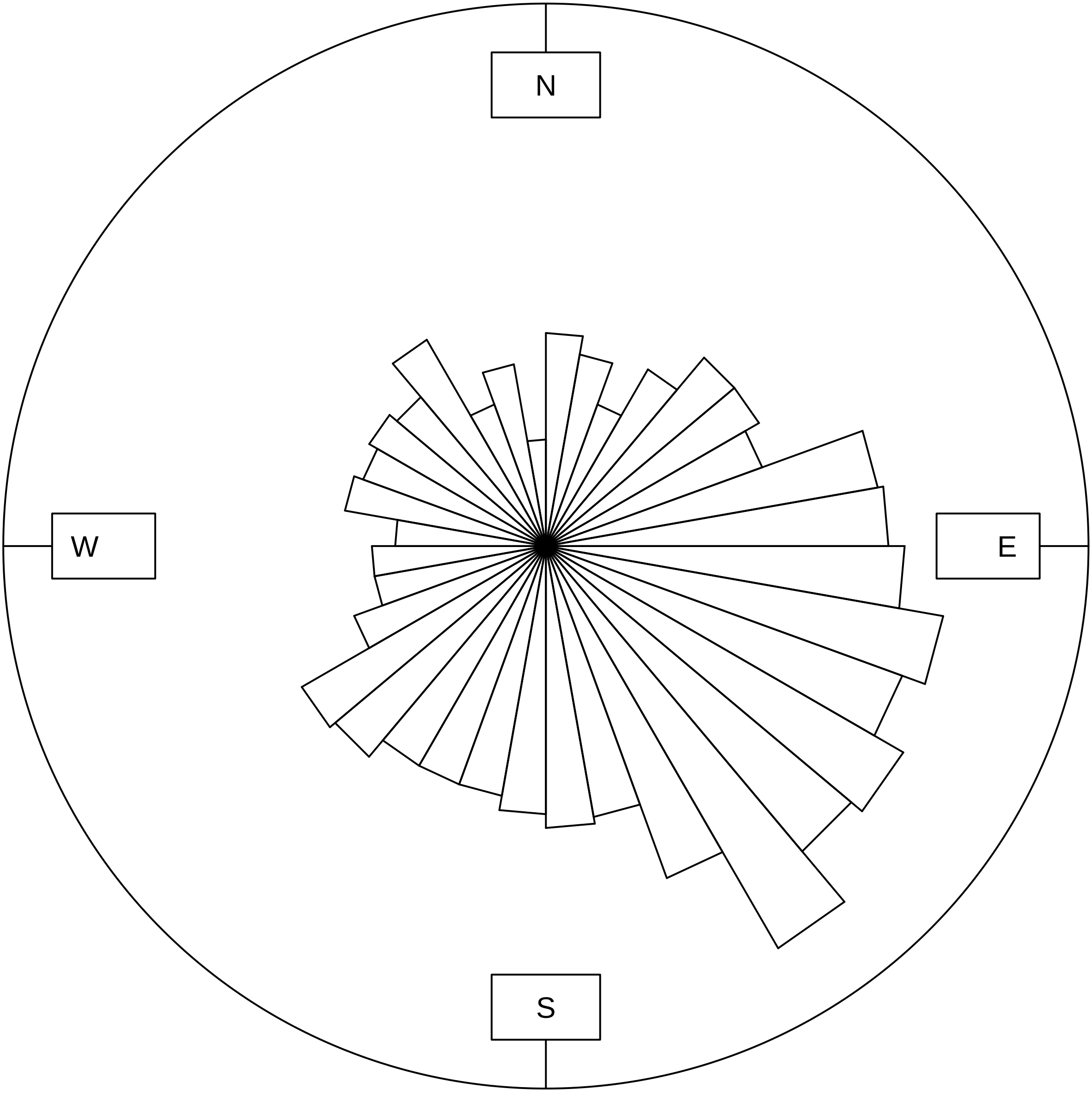

bootstrap 893

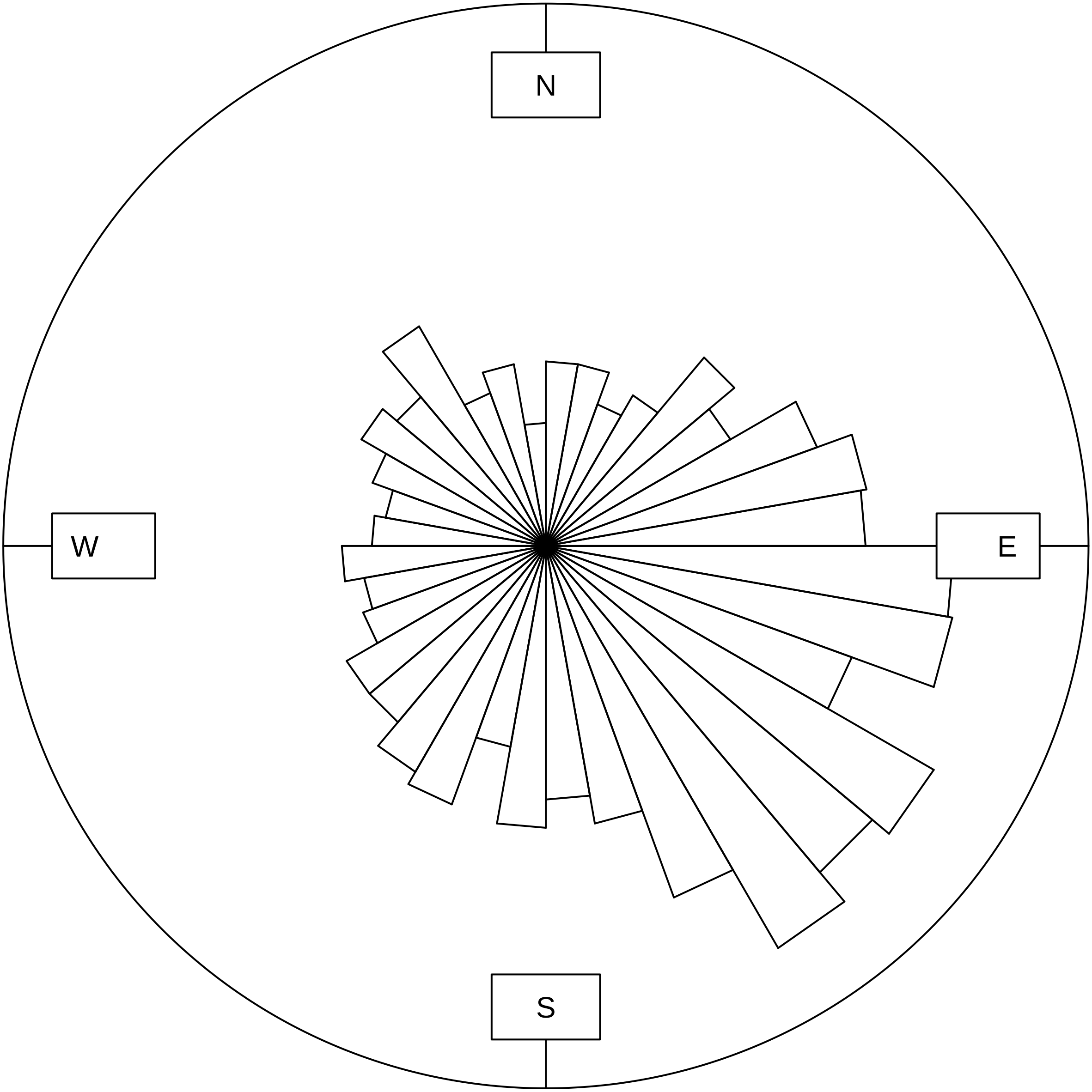

bootstrap 894

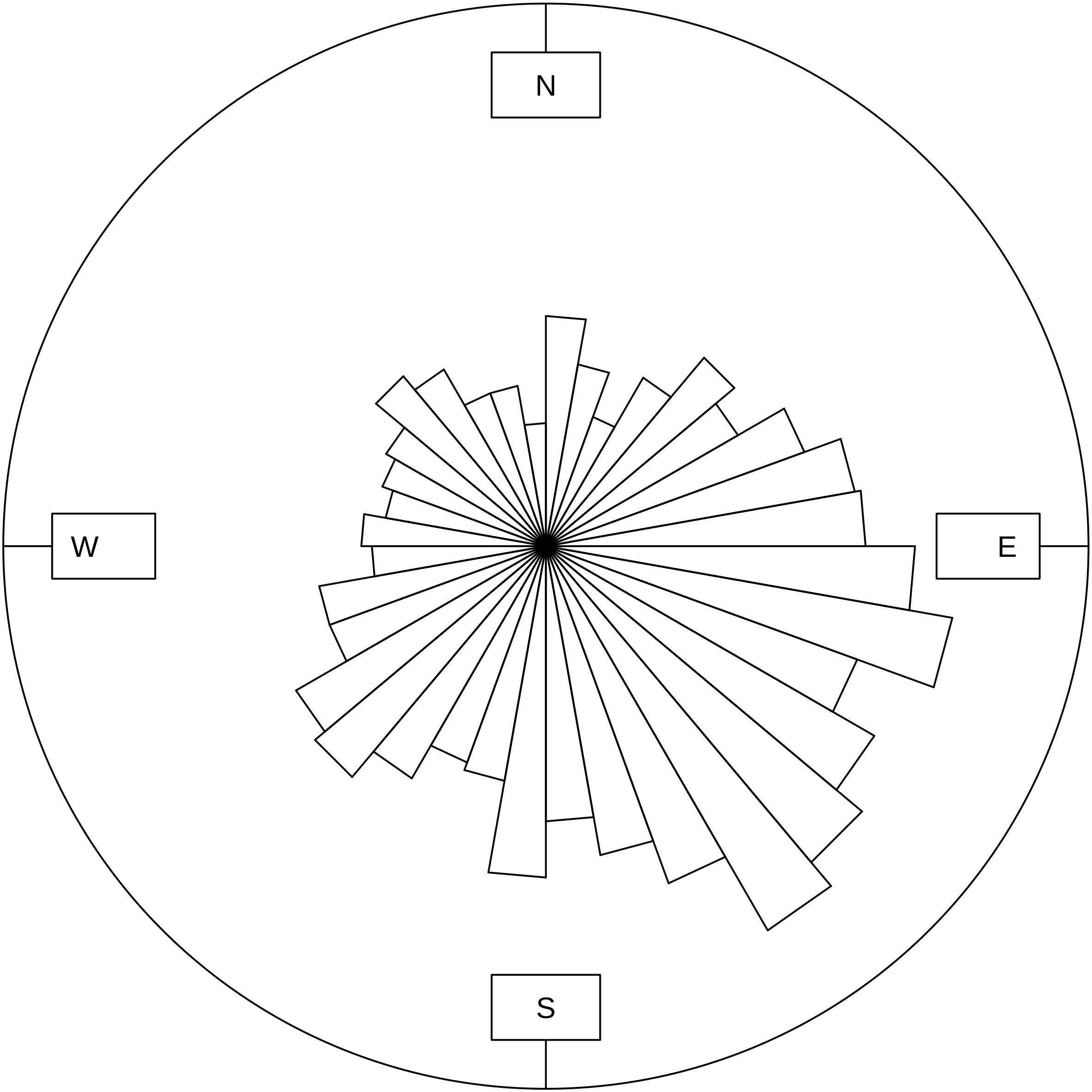

bootstrap 895

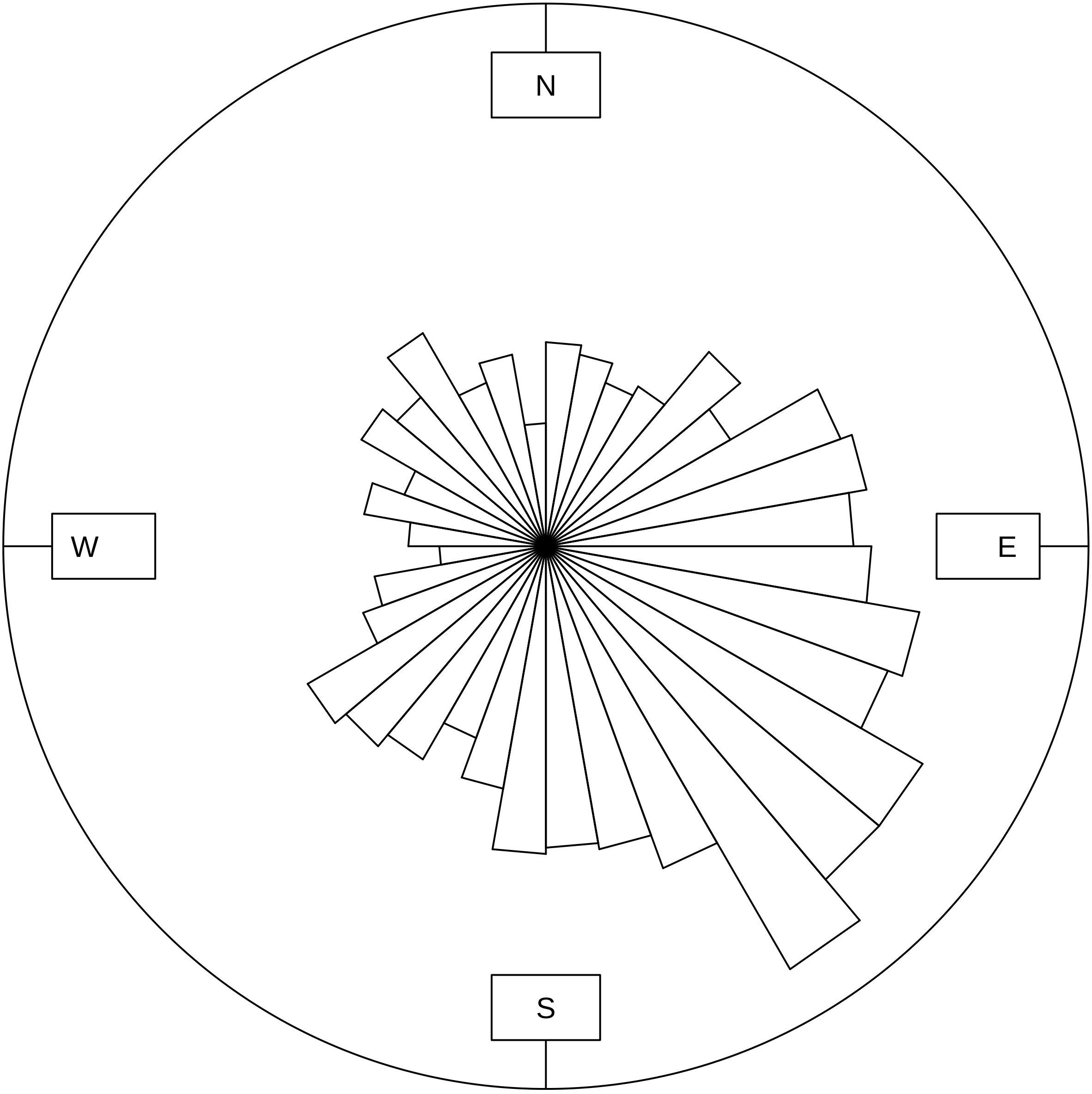

bootstrap 896

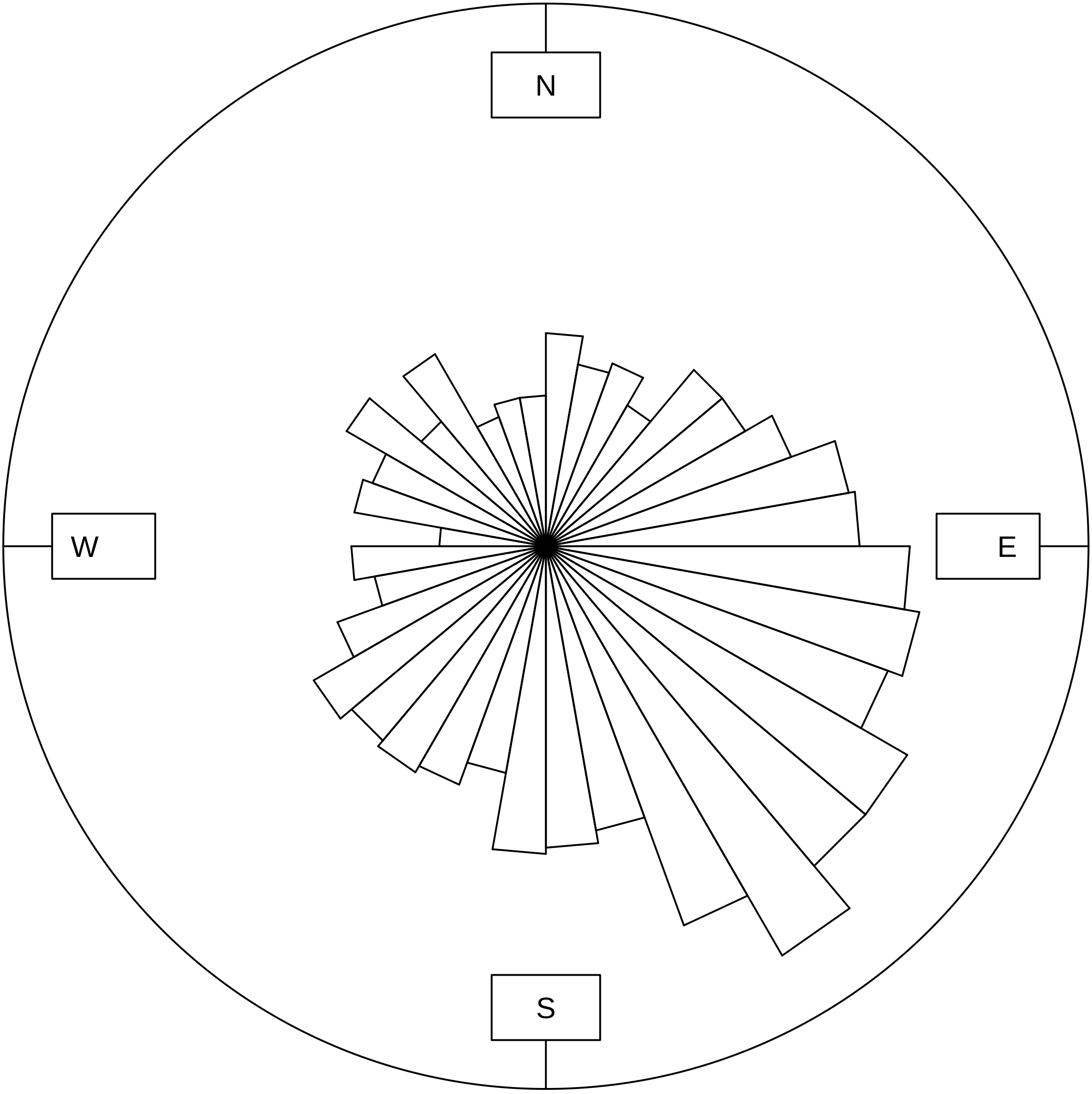

bootstrap 897

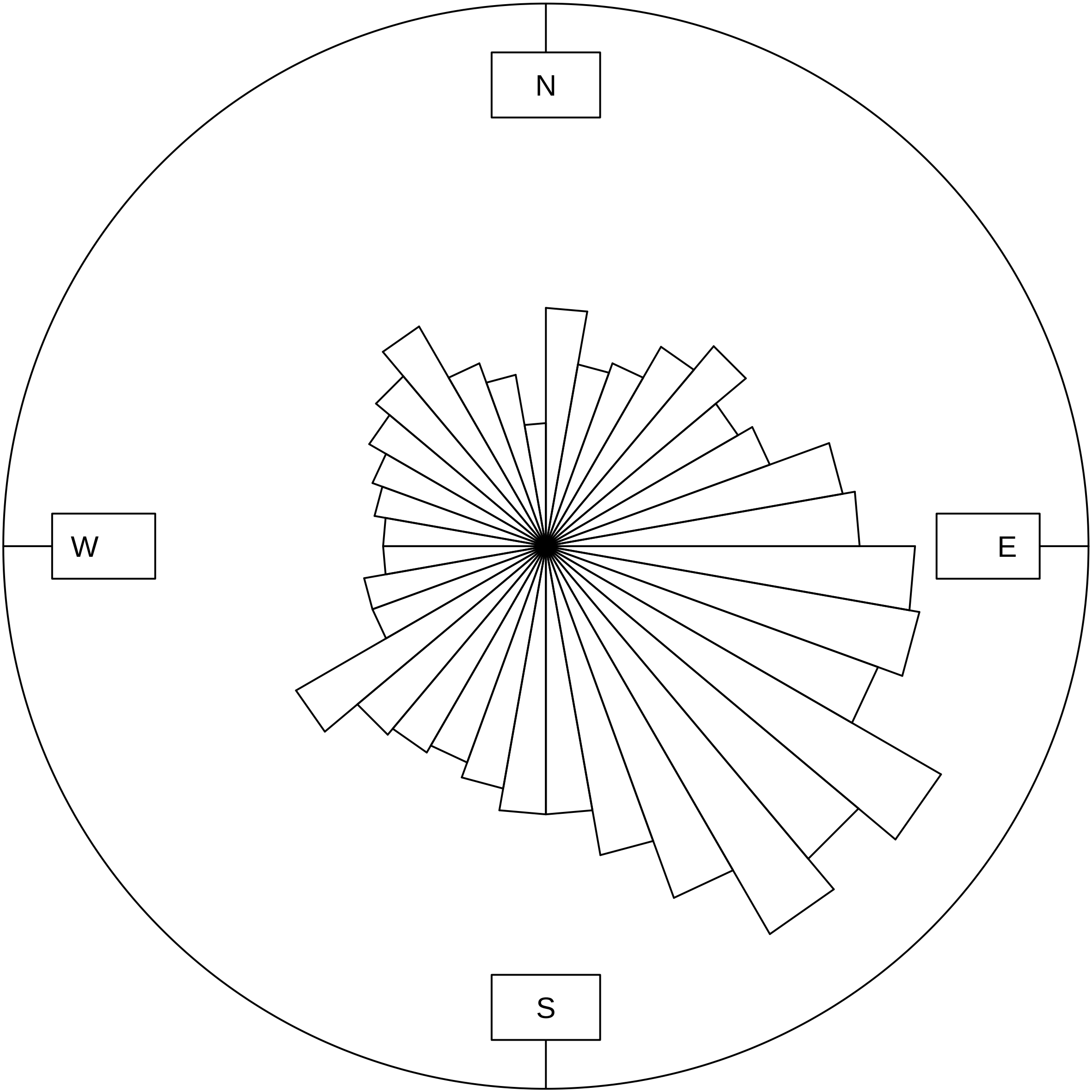

bootstrap 898

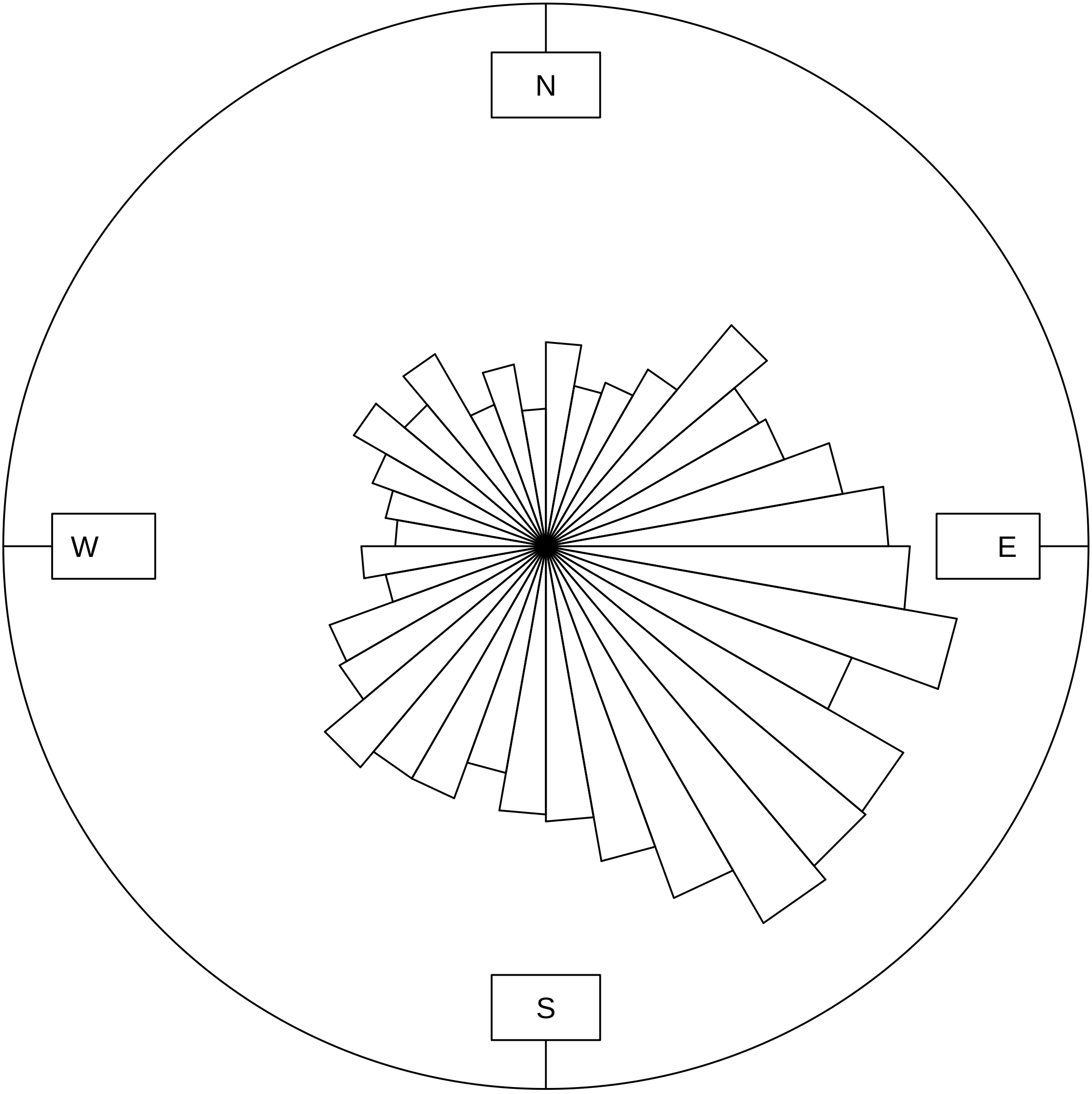

bootstrap 899

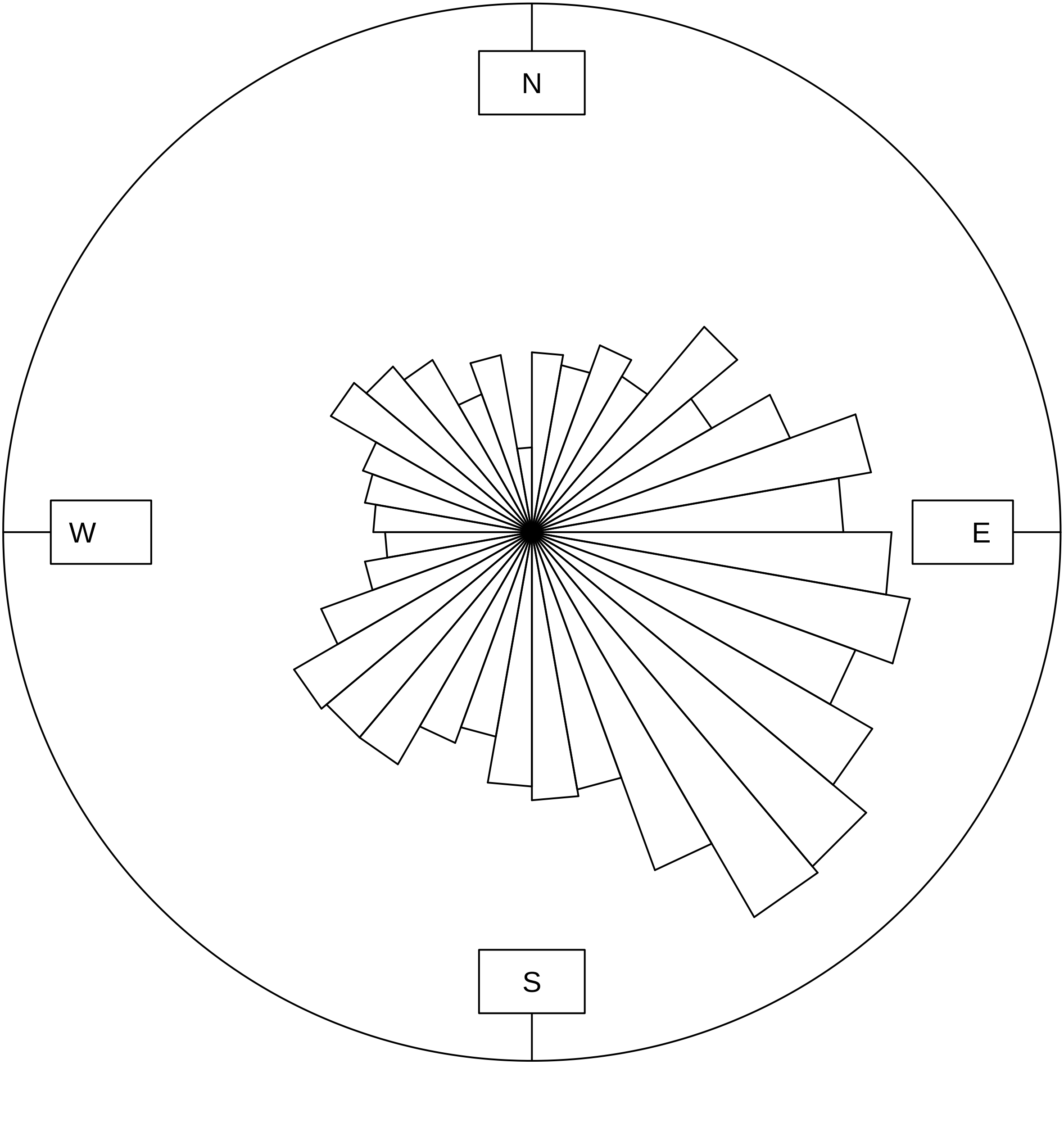

bootstrap 900

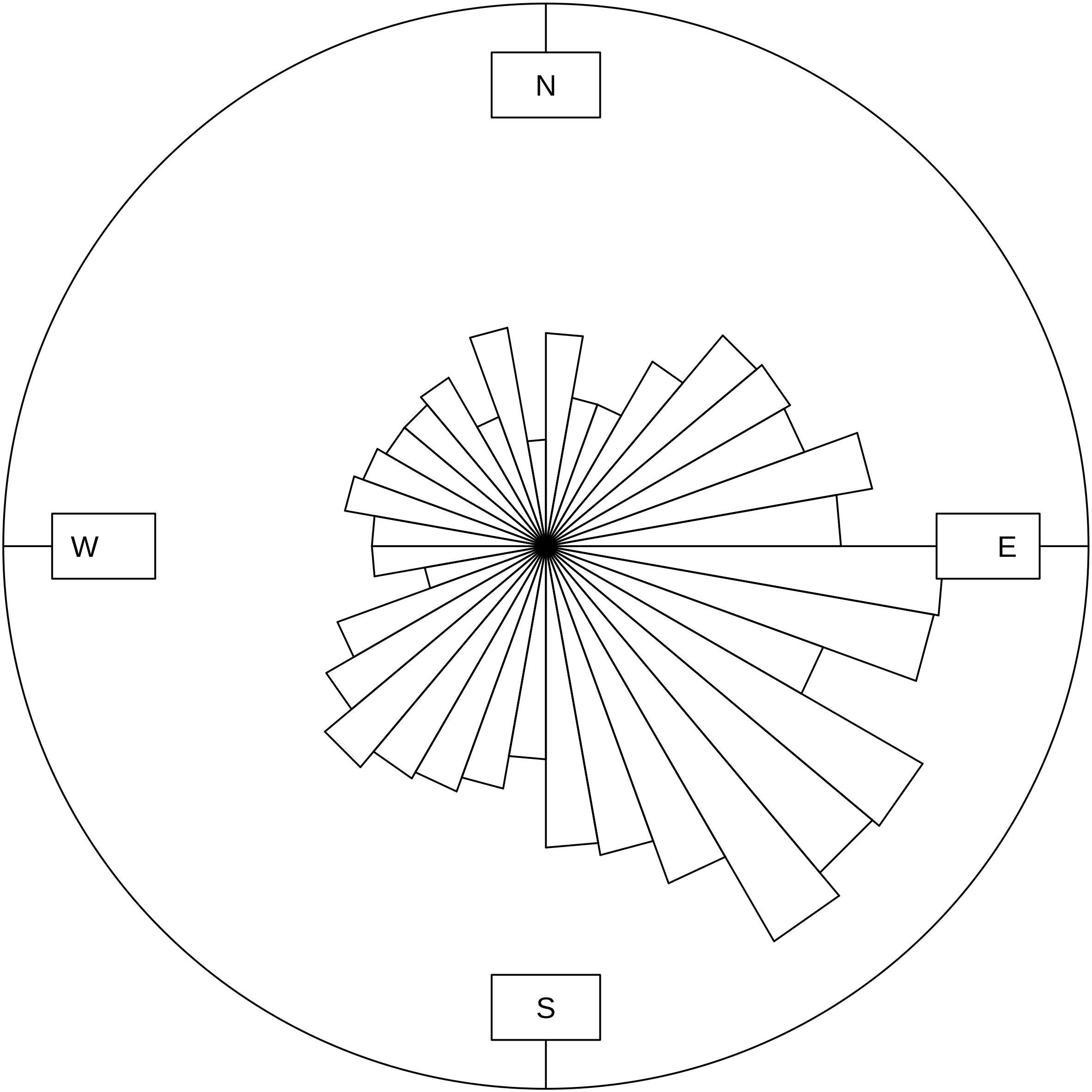

bootstrap 901

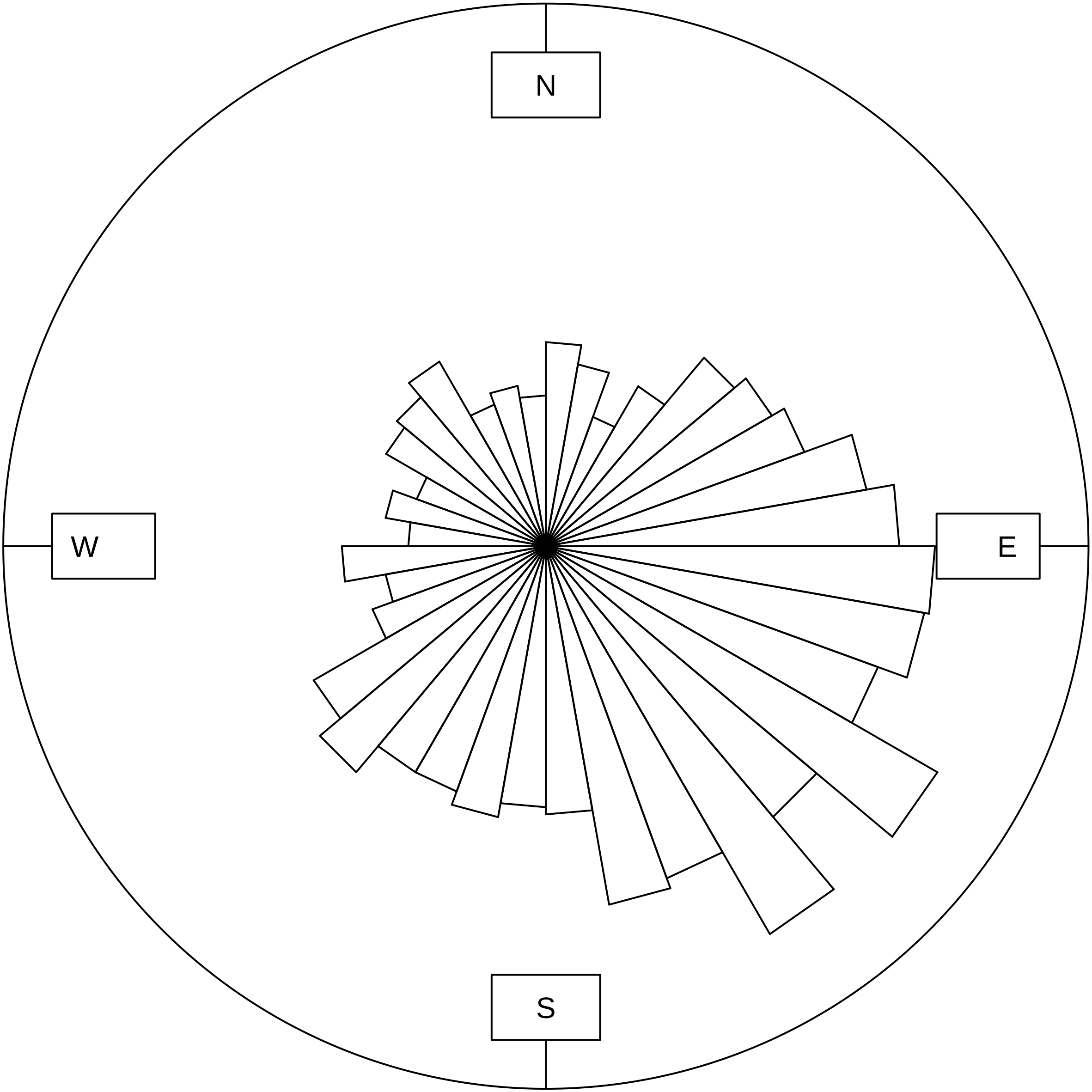

bootstrap 902

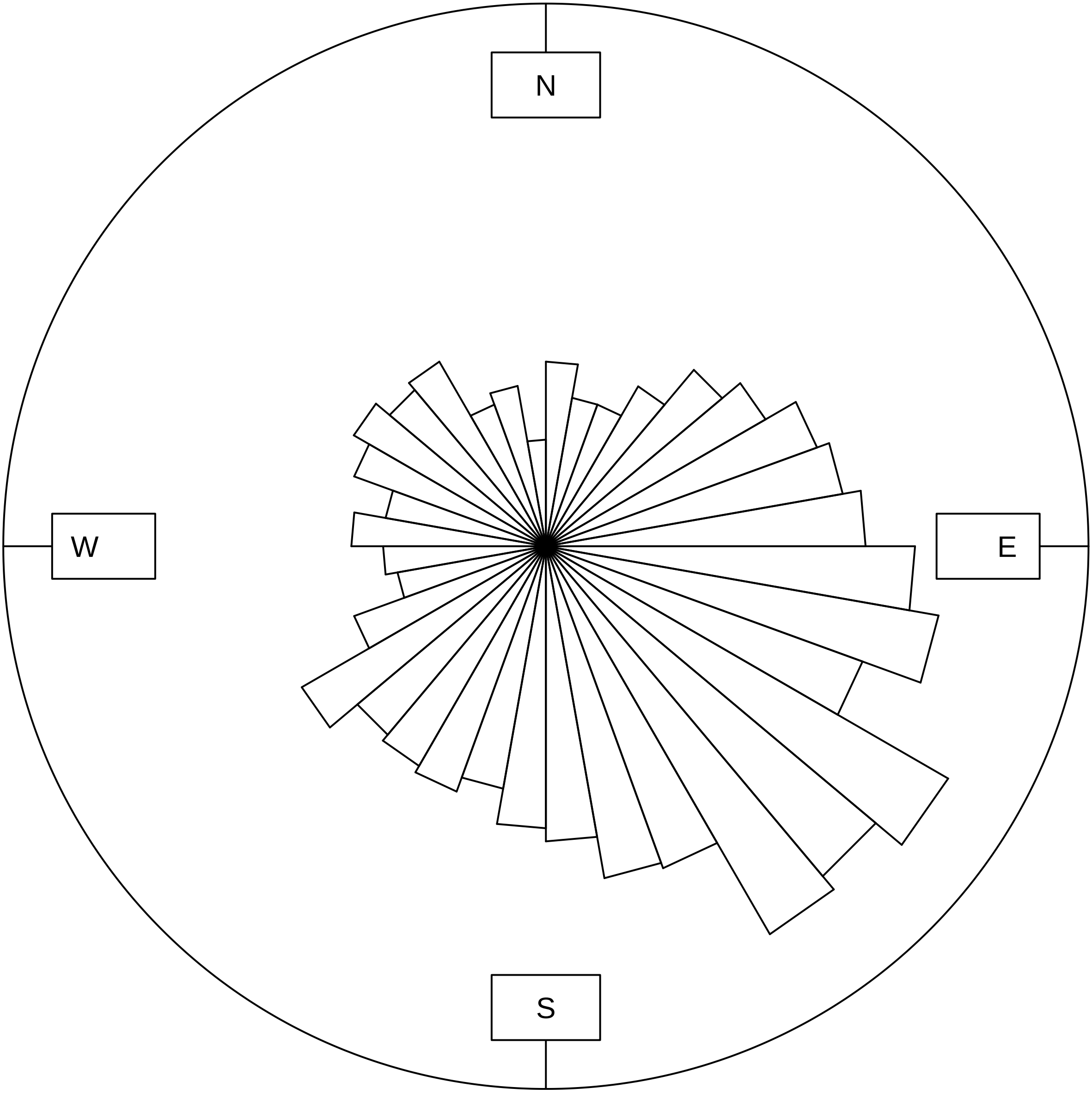

bootstrap 903

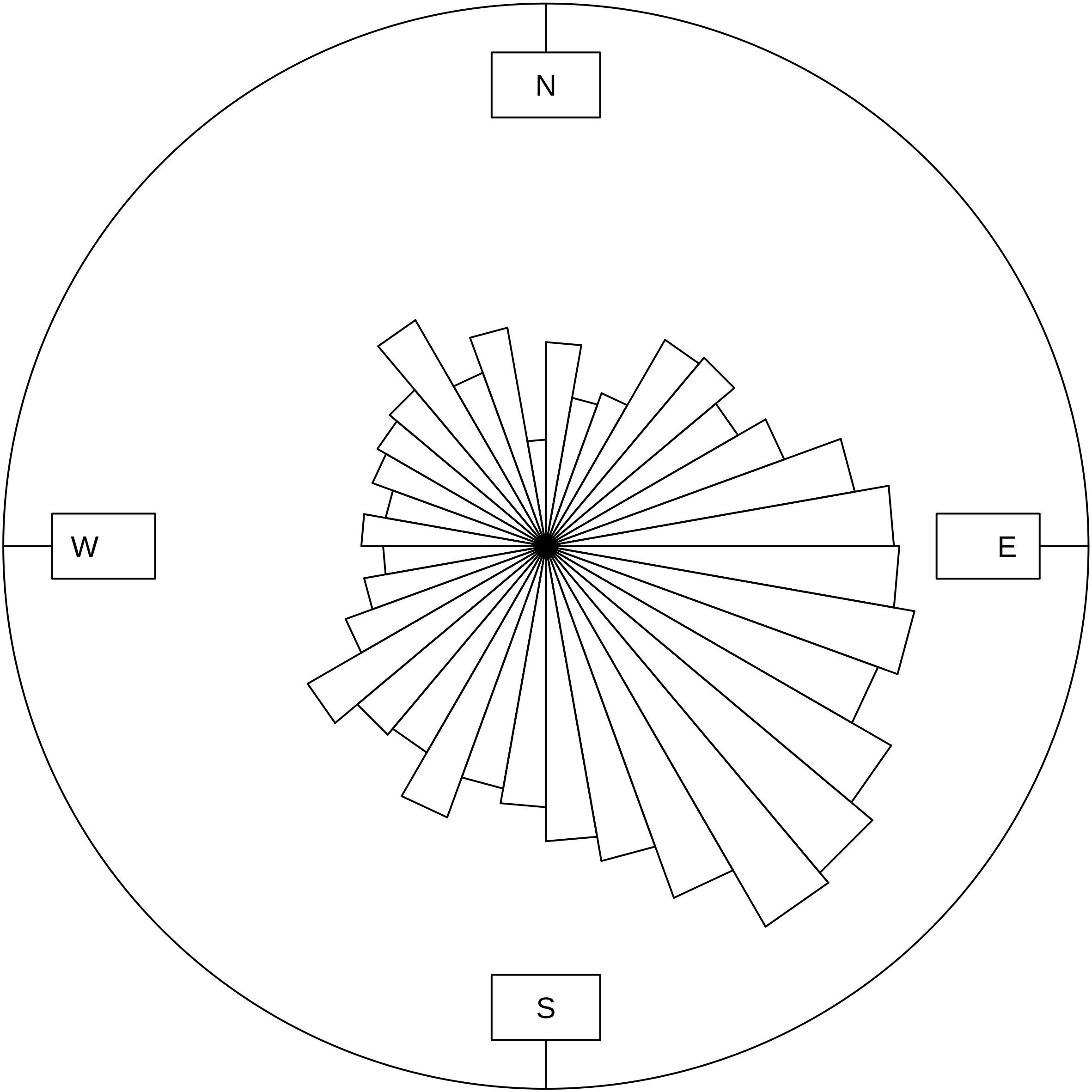

bootstrap 904

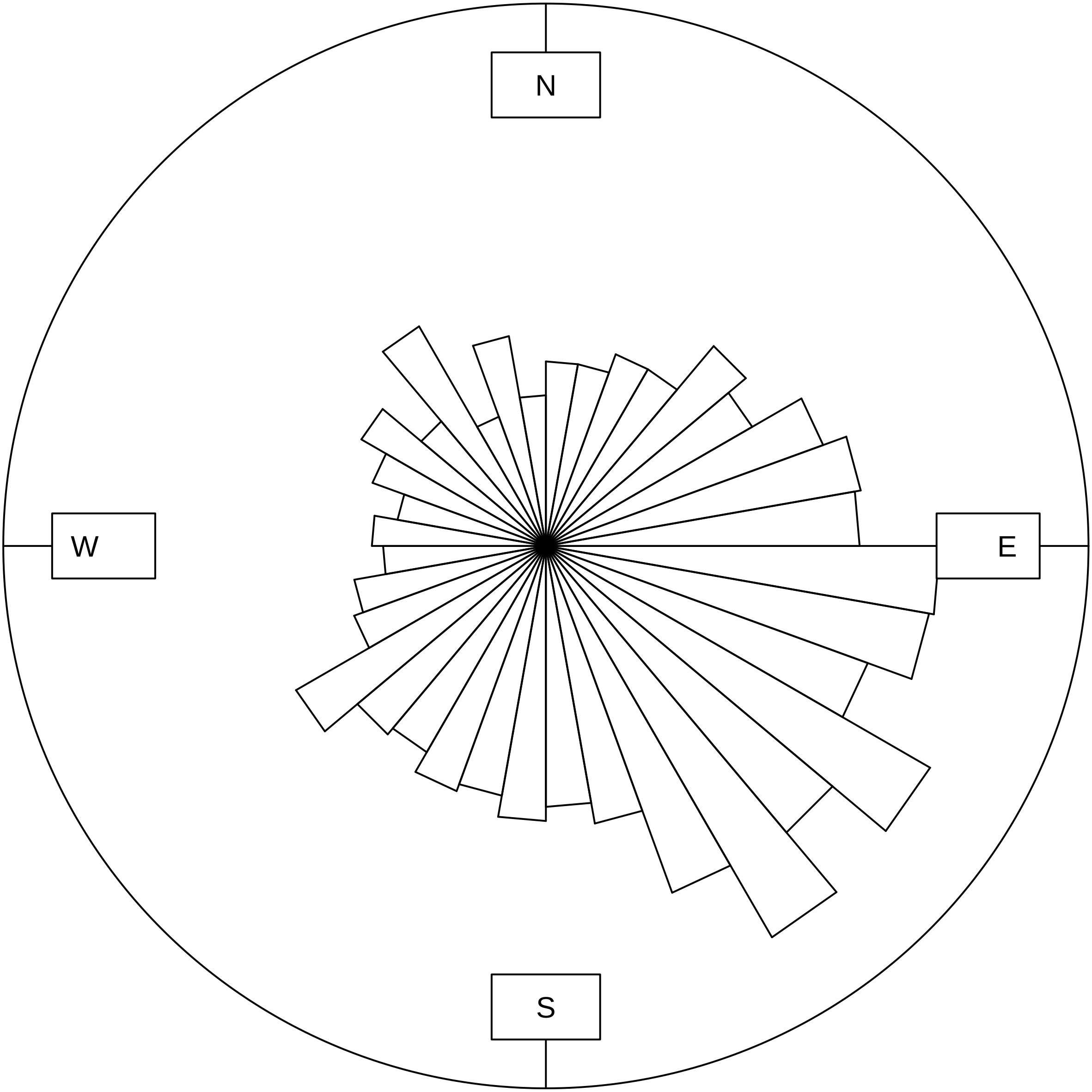

bootstrap 905

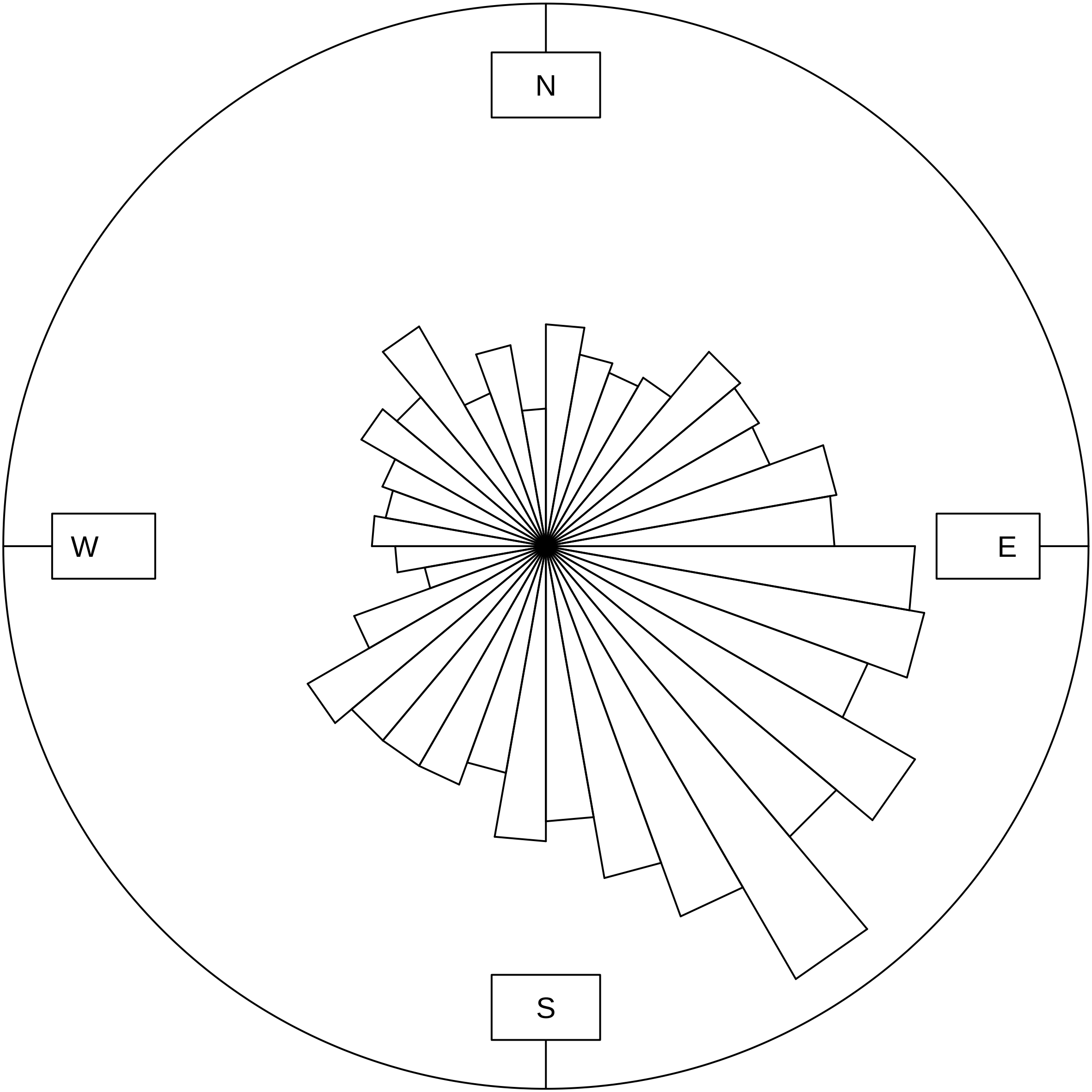

bootstrap 906

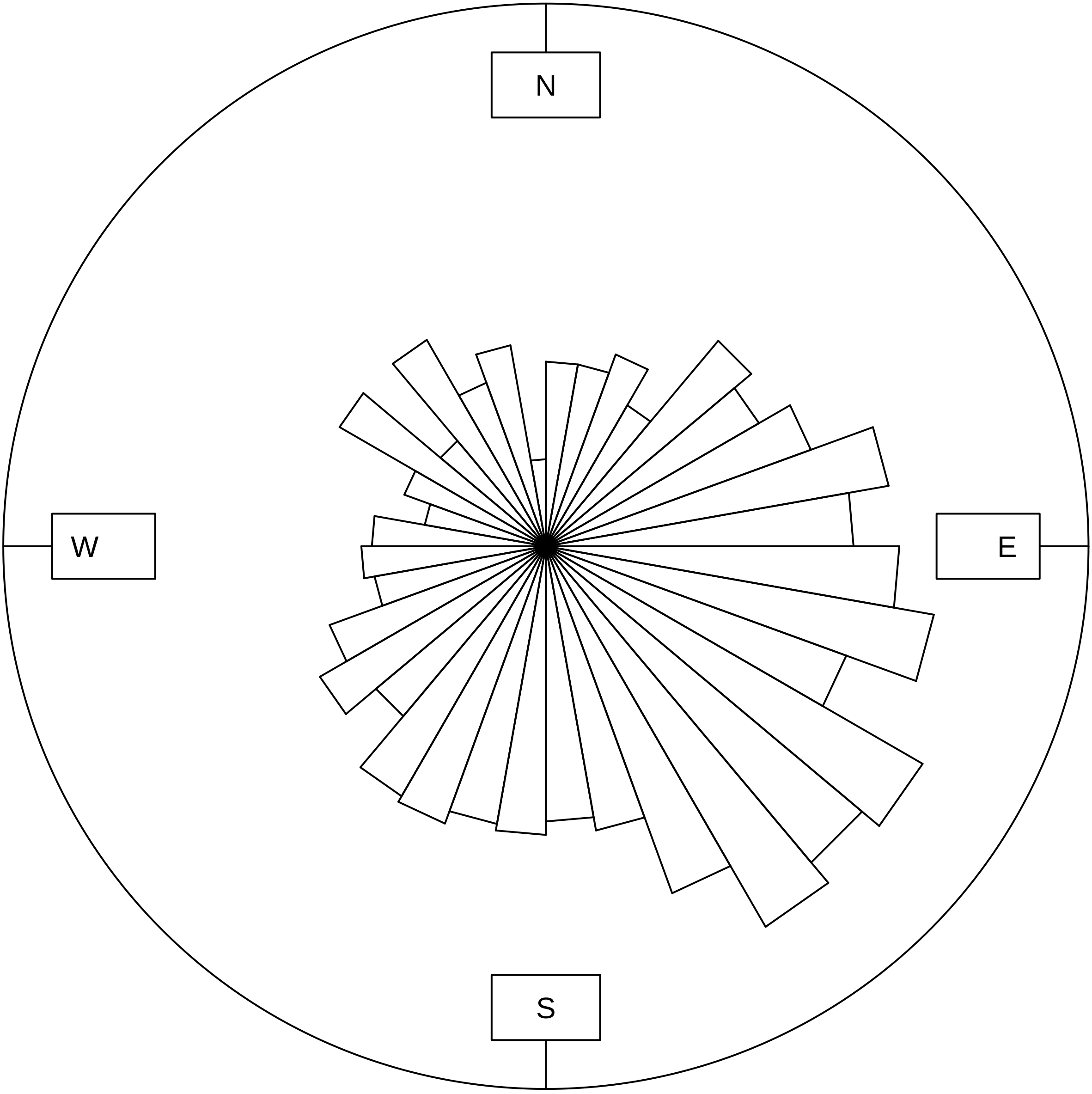

bootstrap 907

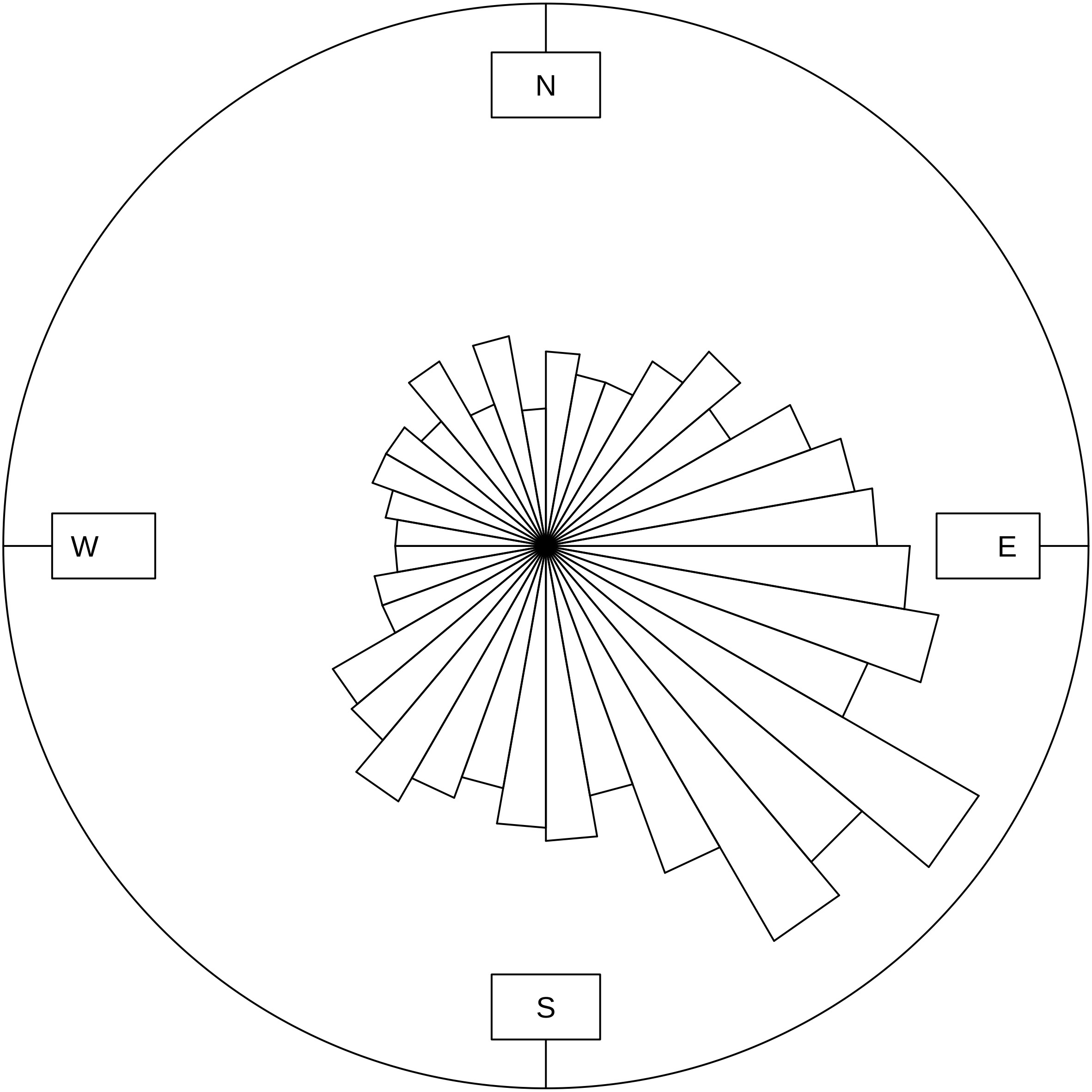

bootstrap 908

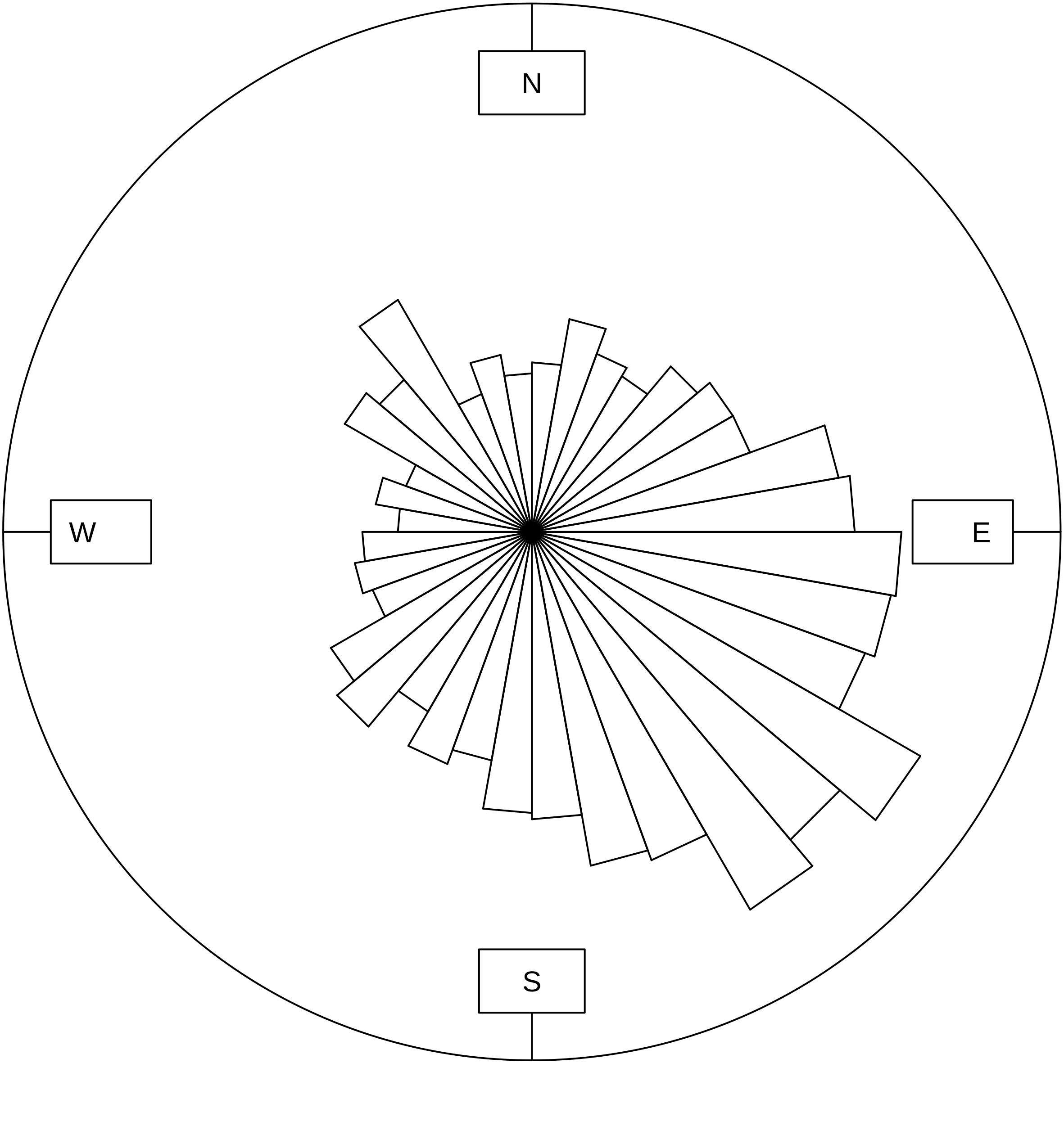

bootstrap 909

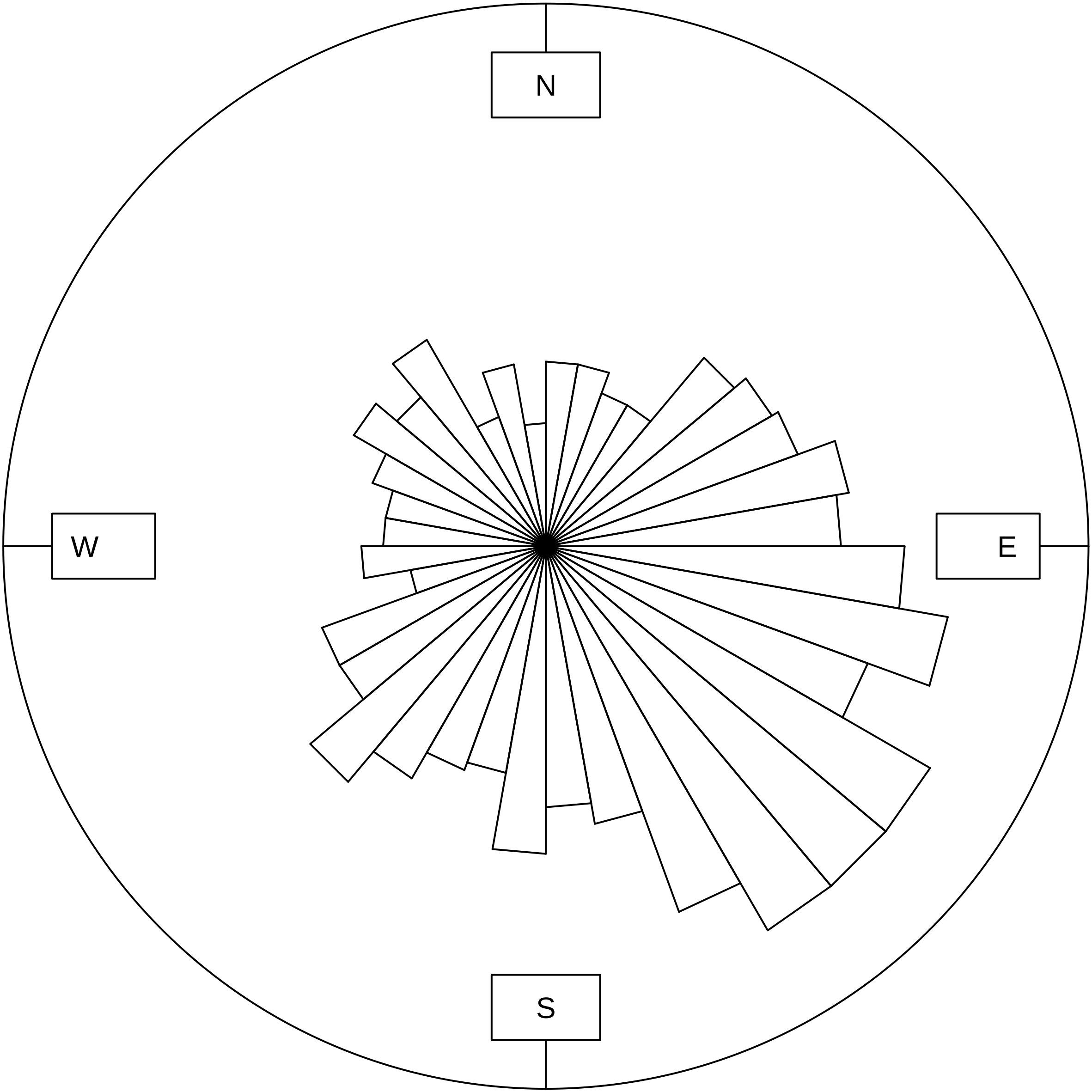

bootstrap 910

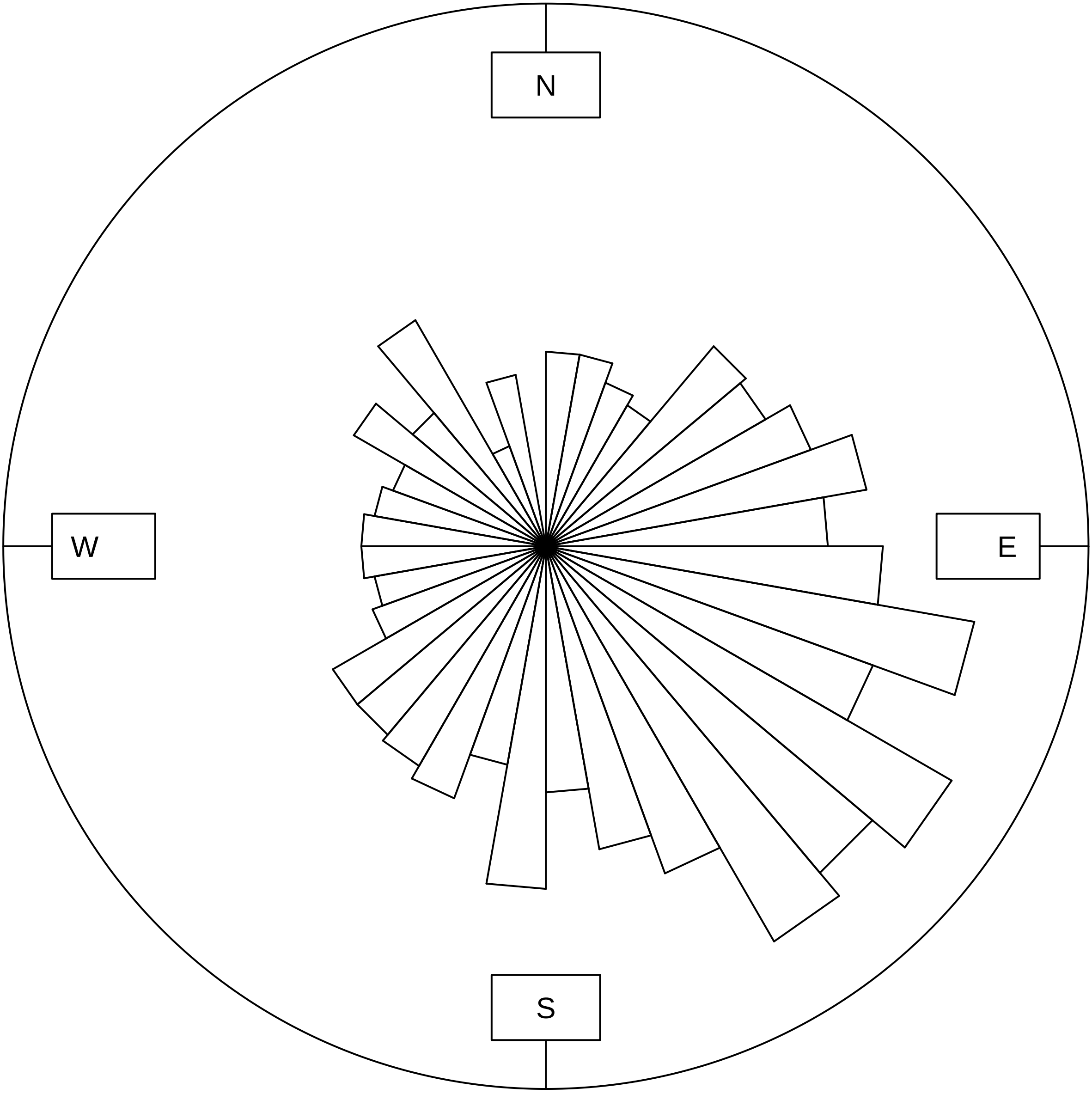

bootstrap 911

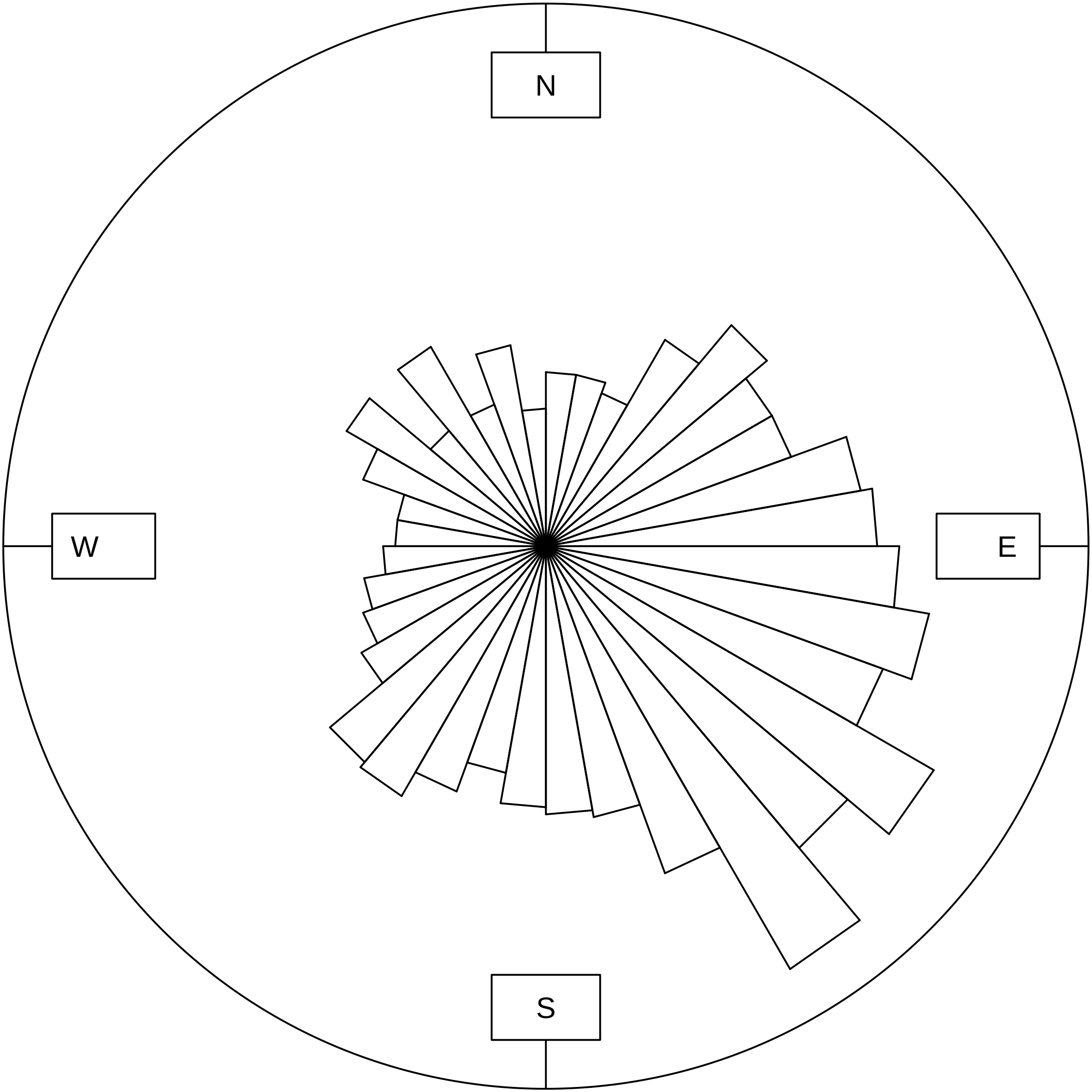

bootstrap 912

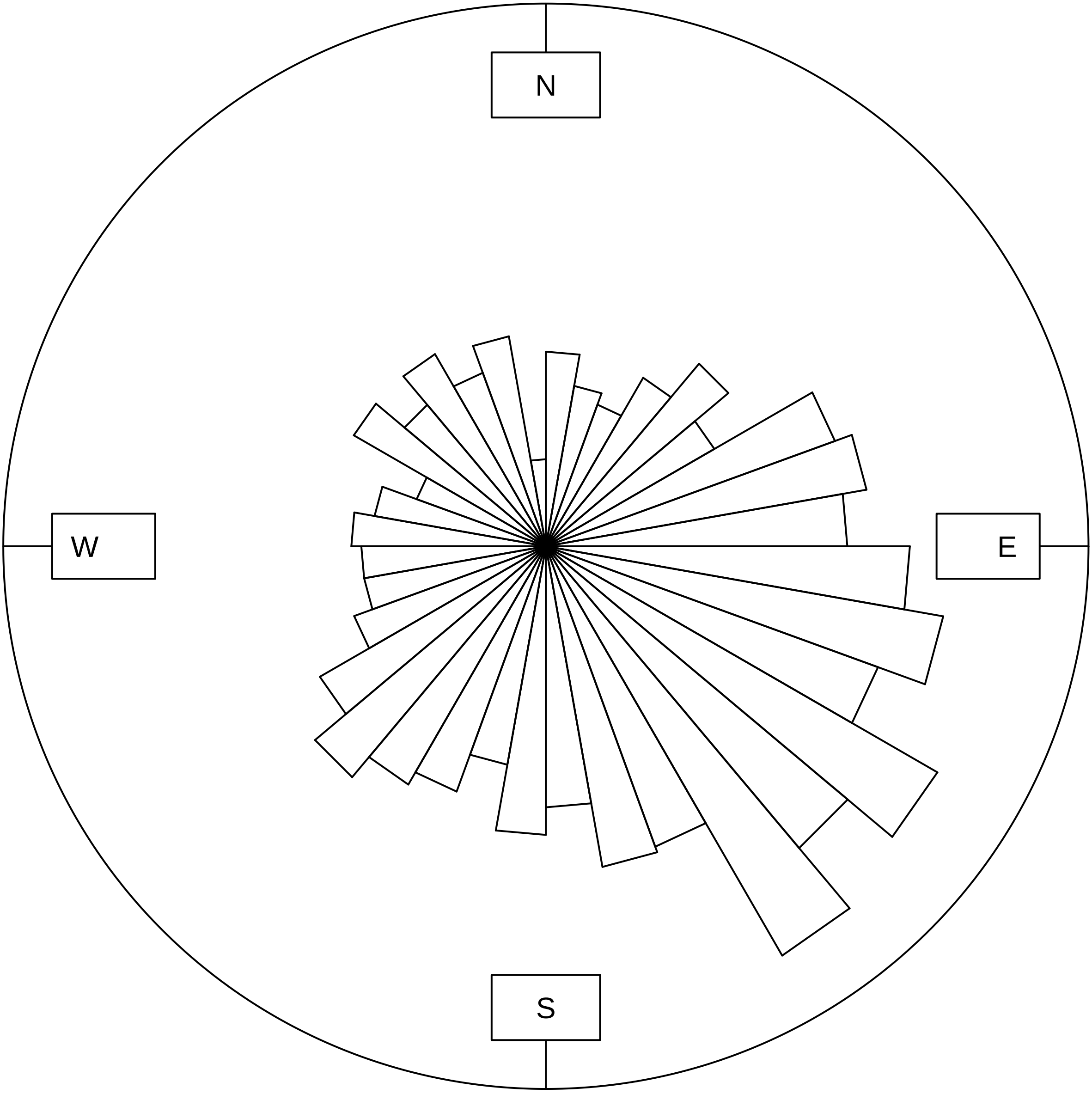

bootstrap 913

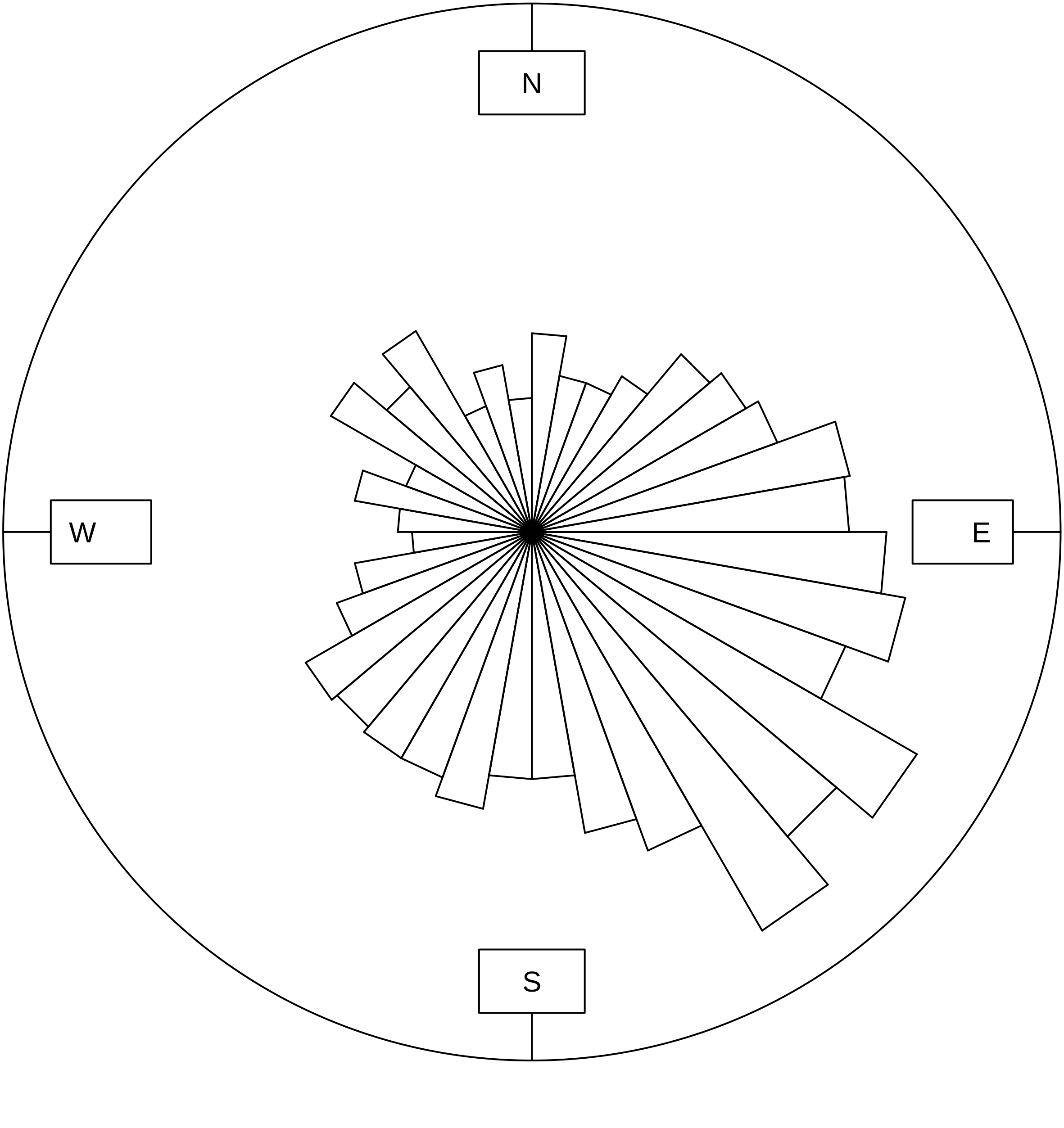

bootstrap 914

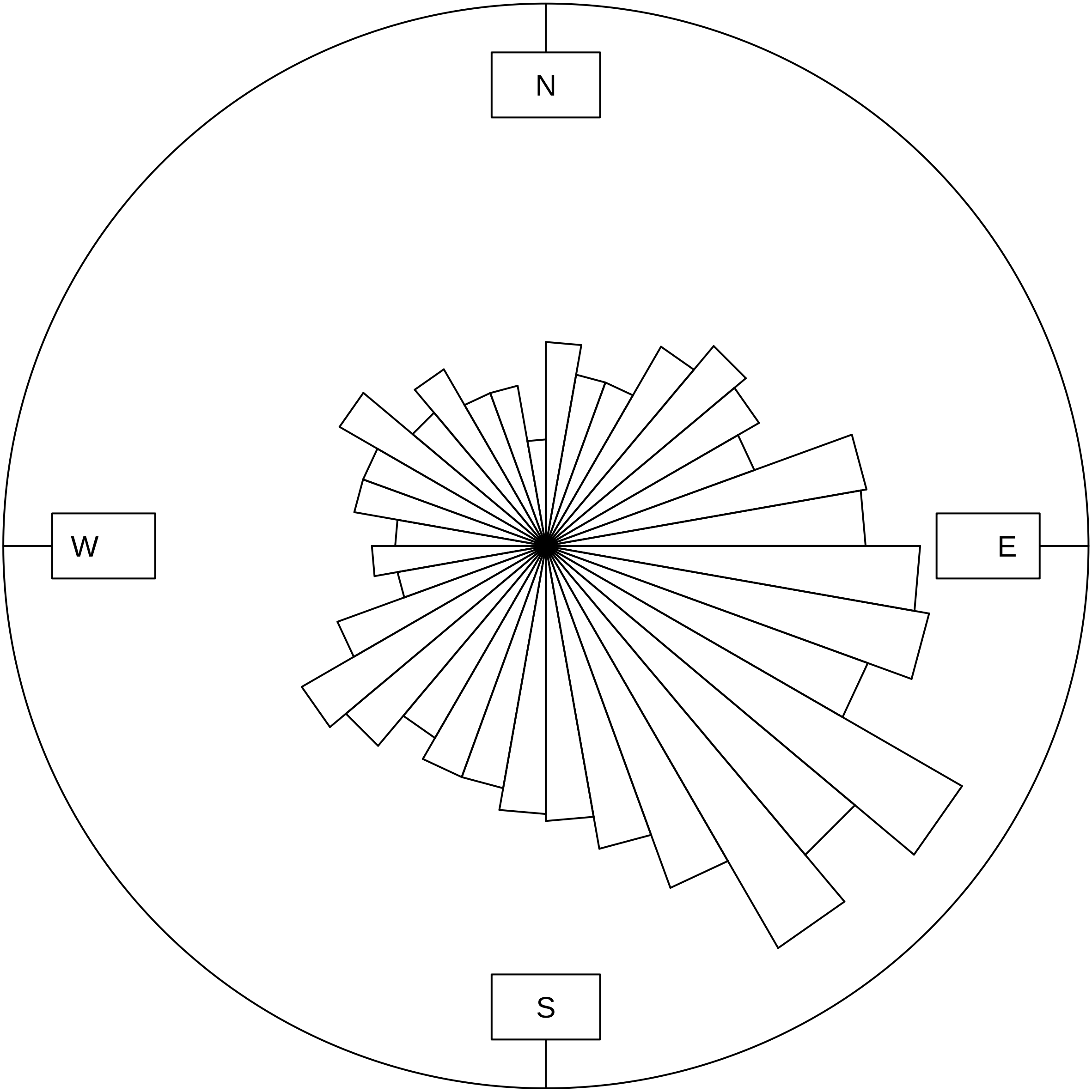

bootstrap 915

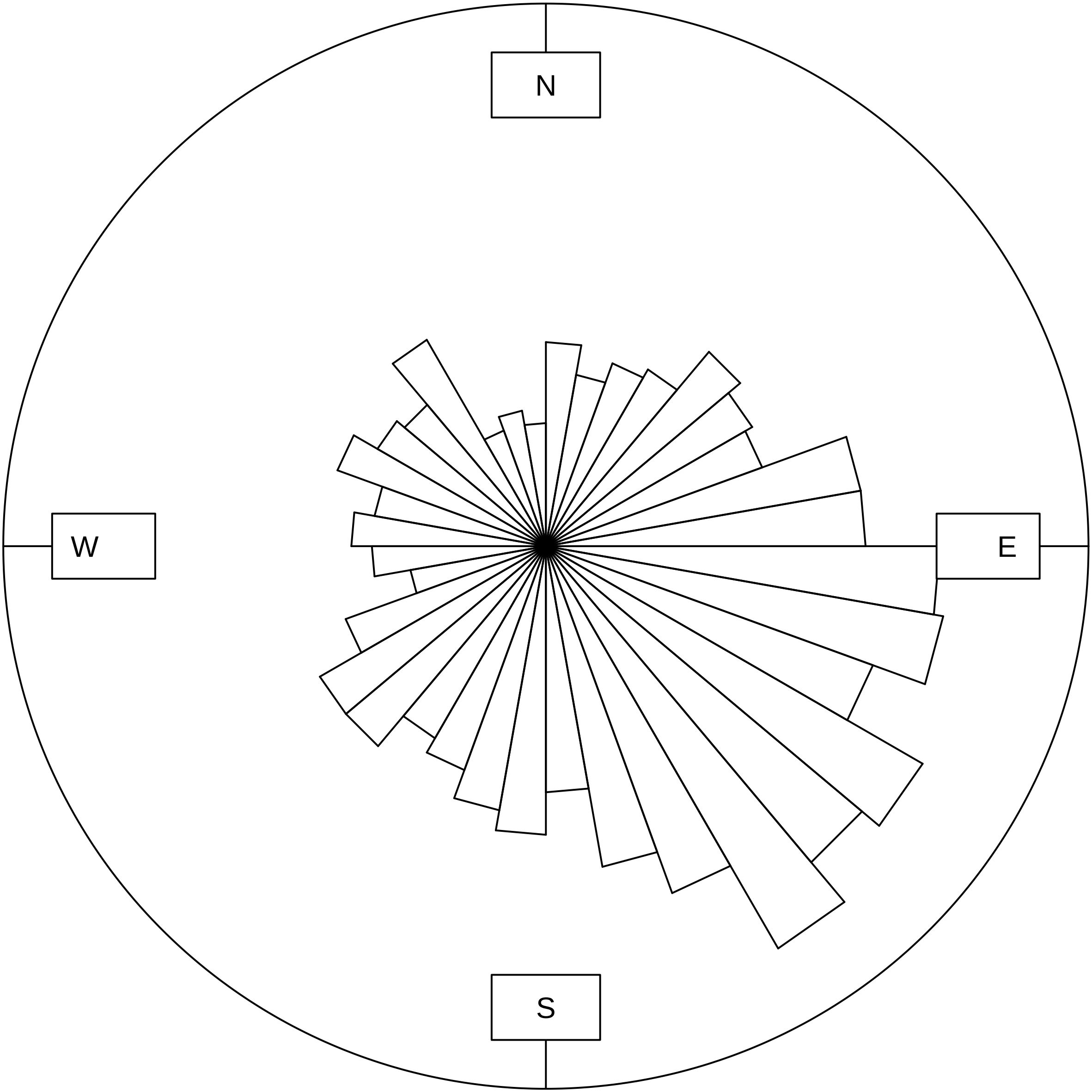

bootstrap 916

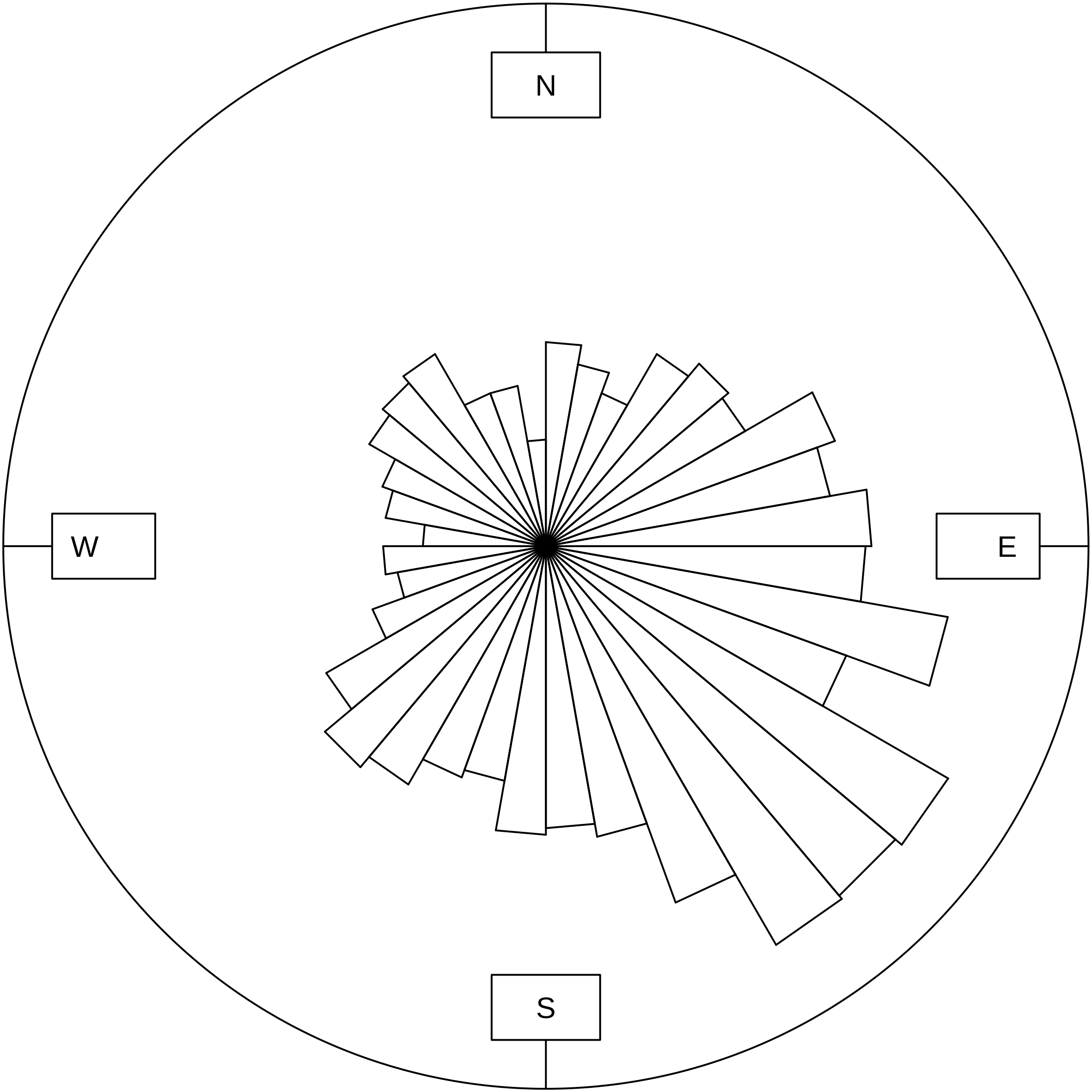

bootstrap 917

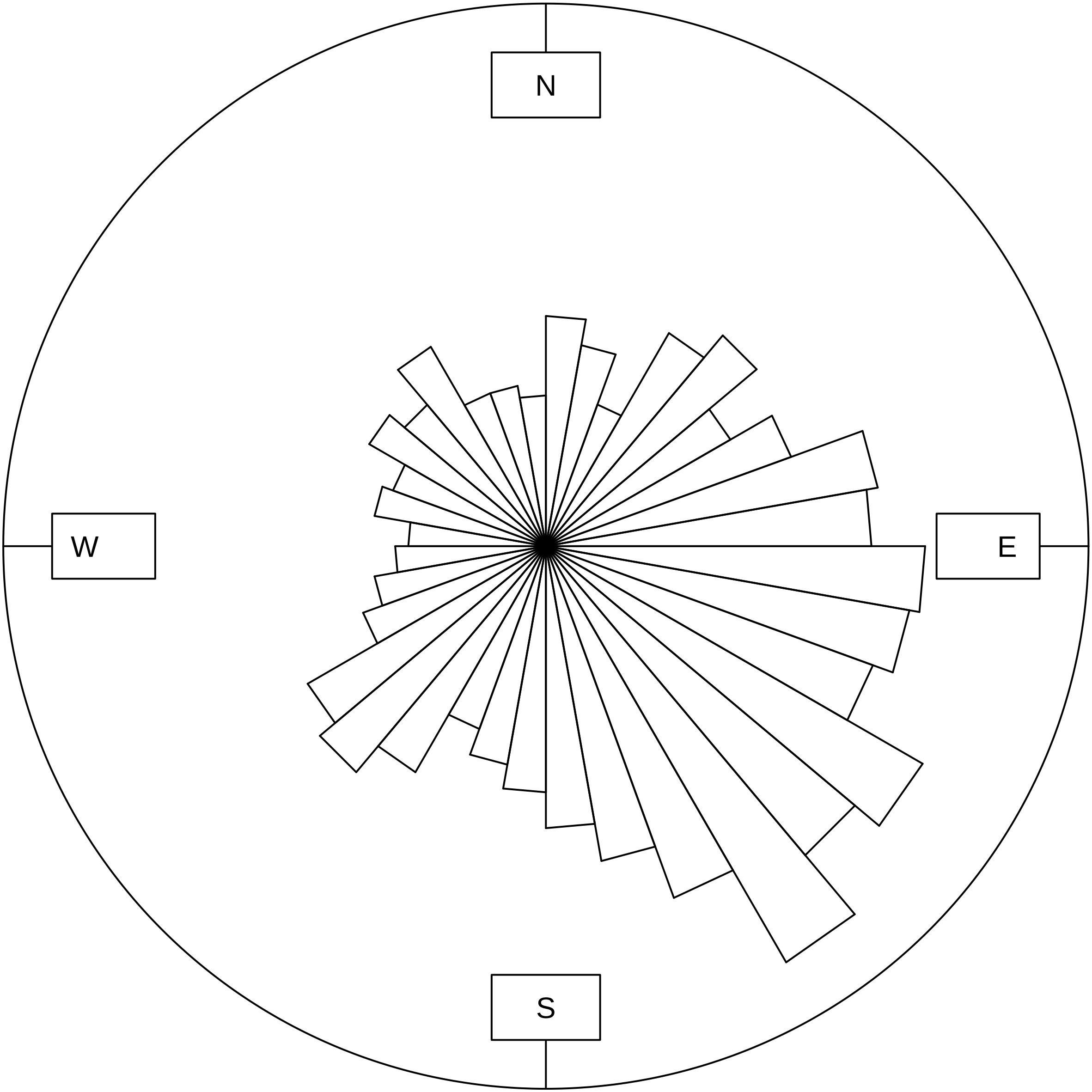

bootstrap 918

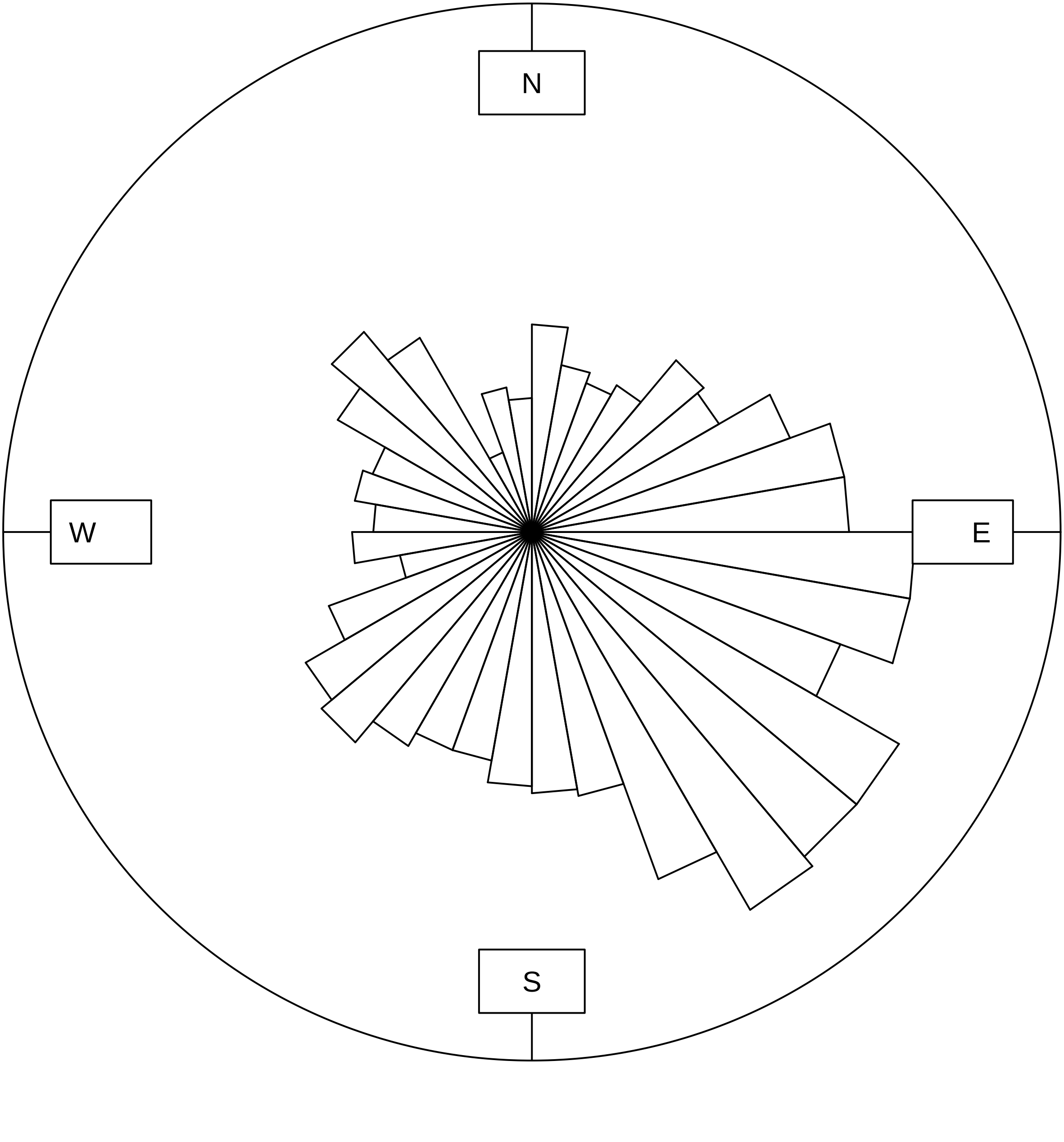

bootstrap 919

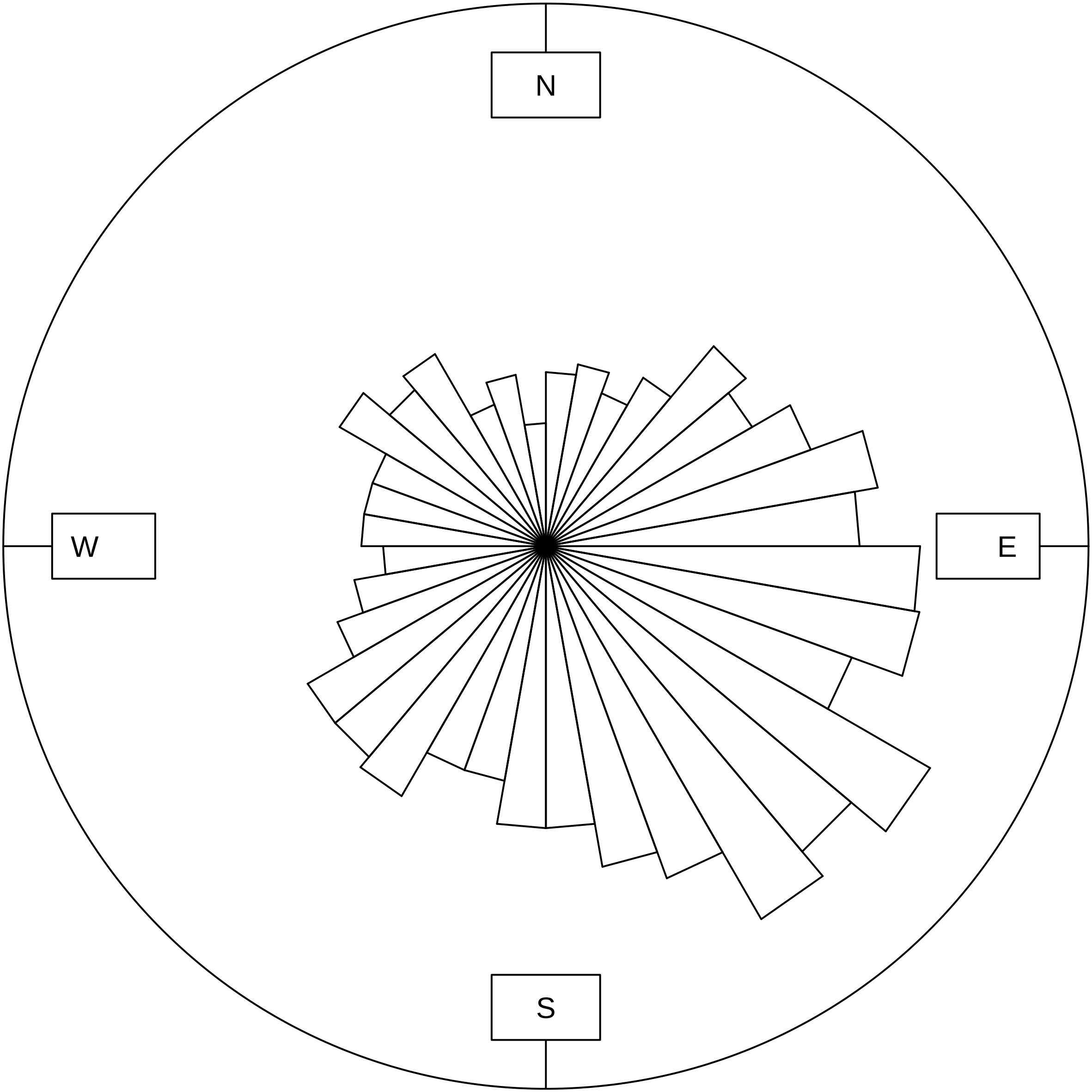

bootstrap 920

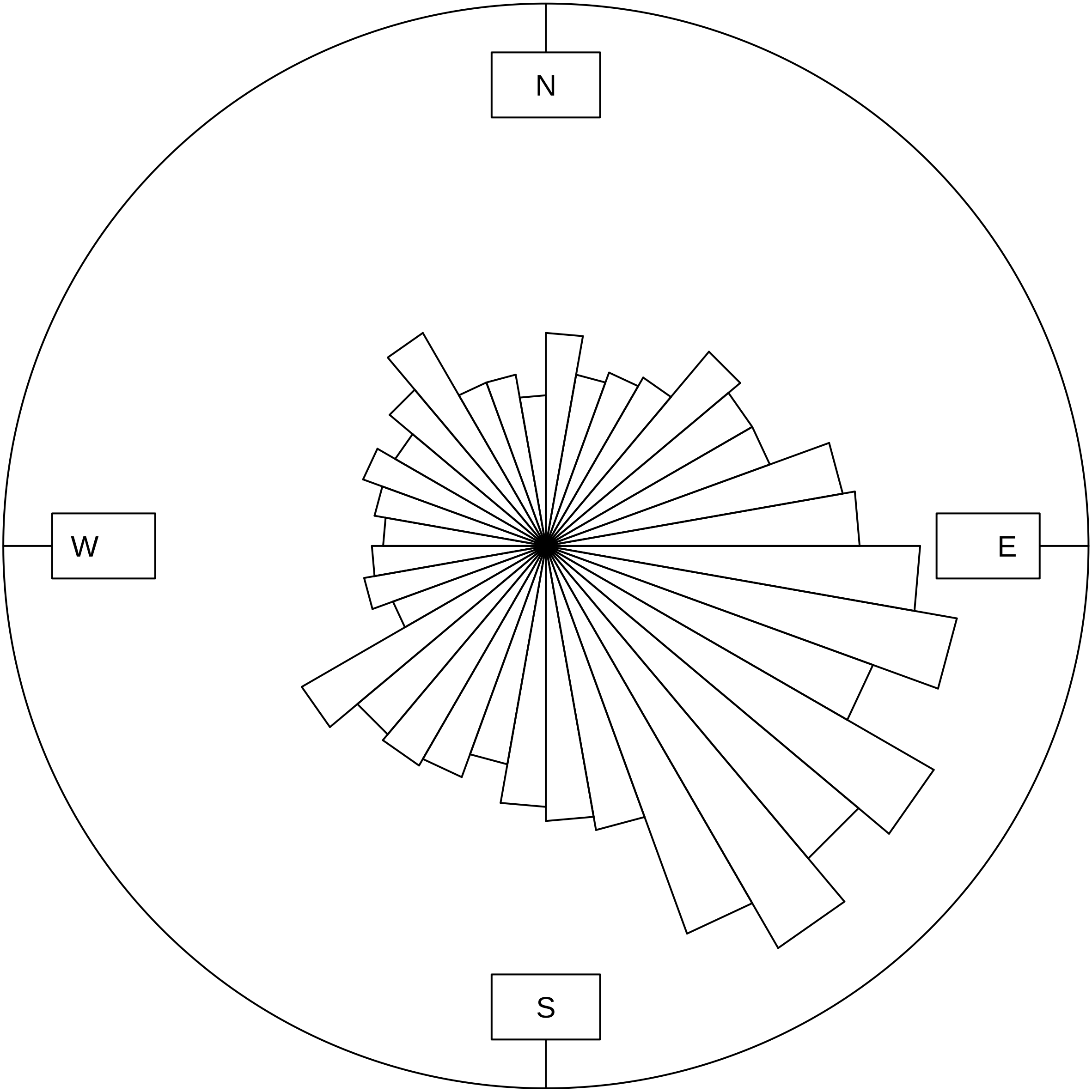

bootstrap 921

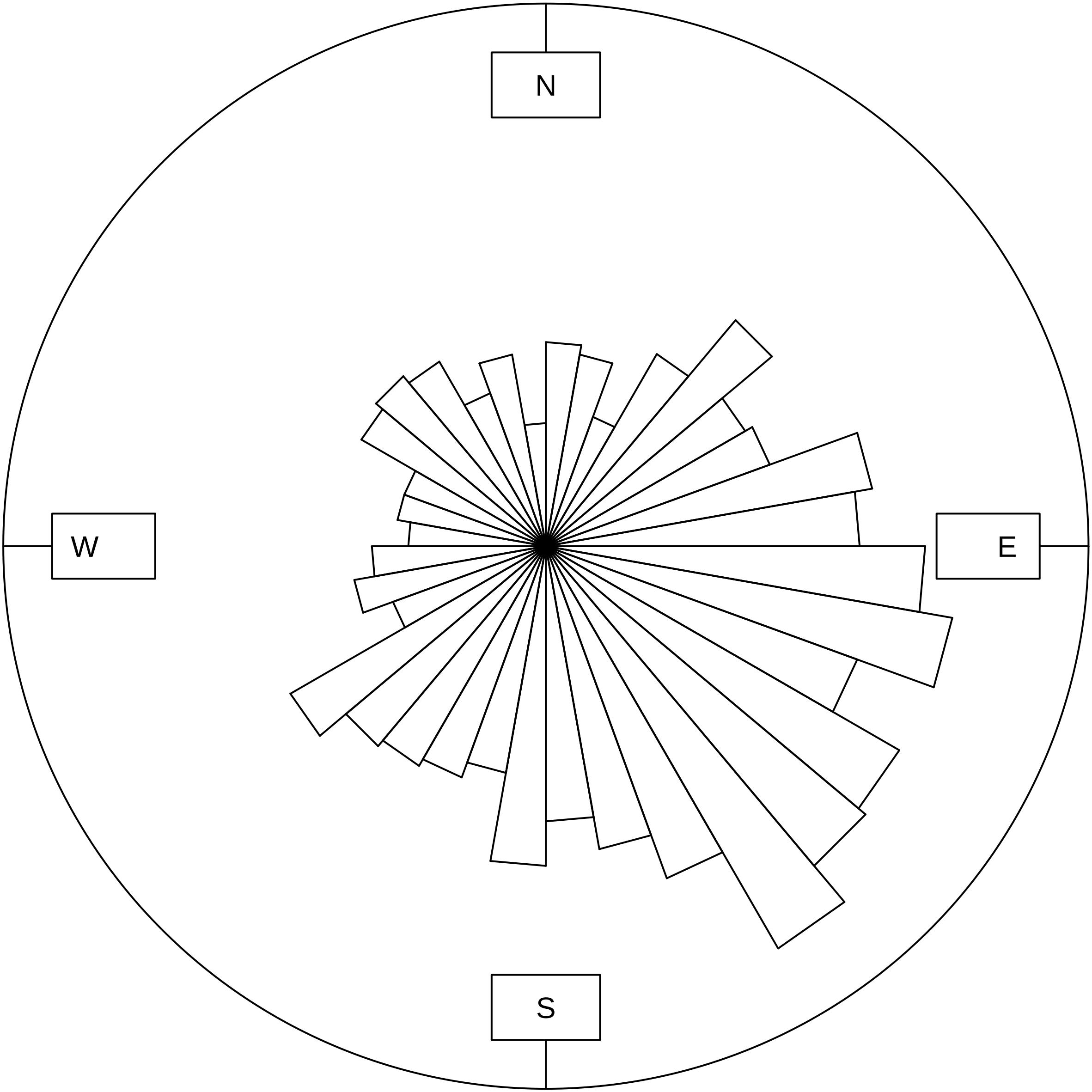

bootstrap 922

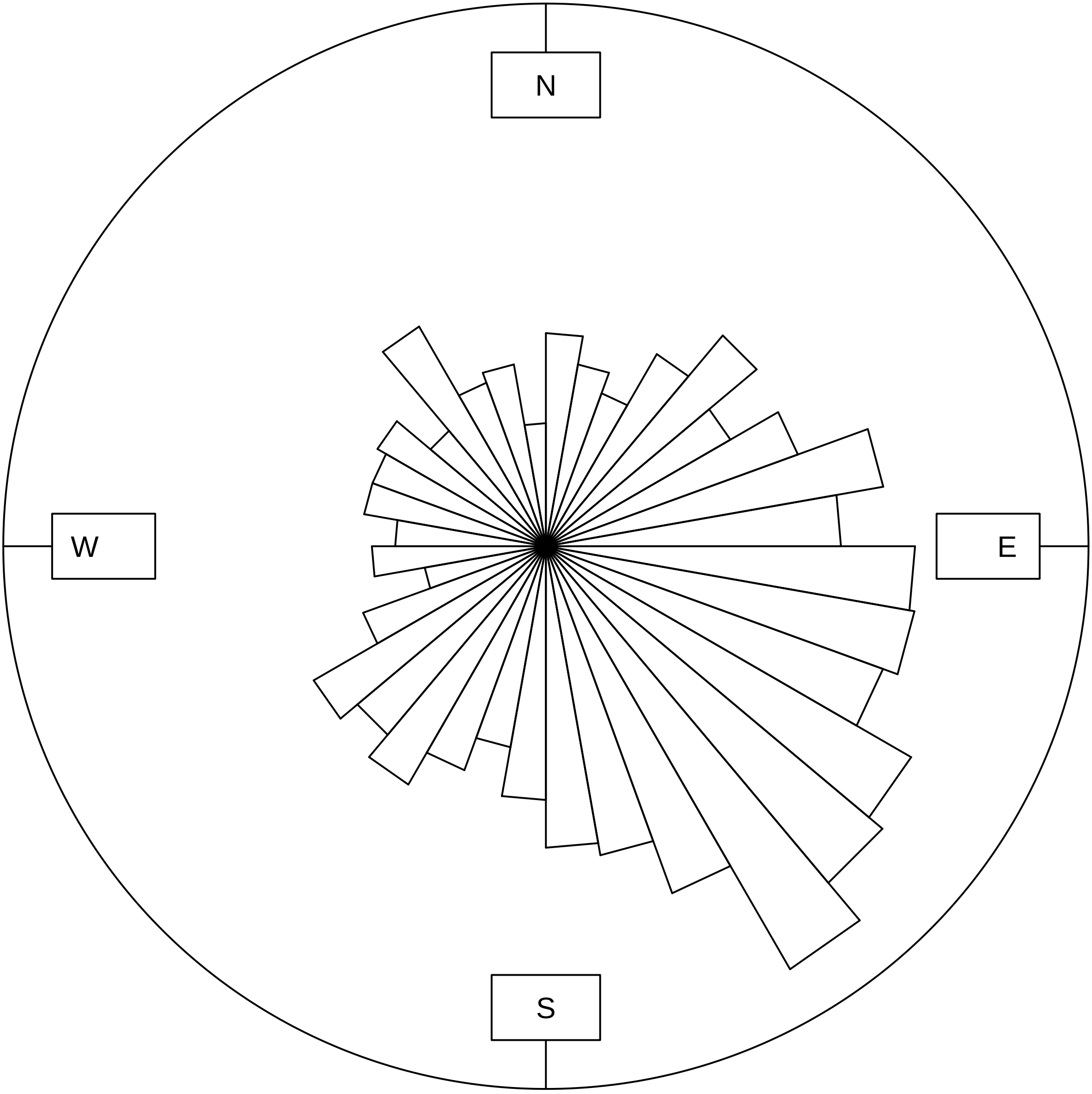

bootstrap 923

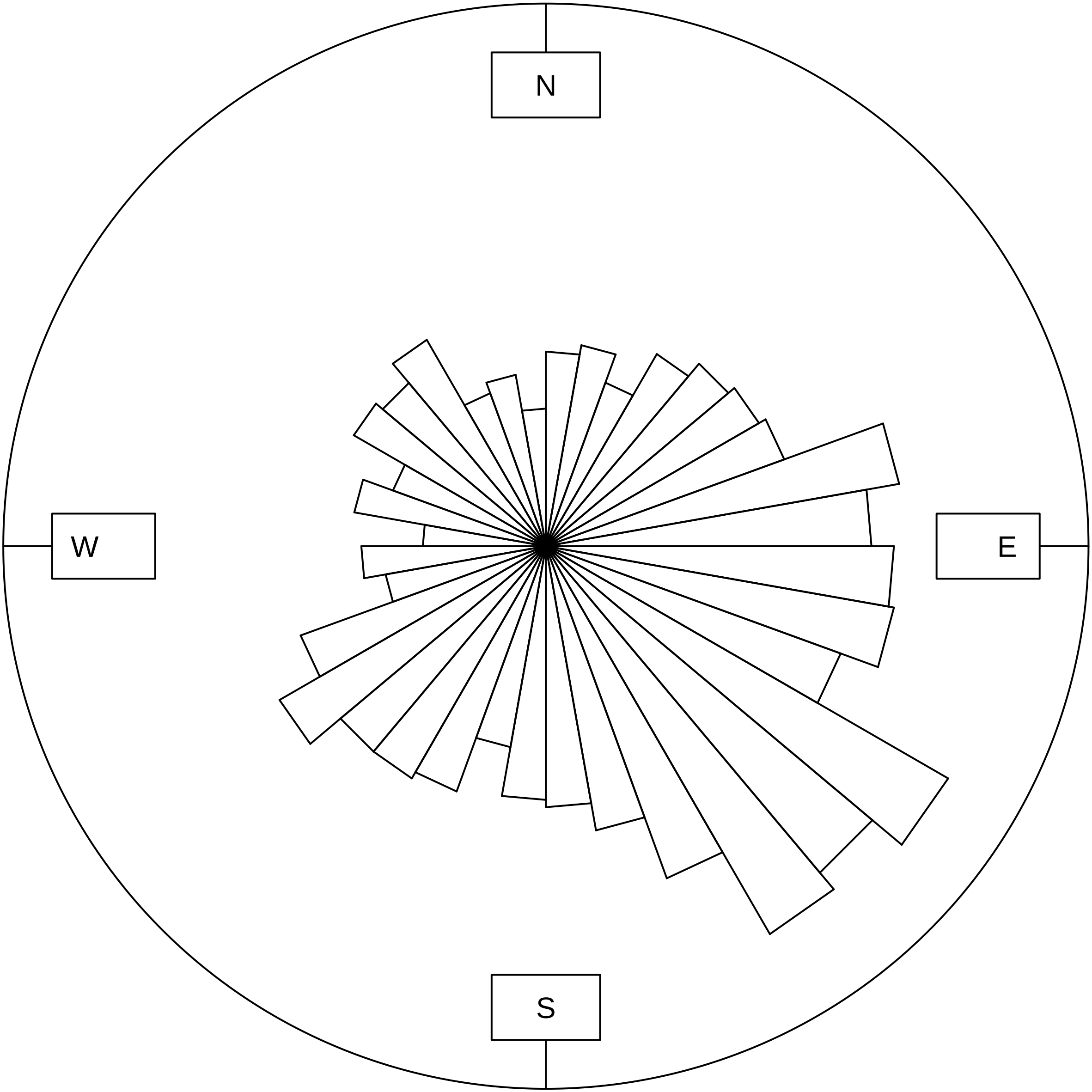

bootstrap 924

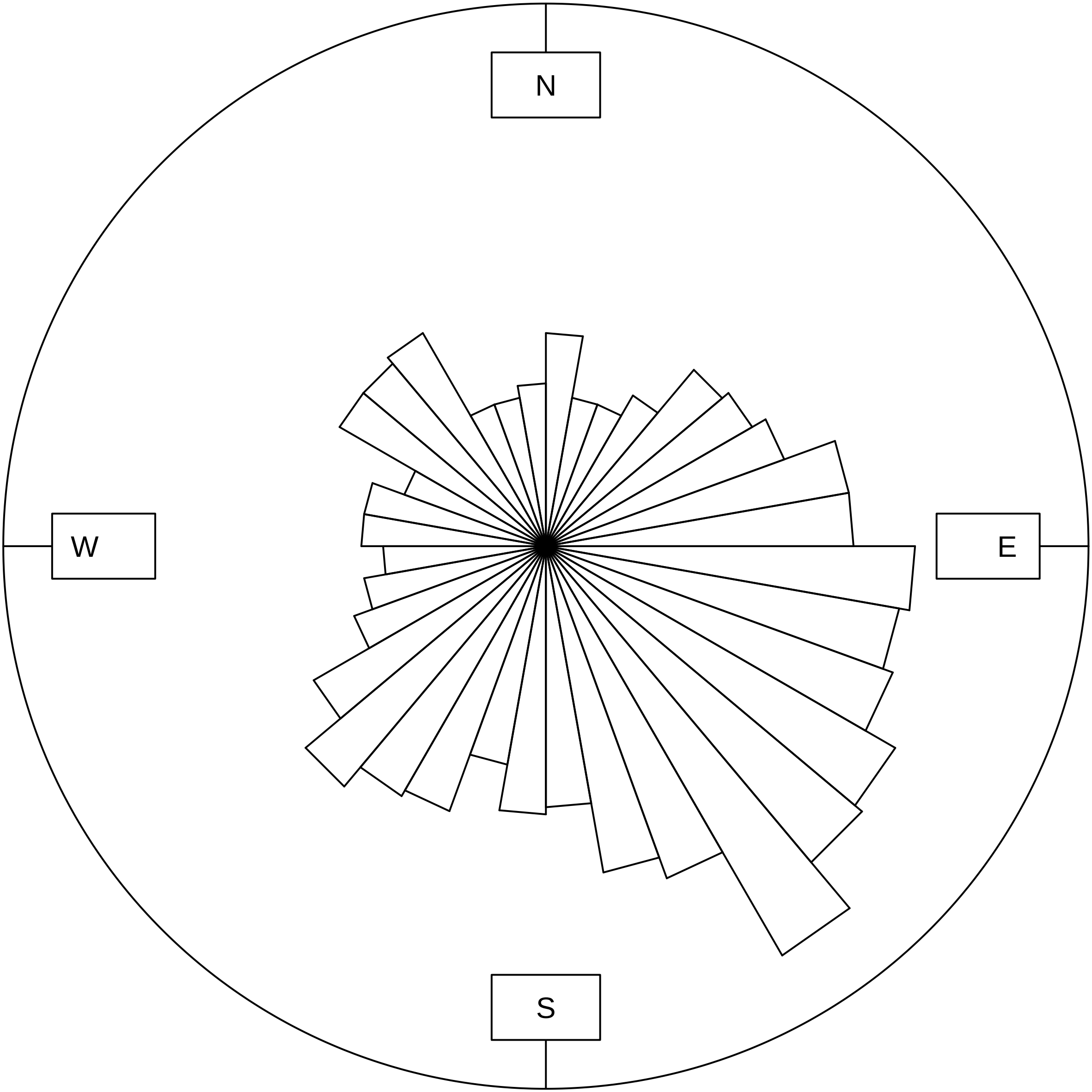

bootstrap 925

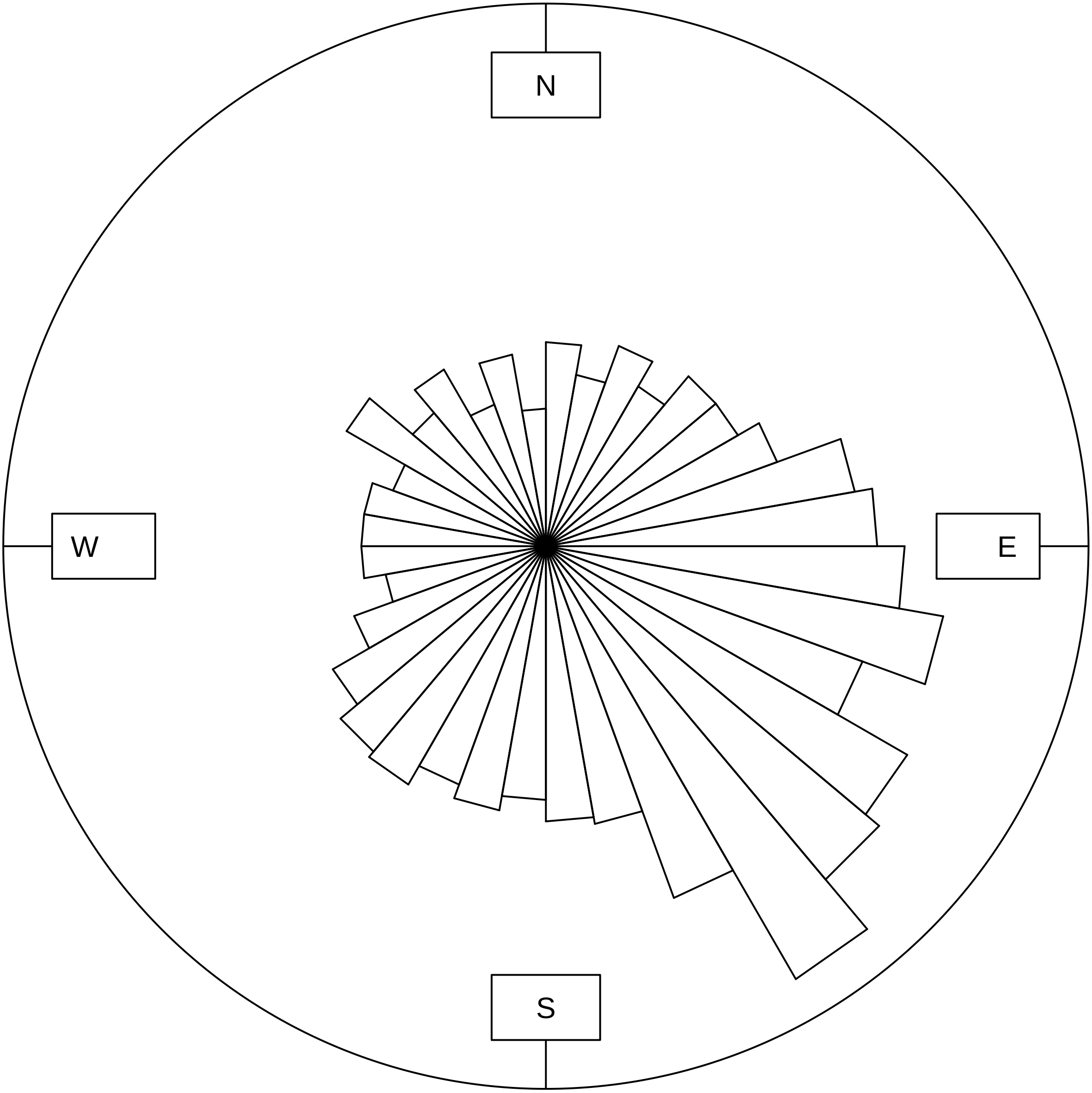

bootstrap 926

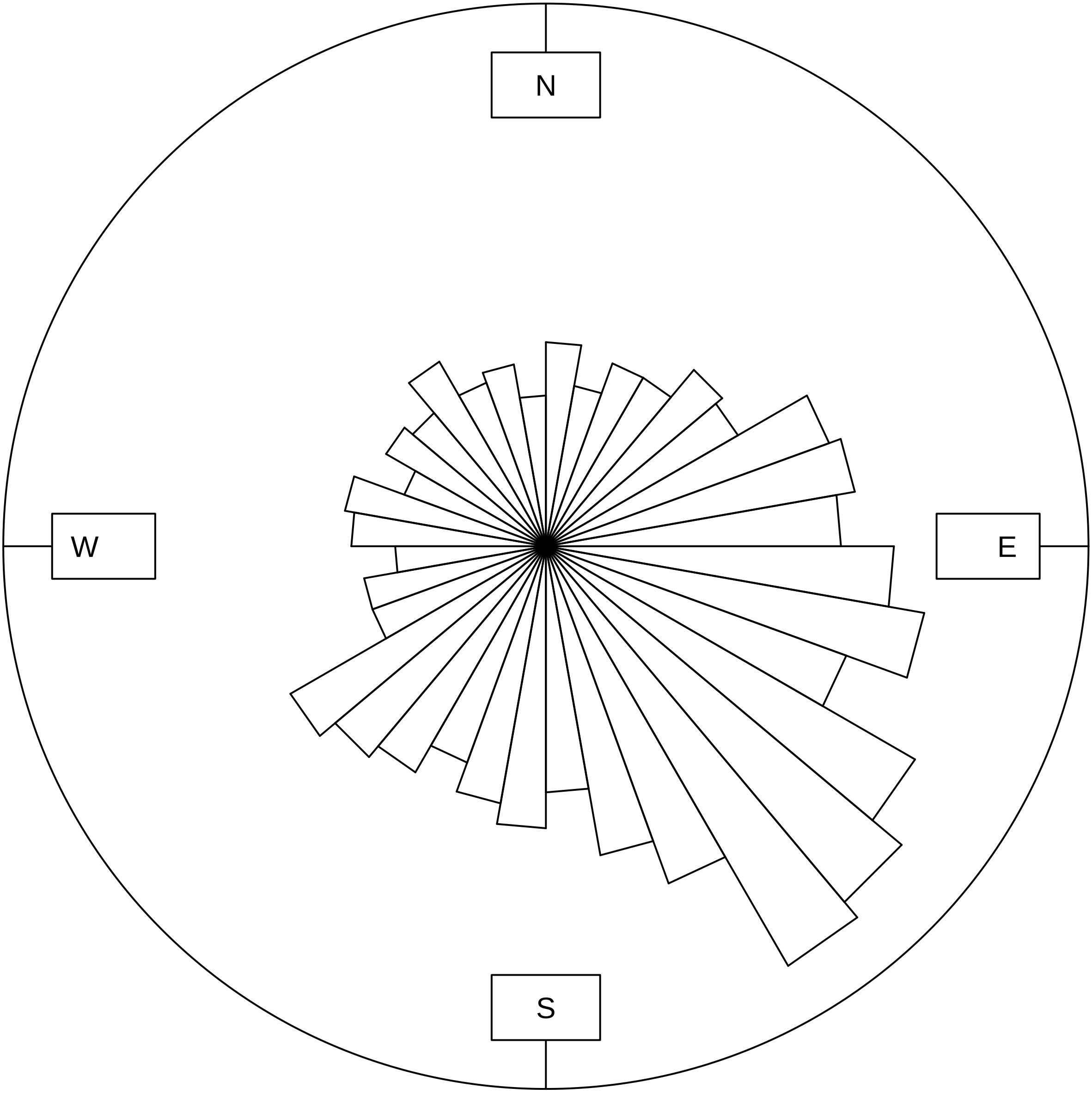

bootstrap 927

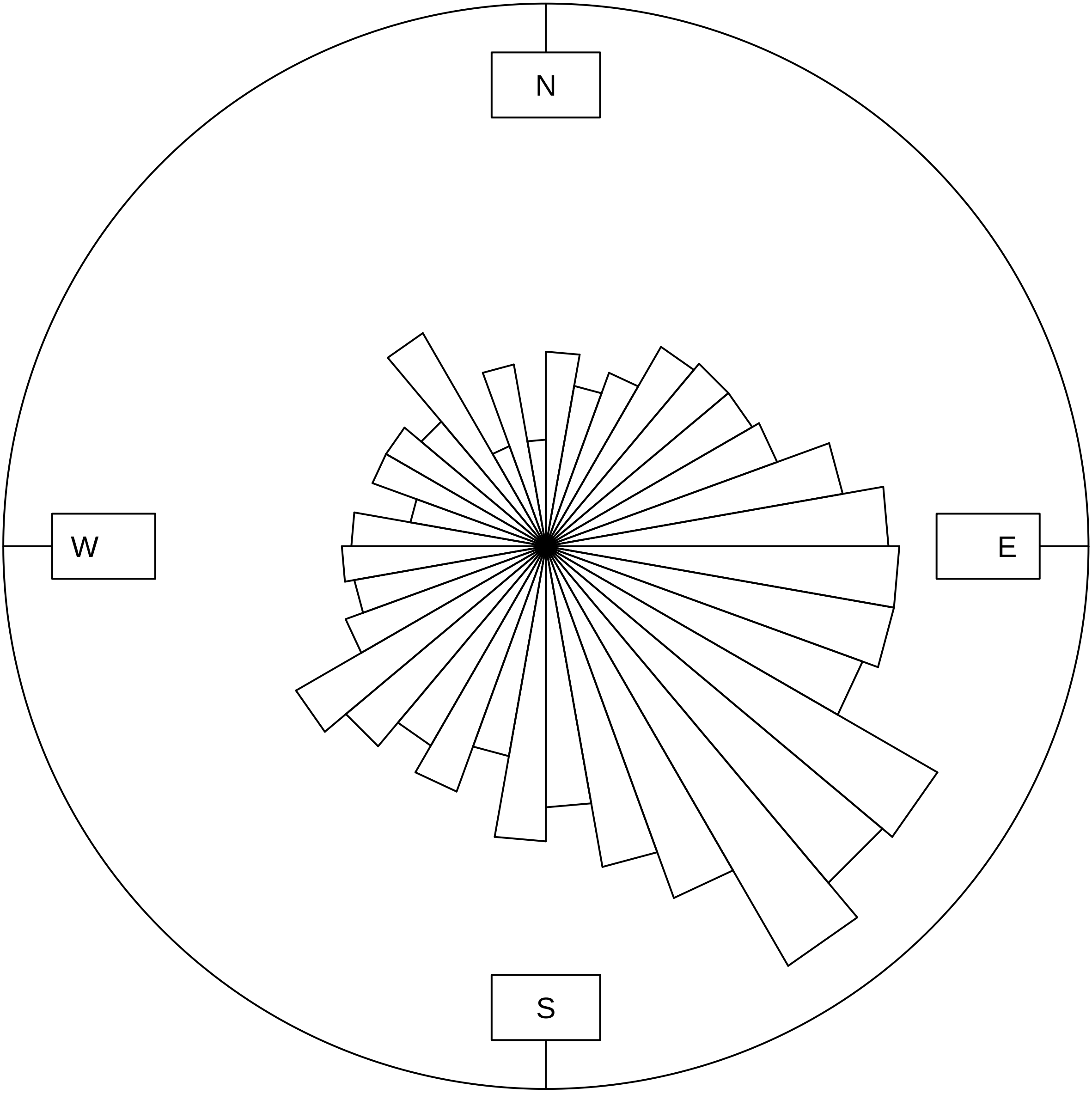

bootstrap 928

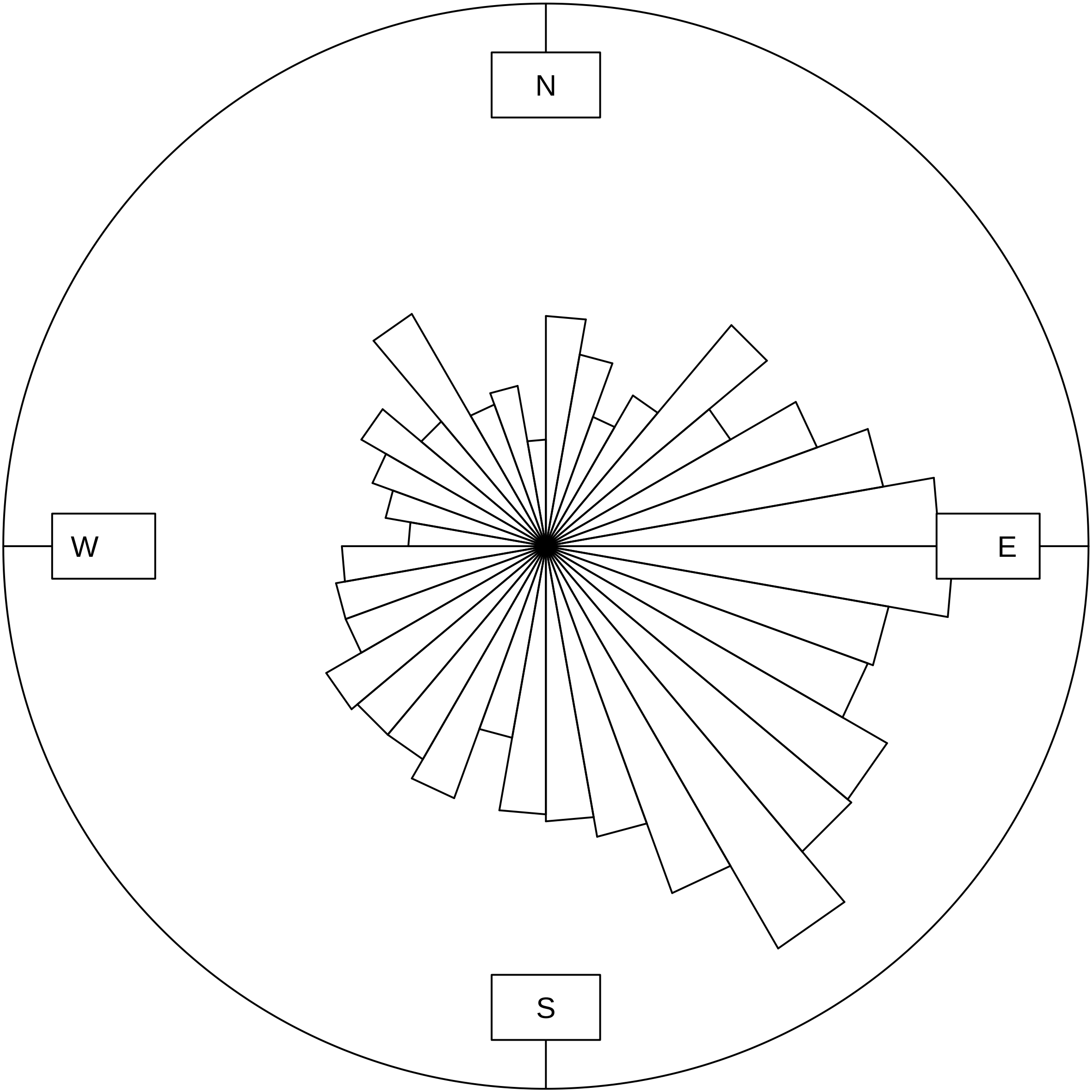

bootstrap 929

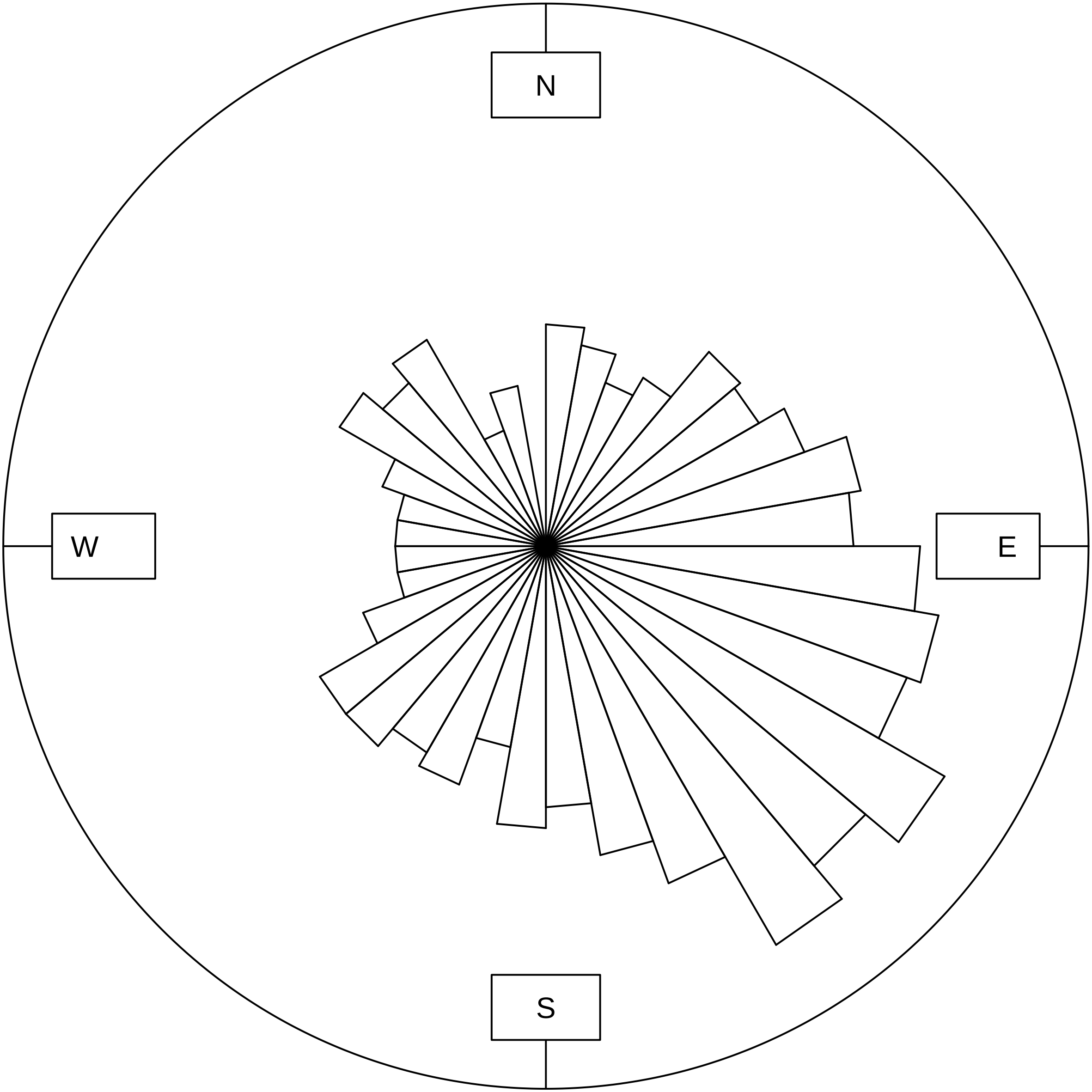

bootstrap 930

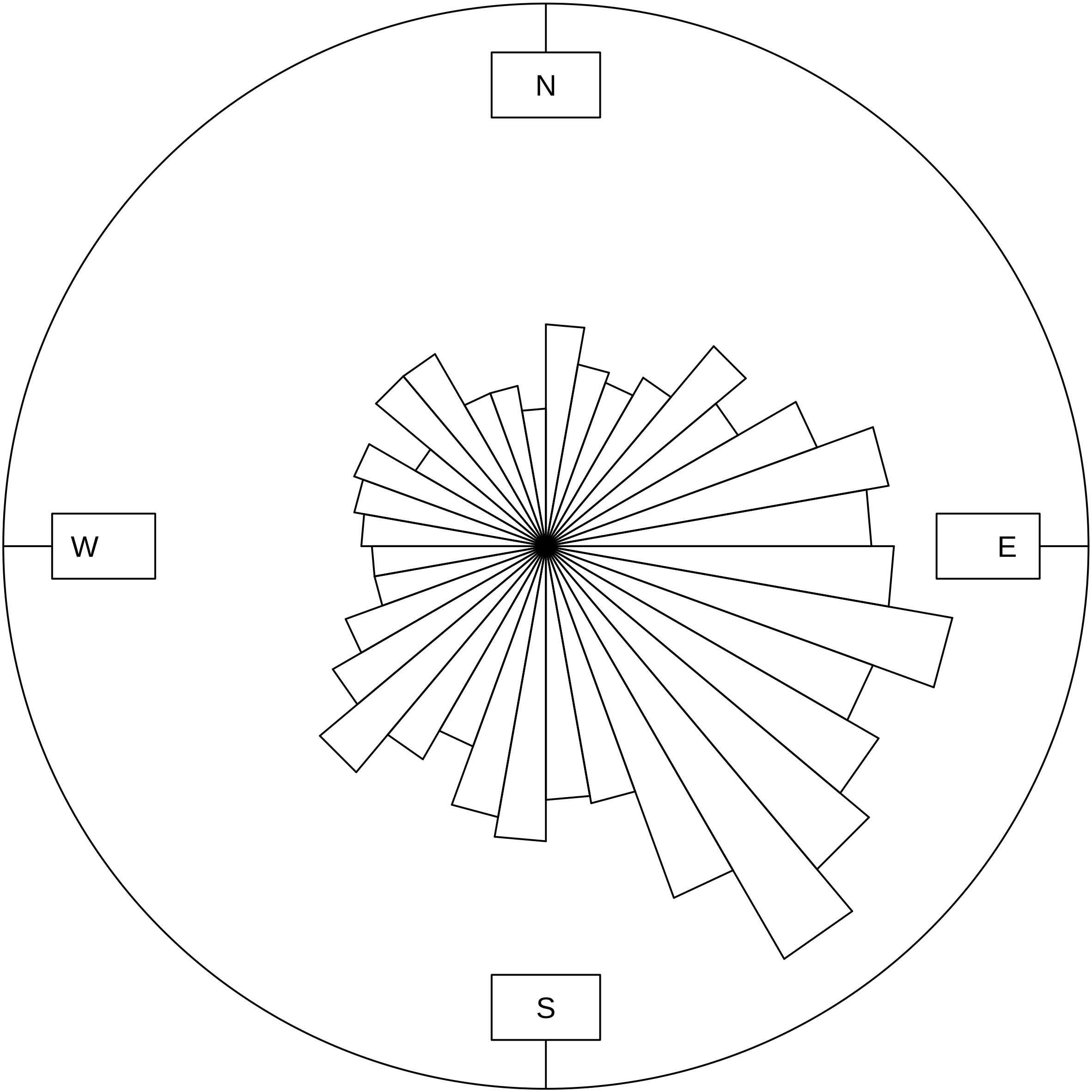

bootstrap 931

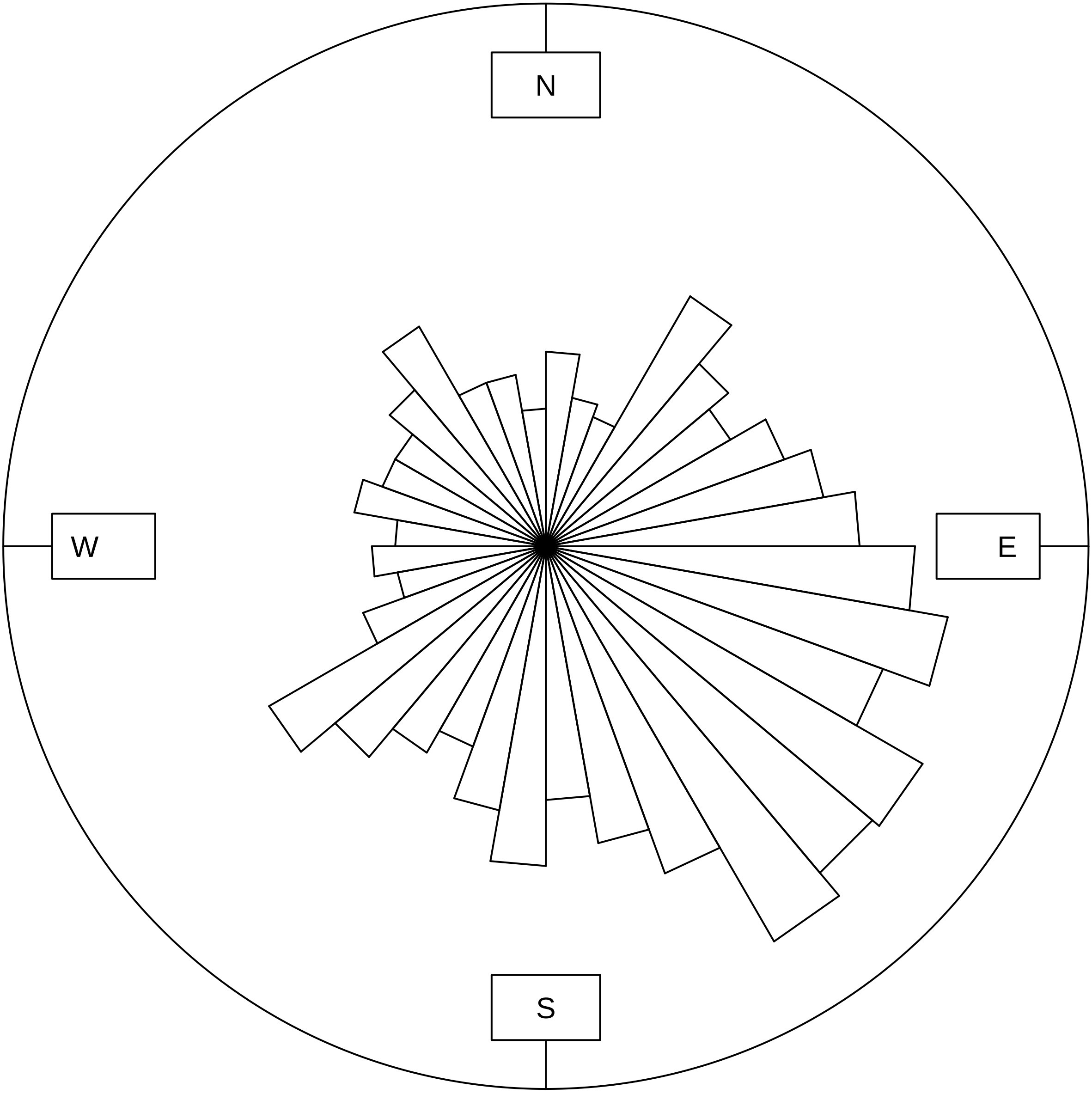

bootstrap 932

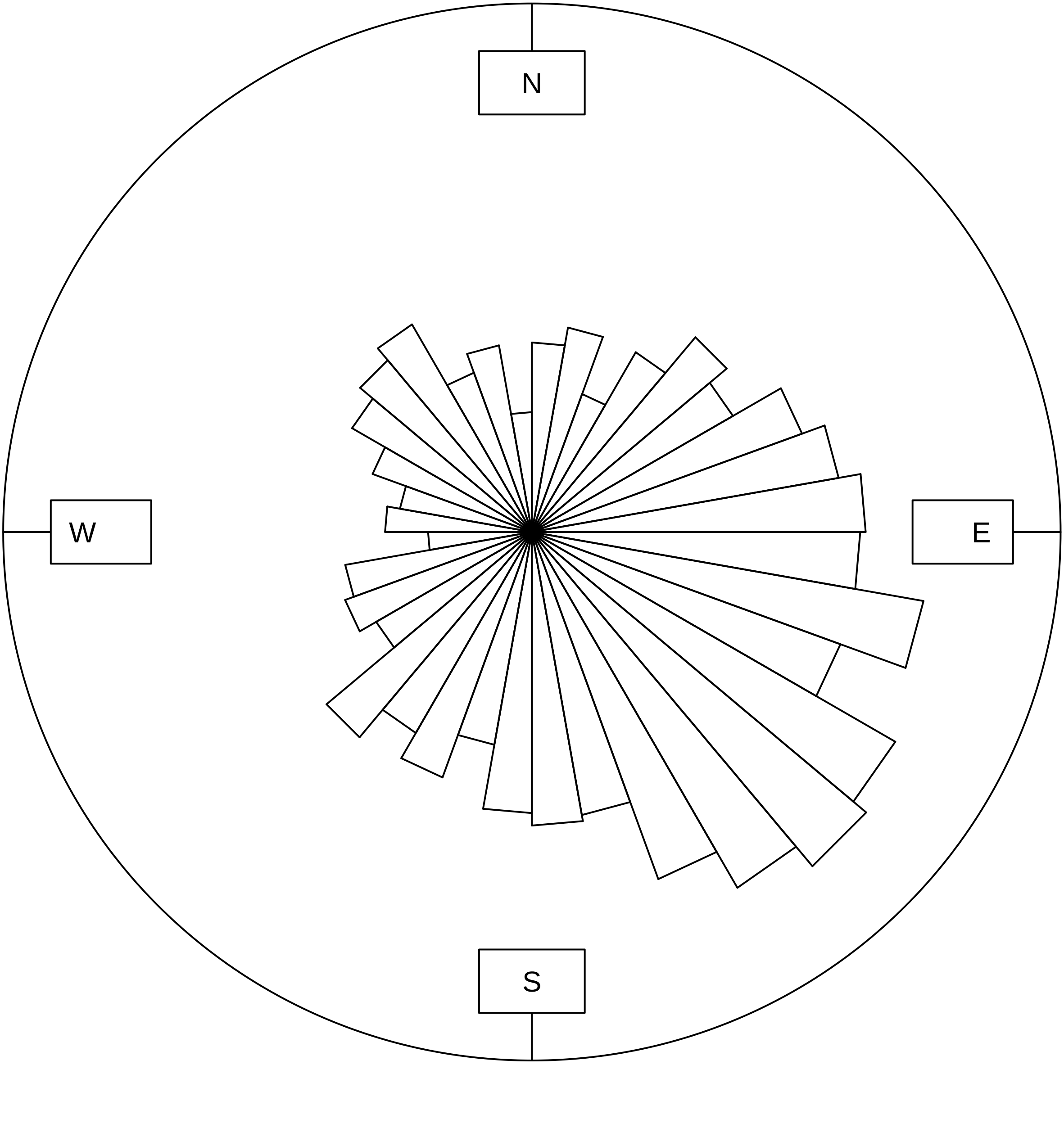

bootstrap 933

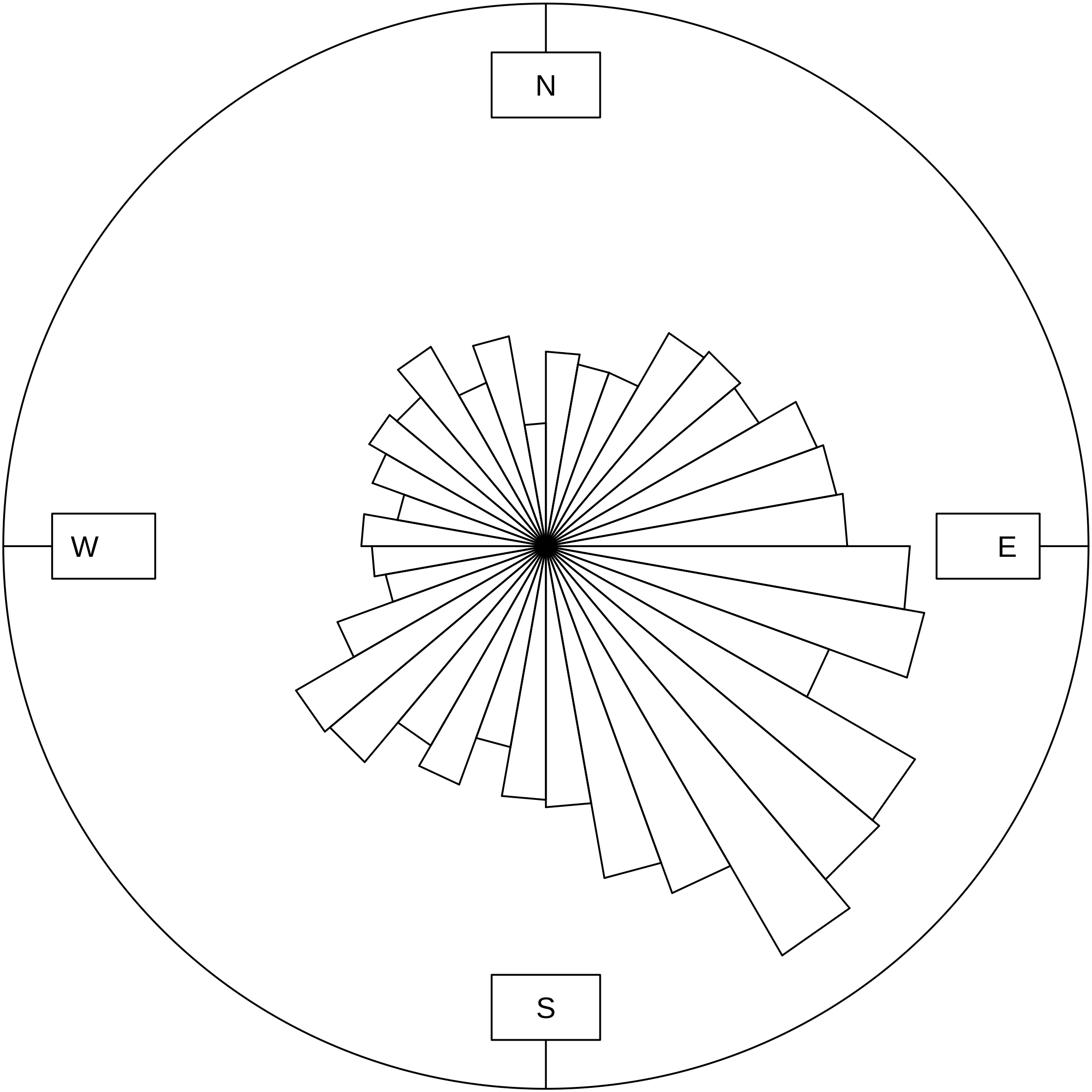

bootstrap 934

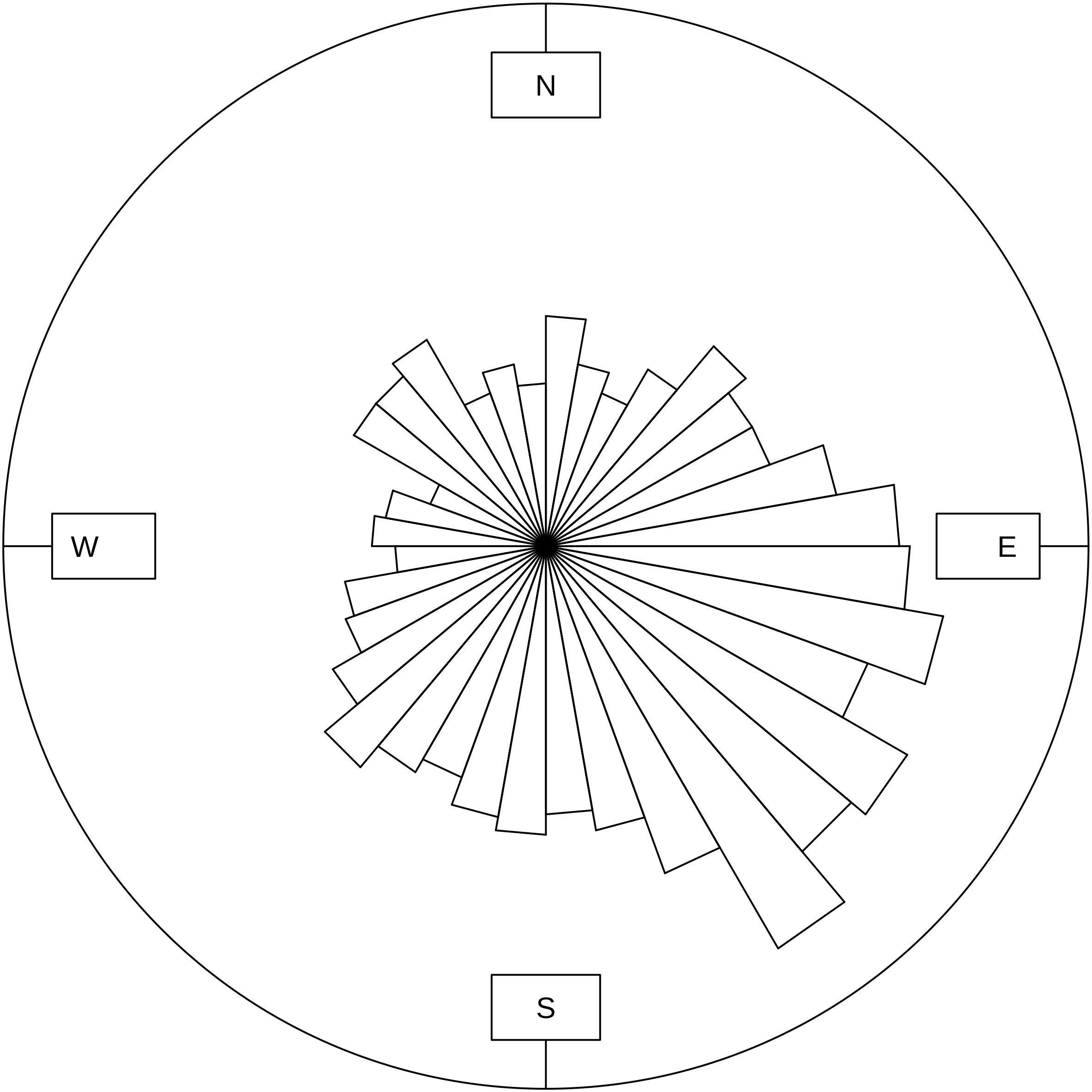

bootstrap 935

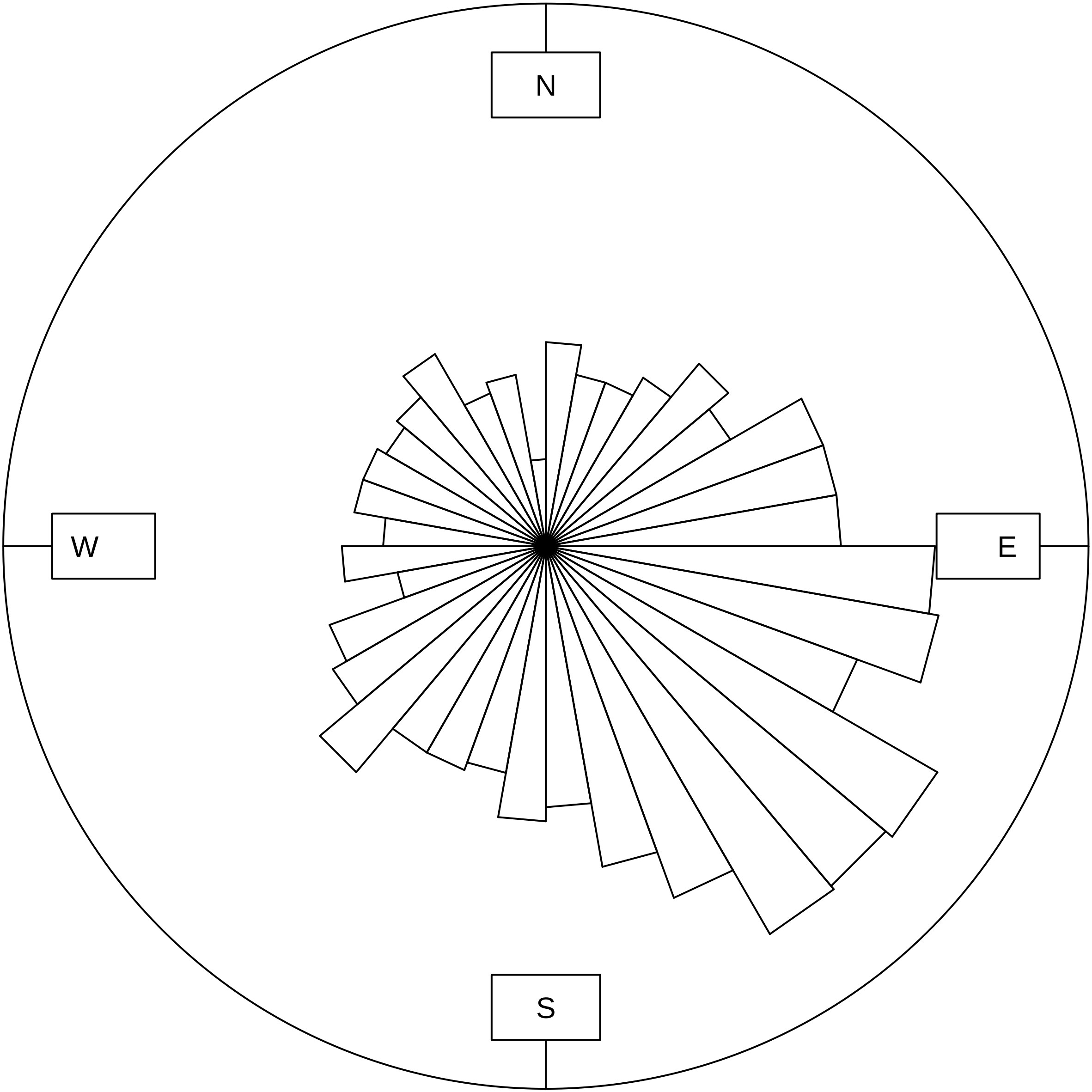

bootstrap 936

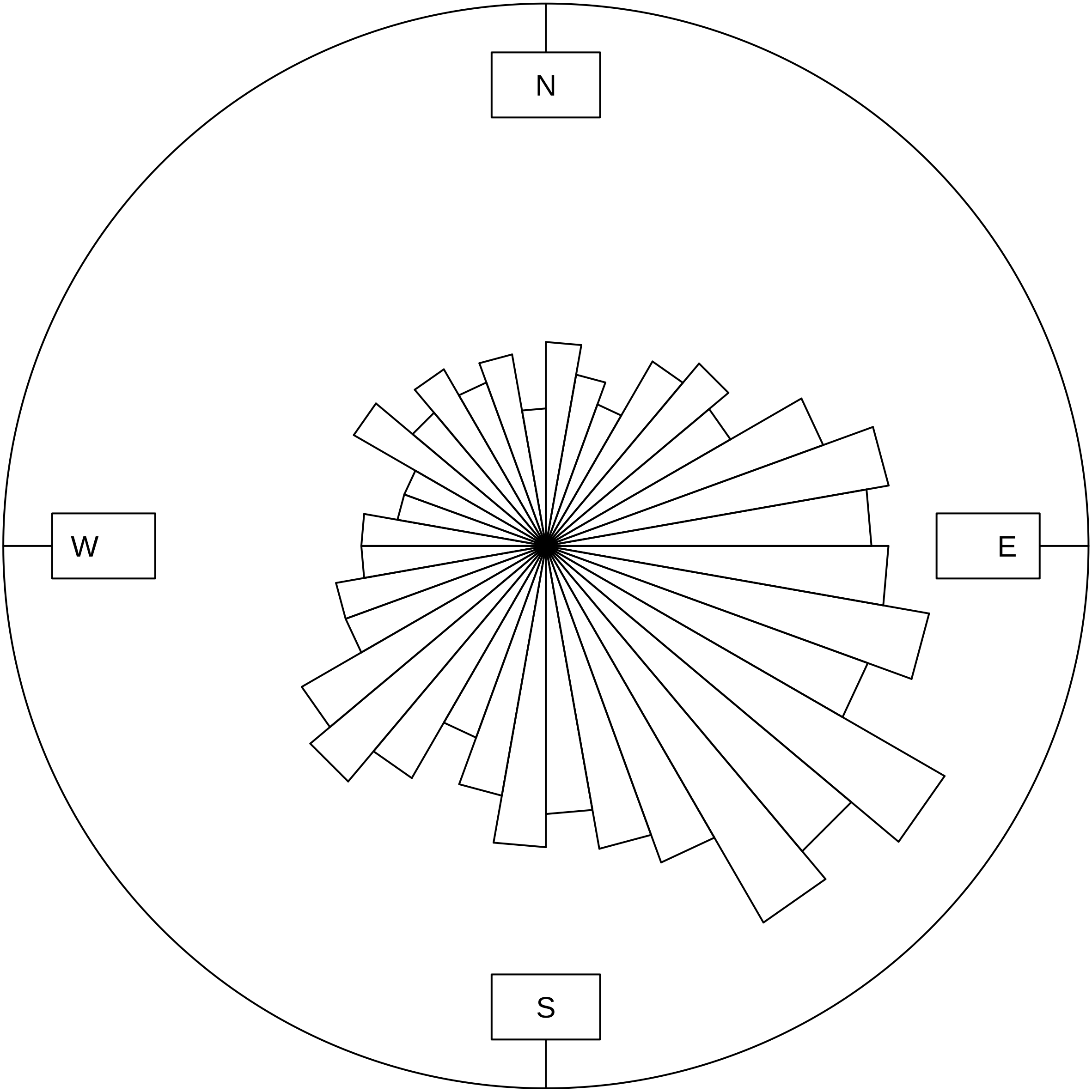

bootstrap 937

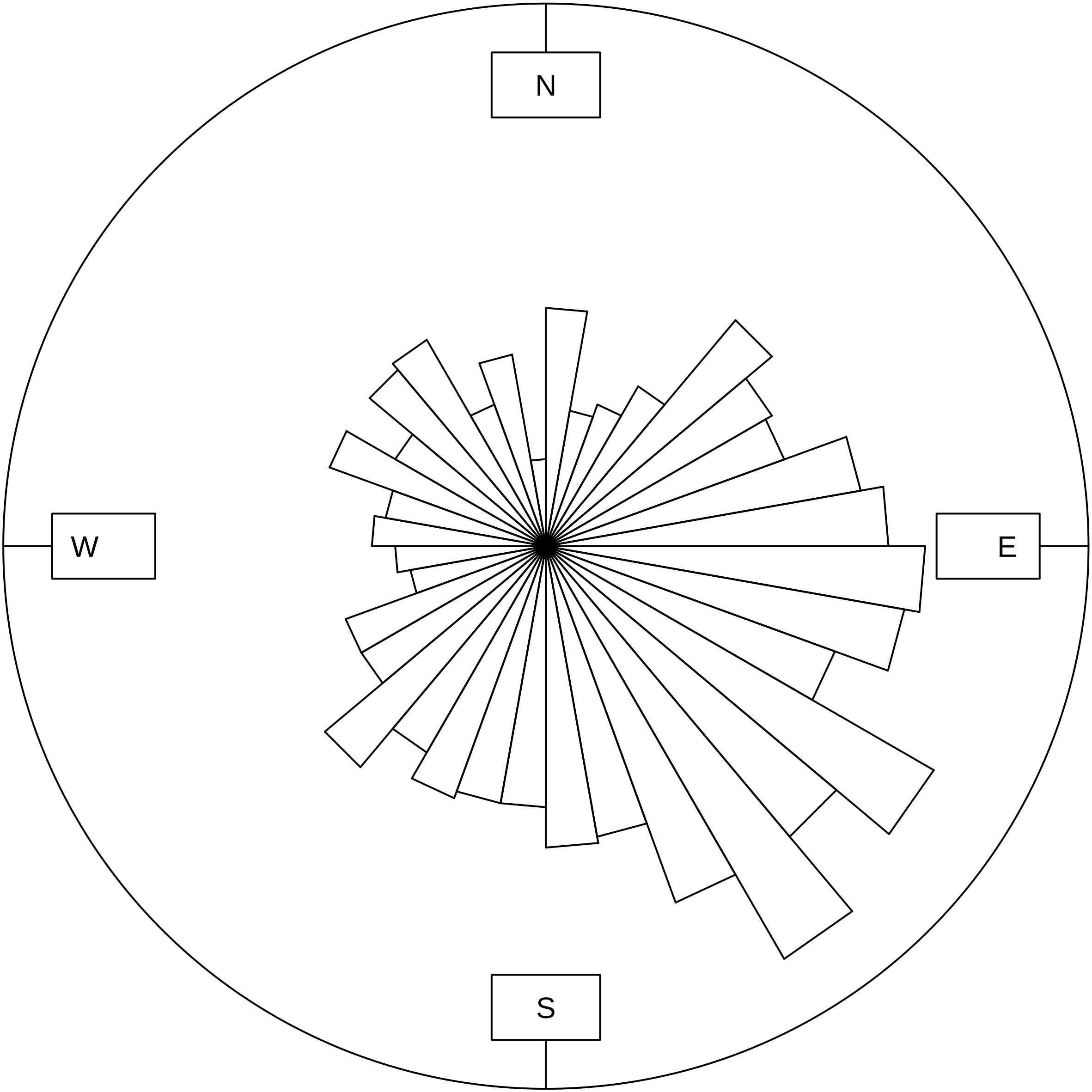

bootstrap 938

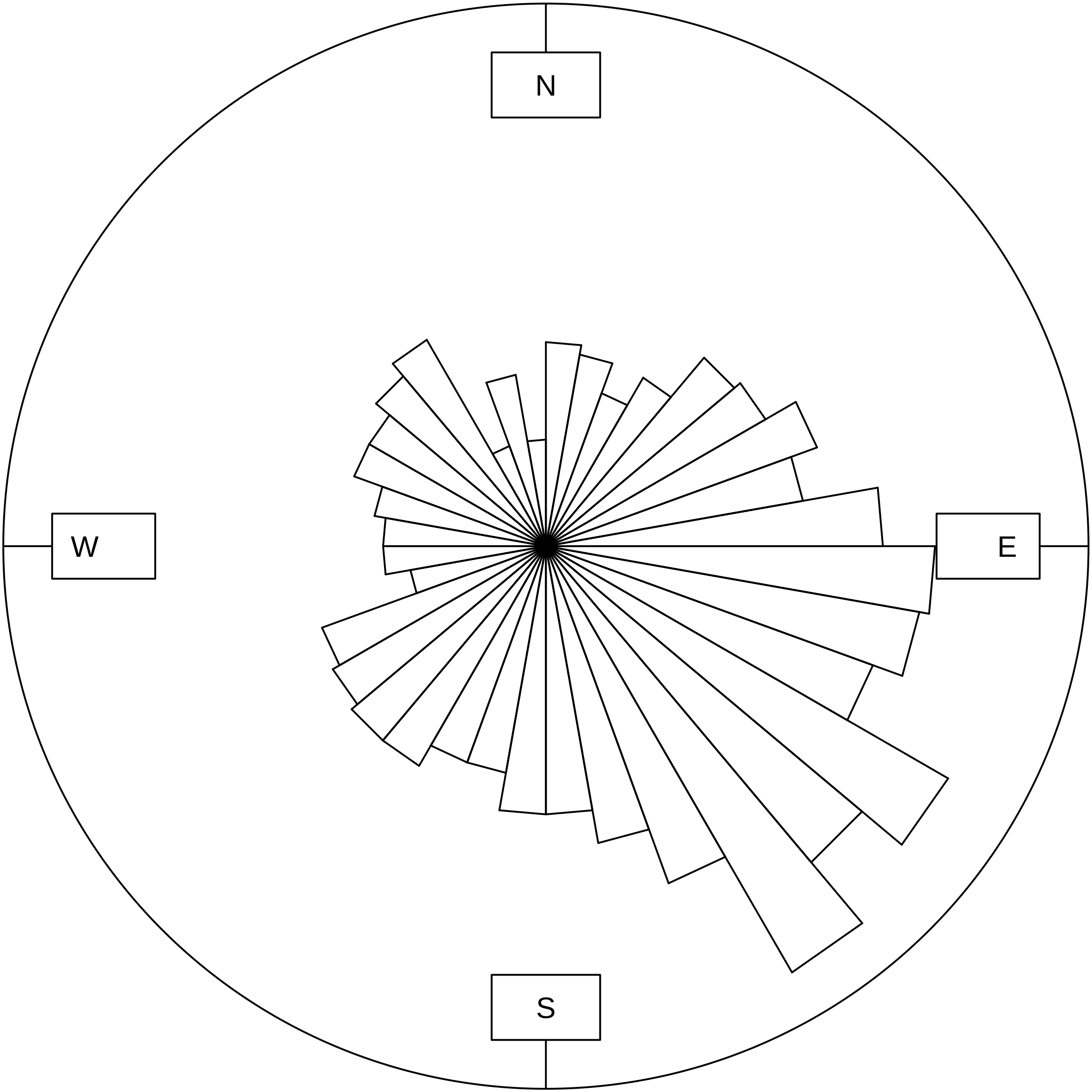

bootstrap 939

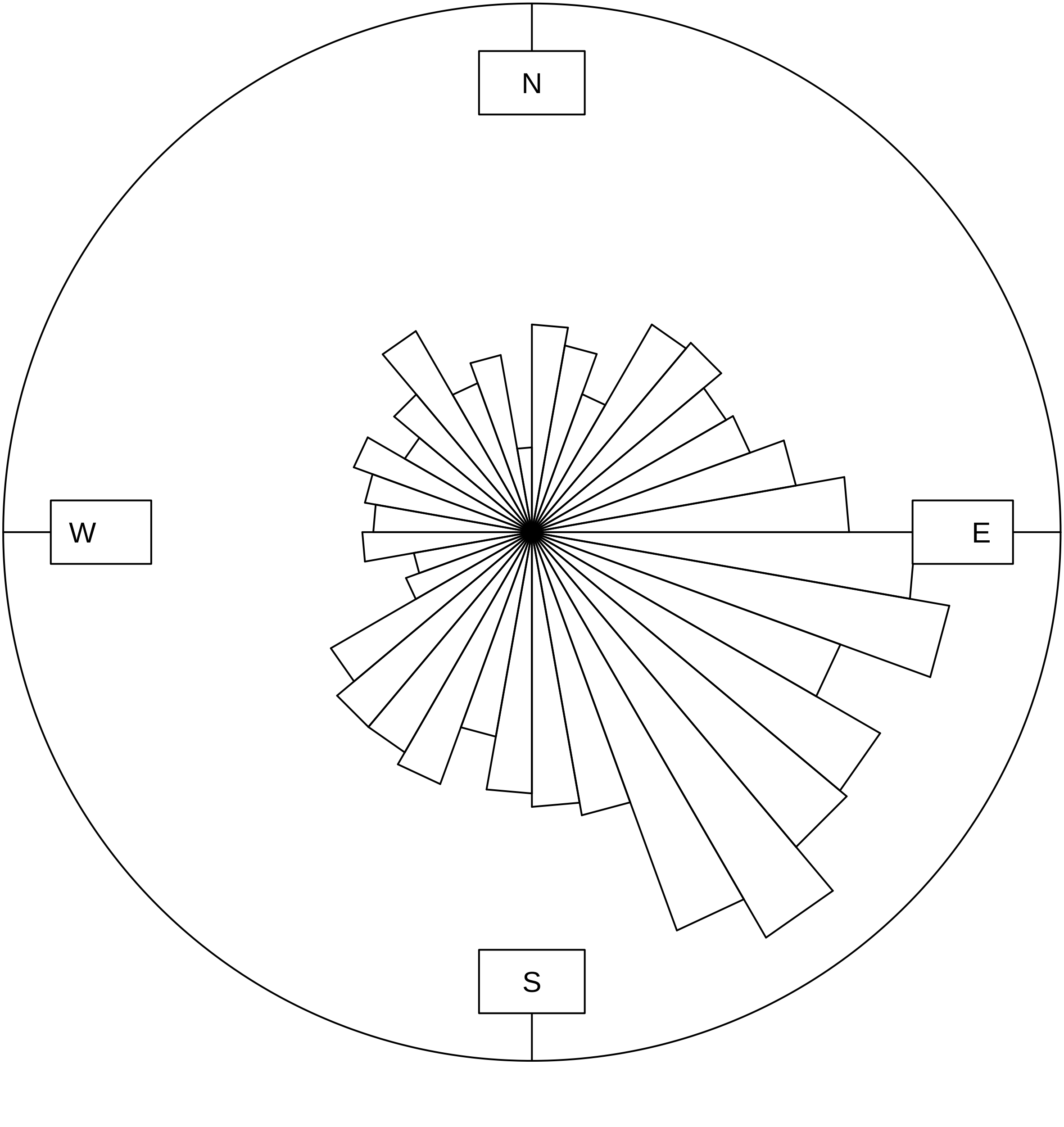

bootstrap 940

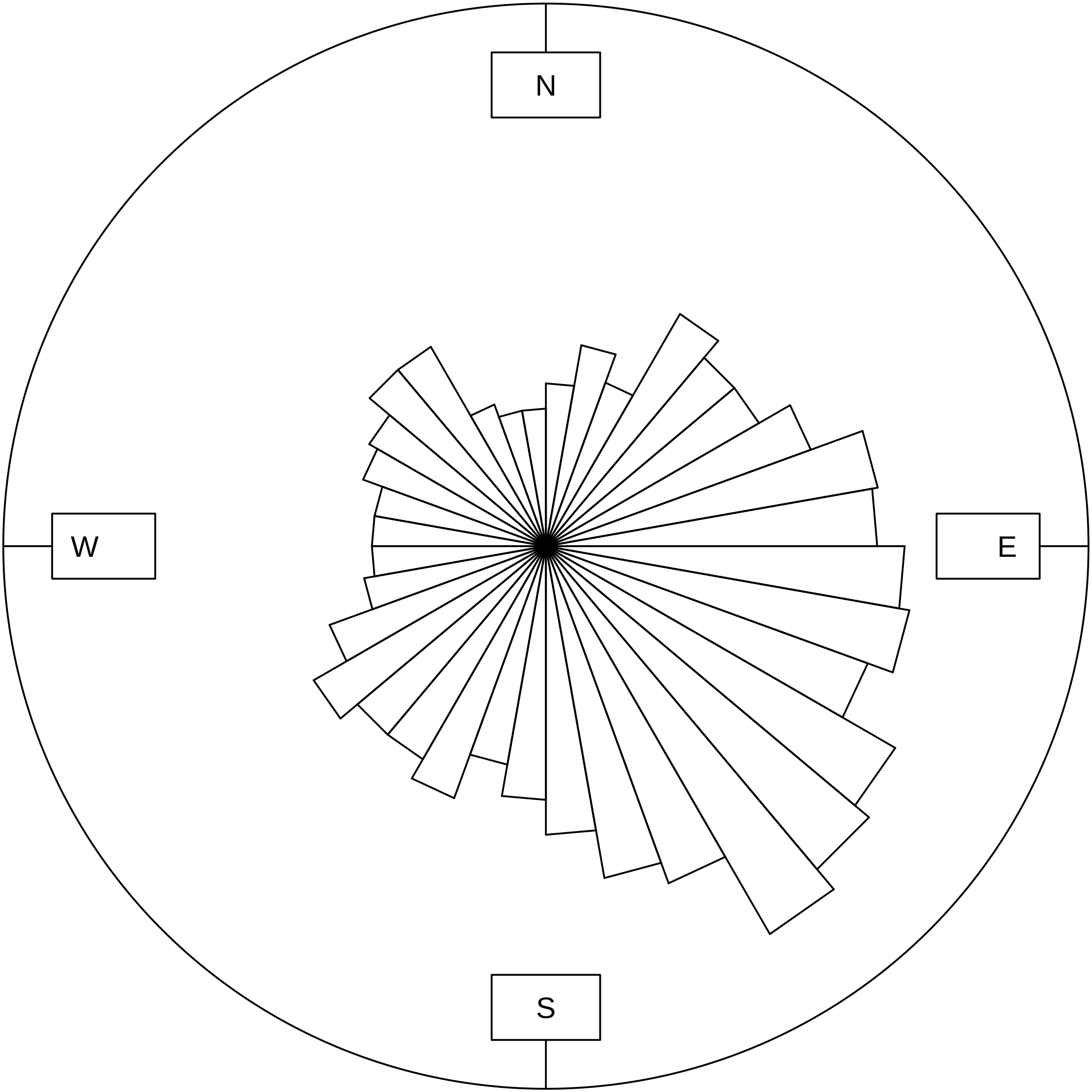

bootstrap 941

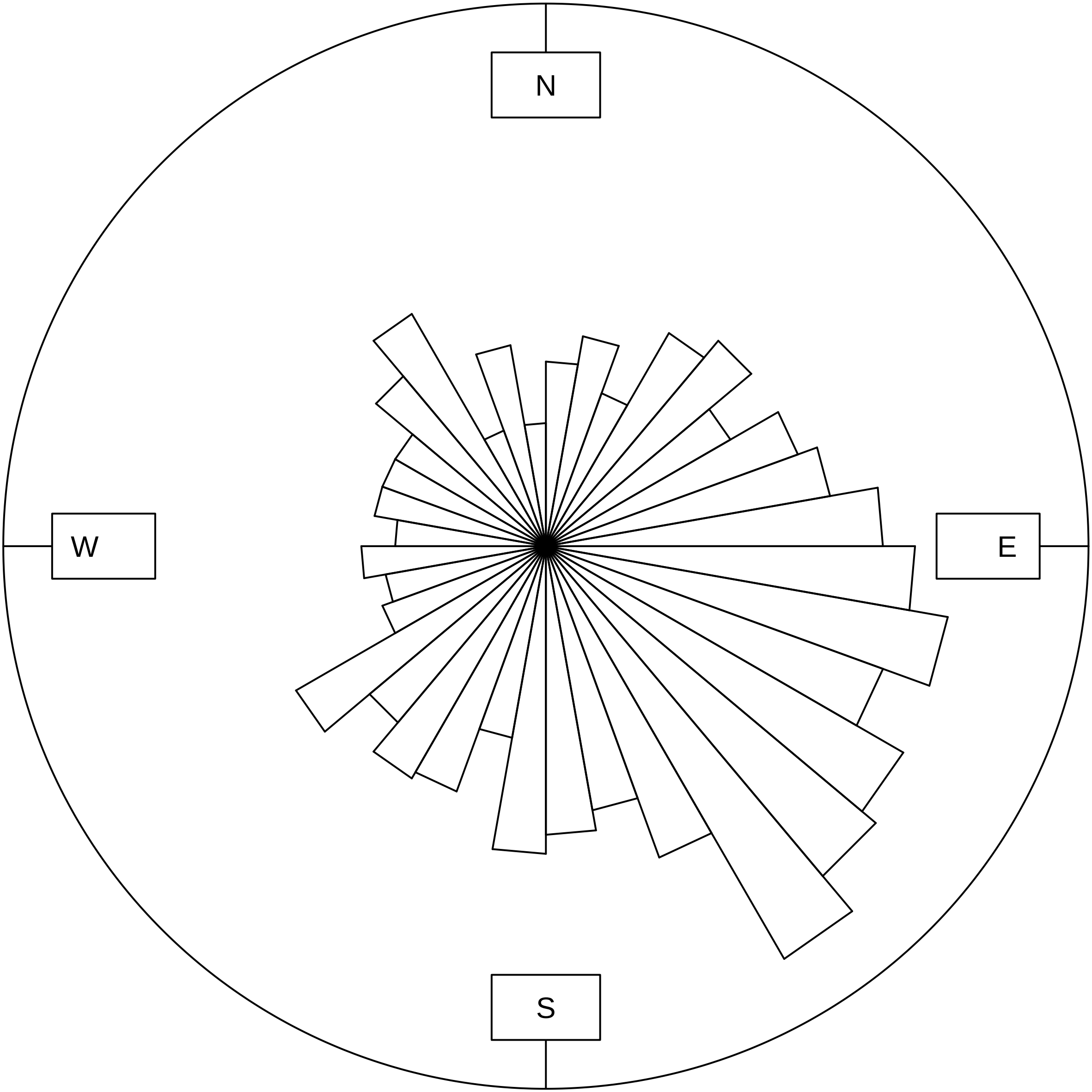

bootstrap 942

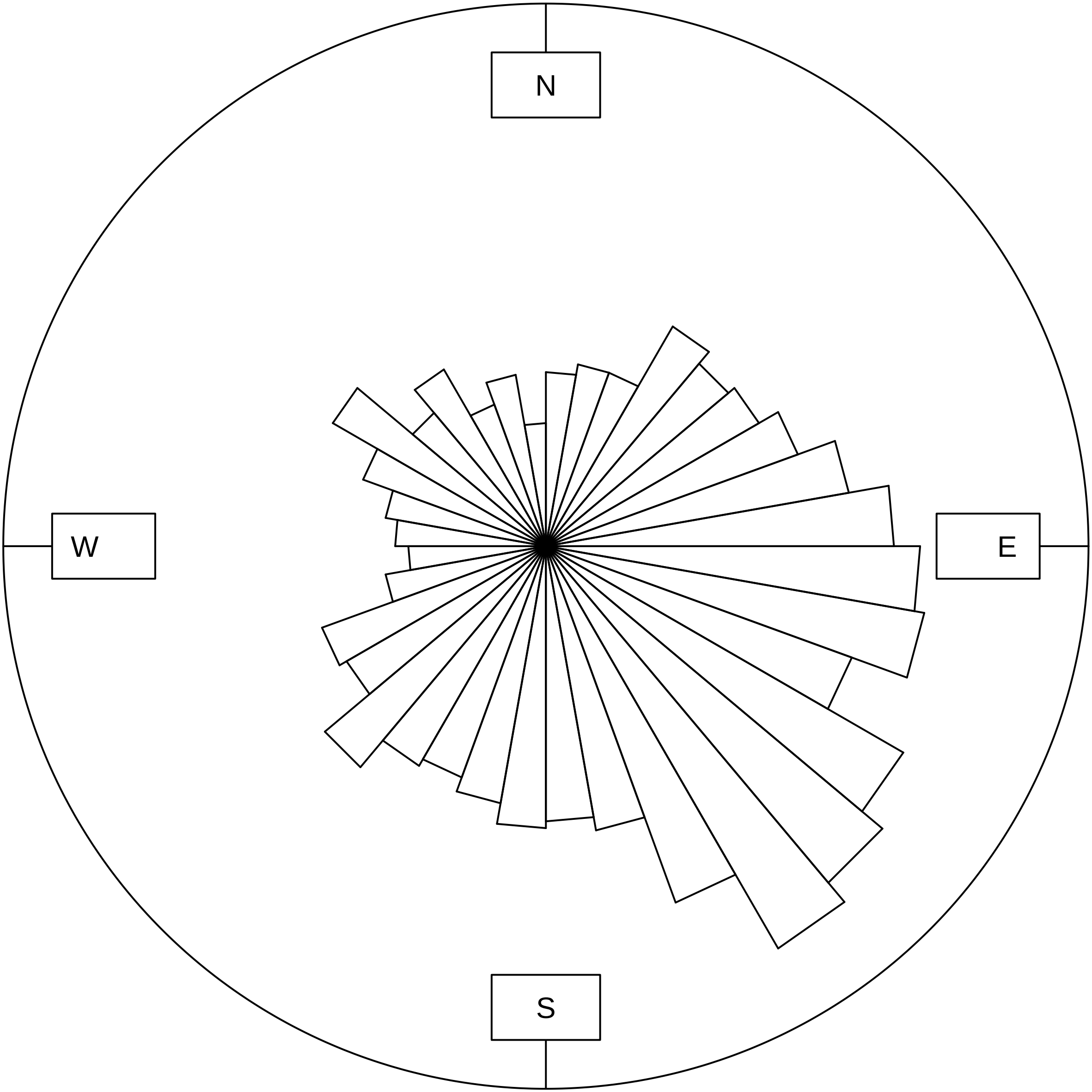

bootstrap 943

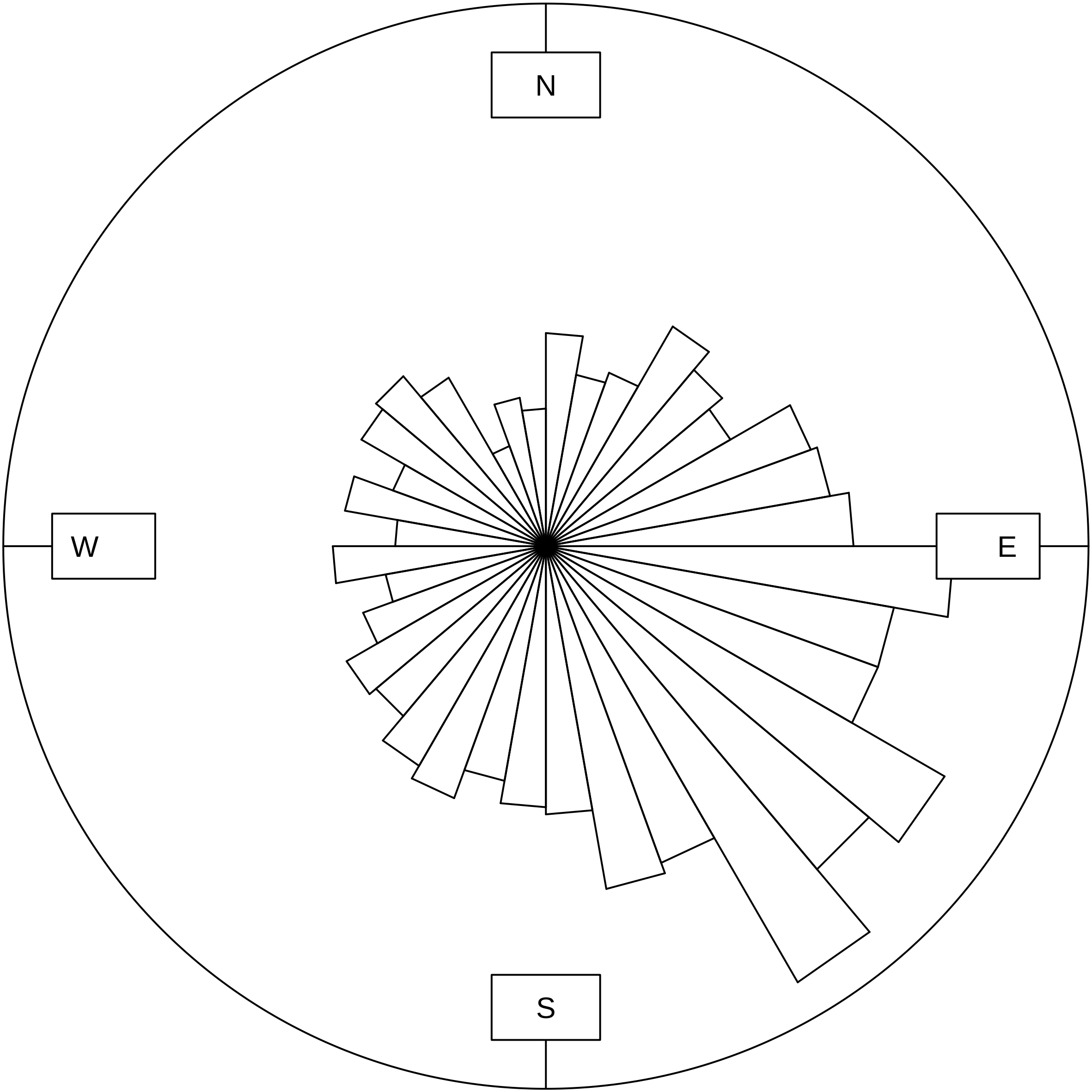

bootstrap 944

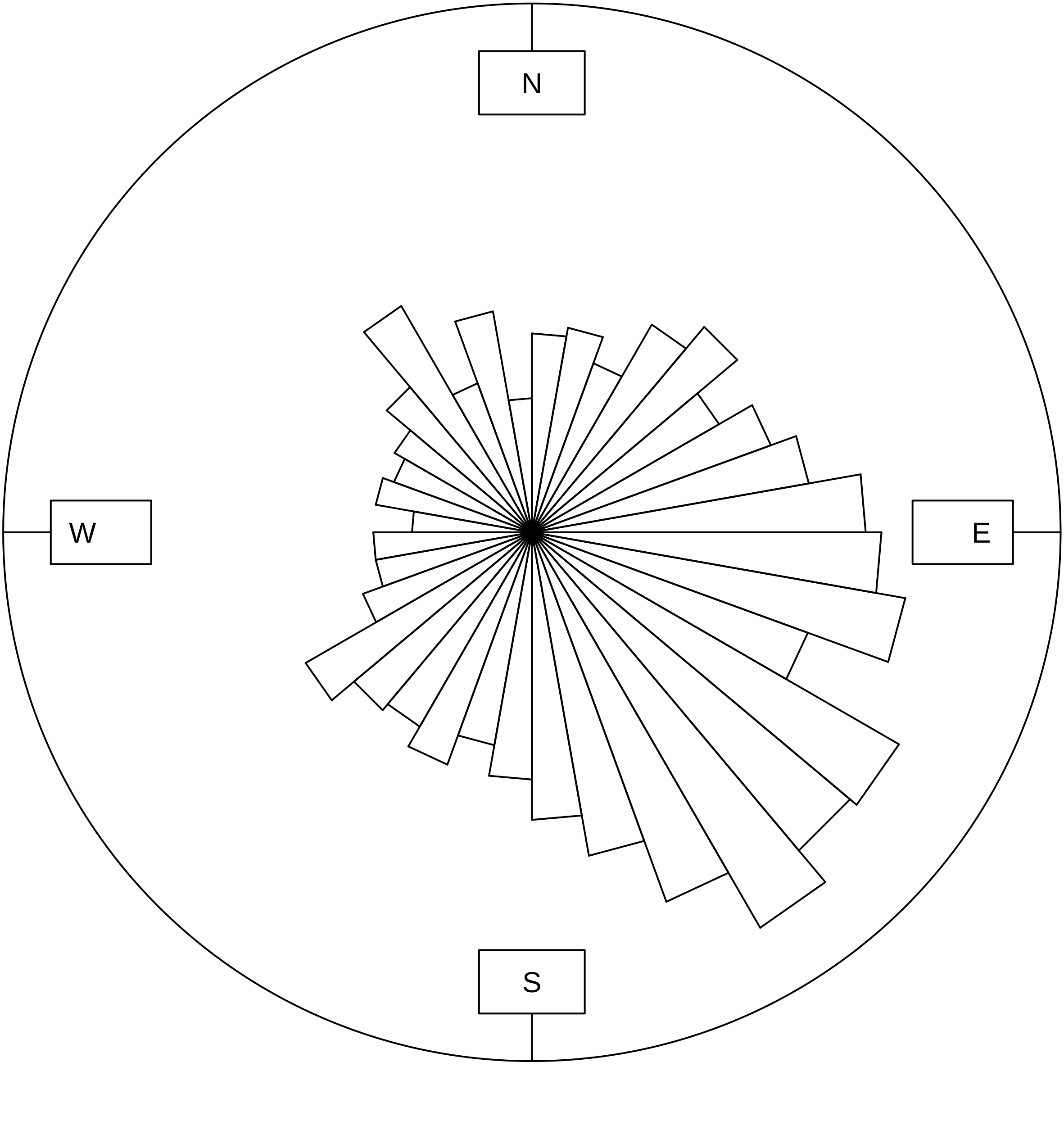

bootstrap 945

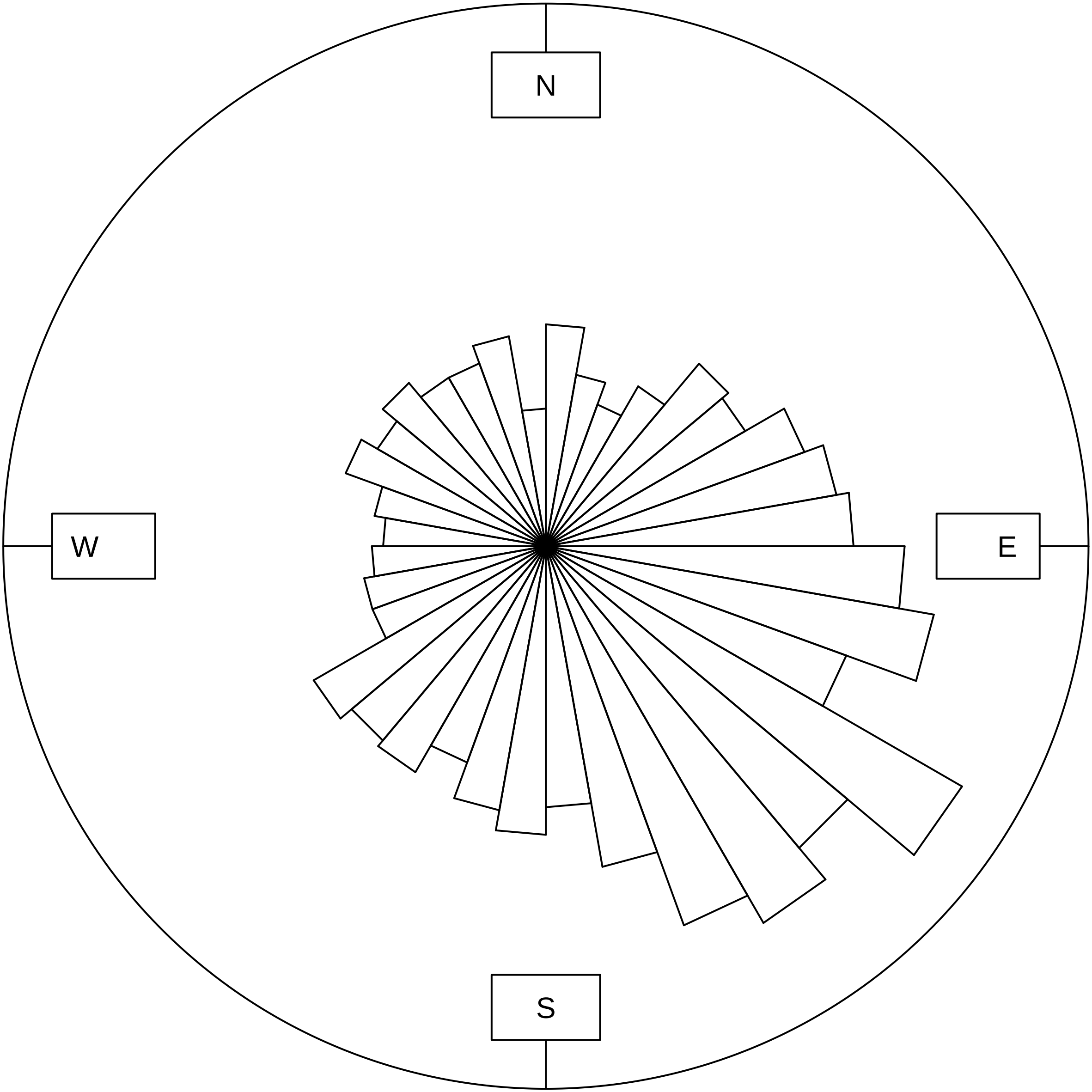

bootstrap 946

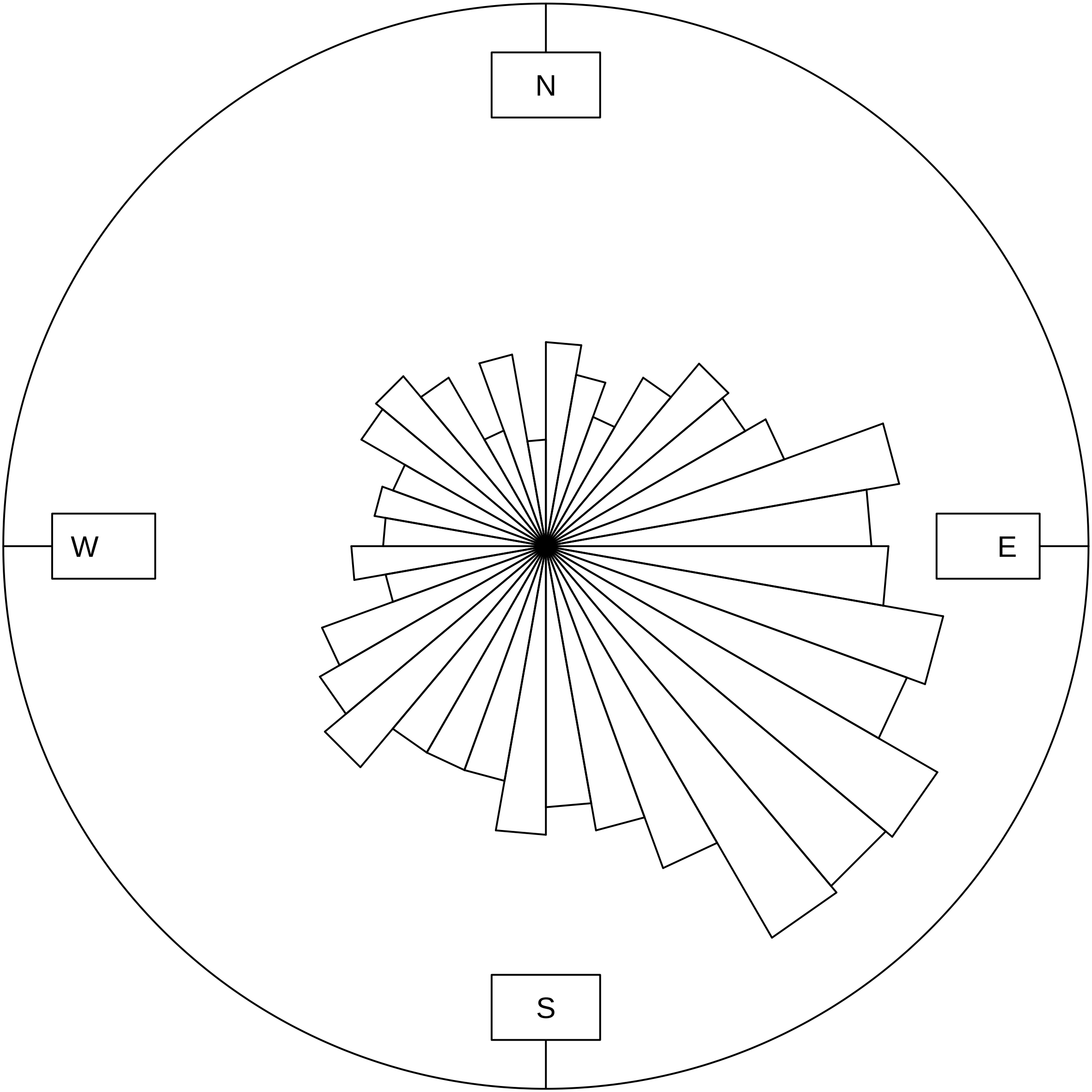

bootstrap 947

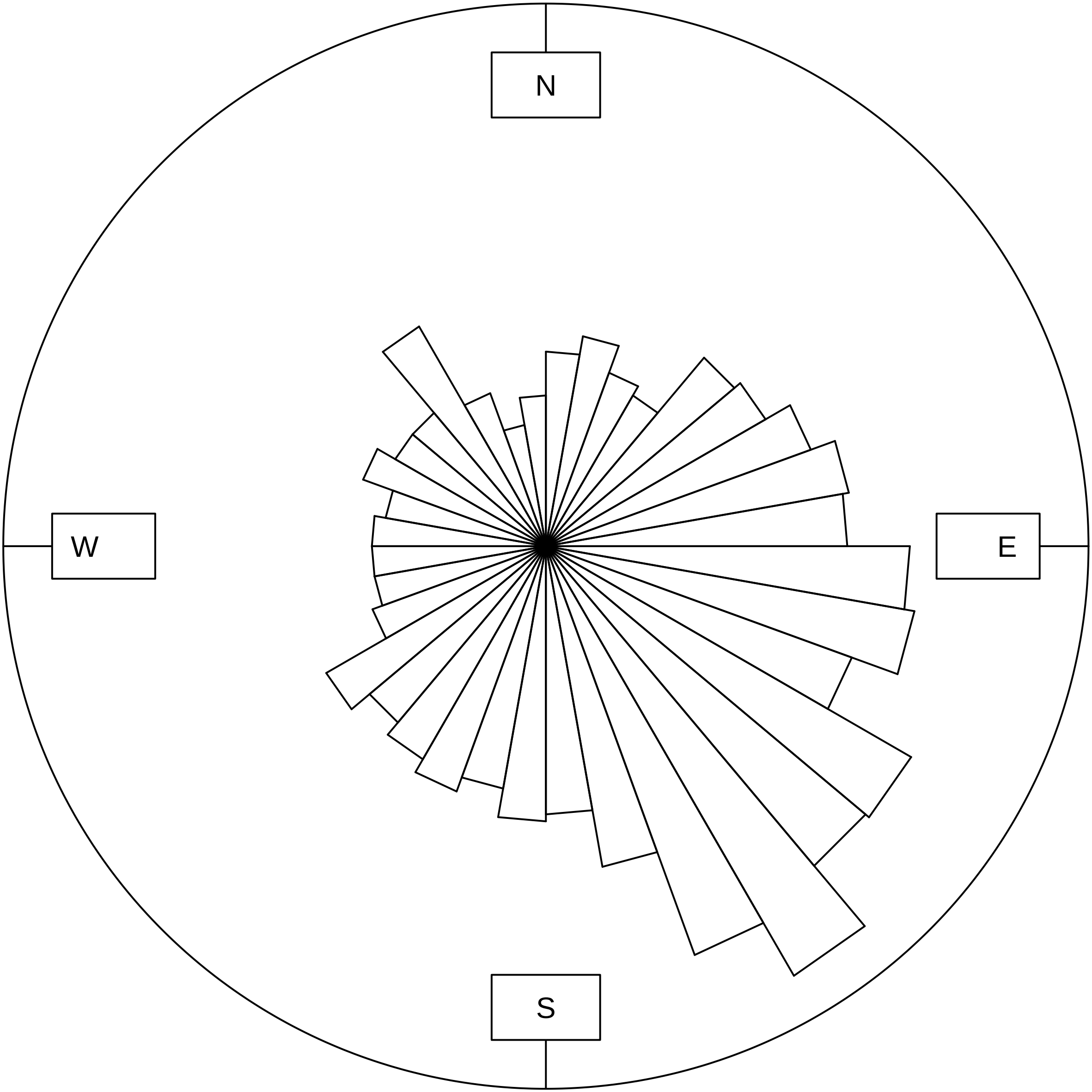

bootstrap 948

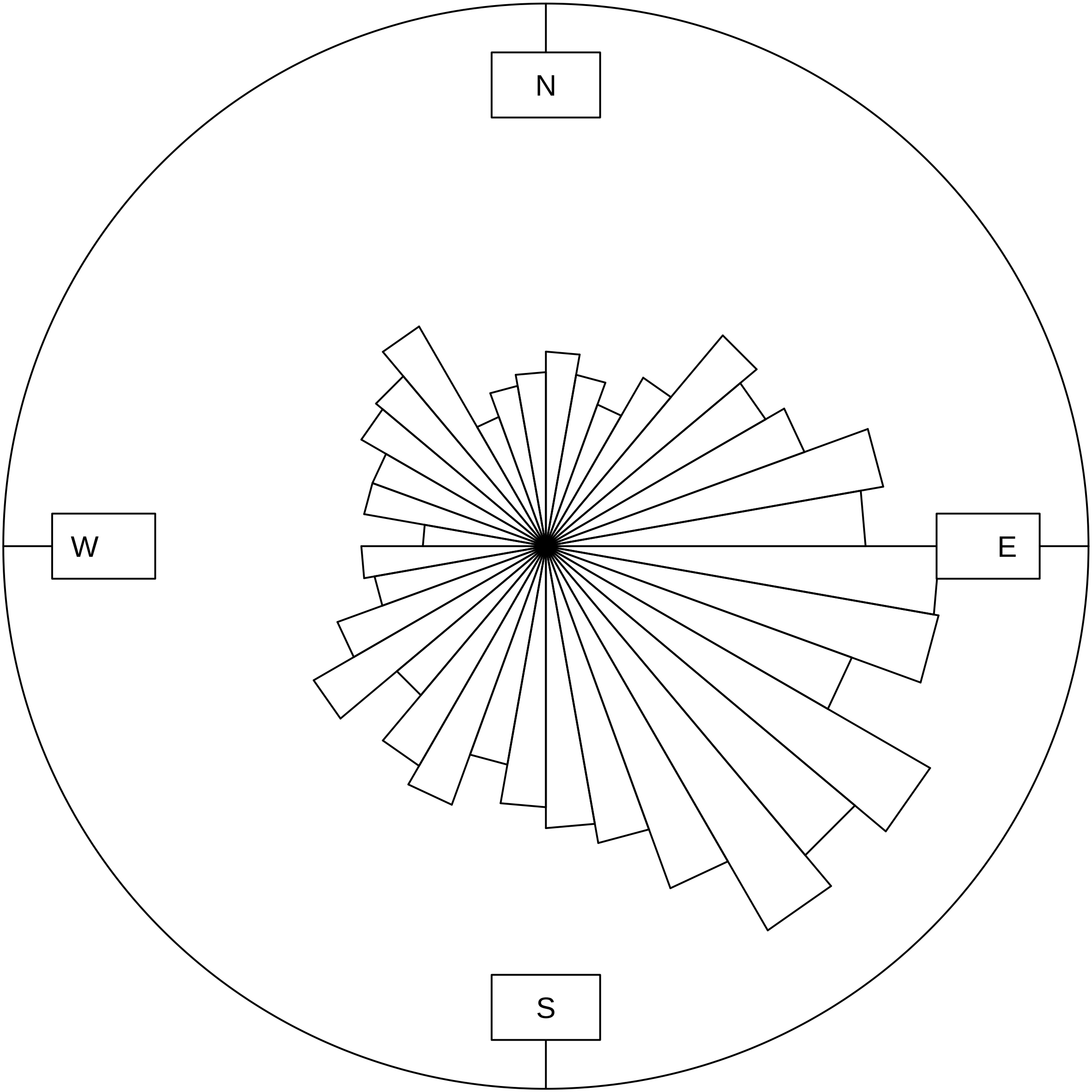

bootstrap 949

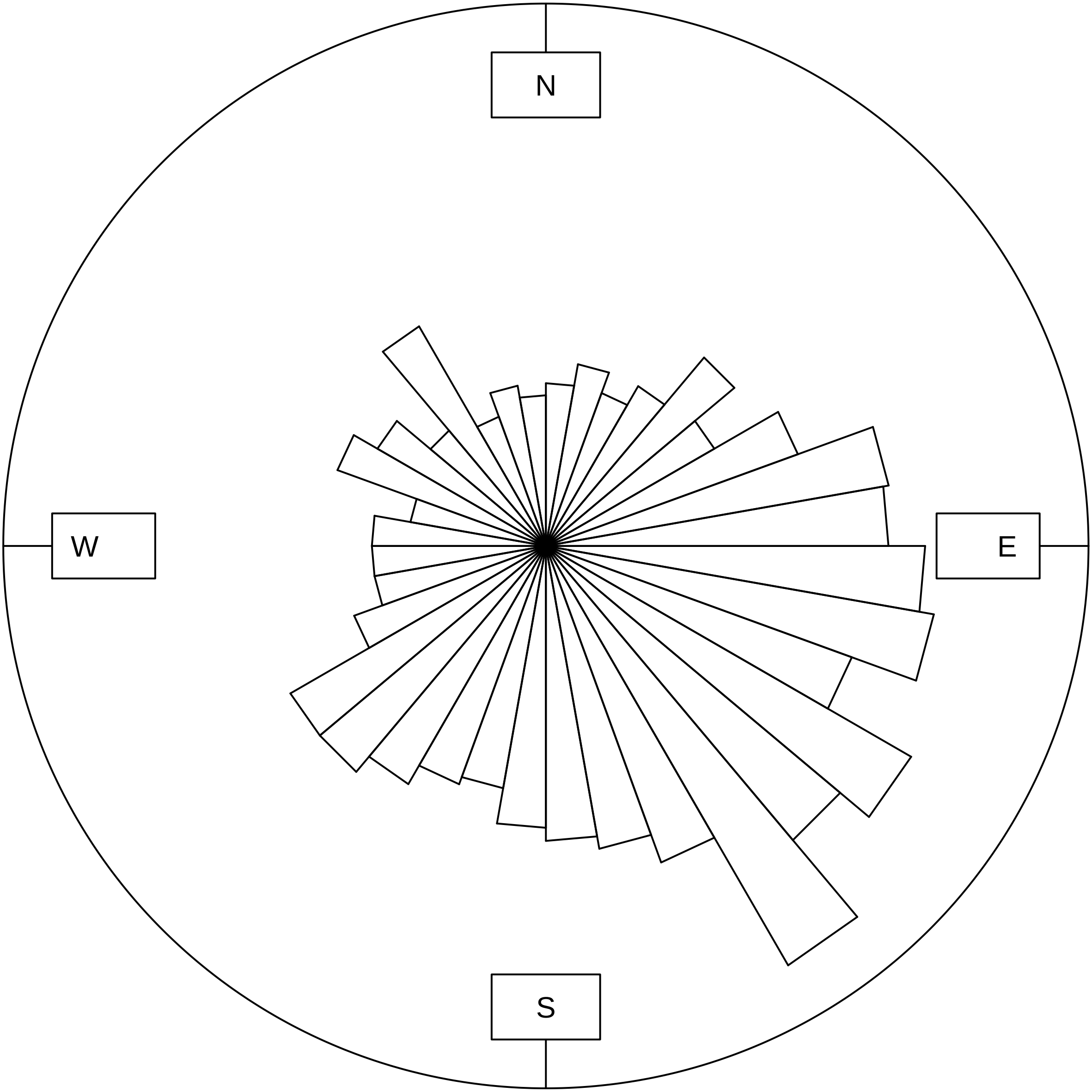

bootstrap 950

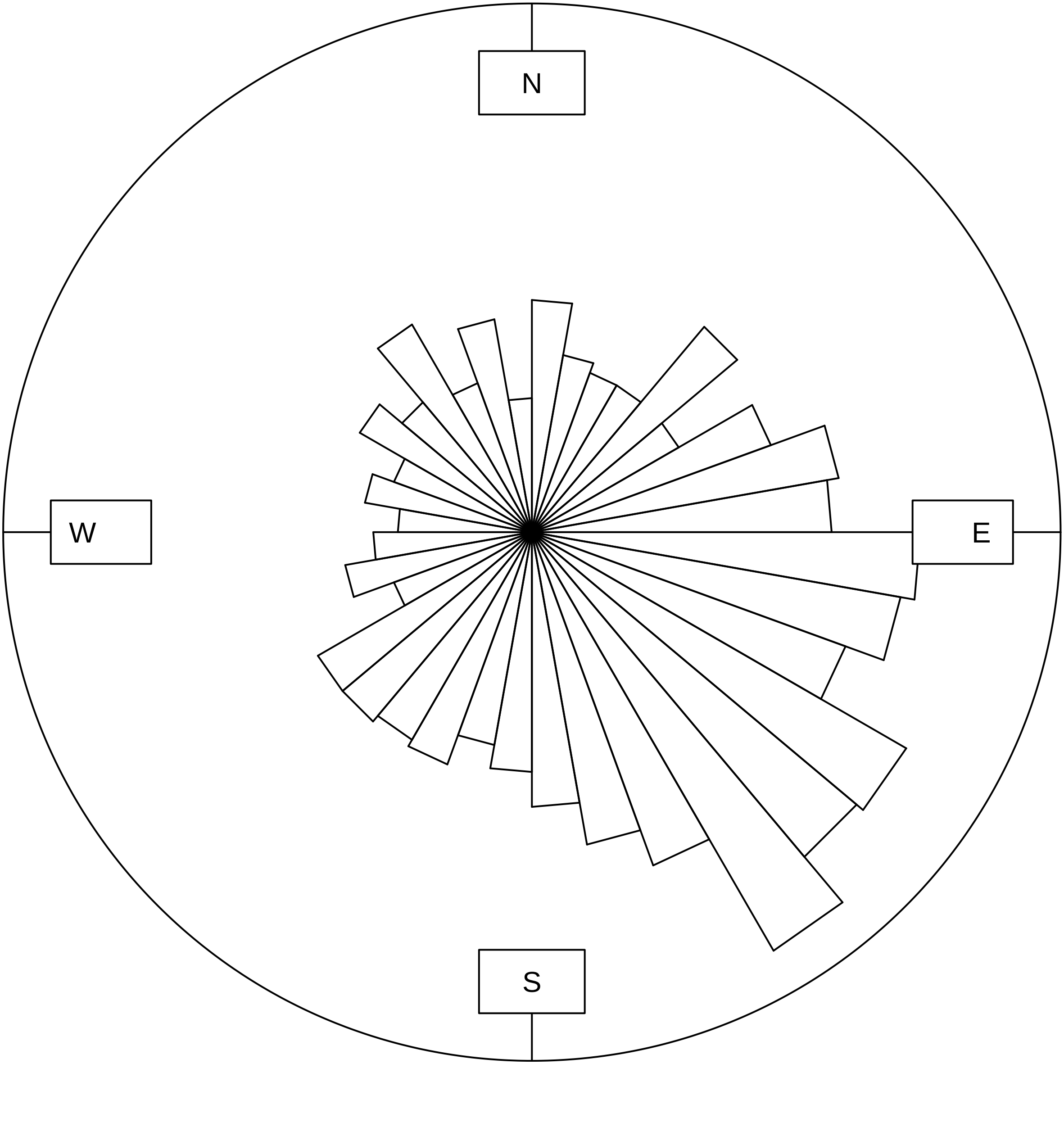

bootstrap 951

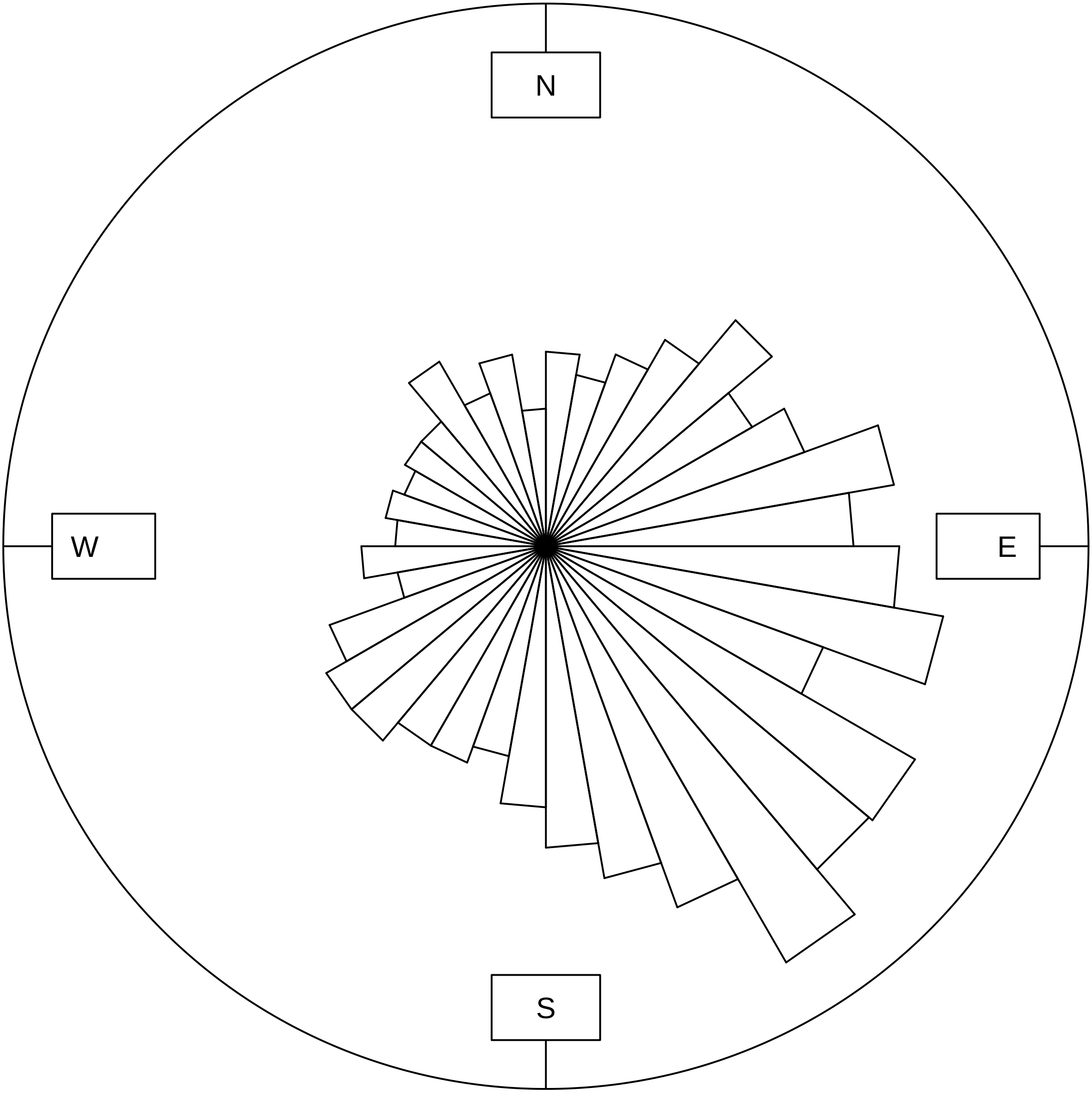

bootstrap 952

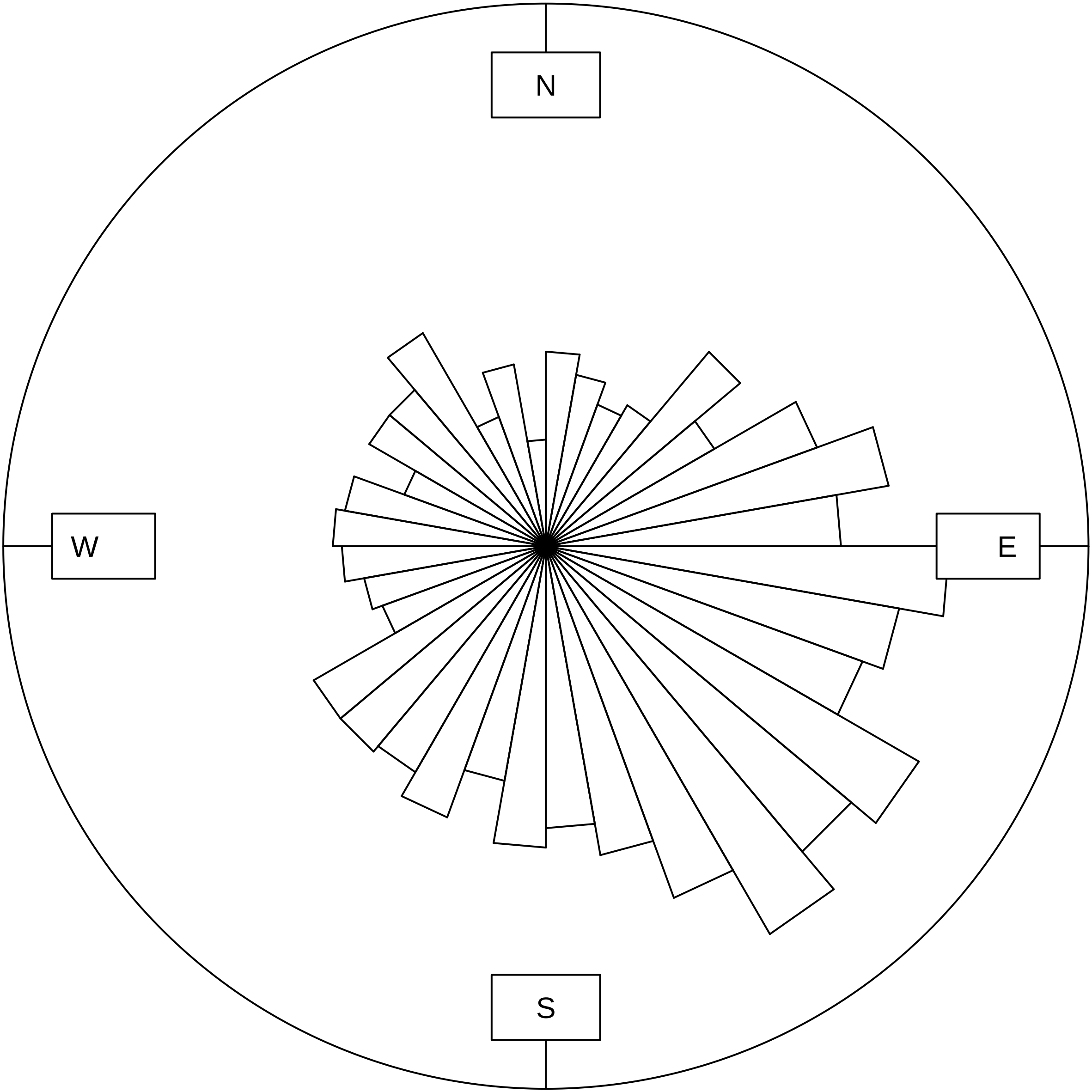

bootstrap 953

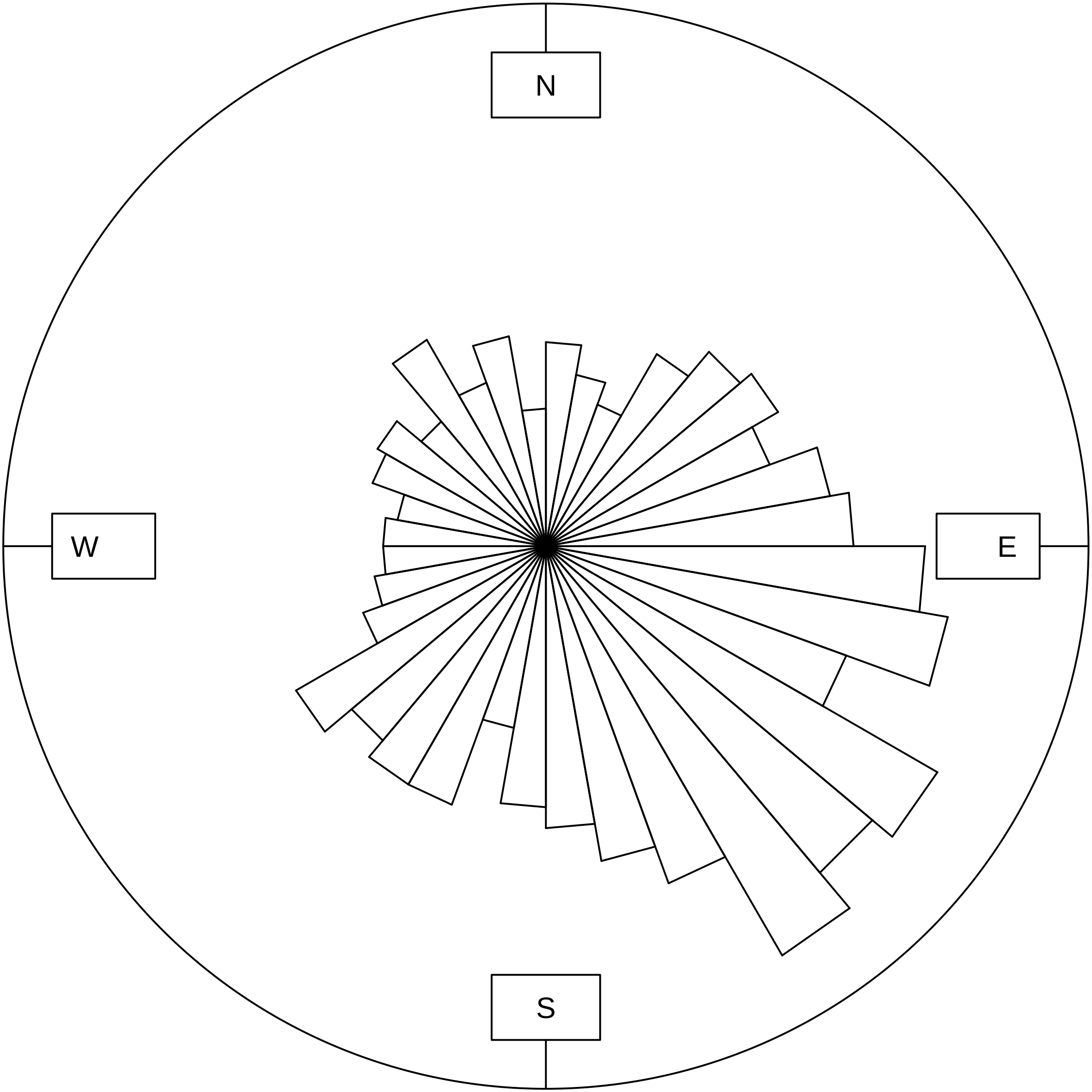

bootstrap 954

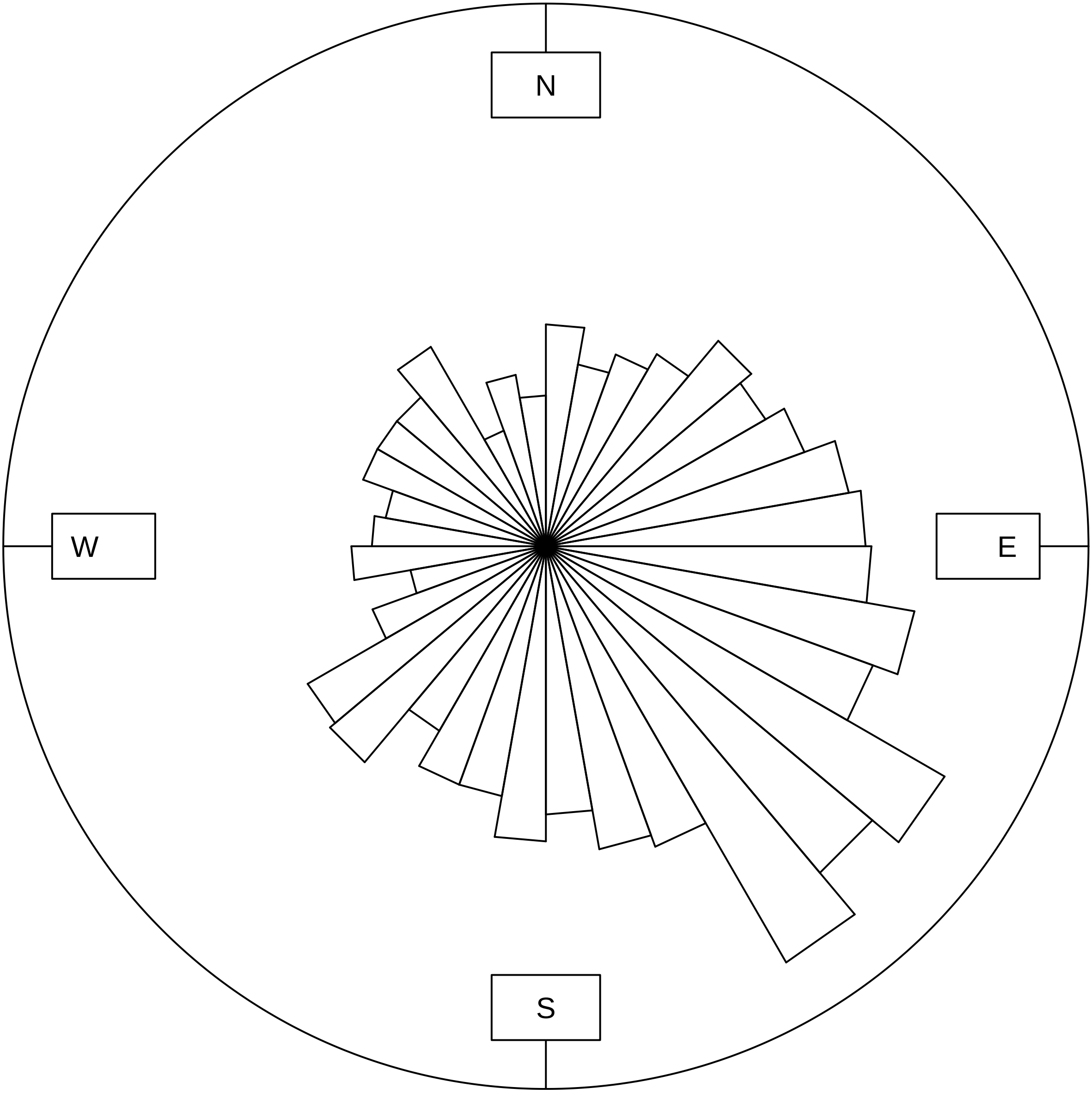

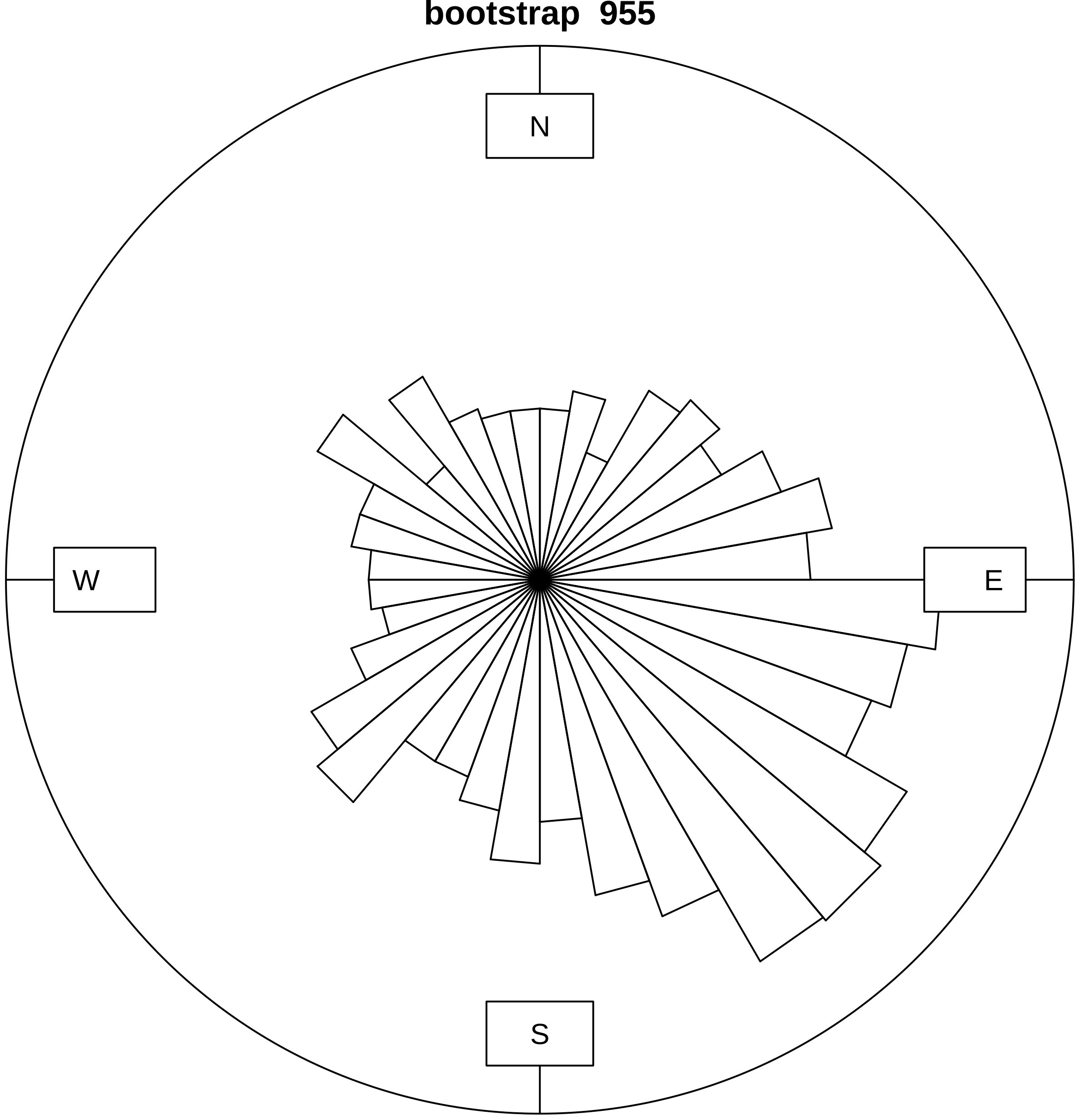
bootstrap 955
N
W
E
S

bootstrap 956

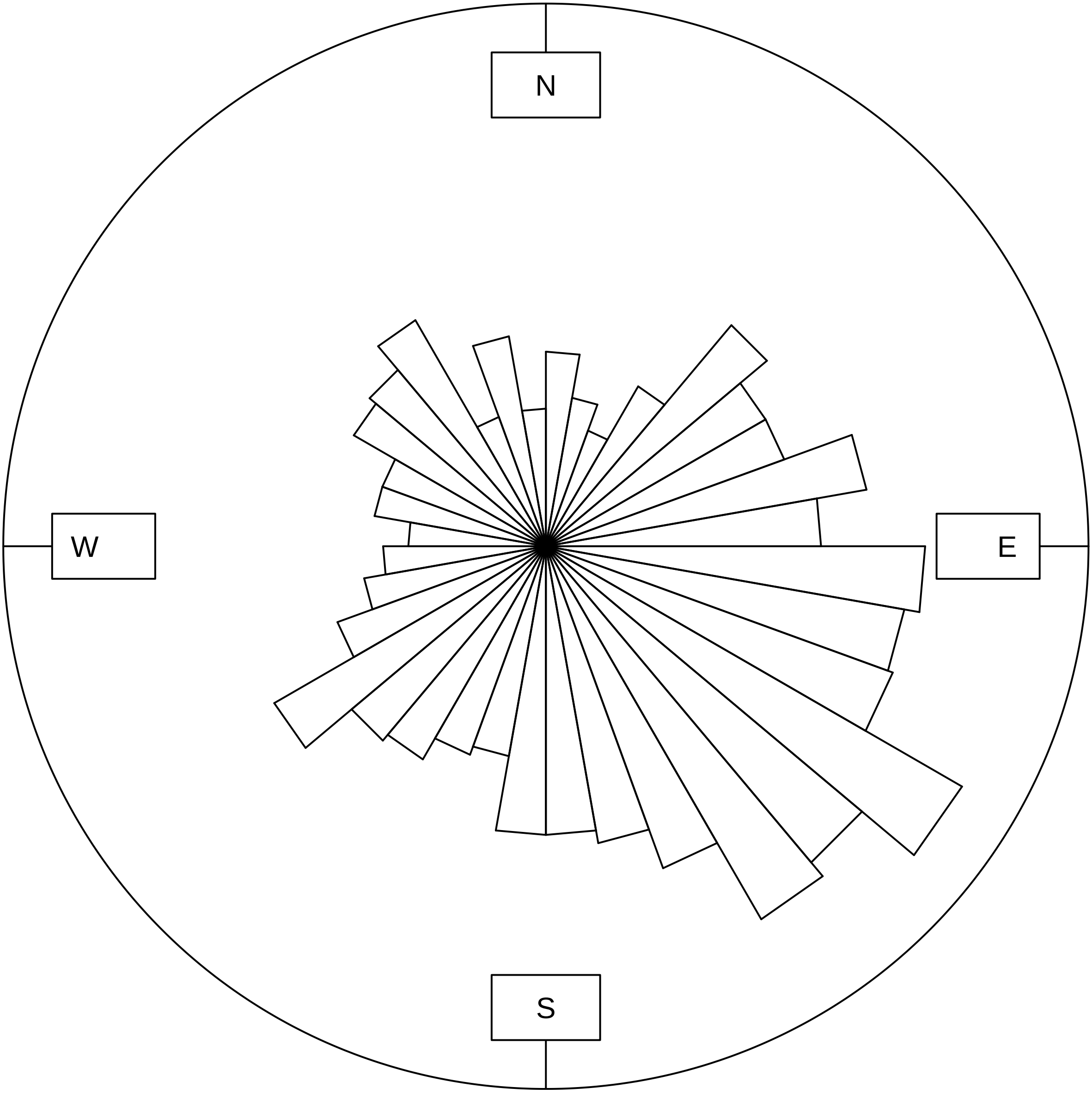

bootstrap 957

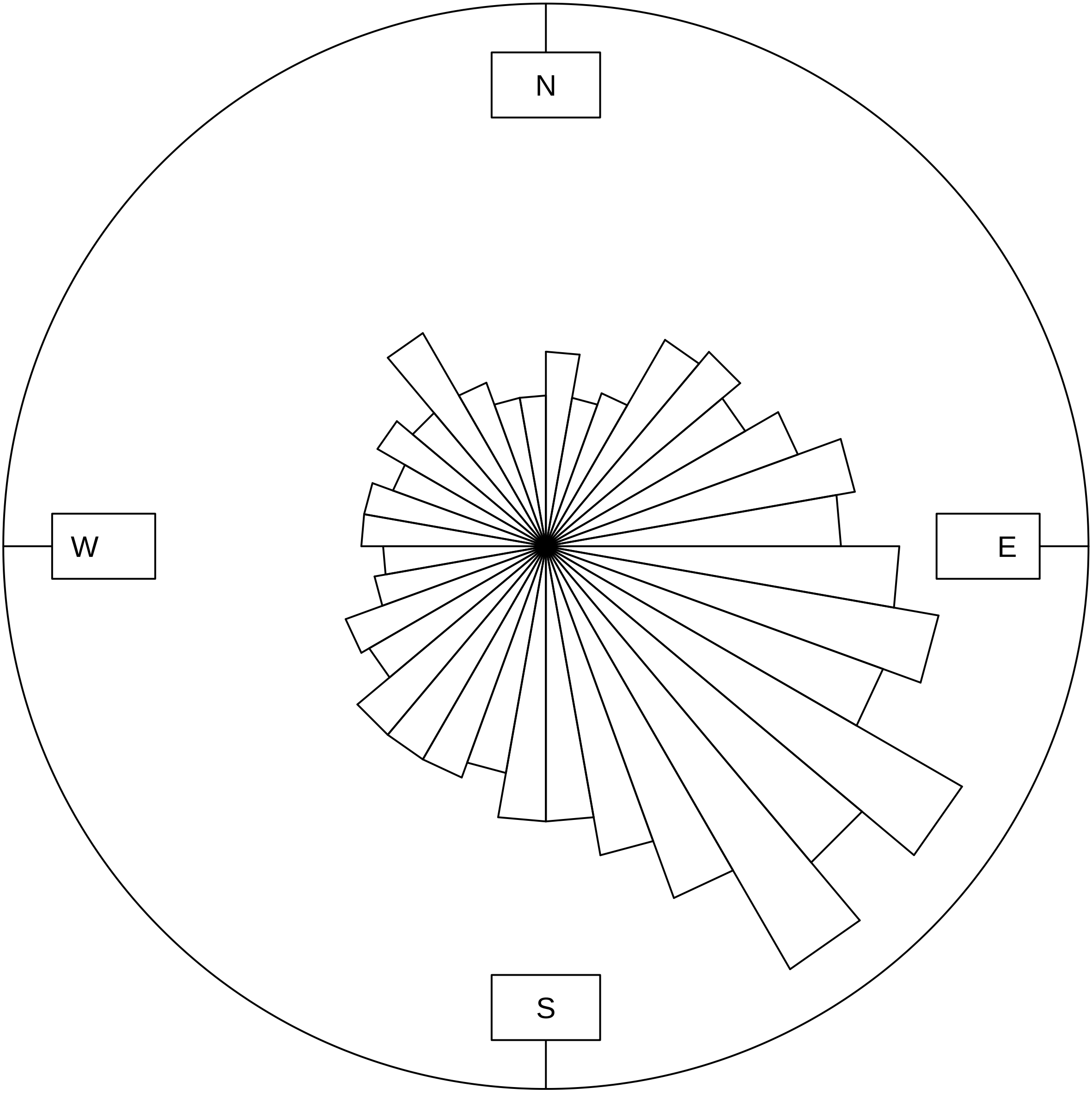

bootstrap 958

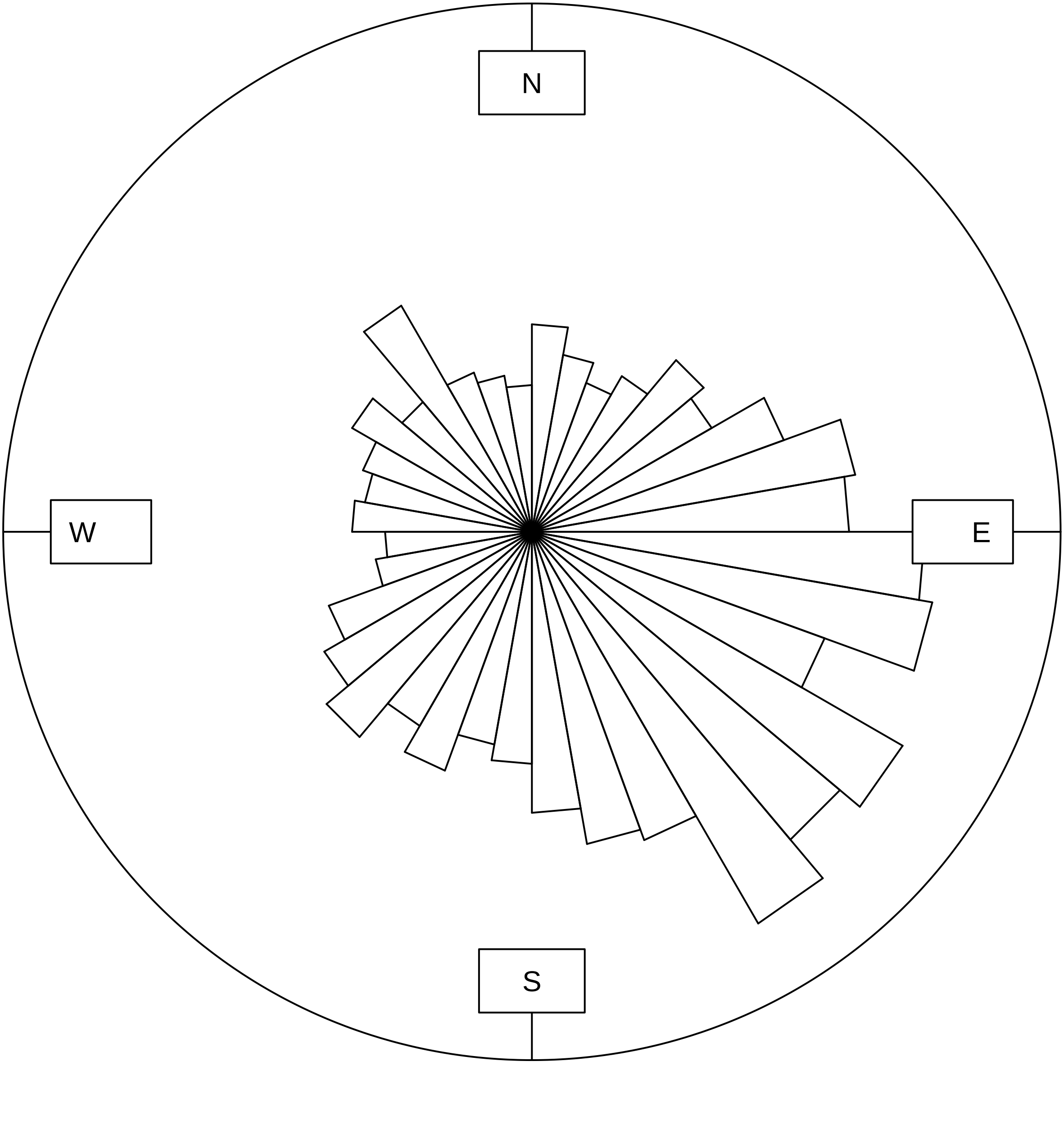

bootstrap 959

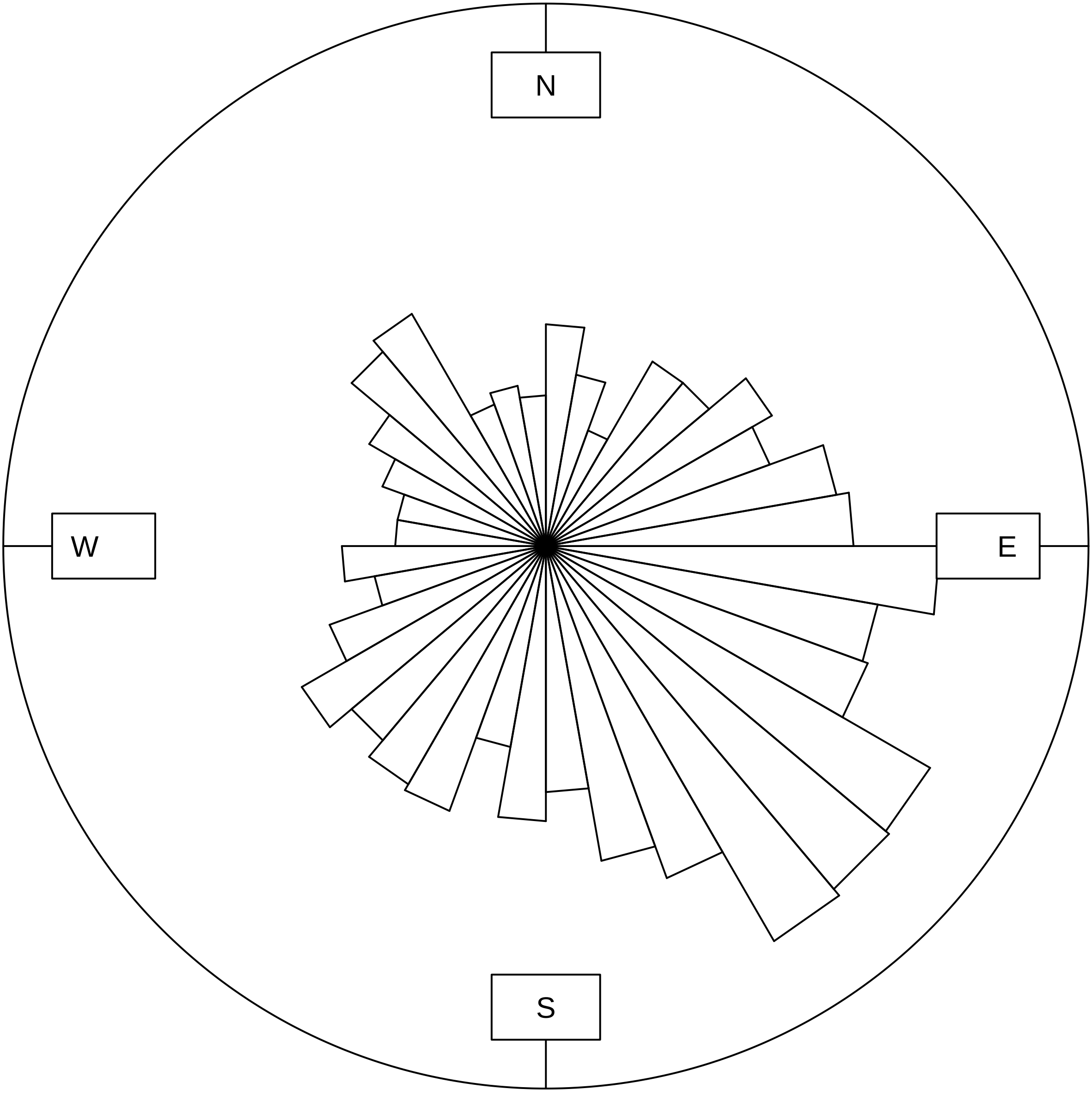

bootstrap 960

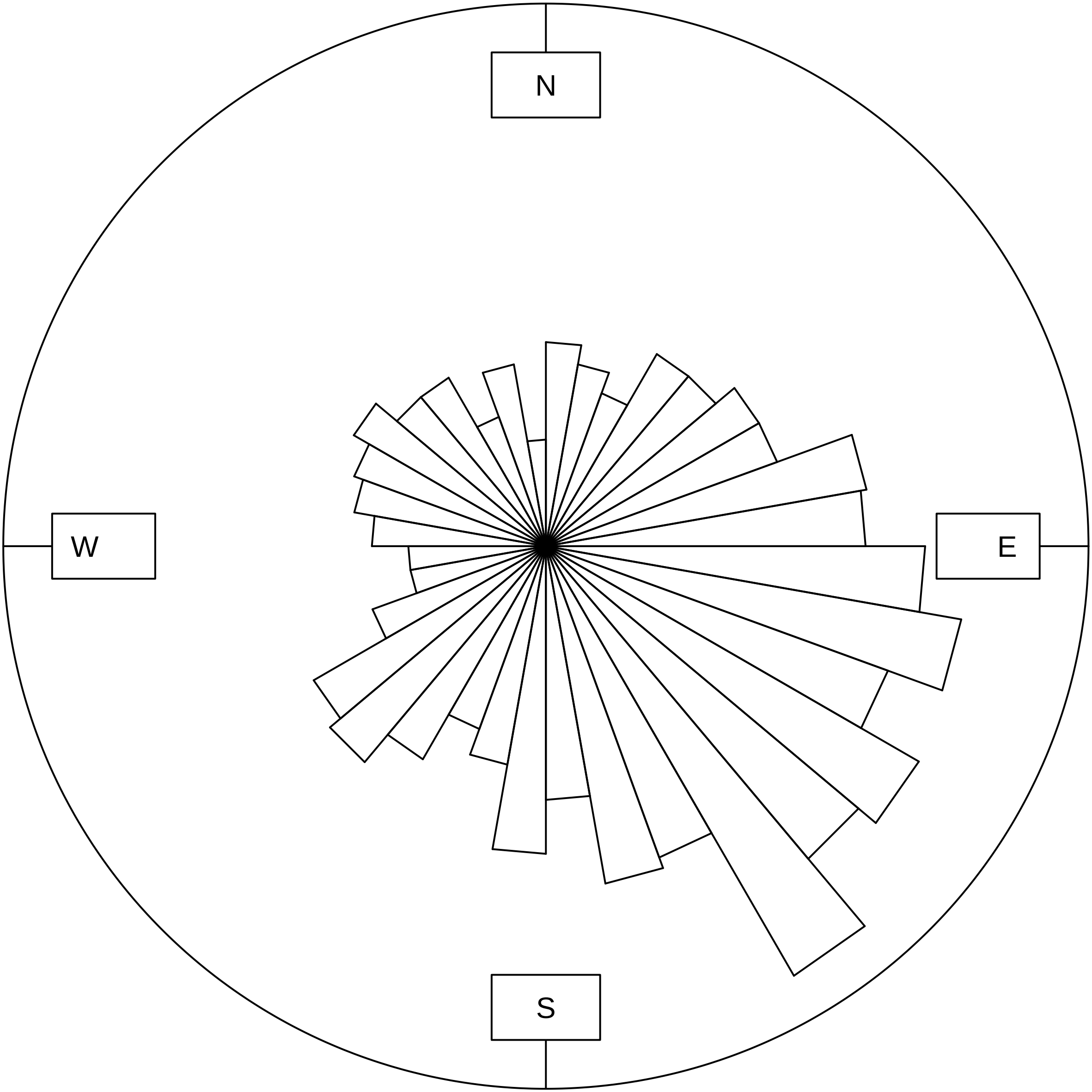

bootstrap 961

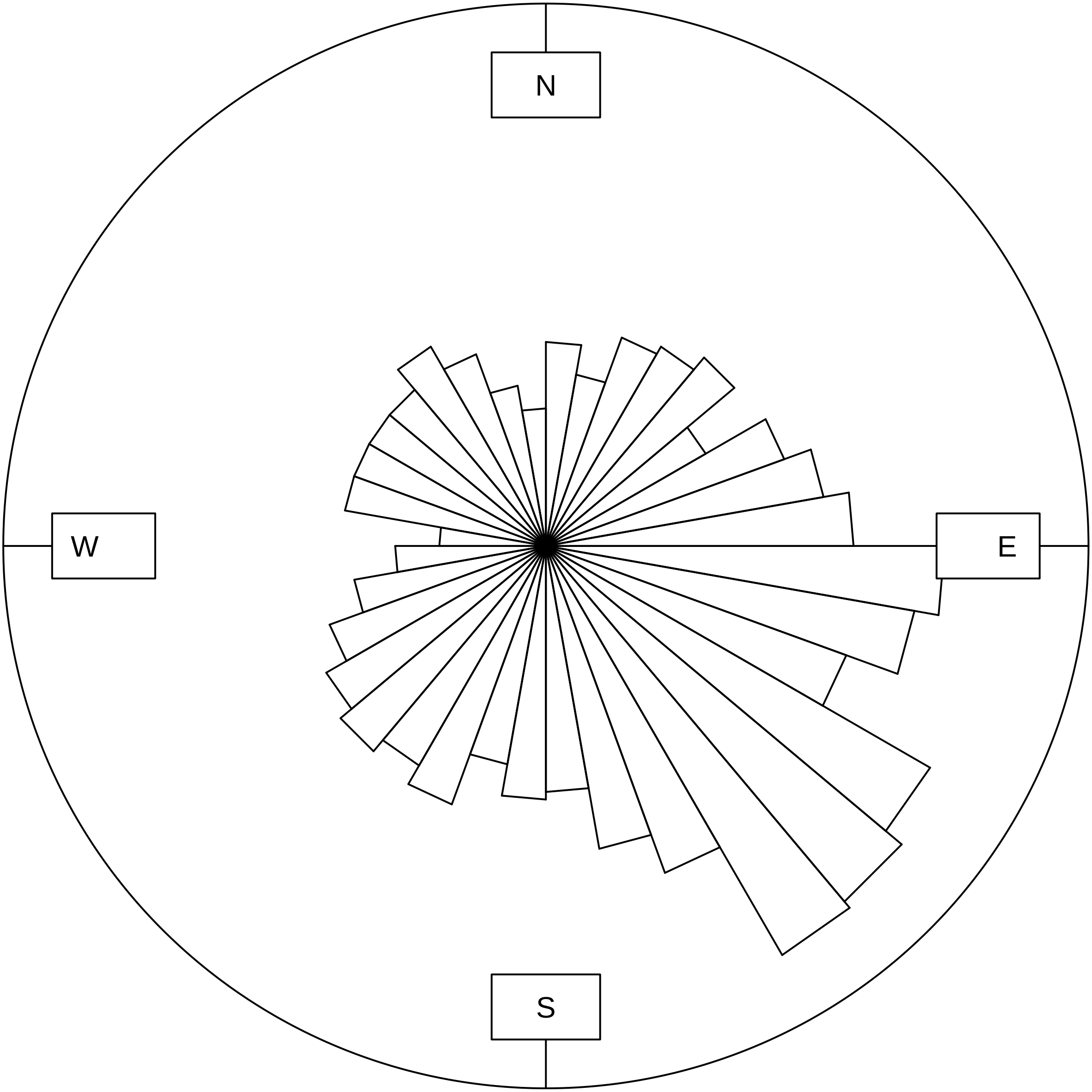

bootstrap 962

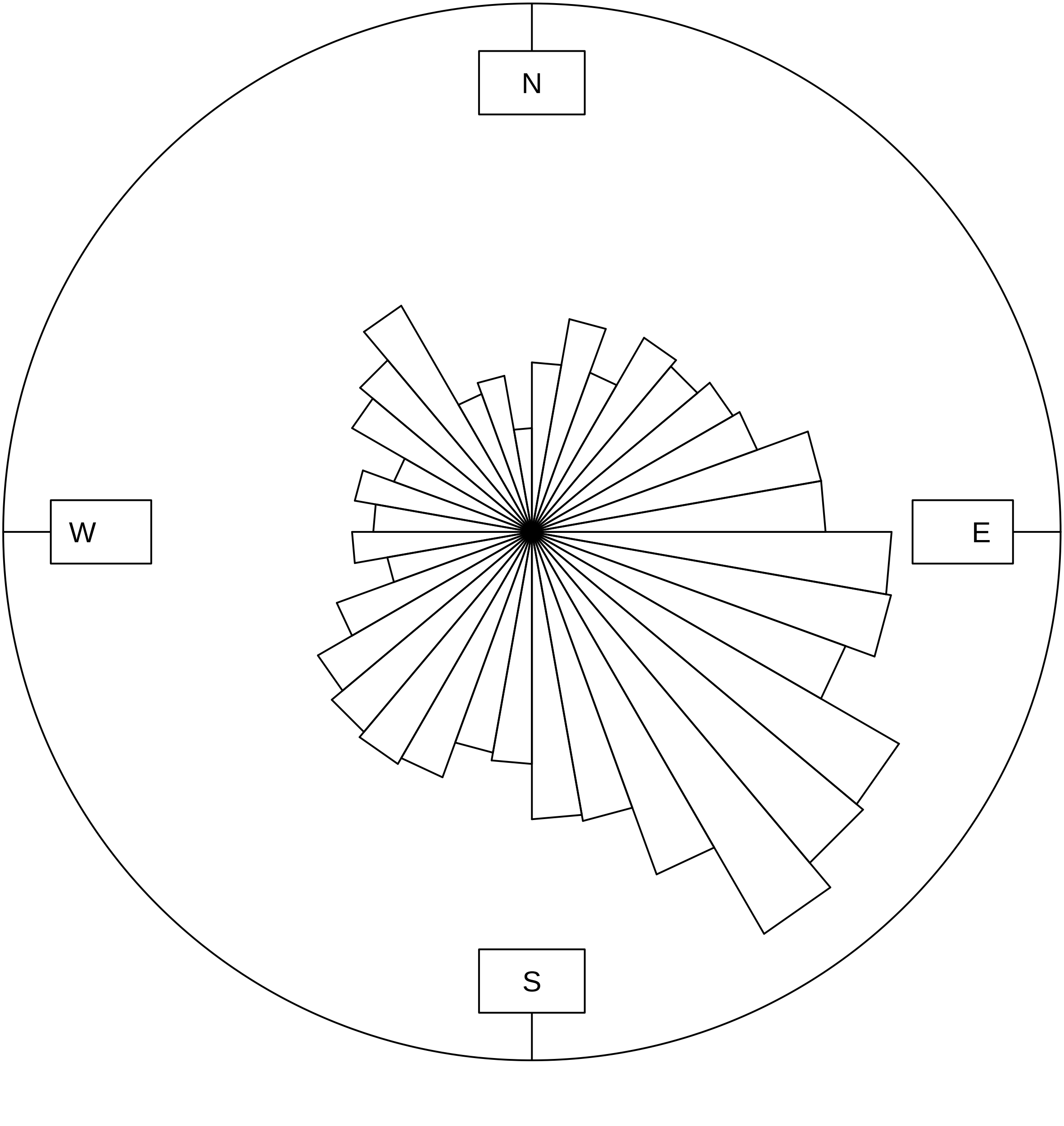

bootstrap 963

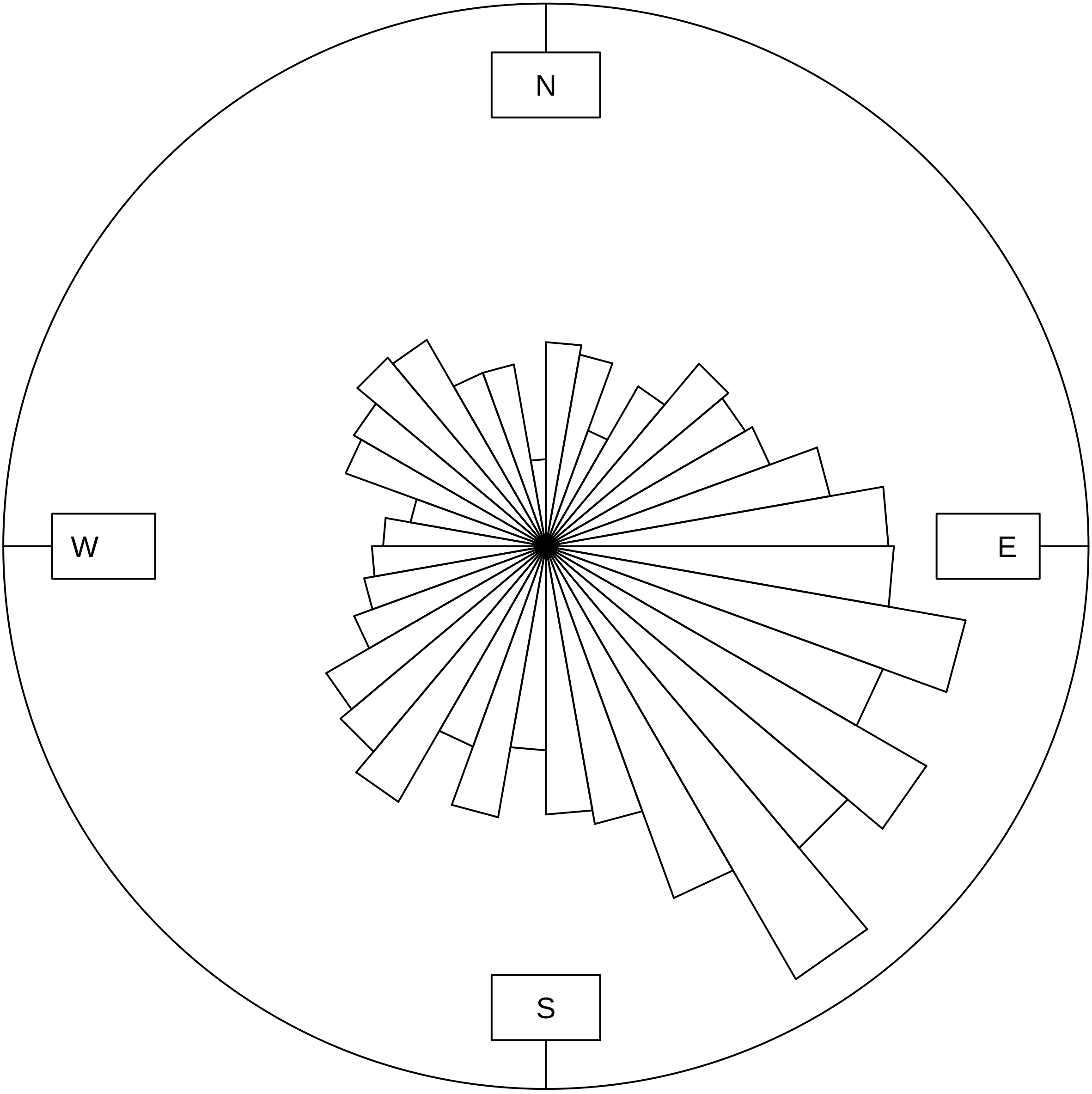

bootstrap 964

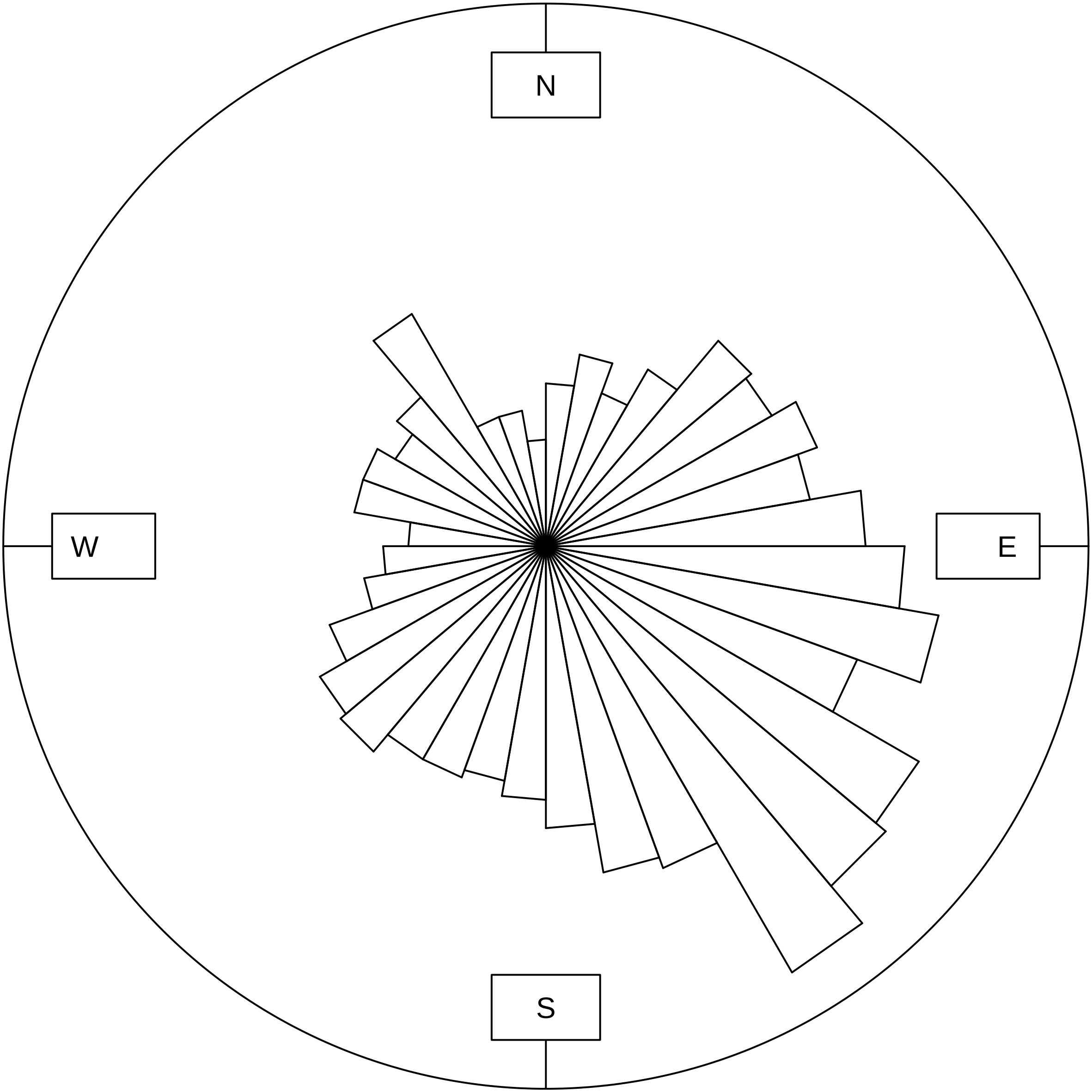

bootstrap 965

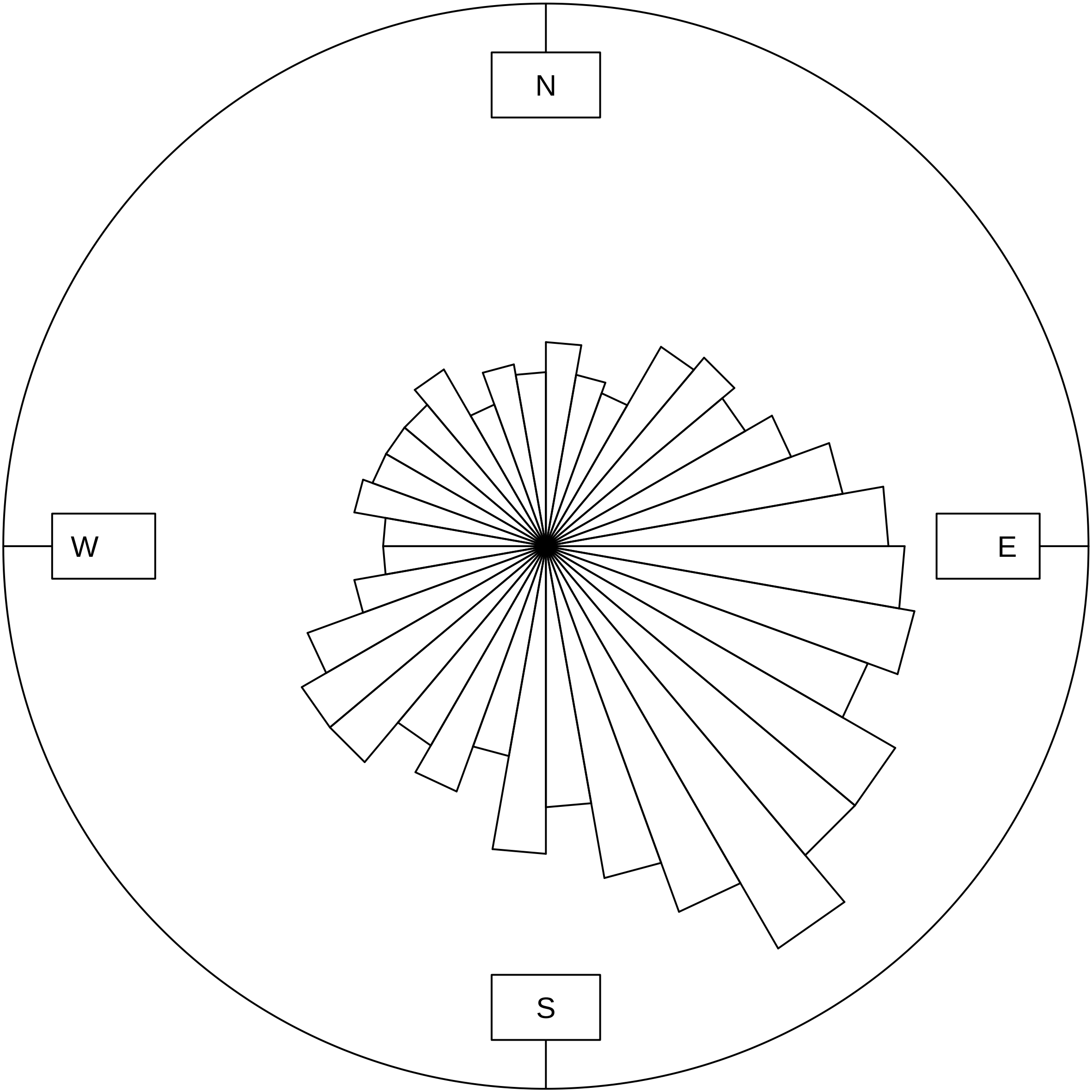

bootstrap 966

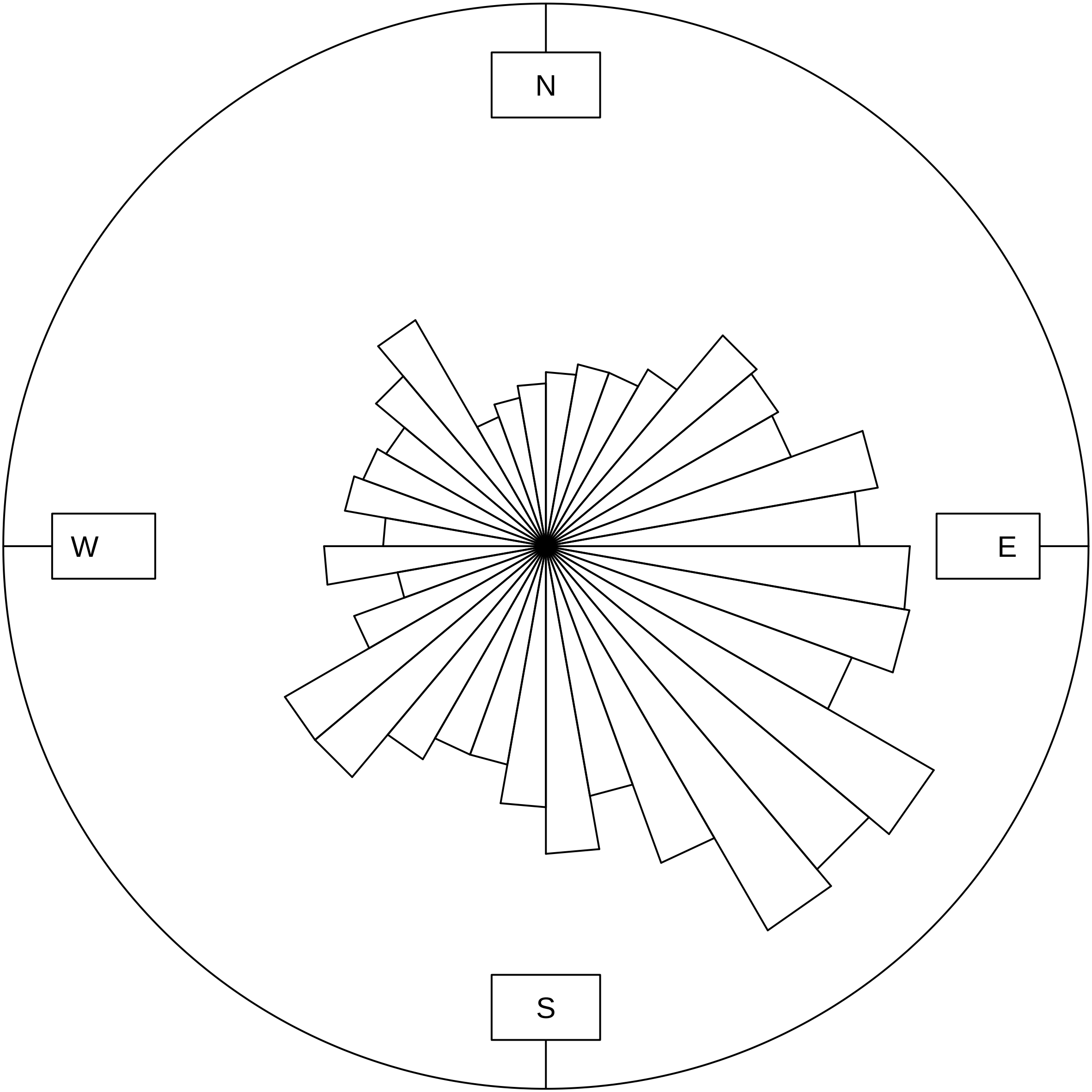

bootstrap 967

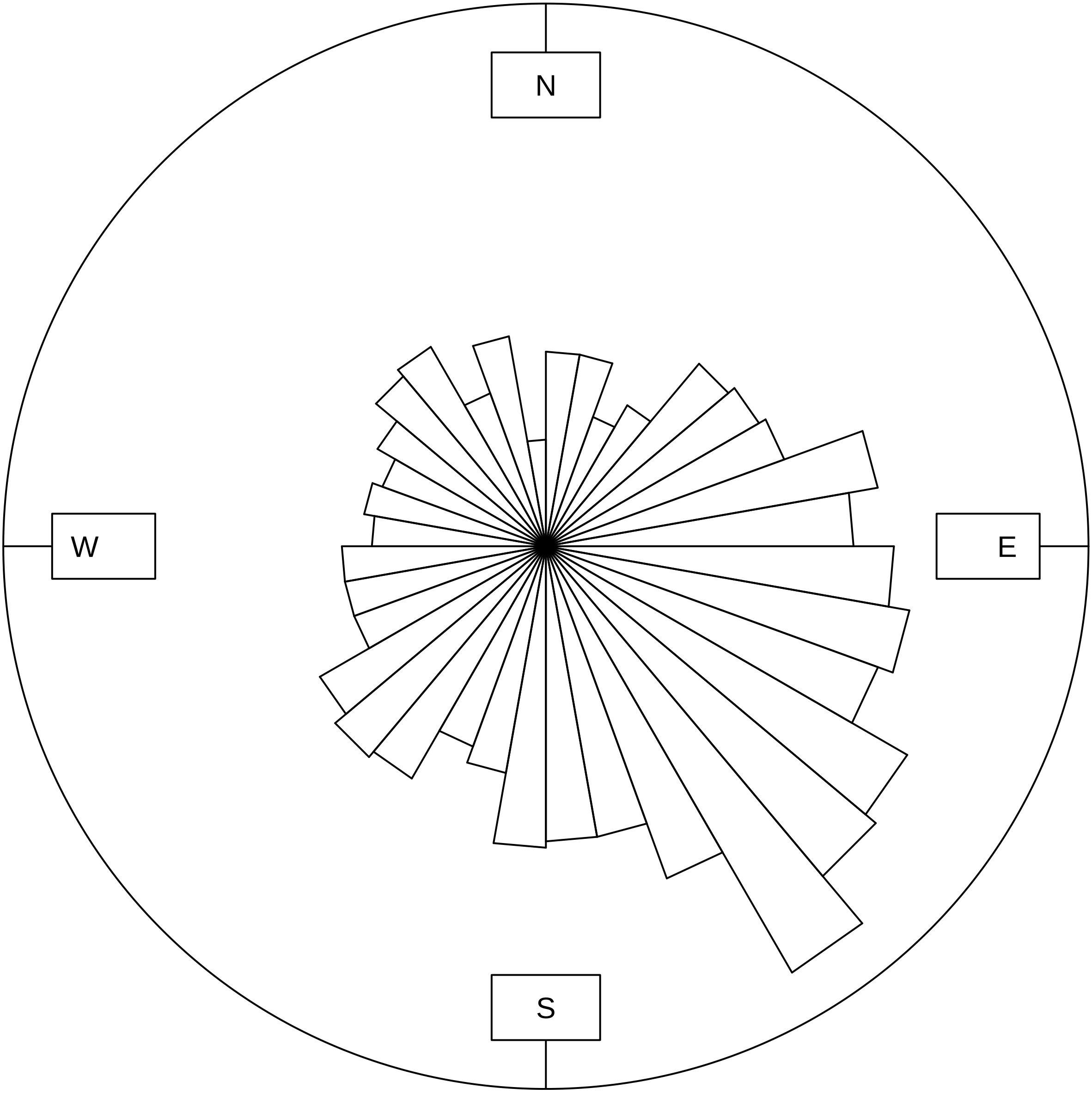

bootstrap 968

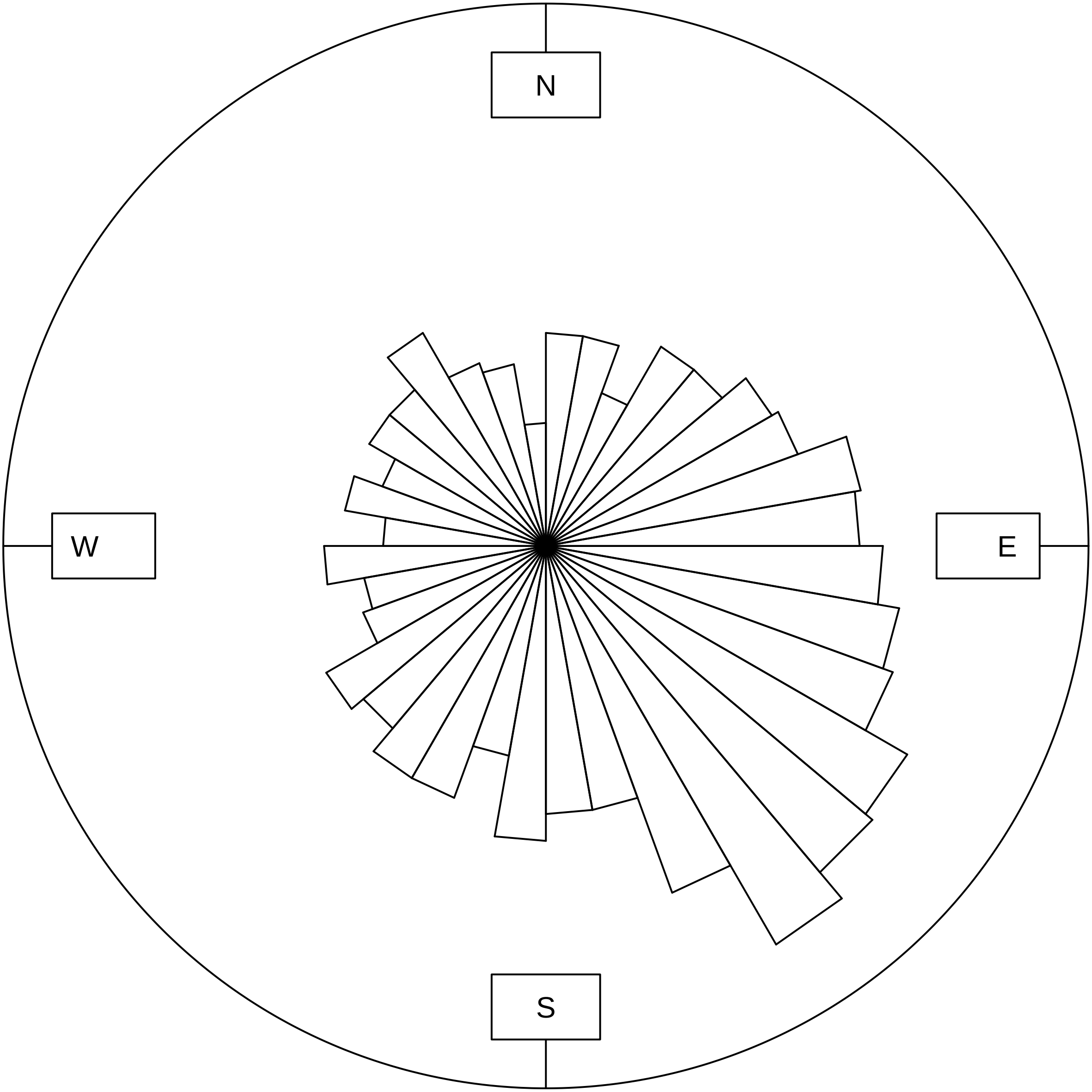

bootstrap 969

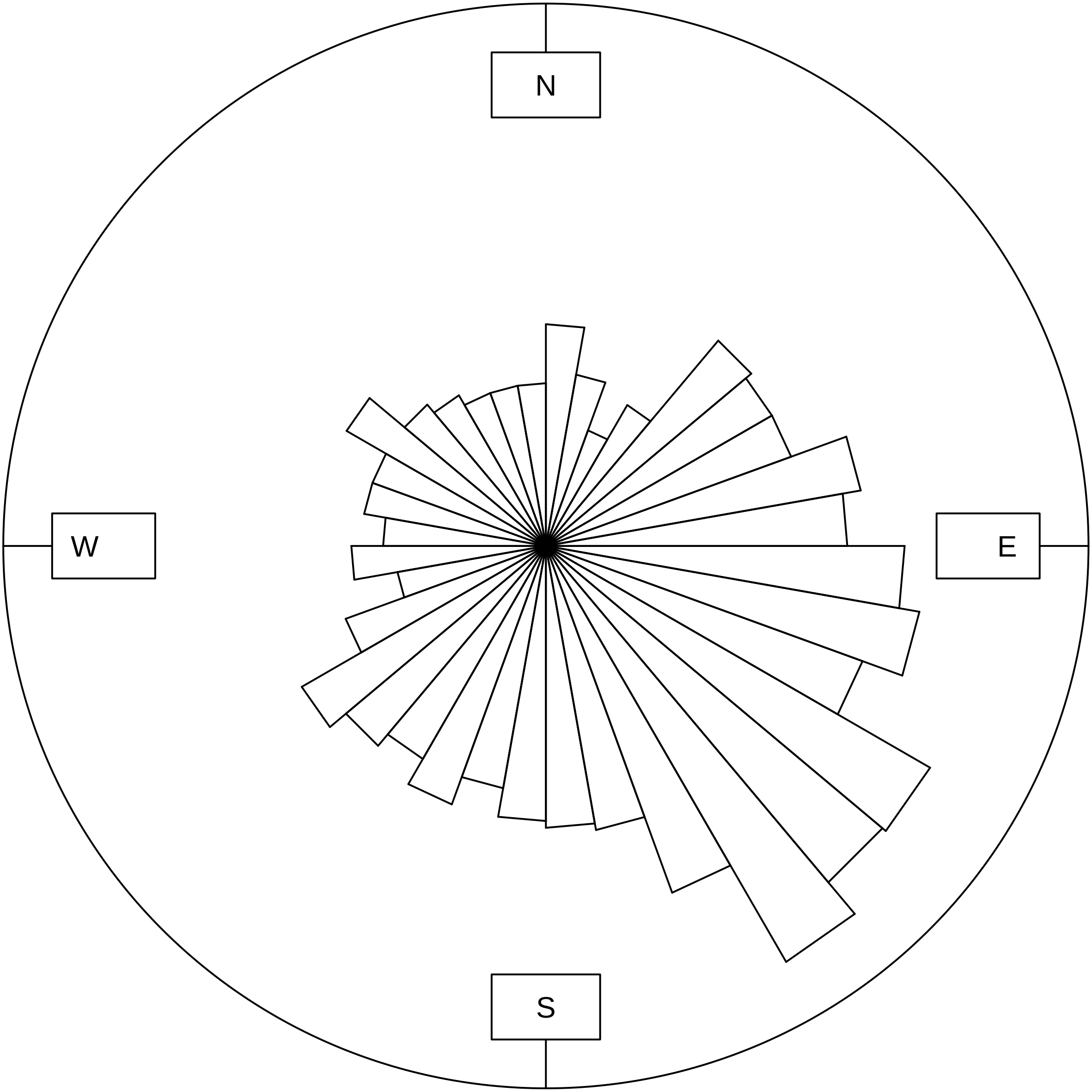

bootstrap 970

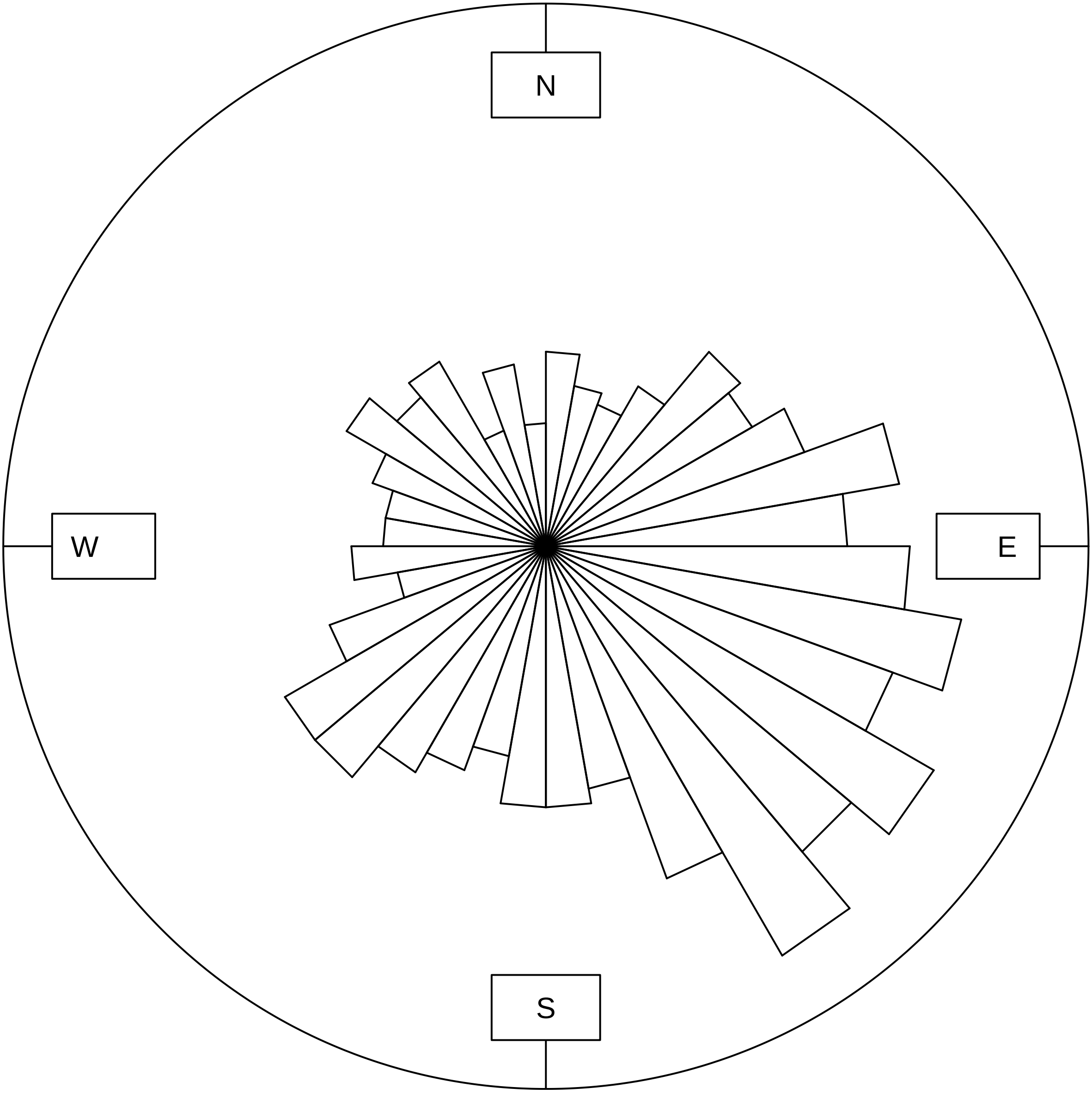

bootstrap 971

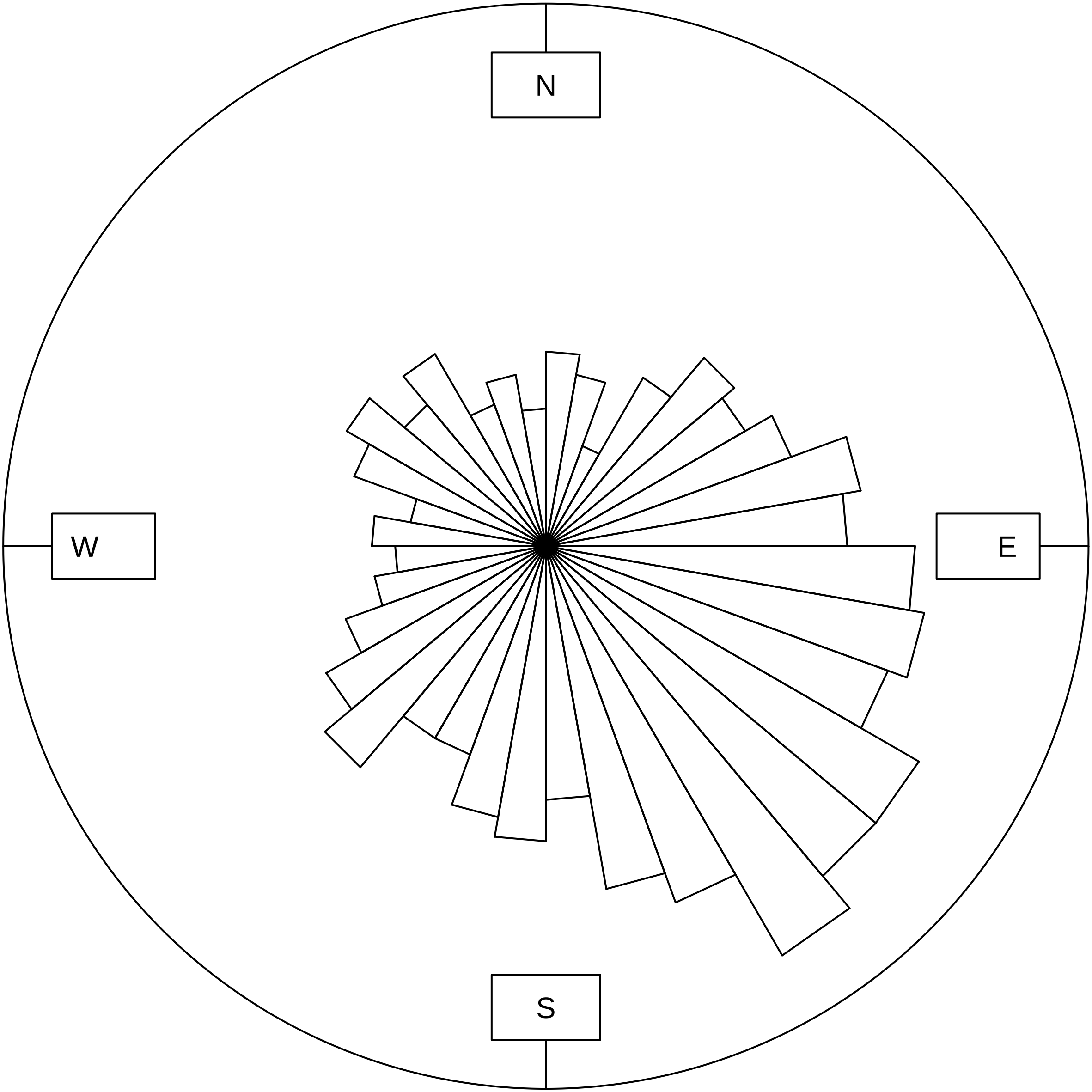

bootstrap 972

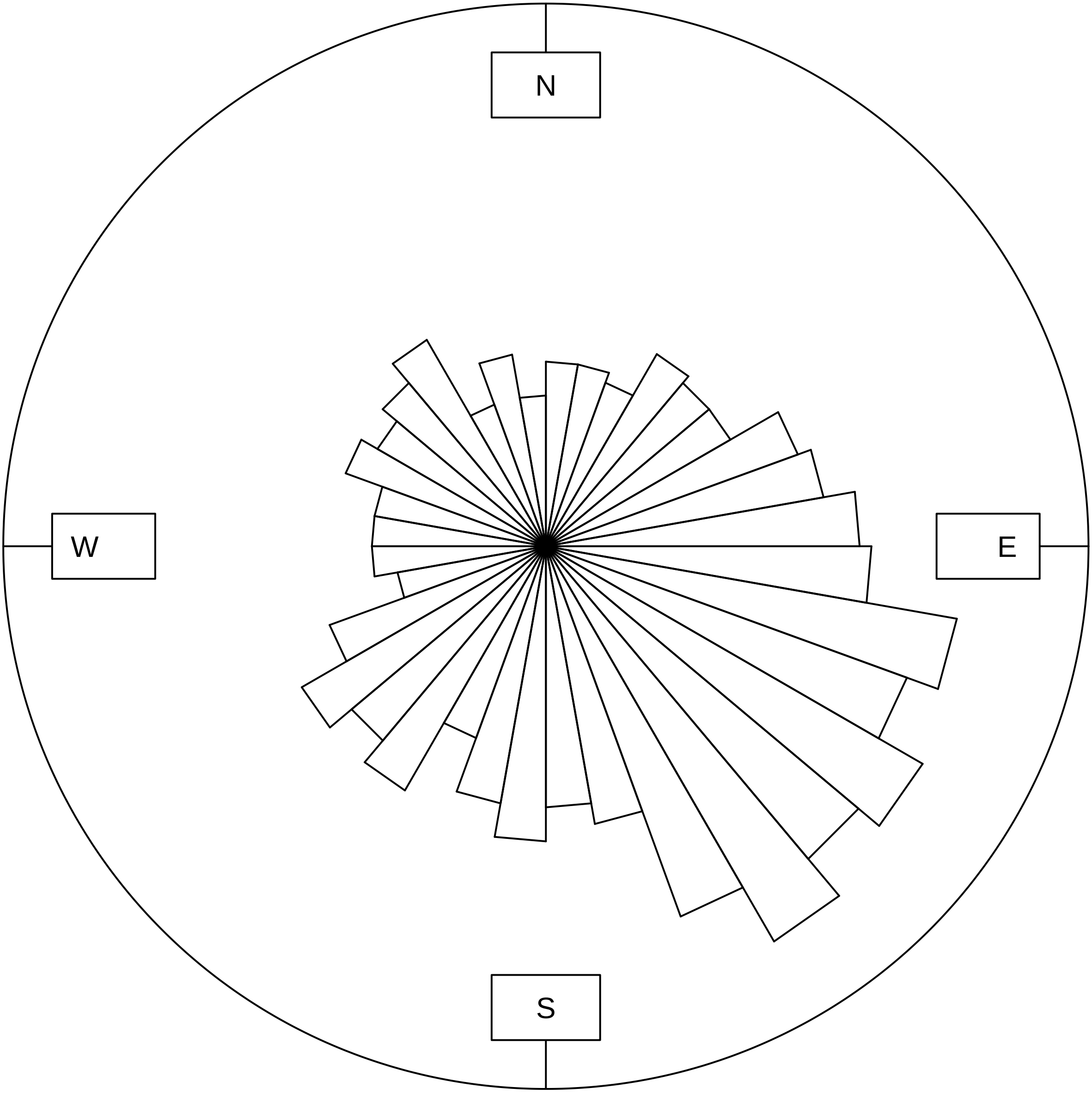

bootstrap 973

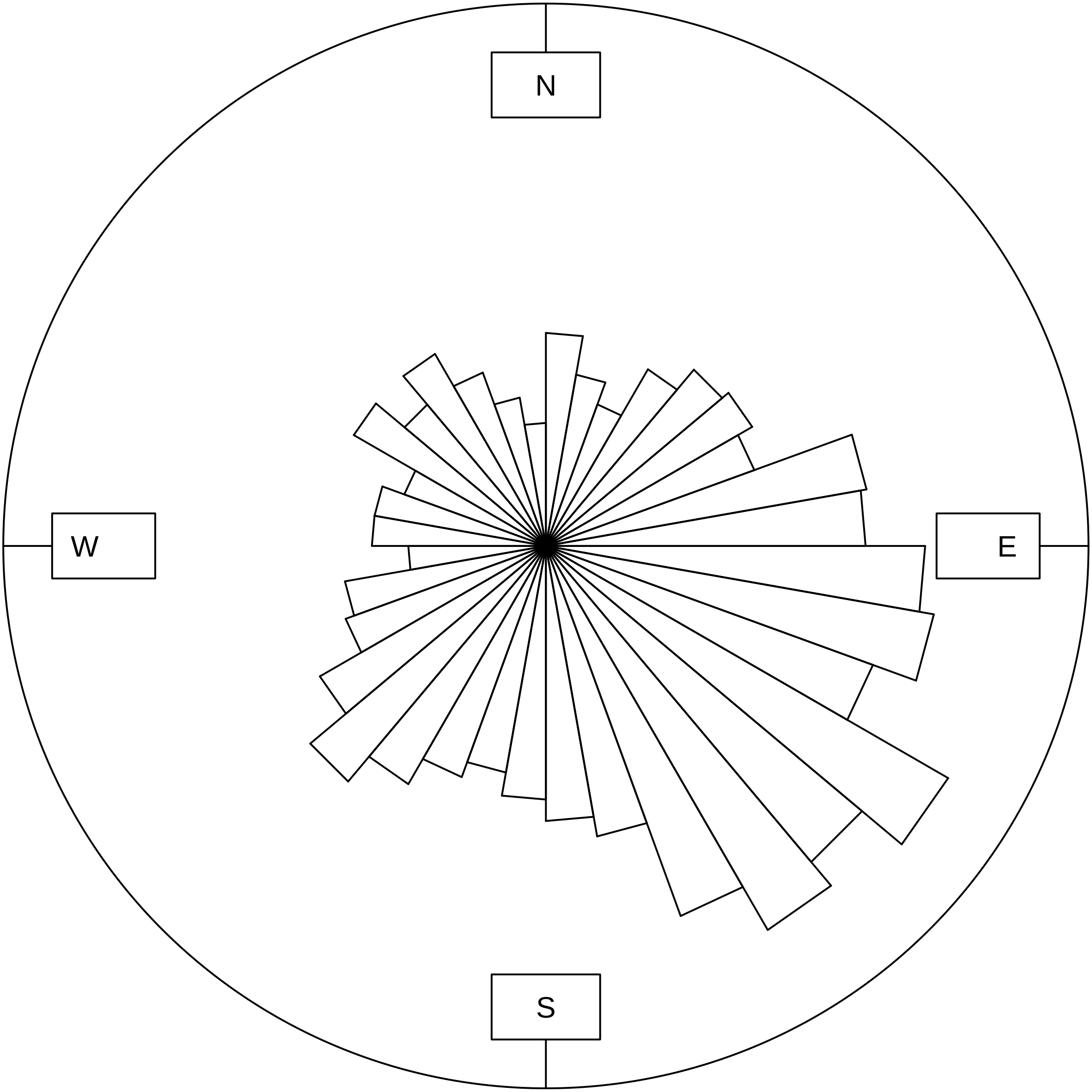

bootstrap 974

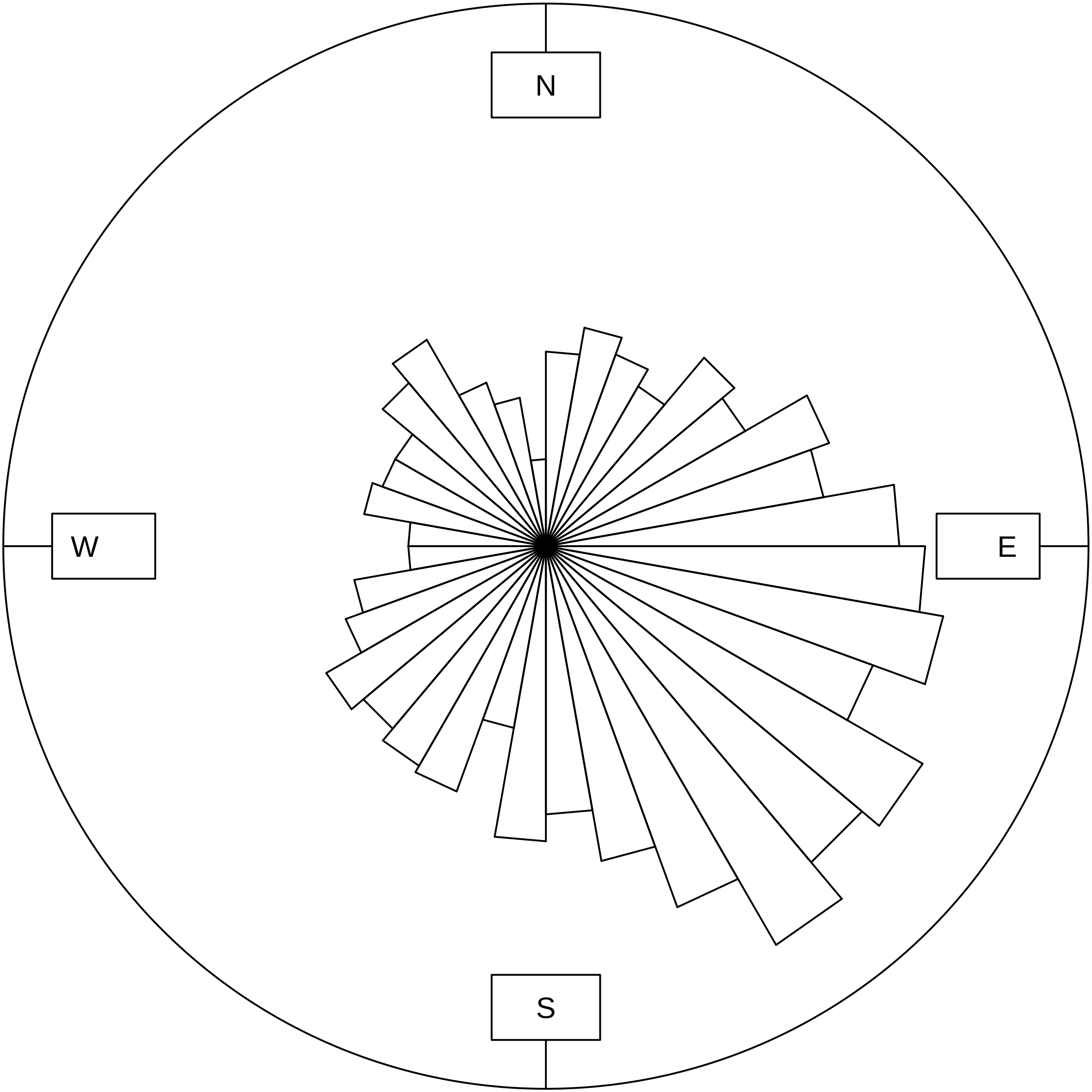

bootstrap 975

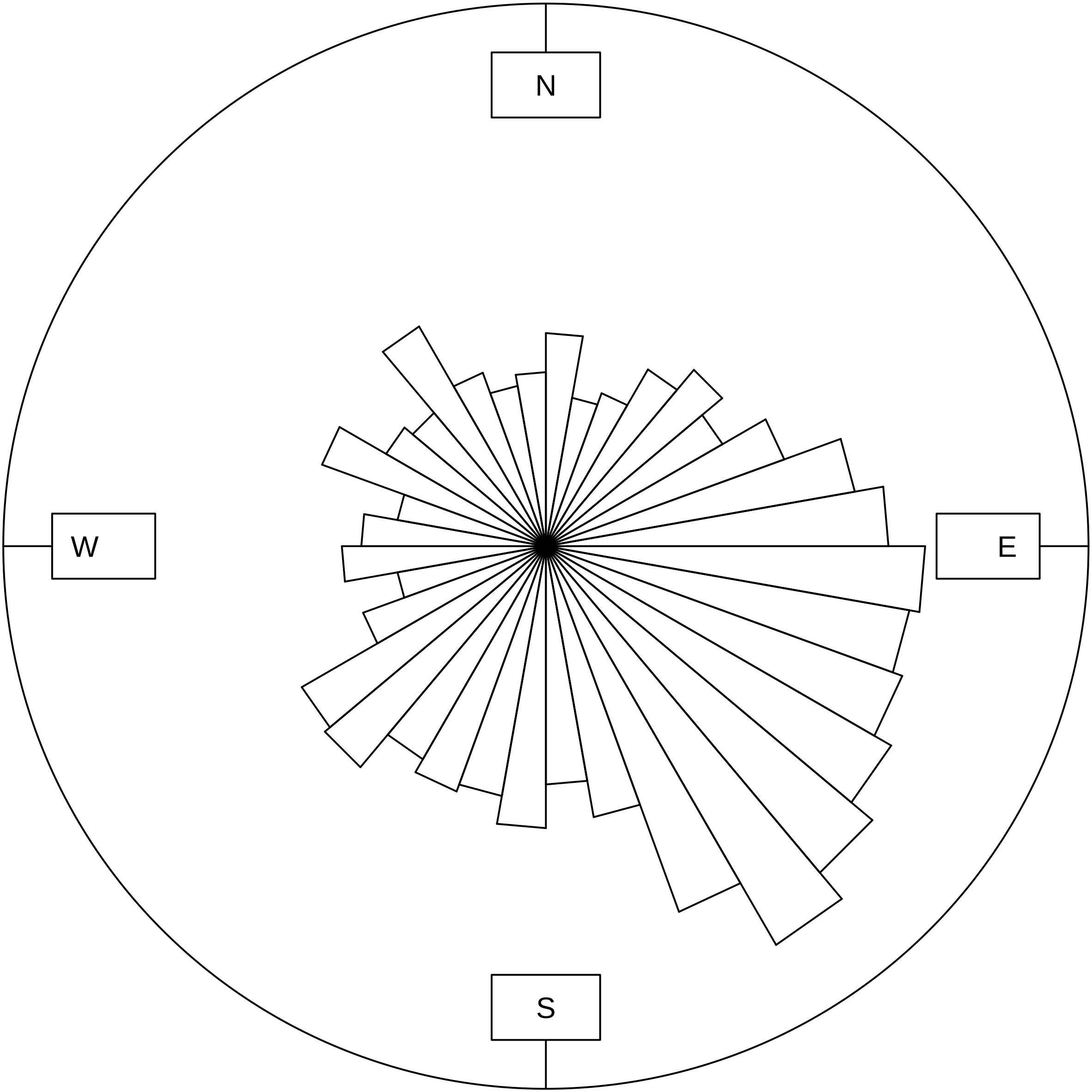

bootstrap 976

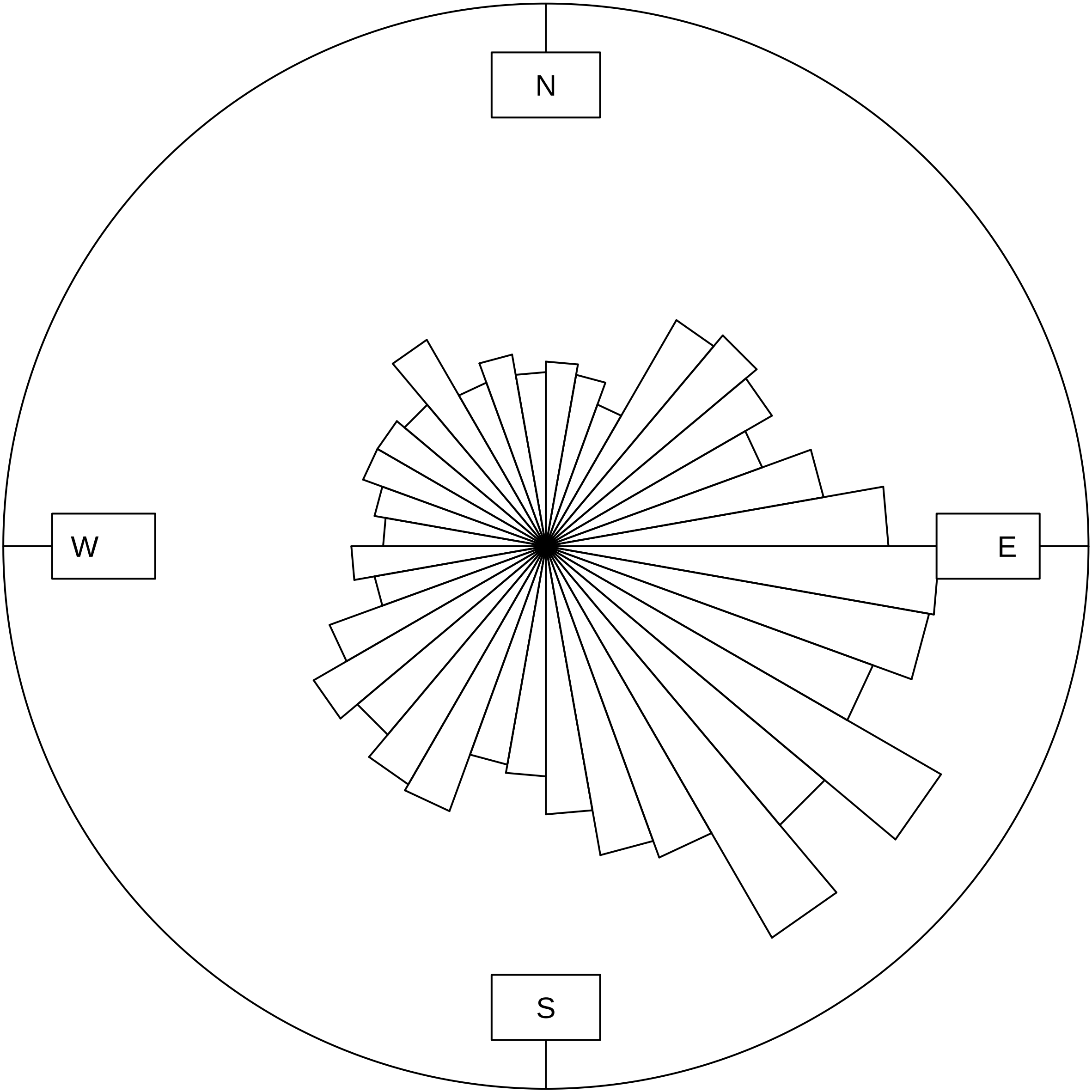

bootstrap 977

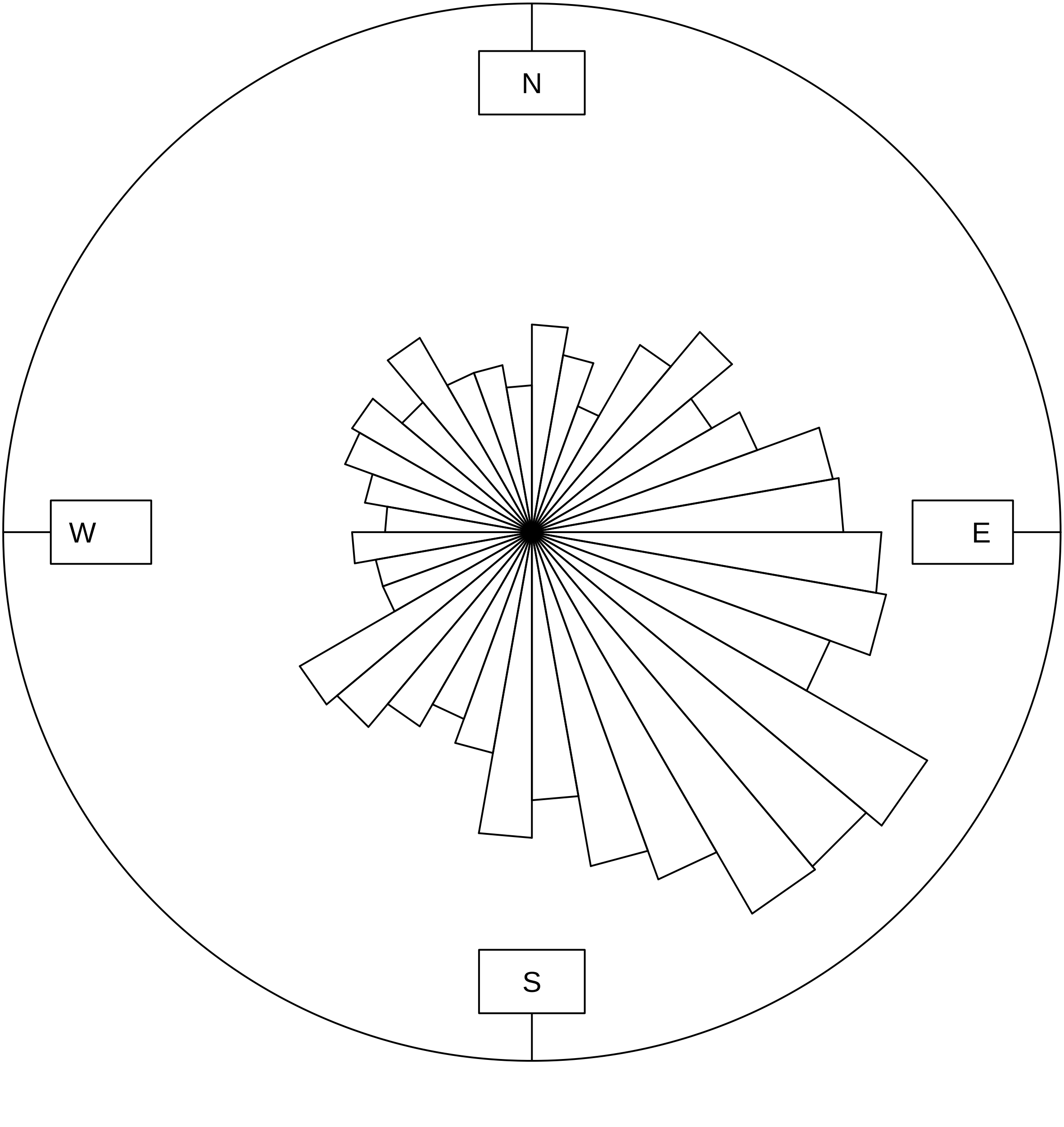

bootstrap 978

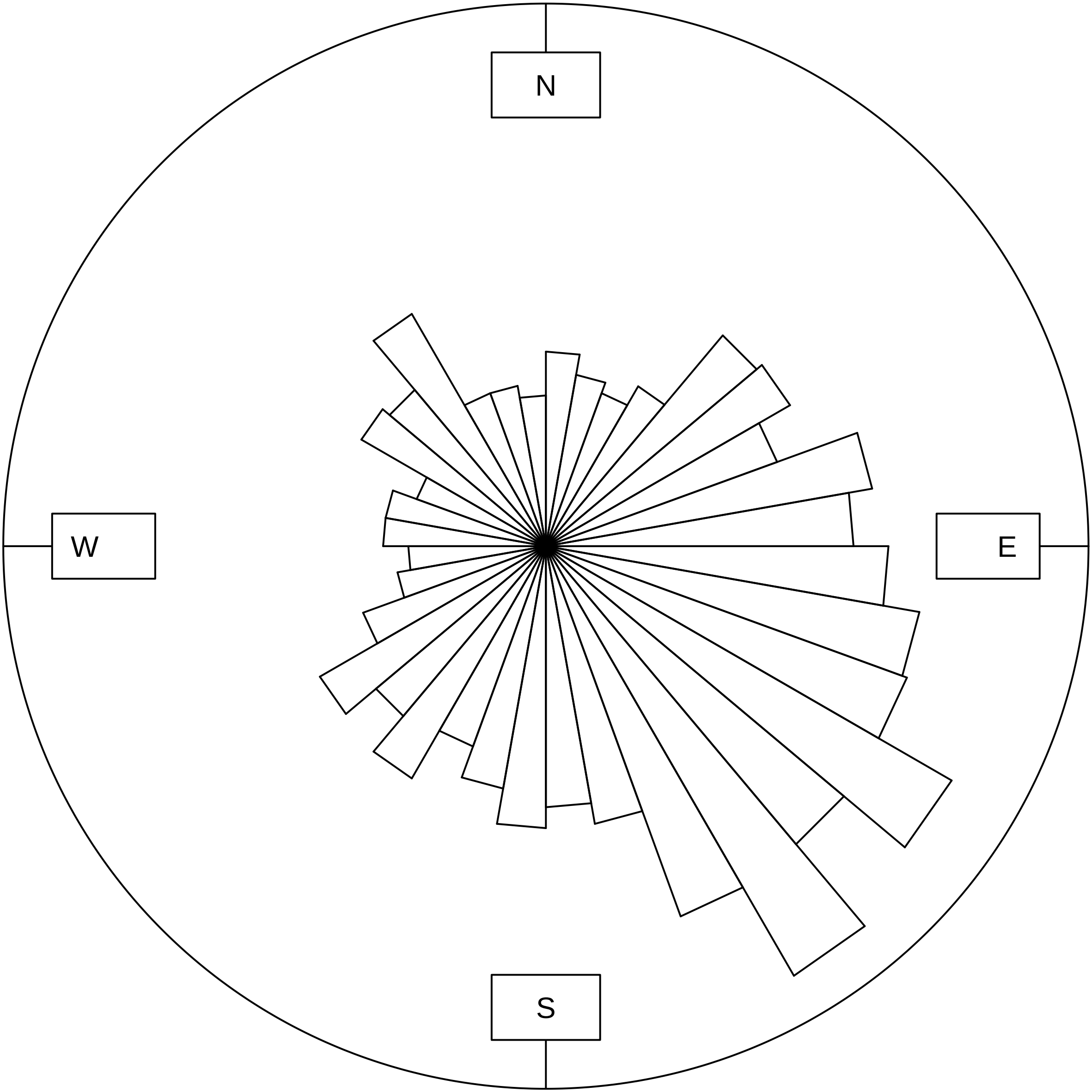

bootstrap 979

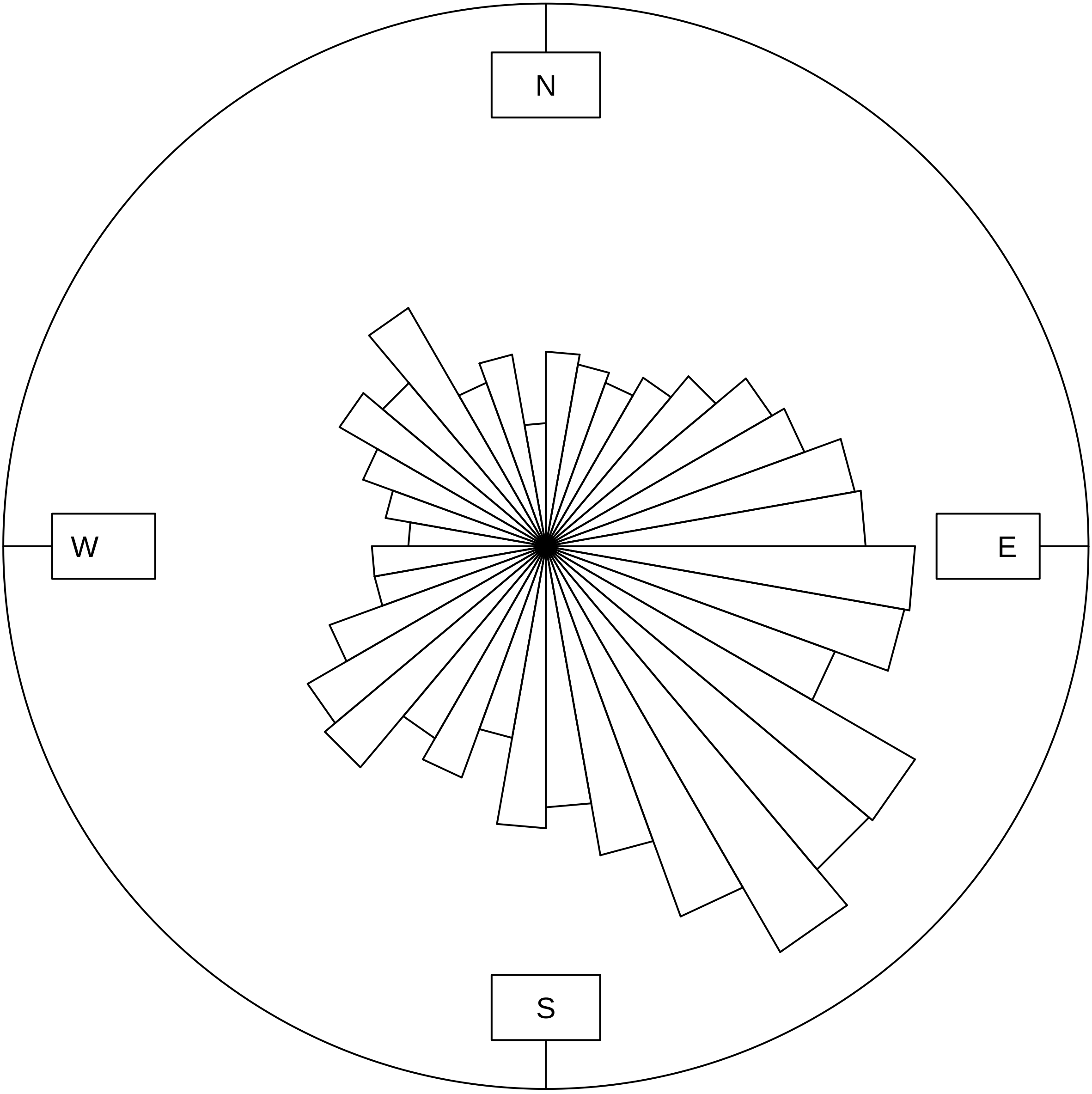

bootstrap 980

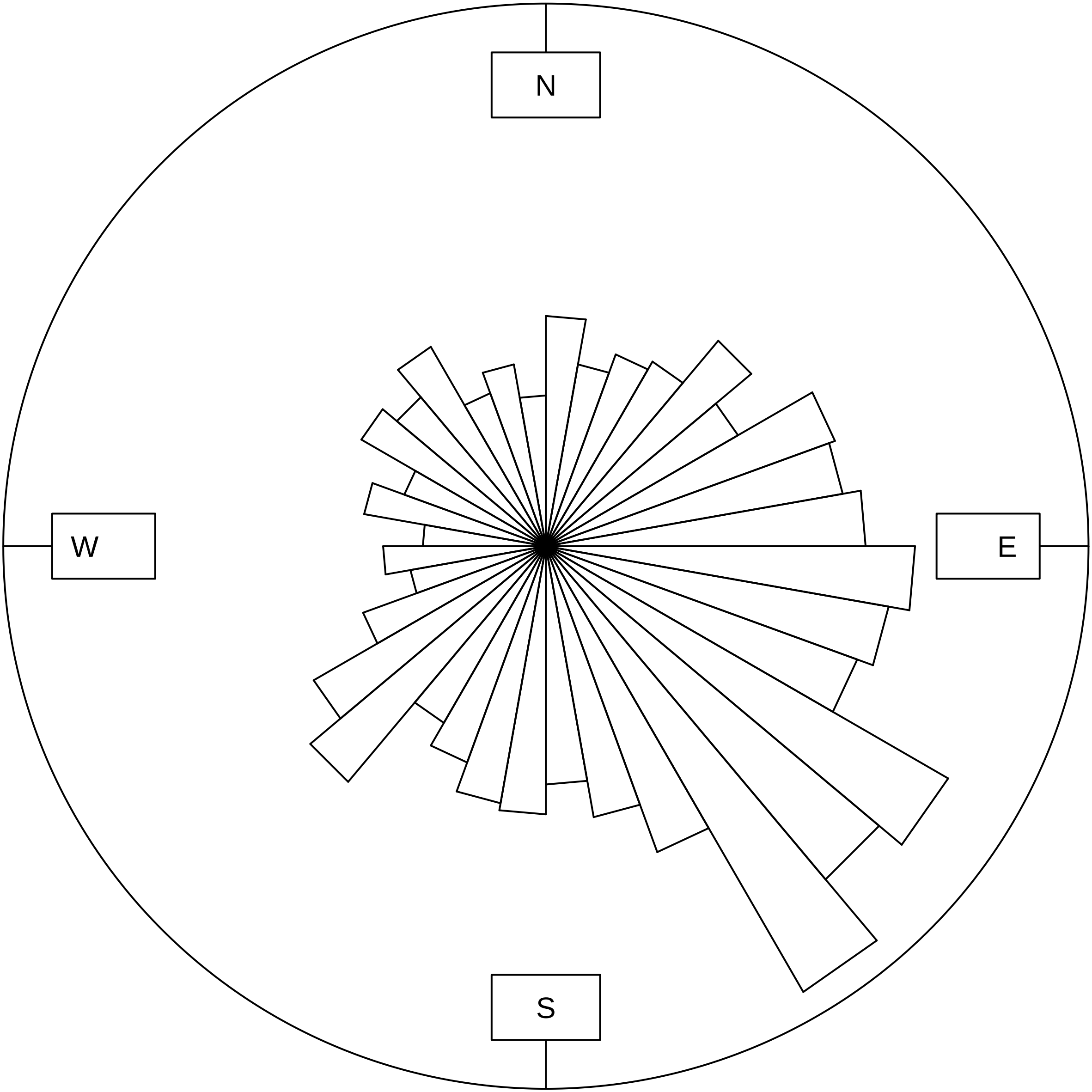

bootstrap 981

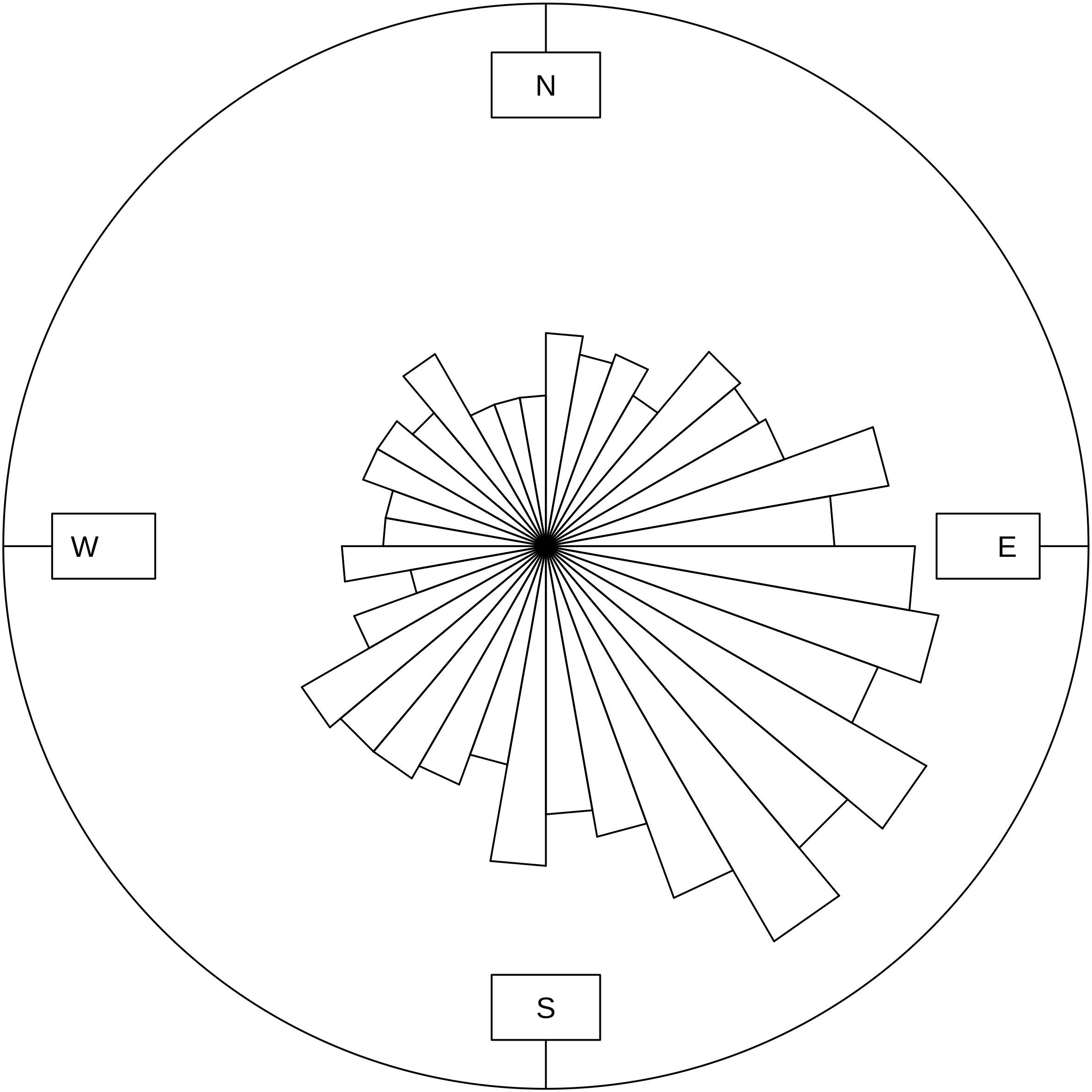

bootstrap 982

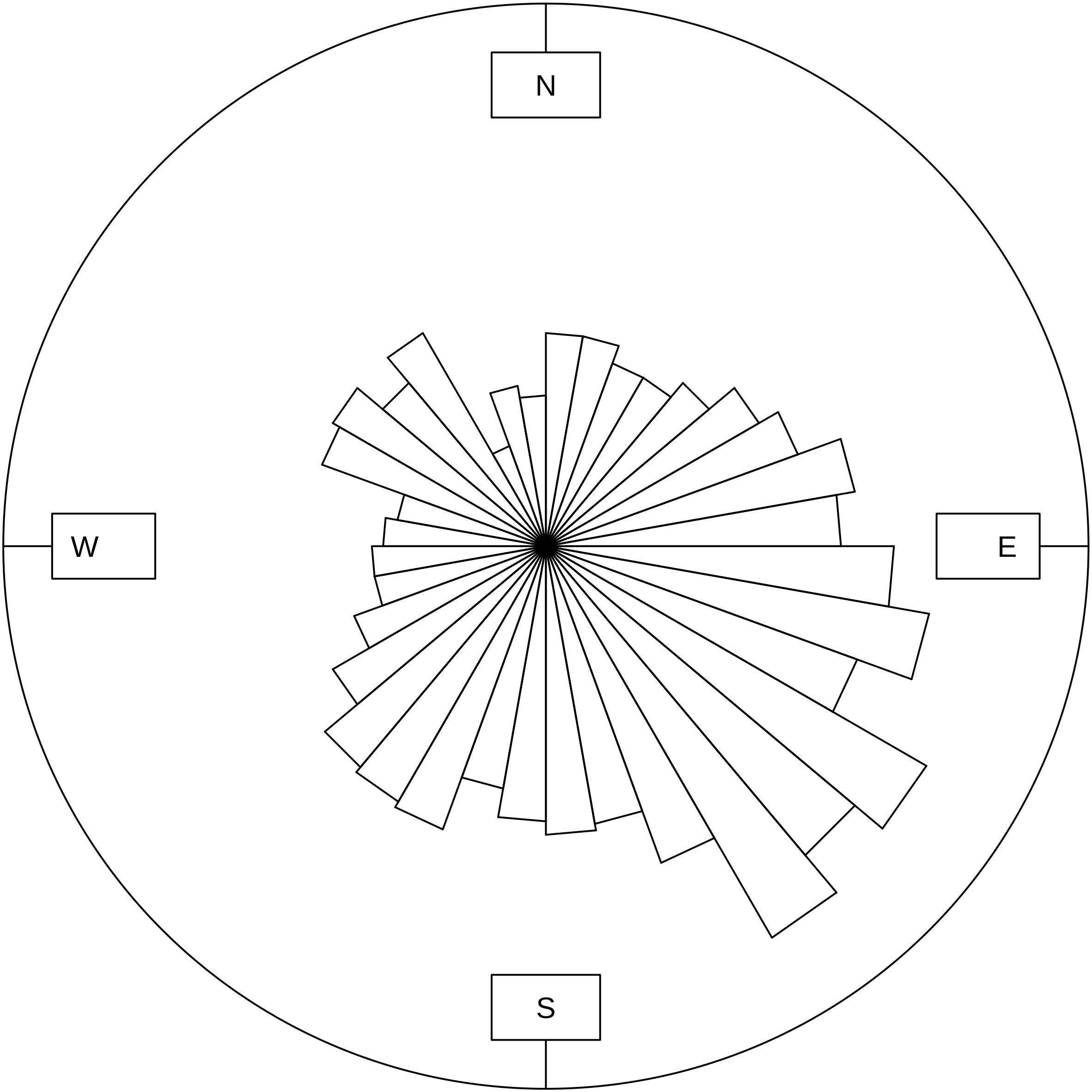

bootstrap 983

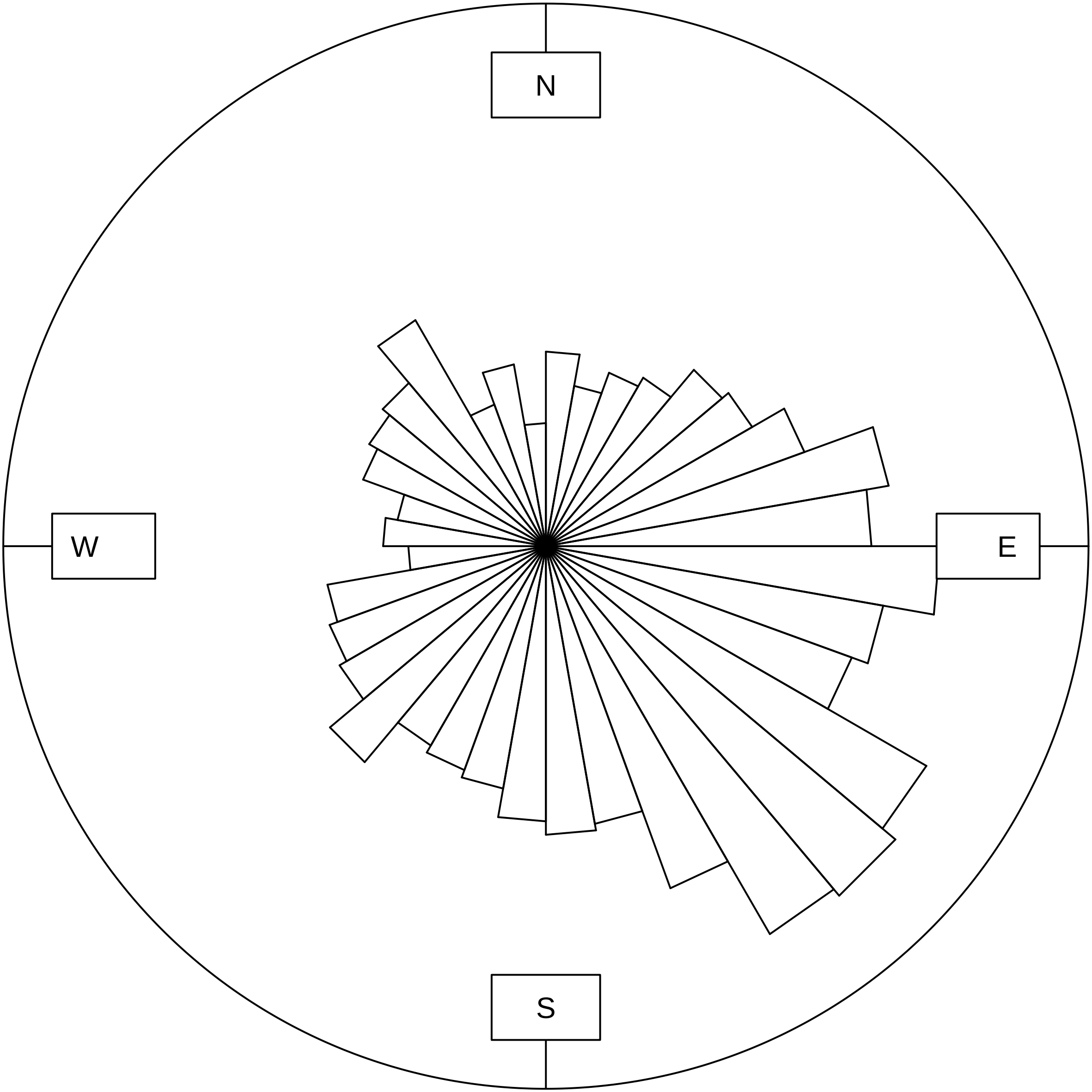

bootstrap 984

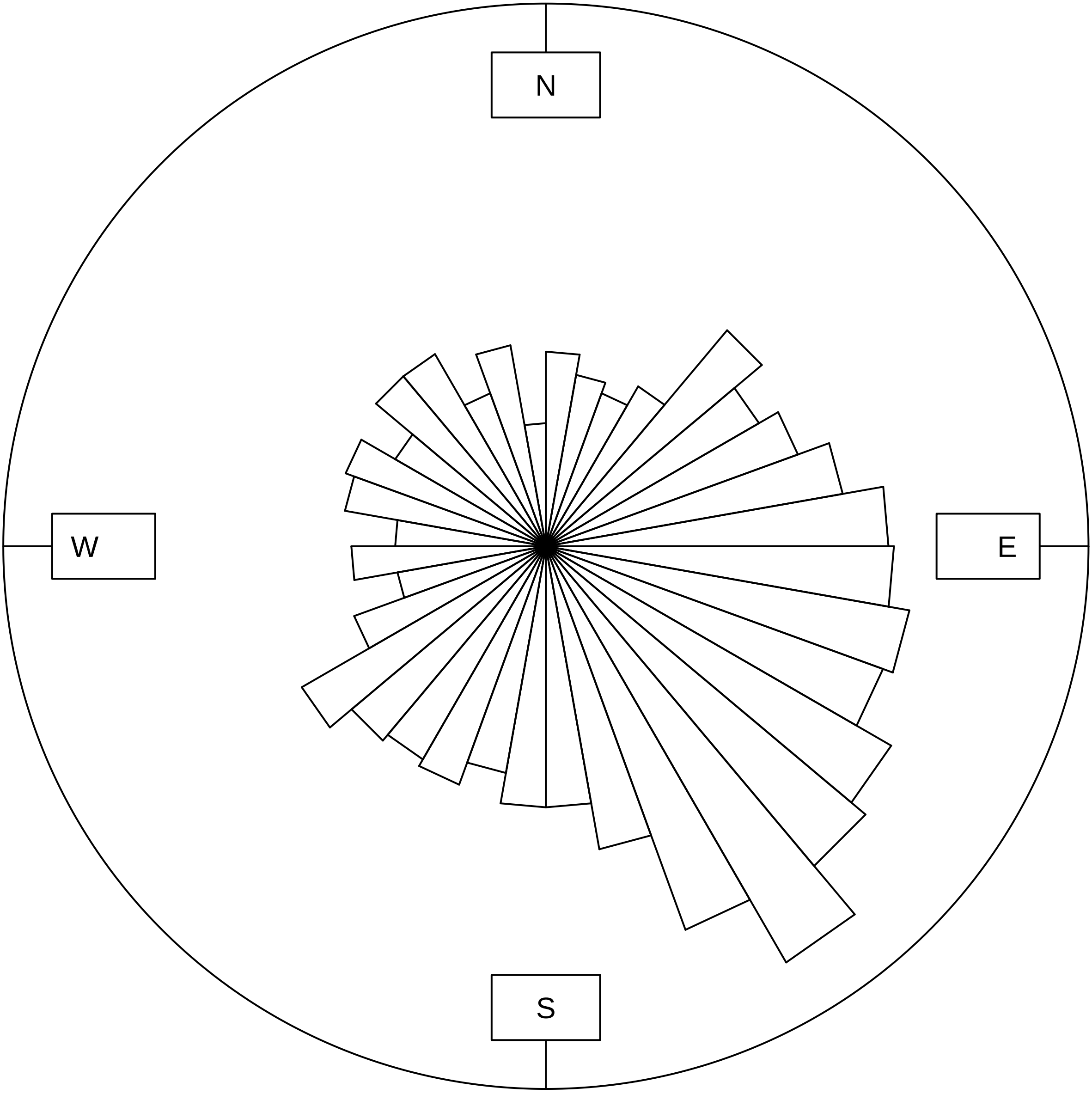

bootstrap 985

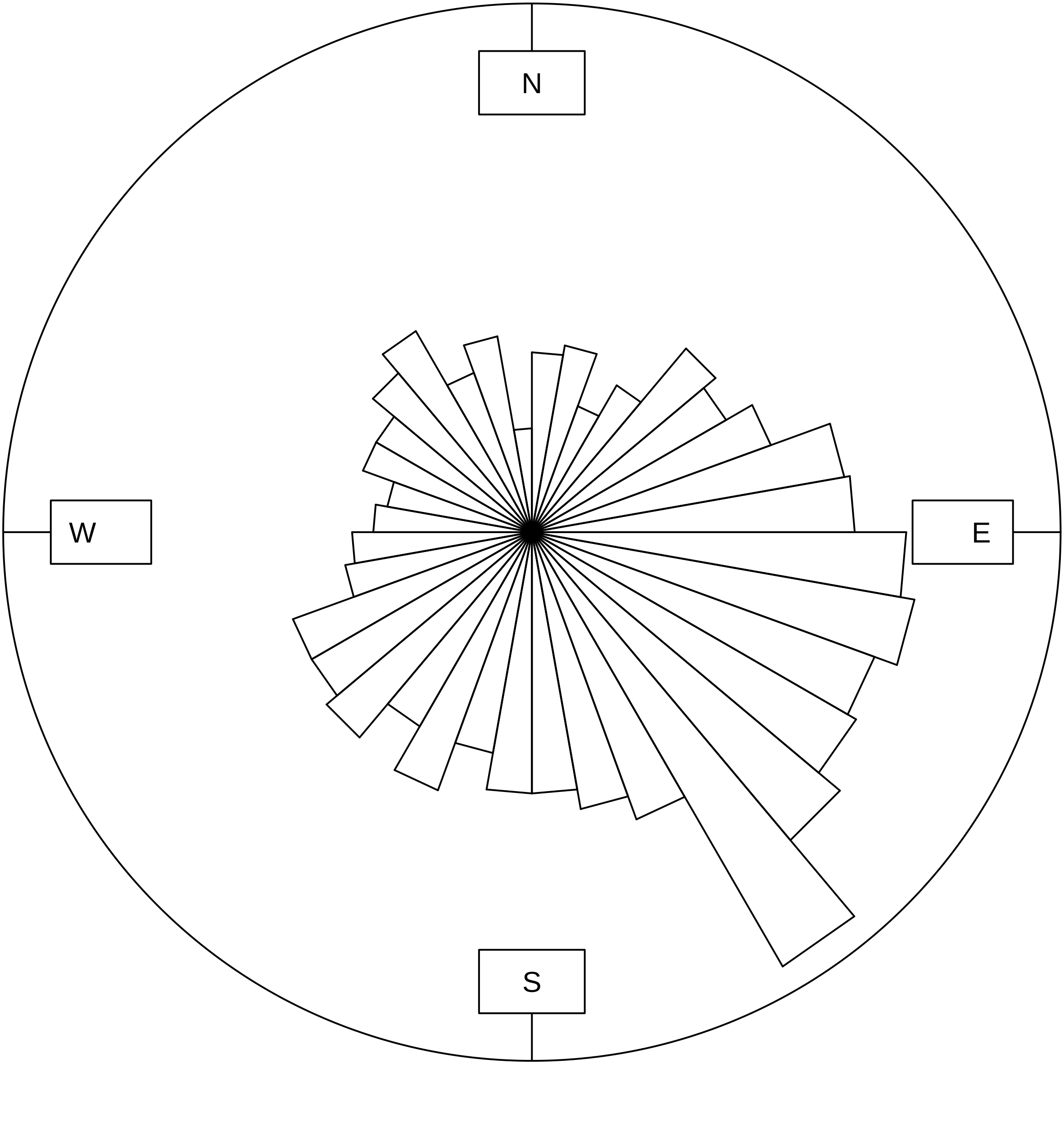

bootstrap 986

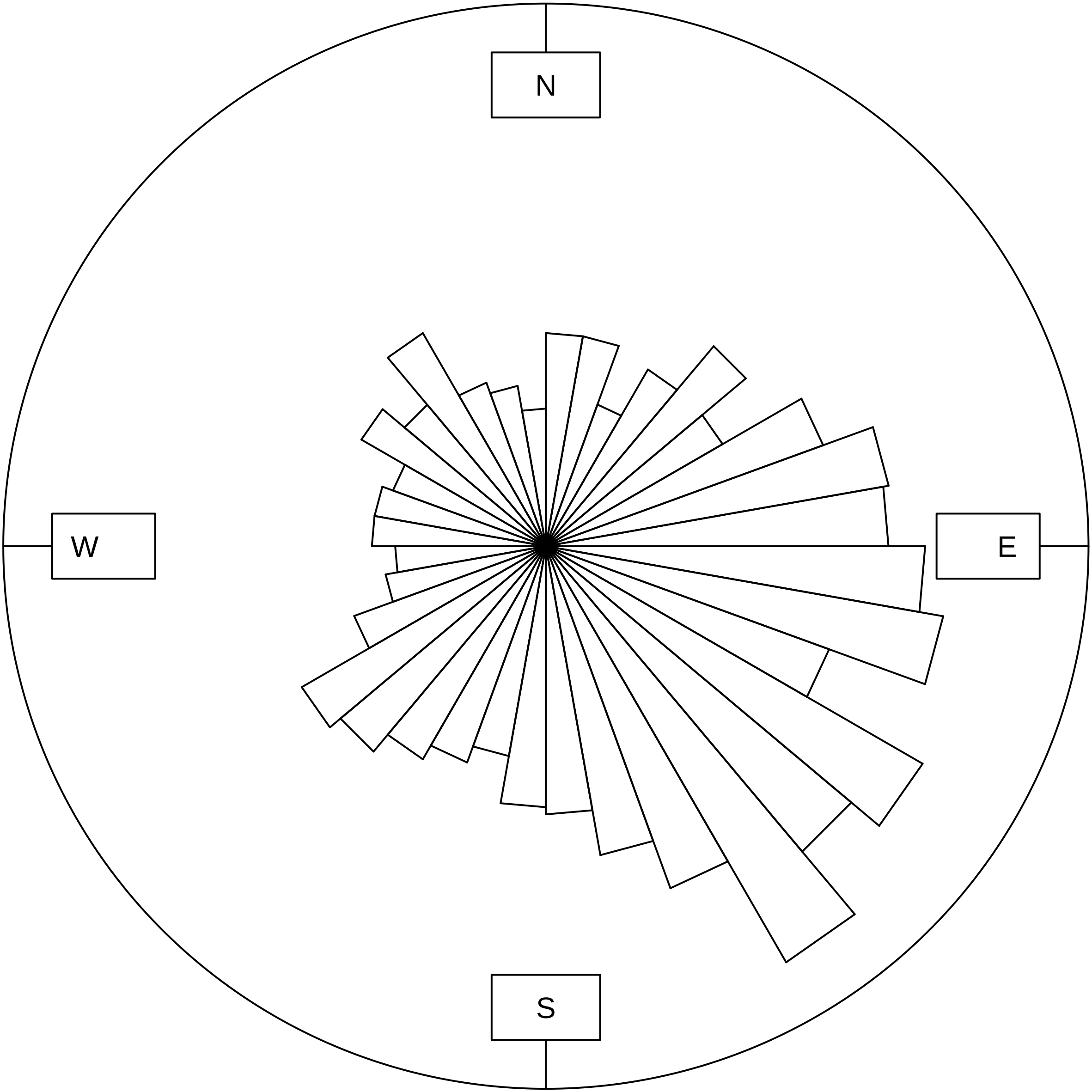

bootstrap 987

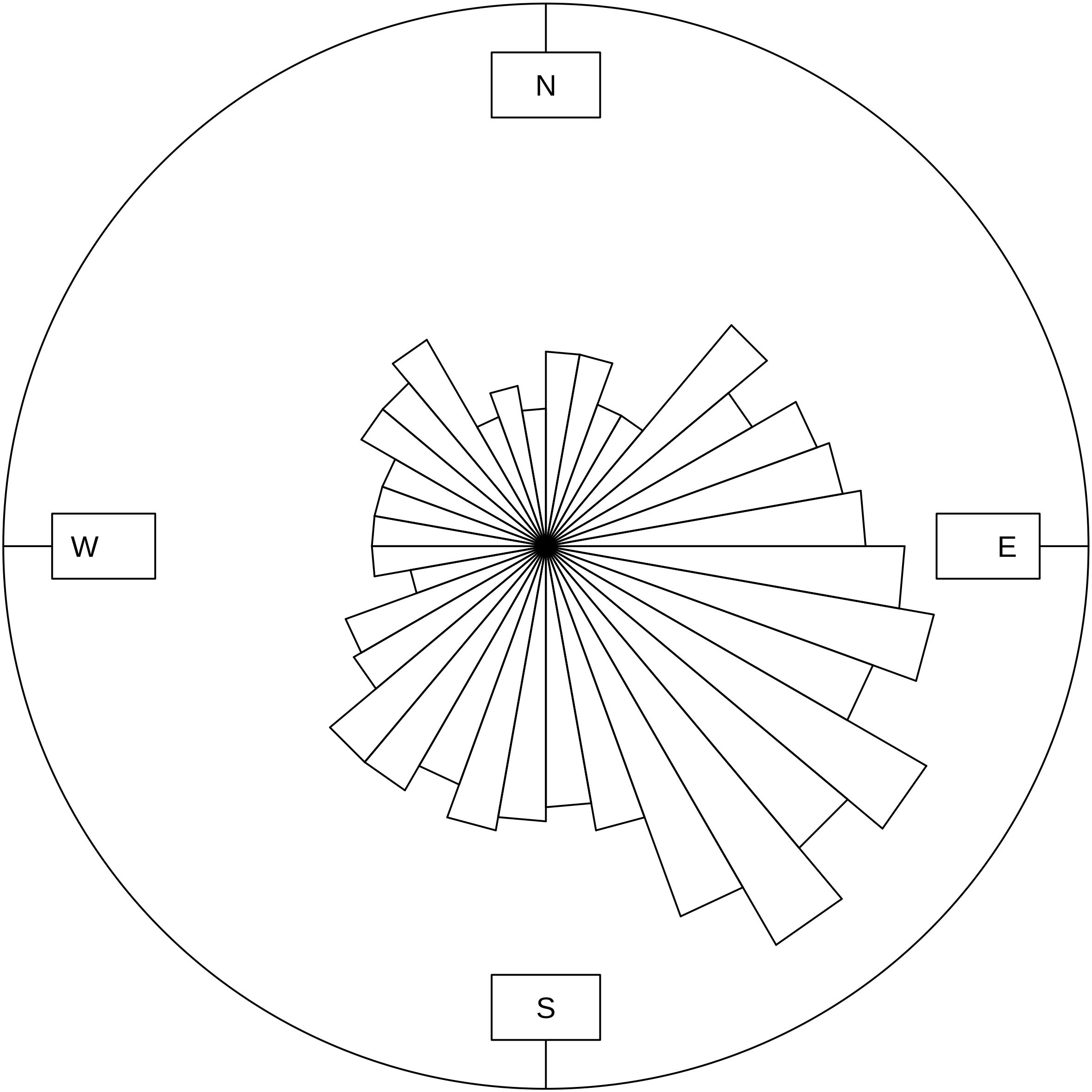

bootstrap 988

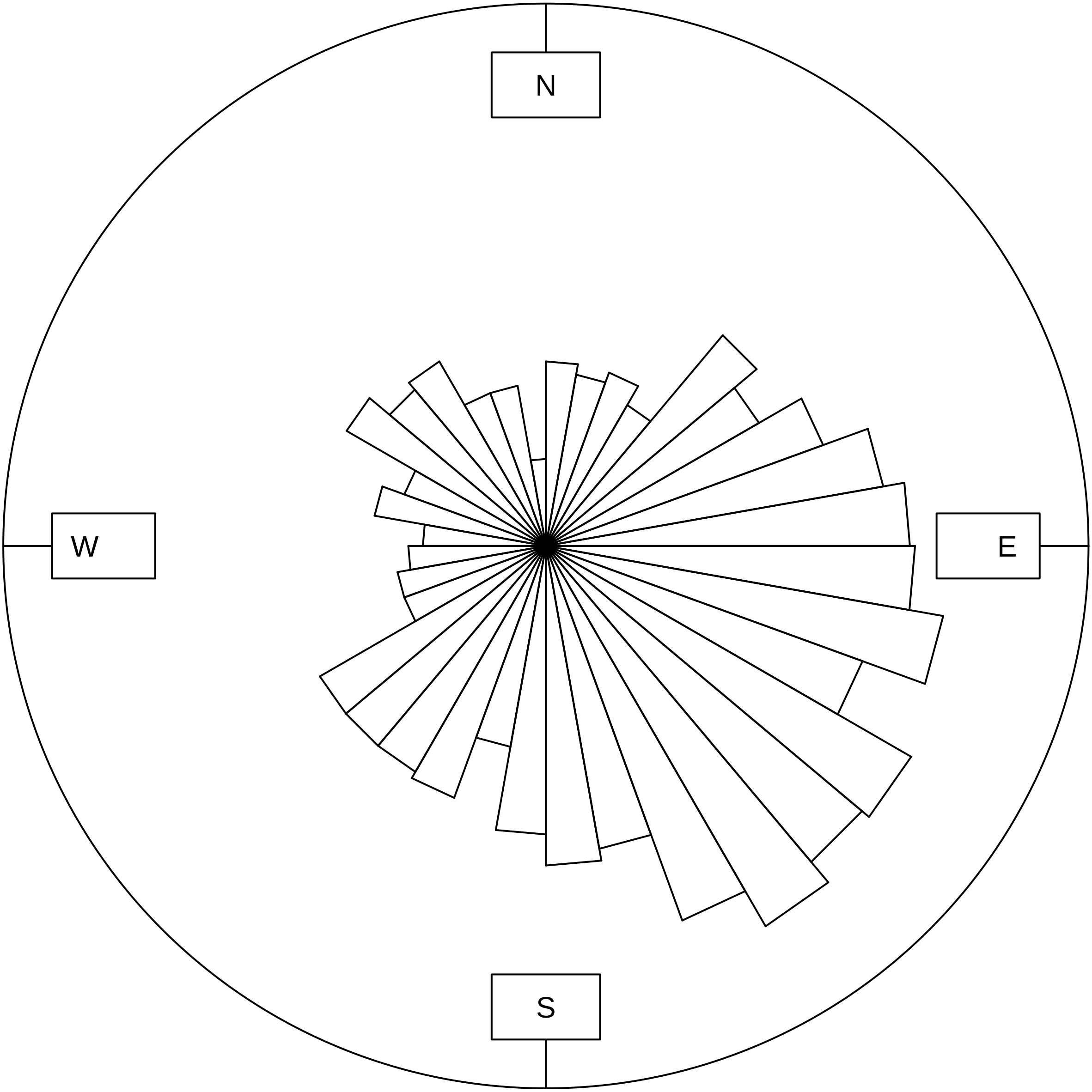

bootstrap 989

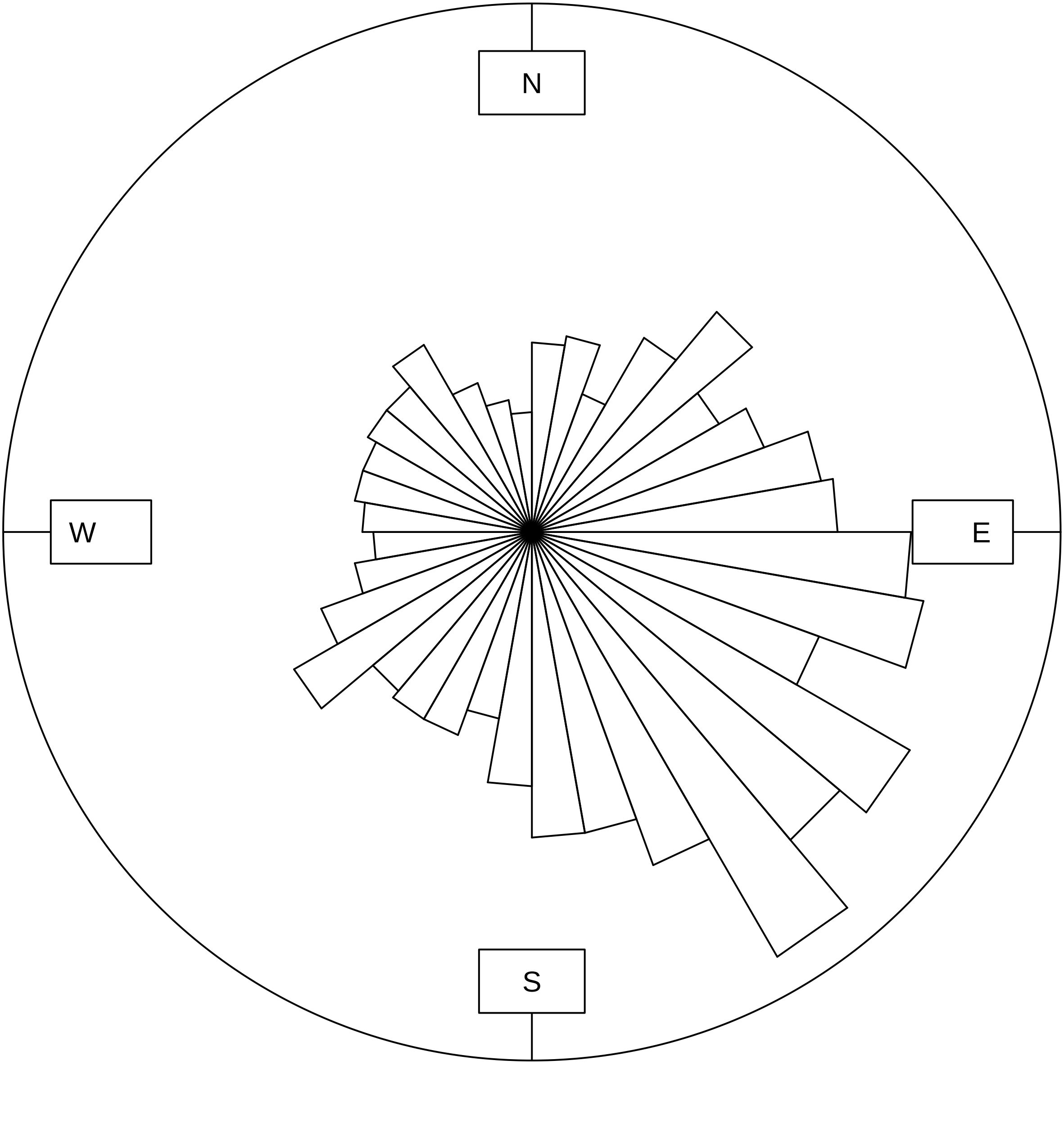

bootstrap 990

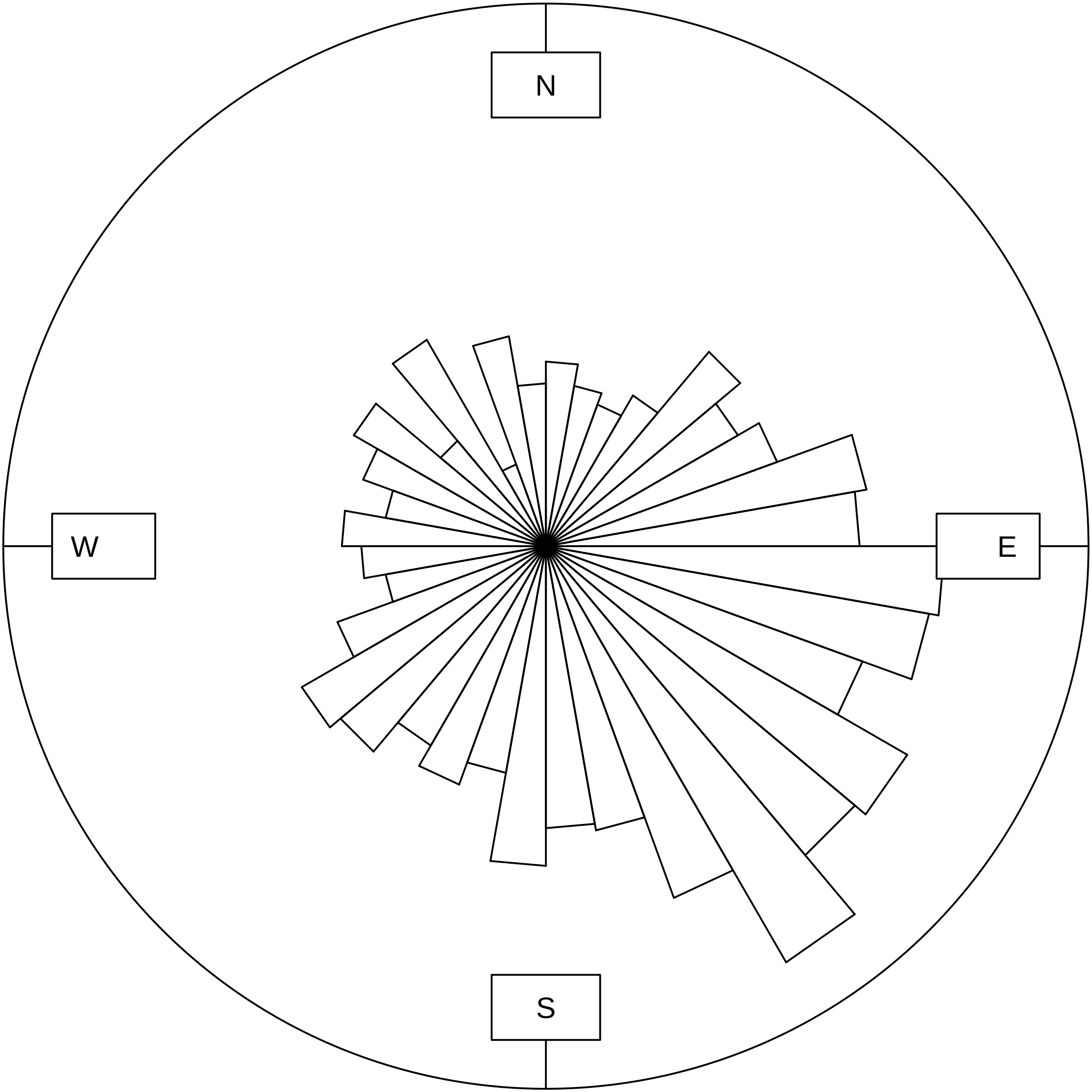

bootstrap 991

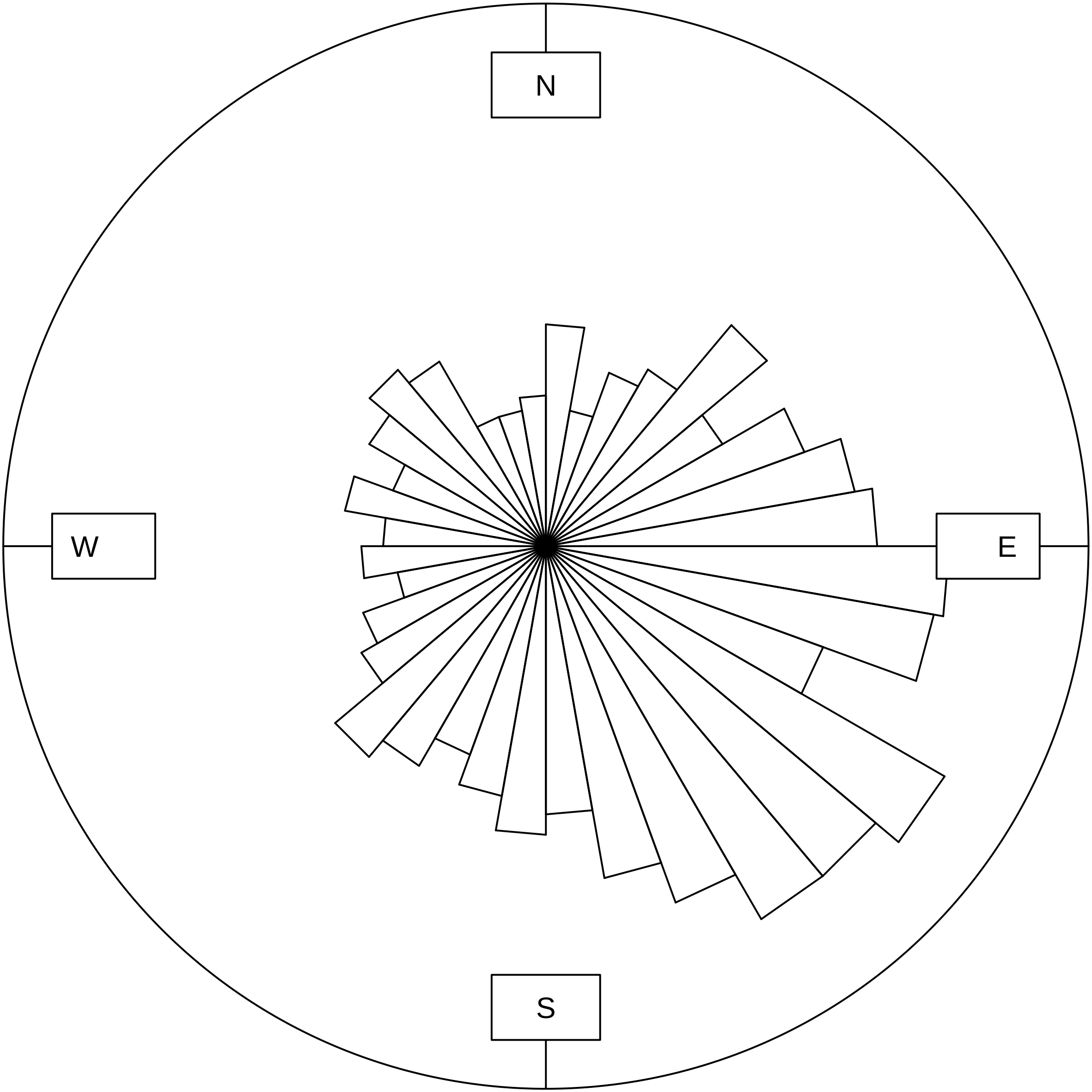

bootstrap 992

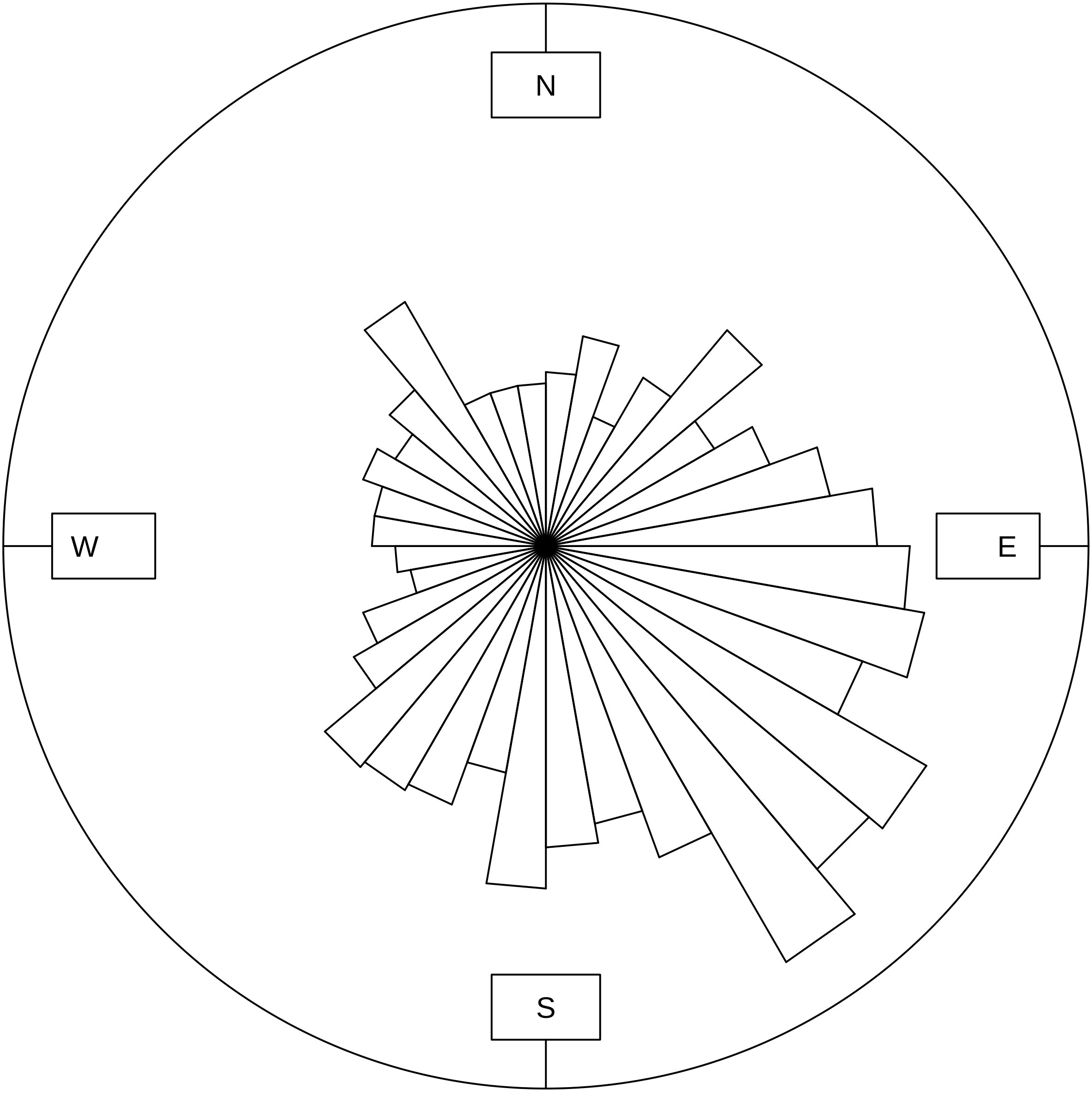

bootstrap 993

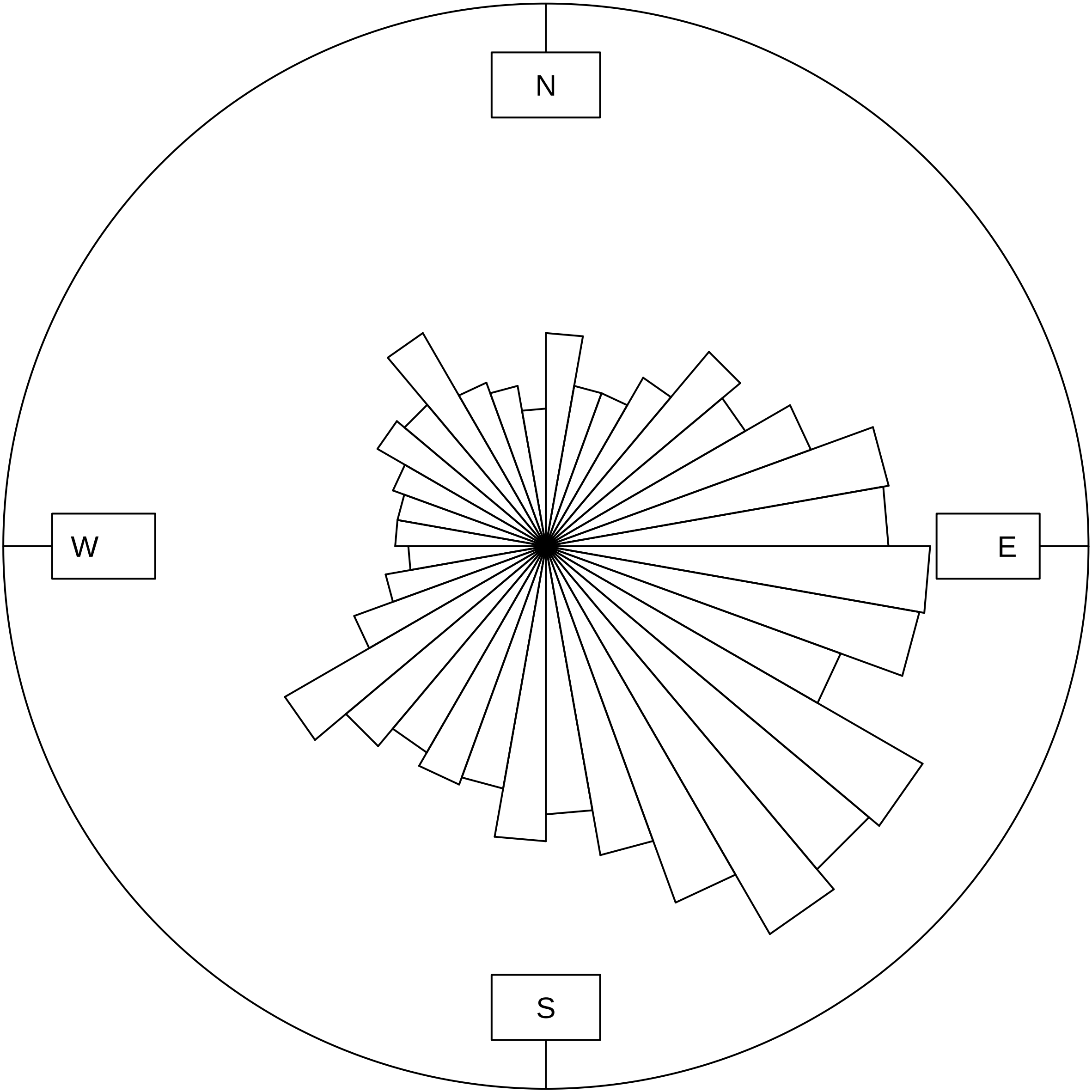

bootstrap 994

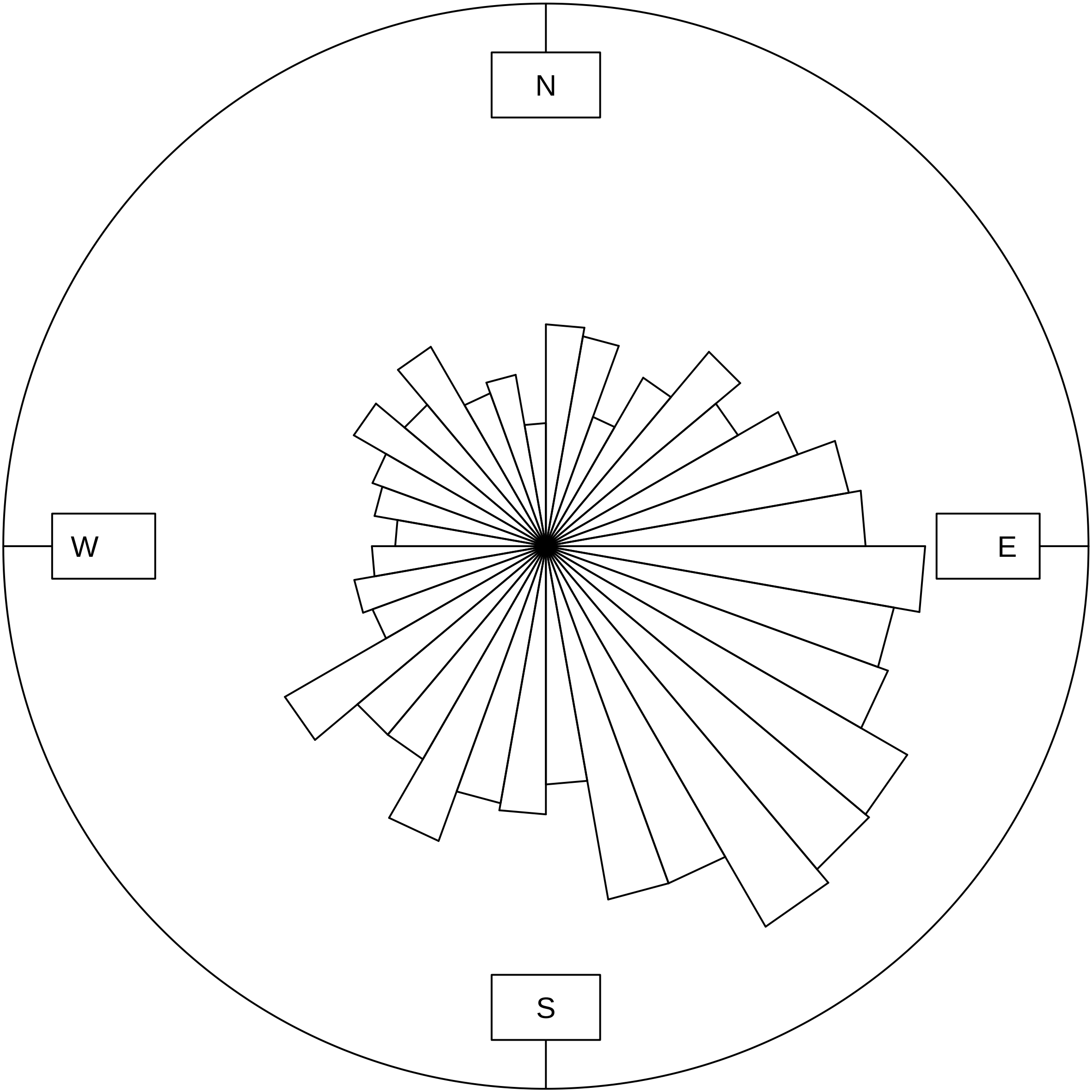

bootstrap 995

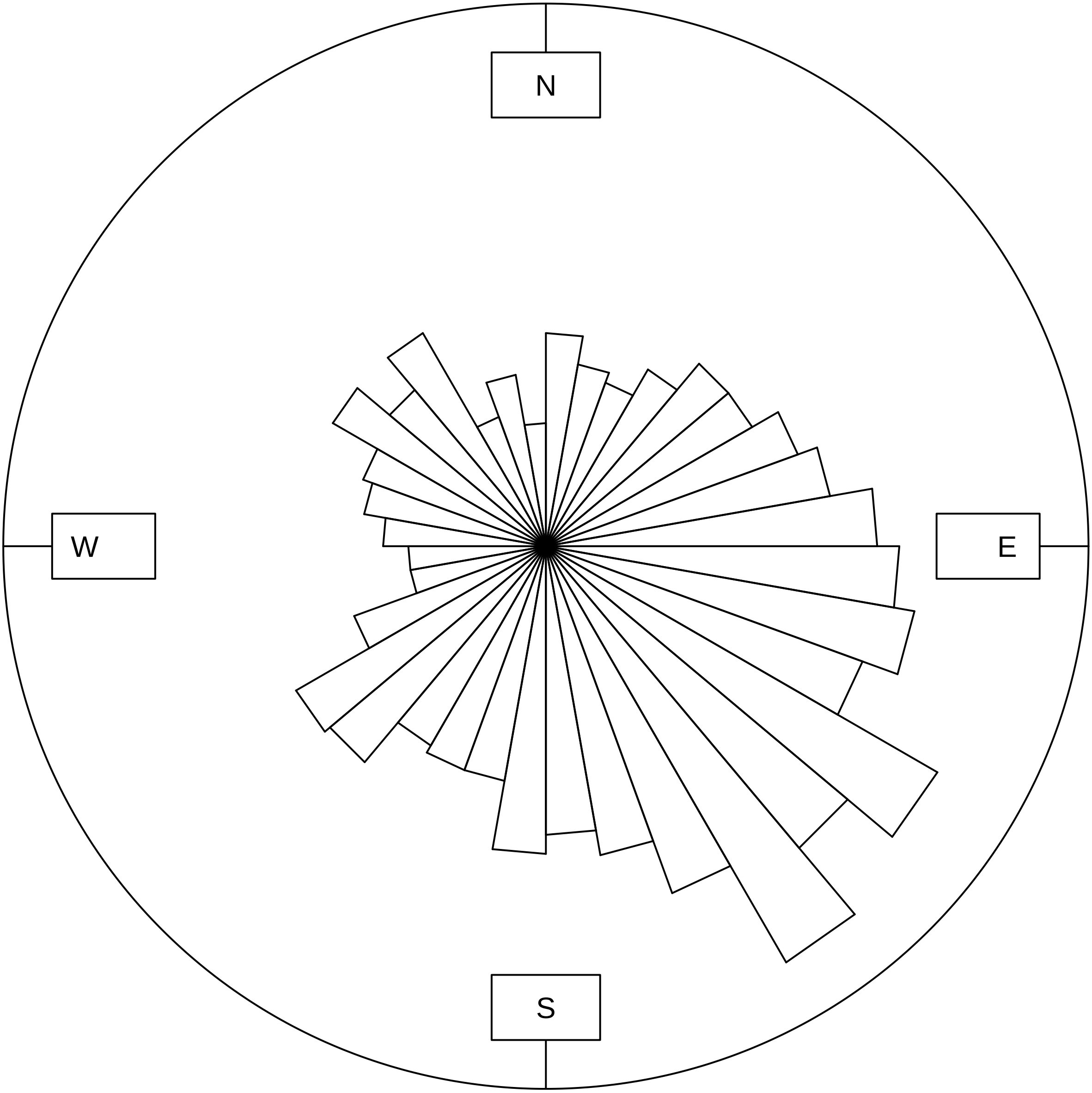

bootstrap 996

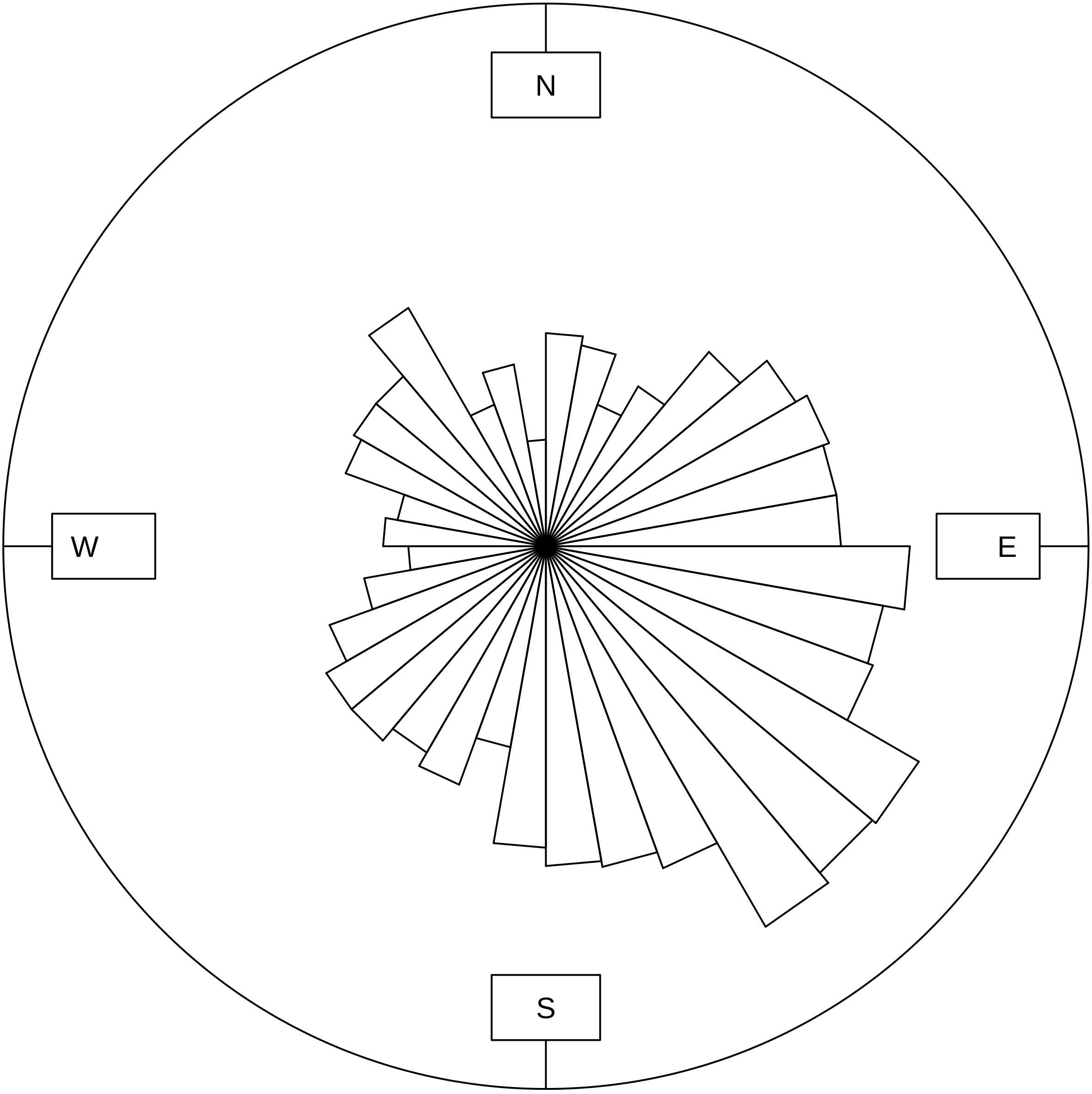

bootstrap 997

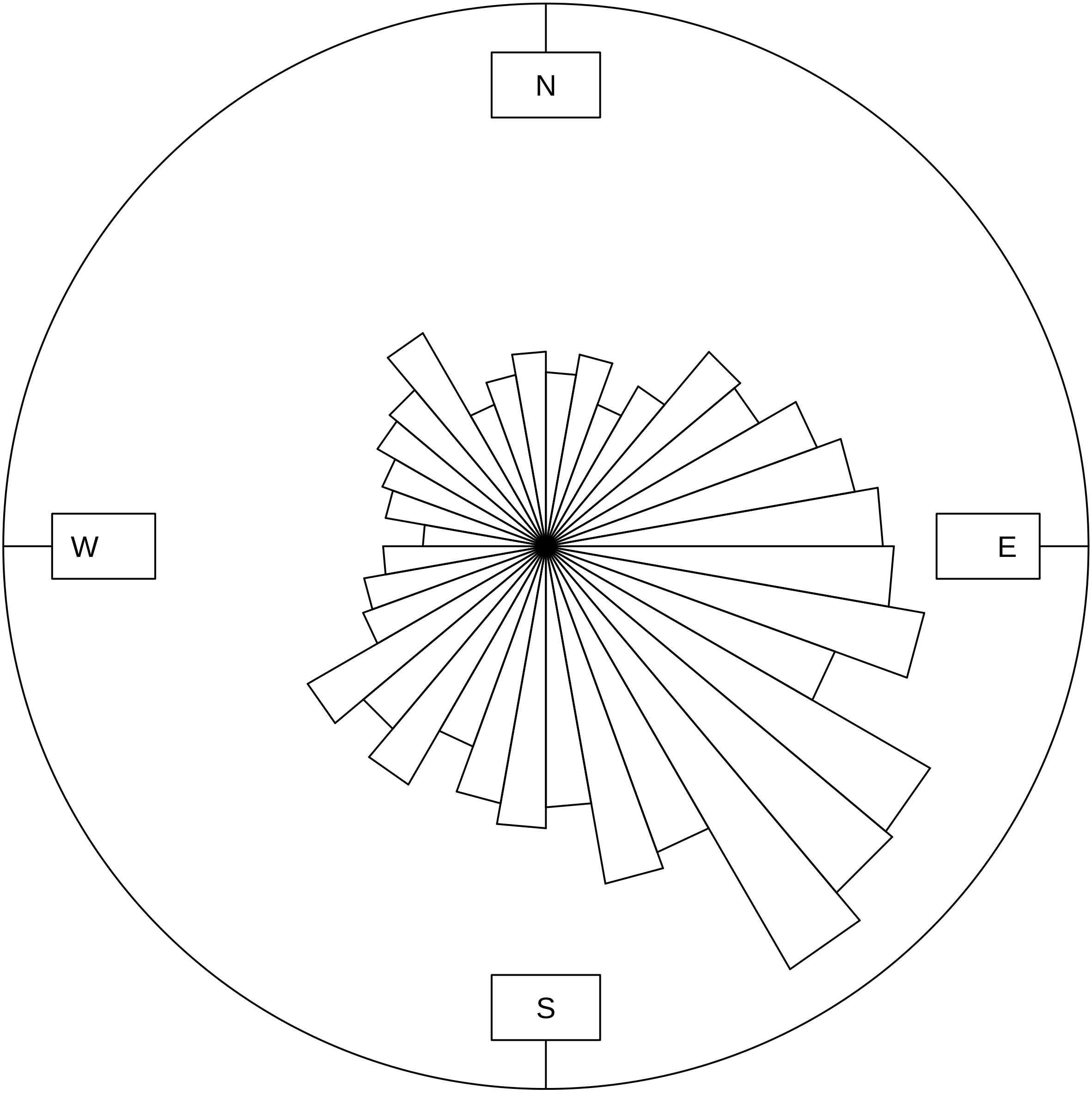

bootstrap 998

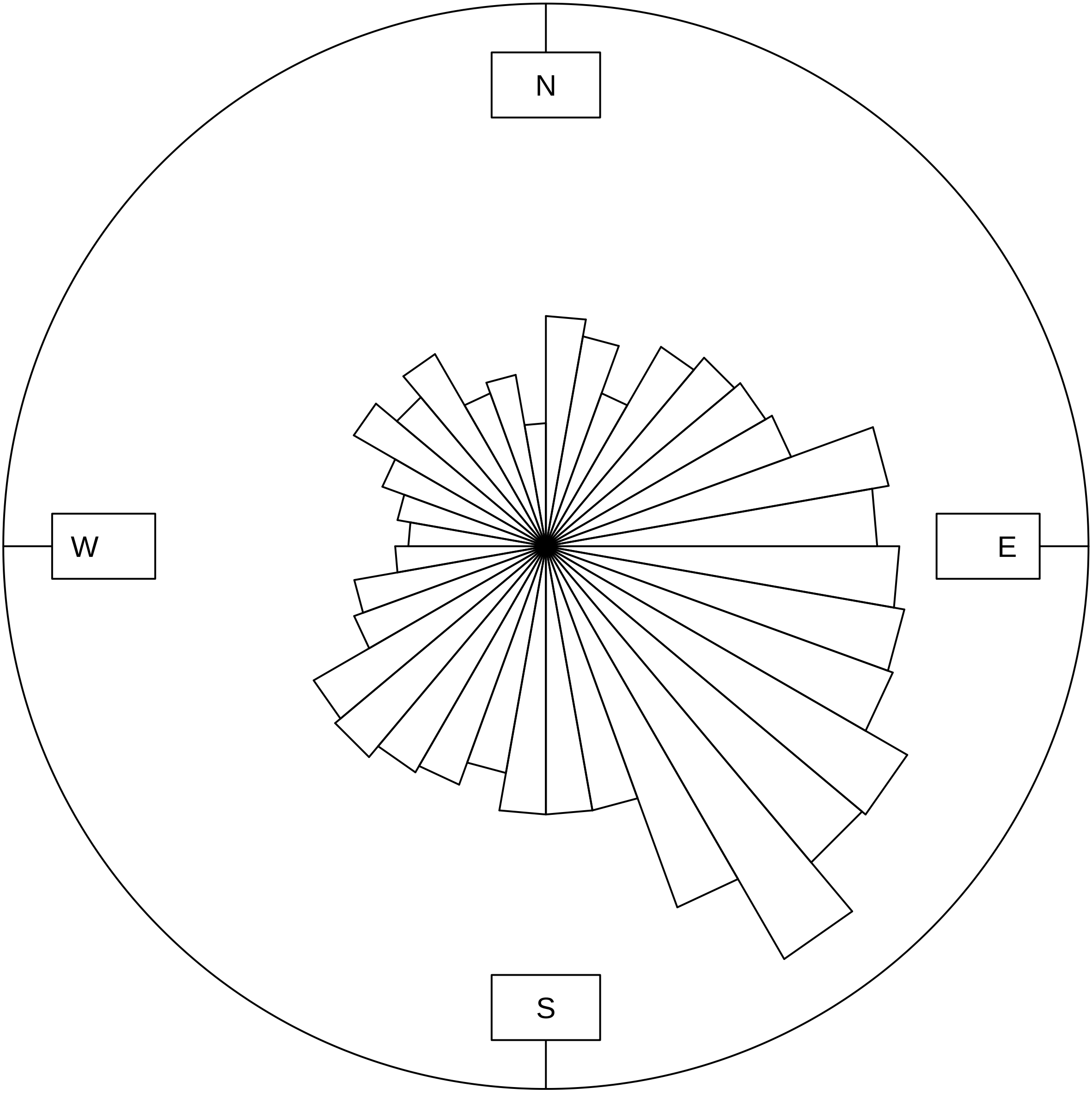

bootstrap 999

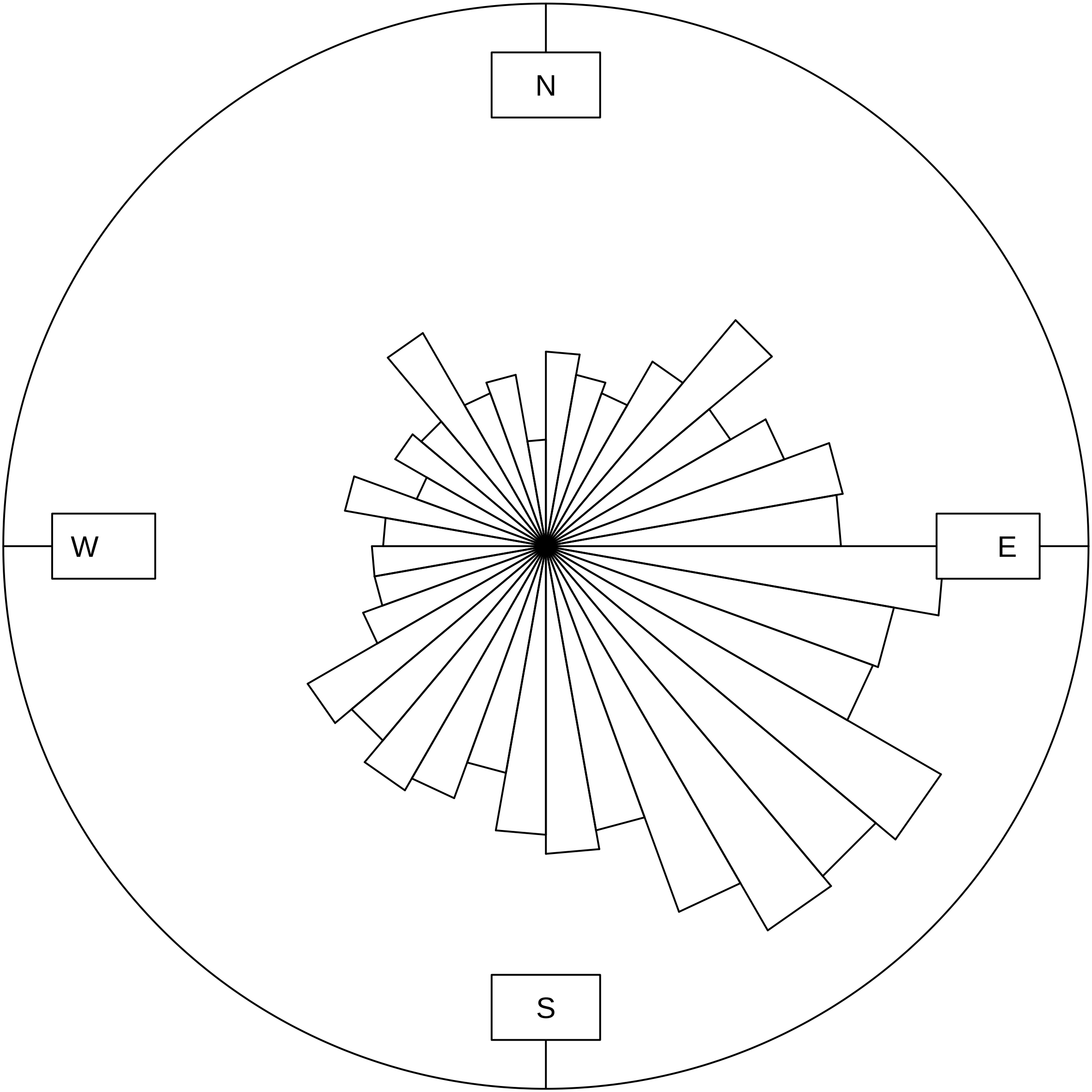

bootstrap 1000

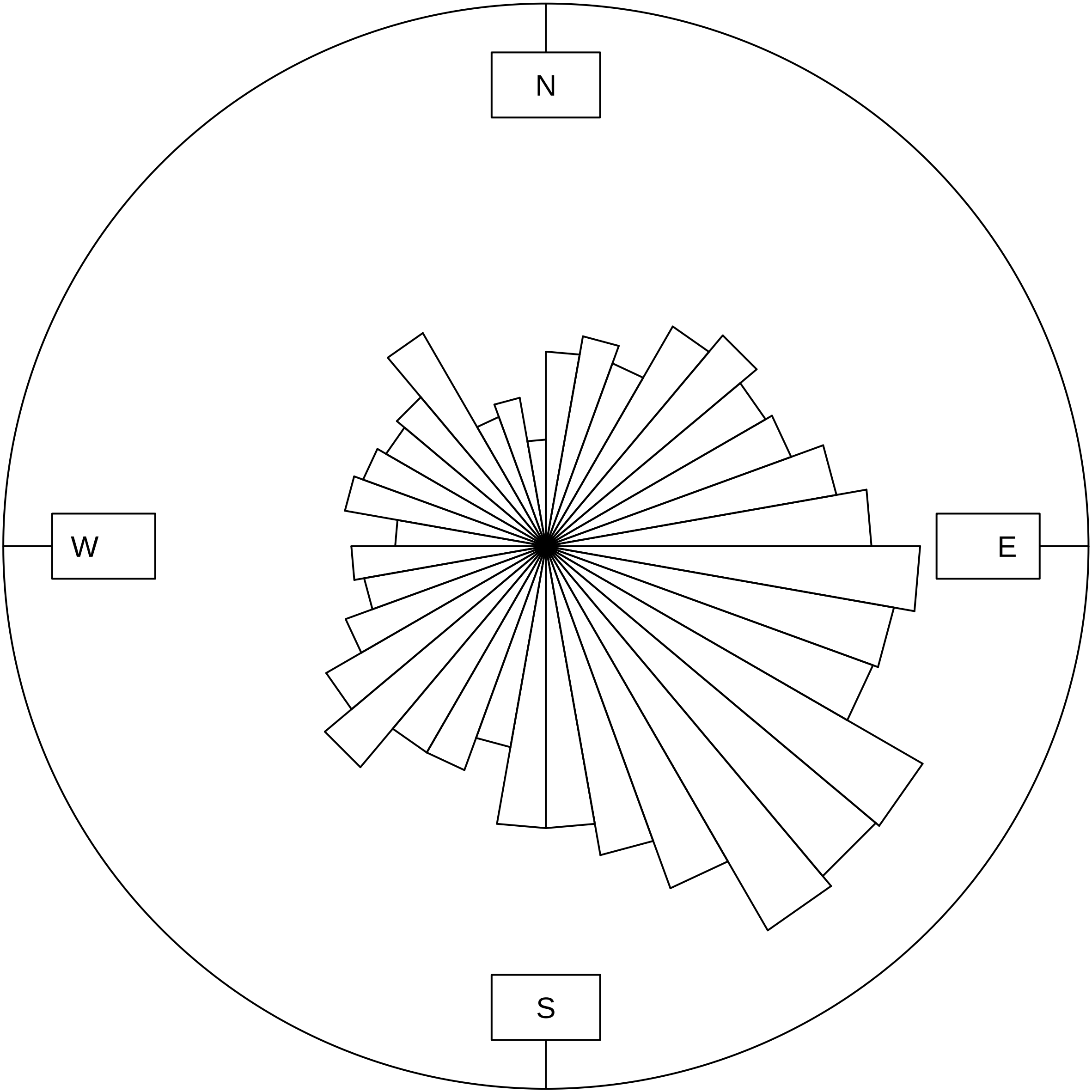

Supplement: S1 File — (PDF) [file pone.0187848.s001.pdf]
